# Comprehensive analysis of miRNAs, lncRNAs and mRNAs profiles in backfat tissue between Daweizi and Yorkshire pigs

**DOI:** 10.5713/ab.22.0165

**Published:** 2022-11-13

**Authors:** Chen Chen, Yitong Chang, Yuan Deng, Qingming Cui, Yingying Liu, Huali Li, Huibo Ren, Ji Zhu, Qi Liu, Yinglin Peng

**Affiliations:** 1 Hunan Institute of Animal and Veterinary Science, Changsha 410131, China; 2 College of Animal Science and Technology, Hunan Agricultural University, Changsha, 410128, China; 3 Hunan Tianfu Ecological Agricultural Limited Company, Changsha, 410144, China

**Keywords:** Backfat, Daweizi (DWZ) Pigs, Pathway, RNA Sequencing, Transcript Profile, Yorkshire Pigs

## Abstract

**Objective:**

Daweizi (DWZ) is a famous indigenous pig breed in China and characterized by tender meat and high fat percentage. However, the expression profiles and functions of transcripts in DWZ pigs is still in infancy. The object of this study was to depict the transcript profiles in DWZ pigs and screen the potential pathway influence adipogenesis and fat deposition,

**Methods:**

Histological analysis of backfat tissue was firstly performed between DWZ and lean-type Yorkshire pigs, and then RNA sequencing technology was utilized to explore miRNAs, lncRNAs and mRNAs profiles in backfat tissue. 18 differentially expressed (DE) transcripts were randomly selected for quantitative real-time polymerase chain reaction (QPCR) to validate the reliability of the sequencing results. Finally, gene ontology (GO) and Kyoto encyclopedia of genes and genomes (KEGG) enrichment analysis were conducted to investigate the potential pathways influence adipocyte differentiation, adipogenesis and lipid metabolism, and a schematic model was further proposed.

**Results:**

A total of 1,625 differentially expressed transcripts were identified in DWZ pigs, including 27 upregulated and 45 downregulated miRNAs, 64 upregulated and 119 downregulated lncRNA, 814 upregulated and 556 downregulated mRNAs. QPCR analysis exhibited strong consistency with the sequencing data. GO and KEGG analysis elucidated that the differentially expressed transcripts were mainly associated with cell growth and death, signal transduction, peroxisome proliferator-activated receptors (PPAR), AMP-activated protein kinase (AMPK), PI3K-Akt, adipocytokine and foxo signaling pathways, all of which are strongly involved in cell development, lipid metabolism and adipogenesis. Further analysis indicated that the BGIR9823_87926/miR-194a-5p/*AQP7* network may be effective in the process of adipocyte differentiation or adipogenesis.

**Conclusion:**

Our study provides comprehensive insights into the regulatory network of backfat deposition and lipid metabolism in pigs from the point of view of miRNAs, lncRNAs and mRNAs.

## INTRODUCTION

Adipose tissue in pigs is associated with important traits of carcass and meat quality [1], and backfat deposition greatly influences porcine growth performance, meat production and final farming profit [2]. Importantly, the pig is emerging as an attractive biomedical model for studying obesity and related diseases in human because of the similar physiology, anatomy, and metabolic features [3,4]. Therefore, understanding the molecular mechanism of backfat deposition in pigs is not only conducive to the progress of genetic breeding, but also expands the comprehension of obesity related metabolic diseases in human.

microRNAs (miRNAs) are a kind of small non-coding RNAs of about 19 to 23 nucleotides in length that negatively regulates gene expression [5]. lncRNAs, a class of non-protein coding RNAs of >200 nucleotides in length, are poorly conserved and expressed in a cell-, tissue-, and stage-specific manner, and lncRNAs can be classified into sense, antisense, intergenic, and bidirectional lncRNAs according to their genomic position [6]. Accumulating evidence indicates that miRNAs play various crucial roles in the processes of adipocyte differentiation, adipogenesis and lipid metabolism [7]. In recent years, studies on identification and functional analysis of porcine lncRNAs were progressively performed, and several differentially expressed lncRNAs and potential regulatory lncRNAs were obtained either in adipose tissue or in the process of adipogenesis from two different pig breeds [8–13].

Daweizi (DWZ), a famous indigenous pig breed in China, is characterized by tender meat, slow growth rate and high fat percentage [14]. In contrast, Yorkshire, a worldwide well-known pig breed, exhibits rapid growth rate with low fat percentage under the intensive selection. In view of the distinctive difference in term of fat content, the two pig breeds can be regarded as the appropriate objects to study the molecular mechanism of fat deposition.

However, the molecular mechanism of the obvious difference in backfat deposition between DWZ and Yorkshire pigs has not yet been studied. In the present investigation, the expression profiles of miRNAs, lncRNAs, and mRNAs were compared in backfat tissue between DWZ and Yorkshire pigs by RNA sequencing (RNA-seq) technology. Then, the differentially expressed miRNAs (DE miRNAs), differentially expressed lncRNAs (DE lncRNAs) and differentially expressed mRNAs (DE mRNAs) associated with porcine adipogenesis were identified, and the functional enrichments and lncRNA-miRNA-mRNA interaction network were further analyzed. The present investigation provides more insights into the mechanism of backfat deposition from the point of view of miRNAs, lncRNAs, and mRNAs.

## MATERIALS AND METHODS

### Experimental animal and tissue collection

Three healthy castrated male DWZ pigs (180-day-old, average slaughter weight 73.83±1.88 kg) and three healthy castrated male Yorkshire pigs (180-day-old, average slaughter weight 121.30±1.33 kg) were raised under the same conditions at Tianfu Ecological Agricultural Limited Company in Hunan province, China. After slaughtered in a commercial slaughter plant, the left side carcass was hung upside down, and the midline backfat thickness at the position of the thickest point in shoulder, last rib and lumbosacral junction was separately measured, and then the average value was calculated. Subsequently, backfat tissues collected at the position of the thickest point in shoulder were divided into two parts. One part was fixed in 4% paraformaldehyde for histological analysis, and the other part was immediately frozen in liquid nitrogen and stored in a −80°C refrigerator. All the experiments in this study were reviewed and approved by the Institutional Animal Care and Use Committee of Hunan Institute of Animal and Veterinary Science (Approval number: 20200110).

### Histological analysis of backfat tissue

The paraformaldehyde-fixed backfat samples were embedded in paraffin. The serial tissue sections were cut using cryostat (Leica RM2235; Leica company, Wetzlar, Germany) and were then stained with hematoxylin/eosin. The sections were viewed at 200× magnification using microscope (Leica DM3000, Germany), and five areas were randomly selected in each sample for measuring the diameter of adipocyte.

### RNA extraction and library construction

Total RNA from backfat tissue was isolated using Trizol (Invitrogen, Carlsbad, CA, USA) according to the manufacturer’s instruction. The quantity and integrity of total RNA were assessed by Agilent 2100 bioanalyzer (Santa Clara, CA, USA), which exhibited that the RNA integrity number was more than 8.0 for each sample. The main processes of small RNA library construction include: i) Total RNA was purified by electrophoretic separation on a 15% urea denaturing polyacrylamide gel electrophoresis (PAGE) gel, and small RNA regions corresponding to the 18 to 30 nt bands were excised and recovered. ii) Small RNAs were ligated to adenylated 3′-adapters annealed to unique molecular identifiers (UMI), followed by ligation of 5′-adapters. iii) The adapter-ligated small RNAs were transcribed into cDNA by SuperScript II Reverse Transcriptase (Invitrogen, USA) and then several rounds of polymerase chain reaction (PCR) amplification were performed to enrich cDNA fragments. iv) PCR products with 110 to 130 bp in length were acquired on PAGE gel and were then purified by QIAquick Gel Extraction Kit (QIAGEN, Düsseldorf, Germany). Finally, these PCR products were circularized and then sequenced using the DNBseq platform (BGI-Shenzhen, China). The brief procedures for construction of mRNAs and lncRNAs libraries were as follows: i) Ribosomal RNA (rRNA) was removed using MGIEasy rRNA kit (BGI, China), and the remained RNA was fragmented into small pieces using divalent cations under elevated temperature. ii) The cleaved RNA fragments were transcribed into first strand cDNA using reverse transcriptase and random primers, followed by second strand cDNA synthesis using DNA polymerase I and RNase H with dUTP instead of dTTP. iii) These cDNA fragments were ligated with an ‘A’ base and sequencing adapter. iv) The products were enriched with PCR and then heat denatured and circularized by the splint oligo sequence. v) The single strand circle DNA was formatted as the final library which was sequenced on DNBseq platform (BGI-Shenzhen, China).

### Sequencing data analysis

For analysis of small RNA sequencing data, the raw reads containing polyA, shorter than 18 nt in length, with low-quality reads (the number of bases with quality score less than 10 is ≤4 and with quality score less than 13 is ≤6), with 5′-adapter contamination, without 3′-adapter or inserted fragments were filtered to obtain clean reads by SOAPnuke (v1.5.0). Subsequently, the clean reads were mapped to porcine reference genome (GCF_000003025.6_Sscrofa11.1) and other small RNA databases including miRNA from miRbase (v22.1), and siRNA, piRNA, and snoRNA from NCBI GenBank by Bowtie2. Particularly, cmsearch was performed for mapping Rfam database (http://rfam.xfam.org/). To make each unique sRNA have a unique annotation, the priority order of miRbase>pirnabank>snoRNA>Rfam>other sRNA was followed to traverse the annotation. The unannotated sequences were used to predict novel miRNA candidates by miRDeep2 program (v0.1.3) based on the secondary structure. The novel miRNAs were aligned to mature miRNAs from other mammals in the miRbase by BLAST.

For analysis of mRNA and lncRNA sequencing data, clean reads were obtained from raw reads by removing adapter pollution, low-quality reads (more than 20% bases with quality score less than 15) and reads whose unknown base ratio was greater than 5% using SOAPnuke. These clean reads were aligned to the porcine reference genome using HISAT2 (v2.0.4), and Bowtie2 (v2.2.5) was applied to align the clean reads to the reference sequence. Then the mapped reads were assembled using StringTie. The transcripts were then screened by Pfam database and three softwares including coding potential calculator (score <0), txCdsPredict (score <500), and coding-non-coding index (score <0). The transcripts that unmatched Pfam database and passed through at least two of the three softwares were considered to be lncRNAs.

### Identification of differentially expressed transcripts

After mapping clean reads to the porcine reference sequence by Bowtie2, the expressions of small RNAs were calculated by counting absolute numbers of molecules using UMI, and then DE miRNAs analysis was performed using DEGseq. The fragments per kilobase of transcript per million (FPKM) reads mapped value was used to estimate the expressions of lncRNAs and mRNAs by RSEM software (v1.2.12), and analysis of DE mRNAs and DE lncRNAs were examined by DEseq2. Q value (adjusted p value) ≤0.05 and |log2(DWZ/Yorkshire)| ≥1 were set as thresholds for significant differential expression. The heatmap of DE transcripts was drawn by pheatmap (v1.0.8) with default parameter.

### Target genes prediction and functional analysis of DE miRNAs and DE lncRNAs

The predicted target genes of miRNAs were implemented by using RNAhybrid, miRanda and TargetScan softwares. lncRNAs can regulate target genes by acting *in cis* and *in trans*. If lncRNAs and genes exhibit similar expression patterns, their biological functions may be highly correlated. Accordingly, the targets genes of DE lncRNAs were predicted as follows. Based on the Spearman and Pearson correlation coefficients of lncRNA-mRNA pair being ≥0.6, mRNAs located within 50 kb upstream and 50 kb downstream of DE lncRNAs were selected for *cis*-acting regulation, and RNAplex was utilized to predict the combination of lncRNA and mRNA for *trans*-acting regulation with binding energy <–20. To explore the potential biological functions of DE transcripts, GO and KEGG analysis were carried out for the DE mRNAs and targets of DE miRNAs and DE lncRNAs based on GO ( http://www.geneontology.org/ ) and KEGG ( https://www.kegg.jp/ ) databases, and then the functional enrichment analysis was performed by Phyper based on Hypergeometric test. The significant levels of terms and pathways were calculated with a rigorous threshold (Q value ≤0.05) by Bonferroni.

### Construction of PPI and lncRNA-miRNA-mRNA network

The STRING was applied to construct protein-protein interaction (PPI) network for DE mRNAs, which was visualized by Cytoscape software. As mentioned above, the target genes of DE miRNAs and DE lncRNAs were predicted, and the interactions of lncRNA-miRNA, lncRNA-mRNA, and miRNA-mRNA were obtained. Consequently, the potential regulatory network of lncRNA-miRNA-mRNA was also visualized using Cytoscape.

### Quantitative real-time polymerase chain reaction analysis

The validation of miRNA expression levels was detected by stem-loop QPCR method [15]. cDNA was synthesized using RevertAid first strand cDNA synthesis kit (K1622, Fermentas) according to the manufacturer’s instructions. QPCR analysis was performed using SYBR Green Supermix (Biomed, Beijing, China) on CFX96 machine (Bio-Rad, Hercules, CA, USA). Porcine glyceraldehyde-3-phosphate dehydrogenase (*GAPDH*) was used as endogenous control for lncRNAs and mRNAs, and U6 snRNA was used for miRNAs. Each QPCR reaction was performed in triplicate, and the relative expression level of transcripts was calculated using the 2^−ΔΔCt^ method. The sequences of QPCR primers for mRNAs, miRNAs and lncRNAs are listed in Supplementary Table S1.

### Statistical analysis

Statistical analysis of the data from adipocyte diameter and QPCR assay was performed by one-way analysis of variance program with SPSS 20.0 software. Mean values and standard error were presented, and p value of less than 0.05 was deemed statistically significant (* p≤0.05 and ** p≤0.01).

## RESULTS

### Histological analysis of backfat tissue

**Figure 1:**
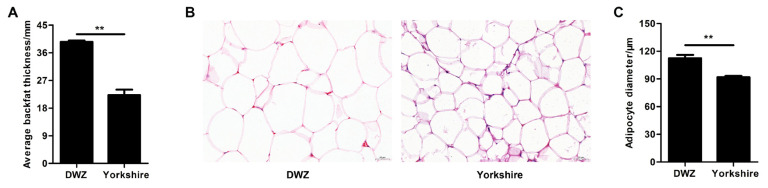
Histological analysis of backfat tissue between Daweizi (DWZ) and Yorkshire pigs. (A) Average backfat thickness of experimental pigs. (B) Representative images of hematoxylin/eosin staining of backfat tissue (magnification: ×200, scale bar = 50 μm). (C) Adipocyte diameter of backfat tissue (n = 3 per group), ** p<0.01.

As shown in Figure 1A, the average backfat thickness in DWZ pigs was notably higher (1.64-fold) than that in Yorkshire pigs. There was an obvious difference in adipocyte phenotype of backfat tissue between DWZ and Yorkshires pigs (Figure 1B), and DWZ pigs had bigger adipocyte diameter that Yorkshire pigs (Figure 1C).

### Overview of sequencing data

To identify small RNA distribution of DWZ and Yorkshire pigs, six small RNA libraries were constructed. After quality filtering and trimming of contaminant and adapter sequences, a total of 23,504,459, 23,631,041, 23,682,253 and 23,528,342, 23,339,095, 23,800,508 clean reads were obtained in DWZ1, DWZ2, DWZ3 and Yorkshire1, Yorkshire2, Yorkshire3, respectively (Supplementary Table S2). About 93.66%, 94.71%, 94.57% and 93.67%, 93.57%, 92.96% of clean reads, respectively, were mapped to the porcine reference genome. For lncRNAs analysis, a total of 122.32Mb, 134.68Mb, 134.34Mb and 134.87Mb, 134.50Mb, 119.25Mb clean reads with greater than 91.55% of Q30 were obtained in DWZ1, DWZ2, DWZ3 and Yorkshire1, Yorkshire2, Yorkshire3, respectively. Among them, 95.53%, 94.70%, 95.40%, 96.43%, 96.31%, 96.38% clean reads were mapped to the porcine reference genome, and 91.16%, 91.66%, 91.77%, 92.47%, 91.14%, 92.15% were uniquely mapped in DWZ1, DWZ2, DWZ3 and Yorkshire1, Yorkshire2, Yorkshire3, respectively (Supplementary Table S3).

The length distribution of reads in small RNA library was analyzed, and the results demonstrated that the majority (74.04% to 82.81%) of reads were between 21 nt and 23 nt in length, which is the typical length of mature miRNA. The 22 nt reads accounted for approximately 50% of the reads in libraries of DWZ and Yorkshire pigs (Supplementary Table S4). In addition, the abundance of mRNA was expectedly higher than that of lncRNA, and both mRNA and lncRNA showed similar distribution in both pig breeds (Supplementary Figure S1).

### Identification and characterization of miRNAs and lncRNAs

**Figure 2:**
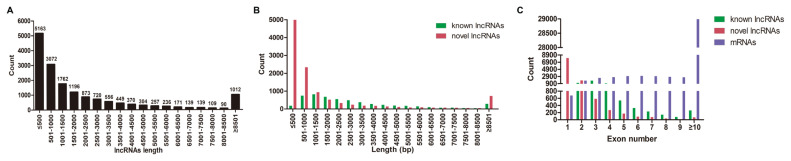
Characterization of lncRNAs and mRNAs. (A) The length distribution of lncRNAs. (B) The length distribution of known and novel lncRNAs. (C) The exon number distribution of known lncRNAs, novel lncRNAs and mRNAs.

A total of 1,740 miRNAs (356 known miRNAs and 1,384 novel miRNAs), 16,618 lncRNAs (5,448 known lncRNAs and 11,170 novel lncRNAs), and 51,123 mRNAs were identified in DWZ and Yorkshire libraries (data not shown). Furthermore, the length and exon number distribution of lncRNAs and mRNAs were analyzed. The most common length of lncRNAs (60.16%) was less than 1,500 bp (Figure 2A). Interestingly, this research also found that the majority (65.55%) of novel lncRNAs were less than 1,000 bp in length, while almost half of the known lncRNAs were between 501 bp and 2,500 bp (Figure 2B). Further analysis demonstrated that the majority of known lncRNAs had two to four exons, while novel lncRNAs mainly had one to three exons, which were significantly less than the exon number of mRNAs (Figure 2C). The present results are highly in line with that in subcutaneous adipocytes from Large White and Chinese Jiaxing Black pigs [16]. Taken together, the biological replicates and matching efficiency demonstrated the desirable sequencing quality for subsequent analysis.

### Analysis of highly abundant miRNAs and lncRNAs

As exhibited in Table 1, the top 20 most abundant miRNAs in DWZ and Yorkshire libraries were the following: ssc-miR-22-3p, ssc-miR-26a, ssc-miR-206, ssc-miR-133a-3p, ssc-miR-126-3p, ssc-miR-378, novel-ssc-miR-857-5p, ssc-miR-499-5p, ssc-miR-451, ssc-miR-191, ssc-miR-99a-5p, ssc-miR-151-5p, novel-ssc-miR-324-3p, ssc-miR-143-3p, ssc-miR-16, ssc-miR-7f-5p, ssc-miR-423-3p, ssc-miR-1, ssc-miR-24-3p, ssc-miR-92a. The top 20 most abundant lncRNAs were the following: BGIR9823_101660, BGIR9823_79653, BGIR9823_ 101661, BGIR9823_101542, BGIR9823_81016, BGIR9823_ 101532, BGIR9823_ 101403, BGIR9823_101535, BGIR9823_ 88517, BGIR9823_101401, BGIR9823_101546, BGIR9823_ 81019, BGIR9823_81018, BGIR9823_91558, BGIR9823_ 81014, BGIR9823_101756, XR_115737.4, BGIR9823_101544, BGIR9823_101672, BGIR9823_101821 (Table 2). Furthermore, the top 20 most abundant miRNAs contributed to 83.30%, 80.60%, and the top 20 most abundant lncRNAs contributed to 77.02%, 74.21%, of the total counts in DWZ and Yorkshire library, respectively. The profiles of the highly abundant miRNAs and lncRNAs were similar in the two libraries, indicating that these miRNAs and lncRNAs may play important roles in maintenance of the physiological state by acting as housekeeping factors.

### Differentially expressed and specifically expressed miRNAs, lncRNAs, and mRNAs

**Figure 3:**
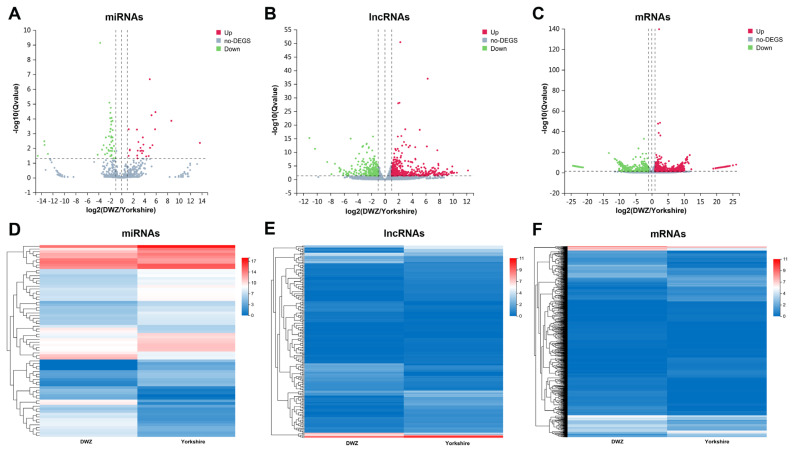
Differentially expressed transcripts and their expression characteristics. The volcano plots of differentially expressed (DE) miRNAs (A), DE lncRNAs (B) and DE mRNAs (C). The X axis represents the fold change of DWZ/Yorkshire after conversion to log2, and the Y axis represents the Q value after conversion to −log10. Red points indicate the upregulated DE transcripts, blue points indicate the downregulated DE transcripts, and gray points indicate the genes without significant changes. The heatmap of the expression profiles of DE miRNAs (D), DE lncRNAs (E) and DE mRNAs (F).

To further understand the difference of regulatory mechanism in backfat deposition between the two pig breeds, comparative transcriptome analysis was performed. Compared with Yorkshire library, 72 miRNAs (27 upregulated and 45 downregulated), 183 lncRNAs (64 upregulated and 119 downregulated) and 1,370 mRNAs (814 upregulated and 556 downregulated) were differentially expressed in DWZ library (Figure 3; Supplementary Table S5). In addition, a total of 225 miRNAs (8 known and 217 novel miRNAs), 1,434 lncRNAs (851 known and 583 novel lncRNAs) and 5,612 mRNAs were specifically expressed (named specifically expressed [SE] miRNAs, SE lncRNAs, and SE mRNAs, respectively) in DWZ library, and 224 SE miRNAs (11 known and 213 novel miRNAs), 1,602 SE lncRNAs (959 known and 643 novel lncRNAs) and 4,756 SE mRNAs were detected in Yorkshire library (Supplementary Table S6).

### Functional analysis and PPI network of DE mRNAs

**Figure 4:**
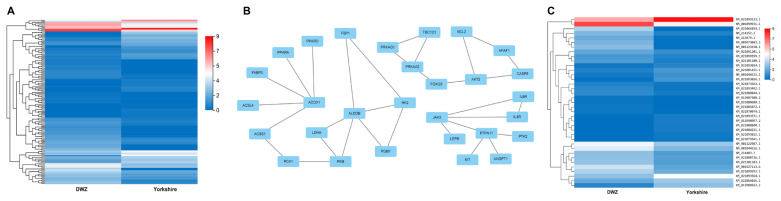
PPI network of DE mRNAs related to fat deposition and lipid metabolism. (A) The heatmap of the expression profile of DE mRNAs. (B) PPI network of DE mRNAs was constructed according to the interaction score. Node represents protein, edge represents interaction between proteins. (C) The heatmap of the expression profile of DE mRNAs in PPI network. PPI, protein-protein interaction; DE, differentially expressed.

A total of 1,370 DE mRNAs were subjected to GO annotation and KEGG enrichment, and the potential functions and signaling pathways were analyzed. GO terms were catalyzed into three main processes (biological process, cellular component, and molecular function) (Supplementary Table S7). In the category of biological process, GO terms were mainly involved in cellular process, biological regulation, and metabolic process. In the category of cellular component, GO terms were closely connected with cell and cell part. In the category of molecular function, DE mRNAs related to binding and catalytic activity were considerably enriched. In addition, the results of KEGG pathway enrichment exhibited that several signaling pathways associated with lipid metabolism were enriched, such as peroxisome proliferator-activated receptor (PPAR), p53, AMP-activated protein kinase (AMPK), and PI3K-Akt signaling pathways. Moreover, arachidonic acid metabolism, glycolysis/gluconeogenesis, retinol metabolism and cellular senescence were significantly enriched (Supplementary Table S7), all of which are strongly associated with lipid metabolism, adipogenesis and cell development. On this basis of the above-mentioned studies, a total of 141 potential DE mRNAs related to fat deposition and lipid metabolism were selected (Figure 4A; Supplementary Table S8), and then a PPI network was constructed. The proteins including acyl-CoA oxidase 1 (ACOX1), aldolase, fructose-bisphosphate B (ALDOB), forkhead box O3 (FOXO3), AKT serine/threonine kinase 2 (AKT2), and Janus kinase 3 (JAK3) were at the hub positions (Figure 4B), and the expression profile of DE mRNAs presented in the PPI network was displayed as heatmap (Figure 4C). Thus, they might be important for regulating fat metabolism and adipogenic differentiation in pigs.

### GO and KEGG analysis of DE miRNAs and DE lncRNAs based on target genes

**Figure 5:**
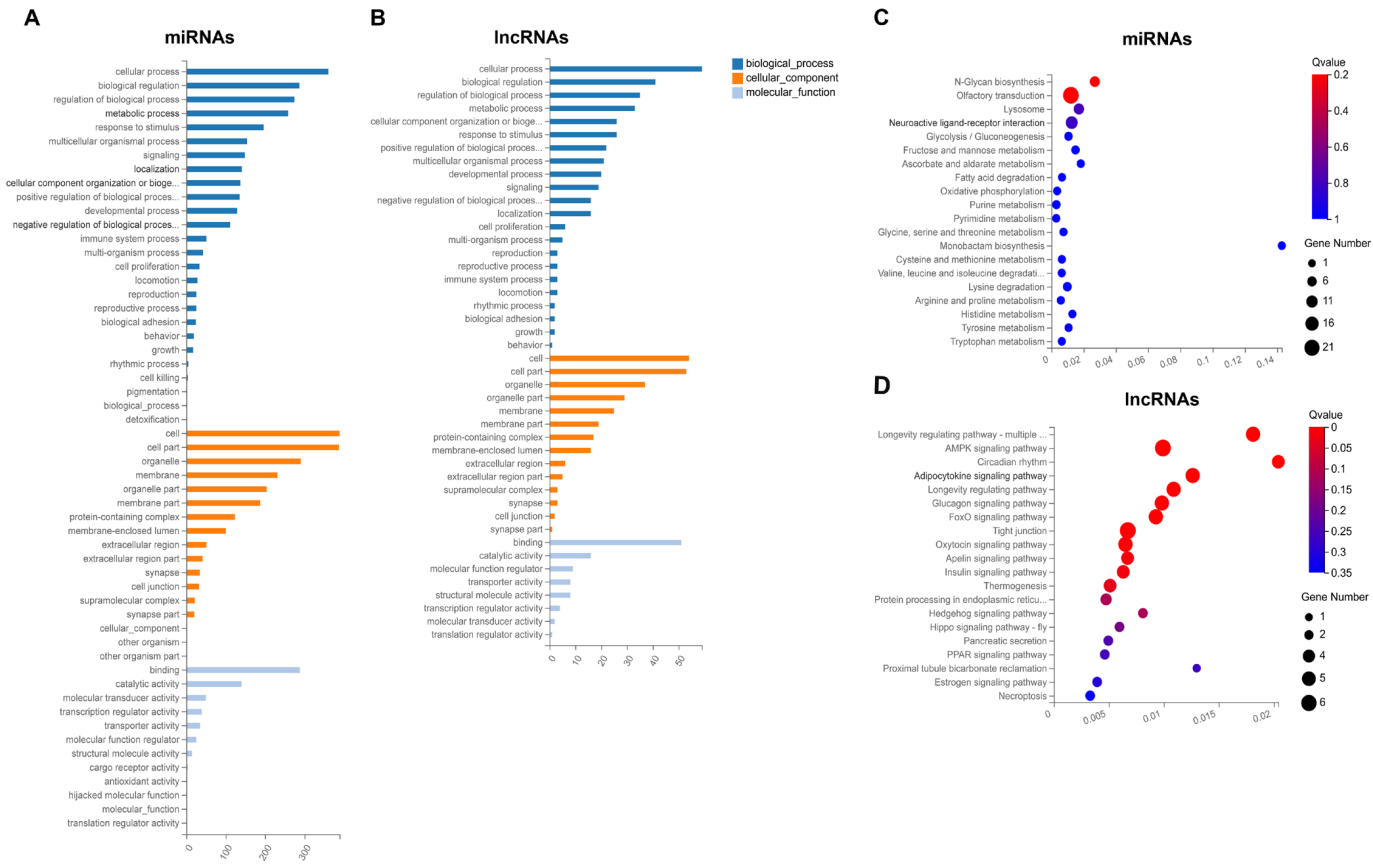
GO annotation and KEGG pathway analysis of targets of DE miRNAs and DE lncRNAs. GO annotation of targets of DE miRNAs (A) and DE lncRNAs (B). The X axis represents the number of genes annotated to the GO entry, and the Y axis represents the GO functional classification. Enrichment of DE miRNAs (C) and DE lncRNAs (D) in signaling pathway. Each bubble represents a term, and the size of bubble indicates the number of involved genes. The color indicates Q value, and the enrichment term was ranked by Q value. GO, gene ontology; KEGG, Kyoto encyclopedia of genes and genomes; DE, differentially expressed.

The potential functions of DE miRNAs and DE lncRNAs were explored by further analysis of their target genes, and the predicted targets of DE miRNAs and DE lncRNAs are listed in Supplementary Table S9 and Supplementary Table S10, respectively. And then, the targets were subjected to GO annotation and KEGG enrichment, and the GO annotation results were similar to that of DE mRNAs (Figure 5A; Supplementary Table S11). In KEGG enrichment analysis, several enriched pathways associated with adipocyte differentiation and proliferation, adipogenesis, and lipid metabolism were obtained, including AMPK, adipocytokine, foxo, apelin, insulin and PPAR signaling pathways, glycolysis/gluconeogenesis, and fatty acid degradation (Figure 5B; Supplementary Table S11). And the signaling pathways, such as AMPK, adipocytokine, insulin and PPAR, were also detected in enrichment analysis of DE mRNAs.

### Construction of lncRNA-miRNA-mRNA network

**Figure 6:**
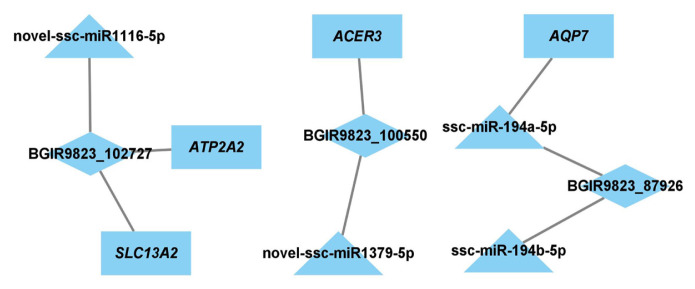
Network of lncRNA-miRNA-mRNA. Rectangle nodes represent mRNAs, triangular nodes represent miRNAs, and diamond nodes represent lncRNA.

To identify the key lncRNAs and mRNAs related to regulation of adipogenesis and lipid metabolism, the target genes of DE miRNAs and DE lncRNAs were predicted, and the lncRNA-miRNA-mRNA network was constructed. There were 11 nodes and 8 connections between 3 lncRNAs, 4 miRNAs, and 4 mRNAs (Figure 6).

### Validation of identified transcripts

**Figure 7:**
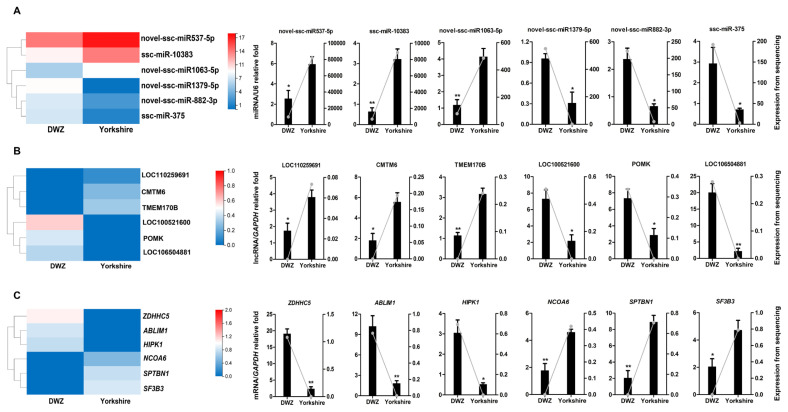
QPCR validation of the expressions of DE transcripts. The heatmap and QPCR validation of the expressions of randomly selected DE transcripts, including 6 DE miRNAs (A), 6 DE lncRNAs (B) and 6 DE mRNAs (C). QPCR results are exhibited as columns labeled on the Y axis on the left, and data from RNA-seq are shown as lines labeled on the Y axis on the right. QPCR, quantitative real-time polymerase chain reaction; DE, differentially expressed.

To validate the reliability of the sequencing results, a total of 18 transcripts, including 6 DE miRNAs (novel-ssc-miR537-5p, ssc-miR-10383, novel-ssc-miR1063-5p, novel-ssc-miR1379-5p, novel-ssc-miR882-3p, ssc-miR-375), 6 DE lncRNAs (LOC110259691, CMTM6, TMEM170B, LOC100521600, POMK, LOC106504881), and 6 DE mRNAs (zinc finger DHHC-type palmitoyltransferase 5 [*ZDHHC5*], actin binding LIM protein 1 [*ABLIM1*], homeodomain interacting protein kinase 1 [*HIPK1*], nuclear receptor coactivator 6 [*NCOA6*], spectrin beta, non-erythrocytic 1 [*SPTBN1*], splicing factor 3b subunit 3 [*SF3B3*]), were randomly selected for QPCR verification (Figure 7). These results manifested strong consistency with the sequencing data.

## DISCUSSION

Fat deposition is closely linked with the genetic background and is influenced by various transcription factors, crucial genes and signaling pathways. To better understand the regulatory network controlling backfat deposition, expression profiles of miRNAs, lncRNAs, and mRNAs in backfat tissue between Chinese indigenous (obese-type) DWZ and foreign lean-type Yorkshire pig breeds were analyzed for the first time by RNA-seq technology. A total of 1,625 DE transcripts were identified, including 72 DE miRNAs, 183 DE lncRNAs and 1,370 DE mRNAs. And the expression levels of several DE transcripts were validated by QPCR analysis, indicating that data of RNA-seq was reliable with strong consistency.

Several of highly abundant miRNAs identified in this study are closely related to adipogenesis. miR-26a, miR-206, miR-378, miR-499, and miR-191 have been reported to participate in adipocyte differentiation and adipogenesis [17–21]. Meanwhile, some of the DE miRNAs are indispensable for adipogenesis, lipid metabolism and adipose development. For instances, miR-375 exerts a positive regulatory effect on adipocyte differentiation. miR 204-5p favors the adipogenic differentiation and lipid synthesis, of human adipose-derived mesenchymal stem cells by suppressing Wnt/β-catenin signaling, of 3T3–L1 cells by targeting KLF transcription factor 3 (*KLF3*). Recently, transcriptome and miRNAome analysis have been used to identify miRNA expression profiles and characterize the possible regulatory relationships for backfat deposition in pigs [1,22–26]. In these researches and our study, miR-26a, miR-206, miR-99a-5p, miR-16, let-7f-5p, miR-1, and miR-24-3p were the top 20 most abundant miRNAs, additionally, miR-375, miR-204–5p, miR-205, miR-192, miR-194a-5p, miR-29b, miR-29c, miR-708–5p, miR-127, miR-369, miR-493–5p, miR-323, miR-432–5p, and miR-493–5p were the identified DE miRNAs. However, some of the DE miRNAs in our study were different from that in previous research, which may be caused by the differences in pig breeds, days of age, or threshold of the fold change.

To identify the differential regulatory networks in backfat tissue between the two pig breeds, GO and KEGG enrichment analysis were fulfilled. For DE mRNAs, several signaling pathways associated with lipid metabolism and adipogenesis were discovered, such as PPAR, p53, AMPK, and PI3K-Akt signaling pathways [8,27–29]. Several hub proteins were identified in PPI network of DE mRNAs. *ACOX1*, *ALDOB*, protein kinase AMP-activated catalytic subunit alpha 2 (*PRKAA2*), *JAK3*, and protein tyrosine phosphatase non-receptor type 11 (*PTPN11*) have been confirmed to be closely related to β-oxidation of fatty acid, lipogenesis, cholesterol metabolism, and lipodystrophy [30–35]. Moreover, several genes, including *PPARδ*, *PPARα*, fatty acid binding protein 3 (*FABP3*), acyl-CoA synthetase long chain family member 4 (*ACSL4*), B cell lymphoma 2 (*BCL2*), *FOXO3* and TBC1 domain family member 1 (*TBC1D1*), are closely associated with fatty acid metabolism, adipogenic differentiation and cell cycle progression. *PPARδ* and *PPARα* are vital regulators in cell differentiation, lipid accumulation, fatty acid oxidation and lipid catabolism. *FABP3* is positively correlated with the backfat thickness in beef cattle, and it regulates transport of fatty acids and lipid deposition. Furthermore, *FABP3* is notably downregulated in subcutaneous adipose tissue from Yorkshire pigs than that in Chinese indigenous Laiwu pigs [8], which is similar to our study. *ACSL4* converts free long-chain fatty acids into fatty acyl-CoA esters which are the key intermediates in the synthesis of complex lipids. Previous study demonstrated that the expression of *ACSL4* gradually increases during adipogenesis of porcine primary intramuscular preadipocytes and *ACSL4* knockdown decreases lipid accumulation. However, another research found that the expression of *ACSL4* was gradually decreased during different differentiation stages of subcutaneous preadipocytes in Erhualian pigs [10], and our result also exhibited that the expression of *ACSL4* in backfat tissue is significantly lower in DWZ pigs than that in Yorkshire pigs. In addition, *AKT2* knockdown remarkably reduces preadipocyte proliferation, adipogenic differentiation and fat mass in human, but the present result showed that *AKT2* expression was drastically lower in DWZ pigs than that in Yorkshire, which is contrary to the result reported in a former article about Bamei and Large White pigs. The discrepancy may be because of the different experimental methods and cell sources. *BCL2* mediates obedience to proliferation and resistance to apoptosis in adipocytes. In addition, *FOXO3* and *TBC1D1* have been shown to be involved in lipid accumulation, fatty acid oxidation and obesity development. In our study, lower expressions of *ACOX1*, *AKT2*, *PRKAA2*, *ACSL4*, *FOXO3* and higher expressions of *ALDOB*, *JAK3*, *PTPN11*, *PPARδ*, *PPARα*, *FABP3*, *BCL2*, *TBC1D1* were observed in DWZ pigs. Taken together, these regulatory relationships may partly illuminate the mechanism of porcine backfat deposition.

The biological function of lncRNAs is generally mediated by their targets. Therefore, the targets of DE lncRNAs were predicted and then underwent enrichment analysis. Several pathways were enriched, among which AMPK, apelin and insulin signaling pathways are closely related to adipocyte differentiation and lipid metabolism. AMPK signaling pathway is believed to act as a key master switch that modulates cholesterol synthesis and lipid metabolism [36,37]. The apelin signaling pathway inhibits adipogenesis and lipolysis through distinct molecular pathways. Insulin is a key regulator and activates the transcription and proteolytic maturation of sterol regulatory element binding transcription factor 1c (SREBP1c), and then SREBP1c induces the expression of a family of genes involves in fatty acid synthesis, such as acetyl-CoA carboxylase (*ACC*) and fatty acid synthase (*FAS*).

The present study indicated that BGIR9823_87741, BGIR 9823_78329, BGIR9823_79919, and BGIR9823_85300 targets *FABP3*, *BCL2*, *FOXO3*, and *PPARα*, respectively. Meanwhile, it is noteworthy that XR_002341917.1, BGIR9823_80765, BGIR9823_79960, BGIR9823_93430, BGIR9823_84443, BGIR9823_80428, BGIR9823_85841 and XR_002340248.1 potentially regulates H2A histone family member Y (*H2AFY*), neuronal regeneration related protein (*NREP*), carboxypeptidase A4 (*CPA4*), optic atrophy 1 (*OPA1*), FKBP prolyl isomerase 5 (*FKBP5*), monoacylglycerol O-acyltransferase 1 (*MGAT1*), angiopoietin like 2 (*ANGPTL2*) and protein kinase AMP-activated non-catalytic subunit gamma 2 (*PRKAG2*), respectively. In our study, *H2AFY*, *NREP*, and *CPA4* are highly expressed in Yorkshire pig. Former research showed that deletion of *H2AFY* results in lipid accumulation in murine liver [38]. Treatment of HepG2 cells with *NREP* knockdown exhibits greater lipid droplet accumulation and increases triglyceride and cholesterol content through TGFβR/PI3K/AKT signaling pathway. *CPA4* knockdown enhances differentiation of human preadipocytes and *CPA4* expression in subcutaneous adipose tissue negatively correlates with indices of insulin sensitivity. These results demonstrated that *H2AFY*, *NREP*, and *CPA4* might inhibit fat deposition in Yorkshire pigs. Moreover, *FABP5* and *MGAT1* were markedly upregulated in backfat tissue in DWZ pigs. *FKBP5* expression in human subcutaneous adipose tissue tends to be increased in type II diabetes subjects and is associated with genes involved in lipid metabolism and adipogenesis. Another research suggested that mice lacking *FKBP5* gene had reduce body weight and were resistant to diet-induced obesity, and knockdown of *FKBP5* in 3T3-L1 cells had a strong anti-adipogenic impact. It has been found that *MGAT1* encodes the enzyme that catalyzes monoacylglycerol and fatty acyl-CoA to form diacylglycerol, which is indispensable for triacylglycerol synthesis, and *MGAT1* knockdown exhibits a significant reduction in lipid accumulation. *ANGPTL2*, predominantly secreted by adipose tissue, enhances fatty acid synthesis and lipid accumulation in mice, and *ANGPTL2* knockdown inhibits adipogenic differentiation of 3T3-L1 cells. On above basis, *FABP5*, *MGAT1*, and *ANGPTL2* might enhance adipogenic differentiation and lipid formation in DWZ pigs. Accordingly, the DE lncRNAs target these genes might play crucial roles in adipogenesis and lipid metabolism in porcine backfat tissue, and further studies are needed to be fulfilled to validate this speculation.

**Figure 8:**
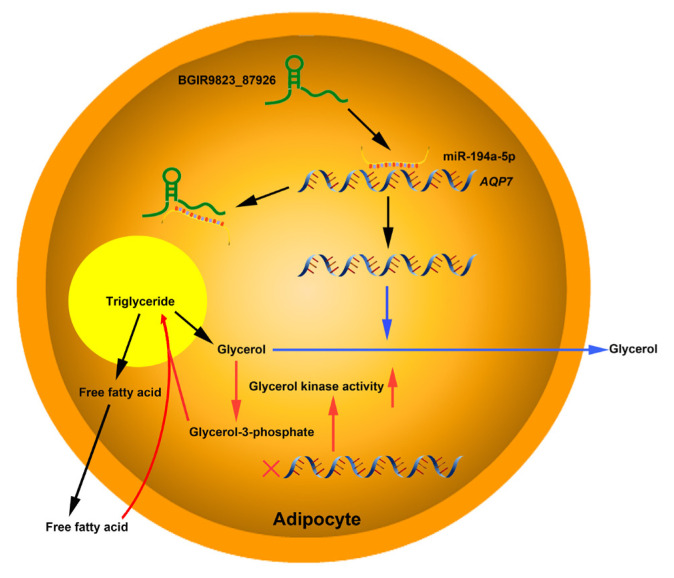
Proposed model of BGIR9823_87926/miR-194a-5p/*AQP7* pathway in adipogenesis. lncRNA BGIR9823_87926 functions as miR-194a-5p sponge, thereby augmenting the expression of *AQP7* which facilitates the release of glycerol and is unfavorable to triglyceride synthesis in adipocytes (Pathway marked by blue lines). On the other hand, low expression of *AQP7* elevates glycerol kinase activity, accelerates glycerol-3-phosphate formation, and finally results in triglyceride accumulation (Pathway marked by red lines).

The target relationships between miRNAs and mRNAs were further analyzed. *ANXA3*, the predicted target of miR-122-3p which plays important roles in cholesterol synthesis and lipogenesis [39,40], is downregulated at an early phase of adipocyte differentiation in 3T3–L1 cells, and suppression of *ANXA3* causes elevation of the *PPARγ2* mRNA level and lipid droplet accumulation. In addition, miR-583-3p, miR-161-3p, miR-671-3p, and miR-817-3p targets activating transcription factor 3 (*ATF3*), triacylglycerol synthase 1 (*TGS1*), protein kinase C and casein kinase substrate in neurons 2 (*PACSIN2*), and ataxin 2 (*ATXN2*), respectively, and these genes have been verified to be associated with fat weight, triglyceride synthase and lipid droplet formation. Furthermore, *AQP7* is the predicted target of miR-194a-5p, and previous report showed that the body weight and fat mass increases significantly in *AQP7* knockout mice, and adipocytes are large and exhibits accumulation of triglyceride by elevating adipose glycerol kinase activity [41]. These results indicated that the BGIR9823_87926/miR-194a-5p/*AQP7* network may affect the process of adipocyte differentiation or adipogenesis (Figure 8), but their functions and interactions needed further validation.

## CONCLUSION

The comparative transcriptome analysis of miRNAs, lncRNAs, and mRNAs in backfat tissue between DWZ and Yorkshire pigs was conducted, and numerous DE miRNAs, DE lncRNAs, and DE mRNAs, which may influence fat development, were further identified. The BGIR9823_87926/miR-194a-5p/*AQP7* network may affect the process of adipocyte differentiation or adipogenesis. The present study provides comprehensive insights into the regulatory network of backfat deposition and lipid metabolism in pigs.

## Figures and Tables

**Table 1:** Top 20 most abundant miRNAs in backfat tissue between Daweizi and Yorkshire pigs

Daweizi		Yorkshire	
	
RNA ID	Average reads	RNA ID	Average reads
ssc-miR-22-3p	1,983,522.667	ssc-miR-22-3p	2,074,014.667
ssc-miR-26a	1,109,601	ssc-miR-26a	1,178,567.667
ssc-miR-206	657,776.667	ssc-miR-206	661,720.333
ssc-miR-133a-3p	413,103.333	ssc-miR-378	554,187.333
ssc-miR-126-3p	353,812	ssc-miR-126-3p	455,123.333
ssc-miR-378	353,190	ssc-miR-133a-3p	263,192.333
novel-ssc-miR857-5p	180,336.333	ssc-miR-99a-5p	167,529
ssc-miR-499-5p	170,159	ssc-miR-191	151,442.333
ssc-miR-451	145,943.667	novel-ssc-miR324-3p	122,949
ssc-miR-191	143,042.333	ssc-miR-143-3p	103,580
ssc-miR-99a-5p	111,548.333	ssc-miR-1	97,816.333
ssc-miR-151-5p	99,942.667	ssc-miR-16	95,581.667
novel-ssc-miR324-3p	99,843	ssc-let-7f-5p	92,016.333
ssc-miR-143-3p	87,045.333	novel-ssc-miR857-5p	80,316
ssc-miR-16	81,623	ssc-miR-151-5p	75,766
ssc-let-7f-5p	75,596.333	ssc-miR-92a	69,724
ssc-miR-423-3p	63,695	ssc-miR-451	66,090.333
ssc-miR-1	62,722.333	ssc-miR-423-3p	57,812
ssc-miR-24-3p	61,382	ssc-miR-24-3p	53,122.667
ssc-miR-92a	61,008	ssc-miR-499-5p	51,642

**Table 2:** Top 20 most abundant lncRNAs in backfat tissue between Daweizi and Yorkshire pigs

Daweizi		Yorkshire	
	
RNA ID	Average reads	RNA ID	Average reads
BGIR9823_101660	1,838,371.06	BGIR9823_79653	971,745.8133
BGIR9823_79653	682,082.0033	BGIR9823_101660	966,481.0833
BGIR9823_101661	330,584.22	BGIR9823_101661	313,130.1167
BGIR9823_101542	220,602.3467	BGIR9823_101542	227,385.9433
BGIR9823_81016	187,836.6067	BGIR9823_101532	219,257.8233
BGIR9823_101532	175,266.8067	BGIR9823_101535	182,488.54
BGIR9823_101403	145,074.2033	BGIR9823_88517	122,587.6333
BGIR9823_101535	107,535.05	BGIR9823_101403	122,400.0433
BGIR9823_88517	101,568.4767	BGIR9823_101546	119,920.8633
BGIR9823_101401	87,428.09667	BGIR9823_101544	110,831.7933
BGIR9823_101546	82,097.83333	BGIR9823_81016	97,270.50667
BGIR9823_81019	71,247.21333	BGIR9823_91558	85,308.18667
BGIR9823_81018	68,523.36	BGIR9823_81019	58,773.41333
BGIR9823_91558	59,872.28	BGIR9823_81014	57,002.03
BGIR9823_81014	48,544.11667	BGIR9823_101756	54,904.6
BGIR9823_101756	45,938.45667	BGIR9823_81018	54,741.92
XR_115737.4	39,352.88667	BGIR9823_101821	35,818.28333
BGIR9823_101544	31,449.29	XR_115737.4	33,244.25
BGIR9823_101672	30,487.78	BGIR9823_101672	32,013.10333
BGIR9823_101821	26,381.69333	BGIR9823_101401	31,828.37667

## SUPPLEMENTARY MATERIAL

**Supplementary Figure S1**. Expression levels of mRNAs and lncRNAs indicated by log(read count+1).

**Supplementary Table S1**. The sequences of primers for QPCR analysis.

**Supplementary Table S2**. Basic data of small RNAs sequencing in backfat tissue between DWZ and Yorkshire pigs.

**Supplementary Table S3**. Basic data of mRNAs and lncRNAs sequencing in backfat tissue between DWZ and Yorkshire pigs.

**Supplementary Table S4**. The length distribution of small RNAs.

**Supplementary Table S5**. Differentially expressed miRNAs, lncRNAs and mRNAs.

**Supplementary Table S6**. Specifically expressed miRNAs, lncRNAs and mRNAs.

**Supplementary Table S7**. GO annotation and KEGG pathway analysis of DE mRNAs.

**Supplementary Table S8**. Potential DE mRNAs related to fat deposition and lipid metabolism.

**Supplementary Table S9**. The predicted targets of DE miRNAs.

**Supplementary Table S10**. The predicted targets of DE lncRNAs.

**Supplementary Table S11**. GO annotation and KEGG pathway analysis of targets of DE miRNAs and DE lncRNAs.
