# Supplementary figures and images for: Comprehensive analysis of miRNAs, lncRNAs and mRNAs profiles in backfat tissue between Daweizi and Yorkshire pigs
Source: Anim Biosci. 2022 Nov 13;36(3):404–16. doi: 10.5713/ab.22.0165 (PMC9996253; doi:10.5713/ab.22.0165)

DWZ

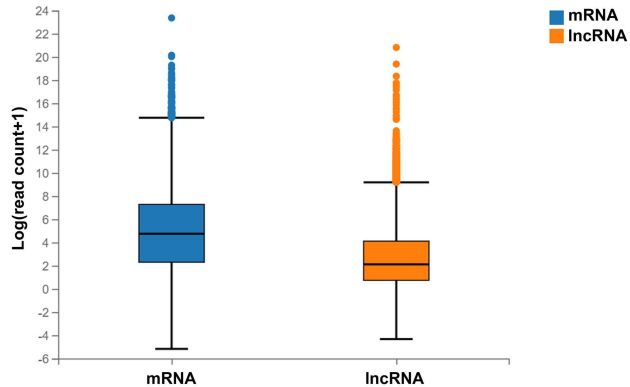

Yorkshire

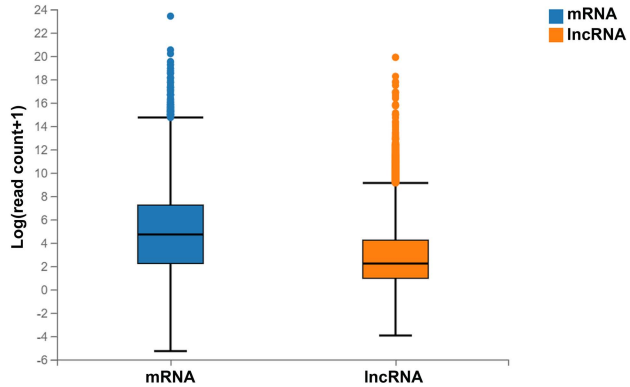

Supplement: Supplementary file 1 [file ab-22-0165-Supplementary-Fig-1.pdf]
