# Supplementary material for: Comprehensive analysis of miRNAs, lncRNAs and mRNAs profiles in backfat tissue between Daweizi and Yorkshire pigs
Source: Anim Biosci. 2022 Nov 13;36(3):404–16. doi: 10.5713/ab.22.0165 (PMC9996253; doi:10.5713/ab.22.0165)
Supplement: Supplementary file 2 [file ab-22-0165-Supplementary-Table-1.pdf]

Supplementary Table S1. The sequences of primers for QPCR analysis.

Name		Primer sequence	Annealing termperature (℃)	Product size (bp)
novel-ssc-miR537-5p	Stem-loop	CTCAACTGGTGTCGTGGAGTCGGCAATTCAGTTGAGAACACTGATTCTGAC	60	63
	Forward	ACACTACTGGACTTGGA		
	Reverse	CTCAACTGGTGTCGTGGA		
ssc-miR-10383	Stem-loop	CTCAACTGGTGTCGTGGAGTCGGCAATTCAGTTGAGAACACTGAACTGCCA	62	66
	Forward	ACACTTGGTGCCTGACGTCT		
	Reverse	CTCAACTGGTGTCGTGGA		
novel-ssc-miR1063-5p	Stem-loop	CTCAACTGGTGTCGTGGAGTCGGCAATTCAGTTGAGAACACTGACGCCCTC	60	66
	Forward	GGGAGGACGGGAAGAGAG		
	Reverse	CTCAACTGGTGTCGTGGA		
novel-ssc-miR1379-5p	Stem-loop	CTCAACTGGTGTCGTGGAGTCGGCAATTCAGTTGAGAACACTGAAGACCTG	62	67
	Forward	ACACTTTGAACCCAGTGAAAC		
	Reverse	CTCAACTGGTGTCGTGGA		
novel-ssc-miR882-3p	Stem-loop	CTCAACTGGTGTCGTGGAGTCGGCAATTCAGTTGAGAACACTGAGAGGAGG	59	64
	Forward	ACACTCCCGGCCTCGCCT		
	Reverse	CTCAACTGGTGTCGTGGA		
ssc-miR-375	Stem-loop	CTCAACTGGTGTCGTGGAGTCGGCAATTCAGTTGAGAACACTGATCACGCG	60	66
	Forward	GGGTTTGTTCGTTCGGCT		
	Reverse	CTCAACTGGTGTCGTGGA		
LOC110259691	Forward	GGGACAAAGCATCAAGGACGA	61	80
	Reverse	CTGGTGAGGGGGTGTTTGTTG		
CMTM6	Forward	CAGACTTTGGATCTGCCCGT	60	132
	Reverse	TAACTCTTCACACCCAGCCG		
TMEM170B	Forward	GGACCCCCTACGTGTTTGTT	59	103
	Reverse	TACAGTCCGCAAAAGGGGTC		
LOC100521600	Forward	TGTGCAGGTCACTGTGTTGG	61	79
	Reverse	TCAGGGGCTGTTTGTCTGTG		
POMK	Forward	ACCTGGCTCGATGCTAAAGG	60	170
	Reverse	TACACCACAAACACCCCGTC		
LOC106504881	Forward	GCACGGAAAGGGACTTAGCA	60	127
	Reverse	CTGCCCAGTTTCCTCTGGAC		
ZDHHC5	Forward	GAGCCGCATGAATGAGAGGA	60	286
	Reverse	GATTACAGTGCCTCACCCCC		
ABLIM1	Forward	ACAAACAGGAGAGGCAGAGC	59	241
	Reverse	TGCTGTTTGTAGATGGGTGGT		
HIPK1	Forward	GGCTTCTGCGTGTGGATTTC	59	213
	Reverse	GTCCAGAAACATCCCAGCCA		
NCOA6	Forward	TAGGAGCAGGACAGGCCAA	60	276
	Reverse	CTTGCATGTTGCCCAGGTTC		
SPTBN1	Forward	GAGGAGCAAAATGGATTCGACC	59	84
	Reverse	GGCTACTGTGGTCGTCATCTT		
SF3B3	Forward	GCTTCAGGTCAACCCTACAA	58	299
	Reverse	CGGATGACACCAAACACTTCC		
U6-snRNA	Forward	GCTTCGGCAGCACATATACT		96
	Reverse	TTCACGAATTTGCGTGTCAT		
GAPDH	Forward	GTCGGAGTGAACGGATTTG		175
	Reverse	TCTCAGCCTTGACTGTGCC		
