# Supplementary material for: Comprehensive analysis of miRNAs, lncRNAs and mRNAs profiles in backfat tissue between Daweizi and Yorkshire pigs
Source: Anim Biosci. 2022 Nov 13;36(3):404–16. doi: 10.5713/ab.22.0165 (PMC9996253; doi:10.5713/ab.22.0165)
Supplement: Supplementary file 3 [file ab-22-0165-Supplementary-Table-2.pdf]

Supplementary Table S2. Basic data of small RNAs sequencing in backfat tissue between DWZ and Yorkshire pigs.

Sample	Low quality number	Invalid adapter number	PolyA number	Short valid length number	Clean reads	Clean reads ratio (%)	Mapping (%)
DWZ1	848893	638567	351	173554	23504459	93.40	93.66
DWZ2	871498	493229	583	169473	23631041	93.90	94.71
DWZ3	772561	492391	334	218285	23682253	94.10	94.57
Yorkshire1	930442	550086	543	156411	23528342	93.49	93.67
Yorkshire2	606367	946161	644	273557	23339095	92.74	93.57
Yorkshire3	398775	769255	1307	195979	23800508	94.57	92.96
