# Supplementary material for: Comprehensive analysis of miRNAs, lncRNAs and mRNAs profiles in backfat tissue between Daweizi and Yorkshire pigs
Source: Anim Biosci. 2022 Nov 13;36(3):404–16. doi: 10.5713/ab.22.0165 (PMC9996253; doi:10.5713/ab.22.0165)
Supplement: Supplementary file 4 [file ab-22-0165-Supplementary-Table-3.pdf]

Supplementary Table S3. Basic data of mRNAs and lncRNAs sequencing in backfat tissue between DWZ and Yorkshire pigs.

Sample	N read number (M)	Adapter read number (M)	Low quality read number (M)	Raw reads (M)	Clean reads (M)	Clean reads Q20 (%)	Clean reads Q30 (%)	Clean reads ratio (%)	Mapping (%)	Uniquely mapping (%)
DWZ1	2.19	0.87	1.62	127.00	122.32	98.03	92.46	96.31	95.53	91.16
DWZ2	2.46	0.93	1.86	139.93	134.68	97.98	92.37	96.25	94.70	91.66
DWZ3	2.40	1.08	2.11	139.93	134.34	97.78	91.55	96.01	95.40	91.77
Yorkshire1	2.37	0.87	1.82	139.93	134.87	97.84	91.75	96.38	96.43	92.47
Yorkshire2	2.46	0.97	2.00	139.93	134.50	97.85	92.06	96.12	96.31	91.14
Yorkshire3	2.10	0.88	1.74	123.97	119.25	97.85	91.82	96.19	96.38	92.15
