# Supplementary material for: Comprehensive analysis of miRNAs, lncRNAs and mRNAs profiles in backfat tissue between Daweizi and Yorkshire pigs
Source: Anim Biosci. 2022 Nov 13;36(3):404–16. doi: 10.5713/ab.22.0165 (PMC9996253; doi:10.5713/ab.22.0165)

Supplementary Table S4. The length distribution of small RNAs.

Length (bp)	DWZ1		DWZ2		DWZ3		Yorkshire1		Yorkshire2		Yorkshire3	
	Count	Percentage	Count	Percentage	Count	Percentage	Count	Percentage	Count	Percentage	Count	Percentage
⩽ 15	114732	0.49%	159779	0.67%	159035	0.67%	121603	0.52%	209300	0.89%	129205	0.54%
16	22515	0.10%	47326	0.20%	28051	0.12%	33002	0.14%	44295	0.19%	30058	0.13%
17	31565	0.14%	173021	0.73%	46496	0.20%	64235	0.27%	82019	0.35%	42710	0.18%
18	51529	0.22%	573291	2.42%	98667	0.42%	196735	0.84%	218849	0.93%	61589	0.26%
19	128737	0.55%	794102	3.35%	293740	1.24%	352795	1.50%	454436	1.94%	161671	0.68%
20	570676	2.45%	1262506	5.33%	936575	3.95%	825783	3.51%	947527	4.05%	814607	3.43%
21	1949755	8.36%	3155043	13.31%	2488436	10.49%	2560400	10.89%	2868312	12.25%	2265494	9.53%
22	11511166	49.33%	10839651	45.73%	12919415	54.46%	12622467	53.71%	12461848	53.24%	13637817	57.34%
23	4585691	19.65%	3555046	15.00%	4221674	17.79%	3751712	15.96%	3262208	13.94%	3791813	15.94%
24	1205401	5.17%	1122070	4.73%	1002496	4.23%	953639	4.06%	805885	3.44%	983890	4.14%
25	467388	2.00%	638424	2.69%	419438	1.77%	507712	2.16%	544308	2.33%	563611	2.37%
26	316342	1.36%	474164	2.00%	217280	0.92%	271398	1.15%	297106	1.27%	338705	1.42%
27	313697	1.34%	408088	1.72%	172062	0.73%	209114	0.89%	257243	1.10%	266578	1.12%
28	403295	1.73%	291251	1.23%	125398	0.53%	191663	0.82%	214923	0.92%	240911	1.01%
29	443190	1.90%	146604	0.62%	138575	0.58%	181828	0.77%	196512	0.84%	227790	0.96%
⩾ 30	1218312	5.22%	61214	0.26%	456734	1.93%	658907	2.80%	541673	2.31%	227026	0.95%

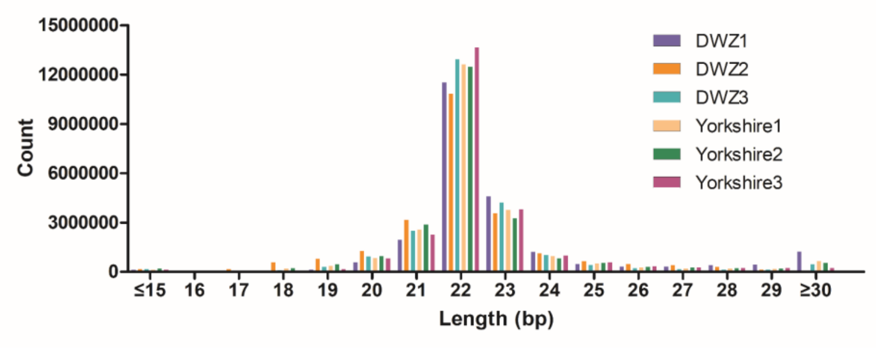

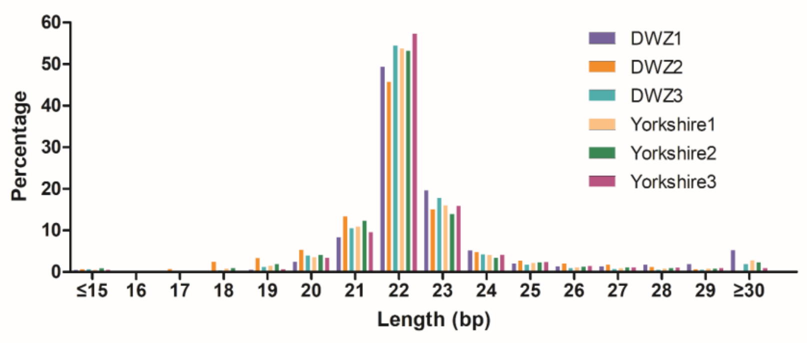

Supplement: Supplementary file 5 [file ab-22-0165-Supplementary-Table-4.pdf]
