# Supplementary material for: Comprehensive analysis of miRNAs, lncRNAs and mRNAs profiles in backfat tissue between Daweizi and Yorkshire pigs
Source: Anim Biosci. 2022 Nov 13;36(3):404–16. doi: 10.5713/ab.22.0165 (PMC9996253; doi:10.5713/ab.22.0165)
Supplement: Supplementary file 6 [file ab-22-0165-Supplementary-Table-5.pdf]

Supplementary Table S5. Differentially expressed miRNAs, lncRNAs and mRNAs.

RNA ID	Sequence/Symbol	log2 (DWZ/Yorkshire)	Q value (DWZ/Yorkshire)
novel-ssc-miR154-5p	AGGGGCCACCGUCGCUCCCGCGCU	13.666224	7.81E-05
novel-ssc-miR1379-5p	UUGAACCCAGUGAAACCAGGUCU	8.68948425	8.11E-07
novel-ssc-miR849-3p	UAAUACUGCCGGGUAAUGAUGGA	5.913086862	1.02E-07
ssc-miR-375	UUUGUUCGUUCGGCUCGCGUGA	5.848141183	5.10E-06
novel-ssc-miR204-5p	UCUCUCCAGCCUCUGCUUCGUGG	5.392317423	1.26E-04
novel-ssc-miR397-5p	UCAGGGCGGAGCAGACGUGCG	5.228097523	2.36E-07
novel-ssc-miR563-3p	GAGUUCUGGGCUGCAGUGCGCU	4.99315822	2.22E-04
novel-ssc-miR627-5p	UCAGGGCGGAGCAGACGUGCGC	4.955593188	2.30E-04
ssc-miR-194b-5p	UGUAACAGCGACUCCAUGUGGA	4.924137206	2.45E-10
novel-ssc-miR882-3p	CCCGGCCUCGCCUCCUCCUC	4.735579886	0.00131818
ssc-miR-205	UCCUUCAUUCCACCGGAGUCUG	4.297705001	0.001440763
novel-ssc-miR1287-5p	GAGCGGGGCGCGCGGUCGC	3.816570946	1.08E-04
novel-ssc-miR480-3p	CCGGCCUCGCCUCCUCCUC	3.77802876	4.76E-04
novel-ssc-miR832-3p	AGGAGUUCUGGGCUGCAGUGCGCU	3.712718048	2.52E-05
novel-ssc-miR802-3p	CCCCGGCCUCGCCUCCUCCUC	3.570575691	7.32E-04
ssc-miR-10386	GUCGUCCUCUCCCUCCCUCCU	3.515590113	4.15E-04
novel-ssc-miR682-3p	UCUCUGCUCACCACCACAGCC	3.307572802	2.24E-04
ssc-miR-122-3p	AACGCCAUUAUCACACUAAAU	3.280107919	4.95E-04
ssc-miR-192	CUGACCUAUGAAUUGACAGCC	2.938350771	6.54E-05
novel-ssc-miR753-5p	AAGGGAACGGGCUUGGCAG	2.815204633	4.21E-04
novel-ssc-miR122-3p	GUUCUCACCUCGCUGGUGUCC	2.765203803	0.002005855
ssc-miR-194a-5p	UGUAACAGCAACUCCAUGUGG	2.72751619	0.001163728
novel-ssc-miR804-3p	GGAGAAGCCGGCGGGAGC	2.686327248	5.65E-06
ssc-miR-24-1-5p	GUGCCUACUGAGCUGAAACACAGU	1.445920207	3.31E-04
ssc-miR-29c	UAGCACCAUUUGAAAUCGGUUA	1.434810733	3.87E-04
ssc-miR-29a-3p	CUAGCACCAUCUGAAAUCGGUUA	1.253756676	4.86E-06
ssc-miR-29b	UAGCACCAUUUGAAAUCAGUGUU	1.240304737	0.001267117
ssc-miR-660	UACCCAUUGCAUAUCGGAGUUG	-1.085598358	0.001916919
ssc-miR-708-5p	AAGGAGCUUACAAUCUAGCUGGG	-1.22448175	5.20E-04
ssc-miR-127	UCGGAUCCGUCUGAGCUUGGCU	-1.329557402	1.47E-04
ssc-miR-874	CUGCCCUGGCCCGAGGGACCGAC	-1.419907154	0.00203604
novel-ssc-miR1140-5p	UGGUAGACUGUGGAACGUAGG	-1.543374176	6.47E-06
novel-ssc-miR161-3p	AAAAAUACGGGUGCACUUCUAU	-1.562755742	2.66E-05
ssc-miR-362	AAUCCUUGGAACCUAGGUGUGAGU	-1.57466003	8.80E-07
novel-ssc-miR16-3p	AGAUAUUGCACGGUUGAUCUC	-1.577719975	4.77E-05
novel-ssc-miR583-3p	CACAAUACACGGUCGGCCUCU	-1.626193645	2.65E-04
novel-ssc-miR568-3p	UGAGUAUUACAUGGCCAAUCUC	-1.653976188	4.97E-04
ssc-miR-7138-5p	UCCCAGCAAGUGUCCAUCCAUCU	-1.689522817	2.34E-04
novel-ssc-miR239-5p	GGUUGAUCAGAGAACAUACAUU	-1.732903033	4.03E-07
novel-ssc-miR1406-3p	AAACAAACAUGGUGCACUUCUUU	-1.750672975	0.001035726
novel-ssc-miR1126-5p	AAGUUGCCCGUGUCUUUUUCG	-1.750689275	0.001204224
novel-ssc-miR407-5p	AGAGGUCUUCCAUGGUGCAUUCG	-1.786382241	1.94E-05
novel-ssc-miR1077-5p	UGGUCGACCAGUUGGAAAGU	-1.791688749	1.08E-06
novel-ssc-miR755-3p	UAUGUAAUAUGGUCCACGUCU	-1.792574288	6.56E-05
novel-ssc-miR671-3p	AGAUAUUGCACGGUUGAUCUCU	-1.811514623	1.87E-06
novel-ssc-miR2-3p	AUCACACAAAGGCAACUUUUGU	-1.824797346	2.80E-05
novel-ssc-miR780-3p	AAUAUUAUACAGUCAACCUCU	-1.853320771	2.33E-04
novel-ssc-miR1086-5p	UGGUCGACCAGUUGGAAAGUAAU	-1.878687886	4.18E-08
novel-ssc-miR620-3p	AUAAUACAUGGUUAACCUCUUU	-1.883476925	4.25E-06
ssc-miR-369	AAUAAUACAUGGUUGAUCUUU	-1.886020915	4.40E-04
ssc-miR-432-5p	UCUUGGAGUAGGUCAUUGGGU	-1.919211182	3.90E-06
novel-ssc-miR1158-3p	AAUAUAACACAGAUGGCCUGU	-1.946082509	1.38E-07
novel-ssc-miR1105-5p	UCUUGUUAAAAGGCAGAUUCU	-1.963401991	0.001370588
ssc-miR-493-5p	UUGUACAUGGUAGGCUUUCAUU	-1.97994347	4.76E-07
novel-ssc-miR9-3p	AUCAUAGAGGAAAAUCCACAU	-1.992573494	8.79E-06
novel-ssc-miR817-3p	UGAAACAUACACGGGAAACCUCU	-2.057030945	1.07E-04
ssc-miR-323	GCACAUUACACGGUCGACCUCU	-2.120680099	1.42E-08

novel-ssc-miR1396-3p	AACACACCUGGUUAACCUCUUU	-2.16999058	4.69E-04
ssc-miR-376a-3p	AUCAUAGAGGAAAAUCCACGU	-2.204655232	1.95E-05
novel-ssc-miR507-5p	AGGUUACCCGAGCAACUUUGCAU	-2.291307231	3.51E-04
novel-ssc-miR710-5p	GGGGCCCUCCUGGUGCUUGCGU	-2.512450001	7.59E-04
novel-ssc-miR1063-5p	AGGACGGGAAGAGAGGAGGGCG	-2.614963303	2.14E-04
novel-ssc-miR512-5p	AGGACGGGAAGAGAGGAGGGCGU	-2.741466986	2.60E-05
novel-ssc-miR16-5p	AGGUUGUCCGUGAUGUAUUUGC	-2.882487176	9.12E-04
novel-ssc-miR537-5p	ACUGGACUUGGAGUCAGAA	-3.13237469	1.30E-04
novel-ssc-miR1116-5p	UUCCCUUUGUCAUCCUUUGCCC	-3.40573107	3.26E-04
ssc-miR-10383	UGGUGCCUGACGUCUUGGCAGU	-3.727214011	4.18E-13
novel-ssc-miR846-3p	UGCUCUGCAGUGCCCCACCUUG	-4.17028572	9.87E-04
novel-ssc-miR1120-5p	UCUUCCACUUAGCCACACGUGG	-12.84018782	8.42E-04
novel-ssc-miR985-5p	CGGGGGGUCGUGUGCCUGCGGU	-13.4252159	1.18E-04
novel-ssc-miR813-5p	CAGCCGCCCCUUUCCUUCUCCG	-13.46824219	5.49E-05
novel-ssc-miR573-5p	CUGCCGUUGGGUCUGGGGUGU	-14.61	0.00
XR_002339910.1	POMK	21.41135874	4.30E-08
XR_001298816.2	LOC106504881	21.35874901	4.64E-08
BGIR9823_101610	LOC100521600	21.19799809	5.86E-08
BGIR9823_101387	LOC106506226	10.87350572	1.12E-18
BGIR9823_84771	TXN2	9.506708195	6.47E-06
XR_002338675.1	LOC110255180	9.132237963	4.74E-07
XR_002346024.1	LOC110261640	8.986807544	0.001231504
BGIR9823_89044	GFOD1	8.727698969	8.95E-06
BGIR9823_78138	ARPP19	6.797748057	7.30E-10
URS0000BC5676	LOC110255218	6.597998768	1.18E-04
BGIR9823_91696	PPP1R15B	6.4913141	5.09E-04
XR_001301217.2	LOC102164998	6.390816127	5.98E-04
XR_002342059.1	LOC110259691	6.273453412	7.88E-04
BGIR9823_101051	LOC110257712	6.139859624	2.06E-06
BGIR9823_97246	GSTO1	5.990244328	6.48E-06
BGIR9823_102282		5.802576565	1.60E-05
XR_002345205.1	LOC110261231	4.786258038	6.32E-05
BGIR9823_101406	LOC110258215	4.210418183	1.15E-09
BGIR9823_101399	LOC110258215	4.109247825	2.65E-12
URS0000BC90E3		4.040744455	2.18E-05
XR_002343884.1	LOC110260634	3.913788436	5.65E-07
BGIR9823_92306	AKR1E2	3.649841971	9.91E-04
BGIR9823_101405	LOC110258214	3.515700923	1.39E-04
BGIR9823_79919	PLPPR1	2.992185707	0.001201119
BGIR9823_87741	FABP3	2.731628182	8.93E-04
BGIR9823_100272	SLPI	2.669315915	7.96E-06
BGIR9823_96973	MCU	2.641616873	2.21E-04
XR_002346030.1	LOC110261643	2.601021448	6.83E-05
BGIR9823_102595		2.430221542	0.001231491
XR_002346232.1	LOC106504299	2.371022931	0.001044637
BGIR9823_92129	PPP1R12B	2.326270253	5.37E-04
XR_002346229.1	LOC106504299	2.220774586	1.30E-07
BGIR9823_96360	RCAN1	2.087396787	2.29E-05
BGIR9823_102727		2.042062835	7.29E-04
BGIR9823_80570	GFPT2	1.975762487	6.55E-06
BGIR9823_100872	MAP3K15	1.912749874	0.001001141
BGIR9823_98003	CNNM2	1.705477555	3.75E-09
BGIR9823_83643	MTFR1	1.69801835	7.23E-04
BGIR9823_88585	SLC41A3	1.693137278	4.91E-04
BGIR9823_78137	ARPP19	1.661102302	3.41E-08
BGIR9823_85300	PPARA	1.598913608	4.13E-04
BGIR9823_83642	PDE7A	1.596781223	8.90E-04
BGIR9823_96972	MCU	1.518581064	5.97E-05
BGIR9823_90771	NEU3	1.508987188	8.37E-06
BGIR9823_92131	PPP1R12B	1.501575052	4.80E-07

BGIR9823_80122	LOC102163816	1.490241602	1.32E-08
BGIR9823_99096	ERBB4	1.41991883	7.38E-04
BGIR9823_100418	LOC106506781	1.349517588	7.74E-09
BGIR9823_99552	RICTOR	1.348062052	9.51E-04
XR_002340248.1	LOC110257516	1.346183857	0.001239855
BGIR9823_78329	BCL2	1.282614884	2.20E-06
BGIR9823_99094	ERBB4	1.276079135	2.93E-06
BGIR9823_99095	ERBB4	1.267544486	8.29E-05
BGIR9823_101854		1.247747233	2.14E-04
BGIR9823_91824	RUNDC3B	1.236845824	4.48E-04
BGIR9823_82386	PSME4	1.233150542	2.86E-04
BGIR9823_80428	ZSWIM4	1.20156263	7.98E-10
XR_002340371.1	LOC110257572	1.198920621	5.67E-05
BGIR9823_83955	CLCC1	1.176241447	1.32E-04
BGIR9823_98456	COQ10B	1.156076609	3.53E-05
BGIR9823_88015	NEXN	1.146744667	1.98E-04
BGIR9823_100321	PMEPA1	1.127541125	3.81E-06
BGIR9823_93572	MSI2	1.039529042	1.83E-05
BGIR9823_93571	MSI2	1.007090934	2.33E-04
BGIR9823_87926	LOC110261182	-1.003453915	1.24E-05
BGIR9823_79650	NEMF	-1.012097783	1.06E-04
BGIR9823_82966	ZC3H6	-1.013255793	2.79E-08
BGIR9823_88780	CCNB1IP1	-1.030030663	1.00E-05
XR_002335844.1	LOC110255479	-1.046878867	7.68E-05
BGIR9823_95770	FHIT	-1.061440436	3.82E-06
XR_002337719.1	LOC106505375	-1.073212981	1.08E-06
BGIR9823_101208	LOC102165633	-1.076882814	2.26E-05
BGIR9823_96364	RUNX1	-1.090698787	1.61E-05
BGIR9823_85841	CRADD	-1.117671884	2.36E-04
BGIR9823_99209	RAMP1	-1.189542878	3.49E-04
BGIR9823_102212		-1.19140003	0.001064522
BGIR9823_88486	PRPH2	-1.195216379	9.41E-06
BGIR9823_87747	TMEM234	-1.21044292	1.39E-07
BGIR9823_93430	ACBD4	-1.211468313	7.87E-05
XR_002335905.1	LOC106504993	-1.221676808	2.75E-04
BGIR9823_81197	ARNTL	-1.319925106	5.32E-04
XR_002336855.1	LOC106508625	-1.325264102	5.01E-05
BGIR9823_88590	SEMA4B	-1.331035491	3.86E-05
BGIR9823_86965	PGM1	-1.341265352	1.41E-06
BGIR9823_83417	MTSS1	-1.341369182	3.25E-06
BGIR9823_90875	BIRC3	-1.379367903	0.001333583
BGIR9823_101185	LOC110257830	-1.385860002	8.71E-04
XR_002342486.1	LOC110259964	-1.390523257	3.05E-06
BGIR9823_88895	RGS6	-1.411355796	2.76E-04
BGIR9823_101989		-1.413902005	7.28E-04
BGIR9823_101566	LOC110258600	-1.428483836	2.43E-05
BGIR9823_98865	NR4A2	-1.432353156	2.95E-10
BGIR9823_87139	LOC106510467	-1.45800243	3.23E-05
BGIR9823_90934	LOC110255441	-1.461161284	1.52E-04
XR_002335693.1	LOC106504896	-1.467039583	5.93E-04
XR_001304283.2	LOC102160368	-1.47908794	4.98E-04
BGIR9823_81968	LOC100626250	-1.491375007	2.81E-06
BGIR9823_102279		-1.514571811	3.18E-05
BGIR9823_90639	LOC110261963	-1.519913391	0.001244564
XR_002346019.1	LOC106504512	-1.557913116	7.52E-04
BGIR9823_81077	PATL1	-1.588918945	5.88E-04
BGIR9823_89592	SYNJ2BP	-1.633880155	0.001100671
BGIR9823_102657		-1.652081307	4.73E-04
XR_002346056.1	LOC110261648	-1.654753004	4.24E-04
BGIR9823_78436	SOS2	-1.660607624	9.65E-04

XR_002341616.1	LOC110259333	-1.669914272	2.18E-07
BGIR9823_98958	NFE2L2	-1.671867137	1.88E-04
BGIR9823_93000	LOC102166871	-1.685651811	5.05E-04
BGIR9823_92999	LOC110255292	-1.710250766	4.16E-05
BGIR9823_101544	LOC110258600	-1.745524765	3.23E-04
BGIR9823_80765	NREP	-1.745993667	7.91E-06
BGIR9823_82709	SUMF2	-1.776152144	3.19E-06
XR_307765.3	LOC102161206	-1.836910662	2.09E-04
BGIR9823_101191	LOC110257830	-1.863599053	1.50E-05
XR_001302653.2	LOC102165633	-1.943475988	8.38E-13
XR_002343346.1	LOC110260299	-1.955574071	2.72E-04
BGIR9823_92415	LOC110255652	-1.960569536	0.001239122
BGIR9823_99751	LOC100155289	-2.078664427	1.53E-08
XR_002335940.1	LOC100514506	-2.124392207	8.14E-05
BGIR9823_85418	LOC102163508	-2.131436721	2.37E-12
XR_002343991.1	LOC106510313	-2.194261008	0.001359171
BGIR9823_89027	LOC102161744	-2.198692476	2.25E-06
BGIR9823_94434	COPS3	-2.228618135	2.61E-04
XR_001297664.2	LOC102161744	-2.248681811	2.81E-06
BGIR9823_89012	LOC110261648	-2.306057453	7.74E-05
BGIR9823_88037	LRRC7	-2.324690794	3.71E-04
BGIR9823_88140	MAST2	-2.375346363	6.05E-05
BGIR9823_102169		-2.521647776	1.77E-07
BGIR9823_101545	LOC110258600	-2.564803531	5.45E-04
XR_002338205.1	LOC110256571	-2.617360515	3.20E-06
BGIR9823_94689	ACOX2	-2.64655891	0.001147009
BGIR9823_102168		-2.687825936	1.61E-14
XR_002343137.1	LOC110260231	-2.705985569	2.03E-12
BGIR9823_102650		-2.865726582	2.71E-04
XR_002335671.1	LOC110255441	-2.911374744	8.22E-06
BGIR9823_101564	LOC110258600	-2.985301731	2.66E-04
XR_002341615.1	LOC110259333	-3.005473783	2.13E-04
XR_002343366.1	LOC106508330	-3.239772335	6.48E-04
BGIR9823_79960	SUSD1	-3.266313264	1.03E-04
BGIR9823_93054	LOC110255759	-3.277664964	4.14E-05
BGIR9823_99528	NPR3	-3.298100792	6.76E-04
XR_002340445.1	LOC100739791	-3.306550194	1.23E-07
BGIR9823_86463	RERE	-3.377991204	2.48E-04
BGIR9823_84443	OAZ3	-3.441247861	2.96E-04
BGIR9823_98543	SPAG16	-3.603516419	3.34E-08
BGIR9823_101781		-3.604871313	6.08E-04
XR_307914.3	LOC102159607	-3.65677597	4.79E-08
XR_002336552.1	LOC110255759	-3.72569871	4.19E-04
BGIR9823_102724		-3.761348191	7.82E-05
XR_002343632.1	LOC102159118	-3.803925687	1.04E-10
XR_002345058.1	LOC110261182	-3.867430437	1.64E-10
XR_002344184.1	LOC110260775	-4.111811541	3.84E-04
BGIR9823_101549	LOC110258600	-4.169881586	1.23E-05
XR_002336149.1	LOC106505119	-4.260795178	6.43E-05
XR_002337059.1	LOC106509220	-4.364773033	9.20E-06
XR_002341917.1	LOC110259485	-4.593322456	5.72E-09
XR_002343891.1	LOC110260642	-4.646704097	6.26E-04
XR_002343215.1	LOC106510024	-4.705267662	1.23E-05
XR_002337660.1	LOC110256287	-4.965438273	9.06E-04
XR_001308019.2	LOC106509966	-5.210727367	1.24E-04
BGIR9823_100551	RAMP3	-5.341996234	3.37E-11
XR_299372.3	LOC102158266	-5.478356501	6.56E-04
URS0000BC5529	LOC106506809	-5.492391673	8.35E-09
XR_002338666.1	LOC110256747	-5.607553907	0.00101011
XR_002342781.1	LOC110260032	-5.749251762	3.00E-04

XR_002343213.1	LOC110260256	-6.040306821	3.44E-07
XR_001303119.2	LOC100628140	-6.200961347	8.28E-04
XR_002343544.1	LOC110260362	-6.648220007	5.47E-04
XR_002341920.1	LOC110259488	-6.700385562	6.85E-06
BGIR9823_100550	RAMP3	-6.702136635	1.06E-08
XR_001298953.2	LOC106504926	-6.774005015	1.53E-04
XR_002345629.1	LOC100155975	-6.96127587	4.71E-05
XR_002346569.1	LOC100627270	-7.325288509	3.62E-05
XR_298148.3	LOC102164588	-7.355109471	5.40E-08
XR_002339623.1	LOC102167708	-7.441572951	3.02E-04
BGIR9823_81015	LOC102163816	-7.932169918	1.77E-07
BGIR9823_101562	LOC110258600	-8.306084089	3.31E-04
XR_002346063.1	LOC102163738	-8.526807304	1.70E-09
XR_002339346.1	FAM126B	-9.027258395	0.001047314
XR_002337772.1	LOC106504926	-10.14089766	1.73E-11
XR_002342057.1	LOC110259691	-21.56818697	3.45E-08
BGIR9823_95558	CMTM6	-21.63539852	3.13E-08
BGIR9823_88211	TMEM170B	-22.68317653	6.46E-09
XM_013998019.2	MYBPC1	26.07323427	2.49E-11
XM_013998022.2	MYBPC1	25.07247446	1.38E-10
XM_021095032.1	LOC100621492	24.10636154	6.83E-10
XM_003353850.4	ZDHHC5	23.60905568	1.52E-09
XM_021090010.1	HIPK1	23.49485944	1.82E-09
XM_021064397.1	ENAH	23.41512063	2.06E-09
XM_021073360.1	ABLIM1	23.07855594	3.49E-09
XM_021079673.1	BRD4	22.99121273	4.00E-09
XR_002338285.1	ATP2A2	22.91795829	4.48E-09
XM_021086578.1	MRTFB	22.89179947	4.67E-09
XM_021102080.1	FERMT2	22.89100724	4.67E-09
XM_021063197.1	ATP2B4	22.84818657	4.99E-09
XM_021079133.1	KMT2C	22.82684719	5.16E-09
XM_013983756.2	ABLIM1	22.7908217	5.45E-09
XM_013988804.2	ZSCAN20	22.69557287	6.31E-09
XM_021073370.1	ABLIM1	22.66519908	6.61E-09
XM_013995290.2	RNF14	22.65870579	6.68E-09
XM_021093688.1	FBXO31	22.64366412	6.84E-09
XM_013980184.2	AQP7	22.60503555	7.25E-09
XM_021072576.1	PPP3CB	22.47669815	8.82E-09
XM_021067269.1	VEZF1	22.40326282	9.86E-09
XM_005672244.3	OBSL1	22.4019093	9.88E-09
XM_021090657.1	LOC100624559	22.37363997	1.03E-08
XM_021085899.1	NCOA1	22.37179951	1.03E-08
XM_013983547.2	PCGF5	22.29482679	1.16E-08
XR_002344506.1	PDPR	22.19216144	1.36E-08
XM_021097027.1	DEF8	22.14203264	1.46E-08
XM_021064419.1	COQ8A	22.00067245	1.80E-08
XM_021073851.1	EP400	21.97092907	1.89E-08
XM_021101962.1	GRB10	21.97092907	1.89E-08
XM_021079417.1	PFKFB1	21.93516869	1.99E-08
XM_021062517.1	ARRB1	21.91908792	2.04E-08
XM_021073351.1	ABLIM1	21.87825147	2.17E-08
XM_021097673.1	EIF4G3	21.87590171	2.17E-08
XM_005654266.3	PRKAG2	21.87227211	2.18E-08
XM_005653504.3	FURIN	21.86913925	2.19E-08
XM_021093897.1	SLC12A3	21.81486385	2.38E-08
XM_021085966.1	MAFK	21.79309788	2.45E-08
XM_021089745.1	ZBTB7B	21.76317035	2.57E-08
XM_021073347.1	ABLIM1	21.76205011	2.57E-08
XM_021064364.1	SMARCA2	21.74170027	2.65E-08
XM_021092270.1	PLEKHA5	21.72998932	2.70E-08

XM_021097995.1	PEAK1	21.72998932	2.70E-08
XM_021082576.1	TSPAN4	21.71373506	2.76E-08
XM_021086266.1	CUX1	21.71373506	2.76E-08
XM_021093854.1	IST1	21.66996916	2.95E-08
XM_021082837.1	EHBP1L1	21.65367568	3.02E-08
XM_013987707.2	ZNF646	21.62904927	3.13E-08
XM_021087703.1	EYA4	21.56468322	3.44E-08
XM_021082960.1	VPS37C	21.55416732	3.49E-08
XM_021084565.1	PAM	21.54939232	3.51E-08
XM_021092242.1	ABCC9	21.50828124	3.71E-08
XM_021095024.1	LOC100621492	21.49034596	3.83E-08
XM_021097909.1	GABBR1	21.44664811	4.08E-08
XM_021084398.1	NSD1	21.43897337	4.13E-08
XM_021094015.1	PDP2	21.41135874	4.30E-08
XM_021076153.1	MYO7B	21.37377793	4.54E-08
XM_021073353.1	ABLIM1	21.33049447	4.83E-08
XM_021090656.1	LOC100624559	21.28806675	5.14E-08
XM_021065837.1	DOCK9	21.28662642	5.15E-08
XM_021082831.1	PCNX3	21.27748316	5.22E-08
XM_021083625.1	WIZ	21.27748316	5.22E-08
XM_021069048.1	SLMAP	21.25826348	5.37E-08
XM_005667404.3	NLRX1	21.25785174	5.37E-08
XM_013987936.2	ROCK2	21.23683133	5.54E-08
XM_021085790.1	ELN	21.23374454	5.56E-08
XM_021095737.1	PUM1	21.23277087	5.57E-08
XM_013991801.2	MGAT1	21.2031531	5.82E-08
XM_021093652.1	PIEZO1	21.1661366	6.13E-08
XM_021097492.1	GNB1	21.16167518	6.17E-08
XM_021079256.1	MPP6	21.1481952	6.29E-08
XM_021066711.1	KCNJ16	21.1424149	6.35E-08
XM_021101612.1	ANKRD50	21.11577302	6.60E-08
XM_021085591.1	NCK2	21.09563034	6.79E-08
XM_021069336.1	ATP2B2	21.07000027	7.04E-08
XM_021075156.1	CLASP1	21.07000027	7.04E-08
XM_021088361.1	ITSN2	21.07000027	7.04E-08
XM_021100629.1	SH3D19	21.06286768	7.12E-08
XM_005663297.2	SSR2	21.04710109	7.28E-08
XM_021091895.1	AGAP2	21.02414961	7.52E-08
XM_021089113.1	ROS1	21.01863293	7.51E-08
XM_013986056.2	BCOR	21.00882448	7.69E-08
XM_021066945.1	NBR1	21.00882448	7.69E-08
XM_021082959.1	VPS37C	20.98703573	7.93E-08
XM_021067204.1	SPAG9	20.96451314	8.19E-08
XM_021064773.1	SVIL	20.96369946	8.20E-08
XM_021101355.1	KIAA1211	20.92186862	8.71E-08
XM_021087694.1	EYA4	20.91604347	8.78E-08
XM_021064751.1	SVIL	20.90717262	8.89E-08
XM_021087497.1	USP34	20.89052668	9.11E-08
XM_021066812.1	MAPT	20.87795336	9.27E-08
XM_003358028.4	NBR1	20.87742961	9.28E-08
XM_021063164.1	IGSF9B	20.86632052	9.42E-08
XM_021066944.1	NBR1	20.8462776	9.70E-08
XM_021088741.1	ST3GAL1	20.83037028	9.92E-08
XM_021063435.1	DNAH11	20.82554525	9.99E-08
XM_021066950.1	NBR1	20.82554525	9.99E-08
XM_021100824.1	PCGF3	20.81882013	1.01E-07
XM_021069032.1	SLMAP	20.81423129	1.02E-07
XM_021070857.1	EIF4E3	20.81423129	1.02E-07
XM_013990907.2	ARHGEF9	20.81179943	1.02E-07
XM_021096943.1	SCMH1	20.81179943	1.02E-07

XR_002342588.1	TIA1	20.81179943	1.02E-07
XM_021091508.1	GGA1	20.78285746	1.06E-07
XM_021094721.1	TEAD2	20.78285746	1.06E-07
XM_003135077.5	GPKOW	20.77636499	1.07E-07
XM_021064013.1	HNRNPK	20.77636499	1.07E-07
XM_021070888.1	BACH1	20.74756797	1.12E-07
XM_021078388.1	PLCG1	20.71542658	1.17E-07
XM_021091832.1	R3HDM2	20.68494485	1.22E-07
XM_021063295.1	SRGAP2	20.66716039	1.25E-07
XM_021068997.1	ASB14	20.66079002	1.26E-07
XM_021087794.1	PUM2	20.66079002	1.26E-07
XM_001929430.6	SLK	20.64534357	1.29E-07
XM_003122570.4	SLC22A12	20.64534357	1.29E-07
XM_013994193.2	SLC22A8	20.64534357	1.29E-07
XM_021069578.1	PAQR9	20.64279984	1.29E-07
XM_021072410.1	HIVEP2	20.62460964	1.33E-07
XM_021075089.1	TRIP12	20.60918853	1.36E-07
XM_021096712.1	EFCAB14	20.60918853	1.36E-07
XM_021095296.1	CLSTN1	20.4665866	1.65E-07
XM_021073127.1	MED13L	20.37076875	1.89E-07
XR_002339780.1	SLC38A9	20.37076875	1.89E-07
NM_001243860.1	TSPAN1	20.26039256	2.19E-07
XR_002339748.1	LOC100737060	20.26039256	2.19E-07
XM_021080861.1	INTS6L	20.08356807	2.78E-07
NM_213999.2	DUOX2	20.07318854	2.83E-07
XM_021075819.1	SLC39A10	20.07318854	2.83E-07
XM_021073598.1	RHOBTB1	20.0719648	2.83E-07
XM_021073352.1	ABLIM1	20.06062601	2.88E-07
XM_021098823.1	PKHD1	20.05226809	2.90E-07
XR_309303.3	IKBKG	20.05226809	2.90E-07
XM_021100955.1	MYO9A	19.67441396	4.85E-07
XM_003355803.5	CIAPIN1	19.00800371	1.16E-06
XM_021084329.1	HNRNPH1	19.00800371	1.16E-06
XM_021086524.1	UMOD	12.22450446	6.63E-06
XM_003128856.5	BOD1L1	11.7331237	1.70E-21
XM_021091479.1	SUN2	11.40734332	8.36E-13
XM_003482030.4	PABPC4	11.09918145	2.85E-14
XM_021086083.1	EIF2AK1	10.9720745	1.25E-19
XM_021070210.1	ZNF148	10.93996147	8.60E-17
XM_013994310.2	ZDHHC5	10.590136	4.10E-16
XM_021064679.1	FAM219A	10.49461003	9.72E-14
XR_002343699.1	LOC100624559	10.48177571	2.46E-17
XM_021083611.1	LOC100739101	10.1881759	4.82E-13
XM_021074868.1	RBPMS	10.11064294	1.21E-04
XM_021072267.1	UTRN	10.091367	1.38E-04
XM_021074020.1	CYP2C36	10.04821267	1.09E-06
NM_214066.2	DAO	9.921752385	1.25E-06
XM_021066854.1	ALDOB	9.908947528	1.90E-09
XM_021078684.1	AOC1	9.902514041	3.10E-04
XM_021069935.1	ABCC5	9.889251559	2.44E-04
XM_003122908.4	SLC5A12	9.874509626	2.68E-07
XM_021093455.1	TNNT1	9.828743608	2.09E-04
XM_021099902.1	SCFD1	9.819588573	2.37E-04
XM_021100310.1	RASGEF1B	9.776815162	1.09E-09
XM_021087392.1	ATP6V1B1	9.770526855	3.04E-06
XM_005660775.2	SLC22A8	9.651085826	3.92E-06
NM_001315734.1	LOC100739741	9.650004263	1.18E-06
XM_003356738.5	MPP5	9.599004294	3.80E-04
XM_001926142.7	SLC12A1	9.589862354	2.86E-06
XR_002337405.1	MIEF2	9.52573152	2.36E-12

XM_021102554.1	PTPN12	9.519831694	1.82E-13
XM_021088357.1	ITSN2	9.490845167	4.89E-04
XM_021089493.1	DDR2	9.490146224	8.06E-14
NM_214277.1	ANPEP	9.475881544	6.34E-04
XM_021073054.1	TAOK3	9.472427516	4.75E-04
NM_001197306.1	STAT6	9.440189487	5.78E-04
XM_021087698.1	EYA4	9.430319353	9.52E-04
XM_021088072.1	RBFOX1	9.426263166	5.31E-04
XM_021097461.1	MIB2	9.415173872	4.80E-04
XM_005671981.3	NFE2L2	9.410288477	1.57E-08
XM_021092803.1	METTL25	9.404974756	1.38E-10
XR_002336593.1	IFT88	9.335164746	6.05E-13
XM_003129745.5	NOX4	9.290644673	5.26E-08
XM_021087700.1	SLC5A6	9.241319163	7.81E-04
XM_003124561.6	ACSM2B	9.238262035	2.15E-06
XM_003356540.3	NRSN1	9.218688185	3.83E-05
XM_013993681.2	TSTD2	9.201512815	2.07E-12
XM_021102384.1	VPS50	9.191688907	8.20E-04
XM_013985327.2	RBM39	9.190323868	0.001121505
XM_013986373.2	SEPT6	9.186505251	2.08E-08
XR_001299971.2	ANKFY1	9.18481092	0.001024077
XM_021078686.1	AOC1	9.179677016	1.71E-05
XM_021069098.1	RPP14	9.171248619	0.001394787
XM_021095507.1	LUZP1	9.153373571	9.04E-04
XM_021079578.1	CLDN2	9.14182487	7.50E-05
XM_021086703.1	SRL	9.129211759	0.001169261
XM_003133513.5	SESTD1	9.107876699	8.98E-04
XM_021075431.1	B3GALT1	9.097714995	3.46E-06
NM_001204771.1	BHMT2	9.091166987	5.60E-06
XM_013993682.2	TSTD2	9.089317223	5.98E-10
XM_013995378.2	ZKSCAN5	9.066787527	1.50E-11
XM_021074889.1	PER2	9.060699541	8.15E-10
XM_021090508.1	ZNF623	9.059263209	9.35E-05
XM_021063812.1	RGL1	9.043338099	1.31E-08
XM_013990499.2	ZNF317	9.030334551	0.00116767
XM_001929254.6	UPB1	9.001771046	3.40E-05
XM_021068597.1	EIF4G1	9.001068855	3.92E-11
XM_021066598.1	RNF157	8.990116517	3.81E-12
XM_013981475.2	WDR48	8.985994557	0.001233851
XM_021087778.1	EYA4	8.982027685	0.001235437
XM_021075273.1	RHBDD1	8.897359299	1.06E-08
XM_021085999.1	LFNG	8.85742506	0.001395789
XR_002338044.1	SMIM11A	8.809491968	1.70E-08
XM_013977857.2	KCTD20	8.802964465	9.84E-10
XM_013995671.2	UBN1	8.754305669	6.98E-11
XM_021065573.1	VPS36	8.737125157	5.79E-11
XM_003125820.4	HAO2	8.73439725	2.20E-05
NM_001244717.1	SLC13A2	8.69624038	6.11E-05
XM_021078046.1	KIF16B	8.682869715	1.37E-06
XM_021070877.1	USP16	8.671608734	5.32E-10
NM_214424.1	CYP4A24	8.62469727	6.73E-05
XM_021090729.1	PHTF1	8.617363654	4.82E-09
XM_021072976.1	CLIP1	8.615477896	2.69E-08
XM_021094023.1	CDH16	8.538281424	4.83E-04
XM_021078191.1	DZANK1	8.537350818	1.34E-06
NM_214108.1	DPEP1	8.423945649	3.90E-04
XM_021071198.1	TBL1XR1	8.405431636	8.33E-10
XM_021067391.1	LOC110256043	8.402721317	1.22E-06
NM_214420.1	CYP2C49	8.397166552	5.83E-04
XM_021071634.1	LRRFIP2	8.392699383	4.71E-06

XM_021079253.1	MPP6	8.392429869	1.33E-05
XM_021079900.1	NRK	8.389189508	4.75E-09
XM_021069837.1	NUP188	8.367172102	2.86E-08
XM_021100323.1	CPEB2	8.363581086	1.83E-09
XM_021067576.1	SMYD4	8.326890335	9.75E-10
XM_005670341.3	KCNJ15	8.295041589	1.01E-04
XM_005663042.3	CSPP1	8.271022228	5.76E-09
XM_021076613.1	MTRR	8.254116884	3.86E-07
XR_002338387.1	ULK1	8.253415417	1.45E-07
XM_021093935.1	KATNB1	8.241130121	6.85E-08
NM_214425.1	CYP4A21	8.227945773	8.82E-05
XM_005661365.3	ARID3A	8.219895972	2.98E-08
XR_308559.3	SAP30L	8.19196291	4.80E-07
XM_005666844.3	LSM6	8.186785099	3.91E-09
XM_021064930.1	MLLT10	8.169221794	7.24E-07
NM_214414.1	CYP2C33	8.165599687	0.001232172
XM_005656555.3	EIF4E	8.120614695	1.30E-06
XM_005653771.3	LOC100627222	8.116212268	3.88E-08
XM_021069067.1	FLNB	8.094712453	3.51E-07
XM_013990980.2	TCEAL1	8.078320253	2.91E-06
XM_005653519.3	HAPLN3	8.073033596	1.01E-05
XM_021084480.1	DMGDH	8.051789652	2.54E-04
XM_005653181.3	MIOX	8.022137169	2.95E-05
XR_002345117.1	TTLL7	8.010958614	5.81E-08
XM_021065007.1	SFMBT2	8.004799624	2.23E-04
XM_021095282.1	SLC2A5	8.001033528	2.31E-11
XM_021071242.1	FXR1	7.995989279	1.48E-08
XM_021079273.1	DNAJB6	7.9474736	5.03E-05
XM_021092608.1	TMEM106C	7.93262588	2.08E-08
XR_002341416.1	ENG	7.91184426	6.47E-08
XM_013987785.2	UNKL	7.907357908	9.76E-08
XM_021100689.1	JADE1	7.896460698	2.01E-04
XM_021066284.1	LOC100736982	7.889244054	3.56E-04
XM_021086300.1	STYXL1	7.882315066	7.72E-06
XM_021074016.1	MIA2	7.869132542	5.55E-04
XM_021091918.1	UBE3D	7.86874952	2.39E-07
XM_021102239.1	HSD11B1	7.863576988	8.60E-08
XM_021076277.1	XRCC5	7.840006914	1.08E-06
XM_021082584.1	MUC2	7.833919223	2.97E-05
XM_013994314.2	CLP1	7.792388789	2.16E-07
XM_013987408.2	TYK2	7.785720556	3.83E-07
XM_021074019.1	CYP2C34	7.783404598	0.001051804
XM_021102465.1	HDAC9	7.780662792	1.63E-07
XM_021087829.1	GREB1	7.774174375	1.63E-05
XM_021091651.1	LARP4	7.741728518	3.19E-07
XM_013990602.2	TNPO2	7.73591737	2.80E-07
NM_213902.1	SLC22A2	7.729388043	3.74E-04
XM_021079779.1	KDM6A	7.718728411	2.67E-04
XR_002344911.1	VPS13D	7.717784358	5.50E-05
XM_021100105.1	EXOC2	7.699942788	1.27E-07
XM_021077863.1	RIPOR3	7.688531481	0.00104041
XM_021073895.1	HNRNPF	7.685480708	2.14E-06
XM_005665059.3	KLHDC7A	7.667638729	0.001368526
XM_021084584.1	PPIP5K2	7.653654664	3.04E-05
XM_021096707.1	LOC100737897	7.634489128	2.15E-04
XM_021066314.1	TSTD2	7.605561018	5.75E-07
XR_002345734.1	CUL9	7.605408955	6.42E-04
XR_002339455.1	DNAJC10	7.602701884	2.27E-07
XR_002342739.1	PLEKHH2	7.601559915	1.98E-05
XM_021094014.1	PDP2	7.594132708	1.67E-06

XM_021085872.1	IL4R	7.552520643	2.22E-05
XM_021089215.1	STAU2	7.527838924	5.00E-06
XR_002344504.1	MLKL	7.526774457	1.76E-05
XM_005673043.3	PCK1	7.516253235	1.58E-05
XM_021082945.1	LOC100519643	7.508623999	1.83E-05
XM_021097263.1	LOC110261291	7.502685589	1.69E-06
XM_013987546.2	ZNF354A	7.489496827	4.91E-06
XM_021072758.1	ARID5B	7.485997342	3.21E-07
NM_001123093.1	CD163L1	7.467957921	1.80E-05
XM_021078011.1	CD151	7.457154377	1.10E-05
XM_013994874.2	CDHR2	7.451601059	6.40E-04
XM_021075386.1	GULP1	7.426365134	3.50E-05
XM_021094476.1	XRCC1	7.42227082	1.34E-06
XM_021074280.1	TAF5L	7.418329433	1.07E-06
XM_021095302.1	CTNNBIP1	7.408915383	2.29E-05
XM_021087028.1	TMEM87B	7.387594654	4.94E-06
XM_021075587.1	PDE1A	7.385528639	8.86E-06
XM_021102193.1	AKAP9	7.378348854	4.17E-05
XM_005666159.3	STRA6	7.373968903	3.18E-04
XM_021074973.1	DNAJB2	7.365120789	2.24E-06
XM_021078830.1	AHCYL2	7.342663359	1.22E-05
NM_001113445.1	G6PC	7.333154638	4.66E-05
XM_021077445.1	COMMD7	7.325787248	3.92E-06
XR_002339040.1	UBE2F	7.314139131	2.74E-05
XM_021099515.1	ENTPD5	7.306321211	9.36E-04
XM_021067731.1	RABEP1	7.276956477	2.59E-06
XM_021090216.1	EVI5	7.275283499	3.66E-05
XM_021062700.1	TRPC6	7.256460085	3.45E-04
XM_021079352.1	TNS3	7.254589501	5.93E-05
XR_002338043.1	SMIM11A	7.237124863	1.65E-06
XM_021088968.1	TBC1D31	7.22956004	0.001028625
XM_021079806.1	RRAGB	7.22134498	8.35E-05
NM_001315766.1	LOC100736962	7.208145416	0.001166781
XM_021067703.1	SPNS2	7.207226752	3.06E-05
XM_013994069.2	YIF1A	7.199253756	2.93E-05
XM_021090995.1	CCDC91	7.168736167	5.89E-06
XM_005661925.3	SLC12A9	7.139927452	6.16E-05
XR_002339429.1	USP40	7.139170708	1.90E-05
XM_003484123.4	DLG3	7.128391434	1.49E-06
XM_021077656.1	RGS19	7.121838379	1.38E-04
XM_013997586.2	ZNF385A	7.091135991	1.77E-05
XM_021092656.1	PAH	7.083124615	2.65E-05
XM_021076436.1	PKP4	7.079470713	2.19E-04
XM_013987480.2	JAK3	7.052183413	2.99E-05
XM_021090726.1	PHTF1	7.035421802	5.94E-05
XM_003359855.4	SLC6A19	7.030740043	9.12E-04
XR_002336297.1	C10H9orf3	7.018805591	8.57E-05
XM_021087338.1	REPS1	7.011677042	1.11E-04
XM_021064107.1	ESRRG	7.008515493	0.001036348
XM_021067233.1	MBTD1	6.990439048	1.77E-05
XM_021080742.1	ADGRE5	6.987119832	2.13E-04
XM_021072725.1	DMTN	6.98634815	7.21E-05
XM_021075384.1	GULP1	6.986128954	3.91E-04
XM_021074330.1	NUDT13	6.984160892	3.98E-04
XM_013994983.2	FAM172A	6.973976335	7.74E-05
XM_021097257.1	LOC110261290	6.950367369	1.83E-05
XM_021064804.1	ARHGAP12	6.949108565	4.21E-04
XM_013997422.2	KYAT3	6.918137143	2.37E-04
XM_013996523.2	ENPP2	6.914181202	4.37E-04
XM_021099613.1	RPS6KA5	6.874088045	2.97E-05

XM_021095301.1	CTNNBIP1	6.802955087	8.83E-05
XM_013990951.2	ARMCX2	6.76751979	2.25E-04
XM_013990160.2	NXPE3	6.766806578	1.49E-04
XM_021083010.1	LOC110259258	6.758950857	2.19E-04
XM_013981389.2	GALNT15	6.757903784	1.49E-04
XM_003124495.4	PRSS36	6.741666892	1.00E-06
XM_005661944.3	RASA4B	6.737341309	7.02E-04
XM_013993662.2	RNF38	6.702504287	1.28E-04
XM_021067390.1	LOC110256043	6.672680615	8.15E-08
XM_013983322.2	CAMK2G	6.667549094	9.05E-05
XM_021078796.1	UBE2H	6.661951018	1.82E-04
XM_021076262.1	C15H2orf88	6.659042119	5.25E-04
XM_005662959.3	CCNE2	6.643013568	6.23E-04
XM_021102295.1	CLPB	6.637440363	1.16E-07
XM_021085758.1	LMAN2L	6.634298712	2.08E-04
NM_001243617.1	ENOSF1	6.616550624	4.75E-04
XM_021078242.1	GID8	6.601848054	4.86E-04
XM_005657158.3	IGSF5	6.59857876	0.001382891
XM_021064973.1	NRP1	6.5864673	1.68E-05
XM_003125581.4	ATP6V0D2	6.57064638	6.45E-04
XM_021094526.1	GEMIN7	6.560497224	3.13E-04
XM_013990993.2	NLRP12L	6.545195833	3.15E-04
XM_021072176.1	CYP2C33	6.524844759	0.001392595
XM_021066920.1	ETV4	6.524268411	2.07E-04
XM_021093260.1	ERCC1	6.523405021	2.60E-04
XM_021095793.1	ADGRB2	6.522278123	2.39E-04
XM_021070327.1	POGLUT1	6.503646747	2.87E-04
XM_021067322.1	ZNHIT3	6.475463864	2.32E-04
XM_021095646.1	FGR	6.466692226	3.68E-04
XM_021078643.1	CAMK2B	6.465262038	0.00102156
XM_005669425.3	HIGD1A	6.456698806	3.80E-04
XM_021066055.1	ADAP2	6.444917326	8.95E-04
XM_021075582.1	SORBS2	6.439408777	0.001248298
XM_021085692.1	APOB	6.425729877	0.001285728
XM_021084636.1	KCNN2	6.389844887	6.51E-04
XM_013998328.2	LIN37	6.382196727	4.61E-04
NM_001123158.1	PCK1	6.369135947	3.94E-07
XM_005669485.3	DHX30	6.355977258	9.26E-07
NM_001243939.1	ADH1C	6.315951874	7.10E-09
XM_003131660.6	RAD51C	6.312769906	5.80E-04
XM_021079731.1	CYP4F55	6.307434669	7.91E-04
XM_021081605.1	PPP2R5C	6.30476285	6.60E-04
XM_005657431.3	LOC102157770	6.282161589	1.43E-06
XM_005654018.3	SRCIN1	6.281989893	0.001157936
XM_021076923.1	BSCL2	6.222121068	8.10E-04
XM_021094409.1	ICE2	6.214292492	0.001367961
XM_021085643.1	PHF10	6.144561819	0.001061848
XM_021083428.1	IYD	6.102301038	1.72E-04
XR_002345567.1	FKBP5	6.063474926	3.29E-04
XM_021078572.1	CHN2	6.032019982	5.55E-07
XM_021064748.1	SVIL	6.021777922	0.001141793
XM_003124061.6	GRXCR2	6.006802863	1.20E-04
XR_002343700.1	LOC100624559	5.998896622	1.22E-04
XM_021062422.1	LOC110255390	5.993306988	1.25E-04
XM_021092390.1	NANOG	5.982815531	1.97E-05
XR_002341590.1	IYD	5.844902174	9.89E-05
XM_021074132.1	SORBS1	5.79154755	5.54E-04
XM_021063976.1	TRDMT1	5.758826456	5.48E-04
XM_021087399.1	TIA1	5.737078966	8.53E-05
XM_021094163.1	CA12	5.73332124	6.34E-04

XM_021063334.1	CR1	5.688398389	3.85E-04
XM_021099522.1	AREL1	5.582457917	1.69E-04
XM_021070181.1	TNK2	5.494503093	9.90E-05
XM_001926978.5	CLCA4	5.441140346	6.01E-04
XM_021064811.1	CUBN	5.438338252	8.56E-07
XR_002336745.1	TPP2	5.347052734	0.001260756
XM_013981430.2	SMPD4	5.288800397	0.0012741
NM_001244806.1	CHRNA9	5.280899614	3.19E-15
XM_003356166.4	PADI2	5.226401876	6.56E-04
XM_005671060.3	DUSP13	5.152313566	8.76E-04
NM_001244653.1	TF	5.105307635	6.83E-04
XM_021097877.1	SLA-2	5.099477482	0.001079961
NM_214394.1	CYP2D25	5.010199757	1.37E-04
XM_021088688.1	ANGPT1	4.992650994	7.55E-04
XM_021093718.1	KIAA0513	4.923239841	7.87E-07
NM_213979.1	FBP1	4.892230367	2.92E-05
XM_021093779.1	BCAR1	4.874035334	0.001148117
XM_013981827.2	JAKMIP3	4.866898441	2.84E-04
XM_021092060.1	DNM1L	4.749904207	0.001251509
XM_021067318.1	PIGW	4.747020891	5.94E-04
XM_021097626.1	LOC100736720	4.742411897	4.85E-05
XM_021078856.1	LOC102162486	4.614108477	3.88E-04
XM_013992909.2	LOC100152206	4.57529946	8.89E-05
NM_214263.1	ABAT	4.472071457	2.54E-07
XM_013983377.2	ANXA11	4.406477992	3.35E-08
XM_021067933.1	AKNA	4.387727349	2.84E-05
XM_021098327.1	TNXB	4.353045528	5.31E-04
XM_021073820.1	SEMA4D	4.329531877	6.83E-05
XM_021083612.1	LOC102167481	4.307329713	1.33E-12
XM_005673453.2	AP1S2	4.27391834	3.34E-10
NM_001243913.1	RCAN1	4.264736223	3.72E-09
NM_001164649.1	SLC1A1	4.261836389	4.73E-05
XM_005672396.3	RETREG1	4.203988684	9.07E-04
XM_021065888.1	TPP2	4.174131117	0.001067319
XM_013989935.2	BRCA1	4.149392283	3.65E-04
XM_005652818.3	SLC39A3	4.113348474	2.04E-12
XM_021086294.1	SRRM3	4.042676601	1.39E-06
XM_021068276.1	GALNT15	3.985779679	2.15E-09
XM_005670521.3	EP400	3.966973773	1.35E-05
XM_001929040.5	LOC100158115	3.940234433	3.26E-04
NM_001128479.1	TRIM39	3.927743213	9.98E-04
XM_013998063.2	FGD6	3.917290108	2.65E-04
XM_021076311.1	LRP2	3.835405532	2.42E-04
NM_214416.1	IYD	3.8303907	5.29E-04
XR_002340566.1	AP1S2	3.827361832	1.80E-08
XM_021075903.1	SLC4A10	3.746436836	2.59E-06
XM_021070373.1	BOC	3.743388106	0.00103227
XM_013994570.2	AMPD3	3.684492108	1.45E-09
XM_013993101.2	TGM5	3.677579035	0.001256836
XM_013984313.2	ZNF385B	3.621807401	3.17E-05
XM_021083369.1	OBSCN	3.580989572	6.07E-08
XM_003133338.5	CENPU	3.558856877	0.001240613
XM_021073384.1	ABLIM1	3.46381257	9.21E-05
XM_021080895.1	LOC102162178	3.452403076	2.08E-07
XM_021073710.1	ABCC2	3.427946782	0.001086554
XM_021092435.1	ANO2	3.416420719	1.65E-04
XM_005662216.3	MMP25	3.388028149	7.42E-06
XM_021083466.1	HOMER3	3.358727196	1.22E-11
NM_001172368.1	CSRP3	3.330774942	6.59E-04
XR_002336387.1	MLLT10	3.310919891	2.25E-05

XM_021078074.1	ACSS1	3.240991487	6.98E-04
XM_021064106.1	ESRRG	3.165441688	0.001065942
XM_021101729.1	C8H4orf54	3.145792805	3.45E-08
XM_021086967.1	LOC100737768	3.122280937	8.16E-05
XM_021079753.1	CYP4F55	3.085266986	4.16E-04
XM_013998020.2	MYBPC1	3.077053295	2.87E-04
XM_021081309.1	LOC106506226	3.01831759	3.76E-04
NM_001206447.1	GALNT15	2.990216514	5.51E-08
XM_021075469.1	CERS6	2.986984813	5.21E-06
XM_013984309.2	ZNF385B	2.986534918	0.001006483
XM_005656367.3	TTC9	2.98637623	3.33E-19
NM_001001546.2	MBP	2.95769955	6.65E-07
XM_005668021.3	PTCH1	2.930334485	5.15E-06
NM_001099931.1	FABP3	2.919151334	5.54E-04
XM_013994568.2	AMPD3	2.899034957	1.00E-13
XM_021074089.1	EXTL3	2.886725402	1.42E-06
XM_021078618.1	PRKAG2	2.830586155	1.04E-08
NM_001243465.1	PM20D1	2.826732625	1.00E-04
NM_214152.2	PPARD	2.825732156	3.89E-08
XM_021070435.1	PRRC2B	2.802400367	2.32E-05
XM_021078951.1	FLNC	2.765335993	1.22E-13
XM_003122877.5	C2H11orf91	2.757766681	3.73E-05
XM_021097872.1	SLA-2	2.756366494	1.41E-06
XM_005667164.3	PAK1	2.706336053	8.07E-04
XM_005653086.3	LOC110255218	2.702897582	1.50E-17
XM_021093903.1	FAM192A	2.673317356	6.95E-06
XM_021064121.1	ESRRG	2.671425511	1.84E-07
XM_003134700.5	FLNC	2.64362394	5.84E-41
NM_001001625.2	DIO3	2.633353133	1.28E-04
XM_005653084.3	LOC110255218	2.63001684	1.74E-07
XM_021090323.1	KYAT3	2.624135628	3.95E-04
XM_001928852.5	PXDC1	2.622581905	4.63E-04
XM_003482749.4	ATF3	2.618174008	7.15E-06
XM_021064126.1	ESRRG	2.58735819	0.001067118
XM_013994566.2	AMPD3	2.568929223	2.09E-05
XM_003359728.5	RETREG1	2.564297572	4.79E-08
XM_021071104.1	THUMPD3	2.558549765	4.92E-04
XM_021083364.1	OBSCN	2.546245498	9.67E-54
XM_021080733.1	FLNA	2.53643537	1.41E-07
XM_021064128.1	ESRRG	2.523068817	4.42E-04
XM_003132123.4	CSRNP1	2.507019056	4.75E-10
XM_021080692.1	PHKA1	2.506151938	3.27E-07
XM_021076885.1	PDE4D	2.494495237	1.41E-07
NM_001128442.1	GATM	2.493279847	0.00120608
XM_013986010.2	ACOT9	2.456600651	0.001320296
XM_021070197.1	LRCH3	2.44673351	6.01E-04
NM_001143928.1	XIRP1	2.411170306	2.24E-17
XM_021067983.1	FLII	2.386571809	1.64E-20
XR_002340567.1	AP1S2	2.368582869	0.001055499
NM_001135960.2	ABRA	2.361625231	4.36E-10
XM_005657042.3	PER1	2.335524222	9.93E-04
XM_021081625.1	TRAF3	2.323780772	4.55E-04
XM_021098329.1	TNXB	2.32106685	8.19E-05
XM_003124245.4	ZFAND2A	2.319421626	1.13E-06
NM_001243388.1	HSPB7	2.307813001	1.93E-09
NM_213910.1	SERPINE1	2.28842103	6.01E-05
XM_001927113.6	ACSS1	2.27019517	1.26E-05
XM_021071028.1	SLC37A1	2.262980527	1.04E-04
NM_213766.1	HSP70.2	2.259542	4.04E-145
XM_021087142.1	MAP4K4	2.249170345	4.05E-05

XM_021097094.1	GNAO1	2.248981865	0.001395886
XM_021064407.1	ENAH	2.244041783	1.61E-05
NM_214339.1	DNAJA4	2.243214006	2.57E-06
XM_021065531.1	KATNAL1	2.237732978	2.47E-04
XM_021093492.1	LEPR	2.228967987	0.001177841
XM_021073156.1	LYST	2.227188417	2.25E-05
XM_021083604.1	LOC110255237	2.224526312	1.97E-04
XM_021080046.1	ARHGAP6	2.218025988	0.001023665
XM_013993244.2	ATP6V1H	2.194954521	4.18E-06
XM_021078573.1	CHN2	2.181775159	0.00117401
XM_003127538.6	SLC25A33	2.174046053	9.78E-05
XM_021072199.1	NRAP	2.164587205	8.54E-13
NM_001201485.1	BAMBI	2.161614152	5.39E-04
XM_013998685.2	VRK3	2.157370132	5.49E-05
NM_001115155.1	GPX3	2.154452184	2.98E-07
XM_005660341.3	WDR31	2.154446997	1.27E-04
XM_021062779.1	CRYAB	2.150372444	1.57E-14
XM_021074134.1	SORBS1	2.141428728	5.81E-06
XM_021073990.1	AGT	2.135598236	1.50E-43
XM_021088451.1	SLC16A1	2.131756699	2.97E-04
XM_005657916.3	FHL1	2.126217372	1.50E-04
XM_003128736.6	LOC100518848	2.108295185	8.56E-08
XM_021079523.1	FHL1	2.107211757	2.96E-06
XM_003355228.4	ZNF697	2.106356238	2.59E-05
XM_021101100.1	TBC1D1	2.074576015	0.00102273
NM_214248.2	HSD11B1	2.073362084	4.66E-05
XM_021086861.1	UNKL	2.071119362	3.49E-04
XM_021087080.1	SHPRH	2.070870327	3.53E-05
XM_021070855.1	EIF4E3	2.064380145	9.44E-08
XM_003127275.4	PPP1R15A	2.063523604	8.46E-06
XM_021065461.1	SGCG	2.04775214	9.08E-04
XR_002346865.1	PDLIM5	2.045054163	2.89E-13
XM_021064550.1	PPP1R12B	2.033549775	3.51E-14
NM_214284.1	SDC4	2.023087266	5.02E-17
XM_021096911.1	P3H1	2.018031891	2.48E-07
XM_021067675.1	P2RX5	2.006727195	8.28E-05
XM_021077487.1	AAR2	1.997622964	1.07E-04
XM_021071989.1	LGALS8	1.978922973	6.53E-04
XM_021091003.1	RBFOX2	1.975351471	1.55E-04
XM_001925989.7	ABHD2	1.970462824	1.25E-07
XM_005660756.3	TRPT1	1.960796267	1.36E-05
XM_021074130.1	SORBS1	1.948759414	7.54E-06
XM_021092511.1	MICAL3	1.947925814	2.73E-06
XM_003134953.5	AP1S2	1.947687203	1.91E-11
XM_021073458.1	BAG3	1.945541912	9.87E-53
XM_021086421.1	ZNF48	1.943286004	3.09E-08
XM_021090294.1	LRRC8D	1.924641386	8.09E-05
XM_013980473.2	VPS36	1.924632079	3.09E-04
XM_021079951.1	TAF1	1.915409216	1.58E-08
XM_013998232.2	LOC100523672	1.912115379	8.08E-11
XM_021068059.1	MAP2K3	1.893675012	1.19E-04
XM_013987795.2	UNKL	1.886373714	3.19E-05
XM_003355593.5	LPCAT3	1.870826079	4.39E-15
XM_021072969.1	CLIP1	1.846555256	1.42E-05
XM_021064561.1	PPP1R12B	1.846206768	1.60E-16
XM_003135260.5	BHLHB9	1.843178274	9.35E-04
XM_001924465.4	MAFF	1.815967756	4.39E-08
NM_001190276.1	NR4A2	1.808113568	3.98E-04
XM_005664689.3	PPP1R15A	1.799895888	1.96E-07
XM_003127724.4	TENT5B	1.796678338	2.73E-10

NM_001142667.1	SLC19A2	1.794701413	1.92E-10
NM_001007518.1	HSPB1	1.78136924	8.40E-09
NM_001044588.1	FBXO32	1.779451685	3.77E-07
XM_005669282.3	BTD	1.779433891	4.46E-04
XM_021091972.1	CPM	1.776945326	3.38E-05
XM_021073816.1	SEMA4D	1.76746377	8.60E-04
XM_001924347.5	SLCO3A1	1.760219096	5.68E-04
NM_001044526.1	PPARA	1.754195949	2.09E-04
XM_021080298.1	KLHL34	1.752965992	1.09E-04
XM_021073998.1	ANXA11	1.740109732	3.76E-06
XM_003359949.4	TP53INP2	1.736832809	4.55E-04
NM_213833.2	CTGF	1.727806053	5.73E-09
XM_021064404.1	ENAH	1.726490854	3.14E-04
XM_003134726.4	LMOD2	1.721135962	2.77E-04
XM_021086791.1	RPL3L	1.72071196	2.23E-04
NM_001244227.1	RGCC	1.716839935	0.001347452
XM_021098332.1	TNXB	1.713106044	5.80E-14
NM_001142839.2	TNFRSF12A	1.709569829	0.001073271
XM_021068593.1	MITF	1.694212752	1.66E-06
XM_005660143.3	TJP2	1.684988096	0.0011628
XM_003126626.6	SLC38A2	1.684045069	2.17E-04
XM_003481472.4	RNF115	1.669908062	2.81E-09
XM_021101862.1	LIN54	1.668227562	1.39E-04
XM_021088564.1	MRPL15	1.662975835	4.52E-04
XM_005657041.3	PER1	1.660407714	1.02E-08
XM_013985297.2	TP53INP2	1.654526465	3.97E-08
XM_003129716.6	DDIAS	1.645375037	9.50E-04
XM_021064562.1	PPP1R12B	1.644698521	1.44E-12
XM_005668414.3	ESD	1.642949931	2.16E-06
XM_003135158.4	FAM155B	1.638208724	4.27E-05
XM_021067563.1	SLC43A2	1.636541749	4.88E-05
XM_021087284.1	IMMT	1.625331448	5.14E-04
XM_021094185.1	CYLD	1.623325297	0.001023987
NM_001204773.1	AKAP1	1.620464113	1.29E-04
XM_005668567.3	METRNL	1.615828386	2.99E-19
XM_021064917.1	OTUD1	1.615574395	8.85E-09
NM_001122987.1	HK2	1.613495229	2.52E-05
NM_214366.1	PPP2CA	1.609945047	6.15E-06
XM_021094073.1	FHOD1	1.604458198	0.001114446
XM_001928774.5	COQ10B	1.601352611	2.27E-06
XM_021099578.1	STON2	1.597452177	4.20E-04
XM_021085028.1	RPS14	1.595309106	1.46E-06
XM_021068635.1	UBE2E2	1.594522129	4.73E-08
XM_005660313.3	FSD1L	1.593501899	3.20E-04
XM_013977487.2	SCN4B	1.592270726	2.29E-04
XM_003122538.5	CDC42EP2	1.585569799	1.11E-06
XM_001929325.5	SLC35E4	1.58287906	5.32E-04
XM_021091396.1	NUP50	1.575253501	8.90E-04
XM_003360439.3	ZDHHC9	1.573649219	4.80E-04
XM_021068448.1	NFKBIZ	1.567620986	9.89E-04
XR_002342763.1	SRSF7	1.567428751	2.39E-04
NM_001005154.1	MYC	1.564948468	1.68E-06
XR_002336825.1	SRSF2	1.559249011	1.46E-10
XM_021089761.1	UBAP2L	1.557213306	1.82E-04
XM_021067740.1	RABEP1	1.556627616	6.90E-06
XM_021101866.1	LIN54	1.556411155	9.95E-07
NM_001044616.1	NRAP	1.551252853	6.49E-08
XM_021075318.1	SESTD1	1.54279372	1.63E-07
XM_003133392.4	DUSP4	1.540420379	7.05E-04
XM_003123638.6	GFPT2	1.531619794	1.42E-05

XM_003126927.4	TPPP3	1.529775635	2.04E-04
XM_021067863.1	MYH10	1.529645684	2.32E-04
XM_005653087.3	LOC110255218	1.52765186	6.21E-09
XM_021101103.1	TBC1D1	1.524432137	5.95E-06
XM_021064473.1	TFB2M	1.522812145	0.001126747
XM_003125930.4	CLCA2	1.521753019	5.51E-05
NM_001244679.1	MFAP5	1.518851256	1.22E-05
XM_021085558.1	RTN4	1.518401945	2.53E-04
XM_013989105.2	MPP5	1.517937969	0.001116453
XM_013988306.2	RNF41	1.508333637	1.07E-04
XM_005659678.3	MAP1A	1.50812276	2.86E-04
XM_021072193.1	NRAP	1.50524567	3.05E-06
XM_003134943.3	PIR	1.505071839	2.90E-04
XM_001925471.7	GPC4	1.501935416	1.69E-04
NM_214320.1	CDKN3	1.497208759	2.60E-04
XM_021089760.1	UBAP2L	1.496851859	1.21E-04
XM_021079521.1	FHL1	1.495513906	2.70E-04
XM_021069692.1	MME	1.493675855	0.001340028
XM_005660449.3	ENG	1.493170008	1.42E-04
NM_001184756.1	TRIM63	1.49151604	3.20E-05
XM_001929654.5	VPS72	1.490468182	5.32E-04
XR_002344356.1	LOC110260827	1.487111328	8.79E-13
NM_001159306.1	PDK4	1.48445086	4.15E-05
NM_001244607.1	MLLT11	1.465661859	3.29E-06
XM_021085920.1	CCL24	1.465068995	4.42E-05
XM_021088844.1	PTK2	1.457558501	1.66E-04
XM_001929515.6	MAD2L1BP	1.457295626	4.42E-04
XM_021086247.1	GIGYF1	1.453978554	0.001120367
XM_021077887.1	DLGAP4	1.451142127	5.46E-08
XM_021096799.1	ERI3	1.449048705	4.28E-04
XM_005664189.3	NT5DC3	1.446611917	2.14E-07
XM_003133324.6	ASB5	1.444763413	0.001092447
XM_021071357.1	RFTN1	1.443944507	1.47E-10
NM_214403.1	IL6R	1.44216946	2.10E-04
XM_003358748.4	BCL6	1.43609603	1.35E-04
XM_005654030.3	PFN2	1.43550212	1.40E-04
XM_021077291.1	SDC4	1.434924974	6.51E-04
XM_021085564.1	LPIN1	1.432221732	3.45E-05
NM_001244377.1	PTPMT1	1.431657653	6.51E-04
XR_002345546.1	SLA-2	1.429697533	1.95E-04
XM_021064558.1	PPP1R12B	1.421477321	3.21E-07
XM_021085033.1	SMIM3	1.419688638	7.63E-04
NM_001031782.2	KLF4	1.419642926	2.65E-05
XM_001927740.5	CYR61	1.40955076	2.03E-05
NM_001097504.1	HSPH1	1.403280572	1.06E-21
XM_021096444.1	NEXN	1.402765948	5.70E-04
XM_021074155.1	SORBS1	1.402747244	1.50E-04
XM_021088294.1	PRKCE	1.401981351	8.17E-06
XM_021075698.1	TRAK2	1.400771109	8.23E-07
XM_005661465.3	RNF44	1.396841795	3.13E-04
XM_021074163.1	SORBS1	1.391927743	2.33E-04
NM_001315701.1	BCL6	1.390170638	1.41E-05
XM_005672807.3	NSFL1C	1.385259829	4.01E-04
XM_021089289.1	PDE7A	1.374274941	2.75E-06
XM_003124781.4	OMD	1.372126543	4.10E-14
XM_013992922.2	RNF111	1.372043993	0.001250011
XM_021064229.1	KIAA1217	1.370485924	1.10E-10
XM_005660427.3	ANGPTL2	1.366457462	2.23E-08
XM_021067339.1	PTPN3	1.366043175	0.001022812
XM_021081915.1	LOC102166678	1.365369162	1.03E-05

XM_021089290.1	PDE7A	1.354140766	5.47E-08
XM_021092095.1	DENND5B	1.34685685	3.12E-04
XM_005672225.3	ABCB6	1.346086289	2.99E-05
NM_001142835.1	UTP3	1.340200184	5.69E-06
NM_001113446.1	CHORDC1	1.334130403	2.49E-04
XM_021080568.1	ARHGEF6	1.329871789	4.01E-07
XM_021070783.1	GBE1	1.329686074	5.85E-04
XM_021088726.1	IL6R	1.327448268	1.73E-04
XM_001926129.6	PTGS1	1.306321694	1.85E-04
XM_003124944.4	RETSAT	1.298125017	5.83E-06
XM_005653121.3	LARP4	1.292541327	7.42E-06
XM_021099593.1	BCL2	1.291763403	2.31E-06
XM_021073562.1	PTPN11	1.282613951	4.62E-05
NM_001038001.1	MITF	1.274865832	5.94E-05
XR_002345409.1	CCNL2	1.274519614	4.83E-04
XM_021099599.1	BCL2	1.273179187	1.90E-09
XM_021062544.1	EMSY	1.272058629	1.49E-04
XM_021064549.1	PPP1R12B	1.271638022	1.02E-08
NM_214322.1	NPC1	1.27046898	2.94E-12
XM_021084109.1	DAPK3	1.270192794	1.56E-04
XM_021100121.1	SLC22A23	1.269558402	1.85E-06
XM_021093339.1	GSK3A	1.264594908	1.08E-04
XM_021067625.1	CLUH	1.26447388	9.65E-06
XM_013982357.2	STRADB	1.264412473	2.75E-04
XM_021093723.1	CRISPLD2	1.262994457	4.35E-05
XM_021084262.1	MIDN	1.261319266	2.39E-12
XM_021078616.1	PRKAG2	1.259927756	0.001013819
XM_003480558.4	LOC100739365	1.258642354	4.17E-05
XM_003124009.4	SPRY4	1.256244309	3.11E-04
XM_003353794.4	STIP1	1.25610226	4.46E-05
NM_001044528.2	RNF4	1.255628423	3.50E-07
XM_021066263.1	SRSF2	1.254058971	6.57E-04
XM_013984988.2	VGLL2	1.248569534	1.24E-05
NM_001348525.1	COX17	1.248391681	4.09E-04
NM_001317081.1	SLC38A2	1.245670157	7.74E-04
XM_021090302.1	LRRC8D	1.24193616	4.83E-04
NM_001130734.1	LPIN1	1.241817373	2.48E-05
XM_021095289.1	SPSB1	1.234084244	2.53E-04
XM_021091201.1	PRKAG1	1.229141864	4.97E-04
XM_005669917.2	PCOLCE2	1.223780884	1.99E-04
XM_021084261.1	MIDN	1.223435751	4.46E-04
NM_001025228.1	NCOA1	1.212724448	1.48E-04
XM_003129731.5	PRSS23	1.212119874	0.001106712
NM_214170.1	CD59	1.210589579	5.02E-06
XM_021084204.1	MKNK2	1.208324646	2.44E-04
XM_003123639.4	SQSTM1	1.204257295	9.79E-05
XR_002338307.1	CAMK2G	1.203152027	5.51E-04
XM_013979005.2	DDIT4L	1.202076886	1.88E-04
XM_021070064.1	OPA1	1.201196574	5.87E-09
XM_021096446.1	DNAJB4	1.199663905	2.20E-05
XM_005661957.3	DTX2	1.198238249	5.04E-04
XM_005661031.3	CD59	1.19675939	7.56E-09
XM_021086676.1	MGRN1	1.195890177	3.53E-04
XM_021086694.1	LOC100513346	1.187603156	2.66E-04
XM_003357589.4	VASH2	1.186376906	3.04E-07
NM_214201.1	GPX1	1.180518799	7.72E-09
XM_003360402.4	MORF4L2	1.166389227	0.00115741
XM_021062259.1	ST5	1.158155679	1.00E-05
XM_021094060.1	HERC1	1.15593636	6.17E-04
XM_021062541.1	EMSY	1.155618613	6.04E-04

XM_021101364.1	AASDH	1.15515596	6.32E-04
XM_005664439.3	RIPOR1	1.154667648	3.61E-10
XM_021068859.1	TEX264	1.152586547	8.12E-04
NM_001287420.1	FUS	1.151015212	3.70E-06
XM_021095311.1	UBE4B	1.145304374	1.72E-07
NM_001258352.1	TACC2	1.140072819	2.08E-09
XM_021095989.1	NT5C1A	1.137575516	4.95E-05
XM_005655271.3	E2F6	1.135547222	4.17E-06
XM_021063797.1	LAMC2	1.127612033	8.79E-04
XM_021086273.1	RASA4B	1.124000519	5.36E-08
XM_021092497.1	FBXL14	1.123144191	1.57E-06
XM_003121345.6	AMD1	1.119816109	5.50E-04
XM_013996945.2	MFGE8	1.114222995	9.25E-13
XM_021068513.1	ITIH4	1.113274873	1.93E-04
XM_003483822.4	PLPP1	1.104799457	3.21E-04
NM_001123107.1	DOK5	1.102579	3.66E-04
XM_005660316.3	KLF4	1.09834184	4.18E-05
XM_021067093.1	NFE2L1	1.09662777	5.39E-04
XM_003134179.4	ANKRD33B	1.094836774	6.07E-04
XM_005663698.3	LRRC8D	1.090758155	1.17E-04
XM_021068586.1	MITF	1.089064557	1.92E-06
XM_003124640.4	PPL	1.087961813	2.64E-07
NM_001244672.1	MYF6	1.083991708	2.66E-05
NM_001190294.1	WIPI1	1.083257946	1.87E-08
XM_021077677.1	KIAA1755	1.080970577	1.43E-06
XM_013995985.2	KDM3A	1.080831352	8.69E-04
XM_021068160.1	BCAS3	1.079467546	2.28E-04
XM_021086702.1	SRL	1.076134132	3.46E-05
XM_003362134.4	BORCS8	1.075939532	1.05E-04
XM_021071712.1	ZNF662	1.07479227	5.69E-04
XM_005670304.3	APP	1.069791435	2.95E-05
XM_021099683.1	PAPOLA	1.065620769	0.001239457
XM_021094966.1	LENG8	1.061671411	2.01E-06
XM_021069037.1	SLMAP	1.061140953	5.46E-04
XM_021088467.1	SOGA3	1.058457583	2.97E-04
XM_013984302.2	AFDN	1.049014734	5.00E-04
XM_021083028.1	ZDHHC5	1.048766706	0.001233681
NM_001134349.2	KLF15	1.04814514	1.55E-06
XM_021096442.1	NEXN	1.046299562	7.36E-04
NM_001244948.1	LETM1	1.039865655	4.52E-05
XM_005657051.3	NDEL1	1.038028142	8.35E-05
XM_003133201.6	ATE1	1.024371849	1.43E-04
XM_021090386.1	PLEC	1.020586416	1.32E-04
NM_001011504.1	KLF9	1.020010461	5.91E-05
XM_021085016.1	SLC26A2	1.013665991	4.04E-05
XM_021102574.1	KMT2E	1.013030752	0.001129563
XM_021077888.1	DLGAP4	1.008255897	1.43E-05
XM_021082580.1	AP2A2	1.00328824	4.92E-04
NM_001243555.1	HNRNPH3	1.003185947	8.97E-04
XM_013999339.2	DNAJB4	1.002792335	6.47E-06
XM_001928898.6	SUPV3L1	1.001182843	1.23E-04
XM_021074107.1	FBRSL1	1.000857408	1.13E-04
XM_001926617.6	MMP16	-1.00973568	1.60E-04
NM_001244498.1	SUOX	-1.012650186	1.83E-06
XM_013980521.2	LRCH1	-1.014428714	0.001373299
XM_021074694.1	COL5A2	-1.017676766	9.32E-04
XM_003128120.6	LOC100514469	-1.02252137	7.23E-04
XM_021075634.1	GIGYF2	-1.023518446	0.001005695
XM_005660302.3	TMEM246	-1.027622882	3.36E-07
XM_013985163.2	LZTS3	-1.027681273	1.42E-06

XM_005669518.3	PFKFB4	-1.028598195	2.25E-04
XM_013979025.2	ABCC5	-1.029086819	4.06E-05
NM_001130736.1	ATIC	-1.033066183	3.88E-05
XM_021076836.1	RBM4B	-1.047037621	6.42E-04
XM_021073205.1	RHOBTB2	-1.047182693	1.83E-04
XM_021075786.1	WRN	-1.050570764	1.00E-03
XM_001927705.5	PRKRA	-1.054884301	2.40E-07
XM_021083408.1	TRIM7	-1.066634636	3.21E-05
XM_013997432.2	PKN2	-1.06853347	1.71E-04
XM_003132600.4	FAM43A	-1.074714376	0.001076391
XM_021100024.1	MALT1	-1.081373567	4.24E-09
XM_021096188.1	OSBPL1A	-1.081758743	6.51E-05
XM_013987679.2	C3H7orf50	-1.084057083	1.60E-05
XM_021098873.1	ADAMTSL3	-1.087224959	9.57E-04
XM_005653940.3	CACNB1	-1.093721064	4.15E-04
XM_021094760.1	AP2A1	-1.095645976	1.78E-04
XM_003129834.4	LOC100512977	-1.096575471	1.42E-05
XM_021065813.1	MBNL2	-1.099504576	4.74E-05
XM_013984460.2	IKZF2	-1.103549064	2.21E-04
XM_021088813.1	PTP4A3	-1.113081383	8.88E-04
XM_001924419.5	PGM3	-1.11471368	1.67E-04
XM_005656828.3	OPTN	-1.117095713	0.001221777
XM_021094302.1	TLN2	-1.129101832	2.78E-04
XM_013977970.2	MAD2L1BP	-1.145559203	4.94E-05
XM_021095392.1	LOC110261101	-1.146818937	2.20E-04
XM_013992761.2	LAMA4	-1.149905214	1.93E-04
XM_021067463.1	KSR1	-1.157009666	0.001024462
XM_021069486.1	PPP2R3A	-1.158642419	1.32E-04
XM_021067121.1	LOC102158609	-1.16441098	3.59E-04
XM_021083344.1	OBSCN	-1.168578204	4.51E-09
XM_021068995.1	ARHGEF3	-1.180368624	1.77E-04
XM_021062754.1	ARHGAP20	-1.18268167	4.64E-04
XM_021101177.1	KLHL28	-1.183167158	2.15E-04
XM_021063165.1	IGSF9B	-1.187694699	6.88E-04
XM_021070388.1	PLPP7	-1.194586606	0.001116412
XM_021072598.1	DHX32	-1.212969253	6.11E-07
XM_021079543.1	DMD	-1.213572527	3.20E-04
XM_021072329.1	FRAT2	-1.214581256	1.37E-04
XR_002337304.1	TLCD2	-1.226880203	8.24E-05
XM_001927321.6	ELOC	-1.227550685	1.28E-04
XM_021093887.1	BBS2	-1.228458509	1.87E-05
XM_005668194.3	LOC102164731	-1.232562469	7.01E-04
XM_021066016.1	ACOX1	-1.233611583	2.10E-07
XM_021090557.1	DEPTOR	-1.242959512	3.80E-05
XM_021098387.1	TTC23	-1.253133201	8.15E-04
XM_021076306.1	OBSL1	-1.257559808	1.39E-04
XM_005654047.3	WDR5B	-1.258206921	0.001262529
XM_003482451.4	SEC24D	-1.263570945	5.69E-04
XM_021071473.1	RXRA	-1.270846026	7.34E-04
XM_013993690.2	ALG2	-1.294106431	4.17E-04
XM_021084212.1	LOC110259374	-1.294590811	2.67E-05
XM_003480856.4	LOC100620238	-1.295892454	6.00E-04
XM_005663542.3	TENT5C	-1.296996478	5.18E-06
XM_021079342.1	LANCL2	-1.302084739	1.47E-04
XM_021096057.1	RAB31	-1.309610347	1.57E-08
XM_021070827.1	USP25	-1.310246402	8.04E-06
NM_001101029.1	ALG2	-1.310373016	1.41E-04
XR_002343817.1	GAPDH	-1.31680227	1.41E-04
XM_021065098.1	SLC46A3	-1.324902142	1.62E-04
XM_013979874.2	TPK1	-1.336456293	8.55E-05

XM_021063175.1	THYN1	-1.336791455	1.03E-04
XM_021096633.1	GPX7	-1.338056783	7.07E-04
NM_001243354.1	DHCR24	-1.34500941	4.52E-05
NM_001001636.1	RPL32	-1.346142816	0.001122795
XM_021069094.1	KCTD6	-1.355360753	2.41E-04
XM_013988572.2	HIPK4	-1.373123645	2.59E-05
XM_021085026.1	RPS14	-1.376610072	4.27E-18
XM_003122487.4	SLC29A2	-1.389727311	1.33E-06
XM_005656682.3	CDCA7L	-1.390379889	2.64E-04
XM_013983837.2	CHST15	-1.395777927	3.87E-04
XR_002338876.1	SGPL1	-1.396789777	1.26E-04
XM_021087279.1	RMND5A	-1.400801288	9.14E-04
XM_013983703.2	NEURL1	-1.401309032	3.32E-08
XM_021092837.1	MRTFA	-1.406776758	8.12E-04
XM_021066359.1	TMOD1	-1.423482501	2.00E-07
XM_003125214.4	GEMIN6	-1.427919954	7.14E-04
XR_002343100.1	TBC1D32	-1.446054398	7.44E-05
XM_021088213.1	EPB41L2	-1.452531092	0.001209048
XM_021098907.1	ZSCAN2	-1.467446606	1.12E-04
XM_005670462.3	HMBOX1	-1.471446089	5.09E-04
XM_021073559.1	TMEM233	-1.471564391	4.39E-04
NM_001243574.1	ACAD8	-1.476719152	7.40E-04
XM_021100881.1	UVSSA	-1.480540089	0.00131444
NM_001098588.1	HPS3	-1.483005301	1.66E-04
XM_021102037.1	BZW2	-1.485209475	2.42E-04
XM_021091964.1	DYRK2	-1.485866296	1.42E-04
XM_003129812.6	LOC100522887	-1.486376	2.44E-05
XM_021092589.1	RPAP3	-1.493945535	4.90E-04
NM_001161636.1	CLDN5	-1.496762012	4.42E-05
XM_003356630.4	POLH	-1.499854932	5.69E-07
XM_003358232.4	RABEP1	-1.514726937	3.02E-04
XM_021088304.1	EPB41L2	-1.519524076	7.34E-04
XM_021099785.1	CDKAL1	-1.539141374	2.27E-07
XR_002343004.1	FRRS1	-1.543376079	5.31E-04
XM_021099094.1	NEO1	-1.553745279	1.74E-05
XM_021100502.1	SLC4A4	-1.555449057	1.35E-05
XM_013982866.2	TCTN1	-1.558175543	1.17E-06
XM_013997719.2	BEST3	-1.563406027	3.10E-05
XR_002340201.1	HUS1	-1.567164014	4.10E-04
XM_021099091.1	NEO1	-1.576109682	2.56E-04
NM_001204395.2	IFIT3	-1.577127782	3.40E-04
XM_003127980.4	CDKN2C	-1.591353553	2.40E-04
XM_021081655.1	LDHA	-1.592368404	7.01E-05
XM_021062565.1	KCTD21	-1.597479869	8.36E-04
XM_021082015.1	APLNR	-1.597666445	2.13E-04
XR_002342617.1	ASPRV1	-1.599595776	5.78E-05
NM_001002824.1	MYOD1	-1.607000104	2.32E-05
XM_021080760.1	TAZ	-1.612016689	3.37E-04
XM_003354188.4	ARL10	-1.61593426	1.15E-11
XM_003483868.4	CNOT7	-1.622281847	4.11E-05
XM_003128523.5	DTD2	-1.635572265	2.43E-04
XM_021067128.1	PHOSPHO1	-1.640831376	9.95E-04
XM_021100941.1	MYO9A	-1.642671375	6.15E-04
NM_001128473.1	GBP1	-1.643456621	2.02E-04
XM_021086847.1	IFT140	-1.66487542	0.001188176
XM_021065460.1	SGCG	-1.670938243	5.42E-08
XM_021087553.1	TTC7A	-1.678277514	1.08E-09
XM_005657128.3	VGLL3	-1.678495472	1.45E-05
XM_003125839.4	BCL2L15	-1.684863303	4.47E-06
XM_005668244.3	LOC100625049	-1.703159762	8.03E-09

NM_001038644.1	ANGPTL4	-1.704947066	1.64E-06
XM_021091117.1	TMBIM6	-1.718490009	6.43E-04
XM_021087550.1	TTC7A	-1.719500383	4.00E-04
XM_021084383.1	PDLIM7	-1.73178155	7.26E-04
XM_005671823.3	PPP1R3B	-1.737570686	7.31E-05
XM_021095197.1	TMEM52	-1.748299409	5.95E-06
XM_021068116.1	MAP2K6	-1.751027008	5.66E-23
XM_021076373.1	TENM3	-1.759290449	8.30E-04
XM_021075038.1	PPP1R3B	-1.762376476	7.99E-04
XM_013980816.2	MAP2K6	-1.770223162	7.66E-10
XM_021095352.1	MFN2	-1.775124268	4.27E-10
XM_021098782.1	TJAP1	-1.781046825	7.12E-04
XM_021069370.1	GRIP2	-1.799877064	5.03E-04
XM_005668283.3	TNFRSF19	-1.839469335	1.31E-10
XM_001924329.5	MTHFD1	-1.839783828	3.22E-12
XR_002346427.1	NUDT6	-1.84172449	6.15E-05
XR_001304378.2	RNF157	-1.849080572	2.87E-10
XM_021074201.1	SYNE2	-1.849226591	0.001164142
XM_013994354.2	DDB2	-1.855766191	3.73E-09
NM_213773.1	CXCR4	-1.868079453	7.32E-05
XM_003132758.4	SMIM11A	-1.869531047	6.71E-12
XM_005660928.3	DDB2	-1.871301977	6.10E-04
XM_021071293.1	ZBTB20	-1.881308293	1.63E-05
XM_021099923.1	ZBTB25	-1.885058908	8.20E-04
NM_001243368.1	PPCDC	-1.891708331	0.001408904
XM_005669860.3	KY	-1.905055761	4.35E-05
XM_021093024.1	APAF1	-1.925932415	5.54E-04
XM_021076507.1	ZEB2	-1.93224714	7.96E-05
XM_021087999.1	ALDOA	-1.93426523	4.38E-12
XM_021084631.1	NREP	-1.934725496	2.06E-04
NM_001164858.2	ENHO	-2.003857061	2.22E-06
XM_021076033.1	UBE2E3	-2.009394268	4.49E-04
XM_021095156.1	SAMD11	-2.028710624	5.73E-05
XM_021089485.1	FAM78B	-2.029946615	2.71E-04
XR_298483.3	LOC102167685	-2.038461299	0.001413945
XM_021088981.1	SNTB1	-2.057797428	5.33E-04
XM_021080575.1	IGF2	-2.066171754	1.87E-11
XM_021068522.1	RHOA	-2.068997439	4.26E-04
XM_021093564.1	PRKAA2	-2.088050702	5.90E-10
XM_021067994.1	MIEF2	-2.092663189	8.23E-11
XM_021096832.1	DPH2	-2.109066977	2.58E-05
XM_021069566.1	TFDP2	-2.11347605	1.59E-05
XM_021096042.1	PRELID3A	-2.12434324	0.001003727
XM_005671503.3	FAM45A	-2.171627958	1.02E-05
XM_021071323.1	NUDT16	-2.172429924	5.47E-06
XM_021080818.1	ST6GALNAC4	-2.178256433	4.29E-04
XM_021070156.1	SMCO1	-2.193823944	4.22E-06
NM_001044525.1	KIT	-2.195084256	1.23E-04
XM_021098999.1	TMEM266	-2.200433477	2.43E-06
XM_003482597.4	PANX1	-2.215654795	3.65E-05
XM_021092770.1	CRADD	-2.222547866	8.46E-06
XR_002335967.1	TSEN15	-2.226828575	9.92E-04
XM_021074943.1	IMP4	-2.230551361	8.49E-04
XM_003128550.6	SLC7A8	-2.231728105	6.16E-04
XM_021100395.1	ART3	-2.233681246	4.98E-15
NM_001348942.1	SERPINA1	-2.250919274	0.001178109
NM_213883.2	IGF2	-2.268181338	8.41E-18
XM_021076369.1	TENM3	-2.268637083	6.90E-04
XM_005667302.3	ACAT1	-2.281318773	2.34E-07
XM_021078824.1	CPA5	-2.283514735	1.37E-04

XM_013988307.2	NABP2	-2.2954391	5.47E-05
XM_021094691.1	MAMSTR	-2.300535106	1.17E-04
XM_021099746.1	LOC110261647	-2.329544968	0.001203275
XM_021076032.1	UBE2E3	-2.335613178	1.23E-05
XM_005669537.3	KLHDC8B	-2.348042454	1.51E-04
XM_021100026.1	LOC110261735	-2.382448507	3.04E-05
XM_021068256.1	COX17	-2.399088878	1.35E-05
XM_021098438.1	LOC100515902	-2.403201879	2.12E-05
NM_001044618.1	AKR1C3	-2.424735154	1.15E-05
NM_001123132.1	ZFP57	-2.427323334	2.73E-04
XM_003355634.4	TMEM106C	-2.431863114	2.96E-10
XM_021100267.1	AIMP1	-2.44252026	1.31E-07
XM_021079567.1	ITGB1BP2	-2.442894373	1.09E-37
XM_021072762.1	SH3PXD2A	-2.462349695	2.40E-07
XM_003356177.4	FAM43B	-2.524026332	3.89E-05
XM_005666276.3	ABHD4	-2.582965954	2.30E-04
XM_021074088.1	EXTL3	-2.605325399	4.72E-07
XR_002337461.1	MASP1	-2.611027796	7.95E-07
XM_021080576.1	IGF2	-2.630210893	1.37E-10
XM_021101480.1	LOC102167454	-2.644998727	1.64E-12
XM_021071494.1	GADL1	-2.650331086	1.23E-04
XM_013988899.2	CDKN2C	-2.657218009	9.31E-06
XM_021084632.1	NREP	-2.665053149	1.51E-14
XR_002336602.1	MICU2	-2.675890951	3.19E-11
XR_002337296.1	SUSD1	-2.680369959	2.07E-04
XM_021089937.1	ANKRD34A	-2.702789024	2.95E-05
XM_021099123.1	PKM	-2.733557111	1.57E-04
XM_021083409.1	TRIM7	-2.760270904	3.22E-06
XM_021084816.1	FAM13B	-2.803725178	1.28E-05
XR_002346855.1	C8H4orf54	-2.833153297	6.35E-23
XM_021065587.1	LRCH1	-2.861125801	8.78E-08
XM_021064299.1	SMARCA2	-2.86553423	1.54E-14
XM_021091815.1	LOC100512656	-2.898929418	4.53E-04
XM_005656496.3	BOD1L1	-2.903123384	1.53E-08
NM_001278755.1	GNLY	-2.916966659	9.59E-04
XM_013988563.2	AKT2	-2.938691008	3.56E-07
XM_013993913.2	LRRC8A	-3.037225766	8.84E-04
XM_021084909.1	PCDHB6	-3.037804506	1.61E-05
XM_021068032.1	MPRIP	-3.04850653	6.71E-04
XM_013993165.2	EXD1	-3.063798654	5.47E-05
NM_214060.1	ESD	-3.078563609	7.24E-15
XM_021089012.1	PKHD1L1	-3.103887413	7.53E-07
XM_021071095.1	LOC100623720	-3.108027055	3.07E-12
XM_021078825.1	CPA4	-3.185646703	3.96E-04
XM_021085918.1	KCNS3	-3.186607356	4.82E-06
XM_021072773.1	NEURL1	-3.192323955	3.60E-14
XM_021082841.1	EHBP1L1	-3.19824794	1.15E-06
XM_013978113.2	GRAMD2A	-3.230597501	5.27E-05
NM_001244296.1	P2RY6	-3.303885172	5.58E-04
XM_013983701.2	NEURL1	-3.484518976	1.84E-19
XM_003122506.5	GAL3ST3	-3.670406546	6.45E-04
XM_021091386.1	ATXN10	-3.686138473	2.29E-08
XM_021095161.1	PERM1	-3.776793678	2.81E-04
XM_021096199.1	CHST9	-3.847331074	2.30E-06
XM_005670072.3	GMNC	-3.867917366	0.001153115
XM_021095613.1	CNKSR1	-3.975284496	7.24E-04
XR_002345229.1	B4GALT2	-4.017668857	1.50E-05
XM_021100501.1	SLC4A4	-4.118801418	2.98E-28
XM_021093738.1	NECAB2	-4.30176751	9.87E-08
XM_021093026.1	APAF1	-4.477990917	2.70E-06

NM_001136511.1	HUS1	-4.573817745	9.26E-05
XM_021101481.1	ANXA3	-4.638190994	6.37E-04
XM_021081809.1	TNNT3	-4.68067757	2.21E-05
XM_021076761.1	MAST4	-4.722738891	9.81E-05
XR_002336272.1	IGFN1	-4.764576469	3.71E-04
XM_021074788.1	LOC110256933	-4.897807315	9.10E-04
XM_013978706.2	TKTL2	-4.901219306	1.86E-06
XM_021102128.1	FERMT2	-4.931631859	9.98E-04
XM_021080585.1	IGF2	-4.961226962	1.14E-19
NM_214094.2	OCA2	-5.02902342	4.68E-11
XM_021084249.1	GAMT	-5.041967379	2.94E-10
XM_021090593.1	OXR1	-5.130005134	3.82E-04
XM_021095957.1	MRPS15	-5.193137638	0.001162743
XM_021083365.1	CILP	-5.244511423	4.53E-04
XM_021067697.1	LOC100520452	-5.316252287	1.19E-04
XM_021073192.1	ADD3	-5.342254306	7.81E-04
XM_021076184.1	ALS2	-5.350052155	2.18E-04
XR_002336047.1	INTS7	-5.61562311	3.72E-04
XR_002337476.1	LOC110256218	-5.741418743	3.45E-17
XM_005669326.3	GADL1	-5.784702237	2.09E-09
XM_003353151.4	DLL1	-6.185610212	9.35E-04
XM_021072575.1	PPP3CB	-6.228823559	3.09E-08
XM_021090994.1	CCDC91	-6.234411426	6.64E-04
XM_021065649.1	DIAPH3	-6.251722803	0.001021061
XM_021084525.1	TMEM161B	-6.25321218	8.47E-04
XM_021063227.1	MDM4	-6.281651299	7.06E-04
XM_021080628.1	FRMPD4	-6.305571109	5.67E-04
XM_021086780.1	NTHL1	-6.325739348	7.79E-04
XM_013996625.2	RIPK2	-6.326842854	0.001140773
XR_002335884.1	LOC106504983	-6.331560217	4.42E-04
XM_013996632.2	WWP1	-6.367917053	3.69E-04
XM_005653068.3	GJA5	-6.370425358	3.66E-04
XM_021098780.1	TJAP1	-6.371915099	4.40E-04
XM_021093392.1	DAPK2	-6.402684753	0.001334461
XM_021098269.1	ARPIN	-6.41399853	5.69E-04
XR_002340795.1	DIAPH2	-6.425992297	3.87E-04
XM_021087441.1	ARFGEF3	-6.431957521	4.17E-04
XM_003128105.4	CTPS1	-6.432122532	8.95E-04
XR_002346872.1	PPM1K	-6.455503847	5.95E-04
XR_002339527.1	SAP30L	-6.457511638	7.70E-06
XM_013994866.2	RGS14	-6.460502872	2.58E-08
NM_001004033.1	SLC52A2	-6.465224155	9.98E-04
XM_021094524.1	GEMIN7	-6.475271077	8.04E-04
XM_013995569.2	TPST1	-6.482897179	2.02E-04
XM_021062379.1	LOC110255376	-6.493203767	2.25E-05
XM_005668096.3	ARHGAP12	-6.495115014	4.06E-04
XM_021073341.1	CCSER2	-6.497124265	9.12E-04
XM_021062721.1	GRIA4	-6.502388451	2.33E-04
XM_005670579.3	TCTN2	-6.507542605	3.49E-04
XM_021085375.1	ADGRV1	-6.519943015	1.28E-05
XM_021094996.1	BRSK1	-6.519978875	3.52E-04
XM_021088206.1	ARID4A	-6.538001574	6.01E-04
XM_021065751.1	MYCBP2	-6.55308149	1.72E-04
XM_013988149.2	CTTNBP2NL	-6.570325137	2.72E-04
XM_013997693.2	LOC106510322	-6.577977246	4.88E-04
XM_021079792.1	CYP4F55	-6.599864824	6.74E-04
XM_013997934.2	KIF21A	-6.604947327	1.68E-04
XR_002343863.1	PACSIN2	-6.622960229	2.78E-04
XM_021068945.1	NEK4	-6.639819603	6.17E-04
XM_003133063.4	SGPL1	-6.641735825	1.17E-05

XM_021102111.1	DRD2	-6.684726065	9.42E-05
XM_021094576.1	FBXO46	-6.702705759	1.13E-04
XM_021074801.1	DOCK10	-6.750333632	2.30E-04
XM_005668974.3	RAD51C	-6.758683004	7.88E-05
XM_021085759.1	LMAN2L	-6.765252662	5.88E-05
XM_021084593.1	NUDT12	-6.769226398	1.78E-04
XM_021100923.1	HAUS3	-6.793865927	9.89E-04
XM_021089935.1	ANKRD34A	-6.837397418	7.46E-05
XR_002339456.1	DNAJC10	-6.841932303	3.85E-06
XM_021098110.1	UROC1	-6.844032524	1.51E-04
NM_001193447.1	ODF2	-6.865327153	1.72E-04
XM_021095795.1	ADGRB2	-6.880449835	3.98E-05
XM_003130090.4	B3GAT1	-6.898790746	2.22E-05
XM_021102507.1	HDAC9	-6.89879826	1.65E-05
XM_021064180.1	SDCCAG8	-6.927635174	2.32E-04
XM_021063581.1	FREM1	-6.92999333	9.45E-05
XM_021094426.1	TMEM145	-6.940975299	6.16E-05
XM_005671336.3	ALDH18A1	-6.942692556	5.80E-05
XM_021099128.1	GRAMD2A	-6.955667856	2.92E-08
XM_005670500.3	LOC100154415	-6.986930264	9.83E-05
XM_003354417.4	PGGHG	-7.009886351	6.70E-04
XM_021070801.1	MAGI1	-7.021449972	5.73E-04
XM_021072600.1	FANK1	-7.032225201	1.19E-04
XM_021067771.1	MINK1	-7.097650584	1.68E-04
XM_003126516.5	CLSTN3	-7.10222096	1.22E-04
XM_021069855.1	ZMAT3	-7.113502883	7.16E-06
XM_021096785.1	TMEM53	-7.12225311	2.53E-05
XM_013989545.2	ZC3H11A	-7.139320611	6.81E-06
XR_002342084.1	LOC110259698	-7.177674899	1.43E-04
XM_021100785.1	ARHGAP24	-7.191621469	2.01E-05
XM_021076157.1	LIMS2	-7.233537031	1.70E-04
XR_002338546.1	ATXN2	-7.238170565	1.41E-06
NM_001123084.1	BLM	-7.243432422	4.33E-05
XM_021096703.1	PPIP5K1	-7.260710457	7.63E-06
XM_003127917.5	FUBP1	-7.261823313	2.65E-05
XM_005660785.3	TTC9C	-7.270269606	5.05E-06
XM_003121797.4	DDHD1	-7.287501132	5.22E-04
XM_021086807.1	MAPK8IP3	-7.302452768	9.18E-05
XM_005663708.3	LOC102161784	-7.313936462	1.00E-05
XM_005660367.3	AKNA	-7.314918257	3.79E-04
XR_002344811.1	PPP1R12C	-7.326384813	2.36E-06
XM_021094701.1	TCF12	-7.338236163	2.22E-06
XM_021099436.1	SIPA1L1	-7.369469144	5.93E-04
XM_021073014.1	ZNF239	-7.37152228	1.73E-04
XM_021100343.1	TRMT10A	-7.401466477	1.86E-06
XM_021066599.1	RNF157	-7.412205539	1.04E-05
XM_021074332.1	NUDT13	-7.412508552	6.96E-07
XM_021081324.1	TNRC6A	-7.422303332	4.70E-06
XM_021098308.1	TSHR	-7.426683416	1.02E-05
XR_002343984.1	LOC100626199	-7.440815787	3.76E-04
XM_021101204.1	DCUN1D4	-7.464209971	2.43E-05
XM_013994161.2	CDK10	-7.469472557	1.88E-05
XM_013982820.2	MED10	-7.474319389	1.64E-04
XM_021100269.1	AIMP1	-7.48331627	7.17E-05
XM_013998997.2	CASP9	-7.494066932	3.52E-06
XM_021100763.1	HERC6	-7.497225114	8.06E-05
XM_021065376.1	FARP1	-7.500696933	4.03E-06
XM_013983480.2	RASSF4	-7.505433692	3.33E-06
XM_021070122.1	PIGZ	-7.50984747	1.23E-05
XM_013977556.2	SLA-1	-7.513247337	1.35E-10

XM_021073375.1	ABLIM1	-7.574409056	3.23E-06
XM_021088253.1	NRXN1	-7.597871473	5.23E-04
XR_002339430.1	USP40	-7.607648644	5.19E-06
XM_021079906.1	NRK	-7.627593932	2.75E-06
XM_021080582.1	IGF2	-7.629361637	4.95E-07
XM_021093439.1	TCF4	-7.691937396	2.54E-06
XM_021070125.1	PIGZ	-7.692898748	2.83E-05
XM_021085181.1	MADD	-7.698834672	8.98E-05
XM_003358694.4	FXR1	-7.717060873	2.36E-07
XM_021078612.1	PRKAG2	-7.72515599	5.17E-06
XR_002336823.1	SRSF2	-7.728240428	3.00E-05
XM_021099615.1	RPS6KA5	-7.736451765	2.94E-07
XR_002343356.1	LOC106510102	-7.737198648	2.77E-06
XM_013978574.2	ARAP2	-7.747300429	4.00E-05
XM_021093571.1	PGM1	-7.764412041	8.19E-04
XM_021085686.1	PTCD3	-7.766128794	1.96E-06
XM_021101859.1	FRMD6	-7.792025823	5.15E-06
XM_005661867.3	ZNF655	-7.798078119	1.06E-07
XM_021070845.1	FOXP1	-7.846731754	1.57E-06
XM_021099953.1	RGS6	-7.878984219	2.55E-05
XR_002342566.1	DUSP11	-7.893072973	8.26E-08
XM_021089019.1	EMC2	-7.991428002	8.75E-08
XM_021072892.1	ZNF140	-8.027899353	3.84E-06
XR_002343352.1	LOC106510102	-8.031986934	1.12E-07
XR_002344898.1	TARDBP	-8.041565523	2.91E-06
XM_021080648.1	IGF2	-8.066590296	1.28E-09
XM_021081835.1	TNNT3	-8.071680547	4.41E-07
XM_013998854.2	ZNF470	-8.083313308	6.18E-08
XM_021079903.1	NRK	-8.090873501	1.20E-08
XM_021088964.1	TBC1D31	-8.126506673	8.23E-09
XM_021063371.1	PPP1R9A	-8.132700638	8.85E-04
XM_021101371.1	AASDH	-8.142341805	2.41E-07
XM_021067679.1	P2RX5	-8.167348516	1.74E-06
XM_013995929.2	MAP4K4	-8.192462276	5.07E-08
XM_013977870.2	C7H6orf89	-8.239431807	1.24E-07
XM_021062829.1	NCAM1	-8.25821249	7.08E-08
XM_021080170.1	ENOX2	-8.26662517	1.68E-06
XM_021091515.1	CARD10	-8.294501437	4.90E-06
XM_021074083.1	KNTC1	-8.2953299	9.54E-10
XR_002339052.1	DNAJB2	-8.313784823	1.12E-05
XM_021088840.1	PTK2	-8.314774955	1.45E-09
XM_021091002.1	RBFOX2	-8.322359434	2.35E-04
NM_001206347.1	POSTN	-8.323196715	1.57E-05
XM_021070376.1	BOC	-8.361711906	2.76E-08
XM_021087634.1	VIT	-8.362760489	7.82E-07
XM_021092369.1	COL12A1	-8.391242676	6.01E-09
XM_021090923.1	C5H12orf4	-8.40269463	1.22E-06
XM_003358761.4	CEP19	-8.426635256	5.59E-09
XM_021076700.1	ZNF622	-8.447663629	1.56E-06
XR_002341441.1	LOC100511545	-8.474558342	1.65E-08
XM_021096052.1	LOC110261162	-8.484763763	5.52E-07
XM_021096457.1	ZZZ3	-8.488063869	2.55E-07
XM_021079618.1	STARD8	-8.50034616	2.78E-08
XM_021097326.1	PLA2G4E	-8.567195919	5.92E-08
XM_005669824.3	ABTB1	-8.622199881	8.01E-08
XM_021100896.1	MYO9A	-8.636933363	4.39E-10
XM_013986424.2	BCORL1	-8.653372071	1.81E-06
XM_021100557.1	CCDC149	-8.65391248	2.69E-10
XM_021089358.1	LYN	-8.683351125	1.22E-08
XM_021076389.1	LOC100525433	-8.727095686	1.62E-09

XM_021075266.1	HDLBP	-8.75368988	1.21E-08
XM_021096653.1	CC2D1B	-8.76135076	3.25E-08
XM_013999427.2	EFCAB14	-8.804853609	2.04E-09
XM_013996610.2	RBM12B	-8.889588631	2.48E-06
XR_002343354.1	LOC106510102	-8.900876632	4.97E-10
XM_021090528.1	TSTA3	-8.907645066	3.17E-09
XM_021070589.1	MAP4	-8.908463269	0.001359457
XM_005656649.3	PRELP	-8.923664959	4.44E-08
XM_021084221.1	FOXO3	-9.00078075	0.001203887
XM_021068494.1	MFN1	-9.008277122	9.62E-08
XM_021071615.1	CLASP2	-9.013899983	2.75E-11
XM_021073984.1	EIF4ENIF1	-9.026130226	1.35E-10
XM_021078375.1	H2AFY	-9.036738523	0.001034241
XM_013988083.2	DDR2	-9.078915445	6.57E-12
XM_005653070.3	BCL9	-9.096452526	2.28E-12
XM_021102050.1	SLC37A4	-9.108877166	4.51E-10
XM_003134660.5	LRGUK	-9.16710064	1.52E-11
XM_021084332.1	HNRNPH1	-9.22695876	3.38E-05
XM_021085584.1	NCK2	-9.230423479	7.09E-04
XM_021090687.1	LOC100624559	-9.273252342	6.66E-04
XM_021075779.1	GPD2	-9.287429702	1.25E-12
XM_021063042.1	SCN3B	-9.287474449	2.31E-10
XM_021101934.1	ATM	-9.332342337	1.90E-12
XM_021101150.1	APBB2	-9.347381142	8.34E-11
XM_021079901.1	NRK	-9.353450082	2.98E-12
NM_001243580.1	MTRF1	-9.484760699	7.33E-14
XM_021073936.1	THSD4	-9.516906253	4.37E-11
XM_021097888.1	BRD2	-9.554643645	1.24E-09
XM_005660225.3	RGP1	-9.561624555	9.09E-13
XM_021076981.1	GPBP1	-9.566660372	4.62E-04
XM_021095294.1	CLSTN1	-9.602149248	1.96E-10
XM_021084318.1	RASGEF1C	-9.650971866	2.39E-11
XM_021080081.1	ATRX	-9.667658651	1.50E-10
XM_005657715.3	LOC100519808	-9.762198567	2.48E-04
XM_003135516.4	LOC100516587	-9.809681587	2.74E-04
XM_021068612.1	EIF4G1	-9.832950513	2.24E-04
XM_021080739.1	FLNA	-9.848592105	1.79E-13
XR_002342933.1	WNK2	-9.883465187	4.63E-12
XM_005670302.3	APP	-9.929989686	6.46E-13
XM_021062325.1	LOC110255360	-9.935496168	6.16E-15
XM_013984766.2	LOC100737060	-10.0393684	1.94E-04
XM_021087675.1	EYA4	-10.0493669	1.45E-04
XM_021068031.1	MPRIP	-10.05896346	1.06E-06
XM_021091966.1	DYRK2	-10.09590565	2.79E-06
XR_002343357.1	LOC106510102	-10.10003258	2.67E-14
XM_021072578.1	PPP3CB	-10.15951945	1.31E-04
XM_021080641.1	IGF2	-10.55693293	1.36E-13
XM_021077880.1	DLGAP4	-10.83478432	4.96E-16
XM_021064406.1	ENAH	-11.27400864	3.29E-17
XM_021066636.1	TMEM94	-11.48144484	1.13E-17
NM_001025218.2	ATP5MC1	-13.26907238	9.18E-24
XM_021083776.1	LOC100738906	-21.2485908	5.49E-08
XM_005659128.3	TIAM2	-21.37898342	4.55E-08
XM_021063618.1	PPFIA4	-21.38370174	4.52E-08
XM_021070587.1	MAP4	-21.39305681	4.45E-08
XM_021070131.1	PIGZ	-21.41247065	4.34E-08
XM_005670893.3	SPECC1L	-21.43856315	4.17E-08
XM_021081348.1	RBBP6	-21.44067571	4.16E-08
XM_021073544.1	CIT	-21.44082266	4.16E-08
XR_002344854.1	FAM214A	-21.47055057	3.98E-08

XR_002337811.1	CCDC50	-21.47099105	3.98E-08
XM_021094296.1	CEBPG	-21.48424374	3.90E-08
XM_021076138.1	ITPRID2	-21.54965931	3.55E-08
XM_021101143.1	APBB2	-21.60015649	3.29E-08
XM_021082575.1	TSPAN4	-21.60071646	3.29E-08
XM_021090660.1	LOC100624559	-21.60071646	3.29E-08
XM_021096709.1	EFCAB14	-21.60071646	3.29E-08
NM_001005157.1	DDX39B	-21.60801811	3.25E-08
XM_021073640.1	JMJD1C	-21.64922127	3.06E-08
XM_013982220.2	ZMAT3	-21.66291004	3.00E-08
XR_002338758.1	PDCD11	-21.66585142	2.98E-08
XM_021064463.1	KANK1	-21.68994624	2.88E-08
XM_021079541.1	ACSL4	-21.69130157	2.88E-08
XM_005669399.3	ACKR2	-21.72146355	2.75E-08
XM_021093863.1	WWP2	-21.7452132	2.66E-08
XM_021100313.1	RASGEF1B	-21.7452132	2.66E-08
XM_021064743.1	JCAD	-21.76978464	2.56E-08
XM_021067917.1	AKNA	-21.76978464	2.56E-08
XM_021070614.1	DHX30	-21.81483063	2.40E-08
XM_021087403.1	TIA1	-21.82036968	2.38E-08
XM_021090072.1	SORT1	-21.82676669	2.35E-08
XM_005656806.3	AGTPBP1	-21.83260737	2.33E-08
XM_021081617.1	BRF1	-21.85145178	2.27E-08
XM_021073985.1	EIF4ENIF1	-21.86036389	2.24E-08
XM_013993939.2	PPP6R2	-21.86182894	2.24E-08
XM_021063444.1	KLHL7	-21.88275099	2.17E-08
XM_013994691.2	SMARCA4	-21.91239752	2.07E-08
XM_021066036.1	MYH4	-21.91553898	2.06E-08
XM_021068286.1	USP19	-21.93213095	2.01E-08
XR_002336185.1	RAB3GAP2	-21.93785037	2.00E-08
XM_021099177.1	ARHGAP5	-21.99949246	1.82E-08
XM_021097493.1	GNB1	-22.00961843	1.79E-08
XM_013984362.2	ANKRD44	-22.0146198	1.78E-08
XM_021083866.1	DOCK6	-22.01756628	1.77E-08
XM_021088884.1	PHF20L1	-22.03946502	1.71E-08
XM_021073600.1	RHOBTB1	-22.04611955	1.70E-08
XM_021094795.1	POLD1	-22.07212969	1.63E-08
XM_021077849.1	HOOK3	-22.08897689	1.59E-08
XM_021097491.1	GNB1	-22.08924085	1.59E-08
XM_021077869.1	CHD6	-22.13649089	1.48E-08
XM_021083436.1	HAPLN4	-22.13649089	1.48E-08
XM_021084995.1	ABLIM3	-22.23020693	1.29E-08
XM_013983649.2	LOC100627422	-22.25041814	1.25E-08
XM_021094209.1	ADCY7	-22.25115875	1.25E-08
NM_001243827.1	HDAC3	-22.25369265	1.24E-08
XM_021074090.1	EXTL3	-22.33020825	1.11E-08
XM_005668715.3	PLEKHM1	-22.36011522	1.06E-08
XM_021093025.1	APAF1	-22.36191102	1.05E-08
XM_005662906.3	OXR1	-22.37396466	1.04E-08
XM_021079134.1	KMT2C	-22.38208355	1.02E-08
XM_021086246.1	GIGYF1	-22.43105316	9.47E-09
XM_021102192.1	AKAP9	-22.55408375	7.86E-09
XM_013995882.2	LOC100511376	-22.66286851	6.67E-09
XM_021073541.1	CIT	-22.69446097	6.35E-09
XM_021080674.1	GANAB	-22.69485959	6.35E-09
XM_001928359.5	DCAF11	-22.72336302	6.07E-09
XM_021076896.1	CREBRF	-22.7291889	6.02E-09
XM_021080391.1	ZNF81	-22.86683786	4.87E-09
XM_021062555.1	ACER3	-23.00691855	3.92E-09
XM_021079063.1	OGDH	-23.17268418	3.03E-09

XM_021092835.1	MRTFA	-23.17409869	3.02E-09
XM_021099032.1	ZNF236	-23.43460426	2.00E-09
XM_021078295.1	NCOA6	-23.51622352	1.76E-09
XM_021093804.1	SF3B3	-23.61983183	1.49E-09
NM_001113702.1	SLA-2	-23.83174519	1.07E-09
XM_021083201.1	HIPK3	-23.95018874	8.80E-10
XM_021083203.1	HIPK3	-23.99488457	8.19E-10
XM_021088220.1	SPTBN1	-24.35712648	4.55E-10
