# Supplementary material for: Comprehensive analysis of miRNAs, lncRNAs and mRNAs profiles in backfat tissue between Daweizi and Yorkshire pigs
Source: Anim Biosci. 2022 Nov 13;36(3):404–16. doi: 10.5713/ab.22.0165 (PMC9996253; doi:10.5713/ab.22.0165)
Supplement: Supplementary file 7 [file ab-22-0165-Supplementary-Table-6.pdf]

Supplementary Table S6. Specifically expressed miRNAs, lncRNAs and mRNAs.

RNA ID	Symbol	DWZ expression	DWZ average read count	Yorkshire expression	Yorkshire average read count	Known/Novel gene
novel-ssc-miR1000-5p		0.333	0.333	0	0	novel
novel-ssc-miR1017-5p		0.333	0.333	0	0	novel
novel-ssc-miR1018-3p		1	1	0	0	novel
novel-ssc-miR1030-5p		0.333	0.333	0	0	novel
novel-ssc-miR1054-3p		0.333	0.333	0	0	novel
novel-ssc-miR1058-3p		0.333	0.333	0	0	novel
novel-ssc-miR1068-5p		0.333	0.333	0	0	novel
novel-ssc-miR1071-3p		0.667	0.667	0	0	novel
novel-ssc-miR1073-3p		0.333	0.333	0	0	novel
novel-ssc-miR109-5p		0.333	0.333	0	0	novel
novel-ssc-miR1097-5p		0.333	0.333	0	0	novel
novel-ssc-miR110-3p		1	1	0	0	novel
novel-ssc-miR1100-3p		0.333	0.333	0	0	novel
novel-ssc-miR1102-3p		0.667	0.667	0	0	novel
novel-ssc-miR1110-5p		0.333	0.333	0	0	novel
novel-ssc-miR1119-3p		1	1	0	0	novel
novel-ssc-miR1133-5p		0.333	0.333	0	0	novel
novel-ssc-miR1134-5p		0.333	0.333	0	0	novel
novel-ssc-miR1138-5p		0.667	0.667	0	0	novel
novel-ssc-miR115-3p		0.333	0.333	0	0	novel
novel-ssc-miR1153-3p		0.667	0.667	0	0	novel
novel-ssc-miR1159-3p		0.333	0.333	0	0	novel
novel-ssc-miR1160-5p		0.333	0.333	0	0	novel
novel-ssc-miR1163-5p		0.667	0.667	0	0	novel
novel-ssc-miR1164-5p		0.333	0.333	0	0	novel
novel-ssc-miR1167-5p		0.333	0.333	0	0	novel
novel-ssc-miR118-3p		0.667	0.667	0	0	novel
novel-ssc-miR1183-3p		0.333	0.333	0	0	novel
novel-ssc-miR1184-3p		0.333	0.333	0	0	novel
novel-ssc-miR1189-5p		0.333	0.333	0	0	novel
novel-ssc-miR119-3p		0.333	0.333	0	0	novel
novel-ssc-miR1194-3p		0.333	0.333	0	0	novel
novel-ssc-miR1194-5p		3.333	3.333	0	0	novel
novel-ssc-miR1198-3p		1	1	0	0	novel
novel-ssc-miR1203-5p		0.333	0.333	0	0	novel
novel-ssc-miR1205-3p		0.333	0.333	0	0	novel
novel-ssc-miR1218-5p		0.333	0.333	0	0	novel
novel-ssc-miR1221-3p		0.333	0.333	0	0	novel
novel-ssc-miR1225-5p		0.333	0.333	0	0	novel
novel-ssc-miR1227-3p		0.667	0.667	0	0	novel
novel-ssc-miR1232-3p		0.333	0.333	0	0	novel
novel-ssc-miR1233-5p		0.333	0.333	0	0	novel
novel-ssc-miR1237-5p		1	1	0	0	novel
novel-ssc-miR1261-3p		3	3	0	0	novel
novel-ssc-miR1282-5p		0.333	0.333	0	0	novel
novel-ssc-miR1286-5p		0.333	0.333	0	0	novel
novel-ssc-miR1291-5p		0.667	0.667	0	0	novel
novel-ssc-miR1293-5p		0.667	0.667	0	0	novel
novel-ssc-miR1294-3p		0.667	0.667	0	0	novel
novel-ssc-miR1322-5p		0.333	0.333	0	0	novel
novel-ssc-miR1330-3p		0.667	0.667	0	0	novel
novel-ssc-miR1339-3p		0.333	0.333	0	0	novel
novel-ssc-miR1348-3p		0.333	0.333	0	0	novel
novel-ssc-miR135-5p		0.333	0.333	0	0	novel
novel-ssc-miR1350-3p		0.333	0.333	0	0	novel
novel-ssc-miR1360-5p		0.333	0.333	0	0	novel

novel-ssc-miR1369-5p	0.667	0.667	0	0	novel
novel-ssc-miR137-5p	0.333	0.333	0	0	novel
novel-ssc-miR1373-3p	0.333	0.333	0	0	novel
novel-ssc-miR1374-3p	0.333	0.333	0	0	novel
novel-ssc-miR1375-5p	0.667	0.667	0	0	novel
novel-ssc-miR1377-3p	0.667	0.667	0	0	novel
novel-ssc-miR1379-3p	9.333	9.333	0	0	novel
novel-ssc-miR1380-5p	0.667	0.667	0	0	novel
novel-ssc-miR1381-3p	0.333	0.333	0	0	novel
novel-ssc-miR1385-3p	1.333	1.333	0	0	novel
novel-ssc-miR1385-5p	0.333	0.333	0	0	novel
novel-ssc-miR1386-3p	0.333	0.333	0	0	novel
novel-ssc-miR1386-5p	0.667	0.667	0	0	novel
novel-ssc-miR1390-5p	0.667	0.667	0	0	novel
novel-ssc-miR1391-5p	1	1	0	0	novel
novel-ssc-miR1408-5p	0.333	0.333	0	0	novel
novel-ssc-miR1413-5p	0.667	0.667	0	0	novel
novel-ssc-miR1414-5p	4	4	0	0	novel
novel-ssc-miR142-3p	0.333	0.333	0	0	novel
novel-ssc-miR142-5p	2	2	0	0	novel
novel-ssc-miR1420-3p	0.667	0.667	0	0	novel
novel-ssc-miR1439-3p	0.333	0.333	0	0	novel
novel-ssc-miR1445-5p	0.333	0.333	0	0	novel
novel-ssc-miR154-5p	13	13	0	0	novel
novel-ssc-miR158-3p	0.333	0.333	0	0	novel
novel-ssc-miR168-3p	0.333	0.333	0	0	novel
novel-ssc-miR170-3p	0.333	0.333	0	0	novel
novel-ssc-miR172-3p	0.333	0.333	0	0	novel
novel-ssc-miR197-3p	0.667	0.667	0	0	novel
novel-ssc-miR209-5p	0.333	0.333	0	0	novel
novel-ssc-miR218-3p	0.667	0.667	0	0	novel
novel-ssc-miR219-3p	0.667	0.667	0	0	novel
novel-ssc-miR236-5p	0.333	0.333	0	0	novel
novel-ssc-miR255-3p	2	2	0	0	novel
novel-ssc-miR260-3p	0.333	0.333	0	0	novel
novel-ssc-miR271-5p	1	1	0	0	novel
novel-ssc-miR273-3p	0.333	0.333	0	0	novel
novel-ssc-miR273-5p	0.667	0.667	0	0	novel
novel-ssc-miR276-5p	0.333	0.333	0	0	novel
novel-ssc-miR278-3p	0.333	0.333	0	0	novel
novel-ssc-miR300-3p	0.333	0.333	0	0	novel
novel-ssc-miR306-5p	0.333	0.333	0	0	novel
novel-ssc-miR308-3p	0.333	0.333	0	0	novel
novel-ssc-miR31-3p	0.667	0.667	0	0	novel
novel-ssc-miR316-5p	0.333	0.333	0	0	novel
novel-ssc-miR32-3p	3	3	0	0	novel
novel-ssc-miR333-5p	0.667	0.667	0	0	novel
novel-ssc-miR341-3p	0.667	0.667	0	0	novel
novel-ssc-miR345-5p	1	1	0	0	novel
novel-ssc-miR354-3p	0.333	0.333	0	0	novel
novel-ssc-miR364-5p	0.333	0.333	0	0	novel
novel-ssc-miR37-3p	0.333	0.333	0	0	novel
novel-ssc-miR378-5p	1.333	1.333	0	0	novel
novel-ssc-miR38-5p	0.333	0.333	0	0	novel
novel-ssc-miR382-3p	0.667	0.667	0	0	novel
novel-ssc-miR385-5p	0.333	0.333	0	0	novel
novel-ssc-miR388-3p	0.667	0.667	0	0	novel
novel-ssc-miR408-5p	0.333	0.333	0	0	novel
novel-ssc-miR414-3p	0.667	0.667	0	0	novel
novel-ssc-miR416-5p	0.333	0.333	0	0	novel

novel-ssc-miR417-5p	0.333	0.333	0	0	novel
novel-ssc-miR420-3p	0.667	0.667	0	0	novel
novel-ssc-miR420-5p	0.667	0.667	0	0	novel
novel-ssc-miR423-3p	0.333	0.333	0	0	novel
novel-ssc-miR427-5p	0.333	0.333	0	0	novel
novel-ssc-miR430-5p	0.667	0.667	0	0	novel
novel-ssc-miR438-5p	0.333	0.333	0	0	novel
novel-ssc-miR443-3p	1.667	1.667	0	0	novel
novel-ssc-miR456-3p	1	1	0	0	novel
novel-ssc-miR462-5p	0.667	0.667	0	0	novel
novel-ssc-miR470-5p	0.667	0.667	0	0	novel
novel-ssc-miR475-3p	0.333	0.333	0	0	novel
novel-ssc-miR477-5p	0.333	0.333	0	0	novel
novel-ssc-miR482-5p	0.333	0.333	0	0	novel
novel-ssc-miR484-3p	0.333	0.333	0	0	novel
novel-ssc-miR491-3p	0.333	0.333	0	0	novel
novel-ssc-miR493-3p	0.333	0.333	0	0	novel
novel-ssc-miR498-3p	0.333	0.333	0	0	novel
novel-ssc-miR499-3p	3	3	0	0	novel
novel-ssc-miR504-5p	0.667	0.667	0	0	novel
novel-ssc-miR51-3p	0.333	0.333	0	0	novel
novel-ssc-miR529-5p	0.333	0.333	0	0	novel
novel-ssc-miR530-3p	0.333	0.333	0	0	novel
novel-ssc-miR532-5p	0.333	0.333	0	0	novel
novel-ssc-miR534-3p	0.333	0.333	0	0	novel
novel-ssc-miR536-3p	0.333	0.333	0	0	novel
novel-ssc-miR54-3p	1.333	1.333	0	0	novel
novel-ssc-miR56-3p	0.667	0.667	0	0	novel
novel-ssc-miR565-5p	0.333	0.333	0	0	novel
novel-ssc-miR566-3p	0.333	0.333	0	0	novel
novel-ssc-miR566-5p	0.333	0.333	0	0	novel
novel-ssc-miR574-3p	0.667	0.667	0	0	novel
novel-ssc-miR575-3p	0.333	0.333	0	0	novel
novel-ssc-miR575-5p	0.667	0.667	0	0	novel
novel-ssc-miR577-3p	0.667	0.667	0	0	novel
novel-ssc-miR582-5p	0.333	0.333	0	0	novel
novel-ssc-miR587-5p	0.333	0.333	0	0	novel
novel-ssc-miR588-5p	1	1	0	0	novel
novel-ssc-miR591-3p	1.333	1.333	0	0	novel
novel-ssc-miR608-5p	0.667	0.667	0	0	novel
novel-ssc-miR611-3p	0.667	0.667	0	0	novel
novel-ssc-miR612-3p	0.333	0.333	0	0	novel
novel-ssc-miR612-5p	1.333	1.333	0	0	novel
novel-ssc-miR62-3p	0.333	0.333	0	0	novel
novel-ssc-miR634-3p	0.333	0.333	0	0	novel
novel-ssc-miR639-3p	0.667	0.667	0	0	novel
novel-ssc-miR643-5p	0.333	0.333	0	0	novel
novel-ssc-miR651-3p	0.333	0.333	0	0	novel
novel-ssc-miR666-5p	1.333	1.333	0	0	novel
novel-ssc-miR672-5p	0.333	0.333	0	0	novel
novel-ssc-miR681-5p	0.333	0.333	0	0	novel
novel-ssc-miR682-5p	3	3	0	0	novel
novel-ssc-miR684-5p	0.333	0.333	0	0	novel
novel-ssc-miR687-5p	0.667	0.667	0	0	novel
novel-ssc-miR688-5p	1	1	0	0	novel
novel-ssc-miR689-3p	0.667	0.667	0	0	novel
novel-ssc-miR69-3p	0.333	0.333	0	0	novel
novel-ssc-miR70-3p	0.333	0.333	0	0	novel
novel-ssc-miR706-3p	0.333	0.333	0	0	novel
novel-ssc-miR726-5p	0.333	0.333	0	0	novel

novel-ssc-miR73-3p	1	1	0	0	novel
novel-ssc-miR734-5p	2.333	2.333	0	0	novel
novel-ssc-miR74-3p	0.333	0.333	0	0	novel
novel-ssc-miR750-3p	0.333	0.333	0	0	novel
novel-ssc-miR751-3p	0.333	0.333	0	0	novel
novel-ssc-miR767-3p	0.333	0.333	0	0	novel
novel-ssc-miR771-3p	0.333	0.333	0	0	novel
novel-ssc-miR772-3p	0.667	0.667	0	0	novel
novel-ssc-miR775-5p	0.333	0.333	0	0	novel
novel-ssc-miR789-3p	0.333	0.333	0	0	novel
novel-ssc-miR795-3p	1	1	0	0	novel
novel-ssc-miR796-3p	0.333	0.333	0	0	novel
novel-ssc-miR806-3p	0.667	0.667	0	0	novel
novel-ssc-miR814-5p	4	4	0	0	novel
novel-ssc-miR82-5p	0.333	0.333	0	0	novel
novel-ssc-miR859-3p	0.333	0.333	0	0	novel
novel-ssc-miR860-5p	0.667	0.667	0	0	novel
novel-ssc-miR867-3p	0.333	0.333	0	0	novel
novel-ssc-miR873-3p	0.333	0.333	0	0	novel
novel-ssc-miR877-5p	0.333	0.333	0	0	novel
novel-ssc-miR88-5p	0.667	0.667	0	0	novel
novel-ssc-miR894-3p	0.333	0.333	0	0	novel
novel-ssc-miR894-5p	1.667	1.667	0	0	novel
novel-ssc-miR895-5p	1	1	0	0	novel
novel-ssc-miR908-3p	0.333	0.333	0	0	novel
novel-ssc-miR915-5p	0.333	0.333	0	0	novel
novel-ssc-miR920-3p	0.333	0.333	0	0	novel
novel-ssc-miR920-5p	0.333	0.333	0	0	novel
novel-ssc-miR922-3p	0.333	0.333	0	0	novel
novel-ssc-miR933-5p	0.333	0.333	0	0	novel
novel-ssc-miR948-3p	0.667	0.667	0	0	novel
novel-ssc-miR949-5p	0.667	0.667	0	0	novel
novel-ssc-miR951-5p	0.667	0.667	0	0	novel
novel-ssc-miR962-3p	0.333	0.333	0	0	novel
novel-ssc-miR965-3p	0.333	0.333	0	0	novel
novel-ssc-miR971-3p	1	1	0	0	novel
novel-ssc-miR979-3p	1.333	1.333	0	0	novel
novel-ssc-miR988-5p	0.333	0.333	0	0	novel
novel-ssc-miR991-3p	0.333	0.333	0	0	novel
novel-ssc-miR994-3p	1	1	0	0	novel
novel-ssc-miR999-3p	0.333	0.333	0	0	novel
ssc-miR-1296-3p	0.333	0.333	0	0	known
ssc-miR-146a-3p	0.333	0.333	0	0	known
ssc-miR-194a-3p	2	2	0	0	known
ssc-miR-194b-3p	0.333	0.333	0	0	known
ssc-miR-215	4	4	0	0	known
ssc-miR-34c	0.333	0.333	0	0	known
ssc-miR-582-3p	0.333	0.333	0	0	known
ssc-miR-802	1.667	1.667	0	0	known
novel-ssc-miR1001-5p	0	0	1.667	1.667	novel
novel-ssc-miR1005-5p	0	0	0.333	0.333	novel
novel-ssc-miR1018-5p	0	0	0.333	0.333	novel
novel-ssc-miR1024-3p	0	0	5.667	5.667	novel
novel-ssc-miR1030-3p	0	0	0.333	0.333	novel
novel-ssc-miR1040-3p	0	0	0.333	0.333	novel
novel-ssc-miR1043-3p	0	0	0.333	0.333	novel
novel-ssc-miR105-5p	0	0	0.667	0.667	novel
novel-ssc-miR1052-5p	0	0	0.333	0.333	novel
novel-ssc-miR1059-5p	0	0	0.333	0.333	novel
novel-ssc-miR1072-3p	0	0	0.333	0.333	novel

novel-ssc-miR1074-5p	0	0	0.333	0.333	novel
novel-ssc-miR1101-5p	0	0	0.667	0.667	novel
novel-ssc-miR1106-3p	0	0	0.333	0.333	novel
novel-ssc-miR1120-3p	0	0	2	2	novel
novel-ssc-miR1120-5p	0	0	7.333	7.333	novel
novel-ssc-miR1135-5p	0	0	0.333	0.333	novel
novel-ssc-miR1139-3p	0	0	0.333	0.333	novel
novel-ssc-miR1149-5p	0	0	0.333	0.333	novel
novel-ssc-miR1151-3p	0	0	0.667	0.667	novel
novel-ssc-miR1161-3p	0	0	1.667	1.667	novel
novel-ssc-miR1165-5p	0	0	0.667	0.667	novel
novel-ssc-miR1178-3p	0	0	0.333	0.333	novel
novel-ssc-miR1181-3p	0	0	0.333	0.333	novel
novel-ssc-miR119-5p	0	0	0.333	0.333	novel
novel-ssc-miR1195-5p	0	0	1.333	1.333	novel
novel-ssc-miR1196-3p	0	0	0.333	0.333	novel
novel-ssc-miR1197-5p	0	0	9.667	9.667	novel
novel-ssc-miR1199-3p	0	0	0.333	0.333	novel
novel-ssc-miR1206-3p	0	0	0.667	0.667	novel
novel-ssc-miR1219-5p	0	0	0.333	0.333	novel
novel-ssc-miR1220-5p	0	0	0.333	0.333	novel
novel-ssc-miR1224-3p	0	0	1.333	1.333	novel
novel-ssc-miR1228-5p	0	0	0.333	0.333	novel
novel-ssc-miR1230-5p	0	0	0.333	0.333	novel
novel-ssc-miR1234-3p	0	0	0.667	0.667	novel
novel-ssc-miR1240-3p	0	0	0.667	0.667	novel
novel-ssc-miR1247-5p	0	0	1	1	novel
novel-ssc-miR1252-5p	0	0	0.333	0.333	novel
novel-ssc-miR1259-5p	0	0	0.333	0.333	novel
novel-ssc-miR1260-3p	0	0	0.333	0.333	novel
novel-ssc-miR1265-3p	0	0	0.667	0.667	novel
novel-ssc-miR1266-5p	0	0	0.333	0.333	novel
novel-ssc-miR1274-3p	0	0	0.667	0.667	novel
novel-ssc-miR1279-3p	0	0	0.333	0.333	novel
novel-ssc-miR1291-3p	0	0	0.333	0.333	novel
novel-ssc-miR1292-3p	0	0	0.333	0.333	novel
novel-ssc-miR1314-3p	0	0	0.667	0.667	novel
novel-ssc-miR1318-5p	0	0	0.667	0.667	novel
novel-ssc-miR1322-3p	0	0	0.333	0.333	novel
novel-ssc-miR1325-5p	0	0	1.667	1.667	novel
novel-ssc-miR1335-5p	0	0	0.667	0.667	novel
novel-ssc-miR1341-3p	0	0	0.667	0.667	novel
novel-ssc-miR135-3p	0	0	0.333	0.333	novel
novel-ssc-miR1351-5p	0	0	0.667	0.667	novel
novel-ssc-miR1357-3p	0	0	0.667	0.667	novel
novel-ssc-miR1368-5p	0	0	0.333	0.333	novel
novel-ssc-miR1371-3p	0	0	0.667	0.667	novel
novel-ssc-miR1374-5p	0	0	0.333	0.333	novel
novel-ssc-miR1380-3p	0	0	0.667	0.667	novel
novel-ssc-miR1382-3p	0	0	0.333	0.333	novel
novel-ssc-miR1388-3p	0	0	0.667	0.667	novel
novel-ssc-miR139-3p	0	0	1.333	1.333	novel
novel-ssc-miR140-5p	0	0	0.333	0.333	novel
novel-ssc-miR1405-3p	0	0	0.333	0.333	novel
novel-ssc-miR141-3p	0	0	0.667	0.667	novel
novel-ssc-miR141-5p	0	0	0.333	0.333	novel
novel-ssc-miR1415-3p	0	0	1.667	1.667	novel
novel-ssc-miR1427-5p	0	0	0.333	0.333	novel
novel-ssc-miR1428-3p	0	0	0.667	0.667	novel
novel-ssc-miR1438-5p	0	0	0.333	0.333	novel

novel-ssc-miR1441-3p	0	0	0.333	0.333	novel
novel-ssc-miR1444-3p	0	0	0.667	0.667	novel
novel-ssc-miR146-3p	0	0	0.667	0.667	novel
novel-ssc-miR154-3p	0	0	1	1	novel
novel-ssc-miR155-5p	0	0	0.667	0.667	novel
novel-ssc-miR163-3p	0	0	0.333	0.333	novel
novel-ssc-miR175-3p	0	0	1.333	1.333	novel
novel-ssc-miR176-5p	0	0	0.333	0.333	novel
novel-ssc-miR185-3p	0	0	0.333	0.333	novel
novel-ssc-miR185-5p	0	0	0.333	0.333	novel
novel-ssc-miR187-3p	0	0	0.667	0.667	novel
novel-ssc-miR192-5p	0	0	0.333	0.667	novel
novel-ssc-miR215-5p	0	0	0.333	0.333	novel
novel-ssc-miR227-5p	0	0	1	1	novel
novel-ssc-miR233-5p	0	0	0.333	0.333	novel
novel-ssc-miR235-3p	0	0	0.333	0.333	novel
novel-ssc-miR237-3p	0	0	1.333	1.333	novel
novel-ssc-miR239-3p	0	0	0.333	0.333	novel
novel-ssc-miR245-3p	0	0	1.667	1.667	novel
novel-ssc-miR246-3p	0	0	0.333	0.333	novel
novel-ssc-miR249-3p	0	0	0.333	0.333	novel
novel-ssc-miR254-3p	0	0	0.333	0.333	novel
novel-ssc-miR263-5p	0	0	3.333	3.333	novel
novel-ssc-miR268-3p	0	0	1	1	novel
novel-ssc-miR268-5p	0	0	1.333	1.333	novel
novel-ssc-miR270-3p	0	0	0.667	0.667	novel
novel-ssc-miR295-3p	0	0	0.333	0.333	novel
novel-ssc-miR296-3p	0	0	0.333	0.333	novel
novel-ssc-miR305-5p	0	0	0.333	0.333	novel
novel-ssc-miR307-5p	0	0	1	1	novel
novel-ssc-miR311-5p	0	0	0.333	0.333	novel
novel-ssc-miR312-5p	0	0	0.667	0.667	novel
novel-ssc-miR315-3p	0	0	0.333	0.333	novel
novel-ssc-miR323-5p	0	0	1	1	novel
novel-ssc-miR329-3p	0	0	0.333	0.333	novel
novel-ssc-miR33-3p	0	0	0.333	0.333	novel
novel-ssc-miR334-3p	0	0	2.333	2.333	novel
novel-ssc-miR336-3p	0	0	0.333	0.333	novel
novel-ssc-miR34-5p	0	0	0.333	0.333	novel
novel-ssc-miR341-5p	0	0	5.333	5.333	novel
novel-ssc-miR342-5p	0	0	0.333	0.333	novel
novel-ssc-miR343-5p	0	0	0.667	0.667	novel
novel-ssc-miR348-5p	0	0	0.333	0.333	novel
novel-ssc-miR351-3p	0	0	0.333	0.333	novel
novel-ssc-miR362-5p	0	0	0.333	0.333	novel
novel-ssc-miR370-3p	0	0	0.667	0.667	novel
novel-ssc-miR376-5p	0	0	0.333	0.333	novel
novel-ssc-miR399-3p	0	0	0.333	0.333	novel
novel-ssc-miR400-5p	0	0	0.333	0.333	novel
novel-ssc-miR409-3p	0	0	0.333	0.333	novel
novel-ssc-miR410-3p	0	0	0.333	0.333	novel
novel-ssc-miR411-3p	0	0	0.333	0.333	novel
novel-ssc-miR418-5p	0	0	0.667	0.667	novel
novel-ssc-miR425-3p	0	0	0.667	0.667	novel
novel-ssc-miR459-5p	0	0	0.333	0.333	novel
novel-ssc-miR488-5p	0	0	0.333	0.333	novel
novel-ssc-miR49-3p	0	0	0.667	0.667	novel
novel-ssc-miR49-5p	0	0	0.333	0.333	novel
novel-ssc-miR520-3p	0	0	1	1	novel
novel-ssc-miR521-3p	0	0	0.333	0.333	novel

novel-ssc-miR521-5p	0	0	0.333	0.333	novel
novel-ssc-miR527-5p	0	0	2.333	2.333	novel
novel-ssc-miR530-5p	0	0	0.333	0.333	novel
novel-ssc-miR536-5p	0	0	0.333	0.333	novel
novel-ssc-miR545-3p	0	0	0.667	0.667	novel
novel-ssc-miR546-3p	0	0	0.333	0.333	novel
novel-ssc-miR549-5p	0	0	0.333	0.333	novel
novel-ssc-miR556-5p	0	0	1	1	novel
novel-ssc-miR564-3p	0	0	0.333	0.333	novel
novel-ssc-miR573-3p	0	0	0.667	0.667	novel
novel-ssc-miR573-5p	0	0	25	25	novel
novel-ssc-miR58-3p	0	0	0.333	0.333	novel
novel-ssc-miR585-3p	0	0	0.667	0.667	novel
novel-ssc-miR586-3p	0	0	0.333	0.333	novel
novel-ssc-miR589-5p	0	0	0.667	0.667	novel
novel-ssc-miR598-3p	0	0	0.667	0.667	novel
novel-ssc-miR6-3p	0	0	0.333	0.333	novel
novel-ssc-miR600-3p	0	0	0.333	0.333	novel
novel-ssc-miR607-5p	0	0	0.333	0.333	novel
novel-ssc-miR610-3p	0	0	0.333	0.333	novel
novel-ssc-miR615-3p	0	0	0.667	0.667	novel
novel-ssc-miR623-3p	0	0	0.667	0.667	novel
novel-ssc-miR625-5p	0	0	2.333	2.333	novel
novel-ssc-miR633-5p	0	0	0.667	0.667	novel
novel-ssc-miR645-3p	0	0	0.333	0.333	novel
novel-ssc-miR648-3p	0	0	0.333	0.333	novel
novel-ssc-miR649-3p	0	0	1.333	1.333	novel
novel-ssc-miR654-3p	0	0	1	1	novel
novel-ssc-miR659-3p	0	0	0.667	0.667	novel
novel-ssc-miR662-3p	0	0	0.333	0.333	novel
novel-ssc-miR666-3p	0	0	0.333	0.333	novel
novel-ssc-miR69-5p	0	0	0.333	0.333	novel
novel-ssc-miR692-3p	0	0	4.667	4.667	novel
novel-ssc-miR692-5p	0	0	0.667	0.667	novel
novel-ssc-miR701-5p	0	0	0.333	0.333	novel
novel-ssc-miR72-3p	0	0	0.333	0.333	novel
novel-ssc-miR730-3p	0	0	0.333	0.333	novel
novel-ssc-miR731-3p	0	0	0.667	0.667	novel
novel-ssc-miR736-5p	0	0	0.333	0.333	novel
novel-ssc-miR737-3p	0	0	0.333	0.333	novel
novel-ssc-miR773-3p	0	0	0.667	0.667	novel
novel-ssc-miR777-5p	0	0	0.333	0.333	novel
novel-ssc-miR798-3p	0	0	0.333	0.333	novel
novel-ssc-miR8-3p	0	0	2.333	2.333	novel
novel-ssc-miR800-3p	0	0	2	2	novel
novel-ssc-miR808-5p	0	0	2.333	2.333	novel
novel-ssc-miR813-5p	0	0	11.333	11.333	novel
novel-ssc-miR816-5p	0	0	0.333	0.333	novel
novel-ssc-miR823-3p	0	0	0.667	0.667	novel
novel-ssc-miR831-5p	0	0	0.333	0.333	novel
novel-ssc-miR833-5p	0	0	1.333	1.333	novel
novel-ssc-miR836-5p	0	0	0.333	0.333	novel
novel-ssc-miR837-3p	0	0	0.333	0.333	novel
novel-ssc-miR839-3p	0	0	0.667	0.667	novel
novel-ssc-miR841-3p	0	0	2.667	2.667	novel
novel-ssc-miR846-5p	0	0	0.333	0.333	novel
novel-ssc-miR847-5p	0	0	0.667	0.667	novel
novel-ssc-miR848-5p	0	0	2.333	2.333	novel
novel-ssc-miR861-3p	0	0	0.333	0.333	novel
novel-ssc-miR874-5p	0	0	1	1	novel

novel-ssc-miR878-3p		0	0	0.333	0.333	novel
novel-ssc-miR881-5p		0	0	0.333	0.333	novel
novel-ssc-miR90-3p		0	0	0.667	0.667	novel
novel-ssc-miR900-5p		0	0	1.667	1.667	novel
novel-ssc-miR906-3p		0	0	0.333	0.333	novel
novel-ssc-miR91-3p		0	0	0.333	0.333	novel
novel-ssc-miR92-3p		0	0	0.333	0.333	novel
novel-ssc-miR932-3p		0	0	0.333	0.333	novel
novel-ssc-miR934-3p		0	0	0.333	0.333	novel
novel-ssc-miR935-5p		0	0	1	1	novel
novel-ssc-miR940-5p		0	0	0.333	0.333	novel
novel-ssc-miR953-5p		0	0	0.333	0.333	novel
novel-ssc-miR954-3p		0	0	2	2	novel
novel-ssc-miR964-3p		0	0	0.667	0.667	novel
novel-ssc-miR967-3p		0	0	0.333	0.333	novel
novel-ssc-miR97-3p		0	0	0.333	0.333	novel
novel-ssc-miR976-3p		0	0	0.667	0.667	novel
novel-ssc-miR977-3p		0	0	1	1	novel
novel-ssc-miR978-5p		0	0	1.333	1.333	novel
novel-ssc-miR981-3p		0	0	1	1	novel
novel-ssc-miR985-5p		0	0	11	11	novel
novel-ssc-miR986-3p		0	0	0.333	0.333	novel
ssc-miR-105-1		0	0	0.333	0.333	known
ssc-miR-196b-3p		0	0	0.667	0.667	known
ssc-miR-301		0	0	0.333	0.333	known
ssc-miR-376a-5p		0	0	1	1	known
ssc-miR-421-5p		0	0	0.333	0.333	known
ssc-miR-432-3p		0	0	0.667	0.667	known
ssc-miR-708-3p		0	0	0.333	0.333	known
ssc-miR-7135-5p		0	0	1.667	1.667	known
ssc-miR-7140-5p		0	0	0.333	0.333	known
ssc-miR-9785-5p		0	0	0.333	0.333	known
ssc-miR-9820-5p		0	0	0.667	0.667	known
BGIR9823_100016	LOC102157885	0.1	0.666666667	0	0	novel
BGIR9823_100115	MKKS	0.04	0.333333333	0	0	novel
BGIR9823_100124	TASP1	0.063333333	0.333333333	0	0	novel
BGIR9823_100170	C17H20orf194	0.01	0.333333333	0	0	novel
BGIR9823_100227	UQCC	0.063333333	0.333333333	0	0	novel
BGIR9823_100247	RBL1	0.053333333	0.333333333	0	0	novel
BGIR9823_100268	LOC102159476	0.003333333	0.663333333	0	0	novel
BGIR9823_100277	PTI	0.006666667	0.36	0	0	novel
BGIR9823_100288	SLC13A3	0.263333333	1.333333333	0	0	novel
BGIR9823_100326	LOC110257473	0.033333333	0.333333333	0	0	novel
BGIR9823_100411	CHCHD3	0.3	2.333333333	0	0	novel
BGIR9823_100420	TMEM209	0.053333333	0.333333333	0	0	novel
BGIR9823_100433	IRF5	0.066666667	0.333333333	0	0	novel
BGIR9823_100455	WASL	0.066666667	0.333333333	0	0	novel
BGIR9823_100475	TFEC	0.003333333	2.676666667	0	0.333333333	novel
BGIR9823_100511	HERPUD2	0.066666667	0.333333333	0	0	novel
BGIR9823_100554	LOC100739791	0.106666667	3.656666667	0	0	novel
BGIR9823_100655	TRIM24	0.06	0.333333333	0	0	novel
BGIR9823_100665	LOC100622227	0.05	7.533333333	0	0	novel
BGIR9823_100719	PTPRZ1	0.043333333	0.333333333	0	0	novel
BGIR9823_100722	LOC100514329	0.016666667	0.333333333	0	0	novel
BGIR9823_100850	TMSB4X	0.023333333	1.213333333	0	0	novel
BGIR9823_100856	ASB11	0.08	0.666666667	0	0	novel
BGIR9823_100859	ZRSR2	0.05	0.333333333	0	0	novel
BGIR9823_100880	MBTPS2	0.056666667	0.333333333	0	0	novel
BGIR9823_100893	IL1RAPL1	0.226666667	1.333333333	0	0	novel
BGIR9823_100894	IL1RAPL1	0.03	1	0	0	novel

BGIR9823_100898	IL1RAPL1	0.03	1	0	0	novel
BGIR9823_100903	IL1RAPL1	0.06	2.33	0	0	novel
BGIR9823_100908	IL1RAPL1	0.04	0.333333333	0	0	novel
BGIR9823_100917	IL1RAPL1	0.04	0.666666667	0	0	novel
BGIR9823_101105	ANOS1	0.026666667	5.74	0	0	novel
BGIR9823_101152	LOC100626655	0.08	0.41	0	0	novel
BGIR9823_101188	LOC110257830	0.036666667	2.476666667	0	0	novel
BGIR9823_101207	LOC100525798	0.013333333	0.633333333	0	0	novel
BGIR9823_101220	MAGT1	0.056666667	0.333333333	0	0	novel
BGIR9823_101221	MAGT1	0.31	1.666666667	0	0	novel
BGIR9823_101324	LOC110257973	0.006666667	0.193333333	0	0	novel
BGIR9823_101340	LOC106505329	0.003333333	0.333333333	0	0	novel
BGIR9823_101387	LOC106506226	1.863333333	346.2833333	0	0	novel
BGIR9823_101395	TNRC6A	0.023333333	0.666666667	0	0	novel
BGIR9823_101409	LOC110258214	0.006666667	0.47	0	0	novel
BGIR9823_101412	LOC110258225	0.033333333	2.656666667	0	0	novel
BGIR9823_101413	LOC110258225	0.01	3.356666667	0	1.356666667	novel
BGIR9823_101416		0.003333333	0.333333333	0	0	novel
BGIR9823_101511	LOC110258473	0.026666667	0.393333333	0	0	novel
BGIR9823_101554	LOC110258600	0.013333333	1.686666667	0	0	novel
BGIR9823_101589	LOC100518053	0.003333333	0.333333333	0	0	novel
BGIR9823_101610	LOC100521600	0.51	45.50666667	0	0	novel
BGIR9823_101613	LOC100521600	0.256666667	17.29666667	0	0	novel
BGIR9823_101637	LOC110259088	0.48	4	0	0	novel
BGIR9823_101653	LSS	0.116666667	0.666666667	0	0	novel
BGIR9823_101664		0.003333333	0.77	0	0	novel
BGIR9823_101716		0.013333333	0.333333333	0	0	novel
BGIR9823_101724		0.02	0.666666667	0	0	novel
BGIR9823_101726		0.01	2.666666667	0	0	novel
BGIR9823_101729		0.1	0.666666667	0	0	novel
BGIR9823_101741		0.096666667	0.666666667	0	0	novel
BGIR9823_101751		0.003333333	0.333333333	0	0	novel
BGIR9823_101752		0.003333333	0.216666667	0	0	novel
BGIR9823_101802		0.093333333	0.666666667	0	0	novel
BGIR9823_101813		0.023333333	0.666666667	0	0	novel
BGIR9823_101847		0.11	1	0	0	novel
BGIR9823_101881		0.043333333	2.333333333	0	0	novel
BGIR9823_101883		0.006666667	0.666666667	0	0	novel
BGIR9823_101913		0.006666667	0.666666667	0	0	novel
BGIR9823_101922		0.013333333	0.333333333	0	0	novel
BGIR9823_101959		0.023333333	0.333333333	0	0	novel
BGIR9823_102021		0.063333333	0.666666667	0	0	novel
BGIR9823_102027		0.043333333	0.333333333	0	0	novel
BGIR9823_102041		0.006666667	0.666666667	0	0	novel
BGIR9823_102101		0.073333333	0.333333333	0	0	novel
BGIR9823_102122		0.01	0.126666667	0	0	novel
BGIR9823_102180		0.01	0.333333333	0	0	novel
BGIR9823_102188		0.07	16.33333333	0	0	novel
BGIR9823_102208		0.03	0.333333333	0	0	novel
BGIR9823_102214		0.006666667	0.333333333	0	0	novel
BGIR9823_102226		0.02	0.333333333	0	0	novel
BGIR9823_102276		0.013333333	0.333333333	0	0	novel
BGIR9823_102277		0.016666667	0.333333333	0	0	novel
BGIR9823_102283		0.103333333	2.18	0	0	novel
BGIR9823_102333		0.046666667	1.666666667	0	0	novel
BGIR9823_102368		0.04	0.333333333	0	0	novel
BGIR9823_102369		0.003333333	1.056666667	0	0.333333333	novel
BGIR9823_102414		0.006666667	0.333333333	0	0	novel
BGIR9823_102458		0.036666667	0.333333333	0	0	novel
BGIR9823_102466		0.1	1.333333333	0	0	novel

BGIR9823_102575		0.006666667	0.333333333	0	0	novel
BGIR9823_102583		0.066666667	0.666666667	0	0	novel
BGIR9823_102584		0.023333333	0.333333333	0	0	novel
BGIR9823_102588		0.03	0.333333333	0	0	novel
BGIR9823_102599		0.013333333	0.333333333	0	0	novel
BGIR9823_102621		0.033333333	0.666666667	0	0	novel
BGIR9823_102714		0.02	0.666666667	0	0	novel
BGIR9823_102725		0.006666667	0.333333333	0	0	novel
BGIR9823_102734		0.073333333	0.333333333	0	0	novel
BGIR9823_102736		0.033333333	0.333333333	0	0	novel
BGIR9823_102743		0.023333333	0.25	0	0	novel
BGIR9823_102750		0.073333333	2.596666667	0	0	novel
BGIR9823_102776		0.173333333	2.336666667	0	0	novel
BGIR9823_77794	MCM9	0.1	1.333333333	0	0	novel
BGIR9823_77824	ADGRB3	0.023333333	0.333333333	0	0	novel
BGIR9823_77828	ADGRB3	0.013333333	0.333333333	0	0	novel
BGIR9823_77903	TSTD3	0.013333333	0.356666667	0	0	novel
BGIR9823_77906	GRIK2	0.02	0.333333333	0	0	novel
BGIR9823_77927	FIG4	0.05	0.333333333	0	0	novel
BGIR9823_77965	PHIP	0.003333333	0.806666667	0	0	novel
BGIR9823_78058	APH1B	0.073333333	0.333333333	0	0	novel
BGIR9823_78069	VPS13C	0.05	0.333333333	0	0	novel
BGIR9823_78105	ADAM10	0.103333333	0.903333333	0	0	novel
BGIR9823_78142	MYO5A	0.053333333	1	0	0	novel
BGIR9823_78156	DMXL2	0.046666667	0.333333333	0	0	novel
BGIR9823_78189	SECISBP2L	0.193333333	1	0	0	novel
BGIR9823_78293	OTUD7A	0.04	0.333333333	0	0	novel
BGIR9823_78410	LRFN5	0.04	0.333333333	0	0	novel
BGIR9823_78415	LRFN5	0.043333333	0.333333333	0	0	novel
BGIR9823_78448	ERO1A	0.013333333	0.346666667	0	0	novel
BGIR9823_78458	SOCS4	0.083333333	0.916666667	0	0	novel
BGIR9823_78461	DLGAP5	0.043333333	0.333333333	0	0	novel
BGIR9823_78529	SGPP1	0.046666667	0.333333333	0	0	novel
BGIR9823_78553	PLAA	0.073333333	0.666666667	0	0	novel
BGIR9823_78555	LOC102163685	0.19	1.666666667	0	0	novel
BGIR9823_78580	MLLT3	0.106666667	0.666666667	0	0	novel
BGIR9823_78581	SLC24A2	0.01	2.493333333	0	0.666666667	novel
BGIR9823_78582	SLC24A2	0.063333333	0.333333333	0	0	novel
BGIR9823_78633	AK3	0.133333333	0.666666667	0	0	novel
BGIR9823_78636	AK3	0.036666667	0.333333333	0	0	novel
BGIR9823_78640	LOC110256886	0.05	1.93	0	0	novel
BGIR9823_78648	LOC100156293	0.056666667	0.333333333	0	0	novel
BGIR9823_78664	GCNT1	0.063333333	1.333333333	0	0	novel
BGIR9823_78689	LOC100514147	0.13	6.763333333	0	0	novel
BGIR9823_78735	TMEM38B	0.153333333	1.333333333	0	0	novel
BGIR9823_78740	ELP1	0.056666667	0.333333333	0	0	novel
BGIR9823_78923	ARID1B	0.113333333	0.666666667	0	0	novel
BGIR9823_78947	SASH1	0.063333333	0.333333333	0	0	novel
BGIR9823_79034	NKAIN2	0.056666667	0.333333333	0	0	novel
BGIR9823_79094	EPHA7	0.053333333	0.333333333	0	0	novel
BGIR9823_79126	CCNC	0.05	1	0	0	novel
BGIR9823_79150	LOC110260563	0.053333333	0.333333333	0	0	novel
BGIR9823_79232	HDHD2	0.08	0.666666667	0	0	novel
BGIR9823_79265	SMAD4	0.04	0.333333333	0	0	novel
BGIR9823_79368	GALK2	0.1	1	0	0	novel
BGIR9823_79400	CASC4	0.073333333	0.666666667	0	0	novel
BGIR9823_79486	MPHOSPH10	0.056666667	0.666666667	0	0	novel
BGIR9823_79524	PHLPP1	0.07	0.333333333	0	0	novel
BGIR9823_79525	PHLPP1	0.136666667	0.666666667	0	0	novel
BGIR9823_79528	PHLPP1	0.253333333	1.333333333	0	0	novel

BGIR9823_79538	RELCH	0.106666667	0.666666667	0	0	novel
BGIR9823_79544	CDH20	0.056666667	0.333333333	0	0	novel
BGIR9823_79714	EXOC5	0.186666667	1.666666667	0	0	novel
BGIR9823_79742	FOCAD	0.04	0.333333333	0	0	novel
BGIR9823_79760	DENND4C	0.12	1.333333333	0	0	novel
BGIR9823_79786	UHRF2	0.04	0.333333333	0	0	novel
BGIR9823_79896	RNF38	0.086666667	0.666666667	0	0	novel
BGIR9823_79976	BRINP1	0.103333333	0.736666667	0	0	novel
BGIR9823_80119	AP5B1	0.006666667	0.666666667	0	0	novel
BGIR9823_80145	LOC100627892	0.023333333	0.67	0	0	novel
BGIR9823_80146	LOC100627892	0.42	9.56	0	0	novel
BGIR9823_80191	CPSF7	0.13	0.666666667	0	0	novel
BGIR9823_80213	LOC100625180	0.036666667	0.413333333	0	0	novel
BGIR9823_80303	GALNT18	0.063333333	0.333333333	0	0	novel
BGIR9823_80316	SBF2	0.053333333	0.333333333	0	0	novel
BGIR9823_80318	SBF2	0.056666667	0.333333333	0	0	novel
BGIR9823_80322	SBF2	0.046666667	0.333333333	0	0	novel
BGIR9823_80370	ELL	0.203333333	1	0	0	novel
BGIR9823_80417	LOC100516957	0.006666667	0.25	0	0	novel
BGIR9823_80442	LOC110255283	0.036666667	0.333333333	0	0	novel
BGIR9823_80477	ELAVL1	0.046666667	0.333333333	0	0	novel
BGIR9823_80538	SF3A2	0.103333333	1	0	0	novel
BGIR9823_80847	KDM3B	0.226666667	1.666666667	0	0	novel
BGIR9823_80849	KDM3B	0.063333333	0.333333333	0	0	novel
BGIR9823_80857	MATR3	0.036666667	3.68	0	0	novel
BGIR9823_81100	ZDHHC5	0.026666667	0.333333333	0	0	novel
BGIR9823_81101	ZDHHC5	0.093333333	0.666666667	0	0	novel
BGIR9823_81172	LOC100737821	0.046666667	0.333333333	0	0	novel
BGIR9823_81311	LOC106508100	0.06	1	0	0	novel
BGIR9823_81317	LOC106508100	0.103333333	1	0	0	novel
BGIR9823_81389	TRIP10	0.063333333	0.333333333	0	0	novel
BGIR9823_81489	PFN3	0.766666667	54.66666667	0	0	novel
BGIR9823_81500	NSD1	0.213333333	1.333333333	0	0	novel
BGIR9823_81537	WDR41	0.05	0.333333333	0	0	novel
BGIR9823_81543	WDR41	0.033333333	0.333333333	0	0	novel
BGIR9823_81597	MBLAC2	0.06	0.333333333	0	0	novel
BGIR9823_81711	ACSL6	0.033333333	1	0	0	novel
BGIR9823_81714	ACSL6	0.056666667	0.333333333	0	0	novel
BGIR9823_81759	LOC106509575	0.026666667	0.333333333	0	0	novel
BGIR9823_81802	PPP2R2B	0.05	0.666666667	0	0	novel
BGIR9823_81805	PPP2R2B	0.04	0.333333333	0	0	novel
BGIR9823_81806	PPP2R2B	0.043333333	0.666666667	0	0	novel
BGIR9823_81816	JAKMIP2	0.036666667	0.333333333	0	0	novel
BGIR9823_82015	GGA2	0.11	1	0	0	novel
BGIR9823_82022	EEF2K	0.063333333	0.333333333	0	0	novel
BGIR9823_82034	SYT17	0.023333333	0.333333333	0	0	novel
BGIR9823_82044	PARN	0.07	0.666666667	0	0	novel
BGIR9823_82151	CENPP	0.07	1.666666667	0	0	novel
BGIR9823_82270	GCFC2	0.136666667	0.666666667	0	0	novel
BGIR9823_82401	NRXN1	0.05	0.333333333	0	0	novel
BGIR9823_82419	SOCS5	0.013333333	0.333333333	0	0	novel
BGIR9823_82429	LRPPRC	0.366666667	1.666666667	0	0	novel
BGIR9823_82505	STRN	0.043333333	0.333333333	0	0	novel
BGIR9823_82516	PPM1G	0.053333333	0.333333333	0	0	novel
BGIR9823_82571	OSR1	0.043333333	0.333333333	0	0	novel
BGIR9823_82596	IAH1	0.073333333	0.666666667	0	0	novel
BGIR9823_82648	USP42	0.033333333	0.333333333	0	0	novel
BGIR9823_82738	KIF22	0.076666667	1	0	0	novel
BGIR9823_82764	NSMCE1	0.1	0.666666667	0	0	novel
BGIR9823_82810	XYLT1	0.09	2	0	0	novel

BGIR9823_82815	XYLT1	0.09	0.666666667	0	0	novel
BGIR9823_82842	C3H16orf72	0.75	4.333333333	0	0	novel
BGIR9823_82855	ALG1	0.06	0.333333333	0	0	novel
BGIR9823_82862	MGRN1	0.073333333	0.333333333	0	0	novel
BGIR9823_82897	MLST8	0.05	0.333333333	0	0	novel
BGIR9823_82978	MALL	0.076666667	1.333333333	0	0	novel
BGIR9823_82999	LOC102166005	0.003333333	0.333333333	0	0	novel
BGIR9823_83008	POU3F3	0.056666667	13.31	0	0	novel
BGIR9823_83009	POU3F3	0.02	3.883333333	0	0	novel
BGIR9823_83018	RNF149	0.223333333	1	0	0	novel
BGIR9823_83022	NPAS2	0.02	0.333333333	0	0	novel
BGIR9823_83120	PNO1	0.093333333	0.663333333	0	0	novel
BGIR9823_83313	HPCAL1	0.313333333	1.666666667	0	0	novel
BGIR9823_83395	LOC110260216	0.013333333	0.333333333	0	0	novel
BGIR9823_83550	MMP16	0.146666667	0.666666667	0	0	novel
BGIR9823_83568	SNX16	0.046666667	0.333333333	0	0	novel
BGIR9823_83571	PAG1	0.106666667	0.666666667	0	0	novel
BGIR9823_83610	NCOA2	0.113333333	1.333333333	0	0	novel
BGIR9823_83615	ARFGEF1	0.07	0.666666667	0	0	novel
BGIR9823_83616	ARFGEF1	0.053333333	0.333333333	0	0	novel
BGIR9823_83622	ARFGEF1	0.103333333	1	0	0	novel
BGIR9823_83626	ARFGEF1	0.136666667	1	0	0	novel
BGIR9823_83683	F5	0.026666667	0.333333333	0	0	novel
BGIR9823_83684	F5	0.023333333	0.333333333	0	0	novel
BGIR9823_83710	ADAMTS4	0.09	1.333333333	0	0	novel
BGIR9823_83924	SLC16A1	0.053333333	0.333333333	0	0	novel
BGIR9823_83927	MOV10	0.066666667	0.666666667	0	0	novel
BGIR9823_83965	DBT	0.046666667	0.333333333	0	0	novel
BGIR9823_84034	ZNHIT6	0.046666667	3.37	0	0	novel
BGIR9823_84087	EFR3A	0.11	0.666666667	0	0	novel
BGIR9823_84186	POP1	0.03	0.333333333	0	0	novel
BGIR9823_84204	NDUFAF6	0.056666667	0.333333333	0	0	novel
BGIR9823_84227	DECR1	0.046666667	0.333333333	0	0	novel
BGIR9823_84278	PREX2	0.193333333	1.333333333	0	0	novel
BGIR9823_84287	PDE7A	0.06	0.333333333	0	0	novel
BGIR9823_84291	LOC106510065	0.003333333	0.666666667	0	0	novel
BGIR9823_84335	MPZL1	0.113333333	0.666666667	0	0	novel
BGIR9823_84348	MGST3	0.043333333	0.333333333	0	0	novel
BGIR9823_84375	FCGR3A	0.03	1	0	0	novel
BGIR9823_84389	LOC106510114	0.01	0.333333333	0	0	novel
BGIR9823_84409	PRCC	0.053333333	0.333333333	0	0	novel
BGIR9823_84484	SV2A	0.053333333	0.333333333	0	0	novel
BGIR9823_84718	ARFGAP3	0.09	1	0	0	novel
BGIR9823_84771	TXN2	1.583333333	526.6633333	0	0.853333333	novel
BGIR9823_84785	FBXO7	0.013333333	3	0	0.333333333	novel
BGIR9823_84874	STAT6	0.113333333	0.666666667	0	0	novel
BGIR9823_84920	TBK1	0.043333333	0.333333333	0	0	novel
BGIR9823_84940	LEMD3	0.29	1.67	0	0	novel
BGIR9823_84960	NUP107	0.03	0.333333333	0	0	novel
BGIR9823_84964	NUP107	0.046666667	0.666666667	0	0	novel
BGIR9823_84978	YEATS4	0.043333333	0.666666667	0	0	novel
BGIR9823_84989	PTPRR	0.1	1	0	0	novel
BGIR9823_84991	THAP2	0.116666667	0.666666667	0	0	novel
BGIR9823_85002	OSBPL8	0.013333333	1	0	0	novel
BGIR9823_85066	LRP6	0.06	0.666666667	0	0	novel
BGIR9823_85126	CCDC77	0.063333333	0.666666667	0	0	novel
BGIR9823_85127	LOC102157617	0.056666667	0.333333333	0	0	novel
BGIR9823_85224	ANKS1B	0.04	0.333333333	0	0	novel
BGIR9823_85226	ANKS1B	0.043333333	0.333333333	0	0	novel
BGIR9823_85229	ANKS1B	0.04	0.333333333	0	0	novel

BGIR9823_85240	IKBIP	0.1	0.666666667	0	0	novel
BGIR9823_85243	NR2C1	0.193333333	1	0	0	novel
BGIR9823_85277	PPFIA2	0.036666667	0.333333333	0	0	novel
BGIR9823_85310	LOC110260622	0.033333333	0.333333333	0	0	novel
BGIR9823_85381	C5H12orf75	0.033333333	1.816666667	0	0	novel
BGIR9823_85488	FAM19A2	0.043333333	0.33	0	0	novel
BGIR9823_85551	OSBPL8	0.073333333	0.666666667	0	0	novel
BGIR9823_85573	KLHL42	0.02	0.333333333	0	0	novel
BGIR9823_85600	ETNK1	0.05	0.333333333	0	0	novel
BGIR9823_85606	ETNK1	0.08	0.586666667	0	0	novel
BGIR9823_85612	PYROXD1	0.033333333	0.333333333	0	0	novel
BGIR9823_85653	LPCAT3	0.05	0.666666667	0	0	novel
BGIR9823_85706	DCP1B	0.056666667	0.666666667	0	0	novel
BGIR9823_85780	UTP20	0.206666667	1.666666667	0	0	novel
BGIR9823_85792	IKBIP	0.223333333	1.333333333	0	0	novel
BGIR9823_85800	LOC100514051	0.036666667	0.33	0	0	novel
BGIR9823_85807	NEDD1	0.03	0.333333333	0	0	novel
BGIR9823_85813	ELK3	0.086666667	1	0	0	novel
BGIR9823_85827	METAP2	0.223333333	1.666666667	0	0	novel
BGIR9823_85849	PLEKHG7	0.03	0.333333333	0	0	novel
BGIR9823_85870	ACSS3	0.046666667	0.666666667	0	0	novel
BGIR9823_85948	GLG1	0.113333333	0.666666667	0	0	novel
BGIR9823_85979	MT1D	0.13	1.083333333	0	0	novel
BGIR9823_86023	C6H16orf70	0.043333333	0.333333333	0	0	novel
BGIR9823_86060	LOC100519366	0.09	14	0	0	novel
BGIR9823_86448	ARHGEF16	0.046666667	0.666666667	0	0	novel
BGIR9823_86465	SLC25A33	0.036666667	0.333333333	0	0	novel
BGIR9823_86501	DNAJC16	0.206666667	1.333333333	0	0	novel
BGIR9823_86511	SPATA21	0.063333333	0.333333333	0	0	novel
BGIR9823_86537	KDM1A	0.07	0.333333333	0	0	novel
BGIR9823_86540	KDM1A	0.123333333	0.666666667	0	0	novel
BGIR9823_86561	CLIC4	0.23	1.333333333	0	0	novel
BGIR9823_86563	CLIC4	0.263333333	1.333333333	0	0	novel
BGIR9823_86591	FAM76A	0.146666667	1	0	0	novel
BGIR9823_86592	FAM76A	0.096666667	0.73	0	0	novel
BGIR9823_86733	PTPN2	0.053333333	0.333333333	0	0	novel
BGIR9823_86772	DLGAP1	0.043333333	0.333333333	0	0	novel
BGIR9823_86781	DLGAP1	0.033333333	0.333333333	0	0	novel
BGIR9823_86785	DLGAP1	0.303333333	2.666666667	0	0	novel
BGIR9823_86791	DLGAP1	0.043333333	0.333333333	0	0	novel
BGIR9823_86794	DLGAP1	0.04	0.333333333	0	0	novel
BGIR9823_86824	RBBP8	0.166666667	1.666666667	0	0	novel
BGIR9823_86828	CABLES1	0.1	0.666666667	0	0	novel
BGIR9823_86829	CABLES1	0.05	0.333333333	0	0	novel
BGIR9823_86832	CABLES1	0.086666667	0.666666667	0	0	novel
BGIR9823_86833	RIOK3	0.233333333	1.333333333	0	0	novel
BGIR9823_86835	RIOK3	0.096666667	0.666666667	0	0	novel
BGIR9823_86837	RIOK3	0.056666667	0.333333333	0	0	novel
BGIR9823_87026	PRPF38A	0.1	0.666666667	0	0	novel
BGIR9823_87052	CYP4A21	0.03	2.43	0	0	novel
BGIR9823_87133	CMIP	0.03	0.223333333	0	0	novel
BGIR9823_87207	CDH11	0.013333333	0.333333333	0	0	novel
BGIR9823_87237	PDP2	0.003333333	0.333333333	0	0	novel
BGIR9823_87300	LONP2	0.263333333	1.666666667	0	0	novel
BGIR9823_87313	C6H19orf12	0.05	0.333333333	0	0	novel
BGIR9823_87529	LOC100513362	0.113333333	0.666666667	0	0	novel
BGIR9823_87598	MTHFR	0.063333333	0.333333333	0	0	novel
BGIR9823_87609	LOC110261103	0.276666667	1.666666667	0	0	novel
BGIR9823_87614	SPATA21	0.056666667	0.333333333	0	0	novel
BGIR9823_87615	CROCC	0.033333333	0.333333333	0	0	novel

BGIR9823_87786	DNALI1	0.106666667	0.666666667	0	0	novel
BGIR9823_87859	SMCHD1	0.03	0.333333333	0	0	novel
BGIR9823_87874	LOC102163958	0.033333333	1	0	0	novel
BGIR9823_87886	ROCK1	0.036666667	0.333333333	0	0	novel
BGIR9823_87887	ROCK1	0.06	1	0	0	novel
BGIR9823_87943	KCTD1	0.043333333	0.333333333	0	0	novel
BGIR9823_87946	KCTD1	0.033333333	0.333333333	0	0	novel
BGIR9823_88069	USP1	0.246666667	1.666666667	0	0	novel
BGIR9823_88131	OSBPL9	0.063333333	0.666666667	0	0	novel
BGIR9823_88133	LOC110261422	0.016666667	0.133333333	0	0	novel
BGIR9823_88218	PHACTR1	0.01	0.743333333	0	0	novel
BGIR9823_88243	LOC100512907	0.006666667	6.066666667	0	0	novel
BGIR9823_88284	ZFP57	0.003333333	0.333333333	0	0	novel
BGIR9823_88368	PRR3	0.013333333	2.106666667	0	0	novel
BGIR9823_88374	LOC100153163	0.073333333	0.666666667	0	0	novel
BGIR9823_88378	LST1	0.033333333	1.203333333	0	0	novel
BGIR9823_88450	MAPK14	0.036666667	0.333333333	0	0	novel
BGIR9823_88523	TBC1D2B	0.026666667	1	0	0	novel
BGIR9823_88532	ZFAND6	0.07	0.333333333	0	0	novel
BGIR9823_88535	ZFAND6	0.136666667	1.666666667	0	0	novel
BGIR9823_88544	ABHD17C	0.053333333	0.333333333	0	0	novel
BGIR9823_88553	HDGFL3	0.28	2	0	0	novel
BGIR9823_88565	WDR73	0.073333333	0.333333333	0	0	novel
BGIR9823_88569	WDR73	0.05	0.333333333	0	0	novel
BGIR9823_88587	ABHD2	0.163333333	1.333333333	0	0	novel
BGIR9823_88602	VPS33B	0.193333333	1	0	0	novel
BGIR9823_88610	RCN2	0.056666667	0.333333333	0	0	novel
BGIR9823_88669	PPP2R3C	0.053333333	0.333333333	0	0	novel
BGIR9823_88768	HNRNPC	0.263333333	1.666666667	0	0	novel
BGIR9823_88822	FUT8	0.063333333	0.333333333	0	0	novel
BGIR9823_88823	FUT8	0.026666667	0.333333333	0	0	novel
BGIR9823_88832	FUT8	0.03	0.333333333	0	0	novel
BGIR9823_88851	SLC39A9	0.066666667	0.333333333	0	0	novel
BGIR9823_88874	PCNX1	0.266666667	2	0	0	novel
BGIR9823_88903	PSEN1	0.106666667	0.666666667	0	0	novel
BGIR9823_88904	PSEN1	0.16	0.966666667	0	0	novel
BGIR9823_88983	PAPOLA	0.306666667	2	0	0	novel
BGIR9823_89003	LOC110261647	0.026666667	0.48	0	0	novel
BGIR9823_89009	LOC110261648	0.193333333	1.353333333	0	0	novel
BGIR9823_89026	PXDC1	0.383333333	3.666666667	0	0	novel
BGIR9823_89044	GFOD1	0.273333333	76.65666667	0	0	novel
BGIR9823_89063	NUP153	0.153333333	1	0	0	novel
BGIR9823_89088	SLC17A4	0.056666667	16	0	0	novel
BGIR9823_89224	PHF1	0.036666667	0.333333333	0	0	novel
BGIR9823_89344	MUT	0.073333333	0.333333333	0	0	novel
BGIR9823_89428	SCAPER	0.073333333	0.333333333	0	0	novel
BGIR9823_89435	SCAPER	0.11	0.666666667	0	0	novel
BGIR9823_89503	DHRS1	0.063333333	0.333333333	0	0	novel
BGIR9823_89564	SLCO3A1	0.156666667	1	0	0	novel
BGIR9823_89629	VIPAS39	0.296666667	1.596666667	0	0	novel
BGIR9823_89641	GTF2A1	0.026666667	0.333333333	0	0	novel
BGIR9823_89730	TNIP2	0.45	2.333333333	0	0	novel
BGIR9823_89749	KIAA0232	0.086666667	0.666666667	0	0	novel
BGIR9823_89754	STX18	0.073333333	0.333333333	0	0	novel
BGIR9823_89779	ZCCHC4	0.47	2.333333333	0	0	novel
BGIR9823_89800	PGM2	0.06	0.65	0	0	novel
BGIR9823_89952	PPBP	0.003333333	0.333333333	0	0	novel
BGIR9823_90021	SLC7A11	0.07	0.64	0	0	novel
BGIR9823_90046	LOC100515290	0.076666667	4.65	0	0	novel
BGIR9823_90082	INTS12	0.073333333	0.666666667	0	0	novel

BGIR9823_90231	RAB28	0.04	0.333333333	0	0	novel
BGIR9823_90272	LOC100737183	0.126666667	0.93	0	0	novel
BGIR9823_90336	APBB2	0.053333333	0.666666667	0	0	novel
BGIR9823_90347	GNPDA2	0.033333333	4.58	0	0	novel
BGIR9823_90361	FRYL	0.036666667	0.333333333	0	0	novel
BGIR9823_90391	NAF1	0.046666667	0.333333333	0	0	novel
BGIR9823_90392	NAF1	0.036666667	0.333333333	0	0	novel
BGIR9823_90494	ABCE1	0.11	1	0	0	novel
BGIR9823_90522	NAA15	0.06	0.333333333	0	0	novel
BGIR9823_90794	LOC102160854	0.016666667	0.333333333	0	0	novel
BGIR9823_90838	MED17	0.043333333	0.333333333	0	0	novel
BGIR9823_90863	ARHGAP42	0.02	0.333333333	0	0	novel
BGIR9823_91155	MALSU1	0.076666667	0.333333333	0	0	novel
BGIR9823_91183	MAGI2	0.06	0.666666667	0	0	novel
BGIR9823_91184	MAGI2	0.043333333	0.333333333	0	0	novel
BGIR9823_91185	MAGI2	0.04	0.333333333	0	0	novel
BGIR9823_91190	PTPN12	0.066666667	0.666666667	0	0	novel
BGIR9823_91247	PRRC2C	0.21	1.333333333	0	0	novel
BGIR9823_91262	RABGAP1L	0.006666667	1.08	0	0	novel
BGIR9823_91294	XPR1	0.043333333	0.333333333	0	0	novel
BGIR9823_91301	XPR1	0.04	0.333333333	0	0	novel
BGIR9823_91308	NPL	0.05	1	0	0	novel
BGIR9823_91320	RGL1	0.093333333	1.333333333	0	0	novel
BGIR9823_91444	ARRB1	0.06	0.333333333	0	0	novel
BGIR9823_91476	ALG8	0.123333333	1	0	0	novel
BGIR9823_91610	PAFAH1B2	0.03	0.333333333	0	0	novel
BGIR9823_91653	HEPACAM	0.096666667	0.666666667	0	0	novel
BGIR9823_91669	KIRREL3	0.033333333	0.333333333	0	0	novel
BGIR9823_91696	PPP1R15B	0.096666667	16.37666667	0	0	novel
BGIR9823_91708	SLC41A1	0.036666667	0.33	0	0	novel
BGIR9823_91745	SGCE	0.05	0.333333333	0	0	novel
BGIR9823_91748	SLC25A13	0.23	1.666666667	0	0	novel
BGIR9823_91809	IGF2BP3	0.023333333	0.333333333	0	0	novel
BGIR9823_91817	DBF4	0.043333333	0.333333333	0	0	novel
BGIR9823_91866	FBXL13	0.01	0.15	0	0	novel
BGIR9823_91938	TOR1AIP2	0.136666667	1	0	0	novel
BGIR9823_92089	SDCCAG8	0.293333333	1.666666667	0	0	novel
BGIR9823_92161	AGTPBP1	0.06	0.333333333	0	0	novel
BGIR9823_92172	DCAF12	0.043333333	0.333333333	0	0	novel
BGIR9823_92248	FRMD4A	0.003333333	1.666666667	0	1.46	novel
BGIR9823_92259	ABI1	0.073333333	0.666666667	0	0	novel
BGIR9823_92383	SCCPDH	0.12	0.666666667	0	0	novel
BGIR9823_92396	ADSS	0.04	0.333333333	0	0	novel
BGIR9823_92420	KIF21B	0.013333333	2.233333333	0	0.24	novel
BGIR9823_92437	LOC102162336	0.12	4.326666667	0	0	novel
BGIR9823_92445	DAPK1	0.05	0.333333333	0	0	novel
BGIR9823_92448	DAPK1	0.123333333	1	0	0	novel
BGIR9823_92457	DAPK1	0.053333333	0.333333333	0	0	novel
BGIR9823_92496	SPINK4	0.023333333	0.333333333	0	0	novel
BGIR9823_92576	ENKUR	0.016666667	0.333333333	0	0	novel
BGIR9823_92690	SKA3	0.04	0.666666667	0	0	novel
BGIR9823_92821	TDRD3	0.106666667	1	0	0	novel
BGIR9823_92925	ING1	0.046666667	0.333333333	0	0	novel
BGIR9823_92962	LATS2	0.08	0.666666667	0	0	novel
BGIR9823_93011	DCLK1	0.056666667	13.74666667	0	0	novel
BGIR9823_93026	STOML3	0.043333333	0.333333333	0	0	novel
BGIR9823_93032	LOC100625564	0.12	1	0	0	novel
BGIR9823_93044	PHF11	0.103333333	0.666666667	0	0	novel
BGIR9823_93222	LOC110255863	0.05	3.653333333	0	0	novel
BGIR9823_93410	CDC27	0.06	0.333333333	0	0	novel

BGIR9823_93447	LSM12	0.15	1	0	0	novel
BGIR9823_93448	LSM12	0.063333333	0.333333333	0	0	novel
BGIR9823_93512	LOC100517731	0.086666667	0.666666667	0	0	novel
BGIR9823_93580	PPM1E	0.016666667	0.333333333	0	0	novel
BGIR9823_93583	PPM1E	0.05	0.333333333	0	0	novel
BGIR9823_93630	APPBP2	0.133333333	0.666666667	0	0	novel
BGIR9823_93651	LOC110256041	0.013333333	1.11	0	0	novel
BGIR9823_93707	TAOK1	0.11	0.666666667	0	0	novel
BGIR9823_93748	ASPA	0.046666667	0.666666667	0	0	novel
BGIR9823_93755	SPNS3	0.02	1	0	0	novel
BGIR9823_93767	NUP88	0.063333333	0.333333333	0	0	novel
BGIR9823_93811	MAP2K4	0.093333333	0.44	0	0	novel
BGIR9823_93921	ARMC7	0.053333333	0.333333333	0	0	novel
BGIR9823_93923	ARMC7	0.106666667	1	0	0	novel
BGIR9823_94065	CDK12	0.043333333	0.333333333	0	0	novel
BGIR9823_94223	RHOT1	0.17	1	0	0	novel
BGIR9823_94257	ERAL1	0.003333333	0.333333333	0	0.333333333	novel
BGIR9823_94270	NUFIP2	0.14	0.666666667	0	0	novel
BGIR9823_94468	RAB5A	0.246666667	1.333333333	0	0	novel
BGIR9823_94525	OXSR1	0.136666667	1	0	0	novel
BGIR9823_94638	SEMA3F	0.133333333	0.666666667	0	0	novel
BGIR9823_94644	LOC110256233	0.15	1.666666667	0	0	novel
BGIR9823_94698	PTPRG	0.113333333	0.666666667	0	0	novel
BGIR9823_94699	PTPRG	0.063333333	0.333333333	0	0	novel
BGIR9823_94712	PTPRG	0.2	1.333333333	0	0	novel
BGIR9823_94725	PTPRG	0.05	0.333333333	0	0	novel
BGIR9823_94749	FAM19A1	0.003333333	0.333333333	0	0	novel
BGIR9823_94772	FAM19A1	0.04	1.33	0	0	novel
BGIR9823_94786	CHL1	0.1	0.666666667	0	0	novel
BGIR9823_94841	GRM7	0.05	0.333333333	0	0	novel
BGIR9823_94860	GRM7	0.046666667	0.333333333	0	0	novel
BGIR9823_94951	UBA5	0.036666667	0.666666667	0	0	novel
BGIR9823_94957	SRPRB	0.063333333	0.33	0	0	novel
BGIR9823_95047	GMPS	0.04	0.333333333	0	0	novel
BGIR9823_95053	KCNAB1	0.1	0.666666667	0	0	novel
BGIR9823_95114	NLGN1	0.15	1	0	0	novel
BGIR9823_95132	MFN1	0.133333333	1	0	0	novel
BGIR9823_95135	ACTL6A	0.05	0.333333333	0	0	novel
BGIR9823_95281	OSBPL11	0.17	1	0	0	novel
BGIR9823_95282	OSBPL11	0.04	0.333333333	0	0	novel
BGIR9823_95289	SNX4	0.17	1	0	0	novel
BGIR9823_95346	NAA50	0.036666667	0.333333333	0	0	novel
BGIR9823_95349	USF3	0.103333333	0.666666667	0	0	novel
BGIR9823_95367	ZBTB11	0.046666667	0.333333333	0	0	novel
BGIR9823_95405	NCAM2	0.026666667	0.333333333	0	0	novel
BGIR9823_95562	DYNC1LI1	0.066666667	0.333333333	0	0	novel
BGIR9823_95581	CLASP2	0.07	0.333333333	0	0	novel
BGIR9823_95617	LZTFL1	0.043333333	0.666666667	0	0	novel
BGIR9823_95656	SLC25A20	0.043333333	0.333333333	0	0	novel
BGIR9823_95754	ARHGEF3	0.05	0.333333333	0	0	novel
BGIR9823_95798	UBA3	0.02	0.333333333	0	0	novel
BGIR9823_95883	CNBP	0.036666667	0.333333333	0	0	novel
BGIR9823_95904	RAB6B	0.003333333	0.826666667	0	0.223333333	novel
BGIR9823_96014	PDCD10	0.04	0.666666667	0	0	novel
BGIR9823_96117	ETV5	0.106666667	0.666666667	0	0	novel
BGIR9823_96158	PAK2	0.043333333	0.333333333	0	0	novel
BGIR9823_96182	ADCY5	0.036666667	0.333333333	0	0	novel
BGIR9823_96351	IFNGR2	0.036666667	0.343333333	0	0	novel
BGIR9823_96465	SCARA3	0.056666667	0.333333333	0	0	novel
BGIR9823_96483	MTMR9	0.053333333	0.333333333	0	0	novel

BGIR9823_96632	LOC100517973	0.056666667	3.596666667	0	0	novel
BGIR9823_96642	CORO1C	0.05	0.333333333	0	0	novel
BGIR9823_96833	ABCB10	0.04	1	0	0	novel
BGIR9823_96843	NUP133	0.046666667	0.333333333	0	0	novel
BGIR9823_96854	ZNF25	0.073333333	0.333333333	0	0	novel
BGIR9823_96870	LOC110256649	0.006666667	2	0	0.666666667	novel
BGIR9823_97004	ADK	0.08	2	0	0	novel
BGIR9823_97045	NRG3	0.003333333	0.333333333	0	0	novel
BGIR9823_97150	5-Mar	0.05	0.333333333	0	0	novel
BGIR9823_97160	LOC100624435	1.62	11.95333333	0	0	novel
BGIR9823_97163	CYP2C36	1.036666667	19.09666667	0	0	novel
BGIR9823_97201	ENTPD7	0.036666667	0.333333333	0	0	novel
BGIR9823_97228	LOC102162056	0.046666667	0.333333333	0	0	novel
BGIR9823_97256	MXI1	0.063333333	0.333333333	0	0	novel
BGIR9823_97294	ATRNL1	0.043333333	0.333333333	0	0	novel
BGIR9823_97296	ATRNL1	0.053333333	0.333333333	0	0	novel
BGIR9823_97300	FAM45A	0.053333333	0.67	0	0	novel
BGIR9823_97319	INPP5F	0.066666667	0.666666667	0	0	novel
BGIR9823_97469	P2RX4	0.09	3	0	0	novel
BGIR9823_97503	OAS1	0.03	0.333333333	0	0	novel
BGIR9823_97624	CABIN1	0.106666667	0.666666667	0	0	novel
BGIR9823_97647	ZNF74	0.103333333	0.666666667	0	0	novel
BGIR9823_97687	TSNAX	0.033333333	0.333333333	0	0	novel
BGIR9823_97774	P4HA1	0.33	1.666666667	0	0	novel
BGIR9823_97807	AP3M1	0.163333333	1	0	0	novel
BGIR9823_97858	NCOA4	0.12	1.333333333	0	0	novel
BGIR9823_97873	PRKG1	0.176666667	1	0	0	novel
BGIR9823_97914	MYOF	0.06	0.333333333	0	0	novel
BGIR9823_97951	PIK3AP1	0.093333333	0.666666667	0	0	novel
BGIR9823_97959	MMS19	0.04	0.333333333	0	0	novel
BGIR9823_97980	CWF19L1	0.073333333	0.333333333	0	0	novel
BGIR9823_98019	STN1	0.173333333	1.333333333	0	0	novel
BGIR9823_98050	PDZD8	0.176666667	1	0	0	novel
BGIR9823_98093	UROS	0.23	1.333333333	0	0	novel
BGIR9823_98193	DLGAP2	0.016666667	0.286666667	0	0	novel
BGIR9823_98206	LOC106506202	0.04	0.333333333	0	0	novel
BGIR9823_98354	TLK1	0.083333333	0.666666667	0	0	novel
BGIR9823_98452	SLC39A10	0.003333333	0.466666667	0	0	novel
BGIR9823_98504	ABI2	0.013333333	0.333333333	0	0	novel
BGIR9823_98517	ADAM23	0.056666667	0.333333333	0	0	novel
BGIR9823_98519	ADAM23	0.043333333	0.333333333	0	0	novel
BGIR9823_98573	LOC102162937	0.003333333	0.333333333	0	0	novel
BGIR9823_98719	CCDC93	0.266666667	1.333333333	0	0	novel
BGIR9823_98757	CSMD1	0.006666667	2	0	0	novel
BGIR9823_98759	CSMD1	0.003333333	4.893333333	0	2.136666667	novel
BGIR9823_98778	GPM6A	0.106666667	0.716666667	0	0	novel
BGIR9823_98854	UGGT1	0.116666667	1	0	0	novel
BGIR9823_99020	TMEFF2	0.05	0.333333333	0	0	novel
BGIR9823_99065	ALS2	0.13	0.666666667	0	0	novel
BGIR9823_99183	FBXO36	0.063333333	0.333333333	0	0	novel
BGIR9823_99356	TRAPPC13	0.206666667	1.666666667	0	0	novel
BGIR9823_99364	SREK1	0.023333333	0.76	0	0	novel
BGIR9823_99389	OCLN	0.033333333	0.333333333	0	0	novel
BGIR9823_99393	BDP1	0.066666667	0.666666667	0	0	novel
BGIR9823_99400	MCCC2	0.136666667	0.666666667	0	0	novel
BGIR9823_99417	CPEB4	0.096666667	0.666666667	0	0	novel
BGIR9823_99450	ATOX1	0.013333333	0.55	0	0	novel
BGIR9823_99468	CTNND2	0.05	0.263333333	0	0	novel
BGIR9823_99477	RETREG1	0.25	1.333333333	0	0	novel
BGIR9823_99513	LOC110257335	0.163333333	1.666666667	0	0	novel

BGIR9823_99530	RAI14	0.04	0.333333333	0	0	novel
BGIR9823_99533	NADK2	0.126666667	1.333333333	0	0	novel
BGIR9823_99580	LOC102159225	0.033333333	1.21	0	0	novel
BGIR9823_99639	SMN1	0.026666667	0.333333333	0	0	novel
BGIR9823_99714	MTRR	0.056666667	0.326666667	0	0	novel
BGIR9823_99716	ADCY2	0.066666667	0.333333333	0	0	novel
BGIR9823_99808	RNF24	0.116666667	1	0	0	novel
BGIR9823_99810	PANK2	0.05	0.666666667	0	0	novel
BGIR9823_99812	PANK2	0.09	0.673333333	0	0	novel
BGIR9823_99866	RALY	0.463333333	3	0	0	novel
BGIR9823_99869	AHCY	0.04	0.333333333	0	0	novel
BGIR9823_99933	LOC100622037	0.01	0.433333333	0	0	novel
BGIR9823_99947	DHX35	0.053333333	0.333333333	0	0	novel
BGIR9823_99983	ZSWIM3	0.043333333	0.333333333	0	0	novel
BGIR9823_99990	ZNF335	0.056666667	0.333333333	0	0	novel
BGIR9823_99992	LOC106506709	0.013333333	0.333333333	0	0	novel
NR_045016.1	LOC100626841	0.07	3.666666667	0	0	known
NR_132429.1	CYP2C91	0.12	6.936666667	0	0	known
URS0000BC489C		0.01	0.333333333	0	0	known
URS0000BC4A4D	LOC106506163	0.016666667	3.486666667	0	0	known
URS0000BC4B7B	LOC110260408	0.02	1	0	0	known
URS0000BC4E55		0.006666667	0.333333333	0	0	known
URS0000BC4E8F	LOC106506869	0.003333333	0.056666667	0	0	known
URS0000BC4FE0		0.003333333	0.333333333	0	0	known
URS0000BC50A3	LOC100516640	0.006666667	0.333333333	0	0	known
URS0000BC5676	LOC110255218	0.1	18.34333333	0	0	known
URS0000BC586D	LOC110259818	0.003333333	0.333333333	0	0	known
URS0000BC59D4	LOC102165590	0.003333333	0.333333333	0	0	known
URS0000BC5B29	LOC110260905	0.003333333	0.343333333	0	0	known
URS0000BC5B6D	LOC102162336	0.046666667	3.56	0	0	known
URS0000BC5D1B		0.006666667	0.983333333	0	0	known
URS0000BC5DA2	LOC102162336	0.003333333	0.333333333	0	0	known
URS0000BC5FFB	LOC110257533	0.006666667	0.83	0	0	known
URS0000BC619E	LOC110261527	0.003333333	0.333333333	0	0	known
URS0000BC6538		0.003333333	0.333333333	0	0	known
URS0000BC6611	LOC110258645	0.073333333	2.496666667	0	0	known
URS0000BC6903	LOC110255422	0.01	0.333333333	0	0	known
URS0000BC6E01	LOC110257548	0.003333333	0.333333333	0	0	known
URS0000BC713E	LOC100624063	0.003333333	0.363333333	0	0	known
URS0000BC7214	LOC102166974	0.003333333	0.666666667	0	0	known
URS0000BC74B9		0.016666667	0.333333333	0	0	known
URS0000BC7C14	LOC100516640	0.006666667	0.333333333	0	0	known
URS0000BC8181	LOC110261620	0.003333333	0.333333333	0	0	known
URS0000BC8281	LOC106506809	0.006666667	0.55	0	0	known
URS0000BC83B2	LOC106506163	0.006666667	1.29	0	0	known
URS0000BC8607		0.006666667	0.333333333	0	0	known
URS0000BC882E		0.006666667	0.666666667	0	0	known
URS0000BC8981	LOC100521600	0.176666667	6.14	0	0	known
URS0000BC8C4F	LOC102164427	0.006666667	0.883333333	0	0	known
URS0000BC8F98	LOC106509894	0.003333333	0.12	0	0	known
URS0000BC9178	LOC102162145	0.01	0.666666667	0	0	known
URS0000BC95C8	LOC106505481	0.006666667	0.333333333	0	0	known
URS0000BC9694		0.013333333	0.333333333	0	0	known
URS0000BC99D3	LOC106510460	0.023333333	0.75	0	0	known
URS0000BC9ABC		0.006666667	0.333333333	0	0	known
URS0000BC9E5C		0.01	0.333333333	0	0	known
URS0000BCA017	LOC110257902	0.006666667	0.666666667	0	0	known
URS0000BCA076	LOC102158641	0.003333333	0.333333333	0	0	known
URS0000BCA109	LOC102159559	0.01	0.883333333	0	0	known
URS0000BCA327	C16H5orf38	0.01	0.333333333	0	0	known

URS0000BCA39D		0.023333333	1.246666667	0	0	known
URS0000BCA3A4	LOC106505240	0.006666667	0.333333333	0	0	known
XR_001297909.2	LOC102166620	0.01	0.333333333	0	0	known
XR_001297958.2	LOC106504468	0.016666667	1.333333333	0	0	known
XR_001298070.2	LOC106504523	0.006666667	0.333333333	0	0	known
XR_001298113.2	LOC106504551	0.036666667	0.666666667	0	0	known
XR_001298146.2	LOC106504564	0.043333333	0.333333333	0	0	known
XR_001298249.2	LOC106504619	0.003333333	0.666666667	0	0.333333333	known
XR_001298322.2	LOC102165777	0.006666667	0.83	0	0	known
XR_001298364.2	LOC106504694	0.003333333	0.333333333	0	0	known
XR_001298539.2	LOC106504772	0.013333333	1.386666667	0	0	known
XR_001298813.2	LOC106504881	0.02	3.966666667	0	0	known
XR_001298814.2	LOC106504881	0.036666667	6.923333333	0	0	known
XR_001298816.2	LOC106504881	0.28	51.14	0	0	known
XR_001298841.2	LOC102161637	0.006666667	0.666666667	0	0.08	known
XR_001298911.2	LOC106504947	0.006666667	0.666666667	0	0	known
XR_001298942.2	LOC102163402	0.036666667	2.226666667	0	0	known
XR_001298943.2	LOC102163402	0.05	1.72	0	0	known
XR_001298964.2	LOC106504962	0.026666667	6.666666667	0	1	known
XR_001299154.2	LOC106505077	0.006666667	0.333333333	0	0	known
XR_001299211.2	LOC102166259	0.01	3	0	0.333333333	known
XR_001299307.2	LOC106505166	0.006666667	0.333333333	0	0	known
XR_001299360.2	LOC106505194	0.006666667	0.666666667	0	0	known
XR_001299381.2	LOC106505206	0.003333333	0.333333333	0	0	known
XR_001299410.2	LOC106505220	0.003333333	0.333333333	0	0	known
XR_001299576.2	LOC106505298	0.016666667	0.333333333	0	0	known
XR_001299694.2	LOC106505361	0.013333333	3.666666667	0	0.333333333	known
XR_001299729.2	LOC106505374	0.013333333	2	0	0	known
XR_001299736.2	LOC106505376	0.003333333	0.333333333	0	0	known
XR_001299926.2	LOC106505472	0.006666667	0.333333333	0	0	known
XR_001299934.2	LOC106505476	0.006666667	0.333333333	0	0	known
XR_001299951.2	LOC106505485	0.006666667	0.166666667	0	0	known
XR_001300053.2	LOC106505533	0.01	0.333333333	0	0	known
XR_001300108.2	LOC102167094	0.01	0.333333333	0	0	known
XR_001300390.2	LOC106505723	0.043333333	1.666666667	0	0	known
XR_001300453.2	LOC106505754	0.02	0.666666667	0	0	known
XR_001300526.2	LOC102157484	0.033333333	4.666666667	0	0	known
XR_001300587.2	LOC106505815	0.003333333	0.333333333	0	0	known
XR_001300710.2	LOC106505875	0.03	0.666666667	0	0	known
XR_001300908.2	LOC106505961	0.026666667	1.333333333	0	0	known
XR_001300933.2	LOC106505983	0.023333333	0.666666667	0	0	known
XR_001301004.2	LOC106506036	0.036666667	0.333333333	0	0	known
XR_001301042.2	LOC106506060	0.016666667	1.666666667	0	0	known
XR_001301043.2	LOC106506062	0.01	1	0	0	known
XR_001301101.2	LOC106506085	0.023333333	1	0	0	known
XR_001301217.2	LOC102164998	0.04	15.62666667	0	0	known
XR_001301285.2	LOC102164652	0.006666667	1.95	0	0	known
XR_001301430.2	LOC106506243	0.003333333	0.333333333	0	0	known
XR_001301824.2	LOC106506503	0.003333333	1.033333333	0	0.333333333	known
XR_001301842.2	LOC102161888	0.013333333	1.96	0	0	known
XR_001301884.2	LOC106506532	0.013333333	0.666666667	0	0	known
XR_001301984.2	LOC106506585	0.006666667	1.666666667	0	0	known
XR_001302150.2	LOC102159476	0.03	3.7	0	0	known
XR_001302153.2	LOC102159476	0.056666667	13.98666667	0	0	known
XR_001302178.2	LOC106506709	0.016666667	0.333333333	0	0	known
XR_001302198.2	LOC106506715	0.013333333	3.666666667	0	0	known
XR_001302236.2	LOC102161909	0.026666667	1.286666667	0	0	known
XR_001302298.2	LOC106506758	0.003333333	0.666666667	0	0.333333333	known
XR_001302325.2	LOC100511483	0.006666667	0.666666667	0	0	known
XR_001302542.2	LOC106506895	0.003333333	0.333333333	0	0	known

XR_001302544.2	LOC106506896	0.006666667	0.753333333	0	0	known
XR_001302908.2	LOC106507150	0.006666667	0.333333333	0	0	known
XR_001303147.2	LOC106507269	0.006666667	0.333333333	0	0	known
XR_001303266.2	LOC106507349	0.016666667	1.64	0	0	known
XR_001303268.2	LOC106507349	0.01	0.36	0	0	known
XR_001303278.2	LOC106507358	0.013333333	0.666666667	0	0	known
XR_001303298.2	LOC102160363	0.03	1.493333333	0	0	known
XR_001303378.2	LOC106507417	0.006666667	0.333333333	0	0	known
XR_001303471.2	LOC100621315	0.003333333	1.57	0	0.666666667	known
XR_001303564.2	LOC100524923	0.006666667	2.423333333	0	0	known
XR_001303602.2	LOC106507552	0.023333333	5.116666667	0	0	known
XR_001303759.2	LOC102162217	0.006666667	0.333333333	0	0	known
XR_001303930.2	CCNB1IP1	0.01	1.113333333	0	0	known
XR_001304125.2	LOC106507833	0.006666667	0.333333333	0	0	known
XR_001304273.2	LOC106507908	0.013333333	0.666666667	0	0	known
XR_001304306.2	LOC102168177	0.043333333	2.003333333	0	0	known
XR_001304442.2	LOC106507985	0.003333333	0.333333333	0	0	known
XR_001304462.2	LOC106507994	0.006666667	0.333333333	0	0	known
XR_001304471.2	LOC106507997	0.02	0.666666667	0	0	known
XR_001304617.2	LOC102158040	0.01	0.666666667	0	0	known
XR_001304656.2	LOC106508097	0.003333333	0.333333333	0	0	known
XR_001304778.2	LOC106508168	0.006666667	0.333333333	0	0	known
XR_001304820.2	LOC106508187	0.013333333	0.333333333	0	0	known
XR_001304929.2	LOC106508222	0.003333333	0.426666667	0	0	known
XR_001305131.2	LOC106508340	0.006666667	0.333333333	0	0	known
XR_001305509.2	LOC106508565	0.02	1.666666667	0	0	known
XR_001305525.2	LOC106508578	0.003333333	0.333333333	0	0	known
XR_001305691.2	LOC102166401	0.003333333	1.333333333	0	0.666666667	known
XR_001305858.2	LOC106508800	0.003333333	0.333333333	0	0	known
XR_001305913.2	LOC100621792	0.013333333	0.666666667	0	0	known
XR_001306050.2	LOC102164763	0.033333333	1.856666667	0	0	known
XR_001306057.2	LOC102164525	0.003333333	0.333333333	0	0	known
XR_001306261.2	LOC106509046	0.003333333	0.393333333	0	0	known
XR_001306638.2	LOC102160504	0.006666667	0.666666667	0	0	known
XR_001307045.2	LOC106509473	0.076666667	1	0	0	known
XR_001307130.2	LOC106509521	0.016666667	1.556666667	0	0	known
XR_001307146.2	LOC106509530	0.003333333	0.333333333	0	0	known
XR_001307168.2	LOC102158429	0.01	0.333333333	0	0	known
XR_001307170.2	LOC106509541	0.003333333	0.666666667	0	0	known
XR_001307305.2	LOC106509627	0.006666667	1	0	0	known
XR_001307332.2	LOC106509641	0.023333333	0.333333333	0	0	known
XR_001307339.2	LOC106509647	0.07	9.44	0	0	known
XR_001307408.2	LOC102162776	0.033333333	0.76	0	0	known
XR_001307581.2	LOC100516649	0.056666667	3.03	0	0	known
XR_001307852.2	LOC102157748	0.003333333	0.113333333	0	0	known
XR_001307888.2	LOC106509905	0.006666667	3.333333333	0	1.333333333	known
XR_001308151.2	LOC106510037	0.01	0.333333333	0	0	known
XR_001308193.2	LOC106510063	0.013333333	0.333333333	0	0	known
XR_001308331.2	LOC106510129	0.003333333	0.45	0	0	known
XR_001308358.2	LOC106510137	0.02	0.333333333	0	0	known
XR_001308418.2	LOC106510188	0.006666667	1	0	0	known
XR_001308620.2	LOC106510256	0.003333333	0.333333333	0	0	known
XR_001308671.2	LOC102159397	0.013333333	0.666666667	0	0	known
XR_001308715.2	LOC102165929	0.01	1.58	0	0	known
XR_001308729.2	LOC106510320	0.006666667	0.666666667	0	0	known
XR_001308846.2	LOC106510400	0.006666667	0.333333333	0	0	known
XR_001309152.2	LOC106510565	0.033333333	4	0	0	known
XR_001309352.2	LOC106510687	0.01	0.333333333	0	0	known
XR_002335538.1	LOC102160761	0.003333333	0.333333333	0	0	known
XR_002335546.1	LOC106504821	0.01	0.333333333	0	0	known

XR_002335585.1	LOC102157509	0.013333333	0.333333333	0	0	known
XR_002335587.1	LOC102157509	0.006666667	0.656666667	0	0	known
XR_002335599.1	LOC110255422	0.01	0.333333333	0	0	known
XR_002335610.1	LOC102166056	0.003333333	0.7	0	0	known
XR_002335613.1	LOC110255424	0.003333333	0.333333333	0	0	known
XR_002335636.1	LOC110255433	0.026666667	3.603333333	0	0	known
XR_002335641.1	LOC102168180	0.133333333	9.006666667	0	0	known
XR_002335649.1	LOC110255434	0.373333333	2.776666667	0	0	known
XR_002335652.1	LOC106504881	0.143333333	27.76	0	0	known
XR_002335653.1	LOC106504881	0.02	3.766666667	0	0	known
XR_002335667.1	LOC106509137	0.003333333	0.666666667	0	0	known
XR_002335692.1	LOC102163364	0.02	3.71	0	0	known
XR_002335730.1	LOC110255457	0.083333333	1.666666667	0	0	known
XR_002335745.1	LOC110255461	0.083333333	1.693333333	0	0	known
XR_002335747.1	LOC106504929	0.013333333	1.666666667	0	0	known
XR_002335760.1	LOC110255465	0.006666667	2	0	0.333333333	known
XR_002335814.1	LOC102163402	0.03	2.286666667	0	0	known
XR_002335826.1	LOC102161092	0.026666667	2.953333333	0	0	known
XR_002335832.1	LOC102161092	0.016666667	0.73	0	0	known
XR_002335877.1	LOC110255491	0.003333333	0.333333333	0	0	known
XR_002335903.1	LOC106507234	0.013333333	1.333333333	0	0	known
XR_002335924.1	LOC110255511	0.05	1.48	0	0	known
XR_002335939.1	LOC106507874	0.003333333	0.333333333	0	0	known
XR_002335948.1	LOC110255517	0.003333333	0.333333333	0	0	known
XR_002335958.1	LOC102161394	0.013333333	2.756666667	0	0	known
XR_002336015.1	LOC102165237	0.013333333	3.696666667	0	0.676666667	known
XR_002336037.1	LOC102164427	0.003333333	0.466666667	0	0	known
XR_002336041.1	LOC102164427	0.006666667	1.186666667	0	0	known
XR_002336042.1	LOC110255533	0.013333333	0.333333333	0	0	known
XR_002336077.1	LOC110255541	0.003333333	0.333333333	0	0	known
XR_002336094.1	LOC110255549	0.003333333	0.333333333	0	0	known
XR_002336099.1	LOC110255552	0.006666667	0.666666667	0	0	known
XR_002336100.1	LOC110255552	0.003333333	0.666666667	0	0.666666667	known
XR_002336128.1	LOC106505102	0.016666667	1.396666667	0	0	known
XR_002336129.1	LOC106505102	0.023333333	0.936666667	0	0	known
XR_002336131.1	LOC106505141	0.003333333	0.666666667	0	0	known
XR_002336135.1	LOC110255585	0.003333333	0.333333333	0	0	known
XR_002336141.1	LOC110255590	0.003333333	1.333333333	0	0	known
XR_002336145.1	LOC102167499	0.016666667	0.666666667	0	0	known
XR_002336153.1	LOC102167241	0.01	0.333333333	0	0	known
XR_002336154.1	LOC106505140	0.01	0.333333333	0	0	known
XR_002336160.1	LOC100737021	0.07	7.87	0	0	known
XR_002336166.1	LOC100737021	0.096666667	10.72666667	0	0	known
XR_002336167.1	LOC102157766	0.013333333	0.333333333	0	0	known
XR_002336181.1	LOC106505078	0.01	1	0	0	known
XR_002336211.1	LOC110255636	0.003333333	0.326666667	0	0	known
XR_002336221.1	LOC106505094	0.006666667	0.666666667	0	0	known
XR_002336226.1	LOC110255645	0.023333333	0.333333333	0	0	known
XR_002336240.1	LOC110255649	0.226666667	8.39	0	0	known
XR_002336241.1	LOC110255649	0.026666667	1.066666667	0	0	known
XR_002336267.1	LOC110255655	0.003333333	0.333333333	0	0	known
XR_002336326.1	LOC110255681	0.003333333	0.333333333	0	0	known
XR_002336342.1	LOC110255687	0.003333333	0.333333333	0	0	known
XR_002336377.1	LOC102166060	0.01	1	0	0	known
XR_002336398.1	LOC106505184	0.003333333	0.913333333	0	0	known
XR_002336413.1	LOC106507622	0.026666667	0.666666667	0	0	known
XR_002336421.1	LOC110255719	0.003333333	0.333333333	0	0	known
XR_002336434.1	LOC102165195	0.006666667	0.333333333	0	0	known
XR_002336439.1	LOC110255728	0.013333333	0.333333333	0	0	known
XR_002336444.1	LOC102157904	0.003333333	1	0	0.333333333	known

XR_002336455.1	LOC106505220	0.003333333	1	0	0	known
XR_002336539.1	LOC110255752	0.01	1	0	0	known
XR_002336544.1	LOC110255754	0.003333333	0.333333333	0	0	known
XR_002336582.1	LOC110255772	0.003333333	0.333333333	0	0	known
XR_002336591.1	LOC110255788	0.016666667	1.333333333	0	0	known
XR_002336603.1	LOC102167456	0.003333333	0.333333333	0	0	known
XR_002336604.1	LOC110255791	0.02	1	0	0	known
XR_002336617.1	LOC106507930	0.066666667	4.816666667	0	0	known
XR_002336620.1	LOC106507930	0.02	1.236666667	0	0	known
XR_002336630.1	LOC106505240	0.003333333	0.333333333	0	0	known
XR_002336633.1	LOC110255799	0.03	7.153333333	0	0	known
XR_002336636.1	LOC110255799	0.056666667	7.26	0	0	known
XR_002336637.1	LOC110255799	0.04	5.29	0	0	known
XR_002336681.1	LOC102167197	0.03	3	0	0	known
XR_002336690.1	LOC110255826	0.003333333	0.333333333	0	0	known
XR_002336691.1	LOC110255827	0.006666667	0.666666667	0	0	known
XR_002336706.1	LOC110255834	0.016666667	0.42	0	0	known
XR_002336707.1	LOC110255834	0.013333333	0.2	0	0.016666667	known
XR_002336713.1	LOC110255837	0.003333333	0.333333333	0	0	known
XR_002336719.1	LOC102164049	0.003333333	0.333333333	0	0	known
XR_002336724.1	LOC110255843	0.006666667	0.333333333	0	0	known
XR_002336727.1	LOC102165694	0.006666667	0.596666667	0	0	known
XR_002336729.1	LOC110255847	0.013333333	1	0	0	known
XR_002336769.1	LOC106505355	0.006666667	0.666666667	0	0	known
XR_002336819.1	LOC110255882	0.006666667	0.666666667	0	0	known
XR_002336820.1	LOC110255882	0.006666667	0.333333333	0	0	known
XR_002336834.1	STX8	0.093333333	3.86	0	0	known
XR_002336843.1	LOC106505520	0.02	0.333333333	0	0	known
XR_002336844.1	LOC110255884	0.016666667	1	0	0	known
XR_002336870.1	LOC110255912	0.013333333	0.666666667	0	0	known
XR_002336887.1	LOC106505369	0.006666667	1.116666667	0	0	known
XR_002336920.1	LOC110255929	0.013333333	0.333333333	0	0	known
XR_002336936.1	LOC102158172	0.003333333	0.333333333	0	0	known
XR_002336960.1	LOC110255960	0.006666667	0.79	0	0	known
XR_002337049.1	LOC102166398	0.023333333	2.666666667	0	0	known
XR_002337078.1	LOC102167870	0.003333333	0.776666667	0	0.143333333	known
XR_002337117.1	LOC102163646	0.046666667	1	0	0	known
XR_002337152.1	LOC106505099	0.033333333	5.413333333	0	0	known
XR_002337165.1	LOC106505435	0.01	0.333333333	0	0	known
XR_002337187.1	LOC110256029	0.003333333	0.333333333	0	0	known
XR_002337218.1	LOC110256041	0.146666667	8.89	0	0	known
XR_002337259.1	LOC106507967	0.003333333	0.333333333	0	0	known
XR_002337263.1	LOC110256047	0.033333333	0.523333333	0	0	known
XR_002337284.1	LOC102161554	0.016666667	0.666666667	0	0	known
XR_002337287.1	LOC106508485	0.026666667	1.666666667	0	0	known
XR_002337344.1	LOC110255314	0.006666667	0.333333333	0	0	known
XR_002337361.1	LOC100525078	0.1	1.89	0	0	known
XR_002337384.1	LOC110256113	0.006666667	0.333333333	0	0	known
XR_002337388.1	LOC110256118	0.146666667	50.47666667	0	0	known
XR_002337416.1	LOC106505263	0.003333333	0.916666667	0	0.513333333	known
XR_002337440.1	LOC110256165	0.006666667	1	0	0	known
XR_002337441.1	LOC110256165	0.01	1.666666667	0	0	known
XR_002337471.1	GSN	0.09	10.37	0	0	known
XR_002337533.1	LOC110256232	0.01	1	0	0	known
XR_002337536.1	LOC106505607	0.03	3.916666667	0	0	known
XR_002337546.1	LOC110256236	0.026666667	0.666666667	0	0	known
XR_002337556.1	SMIM4	0.01	1.176666667	0	0	known
XR_002337586.1	LOC106505618	0.016666667	2.666666667	0	0	known
XR_002337591.1	LOC102162847	0.006666667	0.666666667	0	0	known
XR_002337592.1	LOC102162847	0.006666667	0.333333333	0	0	known

XR_002337595.1	LOC110256251	0.06	1	0	0	known
XR_002337612.1	LOC106505628	0.006666667	0.333333333	0	0	known
XR_002337629.1	LOC110256267	0.016666667	0.333333333	0	0	known
XR_002337634.1	LOC110256273	0.006666667	0.333333333	0	0	known
XR_002337657.1	LOC106505677	0.006666667	0.333333333	0	0	known
XR_002337662.1	LOC106505682	0.036666667	2	0	0	known
XR_002337672.1	LOC110256295	0.026666667	1	0	0	known
XR_002337744.1	LOC110256329	0.006666667	0.333333333	0	0	known
XR_002337750.1	LOC106505741	0.003333333	0.333333333	0	0	known
XR_002337793.1	LOC100519216	0.003333333	0.333333333	0	0	known
XR_002337799.1	LOC110256386	0.01	0.666666667	0	0	known
XR_002337816.1	LOC102162054	0.203333333	3.66	0	0	known
XR_002337817.1	LOC102162054	0.203333333	3.66	0	0	known
XR_002337865.1	LOC110256409	0.03	0.666666667	0	0	known
XR_002337906.1	LOC110256424	0.02	5.16	0	0	known
XR_002337920.1	LOC110256428	0.003333333	0.333333333	0	0	known
XR_002337922.1	LOC110256429	0.006666667	0.333333333	0	0	known
XR_002337930.1	LOC110256433	0.003333333	0.333333333	0	0	known
XR_002337939.1	LOC102162881	0.003333333	0.666666667	0	0	known
XR_002337965.1	LOC110256455	0.003333333	0.666666667	0	0	known
XR_002337975.1	LOC102162007	0.023333333	3.206666667	0	0	known
XR_002337978.1	LOC102162007	0.016666667	2.32	0	0	known
XR_002337984.1	LOC106505633	0.003333333	1.556666667	0	1.913333333	known
XR_002337990.1	LOC106505633	0.003333333	1.423333333	0	0	known
XR_002338005.1	LOC102161811	0.03	2.583333333	0	0	known
XR_002338022.1	LOC102158685	0.006666667	0.333333333	0	0	known
XR_002338058.1	LOC106505851	0.006666667	0.333333333	0	0	known
XR_002338065.1	LOC106505859	0.006666667	0.333333333	0	0	known
XR_002338075.1	LOC110256490	0.006666667	0.333333333	0	0	known
XR_002338092.1	LOC110256500	0.01	1.333333333	0	0	known
XR_002338097.1	LOC110256503	0.003333333	0.333333333	0	0	known
XR_002338113.1	LOC106505748	0.003333333	4	0	0	known
XR_002338195.1	LOC106508320	0.033333333	1.666666667	0	0	known
XR_002338313.1	LOC102160169	0.016666667	4.553333333	0	0	known
XR_002338342.1	ARHGAP19	0.05	12.52666667	0	0	known
XR_002338351.1	LOC110256606	0.003333333	0.333333333	0	0	known
XR_002338354.1	LOC110256609	0.006666667	0.333333333	0	0	known
XR_002338371.1	LOC110256613	0.003333333	0.333333333	0	0	known
XR_002338380.1	LOC106509031	0.003333333	0.333333333	0	0	known
XR_002338401.1	LOC102160044	0.006666667	0.666666667	0	0	known
XR_002338414.1	LOC106505903	0.096666667	9.256666667	0	0	known
XR_002338449.1	LOC106505879	0.006666667	0.483333333	0	0	known
XR_002338477.1	LOC102165722	0.016666667	1.92	0	0	known
XR_002338490.1	LOC110256651	0.003333333	0.333333333	0	0.32	known
XR_002338513.1	LOC110256658	0.03	0.666666667	0	0	known
XR_002338526.1	LOC110256666	0.023333333	0.666666667	0	0	known
XR_002338576.1	LOC110256694	0.003333333	0.333333333	0	0	known
XR_002338640.1	LOC110256730	0.006666667	0.81	0	0	known
XR_002338675.1	LOC110255180	2.21	107.4466667	0	0	known
XR_002338678.1	LOC110255180	0.063333333	3.17	0	0	known
XR_002338700.1	LOC106506052	0.006666667	8.65	0	0	known
XR_002338702.1	LOC106506054	0.016666667	1.666666667	0	0	known
XR_002338732.1	LOC110256774	0.006666667	0.856666667	0	0	known
XR_002338733.1	LOC110256775	0.006666667	2	0	1	known
XR_002338768.1	LOC110256784	0.063333333	5.666666667	0	0	known
XR_002338770.1	LOC110256785	0.003333333	0.513333333	0	0	known
XR_002338772.1	LOC110256785	0.02	2.82	0	0	known
XR_002338782.1	LOC110256790	0.003333333	0.666666667	0	0	known
XR_002338783.1	LOC110256790	0.003333333	0.666666667	0	0	known
XR_002338787.1	LOC110256791	0.003333333	0.333333333	0	0	known

XR_002338789.1	LOC102158750	0.003333333	2.333333333	0	0	known
XR_002338816.1	LOC106506008	0.013333333	0.333333333	0	0	known
XR_002338863.1	LOC110256839	0.01	0.333333333	0	0	known
XR_002338895.1	LOC110256851	0.003333333	1	0	0	known
XR_002338902.1	LOC106508107	0.03	2.333333333	0	0	known
XR_002338903.1	LOC110256852	0.003333333	0.666666667	0	0	known
XR_002338922.1	LOC106505925	0.013333333	0.333333333	0	0	known
XR_002338973.1	LOC110256886	0.003333333	0.403333333	0	0	known
XR_002338980.1	LOC110256901	0.013333333	0.666666667	0	0	known
XR_002339007.1	LOC102165127	0.006666667	1.333333333	0	0	known
XR_002339015.1	LOC110256932	0.016666667	1.28	0	0	known
XR_002339022.1	LOC102157527	0.04	1.333333333	0	0	known
XR_002339055.1	LOC110256963	0.036666667	0.333333333	0	0	known
XR_002339061.1	LOC102158523	0.003333333	0.333333333	0	0	known
XR_002339082.1	LOC102162428	0.04	0.94	0	0	known
XR_002339083.1	LOC102162428	0.026666667	0.603333333	0	0	known
XR_002339095.1	LOC110256990	0.006666667	1	0	0	known
XR_002339096.1	LOC110256990	0.006666667	0.87	0	0	known
XR_002339101.1	LOC110256990	0.003333333	0.793333333	0	0	known
XR_002339104.1	LOC106508120	0.006666667	0.333333333	0	0	known
XR_002339107.1	LOC110256997	0.003333333	0.333333333	0	0	known
XR_002339139.1	LOC102163217	0.013333333	0.666666667	0	0	known
XR_002339141.1	LOC106506229	0.033333333	2.666666667	0	0	known
XR_002339145.1	LOC106506229	0.026666667	1.333333333	0	0	known
XR_002339203.1	LOC106508273	0.013333333	0.666666667	0	0	known
XR_002339243.1	LOC110257068	0.13	5.333333333	0	0	known
XR_002339287.1	LOC100520518	0.013333333	0.666666667	0	0	known
XR_002339306.1	LOC110257080	0.003333333	0.333333333	0	0	known
XR_002339342.1	FAM126B	0.016666667	6.273333333	0	0	known
XR_002339344.1	FAM126B	0.026666667	6.006666667	0	0	known
XR_002339347.1	FAM126B	0.11	44.65333333	0	0	known
XR_002339349.1	FAM126B	0.033333333	12.96666667	0	0	known
XR_002339358.1	LOC110257119	0.026666667	1.333333333	0	0	known
XR_002339365.1	LOC110257124	0.013333333	0.666666667	0	0	known
XR_002339369.1	LOC100516649	0.006666667	0.37	0	0	known
XR_002339370.1	LOC100516649	0.006666667	0.38	0	0	known
XR_002339371.1	LOC100516649	0.02	1.186666667	0	0	known
XR_002339460.1	LOC110257185	0.01	2.666666667	0	1	known
XR_002339475.1	LOC102166560	0.003333333	0.333333333	0	0	known
XR_002339484.1	LOC106506175	0.003333333	0.806666667	0	0	known
XR_002339491.1	LOC110257203	0.003333333	0.333333333	0	0	known
XR_002339525.1	LOC110257218	0.003333333	0.333333333	0	0	known
XR_002339529.1	LOC102160084	0.01	0.333333333	0	0	known
XR_002339566.1	LOC110257233	0.003333333	0.333333333	0	0	known
XR_002339580.1	LOC102168063	0.016666667	3.333333333	0	0	known
XR_002339581.1	LOC102168151	0.02	0.666666667	0	0	known
XR_002339585.1	LOC110257237	0.003333333	0.333333333	0	0	known
XR_002339600.1	LOC102159225	0.003333333	0.333333333	0	0	known
XR_002339609.1	LOC110257247	0.006666667	0.333333333	0	0	known
XR_002339628.1	LOC102164640	0.01	3.75	0	0.073333333	known
XR_002339634.1	LOC102164818	0.04	22.15666667	0	0	known
XR_002339640.1	LOC102164818	0.01	0.523333333	0	0	known
XR_002339644.1	LOC110257262	0.003333333	1.333333333	0	0	known
XR_002339656.1	LOC106506511	0.003333333	0.333333333	0	0	known
XR_002339657.1	LOC106506511	0.003333333	0.333333333	0	0	known
XR_002339681.1	LOC102157413	0.003333333	0.666666667	0	0	known
XR_002339689.1	LOC110257280	0.03	5.863333333	0	0	known
XR_002339703.1	LOC106506539	0.003333333	0.333333333	0	0	known
XR_002339732.1	LOC110257300	0.003333333	0.333333333	0	0	known
XR_002339809.1	LOC110257331	0.01	0.85	0	0	known

XR_002339810.1	LOC110257331	0.003333333	1.15	0	0	known
XR_002339820.1	LOC102161640	0.006666667	0.666666667	0	0	known
XR_002339850.1	LOC110257356	0.003333333	0.333333333	0	0	known
XR_002339856.1	LOC106506707	0.006666667	0.333333333	0	0	known
XR_002339910.1	POMK	0.336666667	53.17	0	0	known
XR_002339916.1	LOC106506661	0.12	2.58	0	0	known
XR_002339917.1	LOC106506661	0.046666667	1.033333333	0	0	known
XR_002339925.1	LOC106506701	0.01	1	0	0	known
XR_002339964.1	LOC100513893	0.013333333	0.666666667	0	0	known
XR_002339976.1	LOC110257414	0.006666667	0.333333333	0	0	known
XR_002339982.1	LOC110257416	0.003333333	0.246666667	0	0	known
XR_002339987.1	LOC102166415	0.023333333	0.333333333	0	0	known
XR_002339995.1	LOC110257422	0.016666667	7.623333333	0	0	known
XR_002340011.1	LOC102165584	0.006666667	0.333333333	0	0	known
XR_002340012.1	LOC102165584	0.006666667	1	0	0	known
XR_002340020.1	LOC110257435	0.006666667	0.333333333	0	0	known
XR_002340032.1	LOC102161214	0.003333333	0.333333333	0	0	known
XR_002340033.1	LOC102161087	0.026666667	1	0	0	known
XR_002340036.1	LOC110257447	0.003333333	0.333333333	0	0	known
XR_002340075.1	LOC110257461	0.003333333	0.333333333	0	0	known
XR_002340081.1	LOC110257463	0.003333333	0.333333333	0	0	known
XR_002340097.1	LOC110257470	0.006666667	0.666666667	0	0	known
XR_002340132.1	LOC106506724	0.006666667	0.333333333	0	0	known
XR_002340214.1	LOC110257509	0.003333333	0.333333333	0	0	known
XR_002340220.1	LOC106508201	0.003333333	0.333333333	0	0	known
XR_002340226.1	LOC100515112	0.023333333	1.046666667	0	0	known
XR_002340232.1	LOC110257513	0.013333333	0.333333333	0	0	known
XR_002340243.1	LOC110257515	0.003333333	0.333333333	0	0	known
XR_002340247.1	LOC102162623	0.03	1.7	0	0	known
XR_002340308.1	LOC102162156	0.003333333	1	0	0	known
XR_002340324.1	LOC110257551	0.033333333	0.77	0	0	known
XR_002340331.1	LOC110257554	0.003333333	0.333333333	0	0	known
XR_002340335.1	LOC102157930	0.026666667	2.34	0	0	known
XR_002340338.1	LOC102157930	0.016666667	0.72	0	0	known
XR_002340355.1	LOC110257569	0.053333333	1.333333333	0	0	known
XR_002340375.1	LOC110257575	0.003333333	0.333333333	0	0	known
XR_002340377.1	LOC110257577	0.006666667	0.333333333	0	0	known
XR_002340414.1	LOC102160006	0.046666667	3.333333333	0	0	known
XR_002340416.1	LOC110257592	0.54	13	0	0	known
XR_002340476.1	LOC102164754	0.023333333	2.306666667	0	0	known
XR_002340477.1	LOC102165006	0.003333333	0.853333333	0	0	known
XR_002340492.1	GIMAP5	0.04	4.923333333	0	0	known
XR_002340504.1	LOC110257621	0.016666667	0.333333333	0	0	known
XR_002340569.1	LOC110257658	0.113333333	4	0	0	known
XR_002340590.1	LOC110257664	0.023333333	1.27	0	0	known
XR_002340616.1	LOC106506983	0.006666667	0.333333333	0	0	known
XR_002340647.1	LOC110257721	0.006666667	1.333333333	0	0	known
XR_002340653.1	LOC110257723	0.023333333	0.333333333	0	0	known
XR_002340675.1	LOC110257735	0.003333333	0.333333333	0	0	known
XR_002340685.1	LOC102166006	0.016666667	1.36	0	0	known
XR_002340688.1	LOC106508240	0.006666667	0.333333333	0	0	known
XR_002340693.1	LOC110257747	0.01	0.333333333	0	0	known
XR_002340703.1	LOC110257753	0.006666667	0.333333333	0	0	known
XR_002340731.1	LOC110257765	0.003333333	0.666666667	0	0.333333333	known
XR_002340752.1	LOC106508228	0.04	0.666666667	0	0	known
XR_002340782.1	LOC110257796	0.013333333	0.666666667	0	0	known
XR_002340798.1	LOC102166211	0.01	1.476666667	0	0	known
XR_002340805.1	LOC110257808	0.01	0.333333333	0	0	known
XR_002340810.1	LOC106506881	0.026666667	2.286666667	0	0	known
XR_002340853.1	LOC106506917	0.016666667	1	0	0	known

XR_002340869.1	LOC100522115	0.036666667	1.22	0	0	known
XR_002340895.1	CD99	0.316666667	24.5	0	0	known
XR_002340905.1	LOC102160243	0.02	2.213333333	0	0	known
XR_002340926.1	LOC110257919	0.006666667	1.32	0	0	known
XR_002340930.1	LOC110257927	0.01	0.333333333	0	0	known
XR_002340949.1	LOC102160834	0.01	0.333333333	0	0	known
XR_002340952.1	LOC110257996	0.003333333	0.333333333	0	0	known
XR_002340993.1	LOC110258138	0.006666667	0.896666667	0	0	known
XR_002341016.1	LOC110258187	0.006666667	0.333333333	0	0	known
XR_002341030.1	IL3RA	0.09	6.78	0	0	known
XR_002341052.1	LOC110258311	0.006666667	0.666666667	0	0	known
XR_002341066.1	LOC102160522	0.08	2.756666667	0	0	known
XR_002341068.1	LOC110258319	0.03	0.366666667	0	0	known
XR_002341114.1	LOC102158111	0.003333333	0.333333333	0	0	known
XR_002341127.1	LOC110258365	0.006666667	0.333333333	0	0	known
XR_002341131.1	LOC110258381	0.12	8.11	0	0	known
XR_002341132.1	LOC110258382	0.31	42.56333333	0	0	known
XR_002341133.1	LOC110258382	0.063333333	8.656666667	0	0	known
XR_002341134.1	LOC110258382	0.146666667	19.18333333	0	0	known
XR_002341136.1	LOC110258382	0.03	3.963333333	0	0	known
XR_002341140.1	LOC110258391	0.026666667	0.56	0	0	known
XR_002341144.1	LOC102165133	0.006666667	1.2	0	0	known
XR_002341145.1	LOC110258406	0.016666667	0.806666667	0	0	known
XR_002341155.1	LOC110258473	0.003333333	0.333333333	0	0	known
XR_002341161.1	LOC110258479	0.116666667	1.373333333	0	0	known
XR_002341176.1	LOC110258560	0.066666667	0.66	0	0	known
XR_002341185.1	LOC102168138	0.01	0.333333333	0	0	known
XR_002341203.1	LOC102166957	0.023333333	6.586666667	0	0	known
XR_002341213.1	LOC110258646	0.036666667	0.45	0	0	known
XR_002341218.1	LOC110258681	0.01	1.723333333	0	0	known
XR_002341243.1	LOC102164320	0.01	2.52	0	0.006666667	known
XR_002341258.1	LOC110258837	0.003333333	0.333333333	0	0	known
XR_002341275.1	LOC110258913	0.706666667	13.42333333	0	0	known
XR_002341283.1	LOC110258921	0.006666667	1	0	0.05	known
XR_002341292.1	LOC110258990	0.023333333	1.993333333	0	0	known
XR_002341304.1	LOC110259015	0.006666667	0.916666667	0	0	known
XR_002341322.1	LOC110259113	0.026666667	0.333333333	0	0	known
XR_002341337.1	LOC110259142	0.003333333	0.333333333	0	0	known
XR_002341411.1	LOC110259227	0.07	8.463333333	0	0	known
XR_002341449.1	LOC110259242	0.043333333	1.666666667	0	0	known
XR_002341460.1	LOC100627892	0.046666667	2.623333333	0	0	known
XR_002341463.1	LOC100627892	0.05	1.953333333	0	0	known
XR_002341475.1	LOC110259253	0.076666667	0.333333333	0	0	known
XR_002341488.1	LOC106509363	0.02	2	0	0	known
XR_002341505.1	LOC106507997	0.006666667	0.406666667	0	0	known
XR_002341508.1	LOC106507997	0.01	0.4	0	0	known
XR_002341515.1	LOC102157601	0.03	5.94	0	0	known
XR_002341517.1	LOC102157601	0.006666667	1.513333333	0	0	known
XR_002341535.1	LOC106509390	0.013333333	1	0	0	known
XR_002341599.1	LOC110259327	0.023333333	0.666666667	0	0	known
XR_002341663.1	LOC110259346	0.006666667	0.333333333	0	0	known
XR_002341699.1	LOC110259368	0.01	0.666666667	0	0	known
XR_002341700.1	LOC110259369	0.013333333	0.666666667	0	0	known
XR_002341706.1	LOC110259375	0.103333333	1.613333333	0	0	known
XR_002341724.1	LOC106507307	0.023333333	0.333333333	0	0	known
XR_002341727.1	LOC110259386	0.01	0.333333333	0	0	known
XR_002341729.1	LOC110259387	0.01	0.333333333	0	0	known
XR_002341735.1	LOC102165038	0.003333333	0.333333333	0	0	known
XR_002341789.1	LOC110259413	0.02	0.333333333	0	0	known
XR_002341790.1	LOC110259414	0.05	0.333333333	0	0	known

XR_002341803.1	LOC110259417	0.006666667	0.333333333	0	0	known
XR_002341812.1	LOC110259421	0.01	1.666666667	0	0	known
XR_002341838.1	LOC106509513	0.016666667	1.666666667	0	0	known
XR_002341858.1	LOC106509534	0.003333333	0.333333333	0	0	known
XR_002341860.1	LOC106509536	0.04	25.93333333	0	0	known
XR_002341875.1	LOC106509537	0.003333333	0.333333333	0	0	known
XR_002341895.1	LOC110259475	0.006666667	0.333333333	0	0	known
XR_002341907.1	LOC110259480	0.013333333	1	0	0	known
XR_002341908.1	LOC110259481	0.003333333	0.333333333	0	0	known
XR_002341916.1	LOC110259484	0.02	0.333333333	0	0	known
XR_002341922.1	LOC110259491	0.003333333	0.333333333	0	0	known
XR_002341930.1	LOC110259496	0.016666667	6.333333333	0	0.333333333	known
XR_002341964.1	LOC110259526	0.006666667	0.333333333	0	0	known
XR_002342024.1	LOC102162290	0.006666667	0.333333333	0	0	known
XR_002342025.1	LOC102162290	0.013333333	0.666666667	0	0	known
XR_002342045.1	LOC110259679	0.003333333	0.933333333	0	0	known
XR_002342059.1	LOC110259691	0.033333333	14.33333333	0	0	known
XR_002342079.1	LOC102158243	0.006666667	0.333333333	0	0	known
XR_002342103.1	LOC110259726	0.08	1	0	0	known
XR_002342112.1	LOC110259772	0.016666667	0.333333333	0	0	known
XR_002342114.1	LOC110259774	0.006666667	0.333333333	0	0	known
XR_002342197.1	LOC110259836	0.003333333	1	0	0	known
XR_002342204.1	LOC110259840	0.02	0.666666667	0	0	known
XR_002342223.1	LOC102157461	0.003333333	0.333333333	0	0.016666667	known
XR_002342227.1	LOC102157461	0.003333333	0.666666667	0	0.333333333	known
XR_002342270.1	LOC110259869	0.013333333	1	0	0	known
XR_002342292.1	LOC106509647	0.043333333	4.92	0	0	known
XR_002342329.1	LOC102167472	0.01	0.666666667	0	0	known
XR_002342352.1	LOC110259902	0.05	0.666666667	0	0	known
XR_002342354.1	ITGAX	0.063333333	13.84666667	0	0	known
XR_002342363.1	LOC110259905	0.053333333	9.793333333	0	0	known
XR_002342365.1	LOC110259904	0.023333333	0.333333333	0	0	known
XR_002342377.1	LOC106506439	0.013333333	1.333333333	0	0	known
XR_002342400.1	LOC110259919	0.003333333	0.333333333	0	0	known
XR_002342403.1	LOC106507143	0.016666667	0.666666667	0	0	known
XR_002342404.1	LOC110259924	0.063333333	5	0	0	known
XR_002342422.1	LOC102166437	0.013333333	0.666666667	0	0	known
XR_002342427.1	LOC110259936	0.006666667	0.333333333	0	0	known
XR_002342444.1	LOC106504252	0.126666667	18.34	0	0	known
XR_002342447.1	LOC106504252	0.026666667	3.873333333	0	0	known
XR_002342456.1	LOC110259949	0.01	0.333333333	0	0	known
XR_002342459.1	LOC106509720	0.01	1.21	0	0	known
XR_002342528.1	LOC106508550	0.243333333	12.45666667	0	0	known
XR_002342541.1	LOC110259986	0.04	0.333333333	0	0	known
XR_002342545.1	LOC102163845	0.003333333	1	0	0	known
XR_002342549.1	LOC106507373	0.01	0.356666667	0	0	known
XR_002342625.1	LOC110259995	0.003333333	0.333333333	0	0	known
XR_002342654.1	LOC106509807	0.02	2.553333333	0	0	known
XR_002342660.1	LOC106509807	0.003333333	0.446666667	0	0	known
XR_002342665.1	LOC106509807	0.04	2.39	0	0	known
XR_002342675.1	LOC106509807	0.01	1.076666667	0	0	known
XR_002342689.1	LOC106509807	0.026666667	2.63	0	0	known
XR_002342690.1	LOC106509807	0.046666667	5.05	0	0	known
XR_002342695.1	LOC106509807	0.01	2.216666667	0	0	known
XR_002342720.1	LOC102167127	0.006666667	0.666666667	0	0	known
XR_002342723.1	LOC110260017	0.006666667	0.666666667	0	0	known
XR_002342742.1	LOC106509841	0.056666667	7.956666667	0	0	known
XR_002342753.1	LOC110260026	0.006666667	0.333333333	0	0	known
XR_002342766.1	LOC102164129	0.016666667	0.663333333	0	0	known
XR_002342787.1	LOC110260036	0.023333333	4.833333333	0	0	known

XR_002342790.1	LOC110260036	0.016666667	4.06	0	0	known
XR_002342798.1	LOC106509865	0.016666667	1.71	0	0	known
XR_002342799.1	LOC106509865	0.006666667	0.656666667	0	0	known
XR_002342833.1	LOC102157748	0.01	0.886666667	0	0	known
XR_002342845.1	LOC106509907	0.003333333	0.333333333	0	0	known
XR_002342846.1	LOC106509907	0.01	0.333333333	0	0	known
XR_002342850.1	LOC106509907	0.003333333	0.333333333	0	0	known
XR_002342864.1	LOC110260057	0.06	0.333333333	0	0	known
XR_002342868.1	LOC102160712	0.006666667	1.666666667	0	0	known
XR_002342873.1	LOC110260061	0.003333333	1	0	0	known
XR_002342935.1	LOC102159571	0.003333333	0.666666667	0	0	known
XR_002342943.1	LOC110260099	0.003333333	0.333333333	0	0	known
XR_002342968.1	LOC106509896	0.013333333	0.333333333	0	0	known
XR_002343014.1	LOC106508854	0.013333333	1.113333333	0	0.036666667	known
XR_002343032.1	LOC110260189	0.013333333	0.666666667	0	0	known
XR_002343065.1	LOC102159040	0.013333333	1.333333333	0	0	known
XR_002343090.1	LOC106509952	0.093333333	6.55	0	0	known
XR_002343108.1	LOC106509961	0.03	1	0	0	known
XR_002343114.1	LOC106507559	0.006666667	0.333333333	0	0	known
XR_002343120.1	LOC110260226	0.046666667	6.27	0	0	known
XR_002343125.1	LOC106507559	0.023333333	1.333333333	0	0	known
XR_002343143.1	LOC102157842	0.01	2.33	0	0.333333333	known
XR_002343162.1	LOC110260238	0.016666667	0.333333333	0	0	known
XR_002343164.1	LOC110260239	0.03	5.406666667	0	0.193333333	known
XR_002343173.1	LOC110260242	0.006666667	0.666666667	0	0	known
XR_002343185.1	LOC110260249	0.03	3.666666667	0	0	known
XR_002343188.1	LOC110260249	0.003333333	0.196666667	0	0	known
XR_002343229.1	LOC110260265	0.036666667	2	0	0	known
XR_002343240.1	LOC106510039	0.006666667	0.64	0	0	known
XR_002343265.1	LOC110260277	0.006666667	0.333333333	0	0	known
XR_002343268.1	LOC102160776	0.016666667	0.666666667	0	0	known
XR_002343293.1	LOC102162563	0.003333333	0.333333333	0	0	known
XR_002343317.1	LOC110260292	0.003333333	0.333333333	0	0	known
XR_002343318.1	LOC110260292	0.003333333	0.333333333	0	0	known
XR_002343362.1	LOC110260300	0.006666667	0.333333333	0	0	known
XR_002343373.1	LOC110260305	0.006666667	1	0	0	known
XR_002343398.1	LOC110260317	0.003333333	0.333333333	0	0	known
XR_002343399.1	LOC102157437	0.033333333	5.806666667	0	0	known
XR_002343400.1	LOC102157437	0.013333333	2.15	0	0	known
XR_002343419.1	LOC106510131	0.003333333	0.98	0	0	known
XR_002343425.1	LOC102165794	0.023333333	0.333333333	0	0	known
XR_002343426.1	LOC102158536	0.053333333	2	0	0	known
XR_002343462.1	LOC110260336	0.003333333	0.333333333	0	0	known
XR_002343473.1	LOC110260339	0.016666667	1.333333333	0	0	known
XR_002343485.1	LOC102165975	0.013333333	0.666666667	0	0	known
XR_002343490.1	LOC110255190	0.006666667	2.906666667	0	0	known
XR_002343492.1	LOC102166460	0.003333333	0.333333333	0	0.333333333	known
XR_002343496.1	LOC110260342	0.003333333	0.333333333	0	0	known
XR_002343500.1	LOC102157897	0.006666667	0.333333333	0	0	known
XR_002343517.1	LOC102165594	0.003333333	0.333333333	0	0	known
XR_002343541.1	LOC110260360	0.006666667	0.666666667	0	0	known
XR_002343547.1	LOC110260363	0.053333333	5.27	0	0	known
XR_002343550.1	LOC110260365	0.013333333	1.666666667	0	0	known
XR_002343568.1	LOC106508363	0.006666667	0.743333333	0	0	known
XR_002343602.1	LOC110260378	0.01	0.666666667	0	0	known
XR_002343607.1	LOC102161901	0.016666667	3.666666667	0	0	known
XR_002343610.1	LOC102161901	0.006666667	1.333333333	0	0	known
XR_002343612.1	LOC102159537	0.01	0.333333333	0	0	known
XR_002343622.1	LOC106510230	0.01	0.49	0	0	known
XR_002343645.1	LOC110260394	0.003333333	0.546666667	0	0.14	known

XR_002343680.1	LOC110260419	0.01	1.07	0	0	known
XR_002343686.1	LOC106510005	0.02	0.333333333	0	0	known
XR_002343705.1	LOC102159561	0.07	6.593333333	0	0	known
XR_002343710.1	LOC102159561	0.016666667	0.333333333	0	0	known
XR_002343711.1	LOC102159995	0.043333333	6.813333333	0	0	known
XR_002343737.1	LOC102158069	0.006666667	0.333333333	0	0	known
XR_002343738.1	LOC110260439	0.003333333	0.333333333	0	0	known
XR_002343740.1	LOC110260439	0.006666667	0.666666667	0	0	known
XR_002343741.1	LOC110260439	0.013333333	1.333333333	0	0	known
XR_002343759.1	LOC102166687	0.003333333	0.333333333	0	0	known
XR_002343798.1	LOC110260563	0.003333333	0.333333333	0	0	known
XR_002343826.1	LOC110260579	0.026666667	1.666666667	0	0	known
XR_002343833.1	LOC102161600	0.006666667	0.97	0	0	known
XR_002343869.1	LOC110260622	0.03	1.186666667	0	0	known
XR_002343871.1	LOC110260622	0.043333333	2.813333333	0	0	known
XR_002343876.1	LOC110260629	0.016666667	3.27	0	0	known
XR_002343879.1	LOC110260629	0.063333333	5.85	0	0	known
XR_002343889.1	LOC110260640	0.003333333	0.333333333	0	0	known
XR_002343926.1	LOC102160723	0.006666667	2	0	0.333333333	known
XR_002343929.1	LOC102160723	0.006666667	0.666666667	0	0	known
XR_002343955.1	RARG	0.126666667	15.9	0	0	known
XR_002343964.1	LOC110260667	0.006666667	0.666666667	0	0	known
XR_002344004.1	LOC106507220	0.006666667	0.333333333	0	0	known
XR_002344006.1	LOC106507220	0.016666667	1.083333333	0	0	known
XR_002344017.1	LOC110260701	0.02	2.666666667	0	0	known
XR_002344019.1	LOC110260701	0.006666667	0.333333333	0	0	known
XR_002344021.1	LOC102168027	0.036666667	4.9	0	0	known
XR_002344022.1	LOC102168027	0.013333333	2.546666667	0	0	known
XR_002344031.1	LOC102168027	0.013333333	2.146666667	0	0	known
XR_002344032.1	LOC102168027	0.006666667	0.873333333	0	0	known
XR_002344035.1	LOC102168027	0.026666667	5.76	0	0	known
XR_002344038.1	LOC102168027	0.01	2.25	0	0	known
XR_002344071.1	LOC110260719	0.006666667	0.95	0	0.333333333	known
XR_002344098.1	LOC110260734	0.006666667	0.666666667	0	0	known
XR_002344107.1	LOC106507529	0.006666667	0.666666667	0	0	known
XR_002344128.1	LOC100524923	0.006666667	3.003333333	0	0	known
XR_002344170.1	LOC110260767	0.013333333	0.666666667	0	0	known
XR_002344248.1	LOC110260791	0.003333333	0.333333333	0	0	known
XR_002344254.1	LOC110260795	0.02	0.333333333	0	0	known
XR_002344262.1	LOC110260799	0.026666667	0.333333333	0	0	known
XR_002344275.1	LOC110260804	0.003333333	1.666666667	0	0.333333333	known
XR_002344327.1	LOC110260820	0.003333333	0.333333333	0	0	known
XR_002344371.1	LOC110260840	0.016666667	0.333333333	0	0	known
XR_002344384.1	LOC110260863	0.016666667	0.333333333	0	0	known
XR_002344398.1	LOC110260876	0.003333333	0.333333333	0	0	known
XR_002344427.1	LOC102168158	0.023333333	1.666666667	0	0	known
XR_002344429.1	LOC102157454	0.006666667	0.666666667	0	0	known
XR_002344472.1	LOC106507552	0.01	0.883333333	0	0	known
XR_002344480.1	LOC110260905	0.003333333	1.656666667	0	0.333333333	known
XR_002344481.1	LOC110260906	0.01	0.666666667	0	0	known
XR_002344484.1	LOC110260909	0.013333333	0.333333333	0	0	known
XR_002344491.1	LOC102159045	0.01	0.333333333	0	0	known
XR_002344534.1	LOC102159752	0.01	0.666666667	0	0	known
XR_002344556.1	LOC110260940	0.02	0.666666667	0	0	known
XR_002344557.1	LOC110260940	0.006666667	0.333333333	0	0	known
XR_002344587.1	LOC102165609	0.02	3.09	0	0	known
XR_002344589.1	LOC102165609	0.07	14.65666667	0	0	known
XR_002344590.1	LOC102165609	0.04	8.733333333	0	0	known
XR_002344594.1	LOC110260952	0.003333333	0.333333333	0	0	known
XR_002344604.1	LOC102158585	0.013333333	0.333333333	0	0	known

XR_002344607.1	LOC110260959	0.003333333	0.333333333	0	0	known
XR_002344612.1	LOC110260962	0.01	1.666666667	0	0	known
XR_002344614.1	LOC110260968	0.003333333	0.333333333	0	0	known
XR_002344615.1	LOC110260967	0.01	0.333333333	0	0	known
XR_002344630.1	LOC102158826	0.003333333	0.633333333	0	0	known
XR_002344674.1	LOC110260996	0.003333333	0.333333333	0	0	known
XR_002344698.1	LOC102158936	0.003333333	0.58	0	0	known
XR_002344700.1	LOC102158936	0.006666667	1.916666667	0	0	known
XR_002344711.1	LOC102160341	0.016666667	1.146666667	0	0	known
XR_002344715.1	LOC102165433	0.02	1	0	0	known
XR_002344728.1	LOC110261022	0.013333333	0.333333333	0	0	known
XR_002344799.1	LOC106510586	0.006666667	0.333333333	0	0	known
XR_002344830.1	LOC110261071	0.006666667	0.666666667	0	0	known
XR_002344844.1	LOC106509643	0.01	1.526666667	0	0	known
XR_002344856.1	LOC110261083	0.003333333	0.666666667	0	0.333333333	known
XR_002344866.1	NPHP4	0.01	2.343333333	0	0	known
XR_002344933.1	LOC102157480	0.01	3	0	0	known
XR_002344950.1	LOC106509758	0.01	1	0	0	known
XR_002344952.1	LOC100621455	0.07	3.473333333	0	0	known
XR_002344961.1	LOC110261129	0.036666667	0.853333333	0	0	known
XR_002344983.1	LOC106510655	0.01	0.666666667	0	0	known
XR_002345003.1	LOC102161909	0.013333333	1.16	0	0	known
XR_002345013.1	LOC110261169	0.006666667	0.333333333	0	0	known
XR_002345075.1	LOC110261188	0.006666667	2	0	0.666666667	known
XR_002345077.1	LOC110261190	0.006666667	0.666666667	0	0	known
XR_002345092.1	LOC110261197	0.006666667	0.333333333	0	0	known
XR_002345099.1	LOC110261198	0.006666667	0.333333333	0	0	known
XR_002345119.1	LOC110261204	0.006666667	0.666666667	0	0	known
XR_002345149.1	LOC106507649	0.03	0.596666667	0	0	known
XR_002345163.1	LOC110261216	0.006666667	1	0	0	known
XR_002345187.1	LOC106510717	0.013333333	0.333333333	0	0	known
XR_002345227.1	LOC110261241	0.033333333	0.333333333	0	0	known
XR_002345241.1	LOC110261245	0.016666667	1	0	0	known
XR_002345295.1	LOC110261258	0.03	4	0	0	known
XR_002345452.1	LOC106510680	0.006666667	0.666666667	0	0	known
XR_002345560.1	LOC110261468	0.01	2	0	0.666666667	known
XR_002345576.1	LOC110261472	0.003333333	0.666666667	0	0.333333333	known
XR_002345597.1	LOC106504348	0.013333333	1.333333333	0	0	known
XR_002345635.1	LOC110261501	0.006666667	0.666666667	0	0	known
XR_002345696.1	LOC102163428	0.01	0.333333333	0	0	known
XR_002345704.1	LOC106504404	0.006666667	0.333333333	0	0	known
XR_002345719.1	LOC106504408	0.006666667	0.333333333	0	0	known
XR_002345745.1	LOC110261531	0.033333333	0.333333333	0	0	known
XR_002345752.1	LOC110261533	0.003333333	0.333333333	0	0	known
XR_002345753.1	LOC102163474	0.01	0.333333333	0	0	known
XR_002345757.1	LOC110261539	0.013333333	0.666666667	0	0	known
XR_002345758.1	LOC106504432	0.01	0.333333333	0	0	known
XR_002345779.1	LOC102161631	0.023333333	0.343333333	0	0	known
XR_002345799.1	LOC100736711	0.02	0.666666667	0	0	known
XR_002345828.1	LOC106509046	0.01	1.273333333	0	0	known
XR_002345858.1	LOC106507710	0.003333333	2	0	0	known
XR_002345862.1	LOC102166186	0.003333333	0.333333333	0	0	known
XR_002345914.1	LOC110261591	0.006666667	0.333333333	0	0	known
XR_002345915.1	LOC110261591	0.02	1	0	0	known
XR_002345972.1	LOC110261615	0.003333333	0.333333333	0	0	known
XR_002346007.1	LOC110261630	0.013333333	0.333333333	0	0	known
XR_002346016.1	LOC110261632	0.026666667	2.666666667	0	0	known
XR_002346024.1	LOC110261640	0.213333333	89.15333333	0	0.24	known
XR_002346037.1	LOC102159128	0.1	4.72	0	0	known
XR_002346058.1	LOC110261648	0.016666667	11.10666667	0	0	known

XR_002346070.1	LOC102163738	0.006666667	3.213333333	0	0	known
XR_002346136.1	LOC110261682	0.003333333	0.333333333	0	0	known
XR_002346139.1	LOC110261683	0.003333333	2	0	0.666666667	known
XR_002346146.1	LOC106507734	0.003333333	1	0	0.333333333	known
XR_002346172.1	LOC110261707	0.013333333	0.333333333	0	0	known
XR_002346219.1	LOC106508466	0.013333333	1.116666667	0	0	known
XR_002346266.1	LOC102162837	0.016666667	1.666666667	0	0	known
XR_002346270.1	LOC106504303	0.02	8.2	0	0	known
XR_002346325.1	LOC110261786	0.013333333	1	0	0	known
XR_002346338.1	LOC110261793	0.02	2.196666667	0	0	known
XR_002346356.1	LOC110261941	0.003333333	1.333333333	0	0.333333333	known
XR_002346405.1	LOC110261980	0.026666667	0.763333333	0	0	known
XR_002346406.1	LOC110261980	0.023333333	0.666666667	0	0	known
XR_002346454.1	LOC110261984	0.013333333	0.666666667	0	0	known
XR_002346457.1	LOC110261986	0.006666667	0.333333333	0	0	known
XR_002346458.1	LOC110261986	0.006666667	0.333333333	0	0	known
XR_002346485.1	LOC110262000	0.003333333	0.333333333	0	0	known
XR_002346520.1	LOC110262038	0.003333333	0.666666667	0	0	known
XR_002346538.1	LOC110262045	0.003333333	1	0	0	known
XR_002346558.1	LOC110262056	0.003333333	0.333333333	0	0	known
XR_002346583.1	LOC106504597	0.013333333	0.333333333	0	0	known
XR_002346601.1	LOC110262076	0.006666667	2.666666667	0	0	known
XR_002346607.1	PDS5A	0.013333333	3.663333333	0	0	known
XR_002346617.1	LOC106504629	0.046666667	1.333333333	0	0	known
XR_002346641.1	LOC100737304	0.126666667	7.826666667	0	0	known
XR_002346649.1	LOC110262097	0.033333333	1.986666667	0	0	known
XR_002346665.1	LOC110262102	0.05	1	0	0	known
XR_002346671.1	LOC102165777	0.003333333	0.436666667	0	0	known
XR_002346673.1	LOC102165777	0.003333333	0.723333333	0	0.666666667	known
XR_002346677.1	LOC110262105	0.006666667	1	0	0	known
XR_002346692.1	LOC102160683	0.006666667	0.666666667	0	0	known
XR_002346704.1	LOC106504691	0.003333333	0.333333333	0	0	known
XR_002346720.1	LOC110262131	0.013333333	0.666666667	0	0	known
XR_002346726.1	LOC110262136	0.01	0.333333333	0	0	known
XR_002346745.1	LOC110262144	0.013333333	0.333333333	0	0	known
XR_002346749.1	LOC110262146	0.003333333	0.1	0	0	known
XR_002346767.1	LOC110262152	0.013333333	0.333333333	0	0	known
XR_002346818.1	LOC106510483	0.02	0.666666667	0	0	known
XR_002346827.1	LOC106504735	0.003333333	0.333333333	0	0	known
XR_002346834.1	LOC100738196	0.003333333	1.666666667	0	0.666666667	known
XR_002346847.1	LOC110262179	0.01	0.333333333	0	0	known
XR_002346891.1	LOC102162767	0.006666667	0.666666667	0	0	known
XR_002346901.1	LOC100737511	0.03	3.953333333	0	0	known
XR_002346919.1	LOC110262202	0.023333333	0.333333333	0	0	known
XR_002346953.1	LOC106509117	0.013333333	0.666666667	0	0	known
XR_002346967.1	LOC110262212	0.006666667	1	0	0	known
XR_002346971.1	LOC110262217	0.003333333	0.333333333	0	0	known
XR_002346983.1	LOC110262219	0.003333333	1	0	0.333333333	known
XR_002347007.1	LOC110262223	0.013333333	0.856666667	0	0	known
XR_002347009.1	LOC110262223	0.06	3.73	0	0	known
XR_002347014.1	LOC110262223	0.006666667	0.386666667	0	0	known
XR_002347068.1	LOC106509119	0.01	1.333333333	0	0	known
XR_002347126.1	LOC110262307	0.013333333	0.333333333	0	0	known
XR_115809.4	LOC100516669	0.06	1.463333333	0	0	known
XR_130524.4	LOC100624063	0.006666667	0.56	0	0	known
XR_135462.4	LOC100622227	0.283333333	7.466666667	0	0	known
XR_297824.3	LOC100523871	0.023333333	0.333333333	0	0	known
XR_298258.3	LOC102157709	0.013333333	0.333333333	0	0	known
XR_298825.3	LOC102163918	0.013333333	0.666666667	0	0	known
XR_299038.3	LOC102162436	0.023333333	1	0	0	known

XR_299365.3	LOC102164637	0.02	1	0	0	known
XR_299492.3	LOC100626264	0.093333333	1.666666667	0	0	known
XR_299673.3	LOC102163484	0.006666667	0.333333333	0	0	known
XR_300762.3	LOC102157779	0.006666667	1	0	0	known
XR_301182.3	LOC102159522	0.006666667	0.333333333	0	0	known
XR_301982.3	LOC102163451	0.013333333	0.333333333	0	0	known
XR_302445.3	LOC102166164	0.053333333	0.573333333	0	0	known
XR_302482.3	LOC102163556	0.026666667	0.666666667	0	0	known
XR_302682.3	LOC102166974	0.006666667	1.666666667	0	0	known
XR_303111.3	LOC100525517	0.046666667	2.156666667	0	0	known
XR_303176.3	LOC102157576	0.006666667	0.34	0	0	known
XR_303532.3	LOC102165305	0.006666667	0.333333333	0	0	known
XR_303622.3	LOC102163647	0.006666667	0.333333333	0	0	known
XR_304466.3	LOC102166347	0.05	1.666666667	0	0	known
XR_305732.3	LOC102166108	0.03	0.333333333	0	0	known
XR_305962.3	LOC102161032	0.02	2	0	0	known
XR_305965.3	LOC102161921	0.003333333	1.333333333	0	0.333333333	known
XR_306176.3	LOC102165785	0.003333333	0.333333333	0	0	known
XR_307292.3	LOC102159603	0.003333333	0.333333333	0	0	known
XR_307378.3	LOC102165957	0.01	0.333333333	0	0	known
XR_307585.3	LOC102164693	0.006666667	0.333333333	0	0	known
XR_307795.3	LOC102163250	0.01	0.333333333	0	0	known
XR_307942.3	LOC102167838	0.036666667	0.333333333	0	0	known
XR_308256.3	LOC102163217	0.003333333	0.333333333	0	0	known
XR_308808.3	LOC102159476	0.013333333	1.586666667	0	0	known
XR_309283.3	LOC102165678	0.026666667	0.333333333	0	0	known
XR_309353.3	LOC102161179	0.006666667	0.333333333	0	0	known
BGIR9823_100051	TPD52L2	0	0	0.05	0.333333333	novel
BGIR9823_100068	SGCZ	0	0	0.02666667	6.746666667	novel
BGIR9823_100097	RNF170	0	0	0.03333333	0.333333333	novel
BGIR9823_100186	SNPH	0	0	0.01333333	0.333333333	novel
BGIR9823_100307	LOC102164659	0	0	0.01666667	0.333333333	novel
BGIR9823_100314	ZFP64	0	0	0.11333333	1	novel
BGIR9823_100403	WDR91	0	0	0.06666667	1	novel
BGIR9823_100440	LOC110257598	0	0	1.28666667	10.77666667	novel
BGIR9823_100464	FAM3C	0	0	0.07333333	0.333333333	novel
BGIR9823_100468	CTTNBP2	0	0	0.13333333	1	novel
BGIR9823_100488	TMEM168	0	0	0.1	1	novel
BGIR9823_100491	DOCK4	0	0	0.05666667	0.666666667	novel
BGIR9823_100607	GALNT11	0	0	0.41666667	3.333333333	novel
BGIR9823_100647	UBN2	0	0	0.02666667	0.333333333	novel
BGIR9823_100700	AHCYL2	0	0	0.04666667	0.333333333	novel
BGIR9823_100727	ING3	0	0	0.08333333	1	novel
BGIR9823_100732	KCND2	0	0	0.03666667	0.333333333	novel
BGIR9823_100773	MINDY4	0	0	0.05666667	0.333333333	novel
BGIR9823_100787	CBX3	0	0	0.05	0.333333333	novel
BGIR9823_100796	LOC110257536	0	0	0.01333333	0.53	novel
BGIR9823_100836	LOC100624032	0	0	0.10666667	0.666666667	novel
BGIR9823_100849	TMSB4X	0	0	0.01	0.576666667	novel
BGIR9823_100860	ZRSR2	0	0	0.08	0.666666667	novel
BGIR9823_100873	CNKSR2	0	0	0.06	0.333333333	novel
BGIR9823_100877	CNKSR2	0	0	0.12	0.666666667	novel
BGIR9823_100892	ZFX	0	0	0.05333333	0.333333333	novel
BGIR9823_100909	IL1RAPL1	0	0	0.1	0.666666667	novel
BGIR9823_100910	IL1RAPL1	0	0	0.05	0.333333333	novel
BGIR9823_100996	PCDH11X	0	0	0.10333333	0.666666667	novel
BGIR9823_100997	PCDH11X	0	0	0.03	0.333333333	novel
BGIR9823_101043	XPNPEP2	0	0	0.00666667	0.463333333	novel
BGIR9823_101073	VMA21	0	0	0.05333333	0.333333333	novel
BGIR9823_101118	TXLNG	0	0	0.09	1	novel

BGIR9823_101252	UPF3B	0	0	0.04	0.333333333	novel
BGIR9823_101334	TRIM27	0	0	0.06666667	0.333333333	novel
BGIR9823_101558	LOC110258600	0	0	0.06	6.513333333	novel
BGIR9823_101594	LOC110258799	0	0	0.04	0.333333333	novel
BGIR9823_101648	LOC102158335	0	0	0.03	0.666666667	novel
BGIR9823_101665		0	0	0.02666667	0.71	novel
BGIR9823_101668		0	0	0.26666667	8.19	novel
BGIR9823_101695		0	0	0.04666667	0.333333333	novel
BGIR9823_101704		0	0	0.04	0.333333333	novel
BGIR9823_101739		0	0	0.07666667	0.666666667	novel
BGIR9823_101747		0	0	0.06	3.47	novel
BGIR9823_101783		0	0	0.01333333	0.333333333	novel
BGIR9823_101787		0	0	0.12333333	0.666666667	novel
BGIR9823_101797		0	0	0.19333333	1.333333333	novel
BGIR9823_101832		0	0	0.02666667	0.666666667	novel
BGIR9823_101837		0	0	0.03333333	1	novel
BGIR9823_101844		0	0	0.20333333	1.333333333	novel
BGIR9823_101866		0	0	0.04	0.666666667	novel
BGIR9823_101901		0	0	0.04	0.333333333	novel
BGIR9823_101916		0	0	0.01666667	0.333333333	novel
BGIR9823_101934		0	0	0.02666667	0.333333333	novel
BGIR9823_101939		0	0	0.03	0.666666667	novel
BGIR9823_101947		0	0	0.06333333	0.333333333	novel
BGIR9823_101961		0	0	0.05	2	novel
BGIR9823_102050		0	0	0.03333333	0.333333333	novel
BGIR9823_102068		0	0	0.13666667	2.333333333	novel
BGIR9823_102106		0	0	0.03333333	1	novel
BGIR9823_102108		0	0	0.05	9.89	novel
BGIR9823_102134		0	0	0.02666667	1	novel
BGIR9823_102156		0	0	0.02	1	novel
BGIR9823_102182		0	0	0.04	0.333333333	novel
BGIR9823_102183		0	0	0.02333333	0.666666667	novel
BGIR9823_102191		0	0	0.15	1	novel
BGIR9823_102216		0	0	0.08666667	0.666666667	novel
BGIR9823_102241		0	0	0.01666667	0.566666667	novel
BGIR9823_102246		0	0	0.05333333	1	novel
BGIR9823_102259		0	0	0.05333333	0.333333333	novel
BGIR9823_102273		0	1.666666667	0.00666667	4	novel
BGIR9823_102285		0	0	0.07	1.873333333	novel
BGIR9823_102331		0	0	0.07	1	novel
BGIR9823_102344		0	0	0.00666667	0.333333333	novel
BGIR9823_102349		0	0	0.01	0.333333333	novel
BGIR9823_102377		0	0	0.04333333	0.333333333	novel
BGIR9823_102389		0	0	0.04333333	0.333333333	novel
BGIR9823_102391		0	0	0.01666667	0.333333333	novel
BGIR9823_102404		0	0	0.01333333	0.333333333	novel
BGIR9823_102412		0	0	0.1	1	novel
BGIR9823_102483		0	0	0.08333333	2	novel
BGIR9823_102487		0	0	0.30333333	3	novel
BGIR9823_102490		0	0	0.04	0.666666667	novel
BGIR9823_102494		0	0	0.02333333	0.333333333	novel
BGIR9823_102557		0	0	0.11666667	1.666666667	novel
BGIR9823_102559		0	0	0.12333333	0.666666667	novel
BGIR9823_102569		0	0	0.06666667	0.333333333	novel
BGIR9823_102570		0	0	0.04666667	0.333333333	novel
BGIR9823_102576		0	0	0.04333333	0.666666667	novel
BGIR9823_102612		0	0	0.05333333	0.333333333	novel
BGIR9823_102643		0	0	0.02	0.333333333	novel
BGIR9823_102687		0	0	0.03333333	0.333333333	novel
BGIR9823_102713		0	0	0.03333333	0.333333333	novel

BGIR9823_102731		0	0	0.10666667	1	novel
BGIR9823_102739		0	0	0.00333333	0.333333333	novel
BGIR9823_102741		0	0	0.01666667	0.666666667	novel
BGIR9823_102796		0	0	0.14666667	0.876666667	novel
BGIR9823_77679	MPC1	0	0	0.04333333	1.79	novel
BGIR9823_77691	TMEM242	0	0	0.1	1	novel
BGIR9823_77732	ADAT2	0	0	0.09666667	0.666666667	novel
BGIR9823_77792	MAN1A1	0	0	0.04	0.333333333	novel
BGIR9823_77801	GOPC	0	0	0.07666667	0.333333333	novel
BGIR9823_77822	ADGRB3	0	0	0.18	1	novel
BGIR9823_77825	ADGRB3	0	0	0.01666667	0.333333333	novel
BGIR9823_77849	ADGRB3	0	0	0.03666667	0.333333333	novel
BGIR9823_77861	ADGRB3	0	0	0.03333333	0.333333333	novel
BGIR9823_77929	AMD1	0	0	0.04333333	0.333333333	novel
BGIR9823_77957	ELOVL4	0	0	0.04666667	0.333333333	novel
BGIR9823_77958	ELOVL4	0	0	0.04333333	0.333333333	novel
BGIR9823_77978	SIGLEC15	0	0	0.09	0.666666667	novel
BGIR9823_78007	WDR7	0	0	0.05666667	0.333333333	novel
BGIR9823_78106	LIPC	0	0	0.03	0.333333333	novel
BGIR9823_78153	DMXL2	0	0	0.03666667	0.426666667	novel
BGIR9823_78154	DMXL2	0	0	0.01666667	0.666666667	novel
BGIR9823_78188	SECISBP2L	0	0	0.06	0.333333333	novel
BGIR9823_78226	LRRC57	0	0	0.06666667	0.333333333	novel
BGIR9823_78250	RASGRP1	0	0	0.02666667	0.73	novel
BGIR9823_78291	OTUD7A	0	0	0.28666667	2.333333333	novel
BGIR9823_78299	FAM189A1	0	0	0.03666667	0.333333333	novel
BGIR9823_78320	TMX3	0	0	0.05333333	0.666666667	novel
BGIR9823_78373	GLCE	0	0	0.24666667	2.333333333	novel
BGIR9823_78431	MIS18BP1	0	0	0.16666667	2.333333333	novel
BGIR9823_78446	GNG2	0	0	0.08666667	0.666666667	novel
BGIR9823_78537	SGPP1	0	0	0.11666667	1	novel
BGIR9823_78541	WDR89	0	0	0.06	0.333333333	novel
BGIR9823_78542	WDR89	0	0	0.05333333	1.666666667	novel
BGIR9823_78598	SNAPC3	0	0	0.08333333	0.666666667	novel
BGIR9823_78635	AK3	0	0	0.12	0.893333333	novel
BGIR9823_78691	LOC100514147	0	0	0.05666667	5.646666667	novel
BGIR9823_78695	LOC110255880	0	0	0.03666667	0.666666667	novel
BGIR9823_78911	WTAP	0	0	0.04	0.333333333	novel
BGIR9823_78916	GTF2H5	0	0	0.05666667	0.333333333	novel
BGIR9823_78972	VTA1	0	0	0.16333333	1.333333333	novel
BGIR9823_78979	ABRACL	0	0	0.01333333	1.53	novel
BGIR9823_79032	NKAIN2	0	0	0.03666667	0.333333333	novel
BGIR9823_79093	EPHA7	0	0	0.01333333	0.333333333	novel
BGIR9823_79116	USP45	0	0	0.1	0.666666667	novel
BGIR9823_79124	CCNC	0	0	0.1	0.666666667	novel
BGIR9823_79196	SH3BGRL2	0	0	0.02333333	0.46	novel
BGIR9823_79231	HDHD2	0	0	0.06666667	1	novel
BGIR9823_79247	DYM	0	0	0.18	1	novel
BGIR9823_79249	C1H18orf32	0	0	0.06333333	0.333333333	novel
BGIR9823_79269	CCDC68	0	0	0.07	0.333333333	novel
BGIR9823_79285	TRIP4	0	0	0.05	0.666666667	novel
BGIR9823_79287	TRIP4	0	0	0.02666667	0.333333333	novel
BGIR9823_79478	UBE3A	0	0	0.04333333	5.216666667	novel
BGIR9823_79479	UBE3A	0	0	0.02666667	3.136666667	novel
BGIR9823_79485	MPHOSPH10	0	0	0.06666667	0.666666667	novel
BGIR9823_79500	TJP1	0	0	0.08333333	1	novel
BGIR9823_79527	PHLPP1	0	0	0.04333333	0.666666667	novel
BGIR9823_79542	RELCH	0	0	0.11	0.666666667	novel
BGIR9823_79629	FBXO33	0	0	0.10333333	1	novel
BGIR9823_79689	ERO1A	0	0	0.03333333	0.333333333	novel

BGIR9823_79719	PSMA3	0	0	0.03333333	0.666666667	novel
BGIR9823_79745	ACER2	0	0	0.07	0.333333333	novel
BGIR9823_79749	ACER2	0	0	0.07333333	0.666666667	novel
BGIR9823_79753	ACER2	0	0	0.03	0.333333333	novel
BGIR9823_79831	PTAR1	0	0	0.08	1	novel
BGIR9823_79926	LOC106509227	0	0	0.07666667	0.306666667	novel
BGIR9823_79942	CTNNAL1	0	0	0.08	1.666666667	novel
BGIR9823_80006	SCAI	0	0	0.03	0.333333333	novel
BGIR9823_80029	URM1	0	0	0.00666667	0.666666667	novel
BGIR9823_80220	LOC110259265	0	0	0.02	2.863333333	novel
BGIR9823_80230	MTCH2	0	0	0.04333333	0.333333333	novel
BGIR9823_80251	PHF21A	0	0	0.05333333	0.836666667	novel
BGIR9823_80295	BTBD10	0	0	0.05	0.333333333	novel
BGIR9823_80305	GALNT18	0	0	0.11333333	0.666666667	novel
BGIR9823_80323	SBF2	0	0	0.14666667	1	novel
BGIR9823_80402	PGLYRP2	0	0	0.01666667	0.333333333	novel
BGIR9823_80474	KANK3	0	0	0.03333333	0.36	novel
BGIR9823_80532	GNG7	0	0	0.05333333	0.333333333	novel
BGIR9823_80590	TBC1D9B	0	0	0.10333333	0.666666667	novel
BGIR9823_80591	TBC1D9B	0	0	0.11	0.666666667	novel
BGIR9823_80641	UTP15	0	0	0.06333333	0.666666667	novel
BGIR9823_80643	UTP15	0	0	0.1	0.716666667	novel
BGIR9823_80655	IQGAP2	0	0	0.06333333	0.333333333	novel
BGIR9823_80656	IQGAP2	0	0	0.08	0.666666667	novel
BGIR9823_80684	RASGRF2	0	0	0.11	0.666666667	novel
BGIR9823_80696	MBLAC2	0	0	0.05666667	1.333333333	novel
BGIR9823_80712	RHOBTB3	0	0	0.11666667	1.333333333	novel
BGIR9823_80764	NREP	0	0	0.03	3.66	novel
BGIR9823_80776	DMXL1	0	0	0.05	1.333333333	novel
BGIR9823_80785	SRFBP1	0	0	0.1	1.333333333	novel
BGIR9823_80848	KDM3B	0	0	0.04333333	0.333333333	novel
BGIR9823_81015	LOC102163816	0	0	0.13666667	45.16333333	novel
BGIR9823_81064	ASRGL1	0	0	0.07333333	0.666666667	novel
BGIR9823_81220	MRPL55	0	0	0.05333333	0.866666667	novel
BGIR9823_81267	TMEM38A	0	0	0.04333333	0.333333333	novel
BGIR9823_81281	ZNF333	0	0	0.33666667	2.666666667	novel
BGIR9823_81313	LOC106508100	0	0	0.08	0.666666667	novel
BGIR9823_81314	LOC106508100	0	0	0.04333333	1	novel
BGIR9823_81316	LOC106508100	0	0	0.08	0.333333333	novel
BGIR9823_81332	TYK2	0	0	0.1	2.036666667	novel
BGIR9823_81334	TYK2	0	0	0.15333333	1	novel
BGIR9823_81349	ELAVL3	0	0	0.00666667	0.333333333	novel
BGIR9823_81499	NSD1	0	0	0.03	0.333333333	novel
BGIR9823_81562	SERINC5	0	0	0.13333333	1	novel
BGIR9823_81628	CHD1	0	0	0.10666667	1	novel
BGIR9823_81630	LOC102162728	0	0	0.05333333	0.333333333	novel
BGIR9823_81654	TRIM36	0	0	0.01333333	0.333333333	novel
BGIR9823_81713	ACSL6	0	0	0.03	0.333333333	novel
BGIR9823_81872	LOC100522670	0	0	0.23	1.333333333	novel
BGIR9823_81927	PRKRIP1	0	0	0.22666667	1.66	novel
BGIR9823_81944	HIP1	0	0	0.05333333	0.333333333	novel
BGIR9823_81957	GTF2I	0	0	0.19666667	1.333333333	novel
BGIR9823_82070	C3H16orf71	0	0	0.11666667	0.666666667	novel
BGIR9823_82180	ST6GAL2	0	0	0.06	0.32	novel
BGIR9823_82239	LOC102164763	0	0	0.04333333	0.666666667	novel
BGIR9823_82244	KRCC1	0	0	0.08666667	1.333333333	novel
BGIR9823_82245	RNF103	0	0	0.06	0.333333333	novel
BGIR9823_82292	FBXO41	0	0	0.06	0.616666667	novel
BGIR9823_82335	SERTAD2	0	0	0.06666667	0.333333333	novel
BGIR9823_82485	ATL2	0	0	0.13	1.67	novel

BGIR9823_82499	STRN	0	0	0.17666667	1	novel
BGIR9823_82597	IAH1	0	0	0.05	0.333333333	novel
BGIR9823_82610	LOC110260066	0	0	0.03333333	0.333333333	novel
BGIR9823_82612	FAM20C	0	0	0.04666667	0.333333333	novel
BGIR9823_82783	MOSMO	0	0	0.15333333	0.666666667	novel
BGIR9823_82803	XYLT1	0	0	0.13	1	novel
BGIR9823_82804	XYLT1	0	0	0.07333333	0.666666667	novel
BGIR9823_82805	XYLT1	0	0	0.06333333	0.333333333	novel
BGIR9823_82807	XYLT1	0	0	0.03	0.333333333	novel
BGIR9823_82835	ATF7IP2	0	0	0.04333333	1	novel
BGIR9823_82864	CLUAP1	0	0	0.05666667	0.333333333	novel
BGIR9823_82951	IPPK	0	0	0.16	1.333333333	novel
BGIR9823_82970	MERTK	0	0	0.06666667	0.333333333	novel
BGIR9823_83021	NPAS2	0	0	0.05333333	0.333333333	novel
BGIR9823_83060	KCMF1	0	0	0.12333333	0.666666667	novel
BGIR9823_83080	CCDC142	0	0	0.32	4	novel
BGIR9823_83246	SLC30A6	0	0	0.04333333	0.333333333	novel
BGIR9823_83278	CENPA	0	0	0.02666667	0.666666667	novel
BGIR9823_83407	FAM49B	0	0	0.05	0.316666667	novel
BGIR9823_83409	FAM49B	0	0	0.04333333	0.333333333	novel
BGIR9823_83461	LRP12	0	0	0.11333333	0.666666667	novel
BGIR9823_83479	UBR5	0	0	0.06333333	0.333333333	novel
BGIR9823_83531	DECR1	0	0	0.05	0.333333333	novel
BGIR9823_83551	MMP16	0	0	0.03666667	0.333333333	novel
BGIR9823_83552	MMP16	0	0	0.04333333	0.333333333	novel
BGIR9823_83554	MMP16	0	0	0.12	0.666666667	novel
BGIR9823_83557	MMP16	0	0	0.01333333	0.333333333	novel
BGIR9823_83584	TPD52	0	0	0.14666667	1.333333333	novel
BGIR9823_83604	TRAM1	0	0	0.12333333	1	novel
BGIR9823_83617	ARFGEF1	0	0	0.07333333	1	novel
BGIR9823_83696	TADA1	0	0	0.04666667	0.666666667	novel
BGIR9823_83722	CD84	0	0	0.02	0.333333333	novel
BGIR9823_83754	LOC106510131	0	0	0.00666667	0.666666667	novel
BGIR9823_83834	PI4KB	0	0	0.10333333	0.666666667	novel
BGIR9823_83908	CSDE1	0	0	0.17	1	novel
BGIR9823_83941	RBM15	0	0	0.04	0.333333333	novel
BGIR9823_84074	GLI4	0	0	0.07666667	2	novel
BGIR9823_84114	TBC1D31	0	0	0.13	2.333333333	novel
BGIR9823_84149	OXR1	0	0	0.11333333	0.666666667	novel
BGIR9823_84188	RPL30	0	0	0.05	0.666666667	novel
BGIR9823_84202	NDUFAF6	0	0	0.02333333	0.333333333	novel
BGIR9823_84258	KCNB2	0	0	0.06666667	0.333333333	novel
BGIR9823_84263	KCNB2	0	0	0.04666667	0.333333333	novel
BGIR9823_84275	PREX2	0	0	0.04333333	0.333333333	novel
BGIR9823_84311	RB1CC1	0	0	0.06	4.013333333	novel
BGIR9823_84334	MPZL1	0	0	0.13333333	1	novel
BGIR9823_84483	ANP32E	0	0	0.08666667	0.666666667	novel
BGIR9823_84505	HAO2	0	0	0.05666667	0.333333333	novel
BGIR9823_84511	MAN1A2	0	0	0.25333333	1.333333333	novel
BGIR9823_84513	MAN1A2	0	0	0.02666667	0.333333333	novel
BGIR9823_84569	WDR47	0	0	0.03333333	0.333333333	novel
BGIR9823_84600	SNX7	0	0	0.10666667	1	novel
BGIR9823_84653	PKN2	0	0	0.04333333	0.333333333	novel
BGIR9823_84697	MLC1	0	0	0.00666667	0.66	novel
BGIR9823_84791	RND1	0	0	0.04333333	0.353333333	novel
BGIR9823_84792	LMBR1L	0	0	0.14333333	1.666666667	novel
BGIR9823_84912	SRGAP1	0	0	0.03666667	0.333333333	novel
BGIR9823_84959	NUP107	0	0	0.09666667	0.666666667	novel
BGIR9823_84963	NUP107	0	0	0.42	2.666666667	novel
BGIR9823_84976	YEATS4	0	0	0.01	0.333333333	novel

BGIR9823_85006	AMN1	0	0	0.12666667	0.666666667	novel
BGIR9823_85188	APPL2	0	0	0.04	0.666666667	novel
BGIR9823_85218	ANKS1B	0	0	0.03333333	0.666666667	novel
BGIR9823_85221	ANKS1B	0	0	0.07666667	0.666666667	novel
BGIR9823_85222	ANKS1B	0	0	0.00666667	0.333333333	novel
BGIR9823_85227	ANKS1B	0	0	0.05666667	0.666666667	novel
BGIR9823_85235	ANKS1B	0	0	0.05333333	0.333333333	novel
BGIR9823_85262	KITLG	0	0	0.13666667	0.666666667	novel
BGIR9823_85270	PPFIA2	0	0	0.01666667	0.333333333	novel
BGIR9823_85329	XPNPEP3	0	0	0.25	1.333333333	novel
BGIR9823_85353	CBY1	0	0	0.02333333	0.333333333	novel
BGIR9823_85375	RIC8B	0	0	0.05	0.333333333	novel
BGIR9823_85477	TSPAN31	0	0	0.04333333	0.333333333	novel
BGIR9823_85512	GLIPR1	0	0	0.04666667	0.33	novel
BGIR9823_85599	ETNK1	0	0	0.03666667	0.333333333	novel
BGIR9823_85615	SLCO1C1	0	0	0.05	0.333333333	novel
BGIR9823_85640	GPR19	0	0	0.09	2.666666667	novel
BGIR9823_85654	LPCAT3	0	0	0.17666667	1.333333333	novel
BGIR9823_85713	BID	0	0	0.17333333	1.333333333	novel
BGIR9823_85720	KIF21A	0	0	0.22666667	1.666666667	novel
BGIR9823_85790	ANKS1B	0	0	0.08666667	1.666666667	novel
BGIR9823_85791	ANKS1B	0	0	0.02666667	0.333333333	novel
BGIR9823_85824	METAP2	0	0	0.28	2	novel
BGIR9823_85924	LOC106510463	0	0	0.00666667	1.333333333	novel
BGIR9823_85944	CTRB2	0	0	0.01666667	0.33	novel
BGIR9823_85963	IST1	0	0	0.05666667	0.666666667	novel
BGIR9823_85968	LOC102158108	0	0	0.07333333	1.333333333	novel
BGIR9823_85989	RSPRY1	0	0	0.12333333	0.666666667	novel
BGIR9823_86007	PLLP	0	0	0.06666667	0.333333333	novel
BGIR9823_86038	TSNAXIP1	0	0	0.03666667	0.333333333	novel
BGIR9823_86177	C6H19orf33	0	0	0.05	1.266666667	novel
BGIR9823_86233	ZNF283	0	0	0.02	0.333333333	novel
BGIR9823_86452	LOC106507613	0	0	0.18666667	1.333333333	novel
BGIR9823_86477	EXOSC10	0	0	0.04333333	0.666666667	novel
BGIR9823_86564	CLIC4	0	0	0.06	0.333333333	novel
BGIR9823_86601	PPP1R8	0	0	0.08666667	0.666666667	novel
BGIR9823_86742	SPIRE1	0	0	0.14333333	1.066666667	novel
BGIR9823_86744	SPIRE1	0	0	0.05666667	0.333333333	novel
BGIR9823_86768	DLGAP1	0	0	0.06666667	0.666666667	novel
BGIR9823_86775	DLGAP1	0	0	0.04666667	0.333333333	novel
BGIR9823_86776	DLGAP1	0	0	0.03666667	0.333333333	novel
BGIR9823_86839	RMC1	0	0	0.05666667	0.333333333	novel
BGIR9823_86878	ELP2	0	0	0.07333333	0.663333333	novel
BGIR9823_86899	RBFA	0	0	0.16666667	1	novel
BGIR9823_86919	NEXN	0	0	0.21	1.666666667	novel
BGIR9823_86994	DAB1	0	0	0.04666667	0.333333333	novel
BGIR9823_87085	ZMYND12	0	0	0.02	0.666666667	novel
BGIR9823_87161	AP1G1	0	0	0.14333333	1	novel
BGIR9823_87251	ENKD1	0	0	0.24	1.333333333	novel
BGIR9823_87257	PSKH1	0	0	0.16	1	novel
BGIR9823_87259	DUS2	0	0	0.09	0.633333333	novel
BGIR9823_87275	RBL2	0	0	0.16333333	1.333333333	novel
BGIR9823_87298	CNEP1R1	0	0	0.23	2.333333333	novel
BGIR9823_87470	LOC100620498	0	0	0.01333333	1.716666667	novel
BGIR9823_87486	LOC102158906	0	0	0.00666667	1.646666667	novel
BGIR9823_87539	LOC102158443	0	0	0.28333333	3	novel
BGIR9823_87542	LOC110261340	0	0	0.06333333	0.333333333	novel
BGIR9823_87593	APITD1	0	0	0.03	0.333333333	novel
BGIR9823_87682	WDTC1	0	0	0.05333333	0.333333333	novel
BGIR9823_87853	SMCHD1	0	0	0.12333333	1	novel

BGIR9823_87882	USP14	0	0	0.38333333	2	novel
BGIR9823_87883	USP14	0	0	0.05666667	0.333333333	novel
BGIR9823_87950	KCTD1	0	0	0.03333333	0.333333333	novel
BGIR9823_87984	TXNL4A	0	0	0.05666667	0.666666667	novel
BGIR9823_87988	PRKACB	0	0	0.01666667	0.333333333	novel
BGIR9823_88021	ST6GALNAC5	0	0	0.15	1.333333333	novel
BGIR9823_88035	SRSF11	0	0	0.10333333	1.333333333	novel
BGIR9823_88089	ACOT11	0	0	0.05	0.333333333	novel
BGIR9823_88211	TMEM170B	0	0	0.24	69.41333333	novel
BGIR9823_88214	TMEM170B	0	0	0.06666667	0.333333333	novel
BGIR9823_88270	LOC110261659	0	0	0.03	1.363333333	novel
BGIR9823_88370	MRPS18B	0	0	0.09	2.82	novel
BGIR9823_88380	AIF1	0	0	0.02666667	1.003333333	novel
BGIR9823_88448	CLPSL2	0	0	0.02666667	0.333333333	novel
BGIR9823_88451	MAPK14	0	0	0.07333333	0.333333333	novel
BGIR9823_88454	KCTD20	0	0	0.12333333	1	novel
BGIR9823_88564	WDR73	0	0	0.10333333	1	novel
BGIR9823_88615	HMG20A	0	0	0.09666667	0.666666667	novel
BGIR9823_88628	SIN3A	0	0	0.15	1	novel
BGIR9823_88656	SEC23A	0	0	0.03333333	0.333333333	novel
BGIR9823_88659	SSTR1	0	0.333333333	0.00666667	2	novel
BGIR9823_88708	STRN3	0	0	0.05	0.333333333	novel
BGIR9823_88826	FUT8	0	0	0.05333333	0.333333333	novel
BGIR9823_88830	FUT8	0	0	0.12	0.666666667	novel
BGIR9823_88877	PCNX1	0	0	0.20333333	1.333333333	novel
BGIR9823_88919	LIN52	0	0	0.04666667	0.333333333	novel
BGIR9823_88946	NRXN3	0	0	0.02666667	0.333333333	novel
BGIR9823_89064	NUP153	0	0	0.10333333	0.666666667	novel
BGIR9823_89237	UHRF1BP1	0	0	0.04333333	0.333333333	novel
BGIR9823_89239	TCP11	0	0	0.03	0.943333333	novel
BGIR9823_89259	STK38	0	0	0.05	0.333333333	novel
BGIR9823_89375	MEX3B	0	0	0.03	0.333333333	novel
BGIR9823_89384	SH3GL3	0	0	0.04666667	0.333333333	novel
BGIR9823_89387	SH3GL3	0	0	0.02666667	0.333333333	novel
BGIR9823_89388	SH3GL3	0	0	0.04	0.333333333	novel
BGIR9823_89394	FSD2	0	0	0.05	0.666666667	novel
BGIR9823_89420	AP3S2	0	0	0.04666667	0.333333333	novel
BGIR9823_89473	BRMS1L	0	0	0.05	0.333333333	novel
BGIR9823_89477	SRP54	0	0	0.03666667	0.333333333	novel
BGIR9823_89491	NUBPL	0	0	0.04333333	0.333333333	novel
BGIR9823_89525	OXA1L	0	0	0.09333333	0.666666667	novel
BGIR9823_89554	CHD2	0	0	0.27	1.993333333	novel
BGIR9823_89644	GTF2A1	0	0	0.04666667	0.333333333	novel
BGIR9823_89751	STX18	0	0	0.15333333	1	novel
BGIR9823_89752	STX18	0	0	0.17666667	1.333333333	novel
BGIR9823_89762	MED28	0	0	0.15666667	14.75	novel
BGIR9823_89873	PDGFRA	0	0	0.03	0.333333333	novel
BGIR9823_89946	ADAMTS3	0	0	0.01333333	0.333333333	novel
BGIR9823_89956	LOC106504698	0	0	0.01333333	0.296666667	novel
BGIR9823_90024	LOC102163618	0	0.68	0.00333333	2.133333333	novel
BGIR9823_90039	NDNF	0	0	0.01	1.08	novel
BGIR9823_90070	LOC100738196	0	0.333333333	0.00666667	1.333333333	novel
BGIR9823_90080	TBCK	0	0	0.04333333	0.333333333	novel
BGIR9823_90083	INTS12	0	0	0.08	1	novel
BGIR9823_90090	TET2	0	0	0.01666667	0.333333333	novel
BGIR9823_90109	DNAJB14	0	0	0.23666667	1.333333333	novel
BGIR9823_90155	WDFY3	0	0	0.01333333	0.366666667	novel
BGIR9823_90227	ZNF518B	0	0	0.05666667	0.333333333	novel
BGIR9823_90228	HS3ST1	0	0	0.11666667	0.666666667	novel
BGIR9823_90292	WDR19	0	0	0.04333333	0.666666667	novel

BGIR9823_90393	NAF1	0	0	0.03666667	0.333333333	novel
BGIR9823_90422	UBA6	0	0	0.09666667	0.666666667	novel
BGIR9823_90450	11-Sep	0	0	0.04	0.333333333	novel
BGIR9823_90464	TRIM2	0	0	0.03	0.333333333	novel
BGIR9823_90511	RAB33B	0	0	0.07666667	0.666666667	novel
BGIR9823_90551	FAT4	0	0	0.00666667	0.04	novel
BGIR9823_90579	USP53	0	0	0.03	0.333333333	novel
BGIR9823_90587	METTL14	0	0	0.08	1	novel
BGIR9823_90616	LOC100518620	0	0	0.08666667	1.333333333	novel
BGIR9823_90640	C8H4orf54	0	0	0.36666667	3.663333333	novel
BGIR9823_90652	SMARCAD1	0	0	0.07333333	0.666666667	novel
BGIR9823_90670	HERC6	0	0	0.14333333	1.666666667	novel
BGIR9823_90685	ARHGAP24	0	0	0.03	0.646666667	novel
BGIR9823_90746	NUP98	0	0	0.01333333	0.333333333	novel
BGIR9823_90761	RELT	0	0	0.04	0.666666667	novel
BGIR9823_90822	RAB38	0	0	0.03333333	0.333333333	novel
BGIR9823_90845	MAML2	0	0	0.01	0.333333333	novel
BGIR9823_90853	ARHGAP42	0	0	0.09666667	1	novel
BGIR9823_90888	AASDHPPT	0	0	0.07666667	0.666666667	novel
BGIR9823_90963	GRAMD1B	0	0	0.07333333	0.333333333	novel
BGIR9823_90972	CCDC15	0	0	0.12333333	0.666666667	novel
BGIR9823_90976	CCDC15	0	0	0.15666667	1.333333333	novel
BGIR9823_90979	CCDC15	0	0	0.06	0.333333333	novel
BGIR9823_91095	ANKIB1	0	0	0.12	0.666666667	novel
BGIR9823_91099	RBM48	0	0	0.06333333	0.666666667	novel
BGIR9823_91224	LAMB1	0	0	0.06333333	0.333333333	novel
BGIR9823_91273	RASAL2	0	0	0.14666667	1	novel
BGIR9823_91291	XPR1	0	0	0.30333333	3	novel
BGIR9823_91318	RGL1	0	0	0.01	0.333333333	novel
BGIR9823_91343	PLA2G4A	0	0	0.02333333	2.796666667	novel
BGIR9823_91489	RAB30	0	0	0.13	1	novel
BGIR9823_91533	SESN3	0	0	0.04333333	0.333333333	novel
BGIR9823_91581	IL18	0	0	0.08666667	1	novel
BGIR9823_91718	CFAP69	0	0	0.05666667	0.666666667	novel
BGIR9823_91788	DNAH11	0	0	0.03333333	0.333333333	novel
BGIR9823_91796	IGF2BP3	0	0	0.05	0.333333333	novel
BGIR9823_91802	IGF2BP3	0	0	0.12666667	1	novel
BGIR9823_91803	IGF2BP3	0	0	0.04	1.333333333	novel
BGIR9823_91805	IGF2BP3	0	0	0.04	0.333333333	novel
BGIR9823_91810	IGF2BP3	0	0	0.01666667	0.333333333	novel
BGIR9823_91882	PUS7	0	0	0.18666667	1.333333333	novel
BGIR9823_91885	ATXN7L1	0	0	0.06	1.003333333	novel
BGIR9823_91893	HBP1	0	0	0.04333333	1.666666667	novel
BGIR9823_91904	CUL1	0	0	0.13333333	0.666666667	novel
BGIR9823_91968	EDEM3	0	0	0.14666667	1.333333333	novel
BGIR9823_92033	BRINP3	0	0	0.04333333	0.333333333	novel
BGIR9823_92061	MIA3	0	0	0.06333333	1.333333333	novel
BGIR9823_92069	LOC110255641	0	0	0.12666667	2	novel
BGIR9823_92078	STUM	0	0	0.08	24.32666667	novel
BGIR9823_92108	FBXO28	0	0	0.17333333	1.333333333	novel
BGIR9823_92180	B4GALT1	0	0	0.11333333	0.666666667	novel
BGIR9823_92197	DDX58	0	0	0.06333333	0.333333333	novel
BGIR9823_92254	OPTN	0	0	0.04666667	0.333333333	novel
BGIR9823_92270	PIP4K2A	0	0	0.07666667	0.666666667	novel
BGIR9823_92364	SDE2	0	0	0.04	0.333333333	novel
BGIR9823_92392	AKT3	0	0	0.10333333	0.666666667	novel
BGIR9823_92422	KIF21B	0	0	0.01	1.29	novel
BGIR9823_92459	DAPK1	0	0	0.02666667	0.333333333	novel
BGIR9823_92470	RMI1	0	0	0.06666667	0.666666667	novel
BGIR9823_92637	PFKFB3	0	0	0.19333333	1.333333333	novel

BGIR9823_92764	NHLRC3	0	0	0.06666667	0.333333333	novel
BGIR9823_92767	COG6	0	0	0.05333333	0.333333333	novel
BGIR9823_92814	CCDC122	0	0	0.13333333	1.333333333	novel
BGIR9823_92829	TDRD3	0	0	0.05	0.333333333	novel
BGIR9823_92866	SLAIN1	0	0	0.07666667	0.333333333	novel
BGIR9823_92892	HS6ST3	0	2.39	0.00666667	6.383333333	novel
BGIR9823_92899	MBNL2	0	0	0.09333333	0.666666667	novel
BGIR9823_92905	TM9SF2	0	0	0.16333333	1	novel
BGIR9823_92985	RNF6	0	0	0.13	1.666666667	novel
BGIR9823_93179	TGDS	0	0	0.02	0.333333333	novel
BGIR9823_93200	FGF14	0	0	0.05666667	1.55	novel
BGIR9823_93207	FGF14	0	0	0.03	1.333333333	novel
BGIR9823_93221	LOC110255863	0	0	0.02	1.303333333	novel
BGIR9823_93289	LOC100516105	0	0	0.14333333	3.476666667	novel
BGIR9823_93325	LOC110255971	0	0	0.02666667	0.333333333	novel
BGIR9823_93342	RGS9	0	0	0.05333333	0.333333333	novel
BGIR9823_93343	RGS9	0	0	0.10333333	0.666666667	novel
BGIR9823_93354	PITPNC1	0	0	0.04	0.333333333	novel
BGIR9823_93449	LSM12	0	0	0.02333333	0.333333333	novel
BGIR9823_93451	PYY	0	0	0.08	1.32	novel
BGIR9823_93476	STAT5B	0	0	0.34666667	2.666666667	novel
BGIR9823_93564	LOC110256032	0	0	0.01666667	0.41	novel
BGIR9823_93582	PPM1E	0	0	0.04	0.333333333	novel
BGIR9823_93595	DHX40	0	0	0.1	0.666666667	novel
BGIR9823_93620	MED13	0	0	0.08333333	0.666666667	novel
BGIR9823_93653	CCL5	0	0	0.17	3.803333333	novel
BGIR9823_93682	TNFAIP1	0	0	0.03666667	0.333333333	novel
BGIR9823_93699	TAOK1	0	0	0.04	0.333333333	novel
BGIR9823_93743	PAFAH1B1	0	0	0.29	1.666666667	novel
BGIR9823_93746	RAP1GAP2	0	0	0.14	1	novel
BGIR9823_93747	RAP1GAP2	0	0	0.04333333	0.333333333	novel
BGIR9823_93775	MED11	0	0	0.05666667	1	novel
BGIR9823_93820	LOC102164714	0	0	0.08666667	0.666666667	novel
BGIR9823_93839	TOP3A	0	0	0.12333333	1.333333333	novel
BGIR9823_93952	GNA13	0	0	0.14333333	1	novel
BGIR9823_94001	LOC100626147	0	0	0.27	5.84	novel
BGIR9823_94054	CASC3	0	0	0.12333333	2	novel
BGIR9823_94077	CBX1	0	0	0.08333333	0.666666667	novel
BGIR9823_94137	PTRH2	0	0	0.07333333	1	novel
BGIR9823_94332	C12H17orf100	0	0	0.11	0.666666667	novel
BGIR9823_94440	MRPL3	0	0	0.04	0.333333333	novel
BGIR9823_94524	OXSR1	0	0	0.05666667	0.333333333	novel
BGIR9823_94545	SEC22C	0	0	0.06	0.666666667	novel
BGIR9823_94694	PTPRG	0	0	0.10333333	0.666666667	novel
BGIR9823_94738	MAGI1	0	0	0.04333333	0.333333333	novel
BGIR9823_94787	CHL1	0	0	0.11666667	1	novel
BGIR9823_94810	GRM7	0	0	0.08	0.666666667	novel
BGIR9823_94818	GRM7	0	0	0.08	0.996666667	novel
BGIR9823_94820	GRM7	0	0	0.04	0.333333333	novel
BGIR9823_94832	GRM7	0	0	0.05333333	0.666666667	novel
BGIR9823_94838	GRM7	0	0	0.03	0.333333333	novel
BGIR9823_94839	GRM7	0	0	0.03333333	0.666666667	novel
BGIR9823_94840	GRM7	0	0	0.03	0.333333333	novel
BGIR9823_94846	GRM7	0	0	0.05	0.333333333	novel
BGIR9823_94863	GRM7	0	0	0.01	0.333333333	novel
BGIR9823_94909	ATG7	0	0	0.1	1.666666667	novel
BGIR9823_94935	RUVBL1	0	0	0.04333333	0.333333333	novel
BGIR9823_95005	U2SURP	0	0	0.05666667	0.666666667	novel
BGIR9823_95008	PLSCR1	0	0	0.02	2	novel
BGIR9823_95022	EIF2A	0	0	0.04666667	0.333333333	novel

BGIR9823_95040	ARHGEF26	0	0	0.06666667	0.333333333	novel
BGIR9823_95041	ARHGEF26	0	0	0.05333333	0.333333333	novel
BGIR9823_95043	ARHGEF26	0	0	0.05	0.333333333	novel
BGIR9823_95046	ARHGEF26	0	0	0.11333333	1	novel
BGIR9823_95057	VEPH1	0	0	0.03666667	0.333333333	novel
BGIR9823_95064	MFSD1	0	0	0.06333333	0.666666667	novel
BGIR9823_95129	LOC110256374	0	0	0.12	1	novel
BGIR9823_95230	UBXN7	0	0	0.20333333	2.333333333	novel
BGIR9823_95286	SNX4	0	0	0.04	0.666666667	novel
BGIR9823_95334	IGSF11	0	0	0.08	0.666666667	novel
BGIR9823_95355	ATG3	0	0	0.05	0.333333333	novel
BGIR9823_95389	RBM11	0	0	0.01666667	0.666666667	novel
BGIR9823_95394	LOC106508546	0	0	0.00666667	1.423333333	novel
BGIR9823_95404	NCAM2	0	0	0.04666667	0.333333333	novel
BGIR9823_95438	RCAN1	0	0	0.07333333	0.666666667	novel
BGIR9823_95558	CMTM6	0	0	0.19333333	32.11	novel
BGIR9823_95616	LZTFL1	0	0	0.02333333	0.333333333	novel
BGIR9823_95632	SMARCC1	0	0	0.04	0.333333333	novel
BGIR9823_95646	SHISA5	0	0	0.03	0.333333333	novel
BGIR9823_95648	PFKFB4	0	0	0.12333333	1	novel
BGIR9823_95651	SLC26A6	0	0	0.06	0.333333333	novel
BGIR9823_95657	SLC25A20	0	0	0.05666667	1	novel
BGIR9823_95680	IP6K1	0	0	0.10333333	0.666666667	novel
BGIR9823_95755	ARHGEF3	0	0	0.07333333	0.333333333	novel
BGIR9823_95758	ARHGEF3	0	0	0.04666667	0.333333333	novel
BGIR9823_95777	PRICKLE2	0	0	0.06	1	novel
BGIR9823_95778	PRICKLE2	0	0	0.06	0.333333333	novel
BGIR9823_95779	PRICKLE2	0	0	0.04666667	0.333333333	novel
BGIR9823_95791	LRIG1	0	0	0.02	0.333333333	novel
BGIR9823_95816	RYBP	0	0	0.03	0.333333333	novel
BGIR9823_95893	EEFSEC	0	0	0.06666667	0.333333333	novel
BGIR9823_95897	MCM2	0	0	0.03	0.666666667	novel
BGIR9823_95953	HLTF	0	0	0.12	1	novel
BGIR9823_95963	LOC110255174	0	0	0.07666667	0.666666667	novel
BGIR9823_95998	LOC100514323	0	0	0.04	0.333333333	novel
BGIR9823_96052	TNIK	0	0	0.03666667	0.333333333	novel
BGIR9823_96080	GNB4	0	0	0.33333333	2.333333333	novel
BGIR9823_96082	GNB4	0	0	0.04333333	0.333333333	novel
BGIR9823_96083	GNB4	0	0	0.04	0.333333333	novel
BGIR9823_96109	LOC110256376	0	0	0.02666667	0.243333333	novel
BGIR9823_96123	P3H2	0	0	0.09333333	1	novel
BGIR9823_96156	SENP5	0	0	0.19666667	2	novel
BGIR9823_96185	PDIA5	0	0	0.08	1	novel
BGIR9823_96212	QTRT2	0	0	0.06666667	0.333333333	novel
BGIR9823_96322	LOC110256457	0	0	0.17333333	1.333333333	novel
BGIR9823_96352	TMEM50B	0	0	0.1	0.666666667	novel
BGIR9823_96368	HLCS	0	0	0.08666667	0.666666667	novel
BGIR9823_96431	SLC39A14	0	0	0.09	1	novel
BGIR9823_96504	ZNF84	0	0	0.03666667	0.333333333	novel
BGIR9823_96552	VPS37B	0	0	0.04666667	0.333333333	novel
BGIR9823_96583	TCTN1	0	0	0.09	0.666666667	novel
BGIR9823_96609	MED13L	0	0	0.02666667	0.333333333	novel
BGIR9823_96687	TCN2	0	0	0.02666667	0.333333333	novel
BGIR9823_96803	TARBP1	0	0	0.04	0.333333333	novel
BGIR9823_96839	NUP133	0	0	0.04	0.333333333	novel
BGIR9823_96840	NUP133	0	0	0.05666667	0.666666667	novel
BGIR9823_96873	PHYHIPL	0	0	0.02666667	0.333333333	novel
BGIR9823_96884	NRBF2	0	0	0.13666667	0.67	novel
BGIR9823_96906	REEP3	0	0	0.06	0.333333333	novel
BGIR9823_96924	CTNNA3	0	0	0.03333333	0.333333333	novel

BGIR9823_96968	PCBD1	0	0	0.08	0.666666667	novel
BGIR9823_97008	ADK	0	0	0.05333333	0.333333333	novel
BGIR9823_97142	TNKS2	0	0	0.06	0.333333333	novel
BGIR9823_97148	5-Mar	0	0	0.03666667	0.416666667	novel
BGIR9823_97154	EXOC6	0	0	0.00666667	0.49	novel
BGIR9823_97194	CNNM1	0	0	0.05	0.333333333	novel
BGIR9823_97197	ENTPD7	0	0	0.03666667	0.333333333	novel
BGIR9823_97198	ENTPD7	0	0	0.04	0.333333333	novel
BGIR9823_97266	LOC102164743	0	0	0.08333333	1.333333333	novel
BGIR9823_97271	SMC3	0	0	0.26	2	novel
BGIR9823_97309	FAM45A	0	0	0.09333333	0.666666667	novel
BGIR9823_97376	LOC100158108	0	0	0.05666667	14.22	novel
BGIR9823_97406	LOC100517194	0	0	0.00666667	0.666666667	novel
BGIR9823_97506	TRAFD1	0	0	0.03333333	0.333333333	novel
BGIR9823_97532	SPPL3	0	0	0.1	0.666666667	novel
BGIR9823_97768	MICU1	0	0	0.25666667	1.333333333	novel
BGIR9823_97833	GRID1	0	0	0.04666667	0.333333333	novel
BGIR9823_97841	WAPL	0	0	0.23666667	2	novel
BGIR9823_97843	WAPL	0	0	0.1	0.666666667	novel
BGIR9823_97913	MYOF	0	0	0.37666667	2.64	novel
BGIR9823_97916	PDE6C	0	0	0.10333333	0.666666667	novel
BGIR9823_97918	PDE6C	0	0	0.04666667	0.333333333	novel
BGIR9823_97924	HELLS	0	0	0.06	0.666666667	novel
BGIR9823_97938	TCTN3	0	0	0.04666667	0.333333333	novel
BGIR9823_97939	ENTPD1	0	0	0.22333333	1.333333333	novel
BGIR9823_97945	TM9SF3	0	0	0.14	0.666666667	novel
BGIR9823_97953	PIK3AP1	0	0	0.04333333	0.333333333	novel
BGIR9823_98045	SHTN1	0	0	0.02333333	0.666666667	novel
BGIR9823_98153	LYPD1	0	1.11	0.05	21.90333333	novel
BGIR9823_98286	IWS1	0	0	0.12666667	1.333333333	novel
BGIR9823_98299	GALNT13	0	0	0.01	0.333333333	novel
BGIR9823_98336	LOC100515171	0	0	0.42333333	2.766666667	novel
BGIR9823_98396	AGPS	0	0	0.03666667	0.333333333	novel
BGIR9823_98468	SPATS2L	0	0	0.06333333	0.333333333	novel
BGIR9823_98484	FAM117B	0	0	0.04	1	novel
BGIR9823_98501	ABI2	0	0	0.07	0.333333333	novel
BGIR9823_98509	ABI2	0	0	0.05333333	0.333333333	novel
BGIR9823_98571	WNT6	0	0	0.03666667	0.333333333	novel
BGIR9823_98641	SH3BP4	0	0	0.12333333	0.666666667	novel
BGIR9823_98667	EPC2	0	0	0.02666667	0.333333333	novel
BGIR9823_98771	GPM6A	0	0	0.07333333	0.333333333	novel
BGIR9823_98776	GPM6A	0	0	0.04	0.333333333	novel
BGIR9823_98789	ENPP6	0	0	0.04333333	0.333333333	novel
BGIR9823_98791	IRF2	0	0	0.13666667	1	novel
BGIR9823_98925	PPIG	0	0	0.03	0.333333333	novel
BGIR9823_98947	CIR1	0	0	0.06333333	0.333333333	novel
BGIR9823_98956	LNPK	0	0	0.07	0.333333333	novel
BGIR9823_99021	TMEFF2	0	0	0.05	0.333333333	novel
BGIR9823_99029	TMEFF2	0	0	0.10333333	1	novel
BGIR9823_99059	TMEM237	0	0	0.03666667	0.666666667	novel
BGIR9823_99060	TMEM237	0	0	0.06333333	0.333333333	novel
BGIR9823_99063	ALS2	0	0	0.24666667	3.666666667	novel
BGIR9823_99126	USP37	0	0	0.02333333	0.333333333	novel
BGIR9823_99186	LOC100517129	0	0	0.03333333	4.666666667	novel
BGIR9823_99224	LOC110257075	0	0	0.06666667	1.483333333	novel
BGIR9823_99244	LOC102167708	0	0	0.08	1	novel
BGIR9823_99345	IPO11	0	0	0.17333333	1.666666667	novel
BGIR9823_99351	LOC110257246	0	0	0.05333333	0.333333333	novel
BGIR9823_99376	PIK3R1	0	0	0.04	0.333333333	novel
BGIR9823_99397	BDP1	0	0	0.04666667	0.333333333	novel

BGIR9823_99474	ANKH	0	0	0.03	0.333333333	novel
BGIR9823_99566	PRKAA1	0	0	0.06333333	0.666666667	novel
BGIR9823_99619	NDUFAF2	0	0	0.02	0.333333333	novel
BGIR9823_99635	MRPS36	0	0	0.08666667	1.333333333	novel
BGIR9823_99739	GINS4	0	0	0.16333333	1	novel
BGIR9823_99875	ITCH	0	0	0.09666667	0.566666667	novel
BGIR9823_99888	ACSS2	0	0	0.28666667	2	novel
BGIR9823_99894	PHF20	0	0	0.08333333	1.666666667	novel
BGIR9823_99964	PLCG1	0	0	0.25	1.666666667	novel
BGIR9823_99974	TTPAL	0	0	0.04666667	1	novel
NR_033706.1	ARL6IP4	0	0	0.06	3.53	known
URS0000BC48FA	LOC102159171	0	0	0.00666667	0.333333333	known
URS0000BC5389	LOC110255457	0	0	0.01333333	0.516666667	known
URS0000BC5412	LOC100525229	0	0	0.00333333	0.18	known
URS0000BC551B	LOC106509907	0	0	0.00333333	0.333333333	known
URS0000BC552D	LOC106506054	0	0	0.01333333	0.44	known
URS0000BC5806	LOC100515582	0	0	0.00333333	0.666666667	known
URS0000BC5A9E	LOC106504574	0	0	0.03666667	5.813333333	known
URS0000BC5DE3	LOC110256465	0	0	0.00333333	0.41	known
URS0000BC631A	LOC110258320	0	0	0.01333333	0.566666667	known
URS0000BC65AD	LOC102164639	0	0	0.00666667	1.666666667	known
URS0000BC6968	LOC110258645	0	0	0.01	0.716666667	known
URS0000BC6EDF	LOC102164131	0	0	0.00666667	0.666666667	known
URS0000BC6FA2		0	0	0.01	1	known
URS0000BC7148	LOC102165655	0	0	0.01	0.666666667	known
URS0000BC77C8	LOC110260289	0	0	0.00333333	0.143333333	known
URS0000BC7B89	LOC110255952	0	0	0.00666667	0.796666667	known
URS0000BC7C7F	CCDC187	0	0.173333333	0.00333333	0.66	known
URS0000BC7E6D	LOC102164631	0	0	0.02333333	0.796666667	known
URS0000BC7FAA	LOC102162336	0	0	0.02666667	1.463333333	known
URS0000BC84FE	LOC102157770	0	0	0.00333333	0.333333333	known
URS0000BC860F	LOC102165655	0	0	0.00666667	0.333333333	known
URS0000BC8617	LOC110255961	0	0	0.01	0.666666667	known
URS0000BC88AB	LOC110262038	0	0	0.03	1.93	known
URS0000BC8970	LOC102164155	0	0	0.00333333	0.256666667	known
URS0000BC8BE6	LOC106505676	0	0	0.00666667	0.333333333	known
URS0000BC8C64	LOC110257768	0	0	0.01	0.333333333	known
URS0000BC8C96	LOC106507506	0	0	0.03333333	6.546666667	known
URS0000BC8F36	CCDC187	0	0	0.00333333	0.6	known
URS0000BC8F5F	LOC106506502	0	0	0.00333333	0.113333333	known
URS0000BC9173	LOC106509516	0	0	0.00666667	0.333333333	known
URS0000BC91DD	LOC110261199	0	0	0.00333333	0.333333333	known
URS0000BC927E	LOC106507406	0	0	0.01666667	2.573333333	known
URS0000BC92F4	LOC102167708	0	0	0.02333333	1.213333333	known
URS0000BC9312	LOC106509843	0	0	0.04666667	5.703333333	known
URS0000BC9AD0	LOC100524954	0	0	0.01666667	0.896666667	known
URS0000BC9BA0	LOC102162879	0	0	0.00333333	0.666666667	known
URS0000BC9F4E	LOC106507666	0	0	0.00333333	0.333333333	known
URS0000BC9F7C	LOC106509123	0	0	0.00666667	0.333333333	known
URS0000BCA38F	LOC106509619	0	0	0.01333333	0.666666667	known
URS0000BCE269	LOC106510124	0	0	0.00333333	0.333333333	known
URS0000BD93CF	CDYL2	0	0	0.01	0.333333333	known
XR_001297633.2	LOC106504277	0	0	0.01333333	0.666666667	known
XR_001297666.2	LOC106504303	0	0	0.01333333	5	known
XR_001297803.2	LOC106504375	0	0	0.00666667	0.333333333	known
XR_001297906.2	LOC102164231	0	0.333333333	0.00333333	1	known
XR_001297957.2	LOC106504467	0	0	0.00666667	0.666666667	known
XR_001298042.2	LOC106504507	0	0	0.00666667	0.333333333	known
XR_001298257.2	LOC106504627	0	0	0.02	0.666666667	known
XR_001298290.2	LOC106504650	0	0	0.17666667	2.666666667	known

XR_001298608.2	LOC106504801	0	0	0.03	0.333333333	known
XR_001298672.2	LOC106504831	0	0	0.00666667	0.666666667	known
XR_001298686.2	LOC106504842	0	0	0.03666667	8.276666667	known
XR_001298812.2	LOC106504881	0	0	0.01333333	3.64	known
XR_001298844.2	LOC102163364	0	0	0.05	8.28	known
XR_001298846.2	LOC102163364	0	0	0.07333333	10.91666667	known
XR_001298850.2	LOC102163364	0	0	0.02	0.803333333	known
XR_001298930.2	LOC102163402	0	0	0.06333333	4.086666667	known
XR_001298940.2	LOC102163402	0	0	0.02666667	6.283333333	known
XR_001298948.2	LOC106504956	0	0	0.01333333	1.666666667	known
XR_001298953.2	LOC106504926	0	0	1.71333333	20.43333333	known
XR_001298963.2	LOC106504961	0	0	0.01	1	known
XR_001299155.2	LOC106505078	0	0	0.01	1	known
XR_001299218.2	LOC106505118	0	0	0.00333333	0.333333333	known
XR_001299338.2	LOC106505183	0	0	0.04666667	2.333333333	known
XR_001299355.2	LOC106505191	0	0	0.00666667	0.333333333	known
XR_001299417.2	LOC102163368	0	0	0.02	2	known
XR_001299630.2	LOC106505322	0	0	0.02666667	1.666666667	known
XR_001299725.2	LOC102167199	0	0	0.00666667	0.333333333	known
XR_001299740.2	LOC106505379	0	0	0.00333333	0.333333333	known
XR_001299821.2	LOC106505407	0	0	0.02	0.333333333	known
XR_001299881.2	LOC106505444	0	0	0.04666667	0.333333333	known
XR_001299919.2	LOC106505468	0	0	0.02	1.333333333	known
XR_001299975.2	LOC106505500	0	0	0.00666667	0.333333333	known
XR_001300041.2	LOC102163370	0	0	0.18	2	known
XR_001300054.2	LOC106505534	0	0	0.01333333	0.333333333	known
XR_001300068.2	LOC106505543	0	0	0.00333333	0.333333333	known
XR_001300091.2	LOC102163243	0	0	0.00333333	0.333333333	known
XR_001300163.2	TMIE	0	0	0.00333333	0.333333333	known
XR_001300300.2	LOC106505675	0	0	0.02333333	1	known
XR_001300369.2	LOC106505712	0	0	0.03666667	2.666666667	known
XR_001300388.2	LOC106505721	0	0	0.02	0.666666667	known
XR_001300433.2	LOC102159375	0	0	0.00333333	0.99	known
XR_001300483.2	LOC106505765	0	0	0.02666667	2.866666667	known
XR_001300528.2	LOC102157484	0	0	0.02666667	3.503333333	known
XR_001300585.2	LOC106505815	0	0	0.00666667	0.666666667	known
XR_001300614.2	LOC106505829	0	0	0.00333333	0.333333333	known
XR_001300738.2	LOC106505895	0	0	0.00666667	0.556666667	known
XR_001300829.2	LOC106505927	0	0	0.04	0.333333333	known
XR_001300877.2	LOC100523337	0	0	0.02	1.406666667	known
XR_001300973.2	LOC106506015	0	0	0.03	0.333333333	known
XR_001301039.2	LOC106506057	0	0	0.00666667	0.333333333	known
XR_001301309.2	LOC106506172	0	0	0.00333333	0.333333333	known
XR_001301393.2	LOC106506223	0	0	0.00666667	0.333333333	known
XR_001301398.2	LOC100520518	0	0	0.01333333	0.803333333	known
XR_001301435.2	LOC106506249	0	0	0.01333333	0.333333333	known
XR_001301501.2	LOC102166504	0	0.666666667	0.00333333	1	known
XR_001301590.2	LOC102160916	0	0	0.02333333	1.333333333	known
XR_001301664.2	LOC102158015	0	0	0.03333333	2.32	known
XR_001301789.2	LOC106506487	0	0	0.00333333	0.333333333	known
XR_001301841.2	LOC102161888	0	0	0.01	1.856666667	known
XR_001301846.2	LOC106506514	0	0	0.12666667	5.333333333	known
XR_001302077.2	LOC106506644	0	0	0.02333333	3.503333333	known
XR_001302202.2	LOC102165584	0	0	0.21666667	26.75333333	known
XR_001302314.2	LOC106506771	0	0	0.01	0.333333333	known
XR_001302329.2	LOC106506780	0	0	0.00333333	1	known
XR_001302370.2	LOC106506806	0	0	0.00666667	1	known
XR_001302468.2	LOC106506857	0	0	0.04333333	1	known
XR_001302522.2	LOC106506885	0	0	0.00333333	0.483333333	known
XR_001302523.2	LOC106506885	0	0	0.00333333	0.333333333	known

XR_001302526.2	LOC106506886	0	0	0.00333333	0.333333333	known
XR_001302555.2	LOC106506903	0	0	0.01333333	0.333333333	known
XR_001302592.2	LOC106506935	0	0	0.00333333	0.333333333	known
XR_001302600.2	LOC106506942	0	0	0.03	0.666666667	known
XR_001303119.2	LOC100628140	0	0	0.07666667	14.08333333	known
XR_001303139.2	LOC100514211	0	0	0.05666667	4.336666667	known
XR_001303159.2	LOC106507279	0	0	0.02	2.333333333	known
XR_001303294.2	LOC106504252	0	0	0.01666667	1.863333333	known
XR_001303303.2	LOC106507373	0	0	0.02	0.876666667	known
XR_001303325.2	LOC106507388	0	0	0.00333333	0.41	known
XR_001303355.2	LOC106507401	0	0	0.00333333	0.333333333	known
XR_001303460.2	LOC106507457	0	0	0.01666667	3.333333333	known
XR_001303606.2	LOC102165143	0	0	0.02333333	0.666666667	known
XR_001303786.2	LOC106507649	0	0	0.03666667	1.61	known
XR_001303895.2	LOC106507704	0	0	0.01	0.666666667	known
XR_001304025.2	LOC102168087	0	0	0.07	1	known
XR_001304032.2	LOC106507771	0	0	0.00666667	0.333333333	known
XR_001304061.2	LOC106507797	0	0	0.01	1	known
XR_001304153.2	LOC106507850	0	0	0.00666667	0.49	known
XR_001304255.2	LOC106507893	0	0	0.01	0.333333333	known
XR_001304381.2	LOC106507954	0	0	0.01	0.333333333	known
XR_001304407.2	LOC106507967	0	0	0.02	1	known
XR_001304410.2	LOC102158363	0	0	0.01333333	0.333333333	known
XR_001304489.2	LOC106508003	0	0	0.01333333	0.78	known
XR_001304490.2	LOC106508004	0	0	0.03	1.6	known
XR_001304557.2	LOC106508030	0	0	0.01666667	0.666666667	known
XR_001304561.2	LOC106508031	0	0	0.00333333	0.333333333	known
XR_001304723.2	LOC102164001	0	0.333333333	0.00333333	0.666666667	known
XR_001304735.2	LOC106508148	0	0	0.01333333	0.333333333	known
XR_001304819.2	LOC106508193	0	0	0.00666667	0.666666667	known
XR_001304958.2	LOC106508240	0	0	0.01	0.666666667	known
XR_001304961.2	LOC106508243	0	0	0.06	3.333333333	known
XR_001305012.2	LOC106508273	0	0	0.01666667	0.333333333	known
XR_001305159.2	LOC106508354	0	0	0.03	2.3	known
XR_001305428.2	LOC102162290	0	0	0.02	0.666666667	known
XR_001305704.2	LOC106508684	0	0	0.02666667	0.666666667	known
XR_001305865.2	LOC100521937	0	0	0.01	0.666666667	known
XR_001305881.2	LOC106508814	0	0	0.00666667	0.333333333	known
XR_001305924.2	LOC106508847	0	0	0.00333333	0.333333333	known
XR_001305928.2	LOC106508854	0	0	0.00666667	0.53	known
XR_001306053.2	LOC106508915	0	0	0.01333333	1.666666667	known
XR_001306104.2	LOC106508944	0	0	0.00666667	0.333333333	known
XR_001306284.2	LOC106509059	0	0	0.04	3.223333333	known
XR_001306319.2	LOC106509078	0	0	0.01	1.156666667	known
XR_001306376.2	LOC106509109	0	0	0.00333333	0.333333333	known
XR_001306506.2	LOC106509193	0	0	0.04666667	1	known
XR_001306623.2	LOC102165127	0	0.333333333	0.00666667	1.666666667	known
XR_001306650.2	LOC102158334	0	0	0.01	1.13	known
XR_001306651.2	LOC102158334	0	0	0.02666667	2.786666667	known
XR_001306847.2	LOC106509364	0	0	0.00666667	0.333333333	known
XR_001306911.2	LOC106509384	0	0	0.01333333	0.666666667	known
XR_001307121.2	LOC106509516	0	0	0.00333333	0.333333333	known
XR_001307143.2	LOC100522542	0	0	0.00666667	0.333333333	known
XR_001307167.2	LOC102158429	0	0	0.03666667	1.846666667	known
XR_001307348.2	LOC106509649	0	0	0.01333333	0.333333333	known
XR_001307391.2	LOC102168016	0	0	0.08	6.7	known
XR_001307760.2	LOC106509839	0	0	0.00333333	0.333333333	known
XR_001307824.2	LOC106509872	0	0	0.00666667	0.666666667	known
XR_001307889.2	LOC106509903	0	0	0.01666667	0.333333333	known
XR_001307915.2	LOC106509914	0	1.333333333	0.01333333	9.333333333	known

XR_001308082.2	LOC106510002	0	0.333333333	0.00666667	1.666666667	known
XR_001308329.2	LOC106510129	0	0.06	0.02	1.24	known
XR_001308431.2	LOC102165975	0	0	0.00333333	0.333333333	known
XR_001308570.2	LOC102161056	0	0	0.02	5.55	known
XR_001308691.2	LOC106510294	0	0.013333333	0.01666667	4.75	known
XR_001308870.2	LOC106510408	0	0	0.00666667	0.666666667	known
XR_001309042.2	LOC102163610	0	0	0.21333333	10.76666667	known
XR_001309252.2	LOC106510625	0	0	0.01333333	0.333333333	known
XR_001309437.2	LOC106510720	0	0	0.02333333	1	known
XR_002335500.1	LOC102164595	0	0	0.01666667	1.343333333	known
XR_002335504.1	LOC102164595	0	0	0.02666667	2.76	known
XR_002335539.1	LOC102161477	0	0	0.01	0.493333333	known
XR_002335548.1	LOC106504821	0	0	0.02	0.666666667	known
XR_002335555.1	LOC110255407	0	0	0.00333333	1.3	known
XR_002335566.1	LOC110255410	0	0	0.01333333	3.853333333	known
XR_002335586.1	LOC102157509	0	0.01	0.02	2.666666667	known
XR_002335590.1	LOC106504859	0	0	0.01666667	0.333333333	known
XR_002335591.1	LOC110255418	0	0	0.00333333	0.333333333	known
XR_002335594.1	LOC110255420	0	0	0.01333333	1.666666667	known
XR_002335618.1	LOC102162684	0	0	0.00666667	0.333333333	known
XR_002335645.1	LOC102168180	0	0	0.01666667	1.493333333	known
XR_002335655.1	LOC110255436	0	0	0.02	3.72	known
XR_002335676.1	LOC106509136	0	0	0.00333333	1.096666667	known
XR_002335680.1	LOC102161637	0	0.333333333	0.00666667	1.586666667	known
XR_002335686.1	LOC102163364	0	0	0.08666667	17.91333333	known
XR_002335687.1	LOC102163364	0	0	0.00333333	0.6	known
XR_002335688.1	LOC102163364	0	0	0.07333333	15.41	known
XR_002335726.1	LOC100736666	0	0	0.02333333	2.34	known
XR_002335738.1	LOC110255460	0	0	0.09	1.06	known
XR_002335739.1	LOC110255460	0	0	0.02333333	0.333333333	known
XR_002335757.1	LOC110255462	0	0	0.00333333	0.333333333	known
XR_002335796.1	LOC110255471	0	0	0.00333333	0.536666667	known
XR_002335797.1	LOC110255471	0	0	0.01333333	1.13	known
XR_002335799.1	LOC106504949	0	0	0.00333333	0.333333333	known
XR_002335810.1	LOC102163402	0	0	0.06	8.306666667	known
XR_002335817.1	LOC102163402	0	0	0.05	2.11	known
XR_002335851.1	LOC110255482	0	0	0.03333333	1.666666667	known
XR_002335873.1	LOC110255490	0	0	0.01	0.333333333	known
XR_002335876.1	LOC110255491	0	0	0.00333333	0.333333333	known
XR_002335878.1	LOC110255492	0	0	0.00666667	0.333333333	known
XR_002335914.1	LOC102157670	0	0	0.00333333	0.333333333	known
XR_002335917.1	LOC110255505	0	0	0.00666667	0.333333333	known
XR_002335933.1	LOC102162878	0	0	0.00333333	0.333333333	known
XR_002335944.1	LOC110255517	0	0	0.00666667	0.333333333	known
XR_002335946.1	LOC110255517	0	0	0.00333333	0.333333333	known
XR_002335963.1	LOC110255525	0	0	0.01333333	0.333333333	known
XR_002336013.1	LOC110255529	0	0	0.00333333	0.333333333	known
XR_002336018.1	LOC106505042	0	0	0.02666667	4.773333333	known
XR_002336033.1	LOC102164427	0	0	0.01	2.113333333	known
XR_002336046.1	LOC106505045	0	0	0.00333333	0.333333333	known
XR_002336055.1	LOC106505054	0	0	0.00333333	1.333333333	known
XR_002336057.1	LOC106505054	0	0	0.00333333	0.333333333	known
XR_002336074.1	LOC102158978	0	0	0.00333333	0.333333333	known
XR_002336088.1	LOC110255546	0	0	0.07333333	9.84	known
XR_002336098.1	LOC110255552	0	0	0.00333333	0.666666667	known
XR_002336136.1	LOC110255585	0	0	0.00333333	0.333333333	known
XR_002336144.1	LOC102167499	0	0	0.00333333	0.333333333	known
XR_002336178.1	LOC100737021	0	0.16	0.09666667	9.85	known
XR_002336201.1	LOC110255631	0	0	0.02	1.666666667	known
XR_002336206.1	LOC110255636	0	0.006666667	0.00666667	0.666666667	known

XR_002336268.1	LOC110255656	0	0	0.01333333	0.333333333	known
XR_002336300.1	LOC110255664	0	0	0.00666667	0.666666667	known
XR_002336310.1	LOC110255670	0	0	0.00666667	0.333333333	known
XR_002336331.1	LOC106509188	0	0	0.02333333	0.333333333	known
XR_002336336.1	LOC110255685	0	0	0.03666667	4.976666667	known
XR_002336362.1	LOC102166141	0	0.333333333	0.01333333	3.666666667	known
XR_002336395.1	LOC102161032	0	0	0.02	2	known
XR_002336404.1	LOC102161921	0	0	0.00333333	1	known
XR_002336409.1	LOC110255709	0	0	0.05	2.896666667	known
XR_002336412.1	LOC110255712	0	0	0.00666667	0.333333333	known
XR_002336414.1	LOC110255713	0	0	0.04	3	known
XR_002336416.1	LOC110255714	0	0	0.02	0.333333333	known
XR_002336422.1	LOC110255720	0	0	0.01666667	0.666666667	known
XR_002336433.1	LOC110255722	0	0	0.00666667	0.333333333	known
XR_002336445.1	LOC102158641	0	0	0.01	1	known
XR_002336446.1	LOC102159132	0	0	0.00333333	0.333333333	known
XR_002336447.1	LOC102159626	0	0	0.02	2	known
XR_002336491.1	LOC102159559	0	0	0.01333333	2.08	known
XR_002336501.1	LOC110255748	0	0	0.01666667	0.333333333	known
XR_002336516.1	NHLRC3	0	0	0.12333333	11.43333333	known
XR_002336523.1	NHLRC3	0	0	0.02333333	2.233333333	known
XR_002336531.1	NHLRC3	0	0	0.08666667	10.77666667	known
XR_002336540.1	LOC110255752	0	0	0.00666667	0.333333333	known
XR_002336559.1	LOC106505309	0	0	0.00333333	0.143333333	known
XR_002336561.1	LOC110255761	0	0	0.00333333	0.333333333	known
XR_002336585.1	LOC102163665	0	0	0.01333333	0.333333333	known
XR_002336618.1	LOC106507930	0	0	0.01333333	0.616666667	known
XR_002336635.1	LOC110255799	0	0	0.05	6.39	known
XR_002336694.1	LOC110255828	0	0	0.00333333	0.333333333	known
XR_002336708.1	LOC106505319	0	0	0.01333333	2	known
XR_002336712.1	LOC110255836	0	0	0.00666667	0.333333333	known
XR_002336717.1	LOC110255838	0	0	0.00666667	0.333333333	known
XR_002336728.1	LOC106509200	0	0	0.01	0.333333333	known
XR_002336771.1	LOC106505360	0	0	0.00333333	0.333333333	known
XR_002336774.1	LOC110255863	0	0	0.04666667	5.03	known
XR_002336778.1	LOC102167979	0	0	0.08	15	known
XR_002336798.1	LOC110255870	0	0	0.01666667	2	known
XR_002336799.1	LOC106504762	0	0.666666667	0.00333333	0.666666667	known
XR_002336851.1	LOC106508625	0	0	0.01666667	14.17666667	known
XR_002336856.1	LOC106508625	0	0	0.01666667	15.39666667	known
XR_002336857.1	LOC110255891	0	0	0.02	1	known
XR_002336941.1	LOC110255946	0	0	0.01	0.333333333	known
XR_002336942.1	LOC110255947	0	0	0.03	0.666666667	known
XR_002336958.1	LOC110255960	0	0	0.02	2.213333333	known
XR_002336962.1	LOC102162292	0	0	0.02333333	2.653333333	known
XR_002336967.1	LOC110255171	0	0	0.00333333	0.333333333	known
XR_002336994.1	LOC102165522	0	0	0.00666667	0.333333333	known
XR_002337061.1	LOC110255986	0	0	0.02333333	0.666666667	known
XR_002337071.1	LOC102165973	0	0	0.05333333	3.333333333	known
XR_002337080.1	LOC102167870	0	0	0.02666667	1.446666667	known
XR_002337096.1	LOC110255996	0	0	0.02	0.333333333	known
XR_002337111.1	LOC106505430	0	0	0.05	0.816666667	known
XR_002337118.1	LOC102163646	0	0	0.01666667	0.333333333	known
XR_002337119.1	LOC106509228	0	0	0.00333333	0.333333333	known
XR_002337122.1	LOC102168162	0	0	0.00666667	0.333333333	known
XR_002337151.1	LOC106505099	0	0	0.04666667	1.1	known
XR_002337176.1	LOC100515340	0	0	0.00666667	1.816666667	known
XR_002337193.1	LOC110256033	0	0	0.02333333	1.72	known
XR_002337194.1	LOC110256033	0	0	0.08333333	4.28	known
XR_002337231.1	LOC110256047	0	0	0.02	4.996666667	known

XR_002337246.1	LOC110256056	0	0.333333333	0.00333333	1.333333333	known
XR_002337314.1	LOC110256075	0	0	0.00666667	0.68	known
XR_002337340.1	CHRNE	0	0	0.25	23.23666667	known
XR_002337346.1	LOC110255314	0	0	0.02666667	1.256666667	known
XR_002337390.1	LOC110256118	0	0	0.01666667	1.19	known
XR_002337395.1	LOC110256125	0	0	0.00666667	0.333333333	known
XR_002337418.1	LOC106505263	0	0	0.01333333	4.566666667	known
XR_002337430.1	LOC102161373	0	0	0.01333333	0.666666667	known
XR_002337433.1	LOC110256163	0	0	0.03	0.333333333	known
XR_002337472.1	GSN	0	0	0.04	3.953333333	known
XR_002337507.1	LOC110256223	0	0	0.00666667	0.666666667	known
XR_002337518.1	LOC110256229	0	0	0.02333333	0.333333333	known
XR_002337555.1	SMIM4	0	0	0.02	1.376666667	known
XR_002337575.1	LOC106509263	0	0	0.00333333	0.333333333	known
XR_002337621.1	LOC110256261	0	0	0.00666667	1	known
XR_002337627.1	LOC110256266	0	0	0.01	0.333333333	known
XR_002337636.1	LOC110256276	0	0	0.00333333	0.333333333	known
XR_002337642.1	LOC106505658	0	0.623333333	0.00333333	0.97	known
XR_002337653.1	LOC106505670	0	0	0.00666667	0.333333333	known
XR_002337709.1	LOC106505711	0	0	0.03666667	0.333333333	known
XR_002337712.1	LOC110256311	0	0	0.00333333	0.333333333	known
XR_002337727.1	LOC110256316	0	0.333333333	0.03	6.496666667	known
XR_002337733.1	LOC110256324	0	0	0.00666667	1	known
XR_002337752.1	LOC106505747	0	0	0.01	1	known
XR_002337754.1	LOC106505747	0	0	0.00333333	0.666666667	known
XR_002337755.1	LOC106505747	0	0	0.01	1	known
XR_002337771.1	LOC110256375	0	0	0.01333333	0.333333333	known
XR_002337772.1	LOC106504926	0	0	5.65333333	208.9	known
XR_002337788.1	LOC102162054	0	0	0.03333333	3.346666667	known
XR_002337790.1	LOC100519216	0	0	0.02	2.333333333	known
XR_002337796.1	LOC110256384	0	0	0.01666667	1	known
XR_002337797.1	LOC102162054	0	0	0.00333333	0.296666667	known
XR_002337800.1	LOC110256387	0	0	0.01333333	0.333333333	known
XR_002337804.1	LOC102162054	0	0	0.01	0.68	known
XR_002337806.1	LOC102162054	0	0	0.06333333	2.02	known
XR_002337807.1	LOC102162054	0	0	0.05333333	1.59	known
XR_002337809.1	LOC102162054	0	0	0.07666667	2.13	known
XR_002337815.1	LOC102162054	0	0	0.03	0.91	known
XR_002337818.1	LOC102162054	0	0	0.01666667	0.46	known
XR_002337832.1	LOC110256392	0	0	0.00333333	0.333333333	known
XR_002337834.1	LOC106508501	0	0	0.02333333	1.333333333	known
XR_002337859.1	LOC102158087	0	0	0.02333333	0.666666667	known
XR_002337863.1	LOC102158087	0	0	0.01	0.333333333	known
XR_002337871.1	LOC102157484	0	0	0.00666667	1.08	known
XR_002337883.1	LOC106505418	0	0	0.02666667	2.333333333	known
XR_002337890.1	LOC110256420	0	0	0.00666667	0.333333333	known
XR_002337894.1	LOC110256422	0	0	0.01	0.666666667	known
XR_002337895.1	LOC110256423	0	0.666666667	0.00333333	1.666666667	known
XR_002337909.1	LOC110256426	0	0	0.00666667	0.333333333	known
XR_002337911.1	LOC110256427	0	0	0.01	1.13	known
XR_002337926.1	LOC106505814	0	0	0.01333333	0.666666667	known
XR_002337940.1	LOC102162881	0	0	0.00666667	0.666666667	known
XR_002337944.1	LOC106507237	0	0	0.02666667	2.376666667	known
XR_002337960.1	LOC110256450	0	0	0.01333333	2	known
XR_002337973.1	LOC102162007	0	0	0.02	3.513333333	known
XR_002337977.1	LOC102162007	0	0	0.02333333	3.58	known
XR_002337982.1	LOC106505633	0	0	0.00333333	0.643333333	known
XR_002337994.1	LOC106505633	0	0	0.00666667	1.783333333	known
XR_002337997.1	LOC102163373	0	0	0.00333333	0.333333333	known
XR_002337999.1	LOC106505825	0	0	0.00333333	1	known

XR_002338004.1	LOC102161811	0	0	0.02666667	3.773333333	known
XR_002338010.1	LOC102164036	0	0	0.00666667	0.666666667	known
XR_002338011.1	LOC110256465	0	0	0.00666667	0.666666667	known
XR_002338059.1	LOC110255178	0	0	0.01666667	0.666666667	known
XR_002338061.1	LOC110256477	0	0	0.00333333	0.333333333	known
XR_002338080.1	LOC110256492	0	0	0.00333333	0.333333333	known
XR_002338096.1	LOC110256503	0	0	0.01333333	0.333333333	known
XR_002338105.1	LOC110256513	0	0	0.23333333	8.666666667	known
XR_002338116.1	LOC106505748	0	0.333333333	0.00333333	1.123333333	known
XR_002338177.1	LOC110256548	0	2	0.01333333	6.996666667	known
XR_002338190.1	LOC110256556	0	0	0.04333333	1.333333333	known
XR_002338198.1	LOC106505563	0	0	0.00333333	0.666666667	known
XR_002338202.1	LOC110256568	0	0	0.01666667	0.666666667	known
XR_002338218.1	LOC110256577	0	0	0.01333333	0.666666667	known
XR_002338246.1	LOC110256581	0	0	0.00333333	1.133333333	known
XR_002338248.1	LOC110256581	0	0	0.01	2.253333333	known
XR_002338255.1	LOC110256584	0	0	0.00666667	0.666666667	known
XR_002338284.1	LOC110256595	0	0	0.01333333	1.333333333	known
XR_002338315.1	LOC102160169	0	0	0.02	1.213333333	known
XR_002338334.1	ARHGAP19	0	0	0.00333333	0.76	known
XR_002338338.1	ARHGAP19	0	0.106666667	0.01	1.83	known
XR_002338348.1	LOC110256604	0	0	0.05666667	11.53666667	known
XR_002338385.1	LOC110256625	0	0	0.01	0.666666667	known
XR_002338426.1	LOC110256639	0	0	0.00666667	0.333333333	known
XR_002338444.1	LOC106505879	0	0	0.02	2.1	known
XR_002338448.1	LOC106505879	0	0	0.01	1.006666667	known
XR_002338461.1	LOC106505937	0	0	0.01	2.333333333	known
XR_002338497.1	LOC100522281	0	0	0.02333333	2.586666667	known
XR_002338506.1	LOC106506106	0	0.066666667	0.00333333	0.333333333	known
XR_002338507.1	LOC110256656	0	0	0.00333333	0.333333333	known
XR_002338508.1	LOC100623931	0	0	0.00666667	1.353333333	known
XR_002338549.1	LOC110256680	0	0	0.00666667	0.333333333	known
XR_002338556.1	LRMDA	0	0	0.24	13.54666667	known
XR_002338574.1	LOC110256691	0	0	0.01	0.333333333	known
XR_002338595.1	LOC110256705	0	0	0.00333333	0.666666667	known
XR_002338650.1	LOC102167253	0	0	0.00333333	0.666666667	known
XR_002338670.1	LOC110256748	0	0	0.02333333	0.333333333	known
XR_002338681.1	LOC110256753	0	0	0.02	0.666666667	known
XR_002338694.1	LOC102160801	0	0	0.00333333	0.413333333	known
XR_002338710.1	LOC110256765	0	0	0.03	3	known
XR_002338716.1	LOC102164998	0	0	0.02666667	9.6	known
XR_002338763.1	LOC110256781	0	0	0.01	0.666666667	known
XR_002338764.1	LOC110256782	0	0	0.01	0.666666667	known
XR_002338765.1	LOC102160539	0	0	0.00666667	0.48	known
XR_002338775.1	LOC110256787	0	0	0.00666667	0.333333333	known
XR_002338781.1	LOC110256790	0	0	0.00333333	0.333333333	known
XR_002338813.1	LOC110256801	0	0	0.01	0.666666667	known
XR_002338814.1	LOC110256801	0	0	0.00333333	0.333333333	known
XR_002338841.1	LOC110256830	0	0	0.00333333	0.333333333	known
XR_002338845.1	LOC106508090	0	0	0.00333333	0.333333333	known
XR_002338878.1	LOC110256848	0	0	0.02666667	0.333333333	known
XR_002338888.1	LOC110256849	0	0	0.00333333	1	known
XR_002338905.1	LOC110256853	0	0	0.02666667	0.333333333	known
XR_002338945.1	LOC106506013	0	0	0.01333333	1	known
XR_002338972.1	LOC106506064	0	0	0.00333333	0.333333333	known
XR_002338975.1	LOC102163174	0	0.06	0.02333333	1	known
XR_002338982.1	LOC102160537	0	0	0.00666667	0.333333333	known
XR_002338994.1	LOC110256903	0	0	0.03666667	0.666666667	known
XR_002339006.1	LOC102165127	0	0	0.01	3.006666667	known
XR_002339034.1	LOC110256949	0	0	0.00333333	0.333333333	known

XR_002339069.1	LOC110256974	0	0	0.00333333	0.343333333	known
XR_002339074.1	LOC106508137	0	0	0.01333333	0.666666667	known
XR_002339081.1	LOC102162428	0	0	0.02666667	0.666666667	known
XR_002339085.1	LOC102162428	0	0	0.05666667	1.366666667	known
XR_002339098.1	LOC110256990	0	0	0.01	2.116666667	known
XR_002339100.1	LOC110256990	0	0	0.00333333	0.55	known
XR_002339144.1	LOC106506229	0	0	0.01	0.666666667	known
XR_002339165.1	LOC106506297	0	0	0.00666667	1.01	known
XR_002339178.1	LOC102164639	0	0	0.03666667	0.666666667	known
XR_002339185.1	LOC110257029	0	0	0.01	1.333333333	known
XR_002339195.1	LOC106506169	0	0	0.00333333	0.333333333	known
XR_002339204.1	LOC102162507	0	0	0.05	0.666666667	known
XR_002339218.1	LOC110257052	0	0	0.00333333	0.666666667	known
XR_002339245.1	LOC110257069	0	0	0.00333333	0.333333333	known
XR_002339249.1	LOC102164446	0	0	0.02	1	known
XR_002339286.1	LOC100520518	0	0	0.01666667	1.083333333	known
XR_002339289.1	LOC100520518	0	0	0.02	1.806666667	known
XR_002339297.1	LOC102165800	0	0	0.04666667	1.666666667	known
XR_002339307.1	LOC106504203	0	0	0.00666667	0.333333333	known
XR_002339308.1	LOC102167218	0	0	0.01333333	0.333333333	known
XR_002339328.1	LOC110257104	0	0	0.01	0.666666667	known
XR_002339346.1	FAM126B	0	0	0.22666667	101.33	known
XR_002339348.1	FAM126B	0	0.023333333	0.05	21.46333333	known
XR_002339436.1	LOC110257175	0	0	0.01666667	1.48	known
XR_002339452.1	LOC102162374	0	0	0.00333333	0.666666667	known
XR_002339458.1	LOC110257183	0	0	0.01666667	0.666666667	known
XR_002339470.1	LOC110257189	0	0	0.00333333	1.666666667	known
XR_002339486.1	LOC110257201	0	0.333333333	0.01	2.333333333	known
XR_002339516.1	LOC102161653	0	0.076666667	0.00666667	0.836666667	known
XR_002339524.1	LOC110257217	0	0	0.00333333	0.333333333	known
XR_002339531.1	LOC110257221	0	0	0.01	2.006666667	known
XR_002339532.1	LOC110257221	0	0	0.01333333	2.66	known
XR_002339552.1	LOC110257230	0	0	0.01333333	2.926666667	known
XR_002339575.1	LOC106508654	0	0	0.01	1	known
XR_002339582.1	LOC106506500	0	0.333333333	0.00333333	1	known
XR_002339604.1	LOC110257244	0	0	0.00333333	0.333333333	known
XR_002339611.1	LOC110257249	0	0	0.00333333	0.333333333	known
XR_002339613.1	LOC110257250	0	0	0.01666667	0.666666667	known
XR_002339614.1	LOC110257250	0	0	0.01666667	1	known
XR_002339615.1	LOC110257251	0	0	0.01	0.333333333	known
XR_002339621.1	LOC110257260	0	0	0.01666667	0.333333333	known
XR_002339637.1	LOC102164818	0	0	0.00666667	2.61	known
XR_002339645.1	LOC110257263	0	0	0.00333333	0.333333333	known
XR_002339652.1	LOC106506503	0	0	0.00333333	0.666666667	known
XR_002339653.1	LOC110257265	0	0	0.02333333	1	known
XR_002339673.1	LOC110257270	0	0	0.00666667	0.333333333	known
XR_002339709.1	LOC110257289	0	0	0.00333333	0.333333333	known
XR_002339712.1	LOC106506544	0	0	0.00666667	0.666666667	known
XR_002339721.1	LOC102167058	0	0	0.03	0.543333333	known
XR_002339755.1	LOC110257303	0	0	0.04	3	known
XR_002339783.1	LOC102165748	0	0	0.00666667	0.666666667	known
XR_002339807.1	LOC106506470	0	0	0.02	1.466666667	known
XR_002339855.1	LOC106506707	0	0	0.00333333	0.333333333	known
XR_002339927.1	LOC106506610	0	0	0.00666667	0.666666667	known
XR_002339928.1	LOC106506610	0	0	0.00333333	0.333333333	known
XR_002339930.1	LOC110257380	0	0	0.00333333	0.333333333	known
XR_002339933.1	LOC106506612	0	0	0.00333333	0.333333333	known
XR_002339950.1	LOC110257400	0	0	0.00666667	0.666666667	known
XR_002339969.1	LOC106506712	0	0	0.00333333	0.666666667	known
XR_002340013.1	LOC110257432	0	0	0.00333333	0.666666667	known

XR_002340014.1	LOC110257432	0	0	0.00333333	1	known
XR_002340018.1	LOC110257434	0	0	0.01	0.333333333	known
XR_002340026.1	LOC110257441	0	0	0.09	1.333333333	known
XR_002340029.1	LOC110257444	0	0	0.02	0.333333333	known
XR_002340050.1	LOC110257450	0	0	0.00666667	0.333333333	known
XR_002340064.1	LOC110257455	0	0	0.00333333	0.333333333	known
XR_002340077.1	LOC102159476	0	0.003333333	0.00333333	0.166666667	known
XR_002340078.1	LOC102159476	0	0.003333333	0.00333333	0.166666667	known
XR_002340102.1	LOC110257474	0	0	0.02	0.333333333	known
XR_002340124.1	LOC106504276	0	0	0.00333333	2.333333333	known
XR_002340149.1	LOC102165363	0	0	0.02333333	5.503333333	known
XR_002340151.1	LOC102165363	0	0	0.01333333	3.693333333	known
XR_002340236.1	LOC100513188	0	0	0.02333333	0.956666667	known
XR_002340252.1	LOC102161969	0	0	0.01666667	1.836666667	known
XR_002340254.1	LOC102161969	0	0	0.02	0.883333333	known
XR_002340302.1	LOC110257543	0	0	0.03	8.826666667	known
XR_002340329.1	LOC106508222	0	0	0.00666667	0.513333333	known
XR_002340360.1	LOC106506781	0	0	0.01333333	1.536666667	known
XR_002340361.1	LOC106506781	0	0	0.01333333	1.663333333	known
XR_002340364.1	LOC106506781	0	0	0.00666667	0.933333333	known
XR_002340366.1	LOC106506781	0	0	0.01333333	1.976666667	known
XR_002340373.1	LOC110257574	0	0	0.01666667	3.666666667	known
XR_002340385.1	LOC110257581	0	0	0.02333333	0.416666667	known
XR_002340435.1	LOC110257596	0	0	0.01	1.213333333	known
XR_002340443.1	LOC100739791	0	0	0.04	2.39	known
XR_002340463.1	LOC106506761	0	0	0.00666667	0.666666667	known
XR_002340479.1	LOC102165006	0	0	0.01	2.666666667	known
XR_002340491.1	GIMAP5	0	0	0.01	0.86	known
XR_002340493.1	GIMAP5	0	0	0.01666667	2.12	known
XR_002340495.1	GIMAP5	0	0	0.00333333	0.233333333	known
XR_002340503.1	LOC106508225	0	0	0.01	1	known
XR_002340514.1	LOC106508214	0	0	0.00333333	0.333333333	known
XR_002340540.1	LOC106506946	0	0	0.00333333	0.333333333	known
XR_002340570.1	LOC110257658	0	0	0.1	3	known
XR_002340589.1	LOC110257664	0	0	0.02666667	1.333333333	known
XR_002340620.1	LOC106507039	0	0	0.01	0.333333333	known
XR_002340627.1	LOC110257717	0	0	0.01	1	known
XR_002340636.1	LOC106506941	0	0	0.00333333	0.333333333	known
XR_002340644.1	LOC102165590	0	0	0.00333333	0.333333333	known
XR_002340666.1	LOC106506903	0	0	0.00666667	0.333333333	known
XR_002340676.1	LOC110257736	0	0	0.00666667	0.333333333	known
XR_002340682.1	LOC102163484	0	0	0.00333333	0.333333333	known
XR_002340683.1	LOC102163484	0	0	0.03333333	1	known
XR_002340694.1	LOC106506947	0	0	0.00666667	0.333333333	known
XR_002340701.1	LOC106506936	0	0	0.00666667	0.333333333	known
XR_002340715.1	LOC110257757	0	0	0.00333333	0.333333333	known
XR_002340722.1	LOC102158739	0	0	0.03666667	2.8	known
XR_002340759.1	LOC110257788	0	0	0.00666667	0.333333333	known
XR_002340761.1	LOC110257791	0	0	0.01	0.333333333	known
XR_002340811.1	LOC106506881	0	0	0.03666667	2.56	known
XR_002340829.1	LOC106507037	0	0	0.00333333	0.333333333	known
XR_002340841.1	LOC110257830	0	0	0.02	1.96	known
XR_002340874.1	LOC110257856	0	0	0.04333333	1.333333333	known
XR_002340901.1	LOC102160243	0	0	0.00666667	0.813333333	known
XR_002340904.1	LOC102160243	0	0	0.01	1.053333333	known
XR_002340919.1	LOC110257911	0	0	0.02	1.433333333	known
XR_002340944.1	LOC110257975	0	0	0.00666667	0.333333333	known
XR_002340946.1	LOC110257976	0	0	0.07	9.736666667	known
XR_002340948.1	LOC110257976	0	0	0.01333333	2.273333333	known
XR_002340994.1	LOC110258139	0	0	0.00333333	0.333333333	known

XR_002341050.1	LOC102164846	0	0	0.03	0.666666667	known
XR_002341138.1	LOC110258382	0	0	0.02	2.356666667	known
XR_002341139.1	LOC110258383	0	0	0.03	0.333333333	known
XR_002341162.1	LOC110258480	0	0	0.01333333	0.666666667	known
XR_002341177.1	LOC110258560	0	0	0.04	0.59	known
XR_002341190.1	LOC102162879	0	0	0.02	0.333333333	known
XR_002341196.1	LOC110258598	0	0	0.01666667	0.103333333	known
XR_002341202.1	LOC102166957	0	0	0.01	3.57	known
XR_002341205.1	LOC102164761	0	0	0.03	2.596666667	known
XR_002341210.1	LOC110258639	0	0	0.00333333	0.333333333	known
XR_002341211.1	LOC110258640	0	0	0.01	0.666666667	known
XR_002341241.1	LOC102164320	0	0	0.00333333	0.65	known
XR_002341242.1	LOC102164320	0	0	0.03	8.203333333	known
XR_002341251.1	LOC110258817	0	0	0.01666667	0.333333333	known
XR_002341263.1	LOC110258845	0	0	0.02	0.336666667	known
XR_002341338.1	LOC110259142	0	0	0.00333333	0.333333333	known
XR_002341339.1	LOC110259143	0	0	0.01333333	1	known
XR_002341352.1	LOC102158335	0	0	0.03333333	1.283333333	known
XR_002341355.1	CUNH21orf58	0	0	0.00333333	0.333333333	known
XR_002341362.1	LOC110259201	0	0	0.00333333	0.13	known
XR_002341385.1	LOC110259219	0	0	0.01	0.666666667	known
XR_002341427.1	LOC102167294	0	0	0.00666667	1.333333333	known
XR_002341448.1	LOC110259241	0	0	0.00333333	0.333333333	known
XR_002341474.1	LOC110259252	0	0	0.00333333	0.333333333	known
XR_002341492.1	LOC110259274	0	0	0.04	2	known
XR_002341510.1	LOC102163604	0	0	0.01333333	0.58	known
XR_002341514.1	LOC102157601	0	0	0.01666667	3.886666667	known
XR_002341520.1	LOC106509384	0	0	0.00333333	0.333333333	known
XR_002341536.1	LOC102165168	0	0	0.00333333	0.333333333	known
XR_002341552.1	LOC100523888	0	0	0.01666667	0.333333333	known
XR_002341575.1	LOC106509423	0	0	0.03333333	0.666666667	known
XR_002341617.1	LOC110259334	0	0	0.02666667	0.666666667	known
XR_002341674.1	LOC106509445	0	0	0.01	1	known
XR_002341675.1	LOC110259350	0	0	0.02666667	0.333333333	known
XR_002341684.1	LOC110259353	0	0	0.00333333	0.333333333	known
XR_002341696.1	LOC110259366	0	0	0.00333333	0.333333333	known
XR_002341701.1	LOC110259369	0	0	0.00666667	0.333333333	known
XR_002341745.1	LOC102161047	0	0	0.00333333	0.666666667	known
XR_002341751.1	LOC110259403	0	0	0.00666667	0.333333333	known
XR_002341794.1	LOC110259416	0	0	0.01333333	0.763333333	known
XR_002341795.1	LOC110259416	0	0	0.01	0.436666667	known
XR_002341824.1	LOC106509500	0	0	0.00333333	0.666666667	known
XR_002341826.1	LOC110259424	0	0	0.00666667	0.666666667	known
XR_002341827.1	LOC110259425	0	0	0.01333333	0.666666667	known
XR_002341830.1	LOC106509504	0	0	0.01666667	2	known
XR_002341840.1	LOC110259432	0	0	0.00333333	0.333333333	known
XR_002341844.1	LOC110259434	0	0	0.03333333	1.333333333	known
XR_002341868.1	LOC110259455	0	0	0.01	0.333333333	known
XR_002341879.1	LOC110259460	0	0	0.01666667	1	known
XR_002341890.1	LOC102162098	0	0	0.01333333	0.65	known
XR_002341891.1	LOC110259470	0	0	0.03	0.333333333	known
XR_002341897.1	LOC110259477	0	0	0.00333333	0.333333333	known
XR_002341904.1	LOC102159960	0	0	0.01666667	0.666666667	known
XR_002341906.1	LOC110259480	0	0	0.01	1	known
XR_002341913.1	LOC106507323	0	0	0.00333333	0.666666667	known
XR_002341914.1	LOC106507323	0	0	0.00666667	0.246666667	known
XR_002341915.1	LOC102164851	0	0	0.09333333	1	known
XR_002341918.1	LOC110259486	0	0	0.01	0.333333333	known
XR_002341923.1	LOC110259492	0	0	0.00333333	0.333333333	known
XR_002341926.1	LOC110259494	0	0	0.00333333	0.333333333	known

XR_002341954.1	LOC110259522	0	0	0.00333333	0.333333333	known
XR_002342017.1	LOC106509607	0	0	0.00666667	0.333333333	known
XR_002342020.1	LOC110259538	0	0	0.01666667	1	known
XR_002342056.1	LOC110259691	0	0	0.03333333	12.35666667	known
XR_002342057.1	LOC110259691	0	0	0.07333333	32.34333333	known
XR_002342058.1	LOC110259691	0	0	0.03333333	15.29	known
XR_002342062.1	LOC106509506	0	0	0.00666667	0.666666667	known
XR_002342064.1	LOC102162145	0	0.333333333	0.00666667	1.666666667	known
XR_002342073.1	LOC106509522	0	0	0.05	1.333333333	known
XR_002342075.1	LOC102158429	0	0	0.04333333	1.666666667	known
XR_002342078.1	LOC102158243	0	0	0.00333333	0.333333333	known
XR_002342096.1	LOC110259703	0	0	0.04333333	0.333333333	known
XR_002342101.1	LOC102166729	0	0	0.00666667	0.333333333	known
XR_002342117.1	LOC110259778	0	0	0.00666667	0.666666667	known
XR_002342205.1	LOC110259840	0	0	0.01666667	0.666666667	known
XR_002342211.1	LOC102160627	0	1.34	0.00666667	2.353333333	known
XR_002342271.1	LOC110259869	0	0	0.00333333	0.666666667	known
XR_002342283.1	LOC106509647	0	0	0.00333333	0.336666667	known
XR_002342286.1	LOC106509647	0	0	0.04333333	6.03	known
XR_002342287.1	LOC106509647	0	0	0.02333333	3.22	known
XR_002342288.1	LOC106509647	0	0	0.00666667	0.84	known
XR_002342291.1	LOC106509647	0	0	0.02666667	3.156666667	known
XR_002342307.1	LOC102166961	0	0	0.00666667	0.666666667	known
XR_002342314.1	LOC110259892	0	0	0.10333333	5.666666667	known
XR_002342317.1	LOC110259895	0	0	0.01	0.333333333	known
XR_002342336.1	LOC100521937	0	0	0.01666667	0.666666667	known
XR_002342382.1	LOC102163149	0	0	0.03	7.87	known
XR_002342388.1	TMEM159	0	0	0.21	14.34666667	known
XR_002342395.1	LOC110259913	0	0	0.05	0.666666667	known
XR_002342396.1	LOC110259915	0	0	0.00333333	0.333333333	known
XR_002342402.1	LOC110259922	0	0	0.01666667	0.333333333	known
XR_002342408.1	LOC110259925	0	0	0.08	2.483333333	known
XR_002342441.1	LOC110259944	0	0	0.02	1	known
XR_002342442.1	LOC102159543	0	0	0.00666667	0.99	known
XR_002342448.1	LOC106504252	0	0	0.02666667	3.186666667	known
XR_002342461.1	LOC110259954	0	0	0.01333333	0.333333333	known
XR_002342478.1	LOC110259959	0	0	0.00333333	0.666666667	known
XR_002342501.1	LOC106509733	0	0	0.00666667	0.5	known
XR_002342507.1	LOC110259968	0	0	0.05	2	known
XR_002342514.1	LOC106509747	0	0	0.01666667	0.333333333	known
XR_002342515.1	LOC110259972	0	0	0.01	0.666666667	known
XR_002342526.1	LOC106508550	0	0	0.1	5.606666667	known
XR_002342536.1	LOC110259983	0	0	0.07333333	0.333333333	known
XR_002342539.1	LOC110259985	0	0	0.00666667	0.333333333	known
XR_002342551.1	LOC110259989	0	0	0.00333333	0.333333333	known
XR_002342656.1	LOC106509807	0	0	0.03	3.88	known
XR_002342661.1	LOC106509807	0	0	0.00666667	0.646666667	known
XR_002342663.1	LOC106509807	0	0	0.00666667	0.966666667	known
XR_002342667.1	LOC106509807	0	0	0.00666667	0.756666667	known
XR_002342669.1	LOC106509807	0	0	0.00666667	0.806666667	known
XR_002342676.1	LOC106509807	0	0	0.02	4.943333333	known
XR_002342680.1	LOC106509807	0	0	0.02	2.29	known
XR_002342692.1	LOC106509807	0	0	0.00666667	0.323333333	known
XR_002342693.1	LOC106509807	0	0	0.00666667	1.133333333	known
XR_002342716.1	LOC110260013	0	0.333333333	0.00333333	0.666666667	known
XR_002342725.1	LOC110260019	0	0	0.01	1.333333333	known
XR_002342736.1	LOC110260024	0	0	0.01333333	1.666666667	known
XR_002342749.1	LOC106509843	0	0	0.01333333	5.916666667	known
XR_002342751.1	LOC106509843	0	0	0.00666667	2.493333333	known
XR_002342755.1	LOC102165712	0	0	0.00666667	0.333333333	known

XR_002342757.1	LOC106507388	0	0.666666667	0.00666667	1.59	known
XR_002342764.1	LOC102164129	0	0	0.08	3.566666667	known
XR_002342781.1	LOC110260032	0	0.666666667	0.16333333	36.33333333	known
XR_002342786.1	LOC110260036	0	0	0.02	4.29	known
XR_002342788.1	LOC110260036	0	0	0.04666667	10.80333333	known
XR_002342802.1	LOC106509865	0	0	0.00333333	0.53	known
XR_002342806.1	LOC106509865	0	0	0.01666667	2.28	known
XR_002342827.1	LOC102165887	0	0	0.00666667	0.79	known
XR_002342849.1	LOC106509907	0	0	0.00333333	0.333333333	known
XR_002342855.1	LOC106509907	0	0	0.00333333	0.333333333	known
XR_002342866.1	LOC102158714	0	0	0.02333333	0.333333333	known
XR_002342871.1	LOC102160712	0	0	0.01	2.32	known
XR_002342878.1	LOC102161617	0	0	0.00333333	0.333333333	known
XR_002342884.1	LOC110260064	0	0	0.00333333	0.333333333	known
XR_002342976.1	LOC106509894	0	0	0.00333333	0.333333333	known
XR_002343031.1	LOC110260189	0	0	0.00666667	0.333333333	known
XR_002343085.1	LOC110260223	0	0	0.01	1	known
XR_002343095.1	LOC106509952	0	0	0.01	0.433333333	known
XR_002343134.1	LOC106509975	0	0	0.01333333	0.333333333	known
XR_002343140.1	LOC110260233	0	0	0.00666667	0.333333333	known
XR_002343155.1	LOC106509999	0	0.333333333	0.02333333	5	known
XR_002343160.1	LOC110260238	0	0	0.01333333	0.666666667	known
XR_002343169.1	LOC106510002	0	0	0.00333333	0.166666667	known
XR_002343171.1	LOC106510002	0	0	0.01	0.333333333	known
XR_002343182.1	LOC102161266	0	0	0.00333333	0.333333333	known
XR_002343184.1	LOC110260247	0	0	0.00333333	0.333333333	known
XR_002343189.1	LOC110260249	0	0	0.01666667	2	known
XR_002343192.1	LOC110260251	0	0	0.00333333	0.333333333	known
XR_002343220.1	LOC106510026	0	0	0.00666667	2	known
XR_002343251.1	LOC106510043	0	0	0.01	1.666666667	known
XR_002343259.1	LOC110260273	0	0	0.00666667	0.333333333	known
XR_002343275.1	LOC102161201	0	0	0.04	1.666666667	known
XR_002343282.1	LOC110260285	0	0	0.00666667	0.333333333	known
XR_002343286.1	LOC106510065	0	0	0.00666667	0.666666667	known
XR_002343303.1	LOC110260288	0	0	0.00333333	0.666666667	known
XR_002343311.1	LOC110260289	0	0	0.00333333	0.486666667	known
XR_002343313.1	LOC102158065	0	0	0.01666667	1.333333333	known
XR_002343315.1	LOC102166303	0	0	0.00666667	0.47	known
XR_002343321.1	LOC110260293	0	0	0.14333333	22.58333333	known
XR_002343325.1	LOC110260293	0	0	0.05666667	8.666666667	known
XR_002343329.1	LOC110260294	0	0	0.00333333	1	known
XR_002343338.1	LOC102162468	0	0	0.01333333	1.226666667	known
XR_002343350.1	LOC102162289	0	0	0.03666667	0.666666667	known
XR_002343359.1	LOC106510109	0	0.333333333	0.01333333	3.666666667	known
XR_002343365.1	LOC106508330	0	0	0.02666667	1.24	known
XR_002343375.1	LOC110260306	0	0	0.01	1	known
XR_002343388.1	LOC106510123	0	0	0.02666667	2	known
XR_002343414.1	LOC106510126	0	0	0.01666667	0.333333333	known
XR_002343437.1	LOC110260323	0	0	0.00666667	0.666666667	known
XR_002343454.1	LOC102165383	0	0	0.00666667	0.666666667	known
XR_002343470.1	LOC110260337	0	0	0.01	0.333333333	known
XR_002343509.1	LOC102160057	0	0	0.00333333	0.333333333	known
XR_002343514.1	LOC106510199	0	1	0.00333333	1.146666667	known
XR_002343523.1	LOC102165301	0	0	0.01333333	1.333333333	known
XR_002343531.1	LOC110260358	0	0	0.01333333	0.333333333	known
XR_002343578.1	LOC110260375	0	0	0.00333333	0.333333333	known
XR_002343580.1	LOC110260376	0	0	0.00333333	0.333333333	known
XR_002343595.1	LOC102161678	0	0	0.00333333	0.333333333	known
XR_002343605.1	LOC102161901	0	0	0.00333333	0.833333333	known
XR_002343609.1	LOC102161901	0	0	0.00333333	0.833333333	known

XR_002343623.1	LOC106510230	0	0	0.06333333	5.723333333	known
XR_002343636.1	LOC102161056	0	0	0.02333333	7.6	known
XR_002343653.1	LOC110260408	0	0	0.00333333	0.333333333	known
XR_002343674.1	LOC102165528	0	0	0.03333333	7.043333333	known
XR_002343676.1	LOC102165528	0	0	0.01666667	3.323333333	known
XR_002343679.1	LOC110260419	0	0	0.01	0.333333333	known
XR_002343688.1	LOC110260424	0	0	0.00666667	0.333333333	known
XR_002343717.1	LOC110260431	0	0	0.05666667	0.666666667	known
XR_002343729.1	LOC110260436	0	0.333333333	0.00666667	1.666666667	known
XR_002343781.1	LOC110260471	0	0	0.00333333	0.333333333	known
XR_002343874.1	LOC110260626	0	0	0.00333333	0.666666667	known
XR_002343877.1	LOC110260629	0	0	0.13666667	13.28666667	known
XR_002343888.1	LOC110260637	0	0	0.03	1	known
XR_002343893.1	LOC110260644	0	0	0.02	0.333333333	known
XR_002343906.1	LOC110260649	0	0	0.00666667	0.333333333	known
XR_002343939.1	LOC102167177	0	0	0.01	2.276666667	known
XR_002343943.1	LOC102167177	0	0	0.06	2.056666667	known
XR_002343946.1	LOC110260662	0	0	0.00666667	0.666666667	known
XR_002343987.1	LOC110260686	0	0	0.01	0.333333333	known
XR_002344000.1	LOC110260694	0	0	0.01333333	0.666666667	known
XR_002344005.1	LOC110260698	0	0	0.01	0.333333333	known
XR_002344023.1	LOC102168027	0	0	0.00666667	1.39	known
XR_002344040.1	LOC102168027	0	0.14	0.05	6.85	known
XR_002344048.1	LOC110260709	0	0	0.00333333	0.333333333	known
XR_002344062.1	LOC110260718	0	0	0.01	0.666666667	known
XR_002344068.1	LOC110260719	0	0	0.00333333	0.666666667	known
XR_002344079.1	LOC110260725	0	0	0.01333333	0.666666667	known
XR_002344106.1	LOC110260743	0	0	0.01333333	1.406666667	known
XR_002344120.1	LOC106506626	0	0	0.03	0.333333333	known
XR_002344122.1	LOC110260752	0	0	0.08333333	5.333333333	known
XR_002344127.1	LOC100524923	0	0	0.01	5.936666667	known
XR_002344129.1	LOC100524923	0	0	0.00333333	1	known
XR_002344134.1	LOC110260755	0	0	0.00333333	0.333333333	known
XR_002344144.1	LOC102157617	0	0	0.17666667	7.123333333	known
XR_002344165.1	LOC102162736	0	0	0.03	2.666666667	known
XR_002344175.1	LOC106510390	0	0	0.01333333	1.333333333	known
XR_002344178.1	LOC110260771	0	0	0.10333333	1.333333333	known
XR_002344193.1	LOC110260785	0	0.333333333	0.00666667	1.666666667	known
XR_002344245.1	LOC110260790	0	0	0.00333333	0.333333333	known
XR_002344246.1	LOC110260790	0	0	0.00333333	0.333333333	known
XR_002344247.1	LOC110260791	0	0	0.00333333	0.666666667	known
XR_002344253.1	LOC110260794	0	0	0.01	0.333333333	known
XR_002344267.1	LOC106507542	0	0	0.00333333	1	known
XR_002344274.1	LOC110260803	0	0	0.00333333	0.333333333	known
XR_002344276.1	LOC106510439	0	0	0.01	0.333333333	known
XR_002344279.1	LOC102161020	0	0	0.00333333	0.333333333	known
XR_002344321.1	LOC110260814	0	0	0.00666667	0.333333333	known
XR_002344322.1	LOC102158467	0	0	0.02666667	0.356666667	known
XR_002344339.1	LOC102159120	0	0	0.01333333	1.69	known
XR_002344350.1	LOC102165555	0	0	0.00666667	0.77	known
XR_002344381.1	LOC110260860	0	0	0.01	0.666666667	known
XR_002344448.1	LOC110260891	0	0	0.01666667	0.666666667	known
XR_002344502.1	ZNRF1	0	0	0.08	4.636666667	known
XR_002344517.1	LOC110260918	0	0	0.00333333	1.093333333	known
XR_002344539.1	LOC110260921	0	0.333333333	0.00333333	0.666666667	known
XR_002344550.1	LOC110260931	0	0	0.00666667	0.333333333	known
XR_002344586.1	LOC102165609	0	0	0.01333333	3.006666667	known
XR_002344618.1	LOC102163023	0	0	0.02	5.4	known
XR_002344640.1	LOC102167359	0	0	0.00333333	0.606666667	known
XR_002344661.1	LOC110260991	0	0	0.01	0.333333333	known

XR_002344719.1	LOC110261016	0	0	0.04	0.666666667	known
XR_002344774.1	LOC102167762	0	0	0.00666667	0.333333333	known
XR_002344779.1	LOC110261039	0	0	0.01	0.333333333	known
XR_002344781.1	LOC110261041	0	0	0.00666667	0.333333333	known
XR_002344795.1	LOC106509560	0	0	0.00666667	0.333333333	known
XR_002344818.1	LOC110261063	0	0	0.00666667	0.666666667	known
XR_002344842.1	LOC106509643	0	0	0.03666667	14.33333333	known
XR_002344864.1	NPHP4	0	0	0.04666667	10.84666667	known
XR_002344865.1	NPHP4	0	0	0.05333333	10.83	known
XR_002344869.1	NPHP4	0	0	0.00666667	1.413333333	known
XR_002344879.1	LOC110261087	0	0	0.07	2.666666667	known
XR_002344899.1	LOC110261096	0	0	0.08333333	3.333333333	known
XR_002344912.1	LOC110261100	0	0	0.01666667	0.333333333	known
XR_002344941.1	LOC110261115	0	0	0.00333333	0.333333333	known
XR_002344953.1	LOC100622481	0	0	0.08	3.006666667	known
XR_002344965.1	LOC110261133	0	0	0.03	0.903333333	known
XR_002344973.1	LOC106510652	0	0	0.03666667	1.333333333	known
XR_002344975.1	LOC110261157	0	0	0.02333333	0.333333333	known
XR_002344992.1	LOC110261160	0	0.666666667	0.03333333	8.333333333	known
XR_002344994.1	LOC110261161	0	1	0.02333333	6	known
XR_002345006.1	LOC102159707	0	0	0.01	0.666666667	known
XR_002345012.1	LOC106507633	0	0	0.03	1.43	known
XR_002345024.1	LOC102165197	0	0	0.02666667	2.186666667	known
XR_002345028.1	LOC102161139	0	0	0.02	4.823333333	known
XR_002345060.1	LOC102162217	0	0	0.00333333	0.333333333	known
XR_002345082.1	LOC102162836	0	0	0.00333333	0.666666667	known
XR_002345083.1	LOC110261192	0	0	0.00333333	0.333333333	known
XR_002345110.1	LOC110261202	0	0	0.01	1.333333333	known
XR_002345153.1	LOC106507649	0	0	0.01666667	0.593333333	known
XR_002345158.1	LOC110261210	0	0	0.00666667	0.333333333	known
XR_002345162.1	LOC110261215	0	0	0.00666667	0.333333333	known
XR_002345177.1	LOC110261223	0	0.333333333	0.00333333	1.333333333	known
XR_002345179.1	LOC106508664	0	0	0.01333333	2.323333333	known
XR_002345181.1	LOC106508664	0	0	0.00333333	0.603333333	known
XR_002345184.1	LOC110261227	0	0	0.04	8.086666667	known
XR_002345224.1	LOC102165936	0	0	0.02666667	1	known
XR_002345253.1	LOC110261250	0	0.31	0.00333333	1.336666667	known
XR_002345300.1	LOC106510470	0	0	0.00333333	1.333333333	known
XR_002345304.1	LOC110261268	0	0	0.00666667	0.333333333	known
XR_002345305.1	LOC110261269	0	0	0.01	0.333333333	known
XR_002345307.1	LOC110261271	0	1.333333333	0.00333333	1.196666667	known
XR_002345313.1	LOC110261275	0	0	0.01333333	2.333333333	known
XR_002345330.1	LOC102161624	0	0	0.02	1	known
XR_002345337.1	LOC106510537	0	0	0.00333333	0.666666667	known
XR_002345338.1	LOC100620402	0	0.02	0.05	1.39	known
XR_002345385.1	LOC110261329	0	0	0.00333333	0.333333333	known
XR_002345416.1	C6H1orf185	0	0	0.03333333	2.333333333	known
XR_002345447.1	LOC106510679	0	0	0.00333333	0.74	known
XR_002345449.1	LOC106510680	0	0	0.00333333	0.333333333	known
XR_002345451.1	LOC106510680	0	0	0.00333333	0.333333333	known
XR_002345468.1	LOC110261418	0	0	0.00333333	0.666666667	known
XR_002345506.1	LOC110261425	0	0	0.01666667	1.333333333	known
XR_002345530.1	LOC110261447	0	0	0.00666667	0.333333333	known
XR_002345629.1	LOC100155975	0	0	0.05333333	23.32333333	known
XR_002345633.1	LOC106504375	0	0	0.00666667	0.333333333	known
XR_002345637.1	LOC110261502	0	0	0.00666667	0.333333333	known
XR_002345651.1	LOC106507687	0	0	0.02666667	0.333333333	known
XR_002345690.1	LOC102161912	0	0	0.00333333	0.333333333	known
XR_002345693.1	LOC102163428	0	0	0.03333333	1.296666667	known
XR_002345700.1	LOC110261513	0	0	0.01666667	0.333333333	known

XR_002345703.1	LOC110261517	0	0	0.10333333	6	known
XR_002345731.1	LOC110261527	0	0	0.00333333	0.333333333	known
XR_002345744.1	LOC110261531	0	0	0.03666667	0.333333333	known
XR_002345749.1	LOC110261532	0	0	0.04333333	0.666666667	known
XR_002345769.1	LOC110261544	0	0	0.00333333	0.333333333	known
XR_002345778.1	LOC106504434	0	0	0.01333333	1.333333333	known
XR_002345814.1	LOC110261561	0	0	0.01666667	0.666666667	known
XR_002345833.1	LOC102164231	0	0.333333333	0.00333333	0.666666667	known
XR_002345837.1	LOC110261566	0	0.406666667	0.02333333	5.666666667	known
XR_002345847.1	LOC102166620	0	0	0.01	0.333333333	known
XR_002345869.1	LOC110261578	0	0	0.00666667	0.666666667	known
XR_002345891.1	LOC110261589	0	0	0.00666667	0.666666667	known
XR_002345933.1	LOC106509052	0	0	0.05666667	8.666666667	known
XR_002345954.1	LOC110261608	0	0	0.01666667	1	known
XR_002345986.1	LOC110261623	0	0.666666667	0.01	2.666666667	known
XR_002346000.1	LOC110261627	0	0	0.01333333	0.333333333	known
XR_002346006.1	LOC110261629	0	0	0.01	1	known
XR_002346013.1	LOC106507740	0	0	0.00333333	0.036666667	known
XR_002346033.1	LOC106507757	0	0	0.04	7.333333333	known
XR_002346039.1	LOC110261645	0	0	0.00666667	0.333333333	known
XR_002346041.1	LOC106504559	0	0	0.02	6.106666667	known
XR_002346057.1	LOC110261648	0	0	0.01	5.246666667	known
XR_002346063.1	LOC102163738	0	0	0.13666667	68.92666667	known
XR_002346065.1	LOC102163738	0	0	0.01666667	6.716666667	known
XR_002346071.1	LOC102163738	0	0	0.02	7.32	known
XR_002346073.1	LOC102163738	0	0	0.00666667	3.67	known
XR_002346124.1	LOC102165284	0	0	0.01	0.333333333	known
XR_002346126.1	LOC110261677	0	0	0.00333333	0.333333333	known
XR_002346130.1	LOC110261678	0	0	0.00333333	0.333333333	known
XR_002346131.1	LOC102162475	0	0	0.02	0.666666667	known
XR_002346143.1	LOC102162169	0	0	0.02	3	known
XR_002346159.1	LOC110261699	0	0	0.06	2	known
XR_002346160.1	LOC110261700	0	0	0.00666667	0.333333333	known
XR_002346192.1	LOC102161104	0	0	0.00666667	0.333333333	known
XR_002346195.1	LOC106504504	0	0	0.03	1.333333333	known
XR_002346218.1	LOC106508466	0	0	0.02666667	2.516666667	known
XR_002346224.1	LOC102158724	0	0	0.01333333	0.666666667	known
XR_002346225.1	LOC106504293	0	0	0.00333333	0.333333333	known
XR_002346281.1	LOC102165101	0	0	0.02	0.666666667	known
XR_002346302.1	LOC110261776	0	0	0.00333333	0.333333333	known
XR_002346312.1	LOC106507125	0	0	0.01	2.336666667	known
XR_002346332.1	LOC110261792	0	0	0.01	0.333333333	known
XR_002346378.1	LOC102161781	0	0	0.00333333	0.333333333	known
XR_002346424.1	LOC102166663	0	0	0.07	3	known
XR_002346440.1	LOC102158976	0	0	0.00333333	0.386666667	known
XR_002346453.1	LOC102158976	0	0	0.00666667	1.083333333	known
XR_002346465.1	LOC106507803	0	0	0.00666667	1	known
XR_002346466.1	LOC106507803	0	0	0.00666667	1.333333333	known
XR_002346471.1	LOC110261991	0	0	0.00666667	0.333333333	known
XR_002346472.1	LOC110261992	0	1	0.01	2.333333333	known
XR_002346490.1	LOC110262000	0	0	0.00666667	0.666666667	known
XR_002346507.1	LOC110262027	0	0	0.01666667	0.333333333	known
XR_002346521.1	LOC110262038	0	0	0.00333333	0.736666667	known
XR_002346525.1	LOC110262042	0	0	0.00333333	0.333333333	known
XR_002346541.1	LOC110262046	0	0.333333333	0.00333333	1	known
XR_002346567.1	LOC100627270	0	0	0.08	2.306666667	known
XR_002346569.1	LOC100627270	0	0	1.31333333	30.55333333	known
XR_002346571.1	LOC100739688	0	0	0.01666667	1.686666667	known
XR_002346573.1	LOC100739688	0	0	0.02666667	4.956666667	known
XR_002346638.1	LOC110262093	0	0	0.01666667	1.666666667	known

XR_002346666.1	LOC110262103	0	0	0.01666667	0.333333333	known
XR_002346680.1	LOC110262108	0	0	0.01	0.666666667	known
XR_002346686.1	LOC110262110	0	0.333333333	0.00333333	1	known
XR_002346689.1	LOC106508499	0	0	0.01	1.316666667	known
XR_002346697.1	LOC106507788	0	0	0.00333333	0.333333333	known
XR_002346722.1	LOC110262134	0	0	0.07333333	2.093333333	known
XR_002346725.1	LOC110262135	0	0	0.01	0.666666667	known
XR_002346752.1	LOC102165892	0	0	0.06	4.166666667	known
XR_002346765.1	LOC110262151	0	0	0.01333333	0.826666667	known
XR_002346778.1	LOC102162389	0	0	0.02666667	0.67	known
XR_002346779.1	LOC110262153	0	0	0.00333333	0.666666667	known
XR_002346784.1	LOC106504725	0	0	0.01333333	1	known
XR_002346806.1	LOC106504728	0	0	0.00666667	0.333333333	known
XR_002346817.1	LOC106510483	0	0	0.00333333	0.333333333	known
XR_002346828.1	LOC110262172	0	0	0.01666667	1	known
XR_002346833.1	LOC102161553	0	0	0.02666667	1.333333333	known
XR_002346841.1	LOC106504749	0	0	0.02	4.333333333	known
XR_002346843.1	LOC110262178	0	0	0.01333333	1.95	known
XR_002346894.1	LOC100737511	0	0	0.04333333	10.73333333	known
XR_002346921.1	LOC106509106	0	0	0.01333333	0.666666667	known
XR_002346961.1	LOC110262210	0	0	0.02	0.333333333	known
XR_002346974.1	LOC102160855	0	0	0.00666667	0.526666667	known
XR_002347008.1	LOC110262223	0	0	0.03666667	1.683333333	known
XR_002347012.1	LOC110262223	0	0	0.01	0.843333333	known
XR_002347021.1	LOC110262226	0	0	0.01666667	0.666666667	known
XR_002347067.1	LOC106509119	0	0	0.01666667	2	known
XR_002347070.1	LOC100737186	0	0	0.02666667	0.866666667	known
XR_002347084.1	LOC110262252	0	0	0.00333333	0.666666667	known
XR_002347127.1	LOC110262308	0	0	0.02	1	known
XR_116026.4	LOC100515492	0	0	0.00333333	0.666666667	known
XR_297444.2	LOC102164080	0	0	0.00333333	0.333333333	known
XR_297579.3	LOC102165845	0	0	0.04	0.953333333	known
XR_297882.2	LOC102158351	0	0	0.00666667	0.683333333	known
XR_297970.3	LOC102161909	0	0	0.02	0.96	known
XR_297971.3	LOC102161909	0	0	0.04666667	2.213333333	known
XR_299114.3	LOC102158497	0	0	0.00333333	0.333333333	known
XR_299180.3	LOC102162556	0	0	0.01666667	1	known
XR_299385.3	LOC102166756	0	0	0.01333333	0.333333333	known
XR_299472.3	LOC102160572	0	0	0.00333333	0.333333333	known
XR_299598.3	LOC102166231	0	0	0.01	0.333333333	known
XR_300618.3	LOC100521937	0	0	0.00666667	0.666666667	known
XR_300753.3	LOC102160378	0	0	0.00666667	0.333333333	known
XR_301708.3	LOC102163715	0	0	0.02	3	known
XR_302699.3	LOC100513921	0	0	0.01333333	0.333333333	known
XR_302794.3	LOC102161379	0	0	0.02666667	0.333333333	known
XR_302848.3	LOC102157842	0	0	0.00333333	1.233333333	known
XR_302933.3	LOC102160555	0	0	0.00666667	0.333333333	known
XR_303325.3	LOC102160181	0	0	0.01	0.333333333	known
XR_303603.3	LOC102164671	0	0	0.02333333	0.333333333	known
XR_304095.3	LOC102163786	0	0	0.06666667	1.666666667	known
XR_304257.3	LOC102162837	0	0	0.00666667	0.333333333	known
XR_304613.2	LOC102158121	0	0	0.00666667	0.666666667	known
XR_304722.3	LOC102163357	0	0	0.01	0.333333333	known
XR_304761.3	LOC100739688	0	0	0.03333333	7.163333333	known
XR_305038.3	LOC102167943	0	0	0.03	1.253333333	known
XR_305150.3	LOC102158037	0	0	0.00666667	0.666666667	known
XR_305327.3	LOC102162684	0	0	0.02333333	0.666666667	known
XR_305334.3	LOC102163572	0	0	0.00333333	1	known
XR_306071.3	LOC102158458	0	0	0.03	1.333333333	known
XR_306159.3	LOC102160118	0	0	0.02333333	0.856666667	known

XR_306327.3	LOC102165115	0	0	0.00333333	0.333333333	known
XR_306474.3	LOC102160528	0	0	0.11	5.073333333	known
XR_306476.3	LOC102160528	0	0	0.02666667	1.223333333	known
XR_306926.3	LOC102159951	0	0	0.01	0.666666667	known
XR_307007.3	LOC102162136	0	0	0.00333333	0.333333333	known
XR_307553.3	LOC102162612	0	0	0.00666667	0.333333333	known
XR_308345.3	LOC102167708	0	0	0.31	20.02666667	known
XR_308458.3	LOC102160395	0	0	0.08333333	2.666666667	known
XR_308810.2	LOC102162503	0	0	0.01333333	0.333333333	known
NM_001001261.1	SLC22A6	0.42	28	0	0	
NM_001001262.1	NECTIN1	0.01	0.593333333	0	0	
NM_001001627.1	DIO1	0.536666667	15.66666667	0	0	
NM_001003926.2	ANGPTL3	0.006666667	0.333333333	0	0	
NM_001004028.1	HAPLN1	0.02	1.093333333	0	0	
NM_001004046.2	FABP1	0.193333333	2.646666667	0	0	
NM_001004049.2	HSD3B1	0.003333333	0.333333333	0	0	
NM_001005155.2	BMP15	0.003333333	0.333333333	0	0	
NM_001007196.1	TAC3	0.01	0.266666667	0	0	
NM_001008684.1	MCHR1	0.006666667	0.333333333	0	0	
NM_001024587.1	LEPR	0.083333333	14.23666667	0	0	
NM_001024695.1	CCL28	0.05	1.666666667	0	0	
NM_001025223.1	SPDYA	0.02	0.666666667	0	0	
NM_001030535.1	BID	0.466666667	9.58	0	0	
NM_001031786.1	SAR1A	0.316666667	6.753333333	0	0	
NM_001032356.1	TCTEX1D4	0.026666667	1	0	0	
NM_001032388.1	HNF1A	0.126666667	17	0	0	
NM_001037150.1	SULT2A1	0.016666667	0.666666667	0	0	
NM_001037996.1	NR1I3	0.013333333	0.666666667	0	0	
NM_001038005.1	NR1I2	0.07	7.146666667	0	0	
NM_001044531.1	CD70	0.016666667	0.333333333	0	0	
NM_001044534.1	CDS1	0.023333333	2.626666667	0	0	
NM_001044568.2	AKR1E2	0.02	1.073333333	0	0	
NM_001044569.1	AKR1C1	0.083333333	4.333333333	0	0	
NM_001044571.1	HNF4A	0.236666667	17.96	0	0	
NM_001044590.2	PLG	0.03	3.333333333	0	0	
NM_001044591.1	F7	0.006666667	0.333333333	0	0	
NM_001044623.1	SLC34A1	2.97	307.0933333	0	0	
NM_001097417.1	SLC2A2	0.06	4.333333333	0	0	
NM_001097449.1	C6	0.013333333	1.666666667	0	0	
NM_001097450.2	C8A	0.006666667	0.666666667	0	0	
NM_001097451.2	C8B	0.016666667	1.666666667	0	0	
NM_001097513.1	ADAM3A	0.003333333	0.333333333	0	0	
NM_001097514.1	SLC15A2	0.106666667	9.9	0	0	
NM_001097520.1	NAGS	0.023333333	1	0	0	
NM_001099927.1	ADRB3	0.003333333	0.146666667	0	0	
NM_001099933.1	SOX10	0.073333333	5.273333333	0	0	
NM_001101818.1	SLC44A4	0.01	0.973333333	0	0	
NM_001102680.2	CD1D	0.033333333	2.666666667	0	0	
NM_001105054.1	SNCAIP	0.063333333	3.983333333	0	0	
NM_001110174.1	CTCFL	0.003333333	0.333333333	0	0	
NM_001110422.1	SLC5A11	0.116666667	10.33333333	0	0	
NM_001110431.2	CASS4	0.003333333	0.363333333	0	0	
NM_001112683.1	AQP8	0.01	0.333333333	0	0	
NM_001112688.1	CIDEB	0.016666667	0.533333333	0	0	
NM_001113064.1	BDKRB1	0.053333333	2.333333333	0	0	
NM_001113218.1	KIR2DL1	0.006666667	0.333333333	0	0	
NM_001113435.1	PTPN1	0.143333333	20.78333333	0	0	
NM_001113703.1	SLA-8	0.073333333	3.026666667	0	0	
NM_001114064.2	SLA-DOB	0.013333333	0.473333333	0	0	
NM_001114285.1	EFNB3	0.01	0.333333333	0	0	

NM_001123093.1	CD163L1	0.32	32.90333333	0	0
NM_001123177.1	RAB33A	0.013333333	0.333333333	0	0
NM_001123207.1	TRIM10	0.013333333	0.666666667	0	0
NM_001123208.1	TRIM15	0.006666667	0.333333333	0	0
NM_001123211.1	UPK1A	0.09	2.666666667	0	0
NM_001128438.1	FOXP3	0.01	0.73	0	0
NM_001128454.1	AQP10	0.003333333	0.133333333	0	0
NM_001128456.1	CBY1	0.186666667	2.8	0	0
NM_001128467.1	AQP6	0.016666667	0.506666667	0	0
NM_001128476.1	AQP2	0.37	11.5	0	0
NM_001129953.2	DMP1	0.01	1.056666667	0	0
NM_001129958.1	SERPINC1	0.09	5.333333333	0	0
NM_001129977.1	SPAG11	0.033333333	0.666666667	0	0
NM_001130226.1	CALB1	0.483333333	16	0	0
NM_001130236.1	IL23A	0.016666667	0.333333333	0	0
NM_001130248.1	SLC26A3	0.003333333	0.333333333	0	0
NM_001134350.2	KLF1	0.006666667	0.333333333	0	0
NM_001136212.1	HAS1	0.003333333	0.333333333	0	0
NM_001136512.1	BCO1	0.033333333	3.266666667	0	0
NM_001142838.1	TMEM173	0.026666667	1.28	0	0
NM_001143690.1	NOS2	0.033333333	5.773333333	0	0
NM_001143713.1	LCK	0.093333333	8.333333333	0	0
NM_001143718.1	MGLL	0.076666667	3.41	0	0
NM_001143725.1	STARD3	0.093333333	7.97	0	0
NM_001144110.1	CRHR1	0.04	2	0	0
NM_001145022.1	C7H6orf52	0.06	1.666666667	0	0
NM_001145219.1	GRIFIN	0.06	1	0	0
NM_001145752.1	SLC22A4	0.003333333	0.22	0	0.08
NM_001145866.1	CLEC7A	0.016666667	0.286666667	0	0
NM_001159310.1	PKMYT1	0.006666667	0.333333333	0	0
NM_001159778.1	CLDN23	0.006666667	0.333333333	0	0
NM_001160074.1	BCL2L11	0.073333333	0.61	0	0
NM_001160075.1	CLDN3	0.066666667	2	0	0
NM_001160273.1	DPPA5	0.016666667	0.333333333	0	0
NM_001160274.1	GUCA2A	0.17	3.333333333	0	0
NM_001160275.1	GUCA2B	0.063333333	1	0	0
NM_001161637.1	CLDN4	0.15	6.02	0	0
NM_001161642.1	CLDN14	0.016666667	0.666666667	0	0
NM_001161646.1	CLDN8	0.16	4.666666667	0	0
NM_001161647.1	CLDN9	0.006666667	0.38	0	0
NM_001162401.1	LPAR2	0.14	9.193333333	0	0
NM_001163411.1	KPNA7	0.043333333	2.83	0	0
NM_001164003.1	F9	0.076666667	8.723333333	0	0
NM_001164006.1	AMBP	0.026666667	1.576666667	0	0
NM_001164021.1	SLC5A1	0.066666667	10.66666667	0	0
NM_001164736.1	HAVCR1	0.023333333	1.093333333	0	0
NM_001166488.1	PGR	0.01	2	0	0.333333333
NM_001167628.1	SSTR3	0.01	0.5	0	0
NM_001167649.1	HSD17B2	0.203333333	9.333333333	0	0
NM_001174132.2	APOC4	0.02	0.333333333	0	0
NM_001177328.2	APOC2	0.02	0.333333333	0	0
NM_001177921.1	SPATA22	0.006666667	0.236666667	0	0
NM_001184753.1	SPATA17	0.113333333	4.816666667	0	0
NM_001185131.1	FDPS	0.183333333	9.733333333	0	0
NM_001185174.1	DAPL1	0.04	0.666666667	0	0
NM_001190215.1	PTI	0.016666667	0.666666667	0	0
NM_001190231.1	SSTR1	0.006666667	0.313333333	0	0.06
NM_001190247.1	SPACA1	0.206666667	7.666666667	0	0
NM_001194971.1	PIWIL2	0.063333333	7.61	0	0
NM_001195105.1	IGFBP1	0.04	1.333333333	0	0

NM_001195332.1	CRHBP	0.01	0.333333333	0	0
NM_001195342.1	HOXA13	0.013333333	0.29	0	0
NM_001195344.1	KLK7	0.01	0.333333333	0	0
NM_001195347.1	LY6G6F	0.006666667	0.333333333	0	0
NM_001195360.1	NAT9	0.3	11.97666667	0	0
NM_001195364.1	PAGE2B	0.016666667	0.333333333	0	0
NM_001197306.1	STAT6	0.803333333	129.67	0	0
NM_001198919.1	PSAP	0.473333333	31.45	0	0
NM_001199808.1	ADCYAP1R1	0.043333333	2.556666667	0	0
NM_001200042.1	BHMT	0.173333333	17.33333333	0	0
NM_001202461.1	ODF4	0.036666667	1.17	0	0.053333333
NM_001204382.1	PRR5	0.09	6.243333333	0	0
NM_001204383.1	ARHGAP8	0.023333333	1.666666667	0	0
NM_001204902.1	SCTR	0.006666667	0.333333333	0	0
NM_001206355.1	ADGRF1	0.036666667	4.253333333	0	0
NM_001206375.1	ODF3B	0.163333333	5.326666667	0	0
NM_001206402.1	TRIM31	0.006666667	0.666666667	0	0
NM_001243022.1	ACSM4	0.133333333	12.33333333	0	0
NM_001243431.1	HAO1	0.083333333	6.333333333	0	0
NM_001243483.1	TFF3	0.043333333	1.333333333	0	0
NM_001243493.1	GNB3	0.02	1.5	0	0
NM_001243690.1	HABP2	0.23	28.33333333	0	0
NM_001243814.1	KRT74	0.003333333	0.333333333	0	0
NM_001243838.1	CRACR2B	0.006666667	0.363333333	0	0
NM_001243841.1	MARVELD3	0.05	4.5	0	0
NM_001243860.1	TSPAN1	0.613333333	31.66666667	0	0
NM_001243870.1	ASPDH	0.17	7	0	0
NM_001243901.1	GPRL15	0.12	5.666666667	0	0
NM_001244077.1	CCDC42	0.006666667	0.333333333	0	0
NM_001244084.1	ASCC1	0.12	6.983333333	0	0
NM_001244109.1	SYAP1	0.043333333	3.25	0	0
NM_001244120.1	ARSE	0.013333333	1.333333333	0	0
NM_001244124.1	UGT2B31	0.01	0.866666667	0	0
NM_001244178.1	PAX6	0.003333333	0.333333333	0	0
NM_001244266.1	SLC51A	0.066666667	4.086666667	0	0
NM_001244285.1	FUBP3	0.093333333	11.65333333	0	0
NM_001244316.1	NAP1L1	0.053333333	6.95	0	0
NM_001244338.1	HOXA10	0.033333333	0.64	0	0
NM_001244361.1	PGLYRP3	0.006666667	0.333333333	0	0
NM_001244410.1	LCN2	0.816666667	31.66666667	0	0
NM_001244453.1	AP2S1	0.033333333	1.066666667	0	0
NM_001244458.1	ASGR1	0.006666667	0.333333333	0	0
NM_001244460.1	SLC39A2	0.016666667	1.016666667	0	0
NM_001244463.1	SLC10A2	0.006666667	0.333333333	0	0
NM_001244470.1	IHH	0.026666667	2.333333333	0	0
NM_001244495.1	ZNF395	0.06	5.03	0	0
NM_001244513.1	SHH	0.006666667	0.333333333	0	0
NM_001244520.1	TFAP2B	0.043333333	2.49	0	0
NM_001244562.1	H2AFY	0.093333333	7.606666667	0	0.03
NM_001244595.1	SLC24A2	0.013333333	1.153333333	0	0
NM_001244639.1	HRG	0.2	15	0	0
NM_001244642.1	LOC100156741	0.163333333	2.163333333	0	0
NM_001244717.1	SLC13A2	0.7	70.33333333	0	0
NM_001244835.1	C2H11orf49	0.02	1.02	0	0
NM_001245009.1	ZNF232	0.07	5.153333333	0	0
NM_001246239.1	GHDC	0.02	1.663333333	0	0
NM_001246240.1	IDO1	0.006666667	0.333333333	0	0
NM_001246266.1	GLP2R	0.003333333	0.2	0	0
NM_001256147.2	CCL17	0.03	0.333333333	0	0
NM_001256365.1	MID1IP1	0.106666667	2.026666667	0	0

NM_001256528.1	PYY	0.07	1	0	0
NM_001258424.1	LMX1B	0.01	0.46	0	0
NM_001260479.1	TRH	0.046666667	1.263333333	0	0
NM_001278750.1	UGT1A6	0.036666667	2.976666667	0	0
NM_001278767.1	GATA1	0.006666667	0.333333333	0	0
NM_001278769.1	CDX2	0.02	1.666666667	0	0
NM_001285974.1	XDH	0.006666667	0.666666667	0	0
NM_001286804.1	G0S2	0.053333333	0.71	0	0
NM_001287417.1	TSSK4	0.006666667	0.24	0	0
NM_001291414.1	SLC5A8	0.093333333	7.053333333	0	0
NM_001293141.1	RSPO2	0.013333333	0.333333333	0	0
NM_001293316.1	TLR4	0.033333333	4.863333333	0	0
NM_001297634.1	BCL11A	0.033333333	3.646666667	0	0
NM_001302088.2	CLEC12B	0.006666667	0.333333333	0	0
NM_001308829.1	SLITRK1	0.03	6	0	0.333333333
NM_001315734.1	LOC100739741	1.436666667	137.78	0	0
NM_001315758.1	LOC106504372	0.05	0.396666667	0	0
NM_001315766.1	LOC100736962	0.49	25.83333333	0	0
NM_001315792.1	CYP26A1	0.006666667	0.666666667	0	0.073333333
NM_001348402.1	SLC6A14	0.003333333	0.333333333	0	0
NM_001348959.1	SFTPA1	0.003333333	0.333333333	0	0
NM_001348964.1	HPD	0.25	14.33333333	0	0
NM_213738.1	PGLYRP2	0.026666667	2	0	0
NM_213744.1	PLET1	0.043333333	2	0	0
NM_213756.1	TREM1	0.013333333	0.666666667	0	0
NM_213787.1	SERPINA3-2	0.016666667	1	0	0
NM_213788.1	LEAP2	0.02	0.333333333	0	0
NM_213799.1	ABO	0.123333333	2	0	0
NM_213802.1	KLK4	0.006666667	0.333333333	0	0
NM_213819.1	PI3	0.153333333	2.383333333	0	0
NM_213825.2	GALP	0.086666667	3.666666667	0	0
NM_213834.1	PTGER3	0.04	2.926666667	0	0
NM_213845.2	UABP-2	0.006666667	0.333333333	0	0
NM_213859.1	LOC396866	0.01	0.333333333	0	0
NM_213860.1	LOC396867	0.1	1.333333333	0	0
NM_213864.1	SPINK4	0.03	0.333333333	0	0
NM_213902.1	SLC22A2	0.29	36.10666667	0	0
NM_213918.1	PROC	0.14	8.333333333	0	0
NM_213956.1	HNF1B	0.166666667	19.44333333	0	0
NM_213957.1	ADAM2	0.003333333	0.333333333	0	0
NM_213988.1	SRD5A2	0.033333333	3.333333333	0	0
NM_213992.1	SULT1E1	0.033333333	2.666666667	0	0
NM_213997.1	IL18	0.026666667	0.666666667	0	0
NM_213999.2	DUOX2	0.106666667	27.62333333	0	0
NM_214008.1	OPTC	0.026666667	1.333333333	0	0
NM_214012.1	UPK2	0.01	0.333333333	0	0
NM_214018.2	KCNN4	0.003333333	0.333333333	0	0
NM_214038.1	PCSK1	0.003333333	0.666666667	0	0.333333333
NM_214066.2	DAO	1.233333333	165.3333333	0	0
NM_214074.1	KLKB1	0.01	1	0	0
NM_214079.1	SAG	0.006666667	0.51	0	0
NM_214102.2	MIOX	0.896666667	30.02333333	0	0
NM_214108.1	DPEP1	0.99	58	0	0
NM_214142.1	HCST	0.053333333	0.816666667	0	0
NM_214169.1	CPB1	0.006666667	0.333333333	0	0
NM_214179.1	NR5A1	0.006666667	0.666666667	0	0
NM_214212.1	TTR	0.193333333	4	0	0
NM_214217.1	HTR2A	0.003333333	0.226666667	0	0
NM_214237.2	CCK	0.08	2.01	0	0
NM_214241.1	ENAM	0.003333333	0.333333333	0	0

NM_214251.1	TGFA	0.013333333	0.21	0	0
NM_214275.1	FTCD	0.16	12	0	0
NM_214277.1	ANPEP	0.86	121.4633333	0	0
NM_214293.1	GATA4	0.01	0.536666667	0	0
NM_214328.2	GATA6	0.043333333	5.443333333	0	0
NM_214344.1	SLC17A1	0.136666667	10.16666667	0	0
NM_214351.1	B3GALNT1	0.083333333	6.433333333	0	0
NM_214380.2	HMGCS2	0.003333333	0.333333333	0	0
NM_214388.1	APOA4	0.006666667	0.333333333	0	0
NM_214390.1	IL15	0.03	0.496666667	0	0
NM_214412.1	CYP1A1	0.016666667	2	0	0
NM_214420.1	CYP2C49	0.706666667	56.81	0	0
NM_214421.1	CYP2E1	0.056666667	4	0	0
NM_214424.1	CYP4A24	0.7	69.30333333	0	0
NM_214425.1	CYP4A21	0.52	51.76333333	0	0
NM_214427.1	CYP11A1	0.003333333	0.333333333	0	0
XM_001924183.4	LOC100153915	0.053333333	2.666666667	0	0
XM_001924471.5	NHS	0.03	11.33333333	0	0
XM_001924770.4	CABCOCO1	0.003333333	0.666666667	0	0
XM_001924824.4	TAAR1	0.006666667	0.666666667	0	0
XM_001925050.1	LOC100152013	0.016666667	0.666666667	0	0
XM_001925212.7	KPNA5	0.006666667	1.836666667	0	0
XM_001925251.5	DPYS	0.476666667	40.46333333	0	0
XM_001925334.3	FBXO43	0.003333333	0.666666667	0	0
XM_001925390.6	LOC100156775	0.046666667	3.153333333	0	0
XM_001925449.4	LMAN1L	0.01	1.116666667	0	0.02
XM_001925579.6	CERCAM	0.026666667	2.873333333	0	0
XM_001925683.5	CDH23	0.006666667	4.726666667	0	0
XM_001925756.5	C17H20orf96	0.003333333	0.333333333	0	0
XM_001926005.2	LOC100156296	0.02	2	0	0
XM_001926142.7	SLC12A1	0.743333333	131.5833333	0	0
XM_001926241.6	POU3F2	0.01	3.333333333	0	0.333333333
XM_001926662.5	TTC13	0.173333333	23.73666667	0	0
XM_001926768.7	MYO5A	0.116666667	58.21333333	0	0
XM_001927159.5	LOC100157581	0.01	0.333333333	0	0
XM_001927556.7	ADCY4	0.103333333	19.43333333	0	0
XM_001927588.5	CPNE5	0.006666667	0.94	0	0.153333333
XM_001927701.5	SLC39A5	0.013333333	2.666666667	0	0
XM_001927772.5	SEM1	0.026666667	0.37	0	0
XM_001927801.6	DPYSL2	0.016666667	3.05	0	0
XM_001927867.7	SLC35F3	0.02	1.963333333	0	0
XM_001927885.6	PRAP1	0.04	1	0	0
XM_001927978.5	TMEM79	0.003333333	0.333333333	0	0
XM_001928096.4	NRSN2	0.003333333	0.333333333	0	0
XM_001928148.5	STRA6	0.013333333	1.476666667	0	0
XM_001928333.4	LOC100156560	0.006666667	0.333333333	0	0
XM_001928385.6	BRPF1	0.046666667	9.636666667	0	0
XM_001928390.5	STOML1	0.01	0.81	0	0
XM_001928493.6	SIPA1L2	0.026666667	7.493333333	0	0
XM_001928546.6	FNBP1L	0.003333333	0.43	0	0.11
XM_001928550.3	LOC100157384	0.01	0.333333333	0	0
XM_001928569.5	SERPINA5	0.013333333	1.333333333	0	0
XM_001928594.4	FMO5	0.126666667	24.52	0	0
XM_001928844.5	LOC100156325	0.056666667	3.683333333	0	0
XM_001928870.6	NFIL3	0.09	7.493333333	0	0
XM_001928882.4	HKDC1	0.013333333	6.666666667	0	0.333333333
XM_001928931.5	KIF20B	0.016666667	4.46	0	0
XM_001929138.5	LCP1	0.083333333	15.39	0	0
XM_001929254.6	UPB1	1.24	86.78666667	0	0
XM_001929276.4	FOXA1	0.003333333	0.666666667	0	0

XM_001929430.6	SLK	0.076666667	25.86666667	0	0
XM_001929519.5	LRRC71	0.01	0.68	0	0
XM_001929566.5	SERPIND1	0.01	1	0	0
XM_003121201.6	L3MBTL3	0.066666667	11.91333333	0	0
XM_003121273.4	COL9A1	0.003333333	0.333333333	0	0
XM_003121288.4	GJB7	0.01	1	0	0
XM_003121290.6	ORC3	0.14	23.95	0	0
XM_003121447.4	C1H18orf54	0.006666667	0.776666667	0	0
XM_003121531.6	TERB2	0.006666667	0.666666667	0	0
XM_003121716.5	SLC51B	0.093333333	3.423333333	0	0
XM_003121826.3	CCDC198	0.116666667	5.69	0	0
XM_003121828.6	SLC35F4	0.01	1	0	0
XM_003121830.4	TOMM20L	0.01	0.333333333	0	0
XM_003121915.5	KIAA2026	0.02	8.583333333	0	0
XM_003121935.4	TMEM252	0.116666667	8.333333333	0	0
XM_003121978.4	PHF24	1.033333333	253.4233333	0	0
XM_003121996.3	LOC100514941	0.006666667	0.333333333	0	0
XM_003122044.5	LOC100515185	0.063333333	9.076666667	0	0
XM_003122142.3	LOC100522178	0.003333333	0.333333333	0	0
XM_003122147.2	LOC100524011	0.006666667	0.333333333	0	0
XM_003122155.6	CRB2	0.023333333	7.666666667	0	0.333333333
XM_003122354.5	ENTPD8	0.003333333	0.893333333	0	0
XM_003122420.3	FGF19	0.01	0.333333333	0	0
XM_003122473.4	SSH3	0.136666667	21.5	0	0
XM_003122551.4	GPHA2	0.006666667	0.666666667	0	0
XM_003122570.4	SLC22A12	0.226666667	25.70666667	0	0
XM_003122615.6	LOC100514700	0.016666667	1.886666667	0	0
XM_003122641.5	TKFC	0.02	2	0	0
XM_003122693.2	LOC100516243	0.003333333	0.083333333	0	0
XM_003122694.3	LOC100516417	0.003333333	0.083333333	0	0
XM_003122871.4	EHF	0.016666667	4.823333333	0	0
XM_003122908.4	SLC5A12	0.786666667	162	0	0
XM_003122929.4	IGSF22	0.003333333	0.333333333	0	0
XM_003122986.4	ELANE	0.016666667	0.666666667	0	0
XM_003123060.6	CREB3L3	0.066666667	5.333333333	0	0
XM_003123197.5	MAP2K7	0.026666667	3.42	0	0
XM_003123246.5	ICAM4	0.04	2	0	0
XM_003123433.3	CYP4F2	0.206666667	15.13333333	0	0
XM_003123434.4	CYP4F2	0.206666667	15.13333333	0	0
XM_003123570.4	CILP2	0.003333333	0.666666667	0	0
XM_003123593.6	LYPD8	0.186666667	13.66666667	0	0
XM_003123621.4	WNT3A	0.006666667	1	0	0
XM_003123640.4	MRNIP	0.03	2	0	0
XM_003123688.4	TMEM174	0.346666667	23.33333333	0	0
XM_003123952.6	KIF20A	0.003333333	0.84	0	0
XM_003123965.6	SLC25A48	0.22	12.99	0	0
XM_003124097.6	SLC6A7	0.003333333	0.666666667	0	0.666666667
XM_003124229.6	IFITM5	0.01	0.333333333	0	0
XM_003124561.6	ACSM2B	0.67	103.6666667	0	0
XM_003124571.4	GP2	0.006666667	1	0	0
XM_003124976.5	LOC100525692	0.01	0.34	0	0
XM_003125331.6	CENPO	0.033333333	3.203333333	0	0
XM_003125387.4	ATP6V1C2	0.023333333	2.31	0	0
XM_003125581.4	ATP6V0D2	0.203333333	17.66666667	0	0
XM_003125718.5	TRIM46	0.003333333	0.333333333	0	0
XM_003125820.4	HAO2	0.72	73.05666667	0	0
XM_003125859.6	SLC16A4	0.113333333	10.66666667	0	0
XM_003125969.6	UPK3A	0.06	2.666666667	0	0
XM_003126024.6	LOC100523244	0.193333333	22.64333333	0	0
XM_003126042.4	KCTD17	0.016666667	1.126666667	0	0

XM_003126101.3	DDN	0.31	47.61	0	0
XM_003126109.4	C1QL4	0.013333333	0.666666667	0	0
XM_003126254.4	RDH5	0.103333333	5.24	0	0
XM_003126273.4	MIP	0.003333333	0.333333333	0	0
XM_003126500.1	TAS2R7	0.01	0.333333333	0	0
XM_003126511.4	AICDA	0.003333333	0.333333333	0	0
XM_003126519.4	APOBEC1	0.006666667	0.333333333	0	0
XM_003126566.4	TULP3	0.03	2.306666667	0	0
XM_003126826.3	ATP2C2	0.003333333	0.773333333	0	0
XM_003126897.6	AP1G1	0.04	11.58	0	0
XM_003126987.4	SALL1	0.096666667	23	0	0.333333333
XM_003127020.3	WDR88	0.006666667	0.333333333	0	0
XM_003127027.6	PDCD2L	0.323333333	14.61333333	0	0
XM_003127416.5	TMC4	0.016666667	1.666666667	0	0
XM_003127459.5	LOC100515837	0.02	1.62	0	0
XM_003127686.5	EPHA8	0.003333333	0.333333333	0	0
XM_003127722.4	KDF1	0.033333333	2.653333333	0	0
XM_003127883.5	MEP1B	0.036666667	5	0	0
XM_003127890.6	KLHL14	0.013333333	2.333333333	0	0.333333333
XM_003128064.3	HYI	0.03	1.026666667	0	0
XM_003128078.4	TMEM125	0.07	5.356666667	0	0
XM_003128089.3	LOC100521802	0.006666667	0.333333333	0	0
XM_003128338.4	DST	0.016666667	11.97666667	0	0
XM_003128432.4	CRISP3	0.003333333	0.333333333	0	0
XM_003128446.6	RASGRF1	0.023333333	5.036666667	0	0
XM_003128497.5	HCN4	0.01	2.333333333	0	0
XM_003128594.2	LOC100512292	0.003333333	0.166666667	0	0
XM_003128772.2	LOC100516627	0.003333333	0.166666667	0	0
XM_003128792.4	LOC100526118	0.006666667	0.333333333	0	0
XM_003128856.5	BOD1L1	1.383333333	620.6	0	0
XM_003128864.4	FGFBP1	0.013333333	0.666666667	0	0
XM_003128879.6	PACRGL	0.003333333	0.173333333	0	0
XM_003128906.4	ARAP2	0.03	11.33333333	0	0
XM_003128939.6	RBM47	0.046666667	9.22	0	0
XM_003128997.6	TDO2	0.013333333	1	0	0
XM_003129067.4	LOC100515394	0.003333333	0.333333333	0	0
XM_003129071.4	LOC100516628	0.026666667	2	0	0
XM_003129438.1	LOC100515042	0.013333333	0.52	0	0
XM_003129461.6	CNGA4	0.06	9.333333333	0	0
XM_003129582.2	LOC100517357	0.01	0.333333333	0	0
XM_003129583.2	LOC100517545	0.04	1.836666667	0	0
XM_003129584.2	LOC100517905	0.123333333	4.53	0	0
XM_003129745.5	NOX4	0.32	120.3433333	0	0
XM_003129804.4	ANGPTL5	0.006666667	0.666666667	0	0
XM_003129995.4	VWA5A	0.076666667	11.11333333	0	0
XM_003130077.5	KCNJ1	0.033333333	3.666666667	0	0
XM_003130080.5	ZBTB44	0.036666667	15.26	0	0
XM_003130089.4	LOC100520582	0.036666667	4.333333333	0	0
XM_003130095.4	LOC100523745	0.003333333	0.333333333	0	0
XM_003130341.4	NPHS2	0.15	11.24333333	0	0
XM_003130409.4	FAM71A	0.02	1.666666667	0	0
XM_003130574.4	CCDC185	0.006666667	0.666666667	0	0
XM_003130597.6	ATP6V1G3	0.066666667	2	0	0
XM_003131219.5	USH1G	0.003333333	0.333333333	0	0
XM_003131266.4	APOH	0.02	1	0	0
XM_003131330.4	CCDC103	0.006666667	0.333333333	0	0
XM_003131347.5	ADAM11	0.013333333	2.99	0	0
XM_003131433.4	KRT32	0.006666667	0.333333333	0	0
XM_003131459.6	KRT23	0.003333333	0.333333333	0	0
XM_003131462.4	KRT20	0.053333333	5	0	0

XM_003131551.3	HOXB13	0.016666667	1	0	0
XM_003131642.3	EPX	0.026666667	3	0	0
XM_003131655.3	MPO	0.013333333	1.333333333	0	0
XM_003131660.6	RAD51C	0.343333333	15.16333333	0	0
XM_003131703.5	GGNBP2	0.05	5.83	0	0
XM_003131706.4	LHX1	0.033333333	4.333333333	0	0
XM_003131843.3	LOC100514488	0.003333333	0.333333333	0	0
XM_003131855.3	LOC100517239	0.003333333	0.333333333	0	0
XM_003131910.6	GLTPD2	0.076666667	3.666666667	0	0
XM_003131911.6	TM4SF5	0.006666667	0.333333333	0	0
XM_003132081.4	EOMES	0.023333333	3.666666667	0	0.323333333
XM_003132276.5	PXK	0.05	6.586666667	0	0
XM_003132414.6	CHST13	0.033333333	3	0	0
XM_003132488.5	P2RY14	0.01	1.69	0	0
XM_003132519.4	SERPINI2	0.003333333	0.666666667	0	0
XM_003132577.4	FETUB	0.006666667	0.333333333	0	0
XM_003132654.4	MAATS1	0.006666667	0.71	0	0.08
XM_003132665.6	GRAMD1C	0.026666667	3.993333333	0	0
XM_003132689.4	LOC100519871	0.15	2.913333333	0	0
XM_003132702.4	LNP1	0.006666667	0.333333333	0	0
XM_003132779.5	FAM3B	0.01	1.326666667	0	0
XM_003132781.4	RIPK4	0.013333333	2	0	0
XM_003132818.5	PTK2B	0.076666667	13.40333333	0	0.183333333
XM_003132944.4	RASL10A	0.006666667	0.333333333	0	0
XM_003132974.4	INPP5J	0.026666667	4.14	0	0
XM_003133000.5	LOC100510930	0.026666667	2.266666667	0	0
XM_003133104.5	GDF2	0.003333333	0.666666667	0	0
XM_003133237.6	INPP5A	0.233333333	28.38333333	0	0.023333333
XM_003133302.5	PTPN18	0.043333333	4.43	0	0
XM_003133513.5	SESTD1	0.246666667	105.6733333	0	0
XM_003133630.5	UNC80	0.006666667	2.23	0	0.666666667
XM_003133648.5	4-Mar	0.003333333	1.333333333	0	0
XM_003133669.4	PTPRN	0.003333333	0.333333333	0	0
XM_003133695.4	SLC19A3	0.046666667	4.666666667	0	0
XM_003133729.4	LOC100522404	0.006666667	0.666666667	0	0
XM_003134066.5	FOXI1	0.07	6.533333333	0	0
XM_003134071.4	LOC100512568	0.006666667	0.666666667	0	0
XM_003134089.6	GABRA1	0.003333333	0.333333333	0	0
XM_003134257.4	SPTLC3	0.033333333	7.666666667	0	0.333333333
XM_003134452.4	WISP2	0.066666667	5.156666667	0	0
XM_003134585.6	GIMAP2	0.003333333	0.333333333	0	0
XM_003134621.4	TMEM213	0.296666667	24.66666667	0	0
XM_003134723.4	TMEM229A	0.006666667	0.666666667	0	0
XM_003134747.3	IQUB	0.003333333	0.886666667	0	0
XM_003134812.4	SCRN1	0.026666667	6.03	0	0
XM_003134850.4	HOXA11	0.013333333	1.333333333	0	0
XM_003134888.4	POLM	0.003333333	0.626666667	0	0
XM_003135077.5	GPKOW	0.42	32.41	0	0
XM_003135152.4	GDPD2	0.04	4	0	0
XM_003135162.4	GJB1	0.146666667	9.796666667	0	0
XM_003135238.4	TAF7L	0.003333333	0.333333333	0	0
XM_003135314.4	NXT2	0.093333333	10.66333333	0	0
XM_003135343.5	SOWAHD	0.003333333	0.333333333	0	0.666666667
XM_003135358.5	TENM1	0.053333333	27.80666667	0	0
XM_003353723.3	LCN15	0.003333333	0.333333333	0	0
XM_003353837.5	MS4A12	0.04	2	0	0
XM_003353850.4	ZDHHC5	1.08	217.46	0	0
XM_003354020.3	ACER1	0.1	3.636666667	0	0
XM_003354287.4	SPOCK1	0.04	9	0	0
XM_003354556.4	QPRT	0.28	17	0	0

XM_003354608.5	TNP2	0.003333333	0.333333333	0	0
XM_003354629.4	C3H16orf89	0.046666667	2.5	0	0
XM_003354711.3	SLC9A4	0.003333333	0.333333333	0	0
XM_003355020.3	ESRP1	0.026666667	4	0	0
XM_003355060.4	NPBWR1	0.003333333	0.333333333	0	0
XM_003355128.5	TSACC	0.016666667	0.333333333	0	0
XM_003355176.4	MRPL9	0.01	0.493333333	0	0
XM_003355378.4	DNAJC22	0.053333333	3.693333333	0	0
XM_003355493.5	INHBC	0.003333333	0.333333333	0	0
XM_003355800.4	DOK4	0.04	4.616666667	0	0
XM_003355928.4	COQ8B	0.096666667	9.66	0	0
XM_003356079.4	ZNF667	0.06	7.986666667	0	0
XM_003356108.4	SDF4	0.09	5.896666667	0	0
XM_003356540.3	NRSN1	1.386666667	115.83	0	0
XM_003356738.5	MPP5	0.653333333	150.1066667	0	0
XM_003356782.5	FAM181A	0.003333333	0.666666667	0	0
XM_003356952.5	LOC100623255	0.003333333	0.666666667	0	0
XM_003356986.4	RBM46	0.003333333	0.666666667	0	0
XM_003357079.3	HPGDS	0.053333333	2	0	0
XM_003357106.4	TMEM150C	0.016666667	1.893333333	0	0
XM_003357180.2	LOC100620193	0.046666667	2.006666667	0	0
XM_003357426.4	ADAM22	0.01	3.88	0	0
XM_003357511.4	SLC26A4	0.01	3	0	0
XM_003357532.2	LOC100525599	0.006666667	0.333333333	0	0
XM_003357643.4	STUM	0.036666667	10.59	0	0
XM_003357809.4	URAD	0.006666667	0.333333333	0	0
XM_003357839.4	MLNR	0.006666667	0.333333333	0	0
XM_003358010.5	LOC100626147	0.003333333	1.436666667	0	0
XM_003358028.4	NBR1	0.133333333	30.34333333	0	0
XM_003358085.4	LOC110255211	0.103333333	3.3	0	0
XM_003358385.4	TRAK1	0.05	10.65666667	0	0
XM_003358406.5	SLC6A20	0.01	2.793333333	0	0
XM_003358614.4	CLRN1	0.003333333	1.666666667	0	0.666666667
XM_003358806.4	SIDT1	0.013333333	2.476666667	0	0
XM_003359330.4	PAX2	0.043333333	7.806666667	0	0
XM_003359332.4	PAX2	0.093333333	17.19333333	0	0
XM_003359398.4	CLRN3	0.086666667	6.333333333	0	0
XM_003359621.4	CPO	0.006666667	0.333333333	0	0
XM_003359779.5	RAB3C	0.023333333	9.666666667	0	0.666666667
XM_003359855.4	SLC6A19	0.156666667	22.66666667	0	0
XM_003360017.5	LOC100513767	0.156666667	5.333333333	0	0
XM_003360392.2	LOC100621480	0.006666667	0.333333333	0	0
XM_003360441.5	SLC25A14	0.13	8.983333333	0	0
XM_003360464.4	SOX3	0.003333333	0.333333333	0	0
XM_003360576.2	LOC100623839	0.006666667	0.333333333	0	0
XM_003361720.4	INSL5	0.003333333	0.333333333	0	0
XM_003361792.4	CCR4	0.006666667	0.666666667	0	0
XM_003361881.4	HGD	0.073333333	5.333333333	0	0
XM_003480551.4	MELK	0.066666667	7.346666667	0	0
XM_003480686.3	LOC100737764	0.106666667	8.333333333	0	0
XM_003480700.4	LOC110255185	0.053333333	2.063333333	0	0
XM_003480735.4	MRVI1	0.046666667	13.22	0	0
XM_003481107.4	BRICD5	0.013333333	0.513333333	0	0
XM_003481255.4	GALNT14	0.016666667	2	0	0
XM_003481575.4	KCNH3	0.003333333	0.666666667	0	0
XM_003481592.4	ZNF385A	0.1	9.686666667	0	0
XM_003481613.3	LOC100738529	0.01	0.333333333	0	0
XM_003482030.4	PABPC4	2.886666667	408.69	0	0
XM_003482125.3	TFAP2A	0.01	1.333333333	0	0
XM_003482193.4	LRRC73	0.02	1	0	0

XM_003482307.4	SLC24A4	0.023333333	8.453333333	0	0.45
XM_003482335.3	HGFAC	0.023333333	2	0	0
XM_003482526.2	LOC100738522	0.113333333	4.31	0	0
XM_003482610.4	DDI1	0.003333333	0.333333333	0	0
XM_003482830.4	CREM	0.03	1.953333333	0	0
XM_003482903.4	OLFM4	0.026666667	3.333333333	0	0
XM_003483042.2	LOC100738790	0.01	0.333333333	0	0
XM_003483116.4	HS3ST3A1	0.006666667	1.15	0	0
XM_003483156.4	ZKSCAN7	0.05	11.24333333	0	0
XM_003483288.3	KNG1	0.32	21.86333333	0	0
XM_003483321.4	LOC100517427	0.073333333	3.333333333	0	0
XM_003483444.3	CRYBB2	0.003333333	0.333333333	0	0
XM_003483501.4	ZSWIM8	0.063333333	17.12	0	0.283333333
XM_003483576.4	DPYSL4	0.003333333	0.333333333	0	0
XM_003483964.2	LOC100739507	0.006666667	0.333333333	0	0
XM_003484131.4	NEXMIF	0.003333333	2.666666667	0	0.333333333
XM_003484152.2	LOC100738042	0.006666667	0.666666667	0	0
XM_005652362.3	TBCCD1	0.063333333	7.55	0	0
XM_005652369.3	AHSG	0.02	2	0	0
XM_005652370.2	KNG1	0.156666667	10.16666667	0	0
XM_005652539.3	PCBP2	0.02	1.546666667	0	0
XM_005652714.3	C8G	0.03	1.496666667	0	0
XM_005652733.3	NOXA1	0.023333333	1.666666667	0	0
XM_005652764.3	CEND1	0.05	3.303333333	0	0
XM_005652781.3	SLC25A22	0.026666667	5.053333333	0	0
XM_005652805.3	PRR5L	0.126666667	11.6	0	0
XM_005652808.3	SGTA	0.003333333	0.296666667	0	0
XM_005652817.3	SLC39A3	0.016666667	3.52	0	0
XM_005652845.3	LOC100516898	0.01	0.333333333	0	0
XM_005652931.3	KXD1	0.173333333	9.743333333	0	0
XM_005652971.3	ZFP62	0.063333333	10.60666667	0	0
XM_005653162.3	PLEKHG6	0.013333333	2.793333333	0	0
XM_005653367.3	HTR1D	0.016666667	1.99	0	0
XM_005653390.3	EPB41	0.036666667	9.436666667	0	0
XM_005653504.3	FURIN	0.603333333	105.25	0	0
XM_005653519.3	HAPLN3	0.286666667	49.14666667	0	0
XM_005653581.3	MND1	0.01	0.333333333	0	0
XM_005653582.3	MND1	0.016666667	0.333333333	0	0
XM_005653603.3	TRMT10A	0.393333333	23.54666667	0	0
XM_005653627.3	NAA11	0.006666667	0.333333333	0	0
XM_005653652.2	FOLR2	0.123333333	6.153333333	0	0
XM_005653653.2	FOLR2	0.076666667	3.876666667	0	0
XM_005653680.3	ANAPC15	0.186666667	6.9	0	0
XM_005653697.3	JAML	0.03	2.076666667	0	0
XM_005653708.3	TMPRSS4	0.02	3.666666667	0	0
XM_005653766.3	GREM2	0.003333333	0.333333333	0	0
XM_005653771.3	LOC100627222	0.346666667	52.34333333	0	0
XM_005653807.3	LOC100524391	0.016666667	1.25	0	0
XM_005653820.3	TGDS	0.03	3.566666667	0	0
XM_005653835.3	TMCO3	0.016666667	2.36	0	0
XM_005653907.3	WIPF2	0.09	25.46666667	0	0
XM_005654018.3	SRCIN1	0.073333333	15.20333333	0	0
XM_005654038.3	CASR	0.023333333	5.666666667	0	0
XM_005654046.3	CD86	0.003333333	0.483333333	0	0
XM_005654048.3	WDR5B	0.13	19.31	0	0
XM_005654050.3	ILDR1	0.02	6.333333333	0	0.333333333
XM_005654266.3	PRKAG2	1.196666667	105.4766667	0	0
XM_005654424.3	SKA1	0.016666667	0.666666667	0	0
XM_005654446.3	SLC12A1	0.1	18.08333333	0	0
XM_005654511.3	PHF24	0.116666667	28.57666667	0	0.053333333

XM_005654514.2	DNAJB5	0.06	6.846666667	0	0.01
XM_005654585.3	POMT1	0.026666667	3.706666667	0	0
XM_005654605.3	SARDH	0.01	1.92	0	0
XM_005654644.3	SYT12	0.09	14.01666667	0	0
XM_005654707.3	DOHH	0.086666667	5.53	0	0
XM_005654769.3	CCDC159	0.01	5.383333333	0	0
XM_005654813.3	ATG4D	0.05	4.763333333	0	0
XM_005654852.3	LOC100517145	0.003333333	1	0	0
XM_005654853.3	ANGPTL6	0.053333333	4.333333333	0	0
XM_005654856.3	LOC100511183	0.003333333	0.666666667	0	0
XM_005654861.3	KRI1	0.076666667	8.866666667	0	0
XM_005654893.3	LOC100736865	0.056666667	1.693333333	0	0
XM_005654898.3	KCNN1	0.063333333	5.796666667	0	0
XM_005654970.3	RMND5B	0.036666667	2.903333333	0	0.08
XM_005654976.3	SFXN1	0.013333333	1.946666667	0	0
XM_005654978.3	SFXN1	0.113333333	15.49333333	0	0
XM_005655063.3	ZFAND2A	0.013333333	0.946666667	0	0
XM_005655133.3	UBE2I	0.206666667	8.956666667	0	0
XM_005655305.3	C4H8orf33	0.133333333	13.74666667	0	0
XM_005655354.3	ERICH5	0.01	1	0	0
XM_005655407.3	HAO2	0.103333333	11.77333333	0	0
XM_005655439.3	FAM19A3	0.003333333	0.333333333	0	0
XM_005655506.3	LOC100523244	0.2	23.9	0	0
XM_005655587.3	MYL6	0.266666667	20.40666667	0	0
XM_005655662.3	LOC100621352	0.006666667	0.666666667	0	0
XM_005655668.3	CLEC7A	0.003333333	0.38	0	0
XM_005655878.3	PRR19	0.033333333	1.993333333	0	0
XM_005656104.3	MPPE1	0.02	1.706666667	0	0
XM_005656126.3	ADGRL2	0.013333333	3.156666667	0	0
XM_005656214.3	STMND1	0.03	4	0	0
XM_005656228.3	ENPP5	0.073333333	13.22333333	0	0
XM_005656419.3	ACYP1	0.023333333	4.833333333	0	0
XM_005656480.3	CDCA4	0.03	1.316666667	0	0
XM_005656509.3	LOC100624788	0.003333333	0.333333333	0	0
XM_005656555.3	EIF4E	0.306666667	53.32333333	0	0
XM_005656626.3	C9H11orf53	0.01	2.333333333	0	0.666666667
XM_005656686.2	RAPGEF5	0.016666667	4.81	0	0
XM_005656701.3	DMTF1	0.026666667	4.503333333	0	0
XM_005656753.3	PROX1	0.053333333	17.85333333	0	0
XM_005656754.3	PROX1	0.053333333	18.24333333	0	0
XM_005656755.3	PROX1	0.023333333	7.52	0	0
XM_005656770.3	GGA2	0.15	19.10333333	0	0
XM_005656813.3	MINDY3	0.006666667	0.603333333	0	0
XM_005656916.3	SPHK1	0.02	2.233333333	0	0
XM_005656927.3	AMZ2	0.03	1.866666667	0	0
XM_005656956.3	BRCA1	0.006666667	2.78	0	0
XM_005656958.3	BRCA1	0.003333333	0.936666667	0	0
XM_005657060.3	ASTE1	0.036666667	3.536666667	0	0
XM_005657158.3	IGSF5	0.16	18.77333333	0	0
XM_005657159.3	IGSF5	0.106666667	10.22666667	0	0
XM_005657244.3	ADRA1A	0.02	13.04	0	0
XM_005657293.3	ZNF605	0.033333333	9.696666667	0	0
XM_005657361.2	RHBDD3	0.003333333	0.45	0	0
XM_005657397.3	AIFM2	0.036666667	2.756666667	0	0
XM_005657407.3	SGPL1	0.09	16.37666667	0	0
XM_005657433.3	TBC1D12	0.016666667	2.383333333	0	0
XM_005657447.3	ZFYVE27	0.02	8.69	0	0
XM_005657469.3	TCF7L2	0.013333333	2.163333333	0	0
XM_005657474.3	TCF7L2	0.06	10.60666667	0	0
XM_005657528.3	MTMR8	0.01	3.19	0	0

XM_005657618.1	LOC102162127	0.006666667	0.11	0	0
XM_005657746.3	TTC26	0.113333333	21.33333333	0	0
XM_005657784.3	CDK16	0.02	4.866666667	0	0
XM_005657795.3	RBM3	0.113333333	5.48	0	0
XM_005657808.2	CCNB3	0.006666667	1.29	0	0
XM_005657817.3	EDA	0.12	27.30333333	0	0
XM_005657826.3	CITED1	0.043333333	1.506666667	0	0
XM_005657828.3	CITED1	0.08	2.616666667	0	0
XM_005657830.3	CITED1	0.12	3.933333333	0	0
XM_005657914.2	FHL1	0.096666667	10.59666667	0	0
XM_005658444.3	ACOD1	0.006666667	0.666666667	0	0
XM_005658570.3	SLC51B	0.073333333	3.243333333	0	0
XM_005659173.3	NHSL1	0.023333333	13.07666667	0	0
XM_005659176.3	NHSL1	0.02	7.3	0	0
XM_005659178.3	HEBP2	0.023333333	3.5	0	0
XM_005659277.3	CGA	0.02	0.666666667	0	0
XM_005659281.3	CFAP206	0.103333333	9.936666667	0	0
XM_005659523.1	TPM1	0.07	5.03	0	0
XM_005659531.2	TPM1	0.093333333	11.44	0	0.02
XM_005659583.3	RAB27A	0.036666667	5.523333333	0	0
XM_005659590.3	ONECUT1	0.003333333	0.253333333	0	0
XM_005659595.3	MYO5A	0.003333333	1.5	0	0
XM_005659734.3	SPINT1	0.02	2.206666667	0	0
XM_005659736.3	PPP1R14D	0.023333333	0.666666667	0	0
XM_005659737.3	EXD1	0.036666667	4.943333333	0	0
XM_005659762.3	RASGRP1	0.02	4.28	0	0
XM_005659763.3	RASGRP1	0.016666667	3.513333333	0	0
XM_005659820.3	CNDP1	0.1	16.98333333	0	0
XM_005659949.3	GNPNAT1	0.016666667	5.276666667	0	0
XM_005659996.2	CCDC198	0.013333333	0.643333333	0	0
XM_005660147.3	TJP2	0.026666667	4.836666667	0	0
XM_005660260.3	TSTD2	0.02	4.006666667	0	0
XM_005660265.3	HEMGN	0.043333333	7.06	0	0
XM_005660297.3	MSANTD3	0.036666667	3.216666667	0	0
XM_005660372.3	ORM1	0.02	0.666666667	0	0
XM_005660383.3	TRIM32	0.023333333	3.25	0	0
XM_005660443.3	STXBP1	0.023333333	4.123333333	0	0
XM_005660472.3	DNM1	0.036666667	5.89	0	0
XM_005660534.3	SPACA9	0.033333333	2.333333333	0	0
XM_005660680.3	SNX32	0.01	0.666666667	0	0
XM_005660719.3	BATF2	0.056666667	6.036666667	0	0
XM_005660743.3	SLC22A12	0.21	15.48333333	0	0
XM_005660775.2	SLC22A8	1.543333333	135.9633333	0	0
XM_005660776.2	SLC22A8	0.05	3.843333333	0	0
XM_005660777.3	SLC22A8	0.046666667	4.153333333	0	0
XM_005660833.3	CD6	0.023333333	3.193333333	0	0
XM_005661131.3	SBF2	0.03	9.183333333	0	0
XM_005661164.3	ZNF672	0.023333333	3.946666667	0	0
XM_005661185.3	OCEL1	0.036666667	2.08	0	0
XM_005661299.3	CAPS	0.006666667	0.333333333	0	0
XM_005661304.3	CATSPERD	0.003333333	0.333333333	0	0
XM_005661351.3	TJP3	0.023333333	3	0	0
XM_005661365.3	ARID3A	0.24	54.30333333	0	0
XM_005661390.3	EFNA2	0.023333333	2.12	0	0
XM_005661394.3	GAMT	0.023333333	1.136666667	0	0
XM_005661395.3	GAMT	0.113333333	5.123333333	0	0
XM_005661400.3	GAMT	0.043333333	1.74	0	0
XM_005661428.3	PLPP2	0.03	1.536666667	0	0
XM_005661449.3	MXD3	0.02	1.19	0	0
XM_005661487.3	GCNT4	0.023333333	5.333333333	0	0

XM_005661521.3	ANKRD34B	0.003333333	0.333333333	0	0
XM_005661540.3	XRCC4	0.073333333	5.14	0	0
XM_005661542.3	EDIL3	0.006666667	1.843333333	0	0
XM_005661655.3	SLC25A48	0.026666667	1.343333333	0	0
XM_005661710.3	MATR3	0.076666667	13.57666667	0	0
XM_005661771.3	RELL2	0.03	2	0	0
XM_005661828.3	AP5Z1	0.146666667	36.82666667	0	0
XM_005661898.3	ZCWPW1	0.006666667	0.71	0	0
XM_005661925.3	SLC12A9	0.186666667	26.30333333	0	0
XM_005661944.3	RASA4B	0.166666667	20.32333333	0	0
XM_005662102.3	GP2	0.003333333	0.333333333	0	0
XM_005662212.3	LOC100517149	0.066666667	8.656666667	0	0
XM_005662332.3	CCDC138	0.02	2.966666667	0	0
XM_005662353.3	POU3F3	0.03	4.47	0	0
XM_005662434.3	C3H2orf81	0.023333333	2	0	0
XM_005662438.2	M1AP	0.003333333	0.333333333	0	0
XM_005662439.3	WBP1	0.22	12.56	0	0
XM_005662613.3	ATL2	0.163333333	23.82	0	0
XM_005662675.3	EMILIN1	0.013333333	1.846666667	0	0
XM_005662702.2	FAM228A	0.1	5.666666667	0	0
XM_005662781.3	COLEC11	0.013333333	0.666666667	0	0
XM_005662905.3	OXR1	0.003333333	0.573333333	0	0.166666667
XM_005662959.3	CCNE2	0.143333333	18.72666667	0	0
XM_005662985.3	WWP1	0.043333333	7.723333333	0	0
XM_005662999.3	LOC100156775	0.01	0.513333333	0	0
XM_005663011.3	HEY1	0.02	2.073333333	0	0
XM_005663013.3	ZC2HC1A	0.033333333	5.46	0	0
XM_005663042.3	CSPP1	0.346666667	57.22	0	0
XM_005663053.3	MTFR1	0.083333333	11.40666667	0	0
XM_005663158.3	FCGR2B	0.033333333	2.153333333	0	0
XM_005663159.3	FCGR2B	0.053333333	3.436666667	0	0
XM_005663219.3	SPTA1	0.003333333	1	0	0
XM_005663230.3	CD1.1	0.013333333	5.08	0	0
XM_005663297.2	SSR2	0.966666667	40.87333333	0	0
XM_005663357.3	S100A4	0.253333333	5.046666667	0	0
XM_005663368.2	TPM3	0.033333333	3.156666667	0	0
XM_005663438.3	S100A10	0.056666667	1.606666667	0	0
XM_005663439.3	C2CD4D	0.006666667	0.333333333	0	0
XM_005663447.3	CGN	0.05	11	0	0
XM_005663567.3	CEPT1	0.003333333	0.306666667	0	0.116666667
XM_005663800.3	SSTR3	0.006666667	1.833333333	0	0
XM_005663861.3	GTSF1	0.003333333	0.333333333	0	0
XM_005663895.3	LOC100620470	0.003333333	0.333333333	0	0
XM_005663899.3	STAT6	0.086666667	15.23	0	0
XM_005663900.3	TAC3	0.006666667	0.733333333	0	0
XM_005663930.3	DTX3	0.01	0.97	0	0
XM_005663957.3	HMGA2	0.006666667	0.333333333	0	0
XM_005664158.3	SCAF11	0.013333333	3.473333333	0	0
XM_005664212.3	ACTR6	0.036666667	2.76	0	0
XM_005664260.3	SOCS2	0.033333333	3.4	0	0
XM_005664379.3	GINS3	0.036666667	3.713333333	0	0
XM_005664617.3	GNG8	0.003333333	0.456666667	0	0.026666667
XM_005664808.3	LOC100626318	0.016666667	3.903333333	0	0.346666667
XM_005664823.3	LOC100622710	0.01	1.153333333	0	0
XM_005664853.3	ZNF581	0.013333333	0.76	0	0
XM_005664952.3	PEX10	0.013333333	0.846666667	0	0
XM_005664985.3	FBXO6	0.026666667	2.17	0	0
XM_005665001.3	KIAA2013	0.03	3.15	0	0
XM_005665059.3	KLHDC7A	0.093333333	34.66666667	0	0
XM_005665196.3	TMEM54	0.04	1.93	0	0

XM_005665258.2	PPIE	0.036666667	6.03	0	0
XM_005665397.3	LRP8	0.006666667	1.41	0	0
XM_005665399.3	LRP8	0.006666667	1.763333333	0	0
XM_005665415.2	ZYG11A	0.003333333	0.666666667	0	0
XM_005665484.3	MED8	0.05	4.45	0	0.03
XM_005665486.3	MED8	0.216666667	18.89	0	0
XM_005665567.3	MAK	0.023333333	2.1	0	0
XM_005665623.3	CDKAL1	0.156666667	15.41	0	0
XM_005665629.3	NRSN1	0.05	4.26	0	0
XM_005665634.3	GMNN	0.063333333	2.746666667	0	0
XM_005665757.2	MUCL3	0.003333333	0.333333333	0	0
XM_005665799.3	SLC44A4	0.393333333	38.27333333	0	0
XM_005665996.3	MRPS10	0.036666667	2.25	0	0
XM_005666097.3	TFAP2B	0.016666667	4.663333333	0	0
XM_005666124.3	CYP1A2	0.003333333	0.333333333	0	0
XM_005666158.3	STRA6	0.11	21.02333333	0	0
XM_005666160.3	ISLR2	0.016666667	3.216666667	0	0
XM_005666250.3	LTB4R	0.013333333	2.793333333	0	0.036666667
XM_005666264.3	SLC7A7	0.196666667	19.00666667	0	0
XM_005666267.3	SLC7A7	0.113333333	10.95	0	0
XM_005666269.3	SLC7A7	0.036666667	3.396666667	0	0
XM_005666281.3	LOC100626606	0.023333333	1	0	0
XM_005666312.3	PLEKHG3	0.016666667	3.283333333	0	0.32
XM_005666335.2	SRSF5	0.073333333	4.776666667	0	0
XM_005666383.3	EFCAB11	0.026666667	3.27	0	0
XM_005666613.3	APBB2	0.02	5.606666667	0	0
XM_005666628.3	GABRA2	0.003333333	1.086666667	0	0
XM_005666641.3	OCIAD2	0.04	4.653333333	0	0
XM_005666740.3	RASSF6	0.04	7.666666667	0	0.333333333
XM_005666747.3	LOC102166306	0.003333333	0.986666667	0	0
XM_005666793.2	FGG	0.01	1.09	0	0
XM_005666841.3	LSM6	0.176666667	35.01333333	0	0
XM_005666844.3	LSM6	0.276666667	53.19333333	0	0
XM_005667001.3	SPARCL1	0.073333333	10.42	0	0
XM_005667045.3	AKIP1	0.063333333	2.62	0	0
XM_005667079.3	LOC100738479	0.013333333	1.08	0	0
XM_005667144.3	EMSY	0.08	20.42333333	0	0
XM_005667256.3	LOC100627380	0.003333333	0.333333333	0	0
XM_005667310.3	ZC3H12C	0.02	7.736666667	0	0
XM_005667369.3	APOA5	0.033333333	1.666666667	0	0
XM_005667372.2	APOC3	0.64	12.66666667	0	0
XM_005667380.2	SIDT2	0.033333333	6.253333333	0	0
XM_005667402.3	SLC37A4	0.053333333	5.943333333	0	0
XM_005667404.3	NLRX1	0.25	40.17	0	0
XM_005667428.3	PDZD3	0.023333333	2.57	0	0
XM_005667471.3	SLC37A2	0.006666667	1.493333333	0	0
XM_005667581.3	CFAP69	0.003333333	0.29	0	0
XM_005667605.3	LOC100519098	0.09	20.35	0	0
XM_005667626.3	SLC25A13	0.126666667	16.63333333	0	0
XM_005667635.3	C1GALT1	0.046666667	15.44	0	0
XM_005667760.3	EZH2	0.006666667	0.683333333	0	0
XM_005667768.3	LOC100522942	0.096666667	25.38	0	0.216666667
XM_005667818.3	RGL1	0.02	4.193333333	0	0
XM_005667893.3	IRF6	0.023333333	2.673333333	0	0
XM_005667949.3	PSEN2	0.033333333	2.87	0	0
XM_005667985.3	LHX9	0.003333333	0.533333333	0	0
XM_005667987.3	LHX9	0.016666667	1.8	0	0
XM_005668034.3	IDNK	0.156666667	8.96	0	0
XM_005668066.3	UBAP2	0.02	6.843333333	0	0
XM_005668082.3	C10H9orf72	0.026666667	3.623333333	0	0

XM_005668177.3	NRP1	0.026666667	6.516666667	0	0
XM_005668179.3	NRP1	0.28	75.68333333	0	0.176666667
XM_005668185.3	CREM	0.063333333	5.35	0	0
XM_005668187.3	CREM	0.09	8.543333333	0	0
XM_005668230.2	AKR1C3	0.016666667	2.213333333	0	0
XM_005668232.3	AKR1C3	0.033333333	4.576666667	0	0
XM_005668258.3	ZMYND11	0.05	9.696666667	0	0
XM_005668317.3	HMGB1	0.09	9.186666667	0	0
XM_005668368.3	LHFPL6	0.03	2.393333333	0	0
XM_005668396.2	DLEU7	0.003333333	0.333333333	0	0
XM_005668407.3	CAB39L	0.086666667	9.816666667	0	0
XM_005668444.3	LACC1	0.066666667	11.04666667	0	0
XM_005668532.3	TNFSF13B	0.013333333	1.666666667	0	0
XM_005668625.3	CD300LF	0.01	0.823333333	0	0
XM_005668662.3	APOH	0.033333333	1.333333333	0	0
XM_005668706.2	GJC1	0.003333333	1.14	0	0
XM_005668770.3	MEOX1	0.09	11.52	0	0
XM_005668838.3	LOC102163700	0.013333333	0.333333333	0	0
XM_005669028.3	GGNBP2	0.016666667	2.163333333	0	0
XM_005669140.3	FBXO39	0.02	3.173333333	0	0
XM_005669150.3	ZNF232	0.033333333	2.716666667	0	0
XM_005669151.3	ZNF232	0.016666667	1.186666667	0	0
XM_005669200.2	ASGR2	0.01	0.666666667	0	0
XM_005669376.3	ENTPD3	0.003333333	0.333333333	0	0
XM_005669402.3	CCK	0.02	0.656666667	0	0
XM_005669406.3	NKTR	0.003333333	0.98	0	0
XM_005669423.3	ZNF662	0.016666667	2.9	0	0
XM_005669425.3	HIGD1A	0.29	16.52333333	0	0
XM_005669433.3	ZNF197	0.016666667	5.88	0	0
XM_005669459.3	XCR1	0.013333333	3.98	0	0
XM_005669488.2	DHX30	0.126666667	21.43	0	0
XM_005669629.3	GLYCTK	0.016666667	1.473333333	0	0
XM_005669660.3	WNT5A	0.083333333	19.78	0	0
XM_005669683.3	KCTD6	0.333333333	51.34	0	0
XM_005669686.3	ACOX2	0.016666667	2.283333333	0	0
XM_005669744.3	CIDEC	1.093333333	62.91	0	0
XM_005669745.3	CIDEC	0.173333333	9.22	0	0
XM_005669795.3	TSEN2	0.07	7.156666667	0	0
XM_005669803.3	TRH	0.01	0.666666667	0	0
XM_005669811.3	GRIP2	0.226666667	52.59	0	0
XM_005669858.3	CEP63	0.006666667	0.7	0	0
XM_005669882.3	RBP1	0.04	1.136666667	0	0
XM_005669924.3	PLSCR4	0.02	2.713333333	0	0
XM_005669961.3	SPTSSB	0.003333333	0.333333333	0	0
XM_005669994.2	SAMD7	0.003333333	0.333333333	0	0
XM_005670037.3	ECE2	0.036666667	4.666666667	0	0
XM_005670133.3	MUC20	0.043333333	3.666666667	0	0
XM_005670241.3	HHLA2	0.026666667	8.23	0	0
XM_005670242.3	HHLA2	0.016666667	5.503333333	0	0
XM_005670255.3	COL8A1	0.076666667	16.73666667	0	0.23
XM_005670262.3	CLDND1	0.04	4.13	0	0
XM_005670312.3	USP16	0.05	6.18	0	0
XM_005670313.3	USP16	0.086666667	11.84	0	0
XM_005670314.3	USP16	0.006666667	0.97	0	0
XM_005670320.3	USP16	0.01	1.443333333	0	0
XM_005670338.3	KCNJ15	0.073333333	18.9	0	0
XM_005670340.3	KCNJ15	0.016666667	4.846666667	0	0
XM_005670341.3	KCNJ15	0.16	54.63333333	0	0
XM_005670524.3	PUS1	0.083333333	5.996666667	0	0
XM_005670634.3	CAMKK2	0.08	17.83333333	0	0

XM_005670688.3	RITA1	0.176666667	11.85666667	0	0
XM_005670702.3	BICDL1	0.043333333	10.14	0	0
XM_005670759.3	SGSM1	0.003333333	1.203333333	0	0
XM_005670802.3	MTMR3	0.006666667	1.4	0	0
XM_005670815.3	SEC14L2	0.066666667	7.846666667	0	0
XM_005670818.3	TCN2	0.43	39.95666667	0	0
XM_005670820.3	TCN2	0.12	11.77666667	0	0
XM_005670906.3	DERL3	0.02	1	0	0
XM_005671078.3	CAMK2G	0.006666667	0.636666667	0	0
XM_005671082.2	CAMK2G	0.016666667	1.63	0	0
XM_005671139.2	PRXL2A	0.056666667	3.366666667	0	0
XM_005671141.3	PRXL2A	0.036666667	2.103333333	0	0
XM_005671145.3	SFTPD	0.003333333	0.723333333	0	0
XM_005671215.3	NCOA4	0.103333333	15.40333333	0	0
XM_005671217.3	NCOA4	0.003333333	0.316666667	0	0.146666667
XM_005671259.3	RNLS	0.033333333	9.513333333	0	0
XM_005671272.3	KIF20B	0.013333333	3.54	0	0
XM_005671335.3	SORBS1	0.036666667	9.233333333	0	0
XM_005671380.3	CNNM1	0.003333333	0.953333333	0	0.333333333
XM_005671382.3	ENTPD7	0.023333333	8.19	0	0
XM_005671431.3	STN1	0.04	4.003333333	0	0
XM_005671442.3	CFAP58	0.003333333	0.333333333	0	0
XM_005671448.3	ADD3	0.053333333	10.46333333	0	0
XM_005671475.3	ABLIM1	0.06	14.70333333	0	0
XM_005671484.3	ENO4	0.003333333	0.51	0	0
XM_005671498.3	FAM204A	0.02	1.34	0	0
XM_005671587.3	MTG1	0.113333333	8.576666667	0	0
XM_005671698.3	STOX2	0.003333333	0.98	0	0
XM_005671700.2	IRF2	0.013333333	1.236666667	0	0
XM_005671711.2	C15H4orf47	0.096666667	5.91	0	0
XM_005671714.3	C15H4orf47	0.07	6.253333333	0	0
XM_005671733.3	SORBS2	0.013333333	2.183333333	0	0
XM_005671871.3	RBMS1	0.176666667	29.91	0	0
XM_005671872.3	RBMS1	0.04	6.933333333	0	0
XM_005671981.3	NFE2L2	0.303333333	118.7266667	0	0
XM_005672059.3	SGO2	0.016666667	2.936666667	0	0
XM_005672244.3	OBSL1	0.38	104.6333333	0	0
XM_005672270.3	ARMC9	0.056666667	7.713333333	0	0
XM_005672318.3	LRRFIP1	0.056666667	9.816666667	0	0.183333333
XM_005672323.3	LRRFIP1	0.2	18.82666667	0	0
XM_005672366.3	OTOS	0.013333333	0.666666667	0	0
XM_005672398.3	CDH12	0.003333333	0.333333333	0	0
XM_005672488.3	NLN	0.06	6.89	0	0
XM_005672514.3	CCDC125	0.03	2.49	0	0
XM_005672566.3	PTTG1	0.036666667	1.163333333	0	0
XM_005672585.3	MED7	0.04	1.97	0	0
XM_005672625.3	SLC6A18	0.79	64.65	0	0
XM_005672626.3	SLC6A18	0.08	6.506666667	0	0
XM_005672637.3	MSR1	0.006666667	0.576666667	0	0
XM_005672697.3	ANKEF1	0.006666667	0.666666667	0	0
XM_005672698.3	ANKEF1	0.01	1	0	0
XM_005672707.3	NDUFAF5	0.026666667	1.14	0	0
XM_005672724.3	ZNF133	0.123333333	14.10666667	0	0
XM_005672791.3	ITPA	0.06	1.166666667	0	0
XM_005672951.3	HNF4A	0.023333333	4.416666667	0	0
XM_005672959.3	TOMM34	0.02	1.776666667	0	0
XM_005672962.3	PABPC1L	0.01	1	0	0
XM_005673107.3	PIP	0.013333333	0.333333333	0	0
XM_005673124.3	PTN	0.046666667	2.923333333	0	0
XM_005673215.3	IQUB	0.01	3.256666667	0	0

XM_005673264.2	LOC100738935	0.003333333	0.333333333	0	0
XM_005673273.3	LSM5	0.023333333	0.426666667	0	0
XM_005673291.3	HOXA10	0.146666667	25.36333333	0	0
XM_005673294.3	HOXA10	0.04	3.996666667	0	0
XM_005673340.3	NPC1L1	0.006666667	2.666666667	0	0
XM_005673390.3	PNPLA4	0.033333333	1.18	0	0
XM_005673427.3	RAB9A	0.223333333	12.69	0	0
XM_005673496.1	PRDX4	0.22	9.34	0	0
XM_005673503.3	PCYT1B	0.013333333	3.11	0	0
XM_005673639.3	GPR173	0.033333333	6.626666667	0	0
XM_005673647.3	RIBC1	0.013333333	1	0	0
XM_005673655.3	PHF8	0.04	10.46	0	0
XM_005673669.3	ALAS2	0.056666667	4.846666667	0	0
XM_005673694.3	HEPH	0.003333333	1.35	0	0.296666667
XM_005673748.3	POF1B	0.003333333	0.333333333	0	0
XM_005673761.3	XKRX	0.01	1.333333333	0	0
XM_005673763.3	DRP2	0.006666667	2.49	0	0
XM_005673801.3	MUM1L1	0.003333333	0.526666667	0	0
XM_005673820.3	ACSL4	0.033333333	7.483333333	0	0
XM_005673891.3	SMARCA1	0.006666667	0.946666667	0	0
XM_005673893.3	SMARCA1	0.01	1.876666667	0	0
XM_005673895.3	SMARCA1	0.01	1.78	0	0
XM_005673897.3	RAB33A	0.003333333	0.666666667	0	0.666666667
XM_005673898.3	XPNPEP2	0.11	22.38666667	0	0
XM_005673939.2	LOC100522722	0.01	0.666666667	0	0
XM_005673958.3	FMR1	0.006666667	1.096666667	0	0.05
XM_005673989.3	ZFP92	0.086666667	6.936666667	0	0
XM_013977400.2	FAM50B	0.006666667	1	0	0.333333333
XM_013977416.2	MAK	0.01	1.53	0	0
XM_013977477.2	GMNN	0.06	2.803333333	0	0
XM_013977486.2	CARMIL1	0.02	4.726666667	0	0
XM_013977531.2	ZSCAN16	0.006666667	1.106666667	0	0
XM_013977534.2	ZSCAN12	0.013333333	3.48	0	0
XM_013977563.2	LOC100154127	0.006666667	0.666666667	0	0
XM_013977604.2	PSORS1C2	0.036666667	1.693333333	0	0
XM_013977651.2	EGFL8	0.013333333	0.806666667	0	0
XM_013977698.2	ADORA1	0.07	5.963333333	0	0
XM_013977729.1	SLC39A7	0.046666667	3.023333333	0	0
XM_013977762.2	BEND6	0.023333333	1.426666667	0	0
XM_013977845.2	C7H6orf222	0.013333333	2.806666667	0	0
XM_013977857.2	KCTD20	0.423333333	82.08666667	0	0
XM_013977868.2	C7H6orf89	0.076666667	23.13	0	0
XM_013977912.2	MDFI	0.006666667	0.333333333	0	0
XM_013977942.2	TRERF1	0.006666667	2.023333333	0	0
XM_013977955.2	DNPH1	0.033333333	0.913333333	0	0
XM_013977979.2	TMEM63B	0.096666667	13.50666667	0	0
XM_013977999.2	PLA2G7	0.273333333	19.08	0	0
XM_013978012.2	ANKRD66	0.006666667	0.333333333	0	0
XM_013978021.2	TFAP2B	0.003333333	1.18	0	0
XM_013978026.2	IL17F	0.006666667	0.333333333	0	0
XM_013978087.2	STRA6	0.11	10.44666667	0	0
XM_013978094.2	ISLR2	0.026666667	4.783333333	0	0
XM_013978134.2	DTD2	0.05	7.776666667	0	0
XM_013978154.2	LOC106504460	0.01	0.333333333	0	0
XM_013978175.2	NFATC4	0.01	0.993333333	0	0
XM_013978206.2	SLC22A17	0.026666667	2.756666667	0	0
XM_013978207.2	SLC22A17	0.006666667	0.816666667	0	0
XM_013978223.2	SLC7A7	0.006666667	1.316666667	0	0
XM_013978224.2	SLC7A7	0.04	3.983333333	0	0
XM_013978236.2	LOC100627222	0.163333333	15.74666667	0	0

XM_013978251.2	CHD8	0.01	3.166666667	0	0
XM_013978319.2	NUMB	0.13	19.05	0	0
XM_013978323.2	HDHD2	0.033333333	3.443333333	0	0
XM_013978337.2	CIPC	0.183333333	46.37666667	0	0
XM_013978361.2	HDHD2	0.06	5.48	0	0
XM_013978400.2	LOC396685	0.083333333	5.67	0	0
XM_013978402.2	LOC396684	0.04	2.46	0	0
XM_013978451.2	EFHC1	0.013333333	1.16	0	0
XM_013978544.2	PROM1	0.073333333	10.3	0	0
XM_013978575.2	LOC100520753	0.04	2.333333333	0	0
XM_013978598.2	RFC1	0.02	6.506666667	0	0
XM_013978641.2	GUF1	0.24	22.54666667	0	0
XM_013978642.2	GNPDA2	0.01	1.203333333	0	0
XM_013978645.2	GABRA2	0.003333333	1	0	0
XM_013978772.2	LOC100520680	0.023333333	1.333333333	0	0
XM_013978857.2	LOC102167861	0.056666667	14.71666667	0	0
XM_013978916.2	NUDT6	0.016666667	0.803333333	0	0
XM_013979019.2	SNCA	0.043333333	1.976666667	0	0
XM_013979156.2	EMSY	0.053333333	13.51333333	0	0
XM_013979177.2	PAK1	0.033333333	4.623333333	0	0
XM_013979215.2	SYTL2	0.053333333	19.35	0	0
XM_013979243.2	UNK	0.056666667	10.42333333	0	0
XM_013979288.2	C9H11orf97	0.016666667	1	0	0
XM_013979325.2	MMP27	0.18	14.51666667	0	0
XM_013979326.2	MMP27	0.05	3.913333333	0	0
XM_013979333.2	GRIA4	0.033333333	6.22	0	0
XM_013979338.2	GRIA4	0.026666667	3.55	0	0
XM_013979361.2	LOC100512977	0.016666667	8.213333333	0	0
XM_013979468.2	ABCG4	0.006666667	1.333333333	0	0
XM_013979478.1	LOC106504894	0.023333333	0.666666667	0	0
XM_013979586.2	SNX19	0.06	26.78333333	0	0
XM_013979647.2	C4BPA	0.12	11.66666667	0	0
XM_013979664.2	STEAP2	0.1	33.20666667	0	0
XM_013979666.2	CFAP69	0.01	1.376666667	0	0
XM_013979728.2	PHF14	0.013333333	2.32	0	0
XM_013979893.2	RASAL2	0.02	8.563333333	0	0.05
XM_013979975.2	LPGAT1	0.083333333	25.48666667	0	0
XM_013980054.2	PSEN2	0.373333333	33.94333333	0	0
XM_013980118.2	PHLDA3	0.046666667	3.816666667	0	0
XM_013980137.2	PTCH1	0.02	6.383333333	0	0
XM_013980152.2	IDNK	0.046666667	2.956666667	0	0
XM_013980184.2	AQP7	1.02	125.18	0	0
XM_013980200.2	C12H17orf97	0.01	0.333333333	0	0
XM_013980293.2	CREM	0.013333333	1.286666667	0	0
XM_013980314.2	USP6NL	0.013333333	6.16	0	0
XM_013980504.2	RCBTB1	0.003333333	0.433333333	0	0
XM_013980506.2	DDAH1	0.13	19.08333333	0	0
XM_013980517.2	HTR2A	0.003333333	0.44	0	0
XM_013980609.2	RBM26	0.06	19.87	0	0
XM_013980615.2	SLITRK5	0.006666667	1.39	0	0
XM_013980746.2	ENPP7	0.003333333	0.333333333	0	0
XM_013980788.2	KIF19	0.003333333	0.383333333	0	0.096666667
XM_013980881.2	ACBD4	0.006666667	0.416666667	0	0
XM_013980883.2	ACBD4	0.013333333	1.03	0	0
XM_013980928.2	ETV4	0.023333333	2.443333333	0	0
XM_013980995.2	LOC110255211	0.026666667	0.56	0	0
XM_013980997.2	PHB	0.09	6.63	0	0
XM_013981021.2	EPN3	0.006666667	1.566666667	0	0
XM_013981068.2	MBTD1	0.03	7.55	0	0
XM_013981087.2	TMEM100	0.053333333	3.24	0	0

XM_013981183.2	KSR1	0.003333333	0.606666667	0	0
XM_013981205.2	SMG6	0.006666667	0.79	0	0
XM_013981240.2	XAF1	0.023333333	1.063333333	0	0
XM_013981242.2	PITPNM3	0.076666667	22.66333333	0	0
XM_013981286.2	ALOX15	0.003333333	0.666666667	0	0
XM_013981380.2	BTD	0.02	3.276666667	0	0
XM_013981389.2	GALNT15	0.183333333	20.47666667	0	0
XM_013981427.2	SLC4A7	0.013333333	4.803333333	0	0.02
XM_013981428.2	SLC4A7	0.026666667	9.25	0	0.066666667
XM_013981475.2	WDR48	0.556666667	89.68666667	0	0
XM_013981480.2	GORASP1	0.19	25.11	0	0
XM_013981484.2	MOBP	0.006666667	0.666666667	0	0
XM_013981501.2	SEC22C	0.04	10.79333333	0	0
XM_013981538.2	LOC100738134	0.023333333	4.826666667	0	0
XM_013981539.2	LOC100738134	0.02	4.783333333	0	0
XM_013981569.2	CCR5	0.046666667	6.6	0	0
XM_013981587.2	TDGF1	0.006666667	0.333333333	0	0
XM_013981588.2	FAM240A	0.146666667	4	0	0
XM_013981691.2	PPYR1	0.003333333	0.333333333	0	0
XM_013981709.2	TRAIP	0.013333333	1.773333333	0	0.016666667
XM_013981756.2	PARP3	0.036666667	3.32	0	0
XM_013981761.2	GLYCTK	0.003333333	0.233333333	0	0
XM_013981825.2	FAM3D	0.003333333	0.446666667	0	0
XM_013981833.2	JAKMIP3	0.026666667	8.866666667	0	0
XM_013981852.2	JAKMIP3	0.023333333	8.226666667	0	0.023333333
XM_013981874.2	FAM19A4	0.003333333	0.666666667	0	0
XM_013981901.2	CHL1	0.016666667	5.02	0	0
XM_013981906.2	IL5RA	0.013333333	2.666666667	0	0
XM_013981921.1	CIDEC	0.033333333	1.84	0	0
XM_013981922.2	CIDEC	0.063333333	3.046666667	0	0
XM_013982003.2	GRIP2	0.11	25.21666667	0	0
XM_013982020.2	PODXL2	0.09	7.343333333	0	0
XM_013982052.2	A4GNT	0.003333333	0.666666667	0	0
XM_013982071.2	PTPN18	0.033333333	3.236666667	0	0
XM_013982075.2	ZBTB38	0.01	4.046666667	0	0
XM_013982094.2	PXYLP1	0.076666667	9.78	0	0
XM_013982169.2	SMC4	0.013333333	5.066666667	0	0
XM_013982266.2	C15H2orf66	0.006666667	0.333333333	0	0
XM_013982302.2	CPN2	0.17	14.33333333	0	0
XM_013982345.2	PCYT1A	1.176666667	246.9566667	0	0.14
XM_013982364.2	ZNF148	0.123333333	16.57666667	0	0
XM_013982428.2	GRAMD1C	0.006666667	1	0	0
XM_013982431.2	ZDHHC23	0.033333333	9.266666667	0	0
XM_013982434.2	ZDHHC23	0.026666667	8.41	0	0
XM_013982435.2	ZDHHC23	0.01	0.873333333	0	0
XM_013982441.2	BOC	0.023333333	7.036666667	0	0
XM_013982469.2	HHLA2	0.003333333	1.176666667	0	0
XM_013982472.2	HHLA2	0.003333333	0.936666667	0	0
XM_013982518.2	ROBO1	0.046666667	16.08333333	0	0
XM_013982532.2	LOC100152428	0.013333333	5.17	0	0
XM_013982566.2	SYNJ1	0.026666667	8.553333333	0	0
XM_013982569.2	SYNJ1	0.043333333	13.48666667	0	0
XM_013982609.2	TMPRSS3	0.003333333	0.666666667	0	0
XM_013982714.2	NEK1	0.066666667	14.65333333	0	0
XM_013982730.2	LOC100154415	0.006666667	0.81	0	0
XM_013982781.2	TCTN2	0.053333333	6.023333333	0	0
XM_013982800.2	CCDC62	0.023333333	3.663333333	0	0
XM_013982801.2	CCDC62	0.213333333	30.87333333	0	0
XM_013982912.2	TBX5	0.01	1	0	0
XM_013982969.2	UBE3B	0.043333333	10.60333333	0	0

XM_013982985.2	SGSM1	0.006666667	1.976666667	0	0
XM_013982998.2	CRYBB1	0.01	0.333333333	0	0
XM_013983026.2	LIF	0.06	14.29	0	0
XM_013983047.2	SEC14L2	0.013333333	1.87	0	0
XM_013983072.2	INPP5J	0.036666667	6.1	0	0
XM_013983137.2	CABIN1	0.12	36.53666667	0	0
XM_013983209.2	TANGO2	0.283333333	16.49333333	0	0
XM_013983234.2	TAF5L	0.06	7.626666667	0	0
XM_013983259.2	FXYD4	0.19	5.333333333	0	0
XM_013983261.2	FXYD4	0.05	1.333333333	0	0
XM_013983302.2	CDH23	0.026666667	7.813333333	0	0
XM_013983303.2	CDH23	0.09	26.35333333	0	0
XM_013983318.2	ASCC1	0.183333333	13.32	0	0
XM_013983322.2	CAMK2G	0.24	19.12333333	0	0
XM_013983327.1	LOC106506035	0.033333333	1.346666667	0	0
XM_013983341.2	CFAP70	0.023333333	4.796666667	0	0
XM_013983348.2	ZSWIM8	0.01	2.47	0	0.44
XM_013983354.2	LOC102160869	0.02	1.1	0	0
XM_013983386.2	RGR	0.143333333	8.333333333	0	0
XM_013983465.2	8-Mar	0.01	2.373333333	0	0
XM_013983466.2	8-Mar	0.006666667	1.92	0	0
XM_013983489.2	TMEM72	0.03	8.666666667	0	0
XM_013983494.2	ZWINT	0.006666667	1.05	0	0
XM_013983528.2	GALNTL5	0.006666667	4.973333333	0	0
XM_013983534.2	GALNTL5	0.006666667	4.853333333	0	0
XM_013983547.2	PCGF5	0.353333333	102.61	0	0
XM_013983592.2	OPALIN	0.01	1.333333333	0	0
XM_013983597.2	CYP2C32	0.166666667	13.35666667	0	0
XM_013983630.2	TCTN3	0.01	0.646666667	0	0
XM_013983651.2	DENND2A	0.076666667	19.88666667	0	0
XM_013983662.2	CWF19L1	0.006666667	0.48	0	0
XM_013983675.2	BTRC	0.016666667	3.353333333	0	0
XM_013983708.2	CFAP43	0.003333333	1.24	0	0.333333333
XM_013983743.2	DCLRE1A	0.113333333	20.61666667	0	0
XM_013983744.2	DCLRE1A	0.19	36.01333333	0	0
XM_013983756.2	ABLIM1	0.593333333	145.01	0	0
XM_013983817.2	ATE1	0.06	13.97333333	0	0
XM_013983858.1	UROS	0.126666667	8.33	0	0
XM_013983862.1	UROS	0.1	6.086666667	0	0
XM_013983889.2	PTPRE	0.013333333	2.676666667	0	0
XM_013983919.2	LYPD6	0.01	1.333333333	0	0
XM_013983952.2	ACMSD	0.073333333	6	0	0
XM_013984028.2	AFF2	0.023333333	8.206666667	0	0.003333333
XM_013984038.2	PRIMPOL	0.07	10.94	0	0
XM_013984041.2	PRIMPOL	0.02	3.206666667	0	0
XM_013984042.2	AFF2	0.003333333	0.803333333	0	0.003333333
XM_013984071.2	NSD3	0.023333333	10.63333333	0	0
XM_013984077.2	DDHD2	0.033333333	6.596666667	0	0
XM_013984155.2	PKP4	0.09	17.39666667	0	0.006666667
XM_013984156.2	PKP4	0.1	19.55	0	0
XM_013984165.2	PKP4	0.026666667	4.82	0	0
XM_013984290.2	MTX2	0.013333333	0.633333333	0	0
XM_013984321.2	LOC106506286	0.016666667	1.726666667	0	0
XM_013984378.2	NIF3L1	0.12	11.08666667	0	0
XM_013984415.2	GPR1	0.03	4	0	0
XM_013984418.2	ZDBF2	0.006666667	3.91	0	0
XM_013984432.2	IDH1	0.043333333	4.506666667	0	0
XM_013984455.2	RPE	0.01	1.83	0	0
XM_013984563.2	HTR2B	0.003333333	0.333333333	0	0
XM_013984576.2	EIF4E2	0.13	4.53	0	0

XM_013984607.2	LOC100525573	0.006666667	0.333333333	0	0
XM_013984621.2	IQCA1	0.003333333	0.333333333	0	0.333333333
XM_013984708.2	CDH12	0.003333333	0.333333333	0	0
XM_013984803.2	SMIM15	0.026666667	2.276666667	0	0
XM_013984804.2	SMIM15	0.053333333	4.53	0	0
XM_013984842.2	CCDC125	0.02	1.85	0	0
XM_013984877.2	GABRB2	0.01	1.06	0	0.063333333
XM_013984949.2	SLC6A18	0.053333333	4.51	0	0
XM_013984956.2	TRMT9B	0.003333333	0.666666667	0	0
XM_013985079.2	TASP1	0.006666667	0.826666667	0	0
XM_013985099.2	BFSP1	0.006666667	0.666666667	0	0
XM_013985267.2	CDK5RAP1	0.133333333	11.99666667	0	0
XM_013985281.2	ASIP	0.003333333	0.333333333	0	0
XM_013985303.2	GGT7	0.03	3.863333333	0	0
XM_013985304.2	GGT7	0.106666667	12.24	0	0
XM_013985327.2	RBM39	0.696666667	107.5733333	0	0
XM_013985338.2	EPB41L1	0.036666667	9.66	0	0
XM_013985367.2	ADIG	0.023333333	0.503333333	0	0
XM_013985427.2	WFDC3	0.03	0.78	0	0
XM_013985433.2	OCSTAMP	0.006666667	0.666666667	0	0
XM_013985494.2	PPP4R1L	0.006666667	2.716666667	0	0
XM_013985496.2	PPP4R1L	0.016666667	4.91	0	0
XM_013985605.2	WDR91	0.05	5.583333333	0	0
XM_013985634.2	POT1	0.126666667	31.86	0	0
XM_013985638.2	TSPAN12	0.023333333	1.15	0	0
XM_013985639.2	AASS	0.156666667	37.72333333	0	0
XM_013985661.2	CADPS2	0.023333333	4.603333333	0	0
XM_013985672.2	CADPS2	0.013333333	2.526666667	0	0
XM_013985673.2	CADPS2	0.026666667	6.19	0	0
XM_013985871.2	PNPLA4	0.046666667	2.06	0	0
XM_013985875.2	SHROOM2	0.02	7.02	0	0.01
XM_013985918.2	GPM6B	0.033333333	2.926666667	0	0
XM_013985920.2	PIGA	0.076666667	12.81666667	0	0
XM_013985923.2	PIGA	0.01	1.403333333	0	0
XM_013985955.2	RS1	0.003333333	1	0	1
XM_013985960.2	PHKA2	0.09	35.13	0	0
XM_013985962.2	PHKA2	0.036666667	13.61333333	0	0
XM_013985969.2	ADGRG2	0.04	7.666666667	0	0
XM_013985975.2	PPEF1	0.023333333	3.473333333	0	0
XM_013986002.2	PHEX	0.013333333	4.076666667	0	0
XM_013986051.2	SYTL5	0.006666667	2.78	0	0
XM_013986056.2	BCOR	0.123333333	33.52	0	0
XM_013986068.2	GPR34	0.02	2.096666667	0	0
XM_013986071.2	MAOB	0.03	3.256666667	0	0
XM_013986138.2	MAGIX	0.043333333	4.856666667	0	0
XM_013986197.2	RRAGB	0.016666667	1.236666667	0	0
XM_013986221.2	VSIG4	0.063333333	6.17	0	0
XM_013986233.2	GJB1	0.016666667	1.303333333	0	0
XM_013986240.2	LOC100621166	0.006666667	0.556666667	0	0.11
XM_013986260.2	GPR174	0.003333333	1	0	0
XM_013986299.2	DRP2	0.01	2.623333333	0	0
XM_013986338.2	DCX	0.01	3.716666667	0	0
XM_013986339.2	DCX	0.003333333	1.736666667	0	0
XM_013986373.2	6-Sep	0.99	110.4933333	0	0.003333333
XM_013986403.2	STAG2	0.006666667	1.876666667	0	0
XM_013986433.2	MBNL3	0.01	3.653333333	0	0
XM_013986494.1	ZNF185	0.036666667	6.566666667	0	0
XM_013986596.2	RABEPK	0.183333333	8.503333333	0	0
XM_013987048.2	SETX	0.02	8.483333333	0	0
XM_013987076.2	SARDH	0.036666667	6.126666667	0	0

XM_013987084.2	VAV2	0.02	4.653333333	0	0
XM_013987096.2	ADAMTSL2	0.023333333	3.92	0	0
XM_013987238.2	C2H11orf86	0.006666667	0.433333333	0	0
XM_013987241.2	RCE1	0.01	1.743333333	0	0
XM_013987275.2	KCNA4	0.003333333	1	0	0.666666667
XM_013987333.2	FCER2	0.006666667	1	0	0
XM_013987368.2	CCDC159	0.016666667	7.996666667	0	0
XM_013987408.2	TYK2	0.173333333	39.79333333	0	0
XM_013987430.2	LOC100511183	0.003333333	0.333333333	0	0
XM_013987439.2	AP1M2	0.026666667	2	0	0
XM_013987479.2	JAK3	0.066666667	13.19	0	0
XM_013987480.2	JAK3	0.123333333	24.81	0	0.076666667
XM_013987485.2	B3GNT3	0.016666667	1.826666667	0	0
XM_013987522.2	HNRNPH1	0.056666667	6.983333333	0	0
XM_013987524.2	HNRNPH1	0.003333333	0.366666667	0	0.03
XM_013987546.2	ZNF354A	0.28	33.84666667	0	0
XM_013987588.2	PRR16	0.01	1.103333333	0	0
XM_013987615.2	ARHGAP26	0.053333333	6.073333333	0	0
XM_013987654.2	PGGHG	0.013333333	1.636666667	0	0
XM_013987690.2	TMEM184A	0.01	1.823333333	0	0
XM_013987691.2	TMEM184A	0.013333333	2.56	0	0
XM_013987707.2	ZNF646	0.246666667	62.20666667	0	0
XM_013987728.2	RHBDF1	0.016666667	2.493333333	0	0
XM_013987739.2	FAM234A	0.063333333	6.66	0	0
XM_013987761.2	MAPK8IP3	0.036666667	8.686666667	0	0
XM_013987768.2	MAPK8IP3	0.063333333	14.99666667	0	0
XM_013987785.2	UNKL	0.236666667	44.52	0	0
XM_013987790.2	UNKL	0.036666667	6.583333333	0	0
XM_013987834.2	BRICD5	0.033333333	1.513333333	0	0
XM_013987837.2	ABCA3	0.013333333	3.953333333	0	0
XM_013987845.2	TEDC2	0.046666667	3.446666667	0	0
XM_013987881.2	ELMOD3	0.04	4.143333333	0	0
XM_013987936.2	ROCK2	0.18	39.58333333	0	0
XM_013987986.2	C4H8orf33	0.126666667	10.66	0	0
XM_013988023.2	SHARPIN	0.023333333	1.553333333	0	0
XM_013988182.2	TTC38	0.04	3.896666667	0	0
XM_013988208.2	KNG1	0.393333333	59.63666667	0	0
XM_013988219.2	BTBD11	0.04	11.68	0	0
XM_013988255.2	TROAP	0.006666667	0.66	0	0
XM_013988274.2	SLC4A8	0.02	7	0	0
XM_013988285.2	VNN3	0.003333333	0.333333333	0	0
XM_013988336.2	FAR2	0.003333333	0.686666667	0	0
XM_013988351.2	PIK3C2G	0.036666667	7.273333333	0	0
XM_013988376.2	LOC106507518	0.003333333	0.333333333	0	0
XM_013988388.2	TMEM52B	0.003333333	0.333333333	0	0
XM_013988418.2	LOC102165642	0.013333333	0.333333333	0	0
XM_013988425.2	LOC100627089	0.003333333	0.333333333	0	0
XM_013988430.2	C5H12orf4	0.126666667	40.95	0	0
XM_013988432.2	AKAP3	0.003333333	0.703333333	0	0
XM_013988435.2	KIAA0408	0.006666667	4.286666667	0	0
XM_013988445.2	TMEM106C	0.293333333	17.61666667	0	0
XM_013988486.2	ADAD2	0.003333333	0.333333333	0	0
XM_013988493.2	SYCE1L	0.006666667	0.336666667	0	0
XM_013988529.2	CCDC102A	0.01	1.076666667	0	0
XM_013988531.2	ADGRG5	0.053333333	9.423333333	0	0
XM_013988587.2	COQ8B	0.07	6.64	0	0
XM_013988596.2	CNFN	0.01	1	0	0
XM_013988597.2	ZNF526	0.03	3.473333333	0	0
XM_013988633.2	LYPD4	0.013333333	0.966666667	0	0
XM_013988641.2	CD177	0.003333333	0.333333333	0	0

XM_013988683.2	LOC100627471	0.003333333	0.226666667	0	0
XM_013988698.2	LOC100517285	0.003333333	0.333333333	0	0
XM_013988725.2	PLCH2	0.003333333	0.333333333	0	0
XM_013988730.2	LOC100624218	0.006666667	0.666666667	0	0
XM_013988745.2	TNFRSF25	0.053333333	3.49	0	0
XM_013988775.2	WNT4	0.183333333	28.11333333	0	0
XM_013988793.2	KDF1	0.006666667	0.68	0	0
XM_013988794.2	KDF1	0.026666667	2	0	0
XM_013988797.2	CD164L2	0.013333333	0.333333333	0	0
XM_013988804.2	ZSCAN20	0.256666667	128.9533333	0	0.07
XM_013988805.2	ZSCAN20	0.04	18.31666667	0	0
XM_013988813.2	MPPE1	0.016666667	1.373333333	0	0
XM_013988855.2	ADGRL2	0.04	10.67666667	0	0
XM_013988882.2	DNAJC6	0.013333333	2.733333333	0	0
XM_013988996.2	ZFAND6	0.103333333	8.386666667	0	0
XM_013989003.2	ARNT2	0.08	16.40666667	0	0.28
XM_013989010.2	CFAP161	0.01	1.063333333	0	0
XM_013989015.2	CFAP161	0.016666667	1.33	0	0
XM_013989029.2	PSTPIP1	0.02	1.416666667	0	0
XM_013989075.2	LOC100153886	0.01	0.333333333	0	0
XM_013989103.2	RDH12	0.003333333	0.333333333	0	0
XM_013989146.2	SIPA1L1	0.036666667	9.386666667	0	0
XM_013989167.2	AREL1	0.043333333	8.876666667	0	0
XM_013989285.2	EXD3	0.003333333	0.303333333	0	0
XM_013989367.2	SLC9B2	0.046666667	13.75	0	0
XM_013989440.2	LIN54	0.023333333	3.603333333	0	0
XM_013989444.2	THAP9	0.033333333	13.86333333	0	0
XM_013989499.2	LOC100518622	0.003333333	0.333333333	0	0
XM_013989550.2	ETNK2	0.006666667	0.666666667	0	0
XM_013989575.2	RAPGEF5	0.01	2.723333333	0	0
XM_013989579.2	FAM126A	0.056666667	17.21666667	0	0
XM_013989596.2	ABCB4	0.016666667	3.963333333	0	0
XM_013989662.2	PROX1	0.04	12.81666667	0	0
XM_013989702.2	CD151	0.07	4.996666667	0	0
XM_013989708.2	TUT7	0.02	4.83	0	0.003333333
XM_013989721.2	ZNF438	0.053333333	7.006666667	0	0
XM_013989725.2	CEND1	0.016666667	1.266666667	0	0
XM_013989728.2	CEND1	0.033333333	2.363333333	0	0
XM_013989870.2	CDHR5	0.303333333	34.7	0	0
XM_013989886.2	CDHR5	0.07	6.88	0	0
XM_013989888.2	RNF157	0.09	19.26666667	0	0
XM_013989919.2	MYL4	0.013333333	0.333333333	0	0
XM_013989959.2	RHOT1	0.02	3	0	0
XM_013990102.2	PRR5L	0.023333333	2.233333333	0	0
XM_013990111.2	PRR5L	0.026666667	2.63	0	0
XM_013990160.2	NXPE3	0.146666667	19.76333333	0	0
XM_013990262.2	DMTN	0.013333333	1.49	0	0
XM_013990264.2	LOC102159280	0.01	0.333333333	0	0
XM_013990271.2	CCDC25	0.02	1.84	0	0
XM_013990298.2	GATA4	0.003333333	0.463333333	0	0
XM_013990380.2	KREMEN1	0.193333333	42.17666667	0	0
XM_013990390.2	AP1B1	0.12	21.54333333	0	0.063333333
XM_013990422.2	COL13A1	0.01	1.236666667	0	0
XM_013990426.2	COL13A1	0.033333333	4.42	0	0.003333333
XM_013990499.2	ZNF317	0.53	90.98	0	0
XM_013990500.2	ZNF317	0.09	15.59	0	0
XM_013990589.2	GPR155	0.036666667	6.936666667	0	0
XM_013990602.2	TNPO2	0.196666667	39.36	0	0
XM_013990625.2	LMBRD2	0.063333333	22.79666667	0	0
XM_013990648.2	ERGIC1	0.17	20.26	0	0

XM_013990680.2	LOC100622460	0.006666667	0.666666667	0	0
XM_013990708.2	IFT52	0.04	3.153333333	0	0
XM_013990726.1	FASTK	0.003333333	0.33	0	0
XM_013990727.2	CCDC130	0.016666667	1.32	0	0
XM_013990730.2	CCDC130	0.006666667	0.386666667	0	0
XM_013990735.2	ASIC3	0.016666667	1.666666667	0	0
XM_013990764.1	TMEM139	0.01	0.806666667	0	0
XM_013990781.2	LOC102162486	0.02	4.453333333	0	0
XM_013990796.2	MKLN1	0.01	6.836666667	0	0
XM_013990801.2	KCP	0.006666667	1.57	0	0
XM_013990844.2	BMPER	0.123333333	17.09333333	0	0
XM_013990846.2	BMPER	0.023333333	2.066666667	0	0
XM_013990852.2	TBX20	0.003333333	1	0	0
XM_013990862.2	CDK16	0.08	9.193333333	0	0.003333333
XM_013990892.2	PORCN	0.03	2.273333333	0	0
XM_013990907.2	ARHGEF9	0.15	34.42	0	0
XM_013990908.2	ARHGEF9	0.03	6.906666667	0	0
XM_013990922.2	CITED1	0.03	1.566666667	0	0
XM_013990951.2	ARMCX2	0.15	19.70666667	0	0
XM_013990952.2	ARMCX2	0.116666667	13.74666667	0	0.023333333
XM_013990953.2	ARMCX2	0.06	7.156666667	0	0
XM_013990954.2	ARMCX2	0.056666667	6.24	0	0
XM_013990957.2	ZMAT1	0.01	1.19	0	0
XM_013990980.2	TCEAL1	1.18	51.60666667	0	0
XM_013990993.2	NLRP12L	0.09	16.98333333	0	0
XM_013991020.2	MAP7D3	0.006666667	1.936666667	0	0
XM_013991030.2	ARHGEF6	0.143333333	49.99666667	0	0
XM_013991314.2	SUGP2	0.066666667	19.50666667	0	0
XM_013991361.2	SUSD3	0.023333333	2.333333333	0	0
XM_013991393.2	ARMCX1	0.023333333	2.136666667	0	0
XM_013991400.2	ARMCX1	0.013333333	1.313333333	0	0
XM_013991460.2	ARL13B	0.026666667	2.19	0	0
XM_013991669.2	TEX30	0.043333333	1.963333333	0	0
XM_013991801.2	MGAT1	0.296666667	38.58666667	0	0
XM_013992416.2	DLL1	0.036666667	7.373333333	0	0
XM_013992509.2	SHPRH	0.03	11.32666667	0	0
XM_013992530.2	TNFAIP3	0.026666667	9.24	0	0
XM_013992580.2	CENPW	0.013333333	0.146666667	0	0
XM_013992660.2	CEP162	0.026666667	6.74	0	0
XM_013992706.2	FUT9	0.003333333	2.666666667	0	0
XM_013992716.2	FBXL4	0.103333333	10.34666667	0	0
XM_013992792.2	SNAP91	0.016666667	3.203333333	0	0
XM_013992810.2	MYO6	0.01	2.203333333	0	0
XM_013992844.2	SMAD7	0.06	11.11666667	0	0
XM_013992980.2	MYO5A	0.016666667	9.383333333	0	0
XM_013993003.1	TRPM7	0.003333333	1.576666667	0	0.336666667
XM_013993029.2	SHC4	0.02	5.096666667	0	0
XM_013993032.2	CEP152	0.013333333	2.966666667	0	0
XM_013993070.2	WDR76	0.003333333	0.68	0	0
XM_013993200.2	SEC31B	0.013333333	3.496666667	0	0
XM_013993205.2	SEC31B	0.07	16.65666667	0	0
XM_013993223.2	TTC23	0.113333333	10.02	0	0
XM_013993284.2	DOK6	0.006666667	0.953333333	0	0
XM_013993286.2	CDH19	0.033333333	4.996666667	0	0
XM_013993317.2	GJA5	0.026666667	4.353333333	0	0
XM_013993332.2	CORO2B	0.003333333	0.873333333	0	0.25
XM_013993543.2	BNC2	0.003333333	1.71	0	0
XM_013993585.2	RIC1	0.03	8.726666667	0	0
XM_013993657.2	RNF38	0.01	1.853333333	0	0
XM_013993662.2	RNF38	0.093333333	19.78333333	0	0

XM_013993681.2	TSTD2	0.543333333	109.05	0	0
XM_013993682.2	TSTD2	0.513333333	102.67	0	0
XM_013993683.2	TSTD2	0.183333333	35.68	0	0
XM_013993765.2	RNF183	0.02	3.883333333	0	0.333333333
XM_013993814.2	GGTA1P	0.103333333	13.61333333	0	0
XM_013993822.2	LOC106504191	0.006666667	0.333333333	0	0
XM_013993826.2	LOC100521885	0.016666667	0.666666667	0	0
XM_013993829.2	LOC100524970	0.01	0.333333333	0	0
XM_013993856.2	NEK6	0.02	2.413333333	0	0
XM_013993926.2	FUBP3	0.063333333	8.366666667	0	0
XM_013993931.2	FUBP3	0.033333333	4.436666667	0	0
XM_013994028.2	TMEM134	0.073333333	2.973333333	0	0
XM_013994039.2	TRABD	0.143333333	15.82333333	0	0
XM_013994043.2	ANKRD13D	0.046666667	4.3	0	0
XM_013994045.2	ANKRD13D	0.023333333	2.063333333	0	0
XM_013994053.2	PELI3	0.076666667	7.053333333	0	0
XM_013994069.2	YIF1A	0.696666667	27.03333333	0	0
XM_013994120.2	ZFPL1	0.296666667	16.94333333	0	0
XM_013994153.2	KNDC1	0.023333333	7	0	0
XM_013994155.2	KCNK4	0.01	0.666666667	0	0
XM_013994192.2	SLC22A8	0.08	7.423333333	0	0
XM_013994193.2	SLC22A8	0.266666667	25.76	0	0
XM_013994194.2	SLC22A8	0.09	7.186666667	0	0
XM_013994298.2	DTX4	0.083333333	22.57333333	0	0
XM_013994302.2	LOC100518109	0.076666667	6.7	0	0
XM_013994303.2	LOC100517803	0.05	3.196666667	0	0
XM_013994310.2	ZDHHC5	1.426666667	282.6233333	0	0
XM_013994314.2	CLP1	0.51	40.19	0	0
XM_013994338.2	C2H11orf49	0.046666667	2.646666667	0	0
XM_013994357.2	DDB2	0.093333333	14.60666667	0	0
XM_013994426.2	CD44	0.01	2.113333333	0	0
XM_013994434.2	EHF	0.026666667	6.663333333	0	0
XM_013994456.2	WT1	0.033333333	4.616666667	0	0
XM_013994496.2	MRGPRX2	0.02	1.333333333	0	0
XM_013994508.2	SAAL1	0.093333333	17.35666667	0	0
XM_013994643.2	IRX6	0.003333333	0.333333333	0	0
XM_013994646.2	LOC100513462	0.006666667	0.333333333	0	0
XM_013994676.2	C2H19orf57	0.006666667	0.89	0	0
XM_013994685.2	YIPF2	0.076666667	6.79	0	0
XM_013994706.2	EVI5L	0.006666667	1.176666667	0	0
XM_013994800.2	PRTN3	0.013333333	2	0	0
XM_013994808.2	ABCA7	0.023333333	6.476666667	0	0
XM_013994840.2	PLK5	0.01	1	0	0
XM_013994862.2	MAPK9	0.116666667	23.49666667	0	0
XM_013994901.2	FAM169A	0.01	2.463333333	0	0
XM_013994957.2	IGSF23	0.036666667	1.59	0	0
XM_013994970.2	CCNH	0.15	21.21666667	0	0.003333333
XM_013994983.2	FAM172A	0.076666667	23.55	0	0
XM_013995028.2	CAMK4	0.006666667	2	0	0
XM_013995037.2	TRIM36	0.01	1.636666667	0	0
XM_013995040.2	COMMD10	0.01	1.17	0	0.096666667
XM_013995046.2	LVRN	0.02	3.523333333	0	0
XM_013995048.2	ARL14EPL	0.016666667	0.683333333	0	0
XM_013995104.2	LOC100515383	0.006666667	0.333333333	0	0
XM_013995150.2	LOC100520183	0.02	0.333333333	0	0
XM_013995235.2	TMCO6	0.113333333	9.61	0	0
XM_013995279.2	LOC100625598	0.006666667	0.333333333	0	0
XM_013995290.2	RNF14	1.563333333	109.5666667	0	0
XM_013995378.2	ZKSCAN5	0.58	99.98	0	0
XM_013995382.2	ZNF655	0.023333333	4.026666667	0	0.243333333

XM_013995388.2	ZNF655	0.033333333	5.556666667	0	0.163333333
XM_013995391.2	ZNF789	0.036666667	7.033333333	0	0
XM_013995395.2	ZNF789	0.02	3.686666667	0	0
XM_013995411.2	C3H7orf43	0.04	4.346666667	0	0
XM_013995436.2	ZCWPW1	0.013333333	1.31	0	0
XM_013995514.2	DTX2	0.013333333	5.536666667	0	0
XM_013995522.2	CLDN4	0.01	0.666666667	0	0
XM_013995535.2	BCL7B	0.066666667	4.603333333	0	0
XM_013995541.2	MLXIPL	0.05	6.846666667	0	0
XM_013995547.2	LOC106509660	0.013333333	1	0	0
XM_013995562.1	LOC100620992	0.05	7.306666667	0	0
XM_013995650.2	TVP23A	0.006666667	0.753333333	0	0
XM_013995664.2	NAGPA	0.016666667	1.233333333	0	0
XM_013995665.2	NAGPA	0.013333333	0.836666667	0	0
XM_013995668.2	C3H16orf89	0.043333333	2.166666667	0	0
XM_013995671.2	UBN1	0.303333333	80.38	0	0
XM_013995672.2	HMOX2	0.12	8.78	0	0
XM_013995694.2	HTR1D	0.013333333	1.73	0	0
XM_013995702.2	HTR1D	0.09	11.25333333	0	0
XM_013995741.2	BICDL2	0.006666667	0.666666667	0	0
XM_013995792.2	BARX1	0.023333333	1.333333333	0	0
XM_013995893.2	LOC100623541	0.106666667	11.66666667	0	0
XM_013995896.2	SLC5A7	0.006666667	1.666666667	0	0
XM_013995935.2	MAP4K4	0.043333333	14.67666667	0	0
XM_013996094.2	PLEK	0.006666667	1.016666667	0	0
XM_013996210.2	PRKD3	0.13	19.85333333	0	0
XM_013996239.2	SLC44A5	0.003333333	0.55	0	0
XM_013996253.2	SLC5A6	0.016666667	2.19	0	0
XM_013996308.2	LDAH	0.086666667	11.81666667	0	0
XM_013996319.2	PUM2	0.133333333	37.85666667	0	0
XM_013996385.2	RECQL4	0.013333333	2.163333333	0	0
XM_013996386.2	RECQL4	0.033333333	5.666666667	0	0
XM_013996393.2	BSND	0.03	3.333333333	0	0
XM_013996523.2	ENPP2	0.143333333	21.95666667	0	0
XM_013996569.2	GRHL2	0.01	2	0	0
XM_013996640.2	SLC7A13	0.01	0.666666667	0	0
XM_013996660.2	ZC2HC1A	0.013333333	2.19	0	0
XM_013996695.2	CSPP1	0.133333333	24.97666667	0	0
XM_013996702.2	CSPP1	0.01	1.743333333	0	0
XM_013996742.2	LOC106510078	0.016666667	4.153333333	0	0.12
XM_013996764.2	EFCAB1	0.026666667	1	0	0
XM_013996772.2	METTL11B	0.02	2.676666667	0	0
XM_013996784.2	SELE	0.01	1.63	0	0
XM_013996798.2	POU2F1	0.02	6.113333333	0	0
XM_013996800.2	TBX19	0.006666667	0.666666667	0	0
XM_013996867.2	CD84	0.006666667	0.756666667	0	0
XM_013996888.2	LOC100517891	0.02	0.666666667	0	0
XM_013996904.2	RCBTB2	0.01	1.73	0	0
XM_013996968.2	PAQR6	0.046666667	4.553333333	0	0
XM_013996984.2	SLC25A44	0.123333333	19.50333333	0	0
XM_013996993.2	SYT11	0.016666667	2.936666667	0	0
XM_013997060.2	UBAP2L	0.083333333	14.55333333	0	0
XM_013997125.2	RORC	0.02	1.816666667	0	0
XM_013997160.2	ANXA9	0.016666667	4.29	0	0
XM_013997185.2	PRUNE1	0.073333333	8.61	0	0
XM_013997190.2	ADAMTSL4	0.12	22.14	0	0
XM_013997193.2	ADAMTSL4	0.033333333	6.346666667	0	0
XM_013997239.2	RCBTB2	0.013333333	1.766666667	0	0
XM_013997245.2	MAB21L3	0.023333333	5.553333333	0	0
XM_013997250.2	MAB21L3	0.003333333	0.396666667	0	0

XM_013997289.2	PSRC1	0.03	2.426666667	0	0
XM_013997298.2	C4H1orf194	0.04	1	0	0
XM_013997304.2	CLCC1	0.05	8.86	0	0
XM_013997355.2	SNX7	0.06	4.583333333	0	0
XM_013997422.2	KYAT3	0.25	22.43333333	0	0
XM_013997424.2	KYAT3	0.19	16.97	0	0
XM_013997425.2	KYAT3	0.19	15.57333333	0	0
XM_013997426.2	KYAT3	0.03	2.406666667	0	0
XM_013997451.2	ODF2L	0.046666667	4.766666667	0	0
XM_013997539.2	MPST	0.146666667	28.61	0	0
XM_013997541.2	TEX33	0.006666667	0.333333333	0	0
XM_013997542.2	NCF4	0.03	4.73	0	0
XM_013997557.2	ISX	0.003333333	0.333333333	0	0
XM_013997572.2	SMUG1	0.023333333	2.823333333	0	0
XM_013997578.2	HOXC4	0.013333333	2.063333333	0	0
XM_013997586.2	ZNF385A	0.253333333	25.63666667	0	0
XM_013997633.2	STAT6	0.286666667	47.37666667	0	0
XM_013997659.2	ARHGEF25	0.16	13.70333333	0	0
XM_013997667.2	GLI1	0.006666667	1.426666667	0	0
XM_013997678.2	SLC16A7	0.01	2.5	0	0
XM_013997707.2	MDM2	0.196666667	32.19	0	0
XM_013997775.2	PPFIBP1	0.07	10.96666667	0	0
XM_013997896.2	ERC1	0.02	7.623333333	0	0
XM_013997932.2	KIF21A	0.02	5.823333333	0	0
XM_013997945.2	CNTN1	0.016666667	4.14	0	0
XM_013998009.2	LOC100515741	0.006666667	0.8	0	0
XM_013998019.2	MYBPC1	9.21	1499.35	0	0
XM_013998022.2	MYBPC1	7.483333333	1207.506667	0	0
XM_013998028.2	ANO4	0.006666667	2.09	0	0
XM_013998029.2	ANO4	0.003333333	0.666666667	0	0
XM_013998033.2	NR1H4	0.023333333	2.373333333	0	0
XM_013998047.2	IKBIP	0.08	13.33666667	0	0
XM_013998082.2	TRIM2	0.006666667	1.676666667	0	0
XM_013998242.2	KCTD19	0.033333333	4.333333333	0	0
XM_013998258.2	FHOD1	0.383333333	62.70333333	0	0
XM_013998283.2	GPT2	0.033333333	6.09	0	0
XM_013998289.2	FFAR1	0.006666667	0.666666667	0	0
XM_013998295.2	ZNF792	0.076666667	11.45333333	0	0
XM_013998304.2	FXYD5	0.043333333	1.736666667	0	0
XM_013998328.2	LIN37	0.4	15.61	0	0
XM_013998346.2	U2AF1L4	0.033333333	2.3	0	0
XM_013998445.2	ZNF529	0.053333333	7.543333333	0	0
XM_013998452.2	ZFP14	0.036666667	7.316666667	0	0
XM_013998456.2	LOC100739425	0.086666667	18.95333333	0	0
XM_013998608.2	BCAT2	0.033333333	3.973333333	0	0
XM_013998683.2	VRK3	0.15	17.32333333	0	0
XM_013998690.2	JOSD2	0.053333333	2.03	0	0
XM_013998748.2	LOC100523969	0.003333333	0.333333333	0	0
XM_013998758.2	MBOAT7	0.02	1.836666667	0	0.076666667
XM_013998893.2	LOC100521386	0.006666667	0.336666667	0	0
XM_013998920.2	LOC100738280	0.026666667	1.846666667	0	0
XM_013998942.2	TNFRSF9	0.003333333	0.333333333	0	0
XM_013999006.2	CPLANE2	0.01	0.556666667	0	0
XM_013999010.2	CPLANE2	0.036666667	3.013333333	0	0
XM_013999054.2	NCMAP	0.036666667	6.643333333	0	0
XM_013999074.2	RCC1	0.06	10.96	0	0
XM_013999096.2	STIM1	0.026666667	5.203333333	0	0
XM_013999144.2	FOLR3	0.226666667	13.47	0	0
XM_013999190.2	FOLR1	0.026666667	2.666666667	0	0
XM_013999288.2	DTNA	0.263333333	42.76333333	0	0

XM_013999356.2	OMA1	0.056666667	6.206666667	0	0
XM_013999360.2	LRP8	0.013333333	3.023333333	0	0
XM_013999369.2	HSPB11	0.006666667	0.483333333	0	0
XM_013999370.2	HSPB11	0.02	0.656666667	0	0
XM_013999390.2	ORC1	0.033333333	3.716666667	0	0
XM_021062223.1	LOC100622524	0.006666667	0.333333333	0	0
XM_021062249.1	TMEM41B	0.103333333	11.05	0	0
XM_021062251.1	LOC100622535	0.006666667	1.666666667	0	0
XM_021062255.1	LOC100622535	0.003333333	1	0	0
XM_021062274.1	LOC100736678	0.003333333	0.33	0	0
XM_021062303.1	PPFIBP2	0.11	11.91333333	0	0
XM_021062328.1	LOC110255361	0.003333333	1.76	0	0
XM_021062329.1	LOC110255361	0.006666667	3.123333333	0	0
XM_021062372.1	LOC110255370	0.01	0.666666667	0	0
XM_021062402.1	C1H14orf39	0.01	1.446666667	0	0
XM_021062421.1	LOC100521672	0.093333333	12.51	0	0
XM_021062422.1	LOC110255390	0.06	23.14666667	0	0.333333333
XM_021062425.1	NUMA1	0.036666667	11.47333333	0	0
XM_021062441.1	PGAP2	0.023333333	2.133333333	0	0
XM_021062446.1	PGAP2	0.053333333	4.323333333	0	0
XM_021062463.1	ANAPC15	0.586666667	39.17	0	0
XM_021062464.1	ANAPC15	0.096666667	3.32	0	0
XM_021062497.1	XRRA1	0.013333333	4.76	0	0
XM_021062502.1	XRRA1	0.01	3.463333333	0	0
XM_021062507.1	RNF169	0.06	25.64	0	0
XM_021062512.1	SNAPC1	0.203333333	27.16666667	0	0
XM_021062517.1	ARRB1	0.223333333	76.49	0	0
XM_021062520.1	GDPD5	0.083333333	12.1	0	0
XM_021062523.1	LOC102161066	0.003333333	0.393333333	0	0.063333333
XM_021062530.1	UVRAG	0.083333333	11.23666667	0	0
XM_021062531.1	UVRAG	0.02	2.996666667	0	0
XM_021062543.1	SYT16	0.013333333	13.33333333	0	0
XM_021062548.1	EMSY	0.066666667	15.36333333	0	0
XM_021062553.1	SYT16	0.006666667	9.333333333	0	2
XM_021062560.1	PAK1	0.006666667	0.666666667	0	0.016666667
XM_021062574.1	PRCP	0.103333333	7.843333333	0	0
XM_021062580.1	ANKRD42	0.123333333	47.31	0	0
XM_021062630.1	DEUP1	0.016666667	2.16	0	0
XM_021062642.1	TAF1D	0.016666667	1.893333333	0	0
XM_021062649.1	TAF1D	0.06	7.493333333	0	0
XM_021062654.1	TAF1D	0.053333333	7.696666667	0	0.07
XM_021062675.1	TAF1D	0.106666667	6.953333333	0	0
XM_021062691.1	MTMR2	0.033333333	4.583333333	0	0
XM_021062695.1	CCDC82	0.136666667	14.38	0	0
XM_021062696.1	CCDC82	0.05	5.74	0	0
XM_021062700.1	TRPC6	0.146666667	29.01666667	0	0
XM_021062701.1	TRPC6	0.026666667	3.703333333	0	0.183333333
XM_021062716.1	PLAA	0.126666667	16.42	0	0
XM_021062724.1	CAAP1	0.006666667	0.67	0	0
XM_021062735.1	ELMOD1	0.12	13.10333333	0	0
XM_021062789.1	BCO2	0.013333333	1.333333333	0	0
XM_021062841.1	USP28	0.096666667	21.02	0	0
XM_021062864.1	USP28	0.013333333	2.32	0	0
XM_021062865.1	USP28	0.263333333	46.35666667	0	0
XM_021062893.1	CEP164	0.006666667	1.633333333	0	0
XM_021062904.1	ELAVL2	0.006666667	1	0	0
XM_021062907.1	IL10RA	0.033333333	4.703333333	0	0
XM_021062915.1	KMT2A	0.02	13.69666667	0	0
XM_021062926.1	PHLDB1	0.013333333	3.606666667	0	0
XM_021062935.1	PHLDB1	0.01	2.703333333	0	0

XM_021062959.1	PDZD3	0.073333333	8.43	0	0
XM_021062979.1	POU2F3	0.03	3.306666667	0	0
XM_021062983.1	ARHGEF12	0.003333333	1.846666667	0	0
XM_021062988.1	TMEM136	0.053333333	7.82	0	0
XM_021063012.1	SORL1	0.036666667	16.37	0	0
XM_021063013.1	SORL1	0.093333333	45.13333333	0	0
XM_021063020.1	UBASH3B	0.016666667	7	0	0
XM_021063022.1	CRTAM	0.006666667	1	0	0
XM_021063023.1	CLMP	0.093333333	16.63	0	0
XM_021063035.1	GRAMD1B	0.036666667	12.72	0	0
XM_021063064.1	NRGN	0.02	1.193333333	0	0
XM_021063067.1	HEPACAM	0.016666667	2.573333333	0	0
XM_021063079.1	CCDC15	0.003333333	0.62	0	0
XM_021063093.1	PKNOX2	0.043333333	6.273333333	0	0
XM_021063126.1	KCNJ1	0.006666667	1.173333333	0	0
XM_021063127.1	KCNJ1	0.103333333	11.67333333	0	0
XM_021063129.1	KCNJ1	0.056666667	6.486666667	0	0
XM_021063138.1	LOC100516455	0.003333333	0.483333333	0	0
XM_021063156.1	TMEM45B	0.006666667	1	0	0
XM_021063164.1	IGSF9B	0.056666667	38.35666667	0	0
XM_021063166.1	IGSF9B	0.01	1.8	0	0
XM_021063172.1	NCAPD3	0.056666667	12.63	0	0
XM_021063182.1	LOC100520582	0.003333333	0.333333333	0	0
XM_021063197.1	ATP2B4	0.396666667	148.7533333	0	0
XM_021063203.1	LAX1	0.006666667	0.936666667	0	0
XM_021063239.1	NFASC	0.003333333	0.8	0	0
XM_021063240.1	NFASC	0.003333333	1.18	0	0
XM_021063242.1	NFASC	0.003333333	1.87	0	0
XM_021063245.1	NFASC	0.003333333	1.246666667	0	0
XM_021063246.1	NFASC	0.003333333	1.563333333	0	0
XM_021063249.1	NFASC	0.006666667	2.666666667	0	0
XM_021063251.1	NFASC	0.003333333	1.536666667	0	0
XM_021063252.1	NFASC	0.003333333	0.79	0	0
XM_021063253.1	NFASC	0.003333333	0.516666667	0	0
XM_021063255.1	NFASC	0.003333333	0.91	0	0
XM_021063258.1	NFASC	0.003333333	1.023333333	0	0
XM_021063261.1	NFASC	0.006666667	1.753333333	0	0
XM_021063266.1	NFASC	0.006666667	2.366666667	0	0
XM_021063274.1	DSTYK	0.026666667	6.59	0	0
XM_021063282.1	CNTLN	0.053333333	12.21666667	0	0
XM_021063291.1	RHEX	0.003333333	0.246666667	0	0
XM_021063294.1	SRGAP2	0.01	3.063333333	0	0
XM_021063295.1	SRGAP2	0.106666667	31.12666667	0	0
XM_021063296.1	SRGAP2	0.033333333	9.796666667	0	0
XM_021063304.1	FAM72A	0.003333333	0.333333333	0	0
XM_021063307.1	IKBKE	0.02	3.333333333	0	0
XM_021063312.1	IKBKE	0.01	1.62	0	0
XM_021063321.1	C9H1orf116	0.083333333	12	0	0
XM_021063323.1	BNC2	0.006666667	3.44	0	0
XM_021063334.1	CR1	0.06	18	0	0.333333333
XM_021063348.1	ADAM22	0.033333333	14.05666667	0	0
XM_021063376.1	PPP1R9A	0.003333333	0.906666667	0	0
XM_021063391.1	C1GALT1	0.026666667	8.9	0	0
XM_021063397.1	COL28A1	0.02	3.333333333	0	0
XM_021063403.1	ETV1	0.016666667	3.806666667	0	0
XM_021063435.1	DNAH11	0.06	34.76	0	0
XM_021063456.1	ABCB4	0.003333333	0.75	0	0
XM_021063460.1	ABCB4	0.006666667	1.326666667	0	0
XM_021063464.1	ABCB4	0.006666667	1.313333333	0	0
XM_021063477.1	SEMA3A	0.006666667	1.693333333	0	0.303333333

XM_021063478.1	SEMA3A	0.013333333	3.13	0	0
XM_021063482.1	SEMA3A	0.006666667	1.026666667	0	0.53
XM_021063486.1	HGF	0.023333333	6	0	0
XM_021063498.1	LHFPL3	0.006666667	2.223333333	0	0
XM_021063530.1	NRCAM	0.016666667	4.616666667	0	0
XM_021063533.1	NRCAM	0.023333333	6.256666667	0	0
XM_021063534.1	NRCAM	0.003333333	1.09	0	0
XM_021063546.1	NRCAM	0.016666667	4.576666667	0	0
XM_021063554.1	NRCAM	0.006666667	1.713333333	0	0.153333333
XM_021063561.1	NRCAM	0.006666667	1.93	0	0
XM_021063569.1	NRCAM	0.01	4.986666667	0	0
XM_021063599.1	ARHGEF5	0.013333333	4.29	0	0
XM_021063602.1	LOC100511828	0.006666667	1.333333333	0	0.333333333
XM_021063608.1	PPFIA4	0.006666667	1.54	0	0
XM_021063611.1	PPFIA4	0.05	14.71	0	0
XM_021063623.1	PPFIA4	0.003333333	0.966666667	0	0
XM_021063624.1	PPFIA4	0.066666667	19.10333333	0	0
XM_021063626.1	LOC100513809	0.043333333	2.55	0	0
XM_021063633.1	PRRC2C	0.076666667	34.78333333	0	0
XM_021063660.1	TNN	0.016666667	3.6	0	0
XM_021063661.1	TNN	0.003333333	0.666666667	0	0
XM_021063664.1	TNN	0.006666667	1.733333333	0	0.666666667
XM_021063672.1	KIAA0040	0.036666667	6.346666667	0	0
XM_021063701.1	SEC16B	0.023333333	4.746666667	0	0
XM_021063704.1	SEC16B	0.02	4.086666667	0	0
XM_021063709.1	SEC16B	0.006666667	1.21	0	0
XM_021063732.1	MPDZ	0.043333333	14.72333333	0	0
XM_021063749.1	FAM163A	0.04	8.353333333	0	0
XM_021063775.1	RGS8	0.003333333	0.893333333	0	0
XM_021063799.1	LAMC2	0.006666667	3.743333333	0	0
XM_021063801.1	LAMC2	0.003333333	1.413333333	0	0
XM_021063808.1	NMNAT2	0.116666667	42.14333333	0	0
XM_021063812.1	RGL1	0.526666667	100.23	0	0
XM_021063817.1	PRG4	0.01	2.65	0	0
XM_021063818.1	PRG4	0.003333333	0.886666667	0	0
XM_021063819.1	PRG4	0.01	2.546666667	0	0
XM_021063823.1	PRG4	0.036666667	8.686666667	0	0
XM_021063826.1	PLA2G4A	0.03	5.366666667	0	0.26
XM_021063827.1	PLA2G4A	0.05	7.526666667	0	0
XM_021063831.1	LOC106507881	0.016666667	0.333333333	0	0
XM_021063835.1	RPS6KC1	0.04	5.813333333	0	0
XM_021063844.1	RPS6KC1	0.043333333	8.47	0	0
XM_021063874.1	RCOR3	0.076666667	14.76666667	0	0
XM_021063880.1	SYT14	0.003333333	1.333333333	0	0
XM_021063903.1	LOC100514786	0.006666667	4.576666667	0	0
XM_021063904.1	LOC100514786	0.006666667	4.726666667	0	0
XM_021063908.1	SPATA48	0.006666667	0.666666667	0	0
XM_021063917.1	IKZF1	0.013333333	3.51	0	0
XM_021063921.1	IKZF1	0.016666667	5.303333333	0	0.046666667
XM_021063922.1	IKZF1	0.036666667	7.89	0	0
XM_021063924.1	IKZF1	0.01	1.84	0	0
XM_021063927.1	DDC	0.63	51.69666667	0	0
XM_021063930.1	DDC	0.043333333	3.22	0	0
XM_021063943.1	LOC100627657	0.003333333	0.333333333	0	0
XM_021063951.1	LOC100524388	0.006666667	0.333333333	0	0
XM_021063980.1	CAMK1D	0.016666667	5.286666667	0	0
XM_021063983.1	CAMK1D	0.023333333	8.82	0	0
XM_021063993.1	PRPF18	0.05	11.02666667	0	0
XM_021064013.1	HNRNPK	0.253333333	32.62	0	0
XM_021064027.1	HNRNPK	0.03	2.456666667	0	0

XM_021064031.1	HNRNPK	0.013333333	1.476666667	0	0
XM_021064062.1	SPATA6L	0.006666667	1.72	0	0
XM_021064068.1	AKR1C4	0.24	11.61333333	0	0
XM_021064071.1	GATA3	0.04	6.32	0	0
XM_021064076.1	AKR1E2	0.023333333	1.676666667	0	0
XM_021064083.1	PSEN2	0.04	4.22	0	0
XM_021064087.1	PSEN2	0.046666667	4.23	0	0
XM_021064103.1	GPATCH2	0.043333333	10.74333333	0	0
XM_021064107.1	ESRRG	0.09	25.14	0	0
XM_021064113.1	ESRRG	0.016666667	5.116666667	0	0
XM_021064120.1	ESRRG	0.003333333	1.093333333	0	0
XM_021064129.1	ESRRG	0.086666667	25.40333333	0	0
XM_021064135.1	MIA3	1.003333333	290.97	0	0
XM_021064147.1	CEP170	0.17	49.31	0	0
XM_021064154.1	CEP170	0.006666667	1.46	0	0
XM_021064164.1	CEP170	0.083333333	23.69333333	0	0
XM_021064181.1	SDCCAG8	0.076666667	9.523333333	0	0
XM_021064191.1	LINGO2	0.003333333	1	0	0
XM_021064200.1	ZNF438	0.03	4.52	0	0
XM_021064209.1	ZNF438	0.05	7.07	0	0
XM_021064210.1	ZNF438	0.213333333	29.51333333	0	0
XM_021064224.1	FRMD4A	0.003333333	0.776666667	0	0
XM_021064238.1	CELF2	0.023333333	7.383333333	0	0
XM_021064248.1	CELF2	0.02	6.016666667	0	0
XM_021064259.1	CELF2	0.023333333	6.413333333	0	0
XM_021064271.1	LOC106507907	0.003333333	0.333333333	0	0
XM_021064280.1	LOC100739603	0.053333333	3.01	0	0
XM_021064357.1	DISP1	0.003333333	0.81	0	0
XM_021064358.1	DISP1	0.003333333	0.94	0	0
XM_021064364.1	SMARCA2	0.273333333	67.32333333	0	0.023333333
XM_021064365.1	WDR64	0.066666667	10.98666667	0	0
XM_021064368.1	WDR64	0.01	1.8	0	0.243333333
XM_021064391.1	ENAH	0.143333333	79.14666667	0	0.01
XM_021064393.1	ENAH	0.013333333	7.263333333	0	0
XM_021064397.1	ENAH	0.92	223.1733333	0	0
XM_021064398.1	ENAH	0.056666667	38.23333333	0	0.053333333
XM_021064403.1	ENAH	0.96	505.69	0	0.176666667
XM_021064419.1	COQ8A	0.653333333	78.19	0	0
XM_021064453.1	PLD5	0.003333333	0.666666667	0	0
XM_021064479.1	KANK1	0.046666667	10.67666667	0	0
XM_021064481.1	ZBTB41	0.05	19.27333333	0	0
XM_021064503.1	INAVA	0.003333333	0.333333333	0	0
XM_021064528.1	ELF3	0.06	4.906666667	0	0
XM_021064529.1	ELF3	0.063333333	4.76	0	0
XM_021064536.1	PTPN7	0.053333333	8.11	0	0
XM_021064541.1	LOC100624894	0.003333333	1.333333333	0	0.666666667
XM_021064543.1	LGR6	0.006666667	1.666666667	0	0.666666667
XM_021064545.1	UBE2T	0.09	3.743333333	0	0
XM_021064566.1	SYT2	0.02	1.323333333	0	0
XM_021064568.1	LOC102162336	0.136666667	8.816666667	0	0
XM_021064570.1	LOC102162336	0.213333333	12.53666667	0	0
XM_021064578.1	CDC14B	0.026666667	6.863333333	0	0
XM_021064581.1	CDC14B	0.01	2.36	0	0
XM_021064582.1	CDC14B	0.03	7.763333333	0	0
XM_021064589.1	CDC14B	0.023333333	6.386666667	0	0
XM_021064613.1	DAPK1	0.05	11.25666667	0	0
XM_021064618.1	DAPK1	0.016666667	4.46	0	0
XM_021064621.1	DAPK1	0.043333333	11.15	0	0
XM_021064645.1	NTRK2	0.003333333	1.506666667	0	0
XM_021064654.1	KIF27	0.013333333	3.116666667	0	0

XM_021064671.1	IL11RA	0.57	42.28666667	0	0
XM_021064679.1	FAM219A	0.743333333	270.4933333	0	0
XM_021064720.1	ARMC4	0.01	2	0	0
XM_021064730.1	MPP7	0.02	3.846666667	0	0
XM_021064751.1	SVIL	0.086666667	31.11666667	0	0
XM_021064773.1	SVIL	0.11	37.20666667	0	0
XM_021064782.1	SVIL	0.246666667	71.49333333	0	0
XM_021064794.1	ARHGAP12	0.056666667	12.49	0	0.026666667
XM_021064804.1	ARHGAP12	0.113333333	23.27666667	0	0
XM_021064838.1	MEIG1	0.006666667	0.333333333	0	0
XM_021064888.1	ACBD5	0.043333333	7.25	0	0
XM_021064891.1	ABI1	0.086666667	12.55333333	0	0
XM_021064895.1	ABI1	0.013333333	2.043333333	0	0
XM_021064898.1	ABI1	0.07	9.77	0	0
XM_021064903.1	ABI1	0.046666667	6.213333333	0	0
XM_021064923.1	MLLT10	0.026666667	6.473333333	0	0
XM_021064930.1	MLLT10	0.236666667	51.18	0	0
XM_021064940.1	MLLT10	0.086666667	17.69333333	0	0
XM_021064950.1	NEBL	0.013333333	4.506666667	0	0
XM_021064951.1	NEBL	0.02	6.653333333	0	0
XM_021064958.1	NEBL	0.016666667	2.51	0	0
XM_021064998.1	PCSK5	0.016666667	5.816666667	0	0
XM_021065007.1	SFMBT2	0.15	45.50666667	0	0
XM_021065040.1	FBH1	0.046666667	12.85	0	0
XM_021065054.1	ASB13	0.053333333	5.9	0	0
XM_021065072.1	LOC100625534	0.026666667	4.803333333	0	0
XM_021065075.1	DIP2C	0.126666667	41.19666667	0	0.006666667
XM_021065094.1	CLDN10	0.206666667	8.793333333	0	0
XM_021065095.1	CLDN10	0.016666667	0.706666667	0	0
XM_021065116.1	KLF12	0.006666667	1.66	0	0
XM_021065119.1	KLF12	0.033333333	9.69	0	0
XM_021065131.1	BIVM	0.48	67.97	0	0
XM_021065142.1	RNASEH2B	0.046666667	12.82333333	0	0.006666667
XM_021065154.1	CRYL1	0.213333333	13.76666667	0	0
XM_021065169.1	MTUS2	0.033333333	9.806666667	0	0
XM_021065178.1	TLE4	0.026666667	4.55	0	0
XM_021065182.1	ZAR1L	0.003333333	0.43	0	0
XM_021065192.1	TLE4	0.006666667	1.28	0	0
XM_021065213.1	DCLK1	0.01	4	0	0
XM_021065224.1	SUPT20H	0.063333333	8.34	0	0
XM_021065227.1	SUPT20H	0.033333333	4.3	0	0
XM_021065230.1	SUPT20H	0.033333333	4.306666667	0	0
XM_021065234.1	SUPT20H	0.136666667	17.45666667	0	0
XM_021065295.1	LOC102163801	0.03	2.896666667	0	0
XM_021065296.1	LOC102163801	0.006666667	0.85	0	0
XM_021065304.1	LOC106505279	0.013333333	0.666666667	0	0
XM_021065308.1	RCBTB1	0.303333333	50.55666667	0	0
XM_021065316.1	CAB39L	0.05	5.66	0	0
XM_021065321.1	LOC106505288	0.006666667	0.333333333	0	0
XM_021065324.1	LOC100525024	0.013333333	8.226666667	0	0
XM_021065333.1	RCBTB2	0.013333333	2.41	0	0
XM_021065357.1	ENOX1	0.003333333	0.81	0	0
XM_021065359.1	ENOX1	0.003333333	0.563333333	0	0
XM_021065378.1	FARP1	0.003333333	0.91	0	0.323333333
XM_021065382.1	FGF14	0.003333333	1	0	0
XM_021065384.1	FAM155A	0.006666667	0.666666667	0	0
XM_021065400.1	LOC100738425	0.046666667	11.82	0	0.336666667
XM_021065402.1	LOC110255297	0.033333333	3.316666667	0	0
XM_021065412.1	CENPJ	0.003333333	0.84	0	0
XM_021065430.1	GJB2	0.18	29.84	0	0.003333333

XM_021065432.1	GJB6	0.003333333	0.333333333	0	0
XM_021065441.1	XPO4	0.033333333	4.29	0	0
XM_021065444.1	SKA3	0.32	68.13666667	0	0
XM_021065454.1	SGCG	0.056666667	3.713333333	0	0
XM_021065456.1	SGCG	0.266666667	18.48	0	0
XM_021065469.1	TNFRSF19	0.006666667	0.62	0	0
XM_021065523.1	UNC13B	0.003333333	2.046666667	0	0
XM_021065528.1	UNC13B	0.026666667	11.21666667	0	0
XM_021065530.1	UNC13B	0.003333333	1.023333333	0	0
XM_021065551.1	LOC102159067	0.013333333	3.333333333	0	0.333333333
XM_021065556.1	RXFP2	0.006666667	1.036666667	0	0
XM_021065573.1	VPS36	0.41	78.66333333	0	0
XM_021065578.1	TRIM13	0.02	1.396666667	0	0
XM_021065592.1	RUBCNL	0.036666667	4.846666667	0	0
XM_021065609.1	KCTD4	0.076666667	7.923333333	0	0
XM_021065647.1	PCDH17	0.056666667	19.24	0	0
XM_021065648.1	DIAPH3	0.023333333	6	0	0
XM_021065655.1	KLHL1	0.006666667	1	0	0
XM_021065662.1	TLN1	0.05	18.05	0	0
XM_021065674.1	TBC1D4	0.05	12.02333333	0	0
XM_021065687.1	LMO7	0.083333333	21.39666667	0	0.08
XM_021065713.1	LMO7	0.146666667	36.87333333	0	0
XM_021065723.1	LMO7	0.013333333	2.55	0	0
XM_021065729.1	ACOD1	0.003333333	0.333333333	0	0
XM_021065733.1	MYCBP2	0.013333333	8.553333333	0	0.01
XM_021065734.1	MYCBP2	0.016666667	12.42	0	0
XM_021065735.1	MYCBP2	0.02	12.68333333	0	0
XM_021065738.1	MYCBP2	0.003333333	2.776666667	0	0.003333333
XM_021065756.1	SCEL	0.006666667	1	0	0
XM_021065761.1	SCEL	0.003333333	0.663333333	0	0
XM_021065767.1	POU4F1	0.003333333	0.333333333	0	0
XM_021065791.1	UGGT2	0.033333333	8.376666667	0	0
XM_021065794.1	OXGR1	0.006666667	0.666666667	0	0
XM_021065830.1	DOCK9	0.003333333	1.416666667	0	0
XM_021065837.1	DOCK9	0.12	46.73666667	0	0
XM_021065846.1	DOCK9	0.056666667	21.24	0	0
XM_021065868.1	GGACT	0.016666667	1.24	0	0
XM_021065869.1	TMEM8B	0.013333333	1.216666667	0	0
XM_021065875.1	GGACT	0.023333333	1.666666667	0	0
XM_021065883.1	TMEM8B	0.01	1.276666667	0	0
XM_021065890.1	TMEM8B	0.006666667	0.663333333	0	0
XM_021065895.1	TEX30	0.143333333	6.086666667	0	0
XM_021065903.1	FAM221B	0.016666667	1.736666667	0	0
XM_021065929.1	ARHGEF7	0.006666667	1	0	0
XM_021065939.1	ATP11A	0.066666667	21.80666667	0	0
XM_021065943.1	ATP11A	0.033333333	10.33666667	0	0
XM_021065946.1	ATP11A	0.006666667	2.52	0	0
XM_021065950.1	LOC100519461	0.01	1	0	0
XM_021065951.1	MCF2L	0.066666667	17.66333333	0	0
XM_021065961.1	MCF2L	0.07	16.08	0	0
XM_021065966.1	MCF2L	0.023333333	5.143333333	0	0
XM_021065971.1	MCF2L	0.02	4.343333333	0	0
XM_021065972.1	MCF2L	0.013333333	3.003333333	0	0
XM_021065973.1	PCID2	0.01	0.81	0	0
XM_021065992.1	C11H13orf46	0.006666667	0.306666667	0	0
XM_021065994.1	UPF3A	0.026666667	3.513333333	0	0.006666667
XM_021065997.1	UPF3A	0.01	0.963333333	0	0
XM_021066001.1	LOC110255871	0.01	0.333333333	0	0
XM_021066009.1	CLTA	0.123333333	5.62	0	0
XM_021066055.1	ADAP2	0.066666667	16.13333333	0	0

XM_021066065.1	SPATA22	0.01	2.666666667	0	0
XM_021066068.1	INPP5K	0.04	5.456666667	0	0
XM_021066072.1	MELK	0.03	3.666666667	0	0
XM_021066086.1	MELK	0.003333333	0.32	0	0
XM_021066090.1	PAX5	0.003333333	1.053333333	0	0
XM_021066095.1	PAX5	0.003333333	0.946666667	0	0
XM_021066111.1	ZNF232	0.03	2.456666667	0	0
XM_021066126.1	TVP23B	0.016666667	3.666666667	0	0
XM_021066129.1	GHDC	0.016666667	1.08	0	0
XM_021066143.1	AP2B1	0.076666667	10.73	0	0
XM_021066148.1	B4GALNT2	0.076666667	5.936666667	0	0
XM_021066150.1	B4GALNT2	0.02	1.116666667	0	0
XM_021066164.1	AMZ2	0.086666667	3.486666667	0	0
XM_021066175.1	MSI2	0.026666667	6.36	0	0
XM_021066194.1	FRMPD1	0.003333333	1	0	0
XM_021066195.1	AURKB	0.106666667	8.003333333	0	0
XM_021066199.1	FRMPD1	0.006666667	1	0	0
XM_021066200.1	LGALS9	0.046666667	1.726666667	0	0
XM_021066203.1	FRMPD1	0.006666667	1	0	0
XM_021066204.1	HNF1B	0.086666667	10.81666667	0	0
XM_021066205.1	HNF1B	0.106666667	12.20333333	0	0
XM_021066206.1	HNF1B	0.113333333	14.27	0	0
XM_021066210.1	PSMC5	0.053333333	3.26	0	0
XM_021066221.1	KCNJ2	0.003333333	1.473333333	0	0.51
XM_021066224.1	KCNJ2	0.01	6.733333333	0	2.413333333
XM_021066230.1	ACACA	0.04	16.87333333	0	0
XM_021066233.1	ACACA	0.043333333	17.34	0	0
XM_021066235.1	ACACA	0.03	10.91666667	0	0
XM_021066238.1	ACACA	0.033333333	12.61333333	0	0
XM_021066252.1	PEMT	0.113333333	3.51	0	0
XM_021066268.1	CFAP52	0.006666667	0.666666667	0	0
XM_021066278.1	LOC110255888	0.003333333	0.666666667	0	0
XM_021066292.1	SPECC1	0.043333333	10.73333333	0	0
XM_021066297.1	SPECC1	0.043333333	6.68	0	0
XM_021066304.1	MEIOC	0.003333333	0.333333333	0	0
XM_021066311.1	LOC110255905	0.013333333	0.333333333	0	0
XM_021066314.1	TSTD2	0.183333333	36.42666667	0	0
XM_021066318.1	LOC102160183	0.003333333	0.333333333	0	0
XM_021066338.1	NARF	0.07	8.533333333	0	0
XM_021066349.1	HEXD	0.01	0.816666667	0	0
XM_021066352.1	C12H17orf62	0.116666667	9.496666667	0	0
XM_021066355.1	UTS2R	0.07	8.01	0	0
XM_021066370.1	CCDC57	0.08	17.66666667	0	0
XM_021066413.1	PYCR1	0.03	2.463333333	0	0
XM_021066441.1	GCGR	0.003333333	1	0	0
XM_021066442.1	GCGR	0.01	1.1	0	0
XM_021066445.1	GCGR	0.026666667	2.343333333	0	0
XM_021066447.1	GCGR	0.006666667	1.89	0	0.333333333
XM_021066456.1	ARL16	0.01	1.596666667	0	0
XM_021066460.1	ARL16	0.05	7.293333333	0	0
XM_021066500.1	CARD14	0.02	3	0	0
XM_021066528.1	TMEM235	0.01	0.333333333	0	0
XM_021066547.1	TMC6	0.073333333	27.21	0	0
XM_021066548.1	TMC8	0.056666667	17.38	0	0
XM_021066567.1	MXRA7	0.03	2.39	0	0
XM_021066571.1	ST6GALNAC1	0.003333333	0.333333333	0	0
XM_021066578.1	RHBDF2	0.05	7.826666667	0	0
XM_021066590.1	SPHK1	0.003333333	0.563333333	0	0
XM_021066592.1	SPHK1	0.103333333	7.543333333	0	0
XM_021066598.1	RNF157	1.033333333	94.06333333	0	0

XM_021066603.1	ZACN	0.026666667	1.333333333	0	0
XM_021066606.1	INVS	0.056666667	12.24666667	0	0
XM_021066609.1	UNC13D	0.01	1.853333333	0	0
XM_021066610.1	UNC13D	0.02	4.48	0	0
XM_021066638.1	INVS	0.03	6.543333333	0	0
XM_021066641.1	TMEM94	0.01	1.936666667	0	0
XM_021066648.1	INVS	0.046666667	10.72333333	0	0
XM_021066650.1	TSEN54	0.026666667	1.57	0	0
XM_021066659.1	SLC16A5	0.06	7.22	0	0
XM_021066660.1	INVS	0.053333333	10.38333333	0	0
XM_021066665.1	LOC110255970	0.013333333	1	0	0
XM_021066681.1	BTBD17	0.003333333	0.333333333	0	0
XM_021066685.1	TTYH2	0.07	9.996666667	0	0
XM_021066700.1	C12H17orf80	0.046666667	4.563333333	0	0
XM_021066704.1	SLC39A11	0.066666667	14.55	0	0
XM_021066711.1	KCNJ16	0.25	36.87333333	0	0
XM_021066724.1	RGS9	0.01	1.336666667	0	0
XM_021066725.1	CEP112	0.056666667	8.78	0	0
XM_021066729.1	CEP112	0.016666667	2.443333333	0	0
XM_021066783.1	STRADA	0.043333333	3.93	0	0
XM_021066794.1	MYL4	0.026666667	1	0	0
XM_021066800.1	MAPT	0.063333333	13.54	0	0
XM_021066810.1	MAPT	0.033333333	7.053333333	0	0
XM_021066812.1	MAPT	0.16	36.06666667	0	0
XM_021066813.1	MAPT	0.093333333	18.90333333	0	0
XM_021066821.1	MAPT	0.09	19.21	0	0
XM_021066831.1	LOC110255172	0.053333333	7.59	0	0
XM_021066834.1	ARHGAP27	0.15	28.29333333	0	0
XM_021066839.1	FMNL1	0.026666667	4.356666667	0	0
XM_021066844.1	LOC100524336	0.013333333	1	0	0
XM_021066852.1	KIF18B	0.003333333	0.28	0	0
XM_021066856.1	ADAM11	0.01	2	0	0
XM_021066877.1	UBTF	0.04	8.103333333	0	0
XM_021066903.1	GRIN3A	0.033333333	7.666666667	0	0
XM_021066912.1	CD300LG	0.133333333	19.19	0	0
XM_021066918.1	ETV4	0.096666667	9.4	0	0
XM_021066920.1	ETV4	0.193333333	17.12	0	0
XM_021066927.1	BRCA1	0.04	13.66666667	0	0
XM_021066933.1	BRCA1	0.03	7.093333333	0	0
XM_021066944.1	NBR1	0.15	29.99666667	0	0
XM_021066945.1	NBR1	0.166666667	33.62666667	0	0
XM_021066950.1	NBR1	0.18	34.92666667	0	0.11
XM_021066951.1	NBR1	0.106666667	22.67666667	0	0
XM_021066953.1	NBR1	0.006666667	1.103333333	0	0
XM_021066963.1	NBR1	0.016666667	3.346666667	0	0
XM_021066999.1	HAP1	0.016666667	3.66	0	0
XM_021067002.1	LOC110255272	0.003333333	0.333333333	0	0
XM_021067012.1	LOC100154081	0.096666667	7.333333333	0	0
XM_021067037.1	GRB7	0.01	1.6	0	0
XM_021067040.1	GRB7	0.11	9.94	0	0
XM_021067041.1	GRB7	0.006666667	0.793333333	0	0
XM_021067059.1	FBXO47	0.003333333	0.666666667	0	0
XM_021067089.1	PRR15L	0.093333333	5.206666667	0	0
XM_021067090.1	PRR15L	0.02	1.793333333	0	0
XM_021067108.1	CBX1	0.013333333	0.583333333	0	0
XM_021067112.1	TTLL6	0.003333333	0.333333333	0	0
XM_021067143.1	DLX4	0.023333333	1.7	0	0
XM_021067184.1	CACNA1G	0.013333333	6.306666667	0	0
XM_021067204.1	SPAG9	0.15	38.51333333	0	0
XM_021067233.1	MBTD1	0.103333333	23.66666667	0	0

XM_021067242.1	CA10	0.006666667	0.613333333	0	0
XM_021067251.1	HLF	0.006666667	1.136666667	0	0.103333333
XM_021067269.1	VEZF1	0.436666667	91.17	0	0
XM_021067289.1	TRIM37	0.083333333	15.79333333	0	0
XM_021067307.1	LOC110256036	0.006666667	0.333333333	0	0
XM_021067321.1	ZNHIT3	0.043333333	6.8	0	0
XM_021067322.1	ZNHIT3	0.11	16.84666667	0	0
XM_021067323.1	GGNBP2	0.03	3.77	0	0
XM_021067337.1	TADA2A	0.056666667	5.666666667	0	0
XM_021067362.1	SYNRG	0.023333333	4.43	0	0
XM_021067374.1	PTPN3	0.053333333	13.06666667	0	0
XM_021067382.1	FBXO34	0.016666667	2.563333333	0	0
XM_021067391.1	LOC110256043	1.146666667	63.22333333	0	0
XM_021067399.1	GAS2L2	0.003333333	0.666666667	0	0.666666667
XM_021067407.1	RASL10B	0.113333333	15.45333333	0	0
XM_021067410.1	SLFN14	0.02	4	0	0.666666667
XM_021067412.1	SLFN11	0.046666667	13.66666667	0	0
XM_021067414.1	RAD51D	0.086666667	9.43	0	0
XM_021067420.1	RFFL	0.043333333	7.126666667	0	0
XM_021067422.1	RFFL	0.043333333	7.31	0	0
XM_021067431.1	RFFL	0.053333333	8.503333333	0	0
XM_021067433.1	RFFL	0.05	7.806666667	0	0
XM_021067435.1	RFFL	0.02	1.196666667	0	0
XM_021067440.1	LOC100627824	0.01	0.863333333	0	0
XM_021067441.1	LOC100627824	0.003333333	0.47	0	0
XM_021067442.1	LOC110256055	0.006666667	0.333333333	0	0
XM_021067446.1	MYO1D	0.01	1.896666667	0	0
XM_021067486.1	PROCA1	0.006666667	0.666666667	0	0
XM_021067503.1	LOC110255229	0.02	2.13	0	0
XM_021067506.1	LOC100515508	0.083333333	7.496666667	0	0
XM_021067507.1	LOC100515508	0.01	1.036666667	0	0
XM_021067519.1	NSRP1	0.02	2.1	0	0
XM_021067542.1	RPH3AL	0.046666667	4.226666667	0	0
XM_021067546.1	RPH3AL	0.01	0.866666667	0	0
XM_021067549.1	RPH3AL	0.096666667	7.313333333	0	0
XM_021067550.1	RPH3AL	0.016666667	1.526666667	0	0
XM_021067576.1	SMYD4	0.29	59.58	0	0
XM_021067578.1	SMYD4	0.066666667	7.87	0	0
XM_021067584.1	TLCD2	0.016666667	5.976666667	0	0
XM_021067595.1	SGSM2	0.05	9.37	0	0
XM_021067638.1	RAP1GAP2	0.03	9.39	0	0
XM_021067643.1	RAP1GAP2	0.013333333	3.59	0	0
XM_021067649.1	RAP1GAP2	0.003333333	1.23	0	0
XM_021067650.1	RAP1GAP2	0.006666667	2.233333333	0	0
XM_021067660.1	WDR31	0.043333333	7.146666667	0	0
XM_021067702.1	MYBBP1A	0.033333333	7.256666667	0	0
XM_021067703.1	SPNS2	0.193333333	28.36	0	0.086666667
XM_021067710.1	CDC26	0.013333333	1.67	0	0
XM_021067713.1	FBXO39	0.07	8.16	0	0
XM_021067722.1	NLRP1	0.01	0.626666667	0	0
XM_021067724.1	BSPRY	0.006666667	0.666666667	0	0
XM_021067726.1	MIS12	0.09	8.356666667	0	0
XM_021067727.1	MIS12	0.086666667	7.89	0	0
XM_021067731.1	RABEP1	0.12	28.7	0	0
XM_021067743.1	NUP88	0.033333333	5.296666667	0	0
XM_021067769.1	MINK1	0.046666667	8.773333333	0	0
XM_021067781.1	MINK1	0.043333333	8.83	0	0
XM_021067800.1	ALOX12	0.02	2	0	0
XM_021067802.1	SLC16A11	0.01	3.69	0	0
XM_021067816.1	KIF12	0.006666667	0.803333333	0	0

XM_021067838.1	CHD3	0.01	3.406666667	0	0
XM_021067853.1	KIF12	0.003333333	0.333333333	0	0
XM_021067855.1	LOC100525835	0.003333333	0.333333333	0	0
XM_021067869.1	PIK3R6	0.036666667	4.96	0	0
XM_021067880.1	GAS7	0.036666667	11.14	0	0
XM_021067883.1	DNAH9	0.006666667	3.706666667	0	0.546666667
XM_021067888.1	DNAH9	0.003333333	3.576666667	0	0
XM_021067899.1	CDRT1	0.03	5.74	0	0
XM_021067903.1	CDRT1	0.006666667	1.706666667	0	0
XM_021067905.1	CDRT1	0.056666667	8.9	0	0
XM_021067909.1	CDRT1	0.03	3.923333333	0	0
XM_021067915.1	ZNF286A	0.053333333	5.04	0	0
XM_021067926.1	ULK2	0.013333333	3.213333333	0	0
XM_021067940.1	LOC102157639	0.006666667	0.666666667	0	0
XM_021067956.1	EPN2	0.12	22.01333333	0	0.056666667
XM_021067969.1	LOC110256121	0.006666667	0.333333333	0	0
XM_021067977.1	PRPSAP2	0.013333333	1.726666667	0	0.043333333
XM_021067997.1	WHRN	0.033333333	6.07	0	0.093333333
XM_021068003.1	DRC3	0.016666667	1.666666667	0	0
XM_021068030.1	PLD6	0.01	0.333333333	0	0
XM_021068043.1	MPRIP	0.033333333	5.176666667	0	0
XM_021068071.1	MYO15B	0.043333333	20	0	0
XM_021068080.1	ABCA6	0.03	7.16	0	0
XM_021068179.1	KCNAB1	0.006666667	1.926666667	0	0
XM_021068205.1	CNTRL	0.01	3.413333333	0	0
XM_021068223.1	RUBCN	0.043333333	10.23	0	0
XM_021068225.1	CNTRL	0.063333333	19.92333333	0	0
XM_021068265.1	ITIH3	0.016666667	1.983333333	0	0
XM_021068266.1	ITIH3	0.016666667	1.966666667	0	0
XM_021068267.1	ITIH3	0.07	8.856666667	0	0
XM_021068270.1	ITIH3	0.013333333	1.553333333	0	0
XM_021068279.1	PDCD10	0.033333333	2.936666667	0	0
XM_021068290.1	USP19	0.02	4.46	0	0
XM_021068304.1	LOC100156618	0.02	1	0	0
XM_021068327.1	AGPAT3	0.066666667	9.236666667	0	0
XM_021068335.1	AGPAT3	0.336666667	44.76	0	0.026666667
XM_021068343.1	CLDN16	0.026666667	2.333333333	0	0
XM_021068362.1	HAUS2	0.08	2.973333333	0	0
XM_021068366.1	MLH1	0.08	8.52	0	0
XM_021068379.1	ATG7	0.013333333	2.74	0	0
XM_021068383.1	ATG7	0.036666667	7.31	0	0
XM_021068388.1	ATG7	0.023333333	5.046666667	0	0
XM_021068410.1	SLC51A	0.006666667	0.58	0	0
XM_021068420.1	TRH	0.023333333	1.736666667	0	0
XM_021068430.1	FAM107A	0.03	4.356666667	0	0
XM_021068437.1	PLD1	0.006666667	2.803333333	0	0
XM_021068456.1	FAIM	0.056666667	2.366666667	0	0
XM_021068461.1	PLSCR1	0.02	1.65	0	0
XM_021068476.1	ABHD14A	0.04	3.143333333	0	0
XM_021068500.1	ACKR2	0.03	8.696666667	0	0
XM_021068505.1	SLC6A6	0.226666667	55.53	0	0
XM_021068523.1	NPG4	0.053333333	2.173333333	0	0
XM_021068533.1	CD80	0.006666667	1.64	0	0.503333333
XM_021068536.1	NR1I2	0.02	2.52	0	0
XM_021068552.1	B3GALNT1	0.006666667	0.863333333	0	0
XM_021068554.1	B3GALNT1	0.006666667	0.936666667	0	0
XM_021068557.1	B3GALNT1	0.006666667	0.91	0	0
XM_021068560.1	B3GALNT1	0.006666667	0.983333333	0	0
XM_021068561.1	B3GALNT1	0.016666667	1.693333333	0	0
XM_021068562.1	B3GALNT1	0.03	3.316666667	0	0

XM_021068570.1	NEK6	0.003333333	0.223333333	0	0
XM_021068597.1	EIF4G1	0.393333333	92.3	0	0
XM_021068620.1	SLC26A6	0.033333333	3.57	0	0
XM_021068626.1	P2RY12	0.013333333	1.43	0	0
XM_021068627.1	ARF4	0.113333333	6.943333333	0	0
XM_021068645.1	ALS2CL	0.033333333	4.21	0	0
XM_021068654.1	NBEAL2	0.006666667	2.84	0	0
XM_021068655.1	NBEAL2	0.006666667	3.633333333	0	0
XM_021068662.1	NBEAL2	0.006666667	3.483333333	0	0.59
XM_021068663.1	NBEAL2	0.016666667	9.41	0	0.623333333
XM_021068664.1	NBEAL2	0.003333333	1.586666667	0	0
XM_021068674.1	NBEAL2	0.003333333	2.143333333	0	0
XM_021068687.1	NBEAL2	0.003333333	3.046666667	0	0
XM_021068690.1	NBEAL2	0.023333333	17.25666667	0	0.153333333
XM_021068708.1	KIF9	0.016666667	3.803333333	0	0
XM_021068712.1	KIF9	0.03	7.543333333	0	0
XM_021068718.1	KIF9	0.016666667	4.16	0	0
XM_021068719.1	KIF9	0.033333333	7.283333333	0	0
XM_021068782.1	GAPVD1	0.05	10.51	0	0
XM_021068787.1	MST1R	0.043333333	10.74	0	0
XM_021068790.1	MST1R	0.006666667	2.433333333	0	0
XM_021068808.1	SEMA3B	0.033333333	3.84	0	0
XM_021068811.1	SEMA3B	0.1	12.50333333	0	0
XM_021068820.1	CACNA2D2	0.026666667	6.776666667	0	0.593333333
XM_021068836.1	DOCK3	0.003333333	1.266666667	0	0.44
XM_021068840.1	DOCK3	0.003333333	1.066666667	0	0
XM_021068856.1	GRM2	0.04	9.013333333	0	0
XM_021068858.1	TEX264	0.056666667	3.8	0	0
XM_021068872.1	PARP3	0.033333333	3.18	0	0
XM_021068894.1	STAB1	0.056666667	20.41	0	0
XM_021068913.1	PBRM1	0.01	3.62	0	0
XM_021068918.1	PBRM1	0.01	2.883333333	0	0
XM_021068972.1	IL17RB	0.01	0.666666667	0	0
XM_021068997.1	ASB14	0.1	26.29333333	0	0
XM_021069000.1	RALGPS1	0.04	5.506666667	0	0
XM_021069007.1	HESX1	0.02	0.836666667	0	0
XM_021069032.1	SLMAP	0.12	29.18333333	0	0
XM_021069048.1	SLMAP	0.243333333	47.38	0	0
XM_021069050.1	RALGPS1	0.063333333	7.11	0	0
XM_021069060.1	RALGPS1	0.22	23.56333333	0	0
XM_021069066.1	FLNB	0.066666667	28.21666667	0	0
XM_021069067.1	FLNB	0.126666667	51.40666667	0	0
XM_021069069.1	RALGPS1	0.026666667	3.8	0	0
XM_021069070.1	ACOX2	0.023333333	2.473333333	0	0
XM_021069072.1	PXK	0.106666667	12.84333333	0	0
XM_021069082.1	PXK	0.01	1.083333333	0	0
XM_021069098.1	RPP14	0.4	112.2933333	0	0
XM_021069102.1	FAM3D	0.02	0.87	0	0
XM_021069104.1	RALGPS1	0.036666667	3.736666667	0	0
XM_021069123.1	RALGPS1	0.01	1.223333333	0	0
XM_021069171.1	SYNPR	0.003333333	0.333333333	0	0
XM_021069210.1	MDFIC2	0.003333333	0.333333333	0	0
XM_021069223.1	CNTN3	0.006666667	1.396666667	0	0
XM_021069228.1	CNTN3	0.01	2.456666667	0	0
XM_021069243.1	CHL1	0.016666667	5.836666667	0	0
XM_021069247.1	CHL1	0.006666667	2.173333333	0	0
XM_021069266.1	CNTN6	0.003333333	0.333333333	0	0
XM_021069292.1	LPAR1	0.036666667	5.393333333	0	0
XM_021069297.1	CPNE9	0.02	1.246666667	0	0
XM_021069306.1	BRPF1	0.033333333	6.95	0	0

XM_021069336.1	ATP2B2	0.153333333	41.63333333	0	0
XM_021069341.1	SLC6A1	0.02	4.236666667	0	0
XM_021069345.1	CFAP157	0.003333333	0.333333333	0	0
XM_021069351.1	RAF1	0.21	29.91666667	0	0
XM_021069374.1	XPC	0.016666667	3.156666667	0	0
XM_021069387.1	FPGS	0.086666667	5.553333333	0	0
XM_021069394.1	IQSEC1	0.086666667	24.09666667	0	0
XM_021069403.1	KIAA1257	0.003333333	0.333333333	0	0
XM_021069410.1	EFCC1	0.03	4.51	0	0
XM_021069454.1	LOC110256296	0.006666667	0.606666667	0	0
XM_021069456.1	ACPP	0.03	4	0	0
XM_021069494.1	GOLGA2	0.05	8.066666667	0	0
XM_021069498.1	DZIP1L	0.016666667	3.756666667	0	0
XM_021069501.1	GOLGA2	0.09	15.43666667	0	0
XM_021069508.1	NME9	0.003333333	0.333333333	0	0
XM_021069527.1	PIK3CB	0.233333333	48.72333333	0	0
XM_021069547.1	LOC100515582	0.003333333	0.333333333	0	0
XM_021069549.1	DNM1	0.056666667	9.483333333	0	0
XM_021069558.1	TFDP2	0.056666667	11.31666667	0	0
XM_021069561.1	TFDP2	0.026666667	7.936666667	0	0
XM_021069562.1	TFDP2	0.016666667	5.363333333	0	0
XM_021069578.1	PAQR9	0.083333333	30.93	0	0
XM_021069603.1	IGSF10	0.013333333	5.116666667	0	0
XM_021069605.1	IGSF10	0.013333333	5.333333333	0	0
XM_021069608.1	MED12L	0.006666667	3.27	0	0
XM_021069617.1	MED12L	0.016666667	7.803333333	0	0
XM_021069625.1	GPR171	0.006666667	1.09	0	0
XM_021069628.1	GPR171	0.026666667	4.376666667	0	0
XM_021069630.1	LOC100623616	0.09	16.99666667	0	0.333333333
XM_021069631.1	LOC100623616	0.013333333	2.67	0	0
XM_021069647.1	MBNL1	0.083333333	19.98666667	0	0
XM_021069649.1	MBNL1	0.036666667	8.663333333	0	0
XM_021069721.1	VEPH1	0.01	2.13	0	0
XM_021069730.1	SMC4	0.023333333	8.026666667	0	0
XM_021069748.1	SI	0.01	2.666666667	0	0
XM_021069761.1	WDR49	0.003333333	0.333333333	0	0
XM_021069766.1	LRRC34	0.01	0.536666667	0	0
XM_021069768.1	LRRIQ4	0.003333333	0.333333333	0	0
XM_021069776.1	LRRC31	0.006666667	0.666666667	0	0
XM_021069791.1	GPR160	0.013333333	3.333333333	0	0
XM_021069792.1	GPR160	0.006666667	1.156666667	0	0
XM_021069799.1	EIF5A2	0.01	2.38	0	0
XM_021069833.1	NUP188	0.026666667	6.153333333	0	0
XM_021069837.1	NUP188	0.25	61.74333333	0	0
XM_021069842.1	NUP188	0.046666667	11.89666667	0	0
XM_021069853.1	ZMAT3	0.036666667	15.09666667	0	0
XM_021069856.1	LOC110256373	0.013333333	0.666666667	0	0
XM_021069868.1	ATP11B	0.03	6.526666667	0	0
XM_021069880.1	ATP11B	0.036666667	7.356666667	0	0
XM_021069921.1	YEATS2	0.023333333	5.833333333	0	0
XM_021069931.1	MAP6D1	0.03	2.586666667	0	0.033333333
XM_021069935.1	ABCC5	0.386666667	180.27	0	0
XM_021069936.1	ABCC5	0.016666667	5.856666667	0	0
XM_021069942.1	LOC102163598	0.003333333	0.333333333	0	0
XM_021069964.1	CHRD	0.033333333	4.456666667	0	0
XM_021069968.1	CHRD	0.026666667	3.306666667	0	0
XM_021069970.1	RBMS3	0.033333333	6.656666667	0	0
XM_021069975.1	VPS8	0.036666667	6.643333333	0	0
XM_021069983.1	MAP3K13	0.003333333	2.293333333	0	0
XM_021069987.1	LIPH	0.006666667	0.666666667	0	0

XM_021070008.1	DGKG	0.003333333	1.56	0	0
XM_021070009.1	DGKG	0.01	2.013333333	0	0
XM_021070010.1	DGKG	0.013333333	2.413333333	0	0
XM_021070021.1	ST6GAL1	0.083333333	18.65666667	0	0
XM_021070033.1	ST6GAL1	0.013333333	2.573333333	0	0
XM_021070045.1	TP63	0.026666667	5.94	0	0
XM_021070050.1	TMEM207	0.023333333	2.333333333	0	0
XM_021070053.1	IL1RAP	0.013333333	2.95	0	0
XM_021070054.1	IL1RAP	0.006666667	1.12	0	0
XM_021070073.1	TMEM44	0.02	2.246666667	0	0
XM_021070082.1	ACAP2	0.173333333	51.48666667	0	0
XM_021070098.1	DLG1	0.04	8.576666667	0	0
XM_021070107.1	DLG1	0.05	9.573333333	0	0
XM_021070126.1	PIGZ	0.02	8.156666667	0	0
XM_021070133.1	PIGZ	0.07	26.45	0	0.186666667
XM_021070134.1	PIGZ	0.003333333	0.703333333	0	0.003333333
XM_021070144.1	WDR53	0.1	5.506666667	0	0
XM_021070147.1	WDR53	0.12	5.773333333	0	0
XM_021070167.1	TNK2	0.096666667	17.99	0	0
XM_021070173.1	TNK2	0.01	1.686666667	0	0
XM_021070200.1	IQCG	0.003333333	0.333333333	0	0
XM_021070210.1	ZNF148	2.773333333	366.9266667	0	0.003333333
XM_021070216.1	ZNF148	0.03	3.246666667	0	0
XM_021070221.1	CCDC14	0.103333333	17.69333333	0	0
XM_021070236.1	MYLK	0.026666667	9.1	0	0
XM_021070244.1	ADCY5	0.046666667	6.543333333	0	0
XM_021070247.1	SEC22A	0.036666667	2.976666667	0	0
XM_021070252.1	SEMA5B	0.006666667	0.97	0	0
XM_021070299.1	EXOSC2	0.046666667	1.673333333	0	0
XM_021070302.1	STXBP5L	0.003333333	0.743333333	0	0
XM_021070322.1	MAATS1	0.016666667	1.923333333	0	0
XM_021070327.1	POGLUT1	0.106666667	17.38666667	0	0
XM_021070342.1	CCDC191	0.016666667	2.56	0	0
XM_021070343.1	CCDC191	0.006666667	1.046666667	0	0
XM_021070352.1	GRAMD1C	0.016666667	2.753333333	0	0
XM_021070366.1	BOC	0.006666667	2.02	0	0
XM_021070382.1	LOC100156381	0.023333333	0.673333333	0	0
XM_021070438.1	HHLA2	0.01	2.533333333	0	0
XM_021070441.1	HHLA2	0.003333333	0.676666667	0	0
XM_021070445.1	HHLA2	0.003333333	1.35	0	0
XM_021070449.1	HHLA2	0.016666667	5.73	0	0
XM_021070454.1	HHLA2	0.006666667	1.646666667	0	0
XM_021070479.1	BBX	0.023333333	5.533333333	0	0
XM_021070484.1	BBX	0.043333333	9.253333333	0	0
XM_021070498.1	ALCAM	0.013333333	3.13	0	0
XM_021070509.1	POMT1	0.02	2.94	0	0
XM_021070514.1	ABI3BP	0.013333333	3.716666667	0	0
XM_021070517.1	ABI3BP	0.023333333	5.863333333	0	0
XM_021070518.1	ABI3BP	0.006666667	2.346666667	0	0
XM_021070519.1	POMT1	0.026666667	3.413333333	0	0
XM_021070527.1	ABI3BP	0.01	3.013333333	0	0
XM_021070560.1	ABI3BP	0.023333333	5.756666667	0	0
XM_021070565.1	ADGRG7	0.01	4.173333333	0	0.333333333
XM_021070566.1	ADGRG7	0.01	1.493333333	0	0
XM_021070601.1	MAP4	0.03	7.36	0	0
XM_021070605.1	MAP4	0.04	9.646666667	0	0
XM_021070612.1	MAP4	0.04	16.46	0	0
XM_021070616.1	DHX30	0.11	19.69	0	0
XM_021070623.1	LOC110255221	0.026666667	0.66	0	0
XM_021070635.1	PFKFB4	0.04	5.633333333	0	0

XM_021070670.1	SETX	0.01	3.763333333	0	0
XM_021070675.1	ST3GAL6	0.036666667	7.7	0	0
XM_021070677.1	ST3GAL6	0.01	2.183333333	0	0
XM_021070684.1	ST3GAL6	0.01	2.23	0	0
XM_021070727.1	ERC2	0.006666667	1.763333333	0	0
XM_021070728.1	ERC2	0.003333333	1.193333333	0	0.463333333
XM_021070735.1	ARL13B	0.06	4.376666667	0	0
XM_021070755.1	FHIT	0.02	3.876666667	0	0
XM_021070756.1	FHIT	0.023333333	4.653333333	0	0
XM_021070762.1	FHIT	0.043333333	7.5	0	0
XM_021070769.1	PTPRG	0.01	3.896666667	0	0
XM_021070777.1	TJP2	0.016666667	3.046666667	0	0
XM_021070789.1	MAGI1	0.03	10.47666667	0	0
XM_021070822.1	USP25	0.03	5.55	0	0
XM_021070841.1	FOXP1	0.05	12.2	0	0
XM_021070844.1	FOXP1	0.03	3.226666667	0	0
XM_021070849.1	FOXP1	0.113333333	61.74333333	0	0
XM_021070857.1	EIF4E3	0.113333333	29.12666667	0	0
XM_021070870.1	USP16	0.03	4.443333333	0	0
XM_021070873.1	USP16	0.04	5.296666667	0	0
XM_021070877.1	USP16	0.566666667	73.90666667	0	0
XM_021070888.1	BACH1	0.106666667	31.84333333	0	0
XM_021070891.1	BACH1	0.076666667	20.97333333	0	0
XM_021070900.1	GFI1B	0.003333333	0.333333333	0	0
XM_021070914.1	MRAP	0.006666667	0.333333333	0	0
XM_021070919.1	EVA1C	0.096666667	6.9	0	0
XM_021070928.1	SYNJ1	0.033333333	10.35333333	0	0
XM_021070937.1	TMEM50B	0.063333333	6.166666667	0	0
XM_021070939.1	TMEM50B	0.086666667	8.193333333	0	0
XM_021070952.1	SMIM34A	0.01	0.666666667	0	0
XM_021070959.1	LOC110256480	0.096666667	5.666666667	0	0
XM_021070979.1	KCNJ15	0.113333333	29.34666667	0	0
XM_021070980.1	GBGT1	0.043333333	3.9	0	0
XM_021070982.1	KCNJ15	0.053333333	13.76	0	0
XM_021070985.1	GBGT1	0.12	10.15666667	0	0
XM_021071003.1	GBGT1	0.046666667	3.616666667	0	0
XM_021071009.1	TMPRSS2	0.126666667	16	0	0
XM_021071026.1	SLC37A1	0.046666667	5.866666667	0	0
XM_021071027.1	SLC37A1	0.073333333	8.896666667	0	0
XM_021071030.1	SLC37A1	0.036666667	3.463333333	0	0
XM_021071034.1	RSPH1	0.006666667	0.333333333	0	0
XM_021071047.1	CBS	0.023333333	2.633333333	0	0
XM_021071072.1	HSF2BP	0.3	23.82333333	0	0
XM_021071091.1	PCBP3	0.023333333	2	0	0
XM_021071105.1	HRH1	0.266666667	36.02	0	0
XM_021071106.1	HRH1	0.113333333	15.53333333	0	0
XM_021071108.1	HRH1	0.04	5.35	0	0
XM_021071134.1	LOC100738971	0.01	0.696666667	0	0
XM_021071167.1	TNIK	0.016666667	8.16	0	0
XM_021071183.1	NLGN1	0.003333333	0.333333333	0	0
XM_021071189.1	NAALADL2	0.003333333	1.636666667	0	0
XM_021071198.1	TBL1XR1	0.213333333	61.99	0	0
XM_021071210.1	TBL1XR1	0.066666667	19.2	0	0
XM_021071229.1	PEX5L	0.006666667	2.666666667	0	0.666666667
XM_021071230.1	STKLD1	0.036666667	3.4	0	0
XM_021071233.1	PEX5L	0.006666667	3	0	0.333333333
XM_021071242.1	FXR1	0.396666667	47.30666667	0	0
XM_021071244.1	C13H3orf56	0.003333333	0.333333333	0	0
XM_021071269.1	ZBTB20	0.023333333	35.51333333	0	0
XM_021071301.1	CACFD1	0.01	2.053333333	0	0

XM_021071308.1	TBC1D23	0.026666667	4.293333333	0	0
XM_021071324.1	ASTE1	0.096666667	9.11	0	0
XM_021071336.1	COL6A5	0.006666667	2.64	0	0
XM_021071364.1	VAV2	0.013333333	3.056666667	0	0
XM_021071374.1	ROBO1	0.013333333	4.516666667	0	0
XM_021071384.1	VAV2	0.023333333	4.336666667	0	0
XM_021071401.1	ROBO2	0.046666667	18.46333333	0	0
XM_021071408.1	ROBO2	0.016666667	5.843333333	0	0
XM_021071410.1	LOC106508546	0.023333333	1.943333333	0	0
XM_021071462.1	SLC4A7	0.016666667	7.233333333	0	0.033333333
XM_021071467.1	SLC4A7	0.02	8.246666667	0	0.05
XM_021071474.1	SLC4A7	0.02	6.406666667	0	0
XM_021071497.1	DSCAM	0.006666667	2.333333333	0	1.666666667
XM_021071520.1	TSPEAR	0.003333333	0.333333333	0	0
XM_021071531.1	COL18A1	0.01	2.693333333	0	0
XM_021071535.1	COL18A1	0.063333333	16.28666667	0	0
XM_021071550.1	CNOT10	0.05	16.84	0	0
XM_021071577.1	CLASP2	0.09	24.68333333	0	0
XM_021071634.1	LRRFIP2	0.406666667	62.55666667	0	0
XM_021071650.1	LRRFIP2	0.1	11.93333333	0	0.106666667
XM_021071666.1	XYLB	0.013333333	1.336666667	0	0
XM_021071669.1	XYLB	0.016666667	2.883333333	0	0
XM_021071733.1	ZKSCAN7	0.05	11.37	0	0
XM_021071738.1	LOC100738134	0.02	1.606666667	0	0
XM_021071739.1	LOC100738134	0.043333333	3.41	0	0
XM_021071747.1	KIF15	0.023333333	5	0	0
XM_021071759.1	SGMS1	0.02	3.1	0	0
XM_021071776.1	TRPV4	0.006666667	1.333333333	0	0
XM_021071780.1	PBK	0.153333333	13.05333333	0	0
XM_021071810.1	CHRM3	0.003333333	1.56	0	0
XM_021071817.1	RHOF	0.006666667	1.186666667	0	0
XM_021071827.1	RHOF	0.01	2.463333333	0	0
XM_021071837.1	RHOF	0.096666667	19.63666667	0	0
XM_021071841.1	RHOF	0.013333333	3.006666667	0	0
XM_021071842.1	RHOF	0.006666667	1.136666667	0	0
XM_021071865.1	ZFAND4	0.013333333	1.813333333	0	0
XM_021071866.1	SEC14L2	0.013333333	1.506666667	0	0
XM_021071873.1	RASSF4	0.036666667	3.993333333	0	0
XM_021071875.1	RASSF4	0.003333333	0.48	0	0
XM_021071883.1	STAMBPL1	0.203333333	15.53	0	0
XM_021071901.1	UROS	0.253333333	15.55	0	0
XM_021071903.1	UROS	0.046666667	2.646666667	0	0
XM_021071918.1	PISD	0.036666667	11.18666667	0	0
XM_021071926.1	DUSP13	0.05	10.6	0	0
XM_021071967.1	NCOA4	0.143333333	16.88666667	0	0
XM_021071972.1	NCOA4	0.003333333	1.06	0	0.113333333
XM_021071975.1	HPGD	0.02	1.083333333	0	0
XM_021071991.1	LGALS8	0.093333333	25.39666667	0	0
XM_021072015.1	ACSL5	0.01	1.27	0	0
XM_021072025.1	STN1	0.003333333	0.906666667	0	0
XM_021072028.1	STN1	0.203333333	17.35333333	0	0
XM_021072041.1	CHEK2	0.05	6.093333333	0	0
XM_021072056.1	OIT3	0.003333333	0.333333333	0	0
XM_021072058.1	PHYHIPL	0.006666667	2.213333333	0	0
XM_021072061.1	SASH1	0.006666667	2.053333333	0	0
XM_021072085.1	CLU	0.08	5.57	0	0
XM_021072121.1	OPALIN	0.006666667	0.666666667	0	0
XM_021072125.1	KCNMA1	0.016666667	8.763333333	0	0
XM_021072126.1	KCNMA1	0.01	5.386666667	0	0
XM_021072141.1	KCNMA1	0.036666667	6.246666667	0	0

XM_021072152.1	KCNMA1	0.02	10.33333333	0	0
XM_021072167.1	KCNMA1	0.076666667	37.43666667	0	0
XM_021072213.1	DDX60	0.033333333	8.666666667	0	0
XM_021072228.1	DNAH10	0.04	22.46666667	0	0
XM_021072235.1	CCDC92	0.05	3.866666667	0	0
XM_021072248.1	ZNF664	0.136666667	26.75666667	0	0
XM_021072251.1	ZNF664	0.023333333	4.63	0	0
XM_021072257.1	TCTN2	0.116666667	11.98333333	0	0
XM_021072259.1	UTRN	0.14	84.35	0	0
XM_021072267.1	UTRN	0.37	198.1466667	0	0
XM_021072276.1	SLC18A1	0.006666667	1.1	0	0
XM_021072300.1	TBC1D10A	0.116666667	8.863333333	0	0
XM_021072306.1	SGSM1	0.006666667	2.426666667	0	0
XM_021072307.1	SGSM1	0.013333333	2.31	0	0
XM_021072339.1	DEPDC5	0.013333333	3.003333333	0	0
XM_021072341.1	DEPDC5	0.056666667	12.64	0	0
XM_021072350.1	LOC100738050	0.016666667	2.443333333	0	0
XM_021072355.1	LOC100738050	0.006666667	1.153333333	0	0
XM_021072364.1	LOC100738050	0.026666667	4.136666667	0	0
XM_021072381.1	LOC106506010	0.046666667	1.65	0	0
XM_021072384.1	LOC106506010	0.03	1.2	0	0
XM_021072410.1	HIVEP2	0.073333333	30.08666667	0	0
XM_021072417.1	MTRF1L	0.056666667	2.846666667	0	0.016666667
XM_021072418.1	ASCC2	0.016666667	3.653333333	0	0
XM_021072435.1	SAP30	0.056666667	2.416666667	0	0
XM_021072456.1	LOC100156470	0.003333333	0.333333333	0	0
XM_021072467.1	AIG1	0.03	1.836666667	0	0
XM_021072474.1	GPR137B	0.016666667	2.72	0	0
XM_021072479.1	SEC31B	0.006666667	1.676666667	0	0.39
XM_021072481.1	SEC31B	0.063333333	14.57	0	0
XM_021072485.1	SEC31B	0.006666667	1.846666667	0	0
XM_021072519.1	CABP1	0.006666667	0.516666667	0	0
XM_021072520.1	CABP1	0.016666667	1.483333333	0	0
XM_021072530.1	CCDC63	0.01	1	0	0
XM_021072535.1	LOC110256626	0.043333333	4.816666667	0	0
XM_021072556.1	USP54	0.03	9.79	0	0
XM_021072563.1	USP54	0.006666667	1.703333333	0	0
XM_021072565.1	USP54	0.04	10.55	0	0
XM_021072572.1	USP54	0.01	3.63	0	0.053333333
XM_021072576.1	PPP3CB	0.296666667	113.81	0	0
XM_021072595.1	DHX32	0.14	18.97	0	0
XM_021072605.1	FANK1	0.02	8.733333333	0	0
XM_021072611.1	MAP7	0.053333333	8.486666667	0	0
XM_021072624.1	LOC100158108	0.006666667	2.063333333	0	0
XM_021072628.1	MAP7	0.013333333	1.743333333	0	0
XM_021072629.1	LOC100158108	0.016666667	4.12	0	0
XM_021072644.1	PRSS55	0.076666667	7	0	0
XM_021072647.1	CCNJ	0.006666667	1.31	0	0
XM_021072659.1	CDH23	0.016666667	7.97	0	0.856666667
XM_021072661.1	CDH23	0.016666667	10.75	0	0
XM_021072678.1	LZTS2	0.043333333	3.956666667	0	0
XM_021072725.1	DMTN	0.216666667	22.90333333	0	0
XM_021072734.1	KCNIP2	0.006666667	0.486666667	0	0
XM_021072772.1	NEURL1	0.026666667	5.013333333	0	0
XM_021072808.1	FBXO16	0.006666667	0.333333333	0	0
XM_021072811.1	TMEM72	0.076666667	21.66666667	0	0
XM_021072820.1	CFAP43	0.01	2.093333333	0	0
XM_021072827.1	GFRA2	0.043333333	5.963333333	0	0
XM_021072843.1	NEK1	0.033333333	7.496666667	0	0
XM_021072844.1	NEK1	0.093333333	21.29	0	0.046666667

XM_021072847.1	NEK1	0.01	2.633333333	0	0
XM_021072853.1	NEK1	0.003333333	1.023333333	0	0
XM_021072858.1	ARMH4	0.016666667	2.523333333	0	0
XM_021072860.1	SH2D4B	0.003333333	0.273333333	0	0.076666667
XM_021072875.1	HERC4	0.17	28.56666667	0	0
XM_021072878.1	LOC100154415	0.04	6.496666667	0	0
XM_021072881.1	LOC100154415	0.04	6.733333333	0	0
XM_021072899.1	RIMS1	0.013333333	3.246666667	0	0
XM_021072915.1	ATXN2	0.016666667	3.383333333	0	0
XM_021072920.1	ATXN2	0.023333333	5.356666667	0	0
XM_021072929.1	ATXN2	0.043333333	8.843333333	0	0
XM_021072930.1	ATXN2	0.046666667	9.436666667	0	0
XM_021072952.1	SLC35E4	0.02	2.5	0	0
XM_021072953.1	DUSP18	0.006666667	2.836666667	0	0.5
XM_021072958.1	CLIP1	0.083333333	20.91333333	0	0
XM_021072976.1	CLIP1	0.29	71.60666667	0	0
XM_021072979.1	LRRC43	0.003333333	0.333333333	0	0
XM_021072995.1	EGR3	0.06	9.96	0	0
XM_021072996.1	OGDHL	0.01	1.666666667	0	0
XM_021073004.1	LOC110256690	0.016666667	0.666666667	0	0
XM_021073005.1	RIMS1	0.02	4.753333333	0	0
XM_021073017.1	ZNF239	0.1	12.74	0	0
XM_021073029.1	CAMKK2	0.126666667	26.54333333	0	0
XM_021073032.1	RIMS1	0.016666667	4.013333333	0	0
XM_021073036.1	CAMKK2	0.08	16.83333333	0	0
XM_021073037.1	CAMKK2	0.02	4.21	0	0
XM_021073052.1	CPN1	0.02	1	0	0
XM_021073053.1	RIMS1	0.003333333	1.04	0	0
XM_021073054.1	TAOK3	0.703333333	127.1966667	0	0
XM_021073055.1	TAOK3	0.166666667	19.78666667	0	0
XM_021073066.1	RIMS1	0.003333333	0.903333333	0	0
XM_021073078.1	ARHGAP22	0.046666667	5.05	0	0
XM_021073127.1	MED13L	0.073333333	30.85333333	0	0
XM_021073137.1	PXN	0.106666667	23.47	0	0
XM_021073173.1	PTK2B	0.003333333	0.446666667	0	0
XM_021073175.1	PTK2B	0.01	1.626666667	0	0
XM_021073177.1	PTK2B	0.013333333	2.14	0	0
XM_021073188.1	MMS22L	0.003333333	0.856666667	0	0
XM_021073193.1	MMS22L	0.01	3.156666667	0	0
XM_021073204.1	RHOBTB2	0.04	8.57	0	0
XM_021073212.1	CNDP1	0.026666667	1.796666667	0	0
XM_021073232.1	KLHL32	0.01	2.666666667	0	0
XM_021073251.1	AADAT	0.596666667	48	0	0
XM_021073252.1	KLHL32	0.003333333	1.333333333	0	0
XM_021073255.1	LOC100156277	0.006666667	1.14	0	0.666666667
XM_021073266.1	TPCN1	0.103333333	16.56666667	0	0
XM_021073280.1	EMID1	0.006666667	0.35	0	0.003333333
XM_021073286.1	ZDHHC6	0.233333333	13.34333333	0	0
XM_021073291.1	TCF7L2	0.02	3.863333333	0	0
XM_021073297.1	TCF7L2	0.01	1.823333333	0	0
XM_021073302.1	TCF7L2	0.006666667	1.376666667	0	0
XM_021073303.1	TCF7L2	0.06	10.68333333	0	0
XM_021073306.1	TCF7L2	0.02	3.256666667	0	0
XM_021073307.1	TCF7L2	0.01	2.143333333	0	0
XM_021073314.1	PALLD	0.013333333	2.163333333	0	0
XM_021073333.1	CCSER2	0.006666667	2.526666667	0	0
XM_021073346.1	GRIK2	0.013333333	2.07	0	0
XM_021073347.1	ABLIM1	0.283333333	68.06666667	0	0
XM_021073351.1	ABLIM1	0.316666667	74.61	0	0
XM_021073352.1	ABLIM1	0.14	32.61333333	0	0

XM_021073353.1	ABLIM1	0.243333333	56.84333333	0	0
XM_021073354.1	ABLIM1	0.06	14.05333333	0	0
XM_021073360.1	ABLIM1	0.766666667	177.6433333	0	0
XM_021073361.1	ABLIM1	0.476666667	110.16	0	0
XM_021073362.1	ABLIM1	0.02	4.953333333	0	0
XM_021073363.1	ABLIM1	0.003333333	0.59	0	0
XM_021073370.1	ABLIM1	0.58	130.45	0	0
XM_021073371.1	ABLIM1	0.053333333	12.72	0	0
XM_021073406.1	HS3ST5	0.006666667	1.106666667	0	0
XM_021073408.1	EMX2	0.003333333	0.243333333	0	0
XM_021073422.1	KDM2B	0.17	38.30666667	0	0
XM_021073433.1	KDM2B	0.026666667	5.303333333	0	0
XM_021073441.1	FAM45A	0.066666667	7.226666667	0	0
XM_021073453.1	TIAL1	0.17	43.29666667	0	0
XM_021073469.1	SFXN2	0.02	2.456666667	0	0
XM_021073479.1	PCGF6	0.113333333	10.63666667	0	0
XM_021073520.1	FAM81A	0.083333333	13.10666667	0	0.136666667
XM_021073527.1	PBLD	0.323333333	25.27	0	0
XM_021073530.1	PBLD	0.096666667	7.693333333	0	0
XM_021073532.1	FAM81A	0.1	15.75666667	0	0
XM_021073537.1	LOC106506082	0.006666667	1	0	0
XM_021073552.1	CIT	0.013333333	4.333333333	0	0
XM_021073589.1	SHLD2	0.053333333	6.416666667	0	0
XM_021073590.1	SLTM	0.006666667	3.036666667	0	0
XM_021073598.1	RHOBTB1	0.19	35.20666667	0	0
XM_021073606.1	CPXM2	0.106666667	12.97	0	0
XM_021073609.1	SLTM	0.01	4.42	0	0
XM_021073613.1	CHST15	0.053333333	10.18666667	0	0
XM_021073616.1	CHST15	0.003333333	0.503333333	0	0.066666667
XM_021073618.1	SLTM	0.01	4.67	0	0.326666667
XM_021073694.1	LOC110256822	0.006666667	0.333333333	0	0
XM_021073709.1	LOC100624435	0.016666667	1.69	0	0
XM_021073714.1	VWA2	0.006666667	2	0	0
XM_021073723.1	MYO1H	0.046666667	13.23666667	0	0
XM_021073724.1	MYO1H	0.003333333	0.683333333	0	0.41
XM_021073739.1	MKI67	0.02	9.236666667	0	0.003333333
XM_021073746.1	MKI67	0.003333333	1.523333333	0	0
XM_021073764.1	MGMT	0.036666667	1.146666667	0	0
XM_021073789.1	LOC100511352	0.03	1.506666667	0	0
XM_021073793.1	EXOC6	0.12	18.24	0	0
XM_021073800.1	SPECC1L	0.06	13.7	0	0
XM_021073845.1	EP400	0.08	43.58333333	0	0
XM_021073851.1	EP400	0.153333333	79.32666667	0	0.023333333
XM_021073864.1	AIFM2	0.203333333	11.96333333	0	0
XM_021073874.1	STK32C	0.003333333	0.333333333	0	0
XM_021073888.1	NEDD4L	0.013333333	4.056666667	0	0
XM_021073895.1	HNRNPF	0.15	37.17	0	0
XM_021073957.1	FUOM	0.053333333	3.05	0	0
XM_021073975.1	MIA2	0.083333333	16.56	0	0.003333333
XM_021073978.1	KSR2	0.003333333	1.316666667	0	0
XM_021073982.1	SFSWAP	0.03	4.116666667	0	0
XM_021074005.1	PLCE1	0.02	7.706666667	0	0
XM_021074008.1	PLCE1	0.023333333	8.83	0	0
XM_021074016.1	MIA2	0.336666667	40.25	0	0.013333333
XM_021074019.1	CYP2C34	0.456666667	38.12	0	0
XM_021074020.1	CYP2C36	2.27	179.97	0	0
XM_021074034.1	AIFM3	0.013333333	1.333333333	0	0
XM_021074035.1	AIFM3	0.003333333	0.333333333	0	0
XM_021074061.1	MPHOSPH9	0.103333333	16.29	0	0
XM_021074077.1	CCDC62	0.02	3.106666667	0	0

XM_021074095.1	UPB1	0.013333333	0.88	0	0
XM_021074100.1	FBRSL1	0.12	25.46666667	0	0
XM_021074117.1	CDC45	0.116666667	10.96333333	0	0
XM_021074138.1	SORBS1	0.01	3.24	0	0
XM_021074214.1	TMEM116	0.013333333	0.716666667	0	0
XM_021074221.1	LOC102160475	0.013333333	0.673333333	0	0
XM_021074227.1	BICDL1	0.006666667	0.62	0	0
XM_021074247.1	WDR66	0.01	1.666666667	0	0
XM_021074261.1	ZNF518A	0.013333333	4.503333333	0	0
XM_021074263.1	RIMBP2	0.023333333	6.386666667	0	0
XM_021074274.1	RIMBP2	0.033333333	6.31	0	0
XM_021074280.1	TAF5L	0.223333333	31.57333333	0	0
XM_021074283.1	TAF5L	0.026666667	3.943333333	0	0
XM_021074286.1	TAF5L	0.126666667	15.99	0	0
XM_021074290.1	ANKRD13A	0.043333333	6.62	0	0
XM_021074324.1	NUDT13	0.026666667	4.11	0	0
XM_021074326.1	NUDT13	0.016666667	2.313333333	0	0
XM_021074330.1	NUDT13	0.183333333	24.26	0	0
XM_021074331.1	NUDT13	0.283333333	25.65333333	0	0
XM_021074346.1	PTPRD	0.166666667	70.1	0	0
XM_021074360.1	HVCN1	0.016666667	2.113333333	0	0
XM_021074369.1	AP1B1	0.056666667	10.21333333	0	0.016666667
XM_021074390.1	GLIS3	0.08	27.4	0	0
XM_021074396.1	GLIS3	0.006666667	2.426666667	0	0
XM_021074418.1	CRTAC1	0.173333333	19.89666667	0	0
XM_021074422.1	ZFYVE27	0.016666667	7.606666667	0	0
XM_021074427.1	GOLGA7B	0.016666667	2.813333333	0	0
XM_021074433.1	LOC100154312	0.003333333	0.333333333	0	0
XM_021074466.1	BIN1	0.47	57.34666667	0	0
XM_021074467.1	BIN1	0.056666667	5.59	0	0
XM_021074472.1	BIN1	0.2	19.54666667	0	0
XM_021074481.1	BIN1	0.023333333	2.19	0	0
XM_021074498.1	MLPH	0.073333333	6.973333333	0	0
XM_021074501.1	MLPH	0.02	1.666666667	0	0
XM_021074511.1	LYPD1	0.003333333	0.48	0	0
XM_021074539.1	LRP2BP	0.01	2.166666667	0	0
XM_021074544.1	HECW2	0.013333333	6.983333333	0	0
XM_021074577.1	SPATS2L	0.006666667	0.67	0	0
XM_021074581.1	SPATS2L	0.313333333	28.27333333	0	0
XM_021074583.1	SPATS2L	0.076666667	6.683333333	0	0
XM_021074586.1	RABGAP1	0.006666667	0.74	0	0
XM_021074599.1	RABGAP1	0.013333333	1.4	0	0
XM_021074608.1	RAB17	0.103333333	6.62	0	0
XM_021074614.1	RAB17	0.023333333	5.713333333	0	0
XM_021074620.1	DLGAP2	0.003333333	0.726666667	0	0
XM_021074642.1	CLN8	0.02	1.06	0	0
XM_021074701.1	CFLAR	0.126666667	32.61	0	0
XM_021074712.1	CASP8	0.053333333	7.02	0	0.116666667
XM_021074724.1	LOC110256907	0.01	0.333333333	0	0
XM_021074741.1	TRPM8	0.013333333	3.333333333	0	0
XM_021074749.1	CYTIP	0.016666667	2.653333333	0	0
XM_021074752.1	DUSP19	0.003333333	0.223333333	0	0
XM_021074753.1	RALB	0.056666667	5.296666667	0	0
XM_021074772.1	TBXT	0.31	24	0	0
XM_021074799.1	DOCK10	0.003333333	2.853333333	0	0
XM_021074808.1	DOCK10	0.01	6.673333333	0	0
XM_021074812.1	DOCK10	0.003333333	1.87	0	0
XM_021074814.1	DOCK10	0.003333333	1.793333333	0	0
XM_021074830.1	CHRNG	0.02	1.993333333	0	0
XM_021074839.1	GTDC1	0.006666667	3.15	0	0

XM_021074843.1	GTDC1	0.013333333	6.67	0	0
XM_021074867.1	RBPMS	0.013333333	1.473333333	0	0
XM_021074868.1	RBPMS	0.49	213.0166667	0	0.003333333
XM_021074889.1	PER2	0.35	94.48	0	0
XM_021074896.1	CKMT1A	0.073333333	5	0	0
XM_021074902.1	AP1S3	0.026666667	3	0	0
XM_021074911.1	CCDC150	0.023333333	3.57	0	0
XM_021074914.1	CCDC150	0.066666667	10.00666667	0	0
XM_021074915.1	CCDC150	0.02	3.08	0	0
XM_021074920.1	CCDC150	0.04	5.973333333	0	0
XM_021074922.1	CCDC150	0.1	14.15	0	0
XM_021074923.1	CCDC150	0.006666667	1.04	0	0
XM_021074940.1	SCN3A	0.003333333	0.666666667	0	0
XM_021074941.1	SCN3A	0.003333333	0.666666667	0	0
XM_021074965.1	RBM45	0.083333333	4.543333333	0	0
XM_021074975.1	ABCA12	0.003333333	1	0	0
XM_021075002.1	MAP2	0.01	4.06	0	0
XM_021075009.1	MAP2	0.003333333	1.84	0	0
XM_021075013.1	MAP2	0.003333333	0.81	0	0
XM_021075015.1	MAP2	0.003333333	0.5	0	0
XM_021075016.1	MAP2	0.013333333	3.116666667	0	0
XM_021075021.1	UNC80	0.003333333	0.77	0	0
XM_021075057.1	TRIP12	0.013333333	6.37	0	0
XM_021075076.1	TRIP12	0.053333333	23.42333333	0	0
XM_021075077.1	TRIP12	0.066666667	29.02	0	0
XM_021075089.1	TRIP12	0.073333333	30.05666667	0	0
XM_021075093.1	TRIP12	0.176666667	80.59	0	0.01
XM_021075102.1	PPP1R1C	0.003333333	0.333333333	0	0
XM_021075131.1	NFE2L2	0.086666667	32.93333333	0	0
XM_021075142.1	EPB41L5	0.003333333	0.49	0	0
XM_021075156.1	CLASP1	0.123333333	41.44333333	0	0
XM_021075176.1	CKMT1A	0.01	0.666666667	0	0
XM_021075179.1	CLASP1	0.033333333	11.77	0	0
XM_021075185.1	MGAT5	0.203333333	72.22333333	0	0
XM_021075191.1	TMEM163	0.006666667	0.646666667	0	0
XM_021075210.1	R3HDM1	0.106666667	21.25666667	0	0
XM_021075250.1	R3HDM1	0.01	1.553333333	0	0
XM_021075273.1	RHBDD1	0.42	86.68	0	0
XM_021075285.1	CXCR2	0.003333333	0.613333333	0	0
XM_021075306.1	CREB1	0.003333333	1.883333333	0	0.436666667
XM_021075309.1	CREB1	0.003333333	0.53	0	0.1
XM_021075336.1	ABI2	0.016666667	6.743333333	0	0
XM_021075357.1	PAX3	0.13	18.01333333	0	0
XM_021075358.1	PAX3	0.023333333	3.32	0	0
XM_021075381.1	GULP1	0.026666667	3.43	0	0
XM_021075384.1	GULP1	0.176666667	23.91666667	0	0
XM_021075386.1	GULP1	0.213333333	30.61333333	0	0
XM_021075422.1	COBLL1	0.016666667	3.33	0	0
XM_021075431.1	B3GALT1	0.463333333	106.16	0	0
XM_021075433.1	B3GALT1	0.013333333	4.53	0	0
XM_021075444.1	CERKL	0.006666667	0.333333333	0	0
XM_021075475.1	LRRFIP1	0.006666667	0.966666667	0	0
XM_021075479.1	LRRFIP1	0.25	47.59666667	0	0
XM_021075481.1	LRRFIP1	0.066666667	12.27333333	0	0
XM_021075486.1	LRRFIP1	0.023333333	4.453333333	0	0
XM_021075489.1	LRRFIP1	0.003333333	0.376666667	0	0
XM_021075495.1	LRRFIP1	0.063333333	10.63	0	0
XM_021075498.1	LRRFIP1	0.06	9.883333333	0	0
XM_021075512.1	LOC110257042	0.01	0.333333333	0	0
XM_021075518.1	SLC25A12	0.1	12.91666667	0	0

XM_021075571.1	SORBS2	0.01	1.863333333	0	0
XM_021075578.1	SORBS2	0.03	5.353333333	0	0
XM_021075582.1	SORBS2	0.1	16.4	0	0
XM_021075587.1	PDE1A	0.16	29.83333333	0	0
XM_021075592.1	PDE1A	0.02	1.483333333	0	0
XM_021075612.1	STOX2	0.003333333	1.043333333	0	0
XM_021075620.1	LOC102158534	0.003333333	0.333333333	0	0
XM_021075623.1	IRF2	0.01	1.063333333	0	0
XM_021075637.1	ZNF385B	0.003333333	0.636666667	0	0
XM_021075639.1	ZNF385B	0.01	3.18	0	0.533333333
XM_021075643.1	SP140	0.02	1.976666667	0	0
XM_021075644.1	SP140	0.016666667	1.866666667	0	0
XM_021075657.1	LOC110257072	0.003333333	0.333333333	0	0
XM_021075663.1	LOC100517129	0.066666667	6.74	0	0
XM_021075665.1	LOC100517129	0.01	1.083333333	0	0
XM_021075688.1	TACC1	0.016666667	1.793333333	0	0
XM_021075694.1	TACC1	0.013333333	0.9	0	0
XM_021075702.1	C2CD6	0.003333333	0.666666667	0	0.276666667
XM_021075712.1	PASK	0.023333333	4.286666667	0	0
XM_021075719.1	MARS2	0.006666667	4.103333333	0	0
XM_021075735.1	RPE	0.033333333	5.343333333	0	0
XM_021075747.1	ARHGEF4	0.02	5.96	0	0
XM_021075780.1	GPD2	0.09	21.30666667	0	0
XM_021075784.1	WRN	0.033333333	10.38666667	0	0
XM_021075791.1	WRN	0.006666667	1.436666667	0	0
XM_021075804.1	MCPH1	0.023333333	3.04	0	0
XM_021075815.1	LOC102166828	0.01	0.333333333	0	0
XM_021075816.1	DNAH7	0.003333333	2	0	1
XM_021075817.1	SLC39A10	0.06	14.87	0	0
XM_021075819.1	SLC39A10	0.123333333	27.46333333	0	0
XM_021075826.1	CSMD1	0.003333333	1.333333333	0	0.666666667
XM_021075833.1	KYNU	0.003333333	0.763333333	0	0
XM_021075840.1	LOC110257105	0.006666667	0.333333333	0	0
XM_021075855.1	PGAP1	0.05	19.23666667	0	0
XM_021075859.1	PGAP1	0.02	7.946666667	0	0
XM_021075879.1	WDR17	0.006666667	1.426666667	0	0
XM_021075884.1	OSBPL6	0.006666667	1.01	0	0
XM_021075891.1	OSBPL6	0.006666667	1.286666667	0	0
XM_021075893.1	LOC110257114	0.006666667	0.333333333	0	0
XM_021075909.1	NSD3	0.016666667	7.083333333	0	0
XM_021075930.1	ZNF804A	0.003333333	1.333333333	0	0
XM_021075933.1	LOC100522040	0.01	0.98	0	0
XM_021075945.1	LOC102160973	0.003333333	0.666666667	0	0
XM_021075955.1	GPR35	0.033333333	4.876666667	0	0
XM_021075958.1	MYO1B	0.083333333	19.51666667	0	0
XM_021075960.1	MYO1B	0.006666667	1.443333333	0	0
XM_021075970.1	IKZF2	0.02	7.823333333	0	0
XM_021075977.1	IKZF2	0.01	4.293333333	0	0
XM_021075979.1	IKZF2	0.003333333	1.173333333	0	0
XM_021075986.1	IKZF2	0.023333333	8.766666667	0	0
XM_021075987.1	IKZF2	0.04	15.43666667	0	0.013333333
XM_021075990.1	IKZF2	0.006666667	2.256666667	0	0
XM_021075991.1	IKZF2	0.01	3.496666667	0	0
XM_021076001.1	IKZF2	0.006666667	2.74	0	0
XM_021076002.1	IKZF2	0.003333333	1.316666667	0	0
XM_021076012.1	FARP2	0.016666667	3.17	0	0
XM_021076015.1	FARP2	0.01	1.99	0	0
XM_021076018.1	FARP2	0.026666667	4.483333333	0	0
XM_021076024.1	STK25	0.106666667	9.566666667	0	0
XM_021076047.1	INO80D	0.016666667	10.27333333	0	0

XM_021076067.1	NEMP2	0.016666667	2.636666667	0	0
XM_021076070.1	SATB2	0.003333333	1.316666667	0	0
XM_021076091.1	SPAG16	0.003333333	1.033333333	0	0.666666667
XM_021076093.1	SPAG16	0.01	0.323333333	0	0
XM_021076127.1	UNC5D	0.006666667	3.12	0	0
XM_021076129.1	UNC5D	0.006666667	3.213333333	0	0
XM_021076135.1	UNC5D	0.003333333	1.666666667	0	0.333333333
XM_021076147.1	ADAM23	0.023333333	6.68	0	0
XM_021076148.1	ADAM23	0.04	10.96666667	0	0
XM_021076153.1	MYO7B	0.146666667	43.58666667	0	0
XM_021076161.1	PIKFYVE	0.146666667	59.34333333	0	0
XM_021076167.1	PIKFYVE	0.12	45.89666667	0	0
XM_021076178.1	SCN9A	0.006666667	1.666666667	0	0.33
XM_021076188.1	CDK15	0.023333333	2	0	0
XM_021076206.1	USP40	0.03	4.943333333	0	0
XM_021076215.1	FBXO25	0.04	13.77333333	0	0
XM_021076230.1	FBXO25	0.01	2.76	0	0
XM_021076232.1	ANKRD44	0.006666667	3.44	0	0
XM_021076262.1	C15H2orf88	0.096666667	18.85666667	0	0
XM_021076272.1	CCDC173	0.016666667	1.333333333	0	0
XM_021076274.1	CCDC173	0.013333333	1.193333333	0	0
XM_021076277.1	XRCC5	0.53	43.82	0	0
XM_021076309.1	TMEM198	0.01	1	0	0
XM_021076324.1	FCER2	0.003333333	0.333333333	0	0
XM_021076341.1	ARHGAP15	0.01	1.396666667	0	0
XM_021076345.1	HOXD10	0.036666667	12.77666667	0	0
XM_021076366.1	TENM3	0.006666667	3.043333333	0	0
XM_021076372.1	UIMC1	0.076666667	25.03	0	0
XM_021076388.1	LOC100525433	0.08	30.57	0	0
XM_021076399.1	NRG1	0.01	3.503333333	0	0
XM_021076400.1	NRG1	0.01	3.166666667	0	0.506666667
XM_021076422.1	IWS1	0.09	11.26333333	0	0
XM_021076423.1	IWS1	0.003333333	0.36	0	0
XM_021076436.1	PKP4	0.13	24.62666667	0	0
XM_021076446.1	PKP4	0.003333333	0.75	0	0.18
XM_021076455.1	ACVR1	0.043333333	4.043333333	0	0
XM_021076462.1	UPP2	0.456666667	45.36333333	0	0
XM_021076463.1	UPP2	0.093333333	7.303333333	0	0
XM_021076491.1	AMMECR1L	0.006666667	1.8	0	0
XM_021076524.1	C1QTNF2	0.02	0.89	0	0
XM_021076535.1	HAVCR1	0.01	1.24	0	0
XM_021076541.1	RGS7BP	0.006666667	0.896666667	0	0.083333333
XM_021076584.1	KCNIP1	0.05	4	0	0
XM_021076613.1	MTRR	0.273333333	54.02666667	0	0
XM_021076614.1	MTRR	0.043333333	7.4	0	0
XM_021076620.1	MTRR	0.013333333	2.766666667	0	0
XM_021076621.1	MTRR	0.1	16.35666667	0	0
XM_021076623.1	MTRR	0.086666667	13.00333333	0	0
XM_021076626.1	TTC23L	0.003333333	1	0	0.333333333
XM_021076646.1	DNAH5	0.003333333	2	0	0.666666667
XM_021076647.1	FYB1	0.026666667	5	0	0
XM_021076650.1	DAB2	0.083333333	16.95666667	0	0
XM_021076680.1	RANBP17	0.09	14.33333333	0	0
XM_021076687.1	ARL15	0.02	0.616666667	0	0
XM_021076693.1	CYFIP2	0.013333333	2.613333333	0	0
XM_021076709.1	FAXDC2	0.046666667	5.233333333	0	0
XM_021076719.1	ZNF366	0.14	34.72333333	0	0
XM_021076721.1	ZNF366	0.026666667	6.573333333	0	0
XM_021076722.1	S1PR5	0.05	6.07	0	0
XM_021076737.1	ANKRD55	0.003333333	0.446666667	0	0

XM_021076748.1	MAST4	0.016666667	7.996666667	0	0
XM_021076764.1	MAST4	0.063333333	66.40666667	0	0
XM_021076782.1	SGTB	0.006666667	0.816666667	0	0.14
XM_021076784.1	CENPK	0.013333333	0.77	0	0
XM_021076787.1	CENPK	0.173333333	5.736666667	0	0
XM_021076790.1	ERBIN	0.01	3.06	0	0
XM_021076792.1	ERBIN	0.013333333	3.403333333	0	0
XM_021076794.1	ERBIN	0.006666667	2.033333333	0	0
XM_021076817.1	LOC100514500	0.013333333	0.556666667	0	0
XM_021076827.1	IRX2	0.04	4.333333333	0	0
XM_021076860.1	FOXI1	0.003333333	0.133333333	0	0
XM_021076867.1	PDE4D	0.043333333	14.00333333	0	0
XM_021076875.1	PDE4D	0.366666667	111.8266667	0	0
XM_021076905.1	ATP10B	0.01	3.666666667	0	0
XM_021076923.1	BSCL2	0.216666667	14.49333333	0	0
XM_021076941.1	GABRP	0.006666667	0.333333333	0	0
XM_021076959.1	C16H5orf47	0.006666667	0.333333333	0	0
XM_021076975.1	FUBP3	0.003333333	0.453333333	0	0
XM_021076986.1	CAPSL	0.02	0.666666667	0	0
XM_021077019.1	TMEM267	0.01	1.26	0	0
XM_021077047.1	SLC38A9	0.013333333	4.026666667	0	0.236666667
XM_021077057.1	WWC1	0.196666667	33.66666667	0	0
XM_021077092.1	RNF180	0.066666667	7.346666667	0	0
XM_021077100.1	LOC110257336	0.006666667	0.11	0	0
XM_021077101.1	LOC110257337	0.006666667	0.11	0	0
XM_021077105.1	CCDC69	0.026666667	6.396666667	0	0
XM_021077193.1	BCAS1	0.01	1.286666667	0	0
XM_021077203.1	ZNF217	0.04	11.93666667	0	0
XM_021077206.1	IL12RB1	0.003333333	0.666666667	0	0
XM_021077232.1	ZHX3	0.013333333	5.606666667	0	0
XM_021077243.1	LOC100158011	0.02	1.236666667	0	0
XM_021077267.1	SDCBP2	0.006666667	0.403333333	0	0
XM_021077270.1	SEC23B	0.026666667	3.226666667	0	0
XM_021077275.1	HAO1	0.01	0.666666667	0	0
XM_021077279.1	SLC23A2	0.063333333	19.43333333	0	0
XM_021077286.1	SLC23A2	0.016666667	4.59	0	0
XM_021077304.1	SIGLEC1	0.01	3.326666667	0	0
XM_021077319.1	BMP7	0.026666667	2.36	0	0
XM_021077331.1	HNF4A	0.133333333	25.79333333	0	0
XM_021077339.1	SNRPB2	0.06	2.473333333	0	0
XM_021077343.1	RBL1	0.083333333	19.41	0	0
XM_021077404.1	PAK5	0.01	1.666666667	0	0
XM_021077409.1	ESRRA	0.003333333	0.26	0	0
XM_021077414.1	CFAP61	0.003333333	0.656666667	0	0
XM_021077445.1	COMMD7	0.373333333	29.37666667	0	0
XM_021077459.1	PLCB4	0.026666667	6.59	0	0
XM_021077464.1	PLCB4	0.013333333	2.69	0	0
XM_021077465.1	PLCB4	0.043333333	9.253333333	0	0
XM_021077531.1	MACROD2	0.01	2	0	0
XM_021077545.1	GOLGA7	0.03	0.493333333	0	0
XM_021077556.1	SLC4A11	0.013333333	2.056666667	0	0
XM_021077557.1	SLC4A11	0.063333333	9.513333333	0	0
XM_021077561.1	SLC4A11	0.03	4.126666667	0	0
XM_021077562.1	SLC4A11	0.056666667	7.976666667	0	0
XM_021077584.1	COL20A1	0.063333333	10.76333333	0	0.173333333
XM_021077595.1	CDK5RAP1	0.186666667	15.25666667	0	0
XM_021077625.1	UCKL1	0.05	13.40666667	0	0
XM_021077627.1	UCKL1	0.01	2.28	0	0
XM_021077656.1	RGS19	0.466666667	26.83666667	0	0
XM_021077660.1	RGS19	0.006666667	0.333333333	0	0

XM_021077662.1	RGS19	0.05	3.81	0	0
XM_021077671.1	ENTPD6	0.253333333	26.46666667	0	0
XM_021077702.1	PTK6	0.013333333	2.346666667	0	0
XM_021077705.1	SRMS	0.01	0.666666667	0	0
XM_021077713.1	CCM2L	0.05	5.976666667	0	0
XM_021077718.1	DLC1	0.046666667	11.98666667	0	0
XM_021077735.1	IDO2	0.05	5.49	0	0
XM_021077739.1	IDO2	0.003333333	0.506666667	0	0
XM_021077740.1	IDO2	0.013333333	1.566666667	0	0
XM_021077741.1	IDO2	0.01	1.436666667	0	0
XM_021077756.1	EBF4	0.02	1.513333333	0	0
XM_021077757.1	EBF4	0.03	2.376666667	0	0
XM_021077775.1	KCNQ2	0.003333333	0.72	0	0.503333333
XM_021077780.1	F11	0.006666667	1.333333333	0	0
XM_021077812.1	RALY	0.05	3.436666667	0	0
XM_021077856.1	TMEM59L	0.01	0.706666667	0	0
XM_021077863.1	RIPOR3	0.243333333	38.73333333	0	0.11
XM_021077872.1	CHD6	0.016666667	7.39	0	0
XM_021077876.1	DLGAP4	0.016666667	3.13	0	0
XM_021077878.1	DLGAP4	0.03	4.06	0	0
XM_021077881.1	DLGAP4	0.06	10.34	0	0
XM_021077891.1	SLA2	0.003333333	0.503333333	0	0.123333333
XM_021077902.1	PCM1	0.003333333	1.313333333	0	0
XM_021077911.1	PCM1	0.066666667	21.60333333	0	0
XM_021077930.1	PCM1	0.053333333	16.59	0	0
XM_021077952.1	FGL1	0.01	1.666666667	0	0
XM_021077956.1	SLC9A8	0.05	9.776666667	0	0
XM_021077972.1	SRC	0.013333333	2.703333333	0	0
XM_021077973.1	SRC	0.006666667	1.136666667	0	0
XM_021078011.1	CD151	0.523333333	33.26666667	0	0
XM_021078012.1	EPB41L1	0.016666667	4.526666667	0	0
XM_021078013.1	EPB41L1	0.143333333	38.64333333	0	0
XM_021078016.1	EPB41L1	0.173333333	48.95	0	0
XM_021078020.1	EPB41L1	0.32	78.39666667	0	0
XM_021078033.1	RALGAPA2	0.036666667	14.54	0	0
XM_021078046.1	KIF16B	0.173333333	71.63666667	0	0
XM_021078062.1	RNF24	0.033333333	10.36	0	0
XM_021078076.1	EFCAB8	0.073333333	19.71333333	0	0
XM_021078086.1	TPX2	0.086666667	10.96	0	0.013333333
XM_021078091.1	TPX2	0.046666667	6.123333333	0	0
XM_021078094.1	TPX2	0.063333333	7.88	0	0
XM_021078098.1	TPX2	0.05	6.453333333	0	0
XM_021078111.1	PTPRT	0.006666667	3.25	0	0
XM_021078120.1	PTPRT	0.003333333	1.53	0	0.79
XM_021078142.1	HCK	0.033333333	3.36	0	0
XM_021078163.1	ARMC6	0.046666667	4.146666667	0	0
XM_021078164.1	SGK2	0.05	4.38	0	0
XM_021078170.1	SGK2	0.01	0.843333333	0	0
XM_021078176.1	SGK2	0.033333333	2.616666667	0	0
XM_021078191.1	DZANK1	0.596666667	70.88	0	0
XM_021078194.1	TP53TG5	0.006666667	0.333333333	0	0
XM_021078236.1	YTHDF1	0.023333333	3.013333333	0	0
XM_021078241.1	GID8	0.11	17.27333333	0	0
XM_021078242.1	GID8	0.16	18.56	0	0
XM_021078264.1	C17H20orf173	0.003333333	0.333333333	0	0
XM_021078305.1	ELMO2	0.046666667	7.64	0	0
XM_021078312.1	NINL	0.026666667	4.216666667	0	0
XM_021078320.1	SLC24A2	0.003333333	0.76	0	0
XM_021078321.1	LOC102165279	0.006666667	0.666666667	0	0
XM_021078328.1	H2AFY	0.313333333	25.21666667	0	0

XM_021078351.1	LOC110257498	0.023333333	1	0	0
XM_021078367.1	RTEL1	0.09	16.98333333	0	0
XM_021078387.1	SLC24A2	0.003333333	1.593333333	0	0.333333333
XM_021078388.1	PLCG1	0.123333333	31.29666667	0	0
XM_021078422.1	FOXP2	0.013333333	1.326666667	0	0
XM_021078427.1	FOXP2	0.01	1.14	0	0
XM_021078452.1	AGK	0.036666667	4.676666667	0	0
XM_021078464.1	LOC100302368	0.003333333	0.47	0	0.036666667
XM_021078465.1	HOXA13	0.003333333	0.376666667	0	0
XM_021078470.1	ADCYAP1R1	0.05	14.24666667	0	0
XM_021078477.1	ADCYAP1R1	0.016666667	4.37	0	0
XM_021078479.1	TSPAN12	0.07	7.296666667	0	0
XM_021078493.1	HOXA10	0.04	8.48	0	0
XM_021078501.1	LEP	0.02	2.483333333	0	0
XM_021078506.1	GHRHR	0.016666667	1.826666667	0	0
XM_021078507.1	GHRHR	0.026666667	2.68	0	0
XM_021078515.1	CHRM2	0.006666667	1.886666667	0	0
XM_021078522.1	CFTR	0.003333333	1.196666667	0	0
XM_021078601.1	SSBP1	0.326666667	10.20666667	0	0
XM_021078605.1	RAB19	0.016666667	1.333333333	0	0
XM_021078610.1	SCRN1	0.013333333	3.97	0	0
XM_021078621.1	PRKAG2	0.02	2.016666667	0	0
XM_021078623.1	GALNTL5	0.013333333	9.773333333	0	0
XM_021078638.1	CAMK2B	0.09	8.943333333	0	0
XM_021078642.1	CAMK2B	0.046666667	4.46	0	0
XM_021078643.1	CAMK2B	0.166666667	15.85666667	0	0
XM_021078651.1	BLVRA	0.32	15.16	0	0
XM_021078655.1	CTTNBP2	0.003333333	1.16	0	0
XM_021078684.1	AOC1	1.22	162.2666667	0	0
XM_021078686.1	AOC1	0.76	97.73333333	0	0
XM_021078692.1	AKR1D1	0.01	0.896666667	0	0
XM_021078702.1	CCDC136	0.033333333	7.426666667	0	0
XM_021078704.1	CCDC136	0.016666667	3.593333333	0	0
XM_021078730.1	STK31	0.003333333	0.666666667	0	0
XM_021078748.1	HNRNPM	0.073333333	7.156666667	0	0.026666667
XM_021078776.1	LOC100525318	0.006666667	1.003333333	0	0
XM_021078781.1	SVOPL	0.003333333	0.333333333	0	0
XM_021078789.1	HNRNPM	0.123333333	12.39666667	0	0.103333333
XM_021078790.1	UPP1	0.026666667	1.113333333	0	0
XM_021078796.1	UBE2H	0.083333333	18.66333333	0	0
XM_021078804.1	CLEC2L	0.01	0.666666667	0	0
XM_021078823.1	CEP41	0.036666667	6.003333333	0	0
XM_021078830.1	AHCYL2	0.136666667	30.47333333	0	0
XM_021078844.1	TAS2R16	0.003333333	2	0	0
XM_021078857.1	LOC102162486	0.066666667	20.25666667	0	0
XM_021078859.1	SLC4A2	0.02	3.466666667	0	0
XM_021078862.1	SLC4A2	0.02	3.156666667	0	0
XM_021078878.1	EPHB6	0.013333333	2.043333333	0	0
XM_021078883.1	EPHB6	0.006666667	1.663333333	0	0
XM_021078898.1	TRPV6	0.016666667	2	0	0
XM_021078904.1	LOC110257558	0.02	1.333333333	0	0
XM_021078906.1	LOC100627175	0.013333333	2.556666667	0	0
XM_021078922.1	NLRP3	0.003333333	0.44	0	0
XM_021078933.1	MKLN1	0.02	13.52666667	0	0
XM_021078963.1	IRF5	0.1	11.98	0	0.013333333
XM_021078966.1	CADPS2	0.066666667	14.63666667	0	0
XM_021078970.1	CADPS2	0.026666667	8.68	0	0.52
XM_021079011.1	DGKI	0.003333333	1	0	0.666666667
XM_021079014.1	DGKI	0.003333333	2	0	0.333333333
XM_021079024.1	WDR60	0.026666667	6.4	0	0

XM_021079041.1	HOXA3	0.01	2.14	0	0
XM_021079045.1	HOXA3	0.073333333	11.56333333	0	0
XM_021079050.1	LOC110257594	0.003333333	0.333333333	0	0
XM_021079068.1	NACAD	0.006666667	2.33	0	0
XM_021079076.1	KIAA0895	0.036666667	6.92	0	0
XM_021079077.1	KIAA0895	0.036666667	6.87	0	0
XM_021079083.1	POU6F2	0.003333333	1	0	0.333333333
XM_021079105.1	ZNF862	0.01	5.116666667	0	0
XM_021079107.1	ZNF862	0.016666667	6.46	0	0
XM_021079120.1	MDFIC	0.01	1.133333333	0	0
XM_021079124.1	KMT2C	0.023333333	16.06333333	0	0
XM_021079133.1	KMT2C	0.17	123.59	0	0
XM_021079152.1	LOC100627175	0.046666667	8.263333333	0	0
XM_021079166.1	CALD1	0.016666667	3.71	0	0
XM_021079183.1	IQCA1L	0.01	1	0	0
XM_021079198.1	ITPRID1	0.006666667	1	0	0
XM_021079206.1	PDE1C	0.006666667	1.073333333	0	0
XM_021079226.1	WDR86	0.023333333	3.106666667	0	0
XM_021079232.1	GIMAP6	0.076666667	6.106666667	0	0
XM_021079238.1	ELMO1	0.006666667	3.213333333	0	0
XM_021079253.1	MPP6	0.25	59.22333333	0	0
XM_021079256.1	MPP6	0.17	42.56333333	0	0
XM_021079261.1	ABCF2	0.066666667	9.33	0	0
XM_021079277.1	DOCK4	0.013333333	5.143333333	0	0
XM_021079296.1	ACTR3B	0.02	1.603333333	0	0
XM_021079301.1	ACTR3B	0.036666667	2.736666667	0	0
XM_021079314.1	DPY19L1	0.026666667	13.32666667	0	0
XM_021079317.1	DPY19L1	0.033333333	16.96333333	0	0
XM_021079319.1	DPY19L1	0.013333333	7.553333333	0	0
XM_021079326.1	NPSR1	0.006666667	1	0	0
XM_021079331.1	BBS9	0.043333333	9.726666667	0	0
XM_021079352.1	TNS3	0.083333333	27.19	0	0
XM_021079358.1	TNS3	0.026666667	8.246666667	0	0
XM_021079360.1	TNS3	0.043333333	14.55666667	0	0
XM_021079361.1	DUOXA1	0.013333333	0.666666667	0	0
XM_021079362.1	CIRBP	0.093333333	26.03	0	0
XM_021079376.1	ACE2	0.05	6.996666667	0	0
XM_021079377.1	ACE2	0.006666667	1.043333333	0	0
XM_021079387.1	TCEAL2	0.093333333	4.033333333	0	0
XM_021079393.1	PHEX	0.003333333	1.226666667	0	0
XM_021079395.1	PHEX	0.01	2.396666667	0	0
XM_021079396.1	ASB11	0.08	4.56	0	0
XM_021079403.1	KLF8	0.026666667	3.483333333	0	0
XM_021079417.1	PFKFB1	0.546666667	77.20666667	0	0
XM_021079426.1	PNPLA4	0.013333333	0.723333333	0	0
XM_021079430.1	RNASEH2A	0.043333333	3.676666667	0	0
XM_021079432.1	PNPLA4	0.033333333	1.67	0	0
XM_021079444.1	RNASEH2A	0.02	1.093333333	0	0
XM_021079448.1	MBNL3	0.016666667	6.576666667	0	0
XM_021079452.1	MBNL3	0.013333333	4.483333333	0	0
XM_021079465.1	GEMIN8	0.07	3.446666667	0	0
XM_021079491.1	TRO	0.003333333	0.666666667	0	0
XM_021079499.1	CYSLTR1	0.003333333	0.826666667	0	0
XM_021079502.1	LHFPL2	0.02	5.003333333	0	0
XM_021079512.1	F9	0.24	28.27666667	0	0
XM_021079519.1	FHL1	0.043333333	4.586666667	0	0
XM_021079535.1	MAGED1	0.05	6.52	0	0
XM_021079536.1	MAGED1	0.03	3.466666667	0	0
XM_021079537.1	FOXP3	0.006666667	1.333333333	0	0
XM_021079544.1	DMD	0.026666667	14.39333333	0	0

XM_021079551.1	DMD	0.006666667	2.896666667	0	0
XM_021079556.1	DMD	0.02	11.23	0	0
XM_021079573.1	CITED1	0.046666667	1.72	0	0
XM_021079575.1	CITED1	0.086666667	2.793333333	0	0
XM_021079578.1	CLDN2	0.783333333	96	0	0
XM_021079584.1	OFD1	0.023333333	3.403333333	0	0
XM_021079587.1	TCEANC	0.05	4.073333333	0	0
XM_021079588.1	TCEANC	0.023333333	1.77	0	0
XM_021079607.1	TRMT2B	0.416666667	29.73	0	0
XM_021079612.1	MAMLD1	0.033333333	5.353333333	0	0
XM_021079625.1	LOC106504240	0.006666667	0.333333333	0	0
XM_021079628.1	LOC100515119	0.003333333	0.55	0	0
XM_021079640.1	LOC100515119	0.016666667	1.796666667	0	0
XM_021079642.1	LOC100515119	0.006666667	0.833333333	0	0
XM_021079661.1	SYTL5	0.03	7.083333333	0	0.426666667
XM_021079669.1	DUSP9	0.053333333	9.496666667	0	0
XM_021079671.1	DUSP9	0.03	2.836666667	0	0
XM_021079673.1	BRD4	0.73	198.74	0	0
XM_021079684.1	IQSEC2	0.026666667	6.993333333	0	0
XM_021079705.1	GLOD5	0.07	2.333333333	0	0
XM_021079711.1	MUM1L1	0.013333333	4.53	0	0
XM_021079723.1	MUM1L1	0.07	14.97333333	0	0
XM_021079726.1	MUM1L1	0.043333333	15.89	0	0
XM_021079731.1	CYP4F55	0.146666667	15.02333333	0	0
XM_021079746.1	NLRP12L	0.133333333	29.23333333	0	0
XM_021079750.1	CYP4F55	0.04	4.096666667	0	0
XM_021079757.1	PCDH19	0.016666667	6.843333333	0	0
XM_021079759.1	CYP4F55	0.01	0.986666667	0	0
XM_021079776.1	HEPH	0.003333333	1.35	0	0.296666667
XM_021079779.1	KDM6A	0.143333333	38.13666667	0	0
XM_021079782.1	KDM6A	0.05	12.44666667	0	0
XM_021079784.1	KDM6A	0.006666667	1.686666667	0	0
XM_021079804.1	ZMAT1	0.013333333	2.92	0	0
XM_021079805.1	ZMAT1	0.003333333	0.72	0	0
XM_021079806.1	RRAGB	0.3	26.42666667	0	0
XM_021079809.1	RRAGB	0.09	5.38	0	0
XM_021079816.1	ARMCX4	0.03	11.90666667	0	0
XM_021079830.1	LOC100621834	0.05	2.383333333	0	0
XM_021079845.1	LOC110257680	0.006666667	0.556666667	0	0.11
XM_021079869.1	LOC110257692	0.01	0.333333333	0	0
XM_021079878.1	GRAMD2B	0.006666667	0.913333333	0	0
XM_021079900.1	NRK	0.18	62.76666667	0	0
XM_021079954.1	HAUS7	0.086666667	14.13666667	0	0
XM_021079955.1	HAUS7	0.06	9.36	0	0
XM_021079974.1	LOC110255258	0.026666667	3.39	0	0
XM_021079982.1	LOC110255258	0.003333333	0.223333333	0	0
XM_021079989.1	STX5	0.08	5.71	0	0
XM_021079990.1	LOC110255258	0.03	3.483333333	0	0
XM_021079994.1	LOC100526148	0.006666667	0.92	0	0
XM_021079997.1	GABRA3	0.03	4.333333333	0	0
XM_021080008.1	GPKOW	0.056666667	4.376666667	0	0
XM_021080013.1	GPKOW	0.056666667	4.413333333	0	0
XM_021080016.1	CCDC120	0.003333333	0.746666667	0	0
XM_021080024.1	CCDC120	0.046666667	9.666666667	0	0
XM_021080032.1	SLC25A14	0.213333333	13.72333333	0	0
XM_021080037.1	WDR13	0.043333333	2.986666667	0	0
XM_021080065.1	ZC3H12B	0.003333333	1	0	0.666666667
XM_021080072.1	EFHC2	0.003333333	0.333333333	0	0
XM_021080080.1	ATRX	0.063333333	30.34666667	0	0
XM_021080093.1	ZFP92	0.006666667	0.68	0	0

XM_021080112.1	NAP1L4	0.06	5.596666667	0	0
XM_021080119.1	PRRG3	0.026666667	6	0	0
XM_021080124.1	ALDH3B1	0.013333333	1.243333333	0	0
XM_021080145.1	SCML1	0.023333333	2.686666667	0	0
XM_021080154.1	LOC110257738	0.006666667	0.556666667	0	0.11
XM_021080160.1	ENOX2	0.076666667	6.163333333	0	0
XM_021080190.1	FRMPD3	0.003333333	0.596666667	0	0.09
XM_021080203.1	ATP11C	0.04	10.32666667	0	0
XM_021080207.1	CALCB	0.003333333	0.44	0	0
XM_021080211.1	CXHXorf67	0.003333333	1	0	0.666666667
XM_021080216.1	TMEM255A	0.026666667	2.056666667	0	0
XM_021080219.1	CACNA1F	0.003333333	1	0	0.666666667
XM_021080241.1	LOC102159844	0.006666667	0.666666667	0	0
XM_021080270.1	TBL1X	0.096666667	19.92	0	0
XM_021080311.1	FAM122B	0.006666667	0.813333333	0	0
XM_021080319.1	FAM122B	0.03	5.473333333	0	0
XM_021080321.1	FAM122B	0.076666667	8.963333333	0	0
XM_021080322.1	FAM122B	0.026666667	5.113333333	0	0
XM_021080328.1	IL13	0.003333333	0.326666667	0	0
XM_021080331.1	FAM122B	0.016666667	0.83	0	0
XM_021080390.1	ZNF81	0.023333333	13.53666667	0	0
XM_021080396.1	ZNF182	0.026666667	3.9	0	0
XM_021080438.1	DCX	0.006666667	3.14	0	0
XM_021080441.1	RTL4	0.006666667	0.666666667	0	0
XM_021080444.1	LDLR	0.043333333	8.216666667	0	0
XM_021080466.1	THOC2	0.08	28.09	0	0
XM_021080494.1	IGSF1	0.063333333	11.94	0	0
XM_021080498.1	FRMD7	0.003333333	0.333333333	0	0
XM_021080500.1	TEX13A	0.006666667	0.333333333	0	0
XM_021080504.1	ZMYM3	0.366666667	89.37	0	0.023333333
XM_021080511.1	CD99L2	0.02	2.32	0	0
XM_021080514.1	FANCB	0.03	3.9	0	0
XM_021080517.1	GLRA2	0.003333333	0.333333333	0	0
XM_021080528.1	SMIM9	0.03	2	0	0
XM_021080544.1	LOC396905	0.003333333	0.186666667	0	0
XM_021080550.1	CDKL5	0.076666667	11.41666667	0	0
XM_021080578.1	LOC110257828	0.046666667	1.376666667	0	0
XM_021080591.1	RAI2	0.013333333	1.553333333	0	0
XM_021080651.1	PHKA2	0.063333333	25.32	0	0
XM_021080656.1	ZFX	0.033333333	7.746666667	0	0
XM_021080676.1	PAK3	0.01	1.376666667	0	0
XM_021080679.1	PAK3	0.003333333	0.586666667	0	0
XM_021080689.1	PAK3	0.04	4.136666667	0	0
XM_021080696.1	PHKA1	0.153333333	41.54333333	0	0.456666667
XM_021080699.1	PHKA1	3.423333333	555.8966667	0	0
XM_021080707.1	CTPS2	0.083333333	8.4	0	0
XM_021080711.1	ABCB7	0.15	22.57666667	0	0
XM_021080728.1	FLNA	0.04	15.79666667	0	0
XM_021080730.1	FLNA	0.053333333	20.76666667	0	0
XM_021080731.1	FLNA	0.076666667	29.80333333	0	0
XM_021080742.1	ADGRE5	0.18	24.38333333	0	0
XM_021080745.1	SLC10A3	0.04	3.123333333	0	0
XM_021080746.1	SLC10A3	0.556666667	44.35666667	0	0
XM_021080781.1	BCLAF3	0.013333333	2.146666667	0	0
XM_021080807.1	MAP3K15	0.016666667	3.333333333	0	0
XM_021080834.1	PPP2R2B	0.006666667	3.686666667	0	1.016666667
XM_021080837.1	MTM1	0.093333333	11.92666667	0	0
XM_021080838.1	MTM1	0.073333333	8.916666667	0	0
XM_021080841.1	MTM1	0.04	4.58	0	0
XM_021080848.1	SMARCA1	0.023333333	3.93	0	0

XM_021080850.1	SMARCA1	0.036666667	6.33	0	0
XM_021080861.1	INTS6L	0.256666667	41.02	0	0
XM_021080863.1	INTS6L	0.046666667	7.603333333	0	0
XM_021080873.1	PNPLA4	0.003333333	0.206666667	0	0
XM_021080916.1	CAST	0.066666667	11.68666667	0	0
XM_021080919.1	CAST	0.033333333	6.283333333	0	0
XM_021080928.1	CAST	0.016666667	3.126666667	0	0
XM_021080933.1	LOC100624329	0.03	7.803333333	0	0
XM_021080948.1	CAST	0.03	5.273333333	0	0
XM_021080989.1	CAST	0.206666667	36.64333333	0	0
XM_021081014.1	ZNF311	0.01	1.333333333	0	0.216666667
XM_021081018.1	LOC110258046	0.103333333	7.743333333	0	0
XM_021081054.1	FAM69B	0.126666667	10.25	0	0
XM_021081063.1	CCDC187	0.016666667	6.826666667	0	0
XM_021081066.1	CCDC187	0.01	4	0	0
XM_021081072.1	PNPLA7	0.003333333	0.37	0	0
XM_021081082.1	NSMF	0.02	2.64	0	0
XM_021081094.1	ENTPD8	0.016666667	3.106666667	0	0
XM_021081110.1	ARRDC1	0.013333333	1.773333333	0	0
XM_021081114.1	WT1	0.01	1.66	0	0
XM_021081148.1	CARD9	0.006666667	0.79	0	0
XM_021081149.1	CARD9	0.006666667	1.093333333	0	0
XM_021081180.1	SLC34A3	0.12	12.56333333	0	0
XM_021081181.1	SLC34A3	0.056666667	5.413333333	0	0.166666667
XM_021081187.1	SLC34A3	0.006666667	0.786666667	0	0
XM_021081188.1	SLC34A3	0.103333333	13.62	0	0
XM_021081201.1	EHMT1	0.006666667	0.8	0	0
XM_021081202.1	EHMT1	0.226666667	47.05	0	0
XM_021081231.1	PAXX	0.053333333	2.016666667	0	0
XM_021081233.1	PAXX	0.223333333	7.72	0	0
XM_021081252.1	NACC2	0.073333333	16.82666667	0	0
XM_021081262.1	ACP5	0.013333333	1.143333333	0	0
XM_021081291.1	LOC110258131	0.013333333	1.333333333	0	0
XM_021081320.1	TNRC6A	0.086666667	27.85	0	0
XM_021081333.1	TNRC6A	0.056666667	16.82	0	0.35
XM_021081352.1	LOC100518090	0.013333333	0.41	0	0
XM_021081386.1	LOC110258194	0.003333333	0.333333333	0	0
XM_021081394.1	LOC102161330	0.02	2	0	0
XM_021081401.1	LOC110258212	0.04	2	0	0
XM_021081403.1	LOC110258215	0.013333333	0.356666667	0	0
XM_021081427.1	LOC100523107	0.01	1.173333333	0	0
XM_021081445.1	LOC100519244	0.01	0.333333333	0	0
XM_021081451.1	LOC100625375	0.026666667	1.016666667	0	0
XM_021081452.1	LOC110258278	0.016666667	0.66	0	0
XM_021081475.1	LOC110258312	0.133333333	5	0	0
XM_021081479.1	HMGXB4	0.01	1.96	0	0
XM_021081502.1	CKB	0.026666667	1.733333333	0	0
XM_021081505.1	PLD4	0.013333333	1.31	0	0
XM_021081517.1	CEP170B	0.023333333	6.56	0	0
XM_021081520.1	CEP170B	0.003333333	1.313333333	0	0
XM_021081539.1	ZFYVE21	0.033333333	1.316666667	0	0
XM_021081555.1	NUDT14	0.026666667	0.986666667	0	0
XM_021081556.1	NUDT14	0.016666667	0.533333333	0	0
XM_021081567.1	TMEM121	0.043333333	6.306666667	0	0
XM_021081579.1	WDR20	0.013333333	0.57	0	0
XM_021081600.1	MARK3	0.726666667	73.02	0	0
XM_021081605.1	PPP2R5C	0.08	14.91	0	0
XM_021081613.1	PACS2	0.393333333	89.90333333	0	0.233333333
XM_021081622.1	CDC42BPB	0.05	13.97666667	0	0
XM_021081627.1	TRAF3	0.056666667	17.87333333	0	0

XM_021081629.1	TRAF3	0.013333333	4.313333333	0	0
XM_021081631.1	AMN	0.023333333	1.603333333	0	0
XM_021081633.1	AMN	0.473333333	30.06333333	0	0
XM_021081665.1	EIF5	0.046666667	8.383333333	0	0
XM_021081672.1	LOC100157908	0.01	1.333333333	0	0
XM_021081673.1	LOC110258386	0.006666667	1.92	0	0
XM_021081687.1	LOC110258402	0.01	0.333333333	0	0
XM_021081701.1	TNNT3	0.146666667	9.116666667	0	0
XM_021081776.1	LOC100512181	0.02	3.986666667	0	0
XM_021081784.1	FUT10	0.013333333	2.913333333	0	0
XM_021081793.1	TTI2	0.743333333	67.27666667	0	0
XM_021081816.1	LOC110258568	0.03	1.666666667	0	0
XM_021081818.1	LOC110258569	0.01	0.436666667	0	0
XM_021081834.1	LOC110258582	0.006666667	0.333333333	0	0
XM_021081840.1	LOC110258589	0.006666667	0.24	0	0
XM_021081848.1	CFH	0.003333333	0.186666667	0	0
XM_021081849.1	CFH	0.006666667	0.72	0	0
XM_021081855.1	KCNT2	0.006666667	1.746666667	0	0
XM_021081872.1	LOC110258596	0.026666667	0.666666667	0	0
XM_021081894.1	LOC110258602	0.08	6.536666667	0	0
XM_021081920.1	LOC110255322	0.003333333	0.333333333	0	0
XM_021081922.1	LOC110258627	0.01	0.666666667	0	0
XM_021081940.1	LOC110258648	0.026666667	1.55	0	0
XM_021081961.1	LOC106505330	0.006666667	0.333333333	0	0
XM_021081962.1	LOC106505330	0.006666667	0.333333333	0	0
XM_021081981.1	LOC110258713	0.01	1.713333333	0	0
XM_021082001.1	LOC100736850	0.016666667	0.666666667	0	0
XM_021082017.1	LOC110258750	0.013333333	0.433333333	0	0
XM_021082040.1	DNMT1	0.046666667	10.84666667	0	0
XM_021082064.1	DNMT1	0.076666667	17.48333333	0	0
XM_021082114.1	LOC110258872	0.006666667	0.333333333	0	0
XM_021082152.1	LOC100521600	0.113333333	11.04	0	0
XM_021082154.1	LOC100521600	0.03	2.24	0	0
XM_021082158.1	LOC100521600	0.063333333	4.693333333	0	0
XM_021082159.1	LOC100521600	0.03	1.026666667	0	0
XM_021082162.1	LOC110258908	0.026666667	5.613333333	0	0
XM_021082163.1	MEF2C	0.01	1.806666667	0	0
XM_021082175.1	SNRPN	0.03	1.853333333	0	0
XM_021082177.1	SNRPN	0.04	2.596666667	0	0
XM_021082178.1	SNRPN	0.086666667	4.693333333	0	0
XM_021082184.1	SNRPN	0.05	17.73	0	0
XM_021082214.1	LOC110258930	0.01	0.333333333	0	0
XM_021082222.1	LOC100739205	0.016666667	0.333333333	0	0
XM_021082230.1	LOC110258939	0.01	0.333333333	0	0
XM_021082258.1	LOC110258970	0.006666667	0.666666667	0	0
XM_021082262.1	LOC106509256	0.016666667	0.666666667	0	0
XM_021082264.1	LOC100153261	0.006666667	0.333333333	0	0
XM_021082268.1	LOC110258977	0.006666667	0.333333333	0	0
XM_021082270.1	LOC100519758	0.006666667	0.333333333	0	0
XM_021082272.1	LOC110258980	0.006666667	0.333333333	0	0
XM_021082276.1	RLN2	0.013333333	0.333333333	0	0
XM_021082280.1	LOC100738075	0.003333333	0.333333333	0	0
XM_021082309.1	LOC100153359	0.023333333	6.046666667	0	0
XM_021082311.1	LOC110259013	0.013333333	1.546666667	0	0
XM_021082337.1	LOC110259044	0.01	2.423333333	0	0.81
XM_021082339.1	SLC34A1	0.15	15.19666667	0	0
XM_021082353.1	SLC34A1	0.143333333	11.50666667	0	0
XM_021082399.1	MGAT1	0.033333333	4.526666667	0	0
XM_021082414.1	LOC110259098	0.003333333	0.333333333	0	0
XM_021082427.1	LOC110259118	0.003333333	0.083333333	0	0

XM_021082433.1	LOC110259120	0.003333333	0.083333333	0	0
XM_021082434.1	SLC22A2	0.07	11.56	0	0
XM_021082437.1	LOC100523228	0.01	0.333333333	0	0
XM_021082449.1	LOC100522225	0.003333333	0.523333333	0	0
XM_021082459.1	LOC110259145	0.023333333	0.666666667	0	0
XM_021082465.1	LOC110259155	0.013333333	0.666666667	0	0
XM_021082473.1	LOC110259163	0.01	0.333333333	0	0
XM_021082480.1	DIP2A	0.046666667	13.28666667	0	0
XM_021082531.1	PGGHG	0.016666667	1.753333333	0	0
XM_021082537.1	B4GALNT4	0.006666667	1.666666667	0	0
XM_021082548.1	SLC22A1	0.01	1	0	0
XM_021082555.1	CDHR5	0.056666667	5.36	0	0
XM_021082557.1	EPS8L2	0.203333333	26.44	0	0
XM_021082561.1	EPS8L2	0.03	3.873333333	0	0
XM_021082562.1	EPS8L2	0.056666667	7.863333333	0	0
XM_021082563.1	EPS8L2	0.02	2.486666667	0	0
XM_021082570.1	PIDD1	0.01	1.383333333	0	0
XM_021082576.1	TSPAN4	0.246666667	65.7	0	0
XM_021082583.1	MUC5AC	0.003333333	1.666666667	0	0
XM_021082584.1	MUC2	0.2	137.3333333	0	0.666666667
XM_021082589.1	BRSK2	0.003333333	0.85	0	0
XM_021082590.1	BRSK2	0.013333333	2	0	0
XM_021082612.1	TSPAN32	0.01	0.853333333	0	0
XM_021082624.1	SLC22A18	0.043333333	2.696666667	0	0
XM_021082625.1	SLC22A18	0.143333333	8.846666667	0	0
XM_021082630.1	SLC22A18	0.103333333	5.963333333	0	0
XM_021082645.1	SHANK2	0.03	11.33333333	0	1
XM_021082677.1	ANO1	0.05	10.00666667	0	0
XM_021082678.1	ANO1	0.073333333	15.04333333	0	0
XM_021082679.1	ANO1	0.04	7.976666667	0	0
XM_021082707.1	PPP6R3	0.02	3.573333333	0	0
XM_021082719.1	LRP5	0.013333333	2.756666667	0	0
XM_021082739.1	ARG1	0.01	0.936666667	0	0
XM_021082747.1	ARG1	0.003333333	0.333333333	0	0
XM_021082754.1	CABP4	0.003333333	0.333333333	0	0
XM_021082759.1	TBC1D10C	0.053333333	4.333333333	0	0
XM_021082769.1	SPTBN2	0.03	10	0	0
XM_021082798.1	PELI3	0.046666667	3.29	0	0
XM_021082801.1	SLC29A2	0.006666667	0.816666667	0	0
XM_021082816.1	CDKN2B	0.01	2.816666667	0	0
XM_021082817.1	CTSW	0.066666667	3.333333333	0	0
XM_021082821.1	SNX32	0.003333333	0.333333333	0	0
XM_021082823.1	AP5B1	0.036666667	4.35	0	0
XM_021082826.1	PCNX3	0.096666667	28.98666667	0	0
XM_021082831.1	PCNX3	0.16	48.44333333	0	0
XM_021082835.1	EHBP1L1	1.173333333	280.9066667	0	0
XM_021082837.1	EHBP1L1	0.233333333	53.30333333	0	0
XM_021082839.1	EHBP1L1	0.02	3.433333333	0	0
XM_021082846.1	EHBP1L1	0.013333333	1.86	0	0
XM_021082858.1	SLC25A45	0.026666667	3.003333333	0	0
XM_021082874.1	SLC22A12	0.243333333	27.47666667	0	0
XM_021082878.1	TRPT1	0.046666667	2.64	0	0
XM_021082880.1	TRPT1	0.043333333	2.186666667	0	0
XM_021082882.1	TRPT1	0.16	7.18	0	0
XM_021082886.1	FLRT1	0.016666667	8.973333333	0	0
XM_021082888.1	FLRT1	0.006666667	3.36	0	0.873333333
XM_021082905.1	LOC102164585	0.023333333	1.783333333	0	0
XM_021082906.1	LOC102164585	0.03	1.933333333	0	0
XM_021082907.1	LOC100513779	0.026666667	2.283333333	0	0
XM_021082917.1	CSKMT	0.133333333	6.14	0	0

XM_021082925.1	SYT7	0.063333333	15.15	0	0
XM_021082927.1	SYT7	0.036666667	7.826666667	0	0
XM_021082936.1	VWCE	0.006666667	1.07	0	0
XM_021082945.1	LOC100519643	0.283333333	35.12	0	0
XM_021082956.1	LOC100524786	0.003333333	0.333333333	0	0
XM_021082959.1	VPS37C	0.29	37.93333333	0	0
XM_021082967.1	MS4A10	0.026666667	0.81	0	0
XM_021082973.1	MS4A4A	0.03	1	0	0
XM_021082978.1	TCN1	0.013333333	0.666666667	0	0
XM_021082988.1	STX3	0.003333333	1.166666667	0	0
XM_021082997.1	FAM111B	0.046666667	5.503333333	0	0
XM_021082998.1	LOC100518109	0.533333333	51.08666667	0	0
XM_021082999.1	LOC100518109	0.036666667	3.573333333	0	0
XM_021083001.1	LOC100518109	0.11	2.933333333	0	0
XM_021083005.1	LOC100518644	0.016666667	0.83	0	0
XM_021083006.1	LOC100518644	0.016666667	1.056666667	0	0
XM_021083010.1	LOC110259258	0.143333333	20.01666667	0	0
XM_021083040.1	LOC110259262	0.01	0.396666667	0	0
XM_021083043.1	LOC100739611	0.06	2.246666667	0	0
XM_021083049.1	LOC110259268	0.01	0.373333333	0	0
XM_021083066.1	AGBL2	0.036666667	5.536666667	0	0
XM_021083072.1	AGBL2	0.016666667	2.416666667	0	0
XM_021083073.1	AGBL2	0.033333333	4.64	0	0
XM_021083103.1	CELF1	0.026666667	10.20666667	0	0
XM_021083118.1	LARGE2	0.03	3.333333333	0	0
XM_021083141.1	PHF21A	0.03	5.616666667	0	0
XM_021083149.1	PRDM11	0.033333333	15.92666667	0	0
XM_021083161.1	ACCS	0.02	1.506666667	0	0
XM_021083187.1	LDLRAD3	0.106666667	19.56666667	0	0
XM_021083188.1	TNC	0.01	3.026666667	0	0
XM_021083196.1	EHF	0.016666667	3.54	0	0
XM_021083219.1	LOC100737821	0.013333333	3.16	0	0
XM_021083221.1	LOC100737821	0.22	48.68	0	0
XM_021083223.1	LOC100737821	0.01	2.33	0	0
XM_021083231.1	NTRK3	0.08	13.61333333	0	0
XM_021083234.1	BBOX1	0.136666667	12.51	0	0
XM_021083236.1	BBOX1	0.066666667	6.156666667	0	0
XM_021083244.1	GAS2	0.016666667	1.73	0	0
XM_021083249.1	SLC17A6	0.006666667	1	0	0
XM_021083273.1	OTOG	0.006666667	4.333333333	0	0.333333333
XM_021083281.1	PLEKHA7	0.026666667	5.873333333	0	0
XM_021083288.1	PLEKHA7	0.023333333	4.7	0	0
XM_021083292.1	CDKN3	0.026666667	1.166666667	0	0
XM_021083295.1	CABP2	0.006666667	0.333333333	0	0
XM_021083296.1	INSC	0.003333333	0.493333333	0	0.093333333
XM_021083307.1	BTBD10	0.106666667	12.11333333	0	0.07
XM_021083312.1	CDKN3	0.18	7.766666667	0	0
XM_021083336.1	AMBP	0.013333333	1.756666667	0	0
XM_021083341.1	GUK1	0.086666667	3.323333333	0	0
XM_021083390.1	PTGR1	0.11	4.73	0	0
XM_021083395.1	TRIM58	0.003333333	0.823333333	0	0
XM_021083396.1	TRIM58	0.006666667	1.176666667	0	0
XM_021083420.1	PBX4	0.053333333	5.943333333	0	0
XM_021083422.1	PBX4	0.056666667	5.866666667	0	0
XM_021083432.1	TM6SF2	0.006666667	0.546666667	0	0
XM_021083448.1	SLC25A42	0.043333333	6.283333333	0	0
XM_021083475.1	CRLF1	0.066666667	4.336666667	0	0
XM_021083479.1	MAST3	0.003333333	1.443333333	0	0
XM_021083485.1	MAST3	0.016666667	4.043333333	0	0
XM_021083493.1	MAST3	0.01	3.5	0	0.003333333

XM_021083513.1	SSBP4	0.033333333	3.02	0	0
XM_021083554.1	UNC13A	0.003333333	0.546666667	0	0.406666667
XM_021083557.1	C5	0.013333333	2.666666667	0	0.333333333
XM_021083591.1	EPS15L1	0.026666667	3.603333333	0	0
XM_021083599.1	LOC110259328	0.02	2.52	0	0
XM_021083609.1	LOC110259329	0.003333333	0.333333333	0	0
XM_021083613.1	LOC102167481	0.183333333	16.15	0	0
XM_021083625.1	WIZ	0.226666667	48.06	0	0
XM_021083628.1	AKAP8L	0.033333333	15.17666667	0	0
XM_021083636.1	LOC100516957	0.01	1.333333333	0	0
XM_021083644.1	LOC100516957	0.003333333	0.476666667	0	0
XM_021083674.1	LOC100518417	0.013333333	3.11	0	0
XM_021083678.1	LOC100518417	0.016666667	3.443333333	0	0
XM_021083687.1	ZNF333	0.16	23.98	0	0
XM_021083688.1	QKI	0.003333333	1.076666667	0	0
XM_021083718.1	SYCE2	0.023333333	1	0	0
XM_021083720.1	MAST1	0.003333333	1	0	0
XM_021083733.1	LOC100521431	0.03	4.826666667	0	0
XM_021083749.1	LOC102167351	0.013333333	3.736666667	0	0
XM_021083759.1	LOC110259338	0.046666667	11.77333333	0	0
XM_021083765.1	LOC110259338	0.006666667	1.806666667	0	0
XM_021083768.1	LOC110259338	0.006666667	1.196666667	0	0
XM_021083780.1	SLC22A11	0.016666667	1.333333333	0	0
XM_021083788.1	LOC100526209	0.003333333	0.486666667	0	0
XM_021083800.1	DNM2	0.003333333	0.603333333	0	0
XM_021083801.1	DNM2	0.01	1.736666667	0	0
XM_021083807.1	DNM2	0.173333333	24.34	0	0
XM_021083814.1	DNM2	0.126666667	19.44666667	0	0
XM_021083819.1	TMED1	0.02	1.33	0	0
XM_021083828.1	SMARCA4	0.03	9.413333333	0	0
XM_021083869.1	DOCK6	0.013333333	1.79	0	0
XM_021083883.1	LOC100624806	0.006666667	1.616666667	0	0
XM_021083889.1	RAB27A	0.02	3.3	0	0
XM_021083893.1	LRRC8E	0.006666667	1.483333333	0	0
XM_021083896.1	TGFBR3L	0.01	0.666666667	0	0
XM_021083909.1	CAMSAP3	0.07	12.43	0	0
XM_021083910.1	CAMSAP3	0.006666667	1	0	0.003333333
XM_021083913.1	RAB27A	0.013333333	1.956666667	0	0
XM_021083916.1	RAB27A	0.026666667	3.86	0	0
XM_021083919.1	MCOLN1	0.096666667	9.663333333	0	0
XM_021083929.1	RAB27A	0.013333333	2.043333333	0	0
XM_021083937.1	PEX11G	0.126666667	4.886666667	0	0
XM_021083946.1	EMR4	0.04	4.776666667	0	0
XM_021083949.1	EMR4	0.02	1.543333333	0	0
XM_021083952.1	ADGRE1	0.003333333	0.55	0	0
XM_021083962.1	ADGRE1	0.003333333	0.626666667	0	0
XM_021083969.1	ADGRE1	0.003333333	0.953333333	0	0
XM_021083979.1	SH2D3A	0.006666667	0.666666667	0	0
XM_021083981.1	SH2D3A	0.01	1.273333333	0	0
XM_021083997.1	RFX2	0.053333333	8.056666667	0	0
XM_021084001.1	RFX2	0.063333333	9.07	0	0
XM_021084003.1	RFX2	0.113333333	16.05	0	0.003333333
XM_021084050.1	TNFAIP8L1	0.016666667	2.173333333	0	0
XM_021084053.1	PLAGL1	0.003333333	1.25	0	0
XM_021084064.1	PLAGL1	0.006666667	2.553333333	0	0.006666667
XM_021084065.1	FSD1	0.006666667	0.333333333	0	0
XM_021084066.1	STAP2	0.03	1.94	0	0
XM_021084096.1	ZFR2	0.003333333	0.663333333	0	0
XM_021084106.1	PLAGL1	0.043333333	15.44333333	0	0
XM_021084123.1	SMIM24	0.133333333	6	0	0

XM_021084142.1	CELF5	0.003333333	0.333333333	0	0
XM_021084147.1	TLE2	0.03	3.233333333	0	0
XM_021084161.1	LOC100516355	0.016666667	5.1	0	0
XM_021084168.1	LOC110259370	0.013333333	1.236666667	0	0
XM_021084217.1	ATP8B3	0.006666667	1.53	0	0
XM_021084222.1	ATP8B3	0.033333333	6.666666667	0	0
XM_021084226.1	TCF3	0.053333333	4.906666667	0	0
XM_021084233.1	PLK5	0.006666667	0.666666667	0	0
XM_021084244.1	LOC100511862	0.113333333	19.68333333	0	0
XM_021084278.1	ABCA7	0.03	8.76	0	0
XM_021084279.1	ABCA7	0.023333333	7.473333333	0	0
XM_021084281.1	ARHGAP45	0.02	3.356666667	0	0
XM_021084283.1	ARHGAP45	0.04	6.49	0	0
XM_021084291.1	MISP	0.046666667	5.423333333	0	0
XM_021084327.1	HNRNPH1	0.006666667	0.613333333	0	0
XM_021084329.1	HNRNPH1	0.373333333	34.03	0	0
XM_021084338.1	LOC100620238	0.003333333	2.17	0	0
XM_021084359.1	PHYKPL	0.03	2.03	0	0
XM_021084396.1	NSD1	0.02	9.566666667	0	0
XM_021084398.1	NSD1	0.123333333	54.31	0	0
XM_021084436.1	LOC110259398	0.103333333	3.113333333	0	0
XM_021084441.1	ARHGEF28	0.106666667	27.45333333	0	0
XM_021084444.1	ARHGEF28	0.02	4.816666667	0	0
XM_021084452.1	ANKRD31	0.003333333	0.666666667	0	0.333333333
XM_021084466.1	PDE8B	0.006666667	0.973333333	0	0
XM_021084472.1	PDE8B	0.033333333	6.2	0	0
XM_021084480.1	DMGDH	0.246666667	45.34333333	0	0
XM_021084481.1	DMGDH	0.026666667	4.99	0	0
XM_021084496.1	EYS	0.01	4	0	1
XM_021084499.1	RASGRF2	0.003333333	1.763333333	0	0
XM_021084506.1	LOC110259411	0.01	0.333333333	0	0
XM_021084518.1	LOC100511846	0.003333333	0.333333333	0	0
XM_021084519.1	TMEM161B	0.03	3.356666667	0	0
XM_021084520.1	TMEM161B	0.06	6.633333333	0	0
XM_021084551.1	PAM	0.093333333	18.89	0	0
XM_021084558.1	PAM	0.066666667	11.94666667	0	0
XM_021084565.1	PAM	0.38	50.22	0	0
XM_021084584.1	PPIP5K2	0.163333333	38.29	0	0
XM_021084609.1	FER	0.043333333	13.77333333	0	0
XM_021084613.1	TMEM232	0.003333333	0.333333333	0	0
XM_021084630.1	NREP	0.026666667	0.57	0	0
XM_021084636.1	KCNN2	0.096666667	15.76666667	0	0
XM_021084643.1	TRIM36	0.006666667	1.06	0	0
XM_021084649.1	FEM1C	0.296666667	70.77333333	0	0
XM_021084659.1	ARL14EPL	0.006666667	0.333333333	0	0
XM_021084663.1	DTWD2	0.013333333	0.846666667	0	0
XM_021084676.1	PRR16	0.01	8.053333333	0	1.163333333
XM_021084687.1	PRDM6	0.006666667	0.643333333	0	0
XM_021084694.1	CSNK1G3	0.16	29.81	0	0
XM_021084707.1	CTXN3	0.033333333	3.063333333	0	0
XM_021084722.1	SLC27A6	0.003333333	1.833333333	0	0
XM_021084744.1	MEIKIN	0.006666667	1	0	0
XM_021084760.1	TCF7	0.013333333	2.126666667	0	0
XM_021084791.1	KLHL3	0.05	13.54666667	0	0
XM_021084795.1	KLHL3	0.006666667	2.073333333	0	0
XM_021084810.1	FAM13B	0.04	10.20666667	0	0.333333333
XM_021084813.1	FAM13B	0.003333333	0.756666667	0	0.036666667
XM_021084814.1	FAM13B	0.016666667	2.646666667	0	0
XM_021084823.1	FAM13B	0.016666667	4.126666667	0	0
XM_021084846.1	SIL1	0.03	2.183333333	0	0

XM_021084862.1	MATR3	0.033333333	3.866666667	0	0
XM_021084867.1	MATR3	0.01	1.38	0	0
XM_021084874.1	PSD2	0.056666667	13.32	0	0
XM_021084879.1	NRG2	0.006666667	1.333333333	0	0.333333333
XM_021084880.1	SLC4A9	0.023333333	5	0	0
XM_021084888.1	LOC100513976	0.006666667	0.666666667	0	0
XM_021084922.1	LOC100515772	0.04	17.11333333	0	0
XM_021084932.1	ARAP3	0.003333333	0.44	0	0.036666667
XM_021085001.1	ABLIM3	0.183333333	29.75333333	0	0
XM_021085019.1	CAMK2A	0.023333333	1.606666667	0	0
XM_021085038.1	LOC100512331	0.006666667	0.333333333	0	0
XM_021085070.1	LOC110259585	0.01	0.333333333	0	0
XM_021085085.1	C1H9orf66	0.006666667	0.333333333	0	0
XM_021085113.1	LOC110259630	0.006666667	0.333333333	0	0
XM_021085122.1	CCDC180	0.013333333	4.666666667	0	1.333333333
XM_021085138.1	TEX45	0.003333333	0.333333333	0	0
XM_021085143.1	LOC110259661	0.01	0.333333333	0	0
XM_021085147.1	THEG	0.006666667	0.333333333	0	0
XM_021085152.1	FAM81B	0.016666667	1	0	0
XM_021085158.1	CCNI2	0.003333333	0.333333333	0	0
XM_021085173.1	MADD	0.046666667	11.16666667	0	0
XM_021085202.1	MADD	0.136666667	29.92	0	0
XM_021085212.1	MADD	0.013333333	2.793333333	0	0
XM_021085214.1	MADD	0.05	11.95666667	0	0
XM_021085226.1	LOC110259676	0.01	0.333333333	0	0
XM_021085252.1	DGKZ	0.39	69.77	0	0
XM_021085270.1	LRRC4C	0.01	2.206666667	0	0
XM_021085288.1	LOC100517025	0.003333333	0.896666667	0	0
XM_021085294.1	LOC100517025	0.016666667	2.34	0	0
XM_021085298.1	NELL1	0.003333333	0.333333333	0	0
XM_021085300.1	NELL1	0.003333333	0.333333333	0	0
XM_021085309.1	NAV2	0.006666667	3.123333333	0	0
XM_021085339.1	SOX6	0.003333333	1.8	0	0
XM_021085356.1	SOX6	0.053333333	13.83333333	0	0
XM_021085373.1	ADGRV1	0.03	28.66666667	0	0
XM_021085378.1	KIAA0825	0.01	2.68	0	0
XM_021085379.1	KIAA0825	0.053333333	13.34666667	0	0
XM_021085386.1	SLF1	0.01	1.526666667	0	0
XM_021085391.1	FAM172A	0.016666667	5.04	0	0
XM_021085406.1	LOC110259697	0.02	0.666666667	0	0
XM_021085423.1	SLC22A5	0.036666667	3.36	0	0
XM_021085446.1	ARHGAP26	0.076666667	29.47	0	0
XM_021085457.1	LOC110259710	0.006666667	0.333333333	0	0
XM_021085459.1	LOC110259712	0.01	0.333333333	0	0
XM_021085522.1	DLL1	0.053333333	10.74666667	0	0
XM_021085533.1	DNMT3A	0.01	3.73	0	0
XM_021085542.1	CLN3	0.043333333	4.053333333	0	0
XM_021085543.1	CLN3	0.02	2.25	0	0
XM_021085552.1	POR	0.213333333	22.22333333	0	0
XM_021085561.1	SLC30A6	0.066666667	12.7	0	0
XM_021085586.1	NCK2	0.006666667	0.803333333	0	0
XM_021085587.1	NCK2	0.05	5.73	0	0
XM_021085589.1	NCK2	0.05	5.73	0	0
XM_021085590.1	NCK2	0.053333333	5.87	0	0
XM_021085591.1	NCK2	0.363333333	40.98666667	0	0
XM_021085603.1	TPRKB	0.006666667	0.506666667	0	0
XM_021085612.1	PKMYT1	0.003333333	0.333333333	0	0
XM_021085616.1	PKMYT1	0.003333333	0.343333333	0	0
XM_021085643.1	PHF10	0.17	13.33333333	0	0
XM_021085653.1	CDIP1	0.02	1.893333333	0	0

XM_021085670.1	HAAO	0.016666667	0.95	0	0
XM_021085677.1	KRCC1	0.076666667	6.396666667	0	0
XM_021085689.1	SDC1	0.023333333	3.31	0	0
XM_021085692.1	APOB	0.026666667	15.43666667	0	0
XM_021085695.1	BCL11A	0.006666667	1.176666667	0	0
XM_021085716.1	FRMD1	0.056666667	8.09	0	0
XM_021085728.1	FRMD1	0.076666667	12.99666667	0	0
XM_021085741.1	ZNF205	0.053333333	4.62	0	0
XM_021085746.1	ZNF205	0.016666667	2.03	0	0
XM_021085750.1	ZNF205	0.013333333	1.1	0	0
XM_021085758.1	LMAN2L	0.136666667	18.99	0	0
XM_021085762.1	FLYWCH2	0.023333333	1.25	0	0
XM_021085772.1	ELN	0.193333333	27.8	0	0
XM_021085790.1	ELN	0.33	46.89333333	0	0
XM_021085794.1	ELN	0.093333333	14.10666667	0	0
XM_021085797.1	ELN	0.056666667	7.49	0	0
XM_021085798.1	ELN	0.12	16.45666667	0	0
XM_021085801.1	RBKS	0.106666667	4.553333333	0	0
XM_021085808.1	CIITA	0.036666667	9.673333333	0	0
XM_021085809.1	CIITA	0.016666667	6.62	0	0
XM_021085832.1	BCL2L11	0.056666667	2.123333333	0	0
XM_021085846.1	IL1A	0.003333333	0.333333333	0	0
XM_021085872.1	IL4R	0.2	33.06333333	0	0
XM_021085891.1	TPO	0.003333333	0.793333333	0	0
XM_021085899.1	NCOA1	0.33	102.2033333	0	0
XM_021085919.1	ITGAL	0.003333333	0.67	0	0
XM_021085923.1	LOC110259820	0.016666667	1	0	0
XM_021085924.1	UNC93A	0.03	2.806666667	0	0
XM_021085930.1	UNC93A	0.016666667	1.756666667	0	0
XM_021085932.1	PRKAR1B	0.05	5.26	0	0
XM_021085933.1	PRKAR1B	0.026666667	2.74	0	0
XM_021085936.1	UNC93A	0.023333333	2.213333333	0	0
XM_021085952.1	UNC93A	0.083333333	6.693333333	0	0
XM_021085961.1	UNC93A	0.01	0.916666667	0	0
XM_021085966.1	MAFK	0.593333333	71.11	0	0
XM_021085995.1	LFNG	0.04	21.40333333	0	0
XM_021085998.1	LFNG	0.06	17.06666667	0	0
XM_021085999.1	LFNG	0.32	89.47666667	0	0
XM_021086004.1	LOC110259835	0.003333333	0.333333333	0	0
XM_021086027.1	RADIL	0.023333333	4.02	0	0
XM_021086034.1	MMD2	0.003333333	0.333333333	0	0
XM_021086065.1	LOC100520903	0.003333333	0.936666667	0	0
XM_021086083.1	EIF2AK1	1.303333333	367.29	0	0
XM_021086092.1	BHLHA15	0.006666667	0.666666667	0	0
XM_021086133.1	ZNF655	0.04	7.05	0	0
XM_021086140.1	ZNF655	0.046666667	8.6	0	0
XM_021086141.1	ZNF655	0.013333333	1.136666667	0	0
XM_021086144.1	ZNF655	0.053333333	2.933333333	0	0
XM_021086154.1	ZNF789	0.073333333	4.516666667	0	0
XM_021086162.1	LOC110259856	0.003333333	0.333333333	0	0
XM_021086167.1	NDE1	0.056666667	6.063333333	0	0
XM_021086171.1	NDE1	0.01	1.236666667	0	0
XM_021086173.1	C3H16orf45	0.016666667	0.54	0	0
XM_021086182.1	LOC110259862	0.003333333	0.333333333	0	0
XM_021086195.1	MCM7	0.123333333	14.47333333	0	0
XM_021086204.1	LOC110255267	0.033333333	2.146666667	0	0
XM_021086214.1	LOC110259877	0.003333333	0.666666667	0	0
XM_021086217.1	STAG3	0.003333333	0.873333333	0	0.333333333
XM_021086251.1	ACHE	0.18	16.11333333	0	0
XM_021086259.1	LOC100624333	0.006666667	0.333333333	0	0

XM_021086266.1	CUX1	0.11	65.89666667	0	0
XM_021086287.1	ORAI2	0.023333333	4.136666667	0	0
XM_021086290.1	ORAI2	0.026666667	3.9	0	0
XM_021086300.1	STYXL1	0.236666667	44.53	0	0
XM_021086301.1	STYXL1	0.023333333	5.466666667	0	0
XM_021086316.1	FZD9	0.02	6.446666667	0	0
XM_021086317.1	FZD9	0.023333333	7.2	0	0
XM_021086319.1	FKBP6	0.003333333	0.333333333	0	0
XM_021086327.1	MLXIPL	0.186666667	22.63333333	0	0
XM_021086339.1	CLIP2	0.046666667	10.63333333	0	0
XM_021086356.1	TYW1	0.453333333	44.57	0	0
XM_021086374.1	AHSP	0.126666667	2	0	0
XM_021086375.1	SLC5A2	0.05	4.693333333	0	0
XM_021086376.1	SLC5A2	0.403333333	37.96666667	0	0
XM_021086377.1	SLC5A2	0.033333333	3.006666667	0	0
XM_021086382.1	ITGAM	0.02	5.066666667	0	0
XM_021086384.1	ITGAM	0.223333333	50.44666667	0	0
XM_021086410.1	HSD3B7	1.356666667	112.6466667	0	0
XM_021086437.1	GSG1L	0.006666667	0.333333333	0	0
XM_021086452.1	NSMCE1	0.17	7.19	0	0
XM_021086464.1	ERN2	0.003333333	0.333333333	0	0
XM_021086473.1	COG7	0.11	10.41	0	0
XM_021086512.1	ERI2	0.003333333	0.396666667	0	0
XM_021086524.1	UMOD	8.08	805.3333333	0	0
XM_021086525.1	ZDHHC14	0.016666667	1.92	0	0
XM_021086535.1	TMC5	0.023333333	3.666666667	0	0
XM_021086578.1	MRTFB	0.373333333	129.5766667	0	0
XM_021086586.1	LOC110259928	0.01	0.333333333	0	0
XM_021086635.1	TVP23A	0.006666667	1.256666667	0	0.85
XM_021086656.1	PMM2	0.103333333	4.023333333	0	0
XM_021086668.1	ROGDI	0.11	7.94	0	0
XM_021086669.1	SMIM22	0.06	3	0	0
XM_021086670.1	LOC110259937	0.006666667	0.433333333	0	0
XM_021086684.1	C3H16orf71	0.01	0.816666667	0	0
XM_021086688.1	NUDT16L1	0.016666667	2.276666667	0	0
XM_021086703.1	SRL	0.48	95.91333333	0	0
XM_021086717.1	LOC100737121	0.006666667	1.703333333	0	0
XM_021086720.1	LOC100737121	0.006666667	1.403333333	0	0
XM_021086730.1	LOC100737121	0.01	2.036666667	0	0.01
XM_021086741.1	PRSS33	0.003333333	0.333333333	0	0
XM_021086763.1	TEDC2	0.016666667	1.19	0	0
XM_021086775.1	TSC2	0.046666667	12.57666667	0	0
XM_021086782.1	SYNGR3	0.006666667	0.786666667	0	0
XM_021086800.1	MEIOB	0.04	2.523333333	0	0
XM_021086843.1	IFT140	0.016666667	3.673333333	0	0
XM_021086851.1	LACTB	0.026666667	2.253333333	0	0
XM_021086864.1	LOC110255206	0.08	4.446666667	0	0
XM_021086865.1	CACNA1H	0.053333333	17.46333333	0	0
XM_021086869.1	CACNA1H	0.036666667	12.54333333	0	0
XM_021086879.1	CHTF18	0.06	7.583333333	0	0
XM_021086881.1	LOC110259953	0.02	0.333333333	0	0
XM_021086884.1	RHBDL1	0.046666667	3.386666667	0	0.033333333
XM_021086888.1	RHBDL1	0.196666667	11.41666667	0	0
XM_021086903.1	WDR90	0.01	2.613333333	0	0
XM_021086911.1	RAB40C	0.533333333	55.06333333	0	0.176666667
XM_021086927.1	AXIN1	0.013333333	3.466666667	0	0
XM_021086934.1	FAM234A	0.09	9.256666667	0	0
XM_021086936.1	FAM234A	0.113333333	11.53333333	0	0
XM_021086941.1	RGS11	0.006666667	0.766666667	0	0
XM_021086943.1	ARHGDIG	0.01	1	0	0

XM_021087015.1	ZNF169	0.003333333	0.936666667	0	0
XM_021087028.1	TMEM87B	0.14	31.41333333	0	0
XM_021087042.1	ACOXL	0.02	2.91	0	0
XM_021087045.1	ACOXL	0.006666667	1.51	0	0
XM_021087067.1	PROM2	0.006666667	1.413333333	0	0
XM_021087110.1	CCDC138	0.003333333	0.396666667	0	0
XM_021087117.1	FHL2	0.016666667	2.223333333	0	0
XM_021087122.1	IL1RL2	0.006666667	0.666666667	0	0
XM_021087130.1	ZC2HC1B	0.01	0.333333333	0	0
XM_021087140.1	MAP4K4	0.013333333	4.033333333	0	0
XM_021087199.1	TSGA10	0.016666667	2.073333333	0	0
XM_021087206.1	TSGA10	0.02	2.393333333	0	0
XM_021087207.1	TSGA10	0.02	2.5	0	0
XM_021087209.1	TSGA10	0.013333333	1.596666667	0	0
XM_021087211.1	LOC110255307	0.04	2.333333333	0	0
XM_021087218.1	KIAA1211L	0.003333333	0.666666667	0	0
XM_021087222.1	KIAA1211L	0.01	1.666666667	0	0
XM_021087238.1	VWA3B	0.003333333	0.733333333	0	0
XM_021087241.1	VWA3B	0.006666667	1.856666667	0	0
XM_021087262.1	SEMA4C	0.02	3.033333333	0	0
XM_021087331.1	DQX1	0.013333333	1.333333333	0	0
XM_021087338.1	REPS1	0.193333333	24	0	0
XM_021087381.1	EMX1	0.013333333	3.333333333	0	0.333333333
XM_021087384.1	ZNF638	0.07	20.84333333	0	0.213333333
XM_021087392.1	ATP6V1B1	1.126666667	148.3333333	0	0
XM_021087394.1	NHSL1	0.02	8.596666667	0	0
XM_021087396.1	ADD2	0.003333333	1	0	0.333333333
XM_021087406.1	NHSL1	0.09	25.40333333	0	0
XM_021087428.1	C1D	0.096666667	10.44333333	0	0
XM_021087452.1	AFTPH	0.006666667	1.556666667	0	0
XM_021087497.1	USP34	0.066666667	36.57	0	0
XM_021087503.1	AHSA2P	0.033333333	6	0	0
XM_021087511.1	IL20RA	0.003333333	0.333333333	0	0
XM_021087517.1	KIAA1841	0.02	3.73	0	0
XM_021087520.1	CCDC88A	0.026666667	11.36666667	0	0
XM_021087582.1	EML4	0.016666667	3.45	0	0
XM_021087587.1	SOS1	0.026666667	8.67	0	0.01
XM_021087593.1	DHX57	0.42	84.87	0	0
XM_021087605.1	HBS1L	0.05	5.966666667	0	0
XM_021087621.1	ALDH8A1	0.083333333	21.66666667	0	0.666666667
XM_021087624.1	RMDN2	0.016666667	3.48	0	0
XM_021087628.1	PRKD3	0.016666667	2.42	0	0
XM_021087661.1	CAPN13	0.013333333	1.666666667	0	0
XM_021087677.1	EYA4	0.03	22.20666667	0	0
XM_021087694.1	EYA4	0.05	37.21666667	0	0
XM_021087695.1	DNAJC5G	0.026666667	2.016666667	0	0
XM_021087698.1	EYA4	0.193333333	132.7366667	0	0
XM_021087700.1	SLC5A6	0.866666667	111.4133333	0	0
XM_021087701.1	SLC5A6	0.123333333	17.22666667	0	0
XM_021087703.1	EYA4	0.08	59.29333333	0	0
XM_021087706.1	EYA4	0.05	14.24666667	0	0
XM_021087748.1	DTNB	0.066666667	6.483333333	0	0
XM_021087749.1	DTNB	0.076666667	7.52	0	0
XM_021087778.1	EYA4	0.116666667	90.18333333	0	0
XM_021087788.1	GDF7	0.003333333	1	0	0
XM_021087789.1	EYA4	0.073333333	20.78666667	0	0
XM_021087794.1	PUM2	0.09	26.25666667	0	0
XM_021087795.1	PUM2	0.126666667	37.61666667	0	0
XM_021087797.1	PUM2	0.036666667	9.756666667	0	0
XM_021087807.1	PUM2	0.073333333	22.01	0	0

XM_021087812.1	OSR1	0.1	10.73666667	0	0
XM_021087817.1	OSR1	0.02	2.556666667	0	0
XM_021087818.1	OSR1	0.11	12.41333333	0	0
XM_021087821.1	VSNL1	0.003333333	0.333333333	0	0
XM_021087827.1	GREB1	0.003333333	0.633333333	0	0
XM_021087829.1	GREB1	0.113333333	41.19333333	0	0
XM_021087875.1	MBOAT2	0.02	2.593333333	0	0
XM_021087958.1	ALKAL2	0.006666667	0.666666667	0	0
XM_021087976.1	LOC110260078	0.01	0.666666667	0	0
XM_021087977.1	MUC12	0.3	27	0	0
XM_021087978.1	LOC106508543	0.01	0.666666667	0	0
XM_021088009.1	INO80E	0.136666667	4.11	0	0
XM_021088012.1	LOC110260082	0.05	1	0	0
XM_021088015.1	TBX6	0.123333333	11.17666667	0	0
XM_021088018.1	TBX6	0.026666667	1.946666667	0	0
XM_021088029.1	CORO1A	0.02	1.546666667	0	0
XM_021088043.1	EPB41L2	0.036666667	7.306666667	0	0
XM_021088072.1	RBFOX1	0.453333333	126.0066667	0	0
XM_021088092.1	NLRC3	0.003333333	1.843333333	0	0
XM_021088104.1	LOC100517149	0.023333333	2.53	0	0
XM_021088106.1	EPB41L2	0.06	12.40333333	0	0
XM_021088116.1	EPB41L2	0.006666667	1.253333333	0	0
XM_021088121.1	LOC106509717	0.006666667	0.406666667	0	0
XM_021088138.1	SUSD3	0.03	1.76	0	0
XM_021088148.1	WNK2	0.043333333	15.86	0	0
XM_021088152.1	WNK2	0.023333333	8.85	0	0
XM_021088163.1	WNK2	0.02	7.013333333	0	0
XM_021088186.1	AFF3	0.003333333	1.31	0	0
XM_021088188.1	AFF3	0.003333333	0.75	0	0
XM_021088194.1	AFF3	0.046666667	16.72333333	0	0
XM_021088198.1	AFF3	0.02	8.073333333	0	0
XM_021088205.1	CTNNA2	0.003333333	0.333333333	0	0
XM_021088221.1	SPTBN1	0.003333333	0.63	0	0.043333333
XM_021088222.1	SPTBN1	0.003333333	1.05	0	0.096666667
XM_021088238.1	NRXN1	0.003333333	1.093333333	0	0
XM_021088246.1	NRXN1	0.016666667	3.73	0	0
XM_021088250.1	NRXN1	0.05	17.70333333	0	0.17
XM_021088265.1	NRXN1	0.03	7.016666667	0	0
XM_021088283.1	NRXN1	0.026666667	2.67	0	0
XM_021088303.1	CAMKMT	0.04	2.346666667	0	0
XM_021088318.1	SLC8A1	0.006666667	5.57	0	0
XM_021088321.1	SLC8A1	0.006666667	6.82	0	0
XM_021088322.1	SLC8A1	0.01	6.873333333	0	0
XM_021088334.1	EPB41L2	0.01	1.443333333	0	0
XM_021088355.1	TMEM200A	0.006666667	2.013333333	0	0
XM_021088357.1	ITSN2	0.483333333	125.1733333	0	0
XM_021088360.1	ITSN2	0.076666667	18.53	0	0
XM_021088361.1	ITSN2	0.133333333	41.50666667	0	0
XM_021088371.1	ZG16	0.02	0.333333333	0	0
XM_021088376.1	PDILT	0.02	2	0	0
XM_021088401.1	L3MBTL3	0.006666667	1.06	0	0.046666667
XM_021088415.1	LOC110260127	0.006666667	0.666666667	0	0
XM_021088447.1	SELL	0.03	1.666666667	0	0
XM_021088471.1	ARHGEF2	0.01	2.746666667	0	0
XM_021088492.1	PMP2	0.04	0.97	0	0
XM_021088495.1	TPD52	0.01	1.85	0	0
XM_021088497.1	TPD52	0.003333333	0.59	0	0
XM_021088506.1	TRIM55	0.06	6.99	0	0
XM_021088527.1	ANXA9	0.003333333	0.843333333	0	0
XM_021088548.1	DCAF8	0.15	25.54	0	0

XM_021088568.1	POGK	0.1	15.22666667	0	0
XM_021088578.1	NCALD	0.06	4.536666667	0	0
XM_021088581.1	NCALD	0.02	1.553333333	0	0
XM_021088582.1	NCALD	0.063333333	4.24	0	0
XM_021088583.1	NCALD	0.013333333	0.903333333	0	0
XM_021088584.1	NCALD	0.016666667	1.156666667	0	0
XM_021088585.1	RPL30	0.1	2.026666667	0	0
XM_021088590.1	USF1	0.04	2.936666667	0	0
XM_021088597.1	RGS4	0.02	0.363333333	0	0
XM_021088645.1	HSF1	0.106666667	9.163333333	0	0
XM_021088680.1	ARNT	0.046666667	8.73	0	0
XM_021088696.1	DPYD	0.02	3.203333333	0	0
XM_021088733.1	TPM3	0.143333333	16.31666667	0	0
XM_021088741.1	ST3GAL1	0.11	29.34	0	0
XM_021088744.1	ST3GAL1	0.05	13.45	0	0
XM_021088745.1	HSD3B1	0.003333333	0.333333333	0	0
XM_021088753.1	S100A6	0.053333333	0.913333333	0	0
XM_021088762.1	LY6L	0.006666667	0.333333333	0	0
XM_021088772.1	LOC110260194	0.006666667	0.666666667	0	0
XM_021088785.1	PSCA	0.003333333	0.333333333	0	0
XM_021088788.1	LOC110260203	0.01	0.46	0	0
XM_021088789.1	LOC110260203	0.01	0.333333333	0	0
XM_021088790.1	LOC110260203	0.016666667	0.54	0	0
XM_021088799.1	TSNARE1	0.01	1.05	0	0
XM_021088800.1	TSNARE1	0.01	1.093333333	0	0
XM_021088806.1	TSNARE1	0.01	0.903333333	0	0
XM_021088823.1	PTK2	0.013333333	2.946666667	0	0
XM_021088829.1	PTK2	0.036666667	7.256666667	0	0
XM_021088832.1	PTK2	0.013333333	3.07	0	0
XM_021088842.1	PTK2	0.023333333	4.403333333	0	0
XM_021088893.1	TMEM71	0.01	1	0	0
XM_021088903.1	ADCY8	0.003333333	0.956666667	0	0
XM_021088953.1	FER1L6	0.01	3.333333333	0	0
XM_021088968.1	TBC1D31	0.2	27.65666667	0	0
XM_021088971.1	TBC1D31	0.036666667	4.746666667	0	0
XM_021088974.1	ZHX2	0.05	11.00666667	0	0
XM_021089000.1	TRPS1	0.013333333	5.786666667	0	0
XM_021089001.1	TRPS1	0.026666667	12.59666667	0	0
XM_021089028.1	DPYS	0.15	17.53666667	0	0
XM_021089038.1	UBR5	0.01	4.183333333	0	0.02
XM_021089040.1	RRM2B	0.02	4.25	0	0
XM_021089043.1	SNX31	0.02	2	0	0
XM_021089048.1	SPAG1	0.123333333	17.31666667	0	0
XM_021089052.1	RGS22	0.03	5.916666667	0	0
XM_021089070.1	TSPYL5	0.04	5.086666667	0	0
XM_021089071.1	TSPYL5	0.106666667	13.03666667	0	0
XM_021089088.1	ESRP1	0.006666667	1	0	0
XM_021089112.1	RBM12B	0.036666667	11.58666667	0	0
XM_021089113.1	ROS1	0.236666667	83.26666667	0	0
XM_021089115.1	TRIQK	0.02	5.983333333	0	0
XM_021089117.1	TRIQK	0.006666667	2.203333333	0	0
XM_021089120.1	TRIQK	0.026666667	7.633333333	0	0
XM_021089126.1	SLC26A7	0.003333333	0.72	0	0
XM_021089163.1	LRRCC1	0.02	4.146666667	0	0
XM_021089164.1	LRRCC1	0.006666667	1.366666667	0	0
XM_021089169.1	LOC100522735	0.286666667	21.35666667	0	0
XM_021089170.1	LOC100522735	0.196666667	13.00666667	0	0
XM_021089171.1	LOC100522735	0.063333333	3.97	0	0
XM_021089182.1	HNF4G	0.043333333	8.813333333	0	0
XM_021089183.1	HNF4G	0.003333333	0.56	0	0.333333333

XM_021089184.1	HNF4G	0.003333333	1.526666667	0	0
XM_021089185.1	HNF4G	0.013333333	2.433333333	0	0.333333333
XM_021089205.1	STAU2	0.406666667	49.86333333	0	0
XM_021089215.1	STAU2	0.27	33.38666667	0	0
XM_021089223.1	RSPH4A	0.006666667	1.333333333	0	0.666666667
XM_021089239.1	LOC110260276	0.003333333	0.333333333	0	0
XM_021089244.1	EYA1	0.053333333	8.876666667	0	0
XM_021089250.1	EYA1	0.02	3.06	0	0
XM_021089272.1	CSPP1	0.073333333	14.2	0	0
XM_021089285.1	LOC100521447	0.083333333	11.96333333	0	0
XM_021089295.1	MTFR1	0.033333333	5.603333333	0	0
XM_021089303.1	YTHDF3	0.083333333	18.10333333	0	0
XM_021089304.1	YTHDF3	0.066666667	14.01666667	0	0
XM_021089319.1	ASPH	0.023333333	5.94	0	0
XM_021089324.1	ASPH	0.003333333	0.613333333	0	0
XM_021089347.1	NSMAF	0.016666667	3.803333333	0	0
XM_021089356.1	LYN	0.053333333	12.35	0	0
XM_021089357.1	LYN	0.02	5	0	0
XM_021089361.1	LYN	0.02	5	0	0
XM_021089362.1	LYN	0.053333333	12.35	0	0
XM_021089376.1	ALKAL1	0.006666667	0.333333333	0	0
XM_021089387.1	FAM135A	0.04	11.20666667	0	0
XM_021089407.1	ST18	0.003333333	1.333333333	0	1.403333333
XM_021089413.1	SNTG1	0.003333333	1	0	0
XM_021089417.1	FAM135A	0.01	2.803333333	0	0
XM_021089431.1	SCYL3	0.07	10.78	0	0
XM_021089437.1	LOC106510097	0.003333333	0.333333333	0	0
XM_021089438.1	FAM135A	0.026666667	6.003333333	0	0.596666667
XM_021089441.1	NME7	0.056666667	3.68	0	0
XM_021089482.1	ILDR2	0.006666667	1.15	0	0
XM_021089489.1	NUF2	0.073333333	5.333333333	0	0
XM_021089493.1	DDR2	0.306666667	132.5133333	0	0.25
XM_021089500.1	DDR2	0.046666667	19.65666667	0	0
XM_021089511.1	OGFRL1	0.083333333	34.83333333	0	0
XM_021089519.1	FCRLA	0.033333333	2.303333333	0	0
XM_021089528.1	PPOX	0.11	8.543333333	0	0
XM_021089543.1	ARHGAP30	0.07	12.67666667	0	0
XM_021089547.1	TSTD1	0.023333333	0.556666667	0	0
XM_021089563.1	LOC100156074	0.003333333	0.333333333	0	0
XM_021089579.1	IGSF9	0.003333333	0.666666667	0	0.333333333
XM_021089582.1	IGSF9	0.013333333	2.666666667	0	0
XM_021089584.1	MRAP2	0.033333333	2.356666667	0	0
XM_021089587.1	CFAP45	0.003333333	0.333333333	0	0
XM_021089589.1	SLAMF8	0.033333333	3.186666667	0	0
XM_021089593.1	LOC100520241	0.003333333	0.333333333	0	0
XM_021089632.1	LOC102162420	0.003333333	0.333333333	0	0.083333333
XM_021089655.1	LRRC71	0.003333333	0.333333333	0	0
XM_021089656.1	LRRC71	0.013333333	1	0	0
XM_021089659.1	LRRC71	0.023333333	1.653333333	0	0
XM_021089668.1	HAPLN2	0.003333333	0.333333333	0	0
XM_021089685.1	SEMA4A	0.11	14	0	0
XM_021089728.1	MUC1	0.04	4.36	0	0
XM_021089745.1	ZBTB7B	0.39	57.53666667	0	0
XM_021089795.1	NUP210L	0.01	2	0	0.333333333
XM_021089799.1	DENND4B	0.02	6.113333333	0	0
XM_021089842.1	THEM5	0.023333333	1	0	0
XM_021089849.1	RORC	0.023333333	3.236666667	0	0
XM_021089852.1	RORC	0.026666667	2.516666667	0	0
XM_021089854.1	RARS2	0.41	32.33666667	0	0
XM_021089878.1	SEMA6C	0.026666667	5.76	0	0

XM_021089897.1	ADAMTSL4	0.006666667	1.273333333	0	0
XM_021089899.1	ADAMTSL4	0.14	23.19333333	0	0
XM_021089948.1	PDZK1	0.923333333	82.90666667	0	0
XM_021089949.1	PDZK1	0.076666667	7.643333333	0	0
XM_021089968.1	LOC110260333	0.013333333	1	0	0
XM_021089972.1	SPAG17	0.003333333	0.666666667	0	0
XM_021089979.1	VTCN1	0.013333333	1.333333333	0	0
XM_021089984.1	ANKRD6	0.013333333	4.396666667	0	0
XM_021089989.1	MAB21L3	0.003333333	0.87	0	0
XM_021089991.1	ANKRD6	0.043333333	12.14333333	0	0
XM_021090003.1	ANKRD6	0.01	2.993333333	0	0
XM_021090010.1	HIPK1	0.693333333	236.2533333	0	0
XM_021090013.1	HIPK1	0.036666667	9.703333333	0	0
XM_021090015.1	SIM1	0.006666667	1.2	0	0
XM_021090021.1	SYT6	0.003333333	0.333333333	0	0
XM_021090031.1	RAP1A	0.046666667	4.91	0	0
XM_021090047.1	CASP8AP2	0.016666667	5.026666667	0	0
XM_021090055.1	EPS8L3	0.01	1.07	0	0
XM_021090066.1	AMPD2	0.03	4.356666667	0	0
XM_021090069.1	CASP8AP2	0.043333333	12.54	0	0
XM_021090091.1	C4H1orf194	0.003333333	0.333333333	0	0
XM_021090119.1	NTNG1	0.003333333	1.766666667	0	0
XM_021090161.1	EPHA7	0.02	5.503333333	0	0
XM_021090171.1	TMEM56	0.036666667	10.90333333	0	0
XM_021090172.1	TMEM56	0.003333333	1.356666667	0	0
XM_021090195.1	CCDC18	0.026666667	5.306666667	0	0
XM_021090203.1	MTF2	0.043333333	4.133333333	0	0
XM_021090211.1	EVI5	0.02	5.96	0	0
XM_021090216.1	EVI5	0.096666667	29.00666667	0	0
XM_021090219.1	EVI5	0.006666667	2.386666667	0	0
XM_021090236.1	GLMN	0.113333333	9.39	0	0
XM_021090241.1	KIAA1107	0.016666667	4.373333333	0	0
XM_021090242.1	KIAA1107	0.03	7.22	0	0
XM_021090246.1	EPHX4	0.003333333	0.333333333	0	0
XM_021090251.1	HFM1	0.006666667	1.623333333	0	0
XM_021090253.1	HFM1	0.023333333	4.89	0	0
XM_021090254.1	HFM1	0.006666667	1.46	0	0
XM_021090255.1	HFM1	0.043333333	9.67	0	0
XM_021090263.1	ZNF644	0.023333333	6.303333333	0	0
XM_021090269.1	ZNF644	0.066666667	16.46	0	0
XM_021090299.1	LRRC8D	0.003333333	3.26	0	0
XM_021090317.1	LOC100523668	0.006666667	1.023333333	0	0
XM_021090350.1	WDR63	0.006666667	0.666666667	0	0
XM_021090402.1	MROH1	0.02	4.526666667	0	0
XM_021090405.1	MROH1	0.006666667	1.656666667	0	0
XM_021090406.1	MROH1	0.08	18.36666667	0	0.003333333
XM_021090469.1	MAPK15	0.006666667	0.666666667	0	0
XM_021090474.1	NRBP2	0.03	4.93	0	0
XM_021090477.1	GPT	0.066666667	7.636666667	0	0
XM_021090505.1	CRYBG1	0.043333333	12.76333333	0	0
XM_021090506.1	GSDMD	0.103333333	7.973333333	0	0
XM_021090508.1	ZNF623	0.493333333	90.13333333	0	0
XM_021090514.1	C4H8orf33	0.116666667	5.99	0	0
XM_021090534.1	FOXH1	0.01	1	0	0
XM_021090535.1	FOXH1	0.006666667	0.666666667	0	0
XM_021090540.1	GRINA	0.026666667	1.77	0	0
XM_021090542.1	SARDH	0.026666667	4.486666667	0	0
XM_021090548.1	SPATC1	0.003333333	0.333333333	0	0
XM_021090558.1	DEPTOR	0.016666667	1.473333333	0	0
XM_021090563.1	COLEC10	0.006666667	0.993333333	0	0

XM_021090564.1	COLEC10	0.003333333	0.443333333	0	0
XM_021090566.1	COLEC10	0.006666667	1.333333333	0	0
XM_021090569.1	COLEC10	0.003333333	0.44	0	0
XM_021090575.1	COLEC10	0.003333333	0.503333333	0	0
XM_021090583.1	CSMD3	0.013333333	7.33	0	0
XM_021090586.1	CSMD3	0.006666667	3.333333333	0	0
XM_021090587.1	CSMD3	0.003333333	1.68	0	0.813333333
XM_021090598.1	GRHL2	0.003333333	0.333333333	0	0
XM_021090640.1	SPIDR	0.053333333	6.736666667	0	0
XM_021090656.1	LOC100624559	0.103333333	41.69666667	0	0
XM_021090657.1	LOC100624559	0.393333333	160.0166667	0	0
XM_021090663.1	LOC100624559	0.013333333	5.2	0	0
XM_021090669.1	LOC100624559	0.036666667	14.43666667	0	0
XM_021090702.1	LOC100519022	0.013333333	2.566666667	0	0
XM_021090707.1	LOC100517759	0.046666667	2.156666667	0	0
XM_021090726.1	PHTF1	0.063333333	24.57333333	0	0
XM_021090729.1	PHTF1	0.126666667	70.26	0	0.14
XM_021090760.1	SCML4	0.006666667	3.063333333	0	0.786666667
XM_021090778.1	ODF2L	0.156666667	15.20666667	0	0
XM_021090789.1	S100A3	0.013333333	0.333333333	0	0
XM_021090795.1	LOC110260467	0.01	0.333333333	0	0
XM_021090800.1	LOC110260475	0.006666667	0.333333333	0	0
XM_021090823.1	SARDH	0.026666667	4.66	0	0
XM_021090827.1	CCDC162P	0.016666667	5.183333333	0	0
XM_021090830.1	C4H1orf146	0.01	0.333333333	0	0
XM_021090838.1	ARMC2	0.016666667	2.15	0	0
XM_021090846.1	ADAMTS20	0.05	16.29666667	0	0
XM_021090849.1	ARMC2	0.003333333	0.33	0	0
XM_021090851.1	SL44-1	0.006666667	2.333333333	0	0.666666667
XM_021090857.1	MDM2	0.04	6.49	0	0
XM_021090858.1	WNT10B	0.013333333	2.666666667	0	0
XM_021090860.1	WNT10B	0.033333333	4.333333333	0	0
XM_021090873.1	LIMA1	0.026666667	4.506666667	0	0
XM_021090888.1	CD163L1	0.023333333	3.113333333	0	0
XM_021090890.1	CD163L1	0.06	7.653333333	0	0
XM_021090894.1	AQP6	0.056666667	5.493333333	0	0
XM_021090895.1	AQP2	0.063333333	3.5	0	0
XM_021090898.1	LGR5	0.006666667	1.246666667	0	0
XM_021090901.1	CPSF6	0.013333333	3.74	0	0
XM_021090903.1	CPSF6	0.033333333	8.816666667	0	0.03
XM_021090905.1	CPSF6	0.046666667	25.09	0	0
XM_021090906.1	PRR5	0.006666667	0.566666667	0	0
XM_021090914.1	GPR84	0.03	2	0	0
XM_021090921.1	C5H12orf4	0.013333333	3.906666667	0	0
XM_021090930.1	CEP57L1	0.02	1.363333333	0	0
XM_021090932.1	NR1H4	0.023333333	2.626666667	0	0
XM_021090939.1	NR1H4	0.006666667	0.666666667	0	0
XM_021090948.1	AMHR2	0.01	0.806666667	0	0
XM_021090955.1	PDE6H	0.006666667	0.333333333	0	0
XM_021090956.1	PDE6H	0.006666667	0.333333333	0	0
XM_021090972.1	CENPM	0.043333333	2.856666667	0	0
XM_021090995.1	CCDC91	0.38	26.41	0	0
XM_021091044.1	PPP6R2	0.14	22.78	0	0
XM_021091062.1	FRS2	0.013333333	3.096666667	0	0
XM_021091076.1	GLI1	0.03	4.573333333	0	0
XM_021091078.1	GLI1	0.013333333	2.636666667	0	0
XM_021091087.1	SLCO1B3	0.006666667	1.173333333	0	0
XM_021091091.1	SLCO1B3	0.003333333	1	0	0
XM_021091096.1	TPM1	0.023333333	3.013333333	0	0
XM_021091107.1	WASF1	0.06	6.586666667	0	0

XM_021091131.1	GDF11	0.013333333	0.62	0	0
XM_021091174.1	PLCZ1	0.01	1.593333333	0	0
XM_021091198.1	CD4	0.086666667	10.35666667	0	0
XM_021091239.1	LOC100522490	0.046666667	1	0	0
XM_021091280.1	PLXNB2	0.046666667	13.08333333	0	0
XM_021091283.1	PLXNB2	0.006666667	2.326666667	0	0
XM_021091303.1	PLXNB2	0.186666667	53.10666667	0	0
XM_021091311.1	DENND6B	0.01	2.06	0	0
XM_021091323.1	MAPK11	0.013333333	1.523333333	0	0
XM_021091327.1	MAPK11	0.123333333	13.22666667	0	0.106666667
XM_021091362.1	GRAMD4	0.013333333	2.196666667	0	0
XM_021091363.1	GRAMD4	0.023333333	3.76	0	0
XM_021091382.1	WISP3	0.003333333	0.333333333	0	0
XM_021091384.1	WNT7B	0.013333333	2.333333333	0	0
XM_021091389.1	SMC1B	0.003333333	0.333333333	0	0
XM_021091410.1	LOC100620827	0.01	0.5	0	0
XM_021091411.1	LOC100620827	0.02	1.076666667	0	0
XM_021091431.1	RRP7A	0.006666667	0.47	0	0
XM_021091437.1	3-Sep	0.006666667	1.743333333	0	0
XM_021091438.1	3-Sep	0.02	2.616666667	0	0
XM_021091479.1	SUN2	3.153333333	507.2466667	0	0
XM_021091498.1	MAFF	0.643333333	54.9	0	0
XM_021091508.1	GGA1	0.24	28.64666667	0	0
XM_021091514.1	CARD10	0.196666667	38.04333333	0	0
XM_021091526.1	IL2RB	0.016666667	3.296666667	0	0
XM_021091531.1	KCTD17	0.016666667	0.616666667	0	0
XM_021091551.1	DSE	0.053333333	9.446666667	0	0
XM_021091572.1	RFX4	0.01	1.776666667	0	0
XM_021091618.1	PRPF40B	0.023333333	4.71	0	0
XM_021091619.1	PRPF40B	0.006666667	1.383333333	0	0
XM_021091626.1	MCRS1	0.046666667	4.146666667	0	0
XM_021091630.1	LOC100152150	0.023333333	1	0	0
XM_021091639.1	ASIC1	0.04	5.473333333	0	0
XM_021091651.1	LARP4	0.143333333	39.92666667	0	0
XM_021091676.1	ATG101	0.053333333	7.09	0	0
XM_021091678.1	KRT80	0.02	3.696666667	0	0
XM_021091697.1	SOAT2	0.03	2.666666667	0	0
XM_021091698.1	ITGB7	0.016666667	1.956666667	0	0
XM_021091701.1	CSAD	0.043333333	5.536666667	0	0
XM_021091704.1	CSAD	0.02	2.723333333	0	0
XM_021091737.1	SNAP91	0.01	1.286666667	0	0
XM_021091749.1	SNAP91	0.013333333	1.81	0	0
XM_021091759.1	LOC100736743	0.013333333	1	0	0
XM_021091764.1	LOC100620469	0.003333333	0.746666667	0	0
XM_021091771.1	LOC110260679	0.01	2.333333333	0	0
XM_021091773.1	LOC110260681	0.006666667	0.986666667	0	0
XM_021091777.1	LOC100523964	0.063333333	6.403333333	0	0
XM_021091781.1	SNAP91	0.026666667	2.803333333	0	0
XM_021091782.1	IKZF4	0.013333333	4.236666667	0	0
XM_021091814.1	LOC100626199	2.113333333	89.58666667	0	0
XM_021091816.1	LOC100512656	0.053333333	3.86	0	0
XM_021091822.1	MYO1A	0.006666667	1.116666667	0	0
XM_021091832.1	R3HDM2	0.176666667	31.34	0	0
XM_021091834.1	R3HDM2	0.036666667	6.5	0	0
XM_021091869.1	ARHGAP9	0.033333333	3.796666667	0	0
XM_021091875.1	ARHGEF25	0.03	3.33	0	0
XM_021091895.1	AGAP2	0.243333333	40.29666667	0	0
XM_021091904.1	ATP23	0.06	2.636666667	0	0
XM_021091909.1	SLC16A7	0.033333333	8.853333333	0	0
XM_021091913.1	FAM19A2	0.02	3.333333333	0	0

XM_021091917.1	USP15	0.09	19.61333333	0	0
XM_021091918.1	UBE3D	0.683333333	42.92333333	0	0
XM_021091945.1	RASSF3	0.033333333	4.923333333	0	0
XM_021091948.1	TBC1D30	0.01	2.536666667	0	0
XM_021092036.1	CAPS2	0.003333333	0.996666667	0	0
XM_021092047.1	GLIPR1	0.013333333	2.04	0	0
XM_021092063.1	DNM1L	0.053333333	8.89	0	0
XM_021092069.1	TTK	0.013333333	2.333333333	0	0
XM_021092098.1	SINHCAF	0.013333333	1.666666667	0	0
XM_021092113.1	CAPRIN2	0.006666667	0.95	0	0
XM_021092118.1	CAPRIN2	0.033333333	4.276666667	0	0
XM_021092126.1	OVCH1	0.003333333	1.333333333	0	1
XM_021092156.1	ARNTL2	0.003333333	0.583333333	0	0
XM_021092162.1	STK38L	0.02	3.57	0	0
XM_021092178.1	LMNTD1	0.016666667	2.663333333	0	0
XM_021092195.1	CASC1	0.02	2.453333333	0	0
XM_021092216.1	C2CD5	0.06	12.71333333	0	0
XM_021092227.1	C2CD5	0.066666667	12.46666667	0	0
XM_021092235.1	ST8SIA1	0.01	4.643333333	0	0.373333333
XM_021092242.1	ABCC9	0.403333333	107.43	0	0
XM_021092266.1	PLEKHA5	0.01	1.76	0	0
XM_021092270.1	PLEKHA5	0.34	66.93333333	0	0
XM_021092283.1	IMPG1	0.006666667	0.666666667	0	0
XM_021092288.1	PIK3C2G	0.006666667	1.666666667	0	0
XM_021092290.1	PIK3C2G	0.023333333	5.06	0	0
XM_021092299.1	EPS8	0.176666667	27.53	0	0
XM_021092316.1	GRIN2B	0.003333333	3.333333333	0	0
XM_021092323.1	GPRC5D	0.013333333	0.81	0	0
XM_021092330.1	GPR19	0.05	5.803333333	0	0
XM_021092348.1	LOC110260741	0.006666667	0.666666667	0	0
XM_021092353.1	STYK1	0.033333333	4	0	0
XM_021092361.1	TMEM52B	0.013333333	1.233333333	0	0
XM_021092362.1	TMEM52B	0.276666667	27.76666667	0	0
XM_021092375.1	PZP	0.016666667	3	0	0
XM_021092380.1	M6PR	0.066666667	6.763333333	0	0
XM_021092385.1	RIMKLB	0.013333333	3.756666667	0	0
XM_021092407.1	CLSTN3	0.096666667	16.33333333	0	0
XM_021092434.1	PLEKHG6	0.016666667	2.54	0	0
XM_021092444.1	GALNT8	0.006666667	0.666666667	0	0
XM_021092458.1	ITFG2	0.08	10.38666667	0	0
XM_021092463.1	DDX11	0.036666667	6.076666667	0	0
XM_021092473.1	DDX11	0.023333333	3.736666667	0	0
XM_021092477.1	DDX11	0.003333333	0.346666667	0	0
XM_021092483.1	SLC6A12	0.013333333	1.49	0	0
XM_021092484.1	SLC6A12	0.01	1.176666667	0	0
XM_021092489.1	CCDC77	0.056666667	6.273333333	0	0
XM_021092530.1	KIF21A	0.003333333	0.856666667	0	0
XM_021092554.1	PPHLN1	0.02	2.663333333	0	0
XM_021092555.1	PPHLN1	0.026666667	3.89	0	0
XM_021092579.1	DBX2	0.003333333	0.55	0	0
XM_021092600.1	RAPGEF3	0.076666667	12.69333333	0	0
XM_021092608.1	TMEM106C	0.756666667	45.18333333	0	0
XM_021092611.1	COL2A1	0.003333333	1	0	0.333333333
XM_021092615.1	ASB8	0.436666667	39.56666667	0	0
XM_021092639.1	GLT8D2	0.056666667	5.73	0	0
XM_021092646.1	GLT8D2	0.053333333	6.483333333	0	0
XM_021092663.1	GNPTAB	0.05	12.5	0	0
XM_021092664.1	GNPTAB	0.02	4.716666667	0	0
XM_021092675.1	GNPTAB	0.08	17.08666667	0	0
XM_021092682.1	MYBPC1	0.16	26.12333333	0	0

XM_021092694.1	SPIC	0.003333333	0.333333333	0	0
XM_021092698.1	ANO4	0.03	6.91	0	0
XM_021092709.1	KATNAL2	0.003333333	0.333333333	0	0
XM_021092716.1	GAS2L3	0.033333333	9.083333333	0	0
XM_021092725.1	UHRF1BP1L	0.066666667	22.98	0	0
XM_021092745.1	SMAD2	0.053333333	22.63666667	0	0
XM_021092802.1	METTL25	0.366666667	33.89666667	0	0
XM_021092803.1	METTL25	0.176666667	120.24	0	0
XM_021092820.1	LOC110260809	0.016666667	0.616666667	0	0
XM_021092842.1	SGSM3	0.043333333	5.366666667	0	0
XM_021092889.1	GLS2	0.003333333	0.333333333	0	0
XM_021092891.1	RBMS2	0.053333333	16.59	0	0
XM_021092894.1	RBMS2	0.036666667	11.66333333	0	0
XM_021092896.1	MIP	0.006666667	0.333333333	0	0
XM_021092903.1	SMAD2	0.03	13.01333333	0	0
XM_021092913.1	GRIP1	0.006666667	2.156666667	0	0
XM_021092918.1	GRIP1	0.006666667	1.516666667	0	0.28
XM_021092927.1	SOX5	0.006666667	2.543333333	0	0
XM_021092928.1	SOX5	0.013333333	4.476666667	0	0
XM_021092930.1	SOX5	0.006666667	1.936666667	0	0
XM_021092939.1	SOX5	0.006666667	1.606666667	0	0
XM_021092947.1	SOX5	0.003333333	0.79	0	0
XM_021092949.1	SOX5	0.003333333	1.27	0	0
XM_021092957.1	SOX5	0.01	4.573333333	0	0
XM_021092966.1	SOX5	0.003333333	0.603333333	0	0.476666667
XM_021092970.1	ERC1	0.033333333	11.83	0	0
XM_021092972.1	ERC1	0.103333333	39.45666667	0	0.003333333
XM_021092973.1	ERC1	0.01	3.333333333	0	0
XM_021092990.1	SCAF11	0.01	2.9	0	0
XM_021093030.1	EPYC	0.003333333	0.54	0	0
XM_021093058.1	LIN7A	0.003333333	1.076666667	0	0
XM_021093066.1	PTPRQ	0.003333333	0.843333333	0	0
XM_021093068.1	PTPRQ	0.026666667	9.403333333	0	0
XM_021093076.1	NAV3	0.083333333	38.49333333	0	0
XM_021093079.1	NAV3	0.013333333	6.816666667	0	0
XM_021093080.1	NAV3	0.006666667	2.966666667	0	0
XM_021093088.1	NAV3	0.02	8.733333333	0	0
XM_021093103.1	FAM227A	0.003333333	0.333333333	0	0
XM_021093110.1	LOC110260841	0.01	0.666666667	0	0
XM_021093126.1	TAS2R8	0.01	0.333333333	0	0
XM_021093139.1	DAB1	0.003333333	1.236666667	0	0
XM_021093142.1	DAB1	0.003333333	0.85	0	0.333333333
XM_021093155.1	DAB1	0.006666667	1.58	0	0
XM_021093160.1	DAB1	0.003333333	0.97	0	0
XM_021093182.1	MBD1	0.003333333	0.326666667	0	0
XM_021093186.1	MBD1	0.023333333	3.086666667	0	0
XM_021093191.1	AQP4	0.026666667	6.44	0	0
XM_021093224.1	MBD1	0.016666667	2.053333333	0	0
XM_021093233.1	LRP8	0.006666667	1.586666667	0	0
XM_021093241.1	LPIN2	0.086666667	21.38333333	0	0
XM_021093245.1	BCO1	0.013333333	1.596666667	0	0
XM_021093247.1	BCO1	0.013333333	1.283333333	0	0
XM_021093259.1	ERCC1	0.043333333	1.856666667	0	0
XM_021093260.1	ERCC1	0.43	17.40666667	0	0
XM_021093265.1	GUCA2B	0.27	6	0	0
XM_021093326.1	TMEM86B	0.003333333	0.236666667	0	0
XM_021093328.1	ADGRG1	0.03	4.646666667	0	0
XM_021093331.1	ADGRG1	0.09	13.49	0	0
XM_021093350.1	FBXO6	0.013333333	1.506666667	0	0
XM_021093352.1	FBXO6	0.036666667	3.636666667	0	0

XM_021093369.1	CTCF	0.01	5.166666667	0	0
XM_021093388.1	PLD3	0.023333333	3.646666667	0	0
XM_021093399.1	LOC100626318	0.006666667	1.366666667	0	0
XM_021093402.1	LOC100626318	0.056666667	12.35	0	0
XM_021093406.1	TMEM51	0.04	3.446666667	0	0
XM_021093414.1	RSRP1	0.05	3.643333333	0	0
XM_021093434.1	PPP1R8	0.016666667	1.356666667	0	0
XM_021093455.1	TNNT1	4.463333333	174.6833333	0	0
XM_021093457.1	TNNT1	14.42	537.7466667	0	0
XM_021093468.1	ST3GAL3	0.16	14.26666667	0	0
XM_021093475.1	ST3GAL3	0.023333333	2.146666667	0	0
XM_021093490.1	LEPR	0.023333333	4.926666667	0	0
XM_021093497.1	AKR1A1	0.05	2.78	0	0
XM_021093507.1	POU2F2	0.006666667	2.186666667	0	0
XM_021093531.1	JAK1	0.063333333	13.81333333	0	0
XM_021093559.1	CES1	0.033333333	3.083333333	0	0
XM_021093569.1	ATP4A	0.02	3.333333333	0	0
XM_021093617.1	RPL13A	0.06	4.01	0	0
XM_021093652.1	PIEZO1	0.11	37.51	0	0
XM_021093654.1	PIEZO1	0.16	54.30666667	0	0
XM_021093661.1	CTU2	0.03	4.776666667	0	0
XM_021093676.1	CA5A	0.046666667	5.52	0	0
XM_021093688.1	FBXO31	0.54	124.1166667	0	0.013333333
XM_021093691.1	FBXO31	0.376666667	85.19333333	0	0.013333333
XM_021093700.1	MTHFSD	0.01	1.523333333	0	0
XM_021093719.1	KIAA0513	0.026666667	5.473333333	0	0
XM_021093728.1	MBTPS1	0.093333333	17.44	0	0
XM_021093735.1	SLC38A8	0.016666667	1.076666667	0	0
XM_021093744.1	PLCG2	0.016666667	5.566666667	0	0
XM_021093773.1	TMEM170A	0.016666667	2.763333333	0	0
XM_021093793.1	LOC100525750	0.006666667	0.333333333	0	0
XM_021093821.1	CHST4	0.016666667	1.333333333	0	0
XM_021093825.1	PHLPP2	0.013333333	4.903333333	0	0
XM_021093854.1	IST1	0.55	53.97	0	0
XM_021093886.1	ZFP90	0.183333333	33.8	0	0
XM_021093894.1	SLC12A3	0.213333333	31.41666667	0	0
XM_021093896.1	SLC12A3	0.023333333	3.346666667	0	0
XM_021093897.1	SLC12A3	0.436666667	63.25333333	0	0
XM_021093898.1	SLC12A3	0.1	14.65333333	0	0
XM_021093912.1	ADGRG5	0.06	10.80666667	0	0
XM_021093913.1	ADGRG5	0.036666667	6.39	0	0
XM_021093916.1	ADGRG5	0.043333333	7.173333333	0	0
XM_021093919.1	ADGRG3	0.02	3.333333333	0	0
XM_021093921.1	DRC7	0.003333333	0.333333333	0	0
XM_021093924.1	KIFC3	0.023333333	3.66	0	0
XM_021093925.1	KIFC3	0.02	2.773333333	0	0
XM_021093935.1	KATNB1	0.513333333	56.91	0	0
XM_021093967.1	NDRG4	0.02	2.726666667	0	0
XM_021093979.1	CDH8	0.003333333	0.666666667	0	0
XM_021093980.1	CDH8	0.006666667	1	0	0
XM_021093981.1	CDH8	0.01	1.666666667	0	0
XM_021093999.1	LOC110260932	0.01	0.666666667	0	0
XM_021094002.1	CMTM2	0.076666667	3.666666667	0	0
XM_021094003.1	CMTM2	0.016666667	0.666666667	0	0
XM_021094015.1	PDP2	0.31	53.16666667	0	0
XM_021094020.1	CA7	0.023333333	1.333333333	0	0
XM_021094022.1	CDH16	0.02	2.333333333	0	0
XM_021094068.1	PLEKHG4	0.006666667	1	0	0
XM_021094080.1	SLC9A5	0.01	2.753333333	0	0
XM_021094088.1	CARMIL2	0.023333333	4	0	0

XM_021094108.1	TSNAXIP1	0.016666667	1.666666667	0	0
XM_021094121.1	LOC100621778	0.023333333	1.333333333	0	0
XM_021094144.1	PRMT7	0.066666667	13.02	0	0
XM_021094158.1	DUS2	0.106666667	9.093333333	0	0
XM_021094173.1	APH1B	0.006666667	0.436666667	0	0
XM_021094176.1	LOC100519366	0.013333333	1.773333333	0	0
XM_021094177.1	TOX3	0.043333333	5.763333333	0	0.096666667
XM_021094181.1	TOX3	0.013333333	1.946666667	0	0
XM_021094182.1	APH1B	0.01	1.12	0	0
XM_021094188.1	APH1B	0.04	2.16	0	0
XM_021094228.1	ABCC11	0.003333333	0.666666667	0	0
XM_021094229.1	ABCC11	0.003333333	0.666666667	0	0
XM_021094257.1	ZNF536	0.003333333	1.153333333	0	0
XM_021094283.1	TLN2	0.15	56.62	0	0
XM_021094287.1	RHPN2	0.036666667	5	0	0
XM_021094294.1	SLC7A10	0.103333333	7.96	0	0
XM_021094309.1	LOC100623157	0.006666667	1.39	0	0
XM_021094332.1	DPF1	0.06	6.063333333	0	0
XM_021094353.1	CCER2	0.043333333	5	0	0
XM_021094361.1	PAK4	0.096666667	11.78666667	0	0
XM_021094363.1	NCCRP1	0.013333333	1	0	0
XM_021094370.1	VPS13C	0.033333333	23.00666667	0	0
XM_021094384.1	LOC100522141	0.04	2.333333333	0	0
XM_021094385.1	LOC100522141	0.046666667	2.666666667	0	0
XM_021094392.1	C6H19orf47	0.056666667	3.823333333	0	0
XM_021094409.1	ICE2	0.07	13.80333333	0	0
XM_021094415.1	ICE2	0.06	12.58	0	0
XM_021094429.1	TMEM145	0.02	1.196666667	0	0
XM_021094434.1	GRIK5	0.026666667	4.816666667	0	0.266666667
XM_021094436.1	GRIK5	0.02	4.246666667	0	0.316666667
XM_021094437.1	GRIK5	0.006666667	0.913333333	0	0.173333333
XM_021094452.1	ARHGEF1	0.08	12.96333333	0	0
XM_021094453.1	ICE2	0.013333333	2.116666667	0	0
XM_021094455.1	ARHGEF1	0.043333333	6.623333333	0	0
XM_021094462.1	DMRTC2	0.003333333	0.333333333	0	0
XM_021094464.1	LOC102158679	0.09	12.53	0	0
XM_021094465.1	LOC102158679	0.026666667	4.613333333	0	0
XM_021094466.1	LOC102158679	0.046666667	5.806666667	0	0
XM_021094467.1	LOC102158679	0.013333333	2.26	0	0
XM_021094476.1	XRCC1	0.386666667	31.99666667	0	0
XM_021094517.1	CEACAM19	0.016666667	1.726666667	0	0
XM_021094522.1	RELB	0.05	5.09	0	0
XM_021094526.1	GEMIN7	0.13	17.76666667	0	0
XM_021094530.1	GEMIN7	0.086666667	4.336666667	0	0
XM_021094531.1	GEMIN7	0.16	8.396666667	0	0
XM_021094532.1	GEMIN7	0.083333333	4.696666667	0	0
XM_021094537.1	EXOC3L2	0.023333333	2.556666667	0	0
XM_021094554.1	KLC3	0.023333333	1.666666667	0	0
XM_021094596.1	CCDC61	0.04	3.263333333	0	0
XM_021094630.1	CCDC9	0.04	4.02	0	0
XM_021094636.1	CCDC9	0.013333333	1.51	0	0
XM_021094666.1	CCDC114	0.006666667	0.936666667	0	0
XM_021094675.1	NTN5	0.006666667	0.46	0	0
XM_021094679.1	NTN5	0.013333333	1.036666667	0	0
XM_021094683.1	NTN5	0.006666667	0.556666667	0	0
XM_021094685.1	CGNL1	0.006666667	2.366666667	0	0
XM_021094694.1	MAMSTR	0.203333333	18.25333333	0	0
XM_021094721.1	TEAD2	0.326666667	28.54666667	0	0
XM_021094724.1	TEAD2	0.003333333	0.163333333	0	0
XM_021094731.1	DKKL1	0.04	1.486666667	0	0

XM_021094733.1	DKKL1	0.113333333	4.513333333	0	0
XM_021094747.1	CPT1C	0.023333333	2.666666667	0	0
XM_021094757.1	FUZ	0.03	3.1	0	0
XM_021094764.1	IL4I1	0.006666667	0.553333333	0	0
XM_021094772.1	ZNF473	0.01	2.546666667	0	0
XM_021094773.1	ZNF280D	0.033333333	5.543333333	0	0
XM_021094781.1	MYH14	0.043333333	13.00666667	0	0
XM_021094786.1	MYH14	0.03	9.36	0	0
XM_021094797.1	LRRC4B	0.043333333	4.603333333	0	0
XM_021094809.1	CLEC11A	0.026666667	1.38	0	0
XM_021094812.1	LOC100620498	0.016666667	2.626666667	0	0.203333333
XM_021094821.1	LOC100620498	0.003333333	0.39	0	0
XM_021094825.1	LOC100620498	0.01	2.266666667	0	0
XM_021094827.1	LOC100620498	0.03	2.476666667	0	0
XM_021094829.1	RFX7	0.013333333	4.983333333	0	0
XM_021094830.1	RFX7	0.006666667	1.743333333	0	0.616666667
XM_021094846.1	LOC110261034	0.023333333	2.446666667	0	0
XM_021094848.1	LOC110261034	0.016666667	1.196666667	0	0
XM_021094855.1	CEACAM18	0.01	1.516666667	0	0
XM_021094856.1	CEACAM18	0.04	2.703333333	0	0
XM_021094858.1	CEACAM18	0.07	4.446666667	0	0
XM_021094862.1	TSEN34	0.01	0.77	0	0
XM_021094863.1	TSEN34	0.033333333	1.94	0	0
XM_021094871.1	TSEN34	0.03	1.936666667	0	0
XM_021094897.1	LOC100622306	0.023333333	1	0	0
XM_021094921.1	PRTG	0.036666667	16.33333333	0	0
XM_021094924.1	LOC100517161	0.023333333	3.34	0	0
XM_021094926.1	LOC100517161	0.073333333	11.19333333	0	0
XM_021094927.1	PRTG	0.006666667	3.563333333	0	0
XM_021094929.1	LOC100514465	0.013333333	0.666666667	0	0
XM_021094930.1	LOC100514465	0.003333333	0.333333333	0	0
XM_021094931.1	LOC100514465	0.033333333	1.666666667	0	0
XM_021094933.1	LOC100625598	0.003333333	0.333333333	0	0.066666667
XM_021094944.1	LOC100515899	0.003333333	0.333333333	0	0
XM_021094958.1	LOC100521529	0.01	1.96	0	0.663333333
XM_021094963.1	LOC100525227	0.006666667	0.61	0	0
XM_021094964.1	LOC100525227	0.016666667	1.39	0	0
XM_021094978.1	LOC100517285	0.02	1.453333333	0	0
XM_021094980.1	LOC110261048	0.01	1.353333333	0	0
XM_021094991.1	PTPRH	0.016666667	2.846666667	0	0
XM_021094992.1	PTPRH	0.013333333	2.153333333	0	0
XM_021094997.1	BRSK1	0.073333333	9.666666667	0	0
XM_021095000.1	TMEM150B	0.023333333	1.516666667	0	0
XM_021095008.1	IL11	0.003333333	0.333333333	0	0
XM_021095024.1	LOC100621492	0.58	56.11333333	0	0
XM_021095032.1	LOC100621492	4.02	368.0166667	0	0
XM_021095036.1	SHISA7	0.003333333	0.666666667	0	0.333333333
XM_021095049.1	ZNF524	0.313333333	13.70333333	0	0
XM_021095077.1	ZNF667	0.04	6.116666667	0	0
XM_021095079.1	UNC13C	0.01	4	0	0
XM_021095084.1	ZNF667	0.163333333	22.73	0	0
XM_021095093.1	WDR72	0.04	10.84	0	0
XM_021095105.1	ZNF582	0.07	6.106666667	0	0
XM_021095106.1	WDR72	0.003333333	0.81	0	0
XM_021095110.1	ZNF787	0.043333333	3.763333333	0	0
XM_021095115.1	WDR72	0.006666667	2.01	0	0
XM_021095116.1	ZNF444	0.113333333	7.47	0	0
XM_021095128.1	LOC110261076	0.046666667	1.496666667	0	0
XM_021095136.1	LOC110261077	0.003333333	0.333333333	0	0
XM_021095164.1	KLHL17	0.033333333	5.136666667	0	0

XM_021095165.1	KLHL17	0.02	3.106666667	0	0
XM_021095167.1	KLHL17	0.013333333	1.756666667	0	0
XM_021095172.1	KLHL17	0.036666667	3.886666667	0	0
XM_021095184.1	RNF223	0.003333333	0.896666667	0	0
XM_021095191.1	LOC110261078	0.013333333	13.66666667	0	0
XM_021095208.1	TTC34	0.003333333	0.666666667	0	0.333333333
XM_021095210.1	LOC110261082	0.003333333	0.333333333	0	0
XM_021095218.1	TP73	0.023333333	2.72	0	0
XM_021095237.1	CHD5	0.01	3.666666667	0	0
XM_021095238.1	CHD5	0.013333333	5.956666667	0	0.333333333
XM_021095251.1	ACOT7	0.023333333	1.333333333	0	0
XM_021095268.1	THAP3	0.053333333	2.44	0	0
XM_021095283.1	MYO5C	0.056666667	15.97666667	0	0
XM_021095292.1	PIK3CD	0.013333333	3.173333333	0	0
XM_021095296.1	CLSTN1	0.173333333	32.76	0	0
XM_021095301.1	CTNNBIP1	0.553333333	21.14	0	0
XM_021095302.1	CTNNBIP1	0.786666667	31.00333333	0	0
XM_021095344.1	MTHFR	0.006666667	1.843333333	0	0
XM_021095389.1	TMEM82	0.02	1.446666667	0	0
XM_021095400.1	ZBTB17	0.023333333	2.94	0	0
XM_021095403.1	CLCNKA	0.026666667	2.926666667	0	0
XM_021095404.1	CLCNKA	0.026666667	2.83	0	0
XM_021095407.1	CLCNKA	0.033333333	3.59	0	0
XM_021095423.1	DMXL2	0.12	55.83333333	0	0
XM_021095453.1	ARHGEF10L	0.003333333	0.543333333	0	0
XM_021095457.1	IGSF21	0.003333333	0.333333333	0	0
XM_021095488.1	HSPG2	0.01	7.28	0	0
XM_021095497.1	LDLRAD2	0.003333333	0.333333333	0	0
XM_021095507.1	LUZP1	0.313333333	101.4566667	0	0
XM_021095511.1	HNRNPR	0.033333333	4.03	0	0.12
XM_021095515.1	HNRNPR	0.003333333	0.556666667	0	0.013333333
XM_021095518.1	ZNF436	0.053333333	10.71666667	0	0
XM_021095538.1	IFNLR1	0.01	2.666666667	0	0.333333333
XM_021095551.1	STPG1	0.003333333	0.756666667	0	0
XM_021095554.1	RCAN3	0.036666667	5.186666667	0	0
XM_021095575.1	SRRM1	0.026666667	8.363333333	0	0
XM_021095592.1	RUNX3	0.023333333	4.666666667	0	0
XM_021095603.1	PAFAH2	0.093333333	19.04666667	0	0.116666667
XM_021095624.1	UBXN11	0.023333333	1.51	0	0
XM_021095646.1	FGR	0.116666667	16.63666667	0	0
XM_021095647.1	USP8	0.053333333	10.17333333	0	0
XM_021095650.1	FGR	0.023333333	3.356666667	0	0
XM_021095682.1	GABPB1	0.013333333	1.6	0	0
XM_021095737.1	PUM1	0.29	48.75666667	0	0
XM_021095776.1	COL16A1	0.03	6.526666667	0	0
XM_021095789.1	ATP8B4	0.003333333	0.553333333	0	0
XM_021095790.1	ADGRB2	0.013333333	2.62	0	0
XM_021095793.1	ADGRB2	0.076666667	17.04666667	0	0
XM_021095812.1	ATP8B4	0.003333333	0.563333333	0	0
XM_021095821.1	S100PBP	0.133333333	40.79666667	0	0
XM_021095838.1	ATP8B4	0.013333333	2.86	0	0
XM_021095852.1	ZNF362	0.053333333	7.393333333	0	0
XM_021095862.1	GJB3	0.006666667	0.666666667	0	0
XM_021095866.1	SMIM12	0.393333333	16.58	0	0
XM_021095892.1	ZMYM1	0.013333333	2.513333333	0	0
XM_021095930.1	SH3D21	0.023333333	2.386666667	0	0
XM_021095947.1	LSM10	0.003333333	0.083333333	0	0.023333333
XM_021095954.1	OSCP1	0.016666667	2.053333333	0	0
XM_021095962.1	GRIK3	0.016666667	9.003333333	0	0.62
XM_021095999.1	CAP1	0.1	13.96666667	0	0

XM_021096007.1	CAP1	0.03	4.333333333	0	0
XM_021096013.1	ZMPSTE24	0.03	3.86	0	0
XM_021096035.1	PRELID3A	0.003333333	0.666666667	0	0
XM_021096038.1	PRELID3A	0.006666667	0.586666667	0	0
XM_021096077.1	ARHGAP28	0.006666667	1.586666667	0	0
XM_021096086.1	EPB41L3	0.006666667	1.79	0	0
XM_021096095.1	EPB41L3	0.043333333	10.35333333	0	0
XM_021096106.1	EPB41L3	0.013333333	2.55	0	0
XM_021096110.1	EPB41L3	0.023333333	4.31	0	0
XM_021096112.1	EPB41L3	0.013333333	2.876666667	0	0
XM_021096115.1	EPB41L3	0.053333333	9.083333333	0	0
XM_021096124.1	EPB41L3	0.093333333	15.81333333	0	0
XM_021096137.1	SMCHD1	0.05	17.82666667	0	0
XM_021096140.1	METTL4	0.076666667	11.08666667	0	0
XM_021096173.1	LOC100525229	0.003333333	0.413333333	0	0
XM_021096186.1	LAMA3	0.043333333	19.51666667	0	0
XM_021096194.1	TTC39C	0.01	1.493333333	0	0
XM_021096217.1	GAREM1	0.053333333	18.05666667	0	0
XM_021096223.1	NOL4	0.003333333	0.48	0	0
XM_021096232.1	DTNA	0.056666667	17.70333333	0	0
XM_021096252.1	DTNA	0.056666667	14.67666667	0	0
XM_021096267.1	LOC100620188	0.003333333	0.62	0	0
XM_021096286.1	KIAA1328	0.046666667	4.206666667	0	0
XM_021096287.1	KIAA1328	0.026666667	2.14	0	0
XM_021096332.1	CELF4	0.003333333	0.333333333	0	0
XM_021096333.1	CELF4	0.003333333	0.333333333	0	0
XM_021096377.1	ADGRL2	0.003333333	1.446666667	0	0
XM_021096383.1	ADGRL2	0.006666667	1.576666667	0	0
XM_021096386.1	ADGRL2	0.13	32.89333333	0	0
XM_021096407.1	ADGRL2	0.006666667	1.466666667	0	0
XM_021096412.1	ADGRL2	0.016666667	4.14	0	0
XM_021096450.1	MIGA1	0.016666667	3.253333333	0	0
XM_021096464.1	SLC44A5	0.01	1.09	0	0
XM_021096475.1	SPG11	0.01	1.02	0	0
XM_021096481.1	LRRIQ3	0.003333333	0.333333333	0	0
XM_021096483.1	SPG11	0.003333333	0.34	0	0
XM_021096492.1	C6H1orf141	0.003333333	0.333333333	0	0
XM_021096507.1	MIER1	0.06	11.70666667	0	0
XM_021096511.1	MIER1	0.02	3.943333333	0	0
XM_021096512.1	MIER1	0.046666667	10.24	0	0
XM_021096523.1	SGIP1	0.003333333	1.533333333	0	0
XM_021096527.1	SGIP1	0.03	14.25666667	0	0.78
XM_021096530.1	SGIP1	0.01	3.903333333	0	0.846666667
XM_021096565.1	LOC100525112	0.036666667	3.333333333	0	0
XM_021096566.1	LOC100621407	0.003333333	0.333333333	0	0
XM_021096573.1	MYSM1	0.05	9.29	0	0
XM_021096575.1	MYSM1	0.013333333	2.446666667	0	0
XM_021096580.1	FYB2	0.013333333	1	0	0
XM_021096591.1	FAM151A	0.086666667	8.333333333	0	0
XM_021096610.1	HSPB11	0.08	2.056666667	0	0
XM_021096622.1	ECHDC2	0.13	9.943333333	0	0
XM_021096626.1	FRMD5	0.006666667	1.086666667	0	0
XM_021096685.1	STIL	0.013333333	3.226666667	0	0
XM_021096699.1	LOC110255311	0.026666667	2.143333333	0	0
XM_021096700.1	LOC110255311	0.043333333	2.506666667	0	0
XM_021096701.1	LOC110255328	0.033333333	2.933333333	0	0
XM_021096704.1	LOC100627285	0.03	1.966666667	0	0
XM_021096706.1	LOC100737897	0.043333333	4.636666667	0	0
XM_021096707.1	LOC100737897	0.353333333	35.38666667	0	0
XM_021096708.1	PPIP5K1	0.053333333	13.92333333	0	0

XM_021096712.1	EFCAB14	0.126666667	29.66666667	0	0
XM_021096732.1	CCDC17	0.006666667	0.666666667	0	0
XM_021096759.1	LOC110261240	0.003333333	0.333333333	0	0
XM_021096768.1	PTCH2	0.02	3.263333333	0	0
XM_021096783.1	ARMH1	0.013333333	1.886666667	0	0
XM_021096796.1	ERI3	0.283333333	18.57	0	0
XM_021096804.1	ERI3	0.033333333	1.33	0	0
XM_021096825.1	SLC6A9	0.06	7.92	0	0
XM_021096842.1	PTPRF	0.003333333	0.633333333	0	0
XM_021096843.1	PTPRF	0.003333333	0.616666667	0	0
XM_021096845.1	PTPRF	0.01	2.8	0	0
XM_021096850.1	PTPRF	0.006666667	1.696666667	0	0
XM_021096857.1	PTPRF	0.046666667	15.70333333	0	0
XM_021096861.1	PTPRF	0.01	3.383333333	0	0
XM_021096873.1	PTPRF	0.03	8.743333333	0	0.226666667
XM_021096882.1	TIE1	0.023333333	4.283333333	0	0
XM_021096903.1	TMEM125	0.023333333	1.643333333	0	0
XM_021096904.1	TMEM125	0.033333333	2	0	0
XM_021096943.1	SCMH1	0.293333333	34.54	0	0
XM_021096947.1	SCMH1	0.046666667	5.686666667	0	0.166666667
XM_021096949.1	SCMH1	0.02	2.17	0	0
XM_021096961.1	SCMH1	0.103333333	8.943333333	0	0
XM_021096982.1	NFYC	0.056666667	4.92	0	0
XM_021096990.1	RIMS3	0.003333333	0.666666667	0	0
XM_021097002.1	FANCA	0.036666667	7	0	0
XM_021097004.1	SPIRE2	0.01	1.203333333	0	0
XM_021097021.1	VPS9D1	0.063333333	4.776666667	0	0
XM_021097027.1	DEF8	0.246666667	86.34666667	0	0
XM_021097078.1	TMEM62	0.393333333	35.73	0	0
XM_021097112.1	PRODH2	0.023333333	3.333333333	0	0
XM_021097113.1	PRODH2	0.093333333	12	0	0
XM_021097145.1	ZNF792	0.043333333	6.796666667	0	0
XM_021097165.1	LOC110255280	0.026666667	2.653333333	0	0
XM_021097167.1	LOC110255280	0.026666667	2.3	0	0
XM_021097178.1	ZBTB32	0.016666667	0.666666667	0	0
XM_021097256.1	LOC110261289	0.013333333	1.333333333	0	0
XM_021097257.1	LOC110261290	0.24	22.66666667	0	0
XM_021097263.1	LOC110261291	0.3	33.79333333	0	0
XM_021097280.1	LOC110261301	0.01	0.333333333	0	0
XM_021097293.1	ZNF304	0.113333333	4.576666667	0	0
XM_021097317.1	LOC110261320	0.03	5.563333333	0	0
XM_021097320.1	LOC110261320	0.026666667	3.346666667	0	0
XM_021097331.1	LOC110261322	0.14	14.81333333	0	0
XM_021097333.1	PLA2G4E	0.016666667	4.47	0	0
XM_021097335.1	LOC110261323	0.003333333	0.333333333	0	0
XM_021097353.1	MZF1	0.026666667	3.096666667	0	0
XM_021097366.1	ZNF135	0.016666667	3.086666667	0	0
XM_021097389.1	ZNF446	0.01	1.51	0	0
XM_021097403.1	LOC110261337	0.113333333	20.34666667	0	0
XM_021097406.1	LOC110261337	0.046666667	6.42	0	0
XM_021097424.1	TTLL10	0.003333333	0.666666667	0	0
XM_021097426.1	TNFRSF18	0.01	0.666666667	0	0
XM_021097428.1	TNFRSF18	0.016666667	1	0	0
XM_021097434.1	SCNN1D	0.036666667	6.33	0	0.206666667
XM_021097436.1	INTS11	0.096666667	9.34	0	0
XM_021097445.1	CCNL2	0.24	24.75666667	0	0
XM_021097461.1	MIB2	0.836666667	131.3566667	0	0.05
XM_021097476.1	SLC35E2B	0.056666667	14.24	0	0
XM_021097492.1	GNB1	0.343333333	42.71	0	0
XM_021097506.1	CAMTA1	0.006666667	2.263333333	0	0

XM_021097508.1	CAMTA1	0.016666667	7.143333333	0	0
XM_021097514.1	CAMTA1	0.006666667	1.8	0	0
XM_021097531.1	CLEC18C	0.12	10.33333333	0	0
XM_021097558.1	LOC110261362	0.003333333	0.333333333	0	0
XM_021097579.1	LOC110255329	0.006666667	0.38	0	0
XM_021097605.1	BMP8A	0.003333333	0.333333333	0	0
XM_021097617.1	ERICH3	0.003333333	0.666666667	0	0
XM_021097627.1	LOC110261409	0.05	3.196666667	0	0
XM_021097652.1	EIF4G3	0.053333333	14.67666667	0	0
XM_021097673.1	EIF4G3	0.24	62.64333333	0	0
XM_021097681.1	ALPL	0.036666667	3.996666667	0	0
XM_021097693.1	RAP1GAP	0.026666667	3.54	0	0
XM_021097695.1	RAP1GAP	0.023333333	3.273333333	0	0
XM_021097696.1	RAP1GAP	0.056666667	7.496666667	0	0
XM_021097708.1	PTPRM	0.01	2.566666667	0	0.38
XM_021097721.1	DLGAP1	0.003333333	1.13	0	0
XM_021097728.1	DLGAP1	0.003333333	0.803333333	0	0
XM_021097739.1	RBBP8	0.073333333	11.50666667	0	0
XM_021097753.1	ST6GALNAC3	0.02	3.856666667	0	0
XM_021097763.1	ROR1	0.006666667	2.243333333	0	0
XM_021097765.1	ROR1	0.07	15.03333333	0	0
XM_021097791.1	PAK6	0.003333333	1.473333333	0	0
XM_021097802.1	PAK6	0.013333333	2.86	0	0
XM_021097824.1	BUB1B	0.01	1.333333333	0	0
XM_021097842.1	BMF	0.053333333	11.37666667	0	0.04
XM_021097846.1	BMF	0.043333333	8.616666667	0	0
XM_021097855.1	BMF	0.03	6.493333333	0	0
XM_021097880.1	SLA-DOB	0.036666667	3.35	0	0
XM_021097882.1	BLM	0.02	3.973333333	0	0
XM_021097884.1	BLM	0.063333333	12.11333333	0	0
XM_021097886.1	EIF2AK4	0.043333333	10.58333333	0	0.136666667
XM_021097907.1	NMB	0.006666667	0.666666667	0	0
XM_021097909.1	GABBR1	0.23	45.94	0	0
XM_021097912.1	ODF2	0.006666667	1.77	0	0
XM_021097915.1	PPP1R10	0.053333333	10.21333333	0	0
XM_021097917.1	PPP1R10	0.02	5.336666667	0	0
XM_021097933.1	DDR1	0.016666667	3.5	0	0
XM_021097944.1	ATAT1	0.063333333	2.176666667	0	0
XM_021097947.1	ATF6B	0.196666667	22.21333333	0	0
XM_021097953.1	ATF6B	0.016666667	1.4	0	0
XM_021097964.1	IRF4	0.013333333	2.41	0	0
XM_021097973.1	PPP1R18	0.026666667	3.26	0	0
XM_021097995.1	PEAK1	0.14	66.80333333	0	0
XM_021098011.1	RASGRP1	0.023333333	4.446666667	0	0
XM_021098040.1	TREML1	0.006666667	0.333333333	0	0
XM_021098054.1	FKBP5	0.386666667	72.20666667	0	0
XM_021098056.1	MEIS2	0.02	3.386666667	0	0
XM_021098065.1	MEIS2	0.06	7.713333333	0	0
XM_021098073.1	ACYP1	0.09	8.536666667	0	0
XM_021098098.1	PRIM2	0.02	1.79	0	0
XM_021098102.1	BTBD9	0.013333333	1.466666667	0	0
XM_021098107.1	UROC1	0.003333333	0.666666667	0	0
XM_021098112.1	SLC25A27	0.01	2.506666667	0	0
XM_021098134.1	ADGRF1	0.023333333	3.226666667	0	0
XM_021098137.1	ADGRF1	0.01	1.183333333	0	0
XM_021098161.1	RNASE1	0.08	2.81	0	0
XM_021098171.1	DICER1	0.033333333	11	0	0
XM_021098179.1	DPH6	0.003333333	2.82	0	0
XM_021098186.1	ODF2	0.006666667	1.576666667	0	0
XM_021098212.1	ALDH1L1	0.296666667	38.22333333	0	0

XM_021098235.1	HNRNPC	0.043333333	18.88666667	0	0
XM_021098247.1	HNRNPC	0.08	36.53666667	0	0
XM_021098252.1	HNRNPC	0.03	13.79333333	0	0
XM_021098266.1	GSTZ1	0.02	0.53	0	0
XM_021098294.1	SLC28A1	0.073333333	8	0	0
XM_021098304.1	ANPEP	0.02	3.236666667	0	0
XM_021098310.1	GREM1	0.033333333	5.333333333	0	0
XM_021098311.1	SLC17A1	0.003333333	0.273333333	0	0
XM_021098314.1	SLC17A1	0.003333333	0.273333333	0	0
XM_021098322.1	CYP21A2	0.01	1	0	0
XM_021098341.1	DDX39B	0.136666667	12.62666667	0	0
XM_021098390.1	NDRG2	0.096666667	8.91	0	0
XM_021098395.1	NDRG2	0.02	1.496666667	0	0
XM_021098415.1	LOC100152878	0.006666667	1	0	0
XM_021098418.1	SLC17A3	0.03	8.733333333	0	0
XM_021098423.1	TRIM38	0.03	2.173333333	0	0
XM_021098449.1	ADAMTS17	0.026666667	7.66	0	0
XM_021098455.1	MPIG6B	0.013333333	0.666666667	0	0
XM_021098480.1	LOC106507398	0.01	0.426666667	0	0
XM_021098503.1	LOC100513097	0.006666667	0.333333333	0	0
XM_021098514.1	TINAG	0.98	81.09	0	0
XM_021098515.1	TINAG	0.02	1.666666667	0	0
XM_021098530.1	MLIP	0.033333333	5.016666667	0	0
XM_021098588.1	COL21A1	0.02	2.16	0	0
XM_021098591.1	DAXX	0.136666667	14.59666667	0	0
XM_021098601.1	LOC100156231	0.056666667	4.323333333	0	0
XM_021098622.1	TCP11	0.003333333	0.336666667	0	0
XM_021098639.1	C7H6orf222	0.003333333	1.193333333	0	0
XM_021098640.1	C7H6orf222	0.003333333	0.666666667	0	0.333333333
XM_021098707.1	TRERF1	0.013333333	4.766666667	0	0
XM_021098713.1	TRERF1	0.01	3.093333333	0	0
XM_021098716.1	TRERF1	0.013333333	5.373333333	0	0
XM_021098717.1	TRERF1	0.02	7.153333333	0	0
XM_021098718.1	TRERF1	0.013333333	5.166666667	0	0
XM_021098737.1	CUL9	0.043333333	16.17666667	0	0
XM_021098757.1	POLH	0.05	8.2	0	0
XM_021098761.1	FAN1	0.06	8.823333333	0	0
XM_021098763.1	TJAP1	0.093333333	16.93333333	0	0
XM_021098769.1	TJAP1	0.026666667	3.636666667	0	0
XM_021098771.1	TJAP1	0.063333333	9.153333333	0	0
XM_021098811.1	GLYATL3	0.003333333	0.333333333	0	0
XM_021098817.1	PKHD1	0.026666667	18.80333333	0	0
XM_021098823.1	PKHD1	0.063333333	34.53	0	0
XM_021098827.1	TJP1	0.11	34.3	0	0
XM_021098829.1	GCM1	0.023333333	1.666666667	0	0
XM_021098843.1	TJP1	0.006666667	2.64	0	0
XM_021098859.1	ARNT2	0.013333333	3.623333333	0	0
XM_021098871.1	SAXO2	0.003333333	0.4	0	0
XM_021098902.1	ZNF592	0.026666667	8.903333333	0	0
XM_021098906.1	SEH1L	0.013333333	1.666666667	0	0
XM_021098917.1	FURIN	0.013333333	2.71	0	0
XM_021098962.1	KIF7	0.07	12.96	0	0
XM_021098963.1	WDR93	0.003333333	0.326666667	0	0
XM_021098973.1	ZNF710	0.046666667	12.53333333	0	0
XM_021098992.1	PRC1	0.05	5.063333333	0	0
XM_021099018.1	SALL3	0.01	4.333333333	0	0
XM_021099028.1	NEIL1	0.033333333	3.283333333	0	0
XM_021099031.1	NEIL1	0.016666667	1.96	0	0
XM_021099046.1	ULK3	0.076666667	8.446666667	0	0
XM_021099047.1	ULK3	0.02	2.163333333	0	0

XM_021099053.1	EDC3	0.23	38.18333333	0	0
XM_021099098.1	NEO1	0.083333333	23.76	0	0
XM_021099105.1	ODF2	0.006666667	1.216666667	0	0
XM_021099138.1	MIPOL1	0.023333333	4.35	0	0
XM_021099170.1	PPP2R3C	0.08	6.426666667	0	0
XM_021099189.1	FBXO15	0.01	1	0	0
XM_021099194.1	LOC100156469	0.01	0.793333333	0	0
XM_021099196.1	NR6A1	0.01	3.056666667	0	0
XM_021099199.1	NETO1	0.003333333	1.05	0	0.666666667
XM_021099203.1	LTB4R2	0.013333333	1.226666667	0	0
XM_021099204.1	LTB4R2	0.013333333	1.35	0	0
XM_021099211.1	NETO1	0.003333333	1.686666667	0	0
XM_021099214.1	EMC9	0.12	1.96	0	0
XM_021099244.1	ACIN1	0.233333333	46.08666667	0	0
XM_021099249.1	RTTN	0.013333333	4.413333333	0	0
XM_021099259.1	RBM23	0.02	2.74	0	0
XM_021099266.1	REM2	0.016666667	2.273333333	0	0
XM_021099270.1	ABHD4	0.08	8.096666667	0	0
XM_021099282.1	RTTN	0.03	9.216666667	0	0
XM_021099286.1	CD226	0.023333333	3.286666667	0	0
XM_021099296.1	CHD8	0.003333333	1.323333333	0	0.003333333
XM_021099302.1	CHD8	0.136666667	43.35666667	0	0
XM_021099315.1	RNASE13	0.023333333	3.333333333	0	0
XM_021099341.1	LOC102160410	0.006666667	1.643333333	0	0
XM_021099342.1	LOC102160410	0.003333333	1.39	0	0
XM_021099346.1	LOC102160410	0.006666667	2.966666667	0	0
XM_021099358.1	MCTP2	0.016666667	2.95	0	0
XM_021099374.1	SV2B	0.003333333	1.333333333	0	0.333333333
XM_021099384.1	RAB15	0.016666667	2.553333333	0	0
XM_021099420.1	PCNX1	0.03	17.29	0	0
XM_021099433.1	SIPA1L1	0.01	3.356666667	0	0
XM_021099443.1	CDH7	0.006666667	1.306666667	0	0
XM_021099450.1	SIPA1L1	0.026666667	7.933333333	0	0
XM_021099455.1	SIPA1L1	0.023333333	6.593333333	0	0
XM_021099456.1	SIPA1L1	0.04	10.28666667	0	0
XM_021099460.1	SIPA1L1	0.006666667	2.623333333	0	0
XM_021099465.1	SIPA1L1	0.023333333	6.113333333	0	0
XM_021099473.1	NUMB	0.013333333	1.86	0	0
XM_021099474.1	NUMB	0.046666667	6.706666667	0	0
XM_021099475.1	NUMB	0.073333333	10.23333333	0	0
XM_021099502.1	ENTPD5	0.026666667	5.773333333	0	0
XM_021099503.1	ENTPD5	0.076666667	17.97666667	0	0
XM_021099505.1	ENTPD5	0.096666667	22.47333333	0	0
XM_021099506.1	ENTPD5	0.06	14.32333333	0	0
XM_021099507.1	ENTPD5	0.013333333	3.653333333	0	0
XM_021099508.1	ENTPD5	0.22	49.37333333	0	0
XM_021099515.1	ENTPD5	0.123333333	30.52	0	0
XM_021099520.1	LTBP2	0.013333333	4.616666667	0	0.023333333
XM_021099526.1	AREL1	0.01	2.203333333	0	0
XM_021099527.1	AREL1	0.04	9.713333333	0	0
XM_021099535.1	JDP2	0.103333333	6.976666667	0	0
XM_021099546.1	LOC110261637	0.003333333	0.333333333	0	0
XM_021099547.1	TMEM63C	0.003333333	1.333333333	0	0.666666667
XM_021099568.1	CEP128	0.016666667	3.3	0	0
XM_021099569.1	CEP128	0.013333333	3.163333333	0	0
XM_021099571.1	CEP128	0.086666667	17.61333333	0	0
XM_021099582.1	STON2	0.046666667	6.426666667	0	0
XM_021099610.1	RPS6KA5	0.063333333	12.09333333	0	0
XM_021099613.1	RPS6KA5	0.14	21.83333333	0	0
XM_021099639.1	UNC79	0.003333333	0.996666667	0	0

XM_021099661.1	PPP4R4	0.026666667	5	0	0
XM_021099670.1	LOC106504547	0.06	3.556666667	0	0
XM_021099671.1	LOC100153899	0.036666667	2.3	0	0
XM_021099672.1	LOC106504545	0.023333333	1.33	0	0
XM_021099677.1	AK7	0.013333333	1.333333333	0	0
XM_021099679.1	PAPOLA	0.08	11.13	0	0
XM_021099682.1	PAPOLA	0.043333333	8.416666667	0	0
XM_021099693.1	EML1	0.076666667	14.49333333	0	0
XM_021099706.1	SLC25A29	0.05	5.556666667	0	0
XM_021099719.1	WDR25	0.036666667	3.063333333	0	0
XM_021099728.1	WDR25	0.076666667	6.043333333	0	0
XM_021099742.1	LOC110261647	0.003333333	0.673333333	0	0
XM_021099745.1	LOC110261647	0.016666667	2.846666667	0	0.006666667
XM_021099754.1	PHACTR1	0.006666667	1.17	0	0
XM_021099780.1	PIGN	0.106666667	22.92333333	0	0
XM_021099791.1	CARMIL1	0.05	12.25	0	0
XM_021099792.1	CARMIL1	0.036666667	9.056666667	0	0
XM_021099809.1	PRSS16	0.003333333	0.333333333	0	0
XM_021099816.1	ZSCAN23	0.01	0.706666667	0	0
XM_021099823.1	LOC100154932	0.01	0.666666667	0	0
XM_021099833.1	TMEM225B	0.01	1.613333333	0	0
XM_021099858.1	CCPG1	0.03	7.033333333	0	0
XM_021099865.1	AKAP6	0.013333333	5.99	0	0
XM_021099866.1	AKAP6	0.023333333	9.923333333	0	0
XM_021099868.1	AKAP6	0.023333333	10.28333333	0	0
XM_021099893.1	STRN3	0.023333333	4.133333333	0	0
XM_021099901.1	CCBE1	0.013333333	3.626666667	0	0
XM_021099902.1	SCFD1	1.92	161.8866667	0	0
XM_021099906.1	SCFD1	0.12	10.37666667	0	0
XM_021099917.1	ZBTB25	0.006666667	0.713333333	0	0
XM_021099924.1	ZBTB25	0.016666667	1.21	0	0
XM_021099927.1	CCPG1	0.11	26.55666667	0	0
XM_021099932.1	RAD51B	0.01	2.07	0	0
XM_021099944.1	RGS6	0.01	2.27	0	0
XM_021099945.1	RGS6	0.026666667	6.846666667	0	0
XM_021099947.1	RGS6	0.023333333	5.353333333	0	0
XM_021099948.1	RGS6	0.013333333	3.33	0	0
XM_021099972.1	NRXN3	0.003333333	1.666666667	0	0
XM_021099977.1	NRXN3	0.003333333	0.666666667	0	0
XM_021099987.1	NRXN3	0.003333333	0.666666667	0	0
XM_021099997.1	LOC110261696	0.026666667	1.013333333	0	0
XM_021100017.1	LOC110261723	0.01	0.333333333	0	0
XM_021100033.1	CLPSL1	0.013333333	0.333333333	0	0
XM_021100068.1	LOC110261756	0.003333333	0.333333333	0	0
XM_021100069.1	CCPG1	0.056666667	7.273333333	0	0
XM_021100105.1	EXOC2	0.276666667	38.25666667	0	0
XM_021100106.1	FOXQ1	0.003333333	0.333333333	0	0
XM_021100149.1	CAGE1	0.023333333	2.87	0	0
XM_021100154.1	CAGE1	0.013333333	1.323333333	0	0
XM_021100172.1	LOC100517166	0.023333333	0.58	0	0
XM_021100191.1	ADTRP	0.006666667	0.333333333	0	0
XM_021100201.1	SLC24A1	0.006666667	2.85	0	0
XM_021100202.1	SLC24A1	0.006666667	2.6	0	0.333333333
XM_021100216.1	KIF13A	0.243333333	74.68	0	0.44
XM_021100218.1	KIF13A	0.02	6.42	0	0.086666667
XM_021100223.1	KIF13A	0.076666667	22.72666667	0	0.103333333
XM_021100225.1	KIF13A	0.006666667	1.743333333	0	0.016666667
XM_021100245.1	MEGF11	0.03	6.476666667	0	0
XM_021100246.1	MEGF11	0.03	4.846666667	0	0
XM_021100253.1	MEGF11	0.013333333	1.87	0	0

XM_021100254.1	MEGF11	0.016666667	2.38	0	0
XM_021100255.1	MEGF11	0.013333333	2.143333333	0	0
XM_021100260.1	SPARCL1	0.576666667	85.83	0	0
XM_021100277.1	LEF1	0.016666667	2.046666667	0	0
XM_021100297.1	LCORL	0.006666667	2.773333333	0	0
XM_021100310.1	RASGEF1B	0.603333333	164.4933333	0	0
XM_021100322.1	CPEB2	0.016666667	5.406666667	0	0
XM_021100323.1	CPEB2	0.2	60.43333333	0	0
XM_021100330.1	UGT8	0.006666667	1.59	0	0
XM_021100332.1	UGT8	0.016666667	2.916666667	0	0
XM_021100334.1	TRMT10A	0.066666667	5.093333333	0	0
XM_021100338.1	TRMT10A	0.026666667	2.02	0	0
XM_021100345.1	RHOH	0.02	4.47	0	0
XM_021100355.1	MRO	0.013333333	2	0	0
XM_021100361.1	ARFIP1	0.14	18.98666667	0	0
XM_021100375.1	ARFIP1	0.08	9.686666667	0	0.056666667
XM_021100376.1	ARFIP1	0.033333333	3.883333333	0	0
XM_021100387.1	ANKRD17	0.026666667	13.73333333	0	0
XM_021100411.1	BMPR1B	0.006666667	4.76	0	0.06
XM_021100414.1	BMPR1B	0.02	5.2	0	0
XM_021100424.1	BMPR1B	0.026666667	6.12	0	0
XM_021100432.1	HOPX	0.023333333	1.166666667	0	0
XM_021100451.1	SMAD1	0.033333333	5.04	0	0
XM_021100455.1	SMAD1	0.166666667	20.59	0	0
XM_021100473.1	PPP3CA	0.053333333	9.273333333	0	0
XM_021100477.1	MAP2K5	0.076666667	7.353333333	0	0
XM_021100482.1	DMP1	0.003333333	0.66	0	0
XM_021100486.1	GLRB	0.006666667	1.063333333	0	0
XM_021100495.1	GUCY1B1	0.016666667	2.146666667	0	0
XM_021100499.1	SLC4A4	0.026666667	5.863333333	0	0
XM_021100519.1	ITGA11	0.063333333	12.92333333	0	0
XM_021100560.1	CCDC149	0.11	21.36666667	0	0
XM_021100569.1	ATP10D	0.036666667	8.97	0	0
XM_021100577.1	CORIN	0.003333333	1	0	0
XM_021100580.1	NFXL1	0.026666667	4.376666667	0	0
XM_021100582.1	NFXL1	0.146666667	23.3	0	0
XM_021100591.1	PAQR5	0.023333333	4.51	0	0
XM_021100600.1	PAQR5	0.03	11.41666667	0	0
XM_021100611.1	PAQR5	0.013333333	3.13	0	0
XM_021100629.1	SH3D19	0.15	34.73666667	0	0
XM_021100641.1	LRBA	0.006666667	1.116666667	0	0
XM_021100652.1	MAB21L2	0.03	3.666666667	0	0
XM_021100655.1	IQCM	0.003333333	0.153333333	0	0
XM_021100681.1	JADE1	0.03	7.343333333	0	0
XM_021100689.1	JADE1	0.18	41.94666667	0	0
XM_021100726.1	TLE3	0.003333333	0.443333333	0	0
XM_021100733.1	TLE3	0.026666667	6.21	0	0
XM_021100737.1	CCSER1	0.036666667	8.926666667	0	0
XM_021100753.1	HERC3	0.06	12.71666667	0	0
XM_021100759.1	HERC6	0.043333333	9.303333333	0	0
XM_021100770.1	MAPK10	0.043333333	11.15666667	0	0.276666667
XM_021100771.1	MAPK10	0.013333333	4.466666667	0	0
XM_021100795.1	PRDM8	0.023333333	2.946666667	0	0
XM_021100800.1	SLC25A51	0.23	50.49	0	0
XM_021100809.1	LOC102165510	0.01	0.666666667	0	0
XM_021100817.1	SLC49A3	0.226666667	23.95333333	0	0
XM_021100820.1	SLC49A3	0.046666667	4.573333333	0	0
XM_021100824.1	PCGF3	0.146666667	33.52	0	0
XM_021100845.1	SLC26A1	0.003333333	0.85	0	0
XM_021100846.1	SLC26A1	0.16	24.69666667	0	0

XM_021100848.1	SLC26A1	0.043333333	7.483333333	0	0
XM_021100862.1	LOC110262034	0.006666667	0.333333333	0	0
XM_021100869.1	CTBP1	0.056666667	5.473333333	0	0
XM_021100870.1	CTBP1	0.04	4.293333333	0	0
XM_021100891.1	FAM53A	0.016666667	1.536666667	0	0
XM_021100895.1	FAM53A	0.02	1.656666667	0	0
XM_021100906.1	FGFR3	0.053333333	9.813333333	0	0
XM_021100908.1	FGFR3	0.003333333	0.843333333	0	0
XM_021100935.1	MYO9A	0.013333333	8.156666667	0	0
XM_021100939.1	MYO9A	0.03	17.29333333	0	0.013333333
XM_021100948.1	MYO9A	0.003333333	1.946666667	0	0
XM_021100955.1	MYO9A	0.053333333	30.23666667	0	0.166666667
XM_021100961.1	SORCS2	0.013333333	1.78	0	0
XM_021100964.1	SORCS2	0.013333333	3.486666667	0	0
XM_021101020.1	FBXL5	0.02	2.643333333	0	0
XM_021101022.1	LOC110262058	0.016666667	0.333333333	0	0
XM_021101046.1	LDB2	0.023333333	3.083333333	0	0
XM_021101057.1	LAP3	0.083333333	7.126666667	0	0
XM_021101070.1	LOC100737183	0.15	15.66666667	0	0
XM_021101081.1	LRFN5	0.013333333	2.81	0	0
XM_021101083.1	CCKAR	0.003333333	1.066666667	0	0
XM_021101084.1	CCKAR	0.003333333	1.09	0	0
XM_021101091.1	ARAP2	0.046666667	15.50666667	0	0
XM_021101096.1	C8H4orf19	0.053333333	6.053333333	0	0
XM_021101114.1	KLB	0.053333333	7.696666667	0	0
XM_021101135.1	RBM47	0.02	4.403333333	0	0
XM_021101149.1	APBB2	0.013333333	3.553333333	0	0.103333333
XM_021101165.1	GABRA2	0.003333333	1.23	0	0
XM_021101167.1	LOC100517408	0.013333333	1	0	0
XM_021101171.1	CNGA1	0.006666667	0.666666667	0	0
XM_021101173.1	TEC	0.01	0.846666667	0	0
XM_021101186.1	FRYL	0.043333333	22.34666667	0	0
XM_021101191.1	OCIAD1	0.01	0.493333333	0	0.01
XM_021101197.1	OCIAD1	0.043333333	2.883333333	0	0.036666667
XM_021101203.1	DCUN1D4	0.03	5.08	0	0
XM_021101218.1	LNX1	0.03	3.54	0	0
XM_021101223.1	LNX1	0.026666667	4.11	0	0
XM_021101224.1	LNX1	0.066666667	9.733333333	0	0
XM_021101235.1	FIP1L1	0.03	6.153333333	0	0
XM_021101237.1	FIP1L1	0.033333333	7.19	0	0
XM_021101240.1	FIP1L1	0.01	2.016666667	0	0
XM_021101242.1	FIP1L1	0.196666667	46.1	0	0
XM_021101243.1	FIP1L1	0.046666667	9.71	0	0
XM_021101253.1	FIP1L1	0.02	4.253333333	0	0
XM_021101259.1	FIP1L1	0.003333333	0.423333333	0	0
XM_021101272.1	LOC110262090	0.073333333	24.95	0	0
XM_021101293.1	RXFP1	0.02	2.396666667	0	0
XM_021101295.1	RXFP1	0.01	1.106666667	0	0
XM_021101297.1	RXFP1	0.026666667	3.546666667	0	0
XM_021101314.1	LOC100519853	0.003333333	0.663333333	0	0
XM_021101315.1	LOC100519853	0.003333333	1.143333333	0	0
XM_021101316.1	APELA	0.006666667	0.333333333	0	0
XM_021101329.1	CLOCK	0.003333333	1.286666667	0	0.263333333
XM_021101344.1	EXOC1	0.27	34.85666667	0	0
XM_021101352.1	CEP135	0.04	7.783333333	0	0
XM_021101355.1	KIAA1211	0.106666667	31.6	0	0
XM_021101359.1	KIAA1211	0.103333333	29.35666667	0	0
XM_021101374.1	ARL9	0.006666667	0.333333333	0	0
XM_021101391.1	STAP1	0.006666667	0.333333333	0	0
XM_021101403.1	KLHDC1	0.1	6.516666667	0	0

XM_021101404.1	LOC100623504	0.066666667	13.33333333	0	0.333333333
XM_021101406.1	LOC110262115	0.01	0.333333333	0	0
XM_021101408.1	LOC100516628	0.016666667	1	0	0
XM_021101410.1	LOC110262116	0.03	2.98	0	0
XM_021101411.1	LOC100515222	0.006666667	0.48	0	0
XM_021101435.1	BTC	0.186666667	25.72333333	0	0
XM_021101448.1	CDKL2	0.016666667	3.273333333	0	0
XM_021101456.1	PPEF2	0.003333333	0.333333333	0	0
XM_021101466.1	CCDC158	0.01	1.333333333	0	0
XM_021101472.1	11-Sep	0.016666667	1.48	0	0
XM_021101479.1	CDKL1	0.13	23.03666667	0	0
XM_021101483.1	FGA	0.05	6.333333333	0	0
XM_021101484.1	FGA	0.033333333	4	0	0
XM_021101488.1	TRIM2	0.023333333	7.663333333	0	0
XM_021101498.1	TRIM2	0.01	3.676666667	0	0
XM_021101520.1	GATB	0.06	10.88666667	0	0
XM_021101524.1	PRMT9	0.016666667	1.886666667	0	0
XM_021101528.1	TTC29	0.003333333	0.333333333	0	0
XM_021101531.1	TTC29	0.003333333	0.333333333	0	0
XM_021101536.1	ZNF827	0.03	7.513333333	0	0
XM_021101573.1	ELF2	0.016666667	3.313333333	0	0
XM_021101574.1	ELF2	0.03	4.393333333	0	0
XM_021101612.1	ANKRD50	0.11	36.28	0	0
XM_021101632.1	NIN	0.03	10.69	0	0
XM_021101640.1	PRDM5	0.033333333	9.703333333	0	0
XM_021101651.1	NIN	0.053333333	19.05666667	0	0.003333333
XM_021101668.1	ZGRF1	0.01	2.706666667	0	0
XM_021101685.1	NIN	0.046666667	13.79	0	0
XM_021101696.1	NIN	0.033333333	9.29	0	0
XM_021101708.1	ARHGEF38	0.003333333	0.666666667	0	0
XM_021101733.1	LOC100512795	0.07	8	0	0
XM_021101734.1	C8H4orf17	0.013333333	1.256666667	0	0
XM_021101735.1	C8H4orf17	0.016666667	1.743333333	0	0
XM_021101742.1	RAP1GDS1	0.146666667	24.04	0	0
XM_021101749.1	PDLIM5	0.006666667	1.72	0	0
XM_021101754.1	ABHD12B	0.003333333	0.333333333	0	0
XM_021101794.1	TRIM9	0.006666667	1	0	0
XM_021101796.1	NKX6-1	0.006666667	0.666666667	0	0
XM_021101800.1	GPAT3	0.07	8.716666667	0	0
XM_021101802.1	HELQ	0.043333333	5.69	0	0
XM_021101808.1	LOC100524999	0.023333333	0.716666667	0	0
XM_021101814.1	SEC31A	0.076666667	14.93333333	0	0
XM_021101818.1	SEC31A	0.046666667	8.613333333	0	0
XM_021101820.1	SEC31A	0.11	20.68	0	0
XM_021101827.1	SEC31A	0.223333333	43.09666667	0	0
XM_021101829.1	SEC31A	0.096666667	18.26333333	0	0
XM_021101832.1	SEC31A	0.046666667	8.636666667	0	0
XM_021101833.1	SEC31A	0.053333333	10.14	0	0
XM_021101892.1	PRKG2	0.003333333	0.666666667	0	0.666666667
XM_021101896.1	GNG2	0.066666667	10.82	0	0
XM_021101904.1	ITGB8	0.026666667	8.973333333	0	0
XM_021101905.1	PON1	0.026666667	4.283333333	0	0
XM_021101913.1	PON1	0.046666667	7.303333333	0	0
XM_021101917.1	PON1	0.046666667	8.836666667	0	0
XM_021101923.1	ATM	0.013333333	7.766666667	0	0
XM_021101926.1	ATM	0.093333333	49.59333333	0	0
XM_021101928.1	ATM	0.02	10.78333333	0	0
XM_021101946.1	SLC26A3	0.033333333	5	0	0
XM_021101962.1	GRB10	0.463333333	79.25	0	0
XM_021101970.1	DBF4	0.033333333	4.543333333	0	0

XM_021101976.1	SGCE	0.033333333	2.613333333	0	0
XM_021101982.1	LMX1B	0.02	4.54	0	0.333333333
XM_021101985.1	ETS1	0.013333333	2.896666667	0	0.186666667
XM_021102000.1	TXNDC16	0.03	6.133333333	0	0
XM_021102006.1	TXNDC16	0.126666667	22.84333333	0	0
XM_021102021.1	C9H11orf54	0.14	14.77333333	0	0
XM_021102026.1	PUS3	0.366666667	27.56666667	0	0
XM_021102035.1	JAML	0.013333333	1.206666667	0	0
XM_021102046.1	SLC37A4	0.046666667	6.163333333	0	0
XM_021102056.1	NLRX1	0.023333333	3.983333333	0	0
XM_021102071.1	PICALM	0.343333333	56.24333333	0	0
XM_021102075.1	PICALM	0.126666667	21.11333333	0	0
XM_021102080.1	FERMT2	1.126666667	153.2833333	0	0.006666667
XM_021102117.1	EZH2	0.006666667	0.663333333	0	0
XM_021102120.1	EZH2	0.03	3.226666667	0	0
XM_021102121.1	EZH2	0.01	1.04	0	0
XM_021102152.1	ST3GAL4	0.016666667	6.823333333	0	0
XM_021102154.1	ST3GAL4	0.023333333	9.923333333	0	0
XM_021102160.1	DDHD1	0.17	39.86333333	0	0
XM_021102162.1	CD55	0.026666667	3.8	0	0
XM_021102163.1	CD55	0.016666667	2.276666667	0	0
XM_021102166.1	CD55	0.016666667	2.37	0	0
XM_021102169.1	CD55	0.033333333	4.63	0	0
XM_021102175.1	FOLR3	0.02	1.23	0	0
XM_021102186.1	AKAP9	0.03	17.46333333	0	0
XM_021102193.1	AKAP9	0.056666667	29.36666667	0	0
XM_021102205.1	PIK3CG	0.033333333	10.17	0	0
XM_021102208.1	PSIP1	0.036666667	2.64	0	0
XM_021102216.1	PIGR	0.163333333	27.20333333	0	0
XM_021102217.1	CGRRF1	0.196666667	18.72333333	0	0
XM_021102237.1	HSD11B1	0.183333333	11.19666667	0	0
XM_021102239.1	HSD11B1	0.743333333	42.19666667	0	0
XM_021102272.1	PON3	0.013333333	6.156666667	0	0
XM_021102320.1	DLG2	0.013333333	4.57	0	1.043333333
XM_021102337.1	CNTN5	0.003333333	1.303333333	0	0.496666667
XM_021102342.1	C9H11orf87	0.003333333	1.146666667	0	0
XM_021102349.1	NTM	0.01	1.26	0	0
XM_021102352.1	NTM	0.01	0.93	0	0
XM_021102384.1	VPS50	0.576666667	111.7333333	0	0
XM_021102390.1	HEPACAM2	0.043333333	4.02	0	0
XM_021102394.1	HEPACAM2	0.02	3.603333333	0	0
XM_021102399.1	SLC25A13	0.006666667	0.736666667	0	0
XM_021102401.1	SLC25A13	0.066666667	8.323333333	0	0
XM_021102403.1	SLC25A13	0.093333333	12.03666667	0	0
XM_021102408.1	DYNC1I1	0.02	2.39	0	0
XM_021102425.1	ICA1	0.023333333	2.42	0	0
XM_021102429.1	ICA1	0.006666667	0.536666667	0	0
XM_021102438.1	DGKB	0.003333333	0.623333333	0	0
XM_021102445.1	DGKB	0.003333333	0.433333333	0	0
XM_021102448.1	DGKB	0.01	1.33	0	0
XM_021102454.1	DGKB	0.01	1.6	0	0
XM_021102456.1	DGKB	0.003333333	0.603333333	0	0
XM_021102461.1	HDAC9	0.01	7.15	0	0
XM_021102465.1	HDAC9	0.056666667	41.44	0	0
XM_021102477.1	HDAC9	0.003333333	0.93	0	0
XM_021102478.1	HDAC9	0.003333333	0.586666667	0	0
XM_021102483.1	HDAC9	0.02	5.396666667	0	0
XM_021102519.1	RUNDC3B	0.006666667	1.666666667	0	0
XM_021102554.1	PTPN12	0.916666667	135.9833333	0	0
XM_021102557.1	PTPN12	0.04	5.4	0	0

XM_021102572.1	KMT2E	0.143333333	44.73333333	0	0
XM_021102579.1	SRPK2	0.006666667	1.213333333	0	0
XM_021102600.1	DUS4L	0.016666667	2.223333333	0	0
XM_021102618.1	CACNA1E	0.01	4.633333333	0	0
XM_021102622.1	CACNA1E	0.01	3.15	0	0
XM_021102644.1	LOC102161316	0.003333333	0.333333333	0	0
XM_021102653.1	LOC100520579	0.003333333	0.333333333	0	0
XM_021102672.1	LOC100739737	0.003333333	0.333333333	0	0
XM_021102704.1	LOC100513421	0.006666667	0.666666667	0	0
XM_021102718.1	JKAMP	0.056666667	4.27	0	0
XM_021102719.1	LOC100520757	0.01	0.48	0	0
XM_021102726.1	LOC110262329	0.01	0.333333333	0	0
XR_001298434.2	LARP1B	0.02	3.326666667	0	0
XR_001298485.2	LOC100518620	0.086666667	16.60333333	0	0
XR_001299173.2	HHIPL2	0.006666667	0.546666667	0	0
XR_001299314.2	C10H10orf67	0.01	2.366666667	0	0
XR_001299773.2	C12H17orf80	0.036666667	3.956666667	0	0
XR_001299971.2	ANKFY1	0.33	110.64	0	0
XR_001300925.2	PRR14L	0.02	5.46	0	0
XR_001301123.2	HTR7	0.006666667	0.543333333	0	0
XR_001301127.2	HECTD2	0.013333333	0.856666667	0	0
XR_001302708.2	THOC2	0.03	9.426666667	0	0
XR_001303420.2	HSD17B7	0.076666667	2.376666667	0	0
XR_001303628.2	LOC100621778	0.01	0.666666667	0	0
XR_001303880.2	MINAR1	0.003333333	1.333333333	0	0.333333333
XR_001304041.2	RUFY3	0.063333333	9.64	0	0
XR_001304683.2	NR4A2	0.03	3.99	0	0
XR_001304688.2	7-Mar	0.11	18.48	0	0
XR_001306592.2	COL27A1	0.05	16.78333333	0	0
XR_001307423.2	ATXN2L	0.066666667	12.51333333	0	0
XR_001308148.2	RMDN1	0.01	0.753333333	0	0
XR_001308304.2	CFAP126	0.13	8.49	0	0
XR_001308305.2	CFAP126	0.046666667	2.946666667	0	0
XR_001308384.2	RIIAD1	0.006666667	0.666666667	0	0
XR_001308425.2	MAB21L3	0.01	1.936666667	0	0
XR_001308467.2	KIAA1324	0.006666667	1	0	0
XR_002335511.1	SYT9	0.083333333	13.81	0	0
XR_002335675.1	HYOU1	0.013333333	2.96	0	0
XR_002335713.1	MSANTD2	0.006666667	1.1	0	0
XR_002335894.1	LOC106504983	0.003333333	0.333333333	0	0
XR_002335920.1	ARHGEF5	0.02	6.376666667	0	0.333333333
XR_002335953.1	NPHS2	0.07	5.423333333	0	0
XR_002335954.1	NPHS2	0.003333333	0.333333333	0	0
XR_002335995.1	LOC106507881	0.03	5.993333333	0	0
XR_002336000.1	LOC106507881	0.01	1.413333333	0	0
XR_002336002.1	LOC106507881	0.003333333	0.403333333	0	0
XR_002336004.1	LOC106507881	0.013333333	3.723333333	0	0
XR_002336005.1	LOC106507881	0.013333333	0.756666667	0	0
XR_002336010.1	LOC106507881	0.01	2.65	0	0.376666667
XR_002336067.1	TRAF3IP3	0.01	2.466666667	0	0
XR_002336069.1	TRAF3IP3	0.036666667	4.423333333	0	0
XR_002336112.1	SPATA6L	0.006666667	1.306666667	0	0
XR_002336255.1	LOC110255652	0.003333333	3.713333333	0	2.72
XR_002336285.1	LOC102162336	0.023333333	1.516666667	0	0
XR_002336294.1	C10H9orf3	0.006666667	0.78	0	0
XR_002336297.1	C10H9orf3	0.16	24.62	0	0
XR_002336329.1	MNAT1	0.046666667	4.093333333	0	0
XR_002336345.1	LOC100524391	0.13	12.05	0	0
XR_002336469.1	GCNT1	0.013333333	0.333333333	0	0
XR_002336475.1	GCNT1	0.013333333	0.333333333	0	0

XR_002336505.1	COG6	0.016666667	3.103333333	0	0
XR_002336508.1	COG6	0.003333333	1.04	0	0
XR_002336513.1	COG6	0.02	5.06	0	0
XR_002336593.1	IFT88	0.586666667	118.0733333	0	0
XR_002336595.1	IFT88	0.036666667	6.83	0	0
XR_002336744.1	TPP2	0.136666667	37.48	0	0
XR_002336785.1	MCF2L	0.013333333	2.91	0	0
XR_002336804.1	UPF3A	0.013333333	1.333333333	0	0
XR_002336875.1	LOC110255247	0.016666667	0.95	0	0.01
XR_002336880.1	LOC110255247	0.02	1.413333333	0	0
XR_002336894.1	B3GNTL1	0.013333333	0.953333333	0	0.106666667
XR_002336901.1	B3GNTL1	0.013333333	1.153333333	0	0
XR_002336952.1	AFMID	0.053333333	7.436666667	0	0
XR_002336955.1	AFMID	0.016666667	1.373333333	0	0
XR_002337019.1	C12H17orf80	0.07	7.733333333	0	0
XR_002337020.1	C12H17orf80	0.143333333	13.29	0	0.09
XR_002337023.1	C12H17orf80	0.043333333	4.83	0	0
XR_002337039.1	LOC102165318	0.01	3.44	0	0
XR_002337093.1	ACBD4	0.05	5.303333333	0	0
XR_002337094.1	ACBD4	0.043333333	4.453333333	0	0
XR_002337101.1	LOC110256000	0.04	6.976666667	0	0
XR_002337131.1	CDK12	0.036666667	15.24	0	0
XR_002337196.1	TSPOAP1	0.016666667	5.4	0	0
XR_002337202.1	MED13	0.046666667	21.27333333	0	0
XR_002337235.1	RAD51D	0.003333333	0.72	0	0
XR_002337315.1	RAP1GAP2	0.006666667	1.646666667	0	0
XR_002337371.1	LOC110256111	0.003333333	0.333333333	0	0
XR_002337399.1	SHMT1	0.016666667	2.843333333	0	0
XR_002337405.1	MIEF2	1.376666667	136.1333333	0	0
XR_002337410.1	TOM1L2	0.03	1.846666667	0	0
XR_002337466.1	SEC22C	0.043333333	2.256666667	0	0
XR_002337479.1	ZBTB38	0.02	2.966666667	0	0
XR_002337530.1	RBM5	0.036666667	8.753333333	0	0
XR_002337573.1	ACTR8	0.063333333	14.16	0	0
XR_002337576.1	ACTR8	0.033333333	7.603333333	0	0
XR_002337596.1	FLNB	0.016666667	5.403333333	0	0
XR_002337618.1	ATXN7	0.126666667	26.87666667	0	0.08
XR_002337682.1	NPHP3	0.03	5.376666667	0	0
XR_002337683.1	NPHP3	0.056666667	10.66	0	0
XR_002337696.1	ESYT3	0.026666667	3.2	0	0
XR_002337742.1	SPTSSB	0.006666667	0.333333333	0	0
XR_002337757.1	KYAT1	0.023333333	2.756666667	0	0.063333333
XR_002337783.1	DGKG	0.036666667	4.11	0	0
XR_002337904.1	CD200	0.033333333	3.646666667	0	0
XR_002337929.1	LOC102157687	0.003333333	1.333333333	0	0
XR_002337959.1	ERC2	0.003333333	1.276666667	0	0
XR_002338044.1	SMIM11A	4.113333333	81.59333333	0	0
XR_002338046.1	SMIM11A	0.37	7.31	0	0
XR_002338048.1	SMIM11A	0.01	0.263333333	0	0
XR_002338049.1	SMIM11A	0.053333333	0.966666667	0	0
XR_002338057.1	SMIM11A	0.046666667	0.853333333	0	0
XR_002338067.1	RALGDS	0.16	22.95	0	0.4
XR_002338120.1	ADAMTS13	0.013333333	5.506666667	0	0
XR_002338208.1	EXOG	0.03	2.8	0	0
XR_002338211.1	EXOG	0.006666667	0.623333333	0	0.106666667
XR_002338229.1	NKTR	0.013333333	4.483333333	0	0
XR_002338231.1	NKTR	0.016666667	7.79	0	0
XR_002338234.1	NKTR	0.036666667	11.42666667	0	0
XR_002338264.1	HPS1	0.423333333	51.38	0	0
XR_002338268.1	ADRA1A	0.01	6.083333333	0	0

XR_002338272.1	LOC110256592	0.026666667	2.333333333	0	0
XR_002338285.1	ATP2A2	0.57	150.76	0	0
XR_002338287.1	RBP4	0.003333333	0.736666667	0	0.343333333
XR_002338293.1	CAMK2G	0.033333333	7.516666667	0	0
XR_002338294.1	CAMK2G	0.003333333	1.063333333	0	0
XR_002338296.1	CAMK2G	0.023333333	5.386666667	0	0
XR_002338327.1	CNNM1	0.016666667	5.046666667	0	0
XR_002338356.1	WSCD2	0.003333333	1.79	0	0
XR_002338387.1	ULK1	0.196666667	54.19	0	0
XR_002338407.1	LOC100158108	0.026666667	6.6	0	0
XR_002338455.1	ZNF488	0.003333333	0.333333333	0	0
XR_002338487.1	ITPRIP	0.013333333	4.73	0	0
XR_002338499.1	NKAIN2	0.003333333	0.333333333	0	0
XR_002338648.1	BBIP1	0.006666667	0.47	0	0
XR_002338668.1	RNLS	0.003333333	1.13	0	0
XR_002338839.1	CLRN3	0.013333333	2.333333333	0	0
XR_002338923.1	HIP1R	0.05	10.03666667	0	0
XR_002339010.1	7-Mar	0.23	68.10333333	0	0
XR_002339027.1	GTDC1	0.016666667	7.223333333	0	0
XR_002339040.1	UBE2F	0.176666667	29.97666667	0	0
XR_002339183.1	ITGA4	0.003333333	0.723333333	0	0
XR_002339224.1	PDE1A	0.033333333	6.003333333	0	0
XR_002339284.1	LOC106509653	0.003333333	0.333333333	0	0
XR_002339298.1	CROCC2	0.003333333	2.333333333	0	0.333333333
XR_002339301.1	PLCL1	0.01	2.056666667	0	0
XR_002339305.1	PLCL1	0.013333333	3.34	0	0
XR_002339389.1	FARP2	0.02	3.44	0	0
XR_002339391.1	FARP2	0.013333333	1.953333333	0	0
XR_002339400.1	NEMP2	0.003333333	0.683333333	0	0
XR_002339403.1	SPAG16	0.02	0.91	0	0
XR_002339429.1	USP40	0.1	25.88333333	0	0
XR_002339455.1	DNAJC10	0.156666667	36.19333333	0	0.003333333
XR_002339523.1	RAD1	0.053333333	13.23666667	0	0.223333333
XR_002339553.1	FAM71B	0.003333333	0.333333333	0	0
XR_002339625.1	RBM4B	0.053333333	4.906666667	0	0
XR_002339748.1	LOC100737060	0.093333333	31.78666667	0	0
XR_002339751.1	LOC100737060	0.013333333	4.86	0	0
XR_002339780.1	SLC38A9	0.056666667	30.91	0	0
XR_002339833.1	CCNH	0.02	5.523333333	0	0
XR_002339837.1	ADIG	0.056666667	2.846666667	0	0
XR_002339879.1	ZNF341	0.13	18.4	0	0
XR_002339883.1	STAU1	0.033333333	9.326666667	0	0
XR_002339886.1	STAU1	0.01	2.726666667	0	0
XR_002339887.1	STAU1	0.14	27.46333333	0	0
XR_002339889.1	STAU1	0.09	22	0	0.003333333
XR_002339892.1	STAU1	0.12	22.07666667	0	0
XR_002339893.1	STAU1	0.03	6.62	0	0
XR_002339899.1	STAU1	0.11	19.63	0	0
XR_002339901.1	STAU1	0.023333333	5.856666667	0	0
XR_002339939.1	UCKL1	0.04	10.47666667	0	0
XR_002339960.1	KCNQ2	0.003333333	0.326666667	0	0
XR_002340089.1	C17H20orf202	0.006666667	0.333333333	0	0
XR_002340108.1	DZANK1	0.04	5.94	0	0
XR_002340110.1	DZANK1	0.023333333	3.656666667	0	0
XR_002340155.1	TUSC3	0.03	3.843333333	0	0
XR_002340173.1	PPP4R1L	0.013333333	4.82	0	0
XR_002340176.1	PPP4R1L	0.026666667	10.32	0	0
XR_002340190.1	PPP4R1L	0.03	8.136666667	0	0
XR_002340199.1	PPP4R1L	0.013333333	3.336666667	0	0
XR_002340203.1	HOXA10	0.04	6.103333333	0	0

XR_002340267.1	NCAPG2	0.01	1.643333333	0	0
XR_002340346.1	EPHB6	0.003333333	0.76	0	0.146666667
XR_002340425.1	LOC106506844	0.003333333	0.45	0	0
XR_002340471.1	KIAA1147	0.036666667	10.28333333	0	0
XR_002340483.1	NUP205	0.013333333	2.89	0	0
XR_002340555.1	LOC100515119	0.05	6.33	0	0
XR_002340597.1	ARMCX4	0.02	1.073333333	0	0
XR_002340617.1	GAB3	0.036666667	6.656666667	0	0
XR_002340747.1	LOC100516891	0.023333333	0.566666667	0	0
XR_002340765.1	LOC100620916	0.026666667	3.09	0	0
XR_002340838.1	LOC110257828	0.006666667	1.356666667	0	0
XR_002340884.1	LOC100519939	0.016666667	1.323333333	0	0
XR_002340973.1	CARD9	0.01	1.066666667	0	0
XR_002340998.1	SLC5A11	0.013333333	1.333333333	0	0
XR_002341077.1	PPP1R13B	0.056666667	12.75333333	0	0
XR_002341078.1	PPP1R13B	0.006666667	1.796666667	0	0
XR_002341089.1	TMEM121	0.153333333	14.72333333	0	0
XR_002341105.1	MOK	0.03	4.916666667	0	0
XR_002341122.1	BRF1	0.013333333	3	0	0
XR_002341170.1	FUT10	0.07	6.353333333	0	0
XR_002341173.1	TTI2	0.136666667	13.50666667	0	0
XR_002341267.1	LOC100521600	0.09	9.706666667	0	0
XR_002341268.1	LOC100521600	0.096666667	9.436666667	0	0
XR_002341282.1	LOC110258920	0.006666667	0.333333333	0	0
XR_002341324.1	LOC100522225	0.006666667	0.643333333	0	0
XR_002341341.1	LOC102159510	0.026666667	8.926666667	0	0
XR_002341342.1	LOC102159510	0.013333333	5.75	0	0
XR_002341375.1	PIDD1	0.023333333	3.13	0	0
XR_002341396.1	SLC22A18	0.02	3.836666667	0	0
XR_002341416.1	ENG	0.26	44.27666667	0	0
XR_002341556.1	PLEKHA7	0.003333333	1.343333333	0	0
XR_002341603.1	C2H19orf44	0.01	1.59	0	0
XR_002341626.1	LOC102166940	0.03	6.593333333	0	0
XR_002341630.1	LOC102167351	0.006666667	1.05	0	0
XR_002341671.1	PRAM1	0.103333333	13.85666667	0	0
XR_002341697.1	TLE2	0.05	5.353333333	0	0
XR_002341773.1	TENT2	0.02	4.546666667	0	0
XR_002341787.1	ANKRD34B	0.006666667	0.333333333	0	0
XR_002341851.1	TMEM232	0.013333333	1.43	0	0
XR_002341938.1	DNAJC18	0.166666667	23.92	0	0
XR_002341949.1	ARAP3	0.03	5.496666667	0	0
XR_002342041.1	MADD	0.016666667	3.393333333	0	0
XR_002342044.1	DGKZ	0.19	37.54333333	0	0
XR_002342083.1	LOC110259698	0.006666667	1.403333333	0	0
XR_002342089.1	LYRM7	0.1	16.76333333	0	0
XR_002342091.1	LYRM7	0.103333333	15.80333333	0	0
XR_002342127.1	DNMT3A	0.013333333	4.473333333	0	0
XR_002342163.1	LOC106506286	0.013333333	4.48	0	0.803333333
XR_002342164.1	LOC106506286	0.01	3.78	0	0
XR_002342165.1	LOC106506286	0.02	8.46	0	0
XR_002342244.1	ZSCAN25	0.06	3.526666667	0	0
XR_002342257.1	LOC100522670	0.003333333	1	0	0
XR_002342279.1	LOC100514951	0.043333333	2.62	0	0
XR_002342297.1	TFR2	0.003333333	0.333333333	0	0
XR_002342351.1	AHSP	0.306666667	5	0	0
XR_002342428.1	GRIN2A	0.003333333	1	0	0
XR_002342469.1	TMEM8A	0.03	7.27	0	0
XR_002342505.1	GPAT2	0.003333333	0.456666667	0	0
XR_002342555.1	SEMA4F	0.033333333	8.51	0	0
XR_002342556.1	SEMA4F	0.006666667	2.05	0	0

XR_002342558.1	LOXL3	0.043333333	4.2	0	0
XR_002342565.1	DUSP11	0.14	9.286666667	0	0
XR_002342569.1	DUSP11	0.01	0.643333333	0	0
XR_002342588.1	TIA1	0.07	34.42	0	0.15
XR_002342590.1	TIA1	0.016666667	8.396666667	0	0
XR_002342596.1	TIA1	0.033333333	17.74666667	0	0
XR_002342602.1	ASPRV1	0.006666667	2.333333333	0	0
XR_002342606.1	ASPRV1	0.053333333	3.443333333	0	0
XR_002342608.1	ASPRV1	0.006666667	1.7	0	0
XR_002342610.1	ASPRV1	0.013333333	3.653333333	0	0
XR_002342611.1	ASPRV1	0.006666667	1.753333333	0	0
XR_002342730.1	ATP6V1E2	0.01	1.216666667	0	0
XR_002342739.1	PLEKHH2	0.2	34.55	0	0
XR_002342776.1	CEBPZOS	0.02	0.613333333	0	0
XR_002342809.1	DNAJC5G	0.006666667	0.42	0	0
XR_002342816.1	DTNB	0.033333333	7.456666667	0	0
XR_002342881.1	RNF144A	0.013333333	0.896666667	0	0
XR_002342899.1	TAOK2	0.056666667	19.86333333	0	0
XR_002342917.1	NLRC3	0.003333333	2.143333333	0	0
XR_002342929.1	LOC100517149	0.056666667	8.103333333	0	0
XR_002342950.1	CCDC85A	0.006666667	0.35	0	0
XR_002342953.1	CCDC85A	0.02	0.71	0	0
XR_002343009.1	ZNHIT6	0.043333333	5.16	0	0
XR_002343040.1	LOC110260191	0.006666667	0.59	0	0
XR_002343046.1	TSNARE1	0.086666667	9.453333333	0	0
XR_002343156.1	EMC2	0.03	1.486666667	0	0
XR_002343194.1	ERICH5	0.003333333	0.333333333	0	0
XR_002343195.1	ERICH5	0.013333333	1.65	0	0
XR_002343205.1	LOC106510020	0.006666667	0.666666667	0	0
XR_002343222.1	ROS1	0.35	110.7733333	0	0.01
XR_002343383.1	LOC100520241	0.003333333	0.333333333	0	0
XR_002343389.1	CEP162	0.03	8.163333333	0	0.066666667
XR_002343394.1	LOC100525572	0.006666667	1.316666667	0	0
XR_002343395.1	LOC100525572	0.013333333	1.27	0	0
XR_002343481.1	MAB21L3	0.01	2.48	0	0
XR_002343529.1	BACH2	0.006666667	1.486666667	0	0
XR_002343590.1	ALG14	0.02	2.503333333	0	0
XR_002343699.1	LOC100624559	0.693333333	259.7266667	0	0
XR_002343703.1	LOC110260430	0.006666667	0.333333333	0	0
XR_002343726.1	SCML4	0.003333333	0.983333333	0	0
XR_002343751.1	ODF2L	0.113333333	8.023333333	0	0
XR_002343807.1	ZBTB24	0.03	4.946666667	0	0
XR_002343919.1	RIC8B	0.083333333	37.71666667	0	0
XR_002343936.1	MCRS1	0.013333333	1.183333333	0	0
XR_002343937.1	MCRS1	0.073333333	7.73	0	0
XR_002343974.1	LOC100523964	0.07	8.176666667	0	0
XR_002344138.1	DDX11	0.006666667	1.066666667	0	0
XR_002344163.1	YAF2	0.006666667	2.6	0	0
XR_002344195.1	PIAS2	0.003333333	0.766666667	0	0.023333333
XR_002344257.1	LOC110260797	0.006666667	0.666666667	0	0
XR_002344291.1	PAN2	0.096666667	19.25333333	0	0
XR_002344313.1	ERC1	0.01	4.123333333	0	0
XR_002344314.1	ERC1	0.02	7.91	0	0
XR_002344370.1	MBD1	0.033333333	11.23	0	0
XR_002344390.1	LPIN2	0.06	16.00666667	0	0.016666667
XR_002344392.1	LPIN2	0.02	5.503333333	0	0
XR_002344416.1	IL34	0.003333333	1.446666667	0	0
XR_002344419.1	CES1	0.073333333	7.766666667	0	0
XR_002344432.1	ACSF3	0.016666667	3.133333333	0	0
XR_002344443.1	MVD	0.05	4.323333333	0	0

XR_002344467.1	MEAK7	0.006666667	1.78	0	0.086666667
XR_002344471.1	ATP2C2	0.013333333	1.893333333	0	0
XR_002344494.1	TMEM170A	0.046666667	6.56	0	0.006666667
XR_002344504.1	MLKL	0.136666667	32.83333333	0	0
XR_002344506.1	PDPR	0.35	92.74333333	0	0
XR_002344552.1	TK2	0.03	1.133333333	0	0
XR_002344560.1	SLC9A5	0.01	2.446666667	0	0
XR_002344575.1	CENPT	0.023333333	2.363333333	0	0
XR_002344636.1	LOC100623157	0.003333333	0.806666667	0	0
XR_002344669.1	LOC102158679	0.016666667	3.17	0	0
XR_002344670.1	LOC102158679	0.026666667	4.36	0	0
XR_002344671.1	LOC102158679	0.063333333	10.25666667	0	0
XR_002344675.1	XRCC1	0.05	4.44	0	0
XR_002344681.1	LOC102167273	0.01	1.376666667	0	0
XR_002344749.1	LOC100620498	0.056666667	16.43666667	0	0.066666667
XR_002344751.1	LOC100620498	0.013333333	3.663333333	0	0
XR_002344758.1	LOC100620498	0.053333333	6.66	0	0.026666667
XR_002344765.1	LOC100620498	0.08	10.93	0	0
XR_002344766.1	LOC100620498	0.023333333	2.58	0	0
XR_002344789.1	LOC100515383	0.006666667	0.666666667	0	0
XR_002344793.1	LOC100515899	0.003333333	0.333333333	0	0
XR_002344810.1	LOC110261048	0.006666667	1.166666667	0	0
XR_002344817.1	ISOC2	0.026666667	0.966666667	0	0
XR_002344848.1	PEX10	0.073333333	12.67	0	0
XR_002344861.1	AJAP1	0.003333333	0.333333333	0	0
XR_002344909.1	VPS13D	0.016666667	11.58333333	0	0
XR_002344911.1	VPS13D	0.073333333	39.83	0	0
XR_002344915.1	RCC2	0.006666667	1.363333333	0	0.333333333
XR_002344986.1	SPIRE1	0.116666667	11.83333333	0	0
XR_002344989.1	PRELID3A	0.003333333	0.333333333	0	0
XR_002345039.1	AKAIN1	0.01	1.443333333	0	0
XR_002345040.1	AKAIN1	0.013333333	1.383333333	0	0
XR_002345049.1	LOC110261176	0.033333333	1.5	0	0
XR_002345063.1	LOC100525229	0.003333333	0.92	0	0
XR_002345108.1	MCOLN2	0.003333333	1.186666667	0	0.503333333
XR_002345112.1	SPATA1	0.043333333	4.956666667	0	0
XR_002345117.1	TTLL7	0.213333333	46.86666667	0	0
XR_002345159.1	MIER1	0.08	9.053333333	0	0
XR_002345198.1	ECHDC2	0.016666667	1.156666667	0	0
XR_002345223.1	CCDC17	0.006666667	0.666666667	0	0
XR_002345225.1	EIF2B3	0.516666667	66.89666667	0	0
XR_002345239.1	C6H1orf210	0.026666667	1.85	0	0
XR_002345249.1	RIMKLA	0.01	1.66	0	0
XR_002345258.1	LOC102166622	0.013333333	2.853333333	0	0
XR_002345260.1	LOC102166622	0.006666667	2.233333333	0	0
XR_002345263.1	LOC102166622	0.006666667	1.6	0	0
XR_002345265.1	LOC102166622	0.043333333	8.75	0	0
XR_002345267.1	LOC102166622	0.006666667	0.98	0	0
XR_002345289.1	TCF25	0.116666667	13.30666667	0	0
XR_002345363.1	ZNF304	0.046666667	1.116666667	0	0
XR_002345366.1	PLA2G4F	0.003333333	0.333333333	0	0
XR_002345399.1	LOC100737218	0.006666667	0.45	0	0
XR_002345404.1	LOC110261337	0.03	3.486666667	0	0
XR_002345431.1	RAP1GAP	0.026666667	3.586666667	0	0
XR_002345536.1	SLC44A4	0.083333333	10.48666667	0	0
XR_002345551.1	EIF2AK4	0.016666667	4.903333333	0	0.076666667
XR_002345569.1	FKBP5	0.013333333	2.443333333	0	0
XR_002345589.1	SLC17A1	0.266666667	31.21333333	0	0
XR_002345599.1	SLC17A2	0.016666667	4.266666667	0	0
XR_002345652.1	TCP11	0.033333333	22.00333333	0	0

XR_002345657.1	TCP11	0.04	25.46333333	0	0
XR_002345660.1	TCP11	0.02	8.64	0	0
XR_002345662.1	TCP11	0.006666667	1.65	0	0
XR_002345664.1	TCP11	0.033333333	10.75333333	0	0
XR_002345675.1	TCP11	0.02	1.256666667	0	0
XR_002345683.1	LHFPL5	0.006666667	0.333333333	0	0
XR_002345721.1	TAF8	0.126666667	17.14333333	0	0
XR_002345734.1	CUL9	0.09	34.13333333	0	0
XR_002345738.1	CUL9	0.02	7.376666667	0	0
XR_002345743.1	LRRC73	0.003333333	0.49	0	0
XR_002345773.1	ZFAND6	0.073333333	5.563333333	0	0
XR_002345786.1	ZSCAN2	0.02	5.24	0	0
XR_002345894.1	LOC100156469	0.023333333	4.04	0	0
XR_002345902.1	LOC100156469	0.003333333	0.573333333	0	0.166666667
XR_002345928.1	PPP1R3E	0.006666667	0.863333333	0	0
XR_002345930.1	PPP1R3E	0.02	1.856666667	0	0
XR_002345931.1	PPP1R3E	0.006666667	0.536666667	0	0
XR_002345977.1	FAM174B	0.05	2.88	0	0
XR_002346009.1	HEATR4	0.026666667	0.333333333	0	0
XR_002346251.1	PRPF4B	0.163333333	42.22666667	0	0
XR_002346272.1	LYRM4	0.01	0.433333333	0	0
XR_002346365.1	ALB	0.01	0.983333333	0	0
XR_002346402.1	DCLK2	0.023333333	2.883333333	0	0
XR_002346530.1	SH3BP2	0.05	7.27	0	0
XR_002346534.1	SH3BP2	0.003333333	0.533333333	0	0
XR_002346873.1	PPM1K	0.083333333	12.80666667	0	0
XR_002346905.1	HNRNPDL	0.023333333	4.74	0	0
XR_002346907.1	HNRNPDL	0.056666667	7.636666667	0	0
XR_002346950.1	TNFSF4	0.056666667	6.533333333	0	0
XR_002346999.1	C9H11orf87	0.02	0.666666667	0	0
XR_002347024.1	MTERF1	0.013333333	1.25	0	0
XR_002347026.1	MTERF1	0.006666667	0.76	0	0
XR_002347129.1	L3HYPDH	0.02	1.2	0	0
XR_297452.3	MAN2A2	0.07	18.49	0	0
XR_298851.3	GSTZ1	0.183333333	11.22666667	0	0
XR_301734.3	SERGEF	0.023333333	1.57	0	0
XR_304982.3	ANAPC10	0.193333333	5.353333333	0	0
XR_306784.3	UBA7	0.093333333	12.7	0	0
XR_307455.3	MVK	0.003333333	0.52	0	0
XR_308559.3	SAP30L	0.223333333	51.84666667	0	0
XR_309303.3	IKBKG	0.356666667	34.47666667	0	0
NM_001001617.1	CCRL2	0	0	0.28	15.42
NM_001001624.1	CCR9	0	0	0.00666667	0.333333333
NM_001001859.1	MGAT4C	0	0	0.04333333	8.666666667
NM_001001908.2	CD4	0	0	0.07	7.473333333
NM_001004033.1	SLC52A2	0	0	0.30333333	16.38
NM_001004040.1	PDYN	0	0	0.00333333	0.333333333
NM_001005157.1	DDX39B	0	0	0.65	31.58
NM_001025218.2	ATP5MC1	0	0	81.2966667	1790.586667
NM_001031777.2	KCNIP1	0	0	0.04	1
NM_001038004.1	MMP9	0	0	0.01666667	1.666666667
NM_001039749.1	DKK3	0	0	0.09	9.796666667
NM_001043346.1	HCRTR1	0	0	0.00666667	0.666666667
NM_001048187.1	DLK1	0	0	0.28666667	11.85333333
NM_001075117.1	VPREB1	0	0	0.01666667	0.333333333
NM_001078680.1	CDC42	0	0	0.37666667	8.643333333
NM_001097428.1	IRF7	0	0	0.10333333	6.74
NM_001097442.1	DAB1	0	0.03	0.00333333	0.153333333
NM_001097491.1	PCD1B	0	0	0.00666667	0.333333333
NM_001097492.1	CD1E	0	0	0.00333333	0.176666667

NM_001097522.1	CYB561	0	0	0.00333333	0.07
NM_001098580.1	TP23	0	0	0.00666667	0.666666667
NM_001098594.1	MLPH	0	0	0.02333333	1.666666667
NM_001099935.1	IKBKB	0	0	0.16666667	17.62333333
NM_001105314.1	KLHDC3	0	0	0.01666667	1.046666667
NM_001113062.1	CRH	0	0	0.00666667	0.333333333
NM_001113702.1	SLA-2	0	0	3.89333333	162.01
NM_001123043.1	HPCAL4	0	0	0.01333333	0.24
NM_001123084.1	BLM	0	0	0.15333333	28.19666667
NM_001123102.1	CREB3L4	0	0	0.20333333	13.49
NM_001123126.1	HPCAL1	0	0	0.24333333	5.55
NM_001123134.1	LIPA	0	0	0.04333333	2.146666667
NM_001123201.1	TFAP2C	0	0	0.00333333	0.333333333
NM_001123212.1	UPK1B	0	0	0.02	0.666666667
NM_001128477.1	FYCO1	0	0	0.00666667	1.063333333
NM_001128488.2	AZIN1	0	0	0.04666667	7.163333333
NM_001129948.1	GULO	0	0	0.00666667	0.666666667
NM_001130241.2	PPARD	0	0	0.00666667	0.76
NM_001137621.1	IL23R	0	0	0.00333333	0.333333333
NM_001141985.1	GP1BB	0	0	0.01333333	0.333333333
NM_001143710.1	GZMB	0	0	0.03666667	1.333333333
NM_001143714.1	LIPC	0	0	0.03666667	2.18
NM_001144845.1	DDIT3	0	0	0.03	0.74
NM_001145384.1	DKK1	0	0	0.02333333	0.666666667
NM_001159308.1	APOA5	0	0	0.00333333	0.333333333
NM_001162891.1	LOC100302368	0	0	0.02333333	0.86
NM_001163649.2	TRIM21	0	0	0.03	2.07
NM_001164511.2	BCL2A1	0	0	0.12333333	3
NM_001164515.1	CCL8	0	0	0.09	1
NM_001166045.1	SELENOV	0	0	0.00666667	0.333333333
NM_001166491.1	CCL1	0	0	0.01666667	0.333333333
NM_001167634.1	ELOVL3	0	0	0.01	0.333333333
NM_001177931.1	SPESP1	0	0	0.00666667	0.333333333
NM_001190220.1	CATIP	0	0	0.01333333	0.666666667
NM_001190289.1	IRGC	0	0	0.01	0.666666667
NM_001190923.1	GPR33	0	0	0.00666667	0.333333333
NM_001193447.1	ODF2	0	0	0.13333333	22.83
NM_001195346.1	LEFTY2	0	0	0.00666667	0.333333333
NM_001195349.1	LY6G6D	0	0	0.01333333	0.333333333
NM_001195354.1	MOSPD1	0	0	0.10666667	4.543333333
NM_001198917.1	OOEP	0	0	0.02333333	0.333333333
NM_001206347.1	POSTN	0	0	0.42	63.26333333
NM_001243303.1	BTBD9	0	0	0.13	16.94
NM_001243402.1	RDH12	0	0	0.02666667	2.666666667
NM_001243411.1	LAT2	0	0	0.11333333	8.533333333
NM_001243580.1	MTRF1	0	0	1.56666667	133.1733333
NM_001243622.1	HSD17B13	0	0.023333333	0.00666667	0.666666667
NM_001243641.1	ZNF205	0	0	0.03666667	3.17
NM_001243677.1	P2RY10	0	0	0.00666667	0.333333333
NM_001243709.1	MORN3	0	0	0.00666667	0.333333333
NM_001243711.1	ELF5	0	0	0.01	0.333333333
NM_001243827.1	HDAC3	0	0	0.67333333	58.49
NM_001243858.1	LIMS1	0	0	0.27333333	30.20666667
NM_001243929.1	ARMC6	0	0	0.22333333	23.39
NM_001243935.1	CAMK1D	0	0	0.01	0.52
NM_001243948.1	MARVELD2	0	0	0.02	2.216666667
NM_001244156.1	HAUS4	0	0	0.03333333	2.043333333
NM_001244205.1	ART3	0	0	0.07666667	5.026666667
NM_001244347.1	ADPRH	0	0	0.03	3.303333333
NM_001244471.1	EDDM3B	0	0	0.01	0.333333333

NM_001244634.1	MSTO1	0	0	0.23333333	17.89333333
NM_001244641.1	RTN1	0	0	0.00333333	0.333333333
NM_001244689.1	CNFN	0	0	0.03	0.573333333
NM_001244863.1	SDCBP2	0	0	0.00666667	0.39
NM_001244899.1	OIT3	0	0	0.00666667	0.666666667
NM_001244971.1	KCTD1	0	0.06	0.02	0.826666667
NM_001244975.1	CAPS	0	0	0.04	2.333333333
NM_001244976.1	CAPS	0	0	0.01333333	0.623333333
NM_001246249.1	TBX4	0	0	0.00333333	0.333333333
NM_001251818.1	GPR12	0	0	0.00666667	0.333333333
NM_001256595.1	SLCO1A2	0	0	0.02333333	2.41
NM_001257225.1	GPR3	0	0	0.00333333	0.333333333
NM_001258356.1	FGFBP3	0	0	0.02	1
NM_001258377.1	CHIA	0	0	0.00666667	0.333333333
NM_001258445.1	DCLRE1C	0	0	0.01333333	1.52
NM_001267893.1	NR5A2	0	0	0.03333333	4.92
NM_001267894.1	TAS1R2	0	0	0.01333333	1.573333333
NM_001278761.1	12-Sep	0	0	0.00666667	0.333333333
NM_001287016.1	WNT10B	0	0	0.01	1
NM_001291682.1	DDX4	0	0	0.00333333	0.333333333
NM_001301406.1	OAZ3	0	0	0.10333333	4
NM_001302771.1	TMIGD3	0	0	0.04	2
NM_001315601.1	FFAR3	0	0	0.00666667	0.333333333
NM_001315666.1	SLC4A5	0	0.333333333	0.00666667	1.666666667
NM_001315668.1	BCL2L14	0	0	0.05	2.596666667
NM_001348936.1	CAV1	0	0	0.04	4.543333333
NM_001348949.1	GALP	0	0	0.04333333	1.666666667
NM_001348961.1	HSD11B1	0	0	0.03333333	1.85
NM_213737.1	PPGRP-S	0	0	0.03333333	0.666666667
NM_213741.2	TRO	0	0	0.00666667	0.666666667
NM_213800.1	AMELY	0	0	0.00666667	0.183333333
NM_213872.1	RLN2	0	0	0.10666667	3
NM_213894.1	GAD1	0	0	0.00333333	0.333333333
NM_213905.1	MMP20	0	0	0.00333333	0.333333333
NM_213941.1	RPN2	0	0.01	0.26333333	27.18
NM_213948.1	IFNG	0	0	0.01	0.333333333
NM_213961.1	OPTN	0	0	0.12333333	8.546666667
NM_213990.1	CLEC5A	0	0	0.01333333	0.333333333
NM_214025.2	PPP2R2B	0	0	0.00333333	0.39
NM_214029.1	IL1A	0	0	0.00666667	0.666666667
NM_214040.1	POU2F2	0	0	0.02666667	2.276666667
NM_214160.2	FCN1	0	0	0.02	0.81
NM_214191.1	MCT7	0	0	0.03	1.333333333
NM_214198.1	TGFB3	0	0	0.03666667	4.17
NM_214309.2	NUDT2	0	0	0.02	0.616666667
NM_214402.2	LIF	0	0	0.03666667	0.86
NM_214451.1	RBP2	0	0	0.01666667	0.333333333
XM_001924803.7	FANK1	0	0	0.05333333	24.51666667
XM_001924885.3	LOC100157018	0	0	0.01	0.333333333
XM_001925183.2	LOC100156374	0	0	0.01	0.333333333
XM_001925360.5	SLC39A4	0	0	0.00333333	0.666666667
XM_001925694.6	TNFSF11	0	0	0.00333333	0.333333333
XM_001925909.7	GPLD1	0	0	0.00333333	1.666666667
XM_001925925.4	PPIL3	0	0	0.15	6.283333333
XM_001925930.5	TMEM106C	0	0	0.04333333	3.036666667
XM_001926086.4	PPP1R42	0	0	0.00666667	0.666666667
XM_001926475.5	LOC100157285	0	0	0.01333333	0.846666667
XM_001926548.7	SKA1	0	0	0.03666667	2
XM_001926953.5	LOC100153093	0	0	0.00333333	1.333333333
XM_001927474.2	TCEANC	0	0	0.08333333	6.993333333

XM_001927487.4	TCEANC	0	0	0.20666667	10.67666667
XM_001927845.5	KIFC1	0	0	0.04666667	4.99
XM_001927979.5	TSPAN2	0	0	0.00333333	0.416666667
XM_001928056.3	LOC100154723	0	0	0.01	0.333333333
XM_001928359.5	DCAF11	0	0	0.38	71.69333333
XM_001928417.6	ZDHHC22	0	0	0.02	4
XM_001928540.5	FAM81A	0	0.003333333	0.05333333	9.333333333
XM_001928730.4	LOC100157159	0	0	0.00333333	0.333333333
XM_001929347.4	FURIN	0	0	0.07	12.95333333
XM_001929447.1	LOC100151929	0	0	0.02	0.666666667
XM_001929525.5	NTRK1	0	0	0.00333333	0.333333333
XM_003121114.4	MAS1	0	0	0.00666667	0.666666667
XM_003121455.6	NARS	0	0	0.03333333	7.223333333
XM_003121544.4	WDR76	0	0	0.08333333	15.87666667
XM_003121797.4	DDHD1	0	0	0.11333333	29.32
XM_003122496.5	NPAS4	0	0	0.00333333	0.333333333
XM_003122527.4	SNX32	0	0	0.00666667	1
XM_003122638.5	LOC100519643	0	0	0.00333333	0.533333333
XM_003122798.5	LOC100525679	0	0	0.02666667	0.333333333
XM_003123173.4	CCDC159	0	0	0.04666667	22.46333333
XM_003123220.4	LOC100511183	0	0	0.01	1.333333333
XM_003123429.5	SLC1A6	0	0	0.00666667	0.666666667
XM_003123473.4	CALR3	0	0	0.06666667	3.333333333
XM_003123492.5	TMEM221	0	0	0.00666667	0.333333333
XM_003123500.4	JAK3	0	0	0.17666667	35.25666667
XM_003123581.3	LOC100520723	0	0	0.00666667	0.333333333
XM_003123655.4	HK3	0	0.206666667	0.13333333	18.33666667
XM_003123706.4	GCNT4	0	0	0.02333333	5.726666667
XM_003123752.6	TENT2	0	0	0.03333333	5.053333333
XM_003123761.4	XRCC4	0	0	0.01666667	1.44
XM_003123768.4	GPR150	0	0	0.01	1.333333333
XM_003123969.4	KLHL3	0	0	0.04666667	14.24666667
XM_003124187.2	LOC100519014	0	0	0.01	0.333333333
XM_003124542.5	CACNG3	0	0	0.00333333	0.333333333
XM_003124710.4	GNG13	0	0	0.00666667	0.333333333
XM_003124904.3	LOC100523177	0	0	0.01	0.666666667
XM_003125122.4	CCDC85A	0	0	0.03333333	6.136666667
XM_003125365.5	FKBP1B	0	0	0.23	9.333333333
XM_003125670.3	SLAMF8	0	0	0.00666667	0.666666667
XM_003125733.4	C4H1orf189	0	0	0.01333333	0.333333333
XM_003126100.5	WNT1	0	0	0.00333333	0.333333333
XM_003126127.5	NCKAP5L	0	0	0.03666667	10.54666667
XM_003126173.4	KRT5	0	0	0.00333333	0.333333333
XM_003126192.6	MAP3K12	0	0	0.04666667	8.763333333
XM_003126205.6	GTSF1	0	0	0.01	2
XM_003126499.2	TAS2R9	0	0	0.01	0.333333333
XM_003126516.5	CLSTN3	0	0	0.14666667	26.33333333
XM_003126523.5	LRRC23	0	0	0.03	4.123333333
XM_003126653.2	LOC100517094	0	0	0.00333333	0.333333333
XM_003126782.4	MLC1	0	0	0.03333333	5.006666667
XM_003126843.3	CLEC3A	0	0	0.00666667	0.333333333
XM_003126929.3	KCTD19	0	0	0.01666667	2.333333333
XM_003127229.4	FOXA3	0	0	0.00666667	0.666666667
XM_003127285.4	IZUMO1	0	0	0.00666667	0.293333333
XM_003127705.6	GRHL3	0	0	0.00333333	0.71
XM_003127726.6	SYTL1	0	0	0.01333333	1.27
XM_003127905.3	LOC100522145	0	0	0.02333333	2.293333333
XM_003127917.5	FUBP1	0	0	0.09	29.12
XM_003127939.6	SLC35D1	0	0	0.03666667	9.903333333
XM_003128044.6	TESK2	0	0	0.05333333	7.58

XM_003128105.4	CTPS1	0	0	0.13	16.32333333
XM_003128450.4	BNC1	0	0	0.00333333	0.666666667
XM_003128671.4	IFT43	0	0	0.02333333	0.723333333
XM_003128672.4	IFT43	0	0	0.11	4.38
XM_003128749.4	TDRD9	0	0.333333333	0.01666667	4
XM_003128777.3	LOC100519495	0	0	0.00666667	0.333333333
XM_003128783.4	LOC100523262	0	0	0.00666667	0.333333333
XM_003128787.2	LOC100524156	0	0	0.02666667	1
XM_003128793.4	LOC100510917	0	0	0.01	0.333333333
XM_003128855.4	TMEM128	0	0	0.04	1.886666667
XM_003128902.4	LOC100520753	0	0	0.05666667	3.666666667
XM_003129044.5	STAP1	0	0	0.03	1.693333333
XM_003129137.6	MND1	0	0	0.01333333	0.483333333
XM_003129146.6	SH3D19	0	0	0.03	6.656666667
XM_003129207.6	SLC25A31	0	0	0.00666667	0.666666667
XM_003129280.6	CXXC4	0	0	0.00333333	0.666666667
XM_003129511.2	LOC100514973	0	0	0.00333333	0.333333333
XM_003129512.2	LOC100515268	0	0	0.00666667	0.333333333
XM_003129570.2	LOC100514307	0	0	0.01666667	1.333333333
XM_003129854.5	BCO2	0	0	0.09333333	10.66666667
XM_003129894.5	RNF214	0	0	0.00333333	0.576666667
XM_003129922.4	HYOU1	0	0.003333333	0.06333333	13.58
XM_003129933.4	PDZD3	0	0	0.00333333	0.333333333
XM_003129967.3	LOC100525476	0	0	0.00666667	0.333333333
XM_003129984.4	LOC100513751	0	0	0.00333333	0.333333333
XM_003129985.2	LOC100513942	0	0	0.00333333	0.333333333
XM_003129997.1	LOC100516283	0	0	0.01	0.333333333
XM_003130016.4	LOC100520398	0	0	0.00666667	0.333333333
XM_003130090.4	B3GAT1	0	0	0.14	22.42333333
XM_003130190.4	PRPS1L1	0	0	0.00666667	0.666666667
XM_003130455.6	C4BPB	0	0	0.01666667	0.666666667
XM_003130541.4	LOC100524517	0	0	0.00666667	0.666666667
XM_003130956.6	CKAP2	0	0	0.06666667	10.96333333
XM_003131203.5	SAP30BP	0	0	0.04666667	5.166666667
XM_003131226.4	TMEM104	0	0	0.33666667	55.87666667
XM_003131296.4	KCNH6	0	0	0.00333333	0.333333333
XM_003131306.2	MYL4	0	0	0.17333333	7.333333333
XM_003131404.4	HSPB9	0	0	0.00666667	0.333333333
XM_003131471.4	CDC6	0	0	0.04333333	9.946666667
XM_003132248.5	GLYCTK	0	0	0.01333333	1.046666667
XM_003132377.2	TMEM40	0	0	0.02333333	1.333333333
XM_003133243.5	LOC100514734	0	0	0.00666667	0.333333333
XM_003133446.5	CSRNP3	0	0	0.01333333	8.32
XM_003134063.5	SPDL1	0	0	0.03	3.33
XM_003134090.4	GABRA6	0	0	0.00666667	0.666666667
XM_003134304.3	LOC100516302	0	0	0.01333333	0.333333333
XM_003134311.6	RNF24	0	0.003333333	0.04333333	13.74666667
XM_003134522.5	MNX1	0	0	0.00333333	0.333333333
XM_003134660.5	LRGUK	0	0	0.35666667	106.5033333
XM_003134735.4	PTPRZ1	0	0	0.01333333	5.17
XM_003135130.4	SPIN2B	0	0	0.01	0.933333333
XM_003135151.6	DLG3	0	0	0.01666667	4.196666667
XM_003135250.4	LOC100520992	0	0	0.11666667	9.933333333
XM_003135273.4	TMSB15B	0	0	0.03333333	1
XM_003135296.5	PIH1D3	0	0	0.00333333	0.333333333
XM_003135360.4	LOC100521308	0	0	0.00333333	1.333333333
XM_003135516.4	LOC100516587	0	0	2.18333333	176.6
XM_003353151.4	DLL1	0	0	0.07	14.41
XM_003353248.3	MRAP2	0	0	0.05	4.573333333
XM_003353266.5	KLHL32	0	0	0.00666667	2.006666667

XM_003353291.3	FAM229B	0	0	0.01666667	0.333333333
XM_003353358.4	LYSMD2	0	0	0.05333333	2.593333333
XM_003353646.2	LOC100621729	0	0	0.00666667	0.333333333
XM_003354125.3	LOC100620265	0	0	0.01	2
XM_003354261.4	C2H5orf63	0	0	0.14	5.06
XM_003354275.5	KIF3A	0	0	0.18666667	41.79333333
XM_003354289.4	NME5	0	0	0.01	0.666666667
XM_003354417.4	PGGHG	0	0	0.21333333	25.27333333
XM_003354670.4	TSC2	0	0	0.29666667	75.84
XM_003354865.5	RASGRP3	0	0	0.03666667	11.26
XM_003354910.4	GDF7	0	0	0.00333333	0.333333333
XM_003355189.3	TCHHL1	0	0	0.00333333	0.333333333
XM_003355416.5	ITGB7	0	0	0.01	1.433333333
XM_003355533.4	FAR2	0	0	0.1	15.89666667
XM_003355617.5	CNTN1	0	0	0.01333333	3.666666667
XM_003355779.5	LOC100621778	0	0	0.01	0.81
XM_003355830.3	VSTM2B	0	0	0.00333333	0.333333333
XM_003355853.4	FAM187B	0	0	0.01	0.666666667
XM_003356326.5	BMP8B	0	0	0.00333333	0.333333333
XM_003356855.5	CYTL1	0	0	0.00666667	0.333333333
XM_003356860.4	NKX3-2	0	0	0.00333333	0.333333333
XM_003356934.4	RXFP1	0	0	0.02666667	3.76
XM_003357156.2	LOC100625080	0	0	0.00666667	0.333333333
XM_003357228.5	DLG2	0	0	0.00666667	2.423333333
XM_003357299.3	TEX12	0	0	0.02666667	1.333333333
XM_003357414.3	RHEX	0	0	0.01	0.86
XM_003357480.4	RUNDC3B	0	0.306666667	0.00666667	2.22
XM_003357551.5	ABL2	0	0.943333333	0.03333333	17.65
XM_003357746.4	MEIG1	0	0	0.04666667	4.443333333
XM_003357886.5	GGACT	0	0	0.01666667	1.176666667
XM_003358030.4	BRCA1	0	0	0.04333333	9.243333333
XM_003358573.5	CEP70	0	0	0.04666667	4.486666667
XM_003358694.4	FXR1	0	0	0.33	39.90666667
XM_003358761.4	CEP19	0	0	0.37	63.57666667
XM_003358822.4	DPPA2	0	0	0.02666667	2
XM_003358997.4	LOC100622629	0	0	0.00666667	0.333333333
XM_003359086.4	DDX55	0	0	0.02333333	3.823333333
XM_003359153.4	DUSP18	0	0	0.01666667	6.443333333
XM_003359241.4	ADK	0	0	0.01	2.39
XM_003359830.5	CCNJL	0	0	0.01	1.666666667
XM_003359872.5	CHRNB3	0	0	0.05333333	6.333333333
XM_003359892.3	LRRN4	0	0	0.00666667	0.666666667
XM_003360003.4	GDAP1L1	0	0	0.02	1
XM_003360338.4	ALAS2	0	0	0.02666667	2.333333333
XM_003360341.4	ZC3H12B	0	0	0.00333333	0.666666667
XM_003360352.4	EDA	0	0	0.04666667	10.69666667
XM_003360630.2	LOC100622230	0	0	0.00666667	0.333333333
XM_003360804.3	PPP3R2	0	0	0.00666667	0.333333333
XM_003360974.4	TRIM52	0	0	0.01333333	1.306666667
XM_003480289.4	EPHA7	0	0	0.03666667	11.24
XM_003480309.4	DOP1A	0	0	0.00333333	1.306666667
XM_003480328.4	SKA1	0	0	0.02333333	1
XM_003480622.4	SPACA9	0	0	0.05333333	2.216666667
XM_003480708.4	SLC1A2	0	0	0.00333333	0.686666667
XM_003480994.4	ORAI2	0	0	0.00666667	1.503333333
XM_003481604.2	LOC110255239	0	0	0.00333333	0.333333333
XM_003481663.4	CAPS2	0	0	0.00666667	0.666666667
XM_003481700.4	PRMT8	0	0	0.00666667	0.666666667
XM_003482032.4	MC2R	0	0	0.00333333	0.333333333
XM_003482217.3	ZFAND6	0	0	0.02	2.136666667

XM_003482253.4	LOC100739080	0	0	0.00666667	0.333333333
XM_003482414.4	ODAPH	0	0	0.00666667	0.333333333
XM_003482543.2	LOC100737744	0	0	0.00666667	0.666666667
XM_003482546.2	LOC100737856	0	0	0.03666667	2.333333333
XM_003482548.3	LOC100738083	0	0	0.00666667	1
XM_003482681.4	CDCA7L	0	0	0.15333333	25.05666667
XM_003482966.4	SPPL2C	0	0	0.00333333	0.536666667
XM_003483921.4	LOC100739707	0	0	0.02333333	1.66
XM_003484018.4	SSMEM1	0	0	0.01666667	0.666666667
XM_005652361.3	DNAJB11	0	0	0.02	1.91
XM_005652423.3	LOC102159965	0	0	0.00666667	1.383333333
XM_005652465.3	CDC40	0	0	0.03666667	5.63
XM_005652914.3	ARMC6	0	0	0.09666667	10.17666667
XM_005652918.2	SUGP2	0	0	0.09666667	13.95666667
XM_005652997.3	PHKG2	0	0	0.12333333	8.886666667
XM_005653017.3	ZNF48	0	0	0.26	34.78
XM_005653049.2	C3H2orf16	0	0	0.00333333	0.666666667
XM_005653068.3	GJA5	0	0	0.09666667	15.64666667
XM_005653070.3	BCL9	0	0	0.37333333	103.2866667
XM_005653074.3	FMO5	0	0	0.03	6
XM_005653151.3	KRAS	0	0	0.03	2.926666667
XM_005653218.3	VPS9D1	0	0	0.03666667	4.69
XM_005653341.3	CPTP	0	0	0.01	1.103333333
XM_005653448.3	RPP40	0	0	0.01333333	2.403333333
XM_005653540.3	RD3L	0	0	0.02	1.333333333
XM_005653544.2	LOC100156196	0	0	0.00666667	0.333333333
XM_005653572.3	LOC100620475	0	0	0.04	0.66
XM_005653578.3	TLR2	0	0	0.02333333	3.18
XM_005653605.2	TRMT10A	0	0	0.03333333	2.556666667
XM_005653654.3	FOLR2	0	0	0.05	2.86
XM_005653678.3	ANAPC15	0	0	0.22	14
XM_005653710.3	VWA5A	0	0	0.07666667	11.18666667
XM_005653741.3	PRRC2C	0	0	0.01333333	6.97
XM_005653830.3	TEX29	0	0	0.07	3
XM_005653885.3	UNK	0	0.266666667	0.03333333	6.056666667
XM_005653890.3	10-Mar	0	0	0.01333333	1.666666667
XM_005654027.3	SLC35G2	0	0	0.04666667	3.136666667
XM_005654173.3	RALB	0	0	0.16333333	16.45333333
XM_005654242.3	REM1	0	0	0.08666667	5.056666667
XM_005654482.3	RELCH	0	0	0.06333333	15.91333333
XM_005654512.3	PHF24	0	0	0.03666667	8.296666667
XM_005654613.3	VAV2	0	0	0.01666667	4.526666667
XM_005654711.2	NCLN	0	0	0.15333333	39.85666667
XM_005654751.3	LOC102167984	0	0	0.00666667	0.666666667
XM_005654827.3	TYK2	0	0	0.09666667	23.63
XM_005654903.3	KCNN1	0	0	0.16333333	11.58666667
XM_005654907.3	JAK3	0	0	0.10666667	23.66666667
XM_005654909.3	FCHO1	0	0	0.00666667	1.22
XM_005654918.3	LPAR2	0	0	0.02333333	1.98
XM_005654941.3	HNRNPH1	0	0	0.05	4.98
XM_005654975.3	SFXN1	0	0	0.02333333	3.336666667
XM_005655064.3	ZFAND2A	0	0	0.02	1.32
XM_005655098.3	ASPHD1	0	0	0.00666667	0.333333333
XM_005655198.3	IL1A	0	0	0.00333333	0.333333333
XM_005655315.3	SHARPIN	0	0	0.10333333	6.763333333
XM_005655438.3	FAM19A3	0	0	0.00666667	0.666666667
XM_005655619.3	PAN2	0	0.006666667	0.11666667	24.99333333
XM_005655643.3	OSBPL8	0	0	0.02	6.686666667
XM_005655670.3	CLEC7A	0	0	0.01333333	1.333333333
XM_005655688.3	LOC100511133	0	0	0.00666667	0.333333333

XM_005655702.3	AKAP3	0	0	0.03666667	9.276666667
XM_005655736.2	C5H12orf50	0	0	0.04	3.096666667
XM_005655968.3	SYT5	0	0	0.09666667	8
XM_005655984.3	LOC100624218	0	0	0.00333333	0.333333333
XM_005656103.3	MPPE1	0	0	0.04333333	4.126666667
XM_005656114.3	GATA6	0	0	0.03666667	5.156666667
XM_005656131.3	PTGER3	0	0	0.00666667	0.8
XM_005656170.3	TAL1	0	0	0.00666667	1.556666667
XM_005656173.3	TAL1	0	0	0.03	5.82
XM_005656220.3	BTN2A2	0	0	0.02	3
XM_005656221.2	TMEM225B	0	0	0.02	1.986666667
XM_005656230.3	ENPP4	0	0	0.03	6.92
XM_005656231.3	ENPP4	0	0	0.01	3.006666667
XM_005656235.3	HYKK	0	0	0.08666667	9.533333333
XM_005656236.3	HYKK	0	0	0.08333333	8.756666667
XM_005656288.3	REC114	0	0	0.03333333	1.256666667
XM_005656320.2	LOC100157169	0	0	0.01	0.333333333
XM_005656347.1	RDH11	0	0	0.06666667	8.57
XM_005656372.2	SIPA1L1	0	0	0.05333333	13.28
XM_005656517.3	RUFY3	0	0	0.02666667	5.523333333
XM_005656614.2	LOC100624993	0	0	0.03	1.073333333
XM_005656649.3	PRELP	0	0	0.52	89.96
XM_005656761.3	LOC102160111	0	0	0.02333333	0.81
XM_005656806.3	AGTPBP1	0	0	0.20666667	37.06333333
XM_005656994.3	PROCA1	0	0	0.01333333	2
XM_005657066.3	ZKSCAN7	0	0	0.03666667	7.626666667
XM_005657121.3	TRMT10C	0	0	0.02333333	2.076666667
XM_005657231.3	DMTN	0	0	0.03	3.333333333
XM_005657235.3	SLC39A14	0	0	0.02	4.523333333
XM_005657398.3	AIFM2	0	0	0.24333333	17.79
XM_005657408.3	SGPL1	0	0	0.09	17.69666667
XM_005657411.3	SGPL1	0	0	0.03	5.603333333
XM_005657465.3	TCF7L2	0	0	0.11	21.04
XM_005657473.3	TCF7L2	0	0.173333333	0.04666667	8.676666667
XM_005657715.3	LOC100519808	0	0	0.66	166.7966667
XM_005657717.3	LOC100519808	0	0	0.04	10.19666667
XM_005657797.3	RBM3	0	0	0.04666667	2.143333333
XM_005657880.3	GPRASP2	0	0	0.03	5.153333333
XM_005657884.3	BHLHB9	0	0	0.21666667	39.88666667
XM_005657885.3	BHLHB9	0	0	0.06666667	12.88333333
XM_005657925.3	MAP7D3	0	0	0.01333333	2.963333333
XM_005657969.2	LOC100623928	0	0	0.02333333	1
XM_005658221.3	ARMCX1	0	0	0.08333333	9.596666667
XM_005658775.3	VSIG1	0	0	0.02	2.333333333
XM_005658814.3	CCR4	0	0	0.01333333	1.333333333
XM_005658930.3	SLC39A2	0	0	0.00666667	0.536666667
XM_005658951.3	PRMT5	0	0	0.28666667	27.80666667
XM_005659128.3	TIAM2	0	0	0.10333333	28.04
XM_005659198.3	L3MBTL3	0	0	0.05666667	9.11
XM_005659300.3	EPHA7	0	0	0.00333333	0.76
XM_005659368.3	CEP57L1	0	0	0.11	7.22
XM_005659632.3	CEP152	0	0	0.02666667	7.486666667
XM_005659640.3	SLC28A2	0	0	0.01666667	1.663333333
XM_005659664.1	CATSPER2	0	0	0.09666667	7.916666667
XM_005659812.3	GALR1	0	0.406666667	0.00666667	2.9
XM_005659909.3	MIA2	0	0	0.04333333	7.043333333
XM_005660023.3	C1H14orf39	0	0	0.01333333	1.616666667
XM_005660055.3	LOC102166359	0	0	0.00333333	0.333333333
XM_005660185.3	CD72	0	0	0.01	1.28
XM_005660225.3	RGP1	0	0	0.96333333	138.5066667

XM_005660248.3	MELK	0	0	0.07	8
XM_005660263.3	TSTD2	0	0	0.10666667	21.32333333
XM_005660296.3	MSANTD3	0	0	0.03	2.796666667
XM_005660298.3	TMEFF1	0	0	0.02666667	2.666666667
XM_005660367.3	AKNA	0	0	0.09333333	30.37
XM_005660368.3	AKNA	0	0	0.02	6.573333333
XM_005660369.3	AKNA	0	0	0.03	8.643333333
XM_005660379.3	TRIM32	0	0	0.12	15.32666667
XM_005660380.3	TRIM32	0	0	0.08333333	12.07666667
XM_005660412.3	ZBTB26	0	0	0.09	17.39666667
XM_005660546.3	PPP1R26	0	0	0.03	7.066666667
XM_005660634.3	TBC1D10C	0	0	0.05	4
XM_005660785.3	TTC9C	0	0	0.17333333	28.71
XM_005660819.3	LOC100519643	0	0	0.07	8.473333333
XM_005660834.3	CD6	0	0	0.00666667	1.113333333
XM_005660879.3	GLYATL2	0	0	0.03666667	3.666666667
XM_005660946.3	ATG13	0	0	0.01333333	2.593333333
XM_005660947.3	ATG13	0	0	0.01333333	2.756666667
XM_005660959.3	MAPK8IP1	0	0	0.1	19.06
XM_005660996.3	SLC1A2	0	0	0.00666667	1.423333333
XM_005661062.3	MPPED2	0	0	0.07	6.756666667
XM_005661081.3	E2F8	0	0	0.00666667	1.39
XM_005661118.3	FAR1	0	0.15	0.06666667	16.88
XM_005661146.3	GJC2	0	0.103333333	0.04666667	4.423333333
XM_005661242.3	C2H19orf57	0	0	0.11	12.27
XM_005661298.3	CAPS	0	0	0.00666667	0.393333333
XM_005661388.3	DAZAP1	0	0	0.12333333	7.37
XM_005661396.3	GAMT	0	0	0.03333333	1.68
XM_005661490.3	GCNT4	0	0	0.03333333	7.89
XM_005661528.3	ATG10	0	0	0.16333333	18.37666667
XM_005661571.3	FER	0	0	0.02666667	9.343333333
XM_005661627.3	CDC42SE2	0	0	0.09333333	16.42
XM_005661640.3	SLC22A4	0	0	0.02666667	3
XM_005661711.3	MATR3	0	0	0.02	3.92
XM_005661730.3	PURA	0	0	0.01333333	9.413333333
XM_005661738.3	APBB3	0	0	0.02	3.683333333
XM_005661767.3	FCHSD1	0	0	0.05666667	11.41333333
XM_005661865.3	ZNF789	0	0	0.00666667	1.403333333
XM_005661867.3	ZNF655	0	0	0.22333333	41.53666667
XM_005661966.3	LAT2	0	0	0.16	14.71
XM_005662048.3	CD19	0	0	0.00333333	0.473333333
XM_005662151.3	NAGPA	0	0	0.04333333	4.256666667
XM_005662208.3	FLYWCH2	0	0	0.01666667	0.496666667
XM_005662331.3	CCDC138	0	0	0.00333333	0.286666667
XM_005662348.3	ST6GAL2	0	0	0.00666667	2
XM_005662394.3	CD8A	0	0	0.03666667	4.213333333
XM_005662436.3	SEMA4F	0	0	0.02666667	6.64
XM_005662442.3	LOC100513982	0	0	0.01	0.333333333
XM_005662666.3	SLC5A6	0	0	0.13666667	17.57333333
XM_005662717.3	LOC100523732	0	0	0.01	1
XM_005662811.3	TONSL	0	0	0.03333333	5.363333333
XM_005662906.3	OXR1	0	0.006666667	0.31666667	55.60333333
XM_005662927.3	NCALD	0	0	0.05333333	4.136666667
XM_005662930.3	NCALD	0	0	0.01666667	1.633333333
XM_005662932.3	NCALD	0	0	0.04	2.73
XM_005662961.3	DPY19L4	0	0	0.25333333	29.22666667
XM_005663015.3	ZC2HC1A	0	0	0.03333333	5.486666667
XM_005663038.3	CSPP1	0	0	0.22	42.37333333
XM_005663135.3	POU2F1	0	0	0.02333333	7.44
XM_005663233.3	CD1E	0	0	0.00333333	0.823333333

XM_005663234.3	CD1D	0	0	0.03	2.666666667
XM_005663277.3	ARHGEF2	0	0	0.02	5.656666667
XM_005663324.3	CLK2	0	0.106666667	0.26666667	25.94
XM_005663335.3	ADAM15	0	0	0.02	1.873333333
XM_005663358.3	TPM3	0	0	0.00666667	0.833333333
XM_005663477.3	GABPB2	0	0	0.04	11.45666667
XM_005663529.3	NUDT17	0	0	0.08	3.88
XM_005663535.3	PDZK1	0	0	0.01	1
XM_005663551.3	MAB21L3	0	0	0.00333333	1.1
XM_005663573.3	SLC16A4	0	0	0.01666667	1.666666667
XM_005663599.3	WDR47	0	0	0.02666667	5.216666667
XM_005663708.3	LOC102161784	0	0	0.07666667	30.81333333
XM_005663721.3	SH3GLB1	0	0	0.09666667	7.026666667
XM_005663829.3	RBFOX2	0	0	0.01333333	4.663333333
XM_005663831.3	RBFOX2	0	0	0.01666667	4.573333333
XM_005663839.3	LOC100156062	0	0	0.02	5.556666667
XM_005664006.3	KIAA1551	0	0	0.00666667	1.866666667
XM_005664011.3	CAPRIN2	0	0	0.04333333	6.743333333
XM_005664042.3	LMO3	0	0	0.00333333	0.416666667
XM_005664049.3	DERA	0	0	0.12	7.403333333
XM_005664071.3	PARP11	0	0	0.07333333	14.06
XM_005664104.3	RAD52	0	0	0.01333333	2.446666667
XM_005664105.3	RAD52	0	0	0.02333333	4.606666667
XM_005664106.3	RAD52	0	0	0.04	6.753333333
XM_005664107.3	RAD52	0	0	0.01666667	3.28
XM_005664338.3	LOC100522551	0	0	0.02	2.286666667
XM_005664359.2	HP	0	0	0.08333333	5
XM_005664411.3	TMEM208	0	0	0.15	4.66
XM_005664425.3	PLEKHG4	0	0	0.00666667	1.333333333
XM_005664436.2	LRRC36	0	0	0.02666667	2.383333333
XM_005664460.3	NETO2	0	0	0.01666667	2.666666667
XM_005664465.3	VSTM2B	0	0	0.00666667	0.666666667
XM_005664518.3	NFKBID	0	0	0.03666667	4.12
XM_005664780.3	KCNC3	0	0	0.06333333	7.483333333
XM_005664786.3	FAM71E1	0	0	0.07	2.666666667
XM_005664876.3	LOC100620869	0	0	0.01333333	1.223333333
XM_005664886.3	ZNF667	0	0	0.03666667	5.336666667
XM_005665008.3	MIIP	0	0	0.04	3.02
XM_005665126.3	PAFAH2	0	0	0.02333333	5.026666667
XM_005665141.3	RCC1	0	0	0.02333333	4.81
XM_005665170.3	HPCA	0	0	0.00333333	0.333333333
XM_005665308.3	RBBP8	0	0	0.14333333	18.30333333
XM_005665426.3	ORC1	0	0	0.01	1.373333333
XM_005665435.3	OSBPL9	0	0	0.41	56.29666667
XM_005665437.3	OSBPL9	0	0	0.16666667	20.95666667
XM_005665487.2	MED8	0	0	0.04	3.24
XM_005665516.3	ZMYND12	0	0	0.01333333	1
XM_005665577.3	MCUR1	0	0	0.01	2.33
XM_005665672.3	ZNF165	0	0	0.11666667	9.696666667
XM_005665674.3	ZSCAN9	0	0	0.11333333	15.33333333
XM_005665736.3	DDR1	0	0	0.05333333	9.5
XM_005665858.3	RXRB	0	0	0.19	19.96
XM_005665963.3	MDFI	0	0	0.03666667	2.183333333
XM_005665984.3	MEA1	0	0	0.08666667	3.466666667
XM_005665993.3	GUCA1B	0	0	0.01333333	1.333333333
XM_005666017.3	KLC4	0	0	0.07	7.7
XM_005666030.3	DLK2	0	0	0.02	1.333333333
XM_005666046.3	RSPH9	0	0	0.00666667	0.553333333
XM_005666134.3	LINGO1	0	0	0.02333333	4.403333333
XM_005666251.3	JPH4	0	0	0.01666667	2

XM_005666277.3	ABHD4	0	0	0.01333333	0.59
XM_005666382.3	EFCAB11	0	0	0.04666667	1.253333333
XM_005666444.3	TRAF3	0	0	0.00333333	1.343333333
XM_005666552.3	PACRGL	0	0	0.00666667	0.563333333
XM_005666554.3	PACRGL	0	0	0.01	1
XM_005666562.3	SLC34A2	0	0	0.03	3
XM_005666610.3	RBM47	0	0	0.00333333	0.78
XM_005666615.3	APBB2	0	0	0.25	75.69666667
XM_005666661.3	TMEM144	0	0	0.02333333	3.54
XM_005666665.3	RXFP1	0	0	0.04333333	6.016666667
XM_005666833.2	LOC102165147	0	0	0.02333333	0.666666667
XM_005666989.3	SNCA	0	0	0.01333333	0.69
XM_005667227.3	DEUP1	0	0	0.01333333	1.48
XM_005667333.3	TEX12	0	0	0.02	1
XM_005667347.2	TTC12	0	0	0.06	6.42
XM_005667371.3	TAGLN	0	0	0.20333333	9.646666667
XM_005667433.3	USP2	0	0	0.14666667	18.04333333
XM_005667448.3	SCN3B	0	0	0.04	8.736666667
XM_005667475.3	LOC100524268	0	0	0.04	1.666666667
XM_005667765.2	EZH2	0	0	0.03333333	4.21
XM_005667786.3	RASAL2	0	0	0.01	4.78
XM_005667825.3	RNF2	0	0	0.14333333	19.25333333
XM_005667844.3	PLA2G4A	0	0	0.00333333	0.633333333
XM_005667951.3	PLD5	0	0	0.00333333	0.333333333
XM_005668035.3	IDNK	0	0	0.12	10.62666667
XM_005668037.3	IDNK	0	0	0.06	3.11
XM_005668094.3	ARHGAP12	0	0	0.18666667	41.70333333
XM_005668096.3	ARHGAP12	0	0	0.07666667	17.70666667
XM_005668198.3	NUDT5	0	0	0.04666667	1.706666667
XM_005668217.3	ASB13	0	0	0.17333333	21.15666667
XM_005668256.3	ZMYND11	0	0	0.05	8.593333333
XM_005668351.3	POSTN	0	0	0.14	21.51
XM_005668384.3	NEK3	0	0	0.01333333	1.183333333
XM_005668398.3	PHF11	0	0	0.05333333	3.673333333
XM_005668531.3	TNFSF13B	0	0	0.03333333	3.923333333
XM_005668624.3	CD300LF	0	0	0.00666667	0.433333333
XM_005668626.3	CD300LF	0	0	0.02333333	1.8
XM_005668628.3	CD300LF	0	0	0.00666667	0.306666667
XM_005668715.3	PLEKHM1	0	0	0.22333333	58.26666667
XM_005668738.2	HIGD1B	0	0	0.04333333	1.103333333
XM_005668741.3	HIGD1B	0	0	0.01666667	0.606666667
XM_005668858.3	LOC110255211	0	0	0.02333333	0.666666667
XM_005668886.3	EPN3	0	0	0.00666667	1.333333333
XM_005668945.3	TOM1L1	0	0	0.07666667	12.32333333
XM_005668950.3	TMEM100	0	0	0.2	10.45333333
XM_005668974.3	RAD51C	0	0	0.45	20.62
XM_005669089.3	IFT20	0	0	0.09333333	3.72
XM_005669090.3	IFT20	0	0	0.02333333	0.633333333
XM_005669160.3	CAMTA2	0	0	0.01666667	3.35
XM_005669173.3	MED11	0	0	0.05333333	1.943333333
XM_005669263.3	COL6A5	0	0	0.00333333	1.743333333
XM_005669315.3	EOMES	0	0	0.04333333	7.343333333
XM_005669399.3	ACKR2	0	0	0.12666667	39.54
XM_005669493.3	MAP4	0	0	0.07666667	21.59333333
XM_005669678.3	PXK	0	0	0.05	7.526666667
XM_005669792.3	HRH1	0	0	0.05666667	8.286666667
XM_005669824.3	ABTB1	0	0	0.54	72.55333333
XM_005669859.3	CEP63	0	0	0.01666667	1.913333333
XM_005670027.3	EIF4G1	0	0	0.08333333	18.66666667
XM_005670210.3	GRAMD1C	0	0.25	0.04333333	7.333333333

XM_005670302.3	APP	0	0	1.22666667	182.9333333
XM_005670326.1	LOC102160250	0	0	0.06666667	0.666666667
XM_005670363.3	BACE2	0	0	0.01333333	5.523333333
XM_005670500.3	LOC100154415	0	0	0.14666667	23.47333333
XM_005670513.3	EP400	0	0	0.00333333	2.903333333
XM_005670515.3	EP400	0	0	0.06666667	37.56666667
XM_005670519.3	EP400	0	0.39	0.04333333	22.57333333
XM_005670525.3	PUS1	0	0	0.06333333	4.9
XM_005670541.3	STX2	0	0	0.08666667	13.48
XM_005670568.2	RFLNA	0	0	0.04	3.136666667
XM_005670579.3	TCTN2	0	0	0.15333333	17.23333333
XM_005670737.3	UBE3B	0	0	0.04333333	11.62333333
XM_005670893.3	SPECC1L	0	0	0.13333333	27.68333333
XM_005670904.3	DERL3	0	0	0.03333333	4.036666667
XM_005670905.3	DERL3	0	0	0.00333333	0.296666667
XM_005670911.3	LOC100523213	0	0	0.14333333	6.333333333
XM_005670983.3	TAF5L	0	0	0.08	10.66666667
XM_005671098.3	CFAP70	0	0	0.10666667	24.69
XM_005671160.1	LRIT2	0	0	0.00666667	0.333333333
XM_005671257.3	ATAD1	0	0	0.04	25.58
XM_005671290.3	FRA10AC1	0	0	0.18333333	11.11666667
XM_005671336.3	ALDH18A1	0	0.003333333	0.15	23.73666667
XM_005671449.3	ADD3	0	0	0.04666667	9.643333333
XM_005671541.3	CHST15	0	0	0.05666667	12.28333333
XM_005671588.3	MTG1	0	0	0.07333333	5.156666667
XM_005671628.3	C1QL2	0	0	0.00333333	0.333333333
XM_005671708.3	ACSL1	0	0	0.05333333	9.55
XM_005671715.2	C15H4orf47	0	0	0.16666667	8.673333333
XM_005671728.3	SORBS2	0	0	0.00333333	0.4
XM_005671730.3	SORBS2	0	0	0.03333333	7.916666667
XM_005671859.3	PKP4	0	0	0.03	5.226666667
XM_005671894.3	CSRNP3	0	0	0.05	29.59
XM_005671915.3	PPIG	0	0	0.03666667	10.90333333
XM_005671924.3	METTL5	0	0	0.06333333	2.656666667
XM_005671961.3	METTL8	0	0	0.05666667	4.436666667
XM_005672075.3	NIF3L1	0	0	0.13	11.43666667
XM_005672125.3	FASTKD2	0	0	0.14	14.36666667
XM_005672146.3	MAP2	0	0	0.00333333	1.133333333
XM_005672235.3	DNAJB2	0	0	0.14333333	12.24
XM_005672269.3	HTR2B	0	0	0.00333333	0.333333333
XM_005672363.3	OTOS	0	0	0.05666667	2.886666667
XM_005672492.3	NLN	0	0	0.02666667	3.38
XM_005672521.3	OCLN	0	0	0.04666667	9.023333333
XM_005672559.3	C1QTNF2	0	0	0.14666667	7.506666667
XM_005672586.3	MED7	0	0	0.02	1.196666667
XM_005672611.3	TNIP1	0	0	0.03	3.85
XM_005672620.3	MTRR	0	0	0.02	3.53
XM_005672630.3	TRMT9B	0	0.333333333	0.00333333	0.666666667
XM_005672721.3	ZNF133	0	0	0.05	6.133333333
XM_005672842.3	LOC102162701	0	0	0.00666667	0.333333333
XM_005672902.3	EPB41L1	0	0	0.00333333	1.606666667
XM_005672914.3	DSN1	0	0	0.02333333	5.263333333
XM_005672935.3	ADIG	0	0	0.03	1.083333333
XM_005672960.3	TOMM34	0	0	0.02333333	2.003333333
XM_005673036.3	AURKA	0	0	0.04333333	3.603333333
XM_005673083.3	RNF32	0	0	0.02	1.483333333
XM_005673097.2	XRCC2	0	0	0.06666667	6.28
XM_005673161.3	MEST	0	0	0.01333333	1.356666667
XM_005673418.3	ASB9	0	0	0.00333333	0.106666667
XM_005673420.3	RAB9A	0	0	0.10666667	7.7

XM_005673447.3	PIR	0	0	0.00666667	0.623333333
XM_005673521.3	CXHXorf21	0	0	0.01	2.75
XM_005673705.3	PJA1	0	0	0.08	12.18
XM_005673764.3	DRP2	0	0	0.01666667	5.416666667
XM_005673785.3	FAM199X	0	0	0.03333333	11.45
XM_005673792.3	MUM1L1	0	0	0.04333333	9.706666667
XM_005673797.3	MUM1L1	0	0	0.01	2.016666667
XM_005673830.3	DCX	0	0	0.02666667	14.97333333
XM_005673843.3	IL13RA2	0	0	0.05666667	3.79
XM_005673847.3	AGTR2	0	0	0.00333333	0.333333333
XM_005673901.3	ELF4	0	0	0.00666667	2.036666667
XM_005673954.3	FMR1	0	0	0.01333333	2.396666667
XM_005673957.3	FMR1	0	0	0.01333333	2.176666667
XM_005673993.3	HAUS7	0	0.096666667	0.09666667	14.72333333
XM_005674036.3	IKBKG	0	0	0.15	28.86
XM_013977540.2	ZNF311	0	0	0.00333333	0.666666667
XM_013977657.2	ADORA1	0	0	0.01333333	1.573333333
XM_013977773.2	BAK1	0	0	0.01333333	1.603333333
XM_013977853.2	LOC106504394	0	0	0.00666667	0.333333333
XM_013977870.2	C7H6orf89	0	0	0.18666667	55.58333333
XM_013977891.2	NFYA	0	0	0.01333333	2.196666667
XM_013977909.2	MDFI	0	0	0.02333333	1.523333333
XM_013977911.2	MDFI	0	0	0.01	0.476666667
XM_013977936.2	TRERF1	0	0	0.00666667	1.976666667
XM_013977947.2	PRPH2	0	0	0.00333333	0.333333333
XM_013977996.2	SLC25A27	0	0.323333333	0.01666667	4.406666667
XM_013978029.2	SH3GL3	0	0	0.02666667	1.666666667
XM_013978157.2	TSSK4	0	0	0.13333333	9.493333333
XM_013978164.2	SDR39U1	0	0	0.07	8.663333333
XM_013978172.2	NFATC4	0	0	0.1	13.50333333
XM_013978174.2	NFATC4	0	0	0.01666667	1.886666667
XM_013978234.2	LOC102166266	0	0	0.00666667	0.333333333
XM_013978276.2	FAM174B	0	0	0.04333333	2.026666667
XM_013978294.2	CHURC1	0	0	0.22666667	3.476666667
XM_013978325.2	IFT43	0	0	0.13333333	4.296666667
XM_013978375.2	EFCAB11	0	0	0.02	0.496666667
XM_013978391.2	HDHD2	0	0	0.00666667	0.59
XM_013978425.2	LOC102168085	0	0	0.00333333	0.333333333
XM_013978534.2	PROM1	0	0	0.11	16.04
XM_013978536.2	PROM1	0	0	0.02	2.9
XM_013978542.2	PROM1	0	0	0.03	4.406666667
XM_013978543.2	PROM1	0	0	0.06333333	8.553333333
XM_013978548.2	PROM1	0	0	0.02	2.56
XM_013978574.2	ARAP2	0	0	0.11333333	39.50333333
XM_013978621.2	APBB2	0	0	0.11666667	31.97
XM_013978637.2	BEND4	0	0	0.01	0.706666667
XM_013978655.2	TEC	0	0	0.01666667	1.893333333
XM_013978683.2	FAM198B	0	0	0.01	2.076666667
XM_013978690.2	RXFP1	0	0	0.01	1.613333333
XM_013978810.2	CCNG2	0	0	0.09	9.73
XM_013978859.2	LOC102167861	0	0	0.01333333	3.75
XM_013978861.2	LSM6	0	0	0.07666667	14.87666667
XM_013978864.2	ANAPC10	0	0	0.16	5.44
XM_013978869.2	IL15	0	0	0.01666667	1.12
XM_013978871.2	IL15	0	0	0.02	1.316666667
XM_013978918.2	TRPC3	0	0	0.01	1.413333333
XM_013978993.2	TET2	0	0	0.02333333	11.94333333
XM_013979064.2	MAPK10	0	0	0.00333333	0.67
XM_013979161.2	LRRC32	0	0	0.14666667	25.03
XM_013979196.2	ANKRD42	0	0	0.07666667	21.47666667

XM_013979226.2	SYTL2	0	0	0.01333333	1.493333333
XM_013979327.2	MMP27	0	0	0.00666667	0.51
XM_013979334.2	GRIA4	0	0	0.05333333	9.343333333
XM_013979348.2	ELMOD1	0	0	0.01666667	2.116666667
XM_013979368.2	PPP2R1B	0	0	0.05333333	8.76
XM_013979385.2	NCAM1	0	0	0.02	5.863333333
XM_013979475.2	MFRP	0	0	0.02666667	3.386666667
XM_013979482.2	ARHGEF12	0	0	0.02666667	11.35666667
XM_013979524.2	LOC106504915	0	0	0.02666667	2.666666667
XM_013979545.2	ZPBP2	0	0	0.02333333	1.333333333
XM_013979562.2	LRRC3C	0	0	0.01333333	1
XM_013979668.2	CFAP69	0	0	0.00333333	0.333333333
XM_013979703.2	IKZF3	0	0	0.01	4.646666667
XM_013979727.2	PHF14	0	0	0.01	2.066666667
XM_013979787.2	HGF	0	0	0.01666667	5.263333333
XM_013979828.2	ATXN7L1	0	0	0.01333333	3.48
XM_013979861.2	EZH2	0	0	0.01	0.926666667
XM_013979863.2	STAC2	0	0	0.02333333	3.003333333
XM_013979903.2	FAM20B	0	0	0.02333333	5.28
XM_013980017.2	BRINP3	0	0	0.00666667	0.666666667
XM_013980061.2	MAP1LC3C	0	0	0.01333333	0.666666667
XM_013980098.2	TP53BP2	0	0	0.09666667	21.16
XM_013980155.2	IDNK	0	0	0.10666667	6.466666667
XM_013980171.2	KIF24	0	0	0.00666667	1.366666667
XM_013980192.2	TMEM215	0	0	0.00666667	1.666666667
XM_013980221.2	ARHGAP12	0	0	0.00333333	0.59
XM_013980271.2	SKIDA1	0	0	0.00333333	1
XM_013980302.2	CREM	0	0	0.01666667	1.47
XM_013980341.2	LOC106505208	0	0	0.00666667	0.91
XM_013980387.2	PIGL	0	0	0.11666667	3.096666667
XM_013980423.2	ALOX5AP	0	0	0.14	6.163333333
XM_013980476.2	NEK3	0	0	0.02666667	2.766666667
XM_013980479.2	NEK3	0	0	0.02666667	2.213333333
XM_013980508.2	SETDB2	0	0	0.02	4.896666667
XM_013980550.2	SMIM2	0	0	0.00333333	0.333333333
XM_013980644.2	TMTC4	0	0	0.02	2.91
XM_013980756.2	SLC16A5	0	0	0.16666667	22.22
XM_013980875.2	FMNL1	0	0	0.01333333	2.213333333
XM_013980900.2	KIF18B	0	0	0.07	10.24
XM_013980966.2	HAP1	0	0	0.00666667	1.666666667
XM_013980999.2	TAC4	0	0	0.01	0.333333333
XM_013981058.1	LUC7L3	0	0	0.02	3.123333333
XM_013981073.2	MBTD1	0	0	0.05333333	14.05
XM_013981108.2	TMEM45A	0	0	0.03	1.666666667
XM_013981255.2	CAMTA2	0	0	0.10666667	21.48
XM_013981268.2	MINK1	0	0	0.01333333	2.746666667
XM_013981289.2	PDE9A	0	0	0.01	0.653333333
XM_013981368.2	COL6A5	0	0	0.00666667	2.62
XM_013981410.2	THRB	0	0	0.04	11.75
XM_013981415.2	THRB	0	0	0.02	4.913333333
XM_013981507.2	VIPR1	0	0.01	0.11	13.23666667
XM_013981529.2	SNRK	0	0.096666667	0.05333333	11.14333333
XM_013981533.2	ZNF197	0	0	0.01333333	4.57
XM_013981541.2	LOC100738134	0	0	0.02	2.183333333
XM_013981543.2	LOC100738134	0	0	0.01	1.136666667
XM_013981547.2	LOC100738134	0	0	0.01	1.08
XM_013981558.2	CDCP1	0	0	0.00666667	0.666666667
XM_013981567.2	CCRL2	0	0	0.05	2.826666667
XM_013981593.2	LOC100515578	0	0	0.03	1.666666667
XM_013981645.2	SLC26A6	0	0	0.12	14.73666667

XM_013981657.2	COL7A1	0	0.02	0.17666667	72.91333333
XM_013981693.2	RNF123	0	0	0.00666667	1.723333333
XM_013981698.2	MST1	0	0	0.02333333	2.806666667
XM_013981703.2	IP6K1	0	0	0.02666667	5.856666667
XM_013981731.2	SEMA3F	0	0	0.05333333	7.98
XM_013981738.2	SEMA3B	0	0	0.16333333	15.38333333
XM_013981818.2	KCTD6	0	0	0.05666667	10.21
XM_013981977.2	ATG7	0	0	0.06666667	7.503333333
XM_013981982.2	PPARG	0	0	0.02	1.933333333
XM_013982037.2	CEP63	0	0	0.17666667	16.73666667
XM_013982062.2	CEP70	0	0	0.02333333	2.633333333
XM_013982114.2	PLSCR4	0	0	0.01666667	2.236666667
XM_013982184.2	GOLIM4	0	0	0.04666667	8.216666667
XM_013982220.2	ZMAT3	0	0	0.09333333	32.95333333
XM_013982232.2	B3GNT5	0	0	0.01666667	2.69
XM_013982236.2	B3GNT5	0	0	0.04666667	6.653333333
XM_013982247.2	EIF4G1	0	0	0.12666667	31.46666667
XM_013982256.2	CLCN2	0	0	0.02	3.27
XM_013982282.2	TPRG1	0	0	0.00666667	0.666666667
XM_013982358.2	TNK2	0	0	0.15	29.72
XM_013982395.2	PARP9	0	0	0.03	4.25
XM_013982432.2	ZDHHC23	0	0	0.05333333	16.69333333
XM_013982433.2	ZDHHC23	0	0	0.01666667	3.85
XM_013982517.2	ROBO1	0	0.156666667	0.01333333	4.95
XM_013982568.2	SYNJ1	0	0	0.02333333	6.936666667
XM_013982570.2	SYNJ1	0	0	0.02666667	9.043333333
XM_013982572.2	SYNJ1	0	0	0.00666667	1.77
XM_013982684.2	FZD3	0	0	0.00333333	0.46
XM_013982715.2	NEK1	0	0	0.04666667	10.04666667
XM_013982720.2	NEK1	0	0	0.04	8.653333333
XM_013982722.2	NEK1	0	0	0.03666667	9.043333333
XM_013982726.2	LOC100154415	0	0	0.01333333	5.48
XM_013982740.2	EP400	0	0	0.12333333	72.45333333
XM_013982742.2	EP400	0	0	0.02	12.88
XM_013982779.2	TCTN2	0	0	0.03666667	4.356666667
XM_013982802.2	CCDC62	0	0.18	0.03333333	5.06
XM_013982820.2	MED10	0	0	0.73333333	33.71666667
XM_013982860.2	ANAPC7	0	0	0.37333333	44.41666667
XM_013982864.2	RAD9B	0	0	0.04333333	3.666666667
XM_013982870.2	HVCN1	0	0	0.03666667	4.436666667
XM_013982988.2	CRYBB2	0	0	0.01666667	0.666666667
XM_013983087.2	PRR14L	0	0	0.09666667	43.38
XM_013983173.2	LOC100155534	0	0	0.04666667	2
XM_013983222.2	C17H20orf96	0	0	0.03	1.666666667
XM_013983227.2	GALNT2	0	0	0.06	10.95333333
XM_013983246.2	ZNF25	0	0	0.03	7.066666667
XM_013983350.2	ZSWIM8	0	0	0.06333333	12.67
XM_013983356.2	KAT6B	0	0	0.02333333	8.38
XM_013983433.2	VSTM4	0	0	0.05	5.126666667
XM_013983439.2	ZFAND4	0	0	0.00333333	0.86
XM_013983472.2	LOC100152956	0	0	0.00333333	0.333333333
XM_013983474.2	LOC100153775	0	0	0.00666667	0.333333333
XM_013983480.2	RASSF4	0	0	0.31333333	34.33333333
XM_013983533.2	LIPM	0	0	0.00333333	0.666666667
XM_013983538.2	PANK1	0	0	0.06	6.77
XM_013983542.2	KIF20B	0	0	0.00666667	1.276666667
XM_013983569.2	FRA10AC1	0	0	0.05333333	2.79
XM_013983574.2	SLC35G1	0	0	0.04	6.04
XM_013983594.2	OPALIN	0	0	0.00666667	0.666666667
XM_013983638.2	ZNF518A	0	0	0.00666667	2.58

XM_013983640.2	ZNF518A	0	0	0.03666667	10.68333333
XM_013983649.2	LOC100627422	0	0	0.12	50.44333333
XM_013983696.2	MFSD13A	0	0	0.00666667	0.59
XM_013983781.2	GFRA1	0	0	0.10333333	44.89666667
XM_013983792.2	MOSPD1	0	0	0.05	6.293333333
XM_013983825.2	LOC100516891	0	0	0.01	0.333333333
XM_013983827.2	CPXM2	0	0	0.19	22.36
XM_013983859.2	UROS	0	0	0.22333333	12.89333333
XM_013983863.2	UROS	0	0	0.24333333	16.7
XM_013983918.2	LYPD6	0	0	0.01666667	2.333333333
XM_013984018.2	DCTD	0	0	0.04	3.3
XM_013984075.2	LETM2	0	0	0.00666667	0.963333333
XM_013984188.2	PSMD14	0	0	0.08333333	4.833333333
XM_013984362.2	ANKRD44	0	0	0.09333333	42.60333333
XM_013984376.2	NIF3L1	0	0	0.02333333	1.916666667
XM_013984420.2	ZDBF2	0	0	0.01333333	9.83
XM_013984435.2	PIKFYVE	0	0	0.12	49.16666667
XM_013984446.2	MAP2	0	0	0.00333333	0.886666667
XM_013984452.2	MAP2	0	0	0.00333333	1.2
XM_013984526.2	DNAJB2	0	0	0.09	4.363333333
XM_013984541.2	DOCK10	0	0	0.01666667	10.78666667
XM_013984543.2	DOCK10	0	0	0.02	11.3
XM_013984544.2	DOCK10	0	0	0.01333333	9.196666667
XM_013984546.2	DOCK10	0	0.243333333	0.00666667	2.643333333
XM_013984553.2	NYAP2	0	0	0.00333333	0.403333333
XM_013984577.2	EIF4E2	0	0	0.04666667	7.106666667
XM_013984602.2	UGT1A6	0	0	0.00666667	0.61
XM_013984766.2	LOC100737060	0	0.016666667	0.34666667	191.3333333
XM_013984813.2	PPWD1	0	0	0.01333333	1.333333333
XM_013984882.2	PTTG1	0	0	0.03	0.84
XM_013984889.2	ZUP1	0	0	0.04	3.756666667
XM_013984911.2	TNIP1	0	0	0.02333333	3.016666667
XM_013984919.2	GM2A	0	0	0.00333333	0.276666667
XM_013984922.2	CCDC69	0	0	0.02333333	5.866666667
XM_013984941.2	C16H5orf38	0	0	0.00333333	0.333333333
XM_013984963.2	TRMT9B	0	0	0.00333333	1
XM_013984976.2	MSR1	0	0	0.01	0.6
XM_013985086.2	NDUFAF5	0	0	0.13666667	5.206666667
XM_013985097.2	SNX5	0	0	0.06333333	7.106666667
XM_013985137.2	SYNDIG1	0	0	0.03333333	3.02
XM_013985184.2	LOC102161654	0	0	0.03	2.783333333
XM_013985247.2	NOL4L	0	0	0.01666667	4.38
XM_013985309.2	CEP250	0	0	0.04	25.18
XM_013985339.2	EPB41L1	0	0	0.02	6.083333333
XM_013985368.2	ADIG	0	0	0.01	0.333333333
XM_013985426.2	WFDC3	0	0	0.05	1.116666667
XM_013985484.2	CTCFL	0	0	0.00333333	0.673333333
XM_013985485.2	CTCFL	0	0	0.00666667	0.993333333
XM_013985518.2	NELFCD	0	0	0.08666667	8.92
XM_013985545.2	LOC106506744	0	0	0.00333333	0.333333333
XM_013985548.2	RNF32	0	0	0.02666667	1.95
XM_013985631.2	SPRED1	0	0	0.01	2.496666667
XM_013985637.2	TSPAN12	0	0	0.01	0.613333333
XM_013985654.2	ASB15	0	0	0.24333333	53.37333333
XM_013985666.2	CADPS2	0	0	0.01	2.22
XM_013985675.2	PTPRZ1	0	0	0.01	2.163333333
XM_013985693.2	GPR85	0	0	0.00666667	2
XM_013985719.2	ITGB7	0	0	0.01	1.33
XM_013985729.2	NT5C3A	0	0	0.09	7.2
XM_013985796.2	TNS3	0	0	0.09333333	27.98

XM_013985816.2	CCM2	0	0	0.04666667	3.416666667
XM_013985830.1	TMED4	0	0	0.04333333	3.733333333
XM_013985885.2	MSL3	0	0	0.12333333	14.00333333
XM_013985896.2	TLR8	0	0	0.01666667	5.583333333
XM_013985909.2	RAB9A	0	0	0.12333333	7.186666667
XM_013985910.2	GEMIN8	0	0	0.09	5.31
XM_013985959.2	PHKA2	0	0	0.01666667	6.09
XM_013985961.2	PHKA2	0	0.003333333	0.06	22.71666667
XM_013986049.2	LOC100624295	0	0	0.00666667	0.166666667
XM_013986055.1	BCOR	0	0	0.08666667	22.22666667
XM_013986085.2	JADE3	0	0	0.02333333	3.18
XM_013986173.2	GPR173	0	0	0.01333333	2.286666667
XM_013986332.2	DCX	0	0	0.00333333	1.316666667
XM_013986335.2	DCX	0	0	0.02	7.71
XM_013986381.2	LONRF3	0	0.01	0.01	1.793333333
XM_013986405.2	TEX13C	0	0	0.00333333	0.333333333
XM_013986424.2	BCORL1	0	0	0.22333333	75.35333333
XM_013986429.2	MBNL3	0	0	0.01333333	7.166666667
XM_013986443.2	IGSF1	0	0	0.05333333	11.18666667
XM_013986472.2	LOC102157658	0	0	0.00333333	0.333333333
XM_013986501.2	ZNF185	0	0	0.04666667	8.043333333
XM_013986506.2	GABRQ	0	1	0.00333333	1.333333333
XM_013986509.2	AVPR2	0	0	0.04333333	3
XM_013986587.2	RABEPK	0	0	0.05333333	2.8
XM_013986981.2	POMT1	0	0	0.11333333	14.24
XM_013987086.2	ATF7	0	0	0.03666667	15.51333333
XM_013987244.2	RCE1	0	0	0.01	1.79
XM_013987247.2	RCE1	0	0.153333333	0.02333333	3.07
XM_013987256.2	LGALS12	0	0	0.02	1.123333333
XM_013987284.2	ANO3	0	0	0.01666667	5.51
XM_013987290.2	ANO3	0	0	0.00333333	0.93
XM_013987376.2	ELOF1	0	0	0.11666667	3.78
XM_013987382.2	LRRC23	0	0	0.01	0.57
XM_013987412.2	GPR162	0	0	0.00666667	1
XM_013987497.2	FCHO1	0	0	0.04	4.27
XM_013987506.2	ZNF496	0	0	0.04666667	10.15333333
XM_013987539.2	LOC100620238	0	0	0.01	2.83
XM_013987557.2	HRH2	0	0	0.03	6.62
XM_013987611.2	ARHGAP26	0	0	0.01666667	2.003333333
XM_013987706.2	ZNF646	0	0	0.03	7.913333333
XM_013987713.2	FBXL19	0	0	0.35333333	48.42
XM_013987740.2	FAM234A	0	0	0.05333333	5.25
XM_013987747.2	TMEM8A	0	0.21	0.04666667	6.303333333
XM_013987788.2	UNKL	0	0	0.04	7.886666667
XM_013987789.2	UNKL	0	0	0.02666667	5.03
XM_013987792.2	UNKL	0	0	0.01333333	2.663333333
XM_013987820.2	NTHL1	0	0	0.00333333	0.14
XM_013987822.2	NTHL1	0	0	0.03	1.49
XM_013987829.2	TRAF7	0	0	0.09333333	9.886666667
XM_013987844.2	CCNF	0	0	0.09666667	14.84333333
XM_013987864.2	UNC50	0	0.07	0.07	3.81
XM_013987886.1	CAPG	0	0	0.02	1.41
XM_013987932.2	GEN1	0	0	0.03333333	4
XM_013988083.2	DDR2	0	0.003333333	0.22333333	101.25
XM_013988107.2	HAO2	0	0	0.00333333	0.65
XM_013988149.2	CTTNBP2NL	0	0	0.08333333	18.64333333
XM_013988161.2	C4H1orf162	0	0	0.02	0.943333333
XM_013988183.2	TTC38	0	0	0.02333333	2.38
XM_013988248.2	LOC100127131	0	0	0.08666667	5.773333333
XM_013988273.2	SLC4A8	0	0	0.01333333	5.666666667

XM_013988275.2	SLC4A8	0	0	0.01	1.95
XM_013988315.2	PAN2	0	0	0.01	1.886666667
XM_013988348.2	SPX	0	0	0.02333333	2.666666667
XM_013988398.2	KLRG1	0	0	0.01666667	0.666666667
XM_013988433.2	AKAP3	0	0	0.00666667	1.33
XM_013988446.2	TMEM106C	0	0	1.06	67.74333333
XM_013988477.2	MEAK7	0	0	0.01	2.04
XM_013988509.2	PDF	0	0	0.01	1.926666667
XM_013988512.1	LOC100621778	0	0	0.06	4.19
XM_013988513.2	DUS2	0	0	0.17666667	16.33
XM_013988561.2	AKT2	0	0	0.00666667	1.78
XM_013988720.2	SNAPC1	0	0	0.10666667	21.08666667
XM_013988743.2	TNFRSF25	0	0	0.00666667	0.496666667
XM_013988814.2	MPPE1	0	0	0.05666667	4.69
XM_013988835.1	LOC100522145	0	0	0.00666667	0.373333333
XM_013988889.2	KANK4	0	0	0.01666667	3.713333333
XM_013988963.2	FCN1	0	0	0.01	0.4
XM_013988982.2	WDR61	0	0	0.02333333	2.763333333
XM_013989063.2	RNASE10	0	0	0.01	0.81
XM_013989064.2	PNP	0	0	0.05333333	3.286666667
XM_013989093.1	LOC100515266	0	0	0.01	0.333333333
XM_013989111.2	ARG2	0	0.03	0.05	2.73
XM_013989142.2	SIPA1L1	0	0	0.02333333	6.536666667
XM_013989153.2	LCN15	0	0	0.01333333	1.363333333
XM_013989193.2	GSTZ1	0	0	0.03	3.036666667
XM_013989283.2	ZBTB49	0	0	0.15	18.57666667
XM_013989301.2	GRIA2	0	0	0.02	3.003333333
XM_013989331.2	JADE1	0	0	0.02333333	6.28
XM_013989415.2	HELQ	0	0	0.02666667	4.12
XM_013989465.1	LOC106507823	0	0	0.02	0.666666667
XM_013989519.2	TMEM25	0	0	0.00666667	0.996666667
XM_013989522.2	TMEM25	0	0	0.02	1.836666667
XM_013989545.2	ZC3H11A	0	0	0.1	26.44333333
XM_013989671.2	LOC102160111	0	0	0.02	0.523333333
XM_013989674.2	PALB2	0	0	0.08	14.51666667
XM_013989711.2	TUT7	0	0	0.11	29.32666667
XM_013989804.2	OGFOD3	0	0	0.12666667	6.333333333
XM_013989852.2	CYTH1	0	0	0.09666667	12.93
XM_013989872.2	PRCD	0	0	0.00666667	0.376666667
XM_013989892.2	RNF157	0	0	0.08	10.75
XM_013989908.2	ARSG	0	0	0.06	10.26333333
XM_013989922.2	KANSL1	0	0.343333333	0.01	2.856666667
XM_013989943.2	C12H17orf78	0	0	0.00666667	0.666666667
XM_013989944.2	TADA2A	0	0	0.07333333	7.31
XM_013989967.2	RAB34	0	0	0.02	1.54
XM_013989985.2	TLCD1	0	0	0.01666667	0.936666667
XM_013990048.2	AURKB	0	0	0.01	1.233333333
XM_013990084.2	ZKSCAN7	0	0	0.02333333	5.606666667
XM_013990095.2	LOC100626731	0	0	0.04666667	5.423333333
XM_013990096.2	LOC100626731	0	0	0.08666667	9.916666667
XM_013990113.2	KIAA1257	0	0	0.00333333	0.333333333
XM_013990129.2	PLD1	0	0	0.04	20.13
XM_013990261.2	DMTN	0	0	0.08666667	9.566666667
XM_013990269.2	ADRA1A	0	0	0.01	8.18
XM_013990340.2	C14H12orf49	0	0	0.07333333	43.65666667
XM_013990342.2	C14H12orf49	0	0	0.07666667	4.82
XM_013990388.2	RHBDD3	0	0	0.02333333	1.59
XM_013990416.2	COL13A1	0	0	0.02333333	3.416666667
XM_013990421.2	COL13A1	0	0	0.02666667	3.85
XM_013990477.2	ZFYVE27	0	0	0.07666667	5.303333333

XM_013990605.2	SP140	0	0	0.13	14.77333333
XM_013990663.2	RNASEH2A	0	0	0.00333333	0.313333333
XM_013990768.2	ZC3HAV1	0	0	0.00666667	2.243333333
XM_013990802.2	KCP	0	0	0.01333333	3.983333333
XM_013990815.2	STRIP2	0	0	0.01	1.866666667
XM_013990822.2	TSPAN33	0	0	0.11	8.213333333
XM_013990885.2	ZNF182	0	0	0.01	1.323333333
XM_013990899.2	CCNB3	0	0.043333333	0.01333333	3.19
XM_013990917.2	PDZD11	0	0	0.10333333	4.783333333
XM_013990918.2	DLG3	0	0	0.05666667	17
XM_013990919.2	DLG3	0	0	0.00666667	1.99
XM_013990972.2	BHLHB9	0	0	0.06666667	11.38
XM_013990981.2	TCEAL8	0	0	0.02	1.043333333
XM_013991006.2	INTS6L	0	0	0.07666667	10.92666667
XM_013991007.2	INTS6L	0	0	0.05333333	8.19
XM_013991013.2	INTS6L	0	0	0.1	15.79666667
XM_013991044.2	MTM1	0	0	0.04333333	5.956666667
XM_013991108.2	LOC106508264	0	0	0.04666667	8
XM_013991111.2	TMEM237	0	0	0.05333333	4.093333333
XM_013991184.2	ANKMY2	0	0	0.22666667	24.30333333
XM_013991405.2	LOC100738003	0	0	0.03	9.433333333
XM_013991805.2	MGAT1	0	0	0.03	4.553333333
XM_013991870.2	ZFP62	0	0	0.08333333	12.68666667
XM_013992129.2	BET1L	0	0	0.04333333	4.94
XM_013992480.2	FBXO5	0	0	0.04	4
XM_013992583.2	TRMT11	0	0	0.03333333	2.666666667
XM_013992659.2	CEP162	0	0	0.00333333	0.476666667
XM_013992665.2	HTR1E	0	0.333333333	0.01	2.333333333
XM_013992782.2	CALHM4	0	0	0.01333333	1.82
XM_013992794.2	SNAP91	0	0	0.00333333	0.96
XM_013992846.2	SMAD7	0	0	0.01666667	1.51
XM_013992889.2	LOC102168143	0	0	0.03	1
XM_013992912.2	ANXA2	0	0	0.22333333	15.63666667
XM_013992966.2	RAB27A	0	0	0.08333333	13.64
XM_013992968.2	RAB27A	0	0	0.04	6.736666667
XM_013993043.2	SHF	0	0	0.03666667	1.78
XM_013993111.2	SNAP23	0	0	0.05	5.47
XM_013993207.2	ZNF770	0	0	0.08666667	16.56
XM_013993339.2	PAQR5	0	0	0.00333333	1
XM_013993391.2	GNPNAT1	0	0	0.03	9.746666667
XM_013993461.2	C1H14orf39	0	0	0.01333333	1.703333333
XM_013993617.2	C1H9orf85	0	0	0.06	7.34
XM_013993711.2	INVS	0	0	0.02333333	5.51
XM_013993714.2	MSANTD3	0	0	0.04333333	3.903333333
XM_013993815.2	GGTA1P	0	0	0.09666667	13.81
XM_013993824.2	TTC13	0	0	0.1	9.933333333
XM_013993838.2	ZBTB26	0	0	0.02666667	5.34
XM_013993841.2	ZBTB26	0	0	0.01333333	2.573333333
XM_013993866.2	LOC102168098	0	0	0.00666667	0.413333333
XM_013993919.2	KYAT1	0	0	0.01666667	1.32
XM_013993939.2	PPP6R2	0	0	0.32333333	43.70333333
XM_013993970.2	SBF1	0	0.063333333	0.03	13.44
XM_013993985.2	CTDP1	0	0	0.02333333	3.68
XM_013994017.2	LOC100522421	0	0	0.03666667	3.333333333
XM_013994029.2	TMEM134	0	0	0.05333333	2.153333333
XM_013994064.2	YIF1A	0	0	0.28	15.43666667
XM_013994066.2	YIF1A	0	0	0.06	2.523333333
XM_013994082.2	SNX32	0	0	0.00666667	0.333333333
XM_013994104.2	SLC25A45	0	0	0.16333333	17.68666667
XM_013994107.2	POLA2	0	0	0.07	9.813333333

XM_013994128.2	BATF2	0	0	0.01666667	1.666666667
XM_013994161.2	CDK10	0	0	0.42666667	32.39333333
XM_013994164.2	NUDT22	0	0	0.24333333	11.35666667
XM_013994166.2	NUDT22	0	0	0.10666667	4.426666667
XM_013994167.2	NUDT22	0	0	0.12	4.696666667
XM_013994204.2	GNG3	0	0	0.00666667	0.333333333
XM_013994300.2	FAM111B	0	0	0.03666667	4.05
XM_013994301.2	FANCA	0	0	0.01	2.156666667
XM_013994340.2	NR1H3	0	0	0.00333333	0.41
XM_013994435.2	EHF	0	0	0.00666667	1.81
XM_013994471.2	LOC100517025	0	0.09	0.13666667	36.39
XM_013994540.2	TSNAXIP1	0	0	0.01666667	1.333333333
XM_013994574.2	LOC106509418	0	0	0.01	0.666666667
XM_013994585.2	SLC6A2	0	0	0.01333333	4
XM_013994597.2	ZNF692	0	0	0.02	1.673333333
XM_013994604.1	LOC100736692	0	0	0.02	0.666666667
XM_013994658.2	CLEC17A	0	0	0.00666667	0.666666667
XM_013994691.2	SMARCA4	0	0	0.18	45.50666667
XM_013994728.2	LOC100513844	0	0	0.01	0.666666667
XM_013994792.2	CEBPG	0	0	0.10333333	19.58666667
XM_013994875.2	GPRIN1	0	0	0.00333333	1
XM_013994895.1	LOC100739644	0	0	0.02	0.333333333
XM_013994911.2	POC5	0	0	0.00666667	1.086666667
XM_013994988.2	FAM172A	0	0	0.03333333	9.64
XM_013995006.2	C2H5orf30	0	0	0.01666667	1.756666667
XM_013995009.2	C2H5orf30	0	0	0.00666667	1.236666667
XM_013995011.2	C2H5orf30	0	0	0.00333333	0.46
XM_013995012.2	C2H5orf30	0	0	0.02666667	2.866666667
XM_013995024.2	TMEM232	0	0	0.03333333	3.55
XM_013995062.2	CLDND2	0	0	0.02	0.333333333
XM_013995072.2	C2H5orf63	0	0	0.02	0.966666667
XM_013995119.2	KIF3A	0	0	0.05333333	11.38666667
XM_013995128.2	SLC25A48	0	0	0.01333333	0.666666667
XM_013995129.2	LOC100514465	0	0	0.1	5.666666667
XM_013995146.2	KLHL3	0	0	0.02	6.306666667
XM_013995167.2	TMEM173	0	0	0.10333333	7.293333333
XM_013995209.2	CYSTM1	0	0	0.24666667	9.626666667
XM_013995245.2	LOC100517161	0	0	0.1	15.81666667
XM_013995412.2	GAL3ST4	0	0	0.04666667	5.026666667
XM_013995478.2	GIGYF1	0	0	0.17	70.31333333
XM_013995487.2	ACHE	0	0	0.05666667	7.133333333
XM_013995525.2	INTS11	0	0	0.03666667	3.593333333
XM_013995569.2	TPST1	0	0	0.19333333	16.98666667
XM_013995590.2	CD19	0	0	0.00333333	0.473333333
XM_013995684.2	C3H16orf71	0	0	0.01666667	1.58
XM_013995693.2	VASN	0	0	0.01333333	2.07
XM_013995707.2	NLRC3	0	0.866666667	0.00333333	2.85
XM_013995728.2	MEFV	0	0	0.00333333	0.333333333
XM_013995795.2	PTPDC1	0	0	0.01	3.29
XM_013995876.2	CCDC138	0	0	0.01666667	1.55
XM_013995880.2	LOC100511376	0	0.01	0.06	26.17333333
XM_013995882.2	LOC100511376	0	0.003333333	0.16	68.40666667
XM_013995889.2	LOC100624389	0	0	0.01666667	1
XM_013995921.2	PTAFR	0	0	0.02333333	4.09
XM_013995929.2	MAP4K4	0	0	0.15333333	53.57666667
XM_013995966.2	TXNDC9	0	0	0.21333333	13.26666667
XM_013996110.2	XPO1	0	0	0.15	24.75
XM_013996148.2	CCDC85A	0	0	0.01666667	3.016666667
XM_013996169.2	AK5	0	0	0.03333333	2.77
XM_013996185.2	EML4	0	0	0.01	3.17

XM_013996211.2	PRKD3	0	0	0.04	5.58
XM_013996322.2	PUM2	0	0	0.09333333	29.75
XM_013996328.2	MATN3	0	0	0.00333333	0.333333333
XM_013996365.2	CFLAR	0	0	0.01	1.323333333
XM_013996382.2	SLC52A2	0	0	0.15	11.78
XM_013996389.2	RECQL4	0	0	0.04333333	6.333333333
XM_013996400.2	FOXH1	0	0	0.01	1.333333333
XM_013996512.2	COL14A1	0	0	0.12666667	39.14333333
XM_013996540.2	KCNV1	0	0	0.00333333	0.333333333
XM_013996559.2	DPYS	0	0	0.00333333	0.333333333
XM_013996593.2	LOC106510020	0	0	0.01333333	0.666666667
XM_013996610.2	RBM12B	0	0.003333333	0.26666667	84.39666667
XM_013996625.2	RIPK2	0	0	0.12	14.89666667
XM_013996632.2	WWP1	0	0	0.07666667	15.82
XM_013996658.2	IL7	0	0.333333333	0.00666667	1.826666667
XM_013996738.2	CLVS1	0	0	0.00333333	1
XM_013996749.2	NSMAF	0	0	0.00666667	1.39
XM_013996819.2	FCGR2B	0	0	0.04	2.836666667
XM_013996824.2	CFAP126	0	0	0.02	1.246666667
XM_013996825.2	CFAP126	0	0	0.13333333	7.706666667
XM_013996918.2	LOC100158069	0	0	0.00333333	1.333333333
XM_013996919.2	LOC100158069	0	0	0.01	2.666666667
XM_013996930.2	FCRL5	0	0	0.00333333	1.006666667
XM_013996957.2	IQGAP3	0	0	0.01333333	4.816666667
XM_013997009.2	RUSC1	0	0	0.01666667	2.58
XM_013997013.2	HCN3	0	0	0.00666667	1.12
XM_013997154.2	MESP2	0	0	0.01	1.343333333
XM_013997166.2	PIP5K1A	0	0	0.08	12.25666667
XM_013997186.1	PRUNE1	0	0	0.08	7.83
XM_013997192.2	ADAMTSL4	0	0	0.03666667	6.68
XM_013997235.2	VTCN1	0	0	0.00333333	0.68
XM_013997252.2	SLC22A15	0	0	0.03	5.263333333
XM_013997305.2	CLCC1	0	0	0.08333333	13.64666667
XM_013997336.2	XRCC3	0	0	0.02	4.123333333
XM_013997357.2	SNX7	0	0	0.03	1.966666667
XM_013997378.2	CDC7	0	0	0.02666667	5.366666667
XM_013997387.2	HFM1	0	0	0.02666667	5.036666667
XM_013997436.2	HS2ST1	0	0	0.01	3.263333333
XM_013997450.2	ODF2L	0	0	0.01666667	1.786666667
XM_013997508.2	MIEF1	0	0	0.07	12.26666667
XM_013997536.2	RAC2	0	0	0.02	1.526666667
XM_013997577.2	HOXC4	0	0	0.02333333	4.683333333
XM_013997618.2	RDH5	0	0	0.02333333	1.286666667
XM_013997666.2	GLI1	0	0	0.02666667	5.12
XM_013997693.2	LOC106510322	0	0	0.29333333	18.51
XM_013997785.2	TM7SF3	0	0	0.02333333	4.073333333
XM_013997789.2	RCBTB2	0	0	0.01	1.466666667
XM_013997865.2	RAD52	0	0	0.01333333	2.716666667
XM_013997934.2	KIF21A	0	0	0.06	18.96
XM_013997935.2	KIF21A	0	0	0.05666667	19.18
XM_013997979.2	SENP1	0	0	0.01	3.723333333
XM_013998066.2	VEZT	0	0	0.00666667	2.543333333
XM_013998067.2	TRIM2	0	0	0.00666667	2.753333333
XM_013998084.2	SOCS2	0	0.063333333	0.29	22.94666667
XM_013998086.2	TRIM2	0	0	0.00333333	0.963333333
XM_013998091.2	MND1	0	0	0.01666667	0.516666667
XM_013998172.2	TMEM170A	0	0	0.01666667	3.283333333
XM_013998268.2	RIPOR1	0	0	0.06	10.99
XM_013998276.2	SIAH1	0	0	0.01666667	1.71
XM_013998301.2	FXYD5	0	0.006666667	0.06333333	3.063333333

XM_013998326.2	LIN37	0	0	0.19666667	8.556666667
XM_013998348.2	U2AF1L4	0	0	0.02333333	1.513333333
XM_013998436.2	ZNF527	0	0	0.07666667	9.78
XM_013998440.2	ZNF527	0	0	0.06333333	7.286666667
XM_013998459.2	LOC100739425	0	0	0.03333333	6.546666667
XM_013998466.2	ZNF829	0	0	0.02666667	2.8
XM_013998471.2	ZNF260	0	0	0.01666667	4.993333333
XM_013998609.2	BCAT2	0	0	0.01666667	1.996666667
XM_013998621.2	PPFIA3	0	0	0.02	4.343333333
XM_013998644.2	RPL13A	0	0	0.03666667	3.47
XM_013998649.2	ALDH16A1	0	0	0.04	4.76
XM_013998681.2	ZNF473	0	0	0.01	1.956666667
XM_013998704.2	SYT3	0	0	0.02	2.006666667
XM_013998824.2	ZNF667	0	0	0.05666667	9.27
XM_013998826.2	ZNF667	0	0	0.09333333	15.63
XM_013998831.2	ZNF583	0	0	0.09666667	8.33
XM_013998832.2	ZNF583	0	0	0.02333333	1.733333333
XM_013998854.2	ZNF470	0	0	0.18666667	49.96
XM_013998858.2	ZNF470	0	0	0.03666667	10.61333333
XM_013998954.2	PIK3CD	0	0	0.01666667	4.856666667
XM_013998997.2	CASP9	0	0	0.33	33.68
XM_013999009.2	CPLANE2	0	0	0.01	0.61
XM_013999077.2	RCC1	0	0	0.08333333	17.09666667
XM_013999083.2	OPRD1	0	0	0.00666667	1
XM_013999113.2	DCDC2B	0	0	0.03333333	2.333333333
XM_013999249.2	NDC80	0	0	0.02333333	2.333333333
XM_013999297.2	LOC100620188	0	0	0.02333333	4.113333333
XM_013999302.2	MOCOS	0	0	0.02666667	2.483333333
XM_013999325.2	PTGFR	0	0	0.00666667	1.893333333
XM_013999329.2	ANAPC15	0	0	0.02	0.633333333
XM_013999361.2	LRP8	0	0	0.03333333	6.456666667
XM_013999427.2	EFCAB14	0	0	0.33	82.31333333
XM_013999448.2	LOC110255224	0	0	0.01	0.746666667
XM_021062227.1	LOC110255335	0	0	0.01	0.433333333
XM_021062237.1	LOC102165268	0	0	0.01333333	0.666666667
XM_021062246.1	ZNF143	0	0	0.12666667	36.53666667
XM_021062267.1	STK33	0	0	0.00333333	0.446666667
XM_021062289.1	PPFIBP2	0	0	0.02333333	3.266666667
XM_021062311.1	NLRP14	0	0	0.00333333	0.333333333
XM_021062325.1	LOC110255360	0	0	0.43333333	185.1533333
XM_021062326.1	LOC110255360	0	0	0.06	23.31333333
XM_021062335.1	ILK	0	0	0.11666667	10.17
XM_021062343.1	APBB1	0	0	0.03666667	3.753333333
XM_021062346.1	APBB1	0	0	0.00333333	0.336666667
XM_021062349.1	CNGA4	0	0	0.03333333	5.333333333
XM_021062355.1	LOC100519674	0	0	0.00666667	0.666666667
XM_021062356.1	LOC100524512	0	0	0.00666667	0.666666667
XM_021062358.1	LOC100622082	0	0	0.01333333	1
XM_021062378.1	LOC100515788	0	0	0.02	0.26
XM_021062379.1	LOC110255376	0	0.333333333	0.07333333	32.99
XM_021062381.1	LOC100522746	0	0	0.03	3.666666667
XM_021062388.1	LOC110255380	0	0	0.00666667	0.523333333
XM_021062389.1	LOC110255381	0	0.333333333	0.03333333	8.16
XM_021062395.1	LOC100737816	0	0	0.00666667	1
XM_021062397.1	LOC100512976	0	0	0.03	4.333333333
XM_021062400.1	LOC100513557	0	0	0.02	10
XM_021062406.1	LOC100511705	0	1	0.01333333	7.333333333
XM_021062407.1	LOC102165149	0	0	0.00666667	1.333333333
XM_021062410.1	LOC110255384	0	0	0.01	3.333333333
XM_021062437.1	PGAP2	0	0	0.02666667	2.15

XM_021062440.1	PGAP2	0	0	0.04	3.38
XM_021062449.1	PGAP2	0	0	0.04666667	4.086666667
XM_021062461.1	ANAPC15	0	0	0.03	1.17
XM_021062484.1	PAAF1	0	0	0.12	8.55
XM_021062499.1	XRRA1	0	0	0.01666667	4.73
XM_021062513.1	ARRB1	0	0	0.03666667	12.09666667
XM_021062545.1	EMSY	0	0	0.05	12.03666667
XM_021062555.1	ACER3	0	0	0.19666667	102.1466667
XM_021062559.1	PAK1	0	0	0.11333333	13.89
XM_021062563.1	KCTD14	0	0	0.00333333	0.626666667
XM_021062570.1	ISG20	0	0	0.01333333	0.666666667
XM_021062579.1	ANKRD42	0	0	0.04	15.81333333
XM_021062588.1	SYTL2	0	0	0.01	3.93
XM_021062605.1	SYTL2	0	0	0.06666667	7.106666667
XM_021062611.1	TMEM135	0	0	0.01	1.346666667
XM_021062615.1	TMEM135	0	0	0.07666667	13.25333333
XM_021062617.1	TMEM135	0	0	0.01666667	2.546666667
XM_021062624.1	LOC100623257	0	0	0.00666667	0.666666667
XM_021062636.1	DEUP1	0	0	0.02	1.9
XM_021062660.1	TAF1D	0	0.02	0.04333333	4.93
XM_021062678.1	TEK	0	0.013333333	0.00666667	1.943333333
XM_021062698.1	CCDC82	0	0	0.04	9.65
XM_021062702.1	CFAP300	0	0	0.02666667	2.17
XM_021062703.1	CFAP300	0	0	0.04	3.24
XM_021062721.1	GRIA4	0	0	0.09333333	17.48666667
XM_021062726.1	KBTBD3	0	0	0.07333333	36.14333333
XM_021062764.1	LOC110255430	0	0	0.00333333	0.666666667
XM_021062786.1	BCO2	0	0	0.01	0.963333333
XM_021062808.1	NCAM1	0	0	0.04666667	13.11666667
XM_021062809.1	NCAM1	0	0	0.03333333	8.63
XM_021062829.1	NCAM1	0	0	0.27	55.98666667
XM_021062838.1	TTC12	0	0	0.05	5.466666667
XM_021062882.1	RNF214	0	0.016666667	0.09333333	11.3
XM_021062891.1	CEP164	0	0	0.02666667	6.296666667
XM_021062901.1	DSCAML1	0	0.666666667	0.00333333	2
XM_021062920.1	TMEM25	0	0	0.01	0.94
XM_021062953.1	HYOU1	0	0	0.02	4.37
XM_021062967.1	CCDC153	0	0	0.00333333	0.506666667
XM_021062992.1	GRIK4	0	0	0.05666667	8.926666667
XM_021062997.1	LOC100523684	0	0	0.00333333	1.356666667
XM_021063002.1	LOC100523684	0	0	0.00666667	1.663333333
XM_021063004.1	LOC100523684	0	0	0.00666667	2.72
XM_021063014.1	SORL1	0	0.04	0.14	62.14333333
XM_021063021.1	CRTAM	0	0	0.03	3
XM_021063024.1	CLMP	0	0	0.00333333	0.453333333
XM_021063034.1	GRAMD1B	0	0	0.02	6.723333333
XM_021063042.1	SCN3B	0	0	0.52666667	120.0366667
XM_021063046.1	VWA5A	0	0	0.00333333	0.503333333
XM_021063057.1	LOC100524098	0	0	0.06	3
XM_021063066.1	MSANTD2	0	0	0.02	1.6
XM_021063069.1	CCDC15	0	0	0.02333333	6.686666667
XM_021063089.1	PKNOX2	0	0	0.02	4.253333333
XM_021063125.1	FOCAD	0	0	0.03666667	13.93666667
XM_021063130.1	ARHGAP32	0	0.01	0.06	24.76
XM_021063143.1	LOC100516455	0	0.333333333	0.00333333	0.466666667
XM_021063174.1	THYN1	0	0	0.21	10.14
XM_021063227.1	MDM4	0	0	0.07666667	14.89666667
XM_021063233.1	NFASC	0	0	0.00666667	2.626666667
XM_021063263.1	NFASC	0	0	0.00666667	3.21
XM_021063278.1	CNTLN	0	0	0.06333333	14.55

XM_021063284.1	CNTLN	0	0	0.01666667	3.476666667
XM_021063285.1	SLC26A9	0	0	0.00666667	1.666666667
XM_021063306.1	FAM72A	0	0	0.01333333	1.333333333
XM_021063327.1	PFKFB2	0	0	0.01333333	2.27
XM_021063333.1	YOD1	0	0	0.03333333	6.273333333
XM_021063343.1	ADAM22	0	0	0.02333333	9.77
XM_021063371.1	PPP1R9A	0	0.49	0.22666667	98.73333333
XM_021063379.1	PPP1R9A	0	0	0.00333333	0.883333333
XM_021063421.1	ABCB5	0	0	0.00333333	0.333333333
XM_021063426.1	ABCB5	0	0	0.00666667	1.333333333
XM_021063444.1	KLHL7	0	0.003333333	0.31	40.84666667
XM_021063450.1	IGF2BP3	0	0	0.01333333	3.146666667
XM_021063455.1	ABCB4	0	0	0.01	2.333333333
XM_021063462.1	ABCB4	0	0	0.00333333	1.09
XM_021063465.1	ABCB4	0	0	0.01	2.12
XM_021063517.1	FREM1	0	0.046666667	0.09333333	31.00333333
XM_021063528.1	NRCAM	0	0	0.00333333	1.356666667
XM_021063536.1	NRCAM	0	0	0.00333333	1.326666667
XM_021063540.1	NRCAM	0	0	0.00333333	0.936666667
XM_021063548.1	NRCAM	0	0	0.00666667	1.866666667
XM_021063579.1	FREM1	0	0	0.05	23.22
XM_021063581.1	FREM1	0	0.033333333	0.08	23.18666667
XM_021063585.1	CUL1	0	0	0.02666667	3.73
XM_021063593.1	TPK1	0	0	0.01333333	1.62
XM_021063601.1	LOC100513612	0	0	0.00333333	0.333333333
XM_021063614.1	PPFIA4	0	0	0.00333333	0.53
XM_021063616.1	PPFIA4	0	0	0.01333333	4.323333333
XM_021063617.1	PPFIA4	0	0	0.07333333	18.97666667
XM_021063618.1	PPFIA4	0	0	0.10333333	30.79333333
XM_021063637.1	DNM3	0	0	0.01	2.656666667
XM_021063641.1	DNM3	0	0	0.04	4.946666667
XM_021063682.1	ASTN1	0	0	0.07666667	25.73333333
XM_021063699.1	SEC16B	0	0	0.02333333	4.953333333
XM_021063706.1	SEC16B	0	0	0.06333333	14.15666667
XM_021063707.1	SEC16B	0	0	0.01666667	3.42
XM_021063724.1	TEX35	0	0	0.00666667	0.666666667
XM_021063725.1	TEX35	0	0	0.02	1
XM_021063734.1	FAM20B	0	0	0.05	7.04
XM_021063766.1	RGS8	0	0	0.00666667	1.606666667
XM_021063771.1	RGS8	0	0	0.00333333	0.87
XM_021063796.1	SMG7	0	0	0.02666667	6.406666667
XM_021063802.1	LAMC2	0	0	0.00333333	2.616666667
XM_021063840.1	RPS6KC1	0	0	0.04	7.17
XM_021063846.1	RPS6KC1	0	0	0.35	64.52333333
XM_021063854.1	ATF3	0	0	0.07666667	6.023333333
XM_021063864.1	RD3	0	0	0.01	0.666666667
XM_021063876.1	KCNH1	0	0	0.00333333	0.666666667
XM_021063881.1	SYT14	0	0	0.00333333	2
XM_021063895.1	LOC102160111	0	0	0.02	0.803333333
XM_021063896.1	LOC102160414	0	0	0.00666667	0.333333333
XM_021063913.1	IKZF1	0	0	0.01666667	4.35
XM_021063916.1	IKZF1	0	0	0.02666667	8.156666667
XM_021063925.1	IKZF1	0	0	0.03	6.993333333
XM_021063958.1	LOC100738575	0	0	0.01	0.333333333
XM_021063961.1	LIPC	0	0	0.05666667	3.563333333
XM_021063984.1	CAMK1D	0	0	0.01	3.5
XM_021063994.1	AKT3	0	0	0.1	29.81333333
XM_021063995.1	AKT3	0	0	0.01333333	4.443333333
XM_021064039.1	SPATA6L	0	0	0.00333333	0.96
XM_021064052.1	UBAP1	0	0	0.01666667	2.403333333

XM_021064066.1	SPATA6L	0	0	0.04333333	6.006666667
XM_021064077.1	AKR1C1	0	0	0.00666667	0.333333333
XM_021064095.1	RGS13	0	0.333333333	0.00333333	0.666666667
XM_021064100.1	GPATCH2	0	0	0.01	2.553333333
XM_021064130.1	AK3	0	0	0.06	15.11666667
XM_021064136.1	MIA3	0	0	0.14333333	39.05333333
XM_021064145.1	CEP170	0	0	0.04	13.25666667
XM_021064149.1	CEP170	0	0	0.02	5.46
XM_021064165.1	CEP170	0	0	0.05666667	17.61333333
XM_021064178.1	SDCCAG8	0	0	0.02	2.306666667
XM_021064180.1	SDCCAG8	0	0	0.17666667	22.34333333
XM_021064199.1	ZNF438	0	0	0.03	4.276666667
XM_021064203.1	RFX3	0	0	0.00666667	1.913333333
XM_021064230.1	RFX3	0	0	0.01333333	4.183333333
XM_021064254.1	CELF2	0	0	0.09333333	25.41333333
XM_021064263.1	LOC110255592	0	0	0.01666667	0.81
XM_021064274.1	LOC110255597	0	0	0.02666667	1
XM_021064281.1	OLAH	0	0	0.01	1
XM_021064303.1	LYPLAL1	0	0	0.03333333	1.24
XM_021064310.1	LOC102164422	0	0	0.04666667	2.093333333
XM_021064320.1	SMARCA2	0	0	0.26333333	65.07666667
XM_021064322.1	BPNT1	0	0	0.02666667	1.84
XM_021064328.1	MARK1	0	0	0.09	18.82333333
XM_021064330.1	MARK1	0	0	0.02	4.426666667
XM_021064334.1	MARK1	0	0	0.05333333	1.516666667
XM_021064366.1	WDR64	0	0	0.01333333	2.516666667
XM_021064367.1	WDR64	0	0	0.00666667	1.09
XM_021064381.1	NVL	0	0	0.02333333	3.09
XM_021064382.1	LOC100627222	0	0	0.09666667	10.13
XM_021064406.1	ENAH	0	0.016666667	0.87333333	472.4
XM_021064411.1	PYCR2	0	0	0.02333333	1.85
XM_021064412.1	KANK1	0	0	0.09333333	23.67666667
XM_021064422.1	CDC42BPA	0	0	0.00666667	4.046666667
XM_021064423.1	CDC42BPA	0	0	0.02666667	13.57
XM_021064444.1	KANK1	0	0	0.17	40.17666667
XM_021064463.1	KANK1	0	0	0.14666667	33.57666667
XM_021064486.1	LHX9	0	0	0.00666667	0.666666667
XM_021064493.1	KANK1	0	0	0.06333333	13.92
XM_021064514.1	NAV1	0	0	0.02333333	11.35333333
XM_021064516.1	NAV1	0	0	0.00333333	2.16
XM_021064534.1	PTPN7	0	0	0.01333333	2.553333333
XM_021064542.1	LGR6	0	0	0.00666667	1.333333333
XM_021064544.1	UBE2T	0	0	0.03333333	1.61
XM_021064564.1	SYT2	0	0	0.01333333	3.643333333
XM_021064572.1	ZNF782	0	0	0.01666667	4.44
XM_021064588.1	CDC14B	0	0	0.03333333	8.143333333
XM_021064628.1	TUT7	0	0	0.02333333	5.443333333
XM_021064635.1	GOLM1	0	0	0.07	13.14333333
XM_021064644.1	NTRK2	0	0	0.01	4.116666667
XM_021064653.1	KIF27	0	0	0.01	2.876666667
XM_021064657.1	KIF27	0	0	0.01333333	3.223333333
XM_021064668.1	FRMD3	0	0	0.04333333	10.66666667
XM_021064678.1	DNAI1	0	0	0.04333333	5
XM_021064688.1	APBA1	0	0	0.04333333	13.03
XM_021064698.1	UBAP2	0	0	0.03333333	12.55
XM_021064706.1	MOB3B	0	0	0.03333333	1.31
XM_021064707.1	ZNF658	0	0	0.02	4.203333333
XM_021064722.1	ARMC4	0	0	0.00333333	0.333333333
XM_021064725.1	C1H9orf135	0	0	0.03333333	1.156666667
XM_021064729.1	MPP7	0	0	0.01	2.236666667

XM_021064740.1	MAP3K8	0	0	0.00333333	0.74
XM_021064743.1	JCAD	0	0	0.14	37.64333333
XM_021064793.1	ARHGAP12	0	0	0.00666667	1.38
XM_021064802.1	ARHGAP12	0	0	0.01333333	3.18
XM_021064824.1	CACNB2	0	0	0.00333333	0.956666667
XM_021064844.1	FAM107B	0	0	0.01333333	1.76
XM_021064855.1	BEND7	0	0	0.02333333	8.653333333
XM_021064857.1	BEND7	0	0	0.05666667	9.063333333
XM_021064870.1	LOC100516390	0	0	0.05	14.21
XM_021064879.1	TMC1	0	0	0.00333333	1.616666667
XM_021064885.1	ACBD5	0	0	0.07	10.84333333
XM_021064904.1	ABI1	0	0	0.03	3.736666667
XM_021064907.1	GPR158	0	0.333333333	0.01333333	4
XM_021064953.1	NEBL	0	0	0.01333333	4.95
XM_021064970.1	NRP1	0	0	0.04333333	12.18666667
XM_021065009.1	PRKCQ	0	0	0.02666667	4.36
XM_021065010.1	PRKCQ	0	0	0.01666667	2.366666667
XM_021065011.1	PCSK5	0	0	0.01	2.67
XM_021065018.1	PRKCQ	0	0	0.06666667	8.89
XM_021065024.1	PRKCQ	0	0	0.10333333	16.27666667
XM_021065034.1	IL15RA	0	0	0.04333333	3.18
XM_021065055.1	LOC106505208	0	0	0.01333333	0.666666667
XM_021065058.1	TUBAL3	0	0	0.01	1
XM_021065059.1	GCNT1	0	0	0.02666667	5.943333333
XM_021065071.1	LOC100625534	0	0.196666667	0.00666667	1
XM_021065087.1	ZMYND11	0	0	0.03	4.803333333
XM_021065101.1	MTRF1	0	0	0.07333333	6.163333333
XM_021065110.1	TRPC4	0	0	0.04333333	6.776666667
XM_021065123.1	KLF12	0	0	0.00333333	1.313333333
XM_021065168.1	MTUS2	0	0	0.08	24.68333333
XM_021065171.1	MTUS2	0	0	0.03333333	11.95
XM_021065188.1	LOC106507939	0	0	0.03	0.816666667
XM_021065201.1	NBEA	0	0.783333333	0.23333333	106.3833333
XM_021065202.1	NBEA	0	0.676666667	0.22666667	108.66
XM_021065211.1	DCLK1	0	0	0.01666667	6.46
XM_021065220.1	SUPT20H	0	0	0.00333333	0.33
XM_021065244.1	SUPT20H	0	0.016666667	0.01333333	1.46
XM_021065249.1	SUPT20H	0	0	0.02333333	3.043333333
XM_021065257.1	SUPT20H	0	0	0.06	6.343333333
XM_021065258.1	SUPT20H	0	0.133333333	0.2	22
XM_021065261.1	SUPT20H	0	0	0.11666667	13.59
XM_021065274.1	STOML3	0	0	0.01666667	1.13
XM_021065289.1	INTS6	0	0	0.06333333	15.32333333
XM_021065293.1	LOC100524475	0	0	0.02333333	3.243333333
XM_021065309.1	RCBTB1	0	0	0.08333333	15.01666667
XM_021065320.1	CAB39L	0	0	0.01333333	1.403333333
XM_021065323.1	LOC106505288	0	0	0.00666667	0.333333333
XM_021065337.1	RCBTB2	0	0	0.01	1.423333333
XM_021065352.1	ENOX1	0	0	0.02	3.076666667
XM_021065364.1	PCDH9	0	0	0.02333333	7.353333333
XM_021065368.1	GPC5	0	0	0.00333333	1.3
XM_021065370.1	GPC5	0	0.423333333	0.00333333	0.593333333
XM_021065376.1	FARP1	0	0	0.16	33.50666667
XM_021065414.1	CENPJ	0	0	0.05666667	13.43333333
XM_021065438.1	XPO4	0	0	0.02666667	4.086666667
XM_021065449.1	ZDHHC20	0	0	0.00333333	0.943333333
XM_021065459.1	SGCG	0	0	4.22666667	293.5533333
XM_021065470.1	TNFRSF19	0	0	0.03333333	2.356666667
XM_021065473.1	AMER2	0	0	0.02	8.666666667
XM_021065496.1	RNF6	0	0	0.29333333	39.84333333

XM_021065499.1	CDK8	0	0	0.01666667	1.98
XM_021065500.1	CDK8	0	0	0.02666667	2.93
XM_021065503.1	WASF3	0	0	0.03333333	2.876666667
XM_021065514.1	RPL21	0	0	0.73	17.58
XM_021065542.1	KATNAL1	0	0	0.11333333	8.396666667
XM_021065571.1	LOC100625564	0	0	0.01	1
XM_021065586.1	UNC13B	0	0	0.07333333	21.62333333
XM_021065616.1	SERP2	0	0	0.04666667	2.463333333
XM_021065637.1	ELF1	0	0.003333333	0.10333333	15.94333333
XM_021065644.1	CNMD	0	0.49	0.00333333	0.496666667
XM_021065646.1	LOC110255823	0	0	0.03333333	2.52
XM_021065649.1	DIAPH3	0	0	0.05	14.33333333
XM_021065657.1	DIS3	0	0	0.09666667	22.17333333
XM_021065664.1	PIBF1	0	0.09	0.08333333	9.706666667
XM_021065669.1	TBC1D4	0	0	0.03666667	9.166666667
XM_021065679.1	LMO7	0	0	0.02	5.743333333
XM_021065680.1	LMO7	0	0	0.02666667	6.666666667
XM_021065684.1	LMO7	0	0	0.02666667	6.853333333
XM_021065688.1	TLN1	0	0	0.06	24.02666667
XM_021065725.1	CA9	0	0	0.01333333	1.03
XM_021065736.1	MYCBP2	0	0	0.01666667	12.61666667
XM_021065739.1	MYCBP2	0	0	0.11	77.82666667
XM_021065745.1	MYCBP2	0	0.556666667	0.11333333	67.26333333
XM_021065751.1	MYCBP2	0	0	0.03333333	17.93666667
XM_021065765.1	SLAIN1	0	0	0.01666667	2.616666667
XM_021065782.1	SLITRK6	0	0	0.00333333	0.84
XM_021065820.1	TMEM8B	0	0	0.02	2.22
XM_021065825.1	DOCK9	0	0	0.02666667	11.31
XM_021065841.1	TMEM8B	0	0	0.01	1.333333333
XM_021065859.1	CLYBL	0	0	0.03333333	2.193333333
XM_021065865.1	ZIC2	0	0	0.01	0.663333333
XM_021065867.1	PCCA	0	0	0.06	6.58
XM_021065870.1	GGACT	0	0	0.02666667	1.996666667
XM_021065879.1	NALCN	0	0	0.04666667	15.25666667
XM_021065913.1	CARS2	0	0	0.10666667	8.246666667
XM_021065944.1	ATP11A	0	0	0.12	37.69333333
XM_021065948.1	MCF2L	0	0	0.02333333	5.733333333
XM_021065967.1	MCF2L	0	0	0.01666667	3.946666667
XM_021065978.1	DCUN1D2	0	0	0.05333333	6.153333333
XM_021065983.1	RECK	0	0	0.05333333	9.77
XM_021065995.1	UPF3A	0	0	0.02	3.356666667
XM_021065998.1	UPF3A	0	0	0.02	1.73
XM_021065999.1	UPF3A	0	0	0.02333333	2.836666667
XM_021066000.1	UPF3A	0	0	0.01333333	1.736666667
XM_021066003.1	LOC110255871	0	0	0.04666667	1.333333333
XM_021066036.1	MYH4	0	0.01	0.15333333	41.99666667
XM_021066054.1	ADAP2	0	0	0.04333333	12.22
XM_021066083.1	ARMC7	0	0	0.00666667	1.693333333
XM_021066097.1	LIMD2	0	0	0.01	1.353333333
XM_021066099.1	LIMD2	0	0	0.01333333	1.88
XM_021066104.1	PAX5	0	0.666666667	0.00333333	2
XM_021066110.1	ZNF232	0	0	0.02666667	2.173333333
XM_021066114.1	ZNF232	0	0	0.01666667	1.16
XM_021066116.1	GLP2R	0	0	0.00333333	0.666666667
XM_021066127.1	GHDC	0	0	0.02333333	1.9
XM_021066131.1	GHDC	0	0	0.02333333	1.54
XM_021066134.1	WIPF2	0	0	0.08	23.84333333
XM_021066179.1	FRMPD1	0	0	0.01	2.333333333
XM_021066193.1	AURKB	0	0	0.05333333	4.203333333
XM_021066223.1	KCNJ2	0	0	0.05	11.92666667

XM_021066232.1	TRMT10B	0	0	0.02333333	3.58
XM_021066244.1	THRA	0	0	0.2	52.84666667
XM_021066251.1	PEMT	0	0	0.04	1.393333333
XM_021066267.1	CFAP52	0	0	0.01333333	1
XM_021066312.1	LOC100517605	0	0	0.00333333	0.333333333
XM_021066347.1	HEXD	0	0	0.02	2.783333333
XM_021066366.1	SLC16A3	0	0	0.03	3.463333333
XM_021066372.1	LRRC45	0	0	0.03	3.786666667
XM_021066386.1	GPS1	0	0	0.04	3.266666667
XM_021066391.1	DUS1L	0	0	0.49333333	39.75
XM_021066404.1	NOTUM	0	0	0.00333333	0.666666667
XM_021066411.1	HEMGN	0	0	0.03333333	5.403333333
XM_021066414.1	PYCR1	0	0	0.03	2.493333333
XM_021066465.1	HEMGN	0	0	0.01	1.18
XM_021066467.1	MCRIP1	0	0	0.10666667	5.15
XM_021066471.1	TSPAN10	0	0	0.15333333	11.81333333
XM_021066489.1	BAIAP2	0	0.006666667	0.03333333	3.08
XM_021066502.1	CCDC40	0	0	0.00333333	1
XM_021066507.1	CBX2	0	0	0.05	10.60333333
XM_021066508.1	CBX2	0	0	0.00666667	1.543333333
XM_021066510.1	ENGASE	0	0	0.01333333	2.983333333
XM_021066524.1	PGS1	0	0	0.1	7.383333333
XM_021066536.1	TNRC6C	0	0	0.02666667	11.60666667
XM_021066583.1	RHBDF2	0	0	0.19	28.22666667
XM_021066591.1	SPHK1	0	0	0.00333333	0.58
XM_021066599.1	RNF157	0	0	0.15333333	31.56666667
XM_021066600.1	INVS	0	0	0.01333333	2.833333333
XM_021066605.1	GALR2	0	0	0.00333333	0.333333333
XM_021066616.1	UNC13D	0	0	0.03	4.326666667
XM_021066624.1	RECQL5	0	0	0.06333333	9.333333333
XM_021066625.1	SMIM5	0	0	0.01333333	0.78
XM_021066632.1	TMEM94	0	0	0.18	42.53
XM_021066635.1	TMEM94	0	0	0.15	41.05
XM_021066636.1	TMEM94	0	0	2.13	528.8966667
XM_021066657.1	SLC16A5	0	0	0.06	8.263333333
XM_021066668.1	OTOP3	0	0	0.00666667	0.666666667
XM_021066675.1	CD300LF	0	0	0.01333333	0.766666667
XM_021066695.1	C12H17orf80	0	0	0.12333333	12.88666667
XM_021066697.1	C12H17orf80	0	0	0.12333333	12.58333333
XM_021066702.1	SLC39A11	0	0	0.00666667	1.61
XM_021066707.1	SLC39A11	0	0	0.01666667	4.04
XM_021066751.1	PLPPR1	0	0	0.02	4.53
XM_021066759.1	PLPPR1	0	0.333333333	0.03	7.443333333
XM_021066764.1	MILR1	0	0	0.01	1.353333333
XM_021066765.1	MILR1	0	0	0.00333333	0.333333333
XM_021066769.1	PLPPR1	0	0	0.01	1.273333333
XM_021066773.1	STRADA	0	0	0.12	12.2
XM_021066778.1	STRADA	0	0	0.03333333	3.32
XM_021066782.1	STRADA	0	0	0.02333333	2.313333333
XM_021066787.1	STRADA	0	0	0.04666667	4.716666667
XM_021066791.1	STRADA	0	0	0.13333333	10.57333333
XM_021066820.1	MAPT	0	0	0.07	17.52
XM_021066841.1	FMNL1	0	0	0.05	9.603333333
XM_021066855.1	ADAM11	0	0	0.00666667	1.333333333
XM_021066866.1	FAM171A2	0	0	0.00666667	1.036666667
XM_021066896.1	GRIN3A	0	0	0.04666667	11.50333333
XM_021066948.1	NBR1	0	0.056666667	0.05	11.00333333
XM_021066955.1	NBR1	0	0	0.04666667	10.11
XM_021066965.1	RND2	0	0	0.03666667	1.92
XM_021067031.1	ORMDL3	0	0	0.05	4.353333333

XM_021067034.1	IKZF3	0	0	0.01	4.113333333
XM_021067075.1	SRCIN1	0	0	0.01333333	2.25
XM_021067101.1	SNX11	0	0	0.02666667	2.883333333
XM_021067110.1	HOXB8	0	0	0.00666667	0.666666667
XM_021067114.1	LOC110255214	0	0	0.01333333	1.753333333
XM_021067122.1	LOC102158609	0	0	0.07	6.08
XM_021067181.1	CACNA1G	0	0	0.01333333	7.046666667
XM_021067221.1	MBTD1	0	0	0.01666667	3.693333333
XM_021067225.1	MBTD1	0	0	0.05	11.32666667
XM_021067244.1	STXBP4	0	0	0.11666667	14.90666667
XM_021067259.1	VEZF1	0	0	0.12	27.56
XM_021067261.1	FBXO34	0	0	0.06	9.606666667
XM_021067281.1	RAD51C	0	0.053333333	0.09333333	5.676666667
XM_021067293.1	RPS6KB1	0	0	0.02333333	5.85
XM_021067304.1	C12H17orf64	0	0	0.01333333	0.666666667
XM_021067312.1	MYO19	0	0	0.00666667	0.75
XM_021067327.1	PTPN3	0	0	0.01	3.006666667
XM_021067405.1	RASL10B	0	0.003333333	0.00666667	1.223333333
XM_021067416.1	RFFL	0	0	0.01333333	2.336666667
XM_021067419.1	RFFL	0	0	0.07333333	20.34
XM_021067421.1	RFFL	0	0	0.02333333	4.103333333
XM_021067438.1	ASIC2	0	0	0.00333333	0.333333333
XM_021067454.1	RHBDL3	0	0	0.01666667	5.536666667
XM_021067484.1	NEK8	0	0	0.10333333	12.04
XM_021067510.1	CRYBA1	0	0	0.00333333	0.333333333
XM_021067548.1	RPH3AL	0	0.04	0.02333333	2
XM_021067555.1	MYO1C	0	0	0.10666667	19.37333333
XM_021067594.1	SGSM2	0	0	0.02666667	5.403333333
XM_021067599.1	SGSM2	0	0	0.08	17.23
XM_021067608.1	DPH1	0	0	0.11666667	5.86
XM_021067617.1	METTL16	0	0	0.00333333	0.506666667
XM_021067620.1	METTL16	0	0	0.08333333	8.126666667
XM_021067635.1	RAP1GAP2	0	0	0.01	3.46
XM_021067640.1	RAP1GAP2	0	0	0.00333333	0.886666667
XM_021067641.1	RAP1GAP2	0	0.443333333	0.00666667	1.816666667
XM_021067645.1	RAP1GAP2	0	0	0.00666667	2.716666667
XM_021067647.1	RAP1GAP2	0	0.35	0.02666667	8.056666667
XM_021067679.1	P2RX5	0	0	0.76333333	53.47666667
XM_021067682.1	CTNS	0	0	0.02	3.243333333
XM_021067685.1	CAMKK1	0	0	0.03333333	5.463333333
XM_021067714.1	CDC26	0	0	0.01	1.366666667
XM_021067738.1	HDHD3	0	0	0.10333333	5.666666667
XM_021067748.1	CAMTA2	0	0	0.03666667	6.61
XM_021067750.1	CAMTA2	0	0	0.01666667	3.326666667
XM_021067771.1	MINK1	0	0	0.12	25.30333333
XM_021067773.1	MINK1	0	0	0.00333333	0.45
XM_021067794.1	ARRB2	0	0	0.05666667	5.246666667
XM_021067804.1	SLC16A11	0	0	0.01666667	5.69
XM_021067849.1	GUCY2D	0	0	0.00333333	0.403333333
XM_021067864.1	PIK3R6	0	0	0.06666667	10.60333333
XM_021067875.1	GAS7	0	0	0.05333333	18.62666667
XM_021067881.1	GAS7	0	0	0.05333333	18.62666667
XM_021067906.1	CDRT1	0	0	0.01	2.226666667
XM_021067912.1	ZNF286A	0	0	0.12333333	10.36333333
XM_021067914.1	ZNF286A	0	0	0.02	1.8
XM_021067917.1	AKNA	0	0	0.15	37.38
XM_021067931.1	ULK2	0	0	0.08	16.40333333
XM_021067944.1	RNF112	0	0	0.00666667	1.163333333
XM_021067957.1	EPN2	0	0	0.1	19.44
XM_021067973.1	PRPSAP2	0	0	0.03666667	4.236666667

XM_021068001.1	DRC3	0	0	0.03666667	3.696666667
XM_021068004.1	DRC3	0	0	0.02	1.95
XM_021068023.1	WHRN	0	0	0.01	1.666666667
XM_021068024.1	RAI1	0	0	0.05666667	16.81
XM_021068031.1	MPRIP	0	0.106666667	0.99	191.61
XM_021068079.1	LOC100516640	0	0	0.00333333	0.333333333
XM_021068101.1	LOC100520512	0	0	0.02666667	9.083333333
XM_021068112.1	ABCA5	0	0	0.02333333	5.173333333
XM_021068122.1	TANC2	0	0	0.00333333	2.123333333
XM_021068148.1	TLK2	0	0.073333333	0.10666667	23.74333333
XM_021068210.1	ANKRD28	0	0	0.01666667	7.286666667
XM_021068220.1	RUBCN	0	0	0.01333333	4.086666667
XM_021068238.1	SEC22C	0	0	0.05333333	15.34
XM_021068252.1	CAMK1	0	0	0.21	12.16
XM_021068261.1	TRPC1	0	0	0.00333333	0.57
XM_021068264.1	TRPC1	0	0	0.00333333	0.553333333
XM_021068269.1	ITIH3	0	0	0.04	4.636666667
XM_021068280.1	PDCD10	0	0	0.01666667	1.263333333
XM_021068286.1	USP19	0	0	0.21	46.22
XM_021068291.1	USP19	0	0	0.00666667	1.073333333
XM_021068319.1	GP9	0	0	0.02	5.083333333
XM_021068346.1	ZBTB38	0	0	0.02333333	8.616666667
XM_021068348.1	ZBTB38	0	0	0.00666667	2.85
XM_021068361.1	ZBTB38	0	0	0.03333333	10.93333333
XM_021068364.1	MLH1	0	0	0.10333333	11.53333333
XM_021068367.1	LHX6	0	0	0.11	17.35
XM_021068389.1	ATG7	0	0	0.08666667	24.40333333
XM_021068396.1	ATG7	0	0	0.02	4.566666667
XM_021068443.1	PLD1	0	0	0.00666667	2.98
XM_021068445.1	PLD1	0	0	0.02333333	8.496666667
XM_021068451.1	IFNAR2	0	0	0.12666667	16.18
XM_021068452.1	IFNAR2	0	0	0.08666667	11.87666667
XM_021068467.1	ETV5	0	0	0.05	9.913333333
XM_021068469.1	ERG	0	0	0.01666667	2.926666667
XM_021068471.1	ERG	0	0	0.00333333	0.29
XM_021068494.1	MFN1	0	0	0.47666667	99.52333333
XM_021068516.1	ITIH4	0	0	0.00666667	0.823333333
XM_021068575.1	PR39	0	0	0.02666667	0.456666667
XM_021068578.1	NEK6	0	0	0.03666667	4.103333333
XM_021068580.1	XCR1	0	0	0.01333333	3.66
XM_021068590.1	MITF	0	0	0.01333333	3.063333333
XM_021068612.1	EIF4G1	0	0	0.77666667	170.0666667
XM_021068619.1	SLC26A6	0	0	0.12	14.12333333
XM_021068622.1	SLC26A6	0	0	0.01	1.266666667
XM_021068624.1	P2RY12	0	0	0.00333333	0.48
XM_021068647.1	LOC100515578	0	0	0.02	1
XM_021068651.1	NBEAL2	0	0	0.01	6.653333333
XM_021068656.1	NBEAL2	0	0	0.02666667	13.64666667
XM_021068666.1	NBEAL2	0	0.73	0.01666667	10.00666667
XM_021068668.1	NBEAL2	0	0.336666667	0.03666667	19.39
XM_021068678.1	NBEAL2	0	0	0.01	5.566666667
XM_021068700.1	NBEAL2	0	0	0.00333333	2.416666667
XM_021068705.1	KIF9	0	0	0.04333333	11.15666667
XM_021068711.1	KIF9	0	0	0.01333333	3.823333333
XM_021068717.1	KIF9	0	0	0.01	2.03
XM_021068756.1	GAPVD1	0	0.003333333	0.20333333	49.48333333
XM_021068773.1	MST1	0	0	0.00666667	0.743333333
XM_021068779.1	MST1R	0	0	0.01666667	4.056666667
XM_021068803.1	GNAT1	0	0	0.01	1.88
XM_021068809.1	GAPVD1	0	0	0.15333333	35.23666667

XM_021068835.1	DOCK3	0	0	0.00333333	1.3
XM_021068842.1	DOCK3	0	0	0.00666667	2.263333333
XM_021068845.1	DOCK3	0	0	0.00333333	1.113333333
XM_021068878.1	POC1A	0	0	0.12	11.04
XM_021068890.1	STAB1	0	0	0.04333333	17.25
XM_021068907.1	PBRM1	0	0	0.01	3.333333333
XM_021068908.1	PBRM1	0	0	0.00333333	1.253333333
XM_021068923.1	PBRM1	0	0	0.03666667	13.63
XM_021068934.1	NEK4	0	0.27	0.00333333	0.52
XM_021068941.1	NEK4	0	0	0.02	2.976666667
XM_021068945.1	NEK4	0	0	0.12666667	19.19333333
XM_021068963.1	PRKCD	0	0	0.12666667	30.20333333
XM_021068964.1	PRKCD	0	0	0.00333333	0.576666667
XM_021068977.1	LRTM1	0	0	0.08333333	7.096666667
XM_021068978.1	LRTM1	0	0	0.06666667	6.236666667
XM_021068989.1	CCDC66	0	0	0.01333333	2.003333333
XM_021069006.1	HESX1	0	0	0.01666667	0.85
XM_021069019.1	SLMAP	0	0	0.04333333	10.19
XM_021069053.1	SLMAP	0	0	0.06333333	13.35
XM_021069073.1	RALGPS1	0	0	0.01666667	2.253333333
XM_021069078.1	PXK	0	0	0.02333333	3.02
XM_021069089.1	ABHD6	0	0	0.07333333	7.966666667
XM_021069090.1	ABHD6	0	0	0.11	9.666666667
XM_021069091.1	ABHD6	0	0	0.11	11.97666667
XM_021069096.1	RALGPS1	0	0	0.15666667	17.84666667
XM_021069108.1	C13H3orf67	0	0	0.00333333	0.43
XM_021069115.1	C13H3orf67	0	0	0.04	5.77
XM_021069159.1	CADPS	0	0	0.00333333	0.333333333
XM_021069175.1	ATXN7	0	0	0.01	4.713333333
XM_021069178.1	ATXN7	0	0	0.03	15.93333333
XM_021069187.1	LOC102168098	0	0	0.00666667	0.92
XM_021069192.1	LOC102168098	0	0	0.00666667	0.91
XM_021069206.1	FRMD4B	0	0	0.03	6.91
XM_021069249.1	CHL1	0	0	0.02	7.536666667
XM_021069250.1	CHL1	0	0	0.01	3.37
XM_021069253.1	CHL1	0	0	0.00666667	2.396666667
XM_021069269.1	TRNT1	0	0	0.02333333	5.33
XM_021069278.1	ITPR1	0	0	0.04333333	16.87
XM_021069279.1	ITPR1	0	0	0.02333333	10.62333333
XM_021069284.1	ITPR1	0	0	0.00333333	0.79
XM_021069299.1	CPNE9	0	0	0.01	0.696666667
XM_021069302.1	BRPF1	0	0	0.00333333	0.7
XM_021069314.1	FANCD2	0	0	0.01333333	3.063333333
XM_021069317.1	FANCD2OS	0	0	0.00666667	0.333333333
XM_021069327.1	TTC16	0	0	0.01666667	2
XM_021069390.1	IQSEC1	0	0	0.03333333	10.04
XM_021069405.1	ACAD9	0	0	0.05	5.206666667
XM_021069411.1	EFCC1	0	0	0.03333333	5.003333333
XM_021069414.1	LOC102162880	0	0	0.29666667	19.45666667
XM_021069459.1	NPHP3	0	0	0.01333333	2.78
XM_021069504.1	NME9	0	0.333333333	0.00333333	0.666666667
XM_021069511.1	MRAS	0	0	0.02333333	4.123333333
XM_021069529.1	PIK3CB	0	0	0.01333333	2.83
XM_021069536.1	NMNAT3	0	0	0.01333333	0.966666667
XM_021069573.1	PLS1	0	0	0.01666667	3.063333333
XM_021069576.1	PLS1	0	0	0.06333333	10.5
XM_021069598.1	GYG1	0	0	0.07	5.786666667
XM_021069604.1	IGSF10	0	0	0.01333333	5.256666667
XM_021069627.1	GPR171	0	0	0.02666667	4
XM_021069664.1	MBNL1	0	0	0.00333333	1.04

XM_021069668.1	MBNL1	0	0	0.02	5.39
XM_021069706.1	LEKR1	0	0	0.00333333	2.76
XM_021069742.1	NMD3	0	0	0.24333333	29.36666667
XM_021069775.1	LRRC31	0	0	0.00333333	0.333333333
XM_021069794.1	GPR160	0	0	0.01666667	3.286666667
XM_021069815.1	LOC110256335	0	0	0.02333333	0.666666667
XM_021069825.1	BTLA	0	0	0.00333333	0.333333333
XM_021069839.1	LIPI	0	0	0.00333333	0.333333333
XM_021069849.1	ZMAT3	0	0	0.03	10.95333333
XM_021069850.1	ZMAT3	0	0	0.02666667	12.02333333
XM_021069851.1	BMP4	0	0	0.02666667	1.913333333
XM_021069855.1	ZMAT3	0	0	0.06666667	26.66
XM_021069869.1	ATP11B	0	0	0.04666667	10.05666667
XM_021069891.1	LAMP3	0	0	0.01666667	1.333333333
XM_021069895.1	MCF2L2	0	0	0.00666667	2.586666667
XM_021069896.1	MCF2L2	0	0	0.01333333	4.973333333
XM_021069913.1	YEATS2	0	0.006666667	0.05	14.29
XM_021069925.1	PARL	0	0	0.01666667	0.96
XM_021069949.1	CLCN2	0	0	0.07	9.006666667
XM_021069955.1	CHRD	0	0	0.01	1.776666667
XM_021069956.1	CHRD	0	0	0.01	1.72
XM_021069958.1	CHRD	0	0	0.03333333	5.51
XM_021069960.1	CHRD	0	0	0.01333333	3.91
XM_021069976.1	VPS8	0	0	0.09333333	18.65333333
XM_021069977.1	VPS8	0	0	0.05333333	8.81
XM_021069982.1	MAP3K13	0	0	0.01	5.443333333
XM_021070000.1	LOC110256379	0	0	0.00333333	0.37
XM_021070001.1	LOC110256379	0	0	0.01666667	0.963333333
XM_021070002.1	LOC110256379	0	0	0.01	0.333333333
XM_021070011.1	DGKG	0	0	0.00333333	1.883333333
XM_021070014.1	DGKG	0	0	0.01333333	1.883333333
XM_021070030.1	ST6GAL1	0	0	0.01	2.26
XM_021070031.1	ST6GAL1	0	0	0.03	7.043333333
XM_021070034.1	ST6GAL1	0	0	0.00666667	1.026666667
XM_021070111.1	DLG1	0	0	0.04333333	8.343333333
XM_021070121.1	PIGZ	0	0	0.00666667	3.063333333
XM_021070122.1	PIGZ	0	0	0.08333333	35.63666667
XM_021070125.1	PIGZ	0	0	0.1	37.81333333
XM_021070129.1	PIGZ	0	0	0.01	3.916666667
XM_021070131.1	PIGZ	0	0	0.08	31.64666667
XM_021070179.1	TNK2	0	0	0.11666667	21.88333333
XM_021070214.1	ZNF148	0	0	0.01666667	2.193333333
XM_021070251.1	SEC22A	0	0	0.12666667	4.9
XM_021070263.1	PARP9	0	0	0.05333333	7.97
XM_021070315.1	GPR156	0	0	0.01	2.006666667
XM_021070324.1	FIBCD1	0	0	0.00333333	0.333333333
XM_021070329.1	ARHGAP31	0	0	0.03	14.06666667
XM_021070350.1	GRAMD1C	0	0	0.05333333	8
XM_021070360.1	CFAP44	0	0.826666667	0.01	4.776666667
XM_021070363.1	CFAP44	0	0	0.00333333	1.29
XM_021070364.1	CFAP44	0	0	0.00333333	0.843333333
XM_021070365.1	BOC	0	0	0.08666667	26.02666667
XM_021070376.1	BOC	0	0	0.18666667	63.13666667
XM_021070379.1	NEPRO	0	0	0.01666667	2.266666667
XM_021070381.1	LOC100156381	0	0	0.01666667	0.533333333
XM_021070410.1	ULK4	0	0	0.05333333	23.30666667
XM_021070415.1	MORC1	0	0	0.02333333	3.666666667
XM_021070416.1	LOC100620198	0	0	0.01	1.146666667
XM_021070419.1	DZIP3	0	0	0.03333333	9.11
XM_021070425.1	DZIP3	0	0	0.02	4.643333333

XM_021070451.1	HHLA2	0	0	0.00333333	1.333333333
XM_021070485.1	CCDC54	0	0	0.00333333	0.333333333
XM_021070511.1	ABI3BP	0	0	0.01	3.27
XM_021070522.1	ABI3BP	0	0	0.01333333	3.52
XM_021070532.1	ABI3BP	0	0.006666667	0.00333333	0.85
XM_021070587.1	MAP4	0	0	0.13666667	30.72
XM_021070589.1	MAP4	0	0.29	0.33666667	91.86666667
XM_021070593.1	MAP4	0	0	0.03	7.763333333
XM_021070614.1	DHX30	0	0	0.20666667	38.81333333
XM_021070627.1	NME6	0	0	0.07	3.166666667
XM_021070641.1	CELSR3	0	0	0.00333333	1
XM_021070644.1	SETX	0	0	0.01333333	6.086666667
XM_021070669.1	ST3GAL6	0	0	0.02666667	4.836666667
XM_021070678.1	ST3GAL6	0	0	0.01	1.8
XM_021070682.1	ST3GAL6	0	0	0.02333333	3.933333333
XM_021070683.1	ST3GAL6	0	0	0.01666667	3.533333333
XM_021070693.1	RIOX2	0	0	0.04	2.686666667
XM_021070746.1	ZNF654	0	0	0.01333333	3.203333333
XM_021070754.1	FHIT	0	0	0.1	18.48
XM_021070770.1	TTF1	0	0	0.07	7.266666667
XM_021070779.1	ADAMTS9	0	0	0.02	7.48
XM_021070781.1	ADAMTS9	0	0	0.01333333	5.456666667
XM_021070788.1	MAGI1	0	0	0.00333333	1.513333333
XM_021070793.1	MAGI1	0	0	0.01666667	5.053333333
XM_021070798.1	MAGI1	0	0	0.07	24.45666667
XM_021070800.1	MAGI1	0	0	0.01	3.483333333
XM_021070801.1	MAGI1	0	0.323333333	0.07	25.60666667
XM_021070826.1	AK8	0	0	0.01	0.713333333
XM_021070845.1	FOXP1	0	0	0.37666667	43.42
XM_021070860.1	SPACA9	0	0	0.02333333	1.68
XM_021070867.1	SPACA9	0	0	0.02	1.623333333
XM_021070903.1	TIAM1	0	0	0.04	11.85333333
XM_021070906.1	TIAM1	0	0	0.00333333	1.423333333
XM_021070907.1	TIAM1	0	0	0.04	13.95666667
XM_021070924.1	SYNJ1	0	0	0.02	6.413333333
XM_021070929.1	SYNJ1	0	0	0.03333333	9.28
XM_021070941.1	TMEM50B	0	0	0.00333333	0.28
XM_021070953.1	RALGDS	0	0	0.01666667	5.446666667
XM_021070995.1	GBGT1	0	0	0.11333333	9.876666667
XM_021070999.1	IGSF5	0	0	0.04	4
XM_021071010.1	PRDM15	0	0.01	0.06	17.04
XM_021071024.1	GBGT1	0	0	0.04666667	3.59
XM_021071032.1	SLC37A1	0	0	0.04333333	5.876666667
XM_021071044.1	GBGT1	0	0	0.19	17.86
XM_021071051.1	GRM7	0	0	0.01	2.856666667
XM_021071052.1	GRM7	0	0	0.00666667	2.83
XM_021071057.1	GRM7	0	0	0.00333333	1.256666667
XM_021071063.1	RRP1B	0	0	0.01333333	3.076666667
XM_021071071.1	GBGT1	0	0	0.14	12.79666667
XM_021071084.1	DNMT3L	0	0.023333333	0.00666667	0.336666667
XM_021071110.1	VGLL4	0	0	0.02333333	8.13
XM_021071192.1	NAALADL2	0	0	0.00333333	1.25
XM_021071223.1	PEX5L	0	0	0.00333333	1.333333333
XM_021071274.1	REXO4	0	0	0.01	0.49
XM_021071311.1	FILIP1L	0	0	0.00666667	2.173333333
XM_021071317.1	CMSS1	0	0	0.05	2.803333333
XM_021071318.1	CMSS1	0	0	0.00666667	0.313333333
XM_021071326.1	NEK11	0	0	0.01	1
XM_021071329.1	NEK11	0	0	0.00333333	0.333333333
XM_021071337.1	COL6A5	0	0	0.01666667	6.85

XM_021071341.1	BTD	0	0	0.25	44.89333333
XM_021071358.1	CADM2	0	0	0.01666667	6.12
XM_021071389.1	ROBO2	0	0	0.01	3.986666667
XM_021071398.1	ROBO2	0	0	0.00666667	2.43
XM_021071402.1	ROBO2	0	0	0.00333333	1.326666667
XM_021071414.1	LOC106508546	0	0	0.00333333	0.446666667
XM_021071415.1	LOC106508546	0	0	0.05	1.943333333
XM_021071429.1	THRB	0	0	0.02333333	5.58
XM_021071438.1	NGLY1	0	0	0.19	21.15333333
XM_021071442.1	GABPA	0	0	0.01666667	3.96
XM_021071459.1	NEK10	0	0	0.00666667	2.303333333
XM_021071476.1	SLC4A7	0	0	0.01666667	6.086666667
XM_021071483.1	ZCWPW2	0	0	0.06666667	8.333333333
XM_021071505.1	TRPM2	0	0	0.02	6.666666667
XM_021071538.1	COL18A1	0	0	0.02666667	5.646666667
XM_021071561.1	FBXL2	0	0.06	0.01	0.58
XM_021071568.1	CLASP2	0	0	0.01	3.473333333
XM_021071600.1	CLASP2	0	0	0.04	10.88333333
XM_021071601.1	CLASP2	0	0	0.02	6.206666667
XM_021071605.1	CLASP2	0	0	0.03666667	9.16
XM_021071607.1	CLASP2	0	0	0.00666667	1.53
XM_021071610.1	CLASP2	0	0	0.01333333	3.45
XM_021071611.1	CLASP2	0	0	0.03333333	9.55
XM_021071613.1	CLASP2	0	0.44	0.03333333	9.74
XM_021071615.1	CLASP2	0	0.02	0.35333333	95.31666667
XM_021071636.1	LRRFIP2	0	0	0.03666667	5.893333333
XM_021071645.1	LRRFIP2	0	0	0.01333333	1.783333333
XM_021071674.1	EXOG	0	0	0.03666667	3.34
XM_021071677.1	SCN10A	0	1.043333333	0.00333333	1.236666667
XM_021071678.1	SCN11A	0	0.333333333	0.00333333	1
XM_021071689.1	TRAK1	0	0	0.1	21.53333333
XM_021071718.1	ANO10	0	0	0.05666667	7.163333333
XM_021071726.1	ZNF445	0	0	0.04666667	15.27
XM_021071746.1	KIF15	0	0	0.09333333	21.33333333
XM_021071775.1	CYP26A1	0	0	0.00666667	0.926666667
XM_021071778.1	PDE6C	0	0	0.00666667	0.923333333
XM_021071779.1	PDE6C	0	0	0.02333333	2.916666667
XM_021071784.1	ADRA1A	0	0	0.02333333	14.68
XM_021071804.1	CHRM3	0	0	0.00333333	1.073333333
XM_021071818.1	RHOF	0	0	0.00333333	0.96
XM_021071853.1	RHOF	0	0	0.01333333	3.15
XM_021071862.1	RHOF	0	0	0.00666667	0.443333333
XM_021071869.1	RASSF4	0	0	0.03666667	2.403333333
XM_021071870.1	RASSF4	0	0	0.01666667	0.87
XM_021071874.1	RASSF4	0	0	0.13666667	16.12333333
XM_021071878.1	RASSF4	0	0	0.13	14.72666667
XM_021071919.1	CDK2AP1	0	0	1.29	54.87333333
XM_021071920.1	ARID1B	0	0	0.00666667	3.17
XM_021071923.1	ARID1B	0	0	0.04333333	16.91666667
XM_021071940.1	PIK3AP1	0	0	0.00333333	0.65
XM_021071976.1	HPGD	0	0	0.02666667	1.46
XM_021072018.1	ARL6IP4	0	0	0.07333333	2.84
XM_021072035.1	CHEK2	0	0	0.06666667	8.9
XM_021072037.1	CHEK2	0	0	0.03666667	3.093333333
XM_021072080.1	CLU	0	0	0.02	1.853333333
XM_021072096.1	PPP2R2A	0	0	0.31666667	28.22
XM_021072098.1	GGT1	0	0	0.06	5.333333333
XM_021072106.1	HTR7	0	0	0.00333333	0.156666667
XM_021072127.1	STXBP5	0	0	0.01333333	5.326666667
XM_021072129.1	KCNMA1	0	0.106666667	0.01	1.65

XM_021072144.1	KCNMA1	0	0	0.07333333	12.49333333
XM_021072146.1	KCNMA1	0	0.003333333	0.00666667	2.596666667
XM_021072147.1	KCNMA1	0	0	0.06	10.03666667
XM_021072150.1	KCNMA1	0	0	0.06333333	10.18333333
XM_021072154.1	KCNMA1	0	0	0.01333333	1.916666667
XM_021072165.1	KCNMA1	0	0	0.01	4.133333333
XM_021072215.1	DDX60	0	0	0.1	30.54666667
XM_021072216.1	ADGRD1	0	0	0.16666667	41.12
XM_021072226.1	DNAH10	0	0	0.00666667	4.656666667
XM_021072246.1	ZNF664	0	0	0.11666667	23.40333333
XM_021072275.1	SLC18A1	0	0	0.01333333	2
XM_021072285.1	MINPP1	0	0	0.01666667	3.446666667
XM_021072291.1	LOC102159834	0	0	0.02666667	3.333333333
XM_021072302.1	CASTOR1	0	0	0.01333333	0.986666667
XM_021072313.1	PYROXD2	0	0	0.00666667	1.093333333
XM_021072333.1	TSPAN15	0	0	0.01	0.603333333
XM_021072343.1	DEPDC5	0	0	0.00333333	0.783333333
XM_021072348.1	LOC100738050	0	0	0.02333333	3.246666667
XM_021072351.1	LOC100738050	0	0	0.01333333	2.29
XM_021072357.1	LOC100738050	0	0	0.03	5.34
XM_021072360.1	LOC100738050	0	0	0.04	6.613333333
XM_021072366.1	LOC100738050	0	0	0.01333333	2.043333333
XM_021072367.1	LOC100738050	0	0	0.07	8.906666667
XM_021072369.1	LOC100738050	0	0	0.02333333	0.82
XM_021072473.1	AIG1	0	0	0.01666667	1.043333333
XM_021072477.1	SEC31B	0	0	0.01	2.943333333
XM_021072499.1	CCAR1	0	0	0.02	4.016666667
XM_021072531.1	CCDC63	0	0.01	0.01333333	1.35
XM_021072544.1	USP54	0	0	0.00333333	0.973333333
XM_021072545.1	USP54	0	0	0.00666667	2.85
XM_021072546.1	USP54	0	0	0.01666667	5.636666667
XM_021072547.1	USP54	0	0	0.00666667	2.273333333
XM_021072552.1	USP54	0	0	0.00666667	1.72
XM_021072557.1	USP54	0	0	0.02666667	10.01666667
XM_021072559.1	USP54	0	0	0.01	3.733333333
XM_021072561.1	USP54	0	0	0.01333333	4.82
XM_021072568.1	USP54	0	0	0.06	17.32
XM_021072578.1	PPP3CB	0	0	0.51	203.1833333
XM_021072580.1	PPP3CB	0	0	0.00666667	3.016666667
XM_021072582.1	MAP7	0	0	0.01	1.383333333
XM_021072584.1	MAP7	0	0	0.00666667	1.523333333
XM_021072600.1	FANK1	0	0	0.05666667	24.21
XM_021072609.1	FANK1	0	0	0.02333333	13.55666667
XM_021072610.1	FANK1	0	0	0.01	5.663333333
XM_021072617.1	KIF20B	0	0	0.01333333	3.583333333
XM_021072620.1	KIF20B	0	0	0.03333333	9.333333333
XM_021072632.1	MAP7	0	0	0.01	0.91
XM_021072645.1	PRSS55	0	0	0.00666667	0.666666667
XM_021072655.1	CDH23	0	0	0.03666667	23.11333333
XM_021072663.1	MAP7	0	0	0.00333333	0.273333333
XM_021072698.1	NOS1	0	0	0.04333333	13.00333333
XM_021072705.1	SPPL3	0	0	0.01666667	2.196666667
XM_021072717.1	PTPRK	0	0	0.12333333	30.99
XM_021072735.1	PTPRK	0	0	0.02333333	5.303333333
XM_021072770.1	NEURL1	0	0	2.81666667	465.5466667
XM_021072794.1	NKAIN2	0	0	0.00333333	0.77
XM_021072810.1	TMEM72	0	0	0.00666667	1.973333333
XM_021072823.1	LOC102166958	0	0	0.00666667	1.616666667
XM_021072849.1	NEK1	0	0	0.03	6.713333333
XM_021072852.1	NEK1	0	0.006666667	0.08	18.32666667

XM_021072862.1	GLT1D1	0	0	0.00333333	0.333333333
XM_021072869.1	ADGRB3	0	0	0.00333333	0.666666667
XM_021072883.1	LOC100154415	0	0	0.08666667	13.02666667
XM_021072886.1	RIMS1	0	0	0.00666667	1.626666667
XM_021072892.1	ZNF140	0	0	0.47666667	47.54666667
XM_021072896.1	ZNF140	0	0	0.05666667	6.236666667
XM_021072905.1	LOC100153205	0	0	0.00333333	0.383333333
XM_021072941.1	DNAJC12	0	0	0.03666667	2.556666667
XM_021072956.1	RIMS1	0	0	0.01666667	4.266666667
XM_021072980.1	LRRC43	0	0	0.00333333	0.666666667
XM_021072987.1	B3GNT4	0	0	0.00666667	0.333333333
XM_021072988.1	RIMS1	0	0	0.00666667	1.893333333
XM_021072992.1	EGR3	0	0	0.03	5.49
XM_021072997.1	RIMS1	0	0	0.00333333	0.82
XM_021073002.1	LOC110256689	0	0	0.00333333	0.333333333
XM_021073014.1	ZNF239	0	0	0.23666667	30.77666667
XM_021073019.1	RIMS1	0	0	0.00333333	1.266666667
XM_021073025.1	RIMS1	0	0	0.00333333	0.73
XM_021073028.1	CAMKK2	0	0	0.08666667	20.98
XM_021073041.1	IFT81	0	0	0.03333333	4.106666667
XM_021073046.1	RIMS1	0	0	0.01	2.273333333
XM_021073069.1	RIMS1	0	0	0.00666667	1.513333333
XM_021073109.1	LOC100627357	0	0	0.00333333	0.333333333
XM_021073164.1	STMN4	0	0	0.01666667	1.666666667
XM_021073183.1	PTK2B	0	0	0.03333333	6.106666667
XM_021073184.1	PTK2B	0	0	0.07666667	14.05333333
XM_021073202.1	RHOBTB2	0	0	0.04333333	6.396666667
XM_021073214.1	C14H8orf74	0	0	0.00333333	0.333333333
XM_021073230.1	LOC100158040	0	0	0.00333333	0.336666667
XM_021073256.1	RNLS	0	0	0.01666667	4.733333333
XM_021073282.1	RHBDD3	0	0	0.06	4.466666667
XM_021073289.1	KLHL32	0	0	0.01333333	3.756666667
XM_021073318.1	CASP7	0	0	0.01	1.466666667
XM_021073319.1	CASP7	0	0	0.02666667	2.723333333
XM_021073330.1	DCLRE1A	0	0	0.21666667	42.52
XM_021073341.1	CCSER2	0	0	0.05	16.81333333
XM_021073365.1	ABLIM1	0	0	0.05666667	14.24666667
XM_021073367.1	ABLIM1	0	0	0.00333333	0.913333333
XM_021073373.1	ABLIM1	0	0	0.02666667	6.36
XM_021073375.1	ABLIM1	0	0	0.14666667	36.39666667
XM_021073376.1	GRIK2	0	0	0.01333333	1.663333333
XM_021073380.1	ABLIM1	0	0	0.00333333	0.77
XM_021073386.1	TRUB1	0	0	0.01	2.203333333
XM_021073394.1	HS3ST5	0	0.023333333	0.00333333	0.63
XM_021073397.1	HSPA12A	0	0	0.00333333	0.536666667
XM_021073407.1	CCDC172	0	0	0.00333333	0.333333333
XM_021073411.1	CBLN2	0	0	0.00333333	0.333333333
XM_021073419.1	CCDC74B	0	0	0.01333333	0.666666667
XM_021073429.1	KDM2B	0	0	0.07	15.19
XM_021073442.1	FAM45A	0	0	0.04	4.726666667
XM_021073443.1	FAM45A	0	0	0.01666667	1.94
XM_021073468.1	SFXN2	0	0	0.02	2.15
XM_021073488.1	LOC100157433	0	0	0.02333333	2.55
XM_021073511.1	CPEB3	0	0	0.23	54.32
XM_021073541.1	CIT	0	0	0.21	70.12
XM_021073542.1	CIT	0	0	0.04333333	11.19333333
XM_021073543.1	CIT	0	0	0.05	20.2
XM_021073544.1	CIT	0	0	0.07666667	29.42
XM_021073548.1	CIT	0	0	0.02666667	9.86
XM_021073557.1	TMEM233	0	0	0.00666667	0.563333333

XM_021073561.1	TMEM233	0	0	0.01	1.036666667
XM_021073578.1	SLTM	0	0	0.01	3.776666667
XM_021073584.1	SLTM	0	0	0.02	8.603333333
XM_021073594.1	SHLD2	0	0	0.06333333	8.136666667
XM_021073600.1	RHOBTB1	0	0	0.25666667	43.61666667
XM_021073614.1	SLTM	0	0	0.01666667	7.61
XM_021073635.1	PPIF	0	0	0.03333333	0.773333333
XM_021073640.1	JMJD1C	0	0	0.09333333	32.33333333
XM_021073656.1	JMJD1C	0	0	0.09	32.63666667
XM_021073671.1	SLTM	0	0	0.00333333	0.88
XM_021073675.1	SLTM	0	0	0.01	4.073333333
XM_021073679.1	IL31	0	0	0.00333333	0.976666667
XM_021073687.1	LOC110256815	0	0	0.01	0.333333333
XM_021073689.1	LOC110256816	0	0	0.01333333	0.886666667
XM_021073690.1	LOC110256817	0	0	0.06	4.956666667
XM_021073691.1	LOC110256818	0	0	0.07333333	3.823333333
XM_021073693.1	LOC110256821	0	0	0.00333333	0.333333333
XM_021073713.1	TDRD1	0	0	0.00333333	0.666666667
XM_021073715.1	PNLIPRP3	0	0	0.00333333	0.333333333
XM_021073716.1	BTBD16	0	0	0.01666667	1.666666667
XM_021073743.1	MKI67	0	0	0.01666667	9.713333333
XM_021073744.1	FAM227B	0	0	0.01666667	1.05
XM_021073745.1	MKI67	0	0	0.18	92.22
XM_021073763.1	DTWD1	0	0	0.14	6.92
XM_021073766.1	IL33	0	0	0.01666667	1.86
XM_021073777.1	GIT2	0	0	0.04	8.173333333
XM_021073778.1	GIT2	0	0	0.09	19.71666667
XM_021073785.1	GIT2	0	0	0.03666667	3.683333333
XM_021073788.1	LOC100511352	0	0	0.02	1.133333333
XM_021073797.1	SPECC1L	0	0	0.19	45.15666667
XM_021073809.1	8-Mar	0	0	0.01333333	2.833333333
XM_021073811.1	8-Mar	0	0	0.01333333	3.003333333
XM_021073821.1	SIPA1L2	0	0	0.04333333	14.28666667
XM_021073822.1	SIPA1L2	0	0	0.04333333	15.13
XM_021073828.1	SIPA1L2	0	0	0.08333333	25.96666667
XM_021073833.1	ACAD10	0	0	0.00666667	0.99
XM_021073837.1	ACAD10	0	0	0.03333333	4.563333333
XM_021073854.1	MYOF	0	0	0.01333333	3.876666667
XM_021073856.1	MYOF	0	0	0.02	5.526666667
XM_021073870.1	LOC100157415	0	0	0.00333333	0.333333333
XM_021073875.1	STK32C	0	0	0.00333333	0.333333333
XM_021073880.1	PWWP2B	0	0	0.04	5.81
XM_021073905.1	CFAP46	0	0	0.00666667	3.656666667
XM_021073908.1	CFAP46	0	0	0.01333333	4.786666667
XM_021073936.1	THSD4	0	0	0.31	139.9866667
XM_021073943.1	THSD4	0	0	0.08	37.56
XM_021073951.1	P2RX2	0	0	0.00333333	0.333333333
XM_021073954.1	LOC102159965	0	0	0.01	2.166666667
XM_021073971.1	SYCE1	0	0	0.00333333	0.333333333
XM_021073974.1	MIA2	0	0	0.00666667	1.436666667
XM_021073984.1	EIF4ENIF1	0	0.016666667	0.61666667	96.17666667
XM_021073985.1	EIF4ENIF1	0	0	0.25	40.32666667
XM_021074044.1	P2RX6	0	0	0.07333333	9.693333333
XM_021074060.1	MPHOSPH9	0	0	0.04	12.49333333
XM_021074078.1	CCDC62	0	0.1	0.08	12.54666667
XM_021074083.1	KNTC1	0	0	0.17	58.7
XM_021074090.1	EXTL3	0	0	0.19666667	56.95333333
XM_021074094.1	MDGA2	0	0	0.00333333	0.796666667
XM_021074103.1	MDGA2	0	0	0.00666667	0.863333333
XM_021074123.1	SYNE2	0	0	0.01333333	14.38

XM_021074198.1	CEP44	0	0	0.05	3.816666667
XM_021074199.1	CEP44	0	0	0.01333333	1.203333333
XM_021074215.1	TMEM116	0	0	0.01	0.623333333
XM_021074216.1	TMEM116	0	0	0.02	1.256666667
XM_021074229.1	CABIN1	0	0	0.04666667	15.91666667
XM_021074235.1	CABIN1	0	0.266666667	0.08666667	26.54666667
XM_021074258.1	ZNF518A	0	0	0.02333333	7.773333333
XM_021074267.1	ADAMTSL1	0	0	0.01333333	5.206666667
XM_021074275.1	RIMBP2	0	0	0.10333333	18.04333333
XM_021074297.1	TLL2	0	0	0.01	3.1
XM_021074307.1	LOC100627422	0	0	0.01	8.666666667
XM_021074318.1	ECD	0	0	0.1	10.75666667
XM_021074332.1	NUDT13	0	0	0.34666667	32.35
XM_021074336.1	NUDT13	0	0	0.09	13.04666667
XM_021074338.1	NUDT13	0	0	0.03	3.446666667
XM_021074354.1	HVCN1	0	0	0.01666667	2.346666667
XM_021074362.1	HVCN1	0	0	0.03	3.9
XM_021074394.1	UBTD1	0	0	0.02333333	1.723333333
XM_021074414.1	CATSPER2	0	0	0.05666667	4.556666667
XM_021074421.1	ZFYVE27	0	0	0.01333333	5.61
XM_021074431.1	GOLGA7B	0	0	0.02333333	3.253333333
XM_021074464.1	BIN1	0	0	0.02	2.16
XM_021074471.1	BIN1	0	0	0.01333333	1.28
XM_021074513.1	ASTN2	0	0	0.00333333	0.5
XM_021074537.1	LRP2BP	0	0	0.01	0.726666667
XM_021074571.1	ERLIN2	0	0	0.02666667	6.506666667
XM_021074585.1	GPR1	0	0	0.03666667	3.213333333
XM_021074587.1	GPR1	0	0	0.01666667	2.206666667
XM_021074621.1	DLGAP2	0	0	0.00666667	1.846666667
XM_021074630.1	PDCD1	0	0	0.02666667	2.333333333
XM_021074637.1	CLN8	0	0	0.04333333	3.366666667
XM_021074638.1	AKIRIN2	0	0	0.00333333	0.186666667
XM_021074645.1	TMEM237	0	0	0.06666667	5.62
XM_021074680.1	CALCRL	0	0	0.01666667	6.356666667
XM_021074693.1	INHA	0	0	0.05	2.666666667
XM_021074698.1	LOC404703	0	0	0.16333333	4
XM_021074729.1	HELT	0	0	0.00666667	0.333333333
XM_021074797.1	TRAPPC11	0	0	0.05	9.88
XM_021074798.1	DOCK10	0	0	0.00333333	1.04
XM_021074801.1	DOCK10	0	0.023333333	0.03	20.41
XM_021074824.1	ARMC9	0	0	0.03	4.58
XM_021074846.1	GTDC1	0	0	0.00666667	3.963333333
XM_021074878.1	SGPP2	0	0	0.00333333	1
XM_021074884.1	SPHKAP	0	0.333333333	0.00666667	2.323333333
XM_021074885.1	SPHKAP	0	0	0.00333333	0.666666667
XM_021074894.1	UBE2F	0	0	0.03666667	2.176666667
XM_021074909.1	CCDC150	0	0	0.02666667	4.36
XM_021074917.1	CCDC150	0	0	0.03333333	5.24
XM_021074918.1	CCDC150	0	0	0.04333333	6.926666667
XM_021074919.1	CCDC150	0	0	0.01666667	2.483333333
XM_021074933.1	SCN3A	0	0.333333333	0.02	6.5
XM_021074935.1	SCN3A	0	0.333333333	0.00666667	2.166666667
XM_021074945.1	IMP4	0	0	0.15	11.95666667
XM_021074948.1	NIF3L1	0	0	0.13	12.14333333
XM_021074986.1	MAP2	0	0	0.00666667	1.706666667
XM_021074989.1	MAP2	0	0	0.00666667	3.17
XM_021075006.1	MAP2	0	0.486666667	0.01666667	8.356666667
XM_021075072.1	TRIP12	0	0	0.00333333	0.8
XM_021075090.1	TRIP12	0	0	0.03666667	16.70333333
XM_021075110.1	NBEAL1	0	0	0.03	14

XM_021075138.1	HNRNPA3	0	0	0.04	9.893333333
XM_021075178.1	CLASP1	0	0	0.02	6.86
XM_021075208.1	R3HDM1	0	0	0.02666667	8.26
XM_021075241.1	R3HDM1	0	0	0.03333333	6.75
XM_021075246.1	R3HDM1	0	0	0.05	9.673333333
XM_021075253.1	ZRANB3	0	0	0.05	10.23666667
XM_021075266.1	HDLBP	0	0	0.40333333	81.6
XM_021075274.1	RHBDD1	0	0	0.08333333	16.72333333
XM_021075277.1	RHBDD1	0	0	0.17666667	10.01666667
XM_021075298.1	SLC38A11	0	0	0.01333333	1.333333333
XM_021075303.1	LOC110257005	0	0	0.03666667	1.333333333
XM_021075310.1	CREB1	0	0	0.00666667	2.61
XM_021075321.1	SESTD1	0	0	0.03	13.08666667
XM_021075329.1	ABI2	0	0	0.01	2.45
XM_021075372.1	GULP1	0	0	0.05666667	8.606666667
XM_021075374.1	GULP1	0	0	0.06	8.686666667
XM_021075377.1	GULP1	0	0	0.44666667	63.55
XM_021075400.1	PMS1	0	0	0.03666667	4.483333333
XM_021075427.1	GRB14	0	0	0.00666667	0.633333333
XM_021075429.1	B3GALT1	0	0	0.00333333	1.01
XM_021075430.1	B3GALT1	0	0	0.03	7.61
XM_021075435.1	CFC1B	0	0	0.24666667	8
XM_021075455.1	RBM44	0	0	0.01666667	3.39
XM_021075464.1	OTOS	0	0	0.03666667	1.113333333
XM_021075465.1	OTOS	0	0	0.01333333	0.333333333
XM_021075468.1	LOC100155087	0	0	0.00333333	0.333333333
XM_021075470.1	CERS6	0	0	0.03	9.806666667
XM_021075483.1	LRRFIP1	0	0	0.00666667	1.33
XM_021075517.1	GPR148	0	0	0.00666667	1.666666667
XM_021075546.1	CARF	0	0	0.02666667	4.303333333
XM_021075570.1	SORBS2	0	0	0.00333333	0.8
XM_021075591.1	PDE1A	0	0	0.04	3.233333333
XM_021075596.1	PDE1A	0	0.166666667	0.00666667	1.746666667
XM_021075600.1	LOC100521754	0	0	0.00666667	0.333333333
XM_021075640.1	ZNF385B	0	0	0.00666667	1.046666667
XM_021075666.1	LOC100517129	0	0	0.01666667	1.513333333
XM_021075685.1	TACC1	0	0	0.02666667	2.683333333
XM_021075701.1	STRADB	0	0	0.35666667	32.09666667
XM_021075703.1	C2CD6	0	0.333333333	0.01333333	4.056666667
XM_021075706.1	CROCC2	0	0	0.00666667	3
XM_021075709.1	PASK	0	0	0.00666667	1.033333333
XM_021075715.1	MAB21L4	0	0	0.00666667	0.666666667
XM_021075716.1	MARS2	0	0	0.01666667	9.556666667
XM_021075720.1	MARS2	0	0	0.01333333	7.126666667
XM_021075722.1	MARS2	0	0	0.00666667	1.293333333
XM_021075729.1	PLCL1	0	0	0.04333333	9.92
XM_021075744.1	ARHGEF4	0	0	0.00666667	1.546666667
XM_021075779.1	GPD2	0	0	0.46	116.9966667
XM_021075782.1	WRN	0	0	0.01333333	4.526666667
XM_021075787.1	WRN	0	0	0.00333333	1.583333333
XM_021075836.1	KYNU	0	0	0.00666667	2.096666667
XM_021075846.1	UBE3A	0	0	0.16	22.62333333
XM_021075857.1	PGAP1	0	0	0.01	4.146666667
XM_021075864.1	AGFG1	0	0	0.02333333	8.483333333
XM_021075878.1	WDR17	0	0	0.02666667	5.65
XM_021075910.1	NSD3	0	0.256666667	0.01666667	8.646666667
XM_021075918.1	LETM2	0	0	0.00333333	0.19
XM_021075929.1	ZNF804A	0	0	0.00333333	0.666666667
XM_021075931.1	LOC100522040	0	0	0.00666667	0.553333333
XM_021075948.1	GPR35	0	0	0.04	6.383333333

XM_021075949.1	GPR35	0	0	0.04666667	7.61
XM_021075951.1	GPR35	0	0	0.01	1.676666667
XM_021075953.1	GPR35	0	0	0.04666667	8.08
XM_021075959.1	MYO1B	0	0	0.05666667	12.71333333
XM_021075972.1	IKZF2	0	0	0.01	4.126666667
XM_021075983.1	IKZF2	0	0	0.01666667	6.293333333
XM_021076000.1	IKZF2	0	0	0.02666667	10.61333333
XM_021076008.1	IKZF2	0	0	0.00666667	3.073333333
XM_021076035.1	NEIL3	0	0	0.01	1
XM_021076063.1	NEMP2	0	0	0.03666667	6.253333333
XM_021076073.1	SATB2	0	0	0.00333333	0.36
XM_021076089.1	SPAG16	0	0	0.00333333	0.333333333
XM_021076096.1	VWC2L	0	0	0.00333333	0.333333333
XM_021076103.1	MBD5	0	0	0.00333333	1.813333333
XM_021076105.1	MBD5	0	0	0.00666667	4.223333333
XM_021076108.1	MBD5	0	0	0.00333333	2.27
XM_021076119.1	ORC4	0	0	0.01666667	2.686666667
XM_021076138.1	ITPRID2	0	0	0.14333333	30.11333333
XM_021076157.1	LIMS2	0	0	0.38666667	27.46
XM_021076169.1	SCN1A	0	0	0.03666667	12.92
XM_021076172.1	SCN1A	0	0	0.01333333	4.693333333
XM_021076176.1	SCN9A	0	0	0.00666667	1.83
XM_021076187.1	CDK15	0	0	0.04333333	3.97
XM_021076191.1	CDK15	0	0	0.01333333	1.633333333
XM_021076192.1	CDK15	0	0	0.00666667	1.013333333
XM_021076194.1	CDK15	0	0	0.01666667	1.726666667
XM_021076197.1	PNKD	0	0	0.04333333	6.216666667
XM_021076233.1	ANKRD44	0	0	0.01666667	5.533333333
XM_021076257.1	NYAP2	0	0.666666667	0.00333333	0.596666667
XM_021076261.1	C15H2orf88	0	0	0.01	2.05
XM_021076279.1	ASB1	0	0.016666667	0.00666667	1.933333333
XM_021076280.1	ASB1	0	0.006666667	0.01	3.466666667
XM_021076283.1	ASB1	0	0	0.07666667	5.606666667
XM_021076290.1	GALNT13	0	0	0.00333333	0.333333333
XM_021076323.1	SAP130	0	0	0.10333333	16.88666667
XM_021076326.1	SAP130	0	0	0.05666667	9.44
XM_021076330.1	SAP130	0	0	0.02	3.503333333
XM_021076362.1	TENM3	0	0	0.00666667	2.916666667
XM_021076364.1	TENM3	0	0	0.00666667	3.64
XM_021076367.1	TENM3	0	0	0.01	5.26
XM_021076379.1	LOC100525433	0	0	0.00333333	0.29
XM_021076389.1	LOC100525433	0	0	0.20333333	78.37333333
XM_021076402.1	NRG1	0	0	0.01	2.45
XM_021076415.1	DTYMK	0	0	0.07333333	1.753333333
XM_021076416.1	PDE11A	0	0	0.00666667	1
XM_021076433.1	PKP4	0	0	0.02333333	5.093333333
XM_021076457.1	ACVR1	0	0	0.03333333	5.673333333
XM_021076460.1	CCDC148	0	0	0.01	1.333333333
XM_021076494.1	POLR2D	0	0	0.12	2.516666667
XM_021076498.1	MYO3B	0	0.086666667	0.01333333	3.303333333
XM_021076499.1	MYO3B	0	0.246666667	0.01666667	4.696666667
XM_021076516.1	IL6ST	0	0	0.08666667	29.10333333
XM_021076539.1	TRIP13	0	0	0.02	1.97
XM_021076583.1	KCNIP1	0	0	0.02	2
XM_021076587.1	KCNIP1	0	0	0.00333333	0.333333333
XM_021076625.1	MTRR	0	0	0.21	33.69333333
XM_021076630.1	CDH10	0	0	0.00333333	0.333333333
XM_021076648.1	FYB1	0	0	0.04	6.783333333
XM_021076649.1	DAB2	0	0	0.09	19.74333333
XM_021076654.1	KEAP1	0	0	0.04333333	5.376666667

XM_021076668.1	CEP72	0	0	0.00333333	0.603333333
XM_021076669.1	CEP72	0	0	0.00666667	0.73
XM_021076674.1	ZNF131	0	0	0.03	3.786666667
XM_021076682.1	RANBP17	0	0	0.01333333	1.866666667
XM_021076692.1	CYFIP2	0	0	0.01666667	3.02
XM_021076696.1	CYFIP2	0	0	0.02	3.733333333
XM_021076700.1	ZNF622	0	0	0.77333333	65.84
XM_021076706.1	MRPL22	0	0	0.23	5.76
XM_021076720.1	ZNF366	0	0	0.07	18.68666667
XM_021076733.1	S1PR5	0	0	0.05	5.436666667
XM_021076734.1	LOC106508653	0	0	0.01333333	0.666666667
XM_021076738.1	ANKRD55	0	0	0.01	1.193333333
XM_021076739.1	ANKRD55	0	0	0.00333333	0.53
XM_021076740.1	ANKRD55	0	0	0.01	0.89
XM_021076744.1	ANKRD55	0	0	0.00333333	0.476666667
XM_021076745.1	S1PR5	0	0	0.06666667	7.496666667
XM_021076747.1	S1PR5	0	0	0.08	9.833333333
XM_021076760.1	MAST4	0	0.183333333	0.01333333	16.18
XM_021076768.1	NSG2	0	0	0.00333333	0.333333333
XM_021076780.1	TRIM23	0	0	0.01333333	2.323333333
XM_021076781.1	TRIM23	0	0	0.03	5.043333333
XM_021076814.1	SPDL1	0	0	0.01666667	1.67
XM_021076815.1	SPDL1	0	0	0.02666667	2.333333333
XM_021076839.1	SLC6A3	0	0	0.00333333	0.333333333
XM_021076862.1	LOC100517502	0	0	0.04	0.8
XM_021076868.1	PDE4D	0	0	0.02	5.376666667
XM_021076896.1	CREBRF	0	0	0.22666667	82.77666667
XM_021076917.1	LOC110255245	0	0	0.01	1
XM_021076919.1	LOC110255245	0	0	0.00666667	0.666666667
XM_021076921.1	ADRA1B	0	0	0.01333333	1.863333333
XM_021076935.1	PDCD6	0	0	0.15333333	7.466666667
XM_021076952.1	MRPS30	0	0	0.14666667	36.56666667
XM_021076965.1	CDH18	0	0	0.01666667	2.863333333
XM_021076971.1	CDH18	0	0	0.00333333	0.713333333
XM_021076981.1	GPBP1	0	0.01	0.94333333	137.17
XM_021077031.1	SREK1	0	0	0.01666667	9.54
XM_021077033.1	SREK1	0	0	0.00666667	2.09
XM_021077038.1	CDH12	0	0	0.00333333	0.666666667
XM_021077058.1	TMEM173	0	0	0.19666667	15.10666667
XM_021077062.1	SLC9A3	0	0	0.00666667	1.666666667
XM_021077076.1	SLC36A3	0	0	0.00666667	0.666666667
XM_021077136.1	SLC12A7	0	0	0.00666667	1.62
XM_021077171.1	IKBKB	0	0	0.01333333	2.366666667
XM_021077178.1	BCAS1	0	0	0.01	1.473333333
XM_021077182.1	BCAS1	0	0	0.02	3.09
XM_021077189.1	BCAS1	0	0	0.02666667	3.776666667
XM_021077199.1	SLC22A4	0	0	0.01333333	1.333333333
XM_021077238.1	SGCZ	0	0.666666667	0.01333333	6.09
XM_021077239.1	SGCZ	0	0.333333333	0.00666667	3.386666667
XM_021077240.1	SGCZ	0	0	0.03	11.8
XM_021077255.1	DNMT3B	0	0	0.02666667	5.06
XM_021077257.1	CDC25B	0	0	0.04333333	6.883333333
XM_021077259.1	CDC25B	0	0	0.15333333	21.53333333
XM_021077263.1	MSR1	0	0	0.13666667	15.01666667
XM_021077300.1	BCL2L1	0	0	0.03333333	3.516666667
XM_021077310.1	PKIG	0	0	0.06333333	3.106666667
XM_021077313.1	ASIP	0	0	0.02	0.666666667
XM_021077355.1	NDRG3	0	0	0.01666667	1.973333333
XM_021077383.1	C17H20orf27	0	0	0.09333333	6.353333333
XM_021077394.1	EYA2	0	0	0.01666667	2.023333333

XM_021077396.1	EYA2	0	0	0.03333333	3.523333333
XM_021077421.1	ZDHHC2	0	0	0.15	25.16666667
XM_021077436.1	COMMD7	0	0	0.10666667	8.576666667
XM_021077443.1	CRHBP	0	0	0.00333333	0.666666667
XM_021077461.1	PLCB4	0	0	0.05666667	10.86666667
XM_021077476.1	TRMT9B	0	0	0.00333333	1
XM_021077507.1	STAU1	0	0	0.12	15.5
XM_021077508.1	STAU1	0	0.12	0.08333333	12.30333333
XM_021077519.1	LOC100524118	0	0	0.00333333	0.333333333
XM_021077522.1	LOC102161654	0	0	0.01	0.76
XM_021077550.1	GGT7	0	0	0.11	14.06
XM_021077565.1	SLC4A11	0	0	0.01	1.343333333
XM_021077570.1	SLC4A11	0	0	0.01333333	2.31
XM_021077575.1	SLC4A11	0	0	0.02	4.986666667
XM_021077580.1	BIRC7	0	0	0.03	2
XM_021077606.1	TPD52L2	0	0	0.02666667	2.416666667
XM_021077621.1	ZNF512B	0	0	0.06	17.41
XM_021077629.1	UCKL1	0	0	0.11333333	8.336666667
XM_021077644.1	TCEA2	0	0	0.36333333	17.69333333
XM_021077646.1	RGS19	0	0	0.07333333	4.506666667
XM_021077649.1	RGS19	0	0	0.04	2.443333333
XM_021077655.1	RGS19	0	0	0.01666667	1.263333333
XM_021077659.1	RGS19	0	0	0.06	3.78
XM_021077665.1	RGS19	0	0	0.05666667	3.03
XM_021077709.1	AP5S1	0	0	0.03	2.646666667
XM_021077717.1	DLC1	0	0	0.06333333	16.98666667
XM_021077720.1	DLC1	0	0	0.02666667	7.47
XM_021077729.1	DLC1	0	0	0.12333333	11.91333333
XM_021077750.1	EBF4	0	0	0.01	1.456666667
XM_021077773.1	KCNQ2	0	0	0.00333333	1.52
XM_021077784.1	LOC110257405	0	0	0.01	0.34
XM_021077808.1	RALY	0	0	0.17	9.196666667
XM_021077842.1	LOC110257420	0	0	0.09333333	2.64
XM_021077849.1	HOOK3	0	0	0.08	47.52666667
XM_021077868.1	CHD6	0	0	0.00666667	2.91
XM_021077869.1	CHD6	0	0	0.10333333	49.53666667
XM_021077873.1	DLGAP4	0	0	0.00666667	1.663333333
XM_021077875.1	DLGAP4	0	0	0.00333333	0.846666667
XM_021077880.1	DLGAP4	0	0	2.66	346.55
XM_021077889.1	SLA2	0	0	0.02666667	5.743333333
XM_021077892.1	SLA2	0	0.003333333	0.02	3.45
XM_021077903.1	PCM1	0	0	0.07	22.98666667
XM_021077905.1	PCM1	0	0.003333333	0.04	15.19333333
XM_021077984.1	WFDC11	0	0	0.00333333	0.166666667
XM_021077986.1	WFDC11	0	0	0.00333333	0.166666667
XM_021077994.1	NKX2-2	0	0	0.00333333	0.333333333
XM_021078000.1	SCP2D1	0	0	0.04666667	3.816666667
XM_021078009.1	EPB41L1	0	0	0.02333333	6.573333333
XM_021078043.1	KIF16B	0	0	0.00666667	3.603333333
XM_021078048.1	RAB24	0	0	0.18666667	8.556666667
XM_021078058.1	PANK2	0	0	0.03	5.996666667
XM_021078060.1	PANK2	0	0	0.03	9.073333333
XM_021078093.1	TPX2	0	0.023333333	0.08	9.326666667
XM_021078097.1	TPX2	0	0	0.16666667	23.04666667
XM_021078116.1	PTPRT	0	0	0.01	3.71
XM_021078140.1	RPRD1B	0	0	0.01	0.7
XM_021078167.1	SGK2	0	0	0.00666667	0.63
XM_021078171.1	SGK2	0	0	0.00666667	0.436666667
XM_021078177.1	SGK2	0	0	0.07333333	5.856666667
XM_021078229.1	TCFL5	0	0.12	0.03666667	4.36

XM_021078249.1	LOC100155289	0	0	0.00333333	0.666666667
XM_021078256.1	CEP250	0	0	0.00666667	5.076666667
XM_021078265.1	RBM12	0	0	0.16333333	61.45333333
XM_021078266.1	RBM12	0	0	0.03333333	10.94333333
XM_021078295.1	NCOA6	0	0.003333333	0.42	136.1366667
XM_021078298.1	NCOA6	0	0.096666667	0.07333333	23.75666667
XM_021078302.1	ELMO2	0	0	0.18	31.02333333
XM_021078304.1	ELMO2	0	0	0.02333333	3.153333333
XM_021078311.1	NINL	0	0	0.05666667	8.666666667
XM_021078324.1	TUSC3	0	0	0.02	3.14
XM_021078340.1	LOC110257498	0	0	0.00666667	0.333333333
XM_021078341.1	LOC110257498	0	0	0.00666667	0.463333333
XM_021078355.1	FASTKD5	0	0	0.07666667	9.613333333
XM_021078371.1	RTEL1	0	0	0.01333333	2.186666667
XM_021078375.1	H2AFY	0	0	1.21666667	100.89
XM_021078401.1	PPP4R1L	0	0	0.01	3.806666667
XM_021078402.1	PPP4R1L	0	0	0.00333333	1.72
XM_021078405.1	PPP4R1L	0	0	0.00666667	2.3
XM_021078414.1	ST7	0	0	0.00666667	2.876666667
XM_021078416.1	ST7	0	0	0.04666667	7.946666667
XM_021078424.1	FOXP2	0	0	0.00333333	0.573333333
XM_021078426.1	FOXP2	0	0	0.00666667	0.913333333
XM_021078431.1	FOXP2	0	0	0.01	1.27
XM_021078444.1	PGAM2	0	0	0.01666667	2.32
XM_021078453.1	AGK	0	0	0.05333333	5.35
XM_021078460.1	LOC100302368	0	1.023333333	0.00666667	2.53
XM_021078461.1	LOC100302368	0	0.53	0.02666667	9.236666667
XM_021078469.1	ADCYAP1R1	0	0	0.01666667	4.666666667
XM_021078490.1	HOXA10	0	0	0.00666667	0.666666667
XM_021078495.1	SLC37A3	0	0	0.01	1.463333333
XM_021078499.1	LEP	0	0	0.00666667	0.333333333
XM_021078517.1	CHRM2	0	0	0.01	2.333333333
XM_021078540.1	ANLN	0	0	0.00333333	1.146666667
XM_021078541.1	GRK2	0	0	0.05666667	9.653333333
XM_021078542.1	ANLN	0	0	0.05	11.01333333
XM_021078578.1	CPVL	0	0	0.00333333	0.333333333
XM_021078589.1	DENND2A	0	0	0.09	24.20666667
XM_021078612.1	PRKAG2	0	0	0.24666667	40.64
XM_021078622.1	GALNTL5	0	0	0.00333333	0.41
XM_021078624.1	GALNTL5	0	0	0.00666667	4.516666667
XM_021078625.1	GALNTL5	0	0	0.00333333	0.483333333
XM_021078626.1	GALNTL5	0	0	0.01333333	8.153333333
XM_021078644.1	CAMK2B	0	0	0.02666667	2.16
XM_021078646.1	CAMK2B	0	0	0.06	5.41
XM_021078664.1	ZMIZ2	0	0	0.05333333	16.05333333
XM_021078665.1	ZMIZ2	0	0	0.02	7.04
XM_021078672.1	ZMIZ2	0	0	0.02	4.47
XM_021078673.1	ZMIZ2	0	0	0.11	26.32333333
XM_021078712.1	KRBA1	0	0	0.02333333	5.99
XM_021078715.1	KRBA1	0	0	0.01333333	3.343333333
XM_021078728.1	ZC3HC1	0	0	0.1	7.483333333
XM_021078736.1	CPA2	0	0	0.00666667	0.333333333
XM_021078739.1	LOC110257524	0	0	0.01666667	0.333333333
XM_021078741.1	HNRNPM	0	0	0.13	13.69
XM_021078750.1	TMEM176A	0	0	0.01	0.333333333
XM_021078764.1	KCNH2	0	0	0.10333333	14.22
XM_021078802.1	HYAL4	0	0.056666667	0.06333333	3.883333333
XM_021078807.1	LOC106506856	0	0	0.00666667	0.666666667
XM_021078808.1	LOC110257542	0	0	0.11	9.603333333
XM_021078836.1	LOC106506836	0	0	0.00666667	1

XM_021078846.1	LOC102162486	0	0	0.09666667	35.40666667
XM_021078866.1	AGAP3	0	0	0.02	2.67
XM_021078867.1	AGAP3	0	0	0.16	19.19666667
XM_021078874.1	EPHB6	0	0	0.00333333	1.093333333
XM_021078882.1	EPHB6	0	0	0.01666667	4.35
XM_021078887.1	EPHB6	0	0	0.00666667	1.67
XM_021078891.1	EPHB6	0	0	0.00333333	0.68
XM_021078913.1	GIMAP4	0	0	0.11666667	12.15333333
XM_021078915.1	GIMAP7	0	0	0.02	1.333333333
XM_021078921.1	GIMAP8	0	0	0.05333333	10.23333333
XM_021078926.1	LOC100519808	0	0	0.00333333	0.936666667
XM_021078940.1	PLXNA4	0	0	0.01666667	9.26
XM_021078942.1	LOC110257573	0	0	0.00666667	1.333333333
XM_021078953.1	KCP	0	0	0.02666667	7.513333333
XM_021078956.1	LOC110257580	0	0	0.02666667	4.553333333
XM_021078964.1	CADPS2	0	0	0.00666667	1.156666667
XM_021078976.1	CADPS2	0	0	0.01666667	3.533333333
XM_021078977.1	NFE2L3	0	0	0.01666667	0.71
XM_021078989.1	CPED1	0	0	0.02666667	6.216666667
XM_021079013.1	DGKI	0	0	0.00333333	1.666666667
XM_021079022.1	LOC110257586	0	0	0.00666667	0.333333333
XM_021079028.1	ESYT2	0	0	0.02333333	5.106666667
XM_021079038.1	HOXA3	0	0	0.00666667	1.72
XM_021079042.1	HOXA3	0	0	0.02	3.753333333
XM_021079063.1	OGDH	0	0.003333333	0.58	99.61333333
XM_021079089.1	IMMP2L	0	0	0.05	1.213333333
XM_021079115.1	KLHDC10	0	0	0.06	17.78333333
XM_021079134.1	KMT2C	0	0	0.08	55.78
XM_021079149.1	HECW1	0	0	0.04	17.68666667
XM_021079192.1	LOC100511166	0	0	0.00333333	0.333333333
XM_021079204.1	ITPRID1	0	0	0.00333333	0.333333333
XM_021079207.1	PDE1C	0	0	0.03666667	7.813333333
XM_021079209.1	TICAM2	0	0	0.09333333	10.54666667
XM_021079211.1	PDE1C	0	0	0.04	8.123333333
XM_021079250.1	MPP6	0	0	0.09333333	20.93333333
XM_021079310.1	DPY19L1	0	0	0.00333333	2.193333333
XM_021079315.1	DPY19L1	0	0	0.02666667	16.82666667
XM_021079320.1	DPY19L1	0	0	0.01	7.29
XM_021079350.1	MRPS24	0	0	0.09	2.523333333
XM_021079379.1	ZC4H2	0	0	0.08	2.056666667
XM_021079397.1	ASB11	0	0	0.09666667	6.263333333
XM_021079409.1	IL13RA2	0	0	0.01666667	1.396666667
XM_021079431.1	PNPLA4	0	0	0.01	0.516666667
XM_021079446.1	MBNL3	0	0	0.02	9.35
XM_021079450.1	MBNL3	0	0	0.00333333	1.9
XM_021079454.1	MBNL3	0	0.006666667	0.00333333	0.273333333
XM_021079466.1	GEMIN8	0	0	0.07333333	3.676666667
XM_021079467.1	GEMIN8	0	0	0.07	3.353333333
XM_021079469.1	LHFPL2	0	0.02	0.09666667	23.34333333
XM_021079476.1	LHFPL2	0	0	0.04333333	12.86333333
XM_021079479.1	LHFPL2	0	0	0.04333333	10.62666667
XM_021079481.1	SLC38A5	0	0	0.03	2.756666667
XM_021079493.1	AMELX	0	0	0.00666667	0.816666667
XM_021079509.1	TLR8	0	0	0.02	6.086666667
XM_021079531.1	PDZD11	0	0	0.03333333	1.26
XM_021079538.1	FOXP3	0	0	0.00666667	1.666666667
XM_021079541.1	ACSL4	0	0	0.15666667	35.55
XM_021079552.1	TNFAIP8	0	0	0.01333333	1.223333333
XM_021079561.1	DMD	0	0	0.01666667	3.14
XM_021079564.1	DMD	0	0	0.01	1.98

XM_021079566.1	ITM2A	0	0	0.02	1.516666667
XM_021079568.1	CITED1	0	0	0.03	1.636666667
XM_021079603.1	LOC106506945	0	0	0.00333333	1.016666667
XM_021079614.1	MAMLD1	0	0	0.01	1.596666667
XM_021079618.1	STARD8	0	0	0.32333333	66.18
XM_021079635.1	DHFR	0	0	0.01	0.736666667
XM_021079637.1	LOC100515119	0	0	0.01666667	2.033333333
XM_021079646.1	SHROOM2	0	0	0.03	11.11666667
XM_021079649.1	ZNF280C	0	0	0.06	10.23666667
XM_021079657.1	SYTL5	0	0	0.01666667	6.41
XM_021079691.1	IQSEC2	0	0	0.03666667	7.856666667
XM_021079709.1	MUM1L1	0	0	0.02333333	7.6
XM_021079712.1	MUM1L1	0	0	0.01	2.563333333
XM_021079715.1	MUM1L1	0	0	0.01666667	3.966666667
XM_021079722.1	MUM1L1	0	0	0.01666667	3.416666667
XM_021079747.1	NLRP12L	0	0	0.04666667	9.86
XM_021079761.1	GYG2	0	0	0.03	4.536666667
XM_021079767.1	GYG2	0	0	0.01333333	1.863333333
XM_021079768.1	GYG2	0	0	0.14333333	22.66666667
XM_021079771.1	SLITRK4	0	0	0.01333333	4.18
XM_021079788.1	MORC4	0	0	0.05	8.23
XM_021079792.1	CYP4F55	0	0	0.19333333	17.79
XM_021079812.1	ARMCX4	0	0	0.04333333	17.79333333
XM_021079818.1	ARMCX4	0	0	0.03333333	12
XM_021079819.1	ARMCX4	0	0	0.01	4.506666667
XM_021079823.1	ARMCX1	0	0	0.02333333	2.52
XM_021079825.1	ARMCX1	0	0	0.04	3.796666667
XM_021079829.1	ARMCX1	0	0	0.01	1.026666667
XM_021079843.1	DGAT2L6	0	0	0.00333333	0.333333333
XM_021079872.1	ADGRG4	0	0	0.00333333	0.333333333
XM_021079887.1	LOC110257705	0	0	0.00666667	0.333333333
XM_021079901.1	NRK	0	0	0.34333333	122.45
XM_021079903.1	NRK	0	0	0.15	51.91666667
XM_021079905.1	NRK	0	0	0.08	30.15666667
XM_021079906.1	NRK	0	0	0.11	38.26333333
XM_021079907.1	NRK	0	0	0.02666667	10.23
XM_021079913.1	ZNF711	0	0	0.00666667	1.82
XM_021079923.1	PAMR1	0	0	0.03666667	4.676666667
XM_021079925.1	GPRASP1	0	0	0.01	2.996666667
XM_021079927.1	GPRASP1	0	0	0.04666667	11.96666667
XM_021079928.1	GPRASP1	0	0	0.01	2.703333333
XM_021079930.1	GPRASP1	0	0	0.00333333	0.76
XM_021079933.1	GPRASP2	0	0	0.03333333	5.503333333
XM_021079935.1	GPRASP2	0	0	0.02333333	3.223333333
XM_021079960.1	ZNF185	0	0	0.02	4.27
XM_021079984.1	LOC110255258	0	0	0.00333333	0.28
XM_021079992.1	SPIN2B	0	0	0.08666667	3.673333333
XM_021079995.1	STX5	0	0	0.10666667	7.493333333
XM_021080007.1	GPKOW	0	0	0.11333333	10.43666667
XM_021080009.1	GPKOW	0	0	0.12666667	10.94333333
XM_021080031.1	POF1B	0	0	0.00333333	0.333333333
XM_021080033.1	SLC25A14	0	0	0.05333333	3.676666667
XM_021080042.1	FAM155B	0	0	0.03666667	6.84
XM_021080063.1	WAS	0	0	0.03333333	3.346666667
XM_021080075.1	LOC106506966	0	0	0.01333333	0.966666667
XM_021080081.1	ATRX	0	0.02	0.29666667	149.94
XM_021080086.1	ATRX	0	0	0.16333333	79.90666667
XM_021080098.1	NUP62CL	0	0	0.01333333	2.31
XM_021080122.1	HMGB3	0	0	0.02333333	3.62
XM_021080130.1	ALDH3B1	0	0	0.03	2.833333333

XM_021080131.1	LOC100520992	0	0	0.02666667	2.093333333
XM_021080132.1	LOC100520992	0	0	0.03	2.283333333
XM_021080138.1	CA5B	0	0	0.04666667	7.453333333
XM_021080143.1	LOC100621626	0	0	0.04333333	1.323333333
XM_021080148.1	USP9X	0	0	0.01	3.746666667
XM_021080167.1	ENOX2	0	0	0.01666667	3.06
XM_021080170.1	ENOX2	0	0.243333333	0.29	56.24333333
XM_021080175.1	ARHGAP36	0	0	0.03666667	4.146666667
XM_021080177.1	ARHGAP36	0	0	0.01666667	1.903333333
XM_021080204.1	CALCB	0	0	0.00666667	0.496666667
XM_021080210.1	IL1RAPL1	0	0	0.00333333	0.69
XM_021080214.1	LPAR4	0	0	0.01	1.313333333
XM_021080252.1	6-Sep	0	0	0.09	13.80666667
XM_021080263.1	LOC100522230	0	0	0.02666667	1.333333333
XM_021080264.1	LOC100522411	0	0	0.02	1.333333333
XM_021080265.1	LOC100623332	0	0	0.00666667	0.363333333
XM_021080275.1	MAGEE2	0	0	0.00333333	0.666666667
XM_021080295.1	CNKSR2	0	0	0.00666667	1.23
XM_021080312.1	FAM122B	0	0	0.00666667	1.426666667
XM_021080313.1	FAM122B	0	0	0.02333333	3.976666667
XM_021080314.1	FAM122B	0	0	0.03333333	5.993333333
XM_021080320.1	FAM122B	0	0	0.03333333	5.533333333
XM_021080325.1	FAM122B	0	0	0.01666667	2.13
XM_021080336.1	PLAC1	0	0	0.02	3.59
XM_021080342.1	ZCCHC13	0	0	0.01333333	0.666666667
XM_021080375.1	RBM10	0	0	0.02333333	2.933333333
XM_021080382.1	JADE3	0	0	0.03333333	4.683333333
XM_021080386.1	ZNF630	0	0	0.01666667	2.333333333
XM_021080391.1	ZNF81	0	0	0.13333333	84.27
XM_021080397.1	ZNF182	0	0	0.06	7.96
XM_021080399.1	CDK16	0	0	0.01333333	3.976666667
XM_021080411.1	PCDH11X	0	0	0.00666667	2.416666667
XM_021080428.1	KLHL13	0	0	0.06333333	6.126666667
XM_021080454.1	SLC16A2	0	0	0.00333333	0.453333333
XM_021080468.1	THOC2	0	0.003333333	0.04	14.89333333
XM_021080471.1	AFF2	0	0	0.00333333	0.946666667
XM_021080481.1	DIAPH2	0	0	0.02333333	9.576666667
XM_021080495.1	LOC100511059	0	0.333333333	0.00333333	1.333333333
XM_021080499.1	FRMD7	0	0	0.00333333	0.666666667
XM_021080510.1	CD99L2	0	0	0.07	8.55
XM_021080523.1	LOC110257816	0	0	0.01333333	1.333333333
XM_021080536.1	PDK3	0	0	0.02666667	16.66666667
XM_021080542.1	LOC100154405	0	0	0.00333333	1
XM_021080569.1	LOC100524727	0	0	0.01333333	6.593333333
XM_021080582.1	IGF2	0	0	0.21666667	37.98666667
XM_021080588.1	RAI2	0	0	0.02	2.033333333
XM_021080602.1	SHROOM4	0	0	0.01	2.393333333
XM_021080604.1	LOC110257838	0	0	0.00666667	0.333333333
XM_021080613.1	BEND2	0	0	0.00666667	1
XM_021080615.1	LOC110257841	0	0	0.00666667	0.166666667
XM_021080628.1	FRMPD4	0	0	0.01666667	14.98333333
XM_021080638.1	CHM	0	0	0.02	4.79
XM_021080641.1	IGF2	0	0	1.68333333	290.0066667
XM_021080642.1	TMSB15B	0	0	0.08333333	2
XM_021080657.1	ZFX	0	0	0.03	8.193333333
XM_021080671.1	TENM1	0	0	0.07	40
XM_021080674.1	GANAB	0	0	0.43	74.14333333
XM_021080687.1	PAK3	0	0	0.03	3.92
XM_021080695.1	PHKA1	0	0	0.22	61.9
XM_021080718.1	ADGRE5	0	0	0.09333333	13.77333333

XM_021080720.1	MTMR8	0	0	0.03333333	11.72
XM_021080739.1	FLNA	0	0	0.56	171.6633333
XM_021080752.1	FAM50A	0	0	0.03666667	2.2
XM_021080766.1	ZDHHC9	0	0	0.26	75.72666667
XM_021080773.1	ARMCX2	0	0	0.04333333	4.983333333
XM_021080795.1	RPGR	0	0	0.00666667	0.926666667
XM_021080798.1	RPGR	0	0	0.08666667	16.48333333
XM_021080836.1	MTM1	0	0	0.02	1.953333333
XM_021080858.1	GPR174	0	0	0.01666667	5
XM_021080874.1	PNPLA4	0	0	0.01666667	0.79
XM_021080876.1	PPP2R2B	0	0	0.00333333	0.35
XM_021080906.1	LOC110257903	0	0	0.01666667	0.92
XM_021080907.1	LOC110257903	0	0	0.02	1.076666667
XM_021080926.1	LOC110257929	0	0	0.01333333	1
XM_021080927.1	LOC110257931	0	0	0.01	1
XM_021080935.1	LOC100624329	0	0	0.02666667	6.583333333
XM_021081034.1	LHX3	0	0	0.00333333	0.333333333
XM_021081058.1	CCDC187	0	0	0.01	4
XM_021081065.1	CCDC187	0	0	0.01333333	5.736666667
XM_021081092.1	NSMF	0	0	0.05666667	5.54
XM_021081106.1	ARRDC1	0	0	0.02333333	3.73
XM_021081108.1	ARRDC1	0	0	0.00666667	0.906666667
XM_021081112.1	ARRDC1	0	0	0.05333333	8.52
XM_021081124.1	LCN15	0	0	0.00333333	0.666666667
XM_021081125.1	LCN15	0	0	0.00333333	0.763333333
XM_021081138.1	SEC16A	0	0	0.01	3.676666667
XM_021081139.1	SEC16A	0	0	0.03	12.08
XM_021081171.1	NDOR1	0	0	0.03333333	3.346666667
XM_021081190.1	SLC34A3	0	0.006666667	0.01333333	2
XM_021081192.1	FAM166A	0	0	0.00666667	0.333333333
XM_021081204.1	UBAC1	0	0	0.16333333	14.59333333
XM_021081236.1	PAXX	0	0	0.04333333	1.516666667
XM_021081253.1	NACC2	0	0	0.10666667	27.14333333
XM_021081264.1	LOC110258110	0	0	0.24	4.15
XM_021081270.1	LOC110258114	0	0	0.00333333	0.333333333
XM_021081319.1	TNRC6A	0	0	0.04	12.15
XM_021081324.1	TNRC6A	0	0	0.10333333	32.65
XM_021081348.1	RBBP6	0	0	0.11666667	32.22333333
XM_021081368.1	F12	0	0	0.01333333	1.333333333
XM_021081381.1	CSF2RA	0	0	0.03333333	2.24
XM_021081382.1	CSF2RA	0	0	0.01666667	1.173333333
XM_021081387.1	LOC110258194	0	0	0.00666667	0.666666667
XM_021081389.1	F12	0	0	0.00666667	0.49
XM_021081391.1	ASMTL	0	0	0.12	18.84666667
XM_021081392.1	ESR2	0	0	0.00333333	0.666666667
XM_021081395.1	LOC102161330	0	0	0.00666667	0.666666667
XM_021081405.1	LOC100519378	0	0	0.01	0.666666667
XM_021081410.1	LOC110258223	0	0	0.00666667	0.333333333
XM_021081425.1	RAG1	0	0	0.00333333	0.666666667
XM_021081456.1	NR1H3	0	0	0.04666667	4.61
XM_021081466.1	LOC110258302	0	0	0.00333333	0.23
XM_021081482.1	HMGXB4	0	0	0.22333333	37.56
XM_021081490.1	LOC110258318	0	0	0.00666667	0.333333333
XM_021081495.1	LOC110258323	0	0	0.13666667	5.956666667
XM_021081498.1	CAT	0	0	0.07666667	7.933333333
XM_021081532.1	AMH	0	0	0.04	3.333333333
XM_021081534.1	KLC1	0	0	0.07333333	8.453333333
XM_021081538.1	KLC1	0	0	0.10333333	10.88666667
XM_021081554.1	NUDT14	0	0	0.02	0.89
XM_021081573.1	LOC110258345	0	0	0.00333333	0.626666667

XM_021081575.1	ASPG	0	0	0.00333333	0.333333333
XM_021081589.1	MARK3	0	0	0.04333333	5.513333333
XM_021081591.1	MARK3	0	0	0.06	8.46
XM_021081599.1	MARK3	0	0	0.04333333	5.52
XM_021081617.1	BRF1	0	0	0.3	43.56666667
XM_021081682.1	LOC110258393	0	0	0.00333333	0.156666667
XM_021081714.1	TNNT3	0	0	0.10666667	6.233333333
XM_021081725.1	GALNT6	0	0	0.02333333	3.333333333
XM_021081739.1	TNNT3	0	0	0.07	4.216666667
XM_021081740.1	TNNT3	0	0	0.09333333	5.18
XM_021081755.1	TNNT3	0	0	0.06333333	3.57
XM_021081758.1	LOC110258507	0	0	0.02333333	0.766666667
XM_021081774.1	LOC106504125	0	0	0.02	1
XM_021081808.1	LOC100155561	0	0	0.00333333	0.333333333
XM_021081864.1	KCNT2	0	0	0.01	2.423333333
XM_021081865.1	KCNT2	0	0	0.00333333	1
XM_021081867.1	KCNT2	0	0	0.00333333	1.706666667
XM_021081900.1	LOC102159001	0	0	0.00666667	0.503333333
XM_021081907.1	LOC100155405	0	0	0.00666667	0.333333333
XM_021081933.1	LOC110258642	0	0	0.00666667	0.58
XM_021081951.1	LOC110258660	0	0	0.00333333	0.333333333
XM_021081971.1	LOC110258709	0	0	0.00333333	0.34
XM_021081977.1	LOC102167556	0	0	0.00666667	1.26
XM_021082042.1	DNMT1	0	0	0.07333333	18.07666667
XM_021082053.1	DNMT1	0	0	0.02666667	6.426666667
XM_021082072.1	LOC110258824	0	0	0.04666667	1.34
XM_021082075.1	LOC100038328	0	0	0.16	4.666666667
XM_021082077.1	LOC110258836	0	0	0.00333333	0.333333333
XM_021082088.1	LOC110258851	0	0	0.01	0.333333333
XM_021082091.1	LOC100153496	0	0	0.01333333	1.333333333
XM_021082095.1	LOC110258854	0	0	0.02666667	0.666666667
XM_021082106.1	LOC110258865	0	0	0.02	0.666666667
XM_021082110.1	LOC110258867	0	0	0.00666667	0.666666667
XM_021082156.1	LOC100521600	0	0	0.00333333	0.353333333
XM_021082164.1	LOC110258908	0	0	0.01333333	2.92
XM_021082186.1	SNRPN	0	0	0.02	0.92
XM_021082219.1	LOC110258934	0	0	0.00333333	0.086666667
XM_021082238.1	MEF2C	0	0.006666667	0.07333333	12.75
XM_021082243.1	MEF2C	0	0	0.04333333	12.12333333
XM_021082310.1	LOC100153359	0	0.333333333	0.00333333	1.333333333
XM_021082340.1	LOC102165831	0	0	0.00666667	0.23
XM_021082354.1	LOC110259059	0	0	0.04666667	3.666666667
XM_021082381.1	MGAT1	0	0	0.06666667	9.346666667
XM_021082389.1	MGAT1	0	0	0.08	12.87333333
XM_021082408.1	LOC110259088	0	0	0.00333333	0.393333333
XM_021082442.1	LOC100522225	0	0	0.00666667	0.843333333
XM_021082475.1	DIP2A	0	0	0.01666667	5.063333333
XM_021082492.1	MCM3AP	0	0	0.05	22.59333333
XM_021082508.1	SYT8	0	0	0.00333333	0.333333333
XM_021082515.1	LOC100626661	0	0	0.09666667	5.886666667
XM_021082533.1	PGGHG	0	0.006666667	0.04666667	4.796666667
XM_021082540.1	RNH1	0	0	0.02	1.466666667
XM_021082551.1	LRRC56	0	0	0.02	2.333333333
XM_021082573.1	TSPAN4	0	0	0.14333333	42.80666667
XM_021082575.1	TSPAN4	0	0	0.11333333	33.21333333
XM_021082592.1	BRSK2	0	0	0.00333333	0.796666667
XM_021082596.1	MOB2	0	0	0.01333333	0.723333333
XM_021082603.1	LSP1	0	0.07	0.04666667	3.96
XM_021082609.1	LOC110259218	0	0	0.00333333	0.333333333
XM_021082614.1	TSPAN32	0	0	0.00333333	0.613333333

XM_021082627.1	SLC22A18	0	0	0.03333333	2.26
XM_021082636.1	LOC100627067	0	0	0.15333333	13.57333333
XM_021082656.1	CTTN	0	0	0.12333333	15.01
XM_021082658.1	CTTN	0	0	0.04666667	4.356666667
XM_021082673.1	ANO1	0	0	0.01333333	2.67
XM_021082734.1	UNC93B1	0	0	0.04333333	4.746666667
XM_021082736.1	LOC100522421	0	0	0.00666667	0.97
XM_021082750.1	TMEM134	0	0	0.02666667	0.933333333
XM_021082770.1	SPTBN2	0	0	0.01333333	6.283333333
XM_021082778.1	C2H11orf80	0	0	0.02	1.556666667
XM_021082780.1	C2H11orf80	0	0	0.01	1
XM_021082781.1	C2H11orf80	0	0	0.00666667	0.443333333
XM_021082800.1	SLC29A2	0	0	0.19333333	22.58666667
XM_021082822.1	CDKN2B	0	0	0.00333333	1.26
XM_021082824.1	LOC100511545	0	0	0.06666667	1.983333333
XM_021082843.1	EHBP1L1	0	0	0.05333333	7.743333333
XM_021082861.1	POLA2	0	0	0.04333333	5.17
XM_021082938.1	VWCE	0	0	0.00666667	0.853333333
XM_021082939.1	VWCE	0	0	0.01	2.866666667
XM_021082948.1	LOC100519643	0	0	0.01333333	2.013333333
XM_021082963.1	CD6	0	0	0.04333333	5.846666667
XM_021082970.1	MS4A10	0	0	0.01	0.333333333
XM_021082981.1	STX3	0	0	0.01666667	1.423333333
XM_021083018.1	ESR1	0	0	0.00333333	0.83
XM_021083033.1	TMX2	0	0	0.1	3.453333333
XM_021083034.1	ESR1	0	0	0.00666667	2.44
XM_021083039.1	SLC43A3	0	0	0.09666667	12.5
XM_021083041.1	ESR1	0	0	0.01333333	1.806666667
XM_021083065.1	AGBL2	0	0	0.05666667	8.893333333
XM_021083068.1	AGBL2	0	0	0.09	14.97
XM_021083069.1	AGBL2	0	0	0.07333333	10.28
XM_021083107.1	TNC	0	0	0.09	31.54333333
XM_021083132.1	PHF21A	0	0	0.05	12.17666667
XM_021083139.1	PHF21A	0	0	0.01333333	4.01
XM_021083151.1	PRDM11	0	0	0.01333333	6.906666667
XM_021083191.1	LDLRAD3	0	0	0.01666667	3.07
XM_021083192.1	LDLRAD3	0	0	0.05333333	9.61
XM_021083201.1	HIPK3	0	0	0.63666667	222.7833333
XM_021083203.1	HIPK3	0	0	0.55333333	193.0266667
XM_021083204.1	HIPK3	0	0	0.00666667	2.243333333
XM_021083216.1	CCDC73	0	0	0.00333333	0.333333333
XM_021083243.1	GAS2	0	0	0.00666667	0.79
XM_021083283.1	PLEKHA7	0	0	0.00333333	0.77
XM_021083286.1	PLEKHA7	0	0	0.00666667	2.06
XM_021083289.1	PLEKHA7	0	0	0.01333333	4.553333333
XM_021083320.1	ZBED5	0	0	0.04666667	7.586666667
XM_021083321.1	ZBED5	0	0	0.04666667	5.723333333
XM_021083340.1	WNT3A	0	0	0.01666667	1.666666667
XM_021083403.1	TRIM41	0	0	0.02	1.66
XM_021083421.1	PBX4	0	0	0.01666667	1.666666667
XM_021083436.1	HAPLN4	0	0	0.3	49.33333333
XM_021083451.1	SUGP2	0	0.003333333	0.13666667	20.61333333
XM_021083480.1	MAST3	0	0	0.00666667	2.073333333
XM_021083482.1	MAST3	0	0	0.00333333	0.956666667
XM_021083483.1	MAST3	0	0	0.02	6.276666667
XM_021083484.1	MAST3	0	0	0.01	3.856666667
XM_021083490.1	MAST3	0	0	0.03666667	11.51333333
XM_021083495.1	MAST3	0	0	0.02666667	7.566666667
XM_021083498.1	MAST3	0	0	0.00666667	1.993333333
XM_021083511.1	CGA	0	0	0.01	0.333333333

XM_021083520.1	SSBP4	0	0	0.15333333	11.12
XM_021083559.1	PLVAP	0	0	0.19333333	19.64333333
XM_021083593.1	EPS15L1	0	0	0.01333333	2.316666667
XM_021083606.1	LOC110259329	0	0	0.01	1.996666667
XM_021083620.1	WIZ	0	0	0.03333333	9.29
XM_021083641.1	LOC100516957	0	0	0.01	1.196666667
XM_021083647.1	LOC100516957	0	0	0.00666667	1.83
XM_021083656.1	LOC100516957	0	0	0.00333333	0.433333333
XM_021083658.1	LOC100516957	0	0	0.00333333	0.643333333
XM_021083661.1	LOC100516957	0	0	0.01	2.393333333
XM_021083666.1	LOC100516957	0	0	0.02	4.52
XM_021083669.1	LOC100516957	0	0	0.01333333	2.753333333
XM_021083685.1	LOC100516420	0	0	0.03	4.216666667
XM_021083705.1	RFX1	0	0	0.03333333	6.183333333
XM_021083731.1	GNG14	0	0	0.02333333	0.333333333
XM_021083734.1	LOC100521431	0	0	0.00333333	0.71
XM_021083735.1	LOC100521431	0	0	0.00333333	0.616666667
XM_021083742.1	LOC106508100	0	0	0.02333333	2.633333333
XM_021083753.1	LOC102167351	0	0	0.00666667	1.84
XM_021083761.1	LOC110259338	0	0	0.01333333	3.8
XM_021083763.1	LOC110259338	0	0	0.04666667	5.563333333
XM_021083767.1	LOC110259338	0	0	0.00333333	0.576666667
XM_021083776.1	LOC100738906	0	0	0.11333333	30.38666667
XM_021083781.1	OLFM2	0	0	0.09	8.66
XM_021083861.1	KANK2	0	0	0.02	3.853333333
XM_021083866.1	DOCK6	0	0	0.16	49.27333333
XM_021083877.1	RGL3	0	0	0.00666667	0.756666667
XM_021083881.1	ZNF653	0	0	0.05333333	4.186666667
XM_021083885.1	MYO1F	0	0	0.00666667	1.336666667
XM_021083897.1	TGFBR3L	0	0	0.00666667	0.333333333
XM_021083902.1	EVI5L	0	0	0.09333333	14.30333333
XM_021083905.1	EVI5L	0	0	0.03	4.366666667
XM_021083922.1	ARHGEF18	0	0	0.01	5.24
XM_021083926.1	ARHGEF18	0	0	0.02333333	6.496666667
XM_021083928.1	ARHGEF18	0	0	0.01	2.68
XM_021083947.1	EMR4	0	0.013333333	0.07666667	8.316666667
XM_021083956.1	ADGRE1	0	0	0.00666667	2.266666667
XM_021083977.1	SH2D3A	0	0	0.02	2.516666667
XM_021083978.1	SH2D3A	0	0	0.03	3.463333333
XM_021083994.1	ACSBG2	0	0	0.00333333	0.333333333
XM_021084074.1	ANKRD24	0	0	0.01	1.423333333
XM_021084084.1	CREB3L3	0	0	0.00333333	0.333333333
XM_021084094.1	ZFR2	0	0	0.00333333	0.333333333
XM_021084100.1	PIP5K1C	0	0	0.02	4.35
XM_021084101.1	PIP5K1C	0	0	0.03333333	6.853333333
XM_021084104.1	PIP5K1C	0	0	0.08	15.75666667
XM_021084108.1	MATK	0	0	0.02333333	1.666666667
XM_021084110.1	TBXA2R	0	0	0.05	6.223333333
XM_021084113.1	TBXA2R	0	0	0.07666667	6.876666667
XM_021084114.1	PLAGL1	0	0	0.04	16.18333333
XM_021084115.1	MFSD12	0	0	0.07333333	13.25333333
XM_021084121.1	PLAGL1	0	0	0.03666667	7.316666667
XM_021084144.1	TLE2	0	0	0.03	3.276666667
XM_021084145.1	TLE2	0	0	0.02	2.77
XM_021084163.1	PLAGL1	0	0	0.01666667	4.956666667
XM_021084176.1	PLAGL1	0	0	0.00333333	0.29
XM_021084192.1	PLAGL1	0	0	0.06666667	9.136666667
XM_021084218.1	ATP8B3	0	0	0.01333333	3
XM_021084220.1	ATP8B3	0	0	0.00666667	1.133333333
XM_021084221.1	FOXO3	0	0	0.29666667	91.66333333

XM_021084230.1	TCF3	0	0	0.75333333	69.57333333
XM_021084234.1	ADAMTSL5	0	0	0.01333333	2.31
XM_021084259.1	CBARP	0	0	0.00666667	0.53
XM_021084284.1	ARHGAP45	0	0	0.03	5.043333333
XM_021084294.1	PRSS57	0	0	0.00333333	0.333333333
XM_021084318.1	RASGEF1C	0	0	0.40666667	154.4266667
XM_021084332.1	HNRNPH1	0	0	1.23666667	112.28
XM_021084339.1	LOC100620238	0	0	0.20666667	67.09333333
XM_021084343.1	LOC100621931	0	0	0.00666667	0.4
XM_021084363.1	COL23A1	0	0	0.03	8.333333333
XM_021084365.1	COL23A1	0	0	0.03666667	9.666666667
XM_021084367.1	DBN1	0	0	0.02	2.63
XM_021084373.1	FYN	0	0	0.16666667	22.73666667
XM_021084400.1	NSD1	0	0	0.02666667	10.75
XM_021084434.1	CPLX2	0	0.333333333	0.01	2
XM_021084458.1	SV2C	0	0	0.00333333	0.333333333
XM_021084467.1	PDE8B	0	0	0.00333333	0.623333333
XM_021084471.1	PDE8B	0	0	0.00666667	1.13
XM_021084489.1	TENT2	0	0	0.00666667	1.176666667
XM_021084491.1	SERINC5	0	0	0.07	19.97
XM_021084497.1	ANKRD34B	0	0	0.02	3.32
XM_021084525.1	TMEM161B	0	0	0.13666667	14.79666667
XM_021084527.1	TMEM161B	0	0	0.06666667	6.94
XM_021084531.1	MCTP1	0	0	0.02333333	4.673333333
XM_021084539.1	MCTP1	0	0	0.01	2.6
XM_021084540.1	MCTP1	0	0	0.03	6.343333333
XM_021084553.1	PAM	0	0	0.04333333	5.65
XM_021084571.1	PPIP5K2	0	0	0.03666667	10.47666667
XM_021084572.1	PPIP5K2	0	0	0.06333333	16.48333333
XM_021084581.1	PPIP5K2	0	0	0.02333333	5.99
XM_021084593.1	NUDT12	0	0	0.12333333	20.43
XM_021084595.1	C2H5orf30	0	0	0.03666667	4.283333333
XM_021084599.1	LOC110259431	0	0	0.00333333	0.333333333
XM_021084612.1	TMEM232	0	0	0.01	1.596666667
XM_021084617.1	CAMK4	0	0	0.01	7
XM_021084622.1	CAMK4	0	0	0.01	6.666666667
XM_021084635.1	KCNN2	0	0	0.01333333	2.443333333
XM_021084642.1	TRIM36	0	0	0.00333333	0.666666667
XM_021084667.1	DMXL1	0	0	0.06666667	42.22333333
XM_021084674.1	PRR16	0	0	0.00666667	6.486666667
XM_021084683.1	PRDM6	0	0	0.00666667	0.803333333
XM_021084684.1	PRDM6	0	0	0.00333333	0.46
XM_021084697.1	CSNK1G3	0	0	0.17	32.36333333
XM_021084709.1	LOC100516424	0	0	0.03	3.296666667
XM_021084711.1	LOC100516424	0	0	0.08666667	11.13333333
XM_021084713.1	LOC100516424	0	0	0.01	0.853333333
XM_021084715.1	LOC100516424	0	0	0.08	9.893333333
XM_021084718.1	LOC100516424	0	0	0.04333333	2.953333333
XM_021084724.1	SLC27A6	0	0	0.00333333	1.513333333
XM_021084725.1	SLC27A6	0	0	0.01333333	3.446666667
XM_021084726.1	SLC27A6	0	0.006666667	0.01	2.803333333
XM_021084730.1	SLC27A6	0	0	0.03666667	6.163333333
XM_021084742.1	ACSL6	0	0	0.00666667	1.096666667
XM_021084763.1	CDKL3	0	0	0.05666667	6.226666667
XM_021084769.1	JADE2	0	0	0.02333333	6.923333333
XM_021084776.1	JADE2	0	0.193333333	0.01333333	3.856666667
XM_021084790.1	KLHL3	0	0	0.04333333	13.10666667
XM_021084797.1	KLHL3	0	0	0.01	2.616666667
XM_021084808.1	FAM13B	0	0	0.00666667	1.456666667
XM_021084822.1	FAM13B	0	0	0.10333333	25.65333333

XM_021084829.1	FAM13B	0	0	0.00666667	1.846666667
XM_021084852.1	MATR3	0	0	0.05	9.153333333
XM_021084860.1	MATR3	0	0	0.01333333	1.723333333
XM_021084873.1	PSD2	0	0	0.01333333	4
XM_021084877.1	PSD2	0	0	0.02333333	5.246666667
XM_021084887.1	LOC100513976	0	0	0.03	2.673333333
XM_021084889.1	LOC100513976	0	0	0.09	7.66
XM_021084891.1	HARS	0	0	0.11333333	9.32
XM_021084925.1	LOC100515772	0	0	0.01	3.51
XM_021084938.1	LOC110259520	0	0	0.01333333	0.743333333
XM_021084949.1	LOC102163334	0	0	0.00333333	0.333333333
XM_021084951.1	KCTD16	0	0	0.01	5.956666667
XM_021084963.1	TRPM1	0	0	0.00333333	1
XM_021084965.1	LOC110259533	0	0	0.00333333	0.333333333
XM_021084966.1	SPINK5	0	0	0.00333333	0.333333333
XM_021084989.1	ABLIM3	0	0	0.03666667	7.39
XM_021084995.1	ABLIM3	0	0	0.30666667	57.53333333
XM_021085000.1	ABLIM3	0	0	0.04333333	7.62
XM_021085002.1	ABLIM3	0	0	0.08333333	14.40333333
XM_021085029.1	IFNGR1	0	0	0.07666667	19.12666667
XM_021085081.1	LOC110259604	0	0	0.00666667	0.333333333
XM_021085082.1	LOC110259605	0	0	0.02333333	1
XM_021085083.1	LOC110259606	0	0	0.01	0.333333333
XM_021085092.1	LOC110259615	0	0	0.00333333	0.166666667
XM_021085093.1	LOC110259616	0	0	0.00333333	0.166666667
XM_021085136.1	LOC100518538	0	0	0.00333333	0.333333333
XM_021085167.1	LOC110259671	0	0	0.01333333	1.333333333
XM_021085180.1	MADD	0	0	0.02333333	5.023333333
XM_021085181.1	MADD	0	0	0.16666667	39.20333333
XM_021085185.1	MADD	0	0	0.01666667	3.846666667
XM_021085195.1	MADD	0	0.003333333	0.08666667	18.59333333
XM_021085203.1	MADD	0	0	0.07333333	17.98
XM_021085207.1	MADD	0	0	0.01	2.316666667
XM_021085213.1	MADD	0	0	0.03	7.126666667
XM_021085271.1	LRRC4C	0	0.243333333	0.03666667	7.783333333
XM_021085276.1	SLC1A2	0	0	0.00666667	1.726666667
XM_021085282.1	CD44	0	0	0.04666667	9.79
XM_021085290.1	LOC100517025	0	0	0.05	13.93333333
XM_021085291.1	LOC100517025	0	0	0.01	3.853333333
XM_021085293.1	LOC100517025	0	0	0.02666667	7.996666667
XM_021085333.1	SOX6	0	0	0.04	18.43666667
XM_021085366.1	SOX6	0	0.013333333	0.09666667	27.53333333
XM_021085369.1	XRCC4	0	0	0.02333333	1.9
XM_021085374.1	ADGRV1	0	0	0.01	11.19333333
XM_021085375.1	ADGRV1	0	0.526666667	0.03333333	33.86666667
XM_021085377.1	KIAA0825	0	0	0.01333333	3.21
XM_021085411.1	RAPGEF6	0	0	0.03333333	11.13
XM_021085419.1	LYRM7	0	0	0.03	0.836666667
XM_021085434.1	P4HA2	0	0	0.01333333	1.58
XM_021085435.1	P4HA2	0	0	0.03	3.656666667
XM_021085438.1	P4HA2	0	0	0.01666667	1.903333333
XM_021085471.1	LOC106507123	0	0	0.00333333	0.333333333
XM_021085579.1	NCK2	0	0	0.30666667	34.10666667
XM_021085580.1	NCK2	0	0	0.72666667	88.40666667
XM_021085584.1	NCK2	0	0.113333333	0.98	115.0533333
XM_021085607.1	GRIFIN	0	0	0.00333333	0.823333333
XM_021085608.1	GRIFIN	0	0	0.00666667	1
XM_021085610.1	PKMYT1	0	0	0.01	1
XM_021085613.1	PKMYT1	0	0	0.04333333	3.77
XM_021085632.1	PSPH	0	0	0.13333333	6.546666667

XM_021085673.1	CLUAP1	0	0	0.02666667	3.206666667
XM_021085679.1	KRCC1	0	0	0.1	7.373333333
XM_021085686.1	PTCD3	0	0	0.26	42.02
XM_021085705.1	UBE2I	0	0.756666667	0.00666667	2.263333333
XM_021085721.1	SH3YL1	0	0	0.04666667	3.686666667
XM_021085745.1	KIF25	0	0	0.00666667	3.333333333
XM_021085759.1	LMAN2L	0	0	0.19	20.63666667
XM_021085814.1	CIITA	0	0	0.03	12.01333333
XM_021085815.1	AFDN	0	0	0.02	5.293333333
XM_021085816.1	CIITA	0	0	0.00666667	3.373333333
XM_021085817.1	CIITA	0	0	0.03666667	16.20333333
XM_021085825.1	BCL2L11	0	0	0.02666667	6.886666667
XM_021085841.1	SRD5A2	0	0	0.00333333	0.333333333
XM_021085851.1	IL18R1	0	0	0.02	3.656666667
XM_021085852.1	KTN1	0	0	0.06666667	12.82666667
XM_021085861.1	LOC106506286	0	0.093333333	0.00333333	0.333333333
XM_021085866.1	IL4R	0	0	0.06	8.61
XM_021085880.1	CD19	0	0	0.00666667	0.383333333
XM_021085885.1	SUCLG1	0	0	0.11333333	6.44
XM_021085886.1	LHCGR	0	0	0.00666667	1.136666667
XM_021085888.1	LHCGR	0	0	0.00666667	0.83
XM_021085896.1	SPDYA	0	0	0.01	0.333333333
XM_021085911.1	KCNS3	0	0	0.03	3.626666667
XM_021085915.1	KCNS3	0	0	0.05333333	5.966666667
XM_021085934.1	PRKAR1B	0	0	0.02666667	2.706666667
XM_021085935.1	PRKAR1B	0	0	0.14	14.84
XM_021085945.1	LOC100522669	0	0	0.00333333	0.333333333
XM_021085964.1	TMEM184A	0	0	0.06666667	13.61
XM_021085967.1	ELFN1	0	0	0.01666667	4.346666667
XM_021085974.1	LOC110259831	0	0	0.01666667	1.823333333
XM_021085977.1	LOC106509613	0	0	0.00666667	1.62
XM_021085980.1	LOC106509613	0	0	0.04	12.30333333
XM_021085981.1	LOC106509613	0	0	0.01666667	4.286666667
XM_021085986.1	SNX8	0	0	0.00333333	0.733333333
XM_021085993.1	LOC110259833	0	0	0.00333333	0.333333333
XM_021085996.1	UNC93A	0	0	0.01333333	1
XM_021086008.1	BRAT1	0	0	0.01666667	1.98
XM_021086016.1	CARD11	0	0	0.00666667	1.356666667
XM_021086019.1	CARD11	0	0	0.00333333	1.11
XM_021086029.1	MMD2	0	0	0.00333333	0.443333333
XM_021086055.1	LOC100520903	0	0	0.02333333	10.61333333
XM_021086057.1	LOC100520903	0	0	0.00666667	1.056666667
XM_021086063.1	LOC100520903	0	0	0.00333333	0.46
XM_021086124.1	ZKSCAN5	0	0	0.09666667	13.87
XM_021086136.1	ZNF655	0	0	0.04	7.463333333
XM_021086138.1	ZNF655	0	0	0.03333333	6.546666667
XM_021086142.1	ZNF655	0	0	0.22333333	16.22666667
XM_021086150.1	ZSCAN25	0	0	0.03666667	6.816666667
XM_021086151.1	ZNF789	0	0	0.01333333	2.823333333
XM_021086157.1	LOC106508762	0	0	0.00666667	1.506666667
XM_021086190.1	ZKSCAN1	0	0	0.14	12.98333333
XM_021086211.1	LOC100514951	0	0	0.21666667	11.64
XM_021086219.1	PILRA	0	0	0.02	1.316666667
XM_021086221.1	PILRA	0	0	0.05666667	2.666666667
XM_021086222.1	PILRA	0	0	0.05333333	2.243333333
XM_021086235.1	TFR2	0	0	0.00666667	0.666666667
XM_021086245.1	GIGYF1	0	0	0.01666667	5.19
XM_021086246.1	GIGYF1	0	0	0.26333333	75.70666667
XM_021086293.1	HEY2	0	0	0.03	3.993333333
XM_021086299.1	STYXL1	0	0	0.04	8.483333333

XM_021086305.1	STYXL1	0	0	0.01666667	3.836666667
XM_021086313.1	RHBDD2	0	0	0.15666667	13.87666667
XM_021086332.1	ABHD11	0	0	0.09666667	4.356666667
XM_021086351.1	CALN1	0	0	0.00333333	1
XM_021086355.1	CALN1	0	0	0.00666667	2.953333333
XM_021086368.1	LOC106509673	0	0	0.02666667	1.623333333
XM_021086419.1	GTF2H5	0	0	0.05333333	1.613333333
XM_021086428.1	PRRT2	0	0	0.03	2.89
XM_021086467.1	PALB2	0	0	0.15666667	27.8
XM_021086471.1	UBFD1	0	0	0.18333333	7.266666667
XM_021086484.1	VWA3A	0	0	0.00666667	1.666666667
XM_021086495.1	LOC100524794	0	0	0.00333333	1
XM_021086501.1	CRYM	0	0	0.00333333	0.076666667
XM_021086502.1	ZDHHC14	0	0	0.05666667	9.246666667
XM_021086518.1	LYRM1	0	0	0.02666667	0.67
XM_021086520.1	ACSM2B	0	0	0.01	1.666666667
XM_021086521.1	ACSM2B	0	0	0.00666667	1
XM_021086625.1	CLEC16A	0	0	0.00333333	0.896666667
XM_021086655.1	PMM2	0	0	0.02666667	0.79
XM_021086671.1	LOC110259937	0	0	0.00666667	0.333333333
XM_021086687.1	NUDT16L1	0	0	0.00666667	0.923333333
XM_021086701.1	TFAP4	0	0	0.01	0.87
XM_021086749.1	PRSS27	0	0	0.03	1.666666667
XM_021086760.1	TBC1D24	0	0	0.15666667	46.70333333
XM_021086765.1	ABCA3	0	0	0.08333333	26.59
XM_021086780.1	NTHL1	0	0	0.32	15.71333333
XM_021086785.1	TBL3	0	0	0.04	4.166666667
XM_021086807.1	MAPK8IP3	0	0	0.12	29.37333333
XM_021086809.1	MAPK8IP3	0	0	0.02	5.446666667
XM_021086831.1	HAGH	0	0	0.05666667	2.74
XM_021086837.1	LOC110259948	0	0	0.02	6.1
XM_021086850.1	TELO2	0	0	0.07333333	9.613333333
XM_021086885.1	RHBDL1	0	0	0.03666667	2.463333333
XM_021086913.1	MCRIP2	0	0	0.01666667	0.416666667
XM_021086966.1	LOC110259958	0	0	0.04	1.673333333
XM_021086969.1	ZNF484	0	0	0.02333333	6.5
XM_021086971.1	ZNF484	0	0	0.02	5.933333333
XM_021086976.1	ZNF484	0	0	0.02	5.076666667
XM_021086977.1	ZNF484	0	0	0.04666667	12.86333333
XM_021086979.1	ZNF484	0	0	0.01333333	3.656666667
XM_021086998.1	UST	0	0	0.06333333	6.216666667
XM_021087009.1	ZNF169	0	0	0.00666667	1.696666667
XM_021087011.1	ZNF169	0	0	0.00333333	0.776666667
XM_021087014.1	ZNF169	0	0	0.01333333	4.426666667
XM_021087019.1	ZNF169	0	0	0.00333333	0.706666667
XM_021087033.1	GRM1	0	0.333333333	0.01	2.666666667
XM_021087061.1	KCNIP3	0	0	0.06	8.176666667
XM_021087065.1	PROM2	0	0	0.00666667	1.666666667
XM_021087078.1	ZNF2	0	0	0.05	8.07
XM_021087174.1	CHST10	0	0	0.06	11.15
XM_021087200.1	TSGA10	0	0	0.03	3.75
XM_021087202.1	ADGRG6	0	0	0.01	2.83
XM_021087212.1	LOC110255307	0	0	0.00666667	0.333333333
XM_021087227.1	ADGRG6	0	0	0.01333333	4.28
XM_021087242.1	VWA3B	0	0.086666667	0.01	2.77
XM_021087256.1	LOC100739325	0	0	0.02666667	3.06
XM_021087259.1	LOC102167341	0	0	0.00666667	0.333333333
XM_021087265.1	ADGRG6	0	0	0.02333333	8.473333333
XM_021087276.1	VTA1	0	0	0.13333333	10.71333333
XM_021087299.1	ELMOD3	0	0	0.10666667	10.02

XM_021087310.1	LRRTM4	0	0	0.00666667	0.666666667
XM_021087329.1	DQX1	0	0	0.00333333	0.333333333
XM_021087344.1	RTKN	0	0	0.07	6.81
XM_021087345.1	RTKN	0	0	0.11333333	10.92
XM_021087352.1	DCTN1	0	0.006666667	0.03	7.386666667
XM_021087358.1	DCTN1	0	0.13	0.02333333	4.563333333
XM_021087359.1	DCTN1	0	0.42	0.04333333	9.753333333
XM_021087368.1	DGUOK	0	0	0.03	1.313333333
XM_021087403.1	TIA1	0	0	0.08	42.36
XM_021087423.1	FBXO48	0	0	0.01333333	1.406666667
XM_021087441.1	ARFGEF3	0	0	0.02333333	16.66333333
XM_021087442.1	SPRED2	0	0	0.03666667	7.486666667
XM_021087487.1	XPO1	0	0	0.02333333	3.646666667
XM_021087504.1	C3H2orf74	0	0	0.00666667	0.996666667
XM_021087513.1	KIAA1841	0	0	0.01333333	2.72
XM_021087522.1	CCDC88A	0	0	0.04	18.15333333
XM_021087533.1	CLHC1	0	0	0.01666667	1.82
XM_021087534.1	CLHC1	0	0	0.00666667	0.473333333
XM_021087590.1	SOS1	0	0	0.03333333	13.86666667
XM_021087614.1	ATL2	0	0.006666667	0.34333333	45.94
XM_021087616.1	ATL2	0	0	0.15333333	19.54666667
XM_021087627.1	RMDN2	0	0	0.07666667	8.116666667
XM_021087634.1	VIT	0	0	0.51333333	62.93666667
XM_021087654.1	SLC2A12	0	0	0.04	4.563333333
XM_021087675.1	EYA4	0	0	0.25666667	200.73
XM_021087678.1	ZNF512	0	0	0.12333333	19
XM_021087681.1	GCKR	0	0	0.02333333	2.29
XM_021087699.1	SLC5A6	0	0	0.00333333	0.61
XM_021087704.1	ABHD1	0	0	0.15666667	9.416666667
XM_021087726.1	EYA4	0	0	0.00666667	1.736666667
XM_021087744.1	DTNB	0	0	0.02333333	2.883333333
XM_021087750.1	DTNB	0	0	0.12	11.17333333
XM_021087760.1	DTNB	0	0	0.07333333	7.416666667
XM_021087769.1	EFR3B	0	0	0.04666667	13.97666667
XM_021087770.1	EFR3B	0	0	0.02666667	8.78
XM_021087787.1	KLHL29	0	0	0.02	4.09
XM_021087815.1	OSR1	0	0	0.05333333	4.456666667
XM_021087816.1	OSR1	0	0	0.08	6.126666667
XM_021087839.1	SLC18B1	0	0	0.02666667	3.713333333
XM_021087859.1	TAF1B	0	0	0.04333333	4.953333333
XM_021087864.1	ASAP2	0	0	0.00333333	0.79
XM_021087867.1	ASAP2	0	0	0.01666667	4.013333333
XM_021087874.1	MBOAT2	0	0	0.07	9.49
XM_021087889.1	KIDINS220	0	0	0.01666667	4.99
XM_021087890.1	KIDINS220	0	0.003333333	0.05666667	13.01
XM_021087900.1	RNF144A	0	0	0.06333333	15.57666667
XM_021087903.1	RNF144A	0	0	0.01333333	3.256666667
XM_021087939.1	MYT1L	0	0	0.00333333	1
XM_021087948.1	MYT1L	0	0.333333333	0.00333333	0.666666667
XM_021087994.1	DOC2A	0	0	0.02333333	2
XM_021088033.1	SGF29	0	0	0.09333333	6.086666667
XM_021088034.1	SGF29	0	0	0.04666667	3.03
XM_021088045.1	ATXN2L	0	0	0.08	16.25666667
XM_021088046.1	ATXN2L	0	0	0.02666667	4.73
XM_021088066.1	RBFOX1	0	0	0.03333333	6.506666667
XM_021088084.1	EPB41L2	0	0	0.03333333	7.06
XM_021088086.1	NLRC3	0	0.213333333	0.00333333	1.343333333
XM_021088088.1	NLRC3	0	0	0.00333333	1.346666667
XM_021088090.1	NLRC3	0	0	0.00333333	1.32
XM_021088094.1	NLRC3	0	0	0.01	6.663333333

XM_021088102.1	LOC100517149	0	0	0.02	2.6
XM_021088107.1	LOC100517149	0	0	0.17666667	9.793333333
XM_021088129.1	LOC106509717	0	0.096666667	0.04666667	2.52
XM_021088130.1	EPB41L2	0	0	0.00666667	0.993333333
XM_021088141.1	SUSD3	0	0	0.03	1.8
XM_021088142.1	SUSD3	0	0	0.07333333	4.65
XM_021088149.1	WNK2	0	0	0.07666667	23.46
XM_021088184.1	AFF3	0	0	0.00666667	1.643333333
XM_021088187.1	AFF3	0	0	0.00333333	1.39
XM_021088195.1	AFF3	0	0	0.02	9.946666667
XM_021088206.1	ARID4A	0	0	0.07333333	17.75
XM_021088209.1	FANCL	0	0	0.02	2.423333333
XM_021088210.1	FANCL	0	0	0.13	9.426666667
XM_021088220.1	SPTBN1	0	0.003333333	0.70666667	237.4033333
XM_021088233.1	NRXN1	0	0	0.01	4.126666667
XM_021088236.1	NRXN1	0	0	0.01666667	4.753333333
XM_021088239.1	NRXN1	0	0	0.04	11.72333333
XM_021088253.1	NRXN1	0	0.15	0.14333333	35.23
XM_021088259.1	NRXN1	0	0.286666667	0.02333333	6.226666667
XM_021088262.1	NRXN1	0	0	0.04333333	11.05333333
XM_021088270.1	NRXN1	0	0	0.11333333	27.52666667
XM_021088285.1	NRXN1	0	0.886666667	0.00333333	2.026666667
XM_021088306.1	SLC8A1	0	0	0.01	9.463333333
XM_021088315.1	SLC8A1	0	0	0.00666667	5.34
XM_021088319.1	SLC8A1	0	0	0.01333333	9.153333333
XM_021088337.1	MAP4K3	0	0	0.06333333	11.21
XM_021088359.1	ITSN2	0	0	0.11666667	28.56333333
XM_021088369.1	PFN4	0	0	0.00333333	0.333333333
XM_021088399.1	L3MBTL3	0	0	0.05333333	10.77333333
XM_021088422.1	TMEM244	0	0	0.00666667	0.666666667
XM_021088433.1	ARHGAP18	0	0	0.03	4.81
XM_021088437.1	ARHGAP18	0	0	0.03	4.136666667
XM_021088439.1	LMNA	0	0	0.12	18.43
XM_021088444.1	LMNA	0	0	0.12	18.38333333
XM_021088470.1	ARHGEF2	0	0	0.01	1.956666667
XM_021088508.1	TRIM55	0	0	0.05	5.533333333
XM_021088522.1	ANXA9	0	0	0.01	3.153333333
XM_021088525.1	ANXA9	0	0	0.00666667	0.783333333
XM_021088526.1	ANXA9	0	0	0.00333333	0.733333333
XM_021088542.1	DCAF8	0	0	0.05	8.48
XM_021088574.1	TG	0	0	0.00333333	0.666666667
XM_021088579.1	NCALD	0	0	0.03333333	2.636666667
XM_021088600.1	PAQR6	0	0	0.00666667	0.533333333
XM_021088604.1	FAM49B	0	0	0.05333333	4.606666667
XM_021088607.1	FAM49B	0	0	0.10333333	9.303333333
XM_021088609.1	FAM49B	0	0	0.03666667	3.02
XM_021088620.1	FAM49B	0	0	0.10333333	7.613333333
XM_021088628.1	DRAM2	0	0	0.24666667	10.56333333
XM_021088630.1	DCSTAMP	0	0	0.00333333	0.583333333
XM_021088683.1	HDDC2	0	0	0.06666667	4.396666667
XM_021088686.1	ANGPT1	0	0	0.02666667	5.666666667
XM_021088698.1	KCNA2	0	0	0.00333333	2.536666667
XM_021088702.1	KCNA2	0	0	0.00333333	2.463333333
XM_021088729.1	TPM3	0	0	0.09	10.32333333
XM_021088751.1	CLVS2	0	0.666666667	0.00666667	3.35
XM_021088771.1	GML	0	0	0.02666667	1
XM_021088776.1	LOC110260198	0	0	0.01333333	0.333333333
XM_021088809.1	TSNARE1	0	0	0.04666667	3.22
XM_021088818.1	PTP4A3	0	0	0.10333333	9.393333333
XM_021088822.1	PTK2	0	0	0.10666667	21.14666667

XM_021088836.1	PTK2	0	0	0.02666667	4.853333333
XM_021088838.1	PTK2	0	0	0.01666667	3.64
XM_021088840.1	PTK2	0	0.003333333	0.30666667	60.27666667
XM_021088878.1	PHF20L1	0	0	0.00666667	1.683333333
XM_021088884.1	PHF20L1	0	0	0.19	45.98333333
XM_021088896.1	TMEM71	0	0	0.01666667	1.963333333
XM_021088897.1	KCNQ3	0	1.333333333	0.02	12
XM_021088906.1	ASAP1	0	0	0.00666667	3.173333333
XM_021088912.1	ASAP1	0	0	0.03333333	11.35666667
XM_021088932.1	WASHC5	0	0	0.38666667	64.24333333
XM_021088939.1	NSMCE2	0	0	0.01	0.526666667
XM_021088942.1	TBC1D32	0	0	0.01333333	3.56
XM_021088949.1	MTSS1	0	0	0.13333333	18.76333333
XM_021088963.1	TBC1D31	0	0	0.04666667	6.146666667
XM_021088964.1	TBC1D31	0	0	0.33666667	52.83
XM_021088972.1	TBC1D31	0	0	0.01666667	2.66
XM_021088988.1	ENPP2	0	0	0.01666667	2.54
XM_021089011.1	FAM184A	0	0	0.00333333	0.666666667
XM_021089014.1	NUDCD1	0	0	0.06	10.67333333
XM_021089019.1	EMC2	0	0	1.08	48.41333333
XM_021089051.1	RGS22	0	0	0.01333333	2.733333333
XM_021089053.1	RGS22	0	0	0.01666667	3.323333333
XM_021089057.1	RGS22	0	0	0.03333333	6.123333333
XM_021089063.1	ERICH5	0	0	0.00333333	0.333333333
XM_021089064.1	ERICH5	0	0	0.01	0.996666667
XM_021089076.1	MTERF3	0	0.096666667	0.00333333	1.02
XM_021089078.1	GDF6	0	0	0.04333333	7
XM_021089086.1	DPY19L4	0	0	0.01666667	1.406666667
XM_021089087.1	ROS1	0	0	0.00666667	3.01
XM_021089102.1	ROS1	0	0	0.00666667	3.53
XM_021089104.1	TMEM67	0	0	0.08666667	15.46666667
XM_021089108.1	ROS1	0	0	0.02333333	11.07333333
XM_021089121.1	TRIQK	0	0	0.01666667	4.356666667
XM_021089125.1	SLC26A7	0	0	0.01333333	2.77
XM_021089147.1	WWP1	0	0.043333333	0.37333333	65.86666667
XM_021089157.1	CPNE3	0	0	0.05	11.01333333
XM_021089168.1	LRRCC1	0	0	0.00666667	0.89
XM_021089212.1	STAU2	0	0	0.03333333	4.35
XM_021089267.1	SULF1	0	0	0.02333333	6.313333333
XM_021089275.1	CSPP1	0	0	0.04666667	8.496666667
XM_021089283.1	LOC100521447	0	0	0.04	6.376666667
XM_021089333.1	ASPH	0	0	0.00666667	1.24
XM_021089335.1	CHD7	0	0	0.02666667	13.65333333
XM_021089343.1	TOX	0	0	0.01333333	4.713333333
XM_021089349.1	FAM110B	0	0	0.19	24.60666667
XM_021089358.1	LYN	0	0	0.33666667	78.16333333
XM_021089372.1	RB1CC1	0	0	0.07333333	23.10333333
XM_021089428.1	KIFAP3	0	0	0.03666667	5.693333333
XM_021089444.1	LOC106510102	0	0	0.09333333	15.54666667
XM_021089475.1	GPR161	0	0	0.01	3.393333333
XM_021089506.1	C4H1orf226	0	0	0.00666667	1.296666667
XM_021089518.1	FCRLA	0	0	0.01	0.88
XM_021089544.1	ARHGAP30	0	0	0.03	6.146666667
XM_021089556.1	LY9	0	0	0.12	15.24333333
XM_021089560.1	LY9	0	0	0.02333333	2.63
XM_021089567.1	CD84	0	0	0.00333333	0.44
XM_021089569.1	CD84	0	0	0.00666667	0.55
XM_021089595.1	CEP162	0	0	0.03	7.88
XM_021089598.1	FCER1A	0	0	0.01666667	0.666666667
XM_021089623.1	LOC110260316	0	0	0.05	4.666666667

XM_021089642.1	ARHGEF11	0	0	0.01333333	3.673333333
XM_021089717.1	RUSC1	0	0	0.07333333	13.51
XM_021089718.1	RUSC1	0	0	0.08	6.073333333
XM_021089732.1	ADAM15	0	0	0.01666667	2.163333333
XM_021089763.1	UBAP2L	0	0	0.05666667	10.34333333
XM_021089906.1	ECM1	0	0	0.20333333	20.33333333
XM_021089907.1	ECM1	0	0	0.11333333	11.23666667
XM_021089940.1	ITGA10	0	0	0.02333333	5.16
XM_021089943.1	ANKRD35	0	0.036666667	0.00666667	0.97
XM_021089945.1	PIAS3	0	0	0.05666667	6.323333333
XM_021089957.1	BCL9	0	0	0.04	9.836666667
XM_021089963.1	CHD1L	0	0	0.02666667	5.756666667
XM_021089980.1	CD101	0	0	0.00666667	1.7
XM_021089985.1	LOC106508382	0	0	0.01666667	1.276666667
XM_021089986.1	LOC106508382	0	0	0.00333333	0.053333333
XM_021089988.1	ANKRD6	0	0	0.01	2.686666667
XM_021090025.1	DDX20	0	0	0.02333333	3.136666667
XM_021090030.1	RAP1A	0	0	0.16666667	16.11666667
XM_021090036.1	CASP8AP2	0	0	0.02333333	6.946666667
XM_021090038.1	LOC100621421	0	0	0.01	0.736666667
XM_021090053.1	CASP8AP2	0	0	0.02	6.403333333
XM_021090070.1	GNAT2	0	0	0.00333333	0.333333333
XM_021090072.1	SORT1	0	0	0.11666667	39.16666667
XM_021090107.1	FNDC7	0	0	0.01	1.333333333
XM_021090123.1	LOC100521789	0	0	0.10333333	8.886666667
XM_021090149.1	SASS6	0	0	0.02	3.626666667
XM_021090174.1	TMEM56	0	0	0.06333333	18.33666667
XM_021090180.1	ALG14	0	0	0.08333333	18.85666667
XM_021090206.1	MTF2	0	0	0.12333333	10.16333333
XM_021090210.1	EVI5	0	0	0.03333333	10.97666667
XM_021090228.1	FAM69A	0	0	0.03333333	3.776666667
XM_021090229.1	GFI1	0	0	0.01333333	1.036666667
XM_021090234.1	GLMN	0	0	0.03666667	3.633333333
XM_021090238.1	KIAA1107	0	0	0.00333333	0.75
XM_021090252.1	HFM1	0	0	0.01	2.35
XM_021090266.1	ZNF644	0	0	0.06333333	18.12666667
XM_021090271.1	ZNF644	0	0	0.07	17.58666667
XM_021090287.1	ZNF644	0	0	0.01333333	1.536666667
XM_021090290.1	ZNF644	0	0	0.03	2.83
XM_021090309.1	LOC102161784	0	0	0.08333333	24.38333333
XM_021090312.1	LOC100523310	0	0	0.07333333	8.18
XM_021090320.1	LOC100523492	0	0	0.02	1.78
XM_021090329.1	KYAT3	0	0	0.06	5.413333333
XM_021090331.1	KYAT3	0	0	0.01333333	1.093333333
XM_021090347.1	WDR63	0	0	0.01333333	2
XM_021090360.1	LOC110260400	0	0	0.00666667	0.333333333
XM_021090435.1	KIFC2	0	0	0.01	1
XM_021090454.1	LOC100621388	0	0	0.01666667	4.94
XM_021090476.1	IQANK1	0	0	0.01	1
XM_021090500.1	LOC106507406	0	0	0.00333333	0.49
XM_021090524.1	SHARPIN	0	0	0.02666667	1.54
XM_021090528.1	TSTA3	0	0	1.72666667	87.46333333
XM_021090529.1	TSTA3	0	0	0.05666667	3.446666667
XM_021090531.1	TSTA3	0	0	0.09666667	4.866666667
XM_021090533.1	FOXH1	0	0	0.00666667	1
XM_021090559.1	DSCC1	0	0	0.00666667	1
XM_021090568.1	COLEC10	0	0	0.00666667	1.593333333
XM_021090571.1	COLEC10	0	0	0.00333333	0.636666667
XM_021090584.1	CSMD3	0	0	0.00333333	2.676666667
XM_021090624.1	RALYL	0	0	0.00333333	0.333333333

XM_021090650.1	LOC110260430	0	0	0.01	0.696666667
XM_021090658.1	LOC100624559	0	0	0.01333333	5.646666667
XM_021090660.1	LOC100624559	0	0	0.07666667	33.04666667
XM_021090671.1	LOC100624559	0	0	0.07	24.38333333
XM_021090675.1	LOC100624559	0	0	0.01	4.426666667
XM_021090687.1	LOC100624559	0	0	0.30666667	118.3533333
XM_021090703.1	LOC100519022	0	0	0.01333333	2.993333333
XM_021090709.1	LOC102159652	0	0	0.01666667	1.17
XM_021090710.1	LOC102159652	0	0	0.03333333	2.946666667
XM_021090716.1	MAGI3	0	0	0.01	2.19
XM_021090724.1	PHTF1	0	0	0.05666667	18.06
XM_021090733.1	SCML4	0	0	0.00666667	4.113333333
XM_021090754.1	SCML4	0	0	0.00666667	3.44
XM_021090762.1	SCML4	0	0	0.02666667	17.54333333
XM_021090782.1	ODF2L	0	0	0.00666667	0.466666667
XM_021090808.1	LOC110260483	0	0	0.00666667	0.333333333
XM_021090836.1	CCDC162P	0	0	0.02333333	6.4
XM_021090867.1	LMO3	0	0	0.00666667	0.543333333
XM_021090871.1	LIMA1	0	0	0.01333333	2.543333333
XM_021090908.1	PRR5	0	0	0.02666667	2.116666667
XM_021090913.1	PRR5	0	0	0.01333333	0.916666667
XM_021090920.1	CEP57L1	0	0	0.00333333	0.12
XM_021090923.1	C5H12orf4	0	0	0.21333333	65.94666667
XM_021090924.1	C5H12orf4	0	0	0.02666667	9.426666667
XM_021090937.1	CEP57L1	0	0	0.05333333	3.196666667
XM_021090957.1	C5H12orf29	0	0	0.03333333	4.88
XM_021090962.1	MICAL1	0	0	0.06666667	11.15666667
XM_021090969.1	CENPM	0	0	0.02333333	0.87
XM_021090971.1	CENPM	0	0	0.02	1.126666667
XM_021090973.1	CENPM	0	0	0.03666667	2.063333333
XM_021090974.1	CENPM	0	0	0.05666667	3.66
XM_021090992.1	CCDC91	0	0	0.03666667	2.476666667
XM_021090994.1	CCDC91	0	0.02	0.20666667	14.53
XM_021090997.1	RBFOX2	0	0	0.00333333	0.936666667
XM_021091001.1	RBFOX2	0	0	0.00333333	0.81
XM_021091002.1	RBFOX2	0	0	0.18666667	61.33666667
XM_021091005.1	RBFOX2	0	0	0.06666667	22.15666667
XM_021091019.1	RBFOX2	0	0	0.1	48.10333333
XM_021091036.1	FAM234B	0	0	0.23333333	52.49333333
XM_021091043.1	AK9	0	0	0.00333333	0.666666667
XM_021091058.1	FRS2	0	0	0.02333333	6.416666667
XM_021091071.1	PARVG	0	0	0.10333333	6.306666667
XM_021091072.1	PARVG	0	0	0.00333333	0.43
XM_021091100.1	WASF1	0	0	0.01333333	1.31
XM_021091126.1	DGKA	0	0	0.02666667	3.506666667
XM_021091150.1	SLCO1A2	0	0	0.01333333	2.393333333
XM_021091153.1	SLCO1A2	0	0	0.02	6.653333333
XM_021091161.1	SLCO1A2	0	0	0.01	3.51
XM_021091173.1	LY49	0	0	0.01	0.813333333
XM_021091186.1	PCBP2	0	0	0.22	21.79666667
XM_021091199.1	CD4	0	0	0.12	14.01
XM_021091203.1	BID	0	0	0.08333333	4.683333333
XM_021091289.1	PLXNB2	0	0	0.06333333	16.47333333
XM_021091300.1	PLXNB2	0	0	0.02	6.67
XM_021091306.1	TRAF3IP2	0	0	0.09333333	4.416666667
XM_021091312.1	DENND6B	0	0	0.03	5.13
XM_021091343.1	MOV10L1	0	0	0.00666667	0.666666667
XM_021091344.1	TTLL8	0	0	0.00666667	0.666666667
XM_021091366.1	GRAMD4	0	0	0.04	7.176666667
XM_021091368.1	GRAMD4	0	0	0.13	23.48666667

XM_021091378.1	WISP3	0	0	0.00333333	0.536666667
XM_021091387.1	WISP3	0	0	0.00333333	0.463333333
XM_021091390.1	FAM118A	0	0	0.03333333	2.61
XM_021091400.1	SHISAL1	0	0	0.01666667	5.333333333
XM_021091416.1	TTLL1	0	0	0.26	19.33
XM_021091451.1	CCDC134	0	0	0.05333333	8.38
XM_021091453.1	CCDC134	0	0	0.20666667	29.38666667
XM_021091470.1	XPNPEP3	0	0	0.04	8.853333333
XM_021091483.1	GTPBP1	0	0	0.06666667	12.07333333
XM_021091496.1	C5H22orf23	0	0	0.01666667	1
XM_021091509.1	CARD10	0	0	0.04666667	9.773333333
XM_021091511.1	CARD10	0	0	0.03	6.456666667
XM_021091515.1	CARD10	0	0	0.29	60.25666667
XM_021091527.1	TMPRSS6	0	0	0.00333333	0.333333333
XM_021091534.1	TEX33	0	0	0.00666667	0.333333333
XM_021091555.1	BTBD11	0	0	0.02	6.58
XM_021091569.1	RFX4	0	0.06	0.00333333	0.333333333
XM_021091583.1	NUAK1	0	0	0.02333333	6.223333333
XM_021091590.1	TRAPPC3L	0	0	0.00333333	0.333333333
XM_021091604.1	KCNH3	0	0	0.01666667	2.333333333
XM_021091614.1	FMNL3	0	0	0.26333333	45.25333333
XM_021091634.1	RACGAP1	0	0	0.20333333	28.76333333
XM_021091650.1	LARP4	0	0	0.01	2.843333333
XM_021091654.1	LARP4	0	0.003333333	0.05	15.48333333
XM_021091655.1	LARP4	0	0	0.01333333	4.08
XM_021091670.1	ANKRD33	0	0	0.00666667	0.666666667
XM_021091684.1	LOC100523670	0	0	0.00333333	0.18
XM_021091714.1	SP7	0	0.666666667	0.00333333	0.666666667
XM_021091715.1	SP7	0	0.333333333	0.00333333	1.333333333
XM_021091718.1	TARBP2	0	0	0.04	2.003333333
XM_021091729.1	HOXC4	0	0.003333333	0.01333333	1.743333333
XM_021091738.1	SMUG1	0	0	0.02	1.003333333
XM_021091741.1	SNAP91	0	0	0.00333333	0.373333333
XM_021091754.1	SNAP91	0	0	0.00333333	0.54
XM_021091792.1	IKZF4	0	0	0.10666667	24.02
XM_021091817.1	LOC100512656	0	0	0.02666667	1.773333333
XM_021091818.1	LOC100512656	0	0	0.10666667	14.25
XM_021091819.1	LOC100512656	0	0	0.66333333	21.50333333
XM_021091845.1	R3HDM2	0	0	0.09333333	15.29333333
XM_021091863.1	DOP1A	0	0	0.01	2.75
XM_021091868.1	ARHGAP9	0	0	0.04666667	6.08
XM_021091906.1	ATP23	0	0	0.25	13.45333333
XM_021091907.1	ATP23	0	0	0.02	1.4
XM_021091937.1	PPM1H	0	0	0.03	7.81
XM_021091958.1	HMGA2	0	0	0.02666667	1.333333333
XM_021091966.1	DYRK2	0	0.086666667	0.53666667	201.32
XM_021091973.1	YEATS4	0	0	0.01666667	0.95
XM_021091981.1	RAB3IP	0	0	0.06666667	5.623333333
XM_021091984.1	RAB3IP	0	0	0.04	2.933333333
XM_021092006.1	KCNC2	0	0	0.00666667	0.89
XM_021092033.1	CAPS2	0	0	0.00333333	0.383333333
XM_021092051.1	TTK	0	0	0.05333333	12
XM_021092052.1	OSBPL8	0	0	0.01	3.38
XM_021092056.1	DNM1L	0	0	0.00333333	0.52
XM_021092062.1	DNM1L	0	0	0.15333333	24.61666667
XM_021092090.1	DENND5B	0	0	0.02333333	8.656666667
XM_021092107.1	ELOVL4	0	0	0.02666667	3.666666667
XM_021092122.1	SH3BGRL2	0	0	0.02	3.126666667
XM_021092125.1	TMTC1	0	0	0.03666667	7.103333333
XM_021092134.1	PPFIBP1	0	0	0.04666667	8.656666667

XM_021092148.1	PPFIBP1	0	0	0.04666667	8.073333333
XM_021092151.1	ARNTL2	0	0	0.00333333	0.5
XM_021092165.1	TM7SF3	0	0	0.07	13.32666667
XM_021092184.1	LMNTD1	0	0	0.05	3.63
XM_021092192.1	CASC1	0	0	0.00666667	1.11
XM_021092198.1	CASC1	0	0	0.01666667	2.346666667
XM_021092199.1	CASC1	0	0	0.01	1.02
XM_021092200.1	CASC1	0	0	0.03	3.85
XM_021092203.1	LRMP	0	0	0.06333333	8.256666667
XM_021092206.1	LRMP	0	0	0.09	9.85
XM_021092215.1	C2CD5	0	0	0.01666667	3.81
XM_021092229.1	C2CD5	0	0	0.00333333	0.46
XM_021092286.1	SQOR	0	0	0.07333333	4.99
XM_021092289.1	PIK3C2G	0	0	0.00666667	1.396666667
XM_021092293.1	PIK3C2G	0	0	0.01333333	2.27
XM_021092303.1	EPS8	0	0	0.04333333	7.053333333
XM_021092358.1	LOC100523789	0	0.333333333	0.01	2.666666667
XM_021092359.1	LOC100523789	0	0	0.00333333	0.333333333
XM_021092369.1	COL12A1	0	0	0.19666667	63.01333333
XM_021092372.1	KLRB1	0	0	0.06	1.666666667
XM_021092373.1	LOC100524679	0	0	0.00333333	0.333333333
XM_021092376.1	A2M	0	0	0.08333333	18.33333333
XM_021092398.1	LOC110260747	0	0	0.00333333	0.193333333
XM_021092409.1	C1RL	0	0	0.01	1
XM_021092432.1	IFFO1	0	0.236666667	0.00666667	1.133333333
XM_021092437.1	KCNA1	0	0.666666667	0.00333333	1.666666667
XM_021092439.1	KCNA6	0	0	0.02	8.863333333
XM_021092459.1	ITFG2	0	0	0.16666667	21.60333333
XM_021092460.1	DDX11	0	0	0.02666667	4.95
XM_021092465.1	DDX11	0	0	0.01666667	3.596666667
XM_021092468.1	DDX11	0	0.28	0.00333333	0.723333333
XM_021092481.1	SLC6A12	0	0	0.03	5
XM_021092500.1	CACNA2D4	0	1	0.00333333	1.666666667
XM_021092507.1	MICAL3	0	0	0.00333333	0.973333333
XM_021092532.1	C5H12orf40	0	0	0.02333333	2
XM_021092562.1	PRICKLE1	0	0	0.04	6.763333333
XM_021092585.1	LOXHD1	0	0.333333333	0.00333333	1.333333333
XM_021092598.1	RAPGEF3	0	0	0.00666667	1.91
XM_021092640.1	GLT8D2	0	0	0.02	2.553333333
XM_021092654.1	ASCL1	0	0	0.01	1
XM_021092665.1	GNPTAB	0	0	0.02	8.056666667
XM_021092676.1	GNPTAB	0	0	0.02666667	6.44
XM_021092683.1	MYBPC1	0	0	0.23333333	42.05333333
XM_021092692.1	KATNAL2	0	0	0.00666667	0.996666667
XM_021092704.1	GAS2L3	0	0	0.00666667	2.083333333
XM_021092710.1	GAS2L3	0	0	0.00666667	1.963333333
XM_021092713.1	GAS2L3	0	0	0.01666667	3.963333333
XM_021092714.1	GAS2L3	0	0	0.01333333	3.91
XM_021092717.1	GAS2L3	0	0	0.00333333	0.733333333
XM_021092733.1	ELK3	0	0	0.09666667	16.64
XM_021092737.1	CCDC38	0	0.02	0.00333333	0.333333333
XM_021092740.1	USP44	0	0.333333333	0.00666667	1.666666667
XM_021092747.1	VEZT	0	0	0.00666667	2.96
XM_021092751.1	HDHD2	0	0	0.04333333	3.913333333
XM_021092766.1	LOC110260797	0	0	0.00333333	0.666666667
XM_021092771.1	CRADD	0	0	0.03	2.63
XM_021092780.1	C5H12orf50	0	0	0.05	3.95
XM_021092816.1	ZDHHC17	0	0	0.03333333	6.993333333
XM_021092824.1	LOC110260809	0	0	0.01666667	0.526666667
XM_021092831.1	LOC110260809	0	0	0.04333333	1.5

XM_021092835.1	MRTFA	0	0	0.51333333	115.27
XM_021092874.1	PAN2	0	0	0.06	11.63
XM_021092884.1	BAZ2A	0	0	0.01333333	5.786666667
XM_021092886.1	BAZ2A	0	0	0.06	25.57
XM_021092923.1	SOX5	0	0	0.00333333	1.563333333
XM_021092936.1	SOX5	0	0	0.00333333	1.28
XM_021092938.1	SOX5	0	0	0.00666667	2.223333333
XM_021092945.1	SOX5	0	0	0.00666667	2.22
XM_021092964.1	SOX5	0	0	0.01	4.746666667
XM_021093004.1	ANKS1B	0	0	0.01	2.446666667
XM_021093010.1	ANKS1B	0	0	0.00666667	0.95
XM_021093018.1	ANKS1B	0	0	0.00333333	0.543333333
XM_021093025.1	APAF1	0	0	0.22333333	63.59666667
XM_021093059.1	MBD1	0	0	0.00333333	0.633333333
XM_021093093.1	MBD1	0	0	0.01	1.196666667
XM_021093108.1	MBD1	0	0	0.04	8.913333333
XM_021093109.1	MBD1	0	0	0.08666667	10.45666667
XM_021093121.1	LOC110260850	0	0	0.00666667	0.333333333
XM_021093136.1	MBD1	0	0	0.02	2.476666667
XM_021093149.1	DAB1	0	0	0.00333333	0.666666667
XM_021093167.1	DAB1	0	0.333333333	0.00333333	0.513333333
XM_021093171.1	MBD1	0	0	0.03	3.336666667
XM_021093198.1	KDM1A	0	0	0.09333333	12.61
XM_021093203.1	MBD1	0	0	0.00333333	0.53
XM_021093222.1	WDR62	0	0	0.02666667	6.153333333
XM_021093223.1	WDR62	0	0	0.04	16.19333333
XM_021093243.1	BCO1	0	0	0.00333333	0.46
XM_021093244.1	BCO1	0	0	0.00333333	0.613333333
XM_021093246.1	BCO1	0	0	0.02	6.72
XM_021093258.1	GNG12	0	0	0.07333333	13.21
XM_021093272.1	ELOVL1	0	0	0.02	1.283333333
XM_021093281.1	MAPK4	0	0	0.05	11.52
XM_021093306.1	CNFN	0	0	0.00333333	0.426666667
XM_021093315.1	POLI	0	0	0.03	4.07
XM_021093343.1	TNFRSF9	0	0	0.02	2.16
XM_021093346.1	C1H18orf54	0	0	0.04333333	6.746666667
XM_021093367.1	C1H18orf54	0	0	0.01666667	2.35
XM_021093379.1	SRSF10	0	0	0.07666667	10.76
XM_021093392.1	DAPK2	0	0	0.21666667	15.57333333
XM_021093407.1	TMEM51	0	0	0.04666667	4.11
XM_021093423.1	ENOSF1	0	0	0.04666667	3.086666667
XM_021093432.1	VPS4A	0	0	0.02	2.193333333
XM_021093439.1	TCF4	0	0	0.30333333	39.77333333
XM_021093440.1	TCTEX1D1	0	0	0.00666667	0.333333333
XM_021093441.1	TCF4	0	0	0.04333333	5.923333333
XM_021093464.1	ST3GAL3	0	0	0.03	2.65
XM_021093469.1	ST3GAL3	0	0	0.00333333	0.323333333
XM_021093485.1	PTGER3	0	0	0.00666667	0.533333333
XM_021093488.1	LEPR	0	0	0.00666667	1.503333333
XM_021093493.1	LEPR	0	0	0.03333333	7.06
XM_021093508.1	POU2F2	0	0	0.01333333	3.64
XM_021093509.1	POU2F2	0	0	0.03	8.923333333
XM_021093519.1	LHB	0	0	0.02	1.973333333
XM_021093521.1	IL12RB2	0	0.433333333	0.00333333	0.6
XM_021093543.1	CKM	0	0	1.42	92.47333333
XM_021093546.1	AGRP	0	0	0.00666667	0.333333333
XM_021093558.1	CES1	0	0.043333333	0.04333333	4.453333333
XM_021093562.1	CES1	0	0	0.01666667	1
XM_021093570.1	ATP4A	0	0	0.00666667	1.333333333
XM_021093571.1	PGM1	0	0	0.33	40.38333333

XM_021093594.1	C1QC	0	0	0.10333333	4.96
XM_021093643.1	APRT	0	0	0.09	3.873333333
XM_021093649.1	PIEZO1	0	0	0.10333333	37.45
XM_021093673.1	CA5A	0	0	0.00333333	0.676666667
XM_021093674.1	CA5A	0	0	0.00333333	0.533333333
XM_021093677.1	CA5A	0	0	0.02666667	2.863333333
XM_021093678.1	CA5A	0	0	0.02	2.583333333
XM_021093682.1	CA5A	0	0	0.06	7.346666667
XM_021093769.1	PIF1	0	0	0.01333333	2
XM_021093804.1	SF3B3	0	0	0.78666667	159.87
XM_021093832.1	ZNF821	0	0	0.05333333	6.856666667
XM_021093840.1	ZNF821	0	0	0.06666667	5.63
XM_021093858.1	PMFBP1	0	0.333333333	0.00333333	1.333333333
XM_021093863.1	WWP2	0	0	0.2	34.92666667
XM_021093870.1	NFAT5	0	0	0.02333333	9.013333333
XM_021093873.1	PDF	0	0	0.02333333	6.046666667
XM_021093891.1	LOC102166944	0	0	0.01666667	0.333333333
XM_021093920.1	ADGRG3	0	0	0.00666667	1.333333333
XM_021093923.1	CIAO2A	0	0	0.12	4.89
XM_021093930.1	KIFC3	0	0	0.00333333	0.516666667
XM_021093945.1	ZNF319	0	0	0.03	4.8
XM_021093968.1	NDRG4	0	0.21	0.03333333	5.396666667
XM_021093972.1	NDRG4	0	0	0.02666667	3.263333333
XM_021093984.1	BEAN1	0	0.333333333	0.00333333	1
XM_021093988.1	TK2	0	0	0.01666667	2.166666667
XM_021094001.1	LOC110260932	0	0	0.00666667	0.333333333
XM_021094007.1	TERB1	0	0	0.01	1.47
XM_021094053.1	CBFB	0	0	0.02	3.123333333
XM_021094061.1	RIPOR1	0	0.003333333	0.29666667	57.72666667
XM_021094091.1	CARMIL2	0	0	0.05	9.83
XM_021094093.1	USP3	0	0	0.01	2.146666667
XM_021094104.1	GFOD2	0	0	0.12666667	11.29333333
XM_021094106.1	GFOD2	0	0	0.10666667	7.906666667
XM_021094134.1	ESRP2	0	0	0.02666667	5.733333333
XM_021094139.1	PRMT7	0	0	0.00666667	1.196666667
XM_021094141.1	PRMT7	0	0	0.01333333	2.323333333
XM_021094199.1	ADCY7	0	0	0.06666667	18.5
XM_021094203.1	ADCY7	0	0.323333333	0.00333333	0.746666667
XM_021094206.1	ADCY7	0	0.323333333	0.00333333	0.746666667
XM_021094208.1	APH1B	0	0	0.01666667	0.81
XM_021094209.1	ADCY7	0	0.04	0.19666667	53.58
XM_021094210.1	ADCY7	0	0.323333333	0.00333333	0.746666667
XM_021094211.1	ADCY7	0	0.323333333	0.00333333	0.746666667
XM_021094248.1	ORC6	0	0	0.02666667	1.903333333
XM_021094256.1	ZNF536	0	0	0.00333333	0.933333333
XM_021094265.1	DPY19L3	0	0	0.01333333	3.39
XM_021094285.1	CEP89	0	0	0.03	4.37
XM_021094293.1	SLC7A10	0	0	0.00333333	0.203333333
XM_021094295.1	TLN2	0	0	0.20666667	76.82
XM_021094296.1	CEBPG	0	0	0.16666667	33.10333333
XM_021094297.1	TLN2	0	0	0.14333333	55.28
XM_021094299.1	CHST8	0	0	0.00666667	0.666666667
XM_021094301.1	LOC100623157	0	0	0.06666667	18.60333333
XM_021094307.1	LOC100623157	0	0	0.01	0.333333333
XM_021094317.1	KCTD15	0	0.45	0.18666667	37.26666667
XM_021094356.1	FBXO17	0	0	0.01333333	0.763333333
XM_021094362.1	VPS13C	0	0	0.03333333	20.91666667
XM_021094388.1	LOC100522141	0	0	0.05333333	3
XM_021094395.1	SPTBN4	0	0	0.00333333	1
XM_021094405.1	COQ8B	0	0	0.08333333	7.32

XM_021094421.1	CEACAM1	0	0	0.10333333	16.47333333
XM_021094426.1	TMEM145	0	0.076666667	0.23	23.99
XM_021094442.1	ARHGEF1	0	0	0.11666667	22.85333333
XM_021094443.1	ARHGEF1	0	0	0.02	3.74
XM_021094449.1	ARHGEF1	0	0	0.08	13.11
XM_021094461.1	DMRTC2	0	0	0.00333333	0.333333333
XM_021094491.1	ZNF45	0	0	0.11333333	22.07666667
XM_021094494.1	ZNF45	0	0	0.01666667	2.963333333
XM_021094495.1	ZNF45	0	0	0.01666667	3.43
XM_021094506.1	LOC100627471	0	0	0.01333333	2.093333333
XM_021094519.1	GCNT3	0	0	0.00666667	1.35
XM_021094524.1	GEMIN7	0	0	0.12666667	17.65333333
XM_021094576.1	FBXO46	0	0	0.14	19.9
XM_021094601.1	HIF3A	0	0	0.00333333	0.78
XM_021094611.1	GNG8	0	0	0.00666667	0.94
XM_021094612.1	GNG8	0	0	0.01666667	2.6
XM_021094624.1	NPAS1	0	0	0.00666667	0.333333333
XM_021094632.1	CCDC9	0	0	0.05333333	5.59
XM_021094633.1	CCDC9	0	0	0.02666667	2.75
XM_021094635.1	CCDC9	0	0	0.06333333	6.273333333
XM_021094639.1	SLC8A2	0	0	0.01333333	2.396666667
XM_021094643.1	ZNF541	0	0	0.01333333	2.666666667
XM_021094662.1	CCDC114	0	0	0.00666667	1
XM_021094681.1	NTN5	0	0	0.01666667	1.45
XM_021094688.1	MAMSTR	0	0	0.16666667	12.55
XM_021094696.1	TCF12	0	0.35	0.00333333	0.893333333
XM_021094701.1	TCF12	0	0	0.11	30.21
XM_021094707.1	TULP2	0	0	0.01333333	0.8
XM_021094726.1	CD37	0	0	0.01333333	1.346666667
XM_021094745.1	TCF12	0	0.19	0.00333333	0.97
XM_021094768.1	ATF5	0	0	0.17333333	14.43
XM_021094776.1	VRK3	0	0	0.06333333	9.056666667
XM_021094777.1	VRK3	0	0	0.13	10.87666667
XM_021094779.1	IZUMO2	0	0	0.00666667	0.333333333
XM_021094780.1	IZUMO2	0	0	0.05	2.666666667
XM_021094795.1	POLD1	0	0	0.31333333	51
XM_021094813.1	LOC100620498	0	0	0.00333333	1.24
XM_021094837.1	KLK10	0	0	0.01	0.666666667
XM_021094875.1	TSEN34	0	0	0.12	6.83
XM_021094882.1	CNOT3	0	0	0.00666667	1.336666667
XM_021094890.1	TARM1	0	0	0.00666667	0.333333333
XM_021094898.1	LOC100622306	0	0	0.01666667	0.65
XM_021094907.1	PRKCG	0	0	0.02	4.37
XM_021094940.1	LOC100515383	0	0	0.00666667	0.666666667
XM_021094945.1	LOC102162204	0	0	0.01	0.333333333
XM_021094954.1	LOC106508173	0	0	0.00666667	0.54
XM_021094959.1	DNAAF4	0	0	0.02333333	4.82
XM_021094960.1	LOC100525227	0	0	0.02	2.333333333
XM_021094996.1	BRSK1	0	0	0.12	17
XM_021094998.1	PIGBOS1	0	0	0.04666667	0.883333333
XM_021095002.1	TMEM150B	0	0	0.13333333	6.486666667
XM_021095050.1	ZNF524	0	0	0.01666667	0.916666667
XM_021095086.1	ZNF470	0	0	0.01	3.36
XM_021095087.1	ZNF470	0	0	0.01	2.923333333
XM_021095089.1	ZNF470	0	0	0.04	6.356666667
XM_021095090.1	ZNF835	0	0	0.05333333	10.88666667
XM_021095102.1	ZNF582	0	0	0.03	2.653333333
XM_021095113.1	ZNF787	0	0	0.01	0.65
XM_021095147.1	LOC100513741	0	0	0.06333333	6.27
XM_021095150.1	LOC100514656	0	0.06	0.15666667	24.23333333

XM_021095171.1	KLHL17	0	0	0.01	1.08
XM_021095193.1	CFAP74	0	0	0.00333333	0.666666667
XM_021095206.1	RNF152	0	0	0.02666667	0.713333333
XM_021095207.1	MMEL1	0	0	0.00666667	0.666666667
XM_021095211.1	ARHGEF16	0	0	0.00666667	1.333333333
XM_021095212.1	MEGF6	0	0	0.01666667	4.666666667
XM_021095215.1	TP73	0	0	0.02333333	3.126666667
XM_021095217.1	TP73	0	0	0.04666667	3.303333333
XM_021095221.1	TP73	0	0	0.02	2.516666667
XM_021095241.1	KCNAB2	0	0	0.00666667	1.283333333
XM_021095244.1	KCNAB2	0	0	0.00666667	0.933333333
XM_021095273.1	PER3	0	0.563333333	0.13333333	66.99
XM_021095291.1	PIK3CD	0	0	0.01666667	4.73
XM_021095294.1	CLSTN1	0	0.066666667	0.73666667	145.91
XM_021095337.1	FBXO44	0	0	0.02666667	2.213333333
XM_021095414.1	ARHGEF19	0	0	0.01666667	3.106666667
XM_021095415.1	ARHGEF19	0	0	0.02666667	3.283333333
XM_021095439.1	ARHGEF10L	0	0	0.09	17.46666667
XM_021095456.1	DMXL2	0	0	0.01	5.42
XM_021095466.1	NBL1	0	0	0.03	2.636666667
XM_021095487.1	HSPG2	0	0	0.03333333	22.92666667
XM_021095502.1	EPHB2	0	0	0.01666667	8.086666667
XM_021095503.1	TNFAIP8L3	0	0	0.03	2.666666667
XM_021095537.1	IFNLR1	0	0	0.01	2
XM_021095548.1	STPG1	0	0	0.01333333	1.333333333
XM_021095556.1	SRRM1	0	0	0.02333333	7.226666667
XM_021095557.1	SRRM1	0	0	0.00333333	1.3
XM_021095571.1	SRRM1	0	0.46	0.07	20.98
XM_021095577.1	SRRM1	0	0	0.05	16.71
XM_021095622.1	UBXN11	0	0	0.01666667	0.976666667
XM_021095645.1	FGR	0	0	0.06333333	9.52
XM_021095649.1	FGR	0	0	0.12	19.03
XM_021095652.1	FGR	0	0	0.02666667	3.98
XM_021095658.1	IFI6	0	0	0.05333333	1.603333333
XM_021095683.1	GMEB1	0	0	0.01333333	2.373333333
XM_021095684.1	GMEB1	0	0	0.03	5.766666667
XM_021095698.1	EPB41	0	0	0.1	25.32666667
XM_021095707.1	EPB41	0	0	0.04333333	12.39666667
XM_021095745.1	PUM1	0	0	0.03666667	8.066666667
XM_021095765.1	PUM1	0	0	0.06333333	10.39333333
XM_021095772.1	ATP8B4	0	0	0.00333333	1.163333333
XM_021095777.1	COL16A1	0	0	0.04333333	11.29666667
XM_021095787.1	ADGRB2	0	0	0.02333333	6.13
XM_021095795.1	ADGRB2	0	0	0.10333333	21.87
XM_021095797.1	TMEM39B	0	0	0.02333333	1.296666667
XM_021095798.1	ATP8B4	0	0	0.00666667	1.42
XM_021095823.1	ATP8B4	0	0	0.00666667	2.086666667
XM_021095871.1	DLGAP3	0	0	0.00333333	0.333333333
XM_021095878.1	ZMYM6	0	0	0.01666667	3.556666667
XM_021095881.1	ZMYM6	0	0	0.03666667	7.763333333
XM_021095933.1	SH3D21	0	0	0.00666667	0.833333333
XM_021095948.1	LSM10	0	0	0.39666667	16.37333333
XM_021095952.1	OSCP1	0	0	0.01666667	2.453333333
XM_021095976.1	MANEAL	0	0	0.00666667	1.61
XM_021095977.1	YRDC	0	0	0.03666667	3.26
XM_021095995.1	MYCL	0	0	0.05	7.333333333
XM_021095996.1	CAP1	0	0	0.04	5.156666667
XM_021095998.1	CAP1	0	0	0.08333333	10.79666667
XM_021096014.1	COL9A2	0	0	0.01333333	1.666666667
XM_021096034.1	PRELID3A	0	0	0.01	1.953333333

XM_021096037.1	PRELID3A	0	0	0.01666667	1.496666667
XM_021096052.1	LOC110261162	0	0	0.98666667	64.94333333
XM_021096070.1	ARHGAP28	0	0.503333333	0.00333333	0.69
XM_021096073.1	ARHGAP28	0	0	0.00333333	2.393333333
XM_021096079.1	ARHGAP28	0	0.473333333	0.00333333	1.356666667
XM_021096088.1	EPB41L3	0	0	0.01	1.86
XM_021096089.1	EPB41L3	0	0	0.01	2.506666667
XM_021096102.1	EPB41L3	0	0	0.00333333	0.61
XM_021096103.1	SEMA6D	0	0	0.00666667	2.26
XM_021096104.1	EPB41L3	0	0	0.00333333	0.826666667
XM_021096105.1	EPB41L3	0	0.086666667	0.04	7.726666667
XM_021096109.1	EPB41L3	0	0	0.01666667	3.123333333
XM_021096114.1	SEMA6D	0	0	0.00666667	2.036666667
XM_021096116.1	EPB41L3	0	0.086666667	0.03666667	6.706666667
XM_021096136.1	EMILIN2	0	0	0.03333333	5.94
XM_021096160.1	SEMA6D	0	0	0.15	31.59333333
XM_021096169.1	ABHD3	0	0	0.01666667	1.503333333
XM_021096184.1	ANKRD29	0	0	0.04666667	4.183333333
XM_021096185.1	ANKRD29	0	0	0.04333333	3.04
XM_021096201.1	CHST9	0	0	0.01	3.636666667
XM_021096245.1	DTNA	0	0	0.00666667	1.83
XM_021096292.1	TPGS2	0	0	0.05333333	6.476666667
XM_021096296.1	TPGS2	0	0	0.01666667	0.453333333
XM_021096337.1	KCNG2	0	0	0.00666667	0.686666667
XM_021096343.1	SSX2IP	0	0	0.14333333	15.43333333
XM_021096360.1	SAMD13	0	0	0.05	3
XM_021096380.1	ADGRL2	0	0	0.01333333	3.516666667
XM_021096381.1	ADGRL2	0	0	0.03333333	9.343333333
XM_021096385.1	ADGRL2	0	0	0.12	33.5
XM_021096387.1	ADGRL2	0	0	0.02666667	8.566666667
XM_021096457.1	ZZZ3	0	0	0.23666667	65.16666667
XM_021096467.1	SLC44A5	0	0	0.02	2.69
XM_021096476.1	LOC102164667	0	0	0.00333333	0.333333333
XM_021096480.1	LRRIQ3	0	0	0.02666667	2.186666667
XM_021096489.1	WLS	0	0	0.02	2.38
XM_021096500.1	MIER1	0	0	0.00333333	0.613333333
XM_021096501.1	MIER1	0	0	0.06666667	14.86333333
XM_021096522.1	SGIP1	0	0	0.00333333	2.583333333
XM_021096528.1	SGIP1	0	0	0.01333333	7.86
XM_021096529.1	SGIP1	0	0	0.01333333	7.223333333
XM_021096536.1	SGIP1	0	0	0.00333333	1.88
XM_021096537.1	SGIP1	0	0.443333333	0.00333333	1.496666667
XM_021096543.1	FOXD3	0	0	0.05333333	4.313333333
XM_021096556.1	KANK4	0	0	0.02333333	3.533333333
XM_021096574.1	MYSM1	0	0	0.01666667	4.886666667
XM_021096590.1	FRMD5	0	0	0.01333333	1.41
XM_021096593.1	SSBP3	0	0	0.02	2.323333333
XM_021096600.1	LOC100622386	0	0	0.05666667	6.73
XM_021096601.1	LOC100622386	0	0	0.15666667	19.15
XM_021096616.1	FRMD5	0	0	0.00333333	0.376666667
XM_021096627.1	ECHDC2	0	0	0.01333333	1.116666667
XM_021096650.1	ORC1	0	0	0.07	8.48
XM_021096653.1	CC2D1B	0	0	0.35666667	84.24333333
XM_021096662.1	OSBPL9	0	0	0.01333333	1.476666667
XM_021096663.1	OSBPL9	0	0	0.13333333	16.87
XM_021096674.1	TTC39A	0	0	0.03333333	4.086666667
XM_021096688.1	STIL	0	0.023333333	0.02666667	6.696666667
XM_021096694.1	PPIP5K1	0	0	0.01666667	4.32
XM_021096703.1	PPIP5K1	0	0	0.11	29.17666667
XM_021096709.1	EFCAB14	0	0	0.12333333	33.01666667

XM_021096718.1	PPIP5K1	0	0	0.01	2.773333333
XM_021096722.1	LOC100511937	0	0	0.2	17.39666667
XM_021096730.1	CCDC17	0	0	0.03	2.956666667
XM_021096733.1	CCDC17	0	0	0.07333333	6.693333333
XM_021096738.1	PPIP5K1	0	0	0.06666667	18.20333333
XM_021096785.1	TMEM53	0	0	0.36333333	26.00666667
XM_021096795.1	PPIP5K1	0	0	0.06333333	16.99
XM_021096816.1	SLC6A9	0	0	0.02	3.103333333
XM_021096819.1	SLC6A9	0	0	0.10333333	13.36666667
XM_021096822.1	SLC6A9	0	0	0.02	2.693333333
XM_021096841.1	PTPRF	0	0	0.01666667	5.303333333
XM_021096868.1	PTPRF	0	0	0.00333333	1.193333333
XM_021096870.1	PTPRF	0	0	0.01333333	3.303333333
XM_021096874.1	PTPRF	0	0	0.01	3.193333333
XM_021096915.1	TP53BP1	0	0	0.02	9.633333333
XM_021096941.1	EDN2	0	0	0.00666667	0.333333333
XM_021096976.1	NFYC	0	0	0.01666667	1.503333333
XM_021096996.1	ZNF643	0	0	0.02	2.693333333
XM_021097006.1	SPIRE2	0	0	0.01333333	2
XM_021097008.1	VPS9D1	0	0	0.01333333	1.716666667
XM_021097009.1	VPS9D1	0	0	0.02	2.53
XM_021097012.1	VPS9D1	0	0	0.01333333	1.533333333
XM_021097013.1	VPS9D1	0	0	0.01333333	1.213333333
XM_021097014.1	VPS9D1	0	0	0.07333333	7.36
XM_021097017.1	VPS9D1	0	0	0.12333333	13.71333333
XM_021097020.1	VPS9D1	0	0	0.09	10.39333333
XM_021097026.1	DEF8	0	0	0.02	7.88
XM_021097037.1	ANKRD11	0	0	0.01666667	7.196666667
XM_021097109.1	PRODH2	0	0	0.00333333	0.333333333
XM_021097115.1	PRODH2	0	0	0.01	1.333333333
XM_021097119.1	PRODH2	0	0	0.00666667	0.666666667
XM_021097127.1	ZNF567	0	0	0.00333333	0.44
XM_021097133.1	APLP1	0	0	0.01333333	1.116666667
XM_021097134.1	APLP1	0	0	0.05	4
XM_021097148.1	HPN	0	0	0.07666667	8.196666667
XM_021097169.1	LOC110255280	0	0	0.05	4.473333333
XM_021097177.1	ZBTB32	0	0	0.01	0.333333333
XM_021097189.1	ZNF829	0	0	0.00666667	0.673333333
XM_021097193.1	ZNF566	0	0	0.07333333	6.666666667
XM_021097215.1	FXYD5	0	0	0.00333333	0.153333333
XM_021097244.1	LOC100627241	0	0	0.03333333	6.073333333
XM_021097247.1	LOC100627241	0	0	0.16666667	29.42666667
XM_021097249.1	LOC100627241	0	0	0.10333333	18.09666667
XM_021097274.1	LOC110261295	0	0	0.01	2.333333333
XM_021097304.1	LOC110261312	0	0	0.02	2.153333333
XM_021097313.1	PLA2G4D	0	0	0.00333333	0.333333333
XM_021097318.1	LOC110261320	0	0	0.00666667	1.023333333
XM_021097324.1	LOC110261321	0	0	0.03	5.376666667
XM_021097326.1	PLA2G4E	0	0	0.25666667	73.44
XM_021097329.1	LOC110261321	0	0	0.02	3.626666667
XM_021097348.1	MZF1	0	0	0.01	1.096666667
XM_021097356.1	MZF1	0	0	0.03666667	3.546666667
XM_021097370.1	SLC27A5	0	0	0.02	2.183333333
XM_021097377.1	LOC110261334	0	0	0.03666667	4.73
XM_021097405.1	LOC110261337	0	0	0.00666667	1.293333333
XM_021097407.1	LOC110261337	0	0	0.01333333	2.106666667
XM_021097409.1	LOC110261337	0	0	0.04	7.47
XM_021097420.1	LOC110261338	0	0.07	0.04333333	2.57
XM_021097429.1	LTK	0	0	0.01	0.666666667
XM_021097435.1	LTK	0	0	0.00333333	0.333333333

XM_021097458.1	NUSAP1	0	0	0.06666667	11.22666667
XM_021097491.1	GNB1	0	0	0.40666667	45.32
XM_021097493.1	GNB1	0	0	0.35333333	44.89666667
XM_021097496.1	LOC110261347	0	0	0.01	0.996666667
XM_021097498.1	CAMTA1	0	0	0.00666667	3.273333333
XM_021097517.1	CAMTA1	0	0	0.00333333	1.626666667
XM_021097518.1	CAMTA1	0	0	0.00666667	3.603333333
XM_021097520.1	CAMTA1	0	0	0.01333333	3.73
XM_021097529.1	CAMTA1	0	0	0.01666667	0.683333333
XM_021097557.1	LOC110261361	0	0	0.01	1.333333333
XM_021097563.1	KAZN	0	0	0.01666667	4.37
XM_021097576.1	LOC110261378	0	0	0.00666667	0.333333333
XM_021097584.1	ZSCAN4	0	0	0.05	2.036666667
XM_021097588.1	CALML6	0	0	0.00666667	0.333333333
XM_021097602.1	SPOCD1	0	0	0.00666667	0.666666667
XM_021097628.1	CCDC32	0	0	0.05	1.126666667
XM_021097651.1	EIF4G3	0	0	0.01666667	4.596666667
XM_021097661.1	EIF4G3	0	0.38	0.02	4.856666667
XM_021097685.1	ALPL	0	0	0.03666667	4.346666667
XM_021097713.1	PTPRM	0	0.09	0.00333333	0.756666667
XM_021097733.1	DLGAP1	0	0	0.00333333	0.54
XM_021097740.1	RBBP8	0	0	0.18666667	26.83
XM_021097742.1	RBBP8	0	0	0.02333333	3.95
XM_021097755.1	ST6GALNAC3	0	0	0.00333333	0.59
XM_021097761.1	ROR1	0	0	0.03666667	9.33
XM_021097766.1	ROR1	0	0	0.06333333	10.98333333
XM_021097790.1	FGGY	0	0	0.08666667	9.546666667
XM_021097798.1	FGGY	0	0	0.02333333	2.25
XM_021097803.1	FGGY	0	0	0.19	16.03
XM_021097829.1	BMF	0	0.03	0.04666667	11.4
XM_021097837.1	BMF	0	0	0.02333333	5.583333333
XM_021097838.1	LOC110261434	0	0	0.01	0.333333333
XM_021097844.1	BMF	0	0.046666667	0.08666667	18.2
XM_021097847.1	BMF	0	0	0.07666667	17.15666667
XM_021097888.1	BRD2	0	0.003333333	0.7	135.92
XM_021097890.1	BRD2	0	0	0.04666667	8.68
XM_021097895.1	BRD2	0	0	0.03666667	7.39
XM_021097954.1	ATF6B	0	0	0.01	0.773333333
XM_021097956.1	ATF6B	0	0	0.09666667	8.76
XM_021097961.1	HSPA1L	0	0	0.04	3.596666667
XM_021097962.1	EGFL8	0	0	0.01	0.733333333
XM_021097970.1	IRF4	0	0	0.00333333	0.973333333
XM_021097972.1	PPP1R18	0	0	0.03333333	5.276666667
XM_021097974.1	PPP1R18	0	0	0.25333333	31.15666667
XM_021097984.1	ODF2	0	0	0.02	4.283333333
XM_021097987.1	ATP6V1G2	0	0	0.05333333	3.693333333
XM_021097998.1	SPDEF	0	0	0.00666667	0.603333333
XM_021098004.1	GPHN	0	0	0.05	8.246666667
XM_021098013.1	GPHN	0	0	0.00666667	1.01
XM_021098015.1	GPHN	0	0.04	0.07666667	12.76
XM_021098025.1	RCN2	0	0	0.28333333	20.05666667
XM_021098057.1	LTB	0	0	0.01666667	0.933333333
XM_021098079.1	ACYP1	0	0	0.01333333	1.16
XM_021098082.1	MEIS2	0	0	0.02666667	3.216666667
XM_021098097.1	PRIM2	0	0	0.05	3.933333333
XM_021098110.1	UROC1	0	0	0.17666667	22.14666667
XM_021098113.1	SLC25A27	0	0	0.06333333	17.80666667
XM_021098131.1	MRPL52	0	0	0.03333333	0.47
XM_021098148.1	MSH5	0	0	0.02	2.743333333
XM_021098165.1	DPH6	0	0.226666667	0.02333333	23.6

XM_021098177.1	DICER1	0	0	0.03666667	12.06666667
XM_021098199.1	DPH6	0	0	0.00333333	2.22
XM_021098211.1	DPH6	0	0	0.00333333	1.45
XM_021098218.1	ANKS1A	0	0	0.02666667	7.78
XM_021098228.1	HAUS4	0	0	0.01	0.74
XM_021098229.1	HAUS4	0	0	0.02	1.333333333
XM_021098269.1	ARPIN	0	0	0.24333333	16.03
XM_021098308.1	TSHR	0	0	0.16666667	32.33
XM_021098318.1	PCK2	0	0	0.01666667	1.666666667
XM_021098345.1	ODF2	0	0	0.01666667	3.766666667
XM_021098350.1	FOXN3	0	0	0.05666667	21.14
XM_021098378.1	TTC23	0	0	0.01666667	3.493333333
XM_021098439.1	CCHCR1	0	0	0.04333333	5.353333333
XM_021098450.1	NFKBIL1	0	0	0.03666667	2.063333333
XM_021098452.1	LST1	0	0	0.01666667	0.686666667
XM_021098460.1	DDAH2	0	0	0.26666667	18.69
XM_021098468.1	LOC100155579	0	0	0.00333333	0.666666667
XM_021098513.1	LINS1	0	0	0.01333333	1.613333333
XM_021098534.1	MLIP	0	0	0.08666667	12.42333333
XM_021098549.1	MLIP	0	0	0.00333333	0.466666667
XM_021098563.1	ODF2	0	0	0.00333333	0.79
XM_021098564.1	ZNF451	0	0	0.05333333	11.44666667
XM_021098598.1	KIFC1	0	0	0.05333333	6.093333333
XM_021098608.1	PACSIN1	0	0	0.01333333	2.596666667
XM_021098610.1	PACSIN1	0	0	0.00666667	2.003333333
XM_021098635.1	MAPK13	0	0	0.01666667	1
XM_021098648.1	CPNE5	0	0	0.02	3.323333333
XM_021098712.1	TRERF1	0	0	0.01	2.843333333
XM_021098729.1	BICRAL	0	0	0.01	3.053333333
XM_021098730.1	BICRAL	0	0	0.3	86.62
XM_021098735.1	KLC4	0	0	0.59333333	66.78666667
XM_021098747.1	CRIP3	0	0	0.02333333	2.333333333
XM_021098755.1	FAN1	0	0	0.06333333	9.946666667
XM_021098773.1	TJAP1	0	0	0.01333333	2.61
XM_021098778.1	TJAP1	0	0	0.04666667	5.943333333
XM_021098780.1	TJAP1	0	0	0.12666667	15.63333333
XM_021098793.1	LOC110261530	0	0	0.21	8.203333333
XM_021098806.1	PTCHD4	0	0	0.01333333	3.333333333
XM_021098824.1	PKHD1	0	0	0.00666667	4
XM_021098852.1	LOC102167410	0	0	0.03	1.223333333
XM_021098853.1	LOC102167410	0	0	0.09666667	3.416666667
XM_021098864.1	CEMIP	0	1.333333333	0.00666667	2.87
XM_021098872.1	SAXO2	0	0	0.00333333	0.446666667
XM_021098914.1	FURIN	0	0.203333333	0.00666667	1.136666667
XM_021098972.1	ZNF710	0	0	0.04666667	13.76666667
XM_021099003.1	TMEM266	0	0	0.04333333	8.683333333
XM_021099029.1	NEIL1	0	0	0.08333333	9.763333333
XM_021099032.1	ZNF236	0	0	0.36666667	121.1966667
XM_021099043.1	ULK3	0	0	0.03333333	4.203333333
XM_021099048.1	LMAN1L	0	0	0.00666667	1
XM_021099061.1	CCDC33	0	0	0.01	0.503333333
XM_021099066.1	ISLR	0	0	0.1	8.803333333
XM_021099071.1	STOML1	0	0	0.12333333	10.77666667
XM_021099129.1	TTC6	0	0	0.00333333	0.666666667
XM_021099146.1	SLC25A21	0	0	0.00666667	1.226666667
XM_021099151.1	RALGAPA1	0	0	0.00666667	2.55
XM_021099177.1	ARHGAP5	0	0	0.12	44.46333333
XM_021099193.1	FBXO15	0	0	0.03666667	4.333333333
XM_021099202.1	NETO1	0	0	0.00333333	0.863333333
XM_021099213.1	CHMP4A	0	0	0.19333333	10.64666667

XM_021099229.1	ZFHX2	0	0	0.02	9.24
XM_021099231.1	ZFHX2	0	0.64	0.00333333	1.613333333
XM_021099254.1	RBM23	0	0	0.13	32.29666667
XM_021099261.1	RBM23	0	0	0.03333333	4.076666667
XM_021099280.1	LOC102160313	0	0	0.05666667	5.603333333
XM_021099287.1	SALL2	0	0	0.03	5.253333333
XM_021099298.1	CHD8	0	0.01	0.04333333	16.67333333
XM_021099300.1	CHD8	0	0.003333333	0.00333333	2.466666667
XM_021099312.1	CD226	0	0	0.01	0.923333333
XM_021099322.1	PNP	0	0	0.05666667	3.826666667
XM_021099327.1	LOC110261611	0	0	0.00333333	0.333333333
XM_021099344.1	LOC102160410	0	0	0.00333333	2.12
XM_021099350.1	LOC102160410	0	0	0.00333333	1.6
XM_021099359.1	MCTP2	0	0	0.00666667	1.346666667
XM_021099367.1	MCTP2	0	0	0.04666667	8.623333333
XM_021099403.1	SLC39A9	0	0	0.02333333	4.713333333
XM_021099416.1	CDH7	0	0	0.01	2
XM_021099432.1	SIPA1L1	0	0	0.01666667	5.49
XM_021099436.1	SIPA1L1	0	0	0.11	30.29
XM_021099437.1	SIPA1L1	0	0.04	0.03	9.07
XM_021099441.1	SIPA1L1	0	0	0.03333333	10.18
XM_021099464.1	CDH7	0	0	0.00666667	1
XM_021099493.1	HEATR4	0	0	0.00333333	0.333333333
XM_021099498.1	PTGR2	0	0	0.03333333	2.32
XM_021099523.1	AREL1	0	0	0.23666667	54.51
XM_021099528.1	AREL1	0	0.106666667	0.00333333	0.466666667
XM_021099537.1	LOC100624918	0	0	0.00333333	0.093333333
XM_021099538.1	LOC110261633	0	0	0.00333333	0.333333333
XM_021099544.1	ESRRB	0	0	0.08666667	17.31666667
XM_021099553.1	ISM2	0	0	0.00333333	0.333333333
XM_021099564.1	CEP128	0	0	0.04666667	8.753333333
XM_021099567.1	CEP128	0	0	0.00666667	1.606666667
XM_021099574.1	CEP128	0	0	0.03333333	7.18
XM_021099595.1	EFCAB11	0	0	0.02333333	0.926666667
XM_021099596.1	EFCAB11	0	0	0.00333333	0.376666667
XM_021099607.1	TTC7B	0	0	0.01333333	1.95
XM_021099612.1	RPS6KA5	0	0	0.02666667	4.53
XM_021099615.1	RPS6KA5	0	0	0.24666667	40.45666667
XM_021099617.1	RPS6KA5	0	0	0.04	5.25
XM_021099620.1	CATSPERB	0	0	0.00666667	0.666666667
XM_021099700.1	DEGS2	0	0	0.00666667	0.616666667
XM_021099709.1	SLC25A29	0	0	0.04	7.906666667
XM_021099710.1	SLC25A29	0	0	0.04	7.986666667
XM_021099713.1	SLC25A29	0	0	0.01	1.513333333
XM_021099744.1	LOC110261647	0	0	0.01	1.966666667
XM_021099747.1	LOC110261647	0	0	0.06333333	11.62
XM_021099764.1	PHACTR1	0	0	0.01	2.173333333
XM_021099777.1	ATXN1	0	0	0.03333333	11.46333333
XM_021099783.1	CDKAL1	0	0	0.09333333	8.613333333
XM_021099800.1	ZNF184	0	0	0.20666667	26.93666667
XM_021099802.1	ZSCAN12	0	0	0.02333333	6.57
XM_021099804.1	ZSCAN12	0	0	0.04	9.746666667
XM_021099861.1	AKAP6	0	0	0.01666667	8.143333333
XM_021099899.1	SCFD1	0	0	0.04666667	4.593333333
XM_021099922.1	ZBTB25	0	0	0.08666667	6.276666667
XM_021099936.1	RAD51B	0	0	0.01333333	2.673333333
XM_021099951.1	LOC100627702	0	0	0.03666667	3.973333333
XM_021099952.1	RGS6	0	0	0.04333333	11.02666667
XM_021099953.1	RGS6	0	0	0.17	42.52666667
XM_021099954.1	RGS6	0	0	0.01	2.973333333

XM_021099955.1	LOC100627702	0	0	0.01	1.003333333
XM_021099957.1	LOC100627702	0	0	0.02	2.023333333
XM_021099984.1	NRXN3	0	0	0.00333333	0.513333333
XM_021099985.1	NRXN3	0	0	0.00333333	1.333333333
XM_021099991.1	NRXN3	0	0	0.00333333	1.153333333
XM_021099996.1	LOC110261695	0	0	0.00666667	0.333333333
XM_021100008.1	LOC100523076	0	0	0.00666667	0.333333333
XM_021100054.1	LOC106507712	0	0	0.01	0.383333333
XM_021100057.1	LOC100620407	0	0	0.02	1
XM_021100072.1	LOC100522152	0	0	0.00666667	0.666666667
XM_021100073.1	LOC102158547	0	0	0.00666667	0.333333333
XM_021100087.1	LOC110261759	0	0	0.01666667	1.333333333
XM_021100094.1	EXOC2	0	0	0.03666667	5.523333333
XM_021100139.1	IGDCC3	0	0	0.01	2.28
XM_021100141.1	LYRM4	0	0	0.01333333	0.356666667
XM_021100155.1	CAGE1	0	0	0.05333333	6.763333333
XM_021100161.1	SLC35B3	0	0	0.02	1.263333333
XM_021100163.1	SLC35B3	0	0	0.15666667	10.70666667
XM_021100166.1	SLC35B3	0	0	0.08	4.95
XM_021100167.1	LOC106504315	0	0	0.00333333	0.333333333
XM_021100168.1	LOC100152566	0	0	0.00666667	0.9
XM_021100169.1	MAK	0	0	0.00666667	1.333333333
XM_021100180.1	ELOVL2	0	0	0.02	3.333333333
XM_021100228.1	KIF13A	0	0.33	0.00333333	0.926666667
XM_021100247.1	MEGF11	0	0	0.02333333	5.64
XM_021100248.1	MEGF11	0	0	0.01	1.733333333
XM_021100257.1	MEGF11	0	0	0.03	3.743333333
XM_021100269.1	AIMP1	0	0	0.64666667	33.37
XM_021100287.1	SLC10A7	0	0	0.07666667	1.496666667
XM_021100312.1	RASGEF1B	0	0	0.03666667	5.386666667
XM_021100313.1	RASGEF1B	0	0	0.29333333	34.86333333
XM_021100314.1	MRO	0	0	0.00666667	1.126666667
XM_021100343.1	TRMT10A	0	0	0.31	32.15333333
XM_021100362.1	ARFIP1	0	0	0.08333333	10.08666667
XM_021100365.1	ARFIP1	0	0.07	0.00333333	0.573333333
XM_021100368.1	ARFIP1	0	0	0.03	4.13
XM_021100391.1	ANKRD17	0	0.713333333	0.00333333	1.706666667
XM_021100430.1	HOPX	0	0	0.03	1.56
XM_021100431.1	HOPX	0	0	0.06	3.133333333
XM_021100434.1	MAP2K5	0	0	0.01	0.836666667
XM_021100438.1	PPARGC1A	0	0	0.01666667	4.973333333
XM_021100449.1	SMAD1	0	0	0.11333333	13.88
XM_021100465.1	TRPC3	0	0	0.01	1.536666667
XM_021100472.1	PPP3CA	0	0	0.09	14.26333333
XM_021100492.1	GUCY1B1	0	0	0.02	2.776666667
XM_021100493.1	GUCY1B1	0	0	0.04666667	6.42
XM_021100510.1	GUCY1A1	0	0	0.04	9.806666667
XM_021100528.1	LOC110261946	0	0	0.00333333	0.333333333
XM_021100557.1	CCDC149	0	0	0.39666667	76.18333333
XM_021100562.1	LGI2	0	0	0.03333333	3.333333333
XM_021100568.1	ATP10D	0	0	0.13	32.75333333
XM_021100596.1	DCK	0	0	0.00333333	0.783333333
XM_021100609.1	CNOT6L	0	0	0.04333333	18.73333333
XM_021100640.1	LRBA	0	0	0.02	5.343333333
XM_021100644.1	DCLK2	0	0	0.02	4.673333333
XM_021100647.1	DCLK2	0	0	0.02666667	5.826666667
XM_021100653.1	IQCM	0	0	0.00333333	0.333333333
XM_021100676.1	KIF23	0	0	0.04	6.72
XM_021100686.1	KIF23	0	0	0.05	7.03
XM_021100707.1	ELOVL6	0	0	0.05333333	12.68

XM_021100713.1	TLE3	0	0	0.00333333	0.843333333
XM_021100721.1	CENPE	0	0.173333333	0.00333333	0.836666667
XM_021100724.1	CENPE	0	0.613333333	0.00333333	1.183333333
XM_021100732.1	SLC9B1	0	0	0.00333333	0.333333333
XM_021100741.1	CCSER1	0	0	0.01666667	3.433333333
XM_021100751.1	HERC3	0	0	0.04666667	10.87333333
XM_021100756.1	HERC3	0	0	0.09666667	21.19666667
XM_021100763.1	HERC6	0	0	0.16333333	35.30333333
XM_021100775.1	MAPK10	0	0	0.00333333	0.666666667
XM_021100785.1	ARHGAP24	0	0	0.15333333	27.63
XM_021100822.1	MYL5	0	0	0.01333333	1
XM_021100834.1	GAK	0	0	0.06666667	12.45333333
XM_021100851.1	SLC26A1	0	0	0.00333333	0.666666667
XM_021100857.1	RNF212	0	0	0.05666667	14.66666667
XM_021100865.1	LRRC49	0	0	0.00333333	0.453333333
XM_021100883.1	UVSSA	0	0	0.03666667	3.266666667
XM_021100896.1	MYO9A	0	0	0.13333333	75.22
XM_021100897.1	TACC3	0	0	0.06666667	7.326666667
XM_021100905.1	FGFR3	0	0	0.01333333	2.333333333
XM_021100923.1	HAUS3	0	0	0.18333333	21.70333333
XM_021100940.1	GRK4	0	0	0.11333333	11.24
XM_021100951.1	ACOX3	0	0	0.11333333	16.19
XM_021100963.1	SORCS2	0	0	0.00333333	0.463333333
XM_021100975.1	C8H4orf50	0	0	0.00333333	1.333333333
XM_021100977.1	JAKMIP1	0	0	0.00666667	1.246666667
XM_021100979.1	JAKMIP1	0	0.333333333	0.00333333	0.753333333
XM_021100986.1	EVC2	0	0	0.01333333	2.223333333
XM_021100999.1	STX18	0	0	0.01333333	1.206666667
XM_021101002.1	STX18	0	0	0.01333333	1.16
XM_021101015.1	ZNF518B	0	0	0.07333333	21.20666667
XM_021101018.1	CLNK	0	0	0.04	3.61
XM_021101023.1	PROM1	0	0	0.03	4.08
XM_021101032.1	GEMIN2	0	0	0.06666667	2.27
XM_021101033.1	LDB2	0	0	0.04333333	8.133333333
XM_021101034.1	LDB2	0	0.053333333	0.02666667	3.903333333
XM_021101035.1	LDB2	0	0	0.02333333	3.706666667
XM_021101044.1	LDB2	0	0.103333333	0.00333333	0.473333333
XM_021101052.1	LDB2	0	0	0.03666667	4.733333333
XM_021101055.1	LDB2	0	0.01	0.05333333	5.903333333
XM_021101071.1	LRFN5	0	0	0.00666667	1.1
XM_021101073.1	PI4K2B	0	0	0.04	8.963333333
XM_021101090.1	ARAP2	0	0	0.03333333	12.30666667
XM_021101112.1	WDR19	0	0	0.06	10.43333333
XM_021101125.1	RBM47	0	0	0.00333333	0.79
XM_021101130.1	RBM47	0	0	0.01	2.926666667
XM_021101134.1	RBM47	0	0.206666667	0.00333333	0.526666667
XM_021101143.1	APBB2	0	0	0.11	36.23333333
XM_021101150.1	APBB2	0	0	0.41	121.44
XM_021101199.1	OCIAD1	0	0	0.12666667	7.046666667
XM_021101204.1	DCUN1D4	0	0	0.18	34.33
XM_021101205.1	DCUN1D4	0	0	0.06	11.92333333
XM_021101206.1	DCUN1D4	0	0	0.05333333	9.686666667
XM_021101233.1	FIP1L1	0	0	0.01	2.663333333
XM_021101263.1	GSX2	0	0	0.00333333	0.333333333
XM_021101268.1	LOC110262090	0	0	0.01	3.393333333
XM_021101276.1	LOC102162630	0	0	0.04666667	2
XM_021101278.1	MAP9	0	0	0.01	1.533333333
XM_021101288.1	TMEM144	0	0	0.04666667	6.786666667
XM_021101323.1	CLOCK	0	0	0.01666667	4.4
XM_021101349.1	EXOC1	0	0	0.03	3.833333333

XM_021101356.1	KIAA1211	0	0.163333333	0.03666667	11.6
XM_021101362.1	KIAA1211	0	0	0.02	5.09
XM_021101371.1	AASDH	0	0	0.14666667	53.26
XM_021101376.1	SPINT1	0	0	0.00666667	1
XM_021101393.1	LOC100513296	0	0	0.00333333	0.666666667
XM_021101429.1	RASSF6	0	0	0.00333333	0.333333333
XM_021101434.1	BTC	0	0	0.03666667	2.24
XM_021101451.1	G3BP2	0	0	0.18	36.62
XM_021101464.1	CCDC158	0	0	0.00666667	1.286666667
XM_021101465.1	CCDC158	0	0	0.00666667	1.066666667
XM_021101487.1	LOC102159783	0	0	0.15333333	5.666666667
XM_021101494.1	TRIM2	0	0	0.00666667	2.15
XM_021101508.1	FBXW7	0	0	0.09	15.03333333
XM_021101525.1	PRMT9	0	0	0.22333333	29.71333333
XM_021101530.1	TTC29	0	0	0.00333333	0.333333333
XM_021101534.1	LSM6	0	0	0.01333333	2.906666667
XM_021101546.1	LOC100523624	0	0	0.01	0.363333333
XM_021101558.1	ATL1	0	0	0.01	2
XM_021101575.1	ELF2	0	0	0.04333333	7.123333333
XM_021101601.1	NIN	0	0.163333333	0.00666667	2.77
XM_021101642.1	PRDM5	0	0	0.01666667	5.18
XM_021101657.1	LOC102165266	0	0	0.01	0.333333333
XM_021101675.1	LOC102157627	0	0	0.01666667	0.666666667
XM_021101694.1	PAPSS1	0	0	0.16333333	19.76666667
XM_021101705.1	NPNT	0	0	0.03333333	6.81
XM_021101737.1	TSPAN5	0	0	0.04666667	11.13333333
XM_021101813.1	SEC31A	0	0	0.01666667	3.673333333
XM_021101817.1	SEC31A	0	0.003333333	0.05	9.563333333
XM_021101821.1	SEC31A	0	0	0.02333333	4.62
XM_021101845.1	SEC31A	0	0.03	0.10333333	19.02333333
XM_021101859.1	FRMD6	0	0	0.20333333	43.05666667
XM_021101871.1	LIN54	0	0	0.02	3.453333333
XM_021101911.1	PON1	0	0	0.00333333	0.333333333
XM_021101934.1	ATM	0	0	0.23333333	121.9
XM_021101936.1	ATM	0	0	0.04333333	24.28666667
XM_021101937.1	LMO1	0	0	0.01	0.333333333
XM_021101943.1	PROX1	0	0	0.03333333	9.996666667
XM_021101945.1	PROX1	0	0	0.04666667	11.95333333
XM_021101954.1	STIM1	0	0	0.03333333	5.536666667
XM_021101960.1	GRB10	0	0	0.06666667	11.29666667
XM_021101967.1	DBF4	0	0	0.07333333	9.713333333
XM_021101990.1	TXNDC16	0	0	0.06333333	13.28
XM_021101996.1	MIOS	0	0	0.03	7.036666667
XM_021102018.1	CAMK1G	0	0	0.00666667	0.753333333
XM_021102019.1	CAMK1G	0	0	0.01	1.233333333
XM_021102024.1	VAMP4	0	0	0.05333333	12.42666667
XM_021102045.1	SLC37A4	0	0	0.21666667	25.74333333
XM_021102050.1	SLC37A4	0	0	0.87666667	101.4666667
XM_021102053.1	PIWIL4	0	0	0.02	3.283333333
XM_021102055.1	PIWIL4	0	0	0.00666667	0.693333333
XM_021102061.1	HBP1	0	0	0.15	18.38333333
XM_021102107.1	ALG9	0	0	0.02666667	5.673333333
XM_021102111.1	DRD2	0	0	0.15666667	19.39666667
XM_021102119.1	EZH2	0	0	0.02666667	2.64
XM_021102135.1	ACAD8	0	0	0.14333333	11.69666667
XM_021102136.1	ACAD8	0	0	0.39666667	33.01
XM_021102149.1	ST3GAL4	0	0	0.02	7.893333333
XM_021102153.1	ST3GAL4	0	0	0.00666667	1.936666667
XM_021102181.1	AKAP9	0	0.583333333	0.38333333	224.4333333
XM_021102192.1	AKAP9	0	0.056666667	0.13333333	77.84

XM_021102204.1	PIK3CG	0	0	0.08666667	23.88666667
XM_021102206.1	PIK3CG	0	0	0.01666667	5.49
XM_021102224.1	CACNA2D1	0	0	0.05333333	8.363333333
XM_021102236.1	SAMD4A	0	0	0.14333333	44.65
XM_021102260.1	ADORA1	0	0	0.01333333	1.466666667
XM_021102262.1	ADORA1	0	0	0.06	5.396666667
XM_021102303.1	DLG2	0	0	0.02333333	9.96
XM_021102309.1	DLG2	0	0	0.01333333	5.28
XM_021102317.1	DLG2	0	0	0.00333333	1.103333333
XM_021102318.1	DLG2	0	0.383333333	0.01	2.49
XM_021102319.1	DLG2	0	0	0.00333333	0.64
XM_021102321.1	DLG2	0	0	0.00333333	0.6
XM_021102323.1	DLG2	0	0	0.00333333	0.65
XM_021102351.1	NTM	0	0	0.01	1.116666667
XM_021102363.1	CDK14	0	0	0.01666667	3.92
XM_021102383.1	LOC100519098	0	0	0.17	34.11333333
XM_021102391.1	HEPACAM2	0	0	0.01333333	1.216666667
XM_021102393.1	HEPACAM2	0	0	0.00666667	1.293333333
XM_021102404.1	DYNC1I1	0	0	0.01333333	1.996666667
XM_021102407.1	DYNC1I1	0	0	0.01333333	1.693333333
XM_021102446.1	DGKB	0	0	0.05666667	10.52666667
XM_021102449.1	DGKB	0	0	0.00333333	0.606666667
XM_021102455.1	DGKB	0	0	0.00666667	0.87
XM_021102459.1	HDAC9	0	0	0.00333333	2.096666667
XM_021102486.1	HDAC9	0	0	0.00333333	1.906666667
XM_021102511.1	HDAC9	0	0.263333333	0.02666667	5.83
XM_021102560.1	NAPEPLD	0	0	0.02333333	5.046666667
XM_021102578.1	SRPK2	0	0	0.01333333	1.31
XM_021102621.1	CACNA1E	0	0	0.01666667	7.923333333
XM_021102635.1	RNF2	0	0	0.07333333	8.433333333
XM_021102652.1	TOMM20L	0	0	0.01	0.333333333
XM_021102674.1	LOC110262271	0	0	0.05666667	4.34
XM_021102675.1	LOC110262272	0	0	0.02666667	0.333333333
XM_021102676.1	UBTFL1	0	0	0.00333333	0.333333333
XM_021102682.1	LOC106504911	0	0	0.00666667	0.333333333
XM_021102683.1	LOC100518799	0	0	0.01	0.333333333
XM_021102686.1	DAAM1	0	0	0.04	9.863333333
XM_021102695.1	LOC110262288	0	0	0.01	0.333333333
XM_021102696.1	LOC110262289	0	0	0.02	1.666666667
XR_001298092.2	TDP1	0	0	0.01666667	1.53
XR_001298111.2	VRK1	0	0	0.01666667	1.506666667
XR_001299387.2	LOC106505208	0	0	0.01666667	2.486666667
XR_001299570.2	TSC22D1	0	0	0.01666667	4.276666667
XR_001301352.2	C15H2orf76	0	0	0.05666667	1.61
XR_001301497.2	LOC106506286	0	0	0.01	0.88
XR_001302710.2	THOC2	0	0	0.13333333	43.28333333
XR_001303253.2	PDGFA	0	0	0.12333333	10.12
XR_001303450.2	FAM19A3	0	0	0.00333333	0.666666667
XR_001303681.2	MEGF8	0	0	0.08666667	53.46666667
XR_001303733.2	SYTL1	0	0	0.01	0.886666667
XR_001304182.2	KIAA0040	0	0	0.00333333	1.283333333
XR_001304377.2	RNF157	0	0	0.03666667	3.076666667
XR_001304500.2	MASP1	0	0	0.07	9.173333333
XR_001306502.2	GCNT1	0	0	0.04333333	2.41
XR_001307151.2	COMMD10	0	0	0.07333333	6.593333333
XR_001307369.2	TBL2	0	0	0.00666667	0.81
XR_001307634.2	DGUOK	0	0	0.09333333	8.383333333
XR_001307920.2	RNF144A	0	0	0.01	0.826666667
XR_001307943.2	LRRC14	0	0	0.03666667	3.7
XR_001308131.2	RBM12B	0	0	0.03	0.833333333

XR_001308219.2	LOC106510078	0	0.003333333	0.00666667	0.563333333
XR_001308301.2	FCGR2B	0	0	0.14	9.556666667
XR_001308362.2	ADAM15	0	0	0.02	2.3
XR_001308430.2	SLC22A15	0	0	0.02	4.75
XR_001309084.2	RASGRP4	0	0	0.02333333	3.023333333
XR_001309222.2	WRAP73	0	0	0.02333333	1.976666667
XR_001309223.2	WRAP73	0	0	0.04333333	2.643333333
XR_001309383.2	FUBP1	0	0	0.02333333	3.143333333
XR_002335743.1	LOC100516455	0	0	0.00333333	0.496666667
XR_002335749.1	SNX19	0	0	0.05666667	31.40333333
XR_002335755.1	SNX19	0	0	0.05333333	8.066666667
XR_002335788.1	CDK18	0	0	0.07333333	8.75
XR_002335867.1	IGF2BP3	0	0	0.00333333	1.103333333
XR_002335884.1	LOC106504983	0	0	0.2	15.21666667
XR_002335889.1	LOC106504983	0	0	0.03	2.233333333
XR_002335893.1	LOC106504983	0	0	0.03666667	2.756666667
XR_002335977.1	LOC106507881	0	0	0.01333333	3.233333333
XR_002335981.1	LOC106507881	0	0	0.00333333	0.736666667
XR_002335985.1	LOC106507881	0	0	0.00666667	1.083333333
XR_002335988.1	LOC106507881	0	0	0.00333333	1.25
XR_002335991.1	LOC106507881	0	0	0.00333333	0.48
XR_002336001.1	LOC106507881	0	0	0.01333333	2.286666667
XR_002336008.1	LOC106507881	0	0	0.00666667	1.29
XR_002336065.1	TRAF3IP3	0	0	0.03666667	4.276666667
XR_002336070.1	TRAF3IP3	0	0	0.01333333	3.053333333
XR_002336185.1	RAB3GAP2	0	0	0.14	40.27666667
XR_002336188.1	RAB3GAP2	0	0	0.02666667	8.096666667
XR_002336275.1	IPO9	0	0	0.07	13.45333333
XR_002336282.1	SYT2	0	0	0.00666667	1.456666667
XR_002336299.1	C10H9orf3	0	0.05	0.01666667	3.04
XR_002336499.1	UFM1	0	0	0.04	7.726666667
XR_002336503.1	COG6	0	0	0.07333333	18.52333333
XR_002336538.1	LOC102163801	0	0	0.01333333	1.99
XR_002336547.1	EBPL	0	0	0.11333333	3.773333333
XR_002336549.1	LOC106505288	0	0	0.01	0.333333333
XR_002336685.1	NAA16	0	0	0.02666667	4.816666667
XR_002336740.1	TPP2	0	0	0.07333333	20.15333333
XR_002336823.1	SRSF2	0	0	0.45333333	38.74666667
XR_002336879.1	LOC110255247	0	0	0.06333333	1.916666667
XR_002336881.1	LOC110255247	0	0	0.00333333	0.223333333
XR_002336883.1	LOC110255247	0	0	0.02666667	1.65
XR_002336892.1	B3GNTL1	0	0	0.11333333	7.633333333
XR_002336898.1	B3GNTL1	0	0	0.02	1.31
XR_002336908.1	C12H17orf62	0	0	0.07666667	5.36
XR_002336925.1	LOC110255939	0	0	0.01	3.083333333
XR_002336951.1	AFMID	0	0	0.03666667	4.99
XR_002337012.1	CPSF4L	0	0	0.01666667	10.44
XR_002337015.1	CPSF4L	0	0.003333333	0.03333333	17.71333333
XR_002337016.1	CPSF4L	0	0	0.01	2.783333333
XR_002337024.1	C12H17orf80	0	0.346666667	0.11	27.17666667
XR_002337033.1	LOC102165318	0	0	0.01	1.626666667
XR_002337037.1	LOC102165318	0	0	0.04666667	11.18333333
XR_002337103.1	VPS25	0	0	0.17333333	8.873333333
XR_002337128.1	CDK12	0	0	0.05333333	23.20333333
XR_002337173.1	LOC100514982	0	0	0.06333333	9.146666667
XR_002337190.1	PCTP	0	0	0.04666667	5.583333333
XR_002337211.1	GGNBP2	0	0	0.01	0.923333333
XR_002337250.1	ZNF207	0	0	0.00666667	2.04
XR_002337302.1	TLCD2	0	0	0.01666667	3.266666667
XR_002337303.1	TLCD2	0	0	0.02666667	5.1

XR_002337400.1	SHMT1	0	0	0.05	9.903333333
XR_002337421.1	FLCN	0	0	0.04333333	11.70333333
XR_002337459.1	MASP1	0	0	0.01333333	2.443333333
XR_002337483.1	CD200R1L	0	0	0.00333333	0.436666667
XR_002337501.1	CCRL2	0	0	0.01333333	1.13
XR_002337520.1	MST1R	0	0.646666667	0.00333333	0.57
XR_002337549.1	LOC100738931	0	0	0.01333333	1.9
XR_002337552.1	LOC100738931	0	0	0.00666667	0.87
XR_002337562.1	RFT1	0	0	0.03333333	6.526666667
XR_002337577.1	ACTR8	0	0	0.05333333	11.07
XR_002337579.1	ACTR8	0	0	0.01666667	4.053333333
XR_002337673.1	KBTBD12	0	0	0.04	3.67
XR_002337676.1	LOC110256296	0	0	0.01333333	2.07
XR_002337680.1	NPHP3	0	0	0.07	13.84666667
XR_002337681.1	NPHP3	0	0	0.02666667	4.66
XR_002337765.1	ATP11B	0	0	0.01666667	4.163333333
XR_002337811.1	CCDC50	0	0	0.08666667	30.06666667
XR_002337885.1	POLQ	0	0	0.02333333	7.536666667
XR_002337958.1	ERC2	0	0	0.00333333	1.036666667
XR_002338045.1	SMIM11A	0	0	0.02333333	0.696666667
XR_002338053.1	SMIM11A	0	0	0.05333333	1.333333333
XR_002338089.1	GRM7	0	0	0.00333333	1.583333333
XR_002338090.1	GRM7	0	0	0.01	2.463333333
XR_002338222.1	NKTR	0	0	0.02333333	9.34
XR_002338225.1	NKTR	0	0	0.01	4.24
XR_002338258.1	LOC100738134	0	0	0.04333333	7.383333333
XR_002338262.1	HPS1	0	0	0.03	4.41
XR_002338269.1	ADRA1A	0	0	0.01666667	9.533333333
XR_002338280.1	SCAF8	0	0	0.02666667	13.05333333
XR_002338297.1	CAMK2G	0	0	0.04333333	10.74333333
XR_002338349.1	LOC100738050	0	0	0.12333333	11.86333333
XR_002338359.1	WSCD2	0	0	0.00666667	1.803333333
XR_002338378.1	SRRM4	0	0	0.00666667	1.836666667
XR_002338392.1	USP54	0	0	0.06	18.42333333
XR_002338412.1	LOC100158108	0	0	0.01666667	4.576666667
XR_002338454.1	ZNF488	0	0	0.00333333	0.333333333
XR_002338456.1	ZNF488	0	0	0.00333333	0.333333333
XR_002338546.1	ATXN2	0	0.376666667	0.17666667	53.71333333
XR_002338751.1	CNNM2	0	0	0.02666667	3.81
XR_002338758.1	PDCD11	0	0	0.10333333	35.27
XR_002338788.1	LOC106506082	0	0	0.00333333	0.333333333
XR_002338873.1	CFAP46	0	0	0.00333333	1.376666667
XR_002338907.1	HELLS	0	0	0.02333333	3.833333333
XR_002338917.1	MPHOSPH9	0	0	0.01	2.126666667
XR_002338934.1	CDC45	0	0	0.03333333	5.573333333
XR_002338940.1	CEP44	0	0	0.01333333	1
XR_002338969.1	ARID4B	0	0	0.02	5.413333333
XR_002338985.1	C15H4orf47	0	0	0.02666667	1.843333333
XR_002339052.1	DNAJB2	0	0	0.47333333	58.27666667
XR_002339072.1	DCTD	0	0	0.03666667	3.816666667
XR_002339169.1	PMS1	0	0	0.05333333	2.17
XR_002339319.1	FAM168B	0	0	0.03666667	9.823333333
XR_002339388.1	FARP2	0	0	0.01	1.37
XR_002339430.1	USP40	0	0	0.21666667	37.79333333
XR_002339740.1	LOC100737060	0	0	0.01	8.67
XR_002339742.1	LOC100737060	0	0	0.00666667	5.306666667
XR_002339812.1	LGALS12	0	0	0.02666667	2.086666667
XR_002339813.1	LGALS12	0	0	0.02333333	1.926666667
XR_002339829.1	DNMT3B	0	0	0.01	2.07
XR_002339838.1	ADIG	0	0	0.04666667	2.393333333

XR_002339854.1	C17H20orf27	0	0	0.13	4.5
XR_002339955.1	EBF4	0	0	0.01	1.146666667
XR_002340051.1	SCP2D1	0	0	0.02333333	0.86
XR_002340104.1	DZANK1	0	0	0.02	2.85
XR_002340157.1	LOC110257498	0	0	0.00666667	0.806666667
XR_002340162.1	DHX35	0	0	0.02	4.233333333
XR_002340163.1	DHX35	0	0	0.02	2.823333333
XR_002340164.1	DHX35	0	0	0.01333333	1.963333333
XR_002340165.1	PPP4R1L	0	0	0.00666667	4.32
XR_002340170.1	PPP4R1L	0	0	0.00666667	3.28
XR_002340187.1	PPP4R1L	0	0.126666667	0.01	3.916666667
XR_002340189.1	PPP4R1L	0	0	0.01	3.003333333
XR_002340268.1	NCAPG2	0	0	0.04	6.636666667
XR_002340269.1	NCAPG2	0	0	0.02333333	4.466666667
XR_002340273.1	RNF32	0	0	0.03	1.776666667
XR_002340274.1	RNF32	0	0	0.02666667	1.81
XR_002340295.1	ASB10	0	0	0.06666667	4.486666667
XR_002340344.1	EPHB6	0	0	0.00666667	1.546666667
XR_002340352.1	TRPV5	0	0	0.00333333	0.333333333
XR_002340389.1	HNRNPA2B1	0	0	0.06333333	10.06
XR_002340429.1	LOC106506844	0	0	0.00333333	0.36
XR_002340474.1	PPP1R17	0	0	0.00333333	0.333333333
XR_002340533.1	NKAP	0	0	0.16333333	10.16666667
XR_002340545.1	LOC106506163	0	0	0.01	4.9
XR_002340594.1	ARMCX4	0	0	0.01666667	1.843333333
XR_002340596.1	ARMCX4	0	0	0.08	4.39
XR_002340724.1	LOC100623332	0	0	0.01666667	1.17
XR_002340746.1	FAM122B	0	0	0.00666667	0.886666667
XR_002340770.1	LOC106507061	0	0	0.00666667	0.333333333
XR_002340774.1	HDAC8	0	0	0.15333333	8.283333333
XR_002340785.1	LOC110257797	0	0	0.00333333	0.666666667
XR_002340795.1	DIAPH2	0	0	0.04	16.38333333
XR_002340845.1	RAI2	0	0	0.03	1.406666667
XR_002340847.1	SHROOM4	0	0	0.00333333	1.64
XR_002340880.1	MCTS1	0	0	0.09666667	6.093333333
XR_002340881.1	MCTS1	0	0	0.02333333	1.573333333
XR_002340910.1	LOC110257900	0	0.846666667	0.01	4.6
XR_002340956.1	EGFL7	0	0	0.04333333	3.37
XR_002340967.1	ARRDC1	0	0	0.04666667	6.016666667
XR_002341073.1	CEP170B	0	0	0.01	2.456666667
XR_002341277.1	SNRPN	0	0	0.01	1.516666667
XR_002341279.1	SNRPN	0	0	0.09	53.76666667
XR_002341281.1	SNRPN	0	0	0.01	2.886666667
XR_002341325.1	LOC100522225	0	0	0.01666667	2.346666667
XR_002341340.1	LOC102159510	0	0	0.04666667	17.51666667
XR_002341344.1	LOC102159510	0	0	0.01666667	9.116666667
XR_002341379.1	BRSK2	0	0	0.01	1.536666667
XR_002341390.1	LOC110259220	0	0	0.01666667	10.70333333
XR_002341391.1	LOC110259220	0	0	0.00333333	1.486666667
XR_002341441.1	LOC100511545	0	0	0.41666667	67.26
XR_002341444.1	FCN1	0	0	0.00333333	0.53
XR_002341604.1	EPS15L1	0	0	0.01666667	2.31
XR_002341610.1	LOC100516957	0	0	0.01666667	3.25
XR_002341613.1	LOC100516957	0	0	0.00666667	1.356666667
XR_002341640.1	YIPF2	0	0	0.01	0.963333333
XR_002341642.1	YIPF2	0	0	0.00666667	0.626666667
XR_002341669.1	MYO1F	0	0	0.10333333	17.89333333
XR_002341676.1	CAMSAP3	0	0	0.00333333	0.333333333
XR_002341689.1	PIP5K1C	0	0	0.00666667	2.603333333
XR_002341690.1	PIP5K1C	0	0.29	0.03666667	13.81333333

XR_002341763.1	TENT2	0	0	0.13666667	38.68
XR_002341767.1	TENT2	0	0	0.05	11.34
XR_002341771.1	TENT2	0	0	0.01	2.18
XR_002341777.1	TENT2	0	0	0.01333333	2.136666667
XR_002341796.1	ATP6AP1L	0	0	0.00666667	1.383333333
XR_002341798.1	ATP6AP1L	0	0	0.01666667	5.993333333
XR_002342009.1	IL17B	0	0.096666667	0.00666667	3.503333333
XR_002342084.1	LOC110259698	0	0	0.14666667	28.49333333
XR_002342085.1	LOC110259698	0	0	0.02	3.94
XR_002342090.1	LYRM7	0	0	0.07	11.06666667
XR_002342143.1	GPR75	0	0.05	0.02	3.5
XR_002342156.1	CD8A	0	0.363333333	0.00333333	1.13
XR_002342187.1	ZFAND2A	0	0	0.06	3.786666667
XR_002342195.1	LOC106509613	0	0	0.02333333	3.5
XR_002342246.1	ZNF789	0	0	0.07333333	5.11
XR_002342251.1	LOC100522670	0	0.796666667	0.00666667	1.6
XR_002342253.1	LOC100522670	0	0	0.00333333	0.8
XR_002342274.1	LOC110255267	0	0	0.00666667	0.333333333
XR_002342276.1	LOC100514951	0	0	0.02	1.193333333
XR_002342318.1	TBL2	0	0	0.05	7.07
XR_002342326.1	CLIP2	0	0	0.02666667	6.306666667
XR_002342339.1	SYTL3	0	0	0.02333333	2.333333333
XR_002342348.1	LOC106509673	0	0	0.00333333	0.043333333
XR_002342390.1	REXO5	0	0	0.03666667	5.256666667
XR_002342393.1	VPS35L	0	0	0.06666667	25.09666667
XR_002342423.1	ZC3H7A	0	0	0.03666667	5.98
XR_002342435.1	NUDT16L1	0	0	0.01333333	1.803333333
XR_002342436.1	NUDT16L1	0	0	0.01	1.426666667
XR_002342473.1	TMEM8A	0	0	0.02333333	3.693333333
XR_002342523.1	REV1	0	0	0.00333333	0.82
XR_002342532.1	VWA3B	0	0	0.01	2.23
XR_002342566.1	DUSP11	0	0	0.26	44.90333333
XR_002342603.1	ASPRV1	0	0	0.01333333	4.623333333
XR_002342609.1	ASPRV1	0	0	0.02333333	7.813333333
XR_002342714.1	CCDC88A	0	0	0.01333333	4.96
XR_002342775.1	GPATCH11	0	0	0.15333333	6.63
XR_002342820.1	DNAJC27	0	0	0.03	6.213333333
XR_002342858.1	GREB1	0	0	0.00333333	0.843333333
XR_002342906.1	AKAP7	0	0	0.00333333	0.743333333
XR_002342918.1	NLRC3	0	0	0.02	11.76
XR_002342933.1	WNK2	0	0	0.43	171.3366667
XR_002342952.1	CCDC85A	0	0	0.03333333	1.353333333
XR_002342954.1	CCDC85A	0	0	0.01	0.426666667
XR_002343044.1	TSNARE1	0	0	0.01	1.133333333
XR_002343352.1	LOC106510102	0	0	0.47666667	48.53
XR_002343353.1	LOC106510102	0	0	0.03333333	5.83
XR_002343354.1	LOC106510102	0	0	0.47666667	89.24
XR_002343355.1	LOC106510102	0	0	0.05333333	6.256666667
XR_002343356.1	LOC106510102	0	0	0.26	39.05333333
XR_002343357.1	LOC106510102	0	0	1.86333333	201.2166667
XR_002343370.1	C4H1orf226	0	0	0.00666667	3.49
XR_002343379.1	LOC100622980	0	0	0.16666667	17.07333333
XR_002343384.1	LOC100154852	0	0	0.01666667	0.416666667
XR_002343411.1	LRRC71	0	0	0.00333333	0.333333333
XR_002343475.1	MAB21L3	0	0	0.00333333	0.956666667
XR_002343479.1	MAB21L3	0	0	0.00333333	0.693333333
XR_002343617.1	GLMN	0	0	0.08666667	6.83
XR_002343638.1	LOC100523492	0	0	0.03333333	2.92
XR_002343658.1	LOC100621388	0	0	0.00666667	1.353333333
XR_002343682.1	RTN4IP1	0	0	0.18333333	25.12

XR_002343704.1	LOC110260430	0	0.026666667	0.05	1.893333333
XR_002343850.1	LOC110260611	0	0	0.06	7.543333333
XR_002343861.1	PACSIN2	0	0	0.03	4.666666667
XR_002343863.1	PACSIN2	0	0	0.13666667	19.02333333
XR_002343932.1	MCRS1	0	0.153333333	0.00666667	1.026666667
XR_002343984.1	LOC100626199	0	0	0.49333333	33.31666667
XR_002343986.1	DOP1A	0	0	0.00333333	1.476666667
XR_002344047.1	KCNC2	0	0	0.01666667	2.776666667
XR_002344073.1	LMNTD1	0	0	0.01	1.726666667
XR_002344111.1	CLEC1A	0	0	0.18666667	10.75666667
XR_002344137.1	DDX11	0	0	0.02333333	3.973333333
XR_002344153.1	MICAL3	0	0	0.03	3.596666667
XR_002344162.1	YAF2	0	0	0.00333333	1.303333333
XR_002344294.1	PAN2	0	0	0.04666667	7.673333333
XR_002344353.1	PTPRQ	0	0	0.00333333	1.14
XR_002344358.1	LOC110260827	0	0	0.02666667	1.9
XR_002344380.1	MBD1	0	0	0.06666667	24.63666667
XR_002344391.1	LPIN2	0	0	0.06666667	30.56666667
XR_002344407.1	ENOSF1	0	0	0.05333333	3.86
XR_002344422.1	FRRS1L	0	0	0.01333333	4.53
XR_002344493.1	TMEM170A	0	0	0.02	3.146666667
XR_002344505.1	PDPR	0	0	0.07333333	25.48333333
XR_002344561.1	SLC9A5	0	0	0.01333333	3.52
XR_002344570.1	LOC102161584	0	0	0.01	0.546666667
XR_002344642.1	CATSPERG	0	0	0.00666667	1.396666667
XR_002344706.1	PRKD2	0	0	0.04666667	8.146666667
XR_002344754.1	LOC100620498	0	0	0.00333333	1.226666667
XR_002344756.1	LOC100620498	0	0	0.01333333	1.39
XR_002344762.1	LOC100620498	0	0	0.00666667	0.953333333
XR_002344791.1	LOC100515899	0	0	0.00333333	0.416666667
XR_002344801.1	LOC110261048	0	0	0.01666667	2.95
XR_002344811.1	PPP1R12C	0	0.146666667	0.20666667	30.40333333
XR_002344839.1	KLHL17	0	0	0.00666667	0.826666667
XR_002344854.1	FAM214A	0	0	0.09	28.59
XR_002344855.1	FAM214A	0	0	0.03	8.47
XR_002344874.1	PLEKHG5	0	0	0.02666667	10.29666667
XR_002344894.1	TARDBP	0	0	0.00333333	1.113333333
XR_002344898.1	TARDBP	0	0	0.42	48.08333333
XR_002344917.1	RCC2	0	0	0.02666667	5.73
XR_002345032.1	AKAIN1	0	0	0.01333333	2.1
XR_002345036.1	AKAIN1	0	0	0.17333333	19.28333333
XR_002345043.1	METTL4	0	0	0.01666667	1.463333333
XR_002345044.1	METTL4	0	0	0.07	6.586666667
XR_002345045.1	LOC110261176	0	0	0.00333333	0.126666667
XR_002345047.1	LOC110261176	0	0	0.01	0.643333333
XR_002345068.1	LOC100525229	0	0	0.00666667	0.82
XR_002345132.1	FUBP1	0	0	0.01333333	2.636666667
XR_002345155.1	DEPDC1	0	0	0.00666667	1.463333333
XR_002345262.1	LOC102166622	0	0	0.02	5.163333333
XR_002345278.1	RIMS3	0	0	0.00666667	1.333333333
XR_002345322.1	GANC	0	0	0.03	4.903333333
XR_002345341.1	LOC102158906	0	0	0.03	15.26333333
XR_002345342.1	LOC102158906	0	0	0.01333333	5.343333333
XR_002345343.1	LOC102158906	0	0	0.01333333	7.296666667
XR_002345344.1	LOC102158906	0	0	0.02666667	11.39
XR_002345362.1	ZNF304	0	0	0.02333333	3.31
XR_002345375.1	LOC110261321	0	0	0.00666667	1.923333333
XR_002345377.1	LOC110261321	0	0	0.01333333	0.39
XR_002345379.1	LOC110261321	0	0.226666667	0.01	1.353333333
XR_002345401.1	LOC100737218	0	0	0.04	1.59

XR_002345402.1	ZNF446	0	0	0.01666667	2.146666667
XR_002345434.1	RAP1GAP	0	0	0.02666667	3.516666667
XR_002345439.1	TMEM241	0	0	0.06	3.103333333
XR_002345543.1	EIF2AK4	0	0	0.01333333	3.88
XR_002345555.1	EIF2AK4	0	0	0.04666667	15.33
XR_002345566.1	LOC106506784	0	0	0.01333333	1.063333333
XR_002345616.1	NCR3	0	0	0.00333333	0.333333333
XR_002345636.1	HMGCLL1	0	0	0.01666667	1.273333333
XR_002345663.1	TCP11	0	0	0.01333333	8.56
XR_002345665.1	TCP11	0	0	0.00333333	2.083333333
XR_002345671.1	TCP11	0	0	0.00666667	0.47
XR_002345677.1	TCP11	0	0	0.00333333	1.613333333
XR_002345785.1	ZSCAN2	0	0	0.02333333	5.686666667
XR_002345789.1	ZSCAN2	0	0	0.05	15.28
XR_002345809.1	VPS33B	0	0	0.13333333	21.74
XR_002345893.1	LOC100156469	0	0	0.00666667	1.233333333
XR_002345898.1	LOC100156469	0	0	0.03333333	6.333333333
XR_002345901.1	LOC100156469	0	0	0.04	13.11666667
XR_002345906.1	LOC100156469	0	0	0.00333333	0.3
XR_002345907.1	LOC100156469	0	0	0.02	2.373333333
XR_002345909.1	LOC100156469	0	0	0.02333333	2.436666667
XR_002345911.1	LOC100156469	0	0	0.02333333	2.193333333
XR_002345952.1	RNASE13	0	0	0.00666667	0.606666667
XR_002346048.1	LOC110261647	0	0	0.01333333	2.143333333
XR_002346217.1	DUSP22	0	0	0.07666667	8.573333333
XR_002346245.1	PRPF4B	0	0	0.09	25.40333333
XR_002346254.1	PRPF4B	0	0	0.03	8.056666667
XR_002346263.1	PRPF4B	0	0	0.03	8.166666667
XR_002346342.1	MEGF11	0	0	0.00666667	1.003333333
XR_002346384.1	LOC100520317	0	0	0.02333333	4.96
XR_002346386.1	LOC100520317	0	0	0.04333333	8.19
XR_002346389.1	LOC100520317	0	0	0.08333333	14.51666667
XR_002346391.1	LOC100520317	0	0.143333333	0.00666667	0.656666667
XR_002346410.1	MGST2	0	0	0.03	6.85
XR_002346411.1	MGST2	0	0	0.01333333	2.786666667
XR_002346412.1	MGST2	0	0	0.04	8.206666667
XR_002346413.1	MGST2	0	0	0.05	9.083333333
XR_002346414.1	MGST2	0	0	0.00666667	1.836666667
XR_002346418.1	MGST2	0	0	0.01333333	2.586666667
XR_002346545.1	ACOX3	0	0	0.03666667	6.006666667
XR_002346552.1	PPP2R2C	0	0	0.04	6.55
XR_002346659.1	LOC100519853	0	0	0.00666667	0.186666667
XR_002346799.1	C8H4orf33	0	0	0.04	3.033333333
XR_002346872.1	PPM1K	0	0	0.10666667	16.79333333
XR_002346934.1	PGR	0	0	0.00333333	0.666666667
XR_002346939.1	NPL	0	0	0.06666667	11.72
XR_002346940.1	KCNE3	0	0	0.04666667	4.78
XR_002347043.1	HEPACAM2	0	0	0.00333333	0.633333333
XR_002347045.1	ASB4	0	0	0.16	34.31333333
XR_002347063.1	SLC25A40	0	0	0.06	8.083333333
XR_002347099.1	TOMM20L	0	0	0.01666667	0.666666667
XR_298704.3	TAL1	0	0.026666667	0.12666667	36.81666667
XR_298748.3	TMEM225B	0	0.053333333	0.00666667	0.68
XR_298986.3	THAP9	0	0	0.03666667	13.44666667
XR_304986.3	LOC100525237	0	0	0.02	1
XR_306538.3	ZNHIT3	0	0	0.16	7.89
XR_309176.3	PNPLA4	0	0	0.01	0.493333333
