# Supplementary material for: Comprehensive analysis of miRNAs, lncRNAs and mRNAs profiles in backfat tissue between Daweizi and Yorkshire pigs
Source: Anim Biosci. 2022 Nov 13;36(3):404–16. doi: 10.5713/ab.22.0165 (PMC9996253; doi:10.5713/ab.22.0165)
Supplement: Supplementary file 8 [file ab-22-0165-Supplementary-Table-7.pdf]

Supplementary Table S7. GO annotation and KEGG pathway analysis of DE mRNAs.

Table S7-1. GO annotation of DE mRNAs.

GO_CFP term level_2	GO_CFP term level_1	Number
cellular process	biological_process	1138
biological regulation	biological_process	847
metabolic process	biological_process	815
regulation of biological process	biological_process	789
response to stimulus	biological_process	594
cellular component organization or biogenesis	biological_process	499
multicellular organismal process	biological_process	486
positive regulation of biological process	biological_process	447
developmental process	biological_process	441
signaling	biological_process	434
localization	biological_process	424
negative regulation of biological process	biological_process	384
locomotion	biological_process	151
immune system process	biological_process	135
cell proliferation	biological_process	132
biological adhesion	biological_process	97
multi-organism process	biological_process	92
growth	biological_process	87
reproduction	biological_process	76
reproductive process	biological_process	76
behavior	biological_process	35
rhythmic process	biological_process	14
cell killing	biological_process	11
detoxification	biological_process	11
pigmentation	biological_process	10
biological_process	biological_process	4
cell aggregation	biological_process	1
cell	cellular_component	1190
cell part	cellular_component	1184

organelle	cellular_component	905
organelle part	cellular_component	624
membrane	cellular_component	570
membrane part	cellular_component	419
protein-containing complex	cellular_component	370
membrane-enclosed lumen	cellular_component	337
extracellular region	cellular_component	115
cell junction	cellular_component	92
supramolecular complex	cellular_component	90
extracellular region part	cellular_component	90
synapse	cellular_component	82
synapse part	cellular_component	59
cellular_component	cellular_component	4
nucleoid	cellular_component	4
other organism	cellular_component	1
other organism part	cellular_component	1
binding	molecular_function	973
catalytic activity	molecular_function	468
molecular function regulator	molecular_function	137
transcription regulator activity	molecular_function	109
transporter activity	molecular_function	84
structural molecule activity	molecular_function	61
molecular transducer activity	molecular_function	59
translation regulator activity	molecular_function	10
antioxidant activity	molecular_function	8
cargo receptor activity	molecular_function	5
molecular carrier activity	molecular_function	4
molecular_function	molecular_function	4
hijacked molecular function	molecular_function	3

Table S7-2. KEGG pathway enrichment of DE mRNAs.

KEGG pathway term ID	KEGG pathway term description	KEGG pathway term level_1	KEGG pathway term level_2	Term candidate gene number	Total candidate gene number	Term gene number	Total gene number	Rich ratio	Q value
590	Arachidonic acid metabolism	Metabolism	Lipid metabolism	17	582	211	27723	0.08056872	6.05E-04
10	Glycolysis/Gluconeogenesis	Metabolism	Carbohydrate metabolism	14	582	189	27723	0.07407407	0.005791549
830	Retinol metabolism	Metabolism	Metabolism of cofactors and vitamins	13	582	216	27723	0.06018519	0.047721002
4218	Cellular senescence	Cellular Processes	Cell growth and death	29	582	727	27723	0.03988996	0.047721002
4360	Axon guidance	Organismal Systems	Development	39	582	1110	27723	0.03513514	0.05006444
4922	Glucagon signaling pathway	Organismal Systems	Endocrine system	22	582	509	27723	0.043222	0.05006444
3320	PPAR signaling pathway	Organismal Systems	Endocrine system	19	582	432	27723	0.04398148	0.062608999
4115	p53 signaling pathway	Cellular Processes	Cell growth and death	14	582	273	27723	0.05128205	0.062608999
4152	AMPK signaling pathway	Environmental Information Processing	Signal transduction	23	582	605	27723	0.03801653	0.113595691
4920	Adipocytokine signaling pathway	Organismal Systems	Endocrine system	17	582	396	27723	0.04292929	0.113595691
4066	HIF-1 signaling pathway	Environmental Information Processing	Signal transduction	17	582	406	27723	0.04187192	0.123195775
4151	PI3K-Akt signaling pathway	Environmental Information Processing	Signal transduction	44	582	1421	27723	0.03096411	0.13170814
51	Fructose and mannose metabolism	Metabolism	Carbohydrate metabolism	8	582	134	27723	0.05970149	0.133302988
4910	Insulin signaling pathway	Organismal Systems	Endocrine system	23	582	634	27723	0.0362776	0.139129903
4726	Serotonergic synapse	Organismal Systems	Nervous system	16	582	403	27723	0.03970223	0.17742896
4921	Oxytocin signaling pathway	Organismal Systems	Endocrine system	26	582	768	27723	0.03385417	0.17742896
4213	Longevity regulating pathway - multiple species	Organismal Systems	Aging	12	582	276	27723	0.04347826	0.195846348
1200	Carbon metabolism	Metabolism	Global and overview maps	14	582	348	27723	0.04022989	0.206785596
1100	Metabolic pathways	Metabolism	Global and overview maps	116	582	4632	27723	0.02504318	0.256074971
4137	Mitophagy - animal	Cellular Processes	Transport and catabolism	13	582	330	27723	0.03939394	0.256074971
4710	Circadian rhythm	Organismal Systems	Environmental adaptation	9	582	196	27723	0.04591837	0.256074971
4744	Phototransduction	Organismal Systems	Sensory system	4	582	55	27723	0.07272727	0.297855098
4977	Vitamin digestion and absorption	Organismal Systems	Digestive system	5	582	85	27723	0.05882353	0.336860974
4510	Focal adhesion	Cellular Processes	Cellular community - eukaryotes	28	582	930	27723	0.03010753	0.358275377
30	Pentose phosphate pathway	Metabolism	Carbohydrate metabolism	5	582	92	27723	0.05434783	0.369117054
71	Fatty acid degradation	Metabolism	Lipid metabolism	7	582	153	27723	0.04575163	0.369117054
311	Penicillin and cephalosporin biosynthesis	Metabolism	Biosynthesis of other secondary metabolites	1	582	2	27723	0.5	0.369117054
472	D-Arginine and D-ornithine metabolism	Metabolism	Metabolism of other amino acids	1	582	2	27723	0.5	0.369117054
4750	Inflammatory mediator regulation of TRP channels	Organismal Systems	Sensory system	17	582	516	27723	0.03294574	0.37245384
620	Pyruvate metabolism	Metabolism	Carbohydrate metabolism	7	582	164	27723	0.04268293	0.443790698
640	Propanoate metabolism	Metabolism	Carbohydrate metabolism	5	582	100	27723	0.05	0.443790698

4370	VEGF signaling pathway	Environmental Information Processing	Signal transduction	10	582	271	27723	0.03690037	0.443790698
4068	FoxO signaling pathway	Environmental Information Processing	Signal transduction	17	582	539	27723	0.03153989	0.452370262
330	Arginine and proline metabolism	Metabolism	Amino acid metabolism	7	582	172	27723	0.04069767	0.487532374
4964	Proximal tubule bicarbonate	Organismal Systems	Excretory system	4	582	77	27723	0.05194805	0.522132969
4010	MAPK signaling pathway	Environmental Information Processing	Signal transduction	36	582	1350	27723	0.02666667	0.548976767
591	Linoleic acid metabolism	Metabolism	Lipid metabolism	4	582	82	27723	0.04878049	0.575637032
4062	Chemokine signaling pathway	Organismal Systems	Immune system	22	582	776	27723	0.02835052	0.575637032
4215	Apoptosis - multiple species	Cellular Processes	Cell growth and death	6	582	149	27723	0.04026846	0.575637032
770	Pantothenate and CoA biosynthesis	Metabolism	Metabolism of cofactors and vitamins	4	582	85	27723	0.04705882	0.60236596
380	Tryptophan metabolism	Metabolism	Amino acid metabolism	6	582	156	27723	0.03846154	0.630172546
52	Galactose metabolism	Metabolism	Carbohydrate metabolism	4	582	94	27723	0.04255319	0.6431272
410	beta-Alanine metabolism	Metabolism	Metabolism of other amino acids	4	582	92	27723	0.04347826	0.6431272
600	Sphingolipid metabolism	Metabolism	Lipid metabolism	6	582	164	27723	0.03658537	0.6431272
630	Glyoxylate and dicarboxylate metabolism	Metabolism	Carbohydrate metabolism	4	582	94	27723	0.04255319	0.6431272
4014	Ras signaling pathway	Environmental Information Processing	Signal transduction	26	582	979	27723	0.02655771	0.6431272
4141	Protein processing in endoplasmic reticulum	Genetic Information Processing	Folding, sorting and degradation	18	582	631	27723	0.02852615	0.6431272
4530	Tight junction	Cellular Processes	Cellular community - eukaryotes	24	582	892	27723	0.02690583	0.6431272
4979	Cholesterol metabolism	Organismal Systems	Digestive system	6	582	161	27723	0.03726708	0.6431272
534	Glycosaminoglycan biosynthesis - heparan sulfate / heparin	Metabolism	Glycan biosynthesis and metabolism	3	582	64	27723	0.046875	0.691860847
4918	Thyroid hormone synthesis	Organismal Systems	Endocrine system	9	582	287	27723	0.03135889	0.691860847
3460	Fanconi anemia pathway	Genetic Information Processing	Replication and repair	7	582	213	27723	0.03286385	0.7136552
4727	GABAergic synapse	Organismal Systems	Nervous system	10	582	332	27723	0.03012048	0.7136552
4728	Dopaminergic synapse	Organismal Systems	Nervous system	18	582	677	27723	0.02658789	0.788259096
563	Glycosylphosphatidylinositol (GPI)-anchor biosynthesis	Metabolism	Glycan biosynthesis and metabolism	4	582	108	27723	0.03703704	0.796622129
4371	Apelin signaling pathway	Environmental Information Processing	Signal transduction	16	582	597	27723	0.02680067	0.796622129
1230	Biosynthesis of amino acids	Metabolism	Global and overview maps	7	582	225	27723	0.03111111	0.797787354
650	Butanoate metabolism	Metabolism	Carbohydrate metabolism	3	582	74	27723	0.04054054	0.8132496
520	Amino sugar and nucleotide sugar metabolism	Metabolism	Carbohydrate metabolism	5	582	150	27723	0.03333333	0.819253342
340	Histidine metabolism	Metabolism	Amino acid metabolism	3	582	77	27723	0.03896104	0.823226929
500	Starch and sucrose metabolism	Metabolism	Carbohydrate metabolism	4	582	113	27723	0.03539823	0.823226929
4974	Protein digestion and absorption	Organismal Systems	Digestive system	10	582	357	27723	0.0280112	0.823226929
140	Steroid hormone biosynthesis	Metabolism	Lipid metabolism	5	582	156	27723	0.03205128	0.837613525

4713	Circadian entrainment	Organismal Systems	Environmental adaptation	13	582	488	27723	0.02663934	0.837613525
400	Phenylalanine, tyrosine and tryptophan biosynthesis	Metabolism	Amino acid metabolism	1	582	14	27723	0.07142857	0.849062644
524	Neomycin, kanamycin and gentamicin biosynthesis	Metabolism	Biosynthesis of other secondary metabolites	1	582	13	27723	0.07692308	0.849062644
3440	Homologous recombination	Genetic Information Processing	Replication and repair	6	582	201	27723	0.02985075	0.849062644
4120	Ubiquitin mediated proteolysis	Genetic Information Processing	Folding, sorting and degradation	18	582	723	27723	0.02489627	0.849062644
4340	Hedgehog signaling pathway	Environmental Information Processing	Signal transduction	7	582	247	27723	0.02834008	0.849062644
4614	Renin-angiotensin system	Organismal Systems	Endocrine system	3	582	84	27723	0.03571429	0.849062644
4630	Jak-STAT signaling pathway	Environmental Information Processing	Signal transduction	15	582	589	27723	0.02546689	0.849062644
4724	Glutamatergic synapse	Organismal Systems	Nervous system	14	582	541	27723	0.025878	0.849062644
20	Citrate cycle (TCA cycle)	Metabolism	Carbohydrate metabolism	3	582	86	27723	0.03488372	0.850328565
430	Taurine and hypotaurine metabolism	Metabolism	Metabolism of other amino acids	2	582	49	27723	0.04081633	0.850328565
4270	Vascular smooth muscle contraction	Organismal Systems	Circulatory system	15	582	596	27723	0.02516779	0.850328565
4114	Oocyte meiosis	Cellular Processes	Cell growth and death	13	582	512	27723	0.02539063	0.859089895
260	Glycine, serine and threonine metabolism	Metabolism	Amino acid metabolism	4	582	134	27723	0.02985075	0.90020437
3013	RNA transport	Genetic Information Processing	Translation	15	582	613	27723	0.02446982	0.90020437
4392	Hippo signaling pathway - multiple species	Environmental Information Processing	Signal transduction	4	582	132	27723	0.03030303	0.90020437
4658	Th1 and Th2 cell differentiation	Organismal Systems	Immune system	10	582	391	27723	0.02557545	0.90020437
480	Glutathione metabolism	Metabolism	Metabolism of other amino acids	4	582	141	27723	0.02836879	0.91803182
604	Glycosphingolipid biosynthesis - ganglio series	Metabolism	Glycan biosynthesis and metabolism	2	582	58	27723	0.03448276	0.91803182
4071	Sphingolipid signaling pathway	Environmental Information Processing	Signal transduction	12	582	493	27723	0.02434077	0.91803182
4610	Complement and coagulation cascades	Organismal Systems	Immune system	6	582	225	27723	0.02666667	0.91803182
4625	C-type lectin receptor signaling pathway	Organismal Systems	Immune system	12	582	497	27723	0.02414487	0.91803182
4640	Hematopoietic cell lineage	Organismal Systems	Immune system	10	582	397	27723	0.02518892	0.91803182
4659	Th17 cell differentiation	Organismal Systems	Immune system	12	582	492	27723	0.02439024	0.91803182
4810	Regulation of actin cytoskeleton	Cellular Processes	Cell motility	21	582	906	27723	0.02317881	0.91803182
4973	Carbohydrate digestion and absorption	Organismal Systems	Digestive system	5	582	180	27723	0.02777778	0.91803182
230	Purine metabolism	Metabolism	Nucleotide metabolism	15	582	654	27723	0.02293578	0.928929353
270	Cysteine and methionine metabolism	Metabolism	Amino acid metabolism	4	582	155	27723	0.02580645	0.928929353
450	Selenocompound metabolism	Metabolism	Metabolism of other amino acids	2	582	70	27723	0.02857143	0.928929353
512	Mucin type O-glycan biosynthesis	Metabolism	Glycan biosynthesis and metabolism	4	582	148	27723	0.02702703	0.928929353

514	Other types of O-glycan biosynthesis	Metabolism	Glycan biosynthesis and metabolism	3	582	119	27723	0.02521008	0.928929353
533	Glycosaminoglycan biosynthesis - keratan sulfate	Metabolism	Glycan biosynthesis and metabolism	2	582	71	27723	0.02816901	0.928929353
592	alpha-Linolenic acid metabolism	Metabolism	Lipid metabolism	2	582	66	27723	0.03030303	0.928929353
670	One carbon pool by folate	Metabolism	Metabolism of cofactors and vitamins	2	582	74	27723	0.02702703	0.928929353
780	Biotin metabolism	Metabolism	Metabolism of cofactors and vitamins	1	582	22	27723	0.04545455	0.928929353
900	Terpenoid backbone biosynthesis	Metabolism	Metabolism of terpenoids and polyketides	2	582	70	27723	0.02857143	0.928929353
1040	Biosynthesis of unsaturated fatty acids	Metabolism	Lipid metabolism	2	582	71	27723	0.02816901	0.928929353
3410	Base excision repair	Genetic Information Processing	Replication and repair	3	582	117	27723	0.02564103	0.928929353
3420	Nucleotide excision repair	Genetic Information Processing	Replication and repair	4	582	156	27723	0.02564103	0.928929353
4024	cAMP signaling pathway	Environmental Information Processing	Signal transduction	20	582	926	27723	0.02159827	0.928929353
4211	Longevity regulating pathway	Organismal Systems	Aging	11	582	459	27723	0.02396514	0.928929353
4217	Necroptosis	Cellular Processes	Cell growth and death	14	582	606	27723	0.02310231	0.928929353
4310	Wnt signaling pathway	Environmental Information Processing	Signal transduction	16	582	727	27723	0.02200825	0.928929353
4380	Osteoclast differentiation	Organismal Systems	Development	13	582	589	27723	0.02207131	0.928929353
4391	Hippo signaling pathway - fly	Environmental Information Processing	Signal transduction	8	582	335	27723	0.0238806	0.928929353
4512	ECM-receptor interaction	Environmental Information Processing	Signaling molecules and interaction	9	582	383	27723	0.02349869	0.928929353
4612	Antigen processing and presentation	Organismal Systems	Immune system	7	582	311	27723	0.02250804	0.928929353
4660	T cell receptor signaling pathway	Organismal Systems	Immune system	11	582	468	27723	0.02350427	0.928929353
4662	B cell receptor signaling pathway	Organismal Systems	Immune system	8	582	353	27723	0.02266289	0.928929353
4664	Fc epsilon RI signaling pathway	Organismal Systems	Immune system	7	582	300	27723	0.02333333	0.928929353
4670	Leukocyte transendothelial migration	Organismal Systems	Immune system	12	582	510	27723	0.02352941	0.928929353
4722	Neurotrophin signaling pathway	Organismal Systems	Nervous system	13	582	580	27723	0.02241379	0.928929353
4745	Phototransduction - fly	Organismal Systems	Sensory system	4	582	164	27723	0.02439024	0.928929353
4924	Renin secretion	Organismal Systems	Endocrine system	8	582	337	27723	0.02373887	0.928929353
4972	Pancreatic secretion	Organismal Systems	Digestive system	9	582	404	27723	0.02227723	0.928929353
4975	Fat digestion and absorption	Organismal Systems	Digestive system	3	582	112	27723	0.02678571	0.928929353
4976	Bile secretion	Organismal Systems	Digestive system	8	582	356	27723	0.02247191	0.928929353
72	Synthesis and degradation of ketone bodies	Metabolism	Lipid metabolism	1	582	33	27723	0.03030303	0.935074144
510	N-Glycan biosynthesis	Metabolism	Glycan biosynthesis and metabolism	5	582	223	27723	0.02242152	0.935074144
4012	ErbB signaling pathway	Environmental Information Processing	Signal transduction	11	582	503	27723	0.02186879	0.935074144
4142	Lysosome	Cellular Processes	Transport and catabolism	9	582	411	27723	0.02189781	0.935074144
4915	Estrogen signaling pathway	Organismal Systems	Endocrine system	11	582	508	27723	0.02165354	0.935074144

4261	Adrenergic signaling in	Organismal Systems	Circulatory system	15	582	707	27723	0.02121641	0.953530961
4916	Melanogenesis	Organismal Systems	Endocrine system	10	582	468	27723	0.02136752	0.953530961
190	Oxidative phosphorylation	Metabolism	Energy metabolism	6	582	284	27723	0.02112676	0.973850823
515	Mannose type O-glycan biosynthesis	Metabolism	Glycan biosynthesis and metabolism	2	582	88	27723	0.02272727	0.973850823
920	Sulfur metabolism	Metabolism	Energy metabolism	1	582	38	27723	0.02631579	0.973850823
4013	MAPK signaling pathway - fly	Environmental Information Processing	Signal transduction	8	582	394	27723	0.02030457	0.973850823
4015	Rap1 signaling pathway	Environmental Information Processing	Signal transduction	21	582	1018	27723	0.02062868	0.973850823
4064	NF-kappa B signaling pathway	Environmental Information Processing	Signal transduction	9	582	437	27723	0.02059497	0.973850823
4150	mTOR signaling pathway	Environmental Information Processing	Signal transduction	14	582	680	27723	0.02058824	0.973850823
4621	NOD-like receptor signaling pathway	Organismal Systems	Immune system	14	582	689	27723	0.0203193	0.973850823
4714	Thermogenesis	Organismal Systems	Environmental adaptation	16	582	782	27723	0.02046036	0.973850823
4720	Long-term potentiation	Organismal Systems	Nervous system	7	582	333	27723	0.02102102	0.973850823
4919	Thyroid hormone signaling pathway	Organismal Systems	Endocrine system	11	582	542	27723	0.0202952	0.973850823
4923	Regulation of lipolysis in adipocytes	Organismal Systems	Endocrine system	5	582	245	27723	0.02040816	0.973850823
4966	Collecting duct acid secretion	Organismal Systems	Excretory system	2	582	88	27723	0.02272727	0.973850823
4214	Apoptosis - fly	Cellular Processes	Cell growth and death	6	582	297	27723	0.02020202	0.977909946
2010	ABC transporters	Environmental Information Processing	Membrane transport	5	582	249	27723	0.02008032	0.982814575
250	Alanine, aspartate and glutamate metabolism	Metabolism	Amino acid metabolism	2	582	98	27723	0.02040816	0.994082036
310	Lysine degradation	Metabolism	Amino acid metabolism	6	582	305	27723	0.01967213	0.998988939
53	Ascorbate and aldarate metabolism	Metabolism	Carbohydrate metabolism	1	582	55	27723	0.01818182	0.9999997
61	Fatty acid biosynthesis	Metabolism	Lipid metabolism	1	582	62	27723	0.01612903	0.9999997
62	Fatty acid elongation	Metabolism	Lipid metabolism	1	582	73	27723	0.01369863	0.9999997
100	Steroid biosynthesis	Metabolism	Lipid metabolism	1	582	52	27723	0.01923077	0.9999997
240	Pyrimidine metabolism	Metabolism	Nucleotide metabolism	5	582	334	27723	0.01497006	0.9999997
280	Valine, leucine and isoleucine degradation	Metabolism	Amino acid metabolism	3	582	157	27723	0.01910828	0.9999997
350	Tyrosine metabolism	Metabolism	Amino acid metabolism	1	582	94	27723	0.0106383	0.9999997
360	Phenylalanine metabolism	Metabolism	Amino acid metabolism	1	582	72	27723	0.01388889	0.9999997
532	Glycosaminoglycan biosynthesis - chondroitin sulfate / dermatan sulfate	Metabolism	Glycan biosynthesis and metabolism	1	582	68	27723	0.01470588	0.9999997
561	Glycerolipid metabolism	Metabolism	Lipid metabolism	3	582	298	27723	0.01006711	0.9999997
562	Inositol phosphate metabolism	Metabolism	Carbohydrate metabolism	2	582	370	27723	0.00540541	0.9999997
564	Glycerophospholipid metabolism	Metabolism	Lipid metabolism	9	582	467	27723	0.01927195	0.9999997
565	Ether lipid metabolism	Metabolism	Lipid metabolism	3	582	201	27723	0.01492537	0.9999997
601	Glycosphingolipid biosynthesis - lacto and neolacto series	Metabolism	Glycan biosynthesis and metabolism	2	582	146	27723	0.01369863	0.9999997
603	Glycosphingolipid biosynthesis - globo and isoglobo series	Metabolism	Glycan biosynthesis and metabolism	1	582	82	27723	0.01219512	0.9999997

730	Thiamine metabolism	Metabolism	Metabolism of cofactors and vitamins	1	582	62	27723	0.01612903	0.9999997
760	Nicotinate and nicotinamide metabolism	Metabolism	Metabolism of cofactors and vitamins	2	582	129	27723	0.01550388	0.9999997
790	Folate biosynthesis	Metabolism	Metabolism of cofactors and vitamins	1	582	92	27723	0.01086957	0.9999997
910	Nitrogen metabolism	Metabolism	Energy metabolism	1	582	55	27723	0.01818182	0.9999997
980	Metabolism of xenobiotics by cytochrome P450	Metabolism	Xenobiotics biodegradation and	2	582	142	27723	0.01408451	0.9999997
982	Drug metabolism - cytochrome P450	Metabolism	Xenobiotics biodegradation and	1	582	146	27723	0.00684932	0.9999997
983	Drug metabolism - other enzymes	Metabolism	Xenobiotics biodegradation and	2	582	225	27723	0.00888889	0.9999997
1212	Fatty acid metabolism	Metabolism	Global and overview maps	3	582	162	27723	0.01851852	0.9999997
3008	Ribosome biogenesis in eukaryotes	Genetic Information Processing	Translation	1	582	201	27723	0.00497512	0.9999997
3010	Ribosome	Genetic Information Processing	Translation	5	582	303	27723	0.01650165	0.9999997
3015	mRNA surveillance pathway	Genetic Information Processing	Translation	7	582	388	27723	0.01804124	0.9999997
3018	RNA degradation	Genetic Information Processing	Folding, sorting and degradation	4	582	306	27723	0.0130719	0.9999997
3022	Basal transcription factors	Genetic Information Processing	Transcription	2	582	155	27723	0.01290323	0.9999997
3030	DNA replication	Genetic Information Processing	Replication and repair	1	582	118	27723	0.00847458	0.9999997
3040	Spliceosome	Genetic Information Processing	Transcription	9	582	512	27723	0.01757813	0.9999997
3050	Proteasome	Genetic Information Processing	Folding, sorting and degradation	1	582	74	27723	0.01351351	0.9999997
3430	Mismatch repair	Genetic Information Processing	Replication and repair	1	582	70	27723	0.01428571	0.9999997
3450	Non-homologous end-joining	Genetic Information Processing	Replication and repair	1	582	51	27723	0.01960784	0.9999997
4020	Calcium signaling pathway	Environmental Information Processing	Signal transduction	17	582	969	27723	0.01754386	0.9999997
4022	cGMP-PKG signaling pathway	Environmental Information Processing	Signal transduction	13	582	794	27723	0.0163728	0.9999997
4060	Cytokine-cytokine receptor interaction	Environmental Information Processing	Signaling molecules and interaction	10	582	779	27723	0.01283697	0.9999997
4070	Phosphatidylinositol signaling system	Environmental Information Processing	Signal transduction	4	582	553	27723	0.00723327	0.9999997
4072	Phospholipase D signaling pathway	Environmental Information Processing	Signal transduction	8	582	813	27723	0.0098401	0.9999997
4080	Neuroactive ligand-receptor interaction	Environmental Information Processing	Signaling molecules and interaction	10	582	874	27723	0.01144165	0.9999997

4110	Cell cycle	Cellular Processes	Cell growth and death	6	582	539	27723	0.01113173	0.9999997
4136	Autophagy - other	Cellular Processes	Transport and catabolism	2	582	157	27723	0.01273885	0.9999997
4140	Autophagy - animal	Cellular Processes	Transport and catabolism	13	582	668	27723	0.01946108	0.9999997
4144	Endocytosis	Cellular Processes	Transport and catabolism	22	582	1203	27723	0.01828761	0.9999997
4145	Phagosome	Cellular Processes	Transport and catabolism	10	582	604	27723	0.01655629	0.9999997
4146	Peroxisome	Cellular Processes	Transport and catabolism	5	582	307	27723	0.01628664	0.9999997
4210	Apoptosis	Cellular Processes	Cell growth and death	9	582	534	27723	0.01685393	0.9999997
4212	Longevity regulating pathway - worm	Organismal Systems	Aging	5	582	281	27723	0.01779359	0.9999997
4216	Ferroptosis	Cellular Processes	Cell growth and death	3	582	216	27723	0.01388889	0.9999997
4260	Cardiac muscle contraction	Organismal Systems	Circulatory system	1	582	257	27723	0.00389105	0.9999997
4320	Dorso-ventral axis formation	Organismal Systems	Development	1	582	128	27723	0.0078125	0.9999997
4330	Notch signaling pathway	Environmental Information Processing	Signal transduction	3	582	214	27723	0.01401869	0.9999997
4341	Hedgehog signaling pathway - fly	Environmental Information Processing	Signal transduction	1	582	147	27723	0.00680272	0.9999997
4350	TGF-beta signaling pathway	Environmental Information Processing	Signal transduction	4	582	294	27723	0.01360544	0.9999997
4390	Hippo signaling pathway	Environmental Information Processing	Signal transduction	9	582	647	27723	0.01391036	0.9999997
4514	Cell adhesion molecules (CAMs)	Environmental Information Processing	Signaling molecules and interaction	14	582	832	27723	0.01682692	0.9999997
4520	Adherens junction	Cellular Processes	Cellular community - eukaryotes	7	582	575	27723	0.01217391	0.9999997
4540	Gap junction	Cellular Processes	Cellular community - eukaryotes	2	582	347	27723	0.00576369	0.9999997
4550	Signaling pathways regulating pluripotency of stem cells	Cellular Processes	Cellular community - eukaryotes	8	582	537	27723	0.01489758	0.9999997
4611	Platelet activation	Organismal Systems	Immune system	8	582	548	27723	0.01459854	0.9999997
4620	Toll-like receptor signaling pathway	Organismal Systems	Immune system	8	582	439	27723	0.01822323	0.9999997
4622	RIG-I-like receptor signaling pathway	Organismal Systems	Immune system	4	582	257	27723	0.0155642	0.9999997
4623	Cytosolic DNA-sensing pathway	Organismal Systems	Immune system	1	582	180	27723	0.00555556	0.9999997
4624	Toll and Imd signaling pathway	Organismal Systems	Immune system	2	582	158	27723	0.01265823	0.9999997
4650	Natural killer cell mediated	Organismal Systems	Immune system	6	582	422	27723	0.01421801	0.9999997
4657	IL-17 signaling pathway	Organismal Systems	Immune system	3	582	299	27723	0.01003344	0.9999997
4666	Fc gamma R-mediated phagocytosis	Organismal Systems	Immune system	6	582	449	27723	0.01336303	0.9999997
4668	TNF signaling pathway	Environmental Information Processing	Signal transduction	8	582	428	27723	0.01869159	0.9999997
4672	Intestinal immune network for IgA production	Organismal Systems	Immune system	1	582	164	27723	0.00609756	0.9999997
4711	Circadian rhythm - fly	Organismal Systems	Environmental adaptation	1	582	70	27723	0.01428571	0.9999997
4721	Synaptic vesicle cycle	Organismal Systems	Nervous system	5	582	322	27723	0.01552795	0.9999997
4723	Retrograde endocannabinoid signaling	Organismal Systems	Nervous system	6	582	541	27723	0.01109057	0.9999997
4725	Cholinergic synapse	Organismal Systems	Nervous system	11	582	558	27723	0.01971326	0.9999997
4730	Long-term depression	Organismal Systems	Nervous system	4	582	246	27723	0.01626016	0.9999997
4740	Olfactory transduction	Organismal Systems	Sensory system	12	582	1738	27723	0.00690449	0.9999997
4742	Taste transduction	Organismal Systems	Sensory system	2	582	229	27723	0.00873362	0.9999997

4911	Insulin secretion	Organismal Systems	Endocrine system	5	582	463	27723	0.01079914	0.9999997
4912	GnRH signaling pathway	Organismal Systems	Endocrine system	9	582	484	27723	0.01859504	0.9999997
4913	Ovarian steroidogenesis	Organismal Systems	Endocrine system	2	582	190	27723	0.01052632	0.9999997
4914	Progesterone-mediated oocyte maturation	Organismal Systems	Endocrine system	3	582	431	27723	0.00696056	0.9999997
4917	Prolactin signaling pathway	Organismal Systems	Endocrine system	2	582	321	27723	0.00623053	0.9999997
4925	Aldosterone synthesis and secretion	Organismal Systems	Endocrine system	9	582	504	27723	0.01785714	0.9999997
4926	Relaxin signaling pathway	Organismal Systems	Endocrine system	7	582	553	27723	0.01265823	0.9999997
4927	Cortisol synthesis and secretion	Organismal Systems	Endocrine system	1	582	305	27723	0.00327869	0.9999997
4928	Parathyroid hormone synthesis, secretion and action	Organismal Systems	Endocrine system	10	582	599	27723	0.01669449	0.9999997
4961	Endocrine and other factor-regulated calcium reabsorption	Organismal Systems	Excretory system	2	582	231	27723	0.00865801	0.9999997
4970	Salivary secretion	Organismal Systems	Digestive system	4	582	400	27723	0.01	0.9999997
4971	Gastric acid secretion	Organismal Systems	Digestive system	7	582	393	27723	0.0178117	0.9999997
4978	Mineral absorption	Organismal Systems	Digestive system	2	582	128	27723	0.015625	0.9999997
