# Supplementary material for: Comprehensive analysis of miRNAs, lncRNAs and mRNAs profiles in backfat tissue between Daweizi and Yorkshire pigs
Source: Anim Biosci. 2022 Nov 13;36(3):404–16. doi: 10.5713/ab.22.0165 (PMC9996253; doi:10.5713/ab.22.0165)
Supplement: Supplementary file 9 [file ab-22-0165-Supplementary-Table-8.pdf]

Supplementary Table S8. Potential DE mRNAs related to fat deposition and lipid metabolism.

RNA ID	Symbol	log2 (DWZ/Yorkshire)	Q value (DWZ/Yorkshire)	KEGG pathway description	GO_C desccrption	GO_F description	GO_P description
NM_213883.2	IGF2	-2.268181338	2.72E-14	04151///PI3K-Akt signaling pathway+++04010///MAPK signaling pathway+++04014///Ras signaling pathway	GO:0016020///membrane+++GO:0005576///extracellular region+++GO:0016021///integral component of membrane+++GO:0005615///extracellular space	GO:0005179///hormone activity+++GO:0005178///integrin binding+++GO:0008083///growth factor activity	GO:0005975///carbohydrate metabolic process+++GO:0008284///positive regulation of cell proliferation+++GO:0001892///embryonic placenta development+++GO:0051781///positive regulation of cell division+++GO:0001503///ossification+++GO:0031056///regulation of histone modification+++GO:0010469///regulation of signaling receptor activity+++GO:0051147///regulation of muscle cell differentiation+++GO:0000122///negative regulation of transcription by RNA polymerase II+++GO:0001701///in utero embryonic development+++GO:0006006///glucose metabolic process+++GO:0060669///embryonic placenta morphogenesis
XM_021080576.1	IGF2	-2.630210893	7.91E-08	04151///PI3K-Akt signaling pathway+++04014///Ras signaling pathway+++04010///MAPK signaling pathway	GO:0016020///membrane+++GO:0005615///extracellular space+++GO:0005576///extracellular region+++GO:0016021///integral component of membrane	GO:0008083///growth factor activity+++GO:0005179///hormone activity+++GO:0005178///integrin binding	GO:0051781///positive regulation of cell division+++GO:0006006///glucose metabolic process+++GO:0001701///in utero embryonic development+++GO:0005975///carbohydrate metabolic process+++GO:0051147///regulation of muscle cell differentiation+++GO:0000122///negative regulation of transcription by RNA polymerase II+++GO:0060669///embryonic placenta morphogenesis+++GO:0001892///embryonic placenta development+++GO:0001503///ossification+++GO:0010469///regulation of signaling receptor activity+++GO:0031056///regulation of histone modification+++GO:0008284///positive regulation of cell proliferation
XM_021097872.1	SLA-2	2.756366494	1.56E-04	04145///Phagosome+++04514///Cell adhesion molecules (CAMs)+++04144///Endocytosis+++04218///Cellular senescence+++04612///Antigen processing and presentation	GO:0016021///integral component of membrane+++GO:0042612///MHC class I protein complex		GO:0002474///antigen processing and presentation of peptide antigen via MHC class I+++GO:0006955///immune response

NM_214201.1	GPX1	1.180518799	2.35E-06	00480///Glutathione metabolism+++04918///Thyroid hormone synthesis+++00590///Arachidonic acid metabolism	GO:0005737///cytoplasm+++GO:0097413///Lewy body+++GO:0005829///cytosol+++GO:0005739///mitochondrion	GO:0004602///glutathione peroxidase activity+++GO:0017124///SH3 domain binding+++GO:0004601///peroxidase activity+++GO:0016491///oxidoreductase activity	GO:0042311///vasodilation+++GO:0090201///negative regulation of release of cytochrome c from mitochondria+++GO:0060055///angiogenesis involved in wound healing+++GO:1902042///negative regulation of extrinsic apoptotic signaling pathway via death domain receptors+++GO:0043523///regulation of neuron apoptotic process+++GO:0051702///interaction with symbiont+++GO:0043534///blood vessel endothelial cell migration+++GO:0042542///response to hydrogen peroxide+++GO:0002862///negative regulation of inflammatory response to antigenic stimulus+++GO:0000302///response to reactive oxygen species+++GO:0001885///endothelial cell development+++GO:0051897///positive regulation of protein kinase B signaling+++GO:0010332///response to gamma radiation+++GO:0018158///protein oxidation+++GO:0048741///skeletal muscle fiber development+++GO:0033194///response to hydroperoxide+++GO:0040029///regulation of gene expression, epigenetic+++GO:0043403///skeletal muscle tissue regeneration+++GO:0055114///oxidation-reduction process+++GO:0061136///regulation of proteasomal protein catabolic process+++GO:0008631///intrinsic apoptotic signaling pathway in response to oxidative stress+++GO:0009650///UV protection+++GO:0043066///negative regulation of apoptotic process+++GO:0009611///response to wounding+++GO:0045444///fat cell differentiation+++GO:0051450///myoblast proliferation+++GO:0008283///cell proliferation+++GO:1902176///negative regulation of oxidative stress-induced intrinsic apoptotic signaling pathway+++GO:0006979///response to oxidative stress+++GO:0001659///temperature homeostasis+++GO:0009410///response to xenobiotic stimulus+++GO:0014902///myotube differentiation+++GO:0043154///negative regulation of cysteine-type endopeptidase activity involved in apoptotic
NM_001115155.1	GPX3	2.154452184	3.74E-05	04918///Thyroid hormone synthesis+++00480///Glutathione metabolism+++00590///Arachidonic acid metabolism	GO:0005615///extracellular space	GO:0004601///peroxidase activity+++GO:0016491///oxidoreductase activity+++GO:0004602///glutathione peroxidase activity+++GO:0008430///selenium binding	GO:0006979///response to oxidative stress+++GO:0098869///cellular oxidant detoxification+++GO:0042744///hydrogen peroxide catabolic process+++GO:0051289///protein homotetramerization+++GO:0055114///oxidation-reduction process

NM_214366.1	PPP2CA	1.609945047	5.59E-04	04152///AMPK signaling pathway+++04013///MAPK signaling pathway - fly+++03015///mRNA surveillance pathway+++04728///Dopaminergic synapse+++04261///Adrenergic signaling in cardiomyocytes+++04350///TGF-beta signaling pathway+++04530///Tight junction+++04151///PI3K-Akt signaling pathway+++04138///Autophagy - yeast+++04140///Autophagy - animal+++04071///Sphingolipid signaling pathway+++04391///Hippo signaling pathway - fly+++04114///Oocyte meiosis+++04390///Hippo signaling pathway+++04730///Long-term depression+++04113///Meiosis - yeast+++04111///Cell cycle - yeast+++04136///Autophagy - other	GO:0000159///protein phosphatase type 2A complex+++GO:0005856///cytoskeleton+++GO:0005886///plasma membrane+++GO:0045121///membrane raft+++GO:0045202///synapse+++GO:0005737///cytoplasm+++GO:0005694///chromosome+++GO:0000922///spindle pole+++GO:0005634///nucleus+++GO:0005829///cytosol+++GO:0000775///chromosome, centromeric region	GO:0046872///metal ion binding+++GO:0046982///protein heterodimerization activity+++GO:0050811///GABA receptor binding+++GO:0016787///hydrolase activity+++GO:0004721///phosphoprotein phosphatase activity+++GO:0008022///protein C-terminus binding	GO:0007498///mesoderm development+++GO:0010719///negative regulation of epithelial to mesenchymal transition+++GO:0006470///protein dephosphorylation+++GO:0071902///positive regulation of protein serine/threonine kinase activity+++GO:0051321///meiotic cell cycle
XR_002345546.1	SLA-2	1.429697533	0.010705227	04612///Antigen processing and presentation+++04514///Cell adhesion molecules (CAMs)+++04218///Cellular senescence+++04144///Endocytosis+++04145///Phagosome	GO:0000139///Golgi membrane+++GO:0005886///plasma membrane+++GO:0005887///integral component of plasma membrane+++GO:0030670///phagocytic vesicle membrane+++GO:0005789///endoplasmic reticulum membrane+++GO:0005783///endoplasmic reticulum+++GO:0055038///recycling endosome membrane+++GO:0071556///integral component of lumenal side of endoplasmic reticulum membrane+++GO:0009986///cell surface+++GO:0012507///ER to Golgi transport vesicle membrane+++GO:0016020///membrane+++GO:0042612///MHC class I protein complex+++GO:0031901///early endosome	GO:0046977///TAP binding+++GO:0030881///beta-2-microglobulin binding+++GO:0042605///peptide antigen binding	GO:0016567///protein ubiquitination+++GO:0006955///immune response+++GO:0060337///type I interferon signaling pathway+++GO:0050776///regulation of immune response+++GO:0002474///antigen processing and presentation of peptide antigen via MHC class I+++GO:0002480///antigen processing and presentation of exogenous peptide antigen via MHC class I, TAP-independent+++GO:0060333///interferon-gamma-mediated signaling pathway+++GO:0002479///antigen processing and presentation of exogenous peptide antigen via MHC class I, TAP-dependent
XM_021099123.1	PKM	-2.733557111	0.008946525	00230///Purine metabolism+++01230///Biosynthesis of amino acids+++01100///Metabolic pathways+++01130///Biosynthesis of antibiotics+++01110///Biosynthesis of secondary metabolites+++00010///Glycolysis / Gluconeogenesis+++04922///Glucagon signaling pathway+++01120///Microbial metabolism in diverse environments+++01200///Carbon metabolism+++00620///Pyruvate metabolism	GO:0043209///myelin sheath+++GO:0005634///nucleus+++GO:0005929///cilium+++GO:0005829///cytosol+++GO:0005737///cytoplasm	GO:0016740///transferase activity+++GO:0000287///magnesium ion binding+++GO:0004743///pyruvate kinase activity+++GO:0030955///potassium ion binding+++GO:0016301///kinase activity+++GO:0003824///catalytic activity	GO:0032869///cellular response to insulin stimulus+++GO:1903672///positive regulation of sprouting angiogenesis+++GO:0012501///programmed cell death+++GO:0016310///phosphorylation+++GO:0006096///glycolytic process

XM_003123639.4	SQSTM1	1.204257295	0.006062215	04380///Osteoclast differentiation+++04137///Mitophagy - animal+++04218///Cellular senescence+++04217///Necroptosis	GO:0000407///phagophore assembly site+++GO:0016235///aggresome+++GO:0016605///PML body+++GO:0016234///inclusion body+++GO:0005737///cytoplasm+++GO:0005776///autophagosome+++GO:0043231///intracellular membrane-bounded organelle+++GO:0005829///cytosol+++GO:0097225///sperm midpiece+++GO:0000932///P-body+++GO:0044754///autolysosome+++GO:0044753///amphisome	GO:0046872///metal ion binding+++GO:0031625///ubiquitin protein ligase binding+++GO:0043130///ubiquitin binding+++GO:0019899///enzyme binding+++GO:0005080///protein kinase C binding+++GO:0019901///protein kinase binding+++GO:0042169///SH2 domain binding+++GO:0008270///zinc ion binding+++GO:0035255///ionotropic glutamate receptor binding+++GO:0070530///K63-linked polyubiquitin modification-dependent protein binding+++GO:0042802///identical protein binding	GO:0061635///regulation of protein complex stability+++GO:0098780///response to mitochondrial depolarisation+++GO:1905719///protein localization to perinuclear region of cytoplasm+++GO:0044130///negative regulation of growth of symbiont in host+++GO:0061912///selective autophagy+++GO:0000122///negative regulation of transcription by RNA polymerase II+++GO:0043122///regulation of I-kappaB kinase/NF-kappaB signaling+++GO:0035973///aggrephagy+++GO:0016236///macroautophagy+++GO:0006914///autophagy+++GO:0007005///mitochondrion organization+++GO:1900273///positive regulation of long-term synaptic potentiation+++GO:0000423///mitophagy+++GO:0007032///endosome organization+++GO:1903078///positive regulation of protein localization to plasma membrane
NM_001099931.1	FABP3	2.919151334	0.024281225	03320///PPAR signaling pathway	GO:0005737///cytoplasm	GO:0008289///lipid binding	
XM_013977556.2	SLA-1	-7.513247337	7.91E-08	04514///Cell adhesion molecules (CAMs)+++04144///Endocytosis+++04612///Antigen processing and presentation+++04145///Phagosome+++04218///Cellular senescence	GO:0042612///MHC class I protein complex+++GO:0016021///integral component of membrane		GO:0006955///immune response+++GO:0002474///antigen processing and presentation of peptide antigen via MHC class I
XM_021080648.1	IGF2	-8.066590296	5.39E-07	04010///MAPK signaling pathway+++04014///Ras signaling pathway+++04151///PI3K-Akt signaling pathway	GO:0016020///membrane+++GO:0016021///integral component of membrane+++GO:0005615///extracellular space+++GO:0005576///extracellular region	GO:0005178///integrin binding+++GO:0008083///growth factor activity+++GO:0005179///hormone activity	GO:0000122///negative regulation of transcription by RNA polymerase II+++GO:0008284///positive regulation of cell proliferation+++GO:0051781///positive regulation of cell division+++GO:0051147///regulation of muscle cell differentiation+++GO:0001503///ossification+++GO:0006006///glucose metabolic process+++GO:0001701///in utero embryonic development+++GO:0060669///embryonic placenta morphogenesis+++GO:0031056///regulation of histone modification+++GO:0005975///carbohydrate metabolic process+++GO:0010469///regulation of signaling receptor activity+++GO:0001892///embryonic placenta development

NM_001113702.1	SLA-2	-23.83174519	4.64E-07	04514///Cell adhesion molecules (CAMs)+++04144///Endocytosis+++04145///Phagosome+++04218///Cellular senescence+++04612///Antigen processing and presentation	GO:0009986///cell surface+++GO:0005789///endoplasmic reticulum membrane+++GO:0000139///Golgi membrane+++GO:0012507///ER to Golgi transport vesicle membrane+++GO:0031901///early endosome membrane+++GO:0005886///plasma membrane+++GO:0030670///phagocytic vesicle membrane+++GO:0005887///integral component of plasma membrane+++GO:0016020///membrane+++GO:0005783///endoplasmic reticulum+++GO:0071556///integral component of lumenal side of endoplasmic reticulum membrane+++GO:0042612///MHC class I protein complex+++GO:0055038///recycling endosome membrane+++GO:0005794///Golgi apparatus	GO:0046977///TAP binding+++GO:0030881///beta-2-microglobulin binding+++GO:0042605///peptide antigen binding	GO:0060333///interferon-gamma-mediated signaling pathway+++GO:0002474///antigen processing and presentation of peptide antigen via MHC class I+++GO:0050776///regulation of immune response+++GO:0002479///antigen processing and presentation of exogenous peptide antigen via MHC class I, TAP-dependent+++GO:0006955///immune response+++GO:0002480///antigen processing and presentation of exogenous peptide antigen via MHC class I, TAP-independent+++GO:0016567///protein ubiquitination+++GO:0060337///type I interferon signaling pathway
NM_001136511.1	HUS1	-4.573817745	0.005775176	04218///Cellular senescence	GO:0035861///site of double-strand break+++GO:0005634///nucleus+++GO:0030896///checkpoint clamp complex+++GO:0005730///nucleolus		GO:0000723///telomere maintenance+++GO:0006289///nucleotide-excision repair+++GO:0007093///mitotic cell cycle checkpoint+++GO:0044778///meiotic DNA integrity checkpoint+++GO:0009792///embryo development ending in birth or egg hatching+++GO:0031573///intra-S DNA damage checkpoint+++GO:0033314///mitotic DNA replication checkpoint+++GO:0000724///double-strand break repair via homologous recombination+++GO:0001932///regulation of protein phosphorylation+++GO:0008156///negative regulation of DNA replication+++GO:0000077///DNA damage checkpoint+++GO:0006974///cellular response to DNA damage stimulus+++GO:0006468///protein phosphorylation+++GO:0000075///cell cycle checkpoint+++GO:0009411///response to UV+++GO:0071479///cellular response to ionizing radiation
XM_021072575.1	PPP3CB	-6.228823559	6.20E-06	04724///Glutamatergic synapse+++04650///Natural killer cell mediated cytotoxicity+++04020///Calcium signaling pathway+++04658///Th1 and Th2 cell differentiation+++04010///MAPK signaling pathway+++04114///Oocyte meiosis+++04662///B cell receptor signaling pathway+++04659///Th17 cell differentiation+++04728///Dopaminergic synapse+++04660///T cell receptor signaling pathway+++04370///VEGF signaling pathway+++04924///Renin secretion+++04921///Oxytocin signaling pathway+++04022///cGMP-PKG signaling pathway+++04218///Cellular senescence+++04360///Axon guidance+++04922///Glucagon signaling pathway+++04380///Osteoclast differentiation+++04625///C-type lectin receptor signaling pathway+++04310///Wnt signaling	GO:0005955///calcineurin complex+++GO:0005886///plasma membrane+++GO:0030018///Z disc+++GO:0098978///glutamatergic synapse+++GO:0030315///T-tubule+++GO:0005737///cytoplasm	GO:0008144///drug binding+++GO:0033192///calmodulin-dependent protein phosphatase activity+++GO:0004722///protein serine/threonine phosphatase activity+++GO:0004721///phosphoprotein phosphatase activity+++GO:0016787///hydrolase activity+++GO:0005516///calmodulin binding+++GO:0019899///enzyme binding+++GO:0005509///calcium ion binding+++GO:0030346///protein phosphatase 2B binding	GO:0035690///cellular response to drug+++GO:1900242///regulation of synaptic vesicle endocytosis+++GO:0006468///protein phosphorylation+++GO:0031987///locomotion involved in locomotory behavior+++GO:0043029///T cell homeostasis+++GO:0030217///T cell differentiation+++GO:0006470///protein dephosphorylation+++GO:0001915///negative regulation of T cell mediated cytotoxicity+++GO:0097720///calcineurin-mediated signaling+++GO:0007507///heart development+++GO:0017156///calcium ion regulated exocytosis+++GO:0035774///positive regulation of insulin secretion involved in cellular response to glucose stimulus+++GO:0001946///lymphangiogenesis+++GO:0034097///response to

XM_013988563.2	AKT2	-2.938691008	4.39E-05	04212///Longevity regulating pathway - worm+++04380///Osteoclast differentiation+++04722///Neurotrophin signaling pathway+++04213///Longevity regulating pathway - multiple species+++04923///Regulation of lipolysis in adipocytes+++04630///Jak-STAT signaling pathway+++04071///Sphingolipid signaling pathway+++04261///Adrenergic signaling in cardiomyocytes+++04725///Cholinergic synapse+++04140///Autophagy - animal+++04917///Prolactin signaling pathway+++04022///cGMP-PKG signaling pathway+++04024///cAMP signaling pathway+++04926///Relaxin signaling pathway+++04668///TNF signaling pathway+++04919///Thyroid hormone signaling pathway+++04914///Progesterone-mediated oocyte maturation+++04915///Estrogen signaling pathway+++04150///mTOR signaling pathway+++04152///AMPK signaling pathway+++04660///T cell receptor signaling pathway+++04371///Apelin signaling pathway+++04066///HIF-1 signaling pathway+++04922///Glucagon signaling pathway+++04218///Cellular senescence+++04370///VEGF signaling pathway+++04211///Longevity regulating pathway+++04015///Rap1 signaling pathway+++04072///Phospholipase D signaling pathway+++04550///Signaling pathways regulating pluripotency of stem cells+++04920///Adipocytokine signaling pathway+++04625///C-type lectin receptor signaling pathway+++04062///Chemokine signaling pathway+++04620///Toll-like receptor signaling pathway+++04510///Focal adhesion+++04010///MAPK signaling pathway+++04666///Fc gamma R-mediated phagocytosis+++04014///Ras signaling pathway+++04664///Fc epsilon RI signaling pathway+++04973///Carbohydrate digestion and absorption+++04662///B cell receptor signaling pathway+++04210///Apoptosis+++04068///FoxO signaling pathway+++04012///ErbB signaling	GO:0005938///cell cortex+++GO:0005829///cytosol+++GO:0032587///ruffle membrane+++GO:0005886///plasma membrane+++GO:0043231///intracellular membrane-bounded organelle+++GO:0032991///protein-containing complex+++GO:0005634///nucleus	GO:0005515///protein binding+++GO:0016740///transferase activity+++GO:0004672///protein kinase activity+++GO:0000166///nucleotide binding+++GO:0004674///protein serine/threonine kinase activity+++GO:0005524///ATP binding+++GO:0016301///kinase activity	GO:0032287///peripheral nervous system myelin maintenance+++GO:0008284///positive regulation of cell proliferation+++GO:0018105///peptidyl-serine phosphorylation+++GO:0065002///intracellular protein transmembrane transport+++GO:0010918///positive regulation of mitochondrial membrane potential+++GO:0016310///phosphorylation+++GO:2000147///positive regulation of cell motility+++GO:0046326///positive regulation of glucose import+++GO:0090314///positive regulation of protein targeting to membrane+++GO:0043066///negative regulation of apoptotic process+++GO:0045725///positive regulation of glycogen biosynthetic process+++GO:0032000///positive regulation of fatty acid beta-oxidation+++GO:1904179///positive regulation of adipose tissue development+++GO:0072659///protein localization to plasma membrane+++GO:0008286///insulin receptor signaling pathway+++GO:0030335///positive regulation of cell migration+++GO:0031340///positive regulation of vesicle fusion+++GO:0006006///glucose metabolic process+++GO:0071486///cellular response to high light intensity+++GO:0097473///retinal rod cell apoptotic process+++GO:0032869///cellular response to insulin stimulus+++GO:0010907///positive regulation of glucose metabolic process+++GO:0006468///protein phosphorylation+++GO:0001934///positive regulation of protein phosphorylation+++GO:0043491///protein kinase B signaling+++GO:0035556///intracellular signal transduction+++GO:0010748///negative regulation of plasma membrane long-chain fatty acid transport+++GO:0090630///activation of GTPase activity
XM_021091815.1	LOC100512656	-2.898929418	0.020724044	00830///Retinol metabolism+++01100///Metabolic pathways	GO:0005789///endoplasmic reticulum membrane+++GO:0043231///intracellular membrane-bounded organelle+++GO:0031090///organelle membrane+++GO:0016021///integral component of membrane	GO:0009055///electron transfer activity+++GO:0004745///retinol dehydrogenase activity	GO:0006629///lipid metabolic process
XM_021071095.1	LOC100623720	-3.108027055	2.55E-09	04510///Focal adhesion+++04512///ECM-receptor interaction+++04151///PI3K-Akt signaling pathway+++04974///Protein digestion and absorption	GO:0005615///extracellular space+++GO:0031012///extracellular matrix+++GO:0005581///collagen trimer+++GO:0062023///collagen-containing extracellular matrix	GO:0005201///extracellular matrix structural constituent+++GO:0030020///extracellular matrix structural constituent conferring tensile strength	GO:0030198///extracellular matrix organization

XM_013980816.2	MAP2K6	-1.770223162	3.54E-07	04218///Cellular senescence+++04015///Rap1 signaling pathway+++04620///Toll-like receptor signaling pathway+++04212///Longevity regulating pathway - worm+++04664///Fc epsilon RI signaling pathway+++04668///TNF signaling pathway+++04010///MAPK signaling pathway+++04380///Osteoclast differentiation+++04912///GnRH signaling pathway+++04750///Inflammatory mediator regulation of TRP channels	GO:0005829///cytosol+++GO:0005634///nucleus	GO:0004674///protein serine/threonine kinase activity+++GO:0000166///nucleotide binding+++GO:0004708///MAP kinase kinase activity+++GO:0019901///protein kinase binding+++GO:0016301///kinase activity+++GO:0005524///ATP binding+++GO:0004672///protein kinase activity	GO:0120163///negative regulation of cold-induced thermogenesis+++GO:0016310///phosphorylation+++GO:0000165///MAPK cascade+++GO:0000187///activation of MAPK activity+++GO:0006468///protein phosphorylation+++GO:0060048///cardiac muscle contraction
XM_021075038.1	PPP1R3B	-1.762376476	0.032147271	04910///Insulin signaling pathway	GO:0000164///protein phosphatase type 1 complex	GO:0019888///protein phosphatase regulator activity	GO:0005977///glycogen metabolic process+++GO:0005981///regulation of glycogen catabolic process+++GO:0043666///regulation of phosphoprotein phosphatase activity+++GO:0005975///carbohydrate metabolic process
XM_005669518.3	PFKFB4	-1.028598195	0.011980256	00051///Fructose and mannose metabolism+++04152///AMPK signaling pathway	GO:0005829///cytosol	GO:0005524///ATP binding+++GO:0004331///fructose-2,6-bisphosphate 2-phosphatase activity+++GO:0003873///6-phosphofructo-2-kinase activity+++GO:0003824///catalytic activity	GO:0006000///fructose metabolic process+++GO:0046835///carbohydrate phosphorylation+++GO:0016311///dephosphorylation+++GO:0006003///fructose 2,6-bisphosphate metabolic process
XM_021096633.1	GPX7	-1.338056783	0.029188595	00590///Arachidonic acid metabolism+++00480///Glutathione metabolism+++04918///Thyroid hormone synthesis	GO:0005783///endoplasmic reticulum+++GO:0005576///extracellular region	GO:0004601///peroxidase activity+++GO:0016491///oxidoreductase activity+++GO:0004602///glutathione peroxidase activity	GO:0098869///cellular oxidant detoxification+++GO:0055114///oxidation-reduction process+++GO:0006979///response to oxidative stress
XM_021080641.1	IGF2	-10.55693293	1.71E-10	04151///PI3K-Akt signaling pathway+++04010///MAPK signaling pathway+++04014///Ras signaling pathway	GO:0005615///extracellular space+++GO:0016020///membrane+++GO:0016021///integral component of membrane+++GO:0005576///extracellular region	GO:0005179///hormone activity+++GO:0008083///growth factor activity+++GO:0005178///integrin binding	GO:0006006///glucose metabolic process+++GO:0008284///positive regulation of cell proliferation+++GO:0000122///negative regulation of transcription by RNA polymerase II+++GO:0060669///embryonic placenta morphogenesis+++GO:0051781///positive regulation of cell division+++GO:0010469///regulation of signaling receptor activity+++GO:0001503///ossification+++GO:0001892///embryonic placenta development+++GO:0005975///carbohydrate metabolic process+++GO:0001701///in utero embryonic development+++GO:0031056///regulation of histone modification+++GO:0051147///regulation of muscle cell differentiation

XM_021093026.1	APAF1	-4.477990917	2.73E-04	04210///Apoptosis+++04215///Apoptosis - multiple species+++04214///Apoptosis - fly+++04115///p53 signaling pathway	GO:0032991///protein-containing complex+++GO:0005634///nucleus+++GO:0005737///cytoplasm+++GO:0043293///apoptosome+++GO:0005829///cytosol	GO:0043531///ADP binding+++GO:0000166///nucleotide binding+++GO:0042802///identical protein binding+++GO:0008233///peptidase activity+++GO:0005524///ATP binding+++GO:0008656///cysteine-type endopeptidase activator activity involved in apoptotic process	GO:0008635///activation of cysteine-type endopeptidase activity involved in apoptotic process by cytochrome c+++GO:0007275///multicellular organism development+++GO:0007420///brain development+++GO:1902510///regulation of apoptotic DNA fragmentation+++GO:0006508///proteolysis+++GO:0042981///regulation of apoptotic process+++GO:0001843///neural tube closure+++GO:0030900///forebrain development+++GO:2001235///positive regulation of apoptotic signaling pathway+++GO:0006919///activation of cysteine-type endopeptidase activity involved in apoptotic process+++GO:0051402///neuron apoptotic process+++GO:0097190///apoptotic signaling pathway+++GO:0006915///apoptotic
XM_013994354.2	DDB2	-1.855766191	1.36E-06	04120///Ubiquitin mediated proteolysis+++03420///Nucleotide excision repair+++04115///p53 signaling pathway	GO:0030054///cell junction+++GO:0080008///Cul4-RING E3 ubiquitin ligase complex+++GO:0032991///protein-containing complex+++GO:0005634///nucleus+++GO:0005654///nucleoplasm+++GO:0031465///Cul4B-RING E3 ubiquitin ligase complex	GO:0004842///ubiquitin-protein transferase activity+++GO:0003684///damaged DNA binding+++GO:0044877///protein-containing complex binding	GO:0051865///protein autoubiquitination+++GO:0009411///response to UV+++GO:0035518///histone H2A monoubiquitination+++GO:0006281///DNA repair+++GO:0010224///response to UV-B+++GO:0070914///UV-damage excision repair+++GO:0000209///protein polyubiquitination+++GO:0006290///pyrimidine dimer repair

NM_001044525.1	KIT	-2.195084256	0.007324293	04014///Ras signaling pathway+++04151///PI3K-Akt signaling pathway+++04072///Phospholipase D signaling pathway+++04640///Hematopoietic cell lineage+++04015///Rap1 signaling pathway+++04916///Melanogenesis+++04010///MAPK signaling pathway	GO:0043235///receptor complex+++GO:0005887///integral component of plasma membrane+++GO:0005886///plasma membrane+++GO:0016021///integral component of membrane+++GO:0005615///extracellular space+++GO:0005737///cytoplasm+++GO:0009897///external side of plasma membrane+++GO:0009986///cell surface+++GO:0016020///membrane+++GO:0005911///cell-cell junction+++GO:0042629///mast cell granule	GO:0002020///protease binding+++GO:0046872///metal ion binding+++GO:0004888///transmembrane signaling receptor activity+++GO:0016301///kinase activity+++GO:0042803///protein homodimerization activity+++GO:0005524///ATP binding+++GO:0004713///protein tyrosine kinase activity+++GO:0004672///protein kinase activity+++GO:0000166///nucleotide binding+++GO:0004714///transmembrane receptor protein tyrosine kinase activity+++GO:0005020///stem cell factor receptor activity+++GO:0019955///cytokine binding+++GO:0016740///transferase activity	GO:0050910///detection of mechanical stimulus involved in sensory perception of sound+++GO:0046777///protein autophosphorylation+++GO:0043410///positive regulation of MAPK cascade+++GO:0030217///T cell differentiation+++GO:0097067///cellular response to thyroid hormone stimulus+++GO:0043473///pigmentation+++GO:0035556///intracellular signal transduction+++GO:0060374///mast cell differentiation+++GO:0035855///megakaryocyte development+++GO:0018108///peptidyl-tyrosine phosphorylation+++GO:0043069///negative regulation of programmed cell death+++GO:0008360///regulation of cell shape+++GO:0007283///spermatogenesis+++GO:0008284///positive regulation of cell proliferation+++GO:0030318///melanocyte differentiation+++GO:0060326///cell chemotaxis+++GO:0043406///positive regulation of MAP kinase activity+++GO:0007286///spermatid development+++GO:0002320///lymphoid progenitor cell differentiation+++GO:0048863///stem cell differentiation+++GO:0042531///positive regulation of tyrosine phosphorylation of STAT protein+++GO:0006954///inflammatory response+++GO:0002327///immature B cell differentiation+++GO:0051091///positive regulation of DNA-binding transcription factor activity+++GO:0002371///dendritic cell cytokine production+++GO:0030032///lamellipodium assembly+++GO:0038109///Kit signaling pathway+++GO:0009314///response to radiation+++GO:0035162///embryonic hemopoiesis+++GO:0030097///hemopoiesis+++GO:0070374///positive regulation of ERK1 and ERK2 cascade+++GO:0006687///glycosphingolipid metabolic process+++GO:0035701///hematopoietic stem cell migration+++GO:0097324///melanocyte migration+++GO:0000187///activation of MAPK activity+++GO:0031532///actin cytoskeleton reorganization+++GO:0032762///mast cell
XM_013997432.2	PKN2	-1.06853347	0.009550899	04151///PI3K-Akt signaling pathway	GO:0005737///cytoplasm+++GO:0030027///lamellipodium+++GO:0030496///midbody+++GO:0016604///nuclear body+++GO:0032991///protein-containing complex+++GO:0045111///intermediate filament cytoskeleton+++GO:0005654///nucleoplasm+++GO:0005634///nucleus+++GO:0005886///plasma membrane+++GO:0005829///cytosol+++GO:0032154///cleavage furrow+++GO:0043296///apical junction complex+++GO:0048471///perinuclear region of cytoplasm+++GO:0005813///centrosome	GO:0004674///protein serine/threonine kinase activity+++GO:0017049///GTP-Rho binding+++GO:0070063///RNA polymerase binding+++GO:0004672///protein kinase activity+++GO:0016740///transferase activity+++GO:0042826///histone deacetylase binding+++GO:0000166///nucleotide binding+++GO:0016301///kinase activity+++GO:0005524///ATP binding	GO:0007165///signal transduction+++GO:2000145///regulation of cell motility+++GO:0016310///phosphorylation+++GO:0006468///protein phosphorylation+++GO:0045931///positive regulation of mitotic cell cycle+++GO:0043297///apical junction assembly+++GO:0010631///epithelial cell migration+++GO:0032467///positive regulation of cytokinesis+++GO:0018105///peptidyl-serine phosphorylation+++GO:0035556///intracellular signal transduction+++GO:0045070///positive regulation of viral genome replication

XM_021071473.1	RXRA	-1.270846026	0.02998124	04919///Thyroid hormone signaling pathway+++04659///Th17 cell differentiation+++04928///Parathyroid hormone synthesis, secretion and action+++03320///PPAR signaling pathway+++04976///Bile secretion+++04151///PI3K-Akt signaling pathway+++04920///Adipocytokine signaling pathway	GO:0090575///RNA polymerase II transcription factor complex+++GO:0000790///nuclear chromatin+++GO:0005634///nucleus+++GO:0043235///receptor complex+++GO:0032991///protein-containing complex	GO:0004886///9-cis retinoic acid receptor activity+++GO:0046982///protein heterodimerization activity+++GO:0043565///sequence-specific DNA binding+++GO:0019899///enzyme binding+++GO:0008270///zinc ion binding+++GO:0008134///transcription factor binding+++GO:0016922///nuclear receptor binding+++GO:0050692///DBD domain binding+++GO:0044323///retinoic acid-responsive element binding+++GO:0000977///RNA polymerase II regulatory region sequence-specific DNA binding+++GO:0003677///DNA binding+++GO:0050693///LBD domain binding+++GO:0046872///metal ion binding+++GO:0042277///peptide binding+++GO:0001972///retinoic acid binding+++GO:0003690///double-stranded DNA binding+++GO:0031490///chromatin DNA binding+++GO:0003700///DNA-binding transcription factor activity+++GO:0044212///transcription regulatory region DNA binding+++GO:0004879///nuclear receptor activity+++GO:0070644///vitamin D response element binding+++GO:0042809///vitamin D receptor binding+++GO:0003707///steroid hormone receptor activity	GO:0055010///ventricular cardiac muscle tissue morphogenesis+++GO:0006357///regulation of transcription by RNA polymerase II+++GO:0007566///embryo implantation+++GO:0060528///secretory columnal luminar epithelial cell differentiation involved in prostate glandular acinus development+++GO:0001890///placenta development+++GO:0001701///in utero embryonic development+++GO:0045893///positive regulation of transcription, DNA-templated+++GO:0007507///heart development+++GO:0032526///response to retinoic acid+++GO:0019048///modulation by virus of host morphology or physiology+++GO:0048384///retinoic acid receptor signaling pathway+++GO:0060038///cardiac muscle cell proliferation+++GO:0045994///positive regulation of translational initiation by iron+++GO:0043401///steroid hormone mediated signaling pathway+++GO:0045944///positive regulation of transcription by RNA polymerase II+++GO:0043010///camera-type eye development+++GO:0001893///maternal placenta development+++GO:0055012///ventricular cardiac muscle cell differentiation+++GO:0051289///protein homotetramerization+++GO:0000122///negative regulation of transcription by RNA polymerase II+++GO:0035357///peroxisome proliferator activated receptor signaling pathway+++GO:0006355///regulation of transcription, DNA-templated+++GO:0060687///regulation of branching involved in prostate gland
XM_013988572.2	HIPK4	-1.373123645	0.001941764	04218///Cellular senescence	GO:0005737///cytoplasm+++GO:0005634///nucleus	GO:0004672///protein kinase activity+++GO:0005524///ATP binding	GO:0016572///histone phosphorylation+++GO:1901796///regulation of signal transduction by p53 class mediator+++GO:0018105///peptidyl-serine phosphorylation+++GO:0006468///protein phosphorylation+++GO:0046777///protein autophosphorylation

XM_021095197.1	TMEM52	-1.748299409	5.42E-04	04912///GnRH signaling pathway+++04728///Dopaminergic synapse+++04745///Phototransduction - fly+++04070///Phosphatidylinositol signaling system+++04916///Melanogenesis+++04713///Circadian entrainment+++04014///Ras signaling pathway+++04218///Cellular senescence+++04024///cAMP signaling pathway+++04915///Estrogen signaling pathway+++04910///Insulin signaling pathway+++04625///C-type lectin receptor signaling pathway+++04750///Inflammatory mediator regulation of TRP channels+++04261///Adrenergic signaling in cardiomyocytes+++04744///Phototransduction+++04925///Aldosterone synthesis and secretion+++04722///Neurotrophin signaling pathway+++04971///Gastric acid secretion+++04921///Oxytocin signaling pathway+++04922///Glucagon signaling pathway+++04016///MAPK signaling pathway - plant+++04740///Olfactory transduction+++04924///Renin secretion+++04020///Calcium signaling pathway+++04015///Rap1 signaling pathway+++04114///Oocyte meiosis+++04022///cGMP-PKG signaling pathway+++04270///Vascular smooth muscle contraction+++04626///Plant-pathogen interaction+++04720///Long-term potentiation+++04970///Salivary	GO:0016020///membrane+++GO:0016021///integral component of membrane		
XR_002340201.1	HUS1	-1.567164014	0.019212631	04218///Cellular senescence	GO:0030896///checkpoint clamp complex+++GO:0005730///nucleolus+++GO:0005634///nucleus+++GO:0035861///site of double-strand break		GO:0001932///regulation of protein phosphorylation+++GO:0031573///intra-S DNA damage checkpoint+++GO:0033314///mitotic DNA replication checkpoint+++GO:0071479///cellular response to ionizing radiation+++GO:0000723///telomere maintenance+++GO:0044778///meiotic DNA integrity checkpoint+++GO:0006289///nucleotide-excision repair+++GO:0000075///cell cycle checkpoint+++GO:0008156///negative regulation of DNA replication+++GO:0006468///protein phosphorylation+++GO:0000724///double-strand break repair via homologous recombination+++GO:0009411///response to UV+++GO:0009792///embryo development ending in birth or egg hatching+++GO:0000077///DNA damage checkpoint+++GO:0007093///mitotic cell cycle checkpoint+++GO:0006974///cellular response to DNA damage stimulus

XM_021098438.1	LOC100515902	-2.403201879	0.00163902	04145///Phagosome+++04612///Antigen processing and presentation+++04218///Cellular senescence+++04514///Cell adhesion molecules (CAMs)+++04144///Endocytosis	GO:0009986///cell surface+++GO:0005887///integral component of plasma membrane+++GO:0016020///membrane+++GO:0055038///recycling endosome membrane+++GO:0012507///ER to Golgi transport vesicle membrane+++GO:0030670///phagocytic vesicle membrane+++GO:0005783///endoplasmic reticulum+++GO:0031901///early endosome membrane+++GO:0042612///MHC class I protein complex+++GO:0005789///endoplasmic reticulum membrane+++GO:0005794///Golgi apparatus+++GO:0005886///plasma membrane+++GO:0071556///integral component of lumenal side of endoplasmic reticulum membrane+++GO:0000139///Golgi membrane	GO:0042605///peptide antigen binding+++GO:0046977///TAP binding+++GO:0030881///beta-2-microglobulin binding	GO:0002474///antigen processing and presentation of peptide antigen via MHC class I+++GO:0060337///type I interferon signaling pathway+++GO:0002479///antigen processing and presentation of exogenous peptide antigen via MHC class I, TAP-dependent+++GO:0002480///antigen processing and presentation of exogenous peptide antigen via MHC class I, TAP-independent+++GO:0050776///regulation of immune response+++GO:0060333///interferon-gamma-mediated signaling pathway+++GO:0006955///immune response+++GO:0016567///protein ubiquitination
XM_005660928.3	DDB2	-1.871301977	0.026124862	03420///Nucleotide excision repair+++04115///p53 signaling pathway+++04120///Ubiquitin mediated proteolysis	GO:0005654///nucleoplasm+++GO:0080008///Cul4-RING E3 ubiquitin ligase complex+++GO:0032991///protein-containing complex+++GO:0030054///cell junction+++GO:0031465///Cul4B-RING E3 ubiquitin ligase complex+++GO:0005634///nucleus	GO:0044877///protein-containing complex binding+++GO:0004842///ubiquitin-protein transferase activity+++GO:0003684///damaged DNA binding	GO:0006290///pyrimidine dimer repair+++GO:0070914///UV-damage excision repair+++GO:0035518///histone H2A monoubiquitination+++GO:0051865///protein autoubiquitination+++GO:0006281///DNA repair+++GO:0009411///response to UV+++GO:0000209///protein polyubiquitination+++GO:0010224///response to UV-B
XM_021081655.1	LDHA	-1.592368404	0.004577741	01120///Microbial metabolism in diverse environments+++01110///Biosynthesis of secondary metabolites+++01100///Metabolic pathways+++00620///Pyruvate metabolism+++00270///Cysteine and methionine metabolism+++04922///Glucagon signaling pathway+++00010///Glycolysis / Gluconeogenesis+++00640///Propanoate metabolism+++01130///Biosynthesis of antibiotics	GO:0005737///cytoplasm	GO:0016616///oxidoreductase activity, acting on the CH-OH group of donors, NAD or NADP as acceptor+++GO:0003824///catalytic activity+++GO:0016491///oxidoreductase activity+++GO:0004459///L-lactate dehydrogenase activity	GO:0019752///carboxylic acid metabolic process+++GO:0055114///oxidation-reduction process+++GO:0005975///carbohydrate metabolic process
XM_005656555.3	EIF4E	8.120614695	1.46E-04	04151///PI3K-Akt signaling pathway+++03013///RNA transport+++04910///Insulin signaling pathway+++04211///Longevity regulating pathway+++04066///HIF-1 signaling pathway+++04150///mTOR signaling pathway	GO:0036464///cytoplasmic ribonucleoprotein granule+++GO:0010494///cytoplasmic stress granule+++GO:0005829///cytosol+++GO:0098978///glutamatergic synapse+++GO:0048471///perinuclear region of cytoplasm+++GO:0000932///P-body+++GO:0005845///mRNA cap binding complex+++GO:0016442///RISC complex+++GO:0005737///cytoplasm+++GO:0098794///postsynapse+++GO:0033391///chromatoid body+++GO:0016281///eukaryotic translation initiation factor 4F complex	GO:0031370///eukaryotic initiation factor 4G binding+++GO:0019899///enzyme binding+++GO:0000340///RNA 7-methylguanosine cap binding+++GO:0003723///RNA binding+++GO:0003743///translation initiation factor activity+++GO:0045182///translation regulator activity+++GO:0070491///repressing transcription factor binding	GO:0099578///regulation of translation at postsynapse, modulating synaptic transmission+++GO:0017148///negative regulation of translation+++GO:0001662///behavioral fear response+++GO:0071549///cellular response to dexamethasone stimulus+++GO:0006413///translational initiation+++GO:0019827///stem cell population maintenance+++GO:0045931///positive regulation of mitotic cell cycle+++GO:0006417///regulation of translation+++GO:0000082///G1/S transition of mitotic cell cycle+++GO:0006412///translation+++GO:0045665///negative regulation of neuron differentiation

XM_021072576.1	PPP3CB	22.47669815	2.61E-06	04010///MAPK signaling pathway+++04218///Cellular senescence+++04625///C-type lectin receptor signaling pathway+++04724///Glutamatergic synapse+++04310///Wnt signaling pathway+++04360///Axon guidance+++04114///Oocyte meiosis+++04662///B cell receptor signaling pathway+++04720///Long-term potentiation+++04658///Th1 and Th2 cell differentiation+++04924///Renin secretion+++04921///Oxytocin signaling pathway+++04728///Dopaminergic synapse+++04659///Th17 cell differentiation+++04022///cGMP-PKG signaling pathway+++04660///T cell receptor signaling pathway+++04380///Osteoclast differentiation+++04922///Glucagon signaling pathway+++04370///VEGF signaling pathway+++04020///Calcium signaling pathway+++04650///Natural killer cell mediated cytotoxicity	GO:0098978///glutamatergic synapse+++GO:0005737///cytoplasm+++GO:0005955///calcineurin complex+++GO:0030315///T-tubule+++GO:0005886///plasma membrane+++GO:0030018///Z disc	GO:0016787///hydrolase activity+++GO:0019899///enzyme binding+++GO:0005516///calmodulin binding+++GO:0004721///phosphoprotein phosphatase activity+++GO:0005509///calcium ion binding+++GO:0033192///calmodulin-dependent protein phosphatase activity+++GO:0008144///drug binding+++GO:0004722///protein serine/threonine phosphatase activity+++GO:0030346///protein phosphatase 2B binding	GO:0006470///protein dephosphorylation+++GO:0001946///lymphangiogenesis+++GO:1900242///regulation of synaptic vesicle endocytosis+++GO:0030217///T cell differentiation+++GO:0035690///cellular response to drug+++GO:0097720///calcineurin-mediated signaling+++GO:0006468///protein phosphorylation+++GO:0035774///positive regulation of insulin secretion involved in cellular response to glucose stimulus+++GO:0010468///regulation of gene expression+++GO:0043029///T cell homeostasis+++GO:0001915///negative regulation of T cell mediated cytotoxicity+++GO:0031987///locomotion involved in locomotory behavior+++GO:0007507///heart development+++GO:0034097///response to cytokine+++GO:0017156///calcium ion regulated exocytosis
XM_021097492.1	GNB1	21.16167518	1.02E-05	04371///Apelin signaling pathway+++04723///Retrograde endocannabinoid signaling+++04744///Phototransduction+++04062///Chemokine signaling pathway+++04727///GABAergic synapse+++04926///Relaxin signaling pathway+++04740///Olfactory transduction+++04724///Glutamatergic synapse+++04151///PI3K-Akt signaling pathway+++04011///MAPK signaling pathway - yeast+++04726///Serotonergic synapse+++04725///Cholinergic synapse+++04728///Dopaminergic synapse+++04014///Ras signaling pathway+++04713///Circadian entrainment	GO:0005834///heterotrimeric G-protein complex+++GO:0043209///myelin sheath+++GO:0001750///photoreceptor outer segment	GO:0003924///GTPase activity+++GO:0044877///protein-containing complex binding+++GO:0051020///GTPase binding	GO:0007186///G protein-coupled receptor signaling pathway+++GO:0007200///phospholipase C-activating G protein-coupled receptor signaling pathway+++GO:0050909///sensory perception of taste+++GO:0060041///retina development in camera-type eye+++GO:0007165///signal transduction+++GO:0008283///cell proliferation
XM_021096707.1	LOC100737897	7.634489128	0.011585609	00590///Arachidonic acid metabolism+++01100///Metabolic pathways+++04270///Vascular smooth muscle contraction+++00830///Retinol metabolism+++04750///Inflammatory mediator regulation of TRP channels+++00071///Fatty acid degradation+++03320///PPAR signaling pathway	GO:0005783///endoplasmic reticulum+++GO:0016020///membrane+++GO:0016021///integral component of membrane+++GO:0005789///endoplasmic reticulum membrane	GO:0004497///monooxygenase activity+++GO:0046872///metal ion binding+++GO:0005506///iron ion binding+++GO:0020037///heme binding+++GO:0016491///oxidoreductase activity+++GO:0016705///oxidoreductase activity, acting on paired donors, with incorporation or reduction of molecular oxygen	GO:0055114///oxidation-reduction process

XR_309303.3	IKBKG	20.05226809	3.65E-05	04010///MAPK signaling pathway+++04622///RIG-I-like receptor signaling pathway+++04668///TNF signaling pathway+++04151///PI3K-Akt signaling pathway+++04380///Osteoclast differentiation+++04625///C-type lectin receptor signaling pathway+++04062///Chemokine signaling pathway+++04620///Toll-like receptor signaling pathway+++04659///Th17 cell differentiation+++04662///B cell receptor signaling pathway+++04210///Apoptosis+++04014///Ras signaling pathway+++04623///Cytosolic DNA-sensing pathway+++04660///T cell receptor signaling pathway+++04064///NF-kappa B signaling pathway+++04621///NOD-like receptor signaling pathway+++04920///Adipocytokine signaling pathway+++04657///IL-17 signaling pathway+++04658///Th1 and Th2 cell differentiation	GO:0000151///ubiquitin ligase complex+++GO:0072686///mitotic spindle+++GO:0005737///cytoplasm+++GO:0000922///spindle pole+++GO:0008385///IkappaB kinase complex+++GO:0005634///nucleus+++GO:0005829///cytosol	GO:0042803///protein homodimerization activity+++GO:0046872///metal ion binding+++GO:1990450///linear polyubiquitin binding+++GO:0046982///protein heterodimerization activity+++GO:0016740///transferase activity+++GO:0031625///ubiquitin protein ligase binding+++GO:0019904///protein domain specific binding+++GO:0016301///kinase activity+++GO:0042802///identical protein binding+++GO:0070530///K63-linked polyubiquitin modification-dependent protein binding	GO:0006974///cellular response to DNA damage stimulus+++GO:0043123///positive regulation of I-kappaB kinase/NF-kappaB signaling+++GO:0051650///establishment of vesicle localization+++GO:0016310///phosphorylation+++GO:0045944///positive regulation of transcription by RNA polymerase II+++GO:0051092///positive regulation of NF-kappaB transcription factor activity
XM_021079417.1	PFKFB1	21.93516869	4.74E-06	04152///AMPK signaling pathway+++04922///Glucagon signaling pathway+++00051///Fructose and mannose metabolism	GO:0043540///6-phosphofructo-2-kinase/fructose-2,6-biphosphatase complex+++GO:0005829///cytosol	GO:0042802///identical protein binding+++GO:0003873///6-phosphofructo-2-kinase activity+++GO:0004331///fructose-2,6-bisphosphate 2-phosphatase activity+++GO:0016301///kinase activity+++GO:0005524///ATP binding+++GO:0003824///catalytic activity	GO:0016310///phosphorylation+++GO:0016311///dephosphorylation+++GO:0006003///fructose 2,6-bisphosphate metabolic process+++GO:0006000///fructose metabolic process+++GO:0046835///carbohydrate phosphorylation
NM_214425.1	CYP4A21	8.227945773	0.005538316	04750///Inflammatory mediator regulation of TRP channels+++00590///Arachidonic acid metabolism+++03320///PPAR signaling pathway+++04270///Vascular smooth muscle contraction+++01100///Metabolic pathways+++00071///Fatty acid degradation+++00830///Retinol metabolism	GO:0016020///membrane+++GO:0016021///integral component of membrane+++GO:0005783///endoplasmic reticulum+++GO:0005789///endoplasmic reticulum membrane	GO:0046872///metal ion binding+++GO:0005506///iron ion binding+++GO:0004497///monooxygenase activity+++GO:0016705///oxidoreductase activity, acting on paired donors, with incorporation or reduction of molecular oxygen+++GO:0020037///heme binding+++GO:0016491///oxidoreductase activity	GO:0055114///oxidation-reduction process
XM_021074019.1	CYP2C34	7.783404598	0.03971261	04726///Serotonergic synapse+++01100///Metabolic pathways+++00830///Retinol metabolism	GO:0031090///organelle membrane+++GO:0005789///endoplasmic reticulum membrane	GO:0016491///oxidoreductase activity+++GO:0046872///metal ion binding+++GO:0004497///monooxygenase activity+++GO:0005506///iron ion binding+++GO:0020037///heme binding+++GO:0016705///oxidoreductase activity, acting on paired donors, with incorporation or reduction of molecular oxygen	GO:0055114///oxidation-reduction process
XM_013998328.2	LIN37	6.382196727	0.021016084	04218///Cellular senescence	GO:0017053///transcriptional repressor complex		GO:0007049///cell cycle

XM_021090010.1	HIPK1	23.49485944	7.31E-07	04218///Cellular senescence	GO:0016605///PML body+++GO:0016607///nuclear speck+++GO:0005737///cytoplasm+++GO:0005654///nucleoplasm+++GO:0005813///centrosome+++GO:0005829///cytosol+++GO:0005634///nucleus	GO:0005524///ATP binding+++GO:0004672///protein kinase activity	GO:0060235///lens induction in camera-type eye+++GO:0042771///intrinsic apoptotic signaling pathway in response to DNA damage by p53 class mediator+++GO:0007224///smoothened signaling pathway+++GO:0008284///positive regulation of cell proliferation+++GO:0060059///embryonic retina morphogenesis in camera-type eye+++GO:0010803///regulation of tumor necrosis factor-mediated signaling pathway+++GO:0097191///extrinsic apoptotic signaling pathway+++GO:0072577///endothelial cell apoptotic process+++GO:0009952///anterior/posterior pattern specification+++GO:0010842///retina layer formation+++GO:0061072///iris morphogenesis+++GO:0030182///neuron differentiation+++GO:0048596///embryonic camera-type eye morphogenesis+++GO:0006468///protein phosphorylation+++GO:0034333///adherens junction assembly
NM_214424.1	CYP4A24	8.62469727	0.004407793	00590///Arachidonic acid metabolism+++04750///Inflammatory mediator regulation of TRP channels+++00830///Retinol metabolism+++04270///Vascular smooth muscle contraction+++03320///PPAR signaling pathway+++00071///Fatty acid degradation+++01100///Metabolic pathways	GO:0005789///endoplasmic reticulum membrane+++GO:0016021///integral component of membrane	GO:0004497///monooxygenase activity+++GO:0020037///heme binding+++GO:0016705///oxidoreductase activity, acting on paired donors, with incorporation or reduction of molecular oxygen+++GO:0005506///iron ion binding	
NM_214420.1	CYP2C49	8.397166552	0.025282449	01100///Metabolic pathways+++00830///Retinol metabolism+++04726///Serotonergic synapse	GO:0005789///endoplasmic reticulum membrane+++GO:0031090///organelle membrane	GO:0070330///aromatase activity+++GO:0005506///iron ion binding+++GO:0004497///monooxygenase activity+++GO:0020037///heme binding+++GO:0008392///arachidonic acid epoxygenase activity+++GO:0008395///steroid hydroxylase activity+++GO:0019825///oxygen binding	GO:0006805///xenobiotic metabolic process+++GO:0019373///epoxygenase P450 pathway
XM_021093492.1	LEPR	2.228967987	0.04327926	04060///Cytokine-cytokine receptor interaction+++04152///AMPK signaling pathway+++04630///Jak-STAT signaling pathway+++04080///Neuroactive ligand-receptor interaction+++04920///Adipocytokine signaling pathway	GO:0016323///basolateral plasma membrane+++GO:0016021///integral component of membrane	GO:0038021///leptin receptor activity	GO:0030217///T cell differentiation+++GO:0060259///regulation of feeding behavior+++GO:0001525///angiogenesis+++GO:0098868///bone growth+++GO:0010507///negative regulation of autophagy+++GO:0046850///regulation of bone remodeling+++GO:0019953///sexual reproduction+++GO:0033210///leptin-mediated signaling pathway+++GO:0042593///glucose homeostasis+++GO:0097009///energy homeostasis+++GO:0044321///response to leptin+++GO:0006909///phagocytosis

XM_021098329.1	TNXB	2.32106685	0.005204574	04151///PI3K-Akt signaling pathway+++04512///ECM-receptor interaction+++04510///Focal adhesion	GO:0031012///extracellular matrix+++GO:0062023///collagen-containing extracellular matrix+++GO:0005576///extracellular region+++GO:0043005///neuron projection+++GO:0005615///extracellular space	GO:0005201///extracellular matrix structural constituent+++GO:0046982///protein heterodimerization activity+++GO:0042803///protein homodimerization activity+++GO:0050839///cell adhesion molecule binding+++GO:0005518///collagen binding+++GO:0008201///heparin binding	GO:0048666///neuron development+++GO:0006631///fatty acid metabolic process+++GO:0098609///cell-cell adhesion+++GO:0043506///regulation of JUN kinase activity+++GO:0030199///collagen fibril organization+++GO:0030198///extracellular matrix organization+++GO:0007160///cell-matrix adhesion+++GO:0007157///heterophilic cell-cell adhesion via plasma membrane cell adhesion molecules+++GO:0006641///triglyceride metabolic process+++GO:0006629///lipid metabolic process
XM_021088844.1	PTK2	1.457558501	0.009348031	04012///ErbB signaling pathway+++04151///PI3K-Akt signaling pathway+++04810///Regulation of actin cytoskeleton+++04670///Leukocyte transendothelial migration+++04360///Axon guidance+++04370///VEGF signaling pathway+++04062///Chemokine signaling pathway+++04510///Focal adhesion	GO:0005856///cytoskeleton+++GO:0005737///cytoplasm+++GO:0005925///focal adhesion+++GO:0005886///plasma membrane+++GO:0005634///nucleus+++GO:0016324///apical plasma membrane+++GO:0005829///cytosol+++GO:0030027///lamellipodium+++GO:0001725///stress fiber	GO:0005524///ATP binding+++GO:0016301///kinase activity+++GO:0004715///non-membrane spanning protein tyrosine kinase activity+++GO:0019901///protein kinase binding+++GO:0003779///actin binding+++GO:0042169///SH2 domain binding+++GO:0004672///protein kinase activity+++GO:0019903///protein phosphatase binding+++GO:0008432///JUN kinase binding+++GO:0004713///protein tyrosine kinase activity	GO:0051964///negative regulation of synapse assembly+++GO:0001568///blood vessel development+++GO:0050771///negative regulation of axonogenesis+++GO:0007229///integrin-mediated signaling pathway+++GO:0007179///transforming growth factor beta receptor signaling pathway+++GO:0051893///regulation of focal adhesion assembly+++GO:0001570///vasculogenesis+++GO:0008284///positive regulation of cell proliferation+++GO:0001932///regulation of protein phosphorylation+++GO:0014068///positive regulation of phosphatidylinositol 3-kinase signaling+++GO:0043542///endothelial cell migration+++GO:2000060///positive regulation of ubiquitin-dependent protein catabolic process+++GO:0060396///growth hormone receptor signaling pathway+++GO:0010632///regulation of epithelial cell migration+++GO:0071560///cellular response to transforming growth factor beta stimulus+++GO:0021955///central nervous system neuron axonogenesis+++GO:0010507///negative regulation of autophagy+++GO:0045860///positive regulation of protein kinase activity+++GO:0018108///peptidyl-tyrosine phosphorylation+++GO:0048013///ephrin receptor signaling pathway+++GO:0043066///negative regulation of apoptotic process+++GO:0046777///protein autophosphorylation+++GO:0045667///regulation of osteoblast differentiation+++GO:0030335///positive regulation of cell migration+++GO:0008360///regulation of cell shape+++GO:0006468///protein phosphorylation+++GO:0046621///negative regulation of organ growth+++GO:0001934///positive regulation of protein phosphorylation+++GO:0051897///positive regulation of protein kinase B signaling+++GO:0001764///neuron migration+++GO:0001525///angiogenesis+++GO:0022408///negative regulation of cell-cell

XM_021101866.1	LIN54	1.556411155	1.16E-04	04218///Cellular senescence	GO:0005634///nucleus	GO:0003677///DNA binding+++GO:0046872///metal ion binding	GO:0007049///cell cycle+++GO:0006355///regulation of transcription, DNA-templated+++GO:0006351///transcription, DNA-templated
XM_021073562.1	PTPN11	1.282613951	0.003171929	04722///Neurotrophin signaling pathway+++04630///Jak-STAT signaling pathway+++04072///Phospholipase D signaling pathway+++04670///Leukocyte transendothelial migration+++04360///Axon guidance+++04013///MAPK signaling pathway - fly+++04920///Adipocytokine signaling pathway+++04650///Natural killer cell mediated cytotoxicity+++04014///Ras signaling pathway+++04625///C-type lectin receptor signaling pathway	GO:0005737///cytoplasm+++GO:0005634///nucleus+++GO:0032991///protein-containing complex	GO:0001784///phosphotyrosine residue binding+++GO:0016791///phosphatase activity+++GO:0051428///peptide hormone receptor binding+++GO:0019901///protein kinase binding+++GO:0050839///cell adhesion molecule binding+++GO:0030971///receptor tyrosine kinase binding+++GO:0004726///non-membrane spanning protein tyrosine phosphatase activity+++GO:0016787///hydrolase activity+++GO:0005070///SH3/SH2 adaptor activity+++GO:0005158///insulin receptor binding+++GO:0004725///protein tyrosine phosphatase activity+++GO:0004721///phosphoprotein phosphatase activity	GO:0046676///negative regulation of insulin secretion+++GO:0046825///regulation of protein export from nucleus+++GO:0046326///positive regulation of glucose import+++GO:0061582///intestinal epithelial cell migration+++GO:0048873///homeostasis of number of cells within a tissue+++GO:0030220///platelet formation+++GO:0048013///ephrin receptor signaling pathway+++GO:0048008///platelet-derived growth factor receptor signaling pathway+++GO:0007173///epidermal growth factor receptor signaling pathway+++GO:0051463///negative regulation of cortisol secretion+++GO:0040014///regulation of multicellular organism growth+++GO:0048609///multicellular organismal reproductive process+++GO:0060125///negative regulation of growth hormone secretion+++GO:0006629///lipid metabolic process+++GO:0033277///abortive mitotic cell cycle+++GO:0009755///hormone-mediated signaling pathway+++GO:0032760///positive regulation of tumor necrosis factor production+++GO:0021697///cerebellar cortex formation+++GO:0048806///genitalia development+++GO:0007507///heart development+++GO:0045931///positive regulation of mitotic cell cycle+++GO:0071364///cellular response to epidermal growth factor stimulus+++GO:0060325///face morphogenesis+++GO:0000187///activation of MAPK activity+++GO:0038127///ERBB signaling pathway+++GO:0042593///glucose homeostasis+++GO:0006470///protein dephosphorylation+++GO:0006641///triglyceride metabolic process+++GO:0007229///integrin-mediated signaling pathway+++GO:0048011///neurotrophin TRK receptor signaling pathway+++GO:0007420///brain development+++GO:0048839///inner ear development+++GO:0046887///positive regulation of hormone secretion+++GO:0032755///positive regulation of interleukin-6 production+++GO:0035264///multicellular

XM_021097491.1	GNB1	-22.08924085	4.01E-06	04728///Dopaminergic synapse+++04727///GABAergic synapse+++04151///PI3K-Akt signaling pathway+++04726///Serotonergic synapse+++04740///Olfactory transduction+++04371///Apelin signaling pathway+++04723///Retrograde endocannabinoid signaling+++04725///Cholinergic synapse+++04014///Ras signaling pathway+++04744///Phototransduction+++04926///Relaxin signaling pathway+++04062///Chemokine signaling pathway+++04713///Circadian entrainment+++04724///Glutamatergic	GO:0043209///myelin sheath+++GO:0001750///photoreceptor outer segment+++GO:0005834///heterotrimeric G-protein complex	GO:0003924///GTPase activity+++GO:0051020///GTPase binding+++GO:0044877///protein-containing complex binding	GO:0007165///signal transduction+++GO:0008283///cell proliferation+++GO:0050909///sensory perception of taste+++GO:0060041///retina development in camera-type eye+++GO:0007200///phospholipase C-activating G protein-coupled receptor signaling pathway+++GO:0007186///G protein-coupled receptor signaling pathway
XM_021088840.1	PTK2	-8.314774955	6.06E-07	04151///PI3K-Akt signaling pathway+++04670///Leukocyte transendothelial migration+++04510///Focal adhesion+++04810///Regulation of actin cytoskeleton+++04062///Chemokine signaling pathway+++04012///ErbB signaling pathway+++04360///Axon guidance+++04370///VEGF signaling pathway	GO:0016324///apical plasma membrane+++GO:0030027///lamellipodium+++GO:0001725///stress fiber+++GO:0005829///cytosol+++GO:0005856///cytoskeleton+++GO:0005886///plasma membrane+++GO:0005737///cytoplasm+++GO:0005925///focal adhesion+++GO:0005634///nucleus	GO:0003779///actin binding+++GO:0019903///protein phosphatase binding+++GO:0008432///JUN kinase binding+++GO:0004715///non-membrane spanning protein tyrosine kinase activity+++GO:0004713///protein tyrosine kinase activity+++GO:0004672///protein kinase activity+++GO:0016301///kinase activity+++GO:0019901///protein kinase binding+++GO:0042169///SH2 domain binding+++GO:0005524///ATP binding	GO:0051897///positive regulation of protein kinase B signaling+++GO:0010613///positive regulation of cardiac muscle hypertrophy+++GO:0051893///regulation of focal adhesion assembly+++GO:0001764///neuron migration+++GO:0051964///negative regulation of synapse assembly+++GO:0030335///positive regulation of cell migration+++GO:0001932///regulation of protein phosphorylation+++GO:0000226///microtubule cytoskeleton organization+++GO:0001934///positive regulation of protein phosphorylation+++GO:0007172///signal complex assembly+++GO:0042127///regulation of cell proliferation+++GO:0001568///blood vessel development+++GO:0010632///regulation of epithelial cell migration+++GO:0038083///peptidyl-tyrosine autophosphorylation+++GO:0048013///ephrin receptor signaling pathway+++GO:0050771///negative regulation of axonogenesis+++GO:0071560///cellular response to transforming growth factor beta stimulus+++GO:0045860///positive regulation of protein kinase activity+++GO:0018108///peptidyl-tyrosine phosphorylation+++GO:0014068///positive regulation of phosphatidylinositol 3-kinase signaling+++GO:0046621///negative regulation of organ growth+++GO:0006468///protein phosphorylation+++GO:0007179///transforming growth factor beta receptor signaling pathway+++GO:0022408///negative regulation of cell-cell adhesion+++GO:0001525///angiogenesis+++GO:0007229///integrin-mediated signaling pathway+++GO:0043066///negative regulation of apoptotic process+++GO:0046777///protein autophosphorylation+++GO:2000060///positive regulation of ubiquitin-dependent protein catabolic process+++GO:1900024///regulation of substrate adhesion-dependent cell spreading+++GO:0033628///regulation of cell adhesion mediated by

XM_021084221.1	FOXO3	-9.00078075	0.044059527	04722///Neurotrophin signaling pathway+++04213///Longevity regulating pathway - multiple species+++04917///Prolactin signaling pathway+++04068///FoxO signaling pathway+++04211///Longevity regulating pathway+++04151///PI3K-Akt signaling pathway+++04218///Cellular senescence+++04137///Mitophagy - animal+++04152///AMPK signaling pathway+++04062///Chemokine signaling pathway+++04212///Longevity regulating pathway - worm	GO:0005737///cytoplasm+++GO:0005634///nucleus+++GO:0005759///mitochondrial matrix+++GO:0005739///mitochondrion+++GO:0032991///protein-containing complex+++GO:0005654///nucleoplasm+++GO:0005829///cytosol+++GO:0005741///mitochondrial outer membrane+++GO:0016020///membrane	GO:0019901///protein kinase binding+++GO:0000978///RNA polymerase II proximal promoter sequence-specific DNA binding+++GO:0000981///DNA-binding transcription factor activity, RNA polymerase II-specific+++GO:0003700///DNA-binding transcription factor activity+++GO:0001227///DNA-binding transcription repressor activity, RNA polymerase II-specific+++GO:0001228///DNA-binding transcription activator activity, RNA polymerase II-specific+++GO:0043565///sequence-specific DNA binding+++GO:0003677///DNA binding+++GO:0008013///beta-catenin binding+++GO:0034246///mitochondrial sequence-specific DNA-binding transcription factor activity+++GO:0031490///chromatin DNA binding+++GO:0008134///transcription factor binding	GO:0042593///glucose homeostasis+++GO:0043525///positive regulation of neuron apoptotic process+++GO:0048854///brain morphogenesis+++GO:0045944///positive regulation of transcription by RNA polymerase II+++GO:0001544///initiation of primordial ovarian follicle growth+++GO:0045893///positive regulation of transcription, DNA-templated+++GO:0043065///positive regulation of apoptotic process+++GO:0006357///regulation of transcription by RNA polymerase II+++GO:0000122///negative regulation of transcription by RNA polymerase II+++GO:0030330///DNA damage response, signal transduction by p53 class mediator+++GO:0045648///positive regulation of erythrocyte differentiation+++GO:0033209///tumor necrosis factor-mediated signaling pathway+++GO:0006390///mitochondrial transcription+++GO:0034599///cellular response to oxidative stress+++GO:0001556///oocyte maturation+++GO:0097192///extrinsic apoptotic signaling pathway in absence of ligand+++GO:0030336///negative regulation of cell migration+++GO:0006355///regulation of transcription, DNA-templated+++GO:0008286///insulin receptor signaling pathway+++GO:0001547///antral ovarian follicle growth+++GO:0097150///neuronal stem cell population maintenance+++GO:0090090///negative regulation of canonical Wnt signaling pathway+++GO:0006417///regulation of translation+++GO:2000177///regulation of neural precursor cell
XM_021097493.1	GNB1	-22.00961843	4.38E-06	04926///Relaxin signaling pathway+++04725///Cholinergic synapse+++04014///Ras signaling pathway+++04371///Apelin signaling pathway+++04740///Olfactory transduction+++04726///Serotonergic synapse+++04011///MAPK signaling pathway - yeast+++04744///Phototransduction+++04723///Retrograde endocannabinoid signaling+++04724///Glutamatergic synapse+++04151///PI3K-Akt signaling pathway+++04062///Chemokine signaling pathway+++04713///Circadian entrainment+++04728///Dopaminergic [illegible]	GO:0005834///heterotrimeric G-protein complex+++GO:0001750///photoreceptor outer segment+++GO:0043209///myelin sheath	GO:0003924///GTPase activity+++GO:0044877///protein-containing complex binding+++GO:0051020///GTPase binding	GO:0007186///G protein-coupled receptor signaling pathway+++GO:0008283///cell proliferation+++GO:0007165///signal transduction+++GO:0007200///phospholipase C-activating G protein-coupled receptor signaling pathway+++GO:0060041///retina development in camera-type eye+++GO:0050909///sensory perception of taste

XM_021093571.1	PGM1	-7.764412041	0.032759186	01100///Metabolic pathways+++01130///Biosynthesis of antibiotics+++01110///Biosynthesis of secondary metabolites+++00230///Purine metabolism+++00520///Amino sugar and nucleotide sugar metabolism+++00030///Pentose phosphate pathway+++00052///Galactose metabolism+++00521///Streptomycin biosynthesis+++00010///Glycolysis / Gluconeogenesis+++01120///Microbial metabolism in diverse environments+++00500///Starch and sucrose metabolism	GO:0015629///actin cytoskeleton+++GO:0005829///cytosol	GO:0004614///phosphoglucomutase activity+++GO:0046872///metal ion binding+++GO:0016868///intramolecular transferase activity, phosphotransferases+++GO:0000287///magnesium ion binding	GO:0019388///galactose catabolic process+++GO:0071704///organic substance metabolic process+++GO:0006006///glucose metabolic process+++GO:0005975///carbohydrate metabolic process+++GO:0005978///glycogen biosynthetic process
XM_013998997.2	CASP9	-7.494066932	3.41E-04	04210///Apoptosis+++04215///Apoptosis - multiple species+++04919///Thyroid hormone signaling pathway+++04370///VEGF signaling pathway+++04115///p53 signaling pathway+++04151///PI3K-Akt signaling pathway	GO:0005829///cytosol+++GO:0032991///protein-containing complex+++GO:0043293///apoptosome+++GO:0005737///cytoplasm+++GO:0005634///nucleus	GO:0008233///peptidase activity+++GO:0017124///SH3 domain binding+++GO:0019901///protein kinase binding+++GO:0097153///cysteine-type endopeptidase activity involved in apoptotic process+++GO:0008234///cysteine-type peptidase activity+++GO:0097199///cysteine-type endopeptidase activity involved in apoptotic signaling pathway+++GO:0004197///cysteine-type endopeptidase activity+++GO:0016787///hydrolase activity	GO:0071549///cellular response to dexamethasone stimulus+++GO:0042770///signal transduction in response to DNA damage+++GO:0006915///apoptotic process+++GO:0043525///positive regulation of neuron apoptotic process+++GO:0009411///response to UV+++GO:0006919///activation of cysteine-type endopeptidase activity involved in apoptotic process+++GO:2001020///regulation of response to DNA damage stimulus+++GO:0043065///positive regulation of apoptotic process+++GO:0008630///intrinsic apoptotic signaling pathway in response to DNA damage+++GO:0006508///proteolysis+++GO:0034644///cellular response to UV+++GO:0006974///cellular response to DNA damage stimulus+++GO:0042981///regulation of apoptotic process
XM_021093024.1	APAF1	-1.925932415	0.024281225	04215///Apoptosis - multiple species+++04214///Apoptosis - fly+++04115///p53 signaling pathway+++04210///Apoptosis	GO:0005829///cytosol+++GO:0005737///cytoplasm+++GO:0043293///apoptosome+++GO:0005634///nucleus+++GO:0032991///protein-containing complex	GO:0043531///ADP binding+++GO:0042802///identical protein binding+++GO:0008233///peptidase activity+++GO:0000166///nucleotide binding+++GO:0005524///ATP binding+++GO:0008656///cysteine-type endopeptidase activator activity involved in apoptotic process	GO:0030900///forebrain development+++GO:0042981///regulation of apoptotic process+++GO:0097190///apoptotic signaling pathway+++GO:0051402///neuron apoptotic process+++GO:0070059///intrinsic apoptotic signaling pathway in response to endoplasmic reticulum stress+++GO:0007275///multicellular organism development+++GO:0006508///proteolysis+++GO:0006915///apoptotic process+++GO:0007420///brain development+++GO:0008635///activation of cysteine-type endopeptidase activity involved in apoptotic process by cytochrome c+++GO:0006919///activation of cysteine-type endopeptidase activity involved in apoptotic process+++GO:2001235///positive regulation of apoptotic signaling
XM_021083201.1	HIPK3	-23.95018874	3.93E-07	04218///Cellular senescence	GO:0005829///cytosol+++GO:0016605///PML body+++GO:0016604///nuclear body+++GO:0005737///cytoplasm	GO:0005524///ATP binding+++GO:0004672///protein kinase activity+++GO:0004674///protein serine/threonine kinase activity	GO:0018107///peptidyl-threonine phosphorylation+++GO:0043508///negative regulation of JUN kinase activity+++GO:0009299///mRNA transcription+++GO:0043066///negative regulation of apoptotic process+++GO:0006468///protein phosphorylation+++GO:0018105///peptidyl-serine phosphorylation

XM_021083203.1	HIPK3	-23.99488457	3.71E-07	04218///Cellular senescence	GO:0016604///nuclear body+++GO:0016605///PML body+++GO:0005737///cytoplasm+++GO:0005829///cytosol	GO:0004674///protein serine/threonine kinase activity+++GO:0004672///protein kinase activity+++GO:0005524///ATP binding	GO:0043508///negative regulation of JUN kinase activity+++GO:0006468///protein phosphorylation+++GO:0043066///negative regulation of apoptotic process+++GO:0018107///peptidyl-threonine phosphorylation+++GO:0018105///peptidyl-serine phosphorylation+++GO:0009299///mRNA transcription
XM_021072578.1	PPP3CB	-10.15951945	0.007719386	04380///Osteoclast differentiation+++04310///Wnt signaling pathway+++04658///Th1 and Th2 cell differentiation+++04218///Cellular senescence+++04922///Glucagon signaling pathway+++04625///C-type lectin receptor signaling pathway+++04921///Oxytocin signaling pathway+++04659///Th17 cell differentiation+++04114///Oocyte meiosis+++04650///Natural killer cell mediated cytotoxicity+++04724///Glutamatergic synapse+++04370///VEGF signaling pathway+++04728///Dopaminergic synapse+++04924///Renin secretion+++04360///Axon guidance+++04022///cGMP-PKG signaling pathway+++04010///MAPK signaling pathway+++04020///Calcium signaling pathway+++04660///T cell receptor signaling pathway+++04720///Long-term	GO:0030315///T-tubule+++GO:0098978///glutamatergic synapse+++GO:0005955///calcineurin complex+++GO:0005886///plasma membrane+++GO:0005737///cytoplasm+++GO:0030018///Z disc	GO:0008144///drug binding+++GO:0005509///calcium ion binding+++GO:0004722///protein serine/threonine phosphatase activity+++GO:0016787///hydrolase activity+++GO:0005516///calmodulin binding+++GO:0019899///enzyme binding+++GO:0033192///calmodulin-dependent protein phosphatase activity+++GO:0004721///phosphoprotein phosphatase activity+++GO:0030346///protein phosphatase 2B binding	GO:0031987///locomotion involved in locomotory behavior+++GO:0097720///calcineurin-mediated signaling+++GO:0035690///cellular response to drug+++GO:0006468///protein phosphorylation+++GO:0035774///positive regulation of insulin secretion involved in cellular response to glucose stimulus+++GO:0034097///response to cytokine+++GO:0043029///T cell homeostasis+++GO:1900242///regulation of synaptic vesicle endocytosis+++GO:0007507///heart development+++GO:0006470///protein dephosphorylation+++GO:0001915///negative regulation of T cell mediated cytotoxicity+++GO:0017156///calcium ion regulated exocytosis+++GO:0010468///regulation of gene expression+++GO:0030217///T cell
XR_002343984.1	LOC100626199	-7.440815787	0.01795358	00830///Retinol metabolism+++01100///Metabolic pathways	GO:0016021///integral component of membrane+++GO:0005789///endoplasmic reticulum membrane+++GO:0031090///organelle membrane+++GO:0043231///intracellular membrane-bounded organelle	GO:0009055///electron transfer activity+++GO:0004745///retinol dehydrogenase activity	GO:0006629///lipid metabolic process
XM_021081605.1	PPP2R5C	6.30476285	0.027589485	04151///PI3K-Akt signaling pathway+++04114///Oocyte meiosis+++04152///AMPK signaling pathway+++04113///Meiosis - yeast+++04728///Dopaminergic synapse+++03015///mRNA surveillance pathway+++04261///Adrenergic signaling in cardiomyocytes+++04071///Sphingolipid signaling pathway	GO:0005794///Golgi apparatus+++GO:0000159///protein phosphatase type 2A complex+++GO:0005654///nucleoplasm+++GO:0005634///nucleus+++GO:0005829///cytosol+++GO:0000775///chromosome, centromeric region	GO:0072542///protein phosphatase activator activity+++GO:0019888///protein phosphatase regulator activity	GO:0007165///signal transduction+++GO:0008285///negative regulation of cell proliferation+++GO:0031952///regulation of protein autophosphorylation+++GO:0043666///regulation of phosphoprotein phosphatase activity+++GO:0043161///proteasome-mediated ubiquitin-dependent protein catabolic process

XM_013987480.2	JAK3	7.052183413	0.002188111	04630///Jak-STAT signaling pathway+++04217///Necroptosis+++04151///PI3K-Akt signaling pathway+++04062///Chemokine signaling pathway+++04550///Signaling pathways regulating pluripotency of stem cells+++04658///Th1 and Th2 cell differentiation+++04659///Th17 cell differentiation	GO:0016020///membrane+++GO:0005856///cytoskeleton	GO:0016301///kinase activity+++GO:0000166///nucleotide binding+++GO:0019903///protein phosphatase binding+++GO:0004715///non-membrane spanning protein tyrosine kinase activity+++GO:0004713///protein tyrosine kinase activity+++GO:0005524///ATP binding+++GO:0016740///transferase activity+++GO:0004672///protein kinase activity	GO:0070670///response to interleukin-4+++GO:0030218///erythrocyte differentiation+++GO:0016310///phosphorylation+++GO:0035771///interleukin-4-mediated signaling pathway+++GO:0043029///T cell homeostasis+++GO:0006468///protein phosphorylation+++GO:0007260///tyrosine phosphorylation of STAT protein+++GO:0030183///B cell differentiation+++GO:0019221///cytokine-mediated signaling pathway+++GO:0018108///peptidyl-tyrosine phosphorylation+++GO:0042102///positive regulation of T cell proliferation+++GO:1903660///negative regulation of complement-dependent cytotoxicity+++GO:0035556///intracellular signal transduction

XM_021088688.1	ANGPT1	4.992650994	0.030651025	04010///MAPK signaling pathway+++04015///Rap1 signaling pathway+++04151///PI3K-Akt signaling pathway+++04066///HIF-1 signaling pathway+++04014///Ras signaling pathway	GO:0005902///microvillus+++GO:0005615///extracellular space+++GO:0045121///membrane raft+++GO:0005886///plasma membrane+++GO:0005576///extracellular region	GO:0005102///signaling receptor binding+++GO:0030971///receptor tyrosine kinase binding	GO:0045785///positive regulation of cell adhesion+++GO:0031589///cell-substrate adhesion+++GO:0007171///activation of transmembrane receptor protein tyrosine kinase activity+++GO:0001701///in utero embryonic development+++GO:0050731///positive regulation of peptidyl-tyrosine phosphorylation+++GO:0002040///sprouting angiogenesis+++GO:0034394///protein localization to cell surface+++GO:0050918///positive chemotaxis+++GO:0010595///positive regulation of endothelial cell migration+++GO:0030154///cell differentiation+++GO:0043066///negative regulation of apoptotic process+++GO:0030097///hemopoiesis+++GO:0032680///regulation of tumor necrosis factor production+++GO:0031398///positive regulation of protein ubiquitination+++GO:0007275///multicellular organism development+++GO:0001525///angiogenesis+++GO:0030210///heparin biosynthetic process+++GO:0033138///positive regulation of peptidyl-serine phosphorylation+++GO:0014068///positive regulation of phosphatidylinositol 3-kinase signaling+++GO:0043536///positive regulation of blood vessel endothelial cell migration+++GO:0070374///positive regulation of ERK1 and ERK2 cascade+++GO:0043122///regulation of I-kappaB kinase/NF-kappaB signaling+++GO:2000446///regulation of macrophage migration inhibitory factor signaling pathway+++GO:0072012///glomerulus vasculature development+++GO:0007162///negative regulation of cell adhesion+++GO:0007169///transmembrane receptor protein tyrosine kinase signaling pathway+++GO:0014842///regulation of skeletal muscle satellite cell proliferation+++GO:0002740///negative regulation of cytokine secretion involved in immune response+++GO:0043116///negative regulation of vascular permeability+++GO:0048014///Tie signaling pathway+++GO:0043393///regulation of [illegible]
XM_021079731.1	CYP4F55	6.307434669	0.031848562	00590///Arachidonic acid metabolism+++01100///Metabolic pathways	GO:0005789///endoplasmic reticulum membrane+++GO:0016021///integral component of membrane+++GO:0031090///organelle membrane	[illegible] GO:0004497///monooxygenase activity+++GO:0005506///iron ion binding+++GO:0097259///20-aldehyde-leukotriene B4 20-monooxygenase activity+++GO:0050051///leukotriene-B4 20-monooxygenase activity+++GO:0097258///20-hydroxy-leukotriene B4 omega oxidase activity+++GO:0020037///heme binding [illegible]	GO:0006690///icosanoid metabolic process+++GO:0006691///leukotriene metabolic process

XM_005662959.3	CCNE2	6.643013568	0.026498754	04151///PI3K-Akt signaling pathway+++04218///Cellular senescence+++04114///Oocyte meiosis+++04115///p53 signaling pathway+++04391///Hippo signaling pathway - fly+++04110///Cell cycle	GO:0000307///cyclin-dependent protein kinase holoenzyme complex+++GO:0097135///cyclin E2-CDK2 complex+++GO:0005634///nucleus+++GO:0005737///cytoplasm+++GO:0005813///centrosome	GO:0004672///protein kinase activity+++GO:0016538///cyclin-dependent protein serine/threonine kinase regulator activity+++GO:0019901///protein kinase binding	GO:0007129///synapsis+++GO:0000278///mitotic cell cycle+++GO:0000082///G1/S transition of mitotic cell cycle+++GO:0045787///positive regulation of cell cycle+++GO:0051726///regulation of cell cycle+++GO:0006270///DNA replication initiation+++GO:0000723///telomere maintenance+++GO:0007088///regulation of mitotic nuclear division+++GO:1903827///regulation of cellular protein localization+++GO:0000079///regulation of cyclin-dependent protein serine/threonine kinase activity+++GO:0070192///chromosome organization involved in meiotic cell cycle+++GO:0008284///positive regulation of cell proliferation+++GO:0006468///protein
XM_021063797.1	LAMC2	1.127612033	0.034671933	04510///Focal adhesion+++04512///ECM-receptor interaction+++04151///PI3K-Akt signaling pathway	GO:0005604///basement membrane	GO:0008201///heparin binding	GO:0048731///system development+++GO:0070831///basement membrane assembly+++GO:0030335///positive regulation of cell migration+++GO:0008284///positive regulation of cell proliferation
XM_021085872.1	IL4R	7.552520643	0.001706112	04630///Jak-STAT signaling pathway+++04658///Th1 and Th2 cell differentiation+++04640///Hematopoietic cell lineage+++04060///Cytokine-cytokine receptor interaction+++04659///Th17 cell differentiation+++04151///PI3K-Akt signaling pathway	GO:0005615///extracellular space+++GO:0043235///receptor complex+++GO:0005576///extracellular region+++GO:0016021///integral component of membrane+++GO:0016020///membrane	GO:0008083///growth factor activity+++GO:0005125///cytokine activity+++GO:0004896///cytokine receptor activity+++GO:0005136///interleukin-4 receptor binding+++GO:0005126///cytokine receptor binding	GO:0019221///cytokine-mediated signaling pathway+++GO:0001934///positive regulation of protein phosphorylation+++GO:0046825///regulation of protein export from nucleus+++GO:0042531///positive regulation of tyrosine phosphorylation of STAT protein+++GO:0042113///B cell activation+++GO:1903142///positive regulation of establishment of endothelial barrier+++GO:0006955///immune response+++GO:0010633///negative regulation of epithelial cell migration+++GO:0042976///activation of Janus kinase activity+++GO:0010469///regulation of signaling receptor activity+++GO:0016239///positive regulation of macroautophagy+++GO:0097028///dendritic cell differentiation+++GO:0046427///positive regulation of JAK-STAT cascade+++GO:0010628///positive regulation of gene expression+++GO:0042110///T cell activation+++GO:0035745///T-helper 2 cell cytokine production+++GO:0045892///negative regulation of transcription, DNA-templated+++GO:0032736///positive regulation of interleukin-13 production+++GO:0045582///positive regulation of T cell differentiation+++GO:0002532///production of molecular mediator involved in inflammatory response+++GO:1903660///negative regulation of complement-dependent cytotoxicity+++GO:2000352///negative regulation

XR_002338387.1	ULK1	8.253415417	2.00E-05	04152///AMPK signaling pathway+++04140///Autophagy - animal+++04137///Mitophagy - animal+++04211///Longevity regulating pathway+++04150///mTOR signaling pathway	GO:1990316///Atg1/ULK1 kinase complex+++GO:0034045///phagophore assembly site membrane+++GO:0097632///extrinsic component of phagophore assembly site membrane+++GO:0097629///extrinsic component of omegasome membrane+++GO:0000407///phagophore assembly site+++GO:0032045///guanyl-nucleotide exchange factor complex+++GO:0016020///membrane+++GO:0097635///extrinsic component of autophagosome membrane+++GO:0005776///autophagosome+++GO:0005829///cytosol	GO:0042802///identical protein binding+++GO:0004674///protein serine/threonine kinase activity+++GO:0016740///transferase activity+++GO:0017137///Rab GTPase binding+++GO:0016301///kinase activity+++GO:0000166///nucleotide binding+++GO:0005524///ATP binding+++GO:0004672///protein kinase activity+++GO:0051020///GTPase binding+++GO:0044877///protein-containing complex binding	GO:0006914///autophagy+++GO:0008104///protein localization+++GO:0031175///neuron projection development+++GO:0006468///protein phosphorylation+++GO:0018105///peptidyl-serine phosphorylation+++GO:0016310///phosphorylation+++GO:0010506///regulation of autophagy+++GO:0000045///autophagosome assembly+++GO:0046777///protein autophosphorylation+++GO:0031333///negative regulation of protein complex assembly+++GO:0018107///peptidyl-threonine phosphorylation+++GO:0048675///axon extension+++GO:0016236///macroautophagy+++GO:0042594///response to starvation+++GO:0075044///autophagy of host cells involved in interaction with symbiont+++GO:0007165///signal

XM_013989935.2	BRCA1	4.149392283	0.017574574	03460///Fanconi anemia pathway+++03440///Homologous recombination+++04120///Ubiquitin mediated proteolysis+++04151///PI3K-Akt signaling pathway	GO:0070531///BRCA1-A complex+++GO:0005737///cytoplasm+++GO:1990904///ribonucleoprotein complex+++GO:0032991///protein-containing complex+++GO:0031436///BRCA1-BARD1 complex+++GO:0000794///condensed nuclear chromosome+++GO:0005886///plasma membrane+++GO:0000800///lateral element+++GO:0005634///nucleus+++GO:0000793///condensed chromosome+++GO:0005694///chromosome	GO:0019899///enzyme binding+++GO:0042802///identical protein binding+++GO:0003723///RNA binding+++GO:0003684///damaged DNA binding+++GO:0070063///RNA polymerase binding+++GO:0003677///DNA binding+++GO:0008270///zinc ion binding+++GO:0003713///transcription coactivator activity+++GO:0046872///metal ion binding+++GO:0044212///transcription regulatory region DNA binding+++GO:0031625///ubiquitin protein ligase binding+++GO:0004842///ubiquitin-protein transferase activity	GO:0045893///positive regulation of transcription, DNA-templated+++GO:0043627///response to estrogen+++GO:2000617///positive regulation of histone H3-K9 acetylation+++GO:0007098///centrosome cycle+++GO:0051574///positive regulation of histone H3-K9 methylation+++GO:0006301///postreplication repair+++GO:0051571///positive regulation of histone H3-K4 methylation+++GO:0007049///cell cycle+++GO:0035067///negative regulation of histone acetylation+++GO:0035066///positive regulation of histone acetylation+++GO:2000620///positive regulation of histone H4-K16 acetylation+++GO:1902042///negative regulation of extrinsic apoptotic signaling pathway via death domain receptors+++GO:0043009///chordate embryonic development+++GO:0072425///signal transduction involved in G2 DNA damage checkpoint+++GO:0044818///mitotic G2/M transition checkpoint+++GO:0006281///DNA repair+++GO:0007059///chromosome segregation+++GO:0044030///regulation of DNA methylation+++GO:2000378///negative regulation of reactive oxygen species metabolic process+++GO:0045766///positive regulation of angiogenesis+++GO:0006310///DNA recombination+++GO:0006357///regulation of transcription by RNA polymerase II+++GO:0045892///negative regulation of transcription, DNA-templated+++GO:0010212///response to ionizing radiation+++GO:0051572///negative regulation of histone H3-K4 methylation+++GO:0033147///negative regulation of intracellular estrogen receptor signaling pathway+++GO:0009048///dosage compensation by inactivation of X chromosome+++GO:0031398///positive regulation of protein ubiquitination+++GO:0006349///regulation of gene expression by genetic imprinting+++GO:0045717///negative regulation of fatty acid biosynthetic [illegible]
XM_021078074.1	ACSS1	3.240991487	0.028933101	00640///Propanoate metabolism+++01200///Carbon metabolism+++00680///Methane metabolism+++01120///Microbial metabolism in diverse environments+++01130///Biosynthesis of antibiotics+++00630///Glyoxylate and dicarboxylate metabolism+++00620///Pyruvate metabolism+++01100///Metabolic pathways+++00010///Glycolysis / Gluconeogenesis+++01110///Biosynthesis of secondary metabolites+++00720///Carbon fixation pathways in prokaryotes	GO:0005759///mitochondrial matrix	GO:0016208///AMP binding+++GO:0003987///acetate-CoA ligase activity+++GO:0003824///catalytic activity+++GO:0005524///ATP binding+++GO:0000166///nucleotide binding+++GO:0016874///ligase activity	GO:0006085///acetyl-CoA biosynthetic process+++GO:0019427///acetyl-CoA biosynthetic process from acetate

XM_013982220.2	ZMAT3	-21.66291004	6.09E-06	04115///p53 signaling pathway	GO:0005886///plasma membrane+++GO:0005654///nucleoplasm	GO:0008270///zinc ion binding+++GO:0003676///nucleic acid binding+++GO:0046872///metal ion binding	GO:0006974///cellular response to DNA damage stimulus+++GO:0072332///intrinsic apoptotic signaling pathway by p53 class mediator+++GO:0015031///protein transport+++GO:0040008///regulation of growth
XM_021063227.1	MDM4	-6.281651299	0.029172568	04115///p53 signaling pathway	GO:0005634///nucleus	GO:0046872///metal ion binding+++GO:0019899///enzyme binding	GO:0030330///DNA damage response, signal transduction by p53 class mediator+++GO:0008283///cell proliferation+++GO:0042177///negative regulation of protein catabolic process+++GO:0065003///protein-containing complex assembly+++GO:0071157///negative regulation of cell cycle arrest+++GO:0071456///cellular response to hypoxia+++GO:0050821///protein stabilization+++GO:0043066///negative regulation of apoptotic process+++GO:0000122///negative regulation of transcription by RNA polymerase II+++GO:0045023///G0 to G1 transition
XM_021069855.1	ZMAT3	-7.113502883	6.35E-04	04115///p53 signaling pathway	GO:0005654///nucleoplasm+++GO:0005886///plasma membrane	GO:0046872///metal ion binding+++GO:0008270///zinc ion binding+++GO:0003676///nucleic acid binding	GO:0006974///cellular response to DNA damage stimulus+++GO:0015031///protein transport+++GO:0072332///intrinsic apoptotic signaling pathway by p53 class mediator+++GO:0040008///regulation of growth
XM_021070801.1	MAGI1	-7.021449972	0.024914946	04015///Rap1 signaling pathway+++04151///PI3K-Akt signaling pathway+++04530///Tight junction	GO:0030054///cell junction+++GO:0005737///cytoplasm+++GO:0016020///membrane+++GO:0005654///nucleoplasm+++GO:0005912///adherens junction+++GO:0042995///cell projection+++GO:0005923///bicellular tight junction+++GO:0005634///nucleus	GO:0005515///protein binding+++GO:0051393///alpha-actinin binding+++GO:0032947///protein-containing complex scaffold activity	GO:0065003///protein-containing complex assembly+++GO:0007166///cell surface receptor signaling pathway+++GO:0070997///neuron death+++GO:0007155///cell adhesion
XM_021078612.1	PRKAG2	-7.72515599	4.78E-04	04710///Circadian rhythm+++04920///Adipocytokine signaling pathway+++04922///Glucagon signaling pathway+++04213///Longevity regulating pathway - multiple species+++04371///Apelin signaling pathway+++04921///Oxytocin signaling pathway+++04910///Insulin signaling pathway+++04714///Thermogenesis+++04211///Longevity regulating pathway+++04068///FoxO signaling pathway+++04530///Tight junction+++04152///AMPK signaling pathway	GO:0031588///nucleotide-activated protein kinase complex	GO:0005524///ATP binding+++GO:0032559///adenyl ribonucleotide binding+++GO:0004862///cAMP-dependent protein kinase inhibitor activity+++GO:0019901///protein kinase binding+++GO:0030295///protein kinase activator activity+++GO:0043531///ADP binding+++GO:0008603///cAMP-dependent protein kinase regulator activity+++GO:0008607///phosphorylase kinase regulator activity	GO:0071900///regulation of protein serine/threonine kinase activity+++GO:0071901///negative regulation of protein serine/threonine kinase activity+++GO:0032147///activation of protein kinase activity+++GO:0019217///regulation of fatty acid metabolic process+++GO:0045860///positive regulation of protein kinase activity+++GO:0005977///glycogen metabolic process+++GO:0006110///regulation of glycolytic process+++GO:0035556///intracellular signal transduction+++GO:0006469///negative regulation of protein kinase activity+++GO:0010800///positive regulation of peptidyl-threonine phosphorylation

XM_021097326.1	PLA2G4E	-8.567195919	9.84E-06	01100///Metabolic pathways+++04010///MAPK signaling pathway+++00590///Arachidonic acid metabolism+++00591///Linoleic acid metabolism+++04730///Long-term depression+++00565///Ether lipid metabolism+++04726///Serotonergic synapse+++04270///Vascular smooth muscle contraction+++04014///Ras signaling pathway+++04611///Platelet activation+++04664///Fc epsilon RI signaling pathway+++04370///VEGF signaling pathway+++01110///Biosynthesis of secondary metabolites+++00592///alpha-Linolenic acid metabolism+++04072///Phospholipase D signaling pathway+++04750///Inflammatory mediator regulation of TRP channels+++04217///Necroptosis+++04724///Glutamatergic synapse+++04912///GnRH signaling pathway+++00564///Glycerophospholipid metabolism+++04913///Ovarian steroidogenesis+++04921///Oxytocin signaling pathway	GO:0005737///cytoplasm	GO:0046872///metal ion binding+++GO:0102568///phospholipase A2 activity consuming 1,2-dioleoylphosphatidylethanolamine)+++GO:0004620///phospholipase activity+++GO:0004623///phospholipase A2 activity+++GO:0016787///hydrolase activity+++GO:0102567///phospholipase A2 activity (consuming 1,2-dipalmitoylphosphatidylcholine)	GO:0006629///lipid metabolic process+++GO:0016042///lipid catabolic process+++GO:0009395///phospholipid catabolic process

XM_021101934.1	ATM	-9.332342337	1.82E-09	04115///p53 signaling pathway+++04068///FoxO signaling pathway+++04210///Apoptosis+++03440///Homologous recombination+++04214///Apoptosis - fly+++04218///Cellular senescence+++04110///Cell cycle+++04064///NF-kappa B signaling pathway	GO:1990391///DNA repair complex+++GO:0005634///nucleus+++GO:0000781///chromosome, telomeric region+++GO:0031410///cytoplasmic vesicle+++GO:0005840///ribosome+++GO:0005730///nucleolus	GO:0016301///kinase activity+++GO:0004674///protein serine/threonine kinase activity+++GO:0016740///transferase activity+++GO:0005524///ATP binding+++GO:0047485///protein N-terminus binding+++GO:0004677///DNA-dependent protein kinase activity+++GO:0003677///DNA binding+++GO:0044877///protein-containing complex binding+++GO:0003735///structural constituent of ribosome+++GO:0046983///protein dimerization activity+++GO:0016303///1-phosphatidylinositol-3-kinase activity+++GO:0000166///nucleotide binding	GO:0071480///cellular response to gamma radiation+++GO:0010506///regulation of autophagy+++GO:0036092///phosphatidylinositol-3-phosphate biosynthetic process+++GO:0046777///protein autophosphorylation+++GO:0000723///telomere maintenance+++GO:0006468///protein phosphorylation+++GO:0000077///DNA damage checkpoint+++GO:0071044///histone mRNA catabolic process+++GO:0097694///establishment of RNA localization to telomere+++GO:1904884///positive regulation of telomerase catalytic core complex assembly+++GO:1904354///negative regulation of telomere capping+++GO:0007050///cell cycle arrest+++GO:0090399///replicative senescence+++GO:0030335///positive regulation of cell migration+++GO:0016310///phosphorylation+++GO:0032210///regulation of telomere maintenance via telomerase+++GO:0018105///peptidyl-serine phosphorylation+++GO:0071481///cellular response to X-ray+++GO:0007049///cell cycle+++GO:0043065///positive regulation of apoptotic process+++GO:0008630///intrinsic apoptotic signaling pathway in response to DNA damage+++GO:0072434///signal transduction involved in mitotic G2 DNA damage checkpoint+++GO:0043517///positive regulation of DNA damage response, signal transduction by p53 class mediator+++GO:0002331///pre-B cell allelic exclusion+++GO:0030889///negative regulation of B cell proliferation+++GO:1904358///positive regulation of telomere maintenance via telomere lengthening+++GO:0016572///histone phosphorylation+++GO:0036289///peptidyl-serine autophosphorylation+++GO:0010628///positive regulation of gene expression+++GO:0006281///DNA repair+++GO:0006975///DNA damage induced protein phosphorylation+++GO:0006412///translation+++GO:1904262///negative regulation of TORC1

XM_021080582.1	IGF2	-7.629361637	5.97E-05	04014///Ras signaling pathway+++04151///PI3K-Akt signaling pathway+++04010///MAPK signaling pathway	GO:0016021///integral component of membrane+++GO:0005576///extracellular region+++GO:0016020///membrane+++GO:0005615///extracellular space	GO:0008083///growth factor activity+++GO:0005179///hormone activity+++GO:0005178///integrin binding	GO:0000122///negative regulation of transcription by RNA polymerase II+++GO:0010469///regulation of signaling receptor activity+++GO:0001892///embryonic placenta development+++GO:0031056///regulation of histone modification+++GO:0060669///embryonic placenta morphogenesis+++GO:0005975///carbohydrate metabolic process+++GO:0006006///glucose metabolic process+++GO:0001503///ossification+++GO:0008284///positive regulation of cell proliferation+++GO:0051147///regulation of muscle cell differentiation+++GO:0001701///in utero embryonic development+++GO:0051781///positive regulation of cell division
XM_021093025.1	APAF1	-22.36191102	2.94E-06	04210///Apoptosis+++04214///Apoptosis - fly+++04115///p53 signaling pathway+++04215///Apoptosis - multiple species	GO:0005829///cytosol+++GO:0005737///cytoplasm+++GO:0043293///apoptosome+++GO:0005634///nucleus+++GO:0032991///protein-containing complex	GO:0042802///identical protein binding+++GO:0008656///cysteine-type endopeptidase activator activity involved in apoptotic process+++GO:0000166///nucleotide binding+++GO:0008233///peptidase activity+++GO:0043531///ADP binding+++GO:0005524///ATP binding	GO:2001235///positive regulation of apoptotic signaling pathway+++GO:0030900///forebrain development+++GO:0007275///multicellular organism development+++GO:0051402///neuron apoptotic process+++GO:0042981///regulation of apoptotic process+++GO:0097190///apoptotic signaling pathway+++GO:0001843///neural tube closure+++GO:1902510///regulation of apoptotic DNA fragmentation+++GO:0070059///intrinsic apoptotic signaling pathway in response to endoplasmic reticulum stress+++GO:0006919///activation of cysteine-type endopeptidase activity involved in apoptotic process+++GO:0006508///proteolysis+++GO:0006915///apoptotic process+++GO:0007420///brain development+++GO:0008635///activation of cysteine-type endopeptidase activity involved in apoptotic process by cytochrome c
XM_021079541.1	ACSL4	-21.69130157	5.94E-06	00071///Fatty acid degradation+++04920///Adipocytokine signaling pathway+++01100///Metabolic pathways+++01212///Fatty acid metabolism+++00061///Fatty acid biosynthesis+++04216///Ferroptosis+++02024///Quorum sensing+++03320///PPAR signaling pathway+++04714///Thermogenesis+++04146///Peroxisome	GO:0005739///mitochondrion+++GO:0016021///integral component of membrane+++GO:0005737///cytoplasm+++GO:0005811///lipid droplet+++GO:0016020///membrane+++GO:0044233///Mitochondria-associated ER Membrane	GO:0016874///ligase activity+++GO:0003824///catalytic activity+++GO:0031957///very long-chain fatty acid-CoA ligase activity+++GO:0004467///long-chain fatty acid-CoA ligase activity+++GO:0102391///decanoate-CoA ligase activity+++GO:0047676///arachidonate-CoA ligase activity	GO:0001676///long-chain fatty acid metabolic process+++GO:0006629///lipid metabolic process+++GO:0060136///embryonic process involved in female pregnancy

XR_002335884.1	LOC106504983	-6.331560217	0.020387217	04920///Adipocytokine signaling pathway+++04979///Cholesterol metabolism+++04152///AMPK signaling pathway+++04975///Fat digestion and absorption+++04512///ECM-receptor interaction+++03320///PPAR signaling pathway+++04640///Hematopoietic cell lineage+++04145///Phagosome	GO:0045121///membrane raft+++GO:0016020///membrane+++GO:0016324///apical plasma membrane+++GO:0005794///Golgi apparatus+++GO:0031526///brush border membrane+++GO:0016021///integral component of membrane		GO:0050909///sensory perception of taste+++GO:0031623///receptor internalization+++GO:0071404///cellular response to low-density lipoprotein particle stimulus+++GO:0007166///cell surface receptor signaling pathway+++GO:0030299///intestinal cholesterol absorption+++GO:0007155///cell adhesion+++GO:0006955///immune response+++GO:0070508///cholesterol import+++GO:1990000///amyloid fibril formation+++GO:0033993///response to lipid+++GO:0044539///long-chain fatty acid import+++GO:0070542///response to fatty acid+++GO:0097009///energy homeostasis+++GO:1900227///positive regulation of NLRP3 inflammasome complex assembly+++GO:0071726///cellular response to diacyl bacterial lipopeptide+++GO:0050702///interleukin-1 beta secretion+++GO:0050892///intestinal absorption+++GO:0007204///positive regulation of cytosolic calcium ion concentration+++GO:0034197///triglyceride transport+++GO:0070374///positive regulation of ERK1 and ERK2 cascade+++GO:0070543///response to linoleic acid
XM_021079792.1	CYP4F55	-6.599864824	0.028008712	00590///Arachidonic acid metabolism+++01100///Metabolic pathways	GO:0005789///endoplasmic reticulum membrane+++GO:0016021///integral component of membrane+++GO:0031090///organelle membrane	GO:0097258///20-hydroxy-leukotriene B4 omega oxidase activity+++GO:0097259///20-aldehyde-leukotriene B4 20-monooxygenase activity+++GO:0050051///leukotriene-B4 20-monooxygenase activity+++GO:0020037///heme binding+++GO:0005506///iron ion binding+++GO:0004497///monooxygenase activity	GO:0006691///leukotriene metabolic process+++GO:0006690///icosanoid metabolic process
XM_021098332.1	TNXB	1.713106044	8.21E-11	04151///PI3K-Akt signaling pathway+++04510///Focal adhesion+++04512///ECM-receptor interaction	GO:0043005///neuron projection+++GO:0005615///extracellular space+++GO:0031012///extracellular matrix+++GO:0005576///extracellular region+++GO:0062023///collagen-containing extracellular matrix	GO:0046982///protein heterodimerization activity+++GO:0042803///protein homodimerization activity+++GO:0050839///cell adhesion molecule binding+++GO:0005201///extracellular matrix structural constituent+++GO:0005518///collagen binding+++GO:0008201///heparin binding	GO:0006631///fatty acid metabolic process+++GO:0007157///heterophilic cell-cell adhesion via plasma membrane cell adhesion molecules+++GO:0030198///extracellular matrix organization+++GO:0006641///triglyceride metabolic process+++GO:0098609///cell-cell adhesion+++GO:0006629///lipid metabolic process+++GO:0048666///neuron development+++GO:0043506///regulation of JUN kinase activity+++GO:0030199///collagen fibril
XM_021101862.1	LIN54	1.668227562	0.008107107	04218///Cellular senescence	GO:0005654///nucleoplasm	GO:0003677///DNA binding+++GO:0046872///metal ion binding	GO:0006355///regulation of transcription, DNA-templated+++GO:0006351///transcription, DNA-templated+++GO:0007049///cell cycle+++GO:0051726///regulation of cell cycle

XM_021078616.1	PRKAG2	1.259927756	0.038686793	04714///Thermogenesis+++04910///Insulin signaling pathway+++04211///Longevity regulating pathway+++04710///Circadian rhythm+++04921///Oxytocin signaling pathway+++04213///Longevity regulating pathway - multiple species+++04068///FoxO signaling pathway+++04920///Adipocytokine signaling pathway+++04152///AMPK signaling pathway+++04371///Apelin signaling pathway+++04530///Tight junction+++04922///Glucagon signaling pathway	GO:0031588///nucleotide-activated protein kinase complex	GO:0019901///protein kinase binding+++GO:0004862///cAMP-dependent protein kinase inhibitor activity+++GO:0008603///cAMP-dependent protein kinase regulator activity+++GO:0032559///adenyl ribonucleotide binding+++GO:0005524///ATP binding+++GO:0030295///protein kinase activator activity+++GO:0043531///ADP binding+++GO:0008607///phosphorylase kinase regulator activity	GO:0035556///intracellular signal transduction+++GO:0071901///negative regulation of protein serine/threonine kinase activity+++GO:0010800///positive regulation of peptidyl-threonine phosphorylation+++GO:0045860///positive regulation of protein kinase activity+++GO:0019217///regulation of fatty acid metabolic process+++GO:0005977///glycogen metabolic process+++GO:0006110///regulation of glycolytic process+++GO:0006469///negative regulation of protein kinase activity+++GO:0032147///activation of protein kinase activity+++GO:0071900///regulation of protein serine/threonine kinase activity
XM_021099599.1	BCL2	1.273179187	7.51E-07	04215///Apoptosis - multiple species+++04725///Cholinergic synapse+++04915///Estrogen signaling pathway+++04151///PI3K-Akt signaling pathway+++04928///Parathyroid hormone synthesis, secretion and action+++04210///Apoptosis+++04510///Focal adhesion+++04261///Adrenergic signaling in cardiomyocytes+++04115///p53 signaling pathway+++04340///Hedgehog signaling pathway+++04722///Neurotrophin signaling pathway+++04140///Autophagy - animal+++04064///NF-kappa B signaling pathway+++04071///Sphingolipid signaling pathway+++04630///Jak-STAT signaling pathway+++04066///HIF-1 signaling pathway+++04141///Protein processing in endoplasmic reticulum+++04621///NOD-like receptor signaling pathway+++04217///Necroptosis	GO:0016020///membrane+++GO:0016021///integral component of membrane	GO:0042803///protein homodimerization activity+++GO:0046982///protein heterodimerization activity	GO:0006915///apoptotic process+++GO:0043066///negative regulation of apoptotic process+++GO:0042981///regulation of apoptotic process
XM_021101100.1	TBC1D1	2.074576015	0.038896377	04152///AMPK signaling pathway	GO:0005623///cell	GO:0005096///GTPase activator activity+++GO:0017137///Rab GTPase binding	GO:0090630///activation of GTPase activity+++GO:0006886///intracellular protein transport
XM_021083604.1	LOC110255237	2.224526312	0.010767769	01100///Metabolic pathways+++00590///Arachidonic acid metabolism	GO:0005789///endoplasmic reticulum membrane+++GO:0031090///organelle membrane	GO:0016705///oxidoreductase activity, acting on paired donors, with incorporation or reduction of molecular oxygen+++GO:0046872///metal ion binding+++GO:0005506///iron ion binding+++GO:0016491///oxidoreductase activity+++GO:0004497///monooxygenase activity+++GO:0020037///heme binding	GO:0055114///oxidation-reduction process
XM_021074130.1	SORBS1	1.948759414	6.66E-04	04910///Insulin signaling pathway+++03320///PPAR signaling pathway+++04520///Adherens junction	GO:0005813///centrosome+++GO:0005924///cell-substrate adherens junction+++GO:0005899///insulin receptor complex+++GO:0005913///cell-cell adherens junction+++GO:0045121///membrane raft+++GO:0016363///nuclear matrix+++GO:0016020///membrane+++GO:0001725///stress fiber+++GO:0005829///cytosol+++GO:0005634///nucleus+++GO:0005856///cytoskeleton+++GO:0005925///focal adhesion+++GO:0005886///plasma membrane	GO:0019901///protein kinase binding+++GO:0031625///ubiquitin protein ligase binding+++GO:0008134///transcription factor binding+++GO:0005070///SH3/SH2 adaptor activity+++GO:0005158///insulin receptor binding	GO:0007015///actin filament organization

XM_021074132.1	SORBS1	5.79154755	0.024281225	04910///Insulin signaling pathway+++03320///PPAR signaling pathway+++04520///Adherens junction	GO:0005813///centrosome+++GO:0005899///insulin receptor complex+++GO:0005829///cytosol+++GO:0045121///membrane raft+++GO:0005634///nucleus+++GO:0016020///membrane+++GO:0001725///stress fiber+++GO:0005856///cytoskeleton+++GO:0005924///cell-substrate adherens junction+++GO:0005925///focal adhesion+++GO:0005913///cell-cell adherens junction+++GO:0016363///nuclear matrix+++GO:0005886///plasma membrane	GO:0019901///protein kinase binding+++GO:0005070///SH3/SH2 adaptor activity+++GO:0005158///insulin receptor binding+++GO:0031625///ubiquitin protein ligase binding+++GO:0008134///transcription factor binding	GO:0007015///actin filament organization
XM_013980184.2	AQP7	22.60503555	2.23E-06	04923///Regulation of lipolysis in adipocytes+++03320///PPAR signaling pathway	GO:0016021///integral component of membrane+++GO:0016020///membrane	GO:0015267///channel activity	GO:0070295///renal water absorption+++GO:0055085///transmembrane transport+++GO:0015793///glycerol transport
XM_021072176.1	CYP2C33	6.524844759	0.049396438	00590///Arachidonic acid metabolism+++00140///Steroid hormone biosynthesis+++04726///Serotonergic synapse+++04750///Inflammatory mediator regulation of TRP channels+++00830///Retinol metabolism+++01100///Metabolic pathways+++00591///Linoleic acid metabolism	GO:0031090///organelle membrane+++GO:0005789///endoplasmic reticulum membrane+++GO:0043231///intracellular membrane-bounded organelle	GO:0020037///heme binding+++GO:0070330///aromatase activity+++GO:0008392///arachidonic acid epoxygenase activity+++GO:0005506///iron ion binding+++GO:0008395///steroid hydroxylase activity	GO:0019369///arachidonic acid metabolic process+++GO:0033574///response to testosterone+++GO:0019373///epoxygenase P450 pathway
NM_214414.1	CYP2C33	8.165599687	0.044853172	04726///Serotonergic synapse+++00140///Steroid hormone biosynthesis+++01100///Metabolic pathways+++04750///Inflammatory mediator regulation of TRP channels+++00591///Linoleic acid metabolism+++00830///Retinol metabolism+++00590///Arachidonic acid metabolism	GO:0005789///endoplasmic reticulum membrane+++GO:0043231///intracellular membrane-bounded organelle+++GO:0031090///organelle membrane	GO:0005506///iron ion binding+++GO:0020037///heme binding+++GO:0008392///arachidonic acid epoxygenase activity+++GO:0070330///aromatase activity+++GO:0008395///steroid hydroxylase activity	GO:0033574///response to testosterone+++GO:0019373///epoxygenase P450 pathway+++GO:0019369///arachidonic acid metabolic process
XM_005654266.3	PRKAG2	21.87227211	4.98E-06	04371///Apelin signaling pathway+++04710///Circadian rhythm+++04530///Tight junction+++04910///Insulin signaling pathway+++04922///Glucagon signaling pathway+++04068///FoxO signaling pathway+++04921///Oxytocin signaling pathway+++04211///Longevity regulating pathway+++04714///Thermogenesis+++04152///AMPK signaling pathway+++04213///Longevity regulating pathway - multiple species+++04920///Adipocytokine signaling pathway	GO:0031588///nucleotide-activated protein kinase complex	GO:0005524///ATP binding+++GO:0019901///protein kinase binding+++GO:0043531///ADP binding+++GO:0008607///phosphorylase kinase regulator activity+++GO:0030295///protein kinase activator activity+++GO:0008603///cAMP-dependent protein kinase regulator activity+++GO:0004862///cAMP-dependent protein kinase inhibitor activity+++GO:0032559///adenyl ribonucleotide binding	GO:0035556///intracellular signal transduction+++GO:0071900///regulation of protein serine/threonine kinase activity+++GO:0005977///glycogen metabolic process+++GO:0071901///negative regulation of protein serine/threonine kinase activity+++GO:0032147///activation of protein kinase activity+++GO:0019217///regulation of fatty acid metabolic process+++GO:0045860///positive regulation of protein kinase activity+++GO:0010800///positive regulation of peptidyl-threonine phosphorylation+++GO:0006469///negative regulation of protein kinase activity+++GO:0006110///regulation of glycolytic process

XM_021098327.1	TNXB	4.353045528	0.023579059	04512///ECM-receptor interaction+++04510///Focal adhesion+++04151///PI3K-Akt signaling pathway	GO:0062023///collagen-containing extracellular matrix+++GO:0043005///neuron projection+++GO:0031012///extracellular matrix+++GO:0005615///extracellular space+++GO:0005576///extracellular region	GO:0005518///collagen binding+++GO:0050839///cell adhesion molecule binding+++GO:0008201///heparin binding+++GO:0005201///extracellular matrix structural constituent+++GO:0046982///protein heterodimerization activity+++GO:0042803///protein homodimerization activity	GO:0048666///neuron development+++GO:0098609///cell-cell adhesion+++GO:0006629///lipid metabolic process+++GO:0006631///fatty acid metabolic process+++GO:0030198///extracellular matrix organization+++GO:0007160///cell-matrix adhesion+++GO:0043506///regulation of JUN kinase activity+++GO:0007157///heterophilic cell-cell adhesion via plasma membrane cell adhesion molecules+++GO:0030199///collagen fibril organization+++GO:0006641///triglyceride metabolic process
NM_001113445.1	G6PC	7.333154638	0.003198036	04151///PI3K-Akt signaling pathway+++04152///AMPK signaling pathway+++04068///FoxO signaling pathway+++04910///Insulin signaling pathway+++00010///Glycolysis / Gluconeogenesis+++01100///Metabolic pathways+++04922///Glucagon signaling pathway+++04973///Carbohydrate digestion and absorption+++00052///Galactose metabolism+++04920///Adipocytokine signaling pathway+++00500///Starch and sucrose metabolism	GO:0005783///endoplasmic reticulum+++GO:0016021///integral component of membrane+++GO:0005789///endoplasmic reticulum membrane+++GO:0016020///membrane	GO:0016773///phosphotransferase activity, alcohol group as acceptor+++GO:0016787///hydrolase activity+++GO:0042301///phosphate ion binding+++GO:0004346///glucose-6-phosphatase activity	GO:0010468///regulation of gene expression+++GO:0006094///gluconeogenesis+++GO:0008202///steroid metabolic process+++GO:0006641///triglyceride metabolic process+++GO:0005980///glycogen catabolic process+++GO:0051156///glucose 6-phosphate metabolic process+++GO:0015760///glucose-6-phosphate transport+++GO:0005977///glycogen metabolic process+++GO:0042593///glucose homeostasis+++GO:0046415///urate metabolic process+++GO:0035264///multicellular organism growth+++GO:0016311///dephosphorylation+++G
NM_001315734.1	LOC100739741	9.650004263	1.34E-04	01100///Metabolic pathways+++00140///Steroid hormone biosynthesis+++00590///Arachidonic acid metabolism+++04726///Serotonergic synapse+++04750///Inflammatory mediator regulation of TRP channels+++00830///Retinol metabolism+++00591///Linoleic acid metabolism	GO:0043231///intracellular membrane-bounded organelle+++GO:0005737///cytoplasm+++GO:0016020///membrane+++GO:0016021///integral component of membrane	GO:0046872///metal ion binding+++GO:0016705///oxidoreductase activity, acting on paired donors, with incorporation or reduction of molecular oxygen+++GO:0005506///iron ion binding+++GO:0019825///oxygen binding+++GO:0020037///heme binding+++GO:0004497///monooxygenase activity+++GO:0016712///oxidoreductase activity, acting on paired donors, with incorporation or reduction of molecular oxygen, reduced flavin or flavoprotein as one donor, and incorporation of one atom of oxygen+++GO:0016491///oxidoreductase activity+++GO:0008395///steroid hydroxylase activity+++GO:0008392///arachidonic acid epoxygenase activity	GO:0042738///exogenous drug catabolic process+++GO:0019373///epoxygenase P450 pathway+++GO:0006082///organic acid metabolic process+++GO:0055114///oxidation-reduction process+++GO:0006805///xenobiotic metabolic process
XM_021083612.1	LOC102167481	4.307329713	1.33E-09	01100///Metabolic pathways+++00590///Arachidonic acid metabolism	GO:0031090///organelle membrane+++GO:0005789///endoplasmic reticulum membrane+++GO:0016021///integral component of membrane	GO:0097258///20-hydroxy-leukotriene B4 omega oxidase activity+++GO:0004497///monooxygenase activity+++GO:0097259///20-aldehyde-leukotriene B4 20-monooxygenase activity+++GO:0050051///leukotriene-B4 20-monooxygenase activity+++GO:0020037///heme binding+++GO:0005506///iron ion binding	GO:0006691///leukotriene metabolic process+++GO:0006690///icosanoid metabolic process

NM_001243939.1	ADH1C	6.315951874	2.19E-06	01100///Metabolic pathways+++00980///Metabolism of xenobiotics by cytochrome P450+++00982///Drug metabolism - cytochrome P450+++00830///Retinol metabolism+++00010///Glycolysis / Gluconeogenesis+++01110///Biosynthesis of secondary metabolites+++00071///Fatty acid degradation+++00350///Tyrosine metabolism	GO:0005737///cytoplasm	GO:0008270///zinc ion binding+++GO:0004022///alcohol dehydrogenase (NAD) activity	
XM_001926129.6	PTGS1	1.306321694	0.010210229	00590///Arachidonic acid metabolism+++01100///Metabolic pathways+++04923///Regulation of lipolysis in adipocytes+++04611///Platelet activation+++04726///Serotonergic synapse	GO:0043231///intracellular membrane-bounded organelle+++GO:0001750///photoreceptor outer segment+++GO:0005737///cytoplasm+++GO:0005794///Golgi apparatus	GO:0020037///heme binding+++GO:0004666///prostaglandin-endoperoxide synthase activity+++GO:0004601///peroxidase activity	GO:0006693///prostaglandin metabolic process+++GO:0055114///oxidation-reduction process+++GO:0006954///inflammatory response+++GO:0042127///regulation of cell proliferation+++GO:0098869///cellular oxidant detoxification+++GO:0060085///smooth muscle relaxation of the bladder outlet+++GO:0001516///prostaglandin biosynthetic process+++GO:0006979///response to oxidative stress+++GO:0008217///regulation of blood pressure+++GO:0019371///cyclooxygenase pathway
XM_013982357.2	STRADB	1.264412473	0.013957128	04152///AMPK signaling pathway+++04150///mTOR signaling pathway	GO:0005737///cytoplasm+++GO:0016235///aggresome+++GO:0005634///nucleus+++GO:0005829///cytosol	GO:0004672///protein kinase activity+++GO:0005524///ATP binding	GO:2001240///negative regulation of extrinsic apoptotic signaling pathway in absence of ligand+++GO:0006468///protein phosphorylation+++GO:0000902///cell morphogenesis+++GO:0032147///activation of protein kinase activity+++GO:0007254///JNK cascade+++GO:0006611///protein export from nucleus
XM_021074163.1	SORBS1	1.391927743	0.012265876	04910///Insulin signaling pathway+++04520///Adherens junction+++03320///PPAR signaling pathway	GO:0005924///cell-substrate adherens junction+++GO:0005913///cell-cell adherens junction+++GO:0005899///insulin receptor complex+++GO:0016020///membrane+++GO:0005856///cytoskeleton+++GO:0005813///centrosome+++GO:0005925///focal adhesion+++GO:0005829///cytosol+++GO:0016363///nuclear matrix+++GO:0005886///plasma membrane+++GO:0005634///nucleus+++GO:0045121///membrane raft+++GO:0001725///stress fiber	GO:0005158///insulin receptor binding+++GO:0019901///protein kinase binding+++GO:0008134///transcription factor binding+++GO:0031625///ubiquitin protein ligase binding+++GO:0005070///SH3/SH2 adaptor activity	GO:0007015///actin filament organization
XM_021091201.1	PRKAG1	1.229141864	0.022333785	04530///Tight junction+++04922///Glucagon signaling pathway+++04211///Longevity regulating pathway+++04910///Insulin signaling pathway+++04920///Adipocytokine signaling pathway+++04714///Thermogenesis+++04921///Oxytocin signaling pathway+++04152///AMPK signaling pathway+++04068///FoxO signaling pathway+++04213///Longevity regulating pathway - multiple species+++04371///Apelin signaling pathway+++04710///Circadian rhythm	GO:0031588///nucleotide-activated protein kinase complex	GO:0004679///AMP-activated protein kinase activity+++GO:0005524///ATP binding+++GO:0016208///AMP binding+++GO:0000166///nucleotide binding+++GO:0016301///kinase activity+++GO:0004672///protein kinase activity+++GO:0019901///protein kinase binding+++GO:0032559///adenyl ribonucleotide binding+++GO:0043531///ADP binding	GO:0071900///regulation of protein serine/threonine kinase activity+++GO:0006631///fatty acid metabolic process+++GO:0006633///fatty acid biosynthetic process+++GO:0006629///lipid metabolic process+++GO:0006468///protein phosphorylation+++GO:0016310///phosphorylation+++GO:0010628///positive regulation of gene expression

XM_021074134.1	SORBS1	2.141428728	5.32E-04	03320///PPAR signaling pathway+++04910///Insulin signaling pathway+++04520///Adherens junction	GO:0005899///insulin receptor complex+++GO:0001725///stress fiber+++GO:0016020///membrane+++GO:0005913///cell-cell adherens junction+++GO:0005924///cell-substrate adherens junction+++GO:0005634///nucleus+++GO:0005925///focal adhesion+++GO:0005829///cytosol+++GO:0005813///centrosome+++GO:0005886///plasma membrane+++GO:0005856///cytoskeleton+++GO:0045121///membrane raft+++GO:0016363///nuclear matrix	GO:0008134///transcription factor binding+++GO:0031625///ubiquitin protein ligase binding+++GO:0019901///protein kinase binding+++GO:0005070///SH3/SH2 adaptor activity+++GO:0005158///insulin receptor binding	GO:0007015///actin filament organization
XM_021068059.1	MAP2K3	1.893675012	0.007165652	04620///Toll-like receptor signaling pathway+++04750///Inflammatory mediator regulation of TRP channels+++04013///MAPK signaling pathway - fly+++04668///TNF signaling pathway+++04912///GnRH signaling pathway+++04218///Cellular senescence+++04714///Thermogenesis+++04015///Rap1 signaling pathway+++04664///Fc epsilon RI signaling pathway+++04010///MAPK signaling pathway+++04624///Toll and Imd signaling pathway	GO:0005737///cytoplasm	GO:0004674///protein serine/threonine kinase activity+++GO:0004708///MAP kinase kinase activity+++GO:0005524///ATP binding+++GO:0004672///protein kinase activity+++GO:0019901///protein kinase binding+++GO:0000166///nucleotide binding+++GO:0016301///kinase activity	GO:0038066///p38MAPK cascade+++GO:0045893///positive regulation of transcription, DNA-templated+++GO:0000165///MAPK cascade+++GO:0045860///positive regulation of protein kinase activity+++GO:0006468///protein phosphorylation+++GO:0016310///phosphorylation+++GO:0000187///activation of MAPK activity+++GO:0042035///regulation of cytokine biosynthetic process+++GO:0032147///activation of protein kinase activity+++GO:0035924///cellular response to vascular endothelial growth factor stimulus+++GO:0023014///signal transduction by protein phosphorylation+++GO:0006954///inflammatory response+++GO:0060048///cardiac muscle contraction+++GO:0043536///positive regulation of blood vessel endothelial cell migration+++GO:0031098///stress-activated protein kinase signaling cascade
XM_021074020.1	CYP2C36	10.04821267	1.25E-04	00830///Retinol metabolism+++04726///Serotonergic synapse+++01100///Metabolic pathways	GO:0043231///intracellular membrane-bounded organelle+++GO:0005737///cytoplasm	GO:0008392///arachidonic acid epoxygenase activity+++GO:0016705///oxidoreductase activity, acting on paired donors, with incorporation or reduction of molecular oxygen+++GO:0016712///oxidoreductase activity, acting on paired donors, with incorporation or reduction of molecular oxygen, reduced flavin or flavoprotein as one donor, and incorporation of one atom of oxygen+++GO:0046872///metal ion binding+++GO:0005506///iron ion binding+++GO:0020037///heme binding+++GO:0016491///oxidoreductase activity+++GO:0008395///steroid hydroxylase activity+++GO:0004497///monooxygenase activity	GO:0006805///xenobiotic metabolic process+++GO:0055114///oxidation-reduction process+++GO:0042738///exogenous drug catabolic process+++GO:0006082///organic acid metabolic process+++GO:0019373///epoxygenase P450 pathway

XM_021097877.1	SLA-2	5.099477482	0.04055044	04145///Phagosome+++04144///Endocytosis+++04218///Cellular senescence+++04612///Antigen processing and presentation+++04514///Cell adhesion molecules (CAMs)	GO:0016020///membrane+++GO:0000139///Golgi membrane+++GO:0071556///integral component of lumenal side of endoplasmic reticulum membrane+++GO:0005794///Golgi apparatus+++GO:0055038///recycling endosome membrane+++GO:0012507///ER to Golgi transport vesicle membrane+++GO:0031901///early endosome membrane+++GO:0042612///MHC class I protein complex+++GO:0005789///endoplasmic reticulum membrane+++GO:0005887///integral component of plasma membrane+++GO:0005886///plasma membrane+++GO:0009986///cell surface+++GO:0030670///phagocytic vesicle membrane+++GO:0005783///endoplasmic reticulum	GO:0046977///TAP binding+++GO:0030881///beta-2-microglobulin binding+++GO:0042605///peptide antigen binding	GO:0002474///antigen processing and presentation of peptide antigen via MHC class I+++GO:0002479///antigen processing and presentation of exogenous peptide antigen via MHC class I, TAP-dependent+++GO:0016567///protein ubiquitination+++GO:0002480///antigen processing and presentation of exogenous peptide antigen via MHC class I, TAP-independent+++GO:0006955///immune response+++GO:0060337///type I interferon signaling pathway+++GO:0060333///interferon-gamma-mediated signaling pathway+++GO:0050776///regulation of immune response
NM_213979.1	FBP1	4.892230367	0.002157843	04152///AMPK signaling pathway+++01130///Biosynthesis of antibiotics+++01100///Metabolic pathways+++01200///Carbon metabolism+++00030///Pentose phosphate pathway+++00010///Glycolysis / Gluconeogenesis+++01110///Biosynthesis of secondary metabolites+++00051///Fructose and mannose metabolism+++01120///Microbial metabolism in diverse environments+++04910///Insulin signaling pathway+++00710///Carbon fixation in photosynthetic organisms+++00680///Methane metabolism	GO:0005634///nucleus+++GO:0005829///cytosol+++GO:0005737///cytoplasm	GO:0048029///monosaccharide binding+++GO:0042802///identical protein binding+++GO:0016208///AMP binding+++GO:0042578///phosphoric ester hydrolase activity+++GO:0016787///hydrolase activity+++GO:0016791///phosphatase activity+++GO:0003824///catalytic activity+++GO:0046872///metal ion binding+++GO:0042132///fructose 1,6-bisphosphate 1-phosphatase activity	GO:0045820///negative regulation of glycolytic process+++GO:0005975///carbohydrate metabolic process+++GO:0006000///fructose metabolic process+++GO:0006094///gluconeogenesis+++GO:0016311///dephosphorylation+++GO:0005986///sucrose biosynthetic process+++GO:0030308///negative regulation of cell growth+++GO:0006111///regulation of gluconeogenesis+++GO:0051289///protein homotetramerization+++GO:0008152///metabolic process+++GO:0030388///fructose 1,6-bisphosphate metabolic process+++GO:0046580///negative regulation of Ras protein signal transduction+++GO:0006002///fructose 6-phosphate metabolic process+++GO:0000122///negative regulation of transcription by RNA polymerase II+++GO:0071286///cellular response to magnesium ion+++GO:0035690///cellular response to drug
XM_005673043.3	PCK1	7.516253235	0.001275788	00620///Pyruvate metabolism+++00010///Glycolysis / Gluconeogenesis+++01100///Metabolic pathways+++04964///Proximal tubule bicarbonate reclamation+++04068///FoxO signaling pathway+++04910///Insulin signaling pathway+++01120///Microbial metabolism in diverse environments+++03320///PPAR signaling pathway+++04920///Adipocytokine signaling pathway+++01130///Biosynthesis of antibiotics+++04151///PI3K-Akt signaling pathway+++00020///Citrate cycle (TCA cycle)+++04922///Glucagon signaling pathway+++04152///AMPK signaling pathway+++01110///Biosynthesis of secondary metabolites	GO:0005739///mitochondrion+++GO:0005829///cytosol	GO:0031406///carboxylic acid binding+++GO:0004613///phosphoenolpyruvate carboxykinase (GTP) activity+++GO:0004611///phosphoenolpyruvate carboxykinase activity+++GO:0000287///magnesium ion binding+++GO:0030145///manganese ion binding+++GO:0016301///kinase activity+++GO:0005525///GTP binding+++GO:0017076///purine nucleotide binding	GO:0006475///internal protein amino acid acetylation+++GO:0016310///phosphorylation+++GO:0033993///response to lipid+++GO:0032869///cellular response to insulin stimulus+++GO:0006006///glucose metabolic process+++GO:0006629///lipid metabolic process+++GO:0071549///cellular response to dexamethasone stimulus+++GO:0051365///cellular response to potassium ion starvation+++GO:0071333///cellular response to glucose stimulus+++GO:0009617///response to bacterium+++GO:0061402///positive regulation of transcription from RNA polymerase II promoter in response to acidic pH+++GO:0042594///response to starvation+++GO:0070365///hepatocyte differentiation+++GO:0046327///glycerol biosynthetic process from pyruvate+++GO:0019543///propionate catabolic process+++GO:0006094///gluconeogenesis+++GO:0032868///response to insulin+++GO:0006090///pyruvate metabolic process

NM_001123158.1	PCK1	6.369135947	4.82E-05	01120///Microbial metabolism in diverse environments+++04964///Proximal tubule bicarbonate reclamation+++00010///Glycolysis / Gluconeogenesis+++00020///Citrate cycle (TCA cycle)+++04151///PI3K-Akt signaling pathway+++04152///AMPK signaling pathway+++03320///PPAR signaling pathway+++04910///Insulin signaling pathway+++04920///Adipocytokine signaling pathway+++01130///Biosynthesis of antibiotics+++01110///Biosynthesis of secondary metabolites+++04922///Glucagon signaling pathway+++04068///FoxO signaling pathway+++00620///Pyruvate metabolism+++01100///Metabolic pathways	GO:0005829///cytosol+++GO:0005739///mitochondrion	GO:0004611///phosphoenolpyruvate carboxykinase activity+++GO:0000287///magnesium ion binding+++GO:0030145///manganese ion binding+++GO:0031406///carboxylic acid binding+++GO:0004613///phosphoenolpyruvate carboxykinase (GTP) activity+++GO:0005525///GTP binding+++GO:0016301///kinase activity+++GO:0017076///purine nucleotide binding	GO:0006475///internal protein amino acid acetylation+++GO:0046327///glycerol biosynthetic process from pyruvate+++GO:0070365///hepatocyte differentiation+++GO:0071549///cellular response to dexamethasone stimulus+++GO:0019543///propionate catabolic process+++GO:0032869///cellular response to insulin stimulus+++GO:0032868///response to insulin+++GO:0033993///response to lipid+++GO:0006094///gluconeogenesis+++GO:0016310///phosphorylation+++GO:0009617///response to bacterium+++GO:0061402///positive regulation of transcription from RNA polymerase II promoter in response to acidic pH+++GO:0006090///pyruvate metabolic process+++GO:0006629///lipid metabolic process+++GO:0051365///cellular response to potassium ion starvation+++GO:0042594///response to starvation+++GO:0071333///cellular response to glucose stimulus+++GO:0006006///glucose metabolic process

XM_021093564.1	PRKAA2	-2.088050702	2.80E-07	04920///Adipocytokine signaling pathway+++04714///Thermogenesis+++04213///Longevity regulating pathway - multiple species+++04710///Circadian rhythm+++04140///Autophagy - animal+++04910///Insulin signaling pathway+++04921///Oxytocin signaling pathway+++04211///Longevity regulating pathway+++04151///PI3K-Akt signaling pathway+++04922///Glucagon signaling pathway+++04068///FoxO signaling pathway+++04150///mTOR signaling pathway+++04152///AMPK signaling pathway+++04371///Apelin signaling pathway+++04530///Tight junction	GO:0043025///neuronal cell body+++GO:0016607///nuclear speck+++GO:0005737///cytoplasm+++GO:0005634///nucleus+++GO:0030425///dendrite+++GO:0031588///nucleotide-activated protein kinase complex+++GO:0005794///Golgi apparatus+++GO:0030424///axon+++GO:0010494///cytoplasmic stress granule	GO:0047322///[hydroxymethylglutaryl-CoA reductase (NADPH)] kinase activity+++GO:0046872///metal ion binding+++GO:0050405///[acetyl-CoA carboxylase] kinase activity+++GO:0004674///protein serine/threonine kinase activity+++GO:0004672///protein kinase activity+++GO:0005524///ATP binding+++GO:0000166///nucleotide binding+++GO:0016740///transferase activity+++GO:0004712///protein serine/threonine/tyrosine kinase activity+++GO:0035174///histone serine kinase activity+++GO:0016301///kinase activity+++GO:0003682///chromatin binding+++GO:0004679///AMP-activated protein kinase activity	GO:0042593///glucose homeostasis+++GO:0035404///histone-serine phosphorylation+++GO:0016055///Wnt signaling pathway+++GO:0010468///regulation of gene expression+++GO:0071380///cellular response to prostaglandin E stimulus+++GO:0071333///cellular response to glucose stimulus+++GO:0014850///response to muscle activity+++GO:0016241///regulation of macroautophagy+++GO:0008610///lipid biosynthetic process+++GO:0006629///lipid metabolic process+++GO:0006914///autophagy+++GO:0016126///sterol biosynthetic process+++GO:0006695///cholesterol biosynthetic process+++GO:0070507///regulation of microtubule cytoskeleton organization+++GO:0062028///regulation of stress granule assembly+++GO:0035556///intracellular signal transduction+++GO:0035690///cellular response to drug+++GO:0042752///regulation of circadian rhythm+++GO:0010629///negative regulation of gene expression+++GO:0032007///negative regulation of TOR signaling+++GO:0031669///cellular response to nutrient levels+++GO:0006631///fatty acid metabolic process+++GO:0048511///rhythmic process+++GO:0071277///cellular response to calcium ion+++GO:0008202///steroid metabolic process+++GO:0043066///negative regulation of apoptotic process+++GO:0016310///phosphorylation+++GO:0034599///cellular response to oxidative stress+++GO:2000758///positive regulation of peptidyl-lysine acetylation+++GO:0006468///protein phosphorylation+++GO:0006694///steroid biosynthetic process+++GO:0010508///positive regulation of autophagy+++GO:1903829///positive regulation of cellular protein localization+++GO:0008203///cholesterol metabolic process+++GO:0042149///cellular response to glucose
XM_021080575.1	IGF2	-2.066171754	1.36E-08	04010///MAPK signaling pathway+++04014///Ras signaling pathway+++04151///PI3K-Akt signaling pathway	GO:0005576///extracellular region+++GO:0016021///integral component of membrane+++GO:0016020///membrane+++GO:0005615///extracellular space	GO:0005178///integrin binding+++GO:0005179///hormone activity+++GO:0008083///growth factor activity	GO:0001503///ossification+++GO:0000122///negative regulation of transcription by RNA polymerase II+++GO:0008284///positive regulation of cell proliferation+++GO:0001701///in utero embryonic development+++GO:0006006///glucose metabolic process+++GO:0051781///positive regulation of cell division+++GO:0051147///regulation of muscle cell differentiation+++GO:0005975///carbohydrate metabolic process+++GO:0060669///embryonic placenta morphogenesis+++GO:0031056///regulation of histone modification+++GO:0010469///regulation of signaling receptor activity+++GO:0001892///embryonic placenta development

NM_001038644.1	ANGPTL4	-1.704947066	1.77E-04	03320///PPAR signaling pathway+++04979///Cholesterol metabolism	GO:0005615///extracellular space+++GO:0005576///extracellular region	GO:0004857///enzyme inhibitor activity+++GO:0042802///identical protein binding	GO:0001525///angiogenesis+++GO:0030154///cell differentiation+++GO:0007275///multicellular organism development+++GO:2000352///negative regulation of endothelial cell apoptotic process+++GO:0051005///negative regulation of lipoprotein lipase activity+++GO:0043066///negative regulation of apoptotic process+++GO:0070328///triglyceride homeostasis
XM_021069486.1	PPP2R3A	-1.158642419	0.007753811	04151///PI3K-Akt signaling pathway+++03015///mRNA surveillance pathway+++04261///Adrenergic signaling in cardiomyocytes+++04152///AMPK signaling pathway+++04728///Dopaminergic synapse+++04071///Sphingolipid signaling pathway	GO:0000159///protein phosphatase type 2A complex	GO:0005509///calcium ion binding	GO:0090263///positive regulation of canonical Wnt signaling pathway+++GO:0090090///negative regulation of canonical Wnt signaling pathway+++GO:0061053///somite development+++GO:0045732///positive regulation of protein catabolic process+++GO:0006470///protein dephosphorylation+++GO:0090244///Wnt signaling pathway involved in somitogenesis+++GO:0090249///regulation of cell motility involved in somitogenic axis elongation+++GO:0007525///somatic muscle development+++GO:0001754///eye photoreceptor cell differentiation
XM_021080585.1	IGF2	-4.961226962	5.52E-16	04151///PI3K-Akt signaling pathway+++04014///Ras signaling pathway+++04010///MAPK signaling pathway	GO:0016021///integral component of membrane+++GO:0005615///extracellular space+++GO:0005576///extracellular region+++GO:0016020///membrane	GO:0005179///hormone activity+++GO:0005178///integrin binding+++GO:0008083///growth factor activity	GO:0060669///embryonic placenta morphogenesis+++GO:0001892///embryonic placenta development+++GO:0001503///ossification+++GO:0051781///positive regulation of cell division+++GO:0001701///in utero embryonic development+++GO:0010469///regulation of signaling receptor activity+++GO:0008284///positive regulation of cell proliferation+++GO:0031056///regulation of histone modification+++GO:0051147///regulation of muscle cell differentiation+++GO:0000122///negative regulation of transcription by RNA polymerase II+++GO:0005975///carbohydrate metabolic process+++GO:0006006///glucose metabolic process
XM_013992761.2	LAMA4	-1.149905214	0.010569534	04510///Focal adhesion+++04512///ECM-receptor interaction+++04151///PI3K-Akt signaling pathway	GO:0031594///neuromuscular junction+++GO:0005604///basement membrane+++GO:0043083///synaptic cleft+++GO:0062023///collagen-containing extracellular matrix	GO:0005102///signaling receptor binding+++GO:0005201///extracellular matrix structural constituent	GO:0030155///regulation of cell adhesion+++GO:0120163///negative regulation of cold-induced thermogenesis+++GO:0001568///blood vessel development+++GO:0030334///regulation of cell migration+++GO:0050873///brown fat cell differentiation+++GO:0045995///regulation of embryonic development+++GO:0007155///cell adhesion

XR_002343817.1	GAPDH	-1.31680227	0.008174347	01110///Biosynthesis of secondary metabolites+++01130///Biosynthesis of antibiotics+++01100///Metabolic pathways+++01200///Carbon metabolism+++04066///HIF-1 signaling pathway+++00710///Carbon fixation in photosynthetic organisms+++01230///Biosynthesis of amino acids+++00010///Glycolysis / Gluconeogenesis+++01120///Microbial metabolism in diverse environments	GO:0005634///nucleus+++GO:0097452///GAIT complex+++GO:0098850///extrinsic component of synaptic vesicle membrane+++GO:0005829///cytosol+++GO:0005737///cytoplasm+++GO:0015630///microtubule cytoskeleton	GO:0097718///disordered domain specific binding+++GO:0035605///peptidyl-cysteine S-nitrosylase activity+++GO:0051287///NAD binding+++GO:0050661///NADP binding+++GO:0008017///microtubule binding+++GO:0004365///glyceraldehyde-3-phosphate dehydrogenase (NAD+) (phosphorylating) activity	GO:0099162///regulation of neurotransmitter loading into synaptic vesicle+++GO:0051402///neuron apoptotic process+++GO:0050821///protein stabilization+++GO:0006417///regulation of translation+++GO:0000226///microtubule cytoskeleton organization+++GO:0006006///glucose metabolic process+++GO:0035606///peptidyl-cysteine S-trans-nitrosylation+++GO:0006096///glycolytic process
XM_021066016.1	ACOX1	-1.233611583	2.81E-05	00592///alpha-Linolenic acid metabolism+++00071///Fatty acid degradation+++01110///Biosynthesis of secondary metabolites+++04024///cAMP signaling pathway+++04146///Peroxisome+++01040///Biosynthesis of unsaturated fatty acids+++01100///Metabolic pathways+++01212///Fatty acid metabolism+++03320///PPAR signaling pathway	GO:0043231///intracellular membrane-bounded organelle+++GO:0005886///plasma membrane+++GO:0005730///nucleolus+++GO:0005654///nucleoplasm+++GO:0005777///peroxisome+++GO:0005737///cytoplasm+++GO:0005778///peroxisomal membrane+++GO:0005634///nucleus	GO:0030165///PDZ domain binding+++GO:0003997///acyl-CoA oxidase activity+++GO:0016401///palmitoyl-CoA oxidase activity+++GO:0050660///flavin adenine dinucleotide binding+++GO:0005102///signaling receptor binding+++GO:0071949///FAD binding+++GO:0005504///fatty acid binding+++GO:0047485///protein N-terminus binding+++GO:0016627///oxidoreductase activity, acting on the CH-CH group of donors	GO:0006635///fatty acid beta-oxidation+++GO:0033540///fatty acid beta-oxidation using acyl-CoA oxidase+++GO:0055088///lipid homeostasis+++GO:0055114///oxidation-reduction process+++GO:0000038///very long-chain fatty acid metabolic process+++GO:0006091///generation of precursor metabolites and energy+++GO:0006629///lipid metabolic process+++GO:0007283///spermatogenesis+++GO:0019395///fatty acid oxidation+++GO:0006631///fatty acid metabolic process+++GO:0006693///prostaglandin metabolic process
XM_005671823.3	PPP1R3B	-1.737570686	0.004752355	04910///Insulin signaling pathway	GO:0000164///protein phosphatase type 1 complex	GO:0019888///protein phosphatase regulator activity	GO:0005975///carbohydrate metabolic process+++GO:0005981///regulation of glycogen catabolic process+++GO:0043666///regulation of phosphoprotein phosphatase activity+++GO:0005977///glycogen metabolic process
XM_021068116.1	MAP2K6	-1.751027008	4.28E-19	04380///Osteoclast differentiation+++04750///Inflammatory mediator regulation of TRP channels+++04912///GnRH signaling pathway+++04010///MAPK signaling pathway+++04218///Cellular senescence+++04212///Longevity regulating pathway - worm+++04015///Rap1 signaling pathway+++04620///Toll-like receptor signaling pathway+++04668///TNF signaling pathway+++04664///Fc epsilon RI signaling pathway	GO:0005829///cytosol+++GO:0005634///nucleus	GO:0000166///nucleotide binding+++GO:0005524///ATP binding+++GO:0019901///protein kinase binding+++GO:0004674///protein serine/threonine kinase activity+++GO:0004708///MAP kinase kinase activity+++GO:0004672///protein kinase activity+++GO:0016301///kinase activity	GO:0060048///cardiac muscle contraction+++GO:0016310///phosphorylation+++GO:0000187///activation of MAPK activity+++GO:0006468///protein phosphorylation+++GO:0120163///negative regulation of cold-induced thermogenesis+++GO:0000165///MAPK cascade

XM_021087999.1	ALDOA	-1.93426523	3.50E-09	00680///Methane metabolism+++00710///Carbon fixation in photosynthetic organisms+++00010///Glycolysis / Gluconeogenesis+++00051///Fructose and mannose metabolism+++01200///Carbon metabolism+++01120///Microbial metabolism in diverse environments+++00030///Pentose phosphate pathway+++01110///Biosynthesis of secondary metabolites+++01230///Biosynthesis of amino acids+++01100///Metabolic pathways+++01130///Biosynthesis of antibiotics	GO:0005737///cytoplasm+++GO:0005720///nuclear heterochromatin	GO:0003824///catalytic activity+++GO:0016829///lyase activity+++GO:0004332///fructose-bisphosphate aldolase activity	GO:0006096///glycolytic process
XM_021080692.1	PHKA1	2.506151938	4.06E-05	04910///Insulin signaling pathway+++04020///Calcium signaling pathway+++04922///Glucagon signaling pathway	GO:0005886///plasma membrane+++GO:0016020///membrane+++GO:0005964///phosphorylase kinase complex	GO:0005516///calmodulin binding+++GO:0004689///phosphorylase kinase activity+++GO:0003824///catalytic activity	GO:0005977///glycogen metabolic process+++GO:0006468///protein phosphorylation+++GO:0005975///carbohydrate metabolic process

NM_001044526.1	PPARA	1.754195949	0.011302082	04922///Glucagon signaling pathway+++04024///cAMP signaling pathway+++04920///Adipocytokine signaling pathway+++03320///PPAR signaling pathway	GO:0005654///nucleoplasm+++GO:0005634///nucleus+++GO:0090575///RNA polymerase II transcription factor complex	GO:0000981///DNA-binding transcription factor activity, RNA polymerase II-specific+++GO:0030374///nuclear receptor transcription coactivator activity+++GO:0043565///sequence-specific DNA binding+++GO:0000978///RNA polymerase II proximal promoter sequence-specific DNA binding+++GO:0008144///drug binding+++GO:0038023///signaling receptor activity+++GO:0003700///DNA-binding transcription factor activity+++GO:0005504///fatty acid binding+++GO:0000977///RNA polymerase II regulatory region sequence-specific DNA binding+++GO:0001228///DNA-binding transcription activator activity, RNA polymerase II-specific+++GO:0004879///nuclear receptor activity+++GO:0003677///DNA binding+++GO:0001227///DNA-binding transcription repressor activity, RNA polymerase II-specific+++GO:0008134///transcription factor binding+++GO:0008270///zinc ion binding+++GO:0000976///transcription regulatory region sequence-specific DNA binding+++GO:0031624///ubiquitin conjugating enzyme binding+++GO:0008289///lipid binding+++GO:0046872///metal ion binding+++GO:0003707///steroid hormone receptor activity+++GO:0001103///RNA polymerase II repressing transcription factor binding	GO:0019216///regulation of lipid metabolic process+++GO:1902894///negative regulation of pri-miRNA transcription by RNA polymerase II+++GO:0010745///negative regulation of macrophage derived foam cell differentiation+++GO:0050728///negative regulation of inflammatory response+++GO:0030522///intracellular receptor signaling pathway+++GO:0006631///fatty acid metabolic process+++GO:0007275///multicellular organism development+++GO:0008544///epidermis development+++GO:0070166///enamel mineralization+++GO:0045893///positive regulation of transcription, DNA-templated+++GO:0030154///cell differentiation+++GO:0019217///regulation of fatty acid metabolic process+++GO:0045722///positive regulation of gluconeogenesis+++GO:0042752///regulation of circadian rhythm+++GO:0072366///regulation of cellular ketone metabolic process by positive regulation of transcription from RNA polymerase II promoter+++GO:0042060///wound healing+++GO:0046321///positive regulation of fatty acid oxidation+++GO:0010468///regulation of gene expression+++GO:0032922///circadian regulation of gene expression+++GO:0032099///negative regulation of appetite+++GO:0045944///positive regulation of transcription by RNA polymerase II+++GO:0010891///negative regulation of sequestering of triglyceride+++GO:0000122///negative regulation of transcription by RNA polymerase II+++GO:1903038///negative regulation of leukocyte cell-cell adhesion+++GO:0010887///negative regulation of cholesterol storage+++GO:0072363///regulation of glycolytic process by positive regulation of transcription from RNA polymerase II promoter+++GO:0045892///negative regulation of transcription, DNA-templated+++GO:0006355///regulation of transcription, DNA-

NM_001122987.1	HK2	1.613495229	0.001906339	04910///Insulin signaling pathway+++01110///Biosynthesis of secondary metabolites+++01120///Microbial metabolism in diverse environments+++01200///Carbon metabolism+++04973///Carbohydrate digestion and absorption+++01130///Biosynthesis of antibiotics+++00500///Starch and sucrose metabolism+++04066///HIF-1 signaling pathway+++01100///Metabolic pathways+++00052///Galactose metabolism+++00010///Glycolysis / Gluconeogenesis+++00051///Fructose and mannose metabolism+++00521///Streptomycin biosynthesis+++00520///Amino sugar and nucleotide sugar metabolism+++00524///Neomycin, kanamycin and gentamicin biosynthesis	GO:0005623///cell+++GO:0005829///cytosol	GO:0016740///transferase activity+++GO:0019158///mannokinase activity+++GO:0004396///hexokinase activity+++GO:0016301///kinase activity+++GO:0004340///glucokinase activity+++GO:0008865///fructokinase activity+++GO:0003824///catalytic activity+++GO:0005524///ATP binding+++GO:0000166///nucleotide binding+++GO:0016773///phosphotransferase activity, alcohol group as acceptor+++GO:0005536///glucose binding	GO:0001678///cellular glucose homeostasis+++GO:0008152///metabolic process+++GO:0046835///carbohydrate phosphorylation+++GO:0016310///phosphorylation+++GO:0051156///glucose 6-phosphate metabolic process+++GO:0019318///hexose metabolic process+++GO:0005975///carbohydrate metabolic process+++GO:0006096///glycolytic process
XM_003124944.4	RETSAT	1.298125017	5.33E-04	00830///Retinol metabolism	GO:0031965///nuclear membrane+++GO:0005789///endoplasmic reticulum membrane+++GO:0005640///nuclear outer membrane	GO:0051786///all-trans-retinol 13,14-reductase activity+++GO:0016491///oxidoreductase activity	GO:0042572///retinol metabolic process+++GO:0055114///oxidation-reduction process
XM_021066854.1	ALDOB	9.908947528	7.51E-07	01110///Biosynthesis of secondary metabolites+++01100///Metabolic pathways+++01230///Biosynthesis of amino acids+++00030///Pentose phosphate pathway+++00710///Carbon fixation in photosynthetic organisms+++00051///Fructose and mannose metabolism+++01200///Carbon metabolism+++00010///Glycolysis / Gluconeogenesis+++01120///Microbial metabolism in diverse environments+++00680///Methane metabolism+++01130///Biosynthesis of antibiotics	GO:0034451///centriolar satellite+++GO:0005815///microtubule organizing center	GO:0016829///lyase activity+++GO:0070061///fructose binding+++GO:0008092///cytoskeletal protein binding+++GO:0051117///ATPase binding+++GO:0061609///fructose-1-phosphate aldolase activity+++GO:0042802///identical protein binding+++GO:0003824///catalytic activity+++GO:0004332///fructose-bisphosphate aldolase activity	GO:0030388///fructose 1,6-bisphosphate metabolic process+++GO:0032781///positive regulation of ATPase activity+++GO:0006000///fructose metabolic process+++GO:0006116///NADH oxidation+++GO:0070072///vacuolar proton-transporting V-type ATPase complex assembly+++GO:0006096///glycolytic process
XM_021083611.1	LOC100739101	10.1881759	5.64E-10	01100///Metabolic pathways+++00590///Arachidonic acid metabolism	GO:0016021///integral component of membrane+++GO:0005789///endoplasmic reticulum membrane+++GO:0031090///organelle membrane	GO:0004497///monooxygenase activity+++GO:0005506///iron ion binding+++GO:0097258///20-hydroxy-leukotriene B4 omega oxidase activity+++GO:0050051///leukotriene-B4 20-monooxygenase activity+++GO:0097259///20-aldehyde-leukotriene B4 20-monooxygenase activity+++GO:0020037///heme binding	GO:0006690///icosanoid metabolic process+++GO:0006691///leukotriene metabolic process
XM_001927113.6	ACSS1	2.27019517	0.001047686	01200///Carbon metabolism+++01110///Biosynthesis of secondary metabolites+++01130///Biosynthesis of antibiotics+++01100///Metabolic pathways+++00630///Glyoxylate and dicarboxylate metabolism+++00010///Glycolysis / Gluconeogenesis+++00640///Propanoate metabolism+++00620///Pyruvate metabolism+++00680///Methane metabolism+++01120///Microbial metabolism in diverse environments+++00720///Carbon fixation pathways in prokaryotes	GO:0005759///mitochondrial matrix	GO:0000166///nucleotide binding+++GO:0003987///acetate-CoA ligase activity+++GO:0016874///ligase activity+++GO:0005524///ATP binding+++GO:0016208///AMP binding+++GO:0003824///catalytic activity	GO:0006085///acetyl-CoA biosynthetic process+++GO:0019427///acetyl-CoA biosynthetic process from acetate

XM_021099593.1	BCL2	1.291763403	2.37E-04	04722///Neurotrophin signaling pathway+++04510///Focal adhesion+++04340///Hedgehog signaling pathway+++04217///Necroptosis+++04071///Sphingolipid signaling pathway+++04630///Jak-STAT signaling pathway+++04064///NF-kappa B signaling pathway+++04210///Apoptosis+++04066///HIF-1 signaling pathway+++04725///Cholinergic synapse+++04928///Parathyroid hormone synthesis, secretion and action+++04621///NOD-like receptor signaling pathway+++04115///p53 signaling pathway+++04915///Estrogen signaling pathway+++04261///Adrenergic signaling in cardiomyocytes+++04141///Protein processing in endoplasmic reticulum+++04151///PI3K-Akt signaling pathway+++04140///Autophagy - animal+++04215///Apoptosis - multiple species	GO:0016021///integral component of membrane+++GO:0016020///membrane	GO:0042803///protein homodimerization activity+++GO:0046982///protein heterodimerization activity	GO:0042981///regulation of apoptotic process+++GO:0006915///apoptotic process+++GO:0043066///negative regulation of apoptotic process
NM_001005154.1	MYC	1.564948468	1.80E-04	04012///ErbB signaling pathway+++04350///TGF-beta signaling pathway+++04310///Wnt signaling pathway+++04390///Hippo signaling pathway+++04630///Jak-STAT signaling pathway+++04218///Cellular senescence+++04919///Thyroid hormone signaling pathway+++04151///PI3K-Akt signaling pathway+++04550///Signaling pathways regulating pluripotency of stem cells+++04391///Hippo signaling pathway - fly+++04110///Cell cycle+++04010///MAPK signaling pathway	GO:0005730///nucleolus+++GO:0005819///spindle+++GO:0005634///nucleus+++GO:0016604///nuclear body+++GO:0005654///nucleoplasm	GO:0070888///E-box binding+++GO:0046983///protein dimerization activity+++GO:0003700///DNA-binding transcription factor activity+++GO:0044877///protein-containing complex binding+++GO:0003677///DNA binding+++GO:0000981///DNA-binding transcription factor activity, RNA polymerase II-specific	GO:0006879///cellular iron ion homeostasis+++GO:0006338///chromatin remodeling+++GO:2000573///positive regulation of DNA biosynthetic process+++GO:0032204///regulation of telomere maintenance+++GO:0000165///MAPK cascade+++GO:0097193///intrinsic apoptotic signaling pathway+++GO:0043280///positive regulation of cysteine-type endopeptidase activity involved in apoptotic process+++GO:0043066///negative regulation of apoptotic process+++GO:2001022///positive regulation of response to DNA damage stimulus+++GO:0006355///regulation of transcription, DNA-templated+++GO:0045656///negative regulation of monocyte differentiation+++GO:0048146///positive regulation of fibroblast proliferation+++GO:0051276///chromosome organization+++GO:0051782///negative regulation of cell division+++GO:0045944///positive regulation of transcription by RNA polymerase II+++GO:0042981///regulation of apoptotic process+++GO:0006974///cellular response to DNA damage stimulus+++GO:0042493///response to drug+++GO:0045893///positive regulation of transcription, DNA-templated+++GO:0010332///response to gamma radiation+++GO:0009314///response to radiation+++GO:0008284///positive regulation of cell proliferation+++GO:0032873///negative regulation of stress-activated MAPK [illegible]

NM_214403.1	IL6R	1.44216946	0.011338353	04066///HIF-1 signaling pathway+++04630///Jak-STAT signaling pathway+++04659///Th17 cell differentiation+++04640///Hematopoietic cell lineage+++04151///PI3K-Akt signaling pathway+++04060///Cytokine-cytokine receptor interaction	GO:0009897///external side of plasma membrane+++GO:0016020///membrane+++GO:0043235///receptor complex+++GO:0005576///extracellular region+++GO:0070110///ciliary neurotrophic factor receptor complex+++GO:0016021///integral component of membrane+++GO:0016324///apical plasma membrane+++GO:0043514///interleukin-12 complex+++GO:0005896///interleukin-6 receptor complex+++GO:0070743///interleukin-23 complex	GO:0042164///interleukin-12 alpha subunit binding+++GO:0004896///cytokine receptor activity+++GO:0019899///enzyme binding+++GO:0008083///growth factor activity+++GO:0004915///interleukin-6 receptor activity+++GO:0045519///interleukin-23 receptor binding+++GO:0005138///interleukin-6 receptor binding+++GO:0042803///protein homodimerization activity+++GO:0019981///interleukin-6 binding+++GO:0004897///ciliary neurotrophic factor receptor activity+++GO:0070119///ciliary neurotrophic factor binding+++GO:0005143///interleukin-12 receptor binding+++GO:0019955///cytokine binding	GO:0032816///positive regulation of natural killer cell activation+++GO:0042104///positive regulation of activated T cell proliferation+++GO:0002827///positive regulation of T-helper 1 type immune response+++GO:0032755///positive regulation of interleukin-6 production+++GO:0048661///positive regulation of smooth muscle cell proliferation+++GO:0070120///ciliary neurotrophic factor-mediated signaling pathway+++GO:0010536///positive regulation of activation of Janus kinase activity+++GO:0042531///positive regulation of tyrosine phosphorylation of STAT protein+++GO:0050671///positive regulation of lymphocyte proliferation+++GO:0031018///endocrine pancreas development+++GO:0019221///cytokine-mediated signaling pathway+++GO:0070102///interleukin-6-mediated signaling pathway+++GO:0051135///positive regulation of NK T cell activation+++GO:0034097///response to cytokine+++GO:0010469///regulation of signaling receptor activity+++GO:0050731///positive regulation of peptidyl-tyrosine phosphorylation+++GO:0032729///positive regulation of interferon-gamma production+++GO:0032722///positive regulation of chemokine production+++GO:0008284///positive regulation of cell proliferation

XM_021088726.1	IL6R	1.327448268	0.009641659	04640///Hematopoietic cell lineage+++04659///Th17 cell differentiation+++04060///Cytokine-cytokine receptor interaction+++04066///HIF-1 signaling pathway+++04151///PI3K-Akt signaling pathway+++04630///Jak-STAT signaling pathway	GO:0005576///extracellular region+++GO:0005896///interleukin-6 receptor complex+++GO:0070743///interleukin-23 complex+++GO:0009897///external side of plasma membrane+++GO:0070110///ciliary neurotrophic factor receptor complex+++GO:0016324///apical plasma membrane+++GO:0043514///interleukin-12 complex+++GO:0016020///membrane+++GO:0016021///integral component of membrane+++GO:0043235///receptor complex	GO:0005143///interleukin-12 receptor binding+++GO:0004896///cytokine receptor activity+++GO:0019981///interleukin-6 binding+++GO:0004897///ciliary neurotrophic factor receptor activity+++GO:0070119///ciliary neurotrophic factor binding+++GO:0045519///interleukin-23 receptor binding+++GO:0042164///interleukin-12 alpha subunit binding+++GO:0005138///interleukin-6 receptor binding+++GO:0004915///interleukin-6 receptor activity+++GO:0019955///cytokine binding+++GO:0019899///enzyme binding+++GO:0008083///growth factor activity+++GO:0042803///protein homodimerization activity	GO:0050671///positive regulation of lymphocyte proliferation+++GO:0042531///positive regulation of tyrosine phosphorylation of STAT protein+++GO:0002827///positive regulation of T-helper 1 type immune response+++GO:0032816///positive regulation of natural killer cell activation+++GO:0051135///positive regulation of NK T cell activation+++GO:0070102///interleukin-6-mediated signaling pathway+++GO:0048661///positive regulation of smooth muscle cell proliferation+++GO:0032729///positive regulation of interferon-gamma production+++GO:0032755///positive regulation of interleukin-6 production+++GO:0019221///cytokine-mediated signaling pathway+++GO:0010469///regulation of signaling receptor activity+++GO:0034097///response to cytokine+++GO:0070120///ciliary neurotrophic factor-mediated signaling pathway+++GO:0010536///positive regulation of activation of Janus kinase activity+++GO:0008284///positive regulation of cell proliferation+++GO:0031018///endocrine pancreas development+++GO:0050731///positive regulation of peptidyl-tyrosine phosphorylation+++GO:0042104///positive regulation of activated T cell proliferation+++GO:0032722///positive regulation of chemokine production
XM_021101103.1	TBC1D1	1.524432137	5.42E-04	04152///AMPK signaling pathway	GO:0005623///cell	GO:0017137///Rab GTPase binding+++GO:0005096///GTPase activator activity	GO:0090630///activation of GTPase activity+++GO:0006886///intracellular protein transport
XM_021074155.1	SORBS1	1.402747244	0.008602096	04910///Insulin signaling pathway+++04520///Adherens junction+++03320///PPAR signaling pathway	GO:0016020///membrane+++GO:0005856///cytoskeleton+++GO:0005813///centrosome+++GO:0005886///plasma membrane+++GO:0001725///stress fiber+++GO:0045121///membrane raft+++GO:0005924///cell-substrate adherens junction+++GO:0005634///nucleus+++GO:0005913///cell-cell adherens junction+++GO:0005899///insulin receptor complex+++GO:0005829///cytosol+++GO:000592	GO:0005070///SH3/SH2 adaptor activity+++GO:0019901///protein kinase binding+++GO:0008134///transcription factor binding+++GO:0005158///insulin receptor binding+++GO:0031625///ubiquitin protein ligase binding	GO:0007015///actin filament organization
XM_021084204.1	MKNK2	1.208324646	0.012681781	04066///HIF-1 signaling pathway+++04010///MAPK signaling pathway+++04910///Insulin signaling pathway	GO:0016604///nuclear body+++GO:0005654///nucleoplasm+++GO:0005737///cytoplasm+++GO:0005634///nucleus	GO:0004683///calmodulin-dependent protein kinase activity+++GO:0004674///protein serine/threonine kinase activity+++GO:0004672///protein kinase activity+++GO:0000166///nucleotide binding+++GO:0009931///calcium-dependent protein serine/threonine kinase activity+++GO:0005524///ATP binding	GO:0071243///cellular response to arsenic-containing substance+++GO:0018105///peptidyl-serine phosphorylation+++GO:0035556///intracellular signal transduction+++GO:0006468///protein phosphorylation+++GO:0030097///hemopoiesis+++GO:0097192///extrinsic apoptotic signaling pathway in absence of ligand

NM_214152.2	PPARD	2.825732156	7.31E-06	03320///PPAR signaling pathway+++04310///Wnt signaling pathway	GO:0005634///nucleus+++GO:0000790///nuclear chromatin+++GO:0005654///nucleoplasm	GO:0003713///transcription coactivator activity+++GO:0004879///nuclear receptor activity+++GO:0001227///DNA-binding transcription repressor activity, RNA polymerase II-specific+++GO:0051059///NF-kappaB binding+++GO:0070539///linoleic acid binding+++GO:0008270///zinc ion binding+++GO:0003677///DNA binding+++GO:0003700///DNA-binding transcription factor activity+++GO:0008134///transcription factor binding+++GO:0000981///DNA-binding transcription factor activity, RNA polymerase II-specific+++GO:0003707///steroid hormone receptor activity+++GO:0046982///protein heterodimerization activity+++GO:0043565///sequence-specific DNA binding+++GO:0008289///lipid binding+++GO:0008144///drug binding	GO:0008654///phospholipid biosynthetic process+++GO:0043415///positive regulation of skeletal muscle tissue regeneration+++GO:0033189///response to vitamin A+++GO:0046697///decidualization+++GO:0008203///cholesterol metabolic process+++GO:0045684///positive regulation of epidermis development+++GO:0043066///negative regulation of apoptotic process+++GO:0045600///positive regulation of fat cell differentiation+++GO:1902894///negative regulation of pri-miRNA transcription by RNA polymerase II+++GO:0032966///negative regulation of collagen biosynthetic process+++GO:0051546///keratinocyte migration+++GO:0014068///positive regulation of phosphatidylinositol 3-kinase signaling+++GO:2000288///positive regulation of myoblast proliferation+++GO:0009749///response to glucose+++GO:0015908///fatty acid transport+++GO:0045892///negative regulation of transcription, DNA-templated+++GO:0045662///negative regulation of myoblast differentiation+++GO:0006091///generation of precursor metabolites and energy+++GO:0009062///fatty acid catabolic process+++GO:0043616///keratinocyte proliferation+++GO:0000122///negative regulation of transcription by RNA polymerase II+++GO:0014823///response to activity+++GO:0032024///positive regulation of insulin secretion+++GO:0042060///wound healing+++GO:0060612///adipose tissue development+++GO:0006357///regulation of transcription by RNA polymerase II+++GO:0031589///cell-substrate adhesion+++GO:0030308///negative regulation of cell growth+++GO:1904659///glucose transmembrane transport+++GO:0009299///mRNA transcription+++GO:0071456///cellular response to hypoxia+++GO:0006029///proteoglycan metabolic process+++GO:0050728///negative regulation of inflammatory response+++GO:0007566///embryo implantation+++GO:0045893///positive regulation
XM_021078618.1	PRKAG2	2.830586155	2.91E-06	04530///Tight junction+++04152///AMPK signaling pathway+++04910///Insulin signaling pathway+++04710///Circadian rhythm+++04211///Longevity regulating pathway+++04213///Longevity regulating pathway - multiple species+++04922///Glucagon signaling pathway+++04920///Adipocytokine signaling pathway+++04714///Thermogenesis+++04921///Oxytocin signaling pathway+++04068///FoxO signaling pathway+++04371///Apelin signaling pathway	GO:0031588///nucleotide-activated protein kinase complex	GO:0019901///protein kinase binding+++GO:0032559///adenyl ribonucleotide binding+++GO:0030295///protein kinase activator activity+++GO:0005524///ATP binding+++GO:0004862///cAMP-dependent protein kinase inhibitor activity+++GO:0008607///phosphorylase kinase regulator activity+++GO:0043531///ADP binding+++GO:0008603///cAMP-dependent protein kinase regulator activity	GO:0035556///intracellular signal transduction+++GO:0045860///positive regulation of protein kinase activity+++GO:0032147///activation of protein kinase activity+++GO:0071901///negative regulation of protein serine/threonine kinase activity+++GO:0006469///negative regulation of protein kinase activity+++GO:0005977///glycogen metabolic process+++GO:0071900///regulation of protein serine/threonine kinase activity+++GO:0006110///regulation of glycolytic process+++GO:0010800///positive regulation of peptidyl-threonine phosphorylation+++GO:0019217///regulation of fatty acid metabolic process

XM_021079753.1	CYP4F55	3.085266986	0.019442856	01100///Metabolic pathways+++00590///Arachidonic acid metabolism	GO:0016021///integral component of membrane+++GO:0031090///organelle membrane+++GO:0005789///endoplasmic reticulum membrane	GO:0020037///heme binding+++GO:0004497///monooxygenase activity+++GO:0050051///leukotriene-B4 20-monooxygenase activity+++GO:0005506///iron ion binding+++GO:0097258///20-hydroxy-leukotriene B4 omega oxidase activity+++GO:0097259///20-aldehyde-leukotriene B4 20-monooxygenase activity	GO:0006691///leukotriene metabolic process+++GO:0006690///icosanoid metabolic process
NM_213910.1	SERPINE1	2.28842103	0.003987346	04218///Cellular senescence+++04371///Apelin signaling pathway+++04390///Hippo signaling pathway+++04115///p53 signaling pathway+++04610///Complement and coagulation cascades+++04066///HIF-1 signaling pathway	GO:0062023///collagen-containing extracellular matrix+++GO:0005615///extracellular space+++GO:0070062///extracellular exosome+++GO:0005576///extracellular region	GO:0005102///signaling receptor binding+++GO:0004867///serine-type endopeptidase inhibitor activity+++GO:0030414///peptidase inhibitor activity+++GO:0002020///protease binding	GO:1901331///positive regulation of odontoblast differentiation+++GO:0001300///chronological cell aging+++GO:0051918///negative regulation of fibrinolysis+++GO:0030194///positive regulation of blood coagulation+++GO:0048260///positive regulation of receptor-mediated endocytosis+++GO:1902042///negative regulation of extrinsic apoptotic signaling pathway via death domain receptors+++GO:0010466///negative regulation of peptidase activity+++GO:2000098///negative regulation of smooth muscle cell-matrix adhesion+++GO:0071222///cellular response to lipopolysaccharide+++GO:0030336///negative regulation of cell migration+++GO:0014912///negative regulation of smooth muscle cell migration+++GO:2000352///negative regulation of endothelial cell apoptotic process+++GO:0001525///angiogenesis+++GO:0090026///positive regulation of monocyte chemotaxis+++GO:0050729///positive regulation of inflammatory response+++GO:0045766///positive regulation of angiogenesis+++GO:0010757///negative regulation of plasminogen activation+++GO:0050829///defense response to Gram-negative bacterium+++GO:0010469///regulation of signaling receptor activity+++GO:0010951///negative regulation of endopeptidase activity+++GO:0090399///replicative senescence+++GO:0032757///positive regulation of interleukin-8 production+++GO:0097187///dentinogenesis+++GO:0061044///negative regulation of vascular wound healing+++GO:0035491///positive
