# Supplementary material for: Comprehensive analysis of miRNAs, lncRNAs and mRNAs profiles in backfat tissue between Daweizi and Yorkshire pigs
Source: Anim Biosci. 2022 Nov 13;36(3):404–16. doi: 10.5713/ab.22.0165 (PMC9996253; doi:10.5713/ab.22.0165)
Supplement: Supplementary file 11 [file ab-22-0165-Supplementary-Table-10.pdf]

Supplementary Table S10. The predicted targe

RNA ID	Symbol	Type	updown_XR_002340371.1	updown_XR_002340445.1	updown_XR_002336855.1	updown_XR_002346030.1	updown_XR_002346056.1	updown_XR_002346229.1	updown_XR_002346569.1
BGIR9823_100361	PRKAG2	mRNA	0	0	0	0	0	0	0
BGIR9823_100362	PRKAG2	mRNA	0	0	0	0	0	0	0
BGIR9823_100363	PRKAG2	mRNA	0	0	0	0	0	0	0
BGIR9823_100364	PRKAG2	mRNA	0	0	0	0	0	0	0
BGIR9823_101535	LOC102158401	lncRNA	0	0	0	0	0	0	0
BGIR9823_101541	LOC102157946	lncRNA	0	0	0	0	0	0	0
BGIR9823_101542	LOC102157946	lncRNA	0	0	0	0	0	0	0
BGIR9823_101560	LOC110258600	lncRNA	0	0	0	0	0	0	0
BGIR9823_101561	LOC110258600	lncRNA	0	0	0	0	0	0	0
BGIR9823_101562	LOC110258600	lncRNA	0	0	0	0	0	0	0
BGIR9823_101563	LOC110258600	lncRNA	0	0	0	0	0	0	0
BGIR9823_101564	LOC110258600	lncRNA	0	0	0	0	0	0	0
BGIR9823_101612	LOC100521600	lncRNA	0	0	0	0	0	0	0
BGIR9823_101853		lncRNA	0	0	0	0	0	0	0
BGIR9823_101854		lncRNA	0	0	0	0	0	0	0
BGIR9823_101855		lncRNA	0	0	0	0	0	0	0
BGIR9823_102146		lncRNA	0	0	0	0	0	1	0
BGIR9823_102180		lncRNA	0	0	0	0	0	0	0
BGIR9823_102279		lncRNA	0	0	0	0	0	0	0
BGIR9823_102282		lncRNA	0	0	0	0	0	0	0
BGIR9823_102361		lncRNA	0	0	0	0	0	0	0
BGIR9823_102362		lncRNA	0	0	0	0	0	0	0
BGIR9823_102363		lncRNA	0	0	0	0	0	0	0
BGIR9823_102727		lncRNA	0	0	0	0	0	0	0
BGIR9823_80121	SSSCA1	lncRNA	0	0	0	0	0	0	0
BGIR9823_80122	LOC102163816	lncRNA	0	0	0	0	0	0	0
BGIR9823_81007	EHBP1L1	mRNA	0	0	0	0	0	0	0
BGIR9823_81008	EHBP1L1	mRNA	0	0	0	0	0	0	0
BGIR9823_81009	EHBP1L1	mRNA	0	0	0	0	0	0	0
BGIR9823_81010	EHBP1L1	mRNA	0	0	0	0	0	0	0
BGIR9823_81011	SSSCA1	mRNA	0	0	0	0	0	0	0
BGIR9823_81012	FRMD8	lncRNA	0	0	0	0	0	0	0
BGIR9823_81013	LOC102163816	lncRNA	0	0	0	0	0	0	0
BGIR9823_81014	LOC102163816	lncRNA	0	0	0	0	0	0	0
BGIR9823_81015	LOC102163816	lncRNA	0	0	0	0	0	0	0
BGIR9823_81016	LOC102163816	lncRNA	0	0	0	0	0	0	0
BGIR9823_81017	LOC102163816	lncRNA	0	0	0	0	0	0	0
BGIR9823_81018	LOC102163816	lncRNA	0	0	0	0	0	0	0
BGIR9823_81019	LOC100513133	lncRNA	0	0	0	0	0	0	0
BGIR9823_84704	LOC102159820	mRNA	0	0	0	0	0	0	0
BGIR9823_84732	RANGAP1	mRNA	0	0	0	0	0	0	0

RNA ID	Symbol	Type	updown_XR_002340371.1	updown_XR_002340445.1	updown_XR_002336855.1	updown_XR_002346030.1	updown_XR_002346056.1	updown_XR_002346229.1	updown_XR_002346569.1
BGIR9823_84733	RANGAP1	mRNA	0	0	0	0	0	0	0
BGIR9823_85245	CRADD	lncRNA	0	0	0	0	0	0	0
BGIR9823_85246	LOC100738154	lncRNA	0	0	0	0	0	0	0
BGIR9823_85293	TRMU	mRNA	0	0	0	0	0	0	0
BGIR9823_85294	TRMU	mRNA	0	0	0	0	0	0	0
BGIR9823_85295	TTC38	mRNA	0	0	0	0	0	0	0
BGIR9823_85296	PKDREJ	lncRNA	0	0	0	0	0	0	0
BGIR9823_85297	PPARA	mRNA	0	0	0	0	0	0	0
BGIR9823_85298	PPARA	mRNA	0	0	0	0	0	0	0
BGIR9823_85299	PPARA	mRNA	0	0	0	0	0	0	0
BGIR9823_85300	PPARA	lncRNA	0	0	0	0	0	0	0
BGIR9823_85301	LOC110260611	mRNA	0	0	0	0	0	0	0
BGIR9823_85317	ACO2	mRNA	0	0	0	0	0	0	0
BGIR9823_85318	LOC110260629	lncRNA	0	0	0	0	0	0	0
BGIR9823_85319	TEF	mRNA	0	0	0	0	0	0	0
BGIR9823_85320	ZC3H7B	mRNA	0	0	0	0	0	0	0
BGIR9823_85345	SYNGR1	mRNA	0	0	0	0	0	0	0
BGIR9823_85835	CRADD	mRNA	0	0	0	0	0	0	0
BGIR9823_85839	CRADD	lncRNA	0	0	0	0	0	0	0
BGIR9823_85840	CRADD	lncRNA	0	0	0	0	0	0	0
BGIR9823_85841	CRADD	lncRNA	0	0	0	0	0	0	0
BGIR9823_85930	LOC106510465	mRNA	0	0	0	0	0	0	0
BGIR9823_85931	MAF	lncRNA	0	0	0	0	0	0	0
BGIR9823_85932	MAF	lncRNA	0	0	0	0	0	0	0
BGIR9823_87139	LOC106510467	lncRNA	0	0	0	0	0	0	0
BGIR9823_88187	WRNIP1	mRNA	0	0	0	0	0	1	0
BGIR9823_88188	NQO2	mRNA	0	0	0	0	0	1	0
BGIR9823_88189	NQO2	mRNA	0	0	0	0	0	1	0
BGIR9823_88190	RIPK1	mRNA	0	0	0	0	0	1	0
BGIR9823_88191	RIPK1	mRNA	0	0	0	0	0	1	0
BGIR9823_88192	RIPK1	mRNA	0	0	0	0	0	1	0
BGIR9823_88193	RIPK1	mRNA	0	0	0	0	0	1	0
BGIR9823_88196	PRPF4B	mRNA	0	0	0	0	0	0	0
BGIR9823_88197	PRPF4B	mRNA	0	0	0	0	0	0	0
BGIR9823_88198	PRPF4B	mRNA	0	0	0	0	0	0	0
BGIR9823_88211	TMEM170B	lncRNA	0	0	0	0	0	0	0
BGIR9823_88212	TMEM170B	lncRNA	0	0	0	0	0	0	0
BGIR9823_88213	TMEM170B	lncRNA	0	0	0	0	0	0	0
BGIR9823_88214	TMEM170B	lncRNA	0	0	0	0	0	0	0
BGIR9823_88217	PHACTR1	mRNA	0	0	0	0	0	0	0
BGIR9823_88218	PHACTR1	lncRNA	0	0	0	0	0	0	0
BGIR9823_89017	MYLK4	lncRNA	0	0	0	0	0	1	0
BGIR9823_89019	SERPINB1	lncRNA	0	0	0	0	0	1	0

RNA ID	Symbol	Type	updown_XR_002340371.1	updown_XR_002340445.1	updown_XR_002336855.1	updown_XR_002346030.1	updown_XR_002346056.1	updown_XR_002346229.1	updown_XR_002346569.1
BGIR9823_89020	SERPINB9	mRNA	0	0	0	0	0	1	0
BGIR9823_89021	RIPK1	lncRNA	0	0	0	0	0	1	0
BGIR9823_89025	PXDC1	mRNA	0	0	0	0	0	0	0
BGIR9823_89026	PXDC1	lncRNA	0	0	0	0	0	0	0
BGIR9823_89027	LOC102161744	lncRNA	0	0	0	0	0	0	0
BGIR9823_89040	TBC1D7	lncRNA	0	0	0	0	0	0	0
BGIR9823_89041	TBC1D7	lncRNA	0	0	0	0	0	0	0
BGIR9823_89042	TBC1D7	lncRNA	0	0	0	0	0	0	0
BGIR9823_89043	TBC1D7	lncRNA	0	0	0	0	0	0	0
BGIR9823_89044	GFOD1	lncRNA	0	0	0	0	0	0	0
BGIR9823_89045	GFOD1	mRNA	0	0	0	0	0	0	0
BGIR9823_89046	GFOD1	mRNA	0	0	0	0	0	0	0
BGIR9823_89047	GFOD1	mRNA	0	0	0	0	0	0	0
BGIR9823_89048	GFOD1	lncRNA	0	0	0	0	0	0	0
BGIR9823_89049	GFOD1	lncRNA	0	0	0	0	0	0	0
BGIR9823_90771	NEU3	lncRNA	0	0	0	0	0	0	0
BGIR9823_90772	SLCO2B1	mRNA	0	0	0	0	0	0	0
BGIR9823_90913	LOC110255435	lncRNA	0	0	0	0	0	0	0
BGIR9823_90934	LOC110255441	lncRNA	0	0	0	0	0	0	0
BGIR9823_90935	CCDC84	mRNA	0	0	0	0	0	0	0
BGIR9823_90936	TRAPPC4	mRNA	0	0	0	0	0	0	0
BGIR9823_90937	HMBS	mRNA	0	0	0	0	0	0	0
BGIR9823_90956	SORL1	mRNA	0	0	0	0	0	0	0
BGIR9823_90957	SORL1	mRNA	0	0	0	0	0	0	0
BGIR9823_90958	SORL1	mRNA	0	0	0	0	0	0	0
BGIR9823_90960	CLMP	lncRNA	0	0	0	0	0	0	0
BGIR9823_91161	LOC100522267	mRNA	0	0	0	0	0	0	0
BGIR9823_91162	LOC100522455	mRNA	0	0	0	0	0	0	0
BGIR9823_91163	ABCB1	mRNA	0	0	0	0	0	0	0
BGIR9823_91402	HBB	mRNA	0	0	0	0	0	0	0
BGIR9823_91403	HBB	mRNA	0	0	0	0	0	0	0
BGIR9823_91404	HBB	mRNA	0	0	0	0	0	0	0
BGIR9823_91405	HBB	mRNA	0	0	0	0	0	0	0
BGIR9823_91406	HBB	mRNA	0	0	0	0	0	0	0
BGIR9823_91407	HBB	mRNA	0	0	0	0	0	0	0
BGIR9823_91408	HBB	mRNA	0	0	0	0	0	0	0
BGIR9823_91409	HBB	mRNA	0	0	0	0	0	0	0
BGIR9823_91625	BCL9L	lncRNA	0	0	0	0	0	0	0
BGIR9823_91626	BCL9L	lncRNA	0	0	0	0	0	0	0
BGIR9823_91627	RPS25	mRNA	0	0	0	0	0	0	0
BGIR9823_91628	RPS25	mRNA	0	0	0	0	0	0	0
BGIR9823_91629	SLC37A4	mRNA	0	0	0	0	0	0	0
BGIR9823_91630	HYOU1	mRNA	0	0	0	0	0	0	0

RNA ID	Symbol	Type	updown_XR_002340371.1	updown_XR_002340445.1	updown_XR_002336855.1	updown_XR_002346030.1	updown_XR_002346056.1	updown_XR_002346229.1	updown_XR_002346569.1
BGIR9823_91640	CLMP	mRNA	0	0	0	0	0	0	0
BGIR9823_91819	RUNDC3B	lncRNA	0	0	0	0	0	0	0
BGIR9823_91820	RUNDC3B	lncRNA	0	0	0	0	0	0	0
BGIR9823_91821	RUNDC3B	lncRNA	0	0	0	0	0	0	0
BGIR9823_91822	RUNDC3B	lncRNA	0	0	0	0	0	0	0
BGIR9823_91823	RUNDC3B	lncRNA	0	0	0	0	0	0	0
BGIR9823_91824	RUNDC3B	lncRNA	0	0	0	0	0	0	0
BGIR9823_91825	RUNDC3B	lncRNA	0	0	0	0	0	0	0
BGIR9823_91826	LOC102164596	lncRNA	0	0	0	0	0	0	0
BGIR9823_92993	KATNAL1	mRNA	0	0	0	0	0	0	0
BGIR9823_92994	KATNAL1	mRNA	0	0	0	0	0	0	0
BGIR9823_92995	KATNAL1	mRNA	0	0	0	0	0	0	0
BGIR9823_92996	KATNAL1	mRNA	0	0	0	0	0	0	0
BGIR9823_92997	KATNAL1	mRNA	0	0	0	0	0	0	0
BGIR9823_92999	LOC110255292	lncRNA	0	0	0	0	0	0	0
BGIR9823_93000	LOC102166871	lncRNA	0	0	0	0	0	0	0
BGIR9823_98051	LOC102164998	lncRNA	0	0	0	0	0	0	0
BGIR9823_99244	LOC102167708	lncRNA	0	0	0	0	0	0	0
NM_001004034.1	HMGB1	mRNA	0	0	0	0	0	0	0
NM_001044526.1	PPARA	mRNA	0	0	0	0	0	0	0
NM_001111256.1	SERPINB9	mRNA	0	0	0	0	0	1	0
NM_001113443.1	ECI2	mRNA	0	0	0	0	0	0	0
NM_001144841.1	HBB	mRNA	0	0	0	0	0	0	0
NM_001199719.1	SLC37A4	mRNA	0	0	0	0	0	0	0
NM_001243907.1	HSPA8	mRNA	0	0	0	0	0	0	0
NM_001244063.1	RPL3	mRNA	0	0	0	0	0	0	0
NM_001244066.1	SYNGR1	mRNA	0	0	0	0	0	0	0
NM_001244396.1	CCDC84	mRNA	0	0	0	0	0	0	0
NM_001287846.1	RHEB	mRNA	0	0	0	0	0	0	0
NM_213954.1	ACO2	mRNA	0	0	0	0	0	0	0
NM_214012.1	UPK2	mRNA	0	0	0	0	0	0	0
URS0000BC92F4	LOC102167708	lncRNA	0	0	0	0	0	0	0
XM_001928309.6	TBC1D7	mRNA	0	0	0	0	0	0	0
XM_001928852.5	PXDC1	mRNA	0	0	0	0	0	0	0
XM_003122534.4	KCNK7	mRNA	0	0	0	0	0	0	0
XM_003122558.5	SCYL1	mRNA	0	0	0	0	0	0	0
XM_003122562.5	FRMD8	mRNA	0	0	0	0	0	0	0
XM_003125951.6	TTC38	mRNA	0	0	0	0	0	0	0
XM_003125953.4	CDPF1	mRNA	0	0	0	0	0	0	0
XM_003126012.6	CBX7	mRNA	0	0	0	0	0	0	0
XM_003128161.4	RIPK1	mRNA	0	0	0	0	0	1	0
XM_003129508.2	LOC100514417	mRNA	0	0	0	0	0	0	0
XM_003129512.2	LOC100515268	mRNA	0	0	0	0	0	0	0

RNA ID	Symbol	Type	updown_XR_002340371.1	updown_XR_002340445.1	updown_XR_002336855.1	updown_XR_002346030.1	updown_XR_002346056.1	updown_XR_002346229.1	updown_XR_002346569.1
XM_003129514.3	LOC100515618	mRNA	0	0	0	0	0	0	0
XM_003129519.2	LOC100517595	mRNA	0	0	0	0	0	0	0
XM_003129529.4	LOC100519910	mRNA	0	0	0	0	0	0	0
XM_003129915.4	CXCR5	mRNA	0	0	0	0	0	0	0
XM_003129916.5	BCL9L	mRNA	0	0	0	0	0	0	0
XM_003129918.4	TRAPPC4	mRNA	0	0	0	0	0	0	0
XM_003129919.5	RPS25	mRNA	0	0	0	0	0	0	0
XM_003129920.3	RPS25	mRNA	0	0	0	0	0	0	0
XM_003129922.4	HYOU1	mRNA	0	0	0	0	0	0	0
XM_003129923.6	VPS11	mRNA	0	0	0	0	0	0	0
XM_003129944.4	SC5D	mRNA	0	0	0	0	0	0	0
XM_003357161.1	LOC100620971	mRNA	0	0	0	0	0	0	0
XM_003482119.4	SERPINB1	mRNA	0	0	0	0	0	1	0
XM_003482132.4	TMEM170B	mRNA	0	0	0	0	0	0	0
XM_003482133.4	GFOD1	mRNA	0	0	0	0	0	0	0
XM_003482563.4	SPCS2	mRNA	0	0	0	0	0	0	0
XM_003482565.4	NEU3	mRNA	0	0	0	0	0	0	0
XM_003482567.1	LOC100739359	mRNA	0	0	0	0	0	0	0
XM_003482630.3	BSX	mRNA	0	0	0	0	0	0	0
XM_005653107.3	CHADL	mRNA	0	0	0	0	0	0	0
XM_005653640.1	LOC100621181	mRNA	0	0	0	0	0	0	0
XM_005654266.3	PRKAG2	mRNA	0	0	0	0	0	0	0
XM_005655470.3	CDPF1	mRNA	0	0	0	0	0	0	0
XM_005660703.3	LTBP3	mRNA	0	0	0	0	0	0	0
XM_005660705.3	SCYL1	mRNA	0	0	0	0	0	0	0
XM_005660707.3	SLC25A45	mRNA	0	0	0	0	0	0	0
XM_005665536.3	RIPK1	mRNA	0	0	0	0	0	1	0
XM_005665537.3	RIPK1	mRNA	0	0	0	0	0	1	0
XM_005665538.3	RIPK1	mRNA	0	0	0	0	0	1	0
XM_005665575.3	PHACTR1	mRNA	0	0	0	0	0	0	0
XM_005665576.3	TBC1D7	mRNA	0	0	0	0	0	0	0
XM_005667128.3	LOC100627340	mRNA	0	0	0	0	0	0	0
XM_005667397.2	CCDC84	mRNA	0	0	0	0	0	0	0
XM_005667398.3	SLC37A4	mRNA	0	0	0	0	0	0	0
XM_005667402.3	SLC37A4	mRNA	0	0	0	0	0	0	0
XM_005667403.3	SLC37A4	mRNA	0	0	0	0	0	0	0
XM_005667413.3	BCL9L	mRNA	0	0	0	0	0	0	0
XM_005667414.3	FOXR1	mRNA	0	0	0	0	0	0	0
XM_005667416.3	HYOU1	mRNA	0	0	0	0	0	0	0
XM_005667419.3	HYOU1	mRNA	0	0	0	0	0	0	0
XM_005667420.3	HYOU1	mRNA	0	0	0	0	0	0	0
XM_005667440.2	JHY	mRNA	0	0	0	0	0	0	0
XM_005668316.3	HMGB1	mRNA	0	0	0	0	0	0	0

RNA ID	Symbol	Type	updown_XR_002340371.1	updown_XR_002340445.1	updown_XR_002336855.1	updown_XR_002346030.1	updown_XR_002346056.1	updown_XR_002346229.1	updown_XR_002346569.1
XM_005668317.3	HMGB1	mRNA	0	0	0	0	0	0	0
XM_005668318.3	HMGB1	mRNA	0	0	0	0	0	0	0
XM_005671489.3	EMX2	mRNA	0	0	0	0	0	0	0
XM_005672397.3	MYO10	mRNA	0	0	0	0	0	0	0
XM_013977400.2	FAM50B	mRNA	0	0	0	0	0	0	0
XM_013977425.2	NEDD9	mRNA	0	0	0	0	0	0	0
XM_013977430.2	TBC1D7	mRNA	0	0	0	0	0	0	0
XM_013977431.2	TBC1D7	mRNA	0	0	0	0	0	0	0
XM_013977432.2	TBC1D7	mRNA	0	0	0	0	0	0	0
XM_013977433.2	GFOD1	mRNA	0	0	0	0	0	0	0
XM_013977434.2	GFOD1	mRNA	0	0	0	0	0	0	0
XM_013979449.2	SLC37A4	mRNA	0	0	0	0	0	0	0
XM_013979456.2	BCL9L	mRNA	0	0	0	0	0	0	0
XM_013979457.2	BCL9L	mRNA	0	0	0	0	0	0	0
XM_013979458.2	BCL9L	mRNA	0	0	0	0	0	0	0
XM_013979459.2	BCL9L	mRNA	0	0	0	0	0	0	0
XM_013979461.2	TRAPPC4	mRNA	0	0	0	0	0	0	0
XM_013979463.2	HYOU1	mRNA	0	0	0	0	0	0	0
XM_013979484.2	LOC100523684	mRNA	0	0	0	0	0	0	0
XM_013979486.2	LOC100523684	mRNA	0	0	0	0	0	0	0
XM_013979491.2	JHY	mRNA	0	0	0	0	0	0	0
XM_013980418.2	KATNAL1	mRNA	0	0	0	0	0	0	0
XM_013988182.2	TTC38	mRNA	0	0	0	0	0	0	0
XM_013988183.2	TTC38	mRNA	0	0	0	0	0	0	0
XM_013989589.2	LOC100522267	mRNA	0	0	0	0	0	0	0
XM_013989592.2	LOC100522455	mRNA	0	0	0	0	0	0	0
XM_013989593.2	LOC100522455	mRNA	0	0	0	0	0	0	0
XM_013994099.2	FRMD8	mRNA	0	0	0	0	0	0	0
XM_013994100.2	SLC25A45	mRNA	0	0	0	0	0	0	0
XM_013994102.2	SLC25A45	mRNA	0	0	0	0	0	0	0
XM_013994103.2	SLC25A45	mRNA	0	0	0	0	0	0	0
XM_013994104.2	SLC25A45	mRNA	0	0	0	0	0	0	0
XM_013997510.2	CBX7	mRNA	0	0	0	0	0	0	0
XM_013999070.2	LOC100520088	mRNA	0	0	0	0	0	0	0
XM_021062230.1	LOC100517771	mRNA	0	0	0	0	0	0	0
XM_021062231.1	LOC100517957	mRNA	0	0	0	0	0	0	0
XM_021062367.1	LOC110255365	mRNA	0	0	0	0	0	0	0
XM_021062369.1	LOC100737110	mRNA	0	0	0	0	0	0	0
XM_021062370.1	LOC110255368	mRNA	0	0	0	0	0	0	0
XM_021062371.1	LOC100515441	mRNA	0	0	0	0	0	0	0
XM_021062374.1	LOC110255371	mRNA	0	0	0	0	0	0	0
XM_021062375.1	LOC100626951	mRNA	0	0	0	0	0	0	0
XM_021062491.1	XRRA1	mRNA	0	0	0	0	0	0	0

RNA ID	Symbol	Type	updown_XR_002340371.1	updown_XR_002340445.1	updown_XR_002336855.1	updown_XR_002346030.1	updown_XR_002346056.1	updown_XR_002346229.1	updown_XR_002346569.1
XM_021062493.1	XRRA1	mRNA	0	0	0	0	0	0	0
XM_021062494.1	XRRA1	mRNA	0	0	0	0	0	0	0
XM_021062495.1	XRRA1	mRNA	0	0	0	0	0	0	0
XM_021062496.1	XRRA1	mRNA	0	0	0	0	0	0	0
XM_021062497.1	XRRA1	mRNA	0	0	0	0	0	0	0
XM_021062498.1	XRRA1	mRNA	0	0	0	0	0	0	0
XM_021062499.1	XRRA1	mRNA	0	0	0	0	0	0	0
XM_021062500.1	XRRA1	mRNA	0	0	0	0	0	0	0
XM_021062501.1	XRRA1	mRNA	0	0	0	0	0	0	0
XM_021062502.1	XRRA1	mRNA	0	0	0	0	0	0	0
XM_021062503.1	XRRA1	mRNA	0	0	0	0	0	0	0
XM_021062511.1	SLCO2B1	mRNA	0	0	0	0	0	0	0
XM_021062948.1	BCL9L	mRNA	0	0	0	0	0	0	0
XM_021062949.1	TRAPPC4	mRNA	0	0	0	0	0	0	0
XM_021062950.1	HYOU1	mRNA	0	0	0	0	0	0	0
XM_021062951.1	HYOU1	mRNA	0	0	0	0	0	0	0
XM_021062952.1	HYOU1	mRNA	0	0	0	0	0	0	0
XM_021062953.1	HYOU1	mRNA	0	0	0	0	0	0	0
XM_021062954.1	HYOU1	mRNA	0	0	0	0	0	0	0
XM_021062955.1	HYOU1	mRNA	0	0	0	0	0	0	0
XM_021062956.1	HYOU1	mRNA	0	0	0	0	0	0	0
XM_021062957.1	VPS11	mRNA	0	0	0	0	0	0	0
XM_021062993.1	LOC100523684	mRNA	0	0	0	0	0	0	0
XM_021062994.1	LOC100523684	mRNA	0	0	0	0	0	0	0
XM_021062995.1	LOC100523684	mRNA	0	0	0	0	0	0	0
XM_021062996.1	LOC100523684	mRNA	0	0	0	0	0	0	0
XM_021062997.1	LOC100523684	mRNA	0	0	0	0	0	0	0
XM_021062998.1	LOC100523684	mRNA	0	0	0	0	0	0	0
XM_021062999.1	LOC100523684	mRNA	0	0	0	0	0	0	0
XM_021063000.1	LOC100523684	mRNA	0	0	0	0	0	0	0
XM_021063001.1	LOC100523684	mRNA	0	0	0	0	0	0	0
XM_021063002.1	LOC100523684	mRNA	0	0	0	0	0	0	0
XM_021063004.1	LOC100523684	mRNA	0	0	0	0	0	0	0
XM_021063005.1	LOC100523684	mRNA	0	0	0	0	0	0	0
XM_021063006.1	LOC100523684	mRNA	0	0	0	0	0	0	0
XM_021063009.1	SORL1	mRNA	0	0	0	0	0	0	0
XM_021063010.1	SORL1	mRNA	0	0	0	0	0	0	0
XM_021063011.1	SORL1	mRNA	0	0	0	0	0	0	0
XM_021063012.1	SORL1	mRNA	0	0	0	0	0	0	0
XM_021063013.1	SORL1	mRNA	0	0	0	0	0	0	0
XM_021063014.1	SORL1	mRNA	0	0	0	0	0	0	0
XM_021063023.1	CLMP	mRNA	0	0	0	0	0	0	0
XM_021063024.1	CLMP	mRNA	0	0	0	0	0	0	0

RNA ID	Symbol	Type	updown_XR_002340371.1	updown_XR_002340445.1	updown_XR_002336855.1	updown_XR_002346030.1	updown_XR_002346056.1	updown_XR_002346229.1	updown_XR_002346569.1
XM_021063025.1	CLMP	mRNA	0	0	0	0	0	0	0
XM_021063026.1	CLMP	mRNA	0	0	0	0	0	0	0
XM_021063950.1	NXPE2	mRNA	0	0	0	0	0	0	0
XM_021063963.1	LOC100623190	mRNA	0	0	0	0	0	0	0
XM_021065159.1	HMGB1	mRNA	0	0	0	0	0	0	0
XM_021065160.1	HMGB1	mRNA	0	0	0	0	0	0	0
XM_021065161.1	HMGB1	mRNA	0	0	0	0	0	0	0
XM_021065529.1	KATNAL1	mRNA	0	0	0	0	0	0	0
XM_021065531.1	KATNAL1	mRNA	0	0	0	0	0	0	0
XM_021065532.1	KATNAL1	mRNA	0	0	0	0	0	0	0
XM_021065533.1	KATNAL1	mRNA	0	0	0	0	0	0	0
XM_021065534.1	KATNAL1	mRNA	0	0	0	0	0	0	0
XM_021065535.1	KATNAL1	mRNA	0	0	0	0	0	0	0
XM_021065536.1	KATNAL1	mRNA	0	0	0	0	0	0	0
XM_021065537.1	KATNAL1	mRNA	0	0	0	0	0	0	0
XM_021065538.1	KATNAL1	mRNA	0	0	0	0	0	0	0
XM_021065539.1	KATNAL1	mRNA	0	0	0	0	0	0	0
XM_021065541.1	KATNAL1	mRNA	0	0	0	0	0	0	0
XM_021065542.1	KATNAL1	mRNA	0	0	0	0	0	0	0
XM_021065543.1	KATNAL1	mRNA	0	0	0	0	0	0	0
XM_021065544.1	KATNAL1	mRNA	0	0	0	0	0	0	0
XM_021073408.1	EMX2	mRNA	0	0	0	0	0	0	0
XM_021078611.1	PRKAG2	mRNA	0	0	0	0	0	0	0
XM_021078612.1	PRKAG2	mRNA	0	0	0	0	0	0	0
XM_021078613.1	PRKAG2	mRNA	0	0	0	0	0	0	0
XM_021078614.1	PRKAG2	mRNA	0	0	0	0	0	0	0
XM_021078615.1	PRKAG2	mRNA	0	0	0	0	0	0	0
XM_021078616.1	PRKAG2	mRNA	0	0	0	0	0	0	0
XM_021078617.1	PRKAG2	mRNA	0	0	0	0	0	0	0
XM_021078618.1	PRKAG2	mRNA	0	0	0	0	0	0	0
XM_021078619.1	PRKAG2	mRNA	0	0	0	0	0	0	0
XM_021078620.1	PRKAG2	mRNA	0	0	0	0	0	0	0
XM_021078621.1	PRKAG2	mRNA	0	0	0	0	0	0	0
XM_021081309.1	LOC106506226	mRNA	0	0	0	0	0	0	0
XM_021081403.1	LOC110258215	mRNA	0	0	0	0	0	0	0
XM_021082153.1	LOC100521600	mRNA	0	0	0	0	0	0	0
XM_021082154.1	LOC100521600	mRNA	0	0	0	0	0	0	0
XM_021082156.1	LOC100521600	mRNA	0	0	0	0	0	0	0
XM_021082157.1	LOC100521600	mRNA	0	0	0	0	0	0	0
XM_021082158.1	LOC100521600	mRNA	0	0	0	0	0	0	0
XM_021082159.1	LOC100521600	mRNA	0	0	0	0	0	0	0
XM_021082835.1	EHBP1L1	mRNA	0	0	0	0	0	0	0
XM_021082836.1	EHBP1L1	mRNA	0	0	0	0	0	0	0

RNA ID	Symbol	Type	updown_XR_002340371.1	updown_XR_002340445.1	updown_XR_002336855.1	updown_XR_002346030.1	updown_XR_002346056.1	updown_XR_002346229.1	updown_XR_002346569.1
XM_021082837.1	EHBP1L1	mRNA	0	0	0	0	0	0	0
XM_021082838.1	EHBP1L1	mRNA	0	0	0	0	0	0	0
XM_021082839.1	EHBP1L1	mRNA	0	0	0	0	0	0	0
XM_021082840.1	EHBP1L1	mRNA	0	0	0	0	0	0	0
XM_021082841.1	EHBP1L1	mRNA	0	0	0	0	0	0	0
XM_021082842.1	EHBP1L1	mRNA	0	0	0	0	0	0	0
XM_021082843.1	EHBP1L1	mRNA	0	0	0	0	0	0	0
XM_021082844.1	EHBP1L1	mRNA	0	0	0	0	0	0	0
XM_021082845.1	EHBP1L1	mRNA	0	0	0	0	0	0	0
XM_021082846.1	EHBP1L1	mRNA	0	0	0	0	0	0	0
XM_021082847.1	EHBP1L1	mRNA	0	0	0	0	0	0	0
XM_021082851.1	MAP3K11	mRNA	0	0	0	0	0	0	0
XM_021082852.1	SSSCA1	mRNA	0	0	0	0	0	0	0
XM_021082853.1	FAM89B	mRNA	0	0	0	0	0	0	0
XM_021082854.1	LTBP3	mRNA	0	0	0	0	0	0	0
XM_021082855.1	LTBP3	mRNA	0	0	0	0	0	0	0
XM_021082856.1	SCYL1	mRNA	0	0	0	0	0	0	0
XM_021082857.1	SLC25A45	mRNA	0	0	0	0	0	0	0
XM_021082858.1	SLC25A45	mRNA	0	0	0	0	0	0	0
XM_021091099.1	SYNGR1	mRNA	0	0	0	0	0	0	0
XM_021091101.1	SYNGR1	mRNA	0	0	0	0	0	0	0
XM_021091102.1	SYNGR1	mRNA	0	0	0	0	0	0	0
XM_021091103.1	SYNGR1	mRNA	0	0	0	0	0	0	0
XM_021091370.1	LOC102159820	mRNA	0	0	0	0	0	0	0
XM_021091373.1	TRMU	mRNA	0	0	0	0	0	0	0
XM_021091374.1	TRMU	mRNA	0	0	0	0	0	0	0
XM_021091375.1	TRMU	mRNA	0	0	0	0	0	0	0
XM_021091376.1	GTSE1	mRNA	0	0	0	0	0	0	0
XM_021091377.1	TTC38	mRNA	0	0	0	0	0	0	0
XM_021091379.1	LOC110260611	mRNA	0	0	0	0	0	0	0
XM_021091381.1	LOC110260611	mRNA	0	0	0	0	0	0	0
XM_021091454.1	TOB2	mRNA	0	0	0	0	0	0	0
XM_021091455.1	TOB2	mRNA	0	0	0	0	0	0	0
XM_021091456.1	PHF5A	mRNA	0	0	0	0	0	0	0
XM_021091457.1	PHF5A	mRNA	0	0	0	0	0	0	0
XM_021091458.1	ZC3H7B	mRNA	0	0	0	0	0	0	0
XM_021091459.1	ZC3H7B	mRNA	0	0	0	0	0	0	0
XM_021091460.1	TEF	mRNA	0	0	0	0	0	0	0
XM_021091461.1	RANGAP1	mRNA	0	0	0	0	0	0	0
XM_021091474.1	CBX7	mRNA	0	0	0	0	0	0	0
XM_021092767.1	CRADD	mRNA	0	0	0	0	0	0	0
XM_021092768.1	CRADD	mRNA	0	0	0	0	0	0	0
XM_021092769.1	CRADD	mRNA	0	0	0	0	0	0	0

RNA ID	Symbol	Type	updown_XR_002340371.1	updown_XR_002340445.1	updown_XR_002336855.1	updown_XR_002346030.1	updown_XR_002346056.1	updown_XR_002346229.1	updown_XR_002346569.1
XM_021092770.1	CRADD	mRNA	0	0	0	0	0	0	0
XM_021092771.1	CRADD	mRNA	0	0	0	0	0	0	0
XM_021092773.1	CRADD	mRNA	0	0	0	0	0	0	0
XM_021093095.1	PKDREJ	mRNA	0	0	0	0	0	0	0
XM_021097064.1	MAF	mRNA	0	0	0	0	0	0	0
XM_021097065.1	MAF	mRNA	0	0	0	0	0	0	0
XM_021097066.1	MAF	mRNA	0	0	0	0	0	0	0
XM_021097870.1	ECI2	mRNA	0	0	0	0	0	0	0
XM_021099751.1	PHACTR1	mRNA	0	0	0	0	0	0	0
XM_021099752.1	PHACTR1	mRNA	0	0	0	0	0	0	0
XM_021099753.1	PHACTR1	mRNA	0	0	0	0	0	0	0
XM_021099754.1	PHACTR1	mRNA	0	0	0	0	0	0	0
XM_021099755.1	PHACTR1	mRNA	0	0	0	0	0	0	0
XM_021099756.1	PHACTR1	mRNA	0	0	0	0	0	0	0
XM_021099757.1	PHACTR1	mRNA	0	0	0	0	0	0	0
XM_021099758.1	PHACTR1	mRNA	0	0	0	0	0	0	0
XM_021099759.1	PHACTR1	mRNA	0	0	0	0	0	0	0
XM_021099760.1	PHACTR1	mRNA	0	0	0	0	0	0	0
XM_021099761.1	PHACTR1	mRNA	0	0	0	0	0	0	0
XM_021099762.1	PHACTR1	mRNA	0	0	0	0	0	0	0
XM_021099763.1	PHACTR1	mRNA	0	0	0	0	0	0	0
XM_021099764.1	PHACTR1	mRNA	0	0	0	0	0	0	0
XM_021099765.1	PHACTR1	mRNA	0	0	0	0	0	0	0
XM_021099766.1	TBC1D7	mRNA	0	0	0	0	0	0	0
XM_021099767.1	TBC1D7	mRNA	0	0	0	0	0	0	0
XM_021099769.1	TBC1D7	mRNA	0	0	0	0	0	0	0
XM_021099770.1	TBC1D7	mRNA	0	0	0	0	0	0	0
XM_021099999.1	LOC110261697	mRNA	0	0	0	0	0	0	0
XM_021100108.1	WRNIP1	mRNA	0	0	0	0	0	1	0
XM_021100109.1	MYLK4	mRNA	0	0	0	0	0	1	0
XM_021100110.1	MYLK4	mRNA	0	0	0	0	0	1	0
XM_021100111.1	MYLK4	mRNA	0	0	0	0	0	1	0
XM_021100112.1	MYLK4	mRNA	0	0	0	0	0	1	0
XM_021100114.1	NQO2	mRNA	0	0	0	0	0	1	0
XM_021100115.1	NQO2	mRNA	0	0	0	0	0	1	0
XM_021100117.1	NQO2	mRNA	0	0	0	0	0	1	0
XM_021100118.1	NQO2	mRNA	0	0	0	0	0	1	0
XM_021100119.1	NQO2	mRNA	0	0	0	0	0	1	0
XM_021100124.1	PRPF4B	mRNA	0	0	0	0	0	0	0
XM_021100126.1	PRPF4B	mRNA	0	0	0	0	0	0	0
XM_021100184.1	NEDD9	mRNA	0	0	0	0	0	0	0
XM_021100188.1	TMEM170B	mRNA	0	0	0	0	0	0	0
XM_021100194.1	GFOD1	mRNA	0	0	0	0	0	0	0

RNA ID	Symbol	Type	updown_XR_002340371.1	updown_XR_002340445.1	updown_XR_002336855.1	updown_XR_002346030.1	updown_XR_002346056.1	updown_XR_002346229.1	updown_XR_002346569.1
XM_021102040.1	SLC37A4	mRNA	0	0	0	0	0	0	0
XM_021102041.1	SLC37A4	mRNA	0	0	0	0	0	0	0
XM_021102042.1	SLC37A4	mRNA	0	0	0	0	0	0	0
XM_021102043.1	SLC37A4	mRNA	0	0	0	0	0	0	0
XM_021102044.1	SLC37A4	mRNA	0	0	0	0	0	0	0
XM_021102045.1	SLC37A4	mRNA	0	0	0	0	0	0	0
XM_021102046.1	SLC37A4	mRNA	0	0	0	0	0	0	0
XM_021102047.1	SLC37A4	mRNA	0	0	0	0	0	0	0
XM_021102048.1	SLC37A4	mRNA	0	0	0	0	0	0	0
XM_021102049.1	SLC37A4	mRNA	0	0	0	0	0	0	0
XM_021102050.1	SLC37A4	mRNA	0	0	0	0	0	0	0
XM_021102051.1	SLC37A4	mRNA	0	0	0	0	0	0	0
XM_021102052.1	SLC37A4	mRNA	0	0	0	0	0	0	0
XM_021102209.1	UPK2	mRNA	0	0	0	0	0	0	0
XM_021102516.1	LOC100522455	mRNA	0	0	0	0	0	0	0
XM_021102517.1	RUNDC3B	mRNA	0	0	0	0	0	0	0
XM_021102518.1	RUNDC3B	mRNA	0	0	0	0	0	0	0
XM_021102519.1	RUNDC3B	mRNA	0	0	0	0	0	0	0
XM_021102522.1	RUNDC3B	mRNA	0	0	0	0	0	0	0
XM_021102523.1	RUNDC3B	mRNA	0	0	0	0	0	0	0
XR_001297664.2	LOC102161744	lncRNA	0	0	0	0	0	0	0
XR_001298807.2	LOC106504881	lncRNA	0	0	0	0	0	0	0
XR_001298808.2	LOC106504881	lncRNA	0	0	0	0	0	0	0
XR_001298810.2	LOC106504881	lncRNA	0	0	0	0	0	0	0
XR_001298811.2	LOC106504881	lncRNA	0	0	0	0	0	0	0
XR_001298812.2	LOC106504881	lncRNA	0	0	0	0	0	0	0
XR_001298813.2	LOC106504881	lncRNA	0	0	0	0	0	0	0
XR_001298814.2	LOC106504881	lncRNA	0	0	0	0	0	0	0
XR_001298816.2	LOC106504881	lncRNA	0	0	0	0	0	0	0
XR_001298817.2	LOC106504881	lncRNA	0	0	0	0	0	0	0
XR_001298844.2	LOC102163364	lncRNA	0	0	0	0	0	0	0
XR_001298846.2	LOC102163364	lncRNA	0	0	0	0	0	0	0
XR_001298850.2	LOC102163364	lncRNA	0	0	0	0	0	0	0
XR_001299456.2	LOC102166670	lncRNA	0	0	0	0	0	0	0
XR_001299457.2	LOC102166670	lncRNA	0	0	0	0	0	0	0
XR_001300715.2	LOC106505879	lncRNA	0	0	0	0	0	0	0
XR_001301078.2	LOC102162623	lncRNA	0	0	0	0	0	0	0
XR_001301087.2	LOC102162623	lncRNA	0	0	0	0	0	0	0
XR_001301217.2	LOC102164998	lncRNA	0	0	0	0	0	0	0
XR_001301305.2	LOC102159607	lncRNA	0	0	0	0	0	0	0
XR_001308967.2	LOC106510467	lncRNA	0	0	0	0	0	0	0
XR_002335533.1	XRRA1	mRNA	0	0	0	0	0	0	0
XR_002335534.1	XRRA1	mRNA	0	0	0	0	0	0	0

RNA ID	Symbol	Type	updown_XR_002340371.1	updown_XR_002340445.1	updown_XR_002336855.1	updown_XR_002346030.1	updown_XR_002346056.1	updown_XR_002346229.1	updown_XR_002346569.1
XR_002335535.1	LOC110255399	lncRNA	0	0	0	0	0	0	0
XR_002335536.1	LOC102160367	lncRNA	0	0	0	0	0	0	0
XR_002335537.1	LOC102160367	lncRNA	0	0	0	0	0	0	0
XR_002335650.1	LOC102157630	lncRNA	0	0	0	0	0	0	0
XR_002335651.1	LOC110255435	lncRNA	0	0	0	0	0	0	0
XR_002335652.1	LOC106504881	lncRNA	0	0	0	0	0	0	0
XR_002335653.1	LOC106504881	lncRNA	0	0	0	0	0	0	0
XR_002335670.1	LOC110255440	lncRNA	0	0	0	0	0	0	0
XR_002335671.1	LOC110255441	lncRNA	0	0	0	0	0	0	0
XR_002335672.1	LOC110255441	lncRNA	0	0	0	0	0	0	0
XR_002335674.1	HYOU1	mRNA	0	0	0	0	0	0	0
XR_002335675.1	HYOU1	mRNA	0	0	0	0	0	0	0
XR_002335685.1	LOC100523684	mRNA	0	0	0	0	0	0	0
XR_002335686.1	LOC102163364	lncRNA	0	0	0	0	0	0	0
XR_002335687.1	LOC102163364	lncRNA	0	0	0	0	0	0	0
XR_002335688.1	LOC102163364	lncRNA	0	0	0	0	0	0	0
XR_002335689.1	LOC102163364	lncRNA	0	0	0	0	0	0	0
XR_002335690.1	LOC102163364	lncRNA	0	0	0	0	0	0	0
XR_002335691.1	LOC102163364	lncRNA	0	0	0	0	0	0	0
XR_002335692.1	LOC102163364	lncRNA	0	0	0	0	0	0	0
XR_002335693.1	LOC106504896	lncRNA	0	0	0	0	0	0	0
XR_002335694.1	SORL1	mRNA	0	0	0	0	0	0	0
XR_002336641.1	LOC106505250	lncRNA	0	0	0	0	0	0	0
XR_002336643.1	LOC110255292	lncRNA	0	0	0	0	0	0	0
XR_002336644.1	LOC102166670	lncRNA	0	0	0	0	0	0	0
XR_002336645.1	LOC102166670	lncRNA	0	0	0	0	0	0	0
XR_002338443.1	LOC106505879	lncRNA	0	0	0	0	0	0	0
XR_002338444.1	LOC106505879	lncRNA	0	0	0	0	0	0	0
XR_002338445.1	LOC106505879	lncRNA	0	0	0	0	0	0	0
XR_002338446.1	LOC106505879	lncRNA	0	0	0	0	0	0	0
XR_002338447.1	LOC106505879	lncRNA	0	0	0	0	0	0	0
XR_002338448.1	LOC106505879	lncRNA	0	0	0	0	0	0	0
XR_002338449.1	LOC106505879	lncRNA	0	0	0	0	0	0	0
XR_002338450.1	LOC106505879	lncRNA	0	0	0	0	0	0	0
XR_002338521.1	LOC110256664	lncRNA	0	0	0	0	0	0	0
XR_002338522.1	LOC110256664	lncRNA	0	0	0	0	0	0	0
XR_002338666.1	LOC110256747	lncRNA	0	0	0	0	0	0	0
XR_002338714.1	LOC102163817	lncRNA	0	0	0	0	0	0	0
XR_002338715.1	LOC102164998	lncRNA	0	0	0	0	0	0	0
XR_002338716.1	LOC102164998	lncRNA	0	0	0	0	0	0	0
XR_002338717.1	LOC110256768	lncRNA	0	0	0	0	0	0	0
XR_002338727.1	LOC110256773	lncRNA	0	0	0	0	0	0	0
XR_002338728.1	LOC110256773	lncRNA	0	0	0	0	0	0	0

RNA ID	Symbol	Type	updown_XR_002340371.1	updown_XR_002340445.1	updown_XR_002336855.1	updown_XR_002346030.1	updown_XR_002346056.1	updown_XR_002346229.1	updown_XR_002346569.1
XR_002338729.1	LOC110256773	lncRNA	0	0	0	0	0	0	0
XR_002338793.1	LOC110256796	lncRNA	0	0	0	0	0	0	0
XR_002339623.1	LOC102167708	lncRNA	0	0	0	0	0	0	0
XR_002340245.1	PRKAG2	mRNA	0	0	0	0	0	0	0
XR_002340246.1	LOC102162623	lncRNA	0	0	0	0	0	0	0
XR_002340247.1	LOC102162623	lncRNA	0	0	0	0	0	0	0
XR_002340248.1	LOC110257516	lncRNA	0	0	0	0	0	0	0
XR_002340249.1	LOC102162071	lncRNA	0	0	0	0	0	0	0
XR_002340901.1	LOC102160243	lncRNA	0	0	0	0	0	0	0
XR_002340902.1	LOC102160243	lncRNA	0	0	0	0	0	0	0
XR_002340903.1	LOC102160243	lncRNA	0	0	0	0	0	0	0
XR_002340904.1	LOC102160243	lncRNA	0	0	0	0	0	0	0
XR_002340905.1	LOC102160243	lncRNA	0	0	0	0	0	0	0
XR_002341237.1	LOC102164320	lncRNA	0	0	0	0	0	0	0
XR_002341238.1	LOC102164320	lncRNA	0	0	0	0	0	0	0
XR_002341239.1	LOC102164320	lncRNA	0	0	0	0	0	0	0
XR_002341240.1	LOC102164320	lncRNA	0	0	0	0	0	0	0
XR_002341241.1	LOC102164320	lncRNA	0	0	0	0	0	0	0
XR_002341242.1	LOC102164320	lncRNA	0	0	0	0	0	0	0
XR_002341243.1	LOC102164320	lncRNA	0	0	0	0	0	0	0
XR_002341244.1	LOC102164320	lncRNA	0	0	0	0	0	0	0
XR_002341270.1	LOC100521600	mRNA	0	0	0	0	0	0	0
XR_002341443.1	LOC102163816	lncRNA	0	0	0	0	0	0	0
XR_002343847.1	LOC110260611	mRNA	0	0	0	0	0	0	0
XR_002343848.1	LOC110260611	mRNA	0	0	0	0	0	0	0
XR_002343849.1	LOC110260611	mRNA	0	0	0	0	0	0	0
XR_002343850.1	LOC110260611	mRNA	0	0	0	0	0	0	0
XR_002343875.1	LOC110260629	lncRNA	0	0	0	0	0	0	0
XR_002343876.1	LOC110260629	lncRNA	0	0	0	0	0	0	0
XR_002343877.1	LOC110260629	lncRNA	0	0	0	0	0	0	0
XR_002343878.1	LOC110260629	lncRNA	0	0	0	0	0	0	0
XR_002343879.1	LOC110260629	lncRNA	0	0	0	0	0	0	0
XR_002343880.1	LOC110260629	lncRNA	0	0	0	0	0	0	0
XR_002343881.1	LOC110260629	lncRNA	0	0	0	0	0	0	0
XR_002343883.1	LOC110260631	lncRNA	0	0	0	0	0	0	0
XR_002343884.1	LOC110260634	lncRNA	0	0	0	0	0	0	0
XR_002343885.1	LOC110260635	lncRNA	0	0	0	0	0	0	0
XR_002343891.1	LOC110260642	lncRNA	0	0	0	0	0	0	0
XR_002343892.1	LOC110260643	lncRNA	0	0	0	0	0	0	0
XR_002345298.1	MAF	mRNA	0	0	0	0	0	0	0
XR_002345300.1	LOC106510470	lncRNA	0	0	0	0	0	0	0
XR_002345301.1	LOC106510468	lncRNA	0	0	0	0	0	0	0
XR_002346166.1	LOC110261703	lncRNA	0	0	0	0	0	0	0

RNA ID	Symbol	Type	updown_XR_002340371.1	updown_XR_002340445.1	updown_XR_002336855.1	updown_XR_002346030.1	updown_XR_002346056.1	updown_XR_002346229.1	updown_XR_002346569.1
XR_002346227.1	LOC106504299	lncRNA	0	0	0	0	0	1	0
XR_002346228.1	LOC106504299	lncRNA	0	0	0	0	0	1	0
XR_002346229.1	LOC106504299	lncRNA	0	0	0	0	0	1	0
XR_002346230.1	LOC106504299	lncRNA	0	0	0	0	0	1	0
XR_002346231.1	LOC106504299	lncRNA	0	0	0	0	0	1	0
XR_002346232.1	LOC106504299	lncRNA	0	0	0	0	0	1	0
XR_002346233.1	NQO2	mRNA	0	0	0	0	0	1	0
XR_002346236.1	PRPF4B	mRNA	0	0	0	0	0	0	0
XR_002346237.1	PRPF4B	mRNA	0	0	0	0	0	0	0
XR_002346238.1	PRPF4B	mRNA	0	0	0	0	0	0	0
XR_002346239.1	PRPF4B	mRNA	0	0	0	0	0	0	0
XR_002346240.1	PRPF4B	mRNA	0	0	0	0	0	0	0
XR_002346241.1	PRPF4B	mRNA	0	0	0	0	0	0	0
XR_002346242.1	PRPF4B	mRNA	0	0	0	0	0	0	0
XR_002346243.1	PRPF4B	mRNA	0	0	0	0	0	0	0
XR_002346244.1	PRPF4B	mRNA	0	0	0	0	0	0	0
XR_002346245.1	PRPF4B	mRNA	0	0	0	0	0	0	0
XR_002346246.1	PRPF4B	mRNA	0	0	0	0	0	0	0
XR_002346247.1	PRPF4B	mRNA	0	0	0	0	0	0	0
XR_002346248.1	PRPF4B	mRNA	0	0	0	0	0	0	0
XR_002346249.1	PRPF4B	mRNA	0	0	0	0	0	0	0
XR_002346250.1	PRPF4B	mRNA	0	0	0	0	0	0	0
XR_002346251.1	PRPF4B	mRNA	0	0	0	0	0	0	0
XR_002346252.1	PRPF4B	mRNA	0	0	0	0	0	0	0
XR_002346253.1	PRPF4B	mRNA	0	0	0	0	0	0	0
XR_002346254.1	PRPF4B	mRNA	0	0	0	0	0	0	0
XR_002346255.1	PRPF4B	mRNA	0	0	0	0	0	0	0
XR_002346256.1	PRPF4B	mRNA	0	0	0	0	0	0	0
XR_002346257.1	PRPF4B	mRNA	0	0	0	0	0	0	0
XR_002346258.1	PRPF4B	mRNA	0	0	0	0	0	0	0
XR_002346259.1	PRPF4B	mRNA	0	0	0	0	0	0	0
XR_002346260.1	PRPF4B	mRNA	0	0	0	0	0	0	0
XR_002346261.1	PRPF4B	mRNA	0	0	0	0	0	0	0
XR_002346262.1	PRPF4B	mRNA	0	0	0	0	0	0	0
XR_002346263.1	PRPF4B	mRNA	0	0	0	0	0	0	0
XR_002346264.1	PRPF4B	mRNA	0	0	0	0	0	0	0
XR_002346265.1	LOC110261764	lncRNA	0	0	0	0	0	0	0
XR_002346266.1	LOC102162837	lncRNA	0	0	0	0	0	0	0
XR_002346299.1	LOC110261774	lncRNA	0	0	0	0	0	0	0
XR_002346300.1	LOC110261774	lncRNA	0	0	0	0	0	0	0
XR_002346302.1	LOC110261776	lncRNA	0	0	0	0	0	0	0
XR_002346303.1	TMEM170B	mRNA	0	0	0	0	0	0	0
XR_002346304.1	TMEM170B	mRNA	0	0	0	0	0	0	0

RNA ID	Symbol	Type	updown_XR_002340371.1	updown_XR_002340445.1	updown_XR_002336855.1	updown_XR_002346030.1	updown_XR_002346056.1	updown_XR_002346229.1	updown_XR_002346569.1
XR_115737.4	LOC100513133	lncRNA	0	0	0	0	0	0	0
XR_299046.2	LOC102164596	lncRNA	0	0	0	0	0	0	0
XR_303634.3	LOC100738154	lncRNA	0	0	0	0	0	0	0
XR_304239.3	LOC102159819	lncRNA	0	0	0	0	0	1	0
XR_304244.3	LOC102161992	lncRNA	0	0	0	0	0	0	0
XR_304257.3	LOC102162837	lncRNA	0	0	0	0	0	0	0
XR_306105.3	LOC102166871	lncRNA	0	0	0	0	0	0	0
XR_307914.3	LOC102159607	lncRNA	0	0	0	0	0	0	0
XR_308345.3	LOC102167708	lncRNA	0	0	0	0	0	0	0
novel-ssc-miR1028-3p		miRNA	0	0	0	0	0	0	0
novel-ssc-miR1028-5p		miRNA	0	0	0	0	0	0	0
novel-ssc-miR1130-3p		miRNA	0	0	0	0	0	0	0
novel-ssc-miR1130-5p		miRNA	0	0	0	0	0	0	0
novel-ssc-miR1162-3p		miRNA	0	0	0	0	0	0	0
novel-ssc-miR1193-3p		miRNA	0	0	0	0	0	0	0
novel-ssc-miR1193-5p		miRNA	0	0	0	0	0	0	0
novel-ssc-miR1217-3p		miRNA	0	0	0	0	0	0	0
novel-ssc-miR1217-5p		miRNA	0	0	0	0	0	0	0
novel-ssc-miR1429-3p		miRNA	0	0	0	0	0	0	0
novel-ssc-miR1429-5p		miRNA	0	0	0	0	0	0	0
novel-ssc-miR259-3p		miRNA	0	0	0	0	0	0	0
novel-ssc-miR259-5p		miRNA	0	0	0	0	0	0	0
novel-ssc-miR324-3p		miRNA	0	0	0	0	0	0	0
novel-ssc-miR324-5p		miRNA	0	0	0	0	0	0	0
novel-ssc-miR42-3p		miRNA	0	0	0	0	0	0	0
novel-ssc-miR42-5p		miRNA	0	0	0	0	0	0	0
novel-ssc-miR433-3p		miRNA	0	0	0	0	0	0	0
novel-ssc-miR433-5p		miRNA	0	0	0	0	0	0	0
novel-ssc-miR557-3p		miRNA	0	0	0	0	0	0	0
novel-ssc-miR557-5p		miRNA	0	0	0	0	0	0	0
novel-ssc-miR619-3p		miRNA	0	0	0	0	0	0	0
novel-ssc-miR619-5p		miRNA	0	0	0	0	0	0	0
novel-ssc-miR719-3p		miRNA	0	0	0	0	0	0	0
novel-ssc-miR719-5p		miRNA	0	0	0	0	0	0	0
novel-ssc-miR966-3p		miRNA	0	0	0	0	0	0	0
novel-ssc-miR966-5p		miRNA	0	0	0	0	0	0	0
ssc-miR-7136-3p		miRNA	0	0	0	0	0	0	0
ssc-miR-7136-5p		miRNA	0	0	0	0	0	0	0
