# Supplementary material for: Comprehensive analysis of miRNAs, lncRNAs and mRNAs profiles in backfat tissue between Daweizi and Yorkshire pigs
Source: Anim Biosci. 2022 Nov 13;36(3):404–16. doi: 10.5713/ab.22.0165 (PMC9996253; doi:10.5713/ab.22.0165)
Supplement: Supplementary file 12 [file ab-22-0165-Supplementary-Table-11.pdf]

Supplementary Table S11. GO annotation and KEGG pathway analysis of targets of DE miRNAs and DE lncRNAs.

Table S11-1. GO annotation of targets of DE miRNAs.

GO_CFP term level_2	GO_CFP term level_1	Number
cellular process	biological_process	362
biological regulation	biological_process	287
regulation of biological process	biological_process	274
metabolic process	biological_process	260
response to stimulus	biological_process	196
multicellular organismal process	biological_process	154
signaling	biological_process	148
localization	biological_process	142
cellular component organization or biogenesis	biological_process	137
positive regulation of biological process	biological_process	135
developmental process	biological_process	129
negative regulation of biological process	biological_process	111
immune system process	biological_process	51
multi-organism process	biological_process	42
cell proliferation	biological_process	33
locomotion	biological_process	28
reproduction	biological_process	25
reproductive process	biological_process	25
biological adhesion	biological_process	24
behavior	biological_process	19
growth	biological_process	17
rhythmic process	biological_process	5
cell killing	biological_process	4
pigmentation	biological_process	1
biological_process	biological_process	1
detoxification	biological_process	1
cell	cellular_component	392
cell part	cellular_component	390
organelle	cellular_component	293
membrane	cellular_component	234
organelle part	cellular_component	206
membrane part	cellular_component	190
protein-containing complex	cellular_component	124
membrane-enclosed lumen	cellular_component	100
extracellular region	cellular_component	51
extracellular region part	cellular_component	41
synapse	cellular_component	34
cell junction	cellular_component	32
supramolecular complex	cellular_component	21
synapse part	cellular_component	20
cellular_component	cellular_component	1
other organism	cellular_component	1
other organism part	cellular_component	1
binding	molecular_function	289
catalytic activity	molecular_function	142
molecular transducer activity	molecular_function	49
transcription regulator activity	molecular_function	39
transporter activity	molecular_function	36
molecular function regulator	molecular_function	25
structural molecule activity	molecular_function	14
cargo receptor activity	molecular_function	4
antioxidant activity	molecular_function	2
hijacked molecular function	molecular_function	2
molecular_function	molecular_function	1
translation regulator activity	molecular_function	1

Table S11-2. GO annotation of targets of DE lncRNAs.

GO_CFP term level_2	GO_CFP term level_1	Number
cellular process	biological_process	59
biological regulation	biological_process	41
regulation of biological process	biological_process	35
metabolic process	biological_process	33
cellular component organization or biogenesis	biological_process	26
response to stimulus	biological_process	26
positive regulation of biological process	biological_process	22
multicellular organismal process	biological_process	21
developmental process	biological_process	20
signaling	biological_process	19
negative regulation of biological process	biological_process	16
localization	biological_process	16
cell proliferation	biological_process	6
multi-organism process	biological_process	5
reproduction	biological_process	3
reproductive process	biological_process	3
immune system process	biological_process	3
locomotion	biological_process	3
rhythmic process	biological_process	2
biological adhesion	biological_process	2
growth	biological_process	2
behavior	biological_process	1
cell	cellular_component	54
cell part	cellular_component	53
organelle	cellular_component	37
organelle part	cellular_component	29
membrane	cellular_component	25
membrane part	cellular_component	19
protein-containing complex	cellular_component	17
membrane-enclosed lumen	cellular_component	16
extracellular region	cellular_component	6
extracellular region part	cellular_component	5
supramolecular complex	cellular_component	3
synapse	cellular_component	3
cell junction	cellular_component	2
synapse part	cellular_component	1
binding	molecular_function	51
catalytic activity	molecular_function	16
molecular function regulator	molecular_function	9
transporter activity	molecular_function	8
structural molecule activity	molecular_function	8
transcription regulator activity	molecular_function	4
molecular transducer activity	molecular_function	2
translation regulator activity	molecular_function	1

Table S11-3. KEGG enrichment of targets of DE miRNAs.

KEGG pathway term ID	KEGG pathway term description	KEGG pathway term level_1	KEGG pathway term level_2	Term candidate gene number	Total candidate gene number	Term gene number	Total gene number	Rich ratio	Q value
510	N-Glycan biosynthesis	Metabolism	Glycan biosynthesis and metabolism	6	170	223	27723	0.02690583	0.2224869
4740	Olfactory transduction	Organismal Systems	Sensory system	21	170	1738	27723	0.012082854	0.2224869
4142	Lysosome	Cellular Processes	Transport and catabolism	7	170	411	27723	0.01703163	0.7825975
4080	Neuroactive ligand-receptor interaction	Environmental Information Processing	Signaling molecules and interaction	11	170	874	27723	0.012585812	0.8242935
10	Glycolysis / Gluconeogenesis	Metabolism	Carbohydrate metabolism	2	170	189	27723	0.010582011	0.9970287
51	Fructose and mannose metabolism	Metabolism	Carbohydrate metabolism	2	170	134	27723	0.014925373	0.9970287
53	Ascorbate and aldarate metabolism	Metabolism	Carbohydrate metabolism	1	170	55	27723	0.018181818	0.9970287
71	Fatty acid degradation	Metabolism	Lipid metabolism	1	170	153	27723	0.006535948	0.9970287
190	Oxidative phosphorylation	Metabolism	Energy metabolism	1	170	284	27723	0.003521127	0.9970287
230	Purine metabolism	Metabolism	Nucleotide metabolism	2	170	654	27723	0.003058104	0.9970287
240	Pyrimidine metabolism	Metabolism	Nucleotide metabolism	1	170	334	27723	0.002994012	0.9970287
260	Glycine, serine and threonine metabolism	Metabolism	Amino acid metabolism	1	170	134	27723	0.007462687	0.9970287
261	Monobactam biosynthesis	Metabolism	Biosynthesis of other secondary metabolites	1	170	7	27723	0.142857143	0.9970287
270	Cysteine and methionine metabolism	Metabolism	Amino acid metabolism	1	170	155	27723	0.006451613	0.9970287
280	Valine, leucine and isoleucine degradation	Metabolism	Amino acid metabolism	1	170	157	27723	0.006369427	0.9970287
310	Lysine degradation	Metabolism	Amino acid metabolism	3	170	305	27723	0.009836066	0.9970287
330	Arginine and proline metabolism	Metabolism	Amino acid metabolism	1	170	172	27723	0.005813953	0.9970287
340	Histidine metabolism	Metabolism	Amino acid metabolism	1	170	77	27723	0.012987013	0.9970287
350	Tyrosine metabolism	Metabolism	Amino acid metabolism	1	170	94	27723	0.010638298	0.9970287
380	Tryptophan metabolism	Metabolism	Amino acid metabolism	1	170	156	27723	0.006410256	0.9970287
410	beta-Alanine metabolism	Metabolism	Metabolism of other amino acids	1	170	92	27723	0.010869565	0.9970287
450	Selenocompound metabolism	Metabolism	Metabolism of other amino acids	1	170	70	27723	0.014285714	0.9970287
480	Glutathione metabolism	Metabolism	Metabolism of other amino acids	1	170	141	27723	0.007092199	0.9970287
511	Other glycan degradation	Metabolism	Glycan biosynthesis and metabolism	1	170	53	27723	0.018867925	0.9970287
512	Mucin type O-glycan biosynthesis	Metabolism	Glycan biosynthesis and metabolism	1	170	148	27723	0.006756757	0.9970287

514	Other types of O-glycan biosynthesis	Metabolism	Glycan biosynthesis and metabolism	1	170	119	27723	0.008403361	0.9970287
515	Mannose type O-glycan biosynthesis	Metabolism	Glycan biosynthesis and metabolism	1	170	88	27723	0.011363636	0.9970287
520	Amino sugar and nucleotide sugar metabolism	Metabolism	Carbohydrate metabolism	1	170	150	27723	0.006666667	0.9970287
531	Glycosaminoglycan degradation	Metabolism	Glycan biosynthesis and metabolism	1	170	52	27723	0.019230769	0.9970287
533	Glycosaminoglycan biosynthesis - keratan sulfate	Metabolism	Glycan biosynthesis and metabolism	1	170	71	27723	0.014084507	0.9970287
561	Glycerolipid metabolism	Metabolism	Lipid metabolism	2	170	298	27723	0.006711409	0.9970287
562	Inositol phosphate metabolism	Metabolism	Carbohydrate metabolism	1	170	370	27723	0.002702703	0.9970287
563	Glycosylphosphatidyl inositol (GPI)-anchor biosynthesis	Metabolism	Glycan biosynthesis and metabolism	1	170	108	27723	0.009259259	0.9970287
564	Glycerophospholipid metabolism	Metabolism	Lipid metabolism	4	170	467	27723	0.00856531	0.9970287
600	Sphingolipid metabolism	Metabolism	Lipid metabolism	3	170	164	27723	0.018292683	0.9970287
601	Glycosphingolipid biosynthesis - lacto and neolacto series	Metabolism	Glycan biosynthesis and metabolism	1	170	146	27723	0.006849315	0.9970287
620	Pyruvate metabolism	Metabolism	Carbohydrate metabolism	1	170	164	27723	0.006097561	0.9970287
790	Folate biosynthesis	Metabolism	Metabolism of cofactors and vitamins	1	170	92	27723	0.010869565	0.9970287
860	Porphyrin and chlorophyll metabolism	Metabolism	Metabolism of cofactors and vitamins	1	170	115	27723	0.008695652	0.9970287
900	Terpenoid backbone biosynthesis	Metabolism	Metabolism of terpenoids and polyketides	2	170	70	27723	0.028571429	0.9970287
920	Sulfur metabolism	Metabolism	Energy metabolism	1	170	38	27723	0.026315789	0.9970287
970	Aminoacyl-tRNA biosynthesis	Genetic Information Processing	Translation	2	170	150	27723	0.013333333	0.9970287
980	Metabolism of xenobiotics by cytochrome P450	Metabolism	Xenobiotics biodegradation and metabolism	1	170	142	27723	0.007042254	0.9970287
982	Drug metabolism - cytochrome P450	Metabolism	Xenobiotics biodegradation and metabolism	1	170	146	27723	0.006849315	0.9970287
983	Drug metabolism - other enzymes	Metabolism	Xenobiotics biodegradation and metabolism	1	170	225	27723	0.004444444	0.9970287
1040	Biosynthesis of unsaturated fatty acids	Metabolism	Lipid metabolism	1	170	71	27723	0.014084507	0.9970287
1100	Metabolic pathways	Metabolism	Global and overview maps	25	170	4632	27723	0.005397237	0.9970287
1200	Carbon metabolism	Metabolism	Global and overview maps	1	170	348	27723	0.002873563	0.9970287

1212	Fatty acid metabolism	Metabolism	Global and overview maps	1	170	162	27723	0.00617284	0.9970287
1230	Biosynthesis of amino acids	Metabolism	Global and overview maps	1	170	225	27723	0.004444444	0.9970287
2010	ABC transporters	Environmental Information Processing	Membrane transport	2	170	249	27723	0.008032129	0.9970287
3008	Ribosome biogenesis in eukaryotes	Genetic Information Processing	Translation	2	170	201	27723	0.009950249	0.9970287
3010	Ribosome	Genetic Information Processing	Translation	2	170	303	27723	0.00660066	0.9970287
3013	RNA transport	Genetic Information Processing	Translation	4	170	613	27723	0.006525285	0.9970287
3018	RNA degradation	Genetic Information Processing	Folding, sorting and degradation	2	170	306	27723	0.006535948	0.9970287
3030	DNA replication	Genetic Information Processing	Replication and repair	2	170	118	27723	0.016949153	0.9970287
3040	Spliceosome	Genetic Information Processing	Transcription	3	170	512	27723	0.005859375	0.9970287
3060	Protein export	Genetic Information Processing	Folding, sorting and degradation	1	170	52	27723	0.019230769	0.9970287
3320	PPAR signaling pathway	Organismal Systems	Endocrine system	3	170	432	27723	0.006944444	0.9970287
3410	Base excision repair	Genetic Information Processing	Replication and repair	2	170	117	27723	0.017094017	0.9970287
3420	Nucleotide excision repair	Genetic Information Processing	Replication and repair	1	170	156	27723	0.006410256	0.9970287
3440	Homologous recombination	Genetic Information Processing	Replication and repair	2	170	201	27723	0.009950249	0.9970287
3460	Fanconi anemia pathway	Genetic Information Processing	Replication and repair	2	170	213	27723	0.009389671	0.9970287
4010	MAPK signaling pathway	Environmental Information Processing	Signal transduction	3	170	1350	27723	0.002222222	0.9970287
4012	ErbB signaling pathway	Environmental Information Processing	Signal transduction	2	170	503	27723	0.003976143	0.9970287
4013	MAPK signaling pathway - fly	Environmental Information Processing	Signal transduction	1	170	394	27723	0.002538071	0.9970287
4014	Ras signaling pathway	Environmental Information Processing	Signal transduction	2	170	979	27723	0.002042901	0.9970287
4015	Rap1 signaling pathway	Environmental Information Processing	Signal transduction	2	170	1018	27723	0.001964637	0.9970287
4020	Calcium signaling pathway	Environmental Information Processing	Signal transduction	4	170	969	27723	0.004127967	0.9970287
4022	cGMP-PKG signaling pathway	Environmental Information Processing	Signal transduction	4	170	794	27723	0.005037783	0.9970287
4024	cAMP signaling pathway	Environmental Information Processing	Signal transduction	4	170	926	27723	0.004319654	0.9970287
4060	Cytokine-cytokine receptor interaction	Environmental Information Processing	Signaling molecules and interaction	2	170	779	27723	0.002567394	0.9970287
4062	Chemokine signaling pathway	Organismal Systems	Immune system	1	170	776	27723	0.00128866	0.9970287

4064	NF-kappa B signaling pathway	Environmental Information Processing	Signal transduction	3	170	437	27723	0.006864989	0.9970287
4066	HIF-1 signaling pathway	Environmental Information Processing	Signal transduction	1	170	406	27723	0.002463054	0.9970287
4068	FoxO signaling pathway	Environmental Information Processing	Signal transduction	1	170	539	27723	0.001855288	0.9970287
4070	Phosphatidylinositol signaling system	Environmental Information Processing	Signal transduction	1	170	553	27723	0.001808318	0.9970287
4071	Sphingolipid signaling pathway	Environmental Information Processing	Signal transduction	5	170	493	27723	0.010141988	0.9970287
4072	Phospholipase D signaling pathway	Environmental Information Processing	Signal transduction	2	170	813	27723	0.002460025	0.9970287
4110	Cell cycle	Cellular Processes	Cell growth and death	1	170	539	27723	0.001855288	0.9970287
4114	Oocyte meiosis	Cellular Processes	Cell growth and death	3	170	512	27723	0.005859375	0.9970287
4115	p53 signaling pathway	Cellular Processes	Cell growth and death	2	170	273	27723	0.007326007	0.9970287
4120	Ubiquitin mediated proteolysis	Genetic Information Processing	Folding, sorting and degradation	3	170	723	27723	0.004149378	0.9970287
4130	SNARE interactions in vesicular transport	Genetic Information Processing	Folding, sorting and degradation	1	170	113	27723	0.008849558	0.9970287
4137	Mitophagy - animal	Cellular Processes	Transport and catabolism	2	170	330	27723	0.006060606	0.9970287
4140	Autophagy - animal	Cellular Processes	Transport and catabolism	2	170	668	27723	0.002994012	0.9970287
4141	Protein processing in endoplasmic reticulum	Genetic Information Processing	Folding, sorting and degradation	7	170	631	27723	0.011093502	0.9970287
4144	Endocytosis	Cellular Processes	Transport and catabolism	8	170	1203	27723	0.006650042	0.9970287
4145	Phagosome	Cellular Processes	Transport and catabolism	5	170	604	27723	0.008278146	0.9970287
4146	Peroxisome	Cellular Processes	Transport and catabolism	2	170	307	27723	0.006514658	0.9970287
4150	mTOR signaling pathway	Environmental Information Processing	Signal transduction	3	170	680	27723	0.004411765	0.9970287
4151	PI3K-Akt signaling pathway	Environmental Information Processing	Signal transduction	3	170	1421	27723	0.002111189	0.9970287
4152	AMPK signaling pathway	Environmental Information Processing	Signal transduction	2	170	605	27723	0.003305785	0.9970287
4210	Apoptosis	Cellular Processes	Cell growth and death	2	170	534	27723	0.003745318	0.9970287
4211	Longevity regulating pathway	Organismal Systems	Aging	2	170	459	27723	0.004357298	0.9970287
4212	Longevity regulating pathway - worm	Organismal Systems	Aging	1	170	281	27723	0.003558719	0.9970287
4213	Longevity regulating pathway - multiple species	Organismal Systems	Aging	1	170	276	27723	0.003623188	0.9970287
4216	Ferroptosis	Cellular Processes	Cell growth and death	1	170	216	27723	0.00462963	0.9970287
4217	Necroptosis	Cellular Processes	Cell growth and death	4	170	606	27723	0.00660066	0.9970287
4218	Cellular senescence	Cellular Processes	Cell growth and death	3	170	727	27723	0.004126547	0.9970287
4260	Cardiac muscle contraction	Organismal Systems	Circulatory system	1	170	257	27723	0.003891051	0.9970287
4261	Adrenergic signaling in cardiomyocytes	Organismal Systems	Circulatory system	1	170	707	27723	0.001414427	0.9970287
4310	Wnt signaling pathway	Environmental Information Processing	Signal transduction	6	170	727	27723	0.008253095	0.9970287

4320	Dorso-ventral axis formation	Organismal Systems	Development	1	170	128	27723	0.0078125	0.9970287
4360	Axon guidance	Organismal Systems	Development	3	170	1110	27723	0.002702703	0.9970287
4370	VEGF signaling pathway	Environmental Information Processing	Signal transduction	1	170	271	27723	0.003690037	0.9970287
4380	Osteoclast differentiation	Organismal Systems	Development	5	170	589	27723	0.008488964	0.9970287
4390	Hippo signaling pathway	Environmental Information Processing	Signal transduction	1	170	647	27723	0.001545595	0.9970287
4391	Hippo signaling pathway - fly	Environmental Information Processing	Signal transduction	1	170	335	27723	0.002985075	0.9970287
4510	Focal adhesion	Cellular Processes	Cellular community - eukaryotes	1	170	930	27723	0.001075269	0.9970287
4512	ECM-receptor interaction	Environmental Information Processing	Signaling molecules and interaction	2	170	383	27723	0.005221932	0.9970287
4514	Cell adhesion molecules (CAMs)	Environmental Information Processing	Signaling molecules and interaction	5	170	832	27723	0.006009615	0.9970287
4530	Tight junction	Cellular Processes	Cellular community - eukaryotes	3	170	892	27723	0.003363229	0.9970287
4550	Signaling pathways regulating pluripotency of stem cells	Cellular Processes	Cellular community - eukaryotes	1	170	537	27723	0.001862197	0.9970287
4610	Complement and coagulation cascades	Organismal Systems	Immune system	4	170	225	27723	0.017777778	0.9970287
4612	Antigen processing and presentation	Organismal Systems	Immune system	2	170	311	27723	0.006430868	0.9970287
4620	Toll-like receptor signaling pathway	Organismal Systems	Immune system	4	170	439	27723	0.009111617	0.9970287
4621	NOD-like receptor signaling pathway	Organismal Systems	Immune system	5	170	689	27723	0.007256894	0.9970287
4622	RIG-I-like receptor signaling pathway	Organismal Systems	Immune system	2	170	257	27723	0.007782101	0.9970287
4623	Cytosolic DNA-sensing pathway	Organismal Systems	Immune system	3	170	180	27723	0.016666667	0.9970287
4625	C-type lectin receptor signaling pathway	Organismal Systems	Immune system	2	170	497	27723	0.004024145	0.9970287
4630	Jak-STAT signaling pathway	Environmental Information Processing	Signal transduction	3	170	589	27723	0.005093379	0.9970287
4640	Hematopoietic cell lineage	Organismal Systems	Immune system	2	170	397	27723	0.005037783	0.9970287
4650	Natural killer cell mediated cytotoxicity	Organismal Systems	Immune system	3	170	422	27723	0.007109005	0.9970287
4657	IL-17 signaling pathway	Organismal Systems	Immune system	2	170	299	27723	0.006688963	0.9970287
4658	Th1 and Th2 cell differentiation	Organismal Systems	Immune system	5	170	391	27723	0.012787724	0.9970287
4659	Th17 cell differentiation	Organismal Systems	Immune system	3	170	492	27723	0.006097561	0.9970287
4660	T cell receptor signaling pathway	Organismal Systems	Immune system	3	170	468	27723	0.006410256	0.9970287
4662	B cell receptor signaling pathway	Organismal Systems	Immune system	2	170	353	27723	0.005665722	0.9970287
4664	Fc epsilon RI signaling pathway	Organismal Systems	Immune system	2	170	300	27723	0.006666667	0.9970287
4666	Fc gamma R-mediated phagocytosis	Organismal Systems	Immune system	2	170	449	27723	0.004454343	0.9970287
4668	TNF signaling pathway	Environmental Information Processing	Signal transduction	3	170	428	27723	0.007009346	0.9970287

4670	Leukocyte transendothelial migration	Organismal Systems	Immune system	1	170	510	27723	0.001960784	0.9970287
4672	Intestinal immune network for IgA production	Organismal Systems	Immune system	2	170	164	27723	0.012195122	0.9970287
4713	Circadian entrainment	Organismal Systems	Environmental adaptation	1	170	488	27723	0.00204918	0.9970287
4714	Thermogenesis	Organismal Systems	Environmental adaptation	3	170	782	27723	0.003836317	0.9970287
4720	Long-term potentiation	Organismal Systems	Nervous system	2	170	333	27723	0.006006006	0.9970287
4722	Neurotrophin signaling pathway	Organismal Systems	Nervous system	1	170	580	27723	0.001724138	0.9970287
4723	Retrograde endocannabinoid signaling	Organismal Systems	Nervous system	1	170	541	27723	0.001848429	0.9970287
4724	Glutamatergic synapse	Organismal Systems	Nervous system	4	170	541	27723	0.007393715	0.9970287
4726	Serotonergic synapse	Organismal Systems	Nervous system	1	170	403	27723	0.00248139	0.9970287
4728	Dopaminergic synapse	Organismal Systems	Nervous system	4	170	677	27723	0.005908419	0.9970287
4742	Taste transduction	Organismal Systems	Sensory system	1	170	229	27723	0.004366812	0.9970287
4810	Regulation of actin cytoskeleton	Cellular Processes	Cell motility	4	170	906	27723	0.004415011	0.9970287
4911	Insulin secretion	Organismal Systems	Endocrine system	1	170	463	27723	0.002159827	0.9970287
4912	GnRH signaling pathway	Organismal Systems	Endocrine system	2	170	484	27723	0.004132231	0.9970287
4914	Progesterone-mediated oocyte maturation	Organismal Systems	Endocrine system	2	170	431	27723	0.004640371	0.9970287
4916	Melanogenesis	Organismal Systems	Endocrine system	2	170	468	27723	0.004273504	0.9970287
4917	Prolactin signaling pathway	Organismal Systems	Endocrine system	2	170	321	27723	0.00623053	0.9970287
4918	Thyroid hormone synthesis	Organismal Systems	Endocrine system	1	170	287	27723	0.003484321	0.9970287
4919	Thyroid hormone signaling pathway	Organismal Systems	Endocrine system	3	170	542	27723	0.005535055	0.9970287
4920	Adipocytokine signaling pathway	Organismal Systems	Endocrine system	1	170	396	27723	0.002525253	0.9970287
4921	Oxytocin signaling pathway	Organismal Systems	Endocrine system	1	170	768	27723	0.001302083	0.9970287
4922	Glucagon signaling pathway	Organismal Systems	Endocrine system	2	170	509	27723	0.003929273	0.9970287
4923	Regulation of lipolysis in adipocytes	Organismal Systems	Endocrine system	1	170	245	27723	0.004081633	0.9970287
4924	Renin secretion	Organismal Systems	Endocrine system	1	170	337	27723	0.002967359	0.9970287
4925	Aldosterone synthesis and secretion	Organismal Systems	Endocrine system	1	170	504	27723	0.001984127	0.9970287
4926	Relaxin signaling pathway	Organismal Systems	Endocrine system	3	170	553	27723	0.005424955	0.9970287
4927	Cortisol synthesis and secretion	Organismal Systems	Endocrine system	1	170	305	27723	0.003278689	0.9970287
4928	Parathyroid hormone synthesis, secretion and action	Organismal Systems	Endocrine system	1	170	599	27723	0.001669449	0.9970287
4960	Aldosterone-regulated sodium reabsorption	Organismal Systems	Excretory system	1	170	144	27723	0.006944444	0.9970287
4961	Endocrine and other factor-regulated calcium reabsorption	Organismal Systems	Excretory system	1	170	231	27723	0.004329004	0.9970287
4964	Proximal tubule bicarbonate reclamation	Organismal Systems	Excretory system	1	170	77	27723	0.012987013	0.9970287
4970	Salivary secretion	Organismal Systems	Digestive system	1	170	400	27723	0.0025	0.9970287

4971	Gastric acid secretion	Organismal Systems	Digestive system	1	170	393	27723	0.002544529	0.9970287
4972	Pancreatic secretion	Organismal Systems	Digestive system	1	170	404	27723	0.002475248	0.9970287
4973	Carbohydrate digestion and absorption	Organismal Systems	Digestive system	1	170	180	27723	0.005555556	0.9970287
4974	Protein digestion and absorption	Organismal Systems	Digestive system	1	170	357	27723	0.00280112	0.9970287
4976	Bile secretion	Organismal Systems	Digestive system	1	170	356	27723	0.002808989	0.9970287
4978	Mineral absorption	Organismal Systems	Digestive system	1	170	128	27723	0.0078125	0.9970287
4979	Cholesterol metabolism	Organismal Systems	Digestive system	3	170	161	27723	0.01863354	0.9970287

Table S11-4. KEGG enrichment of target of DE lncRNAs.

KEGG pathway term ID	KEGG pathway term description	KEGG pathway term level_1	KEGG pathway term level_2	Term candidate gene number	Total candidate gene number	Term gene number	Total gene number	Rich ratio	Q value
4213	Longevity regulating pathway - multiple species	Organismal Systems	Aging	5	27	276	27723	0.018115942	3.69E-04
4152	AMPK signaling pathway	Environmental Information Processing	Signal transduction	6	27	605	27723	0.009917355	5.43E-04
4710	Circadian rhythm	Organismal Systems	Environmental adaptation	4	27	196	27723	0.020408163	5.43E-04
4920	Adipocytokine signaling pathway	Organismal Systems	Endocrine system	5	27	396	27723	0.012626263	5.43E-04
4211	Longevity regulating pathway	Organismal Systems	Aging	5	27	459	27723	0.010893246	8.44E-04
4922	Glucagon signaling pathway	Organismal Systems	Endocrine system	5	27	509	27723	0.009823183	0.001143039
4068	FoxO signaling pathway	Environmental Information Processing	Signal transduction	5	27	539	27723	0.009276438	0.001280198
4530	Tight junction	Cellular Processes	Cellular community - eukaryotes	6	27	892	27723	0.006726457	0.001311637
4921	Oxytocin signaling pathway	Organismal Systems	Endocrine system	5	27	768	27723	0.006510417	0.00504834
4371	Apelin signaling pathway	Environmental Information Processing	Signal transduction	4	27	597	27723	0.006700168	0.014618308
4910	Insulin signaling pathway	Organismal Systems	Endocrine system	4	27	634	27723	0.006309148	0.016502413
4714	Thermogenesis	Organismal Systems	Environmental adaptation	4	27	782	27723	0.00511509	0.03179284
4141	Protein processing in endoplasmic reticulum	Genetic Information Processing	Folding, sorting and degradation	3	27	631	27723	0.004754358	0.099304741
4340	Hedgehog signaling pathway	Environmental Information Processing	Signal transduction	2	27	247	27723	0.008097166	0.099304741
4391	Hippo signaling pathway - fly	Environmental Information Processing	Signal transduction	2	27	335	27723	0.005970149	0.162000883
4972	Pancreatic secretion	Organismal Systems	Digestive system	2	27	404	27723	0.004950495	0.212178464
3320	PPAR signaling pathway	Organismal Systems	Endocrine system	2	27	432	27723	0.00462963	0.224642018
4964	Proximal tubule bicarbonate reclamation	Organismal Systems	Excretory system	1	27	77	27723	0.012987013	0.233219643
4915	Estrogen signaling pathway	Organismal Systems	Endocrine system	2	27	508	27723	0.003937008	0.265907807
4217	Necroptosis	Cellular Processes	Cell growth and death	2	27	606	27723	0.00330033	0.309802518
4320	Dorso-ventral axis formation	Organismal Systems	Development	1	27	128	27723	0.0078125	0.309802518
4722	Neurotrophin signaling pathway	Organismal Systems	Nervous system	2	27	580	27723	0.003448276	0.309802518
4215	Apoptosis - multiple species	Cellular Processes	Cell growth and death	1	27	149	27723	0.006711409	0.327395742
4390	Hippo signaling pathway	Environmental Information Processing	Signal transduction	2	27	647	27723	0.00309119	0.327395742
600	Sphingolipid metabolism	Metabolism	Lipid metabolism	1	27	164	27723	0.006097561	0.330351269
4979	Cholesterol metabolism	Organismal Systems	Digestive system	1	27	161	27723	0.00621118	0.330351269
4218	Cellular senescence	Cellular Processes	Cell growth and death	2	27	727	27723	0.002751032	0.3376006
4330	Notch signaling pathway	Environmental Information Processing	Signal transduction	1	27	214	27723	0.004672897	0.391229264

510	N-Glycan biosynthesis	Metabolism	Glycan biosynthesis and metabolism	1	27	223	27723	0.004484305	0.3920148
4115	p53 signaling pathway	Cellular Processes	Cell growth and death	1	27	273	27723	0.003663004	0.444154394
4212	Longevity regulating pathway - worm	Organismal Systems	Aging	1	27	281	27723	0.003558719	0.444154394
4918	Thyroid hormone synthesis	Organismal Systems	Endocrine system	1	27	287	27723	0.003484321	0.444154394
4137	Mitophagy - animal	Cellular Processes	Transport and catabolism	1	27	330	27723	0.003030303	0.471454024
4917	Prolactin signaling pathway	Organismal Systems	Endocrine system	1	27	321	27723	0.003115265	0.471454024
4976	Bile secretion	Organismal Systems	Digestive system	1	27	356	27723	0.002808989	0.488352874
4066	HIF-1 signaling pathway	Environmental Information Processing	Signal transduction	1	27	406	27723	0.002463054	0.529555789
4064	NF-kappa B signaling pathway	Environmental Information Processing	Signal transduction	1	27	437	27723	0.00228833	0.532635216
4914	Progesterone-mediated oocyte maturation	Organismal Systems	Endocrine system	1	27	431	27723	0.002320186	0.532635216
4071	Sphingolipid signaling pathway	Environmental Information Processing	Signal transduction	1	27	493	27723	0.002028398	0.557087627
4114	Oocyte meiosis	Cellular Processes	Cell growth and death	1	27	512	27723	0.001953125	0.557087627
4151	PI3K-Akt signaling pathway	Environmental Information Processing	Signal transduction	2	27	1421	27723	0.00140746	0.557087627
4210	Apoptosis	Cellular Processes	Cell growth and death	1	27	534	27723	0.001872659	0.557087627
4725	Cholinergic synapse	Organismal Systems	Nervous system	1	27	558	27723	0.001792115	0.557087627
4919	Thyroid hormone signaling pathway	Organismal Systems	Endocrine system	1	27	542	27723	0.001845018	0.557087627
4630	Jak-STAT signaling pathway	Environmental Information Processing	Signal transduction	1	27	589	27723	0.001697793	0.561972087
4928	Parathyroid hormone synthesis, secretion and action	Organismal Systems	Endocrine system	1	27	599	27723	0.001669449	0.561972087
4140	Autophagy - animal	Cellular Processes	Transport and catabolism	1	27	668	27723	0.001497006	0.594599074
4261	Adrenergic signaling in cardiomyocytes	Organismal Systems	Circulatory system	1	27	707	27723	0.001414427	0.594599074
4621	NOD-like receptor signaling pathway	Organismal Systems	Immune system	1	27	689	27723	0.001451379	0.594599074
4022	cGMP-PKG signaling pathway	Environmental Information Processing	Signal transduction	1	27	794	27723	0.001259446	0.618507906
4062	Chemokine signaling pathway	Organismal Systems	Immune system	1	27	776	27723	0.00128866	0.618507906
4024	cAMP signaling pathway	Environmental Information Processing	Signal transduction	1	27	926	27723	0.001079914	0.646773467
4510	Focal adhesion	Cellular Processes	Cellular community - eukaryotes	1	27	930	27723	0.001075269	0.646773467
4810	Regulation of actin cytoskeleton	Cellular Processes	Cell motility	1	27	906	27723	0.001103753	0.646773467
4020	Calcium signaling pathway	Environmental Information Processing	Signal transduction	1	27	969	27723	0.001031992	0.651201538
4144	Endocytosis	Cellular Processes	Transport and catabolism	1	27	1203	27723	8.31E-04	0.723257929
4740	Olfactory transduction	Organismal Systems	Sensory system	1	27	1738	27723	5.75E-04	0.84052786
1100	Metabolic pathways	Metabolism	Global and overview maps	1	27	4632	27723	2.16E-04	0.9928359
