# Supplementary material for: Comprehensive analysis of miRNAs, lncRNAs and mRNAs profiles in backfat tissue between Daweizi and Yorkshire pigs
Source: Anim Biosci. 2022 Nov 13;36(3):404–16. doi: 10.5713/ab.22.0165 (PMC9996253; doi:10.5713/ab.22.0165)
Supplement: Supplementary file 10 [file ab-22-0165-Supplementary-Table-9.pdf]

Supplementary Table S9. The predicted targets of DE miRNAs.

RNA ID	Gene ID	Symbol	Type	target_total	ssc-miR-29a-3p	novel-ssc-miR204-5p	novel-ssc-miR480-3p	novel-ssc-miR537-5p	novel-ssc-miR620-3p	novel-ssc-miR627-5p	novel-ssc-miR671-3p	novel-ssc-miR753-5p	novel-ssc-miR832-3p	novel-ssc-miR1287-5p	novel-ssc-miR1406-3p	ssc-miR-10383	novel-ssc-miR2-3p	ssc-miR-660	novel-ssc-miR9-3p	novel-ssc-miR16-3p	novel-ssc-miR710-5p	novel-ssc-miR802-3p
BGIR9823_100323	100049657	GNAS	lncRNA	17	0	1	1	0	0	1	0	0	1	1	0	1	0	0	0	0	1	1
BGIR9823_100321	100144523	PMEPA1	lncRNA	15	0	1	1	1	0	1	0	0	1	1	0	0	0	0	0	0	1	1
BGIR9823_100436	110257581	LOC110257581	lncRNA	15	0	1	1	0	0	0	0	0	0	0	0	1	0	0	0	0	1	1
BGIR9823_101399	110258215	LOC110258215	lncRNA	14	0	0	1	0	0	1	0	0	0	1	0	1	0	0	0	0	0	1
BGIR9823_100330	100037996	RPS21	lncRNA	14	0	0	1	0	0	1	0	0	0	1	0	1	0	0	0	0	1	1
BGIR9823_101457	110258362	MTA1	lncRNA	14	0	1	0	0	0	1	0	0	0	1	0	1	0	0	0	0	1	1
BGIR9823_101544	110258600	LOC110258600	lncRNA	13	0	0	1	0	0	1	0	0	0	1	0	1	0	0	0	0	0	1
BGIR9823_101531	102158401	LOC102158401	lncRNA	11	0	0	1	0	0	1	0	0	0	1	0	0	0	0	0	0	0	1
BGIR9823_101342	110258067	ARRDC1	lncRNA	11	0	1	1	0	0	0	0	0	0	1	0	1	0	0	0	0	1	1
BGIR9823_101405	110258214	LOC110258214	lncRNA	11	0	0	1	0	0	1	0	0	0	1	0	1	0	0	0	0	0	1
BGIR9823_101472	110258334	LOC110258334	lncRNA	10	0	1	1	0	0	1	0	0	0	1	0	0	0	0	0	0	0	0
BGIR9823_100712	100520873	IMPDH1	lncRNA	10	0	1	0	0	0	1	0	0	0	1	0	0	0	0	0	0	1	1
BGIR9823_101185	110257830	LOC110257830	lncRNA	10	0	1	1	0	0	1	0	0	0	1	0	0	0	0	0	0	1	1
BGIR9823_101542	102157946	LOC102157946	lncRNA	10	0	0	1	0	0	0	0	0	0	1	0	1	0	0	0	0	0	0
BGIR9823_100049	110257373	GMEB2	lncRNA	9	0	0	0	0	0	0	0	0	1	0	0	0	0	0	0	0	1	0
BGIR9823_100378	100514083	ADCK2	lncRNA	9	0	1	0	0	0	0	0	0	1	0	0	0	0	0	0	0	0	1
BGIR9823_100540	100523342	OSBPL3	lncRNA	9	0	0	1	0	0	0	0	0	1	0	0	0	0	0	0	0	1	1
BGIR9823_101051	110257712	LOC110257712	lncRNA	8	0	0	0	0	0	1	0	0	0	0	0	0	0	0	0	0	0	1
BGIR9823_101904	BGIG9823_26581		lncRNA	8	0	0	1	0	0	1	0	0	0	0	0	1	0	0	0	0	0	0
BGIR9823_100561	100516888	GLI3	lncRNA	8	0	1	0	0	0	0	0	0	1	1	0	0	0	0	0	0	1	1
BGIR9823_100665	100622227	LOC100622227	lncRNA	8	0	1	0	0	0	0	0	0	0	0	0	0	0	0	0	0	1	0
BGIR9823_100348	110257384	TCEA2	lncRNA	8	0	1	0	0	0	0	0	1	0	0	0	1	0	0	0	0	1	0
BGIR9823_100834	110257605	LRRC61	lncRNA	8	0	0	1	0	0	0	0	0	0	0	0	0	0	0	0	0	0	1
BGIR9823_100037	110257479	MTG2	lncRNA	8	0	0	0	0	0	0	0	0	0	0	0	1	0	0	0	0	0	1
BGIR9823_100184	106506644	LOC106506644	lncRNA	8	0	0	0	0	0	0	0	0	0	0	0	1	0	0	0	0	1	1
BGIR9823_101820	BGIG9823_26500		lncRNA	7	0	0	0	0	0	1	0	0	0	0	0	0	0	0	0	0	0	0
BGIR9823_100659	100522048	SLC13A4	lncRNA	7	0	1	0	0	0	0	0	0	0	0	0	0	0	0	0	0	1	1
BGIR9823_100223	100627204	MMP24	lncRNA	7	0	1	0	0	0	0	0	0	0	0	0	1	0	0	0	0	1	1
BGIR9823_101137	100152260	PRDX4	lncRNA	7	0	0	0	0	0	0	1	0	0	0	0	0	0	0	0	1	0	0
BGIR9823_100172	100154685	UBOX5	lncRNA	7	0	1	1	0	0	0	0	1	0	0	0	0	0	0	0	0	1	1
BGIR9823_101650	100738659	MCM3AP	lncRNA	6	0	1	0	0	0	1	0	0	0	0	0	0	0	0	0	0	0	0
BGIR9823_101940	BGIG9823_26617		lncRNA	6	0	0	0	0	0	1	0	0	0	0	0	0	0	0	0	0	0	0
BGIR9823_101941	BGIG9823_26618		lncRNA	6	0	0	0	0	0	0	0	1	0	0	0	0	0	0	0	0	0	0
BGIR9823_102278	BGIG9823_26945		lncRNA	6	0	0	0	0	0	1	0	0	0	0	0	0	0	0	0	0	0	0
BGIR9823_101611	100521600	LOC100521600	lncRNA	6	0	1	1	0	0	0	0	0	0	0	0	0	0	0	0	0	0	0
BGIR9823_100544	100627860	SUN3	lncRNA	6	0	0	0	0	0	1	0	0	1	1	0	0	0	0	0	0	0	0
BGIR9823_100360	100514805	KMT2C	lncRNA	6	0	0	0	0	0	0	0	0	1	1	0	1	0	0	0	0	0	0
BGIR9823_100799	397163	RAMP3	lncRNA	6	0	0	1	0	0	0	0	0	0	0	0	0	0	0	0	0	0	0
BGIR9823_101296	100623680	ZFP92	lncRNA	6	0	0	0	0	0	0	0	0	0	1	0	0	0	0	0	0	1	0
BGIR9823_100164	100520054	PANK2	lncRNA	6	0	0	0	0	0	0	0	0	1	0	0	0	0	0	0	0	0	1
BGIR9823_100576	102157930	LOC102157930	lncRNA	6	0	1	0	0	0	0	0	0	1	0	0	0	0	0	0	0	0	1

RNA ID	Gene ID	Symbol	Type	target_total	ssc-miR-29a-3p	novel-ssc-miR204-5p	novel-ssc-miR480-3p	novel-ssc-miR537-5p	novel-ssc-miR620-3p	novel-ssc-miR627-5p	novel-ssc-miR671-3p	novel-ssc-miR753-5p	novel-ssc-miR832-3p	novel-ssc-miR1287-5p	novel-ssc-miR1406-3p	ssc-miR-10383	novel-ssc-miR2-3p	ssc-miR-660	novel-ssc-miR9-3p	novel-ssc-miR16-3p	novel-ssc-miR710-5p	novel-ssc-miR802-3p
BGIR9823_100585	100623772	ZNF862	lncRNA	6	0	0	0	0	0	0	0	1	0	1	0	0	0	0	0	0	1	0
BGIR9823_101341	110258060	LOC110258060	lncRNA	6	0	1	0	0	0	0	0	0	0	0	0	0	0	0	0	0	1	0
BGIR9823_101500	102160467	SMAGP	lncRNA	6	0	0	0	0	0	0	0	0	0	0	0	0	0	0	0	0	1	1
BGIR9823_101475	100521669	BRF1	lncRNA	5	0	0	0	0	0	0	0	1	0	0	0	0	0	0	0	0	0	0
BGIR9823_100400	100523151	NUP205	lncRNA	5	0	1	0	0	0	0	0	0	0	0	0	0	0	0	0	0	0	0
BGIR9823_101360	100512700	LOC100512700	lncRNA	5	0	0	0	0	0	0	0	0	0	0	0	0	0	0	0	0	0	0
BGIR9823_101677	BGIG9823_26364		lncRNA	5	0	0	1	0	0	0	0	0	0	0	0	1	0	0	0	0	0	0
BGIR9823_101879	BGIG9823_26558		lncRNA	5	0	0	0	0	0	0	0	0	0	0	0	0	0	0	0	0	0	0
BGIR9823_102010	BGIG9823_26686		lncRNA	5	0	0	0	0	0	1	0	0	0	0	0	0	0	0	0	0	0	0
BGIR9823_101968	BGIG9823_26645		lncRNA	5	0	0	0	0	0	0	0	0	0	1	0	1	0	1	0	0	0	0
BGIR9823_102338	BGIG9823_27003		lncRNA	5	0	0	0	0	0	0	0	1	0	0	0	0	0	0	0	0	0	0
BGIR9823_100338	BGIG9823_26339		lncRNA	5	0	1	1	0	0	0	0	0	0	0	0	0	0	0	0	0	1	0
BGIR9823_100753	493185	IFRD1	lncRNA	5	0	0	1	0	0	0	0	0	0	0	0	0	0	0	0	0	0	0
BGIR9823_100807	100515808	TMED4	lncRNA	5	0	0	0	0	0	0	0	0	0	1	0	0	0	0	0	0	0	0
BGIR9823_100577	100622254	RALA	lncRNA	5	0	0	0	0	0	0	0	0	1	0	0	0	0	0	0	0	0	0
BGIR9823_101471	100126861	AKT1	lncRNA	5	0	0	0	0	0	0	0	0	0	0	0	1	0	0	0	0	0	1
BGIR9823_100694	100737195	SMKR1	lncRNA	5	0	1	0	0	0	0	0	0	0	0	0	0	0	0	0	0	0	1
BGIR9823_101043	445538	XPNPEP2	lncRNA	5	0	0	0	0	0	1	0	0	0	0	0	1	0	0	0	0	0	0
BGIR9823_101060	102158351	LOC102158351	lncRNA	5	0	1	0	0	0	0	0	0	0	0	0	0	0	0	0	0	0	1
BGIR9823_100405	106506765	LOC106506765	lncRNA	5	0	0	0	0	0	0	0	0	0	0	0	0	0	0	0	0	1	0
BGIR9823_101548	110258600	LOC110258600	lncRNA	5	0	0	0	0	0	0	0	1	0	0	0	0	0	0	0	0	1	0
BGIR9823_100632	100515805	PARP12	lncRNA	5	0	0	0	0	0	0	0	0	0	1	0	0	0	0	0	0	0	1
BGIR9823_100345	100621535	TPD52L2	lncRNA	5	0	0	1	0	0	0	0	0	0	0	0	1	0	0	0	0	0	0
BGIR9823_100658	106506763	LOC106506763	lncRNA	5	0	1	0	0	0	0	0	0	0	0	0	0	0	0	0	0	0	0
BGIR9823_100521	100512260	GARS	lncRNA	5	0	1	0	0	0	0	0	0	0	0	0	1	0	0	0	0	0	0
BGIR9823_100195	106505983	LOC106505983	lncRNA	5	0	1	0	0	0	0	0	0	1	0	0	1	0	0	0	0	0	0
BGIR9823_101416	BGIG9823_26349		lncRNA	4	0	0	0	0	0	0	0	0	0	0	0	0	0	0	0	0	0	1
BGIR9823_102298	BGIG9823_26964		lncRNA	4	0	0	0	1	0	1	0	0	0	0	0	0	0	0	0	0	0	0
BGIR9823_101877	BGIG9823_26556		lncRNA	4	0	0	0	0	0	1	0	1	0	0	0	0	0	0	0	0	0	0
BGIR9823_102235	BGIG9823_26907		lncRNA	4	0	0	0	0	0	1	0	0	0	0	0	0	0	0	0	0	0	0
BGIR9823_102014	BGIG9823_26690		lncRNA	4	0	0	0	0	0	0	0	0	0	0	0	0	0	0	0	0	0	0
BGIR9823_102030	BGIG9823_26705		lncRNA	4	0	0	0	0	0	1	0	0	0	0	0	0	0	0	0	0	0	0
BGIR9823_101900	BGIG9823_26577		lncRNA	4	0	0	1	0	0	1	0	0	0	0	0	0	0	0	0	0	0	0
BGIR9823_101920	BGIG9823_26597		lncRNA	4	0	0	1	0	0	0	0	1	0	0	0	0	0	0	0	0	0	0
BGIR9823_101972	BGIG9823_26649		lncRNA	4	0	0	0	0	0	0	0	0	0	1	0	0	0	0	0	0	0	0
BGIR9823_101815	BGIG9823_26495		lncRNA	4	0	0	0	0	0	1	0	0	0	0	0	0	0	0	0	0	0	0
BGIR9823_101971	BGIG9823_26648		lncRNA	4	0	0	0	0	0	0	0	0	0	1	0	0	0	0	0	0	0	0
BGIR9823_101828	BGIG9823_26507		lncRNA	4	0	0	0	0	0	0	0	0	0	1	0	0	0	0	0	0	0	0
BGIR9823_102358	BGIG9823_27023		lncRNA	4	0	0	0	0	0	1	0	0	0	0	0	0	0	0	0	0	0	0
BGIR9823_101692	BGIG9823_26379		lncRNA	4	0	0	0	0	0	0	0	0	0	1	0	0	0	0	0	0	0	0
BGIR9823_101728	BGIG9823_26410		lncRNA	4	0	1	1	0	0	0	0	0	0	0	0	0	0	0	0	0	0	0
BGIR9823_101871	BGIG9823_26550		lncRNA	4	0	0	0	0	0	1	0	0	0	0	0	1	0	0	0	0	0	0
BGIR9823_101827	BGIG9823_26506		lncRNA	4	0	0	0	0	0	1	0	0	0	0	0	1	0	0	0	0	0	0

RNA ID	Gene ID	Symbol	Type	target_total	ssc-miR-29a-3p	novel-ssc-miR204-5p	novel-ssc-miR480-3p	novel-ssc-miR537-5p	novel-ssc-miR620-3p	novel-ssc-miR627-5p	novel-ssc-miR671-3p	novel-ssc-miR753-5p	novel-ssc-miR832-3p	novel-ssc-miR1287-5p	novel-ssc-miR1406-3p	ssc-miR-10383	novel-ssc-miR2-3p	ssc-miR-660	novel-ssc-miR9-3p	novel-ssc-miR16-3p	novel-ssc-miR710-5p	novel-ssc-miR802-3p
BGIR9823_102129	BGIG9823_26802		lncRNA	4	0	0	0	0	0	0	0	0	0	0	0	0	0	0	0	0	0	0
BGIR9823_102690	BGIG9823_27340		lncRNA	4	0	0	0	0	0	0	0	0	0	0	0	0	0	0	0	0	0	0
BGIR9823_101336	100519166	LOC100519166	lncRNA	4	0	1	0	0	0	0	0	0	0	1	0	1	0	0	0	0	1	0
BGIR9823_101541	102157946	LOC102157946	lncRNA	4	0	0	0	0	0	1	0	0	0	0	0	0	0	0	0	0	0	1
BGIR9823_100268	102159476	LOC102159476	lncRNA	4	0	1	0	0	0	0	0	0	0	0	0	0	0	0	0	0	1	0
BGIR9823_101104	100521877	PUDP	lncRNA	4	0	0	0	0	0	0	0	0	0	0	0	0	0	0	0	0	0	0
BGIR9823_101381	100511434	EGFL7	lncRNA	4	0	0	0	0	0	0	0	0	0	0	0	0	0	0	0	0	0	0
BGIR9823_101015	100520165	TMEM164	lncRNA	4	0	0	0	0	0	0	0	0	0	0	0	0	0	0	0	0	1	0
BGIR9823_100709	100519523	CCDC136	lncRNA	4	0	0	0	0	0	0	0	0	0	0	0	0	0	0	0	0	0	0
BGIR9823_101430	100156979	TRAF3	lncRNA	4	0	1	0	0	0	0	0	0	0	0	0	0	0	0	0	0	1	0
BGIR9823_101450	100623151	INF2	lncRNA	4	0	0	0	0	0	1	0	0	0	0	0	0	0	0	0	0	0	0
BGIR9823_100277	100156830	PTI	lncRNA	4	0	1	0	1	0	0	0	0	0	0	0	0	0	0	0	0	0	0
BGIR9823_101283	100359355	MOSPD1	lncRNA	4	0	0	1	0	0	0	0	0	0	0	0	0	0	0	0	0	1	0
BGIR9823_101340	106505329	LOC106505329	lncRNA	4	0	0	0	0	0	0	0	0	0	1	0	1	0	0	0	0	0	0
BGIR9823_100800	100511907	CCM2	lncRNA	4	0	0	0	0	0	0	0	0	0	0	0	0	0	0	0	0	0	0
BGIR9823_100099	100517562	HGSNAT	lncRNA	4	0	0	1	0	0	0	0	0	0	0	0	1	0	0	0	0	0	0
BGIR9823_100810	100515113	POLM	lncRNA	4	0	0	0	0	0	0	0	0	0	0	0	1	0	0	0	0	0	0
BGIR9823_100578	100622573	POU6F2	lncRNA	4	0	1	0	0	0	0	0	0	0	0	0	0	0	0	0	0	0	0
BGIR9823_78024	100157196	CSNK1G1	lncRNA	4	0	0	0	0	0	0	0	1	0	0	0	0	0	0	0	0	0	0
BGIR9823_100367	100521999	SMARCD3	lncRNA	4	0	0	0	0	0	0	0	0	0	1	0	0	0	0	0	0	0	0
BGIR9823_100389	100627515	HIPK2	lncRNA	4	0	0	0	1	0	0	0	1	0	0	0	0	0	0	0	0	1	0
BGIR9823_100256	100156548	ZHX3	lncRNA	4	0	0	0	0	0	0	0	0	0	1	0	0	0	0	0	0	0	0
BGIR9823_100882	102165056	MBTPS2	lncRNA	3	0	0	0	0	0	0	0	0	0	0	0	0	0	1	0	0	0	0
BGIR9823_100324	100049657	GNAS	lncRNA	3	0	0	0	1	0	0	0	0	0	0	0	0	0	0	0	0	0	0
BGIR9823_100656	100521167	DGKI	lncRNA	3	0	0	0	0	0	0	0	0	0	0	0	0	0	0	0	0	0	0
BGIR9823_101689	BGIG9823_26376		lncRNA	3	0	0	0	0	0	0	0	0	0	0	0	0	0	0	0	0	0	0
BGIR9823_102087	BGIG9823_26761		lncRNA	3	0	0	0	0	0	0	0	0	0	0	0	0	0	0	0	0	0	0
BGIR9823_101951	BGIG9823_26628		lncRNA	3	0	0	0	0	0	0	0	0	0	0	0	1	0	0	0	0	0	0
BGIR9823_101854	BGIG9823_26533		lncRNA	3	0	0	0	1	0	0	0	1	0	0	0	0	0	0	0	0	0	0
BGIR9823_102383	BGIG9823_27048		lncRNA	3	0	0	0	1	0	0	0	0	0	0	0	0	0	0	0	0	0	0
BGIR9823_102727	BGIG9823_27373		lncRNA	3	1	0	0	0	0	0	0	0	0	0	0	0	0	0	0	0	0	0
BGIR9823_102028	BGIG9823_26703		lncRNA	3	0	0	0	1	0	0	0	0	0	0	0	0	0	0	0	0	0	0
BGIR9823_101977	BGIG9823_26653		lncRNA	3	0	0	0	0	0	0	0	0	0	0	0	0	0	0	0	0	0	0
BGIR9823_102406	BGIG9823_27067		lncRNA	3	0	0	0	0	0	0	0	0	0	0	0	0	0	0	0	0	0	0
BGIR9823_101868	BGIG9823_26547		lncRNA	3	0	0	1	0	0	0	0	0	0	1	0	0	0	0	0	0	0	0
BGIR9823_101698	BGIG9823_26385		lncRNA	3	0	0	0	0	0	0	0	0	0	0	0	0	0	0	0	0	0	0
BGIR9823_102438	BGIG9823_27094		lncRNA	3	0	0	0	0	0	0	0	1	0	0	0	0	0	0	0	0	0	0
BGIR9823_102065	BGIG9823_26739		lncRNA	3	0	0	0	0	0	0	0	0	0	0	0	0	0	0	0	0	0	0
BGIR9823_102288	BGIG9823_26954		lncRNA	3	0	0	0	0	0	0	0	0	0	0	0	0	0	0	0	0	0	0
BGIR9823_101990	BGIG9823_26666		lncRNA	3	0	0	0	1	0	0	0	0	0	0	0	0	0	0	0	0	0	0
BGIR9823_101966	BGIG9823_26643		lncRNA	3	0	0	0	0	0	0	0	0	0	1	0	0	0	0	0	0	0	0
BGIR9823_101758	BGIG9823_26438		lncRNA	3	0	0	1	0	0	0	0	0	0	0	0	1	0	0	0	0	0	0
BGIR9823_101861	BGIG9823_26540		lncRNA	3	0	0	0	1	0	0	0	0	0	0	0	0	0	0	0	0	0	0

RNA ID	Gene ID	Symbol	Type	target_total	ssc-miR-29a-3p	novel-ssc-miR204-5p	novel-ssc-miR480-3p	novel-ssc-miR537-5p	novel-ssc-miR620-3p	novel-ssc-miR627-5p	novel-ssc-miR671-3p	novel-ssc-miR753-5p	novel-ssc-miR832-3p	novel-ssc-miR1287-5p	novel-ssc-miR1406-3p	ssc-miR-10383	novel-ssc-miR2-3p	ssc-miR-660	novel-ssc-miR9-3p	novel-ssc-miR16-3p	novel-ssc-miR710-5p	novel-ssc-miR802-3p
BGIR9823_101960	BGIG9823_26637		lncRNA	3	0	0	0	0	0	0	0	0	0	0	0	0	0	0	0	0	0	0
BGIR9823_101964	BGIG9823_26641		lncRNA	3	0	0	0	0	0	0	0	1	0	0	0	1	0	0	0	0	0	0
BGIR9823_102018	BGIG9823_26693		lncRNA	3	0	0	0	0	0	1	0	0	0	0	0	0	0	0	0	0	0	0
BGIR9823_101913	BGIG9823_26590		lncRNA	3	0	0	0	0	0	1	0	0	0	0	0	0	0	0	0	0	0	0
BGIR9823_102617	BGIG9823_27267		lncRNA	3	0	0	0	1	0	0	0	1	0	0	0	0	0	0	0	0	0	0
BGIR9823_102277	BGIG9823_26944		lncRNA	3	0	0	0	0	0	0	0	0	0	0	0	0	0	0	0	0	0	0
BGIR9823_102215	BGIG9823_26887		lncRNA	3	0	0	0	0	0	0	0	0	0	0	0	0	0	0	0	0	0	0
BGIR9823_102525	BGIG9823_27178		lncRNA	3	0	0	0	0	0	0	0	0	0	0	0	0	0	0	0	0	0	0
BGIR9823_102031	BGIG9823_26706		lncRNA	3	0	0	0	0	0	0	0	0	0	0	0	0	0	0	0	0	0	0
BGIR9823_101909	BGIG9823_26586		lncRNA	3	0	0	0	0	0	0	0	0	0	0	0	1	0	0	0	0	0	0
BGIR9823_102249	BGIG9823_26917		lncRNA	3	0	0	0	0	0	0	0	0	0	0	0	0	0	0	0	0	0	0
BGIR9823_102126	BGIG9823_26799		lncRNA	3	0	0	0	0	0	0	0	0	0	0	0	0	0	0	0	0	0	0
BGIR9823_101720	BGIG9823_26404		lncRNA	3	0	0	0	0	0	0	0	1	0	0	0	0	0	0	0	0	0	0
BGIR9823_102111	BGIG9823_26785		lncRNA	3	0	0	0	0	0	0	0	0	0	0	0	0	0	0	0	0	0	0
BGIR9823_101921	BGIG9823_26598		lncRNA	3	0	0	0	0	0	0	0	0	0	0	0	0	0	0	0	0	0	0
BGIR9823_102144	BGIG9823_26817		lncRNA	3	0	0	0	0	0	0	0	0	0	0	0	0	0	0	0	0	0	0
BGIR9823_102006	BGIG9823_26682		lncRNA	3	0	0	0	0	0	1	0	1	0	0	0	0	0	0	0	0	0	0
BGIR9823_102108	BGIG9823_26782		lncRNA	3	0	0	0	0	0	0	0	0	0	0	0	0	0	0	0	0	0	0
BGIR9823_102515	BGIG9823_27169		lncRNA	3	0	0	0	0	0	0	0	1	0	0	0	0	0	0	0	0	0	0
BGIR9823_101872	BGIG9823_26551		lncRNA	3	0	0	0	0	0	1	0	0	0	1	0	0	0	0	0	0	0	0
BGIR9823_101929	BGIG9823_26606		lncRNA	3	0	0	0	0	0	0	0	0	0	0	0	0	0	0	0	0	0	0
BGIR9823_101740	BGIG9823_26421		lncRNA	3	0	1	0	0	0	0	0	0	0	0	0	0	0	0	0	0	0	0
BGIR9823_102035	BGIG9823_26710		lncRNA	3	0	0	0	0	0	0	0	0	0	0	0	0	0	0	0	0	0	0
BGIR9823_102356	BGIG9823_27021		lncRNA	3	0	0	0	0	0	0	0	1	0	0	0	0	0	0	0	0	0	0
BGIR9823_102012	BGIG9823_26688		lncRNA	3	0	0	0	0	0	1	0	0	0	0	0	0	0	0	0	0	0	0
BGIR9823_101980	BGIG9823_26656		lncRNA	3	0	0	0	0	0	1	0	1	0	0	0	0	0	0	0	0	0	0
BGIR9823_102058	BGIG9823_26732		lncRNA	3	0	0	0	0	0	0	0	0	0	0	0	0	0	0	0	0	0	0
BGIR9823_101945	BGIG9823_26622		lncRNA	3	0	0	0	0	0	0	0	0	0	0	0	0	0	0	0	0	0	0
BGIR9823_101770	BGIG9823_26450		lncRNA	3	0	0	0	0	0	0	0	1	0	1	0	0	0	0	0	0	0	0
XR_002341218.1	110258681	LOC110258681	lncRNA	3	0	0	0	0	0	0	0	0	0	0	0	0	0	0	1	0	0	0
BGIR9823_82088	100620215	CREBBP	lncRNA	3	0	0	0	0	0	0	0	0	0	0	0	0	0	0	0	0	0	0
BGIR9823_100016	102157885	LOC102157885	lncRNA	3	0	0	0	0	0	0	0	0	0	0	0	0	0	0	0	0	0	1
BGIR9823_101122	100519810	PHKA2	lncRNA	3	0	0	1	0	0	0	0	0	0	0	0	0	0	0	0	0	0	1
BGIR9823_100002	100627868	LOC100627868	lncRNA	3	0	0	0	0	0	0	0	0	1	0	0	0	0	0	0	0	0	0
BGIR9823_100152	100152012	CST7	lncRNA	3	0	0	1	0	0	0	0	0	0	0	0	0	0	0	0	0	0	1
BGIR9823_100517	100739098	LOC100739098	lncRNA	3	0	0	0	0	0	0	0	1	1	0	0	0	0	0	0	0	0	0
BGIR9823_100137	100155396	ESF1	lncRNA	3	0	0	0	0	0	0	0	0	1	0	0	0	0	0	0	0	0	0
BGIR9823_101191	110257830	LOC110257830	lncRNA	3	0	0	0	0	0	0	0	0	0	0	0	1	0	0	0	0	0	0
BGIR9823_100307	102164659	LOC102164659	lncRNA	3	0	0	0	0	0	0	0	0	0	1	0	1	0	0	0	0	1	0
BGIR9823_101424	100620346	PPP2R5C	lncRNA	3	0	0	0	1	0	0	0	0	0	0	0	0	0	0	0	0	0	0
BGIR9823_100548	100524778	MRPS24	lncRNA	3	0	0	0	0	0	0	0	0	1	0	0	0	0	0	0	0	0	1
BGIR9823_100391	102162486	LOC102162486	lncRNA	3	0	0	0	0	0	0	0	0	0	0	0	0	0	0	0	0	0	1
BGIR9823_81504	100524976	NSD1	lncRNA	3	0	0	0	0	0	0	0	0	0	0	0	0	0	0	0	0	0	0

RNA ID	Gene ID	Symbol	Type	target_total	ssc-miR-29a-3p	novel-ssc-miR204-5p	novel-ssc-miR480-3p	novel-ssc-miR537-5p	novel-ssc-miR620-3p	novel-ssc-miR627-5p	novel-ssc-miR671-3p	novel-ssc-miR753-5p	novel-ssc-miR832-3p	novel-ssc-miR1287-5p	novel-ssc-miR1406-3p	ssc-miR-10383	novel-ssc-miR2-3p	ssc-miR-660	novel-ssc-miR9-3p	novel-ssc-miR16-3p	novel-ssc-miR710-5p	novel-ssc-miR802-3p
BGIR9823_100217	100156283	NCOA6	lncRNA	3	0	1	0	0	0	0	0	0	1	0	0	0	0	0	0	0	0	0
BGIR9823_101364	110258073	SNAPC4	lncRNA	3	0	1	0	0	0	0	0	0	0	0	0	0	0	0	0	0	0	0
BGIR9823_101532	102158401	LOC102158401	lncRNA	3	0	0	0	0	0	0	0	0	0	0	0	1	0	0	0	0	0	0
BGIR9823_100496	100521938	LOC100521938	lncRNA	3	0	0	0	0	0	0	0	0	0	0	0	0	0	0	0	0	0	1
BGIR9823_77776	100158022	HINT3	lncRNA	3	0	0	0	0	0	0	0	0	0	0	0	0	0	0	0	0	0	0
BGIR9823_100235	110257452	LOC110257452	lncRNA	3	0	1	0	0	0	0	0	0	0	0	0	0	0	0	0	0	1	0
BGIR9823_78349	100152069	SMAD6	lncRNA	3	0	0	0	0	0	0	0	0	0	0	0	0	0	0	0	0	0	0
BGIR9823_100688	100515937	COPG2	lncRNA	3	0	0	0	0	0	0	0	0	0	0	0	0	0	0	0	0	0	0
BGIR9823_101393	110258158	TNRC6A	lncRNA	3	0	0	0	0	0	0	0	0	0	0	0	0	0	0	0	0	1	1
BGIR9823_100562	100516888	GLI3	lncRNA	3	0	0	0	0	0	0	0	0	0	0	0	0	0	0	0	0	0	0
BGIR9823_79603	106509080	LOC106509080	lncRNA	3	0	0	0	1	0	0	0	0	0	0	0	0	0	0	0	0	0	0
BGIR9823_100840	102166569	TBL1X	lncRNA	3	0	0	0	0	0	0	0	0	0	0	0	0	0	0	0	0	0	0
BGIR9823_100717	100511294	PTPRZ1	lncRNA	3	0	1	0	0	0	0	0	0	0	0	0	0	0	0	0	0	0	0
BGIR9823_100350	100737248	WDR60	lncRNA	3	0	0	0	0	0	0	0	0	1	0	0	0	0	0	0	0	0	0
BGIR9823_101426	100516626	WDR20	lncRNA	3	0	0	0	0	0	0	0	0	0	0	0	0	0	0	0	0	0	0
BGIR9823_101427	100516626	WDR20	lncRNA	3	0	0	0	0	0	0	0	0	0	0	0	0	0	0	0	0	0	1
BGIR9823_101520	100511414	RNF122	lncRNA	3	0	0	0	0	0	0	0	0	0	0	0	0	0	0	0	0	0	0
BGIR9823_100500	100522109	ELMO1	lncRNA	3	0	0	0	0	0	0	0	0	0	0	0	0	0	0	0	0	0	0
BGIR9823_101384	110258135	LOC110258135	lncRNA	3	0	0	0	0	0	0	0	0	0	1	0	0	0	0	0	0	1	0
BGIR9823_101222	100517568	MAGT1	lncRNA	3	0	0	1	0	0	0	0	0	0	0	0	0	0	0	0	0	0	1
BGIR9823_100838	102166211	LOC102166211	lncRNA	3	0	0	0	0	0	0	0	0	0	0	0	0	0	0	0	0	0	0
BGIR9823_80389	100516724	NR2F6	lncRNA	3	0	0	0	0	0	0	0	0	0	0	0	0	0	0	0	0	0	0
BGIR9823_100923	100233182	GK	lncRNA	3	0	0	1	0	0	0	0	0	0	0	0	0	0	0	0	0	0	1
BGIR9823_100887	397645	SAT1	lncRNA	3	0	1	0	0	0	0	0	0	0	0	0	0	0	0	0	0	0	0
BGIR9823_100119	100519631	TASP1	lncRNA	3	0	1	0	0	0	0	0	0	0	0	0	0	0	0	0	0	0	0
BGIR9823_101408	110258214	LOC110258214	lncRNA	3	0	0	0	0	0	0	0	0	0	0	0	0	0	0	0	0	0	0
BGIR9823_100257	100156548	ZHX3	lncRNA	3	0	0	0	0	0	0	0	0	1	0	0	0	0	0	0	0	1	0
BGIR9823_100095	100738984	SLC20A2	lncRNA	3	0	0	0	0	0	0	0	0	0	0	0	0	0	0	0	0	1	0
BGIR9823_100711	397301	CALU	lncRNA	3	0	0	0	0	0	0	0	0	0	0	0	0	0	0	0	0	1	0
BGIR9823_100252	110257392	LOC110257392	lncRNA	3	0	0	0	0	0	0	0	0	0	0	0	0	0	0	0	0	0	1
BGIR9823_100342	100625428	RTEL1	lncRNA	3	0	0	0	0	0	0	0	0	0	0	0	0	0	0	0	0	1	0
BGIR9823_82688	102164200	LOC102164200	lncRNA	3	1	0	0	0	0	0	0	0	0	0	0	0	0	0	0	0	0	0
BGIR9823_92711	110255798	LOC110255798	lncRNA	3	0	0	0	0	0	0	0	0	0	0	0	0	0	0	0	0	0	0
BGIR9823_82658	100622402	C3H16orf45	mRNA	2	0	0	0	0	0	0	1	0	0	0	0	0	0	0	0	1	0	0
BGIR9823_100282	100153535	PCIF1	lncRNA	2	0	0	0	0	0	0	0	0	0	0	0	0	0	0	0	0	0	1
XR_002341162.1	110258480	LOC110258480	lncRNA	2	0	0	0	0	0	0	0	0	0	0	0	0	0	0	1	0	0	0
BGIR9823_101217	100514440	ATRX	lncRNA	2	0	0	0	0	0	0	0	0	0	0	0	0	0	0	0	0	0	1
BGIR9823_96121	110256382	LOC110256382	lncRNA	2	0	0	0	0	0	0	0	0	0	0	0	0	0	0	0	0	0	0
BGIR9823_83098	100322871	CCT7	mRNA	2	0	0	0	0	0	0	0	0	0	0	0	0	0	0	1	0	0	0
BGIR9823_81464	100516183	CNOT6	lncRNA	2	0	0	0	0	0	0	0	0	0	0	0	0	0	0	0	0	0	0
BGIR9823_81228	100518418	GATAD2A	mRNA	2	0	0	0	0	0	0	0	0	0	0	0	0	0	0	0	0	0	0
BGIR9823_99373	100514689	MAST4	mRNA	2	0	0	0	0	0	0	0	0	0	0	0	0	0	0	1	0	0	0
BGIR9823_101508	110258454	LETMD1	lncRNA	2	0	0	0	0	0	1	0	0	0	0	0	0	0	0	0	0	0	0

RNA ID	Gene ID	Symbol	Type	target_ total	ssc-miR-29a-3p	novel-ssc-miR204-5p	novel-ssc-miR480-3p	novel-ssc-miR537-5p	novel-ssc-miR620-3p	novel-ssc-miR627-5p	novel-ssc-miR671-3p	novel-ssc-miR753-5p	novel-ssc-miR832-3p	novel-ssc-miR1287-5p	novel-ssc-miR1406-3p	ssc-miR-10383	novel-ssc-miR2-3p	ssc-miR-660	novel-ssc-miR9-3p	novel-ssc-miR16-3p	novel-ssc-miR710-5p	novel-ssc-miR802-3p
BGIR9823_100200	100624021	PLAGL2	lncRNA	2	0	0	0	0	0	0	0	0	1	0	0	0	0	0	0	0	0	0
BGIR9823_93132	100174955	KLF12	lncRNA	2	0	0	0	0	0	0	0	0	0	0	0	0	0	0	0	0	0	0
BGIR9823_101233	100739062	LOC100739062	lncRNA	2	0	1	0	0	0	0	0	0	0	0	0	0	0	0	0	0	0	0
BGIR9823_101439	100625932	MARK3	lncRNA	2	0	0	0	0	0	0	0	1	0	0	0	0	0	0	0	0	0	0
BGIR9823_102584	BGIG9823_27234		lncRNA	2	0	0	0	0	0	0	0	1	0	0	0	0	0	0	0	0	0	0
BGIR9823_102601	BGIG9823_27251		lncRNA	2	0	0	0	0	0	0	0	0	0	0	0	0	0	0	0	0	0	0
BGIR9823_101907	BGIG9823_26584		lncRNA	2	0	0	0	0	0	1	0	0	0	0	0	0	0	0	0	0	0	0
BGIR9823_101683	BGIG9823_26370		lncRNA	2	0	0	0	0	0	0	0	0	0	0	0	0	0	0	0	0	0	0
BGIR9823_101932	BGIG9823_26609		lncRNA	2	0	0	0	0	0	1	0	0	0	0	0	0	0	0	0	0	0	0
BGIR9823_102312	BGIG9823_26977		lncRNA	2	0	0	0	0	0	0	0	0	0	0	0	0	0	0	0	0	0	0
BGIR9823_101712	BGIG9823_26396		lncRNA	2	0	0	0	0	0	0	0	0	0	0	0	0	0	0	0	0	0	0
BGIR9823_102749	BGIG9823_27392		lncRNA	2	0	0	0	0	0	0	0	0	0	0	0	0	0	0	0	0	0	0
BGIR9823_101726	BGIG9823_26409		lncRNA	2	0	1	0	0	0	0	0	0	0	0	0	0	0	0	0	0	0	0
BGIR9823_102386	BGIG9823_27051		lncRNA	2	0	0	0	0	0	0	0	0	0	0	0	0	0	0	0	0	0	0
BGIR9823_102032	BGIG9823_26707		lncRNA	2	0	0	0	0	0	1	0	0	0	0	0	0	0	0	0	0	0	0
BGIR9823_102336	BGIG9823_27001		lncRNA	2	0	0	0	0	0	0	0	0	0	0	0	0	0	0	0	0	0	0
BGIR9823_101794	BGIG9823_26474		lncRNA	2	0	0	0	0	0	0	0	0	0	0	0	0	0	0	0	0	0	0
BGIR9823_101752	BGIG9823_26433		lncRNA	2	0	0	0	0	0	0	0	0	0	0	0	0	0	0	0	0	0	0
BGIR9823_101944	BGIG9823_26621		lncRNA	2	0	0	0	0	0	0	0	0	0	0	0	0	0	0	0	0	0	0
BGIR9823_101873	BGIG9823_26552		lncRNA	2	0	0	0	0	0	0	0	0	0	0	0	0	0	0	0	0	0	0
BGIR9823_101671	BGIG9823_26359		lncRNA	2	0	0	0	0	0	0	0	0	0	0	0	0	0	0	0	0	0	0
BGIR9823_101974	BGIG9823_26651		lncRNA	2	0	0	1	0	0	0	0	0	0	0	0	0	0	0	0	0	0	0
BGIR9823_102380	BGIG9823_27045		lncRNA	2	0	0	0	0	0	1	0	0	0	0	0	0	0	0	0	0	0	0
BGIR9823_102232	BGIG9823_26904		lncRNA	2	0	0	0	0	0	0	0	0	0	0	0	0	0	0	0	0	0	0
BGIR9823_102238	BGIG9823_26910		lncRNA	2	0	0	0	0	0	0	0	0	0	0	0	0	0	0	0	0	0	0
BGIR9823_102071	BGIG9823_26745		lncRNA	2	0	0	0	0	0	0	0	0	0	0	0	0	0	0	0	0	0	0
BGIR9823_101784	BGIG9823_26464		lncRNA	2	0	0	0	0	0	0	0	0	0	0	0	0	0	0	0	0	0	0
BGIR9823_102392	BGIG9823_27057		lncRNA	2	0	0	0	0	0	0	0	0	0	0	0	0	0	0	0	0	0	0
BGIR9823_102420	BGIG9823_27081		lncRNA	2	0	0	0	0	0	1	0	0	0	0	0	0	0	0	0	0	0	0
BGIR9823_101988	BGIG9823_26664		lncRNA	2	0	0	0	0	0	0	0	0	0	1	0	0	0	0	0	0	0	0
BGIR9823_101675	BGIG9823_26362		lncRNA	2	0	0	0	0	0	0	0	0	0	0	0	0	0	0	0	0	0	0
BGIR9823_101761	BGIG9823_26441		lncRNA	2	0	0	1	0	0	0	0	0	0	0	0	0	0	0	0	0	0	0
BGIR9823_102382	BGIG9823_27047		lncRNA	2	0	0	0	0	0	0	0	0	0	0	0	0	0	0	0	0	0	0
BGIR9823_102045	BGIG9823_26719		lncRNA	2	0	0	0	0	0	0	0	0	0	0	0	0	0	0	0	0	0	0
BGIR9823_101857	BGIG9823_26536		lncRNA	2	0	0	0	0	0	0	0	0	0	0	0	0	0	0	0	0	0	0
BGIR9823_101775	BGIG9823_26455		lncRNA	2	0	0	0	0	0	0	0	0	0	0	0	1	0	0	0	0	0	0
BGIR9823_102446	BGIG9823_27101		lncRNA	2	0	0	0	0	0	0	0	1	0	0	0	0	0	0	0	0	0	0
BGIR9823_102179	BGIG9823_26851		lncRNA	2	0	0	0	0	0	0	0	1	0	0	0	0	0	0	0	0	0	0
BGIR9823_101678	BGIG9823_26365		lncRNA	2	0	0	0	0	0	0	0	0	0	0	0	0	0	0	0	0	0	0
BGIR9823_102242	BGIG9823_26913		lncRNA	2	0	0	0	0	0	0	0	1	0	0	0	0	0	0	0	0	0	0
BGIR9823_102633	BGIG9823_27283		lncRNA	2	0	0	0	0	0	0	0	0	0	0	0	0	0	0	0	0	0	0
BGIR9823_102205	BGIG9823_26877		lncRNA	2	0	0	0	0	0	0	0	0	0	0	0	0	0	0	0	0	0	0
BGIR9823_101763	BGIG9823_26443		lncRNA	2	0	0	0	1	0	0	0	0	0	0	0	0	0	0	0	0	0	0

RNA ID	Gene ID	Symbol	Type	target_total	ssc-miR-29a-3p	novel-ssc-miR204-5p	novel-ssc-miR480-3p	novel-ssc-miR537-5p	novel-ssc-miR620-3p	novel-ssc-miR627-5p	novel-ssc-miR671-3p	novel-ssc-miR753-5p	novel-ssc-miR832-3p	novel-ssc-miR1287-5p	novel-ssc-miR1406-3p	ssc-miR-10383	novel-ssc-miR2-3p	ssc-miR-660	novel-ssc-miR9-3p	novel-ssc-miR16-3p	novel-ssc-miR710-5p	novel-ssc-miR802-3p
BGIR9823_102754	BGIG9823_27394		lncRNA	2	0	0	0	0	0	0	0	1	0	0	0	0	0	0	0	0	0	0
BGIR9823_102077	BGIG9823_26751		lncRNA	2	0	0	0	0	0	0	0	0	0	0	0	0	0	0	0	0	0	0
BGIR9823_102457	BGIG9823_27111		lncRNA	2	0	0	0	0	0	0	0	0	0	0	0	0	0	0	0	0	0	0
BGIR9823_101881	BGIG9823_26560		lncRNA	2	0	0	1	0	0	0	0	0	0	0	0	0	0	0	0	0	0	0
BGIR9823_102086	BGIG9823_26760		lncRNA	2	0	0	0	0	0	0	0	0	0	0	0	0	0	0	0	0	0	0
BGIR9823_102146	BGIG9823_26819		lncRNA	2	0	0	0	0	0	0	0	0	0	0	0	0	0	0	0	0	0	0
BGIR9823_101735	BGIG9823_26416		lncRNA	2	0	0	0	0	0	0	0	0	0	0	0	0	0	0	0	0	0	0
BGIR9823_102132	BGIG9823_26805		lncRNA	2	0	0	0	0	0	0	0	0	0	0	0	0	0	0	0	0	0	0
BGIR9823_102345	BGIG9823_27010		lncRNA	2	0	0	0	0	0	0	0	1	0	0	0	0	0	0	0	0	0	0
BGIR9823_101715	BGIG9823_26399		lncRNA	2	0	0	0	0	0	0	0	0	0	0	0	0	0	0	0	0	0	0
BGIR9823_102322	BGIG9823_26987		lncRNA	2	0	0	0	0	0	0	0	0	0	0	0	0	0	0	0	0	0	0
BGIR9823_102695	BGIG9823_27345		lncRNA	2	0	0	0	0	0	0	0	0	0	0	0	0	0	0	0	0	0	0
BGIR9823_101684	BGIG9823_26371		lncRNA	2	0	0	0	0	0	0	0	0	0	0	0	0	0	0	0	0	0	0
BGIR9823_102344	BGIG9823_27009		lncRNA	2	0	0	0	0	0	0	0	0	0	0	0	0	0	0	0	0	0	0
BGIR9823_102092	BGIG9823_26766		lncRNA	2	0	0	0	0	0	0	0	0	0	0	0	0	0	0	0	0	0	0
BGIR9823_102073	BGIG9823_26747		lncRNA	2	0	0	0	0	0	0	0	0	0	0	0	0	0	0	0	0	0	0
BGIR9823_102188	BGIG9823_26860		lncRNA	2	0	0	0	0	0	0	0	0	0	0	0	0	0	0	0	0	0	0
BGIR9823_101946	BGIG9823_26623		lncRNA	2	0	0	1	0	0	0	0	0	0	0	0	0	0	0	0	0	0	0
BGIR9823_102176	BGIG9823_26848		lncRNA	2	0	0	0	0	0	0	0	0	0	0	0	0	0	0	0	0	0	0
BGIR9823_102387	BGIG9823_27052		lncRNA	2	0	0	0	0	0	1	0	0	0	0	0	0	0	0	0	0	0	0
BGIR9823_101876	BGIG9823_26555		lncRNA	2	0	0	0	0	0	0	0	0	0	0	0	0	0	0	0	0	0	0
BGIR9823_101888	BGIG9823_26565		lncRNA	2	0	0	1	0	0	0	0	0	0	1	0	0	0	0	0	0	0	0
BGIR9823_101818	BGIG9823_26498		lncRNA	2	0	0	0	0	0	0	0	0	0	1	0	0	0	0	0	0	0	0
BGIR9823_102320	BGIG9823_26985		lncRNA	2	0	0	0	0	0	0	0	1	0	0	0	0	0	0	0	0	0	0
BGIR9823_102080	BGIG9823_26754		lncRNA	2	0	0	0	0	0	1	0	0	0	0	0	0	0	0	0	0	0	0
BGIR9823_101786	BGIG9823_26466		lncRNA	2	0	0	0	0	0	0	0	0	0	1	0	1	0	0	0	0	0	0
BGIR9823_102325	BGIG9823_26990		lncRNA	2	0	0	0	0	0	0	0	0	0	0	0	0	0	0	0	0	0	0
BGIR9823_101989	BGIG9823_26665		lncRNA	2	0	0	0	0	0	0	0	0	0	1	0	0	0	0	0	0	0	0
BGIR9823_101889	BGIG9823_26566		lncRNA	2	0	0	0	0	0	0	0	0	0	0	0	0	0	0	0	0	0	0
BGIR9823_102669	BGIG9823_27319		lncRNA	2	0	0	0	0	0	0	0	0	0	0	0	0	0	0	0	0	0	0
BGIR9823_101878	BGIG9823_26557		lncRNA	2	0	0	0	0	0	0	0	0	0	0	0	0	0	0	0	0	0	0
BGIR9823_101918	BGIG9823_26595		lncRNA	2	0	0	0	0	0	0	0	0	0	0	0	0	0	0	0	0	0	0
BGIR9823_102064	BGIG9823_26738		lncRNA	2	0	0	0	0	0	0	0	0	0	0	0	0	0	0	0	0	0	0
BGIR9823_101985	BGIG9823_26661		lncRNA	2	0	0	0	0	0	1	0	0	0	0	0	0	0	0	0	0	0	0
BGIR9823_101774	BGIG9823_26454		lncRNA	2	0	0	1	0	0	0	0	0	0	1	0	0	0	0	0	0	0	0
BGIR9823_102112	BGIG9823_26786		lncRNA	2	0	0	0	0	0	0	0	0	0	0	0	0	0	0	0	0	0	0
BGIR9823_102585	BGIG9823_27235		lncRNA	2	0	0	0	0	0	0	0	0	0	0	0	0	0	0	0	0	0	0
BGIR9823_101903	BGIG9823_26580		lncRNA	2	0	0	0	0	0	0	0	0	0	1	0	0	0	0	0	0	0	0
BGIR9823_102173	BGIG9823_26846		lncRNA	2	0	0	0	0	0	0	0	0	0	0	0	0	0	0	0	0	0	0
BGIR9823_101912	BGIG9823_26589		lncRNA	2	0	0	0	0	0	1	0	0	0	0	0	0	0	0	0	0	0	0
BGIR9823_101793	BGIG9823_26473		lncRNA	2	0	0	0	0	0	0	0	0	0	0	0	1	0	0	0	0	0	0
BGIR9823_102135	BGIG9823_26808		lncRNA	2	0	0	0	0	0	0	0	0	0	0	0	0	0	0	0	0	0	0
BGIR9823_101911	BGIG9823_26588		lncRNA	2	0	0	0	0	0	0	0	0	0	0	0	0	0	0	0	0	0	0

RNA ID	Gene ID	Symbol	Type	target_total	ssc-miR-29a-3p	novel-ssc-miR204-5p	novel-ssc-miR480-3p	novel-ssc-miR537-5p	novel-ssc-miR620-3p	novel-ssc-miR627-5p	novel-ssc-miR671-3p	novel-ssc-miR753-5p	novel-ssc-miR832-3p	novel-ssc-miR1287-5p	novel-ssc-miR1406-3p	ssc-miR-10383	novel-ssc-miR2-3p	ssc-miR-660	novel-ssc-miR9-3p	novel-ssc-miR16-3p	novel-ssc-miR710-5p	novel-ssc-miR802-3p
BGIR9823_101864	BGIG9823_26543		lncRNA	2	0	0	0	0	0	1	0	0	0	0	0	0	0	0	0	0	0	0
BGIR9823_101851	BGIG9823_26530		lncRNA	2	0	0	1	0	0	0	0	0	0	0	0	0	0	0	0	0	0	0
BGIR9823_102310	BGIG9823_26975		lncRNA	2	0	0	0	0	0	0	0	0	0	0	0	0	0	0	0	0	0	0
BGIR9823_101776	BGIG9823_26456		lncRNA	2	0	0	1	0	0	0	0	0	0	0	0	1	0	0	0	0	0	0
BGIR9823_102381	BGIG9823_27046		lncRNA	2	0	0	0	0	0	0	0	0	0	0	0	0	0	0	0	0	0	0
BGIR9823_101680	BGIG9823_26367		lncRNA	2	0	0	0	0	0	0	0	0	0	0	0	0	0	0	0	0	0	0
BGIR9823_101747	BGIG9823_26428		lncRNA	2	0	0	0	0	0	0	0	0	0	0	0	0	0	0	0	0	0	0
BGIR9823_101830	BGIG9823_26509		lncRNA	2	0	0	0	0	0	0	0	0	0	0	0	0	0	0	0	0	0	0
BGIR9823_102332	BGIG9823_26997		lncRNA	2	0	0	0	0	0	0	0	0	0	0	0	0	0	0	0	0	0	0
XR_002336732.1	102159417	LOC102159417	lncRNA	2	0	0	0	0	0	0	0	0	0	0	0	0	0	0	0	0	0	0
XR_002343571.1	106507458	LOC106507458	lncRNA	2	0	0	0	0	0	0	0	0	0	0	0	0	0	0	1	0	0	0
XR_001304304.2	106507921	LOC106507921	lncRNA	2	0	0	0	0	0	0	0	0	0	0	0	0	0	0	0	0	0	0
XR_002344048.1	110260709	LOC110260709	lncRNA	2	0	0	0	0	0	0	0	0	0	0	0	0	0	0	0	0	0	0
XM_003353597.4	100621832	TMEM246	mRNA	2	0	0	0	0	0	0	0	0	0	0	0	0	0	0	1	0	0	0
XR_002338514.1	102161206	LOC102161206	lncRNA	2	0	0	0	0	0	0	0	0	0	0	0	0	0	0	0	0	0	0
NM_001244791.1	100513225	BAG1	mRNA	2	0	0	0	0	0	0	0	0	0	0	0	0	0	0	1	0	0	0
XR_002341355.1	102167380	CUNH21orf58	lncRNA	2	0	0	0	0	0	0	0	0	0	0	0	0	0	0	0	0	0	0
XR_001299235.2	106505126	LOC106505126	lncRNA	2	0	0	0	0	0	0	0	0	0	0	0	0	0	0	0	0	0	0
XR_304631.3	102167686	LOC102167686	lncRNA	2	0	0	0	0	0	0	0	0	0	0	0	0	0	0	0	0	0	0
XM_021066717.1	100521576	ARSG	mRNA	2	0	0	0	0	0	0	0	0	0	0	0	0	0	0	1	0	0	0
XR_002342261.1	110259855	LOC110259855	lncRNA	2	0	0	0	0	0	0	0	0	0	0	0	0	0	0	0	0	0	0
NM_213891.1	396925	VCAM1	mRNA	2	0	0	0	0	0	0	1	0	0	0	0	0	0	0	0	1	0	0
XM_003129787.4	100516505	VSTM5	mRNA	2	0	0	0	0	0	0	0	0	0	0	0	0	0	0	1	0	0	0
XM_021100543.1	106504722	UCP1	mRNA	2	0	0	0	0	0	0	0	0	0	0	0	0	0	0	1	0	0	0
XR_002345110.1	110261202	LOC110261202	lncRNA	2	0	0	0	0	0	0	0	0	0	0	0	0	0	0	1	0	0	0
XM_013992908.2	102164011	LOC102164011	mRNA	2	0	0	0	0	0	0	0	0	0	0	0	0	0	0	1	0	0	0
XM_021082022.1	100525982	LOC100525982	mRNA	2	0	0	0	0	1	0	0	0	0	0	0	0	0	0	0	0	0	0
NM_214188.1	397385	DDOST	mRNA	2	0	0	0	0	0	0	0	0	0	0	0	0	0	0	1	0	0	0
XM_013983344.2	100156215	MSS51	mRNA	2	0	0	0	0	0	0	0	0	0	0	0	0	0	0	1	0	0	0
NM_001164856.1	100303723	LCAT	mRNA	2	0	0	0	0	0	0	0	0	0	0	0	0	0	0	1	0	0	0
NM_001278748.1	100520980	CASR	mRNA	2	0	0	0	0	0	0	1	0	0	0	0	0	0	0	0	1	0	0
XR_002342824.1	110260048	LOC110260048	lncRNA	2	0	0	0	0	0	0	0	0	0	0	0	0	0	0	0	0	0	0
XM_003481630.4	100738263	SPRYD4	mRNA	2	0	0	0	0	0	0	1	0	0	0	0	0	0	0	0	1	0	0
XR_002340516.1	102163798	LOC102163798	lncRNA	2	0	0	0	0	0	0	0	0	0	0	0	0	0	0	0	0	0	0
XM_021080156.1	102167889	LOC102167889	mRNA	2	0	0	0	0	0	0	1	0	0	0	0	0	0	0	0	1	0	0
XR_002343904.1	110260647	LOC110260647	lncRNA	2	0	0	0	0	0	0	0	0	0	0	0	0	0	0	0	0	0	0
XM_021082023.1	100510954	LOC100510954	mRNA	2	0	0	0	0	1	0	0	0	0	0	0	0	0	0	0	0	0	0
XR_002340992.1	110258138	LOC110258138	lncRNA	2	0	0	0	0	0	0	0	0	0	0	0	0	0	0	0	0	0	0
XM_003129097.6	100523679	CCNI	mRNA	2	0	0	0	0	0	0	0	0	0	0	0	0	0	0	1	0	0	0
XM_021086388.1	110255213	ARMC5	mRNA	2	0	0	0	0	0	0	0	0	0	0	0	0	0	0	1	0	0	0
XM_003133626.3	100522220	LOC100522220	mRNA	2	0	0	0	0	0	0	0	0	0	0	0	0	0	0	1	0	0	0
XR_002341145.1	110258406	LOC110258406	lncRNA	2	0	0	0	0	0	0	0	0	0	0	0	0	0	0	1	0	0	0
XR_002341292.1	110258990	LOC110258990	lncRNA	2	0	0	0	0	0	0	0	0	0	0	0	0	0	0	1	0	0	0

RNA ID	Gene ID	Symbol	Type	target_ total	ssc-miR-29a-3p	novel-ssc-miR204-5p	novel-ssc-miR480-3p	novel-ssc-miR537-5p	novel-ssc-miR620-3p	novel-ssc-miR627-5p	novel-ssc-miR671-3p	novel-ssc-miR753-5p	novel-ssc-miR832-3p	novel-ssc-miR1287-5p	novel-ssc-miR1406-3p	ssc-miR-10383	novel-ssc-miR2-3p	ssc-miR-660	novel-ssc-miR9-3p	novel-ssc-miR16-3p	novel-ssc-miR710-5p	novel-ssc-miR802-3p
NM_001204772.1	100533540	BRD4	mRNA	2	0	0	0	0	0	0	1	0	0	0	0	0	0	0	0	1	0	0
NM_213763.2	396637	UCHL1	mRNA	2	0	0	0	0	0	0	0	0	0	0	0	0	0	0	1	0	0	0
NM_214050.2	397117	GSTO1	mRNA	2	0	0	0	0	0	0	0	0	0	0	0	0	0	0	1	0	0	0
XR_002337239.1	110256047	LOC110256047	lncRNA	2	0	0	0	0	0	0	0	0	0	0	0	0	0	0	0	0	0	0
XR_002343522.1	110260355	LOC110260355	lncRNA	2	0	0	0	0	0	0	0	0	0	0	0	0	0	0	0	0	0	0
XR_001304268.2	106507904	LOC106507904	lncRNA	2	0	0	0	0	0	0	0	0	0	0	0	0	0	0	1	0	0	0
XR_002343855.1	110260615	LOC110260615	lncRNA	2	0	0	0	0	0	0	0	0	0	0	0	0	0	0	0	0	0	0
XR_002336945.1	110255950	LOC110255950	lncRNA	2	0	0	0	0	0	0	0	0	0	0	0	0	0	0	0	0	0	0
XM_021075808.1	110257099	LOC110257099	mRNA	2	0	0	0	0	0	0	0	0	0	0	0	0	0	0	1	0	0	0
XM_021082410.1	110259094	LOC110259094	mRNA	2	0	0	0	0	0	0	0	0	0	0	0	0	0	0	1	0	0	0
XR_002341290.1	110258990	LOC110258990	lncRNA	2	0	0	0	0	0	0	0	0	0	0	0	0	0	0	0	0	0	0
XM_021100189.1	100152943	ADTRP	mRNA	2	0	0	0	0	0	0	0	0	0	0	0	0	0	0	1	0	0	0
XR_002341433.1	106509301	LOC106509301	lncRNA	2	0	0	0	0	0	0	0	0	0	0	0	0	0	0	0	0	0	0
NM_001244722.1	100512802	PUS3	mRNA	2	0	0	0	0	0	0	0	0	0	0	0	0	0	0	1	0	0	0
XM_021092065.1	100624597	YARS2	mRNA	2	0	0	0	0	0	0	1	0	0	0	0	0	0	0	0	1	0	0
XM_021066519.1	100512084	TBC1D2	mRNA	2	0	0	0	0	0	0	0	0	0	0	0	0	0	0	1	0	0	0
XR_002335835.1	110255476	LOC110255476	lncRNA	2	0	0	0	0	0	0	0	0	0	0	0	0	0	0	0	0	0	0
XR_002335866.1	110255489	LOC110255489	lncRNA	2	0	0	0	0	0	0	0	0	0	0	0	0	0	0	0	0	0	0
XR_002337288.1	106509234	LOC106509234	lncRNA	2	0	0	0	0	0	0	0	0	0	0	0	0	0	0	0	0	0	0
XR_002342643.1	106508312	LOC106508312	lncRNA	2	0	0	0	0	0	0	0	0	0	0	0	0	0	0	0	0	0	0
NM_001097492.1	100038008	CD1E	mRNA	2	0	0	0	0	0	0	0	0	0	0	0	0	0	0	1	0	0	0
XR_002336367.1	106507918	LOC106507918	lncRNA	2	0	0	0	0	0	0	0	0	0	0	0	0	0	1	0	0	0	0
XR_002340833.1	110257824	LOC110257824	lncRNA	2	0	0	0	0	0	0	0	0	0	0	0	0	0	0	1	0	0	0
NM_001004028.1	445513	HAPLN1	mRNA	2	0	0	0	0	0	0	1	0	0	0	0	0	0	0	0	1	0	0
XR_001302352.2	106506794	LOC106506794	lncRNA	2	0	0	0	0	0	0	0	0	0	0	0	0	0	0	1	0	0	0
XR_002336622.1	110255794	LOC110255794	lncRNA	2	0	0	0	0	0	0	0	0	0	0	0	0	0	0	0	0	0	0
XM_021070652.1	110256443	QARS	mRNA	2	0	0	0	0	0	0	0	0	0	0	0	0	0	0	1	0	0	0
XM_001928752.5	100154892	LOC100154892	mRNA	2	0	0	0	0	0	0	0	0	0	0	0	0	0	0	1	0	0	0
NM_001243696.1	100158051	DRAM2	mRNA	2	0	0	0	0	0	0	0	0	0	0	0	0	0	0	1	0	0	0
XR_002342202.1	100512593	LOC100512593	lncRNA	2	0	0	0	0	0	0	0	0	0	0	0	0	0	0	0	0	0	0
XR_002343188.1	110260249	LOC110260249	lncRNA	2	0	0	0	0	0	0	0	0	0	0	0	0	0	0	0	0	0	0
XM_021081808.1	100155561	LOC100155561	mRNA	2	0	0	0	0	1	0	0	0	0	0	0	0	0	0	0	0	0	0
XM_003134082.6	100515063	HMMR	mRNA	2	0	0	0	0	0	0	0	0	0	0	0	0	0	0	1	0	0	0
XR_002341327.1	110259136	LOC110259136	lncRNA	2	0	0	0	0	0	0	0	0	0	0	0	0	0	0	0	0	0	0
XR_002346274.1	110261765	LOC110261765	lncRNA	2	0	0	0	0	0	0	0	0	0	0	0	0	0	0	0	0	0	0
URS0000BC4F80	BGIG9823_26330		lncRNA	2	0	0	0	0	0	0	0	0	0	0	0	0	0	0	0	0	0	0
URS0000BC82B3	BGIG9823_26290		lncRNA	2	0	0	0	0	0	0	0	0	0	0	0	0	0	0	0	0	0	0
BGIR9823_100417	106506781	LOC106506781	lncRNA	2	0	0	0	0	0	0	0	0	0	0	0	0	0	0	0	0	0	1
BGIR9823_78908	100157533	WTAP	mRNA	2	0	0	0	0	0	0	0	0	0	0	0	0	0	0	0	0	0	0
BGIR9823_85709	100156112	ATP6V1E1	lncRNA	2	0	0	0	0	0	0	0	0	0	0	0	0	0	0	0	0	0	0
BGIR9823_88195	100152838	SLC22A23	lncRNA	2	0	0	0	0	0	0	0	0	0	0	0	0	0	0	0	0	0	0
BGIR9823_81246	100174943	IFI30	mRNA	2	0	0	0	0	0	0	0	0	0	0	0	0	0	0	0	0	0	0
BGIR9823_100836	100624032	LOC100624032	lncRNA	2	0	0	0	0	0	0	0	0	0	0	0	0	0	0	0	0	0	0

RNA ID	Gene ID	Symbol	Type	target_total	ssc-miR-29a-3p	novel-ssc-miR204-5p	novel-ssc-miR480-3p	novel-ssc-miR537-5p	novel-ssc-miR620-3p	novel-ssc-miR627-5p	novel-ssc-miR671-3p	novel-ssc-miR753-5p	novel-ssc-miR832-3p	novel-ssc-miR1287-5p	novel-ssc-miR1406-3p	ssc-miR-10383	novel-ssc-miR2-3p	ssc-miR-660	novel-ssc-miR9-3p	novel-ssc-miR16-3p	novel-ssc-miR710-5p	novel-ssc-miR802-3p
BGIR9823_84507	100154147	MAN1A2	lncRNA	2	0	0	0	0	0	0	0	0	0	0	0	0	0	0	0	0	0	0
BGIR9823_78933	100516653	CCDC170	lncRNA	2	0	0	0	0	0	0	0	0	0	0	0	0	0	0	0	0	0	0
BGIR9823_85930	106510465	LOC106510465	mRNA	2	0	0	0	0	0	0	0	0	0	0	0	0	0	0	0	0	0	0
BGIR9823_98132	100519056	ORC4	lncRNA	2	0	0	0	0	0	0	0	0	0	0	0	0	0	0	0	0	0	0
BGIR9823_85554	100624497	DNM1L	mRNA	2	0	0	0	0	0	0	0	0	0	0	0	0	0	0	0	0	0	0
BGIR9823_89252	100153410	STK38	mRNA	2	0	0	0	0	0	0	1	0	0	0	0	0	0	0	0	1	0	0
BGIR9823_100096	100738984	SLC20A2	lncRNA	2	0	0	0	0	0	0	0	1	0	0	0	0	0	0	0	0	0	0
BGIR9823_85945	100521092	WDR59	mRNA	2	0	0	0	0	0	0	0	0	0	0	0	0	0	0	0	0	0	0
BGIR9823_85409	100516908	NCKAP5L	lncRNA	2	0	0	0	0	0	0	0	0	0	0	0	0	0	0	0	0	0	0
BGIR9823_100573	100516888	GLI3	lncRNA	2	0	0	0	0	0	0	0	0	0	0	0	0	0	0	0	0	0	0
BGIR9823_101467	100522195	XRCC3	lncRNA	2	0	0	1	0	0	0	0	0	0	0	0	0	0	0	0	0	0	1
BGIR9823_86462	100738753	RERE	lncRNA	2	0	0	0	0	0	0	0	0	0	0	0	0	0	0	0	0	0	0
BGIR9823_100236	106504144	DLGAP4	lncRNA	2	0	0	0	0	0	0	0	0	1	0	0	0	0	0	0	0	0	0
BGIR9823_100421	100626356	UBE2H	lncRNA	2	0	0	0	0	0	0	0	0	0	0	0	0	0	0	0	0	0	0
BGIR9823_101512	110258473	LOC110258473	lncRNA	2	0	1	0	0	0	0	0	0	0	0	0	0	0	0	0	0	0	0
BGIR9823_100354	102167342	CNPY1	lncRNA	2	0	0	0	0	0	0	0	0	0	0	0	0	0	0	0	0	0	1
BGIR9823_100645	100519065	UBN2	lncRNA	2	0	0	0	0	0	0	0	0	0	0	0	0	0	0	0	0	0	0
BGIR9823_100414	414383	CHCHD3	lncRNA	2	0	0	0	0	0	0	0	0	0	0	0	0	0	0	0	0	0	0
BGIR9823_81026	100523886	SF1	lncRNA	2	0	0	0	1	0	0	0	0	0	0	1	0	0	0	0	0	0	0
BGIR9823_100005	106506050	LOC106506050	lncRNA	2	0	0	0	0	0	0	0	0	1	0	0	0	0	0	0	0	0	0
BGIR9823_81229	100518418	GATAD2A	lncRNA	2	0	0	0	0	0	0	0	0	0	0	0	0	0	0	0	0	0	0
BGIR9823_79698	100154949	CNIH1	mRNA	2	0	0	0	0	0	0	0	0	0	0	0	0	0	0	0	0	0	0
BGIR9823_100531	100625903	SKAP2	lncRNA	2	0	0	0	0	0	0	0	0	0	0	0	0	0	0	0	0	0	0
BGIR9823_81900	110259864	LOC110259864	lncRNA	2	0	0	0	0	0	0	0	0	0	0	0	0	0	1	0	0	0	0
BGIR9823_100425	100626356	UBE2H	lncRNA	2	0	0	0	0	0	0	0	0	0	0	0	0	0	0	0	0	0	0
BGIR9823_82216	100511252	ACTR1B	lncRNA	2	0	0	0	0	0	0	0	0	0	0	0	0	0	0	0	0	0	0
BGIR9823_100156	100519522	NINL	lncRNA	2	0	0	1	0	0	0	0	0	0	0	0	0	0	0	0	0	0	1
BGIR9823_101565	110258600	LOC110258600	lncRNA	2	0	0	0	0	0	0	0	0	0	0	0	0	0	0	0	0	0	0
BGIR9823_100677	106504059	EXOC4	lncRNA	2	0	0	0	0	0	0	0	0	0	0	0	0	0	0	0	0	0	0
BGIR9823_100501	100523875	7-Sep	lncRNA	2	0	0	0	0	0	0	0	0	0	0	0	0	0	0	0	0	0	0
BGIR9823_100856	100154431	ASB11	lncRNA	2	0	0	0	0	0	0	0	0	0	0	0	0	0	0	0	0	0	0
BGIR9823_92258	100738420	ABI1	lncRNA	2	0	0	0	0	0	0	0	0	0	0	0	0	0	0	0	0	0	0
BGIR9823_97570	100158098	RHBDD3	lncRNA	2	0	0	0	0	0	0	0	0	0	0	0	0	0	0	0	0	0	0
BGIR9823_101254	106507023	LOC106507023	lncRNA	2	0	0	0	0	0	0	0	0	0	0	0	0	0	0	0	0	0	0
BGIR9823_100601	102159531	ACTR3B	mRNA	2	0	0	0	0	0	0	0	0	0	0	0	0	0	0	0	0	0	0
BGIR9823_83153	110260004	AHSA2P	mRNA	2	0	0	0	0	0	0	0	0	0	0	0	0	0	0	1	0	0	0
BGIR9823_90913	110255435	LOC110255435	lncRNA	2	1	0	0	0	0	0	0	0	0	0	0	0	0	0	0	0	0	0
BGIR9823_100272	396886	SLPI	lncRNA	2	0	1	0	0	0	0	0	0	0	0	0	0	0	0	0	0	0	0
BGIR9823_77799	100520524	GOPC	lncRNA	2	0	0	0	0	0	0	0	0	0	0	0	0	0	0	0	0	0	0
BGIR9823_100990	100153260	APOOL	lncRNA	2	0	0	0	0	0	0	0	0	0	0	0	1	0	0	0	0	0	0
BGIR9823_79735	110255453	LOC110255453	lncRNA	2	0	0	0	0	0	0	0	0	0	0	0	0	0	0	0	0	0	0
BGIR9823_80994	100519350	DPP3	mRNA	2	0	0	0	0	0	0	0	0	0	0	0	0	0	0	0	0	0	0
BGIR9823_100422	100626356	UBE2H	lncRNA	2	0	0	0	0	0	0	0	0	1	0	0	0	0	0	0	0	0	0

RNA ID	Gene ID	Symbol	Type	target_total	ssc-miR-29a-3p	novel-ssc-miR204-5p	novel-ssc-miR480-3p	novel-ssc-miR537-5p	novel-ssc-miR620-3p	novel-ssc-miR627-5p	novel-ssc-miR671-3p	novel-ssc-miR753-5p	novel-ssc-miR832-3p	novel-ssc-miR1287-5p	novel-ssc-miR1406-3p	ssc-miR-10383	novel-ssc-miR2-3p	ssc-miR-660	novel-ssc-miR9-3p	novel-ssc-miR16-3p	novel-ssc-miR710-5p	novel-ssc-miR802-3p
BGIR9823_95751	100627663	ARHGEF3	lncRNA	2	0	0	0	0	0	0	0	0	0	0	0	0	0	0	0	0	0	0
BGIR9823_78308	100154901	ATP9B	lncRNA	2	0	0	0	0	0	0	0	0	0	0	0	0	0	0	0	0	0	0
BGIR9823_89654	733584	FOXN3	lncRNA	2	0	0	0	0	0	0	0	0	0	0	0	0	0	0	0	0	0	0
BGIR9823_94919	100737976	RBSN	lncRNA	2	0	0	0	0	0	0	0	0	0	0	0	0	0	0	0	0	0	0
BGIR9823_83670	100153676	PCMTD1	lncRNA	2	0	0	0	0	0	0	0	0	0	0	0	0	0	0	0	0	0	0
BGIR9823_100051	100621535	TPD52L2	lncRNA	2	0	0	0	0	0	0	0	0	1	0	0	0	0	0	0	0	0	0
BGIR9823_78866	100512264	NTNG2	lncRNA	2	0	0	0	1	0	0	0	0	0	0	0	0	0	0	0	0	0	0
BGIR9823_100572	100516888	GLI3	lncRNA	2	0	0	0	0	0	0	0	0	0	0	0	0	0	0	0	0	0	0
BGIR9823_89017	100157526	MYLK4	lncRNA	2	1	0	0	0	0	0	0	0	0	0	0	0	0	0	0	0	0	0
BGIR9823_78135	100153621	FAM214A	lncRNA	2	0	0	0	0	0	0	0	0	0	0	0	0	0	0	0	0	0	0
BGIR9823_88205	100157926	RIOK1	lncRNA	2	0	0	0	0	0	0	0	0	0	0	0	0	1	0	0	0	0	0
BGIR9823_100635	102162486	LOC102162486	lncRNA	2	0	0	0	0	0	0	0	0	0	0	0	0	0	0	0	0	0	0
BGIR9823_87926	110261182	LOC110261182	lncRNA	2	0	0	0	0	0	0	0	0	0	0	0	0	0	0	0	0	0	0
BGIR9823_96076	110256340	LOC110256340	lncRNA	2	0	0	0	0	0	0	0	0	0	0	0	0	0	0	0	0	0	0
BGIR9823_92591	100521678	MLLT10	lncRNA	2	0	0	0	0	0	0	0	0	0	0	0	0	0	0	0	0	0	0
BGIR9823_78251	106506784	LOC106506784	lncRNA	2	0	0	0	1	0	0	0	1	0	0	0	0	0	0	0	0	0	0
BGIR9823_100897	100621199	IL1RAPL1	lncRNA	2	0	0	0	0	0	0	0	0	0	0	0	0	0	0	0	0	0	0
BGIR9823_100113	110257459	LOC110257459	lncRNA	2	0	0	0	0	0	0	0	0	0	0	0	0	0	0	0	0	0	0
BGIR9823_100197	106506650	LOC106506650	lncRNA	2	0	0	0	0	0	0	0	0	1	0	0	0	0	0	0	0	0	0
BGIR9823_96929	751859	SIRT1	lncRNA	2	0	0	0	0	0	0	0	0	0	0	0	0	0	0	0	0	0	0
BGIR9823_92783	100156695	INTS6	lncRNA	2	0	0	0	0	0	0	0	0	0	0	0	0	0	0	0	0	0	0
BGIR9823_77974	100516051	SLC17A5	lncRNA	2	0	0	0	0	0	0	0	0	0	0	0	0	0	0	0	0	0	0
BGIR9823_100010	397360	CEBPB	lncRNA	2	0	0	1	0	0	0	0	0	0	0	0	0	0	0	0	0	0	0
BGIR9823_101617	110258914	LOC110258914	lncRNA	2	0	0	0	0	0	0	0	0	0	0	0	0	0	0	0	0	0	0
BGIR9823_100936	100154376	KDM6A	lncRNA	2	0	0	0	0	0	0	0	0	0	0	0	0	0	0	0	0	0	0
BGIR9823_79053	100522049	PTP4A1	lncRNA	2	0	0	0	0	0	0	0	0	0	0	0	0	0	0	0	0	0	0
BGIR9823_80764	100037285	NREP	lncRNA	2	0	0	0	0	0	0	0	0	0	0	0	0	0	0	0	0	0	0
BGIR9823_100812	100516527	LOC100516527	lncRNA	2	0	0	0	0	0	0	0	0	0	0	0	0	0	0	0	0	0	0
BGIR9823_100796	110257536	LOC110257536	lncRNA	2	0	0	0	1	0	0	0	0	0	0	0	0	0	0	0	0	0	0
BGIR9823_100878	102165056	MBTPS2	lncRNA	2	0	0	0	0	0	0	0	0	0	0	0	0	0	0	0	0	0	0
BGIR9823_100734	100512382	KCND2	lncRNA	2	0	0	0	0	0	0	0	0	0	0	0	0	0	0	0	0	0	0
BGIR9823_100726	100512026	ING3	lncRNA	2	0	0	0	0	0	0	0	0	0	0	0	0	0	0	0	0	0	0
BGIR9823_84422	100145887	ARHGEF2	lncRNA	2	0	0	0	0	0	0	0	0	0	0	0	0	0	0	0	0	0	0
BGIR9823_84641	100155627	LRRC8B	mRNA	2	0	0	0	0	0	0	0	0	0	0	0	0	0	0	0	0	0	0
BGIR9823_98254	100152049	PLPP5	mRNA	2	0	0	0	0	0	0	0	0	0	0	0	0	0	0	0	0	0	0
BGIR9823_87713	100517038	PUM1	lncRNA	2	0	0	0	0	0	0	0	0	0	0	0	0	0	0	0	0	0	0
BGIR9823_94908	100462749	ATG7	lncRNA	2	0	0	0	0	0	0	0	0	0	0	0	0	0	0	0	0	0	0
BGIR9823_100511	100738688	HERPUD2	lncRNA	2	0	0	0	0	0	0	0	0	1	0	0	0	0	0	0	0	0	0
BGIR9823_80800	100515883	PRRC1	lncRNA	2	0	0	0	0	0	0	0	0	0	0	0	0	0	0	0	0	0	0
BGIR9823_100550	397163	RAMP3	lncRNA	2	0	0	0	0	0	0	0	0	0	0	0	0	0	0	0	0	0	0
BGIR9823_94492	102167379	LOC102167379	lncRNA	2	0	0	0	0	0	0	0	0	0	0	0	0	0	0	0	0	0	0
XM_003126850.6	100520197	VAT1L	mRNA	2	0	0	0	0	0	0	1	0	0	0	0	0	0	0	0	1	0	0
BGIR9823_81847	100739684	FOXK1	lncRNA	2	0	0	0	0	0	0	0	0	0	0	0	0	0	0	0	0	0	0

RNA ID	Gene ID	Symbol	Type	target_total	ssc-miR-29a-3p	novel-ssc-miR204-5p	novel-ssc-miR480-3p	novel-ssc-miR537-5p	novel-ssc-miR620-3p	novel-ssc-miR627-5p	novel-ssc-miR671-3p	novel-ssc-miR753-5p	novel-ssc-miR832-3p	novel-ssc-miR1287-5p	novel-ssc-miR1406-3p	ssc-miR-10383	novel-ssc-miR2-3p	ssc-miR-660	novel-ssc-miR9-3p	novel-ssc-miR16-3p	novel-ssc-miR710-5p	novel-ssc-miR802-3p
BGIR9823_100328	110257483	ADRM1	lncRNA	2	0	0	0	0	0	0	0	0	0	0	0	1	0	0	0	0	0	0
BGIR9823_101062	100524727	LOC100524727	lncRNA	2	0	0	0	0	0	0	0	0	0	0	0	0	0	0	0	0	1	0
BGIR9823_100412	414383	CHCHD3	lncRNA	2	0	0	0	0	0	0	0	0	1	0	0	0	0	0	0	0	0	0
BGIR9823_90505	397683	IL15	lncRNA	2	0	0	0	0	0	0	0	0	0	0	0	0	0	0	0	0	0	0
BGIR9823_77934	102167874	LOC102167874	lncRNA	2	0	0	0	0	0	0	0	0	0	0	0	0	0	0	0	0	0	0
BGIR9823_100539	100622324	NFE2L3	lncRNA	2	0	0	0	0	0	0	0	0	1	0	0	0	0	0	0	0	0	0
BGIR9823_101533	102158401	LOC102158401	lncRNA	2	0	0	0	0	0	0	0	0	0	0	0	0	0	0	0	0	0	0
BGIR9823_100121	100519631	TASP1	lncRNA	2	0	0	0	0	0	0	0	0	0	0	0	0	0	0	0	0	0	0
BGIR9823_100964	110257651	LOC110257651	lncRNA	2	0	0	0	0	0	0	0	0	0	0	0	0	0	0	0	0	1	0
BGIR9823_100069	100157832	SGCZ	lncRNA	2	0	0	0	0	0	0	0	0	0	0	0	0	0	0	0	0	0	0
BGIR9823_101391	110258158	TNRC6A	mRNA	2	0	0	0	0	0	0	0	0	0	0	0	0	0	0	0	0	0	0
BGIR9823_80729	100526040	PAM	mRNA	2	0	0	0	0	0	0	0	0	0	0	1	0	0	0	0	0	0	0
BGIR9823_100266	100157616	IFT52	lncRNA	2	0	0	0	0	0	0	0	0	1	0	0	0	0	0	0	0	0	0
BGIR9823_100743	100125380	ST7	mRNA	2	0	0	0	0	0	0	0	0	0	0	0	0	0	0	0	0	0	0
BGIR9823_80829	100513723	JADE2	lncRNA	2	0	0	0	0	0	0	0	0	0	0	0	0	0	0	0	0	0	0
BGIR9823_91326	100516387	TSEN15	lncRNA	2	0	0	0	0	0	0	0	0	0	0	0	0	0	0	0	0	0	0
BGIR9823_100827	100519067	CDK13	lncRNA	2	0	1	0	0	0	0	0	0	0	0	0	0	0	0	0	0	0	0
BGIR9823_80869	100521956	CYSTM1	lncRNA	2	0	0	0	0	0	0	0	0	0	0	0	0	0	0	0	0	0	0
BGIR9823_89293	100153071	TRERF1	lncRNA	2	0	0	0	0	0	0	0	0	0	0	0	0	0	0	0	0	0	0
BGIR9823_79511	100152487	TSHZ1	mRNA	2	0	0	0	0	0	0	0	0	0	0	0	0	1	0	0	0	0	0
BGIR9823_92832	106505308	PCDH9	lncRNA	2	0	0	0	0	0	0	0	0	0	0	0	0	0	0	1	0	0	0
BGIR9823_82541	100520835	ITSN2	lncRNA	2	0	0	0	0	0	0	0	0	0	0	0	0	0	0	0	0	0	0
BGIR9823_100525	100515288	CPVL	lncRNA	2	0	0	0	0	0	0	0	0	0	0	0	0	0	0	0	0	0	0
BGIR9823_100563	100516888	GLI3	lncRNA	2	0	0	0	0	0	0	0	0	0	0	0	0	1	0	0	0	0	0
BGIR9823_100515	100623924	FKBP9	lncRNA	2	0	0	0	0	0	0	0	0	0	0	0	1	0	0	0	0	0	0
BGIR9823_101109	100524780	ARHGAP6	lncRNA	2	0	0	0	0	0	0	0	0	0	0	0	0	0	0	0	0	1	0
BGIR9823_95657	100524459	SLC25A20	lncRNA	2	0	0	0	0	0	0	0	0	0	0	0	0	0	0	0	0	0	0
XR_001303159.2	106507279	LOC106507279	lncRNA	2	0	0	0	0	0	0	0	0	0	0	0	0	0	0	0	0	0	0
BGIR9823_82132	106509720	LOC106509720	lncRNA	2	0	0	0	0	0	0	0	0	0	0	0	0	0	0	0	0	0	0
BGIR9823_90639	110261963	LOC110261963	lncRNA	2	0	0	0	0	0	0	0	0	0	0	0	0	0	0	0	0	0	0
BGIR9823_92636	100516289	PFKFB3	lncRNA	2	0	0	0	0	0	0	0	0	0	0	0	0	0	0	0	0	0	0
BGIR9823_79919	100154648	PLPPR1	lncRNA	2	0	0	0	1	0	0	0	0	0	0	0	0	0	0	0	0	0	0
BGIR9823_80860	100521716	MATR3	mRNA	2	0	0	0	0	0	0	0	0	0	0	0	0	0	0	0	0	0	0
BGIR9823_100880	102165056	MBTPS2	lncRNA	2	0	0	0	0	0	0	0	0	0	0	0	0	0	0	0	0	0	0
BGIR9823_101396	100521783	RBBP6	mRNA	2	0	0	0	0	0	0	0	0	0	0	0	0	0	0	0	0	0	0
BGIR9823_101310	102159115	ATP6AP1	lncRNA	2	0	0	0	0	0	0	0	0	0	0	0	0	0	0	0	0	0	0
BGIR9823_85863	100512658	METTL25	mRNA	2	0	0	0	0	0	0	0	0	0	0	0	0	0	0	0	0	0	0
BGIR9823_84834	100516439	C5H12orf10	mRNA	2	0	0	0	0	0	0	0	0	0	0	0	0	0	0	1	0	0	0
BGIR9823_98676	397282	ACVR2A	lncRNA	2	1	0	0	0	0	0	0	0	0	0	0	0	0	0	0	0	0	0
BGIR9823_83976	396677	F3	mRNA	2	0	0	0	0	0	0	0	0	0	0	0	0	0	0	0	0	0	0
BGIR9823_90032	102167943	LOC102167943	lncRNA	2	0	0	0	0	0	0	1	0	0	0	0	0	0	0	0	1	0	0
BGIR9823_100671	106506771	LOC106506771	lncRNA	2	0	0	0	0	0	0	0	0	0	0	0	0	0	0	0	0	0	0
BGIR9823_101108	100524780	ARHGAP6	lncRNA	2	0	0	0	0	0	0	0	0	0	0	0	0	0	0	0	0	0	0

RNA ID	Gene ID	Symbol	Type	target_total	ssc-miR-29a-3p	novel-ssc-miR204-5p	novel-ssc-miR480-3p	novel-ssc-miR537-5p	novel-ssc-miR620-3p	novel-ssc-miR627-5p	novel-ssc-miR671-3p	novel-ssc-miR753-5p	novel-ssc-miR832-3p	novel-ssc-miR1287-5p	novel-ssc-miR1406-3p	ssc-miR-10383	novel-ssc-miR2-3p	ssc-miR-660	novel-ssc-miR9-3p	novel-ssc-miR16-3p	novel-ssc-miR710-5p	novel-ssc-miR802-3p
BGIR9823_80923	100515885	SLC26A2	mRNA	2	0	0	0	0	0	0	0	0	0	0	0	0	0	0	0	0	0	0
BGIR9823_100494	100521052	IMMP2L	lncRNA	2	0	0	0	0	0	0	0	0	0	0	0	0	0	0	0	0	0	0
BGIR9823_101344	110258068	DPH7	lncRNA	2	0	0	0	0	0	0	0	0	0	0	0	0	0	0	0	0	0	0
BGIR9823_100314	100155190	ZFP64	lncRNA	2	0	0	0	0	0	0	0	0	0	0	0	0	0	0	0	0	0	0
BGIR9823_83324	100624063	LOC100624063	lncRNA	2	0	0	0	0	0	0	0	0	0	0	0	0	0	0	0	0	0	0
BGIR9823_100237	110257438	LOC110257438	lncRNA	2	0	0	0	1	0	0	0	0	0	0	0	0	0	0	0	0	0	0
BGIR9823_78253	106506784	LOC106506784	lncRNA	2	0	0	0	0	0	0	0	0	0	0	0	0	0	0	0	0	0	0
BGIR9823_77699	100514202	MTRF1L	lncRNA	2	0	0	0	0	0	0	0	0	0	0	0	0	0	0	0	0	0	0
BGIR9823_100849	733606	TMSB4X	lncRNA	2	0	0	0	0	0	0	0	0	0	0	0	0	0	0	0	0	1	0
BGIR9823_101513	102157759	LOC102157759	lncRNA	2	0	0	0	0	0	0	0	0	0	0	0	0	0	0	0	0	0	0
BGIR9823_100598	403162	INSIG1	lncRNA	2	0	0	0	0	0	0	0	0	1	1	0	0	0	0	0	0	0	0
BGIR9823_80828	100513723	JADE2	lncRNA	2	0	0	0	0	0	0	0	0	0	0	0	0	0	0	0	0	0	0
BGIR9823_80639	100514094	FCHO2	lncRNA	2	0	0	0	0	0	0	0	0	0	0	0	0	0	0	0	0	0	0
BGIR9823_85738	100157209	PRICKLE1	mRNA	2	0	0	0	0	0	0	0	0	0	0	0	0	0	0	0	0	0	0
BGIR9823_91822	100522092	RUNDC3B	lncRNA	2	0	0	0	0	0	0	0	0	0	0	0	0	0	0	0	0	0	0
BGIR9823_101413	110258225	LOC110258225	lncRNA	2	0	1	0	0	0	0	0	0	0	0	0	0	0	0	0	0	1	0
BGIR9823_89187	100153386	SLA-DRB1	lncRNA	2	0	0	0	0	0	0	0	0	0	0	0	0	0	0	0	0	0	0
BGIR9823_91714	100521986	MAPKAPK2	lncRNA	2	0	0	0	0	0	0	0	0	0	0	0	0	0	0	0	0	0	0
BGIR9823_83804	100523784	ADAR	mRNA	2	0	0	0	0	0	0	0	0	0	0	0	0	0	0	0	0	0	0
BGIR9823_100308	100141411	ADNP	lncRNA	2	0	0	0	0	0	0	0	0	1	0	0	0	0	0	0	0	1	0
BGIR9823_83998	100156806	HFM1	mRNA	2	0	0	0	0	0	0	0	0	0	0	0	0	0	0	0	0	0	0
BGIR9823_100710	397301	CALU	lncRNA	2	0	0	0	0	0	0	0	0	0	0	0	0	0	0	0	0	0	0
BGIR9823_101105	100154011	ANOS1	lncRNA	2	0	0	0	0	0	1	0	0	0	0	0	0	0	0	0	0	0	0
BGIR9823_88993	100144590	MEG3	lncRNA	2	0	0	0	0	0	0	0	0	0	0	0	0	0	1	0	0	0	0
BGIR9823_80459	100622538	DNM2	mRNA	2	0	0	0	0	0	0	0	0	0	0	0	0	0	0	0	0	0	0
BGIR9823_101097	397339	F8	lncRNA	2	0	0	0	0	0	1	0	0	0	0	0	0	0	0	0	0	0	0
BGIR9823_100022	100144536	VAPB	mRNA	2	0	0	0	0	0	0	0	0	0	0	0	0	0	0	0	0	0	0
BGIR9823_88787	100153736	EMC4	lncRNA	2	0	0	0	0	0	0	1	0	0	0	0	0	0	0	0	1	0	0
BGIR9823_93229	100518091	PCID2	mRNA	2	0	0	0	0	0	0	1	0	0	0	0	0	0	0	0	1	0	0
BGIR9823_100480	100621338	BMT2	lncRNA	2	0	0	0	0	0	0	0	0	0	0	0	0	0	0	0	0	0	0
BGIR9823_81197	100037286	ARNTL	lncRNA	2	1	0	0	1	0	0	0	0	0	0	0	0	0	0	0	0	0	0
BGIR9823_100187	100513829	SNPH	lncRNA	1	0	0	0	0	0	0	0	0	0	0	0	0	0	0	0	0	0	0
BGIR9823_97762	100512075	ASCC1	mRNA	1	0	0	0	0	0	0	0	0	0	0	0	0	0	0	0	0	0	0
BGIR9823_100489	493185	IFRD1	lncRNA	1	0	0	0	0	0	0	0	0	0	0	0	0	0	0	0	0	0	0
BGIR9823_82778	100523894	POLR3E	mRNA	1	0	0	0	0	0	0	0	0	0	0	0	0	0	0	0	0	0	0
BGIR9823_88002	100524685	ADGRL2	lncRNA	1	0	0	0	0	0	0	0	0	0	0	0	0	0	0	0	0	0	0
BGIR9823_97698	106506010	LOC106506010	lncRNA	1	0	0	0	0	0	0	0	0	0	0	0	0	0	0	0	0	0	0
BGIR9823_79746	100155321	ACER2	lncRNA	1	0	0	0	0	0	0	0	0	0	0	0	0	0	0	0	0	0	0
BGIR9823_79823	100626649	KANK1	lncRNA	1	0	0	0	0	0	0	0	0	0	0	0	0	0	0	0	0	0	0
BGIR9823_87325	100513473	ZNF181	lncRNA	1	0	0	0	0	0	0	0	0	0	0	0	0	0	0	0	0	0	0
BGIR9823_88185	100511204	ZNF643	mRNA	1	0	0	0	0	0	0	0	0	0	0	0	0	0	0	0	0	0	0
BGIR9823_81639	100513341	FBXL17	lncRNA	1	0	0	0	0	0	0	0	0	0	0	0	0	0	0	0	0	0	0
BGIR9823_94281	100516758	CORO6	mRNA	1	0	0	0	0	0	0	0	0	0	0	0	0	0	0	0	0	0	0

RNA ID	Gene ID	Symbol	Type	target_total	ssc-miR-29a-3p	novel-ssc-miR204-5p	novel-ssc-miR480-3p	novel-ssc-miR537-5p	novel-ssc-miR620-3p	novel-ssc-miR627-5p	novel-ssc-miR671-3p	novel-ssc-miR753-5p	novel-ssc-miR832-3p	novel-ssc-miR1287-5p	novel-ssc-miR1406-3p	ssc-miR-10383	novel-ssc-miR2-3p	ssc-miR-660	novel-ssc-miR9-3p	novel-ssc-miR16-3p	novel-ssc-miR710-5p	novel-ssc-miR802-3p
BGIR9823_96391	100621858	CFAP410	mRNA	1	0	0	0	0	0	0	0	0	0	0	0	0	0	0	0	0	0	0
BGIR9823_77814	100736796	ADGRB3	lncRNA	1	0	0	0	0	0	0	0	0	0	0	0	0	0	0	0	0	0	0
BGIR9823_84738	100516801	MRTFA	lncRNA	1	0	0	0	0	0	0	0	0	0	0	0	0	0	0	0	0	0	0
BGIR9823_85081	110260749	LOC110260749	lncRNA	1	0	0	0	0	0	0	0	0	0	0	0	0	0	0	0	0	0	0
BGIR9823_83642	100127143	PDE7A	lncRNA	1	0	0	0	0	0	0	0	0	0	0	0	0	0	1	0	0	0	0
BGIR9823_90693	100526062	MRPS18C	lncRNA	1	0	0	0	0	0	0	0	0	0	0	0	0	0	0	0	0	0	0
BGIR9823_94661	100525609	STAB1	mRNA	1	0	0	0	0	0	0	0	0	0	0	0	0	0	0	0	0	0	0
BGIR9823_88204	100157926	RIOK1	mRNA	1	0	0	0	0	0	0	0	0	0	0	0	0	0	0	0	0	0	0
BGIR9823_100214	100154657	PIGU	mRNA	1	0	0	0	0	0	0	0	0	0	0	0	0	0	0	0	0	0	0
BGIR9823_86368	100624191	LOC100624191	mRNA	1	0	0	0	0	0	0	0	0	0	0	0	0	0	0	0	0	0	0
BGIR9823_98692	100525081	R3HDM1	mRNA	1	0	0	0	0	0	0	0	0	0	0	0	0	0	0	0	0	0	0
BGIR9823_94941	110256296	LOC110256296	lncRNA	1	0	0	0	0	0	0	0	0	0	0	0	0	0	0	0	0	0	0
BGIR9823_93186	100519383	DZIP1	mRNA	1	0	0	0	0	0	0	0	0	0	0	0	0	0	0	0	0	0	0
BGIR9823_101339	100519166	LOC100519166	lncRNA	1	0	0	0	0	0	0	0	0	0	0	0	0	0	0	0	0	0	0
BGIR9823_78696	641344	GNE	lncRNA	1	0	0	0	0	0	0	0	0	0	0	0	0	0	0	0	0	0	0
BGIR9823_90334	100514856	APBB2	mRNA	1	0	0	0	0	0	0	0	0	0	0	0	0	0	0	0	0	0	0
BGIR9823_79588	100155833	DPP8	mRNA	1	0	0	0	0	0	0	0	0	0	0	0	0	0	0	1	0	0	0
BGIR9823_93702	100622172	TAOK1	lncRNA	1	1	0	0	0	0	0	0	0	0	0	0	0	0	0	0	0	0	0
BGIR9823_100788	100622432	CBX3	lncRNA	1	0	0	0	0	0	0	0	0	0	0	0	0	0	0	0	0	0	0
BGIR9823_81760	100520896	ETF1	lncRNA	1	0	0	0	0	0	0	0	0	0	0	0	0	0	0	0	0	0	0
BGIR9823_84563	102167065	LOC102167065	lncRNA	1	0	0	0	0	0	0	0	0	0	0	0	0	0	0	0	0	0	0
BGIR9823_100600	100513894	LOC100513894	lncRNA	1	0	0	0	0	0	0	0	0	0	0	0	0	0	0	0	0	0	0
BGIR9823_77938	100156192	NT5DC1	lncRNA	1	0	0	0	0	0	0	0	0	0	0	0	0	0	0	0	0	0	0
BGIR9823_86946	100515670	ANKRD13C	lncRNA	1	0	0	0	0	0	0	0	0	0	0	0	0	0	0	0	0	0	0
BGIR9823_100385	100515632	KDM7A	lncRNA	1	0	0	0	0	0	0	0	0	1	0	0	0	0	0	0	0	0	0
BGIR9823_101073	100515811	VMA21	lncRNA	1	0	0	0	0	0	0	0	0	0	0	0	0	0	0	0	0	0	0
BGIR9823_79171	791125	FYN	lncRNA	1	0	0	0	0	0	0	0	0	0	0	0	0	0	0	0	0	0	0
BGIR9823_91118	106504956	LOC106504956	lncRNA	1	0	0	0	0	0	0	0	0	0	0	0	0	0	0	0	0	0	0
BGIR9823_86164	102164889	LOC102164889	lncRNA	1	0	0	0	0	0	0	0	0	0	0	0	0	0	0	0	0	0	0
BGIR9823_78738	100153669	TMEM38B	lncRNA	1	0	0	0	0	0	0	0	0	0	0	0	0	0	0	0	0	0	0
BGIR9823_88541	100522555	ABHD17C	lncRNA	1	0	0	0	0	0	0	0	0	0	0	0	0	0	0	0	0	0	0
BGIR9823_100267	100624468	OSER1	lncRNA	1	0	0	0	0	0	0	0	0	1	0	0	0	0	0	0	0	0	0
BGIR9823_100299	100625841	DDX27	lncRNA	1	0	0	0	0	0	0	0	0	0	0	0	0	0	0	0	0	0	0
BGIR9823_78983	100622156	TNFAIP3	lncRNA	1	0	0	0	0	0	0	0	0	0	0	0	0	0	0	0	0	0	0
BGIR9823_79796	574058	CD274	lncRNA	1	0	0	0	0	0	0	0	0	0	0	0	0	0	0	0	0	0	0
BGIR9823_82690	100520239	LRWD1	lncRNA	1	0	0	0	0	0	0	0	0	0	0	0	0	0	0	0	0	0	0
BGIR9823_100392	100415778	ZC3HAV1	lncRNA	1	0	0	0	0	0	0	0	0	0	0	0	0	0	0	0	0	0	0
BGIR9823_79386	102162809	CTDSPL2	lncRNA	1	0	0	0	0	0	0	0	0	0	0	0	0	0	0	0	0	0	0
BGIR9823_87847	100627122	SMCHD1	lncRNA	1	1	0	0	0	0	0	0	0	0	0	0	0	0	0	0	0	0	0
BGIR9823_83136	397040	UGP2	mRNA	1	0	0	0	0	0	0	0	0	0	0	0	0	0	0	0	0	0	0
BGIR9823_89310	100151914	MRPL2	lncRNA	1	0	0	0	0	0	0	0	0	0	0	0	0	0	0	0	0	0	0
BGIR9823_82954	106509726	LOC106509726	lncRNA	1	0	0	0	0	0	0	0	0	0	0	0	0	0	0	0	0	0	0
BGIR9823_100019	100141410	ZNF217	lncRNA	1	0	0	0	0	0	0	0	0	1	0	0	0	0	0	0	0	0	0

RNA ID	Gene ID	Symbol	Type	target_ total	ssc-miR-29a-3p	novel-ssc-miR204-5p	novel-ssc-miR480-3p	novel-ssc-miR537-5p	novel-ssc-miR620-3p	novel-ssc-miR627-5p	novel-ssc-miR671-3p	novel-ssc-miR753-5p	novel-ssc-miR832-3p	novel-ssc-miR1287-5p	novel-ssc-miR1406-3p	ssc-miR-10383	novel-ssc-miR2-3p	ssc-miR-660	novel-ssc-miR9-3p	novel-ssc-miR16-3p	novel-ssc-miR710-5p	novel-ssc-miR802-3p
BGIR9823_101550	110258600	LOC110258600	lncRNA	1	0	0	0	0	0	0	0	0	0	0	0	0	0	0	0	0	0	0
BGIR9823_80641	100515474	UTP15	lncRNA	1	0	0	0	1	0	0	0	0	0	0	0	0	0	0	0	0	0	0
BGIR9823_85022	100518251	KRAS	lncRNA	1	0	0	0	0	0	0	0	0	0	0	0	0	1	0	0	0	0	0
BGIR9823_78496	100512450	TMEM260	lncRNA	1	0	0	0	0	0	0	0	0	0	0	0	0	0	0	0	0	0	0
BGIR9823_100476	100621338	BMT2	lncRNA	1	0	0	0	0	0	0	0	0	1	0	0	0	0	0	0	0	0	0
BGIR9823_102668	BGIG9823_27318		lncRNA	1	0	0	0	0	0	0	0	1	0	0	0	0	0	0	0	0	0	0
BGIR9823_101962	BGIG9823_26639		lncRNA	1	0	0	0	0	0	0	0	0	0	0	0	0	0	0	0	0	0	0
BGIR9823_102175	BGIG9823_26847		lncRNA	1	0	0	0	0	0	0	0	0	0	0	0	0	0	0	0	0	0	0
BGIR9823_101847	BGIG9823_26526		lncRNA	1	0	0	0	0	0	0	0	0	0	0	0	0	0	0	0	0	0	0
BGIR9823_101781	BGIG9823_26461		lncRNA	1	0	0	0	0	0	0	0	0	0	0	0	0	0	0	0	0	0	0
BGIR9823_102445	BGIG9823_27101		lncRNA	1	0	0	0	0	0	0	0	0	0	0	0	0	0	0	0	0	0	0
BGIR9823_101840	BGIG9823_26519		lncRNA	1	0	0	0	0	0	0	0	0	0	0	0	0	0	0	0	0	0	0
BGIR9823_102485	BGIG9823_27139		lncRNA	1	0	0	0	0	0	0	0	0	0	0	0	0	0	0	0	0	0	0
BGIR9823_102195	BGIG9823_26867		lncRNA	1	0	0	0	0	0	0	0	0	0	0	0	0	0	0	0	0	0	0
BGIR9823_101771	BGIG9823_26451		lncRNA	1	0	0	0	0	0	0	0	0	0	0	0	0	0	0	0	0	0	0
BGIR9823_101831	BGIG9823_26510		lncRNA	1	0	0	0	0	0	0	0	0	0	0	0	0	0	0	0	0	0	0
BGIR9823_102544	BGIG9823_27197		lncRNA	1	0	0	0	0	0	0	0	0	0	0	0	0	0	0	0	0	0	0
BGIR9823_102359	BGIG9823_27024		lncRNA	1	0	0	0	0	0	0	0	0	0	0	0	0	0	0	0	0	0	0
BGIR9823_102456	BGIG9823_27110		lncRNA	1	0	0	0	0	0	0	0	1	0	0	0	0	0	0	0	0	0	0
BGIR9823_102657	BGIG9823_27307		lncRNA	1	0	0	0	0	0	0	0	0	0	0	0	0	0	0	0	0	0	0
BGIR9823_102305	BGIG9823_26970		lncRNA	1	0	0	0	0	0	0	0	0	0	0	0	0	0	1	0	0	0	0
BGIR9823_102228	BGIG9823_26900		lncRNA	1	0	0	0	0	0	0	0	0	0	0	0	0	0	0	0	0	0	0
BGIR9823_101778	BGIG9823_26458		lncRNA	1	0	0	0	0	0	0	0	0	0	0	0	0	0	0	0	0	0	0
BGIR9823_102408	BGIG9823_27069		lncRNA	1	0	0	0	0	0	0	0	0	0	0	0	0	0	0	0	0	0	0
BGIR9823_101997	BGIG9823_26673		lncRNA	1	0	0	0	0	0	0	0	0	0	0	0	0	0	0	0	0	0	0
BGIR9823_101886	BGIG9823_26563		lncRNA	1	0	0	0	0	0	0	0	0	0	0	0	0	0	0	0	0	0	0
BGIR9823_101714	BGIG9823_26398		lncRNA	1	0	0	1	0	0	0	0	0	0	0	0	0	0	0	0	0	0	0
BGIR9823_102000	BGIG9823_26676		lncRNA	1	0	0	0	0	0	0	0	0	0	0	0	0	0	0	0	0	0	0
BGIR9823_102540	BGIG9823_27193		lncRNA	1	0	0	0	0	0	0	0	0	0	0	0	0	0	0	0	0	0	0
BGIR9823_101852	BGIG9823_26531		lncRNA	1	0	0	0	0	0	0	0	0	0	0	0	0	0	0	0	0	0	0
BGIR9823_102203	BGIG9823_26875		lncRNA	1	0	0	0	0	0	0	0	1	0	0	0	0	0	0	0	0	0	0
BGIR9823_102422	BGIG9823_27083		lncRNA	1	0	0	0	0	0	0	0	0	0	0	0	0	0	0	0	0	0	0
BGIR9823_101937	BGIG9823_26614		lncRNA	1	0	0	0	0	0	0	0	0	0	0	0	0	0	0	0	0	0	0
BGIR9823_102117	BGIG9823_26791		lncRNA	1	0	0	0	0	0	0	0	0	0	0	0	0	0	0	0	0	0	0
BGIR9823_102005	BGIG9823_26681		lncRNA	1	0	0	0	0	0	0	0	0	0	0	0	0	0	0	0	0	0	0
BGIR9823_102177	BGIG9823_26849		lncRNA	1	0	0	0	0	0	0	0	0	0	0	0	0	0	0	0	0	0	0
BGIR9823_102213	BGIG9823_26885		lncRNA	1	0	0	0	0	0	0	0	0	0	0	0	0	0	0	0	0	0	0
BGIR9823_102067	BGIG9823_26741		lncRNA	1	0	0	0	0	0	0	0	0	0	0	0	0	0	0	0	0	0	0
BGIR9823_102357	BGIG9823_27022		lncRNA	1	0	0	0	0	0	0	0	0	0	0	0	0	0	0	0	0	0	0
BGIR9823_102443	BGIG9823_27099		lncRNA	1	0	0	0	0	0	0	0	0	0	0	0	0	0	0	0	0	0	0
BGIR9823_102395	BGIG9823_27060		lncRNA	1	0	0	0	0	0	0	0	0	0	0	0	0	0	0	0	0	0	0
BGIR9823_102615	BGIG9823_27265		lncRNA	1	0	0	0	0	0	0	0	0	0	0	0	0	0	1	0	0	0	0
BGIR9823_102264	BGIG9823_26932		lncRNA	1	0	0	0	1	0	0	0	0	0	0	0	0	0	0	0	0	0	0

RNA ID	Gene ID	Symbol	Type	target_total	ssc-miR-29a-3p	novel-ssc-miR204-5p	novel-ssc-miR480-3p	novel-ssc-miR537-5p	novel-ssc-miR620-3p	novel-ssc-miR627-5p	novel-ssc-miR671-3p	novel-ssc-miR753-5p	novel-ssc-miR832-3p	novel-ssc-miR1287-5p	novel-ssc-miR1406-3p	ssc-miR-10383	novel-ssc-miR2-3p	ssc-miR-660	novel-ssc-miR9-3p	novel-ssc-miR16-3p	novel-ssc-miR710-5p	novel-ssc-miR802-3p
BGIR9823_102052	BGIG9823_26726		lncRNA	1	0	0	0	0	0	0	0	0	0	0	0	0	0	0	0	0	0	0
BGIR9823_102131	BGIG9823_26804		lncRNA	1	0	0	0	0	0	0	0	0	0	0	0	0	0	0	0	0	0	0
BGIR9823_101987	BGIG9823_26663		lncRNA	1	0	0	0	0	0	0	0	0	0	0	0	0	0	1	0	0	0	0
BGIR9823_102764	BGIG9823_27402		lncRNA	1	0	0	0	0	0	0	0	0	0	0	0	0	0	0	0	0	0	0
BGIR9823_102137	BGIG9823_26810		lncRNA	1	0	0	0	0	0	0	0	0	0	0	0	0	0	0	0	0	0	0
BGIR9823_102168	BGIG9823_26841		lncRNA	1	0	0	0	0	0	0	0	0	0	0	0	0	0	0	0	0	0	0
BGIR9823_101967	BGIG9823_26644		lncRNA	1	0	0	0	0	0	0	0	0	0	1	0	0	0	0	0	0	0	0
BGIR9823_102393	BGIG9823_27058		lncRNA	1	0	0	0	0	0	0	0	1	0	0	0	0	0	0	0	0	0	0
BGIR9823_101927	BGIG9823_26604		lncRNA	1	0	0	0	0	0	0	0	0	0	0	0	0	0	0	0	0	0	0
BGIR9823_102343	BGIG9823_27008		lncRNA	1	0	0	0	0	0	0	0	0	0	0	0	0	0	0	0	0	0	0
BGIR9823_102562	BGIG9823_27212		mRNA	1	0	0	0	0	0	0	0	0	0	0	0	0	0	0	0	0	0	0
BGIR9823_102090	BGIG9823_26764		lncRNA	1	0	0	0	0	0	0	0	0	0	0	0	0	0	0	0	0	0	0
BGIR9823_101742	BGIG9823_26423		lncRNA	1	0	0	0	0	0	0	0	0	0	0	0	1	0	0	0	0	0	0
BGIR9823_102398	BGIG9823_27061		lncRNA	1	0	0	0	0	0	0	0	0	0	0	0	0	0	0	0	0	0	0
BGIR9823_101721	BGIG9823_26405		lncRNA	1	0	0	0	0	0	0	1	0	0	0	0	0	0	0	0	0	0	0
BGIR9823_102545	BGIG9823_27198		lncRNA	1	0	0	0	0	0	0	0	0	0	0	0	0	0	0	0	0	0	0
BGIR9823_102138	BGIG9823_26811		lncRNA	1	0	0	0	0	0	0	0	0	0	0	0	0	0	0	0	0	0	0
BGIR9823_102026	BGIG9823_26701		lncRNA	1	0	0	0	0	0	0	0	0	0	0	0	0	0	0	0	0	0	0
BGIR9823_102057	BGIG9823_26731		lncRNA	1	0	0	0	1	0	0	0	0	0	0	0	0	0	0	0	0	0	0
BGIR9823_101995	BGIG9823_26671		lncRNA	1	0	0	0	0	0	0	0	0	0	0	0	0	0	0	0	0	0	0
BGIR9823_102725	BGIG9823_27371		lncRNA	1	0	0	0	0	0	0	0	0	0	0	0	0	0	0	0	0	0	0
BGIR9823_101717	BGIG9823_26401		lncRNA	1	0	0	0	0	0	0	0	0	0	0	0	0	0	0	0	0	0	0
BGIR9823_102441	BGIG9823_27097		lncRNA	1	0	0	0	1	0	0	0	0	0	0	0	0	0	0	0	0	0	0
BGIR9823_102674	BGIG9823_27324		lncRNA	1	0	0	0	0	0	0	0	0	0	0	0	0	0	0	0	0	0	0
BGIR9823_101843	BGIG9823_26522		lncRNA	1	0	0	0	0	0	0	0	0	0	0	0	0	0	0	0	0	0	0
BGIR9823_102119	BGIG9823_26793		lncRNA	1	0	0	0	0	0	0	0	0	0	0	0	0	0	0	0	0	0	0
BGIR9823_101819	BGIG9823_26499		lncRNA	1	0	0	0	0	0	0	0	0	0	0	0	0	0	0	0	0	0	0
BGIR9823_102034	BGIG9823_26709		lncRNA	1	0	0	0	0	0	0	0	0	0	0	0	0	0	0	0	0	0	0
BGIR9823_102373	BGIG9823_27038		lncRNA	1	0	0	0	0	0	0	0	0	0	0	0	0	0	0	0	0	0	0
BGIR9823_101905	BGIG9823_26582		lncRNA	1	0	0	0	0	0	0	0	0	0	0	0	0	0	0	0	0	0	0
BGIR9823_102629	BGIG9823_27279		lncRNA	1	0	0	0	0	0	0	0	0	0	0	0	0	0	0	0	0	0	0
BGIR9823_102328	BGIG9823_26993		lncRNA	1	0	0	0	0	0	0	0	0	0	0	0	0	0	0	0	0	0	0
BGIR9823_102165	BGIG9823_26838		lncRNA	1	0	0	0	0	0	0	0	0	0	0	0	0	0	0	0	0	0	0
BGIR9823_101792	BGIG9823_26472		lncRNA	1	0	0	0	0	0	0	0	0	0	0	0	0	0	0	0	0	0	0
BGIR9823_101992	BGIG9823_26668		lncRNA	1	0	0	0	0	0	0	0	0	0	0	0	0	0	0	0	0	0	0
BGIR9823_101767	BGIG9823_26447		lncRNA	1	0	0	0	0	0	0	0	0	0	0	0	0	0	0	0	0	0	0
BGIR9823_101956	BGIG9823_26633		lncRNA	1	0	0	0	0	0	0	0	0	0	0	0	0	0	0	0	0	0	0
BGIR9823_102186	BGIG9823_26858		lncRNA	1	0	0	0	0	0	0	0	0	0	0	0	0	0	0	0	0	0	0
BGIR9823_102630	BGIG9823_27280		lncRNA	1	0	0	0	0	0	0	0	0	0	0	0	0	0	0	0	0	0	0
BGIR9823_101685	BGIG9823_26372		lncRNA	1	0	0	0	0	0	0	0	0	0	0	0	0	0	0	0	0	0	0
BGIR9823_101700	BGIG9823_26387		lncRNA	1	0	0	0	0	0	0	0	0	0	0	0	0	0	0	0	0	0	0
BGIR9823_102217	BGIG9823_26889		lncRNA	1	0	0	0	0	0	0	0	0	0	0	0	0	0	0	0	0	0	0
BGIR9823_102093	BGIG9823_26767		lncRNA	1	0	0	0	0	0	0	0	0	0	0	0	0	0	0	0	0	0	0

RNA ID	Gene ID	Symbol	Type	target_total	ssc-miR-29a-3p	novel-ssc-miR204-5p	novel-ssc-miR480-3p	novel-ssc-miR537-5p	novel-ssc-miR620-3p	novel-ssc-miR627-5p	novel-ssc-miR671-3p	novel-ssc-miR753-5p	novel-ssc-miR832-3p	novel-ssc-miR1287-5p	novel-ssc-miR1406-3p	ssc-miR-10383	novel-ssc-miR2-3p	ssc-miR-660	novel-ssc-miR9-3p	novel-ssc-miR16-3p	novel-ssc-miR710-5p	novel-ssc-miR802-3p
BGIR9823_101824	BGIG9823_26503		lncRNA	1	0	0	0	0	0	0	0	0	0	0	0	0	0	0	0	0	0	0
BGIR9823_102083	BGIG9823_26757		lncRNA	1	0	0	0	0	0	0	0	1	0	0	0	0	0	0	0	0	0	0
BGIR9823_102805	BGIG9823_27433		lncRNA	1	0	0	0	0	0	0	0	1	0	0	0	0	0	0	0	0	0	0
BGIR9823_101826	BGIG9823_26505		lncRNA	1	0	0	0	0	0	0	0	0	0	0	0	0	0	0	0	0	0	0
BGIR9823_102136	BGIG9823_26809		lncRNA	1	0	0	0	0	0	0	0	0	0	0	0	0	0	0	0	0	0	0
BGIR9823_101982	BGIG9823_26658		lncRNA	1	0	0	0	0	0	0	0	0	0	0	0	0	0	0	0	0	0	0
BGIR9823_101949	BGIG9823_26626		lncRNA	1	0	0	0	0	0	0	0	0	0	1	0	0	0	0	0	0	0	0
BGIR9823_102478	BGIG9823_27132		lncRNA	1	0	0	0	0	0	0	0	0	0	0	0	0	0	0	0	0	0	0
BGIR9823_101746	BGIG9823_26427		lncRNA	1	0	0	0	0	0	0	0	0	0	0	0	0	0	0	0	0	0	0
BGIR9823_101942	BGIG9823_26619		lncRNA	1	0	0	0	0	0	0	0	0	0	0	0	0	0	0	0	0	0	0
BGIR9823_102059	BGIG9823_26733		lncRNA	1	0	0	0	0	0	0	0	0	0	0	0	0	0	0	0	0	0	0
BGIR9823_102156	BGIG9823_26829		lncRNA	1	0	0	0	1	0	0	0	0	0	0	0	0	0	0	0	0	0	0
BGIR9823_101875	BGIG9823_26554		lncRNA	1	1	0	0	0	0	0	0	0	0	0	0	0	0	0	0	0	0	0
BGIR9823_101893	BGIG9823_26570		lncRNA	1	0	0	1	0	0	0	0	0	0	0	0	0	0	0	0	0	0	0
BGIR9823_101659	BGIG9823_26357		lncRNA	1	0	0	0	0	0	0	0	0	0	0	0	0	0	0	0	0	0	0
BGIR9823_102534	BGIG9823_27187		lncRNA	1	0	0	0	0	0	0	0	0	0	0	0	0	0	0	0	0	0	0
BGIR9823_101719	BGIG9823_26403		lncRNA	1	0	0	0	0	0	0	0	0	0	0	0	0	0	0	0	0	0	0
BGIR9823_102665	BGIG9823_27315		lncRNA	1	0	0	0	0	0	0	0	0	0	0	0	0	0	0	0	0	0	0
BGIR9823_102586	BGIG9823_27236		lncRNA	1	0	0	0	0	0	0	0	0	0	0	0	0	0	0	0	0	0	0
BGIR9823_102799	BGIG9823_27428		lncRNA	1	0	0	0	0	0	0	0	0	0	0	0	0	0	0	0	0	0	0
BGIR9823_101782	BGIG9823_26462		lncRNA	1	0	0	0	0	0	0	0	0	0	0	0	0	0	0	0	0	0	0
BGIR9823_102163	BGIG9823_26836		lncRNA	1	0	0	0	0	0	0	0	0	0	0	0	0	0	0	0	0	0	0
BGIR9823_102623	BGIG9823_27273		lncRNA	1	0	0	0	0	0	0	0	0	0	0	0	0	0	0	0	0	0	0
BGIR9823_102550	BGIG9823_27201		lncRNA	1	0	0	0	0	0	0	0	0	0	0	0	0	0	0	0	0	0	0
BGIR9823_101952	BGIG9823_26629		lncRNA	1	0	0	0	1	0	0	0	0	0	0	0	0	0	0	0	0	0	0
BGIR9823_102084	BGIG9823_26758		lncRNA	1	0	0	0	0	0	0	0	0	0	0	0	0	0	0	0	0	0	0
BGIR9823_102746	BGIG9823_27389		lncRNA	1	0	0	0	0	0	0	0	0	0	0	0	0	0	1	0	0	0	0
BGIR9823_102196	BGIG9823_26868		lncRNA	1	0	0	0	0	0	0	0	0	0	0	0	0	0	0	0	0	0	0
BGIR9823_101791	BGIG9823_26471		lncRNA	1	0	0	0	0	0	0	0	0	0	0	0	0	0	0	0	0	0	0
BGIR9823_101707	BGIG9823_26391		lncRNA	1	0	0	0	0	0	0	0	0	0	0	0	0	0	0	0	0	0	0
BGIR9823_102788	BGIG9823_27419		lncRNA	1	0	0	0	0	0	0	0	0	0	0	0	0	0	0	0	0	0	0
BGIR9823_102349	BGIG9823_27014		lncRNA	1	1	0	0	0	0	0	0	0	0	0	0	0	0	0	0	0	0	0
BGIR9823_102713	BGIG9823_27363		lncRNA	1	0	0	0	0	0	0	0	0	0	0	0	0	0	0	0	0	0	0
BGIR9823_101686	BGIG9823_26373		lncRNA	1	0	0	0	0	0	0	0	0	0	0	0	1	0	0	0	0	0	0
BGIR9823_102210	BGIG9823_26882		lncRNA	1	0	0	0	0	0	0	0	0	0	0	0	0	0	0	0	0	0	0
BGIR9823_102323	BGIG9823_26988		lncRNA	1	0	0	0	0	0	0	0	0	0	0	0	0	0	0	0	0	0	0
BGIR9823_102016	BGIG9823_26692		mRNA	1	0	0	0	0	0	0	0	0	0	0	0	0	0	0	0	0	0	0
BGIR9823_102051	BGIG9823_26725		lncRNA	1	0	0	0	0	0	0	0	0	0	0	0	0	0	0	0	0	0	0
BGIR9823_101891	BGIG9823_26568		lncRNA	1	0	0	0	0	0	0	0	0	0	0	0	0	0	0	0	0	0	0
BGIR9823_102043	BGIG9823_26717		lncRNA	1	0	0	0	0	0	0	0	0	0	0	0	0	0	0	0	0	0	0
BGIR9823_102464	BGIG9823_27118		lncRNA	1	0	0	0	0	0	0	0	0	0	0	0	0	0	0	0	0	0	0
BGIR9823_102384	BGIG9823_27049		lncRNA	1	0	0	0	0	0	0	0	0	0	0	0	0	0	0	0	0	0	0
BGIR9823_102347	BGIG9823_27012		lncRNA	1	0	0	0	0	0	0	0	0	0	0	0	0	0	1	0	0	0	0

RNA ID	Gene ID	Symbol	Type	target_total	ssc-miR-29a-3p	novel-ssc-miR204-5p	novel-ssc-miR480-3p	novel-ssc-miR537-5p	novel-ssc-miR620-3p	novel-ssc-miR627-5p	novel-ssc-miR671-3p	novel-ssc-miR753-5p	novel-ssc-miR832-3p	novel-ssc-miR1287-5p	novel-ssc-miR1406-3p	ssc-miR-10383	novel-ssc-miR2-3p	ssc-miR-660	novel-ssc-miR9-3p	novel-ssc-miR16-3p	novel-ssc-miR710-5p	novel-ssc-miR802-3p
BGIR9823_101855	BGIG9823_26534		lncRNA	1	0	0	0	0	0	0	0	0	0	0	0	0	0	0	0	0	0	0
BGIR9823_102597	BGIG9823_27247		lncRNA	1	0	0	0	0	0	0	0	0	0	0	0	0	0	0	0	0	0	0
BGIR9823_101902	BGIG9823_26579		lncRNA	1	0	0	0	0	0	0	0	0	0	0	0	0	0	0	0	0	0	0
BGIR9823_102413	BGIG9823_27074		mRNA	1	0	0	0	0	0	0	0	0	0	0	0	0	0	0	0	0	0	0
BGIR9823_102726	BGIG9823_27372		lncRNA	1	0	0	0	0	0	0	0	0	0	0	0	0	0	0	0	0	0	0
BGIR9823_102647	BGIG9823_27297		lncRNA	1	0	0	0	0	0	0	0	0	0	0	0	0	0	0	0	0	0	0
BGIR9823_101973	BGIG9823_26650		lncRNA	1	0	0	0	0	0	0	0	0	0	0	0	0	0	0	0	0	0	0
BGIR9823_102297	BGIG9823_26963		lncRNA	1	0	0	0	0	0	0	0	0	0	0	0	0	0	0	0	0	0	0
BGIR9823_102389	BGIG9823_27054		lncRNA	1	0	0	0	0	0	0	0	0	0	0	0	0	0	0	0	0	0	0
BGIR9823_101788	BGIG9823_26468		lncRNA	1	0	0	0	0	0	0	0	0	0	0	0	0	0	0	0	0	0	0
BGIR9823_101961	BGIG9823_26638		lncRNA	1	0	0	0	0	0	0	0	0	0	0	0	0	0	0	0	0	0	0
BGIR9823_102423	BGIG9823_27084		lncRNA	1	0	0	0	0	0	0	0	0	0	0	0	0	0	0	0	0	0	0
BGIR9823_102712	BGIG9823_27362		lncRNA	1	0	0	0	0	0	0	0	0	0	0	0	0	0	0	0	0	0	0
BGIR9823_101682	BGIG9823_26369		lncRNA	1	0	0	0	0	0	0	0	0	0	0	0	0	0	0	0	0	0	0
BGIR9823_101865	BGIG9823_26544		lncRNA	1	0	0	0	0	0	0	0	0	0	0	0	0	0	0	0	0	0	0
BGIR9823_102351	BGIG9823_27016		lncRNA	1	0	0	0	0	0	0	0	0	0	0	0	0	0	0	0	0	0	0
BGIR9823_102576	BGIG9823_27226		lncRNA	1	0	0	0	0	0	0	0	0	0	0	0	0	0	0	0	0	0	0
BGIR9823_102428	BGIG9823_27088		lncRNA	1	0	0	0	0	0	0	0	0	0	0	0	0	0	0	0	0	0	0
BGIR9823_102329	BGIG9823_26994		lncRNA	1	0	0	0	0	0	0	0	0	0	0	0	0	0	0	0	0	0	0
BGIR9823_102625	BGIG9823_27275		lncRNA	1	0	0	0	1	0	0	0	0	0	0	0	0	0	0	0	0	0	0
BGIR9823_102075	BGIG9823_26749		lncRNA	1	0	0	0	0	0	0	0	0	0	0	0	0	0	0	0	0	0	0
BGIR9823_102523	BGIG9823_27176		lncRNA	1	0	0	0	0	0	0	0	1	0	0	0	0	0	0	0	0	0	0
BGIR9823_101910	BGIG9823_26587		lncRNA	1	0	0	0	0	0	0	0	0	0	0	0	0	0	0	0	0	0	0
BGIR9823_102748	BGIG9823_27391		lncRNA	1	0	0	0	0	0	0	0	1	0	0	0	0	0	0	0	0	0	0
BGIR9823_101705	BGIG9823_26389		lncRNA	1	0	1	0	0	0	0	0	0	0	0	0	0	0	0	0	0	0	0
BGIR9823_102467	BGIG9823_27121		lncRNA	1	0	0	0	0	0	0	0	0	0	0	0	0	0	0	0	0	0	0
BGIR9823_102501	BGIG9823_27155		lncRNA	1	0	0	0	0	0	0	0	0	0	0	0	0	0	0	0	0	0	0
BGIR9823_101779	BGIG9823_26459		lncRNA	1	0	0	0	0	0	0	0	0	0	0	0	0	0	0	0	0	0	0
BGIR9823_102431	BGIG9823_27090		lncRNA	1	0	0	0	0	0	0	0	0	0	0	0	0	0	0	0	0	0	0
BGIR9823_102628	BGIG9823_27278		lncRNA	1	0	0	0	0	0	0	0	1	0	0	0	0	0	0	0	0	0	0
BGIR9823_101816	BGIG9823_26496		lncRNA	1	0	0	0	0	0	0	0	0	0	0	0	0	0	0	0	0	0	0
BGIR9823_101996	BGIG9823_26672		lncRNA	1	0	0	0	0	0	0	0	1	0	0	0	0	0	0	0	0	0	0
BGIR9823_102304	BGIG9823_26969		lncRNA	1	0	0	0	0	0	0	0	0	0	0	0	0	0	0	0	0	0	0
BGIR9823_101800	BGIG9823_26480		lncRNA	1	0	0	0	0	0	0	0	0	0	0	0	0	0	0	0	0	0	0
BGIR9823_102273	BGIG9823_26940		lncRNA	1	0	0	0	0	0	0	0	0	0	0	0	0	0	0	0	0	0	0
BGIR9823_102407	BGIG9823_27068		lncRNA	1	0	0	0	1	0	0	0	0	0	0	0	0	0	0	0	0	0	0
BGIR9823_102091	BGIG9823_26765		lncRNA	1	0	0	0	0	0	0	0	0	0	0	0	0	0	0	0	0	0	0
BGIR9823_102167	BGIG9823_26840		lncRNA	1	0	0	0	0	0	0	0	0	0	0	0	0	0	0	0	0	0	0
BGIR9823_102166	BGIG9823_26839		lncRNA	1	0	0	0	0	0	0	0	0	0	0	0	0	0	0	0	0	0	0
BGIR9823_102159	BGIG9823_26832		lncRNA	1	0	0	0	0	0	0	0	0	0	0	0	0	0	0	0	0	0	0
BGIR9823_102041	BGIG9823_26715		lncRNA	1	0	0	0	0	0	0	0	0	0	0	0	0	0	0	0	0	0	0
BGIR9823_102369	BGIG9823_27034		lncRNA	1	0	0	0	0	0	0	0	0	0	0	0	0	0	0	0	0	0	0
BGIR9823_101766	BGIG9823_26446		lncRNA	1	0	0	0	0	0	0	0	0	0	0	0	0	0	0	0	0	0	0

RNA ID	Gene ID	Symbol	Type	target_total	ssc-miR-29a-3p	novel-ssc-miR204-5p	novel-ssc-miR480-3p	novel-ssc-miR537-5p	novel-ssc-miR620-3p	novel-ssc-miR627-5p	novel-ssc-miR671-3p	novel-ssc-miR753-5p	novel-ssc-miR832-3p	novel-ssc-miR1287-5p	novel-ssc-miR1406-3p	ssc-miR-10383	novel-ssc-miR2-3p	ssc-miR-660	novel-ssc-miR9-3p	novel-ssc-miR16-3p	novel-ssc-miR710-5p	novel-ssc-miR802-3p
BGIR9823_102029	BGIG9823_26704		lncRNA	1	0	0	0	0	0	0	0	0	0	0	0	0	0	0	0	0	0	0
BGIR9823_102522	BGIG9823_27175		lncRNA	1	0	0	0	0	0	0	0	0	0	0	0	0	0	0	0	0	0	0
BGIR9823_102783	BGIG9823_27414		lncRNA	1	1	0	0	0	0	0	0	0	0	0	0	0	0	0	0	0	0	0
BGIR9823_101935	BGIG9823_26612		lncRNA	1	0	0	0	0	0	0	0	0	0	0	0	0	0	0	0	0	0	0
BGIR9823_102254	BGIG9823_26922		lncRNA	1	0	0	0	0	0	0	0	0	0	0	0	0	0	0	0	0	0	0
BGIR9823_102184	BGIG9823_26856		lncRNA	1	0	0	0	0	0	0	0	0	0	0	0	0	0	0	0	0	0	0
BGIR9823_102246	BGIG9823_26914		lncRNA	1	0	0	0	0	0	0	0	0	0	0	0	0	0	0	0	0	0	0
BGIR9823_101751	BGIG9823_26432		lncRNA	1	0	0	0	0	0	0	0	0	0	1	0	0	0	0	0	0	0	0
BGIR9823_101673	BGIG9823_26360		lncRNA	1	0	0	0	1	0	0	0	0	0	0	0	0	0	0	0	0	0	0
BGIR9823_101811	BGIG9823_26491		lncRNA	1	0	0	0	0	0	0	0	0	0	0	0	0	0	0	0	0	0	0
BGIR9823_102295	BGIG9823_26961		mRNA	1	0	0	0	0	0	0	0	0	0	0	0	0	0	0	0	0	0	0
BGIR9823_101697	BGIG9823_26384		lncRNA	1	0	0	0	0	0	0	0	0	0	0	0	0	0	0	0	0	0	0
BGIR9823_101725	BGIG9823_26409		lncRNA	1	0	0	0	0	0	0	0	0	0	0	0	0	0	0	0	0	0	0
BGIR9823_102033	BGIG9823_26708		lncRNA	1	0	0	0	0	0	0	0	0	0	0	0	0	0	0	0	0	0	0
BGIR9823_102053	BGIG9823_26727		lncRNA	1	0	0	0	0	0	0	0	0	0	0	0	0	0	0	0	0	0	0
BGIR9823_102015	BGIG9823_26691		lncRNA	1	0	0	0	0	0	0	0	0	0	0	0	0	0	0	0	0	0	0
BGIR9823_102489	BGIG9823_27143		lncRNA	1	0	0	0	0	0	0	0	0	0	0	0	0	0	0	0	0	0	0
BGIR9823_102449	BGIG9823_27104		lncRNA	1	0	0	0	0	0	0	0	0	0	0	0	0	0	0	0	0	0	0
BGIR9823_102214	BGIG9823_26886		lncRNA	1	0	0	0	0	0	0	0	0	0	0	0	0	0	0	0	0	0	0
BGIR9823_101834	BGIG9823_26513		lncRNA	1	0	0	0	0	0	0	0	0	0	0	0	0	0	0	0	0	0	0
BGIR9823_102636	BGIG9823_27286		lncRNA	1	0	0	0	0	0	0	0	0	0	0	0	0	0	0	0	0	0	0
BGIR9823_102287	BGIG9823_26954		lncRNA	1	0	0	0	0	0	0	0	0	0	0	0	0	0	0	0	0	0	0
BGIR9823_101906	BGIG9823_26583		lncRNA	1	0	0	0	0	0	0	0	0	0	0	0	0	0	0	0	0	0	0
BGIR9823_102514	BGIG9823_27168		lncRNA	1	0	0	0	0	0	0	0	0	0	0	0	0	0	0	0	0	0	0
BGIR9823_101914	BGIG9823_26591		lncRNA	1	0	0	0	0	0	0	0	0	0	0	0	0	0	0	0	0	0	0
BGIR9823_101768	BGIG9823_26448		lncRNA	1	0	0	0	0	0	0	0	0	0	1	0	0	0	0	0	0	0	0
BGIR9823_102070	BGIG9823_26744		lncRNA	1	0	0	0	0	0	0	0	0	0	0	0	0	0	0	0	0	0	0
BGIR9823_101805	BGIG9823_26485		lncRNA	1	0	0	0	0	0	0	0	0	0	0	0	0	0	0	0	0	0	0
BGIR9823_102103	BGIG9823_26777		lncRNA	1	0	0	0	0	0	0	0	0	0	0	0	0	0	0	0	0	0	0
BGIR9823_102688	BGIG9823_27338		lncRNA	1	0	0	0	0	0	0	0	0	0	0	0	0	0	0	0	0	0	0
BGIR9823_102733	BGIG9823_27379		lncRNA	1	0	0	0	0	0	0	0	0	0	0	0	0	0	0	0	0	0	0
BGIR9823_102411	BGIG9823_27072		lncRNA	1	0	0	0	0	0	0	0	0	0	0	0	0	0	0	0	0	0	0
BGIR9823_102360	BGIG9823_27025		lncRNA	1	0	0	0	0	0	0	0	0	0	0	0	0	0	0	0	0	0	0
BGIR9823_102558	BGIG9823_27208		lncRNA	1	0	0	0	0	0	0	0	0	0	0	0	0	0	0	0	0	0	0
BGIR9823_101837	BGIG9823_26516		lncRNA	1	0	0	0	0	0	0	0	0	0	0	0	0	0	0	0	0	0	0
BGIR9823_101722	BGIG9823_26406		lncRNA	1	0	0	0	0	0	0	0	0	0	0	0	0	0	0	0	0	0	0
BGIR9823_102750	BGIG9823_27392		lncRNA	1	0	0	0	0	0	0	0	0	0	0	0	0	0	0	0	0	0	0
BGIR9823_102692	BGIG9823_27342		lncRNA	1	0	0	0	0	0	0	0	0	0	0	0	0	0	0	0	0	0	0
BGIR9823_102755	BGIG9823_27395		lncRNA	1	0	0	0	0	0	0	0	0	0	0	0	0	0	0	0	0	0	0
BGIR9823_101957	BGIG9823_26634		lncRNA	1	0	0	0	0	0	0	0	1	0	0	0	0	0	0	0	0	0	0
BGIR9823_102097	BGIG9823_26771		lncRNA	1	0	0	0	0	0	0	0	0	0	0	0	0	0	0	0	0	0	0
BGIR9823_102454	BGIG9823_27108		lncRNA	1	0	0	0	0	0	0	0	1	0	0	0	0	0	0	0	0	0	0
BGIR9823_102019	BGIG9823_26694		lncRNA	1	0	0	0	0	0	0	0	0	0	0	0	0	0	0	0	0	0	0

RNA ID	Gene ID	Symbol	Type	target_total	ssc-miR-29a-3p	novel-ssc-miR204-5p	novel-ssc-miR480-3p	novel-ssc-miR537-5p	novel-ssc-miR620-3p	novel-ssc-miR627-5p	novel-ssc-miR671-3p	novel-ssc-miR753-5p	novel-ssc-miR832-3p	novel-ssc-miR1287-5p	novel-ssc-miR1406-3p	ssc-miR-10383	novel-ssc-miR2-3p	ssc-miR-660	novel-ssc-miR9-3p	novel-ssc-miR16-3p	novel-ssc-miR710-5p	novel-ssc-miR802-3p
BGIR9823_101736	BGIG9823_26417		lncRNA	1	0	0	0	0	0	0	0	0	0	0	0	0	0	0	0	0	0	0
BGIR9823_102662	BGIG9823_27312		lncRNA	1	0	0	0	0	0	0	0	0	0	0	0	0	0	0	0	0	0	0
BGIR9823_102303	BGIG9823_26969		lncRNA	1	0	0	0	0	0	0	0	0	0	0	0	0	0	0	0	0	0	0
BGIR9823_101931	BGIG9823_26608		lncRNA	1	0	0	1	0	0	0	0	0	0	0	0	0	0	0	0	0	0	0
BGIR9823_101842	BGIG9823_26521		lncRNA	1	0	0	0	0	0	0	0	0	0	0	0	0	0	0	0	0	0	0
BGIR9823_101783	BGIG9823_26463		lncRNA	1	0	0	0	0	0	0	0	0	0	0	0	0	0	0	0	0	0	0
BGIR9823_102447	BGIG9823_27102		lncRNA	1	0	0	0	0	0	0	0	0	0	0	0	0	0	0	0	0	0	0
BGIR9823_102114	BGIG9823_26788		lncRNA	1	0	0	0	0	0	0	0	0	0	0	0	0	0	0	0	0	0	0
BGIR9823_101753	BGIG9823_26434		lncRNA	1	0	0	0	0	0	0	0	0	0	0	0	0	0	0	0	0	0	0
BGIR9823_102643	BGIG9823_27293		lncRNA	1	0	0	0	0	0	0	0	1	0	0	0	0	0	0	0	0	0	0
BGIR9823_102327	BGIG9823_26992		lncRNA	1	0	0	0	0	0	0	0	0	0	0	0	0	0	0	0	0	0	0
BGIR9823_102216	BGIG9823_26888		lncRNA	1	0	0	0	1	0	0	0	0	0	0	0	0	0	0	0	0	0	0
BGIR9823_102718	BGIG9823_27365		lncRNA	1	0	0	0	0	0	0	0	1	0	0	0	0	0	0	0	0	0	0
BGIR9823_101709	BGIG9823_26393		lncRNA	1	0	0	0	0	0	0	0	0	0	0	0	0	0	0	0	0	0	0
BGIR9823_102241	BGIG9823_26913		lncRNA	1	0	0	0	0	0	0	0	0	0	0	0	0	0	0	0	0	0	0
BGIR9823_102306	BGIG9823_26971		lncRNA	1	0	0	0	0	0	0	0	0	0	0	0	0	0	0	0	0	0	0
BGIR9823_102486	BGIG9823_27140		lncRNA	1	0	0	0	0	0	0	0	0	0	0	0	0	0	0	0	0	0	0
BGIR9823_101724	BGIG9823_26408		lncRNA	1	0	1	0	0	0	0	0	0	0	0	0	0	0	0	0	0	0	0
BGIR9823_101858	BGIG9823_26537		lncRNA	1	0	0	0	0	0	0	0	0	0	0	0	0	0	0	0	0	0	0
BGIR9823_102483	BGIG9823_27137		lncRNA	1	0	0	0	0	0	0	0	1	0	0	0	0	0	0	0	0	0	0
BGIR9823_102027	BGIG9823_26702		lncRNA	1	0	0	0	0	0	0	0	0	0	0	0	0	0	0	0	0	0	0
BGIR9823_101777	BGIG9823_26457		lncRNA	1	0	0	0	0	0	0	0	0	0	0	0	0	0	0	0	0	0	0
BGIR9823_102154	BGIG9823_26827		lncRNA	1	0	0	0	0	0	0	0	0	0	0	0	0	0	0	0	0	0	0
BGIR9823_102426	BGIG9823_27087		lncRNA	1	0	0	0	0	0	0	0	0	0	0	0	0	0	0	0	0	0	0
BGIR9823_101894	BGIG9823_26571		lncRNA	1	0	0	1	0	0	0	0	0	0	0	0	0	0	0	0	0	0	0
BGIR9823_102009	BGIG9823_26685		lncRNA	1	0	0	0	0	0	0	0	0	0	0	0	0	0	0	0	0	0	0
BGIR9823_102250	BGIG9823_26918		lncRNA	1	0	0	0	0	0	0	0	0	0	0	0	0	0	0	0	0	0	0
BGIR9823_101769	BGIG9823_26449		lncRNA	1	0	0	0	0	0	0	0	0	0	0	0	1	0	0	0	0	0	0
XR_002338373.1	110256615	LOC110256615	lncRNA	1	0	0	0	0	0	0	0	0	0	0	0	0	0	0	0	0	0	0
XM_005655448.3	100519711	ST7L	mRNA	1	0	0	0	0	0	0	0	0	0	0	0	0	0	0	0	0	0	0
XR_001308691.2	106510294	LOC106510294	lncRNA	1	0	0	0	0	0	0	0	0	0	0	0	0	0	0	0	0	0	0
XR_002343519.1	110260353	LOC110260353	lncRNA	1	0	0	0	0	0	0	0	0	0	0	0	0	0	0	0	0	0	0
XR_002336962.1	102162292	LOC102162292	lncRNA	1	0	0	0	0	0	0	0	0	0	0	0	0	0	0	0	0	0	0
XM_021087319.1	100517646	POLE4	mRNA	1	0	0	0	0	0	0	0	0	0	0	0	0	0	0	0	0	0	0
XR_002336095.1	110255550	LOC110255550	lncRNA	1	0	0	0	0	0	0	0	0	0	0	0	0	0	0	0	0	0	0
NM_001244786.1	100513209	CFAP20	mRNA	1	0	0	0	0	0	0	0	0	0	0	0	0	0	0	0	0	0	0
XR_002340787.1	110257799	LOC110257799	lncRNA	1	0	0	0	0	0	0	0	0	0	0	1	0	0	0	0	0	0	0
XM_003358899.4	100623886	CCT8	mRNA	1	0	0	0	0	0	0	0	0	0	0	1	0	0	0	0	0	0	0
XR_002337835.1	110256393	LOC110256393	lncRNA	1	0	0	0	0	0	0	0	0	0	0	0	0	0	0	0	0	0	0
XR_002337635.1	106505647	LOC106505647	lncRNA	1	0	0	0	0	0	0	0	0	0	0	0	0	0	0	0	0	0	0
XR_001308778.2	106510350	LOC106510350	lncRNA	1	0	0	0	0	0	0	0	0	0	0	0	0	0	0	0	0	0	0
XR_002346849.1	110262181	LOC110262181	lncRNA	1	0	0	0	0	0	0	0	0	0	0	0	0	0	0	0	0	0	0
XM_005659957.3	102163937	GPR137C	mRNA	1	0	0	0	0	0	0	0	0	0	0	1	0	0	0	0	0	0	0

RNA ID	Gene ID	Symbol	Type	target_total	ssc-miR-29a-3p	novel-ssc-miR204-5p	novel-ssc-miR480-3p	novel-ssc-miR537-5p	novel-ssc-miR620-3p	novel-ssc-miR627-5p	novel-ssc-miR671-3p	novel-ssc-miR753-5p	novel-ssc-miR832-3p	novel-ssc-miR1287-5p	novel-ssc-miR1406-3p	ssc-miR-10383	novel-ssc-miR2-3p	ssc-miR-660	novel-ssc-miR9-3p	novel-ssc-miR16-3p	novel-ssc-miR710-5p	novel-ssc-miR802-3p
XR_001303641.2	106507572	LOC106507572	lncRNA	1	0	0	0	0	0	0	0	0	0	0	0	0	0	0	0	0	0	0
XR_002344010.1	110260700	LOC110260700	lncRNA	1	0	0	0	0	0	0	0	0	0	0	0	0	0	0	0	0	0	0
XM_003359284.4	100623641	ZWINT	mRNA	1	0	0	0	0	0	0	0	0	0	0	0	0	0	0	0	0	0	0
XM_021066040.1	100233199	PITPNC1	mRNA	1	0	0	0	0	0	0	0	0	0	0	0	0	0	0	0	0	0	0
XM_003482526.2	100738522	LOC100738522	mRNA	1	0	0	0	0	0	0	0	0	0	0	0	0	0	0	0	0	0	0
XR_002345706.1	110261519	LOC110261519	lncRNA	1	0	0	0	0	0	0	1	0	0	0	0	0	0	0	0	0	0	0
XM_021066775.1	100513305	STRADA	mRNA	1	0	0	0	0	0	0	0	0	0	0	0	0	0	0	0	0	0	0
XR_303482.2	102164583	LOC102164583	lncRNA	1	0	0	0	0	0	0	0	0	0	0	0	0	0	0	0	0	0	0
XM_013998720.2	100737585	LOC100737585	mRNA	1	0	0	0	0	0	0	0	0	0	0	0	0	0	0	0	0	0	0
XR_002336556.1	102163487	LOC102163487	lncRNA	1	0	0	0	0	0	0	0	0	0	0	0	0	0	0	0	0	0	0
XR_002337553.1	102167511	SMIM4	lncRNA	1	0	0	0	0	0	0	0	0	0	0	0	0	0	0	0	0	0	0
XM_003483492.4	396595	PRF1	mRNA	1	0	0	0	0	0	0	1	0	0	0	0	0	0	0	0	0	0	0
XM_001926866.5	100155931	LOC100155931	mRNA	1	0	0	0	0	0	0	0	0	0	0	0	0	0	0	0	0	0	0
XR_002341737.1	102165038	LOC102165038	lncRNA	1	0	0	0	0	0	0	0	0	0	0	0	0	0	0	0	0	0	0
XR_002337051.1	110255981	LOC110255981	lncRNA	1	0	0	0	0	0	0	0	0	0	0	0	0	0	0	0	0	0	0
XM_021091413.1	100621517	TTLL1	mRNA	1	0	0	0	0	0	0	0	0	0	0	0	0	0	0	0	0	0	0
XR_002344541.1	110260924	LOC110260924	lncRNA	1	0	0	0	0	0	0	0	0	0	0	0	0	0	0	0	0	0	0
NM_213786.1	396680	NPW	mRNA	1	0	0	0	0	0	0	0	0	0	0	0	0	0	0	0	0	0	0
XM_021089665.1	100738802	BCAN	mRNA	1	0	0	0	0	0	0	0	0	0	0	0	0	0	0	0	0	0	0
XR_002340802.1	110257805	LOC110257805	lncRNA	1	0	0	0	0	0	0	0	0	0	0	0	0	0	0	0	0	0	0
XR_002341733.1	102158524	LOC102158524	lncRNA	1	0	0	0	0	0	0	0	0	0	0	0	0	0	0	0	0	0	0
XR_002345869.1	110261578	LOC110261578	lncRNA	1	0	0	0	0	0	0	0	0	0	0	0	0	0	0	0	0	0	0
XR_002338170.1	102161036	LOC102161036	lncRNA	1	0	0	0	0	0	0	0	0	0	0	0	0	0	0	0	0	0	0
XR_002337152.1	106505099	LOC106505099	lncRNA	1	0	0	0	0	0	0	0	0	0	0	0	0	0	0	0	0	0	0
XM_003131571.5	100512492	ANKRD40	mRNA	1	0	0	0	0	0	0	0	0	0	0	0	0	0	0	0	0	0	0
XR_002337061.1	110255986	LOC110255986	lncRNA	1	0	0	0	0	0	0	0	0	0	0	0	0	0	0	0	0	0	0
XR_002341729.1	110259387	LOC110259387	lncRNA	1	0	0	0	0	0	0	0	0	0	0	0	0	0	0	0	0	0	0
XM_005665516.3	100524028	ZMYND12	mRNA	1	0	0	0	0	0	0	0	0	0	0	1	0	0	0	0	0	0	0
XR_002342030.1	110259546	LOC110259546	lncRNA	1	0	0	0	0	0	0	0	0	0	0	0	0	0	0	0	0	0	0
XR_001304742.2	106508150	LOC106508150	lncRNA	1	0	0	0	0	0	0	0	0	0	0	0	0	0	0	0	0	0	0
NM_214322.1	397591	NPC1	mRNA	1	0	0	0	0	0	0	0	0	0	0	0	0	0	0	0	0	0	0
XR_002339061.1	102158523	LOC102158523	lncRNA	1	0	0	0	0	0	0	0	0	0	0	0	0	0	0	0	0	0	0
XR_002335622.1	110255429	LOC110255429	lncRNA	1	0	0	0	0	0	0	0	0	0	0	0	0	0	0	0	0	0	0
XM_003124446.4	100518241	SUMF2	mRNA	1	0	0	0	0	0	0	0	0	0	0	0	0	0	0	0	0	0	0
XR_002337394.1	110256124	LOC110256124	lncRNA	1	0	0	0	0	0	0	0	0	0	0	0	0	0	1	0	0	0	0
XM_003130089.4	100520582	LOC100520582	mRNA	1	0	0	0	0	0	0	0	0	0	0	0	0	0	0	0	0	0	0
XM_021100295.1	100337668	LCORL	mRNA	1	0	0	0	0	0	0	0	0	0	0	0	0	0	0	0	0	0	0
XR_002347096.1	110262257	LOC110262257	lncRNA	1	0	0	0	0	0	0	0	0	0	0	0	0	0	0	0	0	0	0
NM_214272.1	397512	TGFBR3	mRNA	1	0	0	0	0	0	0	0	0	0	0	0	0	0	0	0	0	0	0
XR_002337890.1	110256420	LOC110256420	lncRNA	1	0	0	0	0	0	0	0	0	0	0	0	0	0	0	0	0	0	0
XR_001302346.2	106506787	LOC106506787	lncRNA	1	0	0	0	0	0	0	0	0	0	0	0	0	0	1	0	0	0	0
XR_002340032.1	102161214	LOC102161214	lncRNA	1	0	0	0	0	0	0	0	0	0	0	1	0	0	0	0	0	0	0
XR_002338731.1	110256774	LOC110256774	lncRNA	1	0	0	0	0	0	0	0	0	0	0	0	0	0	0	0	0	0	0

RNA ID	Gene ID	Symbol	Type	target_total	ssc-miR-29a-3p	novel-ssc-miR204-5p	novel-ssc-miR480-3p	novel-ssc-miR537-5p	novel-ssc-miR620-3p	novel-ssc-miR627-5p	novel-ssc-miR671-3p	novel-ssc-miR753-5p	novel-ssc-miR832-3p	novel-ssc-miR1287-5p	novel-ssc-miR1406-3p	ssc-miR-10383	novel-ssc-miR2-3p	ssc-miR-660	novel-ssc-miR9-3p	novel-ssc-miR16-3p	novel-ssc-miR710-5p	novel-ssc-miR802-3p
XR_002342421.1	110259933	LOC110259933	lncRNA	1	0	0	0	0	0	0	0	0	0	0	0	0	0	0	0	0	0	0
XM_021080420.1	110257790	WDR44	mRNA	1	0	0	0	0	0	0	0	0	0	0	0	0	0	0	0	0	0	0
XM_003128412.6	100514413	GTPBP2	mRNA	1	0	0	0	0	0	0	0	0	0	0	0	0	0	0	0	0	0	0
XR_001308377.2	106510160	LOC106510160	lncRNA	1	0	0	0	0	0	0	0	0	0	0	0	0	0	0	0	0	0	0
XM_021091670.1	100522737	ANKRD33	mRNA	1	0	0	0	0	0	0	0	0	0	0	0	0	0	0	0	0	0	0
XM_003134187.6	100512932	ASAH1	mRNA	1	0	0	0	0	0	0	0	0	0	0	0	0	0	0	0	0	0	0
XR_002335568.1	102159627	LOC102159627	lncRNA	1	0	0	0	0	0	0	0	0	0	0	0	0	0	0	0	0	0	0
XR_002345700.1	110261513	LOC110261513	lncRNA	1	0	0	0	0	0	0	0	0	0	0	0	0	0	0	0	0	0	0
NM_001243805.1	100511029	STARD10	mRNA	1	0	0	0	0	0	0	0	0	0	0	0	0	0	0	0	0	0	0
XM_001927894.5	100153448	HENMT1	mRNA	1	0	0	0	0	0	0	0	0	0	0	0	0	0	0	0	0	0	0
NM_214205.1	397409	IL5	mRNA	1	0	0	0	0	0	0	0	0	0	0	0	0	0	0	0	0	0	0
XR_002344130.1	110260754	LOC110260754	lncRNA	1	0	0	0	0	0	0	0	0	0	0	0	0	0	0	0	0	0	0
XM_021075881.1	100157060	WDR17	mRNA	1	0	0	0	0	0	0	0	0	0	0	0	0	0	0	0	0	0	0
XR_002344639.1	110260981	LOC110260981	lncRNA	1	0	0	0	0	0	0	0	0	0	0	0	0	0	0	0	0	0	0
XM_013985613.2	100525844	SLC35B4	mRNA	1	0	0	0	0	0	0	0	0	0	0	0	0	0	0	0	0	0	0
XR_002342231.1	110259849	LOC110259849	lncRNA	1	0	0	0	0	0	0	0	0	0	0	0	0	0	0	0	0	0	0
NM_001004043.1	445532	CFL1	mRNA	1	0	0	0	0	0	0	0	0	0	0	0	0	0	0	0	0	0	0
XM_003132695.5	100521154	ZBTB11	mRNA	1	0	0	0	0	0	0	1	0	0	0	0	0	0	0	0	0	0	0
XM_005657909.3	100519458	TEX13B	mRNA	1	0	0	0	0	0	0	0	0	0	0	0	0	0	0	0	0	0	0
XM_003129582.2	100517357	LOC100517357	mRNA	1	0	0	0	0	0	0	0	0	0	0	0	0	0	0	0	0	0	0
XR_298408.3	102167235	LOC102167235	lncRNA	1	0	0	0	0	0	0	0	0	0	0	0	0	0	0	0	0	0	0
XR_002336226.1	110255645	LOC110255645	lncRNA	1	0	0	0	0	0	0	0	0	0	0	0	0	0	0	0	0	0	0
XM_021077102.1	100515590	CCDC69	mRNA	1	0	0	0	0	0	0	0	0	0	0	0	0	0	0	0	0	0	0
XR_002336343.1	110255688	LOC110255688	lncRNA	1	0	0	0	0	0	0	0	0	0	0	0	0	0	1	0	0	0	0
XR_001309352.2	106510687	LOC106510687	lncRNA	1	0	0	0	0	0	0	0	0	0	0	0	0	0	0	0	0	0	0
XR_001301955.2	106506567	LOC106506567	lncRNA	1	0	0	0	0	0	0	0	0	0	0	0	0	0	0	0	0	0	0
XR_002338652.1	102167906	LOC102167906	lncRNA	1	0	0	0	0	0	0	0	0	0	0	0	0	0	0	0	0	0	0
XR_002336386.1	106506961	LOC106506961	lncRNA	1	0	0	0	0	0	0	0	0	0	0	0	0	0	0	0	0	0	0
XM_021074218.1	110256875	LOC110256875	mRNA	1	0	0	0	0	0	0	0	0	0	0	0	0	0	0	0	0	0	0
XR_002336193.1	100517050	2-Mar	lncRNA	1	0	0	0	0	0	0	0	0	0	0	0	0	0	0	0	0	0	0
XM_021088735.1	414423	GNRHR2	mRNA	1	0	0	0	0	0	0	0	0	0	0	0	0	0	0	0	0	0	0
NM_001146130.2	100271932	HPSE	mRNA	1	0	0	0	0	0	0	0	0	0	0	0	0	0	0	0	0	0	0
XM_013992909.2	100152206	LOC100152206	mRNA	1	0	0	0	0	0	0	0	0	0	0	0	0	0	0	0	0	0	0
XR_001299351.2	106505189	LOC106505189	lncRNA	1	0	0	0	0	0	0	0	0	0	0	0	0	0	0	0	0	0	0
XM_021090795.1	110260467	LOC110260467	mRNA	1	0	0	0	0	0	0	0	0	0	0	0	0	0	0	0	0	0	0
XR_001308232.2	106510085	LOC106510085	lncRNA	1	0	0	0	0	0	0	0	0	0	0	0	0	0	0	0	0	0	0
XR_002346494.1	110262011	LOC110262011	lncRNA	1	0	0	0	0	0	0	0	0	0	0	0	0	0	0	0	0	0	0
XM_005665059.3	100622416	KLHDC7A	mRNA	1	0	0	0	0	0	0	0	0	0	0	1	0	0	0	0	0	0	0
XR_002345769.1	110261544	LOC110261544	lncRNA	1	0	0	0	0	0	0	0	0	0	0	0	0	0	0	0	0	0	0
XR_002341699.1	110259368	LOC110259368	lncRNA	1	0	0	0	0	0	0	0	0	0	0	0	0	0	0	0	0	0	0
XR_001307130.2	106509521	LOC106509521	lncRNA	1	0	0	0	0	0	0	0	0	0	0	0	0	0	0	0	0	0	0
XR_002342847.1	106509907	LOC106509907	lncRNA	1	0	0	0	0	0	0	0	0	0	0	0	0	1	0	0	0	0	0
XR_001309149.2	106510562	LOC106510562	lncRNA	1	0	0	0	0	0	0	0	0	0	0	0	0	0	0	0	0	0	0

RNA ID	Gene ID	Symbol	Type	target_total	ssc-miR-29a-3p	novel-ssc-miR204-5p	novel-ssc-miR480-3p	novel-ssc-miR537-5p	novel-ssc-miR620-3p	novel-ssc-miR627-5p	novel-ssc-miR671-3p	novel-ssc-miR753-5p	novel-ssc-miR832-3p	novel-ssc-miR1287-5p	novel-ssc-miR1406-3p	ssc-miR-10383	novel-ssc-miR2-3p	ssc-miR-660	novel-ssc-miR9-3p	novel-ssc-miR16-3p	novel-ssc-miR710-5p	novel-ssc-miR802-3p
XM_021096012.1	106507628	ZMPSTE24	mRNA	1	0	0	0	0	0	0	0	0	0	0	0	0	0	0	0	0	0	0
XM_021078386.1	110257500	PCMTD2	mRNA	1	0	0	0	0	0	0	0	0	0	0	0	0	0	0	0	0	0	0
XM_003134291.4	100513828	FOXA2	mRNA	1	0	0	0	0	0	0	0	0	0	0	1	0	0	0	0	0	0	0
XR_002339192.1	106506426	LOC106506426	lncRNA	1	0	0	0	0	0	0	0	0	0	0	1	0	0	0	0	0	0	0
XR_002340657.1	106506896	LOC106506896	lncRNA	1	0	0	0	0	0	0	0	0	0	0	0	0	0	0	0	0	0	0
XR_002344578.1	106507567	LOC106507567	lncRNA	1	0	0	0	0	0	0	0	0	0	0	0	0	0	0	0	0	0	0
XR_002337161.1	110256021	LOC110256021	lncRNA	1	0	0	0	0	0	0	0	0	0	0	0	0	0	0	0	0	0	0
XR_002343165.1	110260239	LOC110260239	lncRNA	1	0	0	0	0	0	0	0	0	0	0	0	0	0	0	0	0	0	0
XR_001306470.2	106509172	LOC106509172	lncRNA	1	0	0	0	0	0	0	0	0	0	0	0	0	0	0	0	0	0	0
XM_021087431.1	100622990	ETAA1	mRNA	1	0	0	0	0	0	0	0	0	0	0	0	0	0	0	0	0	0	0
NM_001243677.1	100157702	P2RY10	mRNA	1	0	0	0	0	0	0	0	0	0	0	0	0	0	0	0	0	0	0
NM_001005726.1	406192	ANXA2	mRNA	1	0	0	0	0	0	0	0	0	0	0	0	0	0	0	0	0	0	0
XR_002338199.1	110256565	LOC110256565	lncRNA	1	0	0	0	0	0	0	0	0	0	0	0	0	0	0	0	0	0	0
XR_002346497.1	110262015	LOC110262015	lncRNA	1	0	0	0	0	0	0	0	0	0	0	0	0	1	0	0	0	0	0
XM_021081451.1	100625375	LOC100625375	mRNA	1	0	0	0	0	0	0	0	0	0	0	0	0	0	0	0	0	0	0
NM_001243852.1	100511525	STT3A	mRNA	1	0	0	0	0	0	0	0	0	0	0	0	0	0	0	0	0	0	0
XM_001927799.7	100154361	SRP54	mRNA	1	0	0	0	0	0	0	0	0	0	0	1	0	0	0	0	0	0	0
XR_002339070.1	110256975	LOC110256975	lncRNA	1	0	0	0	0	0	0	0	0	0	0	0	0	0	0	0	0	0	0
XR_002340950.1	110257979	LOC110257979	lncRNA	1	0	0	0	0	0	0	0	0	0	0	0	0	0	0	0	0	0	0
XM_013990143.2	100155468	LOC100155468	mRNA	1	0	0	0	0	0	0	0	0	0	0	1	0	0	0	0	0	0	0
XM_003129448.1	100516817	LOC100516817	mRNA	1	0	0	0	0	0	0	1	0	0	0	0	0	0	0	0	0	0	0
XR_001301393.2	106506223	LOC106506223	lncRNA	1	0	0	0	0	0	0	0	0	0	0	0	0	0	0	0	0	0	0
XR_002337789.1	102162054	LOC102162054	lncRNA	1	0	0	0	0	0	0	0	0	0	0	0	0	0	0	0	0	0	0
XM_021085070.1	110259585	LOC110259585	mRNA	1	0	0	0	0	0	0	0	0	0	0	0	0	0	0	0	0	0	0
XM_005670449.3	100524281	SCARA5	mRNA	1	0	0	0	0	0	0	0	0	0	0	0	0	0	0	0	0	0	0
XM_021081009.1	110257997	LOC110257997	mRNA	1	0	0	0	0	0	0	0	0	0	0	0	0	0	0	0	0	0	0
XR_002343866.1	102166296	LOC102166296	lncRNA	1	0	0	0	0	0	0	0	0	0	0	0	0	0	0	0	0	0	0
XM_003124181.1	100517754	LOC100517754	mRNA	1	0	0	0	0	0	0	0	0	0	0	0	0	0	0	0	0	0	0
XR_001298301.2	102159863	LOC102159863	lncRNA	1	0	0	0	0	0	0	0	0	0	0	0	0	0	0	0	0	0	0
XM_021080605.1	110257839	LOC110257839	mRNA	1	0	0	0	0	0	0	0	0	0	0	0	0	0	0	0	0	0	0
XM_021072297.1	100519392	RAB36	mRNA	1	0	0	0	0	0	0	0	0	0	0	0	0	0	0	0	0	0	0
XM_005657053.3	100621152	RNF222	mRNA	1	0	0	0	0	1	0	0	0	0	0	0	0	0	0	0	0	0	0
XM_021064469.1	100516823	CNST	mRNA	1	0	0	0	0	0	0	0	0	0	0	0	0	0	0	0	0	0	0
XR_002344959.1	110261129	LOC110261129	lncRNA	1	0	0	0	0	0	0	0	0	0	0	0	0	0	0	0	0	0	0
XR_002336671.1	110255811	LOC110255811	lncRNA	1	0	0	0	0	0	0	0	0	0	0	0	0	0	1	0	0	0	0
XR_002346381.1	110261974	LOC110261974	lncRNA	1	0	0	0	0	0	0	0	0	0	0	0	0	0	0	0	0	0	0
XM_013994471.2	100517025	LOC100517025	mRNA	1	0	0	0	0	0	0	0	0	0	0	0	0	0	0	0	0	0	0
NM_001244142.1	100514123	SRI	mRNA	1	0	0	0	0	0	0	0	0	0	0	0	0	0	0	0	0	0	0
NM_001315615.1	100217382	NMUR2	mRNA	1	0	0	0	0	0	0	1	0	0	0	0	0	0	0	0	0	0	0
XR_002345984.1	110261620	LOC110261620	lncRNA	1	0	0	0	0	0	0	0	0	0	0	0	0	0	0	0	0	0	0
XR_002336754.1	110255855	LOC110255855	lncRNA	1	0	0	0	0	0	0	0	0	0	0	0	0	0	0	0	0	0	0
XR_002346598.1	110262073	LOC110262073	lncRNA	1	0	0	0	0	1	0	0	0	0	0	0	0	0	0	0	0	0	0
XR_002335757.1	110255462	LOC110255462	lncRNA	1	0	0	0	0	0	0	0	0	0	0	0	0	0	0	0	0	0	0

RNA ID	Gene ID	Symbol	Type	target_total	ssc-miR-29a-3p	novel-ssc-miR204-5p	novel-ssc-miR480-3p	novel-ssc-miR537-5p	novel-ssc-miR620-3p	novel-ssc-miR627-5p	novel-ssc-miR671-3p	novel-ssc-miR753-5p	novel-ssc-miR832-3p	novel-ssc-miR1287-5p	novel-ssc-miR1406-3p	ssc-miR-10383	novel-ssc-miR2-3p	ssc-miR-660	novel-ssc-miR9-3p	novel-ssc-miR16-3p	novel-ssc-miR710-5p	novel-ssc-miR802-3p
XR_001304351.2	102167865	LOC102167865	lncRNA	1	0	0	0	0	0	0	0	0	0	0	0	0	0	1	0	0	0	0
NM_001011507.1	494565	LAMP1	mRNA	1	0	0	0	0	0	0	0	0	0	0	0	0	0	0	0	0	0	0
XR_002339091.1	106505784	LOC106505784	lncRNA	1	0	0	0	0	0	0	0	0	0	0	0	0	0	0	0	0	0	0
XR_298324.3	102163156	LOC102163156	lncRNA	1	0	0	0	0	0	0	0	0	0	0	0	0	0	0	0	0	0	0
XM_021099319.1	100626159	RNASE9	mRNA	1	0	0	0	0	0	0	0	0	0	0	0	0	0	0	0	0	0	0
XM_021096183.1	100739254	ANKRD29	mRNA	1	0	0	0	0	0	0	0	0	0	0	0	0	0	0	0	0	0	0
XR_002335919.1	110255507	LOC110255507	lncRNA	1	0	0	0	0	0	0	0	0	0	0	0	0	0	0	0	0	0	0
XR_002340438.1	106506789	LOC106506789	lncRNA	1	0	0	0	0	0	0	0	0	0	0	0	0	0	0	0	0	0	0
XM_021099935.1	100519785	RAD51B	mRNA	1	0	0	0	0	0	0	0	0	0	0	0	0	0	0	0	0	0	0
XR_001305518.2	106508572	LOC106508572	lncRNA	1	1	0	0	0	0	0	0	0	0	0	0	0	0	0	0	0	0	0
XM_013992672.2	100520160	CNR1	mRNA	1	0	0	0	0	0	0	0	0	0	0	0	0	0	0	0	1	0	0
NM_001123164.1	100144479	PNMT	mRNA	1	0	0	0	0	0	0	0	0	0	0	0	0	1	0	0	0	0	0
NM_001244365.1	100153444	UROS	mRNA	1	0	0	0	0	0	0	0	0	0	0	0	0	0	0	0	0	0	0
XM_021092559.1	100157209	PRICKLE1	mRNA	1	0	0	0	0	0	0	0	0	0	0	0	0	0	0	0	0	0	0
XM_013979148.2	100521912	WNT11	mRNA	1	0	0	0	0	0	0	0	0	0	0	1	0	0	0	0	0	0	0
XR_002337909.1	110256426	LOC110256426	lncRNA	1	0	0	0	0	0	0	0	0	0	0	0	0	0	0	0	0	0	0
NM_214280.1	397524	VCP	mRNA	1	0	0	0	0	0	0	0	0	0	0	0	0	0	0	0	0	0	0
XM_005671261.3	100155046	RNLS	mRNA	1	0	0	0	0	0	0	0	0	0	0	0	0	0	0	0	0	0	0
XR_002337573.1	100154005	ACTR8	mRNA	1	0	0	0	0	0	0	0	0	0	0	0	0	0	0	0	0	0	0
NM_001244315.1	100514255	ARL4C	mRNA	1	0	0	0	0	0	0	0	0	0	0	0	0	0	0	0	0	0	0
XR_001300300.2	106505675	LOC106505675	lncRNA	1	0	0	0	0	0	0	0	0	0	0	0	0	0	0	0	0	0	0
XR_002341897.1	110259477	LOC110259477	lncRNA	1	0	0	0	0	0	0	0	0	0	0	0	0	0	0	0	0	0	0
XR_002336723.1	110255842	LOC110255842	lncRNA	1	0	0	0	0	0	0	0	0	0	0	0	0	0	0	0	0	0	0
XM_021096057.1	100737127	RAB31	mRNA	1	0	0	0	0	1	0	0	0	0	0	0	0	0	0	0	0	0	0
XR_001299360.2	106505194	LOC106505194	lncRNA	1	0	0	0	0	0	0	0	0	0	0	0	0	0	0	0	0	0	0
XM_001925846.7	106504094	GTF3C3	mRNA	1	0	0	0	0	0	0	0	0	0	0	0	0	0	0	0	0	0	0
XR_002337419.1	106505263	LOC106505263	lncRNA	1	0	0	0	0	0	0	0	0	0	0	0	0	0	0	0	0	0	0
XR_002346301.1	110261775	LOC110261775	lncRNA	1	0	0	0	0	0	0	0	0	0	0	0	0	0	0	0	0	0	0
XR_002339946.1	100627949	LOC100627949	lncRNA	1	0	0	0	0	0	0	0	0	0	0	0	0	0	0	0	0	0	0
XR_002346180.1	110261712	LOC110261712	lncRNA	1	0	0	0	0	0	0	0	0	0	0	0	0	1	0	0	0	0	0
XR_001297665.2	106504303	LOC106504303	lncRNA	1	0	0	0	0	0	0	0	0	0	0	0	0	0	0	0	0	0	0
XR_001298947.2	106504955	LOC106504955	lncRNA	1	0	0	0	0	0	0	0	0	0	0	1	0	0	0	0	0	0	0
XR_002341739.1	110259401	LOC110259401	lncRNA	1	0	0	0	0	0	0	0	0	0	0	0	0	0	0	0	0	0	0
XR_001307225.2	106509575	LOC106509575	lncRNA	1	0	0	0	0	0	0	0	0	0	0	0	0	0	0	0	0	0	0
XR_002338919.1	110256865	LOC110256865	lncRNA	1	0	0	0	0	0	0	0	0	0	0	0	0	1	0	0	0	0	0
XR_001306376.2	106509109	LOC106509109	lncRNA	1	0	0	0	0	0	0	0	0	0	0	0	0	0	0	0	0	0	0
XR_002338130.1	102158325	LOC102158325	lncRNA	1	0	0	0	0	0	0	0	0	0	0	0	0	0	0	0	0	0	0
XR_002337988.1	106505633	LOC106505633	lncRNA	1	0	0	0	0	0	0	0	0	0	0	0	0	0	0	0	0	0	0
XM_021086158.1	102161487	TMEM225B	mRNA	1	0	0	0	0	0	0	0	0	0	0	0	0	0	0	0	0	0	0
XM_003130212.5	100522092	RUNDC3B	mRNA	1	0	0	0	0	0	0	0	0	0	0	0	0	0	0	0	0	0	0
XM_001924771.6	100156540	RPP30	mRNA	1	0	0	0	0	0	0	0	0	0	0	0	0	0	0	0	0	0	0
XM_001928042.4	100152526	MED6	mRNA	1	0	0	0	0	0	0	0	0	0	0	0	0	0	0	0	0	0	0
XM_021095234.1	100738053	CHD5	mRNA	1	0	0	0	0	0	0	0	0	0	0	0	0	0	0	0	0	0	0

RNA ID	Gene ID	Symbol	Type	target_total	ssc-miR-29a-3p	novel-ssc-miR204-5p	novel-ssc-miR480-3p	novel-ssc-miR537-5p	novel-ssc-miR620-3p	novel-ssc-miR627-5p	novel-ssc-miR671-3p	novel-ssc-miR753-5p	novel-ssc-miR832-3p	novel-ssc-miR1287-5p	novel-ssc-miR1406-3p	ssc-miR-10383	novel-ssc-miR2-3p	ssc-miR-660	novel-ssc-miR9-3p	novel-ssc-miR16-3p	novel-ssc-miR710-5p	novel-ssc-miR802-3p
XM_001928146.2	100152920	LOC100152920	mRNA	1	0	0	0	0	0	0	0	0	0	0	0	0	0	0	0	0	0	0
XM_003129758.5	100526065	SESN3	mRNA	1	0	0	0	0	0	0	0	0	0	0	0	0	0	0	0	0	0	0
XR_002339615.1	110257251	LOC110257251	lncRNA	1	0	0	0	0	0	0	0	0	0	0	0	0	0	0	0	0	0	0
XR_002339546.1	102158929	LOC102158929	lncRNA	1	0	0	0	0	0	0	0	0	0	0	0	0	0	0	0	0	0	0
XR_001308433.2	102165975	LOC102165975	lncRNA	1	0	0	0	0	0	0	0	0	0	0	0	0	0	0	0	0	0	0
XR_002341525.1	110259281	LOC110259281	lncRNA	1	0	0	0	0	0	0	0	0	0	0	0	0	0	0	0	0	0	0
NM_001038002.1	414911	EDNRB	mRNA	1	0	0	0	0	0	0	0	0	0	0	0	0	0	0	0	0	0	0
XR_002338445.1	106505879	LOC106505879	lncRNA	1	0	0	0	0	0	0	0	0	0	0	0	0	0	0	0	0	0	0
XR_002341837.1	110259430	LOC110259430	lncRNA	1	0	0	0	0	0	0	0	0	0	0	0	0	0	0	0	0	0	0
XM_021086652.1	100736832	GRIN2A	mRNA	1	0	0	0	0	0	0	0	0	0	0	0	0	0	0	1	0	0	0
XM_013997106.2	106510154	LOC106510154	mRNA	1	0	0	0	0	0	0	0	0	0	0	0	0	0	0	0	0	0	0
XR_002347051.1	110262233	LOC110262233	lncRNA	1	0	0	0	0	0	0	0	0	0	0	0	0	0	0	0	0	0	0
XR_002335588.1	110255417	LOC110255417	lncRNA	1	0	0	0	0	0	0	0	0	0	0	0	0	0	0	0	0	0	0
XR_001302361.2	106506801	LOC106506801	lncRNA	1	0	0	0	0	0	0	0	0	0	0	0	0	0	0	0	0	0	0
XR_002336628.1	110255797	LOC110255797	lncRNA	1	0	0	0	0	0	0	0	0	0	0	0	0	0	0	0	0	0	0
XM_021099547.1	102158165	TMEM63C	mRNA	1	0	0	0	0	0	0	0	0	0	0	0	0	0	0	0	0	0	0
XR_001307739.2	106509824	LOC106509824	lncRNA	1	0	0	0	0	0	0	0	0	0	0	0	0	0	0	0	0	0	0
XM_021084335.1	102162790	GRM6	mRNA	1	0	0	0	0	0	0	0	0	0	0	0	0	0	0	0	0	0	0
XR_002339516.1	102161653	LOC102161653	lncRNA	1	0	0	0	0	0	0	1	0	0	0	0	0	0	0	0	0	0	0
XR_001299377.2	106505203	LOC106505203	lncRNA	1	0	0	0	0	0	0	0	0	0	0	0	0	0	0	0	0	0	0
XM_003481432.4	100738802	BCAN	mRNA	1	0	0	0	0	0	0	0	0	0	0	0	0	0	0	1	0	0	0
XM_021095297.1	100627651	CTNNBIP1	mRNA	1	0	0	0	0	0	0	0	0	0	0	0	0	0	0	0	0	0	0
XM_003134620.5	100517251	TTC26	mRNA	1	0	0	0	0	0	0	0	0	0	0	0	0	0	0	0	0	0	0
XR_002343515.1	102165594	LOC102165594	lncRNA	1	0	0	0	0	0	0	0	0	0	0	0	0	0	0	0	0	0	0
NM_001044607.2	733679	ECH1	mRNA	1	0	0	0	0	0	0	0	0	0	0	0	0	0	0	0	0	0	0
XR_001300056.2	106505536	LOC106505536	lncRNA	1	0	0	0	0	0	0	0	0	0	0	0	0	0	0	0	0	0	0
XR_001301036.2	106506054	LOC106506054	lncRNA	1	0	0	0	0	0	0	0	0	0	0	0	0	0	0	0	0	0	0
XM_005654040.3	397441	CD86	mRNA	1	0	0	0	0	0	0	0	0	0	0	0	0	0	0	0	0	0	0
XR_001298634.2	102163793	LOC102163793	lncRNA	1	0	0	0	0	0	0	0	0	0	0	0	0	0	0	0	0	0	0
XR_002343053.1	110260210	LOC110260210	lncRNA	1	0	0	0	0	0	0	0	0	0	0	0	0	0	0	0	0	0	0
XM_003129012.4	100517956	TMA16	mRNA	1	0	0	0	0	0	0	0	0	0	0	0	0	0	0	0	0	0	0
XM_021076351.1	100515172	HOXD8	mRNA	1	0	0	0	0	0	0	0	0	0	0	0	0	0	0	0	0	0	0
NM_001001636.1	414419	RPL32	mRNA	1	0	0	0	0	0	0	0	0	0	0	0	0	0	0	0	0	0	0
XR_002336222.1	110255642	LOC110255642	lncRNA	1	1	0	0	0	0	0	0	0	0	0	0	0	0	0	0	0	0	0
XR_002337964.1	110256455	LOC110256455	lncRNA	1	0	0	0	0	0	0	0	0	0	0	0	0	0	0	0	0	0	0
XR_002343225.1	110260259	LOC110260259	lncRNA	1	0	0	0	0	0	0	0	0	0	0	1	0	0	0	0	0	0	0
XR_002340084.1	106506691	LOC106506691	lncRNA	1	0	0	0	0	0	0	0	0	0	0	0	0	0	0	0	0	0	0
XM_001928520.6	100153343	RWDD1	mRNA	1	0	0	0	0	0	0	0	0	0	0	1	0	0	0	0	0	0	0
XM_005652653.3	106504062	NLGN3	mRNA	1	0	0	0	0	0	0	0	0	0	0	0	0	0	0	0	0	0	0
XR_002341746.1	102165717	LOC102165717	lncRNA	1	0	0	0	0	0	0	0	0	0	0	0	0	1	0	0	0	0	0
XM_021085042.1	110259549	LOC110259549	mRNA	1	0	0	0	0	0	0	0	0	0	0	0	0	0	0	0	0	0	0
XR_001305221.2	106508400	LOC106508400	lncRNA	1	0	0	0	0	0	0	0	0	0	0	0	0	0	0	0	0	0	0
XR_002343172.1	110260241	LOC110260241	lncRNA	1	0	0	0	0	0	0	0	0	0	0	0	0	0	0	0	0	0	0

RNA ID	Gene ID	Symbol	Type	target_total	ssc-miR-29a-3p	novel-ssc-miR204-5p	novel-ssc-miR480-3p	novel-ssc-miR537-5p	novel-ssc-miR620-3p	novel-ssc-miR627-5p	novel-ssc-miR671-3p	novel-ssc-miR753-5p	novel-ssc-miR832-3p	novel-ssc-miR1287-5p	novel-ssc-miR1406-3p	ssc-miR-10383	novel-ssc-miR2-3p	ssc-miR-660	novel-ssc-miR9-3p	novel-ssc-miR16-3p	novel-ssc-miR710-5p	novel-ssc-miR802-3p
XR_002339487.1	106506400	COPS7B	lncRNA	1	0	0	0	0	0	0	0	0	0	0	0	0	0	0	0	0	0	0
XR_001308351.2	106510133	LOC106510133	lncRNA	1	0	0	0	0	0	0	0	0	0	0	0	0	0	0	0	0	0	0
XM_003124564.4	100511500	GPR139	mRNA	1	0	0	0	0	0	0	1	0	0	0	0	0	0	0	0	0	0	0
NM_001078686.1	780437	ODAM	mRNA	1	0	0	0	0	0	0	0	0	0	0	0	0	0	0	0	0	0	0
XR_298956.3	102160517	LOC102160517	lncRNA	1	0	0	0	0	0	0	0	0	0	0	1	0	0	0	0	0	0	0
XR_002347080.1	110262247	LOC110262247	lncRNA	1	0	0	0	0	0	0	0	0	0	0	0	0	1	0	0	0	0	0
XM_021094930.1	100514465	LOC100514465	mRNA	1	0	0	0	0	0	0	0	0	0	0	0	0	0	0	0	0	0	0
XR_002343951.1	100737981	EIF4B	lncRNA	1	0	0	0	0	0	0	0	0	0	0	0	0	1	0	0	0	0	0
NM_001244614.1	100153985	SIX4	mRNA	1	0	0	0	0	0	0	0	0	0	0	0	0	0	0	0	0	0	0
XM_021066050.1	100302700	SLC25A19	mRNA	1	0	0	0	0	0	0	0	0	0	0	0	0	0	0	0	0	0	0
XM_005653210.3	100522374	GAS8	mRNA	1	0	0	0	0	0	0	0	0	0	0	0	0	0	0	0	0	0	0
XR_302139.2	102161858	LOC102161858	lncRNA	1	0	0	0	0	0	0	0	0	0	0	0	0	0	0	0	0	0	0
XR_002335527.1	110255394	LOC110255394	lncRNA	1	0	0	0	0	0	0	0	0	0	0	0	0	0	0	0	0	0	0
XR_002336548.1	110255755	LOC110255755	lncRNA	1	0	0	0	0	0	0	0	0	0	0	0	0	0	0	0	0	0	0
XR_001307143.2	100522542	LOC100522542	lncRNA	1	0	0	0	0	0	0	0	0	0	0	0	0	0	0	0	0	0	0
XR_002336864.1	110255895	LOC110255895	lncRNA	1	1	0	0	0	0	0	0	0	0	0	0	0	0	0	0	0	0	0
NM_214125.1	397230	MBL2	mRNA	1	0	0	0	0	0	0	1	0	0	0	0	0	0	0	0	0	0	0
XR_002339158.1	106506349	LOC106506349	lncRNA	1	0	0	0	0	0	0	0	0	0	0	0	0	0	0	0	0	0	0
NM_001244309.1	100625497	EZH2	mRNA	1	0	0	0	0	0	0	0	0	0	0	1	0	0	0	0	0	0	0
XM_021102414.1	100523325	ASB4	mRNA	1	0	0	0	0	0	0	0	0	0	0	0	0	0	0	0	0	0	0
XM_021069845.1	110256370	LOC110256370	mRNA	1	0	0	0	0	0	0	0	0	0	0	0	0	0	0	0	0	0	0
XR_309047.3	100519283	LOC100519283	lncRNA	1	0	0	0	0	0	0	0	0	0	0	0	0	0	0	0	0	0	0
XR_001308371.2	106510151	LOC106510151	lncRNA	1	0	0	0	0	0	0	0	0	0	0	0	0	0	0	0	0	0	0
XR_001303230.2	106507324	LOC106507324	lncRNA	1	0	0	0	0	0	0	0	0	0	0	0	0	1	0	0	0	0	0
XR_002338650.1	102167253	LOC102167253	lncRNA	1	1	0	0	0	0	0	0	0	0	0	0	0	0	0	0	0	0	0
XM_003132532.3	100519624	TMEM212	mRNA	1	0	0	0	0	0	0	0	0	0	0	0	0	0	0	0	0	0	0
XM_021086004.1	110259835	LOC110259835	mRNA	1	0	0	0	0	0	0	0	0	0	0	0	0	0	0	0	0	0	0
XM_005663799.3	396643	SSTR3	mRNA	1	0	0	0	0	0	0	0	0	0	0	0	0	0	0	0	0	0	0
XR_002335939.1	106507874	LOC106507874	lncRNA	1	0	0	0	0	0	0	0	0	0	0	0	0	0	0	0	0	0	0
XM_003130663.6	100522820	KIF24	mRNA	1	0	0	0	0	0	0	0	0	0	0	0	0	0	0	1	0	0	0
XM_003124595.4	100518832	RMI2	mRNA	1	0	0	0	0	0	0	0	0	0	0	1	0	0	0	0	0	0	0
XR_002339295.1	106506428	LOC106506428	lncRNA	1	0	0	0	0	0	0	0	0	0	0	0	0	1	0	0	0	0	0
XM_001925784.6	100155342	USP5	mRNA	1	0	0	0	0	0	0	0	0	0	0	0	0	0	0	0	0	0	0
XR_002346720.1	110262131	LOC110262131	lncRNA	1	0	0	0	0	0	0	0	0	0	0	0	0	0	0	0	0	0	0
XR_002339354.1	110257112	LOC110257112	lncRNA	1	0	0	0	0	0	0	0	0	0	0	0	0	0	0	0	0	0	0
XR_002346965.1	110262211	LOC110262211	lncRNA	1	0	0	0	0	0	0	0	0	0	0	0	0	0	0	0	0	0	0
XR_002335796.1	110255471	LOC110255471	lncRNA	1	0	0	0	0	0	0	0	0	0	0	0	0	0	0	0	0	0	0
XM_021101481.1	102166512	ANXA3	mRNA	1	0	0	0	0	0	0	0	0	0	0	0	0	0	0	0	0	0	0
XM_001926306.7	100153723	ABT1	mRNA	1	0	0	0	0	0	0	0	0	0	0	0	0	0	0	0	0	0	0
XR_002336516.1	102158088	NHLRC3	lncRNA	1	0	0	0	0	0	0	0	0	0	0	0	0	0	0	0	0	0	0
XR_002344446.1	102162270	SLC7A5	lncRNA	1	0	0	0	0	0	0	0	0	0	0	1	0	0	0	0	0	0	0
XR_002345296.1	102166616	LOC102166616	lncRNA	1	0	0	0	0	0	0	0	0	0	0	0	0	0	0	0	0	0	0
NM_001001645.1	414435	WDR83OS	mRNA	1	0	0	0	0	0	0	0	0	0	0	0	0	0	0	0	0	0	0

RNA ID	Gene ID	Symbol	Type	target_total	ssc-miR-29a-3p	novel-ssc-miR204-5p	novel-ssc-miR480-3p	novel-ssc-miR537-5p	novel-ssc-miR620-3p	novel-ssc-miR627-5p	novel-ssc-miR671-3p	novel-ssc-miR753-5p	novel-ssc-miR832-3p	novel-ssc-miR1287-5p	novel-ssc-miR1406-3p	ssc-miR-10383	novel-ssc-miR2-3p	ssc-miR-660	novel-ssc-miR9-3p	novel-ssc-miR16-3p	novel-ssc-miR710-5p	novel-ssc-miR802-3p
XR_002337442.1	110256166	LOC110256166	lncRNA	1	0	0	0	0	0	0	0	0	0	0	0	0	0	0	0	0	0	0
XM_021072047.1	100512874	SCAF8	mRNA	1	0	0	0	0	0	0	0	0	0	0	0	0	0	0	0	0	0	0
XR_001298911.2	106504947	LOC106504947	lncRNA	1	0	0	0	0	0	0	0	0	0	0	0	0	0	1	0	0	0	0
XR_002340842.1	110257831	LOC110257831	lncRNA	1	0	0	0	0	0	0	0	0	0	0	0	0	0	0	0	0	0	0
XM_003122534.4	100512393	KCNK7	mRNA	1	0	0	0	0	0	0	0	0	0	0	0	0	0	0	0	0	0	0
XR_002340251.1	110257517	LOC110257517	lncRNA	1	0	0	0	0	0	0	0	0	0	0	0	0	0	0	0	0	0	0
XR_002338712.1	110256766	LOC110256766	lncRNA	1	0	0	0	0	0	0	0	0	0	0	0	0	0	0	0	0	0	0
XM_003129449.2	100516987	LOC100516987	mRNA	1	0	0	0	0	0	0	1	0	0	0	0	0	0	0	0	0	0	0
XR_001305858.2	106508800	LOC106508800	lncRNA	1	0	0	0	0	0	0	0	0	0	0	0	0	0	0	0	0	0	0
XR_001307955.2	102165812	LOC102165812	lncRNA	1	0	0	0	0	0	0	0	0	0	0	0	0	0	0	0	0	0	0
XR_002345578.1	110261473	LOC110261473	lncRNA	1	0	0	0	0	0	0	0	0	0	0	0	0	0	0	0	0	0	0
XR_002340279.1	110257523	LOC110257523	lncRNA	1	0	0	0	0	1	0	0	0	0	0	0	0	0	0	0	0	0	0
XM_021073688.1	100153473	CCDC188	mRNA	1	0	0	0	0	0	0	0	0	0	0	0	0	0	0	0	0	0	0
XR_002347070.1	100737186	LOC100737186	lncRNA	1	0	0	0	0	0	0	0	0	0	0	0	0	0	0	0	0	0	0
XR_002338524.1	110256665	LOC110256665	lncRNA	1	0	0	0	0	0	0	0	0	0	0	0	0	0	0	0	0	0	0
XR_002338128.1	110256525	LOC110256525	lncRNA	1	0	0	0	0	0	0	0	0	0	0	0	0	0	1	0	0	0	0
XR_002338320.1	102160169	LOC102160169	lncRNA	1	0	0	0	0	0	0	0	0	0	0	0	0	0	0	0	0	0	0
XR_002341185.1	102168138	LOC102168138	lncRNA	1	0	0	0	0	0	0	0	0	0	0	0	0	0	0	0	0	0	0
NM_001243681.2	100626539	RNASEH1	mRNA	1	0	0	0	0	0	0	0	0	0	0	0	0	0	0	0	0	0	0
XM_021087419.1	100516073	APLF	mRNA	1	0	0	0	0	1	0	0	0	0	0	0	0	0	0	0	0	0	0
NM_001244932.1	100514188	SUMF1	mRNA	1	0	0	0	0	0	0	0	0	0	0	0	0	0	0	0	0	0	0
XR_002342760.1	110260029	LOC110260029	lncRNA	1	0	0	0	0	0	0	0	0	0	0	0	0	0	0	0	0	0	0
NM_001130735.1	100170853	SMN1	mRNA	1	0	0	0	0	0	0	0	0	0	0	0	0	0	0	0	0	0	0
XR_002337720.1	106505376	LOC106505376	lncRNA	1	0	0	0	0	0	0	0	0	0	0	1	0	0	0	0	0	0	0
XM_021082523.1	100620803	LOC100620803	mRNA	1	0	0	0	0	0	0	0	0	0	0	0	0	0	0	0	0	0	0
XR_002344603.1	102158585	LOC102158585	lncRNA	1	0	0	0	0	0	0	0	0	0	0	0	0	0	0	0	0	0	0
XR_002340236.1	100513188	LOC100513188	lncRNA	1	0	0	0	0	0	0	0	0	0	0	0	0	1	0	0	0	0	0
XR_002335659.1	100622439	LOC100622439	lncRNA	1	0	0	0	0	0	0	0	0	0	0	0	0	0	1	0	0	0	0
XM_003122991.5	100523423	ATP5F1D	mRNA	1	0	0	0	0	0	0	0	0	0	0	0	0	0	0	0	0	0	0
XR_001308436.2	102166460	LOC102166460	lncRNA	1	0	0	0	0	0	0	0	0	0	0	0	0	0	0	0	0	0	0
XM_021070161.1	100620692	UBXN7	mRNA	1	0	0	0	0	0	0	0	0	0	0	0	0	0	0	0	0	0	0
NM_001278777.1	494011	SLC27A2	mRNA	1	0	0	0	0	0	0	0	0	0	0	0	0	0	0	0	0	0	0
XM_013994347.2	397553	NR1H3	mRNA	1	0	0	0	0	0	0	0	0	0	0	0	0	0	0	0	0	0	0
XM_005664733.3	100514292	ALDH16A1	mRNA	1	0	0	0	0	0	0	0	0	0	0	0	0	0	0	0	0	0	0
NM_001099936.1	100101473	BTG1	mRNA	1	0	0	0	0	0	0	0	0	0	0	0	0	0	0	0	0	0	0
XM_021099493.1	100625247	HEATR4	mRNA	1	0	0	0	0	0	0	0	0	0	0	0	0	0	0	0	0	0	0
XR_002335793.1	100523270	PFKFB2	mRNA	1	0	0	0	0	0	0	0	0	0	0	0	0	0	0	0	0	0	0
XR_002343260.1	106510048	LOC106510048	lncRNA	1	0	0	0	0	0	0	0	0	0	0	0	0	0	0	0	0	0	0
XM_021063420.1	100738171	ABCB5	mRNA	1	0	0	0	0	0	0	0	0	0	0	0	0	0	0	0	0	0	0
XM_021098946.1	100155072	CFAP100	mRNA	1	0	0	0	0	0	0	0	0	0	0	0	0	0	0	0	0	0	0
XM_021074210.1	100737404	LOC100737404	mRNA	1	0	0	0	0	0	0	0	0	0	0	0	0	0	0	0	0	0	0
XM_021088107.1	100517149	LOC100517149	mRNA	1	0	0	0	0	0	0	0	0	0	0	0	0	0	0	0	0	0	0
XM_003125346.4	100518190	MYCN	mRNA	1	0	0	0	0	0	0	0	0	0	0	1	0	0	0	0	0	0	0

RNA ID	Gene ID	Symbol	Type	target_total	ssc-miR-29a-3p	novel-ssc-miR204-5p	novel-ssc-miR480-3p	novel-ssc-miR537-5p	novel-ssc-miR620-3p	novel-ssc-miR627-5p	novel-ssc-miR671-3p	novel-ssc-miR753-5p	novel-ssc-miR832-3p	novel-ssc-miR1287-5p	novel-ssc-miR1406-3p	ssc-miR-10383	novel-ssc-miR2-3p	ssc-miR-660	novel-ssc-miR9-3p	novel-ssc-miR16-3p	novel-ssc-miR710-5p	novel-ssc-miR802-3p
XM_003356153.3	100517101	LOC100517101	mRNA	1	0	0	0	0	0	0	1	0	0	0	0	0	0	0	0	0	0	0
XR_002338567.1	110256688	LOC110256688	lncRNA	1	0	0	0	0	0	0	0	0	0	0	0	0	0	0	0	0	0	0
XR_002339778.1	110257320	LOC110257320	lncRNA	1	0	0	0	0	0	0	0	0	0	0	0	0	0	0	0	0	0	0
XM_001927081.6	100156057	TTC8	mRNA	1	0	0	0	0	0	0	0	0	0	0	0	0	0	0	0	0	0	0
XM_021093139.1	100037307	DAB1	mRNA	1	0	0	0	0	0	0	0	0	0	0	0	0	0	0	0	0	0	0
XR_001300917.2	106505968	LOC106505968	lncRNA	1	0	0	0	0	0	0	0	0	0	0	0	0	0	0	0	0	0	0
NM_001190154.1	100157314	ZCCHC8	mRNA	1	0	0	0	0	0	0	0	0	0	0	0	0	0	0	0	0	0	0
XR_002338340.1	102159136	ARHGAP19	lncRNA	1	0	0	0	0	0	0	0	0	0	0	0	0	0	0	0	0	0	0
XR_002340858.1	110257848	LOC110257848	lncRNA	1	0	0	0	0	0	0	0	0	0	0	0	0	0	0	0	0	0	0
XM_021084337.1	102162790	GRM6	mRNA	1	0	0	0	0	0	0	0	0	0	0	0	0	0	0	0	0	0	0
XR_001306165.2	106508978	LOC106508978	lncRNA	1	0	0	0	0	0	0	0	0	0	0	0	0	0	0	0	0	0	0
XM_021085043.1	110259550	LOC110259550	mRNA	1	0	0	0	0	0	0	0	0	0	0	0	0	0	0	0	0	0	0
XR_001302502.2	102164143	LOC102164143	lncRNA	1	0	0	0	0	0	0	0	0	0	0	0	0	0	0	0	0	0	0
XM_021088119.1	100516428	LOC100516428	mRNA	1	0	0	0	0	0	0	0	0	0	0	0	0	0	0	0	0	0	0
XR_002335496.1	110255344	LOC110255344	lncRNA	1	0	0	0	0	0	0	0	0	0	0	0	0	0	0	0	0	0	0
XR_002335923.1	110255511	LOC110255511	lncRNA	1	1	0	0	0	0	0	0	0	0	0	0	0	0	0	0	0	0	0
XR_002336776.1	102168075	LOC102168075	lncRNA	1	0	0	0	0	0	0	0	0	0	0	0	0	0	0	0	0	0	0
XR_304089.2	102160593	LOC102160593	lncRNA	1	0	0	0	0	0	0	0	0	0	0	0	0	0	0	0	0	0	0
XM_003355424.5	100627932	HOXC6	mRNA	1	0	0	0	0	1	0	0	0	0	0	0	0	0	0	0	0	0	0
XR_002335902.1	102161420	LOC102161420	lncRNA	1	0	0	0	0	0	0	0	0	0	0	0	0	0	0	0	0	0	0
XR_002344769.1	110261037	LOC110261037	lncRNA	1	0	0	0	0	0	0	0	0	0	0	0	0	0	0	0	0	0	0
XR_001300967.2	100520338	LOC100520338	lncRNA	1	0	0	0	0	0	0	0	0	0	0	1	0	0	0	0	0	0	0
XR_002338125.1	102167959	LOC102167959	lncRNA	1	0	0	0	0	0	0	0	0	0	0	0	0	0	0	0	0	0	0
XR_002340442.1	102160053	LOC102160053	lncRNA	1	0	0	0	0	0	0	0	0	0	0	0	0	0	0	0	0	0	0
NM_001145752.1	100271724	SLC22A4	mRNA	1	0	0	0	0	0	0	0	0	0	0	0	0	1	0	0	0	0	0
XM_021097098.1	100302694	CES5A	mRNA	1	0	0	0	0	0	0	0	0	0	0	0	0	0	0	0	0	0	0
XR_001299355.2	106505191	LOC106505191	lncRNA	1	0	0	0	0	0	0	0	0	0	0	0	0	0	0	0	0	0	0
XR_001301379.2	106506213	LOC106506213	lncRNA	1	0	0	0	0	0	0	0	0	0	0	0	0	0	0	0	0	0	0
XR_002344182.1	110260775	LOC110260775	lncRNA	1	0	0	0	0	0	0	0	0	0	0	0	0	0	0	0	0	0	0
XR_002337157.1	110256021	LOC110256021	lncRNA	1	0	0	0	0	0	0	0	0	0	0	0	0	1	0	0	0	0	0
XR_002341155.1	110258473	LOC110258473	lncRNA	1	0	0	0	0	0	0	0	0	0	0	0	0	0	0	0	0	0	0
XM_001925364.3	100153329	LOC100153329	mRNA	1	0	0	0	0	0	0	0	0	0	0	0	0	0	0	0	0	0	0
XM_005665343.3	100519720	MAPRE2	mRNA	1	0	0	0	0	0	0	0	0	0	0	0	0	0	0	0	0	0	0
XR_002336444.1	102157904	LOC102157904	lncRNA	1	0	0	0	0	0	0	0	0	0	0	0	0	1	0	0	0	0	0
XM_003132006.5	100520100	MAP2K4	mRNA	1	0	0	0	0	0	0	0	0	0	0	1	0	0	0	0	0	0	0
XR_002343771.1	110260459	LOC110260459	lncRNA	1	0	0	0	0	0	0	0	0	0	0	0	0	0	0	0	0	0	0
XM_003358564.4	100620415	SLC35G2	mRNA	1	0	0	0	0	0	0	0	0	0	0	1	0	0	0	0	0	0	0
NM_001123151.1	100144454	NRM	mRNA	1	0	0	0	0	0	0	0	0	0	0	0	0	0	0	0	0	0	0
XM_005667128.3	100627340	LOC100627340	mRNA	1	0	0	0	0	0	0	0	0	0	0	0	0	0	0	0	0	0	0
NM_001243803.1	100511023	NUDC	mRNA	1	0	0	0	0	0	0	0	0	0	0	0	0	0	0	0	0	0	0
XM_021097456.1	110261343	TMEM240	mRNA	1	0	0	0	0	0	0	0	0	0	0	0	0	0	0	0	0	0	0
XR_001301636.2	106506374	LOC106506374	lncRNA	1	0	0	0	0	0	0	0	0	0	0	0	0	0	0	0	0	0	0
XR_001303107.2	106507246	LOC106507246	lncRNA	1	0	0	0	0	0	0	0	0	0	0	0	0	0	0	0	0	0	0

RNA ID	Gene ID	Symbol	Type	target_total	ssc-miR-29a-3p	novel-ssc-miR204-5p	novel-ssc-miR480-3p	novel-ssc-miR537-5p	novel-ssc-miR620-3p	novel-ssc-miR627-5p	novel-ssc-miR671-3p	novel-ssc-miR753-5p	novel-ssc-miR832-3p	novel-ssc-miR1287-5p	novel-ssc-miR1406-3p	ssc-miR-10383	novel-ssc-miR2-3p	ssc-miR-660	novel-ssc-miR9-3p	novel-ssc-miR16-3p	novel-ssc-miR710-5p	novel-ssc-miR802-3p
XR_001303546.2	106507516	LOC106507516	lncRNA	1	0	0	0	0	0	0	0	0	0	0	0	0	0	0	0	0	0	0
XR_002336300.1	110255664	LOC110255664	lncRNA	1	1	0	0	0	0	0	0	0	0	0	0	0	0	0	0	0	0	0
XR_002336542.1	110255752	LOC110255752	lncRNA	1	0	0	0	0	0	0	0	0	0	0	0	0	1	0	0	0	0	0
XR_002337686.1	110256299	LOC110256299	lncRNA	1	0	0	0	0	0	0	0	0	0	0	0	0	0	0	0	0	0	0
XR_001300145.2	106505585	LOC106505585	lncRNA	1	0	0	0	0	0	0	0	0	0	0	0	0	1	0	0	0	0	0
XR_002344948.1	110261123	LOC110261123	lncRNA	1	0	0	0	0	0	0	0	0	0	0	0	0	0	0	0	0	0	0
XR_002344676.1	110260998	LOC110260998	lncRNA	1	0	0	0	0	0	0	0	0	0	0	0	0	0	0	0	0	0	0
XM_003121205.3	100525016	SMLR1	mRNA	1	0	0	0	0	0	0	0	0	0	0	0	0	0	0	0	0	0	0
XR_002340682.1	102163484	LOC102163484	lncRNA	1	0	0	0	0	0	0	0	0	0	0	0	0	0	0	0	0	0	0
XM_021079561.1	497636	DMD	mRNA	1	0	0	0	0	0	0	0	0	0	0	0	0	0	0	0	0	0	0
XR_002341591.1	102164826	ISYNA1	lncRNA	1	0	0	0	0	0	0	0	0	0	0	0	0	0	0	0	0	0	0
XR_002340822.1	110257819	LOC110257819	lncRNA	1	0	0	0	0	0	0	0	0	0	0	0	0	0	0	0	0	0	0
XR_001298110.1	106504549	LOC106504549	lncRNA	1	0	0	0	0	0	0	0	0	0	0	0	0	0	0	0	0	0	0
XR_002340843.1	110257831	LOC110257831	lncRNA	1	1	0	0	0	0	0	0	0	0	0	0	0	0	0	0	0	0	0
XR_300629.3	102163894	LOC102163894	lncRNA	1	0	0	0	0	0	0	0	0	0	0	0	0	1	0	0	0	0	0
XM_021099082.1	100510914	NEO1	mRNA	1	0	0	0	0	0	0	0	0	0	0	0	0	0	0	0	0	0	0
XR_002345385.1	110261329	LOC110261329	lncRNA	1	0	0	0	0	0	0	0	0	0	0	0	0	0	0	0	0	0	0
XR_002337516.1	110256226	LOC110256226	lncRNA	1	1	0	0	0	0	0	0	0	0	0	0	0	0	0	0	0	0	0
XR_002340123.1	102159962	LOC102159962	lncRNA	1	0	0	0	0	0	0	0	0	0	0	0	0	0	0	0	0	0	0
XM_001926907.5	100156308	PSMD10	mRNA	1	0	0	0	0	0	0	0	0	0	0	1	0	0	0	0	0	0	0
NM_213885.1	396919	MAN1A1	mRNA	1	0	0	0	0	0	0	0	0	0	0	1	0	0	0	0	0	0	0
XR_002338697.1	106506052	LOC106506052	lncRNA	1	0	0	0	0	0	0	0	0	0	0	0	0	0	0	0	0	0	0
XR_002340869.1	100522115	LOC100522115	lncRNA	1	0	0	0	0	0	0	0	0	0	0	0	0	0	0	0	0	0	0
NM_001143692.2	100152339	ITPK1	mRNA	1	0	0	0	0	0	0	0	0	0	0	0	0	1	0	0	0	0	0
NM_001114281.1	100135665	RELA	mRNA	1	0	0	0	0	0	0	0	0	0	0	0	0	0	0	0	0	0	0
XM_021073105.1	100156262	PAPSS2	mRNA	1	0	0	0	0	0	0	0	0	0	0	0	0	0	0	0	0	0	0
XR_002336169.1	110255621	LOC110255621	lncRNA	1	0	0	0	0	0	0	0	0	0	0	0	0	0	0	0	0	0	0
XR_001298531.2	102166545	LOC102166545	lncRNA	1	0	0	0	0	0	0	0	0	0	0	0	0	0	1	0	0	0	0
XR_002339865.1	110257358	LOC110257358	lncRNA	1	0	0	0	0	0	0	0	0	0	0	0	0	0	0	0	0	0	0
XR_002336348.1	110255689	LOC110255689	lncRNA	1	0	0	0	0	0	0	0	0	0	0	0	0	0	0	0	0	0	0
XM_003134418.4	100523033	SPAG4	mRNA	1	0	0	0	0	0	0	0	0	0	0	0	0	0	0	0	0	0	0
XR_001308912.2	106510436	LOC106510436	lncRNA	1	0	0	0	0	0	0	0	0	0	0	0	0	0	0	0	0	0	0
XM_003482567.1	100739359	LOC100739359	mRNA	1	0	0	0	0	0	0	0	0	0	0	0	0	0	0	0	0	0	0
XM_003483535.4	100154776	PGAM1	mRNA	1	0	0	0	0	0	0	0	0	0	0	1	0	0	0	0	0	0	0
XM_003359830.5	100626618	CCNJL	mRNA	1	0	0	0	0	0	0	0	0	0	0	1	0	0	0	0	0	0	0
XR_002339976.1	110257414	LOC110257414	lncRNA	1	1	0	0	0	0	0	0	0	0	0	0	0	0	0	0	0	0	0
NM_001139471.1	100154728	APEX1	mRNA	1	0	0	0	0	0	0	0	0	0	0	0	0	1	0	0	0	0	0
XR_001301667.2	106506397	LOC106506397	lncRNA	1	0	0	0	0	0	0	0	0	0	0	0	0	0	0	0	0	0	0
XR_001307040.2	102159224	LOC102159224	lncRNA	1	0	0	0	0	0	0	0	0	0	0	0	0	0	0	0	0	0	0
XM_021066572.1	100622490	ST6GALNAC1	mRNA	1	0	0	0	0	0	0	0	0	0	0	0	0	0	0	0	0	0	0
XM_001925290.5	100152155	SMPD2	mRNA	1	0	0	0	0	0	0	0	0	0	0	0	0	0	0	0	0	0	0
XR_001306492.2	100737021	LOC100737021	lncRNA	1	1	0	0	0	0	0	0	0	0	0	0	0	0	0	0	0	0	0
XM_013992665.2	102160737	HTR1E	mRNA	1	0	0	0	0	0	0	0	0	0	0	0	0	0	0	0	0	0	0

RNA ID	Gene ID	Symbol	Type	target_total	ssc-miR-29a-3p	novel-ssc-miR204-5p	novel-ssc-miR480-3p	novel-ssc-miR537-5p	novel-ssc-miR620-3p	novel-ssc-miR627-5p	novel-ssc-miR671-3p	novel-ssc-miR753-5p	novel-ssc-miR832-3p	novel-ssc-miR1287-5p	novel-ssc-miR1406-3p	ssc-miR-10383	novel-ssc-miR2-3p	ssc-miR-660	novel-ssc-miR9-3p	novel-ssc-miR16-3p	novel-ssc-miR710-5p	novel-ssc-miR802-3p
XM_021082275.1	110258984	LOC110258984	mRNA	1	0	0	0	0	0	0	0	0	0	0	0	0	0	0	1	0	0	0
XR_001305691.2	102166401	LOC102166401	lncRNA	1	0	0	0	0	0	0	0	0	0	0	0	0	0	1	0	0	0	0
XR_002343757.1	110260447	LOC110260447	lncRNA	1	0	0	0	0	0	0	0	0	0	0	0	0	0	0	0	0	0	0
XR_001306506.2	106509193	LOC106509193	lncRNA	1	0	0	0	0	0	0	0	0	0	0	0	0	0	0	0	0	0	0
XM_021075435.1	100514804	CFC1B	mRNA	1	0	0	0	0	0	0	0	0	0	0	0	0	0	0	0	0	0	0
XR_002337121.1	110256014	LOC110256014	lncRNA	1	0	0	0	0	0	0	0	0	0	0	0	0	0	0	0	0	0	0
XR_002340328.1	106508222	LOC106508222	lncRNA	1	0	0	0	0	0	0	0	0	0	0	1	0	0	0	0	0	0	0
XM_021099830.1	100155756	TMEM225B	mRNA	1	0	0	0	0	0	0	0	0	0	0	0	0	0	0	0	0	0	0
NM_001206441.1	733650	TAP2	mRNA	1	0	0	0	0	0	0	0	0	0	0	0	0	0	0	0	0	0	0
XR_002338713.1	110256767	LOC110256767	lncRNA	1	0	0	0	0	0	0	0	0	0	0	0	0	0	0	0	0	0	0
XR_001307073.2	106509487	LOC106509487	lncRNA	1	0	0	0	0	0	0	0	0	0	0	0	0	0	0	0	0	0	0
XM_003129584.2	100517905	LOC100517905	mRNA	1	0	0	0	0	0	0	0	0	0	0	0	0	0	0	0	0	0	0
XM_003359416.4	100622102	KIF5C	mRNA	1	0	0	0	0	0	0	0	0	0	0	0	0	0	0	1	0	0	0
NM_001348903.1	397672	CILP	mRNA	1	0	0	0	0	0	0	0	0	0	0	0	0	0	0	0	0	0	0
XR_002341788.1	106509487	LOC106509487	lncRNA	1	0	0	0	0	0	0	0	0	0	0	0	0	0	0	0	0	0	0
XR_002337537.1	110256233	LOC110256233	lncRNA	1	0	0	0	0	0	0	0	0	0	0	0	0	0	0	0	0	0	0
XR_002341806.1	110259419	LOC110259419	lncRNA	1	0	0	0	0	0	0	0	0	0	0	0	0	0	0	0	0	0	0
XM_001928015.5	396603	PPP3CB	mRNA	1	0	0	0	0	0	0	0	0	0	0	0	0	0	0	0	0	0	0
XM_001924600.5	100155018	ZNF451	mRNA	1	0	0	0	0	0	0	0	0	0	0	0	0	0	0	0	0	0	0
XM_021089348.1	100153039	NSMAF	mRNA	1	0	0	0	0	0	0	1	0	0	0	0	0	0	0	0	0	0	0
XR_300604.3	102165231	LOC102165231	lncRNA	1	0	0	0	0	0	0	0	0	0	0	0	0	0	0	0	0	0	0
XM_003355060.4	100628123	NPBWR1	mRNA	1	0	0	0	0	0	0	0	0	0	0	0	0	0	0	0	0	0	0
NM_214418.2	403155	SLC25A6	mRNA	1	0	0	0	0	0	0	0	0	0	0	0	0	0	0	0	0	0	0
XR_002335850.1	102161480	LOC102161480	lncRNA	1	0	0	0	0	0	0	0	0	0	0	0	0	0	0	0	0	0	0
XR_002345639.1	110261503	LOC110261503	lncRNA	1	0	0	0	0	0	0	0	0	0	0	0	0	0	0	0	0	0	0
XR_301706.3	102162727	LOC102162727	lncRNA	1	0	0	0	0	0	0	0	0	0	0	0	0	0	0	0	0	0	0
XR_002344083.1	110260726	LOC110260726	lncRNA	1	0	0	0	0	0	0	0	0	0	0	0	0	0	0	0	0	0	0
XR_002336138.1	110255587	LOC110255587	lncRNA	1	0	0	0	0	0	0	0	0	0	0	0	0	0	0	0	0	0	0
NM_001244966.1	100514424	SLC25A39	mRNA	1	0	0	0	0	0	0	0	0	0	0	0	0	0	0	0	0	0	0
XR_001301171.2	106506113	LOC106506113	lncRNA	1	0	0	0	0	0	0	0	0	0	0	0	0	0	0	0	0	0	0
NM_001123177.1	100144495	RAB33A	mRNA	1	0	0	0	0	0	0	0	0	0	0	0	0	0	0	0	0	0	0
XM_021096358.1	102166543	SAMD13	mRNA	1	0	0	0	0	0	0	0	0	0	0	0	0	0	0	0	0	0	0
XM_021086898.1	102157824	GINM1	mRNA	1	0	0	0	0	0	0	0	0	0	0	0	0	0	0	0	0	0	0
XR_002346148.1	110261690	LOC110261690	lncRNA	1	0	0	0	0	0	0	0	0	0	0	0	0	1	0	0	0	0	0
XR_001307288.2	106509619	LOC106509619	lncRNA	1	0	0	0	0	0	0	0	0	0	0	0	0	0	0	0	0	0	0
NM_214253.1	397487	PRM1	mRNA	1	0	0	0	0	0	0	0	0	0	0	0	0	0	0	0	0	0	0
XR_002345199.1	110261229	LOC110261229	lncRNA	1	0	0	0	0	0	0	0	0	0	0	0	0	0	0	0	0	0	0
XM_021091581.1	100154559	CALHM4	mRNA	1	0	0	0	0	0	0	0	0	0	0	0	0	0	0	0	0	0	0
URS0000BC677A	110260766	LOC110260766	lncRNA	1	1	0	0	0	0	0	0	0	0	0	0	0	0	0	0	0	0	0
URS0000BC9805	BGIG9823_26341		lncRNA	1	0	0	0	0	0	0	0	0	0	0	0	0	0	0	0	0	0	0
URS0000BC7DBE	102167218	LOC102167218	lncRNA	1	0	0	0	0	0	0	0	0	0	0	0	0	1	0	0	0	0	0
URS0000BC871E	102165318	LOC102165318	lncRNA	1	1	0	0	0	0	0	0	0	0	0	0	0	0	0	0	0	0	0
URS0000BC6273	BGIG9823_26302		lncRNA	1	0	0	0	0	0	0	0	0	0	0	0	0	1	0	0	0	0	0

RNA ID	Gene ID	Symbol	Type	target_total	ssc-miR-29a-3p	novel-ssc-miR204-5p	novel-ssc-miR480-3p	novel-ssc-miR537-5p	novel-ssc-miR620-3p	novel-ssc-miR627-5p	novel-ssc-miR671-3p	novel-ssc-miR753-5p	novel-ssc-miR832-3p	novel-ssc-miR1287-5p	novel-ssc-miR1406-3p	ssc-miR-10383	novel-ssc-miR2-3p	ssc-miR-660	novel-ssc-miR9-3p	novel-ssc-miR16-3p	novel-ssc-miR710-5p	novel-ssc-miR802-3p
URS0000BC6496	BGIG9823_26337		lncRNA	1	0	0	0	0	0	0	0	0	0	0	0	0	0	0	0	0	0	0
URS0000BC6966	BGIG9823_26316		lncRNA	1	0	0	0	0	0	0	0	0	0	0	0	0	0	0	0	0	0	0
URS0000BC81FE	BGIG9823_26310		lncRNA	1	0	0	0	0	0	0	0	0	0	0	0	0	0	0	0	0	0	0
URS0000BC59B8	100525132	PDZD2	lncRNA	1	0	0	0	0	0	0	0	0	0	0	0	0	0	0	0	0	0	0
URS0000BC725F	106506163	LOC106506163	lncRNA	1	0	0	0	0	0	0	0	0	0	0	0	0	0	1	0	0	0	0
URS0000BC5DE3	110256465	LOC110256465	lncRNA	1	1	0	0	0	0	0	0	0	0	0	0	0	0	0	0	0	0	0
URS0000BC7E35	102157619	LOC102157619	lncRNA	1	0	0	0	0	0	0	0	0	0	0	0	0	0	1	0	0	0	0
URS0000BC4C4F	106510093	LOC106510093	lncRNA	1	0	0	0	0	0	0	0	0	0	0	0	0	0	0	0	0	0	0
URS0000BC46E5	BGIG9823_26314		lncRNA	1	0	0	0	0	0	0	0	0	0	0	1	0	0	0	0	0	0	0
URS0000BC8793	100525132	PDZD2	lncRNA	1	0	0	0	0	0	0	0	0	0	0	0	0	0	0	0	0	0	0
URS0000BC7B31	BGIG9823_26353		lncRNA	1	0	0	0	0	0	0	0	0	0	0	0	0	0	0	0	0	0	0
BGIR9823_88049	100621902	MIER1	mRNA	1	0	0	0	0	0	0	0	0	0	0	1	0	0	0	0	0	0	0
BGIR9823_87964	100737982	GAREM1	lncRNA	1	0	0	0	0	0	0	0	0	0	0	0	0	0	0	0	0	0	0
BGIR9823_81184	100523888	LOC100523888	lncRNA	1	0	0	0	0	0	0	0	0	0	0	0	0	0	0	0	0	0	0
BGIR9823_78564	100737904	LOC100737904	lncRNA	1	0	0	0	1	0	0	0	0	0	0	0	0	0	0	0	0	0	0
BGIR9823_89527	102158035	LOC102158035	mRNA	1	0	0	0	0	0	0	0	0	0	0	0	0	0	0	0	0	0	0
BGIR9823_84122	100152952	ZHX2	lncRNA	1	0	0	0	0	0	0	0	0	0	0	0	0	0	0	0	0	0	0
BGIR9823_79475	100625298	GABRG3	lncRNA	1	1	0	0	0	0	0	0	0	0	0	0	0	0	0	0	0	0	0
XM_021097022.1	100620736	TCF25	mRNA	1	0	0	0	0	0	0	0	0	0	0	0	0	0	0	0	0	0	0
BGIR9823_77873	100513326	SMAP1	lncRNA	1	0	0	0	0	0	0	0	0	0	0	0	0	0	0	0	0	0	0
BGIR9823_80029	100154341	URM1	lncRNA	1	0	0	0	0	0	0	0	0	0	0	0	0	0	0	0	0	0	0
BGIR9823_83646	100154232	GGH	mRNA	1	0	0	0	0	0	0	0	0	0	0	0	0	0	0	1	0	0	0
BGIR9823_97547	100153782	USP30	mRNA	1	0	0	0	0	0	0	0	0	0	0	0	0	1	0	0	0	0	0
BGIR9823_78040	100157196	CSNK1G1	lncRNA	1	0	0	0	0	0	0	0	0	0	0	0	0	0	0	0	0	0	0
BGIR9823_79960	100516174	SUSD1	lncRNA	1	0	0	0	0	0	0	0	0	0	0	0	0	0	0	0	0	0	0
BGIR9823_81702	100521611	FNIP1	lncRNA	1	0	0	0	0	0	0	0	0	0	0	0	0	0	0	0	0	0	0
BGIR9823_78735	100153669	TMEM38B	lncRNA	1	0	0	0	0	0	0	0	0	0	0	0	0	0	0	0	0	0	0
BGIR9823_80417	100516957	LOC100516957	lncRNA	1	0	0	0	0	0	0	0	0	0	0	0	0	0	0	0	0	0	0
BGIR9823_82071	100525811	C3H16orf71	lncRNA	1	0	0	0	0	0	0	0	0	0	0	0	0	0	0	0	0	0	0
BGIR9823_89210	100153184	KHDRBS2	lncRNA	1	0	0	0	0	0	0	0	0	0	0	0	0	0	0	0	0	0	0
BGIR9823_86411	100621774	UBE2M	lncRNA	1	0	0	0	0	0	0	0	0	0	0	0	0	0	0	0	0	0	0
BGIR9823_86823	100621518	RBBP8	mRNA	1	0	0	0	0	0	0	0	0	0	0	0	0	0	0	0	0	0	0
BGIR9823_93974	102164379	LOC102164379	lncRNA	1	0	0	0	0	0	0	0	0	0	0	0	0	0	1	0	0	0	0
BGIR9823_79858	654405	GNAQ	lncRNA	1	0	0	0	0	0	0	0	0	0	0	0	0	0	0	0	0	0	0
BGIR9823_97653	100154344	C14H22orf39	lncRNA	1	0	0	0	0	0	0	0	0	0	0	0	0	0	0	0	0	0	0
BGIR9823_90176	100511210	SEC31A	mRNA	1	0	0	0	0	0	0	0	0	0	0	0	0	0	0	0	0	0	0
BGIR9823_99273	100624231	NIPBL	lncRNA	1	0	0	0	0	0	0	0	0	0	0	0	0	0	0	0	0	0	0
BGIR9823_100693	100739403	KLHDC10	lncRNA	1	0	0	0	0	0	0	0	0	0	0	0	0	0	0	0	0	0	0
BGIR9823_79452	100512576	SPRED1	lncRNA	1	0	0	0	0	0	0	0	0	0	0	0	0	0	0	0	0	0	0
BGIR9823_82923	100525039	MCRIP2	lncRNA	1	0	0	0	0	0	0	0	0	0	0	0	0	0	0	0	0	0	0
BGIR9823_85610	110260725	LOC110260725	lncRNA	1	0	0	0	0	0	0	0	0	0	0	0	0	0	0	0	0	0	0
BGIR9823_79946	100155590	FRRS1L	lncRNA	1	0	0	0	0	0	0	0	0	0	0	0	0	0	0	0	0	0	0
XR_002335729.1	110255457	LOC110255457	lncRNA	1	0	0	0	0	0	0	0	0	0	0	0	0	0	0	0	0	0	0

RNA ID	Gene ID	Symbol	Type	target_total	ssc-miR-29a-3p	novel-ssc-miR204-5p	novel-ssc-miR480-3p	novel-ssc-miR537-5p	novel-ssc-miR620-3p	novel-ssc-miR627-5p	novel-ssc-miR671-3p	novel-ssc-miR753-5p	novel-ssc-miR832-3p	novel-ssc-miR1287-5p	novel-ssc-miR1406-3p	ssc-miR-10383	novel-ssc-miR2-3p	ssc-miR-660	novel-ssc-miR9-3p	novel-ssc-miR16-3p	novel-ssc-miR710-5p	novel-ssc-miR802-3p
BGIR9823_79704	100155666	ATG14	lncRNA	1	0	0	0	0	0	0	0	0	0	0	0	0	0	0	0	0	0	0
BGIR9823_80049	414430	MED27	lncRNA	1	0	0	0	0	0	0	0	0	0	0	0	0	0	0	0	0	0	0
BGIR9823_84572	100519481	STXBP3	lncRNA	1	0	0	0	0	0	0	0	0	0	0	0	0	0	0	0	0	0	0
BGIR9823_87715	100517038	PUM1	lncRNA	1	0	0	0	0	0	0	0	0	0	0	0	0	0	1	0	0	0	0
BGIR9823_84360	100154639	PBX1	lncRNA	1	0	0	0	0	0	0	0	0	0	0	0	0	0	0	0	0	0	0
BGIR9823_101255	100154598	LAMP2	mRNA	1	0	0	0	0	0	0	0	0	0	0	0	0	1	0	0	0	0	0
BGIR9823_98698	100737861	CCNT2	mRNA	1	0	0	0	0	0	0	0	0	0	0	1	0	0	0	0	0	0	0
BGIR9823_81378	100511368	ZNF358	lncRNA	1	0	0	0	0	0	0	0	0	0	0	0	0	0	0	0	0	0	0
BGIR9823_100853	100156439	TCEANC	lncRNA	1	0	0	0	0	0	0	0	0	0	0	0	0	0	0	0	0	0	0
BGIR9823_88507	100153104	POLH	lncRNA	1	0	0	0	0	0	0	0	0	0	0	0	0	0	0	0	0	0	0
BGIR9823_96638	100153422	FAM222A	lncRNA	1	1	0	0	0	0	0	0	0	0	0	0	0	0	0	0	0	0	0
BGIR9823_79570	100512083	NEDD4L	lncRNA	1	0	0	0	0	0	0	0	0	0	0	0	0	0	0	0	0	0	0
BGIR9823_101653	100625138	LSS	lncRNA	1	0	0	0	0	0	0	0	0	0	0	0	0	0	0	0	0	0	0
BGIR9823_101268	100520416	TENM1	lncRNA	1	0	0	0	0	0	0	0	0	0	0	0	0	0	0	0	0	0	0
BGIR9823_78254	106506784	LOC106506784	lncRNA	1	0	0	0	0	0	0	0	0	0	0	0	0	0	0	0	0	0	0
BGIR9823_93514	100514316	CACNB1	lncRNA	1	1	0	0	0	0	0	0	0	0	0	0	0	0	0	0	0	0	0
BGIR9823_78057	100152749	CA12	lncRNA	1	0	0	0	0	0	0	0	0	0	0	0	0	0	0	0	0	0	0
BGIR9823_87011	100621520	YIPF1	lncRNA	1	0	0	0	0	0	0	0	0	0	0	0	0	0	1	0	0	0	0
BGIR9823_79577	100151784	CLPX	lncRNA	1	0	0	0	0	0	0	0	0	0	0	0	0	0	0	0	0	0	0
BGIR9823_83929	100519711	ST7L	lncRNA	1	0	0	0	0	0	0	0	0	0	0	0	0	0	0	0	0	0	0
BGIR9823_78689	100514147	LOC100514147	lncRNA	1	0	0	0	1	0	0	0	0	0	0	0	0	0	0	0	0	0	0
BGIR9823_80847	100515884	KDM3B	lncRNA	1	0	0	0	0	0	0	0	0	0	0	0	0	0	0	0	0	0	0
BGIR9823_100723	100511847	CPED1	mRNA	1	0	0	0	0	0	0	0	0	0	0	0	0	0	0	0	0	0	0
BGIR9823_80653	100521253	IQGAP2	lncRNA	1	0	0	0	0	0	0	0	0	0	0	0	0	0	0	0	0	0	0
BGIR9823_81419	100737330	NFIC	lncRNA	1	0	0	0	0	0	0	0	0	0	0	0	0	0	0	0	0	0	0
BGIR9823_78533	100626032	SGPP1	lncRNA	1	0	0	0	0	0	0	0	0	0	0	0	0	0	0	0	0	0	0
BGIR9823_91477	100511030	ALG8	mRNA	1	0	0	0	0	0	0	0	0	0	0	0	0	0	0	0	0	0	0
BGIR9823_85833	100517653	VEZT	lncRNA	1	0	0	0	0	0	0	0	0	0	0	0	0	0	0	0	0	0	0
BGIR9823_99752	494014	PRNP	mRNA	1	0	0	0	0	0	0	0	0	0	0	0	0	0	0	0	0	0	0
BGIR9823_86172	100620402	LOC100620402	lncRNA	1	0	0	0	0	0	0	0	0	0	0	0	0	0	0	0	0	0	0
BGIR9823_88817	396933	FUT8	lncRNA	1	0	0	0	0	0	0	0	0	0	0	0	0	0	0	0	0	0	0
BGIR9823_94863	100621119	GRM7	lncRNA	1	0	0	0	0	0	0	0	0	0	0	0	0	0	1	0	0	0	0
BGIR9823_82821	110259924	LOC110259924	lncRNA	1	0	0	0	0	0	0	0	0	0	0	0	0	0	0	0	0	0	0
BGIR9823_80743	100513914	FER	lncRNA	1	0	0	0	0	0	0	0	0	0	0	0	0	0	0	0	0	0	0
BGIR9823_86054	100516197	AMFR	mRNA	1	0	0	0	0	0	0	0	0	0	0	0	0	0	0	0	0	0	0
BGIR9823_101027	397245	ATP1B4	mRNA	1	0	0	0	0	0	0	0	0	0	0	0	0	0	0	0	0	0	0
BGIR9823_94279	100520151	GIT1	mRNA	1	0	0	0	0	0	0	0	0	0	0	0	0	1	0	0	0	0	0
BGIR9823_100258	100156548	ZHX3	lncRNA	1	0	0	0	0	0	0	0	0	0	0	0	0	0	0	0	0	0	0
BGIR9823_97925	100516879	CYP2C91	lncRNA	1	1	0	0	0	0	0	0	0	0	0	0	0	0	0	0	0	0	0
BGIR9823_81233	106508326	SLC25A42	lncRNA	1	0	0	0	0	0	0	0	0	0	0	0	0	0	0	0	0	0	0
BGIR9823_100939	100154376	KDM6A	lncRNA	1	0	0	0	0	0	0	0	0	0	0	0	0	0	0	0	0	0	0
BGIR9823_100604	100515515	GALNT11	lncRNA	1	0	0	0	0	0	0	0	0	0	0	0	0	0	0	0	0	0	0
BGIR9823_95197	100626969	LPP	lncRNA	1	0	0	0	0	0	0	0	0	0	0	0	0	0	0	0	0	0	0

RNA ID	Gene ID	Symbol	Type	target_total	ssc-miR-29a-3p	novel-ssc-miR204-5p	novel-ssc-miR480-3p	novel-ssc-miR537-5p	novel-ssc-miR620-3p	novel-ssc-miR627-5p	novel-ssc-miR671-3p	novel-ssc-miR753-5p	novel-ssc-miR832-3p	novel-ssc-miR1287-5p	novel-ssc-miR1406-3p	ssc-miR-10383	novel-ssc-miR2-3p	ssc-miR-660	novel-ssc-miR9-3p	novel-ssc-miR16-3p	novel-ssc-miR710-5p	novel-ssc-miR802-3p
BGIR9823_87581	100514410	ICMT	mRNA	1	0	0	0	0	0	0	0	0	0	0	0	0	0	0	0	0	0	0
BGIR9823_81758	106509575	LOC106509575	lncRNA	1	1	0	0	0	0	0	0	0	0	0	0	0	0	0	0	0	0	0
BGIR9823_79445	100522721	RASGRP1	lncRNA	1	0	0	0	0	0	0	0	0	0	0	0	0	0	0	0	0	0	0
BGIR9823_95280	100623596	OSBPL11	mRNA	1	0	0	0	0	0	0	0	0	0	0	0	0	0	0	0	0	0	0
BGIR9823_88858	100155733	SUSD6	lncRNA	1	0	0	0	0	0	0	0	0	0	0	0	0	0	0	0	0	0	0
BGIR9823_93640	100512984	GGNBP2	lncRNA	1	0	0	0	0	0	0	0	0	0	0	0	0	0	0	0	0	0	0
BGIR9823_82696	100626099	TBL2	mRNA	1	0	0	0	0	0	0	0	0	0	0	0	0	0	0	0	0	0	0
BGIR9823_85289	100519488	MOV10L1	lncRNA	1	0	0	0	0	0	0	0	0	0	0	0	0	0	0	0	0	0	0
BGIR9823_90661	100738411	CCSER1	lncRNA	1	0	0	0	0	0	0	0	0	0	0	0	0	0	0	0	0	0	0
BGIR9823_101099	100624032	LOC100624032	mRNA	1	0	0	0	0	0	0	0	0	0	0	0	0	0	0	0	0	0	0
BGIR9823_83245	100170144	SLC30A6	lncRNA	1	0	0	0	0	0	0	0	0	0	0	0	0	0	0	0	0	0	0
BGIR9823_83495	654326	RNF19A	lncRNA	1	0	0	0	0	0	0	0	0	0	0	0	0	0	0	0	0	0	0
BGIR9823_100642	100519065	UBN2	lncRNA	1	0	0	0	0	0	0	0	0	0	0	0	0	0	0	0	0	0	0
BGIR9823_88851	100158119	SLC39A9	lncRNA	1	0	0	0	0	0	0	0	0	0	0	0	0	0	0	0	0	0	0
BGIR9823_82981	100524615	ARID5A	lncRNA	1	0	0	0	0	0	0	0	0	0	0	0	0	0	0	0	0	0	0
BGIR9823_84758	100157699	TMEM184B	lncRNA	1	0	0	0	0	0	0	0	0	0	0	0	0	0	0	0	0	0	0
BGIR9823_101227	102165634	LOC102165634	lncRNA	1	0	0	0	0	0	0	0	0	0	0	0	0	0	0	0	0	1	0
BGIR9823_82094	100736616	HCFC1R1	lncRNA	1	0	0	0	0	0	0	0	0	0	0	0	0	0	0	0	0	0	0
BGIR9823_87647	100517288	HSPG2	mRNA	1	0	0	0	0	0	0	0	0	0	0	0	0	0	0	0	0	0	0
BGIR9823_85318	110260629	LOC110260629	lncRNA	1	0	0	0	0	0	0	0	0	0	0	0	0	0	0	0	0	0	0
BGIR9823_97476	100513180	FAM216A	lncRNA	1	0	0	0	0	0	0	0	0	0	0	0	0	0	1	0	0	0	0
BGIR9823_100900	100621199	IL1RAPL1	lncRNA	1	0	0	0	0	0	0	0	0	0	0	0	0	0	0	0	0	0	0
BGIR9823_90410	397001	POLR2B	lncRNA	1	1	0	0	0	0	0	0	0	0	0	0	0	0	0	0	0	0	0
XM_005659173.3	100156257	NHSL1	mRNA	1	0	0	0	0	0	0	0	0	0	0	0	0	0	0	0	0	0	0
BGIR9823_90244	100513100	LDB2	lncRNA	1	1	0	0	0	0	0	0	0	0	0	0	0	0	0	0	0	0	0
BGIR9823_78438	100152969	CDKL1	lncRNA	1	0	0	0	0	0	0	0	0	0	0	0	0	0	0	0	0	0	0
BGIR9823_100731	100512382	KCND2	lncRNA	1	0	0	0	0	0	0	0	0	0	0	0	0	0	0	0	0	0	0
BGIR9823_82740	100623146	KIF22	lncRNA	1	0	0	0	0	0	0	0	0	0	0	0	0	0	0	0	0	0	0
BGIR9823_81512	100523721	FAF2	lncRNA	1	0	0	0	0	0	0	0	0	0	0	0	0	0	0	0	0	0	0
BGIR9823_78400	102162695	LOC102162695	lncRNA	1	0	0	0	0	0	0	0	0	0	0	0	0	0	0	0	0	0	0
BGIR9823_100980	110257855	LOC110257855	lncRNA	1	0	0	0	0	0	0	0	0	0	0	0	0	0	0	0	0	1	0
BGIR9823_97780	100037299	P4HA1	lncRNA	1	0	0	0	0	0	0	0	0	0	0	0	0	0	0	0	0	0	0
BGIR9823_79896	100519814	RNF38	lncRNA	1	0	0	0	0	0	0	0	0	0	0	0	0	0	0	0	0	0	0
XM_021065940.1	110255868	ATP11A	mRNA	1	0	0	0	0	0	0	0	0	0	0	0	0	0	0	0	0	0	0
BGIR9823_101616	110258914	LOC110258914	lncRNA	1	0	0	1	0	0	0	0	0	0	0	0	0	0	0	0	0	0	0
BGIR9823_86084	100626752	LOC100626752	lncRNA	1	0	0	0	0	0	0	0	0	0	0	0	0	0	0	0	0	0	0
BGIR9823_78267	100512756	MEIS2	lncRNA	1	0	0	0	0	0	0	0	0	0	0	0	0	0	0	0	0	0	0
BGIR9823_80601	110259385	LOC110259385	lncRNA	1	1	0	0	0	0	0	0	0	0	0	0	0	0	0	0	0	0	0
BGIR9823_87318	100620768	CEP89	lncRNA	1	0	0	0	0	0	0	0	0	0	0	0	0	0	0	0	0	0	0
BGIR9823_80608	100521252	CLK4	lncRNA	1	1	0	0	0	0	0	0	0	0	0	0	0	0	0	0	0	0	0
BGIR9823_100082	100621622	SFRP1	mRNA	1	0	0	0	0	0	0	0	0	0	0	0	0	0	0	0	0	0	0
BGIR9823_80402	396557	PGLYRP2	lncRNA	1	0	0	0	0	0	0	0	0	0	0	0	0	0	0	0	0	0	0
BGIR9823_101117	100518815	TXLNG	lncRNA	1	0	0	0	0	0	0	0	0	0	0	0	0	0	0	0	0	0	0

RNA ID	Gene ID	Symbol	Type	target_total	ssc-miR-29a-3p	novel-ssc-miR204-5p	novel-ssc-miR480-3p	novel-ssc-miR537-5p	novel-ssc-miR620-3p	novel-ssc-miR627-5p	novel-ssc-miR671-3p	novel-ssc-miR753-5p	novel-ssc-miR832-3p	novel-ssc-miR1287-5p	novel-ssc-miR1406-3p	ssc-miR-10383	novel-ssc-miR2-3p	ssc-miR-660	novel-ssc-miR9-3p	novel-ssc-miR16-3p	novel-ssc-miR710-5p	novel-ssc-miR802-3p
BGIR9823_78216	100511488	TTBK2	lncRNA	1	0	0	0	0	0	0	0	0	0	0	0	0	0	0	0	0	0	0
BGIR9823_78392	100526149	PNN	lncRNA	1	0	0	0	0	0	0	0	0	0	0	0	0	0	0	0	0	0	0
BGIR9823_101570	110258602	LOC110258602	lncRNA	1	0	0	0	0	0	0	0	0	0	1	0	0	0	0	0	0	0	0
BGIR9823_93977	100512557	DDX42	mRNA	1	0	0	0	0	0	0	0	0	0	0	1	0	0	0	0	0	0	0
BGIR9823_93667	100294712	SUZ12	lncRNA	1	0	0	0	0	0	0	0	0	0	0	0	0	0	0	0	0	0	0
BGIR9823_94057	397387	THRA	lncRNA	1	0	0	0	0	0	0	0	0	0	0	0	0	0	0	0	0	0	0
BGIR9823_100931	100515940	DDX3X	lncRNA	1	0	0	0	0	0	0	0	0	0	0	0	0	0	0	0	0	0	0
BGIR9823_87442	100518311	NOSIP	lncRNA	1	1	0	0	0	0	0	0	0	0	0	0	0	0	0	0	0	0	0
BGIR9823_92178	100126283	AQP7	mRNA	1	0	0	0	0	0	0	0	0	0	0	0	0	0	0	0	0	0	0
BGIR9823_101406	110258215	LOC110258215	lncRNA	1	0	0	0	0	0	0	0	0	0	0	0	0	0	0	0	0	0	0
BGIR9823_92246	100737056	FRMD4A	lncRNA	1	0	0	0	0	0	0	0	0	0	0	0	0	0	0	0	0	0	0
BGIR9823_82297	100522368	EXOC6B	mRNA	1	0	0	0	0	0	0	0	0	0	0	0	0	0	0	0	0	0	0
BGIR9823_78079	100154204	MYO1E	lncRNA	1	0	0	0	0	0	0	0	0	0	0	0	0	0	0	0	0	0	0
BGIR9823_93720	100517676	GOSR1	lncRNA	1	0	0	0	0	0	0	0	0	0	0	0	0	0	0	0	0	0	0
BGIR9823_95969	100524586	MED12L	lncRNA	1	0	0	0	0	0	0	0	0	0	0	0	0	0	0	0	0	0	0
BGIR9823_95388	100517310	ROBO1	mRNA	1	0	0	0	0	0	0	0	0	0	0	0	0	0	0	0	0	0	0
BGIR9823_92590	100521678	MLLT10	lncRNA	1	0	0	0	0	0	0	0	0	0	0	0	0	0	0	0	0	0	0
BGIR9823_89920	100621006	LOC100621006	lncRNA	1	0	0	0	0	0	0	0	0	0	0	0	0	0	0	0	0	0	0
BGIR9823_87305	100521133	DNAJA2	lncRNA	1	0	0	0	0	0	0	0	0	0	0	0	0	0	0	0	0	0	0
BGIR9823_83514	100153417	C4H8orf37	lncRNA	1	0	0	0	0	0	0	0	0	0	0	0	0	0	0	0	0	0	0
BGIR9823_85521	100525288	OSBPL8	lncRNA	1	0	0	0	0	0	0	0	0	0	0	0	0	0	0	0	0	0	0
BGIR9823_90750	100522939	ART5	mRNA	1	0	0	0	0	0	0	0	0	0	0	0	0	0	0	0	0	0	0
BGIR9823_85923	100579143	HSBP1	lncRNA	1	0	0	0	0	0	0	0	0	0	0	0	0	0	0	0	0	0	0
BGIR9823_91101	100519438	FAM133B	lncRNA	1	0	0	0	0	0	0	0	0	0	0	0	0	0	0	0	0	0	0
BGIR9823_100281	100153535	PCIF1	lncRNA	1	0	0	0	0	0	0	0	0	1	0	0	0	0	0	0	0	0	0
BGIR9823_100276	397528	SDC4	lncRNA	1	0	0	0	0	0	0	0	0	0	0	0	0	0	0	0	0	0	0
BGIR9823_100467	100517505	CTTNBP2	lncRNA	1	0	0	0	0	0	0	0	0	1	0	0	0	0	0	0	0	0	0
BGIR9823_100689	110257546	LOC110257546	lncRNA	1	0	0	0	0	0	0	0	0	0	0	0	0	0	0	0	0	0	0
BGIR9823_94010	100514424	SLC25A39	lncRNA	1	0	0	0	0	0	0	0	0	0	0	0	0	0	0	0	0	0	0
BGIR9823_100247	100153296	RBL1	lncRNA	1	0	0	0	0	0	0	0	0	0	0	0	0	0	0	0	0	0	0
BGIR9823_97926	100516879	CYP2C91	lncRNA	1	0	0	0	0	0	0	0	0	0	0	0	0	0	0	0	0	0	0
BGIR9823_78152	100155516	DMXL2	lncRNA	1	0	0	0	0	0	0	0	0	0	0	0	0	0	0	0	0	0	0
BGIR9823_101623	BGIG9823_26354		lncRNA	1	0	0	0	0	0	0	0	0	0	0	0	0	0	0	0	0	0	0
BGIR9823_98164	110257053	LOC110257053	lncRNA	1	0	0	0	0	0	0	0	0	0	0	0	0	0	1	0	0	0	0
BGIR9823_100903	100621199	IL1RAPL1	lncRNA	1	0	0	0	0	0	0	0	0	0	0	0	0	0	0	0	0	0	0
BGIR9823_77846	100736796	ADGRB3	lncRNA	1	0	0	0	0	0	0	0	0	0	0	0	0	0	0	0	0	0	0
BGIR9823_90432	100520317	LOC100520317	mRNA	1	0	0	0	0	0	0	0	0	0	0	0	0	0	0	0	0	0	0
BGIR9823_95429	396658	IFNAR1	lncRNA	1	0	0	0	0	0	0	0	0	0	0	0	0	0	0	0	0	0	0
BGIR9823_80320	100521123	SBF2	lncRNA	1	0	0	0	0	0	0	0	0	0	0	0	0	0	0	0	0	0	0
BGIR9823_81929	100519894	PRKRIP1	lncRNA	1	0	0	0	0	0	0	0	0	0	0	0	0	0	0	0	0	0	0
BGIR9823_100403	100524054	WDR91	lncRNA	1	0	0	0	0	0	0	0	0	0	0	0	0	0	0	0	0	0	0
BGIR9823_91472	100217384	RSF1	lncRNA	1	0	0	0	0	0	0	0	0	0	0	0	0	0	0	0	0	0	0
BGIR9823_78771	102164650	LOC102164650	lncRNA	1	0	0	0	0	0	0	0	0	0	0	0	0	0	0	0	0	0	0

RNA ID	Gene ID	Symbol	Type	target_total	ssc-miR-29a-3p	novel-ssc-miR204-5p	novel-ssc-miR480-3p	novel-ssc-miR537-5p	novel-ssc-miR620-3p	novel-ssc-miR627-5p	novel-ssc-miR671-3p	novel-ssc-miR753-5p	novel-ssc-miR832-3p	novel-ssc-miR1287-5p	novel-ssc-miR1406-3p	ssc-miR-10383	novel-ssc-miR2-3p	ssc-miR-660	novel-ssc-miR9-3p	novel-ssc-miR16-3p	novel-ssc-miR710-5p	novel-ssc-miR802-3p
BGIR9823_77749	100155211	SLC18B1	lncRNA	1	0	0	0	0	0	0	0	0	0	0	0	0	0	0	0	0	0	0
BGIR9823_80489	100622165	ACER1	lncRNA	1	0	0	0	0	1	0	0	0	0	0	0	0	0	0	0	0	0	0
BGIR9823_100204	100514995	COMMD7	lncRNA	1	0	0	0	0	0	0	0	0	1	0	0	0	0	0	0	0	0	0
BGIR9823_77830	100736796	ADGRB3	lncRNA	1	0	0	0	0	0	0	0	0	0	0	0	0	0	0	0	0	0	0
BGIR9823_78118	100152054	RFX7	lncRNA	1	0	0	0	0	0	0	0	0	0	0	0	0	0	0	0	0	0	0
BGIR9823_80498	106509452	PTPRS	mRNA	1	0	0	0	0	0	0	0	0	0	0	0	0	0	0	0	0	0	0
BGIR9823_83059	100524080	KCMF1	lncRNA	1	0	0	0	0	0	0	0	0	0	0	0	0	0	0	0	0	0	0
BGIR9823_89241	100155423	FKBP5	lncRNA	1	0	0	0	0	0	0	0	0	0	0	0	0	0	0	0	0	0	0
BGIR9823_86626	399532	FABP3	lncRNA	1	1	0	0	0	0	0	0	0	0	0	0	0	0	0	0	0	0	0
BGIR9823_80136	100512584	PLA2G16	lncRNA	1	0	0	0	0	0	0	0	0	0	0	0	0	0	0	0	0	0	0
BGIR9823_78764	100621961	CDC26	lncRNA	1	0	0	0	0	0	0	0	0	0	0	0	0	0	0	0	0	0	0
BGIR9823_100787	100622432	CBX3	lncRNA	1	0	0	0	0	0	0	0	0	0	0	0	0	0	0	0	0	0	0
BGIR9823_81517	100523721	FAF2	lncRNA	1	0	0	0	0	0	0	0	0	0	0	0	0	0	0	0	0	0	0
BGIR9823_94928	100622196	IQSEC1	mRNA	1	0	0	0	0	0	0	0	0	0	0	0	0	0	0	0	0	0	0
BGIR9823_97066	100155963	MMRN2	lncRNA	1	0	0	0	0	0	0	0	0	0	0	0	0	0	0	0	0	0	0
BGIR9823_86005	100515835	RSPRY1	lncRNA	1	0	0	0	0	0	0	0	0	0	0	0	0	0	1	0	0	0	0
BGIR9823_81824	100514516	CSNK1A1	lncRNA	1	0	0	0	0	0	0	0	0	0	0	0	0	0	0	0	0	0	0
BGIR9823_100617	100523293	KCNH2	lncRNA	1	0	0	0	0	0	0	0	0	0	0	0	0	0	0	0	0	0	0
XR_002337475.1	106507029	LOC106507029	lncRNA	1	0	0	0	0	0	0	0	0	0	0	0	0	0	0	0	0	0	0
BGIR9823_99577	100523519	CCDC152	lncRNA	1	0	0	0	0	0	0	0	0	0	0	0	0	0	0	0	0	0	0
BGIR9823_79582	100151784	CLPX	lncRNA	1	0	0	0	0	0	0	0	0	0	0	0	0	0	0	0	0	0	0
BGIR9823_90043	397616	PDE5A	lncRNA	1	0	0	0	0	0	0	0	0	0	0	0	0	0	0	0	0	0	0
BGIR9823_101350	100621019	ANAPC2	mRNA	1	0	0	0	0	0	0	0	0	0	0	0	0	0	0	0	0	0	0
BGIR9823_77999	100512572	WDR7	lncRNA	1	0	0	0	1	0	0	0	0	0	0	0	0	0	0	0	0	0	0
BGIR9823_80986	100294703	KDM2A	lncRNA	1	0	0	0	0	0	0	0	0	0	0	0	0	0	0	0	0	0	0
BGIR9823_82031	110259918	ITPRIPL2	lncRNA	1	0	0	0	0	0	0	0	0	0	0	0	0	0	0	0	0	0	0
BGIR9823_100386	100515632	KDM7A	lncRNA	1	0	0	0	0	0	0	0	0	0	0	0	0	0	0	0	0	0	0
BGIR9823_78769	100157179	ZNF618	lncRNA	1	0	0	0	0	0	0	0	0	0	0	0	0	0	0	0	0	0	0
BGIR9823_77679	100513634	MPC1	lncRNA	1	1	0	0	0	0	0	0	0	0	0	0	0	0	0	0	0	0	0
BGIR9823_101316	100624156	CD99	lncRNA	1	0	0	0	0	0	0	0	0	0	0	0	0	0	0	0	0	0	0
BGIR9823_79248	100738627	DYM	lncRNA	1	0	0	0	0	0	0	0	0	0	0	0	0	0	0	0	0	0	0
BGIR9823_78478	100152019	PELI2	lncRNA	1	0	0	0	0	0	0	0	0	0	0	0	0	0	0	0	0	0	0
BGIR9823_95265	100622532	FYTTD1	lncRNA	1	0	0	0	0	0	0	0	0	0	0	0	0	0	0	0	0	0	0
BGIR9823_84388	100155270	NIT1	mRNA	1	0	0	0	0	0	0	0	0	0	0	0	0	1	0	0	0	0	0
BGIR9823_101285	100622228	ARHGEF6	mRNA	1	0	0	0	0	0	0	0	0	0	0	0	0	0	0	0	0	0	0
BGIR9823_100255	100156548	ZHX3	lncRNA	1	0	0	0	0	0	0	0	0	0	0	0	0	0	0	0	0	0	0
BGIR9823_94455	100151869	OXNAD1	lncRNA	1	0	0	0	0	0	0	0	0	0	0	0	0	0	0	0	0	0	0
BGIR9823_78812	100155133	PTGES2	lncRNA	1	0	0	0	0	0	0	0	0	0	0	0	0	0	0	0	0	0	0
BGIR9823_80806	100519080	LYRM7	lncRNA	1	0	0	0	0	0	0	0	0	0	0	0	0	0	0	0	0	0	0
BGIR9823_101247	100519287	KLHL13	mRNA	1	0	0	0	0	0	0	0	0	0	0	0	0	0	0	0	0	0	0
BGIR9823_79520	102162853	LOC102162853	lncRNA	1	0	0	0	0	0	0	0	0	0	0	0	0	0	0	0	0	0	0
BGIR9823_95143	407739	SOX2	lncRNA	1	0	0	0	0	0	0	0	0	0	0	0	0	0	0	0	0	0	0
BGIR9823_95008	100620055	PLSCR1	lncRNA	1	0	0	0	0	0	0	0	0	0	0	0	0	0	0	0	0	0	0

RNA ID	Gene ID	Symbol	Type	target_total	ssc-miR-29a-3p	novel-ssc-miR204-5p	novel-ssc-miR480-3p	novel-ssc-miR537-5p	novel-ssc-miR620-3p	novel-ssc-miR627-5p	novel-ssc-miR671-3p	novel-ssc-miR753-5p	novel-ssc-miR832-3p	novel-ssc-miR1287-5p	novel-ssc-miR1406-3p	ssc-miR-10383	novel-ssc-miR2-3p	ssc-miR-660	novel-ssc-miR9-3p	novel-ssc-miR16-3p	novel-ssc-miR710-5p	novel-ssc-miR802-3p
BGIR9823_100815	110257576	LOC110257576	lncRNA	1	0	0	0	1	0	0	0	0	0	0	0	0	0	0	0	0	0	0
BGIR9823_83902	110260470	LOC110260470	lncRNA	1	0	0	0	0	0	0	0	0	0	0	0	0	0	1	0	0	0	0
BGIR9823_96518	100623262	SLC15A4	mRNA	1	0	0	0	0	0	0	0	0	0	0	0	0	0	0	0	0	0	0
BGIR9823_101546	110258600	LOC110258600	lncRNA	1	0	0	0	0	0	0	0	0	0	0	0	0	0	0	0	0	0	0
BGIR9823_79803	100154484	RCL1	lncRNA	1	0	0	0	0	0	0	0	0	0	0	0	0	0	0	0	0	0	0
BGIR9823_78229	100154984	TMEM87A	mRNA	1	0	0	0	0	0	0	0	0	0	0	0	0	0	0	0	0	0	0
BGIR9823_78085	100154204	MYO1E	lncRNA	1	0	0	0	0	0	0	0	0	0	0	0	0	0	0	0	0	0	0
BGIR9823_84094	106509960	NSMCE2	lncRNA	1	0	0	0	0	0	0	0	0	0	0	0	0	0	0	0	0	0	0
BGIR9823_80335	102166675	RNF187	lncRNA	1	0	0	0	0	0	0	0	0	0	0	0	0	0	0	0	0	0	0
BGIR9823_101281	100516171	GPC3	lncRNA	1	0	0	0	0	0	0	0	0	0	0	0	0	0	0	0	0	0	0
BGIR9823_83886	102166500	LOC102166500	lncRNA	1	0	0	0	0	0	0	0	0	0	0	0	0	0	0	0	0	0	0
BGIR9823_78367	100520530	CORO2B	lncRNA	1	0	0	0	0	0	0	0	0	0	0	0	0	0	0	0	0	0	0
BGIR9823_81305	102166940	LOC102166940	lncRNA	1	0	0	0	0	0	0	0	0	0	0	0	0	0	0	0	0	0	0
BGIR9823_100832	100520285	ZNF777	lncRNA	1	0	0	0	0	0	0	0	0	0	0	0	0	0	0	0	0	0	0
BGIR9823_100583	102162331	KRBA1	lncRNA	1	0	0	0	0	0	0	0	0	0	0	0	0	0	0	0	0	0	0
BGIR9823_85020	100626912	BHLHE41	mRNA	1	0	0	0	0	0	0	0	0	0	0	0	0	0	0	0	0	0	0
BGIR9823_100375	100514612	BRAF	mRNA	1	0	0	0	0	0	0	0	0	0	0	0	0	0	0	0	0	0	0
BGIR9823_92109	100521742	NEK7	mRNA	1	0	0	0	0	0	0	0	0	0	0	0	0	0	0	0	0	0	0
BGIR9823_100703	100514931	AHCYL2	lncRNA	1	0	0	0	0	0	0	0	0	0	0	0	0	0	0	0	0	0	0
BGIR9823_79724	396697	ESR2	lncRNA	1	0	0	0	0	0	0	0	0	0	0	0	0	0	0	0	0	0	0
BGIR9823_101638	110259113	LOC110259113	lncRNA	1	0	0	0	0	0	0	0	0	0	0	0	0	0	0	0	0	0	0
BGIR9823_92471	100517963	RMI1	lncRNA	1	0	0	0	0	0	0	0	0	0	0	0	0	0	0	0	0	0	0
BGIR9823_100226	654300	UQCC	lncRNA	1	0	0	0	0	0	0	0	0	1	0	0	0	0	0	0	0	0	0
BGIR9823_86749	100624877	PPP4R1	mRNA	1	0	0	0	0	0	0	0	0	0	0	0	0	0	0	0	0	0	0
BGIR9823_97541	100157435	UBE3B	mRNA	1	0	0	0	0	0	0	0	0	0	0	0	0	0	0	0	0	0	0
BGIR9823_80565	102164292	FSTL3	lncRNA	1	0	0	0	1	0	0	0	0	0	0	0	0	0	0	0	0	0	0
BGIR9823_96818	100157092	TTC13	mRNA	1	0	0	0	0	0	0	0	0	0	0	0	0	0	0	0	0	0	0
BGIR9823_84745	100521449	NPTXR	mRNA	1	0	0	0	0	0	0	0	0	0	0	1	0	0	0	0	0	0	0
BGIR9823_79788	102168004	UHRF2	lncRNA	1	0	0	0	0	0	0	0	0	0	0	0	0	0	0	0	0	0	0
BGIR9823_78692	100515356	GLIPR2	lncRNA	1	0	0	0	0	0	0	0	0	0	0	0	0	0	0	0	0	0	0
BGIR9823_79323	100621557	TCF12	lncRNA	1	0	0	0	0	0	0	0	0	0	0	0	0	0	0	0	0	0	0
BGIR9823_101455	110258361	PACS2	lncRNA	1	0	0	0	0	0	0	0	0	0	0	0	0	0	0	0	0	0	0
BGIR9823_94552	100523331	NKTR	lncRNA	1	0	0	0	0	0	0	0	0	0	0	0	0	0	0	0	0	0	0
BGIR9823_100126	100519631	TASP1	lncRNA	1	0	0	0	0	0	0	0	0	0	0	0	0	1	0	0	0	0	0
BGIR9823_100406	106506765	LOC106506765	lncRNA	1	0	0	0	0	0	0	0	0	0	0	0	0	0	0	0	0	0	0
BGIR9823_80256	102168147	LOC102168147	lncRNA	1	0	0	0	0	0	0	0	0	0	0	0	0	0	0	0	0	0	0
BGIR9823_99608	397251	PLK2	mRNA	1	0	0	0	0	0	0	0	0	0	0	0	0	0	0	0	0	0	0
BGIR9823_94040	397550	STAT5A	mRNA	1	0	0	0	0	0	0	0	0	0	0	0	0	0	0	0	0	0	0
BGIR9823_100411	414383	CHCHD3	lncRNA	1	0	0	0	0	0	0	0	0	0	0	0	0	0	0	0	0	0	0
BGIR9823_93144	100174955	KLF12	lncRNA	1	0	0	0	0	0	0	0	0	0	0	0	0	0	1	0	0	0	0
BGIR9823_81306	102166940	LOC102166940	lncRNA	1	0	0	0	0	0	0	0	0	0	0	0	0	0	0	0	0	0	0
BGIR9823_79214	100515594	MTO1	lncRNA	1	0	0	0	0	0	0	0	0	0	0	0	0	0	0	0	0	0	0
XR_001302600.2	106506942	LOC106506942	lncRNA	1	0	0	0	0	0	0	0	0	0	0	0	0	0	0	0	0	0	0

RNA ID	Gene ID	Symbol	Type	target_total	ssc-miR-29a-3p	novel-ssc-miR204-5p	novel-ssc-miR480-3p	novel-ssc-miR537-5p	novel-ssc-miR620-3p	novel-ssc-miR627-5p	novel-ssc-miR671-3p	novel-ssc-miR753-5p	novel-ssc-miR832-3p	novel-ssc-miR1287-5p	novel-ssc-miR1406-3p	ssc-miR-10383	novel-ssc-miR2-3p	ssc-miR-660	novel-ssc-miR9-3p	novel-ssc-miR16-3p	novel-ssc-miR710-5p	novel-ssc-miR802-3p
BGIR9823_99840	100519805	TBC1D20	lncRNA	1	1	0	0	0	0	0	0	0	0	0	0	0	0	0	0	0	0	0
BGIR9823_100155	100519522	NINL	lncRNA	1	0	0	0	0	0	0	0	0	0	0	0	0	0	0	0	0	0	0
BGIR9823_78548	100511733	PLAA	lncRNA	1	0	0	0	0	0	0	0	0	0	0	0	0	0	0	0	0	0	0
BGIR9823_81728	100524487	AFF4	lncRNA	1	0	0	0	0	0	0	0	0	0	0	0	0	0	0	0	0	0	0
BGIR9823_82871	100516601	ZNF597	lncRNA	1	0	0	0	0	0	0	0	0	0	0	0	0	0	1	0	0	0	0
BGIR9823_83578	100157134	ZNF704	lncRNA	1	0	0	0	0	0	0	0	0	0	0	0	0	0	0	0	0	0	0
BGIR9823_78456	100625719	CGRRF1	lncRNA	1	0	0	0	0	0	0	0	0	0	0	0	0	0	0	0	0	0	0
BGIR9823_100241	100154500	SAMHD1	lncRNA	1	0	0	0	0	0	0	0	0	1	0	0	0	0	0	0	0	0	0
XR_002344503.1	110260914	LOC110260914	lncRNA	1	0	0	0	0	0	0	0	0	0	0	0	0	0	0	0	0	0	0
BGIR9823_93124	100174955	KLF12	lncRNA	1	0	0	0	0	0	0	0	0	0	0	0	0	0	0	0	0	0	0
BGIR9823_78918	100627455	SNX9	mRNA	1	0	0	0	0	0	0	0	0	0	0	0	0	0	0	0	0	0	0
BGIR9823_100427	100626356	UBE2H	lncRNA	1	0	0	0	0	0	0	0	0	0	0	0	0	0	0	0	0	0	0
BGIR9823_79882	100156839	FAM166B	mRNA	1	0	0	0	0	0	0	0	0	0	0	0	0	0	0	0	0	0	0
BGIR9823_87816	100626395	NAPG	lncRNA	1	0	0	0	0	0	0	0	0	0	0	0	0	0	0	0	0	0	0
BGIR9823_79529	100154633	PHLPP1	lncRNA	1	0	0	0	0	0	0	0	0	0	0	0	0	0	0	0	0	0	0
BGIR9823_80355	100620218	BORCS8	lncRNA	1	0	0	0	0	0	0	0	0	0	0	0	0	0	0	0	0	0	0
BGIR9823_101515	102157759	LOC102157759	lncRNA	1	0	0	0	0	0	0	0	0	0	0	0	1	0	0	0	0	0	0
BGIR9823_88688	100152987	HEATR5A	mRNA	1	0	0	0	0	0	0	0	0	0	0	0	0	1	0	0	0	0	0
BGIR9823_78932	100514379	FBXO5	lncRNA	1	0	0	0	0	0	0	0	0	0	0	0	0	0	0	0	0	0	0
BGIR9823_78476	100152019	PELI2	lncRNA	1	0	0	0	0	0	0	0	0	0	0	0	0	0	0	0	0	0	0
BGIR9823_92851	100523918	LMO7	mRNA	1	0	0	0	0	0	0	0	0	0	0	0	0	0	0	0	0	0	0
BGIR9823_100326	110257473	LOC110257473	lncRNA	1	0	0	0	0	0	0	0	0	0	0	0	0	0	0	0	0	0	0
BGIR9823_87757	100621544	RNF19B	mRNA	1	0	0	0	0	0	0	0	0	0	0	0	0	1	0	0	0	0	0
BGIR9823_99279	100517617	WDR70	lncRNA	1	0	0	0	0	0	0	0	0	0	0	0	0	0	0	0	0	0	0
BGIR9823_78488	100152019	PELI2	lncRNA	1	0	0	0	0	0	0	0	0	0	0	0	0	0	0	0	0	0	0
BGIR9823_91993	100738612	ATF3	mRNA	1	0	0	0	0	0	0	0	0	0	0	0	0	0	0	0	0	0	0
BGIR9823_94664	102167511	SMIM4	lncRNA	1	0	0	0	0	0	0	0	0	0	0	0	0	0	0	0	0	0	0
BGIR9823_79100	100156082	FBXL4	lncRNA	1	0	0	0	0	0	0	0	0	0	0	0	0	0	0	0	0	0	0
BGIR9823_101197	100156239	VSIG4	mRNA	1	0	0	0	0	0	0	0	0	0	0	0	0	0	0	0	0	0	0
BGIR9823_100442	100192443	POT1	lncRNA	1	0	0	0	0	0	0	0	0	1	0	0	0	0	0	0	0	0	0
BGIR9823_78713	100152263	ANP32B	lncRNA	1	0	0	0	0	0	0	0	0	0	0	0	0	0	0	0	0	0	0
BGIR9823_77769	100153609	PTPRK	lncRNA	1	0	0	0	0	0	0	0	0	0	0	0	0	0	0	0	0	0	0
BGIR9823_78654	100515003	SMC5	mRNA	1	0	0	0	0	0	0	0	0	0	0	0	0	0	0	0	0	0	0
BGIR9823_95994	100512989	VEPH1	lncRNA	1	0	0	0	0	0	0	0	0	0	0	0	0	0	0	0	0	0	0
BGIR9823_100398	100521697	MTPN	lncRNA	1	0	0	0	0	0	0	0	0	0	1	0	0	0	0	0	0	0	0
BGIR9823_77692	110259915	LOC110259915	lncRNA	1	0	0	0	0	0	0	0	0	0	0	0	0	0	0	0	0	0	0
BGIR9823_100510	100738688	HERPUD2	lncRNA	1	0	0	0	0	0	0	0	0	0	0	0	0	0	0	0	0	0	0
BGIR9823_101594	110258799	LOC110258799	lncRNA	1	0	0	0	0	0	0	0	0	0	0	0	0	0	0	0	0	0	0
BGIR9823_100619	100736760	GIMAP8	lncRNA	1	0	0	0	0	0	0	0	0	0	0	0	0	0	0	0	0	0	0
BGIR9823_98052	100153733	RAB11FIP2	mRNA	1	0	0	0	0	0	0	0	0	0	0	0	0	0	0	0	0	0	0
BGIR9823_79578	100151784	CLPX	lncRNA	1	0	0	0	0	0	0	0	0	0	0	0	0	0	0	0	0	0	0
BGIR9823_82021	100628086	METTL9	lncRNA	1	0	0	0	0	0	0	0	0	0	0	0	0	0	0	0	0	0	0
BGIR9823_100265	100520338	LOC100520338	lncRNA	1	0	0	0	0	0	0	0	0	0	0	0	0	0	0	0	0	0	0

RNA ID	Gene ID	Symbol	Type	target_total	ssc-miR-29a-3p	novel-ssc-miR204-5p	novel-ssc-miR480-3p	novel-ssc-miR537-5p	novel-ssc-miR620-3p	novel-ssc-miR627-5p	novel-ssc-miR671-3p	novel-ssc-miR753-5p	novel-ssc-miR832-3p	novel-ssc-miR1287-5p	novel-ssc-miR1406-3p	ssc-miR-10383	novel-ssc-miR2-3p	ssc-miR-660	novel-ssc-miR9-3p	novel-ssc-miR16-3p	novel-ssc-miR710-5p	novel-ssc-miR802-3p
BGIR9823_88390	100124376	SKIV2L	mRNA	1	0	0	0	0	0	0	0	0	0	0	0	0	0	0	0	0	0	0
BGIR9823_78231	100154984	TMEM87A	lncRNA	1	0	0	0	0	0	0	0	0	0	0	0	0	0	0	0	0	0	0
BGIR9823_84096	106509960	NSMCE2	lncRNA	1	0	0	0	0	0	0	0	0	0	0	0	0	0	0	0	0	0	0
BGIR9823_81704	100521611	FNIP1	lncRNA	1	0	0	0	0	0	0	0	0	0	0	0	0	0	0	0	0	0	0
BGIR9823_79498	100736682	TJP1	lncRNA	1	0	0	0	0	0	0	0	0	0	0	0	0	0	0	0	0	0	0
BGIR9823_100070	100157832	SGCZ	lncRNA	1	0	0	0	0	0	0	0	0	1	0	0	0	0	0	0	0	0	0
BGIR9823_87139	106510467	LOC106510467	lncRNA	1	0	0	0	0	0	0	0	0	0	0	0	0	0	0	0	0	0	0
BGIR9823_100722	100514329	LOC100514329	lncRNA	1	0	0	0	0	0	0	0	0	0	0	0	0	0	0	0	0	0	0
BGIR9823_82833	106509697	CIITA	lncRNA	1	0	0	0	0	0	0	0	0	0	0	0	0	0	0	0	0	0	0
BGIR9823_100488	100518941	TMEM168	lncRNA	1	0	0	0	0	0	0	0	0	1	0	0	0	0	0	0	0	0	0
BGIR9823_101179	100517625	FAM120C	lncRNA	1	0	0	0	0	0	0	0	0	0	0	0	0	0	0	0	0	0	0
BGIR9823_100981	100513513	SLC16A2	mRNA	1	0	0	0	0	0	0	0	0	0	0	0	0	0	0	0	0	0	0
BGIR9823_85418	102163508	LOC102163508	lncRNA	1	0	0	0	0	0	0	0	0	0	0	0	0	0	0	0	0	0	0
BGIR9823_100250	100516472	KIAA1755	lncRNA	1	0	0	0	0	0	0	0	1	0	0	0	0	0	0	0	0	0	0
BGIR9823_100877	100523478	CNKSR2	lncRNA	1	0	0	0	0	0	0	0	0	0	0	0	0	0	0	0	0	0	0
BGIR9823_84851	100523964	LOC100523964	mRNA	1	0	0	0	0	0	0	0	0	0	0	0	0	0	0	0	0	0	0
BGIR9823_79979	100737979	CDK5RAP2	mRNA	1	0	0	0	0	0	0	0	0	0	0	0	0	1	0	0	0	0	0
BGIR9823_85878	100622883	NAV3	mRNA	1	0	0	0	0	0	0	0	0	0	0	0	0	0	0	0	0	0	0
BGIR9823_87603	100620358	ZBTB17	mRNA	1	0	0	0	0	0	0	0	0	0	0	0	0	1	0	0	0	0	0
BGIR9823_81340	100622538	DNM2	lncRNA	1	0	0	0	0	0	0	0	0	0	0	0	0	0	0	0	0	0	0
BGIR9823_93496	100521398	SMARCE1	lncRNA	1	0	0	0	0	1	0	0	0	0	0	0	0	0	0	0	0	0	0
BGIR9823_86377	100621391	SSC5D	mRNA	1	0	0	0	0	0	0	0	0	0	0	0	0	1	0	0	0	0	0
BGIR9823_85565	110260712	LOC110260712	lncRNA	1	0	0	0	0	0	0	0	0	0	0	0	0	0	0	0	0	0	0
BGIR9823_95828	100511410	PDZRN3	lncRNA	1	1	0	0	0	0	0	0	0	0	0	0	0	0	0	0	0	0	0
BGIR9823_93148	100174955	KLF12	lncRNA	1	0	0	0	0	0	0	0	0	0	0	0	0	0	0	0	0	0	0
BGIR9823_101568	110258600	LOC110258600	lncRNA	1	0	0	0	0	0	0	0	0	0	0	0	0	0	0	0	0	0	0
BGIR9823_79012	100738102	RNF217	lncRNA	1	0	0	0	0	0	0	0	0	0	0	0	0	0	0	0	0	0	0
XM_021091420.1	100523786	PACSIN2	mRNA	1	0	0	0	0	0	0	1	0	0	0	0	0	0	0	0	0	0	0
BGIR9823_100565	100516888	GLI3	lncRNA	1	0	0	0	0	0	0	0	0	0	0	0	0	0	0	0	0	0	0
BGIR9823_78022	100157196	CSNK1G1	lncRNA	1	0	0	0	0	0	0	0	0	0	0	1	0	0	0	0	0	0	0
BGIR9823_78883	100512199	ERMARD	lncRNA	1	0	0	0	0	0	0	0	0	0	0	0	0	0	0	0	0	0	0
BGIR9823_84618	100157977	FNBP1L	lncRNA	1	0	0	0	0	0	0	0	0	0	0	0	0	0	0	0	0	0	0
BGIR9823_97044	100158106	NRG3	lncRNA	1	0	0	0	0	0	0	0	0	0	0	0	0	0	0	0	0	0	0
BGIR9823_99699	100513766	G3BP1	mRNA	1	0	0	0	0	0	0	0	0	0	0	0	0	1	0	0	0	0	0
BGIR9823_87685	100512789	WASF2	lncRNA	1	0	0	0	0	0	0	0	0	0	0	0	0	0	0	0	0	0	0
BGIR9823_100122	100519631	TASP1	lncRNA	1	0	0	0	0	0	0	0	0	1	0	0	0	0	0	0	0	0	0
BGIR9823_100289	100738810	SLC13A3	lncRNA	1	0	0	0	0	0	0	0	0	1	0	0	0	0	0	0	0	0	0
BGIR9823_80121	100737568	SSSCA1	lncRNA	1	0	0	0	0	0	0	0	0	0	0	0	0	0	0	0	0	0	0
BGIR9823_86301	100628160	SNRNP70	mRNA	1	0	0	0	0	0	0	0	0	0	0	0	0	0	0	0	0	0	0
BGIR9823_100376	100514612	BRAF	lncRNA	1	0	0	0	0	0	0	0	0	0	0	0	1	0	0	0	0	0	0
BGIR9823_80749	100514631	MAN2A1	lncRNA	1	0	0	0	0	0	0	0	0	0	0	0	0	0	0	0	0	0	0
BGIR9823_101511	110258473	LOC110258473	lncRNA	1	0	0	0	0	0	0	0	0	0	0	0	0	0	0	0	0	0	0
BGIR9823_100755	100620722	ZNF277	lncRNA	1	0	0	0	0	0	0	0	0	0	0	0	0	0	0	0	0	0	0

RNA ID	Gene ID	Symbol	Type	target_total	ssc-miR-29a-3p	novel-ssc-miR204-5p	novel-ssc-miR480-3p	novel-ssc-miR537-5p	novel-ssc-miR620-3p	novel-ssc-miR627-5p	novel-ssc-miR671-3p	novel-ssc-miR753-5p	novel-ssc-miR832-3p	novel-ssc-miR1287-5p	novel-ssc-miR1406-3p	ssc-miR-10383	novel-ssc-miR2-3p	ssc-miR-660	novel-ssc-miR9-3p	novel-ssc-miR16-3p	novel-ssc-miR710-5p	novel-ssc-miR802-3p
BGIR9823_82116	100624598	MEIOB	lncRNA	1	0	0	0	0	0	0	0	0	0	0	0	0	0	0	0	0	0	0
BGIR9823_101016	100520165	TMEM164	lncRNA	1	0	0	0	0	0	0	0	0	0	0	0	0	0	0	0	0	0	0
BGIR9823_90026	110262158	LOC110262158	lncRNA	1	0	0	0	0	0	0	0	0	0	0	0	0	0	1	0	0	0	0
BGIR9823_94087	100515340	LOC100515340	lncRNA	1	0	0	0	0	0	0	0	0	0	0	0	0	0	0	0	0	0	0
BGIR9823_89845	100516092	GNPDA2	lncRNA	1	0	0	0	0	0	0	0	0	0	0	0	0	0	0	0	0	0	0
BGIR9823_99243	100521757	ZNF622	lncRNA	1	0	0	0	0	0	0	0	0	0	0	0	0	0	0	0	0	0	0
BGIR9823_91934	100196908	TOR3A	lncRNA	1	0	0	0	0	0	0	0	0	0	0	0	0	0	0	0	0	0	0
BGIR9823_80152	100620658	HNRNPUL2	mRNA	1	0	0	0	0	0	0	0	0	0	0	0	0	0	0	0	0	0	0
BGIR9823_95348	100513438	NAA50	lncRNA	1	0	0	0	0	0	0	0	0	0	0	0	0	0	0	0	0	0	0
BGIR9823_86872	102164512	ZNF397	lncRNA	1	0	0	0	0	0	0	0	0	0	0	0	0	1	0	0	0	0	0
BGIR9823_85545	100525288	OSBPL8	lncRNA	1	0	0	0	0	0	0	0	0	0	0	0	0	1	0	0	0	0	0
BGIR9823_92701	100739805	CDK8	lncRNA	1	0	0	0	0	0	0	0	0	0	0	0	0	0	0	0	0	0	0
BGIR9823_79943	100626033	CTNNAL1	lncRNA	1	0	0	0	1	0	0	0	0	0	0	0	0	0	0	0	0	0	0
BGIR9823_100365	100739295	WDR86	lncRNA	1	0	0	0	0	0	0	0	0	0	0	0	0	0	0	0	0	0	0
BGIR9823_82500	100524371	STRN	lncRNA	1	0	0	0	0	0	0	0	0	0	0	0	0	0	0	0	0	0	0
BGIR9823_83316	397176	TAF1B	lncRNA	1	0	0	0	0	0	0	0	0	0	0	0	0	0	0	0	0	0	0
BGIR9823_82589	110260059	CYS1	lncRNA	1	0	0	0	0	0	0	0	0	0	0	0	0	0	0	0	0	0	0
BGIR9823_100212	100157872	PXMP4	lncRNA	1	0	0	0	0	0	0	0	0	0	0	0	0	0	0	0	0	0	0
BGIR9823_84315	106510091	SPIDR	mRNA	1	0	0	0	0	0	0	0	0	0	0	0	0	1	0	0	0	0	0
BGIR9823_85039	100625270	ST8SIA1	mRNA	1	0	0	0	0	0	0	0	0	0	0	1	0	0	0	0	0	0	0
BGIR9823_79988	396733	GGTA1P	lncRNA	1	0	0	0	0	0	0	0	0	0	0	0	0	0	0	0	0	0	0
BGIR9823_81636	100625008	GIN1	lncRNA	1	0	0	0	0	0	0	0	0	0	0	0	0	0	0	0	0	0	0
BGIR9823_96851	100522707	ZNF25	lncRNA	1	0	0	0	0	0	0	0	0	0	0	0	0	0	0	0	0	0	0
BGIR9823_98026	733691	NRAP	mRNA	1	0	0	0	0	0	0	0	0	0	0	0	0	0	0	0	0	0	0
BGIR9823_91942	100510921	STX6	mRNA	1	0	0	0	0	0	0	0	0	0	0	0	0	0	0	0	0	0	0
BGIR9823_101516	102157759	LOC102157759	lncRNA	1	0	0	0	0	0	0	0	0	0	0	0	0	0	0	0	0	0	0
BGIR9823_79269	100152360	CCDC68	lncRNA	1	0	0	0	0	0	0	0	0	0	0	0	0	0	0	0	0	0	0
BGIR9823_95434	100737549	ITSN1	mRNA	1	0	0	0	0	0	0	0	0	0	0	0	0	0	0	0	0	0	0
BGIR9823_81890	100621608	FOPNL	lncRNA	1	0	0	0	0	0	0	0	0	0	0	0	0	0	0	0	0	0	0
BGIR9823_86166	100739461	ZNF570	lncRNA	1	0	0	0	0	0	0	0	0	0	0	0	0	0	1	0	0	0	0
BGIR9823_81903	102162731	ZKSCAN1	mRNA	1	0	0	0	0	0	0	0	0	0	0	0	0	0	0	0	0	0	0
BGIR9823_84182	100153957	KCNS2	mRNA	1	0	0	0	0	0	0	0	0	0	0	0	0	0	0	0	0	0	0
BGIR9823_78637	100155691	AK3	lncRNA	1	0	0	0	0	0	0	0	0	0	0	0	0	0	0	0	0	0	0
BGIR9823_79359	100157164	GABPB1	lncRNA	1	0	0	0	0	0	0	0	0	0	0	0	0	0	0	0	0	0	0
BGIR9823_81535	100520548	WDR41	lncRNA	1	0	0	0	0	0	0	0	0	0	0	0	0	0	0	0	0	0	0
BGIR9823_86229	100516271	DEDD2	mRNA	1	0	0	0	0	0	0	0	0	0	0	0	0	0	0	0	0	0	0
BGIR9823_81434	110259374	LOC110259374	lncRNA	1	0	0	0	1	0	0	0	0	0	0	0	0	0	0	0	0	0	0
BGIR9823_100426	100626356	UBE2H	lncRNA	1	0	0	0	0	0	0	0	0	0	0	0	0	0	0	0	0	0	0
BGIR9823_100415	100620243	PLXNA4	lncRNA	1	0	0	0	0	0	0	0	0	0	0	0	0	0	0	0	0	0	0
BGIR9823_100894	100621199	IL1RAPL1	lncRNA	1	0	0	0	0	0	0	0	0	0	0	0	0	0	0	0	0	0	0
BGIR9823_95922	100622559	PIK3CB	lncRNA	1	0	0	0	0	0	0	0	0	0	0	0	0	0	0	0	0	0	0
BGIR9823_82064	100736832	GRIN2A	lncRNA	1	0	0	0	0	0	0	0	0	0	0	0	0	0	0	0	0	0	0
BGIR9823_100306	110257429	LOC110257429	lncRNA	1	0	0	0	0	0	0	0	0	0	0	0	0	0	0	0	0	0	0

RNA ID	Gene ID	Symbol	Type	target_total	ssc-miR-29a-3p	novel-ssc-miR204-5p	novel-ssc-miR480-3p	novel-ssc-miR537-5p	novel-ssc-miR620-3p	novel-ssc-miR627-5p	novel-ssc-miR671-3p	novel-ssc-miR753-5p	novel-ssc-miR832-3p	novel-ssc-miR1287-5p	novel-ssc-miR1406-3p	ssc-miR-10383	novel-ssc-miR2-3p	ssc-miR-660	novel-ssc-miR9-3p	novel-ssc-miR16-3p	novel-ssc-miR710-5p	novel-ssc-miR802-3p
BGIR9823_81302	100736972	LOC100736972	lncRNA	1	0	0	0	0	0	0	0	0	0	0	0	0	0	0	0	0	0	0
BGIR9823_100478	100621338	BMT2	lncRNA	1	0	0	0	0	0	0	0	0	0	0	0	0	0	0	0	0	0	0
BGIR9823_86034	100623274	CTCF	mRNA	1	0	0	0	0	0	0	0	0	0	0	0	0	0	0	1	0	0	0
BGIR9823_78972	100155445	VTA1	lncRNA	1	0	0	0	0	0	0	0	0	0	0	0	0	0	0	0	0	0	0
BGIR9823_78078	100154204	MYO1E	lncRNA	1	0	0	0	0	0	0	0	0	0	0	0	0	0	0	0	0	0	0
BGIR9823_82851	100522863	RBFOX1	mRNA	1	0	0	0	0	0	0	0	0	0	0	0	0	0	0	0	0	0	0
BGIR9823_87122	100515140	KIAA0513	lncRNA	1	0	0	0	0	0	0	0	0	0	0	0	0	0	0	0	0	0	0
BGIR9823_97004	100154626	ADK	lncRNA	1	0	0	0	0	0	0	0	0	0	0	0	0	0	0	0	0	0	0
BGIR9823_90785	100524160	ACER3	lncRNA	1	0	0	0	0	0	0	0	0	0	0	0	0	0	0	0	0	0	0
BGIR9823_92442	100513615	C10H9orf3	mRNA	1	0	0	0	0	0	0	0	0	0	0	0	0	0	0	0	0	0	0
BGIR9823_90875	100622590	BIRC3	lncRNA	1	0	0	0	0	0	0	0	0	0	0	0	0	0	0	0	0	0	0
BGIR9823_101409	110258214	LOC110258214	lncRNA	1	0	0	0	0	0	0	0	0	0	0	0	0	0	0	0	0	0	0
BGIR9823_101574	102164761	LOC102164761	lncRNA	1	0	1	0	0	0	0	0	0	0	0	0	0	0	0	0	0	0	0
BGIR9823_81206	100519588	USP47	lncRNA	1	0	0	0	0	0	0	0	0	0	0	0	0	0	0	0	0	0	0
BGIR9823_82146	100737615	LUC7L	lncRNA	1	0	0	0	0	0	0	0	0	0	0	0	0	0	0	0	0	0	0
BGIR9823_88950	100620189	NRXN3	lncRNA	1	0	0	0	0	0	0	0	0	0	0	0	0	0	0	0	0	0	0
BGIR9823_100904	100621199	IL1RAPL1	lncRNA	1	0	0	0	0	0	0	0	0	0	0	0	0	0	0	0	0	0	1
BGIR9823_95076	100622923	PPM1L	lncRNA	1	0	0	0	0	0	0	0	0	0	0	0	0	0	1	0	0	0	0
BGIR9823_100269	102159476	LOC102159476	lncRNA	1	0	0	0	0	0	0	0	0	0	0	0	0	0	0	0	0	0	0
BGIR9823_101628	100519072	LOC100519072	lncRNA	1	0	0	0	0	0	0	0	0	0	0	0	0	0	0	0	0	0	0
BGIR9823_101612	100521600	LOC100521600	lncRNA	1	0	0	0	0	0	0	0	0	0	0	0	0	0	0	0	0	0	0
BGIR9823_101400	110258215	LOC110258215	lncRNA	1	0	0	0	0	0	0	0	0	0	0	0	0	0	0	0	0	0	0
BGIR9823_92255	100514676	ACBD5	lncRNA	1	0	0	0	0	0	0	0	0	0	0	0	0	0	0	0	0	0	0
BGIR9823_85922	100516196	TAF1C	lncRNA	1	0	0	0	0	0	0	0	0	0	0	0	0	0	0	0	0	0	0
BGIR9823_81946	100525688	BUD23	mRNA	1	0	0	0	0	0	0	0	0	0	0	1	0	0	0	0	0	0	0
BGIR9823_80177	106504153	AHNAK	mRNA	1	0	0	0	0	0	0	0	0	0	0	0	0	0	0	0	0	0	0
BGIR9823_88439	106504047	ZNF76	mRNA	1	0	0	0	0	0	0	0	0	0	0	0	0	0	0	0	0	0	0
BGIR9823_81354	100517998	MYO1F	lncRNA	1	0	0	0	0	0	0	0	0	0	0	0	0	0	0	0	0	0	0
BGIR9823_80921	100329221	PPARGC1B	mRNA	1	0	0	0	0	0	0	0	0	0	0	0	0	0	0	0	0	0	0
BGIR9823_77718	100625026	PPIL4	lncRNA	1	0	0	0	0	0	0	0	0	0	0	0	0	0	0	0	0	0	0
BGIR9823_100514	100623678	NT5C3A	lncRNA	1	0	0	0	0	0	0	0	0	1	0	0	0	0	0	0	0	0	0
BGIR9823_78774	396733	GGTA1P	lncRNA	1	0	0	0	0	0	0	0	0	0	0	0	0	0	0	0	0	0	0
BGIR9823_101033	100037300	XIAP	lncRNA	1	0	0	1	0	0	0	0	0	0	0	0	0	0	0	0	0	0	0
BGIR9823_100816	110257601	LOC110257601	lncRNA	1	0	0	0	0	0	0	0	0	0	0	0	0	0	0	0	0	0	0
BGIR9823_90686	733582	CDS1	lncRNA	1	0	0	0	0	0	0	0	0	0	0	0	0	0	0	0	0	0	0
BGIR9823_91751	110255475	LOC110255475	lncRNA	1	0	0	0	0	0	0	0	0	0	0	0	0	0	0	0	0	0	0
NM_001244541.1	100513376	GMPPB	mRNA	1	0	0	0	0	0	0	0	0	0	0	0	0	0	0	0	0	0	0
BGIR9823_81475	100620238	LOC100620238	lncRNA	1	0	0	0	0	0	0	0	0	0	0	0	0	0	0	0	0	0	0
BGIR9823_100582	102162331	KRBA1	lncRNA	1	0	0	0	0	0	0	0	0	0	0	0	0	0	0	0	0	0	0
BGIR9823_101332	100625415	EIF1AY	lncRNA	1	0	0	0	0	0	0	0	0	0	0	0	0	0	0	0	0	0	0
BGIR9823_97502	595128	OAS2	lncRNA	1	0	0	0	0	0	0	0	0	0	0	0	0	0	0	0	0	0	0
BGIR9823_83701	100153809	ALDH9A1	mRNA	1	0	0	0	0	0	0	0	0	0	0	0	0	0	0	0	0	0	0
BGIR9823_80203	100516953	MPEG1	lncRNA	1	0	0	0	0	0	0	0	0	0	0	0	0	0	0	0	0	0	0

RNA ID	Gene ID	Symbol	Type	target_total	ssc-miR-29a-3p	novel-ssc-miR204-5p	novel-ssc-miR480-3p	novel-ssc-miR537-5p	novel-ssc-miR620-3p	novel-ssc-miR627-5p	novel-ssc-miR671-3p	novel-ssc-miR753-5p	novel-ssc-miR832-3p	novel-ssc-miR1287-5p	novel-ssc-miR1406-3p	ssc-miR-10383	novel-ssc-miR2-3p	ssc-miR-660	novel-ssc-miR9-3p	novel-ssc-miR16-3p	novel-ssc-miR710-5p	novel-ssc-miR802-3p
BGIR9823_99394	100517687	BDP1	lncRNA	1	0	0	0	0	0	0	0	0	0	0	0	0	0	0	0	0	0	0
BGIR9823_85850	102159120	LOC102159120	lncRNA	1	1	0	0	0	0	0	0	0	0	0	0	0	0	0	0	0	0	0
BGIR9823_83931	100519711	ST7L	mRNA	1	0	0	0	0	0	0	0	0	0	0	0	0	0	0	0	0	0	0
BGIR9823_93274	100513428	CEP131	lncRNA	1	0	0	0	0	0	0	0	0	0	0	0	0	0	0	0	0	0	0
BGIR9823_78717	100192437	ERP44	lncRNA	1	0	0	0	0	0	0	0	0	0	0	0	0	0	0	0	0	0	0
BGIR9823_81192	100514626	PIK3C2A	lncRNA	1	0	0	0	0	0	0	0	0	0	0	0	0	0	0	0	0	0	0
BGIR9823_77955	100153597	IBTK	lncRNA	1	0	0	0	0	0	0	0	0	0	0	0	0	0	0	0	0	0	0
BGIR9823_89270	100156900	BTBD9	mRNA	1	0	0	0	0	0	0	1	0	0	0	0	0	0	0	0	0	0	0
BGIR9823_98700	100512563	MGAT5	mRNA	1	0	0	0	0	0	0	0	0	0	0	0	0	0	0	0	0	0	0
BGIR9823_88596	110261555	SEMA4B	lncRNA	1	0	0	0	0	0	0	0	0	0	0	0	0	0	0	0	0	0	0
BGIR9823_85735	110260764	YAF2	lncRNA	1	0	0	0	0	0	0	0	0	0	0	0	0	0	0	0	0	0	0
BGIR9823_89296	100153071	TRERF1	lncRNA	1	0	0	0	0	0	0	0	0	0	0	0	0	0	0	0	0	0	0
BGIR9823_79093	100154222	EPHA7	lncRNA	1	0	0	0	0	0	0	0	0	0	0	0	0	0	0	0	0	0	0
BGIR9823_84443	100144448	OAZ3	lncRNA	1	0	0	0	0	0	0	0	0	0	0	0	0	0	0	0	0	0	0
BGIR9823_82885	100192445	PDPK1	lncRNA	1	0	0	0	0	0	0	0	0	0	0	0	0	0	0	0	0	0	0
BGIR9823_88750	100522330	SLC7A8	lncRNA	1	0	0	0	0	0	0	0	0	0	0	0	0	0	1	0	0	0	0
BGIR9823_77991	102159046	LOC102159046	lncRNA	1	0	0	0	0	0	0	0	0	0	0	0	0	0	0	0	0	0	0
BGIR9823_88964	100157303	ZC3H14	mRNA	1	0	0	0	0	0	0	0	0	0	0	0	0	0	0	0	0	0	0
BGIR9823_78191	102161909	LOC102161909	lncRNA	1	0	0	0	0	0	0	0	0	0	0	0	0	0	0	0	0	0	0
BGIR9823_81917	100620791	TRIP6	mRNA	1	0	0	0	0	0	0	0	0	0	0	0	0	0	0	0	0	0	0
BGIR9823_82685	110259888	LOC110259888	lncRNA	1	0	0	0	0	0	0	0	0	0	0	0	0	0	0	0	0	0	0
BGIR9823_84081	100153657	PHF20L1	mRNA	1	0	0	0	0	0	0	0	0	0	0	1	0	0	0	0	0	0	0
BGIR9823_83050	100628224	VAMP5	lncRNA	1	0	0	0	0	0	0	0	0	0	0	0	0	0	0	0	0	0	0
BGIR9823_98423	397285	ITGAV	mRNA	1	0	0	0	0	0	0	0	0	0	0	0	0	0	0	0	0	0	0
BGIR9823_97702	100155475	HNRNPF	mRNA	1	0	0	0	0	0	0	0	0	0	0	0	0	0	0	0	0	0	0
BGIR9823_93940	397293	KCNJ2	lncRNA	1	0	0	0	0	0	0	0	0	0	0	0	0	0	0	0	0	0	0
BGIR9823_87613	100521974	FBXO42	lncRNA	1	0	0	0	0	0	0	0	0	0	0	0	0	0	1	0	0	0	0
BGIR9823_94936	100155306	SEC61A1	lncRNA	1	1	0	0	0	0	0	0	0	0	0	0	0	0	0	0	0	0	0
BGIR9823_100651	100519065	UBN2	lncRNA	1	0	0	0	0	0	0	0	0	0	0	0	0	0	0	0	0	0	0
BGIR9823_84118	100737543	TBC1D31	lncRNA	1	0	0	0	0	0	0	0	0	0	0	0	0	0	1	0	0	0	0
BGIR9823_79608	100523347	UACA	lncRNA	1	0	0	0	0	0	0	0	0	0	0	0	0	0	0	0	0	0	0
BGIR9823_95065	100514494	LOC100514494	lncRNA	1	0	0	0	0	0	0	0	0	0	0	0	0	0	0	0	0	0	0
BGIR9823_89501	100155701	NYNRIN	mRNA	1	0	0	0	0	0	0	0	0	0	0	1	0	0	0	0	0	0	0
BGIR9823_79516	100155619	SOCS6	lncRNA	1	0	0	0	0	0	0	0	0	0	0	0	0	0	0	0	0	0	0
BGIR9823_93273	100626205	SLC38A10	mRNA	1	0	0	0	0	0	0	0	0	0	0	0	0	0	0	0	0	0	0
BGIR9823_82045	100739121	LOC100739121	lncRNA	1	0	0	0	0	0	0	0	0	0	0	0	0	0	0	0	0	0	0
BGIR9823_99804	100519342	RNF24	lncRNA	1	0	0	0	0	0	0	0	0	0	0	1	0	0	0	0	0	0	0
BGIR9823_81050	102164585	LOC102164585	mRNA	1	0	0	0	0	0	0	0	0	0	0	0	0	0	0	0	0	0	0
BGIR9823_83089	100515889	DGUOK	lncRNA	1	0	0	0	0	0	0	0	0	0	0	0	0	0	0	0	0	0	0
BGIR9823_85641	100157726	ETV6	lncRNA	1	0	0	0	0	0	0	0	0	0	0	0	0	0	0	0	0	0	0
BGIR9823_80399	100514037	EPS15L1	lncRNA	1	0	0	0	1	0	0	0	0	0	0	0	0	0	0	0	0	0	0
BGIR9823_100428	100517203	NRF1	lncRNA	1	0	0	0	0	0	0	0	0	0	0	0	0	0	0	0	0	1	0
BGIR9823_79736	110255453	LOC110255453	lncRNA	1	0	0	0	0	0	0	0	0	0	0	0	0	0	0	0	0	0	0

RNA ID	Gene ID	Symbol	Type	target_total	ssc-miR-29a-3p	novel-ssc-miR204-5p	novel-ssc-miR480-3p	novel-ssc-miR537-5p	novel-ssc-miR620-3p	novel-ssc-miR627-5p	novel-ssc-miR671-3p	novel-ssc-miR753-5p	novel-ssc-miR832-3p	novel-ssc-miR1287-5p	novel-ssc-miR1406-3p	ssc-miR-10383	novel-ssc-miR2-3p	ssc-miR-660	novel-ssc-miR9-3p	novel-ssc-miR16-3p	novel-ssc-miR710-5p	novel-ssc-miR802-3p
BGIR9823_88239	100512907	LOC100512907	lncRNA	1	0	0	0	0	0	0	0	0	0	0	0	0	0	0	0	0	0	0
BGIR9823_89801	100524447	TBC1D1	lncRNA	1	1	0	0	0	0	0	0	0	0	0	0	0	0	0	0	0	0	0
BGIR9823_78621	100627072	PTPRD	lncRNA	1	0	0	0	0	0	0	0	0	0	0	0	0	0	0	0	0	0	0
BGIR9823_78489	100152019	PELI2	lncRNA	1	0	0	0	0	0	0	0	0	0	0	0	0	0	0	0	0	0	0
BGIR9823_83940	100155227	RBM15	lncRNA	1	0	0	0	0	0	0	0	0	0	0	0	0	0	0	0	0	0	0
BGIR9823_85932	106510466	MAF	lncRNA	1	0	0	0	0	0	0	0	0	0	0	0	0	0	0	0	0	0	0
BGIR9823_81640	100513341	FBXL17	lncRNA	1	0	0	0	0	0	0	0	0	0	0	0	0	0	0	0	0	0	0
BGIR9823_84349	100154639	PBX1	mRNA	1	0	0	0	0	0	0	0	0	0	0	0	0	0	0	0	0	0	0
BGIR9823_100606	100515515	GALNT11	lncRNA	1	0	0	0	0	0	0	0	0	0	0	0	0	0	0	0	0	0	0
BGIR9823_101514	102157759	LOC102157759	lncRNA	1	0	0	0	0	0	0	0	0	0	0	0	0	0	0	0	0	0	0
BGIR9823_85476	100622365	TSPAN31	lncRNA	1	0	0	0	0	0	0	0	0	0	0	0	0	0	0	0	0	0	0
BGIR9823_89890	100515957	PPID	lncRNA	1	0	0	0	0	0	0	0	0	0	0	0	0	1	0	0	0	0	0
BGIR9823_101279	100153213	HS6ST2	mRNA	1	0	0	0	0	0	0	0	0	0	0	0	0	0	0	0	0	0	0
BGIR9823_92274	100520446	PIP4K2A	lncRNA	1	0	0	0	0	0	0	0	0	0	0	0	0	0	0	0	0	0	0
BGIR9823_83415	106509958	MTSS1	lncRNA	1	0	0	0	0	0	0	0	0	0	0	0	0	0	0	0	0	0	0
BGIR9823_78462	100511360	FBXO34	lncRNA	1	0	0	0	1	0	0	0	0	0	0	0	0	0	0	0	0	0	0
BGIR9823_77832	100736796	ADGRB3	lncRNA	1	0	0	0	0	0	0	0	0	0	0	0	0	0	0	0	0	0	0
BGIR9823_79204	397085	MYO6	lncRNA	1	0	0	0	0	0	0	0	0	0	0	0	0	0	0	0	0	0	0
BGIR9823_83395	110260216	LOC110260216	lncRNA	1	0	0	0	0	0	0	0	0	0	0	0	0	0	0	0	0	0	0
BGIR9823_84473	100153347	GOLPH3L	lncRNA	1	0	0	0	0	0	0	0	0	0	0	0	0	0	1	0	0	0	0
BGIR9823_85369	100518305	LOC100518305	mRNA	1	0	0	0	0	0	0	0	0	0	0	0	0	0	0	0	0	0	0
BGIR9823_84074	100627184	GLI4	lncRNA	1	0	0	0	0	0	0	0	0	0	0	0	0	0	0	0	0	0	0
BGIR9823_92298	100515404	ITIH5	lncRNA	1	0	0	0	0	0	0	0	0	0	0	0	0	0	0	0	0	0	0
BGIR9823_89061	100153581	NUP153	mRNA	1	0	0	0	0	0	0	0	0	0	0	0	0	0	0	0	0	0	0
BGIR9823_96626	100151786	POP5	lncRNA	1	1	0	0	0	0	0	0	0	0	0	0	0	0	0	0	0	0	0
BGIR9823_87721	100517038	PUM1	lncRNA	1	0	0	0	0	0	0	0	0	0	0	0	0	0	0	0	0	0	0
BGIR9823_100728	100512382	KCND2	lncRNA	1	0	0	0	0	0	0	0	0	0	0	0	0	0	0	0	0	0	0
BGIR9823_80555	110259375	LOC110259375	lncRNA	1	0	0	0	0	0	0	0	0	0	0	0	0	0	0	0	0	0	0
BGIR9823_79909	100155492	HEMGN	lncRNA	1	0	0	0	0	0	0	0	0	0	0	0	0	0	0	0	0	0	0
BGIR9823_94689	100516877	ACOX2	lncRNA	1	0	0	0	0	0	0	0	0	0	0	0	0	0	0	0	0	0	0
BGIR9823_92764	102158088	NHLRC3	lncRNA	1	0	0	0	0	0	0	0	0	0	0	0	0	0	0	0	0	0	0
BGIR9823_89682	100152306	LOC100152306	mRNA	1	0	0	0	0	0	0	0	0	0	0	0	0	0	0	0	0	0	0
BGIR9823_96521	100623262	SLC15A4	mRNA	1	0	0	0	0	0	0	0	0	0	0	0	0	0	0	0	0	0	0
BGIR9823_84835	100516439	C5H12orf10	lncRNA	1	0	0	0	0	0	0	0	0	0	0	0	0	0	0	0	0	0	0
BGIR9823_100509	100738688	HERPUD2	lncRNA	1	0	0	0	0	0	0	0	0	0	0	0	0	0	0	0	0	0	0
BGIR9823_86157	100511329	ZNF829	mRNA	1	0	0	0	0	0	0	0	0	0	0	0	0	1	0	0	0	0	0
BGIR9823_82679	100620395	GIGYF1	mRNA	1	0	0	0	0	0	0	0	0	0	0	0	0	0	0	0	0	0	0
BGIR9823_90779	100621182	EMSY	mRNA	1	0	0	0	0	0	0	0	0	0	0	0	0	0	0	0	0	0	0
BGIR9823_90540	100621145	LARP1B	lncRNA	1	0	0	0	0	0	0	0	0	0	0	0	0	0	1	0	0	0	0
NM_001097469.1	100037984	HIGD2A	mRNA	1	0	0	0	0	0	0	0	0	0	0	0	0	1	0	0	0	0	0
BGIR9823_78404	102162695	LOC102162695	lncRNA	1	0	0	0	0	0	0	0	0	0	0	0	0	0	0	0	0	0	0
BGIR9823_84013	102161056	LOC102161056	lncRNA	1	0	0	0	0	0	0	0	0	0	0	0	0	0	1	0	0	0	0
BGIR9823_98214	100512817	TENM3	mRNA	1	0	0	0	0	0	0	0	0	0	0	0	0	0	0	0	0	0	0

RNA ID	Gene ID	Symbol	Type	target_total	ssc-miR-29a-3p	novel-ssc-miR204-5p	novel-ssc-miR480-3p	novel-ssc-miR537-5p	novel-ssc-miR620-3p	novel-ssc-miR627-5p	novel-ssc-miR671-3p	novel-ssc-miR753-5p	novel-ssc-miR832-3p	novel-ssc-miR1287-5p	novel-ssc-miR1406-3p	ssc-miR-10383	novel-ssc-miR2-3p	ssc-miR-660	novel-ssc-miR9-3p	novel-ssc-miR16-3p	novel-ssc-miR710-5p	novel-ssc-miR802-3p
BGIR9823_81705	100521611	FNIP1	lncRNA	1	0	0	0	0	0	0	0	0	0	0	0	0	0	0	0	0	0	0
BGIR9823_79689	100192435	ERO1A	lncRNA	1	0	0	0	0	0	0	0	0	0	0	0	0	0	0	0	0	0	0
BGIR9823_81181	100736787	ZDHHC13	mRNA	1	0	0	0	0	0	0	0	0	0	0	0	0	0	0	0	0	0	0
BGIR9823_95430	396658	IFNAR1	mRNA	1	0	0	0	0	0	0	0	0	0	0	0	0	0	0	0	0	0	0
BGIR9823_77980	100152640	HAUS1	lncRNA	1	0	0	0	0	0	0	0	0	0	0	0	0	0	0	0	0	0	0
BGIR9823_91823	100522092	RUNDC3B	lncRNA	1	0	0	0	0	0	0	0	0	0	0	0	0	0	0	0	0	0	0
BGIR9823_81117	100144442	F2	lncRNA	1	0	0	0	0	0	0	0	0	0	0	0	0	0	0	0	0	0	0
BGIR9823_90627	100624822	GSTCD	mRNA	1	0	0	0	0	0	0	0	0	0	0	0	0	0	0	0	0	0	0
BGIR9823_78165	100169932	TRPM7	lncRNA	1	0	0	0	0	0	0	0	0	0	0	0	0	0	0	0	0	0	0
BGIR9823_80118	100511178	SNX32	lncRNA	1	0	0	0	0	0	0	0	0	0	0	0	0	0	0	0	0	0	0
BGIR9823_84495	102166500	LOC102166500	lncRNA	1	0	0	0	0	0	0	0	0	0	0	0	0	0	0	0	0	0	0
BGIR9823_81367	100127167	CERS4	mRNA	1	0	0	0	0	0	0	0	0	0	0	0	0	0	0	0	0	0	0
BGIR9823_77687	100156632	AGPAT4	lncRNA	1	0	0	0	0	0	0	0	0	0	0	0	0	0	0	0	0	0	0
BGIR9823_80249	100519700	AMBRA1	lncRNA	1	1	0	0	0	0	0	0	0	0	0	0	0	0	0	0	0	0	0
BGIR9823_100622	100521480	GSTK1	lncRNA	1	0	0	0	0	0	0	0	0	0	0	0	0	0	0	0	0	0	0
BGIR9823_86113	100621064	DPY19L3	lncRNA	1	0	0	0	0	0	0	0	0	0	0	0	0	0	0	0	0	0	0
BGIR9823_92205	100516148	MOB3B	mRNA	1	0	0	0	0	0	0	0	0	0	0	0	0	0	0	0	0	0	0
XM_021075409.1	100737004	COBLL1	mRNA	1	0	0	0	0	0	0	0	0	0	0	0	0	0	0	0	0	0	0
BGIR9823_89246	100521322	LOC100521322	lncRNA	1	0	0	0	0	0	0	0	0	0	0	0	0	0	0	0	0	0	0
BGIR9823_78193	100154291	SLC12A1	lncRNA	1	0	0	0	0	0	0	0	0	0	0	0	0	0	0	0	0	0	0
BGIR9823_100566	100516888	GLI3	lncRNA	1	0	0	0	0	0	0	0	0	0	0	0	0	0	0	0	0	0	0
BGIR9823_100446	100192443	POT1	lncRNA	1	0	0	0	0	0	0	0	0	0	0	0	0	0	0	0	0	0	0
BGIR9823_97408	100620430	SAP30	lncRNA	1	0	0	0	0	0	0	0	0	0	0	0	0	0	0	0	0	0	0
BGIR9823_86274	100622144	BICRA	lncRNA	1	0	0	0	0	0	0	0	0	0	0	0	0	0	0	0	0	0	0
BGIR9823_101110	100524780	ARHGAP6	lncRNA	1	0	0	0	0	0	0	0	0	0	0	0	1	0	0	0	0	0	0
BGIR9823_82012	100524333	GGA2	lncRNA	1	0	0	0	0	0	0	0	0	0	0	0	0	0	0	0	0	0	0
BGIR9823_86283	100624752	NOP53	mRNA	1	0	0	0	0	0	0	0	0	0	0	0	0	0	0	0	0	0	0
BGIR9823_98317	110256929	WDSUB1	lncRNA	1	0	0	0	0	0	0	0	0	0	0	0	0	0	0	0	0	0	0
BGIR9823_88517	100511822	ICK	lncRNA	1	0	0	0	0	0	0	0	0	0	0	0	0	0	0	0	0	0	0
BGIR9823_79454	110259520	LOC110259520	lncRNA	1	0	0	0	0	0	0	0	0	0	0	0	0	0	0	0	0	0	0
BGIR9823_81324	606746	DNMT1	mRNA	1	0	0	0	0	0	0	0	0	0	0	0	0	0	0	0	0	0	0
BGIR9823_100702	100514931	AHCYL2	lncRNA	1	0	0	0	0	0	0	0	0	0	0	0	0	0	0	0	0	0	0
BGIR9823_101292	100523658	ATP11C	mRNA	1	0	0	0	0	0	0	0	0	0	0	0	0	0	0	0	0	0	0
BGIR9823_90549	102167943	LOC102167943	lncRNA	1	0	0	0	0	0	0	0	0	0	0	0	0	0	0	0	0	0	0
BGIR9823_84672	100516804	SCO2	mRNA	1	0	0	0	0	0	0	0	0	0	0	0	0	0	0	0	0	0	0
BGIR9823_98499	100514879	ABI2	mRNA	1	0	0	0	0	0	0	0	0	0	0	0	0	0	0	0	0	0	0
BGIR9823_83000	102166005	LOC102166005	lncRNA	1	0	0	0	0	0	0	0	0	0	0	0	0	0	0	0	0	0	0
BGIR9823_79430	100152163	DLL4	lncRNA	1	0	0	0	0	0	0	0	0	0	0	0	0	0	0	0	0	0	0
BGIR9823_80973	100511242	TMEM134	lncRNA	1	1	0	0	0	0	0	0	0	0	0	0	0	0	0	0	0	0	0
BGIR9823_101334	100523617	TRIM27	lncRNA	1	0	0	0	0	0	0	0	0	0	0	0	0	0	0	0	0	0	0
BGIR9823_81536	100520548	WDR41	lncRNA	1	0	0	0	1	0	0	0	0	0	0	0	0	0	0	0	0	0	0
BGIR9823_101625	100519072	LOC100519072	lncRNA	1	0	1	0	0	0	0	0	0	0	0	0	0	0	0	0	0	0	0
BGIR9823_87181	100517402	PDF	lncRNA	1	0	0	0	0	0	0	0	0	0	0	0	0	0	0	0	0	0	0

RNA ID	Gene ID	Symbol	Type	target_total	ssc-miR-29a-3p	novel-ssc-miR204-5p	novel-ssc-miR480-3p	novel-ssc-miR537-5p	novel-ssc-miR620-3p	novel-ssc-miR627-5p	novel-ssc-miR671-3p	novel-ssc-miR753-5p	novel-ssc-miR832-3p	novel-ssc-miR1287-5p	novel-ssc-miR1406-3p	ssc-miR-10383	novel-ssc-miR2-3p	ssc-miR-660	novel-ssc-miR9-3p	novel-ssc-miR16-3p	novel-ssc-miR710-5p	novel-ssc-miR802-3p
BGIR9823_78566	100152132	IFN-OMEGA-3	lncRNA	1	0	0	0	1	0	0	0	0	0	0	0	0	0	0	0	0	0	0
XR_002339179.1	110257024	LOC110257024	lncRNA	1	0	0	0	0	0	0	0	0	0	0	0	0	0	0	0	0	0	0
BGIR9823_100144	100154945	RRBP1	lncRNA	1	0	0	0	0	0	0	0	0	0	0	0	0	0	0	0	0	0	0
BGIR9823_100611	100521999	SMARCD3	lncRNA	1	0	0	0	0	0	0	0	0	0	0	0	0	0	0	0	0	0	0
BGIR9823_95072	100620632	KPNA4	lncRNA	1	1	0	0	0	0	0	0	0	0	0	0	0	0	0	0	0	0	0
BGIR9823_98782	100152203	AGA	mRNA	1	0	0	0	0	0	0	0	0	0	0	0	0	0	0	0	0	0	0
BGIR9823_77731	100154785	ADAT2	lncRNA	1	0	0	0	0	0	0	0	1	0	0	0	0	0	0	0	0	0	0
BGIR9823_96586	100153673	CUX2	mRNA	1	0	0	0	0	0	0	0	0	0	0	0	0	0	0	0	0	0	0
BGIR9823_85978	110260924	LOC110260924	lncRNA	1	0	0	0	0	0	0	0	0	0	0	0	0	0	0	0	0	0	0
BGIR9823_78788	100154489	WDR38	mRNA	1	0	0	0	0	0	0	0	0	0	0	0	0	0	0	0	0	0	0
BGIR9823_86389	110261319	LOC110261319	lncRNA	1	0	0	0	0	0	0	0	0	0	0	0	0	0	0	0	0	0	0
BGIR9823_79497	100736682	TJP1	lncRNA	1	0	0	0	0	0	0	0	0	0	0	0	0	0	0	0	0	0	0
BGIR9823_98375	100522284	METAP1D	mRNA	1	0	0	0	0	0	0	0	0	0	0	0	0	0	0	0	0	0	0
BGIR9823_100000	100523757	ARFGEF2	lncRNA	1	0	0	0	0	0	0	0	0	1	0	0	0	0	0	0	0	0	0
BGIR9823_84378	100524676	SDHC	lncRNA	1	0	0	0	0	0	0	0	0	0	0	0	0	0	0	0	0	0	0
BGIR9823_92192	396723	DDX58	mRNA	1	0	0	0	0	0	0	0	0	0	0	0	0	0	0	0	0	0	0
BGIR9823_88795	102162743	FAM174B	lncRNA	1	0	0	0	0	0	0	0	0	0	0	0	0	0	1	0	0	0	0
BGIR9823_92690	100623356	SKA3	lncRNA	1	0	0	0	0	0	0	0	0	0	0	0	0	0	0	0	0	0	0
XR_002340913.1	110257727	LOC110257727	lncRNA	1	1	0	0	0	0	0	0	0	0	0	0	0	0	0	0	0	0	0
BGIR9823_86991	100037307	DAB1	lncRNA	1	0	0	0	0	0	0	0	0	0	0	0	0	0	0	0	0	0	0
BGIR9823_82075	100513346	LOC100513346	mRNA	1	0	0	0	0	0	0	0	0	0	0	0	0	1	0	0	0	0	0
BGIR9823_86237	100524260	ZNF45	lncRNA	1	0	0	0	0	0	0	0	0	0	0	0	0	0	0	0	0	0	0
BGIR9823_80263	106507997	LOC106507997	lncRNA	1	0	0	0	0	0	0	0	0	0	0	0	0	0	0	0	0	0	0
BGIR9823_93834	110256118	LOC110256118	lncRNA	1	0	0	0	0	0	0	0	0	0	0	0	0	0	0	0	0	0	0
BGIR9823_80349	100738340	ATP13A1	lncRNA	1	0	0	0	0	0	0	0	0	0	0	0	0	0	0	0	0	0	0
BGIR9823_89603	106507740	LOC106507740	lncRNA	1	0	0	0	0	0	0	0	0	0	0	0	0	0	0	0	0	0	0
BGIR9823_77903	100514380	TSTD3	lncRNA	1	0	0	0	0	0	0	0	0	0	0	0	0	0	0	0	0	0	0
BGIR9823_100158	100154020	LOC100154020	lncRNA	1	0	0	0	0	0	0	0	1	0	0	0	0	0	0	0	0	0	0
BGIR9823_89657	733584	FOXN3	lncRNA	1	0	0	0	0	0	0	0	0	0	0	0	0	0	0	0	0	0	0
BGIR9823_98765	100233172	AGPAT5	lncRNA	1	0	0	0	0	0	0	0	0	0	0	0	0	0	0	0	0	0	0
BGIR9823_94610	396679	AMT	lncRNA	1	0	0	0	0	0	0	0	0	0	0	0	0	0	0	0	0	0	0
BGIR9823_78506	100154408	PSMA3	lncRNA	1	0	0	0	0	0	0	0	0	0	0	0	0	0	0	0	0	0	0
BGIR9823_78017	100515517	RBPMS2	lncRNA	1	0	0	0	1	0	0	0	0	0	0	0	0	0	0	0	0	0	0
BGIR9823_98223	100515058	TRAPPC11	mRNA	1	0	0	0	0	0	0	0	0	0	0	0	0	0	0	0	0	0	0
BGIR9823_78148	100155516	DMXL2	mRNA	1	0	0	0	0	0	0	0	0	0	0	0	0	0	0	0	0	0	0
BGIR9823_87377	100521972	LOC100521972	lncRNA	1	0	0	0	0	0	0	0	0	0	0	0	0	0	0	0	0	0	0
BGIR9823_84933	100511508	LEMD3	lncRNA	1	0	0	0	0	0	0	0	0	0	0	0	0	0	0	0	0	0	0
BGIR9823_100906	100621199	IL1RAPL1	lncRNA	1	0	0	0	0	0	0	0	0	0	0	0	0	0	0	0	0	0	0
BGIR9823_89271	100156900	BTBD9	mRNA	1	0	0	0	0	0	0	0	0	0	0	0	0	0	0	0	1	0	0
BGIR9823_90463	100627416	TRIM2	lncRNA	1	0	0	0	0	0	0	0	0	0	0	0	0	0	0	0	0	0	0
BGIR9823_79564	100153830	MALT1	lncRNA	1	0	0	0	0	0	0	0	0	0	0	0	0	0	0	0	0	0	0
BGIR9823_88636	100155770	FAM219B	mRNA	1	0	0	0	0	0	0	0	0	0	0	0	0	0	0	0	0	0	0
BGIR9823_84024	100155974	GTF2B	lncRNA	1	0	0	0	0	0	0	0	0	0	0	0	0	0	0	0	0	0	0

RNA ID	Gene ID	Symbol	Type	target_total	ssc-miR-29a-3p	novel-ssc-miR204-5p	novel-ssc-miR480-3p	novel-ssc-miR537-5p	novel-ssc-miR620-3p	novel-ssc-miR627-5p	novel-ssc-miR671-3p	novel-ssc-miR753-5p	novel-ssc-miR832-3p	novel-ssc-miR1287-5p	novel-ssc-miR1406-3p	ssc-miR-10383	novel-ssc-miR2-3p	ssc-miR-660	novel-ssc-miR9-3p	novel-ssc-miR16-3p	novel-ssc-miR710-5p	novel-ssc-miR802-3p
BGIR9823_100486	100621338	BMT2	lncRNA	1	0	0	0	0	0	0	0	0	0	0	0	0	0	0	0	0	0	0
BGIR9823_86035	100623274	CTCF	mRNA	1	0	0	0	0	0	0	0	0	0	0	0	0	0	0	0	0	0	0
BGIR9823_85003	110260713	AMN1	mRNA	1	0	0	0	0	0	0	0	0	0	0	1	0	0	0	0	0	0	0
BGIR9823_100211	100157872	PXMP4	lncRNA	1	0	0	0	0	0	0	0	0	1	0	0	0	0	0	0	0	0	0
BGIR9823_85764	396681	TXNRD1	mRNA	1	0	0	0	0	0	0	0	0	0	0	0	0	0	0	0	0	0	0
BGIR9823_79047	110260218	LOC110260218	lncRNA	1	0	0	0	0	0	0	0	0	0	0	0	0	0	0	0	0	0	0
BGIR9823_99528	100626363	NPR3	lncRNA	1	0	0	0	0	0	0	0	0	0	0	0	0	0	0	0	0	0	0
BGIR9823_86380	100738636	EPN1	mRNA	1	0	0	0	0	0	0	0	0	0	0	0	0	0	0	0	0	0	0
BGIR9823_101388	100521615	ARHGAP17	mRNA	1	0	0	0	0	0	0	0	0	0	0	1	0	0	0	0	0	0	0
BGIR9823_85180	100625757	ARID2	lncRNA	1	0	0	0	0	0	0	0	0	0	0	0	0	0	1	0	0	0	0
BGIR9823_101519	100511414	RNF122	lncRNA	1	0	0	0	0	0	0	0	0	0	0	0	0	0	0	0	0	0	0
BGIR9823_100416	106506781	LOC106506781	lncRNA	1	0	0	0	0	0	0	0	0	0	0	0	0	0	0	0	0	1	0
BGIR9823_77835	100736796	ADGRB3	lncRNA	1	0	0	0	0	0	0	0	0	0	0	0	0	0	0	0	0	0	0
XM_001928635.2	100156587	LOC100156587	mRNA	1	0	0	0	0	0	0	0	0	0	0	1	0	0	0	0	0	0	0
BGIR9823_91988	100217398	FLVCR1	lncRNA	1	0	0	0	0	0	0	0	0	0	0	0	0	0	0	0	0	0	0
BGIR9823_86369	100517285	LOC100517285	mRNA	1	0	0	0	0	0	0	0	0	0	0	0	0	0	0	0	0	0	0
BGIR9823_101017	100520165	TMEM164	lncRNA	1	0	0	0	0	0	0	0	0	0	0	0	0	0	0	0	0	0	0
BGIR9823_81426	100621275	GNG7	lncRNA	1	0	0	0	1	0	0	0	0	0	0	0	0	0	0	0	0	0	0
BGIR9823_94753	100522704	FAM19A1	lncRNA	1	0	0	0	0	0	0	0	0	0	0	0	0	0	0	0	0	0	0
BGIR9823_87686	100512789	WASF2	lncRNA	1	0	0	0	0	0	0	0	0	0	0	0	0	0	0	0	0	0	0
BGIR9823_88695	100624265	STRN3	mRNA	1	0	0	0	0	0	0	0	0	0	0	0	0	0	0	0	0	0	0
BGIR9823_82270	100625157	GCFC2	lncRNA	1	0	0	0	0	0	0	0	0	0	0	0	0	0	0	0	0	0	0
BGIR9823_93946	397091	PRKAR1A	mRNA	1	0	0	0	0	0	0	0	0	0	0	0	0	0	0	0	0	0	0
BGIR9823_93731	110256070	LOC110256070	lncRNA	1	0	0	0	0	0	0	0	0	0	0	0	0	0	1	0	0	0	0
BGIR9823_80458	100515360	ILF3	lncRNA	1	0	0	0	0	0	0	0	0	0	0	0	0	0	0	0	0	0	0
BGIR9823_80830	100513723	JADE2	lncRNA	1	0	0	0	0	0	0	0	0	0	0	0	0	0	0	0	0	0	0
BGIR9823_87106	100739500	ACSF3	lncRNA	1	0	0	0	0	0	0	0	0	0	0	0	0	0	0	0	0	0	0
BGIR9823_91655	100511341	EI24	lncRNA	1	0	0	0	0	0	0	0	0	0	0	0	0	0	0	0	0	0	0
BGIR9823_84152	100337657	ZFPM2	mRNA	1	0	0	0	0	0	0	0	0	0	0	0	0	1	0	0	0	0	0
BGIR9823_78973	100155445	VTA1	lncRNA	1	0	0	0	0	0	0	0	0	0	0	0	0	0	0	0	0	0	0
BGIR9823_88372	100151743	PPP1R18	lncRNA	1	0	0	0	0	0	0	0	0	0	0	0	0	0	0	0	0	0	0
BGIR9823_97928	100155592	PDLIM1	mRNA	1	0	0	0	0	0	0	0	0	0	0	0	0	1	0	0	0	0	0
BGIR9823_88939	100511141	VASH1	mRNA	1	0	0	0	0	0	0	0	0	0	0	0	0	0	0	0	0	0	0
BGIR9823_93334	100623453	KIF19	lncRNA	1	0	0	0	0	0	0	0	0	0	0	0	0	0	0	0	0	0	0
BGIR9823_79684	100153738	TRIM9	lncRNA	1	0	0	0	0	0	0	0	0	0	0	0	0	0	0	0	0	0	0
BGIR9823_100666	100524964	AGBL3	lncRNA	1	0	0	0	0	0	0	0	0	0	0	0	0	0	0	0	0	0	0
BGIR9823_89830	100515040	LIMCH1	mRNA	1	0	0	0	0	0	0	0	0	0	0	0	0	0	0	0	0	0	0
BGIR9823_85457	100511633	RDH16	lncRNA	1	0	0	0	0	0	0	0	0	0	0	0	0	0	0	0	0	0	0
BGIR9823_81268	100512396	SLC35E1	lncRNA	1	0	0	0	0	0	0	0	0	0	0	0	0	0	0	0	0	0	0
BGIR9823_78329	100049703	BCL2	lncRNA	1	0	0	0	0	0	0	0	0	0	0	0	0	0	0	0	0	0	0
BGIR9823_78260	100512756	MEIS2	lncRNA	1	0	0	0	0	0	0	0	0	0	0	0	0	0	0	0	0	0	0
BGIR9823_89336	100152930	ADGRF5	mRNA	1	0	0	0	0	0	0	0	0	0	0	0	0	0	0	0	0	0	0
BGIR9823_89031	100152689	SSR1	mRNA	1	0	0	0	0	0	0	0	0	0	0	0	0	0	0	0	0	0	0

RNA ID	Gene ID	Symbol	Type	target_total	ssc-miR-29a-3p	novel-ssc-miR204-5p	novel-ssc-miR480-3p	novel-ssc-miR537-5p	novel-ssc-miR620-3p	novel-ssc-miR627-5p	novel-ssc-miR671-3p	novel-ssc-miR753-5p	novel-ssc-miR832-3p	novel-ssc-miR1287-5p	novel-ssc-miR1406-3p	ssc-miR-10383	novel-ssc-miR2-3p	ssc-miR-660	novel-ssc-miR9-3p	novel-ssc-miR16-3p	novel-ssc-miR710-5p	novel-ssc-miR802-3p
BGIR9823_78386	100154444	GLCE	lncRNA	1	0	0	0	0	0	0	0	0	0	0	0	0	0	0	0	0	0	0
BGIR9823_92789	100155063	SPRYD7	mRNA	1	0	0	0	0	0	0	0	0	0	0	0	0	0	0	0	0	0	0
BGIR9823_86252	100622514	CLASRP	lncRNA	1	0	0	0	0	0	0	0	0	0	0	0	0	0	0	0	0	0	0
BGIR9823_101153	100621125	SLC35A2	mRNA	1	0	0	0	0	0	0	0	0	0	0	0	0	0	0	0	0	0	0
BGIR9823_100998	414736	PCDH11X	lncRNA	1	0	0	0	0	0	0	0	0	0	0	0	0	0	0	0	0	0	0
BGIR9823_82762	397614	IL4R	lncRNA	1	0	0	0	0	0	0	0	0	0	0	0	0	0	0	0	0	0	0
BGIR9823_79824	100626649	KANK1	lncRNA	1	0	0	0	0	0	0	0	0	0	0	0	0	0	0	0	0	0	0
BGIR9823_95516	100152333	ANKRD28	lncRNA	1	0	0	0	0	0	0	0	0	0	0	0	0	0	0	0	0	0	0
BGIR9823_81853	106509619	LOC106509619	lncRNA	1	0	0	0	0	0	0	0	0	0	0	0	0	0	0	0	0	0	0
BGIR9823_90521	100513554	NAA15	lncRNA	1	0	0	0	0	0	0	0	0	0	0	0	0	0	0	0	0	0	0
BGIR9823_93604	100520097	TUBD1	mRNA	1	0	0	0	0	0	0	0	0	0	0	0	0	0	0	0	0	0	0
BGIR9823_79674	100157402	NIN	lncRNA	1	0	0	0	0	0	0	0	0	0	0	0	0	0	0	0	0	0	0
BGIR9823_100171	100625861	LZTS3	lncRNA	1	0	0	0	0	0	0	0	0	0	1	0	0	0	0	0	0	0	0
BGIR9823_81709	100521611	FNIP1	lncRNA	1	0	0	0	0	0	0	0	0	0	0	0	0	0	0	0	0	0	0
BGIR9823_82252	100738124	IMMT	lncRNA	1	0	0	0	0	0	0	0	0	0	0	0	0	0	1	0	0	0	0
BGIR9823_80640	102165038	LOC102165038	lncRNA	1	0	0	0	0	0	0	0	0	0	0	0	0	0	0	0	0	0	0
BGIR9823_101575	100737380	CDC25A	lncRNA	1	0	0	0	0	0	0	0	0	0	0	0	0	0	0	0	0	0	0
BGIR9823_84694	100518786	HDAC10	lncRNA	1	0	0	0	0	0	0	0	0	0	0	0	0	0	0	0	0	0	0
BGIR9823_98548	102162936	SPAG16	mRNA	1	0	0	0	0	0	0	0	0	0	0	1	0	0	0	0	0	0	0
BGIR9823_88951	110261640	LOC110261640	lncRNA	1	0	0	0	0	0	0	0	0	0	0	0	0	0	1	0	0	0	0
BGIR9823_101401	110258215	LOC110258215	lncRNA	1	0	0	0	0	0	0	0	0	0	0	0	0	0	0	0	0	0	0
BGIR9823_82476	100518189	SRSF7	lncRNA	1	0	0	0	0	0	0	0	0	0	0	0	0	1	0	0	0	0	0
BGIR9823_80254	100520600	TP53I11	lncRNA	1	0	0	0	0	0	0	0	0	0	0	0	0	0	0	0	0	0	0
BGIR9823_100432	100515455	TNPO3	lncRNA	1	0	0	0	0	0	0	0	0	0	0	0	0	0	0	0	0	0	0
BGIR9823_100491	100520706	DOCK4	lncRNA	1	0	0	0	0	0	0	0	0	0	0	0	0	0	0	0	0	0	0
BGIR9823_101216	100514440	ATRX	lncRNA	1	0	0	0	0	0	0	0	0	0	0	0	0	0	0	0	0	0	0
BGIR9823_94101	100518511	MBTD1	lncRNA	1	0	0	0	0	0	0	0	0	0	0	0	0	1	0	0	0	0	0
BGIR9823_84304	100157730	TGS1	mRNA	1	0	0	0	0	0	0	0	0	0	0	0	0	0	0	0	0	0	0
BGIR9823_98130	100519056	ORC4	lncRNA	1	0	0	0	0	0	0	0	0	0	0	1	0	0	0	0	0	0	0
BGIR9823_95626	100623101	SETD2	mRNA	1	0	0	0	0	0	0	0	0	0	0	1	0	0	0	0	0	0	0
BGIR9823_81836	110259827	LOC110259827	lncRNA	1	0	0	0	0	0	0	0	0	0	0	0	0	0	0	0	0	0	0
BGIR9823_100125	100519631	TASP1	lncRNA	1	0	0	0	0	0	0	0	0	0	0	0	0	0	0	0	0	0	0
BGIR9823_77803	100520524	GOPC	lncRNA	1	0	0	0	0	0	0	0	0	0	0	0	0	0	0	0	0	0	0
BGIR9823_78926	100152904	ARID1B	lncRNA	1	0	0	0	0	0	0	0	0	0	0	0	0	0	0	0	0	0	0
XR_002343327.1	110260295	LOC110260295	lncRNA	1	0	0	0	0	0	0	0	0	0	0	0	0	0	0	0	0	0	0
XM_005661298.3	100514564	CAPS	mRNA	1	0	0	0	0	0	0	0	0	0	0	0	0	0	0	0	0	0	0
BGIR9823_100692	100739403	KLHDC10	lncRNA	1	0	0	0	0	0	0	0	0	0	0	0	0	0	0	0	0	0	0
BGIR9823_95813	100525891	EIF4E3	lncRNA	1	1	0	0	0	0	0	0	0	0	0	0	0	0	0	0	0	0	0
BGIR9823_81281	100516595	ZNF333	lncRNA	1	0	0	0	0	0	0	0	0	0	0	0	0	0	0	0	0	0	0
BGIR9823_100015	100151957	PARD6B	lncRNA	1	0	0	0	0	0	0	0	0	0	0	0	0	0	0	0	0	0	1
BGIR9823_101282	100517991	FAM122B	lncRNA	1	0	0	0	0	0	0	0	0	0	0	0	0	0	0	0	0	0	0
BGIR9823_88581	100151986	UNC45A	lncRNA	1	0	0	0	0	0	0	0	0	0	0	0	0	0	0	0	0	0	0
BGIR9823_88595	110261555	SEMA4B	lncRNA	1	0	0	0	0	0	0	0	0	0	0	0	0	0	0	0	0	0	0

RNA ID	Gene ID	Symbol	Type	target_total	ssc-miR-29a-3p	novel-ssc-miR204-5p	novel-ssc-miR480-3p	novel-ssc-miR537-5p	novel-ssc-miR620-3p	novel-ssc-miR627-5p	novel-ssc-miR671-3p	novel-ssc-miR753-5p	novel-ssc-miR832-3p	novel-ssc-miR1287-5p	novel-ssc-miR1406-3p	ssc-miR-10383	novel-ssc-miR2-3p	ssc-miR-660	novel-ssc-miR9-3p	novel-ssc-miR16-3p	novel-ssc-miR710-5p	novel-ssc-miR802-3p
BGIR9823_91875	106504991	LOC106504991	lncRNA	1	0	0	0	0	0	0	0	0	0	0	0	0	0	0	0	0	0	0
BGIR9823_100454	100525017	WASL	lncRNA	1	0	0	0	0	0	0	0	0	0	0	0	0	0	0	0	0	0	0
BGIR9823_101069	100512515	AFF2	mRNA	1	0	0	0	0	0	0	0	0	0	0	0	0	0	0	0	0	0	0
BGIR9823_101534	102158401	LOC102158401	lncRNA	1	0	0	0	0	0	0	0	0	0	0	0	0	0	0	0	0	0	0
BGIR9823_82897	100738908	MLST8	lncRNA	1	0	0	0	0	0	0	0	0	0	0	0	0	0	0	0	0	0	0
BGIR9823_98106	100158118	CALY	mRNA	1	0	0	0	0	0	0	0	0	0	0	0	0	0	0	0	0	0	0
BGIR9823_80817	100520422	SLC22A5	lncRNA	1	0	0	0	0	0	0	0	0	0	0	0	0	0	0	0	0	0	0
BGIR9823_91425	100511767	ARAP1	mRNA	1	0	0	0	0	0	0	0	0	0	0	0	0	0	0	0	0	0	0
BGIR9823_96623	100154637	SRSF9	lncRNA	1	0	0	0	0	0	0	0	0	0	0	0	0	0	0	0	0	0	0
BGIR9823_81521	100513398	SIMC1	lncRNA	1	0	0	0	0	0	0	0	0	0	0	0	0	0	0	0	0	0	0
BGIR9823_86388	110261319	LOC110261319	lncRNA	1	0	0	0	0	0	0	0	0	0	0	0	0	0	0	0	0	0	0
BGIR9823_81938	100511317	SSC4D	lncRNA	1	0	0	0	0	0	0	0	0	0	0	0	0	0	0	0	0	0	0
BGIR9823_83868	102165219	ANKRD34A	mRNA	1	0	0	0	0	0	0	0	0	0	0	0	0	0	0	0	0	0	0
BGIR9823_85017	100155867	INTS13	lncRNA	1	0	0	0	0	0	0	0	0	0	0	0	0	0	0	0	0	0	0
BGIR9823_91638	100523324	ARHGEF12	lncRNA	1	0	0	0	0	0	0	0	0	0	0	0	0	0	0	0	0	0	0
BGIR9823_78342	100515640	DIS3L	lncRNA	1	1	0	0	0	0	0	0	0	0	0	0	0	0	0	0	0	0	0
BGIR9823_101177	100520413	PHF8	mRNA	1	0	0	0	0	0	0	0	0	0	0	0	0	0	0	0	0	0	0
BGIR9823_100116	100157552	MKKS	lncRNA	1	0	0	0	0	0	0	0	0	0	0	0	0	0	0	0	0	0	0
BGIR9823_82812	100514160	XYLT1	lncRNA	1	0	0	0	0	0	0	0	0	0	0	0	0	0	0	0	0	0	0
BGIR9823_100485	100621338	BMT2	lncRNA	1	0	0	0	0	0	0	0	0	0	0	0	0	0	0	0	0	0	0
BGIR9823_83185	100302367	SOCS5	lncRNA	1	0	0	0	0	0	0	0	0	0	0	0	0	0	0	0	0	0	0
BGIR9823_100135	100519631	TASP1	lncRNA	1	0	0	0	0	0	0	0	0	0	0	0	0	0	0	0	0	0	0
BGIR9823_78629	100155278	ERMP1	mRNA	1	0	0	0	0	0	0	0	0	0	0	0	0	1	0	0	0	0	0
BGIR9823_100610	100517250	NUB1	mRNA	1	0	0	0	0	0	0	0	0	0	0	0	0	0	0	0	0	0	0
BGIR9823_100720	100511294	PTPRZ1	lncRNA	1	0	0	0	0	0	0	0	0	0	0	0	0	0	0	0	0	0	0
BGIR9823_91236	100524699	TPK1	lncRNA	1	0	0	0	0	0	0	0	0	0	0	0	0	0	1	0	0	0	0
BGIR9823_79597	100152069	SMAD6	lncRNA	1	0	0	0	0	0	0	0	0	0	0	0	0	0	0	0	0	0	0
BGIR9823_100727	100512026	ING3	lncRNA	1	0	0	0	0	0	0	0	0	0	0	0	0	0	0	0	0	0	0
BGIR9823_89956	106504698	LOC106504698	lncRNA	1	0	0	0	0	0	0	0	0	0	0	0	0	0	0	0	0	0	0
BGIR9823_100230	100154394	ROMO1	lncRNA	1	0	0	0	0	0	0	0	0	0	0	0	0	0	0	0	0	0	0
BGIR9823_90067	100519324	ETNPPL	mRNA	1	0	0	0	0	0	0	0	0	0	0	0	0	0	0	0	0	0	0
BGIR9823_81199	100216478	TEAD1	lncRNA	1	0	0	0	0	0	0	0	0	0	0	0	0	0	0	0	0	0	0
BGIR9823_100115	100157552	MKKS	lncRNA	1	0	0	0	0	0	0	0	0	1	0	0	0	0	0	0	0	0	0
BGIR9823_97871	733640	PRKG1	lncRNA	1	0	0	0	0	0	0	0	0	0	0	0	0	0	0	0	0	0	0
BGIR9823_79442	106506757	INAFM2	lncRNA	1	0	0	0	0	0	0	0	0	0	0	0	0	0	0	0	0	0	0
BGIR9823_77844	100736796	ADGRB3	lncRNA	1	0	0	0	0	0	0	0	0	0	0	0	0	0	0	0	0	0	0
BGIR9823_81223	110259751	LOC110259751	lncRNA	1	0	0	0	0	0	0	0	0	0	0	0	0	0	0	0	0	0	0
BGIR9823_87714	100517038	PUM1	lncRNA	1	0	0	0	0	0	0	0	0	0	0	0	0	0	0	0	0	0	0
BGIR9823_82332	100517706	CEP68	lncRNA	1	0	0	0	0	0	0	0	0	0	0	0	0	0	1	0	0	0	0
BGIR9823_79033	100624679	NKAIN2	lncRNA	1	0	0	0	0	0	0	0	0	0	0	0	0	0	0	0	0	0	0
BGIR9823_81514	100523721	FAF2	lncRNA	1	0	0	0	1	0	0	0	0	0	0	0	0	0	0	0	0	0	0
BGIR9823_78765	100622501	PRPF4	mRNA	1	0	0	0	0	0	0	0	0	0	0	0	0	0	0	0	0	0	0
BGIR9823_78849	100624075	AIF1L	mRNA	1	0	0	0	0	0	0	0	0	0	0	0	0	0	0	0	0	0	0

RNA ID	Gene ID	Symbol	Type	target_total	ssc-miR-29a-3p	novel-ssc-miR204-5p	novel-ssc-miR480-3p	novel-ssc-miR537-5p	novel-ssc-miR620-3p	novel-ssc-miR627-5p	novel-ssc-miR671-3p	novel-ssc-miR753-5p	novel-ssc-miR832-3p	novel-ssc-miR1287-5p	novel-ssc-miR1406-3p	ssc-miR-10383	novel-ssc-miR2-3p	ssc-miR-660	novel-ssc-miR9-3p	novel-ssc-miR16-3p	novel-ssc-miR710-5p	novel-ssc-miR802-3p
BGIR9823_100607	100515515	GALNT11	lncRNA	1	0	0	0	0	0	0	0	0	1	0	0	0	0	0	0	0	0	0
BGIR9823_83081	100627528	CCDC142	lncRNA	1	0	0	0	0	0	0	0	0	0	0	0	0	0	0	0	0	0	0
XR_002345524.1	102158855	LOC102158855	lncRNA	1	0	0	0	0	0	0	0	0	0	0	0	0	0	0	0	0	0	0
BGIR9823_78387	100154444	GLCE	lncRNA	1	0	0	0	1	0	0	0	0	0	0	0	0	0	0	0	0	0	0
BGIR9823_79739	110255453	LOC110255453	lncRNA	1	0	0	0	0	0	0	0	0	0	0	0	0	0	0	0	0	0	0
BGIR9823_100823	100519067	CDK13	mRNA	1	0	0	0	0	0	0	0	0	0	0	0	0	0	0	0	0	0	0
BGIR9823_80184	397169	BEST1	lncRNA	1	0	0	0	0	0	0	0	0	0	0	0	0	0	0	0	0	0	0
BGIR9823_85074	100144592	KLRF1	lncRNA	1	0	0	0	0	0	0	0	0	0	0	0	0	0	0	0	0	0	0
BGIR9823_80186	444995	FADS1	mRNA	1	0	0	0	0	0	0	0	0	0	0	0	0	0	0	0	0	0	0
BGIR9823_97606	100156356	PRR14L	lncRNA	1	0	0	0	0	0	0	0	0	0	0	0	0	0	0	0	0	0	0
BGIR9823_100902	100621199	IL1RAPL1	lncRNA	1	0	1	0	0	0	0	0	0	0	0	0	0	0	0	0	0	0	0
BGIR9823_78631	100155714	PLGRKT	lncRNA	1	0	0	0	0	0	0	0	0	0	0	0	0	0	0	0	0	0	0
BGIR9823_97867	780419	UBE2D1	mRNA	1	0	0	0	0	0	0	0	0	0	0	1	0	0	0	0	0	0	0
BGIR9823_80393	100512765	SMIM7	lncRNA	1	0	0	0	0	0	0	0	0	0	0	0	0	0	0	0	0	0	0
BGIR9823_83405	100157574	FAM49B	lncRNA	1	0	0	0	0	0	0	0	0	0	0	0	0	0	0	0	0	0	0
BGIR9823_82271	100625157	GCFC2	lncRNA	1	0	0	0	0	0	0	0	0	0	0	0	0	0	0	0	0	0	0
BGIR9823_92748	100154396	PDS5B	lncRNA	1	1	0	0	0	0	0	0	0	0	0	0	0	0	0	0	0	0	0
BGIR9823_81832	100737907	FAM20C	lncRNA	1	0	0	0	0	0	0	0	0	0	0	0	0	0	0	0	0	0	0
BGIR9823_86739	100623382	SPIRE1	mRNA	1	0	0	0	0	0	0	0	0	0	0	0	0	0	0	0	0	0	0
BGIR9823_88779	100515615	CCNB1IP1	lncRNA	1	0	0	0	0	0	0	0	0	0	0	0	0	0	0	0	0	0	0
BGIR9823_82125	100533196	UBE2I	lncRNA	1	0	0	0	0	0	0	0	0	0	0	0	0	0	0	0	0	0	0
BGIR9823_96691	100153895	MORC2	lncRNA	1	0	0	0	0	0	0	0	0	0	0	0	0	0	0	0	0	0	0
BGIR9823_82328	100518067	SPRED2	lncRNA	1	0	0	0	0	0	0	0	0	0	0	0	0	0	0	0	0	0	0
BGIR9823_87494	100625971	LOC100625971	mRNA	1	0	0	0	0	0	0	0	0	0	0	0	0	1	0	0	0	0	0
BGIR9823_79014	100738102	RNF217	lncRNA	1	0	0	0	0	0	0	0	0	0	0	0	0	0	0	0	0	0	0
BGIR9823_79431	100153351	VPS18	mRNA	1	0	0	0	0	0	0	0	0	0	0	0	0	0	0	0	0	0	0
BGIR9823_79792	100154060	RIC1	mRNA	1	0	0	0	0	0	0	0	0	0	0	0	0	0	0	0	0	0	0
BGIR9823_97488	100153708	ATXN2	mRNA	1	0	0	0	0	0	0	0	0	0	0	0	0	0	0	0	0	0	0
BGIR9823_100701	100514931	AHCYL2	lncRNA	1	0	0	0	0	0	0	0	0	0	0	0	0	0	0	0	0	0	0
BGIR9823_83545	100153257	MMP16	lncRNA	1	0	0	0	0	0	0	0	0	0	0	0	0	0	0	0	0	0	0
BGIR9823_82543	100521786	TP53I3	mRNA	1	0	0	0	0	0	0	0	0	0	0	1	0	0	0	0	0	0	0
BGIR9823_81390	100520177	TRIP10	lncRNA	1	0	0	0	0	0	0	0	0	0	0	0	0	0	0	0	0	0	0
BGIR9823_100128	100519631	TASP1	lncRNA	1	0	0	0	0	0	0	0	0	0	0	0	0	0	0	0	0	0	0
BGIR9823_94226	100337675	ADAP2	lncRNA	1	0	0	0	0	0	0	0	0	0	0	0	0	0	0	0	0	0	0
BGIR9823_88221	110261778	NOL7	mRNA	1	0	0	0	0	0	0	0	0	0	0	0	0	0	0	0	0	0	0
BGIR9823_100908	100621199	IL1RAPL1	lncRNA	1	0	0	0	1	0	0	0	0	0	0	0	0	0	0	0	0	0	0
BGIR9823_101335	100523617	TRIM27	lncRNA	1	0	0	0	0	0	0	0	0	0	0	0	0	0	0	0	0	0	0
BGIR9823_101518	106504125	LOC106504125	lncRNA	1	0	0	0	0	0	0	0	0	0	0	0	0	0	0	0	0	0	0
BGIR9823_80840	100624262	SMAD5	mRNA	1	0	0	0	0	0	0	0	0	0	0	0	0	0	0	0	0	0	0
BGIR9823_99908	449527	PHF20	lncRNA	1	0	0	0	0	0	0	0	0	0	0	0	0	0	0	0	0	0	0
BGIR9823_99647	100521758	CPEB4	mRNA	1	0	0	0	0	0	0	0	0	0	0	0	0	0	0	0	0	0	0
BGIR9823_97815	106506044	LOC106506044	lncRNA	1	0	0	0	0	0	0	0	0	0	0	0	0	0	0	0	0	0	0
BGIR9823_77854	100736796	ADGRB3	lncRNA	1	0	0	0	0	0	0	0	0	0	0	0	0	0	0	0	0	0	0

RNA ID	Gene ID	Symbol	Type	target_total	ssc-miR-29a-3p	novel-ssc-miR204-5p	novel-ssc-miR480-3p	novel-ssc-miR537-5p	novel-ssc-miR620-3p	novel-ssc-miR627-5p	novel-ssc-miR671-3p	novel-ssc-miR753-5p	novel-ssc-miR832-3p	novel-ssc-miR1287-5p	novel-ssc-miR1406-3p	ssc-miR-10383	novel-ssc-miR2-3p	ssc-miR-660	novel-ssc-miR9-3p	novel-ssc-miR16-3p	novel-ssc-miR710-5p	novel-ssc-miR802-3p
BGIR9823_92288	110255709	LOC110255709	lncRNA	1	0	0	0	0	0	0	0	0	0	0	0	0	0	0	0	0	0	0
BGIR9823_79245	100738627	DYM	lncRNA	1	0	0	0	0	0	0	0	0	0	0	0	0	0	0	0	0	0	0
BGIR9823_83586	100152963	PEX2	lncRNA	1	0	0	0	0	0	0	0	0	0	0	0	0	0	0	0	0	0	0
BGIR9823_101489	110258382	LOC110258382	lncRNA	1	0	0	0	0	0	0	0	0	0	0	0	0	0	0	0	0	0	0
BGIR9823_100872	100739216	MAP3K15	lncRNA	1	0	0	0	0	0	0	0	0	0	0	0	0	0	0	0	0	1	0
XM_005673239.3	100620722	ZNF277	mRNA	1	0	0	0	0	0	0	0	0	0	0	0	0	0	0	0	0	0	0
BGIR9823_100554	100739791	LOC100739791	lncRNA	1	0	0	0	0	0	0	0	0	0	0	0	1	0	0	0	0	0	0
BGIR9823_83272	100521316	GPN1	lncRNA	1	0	0	0	0	0	0	0	0	0	0	0	0	0	0	0	0	0	0
BGIR9823_77910	110260430	LOC110260430	lncRNA	1	0	0	0	0	0	0	0	0	0	0	0	0	0	0	0	0	0	0
BGIR9823_100153	100152012	CST7	lncRNA	1	0	0	0	0	0	0	0	0	0	0	0	0	0	0	0	0	0	0
BGIR9823_79200	100156851	IRAK1BP1	lncRNA	1	0	0	0	0	0	0	0	0	0	0	0	0	0	0	0	0	0	0
BGIR9823_100475	100517204	TFEC	lncRNA	1	0	0	0	0	0	0	0	0	0	0	0	0	0	0	0	0	1	0
BGIR9823_83002	110259971	C3H2orf49	lncRNA	1	0	0	0	0	0	0	0	0	0	0	0	0	0	0	0	0	0	0
BGIR9823_78732	100152140	SLC44A1	lncRNA	1	0	0	0	0	0	0	0	0	0	0	0	0	0	0	0	0	0	0
BGIR9823_94529	397618	OXSR1	lncRNA	1	0	0	0	0	0	0	0	0	0	0	0	0	0	0	0	0	0	0
BGIR9823_92973	100623901	MICU2	mRNA	1	0	0	0	0	0	0	0	0	0	0	0	0	0	0	0	0	0	0
BGIR9823_80241	100513197	DDB2	lncRNA	1	0	0	0	1	0	0	0	0	0	0	0	0	0	0	0	0	0	0
BGIR9823_96832	100153026	ABCB10	lncRNA	1	0	0	0	0	0	0	0	0	0	0	0	0	0	0	0	0	0	0
BGIR9823_96408	100153797	SECISBP2	mRNA	1	0	0	0	0	0	0	0	0	0	0	0	0	0	0	0	0	0	0
BGIR9823_80734	100526040	PAM	lncRNA	1	0	0	0	1	0	0	0	0	0	0	0	0	0	0	0	0	0	0
BGIR9823_81957	100513345	GTF2I	lncRNA	1	0	0	0	0	0	0	0	0	0	0	0	0	0	1	0	0	0	0
BGIR9823_96796	110256820	LOC110256820	lncRNA	1	0	0	0	0	0	0	0	0	0	0	0	0	0	0	0	0	0	0
BGIR9823_101115	110255263	CXH4orf3	lncRNA	1	0	0	0	0	0	0	0	0	0	0	0	0	0	0	0	0	0	0
BGIR9823_78939	100621167	PLEKHG1	lncRNA	1	0	0	0	1	0	0	0	0	0	0	0	0	0	0	0	0	0	0
BGIR9823_90840	100517358	PIWIL4	lncRNA	1	0	0	0	0	0	0	0	0	0	0	0	0	0	0	0	0	0	0
BGIR9823_94380	396903	MYH10	mRNA	1	0	0	0	0	0	0	0	0	0	0	0	0	0	0	0	0	0	0
BGIR9823_94364	100621720	TNK1	lncRNA	1	0	0	0	0	0	0	0	0	0	0	0	0	0	0	0	0	0	0
BGIR9823_99224	110257075	LOC110257075	lncRNA	1	0	0	0	0	0	0	0	0	0	0	0	0	0	0	0	0	0	0
BGIR9823_88977	102159128	LOC102159128	lncRNA	1	0	0	0	0	0	0	0	0	0	0	0	0	0	0	0	0	0	0
BGIR9823_86907	100623628	PRKACB	lncRNA	1	0	0	0	0	0	0	0	0	0	0	0	0	0	0	0	0	0	0
BGIR9823_83922	100127159	SLC16A1	lncRNA	1	0	0	0	0	0	0	0	0	0	0	0	0	0	0	0	0	0	0
BGIR9823_80165	106504153	AHNAK	mRNA	1	0	0	0	0	0	0	0	0	0	0	0	0	0	0	0	0	0	0
XM_021099336.1	102160410	LOC102160410	mRNA	1	0	0	0	0	0	0	0	0	0	0	0	0	0	0	0	0	0	0
BGIR9823_78034	100157196	CSNK1G1	lncRNA	1	0	0	0	0	0	0	0	0	0	0	0	0	0	0	0	0	0	0
BGIR9823_80409	100517807	SYDE1	lncRNA	1	0	0	0	0	0	0	0	0	0	0	0	0	0	0	0	0	0	0
BGIR9823_82453	100622259	MAP4K3	lncRNA	1	0	0	0	0	0	0	0	0	0	0	0	0	0	0	0	0	0	0
BGIR9823_101610	100521600	LOC100521600	lncRNA	1	0	0	0	0	0	0	0	0	0	0	0	0	0	0	0	0	0	0
BGIR9823_86055	102165609	LOC102165609	lncRNA	1	0	0	0	0	0	0	0	0	0	0	0	0	0	0	0	0	0	0
BGIR9823_92014	396922	CD46	mRNA	1	0	0	0	0	0	0	0	0	0	0	0	0	0	0	0	0	0	0
BGIR9823_100942	100521238	CDK16	mRNA	1	0	0	0	0	0	0	0	0	0	0	0	0	0	0	1	0	0	0
BGIR9823_77933	100513770	SLC16A10	lncRNA	1	0	0	0	0	0	0	0	0	0	0	0	0	0	0	0	0	0	0
BGIR9823_89476	100155589	FAM177A1	lncRNA	1	0	0	0	0	0	0	0	0	0	0	0	0	0	0	0	0	0	0
BGIR9823_101139	100525794	KLHL15	lncRNA	1	0	0	0	0	0	0	0	0	0	0	0	0	0	0	0	0	0	0

RNA ID	Gene ID	Symbol	Type	target_total	ssc-miR-29a-3p	novel-ssc-miR204-5p	novel-ssc-miR480-3p	novel-ssc-miR537-5p	novel-ssc-miR620-3p	novel-ssc-miR627-5p	novel-ssc-miR671-3p	novel-ssc-miR753-5p	novel-ssc-miR832-3p	novel-ssc-miR1287-5p	novel-ssc-miR1406-3p	ssc-miR-10383	novel-ssc-miR2-3p	ssc-miR-660	novel-ssc-miR9-3p	novel-ssc-miR16-3p	novel-ssc-miR710-5p	novel-ssc-miR802-3p
BGIR9823_92285	100512363	CREM	lncRNA	1	0	0	0	0	0	0	0	0	0	0	0	0	0	0	0	0	0	0
BGIR9823_100684	414428	MKLN1	lncRNA	1	0	0	0	0	0	0	0	0	0	0	0	0	0	0	0	0	0	0
BGIR9823_80117	100511000	MUS81	lncRNA	1	0	0	0	0	0	0	0	0	0	0	0	0	0	0	0	0	0	0
BGIR9823_79783	102168004	UHRF2	lncRNA	1	0	0	0	0	0	0	0	0	0	0	0	0	0	0	0	0	0	0
BGIR9823_87527	110261323	LOC110261323	lncRNA	1	0	0	0	0	0	0	0	0	0	0	0	0	0	0	0	0	0	0
BGIR9823_77815	100736796	ADGRB3	lncRNA	1	0	0	0	0	0	0	0	0	0	0	0	0	0	0	0	0	0	0
BGIR9823_100466	100512202	TSPAN12	mRNA	1	0	0	0	0	1	0	0	0	0	0	0	0	0	0	0	0	0	0
BGIR9823_81771	100521956	CYSTM1	lncRNA	1	1	0	0	0	0	0	0	0	0	0	0	0	0	0	0	0	0	0
BGIR9823_93887	100625999	PGS1	mRNA	1	0	0	0	0	0	0	0	0	0	0	0	0	0	0	0	0	0	0
BGIR9823_99449	100312962	ATOX1	lncRNA	1	0	0	0	0	0	0	0	0	0	0	0	0	0	0	0	0	0	0
BGIR9823_82424	100526165	PREPL	lncRNA	1	0	0	0	0	0	0	0	0	0	0	0	0	0	0	0	0	0	0
BGIR9823_78323	100518050	TMX3	lncRNA	1	0	0	0	0	0	0	0	0	0	0	0	0	0	0	0	0	0	0
BGIR9823_81279	110259619	LOC110259619	lncRNA	1	0	0	0	0	0	0	0	0	0	0	0	0	0	0	0	0	0	0
BGIR9823_89593	100514718	SYNJ2BP	mRNA	1	0	0	0	0	0	0	0	0	0	0	0	0	0	0	0	0	0	0
BGIR9823_100641	100519065	UBN2	lncRNA	1	0	0	0	0	0	0	0	0	0	0	0	0	0	0	0	0	0	0
BGIR9823_99284	100518875	EGFLAM	mRNA	1	0	0	0	0	0	0	0	0	0	0	0	0	0	0	0	0	0	0
BGIR9823_97891	100037303	SGMS1	lncRNA	1	0	0	0	0	0	0	0	0	0	0	0	0	0	0	0	0	0	0
BGIR9823_80246	100516314	HARBI1	lncRNA	1	0	0	0	0	0	0	0	0	0	0	0	0	0	0	0	0	0	0
BGIR9823_84831	100154762	ZNF740	lncRNA	1	0	0	0	0	0	0	0	0	0	0	0	0	0	0	0	0	0	0
BGIR9823_100404	100524054	WDR91	lncRNA	1	0	0	0	0	0	0	0	0	0	0	0	0	0	0	0	0	0	0
BGIR9823_89087	100157771	CARMIL1	lncRNA	1	0	0	0	0	0	0	0	0	0	0	0	0	0	0	0	0	0	0
BGIR9823_94449	100737057	BTD	lncRNA	1	0	0	0	0	0	0	0	0	0	0	0	0	0	0	0	0	0	0
BGIR9823_89030	100152689	SSR1	mRNA	1	0	0	0	0	0	0	0	0	0	0	0	0	0	0	0	0	0	0
BGIR9823_100313	100155190	ZFP64	lncRNA	1	0	0	0	0	0	0	0	0	0	0	0	0	0	0	0	0	0	0
BGIR9823_84915	100623414	SRGAP1	lncRNA	1	0	0	0	0	0	0	0	0	0	0	0	0	0	0	0	0	0	0
BGIR9823_100318	100126237	CTCFL	lncRNA	1	0	0	0	0	0	0	0	0	0	0	0	0	0	0	0	0	0	0
BGIR9823_88856	100155733	SUSD6	lncRNA	1	0	0	0	0	0	0	0	0	0	0	0	0	0	0	0	0	0	0
XM_003480892.4	100519890	TRIM36	mRNA	1	0	0	0	0	0	0	0	0	0	0	0	0	0	0	0	0	0	0
BGIR9823_82684	100620890	SRRT	lncRNA	1	0	0	0	0	0	0	0	0	0	0	0	0	0	0	0	0	0	0
BGIR9823_83320	100513350	ASAP2	mRNA	1	0	0	0	0	0	0	0	0	0	0	0	0	0	0	0	0	0	0
BGIR9823_101170	100739016	RIBC1	lncRNA	1	0	0	0	0	0	0	0	0	0	0	0	1	0	0	0	0	0	0
BGIR9823_87081	100521097	ERI3	lncRNA	1	0	0	0	0	0	0	0	0	0	0	1	0	0	0	0	0	0	0
BGIR9823_88961	100152076	PTPN21	lncRNA	1	0	0	0	0	0	0	0	0	0	0	0	0	0	0	0	0	0	0
BGIR9823_89703	100515556	SLC25A47	lncRNA	1	0	0	0	0	0	0	0	0	0	0	0	0	0	0	0	0	0	0
BGIR9823_78067	100625505	VPS13C	lncRNA	1	0	0	0	0	0	0	0	0	0	0	0	0	0	0	0	0	0	0
BGIR9823_78412	100156782	LRFN5	lncRNA	1	0	0	0	0	0	0	0	0	0	0	0	0	0	0	0	0	0	0
BGIR9823_94130	106505457	TRIM37	mRNA	1	0	0	0	0	0	0	0	0	0	0	0	0	0	0	0	0	0	0
BGIR9823_78647	100152831	PUM3	lncRNA	1	0	0	0	0	0	0	0	0	0	0	0	0	0	0	0	0	0	0
BGIR9823_101061	102158351	LOC102158351	lncRNA	1	0	0	0	0	0	0	0	0	0	0	0	0	0	0	0	0	0	0
BGIR9823_101008	100523039	GPRASP1	mRNA	1	0	0	0	0	0	0	0	0	0	0	0	0	0	0	0	0	0	0

Supplementary Table S9. The predicted targets of DE miRN.

RNA ID	Gene ID	Symbol	Type	novel-ssc-miR817-3p	novel-ssc-miR849-3p	novel-ssc-miR882-3p	novel-ssc-miR985-5p	novel-ssc-miR1126-5p	novel-ssc-miR1396-3p	ssc-miR-362	novel-ssc-miR16-5p	ssc-miR-375	ssc-miR-376a-3p	ssc-miR-493-5p	ssc-miR-194a-5p	ssc-miR-205	novel-ssc-miR239-5p	novel-ssc-miR397-5p	novel-ssc-miR507-5p	novel-ssc-miR563-3p	novel-ssc-miR568-3p	novel-ssc-miR682-3p
BGIR9823_100323	100049657	GNAS	lncRNA	0	0	1	1	0	0	0	0	0	0	0	0	0	0	1	0	0	0	1
BGIR9823_100321	100144523	PMEPA1	lncRNA	0	0	0	1	0	1	0	0	0	0	0	0	0	0	1	0	1	0	0
BGIR9823_100436	110257581	LOC110257581	lncRNA	0	0	1	1	0	0	0	0	0	0	0	0	0	0	0	0	0	0	1
BGIR9823_101399	110258215	LOC110258215	lncRNA	0	0	0	1	0	1	0	0	0	0	0	0	1	0	1	0	0	0	0
BGIR9823_100330	100037996	RPS21	lncRNA	0	0	1	1	0	0	0	0	0	0	0	0	0	0	1	0	1	0	0
BGIR9823_101457	110258362	MTA1	lncRNA	0	0	0	1	0	0	0	0	0	0	0	0	0	0	0	0	0	0	0
BGIR9823_101544	110258600	LOC110258600	lncRNA	0	0	0	1	0	0	0	0	0	0	0	0	1	0	0	1	0	0	0
BGIR9823_101531	102158401	LOC102158401	lncRNA	0	0	0	1	0	0	0	0	0	0	0	0	0	0	1	0	0	0	0
BGIR9823_101342	110258067	ARRDC1	lncRNA	0	0	1	0	0	1	0	0	0	0	0	0	0	0	0	0	0	0	1
BGIR9823_101405	110258214	LOC110258214	lncRNA	0	0	0	1	0	1	0	0	0	0	0	0	0	0	1	0	0	0	0
BGIR9823_101472	110258334	LOC110258334	lncRNA	0	0	0	0	0	0	0	0	0	0	0	0	0	0	1	0	0	0	0
BGIR9823_100712	100520873	IMPDH1	lncRNA	0	0	1	0	0	0	0	0	0	0	0	0	0	0	1	0	0	0	0
BGIR9823_101185	110257830	LOC110257830	lncRNA	0	0	1	1	0	0	0	0	0	0	0	0	0	0	1	0	0	0	1
BGIR9823_101542	102157946	LOC102157946	lncRNA	0	0	0	1	0	0	0	0	0	0	0	0	0	0	0	1	0	0	0
BGIR9823_100049	110257373	GMEB2	lncRNA	0	0	0	1	0	0	0	0	0	0	0	0	0	0	0	0	0	0	0
BGIR9823_100378	100514083	ADCK2	lncRNA	0	0	0	1	0	0	1	0	0	0	0	0	0	0	0	0	0	0	0
BGIR9823_100540	100523342	OSBPL3	lncRNA	0	0	1	1	0	0	0	0	0	0	0	0	0	0	1	0	0	0	0
BGIR9823_101051	110257712	LOC110257712	lncRNA	0	0	1	1	0	0	0	0	0	0	0	0	0	0	1	0	0	0	0
BGIR9823_101904	BGIG9823_26581		lncRNA	0	0	0	0	0	0	0	0	0	0	0	0	0	0	1	0	0	0	0
BGIR9823_100561	100516888	GLI3	lncRNA	0	0	0	0	0	0	0	0	0	0	0	0	0	0	0	0	0	0	0
BGIR9823_100665	100622227	LOC100622227	lncRNA	0	0	0	0	0	0	0	0	0	0	0	0	1	0	0	0	1	0	1
BGIR9823_100348	110257384	TCEA2	lncRNA	0	0	0	1	0	0	0	0	0	0	0	0	0	0	0	0	0	0	0
BGIR9823_100834	110257605	LRRC61	lncRNA	0	0	1	1	0	0	0	0	0	0	0	0	0	0	0	0	1	0	1
BGIR9823_100037	110257479	MTG2	lncRNA	0	0	0	0	0	0	0	0	0	0	0	0	0	0	0	0	0	0	0
BGIR9823_100184	106506644	LOC106506644	lncRNA	0	0	1	0	0	0	0	0	0	0	0	0	0	0	0	0	0	0	1
BGIR9823_101820	BGIG9823_26500		lncRNA	0	0	0	0	0	0	0	0	0	0	0	0	0	0	1	0	0	0	1
BGIR9823_100659	100522048	SLC13A4	lncRNA	0	0	1	0	0	0	0	0	0	0	0	0	0	0	0	0	0	0	0
BGIR9823_100223	100627204	MMP24	lncRNA	0	0	0	1	0	0	0	0	0	0	0	0	0	0	0	0	0	0	0
BGIR9823_101137	100152260	PRDX4	lncRNA	0	0	0	0	0	0	0	0	0	0	0	0	0	0	0	0	0	0	1
BGIR9823_100172	100154685	UBOX5	lncRNA	0	0	1	0	0	0	0	0	0	0	0	0	0	0	0	0	0	0	0
BGIR9823_101650	100738659	MCM3AP	lncRNA	0	0	0	0	0	0	0	0	0	0	0	0	0	0	1	0	0	0	0
BGIR9823_101940	BGIG9823_26617		lncRNA	0	0	0	0	0	0	0	0	0	0	0	0	0	0	1	0	0	0	1
BGIR9823_101941	BGIG9823_26618		lncRNA	0	0	0	0	0	0	0	0	0	0	0	0	0	0	0	0	0	0	0
BGIR9823_102278	BGIG9823_26945		lncRNA	0	0	0	0	0	0	0	0	0	0	0	0	0	0	1	0	1	0	0
BGIR9823_101611	100521600	LOC100521600	lncRNA	0	0	0	0	0	0	0	0	0	0	0	0	1	0	0	0	0	0	0
BGIR9823_100544	100627860	SUN3	lncRNA	0	0	0	1	0	0	0	0	0	0	0	0	0	0	0	0	0	0	0
BGIR9823_100360	100514805	KMT2C	lncRNA	0	0	0	0	0	0	0	0	0	0	0	0	0	0	0	0	0	0	0
BGIR9823_100799	397163	RAMP3	lncRNA	0	0	0	0	0	0	0	0	0	0	0	0	0	0	0	0	0	0	0
BGIR9823_101296	100623680	ZFP92	lncRNA	0	0	0	0	0	0	0	0	0	0	0	0	0	0	0	0	0	0	0
BGIR9823_100164	100520054	PANK2	lncRNA	0	0	1	0	0	0	0	0	0	0	0	0	0	0	0	0	0	0	0
BGIR9823_100576	102157930	LOC102157930	lncRNA	0	0	0	0	0	0	0	0	0	0	0	0	0	0	0	0	0	0	0

RNA ID	Gene ID	Symbol	Type	novel-ssc-miR817-3p	novel-ssc-miR849-3p	novel-ssc-miR882-3p	novel-ssc-miR985-5p	novel-ssc-miR1126-5p	novel-ssc-miR1396-3p	ssc-miR-362	novel-ssc-miR16-5p	ssc-miR-375	ssc-miR-376a-3p	ssc-miR-493-5p	ssc-miR-194a-5p	ssc-miR-205	novel-ssc-miR239-5p	novel-ssc-miR397-5p	novel-ssc-miR507-5p	novel-ssc-miR563-3p	novel-ssc-miR568-3p	novel-ssc-miR682-3p
BGIR9823_100585	100623772	ZNF862	lncRNA	0	0	0	0	0	0	0	0	0	0	0	0	0	0	0	0	0	0	0
BGIR9823_101341	110258060	LOC110258060	lncRNA	0	0	0	1	0	0	0	0	0	0	0	0	0	0	0	0	0	0	1
BGIR9823_101500	102160467	SMAGP	lncRNA	0	0	0	0	0	0	0	0	0	0	0	0	0	0	0	0	0	0	1
BGIR9823_101475	100521669	BRF1	lncRNA	0	0	0	0	0	0	0	0	0	0	0	0	0	0	0	0	0	0	0
BGIR9823_100400	100523151	NUP205	lncRNA	0	0	0	1	0	0	0	0	0	0	0	0	0	0	0	0	0	0	0
BGIR9823_101360	100512700	LOC100512700	lncRNA	0	0	0	0	0	0	0	0	0	0	0	1	0	0	0	0	0	0	0
BGIR9823_101677	BGIG9823_26364		lncRNA	0	0	0	0	0	0	0	0	0	0	0	0	0	0	0	0	0	0	1
BGIR9823_101879	BGIG9823_26558		lncRNA	0	0	0	0	0	0	0	0	0	0	0	1	0	0	0	0	0	0	0
BGIR9823_102010	BGIG9823_26686		lncRNA	0	0	0	0	0	0	0	0	0	0	0	0	0	0	1	0	0	0	0
BGIR9823_101968	BGIG9823_26645		lncRNA	0	0	0	0	0	0	0	0	0	0	0	0	0	0	0	0	0	0	0
BGIR9823_102338	BGIG9823_27003		lncRNA	0	0	0	0	0	0	1	0	0	0	0	0	0	0	0	0	1	0	0
BGIR9823_100338	BGIG9823_26339		lncRNA	0	0	0	0	0	0	0	0	0	0	0	0	0	0	0	0	0	0	0
BGIR9823_100753	493185	IFRD1	lncRNA	0	0	1	0	0	0	0	0	0	0	0	0	0	0	0	0	0	0	0
BGIR9823_100807	100515808	TMED4	lncRNA	0	0	0	0	0	0	0	0	0	0	0	0	0	0	0	0	0	0	0
BGIR9823_100577	100622254	RALA	lncRNA	0	0	0	0	0	0	0	0	0	0	0	0	0	0	0	0	0	0	1
BGIR9823_101471	100126861	AKT1	lncRNA	0	0	0	0	0	0	0	0	0	0	0	0	0	0	0	0	0	0	1
BGIR9823_100694	100737195	SMKR1	lncRNA	0	0	0	0	0	0	0	0	0	0	0	0	0	0	0	0	0	0	0
BGIR9823_101043	445538	XPNPEP2	lncRNA	0	0	0	1	0	0	0	0	0	0	0	0	0	0	1	0	0	0	0
BGIR9823_101060	102158351	LOC102158351	lncRNA	0	0	1	0	0	0	0	0	0	0	0	0	0	0	0	0	0	0	0
BGIR9823_100405	106506765	LOC106506765	lncRNA	0	0	0	1	0	0	0	0	0	0	0	0	0	0	0	0	0	0	0
BGIR9823_101548	110258600	LOC110258600	lncRNA	0	0	0	0	0	0	0	0	0	0	0	0	0	0	0	0	0	0	0
BGIR9823_100632	100515805	PARP12	lncRNA	0	0	0	0	0	0	0	0	0	0	0	0	0	0	0	0	0	0	0
BGIR9823_100345	100621535	TPD52L2	lncRNA	0	0	1	0	0	0	0	0	0	0	0	0	0	0	0	0	0	0	1
BGIR9823_100658	106506763	LOC106506763	lncRNA	0	0	0	0	0	0	0	0	0	0	0	0	0	0	0	0	1	0	0
BGIR9823_100521	100512260	GARS	lncRNA	0	0	0	0	0	0	0	0	0	0	0	0	0	0	0	0	0	0	0
BGIR9823_100195	106505983	LOC106505983	lncRNA	0	0	0	0	0	0	0	0	0	0	0	0	0	0	0	0	0	0	0
BGIR9823_101416	BGIG9823_26349		lncRNA	0	0	1	0	0	0	0	0	0	0	0	0	0	0	0	0	0	0	0
BGIR9823_102298	BGIG9823_26964		lncRNA	0	0	0	0	0	0	0	0	0	0	0	0	0	0	1	0	0	0	0
BGIR9823_101877	BGIG9823_26556		lncRNA	0	0	0	0	0	0	0	0	0	0	0	0	0	0	1	0	0	0	0
BGIR9823_102235	BGIG9823_26907		lncRNA	0	0	0	0	0	0	0	0	0	0	0	0	0	0	1	0	0	0	0
BGIR9823_102014	BGIG9823_26690		lncRNA	0	0	0	0	0	0	0	0	0	0	0	0	0	0	0	0	0	0	0
BGIR9823_102030	BGIG9823_26705		lncRNA	0	0	0	0	0	0	0	0	0	0	0	0	0	0	1	0	0	0	0
BGIR9823_101900	BGIG9823_26577		lncRNA	0	0	0	0	0	0	0	0	0	0	0	0	0	0	1	0	0	0	0
BGIR9823_101920	BGIG9823_26597		lncRNA	0	0	0	0	0	0	0	0	0	0	0	0	0	0	0	0	0	0	0
BGIR9823_101972	BGIG9823_26649		lncRNA	0	0	0	0	0	0	0	0	0	0	0	0	0	0	0	0	0	0	0
BGIR9823_101815	BGIG9823_26495		lncRNA	0	0	0	0	0	0	0	0	0	0	0	0	0	0	1	0	0	0	1
BGIR9823_101971	BGIG9823_26648		lncRNA	0	0	0	0	0	0	0	0	0	0	0	0	0	0	0	0	0	0	0
BGIR9823_101828	BGIG9823_26507		lncRNA	0	0	0	0	0	0	0	0	0	0	0	0	0	0	0	0	0	0	0
BGIR9823_102358	BGIG9823_27023		lncRNA	0	0	0	0	0	0	0	0	0	0	0	0	0	0	1	0	0	0	0
BGIR9823_101692	BGIG9823_26379		lncRNA	0	0	0	0	0	0	0	1	0	0	0	0	0	0	0	0	0	0	0
BGIR9823_101728	BGIG9823_26410		lncRNA	0	0	0	0	0	1	0	0	0	0	0	0	0	0	0	0	0	0	0
BGIR9823_101871	BGIG9823_26550		lncRNA	0	0	0	0	0	0	0	0	0	0	0	0	1	0	1	0	0	0	0
BGIR9823_101827	BGIG9823_26506		lncRNA	0	0	0	0	0	0	0	0	0	0	0	0	0	0	1	0	0	0	0

RNA ID	Gene ID	Symbol	Type	novel-ssc-miR817-3p	novel-ssc-miR849-3p	novel-ssc-miR882-3p	novel-ssc-miR985-5p	novel-ssc-miR1126-5p	novel-ssc-miR1396-3p	ssc-miR-362	novel-ssc-miR16-5p	ssc-miR-375	ssc-miR-376a-3p	ssc-miR-493-5p	ssc-miR-194a-5p	ssc-miR-205	novel-ssc-miR239-5p	novel-ssc-miR397-5p	novel-ssc-miR507-5p	novel-ssc-miR563-3p	novel-ssc-miR568-3p	novel-ssc-miR682-3p
BGIR9823_102129	BGIG9823_26802		lncRNA	0	0	0	0	0	1	0	0	0	0	0	0	0	0	0	0	0	0	1
BGIR9823_102690	BGIG9823_27340		lncRNA	0	0	0	0	0	0	0	0	0	0	0	0	0	0	0	0	0	0	0
BGIR9823_101336	100519166	LOC100519166	lncRNA	0	0	0	0	0	0	0	0	0	0	0	0	0	0	0	0	0	0	0
BGIR9823_101541	102157946	LOC102157946	lncRNA	0	0	0	0	0	0	0	0	0	0	0	0	0	0	1	0	0	0	0
BGIR9823_100268	102159476	LOC102159476	lncRNA	0	0	0	0	0	0	0	0	0	0	0	0	0	0	0	0	0	0	0
BGIR9823_101104	100521877	PUDP	lncRNA	0	0	0	0	0	0	0	0	0	0	1	1	0	0	0	0	0	0	0
BGIR9823_101381	100511434	EGFL7	lncRNA	0	0	0	0	0	0	0	0	0	0	0	0	0	0	0	0	0	0	0
BGIR9823_101015	100520165	TMEM164	lncRNA	0	0	1	0	0	0	0	0	0	0	0	0	0	0	0	0	0	0	0
BGIR9823_100709	100519523	CCDC136	lncRNA	0	0	0	1	0	0	0	0	0	0	0	0	0	0	0	0	0	0	1
BGIR9823_101430	100156979	TRAF3	lncRNA	0	0	0	0	0	0	0	0	0	0	0	0	0	0	0	0	0	0	0
BGIR9823_101450	100623151	INF2	lncRNA	0	0	0	0	0	0	0	0	0	0	0	0	0	0	1	0	0	0	1
BGIR9823_100277	100156830	PTI	lncRNA	0	0	0	1	0	0	0	0	0	0	0	0	0	0	0	0	0	0	0
BGIR9823_101283	100359355	MOSPD1	lncRNA	0	0	0	0	0	0	0	0	0	0	0	0	0	0	0	0	0	0	0
BGIR9823_101340	106505329	LOC106505329	lncRNA	0	0	0	1	0	0	0	0	0	0	0	0	0	0	0	0	0	0	1
BGIR9823_100800	100511907	CCM2	lncRNA	0	0	0	0	0	0	1	0	0	0	0	0	0	0	0	0	0	0	0
BGIR9823_100099	100517562	HGSNAT	lncRNA	0	0	1	0	0	0	0	0	0	0	0	0	0	0	0	0	0	0	0
BGIR9823_100810	100515113	POLM	lncRNA	0	0	0	1	0	0	0	0	0	0	0	0	0	0	0	0	0	0	0
BGIR9823_100578	100622573	POU6F2	lncRNA	0	0	0	0	0	0	0	0	0	0	0	0	0	0	0	0	0	0	1
BGIR9823_78024	100157196	CSNK1G1	lncRNA	0	0	0	0	0	0	0	0	0	0	0	0	0	0	0	0	0	0	0
BGIR9823_100367	100521999	SMARCD3	lncRNA	0	0	0	0	0	0	0	0	0	0	0	0	0	0	0	0	0	0	0
BGIR9823_100389	100627515	HIPK2	lncRNA	0	0	0	0	0	0	0	0	0	0	0	0	0	0	0	0	0	0	0
BGIR9823_100256	100156548	ZHX3	lncRNA	0	0	0	1	0	0	0	0	0	0	0	0	0	0	0	0	0	0	1
BGIR9823_100882	102165056	MBTPS2	lncRNA	0	0	0	0	0	0	1	0	0	0	0	0	0	0	0	0	0	0	0
BGIR9823_100324	100049657	GNAS	lncRNA	0	0	0	0	0	0	0	0	0	0	0	0	0	0	0	0	0	0	0
BGIR9823_100656	100521167	DGKI	lncRNA	0	0	0	0	0	0	0	0	0	0	0	0	0	0	0	0	0	0	0
BGIR9823_101689	BGIG9823_26376		lncRNA	0	0	0	0	0	0	0	0	0	0	0	0	0	0	0	0	0	0	0
BGIR9823_102087	BGIG9823_26761		lncRNA	0	0	0	0	0	0	0	0	0	0	0	0	0	0	0	0	0	0	0
BGIR9823_101951	BGIG9823_26628		lncRNA	0	0	0	0	0	0	0	0	0	0	0	0	0	0	0	0	0	0	0
BGIR9823_101854	BGIG9823_26533		lncRNA	0	0	0	0	0	0	0	0	0	0	0	0	0	0	0	0	0	0	0
BGIR9823_102383	BGIG9823_27048		lncRNA	0	0	0	0	0	1	0	0	0	0	0	0	0	0	0	0	0	0	0
BGIR9823_102727	BGIG9823_27373		lncRNA	0	0	0	0	0	0	0	0	0	0	0	0	0	0	0	0	0	0	0
BGIR9823_102028	BGIG9823_26703		lncRNA	0	0	0	0	0	0	0	0	0	0	0	0	0	0	0	0	0	0	0
BGIR9823_101977	BGIG9823_26653		lncRNA	0	0	0	0	0	0	0	0	0	0	0	0	0	0	0	0	1	0	0
BGIR9823_102406	BGIG9823_27067		lncRNA	0	0	0	0	0	0	0	0	0	0	0	0	0	0	0	0	0	0	0
BGIR9823_101868	BGIG9823_26547		lncRNA	0	0	0	0	0	0	0	0	0	0	0	0	0	0	0	0	0	0	0
BGIR9823_101698	BGIG9823_26385		lncRNA	0	0	0	0	0	0	0	0	0	0	0	0	0	0	0	0	0	0	1
BGIR9823_102438	BGIG9823_27094		lncRNA	0	0	0	0	0	0	0	0	0	0	0	0	0	0	0	0	1	0	0
BGIR9823_102065	BGIG9823_26739		lncRNA	0	0	0	0	0	1	0	0	0	0	0	0	0	0	0	0	1	0	1
BGIR9823_102288	BGIG9823_26954		lncRNA	0	0	0	0	0	0	0	0	0	0	0	0	0	0	0	0	0	0	1
BGIR9823_101990	BGIG9823_26666		lncRNA	0	0	0	0	0	0	0	0	0	0	0	0	0	0	0	0	0	0	0
BGIR9823_101966	BGIG9823_26643		lncRNA	0	0	0	0	0	0	0	0	0	0	0	0	0	0	0	0	0	0	0
BGIR9823_101758	BGIG9823_26438		lncRNA	0	0	0	0	0	0	0	0	0	0	0	0	0	0	0	0	1	0	0
BGIR9823_101861	BGIG9823_26540		lncRNA	0	0	0	0	0	0	0	0	0	0	0	0	0	0	0	0	0	0	0

RNA ID	Gene ID	Symbol	Type	novel-ssc-miR817-3p	novel-ssc-miR849-3p	novel-ssc-miR882-3p	novel-ssc-miR985-5p	novel-ssc-miR1126-5p	novel-ssc-miR1396-3p	ssc-miR-362	novel-ssc-miR16-5p	ssc-miR-375	ssc-miR-376a-3p	ssc-miR-493-5p	ssc-miR-194a-5p	ssc-miR-205	novel-ssc-miR239-5p	novel-ssc-miR397-5p	novel-ssc-miR507-5p	novel-ssc-miR563-3p	novel-ssc-miR568-3p	novel-ssc-miR682-3p
BGIR9823_101960	BGIG9823_26637		lncRNA	0	0	0	0	0	0	0	0	0	0	0	0	0	0	0	0	0	0	0
BGIR9823_101964	BGIG9823_26641		lncRNA	0	0	0	0	0	0	0	0	0	0	0	0	0	0	0	0	0	0	0
BGIR9823_102018	BGIG9823_26693		lncRNA	0	0	0	0	0	0	0	0	0	0	0	0	0	0	1	0	0	0	0
BGIR9823_101913	BGIG9823_26590		lncRNA	0	0	0	0	0	0	0	0	0	0	0	0	0	0	0	0	0	0	1
BGIR9823_102617	BGIG9823_27267		lncRNA	0	0	0	0	0	0	0	0	0	0	0	0	0	0	0	0	0	0	0
BGIR9823_102277	BGIG9823_26944		lncRNA	0	0	0	0	0	0	0	1	0	0	0	0	0	0	0	0	0	0	0
BGIR9823_102215	BGIG9823_26887		lncRNA	0	0	0	0	0	0	0	0	0	0	0	0	0	0	0	0	1	0	0
BGIR9823_102525	BGIG9823_27178		lncRNA	0	0	0	0	0	0	1	0	0	0	1	0	0	0	0	0	1	0	0
BGIR9823_102031	BGIG9823_26706		lncRNA	0	0	0	0	0	0	0	1	0	0	0	0	0	0	0	0	0	0	0
BGIR9823_101909	BGIG9823_26586		lncRNA	0	0	0	0	0	0	0	0	0	0	0	0	0	0	0	0	0	0	0
BGIR9823_102249	BGIG9823_26917		lncRNA	0	0	0	0	0	0	0	0	0	0	0	0	0	0	0	0	0	0	0
BGIR9823_102126	BGIG9823_26799		lncRNA	0	0	0	0	0	0	0	0	0	0	0	0	0	0	0	0	0	0	0
BGIR9823_101720	BGIG9823_26404		lncRNA	0	0	0	0	0	0	0	0	0	0	0	0	0	0	0	0	1	0	0
BGIR9823_102111	BGIG9823_26785		lncRNA	0	0	0	0	0	1	0	0	0	0	0	0	0	0	0	0	0	0	0
BGIR9823_101921	BGIG9823_26598		lncRNA	0	0	0	0	0	0	0	0	0	0	0	0	0	0	0	0	0	0	0
BGIR9823_102144	BGIG9823_26817		lncRNA	0	0	0	0	0	0	0	0	0	0	0	0	0	0	0	0	0	0	0
BGIR9823_102006	BGIG9823_26682		lncRNA	0	0	0	0	0	0	0	0	0	0	0	0	0	0	1	0	0	0	0
BGIR9823_102108	BGIG9823_26782		lncRNA	0	0	0	0	0	1	0	0	0	0	0	0	0	0	0	0	0	0	0
BGIR9823_102515	BGIG9823_27169		lncRNA	0	0	0	0	0	0	0	0	0	0	0	0	0	0	0	0	0	0	0
BGIR9823_101872	BGIG9823_26551		lncRNA	0	0	0	0	0	0	0	0	0	0	0	0	0	0	1	0	0	0	0
BGIR9823_101929	BGIG9823_26606		lncRNA	0	0	0	0	0	0	0	0	0	0	0	0	0	0	0	0	1	0	0
BGIR9823_101740	BGIG9823_26421		lncRNA	0	0	0	0	0	0	0	0	0	0	0	0	0	0	0	0	0	0	1
BGIR9823_102035	BGIG9823_26710		lncRNA	0	0	0	0	0	0	1	0	0	0	0	0	0	0	0	0	0	0	0
BGIR9823_102356	BGIG9823_27021		lncRNA	0	0	0	0	0	0	1	0	0	0	0	0	0	0	0	0	0	0	0
BGIR9823_102012	BGIG9823_26688		lncRNA	0	0	0	0	0	0	0	0	0	0	0	0	0	0	0	0	0	0	1
BGIR9823_101980	BGIG9823_26656		lncRNA	0	0	0	0	0	0	0	0	0	0	0	0	0	0	1	0	0	0	0
BGIR9823_102058	BGIG9823_26732		lncRNA	0	0	0	0	0	0	0	0	0	0	0	0	0	0	0	0	0	0	0
BGIR9823_101945	BGIG9823_26622		lncRNA	0	0	0	0	0	0	0	0	0	0	0	0	0	0	0	0	0	0	1
BGIR9823_101770	BGIG9823_26450		lncRNA	0	0	0	0	0	0	0	0	0	0	0	0	0	0	0	0	0	0	0
XR_002341218.1	110258681	LOC110258681	lncRNA	0	0	0	0	0	0	0	0	0	0	0	1	0	0	0	0	0	0	0
BGIR9823_82088	100620215	CREBBP	lncRNA	0	0	0	0	0	1	0	0	0	0	0	0	0	0	0	0	0	0	0
BGIR9823_100016	102157885	LOC102157885	lncRNA	0	0	1	0	0	0	0	0	0	0	0	0	0	0	0	0	0	0	0
BGIR9823_101122	100519810	PHKA2	lncRNA	0	0	1	0	0	0	0	0	0	0	0	0	0	0	0	0	0	0	0
BGIR9823_100002	100627868	LOC100627868	lncRNA	0	0	0	0	0	0	0	0	0	0	0	0	0	0	0	0	0	0	0
BGIR9823_100152	100152012	CST7	lncRNA	0	0	1	0	0	0	0	0	0	0	0	0	0	0	0	0	0	0	0
BGIR9823_100517	100739098	LOC100739098	lncRNA	0	0	1	0	0	0	0	0	0	0	0	0	0	0	0	0	0	0	0
BGIR9823_100137	100155396	ESF1	lncRNA	0	0	0	0	0	0	1	0	0	0	0	0	0	0	0	0	0	0	0
BGIR9823_101191	110257830	LOC110257830	lncRNA	0	0	0	0	0	0	0	0	0	0	0	0	0	0	0	0	0	0	0
BGIR9823_100307	102164659	LOC102164659	lncRNA	0	0	0	0	0	0	0	0	0	0	0	0	0	0	0	0	0	0	0
BGIR9823_101424	100620346	PPP2R5C	lncRNA	0	0	0	1	0	0	0	0	0	0	0	0	0	0	0	0	0	0	0
BGIR9823_100548	100524778	MRPS24	lncRNA	0	0	0	0	0	0	0	0	0	0	0	0	0	0	0	0	0	0	0
BGIR9823_100391	102162486	LOC102162486	lncRNA	0	0	0	1	0	0	0	0	0	0	0	0	0	0	0	0	0	0	0
BGIR9823_81504	100524976	NSD1	lncRNA	0	0	0	0	0	0	0	0	0	0	0	1	0	0	0	0	0	0	0

RNA ID	Gene ID	Symbol	Type	novel-ssc-miR817-3p	novel-ssc-miR849-3p	novel-ssc-miR882-3p	novel-ssc-miR985-5p	novel-ssc-miR1126-5p	novel-ssc-miR1396-3p	ssc-miR-362	novel-ssc-miR16-5p	ssc-miR-375	ssc-miR-376a-3p	ssc-miR-493-5p	ssc-miR-194a-5p	ssc-miR-205	novel-ssc-miR239-5p	novel-ssc-miR397-5p	novel-ssc-miR507-5p	novel-ssc-miR563-3p	novel-ssc-miR568-3p	novel-ssc-miR682-3p
BGIR9823_100217	100156283	NCOA6	lncRNA	0	0	0	0	0	0	0	0	0	0	0	0	0	0	0	0	0	0	0
BGIR9823_101364	110258073	SNAPC4	lncRNA	0	0	1	0	0	0	0	0	0	0	0	0	0	0	0	0	0	0	0
BGIR9823_101532	102158401	LOC102158401	lncRNA	0	0	0	0	0	0	0	0	0	0	0	0	0	0	0	0	0	0	0
BGIR9823_100496	100521938	LOC100521938	lncRNA	0	0	0	1	0	0	0	0	0	0	0	0	0	0	0	0	0	0	0
BGIR9823_77776	100158022	HINT3	lncRNA	0	0	0	0	0	0	0	0	0	0	0	0	0	0	0	0	0	0	0
BGIR9823_100235	110257452	LOC110257452	lncRNA	0	0	0	0	0	0	0	0	0	0	0	0	0	0	0	0	0	0	0
BGIR9823_78349	100152069	SMAD6	lncRNA	0	0	0	0	0	0	0	0	0	0	0	0	1	0	0	0	1	0	0
BGIR9823_100688	100515937	COPG2	lncRNA	0	0	0	0	0	0	0	0	0	0	0	0	0	0	0	0	0	0	0
BGIR9823_101393	110258158	TNRC6A	lncRNA	0	0	0	0	0	0	0	0	0	0	0	0	0	0	0	0	0	0	0
BGIR9823_100562	100516888	GLI3	lncRNA	0	0	0	0	0	0	0	0	0	0	0	0	0	0	0	0	0	0	0
BGIR9823_79603	106509080	LOC106509080	lncRNA	0	0	0	0	0	1	0	0	0	0	0	0	0	0	0	0	0	0	0
BGIR9823_100840	102166569	TBL1X	lncRNA	0	0	0	0	0	0	0	0	0	0	0	0	0	0	0	0	0	0	0
BGIR9823_100717	100511294	PTPRZ1	lncRNA	0	0	1	0	0	0	0	0	0	0	0	0	0	0	0	0	0	0	0
BGIR9823_100350	100737248	WDR60	lncRNA	0	0	0	0	0	0	0	0	0	0	0	0	0	0	0	0	0	0	0
BGIR9823_101426	100516626	WDR20	lncRNA	0	0	0	0	0	0	0	0	0	0	0	0	0	0	0	0	0	0	1
BGIR9823_101427	100516626	WDR20	lncRNA	0	0	0	0	0	0	0	0	0	0	0	0	1	0	0	0	0	0	0
BGIR9823_101520	100511414	RNF122	lncRNA	0	0	0	0	0	0	0	0	0	0	0	0	0	0	0	0	1	0	0
BGIR9823_100500	100522109	ELMO1	lncRNA	0	0	0	1	0	0	0	0	0	0	0	0	0	0	0	0	0	0	0
BGIR9823_101384	110258135	LOC110258135	lncRNA	0	0	0	1	0	0	0	0	0	0	0	0	0	0	0	0	0	0	0
BGIR9823_101222	100517568	MAGT1	lncRNA	0	0	1	0	0	0	0	0	0	0	0	0	0	0	0	0	0	0	0
BGIR9823_100838	102166211	LOC102166211	lncRNA	0	0	0	0	0	0	1	0	0	0	0	0	0	0	0	0	0	0	0
BGIR9823_80389	100516724	NR2F6	lncRNA	0	0	0	0	0	0	0	0	0	0	0	0	0	0	0	0	0	0	0
BGIR9823_100923	100233182	GK	lncRNA	0	0	1	0	0	0	0	0	0	0	0	0	0	0	0	0	0	0	0
BGIR9823_100887	397645	SAT1	lncRNA	0	0	0	0	0	0	0	0	0	0	0	0	0	0	0	0	0	0	0
BGIR9823_100119	100519631	TASP1	lncRNA	0	0	0	0	0	0	0	0	0	0	0	0	0	0	0	0	0	0	0
BGIR9823_101408	110258214	LOC110258214	lncRNA	0	0	0	0	0	0	0	0	0	0	0	0	0	0	0	0	0	0	0
BGIR9823_100257	100156548	ZHX3	lncRNA	0	0	0	0	0	0	0	0	0	0	0	0	0	0	0	0	0	0	0
BGIR9823_100095	100738984	SLC20A2	lncRNA	0	0	0	0	0	0	0	0	0	0	0	0	0	0	0	0	0	0	0
BGIR9823_100711	397301	CALU	lncRNA	0	0	1	0	0	0	0	0	0	0	0	0	0	0	0	0	0	0	0
BGIR9823_100252	110257392	LOC110257392	lncRNA	0	0	1	0	0	0	0	0	0	0	0	0	0	0	0	0	0	0	0
BGIR9823_100342	100625428	RTEL1	lncRNA	0	0	0	1	0	0	0	0	0	0	0	0	0	0	0	0	0	0	0
BGIR9823_82688	102164200	LOC102164200	lncRNA	0	0	0	0	0	0	0	0	0	0	0	0	0	0	0	0	0	0	0
BGIR9823_92711	110255798	LOC110255798	lncRNA	0	0	0	0	0	0	0	0	0	0	0	0	0	0	0	0	0	0	0
BGIR9823_82658	100622402	C3H16orf45	mRNA	0	0	0	0	0	0	0	0	0	0	0	0	0	0	0	0	0	0	0
BGIR9823_100282	100153535	PCIF1	lncRNA	0	0	0	0	0	0	0	0	0	0	0	0	0	0	0	0	0	0	0
XR_002341162.1	110258480	LOC110258480	lncRNA	0	0	0	0	0	0	0	0	0	1	0	0	0	0	0	0	0	0	0
BGIR9823_101217	100514440	ATRX	lncRNA	0	0	0	0	0	0	0	0	0	0	0	0	0	0	0	0	0	0	0
BGIR9823_96121	110256382	LOC110256382	lncRNA	0	0	0	0	0	0	0	0	0	0	0	1	0	0	0	0	0	0	0
BGIR9823_83098	100322871	CCT7	mRNA	0	0	0	0	0	0	0	0	0	1	0	0	0	0	0	0	0	0	0
BGIR9823_81464	100516183	CNOT6	lncRNA	0	0	0	0	0	0	0	0	0	0	0	0	0	0	0	0	0	0	0
BGIR9823_81228	100518418	GATAD2A	mRNA	0	0	0	0	0	0	0	0	0	0	0	0	0	0	0	0	0	0	0
BGIR9823_99373	100514689	MAST4	mRNA	0	0	0	0	0	0	0	0	0	1	0	0	0	0	0	0	0	0	0
BGIR9823_101508	110258454	LETMD1	lncRNA	0	0	0	0	0	0	0	0	0	0	0	0	0	0	1	0	0	0	0

RNA ID	Gene ID	Symbol	Type	novel-ssc-miR817-3p	novel-ssc-miR849-3p	novel-ssc-miR882-3p	novel-ssc-miR985-5p	novel-ssc-miR1126-5p	novel-ssc-miR1396-3p	ssc-miR-362	novel-ssc-miR16-5p	ssc-miR-375	ssc-miR-376a-3p	ssc-miR-493-5p	ssc-miR-194a-5p	ssc-miR-205	novel-ssc-miR239-5p	novel-ssc-miR397-5p	novel-ssc-miR507-5p	novel-ssc-miR563-3p	novel-ssc-miR568-3p	novel-ssc-miR682-3p
BGIR9823_100200	100624021	PLAGL2	lncRNA	0	0	0	0	0	0	0	0	0	0	0	0	0	0	0	0	0	0	0
BGIR9823_93132	100174955	KLF12	lncRNA	0	0	0	0	0	0	0	0	0	0	0	1	0	0	0	0	0	0	0
BGIR9823_101233	100739062	LOC100739062	lncRNA	0	0	0	0	0	0	0	0	0	0	0	0	0	0	0	0	0	0	0
BGIR9823_101439	100625932	MARK3	lncRNA	0	0	0	0	0	0	0	0	0	0	0	0	0	0	0	0	0	0	0
BGIR9823_102584	BGIG9823_27234		lncRNA	0	0	0	0	0	0	0	0	0	0	0	0	0	0	0	0	0	0	0
BGIR9823_102601	BGIG9823_27251		lncRNA	0	0	0	0	0	0	0	0	0	0	0	0	0	0	0	0	0	0	0
BGIR9823_101907	BGIG9823_26584		lncRNA	0	0	0	0	0	0	0	0	0	0	0	0	0	0	1	0	0	0	0
BGIR9823_101683	BGIG9823_26370		lncRNA	0	0	0	1	0	0	0	0	0	0	0	0	0	0	0	0	0	0	0
BGIR9823_101932	BGIG9823_26609		lncRNA	0	0	0	0	0	0	0	0	0	0	0	0	0	0	1	0	0	0	0
BGIR9823_102312	BGIG9823_26977		lncRNA	0	0	0	0	0	0	0	0	0	0	0	0	0	0	0	0	0	0	0
BGIR9823_101712	BGIG9823_26396		lncRNA	0	0	0	0	0	0	0	0	0	0	0	0	0	0	0	0	0	0	0
BGIR9823_102749	BGIG9823_27392		lncRNA	0	0	0	0	0	0	0	0	0	0	0	0	0	0	0	0	0	0	0
BGIR9823_101726	BGIG9823_26409		lncRNA	0	0	0	0	0	0	0	0	0	0	0	0	0	0	0	0	0	0	0
BGIR9823_102386	BGIG9823_27051		lncRNA	0	0	0	0	0	0	0	0	0	0	0	0	1	0	0	0	0	0	0
BGIR9823_102032	BGIG9823_26707		lncRNA	0	0	0	0	0	0	0	0	0	0	0	0	0	0	1	0	0	0	0
BGIR9823_102336	BGIG9823_27001		lncRNA	0	0	0	0	0	0	0	0	0	0	0	0	0	0	0	0	0	0	1
BGIR9823_101794	BGIG9823_26474		lncRNA	0	0	0	0	0	0	0	0	0	0	0	0	0	0	0	0	1	0	0
BGIR9823_101752	BGIG9823_26433		lncRNA	0	0	0	0	0	0	0	0	0	0	0	0	0	0	0	0	0	0	0
BGIR9823_101944	BGIG9823_26621		lncRNA	0	0	0	0	0	0	0	0	0	0	0	0	0	0	0	0	0	0	0
BGIR9823_101873	BGIG9823_26552		lncRNA	0	0	0	0	0	0	0	0	0	0	0	0	0	0	0	0	0	0	0
BGIR9823_101671	BGIG9823_26359		lncRNA	0	0	0	0	0	0	0	0	0	0	0	0	1	0	0	0	0	0	0
BGIR9823_101974	BGIG9823_26651		lncRNA	0	0	0	0	0	0	0	0	0	0	0	0	0	0	0	0	0	0	0
BGIR9823_102380	BGIG9823_27045		lncRNA	0	0	0	0	0	0	0	0	0	0	0	0	0	0	1	0	0	0	0
BGIR9823_102232	BGIG9823_26904		lncRNA	0	0	0	0	0	0	0	0	0	0	0	0	0	0	0	0	0	0	0
BGIR9823_102238	BGIG9823_26910		lncRNA	0	0	0	0	0	0	0	0	0	0	0	0	0	0	0	0	0	0	0
BGIR9823_102071	BGIG9823_26745		lncRNA	0	0	0	0	0	0	1	0	0	0	0	0	0	0	0	0	0	0	0
BGIR9823_101784	BGIG9823_26464		lncRNA	0	0	0	0	0	0	0	0	0	0	0	0	0	0	0	0	0	0	1
BGIR9823_102392	BGIG9823_27057		lncRNA	0	0	0	0	0	0	0	0	0	0	0	0	0	0	0	0	0	0	0
BGIR9823_102420	BGIG9823_27081		lncRNA	0	0	0	0	0	0	0	0	0	0	0	0	0	0	1	0	0	0	0
BGIR9823_101988	BGIG9823_26664		lncRNA	0	0	0	0	0	0	0	0	0	0	0	0	0	0	0	0	0	0	0
BGIR9823_101675	BGIG9823_26362		lncRNA	0	0	0	0	0	0	0	0	0	0	0	0	0	0	0	0	0	0	0
BGIR9823_101761	BGIG9823_26441		lncRNA	0	0	0	0	0	0	0	0	0	0	0	0	0	0	0	0	0	0	0
BGIR9823_102382	BGIG9823_27047		lncRNA	0	0	0	0	0	0	0	0	0	0	0	0	0	0	0	0	0	0	0
BGIR9823_102045	BGIG9823_26719		lncRNA	0	0	0	0	0	0	0	0	0	0	0	0	0	0	0	0	0	0	0
BGIR9823_101857	BGIG9823_26536		lncRNA	0	0	0	0	0	0	0	0	0	0	0	0	0	0	0	0	0	0	0
BGIR9823_101775	BGIG9823_26455		lncRNA	0	0	0	0	0	0	0	0	0	0	0	0	0	0	0	0	0	0	0
BGIR9823_102446	BGIG9823_27101		lncRNA	0	0	0	0	0	0	0	0	0	0	0	0	0	0	0	0	0	0	0
BGIR9823_102179	BGIG9823_26851		lncRNA	0	0	0	0	0	0	0	0	0	0	0	0	0	0	0	0	0	0	0
BGIR9823_101678	BGIG9823_26365		lncRNA	0	0	0	0	0	0	0	0	0	0	0	0	0	0	0	0	0	0	0
BGIR9823_102242	BGIG9823_26913		lncRNA	0	0	0	0	0	0	0	0	0	0	0	0	0	0	0	0	0	0	0
BGIR9823_102633	BGIG9823_27283		lncRNA	0	0	0	0	0	0	0	0	0	0	1	0	0	0	0	0	0	0	0
BGIR9823_102205	BGIG9823_26877		lncRNA	0	0	0	0	0	0	0	0	0	0	0	0	0	0	0	0	0	0	0
BGIR9823_101763	BGIG9823_26443		lncRNA	0	0	0	0	0	0	0	0	0	0	0	0	0	0	0	0	0	0	0

RNA ID	Gene ID	Symbol	Type	novel-ssc-miR817-3p	novel-ssc-miR849-3p	novel-ssc-miR882-3p	novel-ssc-miR985-5p	novel-ssc-miR1126-5p	novel-ssc-miR1396-3p	ssc-miR-362	novel-ssc-miR16-5p	ssc-miR-375	ssc-miR-376a-3p	ssc-miR-493-5p	ssc-miR-194a-5p	ssc-miR-205	novel-ssc-miR239-5p	novel-ssc-miR397-5p	novel-ssc-miR507-5p	novel-ssc-miR563-3p	novel-ssc-miR568-3p	novel-ssc-miR682-3p
BGIR9823_102754	BGIG9823_27394		lncRNA	0	0	0	0	0	0	0	0	0	0	0	0	0	0	0	0	0	0	0
BGIR9823_102077	BGIG9823_26751		lncRNA	0	0	0	0	0	0	0	0	0	0	0	0	0	0	0	0	0	0	0
BGIR9823_102457	BGIG9823_27111		lncRNA	0	0	0	0	0	0	0	0	0	0	0	0	1	0	0	0	0	0	0
BGIR9823_101881	BGIG9823_26560		lncRNA	0	0	0	0	0	0	0	0	0	0	0	0	0	0	0	0	0	0	0
BGIR9823_102086	BGIG9823_26760		lncRNA	0	0	0	0	0	0	0	0	0	0	0	0	0	0	0	0	1	0	0
BGIR9823_102146	BGIG9823_26819		lncRNA	0	0	0	0	0	0	0	0	0	0	0	0	0	0	0	0	0	0	0
BGIR9823_101735	BGIG9823_26416		lncRNA	0	0	0	0	0	0	0	0	0	0	0	0	0	0	0	0	0	0	0
BGIR9823_102132	BGIG9823_26805		lncRNA	0	0	0	0	0	0	0	0	0	0	0	0	0	0	0	0	0	0	0
BGIR9823_102345	BGIG9823_27010		lncRNA	0	0	0	0	0	0	0	0	0	0	0	0	0	0	0	0	1	0	0
BGIR9823_101715	BGIG9823_26399		lncRNA	0	0	0	0	0	0	0	0	0	0	0	0	0	0	0	0	0	0	0
BGIR9823_102322	BGIG9823_26987		lncRNA	0	0	0	0	0	0	0	0	0	0	0	0	0	0	0	0	0	0	0
BGIR9823_102695	BGIG9823_27345		lncRNA	0	0	0	0	0	0	0	0	0	0	0	0	0	0	0	1	0	0	0
BGIR9823_101684	BGIG9823_26371		lncRNA	0	0	0	0	0	0	0	0	0	0	0	0	0	0	0	0	1	0	0
BGIR9823_102344	BGIG9823_27009		lncRNA	0	0	0	0	0	0	0	0	0	0	0	0	0	0	0	0	0	0	0
BGIR9823_102092	BGIG9823_26766		lncRNA	0	0	0	0	0	0	0	0	0	0	0	0	0	0	0	0	0	0	0
BGIR9823_102073	BGIG9823_26747		lncRNA	0	0	0	0	0	0	0	0	0	0	0	0	0	0	0	0	0	0	0
BGIR9823_102188	BGIG9823_26860		lncRNA	0	0	0	0	0	0	0	0	0	0	0	0	0	0	0	0	0	0	0
BGIR9823_101946	BGIG9823_26623		lncRNA	0	0	0	0	0	0	0	0	0	0	0	0	0	0	0	0	0	0	0
BGIR9823_102176	BGIG9823_26848		lncRNA	0	0	0	0	0	0	0	0	0	0	0	0	0	0	0	0	0	0	1
BGIR9823_102387	BGIG9823_27052		lncRNA	0	0	0	0	0	0	0	0	0	0	0	0	0	0	1	0	0	0	0
BGIR9823_101876	BGIG9823_26555		lncRNA	0	0	0	0	0	0	0	0	0	0	0	0	0	0	0	0	0	0	0
BGIR9823_101888	BGIG9823_26565		lncRNA	0	0	0	0	0	0	0	0	0	0	0	0	0	0	0	0	0	0	0
BGIR9823_101818	BGIG9823_26498		lncRNA	0	0	0	0	0	0	0	0	0	0	0	0	0	0	0	0	0	0	0
BGIR9823_102320	BGIG9823_26985		lncRNA	0	0	0	0	0	0	0	0	0	0	0	0	0	0	0	0	0	0	1
BGIR9823_102080	BGIG9823_26754		lncRNA	0	0	0	0	0	0	0	0	0	0	0	0	0	0	1	0	0	0	0
BGIR9823_101786	BGIG9823_26466		lncRNA	0	0	0	0	0	0	0	0	0	0	0	0	0	0	0	0	0	0	0
BGIR9823_102325	BGIG9823_26990		lncRNA	0	0	0	0	0	0	0	0	0	0	0	0	0	0	0	0	0	0	0
BGIR9823_101989	BGIG9823_26665		lncRNA	0	0	0	0	0	0	0	0	0	0	0	0	0	0	0	0	0	0	0
BGIR9823_101889	BGIG9823_26566		lncRNA	0	0	0	0	0	0	0	0	0	0	0	0	0	0	0	0	0	0	0
BGIR9823_102669	BGIG9823_27319		lncRNA	0	0	0	0	0	0	0	0	0	0	0	0	0	0	0	0	0	0	0
BGIR9823_101878	BGIG9823_26557		lncRNA	0	0	0	0	0	0	0	0	0	0	0	0	0	0	0	0	0	0	0
BGIR9823_101918	BGIG9823_26595		lncRNA	0	0	0	0	1	0	0	0	0	0	0	0	0	0	0	0	0	0	0
BGIR9823_102064	BGIG9823_26738		lncRNA	0	0	0	0	0	0	0	0	0	0	0	0	0	0	0	0	0	0	0
BGIR9823_101985	BGIG9823_26661		lncRNA	0	0	0	0	0	0	0	0	0	0	0	0	0	0	1	0	0	0	0
BGIR9823_101774	BGIG9823_26454		lncRNA	0	0	0	0	0	0	0	0	0	0	0	0	0	0	0	0	0	0	0
BGIR9823_102112	BGIG9823_26786		lncRNA	0	0	0	0	0	0	0	0	0	0	0	0	0	0	0	0	0	0	0
BGIR9823_102585	BGIG9823_27235		lncRNA	0	0	0	0	0	0	0	0	0	0	0	0	0	0	0	0	0	0	0
BGIR9823_101903	BGIG9823_26580		lncRNA	0	0	0	0	0	0	0	0	0	0	0	0	0	0	0	0	0	0	0
BGIR9823_102173	BGIG9823_26846		lncRNA	0	0	0	0	0	0	0	0	0	0	0	0	0	0	0	0	0	0	0
BGIR9823_101912	BGIG9823_26589		lncRNA	0	0	0	0	0	0	0	0	0	0	0	0	0	0	1	0	0	0	0
BGIR9823_101793	BGIG9823_26473		lncRNA	0	0	0	0	0	0	0	0	0	0	0	0	0	0	0	0	0	0	0
BGIR9823_102135	BGIG9823_26808		lncRNA	0	0	0	0	0	0	0	0	0	0	0	0	0	0	0	0	0	0	0
BGIR9823_101911	BGIG9823_26588		lncRNA	0	0	0	0	0	0	0	0	0	0	0	0	0	0	0	0	0	0	1

RNA ID	Gene ID	Symbol	Type	novel-ssc-miR817-3p	novel-ssc-miR849-3p	novel-ssc-miR882-3p	novel-ssc-miR985-5p	novel-ssc-miR1126-5p	novel-ssc-miR1396-3p	ssc-miR-362	novel-ssc-miR16-5p	ssc-miR-375	ssc-miR-376a-3p	ssc-miR-493-5p	ssc-miR-194a-5p	ssc-miR-205	novel-ssc-miR239-5p	novel-ssc-miR397-5p	novel-ssc-miR507-5p	novel-ssc-miR563-3p	novel-ssc-miR568-3p	novel-ssc-miR682-3p
BGIR9823_101864	BGIG9823_26543		lncRNA	0	0	0	0	0	0	0	0	0	0	0	0	0	0	1	0	0	0	0
BGIR9823_101851	BGIG9823_26530		lncRNA	0	0	0	0	0	0	0	0	0	0	0	0	0	0	0	0	0	0	0
BGIR9823_102310	BGIG9823_26975		lncRNA	0	0	0	0	0	0	0	0	0	0	0	0	0	0	0	0	0	0	1
BGIR9823_101776	BGIG9823_26456		lncRNA	0	0	0	0	0	0	0	0	0	0	0	0	0	0	0	0	0	0	0
BGIR9823_102381	BGIG9823_27046		lncRNA	0	0	0	0	0	0	0	0	0	0	0	0	0	0	0	0	1	0	0
BGIR9823_101680	BGIG9823_26367		lncRNA	0	0	0	0	0	0	0	0	0	0	0	0	0	0	0	0	0	0	1
BGIR9823_101747	BGIG9823_26428		lncRNA	0	0	0	0	1	0	0	0	0	0	0	0	0	0	0	0	0	0	0
BGIR9823_101830	BGIG9823_26509		lncRNA	0	0	0	0	0	0	0	0	0	0	0	0	0	0	0	0	0	0	0
BGIR9823_102332	BGIG9823_26997		lncRNA	0	0	0	0	0	0	0	0	0	0	0	0	0	0	0	0	0	0	0
XR_002336732.1	102159417	LOC102159417	lncRNA	0	0	0	0	0	0	0	0	0	0	0	0	0	0	0	0	0	0	0
XR_002343571.1	106507458	LOC106507458	lncRNA	0	0	0	0	0	0	0	0	0	1	0	0	0	0	0	0	0	0	0
XR_001304304.2	106507921	LOC106507921	lncRNA	0	0	0	0	0	0	0	0	1	0	1	0	0	0	0	0	0	0	0
XR_002344048.1	110260709	LOC110260709	lncRNA	0	0	0	0	0	0	0	0	0	0	0	1	0	0	0	0	0	0	0
XM_003353597.4	100621832	TMEM246	mRNA	0	0	0	0	0	0	0	0	0	1	0	0	0	0	0	0	0	0	0
XR_002338514.1	102161206	LOC102161206	lncRNA	0	0	0	0	1	0	0	0	1	0	0	0	0	0	0	0	0	0	0
NM_001244791.1	100513225	BAG1	mRNA	0	0	0	0	0	0	0	0	0	1	0	0	0	0	0	0	0	0	0
XR_002341355.1	102167380	CUNH21orf58	lncRNA	0	0	0	0	0	0	0	0	0	0	0	1	0	0	0	0	0	0	0
XR_001299235.2	106505126	LOC106505126	lncRNA	0	0	0	0	1	0	0	0	0	0	0	0	0	0	0	0	0	0	0
XR_304631.3	102167686	LOC102167686	lncRNA	0	0	0	0	0	0	0	0	0	0	0	1	0	0	0	0	0	0	0
XM_021066717.1	100521576	ARSG	mRNA	0	0	0	0	0	0	0	0	0	1	0	0	0	0	0	0	0	0	0
XR_002342261.1	110259855	LOC110259855	lncRNA	0	0	0	0	0	0	0	0	0	0	0	1	0	0	0	0	0	0	0
NM_213891.1	396925	VCAM1	mRNA	0	0	0	0	0	0	0	0	0	0	0	0	0	0	0	0	0	0	0
XM_003129787.4	100516505	VSTM5	mRNA	0	0	0	0	0	0	0	0	0	1	0	0	0	0	0	0	0	0	0
XM_021100543.1	106504722	UCP1	mRNA	0	0	0	0	0	0	0	0	0	1	0	0	0	0	0	0	0	0	0
XR_002345110.1	110261202	LOC110261202	lncRNA	0	0	0	0	0	0	0	0	0	1	0	0	0	0	0	0	0	0	0
XM_013992908.2	102164011	LOC102164011	mRNA	0	0	0	0	0	0	0	0	0	1	0	0	0	0	0	0	0	0	0
XM_021082022.1	100525982	LOC100525982	mRNA	0	0	0	0	0	0	0	0	0	0	0	0	0	0	0	0	0	0	0
NM_214188.1	397385	DDOST	mRNA	0	0	0	0	0	0	0	0	0	1	0	0	0	0	0	0	0	0	0
XM_013983344.2	100156215	MSS51	mRNA	0	0	0	0	0	0	0	0	0	1	0	0	0	0	0	0	0	0	0
NM_001164856.1	100303723	LCAT	mRNA	0	0	0	0	0	0	0	0	0	1	0	0	0	0	0	0	0	0	0
NM_001278748.1	100520980	CASR	mRNA	0	0	0	0	0	0	0	0	0	0	0	0	0	0	0	0	0	0	0
XR_002342824.1	110260048	LOC110260048	lncRNA	0	0	0	0	0	0	0	0	0	0	0	1	0	0	0	0	0	0	0
XM_003481630.4	100738263	SPRYD4	mRNA	0	0	0	0	0	0	0	0	0	0	0	0	0	0	0	0	0	0	0
XR_002340516.1	102163798	LOC102163798	lncRNA	0	1	0	0	0	0	0	0	0	0	0	0	0	0	0	0	0	0	0
XM_021080156.1	102167889	LOC102167889	mRNA	0	0	0	0	0	0	0	0	0	0	0	0	0	0	0	0	0	0	0
XR_002343904.1	110260647	LOC110260647	lncRNA	0	0	0	0	0	0	0	0	0	0	0	0	0	0	0	0	0	0	0
XM_021082023.1	100510954	LOC100510954	mRNA	0	0	0	0	0	0	0	0	0	0	0	0	0	0	0	0	0	0	0
XR_002340992.1	110258138	LOC110258138	lncRNA	0	0	0	0	0	0	0	0	0	0	0	1	0	0	0	0	0	0	0
XM_003129097.6	100523679	CCNI	mRNA	0	0	0	0	0	0	0	0	0	1	0	0	0	0	0	0	0	0	0
XM_021086388.1	110255213	ARMC5	mRNA	0	0	0	0	0	0	0	0	0	1	0	0	0	0	0	0	0	0	0
XM_003133626.3	100522220	LOC100522220	mRNA	0	0	0	0	0	0	0	0	0	1	0	0	0	0	0	0	0	0	0
XR_002341145.1	110258406	LOC110258406	lncRNA	0	0	0	0	0	0	0	0	0	1	0	0	0	0	0	0	0	0	0
XR_002341292.1	110258990	LOC110258990	lncRNA	0	0	0	0	0	0	0	0	0	1	0	0	0	0	0	0	0	0	0

RNA ID	Gene ID	Symbol	Type	novel-ssc-miR817-3p	novel-ssc-miR849-3p	novel-ssc-miR882-3p	novel-ssc-miR985-5p	novel-ssc-miR1126-5p	novel-ssc-miR1396-3p	ssc-miR-362	novel-ssc-miR16-5p	ssc-miR-375	ssc-miR-376a-3p	ssc-miR-493-5p	ssc-miR-194a-5p	ssc-miR-205	novel-ssc-miR239-5p	novel-ssc-miR397-5p	novel-ssc-miR507-5p	novel-ssc-miR563-3p	novel-ssc-miR568-3p	novel-ssc-miR682-3p
NM_001204772.1	100533540	BRD4	mRNA	0	0	0	0	0	0	0	0	0	0	0	0	0	0	0	0	0	0	0
NM_213763.2	396637	UCHL1	mRNA	0	0	0	0	0	0	0	0	0	1	0	0	0	0	0	0	0	0	0
NM_214050.2	397117	GSTO1	mRNA	0	0	0	0	0	0	0	0	0	1	0	0	0	0	0	0	0	0	0
XR_002337239.1	110256047	LOC110256047	lncRNA	0	0	0	0	0	0	0	0	0	0	0	0	0	0	0	0	0	0	0
XR_002343522.1	110260355	LOC110260355	lncRNA	0	0	0	0	0	0	0	0	0	0	0	0	0	0	0	0	0	0	0
XR_001304268.2	106507904	LOC106507904	lncRNA	0	0	0	0	0	0	0	0	0	1	0	0	0	0	0	0	0	0	0
XR_002343855.1	110260615	LOC110260615	lncRNA	0	0	0	0	0	0	0	0	0	0	0	0	0	0	0	0	0	0	0
XR_002336945.1	110255950	LOC110255950	lncRNA	0	0	0	0	0	0	0	0	0	0	0	1	0	0	0	0	0	0	0
XM_021075808.1	110257099	LOC110257099	mRNA	0	0	0	0	0	0	0	0	0	1	0	0	0	0	0	0	0	0	0
XM_021082410.1	110259094	LOC110259094	mRNA	0	0	0	0	0	0	0	0	0	1	0	0	0	0	0	0	0	0	0
XR_002341290.1	110258990	LOC110258990	lncRNA	0	0	0	0	0	0	0	0	0	0	0	1	0	0	0	0	0	0	0
XM_021100189.1	100152943	ADTRP	mRNA	0	0	0	0	0	0	0	0	0	1	0	0	0	0	0	0	0	0	0
XR_002341433.1	106509301	LOC106509301	lncRNA	0	0	0	0	0	0	0	0	1	0	0	0	0	0	0	0	0	0	0
NM_001244722.1	100512802	PUS3	mRNA	0	0	0	0	0	0	0	0	0	1	0	0	0	0	0	0	0	0	0
XM_021092065.1	100624597	YARS2	mRNA	0	0	0	0	0	0	0	0	0	0	0	0	0	0	0	0	0	0	0
XM_021066519.1	100512084	TBC1D2	mRNA	0	0	0	0	0	0	0	0	0	1	0	0	0	0	0	0	0	0	0
XR_002335835.1	110255476	LOC110255476	lncRNA	0	0	0	0	0	0	0	0	0	0	0	0	0	0	0	0	0	0	0
XR_002335866.1	110255489	LOC110255489	lncRNA	0	1	0	0	0	0	0	0	0	0	0	0	0	0	0	1	0	0	0
XR_002337288.1	106509234	LOC106509234	lncRNA	0	0	0	0	0	0	0	0	0	0	0	0	0	0	0	0	0	0	0
XR_002342643.1	106508312	LOC106508312	lncRNA	0	0	0	0	0	0	0	0	0	0	0	0	0	0	0	0	0	0	0
NM_001097492.1	100038008	CD1E	mRNA	0	0	0	0	0	0	0	0	0	1	0	0	0	0	0	0	0	0	0
XR_002336367.1	106507918	LOC106507918	lncRNA	0	0	0	0	0	0	0	0	0	0	1	0	0	0	0	0	0	0	0
XR_002340833.1	110257824	LOC110257824	lncRNA	0	0	0	0	0	0	0	0	0	1	0	0	0	0	0	0	0	0	0
NM_001004028.1	445513	HAPLN1	mRNA	0	0	0	0	0	0	0	0	0	0	0	0	0	0	0	0	0	0	0
XR_001302352.2	106506794	LOC106506794	lncRNA	0	0	0	0	0	0	0	0	0	1	0	0	0	0	0	0	0	0	0
XR_002336622.1	110255794	LOC110255794	lncRNA	0	0	0	0	0	0	0	0	0	0	0	1	0	0	0	0	0	0	0
XM_021070652.1	110256443	QARS	mRNA	0	0	0	0	0	0	0	0	0	1	0	0	0	0	0	0	0	0	0
XM_001928752.5	100154892	LOC100154892	mRNA	0	0	0	0	0	0	0	0	0	1	0	0	0	0	0	0	0	0	0
NM_001243696.1	100158051	DRAM2	mRNA	0	0	0	0	0	0	0	0	0	1	0	0	0	0	0	0	0	0	0
XR_002342202.1	100512593	LOC100512593	lncRNA	0	0	0	0	0	0	0	0	0	0	0	0	0	0	0	0	0	0	0
XR_002343188.1	110260249	LOC110260249	lncRNA	0	0	0	0	0	0	0	0	0	0	0	0	0	0	0	0	0	0	0
XM_021081808.1	100155561	LOC100155561	mRNA	0	0	0	0	0	0	0	0	0	0	0	0	0	0	0	0	0	0	0
XM_003134082.6	100515063	HMMR	mRNA	0	0	0	0	0	0	0	0	0	1	0	0	0	0	0	0	0	0	0
XR_002341327.1	110259136	LOC110259136	lncRNA	0	0	0	0	0	0	0	0	0	0	0	0	0	0	0	0	0	0	0
XR_002346274.1	110261765	LOC110261765	lncRNA	0	0	0	0	0	0	0	0	0	0	0	0	0	0	0	0	0	0	0
URS0000BC4F80	BGIG9823_26330		lncRNA	0	0	0	0	0	0	0	0	0	0	0	0	0	0	0	0	0	0	0
URS0000BC82B3	BGIG9823_26290		lncRNA	0	0	0	0	0	0	0	0	0	0	0	1	0	0	0	0	0	0	0
BGIR9823_100417	106506781	LOC106506781	lncRNA	0	0	0	0	0	1	0	0	0	0	0	0	0	0	0	0	0	0	0
BGIR9823_78908	100157533	WTAP	mRNA	0	0	0	0	0	0	0	0	0	0	0	0	0	0	0	0	0	0	0
BGIR9823_85709	100156112	ATP6V1E1	lncRNA	0	0	0	0	0	1	0	0	0	0	0	0	0	0	0	0	0	0	0
BGIR9823_88195	100152838	SLC22A23	lncRNA	0	0	0	0	0	0	0	0	0	0	0	0	0	0	0	0	0	0	0
BGIR9823_81246	100174943	IFI30	mRNA	0	0	0	0	0	0	0	0	0	0	0	0	0	0	0	0	0	0	0
BGIR9823_100836	100624032	LOC100624032	lncRNA	0	0	0	0	0	0	0	0	0	0	0	0	0	0	0	0	0	0	0

RNA ID	Gene ID	Symbol	Type	novel-ssc-miR817-3p	novel-ssc-miR849-3p	novel-ssc-miR882-3p	novel-ssc-miR985-5p	novel-ssc-miR1126-5p	novel-ssc-miR1396-3p	ssc-miR-362	novel-ssc-miR16-5p	ssc-miR-375	ssc-miR-376a-3p	ssc-miR-493-5p	ssc-miR-194a-5p	ssc-miR-205	novel-ssc-miR239-5p	novel-ssc-miR397-5p	novel-ssc-miR507-5p	novel-ssc-miR563-3p	novel-ssc-miR568-3p	novel-ssc-miR682-3p
BGIR9823_84507	100154147	MAN1A2	lncRNA	0	0	0	0	0	0	0	0	0	0	0	0	0	0	0	0	0	0	0
BGIR9823_78933	100516653	CCDC170	lncRNA	0	0	0	0	0	0	0	0	0	0	0	0	0	0	0	0	0	0	0
BGIR9823_85930	106510465	LOC106510465	mRNA	0	0	0	0	0	0	0	0	0	0	0	0	0	0	0	0	0	0	0
BGIR9823_98132	100519056	ORC4	lncRNA	0	0	0	0	0	0	0	0	0	0	0	0	0	0	0	0	0	0	0
BGIR9823_85554	100624497	DNM1L	mRNA	0	0	0	0	0	0	0	0	1	0	0	0	0	1	0	0	0	0	0
BGIR9823_89252	100153410	STK38	mRNA	0	0	0	0	0	0	0	0	0	0	0	0	0	0	0	0	0	0	0
BGIR9823_100096	100738984	SLC20A2	lncRNA	0	0	0	0	0	0	1	0	0	0	0	0	0	0	0	0	0	0	0
BGIR9823_85945	100521092	WDR59	mRNA	0	0	0	0	0	0	0	0	0	0	0	0	0	0	0	0	0	0	0
BGIR9823_85409	100516908	NCKAP5L	lncRNA	0	0	0	0	0	0	0	0	0	0	0	0	0	0	0	0	0	0	0
BGIR9823_100573	100516888	GLI3	lncRNA	0	0	0	0	0	0	0	0	0	0	0	0	0	0	0	0	0	0	0
BGIR9823_101467	100522195	XRCC3	lncRNA	0	0	0	0	0	0	0	0	0	0	0	0	0	0	0	0	0	0	0
BGIR9823_86462	100738753	RERE	lncRNA	0	0	0	0	0	0	0	0	0	0	0	0	0	0	0	0	0	0	0
BGIR9823_100236	106504144	DLGAP4	lncRNA	0	0	0	0	0	0	0	0	0	0	0	0	0	0	0	0	0	0	1
BGIR9823_100421	100626356	UBE2H	lncRNA	0	0	1	0	0	0	0	0	0	0	0	0	0	0	0	0	0	0	0
BGIR9823_101512	110258473	LOC110258473	lncRNA	0	0	0	0	0	0	0	0	0	0	0	0	0	0	0	0	0	0	0
BGIR9823_100354	102167342	CNPY1	lncRNA	0	0	1	0	0	0	0	0	0	0	0	0	0	0	0	0	0	0	0
BGIR9823_100645	100519065	UBN2	lncRNA	0	0	0	0	0	0	0	0	0	0	0	0	0	0	0	0	0	0	0
BGIR9823_100414	414383	CHCHD3	lncRNA	0	0	0	0	0	0	0	0	0	0	0	0	0	0	0	0	0	0	0
BGIR9823_81026	100523886	SF1	lncRNA	0	0	0	0	0	0	0	0	0	0	0	0	0	0	0	0	0	0	0
BGIR9823_100005	106506050	LOC106506050	lncRNA	0	0	0	0	0	0	0	0	0	0	0	0	0	0	0	0	0	0	0
BGIR9823_81229	100518418	GATAD2A	lncRNA	0	0	0	0	0	0	0	0	0	0	0	0	1	0	0	0	0	0	0
BGIR9823_79698	100154949	CNIH1	mRNA	0	0	0	0	0	0	0	0	0	0	0	1	0	0	0	0	0	0	0
BGIR9823_100531	100625903	SKAP2	lncRNA	0	0	0	0	0	0	0	0	0	0	0	0	0	0	0	0	0	0	0
BGIR9823_81900	110259864	LOC110259864	lncRNA	0	0	0	0	0	0	0	0	0	0	0	0	1	0	0	0	0	0	0
BGIR9823_100425	100626356	UBE2H	lncRNA	0	0	0	0	0	1	0	0	0	0	0	0	0	0	0	0	0	0	0
BGIR9823_82216	100511252	ACTR1B	lncRNA	0	0	0	0	0	1	0	0	0	0	1	0	0	0	0	0	0	0	0
BGIR9823_100156	100519522	NINL	lncRNA	0	0	0	0	0	0	0	0	0	0	0	0	0	0	0	0	0	0	0
BGIR9823_101565	110258600	LOC110258600	lncRNA	0	0	0	0	0	1	0	0	0	0	0	0	0	0	0	0	0	0	0
BGIR9823_100677	106504059	EXOC4	lncRNA	0	0	1	0	0	0	0	0	0	0	0	0	0	0	0	0	0	0	0
BGIR9823_100501	100523875	7-Sep	lncRNA	0	0	0	0	0	0	0	0	0	0	0	0	0	0	0	0	0	0	0
BGIR9823_100856	100154431	ASB11	lncRNA	0	0	1	0	0	0	0	0	0	0	0	0	0	0	0	0	0	0	0
BGIR9823_92258	100738420	ABI1	lncRNA	0	0	0	0	0	0	0	0	0	0	0	0	0	0	0	0	0	0	0
BGIR9823_97570	100158098	RHBDD3	lncRNA	0	0	0	0	0	0	0	0	0	0	0	0	0	0	0	0	0	0	0
BGIR9823_101254	106507023	LOC106507023	lncRNA	0	0	0	0	0	0	0	0	0	0	0	0	0	0	0	0	0	0	0
BGIR9823_100601	102159531	ACTR3B	mRNA	0	0	0	0	0	0	0	0	0	0	0	0	0	0	0	0	0	0	0
BGIR9823_83153	110260004	AHSA2P	mRNA	0	0	0	0	0	0	0	0	0	1	0	0	0	0	0	0	0	0	0
BGIR9823_90913	110255435	LOC110255435	lncRNA	0	0	0	0	0	0	0	0	0	0	0	0	0	0	0	0	0	0	0
BGIR9823_100272	396886	SLPI	lncRNA	0	0	0	1	0	0	0	0	0	0	0	0	0	0	0	0	0	0	0
BGIR9823_77799	100520524	GOPC	lncRNA	0	0	0	0	0	0	0	0	0	0	0	0	0	0	0	0	0	0	0
BGIR9823_100990	100153260	APOOL	lncRNA	0	0	0	0	0	0	0	0	0	0	0	0	0	0	0	0	1	0	0
BGIR9823_79735	110255453	LOC110255453	lncRNA	0	0	0	0	0	0	0	0	0	0	0	0	0	0	0	0	0	0	0
BGIR9823_80994	100519350	DPP3	mRNA	0	0	0	0	0	0	0	0	0	0	0	0	0	0	0	0	0	0	0
BGIR9823_100422	100626356	UBE2H	lncRNA	0	0	0	0	0	0	0	0	0	0	0	0	0	0	0	0	0	0	0

RNA ID	Gene ID	Symbol	Type	novel-ssc-miR817-3p	novel-ssc-miR849-3p	novel-ssc-miR882-3p	novel-ssc-miR985-5p	novel-ssc-miR1126-5p	novel-ssc-miR1396-3p	ssc-miR-362	novel-ssc-miR16-5p	ssc-miR-375	ssc-miR-376a-3p	ssc-miR-493-5p	ssc-miR-194a-5p	ssc-miR-205	novel-ssc-miR239-5p	novel-ssc-miR397-5p	novel-ssc-miR507-5p	novel-ssc-miR563-3p	novel-ssc-miR568-3p	novel-ssc-miR682-3p
BGIR9823_95751	100627663	ARHGEF3	lncRNA	0	0	0	0	0	0	0	0	0	0	0	1	0	0	0	0	0	0	0
BGIR9823_78308	100154901	ATP9B	lncRNA	0	0	0	0	0	0	0	0	0	0	0	0	1	0	0	0	0	0	0
BGIR9823_89654	733584	FOXN3	lncRNA	0	0	0	0	0	0	0	0	0	0	0	1	0	0	0	0	0	0	0
BGIR9823_94919	100737976	RBSN	lncRNA	0	0	0	0	0	0	0	0	0	0	0	0	0	0	0	0	0	0	0
BGIR9823_83670	100153676	PCMTD1	lncRNA	0	0	0	0	0	1	0	0	0	0	0	0	0	0	0	0	0	0	0
BGIR9823_100051	100621535	TPD52L2	lncRNA	0	0	0	0	0	0	0	0	0	0	0	0	0	0	0	0	0	0	0
BGIR9823_78866	100512264	NTNG2	lncRNA	0	0	0	0	1	0	0	0	0	0	0	0	0	0	0	0	0	0	0
BGIR9823_100572	100516888	GLI3	lncRNA	0	0	0	0	0	0	0	0	0	0	0	0	0	0	0	0	0	0	0
BGIR9823_89017	100157526	MYLK4	lncRNA	0	0	0	0	0	0	0	0	0	0	0	0	0	0	0	0	0	0	0
BGIR9823_78135	100153621	FAM214A	lncRNA	0	0	0	0	0	0	0	0	0	0	0	0	0	0	0	0	1	0	0
BGIR9823_88205	100157926	RIOK1	lncRNA	0	0	0	0	0	0	0	0	0	0	0	0	0	0	0	0	0	0	0
BGIR9823_100635	102162486	LOC102162486	lncRNA	0	0	1	0	0	0	0	0	0	0	0	0	0	0	0	0	0	0	0
BGIR9823_87926	110261182	LOC110261182	lncRNA	0	0	0	0	0	0	0	0	0	0	0	1	0	0	0	0	0	0	0
BGIR9823_96076	110256340	LOC110256340	lncRNA	0	0	0	0	0	0	0	0	0	0	0	0	0	0	0	0	0	0	0
BGIR9823_92591	100521678	MLLT10	lncRNA	0	0	0	0	0	0	0	0	0	0	0	0	0	0	0	0	0	0	0
BGIR9823_78251	106506784	LOC106506784	lncRNA	0	0	0	0	0	0	0	0	0	0	0	0	0	0	0	0	0	0	0
BGIR9823_100897	100621199	IL1RAPL1	lncRNA	0	0	0	0	0	0	0	0	0	0	0	0	0	0	0	0	0	0	0
BGIR9823_100113	110257459	LOC110257459	lncRNA	0	0	0	0	0	0	0	0	0	0	0	0	0	0	0	0	0	0	0
BGIR9823_100197	106506650	LOC106506650	lncRNA	0	0	0	0	0	0	0	0	0	0	0	0	0	0	0	0	0	0	0
BGIR9823_96929	751859	SIRT1	lncRNA	0	0	0	0	0	0	0	0	0	0	0	0	0	0	0	0	0	0	0
BGIR9823_92783	100156695	INTS6	lncRNA	0	0	0	0	0	0	0	0	0	0	0	0	0	0	0	0	0	0	0
BGIR9823_77974	100516051	SLC17A5	lncRNA	0	0	0	0	0	1	1	0	0	0	0	0	0	0	0	0	0	0	0
BGIR9823_100010	397360	CEBPB	lncRNA	0	0	0	1	0	0	0	0	0	0	0	0	0	0	0	0	0	0	0
BGIR9823_101617	110258914	LOC110258914	lncRNA	0	0	0	0	0	0	0	0	0	0	0	0	0	0	0	0	0	0	0
BGIR9823_100936	100154376	KDM6A	lncRNA	0	0	1	0	0	0	0	0	0	0	0	0	0	0	0	0	0	0	0
BGIR9823_79053	100522049	PTP4A1	lncRNA	0	0	0	0	1	0	0	0	0	0	0	0	0	0	0	0	0	0	0
BGIR9823_80764	100037285	NREP	lncRNA	0	0	0	0	0	0	0	0	0	0	0	0	0	0	0	0	0	0	0
BGIR9823_100812	100516527	LOC100516527	lncRNA	0	0	0	0	0	0	1	0	0	0	0	0	0	0	0	0	0	0	0
BGIR9823_100796	110257536	LOC110257536	lncRNA	0	0	0	0	0	0	0	0	0	0	0	0	0	0	0	0	0	0	0
BGIR9823_100878	102165056	MBTPS2	lncRNA	0	0	0	0	0	0	0	0	0	0	0	0	0	0	0	0	0	0	0
BGIR9823_100734	100512382	KCND2	lncRNA	0	0	0	0	0	0	0	0	0	0	0	0	1	0	0	0	0	0	0
BGIR9823_100726	100512026	ING3	lncRNA	0	0	0	0	0	0	0	0	0	0	0	0	0	0	0	0	0	0	0
BGIR9823_84422	100145887	ARHGEF2	lncRNA	0	0	0	0	0	0	0	0	1	0	0	0	0	0	0	0	0	0	0
BGIR9823_84641	100155627	LRRC8B	mRNA	0	0	0	0	0	0	0	0	0	0	0	0	0	0	0	0	0	0	0
BGIR9823_98254	100152049	PLPP5	mRNA	0	0	0	0	0	0	0	0	1	0	0	0	0	0	0	0	0	0	0
BGIR9823_87713	100517038	PUM1	lncRNA	0	0	0	0	0	0	0	0	0	0	0	0	0	0	0	0	0	0	0
BGIR9823_94908	100462749	ATG7	lncRNA	0	0	0	0	0	0	0	0	0	0	0	0	0	0	0	0	0	0	0
BGIR9823_100511	100738688	HERPUD2	lncRNA	0	0	0	0	0	0	0	0	0	0	0	0	0	0	0	0	0	0	0
BGIR9823_80800	100515883	PRRC1	lncRNA	0	0	0	0	0	0	0	0	0	0	0	0	0	0	0	0	0	0	0
BGIR9823_100550	397163	RAMP3	lncRNA	0	0	1	0	0	0	0	0	0	0	0	0	0	0	0	0	0	0	0
BGIR9823_94492	102167379	LOC102167379	lncRNA	0	0	0	0	0	0	0	0	0	0	0	0	0	0	0	0	0	0	0
XM_003126850.6	100520197	VAT1L	mRNA	0	0	0	0	0	0	0	0	0	0	0	0	0	0	0	0	0	0	0
BGIR9823_81847	100739684	FOXK1	lncRNA	0	0	0	0	0	0	0	0	0	0	0	1	0	0	0	0	0	0	0

RNA ID	Gene ID	Symbol	Type	novel-ssc-miR817-3p	novel-ssc-miR849-3p	novel-ssc-miR882-3p	novel-ssc-miR985-5p	novel-ssc-miR1126-5p	novel-ssc-miR1396-3p	ssc-miR-362	novel-ssc-miR16-5p	ssc-miR-375	ssc-miR-376a-3p	ssc-miR-493-5p	ssc-miR-194a-5p	ssc-miR-205	novel-ssc-miR239-5p	novel-ssc-miR397-5p	novel-ssc-miR507-5p	novel-ssc-miR563-3p	novel-ssc-miR568-3p	novel-ssc-miR682-3p
BGIR9823_100328	110257483	ADRM1	lncRNA	0	0	0	0	0	0	0	0	0	0	0	0	0	0	0	0	0	0	0
BGIR9823_101062	100524727	LOC100524727	lncRNA	0	0	0	0	0	0	0	0	0	0	0	0	0	0	0	0	0	0	0
BGIR9823_100412	414383	CHCHD3	lncRNA	0	0	0	0	0	0	0	0	0	0	0	0	0	0	0	0	0	0	0
BGIR9823_90505	397683	IL15	lncRNA	0	0	0	0	0	0	0	0	0	0	0	0	0	0	0	0	0	0	0
BGIR9823_77934	102167874	LOC102167874	lncRNA	0	0	0	0	0	0	0	0	0	0	0	0	0	0	0	0	1	0	0
BGIR9823_100539	100622324	NFE2L3	lncRNA	0	0	0	0	0	0	0	0	0	0	0	0	0	0	0	0	1	0	0
BGIR9823_101533	102158401	LOC102158401	lncRNA	0	0	0	0	0	0	0	0	0	0	0	0	1	0	0	0	0	0	0
BGIR9823_100121	100519631	TASP1	lncRNA	0	0	1	0	0	0	0	0	0	0	0	0	0	0	0	0	0	0	0
BGIR9823_100964	110257651	LOC110257651	lncRNA	0	0	0	0	0	0	0	0	0	0	0	0	0	0	0	0	1	0	0
BGIR9823_100069	100157832	SGCZ	lncRNA	0	0	0	0	0	0	1	0	0	0	0	0	0	0	0	0	0	0	0
BGIR9823_101391	110258158	TNRC6A	mRNA	0	1	0	0	0	0	0	0	0	0	0	0	0	0	0	0	0	0	0
BGIR9823_80729	100526040	PAM	mRNA	0	0	0	0	0	0	0	0	1	0	0	0	0	0	0	0	0	0	0
BGIR9823_100266	100157616	IFT52	lncRNA	0	0	0	1	0	0	0	0	0	0	0	0	0	0	0	0	0	0	0
BGIR9823_100743	100125380	ST7	mRNA	0	0	0	0	0	0	0	0	0	0	0	1	0	0	0	0	0	0	0
BGIR9823_80829	100513723	JADE2	lncRNA	0	0	0	0	0	0	1	0	0	0	0	0	1	0	0	0	0	0	0
BGIR9823_91326	100516387	TSEN15	lncRNA	0	0	0	0	0	0	0	0	0	0	0	1	0	0	0	0	0	0	0
BGIR9823_100827	100519067	CDK13	lncRNA	0	0	0	0	0	0	0	0	0	0	0	0	0	0	0	0	0	0	0
BGIR9823_80869	100521956	CYSTM1	lncRNA	1	0	0	0	0	0	0	0	0	0	0	0	0	0	0	0	0	0	0
BGIR9823_89293	100153071	TRERF1	lncRNA	0	0	0	0	0	0	0	0	0	0	0	0	0	0	0	0	0	0	0
BGIR9823_79511	100152487	TSHZ1	mRNA	0	0	0	0	0	0	0	0	0	1	0	0	0	0	0	0	0	0	0
BGIR9823_92832	106505308	PCDH9	lncRNA	0	0	0	0	0	0	0	0	0	1	0	0	0	0	0	0	0	0	0
BGIR9823_82541	100520835	ITSN2	lncRNA	0	0	0	0	0	0	0	0	0	0	0	1	0	0	0	0	0	0	0
BGIR9823_100525	100515288	CPVL	lncRNA	0	0	0	0	0	0	0	0	0	0	0	0	0	0	0	0	0	0	0
BGIR9823_100563	100516888	GLI3	lncRNA	0	0	0	0	0	0	0	0	0	0	0	0	0	0	0	0	1	0	0
BGIR9823_100515	100623924	FKBP9	lncRNA	0	0	0	0	0	0	0	0	0	0	0	0	0	0	0	0	0	0	0
BGIR9823_101109	100524780	ARHGAP6	lncRNA	0	0	0	0	0	0	0	0	0	0	0	0	0	0	0	0	0	0	0
BGIR9823_95657	100524459	SLC25A20	lncRNA	0	0	0	0	0	0	0	0	0	0	0	0	0	0	0	0	0	0	0
XR_001303159.2	106507279	LOC106507279	lncRNA	0	0	0	0	0	0	0	0	0	0	0	0	0	0	0	0	0	0	0
BGIR9823_82132	106509720	LOC106509720	lncRNA	0	0	0	0	0	0	0	0	0	0	0	0	0	0	0	0	0	0	0
BGIR9823_90639	110261963	LOC110261963	lncRNA	0	0	0	0	0	0	0	0	0	0	0	0	0	0	0	0	0	0	0
BGIR9823_92636	100516289	PFKFB3	lncRNA	0	0	0	0	0	0	0	0	0	0	0	0	0	0	0	0	0	0	0
BGIR9823_79919	100154648	PLPPR1	lncRNA	0	0	0	0	0	0	1	0	0	0	0	0	0	0	0	0	0	0	0
BGIR9823_80860	100521716	MATR3	mRNA	0	0	0	0	0	0	0	0	0	0	0	0	0	0	0	0	0	0	0
BGIR9823_100880	102165056	MBTPS2	lncRNA	0	0	1	0	0	0	0	0	0	0	0	0	0	0	0	0	0	0	0
BGIR9823_101396	100521783	RBBP6	mRNA	0	0	0	0	0	0	0	0	0	0	0	0	0	0	0	0	0	0	0
BGIR9823_101310	102159115	ATP6AP1	lncRNA	0	0	0	0	0	0	0	0	0	0	0	0	0	0	0	0	0	0	0
BGIR9823_85863	100512658	METTL25	mRNA	0	0	0	0	0	0	0	0	0	0	0	0	0	0	0	0	0	0	0
BGIR9823_84834	100516439	C5H12orf10	mRNA	0	0	0	0	0	0	0	0	0	1	0	0	0	0	0	0	0	0	0
BGIR9823_98676	397282	ACVR2A	lncRNA	0	0	0	0	0	0	0	0	0	0	0	0	0	0	0	0	0	0	0
BGIR9823_83976	396677	F3	mRNA	0	0	0	0	0	0	0	0	0	0	0	0	0	0	0	0	0	0	0
BGIR9823_90032	102167943	LOC102167943	lncRNA	0	0	0	0	0	0	0	0	0	0	0	0	0	0	0	0	0	0	0
BGIR9823_100671	106506771	LOC106506771	lncRNA	0	0	0	0	0	0	0	0	0	0	0	0	0	0	0	0	0	0	0
BGIR9823_101108	100524780	ARHGAP6	lncRNA	0	0	0	0	0	0	0	0	0	0	0	0	0	0	0	1	0	0	0

RNA ID	Gene ID	Symbol	Type	novel-ssc-miR817-3p	novel-ssc-miR849-3p	novel-ssc-miR882-3p	novel-ssc-miR985-5p	novel-ssc-miR1126-5p	novel-ssc-miR1396-3p	ssc-miR-362	novel-ssc-miR16-5p	ssc-miR-375	ssc-miR-376a-3p	ssc-miR-493-5p	ssc-miR-194a-5p	ssc-miR-205	novel-ssc-miR239-5p	novel-ssc-miR397-5p	novel-ssc-miR507-5p	novel-ssc-miR563-3p	novel-ssc-miR568-3p	novel-ssc-miR682-3p
BGIR9823_80923	100515885	SLC26A2	mRNA	0	0	0	0	0	0	0	0	0	0	0	0	0	0	0	0	0	0	0
BGIR9823_100494	100521052	IMMP2L	lncRNA	0	0	0	0	0	0	0	0	0	0	0	0	0	0	0	0	1	0	0
BGIR9823_101344	110258068	DPH7	lncRNA	0	0	0	0	0	0	0	0	0	0	0	0	0	0	0	0	0	0	0
BGIR9823_100314	100155190	ZFP64	lncRNA	0	0	0	0	0	0	0	0	0	0	0	0	0	0	0	0	0	0	0
BGIR9823_83324	100624063	LOC100624063	lncRNA	0	0	0	0	0	0	0	0	0	0	0	0	1	0	0	0	0	0	0
BGIR9823_100237	110257438	LOC110257438	lncRNA	0	0	0	0	0	0	0	0	0	0	0	0	0	0	0	0	0	0	0
BGIR9823_78253	106506784	LOC106506784	lncRNA	0	0	0	0	0	0	0	0	0	0	0	0	1	0	0	0	0	0	0
BGIR9823_77699	100514202	MTRF1L	lncRNA	0	0	0	0	0	0	0	0	0	0	0	0	0	0	0	0	1	0	0
BGIR9823_100849	733606	TMSB4X	lncRNA	0	0	0	1	0	0	0	0	0	0	0	0	0	0	0	0	0	0	0
BGIR9823_101513	102157759	LOC102157759	lncRNA	0	0	0	1	0	0	0	0	0	0	0	0	0	0	0	0	0	0	0
BGIR9823_100598	403162	INSIG1	lncRNA	0	0	0	0	0	0	0	0	0	0	0	0	0	0	0	0	0	0	0
BGIR9823_80828	100513723	JADE2	lncRNA	0	0	0	0	0	0	0	0	0	0	0	0	0	0	0	0	0	0	0
BGIR9823_80639	100514094	FCHO2	lncRNA	0	0	0	0	0	0	0	1	0	0	0	0	0	0	0	0	0	0	0
BGIR9823_85738	100157209	PRICKLE1	mRNA	0	0	0	0	0	0	0	0	0	0	0	0	0	0	0	0	0	0	0
BGIR9823_91822	100522092	RUNDC3B	lncRNA	0	0	0	0	0	0	0	0	0	0	0	0	0	0	0	0	0	0	0
BGIR9823_101413	110258225	LOC110258225	lncRNA	0	0	0	0	0	0	0	0	0	0	0	0	0	0	0	0	0	0	0
BGIR9823_89187	100153386	SLA-DRB1	lncRNA	0	0	0	0	0	0	0	0	0	0	0	0	0	0	0	0	0	0	0
BGIR9823_91714	100521986	MAPKAPK2	lncRNA	0	0	0	0	0	0	0	0	0	0	0	0	0	0	0	0	0	0	0
BGIR9823_83804	100523784	ADAR	mRNA	0	0	0	0	0	0	0	0	0	0	0	0	0	0	0	0	0	0	0
BGIR9823_100308	100141411	ADNP	lncRNA	0	0	0	0	0	0	0	0	0	0	0	0	0	0	0	0	0	0	0
BGIR9823_83998	100156806	HFM1	mRNA	0	0	0	0	0	0	0	0	0	0	0	0	0	0	0	0	0	0	0
BGIR9823_100710	397301	CALU	lncRNA	0	0	0	0	0	0	0	0	0	0	0	0	0	0	0	0	0	0	0
BGIR9823_101105	100154011	ANOS1	lncRNA	0	0	0	0	0	0	0	0	0	0	0	0	0	0	1	0	0	0	0
BGIR9823_88993	100144590	MEG3	lncRNA	0	0	0	0	0	0	0	0	0	0	0	0	0	0	0	0	0	0	0
BGIR9823_80459	100622538	DNM2	mRNA	0	0	0	0	0	0	0	0	0	0	0	0	0	0	0	0	0	0	0
BGIR9823_101097	397339	F8	lncRNA	0	0	0	0	0	0	0	0	0	0	0	0	0	0	1	0	0	0	0
BGIR9823_100022	100144536	VAPB	mRNA	0	0	0	0	0	0	0	0	0	0	0	0	0	0	0	0	0	0	0
BGIR9823_88787	100153736	EMC4	lncRNA	0	0	0	0	0	0	0	0	0	0	0	0	0	0	0	0	0	0	0
BGIR9823_93229	100518091	PCID2	mRNA	0	0	0	0	0	0	0	0	0	0	0	0	0	0	0	0	0	0	0
BGIR9823_100480	100621338	BMT2	lncRNA	0	0	0	0	0	0	0	0	0	0	0	0	0	0	0	0	0	0	0
BGIR9823_81197	100037286	ARNTL	lncRNA	0	0	0	0	0	0	0	0	0	0	0	0	0	0	0	0	0	0	0
BGIR9823_100187	100513829	SNPH	lncRNA	0	0	0	0	0	0	0	0	0	0	0	0	0	0	0	0	0	0	0
BGIR9823_97762	100512075	ASCC1	mRNA	0	0	0	0	0	0	0	0	0	0	0	0	0	0	0	0	0	1	0
BGIR9823_100489	493185	IFRD1	lncRNA	0	0	0	0	0	0	0	0	0	0	0	0	0	0	0	0	0	0	0
BGIR9823_82778	100523894	POLR3E	mRNA	0	0	0	0	0	0	0	0	0	0	0	0	0	0	0	0	0	0	0
BGIR9823_88002	100524685	ADGRL2	lncRNA	0	0	0	0	1	0	0	0	0	0	0	0	0	0	0	0	0	0	0
BGIR9823_97698	106506010	LOC106506010	lncRNA	0	0	0	0	0	0	0	1	0	0	0	0	0	0	0	0	0	0	0
BGIR9823_79746	100155321	ACER2	lncRNA	0	0	0	0	0	0	0	0	0	0	0	0	0	0	0	0	0	0	0
BGIR9823_79823	100626649	KANK1	lncRNA	0	0	0	0	0	0	0	0	0	0	0	0	0	0	0	0	0	0	0
BGIR9823_87325	100513473	ZNF181	lncRNA	0	0	0	0	0	0	0	0	0	0	0	0	0	0	0	0	0	0	0
BGIR9823_88185	100511204	ZNF643	mRNA	0	0	0	0	0	0	0	0	0	0	0	0	0	0	0	0	0	0	0
BGIR9823_81639	100513341	FBXL17	lncRNA	0	0	0	0	0	0	0	0	0	0	0	0	0	0	0	0	0	0	0
BGIR9823_94281	100516758	CORO6	mRNA	0	0	0	0	0	0	0	0	1	0	0	0	0	0	0	0	0	0	0

RNA ID	Gene ID	Symbol	Type	novel-ssc-miR817-3p	novel-ssc-miR849-3p	novel-ssc-miR882-3p	novel-ssc-miR985-5p	novel-ssc-miR1126-5p	novel-ssc-miR1396-3p	ssc-miR-362	novel-ssc-miR16-5p	ssc-miR-375	ssc-miR-376a-3p	ssc-miR-493-5p	ssc-miR-194a-5p	ssc-miR-205	novel-ssc-miR239-5p	novel-ssc-miR397-5p	novel-ssc-miR507-5p	novel-ssc-miR563-3p	novel-ssc-miR568-3p	novel-ssc-miR682-3p
BGIR9823_96391	100621858	CFAP410	mRNA	0	0	0	0	0	0	0	0	0	0	0	1	0	0	0	0	0	0	0
BGIR9823_77814	100736796	ADGRB3	lncRNA	0	0	0	0	0	0	0	0	0	0	0	0	0	0	0	0	1	0	0
BGIR9823_84738	100516801	MRTFA	lncRNA	0	0	0	0	0	1	0	0	0	0	0	0	0	0	0	0	0	0	0
BGIR9823_85081	110260749	LOC110260749	lncRNA	0	0	0	0	0	0	0	0	0	0	0	0	1	0	0	0	0	0	0
BGIR9823_83642	100127143	PDE7A	lncRNA	0	0	0	0	0	0	0	0	0	0	0	0	0	0	0	0	0	0	0
BGIR9823_90693	100526062	MRPS18C	lncRNA	0	0	0	0	0	0	0	0	0	0	1	0	0	0	0	0	0	0	0
BGIR9823_94661	100525609	STAB1	mRNA	0	0	0	0	0	0	0	0	0	0	0	0	0	0	0	0	0	0	0
BGIR9823_88204	100157926	RIOK1	mRNA	0	0	0	0	0	0	0	0	0	0	0	0	0	0	0	0	0	0	0
BGIR9823_100214	100154657	PIGU	mRNA	0	0	0	0	0	0	0	0	0	0	0	0	0	1	0	0	0	0	0
BGIR9823_86368	100624191	LOC100624191	mRNA	0	0	0	0	0	0	0	0	0	0	0	1	0	0	0	0	0	0	0
BGIR9823_98692	100525081	R3HDM1	mRNA	0	0	0	0	0	0	0	0	0	0	0	1	0	0	0	0	0	0	0
BGIR9823_94941	110256296	LOC110256296	lncRNA	0	0	0	0	0	0	0	0	0	0	1	0	0	0	0	0	0	0	0
BGIR9823_93186	100519383	DZIP1	mRNA	0	1	0	0	0	0	0	0	0	0	0	0	0	0	0	0	0	0	0
BGIR9823_101339	100519166	LOC100519166	lncRNA	0	0	0	0	0	0	0	0	0	0	0	0	0	0	0	0	0	0	0
BGIR9823_78696	641344	GNE	lncRNA	0	0	0	0	0	0	0	0	0	0	0	0	0	0	0	0	0	0	0
BGIR9823_90334	100514856	APBB2	mRNA	0	0	0	0	0	0	0	0	0	0	0	1	0	0	0	0	0	0	0
BGIR9823_79588	100155833	DPP8	mRNA	0	0	0	0	0	0	0	0	0	0	0	0	0	0	0	0	0	0	0
BGIR9823_93702	100622172	TAOK1	lncRNA	0	0	0	0	0	0	0	0	0	0	0	0	0	0	0	0	0	0	0
BGIR9823_100788	100622432	CBX3	lncRNA	0	0	0	0	0	0	0	0	0	0	0	0	0	0	0	0	0	0	0
BGIR9823_81760	100520896	ETF1	lncRNA	0	0	0	0	0	0	0	0	0	0	0	0	0	0	0	0	0	0	0
BGIR9823_84563	102167065	LOC102167065	lncRNA	0	0	0	0	0	0	0	0	0	0	0	0	0	0	0	0	0	0	0
BGIR9823_100600	100513894	LOC100513894	lncRNA	0	0	0	0	0	0	0	0	0	0	0	0	0	0	0	0	0	0	0
BGIR9823_77938	100156192	NT5DC1	lncRNA	0	0	0	0	0	0	0	0	0	0	0	0	0	0	0	0	0	0	0
BGIR9823_86946	100515670	ANKRD13C	lncRNA	0	0	0	0	0	0	0	0	0	0	0	0	0	0	0	0	0	0	0
BGIR9823_100385	100515632	KDM7A	lncRNA	0	0	0	0	0	0	0	0	0	0	0	0	0	0	0	0	0	0	0
BGIR9823_101073	100515811	VMA21	lncRNA	0	0	0	0	0	0	0	0	0	0	0	0	0	0	0	0	0	0	0
BGIR9823_79171	791125	FYN	lncRNA	0	0	0	0	0	0	0	0	0	0	0	0	1	0	0	0	0	0	0
BGIR9823_91118	106504956	LOC106504956	lncRNA	0	0	0	0	0	0	0	0	0	0	0	0	0	0	0	0	0	0	0
BGIR9823_86164	102164889	LOC102164889	lncRNA	0	0	0	0	0	0	0	0	0	0	0	0	1	0	0	0	0	0	0
BGIR9823_78738	100153669	TMEM38B	lncRNA	0	0	0	0	0	0	0	0	0	0	0	0	0	0	0	0	0	0	0
BGIR9823_88541	100522555	ABHD17C	lncRNA	0	0	0	0	0	1	0	0	0	0	0	0	0	0	0	0	0	0	0
BGIR9823_100267	100624468	OSER1	lncRNA	0	0	0	0	0	0	0	0	0	0	0	0	0	0	0	0	0	0	0
BGIR9823_100299	100625841	DDX27	lncRNA	0	0	0	0	0	0	0	0	0	0	0	0	0	0	0	0	0	0	0
BGIR9823_78983	100622156	TNFAIP3	lncRNA	0	0	0	0	0	0	0	0	0	0	0	0	0	0	0	0	0	0	0
BGIR9823_79796	574058	CD274	lncRNA	0	0	0	0	0	0	0	0	0	0	0	0	0	0	0	0	0	0	0
BGIR9823_82690	100520239	LRWD1	lncRNA	0	0	0	0	0	0	0	0	0	0	0	0	1	0	0	0	0	0	0
BGIR9823_100392	100415778	ZC3HAV1	lncRNA	0	0	0	0	0	0	0	0	0	0	0	0	0	0	0	0	0	0	0
BGIR9823_79386	102162809	CTDSPL2	lncRNA	0	0	0	0	0	0	0	1	0	0	0	0	0	0	0	0	0	0	0
BGIR9823_87847	100627122	SMCHD1	lncRNA	0	0	0	0	0	0	0	0	0	0	0	0	0	0	0	0	0	0	0
BGIR9823_83136	397040	UGP2	mRNA	0	1	0	0	0	0	0	0	0	0	0	0	0	0	0	0	0	0	0
BGIR9823_89310	100151914	MRPL2	lncRNA	0	0	0	0	0	0	0	0	0	0	1	0	0	0	0	0	0	0	0
BGIR9823_82954	106509726	LOC106509726	lncRNA	0	0	0	0	0	0	0	0	0	0	0	0	0	0	0	0	0	0	0
BGIR9823_100019	100141410	ZNF217	lncRNA	0	0	0	0	0	0	0	0	0	0	0	0	0	0	0	0	0	0	0

RNA ID	Gene ID	Symbol	Type	novel-ssc-miR817-3p	novel-ssc-miR849-3p	novel-ssc-miR882-3p	novel-ssc-miR985-5p	novel-ssc-miR1126-5p	novel-ssc-miR1396-3p	ssc-miR-362	novel-ssc-miR16-5p	ssc-miR-375	ssc-miR-376a-3p	ssc-miR-493-5p	ssc-miR-194a-5p	ssc-miR-205	novel-ssc-miR239-5p	novel-ssc-miR397-5p	novel-ssc-miR507-5p	novel-ssc-miR563-3p	novel-ssc-miR568-3p	novel-ssc-miR682-3p
BGIR9823_101550	110258600	LOC110258600	lncRNA	0	0	0	0	0	0	0	0	0	0	0	0	0	0	0	0	0	0	0
BGIR9823_80641	100515474	UTP15	lncRNA	0	0	0	0	0	0	0	0	0	0	0	0	0	0	0	0	0	0	0
BGIR9823_85022	100518251	KRAS	lncRNA	0	0	0	0	0	0	0	0	0	0	0	0	0	0	0	0	0	0	0
BGIR9823_78496	100512450	TMEM260	lncRNA	0	0	0	0	0	0	0	0	0	0	0	0	1	0	0	0	0	0	0
BGIR9823_100476	100621338	BMT2	lncRNA	0	0	0	0	0	0	0	0	0	0	0	0	0	0	0	0	0	0	0
BGIR9823_102668	BGIG9823_27318		lncRNA	0	0	0	0	0	0	0	0	0	0	0	0	0	0	0	0	0	0	0
BGIR9823_101962	BGIG9823_26639		lncRNA	0	0	0	0	0	0	0	0	0	0	0	0	0	0	0	0	0	0	0
BGIR9823_102175	BGIG9823_26847		lncRNA	0	0	0	0	0	0	0	0	0	0	0	0	0	0	0	0	0	0	0
BGIR9823_101847	BGIG9823_26526		lncRNA	0	0	0	0	0	0	0	0	0	0	0	0	0	0	0	0	0	0	0
BGIR9823_101781	BGIG9823_26461		lncRNA	0	0	0	0	0	0	0	0	0	0	0	0	0	0	0	0	0	0	0
BGIR9823_102445	BGIG9823_27101		lncRNA	0	0	0	0	0	0	1	0	0	0	0	0	0	0	0	0	0	0	0
BGIR9823_101840	BGIG9823_26519		lncRNA	0	0	0	0	0	0	0	0	0	0	0	0	0	0	0	0	0	0	0
BGIR9823_102485	BGIG9823_27139		lncRNA	0	0	0	0	0	0	0	0	0	0	0	0	0	0	0	0	0	0	0
BGIR9823_102195	BGIG9823_26867		lncRNA	0	0	0	0	0	0	0	0	0	0	0	0	0	0	0	0	0	0	0
BGIR9823_101771	BGIG9823_26451		lncRNA	0	0	0	0	0	0	0	0	0	0	0	0	0	0	0	0	0	0	0
BGIR9823_101831	BGIG9823_26510		lncRNA	0	0	0	0	0	0	0	0	0	0	0	0	0	0	0	0	0	0	0
BGIR9823_102544	BGIG9823_27197		lncRNA	0	0	0	0	0	0	0	0	0	0	0	0	0	0	0	0	1	0	0
BGIR9823_102359	BGIG9823_27024		lncRNA	0	0	0	0	0	0	0	0	0	0	0	0	0	0	0	0	0	0	1
BGIR9823_102456	BGIG9823_27110		lncRNA	0	0	0	0	0	0	0	0	0	0	0	0	0	0	0	0	0	0	0
BGIR9823_102657	BGIG9823_27307		lncRNA	0	0	0	0	0	0	0	0	0	0	0	0	0	0	0	0	0	0	0
BGIR9823_102305	BGIG9823_26970		lncRNA	0	0	0	0	0	0	0	0	0	0	0	0	0	0	0	0	0	0	0
BGIR9823_102228	BGIG9823_26900		lncRNA	0	0	0	0	0	0	0	0	0	0	0	0	0	0	0	0	0	0	1
BGIR9823_101778	BGIG9823_26458		lncRNA	0	0	0	0	0	0	0	0	0	0	0	0	0	0	0	0	0	0	0
BGIR9823_102408	BGIG9823_27069		lncRNA	0	0	0	0	0	0	0	0	0	0	0	0	0	0	0	0	0	0	0
BGIR9823_101997	BGIG9823_26673		lncRNA	0	0	0	0	0	1	0	0	0	0	0	0	0	0	0	0	0	0	0
BGIR9823_101886	BGIG9823_26563		lncRNA	0	0	0	0	0	0	0	0	0	0	0	0	0	0	0	0	0	0	0
BGIR9823_101714	BGIG9823_26398		lncRNA	0	0	0	0	0	0	0	0	0	0	0	0	0	0	0	0	0	0	0
BGIR9823_102000	BGIG9823_26676		lncRNA	0	0	0	0	0	0	0	0	0	0	0	0	0	0	0	0	0	0	0
BGIR9823_102540	BGIG9823_27193		lncRNA	0	0	0	0	0	0	0	0	0	0	0	0	0	0	0	0	0	0	0
BGIR9823_101852	BGIG9823_26531		lncRNA	0	0	0	0	0	0	0	0	0	0	0	0	0	0	0	0	0	0	0
BGIR9823_102203	BGIG9823_26875		lncRNA	0	0	0	0	0	0	0	0	0	0	0	0	0	0	0	0	0	0	0
BGIR9823_102422	BGIG9823_27083		lncRNA	0	0	0	0	0	0	0	0	0	0	0	0	0	0	1	0	0	0	0
BGIR9823_101937	BGIG9823_26614		lncRNA	0	0	0	0	0	0	1	0	0	0	0	0	0	0	0	0	0	0	0
BGIR9823_102117	BGIG9823_26791		lncRNA	0	0	0	0	0	0	0	0	0	0	0	0	0	0	0	0	0	0	0
BGIR9823_102005	BGIG9823_26681		lncRNA	0	0	0	0	0	0	0	0	0	0	0	0	0	0	0	0	0	0	0
BGIR9823_102177	BGIG9823_26849		lncRNA	0	0	0	0	0	0	0	0	0	0	0	0	0	0	0	0	0	0	0
BGIR9823_102213	BGIG9823_26885		lncRNA	0	0	0	0	0	0	0	1	0	0	0	0	0	0	0	0	0	0	0
BGIR9823_102067	BGIG9823_26741		lncRNA	0	0	0	0	0	0	0	0	0	0	0	0	0	0	0	0	0	0	0
BGIR9823_102357	BGIG9823_27022		lncRNA	0	0	0	0	0	0	0	0	0	0	0	0	1	0	0	0	0	0	0
BGIR9823_102443	BGIG9823_27099		lncRNA	0	0	0	0	0	0	0	0	0	0	0	0	0	0	1	0	0	0	0
BGIR9823_102395	BGIG9823_27060		lncRNA	0	0	0	0	0	0	1	0	0	0	0	0	0	0	0	0	0	0	0
BGIR9823_102615	BGIG9823_27265		lncRNA	0	0	0	0	0	0	0	0	0	0	0	0	0	0	0	0	0	0	0
BGIR9823_102264	BGIG9823_26932		lncRNA	0	0	0	0	0	0	0	0	0	0	0	0	0	0	0	0	0	0	0

RNA ID	Gene ID	Symbol	Type	novel-ssc-miR817-3p	novel-ssc-miR849-3p	novel-ssc-miR882-3p	novel-ssc-miR985-5p	novel-ssc-miR1126-5p	novel-ssc-miR1396-3p	ssc-miR-362	novel-ssc-miR16-5p	ssc-miR-375	ssc-miR-376a-3p	ssc-miR-493-5p	ssc-miR-194a-5p	ssc-miR-205	novel-ssc-miR239-5p	novel-ssc-miR397-5p	novel-ssc-miR507-5p	novel-ssc-miR563-3p	novel-ssc-miR568-3p	novel-ssc-miR682-3p
BGIR9823_102052	BGIG9823_26726		lncRNA	0	0	0	0	0	0	0	0	0	0	0	0	0	0	0	0	0	0	0
BGIR9823_102131	BGIG9823_26804		lncRNA	0	0	0	0	0	0	0	0	0	0	0	0	0	0	0	0	1	0	0
BGIR9823_101987	BGIG9823_26663		lncRNA	0	0	0	0	0	0	0	0	0	0	0	0	0	0	0	0	0	0	0
BGIR9823_102764	BGIG9823_27402		lncRNA	0	0	0	0	0	0	0	0	0	0	0	0	0	0	0	0	0	0	0
BGIR9823_102137	BGIG9823_26810		lncRNA	0	0	0	0	0	0	0	0	0	0	0	0	0	0	0	0	0	0	0
BGIR9823_102168	BGIG9823_26841		lncRNA	0	0	0	0	0	0	0	0	0	0	0	0	0	0	0	0	0	0	0
BGIR9823_101967	BGIG9823_26644		lncRNA	0	0	0	0	0	0	0	0	0	0	0	0	0	0	0	0	0	0	0
BGIR9823_102393	BGIG9823_27058		lncRNA	0	0	0	0	0	0	0	0	0	0	0	0	0	0	0	0	0	0	0
BGIR9823_101927	BGIG9823_26604		lncRNA	0	0	0	0	0	0	0	0	0	0	0	0	0	0	0	0	0	0	0
BGIR9823_102343	BGIG9823_27008		lncRNA	0	0	0	0	0	0	0	0	0	0	0	0	0	0	0	0	0	0	0
BGIR9823_102562	BGIG9823_27212		mRNA	0	0	0	0	0	0	0	0	0	0	0	0	0	0	0	0	0	0	0
BGIR9823_102090	BGIG9823_26764		lncRNA	0	0	0	0	0	0	0	0	0	0	0	0	0	0	0	1	0	0	0
BGIR9823_101742	BGIG9823_26423		lncRNA	0	0	0	0	0	0	0	0	0	0	0	0	0	0	0	0	0	0	0
BGIR9823_102398	BGIG9823_27061		lncRNA	0	0	0	0	0	0	0	0	0	0	0	0	0	0	0	0	0	0	0
BGIR9823_101721	BGIG9823_26405		lncRNA	0	0	0	0	0	0	0	0	0	0	0	0	0	0	0	0	0	0	0
BGIR9823_102545	BGIG9823_27198		lncRNA	0	0	0	0	0	0	0	0	0	0	0	0	0	0	0	0	1	0	0
BGIR9823_102138	BGIG9823_26811		lncRNA	0	0	0	0	0	0	0	0	0	0	0	0	0	0	0	0	0	0	0
BGIR9823_102026	BGIG9823_26701		lncRNA	0	0	0	0	0	0	0	0	0	0	0	0	0	0	0	0	0	0	0
BGIR9823_102057	BGIG9823_26731		lncRNA	0	0	0	0	0	0	0	0	0	0	0	0	0	0	0	0	0	0	0
BGIR9823_101995	BGIG9823_26671		lncRNA	0	0	0	0	0	0	0	0	0	0	0	0	0	0	0	0	0	0	0
BGIR9823_102725	BGIG9823_27371		lncRNA	0	0	0	0	0	0	0	0	0	0	0	0	0	0	0	0	0	0	0
BGIR9823_101717	BGIG9823_26401		lncRNA	0	0	0	0	0	0	0	0	0	0	0	0	0	0	0	0	0	0	0
BGIR9823_102441	BGIG9823_27097		lncRNA	0	0	0	0	0	0	0	0	0	0	0	0	0	0	0	0	0	0	0
BGIR9823_102674	BGIG9823_27324		lncRNA	0	1	0	0	0	0	0	0	0	0	0	0	0	0	0	0	0	0	0
BGIR9823_101843	BGIG9823_26522		lncRNA	0	0	0	0	0	0	0	0	0	0	0	0	0	0	0	0	0	0	0
BGIR9823_102119	BGIG9823_26793		lncRNA	0	0	0	0	0	0	0	0	0	0	0	0	0	0	0	0	0	0	0
BGIR9823_101819	BGIG9823_26499		lncRNA	0	0	0	0	0	0	0	0	0	0	0	0	0	0	0	0	0	0	0
BGIR9823_102034	BGIG9823_26709		lncRNA	0	0	0	0	0	0	0	0	0	0	0	0	0	0	0	0	0	0	0
BGIR9823_102373	BGIG9823_27038		lncRNA	0	0	0	0	0	0	0	0	0	0	0	0	0	0	0	0	0	0	0
BGIR9823_101905	BGIG9823_26582		lncRNA	0	0	0	0	0	0	0	0	0	0	0	0	0	0	0	0	0	0	0
BGIR9823_102629	BGIG9823_27279		lncRNA	0	0	0	0	0	0	0	0	0	0	0	0	0	0	0	0	0	0	0
BGIR9823_102328	BGIG9823_26993		lncRNA	0	0	0	0	0	0	0	0	0	0	0	0	0	0	0	0	0	0	0
BGIR9823_102165	BGIG9823_26838		lncRNA	0	0	0	0	0	0	0	0	0	0	0	0	0	0	0	0	0	0	0
BGIR9823_101792	BGIG9823_26472		lncRNA	0	0	0	0	0	0	0	0	0	0	0	0	0	0	0	0	0	0	0
BGIR9823_101992	BGIG9823_26668		lncRNA	0	0	0	0	0	0	0	0	0	0	0	0	0	0	0	0	0	0	0
BGIR9823_101767	BGIG9823_26447		lncRNA	0	0	0	0	0	0	0	0	0	0	0	0	0	0	0	0	0	0	0
BGIR9823_101956	BGIG9823_26633		lncRNA	0	0	0	0	0	0	0	0	0	0	0	0	0	0	0	0	0	0	0
BGIR9823_102186	BGIG9823_26858		lncRNA	0	0	0	0	0	0	0	0	0	0	0	0	0	0	0	0	0	0	0
BGIR9823_102630	BGIG9823_27280		lncRNA	0	0	0	0	0	0	1	0	0	0	0	0	0	0	0	0	0	0	0
BGIR9823_101685	BGIG9823_26372		lncRNA	0	0	0	0	0	0	0	0	0	0	0	0	0	0	0	0	0	0	0
BGIR9823_101700	BGIG9823_26387		lncRNA	0	0	0	0	0	0	0	0	0	0	0	0	0	0	0	0	0	0	0
BGIR9823_102217	BGIG9823_26889		lncRNA	0	0	0	0	0	0	0	0	0	0	0	0	0	0	0	0	0	0	0
BGIR9823_102093	BGIG9823_26767		lncRNA	0	0	0	0	0	0	0	0	0	0	0	0	0	0	0	0	0	0	0

RNA ID	Gene ID	Symbol	Type	novel-ssc-miR817-3p	novel-ssc-miR849-3p	novel-ssc-miR882-3p	novel-ssc-miR985-5p	novel-ssc-miR1126-5p	novel-ssc-miR1396-3p	ssc-miR-362	novel-ssc-miR16-5p	ssc-miR-375	ssc-miR-376a-3p	ssc-miR-493-5p	ssc-miR-194a-5p	ssc-miR-205	novel-ssc-miR239-5p	novel-ssc-miR397-5p	novel-ssc-miR507-5p	novel-ssc-miR563-3p	novel-ssc-miR568-3p	novel-ssc-miR682-3p
BGIR9823_101824	BGIG9823_26503		lncRNA	0	0	0	0	0	0	0	0	0	0	0	0	0	0	0	0	0	0	0
BGIR9823_102083	BGIG9823_26757		lncRNA	0	0	0	0	0	0	0	0	0	0	0	0	0	0	0	0	0	0	0
BGIR9823_102805	BGIG9823_27433		lncRNA	0	0	0	0	0	0	0	0	0	0	0	0	0	0	0	0	0	0	0
BGIR9823_101826	BGIG9823_26505		lncRNA	0	0	0	0	0	0	0	0	0	0	0	0	0	0	0	0	0	0	0
BGIR9823_102136	BGIG9823_26809		lncRNA	0	0	0	0	0	0	0	0	0	0	0	0	0	0	0	0	0	0	0
BGIR9823_101982	BGIG9823_26658		lncRNA	0	0	0	0	0	0	0	0	0	0	0	0	0	0	0	0	0	0	0
BGIR9823_101949	BGIG9823_26626		lncRNA	0	0	0	0	0	0	0	0	0	0	0	0	0	0	0	0	0	0	0
BGIR9823_102478	BGIG9823_27132		lncRNA	0	0	0	0	0	0	0	0	0	0	0	0	0	0	0	0	0	0	0
BGIR9823_101746	BGIG9823_26427		lncRNA	0	0	0	0	0	0	0	0	0	0	0	0	0	0	0	0	0	0	0
BGIR9823_101942	BGIG9823_26619		lncRNA	0	0	0	0	0	0	0	0	0	0	0	0	0	0	0	0	0	0	0
BGIR9823_102059	BGIG9823_26733		lncRNA	0	0	0	0	0	0	0	0	0	0	0	0	0	0	0	0	0	0	0
BGIR9823_102156	BGIG9823_26829		lncRNA	0	0	0	0	0	0	0	0	0	0	0	0	0	0	0	0	0	0	0
BGIR9823_101875	BGIG9823_26554		lncRNA	0	0	0	0	0	0	0	0	0	0	0	0	0	0	0	0	0	0	0
BGIR9823_101893	BGIG9823_26570		lncRNA	0	0	0	0	0	0	0	0	0	0	0	0	0	0	0	0	0	0	0
BGIR9823_101659	BGIG9823_26357		lncRNA	0	0	0	1	0	0	0	0	0	0	0	0	0	0	0	0	0	0	0
BGIR9823_102534	BGIG9823_27187		lncRNA	0	0	0	0	0	0	0	0	0	0	0	0	0	0	0	0	0	0	0
BGIR9823_101719	BGIG9823_26403		lncRNA	0	0	0	0	0	0	0	0	0	0	0	0	0	0	0	0	0	0	0
BGIR9823_102665	BGIG9823_27315		lncRNA	0	0	0	0	0	0	0	0	0	0	0	0	0	0	0	0	0	0	0
BGIR9823_102586	BGIG9823_27236		lncRNA	0	0	0	0	0	0	0	0	0	0	0	0	0	0	0	0	0	0	0
BGIR9823_102799	BGIG9823_27428		lncRNA	0	1	0	0	0	0	0	0	0	0	0	0	0	0	0	0	0	0	0
BGIR9823_101782	BGIG9823_26462		lncRNA	0	0	0	0	0	0	0	0	0	0	0	0	0	0	0	0	0	0	0
BGIR9823_102163	BGIG9823_26836		lncRNA	0	0	0	0	0	0	0	0	0	0	0	0	0	0	0	0	0	0	0
BGIR9823_102623	BGIG9823_27273		lncRNA	0	0	0	0	0	0	0	0	0	0	0	0	0	0	0	0	0	0	0
BGIR9823_102550	BGIG9823_27201		lncRNA	0	0	0	0	0	0	0	0	0	0	0	0	0	0	0	0	0	0	0
BGIR9823_101952	BGIG9823_26629		lncRNA	0	0	0	0	0	0	0	0	0	0	0	0	0	0	0	0	0	0	0
BGIR9823_102084	BGIG9823_26758		lncRNA	0	0	0	0	0	0	0	0	0	0	0	0	0	0	0	0	0	0	1
BGIR9823_102746	BGIG9823_27389		lncRNA	0	0	0	0	0	0	0	0	0	0	0	0	0	0	0	0	0	0	0
BGIR9823_102196	BGIG9823_26868		lncRNA	0	0	0	0	0	0	0	0	0	0	0	0	0	0	0	0	0	0	0
BGIR9823_101791	BGIG9823_26471		lncRNA	0	0	0	0	0	0	0	0	0	0	0	0	0	0	0	0	0	0	0
BGIR9823_101707	BGIG9823_26391		lncRNA	0	0	0	0	0	0	0	0	0	0	0	0	0	0	0	0	0	0	0
BGIR9823_102788	BGIG9823_27419		lncRNA	0	0	0	0	0	0	0	0	0	0	0	0	0	0	0	0	0	0	0
BGIR9823_102349	BGIG9823_27014		lncRNA	0	0	0	0	0	0	0	0	0	0	0	0	0	0	0	0	0	0	0
BGIR9823_102713	BGIG9823_27363		lncRNA	0	0	0	0	0	0	0	0	0	0	0	0	0	0	0	0	0	0	0
BGIR9823_101686	BGIG9823_26373		lncRNA	0	0	0	0	0	0	0	0	0	0	0	0	0	0	0	0	0	0	0
BGIR9823_102210	BGIG9823_26882		lncRNA	0	0	0	0	0	0	0	0	0	0	0	0	0	0	0	0	1	0	0
BGIR9823_102323	BGIG9823_26988		lncRNA	0	0	0	0	0	0	0	0	0	0	0	0	0	0	0	0	1	0	0
BGIR9823_102016	BGIG9823_26692		mRNA	0	0	0	0	0	0	0	0	0	0	0	0	0	0	0	0	0	0	0
BGIR9823_102051	BGIG9823_26725		lncRNA	0	0	0	0	0	0	0	0	0	0	0	0	0	0	0	0	0	0	0
BGIR9823_101891	BGIG9823_26568		lncRNA	0	0	0	0	0	0	0	0	0	0	0	0	0	0	0	0	0	0	0
BGIR9823_102043	BGIG9823_26717		lncRNA	0	0	0	0	0	0	0	0	0	0	0	0	0	0	0	0	0	0	1
BGIR9823_102464	BGIG9823_27118		lncRNA	0	0	0	0	0	0	0	0	0	0	0	0	0	0	0	0	0	0	0
BGIR9823_102384	BGIG9823_27049		lncRNA	0	0	0	0	0	0	0	0	0	0	0	0	0	0	0	0	0	0	0
BGIR9823_102347	BGIG9823_27012		lncRNA	0	0	0	0	0	0	0	0	0	0	0	0	0	0	0	0	0	0	0

RNA ID	Gene ID	Symbol	Type	novel-ssc-miR817-3p	novel-ssc-miR849-3p	novel-ssc-miR882-3p	novel-ssc-miR985-5p	novel-ssc-miR1126-5p	novel-ssc-miR1396-3p	ssc-miR-362	novel-ssc-miR16-5p	ssc-miR-375	ssc-miR-376a-3p	ssc-miR-493-5p	ssc-miR-194a-5p	ssc-miR-205	novel-ssc-miR239-5p	novel-ssc-miR397-5p	novel-ssc-miR507-5p	novel-ssc-miR563-3p	novel-ssc-miR568-3p	novel-ssc-miR682-3p
BGIR9823_101855	BGIG9823_26534		lncRNA	0	0	0	0	0	0	0	0	0	0	0	0	0	0	0	0	0	0	0
BGIR9823_102597	BGIG9823_27247		lncRNA	0	0	0	0	0	0	0	0	0	0	0	0	0	0	0	0	0	0	0
BGIR9823_101902	BGIG9823_26579		lncRNA	0	0	0	0	0	0	0	0	0	0	0	0	0	0	0	0	1	0	0
BGIR9823_102413	BGIG9823_27074		mRNA	0	1	0	0	0	0	0	0	0	0	0	0	0	0	0	0	0	0	0
BGIR9823_102726	BGIG9823_27372		lncRNA	0	0	0	0	0	1	0	0	0	0	0	0	0	0	0	0	0	0	0
BGIR9823_102647	BGIG9823_27297		lncRNA	0	0	0	0	0	0	0	0	0	0	0	0	0	0	0	0	0	0	0
BGIR9823_101973	BGIG9823_26650		lncRNA	0	0	0	0	0	0	0	0	0	0	0	0	0	0	0	0	0	0	0
BGIR9823_102297	BGIG9823_26963		lncRNA	0	0	0	0	0	1	0	0	0	0	0	0	0	0	0	0	0	0	0
BGIR9823_102389	BGIG9823_27054		lncRNA	0	0	0	0	0	0	0	0	0	0	0	0	0	0	0	0	0	0	0
BGIR9823_101788	BGIG9823_26468		lncRNA	0	0	0	0	0	0	0	0	0	0	0	0	0	0	0	0	0	0	1
BGIR9823_101961	BGIG9823_26638		lncRNA	0	0	0	0	0	0	0	0	0	0	0	0	0	0	0	0	0	0	0
BGIR9823_102423	BGIG9823_27084		lncRNA	0	0	0	0	0	0	0	0	0	0	0	0	0	0	0	0	0	0	0
BGIR9823_102712	BGIG9823_27362		lncRNA	0	0	0	0	0	1	0	0	0	0	0	0	0	0	0	0	0	0	0
BGIR9823_101682	BGIG9823_26369		lncRNA	0	0	0	0	0	0	0	0	0	0	0	0	0	0	0	0	0	0	0
BGIR9823_101865	BGIG9823_26544		lncRNA	0	0	0	0	0	0	0	0	0	0	0	0	0	0	0	0	0	0	0
BGIR9823_102351	BGIG9823_27016		lncRNA	0	0	0	0	0	0	0	0	0	0	0	0	0	0	0	0	0	0	0
BGIR9823_102576	BGIG9823_27226		lncRNA	0	0	0	0	0	0	0	0	0	0	0	0	0	0	0	0	0	0	0
BGIR9823_102428	BGIG9823_27088		lncRNA	0	0	0	0	0	0	0	0	0	0	0	0	0	0	1	0	0	0	0
BGIR9823_102329	BGIG9823_26994		lncRNA	0	0	0	0	0	0	0	0	0	0	0	0	1	0	0	0	0	0	0
BGIR9823_102625	BGIG9823_27275		lncRNA	0	0	0	0	0	0	0	0	0	0	0	0	0	0	0	0	0	0	0
BGIR9823_102075	BGIG9823_26749		lncRNA	0	0	0	0	0	0	0	0	0	0	1	0	0	0	0	0	0	0	0
BGIR9823_102523	BGIG9823_27176		lncRNA	0	0	0	0	0	0	0	0	0	0	0	0	0	0	0	0	0	0	0
BGIR9823_101910	BGIG9823_26587		lncRNA	0	0	0	0	0	0	0	0	0	0	0	0	0	0	0	0	0	0	0
BGIR9823_102748	BGIG9823_27391		lncRNA	0	0	0	0	0	0	0	0	0	0	0	0	0	0	0	0	0	0	0
BGIR9823_101705	BGIG9823_26389		lncRNA	0	0	0	0	0	0	0	0	0	0	0	0	0	0	0	0	0	0	0
BGIR9823_102467	BGIG9823_27121		lncRNA	0	0	0	0	0	0	0	0	0	0	0	0	0	0	0	0	0	0	0
BGIR9823_102501	BGIG9823_27155		lncRNA	0	0	0	0	0	0	0	1	0	0	0	0	0	0	0	0	0	0	0
BGIR9823_101779	BGIG9823_26459		lncRNA	0	0	0	0	0	0	0	0	0	0	0	0	0	0	0	0	0	0	0
BGIR9823_102431	BGIG9823_27090		lncRNA	0	0	0	0	0	0	0	0	0	0	0	0	1	0	0	0	0	0	0
BGIR9823_102628	BGIG9823_27278		lncRNA	0	0	0	0	0	0	0	0	0	0	0	0	0	0	0	0	0	0	0
BGIR9823_101816	BGIG9823_26496		lncRNA	0	0	0	0	0	0	0	0	0	0	0	0	0	0	0	0	0	0	0
BGIR9823_101996	BGIG9823_26672		lncRNA	0	0	0	0	0	0	0	0	0	0	0	0	0	0	0	0	0	0	0
BGIR9823_102304	BGIG9823_26969		lncRNA	0	0	0	0	0	0	0	0	0	0	0	0	0	0	0	0	0	0	0
BGIR9823_101800	BGIG9823_26480		lncRNA	0	0	0	0	0	0	0	0	0	0	0	0	0	0	0	0	0	0	0
BGIR9823_102273	BGIG9823_26940		lncRNA	0	0	0	0	0	0	0	0	0	0	0	0	0	0	0	0	0	0	0
BGIR9823_102407	BGIG9823_27068		lncRNA	0	0	0	0	0	0	0	0	0	0	0	0	0	0	0	0	0	0	0
BGIR9823_102091	BGIG9823_26765		lncRNA	0	0	0	0	0	0	0	0	0	0	0	0	0	0	0	0	0	0	0
BGIR9823_102167	BGIG9823_26840		lncRNA	0	0	0	0	0	0	0	0	0	0	0	0	0	0	0	0	0	0	0
BGIR9823_102166	BGIG9823_26839		lncRNA	0	0	0	0	0	0	0	0	0	0	0	0	0	0	0	0	0	0	0
BGIR9823_102159	BGIG9823_26832		lncRNA	0	0	0	0	0	0	0	0	0	0	0	0	0	0	0	0	0	0	0
BGIR9823_102041	BGIG9823_26715		lncRNA	0	0	0	0	0	0	0	0	0	0	0	0	0	0	0	0	0	0	0
BGIR9823_102369	BGIG9823_27034		lncRNA	0	0	0	0	0	0	0	0	0	0	0	0	0	0	0	0	0	0	0
BGIR9823_101766	BGIG9823_26446		lncRNA	0	0	0	0	0	0	0	0	0	0	0	0	0	0	0	0	0	0	0

RNA ID	Gene ID	Symbol	Type	novel-ssc-miR817-3p	novel-ssc-miR849-3p	novel-ssc-miR882-3p	novel-ssc-miR985-5p	novel-ssc-miR1126-5p	novel-ssc-miR1396-3p	ssc-miR-362	novel-ssc-miR16-5p	ssc-miR-375	ssc-miR-376a-3p	ssc-miR-493-5p	ssc-miR-194a-5p	ssc-miR-205	novel-ssc-miR239-5p	novel-ssc-miR397-5p	novel-ssc-miR507-5p	novel-ssc-miR563-3p	novel-ssc-miR568-3p	novel-ssc-miR682-3p
BGIR9823_102029	BGIG9823_26704		lncRNA	0	0	0	0	0	0	0	0	0	0	0	0	0	0	0	0	0	0	0
BGIR9823_102522	BGIG9823_27175		lncRNA	0	0	0	0	0	0	0	0	0	0	0	0	0	0	0	0	0	0	0
BGIR9823_102783	BGIG9823_27414		lncRNA	0	0	0	0	0	0	0	0	0	0	0	0	0	0	0	0	0	0	0
BGIR9823_101935	BGIG9823_26612		lncRNA	0	0	0	0	0	0	0	0	0	0	0	0	0	0	0	0	0	0	0
BGIR9823_102254	BGIG9823_26922		lncRNA	0	0	0	0	0	0	0	0	0	0	0	0	0	0	0	0	0	0	0
BGIR9823_102184	BGIG9823_26856		lncRNA	0	0	0	0	0	0	0	0	0	0	0	0	0	0	0	0	0	0	0
BGIR9823_102246	BGIG9823_26914		lncRNA	0	0	0	0	0	0	0	0	0	0	0	0	0	0	0	0	0	0	0
BGIR9823_101751	BGIG9823_26432		lncRNA	0	0	0	0	0	0	0	0	0	0	0	0	0	0	0	0	0	0	0
BGIR9823_101673	BGIG9823_26360		lncRNA	0	0	0	0	0	0	0	0	0	0	0	0	0	0	0	0	0	0	0
BGIR9823_101811	BGIG9823_26491		lncRNA	0	0	0	0	0	0	0	0	0	0	0	0	0	0	0	0	0	0	0
BGIR9823_102295	BGIG9823_26961		mRNA	0	0	0	0	0	0	0	0	0	0	0	0	0	0	0	0	0	0	0
BGIR9823_101697	BGIG9823_26384		lncRNA	0	0	0	1	0	0	0	0	0	0	0	0	0	0	0	0	0	0	0
BGIR9823_101725	BGIG9823_26409		lncRNA	0	0	0	0	0	0	0	0	0	0	0	0	0	0	0	0	0	0	0
BGIR9823_102033	BGIG9823_26708		lncRNA	0	0	0	0	0	0	0	0	0	0	0	0	0	0	0	0	0	0	0
BGIR9823_102053	BGIG9823_26727		lncRNA	0	0	0	0	0	0	0	0	0	0	0	0	0	0	0	0	0	0	1
BGIR9823_102015	BGIG9823_26691		lncRNA	0	0	0	0	0	0	0	0	0	0	0	0	0	0	0	0	0	0	0
BGIR9823_102489	BGIG9823_27143		lncRNA	0	0	0	0	0	0	0	0	0	0	0	0	0	0	0	0	0	0	0
BGIR9823_102449	BGIG9823_27104		lncRNA	0	0	0	0	0	0	0	0	0	0	0	0	0	0	0	0	0	0	1
BGIR9823_102214	BGIG9823_26886		lncRNA	0	0	0	0	0	0	0	0	0	0	0	0	0	0	0	0	0	0	0
BGIR9823_101834	BGIG9823_26513		lncRNA	0	0	0	0	0	0	0	0	0	0	0	0	0	0	0	0	0	0	0
BGIR9823_102636	BGIG9823_27286		lncRNA	0	0	0	0	0	1	0	0	0	0	0	0	0	0	0	0	0	0	0
BGIR9823_102287	BGIG9823_26954		lncRNA	0	0	0	0	0	0	0	0	0	0	0	0	0	0	0	1	0	0	0
BGIR9823_101906	BGIG9823_26583		lncRNA	0	0	0	0	0	0	0	0	0	0	0	0	0	0	0	0	0	0	0
BGIR9823_102514	BGIG9823_27168		lncRNA	0	0	0	0	0	0	1	0	0	0	0	0	0	0	0	0	0	0	0
BGIR9823_101914	BGIG9823_26591		lncRNA	0	0	0	0	0	0	0	0	0	0	0	0	0	0	0	0	0	0	0
BGIR9823_101768	BGIG9823_26448		lncRNA	0	0	0	0	0	0	0	0	0	0	0	0	0	0	0	0	0	0	0
BGIR9823_102070	BGIG9823_26744		lncRNA	0	0	0	0	0	0	0	0	0	0	0	0	0	0	0	0	0	0	0
BGIR9823_101805	BGIG9823_26485		lncRNA	0	0	0	0	0	0	0	0	0	0	0	0	0	0	0	0	0	0	0
BGIR9823_102103	BGIG9823_26777		lncRNA	0	0	0	0	0	0	0	0	0	0	0	0	0	0	0	0	0	0	0
BGIR9823_102688	BGIG9823_27338		lncRNA	0	0	0	0	0	0	0	0	0	0	0	0	0	0	0	0	0	0	0
BGIR9823_102733	BGIG9823_27379		lncRNA	0	0	0	0	0	0	0	0	0	0	0	0	0	0	0	0	0	0	0
BGIR9823_102411	BGIG9823_27072		lncRNA	0	0	0	0	1	0	0	0	0	0	0	0	0	0	0	0	0	0	0
BGIR9823_102360	BGIG9823_27025		lncRNA	0	0	0	0	0	0	0	0	0	0	1	0	0	0	0	0	0	0	0
BGIR9823_102558	BGIG9823_27208		lncRNA	0	0	0	0	0	0	0	0	0	0	0	0	0	0	0	0	0	0	0
BGIR9823_101837	BGIG9823_26516		lncRNA	0	0	0	0	1	0	0	0	0	0	0	0	0	0	0	0	0	0	0
BGIR9823_101722	BGIG9823_26406		lncRNA	0	0	0	0	0	0	0	0	0	0	0	0	0	0	0	0	1	0	0
BGIR9823_102750	BGIG9823_27392		lncRNA	0	0	0	0	0	0	0	0	0	0	0	0	0	0	0	0	1	0	0
BGIR9823_102692	BGIG9823_27342		lncRNA	0	1	0	0	0	0	0	0	0	0	0	0	0	0	0	0	0	0	0
BGIR9823_102755	BGIG9823_27395		lncRNA	0	0	0	0	0	0	0	0	0	0	0	0	0	0	0	0	0	0	0
BGIR9823_101957	BGIG9823_26634		lncRNA	0	0	0	0	0	0	0	0	0	0	0	0	0	0	0	0	0	0	0
BGIR9823_102097	BGIG9823_26771		lncRNA	0	0	0	0	0	0	0	0	0	0	0	0	0	0	0	0	0	0	0
BGIR9823_102454	BGIG9823_27108		lncRNA	0	0	0	0	0	0	0	0	0	0	0	0	0	0	0	0	0	0	0
BGIR9823_102019	BGIG9823_26694		lncRNA	0	0	0	0	0	0	0	0	0	0	0	0	0	0	0	0	0	0	0

RNA ID	Gene ID	Symbol	Type	novel-ssc-miR817-3p	novel-ssc-miR849-3p	novel-ssc-miR882-3p	novel-ssc-miR985-5p	novel-ssc-miR1126-5p	novel-ssc-miR1396-3p	ssc-miR-362	novel-ssc-miR16-5p	ssc-miR-375	ssc-miR-376a-3p	ssc-miR-493-5p	ssc-miR-194a-5p	ssc-miR-205	novel-ssc-miR239-5p	novel-ssc-miR397-5p	novel-ssc-miR507-5p	novel-ssc-miR563-3p	novel-ssc-miR568-3p	novel-ssc-miR682-3p
BGIR9823_101736	BGIG9823_26417		lncRNA	0	0	0	0	0	0	0	0	0	0	0	0	1	0	0	0	0	0	0
BGIR9823_102662	BGIG9823_27312		lncRNA	0	0	0	0	0	0	0	0	0	0	0	0	0	0	0	0	0	0	0
BGIR9823_102303	BGIG9823_26969		lncRNA	0	0	0	0	0	0	0	0	0	0	0	0	0	0	0	0	0	0	0
BGIR9823_101931	BGIG9823_26608		lncRNA	0	0	0	0	0	0	0	0	0	0	0	0	0	0	0	0	0	0	0
BGIR9823_101842	BGIG9823_26521		lncRNA	0	0	0	0	0	0	0	0	0	0	0	0	0	0	0	0	0	0	0
BGIR9823_101783	BGIG9823_26463		lncRNA	0	0	0	0	0	0	0	0	0	0	0	0	0	0	0	0	0	0	0
BGIR9823_102447	BGIG9823_27102		lncRNA	0	0	0	0	0	0	0	0	0	0	0	0	1	0	0	0	0	0	0
BGIR9823_102114	BGIG9823_26788		lncRNA	0	0	0	0	0	0	0	0	0	0	0	0	0	0	0	0	0	0	0
BGIR9823_101753	BGIG9823_26434		lncRNA	0	0	0	0	0	0	0	0	0	0	0	0	0	0	0	0	0	0	0
BGIR9823_102643	BGIG9823_27293		lncRNA	0	0	0	0	0	0	0	0	0	0	0	0	0	0	0	0	0	0	0
BGIR9823_102327	BGIG9823_26992		lncRNA	0	0	0	0	0	0	0	0	0	0	0	0	0	0	0	0	0	0	0
BGIR9823_102216	BGIG9823_26888		lncRNA	0	0	0	0	0	0	0	0	0	0	0	0	0	0	0	0	0	0	0
BGIR9823_102718	BGIG9823_27365		lncRNA	0	0	0	0	0	0	0	0	0	0	0	0	0	0	0	0	0	0	0
BGIR9823_101709	BGIG9823_26393		lncRNA	0	0	0	0	0	0	0	0	0	0	0	0	0	0	0	0	0	0	0
BGIR9823_102241	BGIG9823_26913		lncRNA	0	0	0	0	0	0	0	0	0	0	0	0	0	0	0	0	0	0	0
BGIR9823_102306	BGIG9823_26971		lncRNA	0	0	0	0	0	0	0	0	0	0	0	0	0	0	0	0	1	0	0
BGIR9823_102486	BGIG9823_27140		lncRNA	0	0	0	0	0	0	0	0	0	0	0	0	0	0	0	0	0	0	0
BGIR9823_101724	BGIG9823_26408		lncRNA	0	0	0	0	0	0	0	0	0	0	0	0	0	0	0	0	0	0	0
BGIR9823_101858	BGIG9823_26537		lncRNA	0	0	0	0	0	0	0	0	0	0	0	0	0	0	0	0	0	0	0
BGIR9823_102483	BGIG9823_27137		lncRNA	0	0	0	0	0	0	0	0	0	0	0	0	0	0	0	0	0	0	0
BGIR9823_102027	BGIG9823_26702		lncRNA	0	0	0	0	0	0	0	0	0	0	0	0	0	0	0	0	0	0	0
BGIR9823_101777	BGIG9823_26457		lncRNA	0	0	0	0	0	0	0	0	0	0	0	0	0	0	0	0	0	1	0
BGIR9823_102154	BGIG9823_26827		lncRNA	0	0	0	0	0	0	0	0	0	0	0	0	0	0	0	0	1	0	0
BGIR9823_102426	BGIG9823_27087		lncRNA	0	0	0	0	0	0	0	0	0	0	0	0	0	0	0	0	0	0	0
BGIR9823_101894	BGIG9823_26571		lncRNA	0	0	0	0	0	0	0	0	0	0	0	0	0	0	0	0	0	0	0
BGIR9823_102009	BGIG9823_26685		lncRNA	0	0	0	0	0	0	0	0	0	0	0	0	0	0	0	0	0	0	0
BGIR9823_102250	BGIG9823_26918		lncRNA	0	0	0	0	0	0	0	0	0	0	0	0	0	0	0	0	0	0	1
BGIR9823_101769	BGIG9823_26449		lncRNA	0	0	0	0	0	0	0	0	0	0	0	0	0	0	0	0	0	0	0
XR_002338373.1	110256615	LOC110256615	lncRNA	0	0	0	0	1	0	0	0	0	0	0	0	0	0	0	0	0	0	0
XM_005655448.3	100519711	ST7L	mRNA	0	0	0	0	0	0	0	0	0	0	0	0	0	0	0	0	0	0	0
XR_001308691.2	106510294	LOC106510294	lncRNA	0	0	0	0	0	0	0	0	0	0	1	0	0	0	0	0	0	0	0
XR_002343519.1	110260353	LOC110260353	lncRNA	0	0	0	0	0	0	0	0	1	0	0	0	0	0	0	0	0	0	0
XR_002336962.1	102162292	LOC102162292	lncRNA	0	0	0	0	0	0	0	0	0	0	0	0	0	0	0	0	0	0	0
XM_021087319.1	100517646	POLE4	mRNA	0	0	0	0	0	0	0	0	0	0	0	0	0	1	0	0	0	0	0
XR_002336095.1	110255550	LOC110255550	lncRNA	1	0	0	0	0	0	0	0	0	0	0	0	0	0	0	0	0	0	0
NM_001244786.1	100513209	CFAP20	mRNA	0	0	0	0	0	0	0	0	0	0	0	0	0	0	0	0	0	1	0
XR_002340787.1	110257799	LOC110257799	lncRNA	0	0	0	0	0	0	0	0	0	0	0	0	0	0	0	0	0	0	0
XM_003358899.4	100623886	CCT8	mRNA	0	0	0	0	0	0	0	0	0	0	0	0	0	0	0	0	0	0	0
XR_002337835.1	110256393	LOC110256393	lncRNA	0	0	0	0	0	0	0	0	0	0	0	0	0	1	0	0	0	0	0
XR_002337635.1	106505647	LOC106505647	lncRNA	0	0	0	0	1	0	0	0	0	0	0	0	0	0	0	0	0	0	0
XR_001308778.2	106510350	LOC106510350	lncRNA	0	0	0	0	0	0	0	0	0	0	1	0	0	0	0	0	0	0	0
XR_002346849.1	110262181	LOC110262181	lncRNA	0	0	0	0	0	0	0	0	0	0	1	0	0	0	0	0	0	0	0
XM_005659957.3	102163937	GPR137C	mRNA	0	0	0	0	0	0	0	0	0	0	0	0	0	0	0	0	0	0	0

RNA ID	Gene ID	Symbol	Type	novel-ssc-miR817-3p	novel-ssc-miR849-3p	novel-ssc-miR882-3p	novel-ssc-miR985-5p	novel-ssc-miR1126-5p	novel-ssc-miR1396-3p	ssc-miR-362	novel-ssc-miR16-5p	ssc-miR-375	ssc-miR-376a-3p	ssc-miR-493-5p	ssc-miR-194a-5p	ssc-miR-205	novel-ssc-miR239-5p	novel-ssc-miR397-5p	novel-ssc-miR507-5p	novel-ssc-miR563-3p	novel-ssc-miR568-3p	novel-ssc-miR682-3p
XR_001303641.2	106507572	LOC106507572	lncRNA	0	0	0	0	0	0	0	1	0	0	0	0	0	0	0	0	0	0	0
XR_002344010.1	110260700	LOC110260700	lncRNA	0	0	0	0	0	0	0	0	0	0	0	0	0	0	0	1	0	0	0
XM_003359284.4	100623641	ZWINT	mRNA	0	0	0	0	0	0	0	0	0	0	0	0	0	0	0	0	0	0	0
XM_021066040.1	100233199	PITPNC1	mRNA	0	0	0	0	0	0	0	0	0	0	0	0	0	0	0	0	0	0	0
XM_003482526.2	100738522	LOC100738522	mRNA	0	0	0	0	0	0	0	0	0	0	0	0	0	0	0	0	0	0	0
XR_002345706.1	110261519	LOC110261519	lncRNA	0	0	0	0	0	0	0	0	0	0	0	0	0	0	0	0	0	0	0
XM_021066775.1	100513305	STRADA	mRNA	0	0	0	0	0	0	0	0	0	0	0	0	0	0	0	0	0	0	0
XR_303482.2	102164583	LOC102164583	lncRNA	0	0	0	0	0	0	0	0	0	0	0	0	0	0	0	0	0	0	0
XM_013998720.2	100737585	LOC100737585	mRNA	0	0	0	0	0	0	0	0	0	0	0	0	0	0	0	0	0	0	0
XR_002336556.1	102163487	LOC102163487	lncRNA	0	1	0	0	0	0	0	0	0	0	0	0	0	0	0	0	0	0	0
XR_002337553.1	102167511	SMIM4	lncRNA	0	0	0	0	0	0	0	0	0	0	0	0	0	0	0	0	0	0	0
XM_003483492.4	396595	PRF1	mRNA	0	0	0	0	0	0	0	0	0	0	0	0	0	0	0	0	0	0	0
XM_001926866.5	100155931	LOC100155931	mRNA	0	0	0	0	0	0	0	0	0	0	0	0	0	0	0	0	0	0	0
XR_002341737.1	102165038	LOC102165038	lncRNA	0	0	0	0	0	0	0	0	0	0	0	0	0	0	0	0	0	1	0
XR_002337051.1	110255981	LOC110255981	lncRNA	0	0	0	0	0	0	0	0	0	0	0	0	0	0	0	0	0	0	0
XM_021091413.1	100621517	TTLL1	mRNA	0	0	0	0	0	0	0	0	0	0	0	0	0	1	0	0	0	0	0
XR_002344541.1	110260924	LOC110260924	lncRNA	0	0	0	0	0	0	0	1	0	0	0	0	0	0	0	0	0	0	0
NM_213786.1	396680	NPW	mRNA	0	0	0	0	0	0	0	0	0	0	0	1	0	0	0	0	0	0	0
XM_021089665.1	100738802	BCAN	mRNA	0	0	0	0	0	0	0	0	0	1	0	0	0	0	0	0	0	0	0
XR_002340802.1	110257805	LOC110257805	lncRNA	0	0	0	0	1	0	0	0	0	0	0	0	0	0	0	0	0	0	0
XR_002341733.1	102158524	LOC102158524	lncRNA	0	0	0	0	0	0	0	0	0	0	1	0	0	0	0	0	0	0	0
XR_002345869.1	110261578	LOC110261578	lncRNA	0	0	0	0	0	0	0	0	0	0	0	0	0	0	0	1	0	0	0
XR_002338170.1	102161036	LOC102161036	lncRNA	0	0	0	0	0	0	0	0	0	0	0	0	0	0	0	0	0	0	0
XR_002337152.1	106505099	LOC106505099	lncRNA	0	0	0	0	0	0	0	0	0	0	0	0	0	0	0	1	0	0	0
XM_003131571.5	100512492	ANKRD40	mRNA	0	0	0	0	0	0	0	0	0	0	0	0	0	0	0	0	0	0	0
XR_002337061.1	110255986	LOC110255986	lncRNA	0	0	0	0	1	0	0	0	0	0	0	0	0	0	0	0	0	0	0
XR_002341729.1	110259387	LOC110259387	lncRNA	1	0	0	0	0	0	0	0	0	0	0	0	0	0	0	0	0	0	0
XM_005665516.3	100524028	ZMYND12	mRNA	0	0	0	0	0	0	0	0	0	0	0	0	0	0	0	0	0	0	0
XR_002342030.1	110259546	LOC110259546	lncRNA	0	0	0	0	0	0	0	0	0	0	0	0	0	0	0	0	0	0	0
XR_001304742.2	106508150	LOC106508150	lncRNA	0	0	0	0	0	0	0	0	0	0	0	0	0	0	0	0	0	0	0
NM_214322.1	397591	NPC1	mRNA	0	0	0	0	0	0	0	0	1	0	0	0	0	0	0	0	0	0	0
XR_002339061.1	102158523	LOC102158523	lncRNA	0	0	0	0	1	0	0	0	0	0	0	0	0	0	0	0	0	0	0
XR_002335622.1	110255429	LOC110255429	lncRNA	0	0	0	0	0	0	0	0	0	0	0	0	0	0	0	0	0	0	0
XM_003124446.4	100518241	SUMF2	mRNA	0	0	0	0	0	0	0	0	0	0	0	0	0	0	0	0	0	1	0
XR_002337394.1	110256124	LOC110256124	lncRNA	0	0	0	0	0	0	0	0	0	0	0	0	0	0	0	0	0	0	0
XM_003130089.4	100520582	LOC100520582	mRNA	0	0	0	0	0	0	0	0	0	0	0	0	0	0	0	0	0	0	0
XM_021100295.1	100337668	LCORL	mRNA	1	0	0	0	0	0	0	0	0	0	0	0	0	0	0	0	0	0	0
XR_002347096.1	110262257	LOC110262257	lncRNA	0	0	0	0	0	0	0	0	0	0	1	0	0	0	0	0	0	0	0
NM_214272.1	397512	TGFBR3	mRNA	0	0	0	0	0	0	0	0	0	0	0	1	0	0	0	0	0	0	0
XR_002337890.1	110256420	LOC110256420	lncRNA	0	0	0	0	0	0	0	0	0	0	0	0	0	0	0	1	0	0	0
XR_001302346.2	106506787	LOC106506787	lncRNA	0	0	0	0	0	0	0	0	0	0	0	0	0	0	0	0	0	0	0
XR_002340032.1	102161214	LOC102161214	lncRNA	0	0	0	0	0	0	0	0	0	0	0	0	0	0	0	0	0	0	0
XR_002338731.1	110256774	LOC110256774	lncRNA	0	0	0	0	1	0	0	0	0	0	0	0	0	0	0	0	0	0	0

RNA ID	Gene ID	Symbol	Type	novel-ssc-miR817-3p	novel-ssc-miR849-3p	novel-ssc-miR882-3p	novel-ssc-miR985-5p	novel-ssc-miR1126-5p	novel-ssc-miR1396-3p	ssc-miR-362	novel-ssc-miR16-5p	ssc-miR-375	ssc-miR-376a-3p	ssc-miR-493-5p	ssc-miR-194a-5p	ssc-miR-205	novel-ssc-miR239-5p	novel-ssc-miR397-5p	novel-ssc-miR507-5p	novel-ssc-miR563-3p	novel-ssc-miR568-3p	novel-ssc-miR682-3p
XR_002342421.1	110259933	LOC110259933	lncRNA	0	0	0	0	0	0	0	0	0	0	0	0	0	0	0	0	0	0	0
XM_021080420.1	110257790	WDR44	mRNA	0	0	0	0	0	0	0	0	0	0	0	0	0	0	0	0	0	0	0
XM_003128412.6	100514413	GTPBP2	mRNA	0	0	0	0	0	0	0	0	0	0	0	0	0	0	0	0	0	0	0
XR_001308377.2	106510160	LOC106510160	lncRNA	0	0	0	0	0	0	0	0	0	0	1	0	0	0	0	0	0	0	0
XM_021091670.1	100522737	ANKRD33	mRNA	0	0	0	0	0	0	0	0	0	0	0	0	0	1	0	0	0	0	0
XM_003134187.6	100512932	ASAH1	mRNA	0	0	0	0	0	0	0	0	0	0	0	0	0	1	0	0	0	0	0
XR_002335568.1	102159627	LOC102159627	lncRNA	0	0	0	0	0	0	0	0	0	0	0	0	0	1	0	0	0	0	0
XR_002345700.1	110261513	LOC110261513	lncRNA	0	0	0	0	0	0	0	0	0	0	1	0	0	0	0	0	0	0	0
NM_001243805.1	100511029	STARD10	mRNA	0	0	0	0	0	0	0	0	1	0	0	0	0	0	0	0	0	0	0
XM_001927894.5	100153448	HENMT1	mRNA	0	0	0	0	0	0	0	0	0	0	0	0	0	0	0	0	0	0	0
NM_214205.1	397409	IL5	mRNA	0	0	0	0	0	0	0	0	0	0	0	0	0	0	0	0	0	0	0
XR_002344130.1	110260754	LOC110260754	lncRNA	0	0	0	0	0	0	0	1	0	0	0	0	0	0	0	0	0	0	0
XM_021075881.1	100157060	WDR17	mRNA	0	0	0	0	0	0	0	0	0	0	0	0	0	1	0	0	0	0	0
XR_002344639.1	110260981	LOC110260981	lncRNA	0	0	0	0	0	0	0	1	0	0	0	0	0	0	0	0	0	0	0
XM_013985613.2	100525844	SLC35B4	mRNA	0	0	0	0	0	0	0	0	0	0	0	0	0	0	0	0	0	0	0
XR_002342231.1	110259849	LOC110259849	lncRNA	0	0	0	0	0	0	0	0	1	0	0	0	0	0	0	0	0	0	0
NM_001004043.1	445532	CFL1	mRNA	0	0	0	0	0	0	0	0	0	0	0	0	0	0	0	0	0	0	0
XM_003132695.5	100521154	ZBTB11	mRNA	0	0	0	0	0	0	0	0	0	0	0	0	0	0	0	0	0	0	0
XM_005657909.3	100519458	TEX13B	mRNA	0	0	0	0	0	0	0	0	0	0	0	0	0	0	0	0	0	0	0
XM_003129582.2	100517357	LOC100517357	mRNA	0	0	0	0	0	0	0	0	0	0	0	0	0	0	0	0	0	0	0
XR_298408.3	102167235	LOC102167235	lncRNA	0	0	0	0	0	0	0	0	0	0	0	0	0	0	0	0	0	0	0
XR_002336226.1	110255645	LOC110255645	lncRNA	0	0	0	0	0	0	0	0	0	0	0	0	0	0	0	0	0	0	0
XM_021077102.1	100515590	CCDC69	mRNA	0	0	0	0	0	0	0	0	0	0	0	0	0	0	0	0	0	0	0
XR_002336343.1	110255688	LOC110255688	lncRNA	0	0	0	0	0	0	0	0	0	0	0	0	0	0	0	0	0	0	0
XR_001309352.2	106510687	LOC106510687	lncRNA	0	0	0	0	0	0	0	1	0	0	0	0	0	0	0	0	0	0	0
XR_001301955.2	106506567	LOC106506567	lncRNA	0	0	0	0	0	0	0	0	0	0	0	0	0	0	0	0	0	0	0
XR_002338652.1	102167906	LOC102167906	lncRNA	0	0	0	0	0	0	0	0	0	0	0	0	0	0	0	0	0	0	0
XR_002336386.1	106506961	LOC106506961	lncRNA	0	0	0	0	0	0	0	0	0	0	1	0	0	0	0	0	0	0	0
XM_021074218.1	110256875	LOC110256875	mRNA	0	0	0	0	0	0	0	0	0	0	0	0	0	0	0	0	0	0	0
XR_002336193.1	100517050	2-Mar	lncRNA	0	0	0	0	0	0	0	0	0	0	0	0	0	0	0	0	0	0	0
XM_021088735.1	414423	GNRHR2	mRNA	1	0	0	0	0	0	0	0	0	0	0	0	0	0	0	0	0	0	0
NM_001146130.2	100271932	HPSE	mRNA	0	0	0	0	0	0	0	0	0	0	0	0	0	0	0	0	0	0	0
XM_013992909.2	100152206	LOC100152206	mRNA	0	0	0	0	0	0	0	0	0	0	0	0	0	0	0	0	0	0	0
XR_001299351.2	106505189	LOC106505189	lncRNA	0	0	0	0	1	0	0	0	0	0	0	0	0	0	0	0	0	0	0
XM_021090795.1	110260467	LOC110260467	mRNA	0	0	0	0	0	0	0	0	0	0	0	0	0	0	0	0	0	0	0
XR_001308232.2	106510085	LOC106510085	lncRNA	0	0	0	0	1	0	0	0	0	0	0	0	0	0	0	0	0	0	0
XR_002346494.1	110262011	LOC110262011	lncRNA	0	0	0	0	0	0	0	0	0	0	0	0	0	0	0	0	0	0	0
XM_005665059.3	100622416	KLHDC7A	mRNA	0	0	0	0	0	0	0	0	0	0	0	0	0	0	0	0	0	0	0
XR_002345769.1	110261544	LOC110261544	lncRNA	0	0	0	0	0	0	0	0	0	0	0	0	0	0	0	0	0	1	0
XR_002341699.1	110259368	LOC110259368	lncRNA	0	0	0	0	1	0	0	0	0	0	0	0	0	0	0	0	0	0	0
XR_001307130.2	106509521	LOC106509521	lncRNA	0	0	0	0	0	0	0	0	0	0	1	0	0	0	0	0	0	0	0
XR_002342847.1	106509907	LOC106509907	lncRNA	0	0	0	0	0	0	0	0	0	0	0	0	0	0	0	0	0	0	0
XR_001309149.2	106510562	LOC106510562	lncRNA	0	0	0	0	0	0	0	0	0	0	0	0	0	0	0	0	0	0	0

RNA ID	Gene ID	Symbol	Type	novel-ssc-miR817-3p	novel-ssc-miR849-3p	novel-ssc-miR882-3p	novel-ssc-miR985-5p	novel-ssc-miR1126-5p	novel-ssc-miR1396-3p	ssc-miR-362	novel-ssc-miR16-5p	ssc-miR-375	ssc-miR-376a-3p	ssc-miR-493-5p	ssc-miR-194a-5p	ssc-miR-205	novel-ssc-miR239-5p	novel-ssc-miR397-5p	novel-ssc-miR507-5p	novel-ssc-miR563-3p	novel-ssc-miR568-3p	novel-ssc-miR682-3p
XM_021096012.1	106507628	ZMPSTE24	mRNA	0	0	0	0	0	0	0	0	0	0	0	0	0	0	0	0	0	0	0
XM_021078386.1	110257500	PCMTD2	mRNA	0	0	0	0	0	0	0	0	0	0	0	0	0	0	0	0	0	0	0
XM_003134291.4	100513828	FOXA2	mRNA	0	0	0	0	0	0	0	0	0	0	0	0	0	0	0	0	0	0	0
XR_002339192.1	106506426	LOC106506426	lncRNA	0	0	0	0	0	0	0	0	0	0	0	0	0	0	0	0	0	0	0
XR_002340657.1	106506896	LOC106506896	lncRNA	0	0	0	0	1	0	0	0	0	0	0	0	0	0	0	0	0	0	0
XR_002344578.1	106507567	LOC106507567	lncRNA	0	0	0	0	0	0	0	1	0	0	0	0	0	0	0	0	0	0	0
XR_002337161.1	110256021	LOC110256021	lncRNA	0	0	0	0	1	0	0	0	0	0	0	0	0	0	0	0	0	0	0
XR_002343165.1	110260239	LOC110260239	lncRNA	0	0	0	0	0	0	0	0	0	0	1	0	0	0	0	0	0	0	0
XR_001306470.2	106509172	LOC106509172	lncRNA	0	0	0	0	0	0	0	0	0	0	1	0	0	0	0	0	0	0	0
XM_021087431.1	100622990	ETAA1	mRNA	0	0	0	0	0	0	0	0	0	0	0	0	0	0	0	0	0	0	0
NM_001243677.1	100157702	P2RY10	mRNA	0	0	0	0	0	0	0	0	0	0	0	0	0	0	0	0	0	0	0
NM_001005726.1	406192	ANXA2	mRNA	0	0	0	0	0	0	0	0	0	0	0	0	0	0	0	0	0	0	0
XR_002338199.1	110256565	LOC110256565	lncRNA	0	0	0	0	0	0	0	0	0	0	1	0	0	0	0	0	0	0	0
XR_002346497.1	110262015	LOC110262015	lncRNA	0	0	0	0	0	0	0	0	0	0	0	0	0	0	0	0	0	0	0
XM_021081451.1	100625375	LOC100625375	mRNA	0	0	0	0	0	0	0	0	0	0	0	0	0	0	0	0	0	0	0
NM_001243852.1	100511525	STT3A	mRNA	0	0	0	0	0	0	0	0	0	0	0	0	0	1	0	0	0	0	0
XM_001927799.7	100154361	SRP54	mRNA	0	0	0	0	0	0	0	0	0	0	0	0	0	0	0	0	0	0	0
XR_002339070.1	110256975	LOC110256975	lncRNA	0	0	0	0	0	0	0	0	0	0	1	0	0	0	0	0	0	0	0
XR_002340950.1	110257979	LOC110257979	lncRNA	0	0	0	0	0	0	0	0	0	0	0	0	0	0	0	0	0	0	0
XM_013990143.2	100155468	LOC100155468	mRNA	0	0	0	0	0	0	0	0	0	0	0	0	0	0	0	0	0	0	0
XM_003129448.1	100516817	LOC100516817	mRNA	0	0	0	0	0	0	0	0	0	0	0	0	0	0	0	0	0	0	0
XR_001301393.2	106506223	LOC106506223	lncRNA	0	0	0	0	1	0	0	0	0	0	0	0	0	0	0	0	0	0	0
XR_002337789.1	102162054	LOC102162054	lncRNA	0	0	0	0	0	0	0	0	0	0	0	0	0	0	0	1	0	0	0
XM_021085070.1	110259585	LOC110259585	mRNA	0	0	0	0	0	0	0	0	0	0	0	0	0	1	0	0	0	0	0
XM_005670449.3	100524281	SCARA5	mRNA	0	0	0	0	0	0	0	0	0	0	0	0	0	1	0	0	0	0	0
XM_021081009.1	110257997	LOC110257997	mRNA	0	0	0	0	0	0	0	0	0	0	0	0	0	1	0	0	0	0	0
XR_002343866.1	102166296	LOC102166296	lncRNA	0	0	0	0	0	0	0	0	0	0	0	0	0	0	0	1	0	0	0
XM_003124181.1	100517754	LOC100517754	mRNA	0	0	0	0	0	0	0	0	0	0	0	0	0	0	0	0	0	0	0
XR_001298301.2	102159863	LOC102159863	lncRNA	0	0	0	0	0	0	0	0	0	0	1	0	0	0	0	0	0	0	0
XM_021080605.1	110257839	LOC110257839	mRNA	1	0	0	0	0	0	0	0	0	0	0	0	0	0	0	0	0	0	0
XM_021072297.1	100519392	RAB36	mRNA	0	0	0	0	0	0	0	0	0	0	0	0	0	0	0	0	0	0	0
XM_005657053.3	100621152	RNF222	mRNA	0	0	0	0	0	0	0	0	0	0	0	0	0	0	0	0	0	0	0
XM_021064469.1	100516823	CNST	mRNA	0	0	0	0	0	0	0	0	0	0	0	0	0	0	0	0	0	0	0
XR_002344959.1	110261129	LOC110261129	lncRNA	0	0	0	0	0	0	0	1	0	0	0	0	0	0	0	0	0	0	0
XR_002336671.1	110255811	LOC110255811	lncRNA	0	0	0	0	0	0	0	0	0	0	0	0	0	0	0	0	0	0	0
XR_002346381.1	110261974	LOC110261974	lncRNA	1	0	0	0	0	0	0	0	0	0	0	0	0	0	0	0	0	0	0
XM_013994471.2	100517025	LOC100517025	mRNA	1	0	0	0	0	0	0	0	0	0	0	0	0	0	0	0	0	0	0
NM_001244142.1	100514123	SRI	mRNA	0	0	0	0	0	0	0	0	0	0	0	0	0	0	0	0	0	0	0
NM_001315615.1	100217382	NMUR2	mRNA	0	0	0	0	0	0	0	0	0	0	0	0	0	0	0	0	0	0	0
XR_002345984.1	110261620	LOC110261620	lncRNA	0	0	0	0	0	0	0	0	0	0	0	0	0	0	0	1	0	0	0
XR_002336754.1	110255855	LOC110255855	lncRNA	0	0	0	0	0	0	0	0	0	0	0	0	0	0	0	0	0	0	0
XR_002346598.1	110262073	LOC110262073	lncRNA	0	0	0	0	0	0	0	0	0	0	0	0	0	0	0	0	0	0	0
XR_002335757.1	110255462	LOC110255462	lncRNA	0	0	0	0	0	0	0	0	0	0	0	0	0	0	0	0	0	0	0

RNA ID	Gene ID	Symbol	Type	novel-ssc-miR817-3p	novel-ssc-miR849-3p	novel-ssc-miR882-3p	novel-ssc-miR985-5p	novel-ssc-miR1126-5p	novel-ssc-miR1396-3p	ssc-miR-362	novel-ssc-miR16-5p	ssc-miR-375	ssc-miR-376a-3p	ssc-miR-493-5p	ssc-miR-194a-5p	ssc-miR-205	novel-ssc-miR239-5p	novel-ssc-miR397-5p	novel-ssc-miR507-5p	novel-ssc-miR563-3p	novel-ssc-miR568-3p	novel-ssc-miR682-3p
XR_001304351.2	102167865	LOC102167865	lncRNA	0	0	0	0	0	0	0	0	0	0	0	0	0	0	0	0	0	0	0
NM_001011507.1	494565	LAMP1	mRNA	0	0	0	0	0	0	0	0	1	0	0	0	0	0	0	0	0	0	0
XR_002339091.1	106505784	LOC106505784	lncRNA	0	0	0	0	0	0	0	0	0	0	0	0	0	0	0	1	0	0	0
XR_298324.3	102163156	LOC102163156	lncRNA	0	0	0	0	0	0	0	1	0	0	0	0	0	0	0	0	0	0	0
XM_021099319.1	100626159	RNASE9	mRNA	0	0	0	0	0	0	0	0	0	0	0	0	0	1	0	0	0	0	0
XM_021096183.1	100739254	ANKRD29	mRNA	0	0	0	0	0	0	0	0	0	0	0	0	0	0	0	0	0	0	0
XR_002335919.1	110255507	LOC110255507	lncRNA	0	0	0	0	0	0	0	0	0	0	0	0	0	0	0	0	0	0	0
XR_002340438.1	106506789	LOC106506789	lncRNA	0	0	0	0	0	0	0	0	0	0	0	0	0	0	0	0	0	0	0
XM_021099935.1	100519785	RAD51B	mRNA	0	0	0	0	0	0	0	0	0	0	0	0	0	0	0	0	0	1	0
XR_001305518.2	106508572	LOC106508572	lncRNA	0	0	0	0	0	0	0	0	0	0	0	0	0	0	0	0	0	0	0
XM_013992672.2	100520160	CNR1	mRNA	0	0	0	0	0	0	0	0	0	0	0	0	0	0	0	0	0	0	0
NM_001123164.1	100144479	PNMT	mRNA	0	0	0	0	0	0	0	0	0	0	0	0	0	0	0	0	0	0	0
NM_001244365.1	100153444	UROS	mRNA	0	0	0	0	0	0	0	0	0	0	0	0	0	0	0	0	0	0	0
XM_021092559.1	100157209	PRICKLE1	mRNA	0	0	0	0	0	0	0	0	0	0	0	0	0	0	0	0	0	1	0
XM_013979148.2	100521912	WNT11	mRNA	0	0	0	0	0	0	0	0	0	0	0	0	0	0	0	0	0	0	0
XR_002337909.1	110256426	LOC110256426	lncRNA	0	0	0	0	1	0	0	0	0	0	0	0	0	0	0	0	0	0	0
NM_214280.1	397524	VCP	mRNA	0	0	0	0	0	0	0	0	0	0	0	0	0	0	0	0	0	0	0
XM_005671261.3	100155046	RNLS	mRNA	0	0	0	0	0	0	0	0	0	0	0	0	0	1	0	0	0	0	0
XR_002337573.1	100154005	ACTR8	mRNA	0	0	0	0	0	0	0	0	0	0	0	0	0	0	0	0	0	0	0
NM_001244315.1	100514255	ARL4C	mRNA	0	0	0	0	0	0	0	0	1	0	0	0	0	0	0	0	0	0	0
XR_001300300.2	106505675	LOC106505675	lncRNA	0	0	0	0	1	0	0	0	0	0	0	0	0	0	0	0	0	0	0
XR_002341897.1	110259477	LOC110259477	lncRNA	0	0	0	0	0	0	0	0	0	0	0	0	0	0	0	1	0	0	0
XR_002336723.1	110255842	LOC110255842	lncRNA	0	0	0	0	1	0	0	0	0	0	0	0	0	0	0	0	0	0	0
XM_021096057.1	100737127	RAB31	mRNA	0	0	0	0	0	0	0	0	0	0	0	0	0	0	0	0	0	0	0
XR_001299360.2	106505194	LOC106505194	lncRNA	0	0	0	0	1	0	0	0	0	0	0	0	0	0	0	0	0	0	0
XM_001925846.7	106504094	GTF3C3	mRNA	0	0	0	0	0	0	0	0	0	0	0	0	0	0	0	0	0	0	0
XR_002337419.1	106505263	LOC106505263	lncRNA	0	0	0	0	0	0	0	0	0	0	1	0	0	0	0	0	0	0	0
XR_002346301.1	110261775	LOC110261775	lncRNA	0	0	0	0	0	0	0	0	0	0	1	0	0	0	0	0	0	0	0
XR_002339946.1	100627949	LOC100627949	lncRNA	0	0	0	0	0	0	0	0	0	0	0	0	0	0	0	1	0	0	0
XR_002346180.1	110261712	LOC110261712	lncRNA	0	0	0	0	0	0	0	0	0	0	0	0	0	0	0	0	0	0	0
XR_001297665.2	106504303	LOC106504303	lncRNA	0	0	0	0	1	0	0	0	0	0	0	0	0	0	0	0	0	0	0
XR_001298947.2	106504955	LOC106504955	lncRNA	0	0	0	0	0	0	0	0	0	0	0	0	0	0	0	0	0	0	0
XR_002341739.1	110259401	LOC110259401	lncRNA	0	0	0	0	1	0	0	0	0	0	0	0	0	0	0	0	0	0	0
XR_001307225.2	106509575	LOC106509575	lncRNA	0	0	0	0	0	0	0	1	0	0	0	0	0	0	0	0	0	0	0
XR_002338919.1	110256865	LOC110256865	lncRNA	0	0	0	0	0	0	0	0	0	0	0	0	0	0	0	0	0	0	0
XR_001306376.2	106509109	LOC106509109	lncRNA	0	1	0	0	0	0	0	0	0	0	0	0	0	0	0	0	0	0	0
XR_002338130.1	102158325	LOC102158325	lncRNA	0	1	0	0	0	0	0	0	0	0	0	0	0	0	0	0	0	0	0
XR_002337988.1	106505633	LOC106505633	lncRNA	0	0	0	0	0	0	0	0	0	0	1	0	0	0	0	0	0	0	0
XM_021086158.1	102161487	TMEM225B	mRNA	1	0	0	0	0	0	0	0	0	0	0	0	0	0	0	0	0	0	0
XM_003130212.5	100522092	RUNDC3B	mRNA	0	0	0	0	0	0	0	0	0	0	0	0	0	0	0	0	0	1	0
XM_001924771.6	100156540	RPP30	mRNA	0	0	0	0	0	0	0	0	0	0	0	0	0	0	0	0	0	0	0
XM_001928042.4	100152526	MED6	mRNA	1	0	0	0	0	0	0	0	0	0	0	0	0	0	0	0	0	0	0
XM_021095234.1	100738053	CHD5	mRNA	0	0	0	0	0	0	0	0	0	0	0	0	0	0	0	0	0	0	0

RNA ID	Gene ID	Symbol	Type	novel-ssc-miR817-3p	novel-ssc-miR849-3p	novel-ssc-miR882-3p	novel-ssc-miR985-5p	novel-ssc-miR1126-5p	novel-ssc-miR1396-3p	ssc-miR-362	novel-ssc-miR16-5p	ssc-miR-375	ssc-miR-376a-3p	ssc-miR-493-5p	ssc-miR-194a-5p	ssc-miR-205	novel-ssc-miR239-5p	novel-ssc-miR397-5p	novel-ssc-miR507-5p	novel-ssc-miR563-3p	novel-ssc-miR568-3p	novel-ssc-miR682-3p
XM_001928146.2	100152920	LOC100152920	mRNA	0	0	0	0	0	0	0	0	0	0	0	0	0	0	0	0	0	0	0
XM_003129758.5	100526065	SESN3	mRNA	0	0	0	0	0	0	0	0	0	0	0	0	0	0	0	0	0	0	0
XR_002339615.1	110257251	LOC110257251	lncRNA	0	0	0	0	0	0	0	1	0	0	0	0	0	0	0	0	0	0	0
XR_002339546.1	102158929	LOC102158929	lncRNA	0	0	0	0	1	0	0	0	0	0	0	0	0	0	0	0	0	0	0
XR_001308433.2	102165975	LOC102165975	lncRNA	0	1	0	0	0	0	0	0	0	0	0	0	0	0	0	0	0	0	0
XR_002341525.1	110259281	LOC110259281	lncRNA	0	1	0	0	0	0	0	0	0	0	0	0	0	0	0	0	0	0	0
NM_001038002.1	414911	EDNRB	mRNA	0	0	0	0	0	0	0	0	0	0	0	0	0	0	0	0	0	0	0
XR_002338445.1	106505879	LOC106505879	lncRNA	0	1	0	0	0	0	0	0	0	0	0	0	0	0	0	0	0	0	0
XR_002341837.1	110259430	LOC110259430	lncRNA	0	0	0	0	0	0	0	0	0	0	0	0	0	0	0	0	0	1	0
XM_021086652.1	100736832	GRIN2A	mRNA	0	0	0	0	0	0	0	0	0	0	0	0	0	0	0	0	0	0	0
XM_013997106.2	106510154	LOC106510154	mRNA	0	0	0	0	0	0	0	0	0	0	0	0	0	0	0	0	0	0	0
XR_002347051.1	110262233	LOC110262233	lncRNA	0	1	0	0	0	0	0	0	0	0	0	0	0	0	0	0	0	0	0
XR_002335588.1	110255417	LOC110255417	lncRNA	0	0	0	0	1	0	0	0	0	0	0	0	0	0	0	0	0	0	0
XR_001302361.2	106506801	LOC106506801	lncRNA	0	0	0	0	0	0	0	0	0	0	0	0	0	0	0	0	0	1	0
XR_002336628.1	110255797	LOC110255797	lncRNA	0	0	0	0	0	0	0	0	0	0	0	0	0	0	0	0	0	0	0
XM_021099547.1	102158165	TMEM63C	mRNA	0	0	0	0	0	0	0	0	0	0	0	0	0	0	0	0	0	0	0
XR_001307739.2	106509824	LOC106509824	lncRNA	0	1	0	0	0	0	0	0	0	0	0	0	0	0	0	0	0	0	0
XM_021084335.1	102162790	GRM6	mRNA	0	0	0	0	0	0	0	0	0	0	0	0	0	0	0	0	0	0	0
XR_002339516.1	102161653	LOC102161653	lncRNA	0	0	0	0	0	0	0	0	0	0	0	0	0	0	0	0	0	0	0
XR_001299377.2	106505203	LOC106505203	lncRNA	0	0	0	0	0	0	0	1	0	0	0	0	0	0	0	0	0	0	0
XM_003481432.4	100738802	BCAN	mRNA	0	0	0	0	0	0	0	0	0	0	0	0	0	0	0	0	0	0	0
XM_021095297.1	100627651	CTNNBIP1	mRNA	0	0	0	0	0	0	0	0	0	0	0	0	0	0	0	0	0	0	0
XM_003134620.5	100517251	TTC26	mRNA	0	0	0	0	0	0	0	0	0	0	0	0	0	0	0	0	0	0	0
XR_002343515.1	102165594	LOC102165594	lncRNA	0	1	0	0	0	0	0	0	0	0	0	0	0	0	0	0	0	0	0
NM_001044607.2	733679	ECH1	mRNA	0	0	0	0	0	0	0	0	0	0	0	0	0	0	0	0	0	0	0
XR_001300056.2	106505536	LOC106505536	lncRNA	0	0	0	0	1	0	0	0	0	0	0	0	0	0	0	0	0	0	0
XR_001301036.2	106506054	LOC106506054	lncRNA	0	0	0	0	0	0	0	0	0	0	0	0	0	0	0	0	0	0	0
XM_005654040.3	397441	CD86	mRNA	0	0	0	0	0	0	0	0	0	0	0	0	0	0	0	0	0	0	0
XR_001298634.2	102163793	LOC102163793	lncRNA	0	0	0	0	0	0	0	1	0	0	0	0	0	0	0	0	0	0	0
XR_002343053.1	110260210	LOC110260210	lncRNA	0	0	0	0	0	0	0	0	0	0	0	0	0	0	0	1	0	0	0
XM_003129012.4	100517956	TMA16	mRNA	0	0	0	0	0	0	0	0	0	0	0	0	0	0	0	0	0	0	0
XM_021076351.1	100515172	HOXD8	mRNA	0	0	0	0	0	0	0	0	0	0	0	0	0	0	0	0	0	0	0
NM_001001636.1	414419	RPL32	mRNA	0	0	0	0	0	0	0	0	0	0	0	0	0	0	0	0	0	0	0
XR_002336222.1	110255642	LOC110255642	lncRNA	0	0	0	0	0	0	0	0	0	0	0	0	0	0	0	0	0	0	0
XR_002337964.1	110256455	LOC110256455	lncRNA	0	0	0	0	0	0	0	0	0	0	0	0	0	0	0	0	0	0	0
XR_002343225.1	110260259	LOC110260259	lncRNA	0	0	0	0	0	0	0	0	0	0	0	0	0	0	0	0	0	0	0
XR_002340084.1	106506691	LOC106506691	lncRNA	0	0	0	0	0	0	0	0	0	0	0	0	0	0	0	1	0	0	0
XM_001928520.6	100153343	RWDD1	mRNA	0	0	0	0	0	0	0	0	0	0	0	0	0	0	0	0	0	0	0
XM_005652653.3	106504062	NLGN3	mRNA	0	0	0	0	0	0	0	0	0	0	0	0	0	0	0	0	0	0	0
XR_002341746.1	102165717	LOC102165717	lncRNA	0	0	0	0	0	0	0	0	0	0	0	0	0	0	0	0	0	0	0
XM_021085042.1	110259549	LOC110259549	mRNA	0	0	0	0	0	0	0	0	0	0	0	0	0	0	0	0	0	0	0
XR_001305221.2	106508400	LOC106508400	lncRNA	0	0	0	0	0	0	0	0	0	0	0	0	0	0	0	0	0	0	0
XR_002343172.1	110260241	LOC110260241	lncRNA	0	0	0	0	0	0	0	0	0	0	1	0	0	0	0	0	0	0	0

RNA ID	Gene ID	Symbol	Type	novel-ssc-miR817-3p	novel-ssc-miR849-3p	novel-ssc-miR882-3p	novel-ssc-miR985-5p	novel-ssc-miR1126-5p	novel-ssc-miR1396-3p	ssc-miR-362	novel-ssc-miR16-5p	ssc-miR-375	ssc-miR-376a-3p	ssc-miR-493-5p	ssc-miR-194a-5p	ssc-miR-205	novel-ssc-miR239-5p	novel-ssc-miR397-5p	novel-ssc-miR507-5p	novel-ssc-miR563-3p	novel-ssc-miR568-3p	novel-ssc-miR682-3p
XR_002339487.1	106506400	COPS7B	lncRNA	1	0	0	0	0	0	0	0	0	0	0	0	0	0	0	0	0	0	0
XR_001308351.2	106510133	LOC106510133	lncRNA	0	0	0	0	1	0	0	0	0	0	0	0	0	0	0	0	0	0	0
XM_003124564.4	100511500	GPR139	mRNA	0	0	0	0	0	0	0	0	0	0	0	0	0	0	0	0	0	0	0
NM_001078686.1	780437	ODAM	mRNA	0	0	0	0	0	0	0	0	0	0	0	0	0	0	0	0	0	0	0
XR_298956.3	102160517	LOC102160517	lncRNA	0	0	0	0	0	0	0	0	0	0	0	0	0	0	0	0	0	0	0
XR_002347080.1	110262247	LOC110262247	lncRNA	0	0	0	0	0	0	0	0	0	0	0	0	0	0	0	0	0	0	0
XM_021094930.1	100514465	LOC100514465	mRNA	0	0	0	0	0	0	0	0	0	0	0	0	0	0	0	0	0	1	0
XR_002343951.1	100737981	EIF4B	lncRNA	0	0	0	0	0	0	0	0	0	0	0	0	0	0	0	0	0	0	0
NM_001244614.1	100153985	SIX4	mRNA	0	0	0	0	0	0	0	0	0	0	0	0	0	0	0	0	0	0	0
XM_021066050.1	100302700	SLC25A19	mRNA	0	0	0	0	0	0	0	0	0	0	0	0	0	1	0	0	0	0	0
XM_005653210.3	100522374	GAS8	mRNA	1	0	0	0	0	0	0	0	0	0	0	0	0	0	0	0	0	0	0
XR_302139.2	102161858	LOC102161858	lncRNA	0	0	0	0	0	0	0	0	0	1	0	0	0	0	0	0	0	0	0
XR_002335527.1	110255394	LOC110255394	lncRNA	0	0	0	0	1	0	0	0	0	0	0	0	0	0	0	0	0	0	0
XR_002336548.1	110255755	LOC110255755	lncRNA	0	0	0	0	0	0	0	0	0	0	0	0	0	0	0	1	0	0	0
XR_001307143.2	100522542	LOC100522542	lncRNA	0	0	0	0	0	0	0	0	0	0	1	0	0	0	0	0	0	0	0
XR_002336864.1	110255895	LOC110255895	lncRNA	0	0	0	0	0	0	0	0	0	0	0	0	0	0	0	0	0	0	0
NM_214125.1	397230	MBL2	mRNA	0	0	0	0	0	0	0	0	0	0	0	0	0	0	0	0	0	0	0
XR_002339158.1	106506349	LOC106506349	lncRNA	0	0	0	0	0	0	0	0	0	0	0	0	0	0	0	0	0	0	0
NM_001244309.1	100625497	EZH2	mRNA	0	0	0	0	0	0	0	0	0	0	0	0	0	0	0	0	0	0	0
XM_021102414.1	100523325	ASB4	mRNA	0	0	0	0	0	0	0	0	0	0	0	0	0	0	0	0	0	0	0
XM_021069845.1	110256370	LOC110256370	mRNA	1	0	0	0	0	0	0	0	0	0	0	0	0	0	0	0	0	0	0
XR_309047.3	100519283	LOC100519283	lncRNA	0	0	0	0	0	0	0	0	0	0	0	0	0	0	0	0	0	0	0
XR_001308371.2	106510151	LOC106510151	lncRNA	0	0	0	0	0	0	0	0	0	0	0	0	0	0	0	0	0	0	0
XR_001303230.2	106507324	LOC106507324	lncRNA	0	0	0	0	0	0	0	0	0	0	0	0	0	0	0	0	0	0	0
XR_002338650.1	102167253	LOC102167253	lncRNA	0	0	0	0	0	0	0	0	0	0	0	0	0	0	0	0	0	0	0
XM_003132532.3	100519624	TMEM212	mRNA	1	0	0	0	0	0	0	0	0	0	0	0	0	0	0	0	0	0	0
XM_021086004.1	110259835	LOC110259835	mRNA	0	0	0	0	0	0	0	0	0	0	0	0	0	0	0	0	0	0	0
XM_005663799.3	396643	SSTR3	mRNA	0	0	0	0	0	0	0	0	0	1	0	0	0	0	0	0	0	0	0
XR_002335939.1	106507874	LOC106507874	lncRNA	0	0	0	0	0	0	0	0	0	0	0	0	0	0	0	0	0	0	0
XM_003130663.6	100522820	KIF24	mRNA	0	0	0	0	0	0	0	0	0	0	0	0	0	0	0	0	0	0	0
XM_003124595.4	100518832	RMI2	mRNA	0	0	0	0	0	0	0	0	0	0	0	0	0	0	0	0	0	0	0
XR_002339295.1	106506428	LOC106506428	lncRNA	0	0	0	0	0	0	0	0	0	0	0	0	0	0	0	0	0	0	0
XM_001925784.6	100155342	USP5	mRNA	0	0	0	0	0	0	0	0	1	0	0	0	0	0	0	0	0	0	0
XR_002346720.1	110262131	LOC110262131	lncRNA	0	0	0	0	0	0	0	1	0	0	0	0	0	0	0	0	0	0	0
XR_002339354.1	110257112	LOC110257112	lncRNA	0	1	0	0	0	0	0	0	0	0	0	0	0	0	0	0	0	0	0
XR_002346965.1	110262211	LOC110262211	lncRNA	0	0	0	0	0	0	0	0	0	0	0	0	0	0	0	0	0	0	0
XR_002335796.1	110255471	LOC110255471	lncRNA	0	0	0	0	0	0	0	0	0	0	0	0	0	0	0	0	0	0	0
XM_021101481.1	102166512	ANXA3	mRNA	0	0	0	0	0	0	0	0	0	0	0	0	0	0	0	0	0	0	0
XM_001926306.7	100153723	ABT1	mRNA	0	0	0	0	0	0	0	0	0	0	0	0	0	0	0	0	0	0	0
XR_002336516.1	102158088	NHLRC3	lncRNA	0	0	0	0	0	0	0	1	0	0	0	0	0	0	0	0	0	0	0
XR_002344446.1	102162270	SLC7A5	lncRNA	0	0	0	0	0	0	0	0	0	0	0	0	0	0	0	0	0	0	0
XR_002345296.1	102166616	LOC102166616	lncRNA	0	0	0	0	0	0	0	0	0	0	0	0	0	0	0	0	0	0	0
NM_001001645.1	414435	WDR83OS	mRNA	1	0	0	0	0	0	0	0	0	0	0	0	0	0	0	0	0	0	0

RNA ID	Gene ID	Symbol	Type	novel-ssc-miR817-3p	novel-ssc-miR849-3p	novel-ssc-miR882-3p	novel-ssc-miR985-5p	novel-ssc-miR1126-5p	novel-ssc-miR1396-3p	ssc-miR-362	novel-ssc-miR16-5p	ssc-miR-375	ssc-miR-376a-3p	ssc-miR-493-5p	ssc-miR-194a-5p	ssc-miR-205	novel-ssc-miR239-5p	novel-ssc-miR397-5p	novel-ssc-miR507-5p	novel-ssc-miR563-3p	novel-ssc-miR568-3p	novel-ssc-miR682-3p
XR_002337442.1	110256166	LOC110256166	lncRNA	0	0	0	0	0	0	0	1	0	0	0	0	0	0	0	0	0	0	0
XM_021072047.1	100512874	SCAF8	mRNA	0	0	0	0	0	0	0	0	0	0	0	0	0	0	0	0	0	0	0
XR_001298911.2	106504947	LOC106504947	lncRNA	0	0	0	0	0	0	0	0	0	0	0	0	0	0	0	0	0	0	0
XR_002340842.1	110257831	LOC110257831	lncRNA	1	0	0	0	0	0	0	0	0	0	0	0	0	0	0	0	0	0	0
XM_003122534.4	100512393	KCNK7	mRNA	0	0	0	0	0	0	0	0	0	0	0	0	0	0	0	0	0	0	0
XR_002340251.1	110257517	LOC110257517	lncRNA	0	0	0	0	0	0	0	0	1	0	0	0	0	0	0	0	0	0	0
XR_002338712.1	110256766	LOC110256766	lncRNA	0	0	0	0	0	0	0	0	0	0	0	0	0	0	0	1	0	0	0
XM_003129449.2	100516987	LOC100516987	mRNA	0	0	0	0	0	0	0	0	0	0	0	0	0	0	0	0	0	0	0
XR_001305858.2	106508800	LOC106508800	lncRNA	0	0	0	0	0	0	0	0	0	0	0	0	0	0	0	1	0	0	0
XR_001307955.2	102165812	LOC102165812	lncRNA	0	0	0	0	0	0	0	0	0	0	0	0	0	0	0	0	0	0	0
XR_002345578.1	110261473	LOC110261473	lncRNA	0	0	0	0	0	0	0	0	0	0	0	0	0	0	0	0	0	0	0
XR_002340279.1	110257523	LOC110257523	lncRNA	0	0	0	0	0	0	0	0	0	0	0	0	0	0	0	0	0	0	0
XM_021073688.1	100153473	CCDC188	mRNA	0	0	0	0	0	0	0	0	0	0	0	0	0	0	0	0	0	0	0
XR_002347070.1	100737186	LOC100737186	lncRNA	0	0	0	0	0	0	0	0	0	0	1	0	0	0	0	0	0	0	0
XR_002338524.1	110256665	LOC110256665	lncRNA	0	0	0	0	0	0	0	1	0	0	0	0	0	0	0	0	0	0	0
XR_002338128.1	110256525	LOC110256525	lncRNA	0	0	0	0	0	0	0	0	0	0	0	0	0	0	0	0	0	0	0
XR_002338320.1	102160169	LOC102160169	lncRNA	0	0	0	0	0	0	0	0	0	0	0	0	0	1	0	0	0	0	0
XR_002341185.1	102168138	LOC102168138	lncRNA	0	0	0	0	0	0	0	0	0	0	0	1	0	0	0	0	0	0	0
NM_001243681.2	100626539	RNASEH1	mRNA	0	0	0	0	0	0	0	0	1	0	0	0	0	0	0	0	0	0	0
XM_021087419.1	100516073	APLF	mRNA	0	0	0	0	0	0	0	0	0	0	0	0	0	0	0	0	0	0	0
NM_001244932.1	100514188	SUMF1	mRNA	0	0	0	0	0	0	0	0	0	0	0	0	0	0	0	0	0	0	0
XR_002342760.1	110260029	LOC110260029	lncRNA	0	0	0	0	0	0	0	0	0	0	0	0	0	0	0	0	0	1	0
NM_001130735.1	100170853	SMN1	mRNA	0	0	0	0	0	0	0	0	0	0	0	0	0	0	0	0	0	0	0
XR_002337720.1	106505376	LOC106505376	lncRNA	0	0	0	0	0	0	0	0	0	0	0	0	0	0	0	0	0	0	0
XM_021082523.1	100620803	LOC100620803	mRNA	0	0	0	0	0	0	0	0	0	0	0	0	0	1	0	0	0	0	0
XR_002344603.1	102158585	LOC102158585	lncRNA	0	0	0	0	0	0	0	1	0	0	0	0	0	0	0	0	0	0	0
XR_002340236.1	100513188	LOC100513188	lncRNA	0	0	0	0	0	0	0	0	0	0	0	0	0	0	0	0	0	0	0
XR_002335659.1	100622439	LOC100622439	lncRNA	0	0	0	0	0	0	0	0	0	0	0	0	0	0	0	0	0	0	0
XM_003122991.5	100523423	ATP5F1D	mRNA	0	0	0	0	0	0	0	0	0	0	0	0	0	0	0	0	0	0	0
XR_001308436.2	102166460	LOC102166460	lncRNA	0	0	0	0	0	0	0	1	0	0	0	0	0	0	0	0	0	0	0
XM_021070161.1	100620692	UBXN7	mRNA	0	0	0	0	0	0	0	0	0	0	0	0	0	0	0	0	0	0	0
NM_001278777.1	494011	SLC27A2	mRNA	0	0	0	0	0	0	0	0	0	0	0	0	0	0	0	0	0	0	0
XM_013994347.2	397553	NR1H3	mRNA	1	0	0	0	0	0	0	0	0	0	0	0	0	0	0	0	0	0	0
XM_005664733.3	100514292	ALDH16A1	mRNA	0	0	0	0	0	0	0	0	0	1	0	0	0	0	0	0	0	0	0
NM_001099936.1	100101473	BTG1	mRNA	0	0	0	0	0	0	0	0	0	0	0	0	0	0	0	0	0	0	0
XM_021099493.1	100625247	HEATR4	mRNA	0	0	0	0	0	0	0	0	0	0	0	0	0	0	0	0	0	0	0
XR_002335793.1	100523270	PFKFB2	mRNA	0	0	0	0	0	0	0	0	0	0	0	0	0	0	0	0	0	0	0
XR_002343260.1	106510048	LOC106510048	lncRNA	0	0	0	0	0	0	0	1	0	0	0	0	0	0	0	0	0	0	0
XM_021063420.1	100738171	ABCB5	mRNA	0	0	0	0	0	0	0	0	0	0	0	0	0	0	0	0	0	0	0
XM_021098946.1	100155072	CFAP100	mRNA	0	0	0	0	0	0	0	0	0	0	0	0	0	0	0	0	0	0	0
XM_021074210.1	100737404	LOC100737404	mRNA	0	0	0	0	0	0	0	0	0	0	0	0	0	0	0	0	0	0	0
XM_021088107.1	100517149	LOC100517149	mRNA	0	0	0	0	0	0	0	0	0	0	0	0	0	0	0	0	0	0	0
XM_003125346.4	100518190	MYCN	mRNA	0	0	0	0	0	0	0	0	0	0	0	0	0	0	0	0	0	0	0

RNA ID	Gene ID	Symbol	Type	novel-ssc-miR817-3p	novel-ssc-miR849-3p	novel-ssc-miR882-3p	novel-ssc-miR985-5p	novel-ssc-miR1126-5p	novel-ssc-miR1396-3p	ssc-miR-362	novel-ssc-miR16-5p	ssc-miR-375	ssc-miR-376a-3p	ssc-miR-493-5p	ssc-miR-194a-5p	ssc-miR-205	novel-ssc-miR239-5p	novel-ssc-miR397-5p	novel-ssc-miR507-5p	novel-ssc-miR563-3p	novel-ssc-miR568-3p	novel-ssc-miR682-3p
XM_003356153.3	100517101	LOC100517101	mRNA	0	0	0	0	0	0	0	0	0	0	0	0	0	0	0	0	0	0	0
XR_002338567.1	110256688	LOC110256688	lncRNA	0	0	0	0	0	0	0	0	0	0	1	0	0	0	0	0	0	0	0
XR_002339778.1	110257320	LOC110257320	lncRNA	0	1	0	0	0	0	0	0	0	0	0	0	0	0	0	0	0	0	0
XM_001927081.6	100156057	TTC8	mRNA	0	0	0	0	0	0	0	0	0	0	0	0	0	1	0	0	0	0	0
XM_021093139.1	100037307	DAB1	mRNA	0	0	0	0	0	0	0	0	0	0	0	0	0	0	0	0	0	0	0
XR_001300917.2	106505968	LOC106505968	lncRNA	0	0	0	0	0	0	0	1	0	0	0	0	0	0	0	0	0	0	0
NM_001190154.1	100157314	ZCCHC8	mRNA	0	0	0	0	0	0	0	0	0	0	0	0	0	0	0	0	0	0	0
XR_002338340.1	102159136	ARHGAP19	lncRNA	0	0	0	0	0	0	0	0	0	0	0	0	0	0	0	0	0	0	0
XR_002340858.1	110257848	LOC110257848	lncRNA	0	0	0	0	0	0	0	0	0	0	0	0	0	0	0	1	0	0	0
XM_021084337.1	102162790	GRM6	mRNA	0	0	0	0	0	0	0	0	0	0	0	0	0	0	0	0	0	0	0
XR_001306165.2	106508978	LOC106508978	lncRNA	0	0	0	0	0	0	0	0	0	0	0	0	0	0	0	0	0	0	0
XM_021085043.1	110259550	LOC110259550	mRNA	0	0	0	0	0	0	0	0	0	0	0	0	0	0	0	0	0	0	0
XR_001302502.2	102164143	LOC102164143	lncRNA	0	0	0	0	1	0	0	0	0	0	0	0	0	0	0	0	0	0	0
XM_021088119.1	100516428	LOC100516428	mRNA	0	0	0	0	0	0	0	0	0	0	0	0	0	0	0	0	0	0	0
XR_002335496.1	110255344	LOC110255344	lncRNA	0	0	0	0	0	0	0	0	0	0	0	0	0	0	0	0	0	0	0
XR_002335923.1	110255511	LOC110255511	lncRNA	0	0	0	0	0	0	0	0	0	0	0	0	0	0	0	0	0	0	0
XR_002336776.1	102168075	LOC102168075	lncRNA	0	0	0	0	0	0	0	0	0	0	0	0	0	0	0	1	0	0	0
XR_304089.2	102160593	LOC102160593	lncRNA	0	0	0	0	0	0	0	0	0	0	0	0	0	0	0	0	0	0	0
XM_003355424.5	100627932	HOXC6	mRNA	0	0	0	0	0	0	0	0	0	0	0	0	0	0	0	0	0	0	0
XR_002335902.1	102161420	LOC102161420	lncRNA	0	0	0	0	0	0	0	0	0	0	0	0	0	0	0	0	0	0	0
XR_002344769.1	110261037	LOC110261037	lncRNA	0	0	0	0	0	0	0	0	0	0	0	0	0	0	0	0	0	0	0
XR_001300967.2	100520338	LOC100520338	lncRNA	0	0	0	0	0	0	0	0	0	0	0	0	0	0	0	0	0	0	0
XR_002338125.1	102167959	LOC102167959	lncRNA	0	0	0	0	0	0	0	0	0	0	0	0	0	0	0	0	0	0	0
XR_002340442.1	102160053	LOC102160053	lncRNA	0	0	0	0	0	0	0	0	0	0	0	0	0	0	0	1	0	0	0
NM_001145752.1	100271724	SLC22A4	mRNA	0	0	0	0	0	0	0	0	0	0	0	0	0	0	0	0	0	0	0
XM_021097098.1	100302694	CES5A	mRNA	0	0	0	0	0	0	0	0	0	0	0	0	0	1	0	0	0	0	0
XR_001299355.2	106505191	LOC106505191	lncRNA	0	0	0	0	0	0	0	0	1	0	0	0	0	0	0	0	0	0	0
XR_001301379.2	106506213	LOC106506213	lncRNA	0	0	0	0	0	0	0	0	0	0	1	0	0	0	0	0	0	0	0
XR_002344182.1	110260775	LOC110260775	lncRNA	0	0	0	0	0	0	0	0	1	0	0	0	0	0	0	0	0	0	0
XR_002337157.1	110256021	LOC110256021	lncRNA	0	0	0	0	0	0	0	0	0	0	0	0	0	0	0	0	0	0	0
XR_002341155.1	110258473	LOC110258473	lncRNA	0	0	0	0	1	0	0	0	0	0	0	0	0	0	0	0	0	0	0
XM_001925364.3	100153329	LOC100153329	mRNA	0	0	0	0	0	0	0	0	0	0	0	0	0	0	0	0	0	0	0
XM_005665343.3	100519720	MAPRE2	mRNA	0	0	0	0	0	0	0	0	0	0	0	0	0	0	0	0	0	0	0
XR_002336444.1	102157904	LOC102157904	lncRNA	0	0	0	0	0	0	0	0	0	0	0	0	0	0	0	0	0	0	0
XM_003132006.5	100520100	MAP2K4	mRNA	0	0	0	0	0	0	0	0	0	0	0	0	0	0	0	0	0	0	0
XR_002343771.1	110260459	LOC110260459	lncRNA	0	1	0	0	0	0	0	0	0	0	0	0	0	0	0	0	0	0	0
XM_003358564.4	100620415	SLC35G2	mRNA	0	0	0	0	0	0	0	0	0	0	0	0	0	0	0	0	0	0	0
NM_001123151.1	100144454	NRM	mRNA	0	0	0	0	0	0	0	0	0	0	0	0	0	0	0	0	0	0	0
XM_005667128.3	100627340	LOC100627340	mRNA	0	0	0	0	0	0	0	0	0	0	0	0	0	1	0	0	0	0	0
NM_001243803.1	100511023	NUDC	mRNA	0	0	0	0	0	0	0	0	0	0	0	1	0	0	0	0	0	0	0
XM_021097456.1	110261343	TMEM240	mRNA	0	0	0	0	0	0	0	0	0	0	0	0	0	0	0	0	0	0	0
XR_001301636.2	106506374	LOC106506374	lncRNA	0	0	0	0	0	0	0	0	0	0	0	0	0	0	0	0	0	0	0
XR_001303107.2	106507246	LOC106507246	lncRNA	0	0	0	0	0	0	0	0	0	0	1	0	0	0	0	0	0	0	0

RNA ID	Gene ID	Symbol	Type	novel-ssc-miR817-3p	novel-ssc-miR849-3p	novel-ssc-miR882-3p	novel-ssc-miR985-5p	novel-ssc-miR1126-5p	novel-ssc-miR1396-3p	ssc-miR-362	novel-ssc-miR16-5p	ssc-miR-375	ssc-miR-376a-3p	ssc-miR-493-5p	ssc-miR-194a-5p	ssc-miR-205	novel-ssc-miR239-5p	novel-ssc-miR397-5p	novel-ssc-miR507-5p	novel-ssc-miR563-3p	novel-ssc-miR568-3p	novel-ssc-miR682-3p
XR_001303546.2	106507516	LOC106507516	lncRNA	0	0	0	0	0	0	0	0	0	0	0	0	0	0	0	0	0	0	0
XR_002336300.1	110255664	LOC110255664	lncRNA	0	0	0	0	0	0	0	0	0	0	0	0	0	0	0	0	0	0	0
XR_002336542.1	110255752	LOC110255752	lncRNA	0	0	0	0	0	0	0	0	0	0	0	0	0	0	0	0	0	0	0
XR_002337686.1	110256299	LOC110256299	lncRNA	0	0	0	0	0	0	0	0	0	0	0	0	0	0	0	0	0	0	0
XR_001300145.2	106505585	LOC106505585	lncRNA	0	0	0	0	0	0	0	0	0	0	0	0	0	0	0	0	0	0	0
XR_002344948.1	110261123	LOC110261123	lncRNA	0	0	0	0	0	0	0	1	0	0	0	0	0	0	0	0	0	0	0
XR_002344676.1	110260998	LOC110260998	lncRNA	0	0	0	0	0	0	0	0	0	0	1	0	0	0	0	0	0	0	0
XM_003121205.3	100525016	SMLR1	mRNA	0	0	0	0	0	0	0	0	0	0	0	0	0	0	0	0	0	0	0
XR_002340682.1	102163484	LOC102163484	lncRNA	0	0	0	0	0	0	0	0	0	0	0	0	0	0	0	1	0	0	0
XM_021079561.1	497636	DMD	mRNA	0	0	0	0	0	0	0	0	0	0	0	0	0	1	0	0	0	0	0
XR_002341591.1	102164826	ISYNA1	lncRNA	0	0	0	0	0	0	0	1	0	0	0	0	0	0	0	0	0	0	0
XR_002340822.1	110257819	LOC110257819	lncRNA	0	0	0	0	0	0	0	0	0	0	1	0	0	0	0	0	0	0	0
XR_001298110.1	106504549	LOC106504549	lncRNA	0	1	0	0	0	0	0	0	0	0	0	0	0	0	0	0	0	0	0
XR_002340843.1	110257831	LOC110257831	lncRNA	0	0	0	0	0	0	0	0	0	0	0	0	0	0	0	0	0	0	0
XR_300629.3	102163894	LOC102163894	lncRNA	0	0	0	0	0	0	0	0	0	0	0	0	0	0	0	0	0	0	0
XM_021099082.1	100510914	NEO1	mRNA	1	0	0	0	0	0	0	0	0	0	0	0	0	0	0	0	0	0	0
XR_002345385.1	110261329	LOC110261329	lncRNA	0	0	0	0	0	0	0	0	0	0	1	0	0	0	0	0	0	0	0
XR_002337516.1	110256226	LOC110256226	lncRNA	0	0	0	0	0	0	0	0	0	0	0	0	0	0	0	0	0	0	0
XR_002340123.1	102159962	LOC102159962	lncRNA	0	0	0	0	0	0	0	0	0	0	0	0	0	0	0	0	0	0	0
XM_001926907.5	100156308	PSMD10	mRNA	0	0	0	0	0	0	0	0	0	0	0	0	0	0	0	0	0	0	0
NM_213885.1	396919	MAN1A1	mRNA	0	0	0	0	0	0	0	0	0	0	0	0	0	0	0	0	0	0	0
XR_002338697.1	106506052	LOC106506052	lncRNA	0	0	0	0	0	0	0	0	0	0	0	0	0	0	0	0	0	0	0
XR_002340869.1	100522115	LOC100522115	lncRNA	0	0	0	0	0	0	0	0	0	0	0	0	0	0	0	1	0	0	0
NM_001143692.2	100152339	ITPK1	mRNA	0	0	0	0	0	0	0	0	0	0	0	0	0	0	0	0	0	0	0
NM_001114281.1	100135665	RELA	mRNA	0	0	0	0	0	0	0	0	0	1	0	0	0	0	0	0	0	0	0
XM_021073105.1	100156262	PAPSS2	mRNA	0	0	0	0	0	0	0	0	0	0	0	0	0	0	0	0	0	0	0
XR_002336169.1	110255621	LOC110255621	lncRNA	1	0	0	0	0	0	0	0	0	0	0	0	0	0	0	0	0	0	0
XR_001298531.2	102166545	LOC102166545	lncRNA	0	0	0	0	0	0	0	0	0	0	0	0	0	0	0	0	0	0	0
XR_002339865.1	110257358	LOC110257358	lncRNA	0	0	0	0	1	0	0	0	0	0	0	0	0	0	0	0	0	0	0
XR_002336348.1	110255689	LOC110255689	lncRNA	0	0	0	0	0	0	0	0	0	0	0	0	0	0	0	0	0	0	0
XM_003134418.4	100523033	SPAG4	mRNA	0	0	0	0	0	0	0	0	0	1	0	0	0	0	0	0	0	0	0
XR_001308912.2	106510436	LOC106510436	lncRNA	0	0	0	0	0	0	0	0	0	0	0	0	0	0	0	0	0	0	0
XM_003482567.1	100739359	LOC100739359	mRNA	0	0	0	0	0	0	0	0	0	0	0	0	0	1	0	0	0	0	0
XM_003483535.4	100154776	PGAM1	mRNA	0	0	0	0	0	0	0	0	0	0	0	0	0	0	0	0	0	0	0
XM_003359830.5	100626618	CCNJL	mRNA	0	0	0	0	0	0	0	0	0	0	0	0	0	0	0	0	0	0	0
XR_002339976.1	110257414	LOC110257414	lncRNA	0	0	0	0	0	0	0	0	0	0	0	0	0	0	0	0	0	0	0
NM_001139471.1	100154728	APEX1	mRNA	0	0	0	0	0	0	0	0	0	0	0	0	0	0	0	0	0	0	0
XR_001301667.2	106506397	LOC106506397	lncRNA	0	0	0	0	0	0	0	0	0	0	0	0	0	0	0	1	0	0	0
XR_001307040.2	102159224	LOC102159224	lncRNA	0	0	0	0	0	0	0	0	0	0	0	0	0	0	0	0	0	0	0
XM_021066572.1	100622490	ST6GALNAC1	mRNA	1	0	0	0	0	0	0	0	0	0	0	0	0	0	0	0	0	0	0
XM_001925290.5	100152155	SMPD2	mRNA	0	0	0	0	0	0	0	0	0	0	0	0	0	0	0	0	0	0	0
XR_001306492.2	100737021	LOC100737021	lncRNA	0	0	0	0	0	0	0	0	0	0	0	0	0	0	0	0	0	0	0
XM_013992665.2	102160737	HTR1E	mRNA	0	0	0	0	0	0	0	0	0	0	0	0	0	1	0	0	0	0	0

RNA ID	Gene ID	Symbol	Type	novel-ssc-miR817-3p	novel-ssc-miR849-3p	novel-ssc-miR882-3p	novel-ssc-miR985-5p	novel-ssc-miR1126-5p	novel-ssc-miR1396-3p	ssc-miR-362	novel-ssc-miR16-5p	ssc-miR-375	ssc-miR-376a-3p	ssc-miR-493-5p	ssc-miR-194a-5p	ssc-miR-205	novel-ssc-miR239-5p	novel-ssc-miR397-5p	novel-ssc-miR507-5p	novel-ssc-miR563-3p	novel-ssc-miR568-3p	novel-ssc-miR682-3p
XM_021082275.1	110258984	LOC110258984	mRNA	0	0	0	0	0	0	0	0	0	0	0	0	0	0	0	0	0	0	0
XR_001305691.2	102166401	LOC102166401	lncRNA	0	0	0	0	0	0	0	0	0	0	0	0	0	0	0	0	0	0	0
XR_002343757.1	110260447	LOC110260447	lncRNA	0	0	0	0	0	0	0	1	0	0	0	0	0	0	0	0	0	0	0
XR_001306506.2	106509193	LOC106509193	lncRNA	0	0	0	0	0	0	0	0	0	0	0	0	0	0	0	0	0	0	0
XM_021075435.1	100514804	CFC1B	mRNA	0	0	0	0	0	0	0	0	0	0	0	0	0	1	0	0	0	0	0
XR_002337121.1	110256014	LOC110256014	lncRNA	0	0	0	0	0	0	0	0	0	0	0	0	0	0	0	0	0	0	0
XR_002340328.1	106508222	LOC106508222	lncRNA	0	0	0	0	0	0	0	0	0	0	0	0	0	0	0	0	0	0	0
XM_021099830.1	100155756	TMEM225B	mRNA	1	0	0	0	0	0	0	0	0	0	0	0	0	0	0	0	0	0	0
NM_001206441.1	733650	TAP2	mRNA	0	0	0	0	0	0	0	0	0	0	0	0	0	0	0	0	0	0	0
XR_002338713.1	110256767	LOC110256767	lncRNA	0	0	0	0	1	0	0	0	0	0	0	0	0	0	0	0	0	0	0
XR_001307073.2	106509487	LOC106509487	lncRNA	0	0	0	0	0	0	0	0	0	0	0	1	0	0	0	0	0	0	0
XM_003129584.2	100517905	LOC100517905	mRNA	0	0	0	0	0	0	0	0	0	0	0	0	0	0	0	0	0	0	0
XM_003359416.4	100622102	KIF5C	mRNA	0	0	0	0	0	0	0	0	0	0	0	0	0	0	0	0	0	0	0
NM_001348903.1	397672	CILP	mRNA	0	0	0	0	0	0	0	0	0	0	0	0	0	0	0	0	0	0	0
XR_002341788.1	106509487	LOC106509487	lncRNA	0	0	0	0	0	0	0	0	0	0	0	0	0	0	0	0	0	0	0
XR_002337537.1	110256233	LOC110256233	lncRNA	0	0	0	0	0	0	0	0	0	0	0	0	0	0	0	1	0	0	0
XR_002341806.1	110259419	LOC110259419	lncRNA	1	0	0	0	0	0	0	0	0	0	0	0	0	0	0	0	0	0	0
XM_001928015.5	396603	PPP3CB	mRNA	0	0	0	0	0	0	0	0	0	1	0	0	0	0	0	0	0	0	0
XM_001924600.5	100155018	ZNF451	mRNA	0	0	0	0	0	0	0	0	1	0	0	0	0	0	0	0	0	0	0
XM_021089348.1	100153039	NSMAF	mRNA	0	0	0	0	0	0	0	0	0	0	0	0	0	0	0	0	0	0	0
XR_300604.3	102165231	LOC102165231	lncRNA	0	0	0	0	0	0	0	0	0	0	0	0	0	0	0	0	0	0	0
XM_003355060.4	100628123	NPBWR1	mRNA	0	0	0	0	0	0	0	0	0	0	0	0	0	0	0	0	0	0	0
NM_214418.2	403155	SLC25A6	mRNA	0	0	0	0	0	0	0	0	0	0	0	1	0	0	0	0	0	0	0
XR_002335850.1	102161480	LOC102161480	lncRNA	0	0	0	0	0	0	0	1	0	0	0	0	0	0	0	0	0	0	0
XR_002345639.1	110261503	LOC110261503	lncRNA	1	0	0	0	0	0	0	0	0	0	0	0	0	0	0	0	0	0	0
XR_301706.3	102162727	LOC102162727	lncRNA	0	0	0	0	0	0	0	0	0	0	0	0	0	0	0	0	0	0	0
XR_002344083.1	110260726	LOC110260726	lncRNA	0	0	0	0	0	0	0	1	0	0	0	0	0	0	0	0	0	0	0
XR_002336138.1	110255587	LOC110255587	lncRNA	0	0	0	0	0	0	0	0	0	0	0	0	0	0	0	0	0	0	0
NM_001244966.1	100514424	SLC25A39	mRNA	0	0	0	0	0	0	0	0	1	0	0	0	0	0	0	0	0	0	0
XR_001301171.2	106506113	LOC106506113	lncRNA	0	0	0	0	1	0	0	0	0	0	0	0	0	0	0	0	0	0	0
NM_001123177.1	100144495	RAB33A	mRNA	0	0	0	0	0	0	0	0	0	1	0	0	0	0	0	0	0	0	0
XM_021096358.1	102166543	SAMD13	mRNA	0	0	0	0	0	0	0	0	0	0	0	0	0	1	0	0	0	0	0
XM_021086898.1	102157824	GINM1	mRNA	0	0	0	0	0	0	0	0	0	0	0	0	0	0	0	0	0	0	0
XR_002346148.1	110261690	LOC110261690	lncRNA	0	0	0	0	0	0	0	0	0	0	0	0	0	0	0	0	0	0	0
XR_001307288.2	106509619	LOC106509619	lncRNA	0	0	0	0	1	0	0	0	0	0	0	0	0	0	0	0	0	0	0
NM_214253.1	397487	PRM1	mRNA	0	0	0	0	0	0	0	0	1	0	0	0	0	0	0	0	0	0	0
XR_002345199.1	110261229	LOC110261229	lncRNA	0	0	0	0	0	0	0	0	0	0	0	0	0	0	0	0	0	0	0
XM_021091581.1	100154559	CALHM4	mRNA	0	0	0	0	0	0	0	0	0	0	0	0	0	0	0	0	0	0	0
URS0000BC677A	110260766	LOC110260766	lncRNA	0	0	0	0	0	0	0	0	0	0	0	0	0	0	0	0	0	0	0
URS0000BC9805	BGIG9823_26341		lncRNA	0	0	0	0	0	0	0	0	0	0	0	0	0	0	0	0	0	0	0
URS0000BC7DBE	102167218	LOC102167218	lncRNA	0	0	0	0	0	0	0	0	0	0	0	0	0	0	0	0	0	0	0
URS0000BC871E	102165318	LOC102165318	lncRNA	0	0	0	0	0	0	0	0	0	0	0	0	0	0	0	0	0	0	0
URS0000BC6273	BGIG9823_26302		lncRNA	0	0	0	0	0	0	0	0	0	0	0	0	0	0	0	0	0	0	0

RNA ID	Gene ID	Symbol	Type	novel-ssc-miR817-3p	novel-ssc-miR849-3p	novel-ssc-miR882-3p	novel-ssc-miR985-5p	novel-ssc-miR1126-5p	novel-ssc-miR1396-3p	ssc-miR-362	novel-ssc-miR16-5p	ssc-miR-375	ssc-miR-376a-3p	ssc-miR-493-5p	ssc-miR-194a-5p	ssc-miR-205	novel-ssc-miR239-5p	novel-ssc-miR397-5p	novel-ssc-miR507-5p	novel-ssc-miR563-3p	novel-ssc-miR568-3p	novel-ssc-miR682-3p
URS0000BC6496	BGIG9823_26337		lncRNA	0	0	0	0	0	0	0	0	1	0	0	0	0	0	0	0	0	0	0
URS0000BC6966	BGIG9823_26316		lncRNA	0	0	0	0	0	0	0	0	0	0	0	0	0	0	0	0	0	0	0
URS0000BC81FE	BGIG9823_26310		lncRNA	0	0	0	0	0	0	0	0	0	0	0	0	0	0	0	0	0	0	0
URS0000BC59B8	100525132	PDZD2	lncRNA	0	0	0	0	0	0	0	0	0	0	1	0	0	0	0	0	0	0	0
URS0000BC725F	106506163	LOC106506163	lncRNA	0	0	0	0	0	0	0	0	0	0	0	0	0	0	0	0	0	0	0
URS0000BC5DE3	110256465	LOC110256465	lncRNA	0	0	0	0	0	0	0	0	0	0	0	0	0	0	0	0	0	0	0
URS0000BC7E35	102157619	LOC102157619	lncRNA	0	0	0	0	0	0	0	0	0	0	0	0	0	0	0	0	0	0	0
URS0000BC4C4F	106510093	LOC106510093	lncRNA	0	0	0	0	0	0	0	1	0	0	0	0	0	0	0	0	0	0	0
URS0000BC46E5	BGIG9823_26314		lncRNA	0	0	0	0	0	0	0	0	0	0	0	0	0	0	0	0	0	0	0
URS0000BC8793	100525132	PDZD2	lncRNA	0	0	0	0	0	0	0	0	0	0	0	0	0	0	0	1	0	0	0
URS0000BC7B31	BGIG9823_26353		lncRNA	0	0	0	0	0	0	0	1	0	0	0	0	0	0	0	0	0	0	0
BGIR9823_88049	100621902	MIER1	mRNA	0	0	0	0	0	0	0	0	0	0	0	0	0	0	0	0	0	0	0
BGIR9823_87964	100737982	GAREM1	lncRNA	0	0	0	0	0	0	0	0	0	0	0	0	0	0	0	0	0	0	0
BGIR9823_81184	100523888	LOC100523888	lncRNA	0	0	0	0	0	0	0	0	0	0	0	0	0	0	0	0	0	0	0
BGIR9823_78564	100737904	LOC100737904	lncRNA	0	0	0	0	0	0	0	0	0	0	0	0	0	0	0	0	0	0	0
BGIR9823_89527	102158035	LOC102158035	mRNA	0	1	0	0	0	0	0	0	0	0	0	0	0	0	0	0	0	0	0
BGIR9823_84122	100152952	ZHX2	lncRNA	0	0	0	0	0	0	0	0	0	0	0	0	0	0	0	0	0	0	0
BGIR9823_79475	100625298	GABRG3	lncRNA	0	0	0	0	0	0	0	0	0	0	0	0	0	0	0	0	0	0	0
XM_021097022.1	100620736	TCF25	mRNA	0	0	0	0	0	0	0	0	0	0	0	0	0	1	0	0	0	0	0
BGIR9823_77873	100513326	SMAP1	lncRNA	0	0	0	0	0	0	0	0	0	0	0	0	0	0	0	0	0	0	0
BGIR9823_80029	100154341	URM1	lncRNA	0	0	0	0	0	0	0	0	0	0	0	0	1	0	0	0	0	0	0
BGIR9823_83646	100154232	GGH	mRNA	0	0	0	0	0	0	0	0	0	0	0	0	0	0	0	0	0	0	0
BGIR9823_97547	100153782	USP30	mRNA	0	0	0	0	0	0	0	0	0	0	0	0	0	0	0	0	0	0	0
BGIR9823_78040	100157196	CSNK1G1	lncRNA	0	0	0	0	0	0	0	0	0	0	0	0	1	0	0	0	0	0	0
BGIR9823_79960	100516174	SUSD1	lncRNA	0	0	0	0	0	0	0	0	0	0	0	0	0	0	0	0	0	0	0
BGIR9823_81702	100521611	FNIP1	lncRNA	0	0	0	0	0	0	0	0	0	0	0	0	0	0	0	0	0	0	0
BGIR9823_78735	100153669	TMEM38B	lncRNA	0	0	0	0	0	0	0	0	0	0	0	0	0	0	0	0	0	0	0
BGIR9823_80417	100516957	LOC100516957	lncRNA	0	0	0	0	0	0	1	0	0	0	0	0	0	0	0	0	0	0	0
BGIR9823_82071	100525811	C3H16orf71	lncRNA	0	0	0	0	0	0	0	0	0	0	0	0	1	0	0	0	0	0	0
BGIR9823_89210	100153184	KHDRBS2	lncRNA	0	0	0	0	0	0	0	0	0	0	0	0	0	0	0	0	0	0	0
BGIR9823_86411	100621774	UBE2M	lncRNA	0	0	0	0	0	0	0	0	0	0	0	0	0	0	0	0	0	0	0
BGIR9823_86823	100621518	RBBP8	mRNA	0	0	0	0	0	0	0	0	1	0	0	0	0	0	0	0	0	0	0
BGIR9823_93974	102164379	LOC102164379	lncRNA	0	0	0	0	0	0	0	0	0	0	0	0	0	0	0	0	0	0	0
BGIR9823_79858	654405	GNAQ	lncRNA	0	0	0	0	0	0	1	0	0	0	0	0	0	0	0	0	0	0	0
BGIR9823_97653	100154344	C14H22orf39	lncRNA	0	1	0	0	0	0	0	0	0	0	0	0	0	0	0	0	0	0	0
BGIR9823_90176	100511210	SEC31A	mRNA	0	1	0	0	0	0	0	0	0	0	0	0	0	0	0	0	0	0	0
BGIR9823_99273	100624231	NIPBL	lncRNA	0	0	0	0	0	0	0	0	0	0	0	0	0	1	0	0	0	0	0
BGIR9823_100693	100739403	KLHDC10	lncRNA	0	0	0	0	0	0	0	0	0	0	0	0	0	0	0	0	0	0	0
BGIR9823_79452	100512576	SPRED1	lncRNA	0	0	0	0	0	0	0	0	0	0	0	0	0	0	0	0	0	0	0
BGIR9823_82923	100525039	MCRIP2	lncRNA	0	0	0	0	0	0	0	0	0	0	0	0	0	0	0	1	0	0	0
BGIR9823_85610	110260725	LOC110260725	lncRNA	0	0	0	0	0	0	0	0	0	0	0	0	0	0	0	0	0	0	0
BGIR9823_79946	100155590	FRRS1L	lncRNA	0	0	0	0	0	0	0	0	0	0	0	0	0	0	0	0	0	0	0
XR_002335729.1	110255457	LOC110255457	lncRNA	0	1	0	0	0	0	0	0	0	0	0	0	0	0	0	0	0	0	0

RNA ID	Gene ID	Symbol	Type	novel-ssc-miR817-3p	novel-ssc-miR849-3p	novel-ssc-miR882-3p	novel-ssc-miR985-5p	novel-ssc-miR1126-5p	novel-ssc-miR1396-3p	ssc-miR-362	novel-ssc-miR16-5p	ssc-miR-375	ssc-miR-376a-3p	ssc-miR-493-5p	ssc-miR-194a-5p	ssc-miR-205	novel-ssc-miR239-5p	novel-ssc-miR397-5p	novel-ssc-miR507-5p	novel-ssc-miR563-3p	novel-ssc-miR568-3p	novel-ssc-miR682-3p
BGIR9823_79704	100155666	ATG14	lncRNA	0	0	0	0	0	1	0	0	0	0	0	0	0	0	0	0	0	0	0
BGIR9823_80049	414430	MED27	lncRNA	0	0	0	0	0	0	1	0	0	0	0	0	0	0	0	0	0	0	0
BGIR9823_84572	100519481	STXBP3	lncRNA	0	0	0	0	0	0	0	0	0	0	0	0	0	0	0	0	0	0	0
BGIR9823_87715	100517038	PUM1	lncRNA	0	0	0	0	0	0	0	0	0	0	0	0	0	0	0	0	0	0	0
BGIR9823_84360	100154639	PBX1	lncRNA	0	0	0	0	0	1	0	0	0	0	0	0	0	0	0	0	0	0	0
BGIR9823_101255	100154598	LAMP2	mRNA	0	0	0	0	0	0	0	0	0	0	0	0	0	0	0	0	0	0	0
BGIR9823_98698	100737861	CCNT2	mRNA	0	0	0	0	0	0	0	0	0	0	0	0	0	0	0	0	0	0	0
BGIR9823_81378	100511368	ZNF358	lncRNA	0	0	0	0	0	0	0	0	0	0	0	0	0	0	0	0	0	0	0
BGIR9823_100853	100156439	TCEANC	lncRNA	0	0	0	0	0	0	0	0	0	0	0	0	0	0	0	0	0	0	0
BGIR9823_88507	100153104	POLH	lncRNA	0	0	0	0	0	0	0	0	0	0	0	0	0	0	0	0	0	0	0
BGIR9823_96638	100153422	FAM222A	lncRNA	0	0	0	0	0	0	0	0	0	0	0	0	0	0	0	0	0	0	0
BGIR9823_79570	100512083	NEDD4L	lncRNA	0	0	0	0	0	0	1	0	0	0	0	0	0	0	0	0	0	0	0
BGIR9823_101653	100625138	LSS	lncRNA	0	0	0	0	0	0	0	0	0	0	0	0	0	0	0	0	0	0	0
BGIR9823_101268	100520416	TENM1	lncRNA	0	0	0	0	0	0	0	0	0	0	0	0	0	0	0	0	0	0	0
BGIR9823_78254	106506784	LOC106506784	lncRNA	0	0	0	0	0	0	0	0	0	0	0	0	0	0	0	0	0	0	0
BGIR9823_93514	100514316	CACNB1	lncRNA	0	0	0	0	0	0	0	0	0	0	0	0	0	0	0	0	0	0	0
BGIR9823_78057	100152749	CA12	lncRNA	0	0	0	0	0	0	0	1	0	0	0	0	0	0	0	0	0	0	0
BGIR9823_87011	100621520	YIPF1	lncRNA	0	0	0	0	0	0	0	0	0	0	0	0	0	0	0	0	0	0	0
BGIR9823_79577	100151784	CLPX	lncRNA	0	0	0	0	0	0	1	0	0	0	0	0	0	0	0	0	0	0	0
BGIR9823_83929	100519711	ST7L	lncRNA	0	0	0	0	0	0	0	0	0	0	0	0	0	0	0	0	0	0	0
BGIR9823_78689	100514147	LOC100514147	lncRNA	0	0	0	0	0	0	0	0	0	0	0	0	0	0	0	0	0	0	0
BGIR9823_80847	100515884	KDM3B	lncRNA	0	0	0	0	0	0	0	0	0	0	0	0	0	0	0	0	0	0	0
BGIR9823_100723	100511847	CPED1	mRNA	0	0	0	0	0	0	0	0	0	0	0	0	0	0	0	0	0	0	0
BGIR9823_80653	100521253	IQGAP2	lncRNA	0	0	0	0	0	0	0	0	0	0	0	0	0	0	0	0	0	0	0
BGIR9823_81419	100737330	NFIC	lncRNA	0	0	0	0	0	0	0	0	0	0	0	0	0	0	0	0	0	0	0
BGIR9823_78533	100626032	SGPP1	lncRNA	0	0	0	0	0	0	0	0	0	0	0	0	0	0	0	0	0	0	0
BGIR9823_91477	100511030	ALG8	mRNA	0	0	0	0	0	0	0	0	0	0	0	0	0	0	0	0	0	0	0
BGIR9823_85833	100517653	VEZT	lncRNA	0	0	0	0	0	0	0	0	0	0	1	0	0	0	0	0	0	0	0
BGIR9823_99752	494014	PRNP	mRNA	0	0	0	0	0	0	0	0	0	0	0	0	0	0	0	0	0	1	0
BGIR9823_86172	100620402	LOC100620402	lncRNA	0	0	0	0	0	1	0	0	0	0	0	0	0	0	0	0	0	0	0
BGIR9823_88817	396933	FUT8	lncRNA	0	0	0	0	0	0	0	0	0	0	0	0	0	0	0	0	0	0	0
BGIR9823_94863	100621119	GRM7	lncRNA	0	0	0	0	0	0	0	0	0	0	0	0	0	0	0	0	0	0	0
BGIR9823_82821	110259924	LOC110259924	lncRNA	0	0	0	0	0	0	0	0	0	0	0	0	1	0	0	0	0	0	0
BGIR9823_80743	100513914	FER	lncRNA	0	0	0	0	0	0	1	0	0	0	0	0	0	0	0	0	0	0	0
BGIR9823_86054	100516197	AMFR	mRNA	0	0	0	0	0	0	0	0	0	0	0	0	0	0	0	0	0	0	0
BGIR9823_101027	397245	ATP1B4	mRNA	0	0	0	0	0	0	0	0	0	0	0	0	0	0	0	0	0	0	0
BGIR9823_94279	100520151	GIT1	mRNA	0	0	0	0	0	0	0	0	0	0	0	0	0	0	0	0	0	0	0
BGIR9823_100258	100156548	ZHX3	lncRNA	0	0	0	0	0	0	0	0	0	0	0	0	0	0	0	0	0	0	0
BGIR9823_97925	100516879	CYP2C91	lncRNA	0	0	0	0	0	0	0	0	0	0	0	0	0	0	0	0	0	0	0
BGIR9823_81233	106508326	SLC25A42	lncRNA	0	0	0	0	0	0	0	0	0	0	0	0	0	0	0	0	0	0	0
BGIR9823_100939	100154376	KDM6A	lncRNA	0	0	0	0	0	0	0	0	0	0	0	0	0	0	0	0	0	0	0
BGIR9823_100604	100515515	GALNT11	lncRNA	0	0	0	0	0	0	0	0	0	0	0	0	0	0	0	0	0	0	0
BGIR9823_95197	100626969	LPP	lncRNA	0	0	0	0	0	0	0	0	0	0	0	0	0	0	0	0	0	0	0

RNA ID	Gene ID	Symbol	Type	novel-ssc-miR817-3p	novel-ssc-miR849-3p	novel-ssc-miR882-3p	novel-ssc-miR985-5p	novel-ssc-miR1126-5p	novel-ssc-miR1396-3p	ssc-miR-362	novel-ssc-miR16-5p	ssc-miR-375	ssc-miR-376a-3p	ssc-miR-493-5p	ssc-miR-194a-5p	ssc-miR-205	novel-ssc-miR239-5p	novel-ssc-miR397-5p	novel-ssc-miR507-5p	novel-ssc-miR563-3p	novel-ssc-miR568-3p	novel-ssc-miR682-3p
BGIR9823_87581	100514410	ICMT	mRNA	0	0	0	0	0	0	0	0	0	0	0	1	0	0	0	0	0	0	0
BGIR9823_81758	106509575	LOC106509575	lncRNA	0	0	0	0	0	0	0	0	0	0	0	0	0	0	0	0	0	0	0
BGIR9823_79445	100522721	RASGRP1	lncRNA	0	0	0	0	0	0	0	0	0	0	0	0	0	0	0	0	0	0	0
BGIR9823_95280	100623596	OSBPL11	mRNA	0	0	0	0	0	0	0	0	0	0	0	0	0	0	0	0	0	0	0
BGIR9823_88858	100155733	SUSD6	lncRNA	0	0	0	0	0	0	0	0	0	0	0	0	0	0	0	0	0	0	0
BGIR9823_93640	100512984	GGNBP2	lncRNA	0	0	0	0	1	0	0	0	0	0	0	0	0	0	0	0	0	0	0
BGIR9823_82696	100626099	TBL2	mRNA	0	0	0	0	0	0	0	0	0	0	0	0	0	0	0	0	0	0	0
BGIR9823_85289	100519488	MOV10L1	lncRNA	0	0	0	0	0	1	0	0	0	0	0	0	0	0	0	0	0	0	0
BGIR9823_90661	100738411	CCSER1	lncRNA	0	0	0	0	0	0	0	0	0	0	0	0	0	0	0	0	0	0	0
BGIR9823_101099	100624032	LOC100624032	mRNA	0	0	0	0	0	0	0	0	1	0	0	0	0	0	0	0	0	0	0
BGIR9823_83245	100170144	SLC30A6	lncRNA	0	0	0	0	0	0	0	0	0	0	0	0	0	0	0	0	0	0	0
BGIR9823_83495	654326	RNF19A	lncRNA	0	0	0	0	0	0	0	0	0	0	0	0	1	0	0	0	0	0	0
BGIR9823_100642	100519065	UBN2	lncRNA	0	0	0	0	0	0	0	0	0	0	0	0	0	0	0	0	0	0	0
BGIR9823_88851	100158119	SLC39A9	lncRNA	0	0	0	0	0	0	0	0	0	0	0	0	0	0	0	0	0	0	0
BGIR9823_82981	100524615	ARID5A	lncRNA	0	0	0	0	0	0	0	0	0	0	0	0	0	0	0	0	0	0	0
BGIR9823_84758	100157699	TMEM184B	lncRNA	0	0	0	0	0	0	0	0	0	0	0	0	0	0	0	0	0	0	0
BGIR9823_101227	102165634	LOC102165634	lncRNA	0	0	0	0	0	0	0	0	0	0	0	0	0	0	0	0	0	0	0
BGIR9823_82094	100736616	HCFC1R1	lncRNA	0	0	0	0	0	1	0	0	0	0	0	0	0	0	0	0	0	0	0
BGIR9823_87647	100517288	HSPG2	mRNA	0	0	0	0	0	0	0	0	0	0	0	0	0	0	0	0	0	0	0
BGIR9823_85318	110260629	LOC110260629	lncRNA	0	0	0	0	0	0	0	0	0	0	0	0	0	0	0	0	0	0	0
BGIR9823_97476	100513180	FAM216A	lncRNA	0	0	0	0	0	0	0	0	0	0	0	0	0	0	0	0	0	0	0
BGIR9823_100900	100621199	IL1RAPL1	lncRNA	0	0	0	0	0	0	0	0	0	0	0	0	0	0	0	0	0	0	0
BGIR9823_90410	397001	POLR2B	lncRNA	0	0	0	0	0	0	0	0	0	0	0	0	0	0	0	0	0	0	0
XM_005659173.3	100156257	NHSL1	mRNA	0	0	0	0	0	0	0	0	0	0	0	0	0	0	0	0	0	0	0
BGIR9823_90244	100513100	LDB2	lncRNA	0	0	0	0	0	0	0	0	0	0	0	0	0	0	0	0	0	0	0
BGIR9823_78438	100152969	CDKL1	lncRNA	0	0	0	0	0	0	0	0	0	0	0	0	0	0	0	0	0	0	0
BGIR9823_100731	100512382	KCND2	lncRNA	0	0	0	0	0	0	0	0	0	0	0	0	0	0	0	0	0	0	0
BGIR9823_82740	100623146	KIF22	lncRNA	0	0	0	0	0	0	0	0	0	0	0	0	0	0	0	0	0	0	0
BGIR9823_81512	100523721	FAF2	lncRNA	0	0	0	0	0	0	0	0	0	0	0	0	0	0	0	0	0	0	0
BGIR9823_78400	102162695	LOC102162695	lncRNA	0	0	0	0	0	0	0	0	0	0	0	0	0	0	0	0	0	0	0
BGIR9823_100980	110257855	LOC110257855	lncRNA	0	0	0	0	0	0	0	0	0	0	0	0	0	0	0	0	0	0	0
BGIR9823_97780	100037299	P4HA1	lncRNA	0	0	0	0	0	0	0	0	0	0	0	0	0	0	0	0	0	0	0
BGIR9823_79896	100519814	RNF38	lncRNA	0	0	0	0	0	0	0	0	0	0	0	0	1	0	0	0	0	0	0
XM_021065940.1	110255868	ATP11A	mRNA	1	0	0	0	0	0	0	0	0	0	0	0	0	0	0	0	0	0	0
BGIR9823_101616	110258914	LOC110258914	lncRNA	0	0	0	0	0	0	0	0	0	0	0	0	0	0	0	0	0	0	0
BGIR9823_86084	100626752	LOC100626752	lncRNA	0	0	0	0	0	0	0	0	0	0	0	0	0	0	0	1	0	0	0
BGIR9823_78267	100512756	MEIS2	lncRNA	0	0	0	0	0	0	0	0	0	0	0	0	0	0	0	0	0	0	0
BGIR9823_80601	110259385	LOC110259385	lncRNA	0	0	0	0	0	0	0	0	0	0	0	0	0	0	0	0	0	0	0
BGIR9823_87318	100620768	CEP89	lncRNA	0	0	0	0	0	0	0	0	0	0	0	0	0	0	0	0	0	0	0
BGIR9823_80608	100521252	CLK4	lncRNA	0	0	0	0	0	0	0	0	0	0	0	0	0	0	0	0	0	0	0
BGIR9823_100082	100621622	SFRP1	mRNA	0	0	0	0	0	0	0	1	0	0	0	0	0	0	0	0	0	0	0
BGIR9823_80402	396557	PGLYRP2	lncRNA	0	0	0	0	0	0	0	0	0	0	0	0	0	0	0	0	0	0	0
BGIR9823_101117	100518815	TXLNG	lncRNA	0	0	0	0	0	0	0	0	0	0	0	0	0	0	0	0	0	0	0

RNA ID	Gene ID	Symbol	Type	novel-ssc-miR817-3p	novel-ssc-miR849-3p	novel-ssc-miR882-3p	novel-ssc-miR985-5p	novel-ssc-miR1126-5p	novel-ssc-miR1396-3p	ssc-miR-362	novel-ssc-miR16-5p	ssc-miR-375	ssc-miR-376a-3p	ssc-miR-493-5p	ssc-miR-194a-5p	ssc-miR-205	novel-ssc-miR239-5p	novel-ssc-miR397-5p	novel-ssc-miR507-5p	novel-ssc-miR563-3p	novel-ssc-miR568-3p	novel-ssc-miR682-3p
BGIR9823_78216	100511488	TTBK2	lncRNA	0	0	0	0	0	0	0	0	0	0	0	0	0	0	0	0	0	0	0
BGIR9823_78392	100526149	PNN	lncRNA	0	0	0	0	0	0	0	0	0	0	0	0	0	0	0	0	0	0	0
BGIR9823_101570	110258602	LOC110258602	lncRNA	0	0	0	0	0	0	0	0	0	0	0	0	0	0	0	0	0	0	0
BGIR9823_93977	100512557	DDX42	mRNA	0	0	0	0	0	0	0	0	0	0	0	0	0	0	0	0	0	0	0
BGIR9823_93667	100294712	SUZ12	lncRNA	0	0	0	0	0	0	0	0	0	0	0	0	0	0	0	0	0	0	0
BGIR9823_94057	397387	THRA	lncRNA	0	0	0	0	1	0	0	0	0	0	0	0	0	0	0	0	0	0	0
BGIR9823_100931	100515940	DDX3X	lncRNA	0	0	0	0	0	0	0	0	0	0	0	0	0	0	0	0	0	0	0
BGIR9823_87442	100518311	NOSIP	lncRNA	0	0	0	0	0	0	0	0	0	0	0	0	0	0	0	0	0	0	0
BGIR9823_92178	100126283	AQP7	mRNA	0	0	0	0	0	0	0	0	0	0	0	1	0	0	0	0	0	0	0
BGIR9823_101406	110258215	LOC110258215	lncRNA	0	0	0	0	0	0	0	0	0	0	0	0	0	0	0	0	0	0	0
BGIR9823_92246	100737056	FRMD4A	lncRNA	0	0	0	0	0	0	0	0	0	0	0	0	0	0	0	0	0	0	0
BGIR9823_82297	100522368	EXOC6B	mRNA	0	0	0	0	0	0	0	0	0	0	0	0	0	0	0	0	0	0	0
BGIR9823_78079	100154204	MYO1E	lncRNA	0	1	0	0	0	0	0	0	0	0	0	0	0	0	0	0	0	0	0
BGIR9823_93720	100517676	GOSR1	lncRNA	0	0	0	0	0	0	0	0	0	0	0	0	0	0	0	0	0	0	0
BGIR9823_95969	100524586	MED12L	lncRNA	0	0	0	0	0	0	0	0	0	0	0	0	0	0	0	1	0	0	0
BGIR9823_95388	100517310	ROBO1	mRNA	0	0	0	0	0	0	0	0	0	0	0	1	0	0	0	0	0	0	0
BGIR9823_92590	100521678	MLLT10	lncRNA	0	0	0	0	0	0	0	0	0	0	0	0	0	0	0	1	0	0	0
BGIR9823_89920	100621006	LOC100621006	lncRNA	0	0	0	0	0	0	0	0	0	0	0	0	0	0	0	0	0	0	0
BGIR9823_87305	100521133	DNAJA2	lncRNA	0	0	0	0	0	0	0	0	0	0	0	0	0	0	0	0	0	0	0
BGIR9823_83514	100153417	C4H8orf37	lncRNA	0	0	0	0	0	0	0	0	0	0	0	0	0	0	0	0	0	0	0
BGIR9823_85521	100525288	OSBPL8	lncRNA	0	0	0	0	0	0	0	0	0	0	0	0	0	0	0	0	0	0	0
BGIR9823_90750	100522939	ART5	mRNA	1	0	0	0	0	0	0	0	0	0	0	0	0	0	0	0	0	0	0
BGIR9823_85923	100579143	HSBP1	lncRNA	0	0	0	0	0	0	0	0	0	0	0	0	0	0	0	1	0	0	0
BGIR9823_91101	100519438	FAM133B	lncRNA	0	0	0	0	0	0	0	0	0	0	0	0	0	0	0	0	0	0	0
BGIR9823_100281	100153535	PCIF1	lncRNA	0	0	0	0	0	0	0	0	0	0	0	0	0	0	0	0	0	0	0
BGIR9823_100276	397528	SDC4	lncRNA	0	0	0	0	0	0	0	0	0	0	0	0	0	0	0	1	0	0	0
BGIR9823_100467	100517505	CTTNBP2	lncRNA	0	0	0	0	0	0	0	0	0	0	0	0	0	0	0	0	0	0	0
BGIR9823_100689	110257546	LOC110257546	lncRNA	0	0	0	0	0	0	0	0	0	0	0	0	0	0	0	0	0	0	0
BGIR9823_94010	100514424	SLC25A39	lncRNA	0	0	0	0	0	0	0	0	0	0	0	0	0	0	0	1	0	0	0
BGIR9823_100247	100153296	RBL1	lncRNA	0	0	0	0	0	0	0	0	0	0	0	0	0	0	0	0	0	0	0
BGIR9823_97926	100516879	CYP2C91	lncRNA	0	0	0	0	0	0	0	0	0	0	0	0	0	0	0	0	0	0	0
BGIR9823_78152	100155516	DMXL2	lncRNA	0	0	0	0	0	0	0	0	0	0	0	0	0	0	0	0	0	0	0
BGIR9823_101623	BGIG9823_26354		lncRNA	0	0	0	0	0	0	0	0	0	0	0	0	0	0	0	0	0	0	0
BGIR9823_98164	110257053	LOC110257053	lncRNA	0	0	0	0	0	0	0	0	0	0	0	0	0	0	0	0	0	0	0
BGIR9823_100903	100621199	IL1RAPL1	lncRNA	0	0	0	0	0	0	1	0	0	0	0	0	0	0	0	0	0	0	0
BGIR9823_77846	100736796	ADGRB3	lncRNA	0	0	0	0	0	0	0	0	0	0	0	0	0	0	0	0	0	0	0
BGIR9823_90432	100520317	LOC100520317	mRNA	0	0	0	0	0	0	0	0	0	0	0	0	0	0	0	0	0	0	0
BGIR9823_95429	396658	IFNAR1	lncRNA	0	0	0	0	0	0	0	0	1	0	0	0	0	0	0	0	0	0	0
BGIR9823_80320	100521123	SBF2	lncRNA	0	0	0	0	0	0	0	0	0	0	0	0	0	0	0	0	0	0	0
BGIR9823_81929	100519894	PRKRIP1	lncRNA	0	0	0	0	0	0	0	0	0	0	0	0	0	0	0	0	0	0	0
BGIR9823_100403	100524054	WDR91	lncRNA	0	0	0	0	0	0	0	0	0	0	0	0	0	0	0	0	0	0	1
BGIR9823_91472	100217384	RSF1	lncRNA	0	0	0	0	0	0	0	0	0	0	0	0	0	0	0	0	0	0	0
BGIR9823_78771	102164650	LOC102164650	lncRNA	0	0	0	0	0	1	0	0	0	0	0	0	0	0	0	0	0	0	0

RNA ID	Gene ID	Symbol	Type	novel-ssc-miR817-3p	novel-ssc-miR849-3p	novel-ssc-miR882-3p	novel-ssc-miR985-5p	novel-ssc-miR1126-5p	novel-ssc-miR1396-3p	ssc-miR-362	novel-ssc-miR16-5p	ssc-miR-375	ssc-miR-376a-3p	ssc-miR-493-5p	ssc-miR-194a-5p	ssc-miR-205	novel-ssc-miR239-5p	novel-ssc-miR397-5p	novel-ssc-miR507-5p	novel-ssc-miR563-3p	novel-ssc-miR568-3p	novel-ssc-miR682-3p
BGIR9823_77749	100155211	SLC18B1	lncRNA	0	0	0	0	0	0	0	0	0	0	0	0	0	0	0	0	1	0	0
BGIR9823_80489	100622165	ACER1	lncRNA	0	0	0	0	0	0	0	0	0	0	0	0	0	0	0	0	0	0	0
BGIR9823_100204	100514995	COMMD7	lncRNA	0	0	0	0	0	0	0	0	0	0	0	0	0	0	0	0	0	0	0
BGIR9823_77830	100736796	ADGRB3	lncRNA	0	0	0	0	0	0	1	0	0	0	0	0	0	0	0	0	0	0	0
BGIR9823_78118	100152054	RFX7	lncRNA	0	0	0	0	0	0	0	0	0	0	0	0	0	0	0	0	0	0	0
BGIR9823_80498	106509452	PTPRS	mRNA	0	0	0	0	0	0	0	0	0	0	0	0	0	0	0	0	0	0	0
BGIR9823_83059	100524080	KCMF1	lncRNA	0	0	0	0	0	0	0	0	0	0	0	0	0	0	0	0	0	0	0
BGIR9823_89241	100155423	FKBP5	lncRNA	0	0	0	0	0	0	0	0	0	0	0	0	0	0	0	0	0	0	0
BGIR9823_86626	399532	FABP3	lncRNA	0	0	0	0	0	0	0	0	0	0	0	0	0	0	0	0	0	0	0
BGIR9823_80136	100512584	PLA2G16	lncRNA	0	0	0	0	0	0	0	0	0	0	0	0	0	0	0	0	0	0	0
BGIR9823_78764	100621961	CDC26	lncRNA	0	0	0	0	0	0	0	0	0	0	0	0	0	0	0	0	0	0	0
BGIR9823_100787	100622432	CBX3	lncRNA	0	0	0	0	0	0	0	0	0	0	0	0	0	0	0	0	0	0	0
BGIR9823_81517	100523721	FAF2	lncRNA	0	0	0	0	0	0	0	0	0	0	0	0	0	0	0	0	0	0	0
BGIR9823_94928	100622196	IQSEC1	mRNA	0	0	0	0	0	0	0	0	0	0	0	0	0	0	0	0	0	0	0
BGIR9823_97066	100155963	MMRN2	lncRNA	0	0	0	0	0	0	0	0	0	0	0	0	0	0	0	0	0	0	0
BGIR9823_86005	100515835	RSPRY1	lncRNA	0	0	0	0	0	0	0	0	0	0	0	0	0	0	0	0	0	0	0
BGIR9823_81824	100514516	CSNK1A1	lncRNA	0	0	0	0	0	0	0	0	0	0	0	0	0	0	0	0	0	0	0
BGIR9823_100617	100523293	KCNH2	lncRNA	0	0	0	0	0	0	0	0	0	0	0	0	0	0	0	0	0	0	0
XR_002337475.1	106507029	LOC106507029	lncRNA	0	0	0	0	0	0	0	0	0	0	0	0	0	0	0	0	0	0	0
BGIR9823_99577	100523519	CCDC152	lncRNA	0	0	0	0	0	0	0	0	0	0	0	0	0	0	0	1	0	0	0
BGIR9823_79582	100151784	CLPX	lncRNA	0	0	0	0	0	0	0	0	0	0	0	0	0	0	0	0	0	0	0
BGIR9823_90043	397616	PDE5A	lncRNA	0	1	0	0	0	0	0	0	0	0	0	0	0	0	0	0	0	0	0
BGIR9823_101350	100621019	ANAPC2	mRNA	0	0	0	0	0	0	0	0	0	0	0	0	0	0	0	0	0	0	0
BGIR9823_77999	100512572	WDR7	lncRNA	0	0	0	0	0	0	0	0	0	0	0	0	0	0	0	0	0	0	0
BGIR9823_80986	100294703	KDM2A	lncRNA	0	0	0	0	0	0	0	0	0	0	0	0	0	0	0	0	0	0	0
BGIR9823_82031	110259918	ITPRIPL2	lncRNA	0	0	0	0	0	0	0	0	0	0	0	0	0	0	0	0	0	0	0
BGIR9823_100386	100515632	KDM7A	lncRNA	0	0	0	1	0	0	0	0	0	0	0	0	0	0	0	0	0	0	0
BGIR9823_78769	100157179	ZNF618	lncRNA	0	0	0	0	0	0	0	0	0	0	0	0	0	0	0	0	0	0	0
BGIR9823_77679	100513634	MPC1	lncRNA	0	0	0	0	0	0	0	0	0	0	0	0	0	0	0	0	0	0	0
BGIR9823_101316	100624156	CD99	lncRNA	0	0	0	0	0	0	0	0	0	0	0	0	0	0	0	0	0	0	0
BGIR9823_79248	100738627	DYM	lncRNA	0	0	0	0	0	0	0	0	0	0	0	0	0	0	0	0	0	0	0
BGIR9823_78478	100152019	PELI2	lncRNA	0	0	0	0	0	0	0	0	0	0	0	0	0	0	0	0	0	0	0
BGIR9823_95265	100622532	FYTTD1	lncRNA	0	1	0	0	0	0	0	0	0	0	0	0	0	0	0	0	0	0	0
BGIR9823_84388	100155270	NIT1	mRNA	0	0	0	0	0	0	0	0	0	0	0	0	0	0	0	0	0	0	0
BGIR9823_101285	100622228	ARHGEF6	mRNA	0	0	0	0	0	0	0	0	0	0	0	0	0	0	0	0	0	0	0
BGIR9823_100255	100156548	ZHX3	lncRNA	0	0	0	0	0	0	0	0	0	0	0	0	0	0	0	0	0	0	0
BGIR9823_94455	100151869	OXNAD1	lncRNA	0	0	0	0	0	0	0	0	1	0	0	0	0	0	0	0	0	0	0
BGIR9823_78812	100155133	PTGES2	lncRNA	0	0	0	0	0	0	0	0	0	0	0	0	0	0	0	0	0	0	0
BGIR9823_80806	100519080	LYRM7	lncRNA	0	0	0	0	0	0	0	0	0	0	0	0	0	0	0	0	0	0	0
BGIR9823_101247	100519287	KLHL13	mRNA	0	0	0	0	0	0	0	0	1	0	0	0	0	0	0	0	0	0	0
BGIR9823_79520	102162853	LOC102162853	lncRNA	0	0	0	0	0	0	0	0	0	0	0	0	0	0	0	0	0	0	0
BGIR9823_95143	407739	SOX2	lncRNA	0	0	0	0	0	0	0	0	1	0	0	0	0	0	0	0	0	0	0
BGIR9823_95008	100620055	PLSCR1	lncRNA	0	1	0	0	0	0	0	0	0	0	0	0	0	0	0	0	0	0	0

RNA ID	Gene ID	Symbol	Type	novel-ssc-miR817-3p	novel-ssc-miR849-3p	novel-ssc-miR882-3p	novel-ssc-miR985-5p	novel-ssc-miR1126-5p	novel-ssc-miR1396-3p	ssc-miR-362	novel-ssc-miR16-5p	ssc-miR-375	ssc-miR-376a-3p	ssc-miR-493-5p	ssc-miR-194a-5p	ssc-miR-205	novel-ssc-miR239-5p	novel-ssc-miR397-5p	novel-ssc-miR507-5p	novel-ssc-miR563-3p	novel-ssc-miR568-3p	novel-ssc-miR682-3p
BGIR9823_100815	110257576	LOC110257576	lncRNA	0	0	0	0	0	0	0	0	0	0	0	0	0	0	0	0	0	0	0
BGIR9823_83902	110260470	LOC110260470	lncRNA	0	0	0	0	0	0	0	0	0	0	0	0	0	0	0	0	0	0	0
BGIR9823_96518	100623262	SLC15A4	mRNA	0	0	0	0	0	0	0	0	0	0	0	0	0	0	0	0	0	0	0
BGIR9823_101546	110258600	LOC110258600	lncRNA	0	0	0	0	0	0	0	0	0	0	0	0	0	0	1	0	0	0	0
BGIR9823_79803	100154484	RCL1	lncRNA	0	0	0	0	0	0	1	0	0	0	0	0	0	0	0	0	0	0	0
BGIR9823_78229	100154984	TMEM87A	mRNA	0	0	0	0	0	0	0	0	0	0	0	0	0	0	0	0	0	0	0
BGIR9823_78085	100154204	MYO1E	lncRNA	0	0	0	0	0	0	0	0	1	0	0	0	0	0	0	0	0	0	0
BGIR9823_84094	106509960	NSMCE2	lncRNA	0	0	0	0	0	0	0	0	0	0	0	0	1	0	0	0	0	0	0
BGIR9823_80335	102166675	RNF187	lncRNA	0	0	0	0	0	0	1	0	0	0	0	0	0	0	0	0	0	0	0
BGIR9823_101281	100516171	GPC3	lncRNA	0	0	0	0	0	0	0	0	0	0	0	0	0	0	0	0	0	0	0
BGIR9823_83886	102166500	LOC102166500	lncRNA	0	0	0	0	0	0	0	0	0	0	0	0	1	0	0	0	0	0	0
BGIR9823_78367	100520530	CORO2B	lncRNA	0	0	0	0	0	0	1	0	0	0	0	0	0	0	0	0	0	0	0
BGIR9823_81305	102166940	LOC102166940	lncRNA	0	0	0	0	0	0	0	0	0	0	0	0	0	0	0	0	0	0	0
BGIR9823_100832	100520285	ZNF777	lncRNA	0	0	0	0	0	0	0	0	0	0	0	0	0	0	0	0	0	0	0
BGIR9823_100583	102162331	KRBA1	lncRNA	0	0	0	0	0	0	0	0	0	0	0	0	0	0	0	0	0	0	0
BGIR9823_85020	100626912	BHLHE41	mRNA	0	0	0	0	0	0	0	0	0	0	0	0	0	1	0	0	0	0	0
BGIR9823_100375	100514612	BRAF	mRNA	0	0	0	0	0	0	0	0	0	0	0	0	0	1	0	0	0	0	0
BGIR9823_92109	100521742	NEK7	mRNA	0	0	0	0	0	0	0	0	0	0	0	1	0	0	0	0	0	0	0
BGIR9823_100703	100514931	AHCYL2	lncRNA	0	0	0	1	0	0	0	0	0	0	0	0	0	0	0	0	0	0	0
BGIR9823_79724	396697	ESR2	lncRNA	0	0	0	0	0	0	0	0	0	0	1	0	0	0	0	0	0	0	0
BGIR9823_101638	110259113	LOC110259113	lncRNA	0	0	0	0	0	0	0	0	0	0	0	0	0	0	0	0	0	0	0
BGIR9823_92471	100517963	RMI1	lncRNA	0	1	0	0	0	0	0	0	0	0	0	0	0	0	0	0	0	0	0
BGIR9823_100226	654300	UQCC	lncRNA	0	0	0	0	0	0	0	0	0	0	0	0	0	0	0	0	0	0	0
BGIR9823_86749	100624877	PPP4R1	mRNA	0	1	0	0	0	0	0	0	0	0	0	0	0	0	0	0	0	0	0
BGIR9823_97541	100157435	UBE3B	mRNA	0	0	0	0	0	0	0	0	0	0	0	0	0	0	0	0	0	0	0
BGIR9823_80565	102164292	FSTL3	lncRNA	0	0	0	0	0	0	0	0	0	0	0	0	0	0	0	0	0	0	0
BGIR9823_96818	100157092	TTC13	mRNA	0	0	0	0	0	0	0	0	0	0	0	0	0	0	0	0	0	0	0
BGIR9823_84745	100521449	NPTXR	mRNA	0	0	0	0	0	0	0	0	0	0	0	0	0	0	0	0	0	0	0
BGIR9823_79788	102168004	UHRF2	lncRNA	0	0	0	0	0	0	0	0	0	0	0	0	1	0	0	0	0	0	0
BGIR9823_78692	100515356	GLIPR2	lncRNA	0	0	0	0	0	0	0	0	0	0	0	0	0	0	0	0	0	0	0
BGIR9823_79323	100621557	TCF12	lncRNA	0	0	0	0	0	0	1	0	0	0	0	0	0	0	0	0	0	0	0
BGIR9823_101455	110258361	PACS2	lncRNA	0	0	0	0	0	0	0	0	0	0	0	0	0	0	0	0	0	0	0
BGIR9823_94552	100523331	NKTR	lncRNA	0	0	0	0	0	0	0	0	0	0	0	0	0	0	0	0	0	0	0
BGIR9823_100126	100519631	TASP1	lncRNA	0	0	0	0	0	0	0	0	0	0	0	0	0	0	0	0	0	0	0
BGIR9823_100406	106506765	LOC106506765	lncRNA	0	0	0	0	0	0	0	0	0	0	0	0	0	0	0	0	0	0	0
BGIR9823_80256	102168147	LOC102168147	lncRNA	0	0	0	0	0	0	0	0	0	0	0	0	0	0	0	0	0	0	0
BGIR9823_99608	397251	PLK2	mRNA	0	0	0	0	0	0	0	0	0	0	0	0	0	0	0	0	0	0	0
BGIR9823_94040	397550	STAT5A	mRNA	0	0	0	0	0	0	0	0	0	0	0	0	0	0	0	0	0	0	0
BGIR9823_100411	414383	CHCHD3	lncRNA	0	0	0	0	0	0	0	0	0	0	0	0	0	0	0	0	0	0	0
BGIR9823_93144	100174955	KLF12	lncRNA	0	0	0	0	0	0	0	0	0	0	0	0	0	0	0	0	0	0	0
BGIR9823_81306	102166940	LOC102166940	lncRNA	0	0	0	0	0	0	0	0	0	0	0	0	0	0	0	0	0	0	0
BGIR9823_79214	100515594	MTO1	lncRNA	0	0	0	0	0	0	0	0	0	0	0	0	0	0	0	0	0	0	0
XR_001302600.2	106506942	LOC106506942	lncRNA	0	0	0	0	0	0	0	0	0	0	0	0	0	0	0	0	0	0	0

RNA ID	Gene ID	Symbol	Type	novel-ssc-miR817-3p	novel-ssc-miR849-3p	novel-ssc-miR882-3p	novel-ssc-miR985-5p	novel-ssc-miR1126-5p	novel-ssc-miR1396-3p	ssc-miR-362	novel-ssc-miR16-5p	ssc-miR-375	ssc-miR-376a-3p	ssc-miR-493-5p	ssc-miR-194a-5p	ssc-miR-205	novel-ssc-miR239-5p	novel-ssc-miR397-5p	novel-ssc-miR507-5p	novel-ssc-miR563-3p	novel-ssc-miR568-3p	novel-ssc-miR682-3p
BGIR9823_99840	100519805	TBC1D20	lncRNA	0	0	0	0	0	0	0	0	0	0	0	0	0	0	0	0	0	0	0
BGIR9823_100155	100519522	NINL	lncRNA	0	0	1	0	0	0	0	0	0	0	0	0	0	0	0	0	0	0	0
BGIR9823_78548	100511733	PLAA	lncRNA	0	0	0	0	0	0	0	0	0	0	0	0	0	0	0	0	0	0	0
BGIR9823_81728	100524487	AFF4	lncRNA	0	0	0	0	0	0	0	0	0	0	0	0	0	0	0	0	0	0	0
BGIR9823_82871	100516601	ZNF597	lncRNA	0	0	0	0	0	0	0	0	0	0	0	0	0	0	0	0	0	0	0
BGIR9823_83578	100157134	ZNF704	lncRNA	0	0	0	0	0	0	0	1	0	0	0	0	0	0	0	0	0	0	0
BGIR9823_78456	100625719	CGRRF1	lncRNA	0	0	0	0	0	0	0	0	0	0	0	0	0	0	0	0	0	0	0
BGIR9823_100241	100154500	SAMHD1	lncRNA	0	0	0	0	0	0	0	0	0	0	0	0	0	0	0	0	0	0	0
XR_002344503.1	110260914	LOC110260914	lncRNA	0	0	0	0	0	0	0	0	0	0	0	0	0	0	0	0	0	0	0
BGIR9823_93124	100174955	KLF12	lncRNA	0	0	0	0	0	0	0	0	0	0	0	0	0	0	0	0	0	0	0
BGIR9823_78918	100627455	SNX9	mRNA	0	0	0	0	0	0	0	0	0	0	0	0	0	0	0	0	0	0	0
BGIR9823_100427	100626356	UBE2H	lncRNA	0	0	0	0	0	0	0	0	0	0	0	0	0	0	0	0	0	0	0
BGIR9823_79882	100156839	FAM166B	mRNA	0	0	0	0	0	0	0	0	0	0	0	0	0	0	0	0	0	0	0
BGIR9823_87816	100626395	NAPG	lncRNA	0	0	0	0	0	1	0	0	0	0	0	0	0	0	0	0	0	0	0
BGIR9823_79529	100154633	PHLPP1	lncRNA	0	0	0	0	0	0	0	0	0	0	0	0	0	0	0	0	0	0	0
BGIR9823_80355	100620218	BORCS8	lncRNA	0	0	0	0	0	0	0	0	0	0	0	0	0	0	0	0	0	0	0
BGIR9823_101515	102157759	LOC102157759	lncRNA	0	0	0	0	0	0	0	0	0	0	0	0	0	0	0	0	0	0	0
BGIR9823_88688	100152987	HEATR5A	mRNA	0	0	0	0	0	0	0	0	0	0	0	0	0	0	0	0	0	0	0
BGIR9823_78932	100514379	FBXO5	lncRNA	0	0	0	0	0	0	0	0	0	0	0	0	1	0	0	0	0	0	0
BGIR9823_78476	100152019	PELI2	lncRNA	0	0	0	0	0	0	0	0	0	0	0	0	0	0	0	0	0	0	0
BGIR9823_92851	100523918	LMO7	mRNA	0	1	0	0	0	0	0	0	0	0	0	0	0	0	0	0	0	0	0
BGIR9823_100326	110257473	LOC110257473	lncRNA	0	0	0	0	0	0	0	0	0	0	0	0	0	0	0	0	0	0	0
BGIR9823_87757	100621544	RNF19B	mRNA	0	0	0	0	0	0	0	0	0	0	0	0	0	0	0	0	0	0	0
BGIR9823_99279	100517617	WDR70	lncRNA	0	1	0	0	0	0	0	0	0	0	0	0	0	0	0	0	0	0	0
BGIR9823_78488	100152019	PELI2	lncRNA	0	0	0	0	0	0	1	0	0	0	0	0	0	0	0	0	0	0	0
BGIR9823_91993	100738612	ATF3	mRNA	0	0	0	0	0	0	0	0	0	0	0	0	0	0	0	0	0	0	0
BGIR9823_94664	102167511	SMIM4	lncRNA	0	0	0	0	0	0	0	0	0	0	0	0	0	1	0	0	0	0	0
BGIR9823_79100	100156082	FBXL4	lncRNA	0	0	0	0	0	0	0	0	0	0	0	0	0	0	0	0	0	0	0
BGIR9823_101197	100156239	VSIG4	mRNA	1	0	0	0	0	0	0	0	0	0	0	0	0	0	0	0	0	0	0
BGIR9823_100442	100192443	POT1	lncRNA	0	0	0	0	0	0	0	0	0	0	0	0	0	0	0	0	0	0	0
BGIR9823_78713	100152263	ANP32B	lncRNA	0	0	0	0	0	0	1	0	0	0	0	0	0	0	0	0	0	0	0
BGIR9823_77769	100153609	PTPRK	lncRNA	0	0	0	0	0	0	0	0	0	0	0	0	0	0	0	0	0	0	0
BGIR9823_78654	100515003	SMC5	mRNA	0	0	0	0	0	0	0	0	0	0	0	0	0	0	0	0	0	0	0
BGIR9823_95994	100512989	VEPH1	lncRNA	0	0	0	0	0	0	0	0	0	0	0	0	0	0	0	0	0	0	0
BGIR9823_100398	100521697	MTPN	lncRNA	0	0	0	0	0	0	0	0	0	0	0	0	0	0	0	0	0	0	0
BGIR9823_77692	110259915	LOC110259915	lncRNA	0	0	0	0	0	0	0	0	0	0	0	0	0	0	0	0	1	0	0
BGIR9823_100510	100738688	HERPUD2	lncRNA	0	0	1	0	0	0	0	0	0	0	0	0	0	0	0	0	0	0	0
BGIR9823_101594	110258799	LOC110258799	lncRNA	0	0	0	0	0	0	0	0	0	0	0	0	0	0	0	0	0	0	0
BGIR9823_100619	100736760	GIMAP8	lncRNA	0	0	0	0	0	0	0	0	0	0	0	0	0	0	0	0	0	0	0
BGIR9823_98052	100153733	RAB11FIP2	mRNA	0	0	0	0	0	0	0	0	0	0	0	1	0	0	0	0	0	0	0
BGIR9823_79578	100151784	CLPX	lncRNA	0	0	0	0	0	0	0	0	0	0	0	0	0	0	0	0	0	0	0
BGIR9823_82021	100628086	METTL9	lncRNA	0	0	0	0	0	0	0	0	0	0	0	0	0	0	0	0	0	0	0
BGIR9823_100265	100520338	LOC100520338	lncRNA	0	0	0	0	0	0	0	0	0	0	0	0	0	0	0	0	0	0	0

RNA ID	Gene ID	Symbol	Type	novel-ssc-miR817-3p	novel-ssc-miR849-3p	novel-ssc-miR882-3p	novel-ssc-miR985-5p	novel-ssc-miR1126-5p	novel-ssc-miR1396-3p	ssc-miR-362	novel-ssc-miR16-5p	ssc-miR-375	ssc-miR-376a-3p	ssc-miR-493-5p	ssc-miR-194a-5p	ssc-miR-205	novel-ssc-miR239-5p	novel-ssc-miR397-5p	novel-ssc-miR507-5p	novel-ssc-miR563-3p	novel-ssc-miR568-3p	novel-ssc-miR682-3p
BGIR9823_88390	100124376	SKIV2L	mRNA	0	0	0	0	0	0	0	0	0	0	0	0	0	0	0	0	0	0	0
BGIR9823_78231	100154984	TMEM87A	lncRNA	0	0	0	0	0	0	0	0	0	0	0	0	0	0	0	0	0	0	0
BGIR9823_84096	106509960	NSMCE2	lncRNA	0	0	0	0	0	0	0	0	0	0	0	0	0	0	0	0	0	0	0
BGIR9823_81704	100521611	FNIP1	lncRNA	0	0	0	0	0	0	0	0	0	0	0	0	0	0	0	0	0	0	0
BGIR9823_79498	100736682	TJP1	lncRNA	0	0	0	0	0	0	0	0	0	0	0	0	0	0	0	0	0	0	0
BGIR9823_100070	100157832	SGCZ	lncRNA	0	0	0	0	0	0	0	0	0	0	0	0	0	0	0	0	0	0	0
BGIR9823_87139	106510467	LOC106510467	lncRNA	0	0	0	0	0	1	0	0	0	0	0	0	0	0	0	0	0	0	0
BGIR9823_100722	100514329	LOC100514329	lncRNA	0	0	0	0	0	0	0	0	0	0	0	0	0	0	0	0	0	0	0
BGIR9823_82833	106509697	CIITA	lncRNA	0	0	0	0	0	0	0	0	0	0	0	0	1	0	0	0	0	0	0
BGIR9823_100488	100518941	TMEM168	lncRNA	0	0	0	0	0	0	0	0	0	0	0	0	0	0	0	0	0	0	0
BGIR9823_101179	100517625	FAM120C	lncRNA	0	0	0	0	0	0	1	0	0	0	0	0	0	0	0	0	0	0	0
BGIR9823_100981	100513513	SLC16A2	mRNA	0	0	0	0	0	0	0	0	1	0	0	0	0	0	0	0	0	0	0
BGIR9823_85418	102163508	LOC102163508	lncRNA	0	0	0	0	0	0	0	0	0	0	0	0	0	0	0	0	0	0	0
BGIR9823_100250	100516472	KIAA1755	lncRNA	0	0	0	0	0	0	0	0	0	0	0	0	0	0	0	0	0	0	0
BGIR9823_100877	100523478	CNKSR2	lncRNA	0	0	0	0	0	0	0	0	0	0	0	0	0	0	0	0	0	0	0
BGIR9823_84851	100523964	LOC100523964	mRNA	0	0	0	0	0	0	0	0	0	0	0	1	0	0	0	0	0	0	0
BGIR9823_79979	100737979	CDK5RAP2	mRNA	0	0	0	0	0	0	0	0	0	0	0	0	0	0	0	0	0	0	0
BGIR9823_85878	100622883	NAV3	mRNA	0	0	0	0	0	0	0	0	1	0	0	0	0	0	0	0	0	0	0
BGIR9823_87603	100620358	ZBTB17	mRNA	0	0	0	0	0	0	0	0	0	0	0	0	0	0	0	0	0	0	0
BGIR9823_81340	100622538	DNM2	lncRNA	0	0	0	0	0	0	0	0	0	0	0	0	0	0	0	0	0	0	0
BGIR9823_93496	100521398	SMARCE1	lncRNA	0	0	0	0	0	0	0	0	0	0	0	0	0	0	0	0	0	0	0
BGIR9823_86377	100621391	SSC5D	mRNA	0	0	0	0	0	0	0	0	0	0	0	0	0	0	0	0	0	0	0
BGIR9823_85565	110260712	LOC110260712	lncRNA	0	0	0	0	0	0	0	0	0	0	1	0	0	0	0	0	0	0	0
BGIR9823_95828	100511410	PDZRN3	lncRNA	0	0	0	0	0	0	0	0	0	0	0	0	0	0	0	0	0	0	0
BGIR9823_93148	100174955	KLF12	lncRNA	0	0	0	0	1	0	0	0	0	0	0	0	0	0	0	0	0	0	0
BGIR9823_101568	110258600	LOC110258600	lncRNA	0	0	0	0	0	0	0	0	0	0	0	0	0	0	0	0	0	0	0
BGIR9823_79012	100738102	RNF217	lncRNA	0	0	0	0	0	0	0	0	0	0	0	0	0	0	0	0	0	0	0
XM_021091420.1	100523786	PACSIN2	mRNA	0	0	0	0	0	0	0	0	0	0	0	0	0	0	0	0	0	0	0
BGIR9823_100565	100516888	GLI3	lncRNA	0	0	0	0	0	0	0	0	0	0	0	0	0	0	0	0	0	0	0
BGIR9823_78022	100157196	CSNK1G1	lncRNA	0	0	0	0	0	0	0	0	0	0	0	0	0	0	0	0	0	0	0
BGIR9823_78883	100512199	ERMARD	lncRNA	0	0	0	0	0	0	0	0	0	0	0	0	0	0	0	0	0	0	0
BGIR9823_84618	100157977	FNBP1L	lncRNA	0	0	0	0	0	1	0	0	0	0	0	0	0	0	0	0	0	0	0
BGIR9823_97044	100158106	NRG3	lncRNA	0	0	0	0	0	0	0	0	0	0	0	0	0	0	0	0	0	0	0
BGIR9823_99699	100513766	G3BP1	mRNA	0	0	0	0	0	0	0	0	0	0	0	0	0	0	0	0	0	0	0
BGIR9823_87685	100512789	WASF2	lncRNA	0	0	0	0	0	0	0	0	0	0	0	0	0	0	0	0	0	0	0
BGIR9823_100122	100519631	TASP1	lncRNA	0	0	0	0	0	0	0	0	0	0	0	0	0	0	0	0	0	0	0
BGIR9823_100289	100738810	SLC13A3	lncRNA	0	0	0	0	0	0	0	0	0	0	0	0	0	0	0	0	0	0	0
BGIR9823_80121	100737568	SSSCA1	lncRNA	0	0	0	0	0	0	0	0	0	0	0	0	0	0	0	0	0	0	0
BGIR9823_86301	100628160	SNRNP70	mRNA	0	0	0	0	0	0	0	0	0	0	0	0	0	0	0	0	0	0	0
BGIR9823_100376	100514612	BRAF	lncRNA	0	0	0	0	0	0	0	0	0	0	0	0	0	0	0	0	0	0	0
BGIR9823_80749	100514631	MAN2A1	lncRNA	0	0	0	0	0	0	1	0	0	0	0	0	0	0	0	0	0	0	0
BGIR9823_101511	110258473	LOC110258473	lncRNA	0	0	0	0	0	0	0	0	0	0	0	0	0	0	0	0	0	0	0
BGIR9823_100755	100620722	ZNF277	lncRNA	0	0	0	0	0	0	0	0	0	0	0	0	0	0	0	0	0	0	0

RNA ID	Gene ID	Symbol	Type	novel-ssc-miR817-3p	novel-ssc-miR849-3p	novel-ssc-miR882-3p	novel-ssc-miR985-5p	novel-ssc-miR1126-5p	novel-ssc-miR1396-3p	ssc-miR-362	novel-ssc-miR16-5p	ssc-miR-375	ssc-miR-376a-3p	ssc-miR-493-5p	ssc-miR-194a-5p	ssc-miR-205	novel-ssc-miR239-5p	novel-ssc-miR397-5p	novel-ssc-miR507-5p	novel-ssc-miR563-3p	novel-ssc-miR568-3p	novel-ssc-miR682-3p
BGIR9823_82116	100624598	MEIOB	lncRNA	0	0	0	0	0	0	0	0	0	0	0	0	0	0	0	0	0	0	0
BGIR9823_101016	100520165	TMEM164	lncRNA	0	0	0	0	0	0	0	0	0	0	0	0	0	0	0	0	0	0	0
BGIR9823_90026	110262158	LOC110262158	lncRNA	0	0	0	0	0	0	0	0	0	0	0	0	0	0	0	0	0	0	0
BGIR9823_94087	100515340	LOC100515340	lncRNA	0	0	0	0	0	0	0	0	0	0	0	0	0	0	0	0	0	0	0
BGIR9823_89845	100516092	GNPDA2	lncRNA	0	0	0	0	0	0	0	0	0	0	1	0	0	0	0	0	0	0	0
BGIR9823_99243	100521757	ZNF622	lncRNA	0	0	0	0	0	0	0	0	0	0	0	0	0	0	0	0	0	0	0
BGIR9823_91934	100196908	TOR3A	lncRNA	0	0	0	0	0	0	0	0	0	0	0	0	0	0	0	0	0	0	0
BGIR9823_80152	100620658	HNRNPUL2	mRNA	0	0	0	0	0	0	0	0	0	0	0	0	0	0	0	0	0	0	0
BGIR9823_95348	100513438	NAA50	lncRNA	0	0	0	0	0	0	0	0	0	0	0	0	0	1	0	0	0	0	0
BGIR9823_86872	102164512	ZNF397	lncRNA	0	0	0	0	0	0	0	0	0	0	0	0	0	0	0	0	0	0	0
BGIR9823_85545	100525288	OSBPL8	lncRNA	0	0	0	0	0	0	0	0	0	0	0	0	0	0	0	0	0	0	0
BGIR9823_92701	100739805	CDK8	lncRNA	0	0	0	0	0	0	0	0	0	0	0	0	0	0	0	0	0	0	0
BGIR9823_79943	100626033	CTNNAL1	lncRNA	0	0	0	0	0	0	0	0	0	0	0	0	0	0	0	0	0	0	0
BGIR9823_100365	100739295	WDR86	lncRNA	0	0	1	0	0	0	0	0	0	0	0	0	0	0	0	0	0	0	0
BGIR9823_82500	100524371	STRN	lncRNA	0	0	0	0	0	0	0	0	0	0	0	0	0	0	0	0	0	0	0
BGIR9823_83316	397176	TAF1B	lncRNA	0	0	0	0	0	0	0	0	0	0	0	0	0	0	0	0	0	0	0
BGIR9823_82589	110260059	CYS1	lncRNA	0	0	0	0	0	0	0	0	1	0	0	0	0	0	0	0	0	0	0
BGIR9823_100212	100157872	PXMP4	lncRNA	0	0	0	0	0	0	0	0	0	0	0	0	0	0	0	0	0	0	0
BGIR9823_84315	106510091	SPIDR	mRNA	0	0	0	0	0	0	0	0	0	0	0	0	0	0	0	0	0	0	0
BGIR9823_85039	100625270	ST8SIA1	mRNA	0	0	0	0	0	0	0	0	0	0	0	0	0	0	0	0	0	0	0
BGIR9823_79988	396733	GGTA1P	lncRNA	0	0	0	0	0	0	0	0	0	0	0	0	0	0	0	1	0	0	0
BGIR9823_81636	100625008	GIN1	lncRNA	0	0	0	0	0	0	0	0	0	0	0	0	1	0	0	0	0	0	0
BGIR9823_96851	100522707	ZNF25	lncRNA	0	0	0	0	0	0	0	0	0	0	0	0	0	0	0	0	0	0	0
BGIR9823_98026	733691	NRAP	mRNA	0	0	0	0	0	0	0	0	0	0	0	0	0	0	0	0	0	0	0
BGIR9823_91942	100510921	STX6	mRNA	1	0	0	0	0	0	0	0	0	0	0	0	0	0	0	0	0	0	0
BGIR9823_101516	102157759	LOC102157759	lncRNA	0	0	0	0	0	0	0	0	0	0	0	0	0	0	0	0	0	0	0
BGIR9823_79269	100152360	CCDC68	lncRNA	0	0	0	0	0	0	0	0	0	0	0	0	0	0	0	0	0	0	0
BGIR9823_95434	100737549	ITSN1	mRNA	1	0	0	0	0	0	0	0	0	0	0	0	0	0	0	0	0	0	0
BGIR9823_81890	100621608	FOPNL	lncRNA	0	0	0	0	0	0	0	1	0	0	0	0	0	0	0	0	0	0	0
BGIR9823_86166	100739461	ZNF570	lncRNA	0	0	0	0	0	0	0	0	0	0	0	0	0	0	0	0	0	0	0
BGIR9823_81903	102162731	ZKSCAN1	mRNA	0	0	0	0	0	0	0	0	0	0	0	0	0	0	0	0	0	1	0
BGIR9823_84182	100153957	KCNS2	mRNA	0	0	0	0	0	0	0	0	0	0	0	0	0	0	0	0	0	0	0
BGIR9823_78637	100155691	AK3	lncRNA	0	0	0	0	0	0	0	0	0	0	0	0	0	0	0	0	0	0	0
BGIR9823_79359	100157164	GABPB1	lncRNA	0	0	0	0	0	0	0	0	0	0	0	0	0	0	0	0	0	0	0
BGIR9823_81535	100520548	WDR41	lncRNA	0	0	0	0	0	0	0	0	0	0	0	0	0	0	0	0	0	0	0
BGIR9823_86229	100516271	DEDD2	mRNA	0	0	0	0	0	0	0	0	0	0	0	0	0	0	0	0	0	0	0
BGIR9823_81434	110259374	LOC110259374	lncRNA	0	0	0	0	0	0	0	0	0	0	0	0	0	0	0	0	0	0	0
BGIR9823_100426	100626356	UBE2H	lncRNA	0	0	0	0	0	0	0	0	0	0	0	0	0	0	0	0	0	0	0
BGIR9823_100415	100620243	PLXNA4	lncRNA	0	0	0	0	0	0	0	0	0	0	0	0	0	0	0	0	0	0	0
BGIR9823_100894	100621199	IL1RAPL1	lncRNA	0	0	1	0	0	0	0	0	0	0	0	0	0	0	0	0	0	0	0
BGIR9823_95922	100622559	PIK3CB	lncRNA	0	0	0	0	0	0	0	0	0	0	0	0	0	0	0	1	0	0	0
BGIR9823_82064	100736832	GRIN2A	lncRNA	0	0	0	0	0	0	0	0	0	0	0	0	0	0	0	0	0	0	0
BGIR9823_100306	110257429	LOC110257429	lncRNA	0	0	0	0	0	0	0	0	0	0	0	0	0	0	0	0	0	0	0

RNA ID	Gene ID	Symbol	Type	novel-ssc-miR817-3p	novel-ssc-miR849-3p	novel-ssc-miR882-3p	novel-ssc-miR985-5p	novel-ssc-miR1126-5p	novel-ssc-miR1396-3p	ssc-miR-362	novel-ssc-miR16-5p	ssc-miR-375	ssc-miR-376a-3p	ssc-miR-493-5p	ssc-miR-194a-5p	ssc-miR-205	novel-ssc-miR239-5p	novel-ssc-miR397-5p	novel-ssc-miR507-5p	novel-ssc-miR563-3p	novel-ssc-miR568-3p	novel-ssc-miR682-3p
BGIR9823_81302	100736972	LOC100736972	lncRNA	0	0	0	0	0	0	0	0	0	0	0	0	0	0	0	0	0	0	0
BGIR9823_100478	100621338	BMT2	lncRNA	0	0	0	0	0	0	0	0	0	0	0	0	0	0	0	0	0	0	0
BGIR9823_86034	100623274	CTCF	mRNA	0	0	0	0	0	0	0	0	0	0	0	0	0	0	0	0	0	0	0
BGIR9823_78972	100155445	VTA1	lncRNA	0	0	0	0	0	0	0	0	0	0	0	0	0	0	0	0	0	0	0
BGIR9823_78078	100154204	MYO1E	lncRNA	0	0	0	0	0	0	0	0	0	0	0	0	0	0	0	0	0	0	0
BGIR9823_82851	100522863	RBFOX1	mRNA	0	1	0	0	0	0	0	0	0	0	0	0	0	0	0	0	0	0	0
BGIR9823_87122	100515140	KIAA0513	lncRNA	0	0	0	0	0	0	0	1	0	0	0	0	0	0	0	0	0	0	0
BGIR9823_97004	100154626	ADK	lncRNA	0	0	0	0	0	0	0	0	0	0	0	0	0	0	0	0	0	0	0
BGIR9823_90785	100524160	ACER3	lncRNA	0	0	0	0	0	0	0	0	0	0	0	0	0	0	0	0	0	0	0
BGIR9823_92442	100513615	C10H9orf3	mRNA	0	0	0	0	0	0	0	0	0	0	0	0	0	0	0	0	0	0	0
BGIR9823_90875	100622590	BIRC3	lncRNA	0	0	0	0	0	0	0	0	0	0	1	0	0	0	0	0	0	0	0
BGIR9823_101409	110258214	LOC110258214	lncRNA	0	0	0	0	0	0	0	0	0	0	0	0	0	0	0	0	0	0	0
BGIR9823_101574	102164761	LOC102164761	lncRNA	0	0	0	0	0	0	0	0	0	0	0	0	0	0	0	0	0	0	0
BGIR9823_81206	100519588	USP47	lncRNA	0	0	0	0	0	0	0	0	0	0	0	0	0	0	0	0	0	0	0
BGIR9823_82146	100737615	LUC7L	lncRNA	0	0	0	0	0	0	0	0	0	0	0	0	0	0	0	0	0	0	0
BGIR9823_88950	100620189	NRXN3	lncRNA	0	0	0	0	0	0	0	0	0	0	0	0	0	0	0	0	0	0	0
BGIR9823_100904	100621199	IL1RAPL1	lncRNA	0	0	0	0	0	0	0	0	0	0	0	0	0	0	0	0	0	0	0
BGIR9823_95076	100622923	PPM1L	lncRNA	0	0	0	0	0	0	0	0	0	0	0	0	0	0	0	0	0	0	0
BGIR9823_100269	102159476	LOC102159476	lncRNA	0	0	0	0	0	0	0	0	0	0	0	0	0	0	0	0	0	0	0
BGIR9823_101628	100519072	LOC100519072	lncRNA	0	0	0	0	0	0	0	0	0	0	0	0	0	0	0	0	0	0	1
BGIR9823_101612	100521600	LOC100521600	lncRNA	0	0	0	0	0	0	0	0	0	0	0	0	0	0	0	0	1	0	0
BGIR9823_101400	110258215	LOC110258215	lncRNA	0	0	1	0	0	0	0	0	0	0	0	0	0	0	0	0	0	0	0
BGIR9823_92255	100514676	ACBD5	lncRNA	0	0	0	0	0	0	0	0	0	0	0	0	0	0	0	0	0	0	0
BGIR9823_85922	100516196	TAF1C	lncRNA	0	0	0	0	0	1	0	0	0	0	0	0	0	0	0	0	0	0	0
BGIR9823_81946	100525688	BUD23	mRNA	0	0	0	0	0	0	0	0	0	0	0	0	0	0	0	0	0	0	0
BGIR9823_80177	106504153	AHNAK	mRNA	0	0	0	0	0	0	0	0	0	0	0	0	0	0	0	0	0	0	0
BGIR9823_88439	106504047	ZNF76	mRNA	0	0	0	0	0	0	0	0	0	0	0	0	0	0	0	0	0	0	0
BGIR9823_81354	100517998	MYO1F	lncRNA	0	0	0	0	0	0	0	0	0	0	0	0	0	0	0	0	0	0	0
BGIR9823_80921	100329221	PPARGC1B	mRNA	0	1	0	0	0	0	0	0	0	0	0	0	0	0	0	0	0	0	0
BGIR9823_77718	100625026	PPIL4	lncRNA	0	0	0	0	0	0	0	0	0	0	0	0	0	0	0	0	0	0	0
BGIR9823_100514	100623678	NT5C3A	lncRNA	0	0	0	0	0	0	0	0	0	0	0	0	0	0	0	0	0	0	0
BGIR9823_78774	396733	GGTA1P	lncRNA	0	0	0	0	0	0	1	0	0	0	0	0	0	0	0	0	0	0	0
BGIR9823_101033	100037300	XIAP	lncRNA	0	0	0	0	0	0	0	0	0	0	0	0	0	0	0	0	0	0	0
BGIR9823_100816	110257601	LOC110257601	lncRNA	0	0	0	0	0	0	0	0	0	0	0	0	0	0	0	0	0	0	0
BGIR9823_90686	733582	CDS1	lncRNA	0	0	0	0	0	0	0	0	0	0	0	0	0	0	0	0	0	0	0
BGIR9823_91751	110255475	LOC110255475	lncRNA	0	0	0	0	0	0	0	0	0	0	0	0	0	0	0	1	0	0	0
NM_001244541.1	100513376	GMPPB	mRNA	0	0	0	0	0	0	0	0	0	0	0	0	0	0	0	0	0	0	0
BGIR9823_81475	100620238	LOC100620238	lncRNA	0	0	0	0	0	0	0	0	0	0	0	0	0	0	0	0	0	0	0
BGIR9823_100582	102162331	KRBA1	lncRNA	0	0	1	0	0	0	0	0	0	0	0	0	0	0	0	0	0	0	0
BGIR9823_101332	100625415	EIF1AY	lncRNA	0	0	0	0	0	0	0	0	0	0	0	0	0	0	0	0	0	0	0
BGIR9823_97502	595128	OAS2	lncRNA	0	0	0	0	0	0	0	1	0	0	0	0	0	0	0	0	0	0	0
BGIR9823_83701	100153809	ALDH9A1	mRNA	0	0	0	0	0	0	0	0	0	0	0	0	0	1	0	0	0	0	0
BGIR9823_80203	100516953	MPEG1	lncRNA	0	0	0	0	0	0	0	0	0	0	0	0	1	0	0	0	0	0	0

RNA ID	Gene ID	Symbol	Type	novel-ssc-miR817-3p	novel-ssc-miR849-3p	novel-ssc-miR882-3p	novel-ssc-miR985-5p	novel-ssc-miR1126-5p	novel-ssc-miR1396-3p	ssc-miR-362	novel-ssc-miR16-5p	ssc-miR-375	ssc-miR-376a-3p	ssc-miR-493-5p	ssc-miR-194a-5p	ssc-miR-205	novel-ssc-miR239-5p	novel-ssc-miR397-5p	novel-ssc-miR507-5p	novel-ssc-miR563-3p	novel-ssc-miR568-3p	novel-ssc-miR682-3p
BGIR9823_99394	100517687	BDP1	lncRNA	0	0	0	0	0	0	0	0	0	0	1	0	0	0	0	0	0	0	0
BGIR9823_85850	102159120	LOC102159120	lncRNA	0	0	0	0	0	0	0	0	0	0	0	0	0	0	0	0	0	0	0
BGIR9823_83931	100519711	ST7L	mRNA	0	0	0	0	0	0	0	0	0	0	0	0	0	0	0	0	0	0	0
BGIR9823_93274	100513428	CEP131	lncRNA	0	0	0	0	1	0	0	0	0	0	0	0	0	0	0	0	0	0	0
BGIR9823_78717	100192437	ERP44	lncRNA	0	0	0	0	0	0	0	0	0	0	0	0	0	0	0	0	0	0	0
BGIR9823_81192	100514626	PIK3C2A	lncRNA	0	0	0	0	0	0	0	0	0	0	0	0	0	0	0	0	0	0	0
BGIR9823_77955	100153597	IBTK	lncRNA	0	0	0	0	0	0	1	0	0	0	0	0	0	0	0	0	0	0	0
BGIR9823_89270	100156900	BTBD9	mRNA	0	0	0	0	0	0	0	0	0	0	0	0	0	0	0	0	0	0	0
BGIR9823_98700	100512563	MGAT5	mRNA	0	0	0	0	0	0	0	0	0	0	0	0	0	0	0	0	0	0	0
BGIR9823_88596	110261555	SEMA4B	lncRNA	0	0	0	0	0	0	0	0	0	0	0	0	0	0	0	0	0	0	0
BGIR9823_85735	110260764	YAF2	lncRNA	0	0	0	0	1	0	0	0	0	0	0	0	0	0	0	0	0	0	0
BGIR9823_89296	100153071	TRERF1	lncRNA	0	0	0	0	0	1	0	0	0	0	0	0	0	0	0	0	0	0	0
BGIR9823_79093	100154222	EPHA7	lncRNA	0	0	0	0	0	0	0	0	0	0	0	0	0	0	0	0	0	0	0
BGIR9823_84443	100144448	OAZ3	lncRNA	0	1	0	0	0	0	0	0	0	0	0	0	0	0	0	0	0	0	0
BGIR9823_82885	100192445	PDPK1	lncRNA	0	0	0	0	0	0	0	0	0	0	0	0	0	0	0	0	0	0	0
BGIR9823_88750	100522330	SLC7A8	lncRNA	0	0	0	0	0	0	0	0	0	0	0	0	0	0	0	0	0	0	0
BGIR9823_77991	102159046	LOC102159046	lncRNA	0	0	0	0	0	0	0	0	0	0	0	0	0	0	0	0	1	0	0
BGIR9823_88964	100157303	ZC3H14	mRNA	0	1	0	0	0	0	0	0	0	0	0	0	0	0	0	0	0	0	0
BGIR9823_78191	102161909	LOC102161909	lncRNA	0	0	0	0	0	0	0	0	0	0	0	0	0	0	0	0	1	0	0
BGIR9823_81917	100620791	TRIP6	mRNA	0	0	0	0	0	0	0	0	0	0	0	0	0	0	0	0	0	0	0
BGIR9823_82685	110259888	LOC110259888	lncRNA	0	0	0	0	0	0	0	0	0	0	1	0	0	0	0	0	0	0	0
BGIR9823_84081	100153657	PHF20L1	mRNA	0	0	0	0	0	0	0	0	0	0	0	0	0	0	0	0	0	0	0
BGIR9823_83050	100628224	VAMP5	lncRNA	0	0	0	0	0	0	0	0	0	0	0	0	0	0	0	0	0	0	0
BGIR9823_98423	397285	ITGAV	mRNA	0	0	0	0	0	0	0	0	0	0	0	0	0	0	0	0	0	0	0
BGIR9823_97702	100155475	HNRNPF	mRNA	0	0	0	0	0	0	0	0	0	0	0	1	0	0	0	0	0	0	0
BGIR9823_93940	397293	KCNJ2	lncRNA	0	0	0	0	0	0	0	0	0	0	0	0	0	0	0	0	0	0	0
BGIR9823_87613	100521974	FBXO42	lncRNA	0	0	0	0	0	0	0	0	0	0	0	0	0	0	0	0	0	0	0
BGIR9823_94936	100155306	SEC61A1	lncRNA	0	0	0	0	0	0	0	0	0	0	0	0	0	0	0	0	0	0	0
BGIR9823_100651	100519065	UBN2	lncRNA	0	0	0	0	0	0	0	0	0	0	0	0	0	0	0	0	0	0	0
BGIR9823_84118	100737543	TBC1D31	lncRNA	0	0	0	0	0	0	0	0	0	0	0	0	0	0	0	0	0	0	0
BGIR9823_79608	100523347	UACA	lncRNA	0	0	0	0	0	0	0	0	0	0	0	0	0	0	0	0	0	0	0
BGIR9823_95065	100514494	LOC100514494	lncRNA	0	0	0	0	0	0	0	0	0	0	0	0	0	0	0	0	0	0	0
BGIR9823_89501	100155701	NYNRIN	mRNA	0	0	0	0	0	0	0	0	0	0	0	0	0	0	0	0	0	0	0
BGIR9823_79516	100155619	SOCS6	lncRNA	0	0	0	0	0	0	1	0	0	0	0	0	0	0	0	0	0	0	0
BGIR9823_93273	100626205	SLC38A10	mRNA	0	0	0	0	0	0	0	0	1	0	0	0	0	0	0	0	0	0	0
BGIR9823_82045	100739121	LOC100739121	lncRNA	0	0	0	0	0	0	0	0	0	0	0	0	0	0	0	0	0	0	0
BGIR9823_99804	100519342	RNF24	lncRNA	0	0	0	0	0	0	0	0	0	0	0	0	0	0	0	0	0	0	0
BGIR9823_81050	102164585	LOC102164585	mRNA	0	0	0	0	0	0	0	0	0	0	0	0	0	1	0	0	0	0	0
BGIR9823_83089	100515889	DGUOK	lncRNA	0	0	0	0	0	0	0	0	0	0	0	0	0	0	0	0	0	0	0
BGIR9823_85641	100157726	ETV6	lncRNA	0	0	0	0	0	0	0	0	0	0	0	0	0	0	0	0	0	0	0
BGIR9823_80399	100514037	EPS15L1	lncRNA	0	0	0	0	0	0	0	0	0	0	0	0	0	0	0	0	0	0	0
BGIR9823_100428	100517203	NRF1	lncRNA	0	0	0	0	0	0	0	0	0	0	0	0	0	0	0	0	0	0	0
BGIR9823_79736	110255453	LOC110255453	lncRNA	0	0	0	0	0	0	0	0	0	0	0	0	0	0	0	0	0	0	0

RNA ID	Gene ID	Symbol	Type	novel-ssc-miR817-3p	novel-ssc-miR849-3p	novel-ssc-miR882-3p	novel-ssc-miR985-5p	novel-ssc-miR1126-5p	novel-ssc-miR1396-3p	ssc-miR-362	novel-ssc-miR16-5p	ssc-miR-375	ssc-miR-376a-3p	ssc-miR-493-5p	ssc-miR-194a-5p	ssc-miR-205	novel-ssc-miR239-5p	novel-ssc-miR397-5p	novel-ssc-miR507-5p	novel-ssc-miR563-3p	novel-ssc-miR568-3p	novel-ssc-miR682-3p
BGIR9823_88239	100512907	LOC100512907	lncRNA	0	0	0	0	0	0	0	0	0	0	0	0	0	0	0	0	0	0	0
BGIR9823_89801	100524447	TBC1D1	lncRNA	0	0	0	0	0	0	0	0	0	0	0	0	0	0	0	0	0	0	0
BGIR9823_78621	100627072	PTPRD	lncRNA	0	0	0	0	0	0	0	0	0	0	0	0	0	0	0	0	0	0	0
BGIR9823_78489	100152019	PELI2	lncRNA	0	0	0	0	0	0	0	0	0	0	0	0	0	0	0	0	0	0	0
BGIR9823_83940	100155227	RBM15	lncRNA	0	0	0	0	0	0	0	0	0	0	1	0	0	0	0	0	0	0	0
BGIR9823_85932	106510466	MAF	lncRNA	0	0	0	0	0	0	0	0	1	0	0	0	0	0	0	0	0	0	0
BGIR9823_81640	100513341	FBXL17	lncRNA	0	0	0	0	0	0	0	0	0	0	0	0	0	0	0	0	0	0	0
BGIR9823_84349	100154639	PBX1	mRNA	1	0	0	0	0	0	0	0	0	0	0	0	0	0	0	0	0	0	0
BGIR9823_100606	100515515	GALNT11	lncRNA	0	0	0	1	0	0	0	0	0	0	0	0	0	0	0	0	0	0	0
BGIR9823_101514	102157759	LOC102157759	lncRNA	0	0	0	0	0	0	0	0	0	0	0	0	0	0	0	0	0	0	0
BGIR9823_85476	100622365	TSPAN31	lncRNA	0	0	0	0	0	1	0	0	0	0	0	0	0	0	0	0	0	0	0
BGIR9823_89890	100515957	PPID	lncRNA	0	0	0	0	0	0	0	0	0	0	0	0	0	0	0	0	0	0	0
BGIR9823_101279	100153213	HS6ST2	mRNA	0	0	0	0	0	0	0	0	0	0	0	0	0	0	0	0	0	0	0
BGIR9823_92274	100520446	PIP4K2A	lncRNA	0	0	0	0	0	0	0	0	0	0	0	0	0	0	0	0	0	0	0
BGIR9823_83415	106509958	MTSS1	lncRNA	0	0	0	0	0	0	0	0	0	0	0	0	0	0	0	0	0	1	0
BGIR9823_78462	100511360	FBXO34	lncRNA	0	0	0	0	0	0	0	0	0	0	0	0	0	0	0	0	0	0	0
BGIR9823_77832	100736796	ADGRB3	lncRNA	0	0	0	0	0	0	0	0	0	0	0	0	0	0	0	0	0	0	0
BGIR9823_79204	397085	MYO6	lncRNA	0	0	0	0	0	0	0	0	0	0	0	0	0	0	0	0	0	0	0
BGIR9823_83395	110260216	LOC110260216	lncRNA	0	0	0	0	0	0	0	0	0	0	0	0	1	0	0	0	0	0	0
BGIR9823_84473	100153347	GOLPH3L	lncRNA	0	0	0	0	0	0	0	0	0	0	0	0	0	0	0	0	0	0	0
BGIR9823_85369	100518305	LOC100518305	mRNA	0	0	0	0	0	0	0	0	0	0	0	0	0	1	0	0	0	0	0
BGIR9823_84074	100627184	GLI4	lncRNA	1	0	0	0	0	0	0	0	0	0	0	0	0	0	0	0	0	0	0
BGIR9823_92298	100515404	ITIH5	lncRNA	0	0	0	0	0	0	0	1	0	0	0	0	0	0	0	0	0	0	0
BGIR9823_89061	100153581	NUP153	mRNA	0	0	0	0	0	0	0	0	0	0	0	0	0	0	0	0	0	0	0
BGIR9823_96626	100151786	POP5	lncRNA	0	0	0	0	0	0	0	0	0	0	0	0	0	0	0	0	0	0	0
BGIR9823_87721	100517038	PUM1	lncRNA	0	0	0	0	0	0	0	1	0	0	0	0	0	0	0	0	0	0	0
BGIR9823_100728	100512382	KCND2	lncRNA	0	0	0	0	0	0	0	0	0	0	0	0	0	0	0	0	0	0	0
BGIR9823_80555	110259375	LOC110259375	lncRNA	0	0	0	0	0	0	0	0	0	0	0	0	0	0	0	0	0	0	0
BGIR9823_79909	100155492	HEMGN	lncRNA	0	0	0	0	0	0	0	0	0	0	0	0	0	0	0	0	0	0	0
BGIR9823_94689	100516877	ACOX2	lncRNA	0	0	0	0	0	0	0	1	0	0	0	0	0	0	0	0	0	0	0
BGIR9823_92764	102158088	NHLRC3	lncRNA	0	0	0	0	0	0	0	0	0	0	0	0	0	0	0	0	0	0	0
BGIR9823_89682	100152306	LOC100152306	mRNA	0	0	0	0	0	0	0	0	0	0	0	0	0	0	0	0	0	0	0
BGIR9823_96521	100623262	SLC15A4	mRNA	0	0	0	0	0	0	0	0	1	0	0	0	0	0	0	0	0	0	0
BGIR9823_84835	100516439	C5H12orf10	lncRNA	0	0	0	0	0	0	0	0	0	0	0	0	0	0	0	0	0	1	0
BGIR9823_100509	100738688	HERPUD2	lncRNA	0	0	0	0	0	0	0	0	0	0	0	0	0	0	0	0	0	0	0
BGIR9823_86157	100511329	ZNF829	mRNA	0	0	0	0	0	0	0	0	0	0	0	0	0	0	0	0	0	0	0
BGIR9823_82679	100620395	GIGYF1	mRNA	0	1	0	0	0	0	0	0	0	0	0	0	0	0	0	0	0	0	0
BGIR9823_90779	100621182	EMSY	mRNA	1	0	0	0	0	0	0	0	0	0	0	0	0	0	0	0	0	0	0
BGIR9823_90540	100621145	LARP1B	lncRNA	0	0	0	0	0	0	0	0	0	0	0	0	0	0	0	0	0	0	0
NM_001097469.1	100037984	HIGD2A	mRNA	0	0	0	0	0	0	0	0	0	0	0	0	0	0	0	0	0	0	0
BGIR9823_78404	102162695	LOC102162695	lncRNA	0	0	0	0	0	0	0	0	0	0	0	0	0	0	0	0	0	0	0
BGIR9823_84013	102161056	LOC102161056	lncRNA	0	0	0	0	0	0	0	0	0	0	0	0	0	0	0	0	0	0	0
BGIR9823_98214	100512817	TENM3	mRNA	0	0	0	0	0	0	0	0	1	0	0	0	0	0	0	0	0	0	0

RNA ID	Gene ID	Symbol	Type	novel-ssc-miR817-3p	novel-ssc-miR849-3p	novel-ssc-miR882-3p	novel-ssc-miR985-5p	novel-ssc-miR1126-5p	novel-ssc-miR1396-3p	ssc-miR-362	novel-ssc-miR16-5p	ssc-miR-375	ssc-miR-376a-3p	ssc-miR-493-5p	ssc-miR-194a-5p	ssc-miR-205	novel-ssc-miR239-5p	novel-ssc-miR397-5p	novel-ssc-miR507-5p	novel-ssc-miR563-3p	novel-ssc-miR568-3p	novel-ssc-miR682-3p
BGIR9823_81705	100521611	FNIP1	lncRNA	0	1	0	0	0	0	0	0	0	0	0	0	0	0	0	0	0	0	0
BGIR9823_79689	100192435	ERO1A	lncRNA	0	0	0	0	0	0	0	0	0	0	0	0	0	0	0	0	0	0	0
BGIR9823_81181	100736787	ZDHHC13	mRNA	0	1	0	0	0	0	0	0	0	0	0	0	0	0	0	0	0	0	0
BGIR9823_95430	396658	IFNAR1	mRNA	0	0	0	0	0	0	0	0	0	0	0	0	0	0	0	0	0	0	0
BGIR9823_77980	100152640	HAUS1	lncRNA	0	0	0	0	0	0	0	0	0	0	0	0	0	0	0	0	0	0	0
BGIR9823_91823	100522092	RUNDC3B	lncRNA	0	0	0	0	0	0	0	1	0	0	0	0	0	0	0	0	0	0	0
BGIR9823_81117	100144442	F2	lncRNA	0	0	0	0	0	0	0	0	0	0	0	0	0	0	0	0	0	0	0
BGIR9823_90627	100624822	GSTCD	mRNA	0	0	0	0	0	0	0	0	0	0	0	1	0	0	0	0	0	0	0
BGIR9823_78165	100169932	TRPM7	lncRNA	0	0	0	0	0	0	0	0	0	0	0	0	0	0	0	0	0	0	0
BGIR9823_80118	100511178	SNX32	lncRNA	0	0	0	0	0	0	0	0	0	0	0	0	0	0	0	0	0	0	0
BGIR9823_84495	102166500	LOC102166500	lncRNA	0	0	0	0	0	0	0	0	0	0	0	0	0	0	0	0	0	0	0
BGIR9823_81367	100127167	CERS4	mRNA	0	0	0	0	0	0	0	0	0	0	0	0	0	0	0	0	0	0	0
BGIR9823_77687	100156632	AGPAT4	lncRNA	0	0	0	0	1	0	0	0	0	0	0	0	0	0	0	0	0	0	0
BGIR9823_80249	100519700	AMBRA1	lncRNA	0	0	0	0	0	0	0	0	0	0	0	0	0	0	0	0	0	0	0
BGIR9823_100622	100521480	GSTK1	lncRNA	0	0	0	0	0	0	0	0	0	0	0	0	0	0	0	0	0	0	0
BGIR9823_86113	100621064	DPY19L3	lncRNA	0	0	0	0	0	0	0	0	0	0	0	0	0	0	0	0	0	0	0
BGIR9823_92205	100516148	MOB3B	mRNA	0	0	0	0	0	0	0	0	0	0	0	0	0	1	0	0	0	0	0
XM_021075409.1	100737004	COBLL1	mRNA	0	0	0	0	0	0	0	0	0	0	0	0	0	1	0	0	0	0	0
BGIR9823_89246	100521322	LOC100521322	lncRNA	0	0	0	0	0	1	0	0	0	0	0	0	0	0	0	0	0	0	0
BGIR9823_78193	100154291	SLC12A1	lncRNA	0	0	0	0	0	0	0	0	0	0	0	0	0	0	0	0	0	0	0
BGIR9823_100566	100516888	GLI3	lncRNA	0	0	1	0	0	0	0	0	0	0	0	0	0	0	0	0	0	0	0
BGIR9823_100446	100192443	POT1	lncRNA	0	0	0	0	0	0	0	0	0	0	0	0	0	0	0	0	0	0	0
BGIR9823_97408	100620430	SAP30	lncRNA	0	0	0	0	1	0	0	0	0	0	0	0	0	0	0	0	0	0	0
BGIR9823_86274	100622144	BICRA	lncRNA	0	0	0	0	0	0	0	0	0	0	0	0	1	0	0	0	0	0	0
BGIR9823_101110	100524780	ARHGAP6	lncRNA	0	0	0	0	0	0	0	0	0	0	0	0	0	0	0	0	0	0	0
BGIR9823_82012	100524333	GGA2	lncRNA	0	0	0	0	0	0	0	0	0	0	0	0	0	0	0	0	0	0	0
BGIR9823_86283	100624752	NOP53	mRNA	0	0	0	0	0	0	0	0	0	0	0	0	0	0	0	0	0	0	0
BGIR9823_98317	110256929	WDSUB1	lncRNA	0	1	0	0	0	0	0	0	0	0	0	0	0	0	0	0	0	0	0
BGIR9823_88517	100511822	ICK	lncRNA	0	0	0	0	0	0	0	0	0	0	0	0	0	0	0	0	0	0	0
BGIR9823_79454	110259520	LOC110259520	lncRNA	0	0	0	0	0	0	0	0	0	0	0	0	0	0	0	0	0	0	0
BGIR9823_81324	606746	DNMT1	mRNA	0	0	0	0	0	0	0	0	0	0	0	0	0	0	0	0	0	0	0
BGIR9823_100702	100514931	AHCYL2	lncRNA	0	0	0	0	0	0	0	0	0	0	0	0	0	0	0	1	0	0	0
BGIR9823_101292	100523658	ATP11C	mRNA	0	1	0	0	0	0	0	0	0	0	0	0	0	0	0	0	0	0	0
BGIR9823_90549	102167943	LOC102167943	lncRNA	0	0	0	0	0	0	0	0	0	0	0	0	0	0	0	0	0	0	0
BGIR9823_84672	100516804	SCO2	mRNA	0	0	0	0	0	0	0	0	0	0	0	0	0	0	0	0	0	0	0
BGIR9823_98499	100514879	ABI2	mRNA	1	0	0	0	0	0	0	0	0	0	0	0	0	0	0	0	0	0	0
BGIR9823_83000	102166005	LOC102166005	lncRNA	0	0	0	0	0	0	0	0	0	0	0	0	0	0	0	0	0	0	0
BGIR9823_79430	100152163	DLL4	lncRNA	0	0	0	0	0	0	0	0	0	0	0	0	0	0	0	0	0	0	0
BGIR9823_80973	100511242	TMEM134	lncRNA	0	0	0	0	0	0	0	0	0	0	0	0	0	0	0	0	0	0	0
BGIR9823_101334	100523617	TRIM27	lncRNA	0	0	0	0	0	0	0	0	0	0	0	0	0	0	0	0	0	0	0
BGIR9823_81536	100520548	WDR41	lncRNA	0	0	0	0	0	0	0	0	0	0	0	0	0	0	0	0	0	0	0
BGIR9823_101625	100519072	LOC100519072	lncRNA	0	0	0	0	0	0	0	0	0	0	0	0	0	0	0	0	0	0	0
BGIR9823_87181	100517402	PDF	lncRNA	0	0	0	0	0	0	0	0	0	0	0	0	0	0	0	0	0	0	0

RNA ID	Gene ID	Symbol	Type	novel-ssc-miR817-3p	novel-ssc-miR849-3p	novel-ssc-miR882-3p	novel-ssc-miR985-5p	novel-ssc-miR1126-5p	novel-ssc-miR1396-3p	ssc-miR-362	novel-ssc-miR16-5p	ssc-miR-375	ssc-miR-376a-3p	ssc-miR-493-5p	ssc-miR-194a-5p	ssc-miR-205	novel-ssc-miR239-5p	novel-ssc-miR397-5p	novel-ssc-miR507-5p	novel-ssc-miR563-3p	novel-ssc-miR568-3p	novel-ssc-miR682-3p
BGIR9823_78566	100152132	IFN-OMEGA-3	lncRNA	0	0	0	0	0	0	0	0	0	0	0	0	0	0	0	0	0	0	0
XR_002339179.1	110257024	LOC110257024	lncRNA	0	0	0	0	0	0	0	0	0	0	0	0	0	0	0	0	0	0	0
BGIR9823_100144	100154945	RRBP1	lncRNA	0	0	0	0	0	0	0	0	0	0	0	0	0	0	0	0	0	0	0
BGIR9823_100611	100521999	SMARCD3	lncRNA	0	0	0	1	0	0	0	0	0	0	0	0	0	0	0	0	0	0	0
BGIR9823_95072	100620632	KPNA4	lncRNA	0	0	0	0	0	0	0	0	0	0	0	0	0	0	0	0	0	0	0
BGIR9823_98782	100152203	AGA	mRNA	0	0	0	0	0	0	0	0	0	0	0	0	0	0	0	0	0	0	0
BGIR9823_77731	100154785	ADAT2	lncRNA	0	0	0	0	0	0	0	0	0	0	0	0	0	0	0	0	0	0	0
BGIR9823_96586	100153673	CUX2	mRNA	0	0	0	0	0	0	0	0	0	0	0	0	0	0	0	0	0	0	0
BGIR9823_85978	110260924	LOC110260924	lncRNA	0	0	0	0	0	0	0	0	0	0	0	0	1	0	0	0	0	0	0
BGIR9823_78788	100154489	WDR38	mRNA	0	0	0	0	0	0	0	0	1	0	0	0	0	0	0	0	0	0	0
BGIR9823_86389	110261319	LOC110261319	lncRNA	0	0	0	0	0	0	0	0	0	0	0	0	0	0	0	0	0	0	0
BGIR9823_79497	100736682	TJP1	lncRNA	0	0	0	0	0	0	0	0	0	0	0	0	0	0	0	0	0	0	0
BGIR9823_98375	100522284	METAP1D	mRNA	1	0	0	0	0	0	0	0	0	0	0	0	0	0	0	0	0	0	0
BGIR9823_100000	100523757	ARFGEF2	lncRNA	0	0	0	0	0	0	0	0	0	0	0	0	0	0	0	0	0	0	0
BGIR9823_84378	100524676	SDHC	lncRNA	0	0	0	0	0	0	0	0	0	0	0	0	1	0	0	0	0	0	0
BGIR9823_92192	396723	DDX58	mRNA	0	1	0	0	0	0	0	0	0	0	0	0	0	0	0	0	0	0	0
BGIR9823_88795	102162743	FAM174B	lncRNA	0	0	0	0	0	0	0	0	0	0	0	0	0	0	0	0	0	0	0
BGIR9823_92690	100623356	SKA3	lncRNA	0	0	0	0	0	0	0	0	0	0	0	0	0	0	0	0	0	0	0
XR_002340913.1	110257727	LOC110257727	lncRNA	0	0	0	0	0	0	0	0	0	0	0	0	0	0	0	0	0	0	0
BGIR9823_86991	100037307	DAB1	lncRNA	0	0	0	0	0	0	0	0	0	0	0	0	0	0	0	0	0	0	0
BGIR9823_82075	100513346	LOC100513346	mRNA	0	0	0	0	0	0	0	0	0	0	0	0	0	0	0	0	0	0	0
BGIR9823_86237	100524260	ZNF45	lncRNA	0	0	0	0	0	0	0	0	0	0	0	0	0	0	0	0	0	0	0
BGIR9823_80263	106507997	LOC106507997	lncRNA	0	0	0	0	0	0	0	0	0	0	0	0	0	0	0	0	0	0	0
BGIR9823_93834	110256118	LOC110256118	lncRNA	0	0	0	0	0	0	0	0	1	0	0	0	0	0	0	0	0	0	0
BGIR9823_80349	100738340	ATP13A1	lncRNA	0	0	0	0	0	0	0	0	0	0	0	0	0	0	0	0	0	0	0
BGIR9823_89603	106507740	LOC106507740	lncRNA	0	0	0	0	1	0	0	0	0	0	0	0	0	0	0	0	0	0	0
BGIR9823_77903	100514380	TSTD3	lncRNA	0	0	0	0	0	0	0	0	0	0	0	0	0	0	0	0	0	0	0
BGIR9823_100158	100154020	LOC100154020	lncRNA	0	0	0	0	0	0	0	0	0	0	0	0	0	0	0	0	0	0	0
BGIR9823_89657	733584	FOXN3	lncRNA	0	0	0	0	1	0	0	0	0	0	0	0	0	0	0	0	0	0	0
BGIR9823_98765	100233172	AGPAT5	lncRNA	0	0	0	0	0	0	0	0	0	0	0	0	0	0	0	1	0	0	0
BGIR9823_94610	396679	AMT	lncRNA	0	0	0	0	0	0	0	0	0	0	0	0	0	0	0	0	0	0	0
BGIR9823_78506	100154408	PSMA3	lncRNA	0	0	0	0	0	0	1	0	0	0	0	0	0	0	0	0	0	0	0
BGIR9823_78017	100515517	RBPMS2	lncRNA	0	0	0	0	0	0	0	0	0	0	0	0	0	0	0	0	0	0	0
BGIR9823_98223	100515058	TRAPPC11	mRNA	1	0	0	0	0	0	0	0	0	0	0	0	0	0	0	0	0	0	0
BGIR9823_78148	100155516	DMXL2	mRNA	0	0	0	0	0	0	0	0	0	0	0	0	0	0	0	0	0	0	0
BGIR9823_87377	100521972	LOC100521972	lncRNA	0	0	0	0	1	0	0	0	0	0	0	0	0	0	0	0	0	0	0
BGIR9823_84933	100511508	LEMD3	lncRNA	0	0	0	0	0	0	0	0	0	0	0	0	1	0	0	0	0	0	0
BGIR9823_100906	100621199	IL1RAPL1	lncRNA	0	0	0	0	0	0	0	0	0	0	0	0	0	0	0	0	1	0	0
BGIR9823_89271	100156900	BTBD9	mRNA	0	0	0	0	0	0	0	0	0	0	0	0	0	0	0	0	0	0	0
BGIR9823_90463	100627416	TRIM2	lncRNA	0	0	0	0	0	0	0	0	0	0	0	0	0	0	0	0	0	0	0
BGIR9823_79564	100153830	MALT1	lncRNA	0	0	0	0	0	0	1	0	0	0	0	0	0	0	0	0	0	0	0
BGIR9823_88636	100155770	FAM219B	mRNA	0	0	0	0	0	0	0	0	0	0	0	1	0	0	0	0	0	0	0
BGIR9823_84024	100155974	GTF2B	lncRNA	0	0	0	0	0	0	0	0	0	0	0	0	0	0	0	0	0	0	0

RNA ID	Gene ID	Symbol	Type	novel-ssc-miR817-3p	novel-ssc-miR849-3p	novel-ssc-miR882-3p	novel-ssc-miR985-5p	novel-ssc-miR1126-5p	novel-ssc-miR1396-3p	ssc-miR-362	novel-ssc-miR16-5p	ssc-miR-375	ssc-miR-376a-3p	ssc-miR-493-5p	ssc-miR-194a-5p	ssc-miR-205	novel-ssc-miR239-5p	novel-ssc-miR397-5p	novel-ssc-miR507-5p	novel-ssc-miR563-3p	novel-ssc-miR568-3p	novel-ssc-miR682-3p
BGIR9823_100486	100621338	BMT2	lncRNA	0	0	0	0	0	0	1	0	0	0	0	0	0	0	0	0	0	0	0
BGIR9823_86035	100623274	CTCF	mRNA	0	0	0	0	0	0	0	0	0	1	0	0	0	0	0	0	0	0	0
BGIR9823_85003	110260713	AMN1	mRNA	0	0	0	0	0	0	0	0	0	0	0	0	0	0	0	0	0	0	0
BGIR9823_100211	100157872	PXMP4	lncRNA	0	0	0	0	0	0	0	0	0	0	0	0	0	0	0	0	0	0	0
BGIR9823_85764	396681	TXNRD1	mRNA	0	0	0	0	0	0	0	0	1	0	0	0	0	0	0	0	0	0	0
BGIR9823_79047	110260218	LOC110260218	lncRNA	0	0	0	0	0	0	0	0	0	0	0	0	0	0	0	0	0	0	0
BGIR9823_99528	100626363	NPR3	lncRNA	0	0	0	0	0	0	0	0	0	0	0	0	0	0	0	0	0	0	0
BGIR9823_86380	100738636	EPN1	mRNA	0	0	0	0	0	0	0	0	0	0	0	0	0	0	0	0	0	0	0
BGIR9823_101388	100521615	ARHGAP17	mRNA	0	0	0	0	0	0	0	0	0	0	0	0	0	0	0	0	0	0	0
BGIR9823_85180	100625757	ARID2	lncRNA	0	0	0	0	0	0	0	0	0	0	0	0	0	0	0	0	0	0	0
BGIR9823_101519	100511414	RNF122	lncRNA	0	0	0	1	0	0	0	0	0	0	0	0	0	0	0	0	0	0	0
BGIR9823_100416	106506781	LOC106506781	lncRNA	0	0	0	0	0	0	0	0	0	0	0	0	0	0	0	0	0	0	0
BGIR9823_77835	100736796	ADGRB3	lncRNA	0	0	0	0	0	1	0	0	0	0	0	0	0	0	0	0	0	0	0
XM_001928635.2	100156587	LOC100156587	mRNA	0	0	0	0	0	0	0	0	0	0	0	0	0	0	0	0	0	0	0
BGIR9823_91988	100217398	FLVCR1	lncRNA	0	0	0	0	0	0	0	0	0	0	0	0	0	0	0	1	0	0	0
BGIR9823_86369	100517285	LOC100517285	mRNA	0	0	0	0	0	0	0	0	0	0	0	1	0	0	0	0	0	0	0
BGIR9823_101017	100520165	TMEM164	lncRNA	0	0	0	1	0	0	0	0	0	0	0	0	0	0	0	0	0	0	0
BGIR9823_81426	100621275	GNG7	lncRNA	0	0	0	0	0	0	0	0	0	0	0	0	0	0	0	0	0	0	0
BGIR9823_94753	100522704	FAM19A1	lncRNA	0	0	0	0	0	0	0	0	0	0	0	0	0	0	0	0	0	0	0
BGIR9823_87686	100512789	WASF2	lncRNA	0	0	0	0	0	0	0	0	0	0	0	0	0	0	0	0	0	0	0
BGIR9823_88695	100624265	STRN3	mRNA	0	0	0	0	0	0	0	0	0	0	0	1	0	0	0	0	0	0	0
BGIR9823_82270	100625157	GCFC2	lncRNA	0	0	0	0	0	0	0	0	0	0	0	0	0	0	0	0	0	0	0
BGIR9823_93946	397091	PRKAR1A	mRNA	0	0	0	0	0	0	0	0	0	0	0	1	0	0	0	0	0	0	0
BGIR9823_93731	110256070	LOC110256070	lncRNA	0	0	0	0	0	0	0	0	0	0	0	0	0	0	0	0	0	0	0
BGIR9823_80458	100515360	ILF3	lncRNA	0	0	0	0	0	0	0	0	0	0	1	0	0	0	0	0	0	0	0
BGIR9823_80830	100513723	JADE2	lncRNA	0	0	0	0	0	0	0	0	0	0	0	0	0	0	0	0	0	0	0
BGIR9823_87106	100739500	ACSF3	lncRNA	0	0	0	0	0	0	0	0	0	0	0	0	0	0	0	0	0	0	0
BGIR9823_91655	100511341	EI24	lncRNA	1	0	0	0	0	0	0	0	0	0	0	0	0	0	0	0	0	0	0
BGIR9823_84152	100337657	ZFPM2	mRNA	0	0	0	0	0	0	0	0	0	0	0	0	0	0	0	0	0	0	0
BGIR9823_78973	100155445	VTA1	lncRNA	0	0	0	0	0	0	0	0	0	0	0	0	0	0	0	0	0	0	0
BGIR9823_88372	100151743	PPP1R18	lncRNA	0	0	0	0	0	1	0	0	0	0	0	0	0	0	0	0	0	0	0
BGIR9823_97928	100155592	PDLIM1	mRNA	0	0	0	0	0	0	0	0	0	0	0	0	0	0	0	0	0	0	0
BGIR9823_88939	100511141	VASH1	mRNA	0	0	0	0	0	0	0	0	1	0	0	0	0	0	0	0	0	0	0
BGIR9823_93334	100623453	KIF19	lncRNA	0	0	0	0	0	0	0	0	0	0	0	0	0	0	0	0	0	0	0
BGIR9823_79684	100153738	TRIM9	lncRNA	0	0	0	0	0	0	0	0	0	0	0	0	0	0	0	0	0	0	0
BGIR9823_100666	100524964	AGBL3	lncRNA	0	0	0	0	0	0	0	0	0	0	0	0	0	0	0	0	0	0	0
BGIR9823_89830	100515040	LIMCH1	mRNA	0	0	0	0	0	0	0	0	0	0	0	0	0	0	0	0	0	0	0
BGIR9823_85457	100511633	RDH16	lncRNA	0	0	0	0	0	1	0	0	0	0	0	0	0	0	0	0	0	0	0
BGIR9823_81268	100512396	SLC35E1	lncRNA	0	0	0	0	0	0	0	0	0	0	0	0	0	0	0	0	0	0	0
BGIR9823_78329	100049703	BCL2	lncRNA	0	0	0	0	0	0	0	0	0	0	0	0	0	0	0	0	1	0	0
BGIR9823_78260	100512756	MEIS2	lncRNA	0	0	0	0	0	0	0	0	0	0	0	0	0	0	0	0	0	0	0
BGIR9823_89336	100152930	ADGRF5	mRNA	0	0	0	0	0	0	0	0	0	0	0	1	0	0	0	0	0	0	0
BGIR9823_89031	100152689	SSR1	mRNA	0	0	0	0	0	0	0	0	0	0	0	0	0	0	0	0	0	0	0

RNA ID	Gene ID	Symbol	Type	novel-ssc-miR817-3p	novel-ssc-miR849-3p	novel-ssc-miR882-3p	novel-ssc-miR985-5p	novel-ssc-miR1126-5p	novel-ssc-miR1396-3p	ssc-miR-362	novel-ssc-miR16-5p	ssc-miR-375	ssc-miR-376a-3p	ssc-miR-493-5p	ssc-miR-194a-5p	ssc-miR-205	novel-ssc-miR239-5p	novel-ssc-miR397-5p	novel-ssc-miR507-5p	novel-ssc-miR563-3p	novel-ssc-miR568-3p	novel-ssc-miR682-3p
BGIR9823_78386	100154444	GLCE	lncRNA	0	0	0	0	0	0	0	0	0	0	0	0	0	0	0	0	0	0	0
BGIR9823_92789	100155063	SPRYD7	mRNA	0	0	0	0	0	0	0	0	0	0	0	0	0	0	0	0	0	0	0
BGIR9823_86252	100622514	CLASRP	lncRNA	0	0	0	0	0	0	0	0	0	0	0	0	0	0	0	0	0	0	0
BGIR9823_101153	100621125	SLC35A2	mRNA	0	0	0	0	0	0	0	0	0	0	0	0	0	0	0	0	0	0	0
BGIR9823_100998	414736	PCDH11X	lncRNA	0	0	1	0	0	0	0	0	0	0	0	0	0	0	0	0	0	0	0
BGIR9823_82762	397614	IL4R	lncRNA	0	0	0	0	0	0	0	0	0	0	0	0	0	1	0	0	0	0	0
BGIR9823_79824	100626649	KANK1	lncRNA	0	0	0	0	0	0	0	0	0	0	0	0	0	0	0	0	0	0	0
BGIR9823_95516	100152333	ANKRD28	lncRNA	0	1	0	0	0	0	0	0	0	0	0	0	0	0	0	0	0	0	0
BGIR9823_81853	106509619	LOC106509619	lncRNA	0	0	0	0	0	1	0	0	0	0	0	0	0	0	0	0	0	0	0
BGIR9823_90521	100513554	NAA15	lncRNA	0	0	0	0	1	0	0	0	0	0	0	0	0	0	0	0	0	0	0
BGIR9823_93604	100520097	TUBD1	mRNA	0	1	0	0	0	0	0	0	0	0	0	0	0	0	0	0	0	0	0
BGIR9823_79674	100157402	NIN	lncRNA	0	0	0	0	0	0	1	0	0	0	0	0	0	0	0	0	0	0	0
BGIR9823_100171	100625861	LZTS3	lncRNA	0	0	0	0	0	0	0	0	0	0	0	0	0	0	0	0	0	0	0
BGIR9823_81709	100521611	FNIP1	lncRNA	0	0	0	0	0	0	0	0	0	0	0	0	0	0	0	0	0	0	0
BGIR9823_82252	100738124	IMMT	lncRNA	0	0	0	0	0	0	0	0	0	0	0	0	0	0	0	0	0	0	0
BGIR9823_80640	102165038	LOC102165038	lncRNA	0	0	0	0	0	0	0	0	0	0	0	0	0	0	0	0	0	0	0
BGIR9823_101575	100737380	CDC25A	lncRNA	0	0	0	0	0	0	0	0	0	0	0	0	0	0	0	0	1	0	0
BGIR9823_84694	100518786	HDAC10	lncRNA	0	0	0	0	0	0	0	1	0	0	0	0	0	0	0	0	0	0	0
BGIR9823_98548	102162936	SPAG16	mRNA	0	0	0	0	0	0	0	0	0	0	0	0	0	0	0	0	0	0	0
BGIR9823_88951	110261640	LOC110261640	lncRNA	0	0	0	0	0	0	0	0	0	0	0	0	0	0	0	0	0	0	0
BGIR9823_101401	110258215	LOC110258215	lncRNA	0	0	0	0	0	0	1	0	0	0	0	0	0	0	0	0	0	0	0
BGIR9823_82476	100518189	SRSF7	lncRNA	0	0	0	0	0	0	0	0	0	0	0	0	0	0	0	0	0	0	0
BGIR9823_80254	100520600	TP53I11	lncRNA	0	0	0	0	0	0	0	1	0	0	0	0	0	0	0	0	0	0	0
BGIR9823_100432	100515455	TNPO3	lncRNA	0	0	1	0	0	0	0	0	0	0	0	0	0	0	0	0	0	0	0
BGIR9823_100491	100520706	DOCK4	lncRNA	0	0	1	0	0	0	0	0	0	0	0	0	0	0	0	0	0	0	0
BGIR9823_101216	100514440	ATRX	lncRNA	0	0	0	0	0	0	0	0	0	0	0	0	0	0	0	0	0	0	0
BGIR9823_94101	100518511	MBTD1	lncRNA	0	0	0	0	0	0	0	0	0	0	0	0	0	0	0	0	0	0	0
BGIR9823_84304	100157730	TGS1	mRNA	0	0	0	0	0	0	0	0	0	0	0	0	0	0	0	0	0	0	0
BGIR9823_98130	100519056	ORC4	lncRNA	0	0	0	0	0	0	0	0	0	0	0	0	0	0	0	0	0	0	0
BGIR9823_95626	100623101	SETD2	mRNA	0	0	0	0	0	0	0	0	0	0	0	0	0	0	0	0	0	0	0
BGIR9823_81836	110259827	LOC110259827	lncRNA	0	0	0	0	0	0	0	0	0	0	0	0	0	0	0	0	0	0	0
BGIR9823_100125	100519631	TASP1	lncRNA	0	0	0	0	0	0	0	0	0	0	0	0	0	0	0	0	0	0	0
BGIR9823_77803	100520524	GOPC	lncRNA	0	0	0	0	0	0	1	0	0	0	0	0	0	0	0	0	0	0	0
BGIR9823_78926	100152904	ARID1B	lncRNA	0	0	0	0	0	0	0	0	0	0	0	0	0	0	0	0	0	0	0
XR_002343327.1	110260295	LOC110260295	lncRNA	0	0	0	0	0	0	0	0	0	0	0	0	0	0	0	0	0	0	0
XM_005661298.3	100514564	CAPS	mRNA	0	0	0	0	0	0	0	0	0	0	0	0	0	0	0	0	0	0	0
BGIR9823_100692	100739403	KLHDC10	lncRNA	0	0	0	0	0	0	0	0	0	0	0	0	0	0	0	0	0	0	0
BGIR9823_95813	100525891	EIF4E3	lncRNA	0	0	0	0	0	0	0	0	0	0	0	0	0	0	0	0	0	0	0
BGIR9823_81281	100516595	ZNF333	lncRNA	0	0	0	0	0	0	0	0	0	0	0	0	0	0	0	0	0	0	0
BGIR9823_100015	100151957	PARD6B	lncRNA	0	0	0	0	0	0	0	0	0	0	0	0	0	0	0	0	0	0	0
BGIR9823_101282	100517991	FAM122B	lncRNA	0	0	0	0	0	0	0	0	0	0	0	0	0	0	0	0	0	0	0
BGIR9823_88581	100151986	UNC45A	lncRNA	0	0	0	0	0	0	0	0	0	0	0	0	0	0	0	0	0	0	0
BGIR9823_88595	110261555	SEMA4B	lncRNA	0	0	0	0	0	0	0	0	0	0	0	0	0	0	0	0	0	0	0

RNA ID	Gene ID	Symbol	Type	novel-ssc-miR817-3p	novel-ssc-miR849-3p	novel-ssc-miR882-3p	novel-ssc-miR985-5p	novel-ssc-miR1126-5p	novel-ssc-miR1396-3p	ssc-miR-362	novel-ssc-miR16-5p	ssc-miR-375	ssc-miR-376a-3p	ssc-miR-493-5p	ssc-miR-194a-5p	ssc-miR-205	novel-ssc-miR239-5p	novel-ssc-miR397-5p	novel-ssc-miR507-5p	novel-ssc-miR563-3p	novel-ssc-miR568-3p	novel-ssc-miR682-3p
BGIR9823_91875	106504991	LOC106504991	lncRNA	0	0	0	0	0	0	0	0	0	0	0	0	0	0	0	1	0	0	0
BGIR9823_100454	100525017	WASL	lncRNA	0	0	0	0	0	0	0	0	0	0	0	0	0	0	0	0	0	0	0
BGIR9823_101069	100512515	AFF2	mRNA	0	0	0	0	0	0	0	0	0	0	0	0	0	0	0	0	0	0	0
BGIR9823_101534	102158401	LOC102158401	lncRNA	0	0	0	0	0	0	0	0	0	0	0	0	0	0	0	0	0	0	0
BGIR9823_82897	100738908	MLST8	lncRNA	0	0	0	0	0	0	0	0	0	0	0	0	1	0	0	0	0	0	0
BGIR9823_98106	100158118	CALY	mRNA	0	0	0	0	0	0	0	0	0	0	0	0	0	0	0	0	0	0	0
BGIR9823_80817	100520422	SLC22A5	lncRNA	0	0	0	0	0	0	0	0	0	0	0	0	0	0	0	0	0	0	0
BGIR9823_91425	100511767	ARAP1	mRNA	0	0	0	0	0	0	0	0	0	0	0	1	0	0	0	0	0	0	0
BGIR9823_96623	100154637	SRSF9	lncRNA	0	0	0	0	0	0	0	0	0	0	0	0	0	0	0	0	0	0	0
BGIR9823_81521	100513398	SIMC1	lncRNA	0	0	0	0	0	0	0	0	0	0	0	0	0	0	0	0	0	0	0
BGIR9823_86388	110261319	LOC110261319	lncRNA	0	0	0	0	0	0	0	0	0	0	0	0	0	0	0	0	0	0	0
BGIR9823_81938	100511317	SSC4D	lncRNA	0	0	0	0	0	0	0	0	0	0	0	0	0	0	0	0	0	0	0
BGIR9823_83868	102165219	ANKRD34A	mRNA	0	0	0	0	0	0	0	0	0	0	0	0	0	0	0	0	0	0	0
BGIR9823_85017	100155867	INTS13	lncRNA	0	0	0	0	0	0	0	0	0	0	0	0	0	0	0	0	0	0	0
BGIR9823_91638	100523324	ARHGEF12	lncRNA	0	0	0	0	0	0	0	0	0	0	0	0	0	0	0	0	0	0	0
BGIR9823_78342	100515640	DIS3L	lncRNA	0	0	0	0	0	0	0	0	0	0	0	0	0	0	0	0	0	0	0
BGIR9823_101177	100520413	PHF8	mRNA	0	0	0	0	0	0	0	0	0	0	0	0	0	0	0	0	0	0	0
BGIR9823_100116	100157552	MKKS	lncRNA	0	0	0	0	0	0	0	0	0	0	0	0	0	0	0	0	0	0	0
BGIR9823_82812	100514160	XYLT1	lncRNA	0	0	0	0	0	1	0	0	0	0	0	0	0	0	0	0	0	0	0
BGIR9823_100485	100621338	BMT2	lncRNA	0	0	0	0	0	0	0	0	0	0	0	0	0	0	0	0	0	0	0
BGIR9823_83185	100302367	SOCS5	lncRNA	0	0	0	0	0	0	0	0	0	0	0	0	0	0	0	0	0	0	0
BGIR9823_100135	100519631	TASP1	lncRNA	0	0	0	0	0	0	0	0	0	0	0	0	0	0	0	0	0	0	0
BGIR9823_78629	100155278	ERMP1	mRNA	0	0	0	0	0	0	0	0	0	0	0	0	0	0	0	0	0	0	0
BGIR9823_100610	100517250	NUB1	mRNA	0	0	0	0	0	0	0	0	0	0	0	0	0	0	0	0	0	0	0
BGIR9823_100720	100511294	PTPRZ1	lncRNA	0	0	0	0	0	0	1	0	0	0	0	0	0	0	0	0	0	0	0
BGIR9823_91236	100524699	TPK1	lncRNA	0	0	0	0	0	0	0	0	0	0	0	0	0	0	0	0	0	0	0
BGIR9823_79597	100152069	SMAD6	lncRNA	0	0	0	0	0	0	0	0	1	0	0	0	0	0	0	0	0	0	0
BGIR9823_100727	100512026	ING3	lncRNA	0	0	0	0	0	0	0	0	0	0	0	0	0	0	0	0	0	0	0
BGIR9823_89956	106504698	LOC106504698	lncRNA	0	0	0	0	0	0	0	1	0	0	0	0	0	0	0	0	0	0	0
BGIR9823_100230	100154394	ROMO1	lncRNA	0	0	0	0	0	0	1	0	0	0	0	0	0	0	0	0	0	0	0
BGIR9823_90067	100519324	ETNPPL	mRNA	0	1	0	0	0	0	0	0	0	0	0	0	0	0	0	0	0	0	0
BGIR9823_81199	100216478	TEAD1	lncRNA	0	0	0	0	0	0	0	0	0	0	0	0	0	0	0	0	0	0	0
BGIR9823_100115	100157552	MKKS	lncRNA	0	0	0	0	0	0	0	0	0	0	0	0	0	0	0	0	0	0	0
BGIR9823_97871	733640	PRKG1	lncRNA	0	0	0	0	0	0	0	0	0	0	0	0	0	0	0	0	0	0	0
BGIR9823_79442	106506757	INAFM2	lncRNA	0	0	0	0	0	0	0	0	0	0	0	0	0	0	0	0	0	0	0
BGIR9823_77844	100736796	ADGRB3	lncRNA	0	0	0	0	0	0	0	0	0	0	0	0	0	0	0	0	0	0	0
BGIR9823_81223	110259751	LOC110259751	lncRNA	0	0	0	0	0	0	0	0	0	0	0	0	0	0	0	0	0	0	0
BGIR9823_87714	100517038	PUM1	lncRNA	0	0	0	0	0	0	0	0	0	0	0	0	0	0	0	0	0	0	0
BGIR9823_82332	100517706	CEP68	lncRNA	0	0	0	0	0	0	0	0	0	0	0	0	0	0	0	0	0	0	0
BGIR9823_79033	100624679	NKAIN2	lncRNA	0	0	0	0	0	0	0	0	0	0	0	0	0	0	0	0	0	0	0
BGIR9823_81514	100523721	FAF2	lncRNA	0	0	0	0	0	0	0	0	0	0	0	0	0	0	0	0	0	0	0
BGIR9823_78765	100622501	PRPF4	mRNA	0	0	0	0	0	0	0	0	0	0	0	0	0	0	0	0	0	0	0
BGIR9823_78849	100624075	AIF1L	mRNA	0	0	0	0	0	0	0	0	0	0	0	0	0	0	0	0	0	0	0

RNA ID	Gene ID	Symbol	Type	novel-ssc-miR817-3p	novel-ssc-miR849-3p	novel-ssc-miR882-3p	novel-ssc-miR985-5p	novel-ssc-miR1126-5p	novel-ssc-miR1396-3p	ssc-miR-362	novel-ssc-miR16-5p	ssc-miR-375	ssc-miR-376a-3p	ssc-miR-493-5p	ssc-miR-194a-5p	ssc-miR-205	novel-ssc-miR239-5p	novel-ssc-miR397-5p	novel-ssc-miR507-5p	novel-ssc-miR563-3p	novel-ssc-miR568-3p	novel-ssc-miR682-3p
BGIR9823_100607	100515515	GALNT11	lncRNA	0	0	0	0	0	0	0	0	0	0	0	0	0	0	0	0	0	0	0
BGIR9823_83081	100627528	CCDC142	lncRNA	0	0	0	0	0	0	0	0	0	0	0	0	0	0	0	1	0	0	0
XR_002345524.1	102158855	LOC102158855	lncRNA	0	0	0	0	0	0	0	0	0	0	0	0	0	1	0	0	0	0	0
BGIR9823_78387	100154444	GLCE	lncRNA	0	0	0	0	0	0	0	0	0	0	0	0	0	0	0	0	0	0	0
BGIR9823_79739	110255453	LOC110255453	lncRNA	0	0	0	0	0	0	0	0	0	0	0	0	0	0	0	0	0	0	0
BGIR9823_100823	100519067	CDK13	mRNA	0	0	0	0	0	0	0	0	1	0	0	0	0	0	0	0	0	0	0
BGIR9823_80184	397169	BEST1	lncRNA	0	0	0	0	0	1	0	0	0	0	0	0	0	0	0	0	0	0	0
BGIR9823_85074	100144592	KLRF1	lncRNA	0	0	0	0	0	0	0	0	0	0	0	0	0	0	0	0	0	0	0
BGIR9823_80186	444995	FADS1	mRNA	0	0	0	0	0	0	0	0	0	0	0	0	0	0	0	0	0	0	0
BGIR9823_97606	100156356	PRR14L	lncRNA	0	0	0	0	0	0	0	0	0	0	0	0	0	0	0	0	0	0	0
BGIR9823_100902	100621199	IL1RAPL1	lncRNA	0	0	0	0	0	0	0	0	0	0	0	0	0	0	0	0	0	0	0
BGIR9823_78631	100155714	PLGRKT	lncRNA	0	0	0	0	0	0	0	0	0	0	0	0	0	0	0	0	0	0	0
BGIR9823_97867	780419	UBE2D1	mRNA	0	0	0	0	0	0	0	0	0	0	0	0	0	0	0	0	0	0	0
BGIR9823_80393	100512765	SMIM7	lncRNA	0	0	0	0	0	0	0	0	0	0	0	0	0	0	0	0	0	0	0
BGIR9823_83405	100157574	FAM49B	lncRNA	0	0	0	0	0	0	0	0	0	0	0	0	1	0	0	0	0	0	0
BGIR9823_82271	100625157	GCFC2	lncRNA	0	0	0	0	0	0	0	0	0	0	0	0	0	0	0	0	0	0	0
BGIR9823_92748	100154396	PDS5B	lncRNA	0	0	0	0	0	0	0	0	0	0	0	0	0	0	0	0	0	0	0
BGIR9823_81832	100737907	FAM20C	lncRNA	0	0	0	0	0	0	0	0	0	0	0	0	0	0	0	0	0	0	0
BGIR9823_86739	100623382	SPIRE1	mRNA	0	0	0	0	0	0	0	0	1	0	0	0	0	0	0	0	0	0	0
BGIR9823_88779	100515615	CCNB1IP1	lncRNA	0	0	0	0	0	0	0	0	0	0	0	0	0	0	0	0	0	0	0
BGIR9823_82125	100533196	UBE2I	lncRNA	0	0	0	0	0	0	0	0	0	0	0	0	1	0	0	0	0	0	0
BGIR9823_96691	100153895	MORC2	lncRNA	0	0	0	0	0	0	0	0	0	0	0	0	0	0	0	0	0	0	0
BGIR9823_82328	100518067	SPRED2	lncRNA	0	0	0	0	0	0	0	0	0	0	0	0	1	0	0	0	0	0	0
BGIR9823_87494	100625971	LOC100625971	mRNA	0	0	0	0	0	0	0	0	0	0	0	0	0	0	0	0	0	0	0
BGIR9823_79014	100738102	RNF217	lncRNA	0	0	0	0	0	0	0	1	0	0	0	0	0	0	0	0	0	0	0
BGIR9823_79431	100153351	VPS18	mRNA	0	0	0	0	0	0	0	0	1	0	0	0	0	0	0	0	0	0	0
BGIR9823_79792	100154060	RIC1	mRNA	0	1	0	0	0	0	0	0	0	0	0	0	0	0	0	0	0	0	0
BGIR9823_97488	100153708	ATXN2	mRNA	1	0	0	0	0	0	0	0	0	0	0	0	0	0	0	0	0	0	0
BGIR9823_100701	100514931	AHCYL2	lncRNA	0	0	0	0	0	0	0	0	0	0	0	0	0	0	0	0	0	0	0
BGIR9823_83545	100153257	MMP16	lncRNA	0	0	0	0	0	0	0	0	0	0	1	0	0	0	0	0	0	0	0
BGIR9823_82543	100521786	TP53I3	mRNA	0	0	0	0	0	0	0	0	0	0	0	0	0	0	0	0	0	0	0
BGIR9823_81390	100520177	TRIP10	lncRNA	0	0	0	0	0	0	0	0	0	0	0	0	0	0	0	0	0	0	0
BGIR9823_100128	100519631	TASP1	lncRNA	0	0	0	0	0	0	0	0	0	0	0	0	0	0	0	0	1	0	0
BGIR9823_94226	100337675	ADAP2	lncRNA	0	0	0	0	0	0	0	0	0	0	0	0	0	0	0	0	0	0	0
BGIR9823_88221	110261778	NOL7	mRNA	0	0	0	0	0	0	0	0	1	0	0	0	0	0	0	0	0	0	0
BGIR9823_100908	100621199	IL1RAPL1	lncRNA	0	0	0	0	0	0	0	0	0	0	0	0	0	0	0	0	0	0	0
BGIR9823_101335	100523617	TRIM27	lncRNA	0	0	1	0	0	0	0	0	0	0	0	0	0	0	0	0	0	0	0
BGIR9823_101518	106504125	LOC106504125	lncRNA	0	0	0	0	0	0	0	0	0	0	0	0	0	0	0	0	0	0	0
BGIR9823_80840	100624262	SMAD5	mRNA	0	1	0	0	0	0	0	0	0	0	0	0	0	0	0	0	0	0	0
BGIR9823_99908	449527	PHF20	lncRNA	0	0	0	0	0	0	0	0	0	0	0	0	0	0	0	0	0	0	0
BGIR9823_99647	100521758	CPEB4	mRNA	0	0	0	0	0	0	0	0	0	0	0	0	0	0	0	0	0	0	0
BGIR9823_97815	106506044	LOC106506044	lncRNA	0	0	0	0	0	0	0	0	0	0	0	0	0	0	0	0	0	0	0
BGIR9823_77854	100736796	ADGRB3	lncRNA	0	0	0	0	0	0	0	0	0	0	0	0	0	0	0	0	0	0	0

RNA ID	Gene ID	Symbol	Type	novel-ssc-miR817-3p	novel-ssc-miR849-3p	novel-ssc-miR882-3p	novel-ssc-miR985-5p	novel-ssc-miR1126-5p	novel-ssc-miR1396-3p	ssc-miR-362	novel-ssc-miR16-5p	ssc-miR-375	ssc-miR-376a-3p	ssc-miR-493-5p	ssc-miR-194a-5p	ssc-miR-205	novel-ssc-miR239-5p	novel-ssc-miR397-5p	novel-ssc-miR507-5p	novel-ssc-miR563-3p	novel-ssc-miR568-3p	novel-ssc-miR682-3p
BGIR9823_92288	110255709	LOC110255709	lncRNA	0	0	0	0	0	0	0	0	0	0	0	0	0	0	0	1	0	0	0
BGIR9823_79245	100738627	DYM	lncRNA	0	0	0	0	0	0	0	0	0	0	0	0	0	0	0	0	0	0	0
BGIR9823_83586	100152963	PEX2	lncRNA	0	0	0	0	0	0	0	0	0	0	0	0	0	0	0	0	0	0	0
BGIR9823_101489	110258382	LOC110258382	lncRNA	0	0	0	0	0	0	0	0	0	0	0	0	0	0	0	0	0	0	0
BGIR9823_100872	100739216	MAP3K15	lncRNA	0	0	0	0	0	0	0	0	0	0	0	0	0	0	0	0	0	0	0
XM_005673239.3	100620722	ZNF277	mRNA	0	0	0	0	0	0	0	0	0	0	0	0	0	0	0	0	0	0	0
BGIR9823_100554	100739791	LOC100739791	lncRNA	0	0	0	0	0	0	0	0	0	0	0	0	0	0	0	0	0	0	0
BGIR9823_83272	100521316	GPN1	lncRNA	0	0	0	0	0	1	0	0	0	0	0	0	0	0	0	0	0	0	0
BGIR9823_77910	110260430	LOC110260430	lncRNA	0	0	0	0	0	0	0	0	0	0	0	0	0	0	0	0	0	0	0
BGIR9823_100153	100152012	CST7	lncRNA	0	0	0	0	0	0	0	0	0	0	0	0	0	0	0	0	0	0	0
BGIR9823_79200	100156851	IRAK1BP1	lncRNA	0	0	0	0	0	0	0	0	0	0	0	0	0	0	0	0	0	0	0
BGIR9823_100475	100517204	TFEC	lncRNA	0	0	0	0	0	0	0	0	0	0	0	0	0	0	0	0	0	0	0
BGIR9823_83002	110259971	C3H2orf49	lncRNA	0	0	0	0	0	0	0	0	0	0	0	0	0	0	0	0	0	0	0
BGIR9823_78732	100152140	SLC44A1	lncRNA	0	0	0	0	0	0	1	0	0	0	0	0	0	0	0	0	0	0	0
BGIR9823_94529	397618	OXSR1	lncRNA	0	0	0	0	0	0	0	0	0	0	0	0	0	0	0	0	0	0	0
BGIR9823_92973	100623901	MICU2	mRNA	0	0	0	0	0	0	0	0	1	0	0	0	0	0	0	0	0	0	0
BGIR9823_80241	100513197	DDB2	lncRNA	0	0	0	0	0	0	0	0	0	0	0	0	0	0	0	0	0	0	0
BGIR9823_96832	100153026	ABCB10	lncRNA	0	0	0	0	0	0	0	0	0	0	0	0	0	0	0	0	0	0	0
BGIR9823_96408	100153797	SECISBP2	mRNA	0	0	0	0	0	0	0	0	0	0	0	0	0	0	0	0	0	0	0
BGIR9823_80734	100526040	PAM	lncRNA	0	0	0	0	0	0	0	0	0	0	0	0	0	0	0	0	0	0	0
BGIR9823_81957	100513345	GTF2I	lncRNA	0	0	0	0	0	0	0	0	0	0	0	0	0	0	0	0	0	0	0
BGIR9823_96796	110256820	LOC110256820	lncRNA	0	0	0	0	0	0	0	0	0	0	0	0	0	0	0	0	0	0	0
BGIR9823_101115	110255263	CXH4orf3	lncRNA	0	0	0	0	0	0	0	0	0	0	0	0	0	0	0	0	0	0	0
BGIR9823_78939	100621167	PLEKHG1	lncRNA	0	0	0	0	0	0	0	0	0	0	0	0	0	0	0	0	0	0	0
BGIR9823_90840	100517358	PIWIL4	lncRNA	1	0	0	0	0	0	0	0	0	0	0	0	0	0	0	0	0	0	0
BGIR9823_94380	396903	MYH10	mRNA	0	0	0	0	0	0	0	0	1	0	0	0	0	0	0	0	0	0	0
BGIR9823_94364	100621720	TNK1	lncRNA	0	0	0	0	0	0	0	0	0	0	0	0	0	0	0	0	0	0	0
BGIR9823_99224	110257075	LOC110257075	lncRNA	0	0	0	0	0	0	0	0	0	0	0	0	0	0	0	0	0	0	0
BGIR9823_88977	102159128	LOC102159128	lncRNA	0	0	0	0	0	0	0	0	0	0	0	0	0	0	0	0	0	0	0
BGIR9823_86907	100623628	PRKACB	lncRNA	0	0	0	0	0	0	0	0	0	0	0	0	0	0	0	1	0	0	0
BGIR9823_83922	100127159	SLC16A1	lncRNA	0	0	0	0	0	0	0	0	1	0	0	0	0	0	0	0	0	0	0
BGIR9823_80165	106504153	AHNAK	mRNA	0	0	0	0	0	0	0	0	0	0	0	0	0	0	0	0	0	0	0
XM_021099336.1	102160410	LOC102160410	mRNA	0	0	0	0	0	0	0	0	0	0	0	0	0	0	0	0	0	0	0
BGIR9823_78034	100157196	CSNK1G1	lncRNA	0	1	0	0	0	0	0	0	0	0	0	0	0	0	0	0	0	0	0
BGIR9823_80409	100517807	SYDE1	lncRNA	0	0	0	0	0	0	0	0	0	0	0	0	0	0	0	0	0	0	0
BGIR9823_82453	100622259	MAP4K3	lncRNA	0	0	0	0	0	0	0	0	0	0	0	0	0	0	0	0	0	0	0
BGIR9823_101610	100521600	LOC100521600	lncRNA	0	0	0	0	0	0	0	0	0	0	0	0	0	0	0	0	0	0	0
BGIR9823_86055	102165609	LOC102165609	lncRNA	0	0	0	0	0	1	0	0	0	0	0	0	0	0	0	0	0	0	0
BGIR9823_92014	396922	CD46	mRNA	0	0	0	0	0	0	0	0	0	0	0	0	0	0	0	0	0	0	0
BGIR9823_100942	100521238	CDK16	mRNA	0	0	0	0	0	0	0	0	0	0	0	0	0	0	0	0	0	0	0
BGIR9823_77933	100513770	SLC16A10	lncRNA	0	0	0	0	0	0	0	0	0	0	0	0	0	0	0	0	0	0	0
BGIR9823_89476	100155589	FAM177A1	lncRNA	0	0	0	0	0	0	0	0	0	0	0	0	0	0	0	0	0	0	0
BGIR9823_101139	100525794	KLHL15	lncRNA	0	0	0	0	0	1	0	0	0	0	0	0	0	0	0	0	0	0	0

RNA ID	Gene ID	Symbol	Type	novel-ssc-miR817-3p	novel-ssc-miR849-3p	novel-ssc-miR882-3p	novel-ssc-miR985-5p	novel-ssc-miR1126-5p	novel-ssc-miR1396-3p	ssc-miR-362	novel-ssc-miR16-5p	ssc-miR-375	ssc-miR-376a-3p	ssc-miR-493-5p	ssc-miR-194a-5p	ssc-miR-205	novel-ssc-miR239-5p	novel-ssc-miR397-5p	novel-ssc-miR507-5p	novel-ssc-miR563-3p	novel-ssc-miR568-3p	novel-ssc-miR682-3p
BGIR9823_92285	100512363	CREM	lncRNA	0	0	0	0	0	0	0	0	0	0	0	0	0	0	0	0	0	0	0
BGIR9823_100684	414428	MKLN1	lncRNA	0	0	0	1	0	0	0	0	0	0	0	0	0	0	0	0	0	0	0
BGIR9823_80117	100511000	MUS81	lncRNA	0	0	0	0	0	0	0	0	0	0	0	0	0	0	0	0	0	0	0
BGIR9823_79783	102168004	UHRF2	lncRNA	0	0	0	0	0	0	0	0	0	0	0	0	0	0	0	0	0	0	0
BGIR9823_87527	110261323	LOC110261323	lncRNA	0	0	0	0	0	0	0	0	0	0	0	0	0	0	0	0	0	0	0
BGIR9823_77815	100736796	ADGRB3	lncRNA	0	0	0	0	0	0	0	0	0	0	0	0	0	0	0	0	0	0	0
BGIR9823_100466	100512202	TSPAN12	mRNA	0	0	0	0	0	0	0	0	0	0	0	0	0	0	0	0	0	0	0
BGIR9823_81771	100521956	CYSTM1	lncRNA	0	0	0	0	0	0	0	0	0	0	0	0	0	0	0	0	0	0	0
BGIR9823_93887	100625999	PGS1	mRNA	0	0	0	0	0	0	0	0	0	0	0	0	0	0	0	0	0	0	0
BGIR9823_99449	100312962	ATOX1	lncRNA	0	0	0	0	0	0	0	1	0	0	0	0	0	0	0	0	0	0	0
BGIR9823_82424	100526165	PREPL	lncRNA	0	0	0	0	0	0	0	0	0	0	0	0	0	0	0	0	0	0	0
BGIR9823_78323	100518050	TMX3	lncRNA	0	0	0	0	0	1	0	0	0	0	0	0	0	0	0	0	0	0	0
BGIR9823_81279	110259619	LOC110259619	lncRNA	0	0	0	0	0	0	0	0	0	0	0	0	0	0	0	0	0	0	0
BGIR9823_89593	100514718	SYNJ2BP	mRNA	0	0	0	0	0	0	0	0	0	0	0	1	0	0	0	0	0	0	0
BGIR9823_100641	100519065	UBN2	lncRNA	0	0	0	0	0	0	0	0	0	0	0	0	0	0	0	0	0	0	0
BGIR9823_99284	100518875	EGFLAM	mRNA	0	0	0	0	0	0	0	0	0	0	0	0	0	0	0	0	0	0	0
BGIR9823_97891	100037303	SGMS1	lncRNA	0	0	0	0	0	0	0	0	0	0	0	0	0	0	0	0	0	0	0
BGIR9823_80246	100516314	HARBI1	lncRNA	0	0	0	0	0	0	1	0	0	0	0	0	0	0	0	0	0	0	0
BGIR9823_84831	100154762	ZNF740	lncRNA	0	0	0	0	0	0	0	0	0	0	0	0	0	0	0	1	0	0	0
BGIR9823_100404	100524054	WDR91	lncRNA	0	0	0	0	0	0	0	0	0	0	0	0	0	0	0	0	0	0	0
BGIR9823_89087	100157771	CARMIL1	lncRNA	0	0	0	0	0	0	0	0	0	0	0	0	0	0	0	0	0	0	0
BGIR9823_94449	100737057	BTD	lncRNA	0	0	0	0	0	0	0	0	0	0	1	0	0	0	0	0	0	0	0
BGIR9823_89030	100152689	SSR1	mRNA	0	0	0	0	0	0	0	0	1	0	0	0	0	0	0	0	0	0	0
BGIR9823_100313	100155190	ZFP64	lncRNA	0	0	0	0	0	0	0	0	0	0	0	0	0	0	0	0	0	0	1
BGIR9823_84915	100623414	SRGAP1	lncRNA	0	0	0	0	0	0	0	0	0	0	0	0	0	0	0	0	0	0	0
BGIR9823_100318	100126237	CTCFL	lncRNA	0	0	1	0	0	0	0	0	0	0	0	0	0	0	0	0	0	0	0
BGIR9823_88856	100155733	SUSD6	lncRNA	0	0	0	0	0	0	0	0	0	0	0	0	0	0	0	1	0	0	0
XM_003480892.4	100519890	TRIM36	mRNA	1	0	0	0	0	0	0	0	0	0	0	0	0	0	0	0	0	0	0
BGIR9823_82684	100620890	SRRT	lncRNA	0	0	0	0	0	0	0	0	0	0	0	0	0	0	0	0	0	0	0
BGIR9823_83320	100513350	ASAP2	mRNA	0	0	0	0	0	0	0	0	0	0	0	0	0	0	0	0	0	0	0
BGIR9823_101170	100739016	RIBC1	lncRNA	0	0	0	0	0	0	0	0	0	0	0	0	0	0	0	0	0	0	0
BGIR9823_87081	100521097	ERI3	lncRNA	0	0	0	0	0	0	0	0	0	0	0	0	0	0	0	0	0	0	0
BGIR9823_88961	100152076	PTPN21	lncRNA	0	0	0	0	1	0	0	0	0	0	0	0	0	0	0	0	0	0	0
BGIR9823_89703	100515556	SLC25A47	lncRNA	0	0	0	0	0	1	0	0	0	0	0	0	0	0	0	0	0	0	0
BGIR9823_78067	100625505	VPS13C	lncRNA	0	0	0	0	0	0	0	0	0	0	0	0	0	0	0	0	0	0	0
BGIR9823_78412	100156782	LRFN5	lncRNA	0	0	0	0	0	0	0	0	0	0	1	0	0	0	0	0	0	0	0
BGIR9823_94130	106505457	TRIM37	mRNA	1	0	0	0	0	0	0	0	0	0	0	0	0	0	0	0	0	0	0
BGIR9823_78647	100152831	PUM3	lncRNA	0	0	0	0	0	0	0	0	0	0	0	0	0	0	0	0	0	0	0
BGIR9823_101061	102158351	LOC102158351	lncRNA	0	0	0	1	0	0	0	0	0	0	0	0	0	0	0	0	0	0	0
BGIR9823_101008	100523039	GPRASP1	mRNA	1	0	0	0	0	0	0	0	0	0	0	0	0	0	0	0	0	0	0

Supplementary Table S9. The predicted targets of DE miRN

RNA ID	Gene ID	Symbol	Type	novel-ssc-miR1063-5p	ssc-miR-192	ssc-miR-432-5p	ssc-miR-7138-5p	ssc-miR-874	ssc-miR-10386	ssc-miR-122-3p	novel-ssc-miR755-3p	novel-ssc-miR780-3p	novel-ssc-miR804-3p	novel-ssc-miR813-5p	novel-ssc-miR407-5p	novel-ssc-miR573-5p	novel-ssc-miR583-3p	novel-ssc-miR1077-5p	novel-ssc-miR122-3p	novel-ssc-miR154-5p	ssc-miR-127	ssc-miR-194b-5p
BGIR9823_100323	100049657	GNAS	lncRNA	1	0	0	0	1	0	0	0	0	1	0	0	0	0	0	0	1	0	0
BGIR9823_100321	100144523	PMEPA1	lncRNA	0	0	0	0	1	0	0	0	0	1	0	0	0	0	0	0	1	0	0
BGIR9823_100436	110257581	LOC110257581	lncRNA	1	0	0	1	1	0	0	0	0	1	1	0	0	0	0	0	1	0	0
BGIR9823_101399	110258215	LOC110258215	lncRNA	1	0	0	0	1	0	0	0	0	0	1	0	0	0	0	0	1	0	0
BGIR9823_100330	100037996	RPS21	lncRNA	0	0	0	0	1	1	0	0	0	0	0	0	0	0	0	0	1	1	0
BGIR9823_101457	110258362	MTA1	lncRNA	1	0	0	0	1	0	0	0	0	0	0	0	1	0	0	1	1	0	0
BGIR9823_101544	110258600	LOC110258600	lncRNA	1	0	0	1	0	0	0	0	0	0	1	0	0	0	0	0	1	0	0
BGIR9823_101531	102158401	LOC102158401	lncRNA	1	0	0	0	0	0	0	0	0	1	1	0	1	0	0	0	0	0	0
BGIR9823_101342	110258067	ARRDC1	lncRNA	0	0	0	0	1	0	0	0	0	0	0	0	0	0	0	0	1	0	0
BGIR9823_101405	110258214	LOC110258214	lncRNA	0	0	0	0	1	0	0	0	0	1	0	0	0	0	0	0	1	0	0
BGIR9823_101472	110258334	LOC110258334	lncRNA	0	0	0	0	1	0	0	0	0	0	1	0	0	0	0	1	1	0	0
BGIR9823_100712	100520873	IMPDH1	lncRNA	0	0	0	0	1	0	0	0	0	0	0	0	0	0	0	0	1	0	0
BGIR9823_101185	110257830	LOC110257830	lncRNA	0	0	0	0	0	0	0	0	0	0	0	0	0	0	0	0	0	0	0
BGIR9823_101542	102157946	LOC102157946	lncRNA	1	0	0	1	0	0	0	0	0	0	1	0	0	0	0	0	1	0	0
BGIR9823_100049	110257373	GMEB2	lncRNA	0	0	0	1	1	0	0	0	0	0	1	0	0	0	0	0	1	1	0
BGIR9823_100378	100514083	ADCK2	lncRNA	0	0	0	0	0	0	0	0	0	0	1	0	0	0	0	0	1	1	0
BGIR9823_100540	100523342	OSBPL3	lncRNA	0	0	0	0	0	0	0	0	0	0	0	0	1	0	0	0	1	0	0
BGIR9823_101051	110257712	LOC110257712	lncRNA	1	0	0	0	0	1	0	0	0	0	0	0	0	0	0	0	0	0	0
BGIR9823_101904	BGIG9823_26581		lncRNA	0	0	0	0	0	1	0	0	0	1	0	1	0	0	0	0	0	0	0
BGIR9823_100561	100516888	GLI3	lncRNA	0	0	0	1	0	0	0	0	0	0	0	0	0	0	0	0	0	1	0
BGIR9823_100665	100622227	LOC100622227	lncRNA	0	0	0	0	0	0	0	0	0	0	0	0	0	0	0	0	1	0	0
BGIR9823_100348	110257384	TCEA2	lncRNA	0	0	0	1	1	0	0	0	0	0	0	1	0	0	0	0	0	0	0
BGIR9823_100834	110257605	LRRC61	lncRNA	0	0	1	0	0	0	0	0	0	1	0	0	0	0	0	0	0	0	0
BGIR9823_100037	110257479	MTG2	lncRNA	1	0	0	0	0	0	0	0	0	1	1	0	0	0	1	0	0	0	0
BGIR9823_100184	106506644	LOC106506644	lncRNA	0	0	0	0	0	0	0	0	0	0	0	0	1	0	0	1	0	0	0
BGIR9823_101820	BGIG9823_26500		lncRNA	1	0	0	0	0	0	0	0	0	0	0	1	1	0	0	0	0	0	0
BGIR9823_100659	100522048	SLC13A4	lncRNA	0	0	0	1	1	0	0	0	0	0	0	0	0	0	0	0	1	0	0
BGIR9823_100223	100627204	MMP24	lncRNA	0	0	0	0	1	0	0	0	0	0	0	0	0	0	0	0	1	0	0
BGIR9823_101137	100152260	PRDX4	lncRNA	1	0	0	0	0	0	0	0	0	0	0	0	0	0	0	0	0	0	0
BGIR9823_100172	100154685	UBOX5	lncRNA	0	0	0	0	1	0	0	0	0	0	0	0	0	0	0	0	0	0	0
BGIR9823_101650	100738659	MCM3AP	lncRNA	1	0	0	0	0	0	0	0	0	0	0	0	0	0	0	1	0	0	0
BGIR9823_101940	BGIG9823_26617		lncRNA	0	1	0	0	0	0	0	0	0	0	1	0	0	0	0	0	0	0	0
BGIR9823_101941	BGIG9823_26618		lncRNA	1	0	1	0	0	0	0	0	0	0	0	0	0	0	0	0	0	0	0
BGIR9823_102278	BGIG9823_26945		lncRNA	1	0	0	0	0	0	0	0	0	0	0	0	0	0	0	0	0	0	0
BGIR9823_101611	100521600	LOC100521600	lncRNA	0	0	0	0	1	1	0	0	0	1	0	0	0	0	0	0	0	0	0
BGIR9823_100544	100627860	SUN3	lncRNA	0	0	0	0	0	0	0	0	0	0	0	0	0	0	0	0	1	1	0
BGIR9823_100360	100514805	KMT2C	lncRNA	0	0	0	0	1	0	0	0	0	0	0	0	0	0	0	0	0	0	0
BGIR9823_100799	397163	RAMP3	lncRNA	1	0	0	1	1	0	0	0	0	0	0	0	0	0	0	0	1	0	0
BGIR9823_101296	100623680	ZFP92	lncRNA	0	0	1	0	0	0	0	0	0	1	1	0	0	0	0	1	0	0	0
BGIR9823_100164	100520054	PANK2	lncRNA	0	0	1	0	0	0	0	0	0	0	0	0	0	0	0	0	0	1	0
BGIR9823_100576	102157930	LOC102157930	lncRNA	0	0	0	1	0	0	0	0	0	0	0	0	0	0	0	0	0	1	0

RNA ID	Gene ID	Symbol	Type	novel-ssc-miR1063-5p	ssc-miR-192	ssc-miR-432-5p	ssc-miR-7138-5p	ssc-miR-874	ssc-miR-10386	ssc-miR-122-3p	novel-ssc-miR755-3p	novel-ssc-miR780-3p	novel-ssc-miR804-3p	novel-ssc-miR813-5p	novel-ssc-miR407-5p	novel-ssc-miR573-5p	novel-ssc-miR583-3p	novel-ssc-miR1077-5p	novel-ssc-miR122-3p	novel-ssc-miR154-5p	ssc-miR-127	ssc-miR-194b-5p
BGIR9823_100585	100623772	ZNF862	lncRNA	1	0	0	0	0	0	0	0	0	0	0	0	0	0	0	0	0	0	0
BGIR9823_101341	110258060	LOC110258060	lncRNA	0	0	0	1	1	0	0	0	0	0	0	0	0	0	0	0	0	0	0
BGIR9823_101500	102160467	SMAGP	lncRNA	1	0	0	0	0	0	0	0	0	0	0	0	0	0	0	0	0	0	0
BGIR9823_101475	100521669	BRF1	lncRNA	1	0	0	0	1	0	0	0	0	0	0	0	0	0	0	0	1	0	0
BGIR9823_100400	100523151	NUP205	lncRNA	0	0	0	0	0	0	0	0	0	0	0	0	0	0	0	0	0	1	0
BGIR9823_101360	100512700	LOC100512700	lncRNA	0	0	0	0	0	0	0	0	0	0	1	0	0	0	0	0	0	0	1
BGIR9823_101677	BGIG9823_26364		lncRNA	0	0	0	1	0	0	0	0	0	0	0	0	0	0	0	0	0	0	0
BGIR9823_101879	BGIG9823_26558		lncRNA	0	0	0	0	0	0	0	0	0	0	0	1	1	0	0	0	0	0	1
BGIR9823_102010	BGIG9823_26686		lncRNA	0	0	0	0	0	0	0	0	0	0	1	0	0	0	0	0	0	0	0
BGIR9823_101968	BGIG9823_26645		lncRNA	0	0	0	0	0	0	0	0	0	1	0	0	0	0	0	0	0	0	0
BGIR9823_102338	BGIG9823_27003		lncRNA	0	0	0	0	0	0	0	0	0	0	0	0	1	0	0	0	0	0	0
BGIR9823_100338	BGIG9823_26339		lncRNA	0	0	0	0	1	0	0	0	0	0	0	0	0	0	0	0	1	0	0
BGIR9823_100753	493185	IFRD1	lncRNA	1	0	0	0	0	0	0	0	0	0	0	0	0	0	0	0	0	0	0
BGIR9823_100807	100515808	TMED4	lncRNA	0	1	0	0	0	0	0	0	0	0	0	0	0	0	0	0	0	1	0
BGIR9823_100577	100622254	RALA	lncRNA	1	0	0	0	0	0	0	0	0	0	0	0	0	0	0	0	1	0	0
BGIR9823_101471	100126861	AKT1	lncRNA	0	0	0	1	0	0	0	0	0	0	0	0	0	0	0	0	0	0	0
BGIR9823_100694	100737195	SMKR1	lncRNA	0	0	0	0	0	0	0	0	0	0	0	1	0	0	0	0	0	0	0
BGIR9823_101043	445538	XPNPEP2	lncRNA	0	0	0	0	0	0	0	0	0	0	0	0	0	0	0	0	0	0	0
BGIR9823_101060	102158351	LOC102158351	lncRNA	0	0	0	0	0	0	0	0	0	0	0	1	1	0	0	0	0	0	0
BGIR9823_100405	106506765	LOC106506765	lncRNA	0	0	0	0	1	0	0	0	0	0	0	0	0	0	0	0	0	0	0
BGIR9823_101548	110258600	LOC110258600	lncRNA	0	0	0	0	0	0	0	0	0	1	0	1	1	0	0	0	0	0	0
BGIR9823_100632	100515805	PARP12	lncRNA	0	1	0	0	1	0	0	0	0	0	1	0	0	0	0	0	0	0	0
BGIR9823_100345	100621535	TPD52L2	lncRNA	0	0	0	0	1	0	0	0	0	0	0	0	0	0	0	0	0	0	0
BGIR9823_100658	106506763	LOC106506763	lncRNA	0	0	0	1	1	0	0	0	0	0	0	0	0	0	0	0	0	0	0
BGIR9823_100521	100512260	GARS	lncRNA	0	0	0	0	1	0	0	0	0	0	1	0	0	0	0	0	1	0	0
BGIR9823_100195	106505983	LOC106505983	lncRNA	0	0	0	1	0	0	0	0	0	0	0	0	0	0	0	0	0	1	0
BGIR9823_101416	BGIG9823_26349		lncRNA	0	0	0	1	0	0	0	0	0	0	0	0	0	0	0	0	1	0	0
BGIR9823_102298	BGIG9823_26964		lncRNA	0	0	0	0	0	1	0	0	0	0	0	0	0	0	0	0	0	0	0
BGIR9823_101877	BGIG9823_26556		lncRNA	0	0	0	0	0	0	0	0	0	0	0	1	0	0	0	0	0	0	0
BGIR9823_102235	BGIG9823_26907		lncRNA	0	0	1	0	0	0	0	0	0	0	0	0	0	0	0	0	0	0	0
BGIR9823_102014	BGIG9823_26690		lncRNA	1	0	0	0	0	0	0	0	0	1	0	0	0	0	0	0	0	0	0
BGIR9823_102030	BGIG9823_26705		lncRNA	0	0	0	0	0	0	0	0	0	1	0	0	0	0	0	0	0	0	0
BGIR9823_101900	BGIG9823_26577		lncRNA	0	0	0	0	0	0	0	0	0	0	0	0	0	0	0	0	0	0	0
BGIR9823_101920	BGIG9823_26597		lncRNA	0	0	0	0	0	0	0	0	0	0	0	0	0	0	0	0	0	0	0
BGIR9823_101972	BGIG9823_26649		lncRNA	0	0	0	1	0	0	0	0	0	0	1	0	0	0	0	0	0	0	0
BGIR9823_101815	BGIG9823_26495		lncRNA	0	0	0	0	0	0	0	0	0	1	0	0	0	0	0	0	0	0	0
BGIR9823_101971	BGIG9823_26648		lncRNA	1	0	0	0	0	0	0	0	0	0	0	1	0	0	0	0	0	0	0
BGIR9823_101828	BGIG9823_26507		lncRNA	1	0	0	0	0	0	0	0	0	0	0	0	0	0	0	0	0	0	0
BGIR9823_102358	BGIG9823_27023		lncRNA	0	0	1	0	0	0	0	0	0	0	0	0	0	0	0	0	0	0	0
BGIR9823_101692	BGIG9823_26379		lncRNA	0	0	0	0	0	0	0	0	0	1	0	0	0	0	0	0	1	0	0
BGIR9823_101728	BGIG9823_26410		lncRNA	0	0	1	0	0	0	0	0	0	0	0	0	0	0	0	0	0	0	0
BGIR9823_101871	BGIG9823_26550		lncRNA	0	0	0	0	0	0	0	0	0	0	0	0	0	0	0	0	0	0	0
BGIR9823_101827	BGIG9823_26506		lncRNA	0	0	0	0	0	0	0	0	0	0	0	0	0	0	0	0	0	0	0

RNA ID	Gene ID	Symbol	Type	novel-ssc-miR1063-5p	ssc-miR-192	ssc-miR-432-5p	ssc-miR-7138-5p	ssc-miR-874	ssc-miR-10386	ssc-miR-122-3p	novel-ssc-miR755-3p	novel-ssc-miR780-3p	novel-ssc-miR804-3p	novel-ssc-miR813-5p	novel-ssc-miR407-5p	novel-ssc-miR573-5p	novel-ssc-miR583-3p	novel-ssc-miR1077-5p	novel-ssc-miR122-3p	novel-ssc-miR154-5p	ssc-miR-127	ssc-miR-194b-5p
BGIR9823_102129	BGIG9823_26802		lncRNA	1	0	0	0	0	0	0	0	0	0	0	0	0	0	0	0	0	0	0
BGIR9823_102690	BGIG9823_27340		lncRNA	0	0	1	0	0	0	0	0	0	0	0	1	0	0	0	1	0	0	0
BGIR9823_101336	100519166	LOC100519166	lncRNA	0	0	0	0	0	0	0	0	0	0	0	0	0	0	0	0	0	0	0
BGIR9823_101541	102157946	LOC102157946	lncRNA	0	0	0	0	0	0	0	0	0	1	0	0	0	0	0	0	0	0	0
BGIR9823_100268	102159476	LOC102159476	lncRNA	0	0	0	0	0	0	0	0	0	0	1	0	0	0	0	0	0	0	0
BGIR9823_101104	100521877	PUDP	lncRNA	0	0	0	0	0	0	0	0	0	0	0	0	0	0	0	0	0	0	1
BGIR9823_101381	100511434	EGFL7	lncRNA	1	0	0	0	0	0	0	0	0	0	0	0	0	0	0	0	0	0	0
BGIR9823_101015	100520165	TMEM164	lncRNA	0	0	0	1	0	0	0	0	0	0	1	0	0	0	0	0	0	0	0
BGIR9823_100709	100519523	CCDC136	lncRNA	0	0	0	0	1	0	0	0	0	0	0	0	0	0	0	0	0	0	0
BGIR9823_101430	100156979	TRAF3	lncRNA	0	0	0	0	1	0	0	0	0	0	1	0	0	0	0	0	0	0	0
BGIR9823_101450	100623151	INF2	lncRNA	0	0	0	0	0	0	0	0	0	0	0	0	0	0	0	0	1	0	0
BGIR9823_100277	100156830	PTI	lncRNA	0	0	0	0	0	0	0	0	0	0	0	0	0	0	0	0	0	1	0
BGIR9823_101283	100359355	MOSPD1	lncRNA	0	0	0	0	0	0	0	0	0	0	1	0	0	0	0	0	0	0	0
BGIR9823_101340	106505329	LOC106505329	lncRNA	0	0	0	0	0	0	0	0	0	0	0	0	0	0	0	0	0	0	0
BGIR9823_100800	100511907	CCM2	lncRNA	1	0	0	0	0	0	0	0	0	0	0	0	0	0	0	0	1	0	0
BGIR9823_100099	100517562	HGSNAT	lncRNA	0	0	0	0	0	0	0	0	0	0	0	0	0	0	0	0	0	0	0
BGIR9823_100810	100515113	POLM	lncRNA	0	0	1	0	0	0	0	0	0	1	0	0	0	0	0	0	0	0	0
BGIR9823_100578	100622573	POU6F2	lncRNA	0	0	0	0	0	0	0	0	0	0	0	0	0	0	0	0	0	0	0
BGIR9823_78024	100157196	CSNK1G1	lncRNA	0	0	0	0	0	0	0	0	0	0	0	0	0	0	0	0	0	0	0
BGIR9823_100367	100521999	SMARCD3	lncRNA	0	0	0	0	1	0	0	0	0	0	0	0	0	0	0	0	1	0	0
BGIR9823_100389	100627515	HIPK2	lncRNA	0	0	0	0	0	0	0	0	0	0	0	0	0	0	0	0	1	0	0
BGIR9823_100256	100156548	ZHX3	lncRNA	0	0	0	0	0	0	0	0	0	0	0	0	0	0	0	0	0	0	0
BGIR9823_100882	102165056	MBTPS2	lncRNA	0	0	0	0	0	0	0	0	0	0	0	0	0	0	0	0	0	0	0
BGIR9823_100324	100049657	GNAS	lncRNA	0	0	0	1	0	0	0	0	0	0	0	0	0	0	0	0	0	0	0
BGIR9823_100656	100521167	DGKI	lncRNA	0	0	0	0	0	0	0	0	0	0	1	0	0	0	0	0	0	1	0
BGIR9823_101689	BGIG9823_26376		lncRNA	0	0	0	0	0	0	0	0	0	0	0	0	1	0	0	1	0	0	0
BGIR9823_102087	BGIG9823_26761		lncRNA	1	0	1	0	0	0	0	0	0	0	0	0	0	0	0	0	0	0	0
BGIR9823_101951	BGIG9823_26628		lncRNA	0	0	1	1	0	0	0	0	0	0	0	0	0	0	0	0	0	0	0
BGIR9823_101854	BGIG9823_26533		lncRNA	0	0	0	0	0	0	0	0	0	0	0	0	0	0	0	0	0	0	0
BGIR9823_102383	BGIG9823_27048		lncRNA	0	0	0	0	0	0	0	0	0	0	0	0	0	0	0	0	0	0	0
BGIR9823_102727	BGIG9823_27373		lncRNA	0	0	0	0	0	0	0	0	0	0	0	1	0	0	0	0	0	0	0
BGIR9823_102028	BGIG9823_26703		lncRNA	0	0	0	0	0	1	0	0	0	0	0	0	0	0	0	0	0	0	0
BGIR9823_101977	BGIG9823_26653		lncRNA	0	0	0	0	0	0	0	0	0	0	0	1	0	0	0	0	0	0	0
BGIR9823_102406	BGIG9823_27067		lncRNA	0	0	0	0	0	0	0	0	0	0	0	0	0	0	0	0	0	0	0
BGIR9823_101868	BGIG9823_26547		lncRNA	0	0	0	0	0	0	0	0	0	0	0	0	0	0	0	0	0	0	0
BGIR9823_101698	BGIG9823_26385		lncRNA	0	0	0	1	0	0	0	0	0	0	0	1	0	0	0	0	0	0	0
BGIR9823_102438	BGIG9823_27094		lncRNA	0	0	0	0	0	0	0	0	0	0	0	0	0	0	0	0	0	0	0
BGIR9823_102065	BGIG9823_26739		lncRNA	0	0	0	0	0	0	0	0	0	0	0	0	0	0	0	0	0	0	0
BGIR9823_102288	BGIG9823_26954		lncRNA	1	0	0	0	0	0	0	0	0	0	0	0	0	0	0	0	0	0	0
BGIR9823_101990	BGIG9823_26666		lncRNA	0	0	0	1	0	0	0	0	0	0	0	0	0	0	0	0	0	0	0
BGIR9823_101966	BGIG9823_26643		lncRNA	0	0	0	0	0	0	0	0	0	0	0	0	0	0	0	0	0	0	0
BGIR9823_101758	BGIG9823_26438		lncRNA	0	0	0	0	0	0	0	0	0	0	0	0	0	0	0	0	0	0	0
BGIR9823_101861	BGIG9823_26540		lncRNA	0	0	0	1	0	0	0	0	0	0	0	0	0	0	0	0	0	0	0

RNA ID	Gene ID	Symbol	Type	novel-ssc-miR1063-5p	ssc-miR-192	ssc-miR-432-5p	ssc-miR-7138-5p	ssc-miR-874	ssc-miR-10386	ssc-miR-122-3p	novel-ssc-miR755-3p	novel-ssc-miR780-3p	novel-ssc-miR804-3p	novel-ssc-miR813-5p	novel-ssc-miR407-5p	novel-ssc-miR573-5p	novel-ssc-miR583-3p	novel-ssc-miR1077-5p	novel-ssc-miR122-3p	novel-ssc-miR154-5p	ssc-miR-127	ssc-miR-194b-5p
BGIR9823_101960	BGIG9823_26637		lncRNA	0	0	0	0	0	0	0	0	0	1	0	0	0	0	0	0	0	0	0
BGIR9823_101964	BGIG9823_26641		lncRNA	0	0	0	0	0	0	0	0	0	0	0	0	0	0	0	0	0	0	0
BGIR9823_102018	BGIG9823_26693		lncRNA	0	0	0	0	0	1	0	0	0	0	0	0	0	0	0	0	0	0	0
BGIR9823_101913	BGIG9823_26590		lncRNA	0	0	1	0	0	0	0	0	0	0	0	0	0	0	0	0	0	0	0
BGIR9823_102617	BGIG9823_27267		lncRNA	0	0	0	0	0	1	0	0	0	0	0	0	0	0	0	0	0	0	0
BGIR9823_102277	BGIG9823_26944		lncRNA	1	0	0	0	0	0	0	0	0	0	0	0	0	0	0	0	0	0	0
BGIR9823_102215	BGIG9823_26887		lncRNA	1	0	0	0	0	0	0	0	0	0	0	0	0	0	0	0	0	0	0
BGIR9823_102525	BGIG9823_27178		lncRNA	0	0	0	0	0	0	0	0	0	0	0	0	0	0	0	0	0	0	0
BGIR9823_102031	BGIG9823_26706		lncRNA	1	0	0	0	0	0	0	0	0	0	0	0	0	0	0	0	0	0	0
BGIR9823_101909	BGIG9823_26586		lncRNA	0	0	0	0	0	0	0	0	0	1	0	0	0	0	0	0	0	0	0
BGIR9823_102249	BGIG9823_26917		lncRNA	0	0	0	0	0	0	0	0	0	0	0	0	0	0	0	0	0	0	0
BGIR9823_102126	BGIG9823_26799		lncRNA	0	0	1	0	0	0	0	0	0	0	0	0	0	0	0	0	0	0	0
BGIR9823_101720	BGIG9823_26404		lncRNA	0	0	0	0	1	0	0	0	0	0	0	0	0	0	0	0	0	0	0
BGIR9823_102111	BGIG9823_26785		lncRNA	0	0	0	0	0	0	0	0	0	1	0	0	0	0	0	0	0	0	0
BGIR9823_101921	BGIG9823_26598		lncRNA	1	0	0	0	0	0	0	0	0	0	0	0	0	0	0	0	0	0	0
BGIR9823_102144	BGIG9823_26817		lncRNA	0	0	1	0	0	0	0	0	0	1	0	1	0	0	0	0	0	0	0
BGIR9823_102006	BGIG9823_26682		lncRNA	0	0	0	0	0	0	0	0	0	0	0	0	0	0	0	0	0	0	0
BGIR9823_102108	BGIG9823_26782		lncRNA	0	0	0	0	0	0	0	0	0	0	0	0	0	0	0	1	0	0	0
BGIR9823_102515	BGIG9823_27169		lncRNA	0	0	0	0	0	0	0	0	0	0	0	1	1	0	0	0	0	0	0
BGIR9823_101872	BGIG9823_26551		lncRNA	0	0	0	0	0	0	0	0	0	0	0	0	0	0	0	0	0	0	0
BGIR9823_101929	BGIG9823_26606		lncRNA	0	0	0	0	0	0	0	0	0	0	0	0	0	0	0	0	0	0	0
BGIR9823_101740	BGIG9823_26421		lncRNA	0	0	0	0	0	0	0	0	0	0	0	0	0	0	0	0	0	0	0
BGIR9823_102035	BGIG9823_26710		lncRNA	0	0	0	0	0	0	0	0	0	1	0	0	0	0	0	0	0	0	0
BGIR9823_102356	BGIG9823_27021		lncRNA	0	0	0	0	0	0	0	0	0	0	0	0	0	0	0	0	0	0	0
BGIR9823_102012	BGIG9823_26688		lncRNA	0	0	0	0	0	0	0	0	0	0	0	0	0	0	0	0	0	0	0
BGIR9823_101980	BGIG9823_26656		lncRNA	0	0	0	0	0	0	0	0	0	0	0	0	0	0	0	0	0	0	0
BGIR9823_102058	BGIG9823_26732		lncRNA	1	0	0	0	0	0	0	0	0	0	0	0	0	0	0	0	0	0	0
BGIR9823_101945	BGIG9823_26622		lncRNA	0	0	0	1	0	0	0	0	0	1	0	0	0	0	0	0	0	0	0
BGIR9823_101770	BGIG9823_26450		lncRNA	0	0	0	0	0	0	0	0	0	0	0	0	0	0	0	0	0	0	0
XR_002341218.1	110258681	LOC110258681	lncRNA	0	0	0	0	0	0	0	0	0	0	0	0	0	0	0	0	0	0	1
BGIR9823_82088	100620215	CREBBP	lncRNA	0	0	0	0	0	0	0	0	0	0	0	0	0	0	1	0	0	0	0
BGIR9823_100016	102157885	LOC102157885	lncRNA	0	0	0	0	0	0	0	0	0	0	0	0	0	0	0	0	0	1	0
BGIR9823_101122	100519810	PHKA2	lncRNA	0	0	0	0	0	0	0	0	0	0	0	0	0	0	0	0	0	0	0
BGIR9823_100002	100627868	LOC100627868	lncRNA	0	0	0	1	0	0	0	0	0	0	0	0	0	0	0	0	0	0	0
BGIR9823_100152	100152012	CST7	lncRNA	0	0	0	0	0	0	0	0	0	0	0	0	0	0	0	0	0	0	0
BGIR9823_100517	100739098	LOC100739098	lncRNA	0	0	0	0	0	0	0	0	0	0	0	0	0	0	0	0	0	0	0
BGIR9823_100137	100155396	ESF1	lncRNA	0	0	0	0	0	0	0	0	0	0	0	0	0	0	0	0	0	1	0
BGIR9823_101191	110257830	LOC110257830	lncRNA	0	0	0	0	0	0	0	0	0	0	0	0	0	0	0	0	1	0	0
BGIR9823_100307	102164659	LOC102164659	lncRNA	0	0	0	0	0	0	0	0	0	0	0	0	0	0	0	0	0	0	0
BGIR9823_101424	100620346	PPP2R5C	lncRNA	0	0	0	0	1	0	0	0	0	0	0	0	0	0	0	0	0	0	0
BGIR9823_100548	100524778	MRPS24	lncRNA	0	0	0	0	1	0	0	0	0	0	0	0	0	0	0	0	0	0	0
BGIR9823_100391	102162486	LOC102162486	lncRNA	0	0	0	0	0	0	0	0	0	0	0	0	0	0	0	0	0	1	0
BGIR9823_81504	100524976	NSD1	lncRNA	0	1	0	0	0	0	0	0	0	0	0	0	0	0	0	0	0	0	1

RNA ID	Gene ID	Symbol	Type	novel-ssc-miR1063-5p	ssc-miR-192	ssc-miR-432-5p	ssc-miR-7138-5p	ssc-miR-874	ssc-miR-10386	ssc-miR-122-3p	novel-ssc-miR755-3p	novel-ssc-miR780-3p	novel-ssc-miR804-3p	novel-ssc-miR813-5p	novel-ssc-miR407-5p	novel-ssc-miR573-5p	novel-ssc-miR583-3p	novel-ssc-miR1077-5p	novel-ssc-miR122-3p	novel-ssc-miR154-5p	ssc-miR-127	ssc-miR-194b-5p
BGIR9823_100217	100156283	NCOA6	lncRNA	0	0	0	0	1	0	0	0	0	0	0	0	0	0	0	0	0	0	0
BGIR9823_101364	110258073	SNAPC4	lncRNA	0	0	0	0	0	0	0	0	0	0	0	0	0	0	0	0	1	0	0
BGIR9823_101532	102158401	LOC102158401	lncRNA	0	0	0	0	0	1	0	0	0	0	0	0	0	0	0	0	1	0	0
BGIR9823_100496	100521938	LOC100521938	lncRNA	0	0	0	0	0	0	0	0	0	0	0	0	0	0	0	0	0	1	0
BGIR9823_77776	100158022	HINT3	lncRNA	0	0	0	0	0	0	0	0	0	0	0	1	0	0	0	0	0	0	0
BGIR9823_100235	110257452	LOC110257452	lncRNA	0	0	0	0	0	0	0	0	0	0	0	0	0	0	0	0	0	0	0
BGIR9823_78349	100152069	SMAD6	lncRNA	0	0	0	0	0	0	0	0	0	0	0	0	0	0	0	0	0	0	0
BGIR9823_100688	100515937	COPG2	lncRNA	0	0	0	0	1	0	0	0	0	0	0	0	0	0	0	0	0	0	0
BGIR9823_101393	110258158	TNRC6A	lncRNA	0	0	0	1	0	0	0	0	0	0	0	0	0	0	0	0	0	0	0
BGIR9823_100562	100516888	GLI3	lncRNA	0	0	0	0	1	0	0	0	0	0	0	0	0	0	0	0	1	0	0
BGIR9823_79603	106509080	LOC106509080	lncRNA	0	0	0	0	0	0	0	0	0	0	0	0	0	0	0	0	0	0	0
BGIR9823_100840	102166569	TBL1X	lncRNA	0	0	0	0	0	1	0	0	0	0	0	0	0	0	0	0	1	0	0
BGIR9823_100717	100511294	PTPRZ1	lncRNA	0	0	0	0	0	0	0	0	0	0	0	0	0	0	0	0	0	1	0
BGIR9823_100350	100737248	WDR60	lncRNA	1	0	0	0	0	0	0	0	0	0	0	0	0	0	0	0	0	0	0
BGIR9823_101426	100516626	WDR20	lncRNA	0	0	0	0	0	0	0	0	0	0	0	0	0	0	0	0	0	0	0
BGIR9823_101427	100516626	WDR20	lncRNA	0	0	0	0	1	0	0	0	0	0	0	0	0	0	0	0	0	0	0
BGIR9823_101520	100511414	RNF122	lncRNA	0	0	0	0	0	0	0	0	0	1	0	0	0	0	0	0	0	0	0
BGIR9823_100500	100522109	ELMO1	lncRNA	0	0	0	0	1	0	0	0	0	0	0	0	0	0	0	0	0	0	0
BGIR9823_101384	110258135	LOC110258135	lncRNA	0	0	0	0	0	0	0	0	0	0	0	0	0	0	0	0	0	0	0
BGIR9823_101222	100517568	MAGT1	lncRNA	0	0	0	0	0	0	0	0	0	0	0	0	0	0	0	0	0	0	0
BGIR9823_100838	102166211	LOC102166211	lncRNA	0	0	0	0	1	0	0	0	0	0	0	0	0	0	0	0	0	0	0
BGIR9823_80389	100516724	NR2F6	lncRNA	0	0	0	0	0	1	0	0	0	0	0	0	0	0	0	0	0	0	0
BGIR9823_100923	100233182	GK	lncRNA	0	0	0	0	0	0	0	0	0	0	0	0	0	0	0	0	0	0	0
BGIR9823_100887	397645	SAT1	lncRNA	0	0	1	0	0	0	0	0	0	0	0	0	0	0	0	0	0	0	0
BGIR9823_100119	100519631	TASP1	lncRNA	0	0	0	0	0	0	0	0	0	0	0	0	0	0	0	0	0	1	0
BGIR9823_101408	110258214	LOC110258214	lncRNA	1	0	0	0	0	0	0	0	0	0	0	0	1	0	0	0	0	0	0
BGIR9823_100257	100156548	ZHX3	lncRNA	0	0	0	0	0	0	0	0	0	1	0	0	0	0	0	0	0	0	0
BGIR9823_100095	100738984	SLC20A2	lncRNA	0	0	0	0	1	0	0	0	0	0	0	1	0	0	0	0	0	0	0
BGIR9823_100711	397301	CALU	lncRNA	0	0	0	0	0	0	0	0	0	0	0	0	0	0	0	0	0	0	0
BGIR9823_100252	110257392	LOC110257392	lncRNA	0	0	0	1	0	0	0	0	0	0	0	0	0	0	0	0	0	0	0
BGIR9823_100342	100625428	RTEL1	lncRNA	0	0	0	0	1	0	0	0	0	0	0	0	0	0	0	0	0	0	0
BGIR9823_82688	102164200	LOC102164200	lncRNA	0	0	0	0	0	0	0	0	0	0	0	0	0	0	0	0	0	0	0
BGIR9823_92711	110255798	LOC110255798	lncRNA	0	0	0	0	0	0	0	0	0	0	0	0	0	0	0	0	0	0	0
BGIR9823_82658	100622402	C3H16orf45	mRNA	0	0	0	0	0	0	0	0	0	0	0	0	0	0	0	0	0	0	0
BGIR9823_100282	100153535	PCIF1	lncRNA	0	0	0	1	0	0	0	0	0	0	0	0	0	0	0	0	0	0	0
XR_002341162.1	110258480	LOC110258480	lncRNA	0	0	0	0	0	0	0	0	0	0	0	0	0	0	0	0	0	0	0
BGIR9823_101217	100514440	ATRX	lncRNA	0	0	0	0	0	0	0	0	0	0	0	0	0	0	0	0	0	0	0
BGIR9823_96121	110256382	LOC110256382	lncRNA	0	0	0	0	0	0	0	0	0	0	0	0	0	0	0	0	0	0	1
BGIR9823_83098	100322871	CCT7	mRNA	0	0	0	0	0	0	0	0	0	0	0	0	0	0	0	0	0	0	0
BGIR9823_81464	100516183	CNOT6	lncRNA	0	0	0	0	0	0	0	0	0	0	0	0	0	0	0	1	0	0	0
BGIR9823_81228	100518418	GATAD2A	mRNA	0	0	0	0	0	0	0	0	0	0	0	0	0	0	1	0	0	0	0
BGIR9823_99373	100514689	MAST4	mRNA	0	0	0	0	0	0	0	0	0	0	0	0	0	0	0	0	0	0	0
BGIR9823_101508	110258454	LETMD1	lncRNA	0	0	0	0	0	0	0	0	0	0	0	0	0	0	0	0	0	0	0

RNA ID	Gene ID	Symbol	Type	novel-ssc-miR1063-5p	ssc-miR-192	ssc-miR-432-5p	ssc-miR-7138-5p	ssc-miR-874	ssc-miR-10386	ssc-miR-122-3p	novel-ssc-miR755-3p	novel-ssc-miR780-3p	novel-ssc-miR804-3p	novel-ssc-miR813-5p	novel-ssc-miR407-5p	novel-ssc-miR573-5p	novel-ssc-miR583-3p	novel-ssc-miR1077-5p	novel-ssc-miR122-3p	novel-ssc-miR154-5p	ssc-miR-127	ssc-miR-194b-5p
BGIR9823_100200	100624021	PLAGL2	lncRNA	0	0	0	0	0	0	0	0	0	0	0	0	0	0	0	0	0	1	0
BGIR9823_93132	100174955	KLF12	lncRNA	0	0	0	0	0	0	0	0	0	0	0	0	0	0	0	0	0	0	1
BGIR9823_101233	100739062	LOC100739062	lncRNA	0	0	0	0	0	0	0	0	0	0	0	0	0	0	0	0	1	0	0
BGIR9823_101439	100625932	MARK3	lncRNA	0	0	0	0	0	0	0	0	0	0	0	0	0	0	0	0	0	0	0
BGIR9823_102584	BGIG9823_27234		lncRNA	0	0	0	0	0	0	0	0	0	0	0	0	0	0	0	0	0	0	0
BGIR9823_102601	BGIG9823_27251		lncRNA	0	0	0	0	0	0	0	0	0	0	0	0	0	0	0	0	0	0	0
BGIR9823_101907	BGIG9823_26584		lncRNA	0	0	0	0	0	0	0	0	0	0	0	0	0	0	0	0	0	0	0
BGIR9823_101683	BGIG9823_26370		lncRNA	0	0	1	0	0	0	0	0	0	0	0	0	0	0	0	0	0	0	0
BGIR9823_101932	BGIG9823_26609		lncRNA	0	0	0	0	0	0	0	0	0	0	0	0	0	0	0	0	0	0	0
BGIR9823_102312	BGIG9823_26977		lncRNA	0	0	1	0	0	0	0	0	0	0	0	0	1	0	0	0	0	0	0
BGIR9823_101712	BGIG9823_26396		lncRNA	0	0	0	0	0	0	0	0	0	0	1	0	0	0	0	0	1	0	0
BGIR9823_102749	BGIG9823_27392		lncRNA	0	0	0	0	0	0	0	0	0	0	0	0	0	0	0	0	0	0	0
BGIR9823_101726	BGIG9823_26409		lncRNA	0	0	0	0	0	0	0	0	0	0	0	0	0	0	0	0	0	0	0
BGIR9823_102386	BGIG9823_27051		lncRNA	0	0	0	0	0	0	0	0	0	0	0	0	0	0	0	0	0	0	0
BGIR9823_102032	BGIG9823_26707		lncRNA	0	0	0	0	0	0	0	0	0	0	0	0	0	0	0	0	0	0	0
BGIR9823_102336	BGIG9823_27001		lncRNA	0	0	0	0	0	0	0	0	0	0	0	0	0	0	0	0	0	0	0
BGIR9823_101794	BGIG9823_26474		lncRNA	0	0	0	0	0	0	0	0	0	1	0	0	0	0	0	0	0	0	0
BGIR9823_101752	BGIG9823_26433		lncRNA	0	0	0	1	0	0	0	0	0	1	0	0	0	0	0	0	0	0	0
BGIR9823_101944	BGIG9823_26621		lncRNA	0	0	0	0	0	0	0	0	0	1	0	0	0	0	0	0	0	0	0
BGIR9823_101873	BGIG9823_26552		lncRNA	1	0	0	0	0	0	0	0	0	0	0	0	0	0	0	0	0	0	0
BGIR9823_101671	BGIG9823_26359		lncRNA	0	0	0	0	0	0	0	0	0	0	0	0	0	0	0	0	1	0	0
BGIR9823_101974	BGIG9823_26651		lncRNA	0	0	0	0	0	0	0	0	0	0	0	0	0	0	0	1	0	0	0
BGIR9823_102380	BGIG9823_27045		lncRNA	0	0	0	0	0	0	0	0	0	0	0	0	0	0	0	0	0	0	0
BGIR9823_102232	BGIG9823_26904		lncRNA	1	0	0	0	0	0	0	0	0	0	0	0	0	0	0	0	0	0	0
BGIR9823_102238	BGIG9823_26910		lncRNA	0	0	0	0	0	0	0	0	0	0	0	0	0	0	0	0	0	0	0
BGIR9823_102071	BGIG9823_26745		lncRNA	0	0	0	0	0	0	0	0	0	0	1	0	0	0	0	0	0	0	0
BGIR9823_101784	BGIG9823_26464		lncRNA	0	0	0	0	0	0	0	0	0	0	0	0	0	0	0	0	0	0	0
BGIR9823_102392	BGIG9823_27057		lncRNA	0	0	0	0	0	1	0	0	0	0	0	0	0	0	0	0	0	0	0
BGIR9823_102420	BGIG9823_27081		lncRNA	0	0	0	0	0	0	0	0	0	0	0	0	0	0	0	0	0	0	0
BGIR9823_101988	BGIG9823_26664		lncRNA	0	0	0	0	0	0	0	0	0	0	0	0	0	0	0	0	0	0	0
BGIR9823_101675	BGIG9823_26362		lncRNA	0	0	0	0	0	0	0	0	0	0	1	0	0	0	0	0	0	0	0
BGIR9823_101761	BGIG9823_26441		lncRNA	0	0	0	0	0	0	0	0	0	0	1	0	0	0	0	0	0	0	0
BGIR9823_102382	BGIG9823_27047		lncRNA	0	0	0	0	0	0	0	0	0	0	0	1	0	0	0	0	0	0	0
BGIR9823_102045	BGIG9823_26719		lncRNA	0	1	0	0	0	0	0	0	0	0	0	0	1	0	0	0	0	0	0
BGIR9823_101857	BGIG9823_26536		lncRNA	0	0	0	0	0	0	0	0	0	1	1	0	0	0	0	0	0	0	0
BGIR9823_101775	BGIG9823_26455		lncRNA	0	0	0	0	0	0	0	0	0	0	0	0	0	0	0	0	0	0	0
BGIR9823_102446	BGIG9823_27101		lncRNA	0	0	0	0	0	0	0	0	0	0	0	0	0	0	0	0	0	0	0
BGIR9823_102179	BGIG9823_26851		lncRNA	0	0	0	0	0	0	0	0	0	0	0	0	0	0	0	0	0	0	0
BGIR9823_101678	BGIG9823_26365		lncRNA	0	0	0	0	1	1	0	0	0	0	0	0	0	0	0	0	0	0	0
BGIR9823_102242	BGIG9823_26913		lncRNA	0	0	0	0	0	0	0	0	0	0	0	0	0	0	0	0	0	0	0
BGIR9823_102633	BGIG9823_27283		lncRNA	0	0	0	0	0	0	0	0	0	0	0	0	0	0	0	0	0	0	0
BGIR9823_102205	BGIG9823_26877		lncRNA	1	0	0	0	0	0	0	0	0	0	0	0	0	0	0	0	0	0	0
BGIR9823_101763	BGIG9823_26443		lncRNA	0	0	0	0	0	0	0	0	0	1	0	0	0	0	0	0	0	0	0

RNA ID	Gene ID	Symbol	Type	novel-ssc-miR1063-5p	ssc-miR-192	ssc-miR-432-5p	ssc-miR-7138-5p	ssc-miR-874	ssc-miR-10386	ssc-miR-122-3p	novel-ssc-miR755-3p	novel-ssc-miR780-3p	novel-ssc-miR804-3p	novel-ssc-miR813-5p	novel-ssc-miR407-5p	novel-ssc-miR573-5p	novel-ssc-miR583-3p	novel-ssc-miR1077-5p	novel-ssc-miR122-3p	novel-ssc-miR154-5p	ssc-miR-127	ssc-miR-194b-5p
BGIR9823_102754	BGIG9823_27394		lncRNA	0	0	0	0	0	0	0	0	0	0	0	1	0	0	0	0	0	0	0
BGIR9823_102077	BGIG9823_26751		lncRNA	0	0	0	0	0	0	0	0	0	0	0	0	0	0	0	0	0	0	0
BGIR9823_102457	BGIG9823_27111		lncRNA	0	0	0	0	0	0	0	0	0	0	0	0	0	0	0	1	0	0	0
BGIR9823_101881	BGIG9823_26560		lncRNA	0	0	0	0	0	0	0	0	0	0	1	0	0	0	0	0	0	0	0
BGIR9823_102086	BGIG9823_26760		lncRNA	0	0	0	0	0	0	0	0	0	1	0	0	0	0	0	0	0	0	0
BGIR9823_102146	BGIG9823_26819		lncRNA	0	0	0	0	0	1	0	0	0	0	0	0	0	0	0	0	0	0	0
BGIR9823_101735	BGIG9823_26416		lncRNA	0	0	1	0	0	0	0	0	0	0	0	0	1	0	0	0	0	0	0
BGIR9823_102132	BGIG9823_26805		lncRNA	1	0	0	0	0	0	0	0	0	0	0	0	0	0	0	0	0	0	0
BGIR9823_102345	BGIG9823_27010		lncRNA	0	0	0	0	0	0	0	0	0	0	0	0	0	0	0	0	0	0	0
BGIR9823_101715	BGIG9823_26399		lncRNA	0	0	0	0	0	0	0	0	0	0	0	0	0	0	0	0	1	0	0
BGIR9823_102322	BGIG9823_26987		lncRNA	1	0	0	0	0	0	0	0	0	0	0	0	0	0	0	0	0	0	0
BGIR9823_102695	BGIG9823_27345		lncRNA	0	0	0	0	0	0	0	0	0	0	0	0	0	0	0	0	0	0	0
BGIR9823_101684	BGIG9823_26371		lncRNA	0	0	0	0	0	0	0	0	0	0	0	0	0	0	0	0	0	0	0
BGIR9823_102344	BGIG9823_27009		lncRNA	0	0	0	0	0	0	0	0	0	0	0	1	0	0	0	0	0	0	0
BGIR9823_102092	BGIG9823_26766		lncRNA	0	0	0	0	0	0	0	0	0	0	0	0	1	0	0	0	0	0	0
BGIR9823_102073	BGIG9823_26747		lncRNA	0	0	0	0	0	0	0	0	0	0	1	1	0	0	0	0	0	0	0
BGIR9823_102188	BGIG9823_26860		lncRNA	0	0	0	0	0	0	0	0	0	1	0	0	0	0	0	1	0	0	0
BGIR9823_101946	BGIG9823_26623		lncRNA	0	0	0	0	0	0	0	0	0	0	1	0	0	0	0	0	0	0	0
BGIR9823_102176	BGIG9823_26848		lncRNA	0	1	0	0	0	0	0	0	0	0	0	0	0	0	0	0	0	0	0
BGIR9823_102387	BGIG9823_27052		lncRNA	0	0	0	0	0	0	0	0	0	0	0	0	0	0	0	0	0	0	0
BGIR9823_101876	BGIG9823_26555		lncRNA	0	0	0	0	0	0	0	0	0	0	1	0	0	0	0	0	0	0	0
BGIR9823_101888	BGIG9823_26565		lncRNA	0	0	0	0	0	0	0	0	0	0	0	0	0	0	0	0	0	0	0
BGIR9823_101818	BGIG9823_26498		lncRNA	0	0	1	0	0	0	0	0	0	0	0	0	0	0	0	0	0	0	0
BGIR9823_102320	BGIG9823_26985		lncRNA	0	0	0	0	0	0	0	0	0	0	0	0	0	0	0	0	0	0	0
BGIR9823_102080	BGIG9823_26754		lncRNA	0	0	0	0	0	0	0	0	0	0	0	0	0	0	0	0	0	0	0
BGIR9823_101786	BGIG9823_26466		lncRNA	0	0	0	0	0	0	0	0	0	0	0	0	0	0	0	0	0	0	0
BGIR9823_102325	BGIG9823_26990		lncRNA	1	0	0	0	0	0	0	0	0	0	0	0	0	0	0	0	0	0	0
BGIR9823_101989	BGIG9823_26665		lncRNA	0	0	0	0	0	0	0	0	0	0	0	0	0	0	0	0	0	0	0
BGIR9823_101889	BGIG9823_26566		lncRNA	0	0	0	0	0	0	0	0	0	0	0	1	0	0	0	0	0	0	0
BGIR9823_102669	BGIG9823_27319		lncRNA	0	0	0	0	0	0	0	0	0	0	0	0	0	0	1	0	0	0	0
BGIR9823_101878	BGIG9823_26557		lncRNA	1	0	0	0	0	0	0	0	0	0	0	0	0	0	0	0	0	0	0
BGIR9823_101918	BGIG9823_26595		lncRNA	0	0	0	0	0	0	0	0	0	0	0	0	0	0	0	0	0	0	0
BGIR9823_102064	BGIG9823_26738		lncRNA	0	0	0	0	0	0	0	0	0	1	0	0	0	0	0	0	0	0	0
BGIR9823_101985	BGIG9823_26661		lncRNA	0	0	0	0	0	0	0	0	0	0	0	0	0	0	0	0	0	0	0
BGIR9823_101774	BGIG9823_26454		lncRNA	0	0	0	0	0	0	0	0	0	0	0	0	0	0	0	0	0	0	0
BGIR9823_102112	BGIG9823_26786		lncRNA	0	0	0	0	0	0	0	0	0	0	0	0	0	0	0	0	0	0	0
BGIR9823_102585	BGIG9823_27235		lncRNA	0	0	0	0	0	0	0	0	0	0	0	0	0	0	0	0	0	0	0
BGIR9823_101903	BGIG9823_26580		lncRNA	0	0	0	0	0	0	0	0	0	0	0	0	0	0	0	0	0	0	0
BGIR9823_102173	BGIG9823_26846		lncRNA	1	0	0	0	0	0	0	0	0	0	0	0	0	0	0	0	0	0	0
BGIR9823_101912	BGIG9823_26589		lncRNA	0	0	0	0	0	0	0	0	0	0	0	0	0	0	0	0	0	0	0
BGIR9823_101793	BGIG9823_26473		lncRNA	0	0	0	0	0	0	0	0	0	0	0	0	0	0	0	0	0	0	0
BGIR9823_102135	BGIG9823_26808		lncRNA	1	0	0	0	0	0	0	0	0	0	0	0	0	0	0	0	0	0	0
BGIR9823_101911	BGIG9823_26588		lncRNA	0	0	0	0	0	0	0	0	0	0	0	0	0	0	0	0	0	0	0

RNA ID	Gene ID	Symbol	Type	novel-ssc-miR1063-5p	ssc-miR-192	ssc-miR-432-5p	ssc-miR-7138-5p	ssc-miR-874	ssc-miR-10386	ssc-miR-122-3p	novel-ssc-miR755-3p	novel-ssc-miR780-3p	novel-ssc-miR804-3p	novel-ssc-miR813-5p	novel-ssc-miR407-5p	novel-ssc-miR573-5p	novel-ssc-miR583-3p	novel-ssc-miR1077-5p	novel-ssc-miR122-3p	novel-ssc-miR154-5p	ssc-miR-127	ssc-miR-194b-5p
BGIR9823_101864	BGIG9823_26543		lncRNA	0	0	0	0	0	0	0	0	0	0	0	0	0	0	0	0	0	0	0
BGIR9823_101851	BGIG9823_26530		lncRNA	0	0	0	0	0	0	0	0	0	0	0	0	0	0	0	0	0	0	0
BGIR9823_102310	BGIG9823_26975		lncRNA	0	0	0	0	0	0	0	0	0	0	0	0	0	0	0	0	0	0	0
BGIR9823_101776	BGIG9823_26456		lncRNA	0	0	0	0	0	0	0	0	0	0	0	0	0	0	0	0	0	0	0
BGIR9823_102381	BGIG9823_27046		lncRNA	0	0	0	0	0	0	0	0	0	0	0	0	0	0	0	1	0	0	0
BGIR9823_101680	BGIG9823_26367		lncRNA	0	0	0	0	0	0	0	0	0	0	0	0	0	0	0	0	0	0	0
BGIR9823_101747	BGIG9823_26428		lncRNA	0	0	0	0	0	0	0	0	0	0	0	0	0	0	0	1	0	0	0
BGIR9823_101830	BGIG9823_26509		lncRNA	0	0	1	0	0	0	0	0	0	0	0	1	0	0	0	0	0	0	0
BGIR9823_102332	BGIG9823_26997		lncRNA	0	0	0	0	0	0	0	0	0	0	0	0	0	0	0	0	0	0	0
XR_002336732.1	102159417	LOC102159417	lncRNA	0	0	0	0	0	0	0	0	0	0	0	0	0	0	1	0	0	0	0
XR_002343571.1	106507458	LOC106507458	lncRNA	0	0	0	0	0	0	0	0	0	0	0	0	0	0	0	0	0	0	0
XR_001304304.2	106507921	LOC106507921	lncRNA	0	0	0	0	0	0	0	0	0	0	0	0	0	0	0	0	0	0	0
XR_002344048.1	110260709	LOC110260709	lncRNA	0	0	0	0	0	0	0	0	0	0	0	0	0	0	0	0	0	0	1
XM_003353597.4	100621832	TMEM246	mRNA	0	0	0	0	0	0	0	0	0	0	0	0	0	0	0	0	0	0	0
XR_002338514.1	102161206	LOC102161206	lncRNA	0	0	0	0	0	0	0	0	0	0	0	0	0	0	0	0	0	0	0
NM_001244791.1	100513225	BAG1	mRNA	0	0	0	0	0	0	0	0	0	0	0	0	0	0	0	0	0	0	0
XR_002341355.1	102167380	CUNH21orf58	lncRNA	0	0	0	0	0	0	0	0	0	0	0	0	0	0	0	0	0	0	1
XR_001299235.2	106505126	LOC106505126	lncRNA	0	0	0	0	0	0	0	0	0	0	0	0	0	0	0	0	0	0	0
XR_304631.3	102167686	LOC102167686	lncRNA	0	0	0	0	0	0	0	0	0	0	0	0	0	0	0	0	0	0	1
XM_021066717.1	100521576	ARSG	mRNA	0	0	0	0	0	0	0	0	0	0	0	0	0	0	0	0	0	0	0
XR_002342261.1	110259855	LOC110259855	lncRNA	0	0	0	0	0	0	0	0	0	0	0	0	0	0	0	0	0	0	1
NM_213891.1	396925	VCAM1	mRNA	0	0	0	0	0	0	0	0	0	0	0	0	0	0	0	0	0	0	0
XM_003129787.4	100516505	VSTM5	mRNA	0	0	0	0	0	0	0	0	0	0	0	0	0	0	0	0	0	0	0
XM_021100543.1	106504722	UCP1	mRNA	0	0	0	0	0	0	0	0	0	0	0	0	0	0	0	0	0	0	0
XR_002345110.1	110261202	LOC110261202	lncRNA	0	0	0	0	0	0	0	0	0	0	0	0	0	0	0	0	0	0	0
XM_013992908.2	102164011	LOC102164011	mRNA	0	0	0	0	0	0	0	0	0	0	0	0	0	0	0	0	0	0	0
XM_021082022.1	100525982	LOC100525982	mRNA	0	0	0	0	0	0	0	0	0	0	0	0	0	0	0	0	0	0	0
NM_214188.1	397385	DDOST	mRNA	0	0	0	0	0	0	0	0	0	0	0	0	0	0	0	0	0	0	0
XM_013983344.2	100156215	MSS51	mRNA	0	0	0	0	0	0	0	0	0	0	0	0	0	0	0	0	0	0	0
NM_001164856.1	100303723	LCAT	mRNA	0	0	0	0	0	0	0	0	0	0	0	0	0	0	0	0	0	0	0
NM_001278748.1	100520980	CASR	mRNA	0	0	0	0	0	0	0	0	0	0	0	0	0	0	0	0	0	0	0
XR_002342824.1	110260048	LOC110260048	lncRNA	0	0	0	0	0	0	0	0	0	0	0	0	0	0	0	0	0	0	1
XM_003481630.4	100738263	SPRYD4	mRNA	0	0	0	0	0	0	0	0	0	0	0	0	0	0	0	0	0	0	0
XR_002340516.1	102163798	LOC102163798	lncRNA	0	0	0	0	0	0	0	0	0	0	0	0	0	0	0	0	0	0	1
XM_021080156.1	102167889	LOC102167889	mRNA	0	0	0	0	0	0	0	0	0	0	0	0	0	0	0	0	0	0	0
XR_002343904.1	110260647	LOC110260647	lncRNA	0	0	0	0	0	0	0	0	0	0	0	0	0	0	1	0	0	0	0
XM_021082023.1	100510954	LOC100510954	mRNA	0	0	0	0	0	0	0	0	0	0	0	0	0	0	0	0	0	0	0
XR_002340992.1	110258138	LOC110258138	lncRNA	0	0	0	0	0	0	0	0	0	0	0	0	0	0	0	0	0	0	1
XM_003129097.6	100523679	CCNI	mRNA	0	0	0	0	0	0	0	0	0	0	0	0	0	0	0	0	0	0	0
XM_021086388.1	110255213	ARMC5	mRNA	0	0	0	0	0	0	0	0	0	0	0	0	0	0	0	0	0	0	0
XM_003133626.3	100522220	LOC100522220	mRNA	0	0	0	0	0	0	0	0	0	0	0	0	0	0	0	0	0	0	0
XR_002341145.1	110258406	LOC110258406	lncRNA	0	0	0	0	0	0	0	0	0	0	0	0	0	0	0	0	0	0	0
XR_002341292.1	110258990	LOC110258990	lncRNA	0	0	0	0	0	0	0	0	0	0	0	0	0	0	0	0	0	0	0

RNA ID	Gene ID	Symbol	Type	novel-ssc-miR1063-5p	ssc-miR-192	ssc-miR-432-5p	ssc-miR-7138-5p	ssc-miR-874	ssc-miR-10386	ssc-miR-122-3p	novel-ssc-miR755-3p	novel-ssc-miR780-3p	novel-ssc-miR804-3p	novel-ssc-miR813-5p	novel-ssc-miR407-5p	novel-ssc-miR573-5p	novel-ssc-miR583-3p	novel-ssc-miR1077-5p	novel-ssc-miR122-3p	novel-ssc-miR154-5p	ssc-miR-127	ssc-miR-194b-5p
NM_001204772.1	100533540	BRD4	mRNA	0	0	0	0	0	0	0	0	0	0	0	0	0	0	0	0	0	0	0
NM_213763.2	396637	UCHL1	mRNA	0	0	0	0	0	0	0	0	0	0	0	0	0	0	0	0	0	0	0
NM_214050.2	397117	GSTO1	mRNA	0	0	0	0	0	0	0	0	0	0	0	0	0	0	0	0	0	0	0
XR_002337239.1	110256047	LOC110256047	lncRNA	0	0	0	0	0	0	0	0	0	0	0	0	0	0	1	0	0	0	0
XR_002343522.1	110260355	LOC110260355	lncRNA	0	0	0	0	0	0	0	0	0	0	0	0	0	0	1	0	0	0	0
XR_001304268.2	106507904	LOC106507904	lncRNA	0	0	0	0	0	0	0	0	0	0	0	0	0	0	0	0	0	0	0
XR_002343855.1	110260615	LOC110260615	lncRNA	0	0	0	0	0	0	0	0	0	0	0	0	0	0	1	0	0	0	0
XR_002336945.1	110255950	LOC110255950	lncRNA	0	0	0	0	0	0	0	0	0	0	0	0	0	0	0	0	0	0	1
XM_021075808.1	110257099	LOC110257099	mRNA	0	0	0	0	0	0	0	0	0	0	0	0	0	0	0	0	0	0	0
XM_021082410.1	110259094	LOC110259094	mRNA	0	0	0	0	0	0	0	0	0	0	0	0	0	0	0	0	0	0	0
XR_002341290.1	110258990	LOC110258990	lncRNA	0	0	0	0	0	0	0	0	0	0	0	0	0	0	0	0	0	0	1
XM_021100189.1	100152943	ADTRP	mRNA	0	0	0	0	0	0	0	0	0	0	0	0	0	0	0	0	0	0	0
XR_002341433.1	106509301	LOC106509301	lncRNA	0	0	0	0	0	0	0	0	0	0	0	0	0	0	0	0	0	0	0
NM_001244722.1	100512802	PUS3	mRNA	0	0	0	0	0	0	0	0	0	0	0	0	0	0	0	0	0	0	0
XM_021092065.1	100624597	YARS2	mRNA	0	0	0	0	0	0	0	0	0	0	0	0	0	0	0	0	0	0	0
XM_021066519.1	100512084	TBC1D2	mRNA	0	0	0	0	0	0	0	0	0	0	0	0	0	0	0	0	0	0	0
XR_002335835.1	110255476	LOC110255476	lncRNA	0	0	0	0	0	0	0	0	0	0	0	0	0	0	1	0	0	0	0
XR_002335866.1	110255489	LOC110255489	lncRNA	0	0	0	0	0	0	0	0	0	0	0	0	0	0	0	0	0	0	0
XR_002337288.1	106509234	LOC106509234	lncRNA	0	0	0	0	0	0	0	0	0	0	0	0	0	0	1	0	0	0	0
XR_002342643.1	106508312	LOC106508312	lncRNA	0	0	0	0	0	0	0	0	0	0	0	0	0	0	1	0	0	0	0
NM_001097492.1	100038008	CD1E	mRNA	0	0	0	0	0	0	0	0	0	0	0	0	0	0	0	0	0	0	0
XR_002336367.1	106507918	LOC106507918	lncRNA	0	0	0	0	0	0	0	0	0	0	0	0	0	0	0	0	0	0	0
XR_002340833.1	110257824	LOC110257824	lncRNA	0	0	0	0	0	0	0	0	0	0	0	0	0	0	0	0	0	0	0
NM_001004028.1	445513	HAPLN1	mRNA	0	0	0	0	0	0	0	0	0	0	0	0	0	0	0	0	0	0	0
XR_001302352.2	106506794	LOC106506794	lncRNA	0	0	0	0	0	0	0	0	0	0	0	0	0	0	0	0	0	0	0
XR_002336622.1	110255794	LOC110255794	lncRNA	0	0	0	0	0	0	0	0	0	0	0	0	0	0	0	0	0	0	1
XM_021070652.1	110256443	QARS	mRNA	0	0	0	0	0	0	0	0	0	0	0	0	0	0	0	0	0	0	0
XM_001928752.5	100154892	LOC100154892	mRNA	0	0	0	0	0	0	0	0	0	0	0	0	0	0	0	0	0	0	0
NM_001243696.1	100158051	DRAM2	mRNA	0	0	0	0	0	0	0	0	0	0	0	0	0	0	0	0	0	0	0
XR_002342202.1	100512593	LOC100512593	lncRNA	0	0	0	0	0	0	0	0	0	0	0	0	0	0	1	0	0	0	0
XR_002343188.1	110260249	LOC110260249	lncRNA	0	0	0	0	0	0	0	0	0	0	0	0	0	0	1	0	0	0	0
XM_021081808.1	100155561	LOC100155561	mRNA	0	0	0	0	0	0	0	0	0	0	0	0	0	0	0	0	0	0	0
XM_003134082.6	100515063	HMMR	mRNA	0	0	0	0	0	0	0	0	0	0	0	0	0	0	0	0	0	0	0
XR_002341327.1	110259136	LOC110259136	lncRNA	0	0	0	0	0	0	0	0	0	0	0	0	0	0	1	0	0	0	0
XR_002346274.1	110261765	LOC110261765	lncRNA	0	0	0	0	0	0	0	0	0	0	0	0	0	0	1	0	0	0	0
URS0000BC4F80	BGIG9823_26330		lncRNA	0	0	0	0	0	0	0	0	0	0	0	0	0	0	1	0	0	0	0
URS0000BC82B3	BGIG9823_26290		lncRNA	0	0	0	0	0	0	0	0	0	0	0	0	0	0	0	0	0	0	1
BGIR9823_100417	106506781	LOC106506781	lncRNA	0	0	0	0	0	0	0	0	0	0	0	0	0	0	0	0	0	0	0
BGIR9823_78908	100157533	WTAP	mRNA	0	0	0	0	0	0	0	0	0	0	0	0	0	0	1	0	0	0	0
BGIR9823_85709	100156112	ATP6V1E1	lncRNA	0	0	0	0	0	0	0	0	0	0	0	0	0	0	0	0	0	0	0
BGIR9823_88195	100152838	SLC22A23	lncRNA	0	0	0	0	0	1	0	0	0	0	0	0	1	0	0	0	0	0	0
BGIR9823_81246	100174943	IFI30	mRNA	0	0	0	0	0	0	0	0	0	0	0	0	0	0	1	0	0	0	0
BGIR9823_100836	100624032	LOC100624032	lncRNA	0	0	0	0	1	0	0	0	0	0	0	0	0	0	0	0	0	0	0

RNA ID	Gene ID	Symbol	Type	novel-ssc-miR1063-5p	ssc-miR-192	ssc-miR-432-5p	ssc-miR-7138-5p	ssc-miR-874	ssc-miR-10386	ssc-miR-122-3p	novel-ssc-miR755-3p	novel-ssc-miR780-3p	novel-ssc-miR804-3p	novel-ssc-miR813-5p	novel-ssc-miR407-5p	novel-ssc-miR573-5p	novel-ssc-miR583-3p	novel-ssc-miR1077-5p	novel-ssc-miR122-3p	novel-ssc-miR154-5p	ssc-miR-127	ssc-miR-194b-5p
BGIR9823_84507	100154147	MAN1A2	lncRNA	0	0	0	0	0	0	0	0	0	0	0	0	0	0	0	0	0	0	0
BGIR9823_78933	100516653	CCDC170	lncRNA	0	0	0	0	0	0	0	0	0	0	0	0	0	0	0	1	0	0	0
BGIR9823_85930	106510465	LOC106510465	mRNA	0	0	0	0	0	0	0	0	0	0	0	0	0	0	1	0	0	0	0
BGIR9823_98132	100519056	ORC4	lncRNA	0	0	0	0	0	0	0	0	0	0	0	0	0	0	1	0	0	0	0
BGIR9823_85554	100624497	DNM1L	mRNA	0	0	0	0	0	0	0	0	0	0	0	0	0	0	0	0	0	0	0
BGIR9823_89252	100153410	STK38	mRNA	0	0	0	0	0	0	0	0	0	0	0	0	0	0	0	0	0	0	0
BGIR9823_100096	100738984	SLC20A2	lncRNA	0	0	0	0	0	0	0	0	0	0	0	0	0	0	0	0	0	0	0
BGIR9823_85945	100521092	WDR59	mRNA	0	0	0	0	0	0	0	0	0	0	0	0	0	0	1	0	0	0	0
BGIR9823_85409	100516908	NCKAP5L	lncRNA	0	0	0	0	0	0	0	0	0	0	0	0	0	0	0	0	0	0	0
BGIR9823_100573	100516888	GLI3	lncRNA	0	0	1	0	0	0	0	0	0	1	0	0	0	0	0	0	0	0	0
BGIR9823_101467	100522195	XRCC3	lncRNA	0	0	0	0	0	0	0	0	0	0	0	0	0	0	0	0	0	0	0
BGIR9823_86462	100738753	RERE	lncRNA	0	0	0	0	0	0	0	0	0	0	0	0	0	0	0	0	0	0	0
BGIR9823_100236	106504144	DLGAP4	lncRNA	0	0	0	0	0	0	0	0	0	0	0	0	0	0	0	0	0	0	0
BGIR9823_100421	100626356	UBE2H	lncRNA	0	0	0	0	0	0	0	0	0	0	1	0	0	0	0	0	0	0	0
BGIR9823_101512	110258473	LOC110258473	lncRNA	0	0	0	0	1	0	0	0	0	0	0	0	0	0	0	0	0	0	0
BGIR9823_100354	102167342	CNPY1	lncRNA	0	0	0	0	0	0	0	0	0	0	0	0	0	0	0	0	0	0	0
BGIR9823_100645	100519065	UBN2	lncRNA	0	0	0	0	0	0	0	0	0	0	1	0	0	0	0	0	0	1	0
BGIR9823_100414	414383	CHCHD3	lncRNA	0	0	0	1	0	0	0	0	0	0	0	0	0	0	0	0	0	1	0
BGIR9823_81026	100523886	SF1	lncRNA	0	0	0	0	0	0	0	0	0	0	0	0	0	0	0	0	0	0	0
BGIR9823_100005	106506050	LOC106506050	lncRNA	0	0	0	0	1	0	0	0	0	0	0	0	0	0	0	0	0	0	0
BGIR9823_81229	100518418	GATAD2A	lncRNA	0	0	0	0	0	0	0	0	0	0	0	0	0	0	0	0	0	0	0
BGIR9823_79698	100154949	CNIH1	mRNA	0	0	0	0	0	0	0	0	0	0	0	0	0	0	0	0	0	0	1
BGIR9823_100531	100625903	SKAP2	lncRNA	0	0	0	0	1	0	0	0	0	0	0	0	0	0	0	0	0	1	0
BGIR9823_81900	110259864	LOC110259864	lncRNA	0	0	0	0	0	0	0	0	0	0	0	0	0	0	0	0	0	0	0
BGIR9823_100425	100626356	UBE2H	lncRNA	0	0	0	0	0	0	0	0	0	1	0	0	0	0	0	0	0	0	0
BGIR9823_82216	100511252	ACTR1B	lncRNA	0	0	0	0	0	0	0	0	0	0	0	0	0	0	0	0	0	0	0
BGIR9823_100156	100519522	NINL	lncRNA	0	0	0	0	0	0	0	0	0	0	0	0	0	0	0	0	0	0	0
BGIR9823_101565	110258600	LOC110258600	lncRNA	0	0	0	0	0	0	0	0	0	0	0	0	0	0	1	0	0	0	0
BGIR9823_100677	106504059	EXOC4	lncRNA	0	0	0	0	0	0	0	0	0	0	1	0	0	0	0	0	0	0	0
BGIR9823_100501	100523875	7-Sep	lncRNA	0	0	0	0	0	0	0	0	0	0	0	0	0	0	0	0	0	0	0
BGIR9823_100856	100154431	ASB11	lncRNA	0	0	0	0	0	0	0	0	0	0	0	0	0	0	0	0	0	1	0
BGIR9823_92258	100738420	ABI1	lncRNA	0	0	0	0	0	0	0	0	0	0	0	0	0	0	1	0	0	0	0
BGIR9823_97570	100158098	RHBDD3	lncRNA	0	0	0	0	0	0	0	0	0	0	0	0	0	0	1	0	0	0	0
BGIR9823_101254	106507023	LOC106507023	lncRNA	0	1	0	0	0	0	0	0	0	0	0	0	0	0	0	0	0	0	0
BGIR9823_100601	102159531	ACTR3B	mRNA	0	0	0	0	0	0	0	0	0	0	0	0	0	0	1	0	0	0	0
BGIR9823_83153	110260004	AHSA2P	mRNA	0	0	0	0	0	0	0	0	0	0	0	0	0	0	0	0	0	0	0
BGIR9823_90913	110255435	LOC110255435	lncRNA	0	0	0	0	0	0	0	0	0	0	0	0	0	0	0	0	0	0	0
BGIR9823_100272	396886	SLPI	lncRNA	0	0	0	0	0	0	0	0	0	0	0	0	0	0	0	0	0	0	0
BGIR9823_77799	100520524	GOPC	lncRNA	0	0	0	0	0	0	0	0	0	0	0	0	0	0	0	0	0	0	0
BGIR9823_100990	100153260	APOOL	lncRNA	0	0	0	0	0	0	0	0	0	0	0	0	0	0	0	0	0	0	0
BGIR9823_79735	110255453	LOC110255453	lncRNA	0	0	0	0	0	1	0	0	0	0	0	0	0	0	0	0	0	0	0
BGIR9823_80994	100519350	DPP3	mRNA	0	0	0	0	0	0	0	0	0	0	0	0	0	0	1	0	0	0	0
BGIR9823_100422	100626356	UBE2H	lncRNA	0	0	0	0	0	0	0	0	0	0	0	0	0	0	0	0	0	1	0

RNA ID	Gene ID	Symbol	Type	novel-ssc-miR1063-5p	ssc-miR-192	ssc-miR-432-5p	ssc-miR-7138-5p	ssc-miR-874	ssc-miR-10386	ssc-miR-122-3p	novel-ssc-miR755-3p	novel-ssc-miR780-3p	novel-ssc-miR804-3p	novel-ssc-miR813-5p	novel-ssc-miR407-5p	novel-ssc-miR573-5p	novel-ssc-miR583-3p	novel-ssc-miR1077-5p	novel-ssc-miR122-3p	novel-ssc-miR154-5p	ssc-miR-127	ssc-miR-194b-5p
BGIR9823_95751	100627663	ARHGEF3	lncRNA	0	0	0	0	0	0	0	0	0	0	0	0	0	0	0	0	0	0	1
BGIR9823_78308	100154901	ATP9B	lncRNA	0	1	0	0	0	0	0	0	0	0	0	0	0	0	0	0	0	0	0
BGIR9823_89654	733584	FOXN3	lncRNA	0	0	0	0	0	0	0	0	0	0	0	0	0	0	0	0	0	0	1
BGIR9823_94919	100737976	RBSN	lncRNA	0	0	0	0	0	0	0	0	0	0	0	0	0	0	0	0	0	0	0
BGIR9823_83670	100153676	PCMTD1	lncRNA	0	0	0	0	0	0	0	0	0	0	0	0	0	0	0	0	0	0	0
BGIR9823_100051	100621535	TPD52L2	lncRNA	0	0	0	0	0	0	0	0	0	0	0	0	0	0	0	0	0	1	0
BGIR9823_78866	100512264	NTNG2	lncRNA	0	0	0	0	0	0	0	0	0	0	0	0	0	0	0	0	0	0	0
BGIR9823_100572	100516888	GLI3	lncRNA	1	0	0	0	0	0	0	0	0	0	0	0	0	0	0	0	0	0	0
BGIR9823_89017	100157526	MYLK4	lncRNA	0	0	0	0	0	0	0	0	0	0	0	0	0	0	0	0	0	0	0
BGIR9823_78135	100153621	FAM214A	lncRNA	0	0	0	0	0	0	0	0	0	0	0	0	0	0	0	0	0	0	0
BGIR9823_88205	100157926	RIOK1	lncRNA	0	0	0	0	0	0	0	0	0	0	0	0	1	0	0	0	0	0	0
BGIR9823_100635	102162486	LOC102162486	lncRNA	0	0	0	0	0	0	0	0	0	0	1	0	0	0	0	0	0	0	0
BGIR9823_87926	110261182	LOC110261182	lncRNA	0	0	0	0	0	0	0	0	0	0	0	0	0	0	0	0	0	0	1
BGIR9823_96076	110256340	LOC110256340	lncRNA	0	0	0	0	0	0	0	0	0	0	0	0	0	0	0	0	0	0	0
BGIR9823_92591	100521678	MLLT10	lncRNA	0	0	0	0	0	0	0	0	0	0	0	0	0	0	0	0	0	0	0
BGIR9823_78251	106506784	LOC106506784	lncRNA	0	0	0	0	0	0	0	0	0	0	0	0	0	0	0	0	0	0	0
BGIR9823_100897	100621199	IL1RAPL1	lncRNA	0	0	0	0	0	0	0	0	0	0	0	1	0	0	0	0	0	0	0
BGIR9823_100113	110257459	LOC110257459	lncRNA	0	0	0	0	0	0	0	0	0	0	0	0	0	0	0	0	0	1	0
BGIR9823_100197	106506650	LOC106506650	lncRNA	0	0	0	0	0	0	0	0	0	0	0	0	0	0	0	0	0	1	0
BGIR9823_96929	751859	SIRT1	lncRNA	0	0	0	0	0	0	0	0	0	0	0	0	0	0	0	0	0	0	0
BGIR9823_92783	100156695	INTS6	lncRNA	0	0	0	0	0	0	0	0	0	0	0	0	0	0	0	0	0	0	0
BGIR9823_77974	100516051	SLC17A5	lncRNA	0	0	0	0	0	0	0	0	0	0	0	0	0	0	0	0	0	0	0
BGIR9823_100010	397360	CEBPB	lncRNA	0	0	0	0	0	0	0	0	0	0	0	0	0	0	0	0	0	0	0
BGIR9823_101617	110258914	LOC110258914	lncRNA	0	0	0	1	0	0	0	0	0	0	0	0	0	0	0	0	0	0	0
BGIR9823_100936	100154376	KDM6A	lncRNA	0	0	0	0	0	0	0	0	0	0	0	0	0	0	0	0	0	0	0
BGIR9823_79053	100522049	PTP4A1	lncRNA	0	0	0	0	0	0	0	0	0	0	0	0	0	0	0	0	0	0	0
BGIR9823_80764	100037285	NREP	lncRNA	0	0	0	0	0	0	0	0	0	0	0	0	0	0	0	0	0	0	0
BGIR9823_100812	100516527	LOC100516527	lncRNA	0	0	0	0	0	0	0	0	0	0	0	0	0	0	0	0	0	0	0
BGIR9823_100796	110257536	LOC110257536	lncRNA	0	0	0	0	0	0	0	0	0	0	0	0	0	0	0	0	1	0	0
BGIR9823_100878	102165056	MBTPS2	lncRNA	0	0	1	0	0	0	0	0	0	0	0	0	0	0	0	0	0	1	0
BGIR9823_100734	100512382	KCND2	lncRNA	0	0	0	0	0	0	0	0	0	0	0	0	0	0	0	0	0	0	0
BGIR9823_100726	100512026	ING3	lncRNA	0	0	0	0	0	0	0	0	0	0	0	0	0	0	0	0	0	1	0
BGIR9823_84422	100145887	ARHGEF2	lncRNA	0	1	0	0	0	0	0	0	0	0	0	0	0	0	0	0	0	0	0
BGIR9823_84641	100155627	LRRC8B	mRNA	0	0	0	0	0	0	0	0	0	0	0	0	0	0	1	0	0	0	0
BGIR9823_98254	100152049	PLPP5	mRNA	0	0	0	0	0	0	0	0	0	0	0	0	0	0	0	0	0	0	0
BGIR9823_87713	100517038	PUM1	lncRNA	0	0	0	0	0	0	0	0	0	0	0	0	0	0	0	0	0	0	0
BGIR9823_94908	100462749	ATG7	lncRNA	0	0	0	0	0	0	0	0	0	0	0	0	0	0	0	0	0	0	0
BGIR9823_100511	100738688	HERPUD2	lncRNA	0	0	1	0	0	0	0	0	0	0	0	0	0	0	0	0	0	0	0
BGIR9823_80800	100515883	PRRC1	lncRNA	0	0	0	0	0	0	0	0	0	0	0	0	0	0	0	0	0	0	0
BGIR9823_100550	397163	RAMP3	lncRNA	0	0	0	0	0	0	0	0	0	0	0	0	0	0	0	0	0	0	0
BGIR9823_94492	102167379	LOC102167379	lncRNA	0	0	0	0	0	0	0	0	0	0	0	0	0	0	0	0	0	0	0
XM_003126850.6	100520197	VAT1L	mRNA	0	0	0	0	0	0	0	0	0	0	0	0	0	0	0	0	0	0	0
BGIR9823_81847	100739684	FOXK1	lncRNA	0	0	0	0	0	0	0	0	0	0	0	0	0	0	0	0	0	0	1

RNA ID	Gene ID	Symbol	Type	novel-ssc-miR1063-5p	ssc-miR-192	ssc-miR-432-5p	ssc-miR-7138-5p	ssc-miR-874	ssc-miR-10386	ssc-miR-122-3p	novel-ssc-miR755-3p	novel-ssc-miR780-3p	novel-ssc-miR804-3p	novel-ssc-miR813-5p	novel-ssc-miR407-5p	novel-ssc-miR573-5p	novel-ssc-miR583-3p	novel-ssc-miR1077-5p	novel-ssc-miR122-3p	novel-ssc-miR154-5p	ssc-miR-127	ssc-miR-194b-5p
BGIR9823_100328	110257483	ADRM1	lncRNA	0	0	0	0	0	0	0	0	0	0	0	0	0	0	0	0	1	0	0
BGIR9823_101062	100524727	LOC100524727	lncRNA	0	0	1	0	0	0	0	0	0	0	0	0	0	0	0	0	0	0	0
BGIR9823_100412	414383	CHCHD3	lncRNA	0	0	0	0	0	0	0	0	0	0	0	0	0	0	0	0	0	0	0
BGIR9823_90505	397683	IL15	lncRNA	0	0	0	0	0	0	0	0	0	0	0	0	0	0	0	0	0	0	0
BGIR9823_77934	102167874	LOC102167874	lncRNA	0	0	0	0	0	0	0	0	0	0	0	0	0	0	0	0	0	0	0
BGIR9823_100539	100622324	NFE2L3	lncRNA	0	0	0	0	0	0	0	0	0	0	0	0	0	0	0	0	0	0	0
BGIR9823_101533	102158401	LOC102158401	lncRNA	0	0	0	0	0	0	0	0	0	0	0	1	0	0	0	0	0	0	0
BGIR9823_100121	100519631	TASP1	lncRNA	0	0	0	0	0	0	0	0	0	0	1	0	0	0	0	0	0	0	0
BGIR9823_100964	110257651	LOC110257651	lncRNA	0	0	0	0	0	0	0	0	0	0	0	0	0	0	0	0	0	0	0
BGIR9823_100069	100157832	SGCZ	lncRNA	0	0	0	1	0	0	0	0	0	0	0	0	0	0	0	0	0	0	0
BGIR9823_101391	110258158	TNRC6A	mRNA	0	0	0	0	0	0	0	0	0	0	0	0	0	0	1	0	0	0	0
BGIR9823_80729	100526040	PAM	mRNA	0	0	0	0	0	0	0	0	0	0	0	0	0	0	0	0	0	0	0
BGIR9823_100266	100157616	IFT52	lncRNA	0	0	0	0	0	0	0	0	0	0	0	0	0	0	0	0	0	0	0
BGIR9823_100743	100125380	ST7	mRNA	0	0	0	0	0	0	0	0	0	0	0	0	0	0	0	0	0	0	1
BGIR9823_80829	100513723	JADE2	lncRNA	0	0	0	0	0	0	0	0	0	0	0	0	0	0	0	0	0	0	0
BGIR9823_91326	100516387	TSEN15	lncRNA	0	0	0	0	0	0	0	0	0	0	0	0	0	0	0	0	0	0	1
BGIR9823_100827	100519067	CDK13	lncRNA	0	0	0	0	0	0	0	0	0	0	1	0	0	0	0	0	0	0	0
BGIR9823_80869	100521956	CYSTM1	lncRNA	0	0	0	0	0	0	0	0	0	0	0	0	0	0	0	0	0	0	0
BGIR9823_89293	100153071	TRERF1	lncRNA	0	0	0	0	0	0	0	0	0	0	0	0	0	0	0	0	0	0	0
BGIR9823_79511	100152487	TSHZ1	mRNA	0	0	0	0	0	0	0	0	0	0	0	0	0	0	0	0	0	0	0
BGIR9823_92832	106505308	PCDH9	lncRNA	0	0	0	0	0	0	0	0	0	0	0	0	0	0	0	0	0	0	0
BGIR9823_82541	100520835	ITSN2	lncRNA	0	0	0	0	0	0	0	0	0	0	0	0	0	0	0	0	0	0	1
BGIR9823_100525	100515288	CPVL	lncRNA	0	0	0	0	0	0	0	0	0	0	0	0	0	0	0	1	0	0	0
BGIR9823_100563	100516888	GLI3	lncRNA	0	0	0	0	0	0	0	0	0	0	0	0	0	0	0	0	0	0	0
BGIR9823_100515	100623924	FKBP9	lncRNA	0	0	0	0	0	0	0	0	0	0	0	0	0	0	0	0	0	1	0
BGIR9823_101109	100524780	ARHGAP6	lncRNA	0	0	0	0	0	0	0	0	0	1	0	0	0	0	0	0	0	0	0
BGIR9823_95657	100524459	SLC25A20	lncRNA	0	0	0	0	0	0	0	0	0	0	0	0	0	0	0	0	0	0	0
XR_001303159.2	106507279	LOC106507279	lncRNA	0	0	0	0	0	0	0	0	0	0	0	0	0	0	1	0	0	0	0
BGIR9823_82132	106509720	LOC106509720	lncRNA	0	0	0	0	0	0	0	0	0	0	0	0	0	0	0	0	0	0	0
BGIR9823_90639	110261963	LOC110261963	lncRNA	0	0	0	0	0	0	0	0	0	0	0	0	0	0	0	0	0	0	0
BGIR9823_92636	100516289	PFKFB3	lncRNA	0	0	0	0	0	0	0	0	0	0	0	0	0	0	1	0	0	0	0
BGIR9823_79919	100154648	PLPPR1	lncRNA	0	0	0	0	0	0	0	0	0	0	0	0	0	0	0	0	0	0	0
BGIR9823_80860	100521716	MATR3	mRNA	0	0	0	0	0	0	0	0	0	0	0	0	0	0	1	0	0	0	0
BGIR9823_100880	102165056	MBTPS2	lncRNA	0	0	0	0	0	0	0	0	0	0	1	0	0	0	0	0	0	0	0
BGIR9823_101396	100521783	RBBP6	mRNA	0	0	0	0	0	0	0	0	0	0	0	0	0	0	1	0	0	0	0
BGIR9823_101310	102159115	ATP6AP1	lncRNA	1	0	0	0	0	0	0	0	0	0	0	0	0	0	0	0	0	0	0
BGIR9823_85863	100512658	METTL25	mRNA	0	0	0	0	0	0	0	0	0	0	0	0	0	0	1	0	0	0	0
BGIR9823_84834	100516439	C5H12orf10	mRNA	0	0	0	0	0	0	0	0	0	0	0	0	0	0	0	0	0	0	0
BGIR9823_98676	397282	ACVR2A	lncRNA	0	0	0	0	0	0	0	0	0	0	0	0	0	0	0	0	0	0	0
BGIR9823_83976	396677	F3	mRNA	0	0	0	0	0	0	0	0	0	0	0	0	0	0	1	0	0	0	0
BGIR9823_90032	102167943	LOC102167943	lncRNA	0	0	0	0	0	0	0	0	0	0	0	0	0	0	0	0	0	0	0
BGIR9823_100671	106506771	LOC106506771	lncRNA	0	0	0	1	0	0	0	0	0	0	0	0	0	0	0	0	0	0	0
BGIR9823_101108	100524780	ARHGAP6	lncRNA	0	0	0	0	1	0	0	0	0	0	0	0	0	0	0	0	0	0	0

RNA ID	Gene ID	Symbol	Type	novel-ssc-miR1063-5p	ssc-miR-192	ssc-miR-432-5p	ssc-miR-7138-5p	ssc-miR-874	ssc-miR-10386	ssc-miR-122-3p	novel-ssc-miR755-3p	novel-ssc-miR780-3p	novel-ssc-miR804-3p	novel-ssc-miR813-5p	novel-ssc-miR407-5p	novel-ssc-miR573-5p	novel-ssc-miR583-3p	novel-ssc-miR1077-5p	novel-ssc-miR122-3p	novel-ssc-miR154-5p	ssc-miR-127	ssc-miR-194b-5p
BGIR9823_80923	100515885	SLC26A2	mRNA	0	0	0	0	0	0	0	0	0	0	0	0	0	0	1	0	0	0	0
BGIR9823_100494	100521052	IMMP2L	lncRNA	0	0	0	0	0	0	0	0	0	0	0	0	0	0	0	0	0	0	0
BGIR9823_101344	110258068	DPH7	lncRNA	1	0	0	0	0	0	0	0	0	0	0	0	0	0	0	0	0	0	0
BGIR9823_100314	100155190	ZFP64	lncRNA	0	0	0	0	0	0	0	0	0	0	0	0	0	0	1	0	0	0	0
BGIR9823_83324	100624063	LOC100624063	lncRNA	0	0	0	0	0	1	0	0	0	0	0	0	0	0	0	0	0	0	0
BGIR9823_100237	110257438	LOC110257438	lncRNA	0	0	1	0	0	0	0	0	0	0	0	0	0	0	0	0	0	0	0
BGIR9823_78253	106506784	LOC106506784	lncRNA	0	0	0	0	0	0	0	0	0	0	0	0	0	0	0	0	0	0	0
BGIR9823_77699	100514202	MTRF1L	lncRNA	0	0	0	0	0	0	0	0	0	0	0	0	0	0	0	1	0	0	0
BGIR9823_100849	733606	TMSB4X	lncRNA	0	0	0	0	0	0	0	0	0	0	0	0	0	0	0	0	0	0	0
BGIR9823_101513	102157759	LOC102157759	lncRNA	0	0	0	0	0	0	0	0	0	0	0	0	0	0	0	0	0	0	0
BGIR9823_100598	403162	INSIG1	lncRNA	0	0	0	0	0	0	0	0	0	0	0	0	0	0	0	0	0	0	0
BGIR9823_80828	100513723	JADE2	lncRNA	0	0	0	0	0	0	0	0	0	0	0	0	0	0	0	0	0	0	0
BGIR9823_80639	100514094	FCHO2	lncRNA	0	0	0	0	0	0	0	0	0	0	0	0	0	0	0	0	0	0	1
BGIR9823_85738	100157209	PRICKLE1	mRNA	0	0	0	0	0	0	0	0	0	0	0	0	0	0	1	0	0	0	0
BGIR9823_91822	100522092	RUNDC3B	lncRNA	0	0	0	0	0	0	0	0	0	0	0	0	0	0	0	0	0	0	0
BGIR9823_101413	110258225	LOC110258225	lncRNA	0	0	0	0	0	0	0	0	0	0	0	0	0	0	0	0	0	0	0
BGIR9823_89187	100153386	SLA-DRB1	lncRNA	0	0	0	0	0	0	0	0	0	0	0	0	0	0	0	0	0	0	0
BGIR9823_91714	100521986	MAPKAPK2	lncRNA	0	0	0	0	0	1	1	0	0	0	0	0	0	0	0	0	0	0	0
BGIR9823_83804	100523784	ADAR	mRNA	0	0	0	0	0	0	0	0	0	0	0	0	0	0	1	0	0	0	0
BGIR9823_100308	100141411	ADNP	lncRNA	0	0	0	0	0	0	0	0	0	0	0	0	0	0	0	0	0	0	0
BGIR9823_83998	100156806	HFM1	mRNA	0	0	0	0	0	0	0	0	0	0	0	0	0	0	1	0	0	0	0
BGIR9823_100710	397301	CALU	lncRNA	0	0	0	1	0	0	0	0	0	0	0	0	0	0	0	0	0	1	0
BGIR9823_101105	100154011	ANOS1	lncRNA	0	0	0	0	0	0	0	0	0	0	0	0	0	0	0	0	0	0	0
BGIR9823_88993	100144590	MEG3	lncRNA	0	0	0	0	0	1	0	0	0	0	0	0	0	0	0	0	0	0	0
BGIR9823_80459	100622538	DNM2	mRNA	0	0	0	0	0	0	0	0	0	0	0	0	0	0	1	0	0	0	0
BGIR9823_101097	397339	F8	lncRNA	0	0	0	0	0	0	0	0	0	0	0	0	0	0	0	0	0	0	0
BGIR9823_100022	100144536	VAPB	mRNA	0	0	0	0	0	0	0	0	0	0	0	0	0	0	1	0	0	0	0
BGIR9823_88787	100153736	EMC4	lncRNA	0	0	0	0	0	0	0	0	0	0	0	0	0	0	0	0	0	0	0
BGIR9823_93229	100518091	PCID2	mRNA	0	0	0	0	0	0	0	0	0	0	0	0	0	0	0	0	0	0	0
BGIR9823_100480	100621338	BMT2	lncRNA	0	0	0	0	0	0	0	0	0	0	0	0	0	0	0	0	0	1	0
BGIR9823_81197	100037286	ARNTL	lncRNA	0	0	0	0	0	0	0	0	0	0	0	0	0	0	0	0	0	0	0
BGIR9823_100187	100513829	SNPH	lncRNA	0	0	0	0	0	0	0	0	0	0	0	0	0	0	0	0	0	0	0
BGIR9823_97762	100512075	ASCC1	mRNA	0	0	0	0	0	0	0	0	0	0	0	0	0	0	0	0	0	0	0
BGIR9823_100489	493185	IFRD1	lncRNA	0	0	0	0	0	0	0	0	0	0	1	0	0	0	0	0	0	0	0
BGIR9823_82778	100523894	POLR3E	mRNA	0	0	0	0	0	0	0	0	0	0	0	0	0	0	0	0	0	0	0
BGIR9823_88002	100524685	ADGRL2	lncRNA	0	0	0	0	0	0	0	0	0	0	0	0	0	0	0	0	0	0	0
BGIR9823_97698	106506010	LOC106506010	lncRNA	0	0	0	0	0	0	0	0	0	0	0	0	0	0	0	0	0	0	0
BGIR9823_79746	100155321	ACER2	lncRNA	0	0	0	0	0	0	0	0	0	0	0	0	0	0	0	0	0	0	0
BGIR9823_79823	100626649	KANK1	lncRNA	0	0	0	0	0	1	0	0	0	0	0	0	0	0	0	0	0	0	0
BGIR9823_87325	100513473	ZNF181	lncRNA	0	0	0	0	0	0	0	0	0	0	0	0	1	0	0	0	0	0	0
BGIR9823_88185	100511204	ZNF643	mRNA	0	0	0	0	0	0	0	0	0	0	0	0	0	1	0	0	0	0	0
BGIR9823_81639	100513341	FBXL17	lncRNA	0	0	0	0	0	0	0	0	0	0	0	0	0	0	0	0	0	0	0
BGIR9823_94281	100516758	CORO6	mRNA	0	0	0	0	0	0	0	0	0	0	0	0	0	0	0	0	0	0	0

RNA ID	Gene ID	Symbol	Type	novel-ssc-miR1063-5p	ssc-miR-192	ssc-miR-432-5p	ssc-miR-7138-5p	ssc-miR-874	ssc-miR-10386	ssc-miR-122-3p	novel-ssc-miR755-3p	novel-ssc-miR780-3p	novel-ssc-miR804-3p	novel-ssc-miR813-5p	novel-ssc-miR407-5p	novel-ssc-miR573-5p	novel-ssc-miR583-3p	novel-ssc-miR1077-5p	novel-ssc-miR122-3p	novel-ssc-miR154-5p	ssc-miR-127	ssc-miR-194b-5p
BGIR9823_96391	100621858	CFAP410	mRNA	0	0	0	0	0	0	0	0	0	0	0	0	0	0	0	0	0	0	0
BGIR9823_77814	100736796	ADGRB3	lncRNA	0	0	0	0	0	0	0	0	0	0	0	0	0	0	0	0	0	0	0
BGIR9823_84738	100516801	MRTFA	lncRNA	0	0	0	0	0	0	0	0	0	0	0	0	0	0	0	0	0	0	0
BGIR9823_85081	110260749	LOC110260749	lncRNA	0	0	0	0	0	0	0	0	0	0	0	0	0	0	0	0	0	0	0
BGIR9823_83642	100127143	PDE7A	lncRNA	0	0	0	0	0	0	0	0	0	0	0	0	0	0	0	0	0	0	0
BGIR9823_90693	100526062	MRPS18C	lncRNA	0	0	0	0	0	0	0	0	0	0	0	0	0	0	0	0	0	0	0
BGIR9823_94661	100525609	STAB1	mRNA	0	0	0	0	0	0	0	0	0	0	0	0	0	1	0	0	0	0	0
BGIR9823_88204	100157926	RIOK1	mRNA	0	0	0	0	0	0	1	0	0	0	0	0	0	0	0	0	0	0	0
BGIR9823_100214	100154657	PIGU	mRNA	0	0	0	0	0	0	0	0	0	0	0	0	0	0	0	0	0	0	0
BGIR9823_86368	100624191	LOC100624191	mRNA	0	0	0	0	0	0	0	0	0	0	0	0	0	0	0	0	0	0	0
BGIR9823_98692	100525081	R3HDM1	mRNA	0	0	0	0	0	0	0	0	0	0	0	0	0	0	0	0	0	0	0
BGIR9823_94941	110256296	LOC110256296	lncRNA	0	0	0	0	0	0	0	0	0	0	0	0	0	0	0	0	0	0	0
BGIR9823_93186	100519383	DZIP1	mRNA	0	0	0	0	0	0	0	0	0	0	0	0	0	0	0	0	0	0	0
BGIR9823_101339	100519166	LOC100519166	lncRNA	0	0	0	0	0	0	0	0	0	0	0	0	0	0	0	0	0	0	0
BGIR9823_78696	641344	GNE	lncRNA	0	0	0	0	0	0	0	0	0	0	0	0	0	0	0	0	0	0	0
BGIR9823_90334	100514856	APBB2	mRNA	0	0	0	0	0	0	0	0	0	0	0	0	0	0	0	0	0	0	0
BGIR9823_79588	100155833	DPP8	mRNA	0	0	0	0	0	0	0	0	0	0	0	0	0	0	0	0	0	0	0
BGIR9823_93702	100622172	TAOK1	lncRNA	0	0	0	0	0	0	0	0	0	0	0	0	0	0	0	0	0	0	0
BGIR9823_100788	100622432	CBX3	lncRNA	0	0	0	0	0	0	0	0	0	0	0	0	0	0	0	0	0	1	0
BGIR9823_81760	100520896	ETF1	lncRNA	0	0	0	0	0	0	0	0	0	0	0	0	0	0	0	0	0	0	0
BGIR9823_84563	102167065	LOC102167065	lncRNA	0	0	0	0	0	0	0	0	0	0	0	0	0	1	0	0	0	0	0
BGIR9823_100600	100513894	LOC100513894	lncRNA	0	0	0	0	0	0	0	0	0	0	0	0	0	0	0	0	0	1	0
BGIR9823_77938	100156192	NT5DC1	lncRNA	0	0	0	0	0	0	0	0	0	0	0	0	0	0	0	0	0	0	0
BGIR9823_86946	100515670	ANKRD13C	lncRNA	0	0	0	0	0	0	0	0	0	0	0	0	0	0	0	0	0	0	0
BGIR9823_100385	100515632	KDM7A	lncRNA	0	0	0	0	0	0	0	0	0	0	0	0	0	0	0	0	0	0	0
BGIR9823_101073	100515811	VMA21	lncRNA	0	0	0	0	0	0	0	0	0	0	1	0	0	0	0	0	0	0	0
BGIR9823_79171	791125	FYN	lncRNA	0	0	0	0	0	0	0	0	0	0	0	0	0	0	0	0	0	0	0
BGIR9823_91118	106504956	LOC106504956	lncRNA	0	0	0	0	0	0	0	0	0	0	0	0	0	0	0	0	0	0	0
BGIR9823_86164	102164889	LOC102164889	lncRNA	0	0	0	0	0	0	0	0	0	0	0	0	0	0	0	0	0	0	0
BGIR9823_78738	100153669	TMEM38B	lncRNA	0	0	0	0	0	0	0	0	0	0	0	0	0	0	0	0	0	0	0
BGIR9823_88541	100522555	ABHD17C	lncRNA	0	0	0	0	0	0	0	0	0	0	0	0	0	0	0	0	0	0	0
BGIR9823_100267	100624468	OSER1	lncRNA	0	0	0	0	0	0	0	0	0	0	0	0	0	0	0	0	0	0	0
BGIR9823_100299	100625841	DDX27	lncRNA	0	0	0	0	0	0	0	0	0	0	0	0	0	0	0	0	1	0	0
BGIR9823_78983	100622156	TNFAIP3	lncRNA	0	0	0	0	0	0	0	0	0	0	0	0	0	0	0	0	0	0	0
BGIR9823_79796	574058	CD274	lncRNA	0	1	0	0	0	0	0	0	0	0	0	0	0	0	0	0	0	0	0
BGIR9823_82690	100520239	LRWD1	lncRNA	0	0	0	0	0	0	0	0	0	0	0	0	0	0	0	0	0	0	0
BGIR9823_100392	100415778	ZC3HAV1	lncRNA	0	0	0	0	1	0	0	0	0	0	0	0	0	0	0	0	0	0	0
BGIR9823_79386	102162809	CTDSPL2	lncRNA	0	0	0	0	0	0	0	0	0	0	0	0	0	0	0	0	0	0	0
BGIR9823_87847	100627122	SMCHD1	lncRNA	0	0	0	0	0	0	0	0	0	0	0	0	0	0	0	0	0	0	0
BGIR9823_83136	397040	UGP2	mRNA	0	0	0	0	0	0	0	0	0	0	0	0	0	0	0	0	0	0	0
BGIR9823_89310	100151914	MRPL2	lncRNA	0	0	0	0	0	0	0	0	0	0	0	0	0	0	0	0	0	0	0
BGIR9823_82954	106509726	LOC106509726	lncRNA	0	0	0	0	0	0	0	0	0	0	0	0	1	0	0	0	0	0	0
BGIR9823_100019	100141410	ZNF217	lncRNA	0	0	0	0	0	0	0	0	0	0	0	0	0	0	0	0	0	0	0

RNA ID	Gene ID	Symbol	Type	novel-ssc-miR1063-5p	ssc-miR-192	ssc-miR-432-5p	ssc-miR-7138-5p	ssc-miR-874	ssc-miR-10386	ssc-miR-122-3p	novel-ssc-miR755-3p	novel-ssc-miR780-3p	novel-ssc-miR804-3p	novel-ssc-miR813-5p	novel-ssc-miR407-5p	novel-ssc-miR573-5p	novel-ssc-miR583-3p	novel-ssc-miR1077-5p	novel-ssc-miR122-3p	novel-ssc-miR154-5p	ssc-miR-127	ssc-miR-194b-5p
BGIR9823_101550	110258600	LOC110258600	lncRNA	0	0	0	0	0	1	0	0	0	0	0	0	0	0	0	0	0	0	0
BGIR9823_80641	100515474	UTP15	lncRNA	0	0	0	0	0	0	0	0	0	0	0	0	0	0	0	0	0	0	0
BGIR9823_85022	100518251	KRAS	lncRNA	0	0	0	0	0	0	0	0	0	0	0	0	0	0	0	0	0	0	0
BGIR9823_78496	100512450	TMEM260	lncRNA	0	0	0	0	0	0	0	0	0	0	0	0	0	0	0	0	0	0	0
BGIR9823_100476	100621338	BMT2	lncRNA	0	0	0	0	0	0	0	0	0	0	0	0	0	0	0	0	0	0	0
BGIR9823_102668	BGIG9823_27318		lncRNA	0	0	0	0	0	0	0	0	0	0	0	0	0	0	0	0	0	0	0
BGIR9823_101962	BGIG9823_26639		lncRNA	0	0	0	1	0	0	0	0	0	0	0	0	0	0	0	0	0	0	0
BGIR9823_102175	BGIG9823_26847		lncRNA	0	0	0	0	0	0	0	0	0	0	0	1	0	0	0	0	0	0	0
BGIR9823_101847	BGIG9823_26526		lncRNA	0	0	0	0	0	0	0	0	0	0	0	0	0	0	0	0	0	0	0
BGIR9823_101781	BGIG9823_26461		lncRNA	0	0	0	1	0	0	0	0	0	0	0	0	0	0	0	0	0	0	0
BGIR9823_102445	BGIG9823_27101		lncRNA	0	0	0	0	0	0	0	0	0	0	0	0	0	0	0	0	0	0	0
BGIR9823_101840	BGIG9823_26519		lncRNA	0	0	0	0	0	0	0	0	0	0	0	0	0	0	0	0	0	0	0
BGIR9823_102485	BGIG9823_27139		lncRNA	0	0	0	0	0	0	0	0	0	0	0	0	0	0	0	0	0	0	0
BGIR9823_102195	BGIG9823_26867		lncRNA	0	0	0	0	0	0	0	0	0	0	0	0	0	0	0	0	0	0	0
BGIR9823_101771	BGIG9823_26451		lncRNA	0	0	0	0	0	0	0	0	0	0	0	0	0	0	0	0	0	0	0
BGIR9823_101831	BGIG9823_26510		lncRNA	0	1	0	0	0	0	0	0	0	0	0	0	0	0	0	0	0	0	0
BGIR9823_102544	BGIG9823_27197		lncRNA	0	0	0	0	0	0	0	0	0	0	0	0	0	0	0	0	0	0	0
BGIR9823_102359	BGIG9823_27024		lncRNA	0	0	0	0	0	0	0	0	0	0	0	0	0	0	0	0	0	0	0
BGIR9823_102456	BGIG9823_27110		lncRNA	0	0	0	0	0	0	0	0	0	0	0	0	0	0	0	0	0	0	0
BGIR9823_102657	BGIG9823_27307		lncRNA	0	0	0	0	0	1	0	0	0	0	0	0	0	0	0	0	0	0	0
BGIR9823_102305	BGIG9823_26970		lncRNA	0	0	0	0	0	0	0	0	0	0	0	0	0	0	0	0	0	0	0
BGIR9823_102228	BGIG9823_26900		lncRNA	0	0	0	0	0	0	0	0	0	0	0	0	0	0	0	0	0	0	0
BGIR9823_101778	BGIG9823_26458		lncRNA	0	0	0	0	0	0	0	0	0	0	0	0	0	0	0	0	0	0	0
BGIR9823_102408	BGIG9823_27069		lncRNA	0	1	0	0	0	0	0	0	0	0	0	0	0	0	0	0	0	0	0
BGIR9823_101997	BGIG9823_26673		lncRNA	0	0	0	0	0	0	0	0	0	0	0	0	0	0	0	0	0	0	0
BGIR9823_101886	BGIG9823_26563		lncRNA	0	0	0	0	0	0	0	0	0	1	0	0	0	0	0	0	0	0	0
BGIR9823_101714	BGIG9823_26398		lncRNA	0	0	0	0	0	0	0	0	0	0	0	0	0	0	0	0	0	0	0
BGIR9823_102000	BGIG9823_26676		lncRNA	0	0	0	0	0	0	0	0	0	0	0	0	0	0	0	0	0	0	0
BGIR9823_102540	BGIG9823_27193		lncRNA	0	0	1	0	0	0	0	0	0	0	0	0	0	0	0	0	0	0	0
BGIR9823_101852	BGIG9823_26531		lncRNA	0	0	0	0	0	0	0	0	0	0	1	0	0	0	0	0	0	0	0
BGIR9823_102203	BGIG9823_26875		lncRNA	0	0	0	0	0	0	0	0	0	0	0	0	0	0	0	0	0	0	0
BGIR9823_102422	BGIG9823_27083		lncRNA	0	0	0	0	0	0	0	0	0	0	0	0	0	0	0	0	0	0	0
BGIR9823_101937	BGIG9823_26614		lncRNA	0	0	0	0	0	0	0	0	0	0	0	0	0	0	0	0	0	0	0
BGIR9823_102117	BGIG9823_26791		lncRNA	0	0	0	0	0	0	0	0	0	1	0	0	0	0	0	0	0	0	0
BGIR9823_102005	BGIG9823_26681		lncRNA	0	0	0	0	0	0	0	0	0	0	0	1	0	0	0	0	0	0	0
BGIR9823_102177	BGIG9823_26849		lncRNA	0	0	0	0	0	0	0	1	0	0	0	0	0	0	0	0	0	0	0
BGIR9823_102213	BGIG9823_26885		lncRNA	0	0	0	0	0	0	0	0	0	0	0	0	0	0	0	0	0	0	0
BGIR9823_102067	BGIG9823_26741		lncRNA	0	0	1	0	0	0	0	0	0	0	0	0	0	0	0	0	0	0	0
BGIR9823_102357	BGIG9823_27022		lncRNA	0	0	0	0	0	0	0	0	0	0	0	0	0	0	0	0	0	0	0
BGIR9823_102443	BGIG9823_27099		lncRNA	0	0	0	0	0	0	0	0	0	0	0	0	0	0	0	0	0	0	0
BGIR9823_102395	BGIG9823_27060		lncRNA	0	0	0	0	0	0	0	0	0	0	0	0	0	0	0	0	0	0	0
BGIR9823_102615	BGIG9823_27265		lncRNA	0	0	0	0	0	0	0	0	0	0	0	0	0	0	0	0	0	0	0
BGIR9823_102264	BGIG9823_26932		lncRNA	0	0	0	0	0	0	0	0	0	0	0	0	0	0	0	0	0	0	0

RNA ID	Gene ID	Symbol	Type	novel-ssc-miR1063-5p	ssc-miR-192	ssc-miR-432-5p	ssc-miR-7138-5p	ssc-miR-874	ssc-miR-10386	ssc-miR-122-3p	novel-ssc-miR755-3p	novel-ssc-miR780-3p	novel-ssc-miR804-3p	novel-ssc-miR813-5p	novel-ssc-miR407-5p	novel-ssc-miR573-5p	novel-ssc-miR583-3p	novel-ssc-miR1077-5p	novel-ssc-miR122-3p	novel-ssc-miR154-5p	ssc-miR-127	ssc-miR-194b-5p
BGIR9823_102052	BGIG9823_26726		lncRNA	0	0	0	0	0	0	0	0	0	0	1	0	0	0	0	0	0	0	0
BGIR9823_102131	BGIG9823_26804		lncRNA	0	0	0	0	0	0	0	0	0	0	0	0	0	0	0	0	0	0	0
BGIR9823_101987	BGIG9823_26663		lncRNA	0	0	0	0	0	0	0	0	0	0	0	0	0	0	0	0	0	0	0
BGIR9823_102764	BGIG9823_27402		lncRNA	0	0	0	0	0	0	0	0	0	0	0	0	0	0	0	0	0	0	0
BGIR9823_102137	BGIG9823_26810		lncRNA	0	0	0	0	0	0	0	0	0	1	0	0	0	0	0	0	0	0	0
BGIR9823_102168	BGIG9823_26841		lncRNA	0	0	1	0	0	0	0	0	0	0	0	0	0	0	0	0	0	0	0
BGIR9823_101967	BGIG9823_26644		lncRNA	0	0	0	0	0	0	0	0	0	0	0	0	0	0	0	0	0	0	0
BGIR9823_102393	BGIG9823_27058		lncRNA	0	0	0	0	0	0	0	0	0	0	0	0	0	0	0	0	0	0	0
BGIR9823_101927	BGIG9823_26604		lncRNA	0	0	0	1	0	0	0	0	0	0	0	0	0	0	0	0	0	0	0
BGIR9823_102343	BGIG9823_27008		lncRNA	0	0	0	0	0	0	0	0	0	0	0	0	0	0	0	0	0	0	0
BGIR9823_102562	BGIG9823_27212		mRNA	0	0	0	0	0	0	0	0	0	0	0	0	0	0	0	0	0	0	1
BGIR9823_102090	BGIG9823_26764		lncRNA	0	0	0	0	0	0	0	0	0	0	0	0	0	0	0	0	0	0	0
BGIR9823_101742	BGIG9823_26423		lncRNA	0	0	0	0	0	0	0	0	0	0	0	0	0	0	0	0	0	0	0
BGIR9823_102398	BGIG9823_27061		lncRNA	0	0	0	0	0	0	0	0	0	0	0	0	0	0	0	0	0	0	0
BGIR9823_101721	BGIG9823_26405		lncRNA	0	0	0	0	0	0	0	0	0	0	0	0	0	0	0	0	0	0	0
BGIR9823_102545	BGIG9823_27198		lncRNA	0	0	0	0	0	0	0	0	0	0	0	0	0	0	0	0	0	0	0
BGIR9823_102138	BGIG9823_26811		lncRNA	0	0	0	0	0	0	0	0	0	0	0	0	0	0	0	0	0	0	0
BGIR9823_102026	BGIG9823_26701		lncRNA	0	0	0	0	0	0	0	0	0	0	0	0	0	0	0	1	0	0	0
BGIR9823_102057	BGIG9823_26731		lncRNA	0	0	0	0	0	0	0	0	0	0	0	0	0	0	0	0	0	0	0
BGIR9823_101995	BGIG9823_26671		lncRNA	0	0	1	0	0	0	0	0	0	0	0	0	0	0	0	0	0	0	0
BGIR9823_102725	BGIG9823_27371		lncRNA	0	0	1	0	0	0	0	0	0	0	0	0	0	0	0	0	0	0	0
BGIR9823_101717	BGIG9823_26401		lncRNA	0	0	0	0	0	0	0	0	0	1	0	0	0	0	0	0	0	0	0
BGIR9823_102441	BGIG9823_27097		lncRNA	0	0	0	0	0	0	0	0	0	0	0	0	0	0	0	0	0	0	0
BGIR9823_102674	BGIG9823_27324		lncRNA	0	0	0	0	0	0	0	0	0	0	0	0	0	0	0	0	0	0	0
BGIR9823_101843	BGIG9823_26522		lncRNA	0	0	0	0	0	0	0	0	0	0	0	0	0	0	0	1	0	0	0
BGIR9823_102119	BGIG9823_26793		lncRNA	0	0	0	0	0	0	0	0	0	0	0	0	0	0	0	0	0	0	0
BGIR9823_101819	BGIG9823_26499		lncRNA	0	1	0	0	0	0	0	0	0	0	0	0	0	0	0	0	0	0	0
BGIR9823_102034	BGIG9823_26709		lncRNA	0	0	0	0	0	0	0	0	0	0	0	0	0	0	0	1	0	0	0
BGIR9823_102373	BGIG9823_27038		lncRNA	0	0	1	0	0	0	0	0	0	0	0	0	0	0	0	0	0	0	0
BGIR9823_101905	BGIG9823_26582		lncRNA	0	0	0	0	0	0	0	0	0	0	1	0	0	0	0	0	0	0	0
BGIR9823_102629	BGIG9823_27279		lncRNA	0	0	1	0	0	0	0	0	0	0	0	0	0	0	0	0	0	0	0
BGIR9823_102328	BGIG9823_26993		lncRNA	1	0	0	0	0	0	0	0	0	0	0	0	0	0	0	0	0	0	0
BGIR9823_102165	BGIG9823_26838		lncRNA	0	0	0	0	0	0	0	0	0	0	0	0	0	0	0	0	0	0	0
BGIR9823_101792	BGIG9823_26472		lncRNA	0	0	0	1	0	0	0	0	0	0	0	0	0	0	0	0	0	0	0
BGIR9823_101992	BGIG9823_26668		lncRNA	0	0	0	0	0	0	0	0	0	0	0	0	0	0	0	1	0	0	0
BGIR9823_101767	BGIG9823_26447		lncRNA	0	0	0	0	0	0	0	0	0	0	0	0	0	0	0	0	0	0	0
BGIR9823_101956	BGIG9823_26633		lncRNA	0	0	0	0	0	0	0	0	0	0	0	0	0	0	0	0	0	0	0
BGIR9823_102186	BGIG9823_26858		lncRNA	0	0	0	0	0	0	0	0	0	0	0	1	0	0	0	0	0	0	0
BGIR9823_102630	BGIG9823_27280		lncRNA	0	0	0	0	0	0	0	0	0	0	0	0	0	0	0	0	0	0	0
BGIR9823_101685	BGIG9823_26372		lncRNA	0	0	0	1	0	0	0	0	0	0	0	0	0	0	0	0	0	0	0
BGIR9823_101700	BGIG9823_26387		lncRNA	0	0	0	0	0	1	0	0	0	0	0	0	0	0	0	0	0	0	0
BGIR9823_102217	BGIG9823_26889		lncRNA	0	0	0	0	0	1	0	0	0	0	0	0	0	0	0	0	0	0	0
BGIR9823_102093	BGIG9823_26767		lncRNA	0	0	0	0	0	0	0	0	0	0	0	0	0	0	0	0	0	0	0

RNA ID	Gene ID	Symbol	Type	novel-ssc-miR1063-5p	ssc-miR-192	ssc-miR-432-5p	ssc-miR-7138-5p	ssc-miR-874	ssc-miR-10386	ssc-miR-122-3p	novel-ssc-miR755-3p	novel-ssc-miR780-3p	novel-ssc-miR804-3p	novel-ssc-miR813-5p	novel-ssc-miR407-5p	novel-ssc-miR573-5p	novel-ssc-miR583-3p	novel-ssc-miR1077-5p	novel-ssc-miR122-3p	novel-ssc-miR154-5p	ssc-miR-127	ssc-miR-194b-5p
BGIR9823_101824	BGIG9823_26503		lncRNA	0	0	0	0	0	0	0	0	0	0	0	0	0	0	0	0	0	0	0
BGIR9823_102083	BGIG9823_26757		lncRNA	0	0	0	0	0	0	0	0	0	0	0	0	0	0	0	0	0	0	0
BGIR9823_102805	BGIG9823_27433		lncRNA	0	0	0	0	0	0	0	0	0	0	0	0	0	0	0	0	0	0	0
BGIR9823_101826	BGIG9823_26505		lncRNA	0	0	1	0	0	0	0	0	0	0	0	0	0	0	0	0	0	0	0
BGIR9823_102136	BGIG9823_26809		lncRNA	0	0	0	0	0	0	0	0	0	0	0	0	0	0	0	0	0	0	0
BGIR9823_101982	BGIG9823_26658		lncRNA	0	0	0	0	0	0	0	0	0	0	0	0	0	0	0	0	0	0	0
BGIR9823_101949	BGIG9823_26626		lncRNA	0	0	0	0	0	0	0	0	0	0	0	0	0	0	0	0	0	0	0
BGIR9823_102478	BGIG9823_27132		lncRNA	0	0	0	0	0	0	0	0	0	0	0	0	0	0	0	0	0	0	0
BGIR9823_101746	BGIG9823_26427		lncRNA	0	0	0	0	0	0	0	0	0	0	0	0	0	0	0	0	0	0	0
BGIR9823_101942	BGIG9823_26619		lncRNA	0	0	0	0	0	0	0	0	0	0	0	0	0	0	0	0	0	0	0
BGIR9823_102059	BGIG9823_26733		lncRNA	0	0	0	0	0	0	0	0	0	0	0	0	0	0	0	1	0	0	0
BGIR9823_102156	BGIG9823_26829		lncRNA	0	0	0	0	0	0	0	0	0	0	0	0	0	0	0	0	0	0	0
BGIR9823_101875	BGIG9823_26554		lncRNA	0	0	0	0	0	0	0	0	0	0	0	0	0	0	0	0	0	0	0
BGIR9823_101893	BGIG9823_26570		lncRNA	0	0	0	0	0	0	0	0	0	0	0	0	0	0	0	0	0	0	0
BGIR9823_101659	BGIG9823_26357		lncRNA	0	0	0	0	0	0	0	0	0	0	0	0	0	0	0	0	0	0	0
BGIR9823_102534	BGIG9823_27187		lncRNA	0	0	0	0	0	0	0	0	0	0	0	0	0	0	0	0	0	0	0
BGIR9823_101719	BGIG9823_26403		lncRNA	0	0	0	0	0	0	0	0	0	0	0	0	0	0	0	0	0	0	0
BGIR9823_102665	BGIG9823_27315		lncRNA	0	0	0	0	0	0	0	0	0	0	0	1	0	0	0	0	0	0	0
BGIR9823_102586	BGIG9823_27236		lncRNA	0	0	1	0	0	0	0	0	0	0	0	0	0	0	0	0	0	0	0
BGIR9823_102799	BGIG9823_27428		lncRNA	0	0	0	0	0	0	0	0	0	0	0	0	0	0	0	0	0	0	0
BGIR9823_101782	BGIG9823_26462		lncRNA	0	0	0	0	0	0	0	0	0	0	0	1	0	0	0	0	0	0	0
BGIR9823_102163	BGIG9823_26836		lncRNA	0	0	1	0	0	0	0	0	0	0	0	0	0	0	0	0	0	0	0
BGIR9823_102623	BGIG9823_27273		lncRNA	0	0	0	0	0	0	0	0	0	0	0	0	0	0	0	0	0	0	0
BGIR9823_102550	BGIG9823_27201		lncRNA	0	0	0	0	0	1	0	0	0	0	0	0	0	0	0	0	0	0	0
BGIR9823_101952	BGIG9823_26629		lncRNA	0	0	0	0	0	0	0	0	0	0	0	0	0	0	0	0	0	0	0
BGIR9823_102084	BGIG9823_26758		lncRNA	0	0	0	0	0	0	0	0	0	0	0	0	0	0	0	0	0	0	0
BGIR9823_102746	BGIG9823_27389		lncRNA	0	0	0	0	0	0	0	0	0	0	0	0	0	0	0	0	0	0	0
BGIR9823_102196	BGIG9823_26868		lncRNA	0	0	0	0	0	0	0	0	0	1	0	0	0	0	0	0	0	0	0
BGIR9823_101791	BGIG9823_26471		lncRNA	0	0	0	0	0	0	0	0	0	0	1	0	0	0	0	0	0	0	0
BGIR9823_101707	BGIG9823_26391		lncRNA	0	0	0	0	0	0	0	0	0	1	0	0	0	0	0	0	0	0	0
BGIR9823_102788	BGIG9823_27419		lncRNA	0	0	1	0	0	0	0	0	0	0	0	0	0	0	0	0	0	0	0
BGIR9823_102349	BGIG9823_27014		lncRNA	0	0	0	0	0	0	0	0	0	0	0	0	0	0	0	0	0	0	0
BGIR9823_102713	BGIG9823_27363		lncRNA	0	0	0	0	0	0	0	0	0	0	0	0	0	0	0	1	0	0	0
BGIR9823_101686	BGIG9823_26373		lncRNA	0	0	0	0	0	0	0	0	0	0	0	0	0	0	0	0	0	0	0
BGIR9823_102210	BGIG9823_26882		lncRNA	0	0	0	0	0	0	0	0	0	0	0	0	0	0	0	0	0	0	0
BGIR9823_102323	BGIG9823_26988		lncRNA	0	0	0	0	0	0	0	0	0	0	0	0	0	0	0	0	0	0	0
BGIR9823_102016	BGIG9823_26692		mRNA	0	0	0	0	0	0	0	0	0	0	0	0	0	0	0	0	0	0	0
BGIR9823_102051	BGIG9823_26725		lncRNA	0	0	0	0	0	0	0	0	0	0	1	0	0	0	0	0	0	0	0
BGIR9823_101891	BGIG9823_26568		lncRNA	0	0	0	1	0	0	0	0	0	0	0	0	0	0	0	0	0	0	0
BGIR9823_102043	BGIG9823_26717		lncRNA	0	0	0	0	0	0	0	0	0	0	0	0	0	0	0	0	0	0	0
BGIR9823_102464	BGIG9823_27118		lncRNA	0	0	0	0	0	0	0	0	0	0	0	1	0	0	0	0	0	0	0
BGIR9823_102384	BGIG9823_27049		lncRNA	0	0	1	0	0	0	0	0	0	0	0	0	0	0	0	0	0	0	0
BGIR9823_102347	BGIG9823_27012		lncRNA	0	0	0	0	0	0	0	0	0	0	0	0	0	0	0	0	0	0	0

RNA ID	Gene ID	Symbol	Type	novel-ssc-miR1063-5p	ssc-miR-192	ssc-miR-432-5p	ssc-miR-7138-5p	ssc-miR-874	ssc-miR-10386	ssc-miR-122-3p	novel-ssc-miR755-3p	novel-ssc-miR780-3p	novel-ssc-miR804-3p	novel-ssc-miR813-5p	novel-ssc-miR407-5p	novel-ssc-miR573-5p	novel-ssc-miR583-3p	novel-ssc-miR1077-5p	novel-ssc-miR122-3p	novel-ssc-miR154-5p	ssc-miR-127	ssc-miR-194b-5p
BGIR9823_101855	BGIG9823_26534		lncRNA	0	0	0	0	0	0	0	0	0	0	0	0	0	0	0	0	0	0	0
BGIR9823_102597	BGIG9823_27247		lncRNA	0	0	0	0	0	0	0	0	0	0	0	0	0	0	0	0	0	0	0
BGIR9823_101902	BGIG9823_26579		lncRNA	0	0	0	0	0	0	0	0	0	0	0	0	0	0	0	0	0	0	0
BGIR9823_102413	BGIG9823_27074		mRNA	0	0	0	0	0	0	0	0	0	0	0	0	0	0	0	0	0	0	0
BGIR9823_102726	BGIG9823_27372		lncRNA	0	0	0	0	0	0	0	0	0	0	0	0	0	0	0	0	0	0	0
BGIR9823_102647	BGIG9823_27297		lncRNA	0	0	0	0	0	0	0	0	0	0	0	1	0	0	0	0	0	0	0
BGIR9823_101973	BGIG9823_26650		lncRNA	0	0	0	0	0	0	0	0	0	0	0	0	0	0	0	0	0	0	0
BGIR9823_102297	BGIG9823_26963		lncRNA	0	0	0	0	0	0	0	0	0	0	0	0	0	0	0	0	0	0	0
BGIR9823_102389	BGIG9823_27054		lncRNA	0	0	0	0	0	0	0	0	0	0	0	0	0	0	0	1	0	0	0
BGIR9823_101788	BGIG9823_26468		lncRNA	0	0	0	0	0	0	0	0	0	0	0	0	0	0	0	0	0	0	0
BGIR9823_101961	BGIG9823_26638		lncRNA	0	0	0	0	0	0	0	0	0	0	0	0	0	0	0	0	0	0	0
BGIR9823_102423	BGIG9823_27084		lncRNA	0	0	0	0	0	0	0	0	0	0	0	0	0	0	0	0	0	0	0
BGIR9823_102712	BGIG9823_27362		lncRNA	0	0	0	0	0	0	0	0	0	0	0	0	0	0	0	0	0	0	0
BGIR9823_101682	BGIG9823_26369		lncRNA	0	0	0	0	0	0	0	0	0	0	0	0	0	0	0	0	0	0	0
BGIR9823_101865	BGIG9823_26544		lncRNA	0	0	0	0	0	0	0	0	0	0	0	0	0	0	0	1	0	0	0
BGIR9823_102351	BGIG9823_27016		lncRNA	1	0	0	0	0	0	0	0	0	0	0	0	0	0	0	0	0	0	0
BGIR9823_102576	BGIG9823_27226		lncRNA	0	0	0	0	0	0	0	0	0	0	0	0	0	0	0	0	0	0	0
BGIR9823_102428	BGIG9823_27088		lncRNA	0	0	0	0	0	0	0	0	0	0	0	0	0	0	0	0	0	0	0
BGIR9823_102329	BGIG9823_26994		lncRNA	0	0	0	0	0	0	0	0	0	0	0	0	0	0	0	0	0	0	0
BGIR9823_102625	BGIG9823_27275		lncRNA	0	0	0	0	0	0	0	0	0	0	0	0	0	0	0	0	0	0	0
BGIR9823_102075	BGIG9823_26749		lncRNA	0	0	0	0	0	0	0	0	0	0	0	0	0	0	0	0	0	0	0
BGIR9823_102523	BGIG9823_27176		lncRNA	0	0	0	0	0	0	0	0	0	0	0	0	0	0	0	0	0	0	0
BGIR9823_101910	BGIG9823_26587		lncRNA	0	0	0	1	0	0	0	0	0	0	0	0	0	0	0	0	0	0	0
BGIR9823_102748	BGIG9823_27391		lncRNA	0	0	0	0	0	0	0	0	0	0	0	0	0	0	0	0	0	0	0
BGIR9823_101705	BGIG9823_26389		lncRNA	0	0	0	0	0	0	0	0	0	0	0	0	0	0	0	0	0	0	0
BGIR9823_102467	BGIG9823_27121		lncRNA	0	0	0	0	0	0	0	0	0	0	0	0	0	0	0	0	0	0	0
BGIR9823_102501	BGIG9823_27155		lncRNA	0	0	0	0	0	0	0	0	0	0	0	0	0	0	0	0	0	0	0
BGIR9823_101779	BGIG9823_26459		lncRNA	0	0	0	0	0	0	0	0	0	1	0	0	0	0	0	0	0	0	0
BGIR9823_102431	BGIG9823_27090		lncRNA	0	0	0	0	0	0	0	0	0	0	0	0	0	0	0	0	0	0	0
BGIR9823_102628	BGIG9823_27278		lncRNA	0	0	0	0	0	0	0	0	0	0	0	0	0	0	0	0	0	0	0
BGIR9823_101816	BGIG9823_26496		lncRNA	0	0	0	1	0	0	0	0	0	0	0	0	0	0	0	0	0	0	0
BGIR9823_101996	BGIG9823_26672		lncRNA	0	0	0	0	0	0	0	0	0	0	0	0	0	0	0	0	0	0	0
BGIR9823_102304	BGIG9823_26969		lncRNA	0	0	0	0	0	0	0	0	0	0	0	0	0	0	0	1	0	0	0
BGIR9823_101800	BGIG9823_26480		lncRNA	0	0	0	1	0	0	0	0	0	0	0	0	0	0	0	0	0	0	0
BGIR9823_102273	BGIG9823_26940		lncRNA	0	0	0	0	0	0	0	0	0	0	0	0	0	0	0	0	0	0	0
BGIR9823_102407	BGIG9823_27068		lncRNA	0	0	0	0	0	0	0	0	0	0	0	0	0	0	0	0	0	0	0
BGIR9823_102091	BGIG9823_26765		lncRNA	0	0	0	0	0	0	0	0	0	0	0	0	0	0	0	0	0	0	0
BGIR9823_102167	BGIG9823_26840		lncRNA	0	0	1	0	0	0	0	0	0	0	0	0	0	0	0	0	0	0	0
BGIR9823_102166	BGIG9823_26839		lncRNA	0	0	0	0	0	0	0	0	0	1	0	0	0	0	0	0	0	0	0
BGIR9823_102159	BGIG9823_26832		lncRNA	0	0	0	0	0	0	0	0	0	0	0	0	0	0	0	0	0	0	0
BGIR9823_102041	BGIG9823_26715		lncRNA	0	0	0	0	0	0	0	0	0	1	0	0	0	0	0	0	0	0	0
BGIR9823_102369	BGIG9823_27034		lncRNA	1	0	0	0	0	0	0	0	0	0	0	0	0	0	0	0	0	0	0
BGIR9823_101766	BGIG9823_26446		lncRNA	0	0	0	0	0	0	0	0	0	0	0	0	0	0	0	0	0	0	0

RNA ID	Gene ID	Symbol	Type	novel-ssc-miR1063-5p	ssc-miR-192	ssc-miR-432-5p	ssc-miR-7138-5p	ssc-miR-874	ssc-miR-10386	ssc-miR-122-3p	novel-ssc-miR755-3p	novel-ssc-miR780-3p	novel-ssc-miR804-3p	novel-ssc-miR813-5p	novel-ssc-miR407-5p	novel-ssc-miR573-5p	novel-ssc-miR583-3p	novel-ssc-miR1077-5p	novel-ssc-miR122-3p	novel-ssc-miR154-5p	ssc-miR-127	ssc-miR-194b-5p
BGIR9823_102029	BGIG9823_26704		lncRNA	0	0	0	0	0	0	0	0	0	0	0	0	0	0	0	0	0	0	0
BGIR9823_102522	BGIG9823_27175		lncRNA	0	0	0	0	0	0	0	0	0	0	0	0	0	0	0	0	0	0	0
BGIR9823_102783	BGIG9823_27414		lncRNA	0	0	0	0	0	0	0	0	0	0	0	0	0	0	0	0	0	0	0
BGIR9823_101935	BGIG9823_26612		lncRNA	0	0	0	0	0	0	0	0	0	0	1	0	0	0	0	0	0	0	0
BGIR9823_102254	BGIG9823_26922		lncRNA	0	0	0	0	0	0	0	0	0	0	0	0	0	0	0	0	0	0	0
BGIR9823_102184	BGIG9823_26856		lncRNA	0	0	0	0	0	0	0	0	0	0	0	0	1	0	0	0	0	0	0
BGIR9823_102246	BGIG9823_26914		lncRNA	0	0	0	0	0	0	0	0	0	0	0	0	0	0	0	0	0	0	0
BGIR9823_101751	BGIG9823_26432		lncRNA	0	0	0	0	0	0	0	0	0	0	0	0	0	0	0	0	0	0	0
BGIR9823_101673	BGIG9823_26360		lncRNA	0	0	0	0	0	0	0	0	0	0	0	0	0	0	0	0	0	0	0
BGIR9823_101811	BGIG9823_26491		lncRNA	0	0	0	0	0	0	0	0	0	0	0	0	0	0	0	0	0	0	0
BGIR9823_102295	BGIG9823_26961		mRNA	0	0	0	0	0	0	0	0	0	0	0	0	0	0	0	0	0	0	1
BGIR9823_101697	BGIG9823_26384		lncRNA	0	0	0	0	0	0	0	0	0	0	0	0	0	0	0	0	0	0	0
BGIR9823_101725	BGIG9823_26409		lncRNA	0	0	0	0	0	0	0	0	0	0	0	0	0	0	0	1	0	0	0
BGIR9823_102033	BGIG9823_26708		lncRNA	0	0	0	0	0	0	0	0	0	0	1	0	0	0	0	0	0	0	0
BGIR9823_102053	BGIG9823_26727		lncRNA	0	0	0	0	0	0	0	0	0	0	0	0	0	0	0	0	0	0	0
BGIR9823_102015	BGIG9823_26691		lncRNA	0	0	0	0	0	0	0	0	0	0	0	0	0	0	0	0	0	0	0
BGIR9823_102489	BGIG9823_27143		lncRNA	0	0	0	0	0	0	0	0	0	0	0	0	0	0	0	0	0	0	0
BGIR9823_102449	BGIG9823_27104		lncRNA	0	0	0	0	0	0	0	0	0	0	0	0	0	0	0	0	0	0	0
BGIR9823_102214	BGIG9823_26886		lncRNA	0	0	0	0	0	0	0	0	0	0	0	0	0	0	0	1	0	0	0
BGIR9823_101834	BGIG9823_26513		lncRNA	0	0	0	1	0	0	0	0	0	0	0	0	0	0	0	0	0	0	0
BGIR9823_102636	BGIG9823_27286		lncRNA	0	0	0	0	0	0	0	0	0	0	0	0	0	0	0	0	0	0	0
BGIR9823_102287	BGIG9823_26954		lncRNA	0	0	0	0	0	0	0	0	0	0	0	0	0	0	0	0	0	0	0
BGIR9823_101906	BGIG9823_26583		lncRNA	0	0	0	0	0	0	0	0	0	0	0	0	0	0	0	0	0	0	0
BGIR9823_102514	BGIG9823_27168		lncRNA	0	0	0	0	0	0	0	0	0	0	0	0	0	0	0	0	0	0	0
BGIR9823_101914	BGIG9823_26591		lncRNA	0	0	0	0	0	0	0	0	0	0	0	0	0	0	0	0	0	0	0
BGIR9823_101768	BGIG9823_26448		lncRNA	0	0	0	0	0	0	0	0	0	0	0	0	0	0	0	0	0	0	0
BGIR9823_102070	BGIG9823_26744		lncRNA	0	0	0	0	0	0	0	0	0	0	0	1	0	0	0	0	0	0	0
BGIR9823_101805	BGIG9823_26485		lncRNA	0	0	1	0	0	0	0	0	0	0	0	0	0	0	0	0	0	0	0
BGIR9823_102103	BGIG9823_26777		lncRNA	0	0	0	0	0	0	0	0	0	1	0	0	0	0	0	0	0	0	0
BGIR9823_102688	BGIG9823_27338		lncRNA	0	0	0	0	0	0	0	0	0	0	0	0	1	0	0	0	0	0	0
BGIR9823_102733	BGIG9823_27379		lncRNA	0	0	0	0	0	0	0	0	0	0	0	0	0	0	0	0	0	0	0
BGIR9823_102411	BGIG9823_27072		lncRNA	0	0	0	0	0	0	0	0	0	0	0	0	0	0	0	0	0	0	0
BGIR9823_102360	BGIG9823_27025		lncRNA	0	0	0	0	0	0	0	0	0	0	0	0	0	0	0	0	0	0	0
BGIR9823_102558	BGIG9823_27208		lncRNA	0	0	0	0	0	0	0	0	0	0	0	0	0	0	0	0	0	0	0
BGIR9823_101837	BGIG9823_26516		lncRNA	0	0	0	0	0	0	0	0	0	0	0	0	0	0	0	0	0	0	0
BGIR9823_101722	BGIG9823_26406		lncRNA	0	0	0	0	0	0	0	0	0	0	0	0	0	0	0	0	0	0	0
BGIR9823_102750	BGIG9823_27392		lncRNA	0	0	0	0	0	0	0	0	0	0	0	0	0	0	0	0	0	0	0
BGIR9823_102692	BGIG9823_27342		lncRNA	0	0	0	0	0	0	0	0	0	0	0	0	0	0	0	0	0	0	0
BGIR9823_102755	BGIG9823_27395		lncRNA	0	0	0	0	0	0	0	0	0	0	0	0	0	0	0	0	0	0	0
BGIR9823_101957	BGIG9823_26634		lncRNA	0	0	0	0	0	0	0	0	0	0	0	0	0	0	0	0	0	0	0
BGIR9823_102097	BGIG9823_26771		lncRNA	0	0	0	0	0	0	0	0	0	0	0	0	0	0	0	0	0	0	0
BGIR9823_102454	BGIG9823_27108		lncRNA	0	0	0	0	0	0	0	0	0	0	0	0	0	0	0	0	0	0	0
BGIR9823_102019	BGIG9823_26694		lncRNA	0	0	0	0	0	0	0	0	0	0	0	0	0	0	0	0	0	0	0

RNA ID	Gene ID	Symbol	Type	novel-ssc-miR1063-5p	ssc-miR-192	ssc-miR-432-5p	ssc-miR-7138-5p	ssc-miR-874	ssc-miR-10386	ssc-miR-122-3p	novel-ssc-miR755-3p	novel-ssc-miR780-3p	novel-ssc-miR804-3p	novel-ssc-miR813-5p	novel-ssc-miR407-5p	novel-ssc-miR573-5p	novel-ssc-miR583-3p	novel-ssc-miR1077-5p	novel-ssc-miR122-3p	novel-ssc-miR154-5p	ssc-miR-127	ssc-miR-194b-5p
BGIR9823_101736	BGIG9823_26417		lncRNA	0	0	0	0	0	0	0	0	0	0	0	0	0	0	0	0	0	0	0
BGIR9823_102662	BGIG9823_27312		lncRNA	0	0	0	0	0	0	0	0	0	0	0	0	0	0	0	0	0	0	0
BGIR9823_102303	BGIG9823_26969		lncRNA	0	0	0	0	0	0	0	0	0	0	0	0	0	0	0	0	0	0	0
BGIR9823_101931	BGIG9823_26608		lncRNA	0	0	0	0	0	0	0	0	0	0	0	0	0	0	0	0	0	0	0
BGIR9823_101842	BGIG9823_26521		lncRNA	0	0	0	0	0	0	0	0	0	0	0	0	0	0	0	0	0	0	0
BGIR9823_101783	BGIG9823_26463		lncRNA	0	0	0	0	0	0	0	0	0	0	0	1	0	0	0	0	0	0	0
BGIR9823_102447	BGIG9823_27102		lncRNA	0	0	0	0	0	0	0	0	0	0	0	0	0	0	0	0	0	0	0
BGIR9823_102114	BGIG9823_26788		lncRNA	0	0	0	0	0	0	0	0	0	0	0	0	0	0	0	0	0	0	0
BGIR9823_101753	BGIG9823_26434		lncRNA	0	0	0	0	0	0	0	0	0	0	0	0	0	0	0	0	0	0	0
BGIR9823_102643	BGIG9823_27293		lncRNA	0	0	0	0	0	0	0	0	0	0	0	0	0	0	0	0	0	0	0
BGIR9823_102327	BGIG9823_26992		lncRNA	0	0	1	0	0	0	0	0	0	0	0	0	0	0	0	0	0	0	0
BGIR9823_102216	BGIG9823_26888		lncRNA	0	0	0	0	0	0	0	0	0	0	0	0	0	0	0	0	0	0	0
BGIR9823_102718	BGIG9823_27365		lncRNA	0	0	0	0	0	0	0	0	0	0	0	0	0	0	0	0	0	0	0
BGIR9823_101709	BGIG9823_26393		lncRNA	0	0	0	0	0	0	0	0	0	0	0	0	0	0	0	0	0	0	0
BGIR9823_102241	BGIG9823_26913		lncRNA	0	0	0	0	0	0	0	0	0	0	0	0	0	0	0	0	0	0	0
BGIR9823_102306	BGIG9823_26971		lncRNA	0	0	0	0	0	0	0	0	0	0	0	0	0	0	0	0	0	0	0
BGIR9823_102486	BGIG9823_27140		lncRNA	0	0	0	0	0	0	0	0	0	0	0	0	0	0	0	0	0	0	0
BGIR9823_101724	BGIG9823_26408		lncRNA	0	0	0	0	0	0	0	0	0	0	0	0	0	0	0	0	0	0	0
BGIR9823_101858	BGIG9823_26537		lncRNA	0	0	0	0	0	0	0	0	0	0	0	0	0	0	0	0	0	0	0
BGIR9823_102483	BGIG9823_27137		lncRNA	0	0	0	0	0	0	0	0	0	0	0	0	0	0	0	0	0	0	0
BGIR9823_102027	BGIG9823_26702		lncRNA	0	1	0	0	0	0	0	0	0	0	0	0	0	0	0	0	0	0	0
BGIR9823_101777	BGIG9823_26457		lncRNA	0	0	0	0	0	0	0	0	0	0	0	0	0	0	0	0	0	0	0
BGIR9823_102154	BGIG9823_26827		lncRNA	0	0	0	0	0	0	0	0	0	0	0	0	0	0	0	0	0	0	0
BGIR9823_102426	BGIG9823_27087		lncRNA	0	0	1	0	0	0	0	0	0	0	0	0	0	0	0	0	0	0	0
BGIR9823_101894	BGIG9823_26571		lncRNA	0	0	0	0	0	0	0	0	0	0	0	0	0	0	0	0	0	0	0
BGIR9823_102009	BGIG9823_26685		lncRNA	0	0	0	0	0	0	0	0	0	0	1	0	0	0	0	0	0	0	0
BGIR9823_102250	BGIG9823_26918		lncRNA	0	0	0	0	0	0	0	0	0	0	0	0	0	0	0	0	0	0	0
BGIR9823_101769	BGIG9823_26449		lncRNA	0	0	0	0	0	0	0	0	0	0	0	0	0	0	0	0	0	0	0
XR_002338373.1	110256615	LOC110256615	lncRNA	0	0	0	0	0	0	0	0	0	0	0	0	0	0	0	0	0	0	0
XM_005655448.3	100519711	ST7L	mRNA	0	0	0	0	0	0	0	0	0	0	0	0	0	0	0	0	0	0	0
XR_001308691.2	106510294	LOC106510294	lncRNA	0	0	0	0	0	0	0	0	0	0	0	0	0	0	0	0	0	0	0
XR_002343519.1	110260353	LOC110260353	lncRNA	0	0	0	0	0	0	0	0	0	0	0	0	0	0	0	0	0	0	0
XR_002336962.1	102162292	LOC102162292	lncRNA	0	0	0	0	0	0	0	0	0	0	0	0	0	0	0	0	0	0	0
XM_021087319.1	100517646	POLE4	mRNA	0	0	0	0	0	0	0	0	0	0	0	0	0	0	0	0	0	0	0
XR_002336095.1	110255550	LOC110255550	lncRNA	0	0	0	0	0	0	0	0	0	0	0	0	0	0	0	0	0	0	0
NM_001244786.1	100513209	CFAP20	mRNA	0	0	0	0	0	0	0	0	0	0	0	0	0	0	0	0	0	0	0
XR_002340787.1	110257799	LOC110257799	lncRNA	0	0	0	0	0	0	0	0	0	0	0	0	0	0	0	0	0	0	0
XM_003358899.4	100623886	CCT8	mRNA	0	0	0	0	0	0	0	0	0	0	0	0	0	0	0	0	0	0	0
XR_002337835.1	110256393	LOC110256393	lncRNA	0	0	0	0	0	0	0	0	0	0	0	0	0	0	0	0	0	0	0
XR_002337635.1	106505647	LOC106505647	lncRNA	0	0	0	0	0	0	0	0	0	0	0	0	0	0	0	0	0	0	0
XR_001308778.2	106510350	LOC106510350	lncRNA	0	0	0	0	0	0	0	0	0	0	0	0	0	0	0	0	0	0	0
XR_002346849.1	110262181	LOC110262181	lncRNA	0	0	0	0	0	0	0	0	0	0	0	0	0	0	0	0	0	0	0
XM_005659957.3	102163937	GPR137C	mRNA	0	0	0	0	0	0	0	0	0	0	0	0	0	0	0	0	0	0	0

RNA ID	Gene ID	Symbol	Type	novel-ssc-miR1063-5p	ssc-miR-192	ssc-miR-432-5p	ssc-miR-7138-5p	ssc-miR-874	ssc-miR-10386	ssc-miR-122-3p	novel-ssc-miR755-3p	novel-ssc-miR780-3p	novel-ssc-miR804-3p	novel-ssc-miR813-5p	novel-ssc-miR407-5p	novel-ssc-miR573-5p	novel-ssc-miR583-3p	novel-ssc-miR1077-5p	novel-ssc-miR122-3p	novel-ssc-miR154-5p	ssc-miR-127	ssc-miR-194b-5p
XR_001303641.2	106507572	LOC106507572	lncRNA	0	0	0	0	0	0	0	0	0	0	0	0	0	0	0	0	0	0	0
XR_002344010.1	110260700	LOC110260700	lncRNA	0	0	0	0	0	0	0	0	0	0	0	0	0	0	0	0	0	0	0
XM_003359284.4	100623641	ZWINT	mRNA	0	0	0	0	0	0	0	0	0	0	0	0	0	0	0	0	0	0	0
XM_021066040.1	100233199	PITPNC1	mRNA	0	0	0	0	0	0	0	0	0	0	0	0	0	0	0	0	0	0	0
XM_003482526.2	100738522	LOC100738522	mRNA	0	0	0	0	0	0	1	0	0	0	0	0	0	0	0	0	0	0	0
XR_002345706.1	110261519	LOC110261519	lncRNA	0	0	0	0	0	0	0	0	0	0	0	0	0	0	0	0	0	0	0
XM_021066775.1	100513305	STRADA	mRNA	0	0	0	0	0	0	0	0	0	0	0	0	0	0	0	0	0	0	0
XR_303482.2	102164583	LOC102164583	lncRNA	0	0	0	0	0	0	0	0	0	0	0	0	0	0	0	0	0	0	0
XM_013998720.2	100737585	LOC100737585	mRNA	0	0	0	0	0	0	0	0	0	0	0	0	0	0	0	0	0	0	0
XR_002336556.1	102163487	LOC102163487	lncRNA	0	0	0	0	0	0	0	0	0	0	0	0	0	0	0	0	0	0	0
XR_002337553.1	102167511	SMIM4	lncRNA	0	0	0	0	0	0	0	0	0	0	0	0	0	0	0	0	0	0	0
XM_003483492.4	396595	PRF1	mRNA	0	0	0	0	0	0	0	0	0	0	0	0	0	0	0	0	0	0	0
XM_001926866.5	100155931	LOC100155931	mRNA	0	0	0	0	0	0	0	0	0	0	0	0	0	0	0	0	0	0	0
XR_002341737.1	102165038	LOC102165038	lncRNA	0	0	0	0	0	0	0	0	0	0	0	0	0	0	0	0	0	0	0
XR_002337051.1	110255981	LOC110255981	lncRNA	0	0	0	0	0	0	0	0	0	0	0	0	0	0	0	0	0	0	0
XM_021091413.1	100621517	TTLL1	mRNA	0	0	0	0	0	0	0	0	0	0	0	0	0	0	0	0	0	0	0
XR_002344541.1	110260924	LOC110260924	lncRNA	0	0	0	0	0	0	0	0	0	0	0	0	0	0	0	0	0	0	0
NM_213786.1	396680	NPW	mRNA	0	0	0	0	0	0	0	0	0	0	0	0	0	0	0	0	0	0	0
XM_021089665.1	100738802	BCAN	mRNA	0	0	0	0	0	0	0	0	0	0	0	0	0	0	0	0	0	0	0
XR_002340802.1	110257805	LOC110257805	lncRNA	0	0	0	0	0	0	0	0	0	0	0	0	0	0	0	0	0	0	0
XR_002341733.1	102158524	LOC102158524	lncRNA	0	0	0	0	0	0	0	0	0	0	0	0	0	0	0	0	0	0	0
XR_002345869.1	110261578	LOC110261578	lncRNA	0	0	0	0	0	0	0	0	0	0	0	0	0	0	0	0	0	0	0
XR_002338170.1	102161036	LOC102161036	lncRNA	0	0	0	0	0	0	0	0	0	0	0	0	0	0	0	0	0	0	0
XR_002337152.1	106505099	LOC106505099	lncRNA	0	0	0	0	0	0	0	0	0	0	0	0	0	0	0	0	0	0	0
XM_003131571.5	100512492	ANKRD40	mRNA	0	0	0	0	0	0	0	0	0	0	0	0	0	1	0	0	0	0	0
XR_002337061.1	110255986	LOC110255986	lncRNA	0	0	0	0	0	0	0	0	0	0	0	0	0	0	0	0	0	0	0
XR_002341729.1	110259387	LOC110259387	lncRNA	0	0	0	0	0	0	0	0	0	0	0	0	0	0	0	0	0	0	0
XM_005665516.3	100524028	ZMYND12	mRNA	0	0	0	0	0	0	0	0	0	0	0	0	0	0	0	0	0	0	0
XR_002342030.1	110259546	LOC110259546	lncRNA	0	0	0	0	0	0	0	0	0	0	0	0	0	0	0	0	0	0	0
XR_001304742.2	106508150	LOC106508150	lncRNA	0	0	0	0	0	0	0	0	0	0	0	0	0	0	0	0	0	0	0
NM_214322.1	397591	NPC1	mRNA	0	0	0	0	0	0	0	0	0	0	0	0	0	0	0	0	0	0	0
XR_002339061.1	102158523	LOC102158523	lncRNA	0	0	0	0	0	0	0	0	0	0	0	0	0	0	0	0	0	0	0
XR_002335622.1	110255429	LOC110255429	lncRNA	0	0	0	0	0	0	0	0	0	0	0	0	0	0	0	0	0	0	1
XM_003124446.4	100518241	SUMF2	mRNA	0	0	0	0	0	0	0	0	0	0	0	0	0	0	0	0	0	0	0
XR_002337394.1	110256124	LOC110256124	lncRNA	0	0	0	0	0	0	0	0	0	0	0	0	0	0	0	0	0	0	0
XM_003130089.4	100520582	LOC100520582	mRNA	0	0	0	0	0	0	0	0	0	0	0	0	0	1	0	0	0	0	0
XM_021100295.1	100337668	LCORL	mRNA	0	0	0	0	0	0	0	0	0	0	0	0	0	0	0	0	0	0	0
XR_002347096.1	110262257	LOC110262257	lncRNA	0	0	0	0	0	0	0	0	0	0	0	0	0	0	0	0	0	0	0
NM_214272.1	397512	TGFBR3	mRNA	0	0	0	0	0	0	0	0	0	0	0	0	0	0	0	0	0	0	0
XR_002337890.1	110256420	LOC110256420	lncRNA	0	0	0	0	0	0	0	0	0	0	0	0	0	0	0	0	0	0	0
XR_001302346.2	106506787	LOC106506787	lncRNA	0	0	0	0	0	0	0	0	0	0	0	0	0	0	0	0	0	0	0
XR_002340032.1	102161214	LOC102161214	lncRNA	0	0	0	0	0	0	0	0	0	0	0	0	0	0	0	0	0	0	0
XR_002338731.1	110256774	LOC110256774	lncRNA	0	0	0	0	0	0	0	0	0	0	0	0	0	0	0	0	0	0	0

RNA ID	Gene ID	Symbol	Type	novel-ssc-miR1063-5p	ssc-miR-192	ssc-miR-432-5p	ssc-miR-7138-5p	ssc-miR-874	ssc-miR-10386	ssc-miR-122-3p	novel-ssc-miR755-3p	novel-ssc-miR780-3p	novel-ssc-miR804-3p	novel-ssc-miR813-5p	novel-ssc-miR407-5p	novel-ssc-miR573-5p	novel-ssc-miR583-3p	novel-ssc-miR1077-5p	novel-ssc-miR122-3p	novel-ssc-miR154-5p	ssc-miR-127	ssc-miR-194b-5p
XR_002342421.1	110259933	LOC110259933	lncRNA	0	0	0	0	0	0	0	1	0	0	0	0	0	0	0	0	0	0	0
XM_021080420.1	110257790	WDR44	mRNA	0	0	0	0	0	0	0	0	0	0	0	0	0	0	0	0	0	0	0
XM_003128412.6	100514413	GTPBP2	mRNA	0	0	0	0	0	0	0	0	0	0	0	0	0	0	0	0	0	0	0
XR_001308377.2	106510160	LOC106510160	lncRNA	0	0	0	0	0	0	0	0	0	0	0	0	0	0	0	0	0	0	0
XM_021091670.1	100522737	ANKRD33	mRNA	0	0	0	0	0	0	0	0	0	0	0	0	0	0	0	0	0	0	0
XM_003134187.6	100512932	ASAH1	mRNA	0	0	0	0	0	0	0	0	0	0	0	0	0	0	0	0	0	0	0
XR_002335568.1	102159627	LOC102159627	lncRNA	0	0	0	0	0	0	0	0	0	0	0	0	0	0	0	0	0	0	0
XR_002345700.1	110261513	LOC110261513	lncRNA	0	0	0	0	0	0	0	0	0	0	0	0	0	0	0	0	0	0	0
NM_001243805.1	100511029	STARD10	mRNA	0	0	0	0	0	0	0	0	0	0	0	0	0	0	0	0	0	0	0
XM_001927894.5	100153448	HENMT1	mRNA	0	0	0	0	0	0	0	0	0	0	0	0	0	0	0	0	0	0	0
NM_214205.1	397409	IL5	mRNA	0	0	0	0	0	0	0	0	0	0	0	0	0	0	0	0	0	0	0
XR_002344130.1	110260754	LOC110260754	lncRNA	0	0	0	0	0	0	0	0	0	0	0	0	0	0	0	0	0	0	0
XM_021075881.1	100157060	WDR17	mRNA	0	0	0	0	0	0	0	0	0	0	0	0	0	0	0	0	0	0	0
XR_002344639.1	110260981	LOC110260981	lncRNA	0	0	0	0	0	0	0	0	0	0	0	0	0	0	0	0	0	0	0
XM_013985613.2	100525844	SLC35B4	mRNA	0	0	0	0	0	0	0	0	0	0	0	0	0	0	0	0	0	0	0
XR_002342231.1	110259849	LOC110259849	lncRNA	0	0	0	0	0	0	0	0	0	0	0	0	0	0	0	0	0	0	0
NM_001004043.1	445532	CFL1	mRNA	0	0	0	0	0	0	0	0	0	0	0	0	0	0	0	0	0	0	0
XM_003132695.5	100521154	ZBTB11	mRNA	0	0	0	0	0	0	0	0	0	0	0	0	0	0	0	0	0	0	0
XM_005657909.3	100519458	TEX13B	mRNA	0	0	0	0	0	0	0	0	0	0	0	0	0	0	0	0	0	0	0
XM_003129582.2	100517357	LOC100517357	mRNA	0	0	0	0	0	0	1	0	0	0	0	0	0	0	0	0	0	0	0
XR_298408.3	102167235	LOC102167235	lncRNA	0	0	0	0	0	0	0	0	0	0	0	0	0	0	0	0	0	0	1
XR_002336226.1	110255645	LOC110255645	lncRNA	0	0	0	0	0	0	0	0	0	0	0	0	0	0	0	0	0	0	0
XM_021077102.1	100515590	CCDC69	mRNA	0	0	0	0	0	0	0	0	0	0	0	0	0	0	0	0	0	0	0
XR_002336343.1	110255688	LOC110255688	lncRNA	0	0	0	0	0	0	0	0	0	0	0	0	0	0	0	0	0	0	0
XR_001309352.2	106510687	LOC106510687	lncRNA	0	0	0	0	0	0	0	0	0	0	0	0	0	0	0	0	0	0	0
XR_001301955.2	106506567	LOC106506567	lncRNA	0	0	0	0	0	0	0	0	0	0	0	0	0	0	0	0	0	0	1
XR_002338652.1	102167906	LOC102167906	lncRNA	0	0	0	0	0	0	0	0	0	0	0	0	0	0	0	0	0	0	0
XR_002336386.1	106506961	LOC106506961	lncRNA	0	0	0	0	0	0	0	0	0	0	0	0	0	0	0	0	0	0	0
XM_021074218.1	110256875	LOC110256875	mRNA	0	0	0	0	0	0	0	0	0	0	0	0	0	0	0	0	0	0	0
XR_002336193.1	100517050	2-Mar	lncRNA	0	0	0	0	0	0	0	0	0	0	0	0	0	0	0	0	0	0	0
XM_021088735.1	414423	GNRHR2	mRNA	0	0	0	0	0	0	0	0	0	0	0	0	0	0	0	0	0	0	0
NM_001146130.2	100271932	HPSE	mRNA	0	0	0	0	0	0	0	0	0	0	0	0	0	0	0	0	0	0	0
XM_013992909.2	100152206	LOC100152206	mRNA	0	0	0	0	0	0	0	0	0	0	0	0	0	0	0	0	0	0	0
XR_001299351.2	106505189	LOC106505189	lncRNA	0	0	0	0	0	0	0	0	0	0	0	0	0	0	0	0	0	0	0
XM_021090795.1	110260467	LOC110260467	mRNA	0	0	0	0	0	0	1	0	0	0	0	0	0	0	0	0	0	0	0
XR_001308232.2	106510085	LOC106510085	lncRNA	0	0	0	0	0	0	0	0	0	0	0	0	0	0	0	0	0	0	0
XR_002346494.1	110262011	LOC110262011	lncRNA	0	0	0	0	0	0	0	0	0	0	0	0	0	1	0	0	0	0	0
XM_005665059.3	100622416	KLHDC7A	mRNA	0	0	0	0	0	0	0	0	0	0	0	0	0	0	0	0	0	0	0
XR_002345769.1	110261544	LOC110261544	lncRNA	0	0	0	0	0	0	0	0	0	0	0	0	0	0	0	0	0	0	0
XR_002341699.1	110259368	LOC110259368	lncRNA	0	0	0	0	0	0	0	0	0	0	0	0	0	0	0	0	0	0	0
XR_001307130.2	106509521	LOC106509521	lncRNA	0	0	0	0	0	0	0	0	0	0	0	0	0	0	0	0	0	0	0
XR_002342847.1	106509907	LOC106509907	lncRNA	0	0	0	0	0	0	0	0	0	0	0	0	0	0	0	0	0	0	0
XR_001309149.2	106510562	LOC106510562	lncRNA	0	0	0	0	0	0	0	0	0	0	0	0	0	0	0	0	0	0	0

RNA ID	Gene ID	Symbol	Type	novel-ssc-miR1063-5p	ssc-miR-192	ssc-miR-432-5p	ssc-miR-7138-5p	ssc-miR-874	ssc-miR-10386	ssc-miR-122-3p	novel-ssc-miR755-3p	novel-ssc-miR780-3p	novel-ssc-miR804-3p	novel-ssc-miR813-5p	novel-ssc-miR407-5p	novel-ssc-miR573-5p	novel-ssc-miR583-3p	novel-ssc-miR1077-5p	novel-ssc-miR122-3p	novel-ssc-miR154-5p	ssc-miR-127	ssc-miR-194b-5p
XM_021096012.1	106507628	ZMPSTE24	mRNA	0	0	0	0	0	0	0	0	0	0	0	0	0	0	0	0	0	0	0
XM_021078386.1	110257500	PCMTD2	mRNA	0	0	0	0	0	0	0	0	0	0	0	0	0	0	0	0	0	0	0
XM_003134291.4	100513828	FOXA2	mRNA	0	0	0	0	0	0	0	0	0	0	0	0	0	0	0	0	0	0	0
XR_002339192.1	106506426	LOC106506426	lncRNA	0	0	0	0	0	0	0	0	0	0	0	0	0	0	0	0	0	0	0
XR_002340657.1	106506896	LOC106506896	lncRNA	0	0	0	0	0	0	0	0	0	0	0	0	0	0	0	0	0	0	0
XR_002344578.1	106507567	LOC106507567	lncRNA	0	0	0	0	0	0	0	0	0	0	0	0	0	0	0	0	0	0	0
XR_002337161.1	110256021	LOC110256021	lncRNA	0	0	0	0	0	0	0	0	0	0	0	0	0	0	0	0	0	0	0
XR_002343165.1	110260239	LOC110260239	lncRNA	0	0	0	0	0	0	0	0	0	0	0	0	0	0	0	0	0	0	0
XR_001306470.2	106509172	LOC106509172	lncRNA	0	0	0	0	0	0	0	0	0	0	0	0	0	0	0	0	0	0	0
XM_021087431.1	100622990	ETAA1	mRNA	0	0	0	0	0	0	0	0	0	0	0	0	0	0	0	0	0	0	0
NM_001243677.1	100157702	P2RY10	mRNA	0	0	0	0	0	0	0	0	0	0	0	0	0	0	0	0	0	0	0
NM_001005726.1	406192	ANXA2	mRNA	0	0	0	0	0	0	0	0	0	0	0	0	0	1	0	0	0	0	0
XR_002338199.1	110256565	LOC110256565	lncRNA	0	0	0	0	0	0	0	0	0	0	0	0	0	0	0	0	0	0	0
XR_002346497.1	110262015	LOC110262015	lncRNA	0	0	0	0	0	0	0	0	0	0	0	0	0	0	0	0	0	0	0
XM_021081451.1	100625375	LOC100625375	mRNA	0	0	0	0	0	0	0	0	0	0	0	0	0	0	0	0	0	0	0
NM_001243852.1	100511525	STT3A	mRNA	0	0	0	0	0	0	0	0	0	0	0	0	0	0	0	0	0	0	0
XM_001927799.7	100154361	SRP54	mRNA	0	0	0	0	0	0	0	0	0	0	0	0	0	0	0	0	0	0	0
XR_002339070.1	110256975	LOC110256975	lncRNA	0	0	0	0	0	0	0	0	0	0	0	0	0	0	0	0	0	0	0
XR_002340950.1	110257979	LOC110257979	lncRNA	0	0	0	0	0	0	1	0	0	0	0	0	0	0	0	0	0	0	0
XM_013990143.2	100155468	LOC100155468	mRNA	0	0	0	0	0	0	0	0	0	0	0	0	0	0	0	0	0	0	0
XM_003129448.1	100516817	LOC100516817	mRNA	0	0	0	0	0	0	0	0	0	0	0	0	0	0	0	0	0	0	0
XR_001301393.2	106506223	LOC106506223	lncRNA	0	0	0	0	0	0	0	0	0	0	0	0	0	0	0	0	0	0	0
XR_002337789.1	102162054	LOC102162054	lncRNA	0	0	0	0	0	0	0	0	0	0	0	0	0	0	0	0	0	0	0
XM_021085070.1	110259585	LOC110259585	mRNA	0	0	0	0	0	0	0	0	0	0	0	0	0	0	0	0	0	0	0
XM_005670449.3	100524281	SCARA5	mRNA	0	0	0	0	0	0	0	0	0	0	0	0	0	0	0	0	0	0	0
XM_021081009.1	110257997	LOC110257997	mRNA	0	0	0	0	0	0	0	0	0	0	0	0	0	0	0	0	0	0	0
XR_002343866.1	102166296	LOC102166296	lncRNA	0	0	0	0	0	0	0	0	0	0	0	0	0	0	0	0	0	0	0
XM_003124181.1	100517754	LOC100517754	mRNA	0	0	0	0	0	0	0	0	0	0	0	0	0	1	0	0	0	0	0
XR_001298301.2	102159863	LOC102159863	lncRNA	0	0	0	0	0	0	0	0	0	0	0	0	0	0	0	0	0	0	0
XM_021080605.1	110257839	LOC110257839	mRNA	0	0	0	0	0	0	0	0	0	0	0	0	0	0	0	0	0	0	0
XM_021072297.1	100519392	RAB36	mRNA	0	0	0	0	0	0	0	0	0	0	0	0	0	0	0	0	0	0	0
XM_005657053.3	100621152	RNF222	mRNA	0	0	0	0	0	0	0	0	0	0	0	0	0	0	0	0	0	0	0
XM_021064469.1	100516823	CNST	mRNA	0	0	0	0	0	0	0	0	0	0	0	0	0	0	0	0	0	0	0
XR_002344959.1	110261129	LOC110261129	lncRNA	0	0	0	0	0	0	0	0	0	0	0	0	0	0	0	0	0	0	0
XR_002336671.1	110255811	LOC110255811	lncRNA	0	0	0	0	0	0	0	0	0	0	0	0	0	0	0	0	0	0	0
XR_002346381.1	110261974	LOC110261974	lncRNA	0	0	0	0	0	0	0	0	0	0	0	0	0	0	0	0	0	0	0
XM_013994471.2	100517025	LOC100517025	mRNA	0	0	0	0	0	0	0	0	0	0	0	0	0	0	0	0	0	0	0
NM_001244142.1	100514123	SRI	mRNA	0	0	0	0	0	0	1	0	0	0	0	0	0	0	0	0	0	0	0
NM_001315615.1	100217382	NMUR2	mRNA	0	0	0	0	0	0	0	0	0	0	0	0	0	0	0	0	0	0	0
XR_002345984.1	110261620	LOC110261620	lncRNA	0	0	0	0	0	0	0	0	0	0	0	0	0	0	0	0	0	0	0
XR_002336754.1	110255855	LOC110255855	lncRNA	0	0	0	0	0	0	0	0	0	0	0	0	0	0	0	0	0	0	0
XR_002346598.1	110262073	LOC110262073	lncRNA	0	0	0	0	0	0	0	0	0	0	0	0	0	0	0	0	0	0	0
XR_002335757.1	110255462	LOC110255462	lncRNA	0	0	0	0	0	0	0	0	0	0	0	0	0	0	0	0	0	0	0

RNA ID	Gene ID	Symbol	Type	novel-ssc-miR1063-5p	ssc-miR-192	ssc-miR-432-5p	ssc-miR-7138-5p	ssc-miR-874	ssc-miR-10386	ssc-miR-122-3p	novel-ssc-miR755-3p	novel-ssc-miR780-3p	novel-ssc-miR804-3p	novel-ssc-miR813-5p	novel-ssc-miR407-5p	novel-ssc-miR573-5p	novel-ssc-miR583-3p	novel-ssc-miR1077-5p	novel-ssc-miR122-3p	novel-ssc-miR154-5p	ssc-miR-127	ssc-miR-194b-5p
XR_001304351.2	102167865	LOC102167865	lncRNA	0	0	0	0	0	0	0	0	0	0	0	0	0	0	0	0	0	0	0
NM_001011507.1	494565	LAMP1	mRNA	0	0	0	0	0	0	0	0	0	0	0	0	0	0	0	0	0	0	0
XR_002339091.1	106505784	LOC106505784	lncRNA	0	0	0	0	0	0	0	0	0	0	0	0	0	0	0	0	0	0	0
XR_298324.3	102163156	LOC102163156	lncRNA	0	0	0	0	0	0	0	0	0	0	0	0	0	0	0	0	0	0	0
XM_021099319.1	100626159	RNASE9	mRNA	0	0	0	0	0	0	0	0	0	0	0	0	0	0	0	0	0	0	0
XM_021096183.1	100739254	ANKRD29	mRNA	0	0	0	0	0	0	1	0	0	0	0	0	0	0	0	0	0	0	0
XR_002335919.1	110255507	LOC110255507	lncRNA	0	0	0	0	0	0	0	0	0	0	0	0	0	0	0	0	0	0	0
XR_002340438.1	106506789	LOC106506789	lncRNA	0	0	0	0	0	0	0	1	0	0	0	0	0	0	0	0	0	0	0
XM_021099935.1	100519785	RAD51B	mRNA	0	0	0	0	0	0	0	0	0	0	0	0	0	0	0	0	0	0	0
XR_001305518.2	106508572	LOC106508572	lncRNA	0	0	0	0	0	0	0	0	0	0	0	0	0	0	0	0	0	0	0
XM_013992672.2	100520160	CNR1	mRNA	0	0	0	0	0	0	0	0	0	0	0	0	0	0	0	0	0	0	0
NM_001123164.1	100144479	PNMT	mRNA	0	0	0	0	0	0	0	0	0	0	0	0	0	0	0	0	0	0	0
NM_001244365.1	100153444	UROS	mRNA	0	0	0	0	0	0	0	0	0	0	0	0	0	1	0	0	0	0	0
XM_021092559.1	100157209	PRICKLE1	mRNA	0	0	0	0	0	0	0	0	0	0	0	0	0	0	0	0	0	0	0
XM_013979148.2	100521912	WNT11	mRNA	0	0	0	0	0	0	0	0	0	0	0	0	0	0	0	0	0	0	0
XR_002337909.1	110256426	LOC110256426	lncRNA	0	0	0	0	0	0	0	0	0	0	0	0	0	0	0	0	0	0	0
NM_214280.1	397524	VCP	mRNA	0	0	0	0	0	0	0	0	0	0	0	0	0	1	0	0	0	0	0
XM_005671261.3	100155046	RNLS	mRNA	0	0	0	0	0	0	0	0	0	0	0	0	0	0	0	0	0	0	0
XR_002337573.1	100154005	ACTR8	mRNA	0	0	0	0	0	0	0	0	0	0	0	0	0	0	0	0	0	0	0
NM_001244315.1	100514255	ARL4C	mRNA	0	0	0	0	0	0	0	0	0	0	0	0	0	0	0	0	0	0	0
XR_001300300.2	106505675	LOC106505675	lncRNA	0	0	0	0	0	0	0	0	0	0	0	0	0	0	0	0	0	0	0
XR_002341897.1	110259477	LOC110259477	lncRNA	0	0	0	0	0	0	0	0	0	0	0	0	0	0	0	0	0	0	0
XR_002336723.1	110255842	LOC110255842	lncRNA	0	0	0	0	0	0	0	0	0	0	0	0	0	0	0	0	0	0	0
XM_021096057.1	100737127	RAB31	mRNA	0	0	0	0	0	0	0	0	0	0	0	0	0	0	0	0	0	0	0
XR_001299360.2	106505194	LOC106505194	lncRNA	0	0	0	0	0	0	0	0	0	0	0	0	0	0	0	0	0	0	0
XM_001925846.7	106504094	GTF3C3	mRNA	0	0	0	0	0	0	0	0	0	0	0	0	0	1	0	0	0	0	0
XR_002337419.1	106505263	LOC106505263	lncRNA	0	0	0	0	0	0	0	0	0	0	0	0	0	0	0	0	0	0	0
XR_002346301.1	110261775	LOC110261775	lncRNA	0	0	0	0	0	0	0	0	0	0	0	0	0	0	0	0	0	0	0
XR_002339946.1	100627949	LOC100627949	lncRNA	0	0	0	0	0	0	0	0	0	0	0	0	0	0	0	0	0	0	0
XR_002346180.1	110261712	LOC110261712	lncRNA	0	0	0	0	0	0	0	0	0	0	0	0	0	0	0	0	0	0	0
XR_001297665.2	106504303	LOC106504303	lncRNA	0	0	0	0	0	0	0	0	0	0	0	0	0	0	0	0	0	0	0
XR_001298947.2	106504955	LOC106504955	lncRNA	0	0	0	0	0	0	0	0	0	0	0	0	0	0	0	0	0	0	0
XR_002341739.1	110259401	LOC110259401	lncRNA	0	0	0	0	0	0	0	0	0	0	0	0	0	0	0	0	0	0	0
XR_001307225.2	106509575	LOC106509575	lncRNA	0	0	0	0	0	0	0	0	0	0	0	0	0	0	0	0	0	0	0
XR_002338919.1	110256865	LOC110256865	lncRNA	0	0	0	0	0	0	0	0	0	0	0	0	0	0	0	0	0	0	0
XR_001306376.2	106509109	LOC106509109	lncRNA	0	0	0	0	0	0	0	0	0	0	0	0	0	0	0	0	0	0	0
XR_002338130.1	102158325	LOC102158325	lncRNA	0	0	0	0	0	0	0	0	0	0	0	0	0	0	0	0	0	0	0
XR_002337988.1	106505633	LOC106505633	lncRNA	0	0	0	0	0	0	0	0	0	0	0	0	0	0	0	0	0	0	0
XM_021086158.1	102161487	TMEM225B	mRNA	0	0	0	0	0	0	0	0	0	0	0	0	0	0	0	0	0	0	0
XM_003130212.5	100522092	RUNDC3B	mRNA	0	0	0	0	0	0	0	0	0	0	0	0	0	0	0	0	0	0	0
XM_001924771.6	100156540	RPP30	mRNA	0	0	0	0	0	0	0	0	0	0	0	0	0	0	0	0	0	0	0
XM_001928042.4	100152526	MED6	mRNA	0	0	0	0	0	0	0	0	0	0	0	0	0	0	0	0	0	0	0
XM_021095234.1	100738053	CHD5	mRNA	0	0	0	0	0	0	0	0	0	0	0	0	0	0	0	0	0	0	0

RNA ID	Gene ID	Symbol	Type	novel-ssc-miR1063-5p	ssc-miR-192	ssc-miR-432-5p	ssc-miR-7138-5p	ssc-miR-874	ssc-miR-10386	ssc-miR-122-3p	novel-ssc-miR755-3p	novel-ssc-miR780-3p	novel-ssc-miR804-3p	novel-ssc-miR813-5p	novel-ssc-miR407-5p	novel-ssc-miR573-5p	novel-ssc-miR583-3p	novel-ssc-miR1077-5p	novel-ssc-miR122-3p	novel-ssc-miR154-5p	ssc-miR-127	ssc-miR-194b-5p
XM_001928146.2	100152920	LOC100152920	mRNA	0	0	0	0	0	0	0	0	0	0	0	0	0	1	0	0	0	0	0
XM_003129758.5	100526065	SESN3	mRNA	0	0	0	0	0	0	0	0	0	0	0	0	0	0	0	0	0	0	0
XR_002339615.1	110257251	LOC110257251	lncRNA	0	0	0	0	0	0	0	0	0	0	0	0	0	0	0	0	0	0	0
XR_002339546.1	102158929	LOC102158929	lncRNA	0	0	0	0	0	0	0	0	0	0	0	0	0	0	0	0	0	0	0
XR_001308433.2	102165975	LOC102165975	lncRNA	0	0	0	0	0	0	0	0	0	0	0	0	0	0	0	0	0	0	0
XR_002341525.1	110259281	LOC110259281	lncRNA	0	0	0	0	0	0	0	0	0	0	0	0	0	0	0	0	0	0	0
NM_001038002.1	414911	EDNRB	mRNA	0	0	0	0	0	0	0	0	0	0	0	0	0	0	0	0	0	0	0
XR_002338445.1	106505879	LOC106505879	lncRNA	0	0	0	0	0	0	0	0	0	0	0	0	0	0	0	0	0	0	0
XR_002341837.1	110259430	LOC110259430	lncRNA	0	0	0	0	0	0	0	0	0	0	0	0	0	0	0	0	0	0	0
XM_021086652.1	100736832	GRIN2A	mRNA	0	0	0	0	0	0	0	0	0	0	0	0	0	0	0	0	0	0	0
XM_013997106.2	106510154	LOC106510154	mRNA	0	0	0	0	0	0	1	0	0	0	0	0	0	0	0	0	0	0	0
XR_002347051.1	110262233	LOC110262233	lncRNA	0	0	0	0	0	0	0	0	0	0	0	0	0	0	0	0	0	0	0
XR_002335588.1	110255417	LOC110255417	lncRNA	0	0	0	0	0	0	0	0	0	0	0	0	0	0	0	0	0	0	0
XR_001302361.2	106506801	LOC106506801	lncRNA	0	0	0	0	0	0	0	0	0	0	0	0	0	0	0	0	0	0	0
XR_002336628.1	110255797	LOC110255797	lncRNA	0	0	0	0	0	0	0	0	0	0	0	0	0	0	0	0	0	0	1
XM_021099547.1	102158165	TMEM63C	mRNA	0	0	0	0	0	0	1	0	0	0	0	0	0	0	0	0	0	0	0
XR_001307739.2	106509824	LOC106509824	lncRNA	0	0	0	0	0	0	0	0	0	0	0	0	0	0	0	0	0	0	0
XM_021084335.1	102162790	GRM6	mRNA	0	0	0	0	0	0	0	0	0	0	0	0	0	0	0	0	0	0	0
XR_002339516.1	102161653	LOC102161653	lncRNA	0	0	0	0	0	0	0	0	0	0	0	0	0	0	0	0	0	0	0
XR_001299377.2	106505203	LOC106505203	lncRNA	0	0	0	0	0	0	0	0	0	0	0	0	0	0	0	0	0	0	0
XM_003481432.4	100738802	BCAN	mRNA	0	0	0	0	0	0	0	0	0	0	0	0	0	0	0	0	0	0	0
XM_021095297.1	100627651	CTNNBIP1	mRNA	0	0	0	0	0	0	1	0	0	0	0	0	0	0	0	0	0	0	0
XM_003134620.5	100517251	TTC26	mRNA	0	0	0	0	0	0	0	0	0	0	0	0	0	0	0	0	0	0	0
XR_002343515.1	102165594	LOC102165594	lncRNA	0	0	0	0	0	0	0	0	0	0	0	0	0	0	0	0	0	0	0
NM_001044607.2	733679	ECH1	mRNA	0	0	0	0	0	0	1	0	0	0	0	0	0	0	0	0	0	0	0
XR_001300056.2	106505536	LOC106505536	lncRNA	0	0	0	0	0	0	0	0	0	0	0	0	0	0	0	0	0	0	0
XR_001301036.2	106506054	LOC106506054	lncRNA	0	0	0	0	0	0	0	0	0	0	0	0	0	0	0	0	0	0	0
XM_005654040.3	397441	CD86	mRNA	0	0	0	0	0	0	0	0	0	0	0	0	0	0	0	0	0	0	0
XR_001298634.2	102163793	LOC102163793	lncRNA	0	0	0	0	0	0	0	0	0	0	0	0	0	0	0	0	0	0	0
XR_002343053.1	110260210	LOC110260210	lncRNA	0	0	0	0	0	0	0	0	0	0	0	0	0	0	0	0	0	0	0
XM_003129012.4	100517956	TMA16	mRNA	0	0	0	0	0	0	0	0	0	0	0	0	0	0	0	0	0	0	0
XM_021076351.1	100515172	HOXD8	mRNA	0	0	0	0	0	0	0	0	0	0	0	0	0	0	0	0	0	0	0
NM_001001636.1	414419	RPL32	mRNA	0	0	0	0	0	0	0	0	0	0	0	0	0	0	0	0	0	0	0
XR_002336222.1	110255642	LOC110255642	lncRNA	0	0	0	0	0	0	0	0	0	0	0	0	0	0	0	0	0	0	0
XR_002337964.1	110256455	LOC110256455	lncRNA	0	0	0	0	0	0	0	0	0	0	0	0	0	0	0	0	0	0	0
XR_002343225.1	110260259	LOC110260259	lncRNA	0	0	0	0	0	0	0	0	0	0	0	0	0	0	0	0	0	0	0
XR_002340084.1	106506691	LOC106506691	lncRNA	0	0	0	0	0	0	0	0	0	0	0	0	0	0	0	0	0	0	0
XM_001928520.6	100153343	RWDD1	mRNA	0	0	0	0	0	0	0	0	0	0	0	0	0	0	0	0	0	0	0
XM_005652653.3	106504062	NLGN3	mRNA	0	0	0	0	0	0	0	0	0	0	0	0	0	0	0	0	0	0	0
XR_002341746.1	102165717	LOC102165717	lncRNA	0	0	0	0	0	0	0	0	0	0	0	0	0	0	0	0	0	0	0
XM_021085042.1	110259549	LOC110259549	mRNA	0	0	0	0	0	0	0	0	0	0	0	0	0	0	0	0	0	0	0
XR_001305221.2	106508400	LOC106508400	lncRNA	0	0	0	0	0	0	0	0	0	0	0	0	0	1	0	0	0	0	0
XR_002343172.1	110260241	LOC110260241	lncRNA	0	0	0	0	0	0	0	0	0	0	0	0	0	0	0	0	0	0	0

RNA ID	Gene ID	Symbol	Type	novel-ssc-miR1063-5p	ssc-miR-192	ssc-miR-432-5p	ssc-miR-7138-5p	ssc-miR-874	ssc-miR-10386	ssc-miR-122-3p	novel-ssc-miR755-3p	novel-ssc-miR780-3p	novel-ssc-miR804-3p	novel-ssc-miR813-5p	novel-ssc-miR407-5p	novel-ssc-miR573-5p	novel-ssc-miR583-3p	novel-ssc-miR1077-5p	novel-ssc-miR122-3p	novel-ssc-miR154-5p	ssc-miR-127	ssc-miR-194b-5p
XR_002339487.1	106506400	COPS7B	lncRNA	0	0	0	0	0	0	0	0	0	0	0	0	0	0	0	0	0	0	0
XR_001308351.2	106510133	LOC106510133	lncRNA	0	0	0	0	0	0	0	0	0	0	0	0	0	0	0	0	0	0	0
XM_003124564.4	100511500	GPR139	mRNA	0	0	0	0	0	0	0	0	0	0	0	0	0	0	0	0	0	0	0
NM_001078686.1	780437	ODAM	mRNA	0	0	0	0	0	0	0	0	0	0	0	0	0	0	0	0	0	0	0
XR_298956.3	102160517	LOC102160517	lncRNA	0	0	0	0	0	0	0	0	0	0	0	0	0	0	0	0	0	0	0
XR_002347080.1	110262247	LOC110262247	lncRNA	0	0	0	0	0	0	0	0	0	0	0	0	0	0	0	0	0	0	0
XM_021094930.1	100514465	LOC100514465	mRNA	0	0	0	0	0	0	0	0	0	0	0	0	0	0	0	0	0	0	0
XR_002343951.1	100737981	EIF4B	lncRNA	0	0	0	0	0	0	0	0	0	0	0	0	0	0	0	0	0	0	0
NM_001244614.1	100153985	SIX4	mRNA	0	0	0	0	0	0	0	0	0	0	0	0	0	1	0	0	0	0	0
XM_021066050.1	100302700	SLC25A19	mRNA	0	0	0	0	0	0	0	0	0	0	0	0	0	0	0	0	0	0	0
XM_005653210.3	100522374	GAS8	mRNA	0	0	0	0	0	0	0	0	0	0	0	0	0	0	0	0	0	0	0
XR_302139.2	102161858	LOC102161858	lncRNA	0	0	0	0	0	0	0	0	0	0	0	0	0	0	0	0	0	0	0
XR_002335527.1	110255394	LOC110255394	lncRNA	0	0	0	0	0	0	0	0	0	0	0	0	0	0	0	0	0	0	0
XR_002336548.1	110255755	LOC110255755	lncRNA	0	0	0	0	0	0	0	0	0	0	0	0	0	0	0	0	0	0	0
XR_001307143.2	100522542	LOC100522542	lncRNA	0	0	0	0	0	0	0	0	0	0	0	0	0	0	0	0	0	0	0
XR_002336864.1	110255895	LOC110255895	lncRNA	0	0	0	0	0	0	0	0	0	0	0	0	0	0	0	0	0	0	0
NM_214125.1	397230	MBL2	mRNA	0	0	0	0	0	0	0	0	0	0	0	0	0	0	0	0	0	0	0
XR_002339158.1	106506349	LOC106506349	lncRNA	0	0	0	0	0	0	0	0	0	0	0	0	0	0	0	0	0	0	1
NM_001244309.1	100625497	EZH2	mRNA	0	0	0	0	0	0	0	0	0	0	0	0	0	0	0	0	0	0	0
XM_021102414.1	100523325	ASB4	mRNA	0	0	0	0	0	0	1	0	0	0	0	0	0	0	0	0	0	0	0
XM_021069845.1	110256370	LOC110256370	mRNA	0	0	0	0	0	0	0	0	0	0	0	0	0	0	0	0	0	0	0
XR_309047.3	100519283	LOC100519283	lncRNA	0	0	0	0	0	0	0	0	0	0	0	0	0	1	0	0	0	0	0
XR_001308371.2	106510151	LOC106510151	lncRNA	0	0	0	0	0	0	1	0	0	0	0	0	0	0	0	0	0	0	0
XR_001303230.2	106507324	LOC106507324	lncRNA	0	0	0	0	0	0	0	0	0	0	0	0	0	0	0	0	0	0	0
XR_002338650.1	102167253	LOC102167253	lncRNA	0	0	0	0	0	0	0	0	0	0	0	0	0	0	0	0	0	0	0
XM_003132532.3	100519624	TMEM212	mRNA	0	0	0	0	0	0	0	0	0	0	0	0	0	0	0	0	0	0	0
XM_021086004.1	110259835	LOC110259835	mRNA	0	0	0	0	0	0	0	0	0	0	0	0	0	0	0	0	0	0	0
XM_005663799.3	396643	SSTR3	mRNA	0	0	0	0	0	0	0	0	0	0	0	0	0	0	0	0	0	0	0
XR_002335939.1	106507874	LOC106507874	lncRNA	0	0	0	0	0	0	0	0	0	0	0	0	0	1	0	0	0	0	0
XM_003130663.6	100522820	KIF24	mRNA	0	0	0	0	0	0	0	0	0	0	0	0	0	0	0	0	0	0	0
XM_003124595.4	100518832	RMI2	mRNA	0	0	0	0	0	0	0	0	0	0	0	0	0	0	0	0	0	0	0
XR_002339295.1	106506428	LOC106506428	lncRNA	0	0	0	0	0	0	0	0	0	0	0	0	0	0	0	0	0	0	0
XM_001925784.6	100155342	USP5	mRNA	0	0	0	0	0	0	0	0	0	0	0	0	0	0	0	0	0	0	0
XR_002346720.1	110262131	LOC110262131	lncRNA	0	0	0	0	0	0	0	0	0	0	0	0	0	0	0	0	0	0	0
XR_002339354.1	110257112	LOC110257112	lncRNA	0	0	0	0	0	0	0	0	0	0	0	0	0	0	0	0	0	0	0
XR_002346965.1	110262211	LOC110262211	lncRNA	0	0	0	0	0	0	0	0	0	0	0	0	0	0	0	0	0	0	1
XR_002335796.1	110255471	LOC110255471	lncRNA	0	0	0	0	0	0	0	0	0	0	0	0	0	0	0	0	0	0	0
XM_021101481.1	102166512	ANXA3	mRNA	0	0	0	0	0	0	1	0	0	0	0	0	0	0	0	0	0	0	0
XM_001926306.7	100153723	ABT1	mRNA	0	0	0	0	0	0	0	0	0	0	0	0	0	1	0	0	0	0	0
XR_002336516.1	102158088	NHLRC3	lncRNA	0	0	0	0	0	0	0	0	0	0	0	0	0	0	0	0	0	0	0
XR_002344446.1	102162270	SLC7A5	lncRNA	0	0	0	0	0	0	0	0	0	0	0	0	0	0	0	0	0	0	0
XR_002345296.1	102166616	LOC102166616	lncRNA	0	0	0	0	0	0	0	0	0	0	0	0	0	0	0	0	0	0	0
NM_001001645.1	414435	WDR83OS	mRNA	0	0	0	0	0	0	0	0	0	0	0	0	0	0	0	0	0	0	0

RNA ID	Gene ID	Symbol	Type	novel-ssc-miR1063-5p	ssc-miR-192	ssc-miR-432-5p	ssc-miR-7138-5p	ssc-miR-874	ssc-miR-10386	ssc-miR-122-3p	novel-ssc-miR755-3p	novel-ssc-miR780-3p	novel-ssc-miR804-3p	novel-ssc-miR813-5p	novel-ssc-miR407-5p	novel-ssc-miR573-5p	novel-ssc-miR583-3p	novel-ssc-miR1077-5p	novel-ssc-miR122-3p	novel-ssc-miR154-5p	ssc-miR-127	ssc-miR-194b-5p
XR_002337442.1	110256166	LOC110256166	lncRNA	0	0	0	0	0	0	0	0	0	0	0	0	0	0	0	0	0	0	0
XM_021072047.1	100512874	SCAF8	mRNA	0	0	0	0	0	0	0	0	0	0	0	0	0	0	0	0	0	0	0
XR_001298911.2	106504947	LOC106504947	lncRNA	0	0	0	0	0	0	0	0	0	0	0	0	0	0	0	0	0	0	0
XR_002340842.1	110257831	LOC110257831	lncRNA	0	0	0	0	0	0	0	0	0	0	0	0	0	0	0	0	0	0	0
XM_003122534.4	100512393	KCNK7	mRNA	0	0	0	0	0	0	1	0	0	0	0	0	0	0	0	0	0	0	0
XR_002340251.1	110257517	LOC110257517	lncRNA	0	0	0	0	0	0	0	0	0	0	0	0	0	0	0	0	0	0	0
XR_002338712.1	110256766	LOC110256766	lncRNA	0	0	0	0	0	0	0	0	0	0	0	0	0	0	0	0	0	0	0
XM_003129449.2	100516987	LOC100516987	mRNA	0	0	0	0	0	0	0	0	0	0	0	0	0	0	0	0	0	0	0
XR_001305858.2	106508800	LOC106508800	lncRNA	0	0	0	0	0	0	0	0	0	0	0	0	0	0	0	0	0	0	0
XR_001307955.2	102165812	LOC102165812	lncRNA	0	0	0	0	0	0	0	0	0	0	0	0	0	0	0	0	0	0	0
XR_002345578.1	110261473	LOC110261473	lncRNA	0	0	0	0	0	0	0	0	0	0	0	0	0	0	0	0	0	0	0
XR_002340279.1	110257523	LOC110257523	lncRNA	0	0	0	0	0	0	0	0	0	0	0	0	0	0	0	0	0	0	0
XM_021073688.1	100153473	CCDC188	mRNA	0	0	0	0	0	0	1	0	0	0	0	0	0	0	0	0	0	0	0
XR_002347070.1	100737186	LOC100737186	lncRNA	0	0	0	0	0	0	0	0	0	0	0	0	0	0	0	0	0	0	0
XR_002338524.1	110256665	LOC110256665	lncRNA	0	0	0	0	0	0	0	0	0	0	0	0	0	0	0	0	0	0	0
XR_002338128.1	110256525	LOC110256525	lncRNA	0	0	0	0	0	0	0	0	0	0	0	0	0	0	0	0	0	0	0
XR_002338320.1	102160169	LOC102160169	lncRNA	0	0	0	0	0	0	0	0	0	0	0	0	0	0	0	0	0	0	0
XR_002341185.1	102168138	LOC102168138	lncRNA	0	0	0	0	0	0	0	0	0	0	0	0	0	0	0	0	0	0	0
NM_001243681.2	100626539	RNASEH1	mRNA	0	0	0	0	0	0	0	0	0	0	0	0	0	0	0	0	0	0	0
XM_021087419.1	100516073	APLF	mRNA	0	0	0	0	0	0	0	0	0	0	0	0	0	0	0	0	0	0	0
NM_001244932.1	100514188	SUMF1	mRNA	0	0	0	0	0	0	0	0	0	0	0	0	0	0	0	0	0	0	0
XR_002342760.1	110260029	LOC110260029	lncRNA	0	0	0	0	0	0	0	0	0	0	0	0	0	0	0	0	0	0	0
NM_001130735.1	100170853	SMN1	mRNA	0	0	0	0	0	0	0	0	0	0	0	0	0	1	0	0	0	0	0
XR_002337720.1	106505376	LOC106505376	lncRNA	0	0	0	0	0	0	0	0	0	0	0	0	0	0	0	0	0	0	0
XM_021082523.1	100620803	LOC100620803	mRNA	0	0	0	0	0	0	0	0	0	0	0	0	0	0	0	0	0	0	0
XR_002344603.1	102158585	LOC102158585	lncRNA	0	0	0	0	0	0	0	0	0	0	0	0	0	0	0	0	0	0	0
XR_002340236.1	100513188	LOC100513188	lncRNA	0	0	0	0	0	0	0	0	0	0	0	0	0	0	0	0	0	0	0
XR_002335659.1	100622439	LOC100622439	lncRNA	0	0	0	0	0	0	0	0	0	0	0	0	0	0	0	0	0	0	0
XM_003122991.5	100523423	ATP5F1D	mRNA	0	0	0	0	0	0	0	0	0	0	0	0	0	0	0	0	0	0	0
XR_001308436.2	102166460	LOC102166460	lncRNA	0	0	0	0	0	0	0	0	0	0	0	0	0	0	0	0	0	0	0
XM_021070161.1	100620692	UBXN7	mRNA	0	0	0	0	0	0	0	0	0	0	0	0	0	0	0	0	0	0	0
NM_001278777.1	494011	SLC27A2	mRNA	0	0	0	0	0	0	0	1	0	0	0	0	0	0	0	0	0	0	0
XM_013994347.2	397553	NR1H3	mRNA	0	0	0	0	0	0	0	0	0	0	0	0	0	0	0	0	0	0	0
XM_005664733.3	100514292	ALDH16A1	mRNA	0	0	0	0	0	0	0	0	0	0	0	0	0	0	0	0	0	0	0
NM_001099936.1	100101473	BTG1	mRNA	0	0	0	0	0	0	0	0	0	0	0	0	0	1	0	0	0	0	0
XM_021099493.1	100625247	HEATR4	mRNA	0	0	0	0	0	0	0	0	0	0	0	0	0	0	0	0	0	0	0
XR_002335793.1	100523270	PFKFB2	mRNA	0	0	0	0	0	0	1	0	0	0	0	0	0	0	0	0	0	0	0
XR_002343260.1	106510048	LOC106510048	lncRNA	0	0	0	0	0	0	0	0	0	0	0	0	0	0	0	0	0	0	0
XM_021063420.1	100738171	ABCB5	mRNA	0	0	0	0	0	0	0	0	0	0	0	0	0	0	0	0	0	0	0
XM_021098946.1	100155072	CFAP100	mRNA	0	0	0	0	0	0	0	0	0	0	0	0	0	0	0	0	0	0	0
XM_021074210.1	100737404	LOC100737404	mRNA	0	0	0	0	0	0	0	0	0	0	0	0	0	0	0	0	0	0	0
XM_021088107.1	100517149	LOC100517149	mRNA	0	0	0	0	0	0	0	0	0	0	0	0	0	0	0	0	0	0	0
XM_003125346.4	100518190	MYCN	mRNA	0	0	0	0	0	0	0	0	0	0	0	0	0	0	0	0	0	0	0

RNA ID	Gene ID	Symbol	Type	novel-ssc-miR1063-5p	ssc-miR-192	ssc-miR-432-5p	ssc-miR-7138-5p	ssc-miR-874	ssc-miR-10386	ssc-miR-122-3p	novel-ssc-miR755-3p	novel-ssc-miR780-3p	novel-ssc-miR804-3p	novel-ssc-miR813-5p	novel-ssc-miR407-5p	novel-ssc-miR573-5p	novel-ssc-miR583-3p	novel-ssc-miR1077-5p	novel-ssc-miR122-3p	novel-ssc-miR154-5p	ssc-miR-127	ssc-miR-194b-5p
XM_003356153.3	100517101	LOC100517101	mRNA	0	0	0	0	0	0	0	0	0	0	0	0	0	0	0	0	0	0	0
XR_002338567.1	110256688	LOC110256688	lncRNA	0	0	0	0	0	0	0	0	0	0	0	0	0	0	0	0	0	0	0
XR_002339778.1	110257320	LOC110257320	lncRNA	0	0	0	0	0	0	0	0	0	0	0	0	0	0	0	0	0	0	0
XM_001927081.6	100156057	TTC8	mRNA	0	0	0	0	0	0	0	0	0	0	0	0	0	0	0	0	0	0	0
XM_021093139.1	100037307	DAB1	mRNA	0	0	0	0	0	0	0	0	0	0	0	0	0	0	0	0	0	0	0
XR_001300917.2	106505968	LOC106505968	lncRNA	0	0	0	0	0	0	0	0	0	0	0	0	0	0	0	0	0	0	0
NM_001190154.1	100157314	ZCCHC8	mRNA	0	0	0	0	0	0	0	0	0	0	0	0	0	0	0	0	0	0	0
XR_002338340.1	102159136	ARHGAP19	lncRNA	0	0	0	0	0	0	0	0	0	0	0	0	0	0	1	0	0	0	0
XR_002340858.1	110257848	LOC110257848	lncRNA	0	0	0	0	0	0	0	0	0	0	0	0	0	0	0	0	0	0	0
XM_021084337.1	102162790	GRM6	mRNA	0	0	0	0	0	0	1	0	0	0	0	0	0	0	0	0	0	0	0
XR_001306165.2	106508978	LOC106508978	lncRNA	0	0	0	0	0	0	0	0	0	0	0	0	0	1	0	0	0	0	0
XM_021085043.1	110259550	LOC110259550	mRNA	0	0	0	0	0	0	0	0	0	0	0	0	0	0	0	0	0	0	0
XR_001302502.2	102164143	LOC102164143	lncRNA	0	0	0	0	0	0	0	0	0	0	0	0	0	0	0	0	0	0	0
XM_021088119.1	100516428	LOC100516428	mRNA	0	0	0	0	0	0	1	0	0	0	0	0	0	0	0	0	0	0	0
XR_002335496.1	110255344	LOC110255344	lncRNA	0	0	0	0	0	0	0	0	0	0	0	0	0	0	0	0	0	0	0
XR_002335923.1	110255511	LOC110255511	lncRNA	0	0	0	0	0	0	0	0	0	0	0	0	0	0	0	0	0	0	0
XR_002336776.1	102168075	LOC102168075	lncRNA	0	0	0	0	0	0	0	0	0	0	0	0	0	0	0	0	0	0	0
XR_304089.2	102160593	LOC102160593	lncRNA	0	0	0	0	0	0	0	0	0	0	0	0	0	1	0	0	0	0	0
XM_003355424.5	100627932	HOXC6	mRNA	0	0	0	0	0	0	0	0	0	0	0	0	0	0	0	0	0	0	0
XR_002335902.1	102161420	LOC102161420	lncRNA	0	0	0	0	0	0	0	0	0	0	0	0	0	0	0	0	0	0	0
XR_002344769.1	110261037	LOC110261037	lncRNA	0	0	0	0	0	0	0	0	0	0	0	0	0	0	0	0	0	0	0
XR_001300967.2	100520338	LOC100520338	lncRNA	0	0	0	0	0	0	0	0	0	0	0	0	0	0	0	0	0	0	0
XR_002338125.1	102167959	LOC102167959	lncRNA	0	0	0	0	0	0	0	0	0	0	0	0	0	0	0	0	0	0	0
XR_002340442.1	102160053	LOC102160053	lncRNA	0	0	0	0	0	0	0	0	0	0	0	0	0	0	0	0	0	0	0
NM_001145752.1	100271724	SLC22A4	mRNA	0	0	0	0	0	0	0	0	0	0	0	0	0	0	0	0	0	0	0
XM_021097098.1	100302694	CES5A	mRNA	0	0	0	0	0	0	0	0	0	0	0	0	0	0	0	0	0	0	0
XR_001299355.2	106505191	LOC106505191	lncRNA	0	0	0	0	0	0	0	0	0	0	0	0	0	0	0	0	0	0	0
XR_001301379.2	106506213	LOC106506213	lncRNA	0	0	0	0	0	0	0	0	0	0	0	0	0	0	0	0	0	0	0
XR_002344182.1	110260775	LOC110260775	lncRNA	0	0	0	0	0	0	0	0	0	0	0	0	0	0	0	0	0	0	0
XR_002337157.1	110256021	LOC110256021	lncRNA	0	0	0	0	0	0	0	0	0	0	0	0	0	0	0	0	0	0	0
XR_002341155.1	110258473	LOC110258473	lncRNA	0	0	0	0	0	0	0	0	0	0	0	0	0	0	0	0	0	0	0
XM_001925364.3	100153329	LOC100153329	mRNA	0	0	0	0	0	0	0	0	0	0	0	0	0	1	0	0	0	0	0
XM_005665343.3	100519720	MAPRE2	mRNA	0	0	0	0	0	0	0	0	0	0	0	0	0	0	0	0	0	0	0
XR_002336444.1	102157904	LOC102157904	lncRNA	0	0	0	0	0	0	0	0	0	0	0	0	0	0	0	0	0	0	0
XM_003132006.5	100520100	MAP2K4	mRNA	0	0	0	0	0	0	0	0	0	0	0	0	0	0	0	0	0	0	0
XR_002343771.1	110260459	LOC110260459	lncRNA	0	0	0	0	0	0	0	0	0	0	0	0	0	0	0	0	0	0	0
XM_003358564.4	100620415	SLC35G2	mRNA	0	0	0	0	0	0	0	0	0	0	0	0	0	0	0	0	0	0	0
NM_001123151.1	100144454	NRM	mRNA	0	0	0	0	0	0	0	0	0	0	0	0	0	1	0	0	0	0	0
XM_005667128.3	100627340	LOC100627340	mRNA	0	0	0	0	0	0	0	0	0	0	0	0	0	0	0	0	0	0	0
NM_001243803.1	100511023	NUDC	mRNA	0	0	0	0	0	0	0	0	0	0	0	0	0	0	0	0	0	0	0
XM_021097456.1	110261343	TMEM240	mRNA	0	0	0	0	0	0	1	0	0	0	0	0	0	0	0	0	0	0	0
XR_001301636.2	106506374	LOC106506374	lncRNA	0	0	0	0	0	0	0	0	0	0	0	0	0	0	0	0	0	0	0
XR_001303107.2	106507246	LOC106507246	lncRNA	0	0	0	0	0	0	0	0	0	0	0	0	0	0	0	0	0	0	0

RNA ID	Gene ID	Symbol	Type	novel-ssc-miR1063-5p	ssc-miR-192	ssc-miR-432-5p	ssc-miR-7138-5p	ssc-miR-874	ssc-miR-10386	ssc-miR-122-3p	novel-ssc-miR755-3p	novel-ssc-miR780-3p	novel-ssc-miR804-3p	novel-ssc-miR813-5p	novel-ssc-miR407-5p	novel-ssc-miR573-5p	novel-ssc-miR583-3p	novel-ssc-miR1077-5p	novel-ssc-miR122-3p	novel-ssc-miR154-5p	ssc-miR-127	ssc-miR-194b-5p
XR_001303546.2	106507516	LOC106507516	lncRNA	0	0	0	0	0	0	0	0	0	0	0	0	0	0	0	0	0	0	0
XR_002336300.1	110255664	LOC110255664	lncRNA	0	0	0	0	0	0	0	0	0	0	0	0	0	0	0	0	0	0	0
XR_002336542.1	110255752	LOC110255752	lncRNA	0	0	0	0	0	0	0	0	0	0	0	0	0	0	0	0	0	0	0
XR_002337686.1	110256299	LOC110256299	lncRNA	0	0	0	0	0	0	0	0	0	0	0	0	0	0	0	0	0	0	1
XR_001300145.2	106505585	LOC106505585	lncRNA	0	0	0	0	0	0	0	0	0	0	0	0	0	0	0	0	0	0	0
XR_002344948.1	110261123	LOC110261123	lncRNA	0	0	0	0	0	0	0	0	0	0	0	0	0	0	0	0	0	0	0
XR_002344676.1	110260998	LOC110260998	lncRNA	0	0	0	0	0	0	0	0	0	0	0	0	0	0	0	0	0	0	0
XM_003121205.3	100525016	SMLR1	mRNA	0	0	0	0	0	0	1	0	0	0	0	0	0	0	0	0	0	0	0
XR_002340682.1	102163484	LOC102163484	lncRNA	0	0	0	0	0	0	0	0	0	0	0	0	0	0	0	0	0	0	0
XM_021079561.1	497636	DMD	mRNA	0	0	0	0	0	0	0	0	0	0	0	0	0	0	0	0	0	0	0
XR_002341591.1	102164826	ISYNA1	lncRNA	0	0	0	0	0	0	0	0	0	0	0	0	0	0	0	0	0	0	0
XR_002340822.1	110257819	LOC110257819	lncRNA	0	0	0	0	0	0	0	0	0	0	0	0	0	0	0	0	0	0	0
XR_001298110.1	106504549	LOC106504549	lncRNA	0	0	0	0	0	0	0	0	0	0	0	0	0	0	0	0	0	0	0
XR_002340843.1	110257831	LOC110257831	lncRNA	0	0	0	0	0	0	0	0	0	0	0	0	0	0	0	0	0	0	0
XR_300629.3	102163894	LOC102163894	lncRNA	0	0	0	0	0	0	0	0	0	0	0	0	0	0	0	0	0	0	0
XM_021099082.1	100510914	NEO1	mRNA	0	0	0	0	0	0	0	0	0	0	0	0	0	0	0	0	0	0	0
XR_002345385.1	110261329	LOC110261329	lncRNA	0	0	0	0	0	0	0	0	0	0	0	0	0	0	0	0	0	0	0
XR_002337516.1	110256226	LOC110256226	lncRNA	0	0	0	0	0	0	0	0	0	0	0	0	0	0	0	0	0	0	0
XR_002340123.1	102159962	LOC102159962	lncRNA	0	0	0	0	0	0	0	0	0	0	0	0	0	0	0	0	0	0	0
XM_001926907.5	100156308	PSMD10	mRNA	0	0	0	0	0	0	0	0	0	0	0	0	0	0	0	0	0	0	0
NM_213885.1	396919	MAN1A1	mRNA	0	0	0	0	0	0	0	0	0	0	0	0	0	0	0	0	0	0	0
XR_002338697.1	106506052	LOC106506052	lncRNA	0	0	0	0	0	0	0	0	0	0	0	0	0	1	0	0	0	0	0
XR_002340869.1	100522115	LOC100522115	lncRNA	0	0	0	0	0	0	0	0	0	0	0	0	0	0	0	0	0	0	0
NM_001143692.2	100152339	ITPK1	mRNA	0	0	0	0	0	0	0	0	0	0	0	0	0	0	0	0	0	0	0
NM_001114281.1	100135665	RELA	mRNA	0	0	0	0	0	0	0	0	0	0	0	0	0	0	0	0	0	0	0
XM_021073105.1	100156262	PAPSS2	mRNA	0	0	0	0	0	0	0	0	0	0	0	0	0	0	0	0	0	0	0
XR_002336169.1	110255621	LOC110255621	lncRNA	0	0	0	0	0	0	0	0	0	0	0	0	0	0	0	0	0	0	0
XR_001298531.2	102166545	LOC102166545	lncRNA	0	0	0	0	0	0	0	0	0	0	0	0	0	0	0	0	0	0	0
XR_002339865.1	110257358	LOC110257358	lncRNA	0	0	0	0	0	0	0	0	0	0	0	0	0	0	0	0	0	0	0
XR_002336348.1	110255689	LOC110255689	lncRNA	0	0	0	0	0	0	0	0	0	0	0	0	0	0	0	0	0	0	0
XM_003134418.4	100523033	SPAG4	mRNA	0	0	0	0	0	0	0	0	0	0	0	0	0	0	0	0	0	0	0
XR_001308912.2	106510436	LOC106510436	lncRNA	0	0	0	0	0	0	1	0	0	0	0	0	0	0	0	0	0	0	0
XM_003482567.1	100739359	LOC100739359	mRNA	0	0	0	0	0	0	0	0	0	0	0	0	0	0	0	0	0	0	0
XM_003483535.4	100154776	PGAM1	mRNA	0	0	0	0	0	0	0	0	0	0	0	0	0	0	0	0	0	0	0
XM_003359830.5	100626618	CCNJL	mRNA	0	0	0	0	0	0	0	0	0	0	0	0	0	0	0	0	0	0	0
XR_002339976.1	110257414	LOC110257414	lncRNA	0	0	0	0	0	0	0	0	0	0	0	0	0	0	0	0	0	0	0
NM_001139471.1	100154728	APEX1	mRNA	0	0	0	0	0	0	0	0	0	0	0	0	0	0	0	0	0	0	0
XR_001301667.2	106506397	LOC106506397	lncRNA	0	0	0	0	0	0	0	0	0	0	0	0	0	0	0	0	0	0	0
XR_001307040.2	102159224	LOC102159224	lncRNA	0	0	0	0	0	0	0	0	0	0	0	0	0	0	0	0	0	0	0
XM_021066572.1	100622490	ST6GALNAC1	mRNA	0	0	0	0	0	0	0	0	0	0	0	0	0	0	0	0	0	0	0
XM_001925290.5	100152155	SMPD2	mRNA	0	0	0	0	0	0	0	0	0	0	0	0	0	0	0	0	0	0	0
XR_001306492.2	100737021	LOC100737021	lncRNA	0	0	0	0	0	0	0	0	0	0	0	0	0	0	0	0	0	0	0
XM_013992665.2	102160737	HTR1E	mRNA	0	0	0	0	0	0	0	0	0	0	0	0	0	0	0	0	0	0	0

RNA ID	Gene ID	Symbol	Type	novel-ssc-miR1063-5p	ssc-miR-192	ssc-miR-432-5p	ssc-miR-7138-5p	ssc-miR-874	ssc-miR-10386	ssc-miR-122-3p	novel-ssc-miR755-3p	novel-ssc-miR780-3p	novel-ssc-miR804-3p	novel-ssc-miR813-5p	novel-ssc-miR407-5p	novel-ssc-miR573-5p	novel-ssc-miR583-3p	novel-ssc-miR1077-5p	novel-ssc-miR122-3p	novel-ssc-miR154-5p	ssc-miR-127	ssc-miR-194b-5p
XM_021082275.1	110258984	LOC110258984	mRNA	0	0	0	0	0	0	0	0	0	0	0	0	0	0	0	0	0	0	0
XR_001305691.2	102166401	LOC102166401	lncRNA	0	0	0	0	0	0	0	0	0	0	0	0	0	0	0	0	0	0	0
XR_002343757.1	110260447	LOC110260447	lncRNA	0	0	0	0	0	0	0	0	0	0	0	0	0	0	0	0	0	0	0
XR_001306506.2	106509193	LOC106509193	lncRNA	0	0	0	0	0	0	0	0	0	0	0	0	0	1	0	0	0	0	0
XM_021075435.1	100514804	CFC1B	mRNA	0	0	0	0	0	0	0	0	0	0	0	0	0	0	0	0	0	0	0
XR_002337121.1	110256014	LOC110256014	lncRNA	0	0	0	0	0	0	0	0	0	0	0	0	0	0	0	0	0	0	0
XR_002340328.1	106508222	LOC106508222	lncRNA	0	0	0	0	0	0	0	0	0	0	0	0	0	0	0	0	0	0	0
XM_021099830.1	100155756	TMEM225B	mRNA	0	0	0	0	0	0	0	0	0	0	0	0	0	0	0	0	0	0	0
NM_001206441.1	733650	TAP2	mRNA	0	0	0	0	0	0	0	0	0	0	0	0	0	1	0	0	0	0	0
XR_002338713.1	110256767	LOC110256767	lncRNA	0	0	0	0	0	0	0	0	0	0	0	0	0	0	0	0	0	0	0
XR_001307073.2	106509487	LOC106509487	lncRNA	0	0	0	0	0	0	0	0	0	0	0	0	0	0	0	0	0	0	0
XM_003129584.2	100517905	LOC100517905	mRNA	0	0	0	0	0	0	1	0	0	0	0	0	0	0	0	0	0	0	0
XM_003359416.4	100622102	KIF5C	mRNA	0	0	0	0	0	0	0	0	0	0	0	0	0	0	0	0	0	0	0
NM_001348903.1	397672	CILP	mRNA	0	0	0	0	0	0	0	0	0	0	0	0	0	0	0	0	0	0	0
XR_002341788.1	106509487	LOC106509487	lncRNA	0	0	0	0	0	0	0	0	0	0	0	0	0	0	0	0	0	0	1
XR_002337537.1	110256233	LOC110256233	lncRNA	0	0	0	0	0	0	0	0	0	0	0	0	0	0	0	0	0	0	0
XR_002341806.1	110259419	LOC110259419	lncRNA	0	0	0	0	0	0	0	0	0	0	0	0	0	0	0	0	0	0	0
XM_001928015.5	396603	PPP3CB	mRNA	0	0	0	0	0	0	0	0	0	0	0	0	0	0	0	0	0	0	0
XM_001924600.5	100155018	ZNF451	mRNA	0	0	0	0	0	0	0	0	0	0	0	0	0	0	0	0	0	0	0
XM_021089348.1	100153039	NSMAF	mRNA	0	0	0	0	0	0	0	0	0	0	0	0	0	0	0	0	0	0	0
XR_300604.3	102165231	LOC102165231	lncRNA	0	0	0	0	0	0	0	0	0	0	0	0	0	0	0	0	0	0	0
XM_003355060.4	100628123	NPBWR1	mRNA	0	0	0	0	0	0	0	0	0	0	0	0	0	0	0	0	0	0	0
NM_214418.2	403155	SLC25A6	mRNA	0	0	0	0	0	0	0	0	0	0	0	0	0	0	0	0	0	0	0
XR_002335850.1	102161480	LOC102161480	lncRNA	0	0	0	0	0	0	0	0	0	0	0	0	0	0	0	0	0	0	0
XR_002345639.1	110261503	LOC110261503	lncRNA	0	0	0	0	0	0	0	0	0	0	0	0	0	0	0	0	0	0	0
XR_301706.3	102162727	LOC102162727	lncRNA	0	0	0	0	0	0	0	0	0	0	0	0	0	0	0	0	0	0	0
XR_002344083.1	110260726	LOC110260726	lncRNA	0	0	0	0	0	0	0	0	0	0	0	0	0	0	0	0	0	0	0
XR_002336138.1	110255587	LOC110255587	lncRNA	0	0	0	0	0	0	0	0	0	0	0	0	0	0	0	0	0	0	0
NM_001244966.1	100514424	SLC25A39	mRNA	0	0	0	0	0	0	0	0	0	0	0	0	0	0	0	0	0	0	0
XR_001301171.2	106506113	LOC106506113	lncRNA	0	0	0	0	0	0	0	0	0	0	0	0	0	0	0	0	0	0	0
NM_001123177.1	100144495	RAB33A	mRNA	0	0	0	0	0	0	0	0	0	0	0	0	0	0	0	0	0	0	0
XM_021096358.1	102166543	SAMD13	mRNA	0	0	0	0	0	0	0	0	0	0	0	0	0	0	0	0	0	0	0
XM_021086898.1	102157824	GINM1	mRNA	0	0	0	0	0	0	0	0	0	0	0	0	0	0	0	0	0	0	0
XR_002346148.1	110261690	LOC110261690	lncRNA	0	0	0	0	0	0	0	0	0	0	0	0	0	0	0	0	0	0	0
XR_001307288.2	106509619	LOC106509619	lncRNA	0	0	0	0	0	0	0	0	0	0	0	0	0	0	0	0	0	0	0
NM_214253.1	397487	PRM1	mRNA	0	0	0	0	0	0	0	0	0	0	0	0	0	0	0	0	0	0	0
XR_002345199.1	110261229	LOC110261229	lncRNA	0	0	0	0	0	0	0	0	0	0	0	0	0	0	0	0	0	0	0
XM_021091581.1	100154559	CALHM4	mRNA	0	0	0	0	0	0	1	0	0	0	0	0	0	0	0	0	0	0	0
URS0000BC677A	110260766	LOC110260766	lncRNA	0	0	0	0	0	0	0	0	0	0	0	0	0	0	0	0	0	0	0
URS0000BC9805	BGIG9823_26341		lncRNA	0	0	0	0	0	0	0	0	0	0	0	0	0	0	0	0	0	0	0
URS0000BC7DBE	102167218	LOC102167218	lncRNA	0	0	0	0	0	0	0	0	0	0	0	0	0	0	0	0	0	0	0
URS0000BC871E	102165318	LOC102165318	lncRNA	0	0	0	0	0	0	0	0	0	0	0	0	0	0	0	0	0	0	0
URS0000BC6273	BGIG9823_26302		lncRNA	0	0	0	0	0	0	0	0	0	0	0	0	0	0	0	0	0	0	0

RNA ID	Gene ID	Symbol	Type	novel-ssc-miR1063-5p	ssc-miR-192	ssc-miR-432-5p	ssc-miR-7138-5p	ssc-miR-874	ssc-miR-10386	ssc-miR-122-3p	novel-ssc-miR755-3p	novel-ssc-miR780-3p	novel-ssc-miR804-3p	novel-ssc-miR813-5p	novel-ssc-miR407-5p	novel-ssc-miR573-5p	novel-ssc-miR583-3p	novel-ssc-miR1077-5p	novel-ssc-miR122-3p	novel-ssc-miR154-5p	ssc-miR-127	ssc-miR-194b-5p
URS0000BC6496	BGIG9823_26337		lncRNA	0	0	0	0	0	0	0	0	0	0	0	0	0	0	0	0	0	0	0
URS0000BC6966	BGIG9823_26316		lncRNA	0	0	0	0	0	0	0	0	0	0	0	0	0	0	0	0	0	0	1
URS0000BC81FE	BGIG9823_26310		lncRNA	0	0	0	0	0	0	0	0	0	0	0	0	0	0	0	0	0	0	0
URS0000BC59B8	100525132	PDZD2	lncRNA	0	0	0	0	0	0	0	0	0	0	0	0	0	0	0	0	0	0	0
URS0000BC725F	106506163	LOC106506163	lncRNA	0	0	0	0	0	0	0	0	0	0	0	0	0	0	0	0	0	0	0
URS0000BC5DE3	110256465	LOC110256465	lncRNA	0	0	0	0	0	0	0	0	0	0	0	0	0	0	0	0	0	0	0
URS0000BC7E35	102157619	LOC102157619	lncRNA	0	0	0	0	0	0	0	0	0	0	0	0	0	0	0	0	0	0	0
URS0000BC4C4F	106510093	LOC106510093	lncRNA	0	0	0	0	0	0	0	0	0	0	0	0	0	0	0	0	0	0	0
URS0000BC46E5	BGIG9823_26314		lncRNA	0	0	0	0	0	0	0	0	0	0	0	0	0	0	0	0	0	0	0
URS0000BC8793	100525132	PDZD2	lncRNA	0	0	0	0	0	0	0	0	0	0	0	0	0	0	0	0	0	0	0
URS0000BC7B31	BGIG9823_26353		lncRNA	0	0	0	0	0	0	0	0	0	0	0	0	0	0	0	0	0	0	0
BGIR9823_88049	100621902	MIER1	mRNA	0	0	0	0	0	0	0	0	0	0	0	0	0	0	0	0	0	0	0
BGIR9823_87964	100737982	GAREM1	lncRNA	0	0	0	0	0	1	0	0	0	0	0	0	0	0	0	0	0	0	0
BGIR9823_81184	100523888	LOC100523888	lncRNA	0	0	0	0	0	0	0	0	0	0	0	0	0	0	0	0	0	0	0
BGIR9823_78564	100737904	LOC100737904	lncRNA	0	0	0	0	0	0	0	0	0	0	0	0	0	0	0	0	0	0	0
BGIR9823_89527	102158035	LOC102158035	mRNA	0	0	0	0	0	0	0	0	0	0	0	0	0	0	0	0	0	0	0
BGIR9823_84122	100152952	ZHX2	lncRNA	0	0	0	0	0	1	0	0	0	0	0	0	0	0	0	0	0	0	0
BGIR9823_79475	100625298	GABRG3	lncRNA	0	0	0	0	0	0	0	0	0	0	0	0	0	0	0	0	0	0	0
XM_021097022.1	100620736	TCF25	mRNA	0	0	0	0	0	0	0	0	0	0	0	0	0	0	0	0	0	0	0
BGIR9823_77873	100513326	SMAP1	lncRNA	0	0	1	0	0	0	0	0	0	0	0	0	0	0	0	0	0	0	0
BGIR9823_80029	100154341	URM1	lncRNA	0	0	0	0	0	0	0	0	0	0	0	0	0	0	0	0	0	0	0
BGIR9823_83646	100154232	GGH	mRNA	0	0	0	0	0	0	0	0	0	0	0	0	0	0	0	0	0	0	0
BGIR9823_97547	100153782	USP30	mRNA	0	0	0	0	0	0	0	0	0	0	0	0	0	0	0	0	0	0	0
BGIR9823_78040	100157196	CSNK1G1	lncRNA	0	0	0	0	0	0	0	0	0	0	0	0	0	0	0	0	0	0	0
BGIR9823_79960	100516174	SUSD1	lncRNA	0	0	0	0	0	0	0	0	0	0	0	1	0	0	0	0	0	0	0
BGIR9823_81702	100521611	FNIP1	lncRNA	0	0	0	0	0	0	0	0	0	0	0	0	0	0	0	0	0	0	0
BGIR9823_78735	100153669	TMEM38B	lncRNA	0	0	0	0	0	0	0	0	0	0	0	0	0	0	0	0	0	0	0
BGIR9823_80417	100516957	LOC100516957	lncRNA	0	0	0	0	0	0	0	0	0	0	0	0	0	0	0	0	0	0	0
BGIR9823_82071	100525811	C3H16orf71	lncRNA	0	0	0	0	0	0	0	0	0	0	0	0	0	0	0	0	0	0	0
BGIR9823_89210	100153184	KHDRBS2	lncRNA	0	0	0	0	0	1	0	0	0	0	0	0	0	0	0	0	0	0	0
BGIR9823_86411	100621774	UBE2M	lncRNA	0	0	0	0	0	1	0	0	0	0	0	0	0	0	0	0	0	0	0
BGIR9823_86823	100621518	RBBP8	mRNA	0	0	0	0	0	0	0	0	0	0	0	0	0	0	0	0	0	0	0
BGIR9823_93974	102164379	LOC102164379	lncRNA	0	0	0	0	0	0	0	0	0	0	0	0	0	0	0	0	0	0	0
BGIR9823_79858	654405	GNAQ	lncRNA	0	0	0	0	0	0	0	0	0	0	0	0	0	0	0	0	0	0	0
BGIR9823_97653	100154344	C14H22orf39	lncRNA	0	0	0	0	0	0	0	0	0	0	0	0	0	0	0	0	0	0	0
BGIR9823_90176	100511210	SEC31A	mRNA	0	0	0	0	0	0	0	0	0	0	0	0	0	0	0	0	0	0	0
BGIR9823_99273	100624231	NIPBL	lncRNA	0	0	0	0	0	0	0	0	0	0	0	0	0	0	0	0	0	0	0
BGIR9823_100693	100739403	KLHDC10	lncRNA	0	0	0	0	0	0	0	0	0	0	0	0	0	0	0	0	0	1	0
BGIR9823_79452	100512576	SPRED1	lncRNA	0	1	0	0	0	0	0	0	0	0	0	0	0	0	0	0	0	0	0
BGIR9823_82923	100525039	MCRIP2	lncRNA	0	0	0	0	0	0	0	0	0	0	0	0	0	0	0	0	0	0	0
BGIR9823_85610	110260725	LOC110260725	lncRNA	0	0	0	0	0	0	0	0	0	0	0	0	0	0	0	0	0	0	0
BGIR9823_79946	100155590	FRRS1L	lncRNA	0	0	0	0	0	0	0	0	0	0	0	0	1	0	0	0	0	0	0
XR_002335729.1	110255457	LOC110255457	lncRNA	0	0	0	0	0	0	0	0	0	0	0	0	0	0	0	0	0	0	0

RNA ID	Gene ID	Symbol	Type	novel-ssc-miR1063-5p	ssc-miR-192	ssc-miR-432-5p	ssc-miR-7138-5p	ssc-miR-874	ssc-miR-10386	ssc-miR-122-3p	novel-ssc-miR755-3p	novel-ssc-miR780-3p	novel-ssc-miR804-3p	novel-ssc-miR813-5p	novel-ssc-miR407-5p	novel-ssc-miR573-5p	novel-ssc-miR583-3p	novel-ssc-miR1077-5p	novel-ssc-miR122-3p	novel-ssc-miR154-5p	ssc-miR-127	ssc-miR-194b-5p
BGIR9823_79704	100155666	ATG14	lncRNA	0	0	0	0	0	0	0	0	0	0	0	0	0	0	0	0	0	0	0
BGIR9823_80049	414430	MED27	lncRNA	0	0	0	0	0	0	0	0	0	0	0	0	0	0	0	0	0	0	0
BGIR9823_84572	100519481	STXBP3	lncRNA	0	0	0	0	0	0	0	0	0	0	0	0	0	0	0	0	0	0	0
BGIR9823_87715	100517038	PUM1	lncRNA	0	0	0	0	0	0	0	0	0	0	0	0	0	0	0	0	0	0	0
BGIR9823_84360	100154639	PBX1	lncRNA	0	0	0	0	0	0	0	0	0	0	0	0	0	0	0	0	0	0	0
BGIR9823_101255	100154598	LAMP2	mRNA	0	0	0	0	0	0	0	0	0	0	0	0	0	0	0	0	0	0	0
BGIR9823_98698	100737861	CCNT2	mRNA	0	0	0	0	0	0	0	0	0	0	0	0	0	0	0	0	0	0	0
BGIR9823_81378	100511368	ZNF358	lncRNA	0	0	0	0	0	0	0	0	0	0	0	1	0	0	0	0	0	0	0
BGIR9823_100853	100156439	TCEANC	lncRNA	0	0	0	0	0	0	0	0	0	0	0	0	0	0	0	0	0	1	0
BGIR9823_88507	100153104	POLH	lncRNA	0	0	0	0	0	0	0	0	0	0	0	0	0	0	0	0	0	0	0
BGIR9823_96638	100153422	FAM222A	lncRNA	0	0	0	0	0	0	0	0	0	0	0	0	0	0	0	0	0	0	0
BGIR9823_79570	100512083	NEDD4L	lncRNA	0	0	0	0	0	0	0	0	0	0	0	0	0	0	0	0	0	0	0
BGIR9823_101653	100625138	LSS	lncRNA	0	0	0	0	0	0	0	0	0	1	0	0	0	0	0	0	0	0	0
BGIR9823_101268	100520416	TENM1	lncRNA	0	0	0	0	0	0	0	0	0	0	0	0	0	0	0	0	0	0	0
BGIR9823_78254	106506784	LOC106506784	lncRNA	0	0	0	0	0	0	0	0	0	0	0	0	0	0	0	1	0	0	0
BGIR9823_93514	100514316	CACNB1	lncRNA	0	0	0	0	0	0	0	0	0	0	0	0	0	0	0	0	0	0	0
BGIR9823_78057	100152749	CA12	lncRNA	0	0	0	0	0	0	0	0	0	0	0	0	0	0	0	0	0	0	0
BGIR9823_87011	100621520	YIPF1	lncRNA	0	0	0	0	0	0	0	0	0	0	0	0	0	0	0	0	0	0	0
BGIR9823_79577	100151784	CLPX	lncRNA	0	0	0	0	0	0	0	0	0	0	0	0	0	0	0	0	0	0	0
BGIR9823_83929	100519711	ST7L	lncRNA	0	0	0	0	0	0	0	0	0	0	0	0	1	0	0	0	0	0	0
BGIR9823_78689	100514147	LOC100514147	lncRNA	0	0	0	0	0	0	0	0	0	0	0	0	0	0	0	0	0	0	0
BGIR9823_80847	100515884	KDM3B	lncRNA	0	0	0	0	0	0	0	0	0	0	0	1	0	0	0	0	0	0	0
BGIR9823_100723	100511847	CPED1	mRNA	0	0	0	0	0	0	0	1	0	0	0	0	0	0	0	0	0	0	0
BGIR9823_80653	100521253	IQGAP2	lncRNA	0	0	0	0	0	0	0	0	0	0	0	0	0	0	0	0	0	0	0
BGIR9823_81419	100737330	NFIC	lncRNA	0	0	0	0	0	0	0	0	0	0	0	0	0	0	0	0	0	0	0
BGIR9823_78533	100626032	SGPP1	lncRNA	0	0	0	0	0	0	0	0	0	0	0	0	0	0	0	0	0	0	0
BGIR9823_91477	100511030	ALG8	mRNA	0	0	0	0	0	0	0	0	0	0	0	0	0	1	0	0	0	0	0
BGIR9823_85833	100517653	VEZT	lncRNA	0	0	0	0	0	0	0	0	0	0	0	0	0	0	0	0	0	0	0
BGIR9823_99752	494014	PRNP	mRNA	0	0	0	0	0	0	0	0	0	0	0	0	0	0	0	0	0	0	0
BGIR9823_86172	100620402	LOC100620402	lncRNA	0	0	0	0	0	0	0	0	0	0	0	0	0	0	0	0	0	0	0
BGIR9823_88817	396933	FUT8	lncRNA	0	0	0	0	0	0	0	0	0	0	0	0	0	0	0	0	0	0	0
BGIR9823_94863	100621119	GRM7	lncRNA	0	0	0	0	0	0	0	0	0	0	0	0	0	0	0	0	0	0	0
BGIR9823_82821	110259924	LOC110259924	lncRNA	0	0	0	0	0	0	0	0	0	0	0	0	0	0	0	0	0	0	0
BGIR9823_80743	100513914	FER	lncRNA	0	0	0	0	0	0	0	0	0	0	0	0	0	0	0	0	0	0	0
BGIR9823_86054	100516197	AMFR	mRNA	0	0	0	0	0	0	0	0	0	0	0	0	0	1	0	0	0	0	0
BGIR9823_101027	397245	ATP1B4	mRNA	0	0	0	0	0	0	0	0	0	0	0	0	0	0	0	0	0	0	0
BGIR9823_94279	100520151	GIT1	mRNA	0	0	0	0	0	0	0	0	0	0	0	0	0	0	0	0	0	0	0
BGIR9823_100258	100156548	ZHX3	lncRNA	0	0	0	0	0	0	0	0	0	0	0	0	0	0	0	0	0	0	0
BGIR9823_97925	100516879	CYP2C91	lncRNA	0	0	0	0	0	0	0	0	0	0	0	0	0	0	0	0	0	0	0
BGIR9823_81233	106508326	SLC25A42	lncRNA	0	0	0	0	0	0	0	0	0	0	0	0	0	0	0	0	0	0	0
BGIR9823_100939	100154376	KDM6A	lncRNA	0	0	0	0	0	0	0	0	0	0	0	0	0	0	0	0	0	0	0
BGIR9823_100604	100515515	GALNT11	lncRNA	0	0	0	0	0	0	0	0	0	0	0	0	0	0	0	0	0	1	0
BGIR9823_95197	100626969	LPP	lncRNA	0	0	0	0	0	0	0	0	0	0	0	0	0	0	0	0	0	0	0

RNA ID	Gene ID	Symbol	Type	novel-ssc-miR1063-5p	ssc-miR-192	ssc-miR-432-5p	ssc-miR-7138-5p	ssc-miR-874	ssc-miR-10386	ssc-miR-122-3p	novel-ssc-miR755-3p	novel-ssc-miR780-3p	novel-ssc-miR804-3p	novel-ssc-miR813-5p	novel-ssc-miR407-5p	novel-ssc-miR573-5p	novel-ssc-miR583-3p	novel-ssc-miR1077-5p	novel-ssc-miR122-3p	novel-ssc-miR154-5p	ssc-miR-127	ssc-miR-194b-5p
BGIR9823_87581	100514410	ICMT	mRNA	0	0	0	0	0	0	0	0	0	0	0	0	0	0	0	0	0	0	0
BGIR9823_81758	106509575	LOC106509575	lncRNA	0	0	0	0	0	0	0	0	0	0	0	0	0	0	0	0	0	0	0
BGIR9823_79445	100522721	RASGRP1	lncRNA	0	0	0	0	0	0	0	0	0	0	0	0	0	0	0	0	0	0	0
BGIR9823_95280	100623596	OSBPL11	mRNA	0	0	0	0	0	0	0	0	0	0	0	0	0	0	0	0	0	0	0
BGIR9823_88858	100155733	SUSD6	lncRNA	0	0	0	0	0	1	0	0	0	0	0	0	0	0	0	0	0	0	0
BGIR9823_93640	100512984	GGNBP2	lncRNA	0	0	0	0	0	0	0	0	0	0	0	0	0	0	0	0	0	0	0
BGIR9823_82696	100626099	TBL2	mRNA	0	0	0	0	0	0	0	0	0	0	0	0	0	0	0	0	0	0	1
BGIR9823_85289	100519488	MOV10L1	lncRNA	0	0	0	0	0	0	0	0	0	0	0	0	0	0	0	0	0	0	0
BGIR9823_90661	100738411	CCSER1	lncRNA	0	0	0	0	0	0	0	0	0	0	0	0	0	0	0	0	0	0	0
BGIR9823_101099	100624032	LOC100624032	mRNA	0	0	0	0	0	0	0	0	0	0	0	0	0	0	0	0	0	0	0
BGIR9823_83245	100170144	SLC30A6	lncRNA	0	1	0	0	0	0	0	0	0	0	0	0	0	0	0	0	0	0	0
BGIR9823_83495	654326	RNF19A	lncRNA	0	0	0	0	0	0	0	0	0	0	0	0	0	0	0	0	0	0	0
BGIR9823_100642	100519065	UBN2	lncRNA	0	0	0	1	0	0	0	0	0	0	0	0	0	0	0	0	0	0	0
BGIR9823_88851	100158119	SLC39A9	lncRNA	0	0	0	0	0	0	0	0	0	0	0	0	0	0	0	0	0	0	1
BGIR9823_82981	100524615	ARID5A	lncRNA	0	0	0	0	0	0	0	0	0	0	0	0	0	0	0	0	0	0	1
BGIR9823_84758	100157699	TMEM184B	lncRNA	0	0	0	0	0	1	0	0	0	0	0	0	0	0	0	0	0	0	0
BGIR9823_101227	102165634	LOC102165634	lncRNA	0	0	0	0	0	0	0	0	0	0	0	0	0	0	0	0	0	0	0
BGIR9823_82094	100736616	HCFC1R1	lncRNA	0	0	0	0	0	0	0	0	0	0	0	0	0	0	0	0	0	0	0
BGIR9823_87647	100517288	HSPG2	mRNA	0	0	0	0	0	0	0	0	0	0	0	0	0	0	0	0	0	0	0
BGIR9823_85318	110260629	LOC110260629	lncRNA	0	0	0	0	0	0	0	0	0	0	0	0	1	0	0	0	0	0	0
BGIR9823_97476	100513180	FAM216A	lncRNA	0	0	0	0	0	0	0	0	0	0	0	0	0	0	0	0	0	0	0
BGIR9823_100900	100621199	IL1RAPL1	lncRNA	0	0	0	1	0	0	0	0	0	0	0	0	0	0	0	0	0	0	0
BGIR9823_90410	397001	POLR2B	lncRNA	0	0	0	0	0	0	0	0	0	0	0	0	0	0	0	0	0	0	0
XM_005659173.3	100156257	NHSL1	mRNA	0	0	0	0	0	0	0	0	0	0	0	0	0	0	0	0	0	0	0
BGIR9823_90244	100513100	LDB2	lncRNA	0	0	0	0	0	0	0	0	0	0	0	0	0	0	0	0	0	0	0
BGIR9823_78438	100152969	CDKL1	lncRNA	0	0	0	0	0	0	0	0	0	0	0	0	0	0	0	0	0	0	0
BGIR9823_100731	100512382	KCND2	lncRNA	0	0	0	0	0	0	0	0	0	0	0	0	0	0	0	0	0	0	0
BGIR9823_82740	100623146	KIF22	lncRNA	0	0	0	0	0	0	0	0	0	0	0	0	0	1	0	0	0	0	0
BGIR9823_81512	100523721	FAF2	lncRNA	0	0	0	0	0	0	0	0	0	0	0	0	0	0	0	0	0	0	0
BGIR9823_78400	102162695	LOC102162695	lncRNA	0	0	0	0	0	0	0	0	0	0	0	0	0	0	0	0	0	0	0
BGIR9823_100980	110257855	LOC110257855	lncRNA	0	0	0	0	0	0	0	0	0	0	0	0	0	0	0	0	0	0	0
BGIR9823_97780	100037299	P4HA1	lncRNA	0	0	0	0	0	0	0	0	0	0	0	0	0	0	0	0	0	0	1
BGIR9823_79896	100519814	RNF38	lncRNA	0	0	0	0	0	0	0	0	0	0	0	0	0	0	0	0	0	0	0
XM_021065940.1	110255868	ATP11A	mRNA	0	0	0	0	0	0	0	0	0	0	0	0	0	0	0	0	0	0	0
BGIR9823_101616	110258914	LOC110258914	lncRNA	0	0	0	0	0	0	0	0	0	0	0	0	0	0	0	0	0	0	0
BGIR9823_86084	100626752	LOC100626752	lncRNA	0	0	0	0	0	0	0	0	0	0	0	0	0	0	0	0	0	0	0
BGIR9823_78267	100512756	MEIS2	lncRNA	0	0	0	0	0	0	0	0	0	0	0	0	0	0	0	0	0	0	0
BGIR9823_80601	110259385	LOC110259385	lncRNA	0	0	0	0	0	0	0	0	0	0	0	0	0	0	0	0	0	0	0
BGIR9823_87318	100620768	CEP89	lncRNA	0	0	0	0	0	0	0	0	0	0	0	0	1	0	0	0	0	0	0
BGIR9823_80608	100521252	CLK4	lncRNA	0	0	0	0	0	0	0	0	0	0	0	0	0	0	0	0	0	0	0
BGIR9823_100082	100621622	SFRP1	mRNA	0	0	0	0	0	0	0	0	0	0	0	0	0	0	0	0	0	0	0
BGIR9823_80402	396557	PGLYRP2	lncRNA	0	0	0	0	0	0	0	0	0	0	0	0	0	0	0	1	0	0	0
BGIR9823_101117	100518815	TXLNG	lncRNA	0	0	0	0	0	0	0	0	0	0	0	0	0	0	0	0	0	0	0

RNA ID	Gene ID	Symbol	Type	novel-ssc-miR1063-5p	ssc-miR-192	ssc-miR-432-5p	ssc-miR-7138-5p	ssc-miR-874	ssc-miR-10386	ssc-miR-122-3p	novel-ssc-miR755-3p	novel-ssc-miR780-3p	novel-ssc-miR804-3p	novel-ssc-miR813-5p	novel-ssc-miR407-5p	novel-ssc-miR573-5p	novel-ssc-miR583-3p	novel-ssc-miR1077-5p	novel-ssc-miR122-3p	novel-ssc-miR154-5p	ssc-miR-127	ssc-miR-194b-5p
BGIR9823_78216	100511488	TTBK2	lncRNA	0	0	0	0	0	0	0	0	0	0	0	0	0	0	0	0	0	0	0
BGIR9823_78392	100526149	PNN	lncRNA	0	0	0	0	0	0	0	0	0	0	0	0	0	0	0	1	0	0	0
BGIR9823_101570	110258602	LOC110258602	lncRNA	0	0	0	0	0	0	0	0	0	0	0	0	0	0	0	0	0	0	0
BGIR9823_93977	100512557	DDX42	mRNA	0	0	0	0	0	0	0	0	0	0	0	0	0	0	0	0	0	0	0
BGIR9823_93667	100294712	SUZ12	lncRNA	0	0	0	0	0	0	0	0	0	0	0	0	0	0	0	0	0	0	0
BGIR9823_94057	397387	THRA	lncRNA	0	0	0	0	0	0	0	0	0	0	0	0	0	0	0	0	0	0	0
BGIR9823_100931	100515940	DDX3X	lncRNA	0	0	0	0	0	0	0	0	0	0	1	0	0	0	0	0	0	0	0
BGIR9823_87442	100518311	NOSIP	lncRNA	0	0	0	0	0	0	0	0	0	0	0	0	0	0	0	0	0	0	0
BGIR9823_92178	100126283	AQP7	mRNA	0	0	0	0	0	0	0	0	0	0	0	0	0	0	0	0	0	0	0
BGIR9823_101406	110258215	LOC110258215	lncRNA	0	0	0	0	0	0	0	0	0	1	0	0	0	0	0	0	0	0	0
BGIR9823_92246	100737056	FRMD4A	lncRNA	0	0	0	0	0	0	0	0	0	0	0	0	0	0	0	0	0	0	0
BGIR9823_82297	100522368	EXOC6B	mRNA	0	0	0	0	0	0	0	0	0	0	0	0	0	0	1	0	0	0	0
BGIR9823_78079	100154204	MYO1E	lncRNA	0	0	0	0	0	0	0	0	0	0	0	0	0	0	0	0	0	0	0
BGIR9823_93720	100517676	GOSR1	lncRNA	0	0	0	0	0	0	0	0	0	0	0	0	0	0	0	0	0	0	0
BGIR9823_95969	100524586	MED12L	lncRNA	0	0	0	0	0	0	0	0	0	0	0	0	0	0	0	0	0	0	0
BGIR9823_95388	100517310	ROBO1	mRNA	0	0	0	0	0	0	0	0	0	0	0	0	0	0	0	0	0	0	0
BGIR9823_92590	100521678	MLLT10	lncRNA	0	0	0	0	0	0	0	0	0	0	0	0	0	0	0	0	0	0	0
BGIR9823_89920	100621006	LOC100621006	lncRNA	0	0	0	0	0	0	0	0	0	0	0	0	0	0	0	0	0	0	0
BGIR9823_87305	100521133	DNAJA2	lncRNA	0	0	0	0	0	0	0	0	0	0	0	0	1	0	0	0	0	0	0
BGIR9823_83514	100153417	C4H8orf37	lncRNA	0	0	0	0	0	0	0	0	0	0	0	0	0	0	0	0	0	0	0
BGIR9823_85521	100525288	OSBPL8	lncRNA	0	0	0	0	0	0	0	0	0	0	0	0	1	0	0	0	0	0	0
BGIR9823_90750	100522939	ART5	mRNA	0	0	0	0	0	0	0	0	0	0	0	0	0	0	0	0	0	0	0
BGIR9823_85923	100579143	HSBP1	lncRNA	0	0	0	0	0	0	0	0	0	0	0	0	0	0	0	0	0	0	0
BGIR9823_91101	100519438	FAM133B	lncRNA	0	0	0	0	0	0	0	0	0	0	0	0	0	0	0	0	0	0	0
BGIR9823_100281	100153535	PCIF1	lncRNA	0	0	0	0	0	0	0	0	0	0	0	0	0	0	0	0	0	0	0
BGIR9823_100276	397528	SDC4	lncRNA	0	0	0	0	0	0	0	0	0	0	0	0	0	0	0	0	0	0	0
BGIR9823_100467	100517505	CTTNBP2	lncRNA	0	0	0	0	0	0	0	0	0	0	0	0	0	0	0	0	0	0	0
BGIR9823_100689	110257546	LOC110257546	lncRNA	0	0	0	0	0	1	0	0	0	0	0	0	0	0	0	0	0	0	0
BGIR9823_94010	100514424	SLC25A39	lncRNA	0	0	0	0	0	0	0	0	0	0	0	0	0	0	0	0	0	0	0
BGIR9823_100247	100153296	RBL1	lncRNA	0	0	0	0	0	0	0	0	0	0	0	0	0	0	0	0	0	0	0
BGIR9823_97926	100516879	CYP2C91	lncRNA	0	0	0	0	0	0	0	0	0	0	0	0	0	0	0	0	0	0	0
BGIR9823_78152	100155516	DMXL2	lncRNA	0	0	0	0	0	0	0	0	0	0	0	0	0	0	0	0	0	0	0
BGIR9823_101623	BGIG9823_26354		lncRNA	0	0	0	0	0	0	0	0	0	0	0	0	0	0	0	0	0	0	0
BGIR9823_98164	110257053	LOC110257053	lncRNA	0	0	0	0	0	0	0	0	0	0	0	0	0	0	0	0	0	0	0
BGIR9823_100903	100621199	IL1RAPL1	lncRNA	0	0	0	0	0	0	0	0	0	0	0	0	0	0	0	0	0	0	0
BGIR9823_77846	100736796	ADGRB3	lncRNA	0	0	0	0	0	0	0	0	0	0	0	0	0	0	0	1	0	0	0
BGIR9823_90432	100520317	LOC100520317	mRNA	0	0	0	0	0	0	0	0	0	0	0	0	0	0	0	0	0	0	0
BGIR9823_95429	396658	IFNAR1	lncRNA	0	0	0	0	0	0	0	0	0	0	0	0	0	0	0	0	0	0	0
BGIR9823_80320	100521123	SBF2	lncRNA	0	1	0	0	0	0	0	0	0	0	0	0	0	0	0	0	0	0	0
BGIR9823_81929	100519894	PRKRIP1	lncRNA	0	1	0	0	0	0	0	0	0	0	0	0	0	0	0	0	0	0	0
BGIR9823_100403	100524054	WDR91	lncRNA	0	0	0	0	0	0	0	0	0	0	0	0	0	0	0	0	0	0	0
BGIR9823_91472	100217384	RSF1	lncRNA	0	0	0	0	0	0	0	0	0	0	0	0	0	0	0	0	0	0	0
BGIR9823_78771	102164650	LOC102164650	lncRNA	0	0	0	0	0	0	0	0	0	0	0	0	0	0	0	0	0	0	0

RNA ID	Gene ID	Symbol	Type	novel-ssc-miR1063-5p	ssc-miR-192	ssc-miR-432-5p	ssc-miR-7138-5p	ssc-miR-874	ssc-miR-10386	ssc-miR-122-3p	novel-ssc-miR755-3p	novel-ssc-miR780-3p	novel-ssc-miR804-3p	novel-ssc-miR813-5p	novel-ssc-miR407-5p	novel-ssc-miR573-5p	novel-ssc-miR583-3p	novel-ssc-miR1077-5p	novel-ssc-miR122-3p	novel-ssc-miR154-5p	ssc-miR-127	ssc-miR-194b-5p
BGIR9823_77749	100155211	SLC18B1	lncRNA	0	0	0	0	0	0	0	0	0	0	0	0	0	0	0	0	0	0	0
BGIR9823_80489	100622165	ACER1	lncRNA	0	0	0	0	0	0	0	0	0	0	0	0	0	0	0	0	0	0	0
BGIR9823_100204	100514995	COMMD7	lncRNA	0	0	0	0	0	0	0	0	0	0	0	0	0	0	0	0	0	0	0
BGIR9823_77830	100736796	ADGRB3	lncRNA	0	0	0	0	0	0	0	0	0	0	0	0	0	0	0	0	0	0	0
BGIR9823_78118	100152054	RFX7	lncRNA	0	0	0	0	0	0	0	0	0	0	0	0	0	0	0	0	0	0	0
BGIR9823_80498	106509452	PTPRS	mRNA	0	0	0	0	0	0	0	0	0	0	0	0	0	0	0	0	0	0	0
BGIR9823_83059	100524080	KCMF1	lncRNA	0	1	0	0	0	0	0	0	0	0	0	0	0	0	0	0	0	0	0
BGIR9823_89241	100155423	FKBP5	lncRNA	0	0	0	0	0	0	0	0	0	0	0	0	1	0	0	0	0	0	0
BGIR9823_86626	399532	FABP3	lncRNA	0	0	0	0	0	0	0	0	0	0	0	0	0	0	0	0	0	0	0
BGIR9823_80136	100512584	PLA2G16	lncRNA	0	0	0	0	0	0	0	0	0	0	0	1	0	0	0	0	0	0	0
BGIR9823_78764	100621961	CDC26	lncRNA	0	0	0	0	0	0	0	0	0	0	0	0	0	0	0	0	0	0	0
BGIR9823_100787	100622432	CBX3	lncRNA	0	0	0	0	0	0	0	0	0	0	0	0	1	0	0	0	0	0	0
BGIR9823_81517	100523721	FAF2	lncRNA	0	1	0	0	0	0	0	0	0	0	0	0	0	0	0	0	0	0	0
BGIR9823_94928	100622196	IQSEC1	mRNA	0	0	0	0	0	0	0	0	0	0	0	0	0	1	0	0	0	0	0
BGIR9823_97066	100155963	MMRN2	lncRNA	0	0	0	0	0	0	0	0	0	0	0	0	0	0	0	0	0	0	0
BGIR9823_86005	100515835	RSPRY1	lncRNA	0	0	0	0	0	0	0	0	0	0	0	0	0	0	0	0	0	0	0
BGIR9823_81824	100514516	CSNK1A1	lncRNA	0	1	0	0	0	0	0	0	0	0	0	0	0	0	0	0	0	0	0
BGIR9823_100617	100523293	KCNH2	lncRNA	0	0	0	0	0	0	0	0	0	0	0	0	0	0	0	0	1	0	0
XR_002337475.1	106507029	LOC106507029	lncRNA	0	0	0	0	0	0	0	0	0	0	0	0	0	0	1	0	0	0	0
BGIR9823_99577	100523519	CCDC152	lncRNA	0	0	0	0	0	0	0	0	0	0	0	0	0	0	0	0	0	0	0
BGIR9823_79582	100151784	CLPX	lncRNA	0	0	0	0	0	0	0	0	0	0	0	0	0	0	0	0	0	0	0
BGIR9823_90043	397616	PDE5A	lncRNA	0	0	0	0	0	0	0	0	0	0	0	0	0	0	0	0	0	0	0
BGIR9823_101350	100621019	ANAPC2	mRNA	0	0	0	0	0	0	0	0	0	0	0	0	0	0	1	0	0	0	0
BGIR9823_77999	100512572	WDR7	lncRNA	0	0	0	0	0	0	0	0	0	0	0	0	0	0	0	0	0	0	0
BGIR9823_80986	100294703	KDM2A	lncRNA	0	0	0	0	0	0	0	0	0	0	0	0	0	0	0	1	0	0	0
BGIR9823_82031	110259918	ITPRIPL2	lncRNA	0	0	0	0	0	0	0	0	0	0	0	0	0	0	0	0	0	0	0
BGIR9823_100386	100515632	KDM7A	lncRNA	0	0	0	0	0	0	0	0	0	0	0	0	0	0	0	0	0	0	0
BGIR9823_78769	100157179	ZNF618	lncRNA	0	0	0	0	0	0	0	0	0	0	0	0	0	0	0	1	0	0	0
BGIR9823_77679	100513634	MPC1	lncRNA	0	0	0	0	0	0	0	0	0	0	0	0	0	0	0	0	0	0	0
BGIR9823_101316	100624156	CD99	lncRNA	0	0	0	0	0	0	0	0	0	0	0	0	0	0	0	0	0	0	0
BGIR9823_79248	100738627	DYM	lncRNA	0	1	0	0	0	0	0	0	0	0	0	0	0	0	0	0	0	0	0
BGIR9823_78478	100152019	PELI2	lncRNA	0	0	0	0	0	0	0	0	0	0	0	0	0	0	0	0	0	0	0
BGIR9823_95265	100622532	FYTTD1	lncRNA	0	0	0	0	0	0	0	0	0	0	0	0	0	0	0	0	0	0	0
BGIR9823_84388	100155270	NIT1	mRNA	0	0	0	0	0	0	0	0	0	0	0	0	0	0	0	0	0	0	0
BGIR9823_101285	100622228	ARHGEF6	mRNA	0	0	0	0	0	0	0	0	0	0	0	0	0	0	0	0	0	0	1
BGIR9823_100255	100156548	ZHX3	lncRNA	0	0	0	0	0	0	0	0	0	0	0	0	0	0	0	0	0	1	0
BGIR9823_94455	100151869	OXNAD1	lncRNA	0	0	0	0	0	0	0	0	0	0	0	0	0	0	0	0	0	0	0
BGIR9823_78812	100155133	PTGES2	lncRNA	0	0	0	0	0	0	0	0	0	0	0	1	0	0	0	0	0	0	0
BGIR9823_80806	100519080	LYRM7	lncRNA	0	0	0	0	0	0	0	0	0	0	0	0	0	0	0	1	0	0	0
BGIR9823_101247	100519287	KLHL13	mRNA	0	0	0	0	0	0	0	0	0	0	0	0	0	0	0	0	0	0	0
BGIR9823_79520	102162853	LOC102162853	lncRNA	0	0	0	0	0	0	0	0	0	0	0	0	0	0	0	0	0	0	0
BGIR9823_95143	407739	SOX2	lncRNA	0	0	0	0	0	0	0	0	0	0	0	0	0	0	0	0	0	0	0
BGIR9823_95008	100620055	PLSCR1	lncRNA	0	0	0	0	0	0	0	0	0	0	0	0	0	0	0	0	0	0	0

RNA ID	Gene ID	Symbol	Type	novel-ssc-miR1063-5p	ssc-miR-192	ssc-miR-432-5p	ssc-miR-7138-5p	ssc-miR-874	ssc-miR-10386	ssc-miR-122-3p	novel-ssc-miR755-3p	novel-ssc-miR780-3p	novel-ssc-miR804-3p	novel-ssc-miR813-5p	novel-ssc-miR407-5p	novel-ssc-miR573-5p	novel-ssc-miR583-3p	novel-ssc-miR1077-5p	novel-ssc-miR122-3p	novel-ssc-miR154-5p	ssc-miR-127	ssc-miR-194b-5p
BGIR9823_100815	110257576	LOC110257576	lncRNA	0	0	0	0	0	0	0	0	0	0	0	0	0	0	0	0	0	0	0
BGIR9823_83902	110260470	LOC110260470	lncRNA	0	0	0	0	0	0	0	0	0	0	0	0	0	0	0	0	0	0	0
BGIR9823_96518	100623262	SLC15A4	mRNA	0	0	0	0	0	0	0	0	0	0	0	0	0	0	0	0	0	0	0
BGIR9823_101546	110258600	LOC110258600	lncRNA	0	0	0	0	0	0	0	0	0	0	0	0	0	0	0	0	0	0	0
BGIR9823_79803	100154484	RCL1	lncRNA	0	0	0	0	0	0	0	0	0	0	0	0	0	0	0	0	0	0	0
BGIR9823_78229	100154984	TMEM87A	mRNA	0	0	0	0	0	0	1	0	0	0	0	0	0	0	0	0	0	0	0
BGIR9823_78085	100154204	MYO1E	lncRNA	0	0	0	0	0	0	0	0	0	0	0	0	0	0	0	0	0	0	0
BGIR9823_84094	106509960	NSMCE2	lncRNA	0	0	0	0	0	0	0	0	0	0	0	0	0	0	0	0	0	0	0
BGIR9823_80335	102166675	RNF187	lncRNA	0	0	0	0	0	0	0	0	0	0	0	0	0	0	0	0	0	0	0
BGIR9823_101281	100516171	GPC3	lncRNA	0	1	0	0	0	0	0	0	0	0	0	0	0	0	0	0	0	0	0
BGIR9823_83886	102166500	LOC102166500	lncRNA	0	0	0	0	0	0	0	0	0	0	0	0	0	0	0	0	0	0	0
BGIR9823_78367	100520530	CORO2B	lncRNA	0	0	0	0	0	0	0	0	0	0	0	0	0	0	0	0	0	0	0
BGIR9823_81305	102166940	LOC102166940	lncRNA	0	0	0	0	0	0	0	0	0	0	0	0	0	0	0	1	0	0	0
BGIR9823_100832	100520285	ZNF777	lncRNA	0	0	0	0	0	0	0	0	0	0	0	0	0	0	0	0	1	0	0
BGIR9823_100583	102162331	KRBA1	lncRNA	0	0	0	0	0	0	0	0	0	0	0	0	0	0	0	1	0	0	0
BGIR9823_85020	100626912	BHLHE41	mRNA	0	0	0	0	0	0	0	0	0	0	0	0	0	0	0	0	0	0	0
BGIR9823_100375	100514612	BRAF	mRNA	0	0	0	0	0	0	0	0	0	0	0	0	0	0	0	0	0	0	0
BGIR9823_92109	100521742	NEK7	mRNA	0	0	0	0	0	0	0	0	0	0	0	0	0	0	0	0	0	0	0
BGIR9823_100703	100514931	AHCYL2	lncRNA	0	0	0	0	0	0	0	0	0	0	0	0	0	0	0	0	0	0	0
BGIR9823_79724	396697	ESR2	lncRNA	0	0	0	0	0	0	0	0	0	0	0	0	0	0	0	0	0	0	0
BGIR9823_101638	110259113	LOC110259113	lncRNA	0	0	0	0	0	0	0	0	0	0	0	0	0	0	0	0	0	0	0
BGIR9823_92471	100517963	RMI1	lncRNA	0	0	0	0	0	0	0	0	0	0	0	0	0	0	0	0	0	0	0
BGIR9823_100226	654300	UQCC	lncRNA	0	0	0	0	0	0	0	0	0	0	0	0	0	0	0	0	0	0	0
BGIR9823_86749	100624877	PPP4R1	mRNA	0	0	0	0	0	0	0	0	0	0	0	0	0	0	0	0	0	0	0
BGIR9823_97541	100157435	UBE3B	mRNA	0	0	0	0	0	0	0	0	0	0	0	0	0	0	0	0	0	0	0
BGIR9823_80565	102164292	FSTL3	lncRNA	0	0	0	0	0	0	0	0	0	0	0	0	0	0	0	0	0	0	0
BGIR9823_96818	100157092	TTC13	mRNA	0	0	0	0	0	0	0	0	0	0	0	0	0	1	0	0	0	0	0
BGIR9823_84745	100521449	NPTXR	mRNA	0	0	0	0	0	0	0	0	0	0	0	0	0	0	0	0	0	0	0
BGIR9823_79788	102168004	UHRF2	lncRNA	0	0	0	0	0	0	0	0	0	0	0	0	0	0	0	0	0	0	0
BGIR9823_78692	100515356	GLIPR2	lncRNA	0	0	0	0	0	0	0	0	0	0	0	0	0	0	0	0	0	0	0
BGIR9823_79323	100621557	TCF12	lncRNA	0	0	0	0	0	0	0	0	0	0	0	0	0	0	0	0	0	0	0
BGIR9823_101455	110258361	PACS2	lncRNA	0	0	1	0	0	0	0	0	0	0	0	0	0	0	0	0	0	0	0
BGIR9823_94552	100523331	NKTR	lncRNA	0	0	0	0	0	0	0	0	0	0	0	0	0	0	0	0	0	0	0
BGIR9823_100126	100519631	TASP1	lncRNA	0	0	0	0	0	0	0	0	0	0	0	0	0	0	0	0	0	0	0
BGIR9823_100406	106506765	LOC106506765	lncRNA	0	0	0	0	0	0	0	0	0	0	0	0	0	0	0	0	0	1	0
BGIR9823_80256	102168147	LOC102168147	lncRNA	0	0	0	0	0	0	0	0	0	0	0	0	0	0	0	0	0	0	0
BGIR9823_99608	397251	PLK2	mRNA	0	0	0	0	0	0	0	0	0	0	0	0	0	1	0	0	0	0	0
BGIR9823_94040	397550	STAT5A	mRNA	0	0	0	0	0	0	0	0	0	0	0	0	0	0	0	0	0	0	0
BGIR9823_100411	414383	CHCHD3	lncRNA	0	1	0	0	0	0	0	0	0	0	0	0	0	0	0	0	0	0	0
BGIR9823_93144	100174955	KLF12	lncRNA	0	0	0	0	0	0	0	0	0	0	0	0	0	0	0	0	0	0	0
BGIR9823_81306	102166940	LOC102166940	lncRNA	0	0	0	0	0	0	0	0	0	0	0	0	0	0	0	0	0	0	0
BGIR9823_79214	100515594	MTO1	lncRNA	0	1	0	0	0	0	0	0	0	0	0	0	0	0	0	0	0	0	0
XR_001302600.2	106506942	LOC106506942	lncRNA	0	0	0	0	0	0	0	0	0	0	0	0	0	1	0	0	0	0	0

RNA ID	Gene ID	Symbol	Type	novel-ssc-miR1063-5p	ssc-miR-192	ssc-miR-432-5p	ssc-miR-7138-5p	ssc-miR-874	ssc-miR-10386	ssc-miR-122-3p	novel-ssc-miR755-3p	novel-ssc-miR780-3p	novel-ssc-miR804-3p	novel-ssc-miR813-5p	novel-ssc-miR407-5p	novel-ssc-miR573-5p	novel-ssc-miR583-3p	novel-ssc-miR1077-5p	novel-ssc-miR122-3p	novel-ssc-miR154-5p	ssc-miR-127	ssc-miR-194b-5p
BGIR9823_99840	100519805	TBC1D20	lncRNA	0	0	0	0	0	0	0	0	0	0	0	0	0	0	0	0	0	0	0
BGIR9823_100155	100519522	NINL	lncRNA	0	0	0	0	0	0	0	0	0	0	0	0	0	0	0	0	0	0	0
BGIR9823_78548	100511733	PLAA	lncRNA	0	0	0	0	0	0	0	0	0	0	0	0	0	0	0	0	0	0	0
BGIR9823_81728	100524487	AFF4	lncRNA	0	1	0	0	0	0	0	0	0	0	0	0	0	0	0	0	0	0	0
BGIR9823_82871	100516601	ZNF597	lncRNA	0	0	0	0	0	0	0	0	0	0	0	0	0	0	0	0	0	0	0
BGIR9823_83578	100157134	ZNF704	lncRNA	0	0	0	0	0	0	0	0	0	0	0	0	0	0	0	0	0	0	0
BGIR9823_78456	100625719	CGRRF1	lncRNA	0	0	0	0	0	0	0	0	0	0	0	0	1	0	0	0	0	0	0
BGIR9823_100241	100154500	SAMHD1	lncRNA	0	0	0	0	0	0	0	0	0	0	0	0	0	0	0	0	0	0	0
XR_002344503.1	110260914	LOC110260914	lncRNA	0	0	0	0	0	0	0	0	0	0	0	0	0	1	0	0	0	0	0
BGIR9823_93124	100174955	KLF12	lncRNA	0	0	0	0	0	0	0	1	0	0	0	0	0	0	0	0	0	0	0
BGIR9823_78918	100627455	SNX9	mRNA	0	0	0	0	0	0	0	0	0	0	0	0	0	0	0	0	0	0	0
BGIR9823_100427	100626356	UBE2H	lncRNA	0	1	0	0	0	0	0	0	0	0	0	0	0	0	0	0	0	0	0
BGIR9823_79882	100156839	FAM166B	mRNA	0	0	0	0	0	0	1	0	0	0	0	0	0	0	0	0	0	0	0
BGIR9823_87816	100626395	NAPG	lncRNA	0	0	0	0	0	0	0	0	0	0	0	0	0	0	0	0	0	0	0
BGIR9823_79529	100154633	PHLPP1	lncRNA	0	0	0	0	0	0	0	0	0	0	0	0	0	0	0	0	0	0	0
BGIR9823_80355	100620218	BORCS8	lncRNA	0	1	0	0	0	0	0	0	0	0	0	0	0	0	0	0	0	0	0
BGIR9823_101515	102157759	LOC102157759	lncRNA	0	0	0	0	0	0	0	0	0	0	0	0	0	0	0	0	0	0	0
BGIR9823_88688	100152987	HEATR5A	mRNA	0	0	0	0	0	0	0	0	0	0	0	0	0	0	0	0	0	0	0
BGIR9823_78932	100514379	FBXO5	lncRNA	0	0	0	0	0	0	0	0	0	0	0	0	0	0	0	0	0	0	0
BGIR9823_78476	100152019	PELI2	lncRNA	0	0	0	0	0	0	0	0	0	0	0	0	0	0	0	0	0	0	0
BGIR9823_92851	100523918	LMO7	mRNA	0	0	0	0	0	0	0	0	0	0	0	0	0	0	0	0	0	0	0
BGIR9823_100326	110257473	LOC110257473	lncRNA	0	0	0	0	0	0	0	0	0	0	0	0	0	0	0	0	0	1	0
BGIR9823_87757	100621544	RNF19B	mRNA	0	0	0	0	0	0	0	0	0	0	0	0	0	0	0	0	0	0	0
BGIR9823_99279	100517617	WDR70	lncRNA	0	0	0	0	0	0	0	0	0	0	0	0	0	0	0	0	0	0	0
BGIR9823_78488	100152019	PELI2	lncRNA	0	0	0	0	0	0	0	0	0	0	0	0	0	0	0	0	0	0	0
BGIR9823_91993	100738612	ATF3	mRNA	0	0	0	0	0	0	0	0	0	0	0	0	0	1	0	0	0	0	0
BGIR9823_94664	102167511	SMIM4	lncRNA	0	0	0	0	0	0	0	0	0	0	0	0	0	0	0	0	0	0	0
BGIR9823_79100	100156082	FBXL4	lncRNA	0	0	0	0	0	0	0	0	0	0	0	1	0	0	0	0	0	0	0
BGIR9823_101197	100156239	VSIG4	mRNA	0	0	0	0	0	0	0	0	0	0	0	0	0	0	0	0	0	0	0
BGIR9823_100442	100192443	POT1	lncRNA	0	0	0	0	0	0	0	0	0	0	0	0	0	0	0	0	0	0	0
BGIR9823_78713	100152263	ANP32B	lncRNA	0	0	0	0	0	0	0	0	0	0	0	0	0	0	0	0	0	0	0
BGIR9823_77769	100153609	PTPRK	lncRNA	0	0	0	0	0	0	0	0	0	0	0	0	0	0	0	0	0	0	0
BGIR9823_78654	100515003	SMC5	mRNA	0	0	0	0	0	0	0	1	0	0	0	0	0	0	0	0	0	0	0
BGIR9823_95994	100512989	VEPH1	lncRNA	0	0	0	0	0	0	0	0	0	0	0	0	0	0	0	0	0	0	0
BGIR9823_100398	100521697	MTPN	lncRNA	0	0	0	0	0	0	0	0	0	0	0	0	0	0	0	0	0	0	0
BGIR9823_77692	110259915	LOC110259915	lncRNA	0	0	0	0	0	0	0	0	0	0	0	0	0	0	0	0	0	0	0
BGIR9823_100510	100738688	HERPUD2	lncRNA	0	0	0	0	0	0	0	0	0	0	0	0	0	0	0	0	0	0	0
BGIR9823_101594	110258799	LOC110258799	lncRNA	0	0	0	0	0	0	0	0	0	0	1	0	0	0	0	0	0	0	0
BGIR9823_100619	100736760	GIMAP8	lncRNA	0	0	0	0	0	0	0	0	0	0	0	0	0	0	0	0	0	1	0
BGIR9823_98052	100153733	RAB11FIP2	mRNA	0	0	0	0	0	0	0	0	0	0	0	0	0	0	0	0	0	0	0
BGIR9823_79578	100151784	CLPX	lncRNA	0	0	0	0	0	0	0	0	0	0	0	1	0	0	0	0	0	0	0
BGIR9823_82021	100628086	METTL9	lncRNA	0	0	0	0	0	0	0	0	0	0	0	0	1	0	0	0	0	0	0
BGIR9823_100265	100520338	LOC100520338	lncRNA	0	0	0	0	0	0	0	0	0	0	0	0	0	0	0	0	0	1	0

RNA ID	Gene ID	Symbol	Type	novel-ssc-miR1063-5p	ssc-miR-192	ssc-miR-432-5p	ssc-miR-7138-5p	ssc-miR-874	ssc-miR-10386	ssc-miR-122-3p	novel-ssc-miR755-3p	novel-ssc-miR780-3p	novel-ssc-miR804-3p	novel-ssc-miR813-5p	novel-ssc-miR407-5p	novel-ssc-miR573-5p	novel-ssc-miR583-3p	novel-ssc-miR1077-5p	novel-ssc-miR122-3p	novel-ssc-miR154-5p	ssc-miR-127	ssc-miR-194b-5p
BGIR9823_88390	100124376	SKIV2L	mRNA	0	0	0	0	0	0	0	0	0	0	0	0	0	0	0	0	0	0	0
BGIR9823_78231	100154984	TMEM87A	lncRNA	0	0	0	0	0	0	0	0	0	0	0	1	0	0	0	0	0	0	0
BGIR9823_84096	106509960	NSMCE2	lncRNA	0	0	0	0	0	0	0	0	0	0	0	0	1	0	0	0	0	0	0
BGIR9823_81704	100521611	FNIP1	lncRNA	0	1	0	0	0	0	0	0	0	0	0	0	0	0	0	0	0	0	0
BGIR9823_79498	100736682	TJP1	lncRNA	0	0	0	0	0	0	0	0	0	0	0	0	1	0	0	0	0	0	0
BGIR9823_100070	100157832	SGCZ	lncRNA	0	0	0	0	0	0	0	0	0	0	0	0	0	0	0	0	0	0	0
BGIR9823_87139	106510467	LOC106510467	lncRNA	0	0	0	0	0	0	0	0	0	0	0	0	0	0	0	0	0	0	0
BGIR9823_100722	100514329	LOC100514329	lncRNA	0	0	0	1	0	0	0	0	0	0	0	0	0	0	0	0	0	0	0
BGIR9823_82833	106509697	CIITA	lncRNA	0	0	0	0	0	0	0	0	0	0	0	0	0	0	0	0	0	0	0
BGIR9823_100488	100518941	TMEM168	lncRNA	0	0	0	0	0	0	0	0	0	0	0	0	0	0	0	0	0	0	0
BGIR9823_101179	100517625	FAM120C	lncRNA	0	0	0	0	0	0	0	0	0	0	0	0	0	0	0	0	0	0	0
BGIR9823_100981	100513513	SLC16A2	mRNA	0	0	0	0	0	0	0	0	0	0	0	0	0	0	0	0	0	0	0
BGIR9823_85418	102163508	LOC102163508	lncRNA	0	0	0	0	0	0	0	0	0	0	0	0	1	0	0	0	0	0	0
BGIR9823_100250	100516472	KIAA1755	lncRNA	0	0	0	0	0	0	0	0	0	0	0	0	0	0	0	0	0	0	0
BGIR9823_100877	100523478	CNKSR2	lncRNA	0	0	0	0	0	0	0	0	0	0	0	0	0	0	0	0	0	1	0
BGIR9823_84851	100523964	LOC100523964	mRNA	0	0	0	0	0	0	0	0	0	0	0	0	0	0	0	0	0	0	0
BGIR9823_79979	100737979	CDK5RAP2	mRNA	0	0	0	0	0	0	0	0	0	0	0	0	0	0	0	0	0	0	0
BGIR9823_85878	100622883	NAV3	mRNA	0	0	0	0	0	0	0	0	0	0	0	0	0	0	0	0	0	0	0
BGIR9823_87603	100620358	ZBTB17	mRNA	0	0	0	0	0	0	0	0	0	0	0	0	0	0	0	0	0	0	0
BGIR9823_81340	100622538	DNM2	lncRNA	0	0	0	0	0	0	0	0	0	0	0	0	0	0	0	0	0	0	0
BGIR9823_93496	100521398	SMARCE1	lncRNA	0	0	0	0	0	0	0	0	0	0	0	0	0	0	0	0	0	0	0
BGIR9823_86377	100621391	SSC5D	mRNA	0	0	0	0	0	0	0	0	0	0	0	0	0	0	0	0	0	0	0
BGIR9823_85565	110260712	LOC110260712	lncRNA	0	0	0	0	0	0	0	0	0	0	0	0	0	0	0	0	0	0	0
BGIR9823_95828	100511410	PDZRN3	lncRNA	0	0	0	0	0	0	0	0	0	0	0	0	0	0	0	0	0	0	0
BGIR9823_93148	100174955	KLF12	lncRNA	0	0	0	0	0	0	0	0	0	0	0	0	0	0	0	0	0	0	0
BGIR9823_101568	110258600	LOC110258600	lncRNA	0	0	0	0	0	0	0	0	0	0	0	0	0	0	0	0	0	0	0
BGIR9823_79012	100738102	RNF217	lncRNA	0	0	0	0	0	0	0	0	0	0	0	0	0	0	0	0	0	0	0
XM_021091420.1	100523786	PACSIN2	mRNA	0	0	0	0	0	0	0	0	0	0	0	0	0	0	0	0	0	0	0
BGIR9823_100565	100516888	GLI3	lncRNA	0	0	0	0	0	0	0	0	0	0	1	0	0	0	0	0	0	0	0
BGIR9823_78022	100157196	CSNK1G1	lncRNA	0	0	0	0	0	0	0	0	0	0	0	0	0	0	0	0	0	0	0
BGIR9823_78883	100512199	ERMARD	lncRNA	0	0	0	0	0	1	0	0	0	0	0	0	0	0	0	0	0	0	0
BGIR9823_84618	100157977	FNBP1L	lncRNA	0	0	0	0	0	0	0	0	0	0	0	0	0	0	0	0	0	0	0
BGIR9823_97044	100158106	NRG3	lncRNA	0	0	0	0	0	0	0	0	0	0	0	0	0	0	0	0	0	0	0
BGIR9823_99699	100513766	G3BP1	mRNA	0	0	0	0	0	0	0	0	0	0	0	0	0	0	0	0	0	0	0
BGIR9823_87685	100512789	WASF2	lncRNA	0	0	0	0	0	0	0	0	0	0	0	0	0	1	0	0	0	0	0
BGIR9823_100122	100519631	TASP1	lncRNA	0	0	0	0	0	0	0	0	0	0	0	0	0	0	0	0	0	0	0
BGIR9823_100289	100738810	SLC13A3	lncRNA	0	0	0	0	0	0	0	0	0	0	0	0	0	0	0	0	0	0	0
BGIR9823_80121	100737568	SSSCA1	lncRNA	0	0	0	0	0	1	0	0	0	0	0	0	0	0	0	0	0	0	0
BGIR9823_86301	100628160	SNRNP70	mRNA	0	0	0	0	0	0	0	0	0	0	0	0	0	1	0	0	0	0	0
BGIR9823_100376	100514612	BRAF	lncRNA	0	0	0	0	0	0	0	0	0	0	0	0	0	0	0	0	0	0	0
BGIR9823_80749	100514631	MAN2A1	lncRNA	0	0	0	0	0	0	0	0	0	0	0	0	0	0	0	0	0	0	0
BGIR9823_101511	110258473	LOC110258473	lncRNA	0	0	0	0	0	0	0	0	0	0	0	0	0	0	0	0	0	0	0
BGIR9823_100755	100620722	ZNF277	lncRNA	0	0	0	0	0	0	0	0	0	0	0	0	0	0	0	0	0	1	0

RNA ID	Gene ID	Symbol	Type	novel-ssc-miR1063-5p	ssc-miR-192	ssc-miR-432-5p	ssc-miR-7138-5p	ssc-miR-874	ssc-miR-10386	ssc-miR-122-3p	novel-ssc-miR755-3p	novel-ssc-miR780-3p	novel-ssc-miR804-3p	novel-ssc-miR813-5p	novel-ssc-miR407-5p	novel-ssc-miR573-5p	novel-ssc-miR583-3p	novel-ssc-miR1077-5p	novel-ssc-miR122-3p	novel-ssc-miR154-5p	ssc-miR-127	ssc-miR-194b-5p
BGIR9823_82116	100624598	MEIOB	lncRNA	0	0	0	0	0	0	0	0	0	0	0	0	1	0	0	0	0	0	0
BGIR9823_101016	100520165	TMEM164	lncRNA	0	0	0	0	1	0	0	0	0	0	0	0	0	0	0	0	0	0	0
BGIR9823_90026	110262158	LOC110262158	lncRNA	0	0	0	0	0	0	0	0	0	0	0	0	0	0	0	0	0	0	0
BGIR9823_94087	100515340	LOC100515340	lncRNA	0	0	0	0	0	0	0	0	0	0	0	0	0	0	0	0	0	0	1
BGIR9823_89845	100516092	GNPDA2	lncRNA	0	0	0	0	0	0	0	0	0	0	0	0	0	0	0	0	0	0	0
BGIR9823_99243	100521757	ZNF622	lncRNA	0	0	0	0	0	0	0	0	0	0	0	0	0	0	0	0	0	0	0
BGIR9823_91934	100196908	TOR3A	lncRNA	0	0	0	0	0	1	0	0	0	0	0	0	0	0	0	0	0	0	0
BGIR9823_80152	100620658	HNRNPUL2	mRNA	0	0	0	0	0	0	0	0	0	0	0	0	0	0	0	0	0	0	0
BGIR9823_95348	100513438	NAA50	lncRNA	0	0	0	0	0	0	0	0	0	0	0	0	0	0	0	0	0	0	0
BGIR9823_86872	102164512	ZNF397	lncRNA	0	0	0	0	0	0	0	0	0	0	0	0	0	0	0	0	0	0	0
BGIR9823_85545	100525288	OSBPL8	lncRNA	0	0	0	0	0	0	0	0	0	0	0	0	0	0	0	0	0	0	0
BGIR9823_92701	100739805	CDK8	lncRNA	0	0	0	0	0	0	0	0	0	0	0	0	0	0	0	0	0	0	0
BGIR9823_79943	100626033	CTNNAL1	lncRNA	0	0	0	0	0	0	0	0	0	0	0	0	0	0	0	0	0	0	0
BGIR9823_100365	100739295	WDR86	lncRNA	0	0	0	0	0	0	0	0	0	0	0	0	0	0	0	0	0	0	0
BGIR9823_82500	100524371	STRN	lncRNA	0	1	0	0	0	0	0	0	0	0	0	0	0	0	0	0	0	0	0
BGIR9823_83316	397176	TAF1B	lncRNA	0	1	0	0	0	0	0	0	0	0	0	0	0	0	0	0	0	0	0
BGIR9823_82589	110260059	CYS1	lncRNA	0	0	0	0	0	0	0	0	0	0	0	0	0	0	0	0	0	0	0
BGIR9823_100212	100157872	PXMP4	lncRNA	0	0	0	0	0	0	0	0	0	0	0	0	0	0	0	0	0	1	0
BGIR9823_84315	106510091	SPIDR	mRNA	0	0	0	0	0	0	0	0	0	0	0	0	0	0	0	0	0	0	0
BGIR9823_85039	100625270	ST8SIA1	mRNA	0	0	0	0	0	0	0	0	0	0	0	0	0	0	0	0	0	0	0
BGIR9823_79988	396733	GGTA1P	lncRNA	0	0	0	0	0	0	0	0	0	0	0	0	0	0	0	0	0	0	0
BGIR9823_81636	100625008	GIN1	lncRNA	0	0	0	0	0	0	0	0	0	0	0	0	0	0	0	0	0	0	0
BGIR9823_96851	100522707	ZNF25	lncRNA	0	0	0	0	0	0	0	0	0	0	0	0	0	0	0	0	0	0	0
BGIR9823_98026	733691	NRAP	mRNA	0	0	0	0	0	0	1	0	0	0	0	0	0	0	0	0	0	0	0
BGIR9823_91942	100510921	STX6	mRNA	0	0	0	0	0	0	0	0	0	0	0	0	0	0	0	0	0	0	0
BGIR9823_101516	102157759	LOC102157759	lncRNA	0	0	0	0	0	1	0	0	0	0	0	0	0	0	0	0	0	0	0
BGIR9823_79269	100152360	CCDC68	lncRNA	0	1	0	0	0	0	0	0	0	0	0	0	0	0	0	0	0	0	0
BGIR9823_95434	100737549	ITSN1	mRNA	0	0	0	0	0	0	0	0	0	0	0	0	0	0	0	0	0	0	0
BGIR9823_81890	100621608	FOPNL	lncRNA	0	0	0	0	0	0	0	0	0	0	0	0	0	0	0	0	0	0	0
BGIR9823_86166	100739461	ZNF570	lncRNA	0	0	0	0	0	0	0	0	0	0	0	0	0	0	0	0	0	0	0
BGIR9823_81903	102162731	ZKSCAN1	mRNA	0	0	0	0	0	0	0	0	0	0	0	0	0	0	0	0	0	0	0
BGIR9823_84182	100153957	KCNS2	mRNA	0	0	0	0	0	0	0	0	0	0	0	0	0	1	0	0	0	0	0
BGIR9823_78637	100155691	AK3	lncRNA	0	1	0	0	0	0	0	0	0	0	0	0	0	0	0	0	0	0	0
BGIR9823_79359	100157164	GABPB1	lncRNA	0	0	0	0	0	1	0	0	0	0	0	0	0	0	0	0	0	0	0
BGIR9823_81535	100520548	WDR41	lncRNA	0	0	0	0	0	0	0	0	0	0	0	0	0	0	0	0	0	0	0
BGIR9823_86229	100516271	DEDD2	mRNA	0	0	0	0	0	0	0	0	0	0	0	0	0	0	0	0	0	0	0
BGIR9823_81434	110259374	LOC110259374	lncRNA	0	0	0	0	0	0	0	0	0	0	0	0	0	0	0	0	0	0	0
BGIR9823_100426	100626356	UBE2H	lncRNA	0	0	0	0	0	0	0	0	0	0	0	0	0	0	0	0	0	0	0
BGIR9823_100415	100620243	PLXNA4	lncRNA	0	0	0	0	0	0	0	0	0	0	0	0	0	0	0	0	0	0	0
BGIR9823_100894	100621199	IL1RAPL1	lncRNA	0	0	0	0	0	0	0	0	0	0	0	0	0	0	0	0	0	0	0
BGIR9823_95922	100622559	PIK3CB	lncRNA	0	0	0	0	0	0	0	0	0	0	0	0	0	0	0	0	0	0	0
BGIR9823_82064	100736832	GRIN2A	lncRNA	0	0	0	0	0	1	0	0	0	0	0	0	0	0	0	0	0	0	0
BGIR9823_100306	110257429	LOC110257429	lncRNA	0	0	0	1	0	0	0	0	0	0	0	0	0	0	0	0	0	0	0

RNA ID	Gene ID	Symbol	Type	novel-ssc-miR1063-5p	ssc-miR-192	ssc-miR-432-5p	ssc-miR-7138-5p	ssc-miR-874	ssc-miR-10386	ssc-miR-122-3p	novel-ssc-miR755-3p	novel-ssc-miR780-3p	novel-ssc-miR804-3p	novel-ssc-miR813-5p	novel-ssc-miR407-5p	novel-ssc-miR573-5p	novel-ssc-miR583-3p	novel-ssc-miR1077-5p	novel-ssc-miR122-3p	novel-ssc-miR154-5p	ssc-miR-127	ssc-miR-194b-5p
BGIR9823_81302	100736972	LOC100736972	lncRNA	0	0	0	0	0	0	0	0	0	0	0	0	0	0	0	1	0	0	0
BGIR9823_100478	100621338	BMT2	lncRNA	0	0	0	0	0	0	0	0	0	0	0	0	0	0	0	0	0	0	0
BGIR9823_86034	100623274	CTCF	mRNA	0	0	0	0	0	0	0	0	0	0	0	0	0	0	0	0	0	0	0
BGIR9823_78972	100155445	VTA1	lncRNA	0	0	0	0	0	0	0	0	0	0	0	0	0	0	0	0	0	0	0
BGIR9823_78078	100154204	MYO1E	lncRNA	0	0	0	0	0	0	0	0	0	0	0	0	0	0	0	0	0	0	0
BGIR9823_82851	100522863	RBFOX1	mRNA	0	0	0	0	0	0	0	0	0	0	0	0	0	0	0	0	0	0	0
BGIR9823_87122	100515140	KIAA0513	lncRNA	0	0	0	0	0	0	0	0	0	0	0	0	0	0	0	0	0	0	0
BGIR9823_97004	100154626	ADK	lncRNA	0	0	0	0	0	0	0	0	0	0	0	0	0	0	0	0	0	0	0
BGIR9823_90785	100524160	ACER3	lncRNA	0	0	0	0	0	0	0	0	0	0	0	0	0	0	0	0	0	0	0
BGIR9823_92442	100513615	C10H9orf3	mRNA	0	0	0	0	0	0	0	0	0	0	0	0	0	0	0	0	0	0	0
BGIR9823_90875	100622590	BIRC3	lncRNA	0	0	0	0	0	0	0	0	0	0	0	0	0	0	0	0	0	0	0
BGIR9823_101409	110258214	LOC110258214	lncRNA	0	0	0	0	0	0	0	0	0	0	0	0	0	0	0	0	0	0	0
BGIR9823_101574	102164761	LOC102164761	lncRNA	0	0	0	0	0	0	0	0	0	0	0	0	0	0	0	0	0	0	0
BGIR9823_81206	100519588	USP47	lncRNA	0	0	0	0	0	0	0	0	0	0	0	0	1	0	0	0	0	0	0
BGIR9823_82146	100737615	LUC7L	lncRNA	0	0	0	0	0	1	0	0	0	0	0	0	0	0	0	0	0	0	0
BGIR9823_88950	100620189	NRXN3	lncRNA	0	0	0	0	0	0	0	0	0	0	0	0	0	0	0	0	0	0	0
BGIR9823_100904	100621199	IL1RAPL1	lncRNA	0	0	0	0	0	0	0	0	0	0	0	0	0	0	0	0	0	0	0
BGIR9823_95076	100622923	PPM1L	lncRNA	0	0	0	0	0	0	0	0	0	0	0	0	0	0	0	0	0	0	0
BGIR9823_100269	102159476	LOC102159476	lncRNA	0	0	0	0	0	0	0	0	0	0	0	0	0	0	0	0	1	0	0
BGIR9823_101628	100519072	LOC100519072	lncRNA	0	0	0	0	0	0	0	0	0	0	0	0	0	0	0	0	0	0	0
BGIR9823_101612	100521600	LOC100521600	lncRNA	0	0	0	0	0	0	0	0	0	0	0	0	0	0	0	0	0	0	0
BGIR9823_101400	110258215	LOC110258215	lncRNA	0	0	0	0	0	0	0	0	0	0	0	0	0	0	0	0	0	0	0
BGIR9823_92255	100514676	ACBD5	lncRNA	0	0	0	0	0	0	0	0	0	0	0	0	0	0	0	0	0	0	0
BGIR9823_85922	100516196	TAF1C	lncRNA	0	0	0	0	0	0	0	0	0	0	0	0	0	0	0	0	0	0	0
BGIR9823_81946	100525688	BUD23	mRNA	0	0	0	0	0	0	0	0	0	0	0	0	0	0	0	0	0	0	0
BGIR9823_80177	106504153	AHNAK	mRNA	0	0	0	0	0	0	0	0	0	0	0	0	0	0	0	0	0	0	0
BGIR9823_88439	106504047	ZNF76	mRNA	0	0	0	0	0	0	0	0	0	0	0	0	0	0	0	0	0	0	0
BGIR9823_81354	100517998	MYO1F	lncRNA	0	0	0	0	0	0	0	0	0	0	0	0	0	0	0	0	0	0	0
BGIR9823_80921	100329221	PPARGC1B	mRNA	0	0	0	0	0	0	0	0	0	0	0	0	0	0	0	0	0	0	0
BGIR9823_77718	100625026	PPIL4	lncRNA	0	0	1	0	0	0	0	0	0	0	0	0	0	0	0	0	0	0	0
BGIR9823_100514	100623678	NT5C3A	lncRNA	0	0	0	0	0	0	0	0	0	0	0	0	0	0	0	0	0	0	0
BGIR9823_78774	396733	GGTA1P	lncRNA	0	0	0	0	0	0	0	0	0	0	0	0	0	0	0	0	0	0	0
BGIR9823_101033	100037300	XIAP	lncRNA	0	0	0	0	0	0	0	0	0	0	0	0	0	0	0	0	0	0	0
BGIR9823_100816	110257601	LOC110257601	lncRNA	0	0	0	0	0	0	0	0	0	0	0	0	0	0	0	0	0	1	0
BGIR9823_90686	733582	CDS1	lncRNA	0	0	0	0	0	0	0	0	0	0	0	0	0	0	0	0	0	0	0
BGIR9823_91751	110255475	LOC110255475	lncRNA	0	0	0	0	0	0	0	0	0	0	0	0	0	0	0	0	0	0	0
NM_001244541.1	100513376	GMPPB	mRNA	0	0	0	0	0	0	1	0	0	0	0	0	0	0	0	0	0	0	0
BGIR9823_81475	100620238	LOC100620238	lncRNA	0	0	0	0	0	0	0	0	0	0	0	0	0	0	0	1	0	0	0
BGIR9823_100582	102162331	KRBA1	lncRNA	0	0	0	0	0	0	0	0	0	0	0	0	0	0	0	0	0	0	0
BGIR9823_101332	100625415	EIF1AY	lncRNA	0	0	1	0	0	0	0	0	0	0	0	0	0	0	0	0	0	0	0
BGIR9823_97502	595128	OAS2	lncRNA	0	0	0	0	0	0	0	0	0	0	0	0	0	0	0	0	0	0	0
BGIR9823_83701	100153809	ALDH9A1	mRNA	0	0	0	0	0	0	0	0	0	0	0	0	0	0	0	0	0	0	0
BGIR9823_80203	100516953	MPEG1	lncRNA	0	0	0	0	0	0	0	0	0	0	0	0	0	0	0	0	0	0	0

RNA ID	Gene ID	Symbol	Type	novel-ssc-miR1063-5p	ssc-miR-192	ssc-miR-432-5p	ssc-miR-7138-5p	ssc-miR-874	ssc-miR-10386	ssc-miR-122-3p	novel-ssc-miR755-3p	novel-ssc-miR780-3p	novel-ssc-miR804-3p	novel-ssc-miR813-5p	novel-ssc-miR407-5p	novel-ssc-miR573-5p	novel-ssc-miR583-3p	novel-ssc-miR1077-5p	novel-ssc-miR122-3p	novel-ssc-miR154-5p	ssc-miR-127	ssc-miR-194b-5p
BGIR9823_99394	100517687	BDP1	lncRNA	0	0	0	0	0	0	0	0	0	0	0	0	0	0	0	0	0	0	0
BGIR9823_85850	102159120	LOC102159120	lncRNA	0	0	0	0	0	0	0	0	0	0	0	0	0	0	0	0	0	0	0
BGIR9823_83931	100519711	ST7L	mRNA	0	0	0	0	0	0	0	0	0	0	0	0	0	0	0	0	0	0	0
BGIR9823_93274	100513428	CEP131	lncRNA	0	0	0	0	0	0	0	0	0	0	0	0	0	0	0	0	0	0	0
BGIR9823_78717	100192437	ERP44	lncRNA	0	1	0	0	0	0	0	0	0	0	0	0	0	0	0	0	0	0	0
BGIR9823_81192	100514626	PIK3C2A	lncRNA	0	1	0	0	0	0	0	0	0	0	0	0	0	0	0	0	0	0	0
BGIR9823_77955	100153597	IBTK	lncRNA	0	0	0	0	0	0	0	0	0	0	0	0	0	0	0	0	0	0	0
BGIR9823_89270	100156900	BTBD9	mRNA	0	0	0	0	0	0	0	0	0	0	0	0	0	0	0	0	0	0	0
BGIR9823_98700	100512563	MGAT5	mRNA	0	0	0	0	0	0	0	0	0	0	0	0	0	0	0	0	0	0	0
BGIR9823_88596	110261555	SEMA4B	lncRNA	0	0	0	0	0	0	0	0	0	0	0	0	0	0	0	0	0	0	1
BGIR9823_85735	110260764	YAF2	lncRNA	0	0	0	0	0	0	0	0	0	0	0	0	0	0	0	0	0	0	0
BGIR9823_89296	100153071	TRERF1	lncRNA	0	0	0	0	0	0	0	0	0	0	0	0	0	0	0	0	0	0	0
BGIR9823_79093	100154222	EPHA7	lncRNA	0	0	0	0	0	0	0	0	0	0	0	1	0	0	0	0	0	0	0
BGIR9823_84443	100144448	OAZ3	lncRNA	0	0	0	0	0	0	0	0	0	0	0	0	0	0	0	0	0	0	0
BGIR9823_82885	100192445	PDPK1	lncRNA	0	0	0	0	0	0	0	0	0	0	0	0	0	0	0	0	0	0	0
BGIR9823_88750	100522330	SLC7A8	lncRNA	0	0	0	0	0	0	0	0	0	0	0	0	0	0	0	0	0	0	0
BGIR9823_77991	102159046	LOC102159046	lncRNA	0	0	0	0	0	0	0	0	0	0	0	0	0	0	0	0	0	0	0
BGIR9823_88964	100157303	ZC3H14	mRNA	0	0	0	0	0	0	0	0	0	0	0	0	0	0	0	0	0	0	0
BGIR9823_78191	102161909	LOC102161909	lncRNA	0	0	0	0	0	0	0	0	0	0	0	0	0	0	0	0	0	0	0
BGIR9823_81917	100620791	TRIP6	mRNA	0	0	0	0	0	0	0	0	0	0	0	0	0	1	0	0	0	0	0
BGIR9823_82685	110259888	LOC110259888	lncRNA	0	0	0	0	0	0	0	0	0	0	0	0	0	0	0	0	0	0	0
BGIR9823_84081	100153657	PHF20L1	mRNA	0	0	0	0	0	0	0	0	0	0	0	0	0	0	0	0	0	0	0
BGIR9823_83050	100628224	VAMP5	lncRNA	0	0	0	0	0	1	0	0	0	0	0	0	0	0	0	0	0	0	0
BGIR9823_98423	397285	ITGAV	mRNA	0	0	0	0	0	0	0	0	0	0	0	0	0	0	0	0	0	0	0
BGIR9823_97702	100155475	HNRNPF	mRNA	0	0	0	0	0	0	0	0	0	0	0	0	0	0	0	0	0	0	0
BGIR9823_93940	397293	KCNJ2	lncRNA	0	0	0	0	0	0	0	0	0	0	0	0	0	1	0	0	0	0	0
BGIR9823_87613	100521974	FBXO42	lncRNA	0	0	0	0	0	0	0	0	0	0	0	0	0	0	0	0	0	0	0
BGIR9823_94936	100155306	SEC61A1	lncRNA	0	0	0	0	0	0	0	0	0	0	0	0	0	0	0	0	0	0	0
BGIR9823_100651	100519065	UBN2	lncRNA	0	0	0	0	0	0	0	0	0	0	0	0	0	0	0	0	0	0	0
BGIR9823_84118	100737543	TBC1D31	lncRNA	0	0	0	0	0	0	0	0	0	0	0	0	0	0	0	0	0	0	0
BGIR9823_79608	100523347	UACA	lncRNA	0	0	0	0	0	0	0	0	0	0	0	0	0	0	0	0	0	0	0
BGIR9823_95065	100514494	LOC100514494	lncRNA	0	0	0	0	0	0	0	0	0	0	0	0	0	0	0	0	0	0	0
BGIR9823_89501	100155701	NYNRIN	mRNA	0	0	0	0	0	0	0	0	0	0	0	0	0	0	0	0	0	0	0
BGIR9823_79516	100155619	SOCS6	lncRNA	0	0	0	0	0	0	0	0	0	0	0	0	0	0	0	0	0	0	0
BGIR9823_93273	100626205	SLC38A10	mRNA	0	0	0	0	0	0	0	0	0	0	0	0	0	0	0	0	0	0	0
BGIR9823_82045	100739121	LOC100739121	lncRNA	0	0	0	0	0	0	0	0	0	0	0	0	1	0	0	0	0	0	0
BGIR9823_99804	100519342	RNF24	lncRNA	0	0	0	0	0	0	0	0	0	0	0	0	0	0	0	0	0	0	0
BGIR9823_81050	102164585	LOC102164585	mRNA	0	0	0	0	0	0	0	0	0	0	0	0	0	0	0	0	0	0	0
BGIR9823_83089	100515889	DGUOK	lncRNA	0	0	0	0	0	0	0	0	0	0	0	0	1	0	0	0	0	0	0
BGIR9823_85641	100157726	ETV6	lncRNA	0	0	0	0	0	1	0	0	0	0	0	0	0	0	0	0	0	0	0
BGIR9823_80399	100514037	EPS15L1	lncRNA	0	0	0	0	0	0	0	0	0	0	0	0	0	0	0	0	0	0	0
BGIR9823_100428	100517203	NRF1	lncRNA	0	0	0	0	0	0	0	0	0	0	0	0	0	0	0	0	0	0	0
BGIR9823_79736	110255453	LOC110255453	lncRNA	0	0	0	0	0	0	0	0	0	0	0	0	1	0	0	0	0	0	0

RNA ID	Gene ID	Symbol	Type	novel-ssc-miR1063-5p	ssc-miR-192	ssc-miR-432-5p	ssc-miR-7138-5p	ssc-miR-874	ssc-miR-10386	ssc-miR-122-3p	novel-ssc-miR755-3p	novel-ssc-miR780-3p	novel-ssc-miR804-3p	novel-ssc-miR813-5p	novel-ssc-miR407-5p	novel-ssc-miR573-5p	novel-ssc-miR583-3p	novel-ssc-miR1077-5p	novel-ssc-miR122-3p	novel-ssc-miR154-5p	ssc-miR-127	ssc-miR-194b-5p
BGIR9823_88239	100512907	LOC100512907	lncRNA	0	0	0	0	0	1	0	0	0	0	0	0	0	0	0	0	0	0	0
BGIR9823_89801	100524447	TBC1D1	lncRNA	0	0	0	0	0	0	0	0	0	0	0	0	0	0	0	0	0	0	0
BGIR9823_78621	100627072	PTPRD	lncRNA	0	0	0	0	0	0	0	0	0	0	0	0	0	0	0	0	0	0	0
BGIR9823_78489	100152019	PELI2	lncRNA	0	1	0	0	0	0	0	0	0	0	0	0	0	0	0	0	0	0	0
BGIR9823_83940	100155227	RBM15	lncRNA	0	0	0	0	0	0	0	0	0	0	0	0	0	0	0	0	0	0	0
BGIR9823_85932	106510466	MAF	lncRNA	0	0	0	0	0	0	0	0	0	0	0	0	0	0	0	0	0	0	0
BGIR9823_81640	100513341	FBXL17	lncRNA	0	0	0	0	0	0	0	0	0	0	0	0	0	0	0	0	0	0	0
BGIR9823_84349	100154639	PBX1	mRNA	0	0	0	0	0	0	0	0	0	0	0	0	0	0	0	0	0	0	0
BGIR9823_100606	100515515	GALNT11	lncRNA	0	0	0	0	0	0	0	0	0	0	0	0	0	0	0	0	0	0	0
BGIR9823_101514	102157759	LOC102157759	lncRNA	0	0	0	0	0	0	0	0	0	0	0	0	0	0	0	0	0	0	0
BGIR9823_85476	100622365	TSPAN31	lncRNA	0	0	0	0	0	0	0	0	0	0	0	0	0	0	0	0	0	0	0
BGIR9823_89890	100515957	PPID	lncRNA	0	0	0	0	0	0	0	0	0	0	0	0	0	0	0	0	0	0	0
BGIR9823_101279	100153213	HS6ST2	mRNA	0	0	0	0	0	0	0	1	0	0	0	0	0	0	0	0	0	0	0
BGIR9823_92274	100520446	PIP4K2A	lncRNA	0	0	0	0	0	0	0	0	0	0	0	0	0	0	0	0	0	0	0
BGIR9823_83415	106509958	MTSS1	lncRNA	0	0	0	0	0	0	0	0	0	0	0	0	0	0	0	0	0	0	0
BGIR9823_78462	100511360	FBXO34	lncRNA	0	0	0	0	0	0	0	0	0	0	0	0	0	0	0	0	0	0	0
BGIR9823_77832	100736796	ADGRB3	lncRNA	0	0	0	0	0	0	0	0	0	0	0	0	0	0	0	0	0	0	0
BGIR9823_79204	397085	MYO6	lncRNA	0	0	0	0	0	0	0	0	0	0	0	0	0	0	0	0	0	0	0
BGIR9823_83395	110260216	LOC110260216	lncRNA	0	0	0	0	0	0	0	0	0	0	0	0	0	0	0	0	0	0	0
BGIR9823_84473	100153347	GOLPH3L	lncRNA	0	0	0	0	0	0	0	0	0	0	0	0	0	0	0	0	0	0	0
BGIR9823_85369	100518305	LOC100518305	mRNA	0	0	0	0	0	0	0	0	0	0	0	0	0	0	0	0	0	0	0
BGIR9823_84074	100627184	GLI4	lncRNA	0	0	0	0	0	0	0	0	0	0	0	0	0	0	0	0	0	0	0
BGIR9823_92298	100515404	ITIH5	lncRNA	0	0	0	0	0	0	0	0	0	0	0	0	0	0	0	0	0	0	0
BGIR9823_89061	100153581	NUP153	mRNA	0	0	0	0	0	0	0	0	0	0	0	0	0	0	0	0	0	0	0
BGIR9823_96626	100151786	POP5	lncRNA	0	0	0	0	0	0	0	0	0	0	0	0	0	0	0	0	0	0	0
BGIR9823_87721	100517038	PUM1	lncRNA	0	0	0	0	0	0	0	0	0	0	0	0	0	0	0	0	0	0	0
BGIR9823_100728	100512382	KCND2	lncRNA	0	0	0	0	0	0	0	0	0	0	0	0	0	0	0	0	0	1	0
BGIR9823_80555	110259375	LOC110259375	lncRNA	0	0	0	0	0	0	0	0	0	0	0	1	0	0	0	0	0	0	0
BGIR9823_79909	100155492	HEMGN	lncRNA	0	0	0	0	0	0	0	0	0	0	0	1	0	0	0	0	0	0	0
BGIR9823_94689	100516877	ACOX2	lncRNA	0	0	0	0	0	0	0	0	0	0	0	0	0	0	0	0	0	0	0
BGIR9823_92764	102158088	NHLRC3	lncRNA	0	0	0	0	0	0	0	0	0	0	0	0	0	0	0	0	0	0	0
BGIR9823_89682	100152306	LOC100152306	mRNA	0	0	0	0	0	0	0	0	0	0	0	0	0	1	0	0	0	0	0
BGIR9823_96521	100623262	SLC15A4	mRNA	0	0	0	0	0	0	0	0	0	0	0	0	0	0	0	0	0	0	0
BGIR9823_84835	100516439	C5H12orf10	lncRNA	0	0	0	0	0	0	0	0	0	0	0	0	0	0	0	0	0	0	0
BGIR9823_100509	100738688	HERPUD2	lncRNA	0	0	0	0	0	0	0	0	0	0	0	0	0	0	0	0	0	1	0
BGIR9823_86157	100511329	ZNF829	mRNA	0	0	0	0	0	0	0	0	0	0	0	0	0	0	0	0	0	0	0
BGIR9823_82679	100620395	GIGYF1	mRNA	0	0	0	0	0	0	0	0	0	0	0	0	0	0	0	0	0	0	0
BGIR9823_90779	100621182	EMSY	mRNA	0	0	0	0	0	0	0	0	0	0	0	0	0	0	0	0	0	0	0
BGIR9823_90540	100621145	LARP1B	lncRNA	0	0	0	0	0	0	0	0	0	0	0	0	0	0	0	0	0	0	0
NM_001097469.1	100037984	HIGD2A	mRNA	0	0	0	0	0	0	0	0	0	0	0	0	0	0	0	0	0	0	0
BGIR9823_78404	102162695	LOC102162695	lncRNA	0	0	0	0	0	0	0	0	0	0	0	0	0	0	0	0	0	0	0
BGIR9823_84013	102161056	LOC102161056	lncRNA	0	0	0	0	0	0	0	0	0	0	0	0	0	0	0	0	0	0	0
BGIR9823_98214	100512817	TENM3	mRNA	0	0	0	0	0	0	0	0	0	0	0	0	0	0	0	0	0	0	0

RNA ID	Gene ID	Symbol	Type	novel-ssc-miR1063-5p	ssc-miR-192	ssc-miR-432-5p	ssc-miR-7138-5p	ssc-miR-874	ssc-miR-10386	ssc-miR-122-3p	novel-ssc-miR755-3p	novel-ssc-miR780-3p	novel-ssc-miR804-3p	novel-ssc-miR813-5p	novel-ssc-miR407-5p	novel-ssc-miR573-5p	novel-ssc-miR583-3p	novel-ssc-miR1077-5p	novel-ssc-miR122-3p	novel-ssc-miR154-5p	ssc-miR-127	ssc-miR-194b-5p
BGIR9823_81705	100521611	FNIP1	lncRNA	0	0	0	0	0	0	0	0	0	0	0	0	0	0	0	0	0	0	0
BGIR9823_79689	100192435	ERO1A	lncRNA	0	0	0	0	0	0	0	0	0	0	0	0	0	0	0	0	0	0	0
BGIR9823_81181	100736787	ZDHHC13	mRNA	0	0	0	0	0	0	0	0	0	0	0	0	0	0	0	0	0	0	0
BGIR9823_95430	396658	IFNAR1	mRNA	0	0	0	0	0	0	0	0	0	0	0	0	0	1	0	0	0	0	0
BGIR9823_77980	100152640	HAUS1	lncRNA	0	0	0	0	0	0	0	0	0	0	0	0	0	0	0	0	0	0	0
BGIR9823_91823	100522092	RUNDC3B	lncRNA	0	0	0	0	0	0	0	0	0	0	0	0	0	0	0	0	0	0	0
BGIR9823_81117	100144442	F2	lncRNA	0	0	0	0	0	0	0	0	0	0	0	0	0	0	0	0	0	0	0
BGIR9823_90627	100624822	GSTCD	mRNA	0	0	0	0	0	0	0	0	0	0	0	0	0	0	0	0	0	0	0
BGIR9823_78165	100169932	TRPM7	lncRNA	0	0	0	0	0	0	1	0	0	0	0	0	0	0	0	0	0	0	0
BGIR9823_80118	100511178	SNX32	lncRNA	0	0	0	0	0	0	0	0	0	0	0	0	0	0	0	0	0	0	0
BGIR9823_84495	102166500	LOC102166500	lncRNA	0	0	0	0	0	1	0	0	0	0	0	0	0	0	0	0	0	0	0
BGIR9823_81367	100127167	CERS4	mRNA	0	0	0	0	0	0	0	0	0	0	0	0	0	0	0	0	0	0	0
BGIR9823_77687	100156632	AGPAT4	lncRNA	0	0	0	0	0	0	0	0	0	0	0	0	0	0	0	0	0	0	0
BGIR9823_80249	100519700	AMBRA1	lncRNA	0	0	0	0	0	0	0	0	0	0	0	0	0	0	0	0	0	0	0
BGIR9823_100622	100521480	GSTK1	lncRNA	0	0	1	0	0	0	0	0	0	0	0	0	0	0	0	0	0	0	0
BGIR9823_86113	100621064	DPY19L3	lncRNA	0	0	0	0	0	0	0	0	0	0	0	0	0	0	0	0	0	0	0
BGIR9823_92205	100516148	MOB3B	mRNA	0	0	0	0	0	0	0	0	0	0	0	0	0	0	0	0	0	0	0
XM_021075409.1	100737004	COBLL1	mRNA	0	0	0	0	0	0	0	0	0	0	0	0	0	0	0	0	0	0	0
BGIR9823_89246	100521322	LOC100521322	lncRNA	0	0	0	0	0	0	0	0	0	0	0	0	0	0	0	0	0	0	0
BGIR9823_78193	100154291	SLC12A1	lncRNA	0	0	0	0	0	0	0	0	0	0	0	0	0	0	0	0	0	0	0
BGIR9823_100566	100516888	GLI3	lncRNA	0	0	0	0	0	0	0	0	0	0	0	0	0	0	0	0	0	0	0
BGIR9823_100446	100192443	POT1	lncRNA	0	0	0	0	0	0	0	0	0	0	0	0	0	0	0	0	0	1	0
BGIR9823_97408	100620430	SAP30	lncRNA	0	0	0	0	0	0	0	0	0	0	0	0	0	0	0	0	0	0	0
BGIR9823_86274	100622144	BICRA	lncRNA	0	0	0	0	0	0	0	0	0	0	0	0	0	0	0	0	0	0	0
BGIR9823_101110	100524780	ARHGAP6	lncRNA	0	0	0	0	0	0	0	0	0	0	0	0	0	0	0	0	0	0	0
BGIR9823_82012	100524333	GGA2	lncRNA	0	0	0	0	0	0	0	0	0	0	0	0	0	0	0	0	0	0	0
BGIR9823_86283	100624752	NOP53	mRNA	0	0	0	0	0	0	0	0	0	0	0	0	0	0	0	0	0	0	0
BGIR9823_98317	110256929	WDSUB1	lncRNA	0	0	0	0	0	0	0	0	0	0	0	0	0	0	0	0	0	0	0
BGIR9823_88517	100511822	ICK	lncRNA	0	0	0	0	0	1	0	0	0	0	0	0	0	0	0	0	0	0	0
BGIR9823_79454	110259520	LOC110259520	lncRNA	0	0	0	0	0	0	0	0	0	0	0	0	0	0	0	1	0	0	0
BGIR9823_81324	606746	DNMT1	mRNA	0	0	0	0	0	0	1	0	0	0	0	0	0	0	0	0	0	0	0
BGIR9823_100702	100514931	AHCYL2	lncRNA	0	0	0	0	0	0	0	0	0	0	0	0	0	0	0	0	0	0	0
BGIR9823_101292	100523658	ATP11C	mRNA	0	0	0	0	0	0	0	0	0	0	0	0	0	0	0	0	0	0	0
BGIR9823_90549	102167943	LOC102167943	lncRNA	0	0	0	0	0	0	0	0	0	0	0	0	0	0	0	0	0	0	0
BGIR9823_84672	100516804	SCO2	mRNA	0	0	0	0	0	0	0	1	0	0	0	0	0	0	0	0	0	0	0
BGIR9823_98499	100514879	ABI2	mRNA	0	0	0	0	0	0	0	0	0	0	0	0	0	0	0	0	0	0	0
BGIR9823_83000	102166005	LOC102166005	lncRNA	0	0	0	0	0	1	0	0	0	0	0	0	0	0	0	0	0	0	0
BGIR9823_79430	100152163	DLL4	lncRNA	0	0	0	0	0	0	0	0	0	0	0	1	0	0	0	0	0	0	0
BGIR9823_80973	100511242	TMEM134	lncRNA	0	0	0	0	0	0	0	0	0	0	0	0	0	0	0	0	0	0	0
BGIR9823_101334	100523617	TRIM27	lncRNA	0	0	1	0	0	0	0	0	0	0	0	0	0	0	0	0	0	0	0
BGIR9823_81536	100520548	WDR41	lncRNA	0	0	0	0	0	0	0	0	0	0	0	0	0	0	0	0	0	0	0
BGIR9823_101625	100519072	LOC100519072	lncRNA	0	0	0	0	0	0	0	0	0	0	0	0	0	0	0	0	0	0	0
BGIR9823_87181	100517402	PDF	lncRNA	0	0	0	0	0	0	0	0	0	0	0	0	0	0	0	0	0	0	0

RNA ID	Gene ID	Symbol	Type	novel-ssc-miR1063-5p	ssc-miR-192	ssc-miR-432-5p	ssc-miR-7138-5p	ssc-miR-874	ssc-miR-10386	ssc-miR-122-3p	novel-ssc-miR755-3p	novel-ssc-miR780-3p	novel-ssc-miR804-3p	novel-ssc-miR813-5p	novel-ssc-miR407-5p	novel-ssc-miR573-5p	novel-ssc-miR583-3p	novel-ssc-miR1077-5p	novel-ssc-miR122-3p	novel-ssc-miR154-5p	ssc-miR-127	ssc-miR-194b-5p
BGIR9823_78566	100152132	IFN-OMEGA-3	lncRNA	0	0	0	0	0	0	0	0	0	0	0	0	0	0	0	0	0	0	0
XR_002339179.1	110257024	LOC110257024	lncRNA	0	0	0	0	0	0	0	0	0	0	0	0	0	0	0	0	0	0	0
BGIR9823_100144	100154945	RRBP1	lncRNA	0	0	0	0	0	0	0	0	0	0	0	0	0	0	0	0	1	0	0
BGIR9823_100611	100521999	SMARCD3	lncRNA	0	0	0	0	0	0	0	0	0	0	0	0	0	0	0	0	0	0	0
BGIR9823_95072	100620632	KPNA4	lncRNA	0	0	0	0	0	0	0	0	0	0	0	0	0	0	0	0	0	0	0
BGIR9823_98782	100152203	AGA	mRNA	0	0	0	0	0	0	0	0	0	0	0	0	0	0	0	0	0	0	0
BGIR9823_77731	100154785	ADAT2	lncRNA	0	0	0	0	0	0	0	0	0	0	0	0	0	0	0	0	0	0	0
BGIR9823_96586	100153673	CUX2	mRNA	0	0	0	0	0	0	0	0	0	0	0	0	0	1	0	0	0	0	0
BGIR9823_85978	110260924	LOC110260924	lncRNA	0	0	0	0	0	0	0	0	0	0	0	0	0	0	0	0	0	0	0
BGIR9823_78788	100154489	WDR38	mRNA	0	0	0	0	0	0	0	0	0	0	0	0	0	0	0	0	0	0	0
BGIR9823_86389	110261319	LOC110261319	lncRNA	0	0	0	0	0	0	0	0	0	0	0	0	0	0	0	0	0	0	1
BGIR9823_79497	100736682	TJP1	lncRNA	0	1	0	0	0	0	0	0	0	0	0	0	0	0	0	0	0	0	0
BGIR9823_98375	100522284	METAP1D	mRNA	0	0	0	0	0	0	0	0	0	0	0	0	0	0	0	0	0	0	0
BGIR9823_100000	100523757	ARFGEF2	lncRNA	0	0	0	0	0	0	0	0	0	0	0	0	0	0	0	0	0	0	0
BGIR9823_84378	100524676	SDHC	lncRNA	0	0	0	0	0	0	0	0	0	0	0	0	0	0	0	0	0	0	0
BGIR9823_92192	396723	DDX58	mRNA	0	0	0	0	0	0	0	0	0	0	0	0	0	0	0	0	0	0	0
BGIR9823_88795	102162743	FAM174B	lncRNA	0	0	0	0	0	0	0	0	0	0	0	0	0	0	0	0	0	0	0
BGIR9823_92690	100623356	SKA3	lncRNA	0	0	0	0	0	0	0	0	0	0	0	0	0	0	0	0	0	0	0
XR_002340913.1	110257727	LOC110257727	lncRNA	0	0	0	0	0	0	0	0	0	0	0	0	0	0	0	0	0	0	0
BGIR9823_86991	100037307	DAB1	lncRNA	0	0	0	0	0	0	0	0	0	0	0	0	0	0	0	0	0	0	0
BGIR9823_82075	100513346	LOC100513346	mRNA	0	0	0	0	0	0	0	0	0	0	0	0	0	0	0	0	0	0	0
BGIR9823_86237	100524260	ZNF45	lncRNA	0	0	0	0	0	1	0	0	0	0	0	0	0	0	0	0	0	0	0
BGIR9823_80263	106507997	LOC106507997	lncRNA	0	0	0	0	0	0	0	0	0	0	0	0	0	0	0	0	0	0	0
BGIR9823_93834	110256118	LOC110256118	lncRNA	0	0	0	0	0	0	0	0	0	0	0	0	0	0	0	0	0	0	0
BGIR9823_80349	100738340	ATP13A1	lncRNA	0	0	0	0	0	0	0	0	0	0	0	0	0	0	0	0	0	0	0
BGIR9823_89603	106507740	LOC106507740	lncRNA	0	0	0	0	0	0	0	0	0	0	0	0	0	0	0	0	0	0	0
BGIR9823_77903	100514380	TSTD3	lncRNA	0	0	0	0	0	0	0	0	0	0	0	0	0	0	0	1	0	0	0
BGIR9823_100158	100154020	LOC100154020	lncRNA	0	0	0	0	0	0	0	0	0	0	0	0	0	0	0	0	0	0	0
BGIR9823_89657	733584	FOXN3	lncRNA	0	0	0	0	0	0	0	0	0	0	0	0	0	0	0	0	0	0	0
BGIR9823_98765	100233172	AGPAT5	lncRNA	0	0	0	0	0	0	0	0	0	0	0	0	0	0	0	0	0	0	0
BGIR9823_94610	396679	AMT	lncRNA	0	0	0	0	0	0	0	0	0	0	0	0	0	0	0	0	0	0	0
BGIR9823_78506	100154408	PSMA3	lncRNA	0	0	0	0	0	0	0	0	0	0	0	0	0	0	0	0	0	0	0
BGIR9823_78017	100515517	RBPMS2	lncRNA	0	0	0	0	0	0	0	0	0	0	0	0	0	0	0	0	0	0	0
BGIR9823_98223	100515058	TRAPPC11	mRNA	0	0	0	0	0	0	0	0	0	0	0	0	0	0	0	0	0	0	0
BGIR9823_78148	100155516	DMXL2	mRNA	0	0	0	0	0	0	0	0	0	0	0	0	0	0	0	0	0	0	0
BGIR9823_87377	100521972	LOC100521972	lncRNA	0	0	0	0	0	0	0	0	0	0	0	0	0	0	0	0	0	0	0
BGIR9823_84933	100511508	LEMD3	lncRNA	0	0	0	0	0	0	0	0	0	0	0	0	0	0	0	0	0	0	0
BGIR9823_100906	100621199	IL1RAPL1	lncRNA	0	0	0	0	0	0	0	0	0	0	0	0	0	0	0	0	0	0	0
BGIR9823_89271	100156900	BTBD9	mRNA	0	0	0	0	0	0	0	0	0	0	0	0	0	0	0	0	0	0	0
BGIR9823_90463	100627416	TRIM2	lncRNA	0	0	0	0	0	0	0	0	0	0	0	0	0	0	0	0	0	0	0
BGIR9823_79564	100153830	MALT1	lncRNA	0	0	0	0	0	0	0	0	0	0	0	0	0	0	0	0	0	0	0
BGIR9823_88636	100155770	FAM219B	mRNA	0	0	0	0	0	0	0	0	0	0	0	0	0	0	0	0	0	0	0
BGIR9823_84024	100155974	GTF2B	lncRNA	0	0	0	0	0	0	0	0	0	0	0	0	0	0	0	0	0	0	0

RNA ID	Gene ID	Symbol	Type	novel-ssc-miR1063-5p	ssc-miR-192	ssc-miR-432-5p	ssc-miR-7138-5p	ssc-miR-874	ssc-miR-10386	ssc-miR-122-3p	novel-ssc-miR755-3p	novel-ssc-miR780-3p	novel-ssc-miR804-3p	novel-ssc-miR813-5p	novel-ssc-miR407-5p	novel-ssc-miR573-5p	novel-ssc-miR583-3p	novel-ssc-miR1077-5p	novel-ssc-miR122-3p	novel-ssc-miR154-5p	ssc-miR-127	ssc-miR-194b-5p
BGIR9823_100486	100621338	BMT2	lncRNA	0	0	0	0	0	0	0	0	0	0	0	0	0	0	0	0	0	0	0
BGIR9823_86035	100623274	CTCF	mRNA	0	0	0	0	0	0	0	0	0	0	0	0	0	0	0	0	0	0	0
BGIR9823_85003	110260713	AMN1	mRNA	0	0	0	0	0	0	0	0	0	0	0	0	0	0	0	0	0	0	0
BGIR9823_100211	100157872	PXMP4	lncRNA	0	0	0	0	0	0	0	0	0	0	0	0	0	0	0	0	0	0	0
BGIR9823_85764	396681	TXNRD1	mRNA	0	0	0	0	0	0	0	0	0	0	0	0	0	0	0	0	0	0	0
BGIR9823_79047	110260218	LOC110260218	lncRNA	0	0	0	0	0	0	0	0	0	0	0	0	0	0	0	0	0	0	0
BGIR9823_99528	100626363	NPR3	lncRNA	0	0	0	0	0	0	0	0	0	0	0	0	0	0	0	0	0	0	0
BGIR9823_86380	100738636	EPN1	mRNA	0	0	0	0	0	0	0	0	0	0	0	0	0	0	0	0	0	0	0
BGIR9823_101388	100521615	ARHGAP17	mRNA	0	0	0	0	0	0	0	0	0	0	0	0	0	0	0	0	0	0	0
BGIR9823_85180	100625757	ARID2	lncRNA	0	0	0	0	0	0	0	0	0	0	0	0	0	0	0	0	0	0	0
BGIR9823_101519	100511414	RNF122	lncRNA	0	0	0	0	0	0	0	0	0	0	0	0	0	0	0	0	0	0	0
BGIR9823_100416	106506781	LOC106506781	lncRNA	0	0	0	0	0	0	0	0	0	0	0	0	0	0	0	0	0	0	0
BGIR9823_77835	100736796	ADGRB3	lncRNA	0	0	0	0	0	0	0	0	0	0	0	0	0	0	0	0	0	0	0
XM_001928635.2	100156587	LOC100156587	mRNA	0	0	0	0	0	0	0	0	0	0	0	0	0	0	0	0	0	0	0
BGIR9823_91988	100217398	FLVCR1	lncRNA	0	0	0	0	0	0	0	0	0	0	0	0	0	0	0	0	0	0	0
BGIR9823_86369	100517285	LOC100517285	mRNA	0	0	0	0	0	0	0	0	0	0	0	0	0	0	0	0	0	0	0
BGIR9823_101017	100520165	TMEM164	lncRNA	0	0	0	0	0	0	0	0	0	0	0	0	0	0	0	0	0	0	0
BGIR9823_81426	100621275	GNG7	lncRNA	0	0	0	0	0	0	0	0	0	0	0	0	0	0	0	0	0	0	0
BGIR9823_94753	100522704	FAM19A1	lncRNA	0	0	0	0	0	0	0	0	0	0	0	0	0	0	0	0	0	0	0
BGIR9823_87686	100512789	WASF2	lncRNA	0	0	0	0	0	0	0	0	0	0	0	0	0	0	0	0	0	0	0
BGIR9823_88695	100624265	STRN3	mRNA	0	0	0	0	0	0	0	0	0	0	0	0	0	0	0	0	0	0	0
BGIR9823_82270	100625157	GCFC2	lncRNA	0	0	0	0	0	0	0	0	0	0	0	0	0	0	0	0	0	0	0
BGIR9823_93946	397091	PRKAR1A	mRNA	0	0	0	0	0	0	0	0	0	0	0	0	0	0	0	0	0	0	0
BGIR9823_93731	110256070	LOC110256070	lncRNA	0	0	0	0	0	0	0	0	0	0	0	0	0	0	0	0	0	0	0
BGIR9823_80458	100515360	ILF3	lncRNA	0	0	0	0	0	0	0	0	0	0	0	0	0	0	0	0	0	0	0
BGIR9823_80830	100513723	JADE2	lncRNA	0	0	0	0	0	0	0	0	0	0	0	0	0	0	0	1	0	0	0
BGIR9823_87106	100739500	ACSF3	lncRNA	0	0	0	0	0	1	0	0	0	0	0	0	0	0	0	0	0	0	0
BGIR9823_91655	100511341	EI24	lncRNA	0	0	0	0	0	0	0	0	0	0	0	0	0	0	0	0	0	0	0
BGIR9823_84152	100337657	ZFPM2	mRNA	0	0	0	0	0	0	0	0	0	0	0	0	0	0	0	0	0	0	0
BGIR9823_78973	100155445	VTA1	lncRNA	0	0	0	0	0	0	0	0	0	0	0	1	0	0	0	0	0	0	0
BGIR9823_88372	100151743	PPP1R18	lncRNA	0	0	0	0	0	0	0	0	0	0	0	0	0	0	0	0	0	0	0
BGIR9823_97928	100155592	PDLIM1	mRNA	0	0	0	0	0	0	0	0	0	0	0	0	0	0	0	0	0	0	0
BGIR9823_88939	100511141	VASH1	mRNA	0	0	0	0	0	0	0	0	0	0	0	0	0	0	0	0	0	0	0
BGIR9823_93334	100623453	KIF19	lncRNA	0	0	0	0	0	0	0	0	0	0	0	0	0	0	0	0	0	0	0
BGIR9823_79684	100153738	TRIM9	lncRNA	0	0	0	0	0	0	0	0	0	0	0	0	0	0	0	1	0	0	0
BGIR9823_100666	100524964	AGBL3	lncRNA	0	0	0	0	0	0	0	0	0	0	0	0	0	0	0	0	1	0	0
BGIR9823_89830	100515040	LIMCH1	mRNA	0	0	0	0	0	0	0	0	0	0	0	0	0	1	0	0	0	0	0
BGIR9823_85457	100511633	RDH16	lncRNA	0	0	0	0	0	0	0	0	0	0	0	0	0	0	0	0	0	0	0
BGIR9823_81268	100512396	SLC35E1	lncRNA	0	0	0	0	0	0	0	0	0	0	0	0	0	0	0	1	0	0	0
BGIR9823_78329	100049703	BCL2	lncRNA	0	0	0	0	0	0	0	0	0	0	0	0	0	0	0	0	0	0	0
BGIR9823_78260	100512756	MEIS2	lncRNA	0	1	0	0	0	0	0	0	0	0	0	0	0	0	0	0	0	0	0
BGIR9823_89336	100152930	ADGRF5	mRNA	0	0	0	0	0	0	0	0	0	0	0	0	0	0	0	0	0	0	0
BGIR9823_89031	100152689	SSR1	mRNA	0	0	0	0	0	0	0	0	0	0	0	0	0	0	0	0	0	0	0

RNA ID	Gene ID	Symbol	Type	novel-ssc-miR1063-5p	ssc-miR-192	ssc-miR-432-5p	ssc-miR-7138-5p	ssc-miR-874	ssc-miR-10386	ssc-miR-122-3p	novel-ssc-miR755-3p	novel-ssc-miR780-3p	novel-ssc-miR804-3p	novel-ssc-miR813-5p	novel-ssc-miR407-5p	novel-ssc-miR573-5p	novel-ssc-miR583-3p	novel-ssc-miR1077-5p	novel-ssc-miR122-3p	novel-ssc-miR154-5p	ssc-miR-127	ssc-miR-194b-5p
BGIR9823_78386	100154444	GLCE	lncRNA	0	1	0	0	0	0	0	0	0	0	0	0	0	0	0	0	0	0	0
BGIR9823_92789	100155063	SPRYD7	mRNA	0	0	0	0	0	0	0	1	0	0	0	0	0	0	0	0	0	0	0
BGIR9823_86252	100622514	CLASRP	lncRNA	0	0	0	0	0	0	0	0	0	0	0	0	0	0	0	0	0	0	0
BGIR9823_101153	100621125	SLC35A2	mRNA	0	0	0	0	0	0	0	0	0	0	0	0	0	0	0	0	0	0	1
BGIR9823_100998	414736	PCDH11X	lncRNA	0	0	0	0	0	0	0	0	0	0	0	0	0	0	0	0	0	0	0
BGIR9823_82762	397614	IL4R	lncRNA	0	0	0	0	0	0	0	0	0	0	0	0	0	0	0	0	0	0	0
BGIR9823_79824	100626649	KANK1	lncRNA	0	1	0	0	0	0	0	0	0	0	0	0	0	0	0	0	0	0	0
BGIR9823_95516	100152333	ANKRD28	lncRNA	0	0	0	0	0	0	0	0	0	0	0	0	0	0	0	0	0	0	0
BGIR9823_81853	106509619	LOC106509619	lncRNA	0	0	0	0	0	0	0	0	0	0	0	0	0	0	0	0	0	0	0
BGIR9823_90521	100513554	NAA15	lncRNA	0	0	0	0	0	0	0	0	0	0	0	0	0	0	0	0	0	0	0
BGIR9823_93604	100520097	TUBD1	mRNA	0	0	0	0	0	0	0	0	0	0	0	0	0	0	0	0	0	0	0
BGIR9823_79674	100157402	NIN	lncRNA	0	0	0	0	0	0	0	0	0	0	0	0	0	0	0	0	0	0	0
BGIR9823_100171	100625861	LZTS3	lncRNA	0	0	0	0	0	0	0	0	0	0	0	0	0	0	0	0	0	0	0
BGIR9823_81709	100521611	FNIP1	lncRNA	0	0	0	0	0	0	0	0	0	0	0	0	0	0	0	0	0	0	0
BGIR9823_82252	100738124	IMMT	lncRNA	0	0	0	0	0	0	0	0	0	0	0	0	0	0	0	0	0	0	0
BGIR9823_80640	102165038	LOC102165038	lncRNA	0	0	0	0	0	0	0	0	0	0	0	0	1	0	0	0	0	0	0
BGIR9823_101575	100737380	CDC25A	lncRNA	0	0	0	0	0	0	0	0	0	0	0	0	0	0	0	0	0	0	0
BGIR9823_84694	100518786	HDAC10	lncRNA	0	0	0	0	0	0	0	0	0	0	0	0	0	0	0	0	0	0	0
BGIR9823_98548	102162936	SPAG16	mRNA	0	0	0	0	0	0	0	0	0	0	0	0	0	0	0	0	0	0	0
BGIR9823_88951	110261640	LOC110261640	lncRNA	0	0	0	0	0	0	0	0	0	0	0	0	0	0	0	0	0	0	0
BGIR9823_101401	110258215	LOC110258215	lncRNA	0	0	0	0	0	0	0	0	0	0	0	0	0	0	0	0	0	0	0
BGIR9823_82476	100518189	SRSF7	lncRNA	0	0	0	0	0	0	0	0	0	0	0	0	0	0	0	0	0	0	0
BGIR9823_80254	100520600	TP53I11	lncRNA	0	0	0	0	0	0	0	0	0	0	0	0	0	0	0	0	0	0	0
BGIR9823_100432	100515455	TNPO3	lncRNA	0	0	0	0	0	0	0	0	0	0	0	0	0	0	0	0	0	0	0
BGIR9823_100491	100520706	DOCK4	lncRNA	0	0	0	0	0	0	0	0	0	0	0	0	0	0	0	0	0	0	0
BGIR9823_101216	100514440	ATRX	lncRNA	0	0	0	1	0	0	0	0	0	0	0	0	0	0	0	0	0	0	0
BGIR9823_94101	100518511	MBTD1	lncRNA	0	0	0	0	0	0	0	0	0	0	0	0	0	0	0	0	0	0	0
BGIR9823_84304	100157730	TGS1	mRNA	0	0	0	0	0	0	0	0	0	0	0	0	0	0	0	0	0	0	0
BGIR9823_98130	100519056	ORC4	lncRNA	0	0	0	0	0	0	0	0	0	0	0	0	0	0	0	0	0	0	0
BGIR9823_95626	100623101	SETD2	mRNA	0	0	0	0	0	0	0	0	0	0	0	0	0	0	0	0	0	0	0
BGIR9823_81836	110259827	LOC110259827	lncRNA	0	0	0	0	0	0	0	0	0	0	0	0	1	0	0	0	0	0	0
BGIR9823_100125	100519631	TASP1	lncRNA	0	0	0	0	1	0	0	0	0	0	0	0	0	0	0	0	0	0	0
BGIR9823_77803	100520524	GOPC	lncRNA	0	0	0	0	0	0	0	0	0	0	0	0	0	0	0	0	0	0	0
BGIR9823_78926	100152904	ARID1B	lncRNA	0	0	0	0	0	1	0	0	0	0	0	0	0	0	0	0	0	0	0
XR_002343327.1	110260295	LOC110260295	lncRNA	0	0	0	0	0	0	0	0	0	0	0	0	0	1	0	0	0	0	0
XM_005661298.3	100514564	CAPS	mRNA	0	0	0	0	0	0	0	0	0	0	0	0	0	0	0	0	0	0	0
BGIR9823_100692	100739403	KLHDC10	lncRNA	0	0	1	0	0	0	0	0	0	0	0	0	0	0	0	0	0	0	0
BGIR9823_95813	100525891	EIF4E3	lncRNA	0	0	0	0	0	0	0	0	0	0	0	0	0	0	0	0	0	0	0
BGIR9823_81281	100516595	ZNF333	lncRNA	0	0	0	0	0	0	0	0	0	0	0	0	0	0	0	0	0	0	0
BGIR9823_100015	100151957	PARD6B	lncRNA	0	0	0	0	0	0	0	0	0	0	0	0	0	0	0	0	0	0	0
BGIR9823_101282	100517991	FAM122B	lncRNA	0	0	0	0	0	0	0	0	0	1	0	0	0	0	0	0	0	0	0
BGIR9823_88581	100151986	UNC45A	lncRNA	0	0	0	0	0	0	0	0	0	0	0	0	0	0	0	0	0	0	0
BGIR9823_88595	110261555	SEMA4B	lncRNA	0	0	0	0	0	0	0	0	0	0	0	0	0	0	0	0	0	0	0

RNA ID	Gene ID	Symbol	Type	novel-ssc-miR1063-5p	ssc-miR-192	ssc-miR-432-5p	ssc-miR-7138-5p	ssc-miR-874	ssc-miR-10386	ssc-miR-122-3p	novel-ssc-miR755-3p	novel-ssc-miR780-3p	novel-ssc-miR804-3p	novel-ssc-miR813-5p	novel-ssc-miR407-5p	novel-ssc-miR573-5p	novel-ssc-miR583-3p	novel-ssc-miR1077-5p	novel-ssc-miR122-3p	novel-ssc-miR154-5p	ssc-miR-127	ssc-miR-194b-5p
BGIR9823_91875	106504991	LOC106504991	lncRNA	0	0	0	0	0	0	0	0	0	0	0	0	0	0	0	0	0	0	0
BGIR9823_100454	100525017	WASL	lncRNA	0	0	1	0	0	0	0	0	0	0	0	0	0	0	0	0	0	0	0
BGIR9823_101069	100512515	AFF2	mRNA	0	0	0	0	0	0	1	0	0	0	0	0	0	0	0	0	0	0	0
BGIR9823_101534	102158401	LOC102158401	lncRNA	0	0	0	0	0	0	0	0	0	0	0	0	0	0	0	0	0	0	0
BGIR9823_82897	100738908	MLST8	lncRNA	0	0	0	0	0	0	0	0	0	0	0	0	0	0	0	0	0	0	0
BGIR9823_98106	100158118	CALY	mRNA	0	0	0	0	0	0	1	0	0	0	0	0	0	0	0	0	0	0	0
BGIR9823_80817	100520422	SLC22A5	lncRNA	0	0	0	0	0	0	0	0	0	0	0	1	0	0	0	0	0	0	0
BGIR9823_91425	100511767	ARAP1	mRNA	0	0	0	0	0	0	0	0	0	0	0	0	0	0	0	0	0	0	0
BGIR9823_96623	100154637	SRSF9	lncRNA	0	0	0	0	0	0	0	0	0	0	0	0	0	0	0	0	0	0	0
BGIR9823_81521	100513398	SIMC1	lncRNA	0	0	0	0	0	0	0	0	0	0	0	0	0	0	0	0	0	0	0
BGIR9823_86388	110261319	LOC110261319	lncRNA	0	0	0	0	0	0	0	0	0	0	0	0	1	0	0	0	0	0	0
BGIR9823_81938	100511317	SSC4D	lncRNA	0	0	0	0	0	0	0	0	0	0	0	0	0	0	0	0	0	0	0
BGIR9823_83868	102165219	ANKRD34A	mRNA	0	0	0	0	0	0	0	0	0	0	0	0	0	0	1	0	0	0	0
BGIR9823_85017	100155867	INTS13	lncRNA	0	0	0	0	0	0	0	0	0	0	0	0	1	0	0	0	0	0	0
BGIR9823_91638	100523324	ARHGEF12	lncRNA	0	0	0	0	0	1	0	0	0	0	0	0	0	0	0	0	0	0	0
BGIR9823_78342	100515640	DIS3L	lncRNA	0	0	0	0	0	0	0	0	0	0	0	0	0	0	0	0	0	0	0
BGIR9823_101177	100520413	PHF8	mRNA	0	0	0	0	0	0	0	0	0	0	0	0	0	0	0	0	0	0	1
BGIR9823_100116	100157552	MKKS	lncRNA	0	0	0	0	0	0	0	0	0	0	0	0	0	0	0	0	0	0	0
BGIR9823_82812	100514160	XYLT1	lncRNA	0	0	0	0	0	0	0	0	0	0	0	0	0	0	0	0	0	0	0
BGIR9823_100485	100621338	BMT2	lncRNA	0	0	1	0	0	0	0	0	0	0	0	0	0	0	0	0	0	0	0
BGIR9823_83185	100302367	SOCS5	lncRNA	0	1	0	0	0	0	0	0	0	0	0	0	0	0	0	0	0	0	0
BGIR9823_100135	100519631	TASP1	lncRNA	0	0	0	0	0	0	0	0	0	0	0	0	0	0	0	0	1	0	0
BGIR9823_78629	100155278	ERMP1	mRNA	0	0	0	0	0	0	0	0	0	0	0	0	0	0	0	0	0	0	0
BGIR9823_100610	100517250	NUB1	mRNA	0	0	0	0	0	0	0	0	0	0	0	0	0	0	0	0	0	0	1
BGIR9823_100720	100511294	PTPRZ1	lncRNA	0	0	0	0	0	0	0	0	0	0	0	0	0	0	0	0	0	0	0
BGIR9823_91236	100524699	TPK1	lncRNA	0	0	0	0	0	0	0	0	0	0	0	0	0	0	0	0	0	0	0
BGIR9823_79597	100152069	SMAD6	lncRNA	0	0	0	0	0	0	0	0	0	0	0	0	0	0	0	0	0	0	0
BGIR9823_100727	100512026	ING3	lncRNA	0	0	0	0	0	0	0	0	0	0	0	0	0	0	0	0	1	0	0
BGIR9823_89956	106504698	LOC106504698	lncRNA	0	0	0	0	0	0	0	0	0	0	0	0	0	0	0	0	0	0	0
BGIR9823_100230	100154394	ROMO1	lncRNA	0	0	0	0	0	0	0	0	0	0	0	0	0	0	0	0	0	0	0
BGIR9823_90067	100519324	ETNPPL	mRNA	0	0	0	0	0	0	0	0	0	0	0	0	0	0	0	0	0	0	0
BGIR9823_81199	100216478	TEAD1	lncRNA	0	0	0	0	0	0	0	0	0	0	0	0	0	0	0	0	0	0	0
BGIR9823_100115	100157552	MKKS	lncRNA	0	0	0	0	0	0	0	0	0	0	0	0	0	0	0	0	0	0	0
BGIR9823_97871	733640	PRKG1	lncRNA	0	0	0	0	0	0	0	0	0	0	0	0	0	0	0	0	0	0	0
BGIR9823_79442	106506757	INAFM2	lncRNA	0	0	0	0	0	0	0	0	0	0	0	0	0	0	0	1	0	0	0
BGIR9823_77844	100736796	ADGRB3	lncRNA	0	1	0	0	0	0	0	0	0	0	0	0	0	0	0	0	0	0	0
BGIR9823_81223	110259751	LOC110259751	lncRNA	0	0	0	0	0	1	0	0	0	0	0	0	0	0	0	0	0	0	0
BGIR9823_87714	100517038	PUM1	lncRNA	0	0	0	0	0	0	0	0	0	0	0	0	0	0	0	0	0	0	1
BGIR9823_82332	100517706	CEP68	lncRNA	0	0	0	0	0	0	0	0	0	0	0	0	0	0	0	0	0	0	0
BGIR9823_79033	100624679	NKAIN2	lncRNA	0	1	0	0	0	0	0	0	0	0	0	0	0	0	0	0	0	0	0
BGIR9823_81514	100523721	FAF2	lncRNA	0	0	0	0	0	0	0	0	0	0	0	0	0	0	0	0	0	0	0
BGIR9823_78765	100622501	PRPF4	mRNA	0	0	0	0	0	0	0	0	0	0	0	0	0	0	0	0	0	0	0
BGIR9823_78849	100624075	AIF1L	mRNA	0	0	0	0	0	0	0	0	0	0	0	0	0	0	0	0	0	0	0

RNA ID	Gene ID	Symbol	Type	novel-ssc-miR1063-5p	ssc-miR-192	ssc-miR-432-5p	ssc-miR-7138-5p	ssc-miR-874	ssc-miR-10386	ssc-miR-122-3p	novel-ssc-miR755-3p	novel-ssc-miR780-3p	novel-ssc-miR804-3p	novel-ssc-miR813-5p	novel-ssc-miR407-5p	novel-ssc-miR573-5p	novel-ssc-miR583-3p	novel-ssc-miR1077-5p	novel-ssc-miR122-3p	novel-ssc-miR154-5p	ssc-miR-127	ssc-miR-194b-5p
BGIR9823_100607	100515515	GALNT11	lncRNA	0	0	0	0	0	0	0	0	0	0	0	0	0	0	0	0	0	0	0
BGIR9823_83081	100627528	CCDC142	lncRNA	0	0	0	0	0	0	0	0	0	0	0	0	0	0	0	0	0	0	0
XR_002345524.1	102158855	LOC102158855	lncRNA	0	0	0	0	0	0	0	0	0	0	0	0	0	0	0	0	0	0	0
BGIR9823_78387	100154444	GLCE	lncRNA	0	0	0	0	0	0	0	0	0	0	0	0	0	0	0	0	0	0	0
BGIR9823_79739	110255453	LOC110255453	lncRNA	0	0	0	0	0	0	0	0	0	0	0	0	0	0	0	0	0	0	0
BGIR9823_100823	100519067	CDK13	mRNA	0	0	0	0	0	0	0	0	0	0	0	0	0	0	0	0	0	0	0
BGIR9823_80184	397169	BEST1	lncRNA	0	0	0	0	0	0	0	0	0	0	0	0	0	0	0	0	0	0	0
BGIR9823_85074	100144592	KLRF1	lncRNA	0	0	0	0	0	0	0	0	0	0	0	0	1	0	0	0	0	0	0
BGIR9823_80186	444995	FADS1	mRNA	0	0	0	0	0	0	0	0	0	0	0	0	0	0	1	0	0	0	0
BGIR9823_97606	100156356	PRR14L	lncRNA	0	0	0	0	0	0	0	0	0	0	0	0	0	1	0	0	0	0	0
BGIR9823_100902	100621199	IL1RAPL1	lncRNA	0	0	0	0	0	0	0	0	0	0	0	0	0	0	0	0	0	0	0
BGIR9823_78631	100155714	PLGRKT	lncRNA	0	0	0	0	0	0	0	0	0	0	0	0	0	0	0	1	0	0	0
BGIR9823_97867	780419	UBE2D1	mRNA	0	0	0	0	0	0	0	0	0	0	0	0	0	0	0	0	0	0	0
BGIR9823_80393	100512765	SMIM7	lncRNA	0	0	0	0	0	0	0	0	0	0	0	0	0	0	0	1	0	0	0
BGIR9823_83405	100157574	FAM49B	lncRNA	0	0	0	0	0	0	0	0	0	0	0	0	0	0	0	0	0	0	0
BGIR9823_82271	100625157	GCFC2	lncRNA	0	1	0	0	0	0	0	0	0	0	0	0	0	0	0	0	0	0	0
BGIR9823_92748	100154396	PDS5B	lncRNA	0	0	0	0	0	0	0	0	0	0	0	0	0	0	0	0	0	0	0
BGIR9823_81832	100737907	FAM20C	lncRNA	0	0	0	0	0	0	0	0	0	0	0	0	0	0	0	0	0	0	0
BGIR9823_86739	100623382	SPIRE1	mRNA	0	0	0	0	0	0	0	0	0	0	0	0	0	0	0	0	0	0	0
BGIR9823_88779	100515615	CCNB1IP1	lncRNA	0	0	0	0	0	0	0	0	0	0	0	0	1	0	0	0	0	0	0
BGIR9823_82125	100533196	UBE2I	lncRNA	0	0	0	0	0	0	0	0	0	0	0	0	0	0	0	0	0	0	0
BGIR9823_96691	100153895	MORC2	lncRNA	0	0	0	0	0	0	1	0	0	0	0	0	0	0	0	0	0	0	0
BGIR9823_82328	100518067	SPRED2	lncRNA	0	0	0	0	0	0	0	0	0	0	0	0	0	0	0	0	0	0	0
BGIR9823_87494	100625971	LOC100625971	mRNA	0	0	0	0	0	0	0	0	0	0	0	0	0	0	0	0	0	0	0
BGIR9823_79014	100738102	RNF217	lncRNA	0	0	0	0	0	0	0	0	0	0	0	0	0	0	0	0	0	0	0
BGIR9823_79431	100153351	VPS18	mRNA	0	0	0	0	0	0	0	0	0	0	0	0	0	0	0	0	0	0	0
BGIR9823_79792	100154060	RIC1	mRNA	0	0	0	0	0	0	0	0	0	0	0	0	0	0	0	0	0	0	0
BGIR9823_97488	100153708	ATXN2	mRNA	0	0	0	0	0	0	0	0	0	0	0	0	0	0	0	0	0	0	0
BGIR9823_100701	100514931	AHCYL2	lncRNA	0	0	0	1	0	0	0	0	0	0	0	0	0	0	0	0	0	0	0
BGIR9823_83545	100153257	MMP16	lncRNA	0	0	0	0	0	0	0	0	0	0	0	0	0	0	0	0	0	0	0
BGIR9823_82543	100521786	TP53I3	mRNA	0	0	0	0	0	0	0	0	0	0	0	0	0	0	0	0	0	0	0
BGIR9823_81390	100520177	TRIP10	lncRNA	0	0	0	0	0	0	0	0	0	0	0	0	1	0	0	0	0	0	0
BGIR9823_100128	100519631	TASP1	lncRNA	0	0	0	0	0	0	0	0	0	0	0	0	0	0	0	0	0	0	0
BGIR9823_94226	100337675	ADAP2	lncRNA	0	0	0	0	0	0	0	0	0	0	0	0	0	0	0	0	0	0	0
BGIR9823_88221	110261778	NOL7	mRNA	0	0	0	0	0	0	0	0	0	0	0	0	0	0	0	0	0	0	0
BGIR9823_100908	100621199	IL1RAPL1	lncRNA	0	0	0	0	0	0	0	0	0	0	0	0	0	0	0	0	0	0	0
BGIR9823_101335	100523617	TRIM27	lncRNA	0	0	0	0	0	0	0	0	0	0	0	0	0	0	0	0	0	0	0
BGIR9823_101518	106504125	LOC106504125	lncRNA	0	0	0	1	0	0	0	0	0	0	0	0	0	0	0	0	0	0	0
BGIR9823_80840	100624262	SMAD5	mRNA	0	0	0	0	0	0	0	0	0	0	0	0	0	0	0	0	0	0	0
BGIR9823_99908	449527	PHF20	lncRNA	0	0	0	0	0	0	0	0	0	0	0	0	0	1	0	0	0	0	0
BGIR9823_99647	100521758	CPEB4	mRNA	0	0	0	0	0	0	0	0	0	0	0	0	0	0	0	0	0	0	0
BGIR9823_97815	106506044	LOC106506044	lncRNA	0	0	0	0	0	0	0	0	0	0	0	0	0	0	0	0	0	0	0
BGIR9823_77854	100736796	ADGRB3	lncRNA	0	0	0	0	0	0	0	0	0	0	0	0	0	0	0	0	0	0	0

RNA ID	Gene ID	Symbol	Type	novel-ssc-miR1063-5p	ssc-miR-192	ssc-miR-432-5p	ssc-miR-7138-5p	ssc-miR-874	ssc-miR-10386	ssc-miR-122-3p	novel-ssc-miR755-3p	novel-ssc-miR780-3p	novel-ssc-miR804-3p	novel-ssc-miR813-5p	novel-ssc-miR407-5p	novel-ssc-miR573-5p	novel-ssc-miR583-3p	novel-ssc-miR1077-5p	novel-ssc-miR122-3p	novel-ssc-miR154-5p	ssc-miR-127	ssc-miR-194b-5p
BGIR9823_92288	110255709	LOC110255709	lncRNA	0	0	0	0	0	0	0	0	0	0	0	0	0	0	0	0	0	0	0
BGIR9823_79245	100738627	DYM	lncRNA	0	0	0	0	0	0	0	0	0	0	0	0	0	1	0	0	0	0	0
BGIR9823_83586	100152963	PEX2	lncRNA	0	1	0	0	0	0	0	0	0	0	0	0	0	0	0	0	0	0	0
BGIR9823_101489	110258382	LOC110258382	lncRNA	0	0	0	0	0	0	0	0	0	0	0	0	0	0	0	0	0	0	0
BGIR9823_100872	100739216	MAP3K15	lncRNA	0	0	0	0	0	0	0	0	0	0	0	0	0	0	0	0	0	0	0
XM_005673239.3	100620722	ZNF277	mRNA	0	0	0	0	0	0	0	0	0	0	0	0	0	0	0	0	0	0	0
BGIR9823_100554	100739791	LOC100739791	lncRNA	0	0	0	0	0	0	0	0	0	0	0	0	0	0	0	0	0	0	0
BGIR9823_83272	100521316	GPN1	lncRNA	0	0	0	0	0	0	0	0	0	0	0	0	0	0	0	0	0	0	0
BGIR9823_77910	110260430	LOC110260430	lncRNA	0	0	0	0	0	0	0	0	0	0	0	0	0	0	0	0	0	0	0
BGIR9823_100153	100152012	CST7	lncRNA	0	0	0	0	0	0	0	0	0	0	0	0	0	0	0	0	1	0	0
BGIR9823_79200	100156851	IRAK1BP1	lncRNA	0	0	0	0	0	0	0	0	0	0	0	0	0	0	0	0	0	0	0
BGIR9823_100475	100517204	TFEC	lncRNA	0	0	0	0	0	0	0	0	0	0	0	0	0	0	0	0	0	0	0
BGIR9823_83002	110259971	C3H2orf49	lncRNA	0	1	0	0	0	0	0	0	0	0	0	0	0	0	0	0	0	0	0
BGIR9823_78732	100152140	SLC44A1	lncRNA	0	0	0	0	0	0	0	0	0	0	0	0	0	0	0	0	0	0	0
BGIR9823_94529	397618	OXSR1	lncRNA	0	0	0	0	0	0	0	0	0	0	0	0	0	0	0	0	0	0	0
BGIR9823_92973	100623901	MICU2	mRNA	0	0	0	0	0	0	0	0	0	0	0	0	0	0	0	0	0	0	0
BGIR9823_80241	100513197	DDB2	lncRNA	0	0	0	0	0	0	0	0	0	0	0	0	0	0	0	0	0	0	0
BGIR9823_96832	100153026	ABCB10	lncRNA	0	0	0	0	0	0	0	0	0	0	0	0	0	0	0	0	0	0	0
BGIR9823_96408	100153797	SECISBP2	mRNA	0	0	0	0	0	0	0	0	0	0	0	0	0	1	0	0	0	0	0
BGIR9823_80734	100526040	PAM	lncRNA	0	0	0	0	0	0	0	0	0	0	0	0	0	0	0	0	0	0	0
BGIR9823_81957	100513345	GTF2I	lncRNA	0	0	0	0	0	0	0	0	0	0	0	0	0	0	0	0	0	0	0
BGIR9823_96796	110256820	LOC110256820	lncRNA	0	0	0	0	0	0	0	0	0	0	0	0	0	0	0	0	0	0	0
BGIR9823_101115	110255263	CXH4orf3	lncRNA	0	0	0	0	1	0	0	0	0	0	0	0	0	0	0	0	0	0	0
BGIR9823_78939	100621167	PLEKHG1	lncRNA	0	0	0	0	0	0	0	0	0	0	0	0	0	0	0	0	0	0	0
BGIR9823_90840	100517358	PIWIL4	lncRNA	0	0	0	0	0	0	0	0	0	0	0	0	0	0	0	0	0	0	0
BGIR9823_94380	396903	MYH10	mRNA	0	0	0	0	0	0	0	0	0	0	0	0	0	0	0	0	0	0	0
BGIR9823_94364	100621720	TNK1	lncRNA	0	0	0	0	0	0	0	0	0	0	0	0	0	0	0	0	0	0	0
BGIR9823_99224	110257075	LOC110257075	lncRNA	0	0	0	0	0	0	0	0	0	0	0	0	0	0	0	0	0	0	0
BGIR9823_88977	102159128	LOC102159128	lncRNA	0	0	0	0	0	0	0	0	0	0	0	0	0	0	0	0	0	0	0
BGIR9823_86907	100623628	PRKACB	lncRNA	0	0	0	0	0	0	0	0	0	0	0	0	0	0	0	0	0	0	0
BGIR9823_83922	100127159	SLC16A1	lncRNA	0	0	0	0	0	0	0	0	0	0	0	0	0	0	0	0	0	0	0
BGIR9823_80165	106504153	AHNAK	mRNA	0	0	0	0	0	0	0	0	0	0	0	0	0	0	1	0	0	0	0
XM_021099336.1	102160410	LOC102160410	mRNA	0	0	0	0	0	0	0	0	0	0	0	0	0	0	0	0	0	0	0
BGIR9823_78034	100157196	CSNK1G1	lncRNA	0	0	0	0	0	0	0	0	0	0	0	0	0	0	0	0	0	0	0
BGIR9823_80409	100517807	SYDE1	lncRNA	0	0	0	0	0	0	0	0	0	0	0	0	0	0	0	0	0	0	0
BGIR9823_82453	100622259	MAP4K3	lncRNA	0	1	0	0	0	0	0	0	0	0	0	0	0	0	0	0	0	0	0
BGIR9823_101610	100521600	LOC100521600	lncRNA	0	0	0	0	0	0	0	0	0	0	0	0	0	0	0	1	0	0	0
BGIR9823_86055	102165609	LOC102165609	lncRNA	0	0	0	0	0	0	0	0	0	0	0	0	0	0	0	0	0	0	0
BGIR9823_92014	396922	CD46	mRNA	0	0	0	0	0	0	0	0	0	0	0	0	0	1	0	0	0	0	0
BGIR9823_100942	100521238	CDK16	mRNA	0	0	0	0	0	0	0	0	0	0	0	0	0	0	0	0	0	0	0
BGIR9823_77933	100513770	SLC16A10	lncRNA	0	0	0	0	0	0	0	0	0	0	0	0	0	0	0	1	0	0	0
BGIR9823_89476	100155589	FAM177A1	lncRNA	0	0	0	0	0	0	0	0	0	0	0	0	0	0	0	0	0	0	0
BGIR9823_101139	100525794	KLHL15	lncRNA	0	0	0	0	0	0	0	0	0	0	0	0	0	0	0	0	0	0	0

RNA ID	Gene ID	Symbol	Type	novel-ssc-miR1063-5p	ssc-miR-192	ssc-miR-432-5p	ssc-miR-7138-5p	ssc-miR-874	ssc-miR-10386	ssc-miR-122-3p	novel-ssc-miR755-3p	novel-ssc-miR780-3p	novel-ssc-miR804-3p	novel-ssc-miR813-5p	novel-ssc-miR407-5p	novel-ssc-miR573-5p	novel-ssc-miR583-3p	novel-ssc-miR1077-5p	novel-ssc-miR122-3p	novel-ssc-miR154-5p	ssc-miR-127	ssc-miR-194b-5p
BGIR9823_92285	100512363	CREM	lncRNA	0	0	0	0	0	0	0	0	0	0	0	0	0	0	0	0	0	0	0
BGIR9823_100684	414428	MKLN1	lncRNA	0	0	0	0	0	0	0	0	0	0	0	0	0	0	0	0	0	0	0
BGIR9823_80117	100511000	MUS81	lncRNA	0	0	0	0	0	0	0	0	0	0	0	0	0	0	0	1	0	0	0
BGIR9823_79783	102168004	UHRF2	lncRNA	0	0	0	0	0	0	0	0	0	0	0	0	1	0	0	0	0	0	0
BGIR9823_87527	110261323	LOC110261323	lncRNA	0	0	0	0	0	0	0	0	0	0	0	0	0	0	0	0	0	0	0
BGIR9823_77815	100736796	ADGRB3	lncRNA	0	0	0	0	0	0	0	0	0	0	0	1	0	0	0	0	0	0	0
BGIR9823_100466	100512202	TSPAN12	mRNA	0	0	0	0	0	0	0	0	0	0	0	0	0	0	0	0	0	0	0
BGIR9823_81771	100521956	CYSTM1	lncRNA	0	0	0	0	0	0	0	0	0	0	0	0	0	0	0	0	0	0	0
BGIR9823_93887	100625999	PGS1	mRNA	0	0	0	0	0	0	1	0	0	0	0	0	0	0	0	0	0	0	0
BGIR9823_99449	100312962	ATOX1	lncRNA	0	0	0	0	0	0	0	0	0	0	0	0	0	0	0	0	0	0	0
BGIR9823_82424	100526165	PREPL	lncRNA	0	1	0	0	0	0	0	0	0	0	0	0	0	0	0	0	0	0	0
BGIR9823_78323	100518050	TMX3	lncRNA	0	0	0	0	0	0	0	0	0	0	0	0	0	0	0	0	0	0	0
BGIR9823_81279	110259619	LOC110259619	lncRNA	0	0	0	0	0	0	0	0	0	0	0	0	0	0	0	0	0	0	0
BGIR9823_89593	100514718	SYNJ2BP	mRNA	0	0	0	0	0	0	0	0	0	0	0	0	0	0	0	0	0	0	0
BGIR9823_100641	100519065	UBN2	lncRNA	0	0	0	0	0	0	0	0	0	0	0	0	1	0	0	0	0	0	0
BGIR9823_99284	100518875	EGFLAM	mRNA	0	0	0	0	0	0	0	1	0	0	0	0	0	0	0	0	0	0	0
BGIR9823_97891	100037303	SGMS1	lncRNA	0	0	0	0	0	0	0	0	0	0	0	0	0	0	0	0	0	0	0
BGIR9823_80246	100516314	HARBI1	lncRNA	0	0	0	0	0	0	0	0	0	0	0	0	0	0	0	0	0	0	0
BGIR9823_84831	100154762	ZNF740	lncRNA	0	0	0	0	0	0	0	0	0	0	0	0	0	0	0	0	0	0	0
BGIR9823_100404	100524054	WDR91	lncRNA	0	0	0	0	0	0	0	0	0	0	0	0	0	0	0	0	0	1	0
BGIR9823_89087	100157771	CARMIL1	lncRNA	0	0	0	0	0	0	0	0	0	0	0	0	0	0	0	0	0	0	0
BGIR9823_94449	100737057	BTD	lncRNA	0	0	0	0	0	0	0	0	0	0	0	0	0	0	0	0	0	0	0
BGIR9823_89030	100152689	SSR1	mRNA	0	0	0	0	0	0	0	0	0	0	0	0	0	0	0	0	0	0	0
BGIR9823_100313	100155190	ZFP64	lncRNA	0	0	0	0	0	0	0	0	0	0	0	0	0	0	0	0	0	0	0
BGIR9823_84915	100623414	SRGAP1	lncRNA	0	1	0	0	0	0	0	0	0	0	0	0	0	0	0	0	0	0	0
BGIR9823_100318	100126237	CTCFL	lncRNA	0	0	0	0	0	0	0	0	0	0	0	0	0	0	0	0	0	0	0
BGIR9823_88856	100155733	SUSD6	lncRNA	0	0	0	0	0	0	0	0	0	0	0	0	0	0	0	0	0	0	0
XM_003480892.4	100519890	TRIM36	mRNA	0	0	0	0	0	0	0	0	0	0	0	0	0	0	0	0	0	0	0
BGIR9823_82684	100620890	SRRT	lncRNA	0	0	0	0	0	0	0	0	0	0	0	0	1	0	0	0	0	0	0
BGIR9823_83320	100513350	ASAP2	mRNA	0	0	0	0	0	0	0	0	0	0	0	0	0	0	1	0	0	0	0
BGIR9823_101170	100739016	RIBC1	lncRNA	0	0	0	0	0	0	0	0	0	0	0	0	0	0	0	0	0	0	0
BGIR9823_87081	100521097	ERI3	lncRNA	0	0	0	0	0	0	0	0	0	0	0	0	0	0	0	0	0	0	0
BGIR9823_88961	100152076	PTPN21	lncRNA	0	0	0	0	0	0	0	0	0	0	0	0	0	0	0	0	0	0	0
BGIR9823_89703	100515556	SLC25A47	lncRNA	0	0	0	0	0	0	0	0	0	0	0	0	0	0	0	0	0	0	0
BGIR9823_78067	100625505	VPS13C	lncRNA	0	1	0	0	0	0	0	0	0	0	0	0	0	0	0	0	0	0	0
BGIR9823_78412	100156782	LRFN5	lncRNA	0	0	0	0	0	0	0	0	0	0	0	0	0	0	0	0	0	0	0
BGIR9823_94130	106505457	TRIM37	mRNA	0	0	0	0	0	0	0	0	0	0	0	0	0	0	0	0	0	0	0
BGIR9823_78647	100152831	PUM3	lncRNA	0	0	0	0	0	0	0	0	0	0	0	1	0	0	0	0	0	0	0
BGIR9823_101061	102158351	LOC102158351	lncRNA	0	0	0	0	0	0	0	0	0	0	0	0	0	0	0	0	0	0	0
BGIR9823_101008	100523039	GPRASP1	mRNA	0	0	0	0	0	0	0	0	0	0	0	0	0	0	0	0	0	0	0

Supplementary Table S9. The predicted targets of DE miRN

RNA ID	Gene ID	Symbol	Type	ssc-miR-24-1-5p	ssc-miR-29b	ssc-miR-29c	ssc-miR-323	ssc-miR-369	ssc-miR-708-5p	novel-ssc-miR161-3p	novel-ssc-miR512-5p	novel-ssc-miR846-3p	novel-ssc-miR1086-5p	novel-ssc-miR1105-5p	novel-ssc-miR1116-5p	novel-ssc-miR1120-5p	novel-ssc-miR1140-5p	novel-ssc-miR1158-3p	novel-ssc-miR1379-5p
BGIR9823_100323	100049657	GNAS	lncRNA	0	0	0	0	0	0	0	1	0	0	0	0	0	0	0	0
BGIR9823_100321	100144523	PMEPA1	lncRNA	0	0	0	0	0	0	0	0	0	0	0	0	0	0	0	0
BGIR9823_100436	110257581	LOC110257581	lncRNA	0	0	0	0	0	0	0	1	0	0	0	0	0	0	0	0
BGIR9823_101399	110258215	LOC110258215	lncRNA	0	0	0	0	0	0	0	1	0	0	0	0	0	0	0	0
BGIR9823_100330	100037996	RPS21	lncRNA	0	0	0	0	0	0	0	0	0	0	0	0	0	0	0	0
BGIR9823_101457	110258362	MTA1	lncRNA	0	0	0	0	0	0	0	1	1	0	0	0	0	0	0	0
BGIR9823_101544	110258600	LOC110258600	lncRNA	0	0	0	0	0	0	0	1	0	0	0	0	0	0	0	0
BGIR9823_101531	102158401	LOC102158401	lncRNA	0	0	0	0	0	0	0	1	0	0	0	0	0	0	0	0
BGIR9823_101342	110258067	ARRDC1	lncRNA	0	0	0	0	0	0	0	0	0	0	0	0	0	0	0	0
BGIR9823_101405	110258214	LOC110258214	lncRNA	0	0	0	0	0	0	0	0	0	0	0	0	0	0	0	0
BGIR9823_101472	110258334	LOC110258334	lncRNA	0	0	0	0	0	0	0	0	0	0	0	1	0	0	0	0
BGIR9823_100712	100520873	IMPDH1	lncRNA	0	0	0	0	0	0	0	0	0	0	0	1	0	0	0	0
BGIR9823_101185	110257830	LOC110257830	lncRNA	0	0	0	0	0	0	0	0	0	0	0	0	0	0	0	0
BGIR9823_101542	102157946	LOC102157946	lncRNA	0	0	0	0	0	0	0	1	0	0	0	0	0	0	0	0
BGIR9823_100049	110257373	GMEB2	lncRNA	0	0	0	0	0	0	0	0	1	0	0	0	0	0	0	0
BGIR9823_100378	100514083	ADCK2	lncRNA	0	0	0	0	0	0	0	0	0	0	0	0	1	0	0	0
BGIR9823_100540	100523342	OSBPL3	lncRNA	0	0	0	0	0	0	0	0	0	0	0	0	0	0	0	0
BGIR9823_101051	110257712	LOC110257712	lncRNA	0	0	0	0	0	0	0	1	0	0	0	0	0	0	0	0
BGIR9823_101904	BGIG9823_26581		lncRNA	0	0	0	0	0	0	0	0	1	0	0	0	0	0	0	0
BGIR9823_100561	100516888	GLI3	lncRNA	0	0	0	0	0	0	0	0	1	0	0	0	0	0	0	0
BGIR9823_100665	100622227	LOC100622227	lncRNA	0	0	0	0	0	0	0	0	0	0	0	1	1	0	0	0
BGIR9823_100348	110257384	TCEA2	lncRNA	0	0	0	0	0	0	0	0	0	0	0	0	0	0	0	0
BGIR9823_100834	110257605	LRRC61	lncRNA	0	0	0	0	0	0	0	0	0	0	0	0	0	0	0	0
BGIR9823_100037	110257479	MTG2	lncRNA	0	0	0	0	0	0	0	1	0	1	0	0	0	0	0	0
BGIR9823_100184	106506644	LOC106506644	lncRNA	0	0	0	0	0	0	0	0	0	0	0	0	1	0	0	0
BGIR9823_101820	BGIG9823_26500		lncRNA	0	0	0	0	0	0	0	1	0	0	0	0	0	0	0	0
BGIR9823_100659	100522048	SLC13A4	lncRNA	0	0	0	0	0	0	0	0	0	0	0	0	0	0	0	0
BGIR9823_100223	100627204	MMP24	lncRNA	0	0	0	0	0	0	0	0	0	0	0	0	0	0	0	0
BGIR9823_101137	100152260	PRDX4	lncRNA	0	0	0	0	0	0	0	1	1	0	0	0	0	1	0	0
BGIR9823_100172	100154685	UBOX5	lncRNA	0	0	0	0	0	0	0	0	0	0	0	0	0	0	0	0
BGIR9823_101650	100738659	MCM3AP	lncRNA	0	0	0	0	0	0	0	1	0	0	0	0	0	0	0	0
BGIR9823_101940	BGIG9823_26617		lncRNA	0	0	0	0	0	0	0	0	0	0	0	0	0	0	0	1
BGIR9823_101941	BGIG9823_26618		lncRNA	0	0	0	0	0	0	0	1	1	0	0	0	0	0	0	1
BGIR9823_102278	BGIG9823_26945		lncRNA	0	0	0	0	0	1	0	1	0	0	0	0	0	0	0	0
BGIR9823_101611	100521600	LOC100521600	lncRNA	0	0	0	0	0	0	0	0	0	0	0	0	0	0	0	0
BGIR9823_100544	100627860	SUN3	lncRNA	0	0	0	0	0	0	0	0	0	0	0	0	0	0	0	0
BGIR9823_100360	100514805	KMT2C	lncRNA	0	0	0	0	0	1	0	0	0	0	0	0	1	0	0	0
BGIR9823_100799	397163	RAMP3	lncRNA	0	0	0	0	0	0	0	1	0	0	0	0	0	0	0	0
BGIR9823_101296	100623680	ZFP92	lncRNA	0	0	0	0	0	0	0	0	0	0	0	0	0	0	0	0
BGIR9823_100164	100520054	PANK2	lncRNA	0	0	0	0	0	0	0	0	1	0	0	0	0	0	0	0
BGIR9823_100576	102157930	LOC102157930	lncRNA	0	0	0	0	0	0	0	0	0	0	0	1	0	0	0	0

RNA ID	Gene ID	Symbol	Type	ssc-miR-24-1-5p	ssc-miR-29b	ssc-miR-29c	ssc-miR-323	ssc-miR-369	ssc-miR-708-5p	novel-ssc-miR161-3p	novel-ssc-miR512-5p	novel-ssc-miR846-3p	novel-ssc-miR1086-5p	novel-ssc-miR1105-5p	novel-ssc-miR1116-5p	novel-ssc-miR1120-5p	novel-ssc-miR1140-5p	novel-ssc-miR1158-3p	novel-ssc-miR1379-5p
BGIR9823_100585	100623772	ZNF862	lncRNA	1	0	0	0	0	0	0	1	0	0	0	0	0	0	0	0
BGIR9823_101341	110258060	LOC110258060	lncRNA	0	0	0	0	0	0	0	0	0	0	0	0	0	0	0	0
BGIR9823_101500	102160467	SMAGP	lncRNA	0	0	0	0	0	0	0	1	1	0	0	0	0	0	0	0
BGIR9823_101475	100521669	BRF1	lncRNA	0	0	0	0	0	0	0	1	0	0	0	0	0	0	0	0
BGIR9823_100400	100523151	NUP205	lncRNA	0	0	0	0	0	1	0	0	1	0	0	0	0	0	0	0
BGIR9823_101360	100512700	LOC100512700	lncRNA	0	0	0	0	0	0	0	0	1	0	0	0	1	0	0	0
BGIR9823_101677	BGIG9823_26364		lncRNA	0	0	0	0	0	0	0	0	1	0	0	0	0	0	0	0
BGIR9823_101879	BGIG9823_26558		lncRNA	0	0	0	1	0	0	0	0	0	0	0	0	0	0	0	0
BGIR9823_102010	BGIG9823_26686		lncRNA	0	0	0	0	0	0	0	1	1	0	0	0	0	0	0	0
BGIR9823_101968	BGIG9823_26645		lncRNA	0	0	0	0	0	0	0	0	0	0	0	1	0	0	0	0
BGIR9823_102338	BGIG9823_27003		lncRNA	0	0	0	0	0	0	0	0	0	0	0	1	0	0	0	0
BGIR9823_100338	BGIG9823_26339		lncRNA	0	0	0	0	0	0	0	0	0	0	0	0	0	0	0	0
BGIR9823_100753	493185	IFRD1	lncRNA	0	0	0	0	0	0	0	1	0	0	0	0	0	0	0	1
BGIR9823_100807	100515808	TMED4	lncRNA	0	0	0	0	0	0	0	0	0	0	0	1	1	0	0	0
BGIR9823_100577	100622254	RALA	lncRNA	0	0	0	0	0	0	0	1	0	0	0	0	0	0	0	0
BGIR9823_101471	100126861	AKT1	lncRNA	0	0	0	0	0	0	0	0	1	0	0	0	0	0	0	0
BGIR9823_100694	100737195	SMKR1	lncRNA	0	0	0	0	0	0	0	0	1	0	0	0	0	0	0	1
BGIR9823_101043	445538	XPNPEP2	lncRNA	0	0	0	0	0	0	0	0	0	0	0	0	1	0	0	0
BGIR9823_101060	102158351	LOC102158351	lncRNA	0	0	0	0	0	0	0	0	0	0	0	0	0	0	0	0
BGIR9823_100405	106506765	LOC106506765	lncRNA	0	0	0	0	0	0	0	0	1	1	0	0	0	0	0	0
BGIR9823_101548	110258600	LOC110258600	lncRNA	0	0	0	0	0	0	0	0	0	0	0	0	0	0	0	0
BGIR9823_100632	100515805	PARP12	lncRNA	0	0	0	0	0	0	0	0	0	0	0	0	0	0	0	0
BGIR9823_100345	100621535	TPD52L2	lncRNA	0	0	0	0	0	0	0	0	0	0	0	0	0	0	0	0
BGIR9823_100658	106506763	LOC106506763	lncRNA	0	0	0	0	0	0	0	0	1	0	0	0	0	0	0	0
BGIR9823_100521	100512260	GARS	lncRNA	0	0	0	0	0	0	0	0	0	0	0	0	0	0	0	0
BGIR9823_100195	106505983	LOC106505983	lncRNA	0	0	0	0	0	0	0	0	0	0	0	0	0	0	0	0
BGIR9823_101416	BGIG9823_26349		lncRNA	0	0	0	0	0	0	0	0	0	0	0	0	0	0	0	0
BGIR9823_102298	BGIG9823_26964		lncRNA	0	0	0	0	0	0	0	0	0	0	0	0	0	0	0	0
BGIR9823_101877	BGIG9823_26556		lncRNA	0	0	0	0	0	0	0	0	0	0	0	0	0	0	0	0
BGIR9823_102235	BGIG9823_26907		lncRNA	1	0	0	0	0	0	0	0	0	0	0	0	0	0	0	0
BGIR9823_102014	BGIG9823_26690		lncRNA	0	0	0	0	0	0	0	1	0	0	0	1	0	0	0	0
BGIR9823_102030	BGIG9823_26705		lncRNA	0	0	0	0	0	1	0	0	0	0	0	0	0	0	0	0
BGIR9823_101900	BGIG9823_26577		lncRNA	0	0	0	0	0	0	0	0	1	0	0	0	0	0	0	0
BGIR9823_101920	BGIG9823_26597		lncRNA	0	0	0	0	0	1	0	0	0	0	0	0	1	0	0	0
BGIR9823_101972	BGIG9823_26649		lncRNA	0	0	0	0	0	1	0	0	0	0	0	0	0	0	0	0
BGIR9823_101815	BGIG9823_26495		lncRNA	0	0	0	0	0	0	0	0	0	0	0	0	0	0	0	0
BGIR9823_101971	BGIG9823_26648		lncRNA	0	0	0	0	0	0	0	1	0	0	0	0	0	0	0	0
BGIR9823_101828	BGIG9823_26507		lncRNA	0	0	0	0	0	0	0	1	0	0	0	0	1	0	0	0
BGIR9823_102358	BGIG9823_27023		lncRNA	0	0	0	0	0	0	0	0	0	0	0	0	0	0	0	1
BGIR9823_101692	BGIG9823_26379		lncRNA	0	0	0	0	0	0	0	0	0	0	0	0	0	0	0	0
BGIR9823_101728	BGIG9823_26410		lncRNA	0	0	0	0	0	0	0	0	0	0	0	0	0	0	0	0
BGIR9823_101871	BGIG9823_26550		lncRNA	0	0	0	0	0	0	0	0	0	0	0	0	0	0	0	0
BGIR9823_101827	BGIG9823_26506		lncRNA	0	0	0	0	0	0	0	0	0	0	0	1	0	0	0	0

RNA ID	Gene ID	Symbol	Type	ssc-miR-24-1-5p	ssc-miR-29b	ssc-miR-29c	ssc-miR-323	ssc-miR-369	ssc-miR-708-5p	novel-ssc-miR161-3p	novel-ssc-miR512-5p	novel-ssc-miR846-3p	novel-ssc-miR1086-5p	novel-ssc-miR1105-5p	novel-ssc-miR1116-5p	novel-ssc-miR1120-5p	novel-ssc-miR1140-5p	novel-ssc-miR1158-3p	novel-ssc-miR1379-5p
BGIR9823_102129	BGIG9823_26802		lncRNA	0	0	0	0	0	0	0	1	0	0	0	0	0	0	0	0
BGIR9823_102690	BGIG9823_27340		lncRNA	0	0	0	0	0	0	0	0	0	0	0	1	0	0	0	0
BGIR9823_101336	100519166	LOC100519166	lncRNA	0	0	0	0	0	0	0	0	0	0	0	0	0	0	0	0
BGIR9823_101541	102157946	LOC102157946	lncRNA	0	0	0	0	0	0	0	0	0	0	0	0	0	0	0	0
BGIR9823_100268	102159476	LOC102159476	lncRNA	0	0	0	0	0	0	0	0	0	0	0	1	0	0	0	0
BGIR9823_101104	100521877	PUDP	lncRNA	0	0	0	0	0	0	0	0	0	0	0	0	1	0	0	0
BGIR9823_101381	100511434	EGFL7	lncRNA	1	0	0	0	0	0	0	1	0	0	0	1	0	0	0	0
BGIR9823_101015	100520165	TMEM164	lncRNA	0	0	0	0	0	0	0	0	0	0	0	0	0	0	0	0
BGIR9823_100709	100519523	CCDC136	lncRNA	0	0	0	0	0	0	0	0	1	0	0	0	0	0	0	0
BGIR9823_101430	100156979	TRAF3	lncRNA	0	0	0	0	0	0	0	0	0	0	0	0	0	0	0	0
BGIR9823_101450	100623151	INF2	lncRNA	0	0	0	0	0	0	0	0	0	0	0	0	0	0	0	0
BGIR9823_100277	100156830	PTI	lncRNA	0	0	0	0	0	0	0	0	0	0	0	0	0	0	0	0
BGIR9823_101283	100359355	MOSPD1	lncRNA	0	0	0	0	0	0	0	0	1	0	0	0	0	0	0	0
BGIR9823_101340	106505329	LOC106505329	lncRNA	0	0	0	0	0	0	0	0	0	0	0	0	0	0	0	0
BGIR9823_100800	100511907	CCM2	lncRNA	0	0	0	0	0	0	0	1	0	0	0	0	0	0	0	0
BGIR9823_100099	100517562	HGSNAT	lncRNA	0	0	0	0	0	1	0	0	0	0	0	0	0	0	0	0
BGIR9823_100810	100515113	POLM	lncRNA	0	0	0	0	0	0	0	0	0	0	0	0	0	0	0	0
BGIR9823_100578	100622573	POU6F2	lncRNA	0	1	1	0	0	0	0	0	0	0	0	0	0	0	0	0
BGIR9823_78024	100157196	CSNK1G1	lncRNA	0	1	1	0	0	0	0	0	0	0	0	0	0	0	0	1
BGIR9823_100367	100521999	SMARCD3	lncRNA	0	0	0	0	0	0	0	0	0	0	0	0	1	0	0	0
BGIR9823_100389	100627515	HIPK2	lncRNA	0	0	0	0	0	0	0	0	0	0	0	0	0	0	0	0
BGIR9823_100256	100156548	ZHX3	lncRNA	0	0	0	0	0	0	0	0	1	0	0	0	0	0	0	0
BGIR9823_100882	102165056	MBTPS2	lncRNA	0	0	0	0	0	0	0	0	0	0	0	0	0	0	0	1
BGIR9823_100324	100049657	GNAS	lncRNA	0	0	0	0	0	0	1	0	0	0	0	0	0	0	0	0
BGIR9823_100656	100521167	DGKI	lncRNA	1	0	0	0	0	0	0	0	0	0	0	0	0	0	0	0
BGIR9823_101689	BGIG9823_26376		lncRNA	1	0	0	0	0	0	0	0	0	0	0	0	0	0	0	0
BGIR9823_102087	BGIG9823_26761		lncRNA	0	0	0	0	0	0	0	1	0	0	0	0	0	0	0	0
BGIR9823_101951	BGIG9823_26628		lncRNA	0	0	0	0	0	0	0	0	0	0	0	0	0	0	0	0
BGIR9823_101854	BGIG9823_26533		lncRNA	0	0	0	0	0	0	0	0	1	0	0	0	0	0	0	0
BGIR9823_102383	BGIG9823_27048		lncRNA	0	0	0	0	0	0	0	0	0	0	0	1	0	0	0	0
BGIR9823_102727	BGIG9823_27373		lncRNA	0	0	0	0	0	0	0	0	0	0	0	1	0	0	0	0
BGIR9823_102028	BGIG9823_26703		lncRNA	0	0	0	0	0	0	0	0	1	0	0	0	0	0	0	0
BGIR9823_101977	BGIG9823_26653		lncRNA	0	0	0	0	0	1	0	0	0	0	0	0	0	0	0	0
BGIR9823_102406	BGIG9823_27067		lncRNA	1	0	0	0	0	1	0	0	0	0	0	1	0	0	0	0
BGIR9823_101868	BGIG9823_26547		lncRNA	0	0	0	0	0	0	0	0	1	0	0	0	0	0	0	0
BGIR9823_101698	BGIG9823_26385		lncRNA	0	0	0	0	0	0	0	0	0	0	0	0	0	0	0	0
BGIR9823_102438	BGIG9823_27094		lncRNA	0	0	0	0	0	0	0	0	0	0	0	0	0	0	0	1
BGIR9823_102065	BGIG9823_26739		lncRNA	0	0	0	0	0	0	0	0	0	0	0	0	0	0	0	0
BGIR9823_102288	BGIG9823_26954		lncRNA	0	0	0	0	0	0	0	1	0	0	0	0	0	0	0	0
BGIR9823_101990	BGIG9823_26666		lncRNA	0	0	0	0	0	0	0	0	1	0	0	0	0	0	0	0
BGIR9823_101966	BGIG9823_26643		lncRNA	0	0	0	0	0	1	0	0	0	0	0	0	1	0	0	0
BGIR9823_101758	BGIG9823_26438		lncRNA	0	0	0	0	0	0	0	0	0	0	0	0	0	0	0	0
BGIR9823_101861	BGIG9823_26540		lncRNA	0	0	0	0	0	0	0	0	1	0	0	0	0	0	0	0

RNA ID	Gene ID	Symbol	Type	ssc-miR-24-1-5p	ssc-miR-29b	ssc-miR-29c	ssc-miR-323	ssc-miR-369	ssc-miR-708-5p	novel-ssc-miR161-3p	novel-ssc-miR512-5p	novel-ssc-miR846-3p	novel-ssc-miR1086-5p	novel-ssc-miR1105-5p	novel-ssc-miR1116-5p	novel-ssc-miR1120-5p	novel-ssc-miR1140-5p	novel-ssc-miR1158-3p	novel-ssc-miR1379-5p
BGIR9823_101960	BGIG9823_26637		lncRNA	0	0	0	0	0	0	0	0	0	0	0	1	0	0	0	1
BGIR9823_101964	BGIG9823_26641		lncRNA	0	0	0	0	0	1	0	0	0	0	0	0	0	0	0	0
BGIR9823_102018	BGIG9823_26693		lncRNA	0	0	0	0	0	0	0	0	0	0	0	0	0	0	0	0
BGIR9823_101913	BGIG9823_26590		lncRNA	0	0	0	0	0	0	0	0	0	0	0	0	0	0	0	0
BGIR9823_102617	BGIG9823_27267		lncRNA	0	0	0	0	0	0	0	0	0	0	0	0	0	0	0	0
BGIR9823_102277	BGIG9823_26944		lncRNA	0	0	0	0	0	0	0	1	0	0	0	0	0	0	0	0
BGIR9823_102215	BGIG9823_26887		lncRNA	0	0	0	0	0	0	0	1	0	0	0	0	0	0	0	0
BGIR9823_102525	BGIG9823_27178		lncRNA	0	0	0	0	0	0	0	0	0	0	0	0	0	0	0	0
BGIR9823_102031	BGIG9823_26706		lncRNA	0	0	0	0	0	0	0	1	0	0	0	0	0	0	0	0
BGIR9823_101909	BGIG9823_26586		lncRNA	0	0	0	0	0	0	0	0	0	0	0	0	0	0	0	1
BGIR9823_102249	BGIG9823_26917		lncRNA	0	1	1	0	0	0	0	0	0	0	0	0	1	0	0	0
BGIR9823_102126	BGIG9823_26799		lncRNA	0	0	0	0	0	1	0	0	0	0	0	0	0	0	0	1
BGIR9823_101720	BGIG9823_26404		lncRNA	0	0	0	0	0	0	0	0	0	0	0	0	0	0	0	0
BGIR9823_102111	BGIG9823_26785		lncRNA	0	0	0	0	0	0	0	0	0	0	0	0	1	0	0	0
BGIR9823_101921	BGIG9823_26598		lncRNA	0	0	0	0	0	0	0	1	0	0	0	1	0	0	0	0
BGIR9823_102144	BGIG9823_26817		lncRNA	0	0	0	0	0	0	0	0	0	0	0	0	0	0	0	0
BGIR9823_102006	BGIG9823_26682		lncRNA	0	0	0	0	0	0	0	0	0	0	0	0	0	0	0	0
BGIR9823_102108	BGIG9823_26782		lncRNA	0	0	0	0	0	1	0	0	0	0	0	0	0	0	0	0
BGIR9823_102515	BGIG9823_27169		lncRNA	0	0	0	0	0	0	0	0	0	0	0	0	0	0	0	0
BGIR9823_101872	BGIG9823_26551		lncRNA	0	0	0	0	0	0	0	0	0	0	0	0	0	0	0	0
BGIR9823_101929	BGIG9823_26606		lncRNA	0	0	0	0	0	0	0	0	1	0	0	1	0	0	0	0
BGIR9823_101740	BGIG9823_26421		lncRNA	1	0	0	0	0	0	0	0	0	0	0	0	0	0	0	0
BGIR9823_102035	BGIG9823_26710		lncRNA	0	0	0	0	0	0	0	0	0	0	0	0	0	0	0	1
BGIR9823_102356	BGIG9823_27021		lncRNA	0	0	0	0	0	0	0	0	0	0	0	0	0	0	0	1
BGIR9823_102012	BGIG9823_26688		lncRNA	0	0	0	0	0	0	0	0	1	0	0	0	0	0	0	0
BGIR9823_101980	BGIG9823_26656		lncRNA	0	0	0	0	0	0	0	0	0	0	0	0	0	0	0	0
BGIR9823_102058	BGIG9823_26732		lncRNA	0	0	0	0	0	0	0	1	0	0	0	0	0	0	0	1
BGIR9823_101945	BGIG9823_26622		lncRNA	0	0	0	0	0	0	0	0	0	0	0	0	0	0	0	0
BGIR9823_101770	BGIG9823_26450		lncRNA	1	0	0	0	0	0	0	0	0	0	0	0	0	0	0	0
XR_002341218.1	110258681	LOC110258681	lncRNA	0	0	0	0	0	0	0	0	0	0	0	0	0	0	0	0
BGIR9823_82088	100620215	CREBBP	lncRNA	0	0	0	0	0	0	0	0	0	1	0	0	0	0	0	0
BGIR9823_100016	102157885	LOC102157885	lncRNA	0	0	0	0	0	0	0	0	0	0	0	0	0	0	0	0
BGIR9823_101122	100519810	PHKA2	lncRNA	0	0	0	0	0	0	0	0	0	0	0	0	0	0	0	0
BGIR9823_100002	100627868	LOC100627868	lncRNA	0	0	0	0	0	0	0	0	0	0	0	0	1	0	0	0
BGIR9823_100152	100152012	CST7	lncRNA	0	0	0	0	0	0	0	0	0	0	0	0	0	0	0	0
BGIR9823_100517	100739098	LOC100739098	lncRNA	0	0	0	0	0	0	0	0	0	0	0	0	0	0	0	0
BGIR9823_100137	100155396	ESF1	lncRNA	0	0	0	0	0	0	0	0	0	0	0	0	0	0	0	0
BGIR9823_101191	110257830	LOC110257830	lncRNA	0	0	0	0	0	0	0	0	1	0	0	0	0	0	0	0
BGIR9823_100307	102164659	LOC102164659	lncRNA	0	0	0	0	0	0	0	0	0	0	0	0	0	0	0	0
BGIR9823_101424	100620346	PPP2R5C	lncRNA	0	0	0	0	0	0	0	0	0	0	0	0	0	0	0	0
BGIR9823_100548	100524778	MRPS24	lncRNA	0	0	0	0	0	0	0	0	0	0	0	0	0	0	0	0
BGIR9823_100391	102162486	LOC102162486	lncRNA	0	0	0	0	0	0	0	0	0	0	0	0	0	0	0	0
BGIR9823_81504	100524976	NSD1	lncRNA	0	0	0	0	0	0	0	0	0	0	0	0	0	0	0	0

RNA ID	Gene ID	Symbol	Type	ssc-miR-24-1-5p	ssc-miR-29b	ssc-miR-29c	ssc-miR-323	ssc-miR-369	ssc-miR-708-5p	novel-ssc-miR161-3p	novel-ssc-miR512-5p	novel-ssc-miR846-3p	novel-ssc-miR1086-5p	novel-ssc-miR1105-5p	novel-ssc-miR1116-5p	novel-ssc-miR1120-5p	novel-ssc-miR1140-5p	novel-ssc-miR1158-3p	novel-ssc-miR1379-5p
BGIR9823_100217	100156283	NCOA6	lncRNA	0	0	0	0	0	0	0	0	0	0	0	0	0	0	0	0
BGIR9823_101364	110258073	SNAPC4	lncRNA	0	0	0	0	0	0	0	0	0	0	0	0	0	0	0	0
BGIR9823_101532	102158401	LOC102158401	lncRNA	0	0	0	0	0	0	0	0	0	0	0	0	0	0	0	0
BGIR9823_100496	100521938	LOC100521938	lncRNA	0	0	0	0	0	0	0	0	0	0	0	0	0	0	0	0
BGIR9823_77776	100158022	HINT3	lncRNA	0	1	1	0	0	0	0	0	0	0	0	0	0	0	0	0
BGIR9823_100235	110257452	LOC110257452	lncRNA	0	0	0	0	0	0	0	0	0	0	0	1	0	0	0	0
BGIR9823_78349	100152069	SMAD6	lncRNA	0	0	0	0	0	1	0	0	0	0	0	0	0	0	0	0
BGIR9823_100688	100515937	COPG2	lncRNA	0	0	0	0	0	0	0	0	0	0	0	1	1	0	0	0
BGIR9823_101393	110258158	TNRC6A	lncRNA	0	0	0	0	0	0	0	0	0	0	0	0	0	0	0	0
BGIR9823_100562	100516888	GLI3	lncRNA	0	0	0	0	0	1	0	0	0	0	0	0	0	0	0	0
BGIR9823_79603	106509080	LOC106509080	lncRNA	0	0	0	0	0	0	0	0	0	0	0	0	0	1	0	0
BGIR9823_100840	102166569	TBL1X	lncRNA	0	0	0	0	0	0	0	0	1	0	0	0	0	0	0	0
BGIR9823_100717	100511294	PTPRZ1	lncRNA	0	0	0	0	0	0	0	0	0	0	0	0	0	0	0	0
BGIR9823_100350	100737248	WDR60	lncRNA	0	0	0	0	0	0	0	1	0	0	0	0	0	0	0	0
BGIR9823_101426	100516626	WDR20	lncRNA	0	0	0	0	0	0	0	0	1	0	0	0	1	0	0	0
BGIR9823_101427	100516626	WDR20	lncRNA	0	0	0	0	0	0	0	0	0	0	0	0	0	0	0	0
BGIR9823_101520	100511414	RNF122	lncRNA	0	0	0	0	0	0	0	1	0	0	0	0	0	0	0	0
BGIR9823_100500	100522109	ELMO1	lncRNA	0	0	0	0	0	0	0	0	1	0	0	0	0	0	0	0
BGIR9823_101384	110258135	LOC110258135	lncRNA	0	0	0	0	0	0	0	0	0	0	0	0	0	0	0	0
BGIR9823_101222	100517568	MAGT1	lncRNA	0	0	0	0	0	0	0	0	0	0	0	0	0	0	0	0
BGIR9823_100838	102166211	LOC102166211	lncRNA	0	0	0	0	0	0	0	0	1	0	0	0	0	0	0	0
BGIR9823_80389	100516724	NR2F6	lncRNA	0	1	1	0	0	0	0	0	0	0	0	0	0	0	0	0
BGIR9823_100923	100233182	GK	lncRNA	0	0	0	0	0	0	0	0	0	0	0	0	0	0	0	0
BGIR9823_100887	397645	SAT1	lncRNA	0	0	0	0	0	0	0	0	0	0	0	1	0	0	0	0
BGIR9823_100119	100519631	TASP1	lncRNA	0	0	0	0	0	0	0	0	0	0	0	1	0	0	0	0
BGIR9823_101408	110258214	LOC110258214	lncRNA	0	0	0	0	0	0	0	1	0	0	0	0	0	0	0	0
BGIR9823_100257	100156548	ZHX3	lncRNA	0	0	0	0	0	0	0	0	0	0	0	0	0	0	0	0
BGIR9823_100095	100738984	SLC20A2	lncRNA	0	0	0	0	0	0	0	0	0	0	0	0	0	0	0	0
BGIR9823_100711	397301	CALU	lncRNA	0	0	0	0	0	0	0	0	0	0	0	0	1	0	0	0
BGIR9823_100252	110257392	LOC110257392	lncRNA	0	0	0	0	0	0	0	0	0	0	0	0	0	0	0	0
BGIR9823_100342	100625428	RTEL1	lncRNA	0	0	0	0	0	0	0	0	0	0	0	0	0	0	0	0
BGIR9823_82688	102164200	LOC102164200	lncRNA	0	1	1	0	0	0	0	0	0	0	0	0	0	0	0	0
BGIR9823_92711	110255798	LOC110255798	lncRNA	0	1	1	0	0	0	0	0	0	0	0	0	0	1	0	0
BGIR9823_82658	100622402	C3H16orf45	mRNA	0	0	0	0	0	0	0	0	0	0	0	0	0	0	0	0
BGIR9823_100282	100153535	PCIF1	lncRNA	0	0	0	0	0	0	0	0	0	0	0	0	0	0	0	0
XR_002341162.1	110258480	LOC110258480	lncRNA	0	0	0	0	0	0	0	0	0	0	0	0	0	0	0	0
BGIR9823_101217	100514440	ATRX	lncRNA	0	0	0	0	0	0	0	0	0	0	0	0	1	0	0	0
BGIR9823_96121	110256382	LOC110256382	lncRNA	0	0	0	0	0	0	0	0	0	0	0	0	0	0	0	0
BGIR9823_83098	100322871	CCT7	mRNA	0	0	0	0	0	0	0	0	0	0	0	0	0	0	0	0
BGIR9823_81464	100516183	CNOT6	lncRNA	0	0	0	0	0	0	0	0	0	0	1	0	0	0	0	0
BGIR9823_81228	100518418	GATAD2A	mRNA	0	0	0	0	0	0	0	0	0	1	0	0	0	0	0	0
BGIR9823_99373	100514689	MAST4	mRNA	0	0	0	0	0	0	0	0	0	0	0	0	0	0	0	0
BGIR9823_101508	110258454	LETMD1	lncRNA	0	0	0	0	0	0	0	0	0	0	0	0	0	0	0	0

RNA ID	Gene ID	Symbol	Type	ssc-miR-24-1-5p	ssc-miR-29b	ssc-miR-29c	ssc-miR-323	ssc-miR-369	ssc-miR-708-5p	novel-ssc-miR161-3p	novel-ssc-miR512-5p	novel-ssc-miR846-3p	novel-ssc-miR1086-5p	novel-ssc-miR1105-5p	novel-ssc-miR1116-5p	novel-ssc-miR1120-5p	novel-ssc-miR1140-5p	novel-ssc-miR1158-3p	novel-ssc-miR1379-5p
BGIR9823_100200	100624021	PLAGL2	lncRNA	0	0	0	0	0	0	0	0	0	0	0	0	0	0	0	0
BGIR9823_93132	100174955	KLF12	lncRNA	0	0	0	0	0	0	0	0	0	0	0	0	0	0	0	0
BGIR9823_101233	100739062	LOC100739062	lncRNA	0	0	0	0	0	0	0	0	0	0	0	0	0	0	0	0
BGIR9823_101439	100625932	MARK3	lncRNA	0	0	0	0	0	0	0	0	1	0	0	0	0	0	0	0
BGIR9823_102584	BGIG9823_27234		lncRNA	0	0	0	1	0	0	0	0	0	0	0	0	0	0	0	0
BGIR9823_102601	BGIG9823_27251		lncRNA	0	0	1	0	0	0	0	0	0	0	0	0	1	0	0	0
BGIR9823_101907	BGIG9823_26584		lncRNA	0	0	0	0	0	0	0	0	0	0	0	0	0	0	0	0
BGIR9823_101683	BGIG9823_26370		lncRNA	0	0	0	0	0	0	0	0	0	0	0	0	0	0	0	0
BGIR9823_101932	BGIG9823_26609		lncRNA	0	0	0	0	0	0	0	0	0	0	0	0	0	0	0	0
BGIR9823_102312	BGIG9823_26977		lncRNA	0	0	0	0	0	0	0	0	0	0	0	0	0	0	0	0
BGIR9823_101712	BGIG9823_26396		lncRNA	0	0	0	0	0	0	0	0	0	0	0	0	0	0	0	0
BGIR9823_102749	BGIG9823_27392		lncRNA	0	0	0	0	0	1	0	0	0	0	0	1	0	0	0	0
BGIR9823_101726	BGIG9823_26409		lncRNA	0	0	0	0	0	0	0	0	0	0	0	0	0	1	0	0
BGIR9823_102386	BGIG9823_27051		lncRNA	0	0	0	0	0	0	0	0	0	0	0	0	1	0	0	0
BGIR9823_102032	BGIG9823_26707		lncRNA	0	0	0	0	0	0	0	0	0	0	0	0	0	0	0	0
BGIR9823_102336	BGIG9823_27001		lncRNA	0	0	0	0	0	0	0	0	0	0	0	0	1	0	0	0
BGIR9823_101794	BGIG9823_26474		lncRNA	0	0	0	0	0	0	0	0	0	0	0	0	0	0	0	0
BGIR9823_101752	BGIG9823_26433		lncRNA	0	0	0	0	0	0	0	0	0	0	0	0	0	0	0	0
BGIR9823_101944	BGIG9823_26621		lncRNA	0	0	0	0	0	1	0	0	0	0	0	0	0	0	0	0
BGIR9823_101873	BGIG9823_26552		lncRNA	0	0	0	0	0	0	0	1	0	0	0	0	0	0	0	0
BGIR9823_101671	BGIG9823_26359		lncRNA	0	0	0	0	0	0	0	0	0	0	0	0	0	0	0	0
BGIR9823_101974	BGIG9823_26651		lncRNA	0	0	0	0	0	0	0	0	0	0	0	0	0	0	0	0
BGIR9823_102380	BGIG9823_27045		lncRNA	0	0	0	0	0	0	0	0	0	0	0	0	0	0	0	0
BGIR9823_102232	BGIG9823_26904		lncRNA	0	0	0	0	0	0	0	1	0	0	0	0	0	0	0	0
BGIR9823_102238	BGIG9823_26910		lncRNA	0	0	0	1	0	1	0	0	0	0	0	0	0	0	0	0
BGIR9823_102071	BGIG9823_26745		lncRNA	0	0	0	0	0	0	0	0	0	0	0	0	0	0	0	0
BGIR9823_101784	BGIG9823_26464		lncRNA	0	0	0	0	0	0	0	0	1	0	0	0	0	0	0	0
BGIR9823_102392	BGIG9823_27057		lncRNA	0	0	0	0	0	0	0	0	0	0	0	1	0	0	0	0
BGIR9823_102420	BGIG9823_27081		lncRNA	0	0	0	0	0	0	0	0	0	0	0	0	0	0	0	0
BGIR9823_101988	BGIG9823_26664		lncRNA	0	0	0	0	0	0	0	0	1	0	0	0	0	0	0	0
BGIR9823_101675	BGIG9823_26362		lncRNA	0	0	0	0	0	0	0	0	1	0	0	0	0	0	0	0
BGIR9823_101761	BGIG9823_26441		lncRNA	0	0	0	0	0	0	0	0	0	0	0	0	0	0	0	0
BGIR9823_102382	BGIG9823_27047		lncRNA	0	0	0	0	0	0	0	0	0	0	0	1	0	0	0	0
BGIR9823_102045	BGIG9823_26719		lncRNA	0	0	0	0	0	0	0	0	0	0	0	0	0	0	0	0
BGIR9823_101857	BGIG9823_26536		lncRNA	0	0	0	0	0	0	0	0	0	0	0	0	0	0	0	0
BGIR9823_101775	BGIG9823_26455		lncRNA	0	0	0	0	0	0	0	0	0	0	0	0	1	0	0	0
BGIR9823_102446	BGIG9823_27101		lncRNA	0	1	0	0	0	0	0	0	0	0	0	0	0	0	0	0
BGIR9823_102179	BGIG9823_26851		lncRNA	0	0	0	0	0	0	0	1	0	0	0	0	0	0	0	0
BGIR9823_101678	BGIG9823_26365		lncRNA	0	0	0	0	0	0	0	0	0	0	0	0	0	0	0	0
BGIR9823_102242	BGIG9823_26913		lncRNA	0	0	0	0	0	0	0	0	0	0	0	1	0	0	0	0
BGIR9823_102633	BGIG9823_27283		lncRNA	0	0	0	0	0	0	0	0	0	0	0	1	0	0	0	0
BGIR9823_102205	BGIG9823_26877		lncRNA	0	0	0	0	0	0	0	1	0	0	0	0	0	0	0	0
BGIR9823_101763	BGIG9823_26443		lncRNA	0	0	0	0	0	0	0	0	0	0	0	0	0	0	0	0

RNA ID	Gene ID	Symbol	Type	ssc-miR-24-1-5p	ssc-miR-29b	ssc-miR-29c	ssc-miR-323	ssc-miR-369	ssc-miR-708-5p	novel-ssc-miR161-3p	novel-ssc-miR512-5p	novel-ssc-miR846-3p	novel-ssc-miR1086-5p	novel-ssc-miR1105-5p	novel-ssc-miR1116-5p	novel-ssc-miR1120-5p	novel-ssc-miR1140-5p	novel-ssc-miR1158-3p	novel-ssc-miR1379-5p
BGIR9823_102754	BGIG9823_27394		lncRNA	0	0	0	0	0	0	0	0	0	0	0	0	0	0	0	0
BGIR9823_102077	BGIG9823_26751		lncRNA	0	1	1	0	0	0	0	0	0	0	0	0	0	0	0	0
BGIR9823_102457	BGIG9823_27111		lncRNA	0	0	0	0	0	0	0	0	0	0	0	0	0	0	0	0
BGIR9823_101881	BGIG9823_26560		lncRNA	0	0	0	0	0	0	0	0	0	0	0	0	0	0	0	0
BGIR9823_102086	BGIG9823_26760		lncRNA	0	0	0	0	0	0	0	0	0	0	0	0	0	0	0	0
BGIR9823_102146	BGIG9823_26819		lncRNA	0	0	0	0	0	0	0	0	0	0	0	0	1	0	0	0
BGIR9823_101735	BGIG9823_26416		lncRNA	0	0	0	0	0	0	0	0	0	0	0	0	0	0	0	0
BGIR9823_102132	BGIG9823_26805		lncRNA	0	0	0	0	0	0	0	1	0	0	0	0	0	0	0	0
BGIR9823_102345	BGIG9823_27010		lncRNA	0	0	0	0	0	0	0	0	0	0	0	0	0	0	0	0
BGIR9823_101715	BGIG9823_26399		lncRNA	0	0	0	0	0	0	0	0	1	0	0	0	0	0	0	0
BGIR9823_102322	BGIG9823_26987		lncRNA	0	0	0	0	0	0	0	1	0	0	0	0	0	0	0	0
BGIR9823_102695	BGIG9823_27345		lncRNA	0	0	0	0	0	1	0	0	0	0	0	0	0	0	0	0
BGIR9823_101684	BGIG9823_26371		lncRNA	0	0	0	0	0	0	0	0	1	0	0	0	0	0	0	0
BGIR9823_102344	BGIG9823_27009		lncRNA	0	0	0	0	0	0	0	0	0	0	0	0	1	0	0	0
BGIR9823_102092	BGIG9823_26766		lncRNA	0	0	0	0	0	0	0	0	0	0	0	0	1	0	0	0
BGIR9823_102073	BGIG9823_26747		lncRNA	0	0	0	0	0	0	0	0	0	0	0	0	0	0	0	0
BGIR9823_102188	BGIG9823_26860		lncRNA	0	0	0	0	0	0	0	0	0	0	0	0	0	0	0	0
BGIR9823_101946	BGIG9823_26623		lncRNA	0	0	0	0	0	0	0	0	0	0	0	0	0	0	0	0
BGIR9823_102176	BGIG9823_26848		lncRNA	0	0	0	0	0	0	0	0	0	0	0	0	0	0	0	0
BGIR9823_102387	BGIG9823_27052		lncRNA	0	0	0	0	0	0	0	0	0	0	0	0	0	0	0	0
BGIR9823_101876	BGIG9823_26555		lncRNA	0	0	0	0	0	0	0	0	0	0	0	0	0	0	0	1
BGIR9823_101888	BGIG9823_26565		lncRNA	0	0	0	0	0	0	0	0	0	0	0	0	0	0	0	0
BGIR9823_101818	BGIG9823_26498		lncRNA	0	0	0	0	0	0	0	0	0	0	0	0	0	0	0	0
BGIR9823_102320	BGIG9823_26985		lncRNA	0	0	0	0	0	0	0	0	0	0	0	0	0	0	0	0
BGIR9823_102080	BGIG9823_26754		lncRNA	0	0	0	0	0	0	0	0	0	0	0	0	0	0	0	0
BGIR9823_101786	BGIG9823_26466		lncRNA	0	0	0	0	0	0	0	0	0	0	0	0	0	0	0	0
BGIR9823_102325	BGIG9823_26990		lncRNA	0	0	0	0	0	0	0	1	0	0	0	0	0	0	0	0
BGIR9823_101989	BGIG9823_26665		lncRNA	1	0	0	0	0	0	0	0	0	0	0	0	0	0	0	0
BGIR9823_101889	BGIG9823_26566		lncRNA	0	0	0	0	0	0	0	0	0	0	0	1	0	0	0	0
BGIR9823_102669	BGIG9823_27319		lncRNA	0	0	0	0	0	0	0	0	0	0	0	0	0	0	0	1
BGIR9823_101878	BGIG9823_26557		lncRNA	0	0	0	0	0	0	0	1	0	0	0	0	0	0	0	0
BGIR9823_101918	BGIG9823_26595		lncRNA	0	0	0	0	0	1	0	0	0	0	0	0	0	0	0	0
BGIR9823_102064	BGIG9823_26738		lncRNA	0	0	0	0	0	0	0	0	0	0	0	0	1	0	0	0
BGIR9823_101985	BGIG9823_26661		lncRNA	0	0	0	0	0	0	0	0	0	0	0	0	0	0	0	0
BGIR9823_101774	BGIG9823_26454		lncRNA	0	0	0	0	0	0	0	0	0	0	0	0	0	0	0	0
BGIR9823_102112	BGIG9823_26786		lncRNA	0	0	0	0	0	0	0	0	0	0	0	0	1	0	0	1
BGIR9823_102585	BGIG9823_27235		lncRNA	0	0	0	0	0	1	0	0	0	0	0	1	0	0	0	0
BGIR9823_101903	BGIG9823_26580		lncRNA	0	0	0	0	0	0	0	0	0	0	0	0	0	0	0	1
BGIR9823_102173	BGIG9823_26846		lncRNA	0	0	0	0	0	0	0	1	0	0	0	0	0	0	0	0
BGIR9823_101912	BGIG9823_26589		lncRNA	0	0	0	0	0	0	0	0	0	0	0	0	0	0	0	0
BGIR9823_101793	BGIG9823_26473		lncRNA	0	0	0	0	0	0	0	0	1	0	0	0	0	0	0	0
BGIR9823_102135	BGIG9823_26808		lncRNA	0	0	0	0	0	0	0	1	0	0	0	0	0	0	0	0
BGIR9823_101911	BGIG9823_26588		lncRNA	0	0	0	0	0	0	0	0	1	0	0	0	0	0	0	0

RNA ID	Gene ID	Symbol	Type	ssc-miR-24-1-5p	ssc-miR-29b	ssc-miR-29c	ssc-miR-323	ssc-miR-369	ssc-miR-708-5p	novel-ssc-miR161-3p	novel-ssc-miR512-5p	novel-ssc-miR846-3p	novel-ssc-miR1086-5p	novel-ssc-miR1105-5p	novel-ssc-miR1116-5p	novel-ssc-miR1120-5p	novel-ssc-miR1140-5p	novel-ssc-miR1158-3p	novel-ssc-miR1379-5p
BGIR9823_101864	BGIG9823_26543		lncRNA	0	0	0	0	0	0	0	0	0	0	0	0	0	0	0	0
BGIR9823_101851	BGIG9823_26530		lncRNA	0	0	0	0	0	0	0	0	0	0	0	0	1	0	0	0
BGIR9823_102310	BGIG9823_26975		lncRNA	0	0	0	0	0	0	0	0	0	0	0	1	0	0	0	0
BGIR9823_101776	BGIG9823_26456		lncRNA	0	0	0	0	0	0	0	0	0	0	0	0	0	0	0	0
BGIR9823_102381	BGIG9823_27046		lncRNA	0	0	0	0	0	0	0	0	0	0	0	0	0	0	0	0
BGIR9823_101680	BGIG9823_26367		lncRNA	0	0	0	0	0	0	0	0	1	0	0	0	0	0	0	0
BGIR9823_101747	BGIG9823_26428		lncRNA	0	0	0	0	0	0	0	0	0	0	0	0	0	0	0	0
BGIR9823_101830	BGIG9823_26509		lncRNA	0	0	0	0	0	0	0	0	0	0	0	0	0	0	0	0
BGIR9823_102332	BGIG9823_26997		lncRNA	0	1	1	0	0	0	0	0	0	0	0	0	0	0	0	0
XR_002336732.1	102159417	LOC102159417	lncRNA	0	0	0	0	0	0	0	0	0	1	0	0	0	0	0	0
XR_002343571.1	106507458	LOC106507458	lncRNA	0	0	0	0	0	0	0	0	0	0	0	0	0	0	0	0
XR_001304304.2	106507921	LOC106507921	lncRNA	0	0	0	0	0	0	0	0	0	0	0	0	0	0	0	0
XR_002344048.1	110260709	LOC110260709	lncRNA	0	0	0	0	0	0	0	0	0	0	0	0	0	0	0	0
XM_003353597.4	100621832	TMEM246	mRNA	0	0	0	0	0	0	0	0	0	0	0	0	0	0	0	0
XR_002338514.1	102161206	LOC102161206	lncRNA	0	0	0	0	0	0	0	0	0	0	0	0	0	0	0	0
NM_001244791.1	100513225	BAG1	mRNA	0	0	0	0	0	0	0	0	0	0	0	0	0	0	0	0
XR_002341355.1	102167380	CUNH21orf58	lncRNA	0	0	0	0	0	0	0	0	0	0	0	0	0	0	0	0
XR_001299235.2	106505126	LOC106505126	lncRNA	0	0	0	0	0	0	0	0	0	0	1	0	0	0	0	0
XR_304631.3	102167686	LOC102167686	lncRNA	0	0	0	0	0	0	0	0	0	0	0	0	0	0	0	0
XM_021066717.1	100521576	ARSG	mRNA	0	0	0	0	0	0	0	0	0	0	0	0	0	0	0	0
XR_002342261.1	110259855	LOC110259855	lncRNA	0	0	0	0	0	0	0	0	0	0	0	0	0	0	0	0
NM_213891.1	396925	VCAM1	mRNA	0	0	0	0	0	0	0	0	0	0	0	0	0	0	0	0
XM_003129787.4	100516505	VSTM5	mRNA	0	0	0	0	0	0	0	0	0	0	0	0	0	0	0	0
XM_021100543.1	106504722	UCP1	mRNA	0	0	0	0	0	0	0	0	0	0	0	0	0	0	0	0
XR_002345110.1	110261202	LOC110261202	lncRNA	0	0	0	0	0	0	0	0	0	0	0	0	0	0	0	0
XM_013992908.2	102164011	LOC102164011	mRNA	0	0	0	0	0	0	0	0	0	0	0	0	0	0	0	0
XM_021082022.1	100525982	LOC100525982	mRNA	0	0	0	0	1	0	0	0	0	0	0	0	0	0	0	0
NM_214188.1	397385	DDOST	mRNA	0	0	0	0	0	0	0	0	0	0	0	0	0	0	0	0
XM_013983344.2	100156215	MSS51	mRNA	0	0	0	0	0	0	0	0	0	0	0	0	0	0	0	0
NM_001164856.1	100303723	LCAT	mRNA	0	0	0	0	0	0	0	0	0	0	0	0	0	0	0	0
NM_001278748.1	100520980	CASR	mRNA	0	0	0	0	0	0	0	0	0	0	0	0	0	0	0	0
XR_002342824.1	110260048	LOC110260048	lncRNA	0	0	0	0	0	0	0	0	0	0	0	0	0	0	0	0
XM_003481630.4	100738263	SPRYD4	mRNA	0	0	0	0	0	0	0	0	0	0	0	0	0	0	0	0
XR_002340516.1	102163798	LOC102163798	lncRNA	0	0	0	0	0	0	0	0	0	0	0	0	0	0	0	0
XM_021080156.1	102167889	LOC102167889	mRNA	0	0	0	0	0	0	0	0	0	0	0	0	0	0	0	0
XR_002343904.1	110260647	LOC110260647	lncRNA	0	0	0	0	0	0	0	0	0	1	0	0	0	0	0	0
XM_021082023.1	100510954	LOC100510954	mRNA	0	0	0	0	1	0	0	0	0	0	0	0	0	0	0	0
XR_002340992.1	110258138	LOC110258138	lncRNA	0	0	0	0	0	0	0	0	0	0	0	0	0	0	0	0
XM_003129097.6	100523679	CCNI	mRNA	0	0	0	0	0	0	0	0	0	0	0	0	0	0	0	0
XM_021086388.1	110255213	ARMC5	mRNA	0	0	0	0	0	0	0	0	0	0	0	0	0	0	0	0
XM_003133626.3	100522220	LOC100522220	mRNA	0	0	0	0	0	0	0	0	0	0	0	0	0	0	0	0
XR_002341145.1	110258406	LOC110258406	lncRNA	0	0	0	0	0	0	0	0	0	0	0	0	0	0	0	0
XR_002341292.1	110258990	LOC110258990	lncRNA	0	0	0	0	0	0	0	0	0	0	0	0	0	0	0	0

RNA ID	Gene ID	Symbol	Type	ssc-miR-24-1-5p	ssc-miR-29b	ssc-miR-29c	ssc-miR-323	ssc-miR-369	ssc-miR-708-5p	novel-ssc-miR161-3p	novel-ssc-miR512-5p	novel-ssc-miR846-3p	novel-ssc-miR1086-5p	novel-ssc-miR1105-5p	novel-ssc-miR1116-5p	novel-ssc-miR1120-5p	novel-ssc-miR1140-5p	novel-ssc-miR1158-3p	novel-ssc-miR1379-5p
NM_001204772.1	100533540	BRD4	mRNA	0	0	0	0	0	0	0	0	0	0	0	0	0	0	0	0
NM_213763.2	396637	UCHL1	mRNA	0	0	0	0	0	0	0	0	0	0	0	0	0	0	0	0
NM_214050.2	397117	GSTO1	mRNA	0	0	0	0	0	0	0	0	0	0	0	0	0	0	0	0
XR_002337239.1	110256047	LOC110256047	lncRNA	0	0	0	0	0	0	0	0	0	1	0	0	0	0	0	0
XR_002343522.1	110260355	LOC110260355	lncRNA	0	0	0	0	0	0	0	0	0	1	0	0	0	0	0	0
XR_001304268.2	106507904	LOC106507904	lncRNA	0	0	0	0	0	0	0	0	0	0	0	0	0	0	0	0
XR_002343855.1	110260615	LOC110260615	lncRNA	0	0	0	0	0	0	0	0	0	1	0	0	0	0	0	0
XR_002336945.1	110255950	LOC110255950	lncRNA	0	0	0	0	0	0	0	0	0	0	0	0	0	0	0	0
XM_021075808.1	110257099	LOC110257099	mRNA	0	0	0	0	0	0	0	0	0	0	0	0	0	0	0	0
XM_021082410.1	110259094	LOC110259094	mRNA	0	0	0	0	0	0	0	0	0	0	0	0	0	0	0	0
XR_002341290.1	110258990	LOC110258990	lncRNA	0	0	0	0	0	0	0	0	0	0	0	0	0	0	0	0
XM_021100189.1	100152943	ADTRP	mRNA	0	0	0	0	0	0	0	0	0	0	0	0	0	0	0	0
XR_002341433.1	106509301	LOC106509301	lncRNA	0	0	0	0	0	0	0	0	0	0	1	0	0	0	0	0
NM_001244722.1	100512802	PUS3	mRNA	0	0	0	0	0	0	0	0	0	0	0	0	0	0	0	0
XM_021092065.1	100624597	YARS2	mRNA	0	0	0	0	0	0	0	0	0	0	0	0	0	0	0	0
XM_021066519.1	100512084	TBC1D2	mRNA	0	0	0	0	0	0	0	0	0	0	0	0	0	0	0	0
XR_002335835.1	110255476	LOC110255476	lncRNA	0	0	0	0	0	0	0	0	0	1	0	0	0	0	0	0
XR_002335866.1	110255489	LOC110255489	lncRNA	0	0	0	0	0	0	0	0	0	0	0	0	0	0	0	0
XR_002337288.1	106509234	LOC106509234	lncRNA	0	0	0	0	0	0	0	0	0	1	0	0	0	0	0	0
XR_002342643.1	106508312	LOC106508312	lncRNA	0	0	0	0	0	0	0	0	0	1	0	0	0	0	0	0
NM_001097492.1	100038008	CD1E	mRNA	0	0	0	0	0	0	0	0	0	0	0	0	0	0	0	0
XR_002336367.1	106507918	LOC106507918	lncRNA	0	0	0	0	0	0	0	0	0	0	0	0	0	0	0	0
XR_002340833.1	110257824	LOC110257824	lncRNA	0	0	0	0	0	0	0	0	0	0	0	0	0	0	0	0
NM_001004028.1	445513	HAPLN1	mRNA	0	0	0	0	0	0	0	0	0	0	0	0	0	0	0	0
XR_001302352.2	106506794	LOC106506794	lncRNA	0	0	0	0	0	0	0	0	0	0	0	0	0	0	0	0
XR_002336622.1	110255794	LOC110255794	lncRNA	0	0	0	0	0	0	0	0	0	0	0	0	0	0	0	0
XM_021070652.1	110256443	QARS	mRNA	0	0	0	0	0	0	0	0	0	0	0	0	0	0	0	0
XM_001928752.5	100154892	LOC100154892	mRNA	0	0	0	0	0	0	0	0	0	0	0	0	0	0	0	0
NM_001243696.1	100158051	DRAM2	mRNA	0	0	0	0	0	0	0	0	0	0	0	0	0	0	0	0
XR_002342202.1	100512593	LOC100512593	lncRNA	0	0	0	0	0	0	0	0	0	1	0	0	0	0	0	0
XR_002343188.1	110260249	LOC110260249	lncRNA	0	0	0	0	0	0	0	0	0	1	0	0	0	0	0	0
XM_021081808.1	100155561	LOC100155561	mRNA	0	0	0	0	1	0	0	0	0	0	0	0	0	0	0	0
XM_003134082.6	100515063	HMMR	mRNA	0	0	0	0	0	0	0	0	0	0	0	0	0	0	0	0
XR_002341327.1	110259136	LOC110259136	lncRNA	0	0	0	0	0	0	0	0	0	1	0	0	0	0	0	0
XR_002346274.1	110261765	LOC110261765	lncRNA	0	0	0	0	0	0	0	0	0	1	0	0	0	0	0	0
URS0000BC4F80	BGIG9823_26330		lncRNA	0	0	0	0	0	0	0	0	0	1	0	0	0	0	0	0
URS0000BC82B3	BGIG9823_26290		lncRNA	0	0	0	0	0	0	0	0	0	0	0	0	0	0	0	0
BGIR9823_100417	106506781	LOC106506781	lncRNA	0	0	0	0	0	0	0	0	0	0	0	0	0	0	0	0
BGIR9823_78908	100157533	WTAP	mRNA	0	0	0	0	0	0	0	0	0	1	0	0	0	0	0	0
BGIR9823_85709	100156112	ATP6V1E1	lncRNA	0	1	0	0	0	0	0	0	0	0	0	0	0	0	0	0
BGIR9823_88195	100152838	SLC22A23	lncRNA	0	0	0	0	0	0	0	0	0	0	0	0	0	0	0	0
BGIR9823_81246	100174943	IFI30	mRNA	0	0	0	0	0	0	0	0	0	1	0	0	0	0	0	0
BGIR9823_100836	100624032	LOC100624032	lncRNA	0	0	0	0	0	1	0	0	0	0	0	0	0	0	0	0

RNA ID	Gene ID	Symbol	Type	ssc-miR-24-1-5p	ssc-miR-29b	ssc-miR-29c	ssc-miR-323	ssc-miR-369	ssc-miR-708-5p	novel-ssc-miR161-3p	novel-ssc-miR512-5p	novel-ssc-miR846-3p	novel-ssc-miR1086-5p	novel-ssc-miR1105-5p	novel-ssc-miR1116-5p	novel-ssc-miR1120-5p	novel-ssc-miR1140-5p	novel-ssc-miR1158-3p	novel-ssc-miR1379-5p
BGIR9823_84507	100154147	MAN1A2	lncRNA	0	1	1	0	0	0	0	0	0	0	0	0	0	0	0	0
BGIR9823_78933	100516653	CCDC170	lncRNA	0	0	0	0	0	1	0	0	0	0	0	0	0	0	0	0
BGIR9823_85930	106510465	LOC106510465	mRNA	0	0	0	0	0	0	0	0	0	1	0	0	0	0	0	0
BGIR9823_98132	100519056	ORC4	lncRNA	0	0	0	0	0	0	0	0	0	1	0	0	0	0	0	0
BGIR9823_85554	100624497	DNM1L	mRNA	0	0	0	0	0	0	0	0	0	0	0	0	0	0	0	0
BGIR9823_89252	100153410	STK38	mRNA	0	0	0	0	0	0	0	0	0	0	0	0	0	0	0	0
BGIR9823_100096	100738984	SLC20A2	lncRNA	0	0	0	0	0	0	0	0	0	0	0	0	0	0	0	0
BGIR9823_85945	100521092	WDR59	mRNA	0	0	0	0	0	0	0	0	0	1	0	0	0	0	0	0
BGIR9823_85409	100516908	NCKAP5L	lncRNA	0	1	1	0	0	0	0	0	0	0	0	0	0	0	0	0
BGIR9823_100573	100516888	GLI3	lncRNA	0	0	0	0	0	0	0	0	0	0	0	0	0	0	0	0
BGIR9823_101467	100522195	XRCC3	lncRNA	0	0	0	0	0	0	0	0	0	0	0	0	0	0	0	0
BGIR9823_86462	100738753	RERE	lncRNA	0	1	1	0	0	0	0	0	0	0	0	0	0	0	0	0
BGIR9823_100236	106504144	DLGAP4	lncRNA	0	0	0	0	0	0	0	0	0	0	0	0	0	0	0	0
BGIR9823_100421	100626356	UBE2H	lncRNA	0	0	0	0	0	0	0	0	0	0	0	0	0	0	0	0
BGIR9823_101512	110258473	LOC110258473	lncRNA	0	0	0	0	0	0	0	0	0	0	0	0	0	0	0	0
BGIR9823_100354	102167342	CNPY1	lncRNA	0	0	0	0	0	0	0	0	0	0	0	0	0	0	0	0
BGIR9823_100645	100519065	UBN2	lncRNA	0	0	0	0	0	0	0	0	0	0	0	0	0	0	0	0
BGIR9823_100414	414383	CHCHD3	lncRNA	0	0	0	0	0	0	0	0	0	0	0	0	0	0	0	0
BGIR9823_81026	100523886	SF1	lncRNA	0	0	0	0	0	0	0	0	0	0	0	0	0	0	0	0
BGIR9823_100005	106506050	LOC106506050	lncRNA	0	0	0	0	0	0	0	0	0	0	0	0	0	0	0	0
BGIR9823_81229	100518418	GATAD2A	lncRNA	0	0	0	0	0	1	0	0	0	0	0	0	0	0	0	0
BGIR9823_79698	100154949	CNIH1	mRNA	0	0	0	0	0	0	0	0	0	0	0	0	0	0	0	0
BGIR9823_100531	100625903	SKAP2	lncRNA	0	0	0	0	0	0	0	0	0	0	0	0	0	0	0	0
BGIR9823_81900	110259864	LOC110259864	lncRNA	0	0	0	0	0	0	0	0	0	0	0	0	0	0	0	0
BGIR9823_100425	100626356	UBE2H	lncRNA	0	0	0	0	0	0	0	0	0	0	0	0	0	0	0	0
BGIR9823_82216	100511252	ACTR1B	lncRNA	0	0	0	0	0	0	0	0	0	0	0	0	0	0	0	0
BGIR9823_100156	100519522	NINL	lncRNA	0	0	0	0	0	0	0	0	0	0	0	0	0	0	0	0
BGIR9823_101565	110258600	LOC110258600	lncRNA	0	0	0	0	0	0	0	0	0	0	0	0	0	0	0	0
BGIR9823_100677	106504059	EXOC4	lncRNA	0	0	0	0	0	0	0	0	0	0	0	0	0	0	0	0
BGIR9823_100501	100523875	7-Sep	lncRNA	0	0	0	0	0	0	0	0	1	0	0	0	0	0	0	1
BGIR9823_100856	100154431	ASB11	lncRNA	0	0	0	0	0	0	0	0	0	0	0	0	0	0	0	0
BGIR9823_92258	100738420	ABI1	lncRNA	0	0	0	0	0	0	0	0	0	1	0	0	0	0	0	0
BGIR9823_97570	100158098	RHBDD3	lncRNA	0	0	0	0	0	0	0	0	0	1	0	0	0	0	0	0
BGIR9823_101254	106507023	LOC106507023	lncRNA	0	0	0	0	0	0	0	0	0	0	0	0	0	0	0	1
BGIR9823_100601	102159531	ACTR3B	mRNA	0	0	0	0	0	0	0	0	0	1	0	0	0	0	0	0
BGIR9823_83153	110260004	AHSA2P	mRNA	0	0	0	0	0	0	0	0	0	0	0	0	0	0	0	0
BGIR9823_90913	110255435	LOC110255435	lncRNA	0	0	1	0	0	0	0	0	0	0	0	0	0	0	0	0
BGIR9823_100272	396886	SLPI	lncRNA	0	0	0	0	0	0	0	0	0	0	0	0	0	0	0	0
BGIR9823_77799	100520524	GOPC	lncRNA	0	0	0	0	0	0	0	0	0	0	0	1	0	0	0	1
BGIR9823_100990	100153260	APOOL	lncRNA	0	0	0	0	0	0	0	0	0	0	0	0	0	0	0	0
BGIR9823_79735	110255453	LOC110255453	lncRNA	0	1	0	0	0	0	0	0	0	0	0	0	0	0	0	0
BGIR9823_80994	100519350	DPP3	mRNA	0	0	0	0	0	0	0	0	0	1	0	0	0	0	0	0
BGIR9823_100422	100626356	UBE2H	lncRNA	0	0	0	0	0	0	0	0	0	0	0	0	0	0	0	0

RNA ID	Gene ID	Symbol	Type	ssc-miR-24-1-5p	ssc-miR-29b	ssc-miR-29c	ssc-miR-323	ssc-miR-369	ssc-miR-708-5p	novel-ssc-miR161-3p	novel-ssc-miR512-5p	novel-ssc-miR846-3p	novel-ssc-miR1086-5p	novel-ssc-miR1105-5p	novel-ssc-miR1116-5p	novel-ssc-miR1120-5p	novel-ssc-miR1140-5p	novel-ssc-miR1158-3p	novel-ssc-miR1379-5p
BGIR9823_95751	100627663	ARHGEF3	lncRNA	0	0	0	0	0	0	0	0	0	0	0	0	0	0	0	0
BGIR9823_78308	100154901	ATP9B	lncRNA	0	0	0	0	0	0	0	0	0	0	0	0	0	0	0	0
BGIR9823_89654	733584	FOXN3	lncRNA	0	0	0	0	0	0	0	0	0	0	0	0	0	0	0	0
BGIR9823_94919	100737976	RBSN	lncRNA	0	1	1	0	0	0	0	0	0	0	0	0	0	0	0	0
BGIR9823_83670	100153676	PCMTD1	lncRNA	0	0	1	0	0	0	0	0	0	0	0	0	0	0	0	0
BGIR9823_100051	100621535	TPD52L2	lncRNA	0	0	0	0	0	0	0	0	0	0	0	0	0	0	0	0
BGIR9823_78866	100512264	NTNG2	lncRNA	0	0	0	0	0	0	0	0	0	0	0	0	0	0	0	0
BGIR9823_100572	100516888	GLI3	lncRNA	0	0	0	0	0	0	0	1	0	0	0	0	0	0	0	0
BGIR9823_89017	100157526	MYLK4	lncRNA	0	0	0	0	0	0	0	0	0	0	1	0	0	0	0	0
BGIR9823_78135	100153621	FAM214A	lncRNA	0	0	0	0	0	0	0	0	0	0	0	0	0	0	0	1
BGIR9823_88205	100157926	RIOK1	lncRNA	0	0	0	0	0	0	0	0	0	0	0	0	0	0	0	0
BGIR9823_100635	102162486	LOC102162486	lncRNA	0	0	0	0	0	0	0	0	0	0	0	0	0	0	0	0
BGIR9823_87926	110261182	LOC110261182	lncRNA	0	0	0	0	0	0	0	0	0	0	0	0	0	0	0	0
BGIR9823_96076	110256340	LOC110256340	lncRNA	0	1	1	0	0	0	0	0	0	0	0	0	0	0	0	0
BGIR9823_92591	100521678	MLLT10	lncRNA	0	1	1	0	0	0	0	0	0	0	0	0	0	0	0	0
BGIR9823_78251	106506784	LOC106506784	lncRNA	0	0	0	0	0	0	0	0	0	0	0	0	0	0	0	0
BGIR9823_100897	100621199	IL1RAPL1	lncRNA	0	0	0	0	0	0	0	0	0	0	0	1	0	0	0	0
BGIR9823_100113	110257459	LOC110257459	lncRNA	0	0	0	0	0	0	0	0	1	0	0	0	0	0	0	0
BGIR9823_100197	106506650	LOC106506650	lncRNA	0	0	0	0	0	0	0	0	0	0	0	0	0	0	0	0
BGIR9823_96929	751859	SIRT1	lncRNA	0	1	1	0	0	0	0	0	0	0	0	0	0	0	0	0
BGIR9823_92783	100156695	INTS6	lncRNA	0	1	1	0	0	0	0	0	0	0	0	0	0	0	0	0
BGIR9823_77974	100516051	SLC17A5	lncRNA	0	0	0	0	0	0	0	0	0	0	0	0	0	0	0	0
BGIR9823_100010	397360	CEBPB	lncRNA	0	0	0	0	0	0	0	0	0	0	0	0	0	0	0	0
BGIR9823_101617	110258914	LOC110258914	lncRNA	0	0	0	0	0	0	0	0	1	0	0	0	0	0	0	0
BGIR9823_100936	100154376	KDM6A	lncRNA	0	0	0	0	0	0	0	0	0	0	0	0	0	1	0	0
BGIR9823_79053	100522049	PTP4A1	lncRNA	1	0	0	0	0	0	0	0	0	0	0	0	0	0	0	0
BGIR9823_80764	100037285	NREP	lncRNA	0	1	1	0	0	0	0	0	0	0	0	0	0	0	0	0
BGIR9823_100812	100516527	LOC100516527	lncRNA	0	0	0	0	0	0	0	0	0	0	0	0	0	0	0	1
BGIR9823_100796	110257536	LOC110257536	lncRNA	0	0	0	0	0	0	0	0	0	0	0	0	0	0	0	0
BGIR9823_100878	102165056	MBTPS2	lncRNA	0	0	0	0	0	0	0	0	0	0	0	0	0	0	0	0
BGIR9823_100734	100512382	KCND2	lncRNA	0	0	0	0	0	0	0	0	0	0	0	0	1	0	0	0
BGIR9823_100726	100512026	ING3	lncRNA	0	0	0	0	0	0	0	0	0	0	1	0	0	0	0	0
BGIR9823_84422	100145887	ARHGEF2	lncRNA	0	0	0	0	0	0	0	0	0	0	0	0	0	0	0	0
BGIR9823_84641	100155627	LRRC8B	mRNA	0	0	0	0	0	0	0	0	0	1	0	0	0	0	0	0
BGIR9823_98254	100152049	PLPP5	mRNA	0	0	0	1	0	0	0	0	0	0	0	0	0	0	0	0
BGIR9823_87713	100517038	PUM1	lncRNA	0	1	1	0	0	0	0	0	0	0	0	0	0	0	0	0
BGIR9823_94908	100462749	ATG7	lncRNA	0	1	1	0	0	0	0	0	0	0	0	0	0	0	0	0
BGIR9823_100511	100738688	HERPUD2	lncRNA	0	0	0	0	0	0	0	0	0	0	0	0	0	0	0	0
BGIR9823_80800	100515883	PRRC1	lncRNA	1	1	0	0	0	0	0	0	0	0	0	0	0	0	0	0
BGIR9823_100550	397163	RAMP3	lncRNA	0	0	0	0	0	0	0	0	0	0	0	0	0	0	0	1
BGIR9823_94492	102167379	LOC102167379	lncRNA	0	1	1	0	0	0	0	0	0	0	0	0	0	0	0	0
XM_003126850.6	100520197	VAT1L	mRNA	0	0	0	0	0	0	0	0	0	0	0	0	0	0	0	0
BGIR9823_81847	100739684	FOXK1	lncRNA	0	0	0	0	0	0	0	0	0	0	0	0	0	0	0	0

RNA ID	Gene ID	Symbol	Type	ssc-miR-24-1-5p	ssc-miR-29b	ssc-miR-29c	ssc-miR-323	ssc-miR-369	ssc-miR-708-5p	novel-ssc-miR161-3p	novel-ssc-miR512-5p	novel-ssc-miR846-3p	novel-ssc-miR1086-5p	novel-ssc-miR1105-5p	novel-ssc-miR1116-5p	novel-ssc-miR1120-5p	novel-ssc-miR1140-5p	novel-ssc-miR1158-3p	novel-ssc-miR1379-5p
BGIR9823_100328	110257483	ADRM1	lncRNA	0	0	0	0	0	0	0	0	0	0	0	0	0	0	0	0
BGIR9823_101062	100524727	LOC100524727	lncRNA	0	0	0	0	0	0	0	0	0	0	0	0	0	0	0	0
BGIR9823_100412	414383	CHCHD3	lncRNA	0	0	0	0	0	0	0	0	0	0	0	0	0	0	0	1
BGIR9823_90505	397683	IL15	lncRNA	0	1	1	0	0	0	0	0	0	0	0	0	0	0	0	0
BGIR9823_77934	102167874	LOC102167874	lncRNA	1	0	0	0	0	0	0	0	0	0	0	0	0	0	0	0
BGIR9823_100539	100622324	NFE2L3	lncRNA	0	0	0	0	0	0	0	0	0	0	0	0	0	0	0	0
BGIR9823_101533	102158401	LOC102158401	lncRNA	0	0	0	0	0	0	0	0	0	0	0	0	0	0	0	0
BGIR9823_100121	100519631	TASP1	lncRNA	0	0	0	0	0	0	0	0	0	0	0	0	0	0	0	0
BGIR9823_100964	110257651	LOC110257651	lncRNA	0	0	0	0	0	0	0	0	0	0	0	0	0	0	0	0
BGIR9823_100069	100157832	SGCZ	lncRNA	0	0	0	0	0	0	0	0	0	0	0	0	0	0	0	0
BGIR9823_101391	110258158	TNRC6A	mRNA	0	0	0	0	0	0	0	0	0	0	0	0	0	0	0	0
BGIR9823_80729	100526040	PAM	mRNA	0	0	0	0	0	0	0	0	0	0	0	0	0	0	0	0
BGIR9823_100266	100157616	IFT52	lncRNA	0	0	0	0	0	0	0	0	0	0	0	0	0	0	0	0
BGIR9823_100743	100125380	ST7	mRNA	0	0	0	0	0	0	0	0	0	0	0	0	0	0	0	0
BGIR9823_80829	100513723	JADE2	lncRNA	0	0	0	0	0	0	0	0	0	0	0	0	0	0	0	0
BGIR9823_91326	100516387	TSEN15	lncRNA	0	0	0	0	0	0	0	0	0	0	0	0	0	0	0	0
BGIR9823_100827	100519067	CDK13	lncRNA	0	0	0	0	0	0	0	0	0	0	0	0	0	0	0	0
BGIR9823_80869	100521956	CYSTM1	lncRNA	0	0	0	0	0	0	0	0	0	0	0	0	0	1	0	0
BGIR9823_89293	100153071	TRERF1	lncRNA	0	1	1	0	0	0	0	0	0	0	0	0	0	0	0	0
BGIR9823_79511	100152487	TSHZ1	mRNA	0	0	0	0	0	0	0	0	0	0	0	0	0	0	0	0
BGIR9823_92832	106505308	PCDH9	lncRNA	0	0	0	0	0	0	0	0	0	0	0	0	0	0	0	0
BGIR9823_82541	100520835	ITSN2	lncRNA	0	0	0	0	0	0	0	0	0	0	0	0	0	0	0	0
BGIR9823_100525	100515288	CPVL	lncRNA	0	1	0	0	0	0	0	0	0	0	0	0	0	0	0	0
BGIR9823_100563	100516888	GLI3	lncRNA	0	0	0	0	0	0	0	0	0	0	0	0	0	0	0	0
BGIR9823_100515	100623924	FKBP9	lncRNA	0	0	0	0	0	0	0	0	0	0	0	0	0	0	0	0
BGIR9823_101109	100524780	ARHGAP6	lncRNA	0	0	0	0	0	0	0	0	0	0	0	0	0	0	0	0
BGIR9823_95657	100524459	SLC25A20	lncRNA	0	1	1	0	0	0	0	0	0	0	0	0	0	0	0	0
XR_001303159.2	106507279	LOC106507279	lncRNA	0	0	0	0	0	0	0	0	0	1	0	0	0	0	0	0
BGIR9823_82132	106509720	LOC106509720	lncRNA	0	1	1	0	0	0	0	0	0	0	0	0	0	0	0	0
BGIR9823_90639	110261963	LOC110261963	lncRNA	0	1	1	0	0	0	0	0	0	0	0	0	0	0	0	0
BGIR9823_92636	100516289	PFKFB3	lncRNA	0	0	0	0	0	0	0	0	0	1	0	0	0	0	0	0
BGIR9823_79919	100154648	PLPPR1	lncRNA	0	0	0	0	0	0	0	0	0	0	0	0	0	0	0	0
BGIR9823_80860	100521716	MATR3	mRNA	0	0	0	0	0	0	0	0	0	1	0	0	0	0	0	0
BGIR9823_100880	102165056	MBTPS2	lncRNA	0	0	0	0	0	0	0	0	0	0	0	0	0	0	0	0
BGIR9823_101396	100521783	RBBP6	mRNA	0	0	0	0	0	0	0	0	0	1	0	0	0	0	0	0
BGIR9823_101310	102159115	ATP6AP1	lncRNA	0	0	0	0	0	0	0	1	0	0	0	0	0	0	0	0
BGIR9823_85863	100512658	METTL25	mRNA	0	0	0	0	0	0	0	0	0	1	0	0	0	0	0	0
BGIR9823_84834	100516439	C5H12orf10	mRNA	0	0	0	0	0	0	0	0	0	0	0	0	0	0	0	0
BGIR9823_98676	397282	ACVR2A	lncRNA	0	0	0	0	0	0	1	0	0	0	0	0	0	0	0	0
BGIR9823_83976	396677	F3	mRNA	0	0	0	0	0	0	0	0	0	1	0	0	0	0	0	0
BGIR9823_90032	102167943	LOC102167943	lncRNA	0	0	0	0	0	0	0	0	0	0	0	0	0	0	0	0
BGIR9823_100671	106506771	LOC106506771	lncRNA	0	0	0	0	0	0	0	0	0	0	0	0	0	0	0	1
BGIR9823_101108	100524780	ARHGAP6	lncRNA	0	0	0	0	0	0	0	0	0	0	0	0	0	0	0	0

RNA ID	Gene ID	Symbol	Type	ssc-miR-24-1-5p	ssc-miR-29b	ssc-miR-29c	ssc-miR-323	ssc-miR-369	ssc-miR-708-5p	novel-ssc-miR161-3p	novel-ssc-miR512-5p	novel-ssc-miR846-3p	novel-ssc-miR1086-5p	novel-ssc-miR1105-5p	novel-ssc-miR1116-5p	novel-ssc-miR1120-5p	novel-ssc-miR1140-5p	novel-ssc-miR1158-3p	novel-ssc-miR1379-5p
BGIR9823_80923	100515885	SLC26A2	mRNA	0	0	0	0	0	0	0	0	0	1	0	0	0	0	0	0
BGIR9823_100494	100521052	IMMP2L	lncRNA	0	0	0	0	0	1	0	0	0	0	0	0	0	0	0	0
BGIR9823_101344	110258068	DPH7	lncRNA	0	0	0	0	0	0	0	1	0	0	0	0	0	0	0	0
BGIR9823_100314	100155190	ZFP64	lncRNA	0	0	0	0	0	0	0	0	0	1	0	0	0	0	0	0
BGIR9823_83324	100624063	LOC100624063	lncRNA	0	0	0	0	0	0	0	0	0	0	0	0	0	0	0	0
BGIR9823_100237	110257438	LOC110257438	lncRNA	0	0	0	0	0	0	0	0	0	0	0	0	0	0	0	0
BGIR9823_78253	106506784	LOC106506784	lncRNA	0	0	0	0	0	0	0	0	0	0	0	0	0	0	0	1
BGIR9823_77699	100514202	MTRF1L	lncRNA	0	0	0	0	0	0	0	0	0	0	0	0	0	0	0	0
BGIR9823_100849	733606	TMSB4X	lncRNA	0	0	0	0	0	0	0	0	0	0	0	0	0	0	0	0
BGIR9823_101513	102157759	LOC102157759	lncRNA	0	0	0	0	0	0	0	0	0	0	0	1	0	0	0	0
BGIR9823_100598	403162	INSIG1	lncRNA	0	0	0	0	0	0	0	0	0	0	0	0	0	0	0	0
BGIR9823_80828	100513723	JADE2	lncRNA	1	0	0	0	0	1	0	0	0	0	0	0	0	0	0	0
BGIR9823_80639	100514094	FCHO2	lncRNA	0	0	0	0	0	0	0	0	0	0	0	0	0	0	0	0
BGIR9823_85738	100157209	PRICKLE1	mRNA	0	0	0	0	0	0	0	0	0	1	0	0	0	0	0	0
BGIR9823_91822	100522092	RUNDC3B	lncRNA	0	1	1	0	0	0	0	0	0	0	0	0	0	0	0	0
BGIR9823_101413	110258225	LOC110258225	lncRNA	0	0	0	0	0	0	0	0	0	0	0	0	0	0	0	0
BGIR9823_89187	100153386	SLA-DRB1	lncRNA	0	1	1	0	0	0	0	0	0	0	0	0	0	0	0	0
BGIR9823_91714	100521986	MAPKAPK2	lncRNA	0	0	0	0	0	0	0	0	0	0	0	0	0	0	0	0
BGIR9823_83804	100523784	ADAR	mRNA	0	0	0	0	0	0	0	0	0	1	0	0	0	0	0	0
BGIR9823_100308	100141411	ADNP	lncRNA	0	0	0	0	0	0	0	0	0	0	0	0	0	0	0	0
BGIR9823_83998	100156806	HFM1	mRNA	0	0	0	0	0	0	0	0	0	1	0	0	0	0	0	0
BGIR9823_100710	397301	CALU	lncRNA	0	0	0	0	0	0	0	0	0	0	0	0	0	0	0	0
BGIR9823_101105	100154011	ANOS1	lncRNA	0	0	0	0	0	0	0	0	0	0	0	0	0	0	0	0
BGIR9823_88993	100144590	MEG3	lncRNA	0	0	0	0	0	0	0	0	0	0	0	0	0	0	0	0
BGIR9823_80459	100622538	DNM2	mRNA	0	0	0	0	0	0	0	0	0	1	0	0	0	0	0	0
BGIR9823_101097	397339	F8	lncRNA	0	0	0	0	0	0	0	0	0	0	0	0	0	0	0	0
BGIR9823_100022	100144536	VAPB	mRNA	0	0	0	0	0	0	0	0	0	1	0	0	0	0	0	0
BGIR9823_88787	100153736	EMC4	lncRNA	0	0	0	0	0	0	0	0	0	0	0	0	0	0	0	0
BGIR9823_93229	100518091	PCID2	mRNA	0	0	0	0	0	0	0	0	0	0	0	0	0	0	0	0
BGIR9823_100480	100621338	BMT2	lncRNA	0	0	0	0	0	0	0	0	0	0	0	0	1	0	0	0
BGIR9823_81197	100037286	ARNTL	lncRNA	0	0	0	0	0	0	0	0	0	0	0	0	0	0	0	0
BGIR9823_100187	100513829	SNPH	lncRNA	0	0	0	0	0	1	0	0	0	0	0	0	0	0	0	0
BGIR9823_97762	100512075	ASCC1	mRNA	0	0	0	0	0	0	0	0	0	0	0	0	0	0	0	0
BGIR9823_100489	493185	IFRD1	lncRNA	0	0	0	0	0	0	0	0	0	0	0	0	0	0	0	0
BGIR9823_82778	100523894	POLR3E	mRNA	0	0	0	0	0	0	0	0	0	0	1	0	0	0	0	0
BGIR9823_88002	100524685	ADGRL2	lncRNA	0	0	0	0	0	0	0	0	0	0	0	0	0	0	0	0
BGIR9823_97698	106506010	LOC106506010	lncRNA	0	0	0	0	0	0	0	0	0	0	0	0	0	0	0	0
BGIR9823_79746	100155321	ACER2	lncRNA	0	0	0	1	0	0	0	0	0	0	0	0	0	0	0	0
BGIR9823_79823	100626649	KANK1	lncRNA	0	0	0	0	0	0	0	0	0	0	0	0	0	0	0	0
BGIR9823_87325	100513473	ZNF181	lncRNA	0	0	0	0	0	0	0	0	0	0	0	0	0	0	0	0
BGIR9823_88185	100511204	ZNF643	mRNA	0	0	0	0	0	0	0	0	0	0	0	0	0	0	0	0
BGIR9823_81639	100513341	FBXL17	lncRNA	1	0	0	0	0	0	0	0	0	0	0	0	0	0	0	0
BGIR9823_94281	100516758	CORO6	mRNA	0	0	0	0	0	0	0	0	0	0	0	0	0	0	0	0

RNA ID	Gene ID	Symbol	Type	ssc-miR-24-1-5p	ssc-miR-29b	ssc-miR-29c	ssc-miR-323	ssc-miR-369	ssc-miR-708-5p	novel-ssc-miR161-3p	novel-ssc-miR512-5p	novel-ssc-miR846-3p	novel-ssc-miR1086-5p	novel-ssc-miR1105-5p	novel-ssc-miR1116-5p	novel-ssc-miR1120-5p	novel-ssc-miR1140-5p	novel-ssc-miR1158-3p	novel-ssc-miR1379-5p
BGIR9823_96391	100621858	CFAP410	mRNA	0	0	0	0	0	0	0	0	0	0	0	0	0	0	0	0
BGIR9823_77814	100736796	ADGRB3	lncRNA	0	0	0	0	0	0	0	0	0	0	0	0	0	0	0	0
BGIR9823_84738	100516801	MRTFA	lncRNA	0	0	0	0	0	0	0	0	0	0	0	0	0	0	0	0
BGIR9823_85081	110260749	LOC110260749	lncRNA	0	0	0	0	0	0	0	0	0	0	0	0	0	0	0	0
BGIR9823_83642	100127143	PDE7A	lncRNA	0	0	0	0	0	0	0	0	0	0	0	0	0	0	0	0
BGIR9823_90693	100526062	MRPS18C	lncRNA	0	0	0	0	0	0	0	0	0	0	0	0	0	0	0	0
BGIR9823_94661	100525609	STAB1	mRNA	0	0	0	0	0	0	0	0	0	0	0	0	0	0	0	0
BGIR9823_88204	100157926	RIOK1	mRNA	0	0	0	0	0	0	0	0	0	0	0	0	0	0	0	0
BGIR9823_100214	100154657	PIGU	mRNA	0	0	0	0	0	0	0	0	0	0	0	0	0	0	0	0
BGIR9823_86368	100624191	LOC100624191	mRNA	0	0	0	0	0	0	0	0	0	0	0	0	0	0	0	0
BGIR9823_98692	100525081	R3HDM1	mRNA	0	0	0	0	0	0	0	0	0	0	0	0	0	0	0	0
BGIR9823_94941	110256296	LOC110256296	lncRNA	0	0	0	0	0	0	0	0	0	0	0	0	0	0	0	0
BGIR9823_93186	100519383	DZIP1	mRNA	0	0	0	0	0	0	0	0	0	0	0	0	0	0	0	0
BGIR9823_101339	100519166	LOC100519166	lncRNA	0	0	0	0	0	0	0	0	0	0	0	1	0	0	0	0
BGIR9823_78696	641344	GNE	lncRNA	1	0	0	0	0	0	0	0	0	0	0	0	0	0	0	0
BGIR9823_90334	100514856	APBB2	mRNA	0	0	0	0	0	0	0	0	0	0	0	0	0	0	0	0
BGIR9823_79588	100155833	DPP8	mRNA	0	0	0	0	0	0	0	0	0	0	0	0	0	0	0	0
BGIR9823_93702	100622172	TAOK1	lncRNA	0	0	0	0	0	0	0	0	0	0	0	0	0	0	0	0
BGIR9823_100788	100622432	CBX3	lncRNA	0	0	0	0	0	0	0	0	0	0	0	0	0	0	0	0
BGIR9823_81760	100520896	ETF1	lncRNA	0	0	0	0	0	0	0	0	0	0	0	0	0	1	0	0
BGIR9823_84563	102167065	LOC102167065	lncRNA	0	0	0	0	0	0	0	0	0	0	0	0	0	0	0	0
BGIR9823_100600	100513894	LOC100513894	lncRNA	0	0	0	0	0	0	0	0	0	0	0	0	0	0	0	0
BGIR9823_77938	100156192	NT5DC1	lncRNA	0	0	0	0	0	1	0	0	0	0	0	0	0	0	0	0
BGIR9823_86946	100515670	ANKRD13C	lncRNA	0	1	0	0	0	0	0	0	0	0	0	0	0	0	0	0
BGIR9823_100385	100515632	KDM7A	lncRNA	0	0	0	0	0	0	0	0	0	0	0	0	0	0	0	0
BGIR9823_101073	100515811	VMA21	lncRNA	0	0	0	0	0	0	0	0	0	0	0	0	0	0	0	0
BGIR9823_79171	791125	FYN	lncRNA	0	0	0	0	0	0	0	0	0	0	0	0	0	0	0	0
BGIR9823_91118	106504956	LOC106504956	lncRNA	0	1	0	0	0	0	0	0	0	0	0	0	0	0	0	0
BGIR9823_86164	102164889	LOC102164889	lncRNA	0	0	0	0	0	0	0	0	0	0	0	0	0	0	0	0
BGIR9823_78738	100153669	TMEM38B	lncRNA	0	0	0	0	0	0	0	0	0	0	0	0	0	0	0	1
BGIR9823_88541	100522555	ABHD17C	lncRNA	0	0	0	0	0	0	0	0	0	0	0	0	0	0	0	0
BGIR9823_100267	100624468	OSER1	lncRNA	0	0	0	0	0	0	0	0	0	0	0	0	0	0	0	0
BGIR9823_100299	100625841	DDX27	lncRNA	0	0	0	0	0	0	0	0	0	0	0	0	0	0	0	0
BGIR9823_78983	100622156	TNFAIP3	lncRNA	1	0	0	0	0	0	0	0	0	0	0	0	0	0	0	0
BGIR9823_79796	574058	CD274	lncRNA	0	0	0	0	0	0	0	0	0	0	0	0	0	0	0	0
BGIR9823_82690	100520239	LRWD1	lncRNA	0	0	0	0	0	0	0	0	0	0	0	0	0	0	0	0
BGIR9823_100392	100415778	ZC3HAV1	lncRNA	0	0	0	0	0	0	0	0	0	0	0	0	0	0	0	0
BGIR9823_79386	102162809	CTDSPL2	lncRNA	0	0	0	0	0	0	0	0	0	0	0	0	0	0	0	0
BGIR9823_87847	100627122	SMCHD1	lncRNA	0	0	0	0	0	0	0	0	0	0	0	0	0	0	0	0
BGIR9823_83136	397040	UGP2	mRNA	0	0	0	0	0	0	0	0	0	0	0	0	0	0	0	0
BGIR9823_89310	100151914	MRPL2	lncRNA	0	0	0	0	0	0	0	0	0	0	0	0	0	0	0	0
BGIR9823_82954	106509726	LOC106509726	lncRNA	0	0	0	0	0	0	0	0	0	0	0	0	0	0	0	0
BGIR9823_100019	100141410	ZNF217	lncRNA	0	0	0	0	0	0	0	0	0	0	0	0	0	0	0	0

RNA ID	Gene ID	Symbol	Type	ssc-miR-24-1-5p	ssc-miR-29b	ssc-miR-29c	ssc-miR-323	ssc-miR-369	ssc-miR-708-5p	novel-ssc-miR161-3p	novel-ssc-miR512-5p	novel-ssc-miR846-3p	novel-ssc-miR1086-5p	novel-ssc-miR1105-5p	novel-ssc-miR1116-5p	novel-ssc-miR1120-5p	novel-ssc-miR1140-5p	novel-ssc-miR1158-3p	novel-ssc-miR1379-5p
BGIR9823_101550	110258600	LOC110258600	lncRNA	0	0	0	0	0	0	0	0	0	0	0	0	0	0	0	0
BGIR9823_80641	100515474	UTP15	lncRNA	0	0	0	0	0	0	0	0	0	0	0	0	0	0	0	0
BGIR9823_85022	100518251	KRAS	lncRNA	0	0	0	0	0	0	0	0	0	0	0	0	0	0	0	0
BGIR9823_78496	100512450	TMEM260	lncRNA	0	0	0	0	0	0	0	0	0	0	0	0	0	0	0	0
BGIR9823_100476	100621338	BMT2	lncRNA	0	0	0	0	0	0	0	0	0	0	0	0	0	0	0	0
BGIR9823_102668	BGIG9823_27318		lncRNA	0	0	0	0	0	0	0	0	0	0	0	0	0	0	0	0
BGIR9823_101962	BGIG9823_26639		lncRNA	0	0	0	0	0	0	0	0	0	0	0	0	0	0	0	0
BGIR9823_102175	BGIG9823_26847		lncRNA	0	0	0	0	0	0	0	0	0	0	0	0	0	0	0	0
BGIR9823_101847	BGIG9823_26526		lncRNA	0	0	0	0	0	0	0	0	0	0	1	0	0	0	0	0
BGIR9823_101781	BGIG9823_26461		lncRNA	0	0	0	0	0	0	0	0	0	0	0	0	0	0	0	0
BGIR9823_102445	BGIG9823_27101		lncRNA	0	0	0	0	0	0	0	0	0	0	0	0	0	0	0	0
BGIR9823_101840	BGIG9823_26519		lncRNA	0	0	0	0	0	0	0	0	0	0	1	0	0	0	0	0
BGIR9823_102485	BGIG9823_27139		lncRNA	0	0	0	0	0	0	0	0	0	0	0	0	1	0	0	0
BGIR9823_102195	BGIG9823_26867		lncRNA	0	0	0	0	0	0	0	0	0	0	0	0	0	0	0	1
BGIR9823_101771	BGIG9823_26451		lncRNA	1	0	0	0	0	0	0	0	0	0	0	0	0	0	0	0
BGIR9823_101831	BGIG9823_26510		lncRNA	0	0	0	0	0	0	0	0	0	0	0	0	0	0	0	0
BGIR9823_102544	BGIG9823_27197		lncRNA	0	0	0	0	0	0	0	0	0	0	0	0	0	0	0	0
BGIR9823_102359	BGIG9823_27024		lncRNA	0	0	0	0	0	0	0	0	0	0	0	0	0	0	0	0
BGIR9823_102456	BGIG9823_27110		lncRNA	0	0	0	0	0	0	0	0	0	0	0	0	0	0	0	0
BGIR9823_102657	BGIG9823_27307		lncRNA	0	0	0	0	0	0	0	0	0	0	0	0	0	0	0	0
BGIR9823_102305	BGIG9823_26970		lncRNA	0	0	0	0	0	0	0	0	0	0	0	0	0	0	0	0
BGIR9823_102228	BGIG9823_26900		lncRNA	0	0	0	0	0	0	0	0	0	0	0	0	0	0	0	0
BGIR9823_101778	BGIG9823_26458		lncRNA	0	0	0	0	0	0	0	0	0	0	0	0	1	0	0	0
BGIR9823_102408	BGIG9823_27069		lncRNA	0	0	0	0	0	0	0	0	0	0	0	0	0	0	0	0
BGIR9823_101997	BGIG9823_26673		lncRNA	0	0	0	0	0	0	0	0	0	0	0	0	0	0	0	0
BGIR9823_101886	BGIG9823_26563		lncRNA	0	0	0	0	0	0	0	0	0	0	0	0	0	0	0	0
BGIR9823_101714	BGIG9823_26398		lncRNA	0	0	0	0	0	0	0	0	0	0	0	0	0	0	0	0
BGIR9823_102000	BGIG9823_26676		lncRNA	0	0	0	0	0	0	0	0	0	0	0	0	1	0	0	0
BGIR9823_102540	BGIG9823_27193		lncRNA	0	0	0	0	0	0	0	0	0	0	0	0	0	0	0	0
BGIR9823_101852	BGIG9823_26531		lncRNA	0	0	0	0	0	0	0	0	0	0	0	0	0	0	0	0
BGIR9823_102203	BGIG9823_26875		lncRNA	0	0	0	0	0	0	0	0	0	0	0	0	0	0	0	0
BGIR9823_102422	BGIG9823_27083		lncRNA	0	0	0	0	0	0	0	0	0	0	0	0	0	0	0	0
BGIR9823_101937	BGIG9823_26614		lncRNA	0	0	0	0	0	0	0	0	0	0	0	0	0	0	0	0
BGIR9823_102117	BGIG9823_26791		lncRNA	0	0	0	0	0	0	0	0	0	0	0	0	0	0	0	0
BGIR9823_102005	BGIG9823_26681		lncRNA	0	0	0	0	0	0	0	0	0	0	0	0	0	0	0	0
BGIR9823_102177	BGIG9823_26849		lncRNA	0	0	0	0	0	0	0	0	0	0	0	0	0	0	0	0
BGIR9823_102213	BGIG9823_26885		lncRNA	0	0	0	0	0	0	0	0	0	0	0	0	0	0	0	0
BGIR9823_102067	BGIG9823_26741		lncRNA	0	0	0	0	0	0	0	0	0	0	0	0	0	0	0	0
BGIR9823_102357	BGIG9823_27022		lncRNA	0	0	0	0	0	0	0	0	0	0	0	0	0	0	0	0
BGIR9823_102443	BGIG9823_27099		lncRNA	0	0	0	0	0	0	0	0	0	0	0	0	0	0	0	0
BGIR9823_102395	BGIG9823_27060		lncRNA	0	0	0	0	0	0	0	0	0	0	0	0	0	0	0	0
BGIR9823_102615	BGIG9823_27265		lncRNA	0	0	0	0	0	0	0	0	0	0	0	0	0	0	0	0
BGIR9823_102264	BGIG9823_26932		lncRNA	0	0	0	0	0	0	0	0	0	0	0	0	0	0	0	0

RNA ID	Gene ID	Symbol	Type	ssc-miR-24-1-5p	ssc-miR-29b	ssc-miR-29c	ssc-miR-323	ssc-miR-369	ssc-miR-708-5p	novel-ssc-miR161-3p	novel-ssc-miR512-5p	novel-ssc-miR846-3p	novel-ssc-miR1086-5p	novel-ssc-miR1105-5p	novel-ssc-miR1116-5p	novel-ssc-miR1120-5p	novel-ssc-miR1140-5p	novel-ssc-miR1158-3p	novel-ssc-miR1379-5p
BGIR9823_102052	BGIG9823_26726		lncRNA	0	0	0	0	0	0	0	0	0	0	0	0	0	0	0	0
BGIR9823_102131	BGIG9823_26804		lncRNA	0	0	0	0	0	0	0	0	0	0	0	0	0	0	0	0
BGIR9823_101987	BGIG9823_26663		lncRNA	0	0	0	0	0	0	0	0	0	0	0	0	0	0	0	0
BGIR9823_102764	BGIG9823_27402		lncRNA	0	0	0	0	0	0	0	0	0	0	0	0	0	1	0	0
BGIR9823_102137	BGIG9823_26810		lncRNA	0	0	0	0	0	0	0	0	0	0	0	0	0	0	0	0
BGIR9823_102168	BGIG9823_26841		lncRNA	0	0	0	0	0	0	0	0	0	0	0	0	0	0	0	0
BGIR9823_101967	BGIG9823_26644		lncRNA	0	0	0	0	0	0	0	0	0	0	0	0	0	0	0	0
BGIR9823_102393	BGIG9823_27058		lncRNA	0	0	0	0	0	0	0	0	0	0	0	0	0	0	0	0
BGIR9823_101927	BGIG9823_26604		lncRNA	0	0	0	0	0	0	0	0	0	0	0	0	0	0	0	0
BGIR9823_102343	BGIG9823_27008		lncRNA	1	0	0	0	0	0	0	0	0	0	0	0	0	0	0	0
BGIR9823_102562	BGIG9823_27212		mRNA	0	0	0	0	0	0	0	0	0	0	0	0	0	0	0	0
BGIR9823_102090	BGIG9823_26764		lncRNA	0	0	0	0	0	0	0	0	0	0	0	0	0	0	0	0
BGIR9823_101742	BGIG9823_26423		lncRNA	0	0	0	0	0	0	0	0	0	0	0	0	0	0	0	0
BGIR9823_102398	BGIG9823_27061		lncRNA	0	0	0	0	0	0	0	0	0	0	0	0	1	0	0	0
BGIR9823_101721	BGIG9823_26405		lncRNA	0	0	0	0	0	0	0	0	0	0	0	0	0	0	0	0
BGIR9823_102545	BGIG9823_27198		lncRNA	0	0	0	0	0	0	0	0	0	0	0	0	0	0	0	0
BGIR9823_102138	BGIG9823_26811		lncRNA	0	0	0	0	0	0	0	0	0	0	0	0	1	0	0	0
BGIR9823_102026	BGIG9823_26701		lncRNA	0	0	0	0	0	0	0	0	0	0	0	0	0	0	0	0
BGIR9823_102057	BGIG9823_26731		lncRNA	0	0	0	0	0	0	0	0	0	0	0	0	0	0	0	0
BGIR9823_101995	BGIG9823_26671		lncRNA	0	0	0	0	0	0	0	0	0	0	0	0	0	0	0	0
BGIR9823_102725	BGIG9823_27371		lncRNA	0	0	0	0	0	0	0	0	0	0	0	0	0	0	0	0
BGIR9823_101717	BGIG9823_26401		lncRNA	0	0	0	0	0	0	0	0	0	0	0	0	0	0	0	0
BGIR9823_102441	BGIG9823_27097		lncRNA	0	0	0	0	0	0	0	0	0	0	0	0	0	0	0	0
BGIR9823_102674	BGIG9823_27324		lncRNA	0	0	0	0	0	0	0	0	0	0	0	0	0	0	0	0
BGIR9823_101843	BGIG9823_26522		lncRNA	0	0	0	0	0	0	0	0	0	0	0	0	0	0	0	0
BGIR9823_102119	BGIG9823_26793		lncRNA	0	0	0	0	0	0	0	0	0	0	0	0	1	0	0	0
BGIR9823_101819	BGIG9823_26499		lncRNA	0	0	0	0	0	0	0	0	0	0	0	0	0	0	0	0
BGIR9823_102034	BGIG9823_26709		lncRNA	0	0	0	0	0	0	0	0	0	0	0	0	0	0	0	0
BGIR9823_102373	BGIG9823_27038		lncRNA	0	0	0	0	0	0	0	0	0	0	0	0	0	0	0	0
BGIR9823_101905	BGIG9823_26582		lncRNA	0	0	0	0	0	0	0	0	0	0	0	0	0	0	0	0
BGIR9823_102629	BGIG9823_27279		lncRNA	0	0	0	0	0	0	0	0	0	0	0	0	0	0	0	0
BGIR9823_102328	BGIG9823_26993		lncRNA	0	0	0	0	0	0	0	0	0	0	0	0	0	0	0	0
BGIR9823_102165	BGIG9823_26838		lncRNA	0	0	0	0	0	0	0	0	0	0	0	0	0	1	0	0
BGIR9823_101792	BGIG9823_26472		lncRNA	0	0	0	0	0	0	0	0	0	0	0	0	0	0	0	0
BGIR9823_101992	BGIG9823_26668		lncRNA	0	0	0	0	0	0	0	0	0	0	0	0	0	0	0	0
BGIR9823_101767	BGIG9823_26447		lncRNA	1	0	0	0	0	0	0	0	0	0	0	0	0	0	0	0
BGIR9823_101956	BGIG9823_26633		lncRNA	0	0	0	0	0	0	0	0	0	0	0	1	0	0	0	0
BGIR9823_102186	BGIG9823_26858		lncRNA	0	0	0	0	0	0	0	0	0	0	0	0	0	0	0	0
BGIR9823_102630	BGIG9823_27280		lncRNA	0	0	0	0	0	0	0	0	0	0	0	0	0	0	0	0
BGIR9823_101685	BGIG9823_26372		lncRNA	0	0	0	0	0	0	0	0	0	0	0	0	0	0	0	0
BGIR9823_101700	BGIG9823_26387		lncRNA	0	0	0	0	0	0	0	0	0	0	0	0	0	0	0	0
BGIR9823_102217	BGIG9823_26889		lncRNA	0	0	0	0	0	0	0	0	0	0	0	0	0	0	0	0
BGIR9823_102093	BGIG9823_26767		lncRNA	0	0	0	0	0	0	0	0	0	0	0	1	0	0	0	0

RNA ID	Gene ID	Symbol	Type	ssc-miR-24-1-5p	ssc-miR-29b	ssc-miR-29c	ssc-miR-323	ssc-miR-369	ssc-miR-708-5p	novel-ssc-miR161-3p	novel-ssc-miR512-5p	novel-ssc-miR846-3p	novel-ssc-miR1086-5p	novel-ssc-miR1105-5p	novel-ssc-miR1116-5p	novel-ssc-miR1120-5p	novel-ssc-miR1140-5p	novel-ssc-miR1158-3p	novel-ssc-miR1379-5p
BGIR9823_101824	BGIG9823_26503		lncRNA	0	0	0	0	0	0	0	0	1	0	0	0	0	0	0	0
BGIR9823_102083	BGIG9823_26757		lncRNA	0	0	0	0	0	0	0	0	0	0	0	0	0	0	0	0
BGIR9823_102805	BGIG9823_27433		lncRNA	0	0	0	0	0	0	0	0	0	0	0	0	0	0	0	0
BGIR9823_101826	BGIG9823_26505		lncRNA	0	0	0	0	0	0	0	0	0	0	0	0	0	0	0	0
BGIR9823_102136	BGIG9823_26809		lncRNA	1	0	0	0	0	0	0	0	0	0	0	0	0	0	0	0
BGIR9823_101982	BGIG9823_26658		lncRNA	1	0	0	0	0	0	0	0	0	0	0	0	0	0	0	0
BGIR9823_101949	BGIG9823_26626		lncRNA	0	0	0	0	0	0	0	0	0	0	0	0	0	0	0	0
BGIR9823_102478	BGIG9823_27132		lncRNA	0	0	0	0	0	1	0	0	0	0	0	0	0	0	0	0
BGIR9823_101746	BGIG9823_26427		lncRNA	0	0	0	0	0	0	0	0	0	0	0	0	0	1	0	0
BGIR9823_101942	BGIG9823_26619		lncRNA	1	0	0	0	0	0	0	0	0	0	0	0	0	0	0	0
BGIR9823_102059	BGIG9823_26733		lncRNA	0	0	0	0	0	0	0	0	0	0	0	0	0	0	0	0
BGIR9823_102156	BGIG9823_26829		lncRNA	0	0	0	0	0	0	0	0	0	0	0	0	0	0	0	0
BGIR9823_101875	BGIG9823_26554		lncRNA	0	0	0	0	0	0	0	0	0	0	0	0	0	0	0	0
BGIR9823_101893	BGIG9823_26570		lncRNA	0	0	0	0	0	0	0	0	0	0	0	0	0	0	0	0
BGIR9823_101659	BGIG9823_26357		lncRNA	0	0	0	0	0	0	0	0	0	0	0	0	0	0	0	0
BGIR9823_102534	BGIG9823_27187		lncRNA	0	0	0	0	0	1	0	0	0	0	0	0	0	0	0	0
BGIR9823_101719	BGIG9823_26403		lncRNA	0	0	0	0	0	0	0	0	0	0	0	0	0	1	0	0
BGIR9823_102665	BGIG9823_27315		lncRNA	0	0	0	0	0	0	0	0	0	0	0	0	0	0	0	0
BGIR9823_102586	BGIG9823_27236		lncRNA	0	0	0	0	0	0	0	0	0	0	0	0	0	0	0	0
BGIR9823_102799	BGIG9823_27428		lncRNA	0	0	0	0	0	0	0	0	0	0	0	0	0	0	0	0
BGIR9823_101782	BGIG9823_26462		lncRNA	0	0	0	0	0	0	0	0	0	0	0	0	0	0	0	0
BGIR9823_102163	BGIG9823_26836		lncRNA	0	0	0	0	0	0	0	0	0	0	0	0	0	0	0	0
BGIR9823_102623	BGIG9823_27273		lncRNA	1	0	0	0	0	0	0	0	0	0	0	0	0	0	0	0
BGIR9823_102550	BGIG9823_27201		lncRNA	0	0	0	0	0	0	0	0	0	0	0	0	0	0	0	0
BGIR9823_101952	BGIG9823_26629		lncRNA	0	0	0	0	0	0	0	0	0	0	0	0	0	0	0	0
BGIR9823_102084	BGIG9823_26758		lncRNA	0	0	0	0	0	0	0	0	0	0	0	0	0	0	0	0
BGIR9823_102746	BGIG9823_27389		lncRNA	0	0	0	0	0	0	0	0	0	0	0	0	0	0	0	0
BGIR9823_102196	BGIG9823_26868		lncRNA	0	0	0	0	0	0	0	0	0	0	0	0	0	0	0	0
BGIR9823_101791	BGIG9823_26471		lncRNA	0	0	0	0	0	0	0	0	0	0	0	0	0	0	0	0
BGIR9823_101707	BGIG9823_26391		lncRNA	0	0	0	0	0	0	0	0	0	0	0	0	0	0	0	0
BGIR9823_102788	BGIG9823_27419		lncRNA	0	0	0	0	0	0	0	0	0	0	0	0	0	0	0	0
BGIR9823_102349	BGIG9823_27014		lncRNA	0	0	0	0	0	0	0	0	0	0	0	0	0	0	0	0
BGIR9823_102713	BGIG9823_27363		lncRNA	0	0	0	0	0	0	0	0	0	0	0	0	0	0	0	0
BGIR9823_101686	BGIG9823_26373		lncRNA	0	0	0	0	0	0	0	0	0	0	0	0	0	0	0	0
BGIR9823_102210	BGIG9823_26882		lncRNA	0	0	0	0	0	0	0	0	0	0	0	0	0	0	0	0
BGIR9823_102323	BGIG9823_26988		lncRNA	0	0	0	0	0	0	0	0	0	0	0	0	0	0	0	0
BGIR9823_102016	BGIG9823_26692		mRNA	0	0	0	0	0	0	0	0	0	0	1	0	0	0	0	0
BGIR9823_102051	BGIG9823_26725		lncRNA	0	0	0	0	0	0	0	0	0	0	0	0	0	0	0	0
BGIR9823_101891	BGIG9823_26568		lncRNA	0	0	0	0	0	0	0	0	0	0	0	0	0	0	0	0
BGIR9823_102043	BGIG9823_26717		lncRNA	0	0	0	0	0	0	0	0	0	0	0	0	0	0	0	0
BGIR9823_102464	BGIG9823_27118		lncRNA	0	0	0	0	0	0	0	0	0	0	0	0	0	0	0	0
BGIR9823_102384	BGIG9823_27049		lncRNA	0	0	0	0	0	0	0	0	0	0	0	0	0	0	0	0
BGIR9823_102347	BGIG9823_27012		lncRNA	0	0	0	0	0	0	0	0	0	0	0	0	0	0	0	0

RNA ID	Gene ID	Symbol	Type	ssc-miR-24-1-5p	ssc-miR-29b	ssc-miR-29c	ssc-miR-323	ssc-miR-369	ssc-miR-708-5p	novel-ssc-miR161-3p	novel-ssc-miR512-5p	novel-ssc-miR846-3p	novel-ssc-miR1086-5p	novel-ssc-miR1105-5p	novel-ssc-miR1116-5p	novel-ssc-miR1120-5p	novel-ssc-miR1140-5p	novel-ssc-miR1158-3p	novel-ssc-miR1379-5p
BGIR9823_101855	BGIG9823_26534		lncRNA	0	0	0	0	0	0	0	0	1	0	0	0	0	0	0	0
BGIR9823_102597	BGIG9823_27247		lncRNA	0	0	0	0	0	0	0	0	0	0	0	0	0	0	0	1
BGIR9823_101902	BGIG9823_26579		lncRNA	0	0	0	0	0	0	0	0	0	0	0	0	0	0	0	0
BGIR9823_102413	BGIG9823_27074		mRNA	0	0	0	0	0	0	0	0	0	0	0	0	0	0	0	0
BGIR9823_102726	BGIG9823_27372		lncRNA	0	0	0	0	0	0	0	0	0	0	0	0	0	0	0	0
BGIR9823_102647	BGIG9823_27297		lncRNA	0	0	0	0	0	0	0	0	0	0	0	0	0	0	0	0
BGIR9823_101973	BGIG9823_26650		lncRNA	0	0	0	0	0	0	0	0	0	0	0	0	1	0	0	0
BGIR9823_102297	BGIG9823_26963		lncRNA	0	0	0	0	0	0	0	0	0	0	0	0	0	0	0	0
BGIR9823_102389	BGIG9823_27054		lncRNA	0	0	0	0	0	0	0	0	0	0	0	0	0	0	0	0
BGIR9823_101788	BGIG9823_26468		lncRNA	0	0	0	0	0	0	0	0	0	0	0	0	0	0	0	0
BGIR9823_101961	BGIG9823_26638		lncRNA	0	0	0	0	0	0	0	0	0	0	0	1	0	0	0	0
BGIR9823_102423	BGIG9823_27084		lncRNA	0	0	0	0	0	0	0	0	0	0	0	0	1	0	0	0
BGIR9823_102712	BGIG9823_27362		lncRNA	0	0	0	0	0	0	0	0	0	0	0	0	0	0	0	0
BGIR9823_101682	BGIG9823_26369		lncRNA	0	0	0	0	0	0	0	0	1	0	0	0	0	0	0	0
BGIR9823_101865	BGIG9823_26544		lncRNA	0	0	0	0	0	0	0	0	0	0	0	0	0	0	0	0
BGIR9823_102351	BGIG9823_27016		lncRNA	0	0	0	0	0	0	0	0	0	0	0	0	0	0	0	0
BGIR9823_102576	BGIG9823_27226		lncRNA	0	0	0	0	0	0	0	0	0	0	0	0	1	0	0	0
BGIR9823_102428	BGIG9823_27088		lncRNA	0	0	0	0	0	0	0	0	0	0	0	0	0	0	0	0
BGIR9823_102329	BGIG9823_26994		lncRNA	0	0	0	0	0	0	0	0	0	0	0	0	0	0	0	0
BGIR9823_102625	BGIG9823_27275		lncRNA	0	0	0	0	0	0	0	0	0	0	0	0	0	0	0	0
BGIR9823_102075	BGIG9823_26749		lncRNA	0	0	0	0	0	0	0	0	0	0	0	0	0	0	0	0
BGIR9823_102523	BGIG9823_27176		lncRNA	0	0	0	0	0	0	0	0	0	0	0	0	0	0	0	0
BGIR9823_101910	BGIG9823_26587		lncRNA	0	0	0	0	0	0	0	0	0	0	0	0	0	0	0	0
BGIR9823_102748	BGIG9823_27391		lncRNA	0	0	0	0	0	0	0	0	0	0	0	0	0	0	0	0
BGIR9823_101705	BGIG9823_26389		lncRNA	0	0	0	0	0	0	0	0	0	0	0	0	0	0	0	0
BGIR9823_102467	BGIG9823_27121		lncRNA	1	0	0	0	0	0	0	0	0	0	0	0	0	0	0	0
BGIR9823_102501	BGIG9823_27155		lncRNA	0	0	0	0	0	0	0	0	0	0	0	0	0	0	0	0
BGIR9823_101779	BGIG9823_26459		lncRNA	0	0	0	0	0	0	0	0	0	0	0	0	0	0	0	0
BGIR9823_102431	BGIG9823_27090		lncRNA	0	0	0	0	0	0	0	0	0	0	0	0	0	0	0	0
BGIR9823_102628	BGIG9823_27278		lncRNA	0	0	0	0	0	0	0	0	0	0	0	0	0	0	0	0
BGIR9823_101816	BGIG9823_26496		lncRNA	0	0	0	0	0	0	0	0	0	0	0	0	0	0	0	0
BGIR9823_101996	BGIG9823_26672		lncRNA	0	0	0	0	0	0	0	0	0	0	0	0	0	0	0	0
BGIR9823_102304	BGIG9823_26969		lncRNA	0	0	0	0	0	0	0	0	0	0	0	0	0	0	0	0
BGIR9823_101800	BGIG9823_26480		lncRNA	0	0	0	0	0	0	0	0	0	0	0	0	0	0	0	0
BGIR9823_102273	BGIG9823_26940		lncRNA	0	0	0	0	0	0	0	0	0	0	0	0	1	0	0	0
BGIR9823_102407	BGIG9823_27068		lncRNA	0	0	0	0	0	0	0	0	0	0	0	0	0	0	0	0
BGIR9823_102091	BGIG9823_26765		lncRNA	0	0	0	0	0	0	0	0	0	0	0	1	0	0	0	0
BGIR9823_102167	BGIG9823_26840		lncRNA	0	0	0	0	0	0	0	0	0	0	0	0	0	0	0	0
BGIR9823_102166	BGIG9823_26839		lncRNA	0	0	0	0	0	0	0	0	0	0	0	0	0	0	0	0
BGIR9823_102159	BGIG9823_26832		lncRNA	0	0	0	0	0	0	0	0	0	0	0	0	1	0	0	0
BGIR9823_102041	BGIG9823_26715		lncRNA	0	0	0	0	0	0	0	0	0	0	0	0	0	0	0	0
BGIR9823_102369	BGIG9823_27034		lncRNA	0	0	0	0	0	0	0	0	0	0	0	0	0	0	0	0
BGIR9823_101766	BGIG9823_26446		lncRNA	0	0	0	0	0	0	0	0	0	0	0	0	1	0	0	0

RNA ID	Gene ID	Symbol	Type	ssc-miR-24-1-5p	ssc-miR-29b	ssc-miR-29c	ssc-miR-323	ssc-miR-369	ssc-miR-708-5p	novel-ssc-miR161-3p	novel-ssc-miR512-5p	novel-ssc-miR846-3p	novel-ssc-miR1086-5p	novel-ssc-miR1105-5p	novel-ssc-miR1116-5p	novel-ssc-miR1120-5p	novel-ssc-miR1140-5p	novel-ssc-miR1158-3p	novel-ssc-miR1379-5p
BGIR9823_102029	BGIG9823_26704		lncRNA	0	0	0	0	0	0	0	0	1	0	0	0	0	0	0	0
BGIR9823_102522	BGIG9823_27175		lncRNA	0	0	0	0	0	0	0	0	0	0	0	1	0	0	0	0
BGIR9823_102783	BGIG9823_27414		lncRNA	0	0	0	0	0	0	0	0	0	0	0	0	0	0	0	0
BGIR9823_101935	BGIG9823_26612		lncRNA	0	0	0	0	0	0	0	0	0	0	0	0	0	0	0	0
BGIR9823_102254	BGIG9823_26922		lncRNA	0	0	0	0	0	0	0	0	0	0	0	0	1	0	0	0
BGIR9823_102184	BGIG9823_26856		lncRNA	0	0	0	0	0	0	0	0	0	0	0	0	0	0	0	0
BGIR9823_102246	BGIG9823_26914		lncRNA	1	0	0	0	0	0	0	0	0	0	0	0	0	0	0	0
BGIR9823_101751	BGIG9823_26432		lncRNA	0	0	0	0	0	0	0	0	0	0	0	0	0	0	0	0
BGIR9823_101673	BGIG9823_26360		lncRNA	0	0	0	0	0	0	0	0	0	0	0	0	0	0	0	0
BGIR9823_101811	BGIG9823_26491		lncRNA	0	0	0	0	0	0	0	0	0	0	0	1	0	0	0	0
BGIR9823_102295	BGIG9823_26961		mRNA	0	0	0	0	0	0	0	0	0	0	0	0	0	0	0	0
BGIR9823_101697	BGIG9823_26384		lncRNA	0	0	0	0	0	0	0	0	0	0	0	0	0	0	0	0
BGIR9823_101725	BGIG9823_26409		lncRNA	0	0	0	0	0	0	0	0	0	0	0	0	0	0	0	0
BGIR9823_102033	BGIG9823_26708		lncRNA	0	0	0	0	0	0	0	0	0	0	0	0	0	0	0	0
BGIR9823_102053	BGIG9823_26727		lncRNA	0	0	0	0	0	0	0	0	0	0	0	0	0	0	0	0
BGIR9823_102015	BGIG9823_26691		lncRNA	0	0	0	0	0	1	0	0	0	0	0	0	0	0	0	0
BGIR9823_102489	BGIG9823_27143		lncRNA	0	0	0	0	0	0	0	0	0	0	0	0	0	0	0	1
BGIR9823_102449	BGIG9823_27104		lncRNA	0	0	0	0	0	0	0	0	0	0	0	0	0	0	0	0
BGIR9823_102214	BGIG9823_26886		lncRNA	0	0	0	0	0	0	0	0	0	0	0	0	0	0	0	0
BGIR9823_101834	BGIG9823_26513		lncRNA	0	0	0	0	0	0	0	0	0	0	0	0	0	0	0	0
BGIR9823_102636	BGIG9823_27286		lncRNA	0	0	0	0	0	0	0	0	0	0	0	0	0	0	0	0
BGIR9823_102287	BGIG9823_26954		lncRNA	0	0	0	0	0	0	0	0	0	0	0	0	0	0	0	0
BGIR9823_101906	BGIG9823_26583		lncRNA	0	0	0	0	0	0	0	0	0	0	0	0	0	0	0	1
BGIR9823_102514	BGIG9823_27168		lncRNA	0	0	0	0	0	0	0	0	0	0	0	0	0	0	0	0
BGIR9823_101914	BGIG9823_26591		lncRNA	1	0	0	0	0	0	0	0	0	0	0	0	0	0	0	0
BGIR9823_101768	BGIG9823_26448		lncRNA	0	0	0	0	0	0	0	0	0	0	0	0	0	0	0	0
BGIR9823_102070	BGIG9823_26744		lncRNA	0	0	0	0	0	0	0	0	0	0	0	0	0	0	0	0
BGIR9823_101805	BGIG9823_26485		lncRNA	0	0	0	0	0	0	0	0	0	0	0	0	0	0	0	0
BGIR9823_102103	BGIG9823_26777		lncRNA	0	0	0	0	0	0	0	0	0	0	0	0	0	0	0	0
BGIR9823_102688	BGIG9823_27338		lncRNA	0	0	0	0	0	0	0	0	0	0	0	0	0	0	0	0
BGIR9823_102733	BGIG9823_27379		lncRNA	0	0	0	0	0	0	0	0	0	0	0	0	0	0	0	1
BGIR9823_102411	BGIG9823_27072		lncRNA	0	0	0	0	0	0	0	0	0	0	0	0	0	0	0	0
BGIR9823_102360	BGIG9823_27025		lncRNA	0	0	0	0	0	0	0	0	0	0	0	0	0	0	0	0
BGIR9823_102558	BGIG9823_27208		lncRNA	0	0	0	0	0	0	0	0	0	0	0	1	0	0	0	0
BGIR9823_101837	BGIG9823_26516		lncRNA	0	0	0	0	0	0	0	0	0	0	0	0	0	0	0	0
BGIR9823_101722	BGIG9823_26406		lncRNA	0	0	0	0	0	0	0	0	0	0	0	0	0	0	0	0
BGIR9823_102750	BGIG9823_27392		lncRNA	0	0	0	0	0	0	0	0	0	0	0	0	0	0	0	0
BGIR9823_102692	BGIG9823_27342		lncRNA	0	0	0	0	0	0	0	0	0	0	0	0	0	0	0	0
BGIR9823_102755	BGIG9823_27395		lncRNA	0	0	0	0	0	0	0	0	0	0	0	0	0	1	0	0
BGIR9823_101957	BGIG9823_26634		lncRNA	0	0	0	0	0	0	0	0	0	0	0	0	0	0	0	0
BGIR9823_102097	BGIG9823_26771		lncRNA	0	0	0	0	0	0	0	0	0	0	0	0	1	0	0	0
BGIR9823_102454	BGIG9823_27108		lncRNA	0	0	0	0	0	0	0	0	0	0	0	0	0	0	0	0
BGIR9823_102019	BGIG9823_26694		lncRNA	0	0	0	0	0	0	0	0	1	0	0	0	0	0	0	0

RNA ID	Gene ID	Symbol	Type	ssc-miR-24-1-5p	ssc-miR-29b	ssc-miR-29c	ssc-miR-323	ssc-miR-369	ssc-miR-708-5p	novel-ssc-miR161-3p	novel-ssc-miR512-5p	novel-ssc-miR846-3p	novel-ssc-miR1086-5p	novel-ssc-miR1105-5p	novel-ssc-miR1116-5p	novel-ssc-miR1120-5p	novel-ssc-miR1140-5p	novel-ssc-miR1158-3p	novel-ssc-miR1379-5p
BGIR9823_101736	BGIG9823_26417		lncRNA	0	0	0	0	0	0	0	0	0	0	0	0	0	0	0	0
BGIR9823_102662	BGIG9823_27312		lncRNA	0	0	0	0	0	1	0	0	0	0	0	0	0	0	0	0
BGIR9823_102303	BGIG9823_26969		lncRNA	0	0	0	0	0	0	0	0	0	0	0	1	0	0	0	0
BGIR9823_101931	BGIG9823_26608		lncRNA	0	0	0	0	0	0	0	0	0	0	0	0	0	0	0	0
BGIR9823_101842	BGIG9823_26521		lncRNA	0	0	0	0	0	0	0	0	0	0	0	0	0	0	0	1
BGIR9823_101783	BGIG9823_26463		lncRNA	0	0	0	0	0	0	0	0	0	0	0	0	0	0	0	0
BGIR9823_102447	BGIG9823_27102		lncRNA	0	0	0	0	0	0	0	0	0	0	0	0	0	0	0	0
BGIR9823_102114	BGIG9823_26788		lncRNA	1	0	0	0	0	0	0	0	0	0	0	0	0	0	0	0
BGIR9823_101753	BGIG9823_26434		lncRNA	0	0	0	0	0	0	0	0	0	0	0	0	1	0	0	0
BGIR9823_102643	BGIG9823_27293		lncRNA	0	0	0	0	0	0	0	0	0	0	0	0	0	0	0	0
BGIR9823_102327	BGIG9823_26992		lncRNA	0	0	0	0	0	0	0	0	0	0	0	0	0	0	0	0
BGIR9823_102216	BGIG9823_26888		lncRNA	0	0	0	0	0	0	0	0	0	0	0	0	0	0	0	0
BGIR9823_102718	BGIG9823_27365		lncRNA	0	0	0	0	0	0	0	0	0	0	0	0	0	0	0	0
BGIR9823_101709	BGIG9823_26393		lncRNA	0	0	0	0	0	0	0	0	0	0	0	0	1	0	0	0
BGIR9823_102241	BGIG9823_26913		lncRNA	0	0	0	0	0	0	0	0	0	0	0	0	0	0	0	1
BGIR9823_102306	BGIG9823_26971		lncRNA	0	0	0	0	0	0	0	0	0	0	0	0	0	0	0	0
BGIR9823_102486	BGIG9823_27140		lncRNA	0	0	0	0	0	0	0	0	0	0	0	0	1	0	0	0
BGIR9823_101724	BGIG9823_26408		lncRNA	0	0	0	0	0	0	0	0	0	0	0	0	0	0	0	0
BGIR9823_101858	BGIG9823_26537		lncRNA	1	0	0	0	0	0	0	0	0	0	0	0	0	0	0	0
BGIR9823_102483	BGIG9823_27137		lncRNA	0	0	0	0	0	0	0	0	0	0	0	0	0	0	0	0
BGIR9823_102027	BGIG9823_26702		lncRNA	0	0	0	0	0	0	0	0	0	0	0	0	0	0	0	0
BGIR9823_101777	BGIG9823_26457		lncRNA	0	0	0	0	0	0	0	0	0	0	0	0	0	0	0	0
BGIR9823_102154	BGIG9823_26827		lncRNA	0	0	0	0	0	0	0	0	0	0	0	0	0	0	0	0
BGIR9823_102426	BGIG9823_27087		lncRNA	0	0	0	0	0	0	0	0	0	0	0	0	0	0	0	0
BGIR9823_101894	BGIG9823_26571		lncRNA	0	0	0	0	0	0	0	0	0	0	0	0	0	0	0	0
BGIR9823_102009	BGIG9823_26685		lncRNA	0	0	0	0	0	0	0	0	0	0	0	0	0	0	0	0
BGIR9823_102250	BGIG9823_26918		lncRNA	0	0	0	0	0	0	0	0	0	0	0	0	0	0	0	0
BGIR9823_101769	BGIG9823_26449		lncRNA	0	0	0	0	0	0	0	0	0	0	0	0	0	0	0	0
XR_002338373.1	110256615	LOC110256615	lncRNA	0	0	0	0	0	0	0	0	0	0	0	0	0	0	0	0
XM_005655448.3	100519711	ST7L	mRNA	0	0	0	1	0	0	0	0	0	0	0	0	0	0	0	0
XR_001308691.2	106510294	LOC106510294	lncRNA	0	0	0	0	0	0	0	0	0	0	0	0	0	0	0	0
XR_002343519.1	110260353	LOC110260353	lncRNA	0	0	0	0	0	0	0	0	0	0	0	0	0	0	0	0
XR_002336962.1	102162292	LOC102162292	lncRNA	0	0	0	0	0	0	1	0	0	0	0	0	0	0	0	0
XM_021087319.1	100517646	POLE4	mRNA	0	0	0	0	0	0	0	0	0	0	0	0	0	0	0	0
XR_002336095.1	110255550	LOC110255550	lncRNA	0	0	0	0	0	0	0	0	0	0	0	0	0	0	0	0
NM_001244786.1	100513209	CFAP20	mRNA	0	0	0	0	0	0	0	0	0	0	0	0	0	0	0	0
XR_002340787.1	110257799	LOC110257799	lncRNA	0	0	0	0	0	0	0	0	0	0	0	0	0	0	0	0
XM_003358899.4	100623886	CCT8	mRNA	0	0	0	0	0	0	0	0	0	0	0	0	0	0	0	0
XR_002337835.1	110256393	LOC110256393	lncRNA	0	0	0	0	0	0	0	0	0	0	0	0	0	0	0	0
XR_002337635.1	106505647	LOC106505647	lncRNA	0	0	0	0	0	0	0	0	0	0	0	0	0	0	0	0
XR_001308778.2	106510350	LOC106510350	lncRNA	0	0	0	0	0	0	0	0	0	0	0	0	0	0	0	0
XR_002346849.1	110262181	LOC110262181	lncRNA	0	0	0	0	0	0	0	0	0	0	0	0	0	0	0	0
XM_005659957.3	102163937	GPR137C	mRNA	0	0	0	0	0	0	0	0	0	0	0	0	0	0	0	0

RNA ID	Gene ID	Symbol	Type	ssc-miR-24-1-5p	ssc-miR-29b	ssc-miR-29c	ssc-miR-323	ssc-miR-369	ssc-miR-708-5p	novel-ssc-miR161-3p	novel-ssc-miR512-5p	novel-ssc-miR846-3p	novel-ssc-miR1086-5p	novel-ssc-miR1105-5p	novel-ssc-miR1116-5p	novel-ssc-miR1120-5p	novel-ssc-miR1140-5p	novel-ssc-miR1158-3p	novel-ssc-miR1379-5p
XR_001303641.2	106507572	LOC106507572	lncRNA	0	0	0	0	0	0	0	0	0	0	0	0	0	0	0	0
XR_002344010.1	110260700	LOC110260700	lncRNA	0	0	0	0	0	0	0	0	0	0	0	0	0	0	0	0
XM_003359284.4	100623641	ZWINT	mRNA	0	0	0	0	0	0	0	0	0	0	0	0	0	0	1	0
XM_021066040.1	100233199	PITPNC1	mRNA	0	0	0	0	0	0	0	0	0	0	1	0	0	0	0	0
XM_003482526.2	100738522	LOC100738522	mRNA	0	0	0	0	0	0	0	0	0	0	0	0	0	0	0	0
XR_002345706.1	110261519	LOC110261519	lncRNA	0	0	0	0	0	0	0	0	0	0	0	0	0	0	0	0
XM_021066775.1	100513305	STRADA	mRNA	0	0	0	0	0	0	0	0	0	0	1	0	0	0	0	0
XR_303482.2	102164583	LOC102164583	lncRNA	0	0	0	0	0	0	0	0	0	0	1	0	0	0	0	0
XM_013998720.2	100737585	LOC100737585	mRNA	0	0	0	1	0	0	0	0	0	0	0	0	0	0	0	0
XR_002336556.1	102163487	LOC102163487	lncRNA	0	0	0	0	0	0	0	0	0	0	0	0	0	0	0	0
XR_002337553.1	102167511	SMIM4	lncRNA	0	0	0	0	0	0	0	0	0	0	0	0	0	1	0	0
XM_003483492.4	396595	PRF1	mRNA	0	0	0	0	0	0	0	0	0	0	0	0	0	0	0	0
XM_001926866.5	100155931	LOC100155931	mRNA	0	0	0	0	0	0	1	0	0	0	0	0	0	0	0	0
XR_002341737.1	102165038	LOC102165038	lncRNA	0	0	0	0	0	0	0	0	0	0	0	0	0	0	0	0
XR_002337051.1	110255981	LOC110255981	lncRNA	0	0	0	0	0	0	0	0	0	0	0	0	0	1	0	0
XM_021091413.1	100621517	TTLL1	mRNA	0	0	0	0	0	0	0	0	0	0	0	0	0	0	0	0
XR_002344541.1	110260924	LOC110260924	lncRNA	0	0	0	0	0	0	0	0	0	0	0	0	0	0	0	0
NM_213786.1	396680	NPW	mRNA	0	0	0	0	0	0	0	0	0	0	0	0	0	0	0	0
XM_021089665.1	100738802	BCAN	mRNA	0	0	0	0	0	0	0	0	0	0	0	0	0	0	0	0
XR_002340802.1	110257805	LOC110257805	lncRNA	0	0	0	0	0	0	0	0	0	0	0	0	0	0	0	0
XR_002341733.1	102158524	LOC102158524	lncRNA	0	0	0	0	0	0	0	0	0	0	0	0	0	0	0	0
XR_002345869.1	110261578	LOC110261578	lncRNA	0	0	0	0	0	0	0	0	0	0	0	0	0	0	0	0
XR_002338170.1	102161036	LOC102161036	lncRNA	0	0	0	0	0	0	0	0	0	0	1	0	0	0	0	0
XR_002337152.1	106505099	LOC106505099	lncRNA	0	0	0	0	0	0	0	0	0	0	0	0	0	0	0	0
XM_003131571.5	100512492	ANKRD40	mRNA	0	0	0	0	0	0	0	0	0	0	0	0	0	0	0	0
XR_002337061.1	110255986	LOC110255986	lncRNA	0	0	0	0	0	0	0	0	0	0	0	0	0	0	0	0
XR_002341729.1	110259387	LOC110259387	lncRNA	0	0	0	0	0	0	0	0	0	0	0	0	0	0	0	0
XM_005665516.3	100524028	ZMYND12	mRNA	0	0	0	0	0	0	0	0	0	0	0	0	0	0	0	0
XR_002342030.1	110259546	LOC110259546	lncRNA	0	0	0	0	0	0	1	0	0	0	0	0	0	0	0	0
XR_001304742.2	106508150	LOC106508150	lncRNA	0	0	0	1	0	0	0	0	0	0	0	0	0	0	0	0
NM_214322.1	397591	NPC1	mRNA	0	0	0	0	0	0	0	0	0	0	0	0	0	0	0	0
XR_002339061.1	102158523	LOC102158523	lncRNA	0	0	0	0	0	0	0	0	0	0	0	0	0	0	0	0
XR_002335622.1	110255429	LOC110255429	lncRNA	0	0	0	0	0	0	0	0	0	0	0	0	0	0	0	0
XM_003124446.4	100518241	SUMF2	mRNA	0	0	0	0	0	0	0	0	0	0	0	0	0	0	0	0
XR_002337394.1	110256124	LOC110256124	lncRNA	0	0	0	0	0	0	0	0	0	0	0	0	0	0	0	0
XM_003130089.4	100520582	LOC100520582	mRNA	0	0	0	0	0	0	0	0	0	0	0	0	0	0	0	0
XM_021100295.1	100337668	LCORL	mRNA	0	0	0	0	0	0	0	0	0	0	0	0	0	0	0	0
XR_002347096.1	110262257	LOC110262257	lncRNA	0	0	0	0	0	0	0	0	0	0	0	0	0	0	0	0
NM_214272.1	397512	TGFBR3	mRNA	0	0	0	0	0	0	0	0	0	0	0	0	0	0	0	0
XR_002337890.1	110256420	LOC110256420	lncRNA	0	0	0	0	0	0	0	0	0	0	0	0	0	0	0	0
XR_001302346.2	106506787	LOC106506787	lncRNA	0	0	0	0	0	0	0	0	0	0	0	0	0	0	0	0
XR_002340032.1	102161214	LOC102161214	lncRNA	0	0	0	0	0	0	0	0	0	0	0	0	0	0	0	0
XR_002338731.1	110256774	LOC110256774	lncRNA	0	0	0	0	0	0	0	0	0	0	0	0	0	0	0	0

RNA ID	Gene ID	Symbol	Type	ssc-miR-24-1-5p	ssc-miR-29b	ssc-miR-29c	ssc-miR-323	ssc-miR-369	ssc-miR-708-5p	novel-ssc-miR161-3p	novel-ssc-miR512-5p	novel-ssc-miR846-3p	novel-ssc-miR1086-5p	novel-ssc-miR1105-5p	novel-ssc-miR1116-5p	novel-ssc-miR1120-5p	novel-ssc-miR1140-5p	novel-ssc-miR1158-3p	novel-ssc-miR1379-5p
XR_002342421.1	110259933	LOC110259933	lncRNA	0	0	0	0	0	0	0	0	0	0	0	0	0	0	0	0
XM_021080420.1	110257790	WDR44	mRNA	0	0	0	0	0	0	1	0	0	0	0	0	0	0	0	0
XM_003128412.6	100514413	GTPBP2	mRNA	0	0	0	0	0	0	0	0	0	0	1	0	0	0	0	0
XR_001308377.2	106510160	LOC106510160	lncRNA	0	0	0	0	0	0	0	0	0	0	0	0	0	0	0	0
XM_021091670.1	100522737	ANKRD33	mRNA	0	0	0	0	0	0	0	0	0	0	0	0	0	0	0	0
XM_003134187.6	100512932	ASAH1	mRNA	0	0	0	0	0	0	0	0	0	0	0	0	0	0	0	0
XR_002335568.1	102159627	LOC102159627	lncRNA	0	0	0	0	0	0	0	0	0	0	0	0	0	0	0	0
XR_002345700.1	110261513	LOC110261513	lncRNA	0	0	0	0	0	0	0	0	0	0	0	0	0	0	0	0
NM_001243805.1	100511029	STARD10	mRNA	0	0	0	0	0	0	0	0	0	0	0	0	0	0	0	0
XM_001927894.5	100153448	HENMT1	mRNA	0	0	0	1	0	0	0	0	0	0	0	0	0	0	0	0
NM_214205.1	397409	IL5	mRNA	0	0	0	1	0	0	0	0	0	0	0	0	0	0	0	0
XR_002344130.1	110260754	LOC110260754	lncRNA	0	0	0	0	0	0	0	0	0	0	0	0	0	0	0	0
XM_021075881.1	100157060	WDR17	mRNA	0	0	0	0	0	0	0	0	0	0	0	0	0	0	0	0
XR_002344639.1	110260981	LOC110260981	lncRNA	0	0	0	0	0	0	0	0	0	0	0	0	0	0	0	0
XM_013985613.2	100525844	SLC35B4	mRNA	0	0	0	1	0	0	0	0	0	0	0	0	0	0	0	0
XR_002342231.1	110259849	LOC110259849	lncRNA	0	0	0	0	0	0	0	0	0	0	0	0	0	0	0	0
NM_001004043.1	445532	CFL1	mRNA	0	0	0	0	0	0	1	0	0	0	0	0	0	0	0	0
XM_003132695.5	100521154	ZBTB11	mRNA	0	0	0	0	0	0	0	0	0	0	0	0	0	0	0	0
XM_005657909.3	100519458	TEX13B	mRNA	0	0	0	0	1	0	0	0	0	0	0	0	0	0	0	0
XM_003129582.2	100517357	LOC100517357	mRNA	0	0	0	0	0	0	0	0	0	0	0	0	0	0	0	0
XR_298408.3	102167235	LOC102167235	lncRNA	0	0	0	0	0	0	0	0	0	0	0	0	0	0	0	0
XR_002336226.1	110255645	LOC110255645	lncRNA	0	0	0	0	0	0	0	0	0	0	0	0	0	1	0	0
XM_021077102.1	100515590	CCDC69	mRNA	0	0	0	0	0	0	0	0	0	0	1	0	0	0	0	0
XR_002336343.1	110255688	LOC110255688	lncRNA	0	0	0	0	0	0	0	0	0	0	0	0	0	0	0	0
XR_001309352.2	106510687	LOC106510687	lncRNA	0	0	0	0	0	0	0	0	0	0	0	0	0	0	0	0
XR_001301955.2	106506567	LOC106506567	lncRNA	0	0	0	0	0	0	0	0	0	0	0	0	0	0	0	0
XR_002338652.1	102167906	LOC102167906	lncRNA	0	0	0	0	0	0	0	0	0	1	0	0	0	0	0	0
XR_002336386.1	106506961	LOC106506961	lncRNA	0	0	0	0	0	0	0	0	0	0	0	0	0	0	0	0
XM_021074218.1	110256875	LOC110256875	mRNA	0	0	0	0	0	0	0	0	0	0	1	0	0	0	0	0
XR_002336193.1	100517050	2-Mar	lncRNA	0	0	0	0	0	0	0	0	0	1	0	0	0	0	0	0
XM_021088735.1	414423	GNRHR2	mRNA	0	0	0	0	0	0	0	0	0	0	0	0	0	0	0	0
NM_001146130.2	100271932	HPSE	mRNA	0	0	0	1	0	0	0	0	0	0	0	0	0	0	0	0
XM_013992909.2	100152206	LOC100152206	mRNA	0	0	0	1	0	0	0	0	0	0	0	0	0	0	0	0
XR_001299351.2	106505189	LOC106505189	lncRNA	0	0	0	0	0	0	0	0	0	0	0	0	0	0	0	0
XM_021090795.1	110260467	LOC110260467	mRNA	0	0	0	0	0	0	0	0	0	0	0	0	0	0	0	0
XR_001308232.2	106510085	LOC106510085	lncRNA	0	0	0	0	0	0	0	0	0	0	0	0	0	0	0	0
XR_002346494.1	110262011	LOC110262011	lncRNA	0	0	0	0	0	0	0	0	0	0	0	0	0	0	0	0
XM_005665059.3	100622416	KLHDC7A	mRNA	0	0	0	0	0	0	0	0	0	0	0	0	0	0	0	0
XR_002345769.1	110261544	LOC110261544	lncRNA	0	0	0	0	0	0	0	0	0	0	0	0	0	0	0	0
XR_002341699.1	110259368	LOC110259368	lncRNA	0	0	0	0	0	0	0	0	0	0	0	0	0	0	0	0
XR_001307130.2	106509521	LOC106509521	lncRNA	0	0	0	0	0	0	0	0	0	0	0	0	0	0	0	0
XR_002342847.1	106509907	LOC106509907	lncRNA	0	0	0	0	0	0	0	0	0	0	0	0	0	0	0	0
XR_001309149.2	106510562	LOC106510562	lncRNA	0	0	0	1	0	0	0	0	0	0	0	0	0	0	0	0

RNA ID	Gene ID	Symbol	Type	ssc-miR-24-1-5p	ssc-miR-29b	ssc-miR-29c	ssc-miR-323	ssc-miR-369	ssc-miR-708-5p	novel-ssc-miR161-3p	novel-ssc-miR512-5p	novel-ssc-miR846-3p	novel-ssc-miR1086-5p	novel-ssc-miR1105-5p	novel-ssc-miR1116-5p	novel-ssc-miR1120-5p	novel-ssc-miR1140-5p	novel-ssc-miR1158-3p	novel-ssc-miR1379-5p
XM_021096012.1	106507628	ZMPSTE24	mRNA	0	0	0	0	0	0	1	0	0	0	0	0	0	0	0	0
XM_021078386.1	110257500	PCMTD2	mRNA	0	0	0	0	0	0	1	0	0	0	0	0	0	0	0	0
XM_003134291.4	100513828	FOXA2	mRNA	0	0	0	0	0	0	0	0	0	0	0	0	0	0	0	0
XR_002339192.1	106506426	LOC106506426	lncRNA	0	0	0	0	0	0	0	0	0	0	0	0	0	0	0	0
XR_002340657.1	106506896	LOC106506896	lncRNA	0	0	0	0	0	0	0	0	0	0	0	0	0	0	0	0
XR_002344578.1	106507567	LOC106507567	lncRNA	0	0	0	0	0	0	0	0	0	0	0	0	0	0	0	0
XR_002337161.1	110256021	LOC110256021	lncRNA	0	0	0	0	0	0	0	0	0	0	0	0	0	0	0	0
XR_002343165.1	110260239	LOC110260239	lncRNA	0	0	0	0	0	0	0	0	0	0	0	0	0	0	0	0
XR_001306470.2	106509172	LOC106509172	lncRNA	0	0	0	0	0	0	0	0	0	0	0	0	0	0	0	0
XM_021087431.1	100622990	ETAA1	mRNA	0	0	0	0	0	0	0	0	0	0	1	0	0	0	0	0
NM_001243677.1	100157702	P2RY10	mRNA	0	0	0	1	0	0	0	0	0	0	0	0	0	0	0	0
NM_001005726.1	406192	ANXA2	mRNA	0	0	0	0	0	0	0	0	0	0	0	0	0	0	0	0
XR_002338199.1	110256565	LOC110256565	lncRNA	0	0	0	0	0	0	0	0	0	0	0	0	0	0	0	0
XR_002346497.1	110262015	LOC110262015	lncRNA	0	0	0	0	0	0	0	0	0	0	0	0	0	0	0	0
XM_021081451.1	100625375	LOC100625375	mRNA	0	0	0	0	0	0	1	0	0	0	0	0	0	0	0	0
NM_001243852.1	100511525	STT3A	mRNA	0	0	0	0	0	0	0	0	0	0	0	0	0	0	0	0
XM_001927799.7	100154361	SRP54	mRNA	0	0	0	0	0	0	0	0	0	0	0	0	0	0	0	0
XR_002339070.1	110256975	LOC110256975	lncRNA	0	0	0	0	0	0	0	0	0	0	0	0	0	0	0	0
XR_002340950.1	110257979	LOC110257979	lncRNA	0	0	0	0	0	0	0	0	0	0	0	0	0	0	0	0
XM_013990143.2	100155468	LOC100155468	mRNA	0	0	0	0	0	0	0	0	0	0	0	0	0	0	0	0
XM_003129448.1	100516817	LOC100516817	mRNA	0	0	0	0	0	0	0	0	0	0	0	0	0	0	0	0
XR_001301393.2	106506223	LOC106506223	lncRNA	0	0	0	0	0	0	0	0	0	0	0	0	0	0	0	0
XR_002337789.1	102162054	LOC102162054	lncRNA	0	0	0	0	0	0	0	0	0	0	0	0	0	0	0	0
XM_021085070.1	110259585	LOC110259585	mRNA	0	0	0	0	0	0	0	0	0	0	0	0	0	0	0	0
XM_005670449.3	100524281	SCARA5	mRNA	0	0	0	0	0	0	0	0	0	0	0	0	0	0	0	0
XM_021081009.1	110257997	LOC110257997	mRNA	0	0	0	0	0	0	0	0	0	0	0	0	0	0	0	0
XR_002343866.1	102166296	LOC102166296	lncRNA	0	0	0	0	0	0	0	0	0	0	0	0	0	0	0	0
XM_003124181.1	100517754	LOC100517754	mRNA	0	0	0	0	0	0	0	0	0	0	0	0	0	0	0	0
XR_001298301.2	102159863	LOC102159863	lncRNA	0	0	0	0	0	0	0	0	0	0	0	0	0	0	0	0
XM_021080605.1	110257839	LOC110257839	mRNA	0	0	0	0	0	0	0	0	0	0	0	0	0	0	0	0
XM_021072297.1	100519392	RAB36	mRNA	0	0	0	0	0	0	0	0	0	0	1	0	0	0	0	0
XM_005657053.3	100621152	RNF222	mRNA	0	0	0	0	0	0	0	0	0	0	0	0	0	0	0	0
XM_021064469.1	100516823	CNST	mRNA	0	0	0	0	0	0	1	0	0	0	0	0	0	0	0	0
XR_002344959.1	110261129	LOC110261129	lncRNA	0	0	0	0	0	0	0	0	0	0	0	0	0	0	0	0
XR_002336671.1	110255811	LOC110255811	lncRNA	0	0	0	0	0	0	0	0	0	0	0	0	0	0	0	0
XR_002346381.1	110261974	LOC110261974	lncRNA	0	0	0	0	0	0	0	0	0	0	0	0	0	0	0	0
XM_013994471.2	100517025	LOC100517025	mRNA	0	0	0	0	0	0	0	0	0	0	0	0	0	0	0	0
NM_001244142.1	100514123	SRI	mRNA	0	0	0	0	0	0	0	0	0	0	0	0	0	0	0	0
NM_001315615.1	100217382	NMUR2	mRNA	0	0	0	0	0	0	0	0	0	0	0	0	0	0	0	0
XR_002345984.1	110261620	LOC110261620	lncRNA	0	0	0	0	0	0	0	0	0	0	0	0	0	0	0	0
XR_002336754.1	110255855	LOC110255855	lncRNA	0	0	0	0	0	0	0	0	0	0	0	0	0	1	0	0
XR_002346598.1	110262073	LOC110262073	lncRNA	0	0	0	0	0	0	0	0	0	0	0	0	0	0	0	0
XR_002335757.1	110255462	LOC110255462	lncRNA	0	0	0	0	0	0	0	0	0	0	0	0	0	1	0	0

RNA ID	Gene ID	Symbol	Type	ssc-miR-24-1-5p	ssc-miR-29b	ssc-miR-29c	ssc-miR-323	ssc-miR-369	ssc-miR-708-5p	novel-ssc-miR161-3p	novel-ssc-miR512-5p	novel-ssc-miR846-3p	novel-ssc-miR1086-5p	novel-ssc-miR1105-5p	novel-ssc-miR1116-5p	novel-ssc-miR1120-5p	novel-ssc-miR1140-5p	novel-ssc-miR1158-3p	novel-ssc-miR1379-5p
XR_001304351.2	102167865	LOC102167865	lncRNA	0	0	0	0	0	0	0	0	0	0	0	0	0	0	0	0
NM_001011507.1	494565	LAMP1	mRNA	0	0	0	0	0	0	0	0	0	0	0	0	0	0	0	0
XR_002339091.1	106505784	LOC106505784	lncRNA	0	0	0	0	0	0	0	0	0	0	0	0	0	0	0	0
XR_298324.3	102163156	LOC102163156	lncRNA	0	0	0	0	0	0	0	0	0	0	0	0	0	0	0	0
XM_021099319.1	100626159	RNASE9	mRNA	0	0	0	0	0	0	0	0	0	0	0	0	0	0	0	0
XM_021096183.1	100739254	ANKRD29	mRNA	0	0	0	0	0	0	0	0	0	0	0	0	0	0	0	0
XR_002335919.1	110255507	LOC110255507	lncRNA	0	0	0	0	0	0	1	0	0	0	0	0	0	0	0	0
XR_002340438.1	106506789	LOC106506789	lncRNA	0	0	0	0	0	0	0	0	0	0	0	0	0	0	0	0
XM_021099935.1	100519785	RAD51B	mRNA	0	0	0	0	0	0	0	0	0	0	0	0	0	0	0	0
XR_001305518.2	106508572	LOC106508572	lncRNA	0	0	0	0	0	0	0	0	0	0	0	0	0	0	0	0
XM_013992672.2	100520160	CNR1	mRNA	0	0	0	0	0	0	0	0	0	0	0	0	0	0	0	0
NM_001123164.1	100144479	PNMT	mRNA	0	0	0	0	0	0	0	0	0	0	0	0	0	0	0	0
NM_001244365.1	100153444	UROS	mRNA	0	0	0	0	0	0	0	0	0	0	0	0	0	0	0	0
XM_021092559.1	100157209	PRICKLE1	mRNA	0	0	0	0	0	0	0	0	0	0	0	0	0	0	0	0
XM_013979148.2	100521912	WNT11	mRNA	0	0	0	0	0	0	0	0	0	0	0	0	0	0	0	0
XR_002337909.1	110256426	LOC110256426	lncRNA	0	0	0	0	0	0	0	0	0	0	0	0	0	0	0	0
NM_214280.1	397524	VCP	mRNA	0	0	0	0	0	0	0	0	0	0	0	0	0	0	0	0
XM_005671261.3	100155046	RNLS	mRNA	0	0	0	0	0	0	0	0	0	0	0	0	0	0	0	0
XR_002337573.1	100154005	ACTR8	mRNA	0	0	0	0	0	0	0	0	0	0	1	0	0	0	0	0
NM_001244315.1	100514255	ARL4C	mRNA	0	0	0	0	0	0	0	0	0	0	0	0	0	0	0	0
XR_001300300.2	106505675	LOC106505675	lncRNA	0	0	0	0	0	0	0	0	0	0	0	0	0	0	0	0
XR_002341897.1	110259477	LOC110259477	lncRNA	0	0	0	0	0	0	0	0	0	0	0	0	0	0	0	0
XR_002336723.1	110255842	LOC110255842	lncRNA	0	0	0	0	0	0	0	0	0	0	0	0	0	0	0	0
XM_021096057.1	100737127	RAB31	mRNA	0	0	0	0	0	0	0	0	0	0	0	0	0	0	0	0
XR_001299360.2	106505194	LOC106505194	lncRNA	0	0	0	0	0	0	0	0	0	0	0	0	0	0	0	0
XM_001925846.7	106504094	GTF3C3	mRNA	0	0	0	0	0	0	0	0	0	0	0	0	0	0	0	0
XR_002337419.1	106505263	LOC106505263	lncRNA	0	0	0	0	0	0	0	0	0	0	0	0	0	0	0	0
XR_002346301.1	110261775	LOC110261775	lncRNA	0	0	0	0	0	0	0	0	0	0	0	0	0	0	0	0
XR_002339946.1	100627949	LOC100627949	lncRNA	0	0	0	0	0	0	0	0	0	0	0	0	0	0	0	0
XR_002346180.1	110261712	LOC110261712	lncRNA	0	0	0	0	0	0	0	0	0	0	0	0	0	0	0	0
XR_001297665.2	106504303	LOC106504303	lncRNA	0	0	0	0	0	0	0	0	0	0	0	0	0	0	0	0
XR_001298947.2	106504955	LOC106504955	lncRNA	0	0	0	0	0	0	0	0	0	0	0	0	0	0	0	0
XR_002341739.1	110259401	LOC110259401	lncRNA	0	0	0	0	0	0	0	0	0	0	0	0	0	0	0	0
XR_001307225.2	106509575	LOC106509575	lncRNA	0	0	0	0	0	0	0	0	0	0	0	0	0	0	0	0
XR_002338919.1	110256865	LOC110256865	lncRNA	0	0	0	0	0	0	0	0	0	0	0	0	0	0	0	0
XR_001306376.2	106509109	LOC106509109	lncRNA	0	0	0	0	0	0	0	0	0	0	0	0	0	0	0	0
XR_002338130.1	102158325	LOC102158325	lncRNA	0	0	0	0	0	0	0	0	0	0	0	0	0	0	0	0
XR_002337988.1	106505633	LOC106505633	lncRNA	0	0	0	0	0	0	0	0	0	0	0	0	0	0	0	0
XM_021086158.1	102161487	TMEM225B	mRNA	0	0	0	0	0	0	0	0	0	0	0	0	0	0	0	0
XM_003130212.5	100522092	RUNDC3B	mRNA	0	0	0	0	0	0	0	0	0	0	0	0	0	0	0	0
XM_001924771.6	100156540	RPP30	mRNA	0	0	0	0	0	0	0	0	0	0	1	0	0	0	0	0
XM_001928042.4	100152526	MED6	mRNA	0	0	0	0	0	0	0	0	0	0	0	0	0	0	0	0
XM_021095234.1	100738053	CHD5	mRNA	0	0	0	0	0	0	1	0	0	0	0	0	0	0	0	0

RNA ID	Gene ID	Symbol	Type	ssc-miR-24-1-5p	ssc-miR-29b	ssc-miR-29c	ssc-miR-323	ssc-miR-369	ssc-miR-708-5p	novel-ssc-miR161-3p	novel-ssc-miR512-5p	novel-ssc-miR846-3p	novel-ssc-miR1086-5p	novel-ssc-miR1105-5p	novel-ssc-miR1116-5p	novel-ssc-miR1120-5p	novel-ssc-miR1140-5p	novel-ssc-miR1158-3p	novel-ssc-miR1379-5p
XM_001928146.2	100152920	LOC100152920	mRNA	0	0	0	0	0	0	0	0	0	0	0	0	0	0	0	0
XM_003129758.5	100526065	SESN3	mRNA	0	0	0	1	0	0	0	0	0	0	0	0	0	0	0	0
XR_002339615.1	110257251	LOC110257251	lncRNA	0	0	0	0	0	0	0	0	0	0	0	0	0	0	0	0
XR_002339546.1	102158929	LOC102158929	lncRNA	0	0	0	0	0	0	0	0	0	0	0	0	0	0	0	0
XR_001308433.2	102165975	LOC102165975	lncRNA	0	0	0	0	0	0	0	0	0	0	0	0	0	0	0	0
XR_002341525.1	110259281	LOC110259281	lncRNA	0	0	0	0	0	0	0	0	0	0	0	0	0	0	0	0
NM_001038002.1	414911	EDNRB	mRNA	0	0	0	0	0	0	1	0	0	0	0	0	0	0	0	0
XR_002338445.1	106505879	LOC106505879	lncRNA	0	0	0	0	0	0	0	0	0	0	0	0	0	0	0	0
XR_002341837.1	110259430	LOC110259430	lncRNA	0	0	0	0	0	0	0	0	0	0	0	0	0	0	0	0
XM_021086652.1	100736832	GRIN2A	mRNA	0	0	0	0	0	0	0	0	0	0	0	0	0	0	0	0
XM_013997106.2	106510154	LOC106510154	mRNA	0	0	0	0	0	0	0	0	0	0	0	0	0	0	0	0
XR_002347051.1	110262233	LOC110262233	lncRNA	0	0	0	0	0	0	0	0	0	0	0	0	0	0	0	0
XR_002335588.1	110255417	LOC110255417	lncRNA	0	0	0	0	0	0	0	0	0	0	0	0	0	0	0	0
XR_001302361.2	106506801	LOC106506801	lncRNA	0	0	0	0	0	0	0	0	0	0	0	0	0	0	0	0
XR_002336628.1	110255797	LOC110255797	lncRNA	0	0	0	0	0	0	0	0	0	0	0	0	0	0	0	0
XM_021099547.1	102158165	TMEM63C	mRNA	0	0	0	0	0	0	0	0	0	0	0	0	0	0	0	0
XR_001307739.2	106509824	LOC106509824	lncRNA	0	0	0	0	0	0	0	0	0	0	0	0	0	0	0	0
XM_021084335.1	102162790	GRM6	mRNA	0	0	0	0	0	0	1	0	0	0	0	0	0	0	0	0
XR_002339516.1	102161653	LOC102161653	lncRNA	0	0	0	0	0	0	0	0	0	0	0	0	0	0	0	0
XR_001299377.2	106505203	LOC106505203	lncRNA	0	0	0	0	0	0	0	0	0	0	0	0	0	0	0	0
XM_003481432.4	100738802	BCAN	mRNA	0	0	0	0	0	0	0	0	0	0	0	0	0	0	0	0
XM_021095297.1	100627651	CTNNBIP1	mRNA	0	0	0	0	0	0	0	0	0	0	0	0	0	0	0	0
XM_003134620.5	100517251	TTC26	mRNA	0	0	0	1	0	0	0	0	0	0	0	0	0	0	0	0
XR_002343515.1	102165594	LOC102165594	lncRNA	0	0	0	0	0	0	0	0	0	0	0	0	0	0	0	0
NM_001044607.2	733679	ECH1	mRNA	0	0	0	0	0	0	0	0	0	0	0	0	0	0	0	0
XR_001300056.2	106505536	LOC106505536	lncRNA	0	0	0	0	0	0	0	0	0	0	0	0	0	0	0	0
XR_001301036.2	106506054	LOC106506054	lncRNA	0	0	0	0	0	0	0	0	0	0	1	0	0	0	0	0
XM_005654040.3	397441	CD86	mRNA	0	0	0	1	0	0	0	0	0	0	0	0	0	0	0	0
XR_001298634.2	102163793	LOC102163793	lncRNA	0	0	0	0	0	0	0	0	0	0	0	0	0	0	0	0
XR_002343053.1	110260210	LOC110260210	lncRNA	0	0	0	0	0	0	0	0	0	0	0	0	0	0	0	0
XM_003129012.4	100517956	TMA16	mRNA	0	0	0	0	0	0	1	0	0	0	0	0	0	0	0	0
XM_021076351.1	100515172	HOXD8	mRNA	0	0	0	0	0	0	0	0	0	0	1	0	0	0	0	0
NM_001001636.1	414419	RPL32	mRNA	0	0	0	1	0	0	0	0	0	0	0	0	0	0	0	0
XR_002336222.1	110255642	LOC110255642	lncRNA	0	0	0	0	0	0	0	0	0	0	0	0	0	0	0	0
XR_002337964.1	110256455	LOC110256455	lncRNA	0	0	0	1	0	0	0	0	0	0	0	0	0	0	0	0
XR_002343225.1	110260259	LOC110260259	lncRNA	0	0	0	0	0	0	0	0	0	0	0	0	0	0	0	0
XR_002340084.1	106506691	LOC106506691	lncRNA	0	0	0	0	0	0	0	0	0	0	0	0	0	0	0	0
XM_001928520.6	100153343	RWDD1	mRNA	0	0	0	0	0	0	0	0	0	0	0	0	0	0	0	0
XM_005652653.3	106504062	NLGN3	mRNA	0	0	0	1	0	0	0	0	0	0	0	0	0	0	0	0
XR_002341746.1	102165717	LOC102165717	lncRNA	0	0	0	0	0	0	0	0	0	0	0	0	0	0	0	0
XM_021085042.1	110259549	LOC110259549	mRNA	0	0	0	0	0	0	1	0	0	0	0	0	0	0	0	0
XR_001305221.2	106508400	LOC106508400	lncRNA	0	0	0	0	0	0	0	0	0	0	0	0	0	0	0	0
XR_002343172.1	110260241	LOC110260241	lncRNA	0	0	0	0	0	0	0	0	0	0	0	0	0	0	0	0

RNA ID	Gene ID	Symbol	Type	ssc-miR-24-1-5p	ssc-miR-29b	ssc-miR-29c	ssc-miR-323	ssc-miR-369	ssc-miR-708-5p	novel-ssc-miR161-3p	novel-ssc-miR512-5p	novel-ssc-miR846-3p	novel-ssc-miR1086-5p	novel-ssc-miR1105-5p	novel-ssc-miR1116-5p	novel-ssc-miR1120-5p	novel-ssc-miR1140-5p	novel-ssc-miR1158-3p	novel-ssc-miR1379-5p
XR_002339487.1	106506400	COPS7B	lncRNA	0	0	0	0	0	0	0	0	0	0	0	0	0	0	0	0
XR_001308351.2	106510133	LOC106510133	lncRNA	0	0	0	0	0	0	0	0	0	0	0	0	0	0	0	0
XM_003124564.4	100511500	GPR139	mRNA	0	0	0	0	0	0	0	0	0	0	0	0	0	0	0	0
NM_001078686.1	780437	ODAM	mRNA	0	0	0	0	0	0	1	0	0	0	0	0	0	0	0	0
XR_298956.3	102160517	LOC102160517	lncRNA	0	0	0	0	0	0	0	0	0	0	0	0	0	0	0	0
XR_002347080.1	110262247	LOC110262247	lncRNA	0	0	0	0	0	0	0	0	0	0	0	0	0	0	0	0
XM_021094930.1	100514465	LOC100514465	mRNA	0	0	0	0	0	0	0	0	0	0	0	0	0	0	0	0
XR_002343951.1	100737981	EIF4B	lncRNA	0	0	0	0	0	0	0	0	0	0	0	0	0	0	0	0
NM_001244614.1	100153985	SIX4	mRNA	0	0	0	0	0	0	0	0	0	0	0	0	0	0	0	0
XM_021066050.1	100302700	SLC25A19	mRNA	0	0	0	0	0	0	0	0	0	0	0	0	0	0	0	0
XM_005653210.3	100522374	GAS8	mRNA	0	0	0	0	0	0	0	0	0	0	0	0	0	0	0	0
XR_302139.2	102161858	LOC102161858	lncRNA	0	0	0	0	0	0	0	0	0	0	0	0	0	0	0	0
XR_002335527.1	110255394	LOC110255394	lncRNA	0	0	0	0	0	0	0	0	0	0	0	0	0	0	0	0
XR_002336548.1	110255755	LOC110255755	lncRNA	0	0	0	0	0	0	0	0	0	0	0	0	0	0	0	0
XR_001307143.2	100522542	LOC100522542	lncRNA	0	0	0	0	0	0	0	0	0	0	0	0	0	0	0	0
XR_002336864.1	110255895	LOC110255895	lncRNA	0	0	0	0	0	0	0	0	0	0	0	0	0	0	0	0
NM_214125.1	397230	MBL2	mRNA	0	0	0	0	0	0	0	0	0	0	0	0	0	0	0	0
XR_002339158.1	106506349	LOC106506349	lncRNA	0	0	0	0	0	0	0	0	0	0	0	0	0	0	0	0
NM_001244309.1	100625497	EZH2	mRNA	0	0	0	0	0	0	0	0	0	0	0	0	0	0	0	0
XM_021102414.1	100523325	ASB4	mRNA	0	0	0	0	0	0	0	0	0	0	0	0	0	0	0	0
XM_021069845.1	110256370	LOC110256370	mRNA	0	0	0	0	0	0	0	0	0	0	0	0	0	0	0	0
XR_309047.3	100519283	LOC100519283	lncRNA	0	0	0	0	0	0	0	0	0	0	0	0	0	0	0	0
XR_001308371.2	106510151	LOC106510151	lncRNA	0	0	0	0	0	0	0	0	0	0	0	0	0	0	0	0
XR_001303230.2	106507324	LOC106507324	lncRNA	0	0	0	0	0	0	0	0	0	0	0	0	0	0	0	0
XR_002338650.1	102167253	LOC102167253	lncRNA	0	0	0	0	0	0	0	0	0	0	0	0	0	0	0	0
XM_003132532.3	100519624	TMEM212	mRNA	0	0	0	0	0	0	0	0	0	0	0	0	0	0	0	0
XM_021086004.1	110259835	LOC110259835	mRNA	0	0	0	0	0	0	0	0	0	0	1	0	0	0	0	0
XM_005663799.3	396643	SSTR3	mRNA	0	0	0	0	0	0	0	0	0	0	0	0	0	0	0	0
XR_002335939.1	106507874	LOC106507874	lncRNA	0	0	0	0	0	0	0	0	0	0	0	0	0	0	0	0
XM_003130663.6	100522820	KIF24	mRNA	0	0	0	0	0	0	0	0	0	0	0	0	0	0	0	0
XM_003124595.4	100518832	RMI2	mRNA	0	0	0	0	0	0	0	0	0	0	0	0	0	0	0	0
XR_002339295.1	106506428	LOC106506428	lncRNA	0	0	0	0	0	0	0	0	0	0	0	0	0	0	0	0
XM_001925784.6	100155342	USP5	mRNA	0	0	0	0	0	0	0	0	0	0	0	0	0	0	0	0
XR_002346720.1	110262131	LOC110262131	lncRNA	0	0	0	0	0	0	0	0	0	0	0	0	0	0	0	0
XR_002339354.1	110257112	LOC110257112	lncRNA	0	0	0	0	0	0	0	0	0	0	0	0	0	0	0	0
XR_002346965.1	110262211	LOC110262211	lncRNA	0	0	0	0	0	0	0	0	0	0	0	0	0	0	0	0
XR_002335796.1	110255471	LOC110255471	lncRNA	0	0	0	1	0	0	0	0	0	0	0	0	0	0	0	0
XM_021101481.1	102166512	ANXA3	mRNA	0	0	0	0	0	0	0	0	0	0	0	0	0	0	0	0
XM_001926306.7	100153723	ABT1	mRNA	0	0	0	0	0	0	0	0	0	0	0	0	0	0	0	0
XR_002336516.1	102158088	NHLRC3	lncRNA	0	0	0	0	0	0	0	0	0	0	0	0	0	0	0	0
XR_002344446.1	102162270	SLC7A5	lncRNA	0	0	0	0	0	0	0	0	0	0	0	0	0	0	0	0
XR_002345296.1	102166616	LOC102166616	lncRNA	0	0	0	0	0	0	0	0	0	0	1	0	0	0	0	0
NM_001001645.1	414435	WDR83OS	mRNA	0	0	0	0	0	0	0	0	0	0	0	0	0	0	0	0

RNA ID	Gene ID	Symbol	Type	ssc-miR-24-1-5p	ssc-miR-29b	ssc-miR-29c	ssc-miR-323	ssc-miR-369	ssc-miR-708-5p	novel-ssc-miR161-3p	novel-ssc-miR512-5p	novel-ssc-miR846-3p	novel-ssc-miR1086-5p	novel-ssc-miR1105-5p	novel-ssc-miR1116-5p	novel-ssc-miR1120-5p	novel-ssc-miR1140-5p	novel-ssc-miR1158-3p	novel-ssc-miR1379-5p
XR_002337442.1	110256166	LOC110256166	lncRNA	0	0	0	0	0	0	0	0	0	0	0	0	0	0	0	0
XM_021072047.1	100512874	SCAF8	mRNA	0	0	0	0	0	0	0	0	0	0	1	0	0	0	0	0
XR_001298911.2	106504947	LOC106504947	lncRNA	0	0	0	0	0	0	0	0	0	0	0	0	0	0	0	0
XR_002340842.1	110257831	LOC110257831	lncRNA	0	0	0	0	0	0	0	0	0	0	0	0	0	0	0	0
XM_003122534.4	100512393	KCNK7	mRNA	0	0	0	0	0	0	0	0	0	0	0	0	0	0	0	0
XR_002340251.1	110257517	LOC110257517	lncRNA	0	0	0	0	0	0	0	0	0	0	0	0	0	0	0	0
XR_002338712.1	110256766	LOC110256766	lncRNA	0	0	0	0	0	0	0	0	0	0	0	0	0	0	0	0
XM_003129449.2	100516987	LOC100516987	mRNA	0	0	0	0	0	0	0	0	0	0	0	0	0	0	0	0
XR_001305858.2	106508800	LOC106508800	lncRNA	0	0	0	0	0	0	0	0	0	0	0	0	0	0	0	0
XR_001307955.2	102165812	LOC102165812	lncRNA	0	0	0	0	0	0	0	0	0	0	0	0	0	1	0	0
XR_002345578.1	110261473	LOC110261473	lncRNA	0	0	0	1	0	0	0	0	0	0	0	0	0	0	0	0
XR_002340279.1	110257523	LOC110257523	lncRNA	0	0	0	0	0	0	0	0	0	0	0	0	0	0	0	0
XM_021073688.1	100153473	CCDC188	mRNA	0	0	0	0	0	0	0	0	0	0	0	0	0	0	0	0
XR_002347070.1	100737186	LOC100737186	lncRNA	0	0	0	0	0	0	0	0	0	0	0	0	0	0	0	0
XR_002338524.1	110256665	LOC110256665	lncRNA	0	0	0	0	0	0	0	0	0	0	0	0	0	0	0	0
XR_002338128.1	110256525	LOC110256525	lncRNA	0	0	0	0	0	0	0	0	0	0	0	0	0	0	0	0
XR_002338320.1	102160169	LOC102160169	lncRNA	0	0	0	0	0	0	0	0	0	0	0	0	0	0	0	0
XR_002341185.1	102168138	LOC102168138	lncRNA	0	0	0	0	0	0	0	0	0	0	0	0	0	0	0	0
NM_001243681.2	100626539	RNASEH1	mRNA	0	0	0	0	0	0	0	0	0	0	0	0	0	0	0	0
XM_021087419.1	100516073	APLF	mRNA	0	0	0	0	0	0	0	0	0	0	0	0	0	0	0	0
NM_001244932.1	100514188	SUMF1	mRNA	0	0	0	1	0	0	0	0	0	0	0	0	0	0	0	0
XR_002342760.1	110260029	LOC110260029	lncRNA	0	0	0	0	0	0	0	0	0	0	0	0	0	0	0	0
NM_001130735.1	100170853	SMN1	mRNA	0	0	0	0	0	0	0	0	0	0	0	0	0	0	0	0
XR_002337720.1	106505376	LOC106505376	lncRNA	0	0	0	0	0	0	0	0	0	0	0	0	0	0	0	0
XM_021082523.1	100620803	LOC100620803	mRNA	0	0	0	0	0	0	0	0	0	0	0	0	0	0	0	0
XR_002344603.1	102158585	LOC102158585	lncRNA	0	0	0	0	0	0	0	0	0	0	0	0	0	0	0	0
XR_002340236.1	100513188	LOC100513188	lncRNA	0	0	0	0	0	0	0	0	0	0	0	0	0	0	0	0
XR_002335659.1	100622439	LOC100622439	lncRNA	0	0	0	0	0	0	0	0	0	0	0	0	0	0	0	0
XM_003122991.5	100523423	ATP5F1D	mRNA	0	0	0	1	0	0	0	0	0	0	0	0	0	0	0	0
XR_001308436.2	102166460	LOC102166460	lncRNA	0	0	0	0	0	0	0	0	0	0	0	0	0	0	0	0
XM_021070161.1	100620692	UBXN7	mRNA	0	0	0	0	1	0	0	0	0	0	0	0	0	0	0	0
NM_001278777.1	494011	SLC27A2	mRNA	0	0	0	0	0	0	0	0	0	0	0	0	0	0	0	0
XM_013994347.2	397553	NR1H3	mRNA	0	0	0	0	0	0	0	0	0	0	0	0	0	0	0	0
XM_005664733.3	100514292	ALDH16A1	mRNA	0	0	0	0	0	0	0	0	0	0	0	0	0	0	0	0
NM_001099936.1	100101473	BTG1	mRNA	0	0	0	0	0	0	0	0	0	0	0	0	0	0	0	0
XM_021099493.1	100625247	HEATR4	mRNA	0	0	0	0	0	0	0	0	0	0	1	0	0	0	0	0
XR_002335793.1	100523270	PFKFB2	mRNA	0	0	0	0	0	0	0	0	0	0	0	0	0	0	0	0
XR_002343260.1	106510048	LOC106510048	lncRNA	0	0	0	0	0	0	0	0	0	0	0	0	0	0	0	0
XM_021063420.1	100738171	ABCB5	mRNA	0	0	0	0	0	0	1	0	0	0	0	0	0	0	0	0
XM_021098946.1	100155072	CFAP100	mRNA	0	0	0	0	0	0	0	0	0	0	1	0	0	0	0	0
XM_021074210.1	100737404	LOC100737404	mRNA	0	0	0	0	0	0	0	0	0	0	1	0	0	0	0	0
XM_021088107.1	100517149	LOC100517149	mRNA	0	0	0	0	0	0	1	0	0	0	0	0	0	0	0	0
XM_003125346.4	100518190	MYCN	mRNA	0	0	0	0	0	0	0	0	0	0	0	0	0	0	0	0

RNA ID	Gene ID	Symbol	Type	ssc-miR-24-1-5p	ssc-miR-29b	ssc-miR-29c	ssc-miR-323	ssc-miR-369	ssc-miR-708-5p	novel-ssc-miR161-3p	novel-ssc-miR512-5p	novel-ssc-miR846-3p	novel-ssc-miR1086-5p	novel-ssc-miR1105-5p	novel-ssc-miR1116-5p	novel-ssc-miR1120-5p	novel-ssc-miR1140-5p	novel-ssc-miR1158-3p	novel-ssc-miR1379-5p
XM_003356153.3	100517101	LOC100517101	mRNA	0	0	0	0	0	0	0	0	0	0	0	0	0	0	0	0
XR_002338567.1	110256688	LOC110256688	lncRNA	0	0	0	0	0	0	0	0	0	0	0	0	0	0	0	0
XR_002339778.1	110257320	LOC110257320	lncRNA	0	0	0	0	0	0	0	0	0	0	0	0	0	0	0	0
XM_001927081.6	100156057	TTC8	mRNA	0	0	0	0	0	0	0	0	0	0	0	0	0	0	0	0
XM_021093139.1	100037307	DAB1	mRNA	0	0	0	0	0	0	0	0	0	0	1	0	0	0	0	0
XR_001300917.2	106505968	LOC106505968	lncRNA	0	0	0	0	0	0	0	0	0	0	0	0	0	0	0	0
NM_001190154.1	100157314	ZCCHC8	mRNA	0	0	0	0	0	0	1	0	0	0	0	0	0	0	0	0
XR_002338340.1	102159136	ARHGAP19	lncRNA	0	0	0	0	0	0	0	0	0	0	0	0	0	0	0	0
XR_002340858.1	110257848	LOC110257848	lncRNA	0	0	0	0	0	0	0	0	0	0	0	0	0	0	0	0
XM_021084337.1	102162790	GRM6	mRNA	0	0	0	0	0	0	0	0	0	0	0	0	0	0	0	0
XR_001306165.2	106508978	LOC106508978	lncRNA	0	0	0	0	0	0	0	0	0	0	0	0	0	0	0	0
XM_021085043.1	110259550	LOC110259550	mRNA	0	0	0	0	0	0	1	0	0	0	0	0	0	0	0	0
XR_001302502.2	102164143	LOC102164143	lncRNA	0	0	0	0	0	0	0	0	0	0	0	0	0	0	0	0
XM_021088119.1	100516428	LOC100516428	mRNA	0	0	0	0	0	0	0	0	0	0	0	0	0	0	0	0
XR_002335496.1	110255344	LOC110255344	lncRNA	0	0	0	0	0	0	1	0	0	0	0	0	0	0	0	0
XR_002335923.1	110255511	LOC110255511	lncRNA	0	0	0	0	0	0	0	0	0	0	0	0	0	0	0	0
XR_002336776.1	102168075	LOC102168075	lncRNA	0	0	0	0	0	0	0	0	0	0	0	0	0	0	0	0
XR_304089.2	102160593	LOC102160593	lncRNA	0	0	0	0	0	0	0	0	0	0	0	0	0	0	0	0
XM_003355424.5	100627932	HOXC6	mRNA	0	0	0	0	0	0	0	0	0	0	0	0	0	0	0	0
XR_002335902.1	102161420	LOC102161420	lncRNA	0	0	0	0	0	0	0	0	0	0	0	0	0	1	0	0
XR_002344769.1	110261037	LOC110261037	lncRNA	0	0	0	0	0	0	1	0	0	0	0	0	0	0	0	0
XR_001300967.2	100520338	LOC100520338	lncRNA	0	0	0	0	0	0	0	0	0	0	0	0	0	0	0	0
XR_002338125.1	102167959	LOC102167959	lncRNA	0	0	0	1	0	0	0	0	0	0	0	0	0	0	0	0
XR_002340442.1	102160053	LOC102160053	lncRNA	0	0	0	0	0	0	0	0	0	0	0	0	0	0	0	0
NM_001145752.1	100271724	SLC22A4	mRNA	0	0	0	0	0	0	0	0	0	0	0	0	0	0	0	0
XM_021097098.1	100302694	CES5A	mRNA	0	0	0	0	0	0	0	0	0	0	0	0	0	0	0	0
XR_001299355.2	106505191	LOC106505191	lncRNA	0	0	0	0	0	0	0	0	0	0	0	0	0	0	0	0
XR_001301379.2	106506213	LOC106506213	lncRNA	0	0	0	0	0	0	0	0	0	0	0	0	0	0	0	0
XR_002344182.1	110260775	LOC110260775	lncRNA	0	0	0	0	0	0	0	0	0	0	0	0	0	0	0	0
XR_002337157.1	110256021	LOC110256021	lncRNA	0	0	0	0	0	0	0	0	0	0	0	0	0	0	0	0
XR_002341155.1	110258473	LOC110258473	lncRNA	0	0	0	0	0	0	0	0	0	0	0	0	0	0	0	0
XM_001925364.3	100153329	LOC100153329	mRNA	0	0	0	0	0	0	0	0	0	0	0	0	0	0	0	0
XM_005665343.3	100519720	MAPRE2	mRNA	0	0	0	1	0	0	0	0	0	0	0	0	0	0	0	0
XR_002336444.1	102157904	LOC102157904	lncRNA	0	0	0	0	0	0	0	0	0	0	0	0	0	0	0	0
XM_003132006.5	100520100	MAP2K4	mRNA	0	0	0	0	0	0	0	0	0	0	0	0	0	0	0	0
XR_002343771.1	110260459	LOC110260459	lncRNA	0	0	0	0	0	0	0	0	0	0	0	0	0	0	0	0
XM_003358564.4	100620415	SLC35G2	mRNA	0	0	0	0	0	0	0	0	0	0	0	0	0	0	0	0
NM_001123151.1	100144454	NRM	mRNA	0	0	0	0	0	0	0	0	0	0	0	0	0	0	0	0
XM_005667128.3	100627340	LOC100627340	mRNA	0	0	0	0	0	0	0	0	0	0	0	0	0	0	0	0
NM_001243803.1	100511023	NUDC	mRNA	0	0	0	0	0	0	0	0	0	0	0	0	0	0	0	0
XM_021097456.1	110261343	TMEM240	mRNA	0	0	0	0	0	0	0	0	0	0	0	0	0	0	0	0
XR_001301636.2	106506374	LOC106506374	lncRNA	0	0	0	0	0	0	0	0	0	0	0	0	0	1	0	0
XR_001303107.2	106507246	LOC106507246	lncRNA	0	0	0	0	0	0	0	0	0	0	0	0	0	0	0	0

RNA ID	Gene ID	Symbol	Type	ssc-miR-24-1-5p	ssc-miR-29b	ssc-miR-29c	ssc-miR-323	ssc-miR-369	ssc-miR-708-5p	novel-ssc-miR161-3p	novel-ssc-miR512-5p	novel-ssc-miR846-3p	novel-ssc-miR1086-5p	novel-ssc-miR1105-5p	novel-ssc-miR1116-5p	novel-ssc-miR1120-5p	novel-ssc-miR1140-5p	novel-ssc-miR1158-3p	novel-ssc-miR1379-5p
XR_001303546.2	106507516	LOC106507516	lncRNA	0	0	0	0	0	0	0	0	0	0	0	0	0	1	0	0
XR_002336300.1	110255664	LOC110255664	lncRNA	0	0	0	0	0	0	0	0	0	0	0	0	0	0	0	0
XR_002336542.1	110255752	LOC110255752	lncRNA	0	0	0	0	0	0	0	0	0	0	0	0	0	0	0	0
XR_002337686.1	110256299	LOC110256299	lncRNA	0	0	0	0	0	0	0	0	0	0	0	0	0	0	0	0
XR_001300145.2	106505585	LOC106505585	lncRNA	0	0	0	0	0	0	0	0	0	0	0	0	0	0	0	0
XR_002344948.1	110261123	LOC110261123	lncRNA	0	0	0	0	0	0	0	0	0	0	0	0	0	0	0	0
XR_002344676.1	110260998	LOC110260998	lncRNA	0	0	0	0	0	0	0	0	0	0	0	0	0	0	0	0
XM_003121205.3	100525016	SMLR1	mRNA	0	0	0	0	0	0	0	0	0	0	0	0	0	0	0	0
XR_002340682.1	102163484	LOC102163484	lncRNA	0	0	0	0	0	0	0	0	0	0	0	0	0	0	0	0
XM_021079561.1	497636	DMD	mRNA	0	0	0	0	0	0	0	0	0	0	0	0	0	0	0	0
XR_002341591.1	102164826	ISYNA1	lncRNA	0	0	0	0	0	0	0	0	0	0	0	0	0	0	0	0
XR_002340822.1	110257819	LOC110257819	lncRNA	0	0	0	0	0	0	0	0	0	0	0	0	0	0	0	0
XR_001298110.1	106504549	LOC106504549	lncRNA	0	0	0	0	0	0	0	0	0	0	0	0	0	0	0	0
XR_002340843.1	110257831	LOC110257831	lncRNA	0	0	0	0	0	0	0	0	0	0	0	0	0	0	0	0
XR_300629.3	102163894	LOC102163894	lncRNA	0	0	0	0	0	0	0	0	0	0	0	0	0	0	0	0
XM_021099082.1	100510914	NEO1	mRNA	0	0	0	0	0	0	0	0	0	0	0	0	0	0	0	0
XR_002345385.1	110261329	LOC110261329	lncRNA	0	0	0	0	0	0	0	0	0	0	0	0	0	0	0	0
XR_002337516.1	110256226	LOC110256226	lncRNA	0	0	0	0	0	0	0	0	0	0	0	0	0	0	0	0
XR_002340123.1	102159962	LOC102159962	lncRNA	0	0	0	1	0	0	0	0	0	0	0	0	0	0	0	0
XM_001926907.5	100156308	PSMD10	mRNA	0	0	0	0	0	0	0	0	0	0	0	0	0	0	0	0
NM_213885.1	396919	MAN1A1	mRNA	0	0	0	0	0	0	0	0	0	0	0	0	0	0	0	0
XR_002338697.1	106506052	LOC106506052	lncRNA	0	0	0	0	0	0	0	0	0	0	0	0	0	0	0	0
XR_002340869.1	100522115	LOC100522115	lncRNA	0	0	0	0	0	0	0	0	0	0	0	0	0	0	0	0
NM_001143692.2	100152339	ITPK1	mRNA	0	0	0	0	0	0	0	0	0	0	0	0	0	0	0	0
NM_001114281.1	100135665	RELA	mRNA	0	0	0	0	0	0	0	0	0	0	0	0	0	0	0	0
XM_021073105.1	100156262	PAPSS2	mRNA	0	0	0	0	0	0	0	0	0	0	1	0	0	0	0	0
XR_002336169.1	110255621	LOC110255621	lncRNA	0	0	0	0	0	0	0	0	0	0	0	0	0	0	0	0
XR_001298531.2	102166545	LOC102166545	lncRNA	0	0	0	0	0	0	0	0	0	0	0	0	0	0	0	0
XR_002339865.1	110257358	LOC110257358	lncRNA	0	0	0	0	0	0	0	0	0	0	0	0	0	0	0	0
XR_002336348.1	110255689	LOC110255689	lncRNA	0	0	0	0	0	0	0	0	0	0	0	0	0	1	0	0
XM_003134418.4	100523033	SPAG4	mRNA	0	0	0	0	0	0	0	0	0	0	0	0	0	0	0	0
XR_001308912.2	106510436	LOC106510436	lncRNA	0	0	0	0	0	0	0	0	0	0	0	0	0	0	0	0
XM_003482567.1	100739359	LOC100739359	mRNA	0	0	0	0	0	0	0	0	0	0	0	0	0	0	0	0
XM_003483535.4	100154776	PGAM1	mRNA	0	0	0	0	0	0	0	0	0	0	0	0	0	0	0	0
XM_003359830.5	100626618	CCNJL	mRNA	0	0	0	0	0	0	0	0	0	0	0	0	0	0	0	0
XR_002339976.1	110257414	LOC110257414	lncRNA	0	0	0	0	0	0	0	0	0	0	0	0	0	0	0	0
NM_001139471.1	100154728	APEX1	mRNA	0	0	0	0	0	0	0	0	0	0	0	0	0	0	0	0
XR_001301667.2	106506397	LOC106506397	lncRNA	0	0	0	0	0	0	0	0	0	0	0	0	0	0	0	0
XR_001307040.2	102159224	LOC102159224	lncRNA	0	0	0	0	0	0	0	0	0	0	0	0	0	1	0	0
XM_021066572.1	100622490	ST6GALNAC1	mRNA	0	0	0	0	0	0	0	0	0	0	0	0	0	0	0	0
XM_001925290.5	100152155	SMPD2	mRNA	0	0	0	1	0	0	0	0	0	0	0	0	0	0	0	0
XR_001306492.2	100737021	LOC100737021	lncRNA	0	0	0	0	0	0	0	0	0	0	0	0	0	0	0	0
XM_013992665.2	102160737	HTR1E	mRNA	0	0	0	0	0	0	0	0	0	0	0	0	0	0	0	0

RNA ID	Gene ID	Symbol	Type	ssc-miR-24-1-5p	ssc-miR-29b	ssc-miR-29c	ssc-miR-323	ssc-miR-369	ssc-miR-708-5p	novel-ssc-miR161-3p	novel-ssc-miR512-5p	novel-ssc-miR846-3p	novel-ssc-miR1086-5p	novel-ssc-miR1105-5p	novel-ssc-miR1116-5p	novel-ssc-miR1120-5p	novel-ssc-miR1140-5p	novel-ssc-miR1158-3p	novel-ssc-miR1379-5p
XM_021082275.1	110258984	LOC110258984	mRNA	0	0	0	0	0	0	0	0	0	0	0	0	0	0	0	0
XR_001305691.2	102166401	LOC102166401	lncRNA	0	0	0	0	0	0	0	0	0	0	0	0	0	0	0	0
XR_002343757.1	110260447	LOC110260447	lncRNA	0	0	0	0	0	0	0	0	0	0	0	0	0	0	0	0
XR_001306506.2	106509193	LOC106509193	lncRNA	0	0	0	0	0	0	0	0	0	0	0	0	0	0	0	0
XM_021075435.1	100514804	CFC1B	mRNA	0	0	0	0	0	0	0	0	0	0	0	0	0	0	0	0
XR_002337121.1	110256014	LOC110256014	lncRNA	0	0	0	0	0	0	0	0	0	0	1	0	0	0	0	0
XR_002340328.1	106508222	LOC106508222	lncRNA	0	0	0	0	0	0	0	0	0	0	0	0	0	0	0	0
XM_021099830.1	100155756	TMEM225B	mRNA	0	0	0	0	0	0	0	0	0	0	0	0	0	0	0	0
NM_001206441.1	733650	TAP2	mRNA	0	0	0	0	0	0	0	0	0	0	0	0	0	0	0	0
XR_002338713.1	110256767	LOC110256767	lncRNA	0	0	0	0	0	0	0	0	0	0	0	0	0	0	0	0
XR_001307073.2	106509487	LOC106509487	lncRNA	0	0	0	0	0	0	0	0	0	0	0	0	0	0	0	0
XM_003129584.2	100517905	LOC100517905	mRNA	0	0	0	0	0	0	0	0	0	0	0	0	0	0	0	0
XM_003359416.4	100622102	KIF5C	mRNA	0	0	0	0	0	0	0	0	0	0	0	0	0	0	0	0
NM_001348903.1	397672	CILP	mRNA	0	0	0	0	0	0	0	0	0	0	1	0	0	0	0	0
XR_002341788.1	106509487	LOC106509487	lncRNA	0	0	0	0	0	0	0	0	0	0	0	0	0	0	0	0
XR_002337537.1	110256233	LOC110256233	lncRNA	0	0	0	0	0	0	0	0	0	0	0	0	0	0	0	0
XR_002341806.1	110259419	LOC110259419	lncRNA	0	0	0	0	0	0	0	0	0	0	0	0	0	0	0	0
XM_001928015.5	396603	PPP3CB	mRNA	0	0	0	0	0	0	0	0	0	0	0	0	0	0	0	0
XM_001924600.5	100155018	ZNF451	mRNA	0	0	0	0	0	0	0	0	0	0	0	0	0	0	0	0
XM_021089348.1	100153039	NSMAF	mRNA	0	0	0	0	0	0	0	0	0	0	0	0	0	0	0	0
XR_300604.3	102165231	LOC102165231	lncRNA	0	0	0	1	0	0	0	0	0	0	0	0	0	0	0	0
XM_003355060.4	100628123	NPBWR1	mRNA	0	0	0	0	0	0	1	0	0	0	0	0	0	0	0	0
NM_214418.2	403155	SLC25A6	mRNA	0	0	0	0	0	0	0	0	0	0	0	0	0	0	0	0
XR_002335850.1	102161480	LOC102161480	lncRNA	0	0	0	0	0	0	0	0	0	0	0	0	0	0	0	0
XR_002345639.1	110261503	LOC110261503	lncRNA	0	0	0	0	0	0	0	0	0	0	0	0	0	0	0	0
XR_301706.3	102162727	LOC102162727	lncRNA	0	0	0	1	0	0	0	0	0	0	0	0	0	0	0	0
XR_002344083.1	110260726	LOC110260726	lncRNA	0	0	0	0	0	0	0	0	0	0	0	0	0	0	0	0
XR_002336138.1	110255587	LOC110255587	lncRNA	0	0	0	0	0	0	0	0	0	0	0	0	0	1	0	0
NM_001244966.1	100514424	SLC25A39	mRNA	0	0	0	0	0	0	0	0	0	0	0	0	0	0	0	0
XR_001301171.2	106506113	LOC106506113	lncRNA	0	0	0	0	0	0	0	0	0	0	0	0	0	0	0	0
NM_001123177.1	100144495	RAB33A	mRNA	0	0	0	0	0	0	0	0	0	0	0	0	0	0	0	0
XM_021096358.1	102166543	SAMD13	mRNA	0	0	0	0	0	0	0	0	0	0	0	0	0	0	0	0
XM_021086898.1	102157824	GINM1	mRNA	0	0	0	0	0	0	1	0	0	0	0	0	0	0	0	0
XR_002346148.1	110261690	LOC110261690	lncRNA	0	0	0	0	0	0	0	0	0	0	0	0	0	0	0	0
XR_001307288.2	106509619	LOC106509619	lncRNA	0	0	0	0	0	0	0	0	0	0	0	0	0	0	0	0
NM_214253.1	397487	PRM1	mRNA	0	0	0	0	0	0	0	0	0	0	0	0	0	0	0	0
XR_002345199.1	110261229	LOC110261229	lncRNA	0	0	0	1	0	0	0	0	0	0	0	0	0	0	0	0
XM_021091581.1	100154559	CALHM4	mRNA	0	0	0	0	0	0	0	0	0	0	0	0	0	0	0	0
URS0000BC677A	110260766	LOC110260766	lncRNA	0	0	0	0	0	0	0	0	0	0	0	0	0	0	0	0
URS0000BC9805	BGIG9823_26341		lncRNA	0	0	0	1	0	0	0	0	0	0	0	0	0	0	0	0
URS0000BC7DBE	102167218	LOC102167218	lncRNA	0	0	0	0	0	0	0	0	0	0	0	0	0	0	0	0
URS0000BC871E	102165318	LOC102165318	lncRNA	0	0	0	0	0	0	0	0	0	0	0	0	0	0	0	0
URS0000BC6273	BGIG9823_26302		lncRNA	0	0	0	0	0	0	0	0	0	0	0	0	0	0	0	0

RNA ID	Gene ID	Symbol	Type	ssc-miR-24-1-5p	ssc-miR-29b	ssc-miR-29c	ssc-miR-323	ssc-miR-369	ssc-miR-708-5p	novel-ssc-miR161-3p	novel-ssc-miR512-5p	novel-ssc-miR846-3p	novel-ssc-miR1086-5p	novel-ssc-miR1105-5p	novel-ssc-miR1116-5p	novel-ssc-miR1120-5p	novel-ssc-miR1140-5p	novel-ssc-miR1158-3p	novel-ssc-miR1379-5p
URS0000BC6496	BGIG9823_26337		lncRNA	0	0	0	0	0	0	0	0	0	0	0	0	0	0	0	0
URS0000BC6966	BGIG9823_26316		lncRNA	0	0	0	0	0	0	0	0	0	0	0	0	0	0	0	0
URS0000BC81FE	BGIG9823_26310		lncRNA	0	0	0	0	0	0	1	0	0	0	0	0	0	0	0	0
URS0000BC59B8	100525132	PDZD2	lncRNA	0	0	0	0	0	0	0	0	0	0	0	0	0	0	0	0
URS0000BC725F	106506163	LOC106506163	lncRNA	0	0	0	0	0	0	0	0	0	0	0	0	0	0	0	0
URS0000BC5DE3	110256465	LOC110256465	lncRNA	0	0	0	0	0	0	0	0	0	0	0	0	0	0	0	0
URS0000BC7E35	102157619	LOC102157619	lncRNA	0	0	0	0	0	0	0	0	0	0	0	0	0	0	0	0
URS0000BC4C4F	106510093	LOC106510093	lncRNA	0	0	0	0	0	0	0	0	0	0	0	0	0	0	0	0
URS0000BC46E5	BGIG9823_26314		lncRNA	0	0	0	0	0	0	0	0	0	0	0	0	0	0	0	0
URS0000BC8793	100525132	PDZD2	lncRNA	0	0	0	0	0	0	0	0	0	0	0	0	0	0	0	0
URS0000BC7B31	BGIG9823_26353		lncRNA	0	0	0	0	0	0	0	0	0	0	0	0	0	0	0	0
BGIR9823_88049	100621902	MIER1	mRNA	0	0	0	0	0	0	0	0	0	0	0	0	0	0	0	0
BGIR9823_87964	100737982	GAREM1	lncRNA	0	0	0	0	0	0	0	0	0	0	0	0	0	0	0	0
BGIR9823_81184	100523888	LOC100523888	lncRNA	0	0	0	0	0	0	0	0	0	0	0	0	0	1	0	0
BGIR9823_78564	100737904	LOC100737904	lncRNA	0	0	0	0	0	0	0	0	0	0	0	0	0	0	0	0
BGIR9823_89527	102158035	LOC102158035	mRNA	0	0	0	0	0	0	0	0	0	0	0	0	0	0	0	0
BGIR9823_84122	100152952	ZHX2	lncRNA	0	0	0	0	0	0	0	0	0	0	0	0	0	0	0	0
BGIR9823_79475	100625298	GABRG3	lncRNA	0	0	0	0	0	0	0	0	0	0	0	0	0	0	0	0
XM_021097022.1	100620736	TCF25	mRNA	0	0	0	0	0	0	0	0	0	0	0	0	0	0	0	0
BGIR9823_77873	100513326	SMAP1	lncRNA	0	0	0	0	0	0	0	0	0	0	0	0	0	0	0	0
BGIR9823_80029	100154341	URM1	lncRNA	0	0	0	0	0	0	0	0	0	0	0	0	0	0	0	0
BGIR9823_83646	100154232	GGH	mRNA	0	0	0	0	0	0	0	0	0	0	0	0	0	0	0	0
BGIR9823_97547	100153782	USP30	mRNA	0	0	0	0	0	0	0	0	0	0	0	0	0	0	0	0
BGIR9823_78040	100157196	CSNK1G1	lncRNA	0	0	0	0	0	0	0	0	0	0	0	0	0	0	0	0
BGIR9823_79960	100516174	SUSD1	lncRNA	0	0	0	0	0	0	0	0	0	0	0	0	0	0	0	0
BGIR9823_81702	100521611	FNIP1	lncRNA	0	1	0	0	0	0	0	0	0	0	0	0	0	0	0	0
BGIR9823_78735	100153669	TMEM38B	lncRNA	0	0	0	0	0	1	0	0	0	0	0	0	0	0	0	0
BGIR9823_80417	100516957	LOC100516957	lncRNA	0	0	0	0	0	0	0	0	0	0	0	0	0	0	0	0
BGIR9823_82071	100525811	C3H16orf71	lncRNA	0	0	0	0	0	0	0	0	0	0	0	0	0	0	0	0
BGIR9823_89210	100153184	KHDRBS2	lncRNA	0	0	0	0	0	0	0	0	0	0	0	0	0	0	0	0
BGIR9823_86411	100621774	UBE2M	lncRNA	0	0	0	0	0	0	0	0	0	0	0	0	0	0	0	0
BGIR9823_86823	100621518	RBBP8	mRNA	0	0	0	0	0	0	0	0	0	0	0	0	0	0	0	0
BGIR9823_93974	102164379	LOC102164379	lncRNA	0	0	0	0	0	0	0	0	0	0	0	0	0	0	0	0
BGIR9823_79858	654405	GNAQ	lncRNA	0	0	0	0	0	0	0	0	0	0	0	0	0	0	0	0
BGIR9823_97653	100154344	C14H22orf39	lncRNA	0	0	0	0	0	0	0	0	0	0	0	0	0	0	0	0
BGIR9823_90176	100511210	SEC31A	mRNA	0	0	0	0	0	0	0	0	0	0	0	0	0	0	0	0
BGIR9823_99273	100624231	NIPBL	lncRNA	0	0	0	0	0	0	0	0	0	0	0	0	0	0	0	0
BGIR9823_100693	100739403	KLHDC10	lncRNA	0	0	0	0	0	0	0	0	0	0	0	0	0	0	0	0
BGIR9823_79452	100512576	SPRED1	lncRNA	0	0	0	0	0	0	0	0	0	0	0	0	0	0	0	0
BGIR9823_82923	100525039	MCRIP2	lncRNA	0	0	0	0	0	0	0	0	0	0	0	0	0	0	0	0
BGIR9823_85610	110260725	LOC110260725	lncRNA	0	0	1	0	0	0	0	0	0	0	0	0	0	0	0	0
BGIR9823_79946	100155590	FRRS1L	lncRNA	0	0	0	0	0	0	0	0	0	0	0	0	0	0	0	0
XR_002335729.1	110255457	LOC110255457	lncRNA	0	0	0	0	0	0	0	0	0	0	0	0	0	0	0	0

RNA ID	Gene ID	Symbol	Type	ssc-miR-24-1-5p	ssc-miR-29b	ssc-miR-29c	ssc-miR-323	ssc-miR-369	ssc-miR-708-5p	novel-ssc-miR161-3p	novel-ssc-miR512-5p	novel-ssc-miR846-3p	novel-ssc-miR1086-5p	novel-ssc-miR1105-5p	novel-ssc-miR1116-5p	novel-ssc-miR1120-5p	novel-ssc-miR1140-5p	novel-ssc-miR1158-3p	novel-ssc-miR1379-5p
BGIR9823_79704	100155666	ATG14	lncRNA	0	0	0	0	0	0	0	0	0	0	0	0	0	0	0	0
BGIR9823_80049	414430	MED27	lncRNA	0	0	0	0	0	0	0	0	0	0	0	0	0	0	0	0
BGIR9823_84572	100519481	STXBP3	lncRNA	0	0	0	0	0	0	0	0	0	0	0	0	0	1	0	0
BGIR9823_87715	100517038	PUM1	lncRNA	0	0	0	0	0	0	0	0	0	0	0	0	0	0	0	0
BGIR9823_84360	100154639	PBX1	lncRNA	0	0	0	0	0	0	0	0	0	0	0	0	0	0	0	0
BGIR9823_101255	100154598	LAMP2	mRNA	0	0	0	0	0	0	0	0	0	0	0	0	0	0	0	0
BGIR9823_98698	100737861	CCNT2	mRNA	0	0	0	0	0	0	0	0	0	0	0	0	0	0	0	0
BGIR9823_81378	100511368	ZNF358	lncRNA	0	0	0	0	0	0	0	0	0	0	0	0	0	0	0	0
BGIR9823_100853	100156439	TCEANC	lncRNA	0	0	0	0	0	0	0	0	0	0	0	0	0	0	0	0
BGIR9823_88507	100153104	POLH	lncRNA	0	0	0	0	0	0	0	0	0	0	0	0	0	1	0	0
BGIR9823_96638	100153422	FAM222A	lncRNA	0	0	0	0	0	0	0	0	0	0	0	0	0	0	0	0
BGIR9823_79570	100512083	NEDD4L	lncRNA	0	0	0	0	0	0	0	0	0	0	0	0	0	0	0	0
BGIR9823_101653	100625138	LSS	lncRNA	0	0	0	0	0	0	0	0	0	0	0	0	0	0	0	0
BGIR9823_101268	100520416	TENM1	lncRNA	0	0	0	0	0	0	0	0	0	0	0	1	0	0	0	0
BGIR9823_78254	106506784	LOC106506784	lncRNA	0	0	0	0	0	0	0	0	0	0	0	0	0	0	0	0
BGIR9823_93514	100514316	CACNB1	lncRNA	0	0	0	0	0	0	0	0	0	0	0	0	0	0	0	0
BGIR9823_78057	100152749	CA12	lncRNA	0	0	0	0	0	0	0	0	0	0	0	0	0	0	0	0
BGIR9823_87011	100621520	YIPF1	lncRNA	0	0	0	0	0	0	0	0	0	0	0	0	0	0	0	0
BGIR9823_79577	100151784	CLPX	lncRNA	0	0	0	0	0	0	0	0	0	0	0	0	0	0	0	0
BGIR9823_83929	100519711	ST7L	lncRNA	0	0	0	0	0	0	0	0	0	0	0	0	0	0	0	0
BGIR9823_78689	100514147	LOC100514147	lncRNA	0	0	0	0	0	0	0	0	0	0	0	0	0	0	0	0
BGIR9823_80847	100515884	KDM3B	lncRNA	0	0	0	0	0	0	0	0	0	0	0	0	0	0	0	0
BGIR9823_100723	100511847	CPED1	mRNA	0	0	0	0	0	0	0	0	0	0	0	0	0	0	0	0
BGIR9823_80653	100521253	IQGAP2	lncRNA	0	1	0	0	0	0	0	0	0	0	0	0	0	0	0	0
BGIR9823_81419	100737330	NFIC	lncRNA	0	0	0	0	0	1	0	0	0	0	0	0	0	0	0	0
BGIR9823_78533	100626032	SGPP1	lncRNA	0	0	0	0	0	0	0	0	0	0	0	0	0	0	0	1
BGIR9823_91477	100511030	ALG8	mRNA	0	0	0	0	0	0	0	0	0	0	0	0	0	0	0	0
BGIR9823_85833	100517653	VEZT	lncRNA	0	0	0	0	0	0	0	0	0	0	0	0	0	0	0	0
BGIR9823_99752	494014	PRNP	mRNA	0	0	0	0	0	0	0	0	0	0	0	0	0	0	0	0
BGIR9823_86172	100620402	LOC100620402	lncRNA	0	0	0	0	0	0	0	0	0	0	0	0	0	0	0	0
BGIR9823_88817	396933	FUT8	lncRNA	0	1	0	0	0	0	0	0	0	0	0	0	0	0	0	0
BGIR9823_94863	100621119	GRM7	lncRNA	0	0	0	0	0	0	0	0	0	0	0	0	0	0	0	0
BGIR9823_82821	110259924	LOC110259924	lncRNA	0	0	0	0	0	0	0	0	0	0	0	0	0	0	0	0
BGIR9823_80743	100513914	FER	lncRNA	0	0	0	0	0	0	0	0	0	0	0	0	0	0	0	0
BGIR9823_86054	100516197	AMFR	mRNA	0	0	0	0	0	0	0	0	0	0	0	0	0	0	0	0
BGIR9823_101027	397245	ATP1B4	mRNA	0	0	0	0	0	0	0	0	0	0	1	0	0	0	0	0
BGIR9823_94279	100520151	GIT1	mRNA	0	0	0	0	0	0	0	0	0	0	0	0	0	0	0	0
BGIR9823_100258	100156548	ZHX3	lncRNA	0	0	0	0	0	0	0	0	0	0	0	0	1	0	0	0
BGIR9823_97925	100516879	CYP2C91	lncRNA	0	0	0	0	0	0	0	0	0	0	0	0	0	0	0	0
BGIR9823_81233	106508326	SLC25A42	lncRNA	0	0	0	0	0	1	0	0	0	0	0	0	0	0	0	0
BGIR9823_100939	100154376	KDM6A	lncRNA	0	0	0	0	0	0	0	0	0	0	0	0	0	0	0	1
BGIR9823_100604	100515515	GALNT11	lncRNA	0	0	0	0	0	0	0	0	0	0	0	0	0	0	0	0
BGIR9823_95197	100626969	LPP	lncRNA	0	0	0	1	0	0	0	0	0	0	0	0	0	0	0	0

RNA ID	Gene ID	Symbol	Type	ssc-miR-24-1-5p	ssc-miR-29b	ssc-miR-29c	ssc-miR-323	ssc-miR-369	ssc-miR-708-5p	novel-ssc-miR161-3p	novel-ssc-miR512-5p	novel-ssc-miR846-3p	novel-ssc-miR1086-5p	novel-ssc-miR1105-5p	novel-ssc-miR1116-5p	novel-ssc-miR1120-5p	novel-ssc-miR1140-5p	novel-ssc-miR1158-3p	novel-ssc-miR1379-5p
BGIR9823_87581	100514410	ICMT	mRNA	0	0	0	0	0	0	0	0	0	0	0	0	0	0	0	0
BGIR9823_81758	106509575	LOC106509575	lncRNA	0	0	0	0	0	0	0	0	0	0	0	0	0	0	0	0
BGIR9823_79445	100522721	RASGRP1	lncRNA	1	0	0	0	0	0	0	0	0	0	0	0	0	0	0	0
BGIR9823_95280	100623596	OSBPL11	mRNA	0	0	0	0	0	0	0	0	0	0	1	0	0	0	0	0
BGIR9823_88858	100155733	SUSD6	lncRNA	0	0	0	0	0	0	0	0	0	0	0	0	0	0	0	0
BGIR9823_93640	100512984	GGNBP2	lncRNA	0	0	0	0	0	0	0	0	0	0	0	0	0	0	0	0
BGIR9823_82696	100626099	TBL2	mRNA	0	0	0	0	0	0	0	0	0	0	0	0	0	0	0	0
BGIR9823_85289	100519488	MOV10L1	lncRNA	0	0	0	0	0	0	0	0	0	0	0	0	0	0	0	0
BGIR9823_90661	100738411	CCSER1	lncRNA	0	0	1	0	0	0	0	0	0	0	0	0	0	0	0	0
BGIR9823_101099	100624032	LOC100624032	mRNA	0	0	0	0	0	0	0	0	0	0	0	0	0	0	0	0
BGIR9823_83245	100170144	SLC30A6	lncRNA	0	0	0	0	0	0	0	0	0	0	0	0	0	0	0	0
BGIR9823_83495	654326	RNF19A	lncRNA	0	0	0	0	0	0	0	0	0	0	0	0	0	0	0	0
BGIR9823_100642	100519065	UBN2	lncRNA	0	0	0	0	0	0	0	0	0	0	0	0	0	0	0	0
BGIR9823_88851	100158119	SLC39A9	lncRNA	0	0	0	0	0	0	0	0	0	0	0	0	0	0	0	0
BGIR9823_82981	100524615	ARID5A	lncRNA	0	0	0	0	0	0	0	0	0	0	0	0	0	0	0	0
BGIR9823_84758	100157699	TMEM184B	lncRNA	0	0	0	0	0	0	0	0	0	0	0	0	0	0	0	0
BGIR9823_101227	102165634	LOC102165634	lncRNA	0	0	0	0	0	0	0	0	0	0	0	0	0	0	0	0
BGIR9823_82094	100736616	HCFC1R1	lncRNA	0	0	0	0	0	0	0	0	0	0	0	0	0	0	0	0
BGIR9823_87647	100517288	HSPG2	mRNA	0	0	0	0	0	0	0	0	0	1	0	0	0	0	0	0
BGIR9823_85318	110260629	LOC110260629	lncRNA	0	0	0	0	0	0	0	0	0	0	0	0	0	0	0	0
BGIR9823_97476	100513180	FAM216A	lncRNA	0	0	0	0	0	0	0	0	0	0	0	0	0	0	0	0
BGIR9823_100900	100621199	IL1RAPL1	lncRNA	0	0	0	0	0	0	0	0	0	0	0	0	0	0	0	0
BGIR9823_90410	397001	POLR2B	lncRNA	0	0	0	0	0	0	0	0	0	0	0	0	0	0	0	0
XM_005659173.3	100156257	NHSL1	mRNA	0	0	0	1	0	0	0	0	0	0	0	0	0	0	0	0
BGIR9823_90244	100513100	LDB2	lncRNA	0	0	0	0	0	0	0	0	0	0	0	0	0	0	0	0
BGIR9823_78438	100152969	CDKL1	lncRNA	0	0	0	0	0	1	0	0	0	0	0	0	0	0	0	0
BGIR9823_100731	100512382	KCND2	lncRNA	0	0	0	0	0	0	0	0	0	0	0	0	0	0	0	1
BGIR9823_82740	100623146	KIF22	lncRNA	0	0	0	0	0	0	0	0	0	0	0	0	0	0	0	0
BGIR9823_81512	100523721	FAF2	lncRNA	0	0	0	0	0	1	0	0	0	0	0	0	0	0	0	0
BGIR9823_78400	102162695	LOC102162695	lncRNA	0	0	0	0	0	0	0	0	0	0	0	0	0	0	0	1
BGIR9823_100980	110257855	LOC110257855	lncRNA	0	0	0	0	0	0	0	0	0	0	0	0	0	0	0	0
BGIR9823_97780	100037299	P4HA1	lncRNA	0	0	0	0	0	0	0	0	0	0	0	0	0	0	0	0
BGIR9823_79896	100519814	RNF38	lncRNA	0	0	0	0	0	0	0	0	0	0	0	0	0	0	0	0
XM_021065940.1	110255868	ATP11A	mRNA	0	0	0	0	0	0	0	0	0	0	0	0	0	0	0	0
BGIR9823_101616	110258914	LOC110258914	lncRNA	0	0	0	0	0	0	0	0	0	0	0	0	0	0	0	0
BGIR9823_86084	100626752	LOC100626752	lncRNA	0	0	0	0	0	0	0	0	0	0	0	0	0	0	0	0
BGIR9823_78267	100512756	MEIS2	lncRNA	0	0	0	0	0	0	0	0	0	0	0	0	0	1	0	0
BGIR9823_80601	110259385	LOC110259385	lncRNA	0	0	0	0	0	0	0	0	0	0	0	0	0	0	0	0
BGIR9823_87318	100620768	CEP89	lncRNA	0	0	0	0	0	0	0	0	0	0	0	0	0	0	0	0
BGIR9823_80608	100521252	CLK4	lncRNA	0	0	0	0	0	0	0	0	0	0	0	0	0	0	0	0
BGIR9823_100082	100621622	SFRP1	mRNA	0	0	0	0	0	0	0	0	0	0	0	0	0	0	0	0
BGIR9823_80402	396557	PGLYRP2	lncRNA	0	0	0	0	0	0	0	0	0	0	0	0	0	0	0	0
BGIR9823_101117	100518815	TXLNG	lncRNA	0	0	1	0	0	0	0	0	0	0	0	0	0	0	0	0

RNA ID	Gene ID	Symbol	Type	ssc-miR-24-1-5p	ssc-miR-29b	ssc-miR-29c	ssc-miR-323	ssc-miR-369	ssc-miR-708-5p	novel-ssc-miR161-3p	novel-ssc-miR512-5p	novel-ssc-miR846-3p	novel-ssc-miR1086-5p	novel-ssc-miR1105-5p	novel-ssc-miR1116-5p	novel-ssc-miR1120-5p	novel-ssc-miR1140-5p	novel-ssc-miR1158-3p	novel-ssc-miR1379-5p
BGIR9823_78216	100511488	TTBK2	lncRNA	0	0	0	0	0	1	0	0	0	0	0	0	0	0	0	0
BGIR9823_78392	100526149	PNN	lncRNA	0	0	0	0	0	0	0	0	0	0	0	0	0	0	0	0
BGIR9823_101570	110258602	LOC110258602	lncRNA	0	0	0	0	0	0	0	0	0	0	0	0	0	0	0	0
BGIR9823_93977	100512557	DDX42	mRNA	0	0	0	0	0	0	0	0	0	0	0	0	0	0	0	0
BGIR9823_93667	100294712	SUZ12	lncRNA	0	0	1	0	0	0	0	0	0	0	0	0	0	0	0	0
BGIR9823_94057	397387	THRA	lncRNA	0	0	0	0	0	0	0	0	0	0	0	0	0	0	0	0
BGIR9823_100931	100515940	DDX3X	lncRNA	0	0	0	0	0	0	0	0	0	0	0	0	0	0	0	0
BGIR9823_87442	100518311	NOSIP	lncRNA	0	0	0	0	0	0	0	0	0	0	0	0	0	0	0	0
BGIR9823_92178	100126283	AQP7	mRNA	0	0	0	0	0	0	0	0	0	0	0	0	0	0	0	0
BGIR9823_101406	110258215	LOC110258215	lncRNA	0	0	0	0	0	0	0	0	0	0	0	0	0	0	0	0
BGIR9823_92246	100737056	FRMD4A	lncRNA	0	0	0	0	0	0	1	0	0	0	0	0	0	0	0	0
BGIR9823_82297	100522368	EXOC6B	mRNA	0	0	0	0	0	0	0	0	0	0	0	0	0	0	0	0
BGIR9823_78079	100154204	MYO1E	lncRNA	0	0	0	0	0	0	0	0	0	0	0	0	0	0	0	0
BGIR9823_93720	100517676	GOSR1	lncRNA	0	0	0	0	0	0	0	0	0	1	0	0	0	0	0	0
BGIR9823_95969	100524586	MED12L	lncRNA	0	0	0	0	0	0	0	0	0	0	0	0	0	0	0	0
BGIR9823_95388	100517310	ROBO1	mRNA	0	0	0	0	0	0	0	0	0	0	0	0	0	0	0	0
BGIR9823_92590	100521678	MLLT10	lncRNA	0	0	0	0	0	0	0	0	0	0	0	0	0	0	0	0
BGIR9823_89920	100621006	LOC100621006	lncRNA	0	0	1	0	0	0	0	0	0	0	0	0	0	0	0	0
BGIR9823_87305	100521133	DNAJA2	lncRNA	0	0	0	0	0	0	0	0	0	0	0	0	0	0	0	0
BGIR9823_83514	100153417	C4H8orf37	lncRNA	0	0	0	0	0	0	0	0	0	0	0	0	0	1	0	0
BGIR9823_85521	100525288	OSBPL8	lncRNA	0	0	0	0	0	0	0	0	0	0	0	0	0	0	0	0
BGIR9823_90750	100522939	ART5	mRNA	0	0	0	0	0	0	0	0	0	0	0	0	0	0	0	0
BGIR9823_85923	100579143	HSBP1	lncRNA	0	0	0	0	0	0	0	0	0	0	0	0	0	0	0	0
BGIR9823_91101	100519438	FAM133B	lncRNA	0	1	0	0	0	0	0	0	0	0	0	0	0	0	0	0
BGIR9823_100281	100153535	PCIF1	lncRNA	0	0	0	0	0	0	0	0	0	0	0	0	0	0	0	0
BGIR9823_100276	397528	SDC4	lncRNA	0	0	0	0	0	0	0	0	0	0	0	0	0	0	0	0
BGIR9823_100467	100517505	CTTNBP2	lncRNA	0	0	0	0	0	0	0	0	0	0	0	0	0	0	0	0
BGIR9823_100689	110257546	LOC110257546	lncRNA	0	0	0	0	0	0	0	0	0	0	0	0	0	0	0	0
BGIR9823_94010	100514424	SLC25A39	lncRNA	0	0	0	0	0	0	0	0	0	0	0	0	0	0	0	0
BGIR9823_100247	100153296	RBL1	lncRNA	0	0	0	0	0	0	0	0	0	0	0	0	0	1	0	0
BGIR9823_97926	100516879	CYP2C91	lncRNA	0	0	1	0	0	0	0	0	0	0	0	0	0	0	0	0
BGIR9823_78152	100155516	DMXL2	lncRNA	0	0	0	0	0	0	0	0	0	0	0	0	0	0	0	1
BGIR9823_101623	BGIG9823_26354		lncRNA	0	0	0	0	0	0	0	0	0	0	0	1	0	0	0	0
BGIR9823_98164	110257053	LOC110257053	lncRNA	0	0	0	0	0	0	0	0	0	0	0	0	0	0	0	0
BGIR9823_100903	100621199	IL1RAPL1	lncRNA	0	0	0	0	0	0	0	0	0	0	0	0	0	0	0	0
BGIR9823_77846	100736796	ADGRB3	lncRNA	0	0	0	0	0	0	0	0	0	0	0	0	0	0	0	0
BGIR9823_90432	100520317	LOC100520317	mRNA	0	0	0	1	0	0	0	0	0	0	0	0	0	0	0	0
BGIR9823_95429	396658	IFNAR1	lncRNA	0	0	0	0	0	0	0	0	0	0	0	0	0	0	0	0
BGIR9823_80320	100521123	SBF2	lncRNA	0	0	0	0	0	0	0	0	0	0	0	0	0	0	0	0
BGIR9823_81929	100519894	PRKRIP1	lncRNA	0	0	0	0	0	0	0	0	0	0	0	0	0	0	0	0
BGIR9823_100403	100524054	WDR91	lncRNA	0	0	0	0	0	0	0	0	0	0	0	0	0	0	0	0
BGIR9823_91472	100217384	RSF1	lncRNA	0	1	0	0	0	0	0	0	0	0	0	0	0	0	0	0
BGIR9823_78771	102164650	LOC102164650	lncRNA	0	0	0	0	0	0	0	0	0	0	0	0	0	0	0	0

RNA ID	Gene ID	Symbol	Type	ssc-miR-24-1-5p	ssc-miR-29b	ssc-miR-29c	ssc-miR-323	ssc-miR-369	ssc-miR-708-5p	novel-ssc-miR161-3p	novel-ssc-miR512-5p	novel-ssc-miR846-3p	novel-ssc-miR1086-5p	novel-ssc-miR1105-5p	novel-ssc-miR1116-5p	novel-ssc-miR1120-5p	novel-ssc-miR1140-5p	novel-ssc-miR1158-3p	novel-ssc-miR1379-5p
BGIR9823_77749	100155211	SLC18B1	lncRNA	0	0	0	0	0	0	0	0	0	0	0	0	0	0	0	0
BGIR9823_80489	100622165	ACER1	lncRNA	0	0	0	0	0	0	0	0	0	0	0	0	0	0	0	0
BGIR9823_100204	100514995	COMMD7	lncRNA	0	0	0	0	0	0	0	0	0	0	0	0	0	0	0	0
BGIR9823_77830	100736796	ADGRB3	lncRNA	0	0	0	0	0	0	0	0	0	0	0	0	0	0	0	0
BGIR9823_78118	100152054	RFX7	lncRNA	0	0	0	0	0	0	0	0	0	0	0	0	0	0	0	1
BGIR9823_80498	106509452	PTPRS	mRNA	0	0	0	0	0	0	0	0	0	0	1	0	0	0	0	0
BGIR9823_83059	100524080	KCMF1	lncRNA	0	0	0	0	0	0	0	0	0	0	0	0	0	0	0	0
BGIR9823_89241	100155423	FKBP5	lncRNA	0	0	0	0	0	0	0	0	0	0	0	0	0	0	0	0
BGIR9823_86626	399532	FABP3	lncRNA	0	0	0	0	0	0	0	0	0	0	0	0	0	0	0	0
BGIR9823_80136	100512584	PLA2G16	lncRNA	0	0	0	0	0	0	0	0	0	0	0	0	0	0	0	0
BGIR9823_78764	100621961	CDC26	lncRNA	0	0	0	0	0	1	0	0	0	0	0	0	0	0	0	0
BGIR9823_100787	100622432	CBX3	lncRNA	0	0	0	0	0	0	0	0	0	0	0	0	0	0	0	0
BGIR9823_81517	100523721	FAF2	lncRNA	0	0	0	0	0	0	0	0	0	0	0	0	0	0	0	0
BGIR9823_94928	100622196	IQSEC1	mRNA	0	0	0	0	0	0	0	0	0	0	0	0	0	0	0	0
BGIR9823_97066	100155963	MMRN2	lncRNA	0	0	1	0	0	0	0	0	0	0	0	0	0	0	0	0
BGIR9823_86005	100515835	RSPRY1	lncRNA	0	0	0	0	0	0	0	0	0	0	0	0	0	0	0	0
BGIR9823_81824	100514516	CSNK1A1	lncRNA	0	0	0	0	0	0	0	0	0	0	0	0	0	0	0	0
BGIR9823_100617	100523293	KCNH2	lncRNA	0	0	0	0	0	0	0	0	0	0	0	0	0	0	0	0
XR_002337475.1	106507029	LOC106507029	lncRNA	0	0	0	0	0	0	0	0	0	0	0	0	0	0	0	0
BGIR9823_99577	100523519	CCDC152	lncRNA	0	0	0	0	0	0	0	0	0	0	0	0	0	0	0	0
BGIR9823_79582	100151784	CLPX	lncRNA	0	0	0	0	0	0	0	0	0	0	1	0	0	0	0	0
BGIR9823_90043	397616	PDE5A	lncRNA	0	0	0	0	0	0	0	0	0	0	0	0	0	0	0	0
BGIR9823_101350	100621019	ANAPC2	mRNA	0	0	0	0	0	0	0	0	0	0	0	0	0	0	0	0
BGIR9823_77999	100512572	WDR7	lncRNA	0	0	0	0	0	0	0	0	0	0	0	0	0	0	0	0
BGIR9823_80986	100294703	KDM2A	lncRNA	0	0	0	0	0	0	0	0	0	0	0	0	0	0	0	0
BGIR9823_82031	110259918	ITPRIPL2	lncRNA	1	0	0	0	0	0	0	0	0	0	0	0	0	0	0	0
BGIR9823_100386	100515632	KDM7A	lncRNA	0	0	0	0	0	0	0	0	0	0	0	0	0	0	0	0
BGIR9823_78769	100157179	ZNF618	lncRNA	0	0	0	0	0	0	0	0	0	0	0	0	0	0	0	0
BGIR9823_77679	100513634	MPC1	lncRNA	0	0	0	0	0	0	0	0	0	0	0	0	0	0	0	0
BGIR9823_101316	100624156	CD99	lncRNA	0	0	0	0	0	0	0	0	0	0	0	0	0	0	0	1
BGIR9823_79248	100738627	DYM	lncRNA	0	0	0	0	0	0	0	0	0	0	0	0	0	0	0	0
BGIR9823_78478	100152019	PELI2	lncRNA	0	0	0	0	0	1	0	0	0	0	0	0	0	0	0	0
BGIR9823_95265	100622532	FYTTD1	lncRNA	0	0	0	0	0	0	0	0	0	0	0	0	0	0	0	0
BGIR9823_84388	100155270	NIT1	mRNA	0	0	0	0	0	0	0	0	0	0	0	0	0	0	0	0
BGIR9823_101285	100622228	ARHGEF6	mRNA	0	0	0	0	0	0	0	0	0	0	0	0	0	0	0	0
BGIR9823_100255	100156548	ZHX3	lncRNA	0	0	0	0	0	0	0	0	0	0	0	0	0	0	0	0
BGIR9823_94455	100151869	OXNAD1	lncRNA	0	0	0	0	0	0	0	0	0	0	0	0	0	0	0	0
BGIR9823_78812	100155133	PTGES2	lncRNA	0	0	0	0	0	0	0	0	0	0	0	0	0	0	0	0
BGIR9823_80806	100519080	LYRM7	lncRNA	0	0	0	0	0	0	0	0	0	0	0	0	0	0	0	0
BGIR9823_101247	100519287	KLHL13	mRNA	0	0	0	0	0	0	0	0	0	0	0	0	0	0	0	0
BGIR9823_79520	102162853	LOC102162853	lncRNA	0	0	1	0	0	0	0	0	0	0	0	0	0	0	0	0
BGIR9823_95143	407739	SOX2	lncRNA	0	0	0	0	0	0	0	0	0	0	0	0	0	0	0	0
BGIR9823_95008	100620055	PLSCR1	lncRNA	0	0	0	0	0	0	0	0	0	0	0	0	0	0	0	0

RNA ID	Gene ID	Symbol	Type	ssc-miR-24-1-5p	ssc-miR-29b	ssc-miR-29c	ssc-miR-323	ssc-miR-369	ssc-miR-708-5p	novel-ssc-miR161-3p	novel-ssc-miR512-5p	novel-ssc-miR846-3p	novel-ssc-miR1086-5p	novel-ssc-miR1105-5p	novel-ssc-miR1116-5p	novel-ssc-miR1120-5p	novel-ssc-miR1140-5p	novel-ssc-miR1158-3p	novel-ssc-miR1379-5p
BGIR9823_100815	110257576	LOC110257576	lncRNA	0	0	0	0	0	0	0	0	0	0	0	0	0	0	0	0
BGIR9823_83902	110260470	LOC110260470	lncRNA	0	0	0	0	0	0	0	0	0	0	0	0	0	0	0	0
BGIR9823_96518	100623262	SLC15A4	mRNA	0	0	0	1	0	0	0	0	0	0	0	0	0	0	0	0
BGIR9823_101546	110258600	LOC110258600	lncRNA	0	0	0	0	0	0	0	0	0	0	0	0	0	0	0	0
BGIR9823_79803	100154484	RCL1	lncRNA	0	0	0	0	0	0	0	0	0	0	0	0	0	0	0	0
BGIR9823_78229	100154984	TMEM87A	mRNA	0	0	0	0	0	0	0	0	0	0	0	0	0	0	0	0
BGIR9823_78085	100154204	MYO1E	lncRNA	0	0	0	0	0	0	0	0	0	0	0	0	0	0	0	0
BGIR9823_84094	106509960	NSMCE2	lncRNA	0	0	0	0	0	0	0	0	0	0	0	0	0	0	0	0
BGIR9823_80335	102166675	RNF187	lncRNA	0	0	0	0	0	0	0	0	0	0	0	0	0	0	0	0
BGIR9823_101281	100516171	GPC3	lncRNA	0	0	0	0	0	0	0	0	0	0	0	0	0	0	0	0
BGIR9823_83886	102166500	LOC102166500	lncRNA	0	0	0	0	0	0	0	0	0	0	0	0	0	0	0	0
BGIR9823_78367	100520530	CORO2B	lncRNA	0	0	0	0	0	0	0	0	0	0	0	0	0	0	0	0
BGIR9823_81305	102166940	LOC102166940	lncRNA	0	0	0	0	0	0	0	0	0	0	0	0	0	0	0	0
BGIR9823_100832	100520285	ZNF777	lncRNA	0	0	0	0	0	0	0	0	0	0	0	0	0	0	0	0
BGIR9823_100583	102162331	KRBA1	lncRNA	0	0	0	0	0	0	0	0	0	0	0	0	0	0	0	0
BGIR9823_85020	100626912	BHLHE41	mRNA	0	0	0	0	0	0	0	0	0	0	0	0	0	0	0	0
BGIR9823_100375	100514612	BRAF	mRNA	0	0	0	0	0	0	0	0	0	0	0	0	0	0	0	0
BGIR9823_92109	100521742	NEK7	mRNA	0	0	0	0	0	0	0	0	0	0	0	0	0	0	0	0
BGIR9823_100703	100514931	AHCYL2	lncRNA	0	0	0	0	0	0	0	0	0	0	0	0	0	0	0	0
BGIR9823_79724	396697	ESR2	lncRNA	0	0	0	0	0	0	0	0	0	0	0	0	0	0	0	0
BGIR9823_101638	110259113	LOC110259113	lncRNA	0	0	0	0	0	0	0	0	0	0	0	1	0	0	0	0
BGIR9823_92471	100517963	RMI1	lncRNA	0	0	0	0	0	0	0	0	0	0	0	0	0	0	0	0
BGIR9823_100226	654300	UQCC	lncRNA	0	0	0	0	0	0	0	0	0	0	0	0	0	0	0	0
BGIR9823_86749	100624877	PPP4R1	mRNA	0	0	0	0	0	0	0	0	0	0	0	0	0	0	0	0
BGIR9823_97541	100157435	UBE3B	mRNA	0	0	0	0	0	0	1	0	0	0	0	0	0	0	0	0
BGIR9823_80565	102164292	FSTL3	lncRNA	0	0	0	0	0	0	0	0	0	0	0	0	0	0	0	0
BGIR9823_96818	100157092	TTC13	mRNA	0	0	0	0	0	0	0	0	0	0	0	0	0	0	0	0
BGIR9823_84745	100521449	NPTXR	mRNA	0	0	0	0	0	0	0	0	0	0	0	0	0	0	0	0
BGIR9823_79788	102168004	UHRF2	lncRNA	0	0	0	0	0	0	0	0	0	0	0	0	0	0	0	0
BGIR9823_78692	100515356	GLIPR2	lncRNA	0	0	0	0	0	0	0	0	0	0	0	0	0	0	0	1
BGIR9823_79323	100621557	TCF12	lncRNA	0	0	0	0	0	0	0	0	0	0	0	0	0	0	0	0
BGIR9823_101455	110258361	PACS2	lncRNA	0	0	0	0	0	0	0	0	0	0	0	0	0	0	0	0
BGIR9823_94552	100523331	NKTR	lncRNA	0	0	0	0	0	0	0	0	0	0	0	0	0	1	0	0
BGIR9823_100126	100519631	TASP1	lncRNA	0	0	0	0	0	0	0	0	0	0	0	0	0	0	0	0
BGIR9823_100406	106506765	LOC106506765	lncRNA	0	0	0	0	0	0	0	0	0	0	0	0	0	0	0	0
BGIR9823_80256	102168147	LOC102168147	lncRNA	1	0	0	0	0	0	0	0	0	0	0	0	0	0	0	0
BGIR9823_99608	397251	PLK2	mRNA	0	0	0	0	0	0	0	0	0	0	0	0	0	0	0	0
BGIR9823_94040	397550	STAT5A	mRNA	0	0	0	1	0	0	0	0	0	0	0	0	0	0	0	0
BGIR9823_100411	414383	CHCHD3	lncRNA	0	0	0	0	0	0	0	0	0	0	0	0	0	0	0	0
BGIR9823_93144	100174955	KLF12	lncRNA	0	0	0	0	0	0	0	0	0	0	0	0	0	0	0	0
BGIR9823_81306	102166940	LOC102166940	lncRNA	0	0	0	0	0	1	0	0	0	0	0	0	0	0	0	0
BGIR9823_79214	100515594	MTO1	lncRNA	0	0	0	0	0	0	0	0	0	0	0	0	0	0	0	0
XR_001302600.2	106506942	LOC106506942	lncRNA	0	0	0	0	0	0	0	0	0	0	0	0	0	0	0	0

RNA ID	Gene ID	Symbol	Type	ssc-miR-24-1-5p	ssc-miR-29b	ssc-miR-29c	ssc-miR-323	ssc-miR-369	ssc-miR-708-5p	novel-ssc-miR161-3p	novel-ssc-miR512-5p	novel-ssc-miR846-3p	novel-ssc-miR1086-5p	novel-ssc-miR1105-5p	novel-ssc-miR1116-5p	novel-ssc-miR1120-5p	novel-ssc-miR1140-5p	novel-ssc-miR1158-3p	novel-ssc-miR1379-5p
BGIR9823_99840	100519805	TBC1D20	lncRNA	0	0	0	0	0	0	0	0	0	0	0	0	0	0	0	0
BGIR9823_100155	100519522	NINL	lncRNA	0	0	0	0	0	0	0	0	0	0	0	0	0	0	0	0
BGIR9823_78548	100511733	PLAA	lncRNA	0	0	0	1	0	0	0	0	0	0	0	0	0	0	0	0
BGIR9823_81728	100524487	AFF4	lncRNA	0	0	0	0	0	0	0	0	0	0	0	0	0	0	0	0
BGIR9823_82871	100516601	ZNF597	lncRNA	0	0	0	0	0	0	0	0	0	0	0	0	0	0	0	0
BGIR9823_83578	100157134	ZNF704	lncRNA	0	0	0	0	0	0	0	0	0	0	0	0	0	0	0	0
BGIR9823_78456	100625719	CGRRF1	lncRNA	0	0	0	0	0	0	0	0	0	0	0	0	0	0	0	0
BGIR9823_100241	100154500	SAMHD1	lncRNA	0	0	0	0	0	0	0	0	0	0	0	0	0	0	0	0
XR_002344503.1	110260914	LOC110260914	lncRNA	0	0	0	0	0	0	0	0	0	0	0	0	0	0	0	0
BGIR9823_93124	100174955	KLF12	lncRNA	0	0	0	0	0	0	0	0	0	0	0	0	0	0	0	0
BGIR9823_78918	100627455	SNX9	mRNA	0	0	0	0	0	0	0	0	0	0	1	0	0	0	0	0
BGIR9823_100427	100626356	UBE2H	lncRNA	0	0	0	0	0	0	0	0	0	0	0	0	0	0	0	0
BGIR9823_79882	100156839	FAM166B	mRNA	0	0	0	0	0	0	0	0	0	0	0	0	0	0	0	0
BGIR9823_87816	100626395	NAPG	lncRNA	0	0	0	0	0	0	0	0	0	0	0	0	0	0	0	0
BGIR9823_79529	100154633	PHLPP1	lncRNA	0	0	0	0	0	1	0	0	0	0	0	0	0	0	0	0
BGIR9823_80355	100620218	BORCS8	lncRNA	0	0	0	0	0	0	0	0	0	0	0	0	0	0	0	0
BGIR9823_101515	102157759	LOC102157759	lncRNA	0	0	0	0	0	0	0	0	0	0	0	0	0	0	0	0
BGIR9823_88688	100152987	HEATR5A	mRNA	0	0	0	0	0	0	0	0	0	0	0	0	0	0	0	0
BGIR9823_78932	100514379	FBXO5	lncRNA	0	0	0	0	0	0	0	0	0	0	0	0	0	0	0	0
BGIR9823_78476	100152019	PELI2	lncRNA	1	0	0	0	0	0	0	0	0	0	0	0	0	0	0	0
BGIR9823_92851	100523918	LMO7	mRNA	0	0	0	0	0	0	0	0	0	0	0	0	0	0	0	0
BGIR9823_100326	110257473	LOC110257473	lncRNA	0	0	0	0	0	0	0	0	0	0	0	0	0	0	0	0
BGIR9823_87757	100621544	RNF19B	mRNA	0	0	0	0	0	0	0	0	0	0	0	0	0	0	0	0
BGIR9823_99279	100517617	WDR70	lncRNA	0	0	0	0	0	0	0	0	0	0	0	0	0	0	0	0
BGIR9823_78488	100152019	PELI2	lncRNA	0	0	0	0	0	0	0	0	0	0	0	0	0	0	0	0
BGIR9823_91993	100738612	ATF3	mRNA	0	0	0	0	0	0	0	0	0	0	0	0	0	0	0	0
BGIR9823_94664	102167511	SMIM4	lncRNA	0	0	0	0	0	0	0	0	0	0	0	0	0	0	0	0
BGIR9823_79100	100156082	FBXL4	lncRNA	0	0	0	0	0	0	0	0	0	0	0	0	0	0	0	0
BGIR9823_101197	100156239	VSIG4	mRNA	0	0	0	0	0	0	0	0	0	0	0	0	0	0	0	0
BGIR9823_100442	100192443	POT1	lncRNA	0	0	0	0	0	0	0	0	0	0	0	0	0	0	0	0
BGIR9823_78713	100152263	ANP32B	lncRNA	0	0	0	0	0	0	0	0	0	0	0	0	0	0	0	0
BGIR9823_77769	100153609	PTPRK	lncRNA	0	0	0	0	0	0	0	0	0	0	0	1	0	0	0	0
BGIR9823_78654	100515003	SMC5	mRNA	0	0	0	0	0	0	0	0	0	0	0	0	0	0	0	0
BGIR9823_95994	100512989	VEPH1	lncRNA	0	0	0	0	0	0	0	0	0	0	0	0	0	1	0	0
BGIR9823_100398	100521697	MTPN	lncRNA	0	0	0	0	0	0	0	0	0	0	0	0	0	0	0	0
BGIR9823_77692	110259915	LOC110259915	lncRNA	0	0	0	0	0	0	0	0	0	0	0	0	0	0	0	0
BGIR9823_100510	100738688	HERPUD2	lncRNA	0	0	0	0	0	0	0	0	0	0	0	0	0	0	0	0
BGIR9823_101594	110258799	LOC110258799	lncRNA	0	0	0	0	0	0	0	0	0	0	0	0	0	0	0	0
BGIR9823_100619	100736760	GIMAP8	lncRNA	0	0	0	0	0	0	0	0	0	0	0	0	0	0	0	0
BGIR9823_98052	100153733	RAB11FIP2	mRNA	0	0	0	0	0	0	0	0	0	0	0	0	0	0	0	0
BGIR9823_79578	100151784	CLPX	lncRNA	0	0	0	0	0	0	0	0	0	0	0	0	0	0	0	0
BGIR9823_82021	100628086	METTL9	lncRNA	0	0	0	0	0	0	0	0	0	0	0	0	0	0	0	0
BGIR9823_100265	100520338	LOC100520338	lncRNA	0	0	0	0	0	0	0	0	0	0	0	0	0	0	0	0

RNA ID	Gene ID	Symbol	Type	ssc-miR-24-1-5p	ssc-miR-29b	ssc-miR-29c	ssc-miR-323	ssc-miR-369	ssc-miR-708-5p	novel-ssc-miR161-3p	novel-ssc-miR512-5p	novel-ssc-miR846-3p	novel-ssc-miR1086-5p	novel-ssc-miR1105-5p	novel-ssc-miR1116-5p	novel-ssc-miR1120-5p	novel-ssc-miR1140-5p	novel-ssc-miR1158-3p	novel-ssc-miR1379-5p
BGIR9823_88390	100124376	SKIV2L	mRNA	0	0	0	0	0	0	0	0	0	1	0	0	0	0	0	0
BGIR9823_78231	100154984	TMEM87A	lncRNA	0	0	0	0	0	0	0	0	0	0	0	0	0	0	0	0
BGIR9823_84096	106509960	NSMCE2	lncRNA	0	0	0	0	0	0	0	0	0	0	0	0	0	0	0	0
BGIR9823_81704	100521611	FNIP1	lncRNA	0	0	0	0	0	0	0	0	0	0	0	0	0	0	0	0
BGIR9823_79498	100736682	TJP1	lncRNA	0	0	0	0	0	0	0	0	0	0	0	0	0	0	0	0
BGIR9823_100070	100157832	SGCZ	lncRNA	0	0	0	0	0	0	0	0	0	0	0	0	0	0	0	0
BGIR9823_87139	106510467	LOC106510467	lncRNA	0	0	0	0	0	0	0	0	0	0	0	0	0	0	0	0
BGIR9823_100722	100514329	LOC100514329	lncRNA	0	0	0	0	0	0	0	0	0	0	0	0	0	0	0	0
BGIR9823_82833	106509697	CIITA	lncRNA	0	0	0	0	0	0	0	0	0	0	0	0	0	0	0	0
BGIR9823_100488	100518941	TMEM168	lncRNA	0	0	0	0	0	0	0	0	0	0	0	0	0	0	0	0
BGIR9823_101179	100517625	FAM120C	lncRNA	0	0	0	0	0	0	0	0	0	0	0	0	0	0	0	0
BGIR9823_100981	100513513	SLC16A2	mRNA	0	0	0	0	0	0	0	0	0	0	0	0	0	0	0	0
BGIR9823_85418	102163508	LOC102163508	lncRNA	0	0	0	0	0	0	0	0	0	0	0	0	0	0	0	0
BGIR9823_100250	100516472	KIAA1755	lncRNA	0	0	0	0	0	0	0	0	0	0	0	0	0	0	0	0
BGIR9823_100877	100523478	CNKSR2	lncRNA	0	0	0	0	0	0	0	0	0	0	0	0	0	0	0	0
BGIR9823_84851	100523964	LOC100523964	mRNA	0	0	0	0	0	0	0	0	0	0	0	0	0	0	0	0
BGIR9823_79979	100737979	CDK5RAP2	mRNA	0	0	0	0	0	0	0	0	0	0	0	0	0	0	0	0
BGIR9823_85878	100622883	NAV3	mRNA	0	0	0	0	0	0	0	0	0	0	0	0	0	0	0	0
BGIR9823_87603	100620358	ZBTB17	mRNA	0	0	0	0	0	0	0	0	0	0	0	0	0	0	0	0
BGIR9823_81340	100622538	DNM2	lncRNA	0	0	0	0	0	1	0	0	0	0	0	0	0	0	0	0
BGIR9823_93496	100521398	SMARCE1	lncRNA	0	0	0	0	0	0	0	0	0	0	0	0	0	0	0	0
BGIR9823_86377	100621391	SSC5D	mRNA	0	0	0	0	0	0	0	0	0	0	0	0	0	0	0	0
BGIR9823_85565	110260712	LOC110260712	lncRNA	0	0	0	0	0	0	0	0	0	0	0	0	0	0	0	0
BGIR9823_95828	100511410	PDZRN3	lncRNA	0	0	0	0	0	0	0	0	0	0	0	0	0	0	0	0
BGIR9823_93148	100174955	KLF12	lncRNA	0	0	0	0	0	0	0	0	0	0	0	0	0	0	0	0
BGIR9823_101568	110258600	LOC110258600	lncRNA	1	0	0	0	0	0	0	0	0	0	0	0	0	0	0	0
BGIR9823_79012	100738102	RNF217	lncRNA	0	0	0	0	0	0	0	0	0	0	0	0	0	0	0	1
XM_021091420.1	100523786	PACSIN2	mRNA	0	0	0	0	0	0	0	0	0	0	0	0	0	0	0	0
BGIR9823_100565	100516888	GLI3	lncRNA	0	0	0	0	0	0	0	0	0	0	0	0	0	0	0	0
BGIR9823_78022	100157196	CSNK1G1	lncRNA	0	0	0	0	0	0	0	0	0	0	0	0	0	0	0	0
BGIR9823_78883	100512199	ERMARD	lncRNA	0	0	0	0	0	0	0	0	0	0	0	0	0	0	0	0
BGIR9823_84618	100157977	FNBP1L	lncRNA	0	0	0	0	0	0	0	0	0	0	0	0	0	0	0	0
BGIR9823_97044	100158106	NRG3	lncRNA	0	0	0	0	0	0	1	0	0	0	0	0	0	0	0	0
BGIR9823_99699	100513766	G3BP1	mRNA	0	0	0	0	0	0	0	0	0	0	0	0	0	0	0	0
BGIR9823_87685	100512789	WASF2	lncRNA	0	0	0	0	0	0	0	0	0	0	0	0	0	0	0	0
BGIR9823_100122	100519631	TASP1	lncRNA	0	0	0	0	0	0	0	0	0	0	0	0	0	0	0	0
BGIR9823_100289	100738810	SLC13A3	lncRNA	0	0	0	0	0	0	0	0	0	0	0	0	0	0	0	0
BGIR9823_80121	100737568	SSSCA1	lncRNA	0	0	0	0	0	0	0	0	0	0	0	0	0	0	0	0
BGIR9823_86301	100628160	SNRNP70	mRNA	0	0	0	0	0	0	0	0	0	0	0	0	0	0	0	0
BGIR9823_100376	100514612	BRAF	lncRNA	0	0	0	0	0	0	0	0	0	0	0	0	0	0	0	0
BGIR9823_80749	100514631	MAN2A1	lncRNA	0	0	0	0	0	0	0	0	0	0	0	0	0	0	0	0
BGIR9823_101511	110258473	LOC110258473	lncRNA	0	0	0	0	0	1	0	0	0	0	0	0	0	0	0	0
BGIR9823_100755	100620722	ZNF277	lncRNA	0	0	0	0	0	0	0	0	0	0	0	0	0	0	0	0

RNA ID	Gene ID	Symbol	Type	ssc-miR-24-1-5p	ssc-miR-29b	ssc-miR-29c	ssc-miR-323	ssc-miR-369	ssc-miR-708-5p	novel-ssc-miR161-3p	novel-ssc-miR512-5p	novel-ssc-miR846-3p	novel-ssc-miR1086-5p	novel-ssc-miR1105-5p	novel-ssc-miR1116-5p	novel-ssc-miR1120-5p	novel-ssc-miR1140-5p	novel-ssc-miR1158-3p	novel-ssc-miR1379-5p
BGIR9823_82116	100624598	MEIOB	lncRNA	0	0	0	0	0	0	0	0	0	0	0	0	0	0	0	0
BGIR9823_101016	100520165	TMEM164	lncRNA	0	0	0	0	0	0	0	0	0	0	0	0	0	0	0	0
BGIR9823_90026	110262158	LOC110262158	lncRNA	0	0	0	0	0	0	0	0	0	0	0	0	0	0	0	0
BGIR9823_94087	100515340	LOC100515340	lncRNA	0	0	0	0	0	0	0	0	0	0	0	0	0	0	0	0
BGIR9823_89845	100516092	GNPDA2	lncRNA	0	0	0	0	0	0	0	0	0	0	0	0	0	0	0	0
BGIR9823_99243	100521757	ZNF622	lncRNA	0	0	0	0	0	0	0	0	0	1	0	0	0	0	0	0
BGIR9823_91934	100196908	TOR3A	lncRNA	0	0	0	0	0	0	0	0	0	0	0	0	0	0	0	0
BGIR9823_80152	100620658	HNRNPUL2	mRNA	0	0	0	1	0	0	0	0	0	0	0	0	0	0	0	0
BGIR9823_95348	100513438	NAA50	lncRNA	0	0	0	0	0	0	0	0	0	0	0	0	0	0	0	0
BGIR9823_86872	102164512	ZNF397	lncRNA	0	0	0	0	0	0	0	0	0	0	0	0	0	0	0	0
BGIR9823_85545	100525288	OSBPL8	lncRNA	0	0	0	0	0	0	0	0	0	0	0	0	0	0	0	0
BGIR9823_92701	100739805	CDK8	lncRNA	0	0	0	0	0	0	0	0	0	0	0	0	0	1	0	0
BGIR9823_79943	100626033	CTNNAL1	lncRNA	0	0	0	0	0	0	0	0	0	0	0	0	0	0	0	0
BGIR9823_100365	100739295	WDR86	lncRNA	0	0	0	0	0	0	0	0	0	0	0	0	0	0	0	0
BGIR9823_82500	100524371	STRN	lncRNA	0	0	0	0	0	0	0	0	0	0	0	0	0	0	0	0
BGIR9823_83316	397176	TAF1B	lncRNA	0	0	0	0	0	0	0	0	0	0	0	0	0	0	0	0
BGIR9823_82589	110260059	CYS1	lncRNA	0	0	0	0	0	0	0	0	0	0	0	0	0	0	0	0
BGIR9823_100212	100157872	PXMP4	lncRNA	0	0	0	0	0	0	0	0	0	0	0	0	0	0	0	0
BGIR9823_84315	106510091	SPIDR	mRNA	0	0	0	0	0	0	0	0	0	0	0	0	0	0	0	0
BGIR9823_85039	100625270	ST8SIA1	mRNA	0	0	0	0	0	0	0	0	0	0	0	0	0	0	0	0
BGIR9823_79988	396733	GGTA1P	lncRNA	0	0	0	0	0	0	0	0	0	0	0	0	0	0	0	0
BGIR9823_81636	100625008	GIN1	lncRNA	0	0	0	0	0	0	0	0	0	0	0	0	0	0	0	0
BGIR9823_96851	100522707	ZNF25	lncRNA	0	0	0	0	0	0	0	0	0	0	0	0	0	1	0	0
BGIR9823_98026	733691	NRAP	mRNA	0	0	0	0	0	0	0	0	0	0	0	0	0	0	0	0
BGIR9823_91942	100510921	STX6	mRNA	0	0	0	0	0	0	0	0	0	0	0	0	0	0	0	0
BGIR9823_101516	102157759	LOC102157759	lncRNA	0	0	0	0	0	0	0	0	0	0	0	0	0	0	0	0
BGIR9823_79269	100152360	CCDC68	lncRNA	0	0	0	0	0	0	0	0	0	0	0	0	0	0	0	0
BGIR9823_95434	100737549	ITSN1	mRNA	0	0	0	0	0	0	0	0	0	0	0	0	0	0	0	0
BGIR9823_81890	100621608	FOPNL	lncRNA	0	0	0	0	0	0	0	0	0	0	0	0	0	0	0	0
BGIR9823_86166	100739461	ZNF570	lncRNA	0	0	0	0	0	0	0	0	0	0	0	0	0	0	0	0
BGIR9823_81903	102162731	ZKSCAN1	mRNA	0	0	0	0	0	0	0	0	0	0	0	0	0	0	0	0
BGIR9823_84182	100153957	KCNS2	mRNA	0	0	0	0	0	0	0	0	0	0	0	0	0	0	0	0
BGIR9823_78637	100155691	AK3	lncRNA	0	0	0	0	0	0	0	0	0	0	0	0	0	0	0	0
BGIR9823_79359	100157164	GABPB1	lncRNA	0	0	0	0	0	0	0	0	0	0	0	0	0	0	0	0
BGIR9823_81535	100520548	WDR41	lncRNA	0	0	0	0	0	0	0	0	0	0	0	0	0	1	0	0
BGIR9823_86229	100516271	DEDD2	mRNA	0	0	0	0	0	0	0	0	0	1	0	0	0	0	0	0
BGIR9823_81434	110259374	LOC110259374	lncRNA	0	0	0	0	0	0	0	0	0	0	0	0	0	0	0	0
BGIR9823_100426	100626356	UBE2H	lncRNA	1	0	0	0	0	0	0	0	0	0	0	0	0	0	0	0
BGIR9823_100415	100620243	PLXNA4	lncRNA	0	0	0	0	0	0	0	0	0	0	0	1	0	0	0	0
BGIR9823_100894	100621199	IL1RAPL1	lncRNA	0	0	0	0	0	0	0	0	0	0	0	0	0	0	0	0
BGIR9823_95922	100622559	PIK3CB	lncRNA	0	0	0	0	0	0	0	0	0	0	0	0	0	0	0	0
BGIR9823_82064	100736832	GRIN2A	lncRNA	0	0	0	0	0	0	0	0	0	0	0	0	0	0	0	0
BGIR9823_100306	110257429	LOC110257429	lncRNA	0	0	0	0	0	0	0	0	0	0	0	0	0	0	0	0

RNA ID	Gene ID	Symbol	Type	ssc-miR-24-1-5p	ssc-miR-29b	ssc-miR-29c	ssc-miR-323	ssc-miR-369	ssc-miR-708-5p	novel-ssc-miR161-3p	novel-ssc-miR512-5p	novel-ssc-miR846-3p	novel-ssc-miR1086-5p	novel-ssc-miR1105-5p	novel-ssc-miR1116-5p	novel-ssc-miR1120-5p	novel-ssc-miR1140-5p	novel-ssc-miR1158-3p	novel-ssc-miR1379-5p
BGIR9823_81302	100736972	LOC100736972	lncRNA	0	0	0	0	0	0	0	0	0	0	0	0	0	0	0	0
BGIR9823_100478	100621338	BMT2	lncRNA	0	0	0	0	0	0	0	0	0	0	0	1	0	0	0	0
BGIR9823_86034	100623274	CTCF	mRNA	0	0	0	0	0	0	0	0	0	0	0	0	0	0	0	0
BGIR9823_78972	100155445	VTA1	lncRNA	0	0	0	0	0	0	0	0	0	0	0	0	0	0	0	1
BGIR9823_78078	100154204	MYO1E	lncRNA	0	0	0	0	0	0	0	0	0	0	0	0	0	0	0	1
BGIR9823_82851	100522863	RBFOX1	mRNA	0	0	0	0	0	0	0	0	0	0	0	0	0	0	0	0
BGIR9823_87122	100515140	KIAA0513	lncRNA	0	0	0	0	0	0	0	0	0	0	0	0	0	0	0	0
BGIR9823_97004	100154626	ADK	lncRNA	0	0	0	0	0	0	0	0	0	0	1	0	0	0	0	0
BGIR9823_90785	100524160	ACER3	lncRNA	0	0	0	0	0	0	0	0	0	0	0	0	0	1	0	0
BGIR9823_92442	100513615	C10H9orf3	mRNA	0	0	0	0	0	0	0	0	0	0	1	0	0	0	0	0
BGIR9823_90875	100622590	BIRC3	lncRNA	0	0	0	0	0	0	0	0	0	0	0	0	0	0	0	0
BGIR9823_101409	110258214	LOC110258214	lncRNA	1	0	0	0	0	0	0	0	0	0	0	0	0	0	0	0
BGIR9823_101574	102164761	LOC102164761	lncRNA	0	0	0	0	0	0	0	0	0	0	0	0	0	0	0	0
BGIR9823_81206	100519588	USP47	lncRNA	0	0	0	0	0	0	0	0	0	0	0	0	0	0	0	0
BGIR9823_82146	100737615	LUC7L	lncRNA	0	0	0	0	0	0	0	0	0	0	0	0	0	0	0	0
BGIR9823_88950	100620189	NRXN3	lncRNA	0	0	0	0	0	0	0	0	0	0	0	0	0	1	0	0
BGIR9823_100904	100621199	IL1RAPL1	lncRNA	0	0	0	0	0	0	0	0	0	0	0	0	0	0	0	0
BGIR9823_95076	100622923	PPM1L	lncRNA	0	0	0	0	0	0	0	0	0	0	0	0	0	0	0	0
BGIR9823_100269	102159476	LOC102159476	lncRNA	0	0	0	0	0	0	0	0	0	0	0	0	0	0	0	0
BGIR9823_101628	100519072	LOC100519072	lncRNA	0	0	0	0	0	0	0	0	0	0	0	0	0	0	0	0
BGIR9823_101612	100521600	LOC100521600	lncRNA	0	0	0	0	0	0	0	0	0	0	0	0	0	0	0	0
BGIR9823_101400	110258215	LOC110258215	lncRNA	0	0	0	0	0	0	0	0	0	0	0	0	0	0	0	0
BGIR9823_92255	100514676	ACBD5	lncRNA	0	0	0	0	0	0	0	0	0	0	0	0	0	0	1	0
BGIR9823_85922	100516196	TAF1C	lncRNA	0	0	0	0	0	0	0	0	0	0	0	0	0	0	0	0
BGIR9823_81946	100525688	BUD23	mRNA	0	0	0	0	0	0	0	0	0	0	0	0	0	0	0	0
BGIR9823_80177	106504153	AHNAK	mRNA	0	0	0	0	0	0	0	0	0	1	0	0	0	0	0	0
BGIR9823_88439	106504047	ZNF76	mRNA	0	0	0	0	0	0	1	0	0	0	0	0	0	0	0	0
BGIR9823_81354	100517998	MYO1F	lncRNA	0	0	0	0	0	0	0	0	0	0	0	0	0	1	0	0
BGIR9823_80921	100329221	PPARGC1B	mRNA	0	0	0	0	0	0	0	0	0	0	0	0	0	0	0	0
BGIR9823_77718	100625026	PPIL4	lncRNA	0	0	0	0	0	0	0	0	0	0	0	0	0	0	0	0
BGIR9823_100514	100623678	NT5C3A	lncRNA	0	0	0	0	0	0	0	0	0	0	0	0	0	0	0	0
BGIR9823_78774	396733	GGTA1P	lncRNA	0	0	0	0	0	0	0	0	0	0	0	0	0	0	0	0
BGIR9823_101033	100037300	XIAP	lncRNA	0	0	0	0	0	0	0	0	0	0	0	0	0	0	0	0
BGIR9823_100816	110257601	LOC110257601	lncRNA	0	0	0	0	0	0	0	0	0	0	0	0	0	0	0	0
BGIR9823_90686	733582	CDS1	lncRNA	0	0	0	0	0	0	0	0	0	0	0	0	0	1	0	0
BGIR9823_91751	110255475	LOC110255475	lncRNA	0	0	0	0	0	0	0	0	0	0	0	0	0	0	0	0
NM_001244541.1	100513376	GMPPB	mRNA	0	0	0	0	0	0	0	0	0	0	0	0	0	0	0	0
BGIR9823_81475	100620238	LOC100620238	lncRNA	0	0	0	0	0	0	0	0	0	0	0	0	0	0	0	0
BGIR9823_100582	102162331	KRBA1	lncRNA	0	0	0	0	0	0	0	0	0	0	0	0	0	0	0	0
BGIR9823_101332	100625415	EIF1AY	lncRNA	0	0	0	0	0	0	0	0	0	0	0	0	0	0	0	0
BGIR9823_97502	595128	OAS2	lncRNA	0	0	0	0	0	0	0	0	0	0	0	0	0	0	0	0
BGIR9823_83701	100153809	ALDH9A1	mRNA	0	0	0	0	0	0	0	0	0	0	0	0	0	0	0	0
BGIR9823_80203	100516953	MPEG1	lncRNA	0	0	0	0	0	0	0	0	0	0	0	0	0	0	0	0

RNA ID	Gene ID	Symbol	Type	ssc-miR-24-1-5p	ssc-miR-29b	ssc-miR-29c	ssc-miR-323	ssc-miR-369	ssc-miR-708-5p	novel-ssc-miR161-3p	novel-ssc-miR512-5p	novel-ssc-miR846-3p	novel-ssc-miR1086-5p	novel-ssc-miR1105-5p	novel-ssc-miR1116-5p	novel-ssc-miR1120-5p	novel-ssc-miR1140-5p	novel-ssc-miR1158-3p	novel-ssc-miR1379-5p
BGIR9823_99394	100517687	BDP1	lncRNA	0	0	0	0	0	0	0	0	0	0	0	0	0	0	0	0
BGIR9823_85850	102159120	LOC102159120	lncRNA	0	0	0	0	0	0	0	0	0	0	0	0	0	0	0	0
BGIR9823_83931	100519711	ST7L	mRNA	0	0	0	0	0	0	0	0	0	0	1	0	0	0	0	0
BGIR9823_93274	100513428	CEP131	lncRNA	0	0	0	0	0	0	0	0	0	0	0	0	0	0	0	0
BGIR9823_78717	100192437	ERP44	lncRNA	0	0	0	0	0	0	0	0	0	0	0	0	0	0	0	0
BGIR9823_81192	100514626	PIK3C2A	lncRNA	0	0	0	0	0	0	0	0	0	0	0	0	0	0	0	0
BGIR9823_77955	100153597	IBTK	lncRNA	0	0	0	0	0	0	0	0	0	0	0	0	0	0	0	0
BGIR9823_89270	100156900	BTBD9	mRNA	0	0	0	0	0	0	0	0	0	0	0	0	0	0	0	0
BGIR9823_98700	100512563	MGAT5	mRNA	0	0	0	0	0	0	0	0	0	0	0	0	0	0	1	0
BGIR9823_88596	110261555	SEMA4B	lncRNA	0	0	0	0	0	0	0	0	0	0	0	0	0	0	0	0
BGIR9823_85735	110260764	YAF2	lncRNA	0	0	0	0	0	0	0	0	0	0	0	0	0	0	0	0
BGIR9823_89296	100153071	TRERF1	lncRNA	0	0	0	0	0	0	0	0	0	0	0	0	0	0	0	0
BGIR9823_79093	100154222	EPHA7	lncRNA	0	0	0	0	0	0	0	0	0	0	0	0	0	0	0	0
BGIR9823_84443	100144448	OAZ3	lncRNA	0	0	0	0	0	0	0	0	0	0	0	0	0	0	0	0
BGIR9823_82885	100192445	PDPK1	lncRNA	0	0	1	0	0	0	0	0	0	0	0	0	0	0	0	0
BGIR9823_88750	100522330	SLC7A8	lncRNA	0	0	0	0	0	0	0	0	0	0	0	0	0	0	0	0
BGIR9823_77991	102159046	LOC102159046	lncRNA	0	0	0	0	0	0	0	0	0	0	0	0	0	0	0	0
BGIR9823_88964	100157303	ZC3H14	mRNA	0	0	0	0	0	0	0	0	0	0	0	0	0	0	0	0
BGIR9823_78191	102161909	LOC102161909	lncRNA	0	0	0	0	0	0	0	0	0	0	0	0	0	0	0	0
BGIR9823_81917	100620791	TRIP6	mRNA	0	0	0	0	0	0	0	0	0	0	0	0	0	0	0	0
BGIR9823_82685	110259888	LOC110259888	lncRNA	0	0	0	0	0	0	0	0	0	0	0	0	0	0	0	0
BGIR9823_84081	100153657	PHF20L1	mRNA	0	0	0	0	0	0	0	0	0	0	0	0	0	0	0	0
BGIR9823_83050	100628224	VAMP5	lncRNA	0	0	0	0	0	0	0	0	0	0	0	0	0	0	0	0
BGIR9823_98423	397285	ITGAV	mRNA	0	0	0	0	0	0	1	0	0	0	0	0	0	0	0	0
BGIR9823_97702	100155475	HNRNPF	mRNA	0	0	0	0	0	0	0	0	0	0	0	0	0	0	0	0
BGIR9823_93940	397293	KCNJ2	lncRNA	0	0	0	0	0	0	0	0	0	0	0	0	0	0	0	0
BGIR9823_87613	100521974	FBXO42	lncRNA	0	0	0	0	0	0	0	0	0	0	0	0	0	0	0	0
BGIR9823_94936	100155306	SEC61A1	lncRNA	0	0	0	0	0	0	0	0	0	0	0	0	0	0	0	0
BGIR9823_100651	100519065	UBN2	lncRNA	0	0	0	0	0	0	0	0	0	0	0	0	1	0	0	0
BGIR9823_84118	100737543	TBC1D31	lncRNA	0	0	0	0	0	0	0	0	0	0	0	0	0	0	0	0
BGIR9823_79608	100523347	UACA	lncRNA	0	0	0	0	0	1	0	0	0	0	0	0	0	0	0	0
BGIR9823_95065	100514494	LOC100514494	lncRNA	0	0	0	0	0	0	0	0	0	0	0	0	0	1	0	0
BGIR9823_89501	100155701	NYNRIN	mRNA	0	0	0	0	0	0	0	0	0	0	0	0	0	0	0	0
BGIR9823_79516	100155619	SOCS6	lncRNA	0	0	0	0	0	0	0	0	0	0	0	0	0	0	0	0
BGIR9823_93273	100626205	SLC38A10	mRNA	0	0	0	0	0	0	0	0	0	0	0	0	0	0	0	0
BGIR9823_82045	100739121	LOC100739121	lncRNA	0	0	0	0	0	0	0	0	0	0	0	0	0	0	0	0
BGIR9823_99804	100519342	RNF24	lncRNA	0	0	0	0	0	0	0	0	0	0	0	0	0	0	0	0
BGIR9823_81050	102164585	LOC102164585	mRNA	0	0	0	0	0	0	0	0	0	0	0	0	0	0	0	0
BGIR9823_83089	100515889	DGUOK	lncRNA	0	0	0	0	0	0	0	0	0	0	0	0	0	0	0	0
BGIR9823_85641	100157726	ETV6	lncRNA	0	0	0	0	0	0	0	0	0	0	0	0	0	0	0	0
BGIR9823_80399	100514037	EPS15L1	lncRNA	0	0	0	0	0	0	0	0	0	0	0	0	0	0	0	0
BGIR9823_100428	100517203	NRF1	lncRNA	0	0	0	0	0	0	0	0	0	0	0	0	0	0	0	0
BGIR9823_79736	110255453	LOC110255453	lncRNA	0	0	0	0	0	0	0	0	0	0	0	0	0	0	0	0

RNA ID	Gene ID	Symbol	Type	ssc-miR-24-1-5p	ssc-miR-29b	ssc-miR-29c	ssc-miR-323	ssc-miR-369	ssc-miR-708-5p	novel-ssc-miR161-3p	novel-ssc-miR512-5p	novel-ssc-miR846-3p	novel-ssc-miR1086-5p	novel-ssc-miR1105-5p	novel-ssc-miR1116-5p	novel-ssc-miR1120-5p	novel-ssc-miR1140-5p	novel-ssc-miR1158-3p	novel-ssc-miR1379-5p
BGIR9823_88239	100512907	LOC100512907	lncRNA	0	0	0	0	0	0	0	0	0	0	0	0	0	0	0	0
BGIR9823_89801	100524447	TBC1D1	lncRNA	0	0	0	0	0	0	0	0	0	0	0	0	0	0	0	0
BGIR9823_78621	100627072	PTPRD	lncRNA	0	0	1	0	0	0	0	0	0	0	0	0	0	0	0	0
BGIR9823_78489	100152019	PELI2	lncRNA	0	0	0	0	0	0	0	0	0	0	0	0	0	0	0	0
BGIR9823_83940	100155227	RBM15	lncRNA	0	0	0	0	0	0	0	0	0	0	0	0	0	0	0	0
BGIR9823_85932	106510466	MAF	lncRNA	0	0	0	0	0	0	0	0	0	0	0	0	0	0	0	0
BGIR9823_81640	100513341	FBXL17	lncRNA	0	0	0	0	0	1	0	0	0	0	0	0	0	0	0	0
BGIR9823_84349	100154639	PBX1	mRNA	0	0	0	0	0	0	0	0	0	0	0	0	0	0	0	0
BGIR9823_100606	100515515	GALNT11	lncRNA	0	0	0	0	0	0	0	0	0	0	0	0	0	0	0	0
BGIR9823_101514	102157759	LOC102157759	lncRNA	0	0	0	0	0	0	0	0	1	0	0	0	0	0	0	0
BGIR9823_85476	100622365	TSPAN31	lncRNA	0	0	0	0	0	0	0	0	0	0	0	0	0	0	0	0
BGIR9823_89890	100515957	PPID	lncRNA	0	0	0	0	0	0	0	0	0	0	0	0	0	0	0	0
BGIR9823_101279	100153213	HS6ST2	mRNA	0	0	0	0	0	0	0	0	0	0	0	0	0	0	0	0
BGIR9823_92274	100520446	PIP4K2A	lncRNA	0	1	0	0	0	0	0	0	0	0	0	0	0	0	0	0
BGIR9823_83415	106509958	MTSS1	lncRNA	0	0	0	0	0	0	0	0	0	0	0	0	0	0	0	0
BGIR9823_78462	100511360	FBXO34	lncRNA	0	0	0	0	0	0	0	0	0	0	0	0	0	0	0	0
BGIR9823_77832	100736796	ADGRB3	lncRNA	1	0	0	0	0	0	0	0	0	0	0	0	0	0	0	0
BGIR9823_79204	397085	MYO6	lncRNA	0	0	0	0	0	0	0	0	0	0	0	0	0	0	0	1
BGIR9823_83395	110260216	LOC110260216	lncRNA	0	0	0	0	0	0	0	0	0	0	0	0	0	0	0	0
BGIR9823_84473	100153347	GOLPH3L	lncRNA	0	0	0	0	0	0	0	0	0	0	0	0	0	0	0	0
BGIR9823_85369	100518305	LOC100518305	mRNA	0	0	0	0	0	0	0	0	0	0	0	0	0	0	0	0
BGIR9823_84074	100627184	GLI4	lncRNA	0	0	0	0	0	0	0	0	0	0	0	0	0	0	0	0
BGIR9823_92298	100515404	ITIH5	lncRNA	0	0	0	0	0	0	0	0	0	0	0	0	0	0	0	0
BGIR9823_89061	100153581	NUP153	mRNA	0	0	0	1	0	0	0	0	0	0	0	0	0	0	0	0
BGIR9823_96626	100151786	POP5	lncRNA	0	0	0	0	0	0	0	0	0	0	0	0	0	0	0	0
BGIR9823_87721	100517038	PUM1	lncRNA	0	0	0	0	0	0	0	0	0	0	0	0	0	0	0	0
BGIR9823_100728	100512382	KCND2	lncRNA	0	0	0	0	0	0	0	0	0	0	0	0	0	0	0	0
BGIR9823_80555	110259375	LOC110259375	lncRNA	0	0	0	0	0	0	0	0	0	0	0	0	0	0	0	0
BGIR9823_79909	100155492	HEMGN	lncRNA	0	0	0	0	0	0	0	0	0	0	0	0	0	0	0	0
BGIR9823_94689	100516877	ACOX2	lncRNA	0	0	0	0	0	0	0	0	0	0	0	0	0	0	0	0
BGIR9823_92764	102158088	NHLRC3	lncRNA	0	1	0	0	0	0	0	0	0	0	0	0	0	0	0	0
BGIR9823_89682	100152306	LOC100152306	mRNA	0	0	0	0	0	0	0	0	0	0	0	0	0	0	0	0
BGIR9823_96521	100623262	SLC15A4	mRNA	0	0	0	0	0	0	0	0	0	0	0	0	0	0	0	0
BGIR9823_84835	100516439	C5H12orf10	lncRNA	0	0	0	0	0	0	0	0	0	0	0	0	0	0	0	0
BGIR9823_100509	100738688	HERPUD2	lncRNA	0	0	0	0	0	0	0	0	0	0	0	0	0	0	0	0
BGIR9823_86157	100511329	ZNF829	mRNA	0	0	0	0	0	0	0	0	0	0	0	0	0	0	0	0
BGIR9823_82679	100620395	GIGYF1	mRNA	0	0	0	0	0	0	0	0	0	0	0	0	0	0	0	0
BGIR9823_90779	100621182	EMSY	mRNA	0	0	0	0	0	0	0	0	0	0	0	0	0	0	0	0
BGIR9823_90540	100621145	LARP1B	lncRNA	0	0	0	0	0	0	0	0	0	0	0	0	0	0	0	0
NM_001097469.1	100037984	HIGD2A	mRNA	0	0	0	0	0	0	0	0	0	0	0	0	0	0	0	0
BGIR9823_78404	102162695	LOC102162695	lncRNA	0	0	0	0	0	1	0	0	0	0	0	0	0	0	0	0
BGIR9823_84013	102161056	LOC102161056	lncRNA	0	0	0	0	0	0	0	0	0	0	0	0	0	0	0	0
BGIR9823_98214	100512817	TENM3	mRNA	0	0	0	0	0	0	0	0	0	0	0	0	0	0	0	0

RNA ID	Gene ID	Symbol	Type	ssc-miR-24-1-5p	ssc-miR-29b	ssc-miR-29c	ssc-miR-323	ssc-miR-369	ssc-miR-708-5p	novel-ssc-miR161-3p	novel-ssc-miR512-5p	novel-ssc-miR846-3p	novel-ssc-miR1086-5p	novel-ssc-miR1105-5p	novel-ssc-miR1116-5p	novel-ssc-miR1120-5p	novel-ssc-miR1140-5p	novel-ssc-miR1158-3p	novel-ssc-miR1379-5p
BGIR9823_81705	100521611	FNIP1	lncRNA	0	0	0	0	0	0	0	0	0	0	0	0	0	0	0	0
BGIR9823_79689	100192435	ERO1A	lncRNA	1	0	0	0	0	0	0	0	0	0	0	0	0	0	0	0
BGIR9823_81181	100736787	ZDHHC13	mRNA	0	0	0	0	0	0	0	0	0	0	0	0	0	0	0	0
BGIR9823_95430	396658	IFNAR1	mRNA	0	0	0	0	0	0	0	0	0	0	0	0	0	0	0	0
BGIR9823_77980	100152640	HAUS1	lncRNA	1	0	0	0	0	0	0	0	0	0	0	0	0	0	0	0
BGIR9823_91823	100522092	RUNDC3B	lncRNA	0	0	0	0	0	0	0	0	0	0	0	0	0	0	0	0
BGIR9823_81117	100144442	F2	lncRNA	1	0	0	0	0	0	0	0	0	0	0	0	0	0	0	0
BGIR9823_90627	100624822	GSTCD	mRNA	0	0	0	0	0	0	0	0	0	0	0	0	0	0	0	0
BGIR9823_78165	100169932	TRPM7	lncRNA	0	0	0	0	0	0	0	0	0	0	0	0	0	0	0	0
BGIR9823_80118	100511178	SNX32	lncRNA	0	0	0	0	0	1	0	0	0	0	0	0	0	0	0	0
BGIR9823_84495	102166500	LOC102166500	lncRNA	0	0	0	0	0	0	0	0	0	0	0	0	0	0	0	0
BGIR9823_81367	100127167	CERS4	mRNA	0	0	0	1	0	0	0	0	0	0	0	0	0	0	0	0
BGIR9823_77687	100156632	AGPAT4	lncRNA	0	0	0	0	0	0	0	0	0	0	0	0	0	0	0	0
BGIR9823_80249	100519700	AMBRA1	lncRNA	0	0	0	0	0	0	0	0	0	0	0	0	0	0	0	0
BGIR9823_100622	100521480	GSTK1	lncRNA	0	0	0	0	0	0	0	0	0	0	0	0	0	0	0	0
BGIR9823_86113	100621064	DPY19L3	lncRNA	0	0	1	0	0	0	0	0	0	0	0	0	0	0	0	0
BGIR9823_92205	100516148	MOB3B	mRNA	0	0	0	0	0	0	0	0	0	0	0	0	0	0	0	0
XM_021075409.1	100737004	COBLL1	mRNA	0	0	0	0	0	0	0	0	0	0	0	0	0	0	0	0
BGIR9823_89246	100521322	LOC100521322	lncRNA	0	0	0	0	0	0	0	0	0	0	0	0	0	0	0	0
BGIR9823_78193	100154291	SLC12A1	lncRNA	0	0	0	0	0	0	0	0	0	0	0	0	0	0	0	1
BGIR9823_100566	100516888	GLI3	lncRNA	0	0	0	0	0	0	0	0	0	0	0	0	0	0	0	0
BGIR9823_100446	100192443	POT1	lncRNA	0	0	0	0	0	0	0	0	0	0	0	0	0	0	0	0
BGIR9823_97408	100620430	SAP30	lncRNA	0	0	0	0	0	0	0	0	0	0	0	0	0	0	0	0
BGIR9823_86274	100622144	BICRA	lncRNA	0	0	0	0	0	0	0	0	0	0	0	0	0	0	0	0
BGIR9823_101110	100524780	ARHGAP6	lncRNA	0	0	0	0	0	0	0	0	0	0	0	0	0	0	0	0
BGIR9823_82012	100524333	GGA2	lncRNA	1	0	0	0	0	0	0	0	0	0	0	0	0	0	0	0
BGIR9823_86283	100624752	NOP53	mRNA	0	0	0	0	0	0	0	0	0	1	0	0	0	0	0	0
BGIR9823_98317	110256929	WDSUB1	lncRNA	0	0	0	0	0	0	0	0	0	0	0	0	0	0	0	0
BGIR9823_88517	100511822	ICK	lncRNA	0	0	0	0	0	0	0	0	0	0	0	0	0	0	0	0
BGIR9823_79454	110259520	LOC110259520	lncRNA	0	0	0	0	0	0	0	0	0	0	0	0	0	0	0	0
BGIR9823_81324	606746	DNMT1	mRNA	0	0	0	0	0	0	0	0	0	0	0	0	0	0	0	0
BGIR9823_100702	100514931	AHCYL2	lncRNA	0	0	0	0	0	0	0	0	0	0	0	0	0	0	0	0
BGIR9823_101292	100523658	ATP11C	mRNA	0	0	0	0	0	0	0	0	0	0	0	0	0	0	0	0
BGIR9823_90549	102167943	LOC102167943	lncRNA	0	0	1	0	0	0	0	0	0	0	0	0	0	0	0	0
BGIR9823_84672	100516804	SCO2	mRNA	0	0	0	0	0	0	0	0	0	0	0	0	0	0	0	0
BGIR9823_98499	100514879	ABI2	mRNA	0	0	0	0	0	0	0	0	0	0	0	0	0	0	0	0
BGIR9823_83000	102166005	LOC102166005	lncRNA	0	0	0	0	0	0	0	0	0	0	0	0	0	0	0	0
BGIR9823_79430	100152163	DLL4	lncRNA	0	0	0	0	0	0	0	0	0	0	0	0	0	0	0	0
BGIR9823_80973	100511242	TMEM134	lncRNA	0	0	0	0	0	0	0	0	0	0	0	0	0	0	0	0
BGIR9823_101334	100523617	TRIM27	lncRNA	0	0	0	0	0	0	0	0	0	0	0	0	0	0	0	0
BGIR9823_81536	100520548	WDR41	lncRNA	0	0	0	0	0	0	0	0	0	0	0	0	0	0	0	0
BGIR9823_101625	100519072	LOC100519072	lncRNA	0	0	0	0	0	0	0	0	0	0	0	0	0	0	0	0
BGIR9823_87181	100517402	PDF	lncRNA	0	0	0	0	0	0	0	0	0	0	0	0	0	1	0	0

RNA ID	Gene ID	Symbol	Type	ssc-miR-24-1-5p	ssc-miR-29b	ssc-miR-29c	ssc-miR-323	ssc-miR-369	ssc-miR-708-5p	novel-ssc-miR161-3p	novel-ssc-miR512-5p	novel-ssc-miR846-3p	novel-ssc-miR1086-5p	novel-ssc-miR1105-5p	novel-ssc-miR1116-5p	novel-ssc-miR1120-5p	novel-ssc-miR1140-5p	novel-ssc-miR1158-3p	novel-ssc-miR1379-5p
BGIR9823_78566	100152132	IFN-OMEGA-3	lncRNA	0	0	0	0	0	0	0	0	0	0	0	0	0	0	0	0
XR_002339179.1	110257024	LOC110257024	lncRNA	0	0	0	0	0	0	0	0	0	0	1	0	0	0	0	0
BGIR9823_100144	100154945	RRBP1	lncRNA	0	0	0	0	0	0	0	0	0	0	0	0	0	0	0	0
BGIR9823_100611	100521999	SMARCD3	lncRNA	0	0	0	0	0	0	0	0	0	0	0	0	0	0	0	0
BGIR9823_95072	100620632	KPNA4	lncRNA	0	0	0	0	0	0	0	0	0	0	0	0	0	0	0	0
BGIR9823_98782	100152203	AGA	mRNA	0	0	0	1	0	0	0	0	0	0	0	0	0	0	0	0
BGIR9823_77731	100154785	ADAT2	lncRNA	0	0	0	0	0	0	0	0	0	0	0	0	0	0	0	0
BGIR9823_96586	100153673	CUX2	mRNA	0	0	0	0	0	0	0	0	0	0	0	0	0	0	0	0
BGIR9823_85978	110260924	LOC110260924	lncRNA	0	0	0	0	0	0	0	0	0	0	0	0	0	0	0	0
BGIR9823_78788	100154489	WDR38	mRNA	0	0	0	0	0	0	0	0	0	0	0	0	0	0	0	0
BGIR9823_86389	110261319	LOC110261319	lncRNA	0	0	0	0	0	0	0	0	0	0	0	0	0	0	0	0
BGIR9823_79497	100736682	TJP1	lncRNA	0	0	0	0	0	0	0	0	0	0	0	0	0	0	0	0
BGIR9823_98375	100522284	METAP1D	mRNA	0	0	0	0	0	0	0	0	0	0	0	0	0	0	0	0
BGIR9823_100000	100523757	ARFGEF2	lncRNA	0	0	0	0	0	0	0	0	0	0	0	0	0	0	0	0
BGIR9823_84378	100524676	SDHC	lncRNA	0	0	0	0	0	0	0	0	0	0	0	0	0	0	0	0
BGIR9823_92192	396723	DDX58	mRNA	0	0	0	0	0	0	0	0	0	0	0	0	0	0	0	0
BGIR9823_88795	102162743	FAM174B	lncRNA	0	0	0	0	0	0	0	0	0	0	0	0	0	0	0	0
BGIR9823_92690	100623356	SKA3	lncRNA	0	1	0	0	0	0	0	0	0	0	0	0	0	0	0	0
XR_002340913.1	110257727	LOC110257727	lncRNA	0	0	0	0	0	0	0	0	0	0	0	0	0	0	0	0
BGIR9823_86991	100037307	DAB1	lncRNA	0	0	1	0	0	0	0	0	0	0	0	0	0	0	0	0
BGIR9823_82075	100513346	LOC100513346	mRNA	0	0	0	0	0	0	0	0	0	0	0	0	0	0	0	0
BGIR9823_86237	100524260	ZNF45	lncRNA	0	0	0	0	0	0	0	0	0	0	0	0	0	0	0	0
BGIR9823_80263	106507997	LOC106507997	lncRNA	1	0	0	0	0	0	0	0	0	0	0	0	0	0	0	0
BGIR9823_93834	110256118	LOC110256118	lncRNA	0	0	0	0	0	0	0	0	0	0	0	0	0	0	0	0
BGIR9823_80349	100738340	ATP13A1	lncRNA	1	0	0	0	0	0	0	0	0	0	0	0	0	0	0	0
BGIR9823_89603	106507740	LOC106507740	lncRNA	0	0	0	0	0	0	0	0	0	0	0	0	0	0	0	0
BGIR9823_77903	100514380	TSTD3	lncRNA	0	0	0	0	0	0	0	0	0	0	0	0	0	0	0	0
BGIR9823_100158	100154020	LOC100154020	lncRNA	0	0	0	0	0	0	0	0	0	0	0	0	0	0	0	0
BGIR9823_89657	733584	FOXN3	lncRNA	0	0	0	0	0	0	0	0	0	0	0	0	0	0	0	0
BGIR9823_98765	100233172	AGPAT5	lncRNA	0	0	0	0	0	0	0	0	0	0	0	0	0	0	0	0
BGIR9823_94610	396679	AMT	lncRNA	0	0	0	0	0	0	0	0	0	0	0	0	0	1	0	0
BGIR9823_78506	100154408	PSMA3	lncRNA	0	0	0	0	0	0	0	0	0	0	0	0	0	0	0	0
BGIR9823_78017	100515517	RBPMS2	lncRNA	0	0	0	0	0	0	0	0	0	0	0	0	0	0	0	0
BGIR9823_98223	100515058	TRAPPC11	mRNA	0	0	0	0	0	0	0	0	0	0	0	0	0	0	0	0
BGIR9823_78148	100155516	DMXL2	mRNA	0	0	0	1	0	0	0	0	0	0	0	0	0	0	0	0
BGIR9823_87377	100521972	LOC100521972	lncRNA	0	0	0	0	0	0	0	0	0	0	0	0	0	0	0	0
BGIR9823_84933	100511508	LEMD3	lncRNA	0	0	0	0	0	0	0	0	0	0	0	0	0	0	0	0
BGIR9823_100906	100621199	IL1RAPL1	lncRNA	0	0	0	0	0	0	0	0	0	0	0	0	0	0	0	0
BGIR9823_89271	100156900	BTBD9	mRNA	0	0	0	0	0	0	0	0	0	0	0	0	0	0	0	0
BGIR9823_90463	100627416	TRIM2	lncRNA	0	0	0	0	0	0	0	0	0	0	0	0	0	1	0	0
BGIR9823_79564	100153830	MALT1	lncRNA	0	0	0	0	0	0	0	0	0	0	0	0	0	0	0	0
BGIR9823_88636	100155770	FAM219B	mRNA	0	0	0	0	0	0	0	0	0	0	0	0	0	0	0	0
BGIR9823_84024	100155974	GTF2B	lncRNA	0	0	0	0	0	0	0	0	0	0	1	0	0	0	0	0

RNA ID	Gene ID	Symbol	Type	ssc-miR-24-1-5p	ssc-miR-29b	ssc-miR-29c	ssc-miR-323	ssc-miR-369	ssc-miR-708-5p	novel-ssc-miR161-3p	novel-ssc-miR512-5p	novel-ssc-miR846-3p	novel-ssc-miR1086-5p	novel-ssc-miR1105-5p	novel-ssc-miR1116-5p	novel-ssc-miR1120-5p	novel-ssc-miR1140-5p	novel-ssc-miR1158-3p	novel-ssc-miR1379-5p
BGIR9823_100486	100621338	BMT2	lncRNA	0	0	0	0	0	0	0	0	0	0	0	0	0	0	0	0
BGIR9823_86035	100623274	CTCF	mRNA	0	0	0	0	0	0	0	0	0	0	0	0	0	0	0	0
BGIR9823_85003	110260713	AMN1	mRNA	0	0	0	0	0	0	0	0	0	0	0	0	0	0	0	0
BGIR9823_100211	100157872	PXMP4	lncRNA	0	0	0	0	0	0	0	0	0	0	0	0	0	0	0	0
BGIR9823_85764	396681	TXNRD1	mRNA	0	0	0	0	0	0	0	0	0	0	0	0	0	0	0	0
BGIR9823_79047	110260218	LOC110260218	lncRNA	0	0	0	0	1	0	0	0	0	0	0	0	0	0	0	0
BGIR9823_99528	100626363	NPR3	lncRNA	0	0	0	0	0	0	0	0	0	0	0	0	0	1	0	0
BGIR9823_86380	100738636	EPN1	mRNA	0	0	0	0	0	0	0	0	0	1	0	0	0	0	0	0
BGIR9823_101388	100521615	ARHGAP17	mRNA	0	0	0	0	0	0	0	0	0	0	0	0	0	0	0	0
BGIR9823_85180	100625757	ARID2	lncRNA	0	0	0	0	0	0	0	0	0	0	0	0	0	0	0	0
BGIR9823_101519	100511414	RNF122	lncRNA	0	0	0	0	0	0	0	0	0	0	0	0	0	0	0	0
BGIR9823_100416	106506781	LOC106506781	lncRNA	0	0	0	0	0	0	0	0	0	0	0	0	0	0	0	0
BGIR9823_77835	100736796	ADGRB3	lncRNA	0	0	0	0	0	0	0	0	0	0	0	0	0	0	0	0
XM_001928635.2	100156587	LOC100156587	mRNA	0	0	0	0	0	0	0	0	0	0	0	0	0	0	0	0
BGIR9823_91988	100217398	FLVCR1	lncRNA	0	0	0	0	0	0	0	0	0	0	0	0	0	0	0	0
BGIR9823_86369	100517285	LOC100517285	mRNA	0	0	0	0	0	0	0	0	0	0	0	0	0	0	0	0
BGIR9823_101017	100520165	TMEM164	lncRNA	0	0	0	0	0	0	0	0	0	0	0	0	0	0	0	0
BGIR9823_81426	100621275	GNG7	lncRNA	0	0	0	0	0	0	0	0	0	0	0	0	0	0	0	0
BGIR9823_94753	100522704	FAM19A1	lncRNA	0	1	0	0	0	0	0	0	0	0	0	0	0	0	0	0
BGIR9823_87686	100512789	WASF2	lncRNA	0	0	0	0	0	0	0	0	0	0	0	0	0	1	0	0
BGIR9823_88695	100624265	STRN3	mRNA	0	0	0	0	0	0	0	0	0	0	0	0	0	0	0	0
BGIR9823_82270	100625157	GCFC2	lncRNA	1	0	0	0	0	0	0	0	0	0	0	0	0	0	0	0
BGIR9823_93946	397091	PRKAR1A	mRNA	0	0	0	0	0	0	0	0	0	0	0	0	0	0	0	0
BGIR9823_93731	110256070	LOC110256070	lncRNA	0	0	0	0	0	0	0	0	0	0	0	0	0	0	0	0
BGIR9823_80458	100515360	ILF3	lncRNA	0	0	0	0	0	0	0	0	0	0	0	0	0	0	0	0
BGIR9823_80830	100513723	JADE2	lncRNA	0	0	0	0	0	0	0	0	0	0	0	0	0	0	0	0
BGIR9823_87106	100739500	ACSF3	lncRNA	0	0	0	0	0	0	0	0	0	0	0	0	0	0	0	0
BGIR9823_91655	100511341	EI24	lncRNA	0	0	0	0	0	0	0	0	0	0	0	0	0	0	0	0
BGIR9823_84152	100337657	ZFPM2	mRNA	0	0	0	0	0	0	0	0	0	0	0	0	0	0	0	0
BGIR9823_78973	100155445	VTA1	lncRNA	0	0	0	0	0	0	0	0	0	0	0	0	0	0	0	0
BGIR9823_88372	100151743	PPP1R18	lncRNA	0	0	0	0	0	0	0	0	0	0	0	0	0	0	0	0
BGIR9823_97928	100155592	PDLIM1	mRNA	0	0	0	0	0	0	0	0	0	0	0	0	0	0	0	0
BGIR9823_88939	100511141	VASH1	mRNA	0	0	0	0	0	0	0	0	0	0	0	0	0	0	0	0
BGIR9823_93334	100623453	KIF19	lncRNA	0	0	1	0	0	0	0	0	0	0	0	0	0	0	0	0
BGIR9823_79684	100153738	TRIM9	lncRNA	0	0	0	0	0	0	0	0	0	0	0	0	0	0	0	0
BGIR9823_100666	100524964	AGBL3	lncRNA	0	0	0	0	0	0	0	0	0	0	0	0	0	0	0	0
BGIR9823_89830	100515040	LIMCH1	mRNA	0	0	0	0	0	0	0	0	0	0	0	0	0	0	0	0
BGIR9823_85457	100511633	RDH16	lncRNA	0	0	0	0	0	0	0	0	0	0	0	0	0	0	0	0
BGIR9823_81268	100512396	SLC35E1	lncRNA	0	0	0	0	0	0	0	0	0	0	0	0	0	0	0	0
BGIR9823_78329	100049703	BCL2	lncRNA	0	0	0	0	0	0	0	0	0	0	0	0	0	0	0	0
BGIR9823_78260	100512756	MEIS2	lncRNA	0	0	0	0	0	0	0	0	0	0	0	0	0	0	0	0
BGIR9823_89336	100152930	ADGRF5	mRNA	0	0	0	0	0	0	0	0	0	0	0	0	0	0	0	0
BGIR9823_89031	100152689	SSR1	mRNA	0	0	0	1	0	0	0	0	0	0	0	0	0	0	0	0

RNA ID	Gene ID	Symbol	Type	ssc-miR-24-1-5p	ssc-miR-29b	ssc-miR-29c	ssc-miR-323	ssc-miR-369	ssc-miR-708-5p	novel-ssc-miR161-3p	novel-ssc-miR512-5p	novel-ssc-miR846-3p	novel-ssc-miR1086-5p	novel-ssc-miR1105-5p	novel-ssc-miR1116-5p	novel-ssc-miR1120-5p	novel-ssc-miR1140-5p	novel-ssc-miR1158-3p	novel-ssc-miR1379-5p
BGIR9823_78386	100154444	GLCE	lncRNA	0	0	0	0	0	0	0	0	0	0	0	0	0	0	0	0
BGIR9823_92789	100155063	SPRYD7	mRNA	0	0	0	0	0	0	0	0	0	0	0	0	0	0	0	0
BGIR9823_86252	100622514	CLASRP	lncRNA	0	1	0	0	0	0	0	0	0	0	0	0	0	0	0	0
BGIR9823_101153	100621125	SLC35A2	mRNA	0	0	0	0	0	0	0	0	0	0	0	0	0	0	0	0
BGIR9823_100998	414736	PCDH11X	lncRNA	0	0	0	0	0	0	0	0	0	0	0	0	0	0	0	0
BGIR9823_82762	397614	IL4R	lncRNA	0	0	0	0	0	0	0	0	0	0	0	0	0	0	0	0
BGIR9823_79824	100626649	KANK1	lncRNA	0	0	0	0	0	0	0	0	0	0	0	0	0	0	0	0
BGIR9823_95516	100152333	ANKRD28	lncRNA	0	0	0	0	0	0	0	0	0	0	0	0	0	0	0	0
BGIR9823_81853	106509619	LOC106509619	lncRNA	0	0	0	0	0	0	0	0	0	0	0	0	0	0	0	0
BGIR9823_90521	100513554	NAA15	lncRNA	0	0	0	0	0	0	0	0	0	0	0	0	0	0	0	0
BGIR9823_93604	100520097	TUBD1	mRNA	0	0	0	0	0	0	0	0	0	0	0	0	0	0	0	0
BGIR9823_79674	100157402	NIN	lncRNA	0	0	0	0	0	0	0	0	0	0	0	0	0	0	0	0
BGIR9823_100171	100625861	LZTS3	lncRNA	0	0	0	0	0	0	0	0	0	0	0	0	0	0	0	0
BGIR9823_81709	100521611	FNIP1	lncRNA	1	0	0	0	0	0	0	0	0	0	0	0	0	0	0	0
BGIR9823_82252	100738124	IMMT	lncRNA	0	0	0	0	0	0	0	0	0	0	0	0	0	0	0	0
BGIR9823_80640	102165038	LOC102165038	lncRNA	0	0	0	0	0	0	0	0	0	0	0	0	0	0	0	0
BGIR9823_101575	100737380	CDC25A	lncRNA	0	0	0	0	0	0	0	0	0	0	0	0	0	0	0	0
BGIR9823_84694	100518786	HDAC10	lncRNA	0	0	0	0	0	0	0	0	0	0	0	0	0	0	0	0
BGIR9823_98548	102162936	SPAG16	mRNA	0	0	0	0	0	0	0	0	0	0	0	0	0	0	0	0
BGIR9823_88951	110261640	LOC110261640	lncRNA	0	0	0	0	0	0	0	0	0	0	0	0	0	0	0	0
BGIR9823_101401	110258215	LOC110258215	lncRNA	0	0	0	0	0	0	0	0	0	0	0	0	0	0	0	0
BGIR9823_82476	100518189	SRSF7	lncRNA	0	0	0	0	0	0	0	0	0	0	0	0	0	0	0	0
BGIR9823_80254	100520600	TP53I11	lncRNA	0	0	0	0	0	0	0	0	0	0	0	0	0	0	0	0
BGIR9823_100432	100515455	TNPO3	lncRNA	0	0	0	0	0	0	0	0	0	0	0	0	0	0	0	0
BGIR9823_100491	100520706	DOCK4	lncRNA	0	0	0	0	0	0	0	0	0	0	0	0	0	0	0	0
BGIR9823_101216	100514440	ATRX	lncRNA	0	0	0	0	0	0	0	0	0	0	0	0	0	0	0	0
BGIR9823_94101	100518511	MBTD1	lncRNA	0	0	0	0	0	0	0	0	0	0	0	0	0	0	0	0
BGIR9823_84304	100157730	TGS1	mRNA	0	0	0	0	0	0	1	0	0	0	0	0	0	0	0	0
BGIR9823_98130	100519056	ORC4	lncRNA	0	0	0	0	0	0	0	0	0	0	0	0	0	0	0	0
BGIR9823_95626	100623101	SETD2	mRNA	0	0	0	0	0	0	0	0	0	0	0	0	0	0	0	0
BGIR9823_81836	110259827	LOC110259827	lncRNA	0	0	0	0	0	0	0	0	0	0	0	0	0	0	0	0
BGIR9823_100125	100519631	TASP1	lncRNA	0	0	0	0	0	0	0	0	0	0	0	0	0	0	0	0
BGIR9823_77803	100520524	GOPC	lncRNA	0	0	0	0	0	0	0	0	0	0	0	0	0	0	0	0
BGIR9823_78926	100152904	ARID1B	lncRNA	0	0	0	0	0	0	0	0	0	0	0	0	0	0	0	0
XR_002343327.1	110260295	LOC110260295	lncRNA	0	0	0	0	0	0	0	0	0	0	0	0	0	0	0	0
XM_005661298.3	100514564	CAPS	mRNA	0	0	0	1	0	0	0	0	0	0	0	0	0	0	0	0
BGIR9823_100692	100739403	KLHDC10	lncRNA	0	0	0	0	0	0	0	0	0	0	0	0	0	0	0	0
BGIR9823_95813	100525891	EIF4E3	lncRNA	0	0	0	0	0	0	0	0	0	0	0	0	0	0	0	0
BGIR9823_81281	100516595	ZNF333	lncRNA	1	0	0	0	0	0	0	0	0	0	0	0	0	0	0	0
BGIR9823_100015	100151957	PARD6B	lncRNA	0	0	0	0	0	0	0	0	0	0	0	0	0	0	0	0
BGIR9823_101282	100517991	FAM122B	lncRNA	0	0	0	0	0	0	0	0	0	0	0	0	0	0	0	0
BGIR9823_88581	100151986	UNC45A	lncRNA	0	0	0	0	0	0	0	0	0	0	1	0	0	0	0	0
BGIR9823_88595	110261555	SEMA4B	lncRNA	0	1	0	0	0	0	0	0	0	0	0	0	0	0	0	0

RNA ID	Gene ID	Symbol	Type	ssc-miR-24-1-5p	ssc-miR-29b	ssc-miR-29c	ssc-miR-323	ssc-miR-369	ssc-miR-708-5p	novel-ssc-miR161-3p	novel-ssc-miR512-5p	novel-ssc-miR846-3p	novel-ssc-miR1086-5p	novel-ssc-miR1105-5p	novel-ssc-miR1116-5p	novel-ssc-miR1120-5p	novel-ssc-miR1140-5p	novel-ssc-miR1158-3p	novel-ssc-miR1379-5p
BGIR9823_91875	106504991	LOC106504991	lncRNA	0	0	0	0	0	0	0	0	0	0	0	0	0	0	0	0
BGIR9823_100454	100525017	WASL	lncRNA	0	0	0	0	0	0	0	0	0	0	0	0	0	0	0	0
BGIR9823_101069	100512515	AFF2	mRNA	0	0	0	0	0	0	0	0	0	0	0	0	0	0	0	0
BGIR9823_101534	102158401	LOC102158401	lncRNA	1	0	0	0	0	0	0	0	0	0	0	0	0	0	0	0
BGIR9823_82897	100738908	MLST8	lncRNA	0	0	0	0	0	0	0	0	0	0	0	0	0	0	0	0
BGIR9823_98106	100158118	CALY	mRNA	0	0	0	0	0	0	0	0	0	0	0	0	0	0	0	0
BGIR9823_80817	100520422	SLC22A5	lncRNA	0	0	0	0	0	0	0	0	0	0	0	0	0	0	0	0
BGIR9823_91425	100511767	ARAP1	mRNA	0	0	0	0	0	0	0	0	0	0	0	0	0	0	0	0
BGIR9823_96623	100154637	SRSF9	lncRNA	0	0	0	0	0	0	0	0	0	0	0	0	0	1	0	0
BGIR9823_81521	100513398	SIMC1	lncRNA	1	0	0	0	0	0	0	0	0	0	0	0	0	0	0	0
BGIR9823_86388	110261319	LOC110261319	lncRNA	0	0	0	0	0	0	0	0	0	0	0	0	0	0	0	0
BGIR9823_81938	100511317	SSC4D	lncRNA	0	0	1	0	0	0	0	0	0	0	0	0	0	0	0	0
BGIR9823_83868	102165219	ANKRD34A	mRNA	0	0	0	0	0	0	0	0	0	0	0	0	0	0	0	0
BGIR9823_85017	100155867	INTS13	lncRNA	0	0	0	0	0	0	0	0	0	0	0	0	0	0	0	0
BGIR9823_91638	100523324	ARHGEF12	lncRNA	0	0	0	0	0	0	0	0	0	0	0	0	0	0	0	0
BGIR9823_78342	100515640	DIS3L	lncRNA	0	0	0	0	0	0	0	0	0	0	0	0	0	0	0	0
BGIR9823_101177	100520413	PHF8	mRNA	0	0	0	0	0	0	0	0	0	0	0	0	0	0	0	0
BGIR9823_100116	100157552	MKKS	lncRNA	0	0	0	0	0	1	0	0	0	0	0	0	0	0	0	0
BGIR9823_82812	100514160	XYLT1	lncRNA	0	0	0	0	0	0	0	0	0	0	0	0	0	0	0	0
BGIR9823_100485	100621338	BMT2	lncRNA	0	0	0	0	0	0	0	0	0	0	0	0	0	0	0	0
BGIR9823_83185	100302367	SOCS5	lncRNA	0	0	0	0	0	0	0	0	0	0	0	0	0	0	0	0
BGIR9823_100135	100519631	TASP1	lncRNA	0	0	0	0	0	0	0	0	0	0	0	0	0	0	0	0
BGIR9823_78629	100155278	ERMP1	mRNA	0	0	0	0	0	0	0	0	0	0	0	0	0	0	0	0
BGIR9823_100610	100517250	NUB1	mRNA	0	0	0	0	0	0	0	0	0	0	0	0	0	0	0	0
BGIR9823_100720	100511294	PTPRZ1	lncRNA	0	0	0	0	0	0	0	0	0	0	0	0	0	0	0	0
BGIR9823_91236	100524699	TPK1	lncRNA	0	0	0	0	0	0	0	0	0	0	0	0	0	0	0	0
BGIR9823_79597	100152069	SMAD6	lncRNA	0	0	0	0	0	0	0	0	0	0	0	0	0	0	0	0
BGIR9823_100727	100512026	ING3	lncRNA	0	0	0	0	0	0	0	0	0	0	0	0	0	0	0	0
BGIR9823_89956	106504698	LOC106504698	lncRNA	0	0	0	0	0	0	0	0	0	0	0	0	0	0	0	0
BGIR9823_100230	100154394	ROMO1	lncRNA	0	0	0	0	0	0	0	0	0	0	0	0	0	0	0	0
BGIR9823_90067	100519324	ETNPPL	mRNA	0	0	0	0	0	0	0	0	0	0	0	0	0	0	0	0
BGIR9823_81199	100216478	TEAD1	lncRNA	1	0	0	0	0	0	0	0	0	0	0	0	0	0	0	0
BGIR9823_100115	100157552	MKKS	lncRNA	0	0	0	0	0	0	0	0	0	0	0	0	0	0	0	0
BGIR9823_97871	733640	PRKG1	lncRNA	0	0	0	1	0	0	0	0	0	0	0	0	0	0	0	0
BGIR9823_79442	106506757	INAFM2	lncRNA	0	0	0	0	0	0	0	0	0	0	0	0	0	0	0	0
BGIR9823_77844	100736796	ADGRB3	lncRNA	0	0	0	0	0	0	0	0	0	0	0	0	0	0	0	0
BGIR9823_81223	110259751	LOC110259751	lncRNA	0	0	0	0	0	0	0	0	0	0	0	0	0	0	0	0
BGIR9823_87714	100517038	PUM1	lncRNA	0	0	0	0	0	0	0	0	0	0	0	0	0	0	0	0
BGIR9823_82332	100517706	CEP68	lncRNA	0	0	0	0	0	0	0	0	0	0	0	0	0	0	0	0
BGIR9823_79033	100624679	NKAIN2	lncRNA	0	0	0	0	0	0	0	0	0	0	0	0	0	0	0	0
BGIR9823_81514	100523721	FAF2	lncRNA	0	0	0	0	0	0	0	0	0	0	0	0	0	0	0	0
BGIR9823_78765	100622501	PRPF4	mRNA	0	0	0	1	0	0	0	0	0	0	0	0	0	0	0	0
BGIR9823_78849	100624075	AIF1L	mRNA	0	0	0	0	0	0	1	0	0	0	0	0	0	0	0	0

RNA ID	Gene ID	Symbol	Type	ssc-miR-24-1-5p	ssc-miR-29b	ssc-miR-29c	ssc-miR-323	ssc-miR-369	ssc-miR-708-5p	novel-ssc-miR161-3p	novel-ssc-miR512-5p	novel-ssc-miR846-3p	novel-ssc-miR1086-5p	novel-ssc-miR1105-5p	novel-ssc-miR1116-5p	novel-ssc-miR1120-5p	novel-ssc-miR1140-5p	novel-ssc-miR1158-3p	novel-ssc-miR1379-5p
BGIR9823_100607	100515515	GALNT11	lncRNA	0	0	0	0	0	0	0	0	0	0	0	0	0	0	0	0
BGIR9823_83081	100627528	CCDC142	lncRNA	0	0	0	0	0	0	0	0	0	0	0	0	0	0	0	0
XR_002345524.1	102158855	LOC102158855	lncRNA	0	0	0	0	0	0	0	0	0	0	0	0	0	0	0	0
BGIR9823_78387	100154444	GLCE	lncRNA	0	0	0	0	0	0	0	0	0	0	0	0	0	0	0	0
BGIR9823_79739	110255453	LOC110255453	lncRNA	0	0	1	0	0	0	0	0	0	0	0	0	0	0	0	0
BGIR9823_100823	100519067	CDK13	mRNA	0	0	0	0	0	0	0	0	0	0	0	0	0	0	0	0
BGIR9823_80184	397169	BEST1	lncRNA	0	0	0	0	0	0	0	0	0	0	0	0	0	0	0	0
BGIR9823_85074	100144592	KLRF1	lncRNA	0	0	0	0	0	0	0	0	0	0	0	0	0	0	0	0
BGIR9823_80186	444995	FADS1	mRNA	0	0	0	0	0	0	0	0	0	0	0	0	0	0	0	0
BGIR9823_97606	100156356	PRR14L	lncRNA	0	0	0	0	0	0	0	0	0	0	0	0	0	0	0	0
BGIR9823_100902	100621199	IL1RAPL1	lncRNA	0	0	0	0	0	0	0	0	0	0	0	0	0	0	0	0
BGIR9823_78631	100155714	PLGRKT	lncRNA	0	0	0	0	0	0	0	0	0	0	0	0	0	0	0	0
BGIR9823_97867	780419	UBE2D1	mRNA	0	0	0	0	0	0	0	0	0	0	0	0	0	0	0	0
BGIR9823_80393	100512765	SMIM7	lncRNA	0	0	0	0	0	0	0	0	0	0	0	0	0	0	0	0
BGIR9823_83405	100157574	FAM49B	lncRNA	0	0	0	0	0	0	0	0	0	0	0	0	0	0	0	0
BGIR9823_82271	100625157	GCFC2	lncRNA	0	0	0	0	0	0	0	0	0	0	0	0	0	0	0	0
BGIR9823_92748	100154396	PDS5B	lncRNA	0	0	0	0	0	0	0	0	0	0	0	0	0	0	0	0
BGIR9823_81832	100737907	FAM20C	lncRNA	0	0	0	0	0	1	0	0	0	0	0	0	0	0	0	0
BGIR9823_86739	100623382	SPIRE1	mRNA	0	0	0	0	0	0	0	0	0	0	0	0	0	0	0	0
BGIR9823_88779	100515615	CCNB1IP1	lncRNA	0	0	0	0	0	0	0	0	0	0	0	0	0	0	0	0
BGIR9823_82125	100533196	UBE2I	lncRNA	0	0	0	0	0	0	0	0	0	0	0	0	0	0	0	0
BGIR9823_96691	100153895	MORC2	lncRNA	0	0	0	0	0	0	0	0	0	0	0	0	0	0	0	0
BGIR9823_82328	100518067	SPRED2	lncRNA	0	0	0	0	0	0	0	0	0	0	0	0	0	0	0	0
BGIR9823_87494	100625971	LOC100625971	mRNA	0	0	0	0	0	0	0	0	0	0	0	0	0	0	0	0
BGIR9823_79014	100738102	RNF217	lncRNA	0	0	0	0	0	0	0	0	0	0	0	0	0	0	0	0
BGIR9823_79431	100153351	VPS18	mRNA	0	0	0	0	0	0	0	0	0	0	0	0	0	0	0	0
BGIR9823_79792	100154060	RIC1	mRNA	0	0	0	0	0	0	0	0	0	0	0	0	0	0	0	0
BGIR9823_97488	100153708	ATXN2	mRNA	0	0	0	0	0	0	0	0	0	0	0	0	0	0	0	0
BGIR9823_100701	100514931	AHCYL2	lncRNA	0	0	0	0	0	0	0	0	0	0	0	0	0	0	0	0
BGIR9823_83545	100153257	MMP16	lncRNA	0	0	0	0	0	0	0	0	0	0	0	0	0	0	0	0
BGIR9823_82543	100521786	TP53I3	mRNA	0	0	0	0	0	0	0	0	0	0	0	0	0	0	0	0
BGIR9823_81390	100520177	TRIP10	lncRNA	0	0	0	0	0	0	0	0	0	0	0	0	0	0	0	0
BGIR9823_100128	100519631	TASP1	lncRNA	0	0	0	0	0	0	0	0	0	0	0	0	0	0	0	0
BGIR9823_94226	100337675	ADAP2	lncRNA	0	0	0	1	0	0	0	0	0	0	0	0	0	0	0	0
BGIR9823_88221	110261778	NOL7	mRNA	0	0	0	0	0	0	0	0	0	0	0	0	0	0	0	0
BGIR9823_100908	100621199	IL1RAPL1	lncRNA	0	0	0	0	0	0	0	0	0	0	0	0	0	0	0	0
BGIR9823_101335	100523617	TRIM27	lncRNA	0	0	0	0	0	0	0	0	0	0	0	0	0	0	0	0
BGIR9823_101518	106504125	LOC106504125	lncRNA	0	0	0	0	0	0	0	0	0	0	0	0	0	0	0	0
BGIR9823_80840	100624262	SMAD5	mRNA	0	0	0	0	0	0	0	0	0	0	0	0	0	0	0	0
BGIR9823_99908	449527	PHF20	lncRNA	0	0	0	0	0	0	0	0	0	0	0	0	0	0	0	0
BGIR9823_99647	100521758	CPEB4	mRNA	0	0	0	0	0	0	0	0	0	0	1	0	0	0	0	0
BGIR9823_97815	106506044	LOC106506044	lncRNA	0	0	1	0	0	0	0	0	0	0	0	0	0	0	0	0
BGIR9823_77854	100736796	ADGRB3	lncRNA	0	0	0	0	0	0	0	0	0	0	0	1	0	0	0	0

RNA ID	Gene ID	Symbol	Type	ssc-miR-24-1-5p	ssc-miR-29b	ssc-miR-29c	ssc-miR-323	ssc-miR-369	ssc-miR-708-5p	novel-ssc-miR161-3p	novel-ssc-miR512-5p	novel-ssc-miR846-3p	novel-ssc-miR1086-5p	novel-ssc-miR1105-5p	novel-ssc-miR1116-5p	novel-ssc-miR1120-5p	novel-ssc-miR1140-5p	novel-ssc-miR1158-3p	novel-ssc-miR1379-5p
BGIR9823_92288	110255709	LOC110255709	lncRNA	0	0	0	0	0	0	0	0	0	0	0	0	0	0	0	0
BGIR9823_79245	100738627	DYM	lncRNA	0	0	0	0	0	0	0	0	0	0	0	0	0	0	0	0
BGIR9823_83586	100152963	PEX2	lncRNA	0	0	0	0	0	0	0	0	0	0	0	0	0	0	0	0
BGIR9823_101489	110258382	LOC110258382	lncRNA	0	0	0	0	0	0	0	0	0	0	0	1	0	0	0	0
BGIR9823_100872	100739216	MAP3K15	lncRNA	0	0	0	0	0	0	0	0	0	0	0	0	0	0	0	0
XM_005673239.3	100620722	ZNF277	mRNA	0	0	0	0	1	0	0	0	0	0	0	0	0	0	0	0
BGIR9823_100554	100739791	LOC100739791	lncRNA	0	0	0	0	0	0	0	0	0	0	0	0	0	0	0	0
BGIR9823_83272	100521316	GPN1	lncRNA	0	0	0	0	0	0	0	0	0	0	0	0	0	0	0	0
BGIR9823_77910	110260430	LOC110260430	lncRNA	0	0	0	0	0	0	0	0	0	0	0	0	0	0	0	1
BGIR9823_100153	100152012	CST7	lncRNA	0	0	0	0	0	0	0	0	0	0	0	0	0	0	0	0
BGIR9823_79200	100156851	IRAK1BP1	lncRNA	0	0	0	0	0	0	0	0	0	0	0	0	0	0	0	1
BGIR9823_100475	100517204	TFEC	lncRNA	0	0	0	0	0	0	0	0	0	0	0	0	0	0	0	0
BGIR9823_83002	110259971	C3H2orf49	lncRNA	0	0	0	0	0	0	0	0	0	0	0	0	0	0	0	0
BGIR9823_78732	100152140	SLC44A1	lncRNA	0	0	0	0	0	0	0	0	0	0	0	0	0	0	0	0
BGIR9823_94529	397618	OXSR1	lncRNA	0	1	0	0	0	0	0	0	0	0	0	0	0	0	0	0
BGIR9823_92973	100623901	MICU2	mRNA	0	0	0	0	0	0	0	0	0	0	0	0	0	0	0	0
BGIR9823_80241	100513197	DDB2	lncRNA	0	0	0	0	0	0	0	0	0	0	0	0	0	0	0	0
BGIR9823_96832	100153026	ABCB10	lncRNA	0	0	0	0	0	0	0	0	0	0	0	0	0	1	0	0
BGIR9823_96408	100153797	SECISBP2	mRNA	0	0	0	0	0	0	0	0	0	0	0	0	0	0	0	0
BGIR9823_80734	100526040	PAM	lncRNA	0	0	0	0	0	0	0	0	0	0	0	0	0	0	0	0
BGIR9823_81957	100513345	GTF2I	lncRNA	0	0	0	0	0	0	0	0	0	0	0	0	0	0	0	0
BGIR9823_96796	110256820	LOC110256820	lncRNA	0	1	0	0	0	0	0	0	0	0	0	0	0	0	0	0
BGIR9823_101115	110255263	CXH4orf3	lncRNA	0	0	0	0	0	0	0	0	0	0	0	0	0	0	0	0
BGIR9823_78939	100621167	PLEKHG1	lncRNA	0	0	0	0	0	0	0	0	0	0	0	0	0	0	0	0
BGIR9823_90840	100517358	PIWIL4	lncRNA	0	0	0	0	0	0	0	0	0	0	0	0	0	0	0	0
BGIR9823_94380	396903	MYH10	mRNA	0	0	0	0	0	0	0	0	0	0	0	0	0	0	0	0
BGIR9823_94364	100621720	TNK1	lncRNA	0	0	0	0	0	0	0	0	0	0	0	0	0	1	0	0
BGIR9823_99224	110257075	LOC110257075	lncRNA	0	0	0	1	0	0	0	0	0	0	0	0	0	0	0	0
BGIR9823_88977	102159128	LOC102159128	lncRNA	0	0	1	0	0	0	0	0	0	0	0	0	0	0	0	0
BGIR9823_86907	100623628	PRKACB	lncRNA	0	0	0	0	0	0	0	0	0	0	0	0	0	0	0	0
BGIR9823_83922	100127159	SLC16A1	lncRNA	0	0	0	0	0	0	0	0	0	0	0	0	0	0	0	0
BGIR9823_80165	106504153	AHNAK	mRNA	0	0	0	0	0	0	0	0	0	0	0	0	0	0	0	0
XM_021099336.1	102160410	LOC102160410	mRNA	0	0	0	0	0	0	0	0	0	0	0	0	0	0	1	0
BGIR9823_78034	100157196	CSNK1G1	lncRNA	0	0	0	0	0	0	0	0	0	0	0	0	0	0	0	0
BGIR9823_80409	100517807	SYDE1	lncRNA	1	0	0	0	0	0	0	0	0	0	0	0	0	0	0	0
BGIR9823_82453	100622259	MAP4K3	lncRNA	0	0	0	0	0	0	0	0	0	0	0	0	0	0	0	0
BGIR9823_101610	100521600	LOC100521600	lncRNA	0	0	0	0	0	0	0	0	0	0	0	0	0	0	0	0
BGIR9823_86055	102165609	LOC102165609	lncRNA	0	0	0	0	0	0	0	0	0	0	0	0	0	0	0	0
BGIR9823_92014	396922	CD46	mRNA	0	0	0	0	0	0	0	0	0	0	0	0	0	0	0	0
BGIR9823_100942	100521238	CDK16	mRNA	0	0	0	0	0	0	0	0	0	0	0	0	0	0	0	0
BGIR9823_77933	100513770	SLC16A10	lncRNA	0	0	0	0	0	0	0	0	0	0	0	0	0	0	0	0
BGIR9823_89476	100155589	FAM177A1	lncRNA	0	0	0	0	0	0	0	0	0	0	0	0	0	1	0	0
BGIR9823_101139	100525794	KLHL15	lncRNA	0	0	0	0	0	0	0	0	0	0	0	0	0	0	0	0

RNA ID	Gene ID	Symbol	Type	ssc-miR-24-1-5p	ssc-miR-29b	ssc-miR-29c	ssc-miR-323	ssc-miR-369	ssc-miR-708-5p	novel-ssc-miR161-3p	novel-ssc-miR512-5p	novel-ssc-miR846-3p	novel-ssc-miR1086-5p	novel-ssc-miR1105-5p	novel-ssc-miR1116-5p	novel-ssc-miR1120-5p	novel-ssc-miR1140-5p	novel-ssc-miR1158-3p	novel-ssc-miR1379-5p
BGIR9823_92285	100512363	CREM	lncRNA	0	1	0	0	0	0	0	0	0	0	0	0	0	0	0	0
BGIR9823_100684	414428	MKLN1	lncRNA	0	0	0	0	0	0	0	0	0	0	0	0	0	0	0	0
BGIR9823_80117	100511000	MUS81	lncRNA	0	0	0	0	0	0	0	0	0	0	0	0	0	0	0	0
BGIR9823_79783	102168004	UHRF2	lncRNA	0	0	0	0	0	0	0	0	0	0	0	0	0	0	0	0
BGIR9823_87527	110261323	LOC110261323	lncRNA	0	1	0	0	0	0	0	0	0	0	0	0	0	0	0	0
BGIR9823_77815	100736796	ADGRB3	lncRNA	0	0	0	0	0	0	0	0	0	0	0	0	0	0	0	0
BGIR9823_100466	100512202	TSPAN12	mRNA	0	0	0	0	0	0	0	0	0	0	0	0	0	0	0	0
BGIR9823_81771	100521956	CYSTM1	lncRNA	0	0	0	0	0	0	0	0	0	0	0	0	0	0	0	0
BGIR9823_93887	100625999	PGS1	mRNA	0	0	0	0	0	0	0	0	0	0	0	0	0	0	0	0
BGIR9823_99449	100312962	ATOX1	lncRNA	0	0	0	0	0	0	0	0	0	0	0	0	0	0	0	0
BGIR9823_82424	100526165	PREPL	lncRNA	0	0	0	0	0	0	0	0	0	0	0	0	0	0	0	0
BGIR9823_78323	100518050	TMX3	lncRNA	0	0	0	0	0	0	0	0	0	0	0	0	0	0	0	0
BGIR9823_81279	110259619	LOC110259619	lncRNA	1	0	0	0	0	0	0	0	0	0	0	0	0	0	0	0
BGIR9823_89593	100514718	SYNJ2BP	mRNA	0	0	0	0	0	0	0	0	0	0	0	0	0	0	0	0
BGIR9823_100641	100519065	UBN2	lncRNA	0	0	0	0	0	0	0	0	0	0	0	0	0	0	0	0
BGIR9823_99284	100518875	EGFLAM	mRNA	0	0	0	0	0	0	0	0	0	0	0	0	0	0	0	0
BGIR9823_97891	100037303	SGMS1	lncRNA	0	0	1	0	0	0	0	0	0	0	0	0	0	0	0	0
BGIR9823_80246	100516314	HARBI1	lncRNA	0	0	0	0	0	0	0	0	0	0	0	0	0	0	0	0
BGIR9823_84831	100154762	ZNF740	lncRNA	0	0	0	0	0	0	0	0	0	0	0	0	0	0	0	0
BGIR9823_100404	100524054	WDR91	lncRNA	0	0	0	0	0	0	0	0	0	0	0	0	0	0	0	0
BGIR9823_89087	100157771	CARMIL1	lncRNA	0	0	0	0	0	0	0	0	0	0	0	0	0	1	0	0
BGIR9823_94449	100737057	BTD	lncRNA	0	0	0	0	0	0	0	0	0	0	0	0	0	0	0	0
BGIR9823_89030	100152689	SSR1	mRNA	0	0	0	0	0	0	0	0	0	0	0	0	0	0	0	0
BGIR9823_100313	100155190	ZFP64	lncRNA	0	0	0	0	0	0	0	0	0	0	0	0	0	0	0	0
BGIR9823_84915	100623414	SRGAP1	lncRNA	0	0	0	0	0	0	0	0	0	0	0	0	0	0	0	0
BGIR9823_100318	100126237	CTCFL	lncRNA	0	0	0	0	0	0	0	0	0	0	0	0	0	0	0	0
BGIR9823_88856	100155733	SUSD6	lncRNA	0	0	0	0	0	0	0	0	0	0	0	0	0	0	0	0
XM_003480892.4	100519890	TRIM36	mRNA	0	0	0	0	0	0	0	0	0	0	0	0	0	0	0	0
BGIR9823_82684	100620890	SRRT	lncRNA	0	0	0	0	0	0	0	0	0	0	0	0	0	0	0	0
BGIR9823_83320	100513350	ASAP2	mRNA	0	0	0	0	0	0	0	0	0	0	0	0	0	0	0	0
BGIR9823_101170	100739016	RIBC1	lncRNA	0	0	0	0	0	0	0	0	0	0	0	0	0	0	0	0
BGIR9823_87081	100521097	ERI3	lncRNA	0	0	0	0	0	0	0	0	0	0	0	0	0	0	0	0
BGIR9823_88961	100152076	PTPN21	lncRNA	0	0	0	0	0	0	0	0	0	0	0	0	0	0	0	0
BGIR9823_89703	100515556	SLC25A47	lncRNA	0	0	0	0	0	0	0	0	0	0	0	0	0	0	0	0
BGIR9823_78067	100625505	VPS13C	lncRNA	0	0	0	0	0	0	0	0	0	0	0	0	0	0	0	0
BGIR9823_78412	100156782	LRFN5	lncRNA	0	0	0	0	0	0	0	0	0	0	0	0	0	0	0	0
BGIR9823_94130	106505457	TRIM37	mRNA	0	0	0	0	0	0	0	0	0	0	0	0	0	0	0	0
BGIR9823_78647	100152831	PUM3	lncRNA	0	0	0	0	0	0	0	0	0	0	0	0	0	0	0	0
BGIR9823_101061	102158351	LOC102158351	lncRNA	0	0	0	0	0	0	0	0	0	0	0	0	0	0	0	0
BGIR9823_101008	100523039	GPRASP1	mRNA	0	0	0	0	0	0	0	0	0	0	0	0	0	0	0	0

Supplementary Table S10. The predicted targets of DE lncRNAs.

RNA ID	Symbol	Type	updown_BGIR9823_80765	updown_BGIR9823_80869	updown_BGIR9823_81015	updown_BGIR9823_81197	updown_BGIR9823_81968	updown_BGIR9823_82765	updown_BGIR9823_78329	updown_BGIR9823_93571	updown_BGIR9823_93054	updown_BGIR9823_96360	updown_XR_002343213.1	updown_XR_002343346.1	updown_XR_002341920.1	updown_XR_002342486.1	updown_XR_307914.3	updown_XR_002341615.1	updown_XR_002343544.1	updown_XR_002343891.1	updown_BGIR9823_87139	updown_BGIR9823_84771	updown_BGIR9823_98958
BGIR9823_100361	PRKAG2	mRNA	0	0	0	0	0	0	0	0	0	0	0	0	0	0	0	0	0	0	0	0	0
BGIR9823_100362	PRKAG2	mRNA	0	0	0	0	0	0	0	0	0	0	0	0	0	0	0	0	0	0	0	0	0
BGIR9823_100363	PRKAG2	mRNA	0	0	0	0	0	0	0	0	0	0	0	0	0	0	0	0	0	0	0	0	0
BGIR9823_100364	PRKAG2	mRNA	0	0	0	0	0	0	0	0	0	0	0	0	0	0	0	0	0	0	0	0	0
BGIR9823_101535	LOC102158401	lncRNA	0	0	0	0	0	0	0	0	0	0	0	0	0	0	0	0	0	0	0	0	0
BGIR9823_101541	LOC102157946	lncRNA	0	0	0	0	0	0	0	0	0	0	0	0	0	0	0	0	0	0	0	0	0
BGIR9823_101542	LOC102157946	lncRNA	0	0	0	0	0	0	0	0	0	0	0	0	0	0	0	0	0	0	0	0	0
BGIR9823_101560	LOC110258600	lncRNA	0	0	0	0	0	0	0	0	0	0	0	0	0	0	0	0	0	0	0	0	0
BGIR9823_101561	LOC110258600	lncRNA	0	0	0	0	0	0	0	0	0	0	0	0	0	0	0	0	0	0	0	0	0
BGIR9823_101562	LOC110258600	lncRNA	0	0	0	0	0	0	0	0	0	0	0	0	0	0	0	0	0	0	0	0	0
BGIR9823_101563	LOC110258600	lncRNA	0	0	0	0	0	0	0	0	0	0	0	0	0	0	0	0	0	0	0	0	0
BGIR9823_101564	LOC110258600	lncRNA	0	0	0	0	0	0	0	0	0	0	0	0	0	0	0	0	0	0	0	0	0
BGIR9823_101612	LOC100521600	lncRNA	0	0	0	0	0	0	0	0	0	0	0	0	0	0	0	0	0	0	0	0	0
BGIR9823_101853		lncRNA	0	0	0	0	0	0	0	0	0	0	0	0	0	0	0	0	0	0	0	0	0
BGIR9823_101854		lncRNA	0	0	0	0	0	0	0	0	0	0	0	0	0	0	0	0	0	0	0	0	0
BGIR9823_101855		lncRNA	0	0	0	0	0	0	0	0	0	0	0	0	0	0	0	0	0	0	0	0	0
BGIR9823_102146		lncRNA	0	0	0	0	0	0	0	0	0	0	0	0	0	0	0	0	0	0	0	0	0
BGIR9823_102180		lncRNA	0	0	0	0	0	0	0	0	0	0	0	0	0	0	0	0	0	0	0	0	0
BGIR9823_102279		lncRNA	0	0	0	0	0	0	0	0	0	0	0	0	0	0	0	0	0	0	0	0	0
BGIR9823_102282		lncRNA	0	0	0	0	0	0	0	0	0	0	0	0	0	0	0	0	0	0	0	0	0
BGIR9823_102361		lncRNA	0	0	0	0	0	0	0	0	0	0	0	0	0	0	0	0	0	0	0	0	0
BGIR9823_102362		lncRNA	0	0	0	0	0	0	0	0	0	0	0	0	0	0	0	0	0	0	0	0	0
BGIR9823_102363		lncRNA	0	0	0	0	0	0	0	0	0	0	0	0	0	0	0	0	0	0	0	0	0
BGIR9823_102727		lncRNA	0	0	0	0	0	0	0	0	0	0	0	0	0	0	0	0	0	0	0	0	0
BGIR9823_80121	SSSCA1	lncRNA	0	0	1	0	0	0	0	0	0	0	0	0	0	0	0	0	0	0	0	0	0
BGIR9823_80122	LOC102163816	lncRNA	0	0	1	0	0	0	0	0	0	0	0	0	0	0	0	0	0	0	0	0	0
BGIR9823_81007	EHBP1L1	mRNA	0	0	1	0	0	0	0	0	0	0	0	0	0	0	0	0	0	0	0	0	0
BGIR9823_81008	EHBP1L1	mRNA	0	0	1	0	0	0	0	0	0	0	0	0	0	0	0	0	0	0	0	0	0
BGIR9823_81009	EHBP1L1	mRNA	0	0	1	0	0	0	0	0	0	0	0	0	0	0	0	0	0	0	0	0	0
BGIR9823_81010	EHBP1L1	mRNA	0	0	1	0	0	0	0	0	0	0	0	0	0	0	0	0	0	0	0	0	0
BGIR9823_81011	SSSCA1	mRNA	0	0	1	0	0	0	0	0	0	0	0	0	0	0	0	0	0	0	0	0	0
BGIR9823_81012	FRMD8	lncRNA	0	0	1	0	0	0	0	0	0	0	0	0	0	0	0	0	0	0	0	0	0
BGIR9823_81013	LOC102163816	lncRNA	0	0	1	0	0	0	0	0	0	0	0	0	0	0	0	0	0	0	0	0	0
BGIR9823_81014	LOC102163816	lncRNA	0	0	1	0	0	0	0	0	0	0	0	0	0	0	0	0	0	0	0	0	0
BGIR9823_81015	LOC102163816	lncRNA	0	0	1	0	0	0	0	0	0	0	0	0	0	0	0	0	0	0	0	0	0
BGIR9823_81016	LOC102163816	lncRNA	0	0	1	0	0	0	0	0	0	0	0	0	0	0	0	0	0	0	0	0	0
BGIR9823_81017	LOC102163816	lncRNA	0	0	1	0	0	0	0	0	0	0	0	0	0	0	0	0	0	0	0	0	0
BGIR9823_81018	LOC102163816	lncRNA	0	0	1	0	0	0	0	0	0	0	0	0	0	0	0	0	0	0	0	0	0
BGIR9823_81019	LOC100513133	lncRNA	0	0	1	0	0	0	0	0	0	0	0	0	0	0	0	0	0	0	0	0	0
BGIR9823_84704	LOC102159820	mRNA	0	0	0	0	0	0	0	0	0	0	0	0	0	0	0	0	0	0	0	0	0
BGIR9823_84732	RANGAP1	mRNA	0	0	0	0	0	0	0	0	0	0	0	0	0	0	0	0	0	0	0	0	0

RNA ID	Symbol	Type	updown_BGIR9823_80765	updown_BGIR9823_80869	updown_BGIR9823_81015	updown_BGIR9823_81197	updown_BGIR9823_81968	updown_BGIR9823_82765	updown_BGIR9823_78329	updown_BGIR9823_93571	updown_BGIR9823_93054	updown_BGIR9823_96360	updown_XR_002343213.1	updown_XR_002343346.1	updown_XR_002341920.1	updown_XR_002342486.1	updown_XR_307914.3	updown_XR_002341615.1	updown_XR_002343544.1	updown_XR_002343891.1	updown_BGIR9823_87139	updown_BGIR9823_84771	updown_BGIR9823_98958
BGIR9823_84733	RANGAP1	mRNA	0	0	0	0	0	0	0	0	0	0	0	0	0	0	0	0	0	0	0	0	0
BGIR9823_85245	CRADD	lncRNA	0	0	0	0	0	0	0	0	0	0	0	0	0	0	0	0	0	0	0	0	0
BGIR9823_85246	LOC100738154	lncRNA	0	0	0	0	0	0	0	0	0	0	0	0	0	0	0	0	0	0	0	0	0
BGIR9823_85293	TRMU	mRNA	0	0	0	0	0	0	0	0	0	0	0	0	0	0	0	0	0	0	0	0	0
BGIR9823_85294	TRMU	mRNA	0	0	0	0	0	0	0	0	0	0	0	0	0	0	0	0	0	0	0	0	0
BGIR9823_85295	TTC38	mRNA	0	0	0	0	0	0	0	0	0	0	0	0	0	0	0	0	0	0	0	0	0
BGIR9823_85296	PKDREJ	lncRNA	0	0	0	0	0	0	0	0	0	0	0	0	0	0	0	0	0	0	0	0	0
BGIR9823_85297	PPARA	mRNA	0	0	0	0	0	0	0	0	0	0	0	0	0	0	0	0	0	0	0	0	0
BGIR9823_85298	PPARA	mRNA	0	0	0	0	0	0	0	0	0	0	0	0	0	0	0	0	0	0	0	0	0
BGIR9823_85299	PPARA	mRNA	0	0	0	0	0	0	0	0	0	0	0	0	0	0	0	0	0	0	0	0	0
BGIR9823_85300	PPARA	lncRNA	0	0	0	0	0	0	0	0	0	0	0	0	0	0	0	0	0	0	0	0	0
BGIR9823_85301	LOC110260611	mRNA	0	0	0	0	0	0	0	0	0	0	0	0	0	0	0	0	0	0	0	0	0
BGIR9823_85317	ACO2	mRNA	0	0	0	0	0	0	0	0	0	0	0	0	0	0	0	0	0	0	0	0	0
BGIR9823_85318	LOC110260629	lncRNA	0	0	0	0	0	0	0	0	0	0	0	0	0	0	0	0	0	0	0	0	0
BGIR9823_85319	TEF	mRNA	0	0	0	0	0	0	0	0	0	0	0	0	0	0	0	0	0	0	0	0	0
BGIR9823_85320	ZC3H7B	mRNA	0	0	0	0	0	0	0	0	0	0	0	0	0	0	0	0	0	0	0	0	0
BGIR9823_85345	SYNGR1	mRNA	0	0	0	0	0	0	0	0	0	0	0	0	0	0	0	0	0	1	0	0	0
BGIR9823_85835	CRADD	mRNA	0	0	0	0	0	0	0	0	0	0	0	0	0	0	0	0	0	0	0	0	0
BGIR9823_85839	CRADD	lncRNA	0	0	0	0	0	0	0	0	0	0	0	0	0	0	0	0	0	0	0	0	0
BGIR9823_85840	CRADD	lncRNA	0	0	0	0	0	0	0	0	0	0	0	0	0	0	0	0	0	0	0	0	0
BGIR9823_85841	CRADD	lncRNA	0	0	0	0	0	0	0	0	0	0	0	0	0	0	0	0	0	0	0	0	0
BGIR9823_85930	LOC106510465	mRNA	0	0	0	0	0	0	0	0	0	0	0	0	0	0	0	0	0	0	1	0	0
BGIR9823_85931	MAF	lncRNA	0	0	0	0	0	0	0	0	0	0	0	0	0	0	0	0	0	0	1	0	0
BGIR9823_85932	MAF	lncRNA	0	0	0	0	0	0	0	0	0	0	0	0	0	0	0	0	0	0	1	0	0
BGIR9823_87139	LOC106510467	lncRNA	0	0	0	0	0	0	0	0	0	0	0	0	0	0	0	0	0	0	1	0	0
BGIR9823_88187	WRNIP1	mRNA	0	0	0	0	0	0	0	0	0	0	0	0	0	0	0	0	0	0	0	0	0
BGIR9823_88188	NQO2	mRNA	0	0	0	0	0	0	0	0	0	0	0	0	0	0	0	0	0	0	0	0	0
BGIR9823_88189	NQO2	mRNA	0	0	0	0	0	0	0	0	0	0	0	0	0	0	0	0	0	0	0	0	0
BGIR9823_88190	RIPK1	mRNA	0	0	0	0	0	0	0	0	0	0	0	0	0	0	0	0	0	0	0	0	0
BGIR9823_88191	RIPK1	mRNA	0	0	0	0	0	0	0	0	0	0	0	0	0	0	0	0	0	0	0	0	0
BGIR9823_88192	RIPK1	mRNA	0	0	0	0	0	0	0	0	0	0	0	0	0	0	0	0	0	0	0	0	0
BGIR9823_88193	RIPK1	mRNA	0	0	0	0	0	0	0	0	0	0	0	0	0	0	0	0	0	0	0	0	0
BGIR9823_88196	PRPF4B	mRNA	0	0	0	0	0	0	0	0	0	0	0	0	0	0	0	0	0	0	0	0	0
BGIR9823_88197	PRPF4B	mRNA	0	0	0	0	0	0	0	0	0	0	0	0	0	0	0	0	0	0	0	0	0
BGIR9823_88198	PRPF4B	mRNA	0	0	0	0	0	0	0	0	0	0	0	0	0	0	0	0	0	0	0	0	0
BGIR9823_88211	TMEM170B	lncRNA	0	0	0	0	0	0	0	0	0	0	0	0	0	0	0	0	0	0	0	0	0
BGIR9823_88212	TMEM170B	lncRNA	0	0	0	0	0	0	0	0	0	0	0	0	0	0	0	0	0	0	0	0	0
BGIR9823_88213	TMEM170B	lncRNA	0	0	0	0	0	0	0	0	0	0	0	0	0	0	0	0	0	0	0	0	0
BGIR9823_88214	TMEM170B	lncRNA	0	0	0	0	0	0	0	0	0	0	0	0	0	0	0	0	0	0	0	0	0
BGIR9823_88217	PHACTR1	mRNA	0	0	0	0	0	0	0	0	0	0	0	0	0	0	0	0	0	0	0	0	0
BGIR9823_88218	PHACTR1	lncRNA	0	0	0	0	0	0	0	0	0	0	0	0	0	0	0	0	0	0	0	0	0
BGIR9823_89017	MYLK4	lncRNA	0	0	0	0	0	0	0	0	0	0	0	0	0	0	0	0	0	0	0	0	0
BGIR9823_89019	SERPINB1	lncRNA	0	0	0	0	0	0	0	0	0	0	0	0	0	0	0	0	0	0	0	0	0

RNA ID	Symbol	Type	updown_BGIR9823_80765	updown_BGIR9823_80869	updown_BGIR9823_81015	updown_BGIR9823_81197	updown_BGIR9823_81968	updown_BGIR9823_82765	updown_BGIR9823_78329	updown_BGIR9823_93571	updown_BGIR9823_93054	updown_BGIR9823_96360	updown_XR_002343213.1	updown_XR_002343346.1	updown_XR_002341920.1	updown_XR_002342486.1	updown_XR_307914.3	updown_XR_002341615.1	updown_XR_002343544.1	updown_XR_002343891.1	updown_BGIR9823_87139	updown_BGIR9823_84771	updown_BGIR9823_98958
BGIR9823_89020	SERPINB9	mRNA	0	0	0	0	0	0	0	0	0	0	0	0	0	0	0	0	0	0	0	0	0
BGIR9823_89021	RIPK1	lncRNA	0	0	0	0	0	0	0	0	0	0	0	0	0	0	0	0	0	0	0	0	0
BGIR9823_89025	PXDC1	mRNA	0	0	0	0	0	0	0	0	0	0	0	0	0	0	0	0	0	0	0	0	0
BGIR9823_89026	PXDC1	lncRNA	0	0	0	0	0	0	0	0	0	0	0	0	0	0	0	0	0	0	0	0	0
BGIR9823_89027	LOC102161744	lncRNA	0	0	0	0	0	0	0	0	0	0	0	0	0	0	0	0	0	0	0	0	0
BGIR9823_89040	TBC1D7	lncRNA	0	0	0	0	0	0	0	0	0	0	0	0	0	0	0	0	0	0	0	0	0
BGIR9823_89041	TBC1D7	lncRNA	0	0	0	0	0	0	0	0	0	0	0	0	0	0	0	0	0	0	0	0	0
BGIR9823_89042	TBC1D7	lncRNA	0	0	0	0	0	0	0	0	0	0	0	0	0	0	0	0	0	0	0	0	0
BGIR9823_89043	TBC1D7	lncRNA	0	0	0	0	0	0	0	0	0	0	0	0	0	0	0	0	0	0	0	0	0
BGIR9823_89044	GFOD1	lncRNA	0	0	0	0	0	0	0	0	0	0	0	0	0	0	0	0	0	0	0	0	0
BGIR9823_89045	GFOD1	mRNA	0	0	0	0	0	0	0	0	0	0	0	0	0	0	0	0	0	0	0	0	0
BGIR9823_89046	GFOD1	mRNA	0	0	0	0	0	0	0	0	0	0	0	0	0	0	0	0	0	0	0	0	0
BGIR9823_89047	GFOD1	mRNA	0	0	0	0	0	0	0	0	0	0	0	0	0	0	0	0	0	0	0	0	0
BGIR9823_89048	GFOD1	lncRNA	0	0	0	0	0	0	0	0	0	0	0	0	0	0	0	0	0	0	0	0	0
BGIR9823_89049	GFOD1	lncRNA	0	0	0	0	0	0	0	0	0	0	0	0	0	0	0	0	0	0	0	0	0
BGIR9823_90771	NEU3	lncRNA	0	0	0	0	0	0	0	0	0	0	0	0	0	0	0	0	0	0	0	0	0
BGIR9823_90772	SLCO2B1	mRNA	0	0	0	0	0	0	0	0	0	0	0	0	0	0	0	0	0	0	0	0	0
BGIR9823_90913	LOC110255435	lncRNA	0	0	0	0	0	0	0	0	0	0	0	0	0	0	0	0	0	0	0	0	0
BGIR9823_90934	LOC110255441	lncRNA	0	0	0	0	0	0	0	0	0	0	0	0	0	0	0	0	0	0	0	0	0
BGIR9823_90935	CCDC84	mRNA	0	0	0	0	0	0	0	0	0	0	0	0	0	0	0	0	0	0	0	0	0
BGIR9823_90936	TRAPPC4	mRNA	0	0	0	0	0	0	0	0	0	0	0	0	0	0	0	0	0	0	0	0	0
BGIR9823_90937	HMBS	mRNA	0	0	0	0	0	0	0	0	0	0	0	0	0	0	0	0	0	0	0	0	0
BGIR9823_90956	SORL1	mRNA	0	0	0	0	0	0	0	0	0	0	0	0	0	0	0	0	0	0	0	0	0
BGIR9823_90957	SORL1	mRNA	0	0	0	0	0	0	0	0	0	0	0	0	0	0	0	0	0	0	0	0	0
BGIR9823_90958	SORL1	mRNA	0	0	0	0	0	0	0	0	0	0	0	0	0	0	0	0	0	0	0	0	0
BGIR9823_90960	CLMP	lncRNA	0	0	0	0	0	0	0	0	0	0	0	0	0	0	0	0	0	0	0	0	0
BGIR9823_91161	LOC100522267	mRNA	0	0	0	0	0	0	0	0	0	0	0	0	0	0	0	0	0	0	0	0	0
BGIR9823_91162	LOC100522455	mRNA	0	0	0	0	0	0	0	0	0	0	0	0	0	0	0	0	0	0	0	0	0
BGIR9823_91163	ABCB1	mRNA	0	0	0	0	0	0	0	0	0	0	0	0	0	0	0	0	0	0	0	0	0
BGIR9823_91402	HBB	mRNA	0	0	0	0	0	0	0	0	0	0	0	0	0	0	0	0	0	0	0	0	0
BGIR9823_91403	HBB	mRNA	0	0	0	0	0	0	0	0	0	0	0	0	0	0	0	0	0	0	0	0	0
BGIR9823_91404	HBB	mRNA	0	0	0	0	0	0	0	0	0	0	0	0	0	0	0	0	0	0	0	0	0
BGIR9823_91405	HBB	mRNA	0	0	0	0	0	0	0	0	0	0	0	0	0	0	0	0	0	0	0	0	0
BGIR9823_91406	HBB	mRNA	0	0	0	0	0	0	0	0	0	0	0	0	0	0	0	0	0	0	0	0	0
BGIR9823_91407	HBB	mRNA	0	0	0	0	0	0	0	0	0	0	0	0	0	0	0	0	0	0	0	0	0
BGIR9823_91408	HBB	mRNA	0	0	0	0	0	0	0	0	0	0	0	0	0	0	0	0	0	0	0	0	0
BGIR9823_91409	HBB	mRNA	0	0	0	0	0	0	0	0	0	0	0	0	0	0	0	0	0	0	0	0	0
BGIR9823_91625	BCL9L	lncRNA	0	0	0	0	0	0	0	0	0	0	0	0	0	0	0	0	0	0	0	0	0
BGIR9823_91626	BCL9L	lncRNA	0	0	0	0	0	0	0	0	0	0	0	0	0	0	0	0	0	0	0	0	0
BGIR9823_91627	RPS25	mRNA	0	0	0	0	0	0	0	0	0	0	0	0	0	0	0	0	0	0	0	0	0
BGIR9823_91628	RPS25	mRNA	0	0	0	0	0	0	0	0	0	0	0	0	0	0	0	0	0	0	0	0	0
BGIR9823_91629	SLC37A4	mRNA	0	0	0	0	0	0	0	0	0	0	0	0	0	0	0	0	0	0	0	0	0
BGIR9823_91630	HYOU1	mRNA	0	0	0	0	0	0	0	0	0	0	0	0	0	0	0	0	0	0	0	0	0

RNA ID	Symbol	Type	updown_BGIR9823_80765	updown_BGIR9823_80869	updown_BGIR9823_81015	updown_BGIR9823_81197	updown_BGIR9823_81968	updown_BGIR9823_82765	updown_BGIR9823_78329	updown_BGIR9823_93571	updown_BGIR9823_93054	updown_BGIR9823_96360	updown_XR_002343213.1	updown_XR_002343346.1	updown_XR_002341920.1	updown_XR_002342486.1	updown_XR_307914.3	updown_XR_002341615.1	updown_XR_002343544.1	updown_XR_002343891.1	updown_BGIR9823_87139	updown_BGIR9823_84771	updown_BGIR9823_98958
BGIR9823_91640	CLMP	mRNA	0	0	0	0	0	0	0	0	0	0	0	0	0	0	0	0	0	0	0	0	0
BGIR9823_91819	RUNDC3B	lncRNA	0	0	0	0	0	0	0	0	0	0	0	0	0	0	0	0	0	0	0	0	0
BGIR9823_91820	RUNDC3B	lncRNA	0	0	0	0	0	0	0	0	0	0	0	0	0	0	0	0	0	0	0	0	0
BGIR9823_91821	RUNDC3B	lncRNA	0	0	0	0	0	0	0	0	0	0	0	0	0	0	0	0	0	0	0	0	0
BGIR9823_91822	RUNDC3B	lncRNA	0	0	0	0	0	0	0	0	0	0	0	0	0	0	0	0	0	0	0	0	0
BGIR9823_91823	RUNDC3B	lncRNA	0	0	0	0	0	0	0	0	0	0	0	0	0	0	0	0	0	0	0	0	0
BGIR9823_91824	RUNDC3B	lncRNA	0	0	0	0	0	0	0	0	0	0	0	0	0	0	0	0	0	0	0	0	0
BGIR9823_91825	RUNDC3B	lncRNA	0	0	0	0	0	0	0	0	0	0	0	0	0	0	0	0	0	0	0	0	0
BGIR9823_91826	LOC102164596	lncRNA	0	0	0	0	0	0	0	0	0	0	0	0	0	0	0	0	0	0	0	0	0
BGIR9823_92993	KATNAL1	mRNA	0	0	0	0	0	0	0	0	0	0	0	0	0	0	0	0	0	0	0	0	0
BGIR9823_92994	KATNAL1	mRNA	0	0	0	0	0	0	0	0	0	0	0	0	0	0	0	0	0	0	0	0	0
BGIR9823_92995	KATNAL1	mRNA	0	0	0	0	0	0	0	0	0	0	0	0	0	0	0	0	0	0	0	0	0
BGIR9823_92996	KATNAL1	mRNA	0	0	0	0	0	0	0	0	0	0	0	0	0	0	0	0	0	0	0	0	0
BGIR9823_92997	KATNAL1	mRNA	0	0	0	0	0	0	0	0	0	0	0	0	0	0	0	0	0	0	0	0	0
BGIR9823_92999	LOC110255292	lncRNA	0	0	0	0	0	0	0	0	0	0	0	0	0	0	0	0	0	0	0	0	0
BGIR9823_93000	LOC102166871	lncRNA	0	0	0	0	0	0	0	0	0	0	0	0	0	0	0	0	0	0	0	0	0
BGIR9823_98051	LOC102164998	lncRNA	0	0	0	0	0	0	0	0	0	0	0	0	0	0	0	0	0	0	0	0	0
BGIR9823_99244	LOC102167708	lncRNA	0	0	0	0	0	0	0	0	0	0	0	0	0	0	0	0	0	0	0	0	0
NM_001004034.1	HMGB1	mRNA	0	0	0	0	0	0	0	0	0	0	0	0	0	0	0	0	0	0	0	0	0
NM_001044526.1	PPARA	mRNA	0	0	0	0	0	0	0	0	0	0	0	0	0	0	0	0	0	0	0	0	0
NM_001111256.1	SERPINB9	mRNA	0	0	0	0	0	0	0	0	0	0	0	0	0	0	0	0	0	0	0	0	0
NM_001113443.1	ECI2	mRNA	0	0	0	0	0	0	0	0	0	0	0	0	0	0	0	0	0	0	0	0	0
NM_001144841.1	HBB	mRNA	0	0	0	0	0	0	0	0	0	0	0	0	0	0	0	0	0	0	0	0	0
NM_001199719.1	SLC37A4	mRNA	0	0	0	0	0	0	0	0	0	0	0	0	0	0	0	0	0	0	0	0	0
NM_001243907.1	HSPA8	mRNA	0	0	0	0	0	0	0	0	0	0	0	0	0	0	0	0	0	0	0	0	0
NM_001244063.1	RPL3	mRNA	0	0	0	0	0	0	0	0	0	0	0	0	0	0	0	0	0	1	0	0	0
NM_001244066.1	SYNGR1	mRNA	0	0	0	0	0	0	0	0	0	0	0	0	0	0	0	0	0	1	0	0	0
NM_001244396.1	CCDC84	mRNA	0	0	0	0	0	0	0	0	0	0	0	0	0	0	0	0	0	0	0	0	0
NM_001287846.1	RHEB	mRNA	0	0	0	0	0	0	0	0	0	0	0	0	0	0	0	0	0	0	0	0	0
NM_213954.1	ACO2	mRNA	0	0	0	0	0	0	0	0	0	0	0	0	0	0	0	0	0	0	0	0	0
NM_214012.1	UPK2	mRNA	0	0	0	0	0	0	0	0	0	0	0	0	0	0	0	0	0	0	0	0	0
URS0000BC92F4	LOC102167708	lncRNA	0	0	0	0	0	0	0	0	0	0	0	0	0	0	0	0	0	0	0	0	0
XM_001928309.6	TBC1D7	mRNA	0	0	0	0	0	0	0	0	0	0	0	0	0	0	0	0	0	0	0	0	0
XM_001928852.5	PXDC1	mRNA	0	0	0	0	0	0	0	0	0	0	0	0	0	0	0	0	0	0	0	0	0
XM_003122534.4	KCNK7	mRNA	0	0	1	0	0	0	0	0	0	0	0	0	0	0	0	0	0	0	0	0	0
XM_003122558.5	SCYL1	mRNA	0	0	1	0	0	0	0	0	0	0	0	0	0	0	0	0	0	0	0	0	0
XM_003122562.5	FRMD8	mRNA	0	0	1	0	0	0	0	0	0	0	0	0	0	0	0	0	0	0	0	0	0
XM_003125951.6	TTC38	mRNA	0	0	0	0	0	0	0	0	0	0	0	0	0	0	0	0	0	0	0	0	0
XM_003125953.4	CDPF1	mRNA	0	0	0	0	0	0	0	0	0	0	0	0	0	0	0	0	0	0	0	0	0
XM_003126012.6	CBX7	mRNA	0	0	0	0	0	0	0	0	0	0	0	0	0	0	0	0	0	1	0	0	0
XM_003128161.4	RIPK1	mRNA	0	0	0	0	0	0	0	0	0	0	0	0	0	0	0	0	0	0	0	0	0
XM_003129508.2	LOC100514417	mRNA	0	0	0	0	0	0	0	0	0	0	0	0	0	0	0	0	0	0	0	0	0
XM_003129512.2	LOC100515268	mRNA	0	0	0	0	0	0	0	0	0	0	0	0	0	0	0	0	0	0	0	0	0

RNA ID	Symbol	Type	updown_BGIR9823_80765	updown_BGIR9823_80869	updown_BGIR9823_81015	updown_BGIR9823_81197	updown_BGIR9823_81968	updown_BGIR9823_82765	updown_BGIR9823_78329	updown_BGIR9823_93571	updown_BGIR9823_93054	updown_BGIR9823_96360	updown_XR_002343213.1	updown_XR_002343346.1	updown_XR_002341920.1	updown_XR_002342486.1	updown_XR_307914.3	updown_XR_002341615.1	updown_XR_002343544.1	updown_XR_002343891.1	updown_BGIR9823_87139	updown_BGIR9823_84771	updown_BGIR9823_98958
XM_003129514.3	LOC100515618	mRNA	0	0	0	0	0	0	0	0	0	0	0	0	0	0	0	0	0	0	0	0	0
XM_003129519.2	LOC100517595	mRNA	0	0	0	0	0	0	0	0	0	0	0	0	0	0	0	0	0	0	0	0	0
XM_003129529.4	LOC100519910	mRNA	0	0	0	0	0	0	0	0	0	0	0	0	0	0	0	0	0	0	0	0	0
XM_003129915.4	CXCR5	mRNA	0	0	0	0	0	0	0	0	0	0	0	0	0	0	0	0	0	0	0	0	0
XM_003129916.5	BCL9L	mRNA	0	0	0	0	0	0	0	0	0	0	0	0	0	0	0	0	0	0	0	0	0
XM_003129918.4	TRAPPC4	mRNA	0	0	0	0	0	0	0	0	0	0	0	0	0	0	0	0	0	0	0	0	0
XM_003129919.5	RPS25	mRNA	0	0	0	0	0	0	0	0	0	0	0	0	0	0	0	0	0	0	0	0	0
XM_003129920.3	RPS25	mRNA	0	0	0	0	0	0	0	0	0	0	0	0	0	0	0	0	0	0	0	0	0
XM_003129922.4	HYOU1	mRNA	0	0	0	0	0	0	0	0	0	0	0	0	0	0	0	0	0	0	0	0	0
XM_003129923.6	VPS11	mRNA	0	0	0	0	0	0	0	0	0	0	0	0	0	0	0	0	0	0	0	0	0
XM_003129944.4	SC5D	mRNA	0	0	0	0	0	0	0	0	0	0	0	0	0	0	0	0	0	0	0	0	0
XM_003357161.1	LOC100620971	mRNA	0	0	0	0	0	0	0	0	0	0	0	0	0	0	0	0	0	0	0	0	0
XM_003482119.4	SERPINB1	mRNA	0	0	0	0	0	0	0	0	0	0	0	0	0	0	0	0	0	0	0	0	0
XM_003482132.4	TMEM170B	mRNA	0	0	0	0	0	0	0	0	0	0	0	0	0	0	0	0	0	0	0	0	0
XM_003482133.4	GFOD1	mRNA	0	0	0	0	0	0	0	0	0	0	0	0	0	0	0	0	0	0	0	0	0
XM_003482563.4	SPCS2	mRNA	0	0	0	0	0	0	0	0	0	0	0	0	0	0	0	0	0	0	0	0	0
XM_003482565.4	NEU3	mRNA	0	0	0	0	0	0	0	0	0	0	0	0	0	0	0	0	0	0	0	0	0
XM_003482567.1	LOC100739359	mRNA	0	0	0	0	0	0	0	0	0	0	0	0	0	0	0	0	0	0	0	0	0
XM_003482630.3	BSX	mRNA	0	0	0	0	0	0	0	0	0	0	0	0	0	0	0	0	0	0	0	0	0
XM_005653107.3	CHADL	mRNA	0	0	0	0	0	0	0	0	0	0	0	0	0	0	0	0	0	0	0	0	0
XM_005653640.1	LOC100621181	mRNA	0	0	0	0	0	0	0	0	0	0	0	0	0	0	0	0	0	0	0	0	0
XM_005654266.3	PRKAG2	mRNA	0	0	0	0	0	0	0	0	0	0	0	0	0	0	0	0	0	0	0	0	0
XM_005655470.3	CDPF1	mRNA	0	0	0	0	0	0	0	0	0	0	0	0	0	0	0	0	0	0	0	0	0
XM_005660703.3	LTBP3	mRNA	0	0	1	0	0	0	0	0	0	0	0	0	0	0	0	0	0	0	0	0	0
XM_005660705.3	SCYL1	mRNA	0	0	1	0	0	0	0	0	0	0	0	0	0	0	0	0	0	0	0	0	0
XM_005660707.3	SLC25A45	mRNA	0	0	1	0	0	0	0	0	0	0	0	0	0	0	0	0	0	0	0	0	0
XM_005665536.3	RIPK1	mRNA	0	0	0	0	0	0	0	0	0	0	0	0	0	0	0	0	0	0	0	0	0
XM_005665537.3	RIPK1	mRNA	0	0	0	0	0	0	0	0	0	0	0	0	0	0	0	0	0	0	0	0	0
XM_005665538.3	RIPK1	mRNA	0	0	0	0	0	0	0	0	0	0	0	0	0	0	0	0	0	0	0	0	0
XM_005665575.3	PHACTR1	mRNA	0	0	0	0	0	0	0	0	0	0	0	0	0	0	0	0	0	0	0	0	0
XM_005665576.3	TBC1D7	mRNA	0	0	0	0	0	0	0	0	0	0	0	0	0	0	0	0	0	0	0	0	0
XM_005667128.3	LOC100627340	mRNA	0	0	0	0	0	0	0	0	0	0	0	0	0	0	0	0	0	0	0	0	0
XM_005667397.2	CCDC84	mRNA	0	0	0	0	0	0	0	0	0	0	0	0	0	0	0	0	0	0	0	0	0
XM_005667398.3	SLC37A4	mRNA	0	0	0	0	0	0	0	0	0	0	0	0	0	0	0	0	0	0	0	0	0
XM_005667402.3	SLC37A4	mRNA	0	0	0	0	0	0	0	0	0	0	0	0	0	0	0	0	0	0	0	0	0
XM_005667403.3	SLC37A4	mRNA	0	0	0	0	0	0	0	0	0	0	0	0	0	0	0	0	0	0	0	0	0
XM_005667413.3	BCL9L	mRNA	0	0	0	0	0	0	0	0	0	0	0	0	0	0	0	0	0	0	0	0	0
XM_005667414.3	FOXR1	mRNA	0	0	0	0	0	0	0	0	0	0	0	0	0	0	0	0	0	0	0	0	0
XM_005667416.3	HYOU1	mRNA	0	0	0	0	0	0	0	0	0	0	0	0	0	0	0	0	0	0	0	0	0
XM_005667419.3	HYOU1	mRNA	0	0	0	0	0	0	0	0	0	0	0	0	0	0	0	0	0	0	0	0	0
XM_005667420.3	HYOU1	mRNA	0	0	0	0	0	0	0	0	0	0	0	0	0	0	0	0	0	0	0	0	0
XM_005667440.2	JHY	mRNA	0	0	0	0	0	0	0	0	0	0	0	0	0	0	0	0	0	0	0	0	0
XM_005668316.3	HMGB1	mRNA	0	0	0	0	0	0	0	0	0	0	0	0	0	0	0	0	0	0	0	0	0

RNA ID	Symbol	Type	updown_BGIR9823_80765	updown_BGIR9823_80869	updown_BGIR9823_81015	updown_BGIR9823_81197	updown_BGIR9823_81968	updown_BGIR9823_82765	updown_BGIR9823_78329	updown_BGIR9823_93571	updown_BGIR9823_93054	updown_BGIR9823_96360	updown_XR_002343213.1	updown_XR_002343346.1	updown_XR_002341920.1	updown_XR_002342486.1	updown_XR_307914.3	updown_XR_002341615.1	updown_XR_002343544.1	updown_XR_002343891.1	updown_BGIR9823_87139	updown_BGIR9823_84771	updown_BGIR9823_98958
XM_005668317.3	HMGB1	mRNA	0	0	0	0	0	0	0	0	0	0	0	0	0	0	0	0	0	0	0	0	0
XM_005668318.3	HMGB1	mRNA	0	0	0	0	0	0	0	0	0	0	0	0	0	0	0	0	0	0	0	0	0
XM_005671489.3	EMX2	mRNA	0	0	0	0	0	0	0	0	0	0	0	0	0	0	0	0	0	0	0	0	0
XM_005672397.3	MYO10	mRNA	0	0	0	0	0	0	0	0	0	0	0	0	0	0	0	0	0	0	0	0	0
XM_013977400.2	FAM50B	mRNA	0	0	0	0	0	0	0	0	0	0	0	0	0	0	0	0	0	0	0	0	0
XM_013977425.2	NEDD9	mRNA	0	0	0	0	0	0	0	0	0	0	0	0	0	0	0	0	0	0	0	0	0
XM_013977430.2	TBC1D7	mRNA	0	0	0	0	0	0	0	0	0	0	0	0	0	0	0	0	0	0	0	0	0
XM_013977431.2	TBC1D7	mRNA	0	0	0	0	0	0	0	0	0	0	0	0	0	0	0	0	0	0	0	0	0
XM_013977432.2	TBC1D7	mRNA	0	0	0	0	0	0	0	0	0	0	0	0	0	0	0	0	0	0	0	0	0
XM_013977433.2	GFOD1	mRNA	0	0	0	0	0	0	0	0	0	0	0	0	0	0	0	0	0	0	0	0	0
XM_013977434.2	GFOD1	mRNA	0	0	0	0	0	0	0	0	0	0	0	0	0	0	0	0	0	0	0	0	0
XM_013979449.2	SLC37A4	mRNA	0	0	0	0	0	0	0	0	0	0	0	0	0	0	0	0	0	0	0	0	0
XM_013979456.2	BCL9L	mRNA	0	0	0	0	0	0	0	0	0	0	0	0	0	0	0	0	0	0	0	0	0
XM_013979457.2	BCL9L	mRNA	0	0	0	0	0	0	0	0	0	0	0	0	0	0	0	0	0	0	0	0	0
XM_013979458.2	BCL9L	mRNA	0	0	0	0	0	0	0	0	0	0	0	0	0	0	0	0	0	0	0	0	0
XM_013979459.2	BCL9L	mRNA	0	0	0	0	0	0	0	0	0	0	0	0	0	0	0	0	0	0	0	0	0
XM_013979461.2	TRAPPC4	mRNA	0	0	0	0	0	0	0	0	0	0	0	0	0	0	0	0	0	0	0	0	0
XM_013979463.2	HYOU1	mRNA	0	0	0	0	0	0	0	0	0	0	0	0	0	0	0	0	0	0	0	0	0
XM_013979484.2	LOC100523684	mRNA	0	0	0	0	0	0	0	0	0	0	0	0	0	0	0	0	0	0	0	0	0
XM_013979486.2	LOC100523684	mRNA	0	0	0	0	0	0	0	0	0	0	0	0	0	0	0	0	0	0	0	0	0
XM_013979491.2	JHY	mRNA	0	0	0	0	0	0	0	0	0	0	0	0	0	0	0	0	0	0	0	0	0
XM_013980418.2	KATNAL1	mRNA	0	0	0	0	0	0	0	0	0	0	0	0	0	0	0	0	0	0	0	0	0
XM_013988182.2	TTC38	mRNA	0	0	0	0	0	0	0	0	0	0	0	0	0	0	0	0	0	0	0	0	0
XM_013988183.2	TTC38	mRNA	0	0	0	0	0	0	0	0	0	0	0	0	0	0	0	0	0	0	0	0	0
XM_013989589.2	LOC100522267	mRNA	0	0	0	0	0	0	0	0	0	0	0	0	0	0	0	0	0	0	0	0	0
XM_013989592.2	LOC100522455	mRNA	0	0	0	0	0	0	0	0	0	0	0	0	0	0	0	0	0	0	0	0	0
XM_013989593.2	LOC100522455	mRNA	0	0	0	0	0	0	0	0	0	0	0	0	0	0	0	0	0	0	0	0	0
XM_013994099.2	FRMD8	mRNA	0	0	1	0	0	0	0	0	0	0	0	0	0	0	0	0	0	0	0	0	0
XM_013994100.2	SLC25A45	mRNA	0	0	1	0	0	0	0	0	0	0	0	0	0	0	0	0	0	0	0	0	0
XM_013994102.2	SLC25A45	mRNA	0	0	1	0	0	0	0	0	0	0	0	0	0	0	0	0	0	0	0	0	0
XM_013994103.2	SLC25A45	mRNA	0	0	1	0	0	0	0	0	0	0	0	0	0	0	0	0	0	0	0	0	0
XM_013994104.2	SLC25A45	mRNA	0	0	1	0	0	0	0	0	0	0	0	0	0	0	0	0	0	0	0	0	0
XM_013997510.2	CBX7	mRNA	0	0	0	0	0	0	0	0	0	0	0	0	0	0	0	0	0	1	0	0	0
XM_013999070.2	LOC100520088	mRNA	0	0	0	0	0	0	0	0	0	0	0	0	0	0	0	0	0	0	0	0	0
XM_021062230.1	LOC100517771	mRNA	0	0	0	0	0	0	0	0	0	0	0	0	0	0	0	0	0	0	0	0	0
XM_021062231.1	LOC100517957	mRNA	0	0	0	0	0	0	0	0	0	0	0	0	0	0	0	0	0	0	0	0	0
XM_021062367.1	LOC110255365	mRNA	0	0	0	0	0	0	0	0	0	0	0	0	0	0	0	0	0	0	0	0	0
XM_021062369.1	LOC100737110	mRNA	0	0	0	0	0	0	0	0	0	0	0	0	0	0	0	0	0	0	0	0	0
XM_021062370.1	LOC110255368	mRNA	0	0	0	0	0	0	0	0	0	0	0	0	0	0	0	0	0	0	0	0	0
XM_021062371.1	LOC100515441	mRNA	0	0	0	0	0	0	0	0	0	0	0	0	0	0	0	0	0	0	0	0	0
XM_021062374.1	LOC110255371	mRNA	0	0	0	0	0	0	0	0	0	0	0	0	0	0	0	0	0	0	0	0	0
XM_021062375.1	LOC100626951	mRNA	0	0	0	0	0	0	0	0	0	0	0	0	0	0	0	0	0	0	0	0	0
XM_021062491.1	XRRA1	mRNA	0	0	0	0	0	0	0	0	0	0	0	0	0	0	0	0	0	0	0	0	0

RNA ID	Symbol	Type	updown_BGIR9823_80765	updown_BGIR9823_80869	updown_BGIR9823_81015	updown_BGIR9823_81197	updown_BGIR9823_81968	updown_BGIR9823_82765	updown_BGIR9823_78329	updown_BGIR9823_93571	updown_BGIR9823_93054	updown_BGIR9823_96360	updown_XR_002343213.1	updown_XR_002343346.1	updown_XR_002341920.1	updown_XR_002342486.1	updown_XR_307914.3	updown_XR_002341615.1	updown_XR_002343544.1	updown_XR_002343891.1	updown_BGIR9823_87139	updown_BGIR9823_84771	updown_BGIR9823_98958
XM_021062493.1	XRRA1	mRNA	0	0	0	0	0	0	0	0	0	0	0	0	0	0	0	0	0	0	0	0	0
XM_021062494.1	XRRA1	mRNA	0	0	0	0	0	0	0	0	0	0	0	0	0	0	0	0	0	0	0	0	0
XM_021062495.1	XRRA1	mRNA	0	0	0	0	0	0	0	0	0	0	0	0	0	0	0	0	0	0	0	0	0
XM_021062496.1	XRRA1	mRNA	0	0	0	0	0	0	0	0	0	0	0	0	0	0	0	0	0	0	0	0	0
XM_021062497.1	XRRA1	mRNA	0	0	0	0	0	0	0	0	0	0	0	0	0	0	0	0	0	0	0	0	0
XM_021062498.1	XRRA1	mRNA	0	0	0	0	0	0	0	0	0	0	0	0	0	0	0	0	0	0	0	0	0
XM_021062499.1	XRRA1	mRNA	0	0	0	0	0	0	0	0	0	0	0	0	0	0	0	0	0	0	0	0	0
XM_021062500.1	XRRA1	mRNA	0	0	0	0	0	0	0	0	0	0	0	0	0	0	0	0	0	0	0	0	0
XM_021062501.1	XRRA1	mRNA	0	0	0	0	0	0	0	0	0	0	0	0	0	0	0	0	0	0	0	0	0
XM_021062502.1	XRRA1	mRNA	0	0	0	0	0	0	0	0	0	0	0	0	0	0	0	0	0	0	0	0	0
XM_021062503.1	XRRA1	mRNA	0	0	0	0	0	0	0	0	0	0	0	0	0	0	0	0	0	0	0	0	0
XM_021062511.1	SLCO2B1	mRNA	0	0	0	0	0	0	0	0	0	0	0	0	0	0	0	0	0	0	0	0	0
XM_021062948.1	BCL9L	mRNA	0	0	0	0	0	0	0	0	0	0	0	0	0	0	0	0	0	0	0	0	0
XM_021062949.1	TRAPPC4	mRNA	0	0	0	0	0	0	0	0	0	0	0	0	0	0	0	0	0	0	0	0	0
XM_021062950.1	HYOU1	mRNA	0	0	0	0	0	0	0	0	0	0	0	0	0	0	0	0	0	0	0	0	0
XM_021062951.1	HYOU1	mRNA	0	0	0	0	0	0	0	0	0	0	0	0	0	0	0	0	0	0	0	0	0
XM_021062952.1	HYOU1	mRNA	0	0	0	0	0	0	0	0	0	0	0	0	0	0	0	0	0	0	0	0	0
XM_021062953.1	HYOU1	mRNA	0	0	0	0	0	0	0	0	0	0	0	0	0	0	0	0	0	0	0	0	0
XM_021062954.1	HYOU1	mRNA	0	0	0	0	0	0	0	0	0	0	0	0	0	0	0	0	0	0	0	0	0
XM_021062955.1	HYOU1	mRNA	0	0	0	0	0	0	0	0	0	0	0	0	0	0	0	0	0	0	0	0	0
XM_021062956.1	HYOU1	mRNA	0	0	0	0	0	0	0	0	0	0	0	0	0	0	0	0	0	0	0	0	0
XM_021062957.1	VPS11	mRNA	0	0	0	0	0	0	0	0	0	0	0	0	0	0	0	0	0	0	0	0	0
XM_021062993.1	LOC100523684	mRNA	0	0	0	0	0	0	0	0	0	0	0	0	0	0	0	0	0	0	0	0	0
XM_021062994.1	LOC100523684	mRNA	0	0	0	0	0	0	0	0	0	0	0	0	0	0	0	0	0	0	0	0	0
XM_021062995.1	LOC100523684	mRNA	0	0	0	0	0	0	0	0	0	0	0	0	0	0	0	0	0	0	0	0	0
XM_021062996.1	LOC100523684	mRNA	0	0	0	0	0	0	0	0	0	0	0	0	0	0	0	0	0	0	0	0	0
XM_021062997.1	LOC100523684	mRNA	0	0	0	0	0	0	0	0	0	0	0	0	0	0	0	0	0	0	0	0	0
XM_021062998.1	LOC100523684	mRNA	0	0	0	0	0	0	0	0	0	0	0	0	0	0	0	0	0	0	0	0	0
XM_021062999.1	LOC100523684	mRNA	0	0	0	0	0	0	0	0	0	0	0	0	0	0	0	0	0	0	0	0	0
XM_021063000.1	LOC100523684	mRNA	0	0	0	0	0	0	0	0	0	0	0	0	0	0	0	0	0	0	0	0	0
XM_021063001.1	LOC100523684	mRNA	0	0	0	0	0	0	0	0	0	0	0	0	0	0	0	0	0	0	0	0	0
XM_021063002.1	LOC100523684	mRNA	0	0	0	0	0	0	0	0	0	0	0	0	0	0	0	0	0	0	0	0	0
XM_021063004.1	LOC100523684	mRNA	0	0	0	0	0	0	0	0	0	0	0	0	0	0	0	0	0	0	0	0	0
XM_021063005.1	LOC100523684	mRNA	0	0	0	0	0	0	0	0	0	0	0	0	0	0	0	0	0	0	0	0	0
XM_021063006.1	LOC100523684	mRNA	0	0	0	0	0	0	0	0	0	0	0	0	0	0	0	0	0	0	0	0	0
XM_021063009.1	SORL1	mRNA	0	0	0	0	0	0	0	0	0	0	0	0	0	0	0	0	0	0	0	0	0
XM_021063010.1	SORL1	mRNA	0	0	0	0	0	0	0	0	0	0	0	0	0	0	0	0	0	0	0	0	0
XM_021063011.1	SORL1	mRNA	0	0	0	0	0	0	0	0	0	0	0	0	0	0	0	0	0	0	0	0	0
XM_021063012.1	SORL1	mRNA	0	0	0	0	0	0	0	0	0	0	0	0	0	0	0	0	0	0	0	0	0
XM_021063013.1	SORL1	mRNA	0	0	0	0	0	0	0	0	0	0	0	0	0	0	0	0	0	0	0	0	0
XM_021063014.1	SORL1	mRNA	0	0	0	0	0	0	0	0	0	0	0	0	0	0	0	0	0	0	0	0	0
XM_021063023.1	CLMP	mRNA	0	0	0	0	0	0	0	0	0	0	0	0	0	0	0	0	0	0	0	0	0
XM_021063024.1	CLMP	mRNA	0	0	0	0	0	0	0	0	0	0	0	0	0	0	0	0	0	0	0	0	0

RNA ID	Symbol	Type	updown_BGIR9823_80765	updown_BGIR9823_80869	updown_BGIR9823_81015	updown_BGIR9823_81197	updown_BGIR9823_81968	updown_BGIR9823_82765	updown_BGIR9823_78329	updown_BGIR9823_93571	updown_BGIR9823_93054	updown_BGIR9823_96360	updown_XR_002343213.1	updown_XR_002343346.1	updown_XR_002341920.1	updown_XR_002342486.1	updown_XR_307914.3	updown_XR_002341615.1	updown_XR_002343544.1	updown_XR_002343891.1	updown_BGIR9823_87139	updown_BGIR9823_84771	updown_BGIR9823_98958
XM_021063025.1	CLMP	mRNA	0	0	0	0	0	0	0	0	0	0	0	0	0	0	0	0	0	0	0	0	0
XM_021063026.1	CLMP	mRNA	0	0	0	0	0	0	0	0	0	0	0	0	0	0	0	0	0	0	0	0	0
XM_021063950.1	NXPE2	mRNA	0	0	0	0	0	0	0	0	0	0	0	0	0	0	0	0	0	0	0	0	0
XM_021063963.1	LOC100623190	mRNA	0	0	0	0	0	0	0	0	0	0	0	0	0	0	0	0	0	0	0	0	0
XM_021065159.1	HMGB1	mRNA	0	0	0	0	0	0	0	0	0	0	0	0	0	0	0	0	0	0	0	0	0
XM_021065160.1	HMGB1	mRNA	0	0	0	0	0	0	0	0	0	0	0	0	0	0	0	0	0	0	0	0	0
XM_021065161.1	HMGB1	mRNA	0	0	0	0	0	0	0	0	0	0	0	0	0	0	0	0	0	0	0	0	0
XM_021065529.1	KATNAL1	mRNA	0	0	0	0	0	0	0	0	0	0	0	0	0	0	0	0	0	0	0	0	0
XM_021065531.1	KATNAL1	mRNA	0	0	0	0	0	0	0	0	0	0	0	0	0	0	0	0	0	0	0	0	0
XM_021065532.1	KATNAL1	mRNA	0	0	0	0	0	0	0	0	0	0	0	0	0	0	0	0	0	0	0	0	0
XM_021065533.1	KATNAL1	mRNA	0	0	0	0	0	0	0	0	0	0	0	0	0	0	0	0	0	0	0	0	0
XM_021065534.1	KATNAL1	mRNA	0	0	0	0	0	0	0	0	0	0	0	0	0	0	0	0	0	0	0	0	0
XM_021065535.1	KATNAL1	mRNA	0	0	0	0	0	0	0	0	0	0	0	0	0	0	0	0	0	0	0	0	0
XM_021065536.1	KATNAL1	mRNA	0	0	0	0	0	0	0	0	0	0	0	0	0	0	0	0	0	0	0	0	0
XM_021065537.1	KATNAL1	mRNA	0	0	0	0	0	0	0	0	0	0	0	0	0	0	0	0	0	0	0	0	0
XM_021065538.1	KATNAL1	mRNA	0	0	0	0	0	0	0	0	0	0	0	0	0	0	0	0	0	0	0	0	0
XM_021065539.1	KATNAL1	mRNA	0	0	0	0	0	0	0	0	0	0	0	0	0	0	0	0	0	0	0	0	0
XM_021065541.1	KATNAL1	mRNA	0	0	0	0	0	0	0	0	0	0	0	0	0	0	0	0	0	0	0	0	0
XM_021065542.1	KATNAL1	mRNA	0	0	0	0	0	0	0	0	0	0	0	0	0	0	0	0	0	0	0	0	0
XM_021065543.1	KATNAL1	mRNA	0	0	0	0	0	0	0	0	0	0	0	0	0	0	0	0	0	0	0	0	0
XM_021065544.1	KATNAL1	mRNA	0	0	0	0	0	0	0	0	0	0	0	0	0	0	0	0	0	0	0	0	0
XM_021073408.1	EMX2	mRNA	0	0	0	0	0	0	0	0	0	0	0	0	0	0	0	0	0	0	0	0	0
XM_021078611.1	PRKAG2	mRNA	0	0	0	0	0	0	0	0	0	0	0	0	0	0	0	0	0	0	0	0	0
XM_021078612.1	PRKAG2	mRNA	0	0	0	0	0	0	0	0	0	0	0	0	0	0	0	0	0	0	0	0	0
XM_021078613.1	PRKAG2	mRNA	0	0	0	0	0	0	0	0	0	0	0	0	0	0	0	0	0	0	0	0	0
XM_021078614.1	PRKAG2	mRNA	0	0	0	0	0	0	0	0	0	0	0	0	0	0	0	0	0	0	0	0	0
XM_021078615.1	PRKAG2	mRNA	0	0	0	0	0	0	0	0	0	0	0	0	0	0	0	0	0	0	0	0	0
XM_021078616.1	PRKAG2	mRNA	0	0	0	0	0	0	0	0	0	0	0	0	0	0	0	0	0	0	0	0	0
XM_021078617.1	PRKAG2	mRNA	0	0	0	0	0	0	0	0	0	0	0	0	0	0	0	0	0	0	0	0	0
XM_021078618.1	PRKAG2	mRNA	0	0	0	0	0	0	0	0	0	0	0	0	0	0	0	0	0	0	0	0	0
XM_021078619.1	PRKAG2	mRNA	0	0	0	0	0	0	0	0	0	0	0	0	0	0	0	0	0	0	0	0	0
XM_021078620.1	PRKAG2	mRNA	0	0	0	0	0	0	0	0	0	0	0	0	0	0	0	0	0	0	0	0	0
XM_021078621.1	PRKAG2	mRNA	0	0	0	0	0	0	0	0	0	0	0	0	0	0	0	0	0	0	0	0	0
XM_021081309.1	LOC106506226	mRNA	0	0	0	0	0	0	0	0	0	0	0	0	0	0	0	0	0	0	0	0	0
XM_021081403.1	LOC110258215	mRNA	0	0	0	0	0	0	0	0	0	0	0	0	0	0	0	0	0	0	0	0	0
XM_021082153.1	LOC100521600	mRNA	0	0	0	0	0	0	0	0	0	0	0	0	0	0	0	0	0	0	0	0	0
XM_021082154.1	LOC100521600	mRNA	0	0	0	0	0	0	0	0	0	0	0	0	0	0	0	0	0	0	0	0	0
XM_021082156.1	LOC100521600	mRNA	0	0	0	0	0	0	0	0	0	0	0	0	0	0	0	0	0	0	0	0	0
XM_021082157.1	LOC100521600	mRNA	0	0	0	0	0	0	0	0	0	0	0	0	0	0	0	0	0	0	0	0	0
XM_021082158.1	LOC100521600	mRNA	0	0	0	0	0	0	0	0	0	0	0	0	0	0	0	0	0	0	0	0	0
XM_021082159.1	LOC100521600	mRNA	0	0	0	0	0	0	0	0	0	0	0	0	0	0	0	0	0	0	0	0	0
XM_021082835.1	EHBP1L1	mRNA	0	0	1	0	0	0	0	0	0	0	0	0	0	0	0	0	0	0	0	0	0
XM_021082836.1	EHBP1L1	mRNA	0	0	1	0	0	0	0	0	0	0	0	0	0	0	0	0	0	0	0	0	0

RNA ID	Symbol	Type	updown_BGIR9823_80765	updown_BGIR9823_80869	updown_BGIR9823_81015	updown_BGIR9823_81197	updown_BGIR9823_81968	updown_BGIR9823_82765	updown_BGIR9823_78329	updown_BGIR9823_93571	updown_BGIR9823_93054	updown_BGIR9823_96360	updown_XR_002343213.1	updown_XR_002343346.1	updown_XR_002341920.1	updown_XR_002342486.1	updown_XR_307914.3	updown_XR_002341615.1	updown_XR_002343544.1	updown_XR_002343891.1	updown_BGIR9823_87139	updown_BGIR9823_84771	updown_BGIR9823_98958
XM_021082837.1	EHBP1L1	mRNA	0	0	1	0	0	0	0	0	0	0	0	0	0	0	0	0	0	0	0	0	0
XM_021082838.1	EHBP1L1	mRNA	0	0	1	0	0	0	0	0	0	0	0	0	0	0	0	0	0	0	0	0	0
XM_021082839.1	EHBP1L1	mRNA	0	0	1	0	0	0	0	0	0	0	0	0	0	0	0	0	0	0	0	0	0
XM_021082840.1	EHBP1L1	mRNA	0	0	1	0	0	0	0	0	0	0	0	0	0	0	0	0	0	0	0	0	0
XM_021082841.1	EHBP1L1	mRNA	0	0	1	0	0	0	0	0	0	0	0	0	0	0	0	0	0	0	0	0	0
XM_021082842.1	EHBP1L1	mRNA	0	0	1	0	0	0	0	0	0	0	0	0	0	0	0	0	0	0	0	0	0
XM_021082843.1	EHBP1L1	mRNA	0	0	1	0	0	0	0	0	0	0	0	0	0	0	0	0	0	0	0	0	0
XM_021082844.1	EHBP1L1	mRNA	0	0	1	0	0	0	0	0	0	0	0	0	0	0	0	0	0	0	0	0	0
XM_021082845.1	EHBP1L1	mRNA	0	0	1	0	0	0	0	0	0	0	0	0	0	0	0	0	0	0	0	0	0
XM_021082846.1	EHBP1L1	mRNA	0	0	1	0	0	0	0	0	0	0	0	0	0	0	0	0	0	0	0	0	0
XM_021082847.1	EHBP1L1	mRNA	0	0	1	0	0	0	0	0	0	0	0	0	0	0	0	0	0	0	0	0	0
XM_021082851.1	MAP3K11	mRNA	0	0	1	0	0	0	0	0	0	0	0	0	0	0	0	0	0	0	0	0	0
XM_021082852.1	SSSCA1	mRNA	0	0	1	0	0	0	0	0	0	0	0	0	0	0	0	0	0	0	0	0	0
XM_021082853.1	FAM89B	mRNA	0	0	1	0	0	0	0	0	0	0	0	0	0	0	0	0	0	0	0	0	0
XM_021082854.1	LTBP3	mRNA	0	0	1	0	0	0	0	0	0	0	0	0	0	0	0	0	0	0	0	0	0
XM_021082855.1	LTBP3	mRNA	0	0	1	0	0	0	0	0	0	0	0	0	0	0	0	0	0	0	0	0	0
XM_021082856.1	SCYL1	mRNA	0	0	1	0	0	0	0	0	0	0	0	0	0	0	0	0	0	0	0	0	0
XM_021082857.1	SLC25A45	mRNA	0	0	1	0	0	0	0	0	0	0	0	0	0	0	0	0	0	0	0	0	0
XM_021082858.1	SLC25A45	mRNA	0	0	1	0	0	0	0	0	0	0	0	0	0	0	0	0	0	0	0	0	0
XM_021091099.1	SYNGR1	mRNA	0	0	0	0	0	0	0	0	0	0	0	0	0	0	0	0	0	1	0	0	0
XM_021091101.1	SYNGR1	mRNA	0	0	0	0	0	0	0	0	0	0	0	0	0	0	0	0	0	1	0	0	0
XM_021091102.1	SYNGR1	mRNA	0	0	0	0	0	0	0	0	0	0	0	0	0	0	0	0	0	1	0	0	0
XM_021091103.1	SYNGR1	mRNA	0	0	0	0	0	0	0	0	0	0	0	0	0	0	0	0	0	1	0	0	0
XM_021091370.1	LOC102159820	mRNA	0	0	0	0	0	0	0	0	0	0	0	0	0	0	0	0	0	0	0	0	0
XM_021091373.1	TRMU	mRNA	0	0	0	0	0	0	0	0	0	0	0	0	0	0	0	0	0	0	0	0	0
XM_021091374.1	TRMU	mRNA	0	0	0	0	0	0	0	0	0	0	0	0	0	0	0	0	0	0	0	0	0
XM_021091375.1	TRMU	mRNA	0	0	0	0	0	0	0	0	0	0	0	0	0	0	0	0	0	0	0	0	0
XM_021091376.1	GTSE1	mRNA	0	0	0	0	0	0	0	0	0	0	0	0	0	0	0	0	0	0	0	0	0
XM_021091377.1	TTC38	mRNA	0	0	0	0	0	0	0	0	0	0	0	0	0	0	0	0	0	0	0	0	0
XM_021091379.1	LOC110260611	mRNA	0	0	0	0	0	0	0	0	0	0	0	0	0	0	0	0	0	0	0	0	0
XM_021091381.1	LOC110260611	mRNA	0	0	0	0	0	0	0	0	0	0	0	0	0	0	0	0	0	0	0	0	0
XM_021091454.1	TOB2	mRNA	0	0	0	0	0	0	0	0	0	0	0	0	0	0	0	0	0	0	0	0	0
XM_021091455.1	TOB2	mRNA	0	0	0	0	0	0	0	0	0	0	0	0	0	0	0	0	0	0	0	0	0
XM_021091456.1	PHF5A	mRNA	0	0	0	0	0	0	0	0	0	0	0	0	0	0	0	0	0	0	0	0	0
XM_021091457.1	PHF5A	mRNA	0	0	0	0	0	0	0	0	0	0	0	0	0	0	0	0	0	0	0	0	0
XM_021091458.1	ZC3H7B	mRNA	0	0	0	0	0	0	0	0	0	0	0	0	0	0	0	0	0	0	0	0	0
XM_021091459.1	ZC3H7B	mRNA	0	0	0	0	0	0	0	0	0	0	0	0	0	0	0	0	0	0	0	0	0
XM_021091460.1	TEF	mRNA	0	0	0	0	0	0	0	0	0	0	0	0	0	0	0	0	0	0	0	0	0
XM_021091461.1	RANGAP1	mRNA	0	0	0	0	0	0	0	0	0	0	0	0	0	0	0	0	0	0	0	0	0
XM_021091474.1	CBX7	mRNA	0	0	0	0	0	0	0	0	0	0	0	0	0	0	0	0	0	1	0	0	0
XM_021092767.1	CRADD	mRNA	0	0	0	0	0	0	0	0	0	0	0	0	0	0	0	0	0	0	0	0	0
XM_021092768.1	CRADD	mRNA	0	0	0	0	0	0	0	0	0	0	0	0	0	0	0	0	0	0	0	0	0
XM_021092769.1	CRADD	mRNA	0	0	0	0	0	0	0	0	0	0	0	0	0	0	0	0	0	0	0	0	0

RNA ID	Symbol	Type	updown_BGIR9823_80765	updown_BGIR9823_80869	updown_BGIR9823_81015	updown_BGIR9823_81197	updown_BGIR9823_81968	updown_BGIR9823_82765	updown_BGIR9823_78329	updown_BGIR9823_93571	updown_BGIR9823_93054	updown_BGIR9823_96360	updown_XR_002343213.1	updown_XR_002343346.1	updown_XR_002341920.1	updown_XR_002342486.1	updown_XR_307914.3	updown_XR_002341615.1	updown_XR_002343544.1	updown_XR_002343891.1	updown_BGIR9823_87139	updown_BGIR9823_84771	updown_BGIR9823_98958
XM_021092770.1	CRADD	mRNA	0	0	0	0	0	0	0	0	0	0	0	0	0	0	0	0	0	0	0	0	0
XM_021092771.1	CRADD	mRNA	0	0	0	0	0	0	0	0	0	0	0	0	0	0	0	0	0	0	0	0	0
XM_021092773.1	CRADD	mRNA	0	0	0	0	0	0	0	0	0	0	0	0	0	0	0	0	0	0	0	0	0
XM_021093095.1	PKDREJ	mRNA	0	0	0	0	0	0	0	0	0	0	0	0	0	0	0	0	0	0	0	0	0
XM_021097064.1	MAF	mRNA	0	0	0	0	0	0	0	0	0	0	0	0	0	0	0	0	0	0	1	0	0
XM_021097065.1	MAF	mRNA	0	0	0	0	0	0	0	0	0	0	0	0	0	0	0	0	0	0	1	0	0
XM_021097066.1	MAF	mRNA	0	0	0	0	0	0	0	0	0	0	0	0	0	0	0	0	0	0	1	0	0
XM_021097870.1	ECI2	mRNA	0	0	0	0	0	0	0	0	0	0	0	0	0	0	0	0	0	0	0	0	0
XM_021099751.1	PHACTR1	mRNA	0	0	0	0	0	0	0	0	0	0	0	0	0	0	0	0	0	0	0	0	0
XM_021099752.1	PHACTR1	mRNA	0	0	0	0	0	0	0	0	0	0	0	0	0	0	0	0	0	0	0	0	0
XM_021099753.1	PHACTR1	mRNA	0	0	0	0	0	0	0	0	0	0	0	0	0	0	0	0	0	0	0	0	0
XM_021099754.1	PHACTR1	mRNA	0	0	0	0	0	0	0	0	0	0	0	0	0	0	0	0	0	0	0	0	0
XM_021099755.1	PHACTR1	mRNA	0	0	0	0	0	0	0	0	0	0	0	0	0	0	0	0	0	0	0	0	0
XM_021099756.1	PHACTR1	mRNA	0	0	0	0	0	0	0	0	0	0	0	0	0	0	0	0	0	0	0	0	0
XM_021099757.1	PHACTR1	mRNA	0	0	0	0	0	0	0	0	0	0	0	0	0	0	0	0	0	0	0	0	0
XM_021099758.1	PHACTR1	mRNA	0	0	0	0	0	0	0	0	0	0	0	0	0	0	0	0	0	0	0	0	0
XM_021099759.1	PHACTR1	mRNA	0	0	0	0	0	0	0	0	0	0	0	0	0	0	0	0	0	0	0	0	0
XM_021099760.1	PHACTR1	mRNA	0	0	0	0	0	0	0	0	0	0	0	0	0	0	0	0	0	0	0	0	0
XM_021099761.1	PHACTR1	mRNA	0	0	0	0	0	0	0	0	0	0	0	0	0	0	0	0	0	0	0	0	0
XM_021099762.1	PHACTR1	mRNA	0	0	0	0	0	0	0	0	0	0	0	0	0	0	0	0	0	0	0	0	0
XM_021099763.1	PHACTR1	mRNA	0	0	0	0	0	0	0	0	0	0	0	0	0	0	0	0	0	0	0	0	0
XM_021099764.1	PHACTR1	mRNA	0	0	0	0	0	0	0	0	0	0	0	0	0	0	0	0	0	0	0	0	0
XM_021099765.1	PHACTR1	mRNA	0	0	0	0	0	0	0	0	0	0	0	0	0	0	0	0	0	0	0	0	0
XM_021099766.1	TBC1D7	mRNA	0	0	0	0	0	0	0	0	0	0	0	0	0	0	0	0	0	0	0	0	0
XM_021099767.1	TBC1D7	mRNA	0	0	0	0	0	0	0	0	0	0	0	0	0	0	0	0	0	0	0	0	0
XM_021099769.1	TBC1D7	mRNA	0	0	0	0	0	0	0	0	0	0	0	0	0	0	0	0	0	0	0	0	0
XM_021099770.1	TBC1D7	mRNA	0	0	0	0	0	0	0	0	0	0	0	0	0	0	0	0	0	0	0	0	0
XM_021099999.1	LOC110261697	mRNA	0	0	0	0	0	0	0	0	0	0	0	0	0	0	0	0	0	0	0	0	0
XM_021100108.1	WRNIP1	mRNA	0	0	0	0	0	0	0	0	0	0	0	0	0	0	0	0	0	0	0	0	0
XM_021100109.1	MYLK4	mRNA	0	0	0	0	0	0	0	0	0	0	0	0	0	0	0	0	0	0	0	0	0
XM_021100110.1	MYLK4	mRNA	0	0	0	0	0	0	0	0	0	0	0	0	0	0	0	0	0	0	0	0	0
XM_021100111.1	MYLK4	mRNA	0	0	0	0	0	0	0	0	0	0	0	0	0	0	0	0	0	0	0	0	0
XM_021100112.1	MYLK4	mRNA	0	0	0	0	0	0	0	0	0	0	0	0	0	0	0	0	0	0	0	0	0
XM_021100114.1	NQO2	mRNA	0	0	0	0	0	0	0	0	0	0	0	0	0	0	0	0	0	0	0	0	0
XM_021100115.1	NQO2	mRNA	0	0	0	0	0	0	0	0	0	0	0	0	0	0	0	0	0	0	0	0	0
XM_021100117.1	NQO2	mRNA	0	0	0	0	0	0	0	0	0	0	0	0	0	0	0	0	0	0	0	0	0
XM_021100118.1	NQO2	mRNA	0	0	0	0	0	0	0	0	0	0	0	0	0	0	0	0	0	0	0	0	0
XM_021100119.1	NQO2	mRNA	0	0	0	0	0	0	0	0	0	0	0	0	0	0	0	0	0	0	0	0	0
XM_021100124.1	PRPF4B	mRNA	0	0	0	0	0	0	0	0	0	0	0	0	0	0	0	0	0	0	0	0	0
XM_021100126.1	PRPF4B	mRNA	0	0	0	0	0	0	0	0	0	0	0	0	0	0	0	0	0	0	0	0	0
XM_021100184.1	NEDD9	mRNA	0	0	0	0	0	0	0	0	0	0	0	0	0	0	0	0	0	0	0	0	0
XM_021100188.1	TMEM170B	mRNA	0	0	0	0	0	0	0	0	0	0	0	0	0	0	0	0	0	0	0	0	0
XM_021100194.1	GFOD1	mRNA	0	0	0	0	0	0	0	0	0	0	0	0	0	0	0	0	0	0	0	0	0

RNA ID	Symbol	Type	updown_BGIR9823_80765	updown_BGIR9823_80869	updown_BGIR9823_81015	updown_BGIR9823_81197	updown_BGIR9823_81968	updown_BGIR9823_82765	updown_BGIR9823_78329	updown_BGIR9823_93571	updown_BGIR9823_93054	updown_BGIR9823_96360	updown_XR_002343213.1	updown_XR_002343346.1	updown_XR_002341920.1	updown_XR_002342486.1	updown_XR_307914.3	updown_XR_002341615.1	updown_XR_002343544.1	updown_XR_002343891.1	updown_BGIR9823_87139	updown_BGIR9823_84771	updown_BGIR9823_98958
XM_021102040.1	SLC37A4	mRNA	0	0	0	0	0	0	0	0	0	0	0	0	0	0	0	0	0	0	0	0	0
XM_021102041.1	SLC37A4	mRNA	0	0	0	0	0	0	0	0	0	0	0	0	0	0	0	0	0	0	0	0	0
XM_021102042.1	SLC37A4	mRNA	0	0	0	0	0	0	0	0	0	0	0	0	0	0	0	0	0	0	0	0	0
XM_021102043.1	SLC37A4	mRNA	0	0	0	0	0	0	0	0	0	0	0	0	0	0	0	0	0	0	0	0	0
XM_021102044.1	SLC37A4	mRNA	0	0	0	0	0	0	0	0	0	0	0	0	0	0	0	0	0	0	0	0	0
XM_021102045.1	SLC37A4	mRNA	0	0	0	0	0	0	0	0	0	0	0	0	0	0	0	0	0	0	0	0	0
XM_021102046.1	SLC37A4	mRNA	0	0	0	0	0	0	0	0	0	0	0	0	0	0	0	0	0	0	0	0	0
XM_021102047.1	SLC37A4	mRNA	0	0	0	0	0	0	0	0	0	0	0	0	0	0	0	0	0	0	0	0	0
XM_021102048.1	SLC37A4	mRNA	0	0	0	0	0	0	0	0	0	0	0	0	0	0	0	0	0	0	0	0	0
XM_021102049.1	SLC37A4	mRNA	0	0	0	0	0	0	0	0	0	0	0	0	0	0	0	0	0	0	0	0	0
XM_021102050.1	SLC37A4	mRNA	0	0	0	0	0	0	0	0	0	0	0	0	0	0	0	0	0	0	0	0	0
XM_021102051.1	SLC37A4	mRNA	0	0	0	0	0	0	0	0	0	0	0	0	0	0	0	0	0	0	0	0	0
XM_021102052.1	SLC37A4	mRNA	0	0	0	0	0	0	0	0	0	0	0	0	0	0	0	0	0	0	0	0	0
XM_021102209.1	UPK2	mRNA	0	0	0	0	0	0	0	0	0	0	0	0	0	0	0	0	0	0	0	0	0
XM_021102516.1	LOC100522455	mRNA	0	0	0	0	0	0	0	0	0	0	0	0	0	0	0	0	0	0	0	0	0
XM_021102517.1	RUNDC3B	mRNA	0	0	0	0	0	0	0	0	0	0	0	0	0	0	0	0	0	0	0	0	0
XM_021102518.1	RUNDC3B	mRNA	0	0	0	0	0	0	0	0	0	0	0	0	0	0	0	0	0	0	0	0	0
XM_021102519.1	RUNDC3B	mRNA	0	0	0	0	0	0	0	0	0	0	0	0	0	0	0	0	0	0	0	0	0
XM_021102522.1	RUNDC3B	mRNA	0	0	0	0	0	0	0	0	0	0	0	0	0	0	0	0	0	0	0	0	0
XM_021102523.1	RUNDC3B	mRNA	0	0	0	0	0	0	0	0	0	0	0	0	0	0	0	0	0	0	0	0	0
XR_001297664.2	LOC102161744	lncRNA	0	0	0	0	0	0	0	0	0	0	0	0	0	0	0	0	0	0	0	0	0
XR_001298807.2	LOC106504881	lncRNA	0	0	0	0	0	0	0	0	0	0	0	0	0	0	0	0	0	0	0	0	0
XR_001298808.2	LOC106504881	lncRNA	0	0	0	0	0	0	0	0	0	0	0	0	0	0	0	0	0	0	0	0	0
XR_001298810.2	LOC106504881	lncRNA	0	0	0	0	0	0	0	0	0	0	0	0	0	0	0	0	0	0	0	0	0
XR_001298811.2	LOC106504881	lncRNA	0	0	0	0	0	0	0	0	0	0	0	0	0	0	0	0	0	0	0	0	0
XR_001298812.2	LOC106504881	lncRNA	0	0	0	0	0	0	0	0	0	0	0	0	0	0	0	0	0	0	0	0	0
XR_001298813.2	LOC106504881	lncRNA	0	0	0	0	0	0	0	0	0	0	0	0	0	0	0	0	0	0	0	0	0
XR_001298814.2	LOC106504881	lncRNA	0	0	0	0	0	0	0	0	0	0	0	0	0	0	0	0	0	0	0	0	0
XR_001298816.2	LOC106504881	lncRNA	0	0	0	0	0	0	0	0	0	0	0	0	0	0	0	0	0	0	0	0	0
XR_001298817.2	LOC106504881	lncRNA	0	0	0	0	0	0	0	0	0	0	0	0	0	0	0	0	0	0	0	0	0
XR_001298844.2	LOC102163364	lncRNA	0	0	0	0	0	0	0	0	0	0	0	0	0	0	0	0	0	0	0	0	0
XR_001298846.2	LOC102163364	lncRNA	0	0	0	0	0	0	0	0	0	0	0	0	0	0	0	0	0	0	0	0	0
XR_001298850.2	LOC102163364	lncRNA	0	0	0	0	0	0	0	0	0	0	0	0	0	0	0	0	0	0	0	0	0
XR_001299456.2	LOC102166670	lncRNA	0	0	0	0	0	0	0	0	0	0	0	0	0	0	0	0	0	0	0	0	0
XR_001299457.2	LOC102166670	lncRNA	0	0	0	0	0	0	0	0	0	0	0	0	0	0	0	0	0	0	0	0	0
XR_001300715.2	LOC106505879	lncRNA	0	0	0	0	0	0	0	0	0	0	0	0	0	0	0	0	0	0	0	0	0
XR_001301078.2	LOC102162623	lncRNA	0	0	0	0	0	0	0	0	0	0	0	0	0	0	0	0	0	0	0	0	0
XR_001301087.2	LOC102162623	lncRNA	0	0	0	0	0	0	0	0	0	0	0	0	0	0	0	0	0	0	0	0	0
XR_001301217.2	LOC102164998	lncRNA	0	0	0	0	0	0	0	0	0	0	0	0	0	0	0	0	0	0	0	0	0
XR_001301305.2	LOC102159607	lncRNA	0	0	0	0	0	0	0	0	0	0	0	0	0	0	1	0	0	0	0	0	0
XR_001308967.2	LOC106510467	lncRNA	0	0	0	0	0	0	0	0	0	0	0	0	0	0	0	0	0	0	1	0	0
XR_002335533.1	XRRA1	mRNA	0	0	0	0	0	0	0	0	0	0	0	0	0	0	0	0	0	0	0	0	0
XR_002335534.1	XRRA1	mRNA	0	0	0	0	0	0	0	0	0	0	0	0	0	0	0	0	0	0	0	0	0

RNA ID	Symbol	Type	updown_BGIR9823_80765	updown_BGIR9823_80869	updown_BGIR9823_81015	updown_BGIR9823_81197	updown_BGIR9823_81968	updown_BGIR9823_82765	updown_BGIR9823_78329	updown_BGIR9823_93571	updown_BGIR9823_93054	updown_BGIR9823_96360	updown_XR_002343213.1	updown_XR_002343346.1	updown_XR_002341920.1	updown_XR_002342486.1	updown_XR_307914.3	updown_XR_002341615.1	updown_XR_002343544.1	updown_XR_002343891.1	updown_BGIR9823_87139	updown_BGIR9823_84771	updown_BGIR9823_98958
XR_002335535.1	LOC110255399	lncRNA	0	0	0	0	0	0	0	0	0	0	0	0	0	0	0	0	0	0	0	0	0
XR_002335536.1	LOC102160367	lncRNA	0	0	0	0	0	0	0	0	0	0	0	0	0	0	0	0	0	0	0	0	0
XR_002335537.1	LOC102160367	lncRNA	0	0	0	0	0	0	0	0	0	0	0	0	0	0	0	0	0	0	0	0	0
XR_002335650.1	LOC102157630	lncRNA	0	0	0	0	0	0	0	0	0	0	0	0	0	0	0	0	0	0	0	0	0
XR_002335651.1	LOC110255435	lncRNA	0	0	0	0	0	0	0	0	0	0	0	0	0	0	0	0	0	0	0	0	0
XR_002335652.1	LOC106504881	lncRNA	0	0	0	0	0	0	0	0	0	0	0	0	0	0	0	0	0	0	0	0	0
XR_002335653.1	LOC106504881	lncRNA	0	0	0	0	0	0	0	0	0	0	0	0	0	0	0	0	0	0	0	0	0
XR_002335670.1	LOC110255440	lncRNA	0	0	0	0	0	0	0	0	0	0	0	0	0	0	0	0	0	0	0	0	0
XR_002335671.1	LOC110255441	lncRNA	0	0	0	0	0	0	0	0	0	0	0	0	0	0	0	0	0	0	0	0	0
XR_002335672.1	LOC110255441	lncRNA	0	0	0	0	0	0	0	0	0	0	0	0	0	0	0	0	0	0	0	0	0
XR_002335674.1	HYOU1	mRNA	0	0	0	0	0	0	0	0	0	0	0	0	0	0	0	0	0	0	0	0	0
XR_002335675.1	HYOU1	mRNA	0	0	0	0	0	0	0	0	0	0	0	0	0	0	0	0	0	0	0	0	0
XR_002335685.1	LOC100523684	mRNA	0	0	0	0	0	0	0	0	0	0	0	0	0	0	0	0	0	0	0	0	0
XR_002335686.1	LOC102163364	lncRNA	0	0	0	0	0	0	0	0	0	0	0	0	0	0	0	0	0	0	0	0	0
XR_002335687.1	LOC102163364	lncRNA	0	0	0	0	0	0	0	0	0	0	0	0	0	0	0	0	0	0	0	0	0
XR_002335688.1	LOC102163364	lncRNA	0	0	0	0	0	0	0	0	0	0	0	0	0	0	0	0	0	0	0	0	0
XR_002335689.1	LOC102163364	lncRNA	0	0	0	0	0	0	0	0	0	0	0	0	0	0	0	0	0	0	0	0	0
XR_002335690.1	LOC102163364	lncRNA	0	0	0	0	0	0	0	0	0	0	0	0	0	0	0	0	0	0	0	0	0
XR_002335691.1	LOC102163364	lncRNA	0	0	0	0	0	0	0	0	0	0	0	0	0	0	0	0	0	0	0	0	0
XR_002335692.1	LOC102163364	lncRNA	0	0	0	0	0	0	0	0	0	0	0	0	0	0	0	0	0	0	0	0	0
XR_002335693.1	LOC106504896	lncRNA	0	0	0	0	0	0	0	0	0	0	0	0	0	0	0	0	0	0	0	0	0
XR_002335694.1	SORL1	mRNA	0	0	0	0	0	0	0	0	0	0	0	0	0	0	0	0	0	0	0	0	0
XR_002336641.1	LOC106505250	lncRNA	0	0	0	0	0	0	0	0	0	0	0	0	0	0	0	0	0	0	0	0	0
XR_002336643.1	LOC110255292	lncRNA	0	0	0	0	0	0	0	0	0	0	0	0	0	0	0	0	0	0	0	0	0
XR_002336644.1	LOC102166670	lncRNA	0	0	0	0	0	0	0	0	0	0	0	0	0	0	0	0	0	0	0	0	0
XR_002336645.1	LOC102166670	lncRNA	0	0	0	0	0	0	0	0	0	0	0	0	0	0	0	0	0	0	0	0	0
XR_002338443.1	LOC106505879	lncRNA	0	0	0	0	0	0	0	0	0	0	0	0	0	0	0	0	0	0	0	0	0
XR_002338444.1	LOC106505879	lncRNA	0	0	0	0	0	0	0	0	0	0	0	0	0	0	0	0	0	0	0	0	0
XR_002338445.1	LOC106505879	lncRNA	0	0	0	0	0	0	0	0	0	0	0	0	0	0	0	0	0	0	0	0	0
XR_002338446.1	LOC106505879	lncRNA	0	0	0	0	0	0	0	0	0	0	0	0	0	0	0	0	0	0	0	0	0
XR_002338447.1	LOC106505879	lncRNA	0	0	0	0	0	0	0	0	0	0	0	0	0	0	0	0	0	0	0	0	0
XR_002338448.1	LOC106505879	lncRNA	0	0	0	0	0	0	0	0	0	0	0	0	0	0	0	0	0	0	0	0	0
XR_002338449.1	LOC106505879	lncRNA	0	0	0	0	0	0	0	0	0	0	0	0	0	0	0	0	0	0	0	0	0
XR_002338450.1	LOC106505879	lncRNA	0	0	0	0	0	0	0	0	0	0	0	0	0	0	0	0	0	0	0	0	0
XR_002338521.1	LOC110256664	lncRNA	0	0	0	0	0	0	0	0	0	0	0	0	0	0	0	0	0	0	0	0	0
XR_002338522.1	LOC110256664	lncRNA	0	0	0	0	0	0	0	0	0	0	0	0	0	0	0	0	0	0	0	0	0
XR_002338666.1	LOC110256747	lncRNA	0	0	0	0	0	0	0	0	0	0	0	0	0	0	0	0	0	0	0	0	0
XR_002338714.1	LOC102163817	lncRNA	0	0	0	0	0	0	0	0	0	0	0	0	0	0	0	0	0	0	0	0	0
XR_002338715.1	LOC102164998	lncRNA	0	0	0	0	0	0	0	0	0	0	0	0	0	0	0	0	0	0	0	0	0
XR_002338716.1	LOC102164998	lncRNA	0	0	0	0	0	0	0	0	0	0	0	0	0	0	0	0	0	0	0	0	0
XR_002338717.1	LOC110256768	lncRNA	0	0	0	0	0	0	0	0	0	0	0	0	0	0	0	0	0	0	0	0	0
XR_002338727.1	LOC110256773	lncRNA	0	0	0	0	0	0	0	0	0	0	0	0	0	0	0	0	0	0	0	0	0
XR_002338728.1	LOC110256773	lncRNA	0	0	0	0	0	0	0	0	0	0	0	0	0	0	0	0	0	0	0	0	0

RNA ID	Symbol	Type	updown_BGIR9823_80765	updown_BGIR9823_80869	updown_BGIR9823_81015	updown_BGIR9823_81197	updown_BGIR9823_81968	updown_BGIR9823_82765	updown_BGIR9823_78329	updown_BGIR9823_93571	updown_BGIR9823_93054	updown_BGIR9823_96360	updown_XR_002343213.1	updown_XR_002343346.1	updown_XR_002341920.1	updown_XR_002342486.1	updown_XR_307914.3	updown_XR_002341615.1	updown_XR_002343544.1	updown_XR_002343891.1	updown_BGIR9823_87139	updown_BGIR9823_84771	updown_BGIR9823_98958
XR_002338729.1	LOC110256773	lncRNA	0	0	0	0	0	0	0	0	0	0	0	0	0	0	0	0	0	0	0	0	0
XR_002338793.1	LOC110256796	lncRNA	0	0	0	0	0	0	0	0	0	0	0	0	0	0	0	0	0	0	0	0	0
XR_002339623.1	LOC102167708	lncRNA	0	0	0	0	0	0	0	0	0	0	0	0	0	0	0	0	0	0	0	0	0
XR_002340245.1	PRKAG2	mRNA	0	0	0	0	0	0	0	0	0	0	0	0	0	0	0	0	0	0	0	0	0
XR_002340246.1	LOC102162623	lncRNA	0	0	0	0	0	0	0	0	0	0	0	0	0	0	0	0	0	0	0	0	0
XR_002340247.1	LOC102162623	lncRNA	0	0	0	0	0	0	0	0	0	0	0	0	0	0	0	0	0	0	0	0	0
XR_002340248.1	LOC110257516	lncRNA	0	0	0	0	0	0	0	0	0	0	0	0	0	0	0	0	0	0	0	0	0
XR_002340249.1	LOC102162071	lncRNA	0	0	0	0	0	0	0	0	0	0	0	0	0	0	0	0	0	0	0	0	0
XR_002340901.1	LOC102160243	lncRNA	0	0	0	0	0	0	0	0	0	0	0	0	0	0	0	0	0	0	0	0	0
XR_002340902.1	LOC102160243	lncRNA	0	0	0	0	0	0	0	0	0	0	0	0	0	0	0	0	0	0	0	0	0
XR_002340903.1	LOC102160243	lncRNA	0	0	0	0	0	0	0	0	0	0	0	0	0	0	0	0	0	0	0	0	0
XR_002340904.1	LOC102160243	lncRNA	0	0	0	0	0	0	0	0	0	0	0	0	0	0	0	0	0	0	0	0	0
XR_002340905.1	LOC102160243	lncRNA	0	0	0	0	0	0	0	0	0	0	0	0	0	0	0	0	0	0	0	0	0
XR_002341237.1	LOC102164320	lncRNA	0	0	0	0	0	0	0	0	0	0	0	0	0	0	0	0	0	0	0	0	0
XR_002341238.1	LOC102164320	lncRNA	0	0	0	0	0	0	0	0	0	0	0	0	0	0	0	0	0	0	0	0	0
XR_002341239.1	LOC102164320	lncRNA	0	0	0	0	0	0	0	0	0	0	0	0	0	0	0	0	0	0	0	0	0
XR_002341240.1	LOC102164320	lncRNA	0	0	0	0	0	0	0	0	0	0	0	0	0	0	0	0	0	0	0	0	0
XR_002341241.1	LOC102164320	lncRNA	0	0	0	0	0	0	0	0	0	0	0	0	0	0	0	0	0	0	0	0	0
XR_002341242.1	LOC102164320	lncRNA	0	0	0	0	0	0	0	0	0	0	0	0	0	0	0	0	0	0	0	0	0
XR_002341243.1	LOC102164320	lncRNA	0	0	0	0	0	0	0	0	0	0	0	0	0	0	0	0	0	0	0	0	0
XR_002341244.1	LOC102164320	lncRNA	0	0	0	0	0	0	0	0	0	0	0	0	0	0	0	0	0	0	0	0	0
XR_002341270.1	LOC100521600	mRNA	0	0	0	0	0	0	0	0	0	0	0	0	0	0	0	0	0	0	0	0	0
XR_002341443.1	LOC102163816	lncRNA	0	0	1	0	0	0	0	0	0	0	0	0	0	0	0	0	0	0	0	0	0
XR_002343847.1	LOC110260611	mRNA	0	0	0	0	0	0	0	0	0	0	0	0	0	0	0	0	0	0	0	0	0
XR_002343848.1	LOC110260611	mRNA	0	0	0	0	0	0	0	0	0	0	0	0	0	0	0	0	0	0	0	0	0
XR_002343849.1	LOC110260611	mRNA	0	0	0	0	0	0	0	0	0	0	0	0	0	0	0	0	0	0	0	0	0
XR_002343850.1	LOC110260611	mRNA	0	0	0	0	0	0	0	0	0	0	0	0	0	0	0	0	0	0	0	0	0
XR_002343875.1	LOC110260629	lncRNA	0	0	0	0	0	0	0	0	0	0	0	0	0	0	0	0	0	0	0	0	0
XR_002343876.1	LOC110260629	lncRNA	0	0	0	0	0	0	0	0	0	0	0	0	0	0	0	0	0	0	0	0	0
XR_002343877.1	LOC110260629	lncRNA	0	0	0	0	0	0	0	0	0	0	0	0	0	0	0	0	0	0	0	0	0
XR_002343878.1	LOC110260629	lncRNA	0	0	0	0	0	0	0	0	0	0	0	0	0	0	0	0	0	0	0	0	0
XR_002343879.1	LOC110260629	lncRNA	0	0	0	0	0	0	0	0	0	0	0	0	0	0	0	0	0	0	0	0	0
XR_002343880.1	LOC110260629	lncRNA	0	0	0	0	0	0	0	0	0	0	0	0	0	0	0	0	0	0	0	0	0
XR_002343881.1	LOC110260629	lncRNA	0	0	0	0	0	0	0	0	0	0	0	0	0	0	0	0	0	0	0	0	0
XR_002343883.1	LOC110260631	lncRNA	0	0	0	0	0	0	0	0	0	0	0	0	0	0	0	0	0	0	0	0	0
XR_002343884.1	LOC110260634	lncRNA	0	0	0	0	0	0	0	0	0	0	0	0	0	0	0	0	0	0	0	0	0
XR_002343885.1	LOC110260635	lncRNA	0	0	0	0	0	0	0	0	0	0	0	0	0	0	0	0	0	0	0	0	0
XR_002343891.1	LOC110260642	lncRNA	0	0	0	0	0	0	0	0	0	0	0	0	0	0	0	0	0	1	0	0	0
XR_002343892.1	LOC110260643	lncRNA	0	0	0	0	0	0	0	0	0	0	0	0	0	0	0	0	0	1	0	0	0
XR_002345298.1	MAF	mRNA	0	0	0	0	0	0	0	0	0	0	0	0	0	0	0	0	0	0	1	0	0
XR_002345300.1	LOC106510470	lncRNA	0	0	0	0	0	0	0	0	0	0	0	0	0	0	0	0	0	0	1	0	0
XR_002345301.1	LOC106510468	lncRNA	0	0	0	0	0	0	0	0	0	0	0	0	0	0	0	0	0	0	1	0	0
XR_002346166.1	LOC110261703	lncRNA	0	0	0	0	0	0	0	0	0	0	0	0	0	0	0	0	0	0	0	0	0

RNA ID	Symbol	Type	updown_BGIR9823_80765	updown_BGIR9823_80869	updown_BGIR9823_81015	updown_BGIR9823_81197	updown_BGIR9823_81968	updown_BGIR9823_82765	updown_BGIR9823_78329	updown_BGIR9823_93571	updown_BGIR9823_93054	updown_BGIR9823_96360	updown_XR_002343213.1	updown_XR_002343346.1	updown_XR_002341920.1	updown_XR_002342486.1	updown_XR_307914.3	updown_XR_002341615.1	updown_XR_002343544.1	updown_XR_002343891.1	updown_BGIR9823_87139	updown_BGIR9823_84771	updown_BGIR9823_98958
XR_002346227.1	LOC106504299	lncRNA	0	0	0	0	0	0	0	0	0	0	0	0	0	0	0	0	0	0	0	0	0
XR_002346228.1	LOC106504299	lncRNA	0	0	0	0	0	0	0	0	0	0	0	0	0	0	0	0	0	0	0	0	0
XR_002346229.1	LOC106504299	lncRNA	0	0	0	0	0	0	0	0	0	0	0	0	0	0	0	0	0	0	0	0	0
XR_002346230.1	LOC106504299	lncRNA	0	0	0	0	0	0	0	0	0	0	0	0	0	0	0	0	0	0	0	0	0
XR_002346231.1	LOC106504299	lncRNA	0	0	0	0	0	0	0	0	0	0	0	0	0	0	0	0	0	0	0	0	0
XR_002346232.1	LOC106504299	lncRNA	0	0	0	0	0	0	0	0	0	0	0	0	0	0	0	0	0	0	0	0	0
XR_002346233.1	NQO2	mRNA	0	0	0	0	0	0	0	0	0	0	0	0	0	0	0	0	0	0	0	0	0
XR_002346236.1	PRPF4B	mRNA	0	0	0	0	0	0	0	0	0	0	0	0	0	0	0	0	0	0	0	0	0
XR_002346237.1	PRPF4B	mRNA	0	0	0	0	0	0	0	0	0	0	0	0	0	0	0	0	0	0	0	0	0
XR_002346238.1	PRPF4B	mRNA	0	0	0	0	0	0	0	0	0	0	0	0	0	0	0	0	0	0	0	0	0
XR_002346239.1	PRPF4B	mRNA	0	0	0	0	0	0	0	0	0	0	0	0	0	0	0	0	0	0	0	0	0
XR_002346240.1	PRPF4B	mRNA	0	0	0	0	0	0	0	0	0	0	0	0	0	0	0	0	0	0	0	0	0
XR_002346241.1	PRPF4B	mRNA	0	0	0	0	0	0	0	0	0	0	0	0	0	0	0	0	0	0	0	0	0
XR_002346242.1	PRPF4B	mRNA	0	0	0	0	0	0	0	0	0	0	0	0	0	0	0	0	0	0	0	0	0
XR_002346243.1	PRPF4B	mRNA	0	0	0	0	0	0	0	0	0	0	0	0	0	0	0	0	0	0	0	0	0
XR_002346244.1	PRPF4B	mRNA	0	0	0	0	0	0	0	0	0	0	0	0	0	0	0	0	0	0	0	0	0
XR_002346245.1	PRPF4B	mRNA	0	0	0	0	0	0	0	0	0	0	0	0	0	0	0	0	0	0	0	0	0
XR_002346246.1	PRPF4B	mRNA	0	0	0	0	0	0	0	0	0	0	0	0	0	0	0	0	0	0	0	0	0
XR_002346247.1	PRPF4B	mRNA	0	0	0	0	0	0	0	0	0	0	0	0	0	0	0	0	0	0	0	0	0
XR_002346248.1	PRPF4B	mRNA	0	0	0	0	0	0	0	0	0	0	0	0	0	0	0	0	0	0	0	0	0
XR_002346249.1	PRPF4B	mRNA	0	0	0	0	0	0	0	0	0	0	0	0	0	0	0	0	0	0	0	0	0
XR_002346250.1	PRPF4B	mRNA	0	0	0	0	0	0	0	0	0	0	0	0	0	0	0	0	0	0	0	0	0
XR_002346251.1	PRPF4B	mRNA	0	0	0	0	0	0	0	0	0	0	0	0	0	0	0	0	0	0	0	0	0
XR_002346252.1	PRPF4B	mRNA	0	0	0	0	0	0	0	0	0	0	0	0	0	0	0	0	0	0	0	0	0
XR_002346253.1	PRPF4B	mRNA	0	0	0	0	0	0	0	0	0	0	0	0	0	0	0	0	0	0	0	0	0
XR_002346254.1	PRPF4B	mRNA	0	0	0	0	0	0	0	0	0	0	0	0	0	0	0	0	0	0	0	0	0
XR_002346255.1	PRPF4B	mRNA	0	0	0	0	0	0	0	0	0	0	0	0	0	0	0	0	0	0	0	0	0
XR_002346256.1	PRPF4B	mRNA	0	0	0	0	0	0	0	0	0	0	0	0	0	0	0	0	0	0	0	0	0
XR_002346257.1	PRPF4B	mRNA	0	0	0	0	0	0	0	0	0	0	0	0	0	0	0	0	0	0	0	0	0
XR_002346258.1	PRPF4B	mRNA	0	0	0	0	0	0	0	0	0	0	0	0	0	0	0	0	0	0	0	0	0
XR_002346259.1	PRPF4B	mRNA	0	0	0	0	0	0	0	0	0	0	0	0	0	0	0	0	0	0	0	0	0
XR_002346260.1	PRPF4B	mRNA	0	0	0	0	0	0	0	0	0	0	0	0	0	0	0	0	0	0	0	0	0
XR_002346261.1	PRPF4B	mRNA	0	0	0	0	0	0	0	0	0	0	0	0	0	0	0	0	0	0	0	0	0
XR_002346262.1	PRPF4B	mRNA	0	0	0	0	0	0	0	0	0	0	0	0	0	0	0	0	0	0	0	0	0
XR_002346263.1	PRPF4B	mRNA	0	0	0	0	0	0	0	0	0	0	0	0	0	0	0	0	0	0	0	0	0
XR_002346264.1	PRPF4B	mRNA	0	0	0	0	0	0	0	0	0	0	0	0	0	0	0	0	0	0	0	0	0
XR_002346265.1	LOC110261764	lncRNA	0	0	0	0	0	0	0	0	0	0	0	0	0	0	0	0	0	0	0	0	0
XR_002346266.1	LOC102162837	lncRNA	0	0	0	0	0	0	0	0	0	0	0	0	0	0	0	0	0	0	0	0	0
XR_002346299.1	LOC110261774	lncRNA	0	0	0	0	0	0	0	0	0	0	0	0	0	0	0	0	0	0	0	0	0
XR_002346300.1	LOC110261774	lncRNA	0	0	0	0	0	0	0	0	0	0	0	0	0	0	0	0	0	0	0	0	0
XR_002346302.1	LOC110261776	lncRNA	0	0	0	0	0	0	0	0	0	0	0	0	0	0	0	0	0	0	0	0	0
XR_002346303.1	TMEM170B	mRNA	0	0	0	0	0	0	0	0	0	0	0	0	0	0	0	0	0	0	0	0	0
XR_002346304.1	TMEM170B	mRNA	0	0	0	0	0	0	0	0	0	0	0	0	0	0	0	0	0	0	0	0	0

RNA ID	Symbol	Type	updown_BGIR9823_80765	updown_BGIR9823_80869	updown_BGIR9823_81015	updown_BGIR9823_81197	updown_BGIR9823_81968	updown_BGIR9823_82765	updown_BGIR9823_78329	updown_BGIR9823_93571	updown_BGIR9823_93054	updown_BGIR9823_96360	updown_XR_002343213.1	updown_XR_002343346.1	updown_XR_002341920.1	updown_XR_002342486.1	updown_XR_307914.3	updown_XR_002341615.1	updown_XR_002343544.1	updown_XR_002343891.1	updown_BGIR9823_87139	updown_BGIR9823_84771	updown_BGIR9823_98958
XR_115737.4	LOC100513133	lncRNA	0	0	1	0	0	0	0	0	0	0	0	0	0	0	0	0	0	0	0	0	0
XR_299046.2	LOC102164596	lncRNA	0	0	0	0	0	0	0	0	0	0	0	0	0	0	0	0	0	0	0	0	0
XR_303634.3	LOC100738154	lncRNA	0	0	0	0	0	0	0	0	0	0	0	0	0	0	0	0	0	0	0	0	0
XR_304239.3	LOC102159819	lncRNA	0	0	0	0	0	0	0	0	0	0	0	0	0	0	0	0	0	0	0	0	0
XR_304244.3	LOC102161992	lncRNA	0	0	0	0	0	0	0	0	0	0	0	0	0	0	0	0	0	0	0	0	0
XR_304257.3	LOC102162837	lncRNA	0	0	0	0	0	0	0	0	0	0	0	0	0	0	0	0	0	0	0	0	0
XR_306105.3	LOC102166871	lncRNA	0	0	0	0	0	0	0	0	0	0	0	0	0	0	0	0	0	0	0	0	0
XR_307914.3	LOC102159607	lncRNA	0	0	0	0	0	0	0	0	0	0	0	0	0	0	1	0	0	0	0	0	0
XR_308345.3	LOC102167708	lncRNA	0	0	0	0	0	0	0	0	0	0	0	0	0	0	0	0	0	0	0	0	0
novel-ssc-miR1028-3p		miRNA	0	0	0	0	0	0	0	0	0	0	0	0	0	0	0	0	0	0	0	0	0
novel-ssc-miR1028-5p		miRNA	0	0	0	0	0	0	0	0	0	0	0	0	0	0	0	0	0	0	0	0	0
novel-ssc-miR1130-3p		miRNA	0	0	0	0	0	0	0	0	0	0	0	0	0	0	0	0	0	0	0	0	0
novel-ssc-miR1130-5p		miRNA	0	0	0	0	0	0	0	0	0	0	0	0	0	0	0	0	0	0	0	0	0
novel-ssc-miR1162-3p		miRNA	0	0	1	0	0	0	0	0	0	0	0	0	0	0	0	0	0	0	0	0	0
novel-ssc-miR1193-3p		miRNA	0	0	0	0	0	0	0	0	0	0	0	0	0	0	0	0	0	0	0	0	0
novel-ssc-miR1193-5p		miRNA	0	0	0	0	0	0	0	0	0	0	0	0	0	0	0	0	0	0	0	0	0
novel-ssc-miR1217-3p		miRNA	0	0	0	0	0	0	0	0	0	0	0	0	0	0	0	0	0	0	0	0	0
novel-ssc-miR1217-5p		miRNA	0	0	0	0	0	0	0	0	0	0	0	0	0	0	0	0	0	0	0	0	0
novel-ssc-miR1429-3p		miRNA	0	0	0	0	0	0	0	0	0	0	0	0	0	0	0	0	0	0	0	0	0
novel-ssc-miR1429-5p		miRNA	0	0	0	0	0	0	0	0	0	0	0	0	0	0	0	0	0	0	0	0	0
novel-ssc-miR259-3p		miRNA	0	0	1	0	0	0	0	0	0	0	0	0	0	0	0	0	0	0	0	0	0
novel-ssc-miR259-5p		miRNA	0	0	1	0	0	0	0	0	0	0	0	0	0	0	0	0	0	0	0	0	0
novel-ssc-miR324-3p		miRNA	0	0	0	0	0	0	0	0	0	0	0	0	0	0	0	0	0	0	0	0	0
novel-ssc-miR324-5p		miRNA	0	0	0	0	0	0	0	0	0	0	0	0	0	0	0	0	0	0	0	0	0
novel-ssc-miR42-3p		miRNA	0	0	0	0	0	0	0	0	0	0	0	0	0	0	0	0	0	0	1	0	0
novel-ssc-miR42-5p		miRNA	0	0	0	0	0	0	0	0	0	0	0	0	0	0	0	0	0	0	1	0	0
novel-ssc-miR433-3p		miRNA	0	0	0	0	0	0	0	0	0	0	0	0	0	0	0	0	0	0	0	0	0
novel-ssc-miR433-5p		miRNA	0	0	0	0	0	0	0	0	0	0	0	0	0	0	0	0	0	0	0	0	0
novel-ssc-miR557-3p		miRNA	0	0	0	0	0	0	0	0	0	0	0	0	0	0	0	0	0	0	0	0	0
novel-ssc-miR557-5p		miRNA	0	0	0	0	0	0	0	0	0	0	0	0	0	0	0	0	0	0	0	0	0
novel-ssc-miR619-3p		miRNA	0	0	0	0	0	0	0	0	0	0	0	0	0	0	0	0	0	0	0	0	0
novel-ssc-miR619-5p		miRNA	0	0	0	0	0	0	0	0	0	0	0	0	0	0	0	0	0	0	0	0	0
novel-ssc-miR719-3p		miRNA	0	0	1	0	0	0	0	0	0	0	0	0	0	0	0	0	0	0	0	0	0
novel-ssc-miR719-5p		miRNA	0	0	1	0	0	0	0	0	0	0	0	0	0	0	0	0	0	0	0	0	0
novel-ssc-miR966-3p		miRNA	0	0	1	0	0	0	0	0	0	0	0	0	0	0	0	0	0	0	0	0	0
novel-ssc-miR966-5p		miRNA	0	0	1	0	0	0	0	0	0	0	0	0	0	0	0	0	0	0	0	0	0
ssc-miR-7136-3p		miRNA	0	0	0	0	0	0	0	0	0	0	0	0	0	0	0	0	0	0	0	0	0
ssc-miR-7136-5p		miRNA	0	0	0	0	0	0	0	0	0	0	0	0	0	0	0	0	0	0	0	0	0

Supplementary Table S10. The predicted targe

RNA ID	Symbol	Type	updown_BGIR9823_99095	updown_BGIR9823_99528	updown_BGIR9823_99552	updown_BGIR9823_96972	updown_URS0000BC5676	updown_XR_001298953.2	updown_BGIR9823_101185	updown_BGIR9823_78435	updown_BGIR9823_101387	updown_BGIR9823_101406	updown_BGIR9823_102168	updown_BGIR9823_102282	updown_BGIR9823_100321	updown_BGIR9823_88590	updown_BGIR9823_89592	updown_BGIR9823_92306	updown_XR_002340248.1	updown_XR_002335693.1	updown_XR_002338205.1	updown_BGIR9823_101399	updown_BGIR9823_101545
BGIR9823_100361	PRKAG2	mRNA	0	0	0	0	0	0	0	0	0	0	0	0	0	0	0	0	1	0	0	0	0
BGIR9823_100362	PRKAG2	mRNA	0	0	0	0	0	0	0	0	0	0	0	0	0	0	0	0	1	0	0	0	0
BGIR9823_100363	PRKAG2	mRNA	0	0	0	0	0	0	0	0	0	0	0	0	0	0	0	0	1	0	0	0	0
BGIR9823_100364	PRKAG2	mRNA	0	0	0	0	0	0	0	0	0	0	0	0	0	0	0	0	1	0	0	0	0
BGIR9823_101535	LOC102158401	lncRNA	0	0	0	0	0	0	0	0	0	0	0	0	0	0	0	0	0	0	0	0	1
BGIR9823_101541	LOC102157946	lncRNA	0	0	0	0	0	0	0	0	0	0	0	0	0	0	0	0	0	0	0	0	1
BGIR9823_101542	LOC102157946	lncRNA	0	0	0	0	0	0	0	0	0	0	0	0	0	0	0	0	0	0	0	0	1
BGIR9823_101560	LOC110258600	lncRNA	0	0	0	0	0	0	0	0	0	0	0	0	0	0	0	0	0	0	0	0	1
BGIR9823_101561	LOC110258600	lncRNA	0	0	0	0	0	0	0	0	0	0	0	0	0	0	0	0	0	0	0	0	1
BGIR9823_101562	LOC110258600	lncRNA	0	0	0	0	0	0	0	0	0	0	0	0	0	0	0	0	0	0	0	0	1
BGIR9823_101563	LOC110258600	lncRNA	0	0	0	0	0	0	0	0	0	0	0	0	0	0	0	0	0	0	0	0	1
BGIR9823_101564	LOC110258600	lncRNA	0	0	0	0	0	0	0	0	0	0	0	0	0	0	0	0	0	0	0	0	1
BGIR9823_101612	LOC100521600	lncRNA	0	0	0	0	0	0	0	0	0	0	0	0	0	0	0	0	0	0	0	0	0
BGIR9823_101853		lncRNA	0	0	0	0	0	0	0	0	0	0	0	0	0	0	0	0	0	0	0	0	0
BGIR9823_101854		lncRNA	0	0	0	0	0	0	0	0	0	0	0	0	0	0	0	0	0	0	0	0	0
BGIR9823_101855		lncRNA	0	0	0	0	0	0	0	0	0	0	0	0	0	0	0	0	0	0	0	0	0
BGIR9823_102146		lncRNA	0	0	0	0	0	0	0	0	0	0	0	0	0	0	0	0	0	0	0	0	0
BGIR9823_102180		lncRNA	0	0	0	0	0	0	0	0	0	0	0	0	0	0	0	0	0	0	0	0	0
BGIR9823_102279		lncRNA	0	0	0	0	0	0	0	0	0	0	0	0	0	0	0	0	0	0	0	0	0
BGIR9823_102282		lncRNA	0	0	0	0	0	0	0	0	0	0	0	1	0	0	0	0	0	0	0	0	0
BGIR9823_102361		lncRNA	0	0	0	0	0	0	0	0	0	0	0	0	0	0	0	0	0	0	0	0	0
BGIR9823_102362		lncRNA	0	0	0	0	0	0	0	0	0	0	0	0	0	0	0	0	0	0	0	0	0
BGIR9823_102363		lncRNA	0	0	0	0	0	0	0	0	0	0	0	0	0	0	0	0	0	0	0	0	0
BGIR9823_102727		lncRNA	0	0	0	0	0	0	0	0	0	0	0	0	0	0	0	0	0	0	0	0	0
BGIR9823_80121	SSSCA1	lncRNA	0	0	0	0	0	0	0	0	0	0	0	0	0	0	0	0	0	0	0	0	0
BGIR9823_80122	LOC102163816	lncRNA	0	0	0	0	0	0	0	0	0	0	0	0	0	0	0	0	0	0	0	0	0
BGIR9823_81007	EHBP1L1	mRNA	0	0	0	0	0	0	0	0	0	0	0	0	0	0	0	0	0	0	0	0	0
BGIR9823_81008	EHBP1L1	mRNA	0	0	0	0	0	0	0	0	0	0	0	0	0	0	0	0	0	0	0	0	0
BGIR9823_81009	EHBP1L1	mRNA	0	0	0	0	0	0	0	0	0	0	0	0	0	0	0	0	0	0	0	0	0
BGIR9823_81010	EHBP1L1	mRNA	0	0	0	0	0	0	0	0	0	0	0	0	0	0	0	0	0	0	0	0	0
BGIR9823_81011	SSSCA1	mRNA	0	0	0	0	0	0	0	0	0	0	0	0	0	0	0	0	0	0	0	0	0
BGIR9823_81012	FRMD8	lncRNA	0	0	0	0	0	0	0	0	0	0	0	0	0	0	0	0	0	0	0	0	0
BGIR9823_81013	LOC102163816	lncRNA	0	0	0	0	0	0	0	0	0	0	0	0	0	0	0	0	0	0	0	0	0
BGIR9823_81014	LOC102163816	lncRNA	0	0	0	0	0	0	0	0	0	0	0	0	0	0	0	0	0	0	0	0	0
BGIR9823_81015	LOC102163816	lncRNA	0	0	0	0	0	0	0	0	0	0	0	0	0	0	0	0	0	0	0	0	0
BGIR9823_81016	LOC102163816	lncRNA	0	0	0	0	0	0	0	0	0	0	0	0	0	0	0	0	0	0	0	0	0
BGIR9823_81017	LOC102163816	lncRNA	0	0	0	0	0	0	0	0	0	0	0	0	0	0	0	0	0	0	0	0	0
BGIR9823_81018	LOC102163816	lncRNA	0	0	0	0	0	0	0	0	0	0	0	0	0	0	0	0	0	0	0	0	0
BGIR9823_81019	LOC100513133	lncRNA	0	0	0	0	0	0	0	0	0	0	0	0	0	0	0	0	0	0	0	0	0
BGIR9823_84704	LOC102159820	mRNA	0	0	0	0	0	0	0	0	0	0	0	0	0	0	0	0	0	0	0	0	0
BGIR9823_84732	RANGAP1	mRNA	0	0	0	0	0	0	0	0	0	0	0	0	0	0	0	0	0	0	0	0	0

RNA ID	Symbol	Type	updown_BGIR9823_99095	updown_BGIR9823_99528	updown_BGIR9823_99552	updown_BGIR9823_96972	updown_URS0000BC5676	updown_XR_001298953.2	updown_BGIR9823_101185	updown_BGIR9823_78435	updown_BGIR9823_101387	updown_BGIR9823_101406	updown_BGIR9823_102168	updown_BGIR9823_102282	updown_BGIR9823_100321	updown_BGIR9823_88590	updown_BGIR9823_89592	updown_BGIR9823_92306	updown_XR_002340248.1	updown_XR_002335693.1	updown_XR_002338205.1	updown_BGIR9823_101399	updown_BGIR9823_101545
BGIR9823_84733	RANGAP1	mRNA	0	0	0	0	0	0	0	0	0	0	0	0	0	0	0	0	0	0	0	0	0
BGIR9823_85245	CRADD	lncRNA	0	0	0	0	0	0	0	0	0	0	0	0	0	0	0	0	0	0	0	0	0
BGIR9823_85246	LOC100738154	lncRNA	0	0	0	0	0	0	0	0	0	0	0	0	0	0	0	0	0	0	0	0	0
BGIR9823_85293	TRMU	mRNA	0	0	0	0	0	0	0	0	0	0	0	0	0	0	0	0	0	0	0	0	0
BGIR9823_85294	TRMU	mRNA	0	0	0	0	0	0	0	0	0	0	0	0	0	0	0	0	0	0	0	0	0
BGIR9823_85295	TTC38	mRNA	0	0	0	0	0	0	0	0	0	0	0	0	0	0	0	0	0	0	0	0	0
BGIR9823_85296	PKDREJ	lncRNA	0	0	0	0	0	0	0	0	0	0	0	0	0	0	0	0	0	0	0	0	0
BGIR9823_85297	PPARA	mRNA	0	0	0	0	0	0	0	0	0	0	0	0	0	0	0	0	0	0	0	0	0
BGIR9823_85298	PPARA	mRNA	0	0	0	0	0	0	0	0	0	0	0	0	0	0	0	0	0	0	0	0	0
BGIR9823_85299	PPARA	mRNA	0	0	0	0	0	0	0	0	0	0	0	0	0	0	0	0	0	0	0	0	0
BGIR9823_85300	PPARA	lncRNA	0	0	0	0	0	0	0	0	0	0	0	0	0	0	0	0	0	0	0	0	0
BGIR9823_85301	LOC110260611	mRNA	0	0	0	0	0	0	0	0	0	0	0	0	0	0	0	0	0	0	0	0	0
BGIR9823_85317	ACO2	mRNA	0	0	0	0	0	0	0	0	0	0	0	0	0	0	0	0	0	0	0	0	0
BGIR9823_85318	LOC110260629	lncRNA	0	0	0	0	0	0	0	0	0	0	0	0	0	0	0	0	0	0	0	0	0
BGIR9823_85319	TEF	mRNA	0	0	0	0	0	0	0	0	0	0	0	0	0	0	0	0	0	0	0	0	0
BGIR9823_85320	ZC3H7B	mRNA	0	0	0	0	0	0	0	0	0	0	0	0	0	0	0	0	0	0	0	0	0
BGIR9823_85345	SYNGR1	mRNA	0	0	0	0	0	0	0	0	0	0	0	0	0	0	0	0	0	0	0	0	0
BGIR9823_85835	CRADD	mRNA	0	0	0	0	0	0	0	0	0	0	0	0	0	0	0	0	0	0	0	0	0
BGIR9823_85839	CRADD	lncRNA	0	0	0	0	0	0	0	0	0	0	0	0	0	0	0	0	0	0	0	0	0
BGIR9823_85840	CRADD	lncRNA	0	0	0	0	0	0	0	0	0	0	0	0	0	0	0	0	0	0	0	0	0
BGIR9823_85841	CRADD	lncRNA	0	0	0	0	0	0	0	0	0	0	0	0	0	0	0	0	0	0	0	0	0
BGIR9823_85930	LOC106510465	mRNA	0	0	0	0	0	0	0	0	0	0	0	0	0	0	0	0	0	0	0	0	0
BGIR9823_85931	MAF	lncRNA	0	0	0	0	0	0	0	0	0	0	0	0	0	0	0	0	0	0	0	0	0
BGIR9823_85932	MAF	lncRNA	0	0	0	0	0	0	0	0	0	0	0	0	0	0	0	0	0	0	0	0	0
BGIR9823_87139	LOC106510467	lncRNA	0	0	0	0	0	0	0	0	0	0	0	0	0	0	0	0	0	0	0	0	0
BGIR9823_88187	WRNIP1	mRNA	0	0	0	0	0	0	0	0	0	0	0	0	0	0	0	0	0	0	0	0	0
BGIR9823_88188	NQO2	mRNA	0	0	0	0	0	0	0	0	0	0	0	0	0	0	0	0	0	0	0	0	0
BGIR9823_88189	NQO2	mRNA	0	0	0	0	0	0	0	0	0	0	0	0	0	0	0	0	0	0	0	0	0
BGIR9823_88190	RIPK1	mRNA	0	0	0	0	0	0	0	0	0	0	0	0	0	0	0	0	0	0	0	0	0
BGIR9823_88191	RIPK1	mRNA	0	0	0	0	0	0	0	0	0	0	0	0	0	0	0	0	0	0	0	0	0
BGIR9823_88192	RIPK1	mRNA	0	0	0	0	0	0	0	0	0	0	0	0	0	0	0	0	0	0	0	0	0
BGIR9823_88193	RIPK1	mRNA	0	0	0	0	0	0	0	0	0	0	0	0	0	0	0	0	0	0	0	0	0
BGIR9823_88196	PRPF4B	mRNA	0	0	0	0	0	0	0	0	0	0	0	0	0	0	0	0	0	0	0	0	0
BGIR9823_88197	PRPF4B	mRNA	0	0	0	0	0	0	0	0	0	0	0	0	0	0	0	0	0	0	0	0	0
BGIR9823_88198	PRPF4B	mRNA	0	0	0	0	0	0	0	0	0	0	0	0	0	0	0	0	0	0	0	0	0
BGIR9823_88211	TMEM170B	lncRNA	0	0	0	0	0	0	0	0	0	0	0	0	0	0	0	0	0	0	0	0	0
BGIR9823_88212	TMEM170B	lncRNA	0	0	0	0	0	0	0	0	0	0	0	0	0	0	0	0	0	0	0	0	0
BGIR9823_88213	TMEM170B	lncRNA	0	0	0	0	0	0	0	0	0	0	0	0	0	0	0	0	0	0	0	0	0
BGIR9823_88214	TMEM170B	lncRNA	0	0	0	0	0	0	0	0	0	0	0	0	0	0	0	0	0	0	0	0	0
BGIR9823_88217	PHACTR1	mRNA	0	0	0	0	0	0	0	0	0	0	0	0	0	0	0	0	0	0	0	0	0
BGIR9823_88218	PHACTR1	lncRNA	0	0	0	0	0	0	0	0	0	0	0	0	0	0	0	0	0	0	0	0	0
BGIR9823_89017	MYLK4	lncRNA	0	0	0	0	0	0	0	0	0	0	0	0	0	0	0	0	0	0	0	0	0
BGIR9823_89019	SERPINB1	lncRNA	0	0	0	0	0	0	0	0	0	0	0	0	0	0	0	0	0	0	0	0	0

RNA ID	Symbol	Type	updown_BGIR9823_99095	updown_BGIR9823_99528	updown_BGIR9823_99552	updown_BGIR9823_96972	updown_URS0000BC5676	updown_XR_001298953.2	updown_BGIR9823_101185	updown_BGIR9823_78435	updown_BGIR9823_101387	updown_BGIR9823_101406	updown_BGIR9823_102168	updown_BGIR9823_102282	updown_BGIR9823_100321	updown_BGIR9823_88590	updown_BGIR9823_89592	updown_BGIR9823_92306	updown_XR_002340248.1	updown_XR_002335693.1	updown_XR_002338205.1	updown_BGIR9823_101399	updown_BGIR9823_101545
BGIR9823_89020	SERPINB9	mRNA	0	0	0	0	0	0	0	0	0	0	0	0	0	0	0	0	0	0	0	0	0
BGIR9823_89021	RIPK1	lncRNA	0	0	0	0	0	0	0	0	0	0	0	0	0	0	0	0	0	0	0	0	0
BGIR9823_89025	PXDC1	mRNA	0	0	0	0	0	0	0	0	0	0	0	0	0	0	0	0	0	0	0	0	0
BGIR9823_89026	PXDC1	lncRNA	0	0	0	0	0	0	0	0	0	0	0	0	0	0	0	0	0	0	0	0	0
BGIR9823_89027	LOC102161744	lncRNA	0	0	0	0	0	0	0	0	0	0	0	0	0	0	0	0	0	0	0	0	0
BGIR9823_89040	TBC1D7	lncRNA	0	0	0	0	0	0	0	0	0	0	0	0	0	0	0	0	0	0	0	0	0
BGIR9823_89041	TBC1D7	lncRNA	0	0	0	0	0	0	0	0	0	0	0	0	0	0	0	0	0	0	0	0	0
BGIR9823_89042	TBC1D7	lncRNA	0	0	0	0	0	0	0	0	0	0	0	0	0	0	0	0	0	0	0	0	0
BGIR9823_89043	TBC1D7	lncRNA	0	0	0	0	0	0	0	0	0	0	0	0	0	0	0	0	0	0	0	0	0
BGIR9823_89044	GFOD1	lncRNA	0	0	0	0	0	0	0	0	0	0	0	0	0	0	0	0	0	0	0	0	0
BGIR9823_89045	GFOD1	mRNA	0	0	0	0	0	0	0	0	0	0	0	0	0	0	0	0	0	0	0	0	0
BGIR9823_89046	GFOD1	mRNA	0	0	0	0	0	0	0	0	0	0	0	0	0	0	0	0	0	0	0	0	0
BGIR9823_89047	GFOD1	mRNA	0	0	0	0	0	0	0	0	0	0	0	0	0	0	0	0	0	0	0	0	0
BGIR9823_89048	GFOD1	lncRNA	0	0	0	0	0	0	0	0	0	0	0	0	0	0	0	0	0	0	0	0	0
BGIR9823_89049	GFOD1	lncRNA	0	0	0	0	0	0	0	0	0	0	0	0	0	0	0	0	0	0	0	0	0
BGIR9823_90771	NEU3	lncRNA	0	0	0	0	0	0	0	0	0	0	0	0	0	0	0	0	0	0	0	0	0
BGIR9823_90772	SLCO2B1	mRNA	0	0	0	0	0	0	0	0	0	0	0	0	0	0	0	0	0	0	0	0	0
BGIR9823_90913	LOC110255435	lncRNA	0	0	0	0	0	0	0	0	0	0	0	0	0	0	0	0	0	0	0	0	0
BGIR9823_90934	LOC110255441	lncRNA	0	0	0	0	0	0	0	0	0	0	0	0	0	0	0	0	0	0	0	0	0
BGIR9823_90935	CCDC84	mRNA	0	0	0	0	0	0	0	0	0	0	0	0	0	0	0	0	0	0	0	0	0
BGIR9823_90936	TRAPPC4	mRNA	0	0	0	0	0	0	0	0	0	0	0	0	0	0	0	0	0	0	0	0	0
BGIR9823_90937	HMBS	mRNA	0	0	0	0	0	0	0	0	0	0	0	0	0	0	0	0	0	0	0	0	0
BGIR9823_90956	SORL1	mRNA	0	0	0	0	0	0	0	0	0	0	0	0	0	0	0	0	0	1	0	0	0
BGIR9823_90957	SORL1	mRNA	0	0	0	0	0	0	0	0	0	0	0	0	0	0	0	0	0	1	0	0	0
BGIR9823_90958	SORL1	mRNA	0	0	0	0	0	0	0	0	0	0	0	0	0	0	0	0	0	1	0	0	0
BGIR9823_90960	CLMP	lncRNA	0	0	0	0	0	0	0	0	0	0	0	1	0	0	0	0	0	0	0	0	0
BGIR9823_91161	LOC100522267	mRNA	0	0	0	0	0	0	0	0	0	0	0	0	0	0	0	0	0	0	0	0	0
BGIR9823_91162	LOC100522455	mRNA	0	0	0	0	0	0	0	0	0	0	0	0	0	0	0	0	0	0	0	0	0
BGIR9823_91163	ABCB1	mRNA	0	0	0	0	0	0	0	0	0	0	0	0	0	0	0	0	0	0	0	0	0
BGIR9823_91402	HBB	mRNA	0	0	0	0	0	0	0	0	0	0	0	0	0	0	0	0	0	0	0	0	0
BGIR9823_91403	HBB	mRNA	0	0	0	0	0	0	0	0	0	0	0	0	0	0	0	0	0	0	0	0	0
BGIR9823_91404	HBB	mRNA	0	0	0	0	0	0	0	0	0	0	0	0	0	0	0	0	0	0	0	0	0
BGIR9823_91405	HBB	mRNA	0	0	0	0	0	0	0	0	0	0	0	0	0	0	0	0	0	0	0	0	0
BGIR9823_91406	HBB	mRNA	0	0	0	0	0	0	0	0	0	0	0	0	0	0	0	0	0	0	0	0	0
BGIR9823_91407	HBB	mRNA	0	0	0	0	0	0	0	0	0	0	0	0	0	0	0	0	0	0	0	0	0
BGIR9823_91408	HBB	mRNA	0	0	0	0	0	0	0	0	0	0	0	0	0	0	0	0	0	0	0	0	0
BGIR9823_91409	HBB	mRNA	0	0	0	0	0	0	0	0	0	0	0	0	0	0	0	0	0	0	0	0	0
BGIR9823_91625	BCL9L	lncRNA	0	0	0	0	0	0	0	0	0	0	0	0	0	0	0	0	0	0	0	0	0
BGIR9823_91626	BCL9L	lncRNA	0	0	0	0	0	0	0	0	0	0	0	0	0	0	0	0	0	0	0	0	0
BGIR9823_91627	RPS25	mRNA	0	0	0	0	0	0	0	0	0	0	0	0	0	0	0	0	0	0	0	0	0
BGIR9823_91628	RPS25	mRNA	0	0	0	0	0	0	0	0	0	0	0	0	0	0	0	0	0	0	0	0	0
BGIR9823_91629	SLC37A4	mRNA	0	0	0	0	0	0	0	0	0	0	0	0	0	0	0	0	0	0	0	0	0
BGIR9823_91630	HYOU1	mRNA	0	0	0	0	0	0	0	0	0	0	0	0	0	0	0	0	0	0	0	0	0

RNA ID	Symbol	Type	updown_BGIR9823_99095	updown_BGIR9823_99528	updown_BGIR9823_99552	updown_BGIR9823_96972	updown_URS0000BC5676	updown_XR_001298953.2	updown_BGIR9823_101185	updown_BGIR9823_78435	updown_BGIR9823_101387	updown_BGIR9823_101406	updown_BGIR9823_102168	updown_BGIR9823_102282	updown_BGIR9823_100321	updown_BGIR9823_88590	updown_BGIR9823_89592	updown_BGIR9823_92306	updown_XR_002340248.1	updown_XR_002335693.1	updown_XR_002338205.1	updown_BGIR9823_101399	updown_BGIR9823_101545
BGIR9823_91640	CLMP	mRNA	0	0	0	0	0	0	0	0	0	0	0	1	0	0	0	0	0	0	0	0	0
BGIR9823_91819	RUNDC3B	lncRNA	0	0	0	0	0	0	0	0	0	0	0	0	0	0	0	0	0	0	0	0	0
BGIR9823_91820	RUNDC3B	lncRNA	0	0	0	0	0	0	0	0	0	0	0	0	0	0	0	0	0	0	0	0	0
BGIR9823_91821	RUNDC3B	lncRNA	0	0	0	0	0	0	0	0	0	0	0	0	0	0	0	0	0	0	0	0	0
BGIR9823_91822	RUNDC3B	lncRNA	0	0	0	0	0	0	0	0	0	0	0	0	0	0	0	0	0	0	0	0	0
BGIR9823_91823	RUNDC3B	lncRNA	0	0	0	0	0	0	0	0	0	0	0	0	0	0	0	0	0	0	0	0	0
BGIR9823_91824	RUNDC3B	lncRNA	0	0	0	0	0	0	0	0	0	0	0	0	0	0	0	0	0	0	0	0	0
BGIR9823_91825	RUNDC3B	lncRNA	0	0	0	0	0	0	0	0	0	0	0	0	0	0	0	0	0	0	0	0	0
BGIR9823_91826	LOC102164596	lncRNA	0	0	0	0	0	0	0	0	0	0	0	0	0	0	0	0	0	0	0	0	0
BGIR9823_92993	KATNAL1	mRNA	0	0	0	0	0	0	0	0	0	0	0	0	0	0	0	0	0	0	0	0	0
BGIR9823_92994	KATNAL1	mRNA	0	0	0	0	0	0	0	0	0	0	0	0	0	0	0	0	0	0	0	0	0
BGIR9823_92995	KATNAL1	mRNA	0	0	0	0	0	0	0	0	0	0	0	0	0	0	0	0	0	0	0	0	0
BGIR9823_92996	KATNAL1	mRNA	0	0	0	0	0	0	0	0	0	0	0	0	0	0	0	0	0	0	0	0	0
BGIR9823_92997	KATNAL1	mRNA	0	0	0	0	0	0	0	0	0	0	0	0	0	0	0	0	0	0	0	0	0
BGIR9823_92999	LOC110255292	lncRNA	0	0	0	0	0	0	0	0	0	0	0	0	0	0	0	0	0	0	0	0	0
BGIR9823_93000	LOC102166871	lncRNA	0	0	0	0	0	0	0	0	0	0	0	0	0	0	0	0	0	0	0	0	0
BGIR9823_98051	LOC102164998	lncRNA	0	0	0	0	0	0	0	0	0	0	0	0	0	0	0	0	0	0	0	0	0
BGIR9823_99244	LOC102167708	lncRNA	0	0	0	0	0	0	0	0	0	0	0	0	0	0	0	0	0	0	0	0	0
NM_001004034.1	HMGB1	mRNA	0	0	0	0	0	0	0	0	0	0	0	0	0	0	0	0	0	0	0	0	0
NM_001044526.1	PPARA	mRNA	0	0	0	0	0	0	0	0	0	0	0	0	0	0	0	0	0	0	0	0	0
NM_001111256.1	SERPINB9	mRNA	0	0	0	0	0	0	0	0	0	0	0	0	0	0	0	0	0	0	0	0	0
NM_001113443.1	ECI2	mRNA	0	0	0	0	0	0	0	0	0	0	0	0	0	0	0	0	0	0	0	0	0
NM_001144841.1	HBB	mRNA	0	0	0	0	0	0	0	0	0	0	0	0	0	0	0	0	0	0	0	0	0
NM_001199719.1	SLC37A4	mRNA	0	0	0	0	0	0	0	0	0	0	0	0	0	0	0	0	0	0	0	0	0
NM_001243907.1	HSPA8	mRNA	0	0	0	0	0	0	0	0	0	0	0	1	0	0	0	0	0	0	0	0	0
NM_001244063.1	RPL3	mRNA	0	0	0	0	0	0	0	0	0	0	0	0	0	0	0	0	0	0	0	0	0
NM_001244066.1	SYNGR1	mRNA	0	0	0	0	0	0	0	0	0	0	0	0	0	0	0	0	0	0	0	0	0
NM_001244396.1	CCDC84	mRNA	0	0	0	0	0	0	0	0	0	0	0	0	0	0	0	0	0	0	0	0	0
NM_001287846.1	RHEB	mRNA	0	0	0	0	0	0	0	0	0	0	0	0	0	0	0	0	1	0	0	0	0
NM_213954.1	ACO2	mRNA	0	0	0	0	0	0	0	0	0	0	0	0	0	0	0	0	0	0	0	0	0
NM_214012.1	UPK2	mRNA	0	0	0	0	0	0	0	0	0	0	0	0	0	0	0	0	0	0	0	0	0
URS0000BC92F4	LOC102167708	lncRNA	0	0	0	0	0	0	0	0	0	0	0	0	0	0	0	0	0	0	0	0	0
XM_001928309.6	TBC1D7	mRNA	0	0	0	0	0	0	0	0	0	0	0	0	0	0	0	0	0	0	0	0	0
XM_001928852.5	PXDC1	mRNA	0	0	0	0	0	0	0	0	0	0	0	0	0	0	0	0	0	0	0	0	0
XM_003122534.4	KCNK7	mRNA	0	0	0	0	0	0	0	0	0	0	0	0	0	0	0	0	0	0	0	0	0
XM_003122558.5	SCYL1	mRNA	0	0	0	0	0	0	0	0	0	0	0	0	0	0	0	0	0	0	0	0	0
XM_003122562.5	FRMD8	mRNA	0	0	0	0	0	0	0	0	0	0	0	0	0	0	0	0	0	0	0	0	0
XM_003125951.6	TTC38	mRNA	0	0	0	0	0	0	0	0	0	0	0	0	0	0	0	0	0	0	0	0	0
XM_003125953.4	CDPF1	mRNA	0	0	0	0	0	0	0	0	0	0	0	0	0	0	0	0	0	0	0	0	0
XM_003126012.6	CBX7	mRNA	0	0	0	0	0	0	0	0	0	0	0	0	0	0	0	0	0	0	0	0	0
XM_003128161.4	RIPK1	mRNA	0	0	0	0	0	0	0	0	0	0	0	0	0	0	0	0	0	0	0	0	0
XM_003129508.2	LOC100514417	mRNA	0	0	0	0	0	0	0	0	0	0	0	0	0	0	0	0	0	0	0	0	0
XM_003129512.2	LOC100515268	mRNA	0	0	0	0	0	0	0	0	0	0	0	0	0	0	0	0	0	0	0	0	0

RNA ID	Symbol	Type	updown_BGIR9823_99095	updown_BGIR9823_99528	updown_BGIR9823_99552	updown_BGIR9823_96972	updown_URS0000BC5676	updown_XR_001298953.2	updown_BGIR9823_101185	updown_BGIR9823_78435	updown_BGIR9823_101387	updown_BGIR9823_101406	updown_BGIR9823_102168	updown_BGIR9823_102282	updown_BGIR9823_100321	updown_BGIR9823_88590	updown_BGIR9823_89592	updown_BGIR9823_92306	updown_XR_002340248.1	updown_XR_002335693.1	updown_XR_002338205.1	updown_BGIR9823_101399	updown_BGIR9823_101545
XM_003129514.3	LOC100515618	mRNA	0	0	0	0	0	0	0	0	0	0	0	0	0	0	0	0	0	0	0	0	0
XM_003129519.2	LOC100517595	mRNA	0	0	0	0	0	0	0	0	0	0	0	0	0	0	0	0	0	0	0	0	0
XM_003129529.4	LOC100519910	mRNA	0	0	0	0	0	0	0	0	0	0	0	0	0	0	0	0	0	0	0	0	0
XM_003129915.4	CXCR5	mRNA	0	0	0	0	0	0	0	0	0	0	0	0	0	0	0	0	0	0	0	0	0
XM_003129916.5	BCL9L	mRNA	0	0	0	0	0	0	0	0	0	0	0	0	0	0	0	0	0	0	0	0	0
XM_003129918.4	TRAPPC4	mRNA	0	0	0	0	0	0	0	0	0	0	0	0	0	0	0	0	0	0	0	0	0
XM_003129919.5	RPS25	mRNA	0	0	0	0	0	0	0	0	0	0	0	0	0	0	0	0	0	0	0	0	0
XM_003129920.3	RPS25	mRNA	0	0	0	0	0	0	0	0	0	0	0	0	0	0	0	0	0	0	0	0	0
XM_003129922.4	HYOU1	mRNA	0	0	0	0	0	0	0	0	0	0	0	0	0	0	0	0	0	0	0	0	0
XM_003129923.6	VPS11	mRNA	0	0	0	0	0	0	0	0	0	0	0	0	0	0	0	0	0	0	0	0	0
XM_003129944.4	SC5D	mRNA	0	0	0	0	0	0	0	0	0	0	0	0	0	0	0	0	0	1	0	0	0
XM_003357161.1	LOC100620971	mRNA	0	0	0	0	0	0	0	0	0	0	0	0	0	0	0	0	0	0	0	0	0
XM_003482119.4	SERPINB1	mRNA	0	0	0	0	0	0	0	0	0	0	0	0	0	0	0	0	0	0	0	0	0
XM_003482132.4	TMEM170B	mRNA	0	0	0	0	0	0	0	0	0	0	0	0	0	0	0	0	0	0	0	0	0
XM_003482133.4	GFOD1	mRNA	0	0	0	0	0	0	0	0	0	0	0	0	0	0	0	0	0	0	0	0	0
XM_003482563.4	SPCS2	mRNA	0	0	0	0	0	0	0	0	0	0	0	0	0	0	0	0	0	0	0	0	0
XM_003482565.4	NEU3	mRNA	0	0	0	0	0	0	0	0	0	0	0	0	0	0	0	0	0	0	0	0	0
XM_003482567.1	LOC100739359	mRNA	0	0	0	0	0	0	0	0	0	0	0	0	0	0	0	0	0	0	0	0	0
XM_003482630.3	BSX	mRNA	0	0	0	0	0	0	0	0	0	0	0	1	0	0	0	0	0	0	0	0	0
XM_005653107.3	CHADL	mRNA	0	0	0	0	0	0	0	0	0	0	0	0	0	0	0	0	0	0	0	0	0
XM_005653640.1	LOC100621181	mRNA	0	0	0	0	0	0	0	0	0	0	0	0	0	0	0	0	0	0	0	0	0
XM_005654266.3	PRKAG2	mRNA	0	0	0	0	0	0	0	0	0	0	0	0	0	0	0	0	1	0	0	0	0
XM_005655470.3	CDPF1	mRNA	0	0	0	0	0	0	0	0	0	0	0	0	0	0	0	0	0	0	0	0	0
XM_005660703.3	LTBP3	mRNA	0	0	0	0	0	0	0	0	0	0	0	0	0	0	0	0	0	0	0	0	0
XM_005660705.3	SCYL1	mRNA	0	0	0	0	0	0	0	0	0	0	0	0	0	0	0	0	0	0	0	0	0
XM_005660707.3	SLC25A45	mRNA	0	0	0	0	0	0	0	0	0	0	0	0	0	0	0	0	0	0	0	0	0
XM_005665536.3	RIPK1	mRNA	0	0	0	0	0	0	0	0	0	0	0	0	0	0	0	0	0	0	0	0	0
XM_005665537.3	RIPK1	mRNA	0	0	0	0	0	0	0	0	0	0	0	0	0	0	0	0	0	0	0	0	0
XM_005665538.3	RIPK1	mRNA	0	0	0	0	0	0	0	0	0	0	0	0	0	0	0	0	0	0	0	0	0
XM_005665575.3	PHACTR1	mRNA	0	0	0	0	0	0	0	0	0	0	0	0	0	0	0	0	0	0	0	0	0
XM_005665576.3	TBC1D7	mRNA	0	0	0	0	0	0	0	0	0	0	0	0	0	0	0	0	0	0	0	0	0
XM_005667128.3	LOC100627340	mRNA	0	0	0	0	0	0	0	0	0	0	0	0	0	0	0	0	0	0	0	0	0
XM_005667397.2	CCDC84	mRNA	0	0	0	0	0	0	0	0	0	0	0	0	0	0	0	0	0	0	0	0	0
XM_005667398.3	SLC37A4	mRNA	0	0	0	0	0	0	0	0	0	0	0	0	0	0	0	0	0	0	0	0	0
XM_005667402.3	SLC37A4	mRNA	0	0	0	0	0	0	0	0	0	0	0	0	0	0	0	0	0	0	0	0	0
XM_005667403.3	SLC37A4	mRNA	0	0	0	0	0	0	0	0	0	0	0	0	0	0	0	0	0	0	0	0	0
XM_005667413.3	BCL9L	mRNA	0	0	0	0	0	0	0	0	0	0	0	0	0	0	0	0	0	0	0	0	0
XM_005667414.3	FOXR1	mRNA	0	0	0	0	0	0	0	0	0	0	0	0	0	0	0	0	0	0	0	0	0
XM_005667416.3	HYOU1	mRNA	0	0	0	0	0	0	0	0	0	0	0	0	0	0	0	0	0	0	0	0	0
XM_005667419.3	HYOU1	mRNA	0	0	0	0	0	0	0	0	0	0	0	0	0	0	0	0	0	0	0	0	0
XM_005667420.3	HYOU1	mRNA	0	0	0	0	0	0	0	0	0	0	0	0	0	0	0	0	0	0	0	0	0
XM_005667440.2	JHY	mRNA	0	0	0	0	0	0	0	0	0	0	0	1	0	0	0	0	0	0	0	0	0
XM_005668316.3	HMGB1	mRNA	0	0	0	0	0	0	0	0	0	0	0	0	0	0	0	0	0	0	0	0	0

RNA ID	Symbol	Type	updown_BGIR9823_99095	updown_BGIR9823_99528	updown_BGIR9823_99552	updown_BGIR9823_96972	updown_URS0000BC5676	updown_XR_001298953.2	updown_BGIR9823_101185	updown_BGIR9823_78435	updown_BGIR9823_101387	updown_BGIR9823_101406	updown_BGIR9823_102168	updown_BGIR9823_102282	updown_BGIR9823_100321	updown_BGIR9823_88590	updown_BGIR9823_89592	updown_BGIR9823_92306	updown_XR_002340248.1	updown_XR_002335693.1	updown_XR_002338205.1	updown_BGIR9823_101399	updown_BGIR9823_101545
XM_005668317.3	HMGB1	mRNA	0	0	0	0	0	0	0	0	0	0	0	0	0	0	0	0	0	0	0	0	0
XM_005668318.3	HMGB1	mRNA	0	0	0	0	0	0	0	0	0	0	0	0	0	0	0	0	0	0	0	0	0
XM_005671489.3	EMX2	mRNA	0	0	0	0	0	0	0	0	0	0	0	0	0	0	0	0	0	0	0	0	0
XM_005672397.3	MYO10	mRNA	0	0	0	0	0	0	0	0	0	0	0	0	0	0	0	0	0	0	0	0	0
XM_013977400.2	FAM50B	mRNA	0	0	0	0	0	0	0	0	0	0	0	0	0	0	0	0	0	0	0	0	0
XM_013977425.2	NEDD9	mRNA	0	0	0	0	0	0	0	0	0	0	0	0	0	0	0	0	0	0	0	0	0
XM_013977430.2	TBC1D7	mRNA	0	0	0	0	0	0	0	0	0	0	0	0	0	0	0	0	0	0	0	0	0
XM_013977431.2	TBC1D7	mRNA	0	0	0	0	0	0	0	0	0	0	0	0	0	0	0	0	0	0	0	0	0
XM_013977432.2	TBC1D7	mRNA	0	0	0	0	0	0	0	0	0	0	0	0	0	0	0	0	0	0	0	0	0
XM_013977433.2	GFOD1	mRNA	0	0	0	0	0	0	0	0	0	0	0	0	0	0	0	0	0	0	0	0	0
XM_013977434.2	GFOD1	mRNA	0	0	0	0	0	0	0	0	0	0	0	0	0	0	0	0	0	0	0	0	0
XM_013979449.2	SLC37A4	mRNA	0	0	0	0	0	0	0	0	0	0	0	0	0	0	0	0	0	0	0	0	0
XM_013979456.2	BCL9L	mRNA	0	0	0	0	0	0	0	0	0	0	0	0	0	0	0	0	0	0	0	0	0
XM_013979457.2	BCL9L	mRNA	0	0	0	0	0	0	0	0	0	0	0	0	0	0	0	0	0	0	0	0	0
XM_013979458.2	BCL9L	mRNA	0	0	0	0	0	0	0	0	0	0	0	0	0	0	0	0	0	0	0	0	0
XM_013979459.2	BCL9L	mRNA	0	0	0	0	0	0	0	0	0	0	0	0	0	0	0	0	0	0	0	0	0
XM_013979461.2	TRAPPC4	mRNA	0	0	0	0	0	0	0	0	0	0	0	0	0	0	0	0	0	0	0	0	0
XM_013979463.2	HYOU1	mRNA	0	0	0	0	0	0	0	0	0	0	0	0	0	0	0	0	0	0	0	0	0
XM_013979484.2	LOC100523684	mRNA	0	0	0	0	0	0	0	0	0	0	0	0	0	0	0	0	0	1	0	0	0
XM_013979486.2	LOC100523684	mRNA	0	0	0	0	0	0	0	0	0	0	0	0	0	0	0	0	0	1	0	0	0
XM_013979491.2	JHY	mRNA	0	0	0	0	0	0	0	0	0	0	0	1	0	0	0	0	0	0	0	0	0
XM_013980418.2	KATNAL1	mRNA	0	0	0	0	0	0	0	0	0	0	0	0	0	0	0	0	0	0	0	0	0
XM_013988182.2	TTC38	mRNA	0	0	0	0	0	0	0	0	0	0	0	0	0	0	0	0	0	0	0	0	0
XM_013988183.2	TTC38	mRNA	0	0	0	0	0	0	0	0	0	0	0	0	0	0	0	0	0	0	0	0	0
XM_013989589.2	LOC100522267	mRNA	0	0	0	0	0	0	0	0	0	0	0	0	0	0	0	0	0	0	0	0	0
XM_013989592.2	LOC100522455	mRNA	0	0	0	0	0	0	0	0	0	0	0	0	0	0	0	0	0	0	0	0	0
XM_013989593.2	LOC100522455	mRNA	0	0	0	0	0	0	0	0	0	0	0	0	0	0	0	0	0	0	0	0	0
XM_013994099.2	FRMD8	mRNA	0	0	0	0	0	0	0	0	0	0	0	0	0	0	0	0	0	0	0	0	0
XM_013994100.2	SLC25A45	mRNA	0	0	0	0	0	0	0	0	0	0	0	0	0	0	0	0	0	0	0	0	0
XM_013994102.2	SLC25A45	mRNA	0	0	0	0	0	0	0	0	0	0	0	0	0	0	0	0	0	0	0	0	0
XM_013994103.2	SLC25A45	mRNA	0	0	0	0	0	0	0	0	0	0	0	0	0	0	0	0	0	0	0	0	0
XM_013994104.2	SLC25A45	mRNA	0	0	0	0	0	0	0	0	0	0	0	0	0	0	0	0	0	0	0	0	0
XM_013997510.2	CBX7	mRNA	0	0	0	0	0	0	0	0	0	0	0	0	0	0	0	0	0	0	0	0	0
XM_013999070.2	LOC100520088	mRNA	0	0	0	0	0	0	0	0	0	0	0	0	0	0	0	0	0	0	0	0	0
XM_021062230.1	LOC100517771	mRNA	0	0	0	0	0	0	0	0	0	0	0	0	0	0	0	0	0	0	0	0	0
XM_021062231.1	LOC100517957	mRNA	0	0	0	0	0	0	0	0	0	0	0	0	0	0	0	0	0	0	0	0	0
XM_021062367.1	LOC110255365	mRNA	0	0	0	0	0	0	0	0	0	0	0	0	0	0	0	0	0	0	0	0	0
XM_021062369.1	LOC100737110	mRNA	0	0	0	0	0	0	0	0	0	0	0	0	0	0	0	0	0	0	0	0	0
XM_021062370.1	LOC110255368	mRNA	0	0	0	0	0	0	0	0	0	0	0	0	0	0	0	0	0	0	0	0	0
XM_021062371.1	LOC100515441	mRNA	0	0	0	0	0	0	0	0	0	0	0	0	0	0	0	0	0	0	0	0	0
XM_021062374.1	LOC110255371	mRNA	0	0	0	0	0	0	0	0	0	0	0	0	0	0	0	0	0	0	0	0	0
XM_021062375.1	LOC100626951	mRNA	0	0	0	0	0	0	0	0	0	0	0	0	0	0	0	0	0	0	0	0	0
XM_021062491.1	XRRA1	mRNA	0	0	0	0	0	0	0	0	0	0	0	0	0	0	0	0	0	0	0	0	0

RNA ID	Symbol	Type	updown_BGIR9823_99095	updown_BGIR9823_99528	updown_BGIR9823_99552	updown_BGIR9823_96972	updown_URS0000BC5676	updown_XR_001298953.2	updown_BGIR9823_101185	updown_BGIR9823_78435	updown_BGIR9823_101387	updown_BGIR9823_101406	updown_BGIR9823_102168	updown_BGIR9823_102282	updown_BGIR9823_100321	updown_BGIR9823_88590	updown_BGIR9823_89592	updown_BGIR9823_92306	updown_XR_002340248.1	updown_XR_002335693.1	updown_XR_002338205.1	updown_BGIR9823_101399	updown_BGIR9823_101545
XM_021062493.1	XRRA1	mRNA	0	0	0	0	0	0	0	0	0	0	0	0	0	0	0	0	0	0	0	0	0
XM_021062494.1	XRRA1	mRNA	0	0	0	0	0	0	0	0	0	0	0	0	0	0	0	0	0	0	0	0	0
XM_021062495.1	XRRA1	mRNA	0	0	0	0	0	0	0	0	0	0	0	0	0	0	0	0	0	0	0	0	0
XM_021062496.1	XRRA1	mRNA	0	0	0	0	0	0	0	0	0	0	0	0	0	0	0	0	0	0	0	0	0
XM_021062497.1	XRRA1	mRNA	0	0	0	0	0	0	0	0	0	0	0	0	0	0	0	0	0	0	0	0	0
XM_021062498.1	XRRA1	mRNA	0	0	0	0	0	0	0	0	0	0	0	0	0	0	0	0	0	0	0	0	0
XM_021062499.1	XRRA1	mRNA	0	0	0	0	0	0	0	0	0	0	0	0	0	0	0	0	0	0	0	0	0
XM_021062500.1	XRRA1	mRNA	0	0	0	0	0	0	0	0	0	0	0	0	0	0	0	0	0	0	0	0	0
XM_021062501.1	XRRA1	mRNA	0	0	0	0	0	0	0	0	0	0	0	0	0	0	0	0	0	0	0	0	0
XM_021062502.1	XRRA1	mRNA	0	0	0	0	0	0	0	0	0	0	0	0	0	0	0	0	0	0	0	0	0
XM_021062503.1	XRRA1	mRNA	0	0	0	0	0	0	0	0	0	0	0	0	0	0	0	0	0	0	0	0	0
XM_021062511.1	SLCO2B1	mRNA	0	0	0	0	0	0	0	0	0	0	0	0	0	0	0	0	0	0	0	0	0
XM_021062948.1	BCL9L	mRNA	0	0	0	0	0	0	0	0	0	0	0	0	0	0	0	0	0	0	0	0	0
XM_021062949.1	TRAPPC4	mRNA	0	0	0	0	0	0	0	0	0	0	0	0	0	0	0	0	0	0	0	0	0
XM_021062950.1	HYOU1	mRNA	0	0	0	0	0	0	0	0	0	0	0	0	0	0	0	0	0	0	0	0	0
XM_021062951.1	HYOU1	mRNA	0	0	0	0	0	0	0	0	0	0	0	0	0	0	0	0	0	0	0	0	0
XM_021062952.1	HYOU1	mRNA	0	0	0	0	0	0	0	0	0	0	0	0	0	0	0	0	0	0	0	0	0
XM_021062953.1	HYOU1	mRNA	0	0	0	0	0	0	0	0	0	0	0	0	0	0	0	0	0	0	0	0	0
XM_021062954.1	HYOU1	mRNA	0	0	0	0	0	0	0	0	0	0	0	0	0	0	0	0	0	0	0	0	0
XM_021062955.1	HYOU1	mRNA	0	0	0	0	0	0	0	0	0	0	0	0	0	0	0	0	0	0	0	0	0
XM_021062956.1	HYOU1	mRNA	0	0	0	0	0	0	0	0	0	0	0	0	0	0	0	0	0	0	0	0	0
XM_021062957.1	VPS11	mRNA	0	0	0	0	0	0	0	0	0	0	0	0	0	0	0	0	0	0	0	0	0
XM_021062993.1	LOC100523684	mRNA	0	0	0	0	0	0	0	0	0	0	0	0	0	0	0	0	0	1	0	0	0
XM_021062994.1	LOC100523684	mRNA	0	0	0	0	0	0	0	0	0	0	0	0	0	0	0	0	0	1	0	0	0
XM_021062995.1	LOC100523684	mRNA	0	0	0	0	0	0	0	0	0	0	0	0	0	0	0	0	0	1	0	0	0
XM_021062996.1	LOC100523684	mRNA	0	0	0	0	0	0	0	0	0	0	0	0	0	0	0	0	0	1	0	0	0
XM_021062997.1	LOC100523684	mRNA	0	0	0	0	0	0	0	0	0	0	0	0	0	0	0	0	0	1	0	0	0
XM_021062998.1	LOC100523684	mRNA	0	0	0	0	0	0	0	0	0	0	0	0	0	0	0	0	0	1	0	0	0
XM_021062999.1	LOC100523684	mRNA	0	0	0	0	0	0	0	0	0	0	0	0	0	0	0	0	0	1	0	0	0
XM_021063000.1	LOC100523684	mRNA	0	0	0	0	0	0	0	0	0	0	0	0	0	0	0	0	0	1	0	0	0
XM_021063001.1	LOC100523684	mRNA	0	0	0	0	0	0	0	0	0	0	0	0	0	0	0	0	0	1	0	0	0
XM_021063002.1	LOC100523684	mRNA	0	0	0	0	0	0	0	0	0	0	0	0	0	0	0	0	0	1	0	0	0
XM_021063004.1	LOC100523684	mRNA	0	0	0	0	0	0	0	0	0	0	0	0	0	0	0	0	0	1	0	0	0
XM_021063005.1	LOC100523684	mRNA	0	0	0	0	0	0	0	0	0	0	0	0	0	0	0	0	0	1	0	0	0
XM_021063006.1	LOC100523684	mRNA	0	0	0	0	0	0	0	0	0	0	0	0	0	0	0	0	0	1	0	0	0
XM_021063009.1	SORL1	mRNA	0	0	0	0	0	0	0	0	0	0	0	0	0	0	0	0	0	1	0	0	0
XM_021063010.1	SORL1	mRNA	0	0	0	0	0	0	0	0	0	0	0	0	0	0	0	0	0	1	0	0	0
XM_021063011.1	SORL1	mRNA	0	0	0	0	0	0	0	0	0	0	0	0	0	0	0	0	0	1	0	0	0
XM_021063012.1	SORL1	mRNA	0	0	0	0	0	0	0	0	0	0	0	0	0	0	0	0	0	1	0	0	0
XM_021063013.1	SORL1	mRNA	0	0	0	0	0	0	0	0	0	0	0	0	0	0	0	0	0	1	0	0	0
XM_021063014.1	SORL1	mRNA	0	0	0	0	0	0	0	0	0	0	0	0	0	0	0	0	0	1	0	0	0
XM_021063023.1	CLMP	mRNA	0	0	0	0	0	0	0	0	0	0	0	1	0	0	0	0	0	0	0	0	0
XM_021063024.1	CLMP	mRNA	0	0	0	0	0	0	0	0	0	0	0	1	0	0	0	0	0	0	0	0	0

RNA ID	Symbol	Type	updown_BGIR9823_99095	updown_BGIR9823_99528	updown_BGIR9823_99552	updown_BGIR9823_96972	updown_URS0000BC5676	updown_XR_001298953.2	updown_BGIR9823_101185	updown_BGIR9823_78435	updown_BGIR9823_101387	updown_BGIR9823_101406	updown_BGIR9823_102168	updown_BGIR9823_102282	updown_BGIR9823_100321	updown_BGIR9823_88590	updown_BGIR9823_89592	updown_BGIR9823_92306	updown_XR_002340248.1	updown_XR_002335693.1	updown_XR_002338205.1	updown_BGIR9823_101399	updown_BGIR9823_101545
XM_021063025.1	CLMP	mRNA	0	0	0	0	0	0	0	0	0	0	0	1	0	0	0	0	0	0	0	0	0
XM_021063026.1	CLMP	mRNA	0	0	0	0	0	0	0	0	0	0	0	1	0	0	0	0	0	0	0	0	0
XM_021063950.1	NXPE2	mRNA	0	0	0	0	0	0	0	0	0	0	0	0	0	0	0	0	0	0	0	0	0
XM_021063963.1	LOC100623190	mRNA	0	0	0	0	0	0	0	0	0	0	0	0	0	0	0	0	0	0	0	0	0
XM_021065159.1	HMGB1	mRNA	0	0	0	0	0	0	0	0	0	0	0	0	0	0	0	0	0	0	0	0	0
XM_021065160.1	HMGB1	mRNA	0	0	0	0	0	0	0	0	0	0	0	0	0	0	0	0	0	0	0	0	0
XM_021065161.1	HMGB1	mRNA	0	0	0	0	0	0	0	0	0	0	0	0	0	0	0	0	0	0	0	0	0
XM_021065529.1	KATNAL1	mRNA	0	0	0	0	0	0	0	0	0	0	0	0	0	0	0	0	0	0	0	0	0
XM_021065531.1	KATNAL1	mRNA	0	0	0	0	0	0	0	0	0	0	0	0	0	0	0	0	0	0	0	0	0
XM_021065532.1	KATNAL1	mRNA	0	0	0	0	0	0	0	0	0	0	0	0	0	0	0	0	0	0	0	0	0
XM_021065533.1	KATNAL1	mRNA	0	0	0	0	0	0	0	0	0	0	0	0	0	0	0	0	0	0	0	0	0
XM_021065534.1	KATNAL1	mRNA	0	0	0	0	0	0	0	0	0	0	0	0	0	0	0	0	0	0	0	0	0
XM_021065535.1	KATNAL1	mRNA	0	0	0	0	0	0	0	0	0	0	0	0	0	0	0	0	0	0	0	0	0
XM_021065536.1	KATNAL1	mRNA	0	0	0	0	0	0	0	0	0	0	0	0	0	0	0	0	0	0	0	0	0
XM_021065537.1	KATNAL1	mRNA	0	0	0	0	0	0	0	0	0	0	0	0	0	0	0	0	0	0	0	0	0
XM_021065538.1	KATNAL1	mRNA	0	0	0	0	0	0	0	0	0	0	0	0	0	0	0	0	0	0	0	0	0
XM_021065539.1	KATNAL1	mRNA	0	0	0	0	0	0	0	0	0	0	0	0	0	0	0	0	0	0	0	0	0
XM_021065541.1	KATNAL1	mRNA	0	0	0	0	0	0	0	0	0	0	0	0	0	0	0	0	0	0	0	0	0
XM_021065542.1	KATNAL1	mRNA	0	0	0	0	0	0	0	0	0	0	0	0	0	0	0	0	0	0	0	0	0
XM_021065543.1	KATNAL1	mRNA	0	0	0	0	0	0	0	0	0	0	0	0	0	0	0	0	0	0	0	0	0
XM_021065544.1	KATNAL1	mRNA	0	0	0	0	0	0	0	0	0	0	0	0	0	0	0	0	0	0	0	0	0
XM_021073408.1	EMX2	mRNA	0	0	0	0	0	0	0	0	0	0	0	0	0	0	0	0	0	0	0	0	0
XM_021078611.1	PRKAG2	mRNA	0	0	0	0	0	0	0	0	0	0	0	0	0	0	0	0	1	0	0	0	0
XM_021078612.1	PRKAG2	mRNA	0	0	0	0	0	0	0	0	0	0	0	0	0	0	0	0	1	0	0	0	0
XM_021078613.1	PRKAG2	mRNA	0	0	0	0	0	0	0	0	0	0	0	0	0	0	0	0	1	0	0	0	0
XM_021078614.1	PRKAG2	mRNA	0	0	0	0	0	0	0	0	0	0	0	0	0	0	0	0	1	0	0	0	0
XM_021078615.1	PRKAG2	mRNA	0	0	0	0	0	0	0	0	0	0	0	0	0	0	0	0	1	0	0	0	0
XM_021078616.1	PRKAG2	mRNA	0	0	0	0	0	0	0	0	0	0	0	0	0	0	0	0	1	0	0	0	0
XM_021078617.1	PRKAG2	mRNA	0	0	0	0	0	0	0	0	0	0	0	0	0	0	0	0	1	0	0	0	0
XM_021078618.1	PRKAG2	mRNA	0	0	0	0	0	0	0	0	0	0	0	0	0	0	0	0	1	0	0	0	0
XM_021078619.1	PRKAG2	mRNA	0	0	0	0	0	0	0	0	0	0	0	0	0	0	0	0	1	0	0	0	0
XM_021078620.1	PRKAG2	mRNA	0	0	0	0	0	0	0	0	0	0	0	0	0	0	0	0	1	0	0	0	0
XM_021078621.1	PRKAG2	mRNA	0	0	0	0	0	0	0	0	0	0	0	0	0	0	0	0	1	0	0	0	0
XM_021081309.1	LOC106506226	mRNA	0	0	0	0	0	0	0	0	1	0	0	0	0	0	0	0	0	0	0	0	0
XM_021081403.1	LOC110258215	mRNA	0	0	0	0	0	0	0	0	0	1	0	0	0	0	0	0	0	0	0	1	0
XM_021082153.1	LOC100521600	mRNA	0	0	0	0	0	0	0	0	0	0	0	0	0	0	0	0	0	0	0	0	0
XM_021082154.1	LOC100521600	mRNA	0	0	0	0	0	0	0	0	0	0	0	0	0	0	0	0	0	0	0	0	0
XM_021082156.1	LOC100521600	mRNA	0	0	0	0	0	0	0	0	0	0	0	0	0	0	0	0	0	0	0	0	0
XM_021082157.1	LOC100521600	mRNA	0	0	0	0	0	0	0	0	0	0	0	0	0	0	0	0	0	0	0	0	0
XM_021082158.1	LOC100521600	mRNA	0	0	0	0	0	0	0	0	0	0	0	0	0	0	0	0	0	0	0	0	0
XM_021082159.1	LOC100521600	mRNA	0	0	0	0	0	0	0	0	0	0	0	0	0	0	0	0	0	0	0	0	0
XM_021082835.1	EHBP1L1	mRNA	0	0	0	0	0	0	0	0	0	0	0	0	0	0	0	0	0	0	0	0	0
XM_021082836.1	EHBP1L1	mRNA	0	0	0	0	0	0	0	0	0	0	0	0	0	0	0	0	0	0	0	0	0

RNA ID	Symbol	Type	updown_BGIR9823_99095	updown_BGIR9823_99528	updown_BGIR9823_99552	updown_BGIR9823_96972	updown_URS0000BC5676	updown_XR_001298953.2	updown_BGIR9823_101185	updown_BGIR9823_78435	updown_BGIR9823_101387	updown_BGIR9823_101406	updown_BGIR9823_102168	updown_BGIR9823_102282	updown_BGIR9823_100321	updown_BGIR9823_88590	updown_BGIR9823_89592	updown_BGIR9823_92306	updown_XR_002340248.1	updown_XR_002335693.1	updown_XR_002338205.1	updown_BGIR9823_101399	updown_BGIR9823_101545
XM_021082837.1	EHBP1L1	mRNA	0	0	0	0	0	0	0	0	0	0	0	0	0	0	0	0	0	0	0	0	0
XM_021082838.1	EHBP1L1	mRNA	0	0	0	0	0	0	0	0	0	0	0	0	0	0	0	0	0	0	0	0	0
XM_021082839.1	EHBP1L1	mRNA	0	0	0	0	0	0	0	0	0	0	0	0	0	0	0	0	0	0	0	0	0
XM_021082840.1	EHBP1L1	mRNA	0	0	0	0	0	0	0	0	0	0	0	0	0	0	0	0	0	0	0	0	0
XM_021082841.1	EHBP1L1	mRNA	0	0	0	0	0	0	0	0	0	0	0	0	0	0	0	0	0	0	0	0	0
XM_021082842.1	EHBP1L1	mRNA	0	0	0	0	0	0	0	0	0	0	0	0	0	0	0	0	0	0	0	0	0
XM_021082843.1	EHBP1L1	mRNA	0	0	0	0	0	0	0	0	0	0	0	0	0	0	0	0	0	0	0	0	0
XM_021082844.1	EHBP1L1	mRNA	0	0	0	0	0	0	0	0	0	0	0	0	0	0	0	0	0	0	0	0	0
XM_021082845.1	EHBP1L1	mRNA	0	0	0	0	0	0	0	0	0	0	0	0	0	0	0	0	0	0	0	0	0
XM_021082846.1	EHBP1L1	mRNA	0	0	0	0	0	0	0	0	0	0	0	0	0	0	0	0	0	0	0	0	0
XM_021082847.1	EHBP1L1	mRNA	0	0	0	0	0	0	0	0	0	0	0	0	0	0	0	0	0	0	0	0	0
XM_021082851.1	MAP3K11	mRNA	0	0	0	0	0	0	0	0	0	0	0	0	0	0	0	0	0	0	0	0	0
XM_021082852.1	SSSCA1	mRNA	0	0	0	0	0	0	0	0	0	0	0	0	0	0	0	0	0	0	0	0	0
XM_021082853.1	FAM89B	mRNA	0	0	0	0	0	0	0	0	0	0	0	0	0	0	0	0	0	0	0	0	0
XM_021082854.1	LTBP3	mRNA	0	0	0	0	0	0	0	0	0	0	0	0	0	0	0	0	0	0	0	0	0
XM_021082855.1	LTBP3	mRNA	0	0	0	0	0	0	0	0	0	0	0	0	0	0	0	0	0	0	0	0	0
XM_021082856.1	SCYL1	mRNA	0	0	0	0	0	0	0	0	0	0	0	0	0	0	0	0	0	0	0	0	0
XM_021082857.1	SLC25A45	mRNA	0	0	0	0	0	0	0	0	0	0	0	0	0	0	0	0	0	0	0	0	0
XM_021082858.1	SLC25A45	mRNA	0	0	0	0	0	0	0	0	0	0	0	0	0	0	0	0	0	0	0	0	0
XM_021091099.1	SYNGR1	mRNA	0	0	0	0	0	0	0	0	0	0	0	0	0	0	0	0	0	0	0	0	0
XM_021091101.1	SYNGR1	mRNA	0	0	0	0	0	0	0	0	0	0	0	0	0	0	0	0	0	0	0	0	0
XM_021091102.1	SYNGR1	mRNA	0	0	0	0	0	0	0	0	0	0	0	0	0	0	0	0	0	0	0	0	0
XM_021091103.1	SYNGR1	mRNA	0	0	0	0	0	0	0	0	0	0	0	0	0	0	0	0	0	0	0	0	0
XM_021091370.1	LOC102159820	mRNA	0	0	0	0	0	0	0	0	0	0	0	0	0	0	0	0	0	0	0	0	0
XM_021091373.1	TRMU	mRNA	0	0	0	0	0	0	0	0	0	0	0	0	0	0	0	0	0	0	0	0	0
XM_021091374.1	TRMU	mRNA	0	0	0	0	0	0	0	0	0	0	0	0	0	0	0	0	0	0	0	0	0
XM_021091375.1	TRMU	mRNA	0	0	0	0	0	0	0	0	0	0	0	0	0	0	0	0	0	0	0	0	0
XM_021091376.1	GTSE1	mRNA	0	0	0	0	0	0	0	0	0	0	0	0	0	0	0	0	0	0	0	0	0
XM_021091377.1	TTC38	mRNA	0	0	0	0	0	0	0	0	0	0	0	0	0	0	0	0	0	0	0	0	0
XM_021091379.1	LOC110260611	mRNA	0	0	0	0	0	0	0	0	0	0	0	0	0	0	0	0	0	0	0	0	0
XM_021091381.1	LOC110260611	mRNA	0	0	0	0	0	0	0	0	0	0	0	0	0	0	0	0	0	0	0	0	0
XM_021091454.1	TOB2	mRNA	0	0	0	0	0	0	0	0	0	0	0	0	0	0	0	0	0	0	0	0	0
XM_021091455.1	TOB2	mRNA	0	0	0	0	0	0	0	0	0	0	0	0	0	0	0	0	0	0	0	0	0
XM_021091456.1	PHF5A	mRNA	0	0	0	0	0	0	0	0	0	0	0	0	0	0	0	0	0	0	0	0	0
XM_021091457.1	PHF5A	mRNA	0	0	0	0	0	0	0	0	0	0	0	0	0	0	0	0	0	0	0	0	0
XM_021091458.1	ZC3H7B	mRNA	0	0	0	0	0	0	0	0	0	0	0	0	0	0	0	0	0	0	0	0	0
XM_021091459.1	ZC3H7B	mRNA	0	0	0	0	0	0	0	0	0	0	0	0	0	0	0	0	0	0	0	0	0
XM_021091460.1	TEF	mRNA	0	0	0	0	0	0	0	0	0	0	0	0	0	0	0	0	0	0	0	0	0
XM_021091461.1	RANGAP1	mRNA	0	0	0	0	0	0	0	0	0	0	0	0	0	0	0	0	0	0	0	0	0
XM_021091474.1	CBX7	mRNA	0	0	0	0	0	0	0	0	0	0	0	0	0	0	0	0	0	0	0	0	0
XM_021092767.1	CRADD	mRNA	0	0	0	0	0	0	0	0	0	0	0	0	0	0	0	0	0	0	0	0	0
XM_021092768.1	CRADD	mRNA	0	0	0	0	0	0	0	0	0	0	0	0	0	0	0	0	0	0	0	0	0
XM_021092769.1	CRADD	mRNA	0	0	0	0	0	0	0	0	0	0	0	0	0	0	0	0	0	0	0	0	0

RNA ID	Symbol	Type	updown_BGIR9823_99095	updown_BGIR9823_99528	updown_BGIR9823_99552	updown_BGIR9823_96972	updown_URS0000BC5676	updown_XR_001298953.2	updown_BGIR9823_101185	updown_BGIR9823_78435	updown_BGIR9823_101387	updown_BGIR9823_101406	updown_BGIR9823_102168	updown_BGIR9823_102282	updown_BGIR9823_100321	updown_BGIR9823_88590	updown_BGIR9823_89592	updown_BGIR9823_92306	updown_XR_002340248.1	updown_XR_002335693.1	updown_XR_002338205.1	updown_BGIR9823_101399	updown_BGIR9823_101545
XM_021092770.1	CRADD	mRNA	0	0	0	0	0	0	0	0	0	0	0	0	0	0	0	0	0	0	0	0	0
XM_021092771.1	CRADD	mRNA	0	0	0	0	0	0	0	0	0	0	0	0	0	0	0	0	0	0	0	0	0
XM_021092773.1	CRADD	mRNA	0	0	0	0	0	0	0	0	0	0	0	0	0	0	0	0	0	0	0	0	0
XM_021093095.1	PKDREJ	mRNA	0	0	0	0	0	0	0	0	0	0	0	0	0	0	0	0	0	0	0	0	0
XM_021097064.1	MAF	mRNA	0	0	0	0	0	0	0	0	0	0	0	0	0	0	0	0	0	0	0	0	0
XM_021097065.1	MAF	mRNA	0	0	0	0	0	0	0	0	0	0	0	0	0	0	0	0	0	0	0	0	0
XM_021097066.1	MAF	mRNA	0	0	0	0	0	0	0	0	0	0	0	0	0	0	0	0	0	0	0	0	0
XM_021097870.1	ECI2	mRNA	0	0	0	0	0	0	0	0	0	0	0	0	0	0	0	0	0	0	0	0	0
XM_021099751.1	PHACTR1	mRNA	0	0	0	0	0	0	0	0	0	0	0	0	0	0	0	0	0	0	0	0	0
XM_021099752.1	PHACTR1	mRNA	0	0	0	0	0	0	0	0	0	0	0	0	0	0	0	0	0	0	0	0	0
XM_021099753.1	PHACTR1	mRNA	0	0	0	0	0	0	0	0	0	0	0	0	0	0	0	0	0	0	0	0	0
XM_021099754.1	PHACTR1	mRNA	0	0	0	0	0	0	0	0	0	0	0	0	0	0	0	0	0	0	0	0	0
XM_021099755.1	PHACTR1	mRNA	0	0	0	0	0	0	0	0	0	0	0	0	0	0	0	0	0	0	0	0	0
XM_021099756.1	PHACTR1	mRNA	0	0	0	0	0	0	0	0	0	0	0	0	0	0	0	0	0	0	0	0	0
XM_021099757.1	PHACTR1	mRNA	0	0	0	0	0	0	0	0	0	0	0	0	0	0	0	0	0	0	0	0	0
XM_021099758.1	PHACTR1	mRNA	0	0	0	0	0	0	0	0	0	0	0	0	0	0	0	0	0	0	0	0	0
XM_021099759.1	PHACTR1	mRNA	0	0	0	0	0	0	0	0	0	0	0	0	0	0	0	0	0	0	0	0	0
XM_021099760.1	PHACTR1	mRNA	0	0	0	0	0	0	0	0	0	0	0	0	0	0	0	0	0	0	0	0	0
XM_021099761.1	PHACTR1	mRNA	0	0	0	0	0	0	0	0	0	0	0	0	0	0	0	0	0	0	0	0	0
XM_021099762.1	PHACTR1	mRNA	0	0	0	0	0	0	0	0	0	0	0	0	0	0	0	0	0	0	0	0	0
XM_021099763.1	PHACTR1	mRNA	0	0	0	0	0	0	0	0	0	0	0	0	0	0	0	0	0	0	0	0	0
XM_021099764.1	PHACTR1	mRNA	0	0	0	0	0	0	0	0	0	0	0	0	0	0	0	0	0	0	0	0	0
XM_021099765.1	PHACTR1	mRNA	0	0	0	0	0	0	0	0	0	0	0	0	0	0	0	0	0	0	0	0	0
XM_021099766.1	TBC1D7	mRNA	0	0	0	0	0	0	0	0	0	0	0	0	0	0	0	0	0	0	0	0	0
XM_021099767.1	TBC1D7	mRNA	0	0	0	0	0	0	0	0	0	0	0	0	0	0	0	0	0	0	0	0	0
XM_021099769.1	TBC1D7	mRNA	0	0	0	0	0	0	0	0	0	0	0	0	0	0	0	0	0	0	0	0	0
XM_021099770.1	TBC1D7	mRNA	0	0	0	0	0	0	0	0	0	0	0	0	0	0	0	0	0	0	0	0	0
XM_021099999.1	LOC110261697	mRNA	0	0	0	0	0	0	0	0	0	0	0	0	0	0	0	0	0	0	0	0	0
XM_021100108.1	WRNIP1	mRNA	0	0	0	0	0	0	0	0	0	0	0	0	0	0	0	0	0	0	0	0	0
XM_021100109.1	MYLK4	mRNA	0	0	0	0	0	0	0	0	0	0	0	0	0	0	0	0	0	0	0	0	0
XM_021100110.1	MYLK4	mRNA	0	0	0	0	0	0	0	0	0	0	0	0	0	0	0	0	0	0	0	0	0
XM_021100111.1	MYLK4	mRNA	0	0	0	0	0	0	0	0	0	0	0	0	0	0	0	0	0	0	0	0	0
XM_021100112.1	MYLK4	mRNA	0	0	0	0	0	0	0	0	0	0	0	0	0	0	0	0	0	0	0	0	0
XM_021100114.1	NQO2	mRNA	0	0	0	0	0	0	0	0	0	0	0	0	0	0	0	0	0	0	0	0	0
XM_021100115.1	NQO2	mRNA	0	0	0	0	0	0	0	0	0	0	0	0	0	0	0	0	0	0	0	0	0
XM_021100117.1	NQO2	mRNA	0	0	0	0	0	0	0	0	0	0	0	0	0	0	0	0	0	0	0	0	0
XM_021100118.1	NQO2	mRNA	0	0	0	0	0	0	0	0	0	0	0	0	0	0	0	0	0	0	0	0	0
XM_021100119.1	NQO2	mRNA	0	0	0	0	0	0	0	0	0	0	0	0	0	0	0	0	0	0	0	0	0
XM_021100124.1	PRPF4B	mRNA	0	0	0	0	0	0	0	0	0	0	0	0	0	0	0	0	0	0	0	0	0
XM_021100126.1	PRPF4B	mRNA	0	0	0	0	0	0	0	0	0	0	0	0	0	0	0	0	0	0	0	0	0
XM_021100184.1	NEDD9	mRNA	0	0	0	0	0	0	0	0	0	0	0	0	0	0	0	0	0	0	0	0	0
XM_021100188.1	TMEM170B	mRNA	0	0	0	0	0	0	0	0	0	0	0	0	0	0	0	0	0	0	0	0	0
XM_021100194.1	GFOD1	mRNA	0	0	0	0	0	0	0	0	0	0	0	0	0	0	0	0	0	0	0	0	0

RNA ID	Symbol	Type	updown_BGIR9823_99095	updown_BGIR9823_99528	updown_BGIR9823_99552	updown_BGIR9823_96972	updown_URS0000BC5676	updown_XR_001298953.2	updown_BGIR9823_101185	updown_BGIR9823_78435	updown_BGIR9823_101387	updown_BGIR9823_101406	updown_BGIR9823_102168	updown_BGIR9823_102282	updown_BGIR9823_100321	updown_BGIR9823_88590	updown_BGIR9823_89592	updown_BGIR9823_92306	updown_XR_002340248.1	updown_XR_002335693.1	updown_XR_002338205.1	updown_BGIR9823_101399	updown_BGIR9823_101545
XM_021102040.1	SLC37A4	mRNA	0	0	0	0	0	0	0	0	0	0	0	0	0	0	0	0	0	0	0	0	0
XM_021102041.1	SLC37A4	mRNA	0	0	0	0	0	0	0	0	0	0	0	0	0	0	0	0	0	0	0	0	0
XM_021102042.1	SLC37A4	mRNA	0	0	0	0	0	0	0	0	0	0	0	0	0	0	0	0	0	0	0	0	0
XM_021102043.1	SLC37A4	mRNA	0	0	0	0	0	0	0	0	0	0	0	0	0	0	0	0	0	0	0	0	0
XM_021102044.1	SLC37A4	mRNA	0	0	0	0	0	0	0	0	0	0	0	0	0	0	0	0	0	0	0	0	0
XM_021102045.1	SLC37A4	mRNA	0	0	0	0	0	0	0	0	0	0	0	0	0	0	0	0	0	0	0	0	0
XM_021102046.1	SLC37A4	mRNA	0	0	0	0	0	0	0	0	0	0	0	0	0	0	0	0	0	0	0	0	0
XM_021102047.1	SLC37A4	mRNA	0	0	0	0	0	0	0	0	0	0	0	0	0	0	0	0	0	0	0	0	0
XM_021102048.1	SLC37A4	mRNA	0	0	0	0	0	0	0	0	0	0	0	0	0	0	0	0	0	0	0	0	0
XM_021102049.1	SLC37A4	mRNA	0	0	0	0	0	0	0	0	0	0	0	0	0	0	0	0	0	0	0	0	0
XM_021102050.1	SLC37A4	mRNA	0	0	0	0	0	0	0	0	0	0	0	0	0	0	0	0	0	0	0	0	0
XM_021102051.1	SLC37A4	mRNA	0	0	0	0	0	0	0	0	0	0	0	0	0	0	0	0	0	0	0	0	0
XM_021102052.1	SLC37A4	mRNA	0	0	0	0	0	0	0	0	0	0	0	0	0	0	0	0	0	0	0	0	0
XM_021102209.1	UPK2	mRNA	0	0	0	0	0	0	0	0	0	0	0	0	0	0	0	0	0	0	0	0	0
XM_021102516.1	LOC100522455	mRNA	0	0	0	0	0	0	0	0	0	0	0	0	0	0	0	0	0	0	0	0	0
XM_021102517.1	RUNDC3B	mRNA	0	0	0	0	0	0	0	0	0	0	0	0	0	0	0	0	0	0	0	0	0
XM_021102518.1	RUNDC3B	mRNA	0	0	0	0	0	0	0	0	0	0	0	0	0	0	0	0	0	0	0	0	0
XM_021102519.1	RUNDC3B	mRNA	0	0	0	0	0	0	0	0	0	0	0	0	0	0	0	0	0	0	0	0	0
XM_021102522.1	RUNDC3B	mRNA	0	0	0	0	0	0	0	0	0	0	0	0	0	0	0	0	0	0	0	0	0
XM_021102523.1	RUNDC3B	mRNA	0	0	0	0	0	0	0	0	0	0	0	0	0	0	0	0	0	0	0	0	0
XR_001297664.2	LOC102161744	lncRNA	0	0	0	0	0	0	0	0	0	0	0	0	0	0	0	0	0	0	0	0	0
XR_001298807.2	LOC106504881	lncRNA	0	0	0	0	0	0	0	0	0	0	0	0	0	0	0	0	0	0	0	0	0
XR_001298808.2	LOC106504881	lncRNA	0	0	0	0	0	0	0	0	0	0	0	0	0	0	0	0	0	0	0	0	0
XR_001298810.2	LOC106504881	lncRNA	0	0	0	0	0	0	0	0	0	0	0	0	0	0	0	0	0	0	0	0	0
XR_001298811.2	LOC106504881	lncRNA	0	0	0	0	0	0	0	0	0	0	0	0	0	0	0	0	0	0	0	0	0
XR_001298812.2	LOC106504881	lncRNA	0	0	0	0	0	0	0	0	0	0	0	0	0	0	0	0	0	0	0	0	0
XR_001298813.2	LOC106504881	lncRNA	0	0	0	0	0	0	0	0	0	0	0	0	0	0	0	0	0	0	0	0	0
XR_001298814.2	LOC106504881	lncRNA	0	0	0	0	0	0	0	0	0	0	0	0	0	0	0	0	0	0	0	0	0
XR_001298816.2	LOC106504881	lncRNA	0	0	0	0	0	0	0	0	0	0	0	0	0	0	0	0	0	0	0	0	0
XR_001298817.2	LOC106504881	lncRNA	0	0	0	0	0	0	0	0	0	0	0	0	0	0	0	0	0	0	0	0	0
XR_001298844.2	LOC102163364	lncRNA	0	0	0	0	0	0	0	0	0	0	0	0	0	0	0	0	0	1	0	0	0
XR_001298846.2	LOC102163364	lncRNA	0	0	0	0	0	0	0	0	0	0	0	0	0	0	0	0	0	1	0	0	0
XR_001298850.2	LOC102163364	lncRNA	0	0	0	0	0	0	0	0	0	0	0	0	0	0	0	0	0	1	0	0	0
XR_001299456.2	LOC102166670	lncRNA	0	0	0	0	0	0	0	0	0	0	0	0	0	0	0	0	0	0	0	0	0
XR_001299457.2	LOC102166670	lncRNA	0	0	0	0	0	0	0	0	0	0	0	0	0	0	0	0	0	0	0	0	0
XR_001300715.2	LOC106505879	lncRNA	0	0	0	0	0	0	0	0	0	0	0	0	0	0	0	0	0	0	0	0	0
XR_001301078.2	LOC102162623	lncRNA	0	0	0	0	0	0	0	0	0	0	0	0	0	0	0	0	1	0	0	0	0
XR_001301087.2	LOC102162623	lncRNA	0	0	0	0	0	0	0	0	0	0	0	0	0	0	0	0	1	0	0	0	0
XR_001301217.2	LOC102164998	lncRNA	0	0	0	0	0	0	0	0	0	0	0	0	0	0	0	0	0	0	0	0	0
XR_001301305.2	LOC102159607	lncRNA	0	0	0	0	0	0	0	0	0	0	0	0	0	0	0	0	0	0	0	0	0
XR_001308967.2	LOC106510467	lncRNA	0	0	0	0	0	0	0	0	0	0	0	0	0	0	0	0	0	0	0	0	0
XR_002335533.1	XRRA1	mRNA	0	0	0	0	0	0	0	0	0	0	0	0	0	0	0	0	0	0	0	0	0
XR_002335534.1	XRRA1	mRNA	0	0	0	0	0	0	0	0	0	0	0	0	0	0	0	0	0	0	0	0	0

RNA ID	Symbol	Type	updown_BGIR9823_99095	updown_BGIR9823_99528	updown_BGIR9823_99552	updown_BGIR9823_96972	updown_URS0000BC5676	updown_XR_001298953.2	updown_BGIR9823_101185	updown_BGIR9823_78435	updown_BGIR9823_101387	updown_BGIR9823_101406	updown_BGIR9823_102168	updown_BGIR9823_102282	updown_BGIR9823_100321	updown_BGIR9823_88590	updown_BGIR9823_89592	updown_BGIR9823_92306	updown_XR_002340248.1	updown_XR_002335693.1	updown_XR_002338205.1	updown_BGIR9823_101399	updown_BGIR9823_101545
XR_002335535.1	LOC110255399	lncRNA	0	0	0	0	0	0	0	0	0	0	0	0	0	0	0	0	0	0	0	0	0
XR_002335536.1	LOC102160367	lncRNA	0	0	0	0	0	0	0	0	0	0	0	0	0	0	0	0	0	0	0	0	0
XR_002335537.1	LOC102160367	lncRNA	0	0	0	0	0	0	0	0	0	0	0	0	0	0	0	0	0	0	0	0	0
XR_002335650.1	LOC102157630	lncRNA	0	0	0	0	0	0	0	0	0	0	0	0	0	0	0	0	0	0	0	0	0
XR_002335651.1	LOC110255435	lncRNA	0	0	0	0	0	0	0	0	0	0	0	0	0	0	0	0	0	0	0	0	0
XR_002335652.1	LOC106504881	lncRNA	0	0	0	0	0	0	0	0	0	0	0	0	0	0	0	0	0	0	0	0	0
XR_002335653.1	LOC106504881	lncRNA	0	0	0	0	0	0	0	0	0	0	0	0	0	0	0	0	0	0	0	0	0
XR_002335670.1	LOC110255440	lncRNA	0	0	0	0	0	0	0	0	0	0	0	0	0	0	0	0	0	0	0	0	0
XR_002335671.1	LOC110255441	lncRNA	0	0	0	0	0	0	0	0	0	0	0	0	0	0	0	0	0	0	0	0	0
XR_002335672.1	LOC110255441	lncRNA	0	0	0	0	0	0	0	0	0	0	0	0	0	0	0	0	0	0	0	0	0
XR_002335674.1	HYOU1	mRNA	0	0	0	0	0	0	0	0	0	0	0	0	0	0	0	0	0	0	0	0	0
XR_002335675.1	HYOU1	mRNA	0	0	0	0	0	0	0	0	0	0	0	0	0	0	0	0	0	0	0	0	0
XR_002335685.1	LOC100523684	mRNA	0	0	0	0	0	0	0	0	0	0	0	0	0	0	0	0	0	1	0	0	0
XR_002335686.1	LOC102163364	lncRNA	0	0	0	0	0	0	0	0	0	0	0	0	0	0	0	0	0	1	0	0	0
XR_002335687.1	LOC102163364	lncRNA	0	0	0	0	0	0	0	0	0	0	0	0	0	0	0	0	0	1	0	0	0
XR_002335688.1	LOC102163364	lncRNA	0	0	0	0	0	0	0	0	0	0	0	0	0	0	0	0	0	1	0	0	0
XR_002335689.1	LOC102163364	lncRNA	0	0	0	0	0	0	0	0	0	0	0	0	0	0	0	0	0	1	0	0	0
XR_002335690.1	LOC102163364	lncRNA	0	0	0	0	0	0	0	0	0	0	0	0	0	0	0	0	0	1	0	0	0
XR_002335691.1	LOC102163364	lncRNA	0	0	0	0	0	0	0	0	0	0	0	0	0	0	0	0	0	1	0	0	0
XR_002335692.1	LOC102163364	lncRNA	0	0	0	0	0	0	0	0	0	0	0	0	0	0	0	0	0	1	0	0	0
XR_002335693.1	LOC106504896	lncRNA	0	0	0	0	0	0	0	0	0	0	0	0	0	0	0	0	0	1	0	0	0
XR_002335694.1	SORL1	mRNA	0	0	0	0	0	0	0	0	0	0	0	0	0	0	0	0	0	1	0	0	0
XR_002336641.1	LOC106505250	lncRNA	0	0	0	0	0	0	0	0	0	0	0	0	0	0	0	0	0	0	0	0	0
XR_002336643.1	LOC110255292	lncRNA	0	0	0	0	0	0	0	0	0	0	0	0	0	0	0	0	0	0	0	0	0
XR_002336644.1	LOC102166670	lncRNA	0	0	0	0	0	0	0	0	0	0	0	0	0	0	0	0	0	0	0	0	0
XR_002336645.1	LOC102166670	lncRNA	0	0	0	0	0	0	0	0	0	0	0	0	0	0	0	0	0	0	0	0	0
XR_002338443.1	LOC106505879	lncRNA	0	0	0	0	0	0	0	0	0	0	0	0	0	0	0	0	0	0	0	0	0
XR_002338444.1	LOC106505879	lncRNA	0	0	0	0	0	0	0	0	0	0	0	0	0	0	0	0	0	0	0	0	0
XR_002338445.1	LOC106505879	lncRNA	0	0	0	0	0	0	0	0	0	0	0	0	0	0	0	0	0	0	0	0	0
XR_002338446.1	LOC106505879	lncRNA	0	0	0	0	0	0	0	0	0	0	0	0	0	0	0	0	0	0	0	0	0
XR_002338447.1	LOC106505879	lncRNA	0	0	0	0	0	0	0	0	0	0	0	0	0	0	0	0	0	0	0	0	0
XR_002338448.1	LOC106505879	lncRNA	0	0	0	0	0	0	0	0	0	0	0	0	0	0	0	0	0	0	0	0	0
XR_002338449.1	LOC106505879	lncRNA	0	0	0	0	0	0	0	0	0	0	0	0	0	0	0	0	0	0	0	0	0
XR_002338450.1	LOC106505879	lncRNA	0	0	0	0	0	0	0	0	0	0	0	0	0	0	0	0	0	0	0	0	0
XR_002338521.1	LOC110256664	lncRNA	0	0	0	0	0	0	0	0	0	0	0	0	0	0	0	0	0	0	0	0	0
XR_002338522.1	LOC110256664	lncRNA	0	0	0	0	0	0	0	0	0	0	0	0	0	0	0	0	0	0	0	0	0
XR_002338666.1	LOC110256747	lncRNA	0	0	0	0	0	0	0	0	0	0	0	0	0	0	0	0	0	0	0	0	0
XR_002338714.1	LOC102163817	lncRNA	0	0	0	0	0	0	0	0	0	0	0	0	0	0	0	0	0	0	0	0	0
XR_002338715.1	LOC102164998	lncRNA	0	0	0	0	0	0	0	0	0	0	0	0	0	0	0	0	0	0	0	0	0
XR_002338716.1	LOC102164998	lncRNA	0	0	0	0	0	0	0	0	0	0	0	0	0	0	0	0	0	0	0	0	0
XR_002338717.1	LOC110256768	lncRNA	0	0	0	0	0	0	0	0	0	0	0	0	0	0	0	0	0	0	0	0	0
XR_002338727.1	LOC110256773	lncRNA	0	0	0	0	0	0	0	0	0	0	0	0	0	0	0	0	0	0	0	0	0
XR_002338728.1	LOC110256773	lncRNA	0	0	0	0	0	0	0	0	0	0	0	0	0	0	0	0	0	0	0	0	0

RNA ID	Symbol	Type	updown_BGIR9823_99095	updown_BGIR9823_99528	updown_BGIR9823_99552	updown_BGIR9823_96972	updown_URS0000BC5676	updown_XR_001298953.2	updown_BGIR9823_101185	updown_BGIR9823_78435	updown_BGIR9823_101387	updown_BGIR9823_101406	updown_BGIR9823_102168	updown_BGIR9823_102282	updown_BGIR9823_100321	updown_BGIR9823_88590	updown_BGIR9823_89592	updown_BGIR9823_92306	updown_XR_002340248.1	updown_XR_002335693.1	updown_XR_002338205.1	updown_BGIR9823_101399	updown_BGIR9823_101545
XR_002338729.1	LOC110256773	lncRNA	0	0	0	0	0	0	0	0	0	0	0	0	0	0	0	0	0	0	0	0	0
XR_002338793.1	LOC110256796	lncRNA	0	0	0	0	0	0	0	0	0	0	0	0	0	0	0	0	0	0	0	0	0
XR_002339623.1	LOC102167708	lncRNA	0	0	0	0	0	0	0	0	0	0	0	0	0	0	0	0	0	0	0	0	0
XR_002340245.1	PRKAG2	mRNA	0	0	0	0	0	0	0	0	0	0	0	0	0	0	0	0	1	0	0	0	0
XR_002340246.1	LOC102162623	lncRNA	0	0	0	0	0	0	0	0	0	0	0	0	0	0	0	0	1	0	0	0	0
XR_002340247.1	LOC102162623	lncRNA	0	0	0	0	0	0	0	0	0	0	0	0	0	0	0	0	1	0	0	0	0
XR_002340248.1	LOC110257516	lncRNA	0	0	0	0	0	0	0	0	0	0	0	0	0	0	0	0	1	0	0	0	0
XR_002340249.1	LOC102162071	lncRNA	0	0	0	0	0	0	0	0	0	0	0	0	0	0	0	0	1	0	0	0	0
XR_002340901.1	LOC102160243	lncRNA	0	0	0	0	0	0	0	0	0	0	0	0	0	0	0	0	0	0	0	0	0
XR_002340902.1	LOC102160243	lncRNA	0	0	0	0	0	0	0	0	0	0	0	0	0	0	0	0	0	0	0	0	0
XR_002340903.1	LOC102160243	lncRNA	0	0	0	0	0	0	0	0	0	0	0	0	0	0	0	0	0	0	0	0	0
XR_002340904.1	LOC102160243	lncRNA	0	0	0	0	0	0	0	0	0	0	0	0	0	0	0	0	0	0	0	0	0
XR_002340905.1	LOC102160243	lncRNA	0	0	0	0	0	0	0	0	0	0	0	0	0	0	0	0	0	0	0	0	0
XR_002341237.1	LOC102164320	lncRNA	0	0	0	0	1	0	0	0	0	0	0	0	0	0	0	0	0	0	0	0	0
XR_002341238.1	LOC102164320	lncRNA	0	0	0	0	1	0	0	0	0	0	0	0	0	0	0	0	0	0	0	0	0
XR_002341239.1	LOC102164320	lncRNA	0	0	0	0	1	0	0	0	0	0	0	0	0	0	0	0	0	0	0	0	0
XR_002341240.1	LOC102164320	lncRNA	0	0	0	0	1	0	0	0	0	0	0	0	0	0	0	0	0	0	0	0	0
XR_002341241.1	LOC102164320	lncRNA	0	0	0	0	1	0	0	0	0	0	0	0	0	0	0	0	0	0	0	0	0
XR_002341242.1	LOC102164320	lncRNA	0	0	0	0	1	0	0	0	0	0	0	0	0	0	0	0	0	0	0	0	0
XR_002341243.1	LOC102164320	lncRNA	0	0	0	0	1	0	0	0	0	0	0	0	0	0	0	0	0	0	0	0	0
XR_002341244.1	LOC102164320	lncRNA	0	0	0	0	1	0	0	0	0	0	0	0	0	0	0	0	0	0	0	0	0
XR_002341270.1	LOC100521600	mRNA	0	0	0	0	0	0	0	0	0	0	0	0	0	0	0	0	0	0	0	0	0
XR_002341443.1	LOC102163816	lncRNA	0	0	0	0	0	0	0	0	0	0	0	0	0	0	0	0	0	0	0	0	0
XR_002343847.1	LOC110260611	mRNA	0	0	0	0	0	0	0	0	0	0	0	0	0	0	0	0	0	0	0	0	0
XR_002343848.1	LOC110260611	mRNA	0	0	0	0	0	0	0	0	0	0	0	0	0	0	0	0	0	0	0	0	0
XR_002343849.1	LOC110260611	mRNA	0	0	0	0	0	0	0	0	0	0	0	0	0	0	0	0	0	0	0	0	0
XR_002343850.1	LOC110260611	mRNA	0	0	0	0	0	0	0	0	0	0	0	0	0	0	0	0	0	0	0	0	0
XR_002343875.1	LOC110260629	lncRNA	0	0	0	0	0	0	0	0	0	0	0	0	0	0	0	0	0	0	0	0	0
XR_002343876.1	LOC110260629	lncRNA	0	0	0	0	0	0	0	0	0	0	0	0	0	0	0	0	0	0	0	0	0
XR_002343877.1	LOC110260629	lncRNA	0	0	0	0	0	0	0	0	0	0	0	0	0	0	0	0	0	0	0	0	0
XR_002343878.1	LOC110260629	lncRNA	0	0	0	0	0	0	0	0	0	0	0	0	0	0	0	0	0	0	0	0	0
XR_002343879.1	LOC110260629	lncRNA	0	0	0	0	0	0	0	0	0	0	0	0	0	0	0	0	0	0	0	0	0
XR_002343880.1	LOC110260629	lncRNA	0	0	0	0	0	0	0	0	0	0	0	0	0	0	0	0	0	0	0	0	0
XR_002343881.1	LOC110260629	lncRNA	0	0	0	0	0	0	0	0	0	0	0	0	0	0	0	0	0	0	0	0	0
XR_002343883.1	LOC110260631	lncRNA	0	0	0	0	0	0	0	0	0	0	0	0	0	0	0	0	0	0	0	0	0
XR_002343884.1	LOC110260634	lncRNA	0	0	0	0	0	0	0	0	0	0	0	0	0	0	0	0	0	0	0	0	0
XR_002343885.1	LOC110260635	lncRNA	0	0	0	0	0	0	0	0	0	0	0	0	0	0	0	0	0	0	0	0	0
XR_002343891.1	LOC110260642	lncRNA	0	0	0	0	0	0	0	0	0	0	0	0	0	0	0	0	0	0	0	0	0
XR_002343892.1	LOC110260643	lncRNA	0	0	0	0	0	0	0	0	0	0	0	0	0	0	0	0	0	0	0	0	0
XR_002345298.1	MAF	mRNA	0	0	0	0	0	0	0	0	0	0	0	0	0	0	0	0	0	0	0	0	0
XR_002345300.1	LOC106510470	lncRNA	0	0	0	0	0	0	0	0	0	0	0	0	0	0	0	0	0	0	0	0	0
XR_002345301.1	LOC106510468	lncRNA	0	0	0	0	0	0	0	0	0	0	0	0	0	0	0	0	0	0	0	0	0
XR_002346166.1	LOC110261703	lncRNA	0	0	0	0	0	0	0	0	0	0	0	0	0	0	0	0	0	0	0	0	0

RNA ID	Symbol	Type	updown_BGIR9823_99095	updown_BGIR9823_99528	updown_BGIR9823_99552	updown_BGIR9823_96972	updown_URS0000BC5676	updown_XR_001298953.2	updown_BGIR9823_101185	updown_BGIR9823_78435	updown_BGIR9823_101387	updown_BGIR9823_101406	updown_BGIR9823_102168	updown_BGIR9823_102282	updown_BGIR9823_100321	updown_BGIR9823_88590	updown_BGIR9823_89592	updown_BGIR9823_92306	updown_XR_002340248.1	updown_XR_002335693.1	updown_XR_002338205.1	updown_BGIR9823_101399	updown_BGIR9823_101545
XR_002346227.1	LOC106504299	lncRNA	0	0	0	0	0	0	0	0	0	0	0	0	0	0	0	0	0	0	0	0	0
XR_002346228.1	LOC106504299	lncRNA	0	0	0	0	0	0	0	0	0	0	0	0	0	0	0	0	0	0	0	0	0
XR_002346229.1	LOC106504299	lncRNA	0	0	0	0	0	0	0	0	0	0	0	0	0	0	0	0	0	0	0	0	0
XR_002346230.1	LOC106504299	lncRNA	0	0	0	0	0	0	0	0	0	0	0	0	0	0	0	0	0	0	0	0	0
XR_002346231.1	LOC106504299	lncRNA	0	0	0	0	0	0	0	0	0	0	0	0	0	0	0	0	0	0	0	0	0
XR_002346232.1	LOC106504299	lncRNA	0	0	0	0	0	0	0	0	0	0	0	0	0	0	0	0	0	0	0	0	0
XR_002346233.1	NQO2	mRNA	0	0	0	0	0	0	0	0	0	0	0	0	0	0	0	0	0	0	0	0	0
XR_002346236.1	PRPF4B	mRNA	0	0	0	0	0	0	0	0	0	0	0	0	0	0	0	0	0	0	0	0	0
XR_002346237.1	PRPF4B	mRNA	0	0	0	0	0	0	0	0	0	0	0	0	0	0	0	0	0	0	0	0	0
XR_002346238.1	PRPF4B	mRNA	0	0	0	0	0	0	0	0	0	0	0	0	0	0	0	0	0	0	0	0	0
XR_002346239.1	PRPF4B	mRNA	0	0	0	0	0	0	0	0	0	0	0	0	0	0	0	0	0	0	0	0	0
XR_002346240.1	PRPF4B	mRNA	0	0	0	0	0	0	0	0	0	0	0	0	0	0	0	0	0	0	0	0	0
XR_002346241.1	PRPF4B	mRNA	0	0	0	0	0	0	0	0	0	0	0	0	0	0	0	0	0	0	0	0	0
XR_002346242.1	PRPF4B	mRNA	0	0	0	0	0	0	0	0	0	0	0	0	0	0	0	0	0	0	0	0	0
XR_002346243.1	PRPF4B	mRNA	0	0	0	0	0	0	0	0	0	0	0	0	0	0	0	0	0	0	0	0	0
XR_002346244.1	PRPF4B	mRNA	0	0	0	0	0	0	0	0	0	0	0	0	0	0	0	0	0	0	0	0	0
XR_002346245.1	PRPF4B	mRNA	0	0	0	0	0	0	0	0	0	0	0	0	0	0	0	0	0	0	0	0	0
XR_002346246.1	PRPF4B	mRNA	0	0	0	0	0	0	0	0	0	0	0	0	0	0	0	0	0	0	0	0	0
XR_002346247.1	PRPF4B	mRNA	0	0	0	0	0	0	0	0	0	0	0	0	0	0	0	0	0	0	0	0	0
XR_002346248.1	PRPF4B	mRNA	0	0	0	0	0	0	0	0	0	0	0	0	0	0	0	0	0	0	0	0	0
XR_002346249.1	PRPF4B	mRNA	0	0	0	0	0	0	0	0	0	0	0	0	0	0	0	0	0	0	0	0	0
XR_002346250.1	PRPF4B	mRNA	0	0	0	0	0	0	0	0	0	0	0	0	0	0	0	0	0	0	0	0	0
XR_002346251.1	PRPF4B	mRNA	0	0	0	0	0	0	0	0	0	0	0	0	0	0	0	0	0	0	0	0	0
XR_002346252.1	PRPF4B	mRNA	0	0	0	0	0	0	0	0	0	0	0	0	0	0	0	0	0	0	0	0	0
XR_002346253.1	PRPF4B	mRNA	0	0	0	0	0	0	0	0	0	0	0	0	0	0	0	0	0	0	0	0	0
XR_002346254.1	PRPF4B	mRNA	0	0	0	0	0	0	0	0	0	0	0	0	0	0	0	0	0	0	0	0	0
XR_002346255.1	PRPF4B	mRNA	0	0	0	0	0	0	0	0	0	0	0	0	0	0	0	0	0	0	0	0	0
XR_002346256.1	PRPF4B	mRNA	0	0	0	0	0	0	0	0	0	0	0	0	0	0	0	0	0	0	0	0	0
XR_002346257.1	PRPF4B	mRNA	0	0	0	0	0	0	0	0	0	0	0	0	0	0	0	0	0	0	0	0	0
XR_002346258.1	PRPF4B	mRNA	0	0	0	0	0	0	0	0	0	0	0	0	0	0	0	0	0	0	0	0	0
XR_002346259.1	PRPF4B	mRNA	0	0	0	0	0	0	0	0	0	0	0	0	0	0	0	0	0	0	0	0	0
XR_002346260.1	PRPF4B	mRNA	0	0	0	0	0	0	0	0	0	0	0	0	0	0	0	0	0	0	0	0	0
XR_002346261.1	PRPF4B	mRNA	0	0	0	0	0	0	0	0	0	0	0	0	0	0	0	0	0	0	0	0	0
XR_002346262.1	PRPF4B	mRNA	0	0	0	0	0	0	0	0	0	0	0	0	0	0	0	0	0	0	0	0	0
XR_002346263.1	PRPF4B	mRNA	0	0	0	0	0	0	0	0	0	0	0	0	0	0	0	0	0	0	0	0	0
XR_002346264.1	PRPF4B	mRNA	0	0	0	0	0	0	0	0	0	0	0	0	0	0	0	0	0	0	0	0	0
XR_002346265.1	LOC110261764	lncRNA	0	0	0	0	0	0	0	0	0	0	0	0	0	0	0	0	0	0	0	0	0
XR_002346266.1	LOC102162837	lncRNA	0	0	0	0	0	0	0	0	0	0	0	0	0	0	0	0	0	0	0	0	0
XR_002346299.1	LOC110261774	lncRNA	0	0	0	0	0	0	0	0	0	0	0	0	0	0	0	0	0	0	0	0	0
XR_002346300.1	LOC110261774	lncRNA	0	0	0	0	0	0	0	0	0	0	0	0	0	0	0	0	0	0	0	0	0
XR_002346302.1	LOC110261776	lncRNA	0	0	0	0	0	0	0	0	0	0	0	0	0	0	0	0	0	0	0	0	0
XR_002346303.1	TMEM170B	mRNA	0	0	0	0	0	0	0	0	0	0	0	0	0	0	0	0	0	0	0	0	0
XR_002346304.1	TMEM170B	mRNA	0	0	0	0	0	0	0	0	0	0	0	0	0	0	0	0	0	0	0	0	0

RNA ID	Symbol	Type	updown_BGIR9823_99095	updown_BGIR9823_99528	updown_BGIR9823_99552	updown_BGIR9823_96972	updown_URS0000BC5676	updown_XR_001298953.2	updown_BGIR9823_101185	updown_BGIR9823_78435	updown_BGIR9823_101387	updown_BGIR9823_101406	updown_BGIR9823_102168	updown_BGIR9823_102282	updown_BGIR9823_100321	updown_BGIR9823_88590	updown_BGIR9823_89592	updown_BGIR9823_92306	updown_XR_002340248.1	updown_XR_002335693.1	updown_XR_002338205.1	updown_BGIR9823_101399	updown_BGIR9823_101545
XR_115737.4	LOC100513133	lncRNA	0	0	0	0	0	0	0	0	0	0	0	0	0	0	0	0	0	0	0	0	0
XR_299046.2	LOC102164596	lncRNA	0	0	0	0	0	0	0	0	0	0	0	0	0	0	0	0	0	0	0	0	0
XR_303634.3	LOC100738154	lncRNA	0	0	0	0	0	0	0	0	0	0	0	0	0	0	0	0	0	0	0	0	0
XR_304239.3	LOC102159819	lncRNA	0	0	0	0	0	0	0	0	0	0	0	0	0	0	0	0	0	0	0	0	0
XR_304244.3	LOC102161992	lncRNA	0	0	0	0	0	0	0	0	0	0	0	0	0	0	0	0	0	0	0	0	0
XR_304257.3	LOC102162837	lncRNA	0	0	0	0	0	0	0	0	0	0	0	0	0	0	0	0	0	0	0	0	0
XR_306105.3	LOC102166871	lncRNA	0	0	0	0	0	0	0	0	0	0	0	0	0	0	0	0	0	0	0	0	0
XR_307914.3	LOC102159607	lncRNA	0	0	0	0	0	0	0	0	0	0	0	0	0	0	0	0	0	0	0	0	0
XR_308345.3	LOC102167708	lncRNA	0	0	0	0	0	0	0	0	0	0	0	0	0	0	0	0	0	0	0	0	0
novel-ssc-miR1028-3p		miRNA	0	0	0	0	0	0	0	0	0	0	0	0	0	0	0	0	0	0	0	0	0
novel-ssc-miR1028-5p		miRNA	0	0	0	0	0	0	0	0	0	0	0	0	0	0	0	0	0	0	0	0	0
novel-ssc-miR1130-3p		miRNA	0	0	0	0	0	0	0	0	0	0	0	0	0	0	0	0	0	0	0	0	0
novel-ssc-miR1130-5p		miRNA	0	0	0	0	0	0	0	0	0	0	0	0	0	0	0	0	0	0	0	0	0
novel-ssc-miR1162-3p		miRNA	0	0	0	0	0	0	0	0	0	0	0	0	0	0	0	0	0	0	0	0	0
novel-ssc-miR1193-3p		miRNA	0	0	0	0	0	0	0	0	0	0	0	0	0	0	0	0	0	0	0	0	0
novel-ssc-miR1193-5p		miRNA	0	0	0	0	0	0	0	0	0	0	0	0	0	0	0	0	0	0	0	0	0
novel-ssc-miR1217-3p		miRNA	0	0	0	0	0	0	0	0	0	0	0	0	0	0	0	0	0	0	0	0	0
novel-ssc-miR1217-5p		miRNA	0	0	0	0	0	0	0	0	0	0	0	0	0	0	0	0	0	0	0	0	0
novel-ssc-miR1429-3p		miRNA	0	0	0	0	0	0	0	0	0	0	0	0	0	0	0	0	0	0	0	0	0
novel-ssc-miR1429-5p		miRNA	0	0	0	0	0	0	0	0	0	0	0	0	0	0	0	0	0	0	0	0	0
novel-ssc-miR259-3p		miRNA	0	0	0	0	0	0	0	0	0	0	0	0	0	0	0	0	0	0	0	0	0
novel-ssc-miR259-5p		miRNA	0	0	0	0	0	0	0	0	0	0	0	0	0	0	0	0	0	0	0	0	0
novel-ssc-miR324-3p		miRNA	0	0	0	0	0	0	0	0	0	0	0	0	0	0	0	0	0	0	0	0	0
novel-ssc-miR324-5p		miRNA	0	0	0	0	0	0	0	0	0	0	0	0	0	0	0	0	0	0	0	0	0
novel-ssc-miR42-3p		miRNA	0	0	0	0	0	0	0	0	0	0	0	0	0	0	0	0	0	0	0	0	0
novel-ssc-miR42-5p		miRNA	0	0	0	0	0	0	0	0	0	0	0	0	0	0	0	0	0	0	0	0	0
novel-ssc-miR433-3p		miRNA	0	0	0	0	0	0	0	0	0	0	0	1	0	0	0	0	0	0	0	0	0
novel-ssc-miR433-5p		miRNA	0	0	0	0	0	0	0	0	0	0	0	1	0	0	0	0	0	0	0	0	0
novel-ssc-miR557-3p		miRNA	0	0	0	0	0	0	0	0	0	0	0	0	0	0	0	0	0	0	0	0	0
novel-ssc-miR557-5p		miRNA	0	0	0	0	0	0	0	0	0	0	0	0	0	0	0	0	0	0	0	0	0
novel-ssc-miR619-3p		miRNA	0	0	0	0	0	0	0	0	0	0	0	0	0	0	0	0	0	0	0	0	0
novel-ssc-miR619-5p		miRNA	0	0	0	0	0	0	0	0	0	0	0	0	0	0	0	0	0	0	0	0	0
novel-ssc-miR719-3p		miRNA	0	0	0	0	0	0	0	0	0	0	0	0	0	0	0	0	0	0	0	0	0
novel-ssc-miR719-5p		miRNA	0	0	0	0	0	0	0	0	0	0	0	0	0	0	0	0	0	0	0	0	0
novel-ssc-miR966-3p		miRNA	0	0	0	0	0	0	0	0	0	0	0	0	0	0	0	0	0	0	0	0	0
novel-ssc-miR966-5p		miRNA	0	0	0	0	0	0	0	0	0	0	0	0	0	0	0	0	0	0	0	0	0
ssc-miR-7136-3p		miRNA	0	0	0	0	0	0	0	0	0	0	0	0	0	0	0	0	0	0	0	0	0
ssc-miR-7136-5p		miRNA	0	0	0	0	0	0	0	0	0	0	0	0	0	0	0	0	0	0	0	0	0

Supplementary Table S10. The predicted targe

RNA ID	Symbol	Type	updown_BGIR9823_101560	updown_BGIR9823_101610	updown_BGIR9823_100872	updown_BGIR9823_101854	updown_BGIR9823_101989	updown_BGIR9823_102727	updown_BGIR9823_79919	updown_BGIR9823_80122	updown_BGIR9823_101217	updown_BGIR9823_83642	updown_BGIR9823_83643	updown_BGIR9823_81077	updown_BGIR9823_86463	updown_BGIR9823_87747	updown_BGIR9823_88211	updown_BGIR9823_88037	updown_BGIR9823_89044	updown_BGIR9823_90934	updown_BGIR9823_95558	updown_BGIR9823_92999	updown_BGIR9823_93000
BGIR9823_100361	PRKAG2	mRNA	0	0	0	0	0	0	0	0	0	0	0	0	0	0	0	0	0	0	0	0	0
BGIR9823_100362	PRKAG2	mRNA	0	0	0	0	0	0	0	0	0	0	0	0	0	0	0	0	0	0	0	0	0
BGIR9823_100363	PRKAG2	mRNA	0	0	0	0	0	0	0	0	0	0	0	0	0	0	0	0	0	0	0	0	0
BGIR9823_100364	PRKAG2	mRNA	0	0	0	0	0	0	0	0	0	0	0	0	0	0	0	0	0	0	0	0	0
BGIR9823_101535	LOC102158401	lncRNA	1	0	0	0	0	0	0	0	0	0	0	0	0	0	0	0	0	0	0	0	0
BGIR9823_101541	LOC102157946	lncRNA	1	0	0	0	0	0	0	0	0	0	0	0	0	0	0	0	0	0	0	0	0
BGIR9823_101542	LOC102157946	lncRNA	1	0	0	0	0	0	0	0	0	0	0	0	0	0	0	0	0	0	0	0	0
BGIR9823_101560	LOC110258600	lncRNA	1	0	0	0	0	0	0	0	0	0	0	0	0	0	0	0	0	0	0	0	0
BGIR9823_101561	LOC110258600	lncRNA	1	0	0	0	0	0	0	0	0	0	0	0	0	0	0	0	0	0	0	0	0
BGIR9823_101562	LOC110258600	lncRNA	1	0	0	0	0	0	0	0	0	0	0	0	0	0	0	0	0	0	0	0	0
BGIR9823_101563	LOC110258600	lncRNA	1	0	0	0	0	0	0	0	0	0	0	0	0	0	0	0	0	0	0	0	0
BGIR9823_101564	LOC110258600	lncRNA	1	0	0	0	0	0	0	0	0	0	0	0	0	0	0	0	0	0	0	0	0
BGIR9823_101612	LOC100521600	lncRNA	0	1	0	0	0	0	0	0	0	0	0	0	0	0	0	0	0	0	0	0	0
BGIR9823_101853		lncRNA	0	0	0	1	0	0	0	0	0	0	0	0	0	0	0	0	0	0	0	0	0
BGIR9823_101854		lncRNA	0	0	0	1	0	0	0	0	0	0	0	0	0	0	0	0	0	0	0	0	0
BGIR9823_101855		lncRNA	0	0	0	1	0	0	0	0	0	0	0	0	0	0	0	0	0	0	0	0	0
BGIR9823_102146		lncRNA	0	0	0	0	0	0	0	0	0	0	0	0	0	0	0	0	0	0	0	0	0
BGIR9823_102180		lncRNA	0	0	0	0	0	0	0	0	0	0	0	0	0	0	1	0	0	0	0	0	0
BGIR9823_102279		lncRNA	0	0	0	0	0	0	0	0	0	0	0	0	0	0	0	0	0	0	0	0	0
BGIR9823_102282		lncRNA	0	0	0	0	0	0	0	0	0	0	0	0	0	0	0	0	0	0	0	0	0
BGIR9823_102361		lncRNA	0	0	0	0	0	0	0	0	0	0	0	0	0	0	0	0	0	0	0	1	1
BGIR9823_102362		lncRNA	0	0	0	0	0	0	0	0	0	0	0	0	0	0	0	0	0	0	0	1	1
BGIR9823_102363		lncRNA	0	0	0	0	0	0	0	0	0	0	0	0	0	0	0	0	0	0	0	1	1
BGIR9823_102727		lncRNA	0	0	0	0	0	1	0	0	0	0	0	0	0	0	0	0	0	0	0	0	0
BGIR9823_80121	SSSCA1	lncRNA	0	0	0	0	0	0	0	1	0	0	0	0	0	0	0	0	0	0	0	0	0
BGIR9823_80122	LOC102163816	lncRNA	0	0	0	0	0	0	0	1	0	0	0	0	0	0	0	0	0	0	0	0	0
BGIR9823_81007	EHBP1L1	mRNA	0	0	0	0	0	0	0	1	0	0	0	0	0	0	0	0	0	0	0	0	0
BGIR9823_81008	EHBP1L1	mRNA	0	0	0	0	0	0	0	1	0	0	0	0	0	0	0	0	0	0	0	0	0
BGIR9823_81009	EHBP1L1	mRNA	0	0	0	0	0	0	0	1	0	0	0	0	0	0	0	0	0	0	0	0	0
BGIR9823_81010	EHBP1L1	mRNA	0	0	0	0	0	0	0	1	0	0	0	0	0	0	0	0	0	0	0	0	0
BGIR9823_81011	SSSCA1	mRNA	0	0	0	0	0	0	0	1	0	0	0	0	0	0	0	0	0	0	0	0	0
BGIR9823_81012	FRMD8	lncRNA	0	0	0	0	0	0	0	1	0	0	0	0	0	0	0	0	0	0	0	0	0
BGIR9823_81013	LOC102163816	lncRNA	0	0	0	0	0	0	0	1	0	0	0	0	0	0	0	0	0	0	0	0	0
BGIR9823_81014	LOC102163816	lncRNA	0	0	0	0	0	0	0	1	0	0	0	0	0	0	0	0	0	0	0	0	0
BGIR9823_81015	LOC102163816	lncRNA	0	0	0	0	0	0	0	1	0	0	0	0	0	0	0	0	0	0	0	0	0
BGIR9823_81016	LOC102163816	lncRNA	0	0	0	0	0	0	0	1	0	0	0	0	0	0	0	0	0	0	0	0	0
BGIR9823_81017	LOC102163816	lncRNA	0	0	0	0	0	0	0	1	0	0	0	0	0	0	0	0	0	0	0	0	0
BGIR9823_81018	LOC102163816	lncRNA	0	0	0	0	0	0	0	1	0	0	0	0	0	0	0	0	0	0	0	0	0
BGIR9823_81019	LOC100513133	lncRNA	0	0	0	0	0	0	0	1	0	0	0	0	0	0	0	0	0	0	0	0	0
BGIR9823_84704	LOC102159820	mRNA	0	0	0	0	0	0	0	0	0	0	0	0	0	0	0	0	0	0	0	0	0
BGIR9823_84732	RANGAP1	mRNA	0	0	0	0	0	0	0	0	0	0	0	0	0	0	0	0	0	0	0	0	0

RNA ID	Symbol	Type	updown_BGIR9823_101560	updown_BGIR9823_101610	updown_BGIR9823_100872	updown_BGIR9823_101854	updown_BGIR9823_101989	updown_BGIR9823_102727	updown_BGIR9823_79919	updown_BGIR9823_80122	updown_BGIR9823_101217	updown_BGIR9823_83642	updown_BGIR9823_83643	updown_BGIR9823_81077	updown_BGIR9823_86463	updown_BGIR9823_87747	updown_BGIR9823_88211	updown_BGIR9823_88037	updown_BGIR9823_89044	updown_BGIR9823_90934	updown_BGIR9823_95558	updown_BGIR9823_92999	updown_BGIR9823_93000
BGIR9823_84733	RANGAP1	mRNA	0	0	0	0	0	0	0	0	0	0	0	0	0	0	0	0	0	0	0	0	0
BGIR9823_85245	CRADD	lncRNA	0	0	0	0	0	0	0	0	0	0	0	0	0	0	0	0	0	0	0	0	0
BGIR9823_85246	LOC100738154	lncRNA	0	0	0	0	0	0	0	0	0	0	0	0	0	0	0	0	0	0	0	0	0
BGIR9823_85293	TRMU	mRNA	0	0	0	0	0	0	0	0	0	0	0	0	0	0	0	0	0	0	0	0	0
BGIR9823_85294	TRMU	mRNA	0	0	0	0	0	0	0	0	0	0	0	0	0	0	0	0	0	0	0	0	0
BGIR9823_85295	TTC38	mRNA	0	0	0	0	0	0	0	0	0	0	0	0	0	0	0	0	0	0	0	0	0
BGIR9823_85296	PKDREJ	lncRNA	0	0	0	0	0	0	0	0	0	0	0	0	0	0	0	0	0	0	0	0	0
BGIR9823_85297	PPARA	mRNA	0	0	0	0	0	0	0	0	0	0	0	0	0	0	0	0	0	0	0	0	0
BGIR9823_85298	PPARA	mRNA	0	0	0	0	0	0	0	0	0	0	0	0	0	0	0	0	0	0	0	0	0
BGIR9823_85299	PPARA	mRNA	0	0	0	0	0	0	0	0	0	0	0	0	0	0	0	0	0	0	0	0	0
BGIR9823_85300	PPARA	lncRNA	0	0	0	0	0	0	0	0	0	0	0	0	0	0	0	0	0	0	0	0	0
BGIR9823_85301	LOC110260611	mRNA	0	0	0	0	0	0	0	0	0	0	0	0	0	0	0	0	0	0	0	0	0
BGIR9823_85317	ACO2	mRNA	0	0	0	0	0	0	0	0	0	0	0	0	0	0	0	0	0	0	0	0	0
BGIR9823_85318	LOC110260629	lncRNA	0	0	0	0	0	0	0	0	0	0	0	0	0	0	0	0	0	0	0	0	0
BGIR9823_85319	TEF	mRNA	0	0	0	0	0	0	0	0	0	0	0	0	0	0	0	0	0	0	0	0	0
BGIR9823_85320	ZC3H7B	mRNA	0	0	0	0	0	0	0	0	0	0	0	0	0	0	0	0	0	0	0	0	0
BGIR9823_85345	SYNGR1	mRNA	0	0	0	0	0	0	0	0	0	0	0	0	0	0	0	0	0	0	0	0	0
BGIR9823_85835	CRADD	mRNA	0	0	0	0	0	0	0	0	0	0	0	0	0	0	0	0	0	0	0	0	0
BGIR9823_85839	CRADD	lncRNA	0	0	0	0	0	0	0	0	0	0	0	0	0	0	0	0	0	0	0	0	0
BGIR9823_85840	CRADD	lncRNA	0	0	0	0	0	0	0	0	0	0	0	0	0	0	0	0	0	0	0	0	0
BGIR9823_85841	CRADD	lncRNA	0	0	0	0	0	0	0	0	0	0	0	0	0	0	0	0	0	0	0	0	0
BGIR9823_85930	LOC106510465	mRNA	0	0	0	0	0	0	0	0	0	0	0	0	0	0	0	0	0	0	0	0	0
BGIR9823_85931	MAF	lncRNA	0	0	0	0	0	0	0	0	0	0	0	0	0	0	0	0	0	0	0	0	0
BGIR9823_85932	MAF	lncRNA	0	0	0	0	0	0	0	0	0	0	0	0	0	0	0	0	0	0	0	0	0
BGIR9823_87139	LOC106510467	lncRNA	0	0	0	0	0	0	0	0	0	0	0	0	0	0	0	0	0	0	0	0	0
BGIR9823_88187	WRNIP1	mRNA	0	0	0	0	0	0	0	0	0	0	0	0	0	0	0	0	0	0	0	0	0
BGIR9823_88188	NQO2	mRNA	0	0	0	0	0	0	0	0	0	0	0	0	0	0	0	0	0	0	0	0	0
BGIR9823_88189	NQO2	mRNA	0	0	0	0	0	0	0	0	0	0	0	0	0	0	0	0	0	0	0	0	0
BGIR9823_88190	RIPK1	mRNA	0	0	0	0	0	0	0	0	0	0	0	0	0	0	0	0	0	0	0	0	0
BGIR9823_88191	RIPK1	mRNA	0	0	0	0	0	0	0	0	0	0	0	0	0	0	0	0	0	0	0	0	0
BGIR9823_88192	RIPK1	mRNA	0	0	0	0	0	0	0	0	0	0	0	0	0	0	0	0	0	0	0	0	0
BGIR9823_88193	RIPK1	mRNA	0	0	0	0	0	0	0	0	0	0	0	0	0	0	0	0	0	0	0	0	0
BGIR9823_88196	PRPF4B	mRNA	0	0	0	0	0	0	0	0	0	0	0	0	0	0	0	0	0	0	0	0	0
BGIR9823_88197	PRPF4B	mRNA	0	0	0	0	0	0	0	0	0	0	0	0	0	0	0	0	0	0	0	0	0
BGIR9823_88198	PRPF4B	mRNA	0	0	0	0	0	0	0	0	0	0	0	0	0	0	0	0	0	0	0	0	0
BGIR9823_88211	TMEM170B	lncRNA	0	0	0	0	0	0	0	0	0	0	0	0	0	0	1	0	0	0	0	0	0
BGIR9823_88212	TMEM170B	lncRNA	0	0	0	0	0	0	0	0	0	0	0	0	0	0	1	0	0	0	0	0	0
BGIR9823_88213	TMEM170B	lncRNA	0	0	0	0	0	0	0	0	0	0	0	0	0	0	1	0	0	0	0	0	0
BGIR9823_88214	TMEM170B	lncRNA	0	0	0	0	0	0	0	0	0	0	0	0	0	0	1	0	0	0	0	0	0
BGIR9823_88217	PHACTR1	mRNA	0	0	0	0	0	0	0	0	0	0	0	0	0	0	0	0	1	0	0	0	0
BGIR9823_88218	PHACTR1	lncRNA	0	0	0	0	0	0	0	0	0	0	0	0	0	0	0	0	1	0	0	0	0
BGIR9823_89017	MYLK4	lncRNA	0	0	0	0	0	0	0	0	0	0	0	0	0	0	0	0	0	0	0	0	0
BGIR9823_89019	SERPINB1	lncRNA	0	0	0	0	0	0	0	0	0	0	0	0	0	0	0	0	0	0	0	0	0

RNA ID	Symbol	Type	updown_BGIR9823_101560	updown_BGIR9823_101610	updown_BGIR9823_100872	updown_BGIR9823_101854	updown_BGIR9823_101989	updown_BGIR9823_102727	updown_BGIR9823_79919	updown_BGIR9823_80122	updown_BGIR9823_101217	updown_BGIR9823_83642	updown_BGIR9823_83643	updown_BGIR9823_81077	updown_BGIR9823_86463	updown_BGIR9823_87747	updown_BGIR9823_88211	updown_BGIR9823_88037	updown_BGIR9823_89044	updown_BGIR9823_90934	updown_BGIR9823_95558	updown_BGIR9823_92999	updown_BGIR9823_93000
BGIR9823_89020	SERPINB9	mRNA	0	0	0	0	0	0	0	0	0	0	0	0	0	0	0	0	0	0	0	0	0
BGIR9823_89021	RIPK1	lncRNA	0	0	0	0	0	0	0	0	0	0	0	0	0	0	0	0	0	0	0	0	0
BGIR9823_89025	PXDC1	mRNA	0	0	0	0	0	0	0	0	0	0	0	0	0	0	0	0	0	0	0	0	0
BGIR9823_89026	PXDC1	lncRNA	0	0	0	0	0	0	0	0	0	0	0	0	0	0	0	0	0	0	0	0	0
BGIR9823_89027	LOC102161744	lncRNA	0	0	0	0	0	0	0	0	0	0	0	0	0	0	0	0	0	0	0	0	0
BGIR9823_89040	TBC1D7	lncRNA	0	0	0	0	0	0	0	0	0	0	0	0	0	0	0	0	1	0	0	0	0
BGIR9823_89041	TBC1D7	lncRNA	0	0	0	0	0	0	0	0	0	0	0	0	0	0	0	0	1	0	0	0	0
BGIR9823_89042	TBC1D7	lncRNA	0	0	0	0	0	0	0	0	0	0	0	0	0	0	0	0	1	0	0	0	0
BGIR9823_89043	TBC1D7	lncRNA	0	0	0	0	0	0	0	0	0	0	0	0	0	0	0	0	1	0	0	0	0
BGIR9823_89044	GFOD1	lncRNA	0	0	0	0	0	0	0	0	0	0	0	0	0	0	0	0	1	0	0	0	0
BGIR9823_89045	GFOD1	mRNA	0	0	0	0	0	0	0	0	0	0	0	0	0	0	0	0	1	0	0	0	0
BGIR9823_89046	GFOD1	mRNA	0	0	0	0	0	0	0	0	0	0	0	0	0	0	0	0	1	0	0	0	0
BGIR9823_89047	GFOD1	mRNA	0	0	0	0	0	0	0	0	0	0	0	0	0	0	0	0	1	0	0	0	0
BGIR9823_89048	GFOD1	lncRNA	0	0	0	0	0	0	0	0	0	0	0	0	0	0	0	0	1	0	0	0	0
BGIR9823_89049	GFOD1	lncRNA	0	0	0	0	0	0	0	0	0	0	0	0	0	0	0	0	1	0	0	0	0
BGIR9823_90771	NEU3	lncRNA	0	0	0	0	0	0	0	0	0	0	0	0	0	0	0	0	0	0	0	0	0
BGIR9823_90772	SLCO2B1	mRNA	0	0	0	0	0	0	0	0	0	0	0	0	0	0	0	0	0	0	0	0	0
BGIR9823_90913	LOC110255435	lncRNA	0	0	0	0	0	0	0	0	0	0	0	0	0	0	0	0	0	0	0	0	0
BGIR9823_90934	LOC110255441	lncRNA	0	0	0	0	0	0	0	0	0	0	0	0	0	0	0	0	0	1	0	0	0
BGIR9823_90935	CCDC84	mRNA	0	0	0	0	0	0	0	0	0	0	0	0	0	0	0	0	0	1	0	0	0
BGIR9823_90936	TRAPPC4	mRNA	0	0	0	0	0	0	0	0	0	0	0	0	0	0	0	0	0	1	0	0	0
BGIR9823_90937	HMBS	mRNA	0	0	0	0	0	0	0	0	0	0	0	0	0	0	0	0	0	1	0	0	0
BGIR9823_90956	SORL1	mRNA	0	0	0	0	0	0	0	0	0	0	0	0	0	0	0	0	0	0	0	0	0
BGIR9823_90957	SORL1	mRNA	0	0	0	0	0	0	0	0	0	0	0	0	0	0	0	0	0	0	0	0	0
BGIR9823_90958	SORL1	mRNA	0	0	0	0	0	0	0	0	0	0	0	0	0	0	0	0	0	0	0	0	0
BGIR9823_90960	CLMP	lncRNA	0	0	0	0	0	0	0	0	0	0	0	0	0	0	0	0	0	0	0	0	0
BGIR9823_91161	LOC100522267	mRNA	0	0	0	0	0	0	0	0	0	0	0	0	0	0	0	0	0	0	0	0	0
BGIR9823_91162	LOC100522455	mRNA	0	0	0	0	0	0	0	0	0	0	0	0	0	0	0	0	0	0	0	0	0
BGIR9823_91163	ABCB1	mRNA	0	0	0	0	0	0	0	0	0	0	0	0	0	0	0	0	0	0	0	0	0
BGIR9823_91402	HBB	mRNA	0	0	0	0	0	0	0	0	0	0	0	0	0	0	0	0	0	0	0	0	0
BGIR9823_91403	HBB	mRNA	0	0	0	0	0	0	0	0	0	0	0	0	0	0	0	0	0	0	0	0	0
BGIR9823_91404	HBB	mRNA	0	0	0	0	0	0	0	0	0	0	0	0	0	0	0	0	0	0	0	0	0
BGIR9823_91405	HBB	mRNA	0	0	0	0	0	0	0	0	0	0	0	0	0	0	0	0	0	0	0	0	0
BGIR9823_91406	HBB	mRNA	0	0	0	0	0	0	0	0	0	0	0	0	0	0	0	0	0	0	0	0	0
BGIR9823_91407	HBB	mRNA	0	0	0	0	0	0	0	0	0	0	0	0	0	0	0	0	0	0	0	0	0
BGIR9823_91408	HBB	mRNA	0	0	0	0	0	0	0	0	0	0	0	0	0	0	0	0	0	0	0	0	0
BGIR9823_91409	HBB	mRNA	0	0	0	0	0	0	0	0	0	0	0	0	0	0	0	0	0	0	0	0	0
BGIR9823_91625	BCL9L	lncRNA	0	0	0	0	0	0	0	0	0	0	0	0	0	0	0	0	0	1	0	0	0
BGIR9823_91626	BCL9L	lncRNA	0	0	0	0	0	0	0	0	0	0	0	0	0	0	0	0	0	1	0	0	0
BGIR9823_91627	RPS25	mRNA	0	0	0	0	0	0	0	0	0	0	0	0	0	0	0	0	0	1	0	0	0
BGIR9823_91628	RPS25	mRNA	0	0	0	0	0	0	0	0	0	0	0	0	0	0	0	0	0	1	0	0	0
BGIR9823_91629	SLC37A4	mRNA	0	0	0	0	0	0	0	0	0	0	0	0	0	0	0	0	0	1	0	0	0
BGIR9823_91630	HYOU1	mRNA	0	0	0	0	0	0	0	0	0	0	0	0	0	0	0	0	0	1	0	0	0

RNA ID	Symbol	Type	updown_BGIR9823_101560	updown_BGIR9823_101610	updown_BGIR9823_100872	updown_BGIR9823_101854	updown_BGIR9823_101989	updown_BGIR9823_102727	updown_BGIR9823_79919	updown_BGIR9823_80122	updown_BGIR9823_101217	updown_BGIR9823_83642	updown_BGIR9823_83643	updown_BGIR9823_81077	updown_BGIR9823_86463	updown_BGIR9823_87747	updown_BGIR9823_88211	updown_BGIR9823_88037	updown_BGIR9823_89044	updown_BGIR9823_90934	updown_BGIR9823_95558	updown_BGIR9823_92999	updown_BGIR9823_93000
BGIR9823_91640	CLMP	mRNA	0	0	0	0	0	0	0	0	0	0	0	0	0	0	0	0	0	0	0	0	0
BGIR9823_91819	RUNDC3B	lncRNA	0	0	0	0	0	0	0	0	0	0	0	0	0	0	0	0	0	0	0	0	0
BGIR9823_91820	RUNDC3B	lncRNA	0	0	0	0	0	0	0	0	0	0	0	0	0	0	0	0	0	0	0	0	0
BGIR9823_91821	RUNDC3B	lncRNA	0	0	0	0	0	0	0	0	0	0	0	0	0	0	0	0	0	0	0	0	0
BGIR9823_91822	RUNDC3B	lncRNA	0	0	0	0	0	0	0	0	0	0	0	0	0	0	0	0	0	0	0	0	0
BGIR9823_91823	RUNDC3B	lncRNA	0	0	0	0	0	0	0	0	0	0	0	0	0	0	0	0	0	0	0	0	0
BGIR9823_91824	RUNDC3B	lncRNA	0	0	0	0	0	0	0	0	0	0	0	0	0	0	0	0	0	0	0	0	0
BGIR9823_91825	RUNDC3B	lncRNA	0	0	0	0	0	0	0	0	0	0	0	0	0	0	0	0	0	0	0	0	0
BGIR9823_91826	LOC102164596	lncRNA	0	0	0	0	0	0	0	0	0	0	0	0	0	0	0	0	0	0	0	0	0
BGIR9823_92993	KATNAL1	mRNA	0	0	0	0	0	0	0	0	0	0	0	0	0	0	0	0	0	0	0	1	1
BGIR9823_92994	KATNAL1	mRNA	0	0	0	0	0	0	0	0	0	0	0	0	0	0	0	0	0	0	0	1	1
BGIR9823_92995	KATNAL1	mRNA	0	0	0	0	0	0	0	0	0	0	0	0	0	0	0	0	0	0	0	1	1
BGIR9823_92996	KATNAL1	mRNA	0	0	0	0	0	0	0	0	0	0	0	0	0	0	0	0	0	0	0	1	1
BGIR9823_92997	KATNAL1	mRNA	0	0	0	0	0	0	0	0	0	0	0	0	0	0	0	0	0	0	0	1	1
BGIR9823_92999	LOC110255292	lncRNA	0	0	0	0	0	0	0	0	0	0	0	0	0	0	0	0	0	0	0	1	1
BGIR9823_93000	LOC102166871	lncRNA	0	0	0	0	0	0	0	0	0	0	0	0	0	0	0	0	0	0	0	1	1
BGIR9823_98051	LOC102164998	lncRNA	0	0	0	0	0	0	0	0	0	0	0	0	0	0	0	0	0	0	0	0	0
BGIR9823_99244	LOC102167708	lncRNA	0	0	0	0	0	0	0	0	0	0	0	0	0	0	0	0	0	0	0	0	0
NM_001004034.1	HMGB1	mRNA	0	0	0	0	0	0	0	0	0	0	0	0	0	0	0	0	0	0	0	1	1
NM_001044526.1	PPARA	mRNA	0	0	0	0	0	0	0	0	0	0	0	0	0	0	0	0	0	0	0	0	0
NM_001111256.1	SERPINB9	mRNA	0	0	0	0	0	0	0	0	0	0	0	0	0	0	0	0	0	0	0	0	0
NM_001113443.1	ECI2	mRNA	0	0	0	0	0	0	0	0	0	0	0	0	0	0	0	0	0	0	0	0	0
NM_001144841.1	HBB	mRNA	0	0	0	0	0	0	0	0	0	0	0	0	0	0	0	0	0	0	0	0	0
NM_001199719.1	SLC37A4	mRNA	0	0	0	0	0	0	0	0	0	0	0	0	0	0	0	0	0	1	0	0	0
NM_001243907.1	HSPA8	mRNA	0	0	0	0	0	0	0	0	0	0	0	0	0	0	0	0	0	0	0	0	0
NM_001244063.1	RPL3	mRNA	0	0	0	0	0	0	0	0	0	0	0	0	0	0	0	0	0	0	0	0	0
NM_001244066.1	SYNGR1	mRNA	0	0	0	0	0	0	0	0	0	0	0	0	0	0	0	0	0	0	0	0	0
NM_001244396.1	CCDC84	mRNA	0	0	0	0	0	0	0	0	0	0	0	0	0	0	0	0	0	1	0	0	0
NM_001287846.1	RHEB	mRNA	0	0	0	0	0	0	0	0	0	0	0	0	0	0	0	0	0	0	0	0	0
NM_213954.1	ACO2	mRNA	0	0	0	0	0	0	0	0	0	0	0	0	0	0	0	0	0	0	0	0	0
NM_214012.1	UPK2	mRNA	0	0	0	0	0	0	0	0	0	0	0	0	0	0	0	0	0	1	0	0	0
URS0000BC92F4	LOC102167708	lncRNA	0	0	0	0	0	0	0	0	0	0	0	0	0	0	0	0	0	0	0	0	0
XM_001928309.6	TBC1D7	mRNA	0	0	0	0	0	0	0	0	0	0	0	0	0	0	0	0	1	0	0	0	0
XM_001928852.5	PXDC1	mRNA	0	0	0	0	0	0	0	0	0	0	0	0	0	0	0	0	0	0	0	0	0
XM_003122534.4	KCNK7	mRNA	0	0	0	0	0	0	0	1	0	0	0	0	0	0	0	0	0	0	0	0	0
XM_003122558.5	SCYL1	mRNA	0	0	0	0	0	0	0	1	0	0	0	0	0	0	0	0	0	0	0	0	0
XM_003122562.5	FRMD8	mRNA	0	0	0	0	0	0	0	1	0	0	0	0	0	0	0	0	0	0	0	0	0
XM_003125951.6	TTC38	mRNA	0	0	0	0	0	0	0	0	0	0	0	0	0	0	0	0	0	0	0	0	0
XM_003125953.4	CDPF1	mRNA	0	0	0	0	0	0	0	0	0	0	0	0	0	0	0	0	0	0	0	0	0
XM_003126012.6	CBX7	mRNA	0	0	0	0	0	0	0	0	0	0	0	0	0	0	0	0	0	0	0	0	0
XM_003128161.4	RIPK1	mRNA	0	0	0	0	0	0	0	0	0	0	0	0	0	0	0	0	0	0	0	0	0
XM_003129508.2	LOC100514417	mRNA	0	0	0	0	0	0	0	0	0	0	0	0	0	0	0	0	0	0	0	0	0
XM_003129512.2	LOC100515268	mRNA	0	0	0	0	0	0	0	0	0	0	0	0	0	0	0	0	0	0	0	0	0

RNA ID	Symbol	Type	updown_BGIR9823_101560	updown_BGIR9823_101610	updown_BGIR9823_100872	updown_BGIR9823_101854	updown_BGIR9823_101989	updown_BGIR9823_102727	updown_BGIR9823_79919	updown_BGIR9823_80122	updown_BGIR9823_101217	updown_BGIR9823_83642	updown_BGIR9823_83643	updown_BGIR9823_81077	updown_BGIR9823_86463	updown_BGIR9823_87747	updown_BGIR9823_88211	updown_BGIR9823_88037	updown_BGIR9823_89044	updown_BGIR9823_90934	updown_BGIR9823_95558	updown_BGIR9823_92999	updown_BGIR9823_93000
XM_003129514.3	LOC100515618	mRNA	0	0	0	0	0	0	0	0	0	0	0	0	0	0	0	0	0	0	0	0	0
XM_003129519.2	LOC100517595	mRNA	0	0	0	0	0	0	0	0	0	0	0	0	0	0	0	0	0	0	0	0	0
XM_003129529.4	LOC100519910	mRNA	0	0	0	0	0	0	0	0	0	0	0	0	0	0	0	0	0	0	0	0	0
XM_003129915.4	CXCR5	mRNA	0	0	0	0	0	0	0	0	0	0	0	0	0	0	0	0	0	1	0	0	0
XM_003129916.5	BCL9L	mRNA	0	0	0	0	0	0	0	0	0	0	0	0	0	0	0	0	0	1	0	0	0
XM_003129918.4	TRAPPC4	mRNA	0	0	0	0	0	0	0	0	0	0	0	0	0	0	0	0	0	1	0	0	0
XM_003129919.5	RPS25	mRNA	0	0	0	0	0	0	0	0	0	0	0	0	0	0	0	0	0	1	0	0	0
XM_003129920.3	RPS25	mRNA	0	0	0	0	0	0	0	0	0	0	0	0	0	0	0	0	0	1	0	0	0
XM_003129922.4	HYOU1	mRNA	0	0	0	0	0	0	0	0	0	0	0	0	0	0	0	0	0	1	0	0	0
XM_003129923.6	VPS11	mRNA	0	0	0	0	0	0	0	0	0	0	0	0	0	0	0	0	0	0	0	0	0
XM_003129944.4	SC5D	mRNA	0	0	0	0	0	0	0	0	0	0	0	0	0	0	0	0	0	0	0	0	0
XM_003357161.1	LOC100620971	mRNA	0	0	0	0	0	0	0	0	0	0	0	0	0	0	0	0	0	0	0	0	0
XM_003482119.4	SERPINB1	mRNA	0	0	0	0	0	0	0	0	0	0	0	0	0	0	0	0	0	0	0	0	0
XM_003482132.4	TMEM170B	mRNA	0	0	0	0	0	0	0	0	0	0	0	0	0	0	1	0	0	0	0	0	0
XM_003482133.4	GFOD1	mRNA	0	0	0	0	0	0	0	0	0	0	0	0	0	0	0	0	1	0	0	0	0
XM_003482563.4	SPCS2	mRNA	0	0	0	0	0	0	0	0	0	0	0	0	0	0	0	0	0	0	0	0	0
XM_003482565.4	NEU3	mRNA	0	0	0	0	0	0	0	0	0	0	0	0	0	0	0	0	0	0	0	0	0
XM_003482567.1	LOC100739359	mRNA	0	0	0	0	0	0	0	0	0	0	0	0	0	0	0	0	0	0	0	0	0
XM_003482630.3	BSX	mRNA	0	0	0	0	0	0	0	0	0	0	0	0	0	0	0	0	0	0	0	0	0
XM_005653107.3	CHADL	mRNA	0	0	0	0	0	0	0	0	0	0	0	0	0	0	0	0	0	0	0	0	0
XM_005653640.1	LOC100621181	mRNA	0	0	0	0	0	0	0	0	0	0	0	0	0	0	0	0	0	0	0	0	0
XM_005654266.3	PRKAG2	mRNA	0	0	0	0	0	0	0	0	0	0	0	0	0	0	0	0	0	0	0	0	0
XM_005655470.3	CDPF1	mRNA	0	0	0	0	0	0	0	0	0	0	0	0	0	0	0	0	0	0	0	0	0
XM_005660703.3	LTBP3	mRNA	0	0	0	0	0	0	0	1	0	0	0	0	0	0	0	0	0	0	0	0	0
XM_005660705.3	SCYL1	mRNA	0	0	0	0	0	0	0	1	0	0	0	0	0	0	0	0	0	0	0	0	0
XM_005660707.3	SLC25A45	mRNA	0	0	0	0	0	0	0	1	0	0	0	0	0	0	0	0	0	0	0	0	0
XM_005665536.3	RIPK1	mRNA	0	0	0	0	0	0	0	0	0	0	0	0	0	0	0	0	0	0	0	0	0
XM_005665537.3	RIPK1	mRNA	0	0	0	0	0	0	0	0	0	0	0	0	0	0	0	0	0	0	0	0	0
XM_005665538.3	RIPK1	mRNA	0	0	0	0	0	0	0	0	0	0	0	0	0	0	0	0	0	0	0	0	0
XM_005665575.3	PHACTR1	mRNA	0	0	0	0	0	0	0	0	0	0	0	0	0	0	0	0	1	0	0	0	0
XM_005665576.3	TBC1D7	mRNA	0	0	0	0	0	0	0	0	0	0	0	0	0	0	0	0	1	0	0	0	0
XM_005667128.3	LOC100627340	mRNA	0	0	0	0	0	0	0	0	0	0	0	0	0	0	0	0	0	0	0	0	0
XM_005667397.2	CCDC84	mRNA	0	0	0	0	0	0	0	0	0	0	0	0	0	0	0	0	0	1	0	0	0
XM_005667398.3	SLC37A4	mRNA	0	0	0	0	0	0	0	0	0	0	0	0	0	0	0	0	0	1	0	0	0
XM_005667402.3	SLC37A4	mRNA	0	0	0	0	0	0	0	0	0	0	0	0	0	0	0	0	0	1	0	0	0
XM_005667403.3	SLC37A4	mRNA	0	0	0	0	0	0	0	0	0	0	0	0	0	0	0	0	0	1	0	0	0
XM_005667413.3	BCL9L	mRNA	0	0	0	0	0	0	0	0	0	0	0	0	0	0	0	0	0	1	0	0	0
XM_005667414.3	FOXR1	mRNA	0	0	0	0	0	0	0	0	0	0	0	0	0	0	0	0	0	1	0	0	0
XM_005667416.3	HYOU1	mRNA	0	0	0	0	0	0	0	0	0	0	0	0	0	0	0	0	0	1	0	0	0
XM_005667419.3	HYOU1	mRNA	0	0	0	0	0	0	0	0	0	0	0	0	0	0	0	0	0	1	0	0	0
XM_005667420.3	HYOU1	mRNA	0	0	0	0	0	0	0	0	0	0	0	0	0	0	0	0	0	1	0	0	0
XM_005667440.2	JHY	mRNA	0	0	0	0	0	0	0	0	0	0	0	0	0	0	0	0	0	0	0	0	0
XM_005668316.3	HMGB1	mRNA	0	0	0	0	0	0	0	0	0	0	0	0	0	0	0	0	0	0	0	1	1

RNA ID	Symbol	Type	updown_BGIR9823_101560	updown_BGIR9823_101610	updown_BGIR9823_100872	updown_BGIR9823_101854	updown_BGIR9823_101989	updown_BGIR9823_102727	updown_BGIR9823_79919	updown_BGIR9823_80122	updown_BGIR9823_101217	updown_BGIR9823_83642	updown_BGIR9823_83643	updown_BGIR9823_81077	updown_BGIR9823_86463	updown_BGIR9823_87747	updown_BGIR9823_88211	updown_BGIR9823_88037	updown_BGIR9823_89044	updown_BGIR9823_90934	updown_BGIR9823_95558	updown_BGIR9823_92999	updown_BGIR9823_93000
XM_005668317.3	HMGB1	mRNA	0	0	0	0	0	0	0	0	0	0	0	0	0	0	0	0	0	0	0	1	1
XM_005668318.3	HMGB1	mRNA	0	0	0	0	0	0	0	0	0	0	0	0	0	0	0	0	0	0	0	1	1
XM_005671489.3	EMX2	mRNA	0	0	0	0	0	0	0	0	0	0	0	0	0	0	0	0	0	0	0	0	0
XM_005672397.3	MYO10	mRNA	0	0	0	0	0	0	0	0	0	0	0	0	0	0	0	0	0	0	0	0	0
XM_013977400.2	FAM50B	mRNA	0	0	0	0	0	0	0	0	0	0	0	0	0	0	0	0	0	0	0	0	0
XM_013977425.2	NEDD9	mRNA	0	0	0	0	0	0	0	0	0	0	0	0	0	0	1	0	0	0	0	0	0
XM_013977430.2	TBC1D7	mRNA	0	0	0	0	0	0	0	0	0	0	0	0	0	0	0	0	1	0	0	0	0
XM_013977431.2	TBC1D7	mRNA	0	0	0	0	0	0	0	0	0	0	0	0	0	0	0	0	1	0	0	0	0
XM_013977432.2	TBC1D7	mRNA	0	0	0	0	0	0	0	0	0	0	0	0	0	0	0	0	1	0	0	0	0
XM_013977433.2	GFOD1	mRNA	0	0	0	0	0	0	0	0	0	0	0	0	0	0	0	0	1	0	0	0	0
XM_013977434.2	GFOD1	mRNA	0	0	0	0	0	0	0	0	0	0	0	0	0	0	0	0	1	0	0	0	0
XM_013979449.2	SLC37A4	mRNA	0	0	0	0	0	0	0	0	0	0	0	0	0	0	0	0	0	1	0	0	0
XM_013979456.2	BCL9L	mRNA	0	0	0	0	0	0	0	0	0	0	0	0	0	0	0	0	0	1	0	0	0
XM_013979457.2	BCL9L	mRNA	0	0	0	0	0	0	0	0	0	0	0	0	0	0	0	0	0	1	0	0	0
XM_013979458.2	BCL9L	mRNA	0	0	0	0	0	0	0	0	0	0	0	0	0	0	0	0	0	1	0	0	0
XM_013979459.2	BCL9L	mRNA	0	0	0	0	0	0	0	0	0	0	0	0	0	0	0	0	0	1	0	0	0
XM_013979461.2	TRAPPC4	mRNA	0	0	0	0	0	0	0	0	0	0	0	0	0	0	0	0	0	1	0	0	0
XM_013979463.2	HYOU1	mRNA	0	0	0	0	0	0	0	0	0	0	0	0	0	0	0	0	0	1	0	0	0
XM_013979484.2	LOC100523684	mRNA	0	0	0	0	0	0	0	0	0	0	0	0	0	0	0	0	0	0	0	0	0
XM_013979486.2	LOC100523684	mRNA	0	0	0	0	0	0	0	0	0	0	0	0	0	0	0	0	0	0	0	0	0
XM_013979491.2	JHY	mRNA	0	0	0	0	0	0	0	0	0	0	0	0	0	0	0	0	0	0	0	0	0
XM_013980418.2	KATNAL1	mRNA	0	0	0	0	0	0	0	0	0	0	0	0	0	0	0	0	0	0	0	1	1
XM_013988182.2	TTC38	mRNA	0	0	0	0	0	0	0	0	0	0	0	0	0	0	0	0	0	0	0	0	0
XM_013988183.2	TTC38	mRNA	0	0	0	0	0	0	0	0	0	0	0	0	0	0	0	0	0	0	0	0	0
XM_013989589.2	LOC100522267	mRNA	0	0	0	0	0	0	0	0	0	0	0	0	0	0	0	0	0	0	0	0	0
XM_013989592.2	LOC100522455	mRNA	0	0	0	0	0	0	0	0	0	0	0	0	0	0	0	0	0	0	0	0	0
XM_013989593.2	LOC100522455	mRNA	0	0	0	0	0	0	0	0	0	0	0	0	0	0	0	0	0	0	0	0	0
XM_013994099.2	FRMD8	mRNA	0	0	0	0	0	0	0	1	0	0	0	0	0	0	0	0	0	0	0	0	0
XM_013994100.2	SLC25A45	mRNA	0	0	0	0	0	0	0	1	0	0	0	0	0	0	0	0	0	0	0	0	0
XM_013994102.2	SLC25A45	mRNA	0	0	0	0	0	0	0	1	0	0	0	0	0	0	0	0	0	0	0	0	0
XM_013994103.2	SLC25A45	mRNA	0	0	0	0	0	0	0	1	0	0	0	0	0	0	0	0	0	0	0	0	0
XM_013994104.2	SLC25A45	mRNA	0	0	0	0	0	0	0	1	0	0	0	0	0	0	0	0	0	0	0	0	0
XM_013997510.2	CBX7	mRNA	0	0	0	0	0	0	0	0	0	0	0	0	0	0	0	0	0	0	0	0	0
XM_013999070.2	LOC100520088	mRNA	0	0	0	0	0	0	0	0	0	0	0	0	0	0	0	0	0	0	0	0	0
XM_021062230.1	LOC100517771	mRNA	0	0	0	0	0	0	0	0	0	0	0	0	0	0	0	0	0	0	0	0	0
XM_021062231.1	LOC100517957	mRNA	0	0	0	0	0	0	0	0	0	0	0	0	0	0	0	0	0	0	0	0	0
XM_021062367.1	LOC110255365	mRNA	0	0	0	0	0	0	0	0	0	0	0	0	0	0	0	0	0	0	0	0	0
XM_021062369.1	LOC100737110	mRNA	0	0	0	0	0	0	0	0	0	0	0	0	0	0	0	0	0	0	0	0	0
XM_021062370.1	LOC110255368	mRNA	0	0	0	0	0	0	0	0	0	0	0	0	0	0	0	0	0	0	0	0	0
XM_021062371.1	LOC100515441	mRNA	0	0	0	0	0	0	0	0	0	0	0	0	0	0	0	0	0	0	0	0	0
XM_021062374.1	LOC110255371	mRNA	0	0	0	0	0	0	0	0	0	0	0	0	0	0	0	0	0	0	0	0	0
XM_021062375.1	LOC100626951	mRNA	0	0	0	0	0	0	0	0	0	0	0	0	0	0	0	0	0	0	0	0	0
XM_021062491.1	XRRA1	mRNA	0	0	0	0	0	0	0	0	0	0	0	0	0	0	0	0	0	0	0	0	0

RNA ID	Symbol	Type	updown_BGIR9823_101560	updown_BGIR9823_101610	updown_BGIR9823_100872	updown_BGIR9823_101854	updown_BGIR9823_101989	updown_BGIR9823_102727	updown_BGIR9823_79919	updown_BGIR9823_80122	updown_BGIR9823_101217	updown_BGIR9823_83642	updown_BGIR9823_83643	updown_BGIR9823_81077	updown_BGIR9823_86463	updown_BGIR9823_87747	updown_BGIR9823_88211	updown_BGIR9823_88037	updown_BGIR9823_89044	updown_BGIR9823_90934	updown_BGIR9823_95558	updown_BGIR9823_92999	updown_BGIR9823_93000
XM_021062493.1	XRRA1	mRNA	0	0	0	0	0	0	0	0	0	0	0	0	0	0	0	0	0	0	0	0	0
XM_021062494.1	XRRA1	mRNA	0	0	0	0	0	0	0	0	0	0	0	0	0	0	0	0	0	0	0	0	0
XM_021062495.1	XRRA1	mRNA	0	0	0	0	0	0	0	0	0	0	0	0	0	0	0	0	0	0	0	0	0
XM_021062496.1	XRRA1	mRNA	0	0	0	0	0	0	0	0	0	0	0	0	0	0	0	0	0	0	0	0	0
XM_021062497.1	XRRA1	mRNA	0	0	0	0	0	0	0	0	0	0	0	0	0	0	0	0	0	0	0	0	0
XM_021062498.1	XRRA1	mRNA	0	0	0	0	0	0	0	0	0	0	0	0	0	0	0	0	0	0	0	0	0
XM_021062499.1	XRRA1	mRNA	0	0	0	0	0	0	0	0	0	0	0	0	0	0	0	0	0	0	0	0	0
XM_021062500.1	XRRA1	mRNA	0	0	0	0	0	0	0	0	0	0	0	0	0	0	0	0	0	0	0	0	0
XM_021062501.1	XRRA1	mRNA	0	0	0	0	0	0	0	0	0	0	0	0	0	0	0	0	0	0	0	0	0
XM_021062502.1	XRRA1	mRNA	0	0	0	0	0	0	0	0	0	0	0	0	0	0	0	0	0	0	0	0	0
XM_021062503.1	XRRA1	mRNA	0	0	0	0	0	0	0	0	0	0	0	0	0	0	0	0	0	0	0	0	0
XM_021062511.1	SLCO2B1	mRNA	0	0	0	0	0	0	0	0	0	0	0	0	0	0	0	0	0	0	0	0	0
XM_021062948.1	BCL9L	mRNA	0	0	0	0	0	0	0	0	0	0	0	0	0	0	0	0	0	1	0	0	0
XM_021062949.1	TRAPPC4	mRNA	0	0	0	0	0	0	0	0	0	0	0	0	0	0	0	0	0	1	0	0	0
XM_021062950.1	HYOU1	mRNA	0	0	0	0	0	0	0	0	0	0	0	0	0	0	0	0	0	1	0	0	0
XM_021062951.1	HYOU1	mRNA	0	0	0	0	0	0	0	0	0	0	0	0	0	0	0	0	0	1	0	0	0
XM_021062952.1	HYOU1	mRNA	0	0	0	0	0	0	0	0	0	0	0	0	0	0	0	0	0	1	0	0	0
XM_021062953.1	HYOU1	mRNA	0	0	0	0	0	0	0	0	0	0	0	0	0	0	0	0	0	1	0	0	0
XM_021062954.1	HYOU1	mRNA	0	0	0	0	0	0	0	0	0	0	0	0	0	0	0	0	0	1	0	0	0
XM_021062955.1	HYOU1	mRNA	0	0	0	0	0	0	0	0	0	0	0	0	0	0	0	0	0	1	0	0	0
XM_021062956.1	HYOU1	mRNA	0	0	0	0	0	0	0	0	0	0	0	0	0	0	0	0	0	1	0	0	0
XM_021062957.1	VPS11	mRNA	0	0	0	0	0	0	0	0	0	0	0	0	0	0	0	0	0	0	0	0	0
XM_021062993.1	LOC100523684	mRNA	0	0	0	0	0	0	0	0	0	0	0	0	0	0	0	0	0	0	0	0	0
XM_021062994.1	LOC100523684	mRNA	0	0	0	0	0	0	0	0	0	0	0	0	0	0	0	0	0	0	0	0	0
XM_021062995.1	LOC100523684	mRNA	0	0	0	0	0	0	0	0	0	0	0	0	0	0	0	0	0	0	0	0	0
XM_021062996.1	LOC100523684	mRNA	0	0	0	0	0	0	0	0	0	0	0	0	0	0	0	0	0	0	0	0	0
XM_021062997.1	LOC100523684	mRNA	0	0	0	0	0	0	0	0	0	0	0	0	0	0	0	0	0	0	0	0	0
XM_021062998.1	LOC100523684	mRNA	0	0	0	0	0	0	0	0	0	0	0	0	0	0	0	0	0	0	0	0	0
XM_021062999.1	LOC100523684	mRNA	0	0	0	0	0	0	0	0	0	0	0	0	0	0	0	0	0	0	0	0	0
XM_021063000.1	LOC100523684	mRNA	0	0	0	0	0	0	0	0	0	0	0	0	0	0	0	0	0	0	0	0	0
XM_021063001.1	LOC100523684	mRNA	0	0	0	0	0	0	0	0	0	0	0	0	0	0	0	0	0	0	0	0	0
XM_021063002.1	LOC100523684	mRNA	0	0	0	0	0	0	0	0	0	0	0	0	0	0	0	0	0	0	0	0	0
XM_021063004.1	LOC100523684	mRNA	0	0	0	0	0	0	0	0	0	0	0	0	0	0	0	0	0	0	0	0	0
XM_021063005.1	LOC100523684	mRNA	0	0	0	0	0	0	0	0	0	0	0	0	0	0	0	0	0	0	0	0	0
XM_021063006.1	LOC100523684	mRNA	0	0	0	0	0	0	0	0	0	0	0	0	0	0	0	0	0	0	0	0	0
XM_021063009.1	SORL1	mRNA	0	0	0	0	0	0	0	0	0	0	0	0	0	0	0	0	0	0	0	0	0
XM_021063010.1	SORL1	mRNA	0	0	0	0	0	0	0	0	0	0	0	0	0	0	0	0	0	0	0	0	0
XM_021063011.1	SORL1	mRNA	0	0	0	0	0	0	0	0	0	0	0	0	0	0	0	0	0	0	0	0	0
XM_021063012.1	SORL1	mRNA	0	0	0	0	0	0	0	0	0	0	0	0	0	0	0	0	0	0	0	0	0
XM_021063013.1	SORL1	mRNA	0	0	0	0	0	0	0	0	0	0	0	0	0	0	0	0	0	0	0	0	0
XM_021063014.1	SORL1	mRNA	0	0	0	0	0	0	0	0	0	0	0	0	0	0	0	0	0	0	0	0	0
XM_021063023.1	CLMP	mRNA	0	0	0	0	0	0	0	0	0	0	0	0	0	0	0	0	0	0	0	0	0
XM_021063024.1	CLMP	mRNA	0	0	0	0	0	0	0	0	0	0	0	0	0	0	0	0	0	0	0	0	0

RNA ID	Symbol	Type	updown_BGIR9823_101560	updown_BGIR9823_101610	updown_BGIR9823_100872	updown_BGIR9823_101854	updown_BGIR9823_101989	updown_BGIR9823_102727	updown_BGIR9823_79919	updown_BGIR9823_80122	updown_BGIR9823_101217	updown_BGIR9823_83642	updown_BGIR9823_83643	updown_BGIR9823_81077	updown_BGIR9823_86463	updown_BGIR9823_87747	updown_BGIR9823_88211	updown_BGIR9823_88037	updown_BGIR9823_89044	updown_BGIR9823_90934	updown_BGIR9823_95558	updown_BGIR9823_92999	updown_BGIR9823_93000
XM_021063025.1	CLMP	mRNA	0	0	0	0	0	0	0	0	0	0	0	0	0	0	0	0	0	0	0	0	0
XM_021063026.1	CLMP	mRNA	0	0	0	0	0	0	0	0	0	0	0	0	0	0	0	0	0	0	0	0	0
XM_021063950.1	NXPE2	mRNA	0	0	0	0	0	0	0	0	0	0	0	0	0	0	0	0	0	0	0	0	0
XM_021063963.1	LOC100623190	mRNA	0	0	0	0	0	0	0	0	0	0	0	0	0	0	0	0	0	0	0	0	0
XM_021065159.1	HMGB1	mRNA	0	0	0	0	0	0	0	0	0	0	0	0	0	0	0	0	0	0	0	1	1
XM_021065160.1	HMGB1	mRNA	0	0	0	0	0	0	0	0	0	0	0	0	0	0	0	0	0	0	0	1	1
XM_021065161.1	HMGB1	mRNA	0	0	0	0	0	0	0	0	0	0	0	0	0	0	0	0	0	0	0	1	1
XM_021065529.1	KATNAL1	mRNA	0	0	0	0	0	0	0	0	0	0	0	0	0	0	0	0	0	0	0	1	1
XM_021065531.1	KATNAL1	mRNA	0	0	0	0	0	0	0	0	0	0	0	0	0	0	0	0	0	0	0	1	1
XM_021065532.1	KATNAL1	mRNA	0	0	0	0	0	0	0	0	0	0	0	0	0	0	0	0	0	0	0	1	1
XM_021065533.1	KATNAL1	mRNA	0	0	0	0	0	0	0	0	0	0	0	0	0	0	0	0	0	0	0	1	1
XM_021065534.1	KATNAL1	mRNA	0	0	0	0	0	0	0	0	0	0	0	0	0	0	0	0	0	0	0	1	1
XM_021065535.1	KATNAL1	mRNA	0	0	0	0	0	0	0	0	0	0	0	0	0	0	0	0	0	0	0	1	1
XM_021065536.1	KATNAL1	mRNA	0	0	0	0	0	0	0	0	0	0	0	0	0	0	0	0	0	0	0	1	1
XM_021065537.1	KATNAL1	mRNA	0	0	0	0	0	0	0	0	0	0	0	0	0	0	0	0	0	0	0	1	1
XM_021065538.1	KATNAL1	mRNA	0	0	0	0	0	0	0	0	0	0	0	0	0	0	0	0	0	0	0	1	1
XM_021065539.1	KATNAL1	mRNA	0	0	0	0	0	0	0	0	0	0	0	0	0	0	0	0	0	0	0	1	1
XM_021065541.1	KATNAL1	mRNA	0	0	0	0	0	0	0	0	0	0	0	0	0	0	0	0	0	0	0	1	1
XM_021065542.1	KATNAL1	mRNA	0	0	0	0	0	0	0	0	0	0	0	0	0	0	0	0	0	0	0	1	1
XM_021065543.1	KATNAL1	mRNA	0	0	0	0	0	0	0	0	0	0	0	0	0	0	0	0	0	0	0	1	1
XM_021065544.1	KATNAL1	mRNA	0	0	0	0	0	0	0	0	0	0	0	0	0	0	0	0	0	0	0	1	1
XM_021073408.1	EMX2	mRNA	0	0	0	0	0	0	0	0	0	0	0	0	0	0	0	0	0	0	0	0	0
XM_021078611.1	PRKAG2	mRNA	0	0	0	0	0	0	0	0	0	0	0	0	0	0	0	0	0	0	0	0	0
XM_021078612.1	PRKAG2	mRNA	0	0	0	0	0	0	0	0	0	0	0	0	0	0	0	0	0	0	0	0	0
XM_021078613.1	PRKAG2	mRNA	0	0	0	0	0	0	0	0	0	0	0	0	0	0	0	0	0	0	0	0	0
XM_021078614.1	PRKAG2	mRNA	0	0	0	0	0	0	0	0	0	0	0	0	0	0	0	0	0	0	0	0	0
XM_021078615.1	PRKAG2	mRNA	0	0	0	0	0	0	0	0	0	0	0	0	0	0	0	0	0	0	0	0	0
XM_021078616.1	PRKAG2	mRNA	0	0	0	0	0	0	0	0	0	0	0	0	0	0	0	0	0	0	0	0	0
XM_021078617.1	PRKAG2	mRNA	0	0	0	0	0	0	0	0	0	0	0	0	0	0	0	0	0	0	0	0	0
XM_021078618.1	PRKAG2	mRNA	0	0	0	0	0	0	0	0	0	0	0	0	0	0	0	0	0	0	0	0	0
XM_021078619.1	PRKAG2	mRNA	0	0	0	0	0	0	0	0	0	0	0	0	0	0	0	0	0	0	0	0	0
XM_021078620.1	PRKAG2	mRNA	0	0	0	0	0	0	0	0	0	0	0	0	0	0	0	0	0	0	0	0	0
XM_021078621.1	PRKAG2	mRNA	0	0	0	0	0	0	0	0	0	0	0	0	0	0	0	0	0	0	0	0	0
XM_021081309.1	LOC106506226	mRNA	0	0	0	0	0	0	0	0	0	0	0	0	0	0	0	0	0	0	0	0	0
XM_021081403.1	LOC110258215	mRNA	0	0	0	0	0	0	0	0	0	0	0	0	0	0	0	0	0	0	0	0	0
XM_021082153.1	LOC100521600	mRNA	0	1	0	0	0	0	0	0	0	0	0	0	0	0	0	0	0	0	0	0	0
XM_021082154.1	LOC100521600	mRNA	0	1	0	0	0	0	0	0	0	0	0	0	0	0	0	0	0	0	0	0	0
XM_021082156.1	LOC100521600	mRNA	0	1	0	0	0	0	0	0	0	0	0	0	0	0	0	0	0	0	0	0	0
XM_021082157.1	LOC100521600	mRNA	0	1	0	0	0	0	0	0	0	0	0	0	0	0	0	0	0	0	0	0	0
XM_021082158.1	LOC100521600	mRNA	0	1	0	0	0	0	0	0	0	0	0	0	0	0	0	0	0	0	0	0	0
XM_021082159.1	LOC100521600	mRNA	0	1	0	0	0	0	0	0	0	0	0	0	0	0	0	0	0	0	0	0	0
XM_021082835.1	EHBP1L1	mRNA	0	0	0	0	0	0	0	1	0	0	0	0	0	0	0	0	0	0	0	0	0
XM_021082836.1	EHBP1L1	mRNA	0	0	0	0	0	0	0	1	0	0	0	0	0	0	0	0	0	0	0	0	0

RNA ID	Symbol	Type	updown_BGIR9823_101560	updown_BGIR9823_101610	updown_BGIR9823_100872	updown_BGIR9823_101854	updown_BGIR9823_101989	updown_BGIR9823_102727	updown_BGIR9823_79919	updown_BGIR9823_80122	updown_BGIR9823_101217	updown_BGIR9823_83642	updown_BGIR9823_83643	updown_BGIR9823_81077	updown_BGIR9823_86463	updown_BGIR9823_87747	updown_BGIR9823_88211	updown_BGIR9823_88037	updown_BGIR9823_89044	updown_BGIR9823_90934	updown_BGIR9823_95558	updown_BGIR9823_92999	updown_BGIR9823_93000
XM_021082837.1	EHBP1L1	mRNA	0	0	0	0	0	0	0	1	0	0	0	0	0	0	0	0	0	0	0	0	0
XM_021082838.1	EHBP1L1	mRNA	0	0	0	0	0	0	0	1	0	0	0	0	0	0	0	0	0	0	0	0	0
XM_021082839.1	EHBP1L1	mRNA	0	0	0	0	0	0	0	1	0	0	0	0	0	0	0	0	0	0	0	0	0
XM_021082840.1	EHBP1L1	mRNA	0	0	0	0	0	0	0	1	0	0	0	0	0	0	0	0	0	0	0	0	0
XM_021082841.1	EHBP1L1	mRNA	0	0	0	0	0	0	0	1	0	0	0	0	0	0	0	0	0	0	0	0	0
XM_021082842.1	EHBP1L1	mRNA	0	0	0	0	0	0	0	1	0	0	0	0	0	0	0	0	0	0	0	0	0
XM_021082843.1	EHBP1L1	mRNA	0	0	0	0	0	0	0	1	0	0	0	0	0	0	0	0	0	0	0	0	0
XM_021082844.1	EHBP1L1	mRNA	0	0	0	0	0	0	0	1	0	0	0	0	0	0	0	0	0	0	0	0	0
XM_021082845.1	EHBP1L1	mRNA	0	0	0	0	0	0	0	1	0	0	0	0	0	0	0	0	0	0	0	0	0
XM_021082846.1	EHBP1L1	mRNA	0	0	0	0	0	0	0	1	0	0	0	0	0	0	0	0	0	0	0	0	0
XM_021082847.1	EHBP1L1	mRNA	0	0	0	0	0	0	0	1	0	0	0	0	0	0	0	0	0	0	0	0	0
XM_021082851.1	MAP3K11	mRNA	0	0	0	0	0	0	0	0	0	0	0	0	0	0	0	0	0	0	0	0	0
XM_021082852.1	SSSCA1	mRNA	0	0	0	0	0	0	0	1	0	0	0	0	0	0	0	0	0	0	0	0	0
XM_021082853.1	FAM89B	mRNA	0	0	0	0	0	0	0	1	0	0	0	0	0	0	0	0	0	0	0	0	0
XM_021082854.1	LTBP3	mRNA	0	0	0	0	0	0	0	1	0	0	0	0	0	0	0	0	0	0	0	0	0
XM_021082855.1	LTBP3	mRNA	0	0	0	0	0	0	0	1	0	0	0	0	0	0	0	0	0	0	0	0	0
XM_021082856.1	SCYL1	mRNA	0	0	0	0	0	0	0	1	0	0	0	0	0	0	0	0	0	0	0	0	0
XM_021082857.1	SLC25A45	mRNA	0	0	0	0	0	0	0	1	0	0	0	0	0	0	0	0	0	0	0	0	0
XM_021082858.1	SLC25A45	mRNA	0	0	0	0	0	0	0	1	0	0	0	0	0	0	0	0	0	0	0	0	0
XM_021091099.1	SYNGR1	mRNA	0	0	0	0	0	0	0	0	0	0	0	0	0	0	0	0	0	0	0	0	0
XM_021091101.1	SYNGR1	mRNA	0	0	0	0	0	0	0	0	0	0	0	0	0	0	0	0	0	0	0	0	0
XM_021091102.1	SYNGR1	mRNA	0	0	0	0	0	0	0	0	0	0	0	0	0	0	0	0	0	0	0	0	0
XM_021091103.1	SYNGR1	mRNA	0	0	0	0	0	0	0	0	0	0	0	0	0	0	0	0	0	0	0	0	0
XM_021091370.1	LOC102159820	mRNA	0	0	0	0	0	0	0	0	0	0	0	0	0	0	0	0	0	0	0	0	0
XM_021091373.1	TRMU	mRNA	0	0	0	0	0	0	0	0	0	0	0	0	0	0	0	0	0	0	0	0	0
XM_021091374.1	TRMU	mRNA	0	0	0	0	0	0	0	0	0	0	0	0	0	0	0	0	0	0	0	0	0
XM_021091375.1	TRMU	mRNA	0	0	0	0	0	0	0	0	0	0	0	0	0	0	0	0	0	0	0	0	0
XM_021091376.1	GTSE1	mRNA	0	0	0	0	0	0	0	0	0	0	0	0	0	0	0	0	0	0	0	0	0
XM_021091377.1	TTC38	mRNA	0	0	0	0	0	0	0	0	0	0	0	0	0	0	0	0	0	0	0	0	0
XM_021091379.1	LOC110260611	mRNA	0	0	0	0	0	0	0	0	0	0	0	0	0	0	0	0	0	0	0	0	0
XM_021091381.1	LOC110260611	mRNA	0	0	0	0	0	0	0	0	0	0	0	0	0	0	0	0	0	0	0	0	0
XM_021091454.1	TOB2	mRNA	0	0	0	0	0	0	0	0	0	0	0	0	0	0	0	0	0	0	0	0	0
XM_021091455.1	TOB2	mRNA	0	0	0	0	0	0	0	0	0	0	0	0	0	0	0	0	0	0	0	0	0
XM_021091456.1	PHF5A	mRNA	0	0	0	0	0	0	0	0	0	0	0	0	0	0	0	0	0	0	0	0	0
XM_021091457.1	PHF5A	mRNA	0	0	0	0	0	0	0	0	0	0	0	0	0	0	0	0	0	0	0	0	0
XM_021091458.1	ZC3H7B	mRNA	0	0	0	0	0	0	0	0	0	0	0	0	0	0	0	0	0	0	0	0	0
XM_021091459.1	ZC3H7B	mRNA	0	0	0	0	0	0	0	0	0	0	0	0	0	0	0	0	0	0	0	0	0
XM_021091460.1	TEF	mRNA	0	0	0	0	0	0	0	0	0	0	0	0	0	0	0	0	0	0	0	0	0
XM_021091461.1	RANGAP1	mRNA	0	0	0	0	0	0	0	0	0	0	0	0	0	0	0	0	0	0	0	0	0
XM_021091474.1	CBX7	mRNA	0	0	0	0	0	0	0	0	0	0	0	0	0	0	0	0	0	0	0	0	0
XM_021092767.1	CRADD	mRNA	0	0	0	0	0	0	0	0	0	0	0	0	0	0	0	0	0	0	0	0	0
XM_021092768.1	CRADD	mRNA	0	0	0	0	0	0	0	0	0	0	0	0	0	0	0	0	0	0	0	0	0
XM_021092769.1	CRADD	mRNA	0	0	0	0	0	0	0	0	0	0	0	0	0	0	0	0	0	0	0	0	0

RNA ID	Symbol	Type	updown_BGIR9823_101560	updown_BGIR9823_101610	updown_BGIR9823_100872	updown_BGIR9823_101854	updown_BGIR9823_101989	updown_BGIR9823_102727	updown_BGIR9823_79919	updown_BGIR9823_80122	updown_BGIR9823_101217	updown_BGIR9823_83642	updown_BGIR9823_83643	updown_BGIR9823_81077	updown_BGIR9823_86463	updown_BGIR9823_87747	updown_BGIR9823_88211	updown_BGIR9823_88037	updown_BGIR9823_89044	updown_BGIR9823_90934	updown_BGIR9823_95558	updown_BGIR9823_92999	updown_BGIR9823_93000
XM_021092770.1	CRADD	mRNA	0	0	0	0	0	0	0	0	0	0	0	0	0	0	0	0	0	0	0	0	0
XM_021092771.1	CRADD	mRNA	0	0	0	0	0	0	0	0	0	0	0	0	0	0	0	0	0	0	0	0	0
XM_021092773.1	CRADD	mRNA	0	0	0	0	0	0	0	0	0	0	0	0	0	0	0	0	0	0	0	0	0
XM_021093095.1	PKDREJ	mRNA	0	0	0	0	0	0	0	0	0	0	0	0	0	0	0	0	0	0	0	0	0
XM_021097064.1	MAF	mRNA	0	0	0	0	0	0	0	0	0	0	0	0	0	0	0	0	0	0	0	0	0
XM_021097065.1	MAF	mRNA	0	0	0	0	0	0	0	0	0	0	0	0	0	0	0	0	0	0	0	0	0
XM_021097066.1	MAF	mRNA	0	0	0	0	0	0	0	0	0	0	0	0	0	0	0	0	0	0	0	0	0
XM_021097870.1	ECI2	mRNA	0	0	0	0	0	0	0	0	0	0	0	0	0	0	0	0	0	0	0	0	0
XM_021099751.1	PHACTR1	mRNA	0	0	0	0	0	0	0	0	0	0	0	0	0	0	0	0	1	0	0	0	0
XM_021099752.1	PHACTR1	mRNA	0	0	0	0	0	0	0	0	0	0	0	0	0	0	0	0	1	0	0	0	0
XM_021099753.1	PHACTR1	mRNA	0	0	0	0	0	0	0	0	0	0	0	0	0	0	0	0	1	0	0	0	0
XM_021099754.1	PHACTR1	mRNA	0	0	0	0	0	0	0	0	0	0	0	0	0	0	0	0	1	0	0	0	0
XM_021099755.1	PHACTR1	mRNA	0	0	0	0	0	0	0	0	0	0	0	0	0	0	0	0	1	0	0	0	0
XM_021099756.1	PHACTR1	mRNA	0	0	0	0	0	0	0	0	0	0	0	0	0	0	0	0	1	0	0	0	0
XM_021099757.1	PHACTR1	mRNA	0	0	0	0	0	0	0	0	0	0	0	0	0	0	0	0	1	0	0	0	0
XM_021099758.1	PHACTR1	mRNA	0	0	0	0	0	0	0	0	0	0	0	0	0	0	0	0	1	0	0	0	0
XM_021099759.1	PHACTR1	mRNA	0	0	0	0	0	0	0	0	0	0	0	0	0	0	0	0	1	0	0	0	0
XM_021099760.1	PHACTR1	mRNA	0	0	0	0	0	0	0	0	0	0	0	0	0	0	0	0	1	0	0	0	0
XM_021099761.1	PHACTR1	mRNA	0	0	0	0	0	0	0	0	0	0	0	0	0	0	0	0	1	0	0	0	0
XM_021099762.1	PHACTR1	mRNA	0	0	0	0	0	0	0	0	0	0	0	0	0	0	0	0	1	0	0	0	0
XM_021099763.1	PHACTR1	mRNA	0	0	0	0	0	0	0	0	0	0	0	0	0	0	0	0	1	0	0	0	0
XM_021099764.1	PHACTR1	mRNA	0	0	0	0	0	0	0	0	0	0	0	0	0	0	0	0	1	0	0	0	0
XM_021099765.1	PHACTR1	mRNA	0	0	0	0	0	0	0	0	0	0	0	0	0	0	0	0	1	0	0	0	0
XM_021099766.1	TBC1D7	mRNA	0	0	0	0	0	0	0	0	0	0	0	0	0	0	0	0	1	0	0	0	0
XM_021099767.1	TBC1D7	mRNA	0	0	0	0	0	0	0	0	0	0	0	0	0	0	0	0	1	0	0	0	0
XM_021099769.1	TBC1D7	mRNA	0	0	0	0	0	0	0	0	0	0	0	0	0	0	0	0	1	0	0	0	0
XM_021099770.1	TBC1D7	mRNA	0	0	0	0	0	0	0	0	0	0	0	0	0	0	0	0	1	0	0	0	0
XM_021099999.1	LOC110261697	mRNA	0	0	0	0	0	0	0	0	0	0	0	0	0	0	0	0	0	0	0	0	0
XM_021100108.1	WRNIP1	mRNA	0	0	0	0	0	0	0	0	0	0	0	0	0	0	0	0	0	0	0	0	0
XM_021100109.1	MYLK4	mRNA	0	0	0	0	0	0	0	0	0	0	0	0	0	0	0	0	0	0	0	0	0
XM_021100110.1	MYLK4	mRNA	0	0	0	0	0	0	0	0	0	0	0	0	0	0	0	0	0	0	0	0	0
XM_021100111.1	MYLK4	mRNA	0	0	0	0	0	0	0	0	0	0	0	0	0	0	0	0	0	0	0	0	0
XM_021100112.1	MYLK4	mRNA	0	0	0	0	0	0	0	0	0	0	0	0	0	0	0	0	0	0	0	0	0
XM_021100114.1	NQO2	mRNA	0	0	0	0	0	0	0	0	0	0	0	0	0	0	0	0	0	0	0	0	0
XM_021100115.1	NQO2	mRNA	0	0	0	0	0	0	0	0	0	0	0	0	0	0	0	0	0	0	0	0	0
XM_021100117.1	NQO2	mRNA	0	0	0	0	0	0	0	0	0	0	0	0	0	0	0	0	0	0	0	0	0
XM_021100118.1	NQO2	mRNA	0	0	0	0	0	0	0	0	0	0	0	0	0	0	0	0	0	0	0	0	0
XM_021100119.1	NQO2	mRNA	0	0	0	0	0	0	0	0	0	0	0	0	0	0	0	0	0	0	0	0	0
XM_021100124.1	PRPF4B	mRNA	0	0	0	0	0	0	0	0	0	0	0	0	0	0	0	0	0	0	0	0	0
XM_021100126.1	PRPF4B	mRNA	0	0	0	0	0	0	0	0	0	0	0	0	0	0	0	0	0	0	0	0	0
XM_021100184.1	NEDD9	mRNA	0	0	0	0	0	0	0	0	0	0	0	0	0	0	1	0	0	0	0	0	0
XM_021100188.1	TMEM170B	mRNA	0	0	0	0	0	0	0	0	0	0	0	0	0	0	1	0	0	0	0	0	0
XM_021100194.1	GFOD1	mRNA	0	0	0	0	0	0	0	0	0	0	0	0	0	0	0	0	1	0	0	0	0

RNA ID	Symbol	Type	updown_BGIR9823_101560	updown_BGIR9823_101610	updown_BGIR9823_100872	updown_BGIR9823_101854	updown_BGIR9823_101989	updown_BGIR9823_102727	updown_BGIR9823_79919	updown_BGIR9823_80122	updown_BGIR9823_101217	updown_BGIR9823_83642	updown_BGIR9823_83643	updown_BGIR9823_81077	updown_BGIR9823_86463	updown_BGIR9823_87747	updown_BGIR9823_88211	updown_BGIR9823_88037	updown_BGIR9823_89044	updown_BGIR9823_90934	updown_BGIR9823_95558	updown_BGIR9823_92999	updown_BGIR9823_93000
XM_021102040.1	SLC37A4	mRNA	0	0	0	0	0	0	0	0	0	0	0	0	0	0	0	0	0	1	0	0	0
XM_021102041.1	SLC37A4	mRNA	0	0	0	0	0	0	0	0	0	0	0	0	0	0	0	0	0	1	0	0	0
XM_021102042.1	SLC37A4	mRNA	0	0	0	0	0	0	0	0	0	0	0	0	0	0	0	0	0	1	0	0	0
XM_021102043.1	SLC37A4	mRNA	0	0	0	0	0	0	0	0	0	0	0	0	0	0	0	0	0	1	0	0	0
XM_021102044.1	SLC37A4	mRNA	0	0	0	0	0	0	0	0	0	0	0	0	0	0	0	0	0	1	0	0	0
XM_021102045.1	SLC37A4	mRNA	0	0	0	0	0	0	0	0	0	0	0	0	0	0	0	0	0	1	0	0	0
XM_021102046.1	SLC37A4	mRNA	0	0	0	0	0	0	0	0	0	0	0	0	0	0	0	0	0	1	0	0	0
XM_021102047.1	SLC37A4	mRNA	0	0	0	0	0	0	0	0	0	0	0	0	0	0	0	0	0	1	0	0	0
XM_021102048.1	SLC37A4	mRNA	0	0	0	0	0	0	0	0	0	0	0	0	0	0	0	0	0	1	0	0	0
XM_021102049.1	SLC37A4	mRNA	0	0	0	0	0	0	0	0	0	0	0	0	0	0	0	0	0	1	0	0	0
XM_021102050.1	SLC37A4	mRNA	0	0	0	0	0	0	0	0	0	0	0	0	0	0	0	0	0	1	0	0	0
XM_021102051.1	SLC37A4	mRNA	0	0	0	0	0	0	0	0	0	0	0	0	0	0	0	0	0	1	0	0	0
XM_021102052.1	SLC37A4	mRNA	0	0	0	0	0	0	0	0	0	0	0	0	0	0	0	0	0	1	0	0	0
XM_021102209.1	UPK2	mRNA	0	0	0	0	0	0	0	0	0	0	0	0	0	0	0	0	0	1	0	0	0
XM_021102516.1	LOC100522455	mRNA	0	0	0	0	0	0	0	0	0	0	0	0	0	0	0	0	0	0	0	0	0
XM_021102517.1	RUNDC3B	mRNA	0	0	0	0	0	0	0	0	0	0	0	0	0	0	0	0	0	0	0	0	0
XM_021102518.1	RUNDC3B	mRNA	0	0	0	0	0	0	0	0	0	0	0	0	0	0	0	0	0	0	0	0	0
XM_021102519.1	RUNDC3B	mRNA	0	0	0	0	0	0	0	0	0	0	0	0	0	0	0	0	0	0	0	0	0
XM_021102522.1	RUNDC3B	mRNA	0	0	0	0	0	0	0	0	0	0	0	0	0	0	0	0	0	0	0	0	0
XM_021102523.1	RUNDC3B	mRNA	0	0	0	0	0	0	0	0	0	0	0	0	0	0	0	0	0	0	0	0	0
XR_001297664.2	LOC102161744	lncRNA	0	0	0	0	0	0	0	0	0	0	0	0	0	0	0	0	0	0	0	0	0
XR_001298807.2	LOC106504881	lncRNA	0	0	0	0	0	0	0	0	0	0	0	0	0	0	0	0	0	0	0	0	0
XR_001298808.2	LOC106504881	lncRNA	0	0	0	0	0	0	0	0	0	0	0	0	0	0	0	0	0	0	0	0	0
XR_001298810.2	LOC106504881	lncRNA	0	0	0	0	0	0	0	0	0	0	0	0	0	0	0	0	0	0	0	0	0
XR_001298811.2	LOC106504881	lncRNA	0	0	0	0	0	0	0	0	0	0	0	0	0	0	0	0	0	0	0	0	0
XR_001298812.2	LOC106504881	lncRNA	0	0	0	0	0	0	0	0	0	0	0	0	0	0	0	0	0	0	0	0	0
XR_001298813.2	LOC106504881	lncRNA	0	0	0	0	0	0	0	0	0	0	0	0	0	0	0	0	0	0	0	0	0
XR_001298814.2	LOC106504881	lncRNA	0	0	0	0	0	0	0	0	0	0	0	0	0	0	0	0	0	0	0	0	0
XR_001298816.2	LOC106504881	lncRNA	0	0	0	0	0	0	0	0	0	0	0	0	0	0	0	0	0	0	0	0	0
XR_001298817.2	LOC106504881	lncRNA	0	0	0	0	0	0	0	0	0	0	0	0	0	0	0	0	0	0	0	0	0
XR_001298844.2	LOC102163364	lncRNA	0	0	0	0	0	0	0	0	0	0	0	0	0	0	0	0	0	0	0	0	0
XR_001298846.2	LOC102163364	lncRNA	0	0	0	0	0	0	0	0	0	0	0	0	0	0	0	0	0	0	0	0	0
XR_001298850.2	LOC102163364	lncRNA	0	0	0	0	0	0	0	0	0	0	0	0	0	0	0	0	0	0	0	0	0
XR_001299456.2	LOC102166670	lncRNA	0	0	0	0	0	0	0	0	0	0	0	0	0	0	0	0	0	0	0	1	1
XR_001299457.2	LOC102166670	lncRNA	0	0	0	0	0	0	0	0	0	0	0	0	0	0	0	0	0	0	0	1	1
XR_001300715.2	LOC106505879	lncRNA	0	0	0	0	0	0	0	0	0	0	0	0	0	0	0	0	0	0	0	0	0
XR_001301078.2	LOC102162623	lncRNA	0	0	0	0	0	0	0	0	0	0	0	0	0	0	0	0	0	0	0	0	0
XR_001301087.2	LOC102162623	lncRNA	0	0	0	0	0	0	0	0	0	0	0	0	0	0	0	0	0	0	0	0	0
XR_001301217.2	LOC102164998	lncRNA	0	0	0	0	0	0	0	0	0	0	0	0	0	0	0	0	0	0	0	0	0
XR_001301305.2	LOC102159607	lncRNA	0	0	0	0	0	0	0	0	0	0	0	0	0	0	0	0	0	0	0	0	0
XR_001308967.2	LOC106510467	lncRNA	0	0	0	0	0	0	0	0	0	0	0	0	0	0	0	0	0	0	0	0	0
XR_002335533.1	XRRA1	mRNA	0	0	0	0	0	0	0	0	0	0	0	0	0	0	0	0	0	0	0	0	0
XR_002335534.1	XRRA1	mRNA	0	0	0	0	0	0	0	0	0	0	0	0	0	0	0	0	0	0	0	0	0

RNA ID	Symbol	Type	updown_BGIR9823_101560	updown_BGIR9823_101610	updown_BGIR9823_100872	updown_BGIR9823_101854	updown_BGIR9823_101989	updown_BGIR9823_102727	updown_BGIR9823_79919	updown_BGIR9823_80122	updown_BGIR9823_101217	updown_BGIR9823_83642	updown_BGIR9823_83643	updown_BGIR9823_81077	updown_BGIR9823_86463	updown_BGIR9823_87747	updown_BGIR9823_88211	updown_BGIR9823_88037	updown_BGIR9823_89044	updown_BGIR9823_90934	updown_BGIR9823_95558	updown_BGIR9823_92999	updown_BGIR9823_93000
XR_002335535.1	LOC110255399	lncRNA	0	0	0	0	0	0	0	0	0	0	0	0	0	0	0	0	0	0	0	0	0
XR_002335536.1	LOC102160367	lncRNA	0	0	0	0	0	0	0	0	0	0	0	0	0	0	0	0	0	0	0	0	0
XR_002335537.1	LOC102160367	lncRNA	0	0	0	0	0	0	0	0	0	0	0	0	0	0	0	0	0	0	0	0	0
XR_002335650.1	LOC102157630	lncRNA	0	0	0	0	0	0	0	0	0	0	0	0	0	0	0	0	0	0	0	0	0
XR_002335651.1	LOC110255435	lncRNA	0	0	0	0	0	0	0	0	0	0	0	0	0	0	0	0	0	0	0	0	0
XR_002335652.1	LOC106504881	lncRNA	0	0	0	0	0	0	0	0	0	0	0	0	0	0	0	0	0	0	0	0	0
XR_002335653.1	LOC106504881	lncRNA	0	0	0	0	0	0	0	0	0	0	0	0	0	0	0	0	0	0	0	0	0
XR_002335670.1	LOC110255440	lncRNA	0	0	0	0	0	0	0	0	0	0	0	0	0	0	0	0	0	1	0	0	0
XR_002335671.1	LOC110255441	lncRNA	0	0	0	0	0	0	0	0	0	0	0	0	0	0	0	0	0	1	0	0	0
XR_002335672.1	LOC110255441	lncRNA	0	0	0	0	0	0	0	0	0	0	0	0	0	0	0	0	0	1	0	0	0
XR_002335674.1	HYOU1	mRNA	0	0	0	0	0	0	0	0	0	0	0	0	0	0	0	0	0	1	0	0	0
XR_002335675.1	HYOU1	mRNA	0	0	0	0	0	0	0	0	0	0	0	0	0	0	0	0	0	1	0	0	0
XR_002335685.1	LOC100523684	mRNA	0	0	0	0	0	0	0	0	0	0	0	0	0	0	0	0	0	0	0	0	0
XR_002335686.1	LOC102163364	lncRNA	0	0	0	0	0	0	0	0	0	0	0	0	0	0	0	0	0	0	0	0	0
XR_002335687.1	LOC102163364	lncRNA	0	0	0	0	0	0	0	0	0	0	0	0	0	0	0	0	0	0	0	0	0
XR_002335688.1	LOC102163364	lncRNA	0	0	0	0	0	0	0	0	0	0	0	0	0	0	0	0	0	0	0	0	0
XR_002335689.1	LOC102163364	lncRNA	0	0	0	0	0	0	0	0	0	0	0	0	0	0	0	0	0	0	0	0	0
XR_002335690.1	LOC102163364	lncRNA	0	0	0	0	0	0	0	0	0	0	0	0	0	0	0	0	0	0	0	0	0
XR_002335691.1	LOC102163364	lncRNA	0	0	0	0	0	0	0	0	0	0	0	0	0	0	0	0	0	0	0	0	0
XR_002335692.1	LOC102163364	lncRNA	0	0	0	0	0	0	0	0	0	0	0	0	0	0	0	0	0	0	0	0	0
XR_002335693.1	LOC106504896	lncRNA	0	0	0	0	0	0	0	0	0	0	0	0	0	0	0	0	0	0	0	0	0
XR_002335694.1	SORL1	mRNA	0	0	0	0	0	0	0	0	0	0	0	0	0	0	0	0	0	0	0	0	0
XR_002336641.1	LOC106505250	lncRNA	0	0	0	0	0	0	0	0	0	0	0	0	0	0	0	0	0	0	0	1	0
XR_002336643.1	LOC110255292	lncRNA	0	0	0	0	0	0	0	0	0	0	0	0	0	0	0	0	0	0	0	1	1
XR_002336644.1	LOC102166670	lncRNA	0	0	0	0	0	0	0	0	0	0	0	0	0	0	0	0	0	0	0	1	1
XR_002336645.1	LOC102166670	lncRNA	0	0	0	0	0	0	0	0	0	0	0	0	0	0	0	0	0	0	0	1	1
XR_002338443.1	LOC106505879	lncRNA	0	0	0	0	0	0	0	0	0	0	0	0	0	0	0	0	0	0	0	0	0
XR_002338444.1	LOC106505879	lncRNA	0	0	0	0	0	0	0	0	0	0	0	0	0	0	0	0	0	0	0	0	0
XR_002338445.1	LOC106505879	lncRNA	0	0	0	0	0	0	0	0	0	0	0	0	0	0	0	0	0	0	0	0	0
XR_002338446.1	LOC106505879	lncRNA	0	0	0	0	0	0	0	0	0	0	0	0	0	0	0	0	0	0	0	0	0
XR_002338447.1	LOC106505879	lncRNA	0	0	0	0	0	0	0	0	0	0	0	0	0	0	0	0	0	0	0	0	0
XR_002338448.1	LOC106505879	lncRNA	0	0	0	0	0	0	0	0	0	0	0	0	0	0	0	0	0	0	0	0	0
XR_002338449.1	LOC106505879	lncRNA	0	0	0	0	0	0	0	0	0	0	0	0	0	0	0	0	0	0	0	0	0
XR_002338450.1	LOC106505879	lncRNA	0	0	0	0	0	0	0	0	0	0	0	0	0	0	0	0	0	0	0	0	0
XR_002338521.1	LOC110256664	lncRNA	0	0	0	0	0	0	0	0	0	0	0	0	0	0	0	0	0	0	0	0	0
XR_002338522.1	LOC110256664	lncRNA	0	0	0	0	0	0	0	0	0	0	0	0	0	0	0	0	0	0	0	0	0
XR_002338666.1	LOC110256747	lncRNA	0	0	0	0	0	0	0	0	0	0	0	0	0	0	0	0	0	0	0	0	0
XR_002338714.1	LOC102163817	lncRNA	0	0	0	0	0	0	0	0	0	0	0	0	0	0	0	0	0	0	0	0	0
XR_002338715.1	LOC102164998	lncRNA	0	0	0	0	0	0	0	0	0	0	0	0	0	0	0	0	0	0	0	0	0
XR_002338716.1	LOC102164998	lncRNA	0	0	0	0	0	0	0	0	0	0	0	0	0	0	0	0	0	0	0	0	0
XR_002338717.1	LOC110256768	lncRNA	0	0	0	0	0	0	0	0	0	0	0	0	0	0	0	0	0	0	0	0	0
XR_002338727.1	LOC110256773	lncRNA	0	0	0	0	0	0	0	0	0	0	0	0	0	0	0	0	0	0	0	0	0
XR_002338728.1	LOC110256773	lncRNA	0	0	0	0	0	0	0	0	0	0	0	0	0	0	0	0	0	0	0	0	0

RNA ID	Symbol	Type	updown_BGIR9823_101560	updown_BGIR9823_101610	updown_BGIR9823_100872	updown_BGIR9823_101854	updown_BGIR9823_101989	updown_BGIR9823_102727	updown_BGIR9823_79919	updown_BGIR9823_80122	updown_BGIR9823_101217	updown_BGIR9823_83642	updown_BGIR9823_83643	updown_BGIR9823_81077	updown_BGIR9823_86463	updown_BGIR9823_87747	updown_BGIR9823_88211	updown_BGIR9823_88037	updown_BGIR9823_89044	updown_BGIR9823_90934	updown_BGIR9823_95558	updown_BGIR9823_92999	updown_BGIR9823_93000
XR_002338729.1	LOC110256773	lncRNA	0	0	0	0	0	0	0	0	0	0	0	0	0	0	0	0	0	0	0	0	0
XR_002338793.1	LOC110256796	lncRNA	0	0	0	0	0	0	0	0	0	0	0	0	0	0	0	0	0	0	0	0	0
XR_002339623.1	LOC102167708	lncRNA	0	0	0	0	0	0	0	0	0	0	0	0	0	0	0	0	0	0	0	0	0
XR_002340245.1	PRKAG2	mRNA	0	0	0	0	0	0	0	0	0	0	0	0	0	0	0	0	0	0	0	0	0
XR_002340246.1	LOC102162623	lncRNA	0	0	0	0	0	0	0	0	0	0	0	0	0	0	0	0	0	0	0	0	0
XR_002340247.1	LOC102162623	lncRNA	0	0	0	0	0	0	0	0	0	0	0	0	0	0	0	0	0	0	0	0	0
XR_002340248.1	LOC110257516	lncRNA	0	0	0	0	0	0	0	0	0	0	0	0	0	0	0	0	0	0	0	0	0
XR_002340249.1	LOC102162071	lncRNA	0	0	0	0	0	0	0	0	0	0	0	0	0	0	0	0	0	0	0	0	0
XR_002340901.1	LOC102160243	lncRNA	0	0	0	0	0	1	0	0	0	0	0	0	0	0	0	0	0	0	0	0	0
XR_002340902.1	LOC102160243	lncRNA	0	0	0	0	0	1	0	0	0	0	0	0	0	0	0	0	0	0	0	0	0
XR_002340903.1	LOC102160243	lncRNA	0	0	0	0	0	1	0	0	0	0	0	0	0	0	0	0	0	0	0	0	0
XR_002340904.1	LOC102160243	lncRNA	0	0	0	0	0	1	0	0	0	0	0	0	0	0	0	0	0	0	0	0	0
XR_002340905.1	LOC102160243	lncRNA	0	0	0	0	0	1	0	0	0	0	0	0	0	0	0	0	0	0	0	0	0
XR_002341237.1	LOC102164320	lncRNA	0	0	0	0	0	0	0	0	0	0	0	0	0	0	0	0	0	0	0	0	0
XR_002341238.1	LOC102164320	lncRNA	0	0	0	0	0	0	0	0	0	0	0	0	0	0	0	0	0	0	0	0	0
XR_002341239.1	LOC102164320	lncRNA	0	0	0	0	0	0	0	0	0	0	0	0	0	0	0	0	0	0	0	0	0
XR_002341240.1	LOC102164320	lncRNA	0	0	0	0	0	0	0	0	0	0	0	0	0	0	0	0	0	0	0	0	0
XR_002341241.1	LOC102164320	lncRNA	0	0	0	0	0	0	0	0	0	0	0	0	0	0	0	0	0	0	0	0	0
XR_002341242.1	LOC102164320	lncRNA	0	0	0	0	0	0	0	0	0	0	0	0	0	0	0	0	0	0	0	0	0
XR_002341243.1	LOC102164320	lncRNA	0	0	0	0	0	0	0	0	0	0	0	0	0	0	0	0	0	0	0	0	0
XR_002341244.1	LOC102164320	lncRNA	0	0	0	0	0	0	0	0	0	0	0	0	0	0	0	0	0	0	0	0	0
XR_002341270.1	LOC100521600	mRNA	0	1	0	0	0	0	0	0	0	0	0	0	0	0	0	0	0	0	0	0	0
XR_002341443.1	LOC102163816	lncRNA	0	0	0	0	0	0	0	1	0	0	0	0	0	0	0	0	0	0	0	0	0
XR_002343847.1	LOC110260611	mRNA	0	0	0	0	0	0	0	0	0	0	0	0	0	0	0	0	0	0	0	0	0
XR_002343848.1	LOC110260611	mRNA	0	0	0	0	0	0	0	0	0	0	0	0	0	0	0	0	0	0	0	0	0
XR_002343849.1	LOC110260611	mRNA	0	0	0	0	0	0	0	0	0	0	0	0	0	0	0	0	0	0	0	0	0
XR_002343850.1	LOC110260611	mRNA	0	0	0	0	0	0	0	0	0	0	0	0	0	0	0	0	0	0	0	0	0
XR_002343875.1	LOC110260629	lncRNA	0	0	0	0	0	0	0	0	0	0	0	0	0	0	0	0	0	0	0	0	0
XR_002343876.1	LOC110260629	lncRNA	0	0	0	0	0	0	0	0	0	0	0	0	0	0	0	0	0	0	0	0	0
XR_002343877.1	LOC110260629	lncRNA	0	0	0	0	0	0	0	0	0	0	0	0	0	0	0	0	0	0	0	0	0
XR_002343878.1	LOC110260629	lncRNA	0	0	0	0	0	0	0	0	0	0	0	0	0	0	0	0	0	0	0	0	0
XR_002343879.1	LOC110260629	lncRNA	0	0	0	0	0	0	0	0	0	0	0	0	0	0	0	0	0	0	0	0	0
XR_002343880.1	LOC110260629	lncRNA	0	0	0	0	0	0	0	0	0	0	0	0	0	0	0	0	0	0	0	0	0
XR_002343881.1	LOC110260629	lncRNA	0	0	0	0	0	0	0	0	0	0	0	0	0	0	0	0	0	0	0	0	0
XR_002343883.1	LOC110260631	lncRNA	0	0	0	0	0	0	0	0	0	0	0	0	0	0	0	0	0	0	0	0	0
XR_002343884.1	LOC110260634	lncRNA	0	0	0	0	0	0	0	0	0	0	0	0	0	0	0	0	0	0	0	0	0
XR_002343885.1	LOC110260635	lncRNA	0	0	0	0	0	0	0	0	0	0	0	0	0	0	0	0	0	0	0	0	0
XR_002343891.1	LOC110260642	lncRNA	0	0	0	0	0	0	0	0	0	0	0	0	0	0	0	0	0	0	0	0	0
XR_002343892.1	LOC110260643	lncRNA	0	0	0	0	0	0	0	0	0	0	0	0	0	0	0	0	0	0	0	0	0
XR_002345298.1	MAF	mRNA	0	0	0	0	0	0	0	0	0	0	0	0	0	0	0	0	0	0	0	0	0
XR_002345300.1	LOC106510470	lncRNA	0	0	0	0	0	0	0	0	0	0	0	0	0	0	0	0	0	0	0	0	0
XR_002345301.1	LOC106510468	lncRNA	0	0	0	0	0	0	0	0	0	0	0	0	0	0	0	0	0	0	0	0	0
XR_002346166.1	LOC110261703	lncRNA	0	0	0	0	0	0	0	0	0	0	0	0	0	0	1	0	0	0	0	0	0

RNA ID	Symbol	Type	updown_BGIR9823_101560	updown_BGIR9823_101610	updown_BGIR9823_100872	updown_BGIR9823_101854	updown_BGIR9823_101989	updown_BGIR9823_102727	updown_BGIR9823_79919	updown_BGIR9823_80122	updown_BGIR9823_101217	updown_BGIR9823_83642	updown_BGIR9823_83643	updown_BGIR9823_81077	updown_BGIR9823_86463	updown_BGIR9823_87747	updown_BGIR9823_88211	updown_BGIR9823_88037	updown_BGIR9823_89044	updown_BGIR9823_90934	updown_BGIR9823_95558	updown_BGIR9823_92999	updown_BGIR9823_93000
XR_002346227.1	LOC106504299	lncRNA	0	0	0	0	0	0	0	0	0	0	0	0	0	0	0	0	0	0	0	0	0
XR_002346228.1	LOC106504299	lncRNA	0	0	0	0	0	0	0	0	0	0	0	0	0	0	0	0	0	0	0	0	0
XR_002346229.1	LOC106504299	lncRNA	0	0	0	0	0	0	0	0	0	0	0	0	0	0	0	0	0	0	0	0	0
XR_002346230.1	LOC106504299	lncRNA	0	0	0	0	0	0	0	0	0	0	0	0	0	0	0	0	0	0	0	0	0
XR_002346231.1	LOC106504299	lncRNA	0	0	0	0	0	0	0	0	0	0	0	0	0	0	0	0	0	0	0	0	0
XR_002346232.1	LOC106504299	lncRNA	0	0	0	0	0	0	0	0	0	0	0	0	0	0	0	0	0	0	0	0	0
XR_002346233.1	NQO2	mRNA	0	0	0	0	0	0	0	0	0	0	0	0	0	0	0	0	0	0	0	0	0
XR_002346236.1	PRPF4B	mRNA	0	0	0	0	0	0	0	0	0	0	0	0	0	0	0	0	0	0	0	0	0
XR_002346237.1	PRPF4B	mRNA	0	0	0	0	0	0	0	0	0	0	0	0	0	0	0	0	0	0	0	0	0
XR_002346238.1	PRPF4B	mRNA	0	0	0	0	0	0	0	0	0	0	0	0	0	0	0	0	0	0	0	0	0
XR_002346239.1	PRPF4B	mRNA	0	0	0	0	0	0	0	0	0	0	0	0	0	0	0	0	0	0	0	0	0
XR_002346240.1	PRPF4B	mRNA	0	0	0	0	0	0	0	0	0	0	0	0	0	0	0	0	0	0	0	0	0
XR_002346241.1	PRPF4B	mRNA	0	0	0	0	0	0	0	0	0	0	0	0	0	0	0	0	0	0	0	0	0
XR_002346242.1	PRPF4B	mRNA	0	0	0	0	0	0	0	0	0	0	0	0	0	0	0	0	0	0	0	0	0
XR_002346243.1	PRPF4B	mRNA	0	0	0	0	0	0	0	0	0	0	0	0	0	0	0	0	0	0	0	0	0
XR_002346244.1	PRPF4B	mRNA	0	0	0	0	0	0	0	0	0	0	0	0	0	0	0	0	0	0	0	0	0
XR_002346245.1	PRPF4B	mRNA	0	0	0	0	0	0	0	0	0	0	0	0	0	0	0	0	0	0	0	0	0
XR_002346246.1	PRPF4B	mRNA	0	0	0	0	0	0	0	0	0	0	0	0	0	0	0	0	0	0	0	0	0
XR_002346247.1	PRPF4B	mRNA	0	0	0	0	0	0	0	0	0	0	0	0	0	0	0	0	0	0	0	0	0
XR_002346248.1	PRPF4B	mRNA	0	0	0	0	0	0	0	0	0	0	0	0	0	0	0	0	0	0	0	0	0
XR_002346249.1	PRPF4B	mRNA	0	0	0	0	0	0	0	0	0	0	0	0	0	0	0	0	0	0	0	0	0
XR_002346250.1	PRPF4B	mRNA	0	0	0	0	0	0	0	0	0	0	0	0	0	0	0	0	0	0	0	0	0
XR_002346251.1	PRPF4B	mRNA	0	0	0	0	0	0	0	0	0	0	0	0	0	0	0	0	0	0	0	0	0
XR_002346252.1	PRPF4B	mRNA	0	0	0	0	0	0	0	0	0	0	0	0	0	0	0	0	0	0	0	0	0
XR_002346253.1	PRPF4B	mRNA	0	0	0	0	0	0	0	0	0	0	0	0	0	0	0	0	0	0	0	0	0
XR_002346254.1	PRPF4B	mRNA	0	0	0	0	0	0	0	0	0	0	0	0	0	0	0	0	0	0	0	0	0
XR_002346255.1	PRPF4B	mRNA	0	0	0	0	0	0	0	0	0	0	0	0	0	0	0	0	0	0	0	0	0
XR_002346256.1	PRPF4B	mRNA	0	0	0	0	0	0	0	0	0	0	0	0	0	0	0	0	0	0	0	0	0
XR_002346257.1	PRPF4B	mRNA	0	0	0	0	0	0	0	0	0	0	0	0	0	0	0	0	0	0	0	0	0
XR_002346258.1	PRPF4B	mRNA	0	0	0	0	0	0	0	0	0	0	0	0	0	0	0	0	0	0	0	0	0
XR_002346259.1	PRPF4B	mRNA	0	0	0	0	0	0	0	0	0	0	0	0	0	0	0	0	0	0	0	0	0
XR_002346260.1	PRPF4B	mRNA	0	0	0	0	0	0	0	0	0	0	0	0	0	0	0	0	0	0	0	0	0
XR_002346261.1	PRPF4B	mRNA	0	0	0	0	0	0	0	0	0	0	0	0	0	0	0	0	0	0	0	0	0
XR_002346262.1	PRPF4B	mRNA	0	0	0	0	0	0	0	0	0	0	0	0	0	0	0	0	0	0	0	0	0
XR_002346263.1	PRPF4B	mRNA	0	0	0	0	0	0	0	0	0	0	0	0	0	0	0	0	0	0	0	0	0
XR_002346264.1	PRPF4B	mRNA	0	0	0	0	0	0	0	0	0	0	0	0	0	0	0	0	0	0	0	0	0
XR_002346265.1	LOC110261764	lncRNA	0	0	0	0	0	0	0	0	0	0	0	0	0	0	0	0	0	0	0	0	0
XR_002346266.1	LOC102162837	lncRNA	0	0	0	0	0	0	0	0	0	0	0	0	0	0	0	0	0	0	0	0	0
XR_002346299.1	LOC110261774	lncRNA	0	0	0	0	0	0	0	0	0	0	0	0	0	0	1	0	0	0	0	0	0
XR_002346300.1	LOC110261774	lncRNA	0	0	0	0	0	0	0	0	0	0	0	0	0	0	1	0	0	0	0	0	0
XR_002346302.1	LOC110261776	lncRNA	0	0	0	0	0	0	0	0	0	0	0	0	0	0	1	0	0	0	0	0	0
XR_002346303.1	TMEM170B	mRNA	0	0	0	0	0	0	0	0	0	0	0	0	0	0	1	0	0	0	0	0	0
XR_002346304.1	TMEM170B	mRNA	0	0	0	0	0	0	0	0	0	0	0	0	0	0	1	0	0	0	0	0	0

RNA ID	Symbol	Type	updown_BGIR9823_101560	updown_BGIR9823_101610	updown_BGIR9823_100872	updown_BGIR9823_101854	updown_BGIR9823_101989	updown_BGIR9823_102727	updown_BGIR9823_79919	updown_BGIR9823_80122	updown_BGIR9823_101217	updown_BGIR9823_83642	updown_BGIR9823_83643	updown_BGIR9823_81077	updown_BGIR9823_86463	updown_BGIR9823_87747	updown_BGIR9823_88211	updown_BGIR9823_88037	updown_BGIR9823_89044	updown_BGIR9823_90934	updown_BGIR9823_95558	updown_BGIR9823_92999	updown_BGIR9823_93000
XR_115737.4	LOC100513133	lncRNA	0	0	0	0	0	0	0	1	0	0	0	0	0	0	0	0	0	0	0	0	0
XR_299046.2	LOC102164596	lncRNA	0	0	0	0	0	0	0	0	0	0	0	0	0	0	0	0	0	0	0	0	0
XR_303634.3	LOC100738154	lncRNA	0	0	0	0	0	0	0	0	0	0	0	0	0	0	0	0	0	0	0	0	0
XR_304239.3	LOC102159819	lncRNA	0	0	0	0	0	0	0	0	0	0	0	0	0	0	0	0	0	0	0	0	0
XR_304244.3	LOC102161992	lncRNA	0	0	0	0	0	0	0	0	0	0	0	0	0	0	0	0	0	0	0	0	0
XR_304257.3	LOC102162837	lncRNA	0	0	0	0	0	0	0	0	0	0	0	0	0	0	0	0	0	0	0	0	0
XR_306105.3	LOC102166871	lncRNA	0	0	0	0	0	0	0	0	0	0	0	0	0	0	0	0	0	0	0	1	1
XR_307914.3	LOC102159607	lncRNA	0	0	0	0	0	0	0	0	0	0	0	0	0	0	0	0	0	0	0	0	0
XR_308345.3	LOC102167708	lncRNA	0	0	0	0	0	0	0	0	0	0	0	0	0	0	0	0	0	0	0	0	0
novel-ssc-miR1028-3p		miRNA	0	0	0	0	0	0	0	0	0	0	0	0	0	0	0	0	0	0	0	0	0
novel-ssc-miR1028-5p		miRNA	0	0	0	0	0	0	0	0	0	0	0	0	0	0	0	0	0	0	0	0	0
novel-ssc-miR1130-3p		miRNA	0	0	0	0	0	0	0	0	0	0	0	0	0	0	0	0	0	1	0	0	0
novel-ssc-miR1130-5p		miRNA	0	0	0	0	0	0	0	0	0	0	0	0	0	0	0	0	0	1	0	0	0
novel-ssc-miR1162-3p		miRNA	0	0	0	0	0	0	0	1	0	0	0	0	0	0	0	0	0	0	0	0	0
novel-ssc-miR1193-3p		miRNA	0	0	0	0	0	0	0	0	0	0	0	0	0	0	0	0	0	1	0	0	0
novel-ssc-miR1193-5p		miRNA	0	0	0	0	0	0	0	0	0	0	0	0	0	0	0	0	0	1	0	0	0
novel-ssc-miR1217-3p		miRNA	0	0	0	0	0	0	0	0	0	0	0	0	0	0	0	0	0	0	0	0	0
novel-ssc-miR1217-5p		miRNA	0	0	0	0	0	0	0	0	0	0	0	0	0	0	0	0	0	0	0	0	0
novel-ssc-miR1429-3p		miRNA	0	0	0	0	0	0	0	0	0	0	0	0	0	0	0	0	0	0	0	0	0
novel-ssc-miR1429-5p		miRNA	0	0	0	0	0	0	0	0	0	0	0	0	0	0	0	0	0	0	0	0	0
novel-ssc-miR259-3p		miRNA	0	0	0	0	0	0	0	1	0	0	0	0	0	0	0	0	0	0	0	0	0
novel-ssc-miR259-5p		miRNA	0	0	0	0	0	0	0	1	0	0	0	0	0	0	0	0	0	0	0	0	0
novel-ssc-miR324-3p		miRNA	0	0	0	0	0	0	0	0	0	0	0	0	0	0	0	0	0	0	0	0	0
novel-ssc-miR324-5p		miRNA	0	0	0	0	0	0	0	0	0	0	0	0	0	0	0	0	0	0	0	0	0
novel-ssc-miR42-3p		miRNA	0	0	0	0	0	0	0	0	0	0	0	0	0	0	0	0	0	0	0	0	0
novel-ssc-miR42-5p		miRNA	0	0	0	0	0	0	0	0	0	0	0	0	0	0	0	0	0	0	0	0	0
novel-ssc-miR433-3p		miRNA	0	0	0	0	0	0	0	0	0	0	0	0	0	0	0	0	0	0	0	0	0
novel-ssc-miR433-5p		miRNA	0	0	0	0	0	0	0	0	0	0	0	0	0	0	0	0	0	0	0	0	0
novel-ssc-miR557-3p		miRNA	0	0	0	0	0	0	0	0	0	0	0	0	0	0	0	0	0	0	0	0	0
novel-ssc-miR557-5p		miRNA	0	0	0	0	0	0	0	0	0	0	0	0	0	0	0	0	0	0	0	0	0
novel-ssc-miR619-3p		miRNA	0	0	0	0	0	0	0	0	0	0	0	0	0	0	0	0	0	1	0	0	0
novel-ssc-miR619-5p		miRNA	0	0	0	0	0	0	0	0	0	0	0	0	0	0	0	0	0	1	0	0	0
novel-ssc-miR719-3p		miRNA	0	0	0	0	0	0	0	1	0	0	0	0	0	0	0	0	0	0	0	0	0
novel-ssc-miR719-5p		miRNA	0	0	0	0	0	0	0	1	0	0	0	0	0	0	0	0	0	0	0	0	0
novel-ssc-miR966-3p		miRNA	0	0	0	0	0	0	0	1	0	0	0	0	0	0	0	0	0	0	0	0	0
novel-ssc-miR966-5p		miRNA	0	0	0	0	0	0	0	1	0	0	0	0	0	0	0	0	0	0	0	0	0
ssc-miR-7136-3p		miRNA	0	0	0	0	0	0	0	0	0	0	0	0	0	0	1	0	0	0	0	0	0
ssc-miR-7136-5p		miRNA	0	0	0	0	0	0	0	0	0	0	0	0	0	0	1	0	0	0	0	0	0

Supplementary Table S10. The predicted targe

RNA ID	Symbol	Type	updown_BGIR9823_91696	updown_BGIR9823_91824	updown_BGIR9823_92415	updown_BGIR9823_88848	updown_BGIR9823_99168	updown_BGIR9823_96364	updown_BGIR9823_96973	updown_XR_001308019.2	updown_XR_002345205.1	updown_XR_002346024.1	updown_XR_002344184.1	updown_BGIR9823_101191	updown_BGIR9823_101562	updown_BGIR9823_102595	updown_BGIR9823_100418	updown_BGIR9823_101051	updown_BGIR9823_78137	updown_BGIR9823_78138	updown_BGIR9823_101781	updown_BGIR9823_81016	updown_BGIR9823_85300
BGIR9823_100361	PRKAG2	mRNA	0	0	0	0	0	0	0	0	0	0	0	0	0	0	0	0	0	0	0	0	0
BGIR9823_100362	PRKAG2	mRNA	0	0	0	0	0	0	0	0	0	0	0	0	0	0	0	0	0	0	0	0	0
BGIR9823_100363	PRKAG2	mRNA	0	0	0	0	0	0	0	0	0	0	0	0	0	0	0	0	0	0	0	0	0
BGIR9823_100364	PRKAG2	mRNA	0	0	0	0	0	0	0	0	0	0	0	0	0	0	0	0	0	0	0	0	0
BGIR9823_101535	LOC102158401	lncRNA	0	0	0	0	0	0	0	0	0	0	0	0	1	0	0	0	0	0	0	0	0
BGIR9823_101541	LOC102157946	lncRNA	0	0	0	0	0	0	0	0	0	0	0	0	1	0	0	0	0	0	0	0	0
BGIR9823_101542	LOC102157946	lncRNA	0	0	0	0	0	0	0	0	0	0	0	0	1	0	0	0	0	0	0	0	0
BGIR9823_101560	LOC110258600	lncRNA	0	0	0	0	0	0	0	0	0	0	0	0	1	0	0	0	0	0	0	0	0
BGIR9823_101561	LOC110258600	lncRNA	0	0	0	0	0	0	0	0	0	0	0	0	1	0	0	0	0	0	0	0	0
BGIR9823_101562	LOC110258600	lncRNA	0	0	0	0	0	0	0	0	0	0	0	0	1	0	0	0	0	0	0	0	0
BGIR9823_101563	LOC110258600	lncRNA	0	0	0	0	0	0	0	0	0	0	0	0	1	0	0	0	0	0	0	0	0
BGIR9823_101564	LOC110258600	lncRNA	0	0	0	0	0	0	0	0	0	0	0	0	1	0	0	0	0	0	0	0	0
BGIR9823_101612	LOC100521600	lncRNA	0	0	0	0	0	0	0	0	0	0	0	0	0	0	0	0	0	0	0	0	0
BGIR9823_101853		lncRNA	0	0	0	0	0	0	0	0	0	0	0	0	0	0	0	0	0	0	0	0	0
BGIR9823_101854		lncRNA	0	0	0	0	0	0	0	0	0	0	0	0	0	0	0	0	0	0	0	0	0
BGIR9823_101855		lncRNA	0	0	0	0	0	0	0	0	0	0	0	0	0	0	0	0	0	0	0	0	0
BGIR9823_102146		lncRNA	0	0	0	0	0	0	0	0	0	0	0	0	0	0	0	0	0	0	0	0	0
BGIR9823_102180		lncRNA	0	0	0	0	0	0	0	0	0	0	0	0	0	0	0	0	0	0	0	0	0
BGIR9823_102279		lncRNA	0	0	0	0	0	0	0	0	0	0	0	0	0	0	0	0	0	0	0	0	0
BGIR9823_102282		lncRNA	0	0	0	0	0	0	0	0	0	0	0	0	0	0	0	0	0	0	0	0	0
BGIR9823_102361		lncRNA	0	0	0	0	0	0	0	0	0	0	0	0	0	0	0	0	0	0	0	0	0
BGIR9823_102362		lncRNA	0	0	0	0	0	0	0	0	0	0	0	0	0	0	0	0	0	0	0	0	0
BGIR9823_102363		lncRNA	0	0	0	0	0	0	0	0	0	0	0	0	0	0	0	0	0	0	0	0	0
BGIR9823_102727		lncRNA	0	0	0	0	0	0	0	0	0	0	0	0	0	0	0	0	0	0	0	0	0
BGIR9823_80121	SSSCA1	lncRNA	0	0	0	0	0	0	0	0	0	0	0	0	0	0	0	0	0	0	0	1	0
BGIR9823_80122	LOC102163816	lncRNA	0	0	0	0	0	0	0	0	0	0	0	0	0	0	0	0	0	0	0	1	0
BGIR9823_81007	EHBP1L1	mRNA	0	0	0	0	0	0	0	0	0	0	0	0	0	0	0	0	0	0	0	1	0
BGIR9823_81008	EHBP1L1	mRNA	0	0	0	0	0	0	0	0	0	0	0	0	0	0	0	0	0	0	0	1	0
BGIR9823_81009	EHBP1L1	mRNA	0	0	0	0	0	0	0	0	0	0	0	0	0	0	0	0	0	0	0	1	0
BGIR9823_81010	EHBP1L1	mRNA	0	0	0	0	0	0	0	0	0	0	0	0	0	0	0	0	0	0	0	1	0
BGIR9823_81011	SSSCA1	mRNA	0	0	0	0	0	0	0	0	0	0	0	0	0	0	0	0	0	0	0	1	0
BGIR9823_81012	FRMD8	lncRNA	0	0	0	0	0	0	0	0	0	0	0	0	0	0	0	0	0	0	0	1	0
BGIR9823_81013	LOC102163816	lncRNA	0	0	0	0	0	0	0	0	0	0	0	0	0	0	0	0	0	0	0	1	0
BGIR9823_81014	LOC102163816	lncRNA	0	0	0	0	0	0	0	0	0	0	0	0	0	0	0	0	0	0	0	1	0
BGIR9823_81015	LOC102163816	lncRNA	0	0	0	0	0	0	0	0	0	0	0	0	0	0	0	0	0	0	0	1	0
BGIR9823_81016	LOC102163816	lncRNA	0	0	0	0	0	0	0	0	0	0	0	0	0	0	0	0	0	0	0	1	0
BGIR9823_81017	LOC102163816	lncRNA	0	0	0	0	0	0	0	0	0	0	0	0	0	0	0	0	0	0	0	1	0
BGIR9823_81018	LOC102163816	lncRNA	0	0	0	0	0	0	0	0	0	0	0	0	0	0	0	0	0	0	0	1	0
BGIR9823_81019	LOC100513133	lncRNA	0	0	0	0	0	0	0	0	0	0	0	0	0	0	0	0	0	0	0	1	0
BGIR9823_84704	LOC102159820	mRNA	0	0	0	0	0	0	0	0	0	0	0	0	0	0	0	0	0	0	0	0	1
BGIR9823_84732	RANGAP1	mRNA	0	0	0	0	0	0	0	0	0	0	0	0	0	0	0	0	0	0	0	0	0

RNA ID	Symbol	Type	updown_BGIR9823_91696	updown_BGIR9823_91824	updown_BGIR9823_92415	updown_BGIR9823_88848	updown_BGIR9823_99168	updown_BGIR9823_96364	updown_BGIR9823_96973	updown_XR_001308019.2	updown_XR_002345205.1	updown_XR_002346024.1	updown_XR_002344184.1	updown_BGIR9823_101191	updown_BGIR9823_101562	updown_BGIR9823_102595	updown_BGIR9823_100418	updown_BGIR9823_101051	updown_BGIR9823_78137	updown_BGIR9823_78138	updown_BGIR9823_101781	updown_BGIR9823_81016	updown_BGIR9823_85300
BGIR9823_84733	RANGAP1	mRNA	0	0	0	0	0	0	0	0	0	0	0	0	0	0	0	0	0	0	0	0	0
BGIR9823_85245	CRADD	lncRNA	0	0	0	0	0	0	0	0	0	0	0	0	0	0	0	0	0	0	0	0	0
BGIR9823_85246	LOC100738154	lncRNA	0	0	0	0	0	0	0	0	0	0	0	0	0	0	0	0	0	0	0	0	0
BGIR9823_85293	TRMU	mRNA	0	0	0	0	0	0	0	0	0	0	0	0	0	0	0	0	0	0	0	0	1
BGIR9823_85294	TRMU	mRNA	0	0	0	0	0	0	0	0	0	0	0	0	0	0	0	0	0	0	0	0	1
BGIR9823_85295	TTC38	mRNA	0	0	0	0	0	0	0	0	0	0	0	0	0	0	0	0	0	0	0	0	1
BGIR9823_85296	PKDREJ	lncRNA	0	0	0	0	0	0	0	0	0	0	0	0	0	0	0	0	0	0	0	0	1
BGIR9823_85297	PPARA	mRNA	0	0	0	0	0	0	0	0	0	0	0	0	0	0	0	0	0	0	0	0	1
BGIR9823_85298	PPARA	mRNA	0	0	0	0	0	0	0	0	0	0	0	0	0	0	0	0	0	0	0	0	1
BGIR9823_85299	PPARA	mRNA	0	0	0	0	0	0	0	0	0	0	0	0	0	0	0	0	0	0	0	0	1
BGIR9823_85300	PPARA	lncRNA	0	0	0	0	0	0	0	0	0	0	0	0	0	0	0	0	0	0	0	0	1
BGIR9823_85301	LOC110260611	mRNA	0	0	0	0	0	0	0	0	0	0	0	0	0	0	0	0	0	0	0	0	1
BGIR9823_85317	ACO2	mRNA	0	0	0	0	0	0	0	0	0	0	0	0	0	0	0	0	0	0	0	0	0
BGIR9823_85318	LOC110260629	lncRNA	0	0	0	0	0	0	0	0	0	0	0	0	0	0	0	0	0	0	0	0	0
BGIR9823_85319	TEF	mRNA	0	0	0	0	0	0	0	0	0	0	0	0	0	0	0	0	0	0	0	0	0
BGIR9823_85320	ZC3H7B	mRNA	0	0	0	0	0	0	0	0	0	0	0	0	0	0	0	0	0	0	0	0	0
BGIR9823_85345	SYNGR1	mRNA	0	0	0	0	0	0	0	0	0	0	0	0	0	0	0	0	0	0	0	0	0
BGIR9823_85835	CRADD	mRNA	0	0	0	0	0	0	0	0	0	0	0	0	0	0	0	0	0	0	0	0	0
BGIR9823_85839	CRADD	lncRNA	0	0	0	0	0	0	0	0	0	0	0	0	0	0	0	0	0	0	0	0	0
BGIR9823_85840	CRADD	lncRNA	0	0	0	0	0	0	0	0	0	0	0	0	0	0	0	0	0	0	0	0	0
BGIR9823_85841	CRADD	lncRNA	0	0	0	0	0	0	0	0	0	0	0	0	0	0	0	0	0	0	0	0	0
BGIR9823_85930	LOC106510465	mRNA	0	0	0	0	0	0	0	0	0	0	0	0	0	0	0	0	0	0	0	0	0
BGIR9823_85931	MAF	lncRNA	0	0	0	0	0	0	0	0	0	0	0	0	0	0	0	0	0	0	0	0	0
BGIR9823_85932	MAF	lncRNA	0	0	0	0	0	0	0	0	0	0	0	0	0	0	0	0	0	0	0	0	0
BGIR9823_87139	LOC106510467	lncRNA	0	0	0	0	0	0	0	0	0	0	0	0	0	0	0	0	0	0	0	0	0
BGIR9823_88187	WRNIP1	mRNA	0	0	0	0	0	0	0	0	0	0	0	0	0	0	0	0	0	0	0	0	0
BGIR9823_88188	NQO2	mRNA	0	0	0	0	0	0	0	0	0	0	0	0	0	0	0	0	0	0	0	0	0
BGIR9823_88189	NQO2	mRNA	0	0	0	0	0	0	0	0	0	0	0	0	0	0	0	0	0	0	0	0	0
BGIR9823_88190	RIPK1	mRNA	0	0	0	0	0	0	0	0	0	0	0	0	0	0	0	0	0	0	0	0	0
BGIR9823_88191	RIPK1	mRNA	0	0	0	0	0	0	0	0	0	0	0	0	0	0	0	0	0	0	0	0	0
BGIR9823_88192	RIPK1	mRNA	0	0	0	0	0	0	0	0	0	0	0	0	0	0	0	0	0	0	0	0	0
BGIR9823_88193	RIPK1	mRNA	0	0	0	0	0	0	0	0	0	0	0	0	0	0	0	0	0	0	0	0	0
BGIR9823_88196	PRPF4B	mRNA	0	0	0	0	0	0	0	0	0	0	0	0	0	0	0	0	0	0	0	0	0
BGIR9823_88197	PRPF4B	mRNA	0	0	0	0	0	0	0	0	0	0	0	0	0	0	0	0	0	0	0	0	0
BGIR9823_88198	PRPF4B	mRNA	0	0	0	0	0	0	0	0	0	0	0	0	0	0	0	0	0	0	0	0	0
BGIR9823_88211	TMEM170B	lncRNA	0	0	0	0	0	0	0	0	0	0	0	0	0	0	0	0	0	0	0	0	0
BGIR9823_88212	TMEM170B	lncRNA	0	0	0	0	0	0	0	0	0	0	0	0	0	0	0	0	0	0	0	0	0
BGIR9823_88213	TMEM170B	lncRNA	0	0	0	0	0	0	0	0	0	0	0	0	0	0	0	0	0	0	0	0	0
BGIR9823_88214	TMEM170B	lncRNA	0	0	0	0	0	0	0	0	0	0	0	0	0	0	0	0	0	0	0	0	0
BGIR9823_88217	PHACTR1	mRNA	0	0	0	0	0	0	0	0	0	0	0	0	0	0	0	0	0	0	0	0	0
BGIR9823_88218	PHACTR1	lncRNA	0	0	0	0	0	0	0	0	0	0	0	0	0	0	0	0	0	0	0	0	0
BGIR9823_89017	MYLK4	lncRNA	0	0	0	0	0	0	0	0	0	0	0	0	0	0	0	0	0	0	0	0	0
BGIR9823_89019	SERPINB1	lncRNA	0	0	0	0	0	0	0	0	0	0	0	0	0	0	0	0	0	0	0	0	0

RNA ID	Symbol	Type	updown_BGIR9823_91696	updown_BGIR9823_91824	updown_BGIR9823_92415	updown_BGIR9823_88848	updown_BGIR9823_99168	updown_BGIR9823_96364	updown_BGIR9823_96973	updown_XR_001308019.2	updown_XR_002345205.1	updown_XR_002346024.1	updown_XR_002344184.1	updown_BGIR9823_101191	updown_BGIR9823_101562	updown_BGIR9823_102595	updown_BGIR9823_100418	updown_BGIR9823_101051	updown_BGIR9823_78137	updown_BGIR9823_78138	updown_BGIR9823_101781	updown_BGIR9823_81016	updown_BGIR9823_85300
BGIR9823_89020	SERPINB9	mRNA	0	0	0	0	0	0	0	0	0	0	0	0	0	0	0	0	0	0	0	0	0
BGIR9823_89021	RIPK1	lncRNA	0	0	0	0	0	0	0	0	0	0	0	0	0	0	0	0	0	0	0	0	0
BGIR9823_89025	PXDC1	mRNA	0	0	0	0	0	0	0	0	0	0	0	0	0	0	0	0	0	0	0	0	0
BGIR9823_89026	PXDC1	lncRNA	0	0	0	0	0	0	0	0	0	0	0	0	0	0	0	0	0	0	0	0	0
BGIR9823_89027	LOC102161744	lncRNA	0	0	0	0	0	0	0	0	0	0	0	0	0	0	0	0	0	0	0	0	0
BGIR9823_89040	TBC1D7	lncRNA	0	0	0	0	0	0	0	0	0	0	0	0	0	0	0	0	0	0	0	0	0
BGIR9823_89041	TBC1D7	lncRNA	0	0	0	0	0	0	0	0	0	0	0	0	0	0	0	0	0	0	0	0	0
BGIR9823_89042	TBC1D7	lncRNA	0	0	0	0	0	0	0	0	0	0	0	0	0	0	0	0	0	0	0	0	0
BGIR9823_89043	TBC1D7	lncRNA	0	0	0	0	0	0	0	0	0	0	0	0	0	0	0	0	0	0	0	0	0
BGIR9823_89044	GFOD1	lncRNA	0	0	0	0	0	0	0	0	0	0	0	0	0	0	0	0	0	0	0	0	0
BGIR9823_89045	GFOD1	mRNA	0	0	0	0	0	0	0	0	0	0	0	0	0	0	0	0	0	0	0	0	0
BGIR9823_89046	GFOD1	mRNA	0	0	0	0	0	0	0	0	0	0	0	0	0	0	0	0	0	0	0	0	0
BGIR9823_89047	GFOD1	mRNA	0	0	0	0	0	0	0	0	0	0	0	0	0	0	0	0	0	0	0	0	0
BGIR9823_89048	GFOD1	lncRNA	0	0	0	0	0	0	0	0	0	0	0	0	0	0	0	0	0	0	0	0	0
BGIR9823_89049	GFOD1	lncRNA	0	0	0	0	0	0	0	0	0	0	0	0	0	0	0	0	0	0	0	0	0
BGIR9823_90771	NEU3	lncRNA	0	0	0	0	0	0	0	0	0	0	0	0	0	0	0	0	0	0	0	0	0
BGIR9823_90772	SLCO2B1	mRNA	0	0	0	0	0	0	0	0	0	0	0	0	0	0	0	0	0	0	0	0	0
BGIR9823_90913	LOC110255435	lncRNA	0	0	0	0	0	0	0	0	0	0	0	0	0	0	0	0	0	0	0	0	0
BGIR9823_90934	LOC110255441	lncRNA	0	0	0	0	0	0	0	0	0	0	0	0	0	0	0	0	0	0	0	0	0
BGIR9823_90935	CCDC84	mRNA	0	0	0	0	0	0	0	0	0	0	0	0	0	0	0	0	0	0	0	0	0
BGIR9823_90936	TRAPPC4	mRNA	0	0	0	0	0	0	0	0	0	0	0	0	0	0	0	0	0	0	0	0	0
BGIR9823_90937	HMBS	mRNA	0	0	0	0	0	0	0	0	0	0	0	0	0	0	0	0	0	0	0	0	0
BGIR9823_90956	SORL1	mRNA	0	0	0	0	0	0	0	0	0	0	0	0	0	0	0	0	0	0	0	0	0
BGIR9823_90957	SORL1	mRNA	0	0	0	0	0	0	0	0	0	0	0	0	0	0	0	0	0	0	0	0	0
BGIR9823_90958	SORL1	mRNA	0	0	0	0	0	0	0	0	0	0	0	0	0	0	0	0	0	0	0	0	0
BGIR9823_90960	CLMP	lncRNA	0	0	0	0	0	0	0	0	0	0	0	0	0	0	0	0	0	0	0	0	0
BGIR9823_91161	LOC100522267	mRNA	0	1	0	0	0	0	0	0	0	0	0	0	0	0	0	0	0	0	0	0	0
BGIR9823_91162	LOC100522455	mRNA	0	1	0	0	0	0	0	0	0	0	0	0	0	0	0	0	0	0	0	0	0
BGIR9823_91163	ABCB1	mRNA	0	1	0	0	0	0	0	0	0	0	0	0	0	0	0	0	0	0	0	0	0
BGIR9823_91402	HBB	mRNA	0	0	0	0	0	0	0	0	0	0	0	0	0	0	0	0	0	0	0	0	0
BGIR9823_91403	HBB	mRNA	0	0	0	0	0	0	0	0	0	0	0	0	0	0	0	0	0	0	0	0	0
BGIR9823_91404	HBB	mRNA	0	0	0	0	0	0	0	0	0	0	0	0	0	0	0	0	0	0	0	0	0
BGIR9823_91405	HBB	mRNA	0	0	0	0	0	0	0	0	0	0	0	0	0	0	0	0	0	0	0	0	0
BGIR9823_91406	HBB	mRNA	0	0	0	0	0	0	0	0	0	0	0	0	0	0	0	0	0	0	0	0	0
BGIR9823_91407	HBB	mRNA	0	0	0	0	0	0	0	0	0	0	0	0	0	0	0	0	0	0	0	0	0
BGIR9823_91408	HBB	mRNA	0	0	0	0	0	0	0	0	0	0	0	0	0	0	0	0	0	0	0	0	0
BGIR9823_91409	HBB	mRNA	0	0	0	0	0	0	0	0	0	0	0	0	0	0	0	0	0	0	0	0	0
BGIR9823_91625	BCL9L	lncRNA	0	0	0	0	0	0	0	0	0	0	0	0	0	0	0	0	0	0	0	0	0
BGIR9823_91626	BCL9L	lncRNA	0	0	0	0	0	0	0	0	0	0	0	0	0	0	0	0	0	0	0	0	0
BGIR9823_91627	RPS25	mRNA	0	0	0	0	0	0	0	0	0	0	0	0	0	0	0	0	0	0	0	0	0
BGIR9823_91628	RPS25	mRNA	0	0	0	0	0	0	0	0	0	0	0	0	0	0	0	0	0	0	0	0	0
BGIR9823_91629	SLC37A4	mRNA	0	0	0	0	0	0	0	0	0	0	0	0	0	0	0	0	0	0	0	0	0
BGIR9823_91630	HYOU1	mRNA	0	0	0	0	0	0	0	0	0	0	0	0	0	0	0	0	0	0	0	0	0

RNA ID	Symbol	Type	updown_BGIR9823_91696	updown_BGIR9823_91824	updown_BGIR9823_92415	updown_BGIR9823_88848	updown_BGIR9823_99168	updown_BGIR9823_96364	updown_BGIR9823_96973	updown_XR_001308019.2	updown_XR_002345205.1	updown_XR_002346024.1	updown_XR_002344184.1	updown_BGIR9823_101191	updown_BGIR9823_101562	updown_BGIR9823_102595	updown_BGIR9823_100418	updown_BGIR9823_101051	updown_BGIR9823_78137	updown_BGIR9823_78138	updown_BGIR9823_101781	updown_BGIR9823_81016	updown_BGIR9823_85300
BGIR9823_91640	CLMP	mRNA	0	0	0	0	0	0	0	0	0	0	0	0	0	0	0	0	0	0	0	0	0
BGIR9823_91819	RUNDC3B	lncRNA	0	1	0	0	0	0	0	0	0	0	0	0	0	0	0	0	0	0	0	0	0
BGIR9823_91820	RUNDC3B	lncRNA	0	1	0	0	0	0	0	0	0	0	0	0	0	0	0	0	0	0	0	0	0
BGIR9823_91821	RUNDC3B	lncRNA	0	1	0	0	0	0	0	0	0	0	0	0	0	0	0	0	0	0	0	0	0
BGIR9823_91822	RUNDC3B	lncRNA	0	1	0	0	0	0	0	0	0	0	0	0	0	0	0	0	0	0	0	0	0
BGIR9823_91823	RUNDC3B	lncRNA	0	1	0	0	0	0	0	0	0	0	0	0	0	0	0	0	0	0	0	0	0
BGIR9823_91824	RUNDC3B	lncRNA	0	1	0	0	0	0	0	0	0	0	0	0	0	0	0	0	0	0	0	0	0
BGIR9823_91825	RUNDC3B	lncRNA	0	1	0	0	0	0	0	0	0	0	0	0	0	0	0	0	0	0	0	0	0
BGIR9823_91826	LOC102164596	lncRNA	0	1	0	0	0	0	0	0	0	0	0	0	0	0	0	0	0	0	0	0	0
BGIR9823_92993	KATNAL1	mRNA	0	0	0	0	0	0	0	0	0	0	0	0	0	0	0	0	0	0	0	0	0
BGIR9823_92994	KATNAL1	mRNA	0	0	0	0	0	0	0	0	0	0	0	0	0	0	0	0	0	0	0	0	0
BGIR9823_92995	KATNAL1	mRNA	0	0	0	0	0	0	0	0	0	0	0	0	0	0	0	0	0	0	0	0	0
BGIR9823_92996	KATNAL1	mRNA	0	0	0	0	0	0	0	0	0	0	0	0	0	0	0	0	0	0	0	0	0
BGIR9823_92997	KATNAL1	mRNA	0	0	0	0	0	0	0	0	0	0	0	0	0	0	0	0	0	0	0	0	0
BGIR9823_92999	LOC110255292	lncRNA	0	0	0	0	0	0	0	0	0	0	0	0	0	0	0	0	0	0	0	0	0
BGIR9823_93000	LOC102166871	lncRNA	0	0	0	0	0	0	0	0	0	0	0	0	0	0	0	0	0	0	0	0	0
BGIR9823_98051	LOC102164998	lncRNA	0	0	0	0	0	0	0	0	0	0	0	0	0	0	0	0	0	0	0	0	0
BGIR9823_99244	LOC102167708	lncRNA	0	0	0	0	0	0	0	0	0	0	0	0	0	0	0	0	0	0	0	0	0
NM_001004034.1	HMGB1	mRNA	0	0	0	0	0	0	0	0	0	0	0	0	0	0	0	0	0	0	0	0	0
NM_001044526.1	PPARA	mRNA	0	0	0	0	0	0	0	0	0	0	0	0	0	0	0	0	0	0	0	0	1
NM_001111256.1	SERPINB9	mRNA	0	0	0	0	0	0	0	0	0	0	0	0	0	0	0	0	0	0	0	0	0
NM_001113443.1	ECI2	mRNA	0	0	0	0	0	0	0	0	0	0	0	0	0	0	0	0	0	0	0	0	0
NM_001144841.1	HBB	mRNA	0	0	0	0	0	0	0	0	0	0	0	0	0	0	0	0	0	0	0	0	0
NM_001199719.1	SLC37A4	mRNA	0	0	0	0	0	0	0	0	0	0	0	0	0	0	0	0	0	0	0	0	0
NM_001243907.1	HSPA8	mRNA	0	0	0	0	0	0	0	0	0	0	0	0	0	0	0	0	0	0	0	0	0
NM_001244063.1	RPL3	mRNA	0	0	0	0	0	0	0	0	0	0	0	0	0	0	0	0	0	0	0	0	0
NM_001244066.1	SYNGR1	mRNA	0	0	0	0	0	0	0	0	0	0	0	0	0	0	0	0	0	0	0	0	0
NM_001244396.1	CCDC84	mRNA	0	0	0	0	0	0	0	0	0	0	0	0	0	0	0	0	0	0	0	0	0
NM_001287846.1	RHEB	mRNA	0	0	0	0	0	0	0	0	0	0	0	0	0	0	0	0	0	0	0	0	0
NM_213954.1	ACO2	mRNA	0	0	0	0	0	0	0	0	0	0	0	0	0	0	0	0	0	0	0	0	0
NM_214012.1	UPK2	mRNA	0	0	0	0	0	0	0	0	0	0	0	0	0	0	0	0	0	0	0	0	0
URS0000BC92F4	LOC102167708	lncRNA	0	0	0	0	0	0	0	0	0	0	0	0	0	0	0	0	0	0	0	0	0
XM_001928309.6	TBC1D7	mRNA	0	0	0	0	0	0	0	0	0	0	0	0	0	0	0	0	0	0	0	0	0
XM_001928852.5	PXDC1	mRNA	0	0	0	0	0	0	0	0	0	0	0	0	0	0	0	0	0	0	0	0	0
XM_003122534.4	KCNK7	mRNA	0	0	0	0	0	0	0	0	0	0	0	0	0	0	0	0	0	0	0	1	0
XM_003122558.5	SCYL1	mRNA	0	0	0	0	0	0	0	0	0	0	0	0	0	0	0	0	0	0	0	1	0
XM_003122562.5	FRMD8	mRNA	0	0	0	0	0	0	0	0	0	0	0	0	0	0	0	0	0	0	0	1	0
XM_003125951.6	TTC38	mRNA	0	0	0	0	0	0	0	0	0	0	0	0	0	0	0	0	0	0	0	0	1
XM_003125953.4	CDPF1	mRNA	0	0	0	0	0	0	0	0	0	0	0	0	0	0	0	0	0	0	0	0	1
XM_003126012.6	CBX7	mRNA	0	0	0	0	0	0	0	0	0	0	0	0	0	0	0	0	0	0	0	0	0
XM_003128161.4	RIPK1	mRNA	0	0	0	0	0	0	0	0	0	0	0	0	0	0	0	0	0	0	0	0	0
XM_003129508.2	LOC100514417	mRNA	0	0	0	0	0	0	0	0	0	0	0	0	0	0	0	0	0	0	0	0	0
XM_003129512.2	LOC100515268	mRNA	0	0	0	0	0	0	0	0	0	0	0	0	0	0	0	0	0	0	0	0	0

RNA ID	Symbol	Type	updown_BGIR9823_91696	updown_BGIR9823_91824	updown_BGIR9823_92415	updown_BGIR9823_88848	updown_BGIR9823_99168	updown_BGIR9823_96364	updown_BGIR9823_96973	updown_XR_001308019.2	updown_XR_002345205.1	updown_XR_002346024.1	updown_XR_002344184.1	updown_BGIR9823_101191	updown_BGIR9823_101562	updown_BGIR9823_102595	updown_BGIR9823_100418	updown_BGIR9823_101051	updown_BGIR9823_78137	updown_BGIR9823_78138	updown_BGIR9823_101781	updown_BGIR9823_81016	updown_BGIR9823_85300
XM_003129514.3	LOC100515618	mRNA	0	0	0	0	0	0	0	0	0	0	0	0	0	0	0	0	0	0	0	0	0
XM_003129519.2	LOC100517595	mRNA	0	0	0	0	0	0	0	0	0	0	0	0	0	0	0	0	0	0	0	0	0
XM_003129529.4	LOC100519910	mRNA	0	0	0	0	0	0	0	0	0	0	0	0	0	0	0	0	0	0	0	0	0
XM_003129915.4	CXCR5	mRNA	0	0	0	0	0	0	0	0	0	0	0	0	0	0	0	0	0	0	0	0	0
XM_003129916.5	BCL9L	mRNA	0	0	0	0	0	0	0	0	0	0	0	0	0	0	0	0	0	0	0	0	0
XM_003129918.4	TRAPPC4	mRNA	0	0	0	0	0	0	0	0	0	0	0	0	0	0	0	0	0	0	0	0	0
XM_003129919.5	RPS25	mRNA	0	0	0	0	0	0	0	0	0	0	0	0	0	0	0	0	0	0	0	0	0
XM_003129920.3	RPS25	mRNA	0	0	0	0	0	0	0	0	0	0	0	0	0	0	0	0	0	0	0	0	0
XM_003129922.4	HYOU1	mRNA	0	0	0	0	0	0	0	0	0	0	0	0	0	0	0	0	0	0	0	0	0
XM_003129923.6	VPS11	mRNA	0	0	0	0	0	0	0	0	0	0	0	0	0	0	0	0	0	0	0	0	0
XM_003129944.4	SC5D	mRNA	0	0	0	0	0	0	0	0	0	0	0	0	0	0	0	0	0	0	0	0	0
XM_003357161.1	LOC100620971	mRNA	0	0	0	0	0	0	0	0	0	0	0	0	0	0	0	0	0	0	0	0	0
XM_003482119.4	SERPINB1	mRNA	0	0	0	0	0	0	0	0	0	0	0	0	0	0	0	0	0	0	0	0	0
XM_003482132.4	TMEM170B	mRNA	0	0	0	0	0	0	0	0	0	0	0	0	0	0	0	0	0	0	0	0	0
XM_003482133.4	GFOD1	mRNA	0	0	0	0	0	0	0	0	0	0	0	0	0	0	0	0	0	0	0	0	0
XM_003482563.4	SPCS2	mRNA	0	0	0	0	0	0	0	0	0	0	0	0	0	0	0	0	0	0	0	0	0
XM_003482565.4	NEU3	mRNA	0	0	0	0	0	0	0	0	0	0	0	0	0	0	0	0	0	0	0	0	0
XM_003482567.1	LOC100739359	mRNA	0	0	0	0	0	0	0	0	0	0	0	0	0	0	0	0	0	0	0	0	0
XM_003482630.3	BSX	mRNA	0	0	0	0	0	0	0	0	0	0	0	0	0	0	0	0	0	0	0	0	0
XM_005653107.3	CHADL	mRNA	0	0	0	0	0	0	0	0	0	0	0	0	0	0	0	0	0	0	0	0	0
XM_005653640.1	LOC100621181	mRNA	0	0	0	0	0	0	0	0	0	0	0	0	0	0	0	0	0	0	0	0	0
XM_005654266.3	PRKAG2	mRNA	0	0	0	0	0	0	0	0	0	0	0	0	0	0	0	0	0	0	0	0	0
XM_005655470.3	CDPF1	mRNA	0	0	0	0	0	0	0	0	0	0	0	0	0	0	0	0	0	0	0	0	1
XM_005660703.3	LTBP3	mRNA	0	0	0	0	0	0	0	0	0	0	0	0	0	0	0	0	0	0	0	1	0
XM_005660705.3	SCYL1	mRNA	0	0	0	0	0	0	0	0	0	0	0	0	0	0	0	0	0	0	0	1	0
XM_005660707.3	SLC25A45	mRNA	0	0	0	0	0	0	0	0	0	0	0	0	0	0	0	0	0	0	0	1	0
XM_005665536.3	RIPK1	mRNA	0	0	0	0	0	0	0	0	0	0	0	0	0	0	0	0	0	0	0	0	0
XM_005665537.3	RIPK1	mRNA	0	0	0	0	0	0	0	0	0	0	0	0	0	0	0	0	0	0	0	0	0
XM_005665538.3	RIPK1	mRNA	0	0	0	0	0	0	0	0	0	0	0	0	0	0	0	0	0	0	0	0	0
XM_005665575.3	PHACTR1	mRNA	0	0	0	0	0	0	0	0	0	0	0	0	0	0	0	0	0	0	0	0	0
XM_005665576.3	TBC1D7	mRNA	0	0	0	0	0	0	0	0	0	0	0	0	0	0	0	0	0	0	0	0	0
XM_005667128.3	LOC100627340	mRNA	0	0	0	0	0	0	0	0	0	0	0	0	0	0	0	0	0	0	0	0	0
XM_005667397.2	CCDC84	mRNA	0	0	0	0	0	0	0	0	0	0	0	0	0	0	0	0	0	0	0	0	0
XM_005667398.3	SLC37A4	mRNA	0	0	0	0	0	0	0	0	0	0	0	0	0	0	0	0	0	0	0	0	0
XM_005667402.3	SLC37A4	mRNA	0	0	0	0	0	0	0	0	0	0	0	0	0	0	0	0	0	0	0	0	0
XM_005667403.3	SLC37A4	mRNA	0	0	0	0	0	0	0	0	0	0	0	0	0	0	0	0	0	0	0	0	0
XM_005667413.3	BCL9L	mRNA	0	0	0	0	0	0	0	0	0	0	0	0	0	0	0	0	0	0	0	0	0
XM_005667414.3	FOXR1	mRNA	0	0	0	0	0	0	0	0	0	0	0	0	0	0	0	0	0	0	0	0	0
XM_005667416.3	HYOU1	mRNA	0	0	0	0	0	0	0	0	0	0	0	0	0	0	0	0	0	0	0	0	0
XM_005667419.3	HYOU1	mRNA	0	0	0	0	0	0	0	0	0	0	0	0	0	0	0	0	0	0	0	0	0
XM_005667420.3	HYOU1	mRNA	0	0	0	0	0	0	0	0	0	0	0	0	0	0	0	0	0	0	0	0	0
XM_005667440.2	JHY	mRNA	0	0	0	0	0	0	0	0	0	0	0	0	0	0	0	0	0	0	0	0	0
XM_005668316.3	HMGB1	mRNA	0	0	0	0	0	0	0	0	0	0	0	0	0	0	0	0	0	0	0	0	0

RNA ID	Symbol	Type	updown_BGIR9823_91696	updown_BGIR9823_91824	updown_BGIR9823_92415	updown_BGIR9823_88848	updown_BGIR9823_99168	updown_BGIR9823_96364	updown_BGIR9823_96973	updown_XR_001308019.2	updown_XR_002345205.1	updown_XR_002346024.1	updown_XR_002344184.1	updown_BGIR9823_101191	updown_BGIR9823_101562	updown_BGIR9823_102595	updown_BGIR9823_100418	updown_BGIR9823_101051	updown_BGIR9823_78137	updown_BGIR9823_78138	updown_BGIR9823_101781	updown_BGIR9823_81016	updown_BGIR9823_85300
XM_005668317.3	HMGB1	mRNA	0	0	0	0	0	0	0	0	0	0	0	0	0	0	0	0	0	0	0	0	0
XM_005668318.3	HMGB1	mRNA	0	0	0	0	0	0	0	0	0	0	0	0	0	0	0	0	0	0	0	0	0
XM_005671489.3	EMX2	mRNA	0	0	0	0	0	0	0	0	0	0	0	0	0	0	0	0	0	0	0	0	0
XM_005672397.3	MYO10	mRNA	0	0	0	0	0	0	0	0	0	0	0	0	0	0	0	0	0	0	0	0	0
XM_013977400.2	FAM50B	mRNA	0	0	0	0	0	0	0	0	0	0	0	0	0	0	0	0	0	0	0	0	0
XM_013977425.2	NEDD9	mRNA	0	0	0	0	0	0	0	0	0	0	0	0	0	0	0	0	0	0	0	0	0
XM_013977430.2	TBC1D7	mRNA	0	0	0	0	0	0	0	0	0	0	0	0	0	0	0	0	0	0	0	0	0
XM_013977431.2	TBC1D7	mRNA	0	0	0	0	0	0	0	0	0	0	0	0	0	0	0	0	0	0	0	0	0
XM_013977432.2	TBC1D7	mRNA	0	0	0	0	0	0	0	0	0	0	0	0	0	0	0	0	0	0	0	0	0
XM_013977433.2	GFOD1	mRNA	0	0	0	0	0	0	0	0	0	0	0	0	0	0	0	0	0	0	0	0	0
XM_013977434.2	GFOD1	mRNA	0	0	0	0	0	0	0	0	0	0	0	0	0	0	0	0	0	0	0	0	0
XM_013979449.2	SLC37A4	mRNA	0	0	0	0	0	0	0	0	0	0	0	0	0	0	0	0	0	0	0	0	0
XM_013979456.2	BCL9L	mRNA	0	0	0	0	0	0	0	0	0	0	0	0	0	0	0	0	0	0	0	0	0
XM_013979457.2	BCL9L	mRNA	0	0	0	0	0	0	0	0	0	0	0	0	0	0	0	0	0	0	0	0	0
XM_013979458.2	BCL9L	mRNA	0	0	0	0	0	0	0	0	0	0	0	0	0	0	0	0	0	0	0	0	0
XM_013979459.2	BCL9L	mRNA	0	0	0	0	0	0	0	0	0	0	0	0	0	0	0	0	0	0	0	0	0
XM_013979461.2	TRAPPC4	mRNA	0	0	0	0	0	0	0	0	0	0	0	0	0	0	0	0	0	0	0	0	0
XM_013979463.2	HYOU1	mRNA	0	0	0	0	0	0	0	0	0	0	0	0	0	0	0	0	0	0	0	0	0
XM_013979484.2	LOC100523684	mRNA	0	0	0	0	0	0	0	0	0	0	0	0	0	0	0	0	0	0	0	0	0
XM_013979486.2	LOC100523684	mRNA	0	0	0	0	0	0	0	0	0	0	0	0	0	0	0	0	0	0	0	0	0
XM_013979491.2	JHY	mRNA	0	0	0	0	0	0	0	0	0	0	0	0	0	0	0	0	0	0	0	0	0
XM_013980418.2	KATNAL1	mRNA	0	0	0	0	0	0	0	0	0	0	0	0	0	0	0	0	0	0	0	0	0
XM_013988182.2	TTC38	mRNA	0	0	0	0	0	0	0	0	0	0	0	0	0	0	0	0	0	0	0	0	1
XM_013988183.2	TTC38	mRNA	0	0	0	0	0	0	0	0	0	0	0	0	0	0	0	0	0	0	0	0	1
XM_013989589.2	LOC100522267	mRNA	0	1	0	0	0	0	0	0	0	0	0	0	0	0	0	0	0	0	0	0	0
XM_013989592.2	LOC100522455	mRNA	0	1	0	0	0	0	0	0	0	0	0	0	0	0	0	0	0	0	0	0	0
XM_013989593.2	LOC100522455	mRNA	0	1	0	0	0	0	0	0	0	0	0	0	0	0	0	0	0	0	0	0	0
XM_013994099.2	FRMD8	mRNA	0	0	0	0	0	0	0	0	0	0	0	0	0	0	0	0	0	0	0	1	0
XM_013994100.2	SLC25A45	mRNA	0	0	0	0	0	0	0	0	0	0	0	0	0	0	0	0	0	0	0	1	0
XM_013994102.2	SLC25A45	mRNA	0	0	0	0	0	0	0	0	0	0	0	0	0	0	0	0	0	0	0	1	0
XM_013994103.2	SLC25A45	mRNA	0	0	0	0	0	0	0	0	0	0	0	0	0	0	0	0	0	0	0	1	0
XM_013994104.2	SLC25A45	mRNA	0	0	0	0	0	0	0	0	0	0	0	0	0	0	0	0	0	0	0	1	0
XM_013997510.2	CBX7	mRNA	0	0	0	0	0	0	0	0	0	0	0	0	0	0	0	0	0	0	0	0	0
XM_013999070.2	LOC100520088	mRNA	0	0	0	0	0	0	0	0	0	0	0	0	0	0	0	0	0	0	0	0	0
XM_021062230.1	LOC100517771	mRNA	0	0	0	0	0	0	0	0	0	0	0	0	0	0	0	0	0	0	0	0	0
XM_021062231.1	LOC100517957	mRNA	0	0	0	0	0	0	0	0	0	0	0	0	0	0	0	0	0	0	0	0	0
XM_021062367.1	LOC110255365	mRNA	0	0	0	0	0	0	0	0	0	0	0	0	0	0	0	0	0	0	0	0	0
XM_021062369.1	LOC100737110	mRNA	0	0	0	0	0	0	0	0	0	0	0	0	0	0	0	0	0	0	0	0	0
XM_021062370.1	LOC110255368	mRNA	0	0	0	0	0	0	0	0	0	0	0	0	0	0	0	0	0	0	0	0	0
XM_021062371.1	LOC100515441	mRNA	0	0	0	0	0	0	0	0	0	0	0	0	0	0	0	0	0	0	0	0	0
XM_021062374.1	LOC110255371	mRNA	0	0	0	0	0	0	0	0	0	0	0	0	0	0	0	0	0	0	0	0	0
XM_021062375.1	LOC100626951	mRNA	0	0	0	0	0	0	0	0	0	0	0	0	0	0	0	0	0	0	0	0	0
XM_021062491.1	XRRA1	mRNA	0	0	0	0	0	0	0	0	0	0	0	0	0	0	0	0	0	0	0	0	0

RNA ID	Symbol	Type	updown_BGIR9823_91696	updown_BGIR9823_91824	updown_BGIR9823_92415	updown_BGIR9823_88848	updown_BGIR9823_99168	updown_BGIR9823_96364	updown_BGIR9823_96973	updown_XR_001308019.2	updown_XR_002345205.1	updown_XR_002346024.1	updown_XR_002344184.1	updown_BGIR9823_101191	updown_BGIR9823_101562	updown_BGIR9823_102595	updown_BGIR9823_100418	updown_BGIR9823_101051	updown_BGIR9823_78137	updown_BGIR9823_78138	updown_BGIR9823_101781	updown_BGIR9823_81016	updown_BGIR9823_85300
XM_021062493.1	XRRA1	mRNA	0	0	0	0	0	0	0	0	0	0	0	0	0	0	0	0	0	0	0	0	0
XM_021062494.1	XRRA1	mRNA	0	0	0	0	0	0	0	0	0	0	0	0	0	0	0	0	0	0	0	0	0
XM_021062495.1	XRRA1	mRNA	0	0	0	0	0	0	0	0	0	0	0	0	0	0	0	0	0	0	0	0	0
XM_021062496.1	XRRA1	mRNA	0	0	0	0	0	0	0	0	0	0	0	0	0	0	0	0	0	0	0	0	0
XM_021062497.1	XRRA1	mRNA	0	0	0	0	0	0	0	0	0	0	0	0	0	0	0	0	0	0	0	0	0
XM_021062498.1	XRRA1	mRNA	0	0	0	0	0	0	0	0	0	0	0	0	0	0	0	0	0	0	0	0	0
XM_021062499.1	XRRA1	mRNA	0	0	0	0	0	0	0	0	0	0	0	0	0	0	0	0	0	0	0	0	0
XM_021062500.1	XRRA1	mRNA	0	0	0	0	0	0	0	0	0	0	0	0	0	0	0	0	0	0	0	0	0
XM_021062501.1	XRRA1	mRNA	0	0	0	0	0	0	0	0	0	0	0	0	0	0	0	0	0	0	0	0	0
XM_021062502.1	XRRA1	mRNA	0	0	0	0	0	0	0	0	0	0	0	0	0	0	0	0	0	0	0	0	0
XM_021062503.1	XRRA1	mRNA	0	0	0	0	0	0	0	0	0	0	0	0	0	0	0	0	0	0	0	0	0
XM_021062511.1	SLCO2B1	mRNA	0	0	0	0	0	0	0	0	0	0	0	0	0	0	0	0	0	0	0	0	0
XM_021062948.1	BCL9L	mRNA	0	0	0	0	0	0	0	0	0	0	0	0	0	0	0	0	0	0	0	0	0
XM_021062949.1	TRAPPC4	mRNA	0	0	0	0	0	0	0	0	0	0	0	0	0	0	0	0	0	0	0	0	0
XM_021062950.1	HYOU1	mRNA	0	0	0	0	0	0	0	0	0	0	0	0	0	0	0	0	0	0	0	0	0
XM_021062951.1	HYOU1	mRNA	0	0	0	0	0	0	0	0	0	0	0	0	0	0	0	0	0	0	0	0	0
XM_021062952.1	HYOU1	mRNA	0	0	0	0	0	0	0	0	0	0	0	0	0	0	0	0	0	0	0	0	0
XM_021062953.1	HYOU1	mRNA	0	0	0	0	0	0	0	0	0	0	0	0	0	0	0	0	0	0	0	0	0
XM_021062954.1	HYOU1	mRNA	0	0	0	0	0	0	0	0	0	0	0	0	0	0	0	0	0	0	0	0	0
XM_021062955.1	HYOU1	mRNA	0	0	0	0	0	0	0	0	0	0	0	0	0	0	0	0	0	0	0	0	0
XM_021062956.1	HYOU1	mRNA	0	0	0	0	0	0	0	0	0	0	0	0	0	0	0	0	0	0	0	0	0
XM_021062957.1	VPS11	mRNA	0	0	0	0	0	0	0	0	0	0	0	0	0	0	0	0	0	0	0	0	0
XM_021062993.1	LOC100523684	mRNA	0	0	0	0	0	0	0	0	0	0	0	0	0	0	0	0	0	0	0	0	0
XM_021062994.1	LOC100523684	mRNA	0	0	0	0	0	0	0	0	0	0	0	0	0	0	0	0	0	0	0	0	0
XM_021062995.1	LOC100523684	mRNA	0	0	0	0	0	0	0	0	0	0	0	0	0	0	0	0	0	0	0	0	0
XM_021062996.1	LOC100523684	mRNA	0	0	0	0	0	0	0	0	0	0	0	0	0	0	0	0	0	0	0	0	0
XM_021062997.1	LOC100523684	mRNA	0	0	0	0	0	0	0	0	0	0	0	0	0	0	0	0	0	0	0	0	0
XM_021062998.1	LOC100523684	mRNA	0	0	0	0	0	0	0	0	0	0	0	0	0	0	0	0	0	0	0	0	0
XM_021062999.1	LOC100523684	mRNA	0	0	0	0	0	0	0	0	0	0	0	0	0	0	0	0	0	0	0	0	0
XM_021063000.1	LOC100523684	mRNA	0	0	0	0	0	0	0	0	0	0	0	0	0	0	0	0	0	0	0	0	0
XM_021063001.1	LOC100523684	mRNA	0	0	0	0	0	0	0	0	0	0	0	0	0	0	0	0	0	0	0	0	0
XM_021063002.1	LOC100523684	mRNA	0	0	0	0	0	0	0	0	0	0	0	0	0	0	0	0	0	0	0	0	0
XM_021063004.1	LOC100523684	mRNA	0	0	0	0	0	0	0	0	0	0	0	0	0	0	0	0	0	0	0	0	0
XM_021063005.1	LOC100523684	mRNA	0	0	0	0	0	0	0	0	0	0	0	0	0	0	0	0	0	0	0	0	0
XM_021063006.1	LOC100523684	mRNA	0	0	0	0	0	0	0	0	0	0	0	0	0	0	0	0	0	0	0	0	0
XM_021063009.1	SORL1	mRNA	0	0	0	0	0	0	0	0	0	0	0	0	0	0	0	0	0	0	0	0	0
XM_021063010.1	SORL1	mRNA	0	0	0	0	0	0	0	0	0	0	0	0	0	0	0	0	0	0	0	0	0
XM_021063011.1	SORL1	mRNA	0	0	0	0	0	0	0	0	0	0	0	0	0	0	0	0	0	0	0	0	0
XM_021063012.1	SORL1	mRNA	0	0	0	0	0	0	0	0	0	0	0	0	0	0	0	0	0	0	0	0	0
XM_021063013.1	SORL1	mRNA	0	0	0	0	0	0	0	0	0	0	0	0	0	0	0	0	0	0	0	0	0
XM_021063014.1	SORL1	mRNA	0	0	0	0	0	0	0	0	0	0	0	0	0	0	0	0	0	0	0	0	0
XM_021063023.1	CLMP	mRNA	0	0	0	0	0	0	0	0	0	0	0	0	0	0	0	0	0	0	0	0	0
XM_021063024.1	CLMP	mRNA	0	0	0	0	0	0	0	0	0	0	0	0	0	0	0	0	0	0	0	0	0

RNA ID	Symbol	Type	updown_BGIR9823_91696	updown_BGIR9823_91824	updown_BGIR9823_92415	updown_BGIR9823_88848	updown_BGIR9823_99168	updown_BGIR9823_96364	updown_BGIR9823_96973	updown_XR_001308019.2	updown_XR_002345205.1	updown_XR_002346024.1	updown_XR_002344184.1	updown_BGIR9823_101191	updown_BGIR9823_101562	updown_BGIR9823_102595	updown_BGIR9823_100418	updown_BGIR9823_101051	updown_BGIR9823_78137	updown_BGIR9823_78138	updown_BGIR9823_101781	updown_BGIR9823_81016	updown_BGIR9823_85300
XM_021063025.1	CLMP	mRNA	0	0	0	0	0	0	0	0	0	0	0	0	0	0	0	0	0	0	0	0	0
XM_021063026.1	CLMP	mRNA	0	0	0	0	0	0	0	0	0	0	0	0	0	0	0	0	0	0	0	0	0
XM_021063950.1	NXPE2	mRNA	0	0	0	0	0	0	0	0	0	0	0	0	0	0	0	0	0	0	0	0	0
XM_021063963.1	LOC100623190	mRNA	0	1	0	0	0	0	0	0	0	0	0	0	0	0	0	0	0	0	0	0	0
XM_021065159.1	HMGB1	mRNA	0	0	0	0	0	0	0	0	0	0	0	0	0	0	0	0	0	0	0	0	0
XM_021065160.1	HMGB1	mRNA	0	0	0	0	0	0	0	0	0	0	0	0	0	0	0	0	0	0	0	0	0
XM_021065161.1	HMGB1	mRNA	0	0	0	0	0	0	0	0	0	0	0	0	0	0	0	0	0	0	0	0	0
XM_021065529.1	KATNAL1	mRNA	0	0	0	0	0	0	0	0	0	0	0	0	0	0	0	0	0	0	0	0	0
XM_021065531.1	KATNAL1	mRNA	0	0	0	0	0	0	0	0	0	0	0	0	0	0	0	0	0	0	0	0	0
XM_021065532.1	KATNAL1	mRNA	0	0	0	0	0	0	0	0	0	0	0	0	0	0	0	0	0	0	0	0	0
XM_021065533.1	KATNAL1	mRNA	0	0	0	0	0	0	0	0	0	0	0	0	0	0	0	0	0	0	0	0	0
XM_021065534.1	KATNAL1	mRNA	0	0	0	0	0	0	0	0	0	0	0	0	0	0	0	0	0	0	0	0	0
XM_021065535.1	KATNAL1	mRNA	0	0	0	0	0	0	0	0	0	0	0	0	0	0	0	0	0	0	0	0	0
XM_021065536.1	KATNAL1	mRNA	0	0	0	0	0	0	0	0	0	0	0	0	0	0	0	0	0	0	0	0	0
XM_021065537.1	KATNAL1	mRNA	0	0	0	0	0	0	0	0	0	0	0	0	0	0	0	0	0	0	0	0	0
XM_021065538.1	KATNAL1	mRNA	0	0	0	0	0	0	0	0	0	0	0	0	0	0	0	0	0	0	0	0	0
XM_021065539.1	KATNAL1	mRNA	0	0	0	0	0	0	0	0	0	0	0	0	0	0	0	0	0	0	0	0	0
XM_021065541.1	KATNAL1	mRNA	0	0	0	0	0	0	0	0	0	0	0	0	0	0	0	0	0	0	0	0	0
XM_021065542.1	KATNAL1	mRNA	0	0	0	0	0	0	0	0	0	0	0	0	0	0	0	0	0	0	0	0	0
XM_021065543.1	KATNAL1	mRNA	0	0	0	0	0	0	0	0	0	0	0	0	0	0	0	0	0	0	0	0	0
XM_021065544.1	KATNAL1	mRNA	0	0	0	0	0	0	0	0	0	0	0	0	0	0	0	0	0	0	0	0	0
XM_021073408.1	EMX2	mRNA	0	0	0	0	0	0	0	0	0	0	0	0	0	0	0	0	0	0	0	0	0
XM_021078611.1	PRKAG2	mRNA	0	0	0	0	0	0	0	0	0	0	0	0	0	0	0	0	0	0	0	0	0
XM_021078612.1	PRKAG2	mRNA	0	0	0	0	0	0	0	0	0	0	0	0	0	0	0	0	0	0	0	0	0
XM_021078613.1	PRKAG2	mRNA	0	0	0	0	0	0	0	0	0	0	0	0	0	0	0	0	0	0	0	0	0
XM_021078614.1	PRKAG2	mRNA	0	0	0	0	0	0	0	0	0	0	0	0	0	0	0	0	0	0	0	0	0
XM_021078615.1	PRKAG2	mRNA	0	0	0	0	0	0	0	0	0	0	0	0	0	0	0	0	0	0	0	0	0
XM_021078616.1	PRKAG2	mRNA	0	0	0	0	0	0	0	0	0	0	0	0	0	0	0	0	0	0	0	0	0
XM_021078617.1	PRKAG2	mRNA	0	0	0	0	0	0	0	0	0	0	0	0	0	0	0	0	0	0	0	0	0
XM_021078618.1	PRKAG2	mRNA	0	0	0	0	0	0	0	0	0	0	0	0	0	0	0	0	0	0	0	0	0
XM_021078619.1	PRKAG2	mRNA	0	0	0	0	0	0	0	0	0	0	0	0	0	0	0	0	0	0	0	0	0
XM_021078620.1	PRKAG2	mRNA	0	0	0	0	0	0	0	0	0	0	0	0	0	0	0	0	0	0	0	0	0
XM_021078621.1	PRKAG2	mRNA	0	0	0	0	0	0	0	0	0	0	0	0	0	0	0	0	0	0	0	0	0
XM_021081309.1	LOC106506226	mRNA	0	0	0	0	0	0	0	0	0	0	0	0	0	0	0	0	0	0	0	0	0
XM_021081403.1	LOC110258215	mRNA	0	0	0	0	0	0	0	0	0	0	0	0	0	0	0	0	0	0	0	0	0
XM_021082153.1	LOC100521600	mRNA	0	0	0	0	0	0	0	0	0	0	0	0	0	0	0	0	0	0	0	0	0
XM_021082154.1	LOC100521600	mRNA	0	0	0	0	0	0	0	0	0	0	0	0	0	0	0	0	0	0	0	0	0
XM_021082156.1	LOC100521600	mRNA	0	0	0	0	0	0	0	0	0	0	0	0	0	0	0	0	0	0	0	0	0
XM_021082157.1	LOC100521600	mRNA	0	0	0	0	0	0	0	0	0	0	0	0	0	0	0	0	0	0	0	0	0
XM_021082158.1	LOC100521600	mRNA	0	0	0	0	0	0	0	0	0	0	0	0	0	0	0	0	0	0	0	0	0
XM_021082159.1	LOC100521600	mRNA	0	0	0	0	0	0	0	0	0	0	0	0	0	0	0	0	0	0	0	0	0
XM_021082835.1	EHBP1L1	mRNA	0	0	0	0	0	0	0	0	0	0	0	0	0	0	0	0	0	0	0	1	0
XM_021082836.1	EHBP1L1	mRNA	0	0	0	0	0	0	0	0	0	0	0	0	0	0	0	0	0	0	0	1	0

RNA ID	Symbol	Type	updown_BGIR9823_91696	updown_BGIR9823_91824	updown_BGIR9823_92415	updown_BGIR9823_88848	updown_BGIR9823_99168	updown_BGIR9823_96364	updown_BGIR9823_96973	updown_XR_001308019.2	updown_XR_002345205.1	updown_XR_002346024.1	updown_XR_002344184.1	updown_BGIR9823_101191	updown_BGIR9823_101562	updown_BGIR9823_102595	updown_BGIR9823_100418	updown_BGIR9823_101051	updown_BGIR9823_78137	updown_BGIR9823_78138	updown_BGIR9823_101781	updown_BGIR9823_81016	updown_BGIR9823_85300
XM_021082837.1	EHBP1L1	mRNA	0	0	0	0	0	0	0	0	0	0	0	0	0	0	0	0	0	0	0	1	0
XM_021082838.1	EHBP1L1	mRNA	0	0	0	0	0	0	0	0	0	0	0	0	0	0	0	0	0	0	0	1	0
XM_021082839.1	EHBP1L1	mRNA	0	0	0	0	0	0	0	0	0	0	0	0	0	0	0	0	0	0	0	1	0
XM_021082840.1	EHBP1L1	mRNA	0	0	0	0	0	0	0	0	0	0	0	0	0	0	0	0	0	0	0	1	0
XM_021082841.1	EHBP1L1	mRNA	0	0	0	0	0	0	0	0	0	0	0	0	0	0	0	0	0	0	0	1	0
XM_021082842.1	EHBP1L1	mRNA	0	0	0	0	0	0	0	0	0	0	0	0	0	0	0	0	0	0	0	1	0
XM_021082843.1	EHBP1L1	mRNA	0	0	0	0	0	0	0	0	0	0	0	0	0	0	0	0	0	0	0	1	0
XM_021082844.1	EHBP1L1	mRNA	0	0	0	0	0	0	0	0	0	0	0	0	0	0	0	0	0	0	0	1	0
XM_021082845.1	EHBP1L1	mRNA	0	0	0	0	0	0	0	0	0	0	0	0	0	0	0	0	0	0	0	1	0
XM_021082846.1	EHBP1L1	mRNA	0	0	0	0	0	0	0	0	0	0	0	0	0	0	0	0	0	0	0	1	0
XM_021082847.1	EHBP1L1	mRNA	0	0	0	0	0	0	0	0	0	0	0	0	0	0	0	0	0	0	0	1	0
XM_021082851.1	MAP3K11	mRNA	0	0	0	0	0	0	0	0	0	0	0	0	0	0	0	0	0	0	0	1	0
XM_021082852.1	SSSCA1	mRNA	0	0	0	0	0	0	0	0	0	0	0	0	0	0	0	0	0	0	0	1	0
XM_021082853.1	FAM89B	mRNA	0	0	0	0	0	0	0	0	0	0	0	0	0	0	0	0	0	0	0	1	0
XM_021082854.1	LTBP3	mRNA	0	0	0	0	0	0	0	0	0	0	0	0	0	0	0	0	0	0	0	1	0
XM_021082855.1	LTBP3	mRNA	0	0	0	0	0	0	0	0	0	0	0	0	0	0	0	0	0	0	0	1	0
XM_021082856.1	SCYL1	mRNA	0	0	0	0	0	0	0	0	0	0	0	0	0	0	0	0	0	0	0	1	0
XM_021082857.1	SLC25A45	mRNA	0	0	0	0	0	0	0	0	0	0	0	0	0	0	0	0	0	0	0	1	0
XM_021082858.1	SLC25A45	mRNA	0	0	0	0	0	0	0	0	0	0	0	0	0	0	0	0	0	0	0	1	0
XM_021091099.1	SYNGR1	mRNA	0	0	0	0	0	0	0	0	0	0	0	0	0	0	0	0	0	0	0	0	0
XM_021091101.1	SYNGR1	mRNA	0	0	0	0	0	0	0	0	0	0	0	0	0	0	0	0	0	0	0	0	0
XM_021091102.1	SYNGR1	mRNA	0	0	0	0	0	0	0	0	0	0	0	0	0	0	0	0	0	0	0	0	0
XM_021091103.1	SYNGR1	mRNA	0	0	0	0	0	0	0	0	0	0	0	0	0	0	0	0	0	0	0	0	0
XM_021091370.1	LOC102159820	mRNA	0	0	0	0	0	0	0	0	0	0	0	0	0	0	0	0	0	0	0	0	1
XM_021091373.1	TRMU	mRNA	0	0	0	0	0	0	0	0	0	0	0	0	0	0	0	0	0	0	0	0	1
XM_021091374.1	TRMU	mRNA	0	0	0	0	0	0	0	0	0	0	0	0	0	0	0	0	0	0	0	0	1
XM_021091375.1	TRMU	mRNA	0	0	0	0	0	0	0	0	0	0	0	0	0	0	0	0	0	0	0	0	1
XM_021091376.1	GTSE1	mRNA	0	0	0	0	0	0	0	0	0	0	0	0	0	0	0	0	0	0	0	0	1
XM_021091377.1	TTC38	mRNA	0	0	0	0	0	0	0	0	0	0	0	0	0	0	0	0	0	0	0	0	1
XM_021091379.1	LOC110260611	mRNA	0	0	0	0	0	0	0	0	0	0	0	0	0	0	0	0	0	0	0	0	1
XM_021091381.1	LOC110260611	mRNA	0	0	0	0	0	0	0	0	0	0	0	0	0	0	0	0	0	0	0	0	1
XM_021091454.1	TOB2	mRNA	0	0	0	0	0	0	0	0	0	0	0	0	0	0	0	0	0	0	0	0	0
XM_021091455.1	TOB2	mRNA	0	0	0	0	0	0	0	0	0	0	0	0	0	0	0	0	0	0	0	0	0
XM_021091456.1	PHF5A	mRNA	0	0	0	0	0	0	0	0	0	0	0	0	0	0	0	0	0	0	0	0	0
XM_021091457.1	PHF5A	mRNA	0	0	0	0	0	0	0	0	0	0	0	0	0	0	0	0	0	0	0	0	0
XM_021091458.1	ZC3H7B	mRNA	0	0	0	0	0	0	0	0	0	0	0	0	0	0	0	0	0	0	0	0	0
XM_021091459.1	ZC3H7B	mRNA	0	0	0	0	0	0	0	0	0	0	0	0	0	0	0	0	0	0	0	0	0
XM_021091460.1	TEF	mRNA	0	0	0	0	0	0	0	0	0	0	0	0	0	0	0	0	0	0	0	0	0
XM_021091461.1	RANGAP1	mRNA	0	0	0	0	0	0	0	0	0	0	0	0	0	0	0	0	0	0	0	0	0
XM_021091474.1	CBX7	mRNA	0	0	0	0	0	0	0	0	0	0	0	0	0	0	0	0	0	0	0	0	0
XM_021092767.1	CRADD	mRNA	0	0	0	0	0	0	0	0	0	0	0	0	0	0	0	0	0	0	0	0	0
XM_021092768.1	CRADD	mRNA	0	0	0	0	0	0	0	0	0	0	0	0	0	0	0	0	0	0	0	0	0
XM_021092769.1	CRADD	mRNA	0	0	0	0	0	0	0	0	0	0	0	0	0	0	0	0	0	0	0	0	0

RNA ID	Symbol	Type	updown_BGIR9823_91696	updown_BGIR9823_91824	updown_BGIR9823_92415	updown_BGIR9823_88848	updown_BGIR9823_99168	updown_BGIR9823_96364	updown_BGIR9823_96973	updown_XR_001308019.2	updown_XR_002345205.1	updown_XR_002346024.1	updown_XR_002344184.1	updown_BGIR9823_101191	updown_BGIR9823_101562	updown_BGIR9823_102595	updown_BGIR9823_100418	updown_BGIR9823_101051	updown_BGIR9823_78137	updown_BGIR9823_78138	updown_BGIR9823_101781	updown_BGIR9823_81016	updown_BGIR9823_85300
XM_021092770.1	CRADD	mRNA	0	0	0	0	0	0	0	0	0	0	0	0	0	0	0	0	0	0	0	0	0
XM_021092771.1	CRADD	mRNA	0	0	0	0	0	0	0	0	0	0	0	0	0	0	0	0	0	0	0	0	0
XM_021092773.1	CRADD	mRNA	0	0	0	0	0	0	0	0	0	0	0	0	0	0	0	0	0	0	0	0	0
XM_021093095.1	PKDREJ	mRNA	0	0	0	0	0	0	0	0	0	0	0	0	0	0	0	0	0	0	0	0	1
XM_021097064.1	MAF	mRNA	0	0	0	0	0	0	0	0	0	0	0	0	0	0	0	0	0	0	0	0	0
XM_021097065.1	MAF	mRNA	0	0	0	0	0	0	0	0	0	0	0	0	0	0	0	0	0	0	0	0	0
XM_021097066.1	MAF	mRNA	0	0	0	0	0	0	0	0	0	0	0	0	0	0	0	0	0	0	0	0	0
XM_021097870.1	ECI2	mRNA	0	0	0	0	0	0	0	0	0	0	0	0	0	0	0	0	0	0	0	0	0
XM_021099751.1	PHACTR1	mRNA	0	0	0	0	0	0	0	0	0	0	0	0	0	0	0	0	0	0	0	0	0
XM_021099752.1	PHACTR1	mRNA	0	0	0	0	0	0	0	0	0	0	0	0	0	0	0	0	0	0	0	0	0
XM_021099753.1	PHACTR1	mRNA	0	0	0	0	0	0	0	0	0	0	0	0	0	0	0	0	0	0	0	0	0
XM_021099754.1	PHACTR1	mRNA	0	0	0	0	0	0	0	0	0	0	0	0	0	0	0	0	0	0	0	0	0
XM_021099755.1	PHACTR1	mRNA	0	0	0	0	0	0	0	0	0	0	0	0	0	0	0	0	0	0	0	0	0
XM_021099756.1	PHACTR1	mRNA	0	0	0	0	0	0	0	0	0	0	0	0	0	0	0	0	0	0	0	0	0
XM_021099757.1	PHACTR1	mRNA	0	0	0	0	0	0	0	0	0	0	0	0	0	0	0	0	0	0	0	0	0
XM_021099758.1	PHACTR1	mRNA	0	0	0	0	0	0	0	0	0	0	0	0	0	0	0	0	0	0	0	0	0
XM_021099759.1	PHACTR1	mRNA	0	0	0	0	0	0	0	0	0	0	0	0	0	0	0	0	0	0	0	0	0
XM_021099760.1	PHACTR1	mRNA	0	0	0	0	0	0	0	0	0	0	0	0	0	0	0	0	0	0	0	0	0
XM_021099761.1	PHACTR1	mRNA	0	0	0	0	0	0	0	0	0	0	0	0	0	0	0	0	0	0	0	0	0
XM_021099762.1	PHACTR1	mRNA	0	0	0	0	0	0	0	0	0	0	0	0	0	0	0	0	0	0	0	0	0
XM_021099763.1	PHACTR1	mRNA	0	0	0	0	0	0	0	0	0	0	0	0	0	0	0	0	0	0	0	0	0
XM_021099764.1	PHACTR1	mRNA	0	0	0	0	0	0	0	0	0	0	0	0	0	0	0	0	0	0	0	0	0
XM_021099765.1	PHACTR1	mRNA	0	0	0	0	0	0	0	0	0	0	0	0	0	0	0	0	0	0	0	0	0
XM_021099766.1	TBC1D7	mRNA	0	0	0	0	0	0	0	0	0	0	0	0	0	0	0	0	0	0	0	0	0
XM_021099767.1	TBC1D7	mRNA	0	0	0	0	0	0	0	0	0	0	0	0	0	0	0	0	0	0	0	0	0
XM_021099769.1	TBC1D7	mRNA	0	0	0	0	0	0	0	0	0	0	0	0	0	0	0	0	0	0	0	0	0
XM_021099770.1	TBC1D7	mRNA	0	0	0	0	0	0	0	0	0	0	0	0	0	0	0	0	0	0	0	0	0
XM_021099999.1	LOC110261697	mRNA	0	0	0	0	0	0	0	0	0	0	0	0	0	0	0	0	0	0	0	0	0
XM_021100108.1	WRNIP1	mRNA	0	0	0	0	0	0	0	0	0	0	0	0	0	0	0	0	0	0	0	0	0
XM_021100109.1	MYLK4	mRNA	0	0	0	0	0	0	0	0	0	0	0	0	0	0	0	0	0	0	0	0	0
XM_021100110.1	MYLK4	mRNA	0	0	0	0	0	0	0	0	0	0	0	0	0	0	0	0	0	0	0	0	0
XM_021100111.1	MYLK4	mRNA	0	0	0	0	0	0	0	0	0	0	0	0	0	0	0	0	0	0	0	0	0
XM_021100112.1	MYLK4	mRNA	0	0	0	0	0	0	0	0	0	0	0	0	0	0	0	0	0	0	0	0	0
XM_021100114.1	NQO2	mRNA	0	0	0	0	0	0	0	0	0	0	0	0	0	0	0	0	0	0	0	0	0
XM_021100115.1	NQO2	mRNA	0	0	0	0	0	0	0	0	0	0	0	0	0	0	0	0	0	0	0	0	0
XM_021100117.1	NQO2	mRNA	0	0	0	0	0	0	0	0	0	0	0	0	0	0	0	0	0	0	0	0	0
XM_021100118.1	NQO2	mRNA	0	0	0	0	0	0	0	0	0	0	0	0	0	0	0	0	0	0	0	0	0
XM_021100119.1	NQO2	mRNA	0	0	0	0	0	0	0	0	0	0	0	0	0	0	0	0	0	0	0	0	0
XM_021100124.1	PRPF4B	mRNA	0	0	0	0	0	0	0	0	0	0	0	0	0	0	0	0	0	0	0	0	0
XM_021100126.1	PRPF4B	mRNA	0	0	0	0	0	0	0	0	0	0	0	0	0	0	0	0	0	0	0	0	0
XM_021100184.1	NEDD9	mRNA	0	0	0	0	0	0	0	0	0	0	0	0	0	0	0	0	0	0	0	0	0
XM_021100188.1	TMEM170B	mRNA	0	0	0	0	0	0	0	0	0	0	0	0	0	0	0	0	0	0	0	0	0
XM_021100194.1	GFOD1	mRNA	0	0	0	0	0	0	0	0	0	0	0	0	0	0	0	0	0	0	0	0	0

RNA ID	Symbol	Type	updown_BGIR9823_91696	updown_BGIR9823_91824	updown_BGIR9823_92415	updown_BGIR9823_88848	updown_BGIR9823_99168	updown_BGIR9823_96364	updown_BGIR9823_96973	updown_XR_001308019.2	updown_XR_002345205.1	updown_XR_002346024.1	updown_XR_002344184.1	updown_BGIR9823_101191	updown_BGIR9823_101562	updown_BGIR9823_102595	updown_BGIR9823_100418	updown_BGIR9823_101051	updown_BGIR9823_78137	updown_BGIR9823_78138	updown_BGIR9823_101781	updown_BGIR9823_81016	updown_BGIR9823_85300
XM_021102040.1	SLC37A4	mRNA	0	0	0	0	0	0	0	0	0	0	0	0	0	0	0	0	0	0	0	0	0
XM_021102041.1	SLC37A4	mRNA	0	0	0	0	0	0	0	0	0	0	0	0	0	0	0	0	0	0	0	0	0
XM_021102042.1	SLC37A4	mRNA	0	0	0	0	0	0	0	0	0	0	0	0	0	0	0	0	0	0	0	0	0
XM_021102043.1	SLC37A4	mRNA	0	0	0	0	0	0	0	0	0	0	0	0	0	0	0	0	0	0	0	0	0
XM_021102044.1	SLC37A4	mRNA	0	0	0	0	0	0	0	0	0	0	0	0	0	0	0	0	0	0	0	0	0
XM_021102045.1	SLC37A4	mRNA	0	0	0	0	0	0	0	0	0	0	0	0	0	0	0	0	0	0	0	0	0
XM_021102046.1	SLC37A4	mRNA	0	0	0	0	0	0	0	0	0	0	0	0	0	0	0	0	0	0	0	0	0
XM_021102047.1	SLC37A4	mRNA	0	0	0	0	0	0	0	0	0	0	0	0	0	0	0	0	0	0	0	0	0
XM_021102048.1	SLC37A4	mRNA	0	0	0	0	0	0	0	0	0	0	0	0	0	0	0	0	0	0	0	0	0
XM_021102049.1	SLC37A4	mRNA	0	0	0	0	0	0	0	0	0	0	0	0	0	0	0	0	0	0	0	0	0
XM_021102050.1	SLC37A4	mRNA	0	0	0	0	0	0	0	0	0	0	0	0	0	0	0	0	0	0	0	0	0
XM_021102051.1	SLC37A4	mRNA	0	0	0	0	0	0	0	0	0	0	0	0	0	0	0	0	0	0	0	0	0
XM_021102052.1	SLC37A4	mRNA	0	0	0	0	0	0	0	0	0	0	0	0	0	0	0	0	0	0	0	0	0
XM_021102209.1	UPK2	mRNA	0	0	0	0	0	0	0	0	0	0	0	0	0	0	0	0	0	0	0	0	0
XM_021102516.1	LOC100522455	mRNA	0	1	0	0	0	0	0	0	0	0	0	0	0	0	0	0	0	0	0	0	0
XM_021102517.1	RUNDC3B	mRNA	0	1	0	0	0	0	0	0	0	0	0	0	0	0	0	0	0	0	0	0	0
XM_021102518.1	RUNDC3B	mRNA	0	1	0	0	0	0	0	0	0	0	0	0	0	0	0	0	0	0	0	0	0
XM_021102519.1	RUNDC3B	mRNA	0	1	0	0	0	0	0	0	0	0	0	0	0	0	0	0	0	0	0	0	0
XM_021102522.1	RUNDC3B	mRNA	0	1	0	0	0	0	0	0	0	0	0	0	0	0	0	0	0	0	0	0	0
XM_021102523.1	RUNDC3B	mRNA	0	1	0	0	0	0	0	0	0	0	0	0	0	0	0	0	0	0	0	0	0
XR_001297664.2	LOC102161744	lncRNA	0	0	0	0	0	0	0	0	0	0	0	0	0	0	0	0	0	0	0	0	0
XR_001298807.2	LOC106504881	lncRNA	0	0	0	0	0	0	0	0	0	0	0	0	0	0	0	0	0	0	0	0	0
XR_001298808.2	LOC106504881	lncRNA	0	0	0	0	0	0	0	0	0	0	0	0	0	0	0	0	0	0	0	0	0
XR_001298810.2	LOC106504881	lncRNA	0	0	0	0	0	0	0	0	0	0	0	0	0	0	0	0	0	0	0	0	0
XR_001298811.2	LOC106504881	lncRNA	0	0	0	0	0	0	0	0	0	0	0	0	0	0	0	0	0	0	0	0	0
XR_001298812.2	LOC106504881	lncRNA	0	0	0	0	0	0	0	0	0	0	0	0	0	0	0	0	0	0	0	0	0
XR_001298813.2	LOC106504881	lncRNA	0	0	0	0	0	0	0	0	0	0	0	0	0	0	0	0	0	0	0	0	0
XR_001298814.2	LOC106504881	lncRNA	0	0	0	0	0	0	0	0	0	0	0	0	0	0	0	0	0	0	0	0	0
XR_001298816.2	LOC106504881	lncRNA	0	0	0	0	0	0	0	0	0	0	0	0	0	0	0	0	0	0	0	0	0
XR_001298817.2	LOC106504881	lncRNA	0	0	0	0	0	0	0	0	0	0	0	0	0	0	0	0	0	0	0	0	0
XR_001298844.2	LOC102163364	lncRNA	0	0	0	0	0	0	0	0	0	0	0	0	0	0	0	0	0	0	0	0	0
XR_001298846.2	LOC102163364	lncRNA	0	0	0	0	0	0	0	0	0	0	0	0	0	0	0	0	0	0	0	0	0
XR_001298850.2	LOC102163364	lncRNA	0	0	0	0	0	0	0	0	0	0	0	0	0	0	0	0	0	0	0	0	0
XR_001299456.2	LOC102166670	lncRNA	0	0	0	0	0	0	0	0	0	0	0	0	0	0	0	0	0	0	0	0	0
XR_001299457.2	LOC102166670	lncRNA	0	0	0	0	0	0	0	0	0	0	0	0	0	0	0	0	0	0	0	0	0
XR_001300715.2	LOC106505879	lncRNA	0	0	0	0	0	0	0	0	0	0	0	0	0	0	0	0	0	0	0	0	0
XR_001301078.2	LOC102162623	lncRNA	0	0	0	0	0	0	0	0	0	0	0	0	0	0	0	0	0	0	0	0	0
XR_001301087.2	LOC102162623	lncRNA	0	0	0	0	0	0	0	0	0	0	0	0	0	0	0	0	0	0	0	0	0
XR_001301217.2	LOC102164998	lncRNA	0	0	0	0	0	0	0	0	0	0	0	0	0	0	0	0	0	0	0	0	0
XR_001301305.2	LOC102159607	lncRNA	0	0	0	0	0	0	0	0	0	0	0	0	0	0	0	0	0	0	0	0	0
XR_001308967.2	LOC106510467	lncRNA	0	0	0	0	0	0	0	0	0	0	0	0	0	0	0	0	0	0	0	0	0
XR_002335533.1	XRRA1	mRNA	0	0	0	0	0	0	0	0	0	0	0	0	0	0	0	0	0	0	0	0	0
XR_002335534.1	XRRA1	mRNA	0	0	0	0	0	0	0	0	0	0	0	0	0	0	0	0	0	0	0	0	0

RNA ID	Symbol	Type	updown_BGIR9823_91696	updown_BGIR9823_91824	updown_BGIR9823_92415	updown_BGIR9823_88848	updown_BGIR9823_99168	updown_BGIR9823_96364	updown_BGIR9823_96973	updown_XR_001308019.2	updown_XR_002345205.1	updown_XR_002346024.1	updown_XR_002344184.1	updown_BGIR9823_101191	updown_BGIR9823_101562	updown_BGIR9823_102595	updown_BGIR9823_100418	updown_BGIR9823_101051	updown_BGIR9823_78137	updown_BGIR9823_78138	updown_BGIR9823_101781	updown_BGIR9823_81016	updown_BGIR9823_85300
XR_002335535.1	LOC110255399	lncRNA	0	0	0	0	0	0	0	0	0	0	0	0	0	0	0	0	0	0	0	0	0
XR_002335536.1	LOC102160367	lncRNA	0	0	0	0	0	0	0	0	0	0	0	0	0	0	0	0	0	0	0	0	0
XR_002335537.1	LOC102160367	lncRNA	0	0	0	0	0	0	0	0	0	0	0	0	0	0	0	0	0	0	0	0	0
XR_002335650.1	LOC102157630	lncRNA	0	0	0	0	0	0	0	0	0	0	0	0	0	0	0	0	0	0	0	0	0
XR_002335651.1	LOC110255435	lncRNA	0	0	0	0	0	0	0	0	0	0	0	0	0	0	0	0	0	0	0	0	0
XR_002335652.1	LOC106504881	lncRNA	0	0	0	0	0	0	0	0	0	0	0	0	0	0	0	0	0	0	0	0	0
XR_002335653.1	LOC106504881	lncRNA	0	0	0	0	0	0	0	0	0	0	0	0	0	0	0	0	0	0	0	0	0
XR_002335670.1	LOC110255440	lncRNA	0	0	0	0	0	0	0	0	0	0	0	0	0	0	0	0	0	0	0	0	0
XR_002335671.1	LOC110255441	lncRNA	0	0	0	0	0	0	0	0	0	0	0	0	0	0	0	0	0	0	0	0	0
XR_002335672.1	LOC110255441	lncRNA	0	0	0	0	0	0	0	0	0	0	0	0	0	0	0	0	0	0	0	0	0
XR_002335674.1	HYOU1	mRNA	0	0	0	0	0	0	0	0	0	0	0	0	0	0	0	0	0	0	0	0	0
XR_002335675.1	HYOU1	mRNA	0	0	0	0	0	0	0	0	0	0	0	0	0	0	0	0	0	0	0	0	0
XR_002335685.1	LOC100523684	mRNA	0	0	0	0	0	0	0	0	0	0	0	0	0	0	0	0	0	0	0	0	0
XR_002335686.1	LOC102163364	lncRNA	0	0	0	0	0	0	0	0	0	0	0	0	0	0	0	0	0	0	0	0	0
XR_002335687.1	LOC102163364	lncRNA	0	0	0	0	0	0	0	0	0	0	0	0	0	0	0	0	0	0	0	0	0
XR_002335688.1	LOC102163364	lncRNA	0	0	0	0	0	0	0	0	0	0	0	0	0	0	0	0	0	0	0	0	0
XR_002335689.1	LOC102163364	lncRNA	0	0	0	0	0	0	0	0	0	0	0	0	0	0	0	0	0	0	0	0	0
XR_002335690.1	LOC102163364	lncRNA	0	0	0	0	0	0	0	0	0	0	0	0	0	0	0	0	0	0	0	0	0
XR_002335691.1	LOC102163364	lncRNA	0	0	0	0	0	0	0	0	0	0	0	0	0	0	0	0	0	0	0	0	0
XR_002335692.1	LOC102163364	lncRNA	0	0	0	0	0	0	0	0	0	0	0	0	0	0	0	0	0	0	0	0	0
XR_002335693.1	LOC106504896	lncRNA	0	0	0	0	0	0	0	0	0	0	0	0	0	0	0	0	0	0	0	0	0
XR_002335694.1	SORL1	mRNA	0	0	0	0	0	0	0	0	0	0	0	0	0	0	0	0	0	0	0	0	0
XR_002336641.1	LOC106505250	lncRNA	0	0	0	0	0	0	0	0	0	0	0	0	0	0	0	0	0	0	0	0	0
XR_002336643.1	LOC110255292	lncRNA	0	0	0	0	0	0	0	0	0	0	0	0	0	0	0	0	0	0	0	0	0
XR_002336644.1	LOC102166670	lncRNA	0	0	0	0	0	0	0	0	0	0	0	0	0	0	0	0	0	0	0	0	0
XR_002336645.1	LOC102166670	lncRNA	0	0	0	0	0	0	0	0	0	0	0	0	0	0	0	0	0	0	0	0	0
XR_002338443.1	LOC106505879	lncRNA	0	0	0	0	0	0	0	0	0	0	0	0	0	0	0	0	0	0	0	0	0
XR_002338444.1	LOC106505879	lncRNA	0	0	0	0	0	0	0	0	0	0	0	0	0	0	0	0	0	0	0	0	0
XR_002338445.1	LOC106505879	lncRNA	0	0	0	0	0	0	0	0	0	0	0	0	0	0	0	0	0	0	0	0	0
XR_002338446.1	LOC106505879	lncRNA	0	0	0	0	0	0	0	0	0	0	0	0	0	0	0	0	0	0	0	0	0
XR_002338447.1	LOC106505879	lncRNA	0	0	0	0	0	0	0	0	0	0	0	0	0	0	0	0	0	0	0	0	0
XR_002338448.1	LOC106505879	lncRNA	0	0	0	0	0	0	0	0	0	0	0	0	0	0	0	0	0	0	0	0	0
XR_002338449.1	LOC106505879	lncRNA	0	0	0	0	0	0	0	0	0	0	0	0	0	0	0	0	0	0	0	0	0
XR_002338450.1	LOC106505879	lncRNA	0	0	0	0	0	0	0	0	0	0	0	0	0	0	0	0	0	0	0	0	0
XR_002338521.1	LOC110256664	lncRNA	0	0	0	0	0	0	0	0	0	0	0	0	0	0	0	0	0	0	0	0	0
XR_002338522.1	LOC110256664	lncRNA	0	0	0	0	0	0	0	0	0	0	0	0	0	0	0	0	0	0	0	0	0
XR_002338666.1	LOC110256747	lncRNA	0	0	0	0	0	0	0	0	0	0	0	0	0	0	0	0	0	0	0	0	0
XR_002338714.1	LOC102163817	lncRNA	0	0	0	0	0	0	0	0	0	0	0	0	0	0	0	0	0	0	0	0	0
XR_002338715.1	LOC102164998	lncRNA	0	0	0	0	0	0	0	0	0	0	0	0	0	0	0	0	0	0	0	0	0
XR_002338716.1	LOC102164998	lncRNA	0	0	0	0	0	0	0	0	0	0	0	0	0	0	0	0	0	0	0	0	0
XR_002338717.1	LOC110256768	lncRNA	0	0	0	0	0	0	0	0	0	0	0	0	0	0	0	0	0	0	0	0	0
XR_002338727.1	LOC110256773	lncRNA	0	0	0	0	0	0	0	0	0	0	0	0	0	0	0	0	0	0	0	0	0
XR_002338728.1	LOC110256773	lncRNA	0	0	0	0	0	0	0	0	0	0	0	0	0	0	0	0	0	0	0	0	0

RNA ID	Symbol	Type	updown_BGIR9823_91696	updown_BGIR9823_91824	updown_BGIR9823_92415	updown_BGIR9823_88848	updown_BGIR9823_99168	updown_BGIR9823_96364	updown_BGIR9823_96973	updown_XR_001308019.2	updown_XR_002345205.1	updown_XR_002346024.1	updown_XR_002344184.1	updown_BGIR9823_101191	updown_BGIR9823_101562	updown_BGIR9823_102595	updown_BGIR9823_100418	updown_BGIR9823_101051	updown_BGIR9823_78137	updown_BGIR9823_78138	updown_BGIR9823_101781	updown_BGIR9823_81016	updown_BGIR9823_85300
XR_002338729.1	LOC110256773	lncRNA	0	0	0	0	0	0	0	0	0	0	0	0	0	0	0	0	0	0	0	0	0
XR_002338793.1	LOC110256796	lncRNA	0	0	0	0	0	0	0	0	0	0	0	0	0	0	0	0	0	0	0	0	0
XR_002339623.1	LOC102167708	lncRNA	0	0	0	0	0	0	0	0	0	0	0	0	0	0	0	0	0	0	0	0	0
XR_002340245.1	PRKAG2	mRNA	0	0	0	0	0	0	0	0	0	0	0	0	0	0	0	0	0	0	0	0	0
XR_002340246.1	LOC102162623	lncRNA	0	0	0	0	0	0	0	0	0	0	0	0	0	0	0	0	0	0	0	0	0
XR_002340247.1	LOC102162623	lncRNA	0	0	0	0	0	0	0	0	0	0	0	0	0	0	0	0	0	0	0	0	0
XR_002340248.1	LOC110257516	lncRNA	0	0	0	0	0	0	0	0	0	0	0	0	0	0	0	0	0	0	0	0	0
XR_002340249.1	LOC102162071	lncRNA	0	0	0	0	0	0	0	0	0	0	0	0	0	0	0	0	0	0	0	0	0
XR_002340901.1	LOC102160243	lncRNA	0	0	0	0	0	0	0	0	0	0	0	0	0	0	0	0	0	0	0	0	0
XR_002340902.1	LOC102160243	lncRNA	0	0	0	0	0	0	0	0	0	0	0	0	0	0	0	0	0	0	0	0	0
XR_002340903.1	LOC102160243	lncRNA	0	0	0	0	0	0	0	0	0	0	0	0	0	0	0	0	0	0	0	0	0
XR_002340904.1	LOC102160243	lncRNA	0	0	0	0	0	0	0	0	0	0	0	0	0	0	0	0	0	0	0	0	0
XR_002340905.1	LOC102160243	lncRNA	0	0	0	0	0	0	0	0	0	0	0	0	0	0	0	0	0	0	0	0	0
XR_002341237.1	LOC102164320	lncRNA	0	0	0	0	0	0	0	0	0	0	0	0	0	0	0	0	0	0	0	0	0
XR_002341238.1	LOC102164320	lncRNA	0	0	0	0	0	0	0	0	0	0	0	0	0	0	0	0	0	0	0	0	0
XR_002341239.1	LOC102164320	lncRNA	0	0	0	0	0	0	0	0	0	0	0	0	0	0	0	0	0	0	0	0	0
XR_002341240.1	LOC102164320	lncRNA	0	0	0	0	0	0	0	0	0	0	0	0	0	0	0	0	0	0	0	0	0
XR_002341241.1	LOC102164320	lncRNA	0	0	0	0	0	0	0	0	0	0	0	0	0	0	0	0	0	0	0	0	0
XR_002341242.1	LOC102164320	lncRNA	0	0	0	0	0	0	0	0	0	0	0	0	0	0	0	0	0	0	0	0	0
XR_002341243.1	LOC102164320	lncRNA	0	0	0	0	0	0	0	0	0	0	0	0	0	0	0	0	0	0	0	0	0
XR_002341244.1	LOC102164320	lncRNA	0	0	0	0	0	0	0	0	0	0	0	0	0	0	0	0	0	0	0	0	0
XR_002341270.1	LOC100521600	mRNA	0	0	0	0	0	0	0	0	0	0	0	0	0	0	0	0	0	0	0	0	0
XR_002341443.1	LOC102163816	lncRNA	0	0	0	0	0	0	0	0	0	0	0	0	0	0	0	0	0	0	0	1	0
XR_002343847.1	LOC110260611	mRNA	0	0	0	0	0	0	0	0	0	0	0	0	0	0	0	0	0	0	0	0	1
XR_002343848.1	LOC110260611	mRNA	0	0	0	0	0	0	0	0	0	0	0	0	0	0	0	0	0	0	0	0	1
XR_002343849.1	LOC110260611	mRNA	0	0	0	0	0	0	0	0	0	0	0	0	0	0	0	0	0	0	0	0	1
XR_002343850.1	LOC110260611	mRNA	0	0	0	0	0	0	0	0	0	0	0	0	0	0	0	0	0	0	0	0	1
XR_002343875.1	LOC110260629	lncRNA	0	0	0	0	0	0	0	0	0	0	0	0	0	0	0	0	0	0	0	0	0
XR_002343876.1	LOC110260629	lncRNA	0	0	0	0	0	0	0	0	0	0	0	0	0	0	0	0	0	0	0	0	0
XR_002343877.1	LOC110260629	lncRNA	0	0	0	0	0	0	0	0	0	0	0	0	0	0	0	0	0	0	0	0	0
XR_002343878.1	LOC110260629	lncRNA	0	0	0	0	0	0	0	0	0	0	0	0	0	0	0	0	0	0	0	0	0
XR_002343879.1	LOC110260629	lncRNA	0	0	0	0	0	0	0	0	0	0	0	0	0	0	0	0	0	0	0	0	0
XR_002343880.1	LOC110260629	lncRNA	0	0	0	0	0	0	0	0	0	0	0	0	0	0	0	0	0	0	0	0	0
XR_002343881.1	LOC110260629	lncRNA	0	0	0	0	0	0	0	0	0	0	0	0	0	0	0	0	0	0	0	0	0
XR_002343883.1	LOC110260631	lncRNA	0	0	0	0	0	0	0	0	0	0	0	0	0	0	0	0	0	0	0	0	0
XR_002343884.1	LOC110260634	lncRNA	0	0	0	0	0	0	0	0	0	0	0	0	0	0	0	0	0	0	0	0	0
XR_002343885.1	LOC110260635	lncRNA	0	0	0	0	0	0	0	0	0	0	0	0	0	0	0	0	0	0	0	0	0
XR_002343891.1	LOC110260642	lncRNA	0	0	0	0	0	0	0	0	0	0	0	0	0	0	0	0	0	0	0	0	0
XR_002343892.1	LOC110260643	lncRNA	0	0	0	0	0	0	0	0	0	0	0	0	0	0	0	0	0	0	0	0	0
XR_002345298.1	MAF	mRNA	0	0	0	0	0	0	0	0	0	0	0	0	0	0	0	0	0	0	0	0	0
XR_002345300.1	LOC106510470	lncRNA	0	0	0	0	0	0	0	0	0	0	0	0	0	0	0	0	0	0	0	0	0
XR_002345301.1	LOC106510468	lncRNA	0	0	0	0	0	0	0	0	0	0	0	0	0	0	0	0	0	0	0	0	0
XR_002346166.1	LOC110261703	lncRNA	0	0	0	0	0	0	0	0	0	0	0	0	0	0	0	0	0	0	0	0	0

RNA ID	Symbol	Type	updown_BGIR9823_91696	updown_BGIR9823_91824	updown_BGIR9823_92415	updown_BGIR9823_88848	updown_BGIR9823_99168	updown_BGIR9823_96364	updown_BGIR9823_96973	updown_XR_001308019.2	updown_XR_002345205.1	updown_XR_002346024.1	updown_XR_002344184.1	updown_BGIR9823_101191	updown_BGIR9823_101562	updown_BGIR9823_102595	updown_BGIR9823_100418	updown_BGIR9823_101051	updown_BGIR9823_78137	updown_BGIR9823_78138	updown_BGIR9823_101781	updown_BGIR9823_81016	updown_BGIR9823_85300
XR_002346227.1	LOC106504299	lncRNA	0	0	0	0	0	0	0	0	0	0	0	0	0	0	0	0	0	0	0	0	0
XR_002346228.1	LOC106504299	lncRNA	0	0	0	0	0	0	0	0	0	0	0	0	0	0	0	0	0	0	0	0	0
XR_002346229.1	LOC106504299	lncRNA	0	0	0	0	0	0	0	0	0	0	0	0	0	0	0	0	0	0	0	0	0
XR_002346230.1	LOC106504299	lncRNA	0	0	0	0	0	0	0	0	0	0	0	0	0	0	0	0	0	0	0	0	0
XR_002346231.1	LOC106504299	lncRNA	0	0	0	0	0	0	0	0	0	0	0	0	0	0	0	0	0	0	0	0	0
XR_002346232.1	LOC106504299	lncRNA	0	0	0	0	0	0	0	0	0	0	0	0	0	0	0	0	0	0	0	0	0
XR_002346233.1	NQO2	mRNA	0	0	0	0	0	0	0	0	0	0	0	0	0	0	0	0	0	0	0	0	0
XR_002346236.1	PRPF4B	mRNA	0	0	0	0	0	0	0	0	0	0	0	0	0	0	0	0	0	0	0	0	0
XR_002346237.1	PRPF4B	mRNA	0	0	0	0	0	0	0	0	0	0	0	0	0	0	0	0	0	0	0	0	0
XR_002346238.1	PRPF4B	mRNA	0	0	0	0	0	0	0	0	0	0	0	0	0	0	0	0	0	0	0	0	0
XR_002346239.1	PRPF4B	mRNA	0	0	0	0	0	0	0	0	0	0	0	0	0	0	0	0	0	0	0	0	0
XR_002346240.1	PRPF4B	mRNA	0	0	0	0	0	0	0	0	0	0	0	0	0	0	0	0	0	0	0	0	0
XR_002346241.1	PRPF4B	mRNA	0	0	0	0	0	0	0	0	0	0	0	0	0	0	0	0	0	0	0	0	0
XR_002346242.1	PRPF4B	mRNA	0	0	0	0	0	0	0	0	0	0	0	0	0	0	0	0	0	0	0	0	0
XR_002346243.1	PRPF4B	mRNA	0	0	0	0	0	0	0	0	0	0	0	0	0	0	0	0	0	0	0	0	0
XR_002346244.1	PRPF4B	mRNA	0	0	0	0	0	0	0	0	0	0	0	0	0	0	0	0	0	0	0	0	0
XR_002346245.1	PRPF4B	mRNA	0	0	0	0	0	0	0	0	0	0	0	0	0	0	0	0	0	0	0	0	0
XR_002346246.1	PRPF4B	mRNA	0	0	0	0	0	0	0	0	0	0	0	0	0	0	0	0	0	0	0	0	0
XR_002346247.1	PRPF4B	mRNA	0	0	0	0	0	0	0	0	0	0	0	0	0	0	0	0	0	0	0	0	0
XR_002346248.1	PRPF4B	mRNA	0	0	0	0	0	0	0	0	0	0	0	0	0	0	0	0	0	0	0	0	0
XR_002346249.1	PRPF4B	mRNA	0	0	0	0	0	0	0	0	0	0	0	0	0	0	0	0	0	0	0	0	0
XR_002346250.1	PRPF4B	mRNA	0	0	0	0	0	0	0	0	0	0	0	0	0	0	0	0	0	0	0	0	0
XR_002346251.1	PRPF4B	mRNA	0	0	0	0	0	0	0	0	0	0	0	0	0	0	0	0	0	0	0	0	0
XR_002346252.1	PRPF4B	mRNA	0	0	0	0	0	0	0	0	0	0	0	0	0	0	0	0	0	0	0	0	0
XR_002346253.1	PRPF4B	mRNA	0	0	0	0	0	0	0	0	0	0	0	0	0	0	0	0	0	0	0	0	0
XR_002346254.1	PRPF4B	mRNA	0	0	0	0	0	0	0	0	0	0	0	0	0	0	0	0	0	0	0	0	0
XR_002346255.1	PRPF4B	mRNA	0	0	0	0	0	0	0	0	0	0	0	0	0	0	0	0	0	0	0	0	0
XR_002346256.1	PRPF4B	mRNA	0	0	0	0	0	0	0	0	0	0	0	0	0	0	0	0	0	0	0	0	0
XR_002346257.1	PRPF4B	mRNA	0	0	0	0	0	0	0	0	0	0	0	0	0	0	0	0	0	0	0	0	0
XR_002346258.1	PRPF4B	mRNA	0	0	0	0	0	0	0	0	0	0	0	0	0	0	0	0	0	0	0	0	0
XR_002346259.1	PRPF4B	mRNA	0	0	0	0	0	0	0	0	0	0	0	0	0	0	0	0	0	0	0	0	0
XR_002346260.1	PRPF4B	mRNA	0	0	0	0	0	0	0	0	0	0	0	0	0	0	0	0	0	0	0	0	0
XR_002346261.1	PRPF4B	mRNA	0	0	0	0	0	0	0	0	0	0	0	0	0	0	0	0	0	0	0	0	0
XR_002346262.1	PRPF4B	mRNA	0	0	0	0	0	0	0	0	0	0	0	0	0	0	0	0	0	0	0	0	0
XR_002346263.1	PRPF4B	mRNA	0	0	0	0	0	0	0	0	0	0	0	0	0	0	0	0	0	0	0	0	0
XR_002346264.1	PRPF4B	mRNA	0	0	0	0	0	0	0	0	0	0	0	0	0	0	0	0	0	0	0	0	0
XR_002346265.1	LOC110261764	lncRNA	0	0	0	0	0	0	0	0	0	0	0	0	0	0	0	0	0	0	0	0	0
XR_002346266.1	LOC102162837	lncRNA	0	0	0	0	0	0	0	0	0	0	0	0	0	0	0	0	0	0	0	0	0
XR_002346299.1	LOC110261774	lncRNA	0	0	0	0	0	0	0	0	0	0	0	0	0	0	0	0	0	0	0	0	0
XR_002346300.1	LOC110261774	lncRNA	0	0	0	0	0	0	0	0	0	0	0	0	0	0	0	0	0	0	0	0	0
XR_002346302.1	LOC110261776	lncRNA	0	0	0	0	0	0	0	0	0	0	0	0	0	0	0	0	0	0	0	0	0
XR_002346303.1	TMEM170B	mRNA	0	0	0	0	0	0	0	0	0	0	0	0	0	0	0	0	0	0	0	0	0
XR_002346304.1	TMEM170B	mRNA	0	0	0	0	0	0	0	0	0	0	0	0	0	0	0	0	0	0	0	0	0

RNA ID	Symbol	Type	updown_BGIR9823_91696	updown_BGIR9823_91824	updown_BGIR9823_92415	updown_BGIR9823_88848	updown_BGIR9823_99168	updown_BGIR9823_96364	updown_BGIR9823_96973	updown_XR_001308019.2	updown_XR_002345205.1	updown_XR_002346024.1	updown_XR_002344184.1	updown_BGIR9823_101191	updown_BGIR9823_101562	updown_BGIR9823_102595	updown_BGIR9823_100418	updown_BGIR9823_101051	updown_BGIR9823_78137	updown_BGIR9823_78138	updown_BGIR9823_101781	updown_BGIR9823_81016	updown_BGIR9823_85300
XR_115737.4	LOC100513133	lncRNA	0	0	0	0	0	0	0	0	0	0	0	0	0	0	0	0	0	0	0	1	0
XR_299046.2	LOC102164596	lncRNA	0	1	0	0	0	0	0	0	0	0	0	0	0	0	0	0	0	0	0	0	0
XR_303634.3	LOC100738154	lncRNA	0	0	0	0	0	0	0	0	0	0	0	0	0	0	0	0	0	0	0	0	0
XR_304239.3	LOC102159819	lncRNA	0	0	0	0	0	0	0	0	0	0	0	0	0	0	0	0	0	0	0	0	0
XR_304244.3	LOC102161992	lncRNA	0	0	0	0	0	0	0	0	0	0	0	0	0	0	0	0	0	0	0	0	0
XR_304257.3	LOC102162837	lncRNA	0	0	0	0	0	0	0	0	0	0	0	0	0	0	0	0	0	0	0	0	0
XR_306105.3	LOC102166871	lncRNA	0	0	0	0	0	0	0	0	0	0	0	0	0	0	0	0	0	0	0	0	0
XR_307914.3	LOC102159607	lncRNA	0	0	0	0	0	0	0	0	0	0	0	0	0	0	0	0	0	0	0	0	0
XR_308345.3	LOC102167708	lncRNA	0	0	0	0	0	0	0	0	0	0	0	0	0	0	0	0	0	0	0	0	0
novel-ssc-miR1028-3p		miRNA	0	0	0	0	0	0	0	0	0	0	0	0	0	0	0	0	0	0	0	0	0
novel-ssc-miR1028-5p		miRNA	0	0	0	0	0	0	0	0	0	0	0	0	0	0	0	0	0	0	0	0	0
novel-ssc-miR1130-3p		miRNA	0	0	0	0	0	0	0	0	0	0	0	0	0	0	0	0	0	0	0	0	0
novel-ssc-miR1130-5p		miRNA	0	0	0	0	0	0	0	0	0	0	0	0	0	0	0	0	0	0	0	0	0
novel-ssc-miR1162-3p		miRNA	0	0	0	0	0	0	0	0	0	0	0	0	0	0	0	0	0	0	0	1	0
novel-ssc-miR1193-3p		miRNA	0	0	0	0	0	0	0	0	0	0	0	0	0	0	0	0	0	0	0	0	0
novel-ssc-miR1193-5p		miRNA	0	0	0	0	0	0	0	0	0	0	0	0	0	0	0	0	0	0	0	0	0
novel-ssc-miR1217-3p		miRNA	0	0	0	0	0	0	0	0	0	0	0	0	0	0	0	0	0	0	0	0	0
novel-ssc-miR1217-5p		miRNA	0	0	0	0	0	0	0	0	0	0	0	0	0	0	0	0	0	0	0	0	0
novel-ssc-miR1429-3p		miRNA	0	0	0	0	0	0	0	0	0	0	0	0	0	0	0	0	0	0	0	0	1
novel-ssc-miR1429-5p		miRNA	0	0	0	0	0	0	0	0	0	0	0	0	0	0	0	0	0	0	0	0	1
novel-ssc-miR259-3p		miRNA	0	0	0	0	0	0	0	0	0	0	0	0	0	0	0	0	0	0	0	1	0
novel-ssc-miR259-5p		miRNA	0	0	0	0	0	0	0	0	0	0	0	0	0	0	0	0	0	0	0	1	0
novel-ssc-miR324-3p		miRNA	0	0	0	0	0	0	0	0	0	0	0	0	0	0	0	0	0	0	0	0	1
novel-ssc-miR324-5p		miRNA	0	0	0	0	0	0	0	0	0	0	0	0	0	0	0	0	0	0	0	0	1
novel-ssc-miR42-3p		miRNA	0	0	0	0	0	0	0	0	0	0	0	0	0	0	0	0	0	0	0	0	0
novel-ssc-miR42-5p		miRNA	0	0	0	0	0	0	0	0	0	0	0	0	0	0	0	0	0	0	0	0	0
novel-ssc-miR433-3p		miRNA	0	0	0	0	0	0	0	0	0	0	0	0	0	0	0	0	0	0	0	0	0
novel-ssc-miR433-5p		miRNA	0	0	0	0	0	0	0	0	0	0	0	0	0	0	0	0	0	0	0	0	0
novel-ssc-miR557-3p		miRNA	0	0	0	0	0	0	0	0	0	0	0	0	0	0	0	0	0	0	0	0	0
novel-ssc-miR557-5p		miRNA	0	0	0	0	0	0	0	0	0	0	0	0	0	0	0	0	0	0	0	0	0
novel-ssc-miR619-3p		miRNA	0	0	0	0	0	0	0	0	0	0	0	0	0	0	0	0	0	0	0	0	0
novel-ssc-miR619-5p		miRNA	0	0	0	0	0	0	0	0	0	0	0	0	0	0	0	0	0	0	0	0	0
novel-ssc-miR719-3p		miRNA	0	0	0	0	0	0	0	0	0	0	0	0	0	0	0	0	0	0	0	1	0
novel-ssc-miR719-5p		miRNA	0	0	0	0	0	0	0	0	0	0	0	0	0	0	0	0	0	0	0	1	0
novel-ssc-miR966-3p		miRNA	0	0	0	0	0	0	0	0	0	0	0	0	0	0	0	0	0	0	0	1	0
novel-ssc-miR966-5p		miRNA	0	0	0	0	0	0	0	0	0	0	0	0	0	0	0	0	0	0	0	1	0
ssc-miR-7136-3p		miRNA	0	0	0	0	0	0	0	0	0	0	0	0	0	0	0	0	0	0	0	0	0
ssc-miR-7136-5p		miRNA	0	0	0	0	0	0	0	0	0	0	0	0	0	0	0	0	0	0	0	0	0

Supplementary Table S10. The predicted targe

RNA ID	Symbol	Type	updown_BGIR9823_84443	updown_BGIR9823_81960	updown_BGIR9823_82315	updown_BGIR9823_82966	updown_BGIR9823_83074	updown_BGIR9823_82386	updown_BGIR9823_82709	updown_BGIR9823_83417	updown_BGIR9823_85418	updown_BGIR9823_87926	updown_BGIR9823_88529	updown_BGIR9823_88698	updown_BGIR9823_86965	updown_BGIR9823_89012	updown_BGIR9823_92131	updown_BGIR9823_90639	updown_BGIR9823_95770	updown_BGIR9823_92788	updown_BGIR9823_93572	updown_BGIR9823_96356	updown_BGIR9823_94689
BGIR9823_100361	PRKAG2	mRNA	0	0	0	0	0	0	0	0	0	0	0	0	0	0	0	0	0	0	0	0	0
BGIR9823_100362	PRKAG2	mRNA	0	0	0	0	0	0	0	0	0	0	0	0	0	0	0	0	0	0	0	0	0
BGIR9823_100363	PRKAG2	mRNA	0	0	0	0	0	0	0	0	0	0	0	0	0	0	0	0	0	0	0	0	0
BGIR9823_100364	PRKAG2	mRNA	0	0	0	0	0	0	0	0	0	0	0	0	0	0	0	0	0	0	0	0	0
BGIR9823_101535	LOC102158401	lncRNA	0	0	0	0	0	0	0	0	0	0	0	0	0	0	0	0	0	0	0	0	0
BGIR9823_101541	LOC102157946	lncRNA	0	0	0	0	0	0	0	0	0	0	0	0	0	0	0	0	0	0	0	0	0
BGIR9823_101542	LOC102157946	lncRNA	0	0	0	0	0	0	0	0	0	0	0	0	0	0	0	0	0	0	0	0	0
BGIR9823_101560	LOC110258600	lncRNA	0	0	0	0	0	0	0	0	0	0	0	0	0	0	0	0	0	0	0	0	0
BGIR9823_101561	LOC110258600	lncRNA	0	0	0	0	0	0	0	0	0	0	0	0	0	0	0	0	0	0	0	0	0
BGIR9823_101562	LOC110258600	lncRNA	0	0	0	0	0	0	0	0	0	0	0	0	0	0	0	0	0	0	0	0	0
BGIR9823_101563	LOC110258600	lncRNA	0	0	0	0	0	0	0	0	0	0	0	0	0	0	0	0	0	0	0	0	0
BGIR9823_101564	LOC110258600	lncRNA	0	0	0	0	0	0	0	0	0	0	0	0	0	0	0	0	0	0	0	0	0
BGIR9823_101612	LOC100521600	lncRNA	0	0	0	0	0	0	0	0	0	0	0	0	0	0	0	0	0	0	0	0	0
BGIR9823_101853		lncRNA	0	0	0	0	0	0	0	0	0	0	0	0	0	0	0	0	0	0	0	0	0
BGIR9823_101854		lncRNA	0	0	0	0	0	0	0	0	0	0	0	0	0	0	0	0	0	0	0	0	0
BGIR9823_101855		lncRNA	0	0	0	0	0	0	0	0	0	0	0	0	0	0	0	0	0	0	0	0	0
BGIR9823_102146		lncRNA	0	0	0	0	0	0	0	0	0	0	0	0	0	0	0	0	0	0	0	0	0
BGIR9823_102180		lncRNA	0	0	0	0	0	0	0	0	0	0	0	0	0	0	0	0	0	0	0	0	0
BGIR9823_102279		lncRNA	0	0	0	0	0	0	0	0	0	0	0	0	0	0	0	0	0	0	0	0	0
BGIR9823_102282		lncRNA	0	0	0	0	0	0	0	0	0	0	0	0	0	0	0	0	0	0	0	0	0
BGIR9823_102361		lncRNA	0	0	0	0	0	0	0	0	0	0	0	0	0	0	0	0	0	0	0	0	0
BGIR9823_102362		lncRNA	0	0	0	0	0	0	0	0	0	0	0	0	0	0	0	0	0	0	0	0	0
BGIR9823_102363		lncRNA	0	0	0	0	0	0	0	0	0	0	0	0	0	0	0	0	0	0	0	0	0
BGIR9823_102727		lncRNA	0	0	0	0	0	0	0	0	0	0	0	0	0	0	0	0	0	0	0	0	0
BGIR9823_80121	SSSCA1	lncRNA	0	0	0	0	0	0	0	0	0	0	0	0	0	0	0	0	0	0	0	0	0
BGIR9823_80122	LOC102163816	lncRNA	0	0	0	0	0	0	0	0	0	0	0	0	0	0	0	0	0	0	0	0	0
BGIR9823_81007	EHBP1L1	mRNA	0	0	0	0	0	0	0	0	0	0	0	0	0	0	0	0	0	0	0	0	0
BGIR9823_81008	EHBP1L1	mRNA	0	0	0	0	0	0	0	0	0	0	0	0	0	0	0	0	0	0	0	0	0
BGIR9823_81009	EHBP1L1	mRNA	0	0	0	0	0	0	0	0	0	0	0	0	0	0	0	0	0	0	0	0	0
BGIR9823_81010	EHBP1L1	mRNA	0	0	0	0	0	0	0	0	0	0	0	0	0	0	0	0	0	0	0	0	0
BGIR9823_81011	SSSCA1	mRNA	0	0	0	0	0	0	0	0	0	0	0	0	0	0	0	0	0	0	0	0	0
BGIR9823_81012	FRMD8	lncRNA	0	0	0	0	0	0	0	0	0	0	0	0	0	0	0	0	0	0	0	0	0
BGIR9823_81013	LOC102163816	lncRNA	0	0	0	0	0	0	0	0	0	0	0	0	0	0	0	0	0	0	0	0	0
BGIR9823_81014	LOC102163816	lncRNA	0	0	0	0	0	0	0	0	0	0	0	0	0	0	0	0	0	0	0	0	0
BGIR9823_81015	LOC102163816	lncRNA	0	0	0	0	0	0	0	0	0	0	0	0	0	0	0	0	0	0	0	0	0
BGIR9823_81016	LOC102163816	lncRNA	0	0	0	0	0	0	0	0	0	0	0	0	0	0	0	0	0	0	0	0	0
BGIR9823_81017	LOC102163816	lncRNA	0	0	0	0	0	0	0	0	0	0	0	0	0	0	0	0	0	0	0	0	0
BGIR9823_81018	LOC102163816	lncRNA	0	0	0	0	0	0	0	0	0	0	0	0	0	0	0	0	0	0	0	0	0
BGIR9823_81019	LOC100513133	lncRNA	0	0	0	0	0	0	0	0	0	0	0	0	0	0	0	0	0	0	0	0	0
BGIR9823_84704	LOC102159820	mRNA	0	0	0	0	0	0	0	0	0	0	0	0	0	0	0	0	0	0	0	0	0
BGIR9823_84732	RANGAP1	mRNA	0	0	0	0	0	0	0	0	0	0	0	0	0	0	0	0	0	0	0	0	0

RNA ID	Symbol	Type	updown_BGIR9823_84443	updown_BGIR9823_81960	updown_BGIR9823_82315	updown_BGIR9823_82966	updown_BGIR9823_83074	updown_BGIR9823_82386	updown_BGIR9823_82709	updown_BGIR9823_83417	updown_BGIR9823_85418	updown_BGIR9823_87926	updown_BGIR9823_88529	updown_BGIR9823_88698	updown_BGIR9823_86965	updown_BGIR9823_89012	updown_BGIR9823_92131	updown_BGIR9823_90639	updown_BGIR9823_95770	updown_BGIR9823_92788	updown_BGIR9823_93572	updown_BGIR9823_96356	updown_BGIR9823_94689
BGIR9823_84733	RANGAP1	mRNA	0	0	0	0	0	0	0	0	0	0	0	0	0	0	0	0	0	0	0	0	0
BGIR9823_85245	CRADD	lncRNA	0	0	0	0	0	0	0	0	0	0	0	0	0	0	0	0	0	0	0	0	0
BGIR9823_85246	LOC100738154	lncRNA	0	0	0	0	0	0	0	0	0	0	0	0	0	0	0	0	0	0	0	0	0
BGIR9823_85293	TRMU	mRNA	0	0	0	0	0	0	0	0	0	0	0	0	0	0	0	0	0	0	0	0	0
BGIR9823_85294	TRMU	mRNA	0	0	0	0	0	0	0	0	0	0	0	0	0	0	0	0	0	0	0	0	0
BGIR9823_85295	TTC38	mRNA	0	0	0	0	0	0	0	0	0	0	0	0	0	0	0	0	0	0	0	0	0
BGIR9823_85296	PKDREJ	lncRNA	0	0	0	0	0	0	0	0	0	0	0	0	0	0	0	0	0	0	0	0	0
BGIR9823_85297	PPARA	mRNA	0	0	0	0	0	0	0	0	0	0	0	0	0	0	0	0	0	0	0	0	0
BGIR9823_85298	PPARA	mRNA	0	0	0	0	0	0	0	0	0	0	0	0	0	0	0	0	0	0	0	0	0
BGIR9823_85299	PPARA	mRNA	0	0	0	0	0	0	0	0	0	0	0	0	0	0	0	0	0	0	0	0	0
BGIR9823_85300	PPARA	lncRNA	0	0	0	0	0	0	0	0	0	0	0	0	0	0	0	0	0	0	0	0	0
BGIR9823_85301	LOC110260611	mRNA	0	0	0	0	0	0	0	0	0	0	0	0	0	0	0	0	0	0	0	0	0
BGIR9823_85317	ACO2	mRNA	0	0	0	0	0	0	0	0	0	0	0	0	0	0	0	0	0	0	0	0	0
BGIR9823_85318	LOC110260629	lncRNA	0	0	0	0	0	0	0	0	0	0	0	0	0	0	0	0	0	0	0	0	0
BGIR9823_85319	TEF	mRNA	0	0	0	0	0	0	0	0	0	0	0	0	0	0	0	0	0	0	0	0	0
BGIR9823_85320	ZC3H7B	mRNA	0	0	0	0	0	0	0	0	0	0	0	0	0	0	0	0	0	0	0	0	0
BGIR9823_85345	SYNGR1	mRNA	0	0	0	0	0	0	0	0	0	0	0	0	0	0	0	0	0	0	0	0	0
BGIR9823_85835	CRADD	mRNA	0	0	0	0	0	0	0	0	0	0	0	0	0	0	0	0	0	0	0	0	0
BGIR9823_85839	CRADD	lncRNA	0	0	0	0	0	0	0	0	0	0	0	0	0	0	0	0	0	0	0	0	0
BGIR9823_85840	CRADD	lncRNA	0	0	0	0	0	0	0	0	0	0	0	0	0	0	0	0	0	0	0	0	0
BGIR9823_85841	CRADD	lncRNA	0	0	0	0	0	0	0	0	0	0	0	0	0	0	0	0	0	0	0	0	0
BGIR9823_85930	LOC106510465	mRNA	0	0	0	0	0	0	0	0	0	0	0	0	0	0	0	0	0	0	0	0	0
BGIR9823_85931	MAF	lncRNA	0	0	0	0	0	0	0	0	0	0	0	0	0	0	0	0	0	0	0	0	0
BGIR9823_85932	MAF	lncRNA	0	0	0	0	0	0	0	0	0	0	0	0	0	0	0	0	0	0	0	0	0
BGIR9823_87139	LOC106510467	lncRNA	0	0	0	0	0	0	0	0	0	0	0	0	0	0	0	0	0	0	0	0	0
BGIR9823_88187	WRNIP1	mRNA	0	0	0	0	0	0	0	0	0	0	0	0	0	0	0	0	0	0	0	0	0
BGIR9823_88188	NQO2	mRNA	0	0	0	0	0	0	0	0	0	0	0	0	0	0	0	0	0	0	0	0	0
BGIR9823_88189	NQO2	mRNA	0	0	0	0	0	0	0	0	0	0	0	0	0	0	0	0	0	0	0	0	0
BGIR9823_88190	RIPK1	mRNA	0	0	0	0	0	0	0	0	0	0	0	0	0	0	0	0	0	0	0	0	0
BGIR9823_88191	RIPK1	mRNA	0	0	0	0	0	0	0	0	0	0	0	0	0	0	0	0	0	0	0	0	0
BGIR9823_88192	RIPK1	mRNA	0	0	0	0	0	0	0	0	0	0	0	0	0	0	0	0	0	0	0	0	0
BGIR9823_88193	RIPK1	mRNA	0	0	0	0	0	0	0	0	0	0	0	0	0	0	0	0	0	0	0	0	0
BGIR9823_88196	PRPF4B	mRNA	0	0	0	0	0	0	0	0	0	0	0	0	0	0	0	0	0	0	0	0	0
BGIR9823_88197	PRPF4B	mRNA	0	0	0	0	0	0	0	0	0	0	0	0	0	0	0	0	0	0	0	0	0
BGIR9823_88198	PRPF4B	mRNA	0	0	0	0	0	0	0	0	0	0	0	0	0	0	0	0	0	0	0	0	0
BGIR9823_88211	TMEM170B	lncRNA	0	0	0	0	0	0	0	0	0	0	0	0	0	0	0	0	0	0	0	0	0
BGIR9823_88212	TMEM170B	lncRNA	0	0	0	0	0	0	0	0	0	0	0	0	0	0	0	0	0	0	0	0	0
BGIR9823_88213	TMEM170B	lncRNA	0	0	0	0	0	0	0	0	0	0	0	0	0	0	0	0	0	0	0	0	0
BGIR9823_88214	TMEM170B	lncRNA	0	0	0	0	0	0	0	0	0	0	0	0	0	0	0	0	0	0	0	0	0
BGIR9823_88217	PHACTR1	mRNA	0	0	0	0	0	0	0	0	0	0	0	0	0	0	0	0	0	0	0	0	0
BGIR9823_88218	PHACTR1	lncRNA	0	0	0	0	0	0	0	0	0	0	0	0	0	0	0	0	0	0	0	0	0
BGIR9823_89017	MYLK4	lncRNA	0	0	0	0	0	0	0	0	0	0	0	0	0	0	0	0	0	0	0	0	0
BGIR9823_89019	SERPINB1	lncRNA	0	0	0	0	0	0	0	0	0	0	0	0	0	0	0	0	0	0	0	0	0

RNA ID	Symbol	Type	updown_BGIR9823_84443	updown_BGIR9823_81960	updown_BGIR9823_82315	updown_BGIR9823_82966	updown_BGIR9823_83074	updown_BGIR9823_82386	updown_BGIR9823_82709	updown_BGIR9823_83417	updown_BGIR9823_85418	updown_BGIR9823_87926	updown_BGIR9823_88529	updown_BGIR9823_88698	updown_BGIR9823_86965	updown_BGIR9823_89012	updown_BGIR9823_92131	updown_BGIR9823_90639	updown_BGIR9823_95770	updown_BGIR9823_92788	updown_BGIR9823_93572	updown_BGIR9823_96356	updown_BGIR9823_94689
BGIR9823_89020	SERPINB9	mRNA	0	0	0	0	0	0	0	0	0	0	0	0	0	0	0	0	0	0	0	0	0
BGIR9823_89021	RIPK1	lncRNA	0	0	0	0	0	0	0	0	0	0	0	0	0	0	0	0	0	0	0	0	0
BGIR9823_89025	PXDC1	mRNA	0	0	0	0	0	0	0	0	0	0	0	0	0	0	0	0	0	0	0	0	0
BGIR9823_89026	PXDC1	lncRNA	0	0	0	0	0	0	0	0	0	0	0	0	0	0	0	0	0	0	0	0	0
BGIR9823_89027	LOC102161744	lncRNA	0	0	0	0	0	0	0	0	0	0	0	0	0	0	0	0	0	0	0	0	0
BGIR9823_89040	TBC1D7	lncRNA	0	0	0	0	0	0	0	0	0	0	0	0	0	0	0	0	0	0	0	0	0
BGIR9823_89041	TBC1D7	lncRNA	0	0	0	0	0	0	0	0	0	0	0	0	0	0	0	0	0	0	0	0	0
BGIR9823_89042	TBC1D7	lncRNA	0	0	0	0	0	0	0	0	0	0	0	0	0	0	0	0	0	0	0	0	0
BGIR9823_89043	TBC1D7	lncRNA	0	0	0	0	0	0	0	0	0	0	0	0	0	0	0	0	0	0	0	0	0
BGIR9823_89044	GFOD1	lncRNA	0	0	0	0	0	0	0	0	0	0	0	0	0	0	0	0	0	0	0	0	0
BGIR9823_89045	GFOD1	mRNA	0	0	0	0	0	0	0	0	0	0	0	0	0	0	0	0	0	0	0	0	0
BGIR9823_89046	GFOD1	mRNA	0	0	0	0	0	0	0	0	0	0	0	0	0	0	0	0	0	0	0	0	0
BGIR9823_89047	GFOD1	mRNA	0	0	0	0	0	0	0	0	0	0	0	0	0	0	0	0	0	0	0	0	0
BGIR9823_89048	GFOD1	lncRNA	0	0	0	0	0	0	0	0	0	0	0	0	0	0	0	0	0	0	0	0	0
BGIR9823_89049	GFOD1	lncRNA	0	0	0	0	0	0	0	0	0	0	0	0	0	0	0	0	0	0	0	0	0
BGIR9823_90771	NEU3	lncRNA	0	0	0	0	0	0	0	0	0	0	0	0	0	0	0	0	0	0	0	0	0
BGIR9823_90772	SLCO2B1	mRNA	0	0	0	0	0	0	0	0	0	0	0	0	0	0	0	0	0	0	0	0	0
BGIR9823_90913	LOC110255435	lncRNA	0	0	0	0	0	0	0	0	0	0	0	0	0	0	0	0	0	0	0	0	0
BGIR9823_90934	LOC110255441	lncRNA	0	0	0	0	0	0	0	0	0	0	0	0	0	0	0	0	0	0	0	0	0
BGIR9823_90935	CCDC84	mRNA	0	0	0	0	0	0	0	0	0	0	0	0	0	0	0	0	0	0	0	0	0
BGIR9823_90936	TRAPPC4	mRNA	0	0	0	0	0	0	0	0	0	0	0	0	0	0	0	0	0	0	0	0	0
BGIR9823_90937	HMBS	mRNA	0	0	0	0	0	0	0	0	0	0	0	0	0	0	0	0	0	0	0	0	0
BGIR9823_90956	SORL1	mRNA	0	0	0	0	0	0	0	0	0	0	0	0	0	0	0	0	0	0	0	0	0
BGIR9823_90957	SORL1	mRNA	0	0	0	0	0	0	0	0	0	0	0	0	0	0	0	0	0	0	0	0	0
BGIR9823_90958	SORL1	mRNA	0	0	0	0	0	0	0	0	0	0	0	0	0	0	0	0	0	0	0	0	0
BGIR9823_90960	CLMP	lncRNA	0	0	0	0	0	0	0	0	0	0	0	0	0	0	0	0	0	0	0	0	0
BGIR9823_91161	LOC100522267	mRNA	0	0	0	0	0	0	0	0	0	0	0	0	0	0	0	0	0	0	0	0	0
BGIR9823_91162	LOC100522455	mRNA	0	0	0	0	0	0	0	0	0	0	0	0	0	0	0	0	0	0	0	0	0
BGIR9823_91163	ABCB1	mRNA	0	0	0	0	0	0	0	0	0	0	0	0	0	0	0	0	0	0	0	0	0
BGIR9823_91402	HBB	mRNA	0	0	0	0	0	0	0	0	0	0	0	0	0	0	0	0	0	0	0	0	0
BGIR9823_91403	HBB	mRNA	0	0	0	0	0	0	0	0	0	0	0	0	0	0	0	0	0	0	0	0	0
BGIR9823_91404	HBB	mRNA	0	0	0	0	0	0	0	0	0	0	0	0	0	0	0	0	0	0	0	0	0
BGIR9823_91405	HBB	mRNA	0	0	0	0	0	0	0	0	0	0	0	0	0	0	0	0	0	0	0	0	0
BGIR9823_91406	HBB	mRNA	0	0	0	0	0	0	0	0	0	0	0	0	0	0	0	0	0	0	0	0	0
BGIR9823_91407	HBB	mRNA	0	0	0	0	0	0	0	0	0	0	0	0	0	0	0	0	0	0	0	0	0
BGIR9823_91408	HBB	mRNA	0	0	0	0	0	0	0	0	0	0	0	0	0	0	0	0	0	0	0	0	0
BGIR9823_91409	HBB	mRNA	0	0	0	0	0	0	0	0	0	0	0	0	0	0	0	0	0	0	0	0	0
BGIR9823_91625	BCL9L	lncRNA	0	0	0	0	0	0	0	0	0	0	0	0	0	0	0	0	0	0	0	0	0
BGIR9823_91626	BCL9L	lncRNA	0	0	0	0	0	0	0	0	0	0	0	0	0	0	0	0	0	0	0	0	0
BGIR9823_91627	RPS25	mRNA	0	0	0	0	0	0	0	0	0	0	0	0	0	0	0	0	0	0	0	0	0
BGIR9823_91628	RPS25	mRNA	0	0	0	0	0	0	0	0	0	0	0	0	0	0	0	0	0	0	0	0	0
BGIR9823_91629	SLC37A4	mRNA	0	0	0	0	0	0	0	0	0	0	0	0	0	0	0	0	0	0	0	0	0
BGIR9823_91630	HYOU1	mRNA	0	0	0	0	0	0	0	0	0	0	0	0	0	0	0	0	0	0	0	0	0

RNA ID	Symbol	Type	updown_BGIR9823_84443	updown_BGIR9823_81960	updown_BGIR9823_82315	updown_BGIR9823_82966	updown_BGIR9823_83074	updown_BGIR9823_82386	updown_BGIR9823_82709	updown_BGIR9823_83417	updown_BGIR9823_85418	updown_BGIR9823_87926	updown_BGIR9823_88529	updown_BGIR9823_88698	updown_BGIR9823_86965	updown_BGIR9823_89012	updown_BGIR9823_92131	updown_BGIR9823_90639	updown_BGIR9823_95770	updown_BGIR9823_92788	updown_BGIR9823_93572	updown_BGIR9823_96356	updown_BGIR9823_94689
BGIR9823_91640	CLMP	mRNA	0	0	0	0	0	0	0	0	0	0	0	0	0	0	0	0	0	0	0	0	0
BGIR9823_91819	RUNDC3B	lncRNA	0	0	0	0	0	0	0	0	0	0	0	0	0	0	0	0	0	0	0	0	0
BGIR9823_91820	RUNDC3B	lncRNA	0	0	0	0	0	0	0	0	0	0	0	0	0	0	0	0	0	0	0	0	0
BGIR9823_91821	RUNDC3B	lncRNA	0	0	0	0	0	0	0	0	0	0	0	0	0	0	0	0	0	0	0	0	0
BGIR9823_91822	RUNDC3B	lncRNA	0	0	0	0	0	0	0	0	0	0	0	0	0	0	0	0	0	0	0	0	0
BGIR9823_91823	RUNDC3B	lncRNA	0	0	0	0	0	0	0	0	0	0	0	0	0	0	0	0	0	0	0	0	0
BGIR9823_91824	RUNDC3B	lncRNA	0	0	0	0	0	0	0	0	0	0	0	0	0	0	0	0	0	0	0	0	0
BGIR9823_91825	RUNDC3B	lncRNA	0	0	0	0	0	0	0	0	0	0	0	0	0	0	0	0	0	0	0	0	0
BGIR9823_91826	LOC102164596	lncRNA	0	0	0	0	0	0	0	0	0	0	0	0	0	0	0	0	0	0	0	0	0
BGIR9823_92993	KATNAL1	mRNA	0	0	0	0	0	0	0	0	0	0	0	0	0	0	0	0	0	0	0	0	0
BGIR9823_92994	KATNAL1	mRNA	0	0	0	0	0	0	0	0	0	0	0	0	0	0	0	0	0	0	0	0	0
BGIR9823_92995	KATNAL1	mRNA	0	0	0	0	0	0	0	0	0	0	0	0	0	0	0	0	0	0	0	0	0
BGIR9823_92996	KATNAL1	mRNA	0	0	0	0	0	0	0	0	0	0	0	0	0	0	0	0	0	0	0	0	0
BGIR9823_92997	KATNAL1	mRNA	0	0	0	0	0	0	0	0	0	0	0	0	0	0	0	0	0	0	0	0	0
BGIR9823_92999	LOC110255292	lncRNA	0	0	0	0	0	0	0	0	0	0	0	0	0	0	0	0	0	0	0	0	0
BGIR9823_93000	LOC102166871	lncRNA	0	0	0	0	0	0	0	0	0	0	0	0	0	0	0	0	0	0	0	0	0
BGIR9823_98051	LOC102164998	lncRNA	0	0	0	0	0	0	0	0	0	0	0	0	0	0	0	0	0	0	0	0	0
BGIR9823_99244	LOC102167708	lncRNA	0	0	0	0	0	0	0	0	0	0	0	0	0	0	0	0	0	0	0	0	0
NM_001004034.1	HMGB1	mRNA	0	0	0	0	0	0	0	0	0	0	0	0	0	0	0	0	0	0	0	0	0
NM_001044526.1	PPARA	mRNA	0	0	0	0	0	0	0	0	0	0	0	0	0	0	0	0	0	0	0	0	0
NM_001111256.1	SERPINB9	mRNA	0	0	0	0	0	0	0	0	0	0	0	0	0	0	0	0	0	0	0	0	0
NM_001113443.1	ECI2	mRNA	0	0	0	0	0	0	0	0	0	0	0	0	0	0	0	0	0	0	0	0	0
NM_001144841.1	HBB	mRNA	0	0	0	0	0	0	0	0	0	0	0	0	0	0	0	0	0	0	0	0	0
NM_001199719.1	SLC37A4	mRNA	0	0	0	0	0	0	0	0	0	0	0	0	0	0	0	0	0	0	0	0	0
NM_001243907.1	HSPA8	mRNA	0	0	0	0	0	0	0	0	0	0	0	0	0	0	0	0	0	0	0	0	0
NM_001244063.1	RPL3	mRNA	0	0	0	0	0	0	0	0	0	0	0	0	0	0	0	0	0	0	0	0	0
NM_001244066.1	SYNGR1	mRNA	0	0	0	0	0	0	0	0	0	0	0	0	0	0	0	0	0	0	0	0	0
NM_001244396.1	CCDC84	mRNA	0	0	0	0	0	0	0	0	0	0	0	0	0	0	0	0	0	0	0	0	0
NM_001287846.1	RHEB	mRNA	0	0	0	0	0	0	0	0	0	0	0	0	0	0	0	0	0	0	0	0	0
NM_213954.1	ACO2	mRNA	0	0	0	0	0	0	0	0	0	0	0	0	0	0	0	0	0	0	0	0	0
NM_214012.1	UPK2	mRNA	0	0	0	0	0	0	0	0	0	0	0	0	0	0	0	0	0	0	0	0	0
URS0000BC92F4	LOC102167708	lncRNA	0	0	0	0	0	0	0	0	0	0	0	0	0	0	0	0	0	0	0	0	0
XM_001928309.6	TBC1D7	mRNA	0	0	0	0	0	0	0	0	0	0	0	0	0	0	0	0	0	0	0	0	0
XM_001928852.5	PXDC1	mRNA	0	0	0	0	0	0	0	0	0	0	0	0	0	0	0	0	0	0	0	0	0
XM_003122534.4	KCNK7	mRNA	0	0	0	0	0	0	0	0	0	0	0	0	0	0	0	0	0	0	0	0	0
XM_003122558.5	SCYL1	mRNA	0	0	0	0	0	0	0	0	0	0	0	0	0	0	0	0	0	0	0	0	0
XM_003122562.5	FRMD8	mRNA	0	0	0	0	0	0	0	0	0	0	0	0	0	0	0	0	0	0	0	0	0
XM_003125951.6	TTC38	mRNA	0	0	0	0	0	0	0	0	0	0	0	0	0	0	0	0	0	0	0	0	0
XM_003125953.4	CDPF1	mRNA	0	0	0	0	0	0	0	0	0	0	0	0	0	0	0	0	0	0	0	0	0
XM_003126012.6	CBX7	mRNA	0	0	0	0	0	0	0	0	0	0	0	0	0	0	0	0	0	0	0	0	0
XM_003128161.4	RIPK1	mRNA	0	0	0	0	0	0	0	0	0	0	0	0	0	0	0	0	0	0	0	0	0
XM_003129508.2	LOC100514417	mRNA	0	0	0	0	0	0	0	0	0	0	0	0	0	0	0	0	0	0	0	0	0
XM_003129512.2	LOC100515268	mRNA	0	0	0	0	0	0	0	0	0	0	0	0	0	0	0	0	0	0	0	0	0

RNA ID	Symbol	Type	updown_BGIR9823_84443	updown_BGIR9823_81960	updown_BGIR9823_82315	updown_BGIR9823_82966	updown_BGIR9823_83074	updown_BGIR9823_82386	updown_BGIR9823_82709	updown_BGIR9823_83417	updown_BGIR9823_85418	updown_BGIR9823_87926	updown_BGIR9823_88529	updown_BGIR9823_88698	updown_BGIR9823_86965	updown_BGIR9823_89012	updown_BGIR9823_92131	updown_BGIR9823_90639	updown_BGIR9823_95770	updown_BGIR9823_92788	updown_BGIR9823_93572	updown_BGIR9823_96356	updown_BGIR9823_94689
XM_003129514.3	LOC100515618	mRNA	0	0	0	0	0	0	0	0	0	0	0	0	0	0	0	0	0	0	0	0	0
XM_003129519.2	LOC100517595	mRNA	0	0	0	0	0	0	0	0	0	0	0	0	0	0	0	0	0	0	0	0	0
XM_003129529.4	LOC100519910	mRNA	0	0	0	0	0	0	0	0	0	0	0	0	0	0	0	0	0	0	0	0	0
XM_003129915.4	CXCR5	mRNA	0	0	0	0	0	0	0	0	0	0	0	0	0	0	0	0	0	0	0	0	0
XM_003129916.5	BCL9L	mRNA	0	0	0	0	0	0	0	0	0	0	0	0	0	0	0	0	0	0	0	0	0
XM_003129918.4	TRAPPC4	mRNA	0	0	0	0	0	0	0	0	0	0	0	0	0	0	0	0	0	0	0	0	0
XM_003129919.5	RPS25	mRNA	0	0	0	0	0	0	0	0	0	0	0	0	0	0	0	0	0	0	0	0	0
XM_003129920.3	RPS25	mRNA	0	0	0	0	0	0	0	0	0	0	0	0	0	0	0	0	0	0	0	0	0
XM_003129922.4	HYOU1	mRNA	0	0	0	0	0	0	0	0	0	0	0	0	0	0	0	0	0	0	0	0	0
XM_003129923.6	VPS11	mRNA	0	0	0	0	0	0	0	0	0	0	0	0	0	0	0	0	0	0	0	0	0
XM_003129944.4	SC5D	mRNA	0	0	0	0	0	0	0	0	0	0	0	0	0	0	0	0	0	0	0	0	0
XM_003357161.1	LOC100620971	mRNA	0	0	0	0	0	0	0	0	0	0	0	0	0	0	0	0	0	0	0	0	0
XM_003482119.4	SERPINB1	mRNA	0	0	0	0	0	0	0	0	0	0	0	0	0	0	0	0	0	0	0	0	0
XM_003482132.4	TMEM170B	mRNA	0	0	0	0	0	0	0	0	0	0	0	0	0	0	0	0	0	0	0	0	0
XM_003482133.4	GFOD1	mRNA	0	0	0	0	0	0	0	0	0	0	0	0	0	0	0	0	0	0	0	0	0
XM_003482563.4	SPCS2	mRNA	0	0	0	0	0	0	0	0	0	0	0	0	0	0	0	0	0	0	0	0	0
XM_003482565.4	NEU3	mRNA	0	0	0	0	0	0	0	0	0	0	0	0	0	0	0	0	0	0	0	0	0
XM_003482567.1	LOC100739359	mRNA	0	0	0	0	0	0	0	0	0	0	0	0	0	0	0	0	0	0	0	0	0
XM_003482630.3	BSX	mRNA	0	0	0	0	0	0	0	0	0	0	0	0	0	0	0	0	0	0	0	0	0
XM_005653107.3	CHADL	mRNA	0	0	0	0	0	0	0	0	0	0	0	0	0	0	0	0	0	0	0	0	0
XM_005653640.1	LOC100621181	mRNA	0	0	0	0	0	0	0	0	0	0	0	0	0	0	0	0	0	0	0	0	0
XM_005654266.3	PRKAG2	mRNA	0	0	0	0	0	0	0	0	0	0	0	0	0	0	0	0	0	0	0	0	0
XM_005655470.3	CDPF1	mRNA	0	0	0	0	0	0	0	0	0	0	0	0	0	0	0	0	0	0	0	0	0
XM_005660703.3	LTBP3	mRNA	0	0	0	0	0	0	0	0	0	0	0	0	0	0	0	0	0	0	0	0	0
XM_005660705.3	SCYL1	mRNA	0	0	0	0	0	0	0	0	0	0	0	0	0	0	0	0	0	0	0	0	0
XM_005660707.3	SLC25A45	mRNA	0	0	0	0	0	0	0	0	0	0	0	0	0	0	0	0	0	0	0	0	0
XM_005665536.3	RIPK1	mRNA	0	0	0	0	0	0	0	0	0	0	0	0	0	0	0	0	0	0	0	0	0
XM_005665537.3	RIPK1	mRNA	0	0	0	0	0	0	0	0	0	0	0	0	0	0	0	0	0	0	0	0	0
XM_005665538.3	RIPK1	mRNA	0	0	0	0	0	0	0	0	0	0	0	0	0	0	0	0	0	0	0	0	0
XM_005665575.3	PHACTR1	mRNA	0	0	0	0	0	0	0	0	0	0	0	0	0	0	0	0	0	0	0	0	0
XM_005665576.3	TBC1D7	mRNA	0	0	0	0	0	0	0	0	0	0	0	0	0	0	0	0	0	0	0	0	0
XM_005667128.3	LOC100627340	mRNA	0	0	0	0	0	0	0	0	0	0	0	0	0	0	0	0	0	0	0	0	0
XM_005667397.2	CCDC84	mRNA	0	0	0	0	0	0	0	0	0	0	0	0	0	0	0	0	0	0	0	0	0
XM_005667398.3	SLC37A4	mRNA	0	0	0	0	0	0	0	0	0	0	0	0	0	0	0	0	0	0	0	0	0
XM_005667402.3	SLC37A4	mRNA	0	0	0	0	0	0	0	0	0	0	0	0	0	0	0	0	0	0	0	0	0
XM_005667403.3	SLC37A4	mRNA	0	0	0	0	0	0	0	0	0	0	0	0	0	0	0	0	0	0	0	0	0
XM_005667413.3	BCL9L	mRNA	0	0	0	0	0	0	0	0	0	0	0	0	0	0	0	0	0	0	0	0	0
XM_005667414.3	FOXR1	mRNA	0	0	0	0	0	0	0	0	0	0	0	0	0	0	0	0	0	0	0	0	0
XM_005667416.3	HYOU1	mRNA	0	0	0	0	0	0	0	0	0	0	0	0	0	0	0	0	0	0	0	0	0
XM_005667419.3	HYOU1	mRNA	0	0	0	0	0	0	0	0	0	0	0	0	0	0	0	0	0	0	0	0	0
XM_005667420.3	HYOU1	mRNA	0	0	0	0	0	0	0	0	0	0	0	0	0	0	0	0	0	0	0	0	0
XM_005667440.2	JHY	mRNA	0	0	0	0	0	0	0	0	0	0	0	0	0	0	0	0	0	0	0	0	0
XM_005668316.3	HMGB1	mRNA	0	0	0	0	0	0	0	0	0	0	0	0	0	0	0	0	0	0	0	0	0

RNA ID	Symbol	Type	updown_BGIR9823_84443	updown_BGIR9823_81960	updown_BGIR9823_82315	updown_BGIR9823_82966	updown_BGIR9823_83074	updown_BGIR9823_82386	updown_BGIR9823_82709	updown_BGIR9823_83417	updown_BGIR9823_85418	updown_BGIR9823_87926	updown_BGIR9823_88529	updown_BGIR9823_88698	updown_BGIR9823_86965	updown_BGIR9823_89012	updown_BGIR9823_92131	updown_BGIR9823_90639	updown_BGIR9823_95770	updown_BGIR9823_92788	updown_BGIR9823_93572	updown_BGIR9823_96356	updown_BGIR9823_94689
XM_005668317.3	HMGB1	mRNA	0	0	0	0	0	0	0	0	0	0	0	0	0	0	0	0	0	0	0	0	0
XM_005668318.3	HMGB1	mRNA	0	0	0	0	0	0	0	0	0	0	0	0	0	0	0	0	0	0	0	0	0
XM_005671489.3	EMX2	mRNA	0	0	0	0	0	0	0	0	0	0	0	0	0	0	0	0	0	0	0	0	0
XM_005672397.3	MYO10	mRNA	0	0	0	0	0	0	0	0	0	0	0	0	0	0	0	0	0	0	0	0	0
XM_013977400.2	FAM50B	mRNA	0	0	0	0	0	0	0	0	0	0	0	0	0	0	0	0	0	0	0	0	0
XM_013977425.2	NEDD9	mRNA	0	0	0	0	0	0	0	0	0	0	0	0	0	0	0	0	0	0	0	0	0
XM_013977430.2	TBC1D7	mRNA	0	0	0	0	0	0	0	0	0	0	0	0	0	0	0	0	0	0	0	0	0
XM_013977431.2	TBC1D7	mRNA	0	0	0	0	0	0	0	0	0	0	0	0	0	0	0	0	0	0	0	0	0
XM_013977432.2	TBC1D7	mRNA	0	0	0	0	0	0	0	0	0	0	0	0	0	0	0	0	0	0	0	0	0
XM_013977433.2	GFOD1	mRNA	0	0	0	0	0	0	0	0	0	0	0	0	0	0	0	0	0	0	0	0	0
XM_013977434.2	GFOD1	mRNA	0	0	0	0	0	0	0	0	0	0	0	0	0	0	0	0	0	0	0	0	0
XM_013979449.2	SLC37A4	mRNA	0	0	0	0	0	0	0	0	0	0	0	0	0	0	0	0	0	0	0	0	0
XM_013979456.2	BCL9L	mRNA	0	0	0	0	0	0	0	0	0	0	0	0	0	0	0	0	0	0	0	0	0
XM_013979457.2	BCL9L	mRNA	0	0	0	0	0	0	0	0	0	0	0	0	0	0	0	0	0	0	0	0	0
XM_013979458.2	BCL9L	mRNA	0	0	0	0	0	0	0	0	0	0	0	0	0	0	0	0	0	0	0	0	0
XM_013979459.2	BCL9L	mRNA	0	0	0	0	0	0	0	0	0	0	0	0	0	0	0	0	0	0	0	0	0
XM_013979461.2	TRAPPC4	mRNA	0	0	0	0	0	0	0	0	0	0	0	0	0	0	0	0	0	0	0	0	0
XM_013979463.2	HYOU1	mRNA	0	0	0	0	0	0	0	0	0	0	0	0	0	0	0	0	0	0	0	0	0
XM_013979484.2	LOC100523684	mRNA	0	0	0	0	0	0	0	0	0	0	0	0	0	0	0	0	0	0	0	0	0
XM_013979486.2	LOC100523684	mRNA	0	0	0	0	0	0	0	0	0	0	0	0	0	0	0	0	0	0	0	0	0
XM_013979491.2	JHY	mRNA	0	0	0	0	0	0	0	0	0	0	0	0	0	0	0	0	0	0	0	0	0
XM_013980418.2	KATNAL1	mRNA	0	0	0	0	0	0	0	0	0	0	0	0	0	0	0	0	0	0	0	0	0
XM_013988182.2	TTC38	mRNA	0	0	0	0	0	0	0	0	0	0	0	0	0	0	0	0	0	0	0	0	0
XM_013988183.2	TTC38	mRNA	0	0	0	0	0	0	0	0	0	0	0	0	0	0	0	0	0	0	0	0	0
XM_013989589.2	LOC100522267	mRNA	0	0	0	0	0	0	0	0	0	0	0	0	0	0	0	0	0	0	0	0	0
XM_013989592.2	LOC100522455	mRNA	0	0	0	0	0	0	0	0	0	0	0	0	0	0	0	0	0	0	0	0	0
XM_013989593.2	LOC100522455	mRNA	0	0	0	0	0	0	0	0	0	0	0	0	0	0	0	0	0	0	0	0	0
XM_013994099.2	FRMD8	mRNA	0	0	0	0	0	0	0	0	0	0	0	0	0	0	0	0	0	0	0	0	0
XM_013994100.2	SLC25A45	mRNA	0	0	0	0	0	0	0	0	0	0	0	0	0	0	0	0	0	0	0	0	0
XM_013994102.2	SLC25A45	mRNA	0	0	0	0	0	0	0	0	0	0	0	0	0	0	0	0	0	0	0	0	0
XM_013994103.2	SLC25A45	mRNA	0	0	0	0	0	0	0	0	0	0	0	0	0	0	0	0	0	0	0	0	0
XM_013994104.2	SLC25A45	mRNA	0	0	0	0	0	0	0	0	0	0	0	0	0	0	0	0	0	0	0	0	0
XM_013997510.2	CBX7	mRNA	0	0	0	0	0	0	0	0	0	0	0	0	0	0	0	0	0	0	0	0	0
XM_013999070.2	LOC100520088	mRNA	0	0	0	0	0	0	0	0	0	0	0	0	0	0	0	0	0	0	0	0	0
XM_021062230.1	LOC100517771	mRNA	0	0	0	0	0	0	0	0	0	0	0	0	0	0	0	0	0	0	0	0	0
XM_021062231.1	LOC100517957	mRNA	0	0	0	0	0	0	0	0	0	0	0	0	0	0	0	0	0	0	0	0	0
XM_021062367.1	LOC110255365	mRNA	0	0	0	0	0	0	0	0	0	0	0	0	0	0	0	0	0	0	0	0	0
XM_021062369.1	LOC100737110	mRNA	0	0	0	0	0	0	0	0	0	0	0	0	0	0	0	0	0	0	0	0	0
XM_021062370.1	LOC110255368	mRNA	0	0	0	0	0	0	0	0	0	0	0	0	0	0	0	0	0	0	0	0	0
XM_021062371.1	LOC100515441	mRNA	0	0	0	0	0	0	0	0	0	0	0	0	0	0	0	0	0	0	0	0	0
XM_021062374.1	LOC110255371	mRNA	0	0	0	0	0	0	0	0	0	0	0	0	0	0	0	0	0	0	0	0	0
XM_021062375.1	LOC100626951	mRNA	0	0	0	0	0	0	0	0	0	0	0	0	0	0	0	0	0	0	0	0	0
XM_021062491.1	XRRA1	mRNA	0	0	0	0	0	0	0	0	0	0	0	0	0	0	0	0	0	0	0	0	0

RNA ID	Symbol	Type	updown_BGIR9823_84443	updown_BGIR9823_81960	updown_BGIR9823_82315	updown_BGIR9823_82966	updown_BGIR9823_83074	updown_BGIR9823_82386	updown_BGIR9823_82709	updown_BGIR9823_83417	updown_BGIR9823_85418	updown_BGIR9823_87926	updown_BGIR9823_88529	updown_BGIR9823_88698	updown_BGIR9823_86965	updown_BGIR9823_89012	updown_BGIR9823_92131	updown_BGIR9823_90639	updown_BGIR9823_95770	updown_BGIR9823_92788	updown_BGIR9823_93572	updown_BGIR9823_96356	updown_BGIR9823_94689
XM_021062493.1	XRRA1	mRNA	0	0	0	0	0	0	0	0	0	0	0	0	0	0	0	0	0	0	0	0	0
XM_021062494.1	XRRA1	mRNA	0	0	0	0	0	0	0	0	0	0	0	0	0	0	0	0	0	0	0	0	0
XM_021062495.1	XRRA1	mRNA	0	0	0	0	0	0	0	0	0	0	0	0	0	0	0	0	0	0	0	0	0
XM_021062496.1	XRRA1	mRNA	0	0	0	0	0	0	0	0	0	0	0	0	0	0	0	0	0	0	0	0	0
XM_021062497.1	XRRA1	mRNA	0	0	0	0	0	0	0	0	0	0	0	0	0	0	0	0	0	0	0	0	0
XM_021062498.1	XRRA1	mRNA	0	0	0	0	0	0	0	0	0	0	0	0	0	0	0	0	0	0	0	0	0
XM_021062499.1	XRRA1	mRNA	0	0	0	0	0	0	0	0	0	0	0	0	0	0	0	0	0	0	0	0	0
XM_021062500.1	XRRA1	mRNA	0	0	0	0	0	0	0	0	0	0	0	0	0	0	0	0	0	0	0	0	0
XM_021062501.1	XRRA1	mRNA	0	0	0	0	0	0	0	0	0	0	0	0	0	0	0	0	0	0	0	0	0
XM_021062502.1	XRRA1	mRNA	0	0	0	0	0	0	0	0	0	0	0	0	0	0	0	0	0	0	0	0	0
XM_021062503.1	XRRA1	mRNA	0	0	0	0	0	0	0	0	0	0	0	0	0	0	0	0	0	0	0	0	0
XM_021062511.1	SLCO2B1	mRNA	0	0	0	0	0	0	0	0	0	0	0	0	0	0	0	0	0	0	0	0	0
XM_021062948.1	BCL9L	mRNA	0	0	0	0	0	0	0	0	0	0	0	0	0	0	0	0	0	0	0	0	0
XM_021062949.1	TRAPPC4	mRNA	0	0	0	0	0	0	0	0	0	0	0	0	0	0	0	0	0	0	0	0	0
XM_021062950.1	HYOU1	mRNA	0	0	0	0	0	0	0	0	0	0	0	0	0	0	0	0	0	0	0	0	0
XM_021062951.1	HYOU1	mRNA	0	0	0	0	0	0	0	0	0	0	0	0	0	0	0	0	0	0	0	0	0
XM_021062952.1	HYOU1	mRNA	0	0	0	0	0	0	0	0	0	0	0	0	0	0	0	0	0	0	0	0	0
XM_021062953.1	HYOU1	mRNA	0	0	0	0	0	0	0	0	0	0	0	0	0	0	0	0	0	0	0	0	0
XM_021062954.1	HYOU1	mRNA	0	0	0	0	0	0	0	0	0	0	0	0	0	0	0	0	0	0	0	0	0
XM_021062955.1	HYOU1	mRNA	0	0	0	0	0	0	0	0	0	0	0	0	0	0	0	0	0	0	0	0	0
XM_021062956.1	HYOU1	mRNA	0	0	0	0	0	0	0	0	0	0	0	0	0	0	0	0	0	0	0	0	0
XM_021062957.1	VPS11	mRNA	0	0	0	0	0	0	0	0	0	0	0	0	0	0	0	0	0	0	0	0	0
XM_021062993.1	LOC100523684	mRNA	0	0	0	0	0	0	0	0	0	0	0	0	0	0	0	0	0	0	0	0	0
XM_021062994.1	LOC100523684	mRNA	0	0	0	0	0	0	0	0	0	0	0	0	0	0	0	0	0	0	0	0	0
XM_021062995.1	LOC100523684	mRNA	0	0	0	0	0	0	0	0	0	0	0	0	0	0	0	0	0	0	0	0	0
XM_021062996.1	LOC100523684	mRNA	0	0	0	0	0	0	0	0	0	0	0	0	0	0	0	0	0	0	0	0	0
XM_021062997.1	LOC100523684	mRNA	0	0	0	0	0	0	0	0	0	0	0	0	0	0	0	0	0	0	0	0	0
XM_021062998.1	LOC100523684	mRNA	0	0	0	0	0	0	0	0	0	0	0	0	0	0	0	0	0	0	0	0	0
XM_021062999.1	LOC100523684	mRNA	0	0	0	0	0	0	0	0	0	0	0	0	0	0	0	0	0	0	0	0	0
XM_021063000.1	LOC100523684	mRNA	0	0	0	0	0	0	0	0	0	0	0	0	0	0	0	0	0	0	0	0	0
XM_021063001.1	LOC100523684	mRNA	0	0	0	0	0	0	0	0	0	0	0	0	0	0	0	0	0	0	0	0	0
XM_021063002.1	LOC100523684	mRNA	0	0	0	0	0	0	0	0	0	0	0	0	0	0	0	0	0	0	0	0	0
XM_021063004.1	LOC100523684	mRNA	0	0	0	0	0	0	0	0	0	0	0	0	0	0	0	0	0	0	0	0	0
XM_021063005.1	LOC100523684	mRNA	0	0	0	0	0	0	0	0	0	0	0	0	0	0	0	0	0	0	0	0	0
XM_021063006.1	LOC100523684	mRNA	0	0	0	0	0	0	0	0	0	0	0	0	0	0	0	0	0	0	0	0	0
XM_021063009.1	SORL1	mRNA	0	0	0	0	0	0	0	0	0	0	0	0	0	0	0	0	0	0	0	0	0
XM_021063010.1	SORL1	mRNA	0	0	0	0	0	0	0	0	0	0	0	0	0	0	0	0	0	0	0	0	0
XM_021063011.1	SORL1	mRNA	0	0	0	0	0	0	0	0	0	0	0	0	0	0	0	0	0	0	0	0	0
XM_021063012.1	SORL1	mRNA	0	0	0	0	0	0	0	0	0	0	0	0	0	0	0	0	0	0	0	0	0
XM_021063013.1	SORL1	mRNA	0	0	0	0	0	0	0	0	0	0	0	0	0	0	0	0	0	0	0	0	0
XM_021063014.1	SORL1	mRNA	0	0	0	0	0	0	0	0	0	0	0	0	0	0	0	0	0	0	0	0	0
XM_021063023.1	CLMP	mRNA	0	0	0	0	0	0	0	0	0	0	0	0	0	0	0	0	0	0	0	0	0
XM_021063024.1	CLMP	mRNA	0	0	0	0	0	0	0	0	0	0	0	0	0	0	0	0	0	0	0	0	0

RNA ID	Symbol	Type	updown_BGIR9823_84443	updown_BGIR9823_81960	updown_BGIR9823_82315	updown_BGIR9823_82966	updown_BGIR9823_83074	updown_BGIR9823_82386	updown_BGIR9823_82709	updown_BGIR9823_83417	updown_BGIR9823_85418	updown_BGIR9823_87926	updown_BGIR9823_88529	updown_BGIR9823_88698	updown_BGIR9823_86965	updown_BGIR9823_89012	updown_BGIR9823_92131	updown_BGIR9823_90639	updown_BGIR9823_95770	updown_BGIR9823_92788	updown_BGIR9823_93572	updown_BGIR9823_96356	updown_BGIR9823_94689
XM_021063025.1	CLMP	mRNA	0	0	0	0	0	0	0	0	0	0	0	0	0	0	0	0	0	0	0	0	0
XM_021063026.1	CLMP	mRNA	0	0	0	0	0	0	0	0	0	0	0	0	0	0	0	0	0	0	0	0	0
XM_021063950.1	NXPE2	mRNA	0	0	0	0	0	0	0	0	0	0	0	0	0	0	0	0	0	0	0	0	0
XM_021063963.1	LOC100623190	mRNA	0	0	0	0	0	0	0	0	0	0	0	0	0	0	0	0	0	0	0	0	0
XM_021065159.1	HMGB1	mRNA	0	0	0	0	0	0	0	0	0	0	0	0	0	0	0	0	0	0	0	0	0
XM_021065160.1	HMGB1	mRNA	0	0	0	0	0	0	0	0	0	0	0	0	0	0	0	0	0	0	0	0	0
XM_021065161.1	HMGB1	mRNA	0	0	0	0	0	0	0	0	0	0	0	0	0	0	0	0	0	0	0	0	0
XM_021065529.1	KATNAL1	mRNA	0	0	0	0	0	0	0	0	0	0	0	0	0	0	0	0	0	0	0	0	0
XM_021065531.1	KATNAL1	mRNA	0	0	0	0	0	0	0	0	0	0	0	0	0	0	0	0	0	0	0	0	0
XM_021065532.1	KATNAL1	mRNA	0	0	0	0	0	0	0	0	0	0	0	0	0	0	0	0	0	0	0	0	0
XM_021065533.1	KATNAL1	mRNA	0	0	0	0	0	0	0	0	0	0	0	0	0	0	0	0	0	0	0	0	0
XM_021065534.1	KATNAL1	mRNA	0	0	0	0	0	0	0	0	0	0	0	0	0	0	0	0	0	0	0	0	0
XM_021065535.1	KATNAL1	mRNA	0	0	0	0	0	0	0	0	0	0	0	0	0	0	0	0	0	0	0	0	0
XM_021065536.1	KATNAL1	mRNA	0	0	0	0	0	0	0	0	0	0	0	0	0	0	0	0	0	0	0	0	0
XM_021065537.1	KATNAL1	mRNA	0	0	0	0	0	0	0	0	0	0	0	0	0	0	0	0	0	0	0	0	0
XM_021065538.1	KATNAL1	mRNA	0	0	0	0	0	0	0	0	0	0	0	0	0	0	0	0	0	0	0	0	0
XM_021065539.1	KATNAL1	mRNA	0	0	0	0	0	0	0	0	0	0	0	0	0	0	0	0	0	0	0	0	0
XM_021065541.1	KATNAL1	mRNA	0	0	0	0	0	0	0	0	0	0	0	0	0	0	0	0	0	0	0	0	0
XM_021065542.1	KATNAL1	mRNA	0	0	0	0	0	0	0	0	0	0	0	0	0	0	0	0	0	0	0	0	0
XM_021065543.1	KATNAL1	mRNA	0	0	0	0	0	0	0	0	0	0	0	0	0	0	0	0	0	0	0	0	0
XM_021065544.1	KATNAL1	mRNA	0	0	0	0	0	0	0	0	0	0	0	0	0	0	0	0	0	0	0	0	0
XM_021073408.1	EMX2	mRNA	0	0	0	0	0	0	0	0	0	0	0	0	0	0	0	0	0	0	0	0	0
XM_021078611.1	PRKAG2	mRNA	0	0	0	0	0	0	0	0	0	0	0	0	0	0	0	0	0	0	0	0	0
XM_021078612.1	PRKAG2	mRNA	0	0	0	0	0	0	0	0	0	0	0	0	0	0	0	0	0	0	0	0	0
XM_021078613.1	PRKAG2	mRNA	0	0	0	0	0	0	0	0	0	0	0	0	0	0	0	0	0	0	0	0	0
XM_021078614.1	PRKAG2	mRNA	0	0	0	0	0	0	0	0	0	0	0	0	0	0	0	0	0	0	0	0	0
XM_021078615.1	PRKAG2	mRNA	0	0	0	0	0	0	0	0	0	0	0	0	0	0	0	0	0	0	0	0	0
XM_021078616.1	PRKAG2	mRNA	0	0	0	0	0	0	0	0	0	0	0	0	0	0	0	0	0	0	0	0	0
XM_021078617.1	PRKAG2	mRNA	0	0	0	0	0	0	0	0	0	0	0	0	0	0	0	0	0	0	0	0	0
XM_021078618.1	PRKAG2	mRNA	0	0	0	0	0	0	0	0	0	0	0	0	0	0	0	0	0	0	0	0	0
XM_021078619.1	PRKAG2	mRNA	0	0	0	0	0	0	0	0	0	0	0	0	0	0	0	0	0	0	0	0	0
XM_021078620.1	PRKAG2	mRNA	0	0	0	0	0	0	0	0	0	0	0	0	0	0	0	0	0	0	0	0	0
XM_021078621.1	PRKAG2	mRNA	0	0	0	0	0	0	0	0	0	0	0	0	0	0	0	0	0	0	0	0	0
XM_021081309.1	LOC106506226	mRNA	0	0	0	0	0	0	0	0	0	0	0	0	0	0	0	0	0	0	0	0	0
XM_021081403.1	LOC110258215	mRNA	0	0	0	0	0	0	0	0	0	0	0	0	0	0	0	0	0	0	0	0	0
XM_021082153.1	LOC100521600	mRNA	0	0	0	0	0	0	0	0	0	0	0	0	0	0	0	0	0	0	0	0	0
XM_021082154.1	LOC100521600	mRNA	0	0	0	0	0	0	0	0	0	0	0	0	0	0	0	0	0	0	0	0	0
XM_021082156.1	LOC100521600	mRNA	0	0	0	0	0	0	0	0	0	0	0	0	0	0	0	0	0	0	0	0	0
XM_021082157.1	LOC100521600	mRNA	0	0	0	0	0	0	0	0	0	0	0	0	0	0	0	0	0	0	0	0	0
XM_021082158.1	LOC100521600	mRNA	0	0	0	0	0	0	0	0	0	0	0	0	0	0	0	0	0	0	0	0	0
XM_021082159.1	LOC100521600	mRNA	0	0	0	0	0	0	0	0	0	0	0	0	0	0	0	0	0	0	0	0	0
XM_021082835.1	EHBP1L1	mRNA	0	0	0	0	0	0	0	0	0	0	0	0	0	0	0	0	0	0	0	0	0
XM_021082836.1	EHBP1L1	mRNA	0	0	0	0	0	0	0	0	0	0	0	0	0	0	0	0	0	0	0	0	0

RNA ID	Symbol	Type	updown_BGIR9823_84443	updown_BGIR9823_81960	updown_BGIR9823_82315	updown_BGIR9823_82966	updown_BGIR9823_83074	updown_BGIR9823_82386	updown_BGIR9823_82709	updown_BGIR9823_83417	updown_BGIR9823_85418	updown_BGIR9823_87926	updown_BGIR9823_88529	updown_BGIR9823_88698	updown_BGIR9823_86965	updown_BGIR9823_89012	updown_BGIR9823_92131	updown_BGIR9823_90639	updown_BGIR9823_95770	updown_BGIR9823_92788	updown_BGIR9823_93572	updown_BGIR9823_96356	updown_BGIR9823_94689
XM_021082837.1	EHBP1L1	mRNA	0	0	0	0	0	0	0	0	0	0	0	0	0	0	0	0	0	0	0	0	0
XM_021082838.1	EHBP1L1	mRNA	0	0	0	0	0	0	0	0	0	0	0	0	0	0	0	0	0	0	0	0	0
XM_021082839.1	EHBP1L1	mRNA	0	0	0	0	0	0	0	0	0	0	0	0	0	0	0	0	0	0	0	0	0
XM_021082840.1	EHBP1L1	mRNA	0	0	0	0	0	0	0	0	0	0	0	0	0	0	0	0	0	0	0	0	0
XM_021082841.1	EHBP1L1	mRNA	0	0	0	0	0	0	0	0	0	0	0	0	0	0	0	0	0	0	0	0	0
XM_021082842.1	EHBP1L1	mRNA	0	0	0	0	0	0	0	0	0	0	0	0	0	0	0	0	0	0	0	0	0
XM_021082843.1	EHBP1L1	mRNA	0	0	0	0	0	0	0	0	0	0	0	0	0	0	0	0	0	0	0	0	0
XM_021082844.1	EHBP1L1	mRNA	0	0	0	0	0	0	0	0	0	0	0	0	0	0	0	0	0	0	0	0	0
XM_021082845.1	EHBP1L1	mRNA	0	0	0	0	0	0	0	0	0	0	0	0	0	0	0	0	0	0	0	0	0
XM_021082846.1	EHBP1L1	mRNA	0	0	0	0	0	0	0	0	0	0	0	0	0	0	0	0	0	0	0	0	0
XM_021082847.1	EHBP1L1	mRNA	0	0	0	0	0	0	0	0	0	0	0	0	0	0	0	0	0	0	0	0	0
XM_021082851.1	MAP3K11	mRNA	0	0	0	0	0	0	0	0	0	0	0	0	0	0	0	0	0	0	0	0	0
XM_021082852.1	SSSCA1	mRNA	0	0	0	0	0	0	0	0	0	0	0	0	0	0	0	0	0	0	0	0	0
XM_021082853.1	FAM89B	mRNA	0	0	0	0	0	0	0	0	0	0	0	0	0	0	0	0	0	0	0	0	0
XM_021082854.1	LTBP3	mRNA	0	0	0	0	0	0	0	0	0	0	0	0	0	0	0	0	0	0	0	0	0
XM_021082855.1	LTBP3	mRNA	0	0	0	0	0	0	0	0	0	0	0	0	0	0	0	0	0	0	0	0	0
XM_021082856.1	SCYL1	mRNA	0	0	0	0	0	0	0	0	0	0	0	0	0	0	0	0	0	0	0	0	0
XM_021082857.1	SLC25A45	mRNA	0	0	0	0	0	0	0	0	0	0	0	0	0	0	0	0	0	0	0	0	0
XM_021082858.1	SLC25A45	mRNA	0	0	0	0	0	0	0	0	0	0	0	0	0	0	0	0	0	0	0	0	0
XM_021091099.1	SYNGR1	mRNA	0	0	0	0	0	0	0	0	0	0	0	0	0	0	0	0	0	0	0	0	0
XM_021091101.1	SYNGR1	mRNA	0	0	0	0	0	0	0	0	0	0	0	0	0	0	0	0	0	0	0	0	0
XM_021091102.1	SYNGR1	mRNA	0	0	0	0	0	0	0	0	0	0	0	0	0	0	0	0	0	0	0	0	0
XM_021091103.1	SYNGR1	mRNA	0	0	0	0	0	0	0	0	0	0	0	0	0	0	0	0	0	0	0	0	0
XM_021091370.1	LOC102159820	mRNA	0	0	0	0	0	0	0	0	0	0	0	0	0	0	0	0	0	0	0	0	0
XM_021091373.1	TRMU	mRNA	0	0	0	0	0	0	0	0	0	0	0	0	0	0	0	0	0	0	0	0	0
XM_021091374.1	TRMU	mRNA	0	0	0	0	0	0	0	0	0	0	0	0	0	0	0	0	0	0	0	0	0
XM_021091375.1	TRMU	mRNA	0	0	0	0	0	0	0	0	0	0	0	0	0	0	0	0	0	0	0	0	0
XM_021091376.1	GTSE1	mRNA	0	0	0	0	0	0	0	0	0	0	0	0	0	0	0	0	0	0	0	0	0
XM_021091377.1	TTC38	mRNA	0	0	0	0	0	0	0	0	0	0	0	0	0	0	0	0	0	0	0	0	0
XM_021091379.1	LOC110260611	mRNA	0	0	0	0	0	0	0	0	0	0	0	0	0	0	0	0	0	0	0	0	0
XM_021091381.1	LOC110260611	mRNA	0	0	0	0	0	0	0	0	0	0	0	0	0	0	0	0	0	0	0	0	0
XM_021091454.1	TOB2	mRNA	0	0	0	0	0	0	0	0	0	0	0	0	0	0	0	0	0	0	0	0	0
XM_021091455.1	TOB2	mRNA	0	0	0	0	0	0	0	0	0	0	0	0	0	0	0	0	0	0	0	0	0
XM_021091456.1	PHF5A	mRNA	0	0	0	0	0	0	0	0	0	0	0	0	0	0	0	0	0	0	0	0	0
XM_021091457.1	PHF5A	mRNA	0	0	0	0	0	0	0	0	0	0	0	0	0	0	0	0	0	0	0	0	0
XM_021091458.1	ZC3H7B	mRNA	0	0	0	0	0	0	0	0	0	0	0	0	0	0	0	0	0	0	0	0	0
XM_021091459.1	ZC3H7B	mRNA	0	0	0	0	0	0	0	0	0	0	0	0	0	0	0	0	0	0	0	0	0
XM_021091460.1	TEF	mRNA	0	0	0	0	0	0	0	0	0	0	0	0	0	0	0	0	0	0	0	0	0
XM_021091461.1	RANGAP1	mRNA	0	0	0	0	0	0	0	0	0	0	0	0	0	0	0	0	0	0	0	0	0
XM_021091474.1	CBX7	mRNA	0	0	0	0	0	0	0	0	0	0	0	0	0	0	0	0	0	0	0	0	0
XM_021092767.1	CRADD	mRNA	0	0	0	0	0	0	0	0	0	0	0	0	0	0	0	0	0	0	0	0	0
XM_021092768.1	CRADD	mRNA	0	0	0	0	0	0	0	0	0	0	0	0	0	0	0	0	0	0	0	0	0
XM_021092769.1	CRADD	mRNA	0	0	0	0	0	0	0	0	0	0	0	0	0	0	0	0	0	0	0	0	0

RNA ID	Symbol	Type	updown_BGIR9823_84443	updown_BGIR9823_81960	updown_BGIR9823_82315	updown_BGIR9823_82966	updown_BGIR9823_83074	updown_BGIR9823_82386	updown_BGIR9823_82709	updown_BGIR9823_83417	updown_BGIR9823_85418	updown_BGIR9823_87926	updown_BGIR9823_88529	updown_BGIR9823_88698	updown_BGIR9823_86965	updown_BGIR9823_89012	updown_BGIR9823_92131	updown_BGIR9823_90639	updown_BGIR9823_95770	updown_BGIR9823_92788	updown_BGIR9823_93572	updown_BGIR9823_96356	updown_BGIR9823_94689
XM_021092770.1	CRADD	mRNA	0	0	0	0	0	0	0	0	0	0	0	0	0	0	0	0	0	0	0	0	0
XM_021092771.1	CRADD	mRNA	0	0	0	0	0	0	0	0	0	0	0	0	0	0	0	0	0	0	0	0	0
XM_021092773.1	CRADD	mRNA	0	0	0	0	0	0	0	0	0	0	0	0	0	0	0	0	0	0	0	0	0
XM_021093095.1	PKDREJ	mRNA	0	0	0	0	0	0	0	0	0	0	0	0	0	0	0	0	0	0	0	0	0
XM_021097064.1	MAF	mRNA	0	0	0	0	0	0	0	0	0	0	0	0	0	0	0	0	0	0	0	0	0
XM_021097065.1	MAF	mRNA	0	0	0	0	0	0	0	0	0	0	0	0	0	0	0	0	0	0	0	0	0
XM_021097066.1	MAF	mRNA	0	0	0	0	0	0	0	0	0	0	0	0	0	0	0	0	0	0	0	0	0
XM_021097870.1	ECI2	mRNA	0	0	0	0	0	0	0	0	0	0	0	0	0	0	0	0	0	0	0	0	0
XM_021099751.1	PHACTR1	mRNA	0	0	0	0	0	0	0	0	0	0	0	0	0	0	0	0	0	0	0	0	0
XM_021099752.1	PHACTR1	mRNA	0	0	0	0	0	0	0	0	0	0	0	0	0	0	0	0	0	0	0	0	0
XM_021099753.1	PHACTR1	mRNA	0	0	0	0	0	0	0	0	0	0	0	0	0	0	0	0	0	0	0	0	0
XM_021099754.1	PHACTR1	mRNA	0	0	0	0	0	0	0	0	0	0	0	0	0	0	0	0	0	0	0	0	0
XM_021099755.1	PHACTR1	mRNA	0	0	0	0	0	0	0	0	0	0	0	0	0	0	0	0	0	0	0	0	0
XM_021099756.1	PHACTR1	mRNA	0	0	0	0	0	0	0	0	0	0	0	0	0	0	0	0	0	0	0	0	0
XM_021099757.1	PHACTR1	mRNA	0	0	0	0	0	0	0	0	0	0	0	0	0	0	0	0	0	0	0	0	0
XM_021099758.1	PHACTR1	mRNA	0	0	0	0	0	0	0	0	0	0	0	0	0	0	0	0	0	0	0	0	0
XM_021099759.1	PHACTR1	mRNA	0	0	0	0	0	0	0	0	0	0	0	0	0	0	0	0	0	0	0	0	0
XM_021099760.1	PHACTR1	mRNA	0	0	0	0	0	0	0	0	0	0	0	0	0	0	0	0	0	0	0	0	0
XM_021099761.1	PHACTR1	mRNA	0	0	0	0	0	0	0	0	0	0	0	0	0	0	0	0	0	0	0	0	0
XM_021099762.1	PHACTR1	mRNA	0	0	0	0	0	0	0	0	0	0	0	0	0	0	0	0	0	0	0	0	0
XM_021099763.1	PHACTR1	mRNA	0	0	0	0	0	0	0	0	0	0	0	0	0	0	0	0	0	0	0	0	0
XM_021099764.1	PHACTR1	mRNA	0	0	0	0	0	0	0	0	0	0	0	0	0	0	0	0	0	0	0	0	0
XM_021099765.1	PHACTR1	mRNA	0	0	0	0	0	0	0	0	0	0	0	0	0	0	0	0	0	0	0	0	0
XM_021099766.1	TBC1D7	mRNA	0	0	0	0	0	0	0	0	0	0	0	0	0	0	0	0	0	0	0	0	0
XM_021099767.1	TBC1D7	mRNA	0	0	0	0	0	0	0	0	0	0	0	0	0	0	0	0	0	0	0	0	0
XM_021099769.1	TBC1D7	mRNA	0	0	0	0	0	0	0	0	0	0	0	0	0	0	0	0	0	0	0	0	0
XM_021099770.1	TBC1D7	mRNA	0	0	0	0	0	0	0	0	0	0	0	0	0	0	0	0	0	0	0	0	0
XM_021099999.1	LOC110261697	mRNA	0	0	0	0	0	0	0	0	0	0	0	0	0	0	0	0	0	0	0	0	0
XM_021100108.1	WRNIP1	mRNA	0	0	0	0	0	0	0	0	0	0	0	0	0	0	0	0	0	0	0	0	0
XM_021100109.1	MYLK4	mRNA	0	0	0	0	0	0	0	0	0	0	0	0	0	0	0	0	0	0	0	0	0
XM_021100110.1	MYLK4	mRNA	0	0	0	0	0	0	0	0	0	0	0	0	0	0	0	0	0	0	0	0	0
XM_021100111.1	MYLK4	mRNA	0	0	0	0	0	0	0	0	0	0	0	0	0	0	0	0	0	0	0	0	0
XM_021100112.1	MYLK4	mRNA	0	0	0	0	0	0	0	0	0	0	0	0	0	0	0	0	0	0	0	0	0
XM_021100114.1	NQO2	mRNA	0	0	0	0	0	0	0	0	0	0	0	0	0	0	0	0	0	0	0	0	0
XM_021100115.1	NQO2	mRNA	0	0	0	0	0	0	0	0	0	0	0	0	0	0	0	0	0	0	0	0	0
XM_021100117.1	NQO2	mRNA	0	0	0	0	0	0	0	0	0	0	0	0	0	0	0	0	0	0	0	0	0
XM_021100118.1	NQO2	mRNA	0	0	0	0	0	0	0	0	0	0	0	0	0	0	0	0	0	0	0	0	0
XM_021100119.1	NQO2	mRNA	0	0	0	0	0	0	0	0	0	0	0	0	0	0	0	0	0	0	0	0	0
XM_021100124.1	PRPF4B	mRNA	0	0	0	0	0	0	0	0	0	0	0	0	0	0	0	0	0	0	0	0	0
XM_021100126.1	PRPF4B	mRNA	0	0	0	0	0	0	0	0	0	0	0	0	0	0	0	0	0	0	0	0	0
XM_021100184.1	NEDD9	mRNA	0	0	0	0	0	0	0	0	0	0	0	0	0	0	0	0	0	0	0	0	0
XM_021100188.1	TMEM170B	mRNA	0	0	0	0	0	0	0	0	0	0	0	0	0	0	0	0	0	0	0	0	0
XM_021100194.1	GFOD1	mRNA	0	0	0	0	0	0	0	0	0	0	0	0	0	0	0	0	0	0	0	0	0

RNA ID	Symbol	Type	updown_BGIR9823_84443	updown_BGIR9823_81960	updown_BGIR9823_82315	updown_BGIR9823_82966	updown_BGIR9823_83074	updown_BGIR9823_82386	updown_BGIR9823_82709	updown_BGIR9823_83417	updown_BGIR9823_85418	updown_BGIR9823_87926	updown_BGIR9823_88529	updown_BGIR9823_88698	updown_BGIR9823_86965	updown_BGIR9823_89012	updown_BGIR9823_92131	updown_BGIR9823_90639	updown_BGIR9823_95770	updown_BGIR9823_92788	updown_BGIR9823_93572	updown_BGIR9823_96356	updown_BGIR9823_94689
XM_021102040.1	SLC37A4	mRNA	0	0	0	0	0	0	0	0	0	0	0	0	0	0	0	0	0	0	0	0	0
XM_021102041.1	SLC37A4	mRNA	0	0	0	0	0	0	0	0	0	0	0	0	0	0	0	0	0	0	0	0	0
XM_021102042.1	SLC37A4	mRNA	0	0	0	0	0	0	0	0	0	0	0	0	0	0	0	0	0	0	0	0	0
XM_021102043.1	SLC37A4	mRNA	0	0	0	0	0	0	0	0	0	0	0	0	0	0	0	0	0	0	0	0	0
XM_021102044.1	SLC37A4	mRNA	0	0	0	0	0	0	0	0	0	0	0	0	0	0	0	0	0	0	0	0	0
XM_021102045.1	SLC37A4	mRNA	0	0	0	0	0	0	0	0	0	0	0	0	0	0	0	0	0	0	0	0	0
XM_021102046.1	SLC37A4	mRNA	0	0	0	0	0	0	0	0	0	0	0	0	0	0	0	0	0	0	0	0	0
XM_021102047.1	SLC37A4	mRNA	0	0	0	0	0	0	0	0	0	0	0	0	0	0	0	0	0	0	0	0	0
XM_021102048.1	SLC37A4	mRNA	0	0	0	0	0	0	0	0	0	0	0	0	0	0	0	0	0	0	0	0	0
XM_021102049.1	SLC37A4	mRNA	0	0	0	0	0	0	0	0	0	0	0	0	0	0	0	0	0	0	0	0	0
XM_021102050.1	SLC37A4	mRNA	0	0	0	0	0	0	0	0	0	0	0	0	0	0	0	0	0	0	0	0	0
XM_021102051.1	SLC37A4	mRNA	0	0	0	0	0	0	0	0	0	0	0	0	0	0	0	0	0	0	0	0	0
XM_021102052.1	SLC37A4	mRNA	0	0	0	0	0	0	0	0	0	0	0	0	0	0	0	0	0	0	0	0	0
XM_021102209.1	UPK2	mRNA	0	0	0	0	0	0	0	0	0	0	0	0	0	0	0	0	0	0	0	0	0
XM_021102516.1	LOC100522455	mRNA	0	0	0	0	0	0	0	0	0	0	0	0	0	0	0	0	0	0	0	0	0
XM_021102517.1	RUNDC3B	mRNA	0	0	0	0	0	0	0	0	0	0	0	0	0	0	0	0	0	0	0	0	0
XM_021102518.1	RUNDC3B	mRNA	0	0	0	0	0	0	0	0	0	0	0	0	0	0	0	0	0	0	0	0	0
XM_021102519.1	RUNDC3B	mRNA	0	0	0	0	0	0	0	0	0	0	0	0	0	0	0	0	0	0	0	0	0
XM_021102522.1	RUNDC3B	mRNA	0	0	0	0	0	0	0	0	0	0	0	0	0	0	0	0	0	0	0	0	0
XM_021102523.1	RUNDC3B	mRNA	0	0	0	0	0	0	0	0	0	0	0	0	0	0	0	0	0	0	0	0	0
XR_001297664.2	LOC102161744	lncRNA	0	0	0	0	0	0	0	0	0	0	0	0	0	0	0	0	0	0	0	0	0
XR_001298807.2	LOC106504881	lncRNA	0	0	0	0	0	0	0	0	0	0	0	0	0	0	0	0	0	0	0	0	0
XR_001298808.2	LOC106504881	lncRNA	0	0	0	0	0	0	0	0	0	0	0	0	0	0	0	0	0	0	0	0	0
XR_001298810.2	LOC106504881	lncRNA	0	0	0	0	0	0	0	0	0	0	0	0	0	0	0	0	0	0	0	0	0
XR_001298811.2	LOC106504881	lncRNA	0	0	0	0	0	0	0	0	0	0	0	0	0	0	0	0	0	0	0	0	0
XR_001298812.2	LOC106504881	lncRNA	0	0	0	0	0	0	0	0	0	0	0	0	0	0	0	0	0	0	0	0	0
XR_001298813.2	LOC106504881	lncRNA	0	0	0	0	0	0	0	0	0	0	0	0	0	0	0	0	0	0	0	0	0
XR_001298814.2	LOC106504881	lncRNA	0	0	0	0	0	0	0	0	0	0	0	0	0	0	0	0	0	0	0	0	0
XR_001298816.2	LOC106504881	lncRNA	0	0	0	0	0	0	0	0	0	0	0	0	0	0	0	0	0	0	0	0	0
XR_001298817.2	LOC106504881	lncRNA	0	0	0	0	0	0	0	0	0	0	0	0	0	0	0	0	0	0	0	0	0
XR_001298844.2	LOC102163364	lncRNA	0	0	0	0	0	0	0	0	0	0	0	0	0	0	0	0	0	0	0	0	0
XR_001298846.2	LOC102163364	lncRNA	0	0	0	0	0	0	0	0	0	0	0	0	0	0	0	0	0	0	0	0	0
XR_001298850.2	LOC102163364	lncRNA	0	0	0	0	0	0	0	0	0	0	0	0	0	0	0	0	0	0	0	0	0
XR_001299456.2	LOC102166670	lncRNA	0	0	0	0	0	0	0	0	0	0	0	0	0	0	0	0	0	0	0	0	0
XR_001299457.2	LOC102166670	lncRNA	0	0	0	0	0	0	0	0	0	0	0	0	0	0	0	0	0	0	0	0	0
XR_001300715.2	LOC106505879	lncRNA	0	0	0	0	0	0	0	0	0	0	0	0	0	0	0	0	0	0	0	0	0
XR_001301078.2	LOC102162623	lncRNA	0	0	0	0	0	0	0	0	0	0	0	0	0	0	0	0	0	0	0	0	0
XR_001301087.2	LOC102162623	lncRNA	0	0	0	0	0	0	0	0	0	0	0	0	0	0	0	0	0	0	0	0	0
XR_001301217.2	LOC102164998	lncRNA	0	0	0	0	0	0	0	0	0	0	0	0	0	0	0	0	0	0	0	0	0
XR_001301305.2	LOC102159607	lncRNA	0	0	0	0	0	0	0	0	0	0	0	0	0	0	0	0	0	0	0	0	0
XR_001308967.2	LOC106510467	lncRNA	0	0	0	0	0	0	0	0	0	0	0	0	0	0	0	0	0	0	0	0	0
XR_002335533.1	XRRA1	mRNA	0	0	0	0	0	0	0	0	0	0	0	0	0	0	0	0	0	0	0	0	0
XR_002335534.1	XRRA1	mRNA	0	0	0	0	0	0	0	0	0	0	0	0	0	0	0	0	0	0	0	0	0

RNA ID	Symbol	Type	updown_BGIR9823_84443	updown_BGIR9823_81960	updown_BGIR9823_82315	updown_BGIR9823_82966	updown_BGIR9823_83074	updown_BGIR9823_82386	updown_BGIR9823_82709	updown_BGIR9823_83417	updown_BGIR9823_85418	updown_BGIR9823_87926	updown_BGIR9823_88529	updown_BGIR9823_88698	updown_BGIR9823_86965	updown_BGIR9823_89012	updown_BGIR9823_92131	updown_BGIR9823_90639	updown_BGIR9823_95770	updown_BGIR9823_92788	updown_BGIR9823_93572	updown_BGIR9823_96356	updown_BGIR9823_94689
XR_002335535.1	LOC110255399	lncRNA	0	0	0	0	0	0	0	0	0	0	0	0	0	0	0	0	0	0	0	0	0
XR_002335536.1	LOC102160367	lncRNA	0	0	0	0	0	0	0	0	0	0	0	0	0	0	0	0	0	0	0	0	0
XR_002335537.1	LOC102160367	lncRNA	0	0	0	0	0	0	0	0	0	0	0	0	0	0	0	0	0	0	0	0	0
XR_002335650.1	LOC102157630	lncRNA	0	0	0	0	0	0	0	0	0	0	0	0	0	0	0	0	0	0	0	0	0
XR_002335651.1	LOC110255435	lncRNA	0	0	0	0	0	0	0	0	0	0	0	0	0	0	0	0	0	0	0	0	0
XR_002335652.1	LOC106504881	lncRNA	0	0	0	0	0	0	0	0	0	0	0	0	0	0	0	0	0	0	0	0	0
XR_002335653.1	LOC106504881	lncRNA	0	0	0	0	0	0	0	0	0	0	0	0	0	0	0	0	0	0	0	0	0
XR_002335670.1	LOC110255440	lncRNA	0	0	0	0	0	0	0	0	0	0	0	0	0	0	0	0	0	0	0	0	0
XR_002335671.1	LOC110255441	lncRNA	0	0	0	0	0	0	0	0	0	0	0	0	0	0	0	0	0	0	0	0	0
XR_002335672.1	LOC110255441	lncRNA	0	0	0	0	0	0	0	0	0	0	0	0	0	0	0	0	0	0	0	0	0
XR_002335674.1	HYOU1	mRNA	0	0	0	0	0	0	0	0	0	0	0	0	0	0	0	0	0	0	0	0	0
XR_002335675.1	HYOU1	mRNA	0	0	0	0	0	0	0	0	0	0	0	0	0	0	0	0	0	0	0	0	0
XR_002335685.1	LOC100523684	mRNA	0	0	0	0	0	0	0	0	0	0	0	0	0	0	0	0	0	0	0	0	0
XR_002335686.1	LOC102163364	lncRNA	0	0	0	0	0	0	0	0	0	0	0	0	0	0	0	0	0	0	0	0	0
XR_002335687.1	LOC102163364	lncRNA	0	0	0	0	0	0	0	0	0	0	0	0	0	0	0	0	0	0	0	0	0
XR_002335688.1	LOC102163364	lncRNA	0	0	0	0	0	0	0	0	0	0	0	0	0	0	0	0	0	0	0	0	0
XR_002335689.1	LOC102163364	lncRNA	0	0	0	0	0	0	0	0	0	0	0	0	0	0	0	0	0	0	0	0	0
XR_002335690.1	LOC102163364	lncRNA	0	0	0	0	0	0	0	0	0	0	0	0	0	0	0	0	0	0	0	0	0
XR_002335691.1	LOC102163364	lncRNA	0	0	0	0	0	0	0	0	0	0	0	0	0	0	0	0	0	0	0	0	0
XR_002335692.1	LOC102163364	lncRNA	0	0	0	0	0	0	0	0	0	0	0	0	0	0	0	0	0	0	0	0	0
XR_002335693.1	LOC106504896	lncRNA	0	0	0	0	0	0	0	0	0	0	0	0	0	0	0	0	0	0	0	0	0
XR_002335694.1	SORL1	mRNA	0	0	0	0	0	0	0	0	0	0	0	0	0	0	0	0	0	0	0	0	0
XR_002336641.1	LOC106505250	lncRNA	0	0	0	0	0	0	0	0	0	0	0	0	0	0	0	0	0	0	0	0	0
XR_002336643.1	LOC110255292	lncRNA	0	0	0	0	0	0	0	0	0	0	0	0	0	0	0	0	0	0	0	0	0
XR_002336644.1	LOC102166670	lncRNA	0	0	0	0	0	0	0	0	0	0	0	0	0	0	0	0	0	0	0	0	0
XR_002336645.1	LOC102166670	lncRNA	0	0	0	0	0	0	0	0	0	0	0	0	0	0	0	0	0	0	0	0	0
XR_002338443.1	LOC106505879	lncRNA	0	0	0	0	0	0	0	0	0	0	0	0	0	0	0	0	0	0	0	0	0
XR_002338444.1	LOC106505879	lncRNA	0	0	0	0	0	0	0	0	0	0	0	0	0	0	0	0	0	0	0	0	0
XR_002338445.1	LOC106505879	lncRNA	0	0	0	0	0	0	0	0	0	0	0	0	0	0	0	0	0	0	0	0	0
XR_002338446.1	LOC106505879	lncRNA	0	0	0	0	0	0	0	0	0	0	0	0	0	0	0	0	0	0	0	0	0
XR_002338447.1	LOC106505879	lncRNA	0	0	0	0	0	0	0	0	0	0	0	0	0	0	0	0	0	0	0	0	0
XR_002338448.1	LOC106505879	lncRNA	0	0	0	0	0	0	0	0	0	0	0	0	0	0	0	0	0	0	0	0	0
XR_002338449.1	LOC106505879	lncRNA	0	0	0	0	0	0	0	0	0	0	0	0	0	0	0	0	0	0	0	0	0
XR_002338450.1	LOC106505879	lncRNA	0	0	0	0	0	0	0	0	0	0	0	0	0	0	0	0	0	0	0	0	0
XR_002338521.1	LOC110256664	lncRNA	0	0	0	0	0	0	0	0	0	0	0	0	0	0	0	0	0	0	0	0	0
XR_002338522.1	LOC110256664	lncRNA	0	0	0	0	0	0	0	0	0	0	0	0	0	0	0	0	0	0	0	0	0
XR_002338666.1	LOC110256747	lncRNA	0	0	0	0	0	0	0	0	0	0	0	0	0	0	0	0	0	0	0	0	0
XR_002338714.1	LOC102163817	lncRNA	0	0	0	0	0	0	0	0	0	0	0	0	0	0	0	0	0	0	0	0	0
XR_002338715.1	LOC102164998	lncRNA	0	0	0	0	0	0	0	0	0	0	0	0	0	0	0	0	0	0	0	0	0
XR_002338716.1	LOC102164998	lncRNA	0	0	0	0	0	0	0	0	0	0	0	0	0	0	0	0	0	0	0	0	0
XR_002338717.1	LOC110256768	lncRNA	0	0	0	0	0	0	0	0	0	0	0	0	0	0	0	0	0	0	0	0	0
XR_002338727.1	LOC110256773	lncRNA	0	0	0	0	0	0	0	0	0	0	0	0	0	0	0	0	0	0	0	0	0
XR_002338728.1	LOC110256773	lncRNA	0	0	0	0	0	0	0	0	0	0	0	0	0	0	0	0	0	0	0	0	0

RNA ID	Symbol	Type	updown_BGIR9823_84443	updown_BGIR9823_81960	updown_BGIR9823_82315	updown_BGIR9823_82966	updown_BGIR9823_83074	updown_BGIR9823_82386	updown_BGIR9823_82709	updown_BGIR9823_83417	updown_BGIR9823_85418	updown_BGIR9823_87926	updown_BGIR9823_88529	updown_BGIR9823_88698	updown_BGIR9823_86965	updown_BGIR9823_89012	updown_BGIR9823_92131	updown_BGIR9823_90639	updown_BGIR9823_95770	updown_BGIR9823_92788	updown_BGIR9823_93572	updown_BGIR9823_96356	updown_BGIR9823_94689
XR_002338729.1	LOC110256773	lncRNA	0	0	0	0	0	0	0	0	0	0	0	0	0	0	0	0	0	0	0	0	0
XR_002338793.1	LOC110256796	lncRNA	0	0	0	0	0	0	0	0	0	0	0	0	0	0	0	0	0	0	0	0	0
XR_002339623.1	LOC102167708	lncRNA	0	0	0	0	0	0	0	0	0	0	0	0	0	0	0	0	0	0	0	0	0
XR_002340245.1	PRKAG2	mRNA	0	0	0	0	0	0	0	0	0	0	0	0	0	0	0	0	0	0	0	0	0
XR_002340246.1	LOC102162623	lncRNA	0	0	0	0	0	0	0	0	0	0	0	0	0	0	0	0	0	0	0	0	0
XR_002340247.1	LOC102162623	lncRNA	0	0	0	0	0	0	0	0	0	0	0	0	0	0	0	0	0	0	0	0	0
XR_002340248.1	LOC110257516	lncRNA	0	0	0	0	0	0	0	0	0	0	0	0	0	0	0	0	0	0	0	0	0
XR_002340249.1	LOC102162071	lncRNA	0	0	0	0	0	0	0	0	0	0	0	0	0	0	0	0	0	0	0	0	0
XR_002340901.1	LOC102160243	lncRNA	0	0	0	0	0	0	0	0	0	0	0	0	0	0	0	0	0	0	0	0	0
XR_002340902.1	LOC102160243	lncRNA	0	0	0	0	0	0	0	0	0	0	0	0	0	0	0	0	0	0	0	0	0
XR_002340903.1	LOC102160243	lncRNA	0	0	0	0	0	0	0	0	0	0	0	0	0	0	0	0	0	0	0	0	0
XR_002340904.1	LOC102160243	lncRNA	0	0	0	0	0	0	0	0	0	0	0	0	0	0	0	0	0	0	0	0	0
XR_002340905.1	LOC102160243	lncRNA	0	0	0	0	0	0	0	0	0	0	0	0	0	0	0	0	0	0	0	0	0
XR_002341237.1	LOC102164320	lncRNA	0	0	0	0	0	0	0	0	0	0	0	0	0	0	0	0	0	0	0	0	0
XR_002341238.1	LOC102164320	lncRNA	0	0	0	0	0	0	0	0	0	0	0	0	0	0	0	0	0	0	0	0	0
XR_002341239.1	LOC102164320	lncRNA	0	0	0	0	0	0	0	0	0	0	0	0	0	0	0	0	0	0	0	0	0
XR_002341240.1	LOC102164320	lncRNA	0	0	0	0	0	0	0	0	0	0	0	0	0	0	0	0	0	0	0	0	0
XR_002341241.1	LOC102164320	lncRNA	0	0	0	0	0	0	0	0	0	0	0	0	0	0	0	0	0	0	0	0	0
XR_002341242.1	LOC102164320	lncRNA	0	0	0	0	0	0	0	0	0	0	0	0	0	0	0	0	0	0	0	0	0
XR_002341243.1	LOC102164320	lncRNA	0	0	0	0	0	0	0	0	0	0	0	0	0	0	0	0	0	0	0	0	0
XR_002341244.1	LOC102164320	lncRNA	0	0	0	0	0	0	0	0	0	0	0	0	0	0	0	0	0	0	0	0	0
XR_002341270.1	LOC100521600	mRNA	0	0	0	0	0	0	0	0	0	0	0	0	0	0	0	0	0	0	0	0	0
XR_002341443.1	LOC102163816	lncRNA	0	0	0	0	0	0	0	0	0	0	0	0	0	0	0	0	0	0	0	0	0
XR_002343847.1	LOC110260611	mRNA	0	0	0	0	0	0	0	0	0	0	0	0	0	0	0	0	0	0	0	0	0
XR_002343848.1	LOC110260611	mRNA	0	0	0	0	0	0	0	0	0	0	0	0	0	0	0	0	0	0	0	0	0
XR_002343849.1	LOC110260611	mRNA	0	0	0	0	0	0	0	0	0	0	0	0	0	0	0	0	0	0	0	0	0
XR_002343850.1	LOC110260611	mRNA	0	0	0	0	0	0	0	0	0	0	0	0	0	0	0	0	0	0	0	0	0
XR_002343875.1	LOC110260629	lncRNA	0	0	0	0	0	0	0	0	0	0	0	0	0	0	0	0	0	0	0	0	0
XR_002343876.1	LOC110260629	lncRNA	0	0	0	0	0	0	0	0	0	0	0	0	0	0	0	0	0	0	0	0	0
XR_002343877.1	LOC110260629	lncRNA	0	0	0	0	0	0	0	0	0	0	0	0	0	0	0	0	0	0	0	0	0
XR_002343878.1	LOC110260629	lncRNA	0	0	0	0	0	0	0	0	0	0	0	0	0	0	0	0	0	0	0	0	0
XR_002343879.1	LOC110260629	lncRNA	0	0	0	0	0	0	0	0	0	0	0	0	0	0	0	0	0	0	0	0	0
XR_002343880.1	LOC110260629	lncRNA	0	0	0	0	0	0	0	0	0	0	0	0	0	0	0	0	0	0	0	0	0
XR_002343881.1	LOC110260629	lncRNA	0	0	0	0	0	0	0	0	0	0	0	0	0	0	0	0	0	0	0	0	0
XR_002343883.1	LOC110260631	lncRNA	0	0	0	0	0	0	0	0	0	0	0	0	0	0	0	0	0	0	0	0	0
XR_002343884.1	LOC110260634	lncRNA	0	0	0	0	0	0	0	0	0	0	0	0	0	0	0	0	0	0	0	0	0
XR_002343885.1	LOC110260635	lncRNA	0	0	0	0	0	0	0	0	0	0	0	0	0	0	0	0	0	0	0	0	0
XR_002343891.1	LOC110260642	lncRNA	0	0	0	0	0	0	0	0	0	0	0	0	0	0	0	0	0	0	0	0	0
XR_002343892.1	LOC110260643	lncRNA	0	0	0	0	0	0	0	0	0	0	0	0	0	0	0	0	0	0	0	0	0
XR_002345298.1	MAF	mRNA	0	0	0	0	0	0	0	0	0	0	0	0	0	0	0	0	0	0	0	0	0
XR_002345300.1	LOC106510470	lncRNA	0	0	0	0	0	0	0	0	0	0	0	0	0	0	0	0	0	0	0	0	0
XR_002345301.1	LOC106510468	lncRNA	0	0	0	0	0	0	0	0	0	0	0	0	0	0	0	0	0	0	0	0	0
XR_002346166.1	LOC110261703	lncRNA	0	0	0	0	0	0	0	0	0	0	0	0	0	0	0	0	0	0	0	0	0

RNA ID	Symbol	Type	updown_BGIR9823_84443	updown_BGIR9823_81960	updown_BGIR9823_82315	updown_BGIR9823_82966	updown_BGIR9823_83074	updown_BGIR9823_82386	updown_BGIR9823_82709	updown_BGIR9823_83417	updown_BGIR9823_85418	updown_BGIR9823_87926	updown_BGIR9823_88529	updown_BGIR9823_88698	updown_BGIR9823_86965	updown_BGIR9823_89012	updown_BGIR9823_92131	updown_BGIR9823_90639	updown_BGIR9823_95770	updown_BGIR9823_92788	updown_BGIR9823_93572	updown_BGIR9823_96356	updown_BGIR9823_94689
XR_002346227.1	LOC106504299	lncRNA	0	0	0	0	0	0	0	0	0	0	0	0	0	0	0	0	0	0	0	0	0
XR_002346228.1	LOC106504299	lncRNA	0	0	0	0	0	0	0	0	0	0	0	0	0	0	0	0	0	0	0	0	0
XR_002346229.1	LOC106504299	lncRNA	0	0	0	0	0	0	0	0	0	0	0	0	0	0	0	0	0	0	0	0	0
XR_002346230.1	LOC106504299	lncRNA	0	0	0	0	0	0	0	0	0	0	0	0	0	0	0	0	0	0	0	0	0
XR_002346231.1	LOC106504299	lncRNA	0	0	0	0	0	0	0	0	0	0	0	0	0	0	0	0	0	0	0	0	0
XR_002346232.1	LOC106504299	lncRNA	0	0	0	0	0	0	0	0	0	0	0	0	0	0	0	0	0	0	0	0	0
XR_002346233.1	NQO2	mRNA	0	0	0	0	0	0	0	0	0	0	0	0	0	0	0	0	0	0	0	0	0
XR_002346236.1	PRPF4B	mRNA	0	0	0	0	0	0	0	0	0	0	0	0	0	0	0	0	0	0	0	0	0
XR_002346237.1	PRPF4B	mRNA	0	0	0	0	0	0	0	0	0	0	0	0	0	0	0	0	0	0	0	0	0
XR_002346238.1	PRPF4B	mRNA	0	0	0	0	0	0	0	0	0	0	0	0	0	0	0	0	0	0	0	0	0
XR_002346239.1	PRPF4B	mRNA	0	0	0	0	0	0	0	0	0	0	0	0	0	0	0	0	0	0	0	0	0
XR_002346240.1	PRPF4B	mRNA	0	0	0	0	0	0	0	0	0	0	0	0	0	0	0	0	0	0	0	0	0
XR_002346241.1	PRPF4B	mRNA	0	0	0	0	0	0	0	0	0	0	0	0	0	0	0	0	0	0	0	0	0
XR_002346242.1	PRPF4B	mRNA	0	0	0	0	0	0	0	0	0	0	0	0	0	0	0	0	0	0	0	0	0
XR_002346243.1	PRPF4B	mRNA	0	0	0	0	0	0	0	0	0	0	0	0	0	0	0	0	0	0	0	0	0
XR_002346244.1	PRPF4B	mRNA	0	0	0	0	0	0	0	0	0	0	0	0	0	0	0	0	0	0	0	0	0
XR_002346245.1	PRPF4B	mRNA	0	0	0	0	0	0	0	0	0	0	0	0	0	0	0	0	0	0	0	0	0
XR_002346246.1	PRPF4B	mRNA	0	0	0	0	0	0	0	0	0	0	0	0	0	0	0	0	0	0	0	0	0
XR_002346247.1	PRPF4B	mRNA	0	0	0	0	0	0	0	0	0	0	0	0	0	0	0	0	0	0	0	0	0
XR_002346248.1	PRPF4B	mRNA	0	0	0	0	0	0	0	0	0	0	0	0	0	0	0	0	0	0	0	0	0
XR_002346249.1	PRPF4B	mRNA	0	0	0	0	0	0	0	0	0	0	0	0	0	0	0	0	0	0	0	0	0
XR_002346250.1	PRPF4B	mRNA	0	0	0	0	0	0	0	0	0	0	0	0	0	0	0	0	0	0	0	0	0
XR_002346251.1	PRPF4B	mRNA	0	0	0	0	0	0	0	0	0	0	0	0	0	0	0	0	0	0	0	0	0
XR_002346252.1	PRPF4B	mRNA	0	0	0	0	0	0	0	0	0	0	0	0	0	0	0	0	0	0	0	0	0
XR_002346253.1	PRPF4B	mRNA	0	0	0	0	0	0	0	0	0	0	0	0	0	0	0	0	0	0	0	0	0
XR_002346254.1	PRPF4B	mRNA	0	0	0	0	0	0	0	0	0	0	0	0	0	0	0	0	0	0	0	0	0
XR_002346255.1	PRPF4B	mRNA	0	0	0	0	0	0	0	0	0	0	0	0	0	0	0	0	0	0	0	0	0
XR_002346256.1	PRPF4B	mRNA	0	0	0	0	0	0	0	0	0	0	0	0	0	0	0	0	0	0	0	0	0
XR_002346257.1	PRPF4B	mRNA	0	0	0	0	0	0	0	0	0	0	0	0	0	0	0	0	0	0	0	0	0
XR_002346258.1	PRPF4B	mRNA	0	0	0	0	0	0	0	0	0	0	0	0	0	0	0	0	0	0	0	0	0
XR_002346259.1	PRPF4B	mRNA	0	0	0	0	0	0	0	0	0	0	0	0	0	0	0	0	0	0	0	0	0
XR_002346260.1	PRPF4B	mRNA	0	0	0	0	0	0	0	0	0	0	0	0	0	0	0	0	0	0	0	0	0
XR_002346261.1	PRPF4B	mRNA	0	0	0	0	0	0	0	0	0	0	0	0	0	0	0	0	0	0	0	0	0
XR_002346262.1	PRPF4B	mRNA	0	0	0	0	0	0	0	0	0	0	0	0	0	0	0	0	0	0	0	0	0
XR_002346263.1	PRPF4B	mRNA	0	0	0	0	0	0	0	0	0	0	0	0	0	0	0	0	0	0	0	0	0
XR_002346264.1	PRPF4B	mRNA	0	0	0	0	0	0	0	0	0	0	0	0	0	0	0	0	0	0	0	0	0
XR_002346265.1	LOC110261764	lncRNA	0	0	0	0	0	0	0	0	0	0	0	0	0	0	0	0	0	0	0	0	0
XR_002346266.1	LOC102162837	lncRNA	0	0	0	0	0	0	0	0	0	0	0	0	0	0	0	0	0	0	0	0	0
XR_002346299.1	LOC110261774	lncRNA	0	0	0	0	0	0	0	0	0	0	0	0	0	0	0	0	0	0	0	0	0
XR_002346300.1	LOC110261774	lncRNA	0	0	0	0	0	0	0	0	0	0	0	0	0	0	0	0	0	0	0	0	0
XR_002346302.1	LOC110261776	lncRNA	0	0	0	0	0	0	0	0	0	0	0	0	0	0	0	0	0	0	0	0	0
XR_002346303.1	TMEM170B	mRNA	0	0	0	0	0	0	0	0	0	0	0	0	0	0	0	0	0	0	0	0	0
XR_002346304.1	TMEM170B	mRNA	0	0	0	0	0	0	0	0	0	0	0	0	0	0	0	0	0	0	0	0	0

RNA ID	Symbol	Type	updown_BGIR9823_84443	updown_BGIR9823_81960	updown_BGIR9823_82315	updown_BGIR9823_82966	updown_BGIR9823_83074	updown_BGIR9823_82386	updown_BGIR9823_82709	updown_BGIR9823_83417	updown_BGIR9823_85418	updown_BGIR9823_87926	updown_BGIR9823_88529	updown_BGIR9823_88698	updown_BGIR9823_86965	updown_BGIR9823_89012	updown_BGIR9823_92131	updown_BGIR9823_90639	updown_BGIR9823_95770	updown_BGIR9823_92788	updown_BGIR9823_93572	updown_BGIR9823_96356	updown_BGIR9823_94689
XR_115737.4	LOC100513133	lncRNA	0	0	0	0	0	0	0	0	0	0	0	0	0	0	0	0	0	0	0	0	0
XR_299046.2	LOC102164596	lncRNA	0	0	0	0	0	0	0	0	0	0	0	0	0	0	0	0	0	0	0	0	0
XR_303634.3	LOC100738154	lncRNA	0	0	0	0	0	0	0	0	0	0	0	0	0	0	0	0	0	0	0	0	0
XR_304239.3	LOC102159819	lncRNA	0	0	0	0	0	0	0	0	0	0	0	0	0	0	0	0	0	0	0	0	0
XR_304244.3	LOC102161992	lncRNA	0	0	0	0	0	0	0	0	0	0	0	0	0	0	0	0	0	0	0	0	0
XR_304257.3	LOC102162837	lncRNA	0	0	0	0	0	0	0	0	0	0	0	0	0	0	0	0	0	0	0	0	0
XR_306105.3	LOC102166871	lncRNA	0	0	0	0	0	0	0	0	0	0	0	0	0	0	0	0	0	0	0	0	0
XR_307914.3	LOC102159607	lncRNA	0	0	0	0	0	0	0	0	0	0	0	0	0	0	0	0	0	0	0	0	0
XR_308345.3	LOC102167708	lncRNA	0	0	0	0	0	0	0	0	0	0	0	0	0	0	0	0	0	0	0	0	0
novel-ssc-miR1028-3p		miRNA	0	0	0	0	0	0	0	0	0	0	0	0	0	0	0	0	0	0	0	0	0
novel-ssc-miR1028-5p		miRNA	0	0	0	0	0	0	0	0	0	0	0	0	0	0	0	0	0	0	0	0	0
novel-ssc-miR1130-3p		miRNA	0	0	0	0	0	0	0	0	0	0	0	0	0	0	0	0	0	0	0	0	0
novel-ssc-miR1130-5p		miRNA	0	0	0	0	0	0	0	0	0	0	0	0	0	0	0	0	0	0	0	0	0
novel-ssc-miR1162-3p		miRNA	0	0	0	0	0	0	0	0	0	0	0	0	0	0	0	0	0	0	0	0	0
novel-ssc-miR1193-3p		miRNA	0	0	0	0	0	0	0	0	0	0	0	0	0	0	0	0	0	0	0	0	0
novel-ssc-miR1193-5p		miRNA	0	0	0	0	0	0	0	0	0	0	0	0	0	0	0	0	0	0	0	0	0
novel-ssc-miR1217-3p		miRNA	0	0	0	0	0	0	0	0	0	0	0	0	0	0	0	0	0	0	0	0	0
novel-ssc-miR1217-5p		miRNA	0	0	0	0	0	0	0	0	0	0	0	0	0	0	0	0	0	0	0	0	0
novel-ssc-miR1429-3p		miRNA	0	0	0	0	0	0	0	0	0	0	0	0	0	0	0	0	0	0	0	0	0
novel-ssc-miR1429-5p		miRNA	0	0	0	0	0	0	0	0	0	0	0	0	0	0	0	0	0	0	0	0	0
novel-ssc-miR259-3p		miRNA	0	0	0	0	0	0	0	0	0	0	0	0	0	0	0	0	0	0	0	0	0
novel-ssc-miR259-5p		miRNA	0	0	0	0	0	0	0	0	0	0	0	0	0	0	0	0	0	0	0	0	0
novel-ssc-miR324-3p		miRNA	0	0	0	0	0	0	0	0	0	0	0	0	0	0	0	0	0	0	0	0	0
novel-ssc-miR324-5p		miRNA	0	0	0	0	0	0	0	0	0	0	0	0	0	0	0	0	0	0	0	0	0
novel-ssc-miR42-3p		miRNA	0	0	0	0	0	0	0	0	0	0	0	0	0	0	0	0	0	0	0	0	0
novel-ssc-miR42-5p		miRNA	0	0	0	0	0	0	0	0	0	0	0	0	0	0	0	0	0	0	0	0	0
novel-ssc-miR433-3p		miRNA	0	0	0	0	0	0	0	0	0	0	0	0	0	0	0	0	0	0	0	0	0
novel-ssc-miR433-5p		miRNA	0	0	0	0	0	0	0	0	0	0	0	0	0	0	0	0	0	0	0	0	0
novel-ssc-miR557-3p		miRNA	0	0	0	0	0	0	0	0	0	0	0	0	0	0	0	0	0	0	0	0	0
novel-ssc-miR557-5p		miRNA	0	0	0	0	0	0	0	0	0	0	0	0	0	0	0	0	0	0	0	0	0
novel-ssc-miR619-3p		miRNA	0	0	0	0	0	0	0	0	0	0	0	0	0	0	0	0	0	0	0	0	0
novel-ssc-miR619-5p		miRNA	0	0	0	0	0	0	0	0	0	0	0	0	0	0	0	0	0	0	0	0	0
novel-ssc-miR719-3p		miRNA	0	0	0	0	0	0	0	0	0	0	0	0	0	0	0	0	0	0	0	0	0
novel-ssc-miR719-5p		miRNA	0	0	0	0	0	0	0	0	0	0	0	0	0	0	0	0	0	0	0	0	0
novel-ssc-miR966-3p		miRNA	0	0	0	0	0	0	0	0	0	0	0	0	0	0	0	0	0	0	0	0	0
novel-ssc-miR966-5p		miRNA	0	0	0	0	0	0	0	0	0	0	0	0	0	0	0	0	0	0	0	0	0
ssc-miR-7136-3p		miRNA	0	0	0	0	0	0	0	0	0	0	0	0	0	0	0	0	0	0	0	0	0
ssc-miR-7136-5p		miRNA	0	0	0	0	0	0	0	0	0	0	0	0	0	0	0	0	0	0	0	0	0

Supplementary Table S10. The predicted targe

RNA ID	Symbol	Type	updown_BGIR9823_94777	updown_BGIR9823_98865	updown_BGIR9823_98901	updown_BGIR9823_99094	updown_BGIR9823_99751	updown_XR_002336552.1	updown_XR_002337059.1	updown_XR_002337719.1	updown_XR_002337772.1	updown_XR_002345058.1	updown_XR_002345629.1	updown_XR_002343991.1	updown_XR_002341917.1	updown_XR_002342057.1	updown_XR_002342059.1	updown_XR_002335940.1	updown_XR_002336149.1	updown_XR_002342781.1	updown_XR_002343137.1	updown_XR_002343215.1	updown_XR_001301217.2
BGIR9823_100361	PRKAG2	mRNA	0	0	0	0	0	0	0	0	0	0	0	0	0	0	0	0	0	0	0	0	0
BGIR9823_100362	PRKAG2	mRNA	0	0	0	0	0	0	0	0	0	0	0	0	0	0	0	0	0	0	0	0	0
BGIR9823_100363	PRKAG2	mRNA	0	0	0	0	0	0	0	0	0	0	0	0	0	0	0	0	0	0	0	0	0
BGIR9823_100364	PRKAG2	mRNA	0	0	0	0	0	0	0	0	0	0	0	0	0	0	0	0	0	0	0	0	0
BGIR9823_101535	LOC102158401	lncRNA	0	0	0	0	0	0	0	0	0	0	0	0	0	0	0	0	0	0	0	0	0
BGIR9823_101541	LOC102157946	lncRNA	0	0	0	0	0	0	0	0	0	0	0	0	0	0	0	0	0	0	0	0	0
BGIR9823_101542	LOC102157946	lncRNA	0	0	0	0	0	0	0	0	0	0	0	0	0	0	0	0	0	0	0	0	0
BGIR9823_101560	LOC110258600	lncRNA	0	0	0	0	0	0	0	0	0	0	0	0	0	0	0	0	0	0	0	0	0
BGIR9823_101561	LOC110258600	lncRNA	0	0	0	0	0	0	0	0	0	0	0	0	0	0	0	0	0	0	0	0	0
BGIR9823_101562	LOC110258600	lncRNA	0	0	0	0	0	0	0	0	0	0	0	0	0	0	0	0	0	0	0	0	0
BGIR9823_101563	LOC110258600	lncRNA	0	0	0	0	0	0	0	0	0	0	0	0	0	0	0	0	0	0	0	0	0
BGIR9823_101564	LOC110258600	lncRNA	0	0	0	0	0	0	0	0	0	0	0	0	0	0	0	0	0	0	0	0	0
BGIR9823_101612	LOC100521600	lncRNA	0	0	0	0	0	0	0	0	0	0	0	0	0	0	0	0	0	0	0	0	0
BGIR9823_101853		lncRNA	0	0	0	0	0	0	0	0	0	0	0	0	0	0	0	0	0	0	0	0	0
BGIR9823_101854		lncRNA	0	0	0	0	0	0	0	0	0	0	0	0	0	0	0	0	0	0	0	0	0
BGIR9823_101855		lncRNA	0	0	0	0	0	0	0	0	0	0	0	0	0	0	0	0	0	0	0	0	0
BGIR9823_102146		lncRNA	0	0	0	0	0	0	0	0	0	0	0	0	0	0	0	0	0	0	0	0	0
BGIR9823_102180		lncRNA	0	0	0	0	0	0	0	0	0	0	0	0	0	0	0	0	0	0	0	0	0
BGIR9823_102279		lncRNA	0	0	0	0	0	0	0	0	0	0	0	0	0	0	0	0	0	0	0	0	0
BGIR9823_102282		lncRNA	0	0	0	0	0	0	0	0	0	0	0	0	0	0	0	0	0	0	0	0	0
BGIR9823_102361		lncRNA	0	0	0	0	0	0	0	0	0	0	0	0	0	0	0	0	0	0	0	0	0
BGIR9823_102362		lncRNA	0	0	0	0	0	0	0	0	0	0	0	0	0	0	0	0	0	0	0	0	0
BGIR9823_102363		lncRNA	0	0	0	0	0	0	0	0	0	0	0	0	0	0	0	0	0	0	0	0	0
BGIR9823_102727		lncRNA	0	0	0	0	0	0	0	0	0	0	0	0	0	0	0	0	0	0	0	0	0
BGIR9823_80121	SSSCA1	lncRNA	0	0	0	0	0	0	0	0	0	0	0	0	0	0	0	0	0	0	0	0	0
BGIR9823_80122	LOC102163816	lncRNA	0	0	0	0	0	0	0	0	0	0	0	0	0	0	0	0	0	0	0	0	0
BGIR9823_81007	EHBP1L1	mRNA	0	0	0	0	0	0	0	0	0	0	0	0	0	0	0	0	0	0	0	0	0
BGIR9823_81008	EHBP1L1	mRNA	0	0	0	0	0	0	0	0	0	0	0	0	0	0	0	0	0	0	0	0	0
BGIR9823_81009	EHBP1L1	mRNA	0	0	0	0	0	0	0	0	0	0	0	0	0	0	0	0	0	0	0	0	0
BGIR9823_81010	EHBP1L1	mRNA	0	0	0	0	0	0	0	0	0	0	0	0	0	0	0	0	0	0	0	0	0
BGIR9823_81011	SSSCA1	mRNA	0	0	0	0	0	0	0	0	0	0	0	0	0	0	0	0	0	0	0	0	0
BGIR9823_81012	FRMD8	lncRNA	0	0	0	0	0	0	0	0	0	0	0	0	0	0	0	0	0	0	0	0	0
BGIR9823_81013	LOC102163816	lncRNA	0	0	0	0	0	0	0	0	0	0	0	0	0	0	0	0	0	0	0	0	0
BGIR9823_81014	LOC102163816	lncRNA	0	0	0	0	0	0	0	0	0	0	0	0	0	0	0	0	0	0	0	0	0
BGIR9823_81015	LOC102163816	lncRNA	0	0	0	0	0	0	0	0	0	0	0	0	0	0	0	0	0	0	0	0	0
BGIR9823_81016	LOC102163816	lncRNA	0	0	0	0	0	0	0	0	0	0	0	0	0	0	0	0	0	0	0	0	0
BGIR9823_81017	LOC102163816	lncRNA	0	0	0	0	0	0	0	0	0	0	0	0	0	0	0	0	0	0	0	0	0
BGIR9823_81018	LOC102163816	lncRNA	0	0	0	0	0	0	0	0	0	0	0	0	0	0	0	0	0	0	0	0	0
BGIR9823_81019	LOC100513133	lncRNA	0	0	0	0	0	0	0	0	0	0	0	0	0	0	0	0	0	0	0	0	0
BGIR9823_84704	LOC102159820	mRNA	0	0	0	0	0	0	0	0	0	0	0	0	0	0	0	0	0	0	0	0	0
BGIR9823_84732	RANGAP1	mRNA	0	0	0	0	0	0	0	0	0	0	0	0	0	0	0	0	0	0	0	0	0

RNA ID	Symbol	Type	updown_BGIR9823_94777	updown_BGIR9823_98865	updown_BGIR9823_98901	updown_BGIR9823_99094	updown_BGIR9823_99751	updown_XR_002336552.1	updown_XR_002337059.1	updown_XR_002337719.1	updown_XR_002337772.1	updown_XR_002345058.1	updown_XR_002345629.1	updown_XR_002343991.1	updown_XR_002341917.1	updown_XR_002342057.1	updown_XR_002342059.1	updown_XR_002335940.1	updown_XR_002336149.1	updown_XR_002342781.1	updown_XR_002343137.1	updown_XR_002343215.1	updown_XR_001301217.2
BGIR9823_84733	RANGAP1	mRNA	0	0	0	0	0	0	0	0	0	0	0	0	0	0	0	0	0	0	0	0	0
BGIR9823_85245	CRADD	lncRNA	0	0	0	0	0	0	0	0	0	0	0	0	0	0	0	0	0	0	0	0	0
BGIR9823_85246	LOC100738154	lncRNA	0	0	0	0	0	0	0	0	0	0	0	0	0	0	0	0	0	0	0	0	0
BGIR9823_85293	TRMU	mRNA	0	0	0	0	0	0	0	0	0	0	0	0	0	0	0	0	0	0	0	0	0
BGIR9823_85294	TRMU	mRNA	0	0	0	0	0	0	0	0	0	0	0	0	0	0	0	0	0	0	0	0	0
BGIR9823_85295	TTC38	mRNA	0	0	0	0	0	0	0	0	0	0	0	0	0	0	0	0	0	0	0	0	0
BGIR9823_85296	PKDREJ	lncRNA	0	0	0	0	0	0	0	0	0	0	0	0	0	0	0	0	0	0	0	0	0
BGIR9823_85297	PPARA	mRNA	0	0	0	0	0	0	0	0	0	0	0	0	0	0	0	0	0	0	0	0	0
BGIR9823_85298	PPARA	mRNA	0	0	0	0	0	0	0	0	0	0	0	0	0	0	0	0	0	0	0	0	0
BGIR9823_85299	PPARA	mRNA	0	0	0	0	0	0	0	0	0	0	0	0	0	0	0	0	0	0	0	0	0
BGIR9823_85300	PPARA	lncRNA	0	0	0	0	0	0	0	0	0	0	0	0	0	0	0	0	0	0	0	0	0
BGIR9823_85301	LOC110260611	mRNA	0	0	0	0	0	0	0	0	0	0	0	0	0	0	0	0	0	0	0	0	0
BGIR9823_85317	ACO2	mRNA	0	0	0	0	0	0	0	0	0	0	0	0	0	0	0	0	0	0	0	0	0
BGIR9823_85318	LOC110260629	lncRNA	0	0	0	0	0	0	0	0	0	0	0	0	0	0	0	0	0	0	0	0	0
BGIR9823_85319	TEF	mRNA	0	0	0	0	0	0	0	0	0	0	0	0	0	0	0	0	0	0	0	0	0
BGIR9823_85320	ZC3H7B	mRNA	0	0	0	0	0	0	0	0	0	0	0	0	0	0	0	0	0	0	0	0	0
BGIR9823_85345	SYNGR1	mRNA	0	0	0	0	0	0	0	0	0	0	0	0	0	0	0	0	0	0	0	0	0
BGIR9823_85835	CRADD	mRNA	0	0	0	0	0	0	0	0	0	0	0	0	0	0	0	0	0	0	0	0	0
BGIR9823_85839	CRADD	lncRNA	0	0	0	0	0	0	0	0	0	0	0	0	0	0	0	0	0	0	0	0	0
BGIR9823_85840	CRADD	lncRNA	0	0	0	0	0	0	0	0	0	0	0	0	0	0	0	0	0	0	0	0	0
BGIR9823_85841	CRADD	lncRNA	0	0	0	0	0	0	0	0	0	0	0	0	0	0	0	0	0	0	0	0	0
BGIR9823_85930	LOC106510465	mRNA	0	0	0	0	0	0	0	0	0	0	0	0	0	0	0	0	0	0	0	0	0
BGIR9823_85931	MAF	lncRNA	0	0	0	0	0	0	0	0	0	0	0	0	0	0	0	0	0	0	0	0	0
BGIR9823_85932	MAF	lncRNA	0	0	0	0	0	0	0	0	0	0	0	0	0	0	0	0	0	0	0	0	0
BGIR9823_87139	LOC106510467	lncRNA	0	0	0	0	0	0	0	0	0	0	0	0	0	0	0	0	0	0	0	0	0
BGIR9823_88187	WRNIP1	mRNA	0	0	0	0	0	0	0	0	0	0	0	0	0	0	0	0	0	0	0	0	0
BGIR9823_88188	NQO2	mRNA	0	0	0	0	0	0	0	0	0	0	0	0	0	0	0	0	0	0	0	0	0
BGIR9823_88189	NQO2	mRNA	0	0	0	0	0	0	0	0	0	0	0	0	0	0	0	0	0	0	0	0	0
BGIR9823_88190	RIPK1	mRNA	0	0	0	0	0	0	0	0	0	0	0	0	0	0	0	0	0	0	0	0	0
BGIR9823_88191	RIPK1	mRNA	0	0	0	0	0	0	0	0	0	0	0	0	0	0	0	0	0	0	0	0	0
BGIR9823_88192	RIPK1	mRNA	0	0	0	0	0	0	0	0	0	0	0	0	0	0	0	0	0	0	0	0	0
BGIR9823_88193	RIPK1	mRNA	0	0	0	0	0	0	0	0	0	0	0	0	0	0	0	0	0	0	0	0	0
BGIR9823_88196	PRPF4B	mRNA	0	0	0	0	0	0	0	0	0	0	0	0	0	0	0	0	0	0	0	0	0
BGIR9823_88197	PRPF4B	mRNA	0	0	0	0	0	0	0	0	0	0	0	0	0	0	0	0	0	0	0	0	0
BGIR9823_88198	PRPF4B	mRNA	0	0	0	0	0	0	0	0	0	0	0	0	0	0	0	0	0	0	0	0	0
BGIR9823_88211	TMEM170B	lncRNA	0	0	0	0	0	0	0	0	0	0	0	0	0	0	0	0	0	0	0	0	0
BGIR9823_88212	TMEM170B	lncRNA	0	0	0	0	0	0	0	0	0	0	0	0	0	0	0	0	0	0	0	0	0
BGIR9823_88213	TMEM170B	lncRNA	0	0	0	0	0	0	0	0	0	0	0	0	0	0	0	0	0	0	0	0	0
BGIR9823_88214	TMEM170B	lncRNA	0	0	0	0	0	0	0	0	0	0	0	0	0	0	0	0	0	0	0	0	0
BGIR9823_88217	PHACTR1	mRNA	0	0	0	0	0	0	0	0	0	0	0	0	0	0	0	0	0	0	0	0	0
BGIR9823_88218	PHACTR1	lncRNA	0	0	0	0	0	0	0	0	0	0	0	0	0	0	0	0	0	0	0	0	0
BGIR9823_89017	MYLK4	lncRNA	0	0	0	0	0	0	0	0	0	0	0	0	0	0	0	0	0	0	0	0	0
BGIR9823_89019	SERPINB1	lncRNA	0	0	0	0	0	0	0	0	0	0	0	0	0	0	0	0	0	0	0	0	0

RNA ID	Symbol	Type	updown_BGIR9823_94777	updown_BGIR9823_98865	updown_BGIR9823_98901	updown_BGIR9823_99094	updown_BGIR9823_99751	updown_XR_002336552.1	updown_XR_002337059.1	updown_XR_002337719.1	updown_XR_002337772.1	updown_XR_002345058.1	updown_XR_002345629.1	updown_XR_002343991.1	updown_XR_002341917.1	updown_XR_002342057.1	updown_XR_002342059.1	updown_XR_002335940.1	updown_XR_002336149.1	updown_XR_002342781.1	updown_XR_002343137.1	updown_XR_002343215.1	updown_XR_001301217.2
BGIR9823_89020	SERPINB9	mRNA	0	0	0	0	0	0	0	0	0	0	0	0	0	0	0	0	0	0	0	0	0
BGIR9823_89021	RIPK1	lncRNA	0	0	0	0	0	0	0	0	0	0	0	0	0	0	0	0	0	0	0	0	0
BGIR9823_89025	PXDC1	mRNA	0	0	0	0	0	0	0	0	0	0	0	0	0	0	0	0	0	0	0	0	0
BGIR9823_89026	PXDC1	lncRNA	0	0	0	0	0	0	0	0	0	0	0	0	0	0	0	0	0	0	0	0	0
BGIR9823_89027	LOC102161744	lncRNA	0	0	0	0	0	0	0	0	0	0	0	0	0	0	0	0	0	0	0	0	0
BGIR9823_89040	TBC1D7	lncRNA	0	0	0	0	0	0	0	0	0	0	0	0	0	0	0	0	0	0	0	0	0
BGIR9823_89041	TBC1D7	lncRNA	0	0	0	0	0	0	0	0	0	0	0	0	0	0	0	0	0	0	0	0	0
BGIR9823_89042	TBC1D7	lncRNA	0	0	0	0	0	0	0	0	0	0	0	0	0	0	0	0	0	0	0	0	0
BGIR9823_89043	TBC1D7	lncRNA	0	0	0	0	0	0	0	0	0	0	0	0	0	0	0	0	0	0	0	0	0
BGIR9823_89044	GFOD1	lncRNA	0	0	0	0	0	0	0	0	0	0	0	0	0	0	0	0	0	0	0	0	0
BGIR9823_89045	GFOD1	mRNA	0	0	0	0	0	0	0	0	0	0	0	0	0	0	0	0	0	0	0	0	0
BGIR9823_89046	GFOD1	mRNA	0	0	0	0	0	0	0	0	0	0	0	0	0	0	0	0	0	0	0	0	0
BGIR9823_89047	GFOD1	mRNA	0	0	0	0	0	0	0	0	0	0	0	0	0	0	0	0	0	0	0	0	0
BGIR9823_89048	GFOD1	lncRNA	0	0	0	0	0	0	0	0	0	0	0	0	0	0	0	0	0	0	0	0	0
BGIR9823_89049	GFOD1	lncRNA	0	0	0	0	0	0	0	0	0	0	0	0	0	0	0	0	0	0	0	0	0
BGIR9823_90771	NEU3	lncRNA	0	0	0	0	0	0	0	0	0	0	0	0	0	0	0	0	0	0	0	0	0
BGIR9823_90772	SLCO2B1	mRNA	0	0	0	0	0	0	0	0	0	0	0	0	0	0	0	0	0	0	0	0	0
BGIR9823_90913	LOC110255435	lncRNA	0	0	0	0	0	0	0	0	0	0	0	0	0	0	0	0	0	0	0	0	0
BGIR9823_90934	LOC110255441	lncRNA	0	0	0	0	0	0	0	0	0	0	0	0	0	0	0	0	0	0	0	0	0
BGIR9823_90935	CCDC84	mRNA	0	0	0	0	0	0	0	0	0	0	0	0	0	0	0	0	0	0	0	0	0
BGIR9823_90936	TRAPPC4	mRNA	0	0	0	0	0	0	0	0	0	0	0	0	0	0	0	0	0	0	0	0	0
BGIR9823_90937	HMBS	mRNA	0	0	0	0	0	0	0	0	0	0	0	0	0	0	0	0	0	0	0	0	0
BGIR9823_90956	SORL1	mRNA	0	0	0	0	0	0	0	0	0	0	0	0	0	0	0	0	0	0	0	0	0
BGIR9823_90957	SORL1	mRNA	0	0	0	0	0	0	0	0	0	0	0	0	0	0	0	0	0	0	0	0	0
BGIR9823_90958	SORL1	mRNA	0	0	0	0	0	0	0	0	0	0	0	0	0	0	0	0	0	0	0	0	0
BGIR9823_90960	CLMP	lncRNA	0	0	0	0	0	0	0	0	0	0	0	0	0	0	0	0	0	0	0	0	0
BGIR9823_91161	LOC100522267	mRNA	0	0	0	0	0	0	0	0	0	0	0	0	0	0	0	0	0	0	0	0	0
BGIR9823_91162	LOC100522455	mRNA	0	0	0	0	0	0	0	0	0	0	0	0	0	0	0	0	0	0	0	0	0
BGIR9823_91163	ABCB1	mRNA	0	0	0	0	0	0	0	0	0	0	0	0	0	0	0	0	0	0	0	0	0
BGIR9823_91402	HBB	mRNA	0	0	0	0	0	0	0	0	0	0	0	0	0	0	0	0	0	0	0	0	0
BGIR9823_91403	HBB	mRNA	0	0	0	0	0	0	0	0	0	0	0	0	0	0	0	0	0	0	0	0	0
BGIR9823_91404	HBB	mRNA	0	0	0	0	0	0	0	0	0	0	0	0	0	0	0	0	0	0	0	0	0
BGIR9823_91405	HBB	mRNA	0	0	0	0	0	0	0	0	0	0	0	0	0	0	0	0	0	0	0	0	0
BGIR9823_91406	HBB	mRNA	0	0	0	0	0	0	0	0	0	0	0	0	0	0	0	0	0	0	0	0	0
BGIR9823_91407	HBB	mRNA	0	0	0	0	0	0	0	0	0	0	0	0	0	0	0	0	0	0	0	0	0
BGIR9823_91408	HBB	mRNA	0	0	0	0	0	0	0	0	0	0	0	0	0	0	0	0	0	0	0	0	0
BGIR9823_91409	HBB	mRNA	0	0	0	0	0	0	0	0	0	0	0	0	0	0	0	0	0	0	0	0	0
BGIR9823_91625	BCL9L	lncRNA	0	0	0	0	0	0	0	0	0	0	0	0	0	0	0	0	0	0	0	0	0
BGIR9823_91626	BCL9L	lncRNA	0	0	0	0	0	0	0	0	0	0	0	0	0	0	0	0	0	0	0	0	0
BGIR9823_91627	RPS25	mRNA	0	0	0	0	0	0	0	0	0	0	0	0	0	0	0	0	0	0	0	0	0
BGIR9823_91628	RPS25	mRNA	0	0	0	0	0	0	0	0	0	0	0	0	0	0	0	0	0	0	0	0	0
BGIR9823_91629	SLC37A4	mRNA	0	0	0	0	0	0	0	0	0	0	0	0	0	0	0	0	0	0	0	0	0
BGIR9823_91630	HYOU1	mRNA	0	0	0	0	0	0	0	0	0	0	0	0	0	0	0	0	0	0	0	0	0

RNA ID	Symbol	Type	updown_BGIR9823_94777	updown_BGIR9823_98865	updown_BGIR9823_98901	updown_BGIR9823_99094	updown_BGIR9823_99751	updown_XR_002336552.1	updown_XR_002337059.1	updown_XR_002337719.1	updown_XR_002337772.1	updown_XR_002345058.1	updown_XR_002345629.1	updown_XR_002343991.1	updown_XR_002341917.1	updown_XR_002342057.1	updown_XR_002342059.1	updown_XR_002335940.1	updown_XR_002336149.1	updown_XR_002342781.1	updown_XR_002343137.1	updown_XR_002343215.1	updown_XR_001301217.2
BGIR9823_91640	CLMP	mRNA	0	0	0	0	0	0	0	0	0	0	0	0	0	0	0	0	0	0	0	0	0
BGIR9823_91819	RUNDC3B	lncRNA	0	0	0	0	0	0	0	0	0	0	0	0	0	0	0	0	0	0	0	0	0
BGIR9823_91820	RUNDC3B	lncRNA	0	0	0	0	0	0	0	0	0	0	0	0	0	0	0	0	0	0	0	0	0
BGIR9823_91821	RUNDC3B	lncRNA	0	0	0	0	0	0	0	0	0	0	0	0	0	0	0	0	0	0	0	0	0
BGIR9823_91822	RUNDC3B	lncRNA	0	0	0	0	0	0	0	0	0	0	0	0	0	0	0	0	0	0	0	0	0
BGIR9823_91823	RUNDC3B	lncRNA	0	0	0	0	0	0	0	0	0	0	0	0	0	0	0	0	0	0	0	0	0
BGIR9823_91824	RUNDC3B	lncRNA	0	0	0	0	0	0	0	0	0	0	0	0	0	0	0	0	0	0	0	0	0
BGIR9823_91825	RUNDC3B	lncRNA	0	0	0	0	0	0	0	0	0	0	0	0	0	0	0	0	0	0	0	0	0
BGIR9823_91826	LOC102164596	lncRNA	0	0	0	0	0	0	0	0	0	0	0	0	0	0	0	0	0	0	0	0	0
BGIR9823_92993	KATNAL1	mRNA	0	0	0	0	0	0	0	0	0	0	0	0	0	0	0	0	0	0	0	0	0
BGIR9823_92994	KATNAL1	mRNA	0	0	0	0	0	0	0	0	0	0	0	0	0	0	0	0	0	0	0	0	0
BGIR9823_92995	KATNAL1	mRNA	0	0	0	0	0	0	0	0	0	0	0	0	0	0	0	0	0	0	0	0	0
BGIR9823_92996	KATNAL1	mRNA	0	0	0	0	0	0	0	0	0	0	0	0	0	0	0	0	0	0	0	0	0
BGIR9823_92997	KATNAL1	mRNA	0	0	0	0	0	0	0	0	0	0	0	0	0	0	0	0	0	0	0	0	0
BGIR9823_92999	LOC110255292	lncRNA	0	0	0	0	0	0	0	0	0	0	0	0	0	0	0	0	0	0	0	0	0
BGIR9823_93000	LOC102166871	lncRNA	0	0	0	0	0	0	0	0	0	0	0	0	0	0	0	0	0	0	0	0	0
BGIR9823_98051	LOC102164998	lncRNA	0	0	0	0	0	0	0	0	0	0	0	0	0	0	0	0	0	0	0	0	1
BGIR9823_99244	LOC102167708	lncRNA	0	0	0	0	0	0	0	0	0	0	0	0	0	0	0	0	0	0	0	0	0
NM_001004034.1	HMGB1	mRNA	0	0	0	0	0	0	0	0	0	0	0	0	0	0	0	0	0	0	0	0	0
NM_001044526.1	PPARA	mRNA	0	0	0	0	0	0	0	0	0	0	0	0	0	0	0	0	0	0	0	0	0
NM_001111256.1	SERPINB9	mRNA	0	0	0	0	0	0	0	0	0	0	0	0	0	0	0	0	0	0	0	0	0
NM_001113443.1	ECI2	mRNA	0	0	0	0	0	0	0	0	0	0	0	0	0	0	0	0	0	0	0	0	0
NM_001144841.1	HBB	mRNA	0	0	0	0	0	0	0	0	0	0	0	0	0	0	0	0	0	0	0	0	0
NM_001199719.1	SLC37A4	mRNA	0	0	0	0	0	0	0	0	0	0	0	0	0	0	0	0	0	0	0	0	0
NM_001243907.1	HSPA8	mRNA	0	0	0	0	0	0	0	0	0	0	0	0	0	0	0	0	0	0	0	0	0
NM_001244063.1	RPL3	mRNA	0	0	0	0	0	0	0	0	0	0	0	0	0	0	0	0	0	0	0	0	0
NM_001244066.1	SYNGR1	mRNA	0	0	0	0	0	0	0	0	0	0	0	0	0	0	0	0	0	0	0	0	0
NM_001244396.1	CCDC84	mRNA	0	0	0	0	0	0	0	0	0	0	0	0	0	0	0	0	0	0	0	0	0
NM_001287846.1	RHEB	mRNA	0	0	0	0	0	0	0	0	0	0	0	0	0	0	0	0	0	0	0	0	0
NM_213954.1	ACO2	mRNA	0	0	0	0	0	0	0	0	0	0	0	0	0	0	0	0	0	0	0	0	0
NM_214012.1	UPK2	mRNA	0	0	0	0	0	0	0	0	0	0	0	0	0	0	0	0	0	0	0	0	0
URS0000BC92F4	LOC102167708	lncRNA	0	0	0	0	0	0	0	0	0	0	0	0	0	0	0	0	0	0	0	0	0
XM_001928309.6	TBC1D7	mRNA	0	0	0	0	0	0	0	0	0	0	0	0	0	0	0	0	0	0	0	0	0
XM_001928852.5	PXDC1	mRNA	0	0	0	0	0	0	0	0	0	0	0	0	0	0	0	0	0	0	0	0	0
XM_003122534.4	KCNK7	mRNA	0	0	0	0	0	0	0	0	0	0	0	0	0	0	0	0	0	0	0	0	0
XM_003122558.5	SCYL1	mRNA	0	0	0	0	0	0	0	0	0	0	0	0	0	0	0	0	0	0	0	0	0
XM_003122562.5	FRMD8	mRNA	0	0	0	0	0	0	0	0	0	0	0	0	0	0	0	0	0	0	0	0	0
XM_003125951.6	TTC38	mRNA	0	0	0	0	0	0	0	0	0	0	0	0	0	0	0	0	0	0	0	0	0
XM_003125953.4	CDPF1	mRNA	0	0	0	0	0	0	0	0	0	0	0	0	0	0	0	0	0	0	0	0	0
XM_003126012.6	CBX7	mRNA	0	0	0	0	0	0	0	0	0	0	0	0	0	0	0	0	0	0	0	0	0
XM_003128161.4	RIPK1	mRNA	0	0	0	0	0	0	0	0	0	0	0	0	0	0	0	0	0	0	0	0	0
XM_003129508.2	LOC100514417	mRNA	0	0	0	0	0	0	0	0	0	0	0	0	0	0	0	0	0	0	0	0	0
XM_003129512.2	LOC100515268	mRNA	0	0	0	0	0	0	0	0	0	0	0	0	0	0	0	0	0	0	0	0	0

RNA ID	Symbol	Type	updown_BGIR9823_94777	updown_BGIR9823_98865	updown_BGIR9823_98901	updown_BGIR9823_99094	updown_BGIR9823_99751	updown_XR_002336552.1	updown_XR_002337059.1	updown_XR_002337719.1	updown_XR_002337772.1	updown_XR_002345058.1	updown_XR_002345629.1	updown_XR_002343991.1	updown_XR_002341917.1	updown_XR_002342057.1	updown_XR_002342059.1	updown_XR_002335940.1	updown_XR_002336149.1	updown_XR_002342781.1	updown_XR_002343137.1	updown_XR_002343215.1	updown_XR_001301217.2
XM_003129514.3	LOC100515618	mRNA	0	0	0	0	0	0	0	0	0	0	0	0	0	0	0	0	0	0	0	0	0
XM_003129519.2	LOC100517595	mRNA	0	0	0	0	0	0	0	0	0	0	0	0	0	0	0	0	0	0	0	0	0
XM_003129529.4	LOC100519910	mRNA	0	0	0	0	0	0	0	0	0	0	0	0	0	0	0	0	0	0	0	0	0
XM_003129915.4	CXCR5	mRNA	0	0	0	0	0	0	0	0	0	0	0	0	0	0	0	0	0	0	0	0	0
XM_003129916.5	BCL9L	mRNA	0	0	0	0	0	0	0	0	0	0	0	0	0	0	0	0	0	0	0	0	0
XM_003129918.4	TRAPPC4	mRNA	0	0	0	0	0	0	0	0	0	0	0	0	0	0	0	0	0	0	0	0	0
XM_003129919.5	RPS25	mRNA	0	0	0	0	0	0	0	0	0	0	0	0	0	0	0	0	0	0	0	0	0
XM_003129920.3	RPS25	mRNA	0	0	0	0	0	0	0	0	0	0	0	0	0	0	0	0	0	0	0	0	0
XM_003129922.4	HYOU1	mRNA	0	0	0	0	0	0	0	0	0	0	0	0	0	0	0	0	0	0	0	0	0
XM_003129923.6	VPS11	mRNA	0	0	0	0	0	0	0	0	0	0	0	0	0	0	0	0	0	0	0	0	0
XM_003129944.4	SC5D	mRNA	0	0	0	0	0	0	0	0	0	0	0	0	0	0	0	0	0	0	0	0	0
XM_003357161.1	LOC100620971	mRNA	0	0	0	0	0	0	0	0	0	0	0	0	0	0	0	0	0	0	0	0	0
XM_003482119.4	SERPINB1	mRNA	0	0	0	0	0	0	0	0	0	0	0	0	0	0	0	0	0	0	0	0	0
XM_003482132.4	TMEM170B	mRNA	0	0	0	0	0	0	0	0	0	0	0	0	0	0	0	0	0	0	0	0	0
XM_003482133.4	GFOD1	mRNA	0	0	0	0	0	0	0	0	0	0	0	0	0	0	0	0	0	0	0	0	0
XM_003482563.4	SPCS2	mRNA	0	0	0	0	0	0	0	0	0	0	0	0	0	0	0	0	0	0	0	0	0
XM_003482565.4	NEU3	mRNA	0	0	0	0	0	0	0	0	0	0	0	0	0	0	0	0	0	0	0	0	0
XM_003482567.1	LOC100739359	mRNA	0	0	0	0	0	0	0	0	0	0	0	0	0	0	0	0	0	0	0	0	0
XM_003482630.3	BSX	mRNA	0	0	0	0	0	0	0	0	0	0	0	0	0	0	0	0	0	0	0	0	0
XM_005653107.3	CHADL	mRNA	0	0	0	0	0	0	0	0	0	0	0	0	0	0	0	0	0	0	0	0	0
XM_005653640.1	LOC100621181	mRNA	0	0	0	0	0	0	0	0	0	0	0	0	0	0	0	0	0	0	0	0	0
XM_005654266.3	PRKAG2	mRNA	0	0	0	0	0	0	0	0	0	0	0	0	0	0	0	0	0	0	0	0	0
XM_005655470.3	CDPF1	mRNA	0	0	0	0	0	0	0	0	0	0	0	0	0	0	0	0	0	0	0	0	0
XM_005660703.3	LTBP3	mRNA	0	0	0	0	0	0	0	0	0	0	0	0	0	0	0	0	0	0	0	0	0
XM_005660705.3	SCYL1	mRNA	0	0	0	0	0	0	0	0	0	0	0	0	0	0	0	0	0	0	0	0	0
XM_005660707.3	SLC25A45	mRNA	0	0	0	0	0	0	0	0	0	0	0	0	0	0	0	0	0	0	0	0	0
XM_005665536.3	RIPK1	mRNA	0	0	0	0	0	0	0	0	0	0	0	0	0	0	0	0	0	0	0	0	0
XM_005665537.3	RIPK1	mRNA	0	0	0	0	0	0	0	0	0	0	0	0	0	0	0	0	0	0	0	0	0
XM_005665538.3	RIPK1	mRNA	0	0	0	0	0	0	0	0	0	0	0	0	0	0	0	0	0	0	0	0	0
XM_005665575.3	PHACTR1	mRNA	0	0	0	0	0	0	0	0	0	0	0	0	0	0	0	0	0	0	0	0	0
XM_005665576.3	TBC1D7	mRNA	0	0	0	0	0	0	0	0	0	0	0	0	0	0	0	0	0	0	0	0	0
XM_005667128.3	LOC100627340	mRNA	0	0	0	0	0	0	0	0	0	0	0	0	0	0	0	0	0	0	0	0	0
XM_005667397.2	CCDC84	mRNA	0	0	0	0	0	0	0	0	0	0	0	0	0	0	0	0	0	0	0	0	0
XM_005667398.3	SLC37A4	mRNA	0	0	0	0	0	0	0	0	0	0	0	0	0	0	0	0	0	0	0	0	0
XM_005667402.3	SLC37A4	mRNA	0	0	0	0	0	0	0	0	0	0	0	0	0	0	0	0	0	0	0	0	0
XM_005667403.3	SLC37A4	mRNA	0	0	0	0	0	0	0	0	0	0	0	0	0	0	0	0	0	0	0	0	0
XM_005667413.3	BCL9L	mRNA	0	0	0	0	0	0	0	0	0	0	0	0	0	0	0	0	0	0	0	0	0
XM_005667414.3	FOXR1	mRNA	0	0	0	0	0	0	0	0	0	0	0	0	0	0	0	0	0	0	0	0	0
XM_005667416.3	HYOU1	mRNA	0	0	0	0	0	0	0	0	0	0	0	0	0	0	0	0	0	0	0	0	0
XM_005667419.3	HYOU1	mRNA	0	0	0	0	0	0	0	0	0	0	0	0	0	0	0	0	0	0	0	0	0
XM_005667420.3	HYOU1	mRNA	0	0	0	0	0	0	0	0	0	0	0	0	0	0	0	0	0	0	0	0	0
XM_005667440.2	JHY	mRNA	0	0	0	0	0	0	0	0	0	0	0	0	0	0	0	0	0	0	0	0	0
XM_005668316.3	HMGB1	mRNA	0	0	0	0	0	0	0	0	0	0	0	0	0	0	0	0	0	0	0	0	0

RNA ID	Symbol	Type	updown_BGIR9823_94777	updown_BGIR9823_98865	updown_BGIR9823_98901	updown_BGIR9823_99094	updown_BGIR9823_99751	updown_XR_002336552.1	updown_XR_002337059.1	updown_XR_002337719.1	updown_XR_002337772.1	updown_XR_002345058.1	updown_XR_002345629.1	updown_XR_002343991.1	updown_XR_002341917.1	updown_XR_002342057.1	updown_XR_002342059.1	updown_XR_002335940.1	updown_XR_002336149.1	updown_XR_002342781.1	updown_XR_002343137.1	updown_XR_002343215.1	updown_XR_001301217.2
XM_005668317.3	HMGB1	mRNA	0	0	0	0	0	0	0	0	0	0	0	0	0	0	0	0	0	0	0	0	0
XM_005668318.3	HMGB1	mRNA	0	0	0	0	0	0	0	0	0	0	0	0	0	0	0	0	0	0	0	0	0
XM_005671489.3	EMX2	mRNA	0	0	0	0	0	0	0	0	0	0	0	0	0	0	0	0	0	0	0	0	1
XM_005672397.3	MYO10	mRNA	0	0	0	0	0	0	0	0	0	0	0	0	0	0	0	0	0	0	0	0	0
XM_013977400.2	FAM50B	mRNA	0	0	0	0	0	0	0	0	0	0	0	0	0	0	0	0	0	0	0	0	0
XM_013977425.2	NEDD9	mRNA	0	0	0	0	0	0	0	0	0	0	0	0	0	0	0	0	0	0	0	0	0
XM_013977430.2	TBC1D7	mRNA	0	0	0	0	0	0	0	0	0	0	0	0	0	0	0	0	0	0	0	0	0
XM_013977431.2	TBC1D7	mRNA	0	0	0	0	0	0	0	0	0	0	0	0	0	0	0	0	0	0	0	0	0
XM_013977432.2	TBC1D7	mRNA	0	0	0	0	0	0	0	0	0	0	0	0	0	0	0	0	0	0	0	0	0
XM_013977433.2	GFOD1	mRNA	0	0	0	0	0	0	0	0	0	0	0	0	0	0	0	0	0	0	0	0	0
XM_013977434.2	GFOD1	mRNA	0	0	0	0	0	0	0	0	0	0	0	0	0	0	0	0	0	0	0	0	0
XM_013979449.2	SLC37A4	mRNA	0	0	0	0	0	0	0	0	0	0	0	0	0	0	0	0	0	0	0	0	0
XM_013979456.2	BCL9L	mRNA	0	0	0	0	0	0	0	0	0	0	0	0	0	0	0	0	0	0	0	0	0
XM_013979457.2	BCL9L	mRNA	0	0	0	0	0	0	0	0	0	0	0	0	0	0	0	0	0	0	0	0	0
XM_013979458.2	BCL9L	mRNA	0	0	0	0	0	0	0	0	0	0	0	0	0	0	0	0	0	0	0	0	0
XM_013979459.2	BCL9L	mRNA	0	0	0	0	0	0	0	0	0	0	0	0	0	0	0	0	0	0	0	0	0
XM_013979461.2	TRAPPC4	mRNA	0	0	0	0	0	0	0	0	0	0	0	0	0	0	0	0	0	0	0	0	0
XM_013979463.2	HYOU1	mRNA	0	0	0	0	0	0	0	0	0	0	0	0	0	0	0	0	0	0	0	0	0
XM_013979484.2	LOC100523684	mRNA	0	0	0	0	0	0	0	0	0	0	0	0	0	0	0	0	0	0	0	0	0
XM_013979486.2	LOC100523684	mRNA	0	0	0	0	0	0	0	0	0	0	0	0	0	0	0	0	0	0	0	0	0
XM_013979491.2	JHY	mRNA	0	0	0	0	0	0	0	0	0	0	0	0	0	0	0	0	0	0	0	0	0
XM_013980418.2	KATNAL1	mRNA	0	0	0	0	0	0	0	0	0	0	0	0	0	0	0	0	0	0	0	0	0
XM_013988182.2	TTC38	mRNA	0	0	0	0	0	0	0	0	0	0	0	0	0	0	0	0	0	0	0	0	0
XM_013988183.2	TTC38	mRNA	0	0	0	0	0	0	0	0	0	0	0	0	0	0	0	0	0	0	0	0	0
XM_013989589.2	LOC100522267	mRNA	0	0	0	0	0	0	0	0	0	0	0	0	0	0	0	0	0	0	0	0	0
XM_013989592.2	LOC100522455	mRNA	0	0	0	0	0	0	0	0	0	0	0	0	0	0	0	0	0	0	0	0	0
XM_013989593.2	LOC100522455	mRNA	0	0	0	0	0	0	0	0	0	0	0	0	0	0	0	0	0	0	0	0	0
XM_013994099.2	FRMD8	mRNA	0	0	0	0	0	0	0	0	0	0	0	0	0	0	0	0	0	0	0	0	0
XM_013994100.2	SLC25A45	mRNA	0	0	0	0	0	0	0	0	0	0	0	0	0	0	0	0	0	0	0	0	0
XM_013994102.2	SLC25A45	mRNA	0	0	0	0	0	0	0	0	0	0	0	0	0	0	0	0	0	0	0	0	0
XM_013994103.2	SLC25A45	mRNA	0	0	0	0	0	0	0	0	0	0	0	0	0	0	0	0	0	0	0	0	0
XM_013994104.2	SLC25A45	mRNA	0	0	0	0	0	0	0	0	0	0	0	0	0	0	0	0	0	0	0	0	0
XM_013997510.2	CBX7	mRNA	0	0	0	0	0	0	0	0	0	0	0	0	0	0	0	0	0	0	0	0	0
XM_013999070.2	LOC100520088	mRNA	0	0	0	0	0	0	0	0	0	0	0	0	0	0	0	0	0	0	0	0	0
XM_021062230.1	LOC100517771	mRNA	0	0	0	0	0	0	0	0	0	0	0	0	0	0	0	0	0	0	0	0	0
XM_021062231.1	LOC100517957	mRNA	0	0	0	0	0	0	0	0	0	0	0	0	0	0	0	0	0	0	0	0	0
XM_021062367.1	LOC110255365	mRNA	0	0	0	0	0	0	0	0	0	0	0	0	0	0	0	0	0	0	0	0	0
XM_021062369.1	LOC100737110	mRNA	0	0	0	0	0	0	0	0	0	0	0	0	0	0	0	0	0	0	0	0	0
XM_021062370.1	LOC110255368	mRNA	0	0	0	0	0	0	0	0	0	0	0	0	0	0	0	0	0	0	0	0	0
XM_021062371.1	LOC100515441	mRNA	0	0	0	0	0	0	0	0	0	0	0	0	0	0	0	0	0	0	0	0	0
XM_021062374.1	LOC110255371	mRNA	0	0	0	0	0	0	0	0	0	0	0	0	0	0	0	0	0	0	0	0	0
XM_021062375.1	LOC100626951	mRNA	0	0	0	0	0	0	0	0	0	0	0	0	0	0	0	0	0	0	0	0	0
XM_021062491.1	XRRA1	mRNA	0	0	0	0	0	0	0	0	0	0	0	0	0	0	0	0	0	0	0	0	0

RNA ID	Symbol	Type	updown_BGIR9823_94777	updown_BGIR9823_98865	updown_BGIR9823_98901	updown_BGIR9823_99094	updown_BGIR9823_99751	updown_XR_002336552.1	updown_XR_002337059.1	updown_XR_002337719.1	updown_XR_002337772.1	updown_XR_002345058.1	updown_XR_002345629.1	updown_XR_002343991.1	updown_XR_002341917.1	updown_XR_002342057.1	updown_XR_002342059.1	updown_XR_002335940.1	updown_XR_002336149.1	updown_XR_002342781.1	updown_XR_002343137.1	updown_XR_002343215.1	updown_XR_001301217.2
XM_021062493.1	XRRA1	mRNA	0	0	0	0	0	0	0	0	0	0	0	0	0	0	0	0	0	0	0	0	0
XM_021062494.1	XRRA1	mRNA	0	0	0	0	0	0	0	0	0	0	0	0	0	0	0	0	0	0	0	0	0
XM_021062495.1	XRRA1	mRNA	0	0	0	0	0	0	0	0	0	0	0	0	0	0	0	0	0	0	0	0	0
XM_021062496.1	XRRA1	mRNA	0	0	0	0	0	0	0	0	0	0	0	0	0	0	0	0	0	0	0	0	0
XM_021062497.1	XRRA1	mRNA	0	0	0	0	0	0	0	0	0	0	0	0	0	0	0	0	0	0	0	0	0
XM_021062498.1	XRRA1	mRNA	0	0	0	0	0	0	0	0	0	0	0	0	0	0	0	0	0	0	0	0	0
XM_021062499.1	XRRA1	mRNA	0	0	0	0	0	0	0	0	0	0	0	0	0	0	0	0	0	0	0	0	0
XM_021062500.1	XRRA1	mRNA	0	0	0	0	0	0	0	0	0	0	0	0	0	0	0	0	0	0	0	0	0
XM_021062501.1	XRRA1	mRNA	0	0	0	0	0	0	0	0	0	0	0	0	0	0	0	0	0	0	0	0	0
XM_021062502.1	XRRA1	mRNA	0	0	0	0	0	0	0	0	0	0	0	0	0	0	0	0	0	0	0	0	0
XM_021062503.1	XRRA1	mRNA	0	0	0	0	0	0	0	0	0	0	0	0	0	0	0	0	0	0	0	0	0
XM_021062511.1	SLCO2B1	mRNA	0	0	0	0	0	0	0	0	0	0	0	0	0	0	0	0	0	0	0	0	0
XM_021062948.1	BCL9L	mRNA	0	0	0	0	0	0	0	0	0	0	0	0	0	0	0	0	0	0	0	0	0
XM_021062949.1	TRAPPC4	mRNA	0	0	0	0	0	0	0	0	0	0	0	0	0	0	0	0	0	0	0	0	0
XM_021062950.1	HYOU1	mRNA	0	0	0	0	0	0	0	0	0	0	0	0	0	0	0	0	0	0	0	0	0
XM_021062951.1	HYOU1	mRNA	0	0	0	0	0	0	0	0	0	0	0	0	0	0	0	0	0	0	0	0	0
XM_021062952.1	HYOU1	mRNA	0	0	0	0	0	0	0	0	0	0	0	0	0	0	0	0	0	0	0	0	0
XM_021062953.1	HYOU1	mRNA	0	0	0	0	0	0	0	0	0	0	0	0	0	0	0	0	0	0	0	0	0
XM_021062954.1	HYOU1	mRNA	0	0	0	0	0	0	0	0	0	0	0	0	0	0	0	0	0	0	0	0	0
XM_021062955.1	HYOU1	mRNA	0	0	0	0	0	0	0	0	0	0	0	0	0	0	0	0	0	0	0	0	0
XM_021062956.1	HYOU1	mRNA	0	0	0	0	0	0	0	0	0	0	0	0	0	0	0	0	0	0	0	0	0
XM_021062957.1	VPS11	mRNA	0	0	0	0	0	0	0	0	0	0	0	0	0	0	0	0	0	0	0	0	0
XM_021062993.1	LOC100523684	mRNA	0	0	0	0	0	0	0	0	0	0	0	0	0	0	0	0	0	0	0	0	0
XM_021062994.1	LOC100523684	mRNA	0	0	0	0	0	0	0	0	0	0	0	0	0	0	0	0	0	0	0	0	0
XM_021062995.1	LOC100523684	mRNA	0	0	0	0	0	0	0	0	0	0	0	0	0	0	0	0	0	0	0	0	0
XM_021062996.1	LOC100523684	mRNA	0	0	0	0	0	0	0	0	0	0	0	0	0	0	0	0	0	0	0	0	0
XM_021062997.1	LOC100523684	mRNA	0	0	0	0	0	0	0	0	0	0	0	0	0	0	0	0	0	0	0	0	0
XM_021062998.1	LOC100523684	mRNA	0	0	0	0	0	0	0	0	0	0	0	0	0	0	0	0	0	0	0	0	0
XM_021062999.1	LOC100523684	mRNA	0	0	0	0	0	0	0	0	0	0	0	0	0	0	0	0	0	0	0	0	0
XM_021063000.1	LOC100523684	mRNA	0	0	0	0	0	0	0	0	0	0	0	0	0	0	0	0	0	0	0	0	0
XM_021063001.1	LOC100523684	mRNA	0	0	0	0	0	0	0	0	0	0	0	0	0	0	0	0	0	0	0	0	0
XM_021063002.1	LOC100523684	mRNA	0	0	0	0	0	0	0	0	0	0	0	0	0	0	0	0	0	0	0	0	0
XM_021063004.1	LOC100523684	mRNA	0	0	0	0	0	0	0	0	0	0	0	0	0	0	0	0	0	0	0	0	0
XM_021063005.1	LOC100523684	mRNA	0	0	0	0	0	0	0	0	0	0	0	0	0	0	0	0	0	0	0	0	0
XM_021063006.1	LOC100523684	mRNA	0	0	0	0	0	0	0	0	0	0	0	0	0	0	0	0	0	0	0	0	0
XM_021063009.1	SORL1	mRNA	0	0	0	0	0	0	0	0	0	0	0	0	0	0	0	0	0	0	0	0	0
XM_021063010.1	SORL1	mRNA	0	0	0	0	0	0	0	0	0	0	0	0	0	0	0	0	0	0	0	0	0
XM_021063011.1	SORL1	mRNA	0	0	0	0	0	0	0	0	0	0	0	0	0	0	0	0	0	0	0	0	0
XM_021063012.1	SORL1	mRNA	0	0	0	0	0	0	0	0	0	0	0	0	0	0	0	0	0	0	0	0	0
XM_021063013.1	SORL1	mRNA	0	0	0	0	0	0	0	0	0	0	0	0	0	0	0	0	0	0	0	0	0
XM_021063014.1	SORL1	mRNA	0	0	0	0	0	0	0	0	0	0	0	0	0	0	0	0	0	0	0	0	0
XM_021063023.1	CLMP	mRNA	0	0	0	0	0	0	0	0	0	0	0	0	0	0	0	0	0	0	0	0	0
XM_021063024.1	CLMP	mRNA	0	0	0	0	0	0	0	0	0	0	0	0	0	0	0	0	0	0	0	0	0

RNA ID	Symbol	Type	updown_BGIR9823_94777	updown_BGIR9823_98865	updown_BGIR9823_98901	updown_BGIR9823_99094	updown_BGIR9823_99751	updown_XR_002336552.1	updown_XR_002337059.1	updown_XR_002337719.1	updown_XR_002337772.1	updown_XR_002345058.1	updown_XR_002345629.1	updown_XR_002343991.1	updown_XR_002341917.1	updown_XR_002342057.1	updown_XR_002342059.1	updown_XR_002335940.1	updown_XR_002336149.1	updown_XR_002342781.1	updown_XR_002343137.1	updown_XR_002343215.1	updown_XR_001301217.2
XM_021063025.1	CLMP	mRNA	0	0	0	0	0	0	0	0	0	0	0	0	0	0	0	0	0	0	0	0	0
XM_021063026.1	CLMP	mRNA	0	0	0	0	0	0	0	0	0	0	0	0	0	0	0	0	0	0	0	0	0
XM_021063950.1	NXPE2	mRNA	0	0	0	0	0	0	0	0	0	0	0	0	0	0	0	0	0	0	0	0	0
XM_021063963.1	LOC100623190	mRNA	0	0	0	0	0	0	0	0	0	0	0	0	0	0	0	0	0	0	0	0	0
XM_021065159.1	HMGB1	mRNA	0	0	0	0	0	0	0	0	0	0	0	0	0	0	0	0	0	0	0	0	0
XM_021065160.1	HMGB1	mRNA	0	0	0	0	0	0	0	0	0	0	0	0	0	0	0	0	0	0	0	0	0
XM_021065161.1	HMGB1	mRNA	0	0	0	0	0	0	0	0	0	0	0	0	0	0	0	0	0	0	0	0	0
XM_021065529.1	KATNAL1	mRNA	0	0	0	0	0	0	0	0	0	0	0	0	0	0	0	0	0	0	0	0	0
XM_021065531.1	KATNAL1	mRNA	0	0	0	0	0	0	0	0	0	0	0	0	0	0	0	0	0	0	0	0	0
XM_021065532.1	KATNAL1	mRNA	0	0	0	0	0	0	0	0	0	0	0	0	0	0	0	0	0	0	0	0	0
XM_021065533.1	KATNAL1	mRNA	0	0	0	0	0	0	0	0	0	0	0	0	0	0	0	0	0	0	0	0	0
XM_021065534.1	KATNAL1	mRNA	0	0	0	0	0	0	0	0	0	0	0	0	0	0	0	0	0	0	0	0	0
XM_021065535.1	KATNAL1	mRNA	0	0	0	0	0	0	0	0	0	0	0	0	0	0	0	0	0	0	0	0	0
XM_021065536.1	KATNAL1	mRNA	0	0	0	0	0	0	0	0	0	0	0	0	0	0	0	0	0	0	0	0	0
XM_021065537.1	KATNAL1	mRNA	0	0	0	0	0	0	0	0	0	0	0	0	0	0	0	0	0	0	0	0	0
XM_021065538.1	KATNAL1	mRNA	0	0	0	0	0	0	0	0	0	0	0	0	0	0	0	0	0	0	0	0	0
XM_021065539.1	KATNAL1	mRNA	0	0	0	0	0	0	0	0	0	0	0	0	0	0	0	0	0	0	0	0	0
XM_021065541.1	KATNAL1	mRNA	0	0	0	0	0	0	0	0	0	0	0	0	0	0	0	0	0	0	0	0	0
XM_021065542.1	KATNAL1	mRNA	0	0	0	0	0	0	0	0	0	0	0	0	0	0	0	0	0	0	0	0	0
XM_021065543.1	KATNAL1	mRNA	0	0	0	0	0	0	0	0	0	0	0	0	0	0	0	0	0	0	0	0	0
XM_021065544.1	KATNAL1	mRNA	0	0	0	0	0	0	0	0	0	0	0	0	0	0	0	0	0	0	0	0	0
XM_021073408.1	EMX2	mRNA	0	0	0	0	0	0	0	0	0	0	0	0	0	0	0	0	0	0	0	0	1
XM_021078611.1	PRKAG2	mRNA	0	0	0	0	0	0	0	0	0	0	0	0	0	0	0	0	0	0	0	0	0
XM_021078612.1	PRKAG2	mRNA	0	0	0	0	0	0	0	0	0	0	0	0	0	0	0	0	0	0	0	0	0
XM_021078613.1	PRKAG2	mRNA	0	0	0	0	0	0	0	0	0	0	0	0	0	0	0	0	0	0	0	0	0
XM_021078614.1	PRKAG2	mRNA	0	0	0	0	0	0	0	0	0	0	0	0	0	0	0	0	0	0	0	0	0
XM_021078615.1	PRKAG2	mRNA	0	0	0	0	0	0	0	0	0	0	0	0	0	0	0	0	0	0	0	0	0
XM_021078616.1	PRKAG2	mRNA	0	0	0	0	0	0	0	0	0	0	0	0	0	0	0	0	0	0	0	0	0
XM_021078617.1	PRKAG2	mRNA	0	0	0	0	0	0	0	0	0	0	0	0	0	0	0	0	0	0	0	0	0
XM_021078618.1	PRKAG2	mRNA	0	0	0	0	0	0	0	0	0	0	0	0	0	0	0	0	0	0	0	0	0
XM_021078619.1	PRKAG2	mRNA	0	0	0	0	0	0	0	0	0	0	0	0	0	0	0	0	0	0	0	0	0
XM_021078620.1	PRKAG2	mRNA	0	0	0	0	0	0	0	0	0	0	0	0	0	0	0	0	0	0	0	0	0
XM_021078621.1	PRKAG2	mRNA	0	0	0	0	0	0	0	0	0	0	0	0	0	0	0	0	0	0	0	0	0
XM_021081309.1	LOC106506226	mRNA	0	0	0	0	0	0	0	0	0	0	0	0	0	0	0	0	0	0	0	0	0
XM_021081403.1	LOC110258215	mRNA	0	0	0	0	0	0	0	0	0	0	0	0	0	0	0	0	0	0	0	0	0
XM_021082153.1	LOC100521600	mRNA	0	0	0	0	0	0	0	0	0	0	0	0	0	0	0	0	0	0	0	0	0
XM_021082154.1	LOC100521600	mRNA	0	0	0	0	0	0	0	0	0	0	0	0	0	0	0	0	0	0	0	0	0
XM_021082156.1	LOC100521600	mRNA	0	0	0	0	0	0	0	0	0	0	0	0	0	0	0	0	0	0	0	0	0
XM_021082157.1	LOC100521600	mRNA	0	0	0	0	0	0	0	0	0	0	0	0	0	0	0	0	0	0	0	0	0
XM_021082158.1	LOC100521600	mRNA	0	0	0	0	0	0	0	0	0	0	0	0	0	0	0	0	0	0	0	0	0
XM_021082159.1	LOC100521600	mRNA	0	0	0	0	0	0	0	0	0	0	0	0	0	0	0	0	0	0	0	0	0
XM_021082835.1	EHBP1L1	mRNA	0	0	0	0	0	0	0	0	0	0	0	0	0	0	0	0	0	0	0	0	0
XM_021082836.1	EHBP1L1	mRNA	0	0	0	0	0	0	0	0	0	0	0	0	0	0	0	0	0	0	0	0	0

RNA ID	Symbol	Type	updown_BGIR9823_94777	updown_BGIR9823_98865	updown_BGIR9823_98901	updown_BGIR9823_99094	updown_BGIR9823_99751	updown_XR_002336552.1	updown_XR_002337059.1	updown_XR_002337719.1	updown_XR_002337772.1	updown_XR_002345058.1	updown_XR_002345629.1	updown_XR_002343991.1	updown_XR_002341917.1	updown_XR_002342057.1	updown_XR_002342059.1	updown_XR_002335940.1	updown_XR_002336149.1	updown_XR_002342781.1	updown_XR_002343137.1	updown_XR_002343215.1	updown_XR_001301217.2
XM_021082837.1	EHBP1L1	mRNA	0	0	0	0	0	0	0	0	0	0	0	0	0	0	0	0	0	0	0	0	0
XM_021082838.1	EHBP1L1	mRNA	0	0	0	0	0	0	0	0	0	0	0	0	0	0	0	0	0	0	0	0	0
XM_021082839.1	EHBP1L1	mRNA	0	0	0	0	0	0	0	0	0	0	0	0	0	0	0	0	0	0	0	0	0
XM_021082840.1	EHBP1L1	mRNA	0	0	0	0	0	0	0	0	0	0	0	0	0	0	0	0	0	0	0	0	0
XM_021082841.1	EHBP1L1	mRNA	0	0	0	0	0	0	0	0	0	0	0	0	0	0	0	0	0	0	0	0	0
XM_021082842.1	EHBP1L1	mRNA	0	0	0	0	0	0	0	0	0	0	0	0	0	0	0	0	0	0	0	0	0
XM_021082843.1	EHBP1L1	mRNA	0	0	0	0	0	0	0	0	0	0	0	0	0	0	0	0	0	0	0	0	0
XM_021082844.1	EHBP1L1	mRNA	0	0	0	0	0	0	0	0	0	0	0	0	0	0	0	0	0	0	0	0	0
XM_021082845.1	EHBP1L1	mRNA	0	0	0	0	0	0	0	0	0	0	0	0	0	0	0	0	0	0	0	0	0
XM_021082846.1	EHBP1L1	mRNA	0	0	0	0	0	0	0	0	0	0	0	0	0	0	0	0	0	0	0	0	0
XM_021082847.1	EHBP1L1	mRNA	0	0	0	0	0	0	0	0	0	0	0	0	0	0	0	0	0	0	0	0	0
XM_021082851.1	MAP3K11	mRNA	0	0	0	0	0	0	0	0	0	0	0	0	0	0	0	0	0	0	0	0	0
XM_021082852.1	SSSCA1	mRNA	0	0	0	0	0	0	0	0	0	0	0	0	0	0	0	0	0	0	0	0	0
XM_021082853.1	FAM89B	mRNA	0	0	0	0	0	0	0	0	0	0	0	0	0	0	0	0	0	0	0	0	0
XM_021082854.1	LTBP3	mRNA	0	0	0	0	0	0	0	0	0	0	0	0	0	0	0	0	0	0	0	0	0
XM_021082855.1	LTBP3	mRNA	0	0	0	0	0	0	0	0	0	0	0	0	0	0	0	0	0	0	0	0	0
XM_021082856.1	SCYL1	mRNA	0	0	0	0	0	0	0	0	0	0	0	0	0	0	0	0	0	0	0	0	0
XM_021082857.1	SLC25A45	mRNA	0	0	0	0	0	0	0	0	0	0	0	0	0	0	0	0	0	0	0	0	0
XM_021082858.1	SLC25A45	mRNA	0	0	0	0	0	0	0	0	0	0	0	0	0	0	0	0	0	0	0	0	0
XM_021091099.1	SYNGR1	mRNA	0	0	0	0	0	0	0	0	0	0	0	0	0	0	0	0	0	0	0	0	0
XM_021091101.1	SYNGR1	mRNA	0	0	0	0	0	0	0	0	0	0	0	0	0	0	0	0	0	0	0	0	0
XM_021091102.1	SYNGR1	mRNA	0	0	0	0	0	0	0	0	0	0	0	0	0	0	0	0	0	0	0	0	0
XM_021091103.1	SYNGR1	mRNA	0	0	0	0	0	0	0	0	0	0	0	0	0	0	0	0	0	0	0	0	0
XM_021091370.1	LOC102159820	mRNA	0	0	0	0	0	0	0	0	0	0	0	0	0	0	0	0	0	0	0	0	0
XM_021091373.1	TRMU	mRNA	0	0	0	0	0	0	0	0	0	0	0	0	0	0	0	0	0	0	0	0	0
XM_021091374.1	TRMU	mRNA	0	0	0	0	0	0	0	0	0	0	0	0	0	0	0	0	0	0	0	0	0
XM_021091375.1	TRMU	mRNA	0	0	0	0	0	0	0	0	0	0	0	0	0	0	0	0	0	0	0	0	0
XM_021091376.1	GTSE1	mRNA	0	0	0	0	0	0	0	0	0	0	0	0	0	0	0	0	0	0	0	0	0
XM_021091377.1	TTC38	mRNA	0	0	0	0	0	0	0	0	0	0	0	0	0	0	0	0	0	0	0	0	0
XM_021091379.1	LOC110260611	mRNA	0	0	0	0	0	0	0	0	0	0	0	0	0	0	0	0	0	0	0	0	0
XM_021091381.1	LOC110260611	mRNA	0	0	0	0	0	0	0	0	0	0	0	0	0	0	0	0	0	0	0	0	0
XM_021091454.1	TOB2	mRNA	0	0	0	0	0	0	0	0	0	0	0	0	0	0	0	0	0	0	0	0	0
XM_021091455.1	TOB2	mRNA	0	0	0	0	0	0	0	0	0	0	0	0	0	0	0	0	0	0	0	0	0
XM_021091456.1	PHF5A	mRNA	0	0	0	0	0	0	0	0	0	0	0	0	0	0	0	0	0	0	0	0	0
XM_021091457.1	PHF5A	mRNA	0	0	0	0	0	0	0	0	0	0	0	0	0	0	0	0	0	0	0	0	0
XM_021091458.1	ZC3H7B	mRNA	0	0	0	0	0	0	0	0	0	0	0	0	0	0	0	0	0	0	0	0	0
XM_021091459.1	ZC3H7B	mRNA	0	0	0	0	0	0	0	0	0	0	0	0	0	0	0	0	0	0	0	0	0
XM_021091460.1	TEF	mRNA	0	0	0	0	0	0	0	0	0	0	0	0	0	0	0	0	0	0	0	0	0
XM_021091461.1	RANGAP1	mRNA	0	0	0	0	0	0	0	0	0	0	0	0	0	0	0	0	0	0	0	0	0
XM_021091474.1	CBX7	mRNA	0	0	0	0	0	0	0	0	0	0	0	0	0	0	0	0	0	0	0	0	0
XM_021092767.1	CRADD	mRNA	0	0	0	0	0	0	0	0	0	0	0	0	0	0	0	0	0	0	0	0	0
XM_021092768.1	CRADD	mRNA	0	0	0	0	0	0	0	0	0	0	0	0	0	0	0	0	0	0	0	0	0
XM_021092769.1	CRADD	mRNA	0	0	0	0	0	0	0	0	0	0	0	0	0	0	0	0	0	0	0	0	0

RNA ID	Symbol	Type	updown_BGIR9823_94777	updown_BGIR9823_98865	updown_BGIR9823_98901	updown_BGIR9823_99094	updown_BGIR9823_99751	updown_XR_002336552.1	updown_XR_002337059.1	updown_XR_002337719.1	updown_XR_002337772.1	updown_XR_002345058.1	updown_XR_002345629.1	updown_XR_002343991.1	updown_XR_002341917.1	updown_XR_002342057.1	updown_XR_002342059.1	updown_XR_002335940.1	updown_XR_002336149.1	updown_XR_002342781.1	updown_XR_002343137.1	updown_XR_002343215.1	updown_XR_001301217.2
XM_021092770.1	CRADD	mRNA	0	0	0	0	0	0	0	0	0	0	0	0	0	0	0	0	0	0	0	0	0
XM_021092771.1	CRADD	mRNA	0	0	0	0	0	0	0	0	0	0	0	0	0	0	0	0	0	0	0	0	0
XM_021092773.1	CRADD	mRNA	0	0	0	0	0	0	0	0	0	0	0	0	0	0	0	0	0	0	0	0	0
XM_021093095.1	PKDREJ	mRNA	0	0	0	0	0	0	0	0	0	0	0	0	0	0	0	0	0	0	0	0	0
XM_021097064.1	MAF	mRNA	0	0	0	0	0	0	0	0	0	0	0	0	0	0	0	0	0	0	0	0	0
XM_021097065.1	MAF	mRNA	0	0	0	0	0	0	0	0	0	0	0	0	0	0	0	0	0	0	0	0	0
XM_021097066.1	MAF	mRNA	0	0	0	0	0	0	0	0	0	0	0	0	0	0	0	0	0	0	0	0	0
XM_021097870.1	ECI2	mRNA	0	0	0	0	0	0	0	0	0	0	0	0	0	0	0	0	0	0	0	0	0
XM_021099751.1	PHACTR1	mRNA	0	0	0	0	0	0	0	0	0	0	0	0	0	0	0	0	0	0	0	0	0
XM_021099752.1	PHACTR1	mRNA	0	0	0	0	0	0	0	0	0	0	0	0	0	0	0	0	0	0	0	0	0
XM_021099753.1	PHACTR1	mRNA	0	0	0	0	0	0	0	0	0	0	0	0	0	0	0	0	0	0	0	0	0
XM_021099754.1	PHACTR1	mRNA	0	0	0	0	0	0	0	0	0	0	0	0	0	0	0	0	0	0	0	0	0
XM_021099755.1	PHACTR1	mRNA	0	0	0	0	0	0	0	0	0	0	0	0	0	0	0	0	0	0	0	0	0
XM_021099756.1	PHACTR1	mRNA	0	0	0	0	0	0	0	0	0	0	0	0	0	0	0	0	0	0	0	0	0
XM_021099757.1	PHACTR1	mRNA	0	0	0	0	0	0	0	0	0	0	0	0	0	0	0	0	0	0	0	0	0
XM_021099758.1	PHACTR1	mRNA	0	0	0	0	0	0	0	0	0	0	0	0	0	0	0	0	0	0	0	0	0
XM_021099759.1	PHACTR1	mRNA	0	0	0	0	0	0	0	0	0	0	0	0	0	0	0	0	0	0	0	0	0
XM_021099760.1	PHACTR1	mRNA	0	0	0	0	0	0	0	0	0	0	0	0	0	0	0	0	0	0	0	0	0
XM_021099761.1	PHACTR1	mRNA	0	0	0	0	0	0	0	0	0	0	0	0	0	0	0	0	0	0	0	0	0
XM_021099762.1	PHACTR1	mRNA	0	0	0	0	0	0	0	0	0	0	0	0	0	0	0	0	0	0	0	0	0
XM_021099763.1	PHACTR1	mRNA	0	0	0	0	0	0	0	0	0	0	0	0	0	0	0	0	0	0	0	0	0
XM_021099764.1	PHACTR1	mRNA	0	0	0	0	0	0	0	0	0	0	0	0	0	0	0	0	0	0	0	0	0
XM_021099765.1	PHACTR1	mRNA	0	0	0	0	0	0	0	0	0	0	0	0	0	0	0	0	0	0	0	0	0
XM_021099766.1	TBC1D7	mRNA	0	0	0	0	0	0	0	0	0	0	0	0	0	0	0	0	0	0	0	0	0
XM_021099767.1	TBC1D7	mRNA	0	0	0	0	0	0	0	0	0	0	0	0	0	0	0	0	0	0	0	0	0
XM_021099769.1	TBC1D7	mRNA	0	0	0	0	0	0	0	0	0	0	0	0	0	0	0	0	0	0	0	0	0
XM_021099770.1	TBC1D7	mRNA	0	0	0	0	0	0	0	0	0	0	0	0	0	0	0	0	0	0	0	0	0
XM_021099999.1	LOC110261697	mRNA	0	0	0	0	0	0	0	0	0	0	0	0	0	0	0	0	0	0	0	0	0
XM_021100108.1	WRNIP1	mRNA	0	0	0	0	0	0	0	0	0	0	0	0	0	0	0	0	0	0	0	0	0
XM_021100109.1	MYLK4	mRNA	0	0	0	0	0	0	0	0	0	0	0	0	0	0	0	0	0	0	0	0	0
XM_021100110.1	MYLK4	mRNA	0	0	0	0	0	0	0	0	0	0	0	0	0	0	0	0	0	0	0	0	0
XM_021100111.1	MYLK4	mRNA	0	0	0	0	0	0	0	0	0	0	0	0	0	0	0	0	0	0	0	0	0
XM_021100112.1	MYLK4	mRNA	0	0	0	0	0	0	0	0	0	0	0	0	0	0	0	0	0	0	0	0	0
XM_021100114.1	NQO2	mRNA	0	0	0	0	0	0	0	0	0	0	0	0	0	0	0	0	0	0	0	0	0
XM_021100115.1	NQO2	mRNA	0	0	0	0	0	0	0	0	0	0	0	0	0	0	0	0	0	0	0	0	0
XM_021100117.1	NQO2	mRNA	0	0	0	0	0	0	0	0	0	0	0	0	0	0	0	0	0	0	0	0	0
XM_021100118.1	NQO2	mRNA	0	0	0	0	0	0	0	0	0	0	0	0	0	0	0	0	0	0	0	0	0
XM_021100119.1	NQO2	mRNA	0	0	0	0	0	0	0	0	0	0	0	0	0	0	0	0	0	0	0	0	0
XM_021100124.1	PRPF4B	mRNA	0	0	0	0	0	0	0	0	0	0	0	0	0	0	0	0	0	0	0	0	0
XM_021100126.1	PRPF4B	mRNA	0	0	0	0	0	0	0	0	0	0	0	0	0	0	0	0	0	0	0	0	0
XM_021100184.1	NEDD9	mRNA	0	0	0	0	0	0	0	0	0	0	0	0	0	0	0	0	0	0	0	0	0
XM_021100188.1	TMEM170B	mRNA	0	0	0	0	0	0	0	0	0	0	0	0	0	0	0	0	0	0	0	0	0
XM_021100194.1	GFOD1	mRNA	0	0	0	0	0	0	0	0	0	0	0	0	0	0	0	0	0	0	0	0	0

RNA ID	Symbol	Type	updown_BGIR9823_94777	updown_BGIR9823_98865	updown_BGIR9823_98901	updown_BGIR9823_99094	updown_BGIR9823_99751	updown_XR_002336552.1	updown_XR_002337059.1	updown_XR_002337719.1	updown_XR_002337772.1	updown_XR_002345058.1	updown_XR_002345629.1	updown_XR_002343991.1	updown_XR_002341917.1	updown_XR_002342057.1	updown_XR_002342059.1	updown_XR_002335940.1	updown_XR_002336149.1	updown_XR_002342781.1	updown_XR_002343137.1	updown_XR_002343215.1	updown_XR_001301217.2
XM_021102040.1	SLC37A4	mRNA	0	0	0	0	0	0	0	0	0	0	0	0	0	0	0	0	0	0	0	0	0
XM_021102041.1	SLC37A4	mRNA	0	0	0	0	0	0	0	0	0	0	0	0	0	0	0	0	0	0	0	0	0
XM_021102042.1	SLC37A4	mRNA	0	0	0	0	0	0	0	0	0	0	0	0	0	0	0	0	0	0	0	0	0
XM_021102043.1	SLC37A4	mRNA	0	0	0	0	0	0	0	0	0	0	0	0	0	0	0	0	0	0	0	0	0
XM_021102044.1	SLC37A4	mRNA	0	0	0	0	0	0	0	0	0	0	0	0	0	0	0	0	0	0	0	0	0
XM_021102045.1	SLC37A4	mRNA	0	0	0	0	0	0	0	0	0	0	0	0	0	0	0	0	0	0	0	0	0
XM_021102046.1	SLC37A4	mRNA	0	0	0	0	0	0	0	0	0	0	0	0	0	0	0	0	0	0	0	0	0
XM_021102047.1	SLC37A4	mRNA	0	0	0	0	0	0	0	0	0	0	0	0	0	0	0	0	0	0	0	0	0
XM_021102048.1	SLC37A4	mRNA	0	0	0	0	0	0	0	0	0	0	0	0	0	0	0	0	0	0	0	0	0
XM_021102049.1	SLC37A4	mRNA	0	0	0	0	0	0	0	0	0	0	0	0	0	0	0	0	0	0	0	0	0
XM_021102050.1	SLC37A4	mRNA	0	0	0	0	0	0	0	0	0	0	0	0	0	0	0	0	0	0	0	0	0
XM_021102051.1	SLC37A4	mRNA	0	0	0	0	0	0	0	0	0	0	0	0	0	0	0	0	0	0	0	0	0
XM_021102052.1	SLC37A4	mRNA	0	0	0	0	0	0	0	0	0	0	0	0	0	0	0	0	0	0	0	0	0
XM_021102209.1	UPK2	mRNA	0	0	0	0	0	0	0	0	0	0	0	0	0	0	0	0	0	0	0	0	0
XM_021102516.1	LOC100522455	mRNA	0	0	0	0	0	0	0	0	0	0	0	0	0	0	0	0	0	0	0	0	0
XM_021102517.1	RUNDC3B	mRNA	0	0	0	0	0	0	0	0	0	0	0	0	0	0	0	0	0	0	0	0	0
XM_021102518.1	RUNDC3B	mRNA	0	0	0	0	0	0	0	0	0	0	0	0	0	0	0	0	0	0	0	0	0
XM_021102519.1	RUNDC3B	mRNA	0	0	0	0	0	0	0	0	0	0	0	0	0	0	0	0	0	0	0	0	0
XM_021102522.1	RUNDC3B	mRNA	0	0	0	0	0	0	0	0	0	0	0	0	0	0	0	0	0	0	0	0	0
XM_021102523.1	RUNDC3B	mRNA	0	0	0	0	0	0	0	0	0	0	0	0	0	0	0	0	0	0	0	0	0
XR_001297664.2	LOC102161744	lncRNA	0	0	0	0	0	0	0	0	0	0	0	0	0	0	0	0	0	0	0	0	0
XR_001298807.2	LOC106504881	lncRNA	0	0	0	0	0	0	0	0	0	0	0	0	0	0	0	0	0	0	0	0	0
XR_001298808.2	LOC106504881	lncRNA	0	0	0	0	0	0	0	0	0	0	0	0	0	0	0	0	0	0	0	0	0
XR_001298810.2	LOC106504881	lncRNA	0	0	0	0	0	0	0	0	0	0	0	0	0	0	0	0	0	0	0	0	0
XR_001298811.2	LOC106504881	lncRNA	0	0	0	0	0	0	0	0	0	0	0	0	0	0	0	0	0	0	0	0	0
XR_001298812.2	LOC106504881	lncRNA	0	0	0	0	0	0	0	0	0	0	0	0	0	0	0	0	0	0	0	0	0
XR_001298813.2	LOC106504881	lncRNA	0	0	0	0	0	0	0	0	0	0	0	0	0	0	0	0	0	0	0	0	0
XR_001298814.2	LOC106504881	lncRNA	0	0	0	0	0	0	0	0	0	0	0	0	0	0	0	0	0	0	0	0	0
XR_001298816.2	LOC106504881	lncRNA	0	0	0	0	0	0	0	0	0	0	0	0	0	0	0	0	0	0	0	0	0
XR_001298817.2	LOC106504881	lncRNA	0	0	0	0	0	0	0	0	0	0	0	0	0	0	0	0	0	0	0	0	0
XR_001298844.2	LOC102163364	lncRNA	0	0	0	0	0	0	0	0	0	0	0	0	0	0	0	0	0	0	0	0	0
XR_001298846.2	LOC102163364	lncRNA	0	0	0	0	0	0	0	0	0	0	0	0	0	0	0	0	0	0	0	0	0
XR_001298850.2	LOC102163364	lncRNA	0	0	0	0	0	0	0	0	0	0	0	0	0	0	0	0	0	0	0	0	0
XR_001299456.2	LOC102166670	lncRNA	0	0	0	0	0	0	0	0	0	0	0	0	0	0	0	0	0	0	0	0	0
XR_001299457.2	LOC102166670	lncRNA	0	0	0	0	0	0	0	0	0	0	0	0	0	0	0	0	0	0	0	0	0
XR_001300715.2	LOC106505879	lncRNA	0	0	0	0	0	0	0	0	0	0	0	0	0	0	0	0	0	0	0	0	0
XR_001301078.2	LOC102162623	lncRNA	0	0	0	0	0	0	0	0	0	0	0	0	0	0	0	0	0	0	0	0	0
XR_001301087.2	LOC102162623	lncRNA	0	0	0	0	0	0	0	0	0	0	0	0	0	0	0	0	0	0	0	0	0
XR_001301217.2	LOC102164998	lncRNA	0	0	0	0	0	0	0	0	0	0	0	0	0	0	0	0	0	0	0	0	1
XR_001301305.2	LOC102159607	lncRNA	0	0	0	0	0	0	0	0	0	0	0	0	0	0	0	0	0	0	0	0	0
XR_001308967.2	LOC106510467	lncRNA	0	0	0	0	0	0	0	0	0	0	0	0	0	0	0	0	0	0	0	0	0
XR_002335533.1	XRRA1	mRNA	0	0	0	0	0	0	0	0	0	0	0	0	0	0	0	0	0	0	0	0	0
XR_002335534.1	XRRA1	mRNA	0	0	0	0	0	0	0	0	0	0	0	0	0	0	0	0	0	0	0	0	0

RNA ID	Symbol	Type	updown_BGIR9823_94777	updown_BGIR9823_98865	updown_BGIR9823_98901	updown_BGIR9823_99094	updown_BGIR9823_99751	updown_XR_002336552.1	updown_XR_002337059.1	updown_XR_002337719.1	updown_XR_002337772.1	updown_XR_002345058.1	updown_XR_002345629.1	updown_XR_002343991.1	updown_XR_002341917.1	updown_XR_002342057.1	updown_XR_002342059.1	updown_XR_002335940.1	updown_XR_002336149.1	updown_XR_002342781.1	updown_XR_002343137.1	updown_XR_002343215.1	updown_XR_001301217.2
XR_002335535.1	LOC110255399	lncRNA	0	0	0	0	0	0	0	0	0	0	0	0	0	0	0	0	0	0	0	0	0
XR_002335536.1	LOC102160367	lncRNA	0	0	0	0	0	0	0	0	0	0	0	0	0	0	0	0	0	0	0	0	0
XR_002335537.1	LOC102160367	lncRNA	0	0	0	0	0	0	0	0	0	0	0	0	0	0	0	0	0	0	0	0	0
XR_002335650.1	LOC102157630	lncRNA	0	0	0	0	0	0	0	0	0	0	0	0	0	0	0	0	0	0	0	0	0
XR_002335651.1	LOC110255435	lncRNA	0	0	0	0	0	0	0	0	0	0	0	0	0	0	0	0	0	0	0	0	0
XR_002335652.1	LOC106504881	lncRNA	0	0	0	0	0	0	0	0	0	0	0	0	0	0	0	0	0	0	0	0	0
XR_002335653.1	LOC106504881	lncRNA	0	0	0	0	0	0	0	0	0	0	0	0	0	0	0	0	0	0	0	0	0
XR_002335670.1	LOC110255440	lncRNA	0	0	0	0	0	0	0	0	0	0	0	0	0	0	0	0	0	0	0	0	0
XR_002335671.1	LOC110255441	lncRNA	0	0	0	0	0	0	0	0	0	0	0	0	0	0	0	0	0	0	0	0	0
XR_002335672.1	LOC110255441	lncRNA	0	0	0	0	0	0	0	0	0	0	0	0	0	0	0	0	0	0	0	0	0
XR_002335674.1	HYOU1	mRNA	0	0	0	0	0	0	0	0	0	0	0	0	0	0	0	0	0	0	0	0	0
XR_002335675.1	HYOU1	mRNA	0	0	0	0	0	0	0	0	0	0	0	0	0	0	0	0	0	0	0	0	0
XR_002335685.1	LOC100523684	mRNA	0	0	0	0	0	0	0	0	0	0	0	0	0	0	0	0	0	0	0	0	0
XR_002335686.1	LOC102163364	lncRNA	0	0	0	0	0	0	0	0	0	0	0	0	0	0	0	0	0	0	0	0	0
XR_002335687.1	LOC102163364	lncRNA	0	0	0	0	0	0	0	0	0	0	0	0	0	0	0	0	0	0	0	0	0
XR_002335688.1	LOC102163364	lncRNA	0	0	0	0	0	0	0	0	0	0	0	0	0	0	0	0	0	0	0	0	0
XR_002335689.1	LOC102163364	lncRNA	0	0	0	0	0	0	0	0	0	0	0	0	0	0	0	0	0	0	0	0	0
XR_002335690.1	LOC102163364	lncRNA	0	0	0	0	0	0	0	0	0	0	0	0	0	0	0	0	0	0	0	0	0
XR_002335691.1	LOC102163364	lncRNA	0	0	0	0	0	0	0	0	0	0	0	0	0	0	0	0	0	0	0	0	0
XR_002335692.1	LOC102163364	lncRNA	0	0	0	0	0	0	0	0	0	0	0	0	0	0	0	0	0	0	0	0	0
XR_002335693.1	LOC106504896	lncRNA	0	0	0	0	0	0	0	0	0	0	0	0	0	0	0	0	0	0	0	0	0
XR_002335694.1	SORL1	mRNA	0	0	0	0	0	0	0	0	0	0	0	0	0	0	0	0	0	0	0	0	0
XR_002336641.1	LOC106505250	lncRNA	0	0	0	0	0	0	0	0	0	0	0	0	0	0	0	0	0	0	0	0	0
XR_002336643.1	LOC110255292	lncRNA	0	0	0	0	0	0	0	0	0	0	0	0	0	0	0	0	0	0	0	0	0
XR_002336644.1	LOC102166670	lncRNA	0	0	0	0	0	0	0	0	0	0	0	0	0	0	0	0	0	0	0	0	0
XR_002336645.1	LOC102166670	lncRNA	0	0	0	0	0	0	0	0	0	0	0	0	0	0	0	0	0	0	0	0	0
XR_002338443.1	LOC106505879	lncRNA	0	0	0	0	0	0	0	0	0	0	0	0	0	0	0	0	0	0	0	0	0
XR_002338444.1	LOC106505879	lncRNA	0	0	0	0	0	0	0	0	0	0	0	0	0	0	0	0	0	0	0	0	0
XR_002338445.1	LOC106505879	lncRNA	0	0	0	0	0	0	0	0	0	0	0	0	0	0	0	0	0	0	0	0	0
XR_002338446.1	LOC106505879	lncRNA	0	0	0	0	0	0	0	0	0	0	0	0	0	0	0	0	0	0	0	0	0
XR_002338447.1	LOC106505879	lncRNA	0	0	0	0	0	0	0	0	0	0	0	0	0	0	0	0	0	0	0	0	0
XR_002338448.1	LOC106505879	lncRNA	0	0	0	0	0	0	0	0	0	0	0	0	0	0	0	0	0	0	0	0	0
XR_002338449.1	LOC106505879	lncRNA	0	0	0	0	0	0	0	0	0	0	0	0	0	0	0	0	0	0	0	0	0
XR_002338450.1	LOC106505879	lncRNA	0	0	0	0	0	0	0	0	0	0	0	0	0	0	0	0	0	0	0	0	0
XR_002338521.1	LOC110256664	lncRNA	0	0	0	0	0	0	0	0	0	0	0	0	0	0	0	0	0	0	0	0	1
XR_002338522.1	LOC110256664	lncRNA	0	0	0	0	0	0	0	0	0	0	0	0	0	0	0	0	0	0	0	0	1
XR_002338666.1	LOC110256747	lncRNA	0	0	0	0	0	0	0	0	0	0	0	0	0	0	0	0	0	0	0	0	0
XR_002338714.1	LOC102163817	lncRNA	0	0	0	0	0	0	0	0	0	0	0	0	0	0	0	0	0	0	0	0	1
XR_002338715.1	LOC102164998	lncRNA	0	0	0	0	0	0	0	0	0	0	0	0	0	0	0	0	0	0	0	0	1
XR_002338716.1	LOC102164998	lncRNA	0	0	0	0	0	0	0	0	0	0	0	0	0	0	0	0	0	0	0	0	1
XR_002338717.1	LOC110256768	lncRNA	0	0	0	0	0	0	0	0	0	0	0	0	0	0	0	0	0	0	0	0	1
XR_002338727.1	LOC110256773	lncRNA	0	0	0	0	0	0	0	0	0	0	0	0	0	0	0	0	0	0	0	0	0
XR_002338728.1	LOC110256773	lncRNA	0	0	0	0	0	0	0	0	0	0	0	0	0	0	0	0	0	0	0	0	0

RNA ID	Symbol	Type	updown_BGIR9823_94777	updown_BGIR9823_98865	updown_BGIR9823_98901	updown_BGIR9823_99094	updown_BGIR9823_99751	updown_XR_002336552.1	updown_XR_002337059.1	updown_XR_002337719.1	updown_XR_002337772.1	updown_XR_002345058.1	updown_XR_002345629.1	updown_XR_002343991.1	updown_XR_002341917.1	updown_XR_002342057.1	updown_XR_002342059.1	updown_XR_002335940.1	updown_XR_002336149.1	updown_XR_002342781.1	updown_XR_002343137.1	updown_XR_002343215.1	updown_XR_001301217.2
XR_002338729.1	LOC110256773	lncRNA	0	0	0	0	0	0	0	0	0	0	0	0	0	0	0	0	0	0	0	0	0
XR_002338793.1	LOC110256796	lncRNA	0	0	0	0	0	0	0	0	0	0	0	0	0	0	0	0	0	0	0	0	1
XR_002339623.1	LOC102167708	lncRNA	0	0	0	0	0	0	0	0	0	0	0	0	0	0	0	0	0	0	0	0	0
XR_002340245.1	PRKAG2	mRNA	0	0	0	0	0	0	0	0	0	0	0	0	0	0	0	0	0	0	0	0	0
XR_002340246.1	LOC102162623	lncRNA	0	0	0	0	0	0	0	0	0	0	0	0	0	0	0	0	0	0	0	0	0
XR_002340247.1	LOC102162623	lncRNA	0	0	0	0	0	0	0	0	0	0	0	0	0	0	0	0	0	0	0	0	0
XR_002340248.1	LOC110257516	lncRNA	0	0	0	0	0	0	0	0	0	0	0	0	0	0	0	0	0	0	0	0	0
XR_002340249.1	LOC102162071	lncRNA	0	0	0	0	0	0	0	0	0	0	0	0	0	0	0	0	0	0	0	0	0
XR_002340901.1	LOC102160243	lncRNA	0	0	0	0	0	0	0	0	0	0	0	0	0	0	0	0	0	0	0	0	0
XR_002340902.1	LOC102160243	lncRNA	0	0	0	0	0	0	0	0	0	0	0	0	0	0	0	0	0	0	0	0	0
XR_002340903.1	LOC102160243	lncRNA	0	0	0	0	0	0	0	0	0	0	0	0	0	0	0	0	0	0	0	0	0
XR_002340904.1	LOC102160243	lncRNA	0	0	0	0	0	0	0	0	0	0	0	0	0	0	0	0	0	0	0	0	0
XR_002340905.1	LOC102160243	lncRNA	0	0	0	0	0	0	0	0	0	0	0	0	0	0	0	0	0	0	0	0	0
XR_002341237.1	LOC102164320	lncRNA	0	0	0	0	0	0	0	0	0	0	0	0	0	0	0	0	0	0	0	0	0
XR_002341238.1	LOC102164320	lncRNA	0	0	0	0	0	0	0	0	0	0	0	0	0	0	0	0	0	0	0	0	0
XR_002341239.1	LOC102164320	lncRNA	0	0	0	0	0	0	0	0	0	0	0	0	0	0	0	0	0	0	0	0	0
XR_002341240.1	LOC102164320	lncRNA	0	0	0	0	0	0	0	0	0	0	0	0	0	0	0	0	0	0	0	0	0
XR_002341241.1	LOC102164320	lncRNA	0	0	0	0	0	0	0	0	0	0	0	0	0	0	0	0	0	0	0	0	0
XR_002341242.1	LOC102164320	lncRNA	0	0	0	0	0	0	0	0	0	0	0	0	0	0	0	0	0	0	0	0	0
XR_002341243.1	LOC102164320	lncRNA	0	0	0	0	0	0	0	0	0	0	0	0	0	0	0	0	0	0	0	0	0
XR_002341244.1	LOC102164320	lncRNA	0	0	0	0	0	0	0	0	0	0	0	0	0	0	0	0	0	0	0	0	0
XR_002341270.1	LOC100521600	mRNA	0	0	0	0	0	0	0	0	0	0	0	0	0	0	0	0	0	0	0	0	0
XR_002341443.1	LOC102163816	lncRNA	0	0	0	0	0	0	0	0	0	0	0	0	0	0	0	0	0	0	0	0	0
XR_002343847.1	LOC110260611	mRNA	0	0	0	0	0	0	0	0	0	0	0	0	0	0	0	0	0	0	0	0	0
XR_002343848.1	LOC110260611	mRNA	0	0	0	0	0	0	0	0	0	0	0	0	0	0	0	0	0	0	0	0	0
XR_002343849.1	LOC110260611	mRNA	0	0	0	0	0	0	0	0	0	0	0	0	0	0	0	0	0	0	0	0	0
XR_002343850.1	LOC110260611	mRNA	0	0	0	0	0	0	0	0	0	0	0	0	0	0	0	0	0	0	0	0	0
XR_002343875.1	LOC110260629	lncRNA	0	0	0	0	0	0	0	0	0	0	0	0	0	0	0	0	0	0	0	0	0
XR_002343876.1	LOC110260629	lncRNA	0	0	0	0	0	0	0	0	0	0	0	0	0	0	0	0	0	0	0	0	0
XR_002343877.1	LOC110260629	lncRNA	0	0	0	0	0	0	0	0	0	0	0	0	0	0	0	0	0	0	0	0	0
XR_002343878.1	LOC110260629	lncRNA	0	0	0	0	0	0	0	0	0	0	0	0	0	0	0	0	0	0	0	0	0
XR_002343879.1	LOC110260629	lncRNA	0	0	0	0	0	0	0	0	0	0	0	0	0	0	0	0	0	0	0	0	0
XR_002343880.1	LOC110260629	lncRNA	0	0	0	0	0	0	0	0	0	0	0	0	0	0	0	0	0	0	0	0	0
XR_002343881.1	LOC110260629	lncRNA	0	0	0	0	0	0	0	0	0	0	0	0	0	0	0	0	0	0	0	0	0
XR_002343883.1	LOC110260631	lncRNA	0	0	0	0	0	0	0	0	0	0	0	0	0	0	0	0	0	0	0	0	0
XR_002343884.1	LOC110260634	lncRNA	0	0	0	0	0	0	0	0	0	0	0	0	0	0	0	0	0	0	0	0	0
XR_002343885.1	LOC110260635	lncRNA	0	0	0	0	0	0	0	0	0	0	0	0	0	0	0	0	0	0	0	0	0
XR_002343891.1	LOC110260642	lncRNA	0	0	0	0	0	0	0	0	0	0	0	0	0	0	0	0	0	0	0	0	0
XR_002343892.1	LOC110260643	lncRNA	0	0	0	0	0	0	0	0	0	0	0	0	0	0	0	0	0	0	0	0	0
XR_002345298.1	MAF	mRNA	0	0	0	0	0	0	0	0	0	0	0	0	0	0	0	0	0	0	0	0	0
XR_002345300.1	LOC106510470	lncRNA	0	0	0	0	0	0	0	0	0	0	0	0	0	0	0	0	0	0	0	0	0
XR_002345301.1	LOC106510468	lncRNA	0	0	0	0	0	0	0	0	0	0	0	0	0	0	0	0	0	0	0	0	0
XR_002346166.1	LOC110261703	lncRNA	0	0	0	0	0	0	0	0	0	0	0	0	0	0	0	0	0	0	0	0	0

RNA ID	Symbol	Type	updown_BGIR9823_94777	updown_BGIR9823_98865	updown_BGIR9823_98901	updown_BGIR9823_99094	updown_BGIR9823_99751	updown_XR_002336552.1	updown_XR_002337059.1	updown_XR_002337719.1	updown_XR_002337772.1	updown_XR_002345058.1	updown_XR_002345629.1	updown_XR_002343991.1	updown_XR_002341917.1	updown_XR_002342057.1	updown_XR_002342059.1	updown_XR_002335940.1	updown_XR_002336149.1	updown_XR_002342781.1	updown_XR_002343137.1	updown_XR_002343215.1	updown_XR_001301217.2
XR_002346227.1	LOC106504299	lncRNA	0	0	0	0	0	0	0	0	0	0	0	0	0	0	0	0	0	0	0	0	0
XR_002346228.1	LOC106504299	lncRNA	0	0	0	0	0	0	0	0	0	0	0	0	0	0	0	0	0	0	0	0	0
XR_002346229.1	LOC106504299	lncRNA	0	0	0	0	0	0	0	0	0	0	0	0	0	0	0	0	0	0	0	0	0
XR_002346230.1	LOC106504299	lncRNA	0	0	0	0	0	0	0	0	0	0	0	0	0	0	0	0	0	0	0	0	0
XR_002346231.1	LOC106504299	lncRNA	0	0	0	0	0	0	0	0	0	0	0	0	0	0	0	0	0	0	0	0	0
XR_002346232.1	LOC106504299	lncRNA	0	0	0	0	0	0	0	0	0	0	0	0	0	0	0	0	0	0	0	0	0
XR_002346233.1	NQO2	mRNA	0	0	0	0	0	0	0	0	0	0	0	0	0	0	0	0	0	0	0	0	0
XR_002346236.1	PRPF4B	mRNA	0	0	0	0	0	0	0	0	0	0	0	0	0	0	0	0	0	0	0	0	0
XR_002346237.1	PRPF4B	mRNA	0	0	0	0	0	0	0	0	0	0	0	0	0	0	0	0	0	0	0	0	0
XR_002346238.1	PRPF4B	mRNA	0	0	0	0	0	0	0	0	0	0	0	0	0	0	0	0	0	0	0	0	0
XR_002346239.1	PRPF4B	mRNA	0	0	0	0	0	0	0	0	0	0	0	0	0	0	0	0	0	0	0	0	0
XR_002346240.1	PRPF4B	mRNA	0	0	0	0	0	0	0	0	0	0	0	0	0	0	0	0	0	0	0	0	0
XR_002346241.1	PRPF4B	mRNA	0	0	0	0	0	0	0	0	0	0	0	0	0	0	0	0	0	0	0	0	0
XR_002346242.1	PRPF4B	mRNA	0	0	0	0	0	0	0	0	0	0	0	0	0	0	0	0	0	0	0	0	0
XR_002346243.1	PRPF4B	mRNA	0	0	0	0	0	0	0	0	0	0	0	0	0	0	0	0	0	0	0	0	0
XR_002346244.1	PRPF4B	mRNA	0	0	0	0	0	0	0	0	0	0	0	0	0	0	0	0	0	0	0	0	0
XR_002346245.1	PRPF4B	mRNA	0	0	0	0	0	0	0	0	0	0	0	0	0	0	0	0	0	0	0	0	0
XR_002346246.1	PRPF4B	mRNA	0	0	0	0	0	0	0	0	0	0	0	0	0	0	0	0	0	0	0	0	0
XR_002346247.1	PRPF4B	mRNA	0	0	0	0	0	0	0	0	0	0	0	0	0	0	0	0	0	0	0	0	0
XR_002346248.1	PRPF4B	mRNA	0	0	0	0	0	0	0	0	0	0	0	0	0	0	0	0	0	0	0	0	0
XR_002346249.1	PRPF4B	mRNA	0	0	0	0	0	0	0	0	0	0	0	0	0	0	0	0	0	0	0	0	0
XR_002346250.1	PRPF4B	mRNA	0	0	0	0	0	0	0	0	0	0	0	0	0	0	0	0	0	0	0	0	0
XR_002346251.1	PRPF4B	mRNA	0	0	0	0	0	0	0	0	0	0	0	0	0	0	0	0	0	0	0	0	0
XR_002346252.1	PRPF4B	mRNA	0	0	0	0	0	0	0	0	0	0	0	0	0	0	0	0	0	0	0	0	0
XR_002346253.1	PRPF4B	mRNA	0	0	0	0	0	0	0	0	0	0	0	0	0	0	0	0	0	0	0	0	0
XR_002346254.1	PRPF4B	mRNA	0	0	0	0	0	0	0	0	0	0	0	0	0	0	0	0	0	0	0	0	0
XR_002346255.1	PRPF4B	mRNA	0	0	0	0	0	0	0	0	0	0	0	0	0	0	0	0	0	0	0	0	0
XR_002346256.1	PRPF4B	mRNA	0	0	0	0	0	0	0	0	0	0	0	0	0	0	0	0	0	0	0	0	0
XR_002346257.1	PRPF4B	mRNA	0	0	0	0	0	0	0	0	0	0	0	0	0	0	0	0	0	0	0	0	0
XR_002346258.1	PRPF4B	mRNA	0	0	0	0	0	0	0	0	0	0	0	0	0	0	0	0	0	0	0	0	0
XR_002346259.1	PRPF4B	mRNA	0	0	0	0	0	0	0	0	0	0	0	0	0	0	0	0	0	0	0	0	0
XR_002346260.1	PRPF4B	mRNA	0	0	0	0	0	0	0	0	0	0	0	0	0	0	0	0	0	0	0	0	0
XR_002346261.1	PRPF4B	mRNA	0	0	0	0	0	0	0	0	0	0	0	0	0	0	0	0	0	0	0	0	0
XR_002346262.1	PRPF4B	mRNA	0	0	0	0	0	0	0	0	0	0	0	0	0	0	0	0	0	0	0	0	0
XR_002346263.1	PRPF4B	mRNA	0	0	0	0	0	0	0	0	0	0	0	0	0	0	0	0	0	0	0	0	0
XR_002346264.1	PRPF4B	mRNA	0	0	0	0	0	0	0	0	0	0	0	0	0	0	0	0	0	0	0	0	0
XR_002346265.1	LOC110261764	lncRNA	0	0	0	0	0	0	0	0	0	0	0	0	0	0	0	0	0	0	0	0	0
XR_002346266.1	LOC102162837	lncRNA	0	0	0	0	0	0	0	0	0	0	0	0	0	0	0	0	0	0	0	0	0
XR_002346299.1	LOC110261774	lncRNA	0	0	0	0	0	0	0	0	0	0	0	0	0	0	0	0	0	0	0	0	0
XR_002346300.1	LOC110261774	lncRNA	0	0	0	0	0	0	0	0	0	0	0	0	0	0	0	0	0	0	0	0	0
XR_002346302.1	LOC110261776	lncRNA	0	0	0	0	0	0	0	0	0	0	0	0	0	0	0	0	0	0	0	0	0
XR_002346303.1	TMEM170B	mRNA	0	0	0	0	0	0	0	0	0	0	0	0	0	0	0	0	0	0	0	0	0
XR_002346304.1	TMEM170B	mRNA	0	0	0	0	0	0	0	0	0	0	0	0	0	0	0	0	0	0	0	0	0

RNA ID	Symbol	Type	updown_BGIR9823_94777	updown_BGIR9823_98865	updown_BGIR9823_98901	updown_BGIR9823_99094	updown_BGIR9823_99751	updown_XR_002336552.1	updown_XR_002337059.1	updown_XR_002337719.1	updown_XR_002337772.1	updown_XR_002345058.1	updown_XR_002345629.1	updown_XR_002343991.1	updown_XR_002341917.1	updown_XR_002342057.1	updown_XR_002342059.1	updown_XR_002335940.1	updown_XR_002336149.1	updown_XR_002342781.1	updown_XR_002343137.1	updown_XR_002343215.1	updown_XR_001301217.2
XR_115737.4	LOC100513133	lncRNA	0	0	0	0	0	0	0	0	0	0	0	0	0	0	0	0	0	0	0	0	0
XR_299046.2	LOC102164596	lncRNA	0	0	0	0	0	0	0	0	0	0	0	0	0	0	0	0	0	0	0	0	0
XR_303634.3	LOC100738154	lncRNA	0	0	0	0	0	0	0	0	0	0	0	0	0	0	0	0	0	0	0	0	0
XR_304239.3	LOC102159819	lncRNA	0	0	0	0	0	0	0	0	0	0	0	0	0	0	0	0	0	0	0	0	0
XR_304244.3	LOC102161992	lncRNA	0	0	0	0	0	0	0	0	0	0	0	0	0	0	0	0	0	0	0	0	0
XR_304257.3	LOC102162837	lncRNA	0	0	0	0	0	0	0	0	0	0	0	0	0	0	0	0	0	0	0	0	0
XR_306105.3	LOC102166871	lncRNA	0	0	0	0	0	0	0	0	0	0	0	0	0	0	0	0	0	0	0	0	0
XR_307914.3	LOC102159607	lncRNA	0	0	0	0	0	0	0	0	0	0	0	0	0	0	0	0	0	0	0	0	0
XR_308345.3	LOC102167708	lncRNA	0	0	0	0	0	0	0	0	0	0	0	0	0	0	0	0	0	0	0	0	0
novel-ssc-miR1028-3p		miRNA	0	0	0	0	0	0	0	0	0	0	0	0	0	0	0	0	0	0	0	0	0
novel-ssc-miR1028-5p		miRNA	0	0	0	0	0	0	0	0	0	0	0	0	0	0	0	0	0	0	0	0	0
novel-ssc-miR1130-3p		miRNA	0	0	0	0	0	0	0	0	0	0	0	0	0	0	0	0	0	0	0	0	0
novel-ssc-miR1130-5p		miRNA	0	0	0	0	0	0	0	0	0	0	0	0	0	0	0	0	0	0	0	0	0
novel-ssc-miR1162-3p		miRNA	0	0	0	0	0	0	0	0	0	0	0	0	0	0	0	0	0	0	0	0	0
novel-ssc-miR1193-3p		miRNA	0	0	0	0	0	0	0	0	0	0	0	0	0	0	0	0	0	0	0	0	0
novel-ssc-miR1193-5p		miRNA	0	0	0	0	0	0	0	0	0	0	0	0	0	0	0	0	0	0	0	0	0
novel-ssc-miR1217-3p		miRNA	0	0	0	0	0	0	0	0	0	0	0	0	0	0	0	0	0	0	0	0	0
novel-ssc-miR1217-5p		miRNA	0	0	0	0	0	0	0	0	0	0	0	0	0	0	0	0	0	0	0	0	0
novel-ssc-miR1429-3p		miRNA	0	0	0	0	0	0	0	0	0	0	0	0	0	0	0	0	0	0	0	0	0
novel-ssc-miR1429-5p		miRNA	0	0	0	0	0	0	0	0	0	0	0	0	0	0	0	0	0	0	0	0	0
novel-ssc-miR259-3p		miRNA	0	0	0	0	0	0	0	0	0	0	0	0	0	0	0	0	0	0	0	0	0
novel-ssc-miR259-5p		miRNA	0	0	0	0	0	0	0	0	0	0	0	0	0	0	0	0	0	0	0	0	0
novel-ssc-miR324-3p		miRNA	0	0	0	0	0	0	0	0	0	0	0	0	0	0	0	0	0	0	0	0	0
novel-ssc-miR324-5p		miRNA	0	0	0	0	0	0	0	0	0	0	0	0	0	0	0	0	0	0	0	0	0
novel-ssc-miR42-3p		miRNA	0	0	0	0	0	0	0	0	0	0	0	0	0	0	0	0	0	0	0	0	0
novel-ssc-miR42-5p		miRNA	0	0	0	0	0	0	0	0	0	0	0	0	0	0	0	0	0	0	0	0	0
novel-ssc-miR433-3p		miRNA	0	0	0	0	0	0	0	0	0	0	0	0	0	0	0	0	0	0	0	0	0
novel-ssc-miR433-5p		miRNA	0	0	0	0	0	0	0	0	0	0	0	0	0	0	0	0	0	0	0	0	0
novel-ssc-miR557-3p		miRNA	0	0	0	0	0	0	0	0	0	0	0	0	0	0	0	0	0	0	0	0	0
novel-ssc-miR557-5p		miRNA	0	0	0	0	0	0	0	0	0	0	0	0	0	0	0	0	0	0	0	0	0
novel-ssc-miR619-3p		miRNA	0	0	0	0	0	0	0	0	0	0	0	0	0	0	0	0	0	0	0	0	0
novel-ssc-miR619-5p		miRNA	0	0	0	0	0	0	0	0	0	0	0	0	0	0	0	0	0	0	0	0	0
novel-ssc-miR719-3p		miRNA	0	0	0	0	0	0	0	0	0	0	0	0	0	0	0	0	0	0	0	0	0
novel-ssc-miR719-5p		miRNA	0	0	0	0	0	0	0	0	0	0	0	0	0	0	0	0	0	0	0	0	0
novel-ssc-miR966-3p		miRNA	0	0	0	0	0	0	0	0	0	0	0	0	0	0	0	0	0	0	0	0	0
novel-ssc-miR966-5p		miRNA	0	0	0	0	0	0	0	0	0	0	0	0	0	0	0	0	0	0	0	0	0
ssc-miR-7136-3p		miRNA	0	0	0	0	0	0	0	0	0	0	0	0	0	0	0	0	0	0	0	0	0
ssc-miR-7136-5p		miRNA	0	0	0	0	0	0	0	0	0	0	0	0	0	0	0	0	0	0	0	0	0

Supplementary Table S10. The predicted targe

RNA ID	Symbol	Type	updown_XR_001303119.2	updown_XR_002341616.1	updown_XR_299372.3	updown_XR_298148.3	updown_XR_002346063.1	updown_BGIR9823_100550	updown_BGIR9823_100272	updown_BGIR9823_101549	updown_BGIR9823_101564	updown_BGIR9823_101567	updown_BGIR9823_102169	updown_BGIR9823_102657	updown_BGIR9823_102650	updown_BGIR9823_79650	updown_BGIR9823_79960	updown_BGIR9823_80428	updown_BGIR9823_80570	updown_BGIR9823_84360	updown_BGIR9823_84364	updown_BGIR9823_81795	updown_BGIR9823_87741
BGIR9823_100361	PRKAG2	mRNA	0	0	0	0	0	0	0	0	0	0	0	0	0	0	0	0	0	0	0	0	0
BGIR9823_100362	PRKAG2	mRNA	0	0	0	0	0	0	0	0	0	0	0	0	0	0	0	0	0	0	0	0	0
BGIR9823_100363	PRKAG2	mRNA	0	0	0	0	0	0	0	0	0	0	0	0	0	0	0	0	0	0	0	0	0
BGIR9823_100364	PRKAG2	mRNA	0	0	0	0	0	0	0	0	0	0	0	0	0	0	0	0	0	0	0	0	0
BGIR9823_101535	LOC102158401	lncRNA	0	0	0	0	0	0	0	1	1	1	0	0	0	0	0	0	0	0	0	0	0
BGIR9823_101541	LOC102157946	lncRNA	0	0	0	0	0	0	0	1	1	1	0	0	0	0	0	0	0	0	0	0	0
BGIR9823_101542	LOC102157946	lncRNA	0	0	0	0	0	0	0	1	1	1	0	0	0	0	0	0	0	0	0	0	0
BGIR9823_101560	LOC110258600	lncRNA	0	0	0	0	0	0	0	1	1	1	0	0	0	0	0	0	0	0	0	0	0
BGIR9823_101561	LOC110258600	lncRNA	0	0	0	0	0	0	0	1	1	1	0	0	0	0	0	0	0	0	0	0	0
BGIR9823_101562	LOC110258600	lncRNA	0	0	0	0	0	0	0	1	1	1	0	0	0	0	0	0	0	0	0	0	0
BGIR9823_101563	LOC110258600	lncRNA	0	0	0	0	0	0	0	1	1	1	0	0	0	0	0	0	0	0	0	0	0
BGIR9823_101564	LOC110258600	lncRNA	0	0	0	0	0	0	0	1	1	1	0	0	0	0	0	0	0	0	0	0	0
BGIR9823_101612	LOC100521600	lncRNA	0	0	0	0	0	0	0	0	0	0	0	0	0	0	0	0	0	0	0	0	0
BGIR9823_101853		lncRNA	0	0	0	0	0	0	0	0	0	0	0	0	0	0	0	0	0	0	0	0	0
BGIR9823_101854		lncRNA	0	0	0	0	0	0	0	0	0	0	0	0	0	0	0	0	0	0	0	0	0
BGIR9823_101855		lncRNA	0	0	0	0	0	0	0	0	0	0	0	0	0	0	0	0	0	0	0	0	0
BGIR9823_102146		lncRNA	0	0	0	0	0	0	0	0	0	0	0	0	0	0	0	0	0	0	0	0	0
BGIR9823_102180		lncRNA	0	0	0	0	0	0	0	0	0	0	0	0	0	0	0	0	0	0	0	0	0
BGIR9823_102279		lncRNA	0	0	0	0	0	0	0	0	0	0	0	0	0	0	0	0	0	0	0	0	0
BGIR9823_102282		lncRNA	0	0	0	0	0	0	0	0	0	0	0	0	0	0	0	0	0	0	0	0	0
BGIR9823_102361		lncRNA	0	0	0	0	0	0	0	0	0	0	0	0	0	0	0	0	0	0	0	0	0
BGIR9823_102362		lncRNA	0	0	0	0	0	0	0	0	0	0	0	0	0	0	0	0	0	0	0	0	0
BGIR9823_102363		lncRNA	0	0	0	0	0	0	0	0	0	0	0	0	0	0	0	0	0	0	0	0	0
BGIR9823_102727		lncRNA	0	0	0	0	0	0	0	0	0	0	0	0	0	0	0	0	0	0	0	0	0
BGIR9823_80121	SSSCA1	lncRNA	0	0	0	0	0	0	0	0	0	0	0	0	0	0	0	0	0	0	0	0	0
BGIR9823_80122	LOC102163816	lncRNA	0	0	0	0	0	0	0	0	0	0	0	0	0	0	0	0	0	0	0	0	0
BGIR9823_81007	EHBP1L1	mRNA	0	0	0	0	0	0	0	0	0	0	0	0	0	0	0	0	0	0	0	0	0
BGIR9823_81008	EHBP1L1	mRNA	0	0	0	0	0	0	0	0	0	0	0	0	0	0	0	0	0	0	0	0	0
BGIR9823_81009	EHBP1L1	mRNA	0	0	0	0	0	0	0	0	0	0	0	0	0	0	0	0	0	0	0	0	0
BGIR9823_81010	EHBP1L1	mRNA	0	0	0	0	0	0	0	0	0	0	0	0	0	0	0	0	0	0	0	0	0
BGIR9823_81011	SSSCA1	mRNA	0	0	0	0	0	0	0	0	0	0	0	0	0	0	0	0	0	0	0	0	0
BGIR9823_81012	FRMD8	lncRNA	0	0	0	0	0	0	0	0	0	0	0	0	0	0	0	0	0	0	0	0	0
BGIR9823_81013	LOC102163816	lncRNA	0	0	0	0	0	0	0	0	0	0	0	0	0	0	0	0	0	0	0	0	0
BGIR9823_81014	LOC102163816	lncRNA	0	0	0	0	0	0	0	0	0	0	0	0	0	0	0	0	0	0	0	0	0
BGIR9823_81015	LOC102163816	lncRNA	0	0	0	0	0	0	0	0	0	0	0	0	0	0	0	0	0	0	0	0	0
BGIR9823_81016	LOC102163816	lncRNA	0	0	0	0	0	0	0	0	0	0	0	0	0	0	0	0	0	0	0	0	0
BGIR9823_81017	LOC102163816	lncRNA	0	0	0	0	0	0	0	0	0	0	0	0	0	0	0	0	0	0	0	0	0
BGIR9823_81018	LOC102163816	lncRNA	0	0	0	0	0	0	0	0	0	0	0	0	0	0	0	0	0	0	0	0	0
BGIR9823_81019	LOC100513133	lncRNA	0	0	0	0	0	0	0	0	0	0	0	0	0	0	0	0	0	0	0	0	0
BGIR9823_84704	LOC102159820	mRNA	0	0	0	0	0	0	0	0	0	0	0	0	0	0	0	0	0	0	0	0	0
BGIR9823_84732	RANGAP1	mRNA	0	0	0	0	0	0	0	0	0	0	0	0	0	0	0	0	0	0	0	0	0

RNA ID	Symbol	Type	updown_XR_001303119.2	updown_XR_002341616.1	updown_XR_299372.3	updown_XR_298148.3	updown_XR_002346063.1	updown_BGIR9823_100550	updown_BGIR9823_100272	updown_BGIR9823_101549	updown_BGIR9823_101564	updown_BGIR9823_101567	updown_BGIR9823_102169	updown_BGIR9823_102657	updown_BGIR9823_102650	updown_BGIR9823_79650	updown_BGIR9823_79960	updown_BGIR9823_80428	updown_BGIR9823_80570	updown_BGIR9823_84360	updown_BGIR9823_84364	updown_BGIR9823_81795	updown_BGIR9823_87741
BGIR9823_84733	RANGAP1	mRNA	0	0	0	0	0	0	0	0	0	0	0	0	0	0	0	0	0	0	0	0	0
BGIR9823_85245	CRADD	lncRNA	0	0	0	0	0	0	0	0	0	0	0	0	0	0	0	0	0	0	0	0	0
BGIR9823_85246	LOC100738154	lncRNA	0	0	0	0	0	0	0	0	0	0	0	0	0	0	0	0	0	0	0	0	0
BGIR9823_85293	TRMU	mRNA	0	0	0	0	0	0	0	0	0	0	0	0	0	0	0	0	0	0	0	0	0
BGIR9823_85294	TRMU	mRNA	0	0	0	0	0	0	0	0	0	0	0	0	0	0	0	0	0	0	0	0	0
BGIR9823_85295	TTC38	mRNA	0	0	0	0	0	0	0	0	0	0	0	0	0	0	0	0	0	0	0	0	0
BGIR9823_85296	PKDREJ	lncRNA	0	0	0	0	0	0	0	0	0	0	0	0	0	0	0	0	0	0	0	0	0
BGIR9823_85297	PPARA	mRNA	0	0	0	0	0	0	0	0	0	0	0	0	0	0	0	0	0	0	0	0	0
BGIR9823_85298	PPARA	mRNA	0	0	0	0	0	0	0	0	0	0	0	0	0	0	0	0	0	0	0	0	0
BGIR9823_85299	PPARA	mRNA	0	0	0	0	0	0	0	0	0	0	0	0	0	0	0	0	0	0	0	0	0
BGIR9823_85300	PPARA	lncRNA	0	0	0	0	0	0	0	0	0	0	0	0	0	0	0	0	0	0	0	0	0
BGIR9823_85301	LOC110260611	mRNA	0	0	0	0	0	0	0	0	0	0	0	0	0	0	0	0	0	0	0	0	0
BGIR9823_85317	ACO2	mRNA	0	0	0	0	0	0	0	0	0	0	0	0	0	0	0	0	0	0	0	0	0
BGIR9823_85318	LOC110260629	lncRNA	0	0	0	0	0	0	0	0	0	0	0	0	0	0	0	0	0	0	0	0	0
BGIR9823_85319	TEF	mRNA	0	0	0	0	0	0	0	0	0	0	0	0	0	0	0	0	0	0	0	0	0
BGIR9823_85320	ZC3H7B	mRNA	0	0	0	0	0	0	0	0	0	0	0	0	0	0	0	0	0	0	0	0	0
BGIR9823_85345	SYNGR1	mRNA	0	0	0	0	0	0	0	0	0	0	0	0	0	0	0	0	0	0	0	0	0
BGIR9823_85835	CRADD	mRNA	0	0	0	0	0	0	0	0	0	0	0	0	0	0	0	0	0	0	0	0	0
BGIR9823_85839	CRADD	lncRNA	0	0	0	0	0	0	0	0	0	0	0	0	0	0	0	0	0	0	0	0	0
BGIR9823_85840	CRADD	lncRNA	0	0	0	0	0	0	0	0	0	0	0	0	0	0	0	0	0	0	0	0	0
BGIR9823_85841	CRADD	lncRNA	0	0	0	0	0	0	0	0	0	0	0	0	0	0	0	0	0	0	0	0	0
BGIR9823_85930	LOC106510465	mRNA	0	0	0	0	0	0	0	0	0	0	0	0	0	0	0	0	0	0	0	0	0
BGIR9823_85931	MAF	lncRNA	0	0	0	0	0	0	0	0	0	0	0	0	0	0	0	0	0	0	0	0	0
BGIR9823_85932	MAF	lncRNA	0	0	0	0	0	0	0	0	0	0	0	0	0	0	0	0	0	0	0	0	0
BGIR9823_87139	LOC106510467	lncRNA	0	0	0	0	0	0	0	0	0	0	0	0	0	0	0	0	0	0	0	0	0
BGIR9823_88187	WRNIP1	mRNA	0	0	0	0	0	0	0	0	0	0	0	0	0	0	0	0	0	0	0	0	0
BGIR9823_88188	NQO2	mRNA	0	0	0	0	0	0	0	0	0	0	0	0	0	0	0	0	0	0	0	0	0
BGIR9823_88189	NQO2	mRNA	0	0	0	0	0	0	0	0	0	0	0	0	0	0	0	0	0	0	0	0	0
BGIR9823_88190	RIPK1	mRNA	0	0	0	0	0	0	0	0	0	0	0	0	0	0	0	0	0	0	0	0	0
BGIR9823_88191	RIPK1	mRNA	0	0	0	0	0	0	0	0	0	0	0	0	0	0	0	0	0	0	0	0	0
BGIR9823_88192	RIPK1	mRNA	0	0	0	0	0	0	0	0	0	0	0	0	0	0	0	0	0	0	0	0	0
BGIR9823_88193	RIPK1	mRNA	0	0	0	0	0	0	0	0	0	0	0	0	0	0	0	0	0	0	0	0	0
BGIR9823_88196	PRPF4B	mRNA	0	0	0	0	0	0	0	0	0	0	0	0	0	0	0	0	0	0	0	0	0
BGIR9823_88197	PRPF4B	mRNA	0	0	0	0	0	0	0	0	0	0	0	0	0	0	0	0	0	0	0	0	0
BGIR9823_88198	PRPF4B	mRNA	0	0	0	0	0	0	0	0	0	0	0	0	0	0	0	0	0	0	0	0	0
BGIR9823_88211	TMEM170B	lncRNA	0	0	0	0	0	0	0	0	0	0	0	0	0	0	0	0	0	0	0	0	0
BGIR9823_88212	TMEM170B	lncRNA	0	0	0	0	0	0	0	0	0	0	0	0	0	0	0	0	0	0	0	0	0
BGIR9823_88213	TMEM170B	lncRNA	0	0	0	0	0	0	0	0	0	0	0	0	0	0	0	0	0	0	0	0	0
BGIR9823_88214	TMEM170B	lncRNA	0	0	0	0	0	0	0	0	0	0	0	0	0	0	0	0	0	0	0	0	0
BGIR9823_88217	PHACTR1	mRNA	0	0	0	0	0	0	0	0	0	0	0	0	0	0	0	0	0	0	0	0	0
BGIR9823_88218	PHACTR1	lncRNA	0	0	0	0	0	0	0	0	0	0	0	0	0	0	0	0	0	0	0	0	0
BGIR9823_89017	MYLK4	lncRNA	0	0	0	0	0	0	0	0	0	0	0	0	0	0	0	0	0	0	0	0	0
BGIR9823_89019	SERPINB1	lncRNA	0	0	0	0	0	0	0	0	0	0	0	0	0	0	0	0	0	0	0	0	0

RNA ID	Symbol	Type	updown_XR_001303119.2	updown_XR_002341616.1	updown_XR_299372.3	updown_XR_298148.3	updown_XR_002346063.1	updown_BGIR9823_100550	updown_BGIR9823_100272	updown_BGIR9823_101549	updown_BGIR9823_101564	updown_BGIR9823_101567	updown_BGIR9823_102169	updown_BGIR9823_102657	updown_BGIR9823_102650	updown_BGIR9823_79650	updown_BGIR9823_79960	updown_BGIR9823_80428	updown_BGIR9823_80570	updown_BGIR9823_84360	updown_BGIR9823_84364	updown_BGIR9823_81795	updown_BGIR9823_87741
BGIR9823_89020	SERPINB9	mRNA	0	0	0	0	0	0	0	0	0	0	0	0	0	0	0	0	0	0	0	0	0
BGIR9823_89021	RIPK1	lncRNA	0	0	0	0	0	0	0	0	0	0	0	0	0	0	0	0	0	0	0	0	0
BGIR9823_89025	PXDC1	mRNA	0	0	0	0	0	0	0	0	0	0	0	0	0	0	0	0	0	0	0	0	0
BGIR9823_89026	PXDC1	lncRNA	0	0	0	0	0	0	0	0	0	0	0	0	0	0	0	0	0	0	0	0	0
BGIR9823_89027	LOC102161744	lncRNA	0	0	0	0	0	0	0	0	0	0	0	0	0	0	0	0	0	0	0	0	0
BGIR9823_89040	TBC1D7	lncRNA	0	0	0	0	0	0	0	0	0	0	0	0	0	0	0	0	0	0	0	0	0
BGIR9823_89041	TBC1D7	lncRNA	0	0	0	0	0	0	0	0	0	0	0	0	0	0	0	0	0	0	0	0	0
BGIR9823_89042	TBC1D7	lncRNA	0	0	0	0	0	0	0	0	0	0	0	0	0	0	0	0	0	0	0	0	0
BGIR9823_89043	TBC1D7	lncRNA	0	0	0	0	0	0	0	0	0	0	0	0	0	0	0	0	0	0	0	0	0
BGIR9823_89044	GFOD1	lncRNA	0	0	0	0	0	0	0	0	0	0	0	0	0	0	0	0	0	0	0	0	0
BGIR9823_89045	GFOD1	mRNA	0	0	0	0	0	0	0	0	0	0	0	0	0	0	0	0	0	0	0	0	0
BGIR9823_89046	GFOD1	mRNA	0	0	0	0	0	0	0	0	0	0	0	0	0	0	0	0	0	0	0	0	0
BGIR9823_89047	GFOD1	mRNA	0	0	0	0	0	0	0	0	0	0	0	0	0	0	0	0	0	0	0	0	0
BGIR9823_89048	GFOD1	lncRNA	0	0	0	0	0	0	0	0	0	0	0	0	0	0	0	0	0	0	0	0	0
BGIR9823_89049	GFOD1	lncRNA	0	0	0	0	0	0	0	0	0	0	0	0	0	0	0	0	0	0	0	0	0
BGIR9823_90771	NEU3	lncRNA	0	0	0	0	0	0	0	0	0	0	0	0	0	0	0	0	0	0	0	0	0
BGIR9823_90772	SLCO2B1	mRNA	0	0	0	0	0	0	0	0	0	0	0	0	0	0	0	0	0	0	0	0	0
BGIR9823_90913	LOC110255435	lncRNA	0	0	0	0	0	0	0	0	0	0	0	0	0	0	0	0	0	0	0	0	0
BGIR9823_90934	LOC110255441	lncRNA	0	0	0	0	0	0	0	0	0	0	0	0	0	0	0	0	0	0	0	0	0
BGIR9823_90935	CCDC84	mRNA	0	0	0	0	0	0	0	0	0	0	0	0	0	0	0	0	0	0	0	0	0
BGIR9823_90936	TRAPPC4	mRNA	0	0	0	0	0	0	0	0	0	0	0	0	0	0	0	0	0	0	0	0	0
BGIR9823_90937	HMBS	mRNA	0	0	0	0	0	0	0	0	0	0	0	0	0	0	0	0	0	0	0	0	0
BGIR9823_90956	SORL1	mRNA	0	0	0	0	0	0	0	0	0	0	0	0	0	0	0	0	0	0	0	0	0
BGIR9823_90957	SORL1	mRNA	0	0	0	0	0	0	0	0	0	0	0	0	0	0	0	0	0	0	0	0	0
BGIR9823_90958	SORL1	mRNA	0	0	0	0	0	0	0	0	0	0	0	0	0	0	0	0	0	0	0	0	0
BGIR9823_90960	CLMP	lncRNA	0	0	0	0	0	0	0	0	0	0	0	0	0	0	0	0	0	0	0	0	0
BGIR9823_91161	LOC100522267	mRNA	0	0	0	0	0	0	0	0	0	0	0	0	0	0	0	0	0	0	0	0	0
BGIR9823_91162	LOC100522455	mRNA	0	0	0	0	0	0	0	0	0	0	0	0	0	0	0	0	0	0	0	0	0
BGIR9823_91163	ABCB1	mRNA	0	0	0	0	0	0	0	0	0	0	0	0	0	0	0	0	0	0	0	0	0
BGIR9823_91402	HBB	mRNA	0	0	0	0	0	0	0	0	0	0	0	0	0	0	0	0	0	0	0	0	0
BGIR9823_91403	HBB	mRNA	0	0	0	0	0	0	0	0	0	0	0	0	0	0	0	0	0	0	0	0	0
BGIR9823_91404	HBB	mRNA	0	0	0	0	0	0	0	0	0	0	0	0	0	0	0	0	0	0	0	0	0
BGIR9823_91405	HBB	mRNA	0	0	0	0	0	0	0	0	0	0	0	0	0	0	0	0	0	0	0	0	0
BGIR9823_91406	HBB	mRNA	0	0	0	0	0	0	0	0	0	0	0	0	0	0	0	0	0	0	0	0	0
BGIR9823_91407	HBB	mRNA	0	0	0	0	0	0	0	0	0	0	0	0	0	0	0	0	0	0	0	0	0
BGIR9823_91408	HBB	mRNA	0	0	0	0	0	0	0	0	0	0	0	0	0	0	0	0	0	0	0	0	0
BGIR9823_91409	HBB	mRNA	0	0	0	0	0	0	0	0	0	0	0	0	0	0	0	0	0	0	0	0	0
BGIR9823_91625	BCL9L	lncRNA	0	0	0	0	0	0	0	0	0	0	0	0	0	0	0	0	0	0	0	0	0
BGIR9823_91626	BCL9L	lncRNA	0	0	0	0	0	0	0	0	0	0	0	0	0	0	0	0	0	0	0	0	0
BGIR9823_91627	RPS25	mRNA	0	0	0	0	0	0	0	0	0	0	0	0	0	0	0	0	0	0	0	0	0
BGIR9823_91628	RPS25	mRNA	0	0	0	0	0	0	0	0	0	0	0	0	0	0	0	0	0	0	0	0	0
BGIR9823_91629	SLC37A4	mRNA	0	0	0	0	0	0	0	0	0	0	0	0	0	0	0	0	0	0	0	0	0
BGIR9823_91630	HYOU1	mRNA	0	0	0	0	0	0	0	0	0	0	0	0	0	0	0	0	0	0	0	0	0

RNA ID	Symbol	Type	updown_XR_001303119.2	updown_XR_002341616.1	updown_XR_299372.3	updown_XR_298148.3	updown_XR_002346063.1	updown_BGIR9823_100550	updown_BGIR9823_100272	updown_BGIR9823_101549	updown_BGIR9823_101564	updown_BGIR9823_101567	updown_BGIR9823_102169	updown_BGIR9823_102657	updown_BGIR9823_102650	updown_BGIR9823_79650	updown_BGIR9823_79960	updown_BGIR9823_80428	updown_BGIR9823_80570	updown_BGIR9823_84360	updown_BGIR9823_84364	updown_BGIR9823_81795	updown_BGIR9823_87741
BGIR9823_91640	CLMP	mRNA	0	0	0	0	0	0	0	0	0	0	0	0	0	0	0	0	0	0	0	0	0
BGIR9823_91819	RUNDC3B	lncRNA	0	0	0	0	0	0	0	0	0	0	0	0	0	0	0	0	0	0	0	0	0
BGIR9823_91820	RUNDC3B	lncRNA	0	0	0	0	0	0	0	0	0	0	0	0	0	0	0	0	0	0	0	0	0
BGIR9823_91821	RUNDC3B	lncRNA	0	0	0	0	0	0	0	0	0	0	0	0	0	0	0	0	0	0	0	0	0
BGIR9823_91822	RUNDC3B	lncRNA	0	0	0	0	0	0	0	0	0	0	0	0	0	0	0	0	0	0	0	0	0
BGIR9823_91823	RUNDC3B	lncRNA	0	0	0	0	0	0	0	0	0	0	0	0	0	0	0	0	0	0	0	0	0
BGIR9823_91824	RUNDC3B	lncRNA	0	0	0	0	0	0	0	0	0	0	0	0	0	0	0	0	0	0	0	0	0
BGIR9823_91825	RUNDC3B	lncRNA	0	0	0	0	0	0	0	0	0	0	0	0	0	0	0	0	0	0	0	0	0
BGIR9823_91826	LOC102164596	lncRNA	0	0	0	0	0	0	0	0	0	0	0	0	0	0	0	0	0	0	0	0	0
BGIR9823_92993	KATNAL1	mRNA	0	0	0	0	0	0	0	0	0	0	0	0	0	0	0	0	0	0	0	0	0
BGIR9823_92994	KATNAL1	mRNA	0	0	0	0	0	0	0	0	0	0	0	0	0	0	0	0	0	0	0	0	0
BGIR9823_92995	KATNAL1	mRNA	0	0	0	0	0	0	0	0	0	0	0	0	0	0	0	0	0	0	0	0	0
BGIR9823_92996	KATNAL1	mRNA	0	0	0	0	0	0	0	0	0	0	0	0	0	0	0	0	0	0	0	0	0
BGIR9823_92997	KATNAL1	mRNA	0	0	0	0	0	0	0	0	0	0	0	0	0	0	0	0	0	0	0	0	0
BGIR9823_92999	LOC110255292	lncRNA	0	0	0	0	0	0	0	0	0	0	0	0	0	0	0	0	0	0	0	0	0
BGIR9823_93000	LOC102166871	lncRNA	0	0	0	0	0	0	0	0	0	0	0	0	0	0	0	0	0	0	0	0	0
BGIR9823_98051	LOC102164998	lncRNA	0	0	0	0	0	0	0	0	0	0	0	0	0	0	0	0	0	0	0	0	0
BGIR9823_99244	LOC102167708	lncRNA	0	0	0	0	0	0	0	0	0	0	0	0	0	0	0	0	0	0	0	0	0
NM_001004034.1	HMGB1	mRNA	0	0	0	0	0	0	0	0	0	0	0	0	0	0	0	0	0	0	0	0	0
NM_001044526.1	PPARA	mRNA	0	0	0	0	0	0	0	0	0	0	0	0	0	0	0	0	0	0	0	0	0
NM_001111256.1	SERPINB9	mRNA	0	0	0	0	0	0	0	0	0	0	0	0	0	0	0	0	0	0	0	0	0
NM_001113443.1	ECI2	mRNA	0	0	0	0	0	0	0	0	0	0	0	0	0	0	0	0	0	0	0	0	0
NM_001144841.1	HBB	mRNA	0	0	0	0	0	0	0	0	0	0	0	0	0	0	0	0	0	0	0	0	0
NM_001199719.1	SLC37A4	mRNA	0	0	0	0	0	0	0	0	0	0	0	0	0	0	0	0	0	0	0	0	0
NM_001243907.1	HSPA8	mRNA	0	0	0	0	0	0	0	0	0	0	0	0	0	0	0	0	0	0	0	0	0
NM_001244063.1	RPL3	mRNA	0	0	0	0	0	0	0	0	0	0	0	0	0	0	0	0	0	0	0	0	0
NM_001244066.1	SYNGR1	mRNA	0	0	0	0	0	0	0	0	0	0	0	0	0	0	0	0	0	0	0	0	0
NM_001244396.1	CCDC84	mRNA	0	0	0	0	0	0	0	0	0	0	0	0	0	0	0	0	0	0	0	0	0
NM_001287846.1	RHEB	mRNA	0	0	0	0	0	0	0	0	0	0	0	0	0	0	0	0	0	0	0	0	0
NM_213954.1	ACO2	mRNA	0	0	0	0	0	0	0	0	0	0	0	0	0	0	0	0	0	0	0	0	0
NM_214012.1	UPK2	mRNA	0	0	0	0	0	0	0	0	0	0	0	0	0	0	0	0	0	0	0	0	0
URS0000BC92F4	LOC102167708	lncRNA	0	0	0	0	0	0	0	0	0	0	0	0	0	0	0	0	0	0	0	0	0
XM_001928309.6	TBC1D7	mRNA	0	0	0	0	0	0	0	0	0	0	0	0	0	0	0	0	0	0	0	0	0
XM_001928852.5	PXDC1	mRNA	0	0	0	0	0	0	0	0	0	0	0	0	0	0	0	0	0	0	0	0	0
XM_003122534.4	KCNK7	mRNA	0	0	0	0	0	0	0	0	0	0	0	0	0	0	0	0	0	0	0	0	0
XM_003122558.5	SCYL1	mRNA	0	0	0	0	0	0	0	0	0	0	0	0	0	0	0	0	0	0	0	0	0
XM_003122562.5	FRMD8	mRNA	0	0	0	0	0	0	0	0	0	0	0	0	0	0	0	0	0	0	0	0	0
XM_003125951.6	TTC38	mRNA	0	0	0	0	0	0	0	0	0	0	0	0	0	0	0	0	0	0	0	0	0
XM_003125953.4	CDPF1	mRNA	0	0	0	0	0	0	0	0	0	0	0	0	0	0	0	0	0	0	0	0	0
XM_003126012.6	CBX7	mRNA	0	0	0	0	0	0	0	0	0	0	0	0	0	0	0	0	0	0	0	0	0
XM_003128161.4	RIPK1	mRNA	0	0	0	0	0	0	0	0	0	0	0	0	0	0	0	0	0	0	0	0	0
XM_003129508.2	LOC100514417	mRNA	0	0	0	0	0	0	0	0	0	0	0	0	0	0	0	0	0	0	0	0	0
XM_003129512.2	LOC100515268	mRNA	0	0	0	0	0	0	0	0	0	0	0	0	0	0	0	0	0	0	0	0	0

RNA ID	Symbol	Type	updown_XR_001303119.2	updown_XR_002341616.1	updown_XR_299372.3	updown_XR_298148.3	updown_XR_002346063.1	updown_BGIR9823_100550	updown_BGIR9823_100272	updown_BGIR9823_101549	updown_BGIR9823_101564	updown_BGIR9823_101567	updown_BGIR9823_102169	updown_BGIR9823_102657	updown_BGIR9823_102650	updown_BGIR9823_79650	updown_BGIR9823_79960	updown_BGIR9823_80428	updown_BGIR9823_80570	updown_BGIR9823_84360	updown_BGIR9823_84364	updown_BGIR9823_81795	updown_BGIR9823_87741
XM_003129514.3	LOC100515618	mRNA	0	0	0	0	0	0	0	0	0	0	0	0	0	0	0	0	0	0	0	0	0
XM_003129519.2	LOC100517595	mRNA	0	0	0	0	0	0	0	0	0	0	0	0	0	0	0	0	0	0	0	0	0
XM_003129529.4	LOC100519910	mRNA	0	0	0	0	0	0	0	0	0	0	0	0	0	0	0	0	0	0	0	0	0
XM_003129915.4	CXCR5	mRNA	0	0	0	0	0	0	0	0	0	0	0	0	0	0	0	0	0	0	0	0	0
XM_003129916.5	BCL9L	mRNA	0	0	0	0	0	0	0	0	0	0	0	0	0	0	0	0	0	0	0	0	0
XM_003129918.4	TRAPPC4	mRNA	0	0	0	0	0	0	0	0	0	0	0	0	0	0	0	0	0	0	0	0	0
XM_003129919.5	RPS25	mRNA	0	0	0	0	0	0	0	0	0	0	0	0	0	0	0	0	0	0	0	0	0
XM_003129920.3	RPS25	mRNA	0	0	0	0	0	0	0	0	0	0	0	0	0	0	0	0	0	0	0	0	0
XM_003129922.4	HYOU1	mRNA	0	0	0	0	0	0	0	0	0	0	0	0	0	0	0	0	0	0	0	0	0
XM_003129923.6	VPS11	mRNA	0	0	0	0	0	0	0	0	0	0	0	0	0	0	0	0	0	0	0	0	0
XM_003129944.4	SC5D	mRNA	0	0	0	0	0	0	0	0	0	0	0	0	0	0	0	0	0	0	0	0	0
XM_003357161.1	LOC100620971	mRNA	0	0	0	0	0	0	0	0	0	0	0	0	0	0	0	0	0	0	0	0	0
XM_003482119.4	SERPINB1	mRNA	0	0	0	0	0	0	0	0	0	0	0	0	0	0	0	0	0	0	0	0	0
XM_003482132.4	TMEM170B	mRNA	0	0	0	0	0	0	0	0	0	0	0	0	0	0	0	0	0	0	0	0	0
XM_003482133.4	GFOD1	mRNA	0	0	0	0	0	0	0	0	0	0	0	0	0	0	0	0	0	0	0	0	0
XM_003482563.4	SPCS2	mRNA	0	0	0	0	0	0	0	0	0	0	0	0	0	0	0	0	0	0	0	0	0
XM_003482565.4	NEU3	mRNA	0	0	0	0	0	0	0	0	0	0	0	0	0	0	0	0	0	0	0	0	0
XM_003482567.1	LOC100739359	mRNA	0	0	0	0	0	0	0	0	0	0	0	0	0	0	0	0	0	0	0	0	0
XM_003482630.3	BSX	mRNA	0	0	0	0	0	0	0	0	0	0	0	0	0	0	0	0	0	0	0	0	0
XM_005653107.3	CHADL	mRNA	0	0	0	0	0	0	0	0	0	0	0	0	0	0	0	0	0	0	0	0	0
XM_005653640.1	LOC100621181	mRNA	0	0	0	0	0	0	0	0	0	0	0	0	0	0	0	0	0	0	0	0	0
XM_005654266.3	PRKAG2	mRNA	0	0	0	0	0	0	0	0	0	0	0	0	0	0	0	0	0	0	0	0	0
XM_005655470.3	CDPF1	mRNA	0	0	0	0	0	0	0	0	0	0	0	0	0	0	0	0	0	0	0	0	0
XM_005660703.3	LTBP3	mRNA	0	0	0	0	0	0	0	0	0	0	0	0	0	0	0	0	0	0	0	0	0
XM_005660705.3	SCYL1	mRNA	0	0	0	0	0	0	0	0	0	0	0	0	0	0	0	0	0	0	0	0	0
XM_005660707.3	SLC25A45	mRNA	0	0	0	0	0	0	0	0	0	0	0	0	0	0	0	0	0	0	0	0	0
XM_005665536.3	RIPK1	mRNA	0	0	0	0	0	0	0	0	0	0	0	0	0	0	0	0	0	0	0	0	0
XM_005665537.3	RIPK1	mRNA	0	0	0	0	0	0	0	0	0	0	0	0	0	0	0	0	0	0	0	0	0
XM_005665538.3	RIPK1	mRNA	0	0	0	0	0	0	0	0	0	0	0	0	0	0	0	0	0	0	0	0	0
XM_005665575.3	PHACTR1	mRNA	0	0	0	0	0	0	0	0	0	0	0	0	0	0	0	0	0	0	0	0	0
XM_005665576.3	TBC1D7	mRNA	0	0	0	0	0	0	0	0	0	0	0	0	0	0	0	0	0	0	0	0	0
XM_005667128.3	LOC100627340	mRNA	0	0	0	0	0	0	0	0	0	0	0	0	0	0	0	0	0	0	0	0	0
XM_005667397.2	CCDC84	mRNA	0	0	0	0	0	0	0	0	0	0	0	0	0	0	0	0	0	0	0	0	0
XM_005667398.3	SLC37A4	mRNA	0	0	0	0	0	0	0	0	0	0	0	0	0	0	0	0	0	0	0	0	0
XM_005667402.3	SLC37A4	mRNA	0	0	0	0	0	0	0	0	0	0	0	0	0	0	0	0	0	0	0	0	0
XM_005667403.3	SLC37A4	mRNA	0	0	0	0	0	0	0	0	0	0	0	0	0	0	0	0	0	0	0	0	0
XM_005667413.3	BCL9L	mRNA	0	0	0	0	0	0	0	0	0	0	0	0	0	0	0	0	0	0	0	0	0
XM_005667414.3	FOXR1	mRNA	0	0	0	0	0	0	0	0	0	0	0	0	0	0	0	0	0	0	0	0	0
XM_005667416.3	HYOU1	mRNA	0	0	0	0	0	0	0	0	0	0	0	0	0	0	0	0	0	0	0	0	0
XM_005667419.3	HYOU1	mRNA	0	0	0	0	0	0	0	0	0	0	0	0	0	0	0	0	0	0	0	0	0
XM_005667420.3	HYOU1	mRNA	0	0	0	0	0	0	0	0	0	0	0	0	0	0	0	0	0	0	0	0	0
XM_005667440.2	JHY	mRNA	0	0	0	0	0	0	0	0	0	0	0	0	0	0	0	0	0	0	0	0	0
XM_005668316.3	HMGB1	mRNA	0	0	0	0	0	0	0	0	0	0	0	0	0	0	0	0	0	0	0	0	0

RNA ID	Symbol	Type	updown_XR_001303119.2	updown_XR_002341616.1	updown_XR_299372.3	updown_XR_298148.3	updown_XR_002346063.1	updown_BGIR9823_100550	updown_BGIR9823_100272	updown_BGIR9823_101549	updown_BGIR9823_101564	updown_BGIR9823_101567	updown_BGIR9823_102169	updown_BGIR9823_102657	updown_BGIR9823_102650	updown_BGIR9823_79650	updown_BGIR9823_79960	updown_BGIR9823_80428	updown_BGIR9823_80570	updown_BGIR9823_84360	updown_BGIR9823_84364	updown_BGIR9823_81795	updown_BGIR9823_87741
XM_005668317.3	HMGB1	mRNA	0	0	0	0	0	0	0	0	0	0	0	0	0	0	0	0	0	0	0	0	0
XM_005668318.3	HMGB1	mRNA	0	0	0	0	0	0	0	0	0	0	0	0	0	0	0	0	0	0	0	0	0
XM_005671489.3	EMX2	mRNA	0	0	0	0	0	0	0	0	0	0	0	0	0	0	0	0	0	0	0	0	0
XM_005672397.3	MYO10	mRNA	0	0	0	0	0	0	0	0	0	0	0	0	0	0	0	0	0	0	0	0	0
XM_013977400.2	FAM50B	mRNA	0	0	0	0	0	0	0	0	0	0	0	0	0	0	0	0	0	0	0	0	0
XM_013977425.2	NEDD9	mRNA	0	0	0	0	0	0	0	0	0	0	0	0	0	0	0	0	0	0	0	0	0
XM_013977430.2	TBC1D7	mRNA	0	0	0	0	0	0	0	0	0	0	0	0	0	0	0	0	0	0	0	0	0
XM_013977431.2	TBC1D7	mRNA	0	0	0	0	0	0	0	0	0	0	0	0	0	0	0	0	0	0	0	0	0
XM_013977432.2	TBC1D7	mRNA	0	0	0	0	0	0	0	0	0	0	0	0	0	0	0	0	0	0	0	0	0
XM_013977433.2	GFOD1	mRNA	0	0	0	0	0	0	0	0	0	0	0	0	0	0	0	0	0	0	0	0	0
XM_013977434.2	GFOD1	mRNA	0	0	0	0	0	0	0	0	0	0	0	0	0	0	0	0	0	0	0	0	0
XM_013979449.2	SLC37A4	mRNA	0	0	0	0	0	0	0	0	0	0	0	0	0	0	0	0	0	0	0	0	0
XM_013979456.2	BCL9L	mRNA	0	0	0	0	0	0	0	0	0	0	0	0	0	0	0	0	0	0	0	0	0
XM_013979457.2	BCL9L	mRNA	0	0	0	0	0	0	0	0	0	0	0	0	0	0	0	0	0	0	0	0	0
XM_013979458.2	BCL9L	mRNA	0	0	0	0	0	0	0	0	0	0	0	0	0	0	0	0	0	0	0	0	0
XM_013979459.2	BCL9L	mRNA	0	0	0	0	0	0	0	0	0	0	0	0	0	0	0	0	0	0	0	0	0
XM_013979461.2	TRAPPC4	mRNA	0	0	0	0	0	0	0	0	0	0	0	0	0	0	0	0	0	0	0	0	0
XM_013979463.2	HYOU1	mRNA	0	0	0	0	0	0	0	0	0	0	0	0	0	0	0	0	0	0	0	0	0
XM_013979484.2	LOC100523684	mRNA	0	0	0	0	0	0	0	0	0	0	0	0	0	0	0	0	0	0	0	0	0
XM_013979486.2	LOC100523684	mRNA	0	0	0	0	0	0	0	0	0	0	0	0	0	0	0	0	0	0	0	0	0
XM_013979491.2	JHY	mRNA	0	0	0	0	0	0	0	0	0	0	0	0	0	0	0	0	0	0	0	0	0
XM_013980418.2	KATNAL1	mRNA	0	0	0	0	0	0	0	0	0	0	0	0	0	0	0	0	0	0	0	0	0
XM_013988182.2	TTC38	mRNA	0	0	0	0	0	0	0	0	0	0	0	0	0	0	0	0	0	0	0	0	0
XM_013988183.2	TTC38	mRNA	0	0	0	0	0	0	0	0	0	0	0	0	0	0	0	0	0	0	0	0	0
XM_013989589.2	LOC100522267	mRNA	0	0	0	0	0	0	0	0	0	0	0	0	0	0	0	0	0	0	0	0	0
XM_013989592.2	LOC100522455	mRNA	0	0	0	0	0	0	0	0	0	0	0	0	0	0	0	0	0	0	0	0	0
XM_013989593.2	LOC100522455	mRNA	0	0	0	0	0	0	0	0	0	0	0	0	0	0	0	0	0	0	0	0	0
XM_013994099.2	FRMD8	mRNA	0	0	0	0	0	0	0	0	0	0	0	0	0	0	0	0	0	0	0	0	0
XM_013994100.2	SLC25A45	mRNA	0	0	0	0	0	0	0	0	0	0	0	0	0	0	0	0	0	0	0	0	0
XM_013994102.2	SLC25A45	mRNA	0	0	0	0	0	0	0	0	0	0	0	0	0	0	0	0	0	0	0	0	0
XM_013994103.2	SLC25A45	mRNA	0	0	0	0	0	0	0	0	0	0	0	0	0	0	0	0	0	0	0	0	0
XM_013994104.2	SLC25A45	mRNA	0	0	0	0	0	0	0	0	0	0	0	0	0	0	0	0	0	0	0	0	0
XM_013997510.2	CBX7	mRNA	0	0	0	0	0	0	0	0	0	0	0	0	0	0	0	0	0	0	0	0	0
XM_013999070.2	LOC100520088	mRNA	0	0	0	0	0	0	0	0	0	0	0	0	0	0	0	0	0	0	0	0	0
XM_021062230.1	LOC100517771	mRNA	0	0	0	0	0	0	0	0	0	0	0	0	0	0	0	0	0	0	0	0	0
XM_021062231.1	LOC100517957	mRNA	0	0	0	0	0	0	0	0	0	0	0	0	0	0	0	0	0	0	0	0	0
XM_021062367.1	LOC110255365	mRNA	0	0	0	0	0	0	0	0	0	0	0	0	0	0	0	0	0	0	0	0	0
XM_021062369.1	LOC100737110	mRNA	0	0	0	0	0	0	0	0	0	0	0	0	0	0	0	0	0	0	0	0	0
XM_021062370.1	LOC110255368	mRNA	0	0	0	0	0	0	0	0	0	0	0	0	0	0	0	0	0	0	0	0	0
XM_021062371.1	LOC100515441	mRNA	0	0	0	0	0	0	0	0	0	0	0	0	0	0	0	0	0	0	0	0	0
XM_021062374.1	LOC110255371	mRNA	0	0	0	0	0	0	0	0	0	0	0	0	0	0	0	0	0	0	0	0	0
XM_021062375.1	LOC100626951	mRNA	0	0	0	0	0	0	0	0	0	0	0	0	0	0	0	0	0	0	0	0	0
XM_021062491.1	XRRA1	mRNA	0	0	0	0	0	0	0	0	0	0	0	0	0	0	0	0	0	0	0	0	0

RNA ID	Symbol	Type	updown_XR_001303119.2	updown_XR_002341616.1	updown_XR_299372.3	updown_XR_298148.3	updown_XR_002346063.1	updown_BGIR9823_100550	updown_BGIR9823_100272	updown_BGIR9823_101549	updown_BGIR9823_101564	updown_BGIR9823_101567	updown_BGIR9823_102169	updown_BGIR9823_102657	updown_BGIR9823_102650	updown_BGIR9823_79650	updown_BGIR9823_79960	updown_BGIR9823_80428	updown_BGIR9823_80570	updown_BGIR9823_84360	updown_BGIR9823_84364	updown_BGIR9823_81795	updown_BGIR9823_87741
XM_021062493.1	XRRA1	mRNA	0	0	0	0	0	0	0	0	0	0	0	0	0	0	0	0	0	0	0	0	0
XM_021062494.1	XRRA1	mRNA	0	0	0	0	0	0	0	0	0	0	0	0	0	0	0	0	0	0	0	0	0
XM_021062495.1	XRRA1	mRNA	0	0	0	0	0	0	0	0	0	0	0	0	0	0	0	0	0	0	0	0	0
XM_021062496.1	XRRA1	mRNA	0	0	0	0	0	0	0	0	0	0	0	0	0	0	0	0	0	0	0	0	0
XM_021062497.1	XRRA1	mRNA	0	0	0	0	0	0	0	0	0	0	0	0	0	0	0	0	0	0	0	0	0
XM_021062498.1	XRRA1	mRNA	0	0	0	0	0	0	0	0	0	0	0	0	0	0	0	0	0	0	0	0	0
XM_021062499.1	XRRA1	mRNA	0	0	0	0	0	0	0	0	0	0	0	0	0	0	0	0	0	0	0	0	0
XM_021062500.1	XRRA1	mRNA	0	0	0	0	0	0	0	0	0	0	0	0	0	0	0	0	0	0	0	0	0
XM_021062501.1	XRRA1	mRNA	0	0	0	0	0	0	0	0	0	0	0	0	0	0	0	0	0	0	0	0	0
XM_021062502.1	XRRA1	mRNA	0	0	0	0	0	0	0	0	0	0	0	0	0	0	0	0	0	0	0	0	0
XM_021062503.1	XRRA1	mRNA	0	0	0	0	0	0	0	0	0	0	0	0	0	0	0	0	0	0	0	0	0
XM_021062511.1	SLCO2B1	mRNA	0	0	0	0	0	0	0	0	0	0	0	0	0	0	0	0	0	0	0	0	0
XM_021062948.1	BCL9L	mRNA	0	0	0	0	0	0	0	0	0	0	0	0	0	0	0	0	0	0	0	0	0
XM_021062949.1	TRAPPC4	mRNA	0	0	0	0	0	0	0	0	0	0	0	0	0	0	0	0	0	0	0	0	0
XM_021062950.1	HYOU1	mRNA	0	0	0	0	0	0	0	0	0	0	0	0	0	0	0	0	0	0	0	0	0
XM_021062951.1	HYOU1	mRNA	0	0	0	0	0	0	0	0	0	0	0	0	0	0	0	0	0	0	0	0	0
XM_021062952.1	HYOU1	mRNA	0	0	0	0	0	0	0	0	0	0	0	0	0	0	0	0	0	0	0	0	0
XM_021062953.1	HYOU1	mRNA	0	0	0	0	0	0	0	0	0	0	0	0	0	0	0	0	0	0	0	0	0
XM_021062954.1	HYOU1	mRNA	0	0	0	0	0	0	0	0	0	0	0	0	0	0	0	0	0	0	0	0	0
XM_021062955.1	HYOU1	mRNA	0	0	0	0	0	0	0	0	0	0	0	0	0	0	0	0	0	0	0	0	0
XM_021062956.1	HYOU1	mRNA	0	0	0	0	0	0	0	0	0	0	0	0	0	0	0	0	0	0	0	0	0
XM_021062957.1	VPS11	mRNA	0	0	0	0	0	0	0	0	0	0	0	0	0	0	0	0	0	0	0	0	0
XM_021062993.1	LOC100523684	mRNA	0	0	0	0	0	0	0	0	0	0	0	0	0	0	0	0	0	0	0	0	0
XM_021062994.1	LOC100523684	mRNA	0	0	0	0	0	0	0	0	0	0	0	0	0	0	0	0	0	0	0	0	0
XM_021062995.1	LOC100523684	mRNA	0	0	0	0	0	0	0	0	0	0	0	0	0	0	0	0	0	0	0	0	0
XM_021062996.1	LOC100523684	mRNA	0	0	0	0	0	0	0	0	0	0	0	0	0	0	0	0	0	0	0	0	0
XM_021062997.1	LOC100523684	mRNA	0	0	0	0	0	0	0	0	0	0	0	0	0	0	0	0	0	0	0	0	0
XM_021062998.1	LOC100523684	mRNA	0	0	0	0	0	0	0	0	0	0	0	0	0	0	0	0	0	0	0	0	0
XM_021062999.1	LOC100523684	mRNA	0	0	0	0	0	0	0	0	0	0	0	0	0	0	0	0	0	0	0	0	0
XM_021063000.1	LOC100523684	mRNA	0	0	0	0	0	0	0	0	0	0	0	0	0	0	0	0	0	0	0	0	0
XM_021063001.1	LOC100523684	mRNA	0	0	0	0	0	0	0	0	0	0	0	0	0	0	0	0	0	0	0	0	0
XM_021063002.1	LOC100523684	mRNA	0	0	0	0	0	0	0	0	0	0	0	0	0	0	0	0	0	0	0	0	0
XM_021063004.1	LOC100523684	mRNA	0	0	0	0	0	0	0	0	0	0	0	0	0	0	0	0	0	0	0	0	0
XM_021063005.1	LOC100523684	mRNA	0	0	0	0	0	0	0	0	0	0	0	0	0	0	0	0	0	0	0	0	0
XM_021063006.1	LOC100523684	mRNA	0	0	0	0	0	0	0	0	0	0	0	0	0	0	0	0	0	0	0	0	0
XM_021063009.1	SORL1	mRNA	0	0	0	0	0	0	0	0	0	0	0	0	0	0	0	0	0	0	0	0	0
XM_021063010.1	SORL1	mRNA	0	0	0	0	0	0	0	0	0	0	0	0	0	0	0	0	0	0	0	0	0
XM_021063011.1	SORL1	mRNA	0	0	0	0	0	0	0	0	0	0	0	0	0	0	0	0	0	0	0	0	0
XM_021063012.1	SORL1	mRNA	0	0	0	0	0	0	0	0	0	0	0	0	0	0	0	0	0	0	0	0	0
XM_021063013.1	SORL1	mRNA	0	0	0	0	0	0	0	0	0	0	0	0	0	0	0	0	0	0	0	0	0
XM_021063014.1	SORL1	mRNA	0	0	0	0	0	0	0	0	0	0	0	0	0	0	0	0	0	0	0	0	0
XM_021063023.1	CLMP	mRNA	0	0	0	0	0	0	0	0	0	0	0	0	0	0	0	0	0	0	0	0	0
XM_021063024.1	CLMP	mRNA	0	0	0	0	0	0	0	0	0	0	0	0	0	0	0	0	0	0	0	0	0

RNA ID	Symbol	Type	updown_XR_001303119.2	updown_XR_002341616.1	updown_XR_299372.3	updown_XR_298148.3	updown_XR_002346063.1	updown_BGIR9823_100550	updown_BGIR9823_100272	updown_BGIR9823_101549	updown_BGIR9823_101564	updown_BGIR9823_101567	updown_BGIR9823_102169	updown_BGIR9823_102657	updown_BGIR9823_102650	updown_BGIR9823_79650	updown_BGIR9823_79960	updown_BGIR9823_80428	updown_BGIR9823_80570	updown_BGIR9823_84360	updown_BGIR9823_84364	updown_BGIR9823_81795	updown_BGIR9823_87741
XM_021063025.1	CLMP	mRNA	0	0	0	0	0	0	0	0	0	0	0	0	0	0	0	0	0	0	0	0	0
XM_021063026.1	CLMP	mRNA	0	0	0	0	0	0	0	0	0	0	0	0	0	0	0	0	0	0	0	0	0
XM_021063950.1	NXPE2	mRNA	0	0	0	0	0	0	0	0	0	0	0	0	0	0	0	0	0	0	0	0	0
XM_021063963.1	LOC100623190	mRNA	0	0	0	0	0	0	0	0	0	0	0	0	0	0	0	0	0	0	0	0	0
XM_021065159.1	HMGB1	mRNA	0	0	0	0	0	0	0	0	0	0	0	0	0	0	0	0	0	0	0	0	0
XM_021065160.1	HMGB1	mRNA	0	0	0	0	0	0	0	0	0	0	0	0	0	0	0	0	0	0	0	0	0
XM_021065161.1	HMGB1	mRNA	0	0	0	0	0	0	0	0	0	0	0	0	0	0	0	0	0	0	0	0	0
XM_021065529.1	KATNAL1	mRNA	0	0	0	0	0	0	0	0	0	0	0	0	0	0	0	0	0	0	0	0	0
XM_021065531.1	KATNAL1	mRNA	0	0	0	0	0	0	0	0	0	0	0	0	0	0	0	0	0	0	0	0	0
XM_021065532.1	KATNAL1	mRNA	0	0	0	0	0	0	0	0	0	0	0	0	0	0	0	0	0	0	0	0	0
XM_021065533.1	KATNAL1	mRNA	0	0	0	0	0	0	0	0	0	0	0	0	0	0	0	0	0	0	0	0	0
XM_021065534.1	KATNAL1	mRNA	0	0	0	0	0	0	0	0	0	0	0	0	0	0	0	0	0	0	0	0	0
XM_021065535.1	KATNAL1	mRNA	0	0	0	0	0	0	0	0	0	0	0	0	0	0	0	0	0	0	0	0	0
XM_021065536.1	KATNAL1	mRNA	0	0	0	0	0	0	0	0	0	0	0	0	0	0	0	0	0	0	0	0	0
XM_021065537.1	KATNAL1	mRNA	0	0	0	0	0	0	0	0	0	0	0	0	0	0	0	0	0	0	0	0	0
XM_021065538.1	KATNAL1	mRNA	0	0	0	0	0	0	0	0	0	0	0	0	0	0	0	0	0	0	0	0	0
XM_021065539.1	KATNAL1	mRNA	0	0	0	0	0	0	0	0	0	0	0	0	0	0	0	0	0	0	0	0	0
XM_021065541.1	KATNAL1	mRNA	0	0	0	0	0	0	0	0	0	0	0	0	0	0	0	0	0	0	0	0	0
XM_021065542.1	KATNAL1	mRNA	0	0	0	0	0	0	0	0	0	0	0	0	0	0	0	0	0	0	0	0	0
XM_021065543.1	KATNAL1	mRNA	0	0	0	0	0	0	0	0	0	0	0	0	0	0	0	0	0	0	0	0	0
XM_021065544.1	KATNAL1	mRNA	0	0	0	0	0	0	0	0	0	0	0	0	0	0	0	0	0	0	0	0	0
XM_021073408.1	EMX2	mRNA	0	0	0	0	0	0	0	0	0	0	0	0	0	0	0	0	0	0	0	0	0
XM_021078611.1	PRKAG2	mRNA	0	0	0	0	0	0	0	0	0	0	0	0	0	0	0	0	0	0	0	0	0
XM_021078612.1	PRKAG2	mRNA	0	0	0	0	0	0	0	0	0	0	0	0	0	0	0	0	0	0	0	0	0
XM_021078613.1	PRKAG2	mRNA	0	0	0	0	0	0	0	0	0	0	0	0	0	0	0	0	0	0	0	0	0
XM_021078614.1	PRKAG2	mRNA	0	0	0	0	0	0	0	0	0	0	0	0	0	0	0	0	0	0	0	0	0
XM_021078615.1	PRKAG2	mRNA	0	0	0	0	0	0	0	0	0	0	0	0	0	0	0	0	0	0	0	0	0
XM_021078616.1	PRKAG2	mRNA	0	0	0	0	0	0	0	0	0	0	0	0	0	0	0	0	0	0	0	0	0
XM_021078617.1	PRKAG2	mRNA	0	0	0	0	0	0	0	0	0	0	0	0	0	0	0	0	0	0	0	0	0
XM_021078618.1	PRKAG2	mRNA	0	0	0	0	0	0	0	0	0	0	0	0	0	0	0	0	0	0	0	0	0
XM_021078619.1	PRKAG2	mRNA	0	0	0	0	0	0	0	0	0	0	0	0	0	0	0	0	0	0	0	0	0
XM_021078620.1	PRKAG2	mRNA	0	0	0	0	0	0	0	0	0	0	0	0	0	0	0	0	0	0	0	0	0
XM_021078621.1	PRKAG2	mRNA	0	0	0	0	0	0	0	0	0	0	0	0	0	0	0	0	0	0	0	0	0
XM_021081309.1	LOC106506226	mRNA	0	0	0	0	0	0	0	0	0	0	0	0	0	0	0	0	0	0	0	0	0
XM_021081403.1	LOC110258215	mRNA	0	0	0	0	0	0	0	0	0	0	0	0	0	0	0	0	0	0	0	0	0
XM_021082153.1	LOC100521600	mRNA	0	0	0	0	0	0	0	0	0	0	0	0	0	0	0	0	0	0	0	0	0
XM_021082154.1	LOC100521600	mRNA	0	0	0	0	0	0	0	0	0	0	0	0	0	0	0	0	0	0	0	0	0
XM_021082156.1	LOC100521600	mRNA	0	0	0	0	0	0	0	0	0	0	0	0	0	0	0	0	0	0	0	0	0
XM_021082157.1	LOC100521600	mRNA	0	0	0	0	0	0	0	0	0	0	0	0	0	0	0	0	0	0	0	0	0
XM_021082158.1	LOC100521600	mRNA	0	0	0	0	0	0	0	0	0	0	0	0	0	0	0	0	0	0	0	0	0
XM_021082159.1	LOC100521600	mRNA	0	0	0	0	0	0	0	0	0	0	0	0	0	0	0	0	0	0	0	0	0
XM_021082835.1	EHBP1L1	mRNA	0	0	0	0	0	0	0	0	0	0	0	0	0	0	0	0	0	0	0	0	0
XM_021082836.1	EHBP1L1	mRNA	0	0	0	0	0	0	0	0	0	0	0	0	0	0	0	0	0	0	0	0	0

RNA ID	Symbol	Type	updown_XR_001303119.2	updown_XR_002341616.1	updown_XR_299372.3	updown_XR_298148.3	updown_XR_002346063.1	updown_BGIR9823_100550	updown_BGIR9823_100272	updown_BGIR9823_101549	updown_BGIR9823_101564	updown_BGIR9823_101567	updown_BGIR9823_102169	updown_BGIR9823_102657	updown_BGIR9823_102650	updown_BGIR9823_79650	updown_BGIR9823_79960	updown_BGIR9823_80428	updown_BGIR9823_80570	updown_BGIR9823_84360	updown_BGIR9823_84364	updown_BGIR9823_81795	updown_BGIR9823_87741
XM_021082837.1	EHBP1L1	mRNA	0	0	0	0	0	0	0	0	0	0	0	0	0	0	0	0	0	0	0	0	0
XM_021082838.1	EHBP1L1	mRNA	0	0	0	0	0	0	0	0	0	0	0	0	0	0	0	0	0	0	0	0	0
XM_021082839.1	EHBP1L1	mRNA	0	0	0	0	0	0	0	0	0	0	0	0	0	0	0	0	0	0	0	0	0
XM_021082840.1	EHBP1L1	mRNA	0	0	0	0	0	0	0	0	0	0	0	0	0	0	0	0	0	0	0	0	0
XM_021082841.1	EHBP1L1	mRNA	0	0	0	0	0	0	0	0	0	0	0	0	0	0	0	0	0	0	0	0	0
XM_021082842.1	EHBP1L1	mRNA	0	0	0	0	0	0	0	0	0	0	0	0	0	0	0	0	0	0	0	0	0
XM_021082843.1	EHBP1L1	mRNA	0	0	0	0	0	0	0	0	0	0	0	0	0	0	0	0	0	0	0	0	0
XM_021082844.1	EHBP1L1	mRNA	0	0	0	0	0	0	0	0	0	0	0	0	0	0	0	0	0	0	0	0	0
XM_021082845.1	EHBP1L1	mRNA	0	0	0	0	0	0	0	0	0	0	0	0	0	0	0	0	0	0	0	0	0
XM_021082846.1	EHBP1L1	mRNA	0	0	0	0	0	0	0	0	0	0	0	0	0	0	0	0	0	0	0	0	0
XM_021082847.1	EHBP1L1	mRNA	0	0	0	0	0	0	0	0	0	0	0	0	0	0	0	0	0	0	0	0	0
XM_021082851.1	MAP3K11	mRNA	0	0	0	0	0	0	0	0	0	0	0	0	0	0	0	0	0	0	0	0	0
XM_021082852.1	SSSCA1	mRNA	0	0	0	0	0	0	0	0	0	0	0	0	0	0	0	0	0	0	0	0	0
XM_021082853.1	FAM89B	mRNA	0	0	0	0	0	0	0	0	0	0	0	0	0	0	0	0	0	0	0	0	0
XM_021082854.1	LTBP3	mRNA	0	0	0	0	0	0	0	0	0	0	0	0	0	0	0	0	0	0	0	0	0
XM_021082855.1	LTBP3	mRNA	0	0	0	0	0	0	0	0	0	0	0	0	0	0	0	0	0	0	0	0	0
XM_021082856.1	SCYL1	mRNA	0	0	0	0	0	0	0	0	0	0	0	0	0	0	0	0	0	0	0	0	0
XM_021082857.1	SLC25A45	mRNA	0	0	0	0	0	0	0	0	0	0	0	0	0	0	0	0	0	0	0	0	0
XM_021082858.1	SLC25A45	mRNA	0	0	0	0	0	0	0	0	0	0	0	0	0	0	0	0	0	0	0	0	0
XM_021091099.1	SYNGR1	mRNA	0	0	0	0	0	0	0	0	0	0	0	0	0	0	0	0	0	0	0	0	0
XM_021091101.1	SYNGR1	mRNA	0	0	0	0	0	0	0	0	0	0	0	0	0	0	0	0	0	0	0	0	0
XM_021091102.1	SYNGR1	mRNA	0	0	0	0	0	0	0	0	0	0	0	0	0	0	0	0	0	0	0	0	0
XM_021091103.1	SYNGR1	mRNA	0	0	0	0	0	0	0	0	0	0	0	0	0	0	0	0	0	0	0	0	0
XM_021091370.1	LOC102159820	mRNA	0	0	0	0	0	0	0	0	0	0	0	0	0	0	0	0	0	0	0	0	0
XM_021091373.1	TRMU	mRNA	0	0	0	0	0	0	0	0	0	0	0	0	0	0	0	0	0	0	0	0	0
XM_021091374.1	TRMU	mRNA	0	0	0	0	0	0	0	0	0	0	0	0	0	0	0	0	0	0	0	0	0
XM_021091375.1	TRMU	mRNA	0	0	0	0	0	0	0	0	0	0	0	0	0	0	0	0	0	0	0	0	0
XM_021091376.1	GTSE1	mRNA	0	0	0	0	0	0	0	0	0	0	0	0	0	0	0	0	0	0	0	0	0
XM_021091377.1	TTC38	mRNA	0	0	0	0	0	0	0	0	0	0	0	0	0	0	0	0	0	0	0	0	0
XM_021091379.1	LOC110260611	mRNA	0	0	0	0	0	0	0	0	0	0	0	0	0	0	0	0	0	0	0	0	0
XM_021091381.1	LOC110260611	mRNA	0	0	0	0	0	0	0	0	0	0	0	0	0	0	0	0	0	0	0	0	0
XM_021091454.1	TOB2	mRNA	0	0	0	0	0	0	0	0	0	0	0	0	0	0	0	0	0	0	0	0	0
XM_021091455.1	TOB2	mRNA	0	0	0	0	0	0	0	0	0	0	0	0	0	0	0	0	0	0	0	0	0
XM_021091456.1	PHF5A	mRNA	0	0	0	0	0	0	0	0	0	0	0	0	0	0	0	0	0	0	0	0	0
XM_021091457.1	PHF5A	mRNA	0	0	0	0	0	0	0	0	0	0	0	0	0	0	0	0	0	0	0	0	0
XM_021091458.1	ZC3H7B	mRNA	0	0	0	0	0	0	0	0	0	0	0	0	0	0	0	0	0	0	0	0	0
XM_021091459.1	ZC3H7B	mRNA	0	0	0	0	0	0	0	0	0	0	0	0	0	0	0	0	0	0	0	0	0
XM_021091460.1	TEF	mRNA	0	0	0	0	0	0	0	0	0	0	0	0	0	0	0	0	0	0	0	0	0
XM_021091461.1	RANGAP1	mRNA	0	0	0	0	0	0	0	0	0	0	0	0	0	0	0	0	0	0	0	0	0
XM_021091474.1	CBX7	mRNA	0	0	0	0	0	0	0	0	0	0	0	0	0	0	0	0	0	0	0	0	0
XM_021092767.1	CRADD	mRNA	0	0	0	0	0	0	0	0	0	0	0	0	0	0	0	0	0	0	0	0	0
XM_021092768.1	CRADD	mRNA	0	0	0	0	0	0	0	0	0	0	0	0	0	0	0	0	0	0	0	0	0
XM_021092769.1	CRADD	mRNA	0	0	0	0	0	0	0	0	0	0	0	0	0	0	0	0	0	0	0	0	0

RNA ID	Symbol	Type	updown_XR_001303119.2	updown_XR_002341616.1	updown_XR_299372.3	updown_XR_298148.3	updown_XR_002346063.1	updown_BGIR9823_100550	updown_BGIR9823_100272	updown_BGIR9823_101549	updown_BGIR9823_101564	updown_BGIR9823_101567	updown_BGIR9823_102169	updown_BGIR9823_102657	updown_BGIR9823_102650	updown_BGIR9823_79650	updown_BGIR9823_79960	updown_BGIR9823_80428	updown_BGIR9823_80570	updown_BGIR9823_84360	updown_BGIR9823_84364	updown_BGIR9823_81795	updown_BGIR9823_87741
XM_021092770.1	CRADD	mRNA	0	0	0	0	0	0	0	0	0	0	0	0	0	0	0	0	0	0	0	0	0
XM_021092771.1	CRADD	mRNA	0	0	0	0	0	0	0	0	0	0	0	0	0	0	0	0	0	0	0	0	0
XM_021092773.1	CRADD	mRNA	0	0	0	0	0	0	0	0	0	0	0	0	0	0	0	0	0	0	0	0	0
XM_021093095.1	PKDREJ	mRNA	0	0	0	0	0	0	0	0	0	0	0	0	0	0	0	0	0	0	0	0	0
XM_021097064.1	MAF	mRNA	0	0	0	0	0	0	0	0	0	0	0	0	0	0	0	0	0	0	0	0	0
XM_021097065.1	MAF	mRNA	0	0	0	0	0	0	0	0	0	0	0	0	0	0	0	0	0	0	0	0	0
XM_021097066.1	MAF	mRNA	0	0	0	0	0	0	0	0	0	0	0	0	0	0	0	0	0	0	0	0	0
XM_021097870.1	ECI2	mRNA	0	0	0	0	0	0	0	0	0	0	0	0	0	0	0	0	0	0	0	0	0
XM_021099751.1	PHACTR1	mRNA	0	0	0	0	0	0	0	0	0	0	0	0	0	0	0	0	0	0	0	0	0
XM_021099752.1	PHACTR1	mRNA	0	0	0	0	0	0	0	0	0	0	0	0	0	0	0	0	0	0	0	0	0
XM_021099753.1	PHACTR1	mRNA	0	0	0	0	0	0	0	0	0	0	0	0	0	0	0	0	0	0	0	0	0
XM_021099754.1	PHACTR1	mRNA	0	0	0	0	0	0	0	0	0	0	0	0	0	0	0	0	0	0	0	0	0
XM_021099755.1	PHACTR1	mRNA	0	0	0	0	0	0	0	0	0	0	0	0	0	0	0	0	0	0	0	0	0
XM_021099756.1	PHACTR1	mRNA	0	0	0	0	0	0	0	0	0	0	0	0	0	0	0	0	0	0	0	0	0
XM_021099757.1	PHACTR1	mRNA	0	0	0	0	0	0	0	0	0	0	0	0	0	0	0	0	0	0	0	0	0
XM_021099758.1	PHACTR1	mRNA	0	0	0	0	0	0	0	0	0	0	0	0	0	0	0	0	0	0	0	0	0
XM_021099759.1	PHACTR1	mRNA	0	0	0	0	0	0	0	0	0	0	0	0	0	0	0	0	0	0	0	0	0
XM_021099760.1	PHACTR1	mRNA	0	0	0	0	0	0	0	0	0	0	0	0	0	0	0	0	0	0	0	0	0
XM_021099761.1	PHACTR1	mRNA	0	0	0	0	0	0	0	0	0	0	0	0	0	0	0	0	0	0	0	0	0
XM_021099762.1	PHACTR1	mRNA	0	0	0	0	0	0	0	0	0	0	0	0	0	0	0	0	0	0	0	0	0
XM_021099763.1	PHACTR1	mRNA	0	0	0	0	0	0	0	0	0	0	0	0	0	0	0	0	0	0	0	0	0
XM_021099764.1	PHACTR1	mRNA	0	0	0	0	0	0	0	0	0	0	0	0	0	0	0	0	0	0	0	0	0
XM_021099765.1	PHACTR1	mRNA	0	0	0	0	0	0	0	0	0	0	0	0	0	0	0	0	0	0	0	0	0
XM_021099766.1	TBC1D7	mRNA	0	0	0	0	0	0	0	0	0	0	0	0	0	0	0	0	0	0	0	0	0
XM_021099767.1	TBC1D7	mRNA	0	0	0	0	0	0	0	0	0	0	0	0	0	0	0	0	0	0	0	0	0
XM_021099769.1	TBC1D7	mRNA	0	0	0	0	0	0	0	0	0	0	0	0	0	0	0	0	0	0	0	0	0
XM_021099770.1	TBC1D7	mRNA	0	0	0	0	0	0	0	0	0	0	0	0	0	0	0	0	0	0	0	0	0
XM_021099999.1	LOC110261697	mRNA	0	0	0	0	0	0	0	0	0	0	0	0	0	0	0	0	0	0	0	0	0
XM_021100108.1	WRNIP1	mRNA	0	0	0	0	0	0	0	0	0	0	0	0	0	0	0	0	0	0	0	0	0
XM_021100109.1	MYLK4	mRNA	0	0	0	0	0	0	0	0	0	0	0	0	0	0	0	0	0	0	0	0	0
XM_021100110.1	MYLK4	mRNA	0	0	0	0	0	0	0	0	0	0	0	0	0	0	0	0	0	0	0	0	0
XM_021100111.1	MYLK4	mRNA	0	0	0	0	0	0	0	0	0	0	0	0	0	0	0	0	0	0	0	0	0
XM_021100112.1	MYLK4	mRNA	0	0	0	0	0	0	0	0	0	0	0	0	0	0	0	0	0	0	0	0	0
XM_021100114.1	NQO2	mRNA	0	0	0	0	0	0	0	0	0	0	0	0	0	0	0	0	0	0	0	0	0
XM_021100115.1	NQO2	mRNA	0	0	0	0	0	0	0	0	0	0	0	0	0	0	0	0	0	0	0	0	0
XM_021100117.1	NQO2	mRNA	0	0	0	0	0	0	0	0	0	0	0	0	0	0	0	0	0	0	0	0	0
XM_021100118.1	NQO2	mRNA	0	0	0	0	0	0	0	0	0	0	0	0	0	0	0	0	0	0	0	0	0
XM_021100119.1	NQO2	mRNA	0	0	0	0	0	0	0	0	0	0	0	0	0	0	0	0	0	0	0	0	0
XM_021100124.1	PRPF4B	mRNA	0	0	0	0	0	0	0	0	0	0	0	0	0	0	0	0	0	0	0	0	0
XM_021100126.1	PRPF4B	mRNA	0	0	0	0	0	0	0	0	0	0	0	0	0	0	0	0	0	0	0	0	0
XM_021100184.1	NEDD9	mRNA	0	0	0	0	0	0	0	0	0	0	0	0	0	0	0	0	0	0	0	0	0
XM_021100188.1	TMEM170B	mRNA	0	0	0	0	0	0	0	0	0	0	0	0	0	0	0	0	0	0	0	0	0
XM_021100194.1	GFOD1	mRNA	0	0	0	0	0	0	0	0	0	0	0	0	0	0	0	0	0	0	0	0	0

RNA ID	Symbol	Type	updown_XR_001303119.2	updown_XR_002341616.1	updown_XR_299372.3	updown_XR_298148.3	updown_XR_002346063.1	updown_BGIR9823_100550	updown_BGIR9823_100272	updown_BGIR9823_101549	updown_BGIR9823_101564	updown_BGIR9823_101567	updown_BGIR9823_102169	updown_BGIR9823_102657	updown_BGIR9823_102650	updown_BGIR9823_79650	updown_BGIR9823_79960	updown_BGIR9823_80428	updown_BGIR9823_80570	updown_BGIR9823_84360	updown_BGIR9823_84364	updown_BGIR9823_81795	updown_BGIR9823_87741
XM_021102040.1	SLC37A4	mRNA	0	0	0	0	0	0	0	0	0	0	0	0	0	0	0	0	0	0	0	0	0
XM_021102041.1	SLC37A4	mRNA	0	0	0	0	0	0	0	0	0	0	0	0	0	0	0	0	0	0	0	0	0
XM_021102042.1	SLC37A4	mRNA	0	0	0	0	0	0	0	0	0	0	0	0	0	0	0	0	0	0	0	0	0
XM_021102043.1	SLC37A4	mRNA	0	0	0	0	0	0	0	0	0	0	0	0	0	0	0	0	0	0	0	0	0
XM_021102044.1	SLC37A4	mRNA	0	0	0	0	0	0	0	0	0	0	0	0	0	0	0	0	0	0	0	0	0
XM_021102045.1	SLC37A4	mRNA	0	0	0	0	0	0	0	0	0	0	0	0	0	0	0	0	0	0	0	0	0
XM_021102046.1	SLC37A4	mRNA	0	0	0	0	0	0	0	0	0	0	0	0	0	0	0	0	0	0	0	0	0
XM_021102047.1	SLC37A4	mRNA	0	0	0	0	0	0	0	0	0	0	0	0	0	0	0	0	0	0	0	0	0
XM_021102048.1	SLC37A4	mRNA	0	0	0	0	0	0	0	0	0	0	0	0	0	0	0	0	0	0	0	0	0
XM_021102049.1	SLC37A4	mRNA	0	0	0	0	0	0	0	0	0	0	0	0	0	0	0	0	0	0	0	0	0
XM_021102050.1	SLC37A4	mRNA	0	0	0	0	0	0	0	0	0	0	0	0	0	0	0	0	0	0	0	0	0
XM_021102051.1	SLC37A4	mRNA	0	0	0	0	0	0	0	0	0	0	0	0	0	0	0	0	0	0	0	0	0
XM_021102052.1	SLC37A4	mRNA	0	0	0	0	0	0	0	0	0	0	0	0	0	0	0	0	0	0	0	0	0
XM_021102209.1	UPK2	mRNA	0	0	0	0	0	0	0	0	0	0	0	0	0	0	0	0	0	0	0	0	0
XM_021102516.1	LOC100522455	mRNA	0	0	0	0	0	0	0	0	0	0	0	0	0	0	0	0	0	0	0	0	0
XM_021102517.1	RUNDC3B	mRNA	0	0	0	0	0	0	0	0	0	0	0	0	0	0	0	0	0	0	0	0	0
XM_021102518.1	RUNDC3B	mRNA	0	0	0	0	0	0	0	0	0	0	0	0	0	0	0	0	0	0	0	0	0
XM_021102519.1	RUNDC3B	mRNA	0	0	0	0	0	0	0	0	0	0	0	0	0	0	0	0	0	0	0	0	0
XM_021102522.1	RUNDC3B	mRNA	0	0	0	0	0	0	0	0	0	0	0	0	0	0	0	0	0	0	0	0	0
XM_021102523.1	RUNDC3B	mRNA	0	0	0	0	0	0	0	0	0	0	0	0	0	0	0	0	0	0	0	0	0
XR_001297664.2	LOC102161744	lncRNA	0	0	0	0	0	0	0	0	0	0	0	0	0	0	0	0	0	0	0	0	0
XR_001298807.2	LOC106504881	lncRNA	0	0	0	0	0	0	0	0	0	0	0	0	0	0	0	0	0	0	0	0	0
XR_001298808.2	LOC106504881	lncRNA	0	0	0	0	0	0	0	0	0	0	0	0	0	0	0	0	0	0	0	0	0
XR_001298810.2	LOC106504881	lncRNA	0	0	0	0	0	0	0	0	0	0	0	0	0	0	0	0	0	0	0	0	0
XR_001298811.2	LOC106504881	lncRNA	0	0	0	0	0	0	0	0	0	0	0	0	0	0	0	0	0	0	0	0	0
XR_001298812.2	LOC106504881	lncRNA	0	0	0	0	0	0	0	0	0	0	0	0	0	0	0	0	0	0	0	0	0
XR_001298813.2	LOC106504881	lncRNA	0	0	0	0	0	0	0	0	0	0	0	0	0	0	0	0	0	0	0	0	0
XR_001298814.2	LOC106504881	lncRNA	0	0	0	0	0	0	0	0	0	0	0	0	0	0	0	0	0	0	0	0	0
XR_001298816.2	LOC106504881	lncRNA	0	0	0	0	0	0	0	0	0	0	0	0	0	0	0	0	0	0	0	0	0
XR_001298817.2	LOC106504881	lncRNA	0	0	0	0	0	0	0	0	0	0	0	0	0	0	0	0	0	0	0	0	0
XR_001298844.2	LOC102163364	lncRNA	0	0	0	0	0	0	0	0	0	0	0	0	0	0	0	0	0	0	0	0	0
XR_001298846.2	LOC102163364	lncRNA	0	0	0	0	0	0	0	0	0	0	0	0	0	0	0	0	0	0	0	0	0
XR_001298850.2	LOC102163364	lncRNA	0	0	0	0	0	0	0	0	0	0	0	0	0	0	0	0	0	0	0	0	0
XR_001299456.2	LOC102166670	lncRNA	0	0	0	0	0	0	0	0	0	0	0	0	0	0	0	0	0	0	0	0	0
XR_001299457.2	LOC102166670	lncRNA	0	0	0	0	0	0	0	0	0	0	0	0	0	0	0	0	0	0	0	0	0
XR_001300715.2	LOC106505879	lncRNA	0	0	0	0	0	0	0	0	0	0	0	0	0	0	0	0	0	0	0	0	0
XR_001301078.2	LOC102162623	lncRNA	0	0	0	0	0	0	0	0	0	0	0	0	0	0	0	0	0	0	0	0	0
XR_001301087.2	LOC102162623	lncRNA	0	0	0	0	0	0	0	0	0	0	0	0	0	0	0	0	0	0	0	0	0
XR_001301217.2	LOC102164998	lncRNA	0	0	0	0	0	0	0	0	0	0	0	0	0	0	0	0	0	0	0	0	0
XR_001301305.2	LOC102159607	lncRNA	0	0	0	0	0	0	0	0	0	0	0	0	0	0	0	0	0	0	0	0	0
XR_001308967.2	LOC106510467	lncRNA	0	0	0	0	0	0	0	0	0	0	0	0	0	0	0	0	0	0	0	0	0
XR_002335533.1	XRRA1	mRNA	0	0	0	0	0	0	0	0	0	0	0	0	0	0	0	0	0	0	0	0	0
XR_002335534.1	XRRA1	mRNA	0	0	0	0	0	0	0	0	0	0	0	0	0	0	0	0	0	0	0	0	0

RNA ID	Symbol	Type	updown_XR_001303119.2	updown_XR_002341616.1	updown_XR_299372.3	updown_XR_298148.3	updown_XR_002346063.1	updown_BGIR9823_100550	updown_BGIR9823_100272	updown_BGIR9823_101549	updown_BGIR9823_101564	updown_BGIR9823_101567	updown_BGIR9823_102169	updown_BGIR9823_102657	updown_BGIR9823_102650	updown_BGIR9823_79650	updown_BGIR9823_79960	updown_BGIR9823_80428	updown_BGIR9823_80570	updown_BGIR9823_84360	updown_BGIR9823_84364	updown_BGIR9823_81795	updown_BGIR9823_87741
XR_002335535.1	LOC110255399	lncRNA	0	0	0	0	0	0	0	0	0	0	0	0	0	0	0	0	0	0	0	0	0
XR_002335536.1	LOC102160367	lncRNA	0	0	0	0	0	0	0	0	0	0	0	0	0	0	0	0	0	0	0	0	0
XR_002335537.1	LOC102160367	lncRNA	0	0	0	0	0	0	0	0	0	0	0	0	0	0	0	0	0	0	0	0	0
XR_002335650.1	LOC102157630	lncRNA	0	0	0	0	0	0	0	0	0	0	0	0	0	0	0	0	0	0	0	0	0
XR_002335651.1	LOC110255435	lncRNA	0	0	0	0	0	0	0	0	0	0	0	0	0	0	0	0	0	0	0	0	0
XR_002335652.1	LOC106504881	lncRNA	0	0	0	0	0	0	0	0	0	0	0	0	0	0	0	0	0	0	0	0	0
XR_002335653.1	LOC106504881	lncRNA	0	0	0	0	0	0	0	0	0	0	0	0	0	0	0	0	0	0	0	0	0
XR_002335670.1	LOC110255440	lncRNA	0	0	0	0	0	0	0	0	0	0	0	0	0	0	0	0	0	0	0	0	0
XR_002335671.1	LOC110255441	lncRNA	0	0	0	0	0	0	0	0	0	0	0	0	0	0	0	0	0	0	0	0	0
XR_002335672.1	LOC110255441	lncRNA	0	0	0	0	0	0	0	0	0	0	0	0	0	0	0	0	0	0	0	0	0
XR_002335674.1	HYOU1	mRNA	0	0	0	0	0	0	0	0	0	0	0	0	0	0	0	0	0	0	0	0	0
XR_002335675.1	HYOU1	mRNA	0	0	0	0	0	0	0	0	0	0	0	0	0	0	0	0	0	0	0	0	0
XR_002335685.1	LOC100523684	mRNA	0	0	0	0	0	0	0	0	0	0	0	0	0	0	0	0	0	0	0	0	0
XR_002335686.1	LOC102163364	lncRNA	0	0	0	0	0	0	0	0	0	0	0	0	0	0	0	0	0	0	0	0	0
XR_002335687.1	LOC102163364	lncRNA	0	0	0	0	0	0	0	0	0	0	0	0	0	0	0	0	0	0	0	0	0
XR_002335688.1	LOC102163364	lncRNA	0	0	0	0	0	0	0	0	0	0	0	0	0	0	0	0	0	0	0	0	0
XR_002335689.1	LOC102163364	lncRNA	0	0	0	0	0	0	0	0	0	0	0	0	0	0	0	0	0	0	0	0	0
XR_002335690.1	LOC102163364	lncRNA	0	0	0	0	0	0	0	0	0	0	0	0	0	0	0	0	0	0	0	0	0
XR_002335691.1	LOC102163364	lncRNA	0	0	0	0	0	0	0	0	0	0	0	0	0	0	0	0	0	0	0	0	0
XR_002335692.1	LOC102163364	lncRNA	0	0	0	0	0	0	0	0	0	0	0	0	0	0	0	0	0	0	0	0	0
XR_002335693.1	LOC106504896	lncRNA	0	0	0	0	0	0	0	0	0	0	0	0	0	0	0	0	0	0	0	0	0
XR_002335694.1	SORL1	mRNA	0	0	0	0	0	0	0	0	0	0	0	0	0	0	0	0	0	0	0	0	0
XR_002336641.1	LOC106505250	lncRNA	0	0	0	0	0	0	0	0	0	0	0	0	0	0	0	0	0	0	0	0	0
XR_002336643.1	LOC110255292	lncRNA	0	0	0	0	0	0	0	0	0	0	0	0	0	0	0	0	0	0	0	0	0
XR_002336644.1	LOC102166670	lncRNA	0	0	0	0	0	0	0	0	0	0	0	0	0	0	0	0	0	0	0	0	0
XR_002336645.1	LOC102166670	lncRNA	0	0	0	0	0	0	0	0	0	0	0	0	0	0	0	0	0	0	0	0	0
XR_002338443.1	LOC106505879	lncRNA	0	0	0	0	0	0	0	0	0	0	0	0	0	0	0	0	0	0	0	0	0
XR_002338444.1	LOC106505879	lncRNA	0	0	0	0	0	0	0	0	0	0	0	0	0	0	0	0	0	0	0	0	0
XR_002338445.1	LOC106505879	lncRNA	0	0	0	0	0	0	0	0	0	0	0	0	0	0	0	0	0	0	0	0	0
XR_002338446.1	LOC106505879	lncRNA	0	0	0	0	0	0	0	0	0	0	0	0	0	0	0	0	0	0	0	0	0
XR_002338447.1	LOC106505879	lncRNA	0	0	0	0	0	0	0	0	0	0	0	0	0	0	0	0	0	0	0	0	0
XR_002338448.1	LOC106505879	lncRNA	0	0	0	0	0	0	0	0	0	0	0	0	0	0	0	0	0	0	0	0	0
XR_002338449.1	LOC106505879	lncRNA	0	0	0	0	0	0	0	0	0	0	0	0	0	0	0	0	0	0	0	0	0
XR_002338450.1	LOC106505879	lncRNA	0	0	0	0	0	0	0	0	0	0	0	0	0	0	0	0	0	0	0	0	0
XR_002338521.1	LOC110256664	lncRNA	0	0	0	0	0	0	0	0	0	0	0	0	0	0	0	0	0	0	0	0	0
XR_002338522.1	LOC110256664	lncRNA	0	0	0	0	0	0	0	0	0	0	0	0	0	0	0	0	0	0	0	0	0
XR_002338666.1	LOC110256747	lncRNA	0	0	0	0	0	0	0	0	0	0	0	0	0	0	0	0	0	0	0	0	0
XR_002338714.1	LOC102163817	lncRNA	0	0	0	0	0	0	0	0	0	0	0	0	0	0	0	0	0	0	0	0	0
XR_002338715.1	LOC102164998	lncRNA	0	0	0	0	0	0	0	0	0	0	0	0	0	0	0	0	0	0	0	0	0
XR_002338716.1	LOC102164998	lncRNA	0	0	0	0	0	0	0	0	0	0	0	0	0	0	0	0	0	0	0	0	0
XR_002338717.1	LOC110256768	lncRNA	0	0	0	0	0	0	0	0	0	0	0	0	0	0	0	0	0	0	0	0	0
XR_002338727.1	LOC110256773	lncRNA	0	0	0	0	0	0	0	0	0	0	0	0	0	0	0	0	0	0	0	0	0
XR_002338728.1	LOC110256773	lncRNA	0	0	0	0	0	0	0	0	0	0	0	0	0	0	0	0	0	0	0	0	0

RNA ID	Symbol	Type	updown_XR_001303119.2	updown_XR_002341616.1	updown_XR_299372.3	updown_XR_298148.3	updown_XR_002346063.1	updown_BGIR9823_100550	updown_BGIR9823_100272	updown_BGIR9823_101549	updown_BGIR9823_101564	updown_BGIR9823_101567	updown_BGIR9823_102169	updown_BGIR9823_102657	updown_BGIR9823_102650	updown_BGIR9823_79650	updown_BGIR9823_79960	updown_BGIR9823_80428	updown_BGIR9823_80570	updown_BGIR9823_84360	updown_BGIR9823_84364	updown_BGIR9823_81795	updown_BGIR9823_87741
XR_002338729.1	LOC110256773	lncRNA	0	0	0	0	0	0	0	0	0	0	0	0	0	0	0	0	0	0	0	0	0
XR_002338793.1	LOC110256796	lncRNA	0	0	0	0	0	0	0	0	0	0	0	0	0	0	0	0	0	0	0	0	0
XR_002339623.1	LOC102167708	lncRNA	0	0	0	0	0	0	0	0	0	0	0	0	0	0	0	0	0	0	0	0	0
XR_002340245.1	PRKAG2	mRNA	0	0	0	0	0	0	0	0	0	0	0	0	0	0	0	0	0	0	0	0	0
XR_002340246.1	LOC102162623	lncRNA	0	0	0	0	0	0	0	0	0	0	0	0	0	0	0	0	0	0	0	0	0
XR_002340247.1	LOC102162623	lncRNA	0	0	0	0	0	0	0	0	0	0	0	0	0	0	0	0	0	0	0	0	0
XR_002340248.1	LOC110257516	lncRNA	0	0	0	0	0	0	0	0	0	0	0	0	0	0	0	0	0	0	0	0	0
XR_002340249.1	LOC102162071	lncRNA	0	0	0	0	0	0	0	0	0	0	0	0	0	0	0	0	0	0	0	0	0
XR_002340901.1	LOC102160243	lncRNA	0	0	0	0	0	0	0	0	0	0	0	0	0	0	0	0	0	0	0	0	0
XR_002340902.1	LOC102160243	lncRNA	0	0	0	0	0	0	0	0	0	0	0	0	0	0	0	0	0	0	0	0	0
XR_002340903.1	LOC102160243	lncRNA	0	0	0	0	0	0	0	0	0	0	0	0	0	0	0	0	0	0	0	0	0
XR_002340904.1	LOC102160243	lncRNA	0	0	0	0	0	0	0	0	0	0	0	0	0	0	0	0	0	0	0	0	0
XR_002340905.1	LOC102160243	lncRNA	0	0	0	0	0	0	0	0	0	0	0	0	0	0	0	0	0	0	0	0	0
XR_002341237.1	LOC102164320	lncRNA	0	0	0	0	0	0	0	0	0	0	0	0	0	0	0	0	0	0	0	0	0
XR_002341238.1	LOC102164320	lncRNA	0	0	0	0	0	0	0	0	0	0	0	0	0	0	0	0	0	0	0	0	0
XR_002341239.1	LOC102164320	lncRNA	0	0	0	0	0	0	0	0	0	0	0	0	0	0	0	0	0	0	0	0	0
XR_002341240.1	LOC102164320	lncRNA	0	0	0	0	0	0	0	0	0	0	0	0	0	0	0	0	0	0	0	0	0
XR_002341241.1	LOC102164320	lncRNA	0	0	0	0	0	0	0	0	0	0	0	0	0	0	0	0	0	0	0	0	0
XR_002341242.1	LOC102164320	lncRNA	0	0	0	0	0	0	0	0	0	0	0	0	0	0	0	0	0	0	0	0	0
XR_002341243.1	LOC102164320	lncRNA	0	0	0	0	0	0	0	0	0	0	0	0	0	0	0	0	0	0	0	0	0
XR_002341244.1	LOC102164320	lncRNA	0	0	0	0	0	0	0	0	0	0	0	0	0	0	0	0	0	0	0	0	0
XR_002341270.1	LOC100521600	mRNA	0	0	0	0	0	0	0	0	0	0	0	0	0	0	0	0	0	0	0	0	0
XR_002341443.1	LOC102163816	lncRNA	0	0	0	0	0	0	0	0	0	0	0	0	0	0	0	0	0	0	0	0	0
XR_002343847.1	LOC110260611	mRNA	0	0	0	0	0	0	0	0	0	0	0	0	0	0	0	0	0	0	0	0	0
XR_002343848.1	LOC110260611	mRNA	0	0	0	0	0	0	0	0	0	0	0	0	0	0	0	0	0	0	0	0	0
XR_002343849.1	LOC110260611	mRNA	0	0	0	0	0	0	0	0	0	0	0	0	0	0	0	0	0	0	0	0	0
XR_002343850.1	LOC110260611	mRNA	0	0	0	0	0	0	0	0	0	0	0	0	0	0	0	0	0	0	0	0	0
XR_002343875.1	LOC110260629	lncRNA	0	0	0	0	0	0	0	0	0	0	0	0	0	0	0	0	0	0	0	0	0
XR_002343876.1	LOC110260629	lncRNA	0	0	0	0	0	0	0	0	0	0	0	0	0	0	0	0	0	0	0	0	0
XR_002343877.1	LOC110260629	lncRNA	0	0	0	0	0	0	0	0	0	0	0	0	0	0	0	0	0	0	0	0	0
XR_002343878.1	LOC110260629	lncRNA	0	0	0	0	0	0	0	0	0	0	0	0	0	0	0	0	0	0	0	0	0
XR_002343879.1	LOC110260629	lncRNA	0	0	0	0	0	0	0	0	0	0	0	0	0	0	0	0	0	0	0	0	0
XR_002343880.1	LOC110260629	lncRNA	0	0	0	0	0	0	0	0	0	0	0	0	0	0	0	0	0	0	0	0	0
XR_002343881.1	LOC110260629	lncRNA	0	0	0	0	0	0	0	0	0	0	0	0	0	0	0	0	0	0	0	0	0
XR_002343883.1	LOC110260631	lncRNA	0	0	0	0	0	0	0	0	0	0	0	0	0	0	0	0	0	0	0	0	0
XR_002343884.1	LOC110260634	lncRNA	0	0	0	0	0	0	0	0	0	0	0	0	0	0	0	0	0	0	0	0	0
XR_002343885.1	LOC110260635	lncRNA	0	0	0	0	0	0	0	0	0	0	0	0	0	0	0	0	0	0	0	0	0
XR_002343891.1	LOC110260642	lncRNA	0	0	0	0	0	0	0	0	0	0	0	0	0	0	0	0	0	0	0	0	0
XR_002343892.1	LOC110260643	lncRNA	0	0	0	0	0	0	0	0	0	0	0	0	0	0	0	0	0	0	0	0	0
XR_002345298.1	MAF	mRNA	0	0	0	0	0	0	0	0	0	0	0	0	0	0	0	0	0	0	0	0	0
XR_002345300.1	LOC106510470	lncRNA	0	0	0	0	0	0	0	0	0	0	0	0	0	0	0	0	0	0	0	0	0
XR_002345301.1	LOC106510468	lncRNA	0	0	0	0	0	0	0	0	0	0	0	0	0	0	0	0	0	0	0	0	0
XR_002346166.1	LOC110261703	lncRNA	0	0	0	0	0	0	0	0	0	0	0	0	0	0	0	0	0	0	0	0	0

RNA ID	Symbol	Type	updown_XR_001303119.2	updown_XR_002341616.1	updown_XR_299372.3	updown_XR_298148.3	updown_XR_002346063.1	updown_BGIR9823_100550	updown_BGIR9823_100272	updown_BGIR9823_101549	updown_BGIR9823_101564	updown_BGIR9823_101567	updown_BGIR9823_102169	updown_BGIR9823_102657	updown_BGIR9823_102650	updown_BGIR9823_79650	updown_BGIR9823_79960	updown_BGIR9823_80428	updown_BGIR9823_80570	updown_BGIR9823_84360	updown_BGIR9823_84364	updown_BGIR9823_81795	updown_BGIR9823_87741
XR_002346227.1	LOC106504299	lncRNA	0	0	0	0	0	0	0	0	0	0	0	0	0	0	0	0	0	0	0	0	0
XR_002346228.1	LOC106504299	lncRNA	0	0	0	0	0	0	0	0	0	0	0	0	0	0	0	0	0	0	0	0	0
XR_002346229.1	LOC106504299	lncRNA	0	0	0	0	0	0	0	0	0	0	0	0	0	0	0	0	0	0	0	0	0
XR_002346230.1	LOC106504299	lncRNA	0	0	0	0	0	0	0	0	0	0	0	0	0	0	0	0	0	0	0	0	0
XR_002346231.1	LOC106504299	lncRNA	0	0	0	0	0	0	0	0	0	0	0	0	0	0	0	0	0	0	0	0	0
XR_002346232.1	LOC106504299	lncRNA	0	0	0	0	0	0	0	0	0	0	0	0	0	0	0	0	0	0	0	0	0
XR_002346233.1	NQO2	mRNA	0	0	0	0	0	0	0	0	0	0	0	0	0	0	0	0	0	0	0	0	0
XR_002346236.1	PRPF4B	mRNA	0	0	0	0	0	0	0	0	0	0	0	0	0	0	0	0	0	0	0	0	0
XR_002346237.1	PRPF4B	mRNA	0	0	0	0	0	0	0	0	0	0	0	0	0	0	0	0	0	0	0	0	0
XR_002346238.1	PRPF4B	mRNA	0	0	0	0	0	0	0	0	0	0	0	0	0	0	0	0	0	0	0	0	0
XR_002346239.1	PRPF4B	mRNA	0	0	0	0	0	0	0	0	0	0	0	0	0	0	0	0	0	0	0	0	0
XR_002346240.1	PRPF4B	mRNA	0	0	0	0	0	0	0	0	0	0	0	0	0	0	0	0	0	0	0	0	0
XR_002346241.1	PRPF4B	mRNA	0	0	0	0	0	0	0	0	0	0	0	0	0	0	0	0	0	0	0	0	0
XR_002346242.1	PRPF4B	mRNA	0	0	0	0	0	0	0	0	0	0	0	0	0	0	0	0	0	0	0	0	0
XR_002346243.1	PRPF4B	mRNA	0	0	0	0	0	0	0	0	0	0	0	0	0	0	0	0	0	0	0	0	0
XR_002346244.1	PRPF4B	mRNA	0	0	0	0	0	0	0	0	0	0	0	0	0	0	0	0	0	0	0	0	0
XR_002346245.1	PRPF4B	mRNA	0	0	0	0	0	0	0	0	0	0	0	0	0	0	0	0	0	0	0	0	0
XR_002346246.1	PRPF4B	mRNA	0	0	0	0	0	0	0	0	0	0	0	0	0	0	0	0	0	0	0	0	0
XR_002346247.1	PRPF4B	mRNA	0	0	0	0	0	0	0	0	0	0	0	0	0	0	0	0	0	0	0	0	0
XR_002346248.1	PRPF4B	mRNA	0	0	0	0	0	0	0	0	0	0	0	0	0	0	0	0	0	0	0	0	0
XR_002346249.1	PRPF4B	mRNA	0	0	0	0	0	0	0	0	0	0	0	0	0	0	0	0	0	0	0	0	0
XR_002346250.1	PRPF4B	mRNA	0	0	0	0	0	0	0	0	0	0	0	0	0	0	0	0	0	0	0	0	0
XR_002346251.1	PRPF4B	mRNA	0	0	0	0	0	0	0	0	0	0	0	0	0	0	0	0	0	0	0	0	0
XR_002346252.1	PRPF4B	mRNA	0	0	0	0	0	0	0	0	0	0	0	0	0	0	0	0	0	0	0	0	0
XR_002346253.1	PRPF4B	mRNA	0	0	0	0	0	0	0	0	0	0	0	0	0	0	0	0	0	0	0	0	0
XR_002346254.1	PRPF4B	mRNA	0	0	0	0	0	0	0	0	0	0	0	0	0	0	0	0	0	0	0	0	0
XR_002346255.1	PRPF4B	mRNA	0	0	0	0	0	0	0	0	0	0	0	0	0	0	0	0	0	0	0	0	0
XR_002346256.1	PRPF4B	mRNA	0	0	0	0	0	0	0	0	0	0	0	0	0	0	0	0	0	0	0	0	0
XR_002346257.1	PRPF4B	mRNA	0	0	0	0	0	0	0	0	0	0	0	0	0	0	0	0	0	0	0	0	0
XR_002346258.1	PRPF4B	mRNA	0	0	0	0	0	0	0	0	0	0	0	0	0	0	0	0	0	0	0	0	0
XR_002346259.1	PRPF4B	mRNA	0	0	0	0	0	0	0	0	0	0	0	0	0	0	0	0	0	0	0	0	0
XR_002346260.1	PRPF4B	mRNA	0	0	0	0	0	0	0	0	0	0	0	0	0	0	0	0	0	0	0	0	0
XR_002346261.1	PRPF4B	mRNA	0	0	0	0	0	0	0	0	0	0	0	0	0	0	0	0	0	0	0	0	0
XR_002346262.1	PRPF4B	mRNA	0	0	0	0	0	0	0	0	0	0	0	0	0	0	0	0	0	0	0	0	0
XR_002346263.1	PRPF4B	mRNA	0	0	0	0	0	0	0	0	0	0	0	0	0	0	0	0	0	0	0	0	0
XR_002346264.1	PRPF4B	mRNA	0	0	0	0	0	0	0	0	0	0	0	0	0	0	0	0	0	0	0	0	0
XR_002346265.1	LOC110261764	lncRNA	0	0	0	0	0	0	0	0	0	0	0	0	0	0	0	0	0	0	0	0	0
XR_002346266.1	LOC102162837	lncRNA	0	0	0	0	0	0	0	0	0	0	0	0	0	0	0	0	0	0	0	0	0
XR_002346299.1	LOC110261774	lncRNA	0	0	0	0	0	0	0	0	0	0	0	0	0	0	0	0	0	0	0	0	0
XR_002346300.1	LOC110261774	lncRNA	0	0	0	0	0	0	0	0	0	0	0	0	0	0	0	0	0	0	0	0	0
XR_002346302.1	LOC110261776	lncRNA	0	0	0	0	0	0	0	0	0	0	0	0	0	0	0	0	0	0	0	0	0
XR_002346303.1	TMEM170B	mRNA	0	0	0	0	0	0	0	0	0	0	0	0	0	0	0	0	0	0	0	0	0
XR_002346304.1	TMEM170B	mRNA	0	0	0	0	0	0	0	0	0	0	0	0	0	0	0	0	0	0	0	0	0

RNA ID	Symbol	Type	updown_XR_001303119.2	updown_XR_002341616.1	updown_XR_299372.3	updown_XR_298148.3	updown_XR_002346063.1	updown_BGIR9823_100550	updown_BGIR9823_100272	updown_BGIR9823_101549	updown_BGIR9823_101564	updown_BGIR9823_101567	updown_BGIR9823_102169	updown_BGIR9823_102657	updown_BGIR9823_102650	updown_BGIR9823_79650	updown_BGIR9823_79960	updown_BGIR9823_80428	updown_BGIR9823_80570	updown_BGIR9823_84360	updown_BGIR9823_84364	updown_BGIR9823_81795	updown_BGIR9823_87741
XR_115737.4	LOC100513133	lncRNA	0	0	0	0	0	0	0	0	0	0	0	0	0	0	0	0	0	0	0	0	0
XR_299046.2	LOC102164596	lncRNA	0	0	0	0	0	0	0	0	0	0	0	0	0	0	0	0	0	0	0	0	0
XR_303634.3	LOC100738154	lncRNA	0	0	0	0	0	0	0	0	0	0	0	0	0	0	0	0	0	0	0	0	0
XR_304239.3	LOC102159819	lncRNA	0	0	0	0	0	0	0	0	0	0	0	0	0	0	0	0	0	0	0	0	0
XR_304244.3	LOC102161992	lncRNA	0	0	0	0	0	0	0	0	0	0	0	0	0	0	0	0	0	0	0	0	0
XR_304257.3	LOC102162837	lncRNA	0	0	0	0	0	0	0	0	0	0	0	0	0	0	0	0	0	0	0	0	0
XR_306105.3	LOC102166871	lncRNA	0	0	0	0	0	0	0	0	0	0	0	0	0	0	0	0	0	0	0	0	0
XR_307914.3	LOC102159607	lncRNA	0	0	0	0	0	0	0	0	0	0	0	0	0	0	0	0	0	0	0	0	0
XR_308345.3	LOC102167708	lncRNA	0	0	0	0	0	0	0	0	0	0	0	0	0	0	0	0	0	0	0	0	0
novel-ssc-miR1028-3p		miRNA	0	0	0	0	0	0	0	0	0	0	0	0	0	0	0	0	0	0	0	0	0
novel-ssc-miR1028-5p		miRNA	0	0	0	0	0	0	0	0	0	0	0	0	0	0	0	0	0	0	0	0	0
novel-ssc-miR1130-3p		miRNA	0	0	0	0	0	0	0	0	0	0	0	0	0	0	0	0	0	0	0	0	0
novel-ssc-miR1130-5p		miRNA	0	0	0	0	0	0	0	0	0	0	0	0	0	0	0	0	0	0	0	0	0
novel-ssc-miR1162-3p		miRNA	0	0	0	0	0	0	0	0	0	0	0	0	0	0	0	0	0	0	0	0	0
novel-ssc-miR1193-3p		miRNA	0	0	0	0	0	0	0	0	0	0	0	0	0	0	0	0	0	0	0	0	0
novel-ssc-miR1193-5p		miRNA	0	0	0	0	0	0	0	0	0	0	0	0	0	0	0	0	0	0	0	0	0
novel-ssc-miR1217-3p		miRNA	0	0	0	0	0	0	0	0	0	0	0	0	0	0	0	0	0	0	0	0	0
novel-ssc-miR1217-5p		miRNA	0	0	0	0	0	0	0	0	0	0	0	0	0	0	0	0	0	0	0	0	0
novel-ssc-miR1429-3p		miRNA	0	0	0	0	0	0	0	0	0	0	0	0	0	0	0	0	0	0	0	0	0
novel-ssc-miR1429-5p		miRNA	0	0	0	0	0	0	0	0	0	0	0	0	0	0	0	0	0	0	0	0	0
novel-ssc-miR259-3p		miRNA	0	0	0	0	0	0	0	0	0	0	0	0	0	0	0	0	0	0	0	0	0
novel-ssc-miR259-5p		miRNA	0	0	0	0	0	0	0	0	0	0	0	0	0	0	0	0	0	0	0	0	0
novel-ssc-miR324-3p		miRNA	0	0	0	0	0	0	0	0	0	0	0	0	0	0	0	0	0	0	0	0	0
novel-ssc-miR324-5p		miRNA	0	0	0	0	0	0	0	0	0	0	0	0	0	0	0	0	0	0	0	0	0
novel-ssc-miR42-3p		miRNA	0	0	0	0	0	0	0	0	0	0	0	0	0	0	0	0	0	0	0	0	0
novel-ssc-miR42-5p		miRNA	0	0	0	0	0	0	0	0	0	0	0	0	0	0	0	0	0	0	0	0	0
novel-ssc-miR433-3p		miRNA	0	0	0	0	0	0	0	0	0	0	0	0	0	0	0	0	0	0	0	0	0
novel-ssc-miR433-5p		miRNA	0	0	0	0	0	0	0	0	0	0	0	0	0	0	0	0	0	0	0	0	0
novel-ssc-miR557-3p		miRNA	0	0	0	0	0	0	0	0	0	0	0	0	0	0	0	0	0	0	0	0	0
novel-ssc-miR557-5p		miRNA	0	0	0	0	0	0	0	0	0	0	0	0	0	0	0	0	0	0	0	0	0
novel-ssc-miR619-3p		miRNA	0	0	0	0	0	0	0	0	0	0	0	0	0	0	0	0	0	0	0	0	0
novel-ssc-miR619-5p		miRNA	0	0	0	0	0	0	0	0	0	0	0	0	0	0	0	0	0	0	0	0	0
novel-ssc-miR719-3p		miRNA	0	0	0	0	0	0	0	0	0	0	0	0	0	0	0	0	0	0	0	0	0
novel-ssc-miR719-5p		miRNA	0	0	0	0	0	0	0	0	0	0	0	0	0	0	0	0	0	0	0	0	0
novel-ssc-miR966-3p		miRNA	0	0	0	0	0	0	0	0	0	0	0	0	0	0	0	0	0	0	0	0	0
novel-ssc-miR966-5p		miRNA	0	0	0	0	0	0	0	0	0	0	0	0	0	0	0	0	0	0	0	0	0
ssc-miR-7136-3p		miRNA	0	0	0	0	0	0	0	0	0	0	0	0	0	0	0	0	0	0	0	0	0
ssc-miR-7136-5p		miRNA	0	0	0	0	0	0	0	0	0	0	0	0	0	0	0	0	0	0	0	0	0

Supplementary Table S10. The predicted targe

RNA ID	Symbol	Type	updown_BGIR9823_88140	updown_BGIR9823_86964	updown_BGIR9823_90875	updown_URS0000BC5529	updown_URS0000BC90E3	updown_XR_002335844.1	updown_XR_001302653.2	updown_XR_002335671.1	updown_XR_002337554.1	updown_XR_001298816.2	updown_BGIR9823_99209	updown_BGIR9823_98003	updown_BGIR9823_98456	updown_BGIR9823_98543	updown_BGIR9823_88486	updown_BGIR9823_88585	updown_BGIR9823_97246	updown_XR_002346019.1	updown_XR_002346232.1	updown_XR_309047.3	updown_BGIR9823_93223
BGIR9823_100361	PRKAG2	mRNA	0	0	0	0	0	0	0	0	0	0	0	0	0	0	0	0	0	0	0	0	0
BGIR9823_100362	PRKAG2	mRNA	0	0	0	0	0	0	0	0	0	0	0	0	0	0	0	0	0	0	0	0	0
BGIR9823_100363	PRKAG2	mRNA	0	0	0	0	0	0	0	0	0	0	0	0	0	0	0	0	0	0	0	0	0
BGIR9823_100364	PRKAG2	mRNA	0	0	0	0	0	0	0	0	0	0	0	0	0	0	0	0	0	0	0	0	0
BGIR9823_101535	LOC102158401	lncRNA	0	0	0	0	0	0	0	0	0	0	0	0	0	0	0	0	0	0	0	0	0
BGIR9823_101541	LOC102157946	lncRNA	0	0	0	0	0	0	0	0	0	0	0	0	0	0	0	0	0	0	0	0	0
BGIR9823_101542	LOC102157946	lncRNA	0	0	0	0	0	0	0	0	0	0	0	0	0	0	0	0	0	0	0	0	0
BGIR9823_101560	LOC110258600	lncRNA	0	0	0	0	0	0	0	0	0	0	0	0	0	0	0	0	0	0	0	0	0
BGIR9823_101561	LOC110258600	lncRNA	0	0	0	0	0	0	0	0	0	0	0	0	0	0	0	0	0	0	0	0	0
BGIR9823_101562	LOC110258600	lncRNA	0	0	0	0	0	0	0	0	0	0	0	0	0	0	0	0	0	0	0	0	0
BGIR9823_101563	LOC110258600	lncRNA	0	0	0	0	0	0	0	0	0	0	0	0	0	0	0	0	0	0	0	0	0
BGIR9823_101564	LOC110258600	lncRNA	0	0	0	0	0	0	0	0	0	0	0	0	0	0	0	0	0	0	0	0	0
BGIR9823_101612	LOC100521600	lncRNA	0	0	0	0	0	0	0	0	0	0	0	0	0	0	0	0	0	0	0	0	0
BGIR9823_101853		lncRNA	0	0	0	0	0	0	0	0	0	0	0	0	0	0	0	0	0	0	0	0	0
BGIR9823_101854		lncRNA	0	0	0	0	0	0	0	0	0	0	0	0	0	0	0	0	0	0	0	0	0
BGIR9823_101855		lncRNA	0	0	0	0	0	0	0	0	0	0	0	0	0	0	0	0	0	0	0	0	0
BGIR9823_102146		lncRNA	0	0	0	0	0	0	0	0	0	0	0	0	0	0	0	0	0	0	1	0	0
BGIR9823_102180		lncRNA	0	0	0	0	0	0	0	0	0	0	0	0	0	0	0	0	0	0	0	0	0
BGIR9823_102279		lncRNA	0	0	0	0	0	0	0	0	0	0	0	0	0	0	0	0	0	0	0	0	0
BGIR9823_102282		lncRNA	0	0	0	0	0	0	0	0	0	0	0	0	0	0	0	0	0	0	0	0	0
BGIR9823_102361		lncRNA	0	0	0	0	0	0	0	0	0	0	0	0	0	0	0	0	0	0	0	0	0
BGIR9823_102362		lncRNA	0	0	0	0	0	0	0	0	0	0	0	0	0	0	0	0	0	0	0	0	0
BGIR9823_102363		lncRNA	0	0	0	0	0	0	0	0	0	0	0	0	0	0	0	0	0	0	0	0	0
BGIR9823_102727		lncRNA	0	0	0	0	0	0	0	0	0	0	0	0	0	0	0	0	0	0	0	0	0
BGIR9823_80121	SSSCA1	lncRNA	0	0	0	0	0	0	0	0	0	0	0	0	0	0	0	0	0	0	0	0	0
BGIR9823_80122	LOC102163816	lncRNA	0	0	0	0	0	0	0	0	0	0	0	0	0	0	0	0	0	0	0	0	0
BGIR9823_81007	EHBP1L1	mRNA	0	0	0	0	0	0	0	0	0	0	0	0	0	0	0	0	0	0	0	0	0
BGIR9823_81008	EHBP1L1	mRNA	0	0	0	0	0	0	0	0	0	0	0	0	0	0	0	0	0	0	0	0	0
BGIR9823_81009	EHBP1L1	mRNA	0	0	0	0	0	0	0	0	0	0	0	0	0	0	0	0	0	0	0	0	0
BGIR9823_81010	EHBP1L1	mRNA	0	0	0	0	0	0	0	0	0	0	0	0	0	0	0	0	0	0	0	0	0
BGIR9823_81011	SSSCA1	mRNA	0	0	0	0	0	0	0	0	0	0	0	0	0	0	0	0	0	0	0	0	0
BGIR9823_81012	FRMD8	lncRNA	0	0	0	0	0	0	0	0	0	0	0	0	0	0	0	0	0	0	0	0	0
BGIR9823_81013	LOC102163816	lncRNA	0	0	0	0	0	0	0	0	0	0	0	0	0	0	0	0	0	0	0	0	0
BGIR9823_81014	LOC102163816	lncRNA	0	0	0	0	0	0	0	0	0	0	0	0	0	0	0	0	0	0	0	0	0
BGIR9823_81015	LOC102163816	lncRNA	0	0	0	0	0	0	0	0	0	0	0	0	0	0	0	0	0	0	0	0	0
BGIR9823_81016	LOC102163816	lncRNA	0	0	0	0	0	0	0	0	0	0	0	0	0	0	0	0	0	0	0	0	0
BGIR9823_81017	LOC102163816	lncRNA	0	0	0	0	0	0	0	0	0	0	0	0	0	0	0	0	0	0	0	0	0
BGIR9823_81018	LOC102163816	lncRNA	0	0	0	0	0	0	0	0	0	0	0	0	0	0	0	0	0	0	0	0	0
BGIR9823_81019	LOC100513133	lncRNA	0	0	0	0	0	0	0	0	0	0	0	0	0	0	0	0	0	0	0	0	0
BGIR9823_84704	LOC102159820	mRNA	0	0	0	0	0	0	0	0	0	0	0	0	0	0	0	0	0	0	0	0	0
BGIR9823_84732	RANGAP1	mRNA	0	0	0	0	0	0	0	0	0	0	0	0	0	0	0	0	0	0	0	0	0

RNA ID	Symbol	Type	updown_BGIR9823_88140	updown_BGIR9823_86964	updown_BGIR9823_90875	updown_URS0000BC5529	updown_URS0000BC90E3	updown_XR_002335844.1	updown_XR_001302653.2	updown_XR_002335671.1	updown_XR_002337554.1	updown_XR_001298816.2	updown_BGIR9823_99209	updown_BGIR9823_98003	updown_BGIR9823_98456	updown_BGIR9823_98543	updown_BGIR9823_88486	updown_BGIR9823_88585	updown_BGIR9823_97246	updown_XR_002346019.1	updown_XR_002346232.1	updown_XR_309047.3	updown_BGIR9823_93223
BGIR9823_84733	RANGAP1	mRNA	0	0	0	0	0	0	0	0	0	0	0	0	0	0	0	0	0	0	0	0	0
BGIR9823_85245	CRADD	lncRNA	0	0	0	0	0	0	0	0	0	0	0	0	0	0	0	0	0	0	0	0	0
BGIR9823_85246	LOC100738154	lncRNA	0	0	0	0	0	0	0	0	0	0	0	0	0	0	0	0	0	0	0	0	0
BGIR9823_85293	TRMU	mRNA	0	0	0	0	0	0	0	0	0	0	0	0	0	0	0	0	0	0	0	0	0
BGIR9823_85294	TRMU	mRNA	0	0	0	0	0	0	0	0	0	0	0	0	0	0	0	0	0	0	0	0	0
BGIR9823_85295	TTC38	mRNA	0	0	0	0	0	0	0	0	0	0	0	0	0	0	0	0	0	0	0	0	0
BGIR9823_85296	PKDREJ	lncRNA	0	0	0	0	0	0	0	0	0	0	0	0	0	0	0	0	0	0	0	0	0
BGIR9823_85297	PPARA	mRNA	0	0	0	0	0	0	0	0	0	0	0	0	0	0	0	0	0	0	0	0	0
BGIR9823_85298	PPARA	mRNA	0	0	0	0	0	0	0	0	0	0	0	0	0	0	0	0	0	0	0	0	0
BGIR9823_85299	PPARA	mRNA	0	0	0	0	0	0	0	0	0	0	0	0	0	0	0	0	0	0	0	0	0
BGIR9823_85300	PPARA	lncRNA	0	0	0	0	0	0	0	0	0	0	0	0	0	0	0	0	0	0	0	0	0
BGIR9823_85301	LOC110260611	mRNA	0	0	0	0	0	0	0	0	0	0	0	0	0	0	0	0	0	0	0	0	0
BGIR9823_85317	ACO2	mRNA	0	0	0	0	0	0	0	0	0	0	0	0	0	0	0	0	0	0	0	0	0
BGIR9823_85318	LOC110260629	lncRNA	0	0	0	0	0	0	0	0	0	0	0	0	0	0	0	0	0	0	0	0	0
BGIR9823_85319	TEF	mRNA	0	0	0	0	0	0	0	0	0	0	0	0	0	0	0	0	0	0	0	0	0
BGIR9823_85320	ZC3H7B	mRNA	0	0	0	0	0	0	0	0	0	0	0	0	0	0	0	0	0	0	0	0	0
BGIR9823_85345	SYNGR1	mRNA	0	0	0	0	0	0	0	0	0	0	0	0	0	0	0	0	0	0	0	0	0
BGIR9823_85835	CRADD	mRNA	0	0	0	0	0	0	0	0	0	0	0	0	0	0	0	0	0	0	0	0	0
BGIR9823_85839	CRADD	lncRNA	0	0	0	0	0	0	0	0	0	0	0	0	0	0	0	0	0	0	0	0	0
BGIR9823_85840	CRADD	lncRNA	0	0	0	0	0	0	0	0	0	0	0	0	0	0	0	0	0	0	0	0	0
BGIR9823_85841	CRADD	lncRNA	0	0	0	0	0	0	0	0	0	0	0	0	0	0	0	0	0	0	0	0	0
BGIR9823_85930	LOC106510465	mRNA	0	0	0	0	0	0	0	0	0	0	0	0	0	0	0	0	0	0	0	0	0
BGIR9823_85931	MAF	lncRNA	0	0	0	0	0	0	0	0	0	0	0	0	0	0	0	0	0	0	0	0	0
BGIR9823_85932	MAF	lncRNA	0	0	0	0	0	0	0	0	0	0	0	0	0	0	0	0	0	0	0	0	0
BGIR9823_87139	LOC106510467	lncRNA	0	0	0	0	0	0	0	0	0	0	0	0	0	0	0	0	0	0	0	0	0
BGIR9823_88187	WRNIP1	mRNA	0	0	0	0	0	0	0	0	0	0	0	0	0	0	0	0	0	0	1	0	0
BGIR9823_88188	NQO2	mRNA	0	0	0	0	0	0	0	0	0	0	0	0	0	0	0	0	0	0	1	0	0
BGIR9823_88189	NQO2	mRNA	0	0	0	0	0	0	0	0	0	0	0	0	0	0	0	0	0	0	1	0	0
BGIR9823_88190	RIPK1	mRNA	0	0	0	0	0	0	0	0	0	0	0	0	0	0	0	0	0	0	1	0	0
BGIR9823_88191	RIPK1	mRNA	0	0	0	0	0	0	0	0	0	0	0	0	0	0	0	0	0	0	1	0	0
BGIR9823_88192	RIPK1	mRNA	0	0	0	0	0	0	0	0	0	0	0	0	0	0	0	0	0	0	1	0	0
BGIR9823_88193	RIPK1	mRNA	0	0	0	0	0	0	0	0	0	0	0	0	0	0	0	0	0	0	1	0	0
BGIR9823_88196	PRPF4B	mRNA	0	0	0	0	0	0	0	0	0	0	0	0	0	0	0	0	0	0	0	0	0
BGIR9823_88197	PRPF4B	mRNA	0	0	0	0	0	0	0	0	0	0	0	0	0	0	0	0	0	0	0	0	0
BGIR9823_88198	PRPF4B	mRNA	0	0	0	0	0	0	0	0	0	0	0	0	0	0	0	0	0	0	0	0	0
BGIR9823_88211	TMEM170B	lncRNA	0	0	0	0	0	0	0	0	0	0	0	0	0	0	0	0	0	0	0	0	0
BGIR9823_88212	TMEM170B	lncRNA	0	0	0	0	0	0	0	0	0	0	0	0	0	0	0	0	0	0	0	0	0
BGIR9823_88213	TMEM170B	lncRNA	0	0	0	0	0	0	0	0	0	0	0	0	0	0	0	0	0	0	0	0	0
BGIR9823_88214	TMEM170B	lncRNA	0	0	0	0	0	0	0	0	0	0	0	0	0	0	0	0	0	0	0	0	0
BGIR9823_88217	PHACTR1	mRNA	0	0	0	0	0	0	0	0	0	0	0	0	0	0	0	0	0	0	0	0	0
BGIR9823_88218	PHACTR1	lncRNA	0	0	0	0	0	0	0	0	0	0	0	0	0	0	0	0	0	0	0	0	0
BGIR9823_89017	MYLK4	lncRNA	0	0	0	0	0	0	0	0	0	0	0	0	0	0	0	0	0	0	1	0	0
BGIR9823_89019	SERPINB1	lncRNA	0	0	0	0	0	0	0	0	0	0	0	0	0	0	0	0	0	0	1	0	0

RNA ID	Symbol	Type	updown_BGIR9823_88140	updown_BGIR9823_86964	updown_BGIR9823_90875	updown_URS0000BC5529	updown_URS0000BC90E3	updown_XR_002335844.1	updown_XR_001302653.2	updown_XR_002335671.1	updown_XR_002337554.1	updown_XR_001298816.2	updown_BGIR9823_99209	updown_BGIR9823_98003	updown_BGIR9823_98456	updown_BGIR9823_98543	updown_BGIR9823_88486	updown_BGIR9823_88585	updown_BGIR9823_97246	updown_XR_002346019.1	updown_XR_002346232.1	updown_XR_309047.3	updown_BGIR9823_93223
BGIR9823_89020	SERPINB9	mRNA	0	0	0	0	0	0	0	0	0	0	0	0	0	0	0	0	0	0	1	0	0
BGIR9823_89021	RIPK1	lncRNA	0	0	0	0	0	0	0	0	0	0	0	0	0	0	0	0	0	0	1	0	0
BGIR9823_89025	PXDC1	mRNA	0	0	0	0	0	0	0	0	0	0	0	0	0	0	0	0	0	0	0	0	0
BGIR9823_89026	PXDC1	lncRNA	0	0	0	0	0	0	0	0	0	0	0	0	0	0	0	0	0	0	0	0	0
BGIR9823_89027	LOC102161744	lncRNA	0	0	0	0	0	0	0	0	0	0	0	0	0	0	0	0	0	0	0	0	0
BGIR9823_89040	TBC1D7	lncRNA	0	0	0	0	0	0	0	0	0	0	0	0	0	0	0	0	0	0	0	0	0
BGIR9823_89041	TBC1D7	lncRNA	0	0	0	0	0	0	0	0	0	0	0	0	0	0	0	0	0	0	0	0	0
BGIR9823_89042	TBC1D7	lncRNA	0	0	0	0	0	0	0	0	0	0	0	0	0	0	0	0	0	0	0	0	0
BGIR9823_89043	TBC1D7	lncRNA	0	0	0	0	0	0	0	0	0	0	0	0	0	0	0	0	0	0	0	0	0
BGIR9823_89044	GFOD1	lncRNA	0	0	0	0	0	0	0	0	0	0	0	0	0	0	0	0	0	0	0	0	0
BGIR9823_89045	GFOD1	mRNA	0	0	0	0	0	0	0	0	0	0	0	0	0	0	0	0	0	0	0	0	0
BGIR9823_89046	GFOD1	mRNA	0	0	0	0	0	0	0	0	0	0	0	0	0	0	0	0	0	0	0	0	0
BGIR9823_89047	GFOD1	mRNA	0	0	0	0	0	0	0	0	0	0	0	0	0	0	0	0	0	0	0	0	0
BGIR9823_89048	GFOD1	lncRNA	0	0	0	0	0	0	0	0	0	0	0	0	0	0	0	0	0	0	0	0	0
BGIR9823_89049	GFOD1	lncRNA	0	0	0	0	0	0	0	0	0	0	0	0	0	0	0	0	0	0	0	0	0
BGIR9823_90771	NEU3	lncRNA	0	0	0	0	0	0	0	0	0	0	0	0	0	0	0	0	0	0	0	0	0
BGIR9823_90772	SLCO2B1	mRNA	0	0	0	0	0	0	0	0	0	0	0	0	0	0	0	0	0	0	0	0	0
BGIR9823_90913	LOC110255435	lncRNA	0	0	0	0	0	0	0	0	0	1	0	0	0	0	0	0	0	0	0	0	0
BGIR9823_90934	LOC110255441	lncRNA	0	0	0	0	0	0	0	1	0	0	0	0	0	0	0	0	0	0	0	0	0
BGIR9823_90935	CCDC84	mRNA	0	0	0	0	0	0	0	1	0	0	0	0	0	0	0	0	0	0	0	0	0
BGIR9823_90936	TRAPPC4	mRNA	0	0	0	0	0	0	0	1	0	0	0	0	0	0	0	0	0	0	0	0	0
BGIR9823_90937	HMBS	mRNA	0	0	0	0	0	0	0	1	0	0	0	0	0	0	0	0	0	0	0	0	0
BGIR9823_90956	SORL1	mRNA	0	0	0	0	0	0	0	0	0	0	0	0	0	0	0	0	0	0	0	0	0
BGIR9823_90957	SORL1	mRNA	0	0	0	0	0	0	0	0	0	0	0	0	0	0	0	0	0	0	0	0	0
BGIR9823_90958	SORL1	mRNA	0	0	0	0	0	0	0	0	0	0	0	0	0	0	0	0	0	0	0	0	0
BGIR9823_90960	CLMP	lncRNA	0	0	0	0	0	0	0	0	0	0	0	0	0	0	0	0	0	0	0	0	0
BGIR9823_91161	LOC100522267	mRNA	0	0	0	0	0	0	0	0	0	0	0	0	0	0	0	0	0	0	0	0	0
BGIR9823_91162	LOC100522455	mRNA	0	0	0	0	0	0	0	0	0	0	0	0	0	0	0	0	0	0	0	0	0
BGIR9823_91163	ABCB1	mRNA	0	0	0	0	0	0	0	0	0	0	0	0	0	0	0	0	0	0	0	0	0
BGIR9823_91402	HBB	mRNA	0	0	0	0	0	0	0	0	0	0	0	0	0	0	0	0	0	0	0	0	0
BGIR9823_91403	HBB	mRNA	0	0	0	0	0	0	0	0	0	0	0	0	0	0	0	0	0	0	0	0	0
BGIR9823_91404	HBB	mRNA	0	0	0	0	0	0	0	0	0	0	0	0	0	0	0	0	0	0	0	0	0
BGIR9823_91405	HBB	mRNA	0	0	0	0	0	0	0	0	0	0	0	0	0	0	0	0	0	0	0	0	0
BGIR9823_91406	HBB	mRNA	0	0	0	0	0	0	0	0	0	0	0	0	0	0	0	0	0	0	0	0	0
BGIR9823_91407	HBB	mRNA	0	0	0	0	0	0	0	0	0	0	0	0	0	0	0	0	0	0	0	0	0
BGIR9823_91408	HBB	mRNA	0	0	0	0	0	0	0	0	0	0	0	0	0	0	0	0	0	0	0	0	0
BGIR9823_91409	HBB	mRNA	0	0	0	0	0	0	0	0	0	0	0	0	0	0	0	0	0	0	0	0	0
BGIR9823_91625	BCL9L	lncRNA	0	0	0	0	0	0	0	1	0	0	0	0	0	0	0	0	0	0	0	0	0
BGIR9823_91626	BCL9L	lncRNA	0	0	0	0	0	0	0	1	0	0	0	0	0	0	0	0	0	0	0	0	0
BGIR9823_91627	RPS25	mRNA	0	0	0	0	0	0	0	1	0	0	0	0	0	0	0	0	0	0	0	0	0
BGIR9823_91628	RPS25	mRNA	0	0	0	0	0	0	0	1	0	0	0	0	0	0	0	0	0	0	0	0	0
BGIR9823_91629	SLC37A4	mRNA	0	0	0	0	0	0	0	1	0	0	0	0	0	0	0	0	0	0	0	0	0
BGIR9823_91630	HYOU1	mRNA	0	0	0	0	0	0	0	1	0	0	0	0	0	0	0	0	0	0	0	0	0

RNA ID	Symbol	Type	updown_BGIR9823_88140	updown_BGIR9823_86964	updown_BGIR9823_90875	updown_URS0000BC5529	updown_URS0000BC90E3	updown_XR_002335844.1	updown_XR_001302653.2	updown_XR_002335671.1	updown_XR_002337554.1	updown_XR_001298816.2	updown_BGIR9823_99209	updown_BGIR9823_98003	updown_BGIR9823_98456	updown_BGIR9823_98543	updown_BGIR9823_88486	updown_BGIR9823_88585	updown_BGIR9823_97246	updown_XR_002346019.1	updown_XR_002346232.1	updown_XR_309047.3	updown_BGIR9823_93223
BGIR9823_91640	CLMP	mRNA	0	0	0	0	0	0	0	0	0	0	0	0	0	0	0	0	0	0	0	0	0
BGIR9823_91819	RUNDC3B	lncRNA	0	0	0	0	0	0	0	0	0	0	0	0	0	0	0	0	0	0	0	0	0
BGIR9823_91820	RUNDC3B	lncRNA	0	0	0	0	0	0	0	0	0	0	0	0	0	0	0	0	0	0	0	0	0
BGIR9823_91821	RUNDC3B	lncRNA	0	0	0	0	0	0	0	0	0	0	0	0	0	0	0	0	0	0	0	0	0
BGIR9823_91822	RUNDC3B	lncRNA	0	0	0	0	0	0	0	0	0	0	0	0	0	0	0	0	0	0	0	0	0
BGIR9823_91823	RUNDC3B	lncRNA	0	0	0	0	0	0	0	0	0	0	0	0	0	0	0	0	0	0	0	0	0
BGIR9823_91824	RUNDC3B	lncRNA	0	0	0	0	0	0	0	0	0	0	0	0	0	0	0	0	0	0	0	0	0
BGIR9823_91825	RUNDC3B	lncRNA	0	0	0	0	0	0	0	0	0	0	0	0	0	0	0	0	0	0	0	0	0
BGIR9823_91826	LOC102164596	lncRNA	0	0	0	0	0	0	0	0	0	0	0	0	0	0	0	0	0	0	0	0	0
BGIR9823_92993	KATNAL1	mRNA	0	0	0	0	0	0	0	0	0	0	0	0	0	0	0	0	0	0	0	0	0
BGIR9823_92994	KATNAL1	mRNA	0	0	0	0	0	0	0	0	0	0	0	0	0	0	0	0	0	0	0	0	0
BGIR9823_92995	KATNAL1	mRNA	0	0	0	0	0	0	0	0	0	0	0	0	0	0	0	0	0	0	0	0	0
BGIR9823_92996	KATNAL1	mRNA	0	0	0	0	0	0	0	0	0	0	0	0	0	0	0	0	0	0	0	0	0
BGIR9823_92997	KATNAL1	mRNA	0	0	0	0	0	0	0	0	0	0	0	0	0	0	0	0	0	0	0	0	0
BGIR9823_92999	LOC110255292	lncRNA	0	0	0	0	0	0	0	0	0	0	0	0	0	0	0	0	0	0	0	0	0
BGIR9823_93000	LOC102166871	lncRNA	0	0	0	0	0	0	0	0	0	0	0	0	0	0	0	0	0	0	0	0	0
BGIR9823_98051	LOC102164998	lncRNA	0	0	0	0	0	0	0	0	0	0	0	0	0	0	0	0	0	0	0	0	0
BGIR9823_99244	LOC102167708	lncRNA	0	0	0	0	0	0	0	0	0	0	0	0	0	0	0	0	0	0	0	0	0
NM_001004034.1	HMGB1	mRNA	0	0	0	0	0	0	0	0	0	0	0	0	0	0	0	0	0	0	0	0	0
NM_001044526.1	PPARA	mRNA	0	0	0	0	0	0	0	0	0	0	0	0	0	0	0	0	0	0	0	0	0
NM_001111256.1	SERPINB9	mRNA	0	0	0	0	0	0	0	0	0	0	0	0	0	0	0	0	0	0	1	0	0
NM_001113443.1	ECI2	mRNA	0	0	0	0	0	0	0	0	0	0	0	0	0	0	0	0	0	0	0	0	0
NM_001144841.1	HBB	mRNA	0	0	0	0	0	0	0	0	0	0	0	0	0	0	0	0	0	0	0	0	0
NM_001199719.1	SLC37A4	mRNA	0	0	0	0	0	0	0	1	0	0	0	0	0	0	0	0	0	0	0	0	0
NM_001243907.1	HSPA8	mRNA	0	0	0	0	0	0	0	0	0	0	0	0	0	0	0	0	0	0	0	0	0
NM_001244063.1	RPL3	mRNA	0	0	0	0	0	0	0	0	0	0	0	0	0	0	0	0	0	0	0	0	0
NM_001244066.1	SYNGR1	mRNA	0	0	0	0	0	0	0	0	0	0	0	0	0	0	0	0	0	0	0	0	0
NM_001244396.1	CCDC84	mRNA	0	0	0	0	0	0	0	1	0	0	0	0	0	0	0	0	0	0	0	0	0
NM_001287846.1	RHEB	mRNA	0	0	0	0	0	0	0	0	0	0	0	0	0	0	0	0	0	0	0	0	0
NM_213954.1	ACO2	mRNA	0	0	0	0	0	0	0	0	0	0	0	0	0	0	0	0	0	0	0	0	0
NM_214012.1	UPK2	mRNA	0	0	0	0	0	0	0	1	0	0	0	0	0	0	0	0	0	0	0	0	0
URS0000BC92F4	LOC102167708	lncRNA	0	0	0	0	0	0	0	0	0	0	0	0	0	0	0	0	0	0	0	0	0
XM_001928309.6	TBC1D7	mRNA	0	0	0	0	0	0	0	0	0	0	0	0	0	0	0	0	0	0	0	0	0
XM_001928852.5	PXDC1	mRNA	0	0	0	0	0	0	0	0	0	0	0	0	0	0	0	0	0	0	0	0	0
XM_003122534.4	KCNK7	mRNA	0	0	0	0	0	0	0	0	0	0	0	0	0	0	0	0	0	0	0	0	0
XM_003122558.5	SCYL1	mRNA	0	0	0	0	0	0	0	0	0	0	0	0	0	0	0	0	0	0	0	0	0
XM_003122562.5	FRMD8	mRNA	0	0	0	0	0	0	0	0	0	0	0	0	0	0	0	0	0	0	0	0	0
XM_003125951.6	TTC38	mRNA	0	0	0	0	0	0	0	0	0	0	0	0	0	0	0	0	0	0	0	0	0
XM_003125953.4	CDPF1	mRNA	0	0	0	0	0	0	0	0	0	0	0	0	0	0	0	0	0	0	0	0	0
XM_003126012.6	CBX7	mRNA	0	0	0	0	0	0	0	0	0	0	0	0	0	0	0	0	0	0	0	0	0
XM_003128161.4	RIPK1	mRNA	0	0	0	0	0	0	0	0	0	0	0	0	0	0	0	0	0	0	1	0	0
XM_003129508.2	LOC100514417	mRNA	0	0	0	0	0	0	0	0	0	0	0	0	0	0	0	0	0	0	0	0	0
XM_003129512.2	LOC100515268	mRNA	0	0	0	0	0	0	0	0	0	0	0	0	0	0	0	0	0	0	0	0	0

RNA ID	Symbol	Type	updown_BGIR9823_88140	updown_BGIR9823_86964	updown_BGIR9823_90875	updown_URS0000BC5529	updown_URS0000BC90E3	updown_XR_002335844.1	updown_XR_001302653.2	updown_XR_002335671.1	updown_XR_002337554.1	updown_XR_001298816.2	updown_BGIR9823_99209	updown_BGIR9823_98003	updown_BGIR9823_98456	updown_BGIR9823_98543	updown_BGIR9823_88486	updown_BGIR9823_88585	updown_BGIR9823_97246	updown_XR_002346019.1	updown_XR_002346232.1	updown_XR_309047.3	updown_BGIR9823_93223
XM_003129514.3	LOC100515618	mRNA	0	0	0	0	0	0	0	0	0	0	0	0	0	0	0	0	0	0	0	0	0
XM_003129519.2	LOC100517595	mRNA	0	0	0	0	0	0	0	0	0	0	0	0	0	0	0	0	0	0	0	0	0
XM_003129529.4	LOC100519910	mRNA	0	0	0	0	0	0	0	0	0	0	0	0	0	0	0	0	0	0	0	0	0
XM_003129915.4	CXCR5	mRNA	0	0	0	0	0	0	0	1	0	0	0	0	0	0	0	0	0	0	0	0	0
XM_003129916.5	BCL9L	mRNA	0	0	0	0	0	0	0	1	0	0	0	0	0	0	0	0	0	0	0	0	0
XM_003129918.4	TRAPPC4	mRNA	0	0	0	0	0	0	0	1	0	0	0	0	0	0	0	0	0	0	0	0	0
XM_003129919.5	RPS25	mRNA	0	0	0	0	0	0	0	1	0	0	0	0	0	0	0	0	0	0	0	0	0
XM_003129920.3	RPS25	mRNA	0	0	0	0	0	0	0	1	0	0	0	0	0	0	0	0	0	0	0	0	0
XM_003129922.4	HYOU1	mRNA	0	0	0	0	0	0	0	1	0	0	0	0	0	0	0	0	0	0	0	0	0
XM_003129923.6	VPS11	mRNA	0	0	0	0	0	0	0	1	0	0	0	0	0	0	0	0	0	0	0	0	0
XM_003129944.4	SC5D	mRNA	0	0	0	0	0	0	0	0	0	0	0	0	0	0	0	0	0	0	0	0	0
XM_003357161.1	LOC100620971	mRNA	0	0	0	0	0	0	0	0	0	0	0	0	0	0	0	0	0	0	0	0	0
XM_003482119.4	SERPINB1	mRNA	0	0	0	0	0	0	0	0	0	0	0	0	0	0	0	0	0	0	1	0	0
XM_003482132.4	TMEM170B	mRNA	0	0	0	0	0	0	0	0	0	0	0	0	0	0	0	0	0	0	0	0	0
XM_003482133.4	GFOD1	mRNA	0	0	0	0	0	0	0	0	0	0	0	0	0	0	0	0	0	0	0	0	0
XM_003482563.4	SPCS2	mRNA	0	0	0	0	0	0	0	0	0	0	0	0	0	0	0	0	0	0	0	0	0
XM_003482565.4	NEU3	mRNA	0	0	0	0	0	0	0	0	0	0	0	0	0	0	0	0	0	0	0	0	0
XM_003482567.1	LOC100739359	mRNA	0	0	0	0	0	0	0	0	0	0	0	0	0	0	0	0	0	0	0	0	0
XM_003482630.3	BSX	mRNA	0	0	0	0	0	0	0	0	0	0	0	0	0	0	0	0	0	0	0	0	0
XM_005653107.3	CHADL	mRNA	0	0	0	0	0	0	0	0	0	0	0	0	0	0	0	0	0	0	0	0	0
XM_005653640.1	LOC100621181	mRNA	0	0	0	0	0	0	0	0	0	0	0	0	0	0	0	0	0	0	0	0	0
XM_005654266.3	PRKAG2	mRNA	0	0	0	0	0	0	0	0	0	0	0	0	0	0	0	0	0	0	0	0	0
XM_005655470.3	CDPF1	mRNA	0	0	0	0	0	0	0	0	0	0	0	0	0	0	0	0	0	0	0	0	0
XM_005660703.3	LTBP3	mRNA	0	0	0	0	0	0	0	0	0	0	0	0	0	0	0	0	0	0	0	0	0
XM_005660705.3	SCYL1	mRNA	0	0	0	0	0	0	0	0	0	0	0	0	0	0	0	0	0	0	0	0	0
XM_005660707.3	SLC25A45	mRNA	0	0	0	0	0	0	0	0	0	0	0	0	0	0	0	0	0	0	0	0	0
XM_005665536.3	RIPK1	mRNA	0	0	0	0	0	0	0	0	0	0	0	0	0	0	0	0	0	0	1	0	0
XM_005665537.3	RIPK1	mRNA	0	0	0	0	0	0	0	0	0	0	0	0	0	0	0	0	0	0	1	0	0
XM_005665538.3	RIPK1	mRNA	0	0	0	0	0	0	0	0	0	0	0	0	0	0	0	0	0	0	1	0	0
XM_005665575.3	PHACTR1	mRNA	0	0	0	0	0	0	0	0	0	0	0	0	0	0	0	0	0	0	0	0	0
XM_005665576.3	TBC1D7	mRNA	0	0	0	0	0	0	0	0	0	0	0	0	0	0	0	0	0	0	0	0	0
XM_005667128.3	LOC100627340	mRNA	0	0	0	0	0	0	0	0	0	0	0	0	0	0	0	0	0	0	0	0	0
XM_005667397.2	CCDC84	mRNA	0	0	0	0	0	0	0	1	0	0	0	0	0	0	0	0	0	0	0	0	0
XM_005667398.3	SLC37A4	mRNA	0	0	0	0	0	0	0	1	0	0	0	0	0	0	0	0	0	0	0	0	0
XM_005667402.3	SLC37A4	mRNA	0	0	0	0	0	0	0	1	0	0	0	0	0	0	0	0	0	0	0	0	0
XM_005667403.3	SLC37A4	mRNA	0	0	0	0	0	0	0	1	0	0	0	0	0	0	0	0	0	0	0	0	0
XM_005667413.3	BCL9L	mRNA	0	0	0	0	0	0	0	1	0	0	0	0	0	0	0	0	0	0	0	0	0
XM_005667414.3	FOXR1	mRNA	0	0	0	0	0	0	0	1	0	0	0	0	0	0	0	0	0	0	0	0	0
XM_005667416.3	HYOU1	mRNA	0	0	0	0	0	0	0	1	0	0	0	0	0	0	0	0	0	0	0	0	0
XM_005667419.3	HYOU1	mRNA	0	0	0	0	0	0	0	1	0	0	0	0	0	0	0	0	0	0	0	0	0
XM_005667420.3	HYOU1	mRNA	0	0	0	0	0	0	0	1	0	0	0	0	0	0	0	0	0	0	0	0	0
XM_005667440.2	JHY	mRNA	0	0	0	0	0	0	0	0	0	0	0	0	0	0	0	0	0	0	0	0	0
XM_005668316.3	HMGB1	mRNA	0	0	0	0	0	0	0	0	0	0	0	0	0	0	0	0	0	0	0	0	0

RNA ID	Symbol	Type	updown_BGIR9823_88140	updown_BGIR9823_86964	updown_BGIR9823_90875	updown_URS0000BC5529	updown_URS0000BC90E3	updown_XR_002335844.1	updown_XR_001302653.2	updown_XR_002335671.1	updown_XR_002337554.1	updown_XR_001298816.2	updown_BGIR9823_99209	updown_BGIR9823_98003	updown_BGIR9823_98456	updown_BGIR9823_98543	updown_BGIR9823_88486	updown_BGIR9823_88585	updown_BGIR9823_97246	updown_XR_002346019.1	updown_XR_002346232.1	updown_XR_309047.3	updown_BGIR9823_93223
XM_005668317.3	HMGB1	mRNA	0	0	0	0	0	0	0	0	0	0	0	0	0	0	0	0	0	0	0	0	0
XM_005668318.3	HMGB1	mRNA	0	0	0	0	0	0	0	0	0	0	0	0	0	0	0	0	0	0	0	0	0
XM_005671489.3	EMX2	mRNA	0	0	0	0	0	0	0	0	0	0	0	0	0	0	0	0	0	0	0	0	0
XM_005672397.3	MYO10	mRNA	0	0	0	0	0	0	0	0	0	0	0	0	0	0	0	0	0	0	0	0	0
XM_013977400.2	FAM50B	mRNA	0	0	0	0	0	0	0	0	0	0	0	0	0	0	0	0	0	0	0	0	0
XM_013977425.2	NEDD9	mRNA	0	0	0	0	0	0	0	0	0	0	0	0	0	0	0	0	0	0	0	0	0
XM_013977430.2	TBC1D7	mRNA	0	0	0	0	0	0	0	0	0	0	0	0	0	0	0	0	0	0	0	0	0
XM_013977431.2	TBC1D7	mRNA	0	0	0	0	0	0	0	0	0	0	0	0	0	0	0	0	0	0	0	0	0
XM_013977432.2	TBC1D7	mRNA	0	0	0	0	0	0	0	0	0	0	0	0	0	0	0	0	0	0	0	0	0
XM_013977433.2	GFOD1	mRNA	0	0	0	0	0	0	0	0	0	0	0	0	0	0	0	0	0	0	0	0	0
XM_013977434.2	GFOD1	mRNA	0	0	0	0	0	0	0	0	0	0	0	0	0	0	0	0	0	0	0	0	0
XM_013979449.2	SLC37A4	mRNA	0	0	0	0	0	0	0	1	0	0	0	0	0	0	0	0	0	0	0	0	0
XM_013979456.2	BCL9L	mRNA	0	0	0	0	0	0	0	1	0	0	0	0	0	0	0	0	0	0	0	0	0
XM_013979457.2	BCL9L	mRNA	0	0	0	0	0	0	0	1	0	0	0	0	0	0	0	0	0	0	0	0	0
XM_013979458.2	BCL9L	mRNA	0	0	0	0	0	0	0	1	0	0	0	0	0	0	0	0	0	0	0	0	0
XM_013979459.2	BCL9L	mRNA	0	0	0	0	0	0	0	1	0	0	0	0	0	0	0	0	0	0	0	0	0
XM_013979461.2	TRAPPC4	mRNA	0	0	0	0	0	0	0	1	0	0	0	0	0	0	0	0	0	0	0	0	0
XM_013979463.2	HYOU1	mRNA	0	0	0	0	0	0	0	1	0	0	0	0	0	0	0	0	0	0	0	0	0
XM_013979484.2	LOC100523684	mRNA	0	0	0	0	0	0	0	0	0	0	0	0	0	0	0	0	0	0	0	0	0
XM_013979486.2	LOC100523684	mRNA	0	0	0	0	0	0	0	0	0	0	0	0	0	0	0	0	0	0	0	0	0
XM_013979491.2	JHY	mRNA	0	0	0	0	0	0	0	0	0	0	0	0	0	0	0	0	0	0	0	0	0
XM_013980418.2	KATNAL1	mRNA	0	0	0	0	0	0	0	0	0	0	0	0	0	0	0	0	0	0	0	0	0
XM_013988182.2	TTC38	mRNA	0	0	0	0	0	0	0	0	0	0	0	0	0	0	0	0	0	0	0	0	0
XM_013988183.2	TTC38	mRNA	0	0	0	0	0	0	0	0	0	0	0	0	0	0	0	0	0	0	0	0	0
XM_013989589.2	LOC100522267	mRNA	0	0	0	0	0	0	0	0	0	0	0	0	0	0	0	0	0	0	0	0	0
XM_013989592.2	LOC100522455	mRNA	0	0	0	0	0	0	0	0	0	0	0	0	0	0	0	0	0	0	0	0	0
XM_013989593.2	LOC100522455	mRNA	0	0	0	0	0	0	0	0	0	0	0	0	0	0	0	0	0	0	0	0	0
XM_013994099.2	FRMD8	mRNA	0	0	0	0	0	0	0	0	0	0	0	0	0	0	0	0	0	0	0	0	0
XM_013994100.2	SLC25A45	mRNA	0	0	0	0	0	0	0	0	0	0	0	0	0	0	0	0	0	0	0	0	0
XM_013994102.2	SLC25A45	mRNA	0	0	0	0	0	0	0	0	0	0	0	0	0	0	0	0	0	0	0	0	0
XM_013994103.2	SLC25A45	mRNA	0	0	0	0	0	0	0	0	0	0	0	0	0	0	0	0	0	0	0	0	0
XM_013994104.2	SLC25A45	mRNA	0	0	0	0	0	0	0	0	0	0	0	0	0	0	0	0	0	0	0	0	0
XM_013997510.2	CBX7	mRNA	0	0	0	0	0	0	0	0	0	0	0	0	0	0	0	0	0	0	0	0	0
XM_013999070.2	LOC100520088	mRNA	0	0	0	0	0	0	0	0	0	0	0	0	0	0	0	0	0	0	0	0	0
XM_021062230.1	LOC100517771	mRNA	0	0	0	0	0	0	0	0	0	0	0	0	0	0	0	0	0	0	0	0	0
XM_021062231.1	LOC100517957	mRNA	0	0	0	0	0	0	0	0	0	0	0	0	0	0	0	0	0	0	0	0	0
XM_021062367.1	LOC110255365	mRNA	0	0	0	0	0	0	0	0	0	0	0	0	0	0	0	0	0	0	0	0	0
XM_021062369.1	LOC100737110	mRNA	0	0	0	0	0	0	0	0	0	0	0	0	0	0	0	0	0	0	0	0	0
XM_021062370.1	LOC110255368	mRNA	0	0	0	0	0	0	0	0	0	0	0	0	0	0	0	0	0	0	0	0	0
XM_021062371.1	LOC100515441	mRNA	0	0	0	0	0	0	0	0	0	0	0	0	0	0	0	0	0	0	0	0	0
XM_021062374.1	LOC110255371	mRNA	0	0	0	0	0	0	0	0	0	0	0	0	0	0	0	0	0	0	0	0	0
XM_021062375.1	LOC100626951	mRNA	0	0	0	0	0	0	0	0	0	0	0	0	0	0	0	0	0	0	0	0	0
XM_021062491.1	XRRA1	mRNA	0	0	0	0	0	0	0	0	0	0	0	0	0	0	0	0	0	0	0	0	0

RNA ID	Symbol	Type	updown_BGIR9823_88140	updown_BGIR9823_86964	updown_BGIR9823_90875	updown_URS0000BC5529	updown_URS0000BC90E3	updown_XR_002335844.1	updown_XR_001302653.2	updown_XR_002335671.1	updown_XR_002337554.1	updown_XR_001298816.2	updown_BGIR9823_99209	updown_BGIR9823_98003	updown_BGIR9823_98456	updown_BGIR9823_98543	updown_BGIR9823_88486	updown_BGIR9823_88585	updown_BGIR9823_97246	updown_XR_002346019.1	updown_XR_002346232.1	updown_XR_309047.3	updown_BGIR9823_93223
XM_021062493.1	XRRA1	mRNA	0	0	0	0	0	0	0	0	0	0	0	0	0	0	0	0	0	0	0	0	0
XM_021062494.1	XRRA1	mRNA	0	0	0	0	0	0	0	0	0	0	0	0	0	0	0	0	0	0	0	0	0
XM_021062495.1	XRRA1	mRNA	0	0	0	0	0	0	0	0	0	0	0	0	0	0	0	0	0	0	0	0	0
XM_021062496.1	XRRA1	mRNA	0	0	0	0	0	0	0	0	0	0	0	0	0	0	0	0	0	0	0	0	0
XM_021062497.1	XRRA1	mRNA	0	0	0	0	0	0	0	0	0	0	0	0	0	0	0	0	0	0	0	0	0
XM_021062498.1	XRRA1	mRNA	0	0	0	0	0	0	0	0	0	0	0	0	0	0	0	0	0	0	0	0	0
XM_021062499.1	XRRA1	mRNA	0	0	0	0	0	0	0	0	0	0	0	0	0	0	0	0	0	0	0	0	0
XM_021062500.1	XRRA1	mRNA	0	0	0	0	0	0	0	0	0	0	0	0	0	0	0	0	0	0	0	0	0
XM_021062501.1	XRRA1	mRNA	0	0	0	0	0	0	0	0	0	0	0	0	0	0	0	0	0	0	0	0	0
XM_021062502.1	XRRA1	mRNA	0	0	0	0	0	0	0	0	0	0	0	0	0	0	0	0	0	0	0	0	0
XM_021062503.1	XRRA1	mRNA	0	0	0	0	0	0	0	0	0	0	0	0	0	0	0	0	0	0	0	0	0
XM_021062511.1	SLCO2B1	mRNA	0	0	0	0	0	0	0	0	0	0	0	0	0	0	0	0	0	0	0	0	0
XM_021062948.1	BCL9L	mRNA	0	0	0	0	0	0	0	1	0	0	0	0	0	0	0	0	0	0	0	0	0
XM_021062949.1	TRAPPC4	mRNA	0	0	0	0	0	0	0	1	0	0	0	0	0	0	0	0	0	0	0	0	0
XM_021062950.1	HYOU1	mRNA	0	0	0	0	0	0	0	1	0	0	0	0	0	0	0	0	0	0	0	0	0
XM_021062951.1	HYOU1	mRNA	0	0	0	0	0	0	0	1	0	0	0	0	0	0	0	0	0	0	0	0	0
XM_021062952.1	HYOU1	mRNA	0	0	0	0	0	0	0	1	0	0	0	0	0	0	0	0	0	0	0	0	0
XM_021062953.1	HYOU1	mRNA	0	0	0	0	0	0	0	1	0	0	0	0	0	0	0	0	0	0	0	0	0
XM_021062954.1	HYOU1	mRNA	0	0	0	0	0	0	0	1	0	0	0	0	0	0	0	0	0	0	0	0	0
XM_021062955.1	HYOU1	mRNA	0	0	0	0	0	0	0	1	0	0	0	0	0	0	0	0	0	0	0	0	0
XM_021062956.1	HYOU1	mRNA	0	0	0	0	0	0	0	1	0	0	0	0	0	0	0	0	0	0	0	0	0
XM_021062957.1	VPS11	mRNA	0	0	0	0	0	0	0	1	0	0	0	0	0	0	0	0	0	0	0	0	0
XM_021062993.1	LOC100523684	mRNA	0	0	0	0	0	0	0	0	0	0	0	0	0	0	0	0	0	0	0	0	0
XM_021062994.1	LOC100523684	mRNA	0	0	0	0	0	0	0	0	0	0	0	0	0	0	0	0	0	0	0	0	0
XM_021062995.1	LOC100523684	mRNA	0	0	0	0	0	0	0	0	0	0	0	0	0	0	0	0	0	0	0	0	0
XM_021062996.1	LOC100523684	mRNA	0	0	0	0	0	0	0	0	0	0	0	0	0	0	0	0	0	0	0	0	0
XM_021062997.1	LOC100523684	mRNA	0	0	0	0	0	0	0	0	0	0	0	0	0	0	0	0	0	0	0	0	0
XM_021062998.1	LOC100523684	mRNA	0	0	0	0	0	0	0	0	0	0	0	0	0	0	0	0	0	0	0	0	0
XM_021062999.1	LOC100523684	mRNA	0	0	0	0	0	0	0	0	0	0	0	0	0	0	0	0	0	0	0	0	0
XM_021063000.1	LOC100523684	mRNA	0	0	0	0	0	0	0	0	0	0	0	0	0	0	0	0	0	0	0	0	0
XM_021063001.1	LOC100523684	mRNA	0	0	0	0	0	0	0	0	0	0	0	0	0	0	0	0	0	0	0	0	0
XM_021063002.1	LOC100523684	mRNA	0	0	0	0	0	0	0	0	0	0	0	0	0	0	0	0	0	0	0	0	0
XM_021063004.1	LOC100523684	mRNA	0	0	0	0	0	0	0	0	0	0	0	0	0	0	0	0	0	0	0	0	0
XM_021063005.1	LOC100523684	mRNA	0	0	0	0	0	0	0	0	0	0	0	0	0	0	0	0	0	0	0	0	0
XM_021063006.1	LOC100523684	mRNA	0	0	0	0	0	0	0	0	0	0	0	0	0	0	0	0	0	0	0	0	0
XM_021063009.1	SORL1	mRNA	0	0	0	0	0	0	0	0	0	0	0	0	0	0	0	0	0	0	0	0	0
XM_021063010.1	SORL1	mRNA	0	0	0	0	0	0	0	0	0	0	0	0	0	0	0	0	0	0	0	0	0
XM_021063011.1	SORL1	mRNA	0	0	0	0	0	0	0	0	0	0	0	0	0	0	0	0	0	0	0	0	0
XM_021063012.1	SORL1	mRNA	0	0	0	0	0	0	0	0	0	0	0	0	0	0	0	0	0	0	0	0	0
XM_021063013.1	SORL1	mRNA	0	0	0	0	0	0	0	0	0	0	0	0	0	0	0	0	0	0	0	0	0
XM_021063014.1	SORL1	mRNA	0	0	0	0	0	0	0	0	0	0	0	0	0	0	0	0	0	0	0	0	0
XM_021063023.1	CLMP	mRNA	0	0	0	0	0	0	0	0	0	0	0	0	0	0	0	0	0	0	0	0	0
XM_021063024.1	CLMP	mRNA	0	0	0	0	0	0	0	0	0	0	0	0	0	0	0	0	0	0	0	0	0

RNA ID	Symbol	Type	updown_BGIR9823_88140	updown_BGIR9823_86964	updown_BGIR9823_90875	updown_URS0000BC5529	updown_URS0000BC90E3	updown_XR_002335844.1	updown_XR_001302653.2	updown_XR_002335671.1	updown_XR_002337554.1	updown_XR_001298816.2	updown_BGIR9823_99209	updown_BGIR9823_98003	updown_BGIR9823_98456	updown_BGIR9823_98543	updown_BGIR9823_88486	updown_BGIR9823_88585	updown_BGIR9823_97246	updown_XR_002346019.1	updown_XR_002346232.1	updown_XR_309047.3	updown_BGIR9823_93223
XM_021063025.1	CLMP	mRNA	0	0	0	0	0	0	0	0	0	0	0	0	0	0	0	0	0	0	0	0	0
XM_021063026.1	CLMP	mRNA	0	0	0	0	0	0	0	0	0	0	0	0	0	0	0	0	0	0	0	0	0
XM_021063950.1	NXPE2	mRNA	0	0	0	0	0	0	0	0	0	1	0	0	0	0	0	0	0	0	0	0	0
XM_021063963.1	LOC100623190	mRNA	0	0	0	0	0	0	0	0	0	0	0	0	0	0	0	0	0	0	0	0	0
XM_021065159.1	HMGB1	mRNA	0	0	0	0	0	0	0	0	0	0	0	0	0	0	0	0	0	0	0	0	0
XM_021065160.1	HMGB1	mRNA	0	0	0	0	0	0	0	0	0	0	0	0	0	0	0	0	0	0	0	0	0
XM_021065161.1	HMGB1	mRNA	0	0	0	0	0	0	0	0	0	0	0	0	0	0	0	0	0	0	0	0	0
XM_021065529.1	KATNAL1	mRNA	0	0	0	0	0	0	0	0	0	0	0	0	0	0	0	0	0	0	0	0	0
XM_021065531.1	KATNAL1	mRNA	0	0	0	0	0	0	0	0	0	0	0	0	0	0	0	0	0	0	0	0	0
XM_021065532.1	KATNAL1	mRNA	0	0	0	0	0	0	0	0	0	0	0	0	0	0	0	0	0	0	0	0	0
XM_021065533.1	KATNAL1	mRNA	0	0	0	0	0	0	0	0	0	0	0	0	0	0	0	0	0	0	0	0	0
XM_021065534.1	KATNAL1	mRNA	0	0	0	0	0	0	0	0	0	0	0	0	0	0	0	0	0	0	0	0	0
XM_021065535.1	KATNAL1	mRNA	0	0	0	0	0	0	0	0	0	0	0	0	0	0	0	0	0	0	0	0	0
XM_021065536.1	KATNAL1	mRNA	0	0	0	0	0	0	0	0	0	0	0	0	0	0	0	0	0	0	0	0	0
XM_021065537.1	KATNAL1	mRNA	0	0	0	0	0	0	0	0	0	0	0	0	0	0	0	0	0	0	0	0	0
XM_021065538.1	KATNAL1	mRNA	0	0	0	0	0	0	0	0	0	0	0	0	0	0	0	0	0	0	0	0	0
XM_021065539.1	KATNAL1	mRNA	0	0	0	0	0	0	0	0	0	0	0	0	0	0	0	0	0	0	0	0	0
XM_021065541.1	KATNAL1	mRNA	0	0	0	0	0	0	0	0	0	0	0	0	0	0	0	0	0	0	0	0	0
XM_021065542.1	KATNAL1	mRNA	0	0	0	0	0	0	0	0	0	0	0	0	0	0	0	0	0	0	0	0	0
XM_021065543.1	KATNAL1	mRNA	0	0	0	0	0	0	0	0	0	0	0	0	0	0	0	0	0	0	0	0	0
XM_021065544.1	KATNAL1	mRNA	0	0	0	0	0	0	0	0	0	0	0	0	0	0	0	0	0	0	0	0	0
XM_021073408.1	EMX2	mRNA	0	0	0	0	0	0	0	0	0	0	0	0	0	0	0	0	0	0	0	0	0
XM_021078611.1	PRKAG2	mRNA	0	0	0	0	0	0	0	0	0	0	0	0	0	0	0	0	0	0	0	0	0
XM_021078612.1	PRKAG2	mRNA	0	0	0	0	0	0	0	0	0	0	0	0	0	0	0	0	0	0	0	0	0
XM_021078613.1	PRKAG2	mRNA	0	0	0	0	0	0	0	0	0	0	0	0	0	0	0	0	0	0	0	0	0
XM_021078614.1	PRKAG2	mRNA	0	0	0	0	0	0	0	0	0	0	0	0	0	0	0	0	0	0	0	0	0
XM_021078615.1	PRKAG2	mRNA	0	0	0	0	0	0	0	0	0	0	0	0	0	0	0	0	0	0	0	0	0
XM_021078616.1	PRKAG2	mRNA	0	0	0	0	0	0	0	0	0	0	0	0	0	0	0	0	0	0	0	0	0
XM_021078617.1	PRKAG2	mRNA	0	0	0	0	0	0	0	0	0	0	0	0	0	0	0	0	0	0	0	0	0
XM_021078618.1	PRKAG2	mRNA	0	0	0	0	0	0	0	0	0	0	0	0	0	0	0	0	0	0	0	0	0
XM_021078619.1	PRKAG2	mRNA	0	0	0	0	0	0	0	0	0	0	0	0	0	0	0	0	0	0	0	0	0
XM_021078620.1	PRKAG2	mRNA	0	0	0	0	0	0	0	0	0	0	0	0	0	0	0	0	0	0	0	0	0
XM_021078621.1	PRKAG2	mRNA	0	0	0	0	0	0	0	0	0	0	0	0	0	0	0	0	0	0	0	0	0
XM_021081309.1	LOC106506226	mRNA	0	0	0	0	0	0	0	0	0	0	0	0	0	0	0	0	0	0	0	0	0
XM_021081403.1	LOC110258215	mRNA	0	0	0	0	0	0	0	0	0	0	0	0	0	0	0	0	0	0	0	0	0
XM_021082153.1	LOC100521600	mRNA	0	0	0	0	0	0	0	0	0	0	0	0	0	0	0	0	0	0	0	0	0
XM_021082154.1	LOC100521600	mRNA	0	0	0	0	0	0	0	0	0	0	0	0	0	0	0	0	0	0	0	0	0
XM_021082156.1	LOC100521600	mRNA	0	0	0	0	0	0	0	0	0	0	0	0	0	0	0	0	0	0	0	0	0
XM_021082157.1	LOC100521600	mRNA	0	0	0	0	0	0	0	0	0	0	0	0	0	0	0	0	0	0	0	0	0
XM_021082158.1	LOC100521600	mRNA	0	0	0	0	0	0	0	0	0	0	0	0	0	0	0	0	0	0	0	0	0
XM_021082159.1	LOC100521600	mRNA	0	0	0	0	0	0	0	0	0	0	0	0	0	0	0	0	0	0	0	0	0
XM_021082835.1	EHBP1L1	mRNA	0	0	0	0	0	0	0	0	0	0	0	0	0	0	0	0	0	0	0	0	0
XM_021082836.1	EHBP1L1	mRNA	0	0	0	0	0	0	0	0	0	0	0	0	0	0	0	0	0	0	0	0	0

RNA ID	Symbol	Type	updown_BGIR9823_88140	updown_BGIR9823_86964	updown_BGIR9823_90875	updown_URS0000BC5529	updown_URS0000BC90E3	updown_XR_002335844.1	updown_XR_001302653.2	updown_XR_002335671.1	updown_XR_002337554.1	updown_XR_001298816.2	updown_BGIR9823_99209	updown_BGIR9823_98003	updown_BGIR9823_98456	updown_BGIR9823_98543	updown_BGIR9823_88486	updown_BGIR9823_88585	updown_BGIR9823_97246	updown_XR_002346019.1	updown_XR_002346232.1	updown_XR_309047.3	updown_BGIR9823_93223
XM_021082837.1	EHBP1L1	mRNA	0	0	0	0	0	0	0	0	0	0	0	0	0	0	0	0	0	0	0	0	0
XM_021082838.1	EHBP1L1	mRNA	0	0	0	0	0	0	0	0	0	0	0	0	0	0	0	0	0	0	0	0	0
XM_021082839.1	EHBP1L1	mRNA	0	0	0	0	0	0	0	0	0	0	0	0	0	0	0	0	0	0	0	0	0
XM_021082840.1	EHBP1L1	mRNA	0	0	0	0	0	0	0	0	0	0	0	0	0	0	0	0	0	0	0	0	0
XM_021082841.1	EHBP1L1	mRNA	0	0	0	0	0	0	0	0	0	0	0	0	0	0	0	0	0	0	0	0	0
XM_021082842.1	EHBP1L1	mRNA	0	0	0	0	0	0	0	0	0	0	0	0	0	0	0	0	0	0	0	0	0
XM_021082843.1	EHBP1L1	mRNA	0	0	0	0	0	0	0	0	0	0	0	0	0	0	0	0	0	0	0	0	0
XM_021082844.1	EHBP1L1	mRNA	0	0	0	0	0	0	0	0	0	0	0	0	0	0	0	0	0	0	0	0	0
XM_021082845.1	EHBP1L1	mRNA	0	0	0	0	0	0	0	0	0	0	0	0	0	0	0	0	0	0	0	0	0
XM_021082846.1	EHBP1L1	mRNA	0	0	0	0	0	0	0	0	0	0	0	0	0	0	0	0	0	0	0	0	0
XM_021082847.1	EHBP1L1	mRNA	0	0	0	0	0	0	0	0	0	0	0	0	0	0	0	0	0	0	0	0	0
XM_021082851.1	MAP3K11	mRNA	0	0	0	0	0	0	0	0	0	0	0	0	0	0	0	0	0	0	0	0	0
XM_021082852.1	SSSCA1	mRNA	0	0	0	0	0	0	0	0	0	0	0	0	0	0	0	0	0	0	0	0	0
XM_021082853.1	FAM89B	mRNA	0	0	0	0	0	0	0	0	0	0	0	0	0	0	0	0	0	0	0	0	0
XM_021082854.1	LTBP3	mRNA	0	0	0	0	0	0	0	0	0	0	0	0	0	0	0	0	0	0	0	0	0
XM_021082855.1	LTBP3	mRNA	0	0	0	0	0	0	0	0	0	0	0	0	0	0	0	0	0	0	0	0	0
XM_021082856.1	SCYL1	mRNA	0	0	0	0	0	0	0	0	0	0	0	0	0	0	0	0	0	0	0	0	0
XM_021082857.1	SLC25A45	mRNA	0	0	0	0	0	0	0	0	0	0	0	0	0	0	0	0	0	0	0	0	0
XM_021082858.1	SLC25A45	mRNA	0	0	0	0	0	0	0	0	0	0	0	0	0	0	0	0	0	0	0	0	0
XM_021091099.1	SYNGR1	mRNA	0	0	0	0	0	0	0	0	0	0	0	0	0	0	0	0	0	0	0	0	0
XM_021091101.1	SYNGR1	mRNA	0	0	0	0	0	0	0	0	0	0	0	0	0	0	0	0	0	0	0	0	0
XM_021091102.1	SYNGR1	mRNA	0	0	0	0	0	0	0	0	0	0	0	0	0	0	0	0	0	0	0	0	0
XM_021091103.1	SYNGR1	mRNA	0	0	0	0	0	0	0	0	0	0	0	0	0	0	0	0	0	0	0	0	0
XM_021091370.1	LOC102159820	mRNA	0	0	0	0	0	0	0	0	0	0	0	0	0	0	0	0	0	0	0	0	0
XM_021091373.1	TRMU	mRNA	0	0	0	0	0	0	0	0	0	0	0	0	0	0	0	0	0	0	0	0	0
XM_021091374.1	TRMU	mRNA	0	0	0	0	0	0	0	0	0	0	0	0	0	0	0	0	0	0	0	0	0
XM_021091375.1	TRMU	mRNA	0	0	0	0	0	0	0	0	0	0	0	0	0	0	0	0	0	0	0	0	0
XM_021091376.1	GTSE1	mRNA	0	0	0	0	0	0	0	0	0	0	0	0	0	0	0	0	0	0	0	0	0
XM_021091377.1	TTC38	mRNA	0	0	0	0	0	0	0	0	0	0	0	0	0	0	0	0	0	0	0	0	0
XM_021091379.1	LOC110260611	mRNA	0	0	0	0	0	0	0	0	0	0	0	0	0	0	0	0	0	0	0	0	0
XM_021091381.1	LOC110260611	mRNA	0	0	0	0	0	0	0	0	0	0	0	0	0	0	0	0	0	0	0	0	0
XM_021091454.1	TOB2	mRNA	0	0	0	0	0	0	0	0	0	0	0	0	0	0	0	0	0	0	0	0	0
XM_021091455.1	TOB2	mRNA	0	0	0	0	0	0	0	0	0	0	0	0	0	0	0	0	0	0	0	0	0
XM_021091456.1	PHF5A	mRNA	0	0	0	0	0	0	0	0	0	0	0	0	0	0	0	0	0	0	0	0	0
XM_021091457.1	PHF5A	mRNA	0	0	0	0	0	0	0	0	0	0	0	0	0	0	0	0	0	0	0	0	0
XM_021091458.1	ZC3H7B	mRNA	0	0	0	0	0	0	0	0	0	0	0	0	0	0	0	0	0	0	0	0	0
XM_021091459.1	ZC3H7B	mRNA	0	0	0	0	0	0	0	0	0	0	0	0	0	0	0	0	0	0	0	0	0
XM_021091460.1	TEF	mRNA	0	0	0	0	0	0	0	0	0	0	0	0	0	0	0	0	0	0	0	0	0
XM_021091461.1	RANGAP1	mRNA	0	0	0	0	0	0	0	0	0	0	0	0	0	0	0	0	0	0	0	0	0
XM_021091474.1	CBX7	mRNA	0	0	0	0	0	0	0	0	0	0	0	0	0	0	0	0	0	0	0	0	0
XM_021092767.1	CRADD	mRNA	0	0	0	0	0	0	0	0	0	0	0	0	0	0	0	0	0	0	0	0	0
XM_021092768.1	CRADD	mRNA	0	0	0	0	0	0	0	0	0	0	0	0	0	0	0	0	0	0	0	0	0
XM_021092769.1	CRADD	mRNA	0	0	0	0	0	0	0	0	0	0	0	0	0	0	0	0	0	0	0	0	0

RNA ID	Symbol	Type	updown_BGIR9823_88140	updown_BGIR9823_86964	updown_BGIR9823_90875	updown_URS0000BC5529	updown_URS0000BC90E3	updown_XR_002335844.1	updown_XR_001302653.2	updown_XR_002335671.1	updown_XR_002337554.1	updown_XR_001298816.2	updown_BGIR9823_99209	updown_BGIR9823_98003	updown_BGIR9823_98456	updown_BGIR9823_98543	updown_BGIR9823_88486	updown_BGIR9823_88585	updown_BGIR9823_97246	updown_XR_002346019.1	updown_XR_002346232.1	updown_XR_309047.3	updown_BGIR9823_93223
XM_021092770.1	CRADD	mRNA	0	0	0	0	0	0	0	0	0	0	0	0	0	0	0	0	0	0	0	0	0
XM_021092771.1	CRADD	mRNA	0	0	0	0	0	0	0	0	0	0	0	0	0	0	0	0	0	0	0	0	0
XM_021092773.1	CRADD	mRNA	0	0	0	0	0	0	0	0	0	0	0	0	0	0	0	0	0	0	0	0	0
XM_021093095.1	PKDREJ	mRNA	0	0	0	0	0	0	0	0	0	0	0	0	0	0	0	0	0	0	0	0	0
XM_021097064.1	MAF	mRNA	0	0	0	0	0	0	0	0	0	0	0	0	0	0	0	0	0	0	0	0	0
XM_021097065.1	MAF	mRNA	0	0	0	0	0	0	0	0	0	0	0	0	0	0	0	0	0	0	0	0	0
XM_021097066.1	MAF	mRNA	0	0	0	0	0	0	0	0	0	0	0	0	0	0	0	0	0	0	0	0	0
XM_021097870.1	ECI2	mRNA	0	0	0	0	0	0	0	0	0	0	0	0	0	0	0	0	0	0	0	0	0
XM_021099751.1	PHACTR1	mRNA	0	0	0	0	0	0	0	0	0	0	0	0	0	0	0	0	0	0	0	0	0
XM_021099752.1	PHACTR1	mRNA	0	0	0	0	0	0	0	0	0	0	0	0	0	0	0	0	0	0	0	0	0
XM_021099753.1	PHACTR1	mRNA	0	0	0	0	0	0	0	0	0	0	0	0	0	0	0	0	0	0	0	0	0
XM_021099754.1	PHACTR1	mRNA	0	0	0	0	0	0	0	0	0	0	0	0	0	0	0	0	0	0	0	0	0
XM_021099755.1	PHACTR1	mRNA	0	0	0	0	0	0	0	0	0	0	0	0	0	0	0	0	0	0	0	0	0
XM_021099756.1	PHACTR1	mRNA	0	0	0	0	0	0	0	0	0	0	0	0	0	0	0	0	0	0	0	0	0
XM_021099757.1	PHACTR1	mRNA	0	0	0	0	0	0	0	0	0	0	0	0	0	0	0	0	0	0	0	0	0
XM_021099758.1	PHACTR1	mRNA	0	0	0	0	0	0	0	0	0	0	0	0	0	0	0	0	0	0	0	0	0
XM_021099759.1	PHACTR1	mRNA	0	0	0	0	0	0	0	0	0	0	0	0	0	0	0	0	0	0	0	0	0
XM_021099760.1	PHACTR1	mRNA	0	0	0	0	0	0	0	0	0	0	0	0	0	0	0	0	0	0	0	0	0
XM_021099761.1	PHACTR1	mRNA	0	0	0	0	0	0	0	0	0	0	0	0	0	0	0	0	0	0	0	0	0
XM_021099762.1	PHACTR1	mRNA	0	0	0	0	0	0	0	0	0	0	0	0	0	0	0	0	0	0	0	0	0
XM_021099763.1	PHACTR1	mRNA	0	0	0	0	0	0	0	0	0	0	0	0	0	0	0	0	0	0	0	0	0
XM_021099764.1	PHACTR1	mRNA	0	0	0	0	0	0	0	0	0	0	0	0	0	0	0	0	0	0	0	0	0
XM_021099765.1	PHACTR1	mRNA	0	0	0	0	0	0	0	0	0	0	0	0	0	0	0	0	0	0	0	0	0
XM_021099766.1	TBC1D7	mRNA	0	0	0	0	0	0	0	0	0	0	0	0	0	0	0	0	0	0	0	0	0
XM_021099767.1	TBC1D7	mRNA	0	0	0	0	0	0	0	0	0	0	0	0	0	0	0	0	0	0	0	0	0
XM_021099769.1	TBC1D7	mRNA	0	0	0	0	0	0	0	0	0	0	0	0	0	0	0	0	0	0	0	0	0
XM_021099770.1	TBC1D7	mRNA	0	0	0	0	0	0	0	0	0	0	0	0	0	0	0	0	0	0	0	0	0
XM_021099999.1	LOC110261697	mRNA	0	0	0	0	0	0	0	0	0	0	0	0	0	0	0	0	0	0	0	0	0
XM_021100108.1	WRNIP1	mRNA	0	0	0	0	0	0	0	0	0	0	0	0	0	0	0	0	0	0	1	0	0
XM_021100109.1	MYLK4	mRNA	0	0	0	0	0	0	0	0	0	0	0	0	0	0	0	0	0	0	1	0	0
XM_021100110.1	MYLK4	mRNA	0	0	0	0	0	0	0	0	0	0	0	0	0	0	0	0	0	0	1	0	0
XM_021100111.1	MYLK4	mRNA	0	0	0	0	0	0	0	0	0	0	0	0	0	0	0	0	0	0	1	0	0
XM_021100112.1	MYLK4	mRNA	0	0	0	0	0	0	0	0	0	0	0	0	0	0	0	0	0	0	1	0	0
XM_021100114.1	NQO2	mRNA	0	0	0	0	0	0	0	0	0	0	0	0	0	0	0	0	0	0	1	0	0
XM_021100115.1	NQO2	mRNA	0	0	0	0	0	0	0	0	0	0	0	0	0	0	0	0	0	0	1	0	0
XM_021100117.1	NQO2	mRNA	0	0	0	0	0	0	0	0	0	0	0	0	0	0	0	0	0	0	1	0	0
XM_021100118.1	NQO2	mRNA	0	0	0	0	0	0	0	0	0	0	0	0	0	0	0	0	0	0	1	0	0
XM_021100119.1	NQO2	mRNA	0	0	0	0	0	0	0	0	0	0	0	0	0	0	0	0	0	0	1	0	0
XM_021100124.1	PRPF4B	mRNA	0	0	0	0	0	0	0	0	0	0	0	0	0	0	0	0	0	0	0	0	0
XM_021100126.1	PRPF4B	mRNA	0	0	0	0	0	0	0	0	0	0	0	0	0	0	0	0	0	0	0	0	0
XM_021100184.1	NEDD9	mRNA	0	0	0	0	0	0	0	0	0	0	0	0	0	0	0	0	0	0	0	0	0
XM_021100188.1	TMEM170B	mRNA	0	0	0	0	0	0	0	0	0	0	0	0	0	0	0	0	0	0	0	0	0
XM_021100194.1	GFOD1	mRNA	0	0	0	0	0	0	0	0	0	0	0	0	0	0	0	0	0	0	0	0	0

RNA ID	Symbol	Type	updown_BGIR9823_88140	updown_BGIR9823_86964	updown_BGIR9823_90875	updown_URS0000BC5529	updown_URS0000BC90E3	updown_XR_002335844.1	updown_XR_001302653.2	updown_XR_002335671.1	updown_XR_002337554.1	updown_XR_001298816.2	updown_BGIR9823_99209	updown_BGIR9823_98003	updown_BGIR9823_98456	updown_BGIR9823_98543	updown_BGIR9823_88486	updown_BGIR9823_88585	updown_BGIR9823_97246	updown_XR_002346019.1	updown_XR_002346232.1	updown_XR_309047.3	updown_BGIR9823_93223
XM_021102040.1	SLC37A4	mRNA	0	0	0	0	0	0	0	1	0	0	0	0	0	0	0	0	0	0	0	0	0
XM_021102041.1	SLC37A4	mRNA	0	0	0	0	0	0	0	1	0	0	0	0	0	0	0	0	0	0	0	0	0
XM_021102042.1	SLC37A4	mRNA	0	0	0	0	0	0	0	1	0	0	0	0	0	0	0	0	0	0	0	0	0
XM_021102043.1	SLC37A4	mRNA	0	0	0	0	0	0	0	1	0	0	0	0	0	0	0	0	0	0	0	0	0
XM_021102044.1	SLC37A4	mRNA	0	0	0	0	0	0	0	1	0	0	0	0	0	0	0	0	0	0	0	0	0
XM_021102045.1	SLC37A4	mRNA	0	0	0	0	0	0	0	1	0	0	0	0	0	0	0	0	0	0	0	0	0
XM_021102046.1	SLC37A4	mRNA	0	0	0	0	0	0	0	1	0	0	0	0	0	0	0	0	0	0	0	0	0
XM_021102047.1	SLC37A4	mRNA	0	0	0	0	0	0	0	1	0	0	0	0	0	0	0	0	0	0	0	0	0
XM_021102048.1	SLC37A4	mRNA	0	0	0	0	0	0	0	1	0	0	0	0	0	0	0	0	0	0	0	0	0
XM_021102049.1	SLC37A4	mRNA	0	0	0	0	0	0	0	1	0	0	0	0	0	0	0	0	0	0	0	0	0
XM_021102050.1	SLC37A4	mRNA	0	0	0	0	0	0	0	1	0	0	0	0	0	0	0	0	0	0	0	0	0
XM_021102051.1	SLC37A4	mRNA	0	0	0	0	0	0	0	1	0	0	0	0	0	0	0	0	0	0	0	0	0
XM_021102052.1	SLC37A4	mRNA	0	0	0	0	0	0	0	1	0	0	0	0	0	0	0	0	0	0	0	0	0
XM_021102209.1	UPK2	mRNA	0	0	0	0	0	0	0	1	0	0	0	0	0	0	0	0	0	0	0	0	0
XM_021102516.1	LOC100522455	mRNA	0	0	0	0	0	0	0	0	0	0	0	0	0	0	0	0	0	0	0	0	0
XM_021102517.1	RUNDC3B	mRNA	0	0	0	0	0	0	0	0	0	0	0	0	0	0	0	0	0	0	0	0	0
XM_021102518.1	RUNDC3B	mRNA	0	0	0	0	0	0	0	0	0	0	0	0	0	0	0	0	0	0	0	0	0
XM_021102519.1	RUNDC3B	mRNA	0	0	0	0	0	0	0	0	0	0	0	0	0	0	0	0	0	0	0	0	0
XM_021102522.1	RUNDC3B	mRNA	0	0	0	0	0	0	0	0	0	0	0	0	0	0	0	0	0	0	0	0	0
XM_021102523.1	RUNDC3B	mRNA	0	0	0	0	0	0	0	0	0	0	0	0	0	0	0	0	0	0	0	0	0
XR_001297664.2	LOC102161744	lncRNA	0	0	0	0	0	0	0	0	0	0	0	0	0	0	0	0	0	0	0	0	0
XR_001298807.2	LOC106504881	lncRNA	0	0	0	0	0	0	0	0	0	1	0	0	0	0	0	0	0	0	0	0	0
XR_001298808.2	LOC106504881	lncRNA	0	0	0	0	0	0	0	0	0	1	0	0	0	0	0	0	0	0	0	0	0
XR_001298810.2	LOC106504881	lncRNA	0	0	0	0	0	0	0	0	0	1	0	0	0	0	0	0	0	0	0	0	0
XR_001298811.2	LOC106504881	lncRNA	0	0	0	0	0	0	0	0	0	1	0	0	0	0	0	0	0	0	0	0	0
XR_001298812.2	LOC106504881	lncRNA	0	0	0	0	0	0	0	0	0	1	0	0	0	0	0	0	0	0	0	0	0
XR_001298813.2	LOC106504881	lncRNA	0	0	0	0	0	0	0	0	0	1	0	0	0	0	0	0	0	0	0	0	0
XR_001298814.2	LOC106504881	lncRNA	0	0	0	0	0	0	0	0	0	1	0	0	0	0	0	0	0	0	0	0	0
XR_001298816.2	LOC106504881	lncRNA	0	0	0	0	0	0	0	0	0	1	0	0	0	0	0	0	0	0	0	0	0
XR_001298817.2	LOC106504881	lncRNA	0	0	0	0	0	0	0	0	0	1	0	0	0	0	0	0	0	0	0	0	0
XR_001298844.2	LOC102163364	lncRNA	0	0	0	0	0	0	0	0	0	0	0	0	0	0	0	0	0	0	0	0	0
XR_001298846.2	LOC102163364	lncRNA	0	0	0	0	0	0	0	0	0	0	0	0	0	0	0	0	0	0	0	0	0
XR_001298850.2	LOC102163364	lncRNA	0	0	0	0	0	0	0	0	0	0	0	0	0	0	0	0	0	0	0	0	0
XR_001299456.2	LOC102166670	lncRNA	0	0	0	0	0	0	0	0	0	0	0	0	0	0	0	0	0	0	0	0	0
XR_001299457.2	LOC102166670	lncRNA	0	0	0	0	0	0	0	0	0	0	0	0	0	0	0	0	0	0	0	0	0
XR_001300715.2	LOC106505879	lncRNA	0	0	0	0	0	0	0	0	0	0	0	0	0	0	0	0	0	0	0	0	0
XR_001301078.2	LOC102162623	lncRNA	0	0	0	0	0	0	0	0	0	0	0	0	0	0	0	0	0	0	0	0	0
XR_001301087.2	LOC102162623	lncRNA	0	0	0	0	0	0	0	0	0	0	0	0	0	0	0	0	0	0	0	0	0
XR_001301217.2	LOC102164998	lncRNA	0	0	0	0	0	0	0	0	0	0	0	0	0	0	0	0	0	0	0	0	0
XR_001301305.2	LOC102159607	lncRNA	0	0	0	0	0	0	0	0	0	0	0	0	0	0	0	0	0	0	0	0	0
XR_001308967.2	LOC106510467	lncRNA	0	0	0	0	0	0	0	0	0	0	0	0	0	0	0	0	0	0	0	0	0
XR_002335533.1	XRRA1	mRNA	0	0	0	0	0	0	0	0	0	0	0	0	0	0	0	0	0	0	0	0	0
XR_002335534.1	XRRA1	mRNA	0	0	0	0	0	0	0	0	0	0	0	0	0	0	0	0	0	0	0	0	0

RNA ID	Symbol	Type	updown_BGIR9823_88140	updown_BGIR9823_86964	updown_BGIR9823_90875	updown_URS0000BC5529	updown_URS0000BC90E3	updown_XR_002335844.1	updown_XR_001302653.2	updown_XR_002335671.1	updown_XR_002337554.1	updown_XR_001298816.2	updown_BGIR9823_99209	updown_BGIR9823_98003	updown_BGIR9823_98456	updown_BGIR9823_98543	updown_BGIR9823_88486	updown_BGIR9823_88585	updown_BGIR9823_97246	updown_XR_002346019.1	updown_XR_002346232.1	updown_XR_309047.3	updown_BGIR9823_93223
XR_002335535.1	LOC110255399	lncRNA	0	0	0	0	0	0	0	0	0	0	0	0	0	0	0	0	0	0	0	0	0
XR_002335536.1	LOC102160367	lncRNA	0	0	0	0	0	0	0	0	0	0	0	0	0	0	0	0	0	0	0	0	0
XR_002335537.1	LOC102160367	lncRNA	0	0	0	0	0	0	0	0	0	0	0	0	0	0	0	0	0	0	0	0	0
XR_002335650.1	LOC102157630	lncRNA	0	0	0	0	0	0	0	0	0	1	0	0	0	0	0	0	0	0	0	0	0
XR_002335651.1	LOC110255435	lncRNA	0	0	0	0	0	0	0	0	0	1	0	0	0	0	0	0	0	0	0	0	0
XR_002335652.1	LOC106504881	lncRNA	0	0	0	0	0	0	0	0	0	1	0	0	0	0	0	0	0	0	0	0	0
XR_002335653.1	LOC106504881	lncRNA	0	0	0	0	0	0	0	0	0	1	0	0	0	0	0	0	0	0	0	0	0
XR_002335670.1	LOC110255440	lncRNA	0	0	0	0	0	0	0	1	0	0	0	0	0	0	0	0	0	0	0	0	0
XR_002335671.1	LOC110255441	lncRNA	0	0	0	0	0	0	0	1	0	0	0	0	0	0	0	0	0	0	0	0	0
XR_002335672.1	LOC110255441	lncRNA	0	0	0	0	0	0	0	1	0	0	0	0	0	0	0	0	0	0	0	0	0
XR_002335674.1	HYOU1	mRNA	0	0	0	0	0	0	0	1	0	0	0	0	0	0	0	0	0	0	0	0	0
XR_002335675.1	HYOU1	mRNA	0	0	0	0	0	0	0	1	0	0	0	0	0	0	0	0	0	0	0	0	0
XR_002335685.1	LOC100523684	mRNA	0	0	0	0	0	0	0	0	0	0	0	0	0	0	0	0	0	0	0	0	0
XR_002335686.1	LOC102163364	lncRNA	0	0	0	0	0	0	0	0	0	0	0	0	0	0	0	0	0	0	0	0	0
XR_002335687.1	LOC102163364	lncRNA	0	0	0	0	0	0	0	0	0	0	0	0	0	0	0	0	0	0	0	0	0
XR_002335688.1	LOC102163364	lncRNA	0	0	0	0	0	0	0	0	0	0	0	0	0	0	0	0	0	0	0	0	0
XR_002335689.1	LOC102163364	lncRNA	0	0	0	0	0	0	0	0	0	0	0	0	0	0	0	0	0	0	0	0	0
XR_002335690.1	LOC102163364	lncRNA	0	0	0	0	0	0	0	0	0	0	0	0	0	0	0	0	0	0	0	0	0
XR_002335691.1	LOC102163364	lncRNA	0	0	0	0	0	0	0	0	0	0	0	0	0	0	0	0	0	0	0	0	0
XR_002335692.1	LOC102163364	lncRNA	0	0	0	0	0	0	0	0	0	0	0	0	0	0	0	0	0	0	0	0	0
XR_002335693.1	LOC106504896	lncRNA	0	0	0	0	0	0	0	0	0	0	0	0	0	0	0	0	0	0	0	0	0
XR_002335694.1	SORL1	mRNA	0	0	0	0	0	0	0	0	0	0	0	0	0	0	0	0	0	0	0	0	0
XR_002336641.1	LOC106505250	lncRNA	0	0	0	0	0	0	0	0	0	0	0	0	0	0	0	0	0	0	0	0	0
XR_002336643.1	LOC110255292	lncRNA	0	0	0	0	0	0	0	0	0	0	0	0	0	0	0	0	0	0	0	0	0
XR_002336644.1	LOC102166670	lncRNA	0	0	0	0	0	0	0	0	0	0	0	0	0	0	0	0	0	0	0	0	0
XR_002336645.1	LOC102166670	lncRNA	0	0	0	0	0	0	0	0	0	0	0	0	0	0	0	0	0	0	0	0	0
XR_002338443.1	LOC106505879	lncRNA	0	0	0	0	0	0	0	0	0	0	0	0	0	0	0	0	0	0	0	0	0
XR_002338444.1	LOC106505879	lncRNA	0	0	0	0	0	0	0	0	0	0	0	0	0	0	0	0	0	0	0	0	0
XR_002338445.1	LOC106505879	lncRNA	0	0	0	0	0	0	0	0	0	0	0	0	0	0	0	0	0	0	0	0	0
XR_002338446.1	LOC106505879	lncRNA	0	0	0	0	0	0	0	0	0	0	0	0	0	0	0	0	0	0	0	0	0
XR_002338447.1	LOC106505879	lncRNA	0	0	0	0	0	0	0	0	0	0	0	0	0	0	0	0	0	0	0	0	0
XR_002338448.1	LOC106505879	lncRNA	0	0	0	0	0	0	0	0	0	0	0	0	0	0	0	0	0	0	0	0	0
XR_002338449.1	LOC106505879	lncRNA	0	0	0	0	0	0	0	0	0	0	0	0	0	0	0	0	0	0	0	0	0
XR_002338450.1	LOC106505879	lncRNA	0	0	0	0	0	0	0	0	0	0	0	0	0	0	0	0	0	0	0	0	0
XR_002338521.1	LOC110256664	lncRNA	0	0	0	0	0	0	0	0	0	0	0	0	0	0	0	0	0	0	0	0	0
XR_002338522.1	LOC110256664	lncRNA	0	0	0	0	0	0	0	0	0	0	0	0	0	0	0	0	0	0	0	0	0
XR_002338666.1	LOC110256747	lncRNA	0	0	0	0	0	0	0	0	0	0	0	0	0	0	0	0	0	0	0	0	0
XR_002338714.1	LOC102163817	lncRNA	0	0	0	0	0	0	0	0	0	0	0	0	0	0	0	0	0	0	0	0	0
XR_002338715.1	LOC102164998	lncRNA	0	0	0	0	0	0	0	0	0	0	0	0	0	0	0	0	0	0	0	0	0
XR_002338716.1	LOC102164998	lncRNA	0	0	0	0	0	0	0	0	0	0	0	0	0	0	0	0	0	0	0	0	0
XR_002338717.1	LOC110256768	lncRNA	0	0	0	0	0	0	0	0	0	0	0	0	0	0	0	0	0	0	0	0	0
XR_002338727.1	LOC110256773	lncRNA	0	0	0	0	0	0	0	0	0	0	0	0	0	0	0	0	0	0	0	0	0
XR_002338728.1	LOC110256773	lncRNA	0	0	0	0	0	0	0	0	0	0	0	0	0	0	0	0	0	0	0	0	0

RNA ID	Symbol	Type	updown_BGIR9823_88140	updown_BGIR9823_86964	updown_BGIR9823_90875	updown_URS0000BC5529	updown_URS0000BC90E3	updown_XR_002335844.1	updown_XR_001302653.2	updown_XR_002335671.1	updown_XR_002337554.1	updown_XR_001298816.2	updown_BGIR9823_99209	updown_BGIR9823_98003	updown_BGIR9823_98456	updown_BGIR9823_98543	updown_BGIR9823_88486	updown_BGIR9823_88585	updown_BGIR9823_97246	updown_XR_002346019.1	updown_XR_002346232.1	updown_XR_309047.3	updown_BGIR9823_93223
XR_002338729.1	LOC110256773	lncRNA	0	0	0	0	0	0	0	0	0	0	0	0	0	0	0	0	0	0	0	0	0
XR_002338793.1	LOC110256796	lncRNA	0	0	0	0	0	0	0	0	0	0	0	0	0	0	0	0	0	0	0	0	0
XR_002339623.1	LOC102167708	lncRNA	0	0	0	0	0	0	0	0	0	0	0	0	0	0	0	0	0	0	0	0	0
XR_002340245.1	PRKAG2	mRNA	0	0	0	0	0	0	0	0	0	0	0	0	0	0	0	0	0	0	0	0	0
XR_002340246.1	LOC102162623	lncRNA	0	0	0	0	0	0	0	0	0	0	0	0	0	0	0	0	0	0	0	0	0
XR_002340247.1	LOC102162623	lncRNA	0	0	0	0	0	0	0	0	0	0	0	0	0	0	0	0	0	0	0	0	0
XR_002340248.1	LOC110257516	lncRNA	0	0	0	0	0	0	0	0	0	0	0	0	0	0	0	0	0	0	0	0	0
XR_002340249.1	LOC102162071	lncRNA	0	0	0	0	0	0	0	0	0	0	0	0	0	0	0	0	0	0	0	0	0
XR_002340901.1	LOC102160243	lncRNA	0	0	0	0	0	0	0	0	0	0	0	0	0	0	0	0	0	0	0	0	0
XR_002340902.1	LOC102160243	lncRNA	0	0	0	0	0	0	0	0	0	0	0	0	0	0	0	0	0	0	0	0	0
XR_002340903.1	LOC102160243	lncRNA	0	0	0	0	0	0	0	0	0	0	0	0	0	0	0	0	0	0	0	0	0
XR_002340904.1	LOC102160243	lncRNA	0	0	0	0	0	0	0	0	0	0	0	0	0	0	0	0	0	0	0	0	0
XR_002340905.1	LOC102160243	lncRNA	0	0	0	0	0	0	0	0	0	0	0	0	0	0	0	0	0	0	0	0	0
XR_002341237.1	LOC102164320	lncRNA	0	0	0	0	0	0	0	0	0	0	0	0	0	0	0	0	0	0	0	0	0
XR_002341238.1	LOC102164320	lncRNA	0	0	0	0	0	0	0	0	0	0	0	0	0	0	0	0	0	0	0	0	0
XR_002341239.1	LOC102164320	lncRNA	0	0	0	0	0	0	0	0	0	0	0	0	0	0	0	0	0	0	0	0	0
XR_002341240.1	LOC102164320	lncRNA	0	0	0	0	0	0	0	0	0	0	0	0	0	0	0	0	0	0	0	0	0
XR_002341241.1	LOC102164320	lncRNA	0	0	0	0	0	0	0	0	0	0	0	0	0	0	0	0	0	0	0	0	0
XR_002341242.1	LOC102164320	lncRNA	0	0	0	0	0	0	0	0	0	0	0	0	0	0	0	0	0	0	0	0	0
XR_002341243.1	LOC102164320	lncRNA	0	0	0	0	0	0	0	0	0	0	0	0	0	0	0	0	0	0	0	0	0
XR_002341244.1	LOC102164320	lncRNA	0	0	0	0	0	0	0	0	0	0	0	0	0	0	0	0	0	0	0	0	0
XR_002341270.1	LOC100521600	mRNA	0	0	0	0	0	0	0	0	0	0	0	0	0	0	0	0	0	0	0	0	0
XR_002341443.1	LOC102163816	lncRNA	0	0	0	0	0	0	0	0	0	0	0	0	0	0	0	0	0	0	0	0	0
XR_002343847.1	LOC110260611	mRNA	0	0	0	0	0	0	0	0	0	0	0	0	0	0	0	0	0	0	0	0	0
XR_002343848.1	LOC110260611	mRNA	0	0	0	0	0	0	0	0	0	0	0	0	0	0	0	0	0	0	0	0	0
XR_002343849.1	LOC110260611	mRNA	0	0	0	0	0	0	0	0	0	0	0	0	0	0	0	0	0	0	0	0	0
XR_002343850.1	LOC110260611	mRNA	0	0	0	0	0	0	0	0	0	0	0	0	0	0	0	0	0	0	0	0	0
XR_002343875.1	LOC110260629	lncRNA	0	0	0	0	0	0	0	0	0	0	0	0	0	0	0	0	0	0	0	0	0
XR_002343876.1	LOC110260629	lncRNA	0	0	0	0	0	0	0	0	0	0	0	0	0	0	0	0	0	0	0	0	0
XR_002343877.1	LOC110260629	lncRNA	0	0	0	0	0	0	0	0	0	0	0	0	0	0	0	0	0	0	0	0	0
XR_002343878.1	LOC110260629	lncRNA	0	0	0	0	0	0	0	0	0	0	0	0	0	0	0	0	0	0	0	0	0
XR_002343879.1	LOC110260629	lncRNA	0	0	0	0	0	0	0	0	0	0	0	0	0	0	0	0	0	0	0	0	0
XR_002343880.1	LOC110260629	lncRNA	0	0	0	0	0	0	0	0	0	0	0	0	0	0	0	0	0	0	0	0	0
XR_002343881.1	LOC110260629	lncRNA	0	0	0	0	0	0	0	0	0	0	0	0	0	0	0	0	0	0	0	0	0
XR_002343883.1	LOC110260631	lncRNA	0	0	0	0	0	0	0	0	0	0	0	0	0	0	0	0	0	0	0	0	0
XR_002343884.1	LOC110260634	lncRNA	0	0	0	0	0	0	0	0	0	0	0	0	0	0	0	0	0	0	0	0	0
XR_002343885.1	LOC110260635	lncRNA	0	0	0	0	0	0	0	0	0	0	0	0	0	0	0	0	0	0	0	0	0
XR_002343891.1	LOC110260642	lncRNA	0	0	0	0	0	0	0	0	0	0	0	0	0	0	0	0	0	0	0	0	0
XR_002343892.1	LOC110260643	lncRNA	0	0	0	0	0	0	0	0	0	0	0	0	0	0	0	0	0	0	0	0	0
XR_002345298.1	MAF	mRNA	0	0	0	0	0	0	0	0	0	0	0	0	0	0	0	0	0	0	0	0	0
XR_002345300.1	LOC106510470	lncRNA	0	0	0	0	0	0	0	0	0	0	0	0	0	0	0	0	0	0	0	0	0
XR_002345301.1	LOC106510468	lncRNA	0	0	0	0	0	0	0	0	0	0	0	0	0	0	0	0	0	0	0	0	0
XR_002346166.1	LOC110261703	lncRNA	0	0	0	0	0	0	0	0	0	0	0	0	0	0	0	0	0	0	0	0	0

RNA ID	Symbol	Type	updown_BGIR9823_88140	updown_BGIR9823_86964	updown_BGIR9823_90875	updown_URS0000BC5529	updown_URS0000BC90E3	updown_XR_002335844.1	updown_XR_001302653.2	updown_XR_002335671.1	updown_XR_002337554.1	updown_XR_001298816.2	updown_BGIR9823_99209	updown_BGIR9823_98003	updown_BGIR9823_98456	updown_BGIR9823_98543	updown_BGIR9823_88486	updown_BGIR9823_88585	updown_BGIR9823_97246	updown_XR_002346019.1	updown_XR_002346232.1	updown_XR_309047.3	updown_BGIR9823_93223
XR_002346227.1	LOC106504299	lncRNA	0	0	0	0	0	0	0	0	0	0	0	0	0	0	0	0	0	0	1	0	0
XR_002346228.1	LOC106504299	lncRNA	0	0	0	0	0	0	0	0	0	0	0	0	0	0	0	0	0	0	1	0	0
XR_002346229.1	LOC106504299	lncRNA	0	0	0	0	0	0	0	0	0	0	0	0	0	0	0	0	0	0	1	0	0
XR_002346230.1	LOC106504299	lncRNA	0	0	0	0	0	0	0	0	0	0	0	0	0	0	0	0	0	0	1	0	0
XR_002346231.1	LOC106504299	lncRNA	0	0	0	0	0	0	0	0	0	0	0	0	0	0	0	0	0	0	1	0	0
XR_002346232.1	LOC106504299	lncRNA	0	0	0	0	0	0	0	0	0	0	0	0	0	0	0	0	0	0	1	0	0
XR_002346233.1	NQO2	mRNA	0	0	0	0	0	0	0	0	0	0	0	0	0	0	0	0	0	0	1	0	0
XR_002346236.1	PRPF4B	mRNA	0	0	0	0	0	0	0	0	0	0	0	0	0	0	0	0	0	0	0	0	0
XR_002346237.1	PRPF4B	mRNA	0	0	0	0	0	0	0	0	0	0	0	0	0	0	0	0	0	0	0	0	0
XR_002346238.1	PRPF4B	mRNA	0	0	0	0	0	0	0	0	0	0	0	0	0	0	0	0	0	0	0	0	0
XR_002346239.1	PRPF4B	mRNA	0	0	0	0	0	0	0	0	0	0	0	0	0	0	0	0	0	0	0	0	0
XR_002346240.1	PRPF4B	mRNA	0	0	0	0	0	0	0	0	0	0	0	0	0	0	0	0	0	0	0	0	0
XR_002346241.1	PRPF4B	mRNA	0	0	0	0	0	0	0	0	0	0	0	0	0	0	0	0	0	0	0	0	0
XR_002346242.1	PRPF4B	mRNA	0	0	0	0	0	0	0	0	0	0	0	0	0	0	0	0	0	0	0	0	0
XR_002346243.1	PRPF4B	mRNA	0	0	0	0	0	0	0	0	0	0	0	0	0	0	0	0	0	0	0	0	0
XR_002346244.1	PRPF4B	mRNA	0	0	0	0	0	0	0	0	0	0	0	0	0	0	0	0	0	0	0	0	0
XR_002346245.1	PRPF4B	mRNA	0	0	0	0	0	0	0	0	0	0	0	0	0	0	0	0	0	0	0	0	0
XR_002346246.1	PRPF4B	mRNA	0	0	0	0	0	0	0	0	0	0	0	0	0	0	0	0	0	0	0	0	0
XR_002346247.1	PRPF4B	mRNA	0	0	0	0	0	0	0	0	0	0	0	0	0	0	0	0	0	0	0	0	0
XR_002346248.1	PRPF4B	mRNA	0	0	0	0	0	0	0	0	0	0	0	0	0	0	0	0	0	0	0	0	0
XR_002346249.1	PRPF4B	mRNA	0	0	0	0	0	0	0	0	0	0	0	0	0	0	0	0	0	0	0	0	0
XR_002346250.1	PRPF4B	mRNA	0	0	0	0	0	0	0	0	0	0	0	0	0	0	0	0	0	0	0	0	0
XR_002346251.1	PRPF4B	mRNA	0	0	0	0	0	0	0	0	0	0	0	0	0	0	0	0	0	0	0	0	0
XR_002346252.1	PRPF4B	mRNA	0	0	0	0	0	0	0	0	0	0	0	0	0	0	0	0	0	0	0	0	0
XR_002346253.1	PRPF4B	mRNA	0	0	0	0	0	0	0	0	0	0	0	0	0	0	0	0	0	0	0	0	0
XR_002346254.1	PRPF4B	mRNA	0	0	0	0	0	0	0	0	0	0	0	0	0	0	0	0	0	0	0	0	0
XR_002346255.1	PRPF4B	mRNA	0	0	0	0	0	0	0	0	0	0	0	0	0	0	0	0	0	0	0	0	0
XR_002346256.1	PRPF4B	mRNA	0	0	0	0	0	0	0	0	0	0	0	0	0	0	0	0	0	0	0	0	0
XR_002346257.1	PRPF4B	mRNA	0	0	0	0	0	0	0	0	0	0	0	0	0	0	0	0	0	0	0	0	0
XR_002346258.1	PRPF4B	mRNA	0	0	0	0	0	0	0	0	0	0	0	0	0	0	0	0	0	0	0	0	0
XR_002346259.1	PRPF4B	mRNA	0	0	0	0	0	0	0	0	0	0	0	0	0	0	0	0	0	0	0	0	0
XR_002346260.1	PRPF4B	mRNA	0	0	0	0	0	0	0	0	0	0	0	0	0	0	0	0	0	0	0	0	0
XR_002346261.1	PRPF4B	mRNA	0	0	0	0	0	0	0	0	0	0	0	0	0	0	0	0	0	0	0	0	0
XR_002346262.1	PRPF4B	mRNA	0	0	0	0	0	0	0	0	0	0	0	0	0	0	0	0	0	0	0	0	0
XR_002346263.1	PRPF4B	mRNA	0	0	0	0	0	0	0	0	0	0	0	0	0	0	0	0	0	0	0	0	0
XR_002346264.1	PRPF4B	mRNA	0	0	0	0	0	0	0	0	0	0	0	0	0	0	0	0	0	0	0	0	0
XR_002346265.1	LOC110261764	lncRNA	0	0	0	0	0	0	0	0	0	0	0	0	0	0	0	0	0	0	0	0	0
XR_002346266.1	LOC102162837	lncRNA	0	0	0	0	0	0	0	0	0	0	0	0	0	0	0	0	0	0	0	0	0
XR_002346299.1	LOC110261774	lncRNA	0	0	0	0	0	0	0	0	0	0	0	0	0	0	0	0	0	0	0	0	0
XR_002346300.1	LOC110261774	lncRNA	0	0	0	0	0	0	0	0	0	0	0	0	0	0	0	0	0	0	0	0	0
XR_002346302.1	LOC110261776	lncRNA	0	0	0	0	0	0	0	0	0	0	0	0	0	0	0	0	0	0	0	0	0
XR_002346303.1	TMEM170B	mRNA	0	0	0	0	0	0	0	0	0	0	0	0	0	0	0	0	0	0	0	0	0
XR_002346304.1	TMEM170B	mRNA	0	0	0	0	0	0	0	0	0	0	0	0	0	0	0	0	0	0	0	0	0

RNA ID	Symbol	Type	updown_BGIR9823_88140	updown_BGIR9823_86964	updown_BGIR9823_90875	updown_URS0000BC5529	updown_URS0000BC90E3	updown_XR_002335844.1	updown_XR_001302653.2	updown_XR_002335671.1	updown_XR_002337554.1	updown_XR_001298816.2	updown_BGIR9823_99209	updown_BGIR9823_98003	updown_BGIR9823_98456	updown_BGIR9823_98543	updown_BGIR9823_88486	updown_BGIR9823_88585	updown_BGIR9823_97246	updown_XR_002346019.1	updown_XR_002346232.1	updown_XR_309047.3	updown_BGIR9823_93223
XR_115737.4	LOC100513133	lncRNA	0	0	0	0	0	0	0	0	0	0	0	0	0	0	0	0	0	0	0	0	0
XR_299046.2	LOC102164596	lncRNA	0	0	0	0	0	0	0	0	0	0	0	0	0	0	0	0	0	0	0	0	0
XR_303634.3	LOC100738154	lncRNA	0	0	0	0	0	0	0	0	0	0	0	0	0	0	0	0	0	0	0	0	0
XR_304239.3	LOC102159819	lncRNA	0	0	0	0	0	0	0	0	0	0	0	0	0	0	0	0	0	0	1	0	0
XR_304244.3	LOC102161992	lncRNA	0	0	0	0	0	0	0	0	0	0	0	0	0	0	0	0	0	0	0	0	0
XR_304257.3	LOC102162837	lncRNA	0	0	0	0	0	0	0	0	0	0	0	0	0	0	0	0	0	0	0	0	0
XR_306105.3	LOC102166871	lncRNA	0	0	0	0	0	0	0	0	0	0	0	0	0	0	0	0	0	0	0	0	0
XR_307914.3	LOC102159607	lncRNA	0	0	0	0	0	0	0	0	0	0	0	0	0	0	0	0	0	0	0	0	0
XR_308345.3	LOC102167708	lncRNA	0	0	0	0	0	0	0	0	0	0	0	0	0	0	0	0	0	0	0	0	0
novel-ssc-miR1028-3p		miRNA	0	0	0	0	0	0	0	0	0	0	0	0	0	0	0	0	0	0	0	0	0
novel-ssc-miR1028-5p		miRNA	0	0	0	0	0	0	0	0	0	0	0	0	0	0	0	0	0	0	0	0	0
novel-ssc-miR1130-3p		miRNA	0	0	0	0	0	0	0	1	0	0	0	0	0	0	0	0	0	0	0	0	0
novel-ssc-miR1130-5p		miRNA	0	0	0	0	0	0	0	1	0	0	0	0	0	0	0	0	0	0	0	0	0
novel-ssc-miR1162-3p		miRNA	0	0	0	0	0	0	0	0	0	0	0	0	0	0	0	0	0	0	0	0	0
novel-ssc-miR1193-3p		miRNA	0	0	0	0	0	0	0	1	0	0	0	0	0	0	0	0	0	0	0	0	0
novel-ssc-miR1193-5p		miRNA	0	0	0	0	0	0	0	1	0	0	0	0	0	0	0	0	0	0	0	0	0
novel-ssc-miR1217-3p		miRNA	0	0	0	0	0	0	0	0	0	0	0	0	0	0	0	0	0	0	0	0	0
novel-ssc-miR1217-5p		miRNA	0	0	0	0	0	0	0	0	0	0	0	0	0	0	0	0	0	0	0	0	0
novel-ssc-miR1429-3p		miRNA	0	0	0	0	0	0	0	0	0	0	0	0	0	0	0	0	0	0	0	0	0
novel-ssc-miR1429-5p		miRNA	0	0	0	0	0	0	0	0	0	0	0	0	0	0	0	0	0	0	0	0	0
novel-ssc-miR259-3p		miRNA	0	0	0	0	0	0	0	0	0	0	0	0	0	0	0	0	0	0	0	0	0
novel-ssc-miR259-5p		miRNA	0	0	0	0	0	0	0	0	0	0	0	0	0	0	0	0	0	0	0	0	0
novel-ssc-miR324-3p		miRNA	0	0	0	0	0	0	0	0	0	0	0	0	0	0	0	0	0	0	0	0	0
novel-ssc-miR324-5p		miRNA	0	0	0	0	0	0	0	0	0	0	0	0	0	0	0	0	0	0	0	0	0
novel-ssc-miR42-3p		miRNA	0	0	0	0	0	0	0	0	0	0	0	0	0	0	0	0	0	0	0	0	0
novel-ssc-miR42-5p		miRNA	0	0	0	0	0	0	0	0	0	0	0	0	0	0	0	0	0	0	0	0	0
novel-ssc-miR433-3p		miRNA	0	0	0	0	0	0	0	0	0	0	0	0	0	0	0	0	0	0	0	0	0
novel-ssc-miR433-5p		miRNA	0	0	0	0	0	0	0	0	0	0	0	0	0	0	0	0	0	0	0	0	0
novel-ssc-miR557-3p		miRNA	0	0	0	0	0	0	0	0	0	0	0	0	0	0	0	0	0	0	0	0	0
novel-ssc-miR557-5p		miRNA	0	0	0	0	0	0	0	0	0	0	0	0	0	0	0	0	0	0	0	0	0
novel-ssc-miR619-3p		miRNA	0	0	0	0	0	0	0	1	0	0	0	0	0	0	0	0	0	0	0	0	0
novel-ssc-miR619-5p		miRNA	0	0	0	0	0	0	0	1	0	0	0	0	0	0	0	0	0	0	0	0	0
novel-ssc-miR719-3p		miRNA	0	0	0	0	0	0	0	0	0	0	0	0	0	0	0	0	0	0	0	0	0
novel-ssc-miR719-5p		miRNA	0	0	0	0	0	0	0	0	0	0	0	0	0	0	0	0	0	0	0	0	0
novel-ssc-miR966-3p		miRNA	0	0	0	0	0	0	0	0	0	0	0	0	0	0	0	0	0	0	0	0	0
novel-ssc-miR966-5p		miRNA	0	0	0	0	0	0	0	0	0	0	0	0	0	0	0	0	0	0	0	0	0
ssc-miR-7136-3p		miRNA	0	0	0	0	0	0	0	0	0	0	0	0	0	0	0	0	0	0	0	0	0
ssc-miR-7136-5p		miRNA	0	0	0	0	0	0	0	0	0	0	0	0	0	0	0	0	0	0	0	0	0

Supplementary Table S10. The predicted targe

RNA ID	Symbol	Type	updown_BGIR9823_88895	updown_BGIR9823_93430	updown_BGIR9823_94434	updown_BGIR9823_94471	updown_XR_002339623.1	updown_XR_002339910.1	updown_XR_002340372.1	updown_XR_002339346.1	updown_XR_002343632.1	updown_XR_002343884.1	updown_BGIR9823_102724	updown_BGIR9823_102212	updown_BGIR9823_102279	updown_BGIR9823_78573	updown_BGIR9823_101405	updown_BGIR9823_101544	updown_BGIR9823_101566	updown_BGIR9823_100548	updown_BGIR9823_100551	updown_BGIR9823_101208	updown_BGIR9823_78436
BGIR9823_100361	PRKAG2	mRNA	0	0	0	0	0	0	0	0	0	0	0	0	0	0	0	0	0	0	0	0	0
BGIR9823_100362	PRKAG2	mRNA	0	0	0	0	0	0	0	0	0	0	0	0	0	0	0	0	0	0	0	0	0
BGIR9823_100363	PRKAG2	mRNA	0	0	0	0	0	0	0	0	0	0	0	0	0	0	0	0	0	0	0	0	0
BGIR9823_100364	PRKAG2	mRNA	0	0	0	0	0	0	0	0	0	0	0	0	0	0	0	0	0	0	0	0	0
BGIR9823_101535	LOC102158401	lncRNA	0	0	0	0	0	0	0	0	0	0	0	0	0	0	0	1	1	0	0	0	0
BGIR9823_101541	LOC102157946	lncRNA	0	0	0	0	0	0	0	0	0	0	0	0	0	0	0	1	1	0	0	0	0
BGIR9823_101542	LOC102157946	lncRNA	0	0	0	0	0	0	0	0	0	0	0	0	0	0	0	1	1	0	0	0	0
BGIR9823_101560	LOC110258600	lncRNA	0	0	0	0	0	0	0	0	0	0	0	0	0	0	0	1	1	0	0	0	0
BGIR9823_101561	LOC110258600	lncRNA	0	0	0	0	0	0	0	0	0	0	0	0	0	0	0	1	1	0	0	0	0
BGIR9823_101562	LOC110258600	lncRNA	0	0	0	0	0	0	0	0	0	0	0	0	0	0	0	1	1	0	0	0	0
BGIR9823_101563	LOC110258600	lncRNA	0	0	0	0	0	0	0	0	0	0	0	0	0	0	0	1	1	0	0	0	0
BGIR9823_101564	LOC110258600	lncRNA	0	0	0	0	0	0	0	0	0	0	0	0	0	0	0	1	1	0	0	0	0
BGIR9823_101612	LOC100521600	lncRNA	0	0	0	0	0	0	0	0	0	0	0	0	0	0	0	0	0	0	0	0	0
BGIR9823_101853		lncRNA	0	0	0	0	0	0	0	0	0	0	0	0	0	0	0	0	0	0	0	0	0
BGIR9823_101854		lncRNA	0	0	0	0	0	0	0	0	0	0	0	0	0	0	0	0	0	0	0	0	0
BGIR9823_101855		lncRNA	0	0	0	0	0	0	0	0	0	0	0	0	0	0	0	0	0	0	0	0	0
BGIR9823_102146		lncRNA	0	0	0	0	0	0	0	0	0	0	0	0	0	0	0	0	0	0	0	0	0
BGIR9823_102180		lncRNA	0	0	0	0	0	0	0	0	0	0	0	0	0	0	0	0	0	0	0	0	0
BGIR9823_102279		lncRNA	0	0	0	0	0	0	0	0	0	0	0	0	1	0	0	0	0	0	0	0	0
BGIR9823_102282		lncRNA	0	0	0	0	0	0	0	0	0	0	0	0	0	0	0	0	0	0	0	0	0
BGIR9823_102361		lncRNA	0	0	0	0	0	0	0	0	0	0	0	0	0	0	0	0	0	0	0	0	0
BGIR9823_102362		lncRNA	0	0	0	0	0	0	0	0	0	0	0	0	0	0	0	0	0	0	0	0	0
BGIR9823_102363		lncRNA	0	0	0	0	0	0	0	0	0	0	0	0	0	0	0	0	0	0	0	0	0
BGIR9823_102727		lncRNA	0	0	0	0	0	0	0	0	0	0	0	0	0	0	0	0	0	0	0	0	0
BGIR9823_80121	SSSCA1	lncRNA	0	0	0	0	0	0	0	0	0	0	0	0	0	0	0	0	0	0	0	0	0
BGIR9823_80122	LOC102163816	lncRNA	0	0	0	0	0	0	0	0	0	0	0	0	0	0	0	0	0	0	0	0	0
BGIR9823_81007	EHBP1L1	mRNA	0	0	0	0	0	0	0	0	0	0	0	0	0	0	0	0	0	0	0	0	0
BGIR9823_81008	EHBP1L1	mRNA	0	0	0	0	0	0	0	0	0	0	0	0	0	0	0	0	0	0	0	0	0
BGIR9823_81009	EHBP1L1	mRNA	0	0	0	0	0	0	0	0	0	0	0	0	0	0	0	0	0	0	0	0	0
BGIR9823_81010	EHBP1L1	mRNA	0	0	0	0	0	0	0	0	0	0	0	0	0	0	0	0	0	0	0	0	0
BGIR9823_81011	SSSCA1	mRNA	0	0	0	0	0	0	0	0	0	0	0	0	0	0	0	0	0	0	0	0	0
BGIR9823_81012	FRMD8	lncRNA	0	0	0	0	0	0	0	0	0	0	0	0	0	0	0	0	0	0	0	0	0
BGIR9823_81013	LOC102163816	lncRNA	0	0	0	0	0	0	0	0	0	0	0	0	0	0	0	0	0	0	0	0	0
BGIR9823_81014	LOC102163816	lncRNA	0	0	0	0	0	0	0	0	0	0	0	0	0	0	0	0	0	0	0	0	0
BGIR9823_81015	LOC102163816	lncRNA	0	0	0	0	0	0	0	0	0	0	0	0	0	0	0	0	0	0	0	0	0
BGIR9823_81016	LOC102163816	lncRNA	0	0	0	0	0	0	0	0	0	0	0	0	0	0	0	0	0	0	0	0	0
BGIR9823_81017	LOC102163816	lncRNA	0	0	0	0	0	0	0	0	0	0	0	0	0	0	0	0	0	0	0	0	0
BGIR9823_81018	LOC102163816	lncRNA	0	0	0	0	0	0	0	0	0	0	0	0	0	0	0	0	0	0	0	0	0
BGIR9823_81019	LOC100513133	lncRNA	0	0	0	0	0	0	0	0	0	0	0	0	0	0	0	0	0	0	0	0	0
BGIR9823_84704	LOC102159820	mRNA	0	0	0	0	0	0	0	0	0	0	0	0	0	0	0	0	0	0	0	0	0
BGIR9823_84732	RANGAP1	mRNA	0	0	0	0	0	0	0	0	0	1	0	0	0	0	0	0	0	0	0	0	0

RNA ID	Symbol	Type	updown_BGIR9823_88895	updown_BGIR9823_93430	updown_BGIR9823_94434	updown_BGIR9823_94471	updown_XR_002339623.1	updown_XR_002339910.1	updown_XR_002340372.1	updown_XR_002339346.1	updown_XR_002343632.1	updown_XR_002343884.1	updown_BGIR9823_102724	updown_BGIR9823_102212	updown_BGIR9823_102279	updown_BGIR9823_78573	updown_BGIR9823_101405	updown_BGIR9823_101544	updown_BGIR9823_101566	updown_BGIR9823_100548	updown_BGIR9823_100551	updown_BGIR9823_101208	updown_BGIR9823_78436
BGIR9823_84733	RANGAP1	mRNA	0	0	0	0	0	0	0	0	0	1	0	0	0	0	0	0	0	0	0	0	0
BGIR9823_85245	CRADD	lncRNA	0	0	0	0	0	0	0	0	0	0	0	0	0	0	0	0	0	0	0	0	0
BGIR9823_85246	LOC100738154	lncRNA	0	0	0	0	0	0	0	0	0	0	0	0	0	0	0	0	0	0	0	0	0
BGIR9823_85293	TRMU	mRNA	0	0	0	0	0	0	0	0	0	0	0	0	0	0	0	0	0	0	0	0	0
BGIR9823_85294	TRMU	mRNA	0	0	0	0	0	0	0	0	0	0	0	0	0	0	0	0	0	0	0	0	0
BGIR9823_85295	TTC38	mRNA	0	0	0	0	0	0	0	0	0	0	0	0	0	0	0	0	0	0	0	0	0
BGIR9823_85296	PKDREJ	lncRNA	0	0	0	0	0	0	0	0	0	0	0	0	0	0	0	0	0	0	0	0	0
BGIR9823_85297	PPARA	mRNA	0	0	0	0	0	0	0	0	0	0	0	0	0	0	0	0	0	0	0	0	0
BGIR9823_85298	PPARA	mRNA	0	0	0	0	0	0	0	0	0	0	0	0	0	0	0	0	0	0	0	0	0
BGIR9823_85299	PPARA	mRNA	0	0	0	0	0	0	0	0	0	0	0	0	0	0	0	0	0	0	0	0	0
BGIR9823_85300	PPARA	lncRNA	0	0	0	0	0	0	0	0	0	0	0	0	0	0	0	0	0	0	0	0	0
BGIR9823_85301	LOC110260611	mRNA	0	0	0	0	0	0	0	0	0	0	0	0	0	0	0	0	0	0	0	0	0
BGIR9823_85317	ACO2	mRNA	0	0	0	0	0	0	0	0	0	1	0	0	0	0	0	0	0	0	0	0	0
BGIR9823_85318	LOC110260629	lncRNA	0	0	0	0	0	0	0	0	0	1	0	0	0	0	0	0	0	0	0	0	0
BGIR9823_85319	TEF	mRNA	0	0	0	0	0	0	0	0	0	1	0	0	0	0	0	0	0	0	0	0	0
BGIR9823_85320	ZC3H7B	mRNA	0	0	0	0	0	0	0	0	0	1	0	0	0	0	0	0	0	0	0	0	0
BGIR9823_85345	SYNGR1	mRNA	0	0	0	0	0	0	0	0	0	0	0	0	0	0	0	0	0	0	0	0	0
BGIR9823_85835	CRADD	mRNA	0	0	0	0	0	0	0	0	0	0	0	0	0	0	0	0	0	0	0	0	0
BGIR9823_85839	CRADD	lncRNA	0	0	0	0	0	0	0	0	0	0	0	0	0	0	0	0	0	0	0	0	0
BGIR9823_85840	CRADD	lncRNA	0	0	0	0	0	0	0	0	0	0	0	0	0	0	0	0	0	0	0	0	0
BGIR9823_85841	CRADD	lncRNA	0	0	0	0	0	0	0	0	0	0	0	0	0	0	0	0	0	0	0	0	0
BGIR9823_85930	LOC106510465	mRNA	0	0	0	0	0	0	0	0	0	0	0	0	0	0	0	0	0	0	0	0	0
BGIR9823_85931	MAF	lncRNA	0	0	0	0	0	0	0	0	0	0	0	0	0	0	0	0	0	0	0	0	0
BGIR9823_85932	MAF	lncRNA	0	0	0	0	0	0	0	0	0	0	0	0	0	0	0	0	0	0	0	0	0
BGIR9823_87139	LOC106510467	lncRNA	0	0	0	0	0	0	0	0	0	0	0	0	0	0	0	0	0	0	0	0	0
BGIR9823_88187	WRNIP1	mRNA	0	0	0	0	0	0	0	0	0	0	0	0	0	0	0	0	0	0	0	0	0
BGIR9823_88188	NQO2	mRNA	0	0	0	0	0	0	0	0	0	0	0	0	0	0	0	0	0	0	0	0	0
BGIR9823_88189	NQO2	mRNA	0	0	0	0	0	0	0	0	0	0	0	0	0	0	0	0	0	0	0	0	0
BGIR9823_88190	RIPK1	mRNA	0	0	0	0	0	0	0	0	0	0	0	0	0	0	0	0	0	0	0	0	0
BGIR9823_88191	RIPK1	mRNA	0	0	0	0	0	0	0	0	0	0	0	0	0	0	0	0	0	0	0	0	0
BGIR9823_88192	RIPK1	mRNA	0	0	0	0	0	0	0	0	0	0	0	0	0	0	0	0	0	0	0	0	0
BGIR9823_88193	RIPK1	mRNA	0	0	0	0	0	0	0	0	0	0	0	0	0	0	0	0	0	0	0	0	0
BGIR9823_88196	PRPF4B	mRNA	0	0	0	0	0	0	0	0	0	0	0	0	0	0	0	0	0	0	0	0	0
BGIR9823_88197	PRPF4B	mRNA	0	0	0	0	0	0	0	0	0	0	0	0	0	0	0	0	0	0	0	0	0
BGIR9823_88198	PRPF4B	mRNA	0	0	0	0	0	0	0	0	0	0	0	0	0	0	0	0	0	0	0	0	0
BGIR9823_88211	TMEM170B	lncRNA	0	0	0	0	0	0	0	0	0	0	0	0	0	0	0	0	0	0	0	0	0
BGIR9823_88212	TMEM170B	lncRNA	0	0	0	0	0	0	0	0	0	0	0	0	0	0	0	0	0	0	0	0	0
BGIR9823_88213	TMEM170B	lncRNA	0	0	0	0	0	0	0	0	0	0	0	0	0	0	0	0	0	0	0	0	0
BGIR9823_88214	TMEM170B	lncRNA	0	0	0	0	0	0	0	0	0	0	0	0	0	0	0	0	0	0	0	0	0
BGIR9823_88217	PHACTR1	mRNA	0	0	0	0	0	0	0	0	0	0	0	0	0	0	0	0	0	0	0	0	0
BGIR9823_88218	PHACTR1	lncRNA	0	0	0	0	0	0	0	0	0	0	0	0	0	0	0	0	0	0	0	0	0
BGIR9823_89017	MYLK4	lncRNA	0	0	0	0	0	0	0	0	0	0	0	0	0	0	0	0	0	0	0	0	0
BGIR9823_89019	SERPINB1	lncRNA	0	0	0	0	0	0	0	0	0	0	0	0	0	0	0	0	0	0	0	0	0

RNA ID	Symbol	Type	updown_BGIR9823_88895	updown_BGIR9823_93430	updown_BGIR9823_94434	updown_BGIR9823_94471	updown_XR_002339623.1	updown_XR_002339910.1	updown_XR_002340372.1	updown_XR_002339346.1	updown_XR_002343632.1	updown_XR_002343884.1	updown_BGIR9823_102724	updown_BGIR9823_102212	updown_BGIR9823_102279	updown_BGIR9823_78573	updown_BGIR9823_101405	updown_BGIR9823_101544	updown_BGIR9823_101566	updown_BGIR9823_100548	updown_BGIR9823_100551	updown_BGIR9823_101208	updown_BGIR9823_78436
BGIR9823_89020	SERPINB9	mRNA	0	0	0	0	0	0	0	0	0	0	0	0	0	0	0	0	0	0	0	0	0
BGIR9823_89021	RIPK1	lncRNA	0	0	0	0	0	0	0	0	0	0	0	0	0	0	0	0	0	0	0	0	0
BGIR9823_89025	PXDC1	mRNA	0	0	0	0	0	0	0	0	0	0	0	0	0	0	0	0	0	0	0	0	0
BGIR9823_89026	PXDC1	lncRNA	0	0	0	0	0	0	0	0	0	0	0	0	0	0	0	0	0	0	0	0	0
BGIR9823_89027	LOC102161744	lncRNA	0	0	0	0	0	0	0	0	0	0	0	0	0	0	0	0	0	0	0	0	0
BGIR9823_89040	TBC1D7	lncRNA	0	0	0	0	0	0	0	0	0	0	0	0	0	0	0	0	0	0	0	0	0
BGIR9823_89041	TBC1D7	lncRNA	0	0	0	0	0	0	0	0	0	0	0	0	0	0	0	0	0	0	0	0	0
BGIR9823_89042	TBC1D7	lncRNA	0	0	0	0	0	0	0	0	0	0	0	0	0	0	0	0	0	0	0	0	0
BGIR9823_89043	TBC1D7	lncRNA	0	0	0	0	0	0	0	0	0	0	0	0	0	0	0	0	0	0	0	0	0
BGIR9823_89044	GFOD1	lncRNA	0	0	0	0	0	0	0	0	0	0	0	0	0	0	0	0	0	0	0	0	0
BGIR9823_89045	GFOD1	mRNA	0	0	0	0	0	0	0	0	0	0	0	0	0	0	0	0	0	0	0	0	0
BGIR9823_89046	GFOD1	mRNA	0	0	0	0	0	0	0	0	0	0	0	0	0	0	0	0	0	0	0	0	0
BGIR9823_89047	GFOD1	mRNA	0	0	0	0	0	0	0	0	0	0	0	0	0	0	0	0	0	0	0	0	0
BGIR9823_89048	GFOD1	lncRNA	0	0	0	0	0	0	0	0	0	0	0	0	0	0	0	0	0	0	0	0	0
BGIR9823_89049	GFOD1	lncRNA	0	0	0	0	0	0	0	0	0	0	0	0	0	0	0	0	0	0	0	0	0
BGIR9823_90771	NEU3	lncRNA	0	0	0	0	0	0	0	0	0	0	0	0	0	0	0	0	0	0	0	0	0
BGIR9823_90772	SLCO2B1	mRNA	0	0	0	0	0	0	0	0	0	0	0	0	0	0	0	0	0	0	0	0	0
BGIR9823_90913	LOC110255435	lncRNA	0	0	0	0	0	0	0	0	0	0	0	0	0	0	0	0	0	0	0	0	0
BGIR9823_90934	LOC110255441	lncRNA	0	0	0	0	0	0	0	0	0	0	0	0	0	0	0	0	0	0	0	0	0
BGIR9823_90935	CCDC84	mRNA	0	0	0	0	0	0	0	0	0	0	0	0	0	0	0	0	0	0	0	0	0
BGIR9823_90936	TRAPPC4	mRNA	0	0	0	0	0	0	0	0	0	0	0	0	0	0	0	0	0	0	0	0	0
BGIR9823_90937	HMBS	mRNA	0	0	0	0	0	0	0	0	0	0	0	0	0	0	0	0	0	0	0	0	0
BGIR9823_90956	SORL1	mRNA	0	0	0	0	0	0	0	0	0	0	0	0	0	0	0	0	0	0	0	0	0
BGIR9823_90957	SORL1	mRNA	0	0	0	0	0	0	0	0	0	0	0	0	0	0	0	0	0	0	0	0	0
BGIR9823_90958	SORL1	mRNA	0	0	0	0	0	0	0	0	0	0	0	0	0	0	0	0	0	0	0	0	0
BGIR9823_90960	CLMP	lncRNA	0	0	0	0	0	0	0	0	0	0	0	0	0	0	0	0	0	0	0	0	0
BGIR9823_91161	LOC100522267	mRNA	0	0	0	0	0	0	0	0	0	0	0	0	0	0	0	0	0	0	0	0	0
BGIR9823_91162	LOC100522455	mRNA	0	0	0	0	0	0	0	0	0	0	0	0	0	0	0	0	0	0	0	0	0
BGIR9823_91163	ABCB1	mRNA	0	0	0	0	0	0	0	0	0	0	0	0	0	0	0	0	0	0	0	0	0
BGIR9823_91402	HBB	mRNA	0	0	0	0	0	0	0	0	0	0	0	0	1	0	0	0	0	0	0	0	0
BGIR9823_91403	HBB	mRNA	0	0	0	0	0	0	0	0	0	0	0	0	1	0	0	0	0	0	0	0	0
BGIR9823_91404	HBB	mRNA	0	0	0	0	0	0	0	0	0	0	0	0	1	0	0	0	0	0	0	0	0
BGIR9823_91405	HBB	mRNA	0	0	0	0	0	0	0	0	0	0	0	0	1	0	0	0	0	0	0	0	0
BGIR9823_91406	HBB	mRNA	0	0	0	0	0	0	0	0	0	0	0	0	1	0	0	0	0	0	0	0	0
BGIR9823_91407	HBB	mRNA	0	0	0	0	0	0	0	0	0	0	0	0	1	0	0	0	0	0	0	0	0
BGIR9823_91408	HBB	mRNA	0	0	0	0	0	0	0	0	0	0	0	0	1	0	0	0	0	0	0	0	0
BGIR9823_91409	HBB	mRNA	0	0	0	0	0	0	0	0	0	0	0	0	1	0	0	0	0	0	0	0	0
BGIR9823_91625	BCL9L	lncRNA	0	0	0	0	0	0	0	0	0	0	0	0	0	0	0	0	0	0	0	0	0
BGIR9823_91626	BCL9L	lncRNA	0	0	0	0	0	0	0	0	0	0	0	0	0	0	0	0	0	0	0	0	0
BGIR9823_91627	RPS25	mRNA	0	0	0	0	0	0	0	0	0	0	0	0	0	0	0	0	0	0	0	0	0
BGIR9823_91628	RPS25	mRNA	0	0	0	0	0	0	0	0	0	0	0	0	0	0	0	0	0	0	0	0	0
BGIR9823_91629	SLC37A4	mRNA	0	0	0	0	0	0	0	0	0	0	0	0	0	0	0	0	0	0	0	0	0
BGIR9823_91630	HYOU1	mRNA	0	0	0	0	0	0	0	0	0	0	0	0	0	0	0	0	0	0	0	0	0

RNA ID	Symbol	Type	updown_BGIR9823_88895	updown_BGIR9823_93430	updown_BGIR9823_94434	updown_BGIR9823_94471	updown_XR_002339623.1	updown_XR_002339910.1	updown_XR_002340372.1	updown_XR_002339346.1	updown_XR_002343632.1	updown_XR_002343884.1	updown_BGIR9823_102724	updown_BGIR9823_102212	updown_BGIR9823_102279	updown_BGIR9823_78573	updown_BGIR9823_101405	updown_BGIR9823_101544	updown_BGIR9823_101566	updown_BGIR9823_100548	updown_BGIR9823_100551	updown_BGIR9823_101208	updown_BGIR9823_78436
BGIR9823_91640	CLMP	mRNA	0	0	0	0	0	0	0	0	0	0	0	0	0	0	0	0	0	0	0	0	0
BGIR9823_91819	RUNDC3B	lncRNA	0	0	0	0	0	0	0	0	0	0	0	0	0	0	0	0	0	0	0	0	0
BGIR9823_91820	RUNDC3B	lncRNA	0	0	0	0	0	0	0	0	0	0	0	0	0	0	0	0	0	0	0	0	0
BGIR9823_91821	RUNDC3B	lncRNA	0	0	0	0	0	0	0	0	0	0	0	0	0	0	0	0	0	0	0	0	0
BGIR9823_91822	RUNDC3B	lncRNA	0	0	0	0	0	0	0	0	0	0	0	0	0	0	0	0	0	0	0	0	0
BGIR9823_91823	RUNDC3B	lncRNA	0	0	0	0	0	0	0	0	0	0	0	0	0	0	0	0	0	0	0	0	0
BGIR9823_91824	RUNDC3B	lncRNA	0	0	0	0	0	0	0	0	0	0	0	0	0	0	0	0	0	0	0	0	0
BGIR9823_91825	RUNDC3B	lncRNA	0	0	0	0	0	0	0	0	0	0	0	0	0	0	0	0	0	0	0	0	0
BGIR9823_91826	LOC102164596	lncRNA	0	0	0	0	0	0	0	0	0	0	0	0	0	0	0	0	0	0	0	0	0
BGIR9823_92993	KATNAL1	mRNA	0	0	0	0	0	0	0	0	0	0	0	0	0	0	0	0	0	0	0	0	0
BGIR9823_92994	KATNAL1	mRNA	0	0	0	0	0	0	0	0	0	0	0	0	0	0	0	0	0	0	0	0	0
BGIR9823_92995	KATNAL1	mRNA	0	0	0	0	0	0	0	0	0	0	0	0	0	0	0	0	0	0	0	0	0
BGIR9823_92996	KATNAL1	mRNA	0	0	0	0	0	0	0	0	0	0	0	0	0	0	0	0	0	0	0	0	0
BGIR9823_92997	KATNAL1	mRNA	0	0	0	0	0	0	0	0	0	0	0	0	0	0	0	0	0	0	0	0	0
BGIR9823_92999	LOC110255292	lncRNA	0	0	0	0	0	0	0	0	0	0	0	0	0	0	0	0	0	0	0	0	0
BGIR9823_93000	LOC102166871	lncRNA	0	0	0	0	0	0	0	0	0	0	0	0	0	0	0	0	0	0	0	0	0
BGIR9823_98051	LOC102164998	lncRNA	0	0	0	0	0	0	0	0	0	0	0	0	0	0	0	0	0	0	0	0	0
BGIR9823_99244	LOC102167708	lncRNA	0	0	0	0	1	0	0	0	0	0	0	0	0	0	0	0	0	0	0	0	0
NM_001004034.1	HMGB1	mRNA	0	0	0	0	0	0	0	0	0	0	0	0	0	0	0	0	0	0	0	0	0
NM_001044526.1	PPARA	mRNA	0	0	0	0	0	0	0	0	0	0	0	0	0	0	0	0	0	0	0	0	0
NM_001111256.1	SERPINB9	mRNA	0	0	0	0	0	0	0	0	0	0	0	0	0	0	0	0	0	0	0	0	0
NM_001113443.1	ECI2	mRNA	0	0	0	0	0	0	0	0	0	0	0	0	0	0	0	0	0	0	0	0	0
NM_001144841.1	HBB	mRNA	0	0	0	0	0	0	0	0	0	0	0	0	1	0	0	0	0	0	0	0	0
NM_001199719.1	SLC37A4	mRNA	0	0	0	0	0	0	0	0	0	0	0	0	0	0	0	0	0	0	0	0	0
NM_001243907.1	HSPA8	mRNA	0	0	0	0	0	0	0	0	0	0	0	0	0	0	0	0	0	0	0	0	0
NM_001244063.1	RPL3	mRNA	0	0	0	0	0	0	0	0	0	0	0	0	0	0	0	0	0	0	0	0	0
NM_001244066.1	SYNGR1	mRNA	0	0	0	0	0	0	0	0	0	0	0	0	0	0	0	0	0	0	0	0	0
NM_001244396.1	CCDC84	mRNA	0	0	0	0	0	0	0	0	0	0	0	0	0	0	0	0	0	0	0	0	0
NM_001287846.1	RHEB	mRNA	0	0	0	0	0	0	0	0	0	0	0	0	0	0	0	0	0	0	0	0	0
NM_213954.1	ACO2	mRNA	0	0	0	0	0	0	0	0	0	1	0	0	0	0	0	0	0	0	0	0	0
NM_214012.1	UPK2	mRNA	0	0	0	0	0	0	0	0	0	0	0	0	0	0	0	0	0	0	0	0	0
URS0000BC92F4	LOC102167708	lncRNA	0	0	0	0	1	0	0	0	0	0	0	0	0	0	0	0	0	0	0	0	0
XM_001928309.6	TBC1D7	mRNA	0	0	0	0	0	0	0	0	0	0	0	0	0	0	0	0	0	0	0	0	0
XM_001928852.5	PXDC1	mRNA	0	0	0	0	0	0	0	0	0	0	0	0	0	0	0	0	0	0	0	0	0
XM_003122534.4	KCNK7	mRNA	0	0	0	0	0	0	0	0	0	0	0	0	0	0	0	0	0	0	0	0	0
XM_003122558.5	SCYL1	mRNA	0	0	0	0	0	0	0	0	0	0	0	0	0	0	0	0	0	0	0	0	0
XM_003122562.5	FRMD8	mRNA	0	0	0	0	0	0	0	0	0	0	0	0	0	0	0	0	0	0	0	0	0
XM_003125951.6	TTC38	mRNA	0	0	0	0	0	0	0	0	0	0	0	0	0	0	0	0	0	0	0	0	0
XM_003125953.4	CDPF1	mRNA	0	0	0	0	0	0	0	0	0	0	0	0	0	0	0	0	0	0	0	0	0
XM_003126012.6	CBX7	mRNA	0	0	0	0	0	0	0	0	0	0	0	0	0	0	0	0	0	0	0	0	0
XM_003128161.4	RIPK1	mRNA	0	0	0	0	0	0	0	0	0	0	0	0	0	0	0	0	0	0	0	0	0
XM_003129508.2	LOC100514417	mRNA	0	0	0	0	0	0	0	0	0	0	0	0	1	0	0	0	0	0	0	0	0
XM_003129512.2	LOC100515268	mRNA	0	0	0	0	0	0	0	0	0	0	0	0	1	0	0	0	0	0	0	0	0

RNA ID	Symbol	Type	updown_BGIR9823_88895	updown_BGIR9823_93430	updown_BGIR9823_94434	updown_BGIR9823_94471	updown_XR_002339623.1	updown_XR_002339910.1	updown_XR_002340372.1	updown_XR_002339346.1	updown_XR_002343632.1	updown_XR_002343884.1	updown_BGIR9823_102724	updown_BGIR9823_102212	updown_BGIR9823_102279	updown_BGIR9823_78573	updown_BGIR9823_101405	updown_BGIR9823_101544	updown_BGIR9823_101566	updown_BGIR9823_100548	updown_BGIR9823_100551	updown_BGIR9823_101208	updown_BGIR9823_78436
XM_003129514.3	LOC100515618	mRNA	0	0	0	0	0	0	0	0	0	0	0	0	1	0	0	0	0	0	0	0	0
XM_003129519.2	LOC100517595	mRNA	0	0	0	0	0	0	0	0	0	0	0	0	1	0	0	0	0	0	0	0	0
XM_003129529.4	LOC100519910	mRNA	0	0	0	0	0	0	0	0	0	0	0	0	1	0	0	0	0	0	0	0	0
XM_003129915.4	CXCR5	mRNA	0	0	0	0	0	0	0	0	0	0	0	0	0	0	0	0	0	0	0	0	0
XM_003129916.5	BCL9L	mRNA	0	0	0	0	0	0	0	0	0	0	0	0	0	0	0	0	0	0	0	0	0
XM_003129918.4	TRAPPC4	mRNA	0	0	0	0	0	0	0	0	0	0	0	0	0	0	0	0	0	0	0	0	0
XM_003129919.5	RPS25	mRNA	0	0	0	0	0	0	0	0	0	0	0	0	0	0	0	0	0	0	0	0	0
XM_003129920.3	RPS25	mRNA	0	0	0	0	0	0	0	0	0	0	0	0	0	0	0	0	0	0	0	0	0
XM_003129922.4	HYOU1	mRNA	0	0	0	0	0	0	0	0	0	0	0	0	0	0	0	0	0	0	0	0	0
XM_003129923.6	VPS11	mRNA	0	0	0	0	0	0	0	0	0	0	0	0	0	0	0	0	0	0	0	0	0
XM_003129944.4	SC5D	mRNA	0	0	0	0	0	0	0	0	0	0	0	0	0	0	0	0	0	0	0	0	0
XM_003357161.1	LOC100620971	mRNA	0	0	0	0	0	0	0	0	0	0	0	0	1	0	0	0	0	0	0	0	0
XM_003482119.4	SERPINB1	mRNA	0	0	0	0	0	0	0	0	0	0	0	0	0	0	0	0	0	0	0	0	0
XM_003482132.4	TMEM170B	mRNA	0	0	0	0	0	0	0	0	0	0	0	0	0	0	0	0	0	0	0	0	0
XM_003482133.4	GFOD1	mRNA	0	0	0	0	0	0	0	0	0	0	0	0	0	0	0	0	0	0	0	0	0
XM_003482563.4	SPCS2	mRNA	0	0	0	0	0	0	0	0	0	0	0	0	0	0	0	0	0	0	0	0	0
XM_003482565.4	NEU3	mRNA	0	0	0	0	0	0	0	0	0	0	0	0	0	0	0	0	0	0	0	0	0
XM_003482567.1	LOC100739359	mRNA	0	0	0	0	0	0	0	0	0	0	0	0	0	0	0	0	0	0	0	0	0
XM_003482630.3	BSX	mRNA	0	0	0	0	0	0	0	0	0	0	0	0	0	0	0	0	0	0	0	0	0
XM_005653107.3	CHADL	mRNA	0	0	0	0	0	0	0	0	0	1	0	0	0	0	0	0	0	0	0	0	0
XM_005653640.1	LOC100621181	mRNA	0	0	0	0	0	0	0	0	0	0	0	0	1	0	0	0	0	0	0	0	0
XM_005654266.3	PRKAG2	mRNA	0	0	0	0	0	0	0	0	0	0	0	0	0	0	0	0	0	0	0	0	0
XM_005655470.3	CDPF1	mRNA	0	0	0	0	0	0	0	0	0	0	0	0	0	0	0	0	0	0	0	0	0
XM_005660703.3	LTBP3	mRNA	0	0	0	0	0	0	0	0	0	0	0	0	0	0	0	0	0	0	0	0	0
XM_005660705.3	SCYL1	mRNA	0	0	0	0	0	0	0	0	0	0	0	0	0	0	0	0	0	0	0	0	0
XM_005660707.3	SLC25A45	mRNA	0	0	0	0	0	0	0	0	0	0	0	0	0	0	0	0	0	0	0	0	0
XM_005665536.3	RIPK1	mRNA	0	0	0	0	0	0	0	0	0	0	0	0	0	0	0	0	0	0	0	0	0
XM_005665537.3	RIPK1	mRNA	0	0	0	0	0	0	0	0	0	0	0	0	0	0	0	0	0	0	0	0	0
XM_005665538.3	RIPK1	mRNA	0	0	0	0	0	0	0	0	0	0	0	0	0	0	0	0	0	0	0	0	0
XM_005665575.3	PHACTR1	mRNA	0	0	0	0	0	0	0	0	0	0	0	0	0	0	0	0	0	0	0	0	0
XM_005665576.3	TBC1D7	mRNA	0	0	0	0	0	0	0	0	0	0	0	0	0	0	0	0	0	0	0	0	0
XM_005667128.3	LOC100627340	mRNA	0	0	0	0	0	0	0	0	0	0	0	0	0	0	0	0	0	0	0	0	0
XM_005667397.2	CCDC84	mRNA	0	0	0	0	0	0	0	0	0	0	0	0	0	0	0	0	0	0	0	0	0
XM_005667398.3	SLC37A4	mRNA	0	0	0	0	0	0	0	0	0	0	0	0	0	0	0	0	0	0	0	0	0
XM_005667402.3	SLC37A4	mRNA	0	0	0	0	0	0	0	0	0	0	0	0	0	0	0	0	0	0	0	0	0
XM_005667403.3	SLC37A4	mRNA	0	0	0	0	0	0	0	0	0	0	0	0	0	0	0	0	0	0	0	0	0
XM_005667413.3	BCL9L	mRNA	0	0	0	0	0	0	0	0	0	0	0	0	0	0	0	0	0	0	0	0	0
XM_005667414.3	FOXR1	mRNA	0	0	0	0	0	0	0	0	0	0	0	0	0	0	0	0	0	0	0	0	0
XM_005667416.3	HYOU1	mRNA	0	0	0	0	0	0	0	0	0	0	0	0	0	0	0	0	0	0	0	0	0
XM_005667419.3	HYOU1	mRNA	0	0	0	0	0	0	0	0	0	0	0	0	0	0	0	0	0	0	0	0	0
XM_005667420.3	HYOU1	mRNA	0	0	0	0	0	0	0	0	0	0	0	0	0	0	0	0	0	0	0	0	0
XM_005667440.2	JHY	mRNA	0	0	0	0	0	0	0	0	0	0	0	0	0	0	0	0	0	0	0	0	0
XM_005668316.3	HMGB1	mRNA	0	0	0	0	0	0	0	0	0	0	0	0	0	0	0	0	0	0	0	0	0

RNA ID	Symbol	Type	updown_BGIR9823_88895	updown_BGIR9823_93430	updown_BGIR9823_94434	updown_BGIR9823_94471	updown_XR_002339623.1	updown_XR_002339910.1	updown_XR_002340372.1	updown_XR_002339346.1	updown_XR_002343632.1	updown_XR_002343884.1	updown_BGIR9823_102724	updown_BGIR9823_102212	updown_BGIR9823_102279	updown_BGIR9823_78573	updown_BGIR9823_101405	updown_BGIR9823_101544	updown_BGIR9823_101566	updown_BGIR9823_100548	updown_BGIR9823_100551	updown_BGIR9823_101208	updown_BGIR9823_78436
XM_005668317.3	HMGB1	mRNA	0	0	0	0	0	0	0	0	0	0	0	0	0	0	0	0	0	0	0	0	0
XM_005668318.3	HMGB1	mRNA	0	0	0	0	0	0	0	0	0	0	0	0	0	0	0	0	0	0	0	0	0
XM_005671489.3	EMX2	mRNA	0	0	0	0	0	0	0	0	0	0	0	0	0	0	0	0	0	0	0	0	0
XM_005672397.3	MYO10	mRNA	0	0	0	0	1	0	0	0	0	0	0	0	0	0	0	0	0	0	0	0	0
XM_013977400.2	FAM50B	mRNA	0	0	0	0	0	0	0	0	0	0	0	0	0	0	0	0	0	0	0	0	0
XM_013977425.2	NEDD9	mRNA	0	0	0	0	0	0	0	0	0	0	0	0	0	0	0	0	0	0	0	0	0
XM_013977430.2	TBC1D7	mRNA	0	0	0	0	0	0	0	0	0	0	0	0	0	0	0	0	0	0	0	0	0
XM_013977431.2	TBC1D7	mRNA	0	0	0	0	0	0	0	0	0	0	0	0	0	0	0	0	0	0	0	0	0
XM_013977432.2	TBC1D7	mRNA	0	0	0	0	0	0	0	0	0	0	0	0	0	0	0	0	0	0	0	0	0
XM_013977433.2	GFOD1	mRNA	0	0	0	0	0	0	0	0	0	0	0	0	0	0	0	0	0	0	0	0	0
XM_013977434.2	GFOD1	mRNA	0	0	0	0	0	0	0	0	0	0	0	0	0	0	0	0	0	0	0	0	0
XM_013979449.2	SLC37A4	mRNA	0	0	0	0	0	0	0	0	0	0	0	0	0	0	0	0	0	0	0	0	0
XM_013979456.2	BCL9L	mRNA	0	0	0	0	0	0	0	0	0	0	0	0	0	0	0	0	0	0	0	0	0
XM_013979457.2	BCL9L	mRNA	0	0	0	0	0	0	0	0	0	0	0	0	0	0	0	0	0	0	0	0	0
XM_013979458.2	BCL9L	mRNA	0	0	0	0	0	0	0	0	0	0	0	0	0	0	0	0	0	0	0	0	0
XM_013979459.2	BCL9L	mRNA	0	0	0	0	0	0	0	0	0	0	0	0	0	0	0	0	0	0	0	0	0
XM_013979461.2	TRAPPC4	mRNA	0	0	0	0	0	0	0	0	0	0	0	0	0	0	0	0	0	0	0	0	0
XM_013979463.2	HYOU1	mRNA	0	0	0	0	0	0	0	0	0	0	0	0	0	0	0	0	0	0	0	0	0
XM_013979484.2	LOC100523684	mRNA	0	0	0	0	0	0	0	0	0	0	0	0	0	0	0	0	0	0	0	0	0
XM_013979486.2	LOC100523684	mRNA	0	0	0	0	0	0	0	0	0	0	0	0	0	0	0	0	0	0	0	0	0
XM_013979491.2	JHY	mRNA	0	0	0	0	0	0	0	0	0	0	0	0	0	0	0	0	0	0	0	0	0
XM_013980418.2	KATNAL1	mRNA	0	0	0	0	0	0	0	0	0	0	0	0	0	0	0	0	0	0	0	0	0
XM_013988182.2	TTC38	mRNA	0	0	0	0	0	0	0	0	0	0	0	0	0	0	0	0	0	0	0	0	0
XM_013988183.2	TTC38	mRNA	0	0	0	0	0	0	0	0	0	0	0	0	0	0	0	0	0	0	0	0	0
XM_013989589.2	LOC100522267	mRNA	0	0	0	0	0	0	0	0	0	0	0	0	0	0	0	0	0	0	0	0	0
XM_013989592.2	LOC100522455	mRNA	0	0	0	0	0	0	0	0	0	0	0	0	0	0	0	0	0	0	0	0	0
XM_013989593.2	LOC100522455	mRNA	0	0	0	0	0	0	0	0	0	0	0	0	0	0	0	0	0	0	0	0	0
XM_013994099.2	FRMD8	mRNA	0	0	0	0	0	0	0	0	0	0	0	0	0	0	0	0	0	0	0	0	0
XM_013994100.2	SLC25A45	mRNA	0	0	0	0	0	0	0	0	0	0	0	0	0	0	0	0	0	0	0	0	0
XM_013994102.2	SLC25A45	mRNA	0	0	0	0	0	0	0	0	0	0	0	0	0	0	0	0	0	0	0	0	0
XM_013994103.2	SLC25A45	mRNA	0	0	0	0	0	0	0	0	0	0	0	0	0	0	0	0	0	0	0	0	0
XM_013994104.2	SLC25A45	mRNA	0	0	0	0	0	0	0	0	0	0	0	0	0	0	0	0	0	0	0	0	0
XM_013997510.2	CBX7	mRNA	0	0	0	0	0	0	0	0	0	0	0	0	0	0	0	0	0	0	0	0	0
XM_013999070.2	LOC100520088	mRNA	0	0	0	0	0	0	0	0	0	0	0	0	1	0	0	0	0	0	0	0	0
XM_021062230.1	LOC100517771	mRNA	0	0	0	0	0	0	0	0	0	0	0	0	1	0	0	0	0	0	0	0	0
XM_021062231.1	LOC100517957	mRNA	0	0	0	0	0	0	0	0	0	0	0	0	1	0	0	0	0	0	0	0	0
XM_021062367.1	LOC110255365	mRNA	0	0	0	0	0	0	0	0	0	0	0	0	1	0	0	0	0	0	0	0	0
XM_021062369.1	LOC100737110	mRNA	0	0	0	0	0	0	0	0	0	0	0	0	1	0	0	0	0	0	0	0	0
XM_021062370.1	LOC110255368	mRNA	0	0	0	0	0	0	0	0	0	0	0	0	1	0	0	0	0	0	0	0	0
XM_021062371.1	LOC100515441	mRNA	0	0	0	0	0	0	0	0	0	0	0	0	1	0	0	0	0	0	0	0	0
XM_021062374.1	LOC110255371	mRNA	0	0	0	0	0	0	0	0	0	0	0	0	1	0	0	0	0	0	0	0	0
XM_021062375.1	LOC100626951	mRNA	0	0	0	0	0	0	0	0	0	0	0	0	1	0	0	0	0	0	0	0	0
XM_021062491.1	XRRA1	mRNA	0	0	0	0	0	0	0	0	0	0	0	0	0	0	0	0	0	0	0	0	0

RNA ID	Symbol	Type	updown_BGIR9823_88895	updown_BGIR9823_93430	updown_BGIR9823_94434	updown_BGIR9823_94471	updown_XR_002339623.1	updown_XR_002339910.1	updown_XR_002340372.1	updown_XR_002339346.1	updown_XR_002343632.1	updown_XR_002343884.1	updown_BGIR9823_102724	updown_BGIR9823_102212	updown_BGIR9823_102279	updown_BGIR9823_78573	updown_BGIR9823_101405	updown_BGIR9823_101544	updown_BGIR9823_101566	updown_BGIR9823_100548	updown_BGIR9823_100551	updown_BGIR9823_101208	updown_BGIR9823_78436
XM_021062493.1	XRRA1	mRNA	0	0	0	0	0	0	0	0	0	0	0	0	0	0	0	0	0	0	0	0	0
XM_021062494.1	XRRA1	mRNA	0	0	0	0	0	0	0	0	0	0	0	0	0	0	0	0	0	0	0	0	0
XM_021062495.1	XRRA1	mRNA	0	0	0	0	0	0	0	0	0	0	0	0	0	0	0	0	0	0	0	0	0
XM_021062496.1	XRRA1	mRNA	0	0	0	0	0	0	0	0	0	0	0	0	0	0	0	0	0	0	0	0	0
XM_021062497.1	XRRA1	mRNA	0	0	0	0	0	0	0	0	0	0	0	0	0	0	0	0	0	0	0	0	0
XM_021062498.1	XRRA1	mRNA	0	0	0	0	0	0	0	0	0	0	0	0	0	0	0	0	0	0	0	0	0
XM_021062499.1	XRRA1	mRNA	0	0	0	0	0	0	0	0	0	0	0	0	0	0	0	0	0	0	0	0	0
XM_021062500.1	XRRA1	mRNA	0	0	0	0	0	0	0	0	0	0	0	0	0	0	0	0	0	0	0	0	0
XM_021062501.1	XRRA1	mRNA	0	0	0	0	0	0	0	0	0	0	0	0	0	0	0	0	0	0	0	0	0
XM_021062502.1	XRRA1	mRNA	0	0	0	0	0	0	0	0	0	0	0	0	0	0	0	0	0	0	0	0	0
XM_021062503.1	XRRA1	mRNA	0	0	0	0	0	0	0	0	0	0	0	0	0	0	0	0	0	0	0	0	0
XM_021062511.1	SLCO2B1	mRNA	0	0	0	0	0	0	0	0	0	0	0	0	0	0	0	0	0	0	0	0	0
XM_021062948.1	BCL9L	mRNA	0	0	0	0	0	0	0	0	0	0	0	0	0	0	0	0	0	0	0	0	0
XM_021062949.1	TRAPPC4	mRNA	0	0	0	0	0	0	0	0	0	0	0	0	0	0	0	0	0	0	0	0	0
XM_021062950.1	HYOU1	mRNA	0	0	0	0	0	0	0	0	0	0	0	0	0	0	0	0	0	0	0	0	0
XM_021062951.1	HYOU1	mRNA	0	0	0	0	0	0	0	0	0	0	0	0	0	0	0	0	0	0	0	0	0
XM_021062952.1	HYOU1	mRNA	0	0	0	0	0	0	0	0	0	0	0	0	0	0	0	0	0	0	0	0	0
XM_021062953.1	HYOU1	mRNA	0	0	0	0	0	0	0	0	0	0	0	0	0	0	0	0	0	0	0	0	0
XM_021062954.1	HYOU1	mRNA	0	0	0	0	0	0	0	0	0	0	0	0	0	0	0	0	0	0	0	0	0
XM_021062955.1	HYOU1	mRNA	0	0	0	0	0	0	0	0	0	0	0	0	0	0	0	0	0	0	0	0	0
XM_021062956.1	HYOU1	mRNA	0	0	0	0	0	0	0	0	0	0	0	0	0	0	0	0	0	0	0	0	0
XM_021062957.1	VPS11	mRNA	0	0	0	0	0	0	0	0	0	0	0	0	0	0	0	0	0	0	0	0	0
XM_021062993.1	LOC100523684	mRNA	0	0	0	0	0	0	0	0	0	0	0	0	0	0	0	0	0	0	0	0	0
XM_021062994.1	LOC100523684	mRNA	0	0	0	0	0	0	0	0	0	0	0	0	0	0	0	0	0	0	0	0	0
XM_021062995.1	LOC100523684	mRNA	0	0	0	0	0	0	0	0	0	0	0	0	0	0	0	0	0	0	0	0	0
XM_021062996.1	LOC100523684	mRNA	0	0	0	0	0	0	0	0	0	0	0	0	0	0	0	0	0	0	0	0	0
XM_021062997.1	LOC100523684	mRNA	0	0	0	0	0	0	0	0	0	0	0	0	0	0	0	0	0	0	0	0	0
XM_021062998.1	LOC100523684	mRNA	0	0	0	0	0	0	0	0	0	0	0	0	0	0	0	0	0	0	0	0	0
XM_021062999.1	LOC100523684	mRNA	0	0	0	0	0	0	0	0	0	0	0	0	0	0	0	0	0	0	0	0	0
XM_021063000.1	LOC100523684	mRNA	0	0	0	0	0	0	0	0	0	0	0	0	0	0	0	0	0	0	0	0	0
XM_021063001.1	LOC100523684	mRNA	0	0	0	0	0	0	0	0	0	0	0	0	0	0	0	0	0	0	0	0	0
XM_021063002.1	LOC100523684	mRNA	0	0	0	0	0	0	0	0	0	0	0	0	0	0	0	0	0	0	0	0	0
XM_021063004.1	LOC100523684	mRNA	0	0	0	0	0	0	0	0	0	0	0	0	0	0	0	0	0	0	0	0	0
XM_021063005.1	LOC100523684	mRNA	0	0	0	0	0	0	0	0	0	0	0	0	0	0	0	0	0	0	0	0	0
XM_021063006.1	LOC100523684	mRNA	0	0	0	0	0	0	0	0	0	0	0	0	0	0	0	0	0	0	0	0	0
XM_021063009.1	SORL1	mRNA	0	0	0	0	0	0	0	0	0	0	0	0	0	0	0	0	0	0	0	0	0
XM_021063010.1	SORL1	mRNA	0	0	0	0	0	0	0	0	0	0	0	0	0	0	0	0	0	0	0	0	0
XM_021063011.1	SORL1	mRNA	0	0	0	0	0	0	0	0	0	0	0	0	0	0	0	0	0	0	0	0	0
XM_021063012.1	SORL1	mRNA	0	0	0	0	0	0	0	0	0	0	0	0	0	0	0	0	0	0	0	0	0
XM_021063013.1	SORL1	mRNA	0	0	0	0	0	0	0	0	0	0	0	0	0	0	0	0	0	0	0	0	0
XM_021063014.1	SORL1	mRNA	0	0	0	0	0	0	0	0	0	0	0	0	0	0	0	0	0	0	0	0	0
XM_021063023.1	CLMP	mRNA	0	0	0	0	0	0	0	0	0	0	0	0	0	0	0	0	0	0	0	0	0
XM_021063024.1	CLMP	mRNA	0	0	0	0	0	0	0	0	0	0	0	0	0	0	0	0	0	0	0	0	0

RNA ID	Symbol	Type	updown_BGIR9823_88895	updown_BGIR9823_93430	updown_BGIR9823_94434	updown_BGIR9823_94471	updown_XR_002339623.1	updown_XR_002339910.1	updown_XR_002340372.1	updown_XR_002339346.1	updown_XR_002343632.1	updown_XR_002343884.1	updown_BGIR9823_102724	updown_BGIR9823_102212	updown_BGIR9823_102279	updown_BGIR9823_78573	updown_BGIR9823_101405	updown_BGIR9823_101544	updown_BGIR9823_101566	updown_BGIR9823_100548	updown_BGIR9823_100551	updown_BGIR9823_101208	updown_BGIR9823_78436
XM_021063025.1	CLMP	mRNA	0	0	0	0	0	0	0	0	0	0	0	0	0	0	0	0	0	0	0	0	0
XM_021063026.1	CLMP	mRNA	0	0	0	0	0	0	0	0	0	0	0	0	0	0	0	0	0	0	0	0	0
XM_021063950.1	NXPE2	mRNA	0	0	0	0	0	0	0	0	0	0	0	0	0	0	0	0	0	0	0	0	0
XM_021063963.1	LOC100623190	mRNA	0	0	0	0	0	0	0	0	0	0	0	0	0	0	0	0	0	0	0	0	0
XM_021065159.1	HMGB1	mRNA	0	0	0	0	0	0	0	0	0	0	0	0	0	0	0	0	0	0	0	0	0
XM_021065160.1	HMGB1	mRNA	0	0	0	0	0	0	0	0	0	0	0	0	0	0	0	0	0	0	0	0	0
XM_021065161.1	HMGB1	mRNA	0	0	0	0	0	0	0	0	0	0	0	0	0	0	0	0	0	0	0	0	0
XM_021065529.1	KATNAL1	mRNA	0	0	0	0	0	0	0	0	0	0	0	0	0	0	0	0	0	0	0	0	0
XM_021065531.1	KATNAL1	mRNA	0	0	0	0	0	0	0	0	0	0	0	0	0	0	0	0	0	0	0	0	0
XM_021065532.1	KATNAL1	mRNA	0	0	0	0	0	0	0	0	0	0	0	0	0	0	0	0	0	0	0	0	0
XM_021065533.1	KATNAL1	mRNA	0	0	0	0	0	0	0	0	0	0	0	0	0	0	0	0	0	0	0	0	0
XM_021065534.1	KATNAL1	mRNA	0	0	0	0	0	0	0	0	0	0	0	0	0	0	0	0	0	0	0	0	0
XM_021065535.1	KATNAL1	mRNA	0	0	0	0	0	0	0	0	0	0	0	0	0	0	0	0	0	0	0	0	0
XM_021065536.1	KATNAL1	mRNA	0	0	0	0	0	0	0	0	0	0	0	0	0	0	0	0	0	0	0	0	0
XM_021065537.1	KATNAL1	mRNA	0	0	0	0	0	0	0	0	0	0	0	0	0	0	0	0	0	0	0	0	0
XM_021065538.1	KATNAL1	mRNA	0	0	0	0	0	0	0	0	0	0	0	0	0	0	0	0	0	0	0	0	0
XM_021065539.1	KATNAL1	mRNA	0	0	0	0	0	0	0	0	0	0	0	0	0	0	0	0	0	0	0	0	0
XM_021065541.1	KATNAL1	mRNA	0	0	0	0	0	0	0	0	0	0	0	0	0	0	0	0	0	0	0	0	0
XM_021065542.1	KATNAL1	mRNA	0	0	0	0	0	0	0	0	0	0	0	0	0	0	0	0	0	0	0	0	0
XM_021065543.1	KATNAL1	mRNA	0	0	0	0	0	0	0	0	0	0	0	0	0	0	0	0	0	0	0	0	0
XM_021065544.1	KATNAL1	mRNA	0	0	0	0	0	0	0	0	0	0	0	0	0	0	0	0	0	0	0	0	0
XM_021073408.1	EMX2	mRNA	0	0	0	0	0	0	0	0	0	0	0	0	0	0	0	0	0	0	0	0	0
XM_021078611.1	PRKAG2	mRNA	0	0	0	0	0	0	0	0	0	0	0	0	0	0	0	0	0	0	0	0	0
XM_021078612.1	PRKAG2	mRNA	0	0	0	0	0	0	0	0	0	0	0	0	0	0	0	0	0	0	0	0	0
XM_021078613.1	PRKAG2	mRNA	0	0	0	0	0	0	0	0	0	0	0	0	0	0	0	0	0	0	0	0	0
XM_021078614.1	PRKAG2	mRNA	0	0	0	0	0	0	0	0	0	0	0	0	0	0	0	0	0	0	0	0	0
XM_021078615.1	PRKAG2	mRNA	0	0	0	0	0	0	0	0	0	0	0	0	0	0	0	0	0	0	0	0	0
XM_021078616.1	PRKAG2	mRNA	0	0	0	0	0	0	0	0	0	0	0	0	0	0	0	0	0	0	0	0	0
XM_021078617.1	PRKAG2	mRNA	0	0	0	0	0	0	0	0	0	0	0	0	0	0	0	0	0	0	0	0	0
XM_021078618.1	PRKAG2	mRNA	0	0	0	0	0	0	0	0	0	0	0	0	0	0	0	0	0	0	0	0	0
XM_021078619.1	PRKAG2	mRNA	0	0	0	0	0	0	0	0	0	0	0	0	0	0	0	0	0	0	0	0	0
XM_021078620.1	PRKAG2	mRNA	0	0	0	0	0	0	0	0	0	0	0	0	0	0	0	0	0	0	0	0	0
XM_021078621.1	PRKAG2	mRNA	0	0	0	0	0	0	0	0	0	0	0	0	0	0	0	0	0	0	0	0	0
XM_021081309.1	LOC106506226	mRNA	0	0	0	0	0	0	0	0	0	0	0	0	0	0	0	0	0	0	0	0	0
XM_021081403.1	LOC110258215	mRNA	0	0	0	0	0	0	0	0	0	0	0	0	0	0	1	0	0	0	0	0	0
XM_021082153.1	LOC100521600	mRNA	0	0	0	0	0	0	0	0	0	0	0	0	0	0	0	0	0	0	0	0	0
XM_021082154.1	LOC100521600	mRNA	0	0	0	0	0	0	0	0	0	0	0	0	0	0	0	0	0	0	0	0	0
XM_021082156.1	LOC100521600	mRNA	0	0	0	0	0	0	0	0	0	0	0	0	0	0	0	0	0	0	0	0	0
XM_021082157.1	LOC100521600	mRNA	0	0	0	0	0	0	0	0	0	0	0	0	0	0	0	0	0	0	0	0	0
XM_021082158.1	LOC100521600	mRNA	0	0	0	0	0	0	0	0	0	0	0	0	0	0	0	0	0	0	0	0	0
XM_021082159.1	LOC100521600	mRNA	0	0	0	0	0	0	0	0	0	0	0	0	0	0	0	0	0	0	0	0	0
XM_021082835.1	EHBP1L1	mRNA	0	0	0	0	0	0	0	0	0	0	0	0	0	0	0	0	0	0	0	0	0
XM_021082836.1	EHBP1L1	mRNA	0	0	0	0	0	0	0	0	0	0	0	0	0	0	0	0	0	0	0	0	0

RNA ID	Symbol	Type	updown_BGIR9823_88895	updown_BGIR9823_93430	updown_BGIR9823_94434	updown_BGIR9823_94471	updown_XR_002339623.1	updown_XR_002339910.1	updown_XR_002340372.1	updown_XR_002339346.1	updown_XR_002343632.1	updown_XR_002343884.1	updown_BGIR9823_102724	updown_BGIR9823_102212	updown_BGIR9823_102279	updown_BGIR9823_78573	updown_BGIR9823_101405	updown_BGIR9823_101544	updown_BGIR9823_101566	updown_BGIR9823_100548	updown_BGIR9823_100551	updown_BGIR9823_101208	updown_BGIR9823_78436
XM_021082837.1	EHBP1L1	mRNA	0	0	0	0	0	0	0	0	0	0	0	0	0	0	0	0	0	0	0	0	0
XM_021082838.1	EHBP1L1	mRNA	0	0	0	0	0	0	0	0	0	0	0	0	0	0	0	0	0	0	0	0	0
XM_021082839.1	EHBP1L1	mRNA	0	0	0	0	0	0	0	0	0	0	0	0	0	0	0	0	0	0	0	0	0
XM_021082840.1	EHBP1L1	mRNA	0	0	0	0	0	0	0	0	0	0	0	0	0	0	0	0	0	0	0	0	0
XM_021082841.1	EHBP1L1	mRNA	0	0	0	0	0	0	0	0	0	0	0	0	0	0	0	0	0	0	0	0	0
XM_021082842.1	EHBP1L1	mRNA	0	0	0	0	0	0	0	0	0	0	0	0	0	0	0	0	0	0	0	0	0
XM_021082843.1	EHBP1L1	mRNA	0	0	0	0	0	0	0	0	0	0	0	0	0	0	0	0	0	0	0	0	0
XM_021082844.1	EHBP1L1	mRNA	0	0	0	0	0	0	0	0	0	0	0	0	0	0	0	0	0	0	0	0	0
XM_021082845.1	EHBP1L1	mRNA	0	0	0	0	0	0	0	0	0	0	0	0	0	0	0	0	0	0	0	0	0
XM_021082846.1	EHBP1L1	mRNA	0	0	0	0	0	0	0	0	0	0	0	0	0	0	0	0	0	0	0	0	0
XM_021082847.1	EHBP1L1	mRNA	0	0	0	0	0	0	0	0	0	0	0	0	0	0	0	0	0	0	0	0	0
XM_021082851.1	MAP3K11	mRNA	0	0	0	0	0	0	0	0	0	0	0	0	0	0	0	0	0	0	0	0	0
XM_021082852.1	SSSCA1	mRNA	0	0	0	0	0	0	0	0	0	0	0	0	0	0	0	0	0	0	0	0	0
XM_021082853.1	FAM89B	mRNA	0	0	0	0	0	0	0	0	0	0	0	0	0	0	0	0	0	0	0	0	0
XM_021082854.1	LTBP3	mRNA	0	0	0	0	0	0	0	0	0	0	0	0	0	0	0	0	0	0	0	0	0
XM_021082855.1	LTBP3	mRNA	0	0	0	0	0	0	0	0	0	0	0	0	0	0	0	0	0	0	0	0	0
XM_021082856.1	SCYL1	mRNA	0	0	0	0	0	0	0	0	0	0	0	0	0	0	0	0	0	0	0	0	0
XM_021082857.1	SLC25A45	mRNA	0	0	0	0	0	0	0	0	0	0	0	0	0	0	0	0	0	0	0	0	0
XM_021082858.1	SLC25A45	mRNA	0	0	0	0	0	0	0	0	0	0	0	0	0	0	0	0	0	0	0	0	0
XM_021091099.1	SYNGR1	mRNA	0	0	0	0	0	0	0	0	0	0	0	0	0	0	0	0	0	0	0	0	0
XM_021091101.1	SYNGR1	mRNA	0	0	0	0	0	0	0	0	0	0	0	0	0	0	0	0	0	0	0	0	0
XM_021091102.1	SYNGR1	mRNA	0	0	0	0	0	0	0	0	0	0	0	0	0	0	0	0	0	0	0	0	0
XM_021091103.1	SYNGR1	mRNA	0	0	0	0	0	0	0	0	0	0	0	0	0	0	0	0	0	0	0	0	0
XM_021091370.1	LOC102159820	mRNA	0	0	0	0	0	0	0	0	0	0	0	0	0	0	0	0	0	0	0	0	0
XM_021091373.1	TRMU	mRNA	0	0	0	0	0	0	0	0	0	0	0	0	0	0	0	0	0	0	0	0	0
XM_021091374.1	TRMU	mRNA	0	0	0	0	0	0	0	0	0	0	0	0	0	0	0	0	0	0	0	0	0
XM_021091375.1	TRMU	mRNA	0	0	0	0	0	0	0	0	0	0	0	0	0	0	0	0	0	0	0	0	0
XM_021091376.1	GTSE1	mRNA	0	0	0	0	0	0	0	0	0	0	0	0	0	0	0	0	0	0	0	0	0
XM_021091377.1	TTC38	mRNA	0	0	0	0	0	0	0	0	0	0	0	0	0	0	0	0	0	0	0	0	0
XM_021091379.1	LOC110260611	mRNA	0	0	0	0	0	0	0	0	0	0	0	0	0	0	0	0	0	0	0	0	0
XM_021091381.1	LOC110260611	mRNA	0	0	0	0	0	0	0	0	0	0	0	0	0	0	0	0	0	0	0	0	0
XM_021091454.1	TOB2	mRNA	0	0	0	0	0	0	0	0	0	1	0	0	0	0	0	0	0	0	0	0	0
XM_021091455.1	TOB2	mRNA	0	0	0	0	0	0	0	0	0	1	0	0	0	0	0	0	0	0	0	0	0
XM_021091456.1	PHF5A	mRNA	0	0	0	0	0	0	0	0	0	1	0	0	0	0	0	0	0	0	0	0	0
XM_021091457.1	PHF5A	mRNA	0	0	0	0	0	0	0	0	0	1	0	0	0	0	0	0	0	0	0	0	0
XM_021091458.1	ZC3H7B	mRNA	0	0	0	0	0	0	0	0	0	1	0	0	0	0	0	0	0	0	0	0	0
XM_021091459.1	ZC3H7B	mRNA	0	0	0	0	0	0	0	0	0	1	0	0	0	0	0	0	0	0	0	0	0
XM_021091460.1	TEF	mRNA	0	0	0	0	0	0	0	0	0	1	0	0	0	0	0	0	0	0	0	0	0
XM_021091461.1	RANGAP1	mRNA	0	0	0	0	0	0	0	0	0	1	0	0	0	0	0	0	0	0	0	0	0
XM_021091474.1	CBX7	mRNA	0	0	0	0	0	0	0	0	0	0	0	0	0	0	0	0	0	0	0	0	0
XM_021092767.1	CRADD	mRNA	0	0	0	0	0	0	0	0	0	0	0	0	0	0	0	0	0	0	0	0	0
XM_021092768.1	CRADD	mRNA	0	0	0	0	0	0	0	0	0	0	0	0	0	0	0	0	0	0	0	0	0
XM_021092769.1	CRADD	mRNA	0	0	0	0	0	0	0	0	0	0	0	0	0	0	0	0	0	0	0	0	0

RNA ID	Symbol	Type	updown_BGIR9823_88895	updown_BGIR9823_93430	updown_BGIR9823_94434	updown_BGIR9823_94471	updown_XR_002339623.1	updown_XR_002339910.1	updown_XR_002340372.1	updown_XR_002339346.1	updown_XR_002343632.1	updown_XR_002343884.1	updown_BGIR9823_102724	updown_BGIR9823_102212	updown_BGIR9823_102279	updown_BGIR9823_78573	updown_BGIR9823_101405	updown_BGIR9823_101544	updown_BGIR9823_101566	updown_BGIR9823_100548	updown_BGIR9823_100551	updown_BGIR9823_101208	updown_BGIR9823_78436
XM_021092770.1	CRADD	mRNA	0	0	0	0	0	0	0	0	0	0	0	0	0	0	0	0	0	0	0	0	0
XM_021092771.1	CRADD	mRNA	0	0	0	0	0	0	0	0	0	0	0	0	0	0	0	0	0	0	0	0	0
XM_021092773.1	CRADD	mRNA	0	0	0	0	0	0	0	0	0	0	0	0	0	0	0	0	0	0	0	0	0
XM_021093095.1	PKDREJ	mRNA	0	0	0	0	0	0	0	0	0	0	0	0	0	0	0	0	0	0	0	0	0
XM_021097064.1	MAF	mRNA	0	0	0	0	0	0	0	0	0	0	0	0	0	0	0	0	0	0	0	0	0
XM_021097065.1	MAF	mRNA	0	0	0	0	0	0	0	0	0	0	0	0	0	0	0	0	0	0	0	0	0
XM_021097066.1	MAF	mRNA	0	0	0	0	0	0	0	0	0	0	0	0	0	0	0	0	0	0	0	0	0
XM_021097870.1	ECI2	mRNA	0	0	0	0	0	0	0	0	0	0	0	0	0	0	0	0	0	0	0	0	0
XM_021099751.1	PHACTR1	mRNA	0	0	0	0	0	0	0	0	0	0	0	0	0	0	0	0	0	0	0	0	0
XM_021099752.1	PHACTR1	mRNA	0	0	0	0	0	0	0	0	0	0	0	0	0	0	0	0	0	0	0	0	0
XM_021099753.1	PHACTR1	mRNA	0	0	0	0	0	0	0	0	0	0	0	0	0	0	0	0	0	0	0	0	0
XM_021099754.1	PHACTR1	mRNA	0	0	0	0	0	0	0	0	0	0	0	0	0	0	0	0	0	0	0	0	0
XM_021099755.1	PHACTR1	mRNA	0	0	0	0	0	0	0	0	0	0	0	0	0	0	0	0	0	0	0	0	0
XM_021099756.1	PHACTR1	mRNA	0	0	0	0	0	0	0	0	0	0	0	0	0	0	0	0	0	0	0	0	0
XM_021099757.1	PHACTR1	mRNA	0	0	0	0	0	0	0	0	0	0	0	0	0	0	0	0	0	0	0	0	0
XM_021099758.1	PHACTR1	mRNA	0	0	0	0	0	0	0	0	0	0	0	0	0	0	0	0	0	0	0	0	0
XM_021099759.1	PHACTR1	mRNA	0	0	0	0	0	0	0	0	0	0	0	0	0	0	0	0	0	0	0	0	0
XM_021099760.1	PHACTR1	mRNA	0	0	0	0	0	0	0	0	0	0	0	0	0	0	0	0	0	0	0	0	0
XM_021099761.1	PHACTR1	mRNA	0	0	0	0	0	0	0	0	0	0	0	0	0	0	0	0	0	0	0	0	0
XM_021099762.1	PHACTR1	mRNA	0	0	0	0	0	0	0	0	0	0	0	0	0	0	0	0	0	0	0	0	0
XM_021099763.1	PHACTR1	mRNA	0	0	0	0	0	0	0	0	0	0	0	0	0	0	0	0	0	0	0	0	0
XM_021099764.1	PHACTR1	mRNA	0	0	0	0	0	0	0	0	0	0	0	0	0	0	0	0	0	0	0	0	0
XM_021099765.1	PHACTR1	mRNA	0	0	0	0	0	0	0	0	0	0	0	0	0	0	0	0	0	0	0	0	0
XM_021099766.1	TBC1D7	mRNA	0	0	0	0	0	0	0	0	0	0	0	0	0	0	0	0	0	0	0	0	0
XM_021099767.1	TBC1D7	mRNA	0	0	0	0	0	0	0	0	0	0	0	0	0	0	0	0	0	0	0	0	0
XM_021099769.1	TBC1D7	mRNA	0	0	0	0	0	0	0	0	0	0	0	0	0	0	0	0	0	0	0	0	0
XM_021099770.1	TBC1D7	mRNA	0	0	0	0	0	0	0	0	0	0	0	0	0	0	0	0	0	0	0	0	0
XM_021099999.1	LOC110261697	mRNA	0	0	0	0	0	0	0	0	0	0	0	0	0	0	0	0	0	0	0	0	0
XM_021100108.1	WRNIP1	mRNA	0	0	0	0	0	0	0	0	0	0	0	0	0	0	0	0	0	0	0	0	0
XM_021100109.1	MYLK4	mRNA	0	0	0	0	0	0	0	0	0	0	0	0	0	0	0	0	0	0	0	0	0
XM_021100110.1	MYLK4	mRNA	0	0	0	0	0	0	0	0	0	0	0	0	0	0	0	0	0	0	0	0	0
XM_021100111.1	MYLK4	mRNA	0	0	0	0	0	0	0	0	0	0	0	0	0	0	0	0	0	0	0	0	0
XM_021100112.1	MYLK4	mRNA	0	0	0	0	0	0	0	0	0	0	0	0	0	0	0	0	0	0	0	0	0
XM_021100114.1	NQO2	mRNA	0	0	0	0	0	0	0	0	0	0	0	0	0	0	0	0	0	0	0	0	0
XM_021100115.1	NQO2	mRNA	0	0	0	0	0	0	0	0	0	0	0	0	0	0	0	0	0	0	0	0	0
XM_021100117.1	NQO2	mRNA	0	0	0	0	0	0	0	0	0	0	0	0	0	0	0	0	0	0	0	0	0
XM_021100118.1	NQO2	mRNA	0	0	0	0	0	0	0	0	0	0	0	0	0	0	0	0	0	0	0	0	0
XM_021100119.1	NQO2	mRNA	0	0	0	0	0	0	0	0	0	0	0	0	0	0	0	0	0	0	0	0	0
XM_021100124.1	PRPF4B	mRNA	0	0	0	0	0	0	0	0	0	0	0	0	0	0	0	0	0	0	0	0	0
XM_021100126.1	PRPF4B	mRNA	0	0	0	0	0	0	0	0	0	0	0	0	0	0	0	0	0	0	0	0	0
XM_021100184.1	NEDD9	mRNA	0	0	0	0	0	0	0	0	0	0	0	0	0	0	0	0	0	0	0	0	0
XM_021100188.1	TMEM170B	mRNA	0	0	0	0	0	0	0	0	0	0	0	0	0	0	0	0	0	0	0	0	0
XM_021100194.1	GFOD1	mRNA	0	0	0	0	0	0	0	0	0	0	0	0	0	0	0	0	0	0	0	0	0

RNA ID	Symbol	Type	updown_BGIR9823_88895	updown_BGIR9823_93430	updown_BGIR9823_94434	updown_BGIR9823_94471	updown_XR_002339623.1	updown_XR_002339910.1	updown_XR_002340372.1	updown_XR_002339346.1	updown_XR_002343632.1	updown_XR_002343884.1	updown_BGIR9823_102724	updown_BGIR9823_102212	updown_BGIR9823_102279	updown_BGIR9823_78573	updown_BGIR9823_101405	updown_BGIR9823_101544	updown_BGIR9823_101566	updown_BGIR9823_100548	updown_BGIR9823_100551	updown_BGIR9823_101208	updown_BGIR9823_78436
XM_021102040.1	SLC37A4	mRNA	0	0	0	0	0	0	0	0	0	0	0	0	0	0	0	0	0	0	0	0	0
XM_021102041.1	SLC37A4	mRNA	0	0	0	0	0	0	0	0	0	0	0	0	0	0	0	0	0	0	0	0	0
XM_021102042.1	SLC37A4	mRNA	0	0	0	0	0	0	0	0	0	0	0	0	0	0	0	0	0	0	0	0	0
XM_021102043.1	SLC37A4	mRNA	0	0	0	0	0	0	0	0	0	0	0	0	0	0	0	0	0	0	0	0	0
XM_021102044.1	SLC37A4	mRNA	0	0	0	0	0	0	0	0	0	0	0	0	0	0	0	0	0	0	0	0	0
XM_021102045.1	SLC37A4	mRNA	0	0	0	0	0	0	0	0	0	0	0	0	0	0	0	0	0	0	0	0	0
XM_021102046.1	SLC37A4	mRNA	0	0	0	0	0	0	0	0	0	0	0	0	0	0	0	0	0	0	0	0	0
XM_021102047.1	SLC37A4	mRNA	0	0	0	0	0	0	0	0	0	0	0	0	0	0	0	0	0	0	0	0	0
XM_021102048.1	SLC37A4	mRNA	0	0	0	0	0	0	0	0	0	0	0	0	0	0	0	0	0	0	0	0	0
XM_021102049.1	SLC37A4	mRNA	0	0	0	0	0	0	0	0	0	0	0	0	0	0	0	0	0	0	0	0	0
XM_021102050.1	SLC37A4	mRNA	0	0	0	0	0	0	0	0	0	0	0	0	0	0	0	0	0	0	0	0	0
XM_021102051.1	SLC37A4	mRNA	0	0	0	0	0	0	0	0	0	0	0	0	0	0	0	0	0	0	0	0	0
XM_021102052.1	SLC37A4	mRNA	0	0	0	0	0	0	0	0	0	0	0	0	0	0	0	0	0	0	0	0	0
XM_021102209.1	UPK2	mRNA	0	0	0	0	0	0	0	0	0	0	0	0	0	0	0	0	0	0	0	0	0
XM_021102516.1	LOC100522455	mRNA	0	0	0	0	0	0	0	0	0	0	0	0	0	0	0	0	0	0	0	0	0
XM_021102517.1	RUNDC3B	mRNA	0	0	0	0	0	0	0	0	0	0	0	0	0	0	0	0	0	0	0	0	0
XM_021102518.1	RUNDC3B	mRNA	0	0	0	0	0	0	0	0	0	0	0	0	0	0	0	0	0	0	0	0	0
XM_021102519.1	RUNDC3B	mRNA	0	0	0	0	0	0	0	0	0	0	0	0	0	0	0	0	0	0	0	0	0
XM_021102522.1	RUNDC3B	mRNA	0	0	0	0	0	0	0	0	0	0	0	0	0	0	0	0	0	0	0	0	0
XM_021102523.1	RUNDC3B	mRNA	0	0	0	0	0	0	0	0	0	0	0	0	0	0	0	0	0	0	0	0	0
XR_001297664.2	LOC102161744	lncRNA	0	0	0	0	0	0	0	0	0	0	0	0	0	0	0	0	0	0	0	0	0
XR_001298807.2	LOC106504881	lncRNA	0	0	0	0	0	0	0	0	0	0	0	0	0	0	0	0	0	0	0	0	0
XR_001298808.2	LOC106504881	lncRNA	0	0	0	0	0	0	0	0	0	0	0	0	0	0	0	0	0	0	0	0	0
XR_001298810.2	LOC106504881	lncRNA	0	0	0	0	0	0	0	0	0	0	0	0	0	0	0	0	0	0	0	0	0
XR_001298811.2	LOC106504881	lncRNA	0	0	0	0	0	0	0	0	0	0	0	0	0	0	0	0	0	0	0	0	0
XR_001298812.2	LOC106504881	lncRNA	0	0	0	0	0	0	0	0	0	0	0	0	0	0	0	0	0	0	0	0	0
XR_001298813.2	LOC106504881	lncRNA	0	0	0	0	0	0	0	0	0	0	0	0	0	0	0	0	0	0	0	0	0
XR_001298814.2	LOC106504881	lncRNA	0	0	0	0	0	0	0	0	0	0	0	0	0	0	0	0	0	0	0	0	0
XR_001298816.2	LOC106504881	lncRNA	0	0	0	0	0	0	0	0	0	0	0	0	0	0	0	0	0	0	0	0	0
XR_001298817.2	LOC106504881	lncRNA	0	0	0	0	0	0	0	0	0	0	0	0	0	0	0	0	0	0	0	0	0
XR_001298844.2	LOC102163364	lncRNA	0	0	0	0	0	0	0	0	0	0	0	0	0	0	0	0	0	0	0	0	0
XR_001298846.2	LOC102163364	lncRNA	0	0	0	0	0	0	0	0	0	0	0	0	0	0	0	0	0	0	0	0	0
XR_001298850.2	LOC102163364	lncRNA	0	0	0	0	0	0	0	0	0	0	0	0	0	0	0	0	0	0	0	0	0
XR_001299456.2	LOC102166670	lncRNA	0	0	0	0	0	0	0	0	0	0	0	0	0	0	0	0	0	0	0	0	0
XR_001299457.2	LOC102166670	lncRNA	0	0	0	0	0	0	0	0	0	0	0	0	0	0	0	0	0	0	0	0	0
XR_001300715.2	LOC106505879	lncRNA	0	0	0	0	0	0	0	0	0	0	0	0	0	0	0	0	0	0	0	0	0
XR_001301078.2	LOC102162623	lncRNA	0	0	0	0	0	0	0	0	0	0	0	0	0	0	0	0	0	0	0	0	0
XR_001301087.2	LOC102162623	lncRNA	0	0	0	0	0	0	0	0	0	0	0	0	0	0	0	0	0	0	0	0	0
XR_001301217.2	LOC102164998	lncRNA	0	0	0	0	0	0	0	0	0	0	0	0	0	0	0	0	0	0	0	0	0
XR_001301305.2	LOC102159607	lncRNA	0	0	0	0	0	0	0	0	0	0	0	0	0	0	0	0	0	0	0	0	0
XR_001308967.2	LOC106510467	lncRNA	0	0	0	0	0	0	0	0	0	0	0	0	0	0	0	0	0	0	0	0	0
XR_002335533.1	XRRA1	mRNA	0	0	0	0	0	0	0	0	0	0	0	0	0	0	0	0	0	0	0	0	0
XR_002335534.1	XRRA1	mRNA	0	0	0	0	0	0	0	0	0	0	0	0	0	0	0	0	0	0	0	0	0

RNA ID	Symbol	Type	updown_BGIR9823_88895	updown_BGIR9823_93430	updown_BGIR9823_94434	updown_BGIR9823_94471	updown_XR_002339623.1	updown_XR_002339910.1	updown_XR_002340372.1	updown_XR_002339346.1	updown_XR_002343632.1	updown_XR_002343884.1	updown_BGIR9823_102724	updown_BGIR9823_102212	updown_BGIR9823_102279	updown_BGIR9823_78573	updown_BGIR9823_101405	updown_BGIR9823_101544	updown_BGIR9823_101566	updown_BGIR9823_100548	updown_BGIR9823_100551	updown_BGIR9823_101208	updown_BGIR9823_78436
XR_002335535.1	LOC110255399	lncRNA	0	0	0	0	0	0	0	0	0	0	0	0	0	0	0	0	0	0	0	0	0
XR_002335536.1	LOC102160367	lncRNA	0	0	0	0	0	0	0	0	0	0	0	0	0	0	0	0	0	0	0	0	0
XR_002335537.1	LOC102160367	lncRNA	0	0	0	0	0	0	0	0	0	0	0	0	0	0	0	0	0	0	0	0	0
XR_002335650.1	LOC102157630	lncRNA	0	0	0	0	0	0	0	0	0	0	0	0	0	0	0	0	0	0	0	0	0
XR_002335651.1	LOC110255435	lncRNA	0	0	0	0	0	0	0	0	0	0	0	0	0	0	0	0	0	0	0	0	0
XR_002335652.1	LOC106504881	lncRNA	0	0	0	0	0	0	0	0	0	0	0	0	0	0	0	0	0	0	0	0	0
XR_002335653.1	LOC106504881	lncRNA	0	0	0	0	0	0	0	0	0	0	0	0	0	0	0	0	0	0	0	0	0
XR_002335670.1	LOC110255440	lncRNA	0	0	0	0	0	0	0	0	0	0	0	0	0	0	0	0	0	0	0	0	0
XR_002335671.1	LOC110255441	lncRNA	0	0	0	0	0	0	0	0	0	0	0	0	0	0	0	0	0	0	0	0	0
XR_002335672.1	LOC110255441	lncRNA	0	0	0	0	0	0	0	0	0	0	0	0	0	0	0	0	0	0	0	0	0
XR_002335674.1	HYOU1	mRNA	0	0	0	0	0	0	0	0	0	0	0	0	0	0	0	0	0	0	0	0	0
XR_002335675.1	HYOU1	mRNA	0	0	0	0	0	0	0	0	0	0	0	0	0	0	0	0	0	0	0	0	0
XR_002335685.1	LOC100523684	mRNA	0	0	0	0	0	0	0	0	0	0	0	0	0	0	0	0	0	0	0	0	0
XR_002335686.1	LOC102163364	lncRNA	0	0	0	0	0	0	0	0	0	0	0	0	0	0	0	0	0	0	0	0	0
XR_002335687.1	LOC102163364	lncRNA	0	0	0	0	0	0	0	0	0	0	0	0	0	0	0	0	0	0	0	0	0
XR_002335688.1	LOC102163364	lncRNA	0	0	0	0	0	0	0	0	0	0	0	0	0	0	0	0	0	0	0	0	0
XR_002335689.1	LOC102163364	lncRNA	0	0	0	0	0	0	0	0	0	0	0	0	0	0	0	0	0	0	0	0	0
XR_002335690.1	LOC102163364	lncRNA	0	0	0	0	0	0	0	0	0	0	0	0	0	0	0	0	0	0	0	0	0
XR_002335691.1	LOC102163364	lncRNA	0	0	0	0	0	0	0	0	0	0	0	0	0	0	0	0	0	0	0	0	0
XR_002335692.1	LOC102163364	lncRNA	0	0	0	0	0	0	0	0	0	0	0	0	0	0	0	0	0	0	0	0	0
XR_002335693.1	LOC106504896	lncRNA	0	0	0	0	0	0	0	0	0	0	0	0	0	0	0	0	0	0	0	0	0
XR_002335694.1	SORL1	mRNA	0	0	0	0	0	0	0	0	0	0	0	0	0	0	0	0	0	0	0	0	0
XR_002336641.1	LOC106505250	lncRNA	0	0	0	0	0	0	0	0	0	0	0	0	0	0	0	0	0	0	0	0	0
XR_002336643.1	LOC110255292	lncRNA	0	0	0	0	0	0	0	0	0	0	0	0	0	0	0	0	0	0	0	0	0
XR_002336644.1	LOC102166670	lncRNA	0	0	0	0	0	0	0	0	0	0	0	0	0	0	0	0	0	0	0	0	0
XR_002336645.1	LOC102166670	lncRNA	0	0	0	0	0	0	0	0	0	0	0	0	0	0	0	0	0	0	0	0	0
XR_002338443.1	LOC106505879	lncRNA	0	0	0	0	0	0	0	0	0	0	0	0	0	0	0	0	0	0	0	0	0
XR_002338444.1	LOC106505879	lncRNA	0	0	0	0	0	0	0	0	0	0	0	0	0	0	0	0	0	0	0	0	0
XR_002338445.1	LOC106505879	lncRNA	0	0	0	0	0	0	0	0	0	0	0	0	0	0	0	0	0	0	0	0	0
XR_002338446.1	LOC106505879	lncRNA	0	0	0	0	0	0	0	0	0	0	0	0	0	0	0	0	0	0	0	0	0
XR_002338447.1	LOC106505879	lncRNA	0	0	0	0	0	0	0	0	0	0	0	0	0	0	0	0	0	0	0	0	0
XR_002338448.1	LOC106505879	lncRNA	0	0	0	0	0	0	0	0	0	0	0	0	0	0	0	0	0	0	0	0	0
XR_002338449.1	LOC106505879	lncRNA	0	0	0	0	0	0	0	0	0	0	0	0	0	0	0	0	0	0	0	0	0
XR_002338450.1	LOC106505879	lncRNA	0	0	0	0	0	0	0	0	0	0	0	0	0	0	0	0	0	0	0	0	0
XR_002338521.1	LOC110256664	lncRNA	0	0	0	0	0	0	0	0	0	0	0	0	0	0	0	0	0	0	0	0	0
XR_002338522.1	LOC110256664	lncRNA	0	0	0	0	0	0	0	0	0	0	0	0	0	0	0	0	0	0	0	0	0
XR_002338666.1	LOC110256747	lncRNA	0	0	0	0	0	0	0	0	0	0	0	0	0	0	0	0	0	0	0	0	0
XR_002338714.1	LOC102163817	lncRNA	0	0	0	0	0	0	0	0	0	0	0	0	0	0	0	0	0	0	0	0	0
XR_002338715.1	LOC102164998	lncRNA	0	0	0	0	0	0	0	0	0	0	0	0	0	0	0	0	0	0	0	0	0
XR_002338716.1	LOC102164998	lncRNA	0	0	0	0	0	0	0	0	0	0	0	0	0	0	0	0	0	0	0	0	0
XR_002338717.1	LOC110256768	lncRNA	0	0	0	0	0	0	0	0	0	0	0	0	0	0	0	0	0	0	0	0	0
XR_002338727.1	LOC110256773	lncRNA	0	0	0	0	0	0	0	0	0	0	0	0	0	0	0	0	0	0	0	0	0
XR_002338728.1	LOC110256773	lncRNA	0	0	0	0	0	0	0	0	0	0	0	0	0	0	0	0	0	0	0	0	0

RNA ID	Symbol	Type	updown_BGIR9823_88895	updown_BGIR9823_93430	updown_BGIR9823_94434	updown_BGIR9823_94471	updown_XR_002339623.1	updown_XR_002339910.1	updown_XR_002340372.1	updown_XR_002339346.1	updown_XR_002343632.1	updown_XR_002343884.1	updown_BGIR9823_102724	updown_BGIR9823_102212	updown_BGIR9823_102279	updown_BGIR9823_78573	updown_BGIR9823_101405	updown_BGIR9823_101544	updown_BGIR9823_101566	updown_BGIR9823_100548	updown_BGIR9823_100551	updown_BGIR9823_101208	updown_BGIR9823_78436
XR_002338729.1	LOC110256773	lncRNA	0	0	0	0	0	0	0	0	0	0	0	0	0	0	0	0	0	0	0	0	0
XR_002338793.1	LOC110256796	lncRNA	0	0	0	0	0	0	0	0	0	0	0	0	0	0	0	0	0	0	0	0	0
XR_002339623.1	LOC102167708	lncRNA	0	0	0	0	1	0	0	0	0	0	0	0	0	0	0	0	0	0	0	0	0
XR_002340245.1	PRKAG2	mRNA	0	0	0	0	0	0	0	0	0	0	0	0	0	0	0	0	0	0	0	0	0
XR_002340246.1	LOC102162623	lncRNA	0	0	0	0	0	0	0	0	0	0	0	0	0	0	0	0	0	0	0	0	0
XR_002340247.1	LOC102162623	lncRNA	0	0	0	0	0	0	0	0	0	0	0	0	0	0	0	0	0	0	0	0	0
XR_002340248.1	LOC110257516	lncRNA	0	0	0	0	0	0	0	0	0	0	0	0	0	0	0	0	0	0	0	0	0
XR_002340249.1	LOC102162071	lncRNA	0	0	0	0	0	0	0	0	0	0	0	0	0	0	0	0	0	0	0	0	0
XR_002340901.1	LOC102160243	lncRNA	0	0	0	0	0	0	0	0	0	0	0	0	0	0	0	0	0	0	0	0	0
XR_002340902.1	LOC102160243	lncRNA	0	0	0	0	0	0	0	0	0	0	0	0	0	0	0	0	0	0	0	0	0
XR_002340903.1	LOC102160243	lncRNA	0	0	0	0	0	0	0	0	0	0	0	0	0	0	0	0	0	0	0	0	0
XR_002340904.1	LOC102160243	lncRNA	0	0	0	0	0	0	0	0	0	0	0	0	0	0	0	0	0	0	0	0	0
XR_002340905.1	LOC102160243	lncRNA	0	0	0	0	0	0	0	0	0	0	0	0	0	0	0	0	0	0	0	0	0
XR_002341237.1	LOC102164320	lncRNA	0	0	0	0	0	0	0	0	0	0	0	0	0	0	0	0	0	0	0	0	0
XR_002341238.1	LOC102164320	lncRNA	0	0	0	0	0	0	0	0	0	0	0	0	0	0	0	0	0	0	0	0	0
XR_002341239.1	LOC102164320	lncRNA	0	0	0	0	0	0	0	0	0	0	0	0	0	0	0	0	0	0	0	0	0
XR_002341240.1	LOC102164320	lncRNA	0	0	0	0	0	0	0	0	0	0	0	0	0	0	0	0	0	0	0	0	0
XR_002341241.1	LOC102164320	lncRNA	0	0	0	0	0	0	0	0	0	0	0	0	0	0	0	0	0	0	0	0	0
XR_002341242.1	LOC102164320	lncRNA	0	0	0	0	0	0	0	0	0	0	0	0	0	0	0	0	0	0	0	0	0
XR_002341243.1	LOC102164320	lncRNA	0	0	0	0	0	0	0	0	0	0	0	0	0	0	0	0	0	0	0	0	0
XR_002341244.1	LOC102164320	lncRNA	0	0	0	0	0	0	0	0	0	0	0	0	0	0	0	0	0	0	0	0	0
XR_002341270.1	LOC100521600	mRNA	0	0	0	0	0	0	0	0	0	0	0	0	0	0	0	0	0	0	0	0	0
XR_002341443.1	LOC102163816	lncRNA	0	0	0	0	0	0	0	0	0	0	0	0	0	0	0	0	0	0	0	0	0
XR_002343847.1	LOC110260611	mRNA	0	0	0	0	0	0	0	0	0	0	0	0	0	0	0	0	0	0	0	0	0
XR_002343848.1	LOC110260611	mRNA	0	0	0	0	0	0	0	0	0	0	0	0	0	0	0	0	0	0	0	0	0
XR_002343849.1	LOC110260611	mRNA	0	0	0	0	0	0	0	0	0	0	0	0	0	0	0	0	0	0	0	0	0
XR_002343850.1	LOC110260611	mRNA	0	0	0	0	0	0	0	0	0	0	0	0	0	0	0	0	0	0	0	0	0
XR_002343875.1	LOC110260629	lncRNA	0	0	0	0	0	0	0	0	0	1	0	0	0	0	0	0	0	0	0	0	0
XR_002343876.1	LOC110260629	lncRNA	0	0	0	0	0	0	0	0	0	1	0	0	0	0	0	0	0	0	0	0	0
XR_002343877.1	LOC110260629	lncRNA	0	0	0	0	0	0	0	0	0	1	0	0	0	0	0	0	0	0	0	0	0
XR_002343878.1	LOC110260629	lncRNA	0	0	0	0	0	0	0	0	0	1	0	0	0	0	0	0	0	0	0	0	0
XR_002343879.1	LOC110260629	lncRNA	0	0	0	0	0	0	0	0	0	1	0	0	0	0	0	0	0	0	0	0	0
XR_002343880.1	LOC110260629	lncRNA	0	0	0	0	0	0	0	0	0	1	0	0	0	0	0	0	0	0	0	0	0
XR_002343881.1	LOC110260629	lncRNA	0	0	0	0	0	0	0	0	0	1	0	0	0	0	0	0	0	0	0	0	0
XR_002343883.1	LOC110260631	lncRNA	0	0	0	0	0	0	0	0	0	1	0	0	0	0	0	0	0	0	0	0	0
XR_002343884.1	LOC110260634	lncRNA	0	0	0	0	0	0	0	0	0	1	0	0	0	0	0	0	0	0	0	0	0
XR_002343885.1	LOC110260635	lncRNA	0	0	0	0	0	0	0	0	0	1	0	0	0	0	0	0	0	0	0	0	0
XR_002343891.1	LOC110260642	lncRNA	0	0	0	0	0	0	0	0	0	0	0	0	0	0	0	0	0	0	0	0	0
XR_002343892.1	LOC110260643	lncRNA	0	0	0	0	0	0	0	0	0	0	0	0	0	0	0	0	0	0	0	0	0
XR_002345298.1	MAF	mRNA	0	0	0	0	0	0	0	0	0	0	0	0	0	0	0	0	0	0	0	0	0
XR_002345300.1	LOC106510470	lncRNA	0	0	0	0	0	0	0	0	0	0	0	0	0	0	0	0	0	0	0	0	0
XR_002345301.1	LOC106510468	lncRNA	0	0	0	0	0	0	0	0	0	0	0	0	0	0	0	0	0	0	0	0	0
XR_002346166.1	LOC110261703	lncRNA	0	0	0	0	0	0	0	0	0	0	0	0	0	0	0	0	0	0	0	0	0

RNA ID	Symbol	Type	updown_BGIR9823_88895	updown_BGIR9823_93430	updown_BGIR9823_94434	updown_BGIR9823_94471	updown_XR_002339623.1	updown_XR_002339910.1	updown_XR_002340372.1	updown_XR_002339346.1	updown_XR_002343632.1	updown_XR_002343884.1	updown_BGIR9823_102724	updown_BGIR9823_102212	updown_BGIR9823_102279	updown_BGIR9823_78573	updown_BGIR9823_101405	updown_BGIR9823_101544	updown_BGIR9823_101566	updown_BGIR9823_100548	updown_BGIR9823_100551	updown_BGIR9823_101208	updown_BGIR9823_78436
XR_002346227.1	LOC106504299	lncRNA	0	0	0	0	0	0	0	0	0	0	0	0	0	0	0	0	0	0	0	0	0
XR_002346228.1	LOC106504299	lncRNA	0	0	0	0	0	0	0	0	0	0	0	0	0	0	0	0	0	0	0	0	0
XR_002346229.1	LOC106504299	lncRNA	0	0	0	0	0	0	0	0	0	0	0	0	0	0	0	0	0	0	0	0	0
XR_002346230.1	LOC106504299	lncRNA	0	0	0	0	0	0	0	0	0	0	0	0	0	0	0	0	0	0	0	0	0
XR_002346231.1	LOC106504299	lncRNA	0	0	0	0	0	0	0	0	0	0	0	0	0	0	0	0	0	0	0	0	0
XR_002346232.1	LOC106504299	lncRNA	0	0	0	0	0	0	0	0	0	0	0	0	0	0	0	0	0	0	0	0	0
XR_002346233.1	NQO2	mRNA	0	0	0	0	0	0	0	0	0	0	0	0	0	0	0	0	0	0	0	0	0
XR_002346236.1	PRPF4B	mRNA	0	0	0	0	0	0	0	0	0	0	0	0	0	0	0	0	0	0	0	0	0
XR_002346237.1	PRPF4B	mRNA	0	0	0	0	0	0	0	0	0	0	0	0	0	0	0	0	0	0	0	0	0
XR_002346238.1	PRPF4B	mRNA	0	0	0	0	0	0	0	0	0	0	0	0	0	0	0	0	0	0	0	0	0
XR_002346239.1	PRPF4B	mRNA	0	0	0	0	0	0	0	0	0	0	0	0	0	0	0	0	0	0	0	0	0
XR_002346240.1	PRPF4B	mRNA	0	0	0	0	0	0	0	0	0	0	0	0	0	0	0	0	0	0	0	0	0
XR_002346241.1	PRPF4B	mRNA	0	0	0	0	0	0	0	0	0	0	0	0	0	0	0	0	0	0	0	0	0
XR_002346242.1	PRPF4B	mRNA	0	0	0	0	0	0	0	0	0	0	0	0	0	0	0	0	0	0	0	0	0
XR_002346243.1	PRPF4B	mRNA	0	0	0	0	0	0	0	0	0	0	0	0	0	0	0	0	0	0	0	0	0
XR_002346244.1	PRPF4B	mRNA	0	0	0	0	0	0	0	0	0	0	0	0	0	0	0	0	0	0	0	0	0
XR_002346245.1	PRPF4B	mRNA	0	0	0	0	0	0	0	0	0	0	0	0	0	0	0	0	0	0	0	0	0
XR_002346246.1	PRPF4B	mRNA	0	0	0	0	0	0	0	0	0	0	0	0	0	0	0	0	0	0	0	0	0
XR_002346247.1	PRPF4B	mRNA	0	0	0	0	0	0	0	0	0	0	0	0	0	0	0	0	0	0	0	0	0
XR_002346248.1	PRPF4B	mRNA	0	0	0	0	0	0	0	0	0	0	0	0	0	0	0	0	0	0	0	0	0
XR_002346249.1	PRPF4B	mRNA	0	0	0	0	0	0	0	0	0	0	0	0	0	0	0	0	0	0	0	0	0
XR_002346250.1	PRPF4B	mRNA	0	0	0	0	0	0	0	0	0	0	0	0	0	0	0	0	0	0	0	0	0
XR_002346251.1	PRPF4B	mRNA	0	0	0	0	0	0	0	0	0	0	0	0	0	0	0	0	0	0	0	0	0
XR_002346252.1	PRPF4B	mRNA	0	0	0	0	0	0	0	0	0	0	0	0	0	0	0	0	0	0	0	0	0
XR_002346253.1	PRPF4B	mRNA	0	0	0	0	0	0	0	0	0	0	0	0	0	0	0	0	0	0	0	0	0
XR_002346254.1	PRPF4B	mRNA	0	0	0	0	0	0	0	0	0	0	0	0	0	0	0	0	0	0	0	0	0
XR_002346255.1	PRPF4B	mRNA	0	0	0	0	0	0	0	0	0	0	0	0	0	0	0	0	0	0	0	0	0
XR_002346256.1	PRPF4B	mRNA	0	0	0	0	0	0	0	0	0	0	0	0	0	0	0	0	0	0	0	0	0
XR_002346257.1	PRPF4B	mRNA	0	0	0	0	0	0	0	0	0	0	0	0	0	0	0	0	0	0	0	0	0
XR_002346258.1	PRPF4B	mRNA	0	0	0	0	0	0	0	0	0	0	0	0	0	0	0	0	0	0	0	0	0
XR_002346259.1	PRPF4B	mRNA	0	0	0	0	0	0	0	0	0	0	0	0	0	0	0	0	0	0	0	0	0
XR_002346260.1	PRPF4B	mRNA	0	0	0	0	0	0	0	0	0	0	0	0	0	0	0	0	0	0	0	0	0
XR_002346261.1	PRPF4B	mRNA	0	0	0	0	0	0	0	0	0	0	0	0	0	0	0	0	0	0	0	0	0
XR_002346262.1	PRPF4B	mRNA	0	0	0	0	0	0	0	0	0	0	0	0	0	0	0	0	0	0	0	0	0
XR_002346263.1	PRPF4B	mRNA	0	0	0	0	0	0	0	0	0	0	0	0	0	0	0	0	0	0	0	0	0
XR_002346264.1	PRPF4B	mRNA	0	0	0	0	0	0	0	0	0	0	0	0	0	0	0	0	0	0	0	0	0
XR_002346265.1	LOC110261764	lncRNA	0	0	0	0	0	0	0	0	0	0	0	0	0	0	0	0	0	0	0	0	0
XR_002346266.1	LOC102162837	lncRNA	0	0	0	0	0	0	0	0	0	0	0	0	0	0	0	0	0	0	0	0	0
XR_002346299.1	LOC110261774	lncRNA	0	0	0	0	0	0	0	0	0	0	0	0	0	0	0	0	0	0	0	0	0
XR_002346300.1	LOC110261774	lncRNA	0	0	0	0	0	0	0	0	0	0	0	0	0	0	0	0	0	0	0	0	0
XR_002346302.1	LOC110261776	lncRNA	0	0	0	0	0	0	0	0	0	0	0	0	0	0	0	0	0	0	0	0	0
XR_002346303.1	TMEM170B	mRNA	0	0	0	0	0	0	0	0	0	0	0	0	0	0	0	0	0	0	0	0	0
XR_002346304.1	TMEM170B	mRNA	0	0	0	0	0	0	0	0	0	0	0	0	0	0	0	0	0	0	0	0	0

RNA ID	Symbol	Type	updown_BGIR9823_88895	updown_BGIR9823_93430	updown_BGIR9823_94434	updown_BGIR9823_94471	updown_XR_002339623.1	updown_XR_002339910.1	updown_XR_002340372.1	updown_XR_002339346.1	updown_XR_002343632.1	updown_XR_002343884.1	updown_BGIR9823_102724	updown_BGIR9823_102212	updown_BGIR9823_102279	updown_BGIR9823_78573	updown_BGIR9823_101405	updown_BGIR9823_101544	updown_BGIR9823_101566	updown_BGIR9823_100548	updown_BGIR9823_100551	updown_BGIR9823_101208	updown_BGIR9823_78436
XR_115737.4	LOC100513133	lncRNA	0	0	0	0	0	0	0	0	0	0	0	0	0	0	0	0	0	0	0	0	0
XR_299046.2	LOC102164596	lncRNA	0	0	0	0	0	0	0	0	0	0	0	0	0	0	0	0	0	0	0	0	0
XR_303634.3	LOC100738154	lncRNA	0	0	0	0	0	0	0	0	0	0	0	0	0	0	0	0	0	0	0	0	0
XR_304239.3	LOC102159819	lncRNA	0	0	0	0	0	0	0	0	0	0	0	0	0	0	0	0	0	0	0	0	0
XR_304244.3	LOC102161992	lncRNA	0	0	0	0	0	0	0	0	0	0	0	0	0	0	0	0	0	0	0	0	0
XR_304257.3	LOC102162837	lncRNA	0	0	0	0	0	0	0	0	0	0	0	0	0	0	0	0	0	0	0	0	0
XR_306105.3	LOC102166871	lncRNA	0	0	0	0	0	0	0	0	0	0	0	0	0	0	0	0	0	0	0	0	0
XR_307914.3	LOC102159607	lncRNA	0	0	0	0	0	0	0	0	0	0	0	0	0	0	0	0	0	0	0	0	0
XR_308345.3	LOC102167708	lncRNA	0	0	0	0	1	0	0	0	0	0	0	0	0	0	0	0	0	0	0	0	0
novel-ssc-miR1028-3p		miRNA	0	0	0	0	1	0	0	0	0	0	0	0	0	0	0	0	0	0	0	0	0
novel-ssc-miR1028-5p		miRNA	0	0	0	0	1	0	0	0	0	0	0	0	0	0	0	0	0	0	0	0	0
novel-ssc-miR1130-3p		miRNA	0	0	0	0	0	0	0	0	0	0	0	0	0	0	0	0	0	0	0	0	0
novel-ssc-miR1130-5p		miRNA	0	0	0	0	0	0	0	0	0	0	0	0	0	0	0	0	0	0	0	0	0
novel-ssc-miR1162-3p		miRNA	0	0	0	0	0	0	0	0	0	0	0	0	0	0	0	0	0	0	0	0	0
novel-ssc-miR1193-3p		miRNA	0	0	0	0	0	0	0	0	0	0	0	0	0	0	0	0	0	0	0	0	0
novel-ssc-miR1193-5p		miRNA	0	0	0	0	0	0	0	0	0	0	0	0	0	0	0	0	0	0	0	0	0
novel-ssc-miR1217-3p		miRNA	0	0	0	0	0	0	0	0	0	1	0	0	0	0	0	0	0	0	0	0	0
novel-ssc-miR1217-5p		miRNA	0	0	0	0	0	0	0	0	0	1	0	0	0	0	0	0	0	0	0	0	0
novel-ssc-miR1429-3p		miRNA	0	0	0	0	0	0	0	0	0	0	0	0	0	0	0	0	0	0	0	0	0
novel-ssc-miR1429-5p		miRNA	0	0	0	0	0	0	0	0	0	0	0	0	0	0	0	0	0	0	0	0	0
novel-ssc-miR259-3p		miRNA	0	0	0	0	0	0	0	0	0	0	0	0	0	0	0	0	0	0	0	0	0
novel-ssc-miR259-5p		miRNA	0	0	0	0	0	0	0	0	0	0	0	0	0	0	0	0	0	0	0	0	0
novel-ssc-miR324-3p		miRNA	0	0	0	0	0	0	0	0	0	0	0	0	0	0	0	0	0	0	0	0	0
novel-ssc-miR324-5p		miRNA	0	0	0	0	0	0	0	0	0	0	0	0	0	0	0	0	0	0	0	0	0
novel-ssc-miR42-3p		miRNA	0	0	0	0	0	0	0	0	0	0	0	0	0	0	0	0	0	0	0	0	0
novel-ssc-miR42-5p		miRNA	0	0	0	0	0	0	0	0	0	0	0	0	0	0	0	0	0	0	0	0	0
novel-ssc-miR433-3p		miRNA	0	0	0	0	0	0	0	0	0	0	0	0	0	0	0	0	0	0	0	0	0
novel-ssc-miR433-5p		miRNA	0	0	0	0	0	0	0	0	0	0	0	0	0	0	0	0	0	0	0	0	0
novel-ssc-miR557-3p		miRNA	0	0	0	0	0	0	0	0	0	1	0	0	0	0	0	0	0	0	0	0	0
novel-ssc-miR557-5p		miRNA	0	0	0	0	0	0	0	0	0	1	0	0	0	0	0	0	0	0	0	0	0
novel-ssc-miR619-3p		miRNA	0	0	0	0	0	0	0	0	0	0	0	0	0	0	0	0	0	0	0	0	0
novel-ssc-miR619-5p		miRNA	0	0	0	0	0	0	0	0	0	0	0	0	0	0	0	0	0	0	0	0	0
novel-ssc-miR719-3p		miRNA	0	0	0	0	0	0	0	0	0	0	0	0	0	0	0	0	0	0	0	0	0
novel-ssc-miR719-5p		miRNA	0	0	0	0	0	0	0	0	0	0	0	0	0	0	0	0	0	0	0	0	0
novel-ssc-miR966-3p		miRNA	0	0	0	0	0	0	0	0	0	0	0	0	0	0	0	0	0	0	0	0	0
novel-ssc-miR966-5p		miRNA	0	0	0	0	0	0	0	0	0	0	0	0	0	0	0	0	0	0	0	0	0
ssc-miR-7136-3p		miRNA	0	0	0	0	0	0	0	0	0	0	0	0	0	0	0	0	0	0	0	0	0
ssc-miR-7136-5p		miRNA	0	0	0	0	0	0	0	0	0	0	0	0	0	0	0	0	0	0	0	0	0

Supplementary Table S10. The predicted targe

RNA ID	Symbol	Type	updown_BGIR9823_85841	updown_BGIR9823_83955	updown_BGIR9823_84358	updown_BGIR9823_88015	updown_BGIR9823_88124	updown_BGIR9823_88780	updown_BGIR9823_92129	updown_BGIR9823_89027	updown_BGIR9823_89210	updown_BGIR9823_90771	updown_BGIR9823_95037	updown_BGIR9823_99096	updown_XR_001297664.2	updown_XR_002338666.1	updown_XR_002338675.1	updown_XR_002335905.1	updown_XR_002336236.1	updown_XR_001304283.2	updown_XR_002337660.1	updown_XR_307765.3	updown_XR_002343366.1
BGIR9823_100361	PRKAG2	mRNA	0	0	0	0	0	0	0	0	0	0	0	0	0	0	0	0	0	0	0	0	0
BGIR9823_100362	PRKAG2	mRNA	0	0	0	0	0	0	0	0	0	0	0	0	0	0	0	0	0	0	0	0	0
BGIR9823_100363	PRKAG2	mRNA	0	0	0	0	0	0	0	0	0	0	0	0	0	0	0	0	0	0	0	0	0
BGIR9823_100364	PRKAG2	mRNA	0	0	0	0	0	0	0	0	0	0	0	0	0	0	0	0	0	0	0	0	0
BGIR9823_101535	LOC102158401	lncRNA	0	0	0	0	0	0	0	0	0	0	0	0	0	0	0	0	0	0	0	0	0
BGIR9823_101541	LOC102157946	lncRNA	0	0	0	0	0	0	0	0	0	0	0	0	0	0	0	0	0	0	0	0	0
BGIR9823_101542	LOC102157946	lncRNA	0	0	0	0	0	0	0	0	0	0	0	0	0	0	0	0	0	0	0	0	0
BGIR9823_101560	LOC110258600	lncRNA	0	0	0	0	0	0	0	0	0	0	0	0	0	0	0	0	0	0	0	0	0
BGIR9823_101561	LOC110258600	lncRNA	0	0	0	0	0	0	0	0	0	0	0	0	0	0	0	0	0	0	0	0	0
BGIR9823_101562	LOC110258600	lncRNA	0	0	0	0	0	0	0	0	0	0	0	0	0	0	0	0	0	0	0	0	0
BGIR9823_101563	LOC110258600	lncRNA	0	0	0	0	0	0	0	0	0	0	0	0	0	0	0	0	0	0	0	0	0
BGIR9823_101564	LOC110258600	lncRNA	0	0	0	0	0	0	0	0	0	0	0	0	0	0	0	0	0	0	0	0	0
BGIR9823_101612	LOC100521600	lncRNA	0	0	0	0	0	0	0	0	0	0	0	0	0	0	0	0	0	0	0	0	0
BGIR9823_101853		lncRNA	0	0	0	0	0	0	0	0	0	0	0	0	0	0	0	0	0	0	0	0	0
BGIR9823_101854		lncRNA	0	0	0	0	0	0	0	0	0	0	0	0	0	0	0	0	0	0	0	0	0
BGIR9823_101855		lncRNA	0	0	0	0	0	0	0	0	0	0	0	0	0	0	0	0	0	0	0	0	0
BGIR9823_102146		lncRNA	0	0	0	0	0	0	0	0	0	0	0	0	0	0	0	0	0	0	0	0	0
BGIR9823_102180		lncRNA	0	0	0	0	0	0	0	0	0	0	0	0	0	0	0	0	0	0	0	0	0
BGIR9823_102279		lncRNA	0	0	0	0	0	0	0	0	0	0	0	0	0	0	0	0	0	0	0	0	0
BGIR9823_102282		lncRNA	0	0	0	0	0	0	0	0	0	0	0	0	0	0	0	0	0	0	0	0	0
BGIR9823_102361		lncRNA	0	0	0	0	0	0	0	0	0	0	0	0	0	0	0	0	0	0	0	0	0
BGIR9823_102362		lncRNA	0	0	0	0	0	0	0	0	0	0	0	0	0	0	0	0	0	0	0	0	0
BGIR9823_102363		lncRNA	0	0	0	0	0	0	0	0	0	0	0	0	0	0	0	0	0	0	0	0	0
BGIR9823_102727		lncRNA	0	0	0	0	0	0	0	0	0	0	0	0	0	0	0	0	0	0	0	0	0
BGIR9823_80121	SSSCA1	lncRNA	0	0	0	0	0	0	0	0	0	0	0	0	0	0	0	0	0	0	0	0	0
BGIR9823_80122	LOC102163816	lncRNA	0	0	0	0	0	0	0	0	0	0	0	0	0	0	0	0	0	0	0	0	0
BGIR9823_81007	EHBP1L1	mRNA	0	0	0	0	0	0	0	0	0	0	0	0	0	0	0	0	0	0	0	0	0
BGIR9823_81008	EHBP1L1	mRNA	0	0	0	0	0	0	0	0	0	0	0	0	0	0	0	0	0	0	0	0	0
BGIR9823_81009	EHBP1L1	mRNA	0	0	0	0	0	0	0	0	0	0	0	0	0	0	0	0	0	0	0	0	0
BGIR9823_81010	EHBP1L1	mRNA	0	0	0	0	0	0	0	0	0	0	0	0	0	0	0	0	0	0	0	0	0
BGIR9823_81011	SSSCA1	mRNA	0	0	0	0	0	0	0	0	0	0	0	0	0	0	0	0	0	0	0	0	0
BGIR9823_81012	FRMD8	lncRNA	0	0	0	0	0	0	0	0	0	0	0	0	0	0	0	0	0	0	0	0	0
BGIR9823_81013	LOC102163816	lncRNA	0	0	0	0	0	0	0	0	0	0	0	0	0	0	0	0	0	0	0	0	0
BGIR9823_81014	LOC102163816	lncRNA	0	0	0	0	0	0	0	0	0	0	0	0	0	0	0	0	0	0	0	0	0
BGIR9823_81015	LOC102163816	lncRNA	0	0	0	0	0	0	0	0	0	0	0	0	0	0	0	0	0	0	0	0	0
BGIR9823_81016	LOC102163816	lncRNA	0	0	0	0	0	0	0	0	0	0	0	0	0	0	0	0	0	0	0	0	0
BGIR9823_81017	LOC102163816	lncRNA	0	0	0	0	0	0	0	0	0	0	0	0	0	0	0	0	0	0	0	0	0
BGIR9823_81018	LOC102163816	lncRNA	0	0	0	0	0	0	0	0	0	0	0	0	0	0	0	0	0	0	0	0	0
BGIR9823_81019	LOC100513133	lncRNA	0	0	0	0	0	0	0	0	0	0	0	0	0	0	0	0	0	0	0	0	0
BGIR9823_84704	LOC102159820	mRNA	0	0	0	0	0	0	0	0	0	0	0	0	0	0	0	0	0	0	0	0	0
BGIR9823_84732	RANGAP1	mRNA	0	0	0	0	0	0	0	0	0	0	0	0	0	0	0	0	0	0	0	0	0

RNA ID	Symbol	Type	updown_BGIR9823_85841	updown_BGIR9823_83955	updown_BGIR9823_84358	updown_BGIR9823_88015	updown_BGIR9823_88124	updown_BGIR9823_88780	updown_BGIR9823_92129	updown_BGIR9823_89027	updown_BGIR9823_89210	updown_BGIR9823_90771	updown_BGIR9823_95037	updown_BGIR9823_99096	updown_XR_001297664.2	updown_XR_002338666.1	updown_XR_002338675.1	updown_XR_002335905.1	updown_XR_002336236.1	updown_XR_001304283.2	updown_XR_002337660.1	updown_XR_307765.3	updown_XR_002343366.1
BGIR9823_84733	RANGAP1	mRNA	0	0	0	0	0	0	0	0	0	0	0	0	0	0	0	0	0	0	0	0	0
BGIR9823_85245	CRADD	lncRNA	1	0	0	0	0	0	0	0	0	0	0	0	0	0	0	0	0	0	0	0	0
BGIR9823_85246	LOC100738154	lncRNA	1	0	0	0	0	0	0	0	0	0	0	0	0	0	0	0	0	0	0	0	0
BGIR9823_85293	TRMU	mRNA	0	0	0	0	0	0	0	0	0	0	0	0	0	0	0	0	0	0	0	0	0
BGIR9823_85294	TRMU	mRNA	0	0	0	0	0	0	0	0	0	0	0	0	0	0	0	0	0	0	0	0	0
BGIR9823_85295	TTC38	mRNA	0	0	0	0	0	0	0	0	0	0	0	0	0	0	0	0	0	0	0	0	0
BGIR9823_85296	PKDREJ	lncRNA	0	0	0	0	0	0	0	0	0	0	0	0	0	0	0	0	0	0	0	0	0
BGIR9823_85297	PPARA	mRNA	0	0	0	0	0	0	0	0	0	0	0	0	0	0	0	0	0	0	0	0	0
BGIR9823_85298	PPARA	mRNA	0	0	0	0	0	0	0	0	0	0	0	0	0	0	0	0	0	0	0	0	0
BGIR9823_85299	PPARA	mRNA	0	0	0	0	0	0	0	0	0	0	0	0	0	0	0	0	0	0	0	0	0
BGIR9823_85300	PPARA	lncRNA	0	0	0	0	0	0	0	0	0	0	0	0	0	0	0	0	0	0	0	0	0
BGIR9823_85301	LOC110260611	mRNA	0	0	0	0	0	0	0	0	0	0	0	0	0	0	0	0	0	0	0	0	0
BGIR9823_85317	ACO2	mRNA	0	0	0	0	0	0	0	0	0	0	0	0	0	0	0	0	0	0	0	0	0
BGIR9823_85318	LOC110260629	lncRNA	0	0	0	0	0	0	0	0	0	0	0	0	0	0	0	0	0	0	0	0	0
BGIR9823_85319	TEF	mRNA	0	0	0	0	0	0	0	0	0	0	0	0	0	0	0	0	0	0	0	0	0
BGIR9823_85320	ZC3H7B	mRNA	0	0	0	0	0	0	0	0	0	0	0	0	0	0	0	0	0	0	0	0	0
BGIR9823_85345	SYNGR1	mRNA	0	0	0	0	0	0	0	0	0	0	0	0	0	0	0	0	0	0	0	0	0
BGIR9823_85835	CRADD	mRNA	1	0	0	0	0	0	0	0	0	0	0	0	0	0	0	0	0	0	0	0	0
BGIR9823_85839	CRADD	lncRNA	1	0	0	0	0	0	0	0	0	0	0	0	0	0	0	0	0	0	0	0	0
BGIR9823_85840	CRADD	lncRNA	1	0	0	0	0	0	0	0	0	0	0	0	0	0	0	0	0	0	0	0	0
BGIR9823_85841	CRADD	lncRNA	1	0	0	0	0	0	0	0	0	0	0	0	0	0	0	0	0	0	0	0	0
BGIR9823_85930	LOC106510465	mRNA	0	0	0	0	0	0	0	0	0	0	0	0	0	0	0	0	0	0	0	0	0
BGIR9823_85931	MAF	lncRNA	0	0	0	0	0	0	0	0	0	0	0	0	0	0	0	0	0	0	0	0	0
BGIR9823_85932	MAF	lncRNA	0	0	0	0	0	0	0	0	0	0	0	0	0	0	0	0	0	0	0	0	0
BGIR9823_87139	LOC106510467	lncRNA	0	0	0	0	0	0	0	0	0	0	0	0	0	0	0	0	0	0	0	0	0
BGIR9823_88187	WRNIP1	mRNA	0	0	0	0	0	0	0	0	0	0	0	0	0	0	0	0	0	0	0	0	0
BGIR9823_88188	NQO2	mRNA	0	0	0	0	0	0	0	0	0	0	0	0	0	0	0	0	0	0	0	0	0
BGIR9823_88189	NQO2	mRNA	0	0	0	0	0	0	0	0	0	0	0	0	0	0	0	0	0	0	0	0	0
BGIR9823_88190	RIPK1	mRNA	0	0	0	0	0	0	0	0	0	0	0	0	0	0	0	0	0	0	0	0	0
BGIR9823_88191	RIPK1	mRNA	0	0	0	0	0	0	0	0	0	0	0	0	0	0	0	0	0	0	0	0	0
BGIR9823_88192	RIPK1	mRNA	0	0	0	0	0	0	0	0	0	0	0	0	0	0	0	0	0	0	0	0	0
BGIR9823_88193	RIPK1	mRNA	0	0	0	0	0	0	0	0	0	0	0	0	0	0	0	0	0	0	0	0	0
BGIR9823_88196	PRPF4B	mRNA	0	0	0	0	0	0	0	1	0	0	0	0	1	0	0	0	0	0	0	0	0
BGIR9823_88197	PRPF4B	mRNA	0	0	0	0	0	0	0	1	0	0	0	0	1	0	0	0	0	0	0	0	0
BGIR9823_88198	PRPF4B	mRNA	0	0	0	0	0	0	0	1	0	0	0	0	1	0	0	0	0	0	0	0	0
BGIR9823_88211	TMEM170B	lncRNA	0	0	0	0	0	0	0	0	0	0	0	0	0	0	0	0	0	0	0	0	0
BGIR9823_88212	TMEM170B	lncRNA	0	0	0	0	0	0	0	0	0	0	0	0	0	0	0	0	0	0	0	0	0
BGIR9823_88213	TMEM170B	lncRNA	0	0	0	0	0	0	0	0	0	0	0	0	0	0	0	0	0	0	0	0	0
BGIR9823_88214	TMEM170B	lncRNA	0	0	0	0	0	0	0	0	0	0	0	0	0	0	0	0	0	0	0	0	0
BGIR9823_88217	PHACTR1	mRNA	0	0	0	0	0	0	0	0	0	0	0	0	0	0	0	0	0	0	0	0	0
BGIR9823_88218	PHACTR1	lncRNA	0	0	0	0	0	0	0	0	0	0	0	0	0	0	0	0	0	0	0	0	0
BGIR9823_89017	MYLK4	lncRNA	0	0	0	0	0	0	0	0	0	0	0	0	0	0	0	0	0	0	0	0	0
BGIR9823_89019	SERPINB1	lncRNA	0	0	0	0	0	0	0	0	0	0	0	0	0	0	0	0	0	0	0	0	0

RNA ID	Symbol	Type	updown_BGIR9823_85841	updown_BGIR9823_83955	updown_BGIR9823_84358	updown_BGIR9823_88015	updown_BGIR9823_88124	updown_BGIR9823_88780	updown_BGIR9823_92129	updown_BGIR9823_89027	updown_BGIR9823_89210	updown_BGIR9823_90771	updown_BGIR9823_95037	updown_BGIR9823_99096	updown_XR_001297664.2	updown_XR_002338666.1	updown_XR_002338675.1	updown_XR_002335905.1	updown_XR_002336236.1	updown_XR_001304283.2	updown_XR_002337660.1	updown_XR_307765.3	updown_XR_002343366.1
BGIR9823_89020	SERPINB9	mRNA	0	0	0	0	0	0	0	0	0	0	0	0	0	0	0	0	0	0	0	0	0
BGIR9823_89021	RIPK1	lncRNA	0	0	0	0	0	0	0	0	0	0	0	0	0	0	0	0	0	0	0	0	0
BGIR9823_89025	PXDC1	mRNA	0	0	0	0	0	0	0	1	0	0	0	0	1	0	0	0	0	0	0	0	0
BGIR9823_89026	PXDC1	lncRNA	0	0	0	0	0	0	0	1	0	0	0	0	0	0	0	0	0	0	0	0	0
BGIR9823_89027	LOC102161744	lncRNA	0	0	0	0	0	0	0	1	0	0	0	0	1	0	0	0	0	0	0	0	0
BGIR9823_89040	TBC1D7	lncRNA	0	0	0	0	0	0	0	0	0	0	0	0	0	0	0	0	0	0	0	0	0
BGIR9823_89041	TBC1D7	lncRNA	0	0	0	0	0	0	0	0	0	0	0	0	0	0	0	0	0	0	0	0	0
BGIR9823_89042	TBC1D7	lncRNA	0	0	0	0	0	0	0	0	0	0	0	0	0	0	0	0	0	0	0	0	0
BGIR9823_89043	TBC1D7	lncRNA	0	0	0	0	0	0	0	0	0	0	0	0	0	0	0	0	0	0	0	0	0
BGIR9823_89044	GFOD1	lncRNA	0	0	0	0	0	0	0	0	0	0	0	0	0	0	0	0	0	0	0	0	0
BGIR9823_89045	GFOD1	mRNA	0	0	0	0	0	0	0	0	0	0	0	0	0	0	0	0	0	0	0	0	0
BGIR9823_89046	GFOD1	mRNA	0	0	0	0	0	0	0	0	0	0	0	0	0	0	0	0	0	0	0	0	0
BGIR9823_89047	GFOD1	mRNA	0	0	0	0	0	0	0	0	0	0	0	0	0	0	0	0	0	0	0	0	0
BGIR9823_89048	GFOD1	lncRNA	0	0	0	0	0	0	0	0	0	0	0	0	0	0	0	0	0	0	0	0	0
BGIR9823_89049	GFOD1	lncRNA	0	0	0	0	0	0	0	0	0	0	0	0	0	0	0	0	0	0	0	0	0
BGIR9823_90771	NEU3	lncRNA	0	0	0	0	0	0	0	0	0	1	0	0	0	0	0	0	0	0	0	0	0
BGIR9823_90772	SLCO2B1	mRNA	0	0	0	0	0	0	0	0	0	1	0	0	0	0	0	0	0	0	0	0	0
BGIR9823_90913	LOC110255435	lncRNA	0	0	0	0	0	0	0	0	0	0	0	0	0	0	0	0	0	0	0	0	0
BGIR9823_90934	LOC110255441	lncRNA	0	0	0	0	0	0	0	0	0	0	0	0	0	0	0	0	0	0	0	0	0
BGIR9823_90935	CCDC84	mRNA	0	0	0	0	0	0	0	0	0	0	0	0	0	0	0	0	0	0	0	0	0
BGIR9823_90936	TRAPPC4	mRNA	0	0	0	0	0	0	0	0	0	0	0	0	0	0	0	0	0	0	0	0	0
BGIR9823_90937	HMBS	mRNA	0	0	0	0	0	0	0	0	0	0	0	0	0	0	0	0	0	0	0	0	0
BGIR9823_90956	SORL1	mRNA	0	0	0	0	0	0	0	0	0	0	0	0	0	0	0	0	0	0	0	0	0
BGIR9823_90957	SORL1	mRNA	0	0	0	0	0	0	0	0	0	0	0	0	0	0	0	0	0	0	0	0	0
BGIR9823_90958	SORL1	mRNA	0	0	0	0	0	0	0	0	0	0	0	0	0	0	0	0	0	0	0	0	0
BGIR9823_90960	CLMP	lncRNA	0	0	0	0	0	0	0	0	0	0	0	0	0	0	0	0	0	0	0	0	0
BGIR9823_91161	LOC100522267	mRNA	0	0	0	0	0	0	0	0	0	0	0	0	0	0	0	0	0	0	0	0	0
BGIR9823_91162	LOC100522455	mRNA	0	0	0	0	0	0	0	0	0	0	0	0	0	0	0	0	0	0	0	0	0
BGIR9823_91163	ABCB1	mRNA	0	0	0	0	0	0	0	0	0	0	0	0	0	0	0	0	0	0	0	0	0
BGIR9823_91402	HBB	mRNA	0	0	0	0	0	0	0	0	0	0	0	0	0	0	0	0	0	0	0	0	0
BGIR9823_91403	HBB	mRNA	0	0	0	0	0	0	0	0	0	0	0	0	0	0	0	0	0	0	0	0	0
BGIR9823_91404	HBB	mRNA	0	0	0	0	0	0	0	0	0	0	0	0	0	0	0	0	0	0	0	0	0
BGIR9823_91405	HBB	mRNA	0	0	0	0	0	0	0	0	0	0	0	0	0	0	0	0	0	0	0	0	0
BGIR9823_91406	HBB	mRNA	0	0	0	0	0	0	0	0	0	0	0	0	0	0	0	0	0	0	0	0	0
BGIR9823_91407	HBB	mRNA	0	0	0	0	0	0	0	0	0	0	0	0	0	0	0	0	0	0	0	0	0
BGIR9823_91408	HBB	mRNA	0	0	0	0	0	0	0	0	0	0	0	0	0	0	0	0	0	0	0	0	0
BGIR9823_91409	HBB	mRNA	0	0	0	0	0	0	0	0	0	0	0	0	0	0	0	0	0	0	0	0	0
BGIR9823_91625	BCL9L	lncRNA	0	0	0	0	0	0	0	0	0	0	0	0	0	0	0	0	0	0	0	0	0
BGIR9823_91626	BCL9L	lncRNA	0	0	0	0	0	0	0	0	0	0	0	0	0	0	0	0	0	0	0	0	0
BGIR9823_91627	RPS25	mRNA	0	0	0	0	0	0	0	0	0	0	0	0	0	0	0	0	0	0	0	0	0
BGIR9823_91628	RPS25	mRNA	0	0	0	0	0	0	0	0	0	0	0	0	0	0	0	0	0	0	0	0	0
BGIR9823_91629	SLC37A4	mRNA	0	0	0	0	0	0	0	0	0	0	0	0	0	0	0	0	0	0	0	0	0
BGIR9823_91630	HYOU1	mRNA	0	0	0	0	0	0	0	0	0	0	0	0	0	0	0	0	0	0	0	0	0

RNA ID	Symbol	Type	updown_BGIR9823_85841	updown_BGIR9823_83955	updown_BGIR9823_84358	updown_BGIR9823_88015	updown_BGIR9823_88124	updown_BGIR9823_88780	updown_BGIR9823_92129	updown_BGIR9823_89027	updown_BGIR9823_89210	updown_BGIR9823_90771	updown_BGIR9823_95037	updown_BGIR9823_99096	updown_XR_001297664.2	updown_XR_002338666.1	updown_XR_002338675.1	updown_XR_002335905.1	updown_XR_002336236.1	updown_XR_001304283.2	updown_XR_002337660.1	updown_XR_307765.3	updown_XR_002343366.1
BGIR9823_91640	CLMP	mRNA	0	0	0	0	0	0	0	0	0	0	0	0	0	0	0	0	0	0	0	0	0
BGIR9823_91819	RUNDC3B	lncRNA	0	0	0	0	0	0	0	0	0	0	0	0	0	0	0	0	0	0	0	0	0
BGIR9823_91820	RUNDC3B	lncRNA	0	0	0	0	0	0	0	0	0	0	0	0	0	0	0	0	0	0	0	0	0
BGIR9823_91821	RUNDC3B	lncRNA	0	0	0	0	0	0	0	0	0	0	0	0	0	0	0	0	0	0	0	0	0
BGIR9823_91822	RUNDC3B	lncRNA	0	0	0	0	0	0	0	0	0	0	0	0	0	0	0	0	0	0	0	0	0
BGIR9823_91823	RUNDC3B	lncRNA	0	0	0	0	0	0	0	0	0	0	0	0	0	0	0	0	0	0	0	0	0
BGIR9823_91824	RUNDC3B	lncRNA	0	0	0	0	0	0	0	0	0	0	0	0	0	0	0	0	0	0	0	0	0
BGIR9823_91825	RUNDC3B	lncRNA	0	0	0	0	0	0	0	0	0	0	0	0	0	0	0	0	0	0	0	0	0
BGIR9823_91826	LOC102164596	lncRNA	0	0	0	0	0	0	0	0	0	0	0	0	0	0	0	0	0	0	0	0	0
BGIR9823_92993	KATNAL1	mRNA	0	0	0	0	0	0	0	0	0	0	0	0	0	0	0	0	0	0	0	0	0
BGIR9823_92994	KATNAL1	mRNA	0	0	0	0	0	0	0	0	0	0	0	0	0	0	0	0	0	0	0	0	0
BGIR9823_92995	KATNAL1	mRNA	0	0	0	0	0	0	0	0	0	0	0	0	0	0	0	0	0	0	0	0	0
BGIR9823_92996	KATNAL1	mRNA	0	0	0	0	0	0	0	0	0	0	0	0	0	0	0	0	0	0	0	0	0
BGIR9823_92997	KATNAL1	mRNA	0	0	0	0	0	0	0	0	0	0	0	0	0	0	0	0	0	0	0	0	0
BGIR9823_92999	LOC110255292	lncRNA	0	0	0	0	0	0	0	0	0	0	0	0	0	0	0	0	0	0	0	0	0
BGIR9823_93000	LOC102166871	lncRNA	0	0	0	0	0	0	0	0	0	0	0	0	0	0	0	0	0	0	0	0	0
BGIR9823_98051	LOC102164998	lncRNA	0	0	0	0	0	0	0	0	0	0	0	0	0	0	0	0	0	0	0	0	0
BGIR9823_99244	LOC102167708	lncRNA	0	0	0	0	0	0	0	0	0	0	0	0	0	0	0	0	0	0	0	0	0
NM_001004034.1	HMGB1	mRNA	0	0	0	0	0	0	0	0	0	0	0	0	0	0	0	0	0	0	0	0	0
NM_001044526.1	PPARA	mRNA	0	0	0	0	0	0	0	0	0	0	0	0	0	0	0	0	0	0	0	0	0
NM_001111256.1	SERPINB9	mRNA	0	0	0	0	0	0	0	0	0	0	0	0	0	0	0	0	0	0	0	0	0
NM_001113443.1	ECI2	mRNA	0	0	0	0	0	0	0	1	0	0	0	0	1	0	0	0	0	0	0	0	0
NM_001144841.1	HBB	mRNA	0	0	0	0	0	0	0	0	0	0	0	0	0	0	0	0	0	0	0	0	0
NM_001199719.1	SLC37A4	mRNA	0	0	0	0	0	0	0	0	0	0	0	0	0	0	0	0	0	0	0	0	0
NM_001243907.1	HSPA8	mRNA	0	0	0	0	0	0	0	0	0	0	0	0	0	0	0	0	0	0	0	0	0
NM_001244063.1	RPL3	mRNA	0	0	0	0	0	0	0	0	0	0	0	0	0	0	0	0	0	0	0	0	0
NM_001244066.1	SYNGR1	mRNA	0	0	0	0	0	0	0	0	0	0	0	0	0	0	0	0	0	0	0	0	0
NM_001244396.1	CCDC84	mRNA	0	0	0	0	0	0	0	0	0	0	0	0	0	0	0	0	0	0	0	0	0
NM_001287846.1	RHEB	mRNA	0	0	0	0	0	0	0	0	0	0	0	0	0	0	0	0	0	0	0	0	0
NM_213954.1	ACO2	mRNA	0	0	0	0	0	0	0	0	0	0	0	0	0	0	0	0	0	0	0	0	0
NM_214012.1	UPK2	mRNA	0	0	0	0	0	0	0	0	0	0	0	0	0	0	0	0	0	0	0	0	0
URS0000BC92F4	LOC102167708	lncRNA	0	0	0	0	0	0	0	0	0	0	0	0	0	0	0	0	0	0	0	0	0
XM_001928309.6	TBC1D7	mRNA	0	0	0	0	0	0	0	0	0	0	0	0	0	0	0	0	0	0	0	0	0
XM_001928852.5	PXDC1	mRNA	0	0	0	0	0	0	0	1	0	0	0	0	0	0	0	0	0	0	0	0	0
XM_003122534.4	KCNK7	mRNA	0	0	0	0	0	0	0	0	0	0	0	0	0	0	0	0	0	0	0	0	0
XM_003122558.5	SCYL1	mRNA	0	0	0	0	0	0	0	0	0	0	0	0	0	0	0	0	0	0	0	0	0
XM_003122562.5	FRMD8	mRNA	0	0	0	0	0	0	0	0	0	0	0	0	0	0	0	0	0	0	0	0	0
XM_003125951.6	TTC38	mRNA	0	0	0	0	0	0	0	0	0	0	0	0	0	0	0	0	0	0	0	0	0
XM_003125953.4	CDPF1	mRNA	0	0	0	0	0	0	0	0	0	0	0	0	0	0	0	0	0	0	0	0	0
XM_003126012.6	CBX7	mRNA	0	0	0	0	0	0	0	0	0	0	0	0	0	0	0	0	0	0	0	0	0
XM_003128161.4	RIPK1	mRNA	0	0	0	0	0	0	0	0	0	0	0	0	0	0	0	0	0	0	0	0	0
XM_003129508.2	LOC100514417	mRNA	0	0	0	0	0	0	0	0	0	0	0	0	0	0	0	0	0	0	0	0	0
XM_003129512.2	LOC100515268	mRNA	0	0	0	0	0	0	0	0	0	0	0	0	0	0	0	0	0	0	0	0	0

RNA ID	Symbol	Type	updown_BGIR9823_85841	updown_BGIR9823_83955	updown_BGIR9823_84358	updown_BGIR9823_88015	updown_BGIR9823_88124	updown_BGIR9823_88780	updown_BGIR9823_92129	updown_BGIR9823_89027	updown_BGIR9823_89210	updown_BGIR9823_90771	updown_BGIR9823_95037	updown_BGIR9823_99096	updown_XR_001297664.2	updown_XR_002338666.1	updown_XR_002338675.1	updown_XR_002335905.1	updown_XR_002336236.1	updown_XR_001304283.2	updown_XR_002337660.1	updown_XR_307765.3	updown_XR_002343366.1
XM_003129514.3	LOC100515618	mRNA	0	0	0	0	0	0	0	0	0	0	0	0	0	0	0	0	0	0	0	0	0
XM_003129519.2	LOC100517595	mRNA	0	0	0	0	0	0	0	0	0	0	0	0	0	0	0	0	0	0	0	0	0
XM_003129529.4	LOC100519910	mRNA	0	0	0	0	0	0	0	0	0	0	0	0	0	0	0	0	0	0	0	0	0
XM_003129915.4	CXCR5	mRNA	0	0	0	0	0	0	0	0	0	0	0	0	0	0	0	0	0	0	0	0	0
XM_003129916.5	BCL9L	mRNA	0	0	0	0	0	0	0	0	0	0	0	0	0	0	0	0	0	0	0	0	0
XM_003129918.4	TRAPPC4	mRNA	0	0	0	0	0	0	0	0	0	0	0	0	0	0	0	0	0	0	0	0	0
XM_003129919.5	RPS25	mRNA	0	0	0	0	0	0	0	0	0	0	0	0	0	0	0	0	0	0	0	0	0
XM_003129920.3	RPS25	mRNA	0	0	0	0	0	0	0	0	0	0	0	0	0	0	0	0	0	0	0	0	0
XM_003129922.4	HYOU1	mRNA	0	0	0	0	0	0	0	0	0	0	0	0	0	0	0	0	0	0	0	0	0
XM_003129923.6	VPS11	mRNA	0	0	0	0	0	0	0	0	0	0	0	0	0	0	0	0	0	0	0	0	0
XM_003129944.4	SC5D	mRNA	0	0	0	0	0	0	0	0	0	0	0	0	0	0	0	0	0	0	0	0	0
XM_003357161.1	LOC100620971	mRNA	0	0	0	0	0	0	0	0	0	0	0	0	0	0	0	0	0	0	0	0	0
XM_003482119.4	SERPINB1	mRNA	0	0	0	0	0	0	0	0	0	0	0	0	0	0	0	0	0	0	0	0	0
XM_003482132.4	TMEM170B	mRNA	0	0	0	0	0	0	0	0	0	0	0	0	0	0	0	0	0	0	0	0	0
XM_003482133.4	GFOD1	mRNA	0	0	0	0	0	0	0	0	0	0	0	0	0	0	0	0	0	0	0	0	0
XM_003482563.4	SPCS2	mRNA	0	0	0	0	0	0	0	0	0	1	0	0	0	0	0	0	0	0	0	0	0
XM_003482565.4	NEU3	mRNA	0	0	0	0	0	0	0	0	0	1	0	0	0	0	0	0	0	0	0	0	0
XM_003482567.1	LOC100739359	mRNA	0	0	0	0	0	0	0	0	0	1	0	0	0	0	0	0	0	0	0	0	0
XM_003482630.3	BSX	mRNA	0	0	0	0	0	0	0	0	0	0	0	0	0	0	0	0	0	0	0	0	0
XM_005653107.3	CHADL	mRNA	0	0	0	0	0	0	0	0	0	0	0	0	0	0	0	0	0	0	0	0	0
XM_005653640.1	LOC100621181	mRNA	0	0	0	0	0	0	0	0	0	0	0	0	0	0	0	0	0	0	0	0	0
XM_005654266.3	PRKAG2	mRNA	0	0	0	0	0	0	0	0	0	0	0	0	0	0	0	0	0	0	0	0	0
XM_005655470.3	CDPF1	mRNA	0	0	0	0	0	0	0	0	0	0	0	0	0	0	0	0	0	0	0	0	0
XM_005660703.3	LTBP3	mRNA	0	0	0	0	0	0	0	0	0	0	0	0	0	0	0	0	0	0	0	0	0
XM_005660705.3	SCYL1	mRNA	0	0	0	0	0	0	0	0	0	0	0	0	0	0	0	0	0	0	0	0	0
XM_005660707.3	SLC25A45	mRNA	0	0	0	0	0	0	0	0	0	0	0	0	0	0	0	0	0	0	0	0	0
XM_005665536.3	RIPK1	mRNA	0	0	0	0	0	0	0	0	0	0	0	0	0	0	0	0	0	0	0	0	0
XM_005665537.3	RIPK1	mRNA	0	0	0	0	0	0	0	0	0	0	0	0	0	0	0	0	0	0	0	0	0
XM_005665538.3	RIPK1	mRNA	0	0	0	0	0	0	0	0	0	0	0	0	0	0	0	0	0	0	0	0	0
XM_005665575.3	PHACTR1	mRNA	0	0	0	0	0	0	0	0	0	0	0	0	0	0	0	0	0	0	0	0	0
XM_005665576.3	TBC1D7	mRNA	0	0	0	0	0	0	0	0	0	0	0	0	0	0	0	0	0	0	0	0	0
XM_005667128.3	LOC100627340	mRNA	0	0	0	0	0	0	0	0	0	1	0	0	0	0	0	0	0	0	0	0	0
XM_005667397.2	CCDC84	mRNA	0	0	0	0	0	0	0	0	0	0	0	0	0	0	0	0	0	0	0	0	0
XM_005667398.3	SLC37A4	mRNA	0	0	0	0	0	0	0	0	0	0	0	0	0	0	0	0	0	0	0	0	0
XM_005667402.3	SLC37A4	mRNA	0	0	0	0	0	0	0	0	0	0	0	0	0	0	0	0	0	0	0	0	0
XM_005667403.3	SLC37A4	mRNA	0	0	0	0	0	0	0	0	0	0	0	0	0	0	0	0	0	0	0	0	0
XM_005667413.3	BCL9L	mRNA	0	0	0	0	0	0	0	0	0	0	0	0	0	0	0	0	0	0	0	0	0
XM_005667414.3	FOXR1	mRNA	0	0	0	0	0	0	0	0	0	0	0	0	0	0	0	0	0	0	0	0	0
XM_005667416.3	HYOU1	mRNA	0	0	0	0	0	0	0	0	0	0	0	0	0	0	0	0	0	0	0	0	0
XM_005667419.3	HYOU1	mRNA	0	0	0	0	0	0	0	0	0	0	0	0	0	0	0	0	0	0	0	0	0
XM_005667420.3	HYOU1	mRNA	0	0	0	0	0	0	0	0	0	0	0	0	0	0	0	0	0	0	0	0	0
XM_005667440.2	JHY	mRNA	0	0	0	0	0	0	0	0	0	0	0	0	0	0	0	0	0	0	0	0	0
XM_005668316.3	HMGB1	mRNA	0	0	0	0	0	0	0	0	0	0	0	0	0	0	0	0	0	0	0	0	0

RNA ID	Symbol	Type	updown_BGIR9823_85841	updown_BGIR9823_83955	updown_BGIR9823_84358	updown_BGIR9823_88015	updown_BGIR9823_88124	updown_BGIR9823_88780	updown_BGIR9823_92129	updown_BGIR9823_89027	updown_BGIR9823_89210	updown_BGIR9823_90771	updown_BGIR9823_95037	updown_BGIR9823_99096	updown_XR_001297664.2	updown_XR_002338666.1	updown_XR_002338675.1	updown_XR_002335905.1	updown_XR_002336236.1	updown_XR_001304283.2	updown_XR_002337660.1	updown_XR_307765.3	updown_XR_002343366.1
XM_005668317.3	HMGB1	mRNA	0	0	0	0	0	0	0	0	0	0	0	0	0	0	0	0	0	0	0	0	0
XM_005668318.3	HMGB1	mRNA	0	0	0	0	0	0	0	0	0	0	0	0	0	0	0	0	0	0	0	0	0
XM_005671489.3	EMX2	mRNA	0	0	0	0	0	0	0	0	0	0	0	0	0	0	0	0	0	0	0	0	0
XM_005672397.3	MYO10	mRNA	0	0	0	0	0	0	0	0	0	0	0	0	0	0	0	0	0	0	0	0	0
XM_013977400.2	FAM50B	mRNA	0	0	0	0	0	0	0	1	0	0	0	0	1	0	0	0	0	0	0	0	0
XM_013977425.2	NEDD9	mRNA	0	0	0	0	0	0	0	0	0	0	0	0	0	0	0	0	0	0	0	0	0
XM_013977430.2	TBC1D7	mRNA	0	0	0	0	0	0	0	0	0	0	0	0	0	0	0	0	0	0	0	0	0
XM_013977431.2	TBC1D7	mRNA	0	0	0	0	0	0	0	0	0	0	0	0	0	0	0	0	0	0	0	0	0
XM_013977432.2	TBC1D7	mRNA	0	0	0	0	0	0	0	0	0	0	0	0	0	0	0	0	0	0	0	0	0
XM_013977433.2	GFOD1	mRNA	0	0	0	0	0	0	0	0	0	0	0	0	0	0	0	0	0	0	0	0	0
XM_013977434.2	GFOD1	mRNA	0	0	0	0	0	0	0	0	0	0	0	0	0	0	0	0	0	0	0	0	0
XM_013979449.2	SLC37A4	mRNA	0	0	0	0	0	0	0	0	0	0	0	0	0	0	0	0	0	0	0	0	0
XM_013979456.2	BCL9L	mRNA	0	0	0	0	0	0	0	0	0	0	0	0	0	0	0	0	0	0	0	0	0
XM_013979457.2	BCL9L	mRNA	0	0	0	0	0	0	0	0	0	0	0	0	0	0	0	0	0	0	0	0	0
XM_013979458.2	BCL9L	mRNA	0	0	0	0	0	0	0	0	0	0	0	0	0	0	0	0	0	0	0	0	0
XM_013979459.2	BCL9L	mRNA	0	0	0	0	0	0	0	0	0	0	0	0	0	0	0	0	0	0	0	0	0
XM_013979461.2	TRAPPC4	mRNA	0	0	0	0	0	0	0	0	0	0	0	0	0	0	0	0	0	0	0	0	0
XM_013979463.2	HYOU1	mRNA	0	0	0	0	0	0	0	0	0	0	0	0	0	0	0	0	0	0	0	0	0
XM_013979484.2	LOC100523684	mRNA	0	0	0	0	0	0	0	0	0	0	0	0	0	0	0	0	0	0	0	0	0
XM_013979486.2	LOC100523684	mRNA	0	0	0	0	0	0	0	0	0	0	0	0	0	0	0	0	0	0	0	0	0
XM_013979491.2	JHY	mRNA	0	0	0	0	0	0	0	0	0	0	0	0	0	0	0	0	0	0	0	0	0
XM_013980418.2	KATNAL1	mRNA	0	0	0	0	0	0	0	0	0	0	0	0	0	0	0	0	0	0	0	0	0
XM_013988182.2	TTC38	mRNA	0	0	0	0	0	0	0	0	0	0	0	0	0	0	0	0	0	0	0	0	0
XM_013988183.2	TTC38	mRNA	0	0	0	0	0	0	0	0	0	0	0	0	0	0	0	0	0	0	0	0	0
XM_013989589.2	LOC100522267	mRNA	0	0	0	0	0	0	0	0	0	0	0	0	0	0	0	0	0	0	0	0	0
XM_013989592.2	LOC100522455	mRNA	0	0	0	0	0	0	0	0	0	0	0	0	0	0	0	0	0	0	0	0	0
XM_013989593.2	LOC100522455	mRNA	0	0	0	0	0	0	0	0	0	0	0	0	0	0	0	0	0	0	0	0	0
XM_013994099.2	FRMD8	mRNA	0	0	0	0	0	0	0	0	0	0	0	0	0	0	0	0	0	0	0	0	0
XM_013994100.2	SLC25A45	mRNA	0	0	0	0	0	0	0	0	0	0	0	0	0	0	0	0	0	0	0	0	0
XM_013994102.2	SLC25A45	mRNA	0	0	0	0	0	0	0	0	0	0	0	0	0	0	0	0	0	0	0	0	0
XM_013994103.2	SLC25A45	mRNA	0	0	0	0	0	0	0	0	0	0	0	0	0	0	0	0	0	0	0	0	0
XM_013994104.2	SLC25A45	mRNA	0	0	0	0	0	0	0	0	0	0	0	0	0	0	0	0	0	0	0	0	0
XM_013997510.2	CBX7	mRNA	0	0	0	0	0	0	0	0	0	0	0	0	0	0	0	0	0	0	0	0	0
XM_013999070.2	LOC100520088	mRNA	0	0	0	0	0	0	0	0	0	0	0	0	0	0	0	0	0	0	0	0	0
XM_021062230.1	LOC100517771	mRNA	0	0	0	0	0	0	0	0	0	0	0	0	0	0	0	0	0	0	0	0	0
XM_021062231.1	LOC100517957	mRNA	0	0	0	0	0	0	0	0	0	0	0	0	0	0	0	0	0	0	0	0	0
XM_021062367.1	LOC110255365	mRNA	0	0	0	0	0	0	0	0	0	0	0	0	0	0	0	0	0	0	0	0	0
XM_021062369.1	LOC100737110	mRNA	0	0	0	0	0	0	0	0	0	0	0	0	0	0	0	0	0	0	0	0	0
XM_021062370.1	LOC110255368	mRNA	0	0	0	0	0	0	0	0	0	0	0	0	0	0	0	0	0	0	0	0	0
XM_021062371.1	LOC100515441	mRNA	0	0	0	0	0	0	0	0	0	0	0	0	0	0	0	0	0	0	0	0	0
XM_021062374.1	LOC110255371	mRNA	0	0	0	0	0	0	0	0	0	0	0	0	0	0	0	0	0	0	0	0	0
XM_021062375.1	LOC100626951	mRNA	0	0	0	0	0	0	0	0	0	0	0	0	0	0	0	0	0	0	0	0	0
XM_021062491.1	XRRA1	mRNA	0	0	0	0	0	0	0	0	0	1	0	0	0	0	0	0	0	0	0	0	0

RNA ID	Symbol	Type	updown_BGIR9823_85841	updown_BGIR9823_83955	updown_BGIR9823_84358	updown_BGIR9823_88015	updown_BGIR9823_88124	updown_BGIR9823_88780	updown_BGIR9823_92129	updown_BGIR9823_89027	updown_BGIR9823_89210	updown_BGIR9823_90771	updown_BGIR9823_95037	updown_BGIR9823_99096	updown_XR_001297664.2	updown_XR_002338666.1	updown_XR_002338675.1	updown_XR_002335905.1	updown_XR_002336236.1	updown_XR_001304283.2	updown_XR_002337660.1	updown_XR_307765.3	updown_XR_002343366.1
XM_021062493.1	XRRA1	mRNA	0	0	0	0	0	0	0	0	0	1	0	0	0	0	0	0	0	0	0	0	0
XM_021062494.1	XRRA1	mRNA	0	0	0	0	0	0	0	0	0	1	0	0	0	0	0	0	0	0	0	0	0
XM_021062495.1	XRRA1	mRNA	0	0	0	0	0	0	0	0	0	1	0	0	0	0	0	0	0	0	0	0	0
XM_021062496.1	XRRA1	mRNA	0	0	0	0	0	0	0	0	0	1	0	0	0	0	0	0	0	0	0	0	0
XM_021062497.1	XRRA1	mRNA	0	0	0	0	0	0	0	0	0	1	0	0	0	0	0	0	0	0	0	0	0
XM_021062498.1	XRRA1	mRNA	0	0	0	0	0	0	0	0	0	1	0	0	0	0	0	0	0	0	0	0	0
XM_021062499.1	XRRA1	mRNA	0	0	0	0	0	0	0	0	0	1	0	0	0	0	0	0	0	0	0	0	0
XM_021062500.1	XRRA1	mRNA	0	0	0	0	0	0	0	0	0	1	0	0	0	0	0	0	0	0	0	0	0
XM_021062501.1	XRRA1	mRNA	0	0	0	0	0	0	0	0	0	1	0	0	0	0	0	0	0	0	0	0	0
XM_021062502.1	XRRA1	mRNA	0	0	0	0	0	0	0	0	0	1	0	0	0	0	0	0	0	0	0	0	0
XM_021062503.1	XRRA1	mRNA	0	0	0	0	0	0	0	0	0	1	0	0	0	0	0	0	0	0	0	0	0
XM_021062511.1	SLCO2B1	mRNA	0	0	0	0	0	0	0	0	0	1	0	0	0	0	0	0	0	0	0	0	0
XM_021062948.1	BCL9L	mRNA	0	0	0	0	0	0	0	0	0	0	0	0	0	0	0	0	0	0	0	0	0
XM_021062949.1	TRAPPC4	mRNA	0	0	0	0	0	0	0	0	0	0	0	0	0	0	0	0	0	0	0	0	0
XM_021062950.1	HYOU1	mRNA	0	0	0	0	0	0	0	0	0	0	0	0	0	0	0	0	0	0	0	0	0
XM_021062951.1	HYOU1	mRNA	0	0	0	0	0	0	0	0	0	0	0	0	0	0	0	0	0	0	0	0	0
XM_021062952.1	HYOU1	mRNA	0	0	0	0	0	0	0	0	0	0	0	0	0	0	0	0	0	0	0	0	0
XM_021062953.1	HYOU1	mRNA	0	0	0	0	0	0	0	0	0	0	0	0	0	0	0	0	0	0	0	0	0
XM_021062954.1	HYOU1	mRNA	0	0	0	0	0	0	0	0	0	0	0	0	0	0	0	0	0	0	0	0	0
XM_021062955.1	HYOU1	mRNA	0	0	0	0	0	0	0	0	0	0	0	0	0	0	0	0	0	0	0	0	0
XM_021062956.1	HYOU1	mRNA	0	0	0	0	0	0	0	0	0	0	0	0	0	0	0	0	0	0	0	0	0
XM_021062957.1	VPS11	mRNA	0	0	0	0	0	0	0	0	0	0	0	0	0	0	0	0	0	0	0	0	0
XM_021062993.1	LOC100523684	mRNA	0	0	0	0	0	0	0	0	0	0	0	0	0	0	0	0	0	0	0	0	0
XM_021062994.1	LOC100523684	mRNA	0	0	0	0	0	0	0	0	0	0	0	0	0	0	0	0	0	0	0	0	0
XM_021062995.1	LOC100523684	mRNA	0	0	0	0	0	0	0	0	0	0	0	0	0	0	0	0	0	0	0	0	0
XM_021062996.1	LOC100523684	mRNA	0	0	0	0	0	0	0	0	0	0	0	0	0	0	0	0	0	0	0	0	0
XM_021062997.1	LOC100523684	mRNA	0	0	0	0	0	0	0	0	0	0	0	0	0	0	0	0	0	0	0	0	0
XM_021062998.1	LOC100523684	mRNA	0	0	0	0	0	0	0	0	0	0	0	0	0	0	0	0	0	0	0	0	0
XM_021062999.1	LOC100523684	mRNA	0	0	0	0	0	0	0	0	0	0	0	0	0	0	0	0	0	0	0	0	0
XM_021063000.1	LOC100523684	mRNA	0	0	0	0	0	0	0	0	0	0	0	0	0	0	0	0	0	0	0	0	0
XM_021063001.1	LOC100523684	mRNA	0	0	0	0	0	0	0	0	0	0	0	0	0	0	0	0	0	0	0	0	0
XM_021063002.1	LOC100523684	mRNA	0	0	0	0	0	0	0	0	0	0	0	0	0	0	0	0	0	0	0	0	0
XM_021063004.1	LOC100523684	mRNA	0	0	0	0	0	0	0	0	0	0	0	0	0	0	0	0	0	0	0	0	0
XM_021063005.1	LOC100523684	mRNA	0	0	0	0	0	0	0	0	0	0	0	0	0	0	0	0	0	0	0	0	0
XM_021063006.1	LOC100523684	mRNA	0	0	0	0	0	0	0	0	0	0	0	0	0	0	0	0	0	0	0	0	0
XM_021063009.1	SORL1	mRNA	0	0	0	0	0	0	0	0	0	0	0	0	0	0	0	0	0	0	0	0	0
XM_021063010.1	SORL1	mRNA	0	0	0	0	0	0	0	0	0	0	0	0	0	0	0	0	0	0	0	0	0
XM_021063011.1	SORL1	mRNA	0	0	0	0	0	0	0	0	0	0	0	0	0	0	0	0	0	0	0	0	0
XM_021063012.1	SORL1	mRNA	0	0	0	0	0	0	0	0	0	0	0	0	0	0	0	0	0	0	0	0	0
XM_021063013.1	SORL1	mRNA	0	0	0	0	0	0	0	0	0	0	0	0	0	0	0	0	0	0	0	0	0
XM_021063014.1	SORL1	mRNA	0	0	0	0	0	0	0	0	0	0	0	0	0	0	0	0	0	0	0	0	0
XM_021063023.1	CLMP	mRNA	0	0	0	0	0	0	0	0	0	0	0	0	0	0	0	0	0	0	0	0	0
XM_021063024.1	CLMP	mRNA	0	0	0	0	0	0	0	0	0	0	0	0	0	0	0	0	0	0	0	0	0

RNA ID	Symbol	Type	updown_BGIR9823_85841	updown_BGIR9823_83955	updown_BGIR9823_84358	updown_BGIR9823_88015	updown_BGIR9823_88124	updown_BGIR9823_88780	updown_BGIR9823_92129	updown_BGIR9823_89027	updown_BGIR9823_89210	updown_BGIR9823_90771	updown_BGIR9823_95037	updown_BGIR9823_99096	updown_XR_001297664.2	updown_XR_002338666.1	updown_XR_002338675.1	updown_XR_002335905.1	updown_XR_002336236.1	updown_XR_001304283.2	updown_XR_002337660.1	updown_XR_307765.3	updown_XR_002343366.1
XM_021063025.1	CLMP	mRNA	0	0	0	0	0	0	0	0	0	0	0	0	0	0	0	0	0	0	0	0	0
XM_021063026.1	CLMP	mRNA	0	0	0	0	0	0	0	0	0	0	0	0	0	0	0	0	0	0	0	0	0
XM_021063950.1	NXPE2	mRNA	0	0	0	0	0	0	0	0	0	0	0	0	0	0	0	0	0	0	0	0	0
XM_021063963.1	LOC100623190	mRNA	0	0	0	0	0	0	0	0	0	0	0	0	0	0	0	0	0	0	0	0	0
XM_021065159.1	HMGB1	mRNA	0	0	0	0	0	0	0	0	0	0	0	0	0	0	0	0	0	0	0	0	0
XM_021065160.1	HMGB1	mRNA	0	0	0	0	0	0	0	0	0	0	0	0	0	0	0	0	0	0	0	0	0
XM_021065161.1	HMGB1	mRNA	0	0	0	0	0	0	0	0	0	0	0	0	0	0	0	0	0	0	0	0	0
XM_021065529.1	KATNAL1	mRNA	0	0	0	0	0	0	0	0	0	0	0	0	0	0	0	0	0	0	0	0	0
XM_021065531.1	KATNAL1	mRNA	0	0	0	0	0	0	0	0	0	0	0	0	0	0	0	0	0	0	0	0	0
XM_021065532.1	KATNAL1	mRNA	0	0	0	0	0	0	0	0	0	0	0	0	0	0	0	0	0	0	0	0	0
XM_021065533.1	KATNAL1	mRNA	0	0	0	0	0	0	0	0	0	0	0	0	0	0	0	0	0	0	0	0	0
XM_021065534.1	KATNAL1	mRNA	0	0	0	0	0	0	0	0	0	0	0	0	0	0	0	0	0	0	0	0	0
XM_021065535.1	KATNAL1	mRNA	0	0	0	0	0	0	0	0	0	0	0	0	0	0	0	0	0	0	0	0	0
XM_021065536.1	KATNAL1	mRNA	0	0	0	0	0	0	0	0	0	0	0	0	0	0	0	0	0	0	0	0	0
XM_021065537.1	KATNAL1	mRNA	0	0	0	0	0	0	0	0	0	0	0	0	0	0	0	0	0	0	0	0	0
XM_021065538.1	KATNAL1	mRNA	0	0	0	0	0	0	0	0	0	0	0	0	0	0	0	0	0	0	0	0	0
XM_021065539.1	KATNAL1	mRNA	0	0	0	0	0	0	0	0	0	0	0	0	0	0	0	0	0	0	0	0	0
XM_021065541.1	KATNAL1	mRNA	0	0	0	0	0	0	0	0	0	0	0	0	0	0	0	0	0	0	0	0	0
XM_021065542.1	KATNAL1	mRNA	0	0	0	0	0	0	0	0	0	0	0	0	0	0	0	0	0	0	0	0	0
XM_021065543.1	KATNAL1	mRNA	0	0	0	0	0	0	0	0	0	0	0	0	0	0	0	0	0	0	0	0	0
XM_021065544.1	KATNAL1	mRNA	0	0	0	0	0	0	0	0	0	0	0	0	0	0	0	0	0	0	0	0	0
XM_021073408.1	EMX2	mRNA	0	0	0	0	0	0	0	0	0	0	0	0	0	0	0	0	0	0	0	0	0
XM_021078611.1	PRKAG2	mRNA	0	0	0	0	0	0	0	0	0	0	0	0	0	0	0	0	0	0	0	0	0
XM_021078612.1	PRKAG2	mRNA	0	0	0	0	0	0	0	0	0	0	0	0	0	0	0	0	0	0	0	0	0
XM_021078613.1	PRKAG2	mRNA	0	0	0	0	0	0	0	0	0	0	0	0	0	0	0	0	0	0	0	0	0
XM_021078614.1	PRKAG2	mRNA	0	0	0	0	0	0	0	0	0	0	0	0	0	0	0	0	0	0	0	0	0
XM_021078615.1	PRKAG2	mRNA	0	0	0	0	0	0	0	0	0	0	0	0	0	0	0	0	0	0	0	0	0
XM_021078616.1	PRKAG2	mRNA	0	0	0	0	0	0	0	0	0	0	0	0	0	0	0	0	0	0	0	0	0
XM_021078617.1	PRKAG2	mRNA	0	0	0	0	0	0	0	0	0	0	0	0	0	0	0	0	0	0	0	0	0
XM_021078618.1	PRKAG2	mRNA	0	0	0	0	0	0	0	0	0	0	0	0	0	0	0	0	0	0	0	0	0
XM_021078619.1	PRKAG2	mRNA	0	0	0	0	0	0	0	0	0	0	0	0	0	0	0	0	0	0	0	0	0
XM_021078620.1	PRKAG2	mRNA	0	0	0	0	0	0	0	0	0	0	0	0	0	0	0	0	0	0	0	0	0
XM_021078621.1	PRKAG2	mRNA	0	0	0	0	0	0	0	0	0	0	0	0	0	0	0	0	0	0	0	0	0
XM_021081309.1	LOC106506226	mRNA	0	0	0	0	0	0	0	0	0	0	0	0	0	0	0	0	0	0	0	0	0
XM_021081403.1	LOC110258215	mRNA	0	0	0	0	0	0	0	0	0	0	0	0	0	0	0	0	0	0	0	0	0
XM_021082153.1	LOC100521600	mRNA	0	0	0	0	0	0	0	0	0	0	0	0	0	0	0	0	0	0	0	0	0
XM_021082154.1	LOC100521600	mRNA	0	0	0	0	0	0	0	0	0	0	0	0	0	0	0	0	0	0	0	0	0
XM_021082156.1	LOC100521600	mRNA	0	0	0	0	0	0	0	0	0	0	0	0	0	0	0	0	0	0	0	0	0
XM_021082157.1	LOC100521600	mRNA	0	0	0	0	0	0	0	0	0	0	0	0	0	0	0	0	0	0	0	0	0
XM_021082158.1	LOC100521600	mRNA	0	0	0	0	0	0	0	0	0	0	0	0	0	0	0	0	0	0	0	0	0
XM_021082159.1	LOC100521600	mRNA	0	0	0	0	0	0	0	0	0	0	0	0	0	0	0	0	0	0	0	0	0
XM_021082835.1	EHBP1L1	mRNA	0	0	0	0	0	0	0	0	0	0	0	0	0	0	0	0	0	0	0	0	0
XM_021082836.1	EHBP1L1	mRNA	0	0	0	0	0	0	0	0	0	0	0	0	0	0	0	0	0	0	0	0	0

RNA ID	Symbol	Type	updown_BGIR9823_85841	updown_BGIR9823_83955	updown_BGIR9823_84358	updown_BGIR9823_88015	updown_BGIR9823_88124	updown_BGIR9823_88780	updown_BGIR9823_92129	updown_BGIR9823_89027	updown_BGIR9823_89210	updown_BGIR9823_90771	updown_BGIR9823_95037	updown_BGIR9823_99096	updown_XR_001297664.2	updown_XR_002338666.1	updown_XR_002338675.1	updown_XR_002335905.1	updown_XR_002336236.1	updown_XR_001304283.2	updown_XR_002337660.1	updown_XR_307765.3	updown_XR_002343366.1
XM_021082837.1	EHBP1L1	mRNA	0	0	0	0	0	0	0	0	0	0	0	0	0	0	0	0	0	0	0	0	0
XM_021082838.1	EHBP1L1	mRNA	0	0	0	0	0	0	0	0	0	0	0	0	0	0	0	0	0	0	0	0	0
XM_021082839.1	EHBP1L1	mRNA	0	0	0	0	0	0	0	0	0	0	0	0	0	0	0	0	0	0	0	0	0
XM_021082840.1	EHBP1L1	mRNA	0	0	0	0	0	0	0	0	0	0	0	0	0	0	0	0	0	0	0	0	0
XM_021082841.1	EHBP1L1	mRNA	0	0	0	0	0	0	0	0	0	0	0	0	0	0	0	0	0	0	0	0	0
XM_021082842.1	EHBP1L1	mRNA	0	0	0	0	0	0	0	0	0	0	0	0	0	0	0	0	0	0	0	0	0
XM_021082843.1	EHBP1L1	mRNA	0	0	0	0	0	0	0	0	0	0	0	0	0	0	0	0	0	0	0	0	0
XM_021082844.1	EHBP1L1	mRNA	0	0	0	0	0	0	0	0	0	0	0	0	0	0	0	0	0	0	0	0	0
XM_021082845.1	EHBP1L1	mRNA	0	0	0	0	0	0	0	0	0	0	0	0	0	0	0	0	0	0	0	0	0
XM_021082846.1	EHBP1L1	mRNA	0	0	0	0	0	0	0	0	0	0	0	0	0	0	0	0	0	0	0	0	0
XM_021082847.1	EHBP1L1	mRNA	0	0	0	0	0	0	0	0	0	0	0	0	0	0	0	0	0	0	0	0	0
XM_021082851.1	MAP3K11	mRNA	0	0	0	0	0	0	0	0	0	0	0	0	0	0	0	0	0	0	0	0	0
XM_021082852.1	SSSCA1	mRNA	0	0	0	0	0	0	0	0	0	0	0	0	0	0	0	0	0	0	0	0	0
XM_021082853.1	FAM89B	mRNA	0	0	0	0	0	0	0	0	0	0	0	0	0	0	0	0	0	0	0	0	0
XM_021082854.1	LTBP3	mRNA	0	0	0	0	0	0	0	0	0	0	0	0	0	0	0	0	0	0	0	0	0
XM_021082855.1	LTBP3	mRNA	0	0	0	0	0	0	0	0	0	0	0	0	0	0	0	0	0	0	0	0	0
XM_021082856.1	SCYL1	mRNA	0	0	0	0	0	0	0	0	0	0	0	0	0	0	0	0	0	0	0	0	0
XM_021082857.1	SLC25A45	mRNA	0	0	0	0	0	0	0	0	0	0	0	0	0	0	0	0	0	0	0	0	0
XM_021082858.1	SLC25A45	mRNA	0	0	0	0	0	0	0	0	0	0	0	0	0	0	0	0	0	0	0	0	0
XM_021091099.1	SYNGR1	mRNA	0	0	0	0	0	0	0	0	0	0	0	0	0	0	0	0	0	0	0	0	0
XM_021091101.1	SYNGR1	mRNA	0	0	0	0	0	0	0	0	0	0	0	0	0	0	0	0	0	0	0	0	0
XM_021091102.1	SYNGR1	mRNA	0	0	0	0	0	0	0	0	0	0	0	0	0	0	0	0	0	0	0	0	0
XM_021091103.1	SYNGR1	mRNA	0	0	0	0	0	0	0	0	0	0	0	0	0	0	0	0	0	0	0	0	0
XM_021091370.1	LOC102159820	mRNA	0	0	0	0	0	0	0	0	0	0	0	0	0	0	0	0	0	0	0	0	0
XM_021091373.1	TRMU	mRNA	0	0	0	0	0	0	0	0	0	0	0	0	0	0	0	0	0	0	0	0	0
XM_021091374.1	TRMU	mRNA	0	0	0	0	0	0	0	0	0	0	0	0	0	0	0	0	0	0	0	0	0
XM_021091375.1	TRMU	mRNA	0	0	0	0	0	0	0	0	0	0	0	0	0	0	0	0	0	0	0	0	0
XM_021091376.1	GTSE1	mRNA	0	0	0	0	0	0	0	0	0	0	0	0	0	0	0	0	0	0	0	0	0
XM_021091377.1	TTC38	mRNA	0	0	0	0	0	0	0	0	0	0	0	0	0	0	0	0	0	0	0	0	0
XM_021091379.1	LOC110260611	mRNA	0	0	0	0	0	0	0	0	0	0	0	0	0	0	0	0	0	0	0	0	0
XM_021091381.1	LOC110260611	mRNA	0	0	0	0	0	0	0	0	0	0	0	0	0	0	0	0	0	0	0	0	0
XM_021091454.1	TOB2	mRNA	0	0	0	0	0	0	0	0	0	0	0	0	0	0	0	0	0	0	0	0	0
XM_021091455.1	TOB2	mRNA	0	0	0	0	0	0	0	0	0	0	0	0	0	0	0	0	0	0	0	0	0
XM_021091456.1	PHF5A	mRNA	0	0	0	0	0	0	0	0	0	0	0	0	0	0	0	0	0	0	0	0	0
XM_021091457.1	PHF5A	mRNA	0	0	0	0	0	0	0	0	0	0	0	0	0	0	0	0	0	0	0	0	0
XM_021091458.1	ZC3H7B	mRNA	0	0	0	0	0	0	0	0	0	0	0	0	0	0	0	0	0	0	0	0	0
XM_021091459.1	ZC3H7B	mRNA	0	0	0	0	0	0	0	0	0	0	0	0	0	0	0	0	0	0	0	0	0
XM_021091460.1	TEF	mRNA	0	0	0	0	0	0	0	0	0	0	0	0	0	0	0	0	0	0	0	0	0
XM_021091461.1	RANGAP1	mRNA	0	0	0	0	0	0	0	0	0	0	0	0	0	0	0	0	0	0	0	0	0
XM_021091474.1	CBX7	mRNA	0	0	0	0	0	0	0	0	0	0	0	0	0	0	0	0	0	0	0	0	0
XM_021092767.1	CRADD	mRNA	1	0	0	0	0	0	0	0	0	0	0	0	0	0	0	0	0	0	0	0	0
XM_021092768.1	CRADD	mRNA	1	0	0	0	0	0	0	0	0	0	0	0	0	0	0	0	0	0	0	0	0
XM_021092769.1	CRADD	mRNA	1	0	0	0	0	0	0	0	0	0	0	0	0	0	0	0	0	0	0	0	0

RNA ID	Symbol	Type	updown_BGIR9823_85841	updown_BGIR9823_83955	updown_BGIR9823_84358	updown_BGIR9823_88015	updown_BGIR9823_88124	updown_BGIR9823_88780	updown_BGIR9823_92129	updown_BGIR9823_89027	updown_BGIR9823_89210	updown_BGIR9823_90771	updown_BGIR9823_95037	updown_BGIR9823_99096	updown_XR_001297664.2	updown_XR_002338666.1	updown_XR_002338675.1	updown_XR_002335905.1	updown_XR_002336236.1	updown_XR_001304283.2	updown_XR_002337660.1	updown_XR_307765.3	updown_XR_002343366.1
XM_021092770.1	CRADD	mRNA	1	0	0	0	0	0	0	0	0	0	0	0	0	0	0	0	0	0	0	0	0
XM_021092771.1	CRADD	mRNA	1	0	0	0	0	0	0	0	0	0	0	0	0	0	0	0	0	0	0	0	0
XM_021092773.1	CRADD	mRNA	1	0	0	0	0	0	0	0	0	0	0	0	0	0	0	0	0	0	0	0	0
XM_021093095.1	PKDREJ	mRNA	0	0	0	0	0	0	0	0	0	0	0	0	0	0	0	0	0	0	0	0	0
XM_021097064.1	MAF	mRNA	0	0	0	0	0	0	0	0	0	0	0	0	0	0	0	0	0	0	0	0	0
XM_021097065.1	MAF	mRNA	0	0	0	0	0	0	0	0	0	0	0	0	0	0	0	0	0	0	0	0	0
XM_021097066.1	MAF	mRNA	0	0	0	0	0	0	0	0	0	0	0	0	0	0	0	0	0	0	0	0	0
XM_021097870.1	ECI2	mRNA	0	0	0	0	0	0	0	1	0	0	0	0	1	0	0	0	0	0	0	0	0
XM_021099751.1	PHACTR1	mRNA	0	0	0	0	0	0	0	0	0	0	0	0	0	0	0	0	0	0	0	0	0
XM_021099752.1	PHACTR1	mRNA	0	0	0	0	0	0	0	0	0	0	0	0	0	0	0	0	0	0	0	0	0
XM_021099753.1	PHACTR1	mRNA	0	0	0	0	0	0	0	0	0	0	0	0	0	0	0	0	0	0	0	0	0
XM_021099754.1	PHACTR1	mRNA	0	0	0	0	0	0	0	0	0	0	0	0	0	0	0	0	0	0	0	0	0
XM_021099755.1	PHACTR1	mRNA	0	0	0	0	0	0	0	0	0	0	0	0	0	0	0	0	0	0	0	0	0
XM_021099756.1	PHACTR1	mRNA	0	0	0	0	0	0	0	0	0	0	0	0	0	0	0	0	0	0	0	0	0
XM_021099757.1	PHACTR1	mRNA	0	0	0	0	0	0	0	0	0	0	0	0	0	0	0	0	0	0	0	0	0
XM_021099758.1	PHACTR1	mRNA	0	0	0	0	0	0	0	0	0	0	0	0	0	0	0	0	0	0	0	0	0
XM_021099759.1	PHACTR1	mRNA	0	0	0	0	0	0	0	0	0	0	0	0	0	0	0	0	0	0	0	0	0
XM_021099760.1	PHACTR1	mRNA	0	0	0	0	0	0	0	0	0	0	0	0	0	0	0	0	0	0	0	0	0
XM_021099761.1	PHACTR1	mRNA	0	0	0	0	0	0	0	0	0	0	0	0	0	0	0	0	0	0	0	0	0
XM_021099762.1	PHACTR1	mRNA	0	0	0	0	0	0	0	0	0	0	0	0	0	0	0	0	0	0	0	0	0
XM_021099763.1	PHACTR1	mRNA	0	0	0	0	0	0	0	0	0	0	0	0	0	0	0	0	0	0	0	0	0
XM_021099764.1	PHACTR1	mRNA	0	0	0	0	0	0	0	0	0	0	0	0	0	0	0	0	0	0	0	0	0
XM_021099765.1	PHACTR1	mRNA	0	0	0	0	0	0	0	0	0	0	0	0	0	0	0	0	0	0	0	0	0
XM_021099766.1	TBC1D7	mRNA	0	0	0	0	0	0	0	0	0	0	0	0	0	0	0	0	0	0	0	0	0
XM_021099767.1	TBC1D7	mRNA	0	0	0	0	0	0	0	0	0	0	0	0	0	0	0	0	0	0	0	0	0
XM_021099769.1	TBC1D7	mRNA	0	0	0	0	0	0	0	0	0	0	0	0	0	0	0	0	0	0	0	0	0
XM_021099770.1	TBC1D7	mRNA	0	0	0	0	0	0	0	0	0	0	0	0	0	0	0	0	0	0	0	0	0
XM_021099999.1	LOC110261697	mRNA	0	0	0	0	0	0	0	1	0	0	0	0	1	0	0	0	0	0	0	0	0
XM_021100108.1	WRNIP1	mRNA	0	0	0	0	0	0	0	0	0	0	0	0	0	0	0	0	0	0	0	0	0
XM_021100109.1	MYLK4	mRNA	0	0	0	0	0	0	0	0	0	0	0	0	0	0	0	0	0	0	0	0	0
XM_021100110.1	MYLK4	mRNA	0	0	0	0	0	0	0	0	0	0	0	0	0	0	0	0	0	0	0	0	0
XM_021100111.1	MYLK4	mRNA	0	0	0	0	0	0	0	0	0	0	0	0	0	0	0	0	0	0	0	0	0
XM_021100112.1	MYLK4	mRNA	0	0	0	0	0	0	0	0	0	0	0	0	0	0	0	0	0	0	0	0	0
XM_021100114.1	NQO2	mRNA	0	0	0	0	0	0	0	0	0	0	0	0	0	0	0	0	0	0	0	0	0
XM_021100115.1	NQO2	mRNA	0	0	0	0	0	0	0	0	0	0	0	0	0	0	0	0	0	0	0	0	0
XM_021100117.1	NQO2	mRNA	0	0	0	0	0	0	0	0	0	0	0	0	0	0	0	0	0	0	0	0	0
XM_021100118.1	NQO2	mRNA	0	0	0	0	0	0	0	0	0	0	0	0	0	0	0	0	0	0	0	0	0
XM_021100119.1	NQO2	mRNA	0	0	0	0	0	0	0	0	0	0	0	0	0	0	0	0	0	0	0	0	0
XM_021100124.1	PRPF4B	mRNA	0	0	0	0	0	0	0	1	0	0	0	0	1	0	0	0	0	0	0	0	0
XM_021100126.1	PRPF4B	mRNA	0	0	0	0	0	0	0	1	0	0	0	0	1	0	0	0	0	0	0	0	0
XM_021100184.1	NEDD9	mRNA	0	0	0	0	0	0	0	0	0	0	0	0	0	0	0	0	0	0	0	0	0
XM_021100188.1	TMEM170B	mRNA	0	0	0	0	0	0	0	0	0	0	0	0	0	0	0	0	0	0	0	0	0
XM_021100194.1	GFOD1	mRNA	0	0	0	0	0	0	0	0	0	0	0	0	0	0	0	0	0	0	0	0	0

RNA ID	Symbol	Type	updown_BGIR9823_85841	updown_BGIR9823_83955	updown_BGIR9823_84358	updown_BGIR9823_88015	updown_BGIR9823_88124	updown_BGIR9823_88780	updown_BGIR9823_92129	updown_BGIR9823_89027	updown_BGIR9823_89210	updown_BGIR9823_90771	updown_BGIR9823_95037	updown_BGIR9823_99096	updown_XR_001297664.2	updown_XR_002338666.1	updown_XR_002338675.1	updown_XR_002335905.1	updown_XR_002336236.1	updown_XR_001304283.2	updown_XR_002337660.1	updown_XR_307765.3	updown_XR_002343366.1
XM_021102040.1	SLC37A4	mRNA	0	0	0	0	0	0	0	0	0	0	0	0	0	0	0	0	0	0	0	0	0
XM_021102041.1	SLC37A4	mRNA	0	0	0	0	0	0	0	0	0	0	0	0	0	0	0	0	0	0	0	0	0
XM_021102042.1	SLC37A4	mRNA	0	0	0	0	0	0	0	0	0	0	0	0	0	0	0	0	0	0	0	0	0
XM_021102043.1	SLC37A4	mRNA	0	0	0	0	0	0	0	0	0	0	0	0	0	0	0	0	0	0	0	0	0
XM_021102044.1	SLC37A4	mRNA	0	0	0	0	0	0	0	0	0	0	0	0	0	0	0	0	0	0	0	0	0
XM_021102045.1	SLC37A4	mRNA	0	0	0	0	0	0	0	0	0	0	0	0	0	0	0	0	0	0	0	0	0
XM_021102046.1	SLC37A4	mRNA	0	0	0	0	0	0	0	0	0	0	0	0	0	0	0	0	0	0	0	0	0
XM_021102047.1	SLC37A4	mRNA	0	0	0	0	0	0	0	0	0	0	0	0	0	0	0	0	0	0	0	0	0
XM_021102048.1	SLC37A4	mRNA	0	0	0	0	0	0	0	0	0	0	0	0	0	0	0	0	0	0	0	0	0
XM_021102049.1	SLC37A4	mRNA	0	0	0	0	0	0	0	0	0	0	0	0	0	0	0	0	0	0	0	0	0
XM_021102050.1	SLC37A4	mRNA	0	0	0	0	0	0	0	0	0	0	0	0	0	0	0	0	0	0	0	0	0
XM_021102051.1	SLC37A4	mRNA	0	0	0	0	0	0	0	0	0	0	0	0	0	0	0	0	0	0	0	0	0
XM_021102052.1	SLC37A4	mRNA	0	0	0	0	0	0	0	0	0	0	0	0	0	0	0	0	0	0	0	0	0
XM_021102209.1	UPK2	mRNA	0	0	0	0	0	0	0	0	0	0	0	0	0	0	0	0	0	0	0	0	0
XM_021102516.1	LOC100522455	mRNA	0	0	0	0	0	0	0	0	0	0	0	0	0	0	0	0	0	0	0	0	0
XM_021102517.1	RUNDC3B	mRNA	0	0	0	0	0	0	0	0	0	0	0	0	0	0	0	0	0	0	0	0	0
XM_021102518.1	RUNDC3B	mRNA	0	0	0	0	0	0	0	0	0	0	0	0	0	0	0	0	0	0	0	0	0
XM_021102519.1	RUNDC3B	mRNA	0	0	0	0	0	0	0	0	0	0	0	0	0	0	0	0	0	0	0	0	0
XM_021102522.1	RUNDC3B	mRNA	0	0	0	0	0	0	0	0	0	0	0	0	0	0	0	0	0	0	0	0	0
XM_021102523.1	RUNDC3B	mRNA	0	0	0	0	0	0	0	0	0	0	0	0	0	0	0	0	0	0	0	0	0
XR_001297664.2	LOC102161744	lncRNA	0	0	0	0	0	0	0	1	0	0	0	0	1	0	0	0	0	0	0	0	0
XR_001298807.2	LOC106504881	lncRNA	0	0	0	0	0	0	0	0	0	0	0	0	0	0	0	0	0	0	0	0	0
XR_001298808.2	LOC106504881	lncRNA	0	0	0	0	0	0	0	0	0	0	0	0	0	0	0	0	0	0	0	0	0
XR_001298810.2	LOC106504881	lncRNA	0	0	0	0	0	0	0	0	0	0	0	0	0	0	0	0	0	0	0	0	0
XR_001298811.2	LOC106504881	lncRNA	0	0	0	0	0	0	0	0	0	0	0	0	0	0	0	0	0	0	0	0	0
XR_001298812.2	LOC106504881	lncRNA	0	0	0	0	0	0	0	0	0	0	0	0	0	0	0	0	0	0	0	0	0
XR_001298813.2	LOC106504881	lncRNA	0	0	0	0	0	0	0	0	0	0	0	0	0	0	0	0	0	0	0	0	0
XR_001298814.2	LOC106504881	lncRNA	0	0	0	0	0	0	0	0	0	0	0	0	0	0	0	0	0	0	0	0	0
XR_001298816.2	LOC106504881	lncRNA	0	0	0	0	0	0	0	0	0	0	0	0	0	0	0	0	0	0	0	0	0
XR_001298817.2	LOC106504881	lncRNA	0	0	0	0	0	0	0	0	0	0	0	0	0	0	0	0	0	0	0	0	0
XR_001298844.2	LOC102163364	lncRNA	0	0	0	0	0	0	0	0	0	0	0	0	0	0	0	0	0	0	0	0	0
XR_001298846.2	LOC102163364	lncRNA	0	0	0	0	0	0	0	0	0	0	0	0	0	0	0	0	0	0	0	0	0
XR_001298850.2	LOC102163364	lncRNA	0	0	0	0	0	0	0	0	0	0	0	0	0	0	0	0	0	0	0	0	0
XR_001299456.2	LOC102166670	lncRNA	0	0	0	0	0	0	0	0	0	0	0	0	0	0	0	0	0	0	0	0	0
XR_001299457.2	LOC102166670	lncRNA	0	0	0	0	0	0	0	0	0	0	0	0	0	0	0	0	0	0	0	0	0
XR_001300715.2	LOC106505879	lncRNA	0	0	0	0	0	0	0	0	0	0	0	0	0	1	0	0	0	0	0	0	0
XR_001301078.2	LOC102162623	lncRNA	0	0	0	0	0	0	0	0	0	0	0	0	0	0	0	0	0	0	0	0	0
XR_001301087.2	LOC102162623	lncRNA	0	0	0	0	0	0	0	0	0	0	0	0	0	0	0	0	0	0	0	0	0
XR_001301217.2	LOC102164998	lncRNA	0	0	0	0	0	0	0	0	0	0	0	0	0	0	0	0	0	0	0	0	0
XR_001301305.2	LOC102159607	lncRNA	0	0	0	0	0	0	0	0	0	0	0	0	0	0	0	0	0	0	0	0	0
XR_001308967.2	LOC106510467	lncRNA	0	0	0	0	0	0	0	0	0	0	0	0	0	0	0	0	0	0	0	0	0
XR_002335533.1	XRRA1	mRNA	0	0	0	0	0	0	0	0	0	1	0	0	0	0	0	0	0	0	0	0	0
XR_002335534.1	XRRA1	mRNA	0	0	0	0	0	0	0	0	0	1	0	0	0	0	0	0	0	0	0	0	0

RNA ID	Symbol	Type	updown_BGIR9823_85841	updown_BGIR9823_83955	updown_BGIR9823_84358	updown_BGIR9823_88015	updown_BGIR9823_88124	updown_BGIR9823_88780	updown_BGIR9823_92129	updown_BGIR9823_89027	updown_BGIR9823_89210	updown_BGIR9823_90771	updown_BGIR9823_95037	updown_BGIR9823_99096	updown_XR_001297664.2	updown_XR_002338666.1	updown_XR_002338675.1	updown_XR_002335905.1	updown_XR_002336236.1	updown_XR_001304283.2	updown_XR_002337660.1	updown_XR_307765.3	updown_XR_002343366.1
XR_002335535.1	LOC110255399	lncRNA	0	0	0	0	0	0	0	0	0	1	0	0	0	0	0	0	0	0	0	0	0
XR_002335536.1	LOC102160367	lncRNA	0	0	0	0	0	0	0	0	0	1	0	0	0	0	0	0	0	0	0	0	0
XR_002335537.1	LOC102160367	lncRNA	0	0	0	0	0	0	0	0	0	1	0	0	0	0	0	0	0	0	0	0	0
XR_002335650.1	LOC102157630	lncRNA	0	0	0	0	0	0	0	0	0	0	0	0	0	0	0	0	0	0	0	0	0
XR_002335651.1	LOC110255435	lncRNA	0	0	0	0	0	0	0	0	0	0	0	0	0	0	0	0	0	0	0	0	0
XR_002335652.1	LOC106504881	lncRNA	0	0	0	0	0	0	0	0	0	0	0	0	0	0	0	0	0	0	0	0	0
XR_002335653.1	LOC106504881	lncRNA	0	0	0	0	0	0	0	0	0	0	0	0	0	0	0	0	0	0	0	0	0
XR_002335670.1	LOC110255440	lncRNA	0	0	0	0	0	0	0	0	0	0	0	0	0	0	0	0	0	0	0	0	0
XR_002335671.1	LOC110255441	lncRNA	0	0	0	0	0	0	0	0	0	0	0	0	0	0	0	0	0	0	0	0	0
XR_002335672.1	LOC110255441	lncRNA	0	0	0	0	0	0	0	0	0	0	0	0	0	0	0	0	0	0	0	0	0
XR_002335674.1	HYOU1	mRNA	0	0	0	0	0	0	0	0	0	0	0	0	0	0	0	0	0	0	0	0	0
XR_002335675.1	HYOU1	mRNA	0	0	0	0	0	0	0	0	0	0	0	0	0	0	0	0	0	0	0	0	0
XR_002335685.1	LOC100523684	mRNA	0	0	0	0	0	0	0	0	0	0	0	0	0	0	0	0	0	0	0	0	0
XR_002335686.1	LOC102163364	lncRNA	0	0	0	0	0	0	0	0	0	0	0	0	0	0	0	0	0	0	0	0	0
XR_002335687.1	LOC102163364	lncRNA	0	0	0	0	0	0	0	0	0	0	0	0	0	0	0	0	0	0	0	0	0
XR_002335688.1	LOC102163364	lncRNA	0	0	0	0	0	0	0	0	0	0	0	0	0	0	0	0	0	0	0	0	0
XR_002335689.1	LOC102163364	lncRNA	0	0	0	0	0	0	0	0	0	0	0	0	0	0	0	0	0	0	0	0	0
XR_002335690.1	LOC102163364	lncRNA	0	0	0	0	0	0	0	0	0	0	0	0	0	0	0	0	0	0	0	0	0
XR_002335691.1	LOC102163364	lncRNA	0	0	0	0	0	0	0	0	0	0	0	0	0	0	0	0	0	0	0	0	0
XR_002335692.1	LOC102163364	lncRNA	0	0	0	0	0	0	0	0	0	0	0	0	0	0	0	0	0	0	0	0	0
XR_002335693.1	LOC106504896	lncRNA	0	0	0	0	0	0	0	0	0	0	0	0	0	0	0	0	0	0	0	0	0
XR_002335694.1	SORL1	mRNA	0	0	0	0	0	0	0	0	0	0	0	0	0	0	0	0	0	0	0	0	0
XR_002336641.1	LOC106505250	lncRNA	0	0	0	0	0	0	0	0	0	0	0	0	0	0	0	0	0	0	0	0	0
XR_002336643.1	LOC110255292	lncRNA	0	0	0	0	0	0	0	0	0	0	0	0	0	0	0	0	0	0	0	0	0
XR_002336644.1	LOC102166670	lncRNA	0	0	0	0	0	0	0	0	0	0	0	0	0	0	0	0	0	0	0	0	0
XR_002336645.1	LOC102166670	lncRNA	0	0	0	0	0	0	0	0	0	0	0	0	0	0	0	0	0	0	0	0	0
XR_002338443.1	LOC106505879	lncRNA	0	0	0	0	0	0	0	0	0	0	0	0	0	1	0	0	0	0	0	0	0
XR_002338444.1	LOC106505879	lncRNA	0	0	0	0	0	0	0	0	0	0	0	0	0	1	0	0	0	0	0	0	0
XR_002338445.1	LOC106505879	lncRNA	0	0	0	0	0	0	0	0	0	0	0	0	0	1	0	0	0	0	0	0	0
XR_002338446.1	LOC106505879	lncRNA	0	0	0	0	0	0	0	0	0	0	0	0	0	1	0	0	0	0	0	0	0
XR_002338447.1	LOC106505879	lncRNA	0	0	0	0	0	0	0	0	0	0	0	0	0	1	0	0	0	0	0	0	0
[truncated: 18,583 more chars]
